# Genome-wide identification of long non-coding RNA genes and their association with insecticide resistance and metamorphosis in diamondback moth, *Plutella xylostella*

**DOI:** 10.1038/s41598-017-16057-2

**Published:** 2017-11-20

**Authors:** Feiling Liu, Dianhao Guo, Zhuting Yuan, Chen Chen, Huamei Xiao

**Affiliations:** 1 College of Life Sciences and Resource Environment, Yichun University, Yichun, 336000 China; 2 Department of Entomology, College of Plant Protection, Nanjing Agricultural University, Nanjing, 210095 China; 3 The Center for Translational Medicine, Yichun University, Yichun, 336000 China

## Abstract

Long non-coding RNA (lncRNA) is a class of noncoding RNA >200 bp in length that has essential roles in regulating a variety of biological processes. Here, we constructed a computational pipeline to identify lncRNA genes in the diamondback moth (*Plutella xylostella*), a major insect pest of cruciferous vegetables. In total, 3,324 lncRNAs corresponding to 2,475 loci were identified from 13 RNA-Seq datasets, including samples from parasitized, insecticide-resistant strains and different developmental stages. The identified *P. xylostella* lncRNAs had shorter transcripts and fewer exons than protein-coding genes. Seven out of nine randomly selected lncRNAs were validated by strand-specific RT-PCR. In total, 54–172 lncRNAs were specifically expressed in the insecticide resistant strains, among which one lncRNA was located adjacent to the sodium channel gene. In addition, 63–135 lncRNAs were specifically expressed in different developmental stages, among which three lncRNAs overlapped or were located adjacent to the metamorphosis-associated genes. These lncRNAs were either strongly or weakly co-expressed with their overlapping or neighboring mRNA genes. In summary, we identified thousands of lncRNAs and presented evidence that lncRNAs might have key roles in conferring insecticide resistance and regulating the metamorphosis development in *P. xylostella*.

## Introduction

Given that the cost of whole-genome sequencing has decreased dramatically, numerous genome and transcriptome-sequencing projects in insects have been initiated in recent years, leading to the rapid accumulation of insect gene data. Currently, the genomes of 156 insects, including those of Diptera, Lepidoptera, and Hymenoptera^1^ insects, have been sequenced and deposited in public databases. Tens of thousands of insect transcriptomes have been submitted to the NCBI SRA database^2^, providing valuable resources for gene analysis^3^. However, most studies involving insect RNA-Seq data were limited to protein-coding genes. Information regarding noncoding RNA has not been fully explored.

The Encyclopedia of DNA Elements (ENCODE) project revealed that 80% of the human genome serves some purpose, and 76% of the bases in the full genome were transcribed into RNA^4^. Increasing evidence indicated that noncoding RNA (ncRNA) genes exist widely in the genomes of almost all organisms^5,6^. Greater than half of the mammalian transcriptome is comprised of ncRNAs^7^, consisting of small ncRNAs (microRNAs and piRNAs) and long ncRNAs (lncRNA, with transcripts ≥200 nucleotides that do not contain an open reading frame of longer than 100 amino acids)^8^. Based on their genome locations, lncRNAs can be classified into long intergenic ncRNA (lincRNA), intronic lncRNA, antisense lncRNA and enhancer RNA^8^.

The discovery and annotation of lncRNAs in insects has attracted increasing attentions in recent years^9–11^. Transcriptome data from 27 *Drosophila melanogaster* samples obtained at different developmental stages ranging from embryo to adult were analyzed by Chen *et al*.; in total, 21% and 42% lncRNAs were significantly upregulated at the late embryonic and larval stage, respectively, indicating that lncRNAs may participate in the development transition during metamorphosis^12^. Etebari *et al*. found that DENV-2 infection increased the expression of a number of host lincRNAs. RNA interference of some lincRNAs induced the suppression of viral replication, indicating that lncRNAs may be involved in the anti-viral defense^13^. Xiao *et al*. used a computational pipeline to identify lncRNAs from multiple *Nilaparvata lugens* RNA-Seq data, yielding 1,882 lncRNA genes. Numerous lncRNAs were specifically expressed in the high and low fecundity population, and 3 lncRNAs overlapped with three fertility-related protein-coding gene, separately, suggesting that lncRNAs might have key roles in fecundity in *N. lugens* ^14^.

Increasing evidence suggests that lncRNAs have important roles in a variety of biological processes^15–17^. LncRNAs are involved in dosage compensation, genomic imprinting, epigenetic and gene expression regulation^15,18,19^. The function of numerous lncRNAs have been experimentally confirmed in insects^20^. For instance, lncRNAs produced by the *hsw-ω* gene forms perinuclear omega-speckles in nuclei in response to heat shock^21^. Two male-specific lncRNAs, *roX1* and *roX2*, play pivotal roles in targeting chromosome-wide modification for dosage compensation in *Drosophila* ^22^. Yellow-achaete intergenic RNA (*yar*), the neural-specific *CRG* and the chemosensory organs-specific *sphinx* serves as regulators of sleeping behavior, locomotion and climbing behavior and male courtship behavior in *Drosophila* ^23–25^. *acal* is a recently identificated lncRNA that functions in JNK signaling involved in epithelial shape changes during *Drosophila* dorsal closure^26^. In *Apis mellifera*, four lncRNA (*Nb-1*, *Ks-1*, *AncR-1*, and *kakusei*) are preferentially expressed in the brain and related to behavior and the other two lncRNAs (*lncov1* and *lncov2*) are expressed in the ovaries^27–30^. *lncov1* is overexpressed in the ovaries of worker bees and regulates transgressive ovary size^31^.

The diamondback moth *Plutella xylostella* (L.) (Lepidoptera: Plutellidae) is a major pest of cruciferous vegetables and has developed resistance to numerous insecticides given the long-term use of chemical control coupled with the intensive and irrational use of insecticides^32^. In 1990, *P. xylostella* became the first reported insect species to have field-evolved Bt resistance^33^. In 2000, resistance to fipronil was reported for the first time in *P. xylostella* ^34^. *P. xylostella* is one of the most resistant pests in the world and the annual worldwide costs for controlling this insect pest are estimated at 4–5 billion dollars^35^.

Several studies have been performed to identify the lncRNAs in *P. xylostella*. Etebari *et al*. identified highly expressed lncRNAs in different insecticide-resistant strains^36^, and Zhu *et al*. identified lncRNAs associated with chlorantraniliprole resistance in diamondback moth by analyzing the high-throughput sequencing data^37^. Wang *et al*. identified many lncRNAs were microRNA precursors or competing endogenous RNA^38^. Understanding the role of lncRNAs in conferring insecticide resistance is important for studying the regulatory mechanisms to develop alternative pest control strategies.

Here, we developed a computational pipeline to identify lncRNAs from 13 RNA-Seq datasets from diamondback moth. We identified specifically or differentially expressed lncRNAs in different strain of diamondback moth that are resistant to insecticides fipronil, Bt, and chlorpyrifos and in samples obtained from different developmental stages. The results indicate that lncRNAs potentially have key roles in conferring insecticide resistance and regulating metamorphosis in insects.

## Results

### Identification and validation of lncRNAs in *P. xylostella*

**Figure 1:**
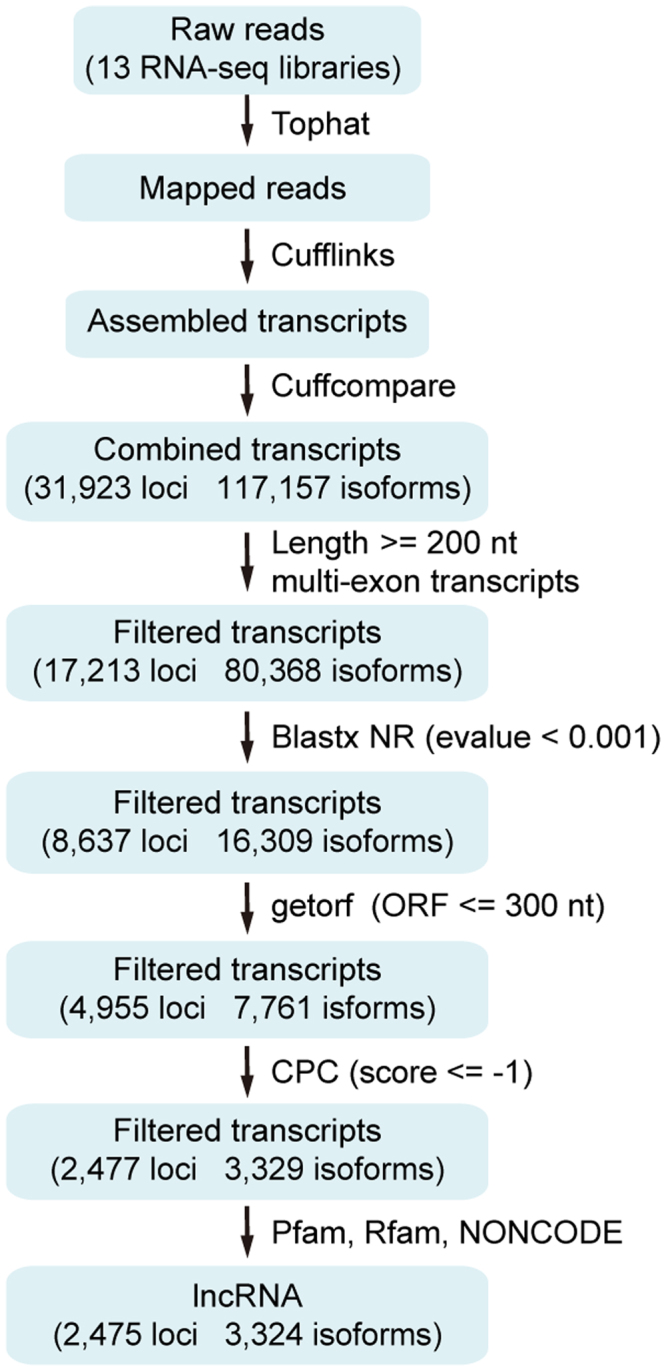
The pipeline for identifying lncRNAs in *P. xylostella* based on transcriptome data.

A computational pipeline was developed to identify lncRNA genes from 13* P. xylostella* transcriptomes, yielding 3,324 transcripts corresponding to 2,475 loci (Fig. 1, Supplementary file: Text file containing identified lncRNAs sequences). We divided these lncRNA transcripts into seven types based on their genome locations (Table 1). In total, 25.48% of lncRNAs are located in the intergenic region, whereas less than 1% of lncRNAs overlapped with a reference intron on the opposite strand. The total number of unclassified lncRNA was 756, accounting for 22.74%.

**Table 1:** The numbers of lncRNA in 13 transcriptomes of *P. xylostella*.

lncRNA types^a^	Number of lncRNA	Parasitism		Midgut				Resistance			Development			
		PDse^b^ larvae 3^rd^ instar	Control larvae 3^rd^ instar	CAS^c^ larvae 4^th^ instar (MM)	CAR^c^ larvae 4^th^ instar (GK)	CAR^c^ larvae 4^th^ instar (MK)	Common strain	PXS^d^ larvae 4^th^ instar	FR^d^ larvae 3^rd^ instar	CR^d^ Larvae 3^rd^ instar	Adults	Pupae	Larvae 3^rd^ instar	Eggs
Intergenic^1^	847	39	35	19	17	20	34	155	43	45	181	93	54	112
Intronic^2^	280	35	33	23		16	26	61	62	43	99	75	48	66
Intronic overlap^3^ (−)	11	3	3	3	3	3	4	5	2	4	1	0	4	0
Exonic overlap^4^ (+)	517	72	68	42	44	51	58	144	90	102	148	135	91	108
Exonic overlap^5^ (−)	284	29	39	15	26	23	20	72	40	44	73	51	40	52
Splice junction overlap^6^	629	77	89	36	55	54	59	140	132	115	190	186	108	130
Unclassified^7^	756	202	218	104	111	122	125	339	321	289	401	391	285	308
Total	3,324	457	485	242	274	289	326	916	690	642	1,093	931	630	776

^a^LncRNA types.

(1) Intergenic transcript; (2) Located completely within a reference intron; (3) Overlaps with a reference intron on the opposite strand; (4) Overlaps with a reference exon.

(5) Overlaps with a reference exon on the opposite strand; (6) At least one splice junction is shared with a reference transcript; (7) Unclassified.

^b^PDse: parasitized by *D. semiclausum*.

^c^CAS: Cry1Ac-susceptible, CAR: Cry1Ac-resistant; MM: susceptible strain DBM1Ac-S; GK: Cry1Ac-resistant strain T2-R; MK: Cry1Ac-resistant strain DBM1Ac-R.

^d^PXS: Bt toxin susceptible, FR: fipronil-resistant strain, CR: chlorpyrifos-resistant strain.

In total, 457 and 485 lncRNA genes were identified in the 3^rd^ instar larvae parasitized by *Diadegma semiclausum* and the unparasitized larvae, respectively (Table 1). In addition, 326, 242, 274 and 289 lncRNA genes were discovered in the midgut of control, Bt susceptible strain DBM1Ac-S (MM), Cry1Ac-resistant strain T2-R (GK) and Cry1Ac-resistant strain DBM1Ac-R (MK), respectively. Moreover, 916, 690 and 642 lncRNA were found in the Bt toxin susceptible, fipronil- and chlorpyrifos-resistant strains, respectively. In total, 776–1,093 lncRNA genes were identified in the egg, the 3^rd^ instar larvae, pupae and adult (Table 1).

**Figure 2:**
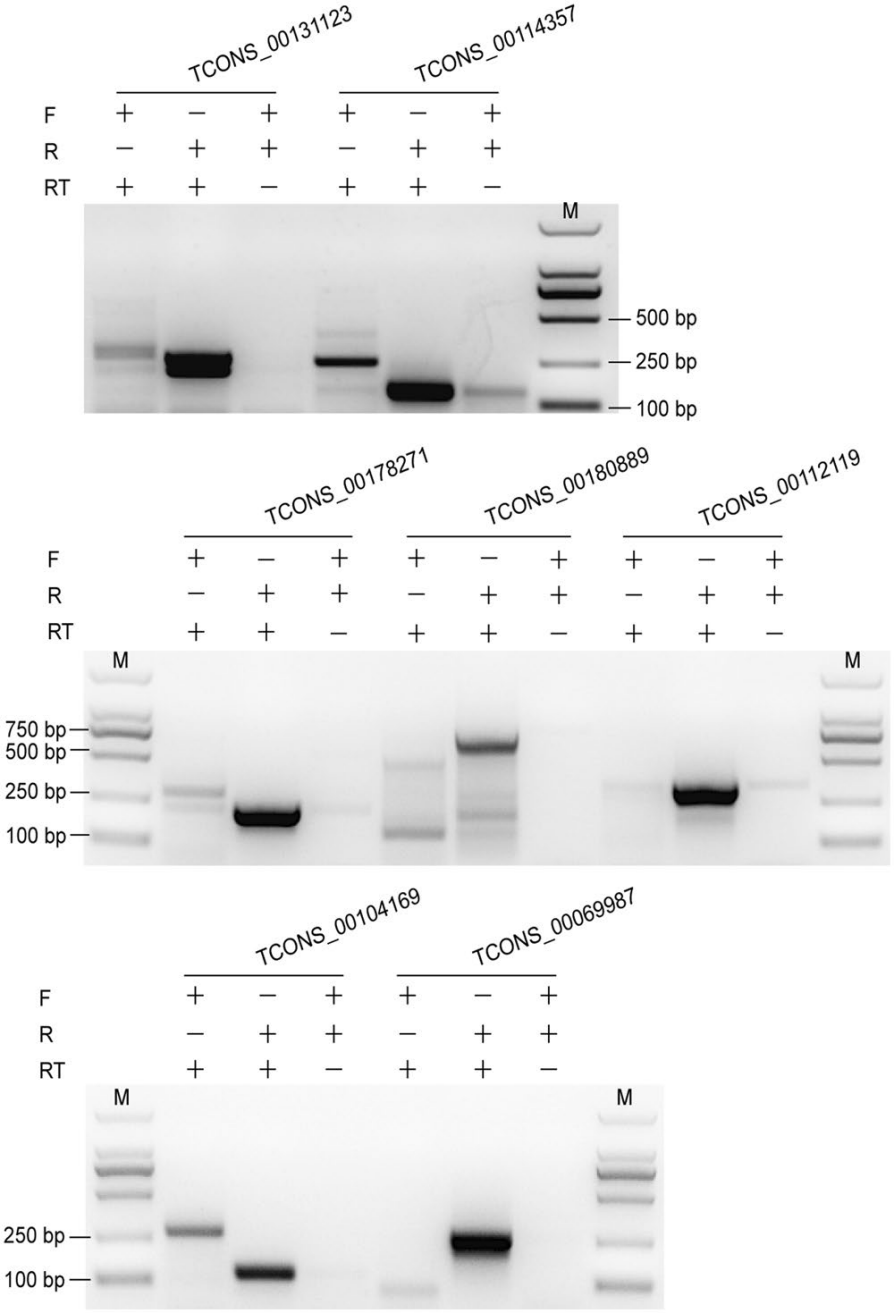
Strand-specific RT-PCR of nine randomly selected lncRNAs to determine the transcription orientation. Seven lncRNAs were successfully amplified and confirmed by sequencing. The results indicated that 7 lncRNAs were transcribed from the antisense strand. F: Forward primer; R: Reverse primer; RT: Reverse transcriptase. The full-length gels for (**A**, **B** and **C**) were presented in Supplementary Figs 1, 2 and 3.

To verify the reliability of the identified lncRNA genes, we randomly selected 9 lncRNAs for RT-PCR validation. Strand-specific RT-PCR was used to validate and confirm the transcription orientation of these lncRNAs. Seven lncRNAs were successfully amplified and confirmed to be transcribed from the antisense strand, demonstrating the high reliability of identified lncRNAs in terms of expression (Fig. 2).

### Structural features of lncRNAs in *P. xylostella*

**Figure 3:**
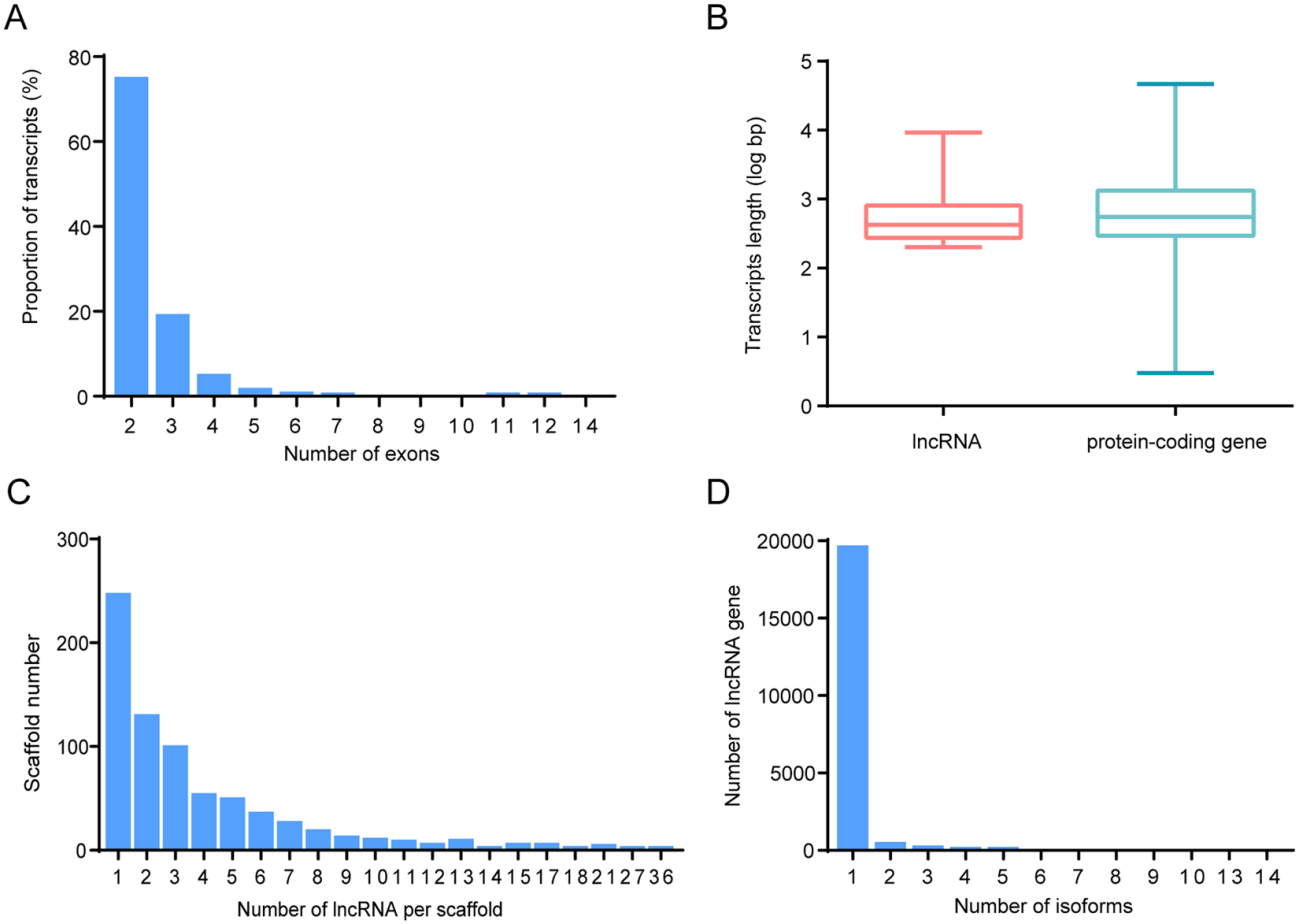
Structural gene features of *P. xylostella* lncRNAs. (**A**) Distribution of lncRNAs exon number in transcripts. The majority of lncRNAs have only two exons. (**B**) Length comparison of lncRNAs and protein-coding gene. On average, lncRNAs have shorter transcripts. (**C**) Distribution of lncRNAs among different scaffolds. The majority of scaffolds (74.5%) contain only 1–4 lncRNAs, whereas only 35* P. xylostella* genome scaffolds contain greater than 10 lncRNAs (5.0%). (**D**) Distribution of alternative spliced lncRNAs. Approximately 21% of lncRNAs exhibited alternative splicing.

The structural features of *P. xylostella* lncRNA genes were analyzed, suggesting that insect lncRNAs share similar features with their counterparts in mammals. In total, 74.49% of *P. xylostella* lncRNAs had only two exons, 4.57% had four exons and only 2.29% had greater than four exons (Fig. 3a). The average transcript length of *P. xylostella* lncRNAs was 912 bp whereas that of protein-coding genes was 1,385 bp (Fig. 3b). The majority of *P. xylostella* genome scaffolds (74.5%) contain less than five lncRNA loci. Only 35 of scaffold (5.0%) were enriched with greater than 10 lncRNA loci. One scaffold contained 36 lncRNA loci and 245 scaffolds contained only one lncRNA loci (Fig. 3c).

**Figure 4:**
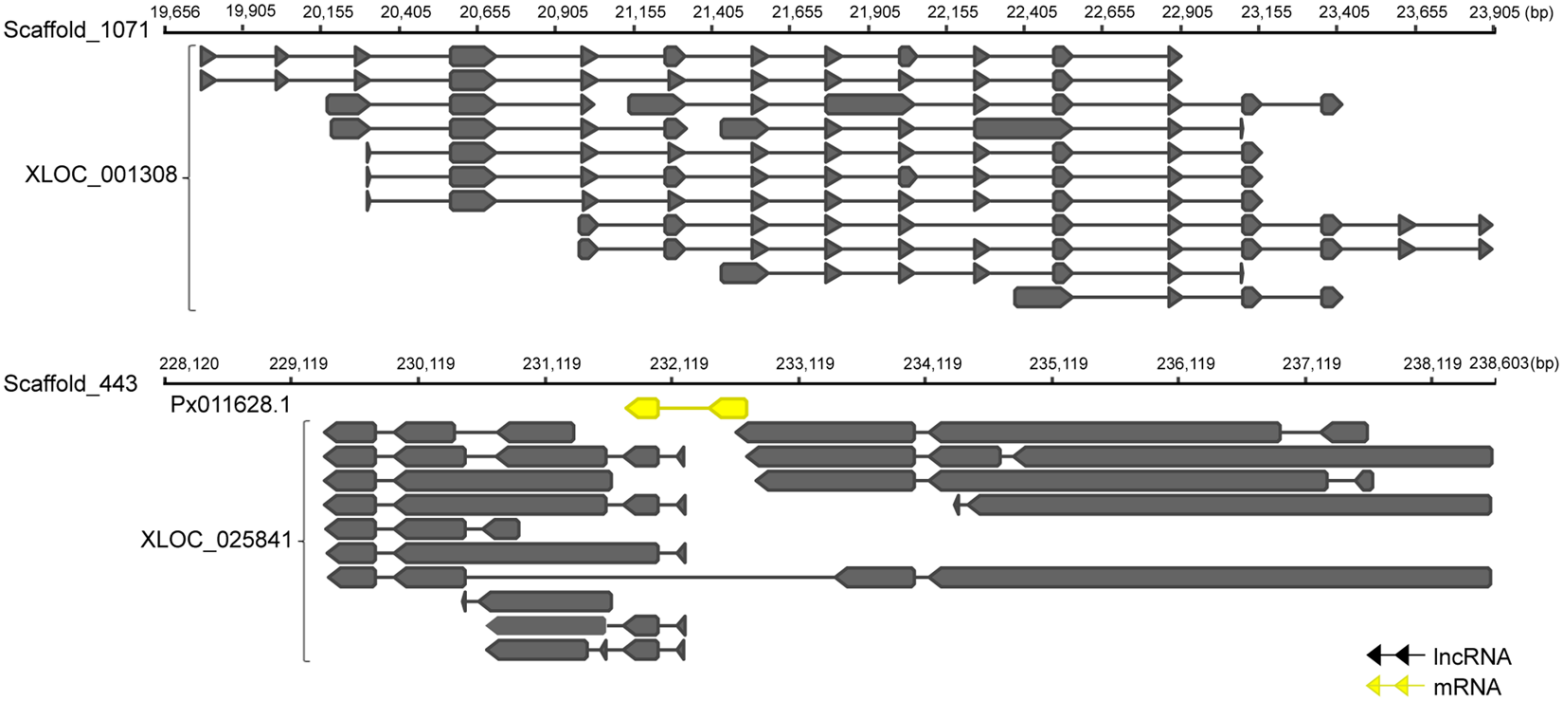
Gene structures of two lncRNAs that had the most alternatively spliced isoforms. XLOC_001308 has 13 spliced isoforms and XLOC_025841 has 14 spliced isoforms which overlapped with the protein-coding gene Px011628.1.

Only 21.17% of *P. xylostella* lncRNA genes exhibited alternative spliced isoforms (Fig. 3d), suggesting that alternative splicing (AS) was not abundant in lncRNA. XLOC_001308 which is located in the intergenic region of scaffold_1071, serves as an exception with 13 isoforms. XLOC_025841 which overlapped with the protein-coding gene Px011628.1 in the scaffold_443 had 14 isoforms (Fig. 4).

### LncRNAs potentially have important roles in conferring insecticide resistance

**Figure 5:**
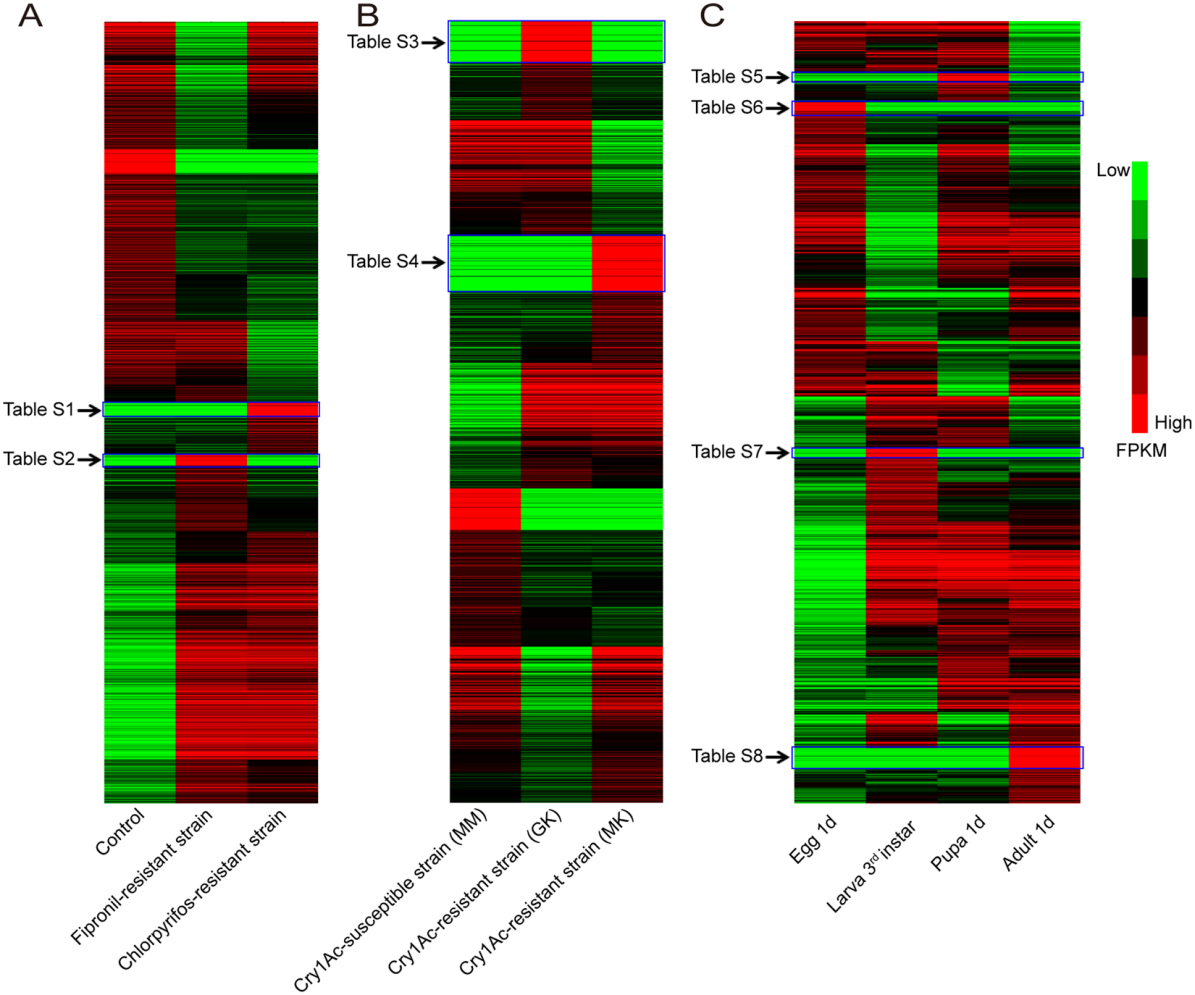
Heatmap of the lncRNA expression patterns in fipronil, chlorpyrifos, Bt-resistance strain and different developmental stage. (**A**) Expression profile changes in lncRNA transcripts across control and fipronil- and chlorpyrifos-resistant strains. (**B**) Expression profile changes of lncRNA transcripts across Bt-resistance strains (MM, GK and MK). (**C**) Hierarchical clustering of expressional abundance of lncRNA transcripts in egg, larvae, pupae and adult. LncRNAs specifically expressed in the fipronil- and chlorpyrifos-strains are listed in Supplementary Tables S1 and S2. The lncRNAs specifically expressed in the Bt-resistance strains are listed in Supplementary Tables S3 and S4. The lncRNAs specifically expressed in different developmental stages are listed in Supplementary Tables S5, S6, S7 and S8.

**Figure 6:**
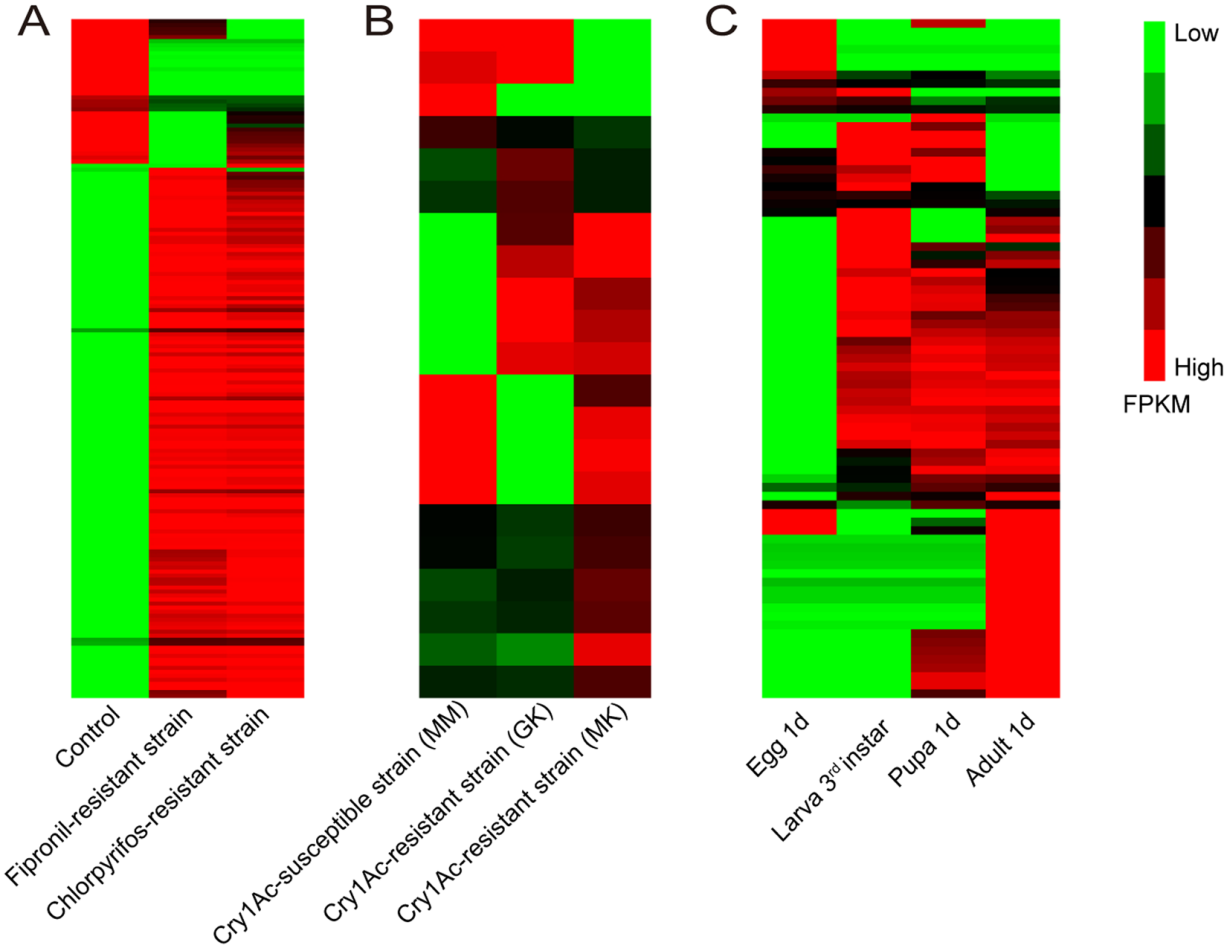
The heatmap of the differentially expressed lncRNAs in fipronil-, chlorpyrifos-, Bt-resistance strains and different developmental stages. (**A**) Clustering analysis of differentially expressed lncRNAs in the control and fipronil- and chlorpyrifos-resistant strains. (**B**) Clustering analysis of differentially expressed lncRNAs in different Bt-resistant strains. (**C**) Clustering analysis of differentially expressed lncRNAs in different developmental stages.

The transcript abundance of lncRNAs was estimated, indicating that most lncRNAs were ubiquitously expressed in all samples (Fig. 5). We analyzed the specifically and differentially expressed lncRNAs in insecticide-resistant strains. In the chlorpyrifos and fipronil resistant strain, 63 and 54 lncRNAs were specifically expressed, respectively (Fig. 5A, Supplementary Tables S1 and S2). In addition, 135 and 172 lncRNAs were specifically expressed in the Bt-resistant GK strain and MK strain, respectively (Fig. 5B, Supplementary Tables S3 and S4). In total, 152 and 127 lncRNAs were differentially expressed in the fipronil-resistant strain and in the chlorpyrifos-resistant strain, respectively (Fig. 6A). Ten lncRNAs were differentially expressed in the GK and MM strain and ten were in the MK and MM strain (Fig. 6B). The high number of specifically and differentially expressed lncRNAs in the chlorpyrifos-, fipronil- and Bt-resistant strains suggests that lncRNAs might play key roles in developing insecticide resistance in *P. xylostella*.

**Figure 7:**
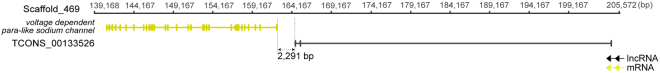
Relative genome position for the specifically expressed lncRNA and the closest protein-coding gene. TCONS_00133526 was specifically expressed in the fipronil-resistance strain, and the neighboring gene is voltage dependent *para*-like sodium channel.

We also analyzed overlapping or adjacent mRNA genes of specifically or differentially expressed lncRNAs. In the chlorpyrifos resistant strain, 28 mRNA genes overlapped with the specifically-expressed lncRNAs and 12 genes were located within ≤10 kb of these lncRNAs. In the fipronil resistant strain, 16 protein coding gene overlapped with the specifically or differentially expressed lncRNAs, and 15 genes were located within ≤10 kb of these lncRNAs. Among the overlapping or adjacent mRNA genes, we did not identify any known chlorpyrifos and fipronil detoxification or target genes. Interestingly, a voltage dependent *para*-like sodium channel gene was located at 2,291 bp upstream of TCONS_00133526 in the fipronil-resistant strain (Fig. 7, Pearson correlation r = 0.4, p = 0.2, *t*-test).The *para*-like voltage dependent-sodium channel is the target of pyrethroid insecticides. This interesting discovery is worthy of further investigation.

### Development-associated lncRNAs in *P. xylostella*

LncRNAs regulate insect metamorphosis development^12,39^. In *P. xylostella*, 63–135 lncRNAs were specifically expressed from the egg to adult (Fig. 5C, Supplementary Tables S5, S6, S7 and S8). Twenty-nine lncRNAs were differentially expressed between the egg and the 3^rd^ instar larvae. Specifically, 27 were identified in the egg and pupa, 40 in the egg and adult, 26 in the larvae and adult, and 27 in the pupa and adult. In total, 79 lncRNAs were differentially expressed (Fig. 6C).

**Figure 8:**
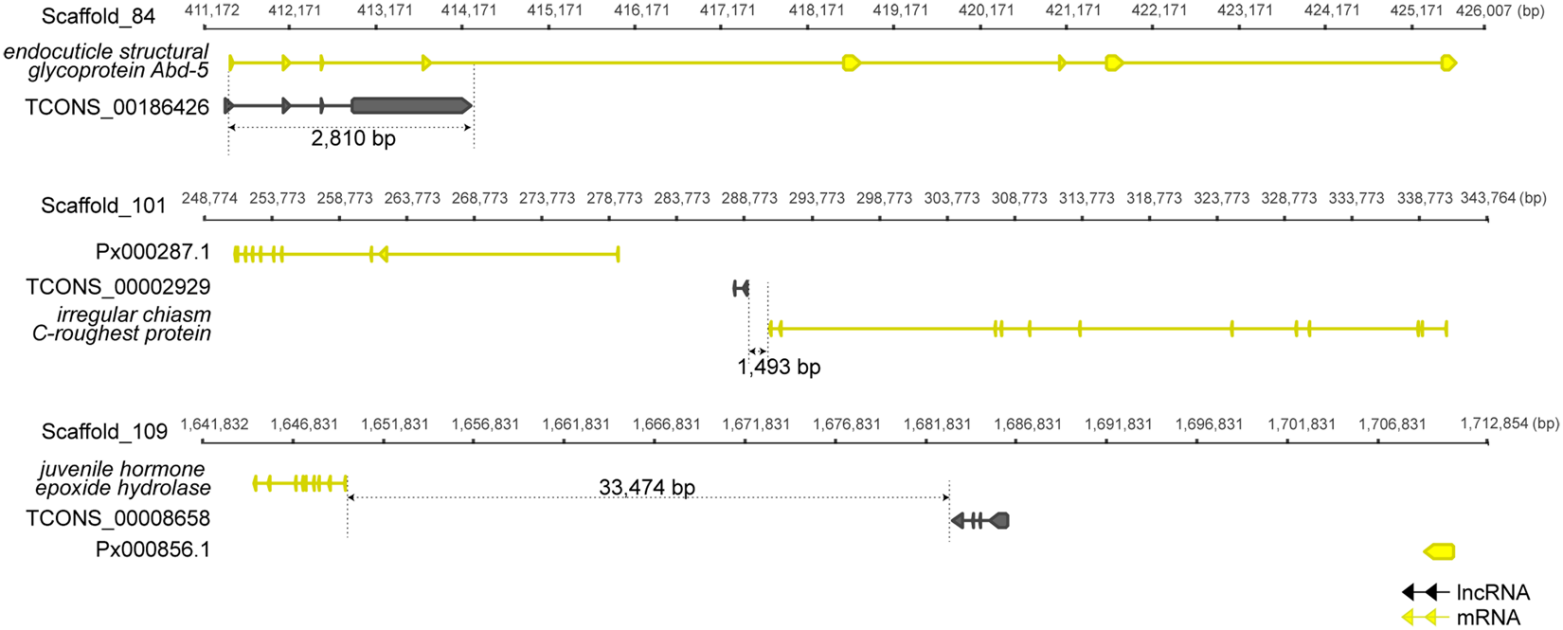
The exon and intron structures for three lncRNA genes that were specifically expressed in different developmental stages. These lncRNAs were overlapped with or were located adjacently to the metamorphosis-associated protein-coding genes. TCONS_00186426 was specifically expressed in larvae and overlapped with the endocuticle structural glycoprotein *Abd-5*. TCONS_00002929 and TCONS_00008658 were specifically expressed in adults, and located close to the irregular chiasm C-roughest protein and juvenile hormone epoxide hydrolase, respectively.

Some specifically or differentially expressed lncRNA overlapped or were adjacent to metamorphosis associated genes (Fig. 8). LncRNA TCONS_00186426 was specifically expressed in the larvae and differentially expressed in pupa and adult. This lncRNA overlapped with the endocuticle structural glycoprotein *Abd-5* at the 5′ UTR. The overlapping region was 2,810 bp in length. *Abd-5* is important in cuticle formation in insects. In addition, TCONS_00186426 was strongly co-expressed with *Abd-5* (Pearson correlation r = 0.91, p < 0.01, *t*-test). TCONS_00008658 was located in the intergenic region adjacent to juvenile hormone epoxide hydrolase (*JHEH*) at a distance of 33,474 bp. This lncRNA was weakly co-expressed with *JHEH*, an enzyme that inactivates insect juvenile hormones (Pearson correlation r = −0.03, p = 0.9, *t*-test). TCONS_0002929 was located in the intergenic region adjacent to irregular chiasm C-roughest protein (*rst*) at a distance of 1,493 bp. TCONS_0002929 and *rst* exhibits a weak expression correlation (Pearson correlation r = −0.3, p = 0.3, *t*-test). *rst* has been reported to participates in eye morphogenesis and development in *D. melanogaster*. These results indicated that lncRNAs might participated in regulating metamorphosis in *P. xylostella.*

## Discussion

Given the rapid development of high-throughput techniques, numerous lncRNAs have been identified in insect species, such as *D. melanogaster* ^12,40^, *Drosophila pseudoobscura* ^41^, *Anopheles gambiae* ^42^, *Aedes aegypti* ^13^, *Phlebotominae perniciosus* ^43^, *Apis cerana* ^44^, *A. mellifera* ^44^, *N. lugens* ^14^, *Bombyx mori* ^45^ and *P. xylostella* ^36,37^. In *P. xylostella*, several studies have been performed to date. Etebari *et al*. identified 3,844 lincRNAs from 7 diamondback moth RNA-Seq libraries^36^. Zhu *et al*. identified 1,309 lncRNAs from 9 chlorantraniliprole-resistance diamondback moth RNA-Seq samples^37^. Wang *et al*. identified 8,906 lncRNAs from 6 diamondback moth RNA-Seq samples^38^. Here, we identified 3,324 lncRNA transcripts corresponding to 2,475 loci from 13*P. xylostella* samples. We found that the gene features of *P. xylostella* lncRNAs are similar to mammalian counterparts. The lncRNA transcripts are shorter than protein-coding genes. Most *P. xylostella* lncRNAs have only two exons and exist in one scaffold. In total, 747 lncRNAs partially overlapped (based on the similarity of sequences) with the lncRNAs identified by Wang *et al*.^38^ according to the lncRNA sequences supplied by the authors, 478 lncRNAs partially overlapped with the lncRNAs identified by Zhu *et al*.^37^, 310 lncRNAs partially overlapped with the lincRNAs identified by Etebari *et al*.^36^. The total number of novel lncRNAs identified in this research was 2,146 (Supplementary Table S11).

Hundreds of *P. xylostella* lncRNAs were specifically expressed in the chlorpyrifos-resistant, fipronil-resistant, GK, MK strains and also in the different developmental stage. In addition, different numbers of lncRNAs were differentially expressed in various samples. But only 5 lncRNAs were identified to be differentially expressed in the parasitized and unparasitized samples. The differentially and specifically expressed lncRNAs in the insecticide associated strains and different developmental stage, suggested that lncRNAs may play key roles in regulating insecticide resistance and development. Etebari *et al*.^36^ found 358, 280, 162, 191 lincRNAs genes differentially expressed in chlorpyrifos-resistant, fipronil-resistant, GK and MK strains, respectively. The same and different number of insecticide resistance strain differentially expressed lncRNAs were analyzed, and 5, 9, 2, 2 lncRNAs were overlapped with the chlorpyrifos-resistant, fipronil-resistant, GK and MK strains that identified by Etebari *et al*.^36^, respectively (Supplementary Table S12). In addition, Wang *et al*.^38^ found 114 differentially expressed lncRNAs during the diamondback moth development, and the same lncRNAs number for our research was only 6 (Supplementary Table S13). The number of the same differentially expressed lncRNAs in the insecticide resistance strain and developmental stage was low between our research and that reported by Etebari *et al*.^36^ and Wang *et al*.^38^, mainly because only multiple exon transcripts were kept in our research, but almost all of the transcripts including the single exon lncRNAs were retained in their research, and only the intergenic lncRNAs were kept in the research reported by Etebari *et al*.^36^.

The functions of lncRNAs can be deduced by analyzing their co-expressed mRNAs or their genome locations^46,47^. Chlorpyrifos is an organophosphorus pesticide, and its target gene is acetylcholinesterase. Fipronil is phenylpyrazole insecticide that target the gamma-aminobutyric acid (GABA) receptor. Cry1AC belongs to the class of Bt endotoxins, which lyse midgut cells. We analyzed the overlapping and adjacent protein coding genes of specifically or differentially expressed lncRNAs in insecticide-resistant strains, however, we did not identify any detoxification or related target genes potentially due to the number of lncRNAs identified in the current study. According to the statistics presented in the NONCODE 2016^48^, 144,134 and 14,848 lncRNAs genes were identified in human and fly, respectively. Thus, more lncRNAs in diamondback moth are expected to be discovered in different tissues or individuals exposed to different insecticides. Nevertheless, we identified one fipronil-specifically expressed lncRNA, TCONS_00133526 lies in scaffold_469 with 2,291 bp of voltage dependent *para*-like sodium channel. The resistance of insects to pyrethroids insecticide was linked to the *para*-like voltage dependent sodium channel^49^. This result further indicated the possible function of lncRNAs in the regulation of insecticide resistance.

In addition, three lncRNAs overlapped or were located adjacent to a metamorphosis-associated gene. The larvae-specific lncRNA TCONS_00186426 overlapped with a gene involved in cuticle formation. The adult-specific lncRNA TCONS_00008658 and TCONS_00002929 were located in the intergenic region of juvenile hormones synthesis and eye development associated gene. Based on genome location and co-expression data, these lncRNAs might have important roles in regulating metamorphosis in the diamondback moth. Rapidly development of high resistance to insecticide and high fecundity are two main factors that make *P. xylostella* the most destructive insect pests^35,50^. In conclusion, we present evidence that lncRNAs might participate in conferring insecticide resistant and regulating development, which should provide new insights into developing alternative eco-friendly pest-control policies for this notorious insect pest.

## Methods

### Insects

The *P. xylostella* insects were kindly provided by Professor Yidong Wu in Nanjing Agricultural University. Insects were fed in the laboratory nursery room which was maintained at a temperature of 28 ± 1 °C, and 70–80% humidity with a 16-h light/8-h dark photoperiod. All collected *P. xylostella* were stored in a −70 °C refrigerator.

### Data

Transcriptome data of *P. xylostella* were downloaded from the NCBI SRA database. Nine samples were obtained from the whole body and the other four samples were collected from the midgut (Supplementary: Table S9). The sample included four different developmental periods of *P. xylostella*, different resistant strains, and parasitized and unparasitized samples. Genome data were downloaded from InsectBase (http://www.insect-genome.com/). Rfam 12.0 was downloaded from the website (http://rfam.xfam.org/). Non-redundant protein sequence were downloaded from NCBI (ftp://ftp.ncbi.nlm.gov/blast/db/). Pfam 30.0 was downloaded from the website (http://pfam.xfam.org/).

### Developing a computational pipeline to identify lncRNAs

To identify lncRNA genes from the transcriptome data, we constructed a computational pipeline following the protocol of Xiao *et al*.,^14^ with minor modification. First of all, we used the software Trimmomatic^51^ to filter low-quality reads. Then the raw reads from all 13 RNA-Seq data were mapped to the *P. xylostella* genome using TopHat^52^. First, the reads of each transcriptome were mapped to the scaffolds. The junctions outputs from each RNA-Seq datasets were combined to produce a Pooled Junction Set. Second, TopHat was used to map all the reads of each RNA-Seq datasets to the scaffolds using the Pooled Junction Set. This step produced a final junction set for Cufflinks^3^. Then, 13 transcriptome datasets were integrated into a complete transcriptome with Cuffcompare using the genome-annotation information. The transcripts that satisfied two criteria were reserved: length ≥200 nt and exon numbers ≥2. We obtained 80,368 transcripts corresponding to 17,213 loci in this step. Third, the potential protein coding genes were removed by NCBI Blast to the NR database (e-value <0.001). Fourth, open reading frames longer than 300 nt were deleted using getorf (http://emboss.sourceforge.net/apps/cvs/emboss/apps/getorf.html) software. Fifth, Coding Potential Calculator software (CPC, http://cpc.cbi.pku.edu.cn/) was used to predict the protein-coding potential for transcripts. Only transcripts with a CPC score ≤−1 were retained. Sixth, the remaining transcripts were used to search the Pfam database by using Hmmer software^53^. The transcripts that do not have the potential to encode conserved domains or motifs were reserved, and the known tRNAs, small nuclear RNAs (snRNAs), snoRNA, ribosomal RNAs (rRNAs) and other noncoding RNA except lncRNA were removed by searching the Rfam database using Infernal^54^ and BLASTN against the NONCODE database^48^, producing the final lncRNA gene sets.

### Structural gene features of *P. xylostella* lncRNAs

Gene structures of lncRNAs were constructed by aligning lncRNAs with the *P. xylostella* genome. We analyzed the number and length of lncRNA exon and the distribution of lncRNA among the scaffolds of the *P. xylostella* genome. The exon-intron structures of lncRNAs and protein-coding genes were showed by the software Geneious^55^.

### LncRNA gene expression analysis of 13 transcriptome datasets in *P. xylostella*

The transcript abundance of the identified lncRNA genes from populations at different developmental stages, parasitic populations and insecticide-resistant strains were estimated by counting reads and normalizing with the software Cuffdiff, which used *t*-test (*p*-value < 0.05) to measure the significance of the expressional difference. A heatmap was produced by analyzing the expression abundance of lncRNA genes using Clustering^56^. The average linkage method was used and the results were viewed using Java Treeview^57^. A lncRNA was defined as specifically expressed based on the following criteria: 1) the expression is >3 FPKM in one sample and <1 FPKM in other samples; 2) 10-fold increased expression in one sample compared with others. The cutoff was p-value < 0.01 and q-value < 0.05. q-value is the FDR-adjusted p-value. An R script was used to estimate the Pearson product–moment correlation coefficient for lncRNAs and the protein-coding gene. LncRNA and mRNA co-expression with |r| > 0.8 was treated as a strong correlation.

### Total RNA isolation

We extracted RNA from 30 mg mixture samples that included 1–5 instar larvae and adults of *P. xylostella*. Using the TRIzol® reagent and following manufacturer’s instructions (Life Technologies, CA, USA), we obtained the total RNA from *P. xylostella*. Then, the RNA integrity was detected by electrophoresis of 1.2% agarose gels, and purity was assessed by using the NanoDrop spectrophotometer (Thermo Fisher Scientific, Waltham, MA, USA).

### cDNA synthesis and RT-PCR

cDNA synthesis was performed following the manual of the PrimeScriptTM II 1st Strand cDNA Synthesis Kit (Takara, Kyoto, Japan). In this step, we substituted random primers with specific primers that we designed for the following strand-specific RT-PCR. In the cDNA synthesis, three reactions were used: forward (F) primer with reverse transcriptase (RT), reverse (R) primer with RT, both F and R primers without RT. We randomly selected 9 lncRNA genes for strand-specific RT-PCR validation to determine the transcript orientation.

We use an Integrated DNA Technologies online tool (IDT, Coralville, IA, USA; http://www.idtdna.com/Primerquest/Home/Index) to design our primers. Detail primer sequences are presented in an additional file (Supplemental: Table S10). The PCR reactions were performed in a T100 thermal cycler (Bio-Rad, Hercules, CA, USA) using the Premix Taq® Version 2.0 kit (Takara). Setting conditions were as follows: 94 °C for 3 min; followed by 35 cycles of 94 °C for 30 s, 58–48 °C (reduced by 1 °C/cycle) for 30 s and 72 °C for 1 min; and final extension at 72 °C for 10 min. Then, PCR products were abalyzed by electrophoresis using 1.2% agarose gels. The PCR products were purified by Wizard® SV Gel and PCR Clean-Up System (Promega, Madison, WI, USA), following the manufacturer’s instructions. The PCR products were sequenced by the GeneScript Company (Nanjing, China).

### Data availability

All data generated or analyzed during this study are included in this published article (and its Supplementary Information files).

## Electronic supplementary material

Supplementary Information

Dataset 1

Dataset 2

Dataset 3

Dataset 4

Dataset 5

Dataset 6

Dataset 7

Dataset 8

Dataset 9

Dataset 10

Dataset 11
