# Supplementary material for: Genome-wide identification of long non-coding RNA genes and their association with insecticide resistance and metamorphosis in diamondback moth, Plutella xylostella
Source: Sci Rep. 2017 Nov 20;7:15870. doi: 10.1038/s41598-017-16057-2 (PMC5696462; doi:10.1038/s41598-017-16057-2)
Supplement: Supplementary file 1 — Supplementary Information [file 41598_2017_16057_MOESM1_ESM.pdf]

**Supplementary Information**

# Genome-wide identification of long non-coding RNA genes and their association with insecticide resistance and metamorphosis in diamondback moth, *Plutella xylostella*

Feiling Liu[1,†], Dianhao Guo[2,†], Zhuting Yuan[2], Chen Chen[1], Huamei Xiao[1,*]

[1]College of Life Sciences and Resource Environment, Yichun University, Yichun 336000, China

[2]Department of Entomology, College of Plant protection, Nanjing Agricultural University, Nanjing 210095, China

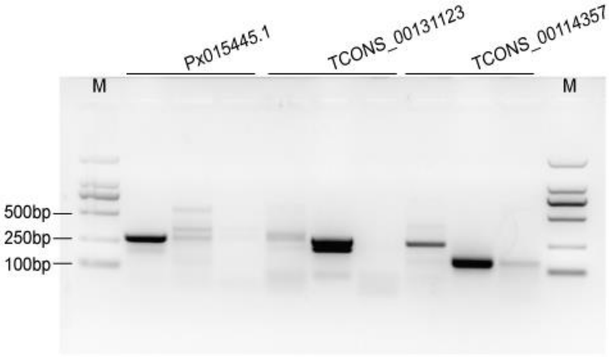


**Supplementary Figure 1:** The full-length gels of strand-specific RT-PCR for lncRNAs TCONS_00131123 and TCONS_00114357. The lanes 2-4 in the electrophoretic gels were from a protein-coding gene Px015445.1, so these three bands were cropped in the Figure 2A.

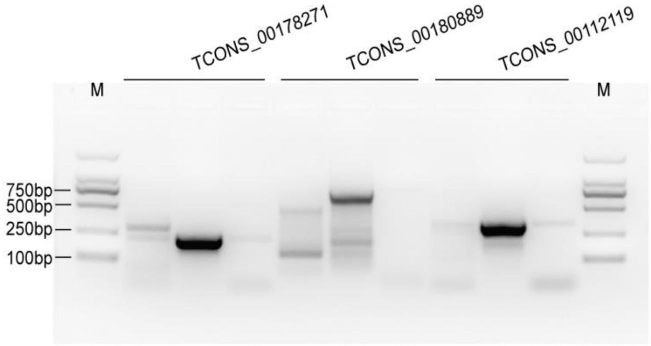


**Supplementary Figure 2:** The full-length gels of strand-specific RT-PCR for lncRNAs TCONS_00178271, TCONS_00180889 and TCONS_00112119. This figure is the original picture of Figure 2B.

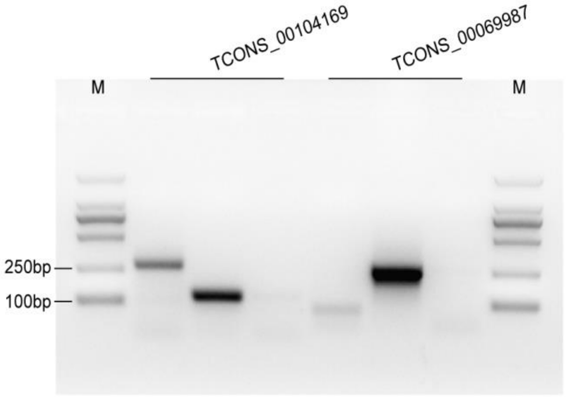


**Supplementary Figure 3:** The full-length gels of strand-specific RT-PCR for lncRNAs TCONS_00104169 and TCONS_00069987. This figure is the original picture of Figure 2C.

**Table S9** Transcriptome samples of *P. xylostella*

| SRA number | Sample | Tissue |
|---|---|---|
| SRR2062281 | 3rd instar larvae parasitized by *Diadegma semiclausum* | Whole body |
| SRR2062280 | 3rd instar larvae control | Whole body |
| DRR002645 | RNA sequencing of Bt toxin susceptible (PXS) *Plutella xylostella* strain 4th instar larvae | Whole body |
| SRR835317 | fourth-instar larvae Cry1Ac-susceptible (MM strain) | Midgut |
| SRR835316 | fourth-instar larvae Cry1Ac-resistant (GK strain) | Midgut |
| SRR835315 | fourth-instar larvae Cry1Ac-resistant (MK strain) | Midgut |
| SRR353499 | Major putative pesticide receptors, detoxification enzymes, and transcriptional profile of the midgut | Midgut |
| SRR179587 | The third instar of fipronil-resistant larvae | whole body |
| SRR179511 | The third instar of chlorpyrifos-resistant larvae | whole body |
| SRR179510 | Adults of day 1 after eclosion were selected randomly, regardless of sexuality | whole body |
| SRR179509 | The pupae of day 1 or 2 after pupation were collected | whole body |
| SRR179508 | The third instar larvae were used | whole body |
| SRR179062 | The eggs of day 1 after oviposition were collected | whole body |

**Table S10** Primers used for qRT-PCR

| Index | lncRNA genes | Sense (5’-3’) | Antisense (5’-3’) |
|---|---|---|---|
| 1 | TCONS_00131123 | ATGGTGGAAAGGCATCCTAAA | TGCAAACTGAGTCTTACCTACG |
| 2 | TCONS_00114357 | AGGTCCAGTACCACGCT | AGACGTGTCCGAGGTTCT |
| 3 | TCONS_00178271 | CCGACAGGATTTAGCGGATAAG | CATGGACAGTTGGCTGTAAGA |
| 4 | TCONS_00180889 | CCAAGCCGTAGTGTAGAAGTAG | GAACAAGGGAACTGGGATACA |
| 5 | TCONS_00104169 | ACACACGTGCGTGACTC | TCCTCAGTCCGGTCGAAA |
| 6 | TCONS_00069987 | TGACACCGCGACAACAATA | GTGCTTGGTGAGTCAGATCTATAA |
| 7 | TCONS_00112119 | CGGCGTATGTACAGACATTCA | CCGAACGTATTTCTTCGTTGC |
| 8 | TCONS_00014403 | CGGCAGCGGCATAGATAAA | GAGTGCCTTGCCTCCAATAA |
| 9 | TCONS_00155598 | CCACATGGATCAAGGCTACTC | TGCAAACTGAGTCTTACCTACG |

**Supplementary File 1.** The sequences of identified lncRNAs

>TCONS_00000265 geneID=XLOC_000008 scaffold_1:316698-319576(+) Exonic overlap (+)

CAGCCACCCACCATCGAATACAAACCTTCTCAGTGACTGCTGAACAATCTGTACCATAGTGTGCTAAG
AGTAAAGTGTTGTTTTGAGTGGACATCCTTAGTTAAGAGGTACGGTAGTTGTTTTAACTATTCAACTTT
TTCGTTTCTAGACAGTAGCTGCGAGTGAAATGTTTCTTTCTTCGTCGGAATGTCACATTGACAGTGTAT
TTGTTTTTTTTTAAATTTTCAATGACTCAGTGACTAAGCAAATAGATTTATTGATTTAAGAGTGTATGAA
ATATGCCTGATTGGATAGAATGGAAAGTGCAATGTTTTATTTTTACAGGCTAATAATCGTTATATAATTAT
TTAAAAGTAATAATTGTAGAGAGGACATTGTTGCTTTTAATGTTTATTTGATGAGGAATCTGCTTACGGA
ACGATCTTTTAATGATTTCGTGTAATCTTACAAACTGTTCAACTGATGTAACTGGGTACTACGAATAAAT
TTTATTCAAGTATCATCCTTCTTTTTTCTTTATGTGGCCAGTATGCAAAAACTCGAACTATTTAAAATTAT
AAACAAAAAATAAACTTCAATATTACGAGGAAAAATGGAAGCTGGATAACATTTTCTTCTATTTGCCCT
GTTGCAATTTTCTTAACAATCATAAAACTATCACTAACTGGCGATTATCCCTCTTGTAAATAACGTTAGG
ATGGCGGCGACTTTAGATTCAATTTTTGTCTAAAACTGGCCAATAGAACATCAATAAGGGGCCGGCTGA
ATCTTACTACATGTACATTGGACAAAGATTTGGGCCTGTTTAAGCTAACATAAAACTCTATACAATTAAA
ATTTATTCATAAACTATTTACATTATAGATTTAGATATTCTAATTAATTCCGTACCTAATAGAAGATGAAAT
AAAGCGAAATTTTGACTGTACTATTCTAACGAATTTCATATCTCAGTACAACTTAACATAAATGTATTCC
CAATTTCATTGAAATGCTGGGAGGTTTTAATATTGAACTTTTATGTCATTTGATTACAATGAGTCACTGA
CTGAGGACAAAATAGTTCAATATCTAAATTTACTGTGACTTTTCTATGTATTTGGGGTATTCATTATTTGT
TAAAACTTGTAGGGGTTTTAGACAAATATACAATGTTTACGTTGAAACTTAGATAATCCCATTTGCGATT
GCGAAAAATGGTTGATGTGTACCATTTCTTTCGGTGCAATAAATTATAGGTATTTTTGGAATGTCCCTTA
TCGTACTTACATACCGTAATTTACAGTTATATACCTATTATCATATAAAAATAAATGCAAAAGTACGATCT
GTACATTGAAAGCCACAGTCCTAAGTGAAAATAACGACAACATTGTTGAATTACAGTGTATTAATTCAA
ACGTTTCATGAGTTTGACAATTTACAATAGTAGGTACTTGGAGGATATTATGTATTACGAAATGTCTGTT
ATAGTTTAGATGTTCTCTTTGAATTTTTAACCGAATTCGAAAAAAGGAGGAAGTTCACAATTCGTCTTC
ATGGTTACCCAAGTTTTAGGCAATACATCTAAAATGACGGATTTATACATTAATCTAGAATAGTTAAAGA
GTACCCGTTTAAAACACTATTGATTTTATAATAATCCAAGGACAGCATCTCGGTGGAAAGTAGTCATAAT
ATATTCAAATAAAGTTCCTTTTTTTTGCCACATAACCGGTTTGTTATAAGTTAGATATTGCTAAATTAGTGT
AAAAATGCTCAATACTGAATGTGTTATTCTGAATCCTAGCATCAAGAACACCAATAACCAGTACATTTA
GAATAGAAGCACACGTGTAATAAGTAATAGTTATTATGTAAATAATATATATCTCCAAATTAATAGACGAA

TATAATACTATTCTTTCCTTCCGTGTCCTGTTTGGAATATAGGTTTTACACGTTAGTGAACATAATTAACT
CCATCCGTATTCATTCTAGAACACAACCTCTGTTCAAATTCGTATTTACAATATTTGAGAACCGCCTACT
ATAGGGTGTTGGTTGTACAAAGTGGTATTAGATCTCGTTACTTGCTGTTAACTCAACACCCTGTATGTGA
TGGTGTGGGCTATGTGCCATGGACTTTATGTAACAACTATATTATGCAATAAAAAATCTTGTATAAATTAC
TATTGGTATGCACATGACATGACTAAATATAAAACTGCATAATCACACACCACCAAAATGTTGAAGGAA
GCAGCGAATCTTTAGCATTTGGCACTATACCTACATCTTTGCCGAATGAAAGTTTTCAGGTGCATTGGG
AACCGTAAACTCTGGGTCAAAATAATGTTCACTCTGGTGGGAAAACTTACCCCTCCCAGGTAACAATG
TCACTCTACATACATTAGGACAGTGCAGTCGAGGCTTCCAATGTGACTTCATGTGACACATGTGCTGAG
TGCTCCTAGCATTACTTCTCTCATGTGCAAACTCAAATATGTAATTTGACATGATTATTTTATGTTTGCTC
TGTAACTAAATAAAGTCTTACAAAAATGTAACATGAGCATATTTCAACTCAAAATGATTACCCTTTCCAC
GGTGGCGGTACAACTAGAATAAAAATACTTTACATGAAATTATCGCATTATATTTCCTCATCAAAATACTA
TATTAAATTTAATTGTAC
>TCONS_00000597 geneID=XLOC_000059 scaffold_1:371179-372461(-) Splice junction overlap
TCTGATCCTTTCCAAACGATTACCAACACTTTAAACAATTTTAGAAGAAGCAAGATGAAGTTCTTCGGC
GTGCTATTCTCGCTGGTGGCAGCCCTGGCGACCGTCTCCGCGGGCTACATCGGCTGGTCGTCCCCGCA
CTACTACTCGCGCCCAGTGGTAGTCCGCCGCGTGGTCTACCCCTCGTCTGGCTGGAGCTCTGGCTGGC
CAAGCTCTGGCTGGCCAAGCTCTGGCTGGAGCTCTGGCTGGCGCTCTGGCTGGCCAAGCTACAGGAA
GTCTGGCTGGAGCAGCGGATGGTGGTAAATTGGGGAAATGTTGAGAACCGATTGCTTTTTTTTGACAT
GCTCTTTTTGTATTGGTTATTTAGAGATGGTTAATGGTTTTTTCAAGCGTATATTTAATGAATATTATTGTA
ATGATAACTGTGATAGTAAATAAATTATTAAAATAAAGGCTTAAAGTGACAGAAGTTGAATTTAAAGTC
CAATAAAAAGATATAAAATTCGTTACTCAATCTGCGAAATGTCAATGTGGAATTTGCAAAATAAAGTGC
AAGCCATAAAACTCGTAAACATTCCTTAAAAGGAGATAAATACATATTTAGTTGTGGAGAAAAAACTAT
CCATCTAAGTGTTCACCTACCTGCTTCAAATATTATTTATATACCTGATTGTGATAAATTTGAAAGTACCT
ATGTATTACCAATAAACTATGTATTTAACCAATT
>TCONS_00001021 geneID=XLOC_000061 scaffold_1:394969-398756(-) Intergenic
CAGGAAATTATTAAAGAAGTACTACATTGGAGATCTCCCAGTGTCTGAAGCAGGAGAAGGAAGCAGTT
GGTGGTGGGTGAAATACGCTATCGGAGGCGTGGTCATCGCCATAATAGGAGGCCTGGTCTACCGCAAG
CTGTCTTCACCCTCGCCCCCCGAGCCCCTCATAGAGCCCGCCGCGCCCGCCGCGCCGGCCGCCACGTA
GACCACCAGGCTGGGACTCGATGCACGGAAGTAAATGAAGATGGTGTTTTAGCGACTCACATTTTTTA
CGTCAGACTTCCTAAGAATAGTAGAAGTGAGGCACACACGGTAAGTTGCTTTAAAGAAACTTTGACTT
CCTTTTACTTATAATGATCTATGTGCTACTCCACTTCGTTCACTCACGATTTAGTTTTTTTAGGCGTAATG
TGCGTGAAAATGCGAGTCGCTGAGATGCCGCTATCGTTTTGCTCGTGAACTGAGGGGTAGATGTAGGA
AAATAACAATGGGTTTTATAGTGTCGCCGTATCGCCCTCGAGAAGGCATTTCGTAGATACATGTTAATTT
TAAGCAACA
>TCONS_00000184 geneID=XLOC_000079 scaffold_1:710238-718755(-) Intergenic
ATTAAATAAGTAACACTATTTTCCATTATAATGATCTTATTGTAATTTAGCTATTATTCTGAGATCTGGGAT
TGACCAGAGTATAGCTGGAACGGAGCAATCGCTCATTGTCTATGGTTTGTCTGTGATTCGAAAGAGGA
AGTGAGATCCGGTGCCGGCCATCTTAAAAAAAAAATAAATAGAAACGACAGATTGTTTGGTTGGATTGA
AAATTGCAATTTATTATTGTTAGTTGGTGATTCGGTGCCTTTATCTGCTTCCTTTCTCTCAAATTATCATC
AGTGTTATTGAGCATCTGTTAAGGTATAATTTTGTTTATATCAGAAGTGAAAGTGGTTCGCGCCTACCGG
ATAAAAATGTTTAAATATAAATTATAAAGAAAATGTTGATTAATATGAAGACAAGATGATGATGATTATAA
TATAAGAAATAAAATTGAAGAATTGAAATACATGATGAGAATATCGAAAGAAGATACTTACATTATATAT
ATATATACATATTATGAGAATCGCTGGCTGAACAGTATTGTTTTAATAGATCAAGATATTTAAATTGTTATA
ATAATAAATGATGATACAAGAATATCATATATAAAGCAGAAGAATTGGAATATATTACATATGAAAACAA

GATTATAAATGTATAAGGAGAATGGCTGGCCGAACAGTATTGTTCTGATATATATTGATCTGATATATGAA
GACGATTCTCCGAGCAACAACGAATGCAGCGTAGCAGCATCACATCCATAAGAACAACCGGAATATGG
TTTACAAATTTAATTATCTAGGAACTTCGTTTAGTCCTATTTTTAAATATTTCTCAGAGAAGATTGTGTTT
CAATGTTTGCTAAGGGCACTTGAGAATAACGCATATATTAGTAACTAAATTAATAGGCTTAAGTATAATTT
AGTTGATTCATTTGGTTTGTTAACAAATTAGAAGTTTTTAGCTTGCGTTCGAATGGCCGACAGGGAATG
GGGACATTCCTAATAACAGGACGGCCGACTGTTAGCGTTTACATTTTTAAATACTATTCTTGTATTAAAT
AAGTAACACTATTTTCCATTATAATGATCTTATTGTAATTTAGCTATTATTCTGAGATCTGGGATTGACCA
GAGTATAGCTGGAACGGAGCAATCGCTCATTGTCTATGGTTTGTCTGTGATTCGAAAGAGGAAGTGAG
ATCCGGTGCCGGCCATCTTAAAAAAAAAAAAATAGAAACGACAGATTGTTTGGTTGGATTGAAAATTGC
AATTTATTATTGTTAGTTGGTGATTCGGTGCCTTTATCTGCTTCCTTTCTCTCAAATTATCATCAGTGTTAT
TGAGCAT
>TCONS_00000289 geneID=XLOC_000029 scaffold_1:839752-842656(+) Splice junction overlap
CTCAGTATCTCCCCATACCCCTACCTACTTCAGCTCTGCGTACAAAACCCGACCTTATATCACCTGTCAC
TAAAGATGAGTGTGTGTTGCAGCCGCGACGACAAGGGGCGACACGACGACCGCGACAAGGAGCGCG
AGCGCGACCGCGAGCGACGACACCGCAAGGACCGACGTTCTTCCCACGAGCGTTCATCCCGCCGGCG
CTCCCGCGAACGCCACTAGCACTCTGAGGTATCACATGCACCGCACCCGTTGGCAGCGTCGCGTCGCC
ATAAAGAGGTACCTTTCACTTTAAGAACTCAGAAGAAGGCAGACGCAAGCGAAGCCGCCAGACTCGA
CCCCCAACGGCCTTGAGGTGACTTAGAATAATAATTTACAATCTGTGAAACTTGATTATATAATAAATGTTA
GCTCGTTAATCCCAATTAGACTTAAATATGCATGTAGATTGCACCAAAATGGACCTGTTCTTATTTTCGC
TTAAATGGTAAATGTTTGTATACATCTAGCTACCTCTTGAATATTGTTTGAAGCTGACACTGTTACTATTG
TACCGAGAATCCATCGCGGAGTGATTCAGGTATTTATCCAAGTTATTTCTTAATTTTTAATGTAAATGATG
TCACGTGAGAATAATGTTAGTATGAATTGCATACGTACGTGTGAACCTGTAGGCGACTAGGCGATACAT
TGAGAAGAATAAAC
>TCONS_00000559 geneID=XLOC_000031 scaffold_1:1074939-1075742(+) Exonic overlap (+)
GTAAACTATAGAAACAAGATAGAAACTATATTACCTACTCTGTGGATCTATGCAAAACTGAGATGCAAA
ACTAAACAACACCAAAAAAATTTGAATAATTAATATCAATAATGAAAATTTCACGTCACGTAGGTATTTA
CGTATTTTCATCAAGATGGTGAGATAAATTATCAATATACAATACCGATCACAAAATAGTTAATACTTAAA
TATGACTGTGATGTAGGTTTTATAATATTATAAAGAGCAAAACTACCAAATTGGCATTATGACAACGAGT
TTCCGGAAAGTGGAATCACCGAAACGGGATCGTTCCCCGGTGCCAGCCCTCCACACCCCGCACTCCA
CACTCCTCAAACCGCCCGCAGCCTGCCTCCCGCCCCCCACCCCGCGAAAGCCCCG
>TCONS_00000085 geneID=XLOC_000034 scaffold_1:1124406-1126251(+) Unclassified
ATAACAAACCTGAAATATTATTAGGGAAATGGCAATTTTCACGAACAGCCTTTGGTATTTGGAATTTTTT
GCTTTCGCTATAATTTTGATTGGATTTTTCTTGTACGTGGTTTATTTAGGGGCTAACTCCCCTCCCTACAA
CACTCGGTACGACATGAAAAGAAAATACCTCTTTGACGGAACATGGCTAGCTCAGTGAAAAAGCATTG
TATAGAGAAGAGAACGAGTTCTCATGTCTGATCAACGAAATCGACAAAATATAGATATCAGGAATCCA
GAAGATGTGATTAAACTTCAGAAGCTGAAAGTAAAAGTGGACACCCGGCCATTCTATTGTGACCCTAG
TTGCATATGGTTTTTAAGACTATTCTTCAGTATAGCTACCATTGCCGTGTGTTATTTTTTCGCTTACACTT
TCATCACACATACAAAGTTACGATTTGGACTATTTATTCCTTGAGTGGCTGAAAATTTATTCATATAGGTA
CAGATAAAACCTAATTGCGCATTTTATTAGATGCGAAATGTGTTTGCAAAAATTTTAAC
>TCONS_00000752 geneID=XLOC_000035 scaffold_1:1131322-1158609(+) Intergenic
AGCCGGCCCAGTGACGCGCACACCGCGCTTGCGCACTCCGCGACTCACCCGCAACTCACTCGCGACT
CACGTGATAATTCTGTGCTAAAAAACAGTGACAAAACGAATCGAACAACCCAAATCTCGGTGCGTGC
GCAGCAGAATTGGAGATCGCAGCTTGGAGGTGGGAACTGTAGCGGGCGGCCTTTCCTGATTGGTGTC
AGACTGC

>TCONS_00000929 geneID=XLOC_000090 scaffold_1:1256998-1257300(-) Intronic
CAACAAAGTTTCTTAGTATACCCTTTTTGCAACACCCTGTATAGTTATTATAGGTAATAGTAGTAATATCT
TCAGTATTTAAGGTAGGTACTTTCCCTTCGTGTAGCCCTGGTACACCCTGTGGGGCTCGTGTTGTTCTTT
AGTTGACAACAGGTGCATTATGCGATAGGTGCATTATAAGATGGGTGCATTTTGAGAAAGGTGCAT
>TCONS_00000692 geneID=XLOC_000091 scaffold_1:1356683-1417992(-) Exonic overlap (+)
TTTCCGTAATAATCCTAAATTAGTCTTAGCACTTTTAAACATAGGATCTAATTTAAAGCATATAAATACA
GTTCTTCTGCTAGGTGATATTTTTTCCATCGATGATACAGCACACCTGAAAAAAGATATCATTTTAACTC
AGTAGGGGTTAGTAAACTAGGTAAAAAACACCATAAGGCCAGGCAGTATGGGATAATGCCCTTAGCCT
TCGTTCAAAGAAAATAAACCTACAAAAAATATTAATAAAATAATGGGGGTAGTATGAAAACAAAACCA
CATTTTGTGAAGTTACTTCCATAGATGACATAAAAGGCCAAAGTGATCGCGTATACCCATATAAGGGCC
TCGGGTTCGTTTATCACCCGTGTTATTTAATCTTAGTGTTGTGTGCCTCTTTTTTATCAAACTTACTTAAC
CTATAATTTGATTAAGTATCCAAATCCAGACTTTCATTTAAGTGTAAAAAGTGTCATTATCCAGTTTTACA
TAGATGTAAAATGTGTCTCACCAAGATTAGTGTAGTGTATAGCTTGCGCATTTCTGCCGAACAGAGTGA
GCGCTCGCTCGAAGTGCGCTTCGGCGTCCTCGAAGCGGCCGAGCTTGCCGTACACGTTGGCGACCGC
GAAGTGCACGGGCGCGGACTGCGGCAGTTGGGCCAATGCTTCGGGCGCCAACTGCAGGGCGCGCTCT
AGTTGCCCTGCGAATTTGAGTACTCTACGGGTCAGGCTCTCACGGGATCTTCGCTGCACAAGACACGC
AGATTTTCATCACCCTGCTTCGCATTCAGAACTTCGGATCAAGCATGGT
>TCONS_00000985 geneID=XLOC_000091 scaffold_1:1356688-1398008(-) Intergenic
CGCCTCCTCAGCTCCTAACAGCTCCGCCACCACCCGAGGAAACCAACCTAAACTTCCTGTCCAGTGAC
TTGTGAATTTCCTCAGTGTAATTCGTCGTTTGTTTCCCGCGCCGGGCCAGCCGTCTCAATTCTTCGGAA
AAGTACTCTACGGGTCAGGCTCTCACGGGATCTTCGCTGCACAAGACACGCAGATTTTCATCACCCTG
CTTCGCATTCAGAACTTCGGATCAAGC
>TCONS_00000102 geneID=XLOC_000039 scaffold_1:1405987-1407791(+) Unclassified
GTAAAATATAATATCAGTTCATCAGCTCATCAATCAGCCAAATCCTAAAATAAACATATTTAATATACCGG
CCAGTTTTCTACTTACTATTTCAGCACCACTACGACCACTTAACATTTCTCTACATCAAGAAATAATCTA
ATATGTACTTATAATAAAATATACAGCATCATCCTCAAGAAGCATGGGGCAGTATAAAATGATAAAAGCG
ATCCCAGTACAGGCGGCCGACACCACCACCCACACGTTTTTGGTTGTGTCCACCGCGGTGGTGGTGGT
AGCAGCCATCGTCTTCTACCTCGCCACCAGGGACTGGAAGGGATGCACGTGCCCCGAGGAAGTGGAC
CTCGGCACCTTCCGAAGGAATATGAACAACGGCACACGCAAGAAAACCATCGAGTACCAAGTATTACC
CCCCCCCCGTATTACTCTTAATGCTCATTTGAAACAGAATAAATCCAATTGAAACCACAAGACTTGTGC
AAGTATTTTATTGGACGGTTTCAGCCTTACTCTCCAGCATTTCCAGGACTTGTTTTAAGTTTAGAAACG
GGTATTTACCCGTGCTTGGTGTGTTCTCGTCACGATCGAGAGCTATACCGTACAAGCTGCCGTCTTGGG
TCAGCAGGTCACTCGCCTGAACTGGGCATTTGGTTAATTCTTGGACACTAATGATCGAT
>TCONS_00000109 geneID=XLOC_000041 scaffold_1:1422507-1423706(+) Unclassified
GCTAATCCGTGTGTCACTTTCATCAACAAATTCTTGCTTTTACTCCAATGTTTTGATAATTTTACTAATTT
TTATACAAAATGTTCCTGTGTGGTTGTGAAGAGAAGCCGGAACCGGATCCACAGGGCTCAGAAGAAG
CGCCCACGACGCTGCTGGTGGGCACGGCGGTGGGGACTGCGCTGTTGTTTGTGGCTCTCATGTACCTG
GCTTATACGGCAGTAAAAGCCAAAATGAGTCGGCGACAGAGAAACTATGAAGCACCAAGTTGGACAC
TGCCTCGGCGAAGGAACTTCACGTGAACTGAGAAAAAAACACAAATTTAATATTTCATTAAAAATATAT
TCGAGTATAATGAAAAACTTAGTATAACATTTTATGAGTATATATGAGCACCGCTCCCACTGCTGTACCA
CGTAATTTAAATGATAAAAAAAATGTGCTGATATATTTTGAATTTGGTAAATAACTTCCTCTGAGAAGTA
TTATTCAACTGGTATGGCTTGCTAGGTTCCTGCGGCGTGTATACAAATAAAAACGTTATAGGTAGTTATA
GCATTCATATCGCACAGACTCGACAGCAAACGCGATAAAATATTTTGTCACAGCTAGTCGTATGCGTTA
AAAAGTACATGAAAACTGGCTCTCTTGCAGTGCTCGTATCTACTCCGTCATAACGTTAAAATGATCTAA

ACATTCCGAATAATAACAACATAAAATTCTCAAGTAATTTTGAAACATTTTTGTATGATTTAATTTGCTTA
AGGTTAAACCTACTTAATAATAAATTGATTATTGAGCAACAGCTTTATAATATTTATCATAAAATCTATGT
AATTAATATCATAAGATCTATGTATGTATGTATGTATAAAAATTGCAGTCTTATAATAAATTTCATAATGTA
CTAAAAATATATGGCATTTGTTAGATAC
>TCONS_00000932 geneID=XLOC_000095 scaffold_1:1605163-1621566(-) Exonic overlap (-)
CACATTTGTTTACAATCTATAGCTTACTTGAATAAGGTTGATTTTCGCATAGCAGAAACCATTAGACTGT
AATGAGACGCGTTATTATTACGCTGCTCATTCATACTAGTCGATGTCGTAGTAACCTTGAAAGATACTCA
CAGGCTGACGTAACTATGAGTTCGTGACGTTCGACACCGTAGGATACTTAAGTCCGTGCTACGAATTTT
TATAGCTAGATACAGAACAATGTCAGTGATGAACAAAATAAACACCTAAATATAAGAACATGGGTATTT
TTAAGAAAAACATAATATATGTAAGTACTCTTATGGTGAAGGAAAACATCATGGAAAAACCTGCACATC
TAGATTCTAGATGTATTGTATATCCTAAATTAAAAAAGGCCATACCGTTAATGACGCACGAGCGTAATAT
ACAGAGAAAAGCGGGTGGCTCGCTTAGGAGTCCATGCAAGGTCTTCAAAATTGAGATTCCAATCAAC
AATTTTTCTTTTCAGGTGCACGTCTAGGACAAATGCAGTCGATGGCTGGACTGGCGGCTGTACTTCAG
AAGTTCACATTCGAGCCGGCTGCCAACTCTAAGAAGGTCCCTACTCCCGACCCCAAGGCCATAGTATC
AGAGAACATCGTCGGAGGACTGCCACTTCTTATCAAGAAACGAAACTTATAAATTATTATTAGTTGTTA
ACGAGTTATTTCTGGACGGACTTTGGACTTTGTATCGTTTAAAATGTATTTATAGGTTATTGTATGCTGCT
TTTACGTTTTTGTTACTTTATTAATAGATTTTGTAAATAAATATCTTGAGAGTAC
>TCONS_00001487 geneID=XLOC_000247 scaffold_10:202150-204260(-) Splice junction overlap
CACCGCGACCACCACAAGAAGGACTCCGTCTACGACAAGATCAGCCAACTCCTCGAATCCGAAGCGA
AAACACACCCACAAAAGGAGACGAAGGCGCGCAGGATCGACAAGCGGTTCGTGGCGACGGACGGCG
GGCTGGTGGCCGAGCTGCGCGGCCTCAAGCACCGCATCCGGACCTAGACCACCTCCCCCACCCGCTC
ACTCCACACTCAAGAGACTGAATCTTAAATATAGACAATGTAAATAAAAAATACGAGGGCTGAGATCG
TCAATTCGATCAAGCAAGCTGGGCAAACTAAAAGAAAACCCAAAAAAAAAGTTACAATTCTAAAGAA
TGAATACCGCGATGCCCGCTGTAAATTATAATTAGAAAACTTGTCTCCTAATCATTTTAGAGATCGGTTT
ATCTTCTCTTTCCGAACTTTACTTCGTTATTTTAGGTGATAGGTAATTGTAAGCACATACGGGTATACATG
ATCGTACAAAGTGTGACTATAATCGTTAAAAGGAAGCTTGTTTGTTGAGAGTTTTTGCATGAAATTATT
TTATAGGAAGAAGAAATGTTGTACCCTTCGGTCGGCAGCTAAAATGAATGCACATGTTTAAAAAAACG
TGCGACTTGTAAATATTTGTACTGATTATGTCTAAGTCTTTCTTTAGTTAGATAGTTACGTGTCTATTTAA
ATATATGTACTTCTGTTTAGGCGCAAGCTATTGAGAAGTTAACTGATGTCTAGCTGTAAAAGAGAAAAT
ACATTTTCTAATCTCTTTTTGTAAATTTGGCCTAAATAATTTGAAACGAAGGCGCTGAGTATTGTTTAAA
ACTTTAGCTCTCTGGTTCTGCGGACAATAGTCTCATTTTGCGAAGCAACACGGGAGGGAGTCGCACTT
CTCAAATGAATTTGATGATTCTCTCGAAGGGCGGAGGATTCGGTGATATTGTAGCTTTACAGCTTTAAA
ATGTACTTTTTATATACTTCAGAAGGTCTGTAAGACTTTATATAGAGATGAAAACTTTCGTAGTTCTTTCT
AAGATAATTTTTATTCCCTCCAAAACGTCTCGGTCCCAGATTACAACACTAGTCCCTGAAGGACACAAG
ACTTTTTCTATTCTTTTTTCTATATAAATATAAGACTAAGATAACTTCATCCGAACTATCACGTATAAAATA
CATATCTGTTTCAAGTAATGTCATTATCCCATTGGGTTTAAAATTCTTACGTATTTATTCAAAATTATACCT
AATGAAAAAGTAGCCTAATAAATGTGACCTGTAAAGTATTATTAGATAAAGCAATTATTAAAATTAATTA
CTGTCGTCAAAACAGTAATGTATTCGTTCGTTTTAAGCCAAATTTTGTATGATTAAATTTTTCCATAAGTA
TACCTACCTATTTGAAATAATATTAGGACCTACCTACTTACCTACTACGAACTTGATGCAATTATCCAGTC
CATGCTGTAGTAACAATACCATGTATGGTGTAATACAAAAAAATATAGAGAGCATTATTGTAAATCTTTAT
ATTTATAAAGACAGGCCGAGACAGGCAAGATGAGTTGGACAAGTAACTCGACTCTCTGTTCAAATAAA
ATGGCAGATAATAATTATGATATACTTATAATAGAATGGATGCTCTTACCTTGCTGTGCTTGTAATATACCT
ATTAGATATACAATAATACAAACTGAAAATAGCCTAAAGTATATTTTATATAGAAATGTCTAGTAACGTTC
CTGTATTTTAATATTAGAAGTTTATTTTTCAACTACAGTGAAATATAACAATGTACTTTAGATAGAACTTT

TGTTGAAAGATAATATACGAATACATAGAGCTATTAAATAC
>TCONS_00001899 geneID=XLOC_000247 scaffold_10:202299-204259(-) Exonic overlap (+)
ACCGCGACCACCACAAGAAGGACTCCGTCTACGACAAGATCAGCCAACTCCTCGAATCCGAAGCGAA
AACACACCCACAAGAGACGAAGGCGCGCAGGATCGACAAGCGGTTCGTGGCGACGGACGGCGGGCT
GGTGGCCGAGCTGCGCGGCCTCAAGCACCGCATCCGGACCTAGACCACCTCCCCCACCCGCTCACTC
CACACTCAAGAGACTGAATCTTAAATATAGACAATGTAAATAAAAAATACGAGGGCTGAGATCGTCAA
TTCGATCAAGCAAGCTGGGCAAACTAAAAGAAAACCCAAAAAAAAAGTTACAATTCTAAAGAATGAA
TACCGCGATGCCCGCTGTAAATTATAATTAGAAAACTTGTCTCCTAATCATTTTAGAGATCGGTTTATCTT
CTCTTTCCGAACTTTACTTCGTTATTTTAGGTGATAGGTAATTGTAAGCACATACGGGTATACATGATCGT
ACAAAGTGTGACTATAATCGTTAAAAGGAAGCTTGTTTGTTGAGAGTTTTTGCATGAAATTATTTTATAG
GAAGAAGAAATGTTGTACCCTTCGGTCGGCAGCTAAAATGAATGCACATGTTTAAAAAAACGTGCGAC
TTGTAAATATTTGTACTGATTATGTCTAAGTCTTTCTTTAGTTAGATAGTTACGTGTCTATTTAAATATATG
TACTTCTGTTTAGGCGCAAGCTATTGAGAAGTTAACTGATGTCTAGCTGTAAAAGAGAAAATACATTTT
CTAATCTCTTTTTGTAAATTTGGCCTAAATAATTTGAAACGAAGGCGCTGAGTATTGTTTAAAACTTTAG
CTCTCTGGTTCTGCGGACAATAGTCTCATTTTGCGAAGCAACACGGGAGGGAGTCGCACTTCTCAAAT
GAATTTGATGATTCTCTCGAAGGGCGGAGGATTCGGTGATATTGTAGCTTTACAGCTTTAAAATGTACTT
TTTATATACTTCAGAAGGTCTGTAAGACTTTATATAGAGATGAAAACTTTCGTAGTTCTTTCTAAGATAAT
TTTTATTCCCTCCAAAACGTCTCGGTCCCAGATTACAACACTAGTCCCTGAAGGACACAAGACTTTTTC
TATTCTTTTTTCTATATAAATATAAGACTAAGATAACTTCATCCGAACTATCACGTATAAAATACATATCTG
TTTCAAGTAATGTCATTATCCCATTGGGTTTAAAATTCTTACGTATTTATTCAAAATTATACCTAATGAAA
AAGTAGCCTAATAAATGTGACCTGTAAAGTATTATTAGATAAAGCAATTATTAAAATTAATTACTGTCGT
CAAAACAGTAATGTATTCGTTCGTTTTAAGCCAAATTTTGTATGATTAAATTTTTCCATAAGTATACCTAC
CTATTTGAAATAATATTAGGACCTACCTACTTACCTACTACGAACTTGATGCAATTATCCAGTCCATGCTG
TAGTAACAATACCATGTATGGTGTAATACAAAAAAATATAGAGAGCATTATTGTAAATCTTTATATTTATA
AAGACAGGCCGAGACAGGCAAGATGAGTTGGACAAGTAACTCGACTCTCTGTTCAAATAAAATGGCA
GATAATAATTATGATATACTTATAATAGAATGGATGCTCTTACCTTGCTGTGCTTGTAATATACCTATTAGA
TATACAATAATACAAACTGAAAATAGCC
>TCONS_00001052 geneID=XLOC_000196 scaffold_10:389436-389994(+) Exonic overlap (-)
TGGACCCTTTAATAAAAACATAGCTTGGATTCTTAACTACTCACATAGCATATCTAGGTACTATACGCTA
AGAATTCAGATAAAAGAACTGTTTCATCTTAGTGACTTGCGTGAACAGCACGACGTCCTTGCTGCAAA
CGTCTCCGTTAAAGAGCATGCTGGAGGCCACGCCGCGCAGGTAGTGCCGGGTCACGCCCTTCTCCAC
GGCGGGGAACACCAGCCCCGCGCCGCTGTCGCCCTTGCACACCAGCCCT
>TCONS_00001686 geneID=XLOC_000211 scaffold_10:820145-828219(+) Splice junction overlap
GAGACAAGCTAGACGTGACGGAGAAGAACGTGGCGGACGTGGTGGTGGCCAAGCTGGCGGAGCTGG
ACGCCTCGCGGCGAATGCTGGACCAGTACCGCGGCTCCGTCGAGAGCGCTAAGGTTCAAGATGGCGT
GAACCCTCTGCAGTGCTGCCAACTGTTGCGAGCAAAAATACAGGAGTGCACTGAGTTCAAGATGTATG
TAAAATGAGACAAAAAATATTGCTTGTTTCTTTATTGAATTGTCGCTAATCTCCTAATATTTGTGCCGTTT
TCCCAACAGTTTGTATATTTTGGCCCGGTGTAGGCATTTTTTATGTTTTTGTGCTGTCTGGGGATGTTTG
GGAGAAACAGATTTCATAATTATGTTGTTTTATTCTAACATAGCTCTAGATGTAAAGCTAGTTCACTTAA
CCTTCTGTGTGTTATGATTATGGATCTTCGTGCACTGTAAATACATATCAAAATCACTTTTACTTAACTTG
GC
>TCONS_00001797 geneID=XLOC_000260 scaffold_10:820149-823331(-) Exonic overlap (-)
CGACCGCTGGAGTAGAAAGGTTCTGGAGTGGAGACCCCGTGTCGGCAAACGGCGTGTCGGTCGCCCC
CCAACCCGTTGGTCTGATGATCTGCGGAAGGTAGCGGGAAGCCGCTGGATGCAGATGGCGGGTGACC

GTTTGGGGTGGCGATCGTTAGGAGAGGCCTATGTCCAACAGTGGACTACAGAAGGCTGAGAGAGAGA
GAGATTGAAAAATCAGCCCTTAATCAATTATTTAATCCTTATTTCCTCCATTTAAGCATCGAACAGTCCA
CGCTACGGGTGCACACACATCTCCACCGACTGCCCCTCATCATCAACTACACACTGACCTCGACGGAG
CCGCGGTACTGGTCGAGCATGCGGCGCGAGGCGTCCAGCTCCGCCAGCTTGGCCACCACCACGTCCG
CCACGTTCTTCTCCGTCACGTCTAGCTTG
>TCONS_00001685 geneID=XLOC_000211 scaffold_10:820167-821774(+) Splice junction overlap
GAAGAACGTGGCGGACGTGGTGGTGGCCAAGCTGGCGGAGCTGGACGCCTCGCGGCGAATGCTGGA
CCAGTACCGCGGCTCCGTCGAGAGCGCTAAGGTTCAAGATGGCGTGAACCCTCTGCAGTGCTGCCAA
CTGTTGCGAGCAAAAATACAGGAGTGCACTGAGTTCAAGATGTATGTAAAATGAGACAAAAAATATTG
CTTGTTCGTTATTAAATTGTCGCTAATCTCCTAATATTTGTGCCGTTTTCCCGAGAGTTTGTATATTATGG
CCCGGTGTAGGCATTTTTATGTTTTTGTGCTGTCTGGG
>TCONS_00001301 geneID=XLOC_000211 scaffold_10:822039-827945(+) Splice junction overlap
GGCGGCGGCGGCGGCCGCGCACTCCGACGACGCTGACTCTATGCGCCTTGCCAACGCTCTGAGAGAC
AAGCTCGACGTGACGGAGAAGAACGTGGCGGACGTGGTAGTGGCCAAGCTGGGGGGGCGGGGCGCC
CCGCGCCGCCTGCCTGGCGGAGCTGGACGCCTCGCGCCGCATGCTCGACCAGTACCGCGGCTCCGTC
GAGAGCGCTAAGGTTCAAGATGGCGTGAACCCTCTGCAGTGCTGCCAACTGTTGCGAGCAAAAATAC
AGGAGTGCACTGAGTTCAAGATGTATGTAAAATGAGA
>TCONS_00001930 geneID=XLOC_000211 scaffold_10:822950-828178(+) Splice junction overlap
CGGAGCTGGACGCCTCGCGCCGCATGCTCGACCAGTACCGCGGCTCCGTCGAGAGCGCTAAGGTTCA
AGATGGCGTGAACCCTCTGCAGTGCTGCCAACTGTTGCGAGCAAAAATACAGGAGTGCACTGAGTTC
AAGATGTATGTAAAATGAGACAAAAAATATTGCTTGTTTCTTTATTGAATTGTCGCTAATCTCCTAATATT
TGTGCCGTTTTCCCAACAGTTTGTATATTTTGGCCCGGTGTAGGCATTTTTATGTTTTTGTGCTGTCTG
GGGATGTTTGGGAGAAACAGATTTCATAATTATGTTGTTTTATTCTAACATAGCTCTAGATGTAAAGCTA
GTTCACTTAACCTTCTGTGTGTTATGATTATGGATCTTCGTG
>TCONS_00001909 geneID=XLOC_000263 scaffold_10:865748-867881(-) Intergenic
AGCACTTACGTCAACGGAAAATCTCGAGTTTATTTTATGGATTATATTTTTGTGTTCAAATAGGAACATA
ACATAACGTTGAACCGCGACTTAACGTTGGTGTCATTGTGTAACACTGAAATCCTATTGTGTAAAACTG
AAATTAAAGTAATACATACAATACACACGCACTTTTTTTTTTACTAGTTATCGATAAAAATATCCATAAGA
AGCACATCACTATTTCAGCGGGGCTATGTTGGCAGTTTTGTACGTCATAATTTTTGTTTAGTTGGTACCC
TCTGTTAGTACGCGTGAATAAAAAAAAATAATACAAGTATTTTTTTCTTTTTTTTCCCTTGTACTCCCATC
TCTTAAAACGTAGTGTTCTTTTCTCTATGCGTAGTATCTCTATTTAGTCTGTGGCATAAACCAATATTACT
ATTTTAAAATAAGATAGTTTTGTATATTATAATTAGGTTATTGAAAATTTAAGTATAAAAACATTATAAGG
GAAATTGCACATCTAGGACAACAAAATGAAGTAGGAAGTCATGGTTGTGCTGTTCACCATCACAGTAG
GATATGGCCTTTGGGCCTAGGTTTGTTACTGGTACAATAGCAGGAGCAAAAGTTATTATTATAATACCTA
TGTATTCATGTATACAATAAAGTATTAAAATAAAAATACTAAGTAGGTATAATTATATTGCATGGTACAAA
CTACTTTATTTACTTGTACAATAACTCCCAACTATCTAGTAGTTTGCAATAGAAACCAGCAATCATGTAA
ACAAACCAAACTGTCACGATTACTCCGTAATCACATACATTTGACGATCAATTATGAACATAGTCTGATT
TCAACGAACGCACCATTGTTTTCACATTATCTTCAACACGATTTAACTTGTATTTATAAGTTAAATTTGTT
AAAACATTTGCAACGTAAAAATTTCTAAGTATTTGACAAGCTGTCACAGTCTGTAGCAACTTATAACTT
CAAACGAGCTTCAAACACAAATGTATGGAGTTTGGATAGTTTTGTTTATGGTGGAGTCATTCTATCTCA
CTCACTTCCTCTAACCTTTCATGTCTCATAAAATTACCTACTCTGTATAAAATGTCATCTGTCATTTACCT
ACTCTGTGCTATATTACCTACTCTGTGGTCTGTGTCACTCACTGCTGTCAGTCGTTTTGTTCAATGTCGG
TTATATTTAAATTTTCAATTTGTTATAAGTACAATAATACCATGTTAATGAAAAAGTGGAAAGAATCAGT
TTCCTTTTAAGTGTATGTGACATCTAAAACAATAAAATCACTACCAAAATGTTAACAAGAAAGTGCGAA

ATATGTGGACTGTAATCGAATTGTAAAGACGGCCAAAGACGTTTTTTCTATGGCCAGATTTCCTTTGGA
CGTATCTCGGTAAGTAAAAAATAAGTAGCTATAAGTAAAGCAACGTATCTTCCTCGATATAAAGGGATAT
TTCTGTAAGAAAACATAATTTGCTCCATTTCATTTCAAAATATAGTGTTGGGATGAGACAATTTTAAAAC
TATCGATATTTTTCTCAGGTGTAAAAAATGGGTAATAGCTACGGGCTAGGAAGATTTAGCATATGTTCCC
ATTGAAAAACTGCACCAGCTAAAATTTATATGTGGAAATCACTTCAATGCTAGAGATTTTAATACGAAG
AAGAGTCGGTTGAAAAAACTGCGGTTCCAAGGCTAAGACTAACTCTAAAGCCACTCTCTGATGAAATA
CTAAAAGAATTTCCTTTGAAACTTATGAAAGAAGAAACAAGGTGCATCATGTTCTACAGATAATAATAT
GACCAATATTCCACCAGTACAATGTGCTGTAGAAGTACACACGACACGACAGGACTTCAACTGCTGAA
ATCAAGGGGATATGTACAAAGGTTCCACTCGAGAGAGCCACGTACAGGATCTTCACCCCCTACCAGAA
TA
>TCONS_00001199 geneID=XLOC_000267 scaffold_10:1080812-1081883(-) Intergenic
CTGCTGCTGTCCGTGTTCCGGGATAAGACGCAGGGGTATCCTTACAGCTTCCTGTTCAAATAACAAGCT
GCGTCGAACCAATATTATACATAAGATGTGAGTGAATGTGTTTTAATACAGTGATTATTTAATTATTTGTT
GGATACAATTAGCAACACCTAAGTAAAAAATATGTTTTTATTTAAGCACTTCATTACTGAATTTGAGTGT
TAATAAAATAGGA
>TCONS_00001520 geneID=XLOC_000219 scaffold_10:1166981-1171162(+) Exonic overlap (+)
CTGATATTTCAAAAATGGGGATTGGTACAAAAGTAACAGCTCGCGGCAGGACAAAAGTAACGAATTAC
TTACGTAAGTTTGCCATGTCACATTATACATTTTTATTTTTTTATTTTCTTCAGACAGTTTTTATTTACTCT
TCTGAATTAGTATCATCGTCATCAGTCAACATCTGTTGTCCTTCTCAACCCGTGTTGCAAATATCAACAT
TTTATACATTATGTTTTAAAATTGATTGTCGGATTCATTGACATAAATAAATCATTATAATATAATATTTTAT
TAGGTAAATATATTTCAGTAGACAAGCATAATACATAGTATGTTTTACACCACCATTGGTAAAAATGTATA
TCTACAATATTCCTACTATAGGAGCATTTCTCACGGTTTTATCATAATTAATAAATAGGTATATCGATCTAC
AATTGTCTAGAGTTTAGATTAAAATGTTAATGCGTAAGGACATTTTCGTAAGCTAATAAAATTTACCTAC
AAAATGCCTAAATTGAGCGTCTGCTAGTAAATTGTGCGCGACTACTATAATTCAGGATTTCATAACATTT
ACAGTGATTTATTCAACTCTACAAACACAAAATCAAACTTATTAAAGATTCTATACACTGTGAACACTC
GTGTGTCTTATAACTTACAATGGAAACATAAAATAAGATAAAAACATGCCGGTACTTTATCAACCCATTT
CAGGTTCGAACTTTACATTTCAGTAGATAATTAAAATGTTTACCAGCCAGATATTTTTTTAAAAGAACTA
GTTAACCAATCGTACAATAAAGACAAAGATA
>TCONS_00001201 geneID=XLOC_000268 scaffold_10:1170816-1172304(-) Unclassified
GCTGAATGTAAGTTTCGATTATGATTTCATCTCTTAATACCACTAGACAGATACAACAAACCATATGGCC
GCCGTTACATAAGTAGGAGCATTATGATTATTGACAGACCTACCAATAGGATGAGACAGCATGATTTCG
GCTCCAATTATCACACTATTTCTTTATATGCGTGGCGTATACTATAATTATATTCAACATTGTATATGTCTTA
TTTCACGATAGTTCAAACTGGGCCTACATGGTTCATATAAAAATAGGTAGGTATACACTATCTATTAAAC
TCAGACTATCCTTACGTTAGCTCCAAGAGGTCATACGCGATCACCATCGTATTTAACGCAGTCAATTCGT
TCTTGTTCACCTTTTATTGTAATTTAGGCTTTTTAAATTTTCAATACCTTTTTGTTTGTATTTCTCTTCATT
GACCGTTTTGATAAACTATTTATTTCTCTTATATTTACCTAATAACTAAGTACCTATCTTTGTCTTTATTGT
ACGATTGGTTAACTAGTTCTTTTAAAAAAATATCTGGCTGGTAAACATTTTAATTATCTACTGAAATGTA
AAGTTCGAACCTGAAATGGGTTGATAAAGTACCGGCATGTTTTTATCTTATTTTATGTTTCCATTGTAAG
TTATAAGACACACGAGTGTTCACAGTGTATAGAATCTTTAATAAGTTTGATTTTGTGTTTGTAGAGTTGA
ATAAATCACTGTAAATGTTATGAAATCCTGAATTATAGTAGTCGCGCACAATTTACTAGCAGACGCTCAA
TTTAGGCATTTTGTAGGTAAATTTTATTAGCTTACGAAAATGTCCTTAC
>TCONS_00001885 geneID=XLOC_000220 scaffold_10:1178333-1184960(+) Intergenic
TGCCCTATAAATACGACCGCAGGCGTAAGGCCACTCCCACTTTTGTGACAGCAGCCGACGAGTGAACA
CCTCATTAAGATAAGTACACCCGATTGTTATTTGTTAATTAAATTACTTATGCTTTGGTTAAAACGATAGC

AAAACGTTTTATGTACTCTGTTTTCAAGTTCTTGTAAATTGGTGTATTTTGGTTATGTAATTTGTATGGTA
ACCACATATTTATAATAACTTACATAAATAGCTAGATATAATGAATAATTGTGTGTTATTATTGTGTCACTG
TTGTTGGTTATGTTTCCTACCCTTAATAAAGTTGCTAATACGTTATTCCCTGTTTCTTTTTAAAATCATGAT
TTTCTTATGAGAATTATTTTTAACATTTTGGTAACCACATATTTATAAAAACTTACATAAATAGCTAGATAT
AATGAATAATTGTGTGTTATTATTGTTTCACGGTTGTTGGTTATGTTTCCTACCCTTAATAAAGTTGCTTA
TACGTTATTCCCTGTTTCATTTTAAAATCAACATGTTCTTATAGGAATTATTTTTAACATTTTGGTAACCA
CATATTTATAAAAACTTACATAAATAGCTAGATATAATGAATAATTGTGTGTTATTATTGTTTCACTGTTGT
TGGTTATGTTTCCTACCCTTAATAAAGTTGCTTATACGTTATTCCCTGTTTCATTTTAAAATCAACATGTT
CTTATAGGAATTAT
>TCONS_00001212 geneID=XLOC_000275 scaffold_10:1304932-1311374(-) Exonic overlap (+)
CCTCCTCGCCTCTTTATCCTCTTTCCATTGTCATTGCCATTTTATTTAGAAGCTATTGTTAATTATCACTAC
AGCTTGCCTTCACTTCGTATCTGCCGAGTTGTCCTGAATATCGCACATTTCAGTTGGAATGGTGCCGTA
GAAGTGAGTCGCGCACTATTAATAGCGACCAACTCGCCAAAGTTTGGTGCAAAGTACAAGATTTAGGC
GATTTACTAAAAATTATTTAGAATATACCAAGTTTAATAATTATTTTTATTGTCTACTGGAATCTACTCAG
ACTATGGAATCTTCTACGTACTGGGCTTCTCGTTAGAGAAGTAAGTACCTACTTACTTATGTTCCTTGGT
ATTGGTTTTGGGAGGTAGATTCTACGTACAAATTGGAATTACATAGTATGAAGAGGGTGCAAACGTGGC
AGTTTTGGGAAAGTTTTGGGGTTGGCCTTTCACTTCACCTTGAGTCTATCAATCTGTCTACAGCTGGCG
AAACGCTATTGATCTAAGTTGTGATGTCGTATGAATAAATTTACAACTGCACATATTACATTTACGGTGC
ATTTCGCTGTACATATCTACTATAATAGGGCTAACGAGGGATGATAACTAGTAGTAAATAAAACATATTTC
ACATGTTTGTCAAGTATAAGAGGAATAACCTTTATATTCTTTGCATCATAGTTTTTCACCTTAAAGCTATA
CTTAGACCTATTTCTGCACTCATTGGAGCCGATTCTGAAGGCACATATTTTAATTTTAGCGATGACAAAA
TTGCCCAACCTTGAATGTAAAATATGTGAAATGTGATTTACACTGGCAATCCTAAAGCCTTAAATTTAA
AATTGGTGCCTTCAGAATTACTACCATTATTTAACTTTATAGAAACGAACATAATGACATTGTCTGTATGT
CTGTTACAAATTACAATACAAAATTAGAACTTCTAATAGGAAACACCTTGGCAGTACCTGTTGCTATTAT
TATTATAGTTTATAAAATAAAATGCATGTGATGGCACGATGGTACGATACAAACAATGACAGTGTGTCCA
TATTTTTGTTTTGGTCATGTTGCAATAAAATATAGTTTTTGCATTGAATACTGGTGTACATTGTAGCGACA
CACGCAGGTCGTCCTCGTACCTGATTGAAGTTTGATTGCTTTAGCATCTCACCCAATATTATACTTGATT
TTATGTAAAATACAATGTACACCAACATCAATTTCTACAAAGGTAATTATTACAGTCAAACAAAAACTT
AGATAATATAATGCACAAAAGATTAATATAACATTACGTGCACGTAATTTGAACTTTTCAATTATTGCTTA
GAATATCTTAGAAGAATAATAAATAGCCCGACACGCTAAAACGGACATATTATACGCAAAACAAAATTT
CCGCGCTGTACTATGGAAACTACACGATTTTTAGACAATATTTTCTAAAAGGGAGCAGATTGTGTTAAA
ACAATACTAATGAACCAACGTATTATTTTATCAAAATAAGTGCTAAAGCTTTTTTAATATAACTGTTTGAT
AATTTACTGAAATTGCG
>TCONS_00001217 geneID=XLOC_000281 scaffold_10:1675605-1676657(-) Unclassified
GTACTTTGGCAACATTGCTGGAAAATTAGTAAACATTTCTTTTACAGTCTTCCTAAGAAACTATAAAGC
GACTTTCCGATGCCATGTTAGTCTCATATTAGTCGCACTGGCAACGAGGTAGACATACTTACATATATTA
TGTTCTCAAAAGTGTATTGTATGTATGTTATTCTTTTATTTTTTATGTTACAACAATGCTCTTGTTATACGG
GTTATAATTTAAATGACTACAATATTTTGCCGAAGGATAAAGAACTGAGTAAAGTTGAAAATGGCTTAA
TGGAGGTGTGGTTTAAAAACAAGAGTTTAGCGAGTGTTTTGTACCAATCTATTTCAGATATAGTCGGAA
GTTTGATGGGAACCAAAACAGGTGACAGTGAAAAAGTTTAAAAGGATAATTGGTG
>TCONS_00001815 geneID=XLOC_000289 scaffold_10:2182201-2257239(-) Splice junction overlap
CAGACGCCCGCAGGGAGGGCACGCGCGGGCACAATCGATCGATTTAAATCAACAGAATCGAATGAAT
CGAACACCGATTTAACCTAATACCTCACATAAAACTCCAATTCTATTGTTCCATATTTGAATTTTGTGTTC
CGTCTTAATTTTGCAAGTTTGGTGTGTGTATTCCTTTTTTAAAATGAAAGTAGGTTGTGCGAGTGCAGA

AAAGAGGGAAATCGGGACAAAACAAGGATATTAATAAAAAGTCTGTTTGCTGAGTGGCAGACTTGCG
ATGCCATTGGTCCAATCGTTGTGACGTCAAACGTCGGCAGCGACCTCTGTGTGCCTGGAAGAAGTTGC
TATGAGCGTTAAGCTCACCCGTGACAGTGTCTAAGAAAGTGACAAAGAAAGTGACGATGGCGGACGC
GGATGCCTTCGATGATGAGAAGTTGGTGGTCGGCACCGCTCAGCAGCGCAACTGGCGCGGGATCCTCA
TAGCACTGCTGGTCATCGTGGCAGTGCTGGCACTCATCGTGACCTCAGTGGTGCTGCTGACGCCACCG
GACGAAGGGCCCAGGGTCAAGGGCAGGAGGCTGAGGA
>TCONS_00001952 geneID=XLOC_000239 scaffold_10:2204511-2210184(+) Splice junction overlap
ATTTAGGAAGCATATTTACAAATCCCCACTACTTTTTCCGTTCGCTATCGAAAGTTAAACACAAAACAA
TTGAAACGAGATAATGCAACCTTTTTTCAGTTGCGGACCTCAGGTTAAAGAAAAATGTACTTTAACAA
GTTAAGTTGTGGTTTTTTCTTAGTATTATTTTATTTGGTGAATGTGCAAAGTACTGAAACAACTGAAGAT
CATGAATCTAATGACCAATCAGACAAGGAAGCCAGCTCCTCTCACCTGGGGCCCGCAGTGACCCAGG
TGGAACAGCATC
>TCONS_00002023 geneID=XLOC_000383 scaffold_100:87716-130077(+) Exonic overlap (+)
AATGAATGAAACTACCATATTATTTTTTGAGTTTACTTATTCGTTTCAGATGCAAATGTTGTATTATAGAT
ACTTACATCAAATCAGTCCTCTTAATAATAGATTTTATAAACCATATTCTATTGACATAATGTCTATGCATC
TATCCAACCGATAACCTGTCGAGTCGATACTCACAGTTCTAGAAGCTTCTAAGAAATTAAGATATGAGG
ATATGAGCGCCAGAACTCGTTACTGAACTACTAAGTACGTTGTTTTAACGACCCGGCTCATTTTATGCC
GTCCTCAGTACAGCGGGACAGATGTTCAGACGTTTTCAGTTTAGTGTCTTCCTACTCCTCCCCTTTTCG
TCTTTATTCAGTTGCTATATTTGTAAATCCCTATCGAAAACCAGAGGACTTGTAAGTTTGACATGTGTAA
TGATGTTGTGCGTGATCTTTGGCCGCAGAATTTGTGGCAACTCCCAAAGGGACGAATCAATTTGAATG
CTGAAAGTTTCCTGAGATATTTTGTTTAATCAAAAACTGTTAATCCATTTATAAGTTAAGCAATGATACC
ATGAGGACCTAAGTTAATACCACTAAAATCGTCTTATTTTTTCATGGATCTATTATACCTAAGAGTGAATA
CTGACCTCGCTGAAAAGAAAGTTGATTGCCGCATCAGAGACAGAAGTAAAGGACGATCAACCCTTCC
TGGTTTTCCTACCTCAAAATTCCTGAGTGTTCAATATATTTCCATTTTCCATCACAAGTTGCCTACATCAC
TTAGATAACTCCTGCATTCCAGCTTCCCGAACATGCTACTCTATAAATTGCAGCCTCGATAGTAAAATGA
CAATAGCATACTAGTAGTGTAGCAACATATTCTCCTCAAGTATAAGCGCGGTAGACGCATTATTAAAGTT
AGCGCTACCGTCCAATATAATACGGCTGTGCCGATGAGGTGGACGCACTTGTGTGAATTTCTATATAAA
GAATACCTCAATCCTTTTGATGCGACGCGTGCGATACTTAGGATGCGGAAAGGTGGACGGGAGCCCTA
AGTGTTCTTCTAGTGCAGTGTCACGCTGGCGTTGGCAGGACGCCAGGGGGTTATTCTGATTAACACAA
TCACCGATCAGTTGAACTCTACGATGCAGCTGGTTTATTCCTGATAGCTCCTGGGGTGATACTTCTTCTC
TAGTTTTCACATTTTTGGTAAACGGTTTGGGATGGATAAAAATTATCACATCTTTCAATAATGGCATCAT
TTCTACAAAGAAAATTTATCTTAATCCTTCAGTATTGCACGCGTCCCCTCAAAGTATTTGAATATTGGTA
GCTCTGATTTCTACAGTATGATCACAACCAGTGCAACACATTCCAATTATATCAGTTTTTTCACTTATCGT
AGTAGACGCATTATTCTACGTATCATAGTCTGTTCATGTACAGTCTGATTAAATAGTTTGTCATAATATAG
AGTTTACTTTCTCTCTACTTTGATCAAGGCAAATTATGACAATCGTCTAAAATCTTGTTCGTTTTGTTCC
AATAAATCTATGACGGTGGCCCATTGTCGCATGTGGGACACAAATAGTAAAACAAGCGTTGTCAAACG
GTCATGCTAGTTTAATTATTGTGTCACAAATGCTGCGCTTGATTACAGGAATAGAATCTATTTCTTGCCA
CTATTAAGGCATGTGGTTTCGCAGACGGCGAATAAATCTTGGTAGATATGTCTTTATTGTTCCTGCCATT
CAATTCCTAGGAAGAATTAAATTGTCCGCGGCATTGCATTCAGTTTGCAAGCTTTATGACATTATGACTC
TTCTGTAAAATGTGGTCGGTAATACACCGATTCGTTTAGGAGTTTTGTGTCTAATGGCCGTTATTAACTG
AACTGTAAAAACTTTAAGACAATGAAACGAATGGATAGCATAACATATAAAACAGTGATATCAAAATCC
CAGTTGCCGACTTTCAATTAAGCTTTCATTGTGTATTGCTTGTGACATCATCGCATCATTAAGCATGCCA
TAAACTGCTTACACAGCCCAACTTACAATGTCGTCCAGATAAGATAGACTTGGCAAATTCAATTTCACA
AAACAATCACTGACATTACACACCCAGTACAAACATGCACTCGGACAACTAAGTAGCACAAAATACTT

AGTCAGGGAAGCCGGTGCCTGATGGCAGCCAGCCTCTAACCAGTAGTGATTTCCACTTTCCAGCGACC
GGACGAGGGTGGAGAAACTCGAAATTTAAAAGTGACATTTCGAGTGCTCCCAGTTTTCAAATATCGAA
TCCCCGGCTGCTAGTGCTGTGTTATGTGCCAGTGTTTTTAAAAATAATTTCTCTTGTTTGTAGGAAAATG
CTGAGCGAAATAAATAACATTAGTGGTGTACTATCGTCTTCGATGAAATTGTTTTGCGAATGATACTTTT
TGTTTAGTGTGTGTGTGTGGGACCCAAGTGGGGCTGTGCTAACTATAGTGCGTGTGCGCGCACCGCCG
CCGTCGCGACGCCGTCGGCGCACTGGGTGGTGCACCAATGGCGGCTGTACAAGCGGTCGCTGTGCTC
GCTGCTGGACACGTACTCGCTGTGCT
>TCONS_00002025 geneID=XLOC_000384 scaffold_100:155847-175342(+) Exonic overlap (+)
CCGCGCGCGCCTAGCGCCCCACGCTCGCCCCACGCCCCGCGCGCGCCCAGCGCCCCACGGTGGGACC
ACGGCCCCACTCTCAGCCCCGGCAGTGATATAAGACATTCAAACTAGCAGTAACGAGGTACTACGATT
CGTGTTATTTTTGTAGTATTTTTTTGTCTGTTCATATTTAATTGCTAACGTACGTCCGTGTTTCAGTGGAT
CGTTCATTCTATTTGTACTGTAAAGTGTTAAACTTTGAATGTGTCGGTTTGTAAGTGGCCGAAGTATGG
AGACAATCGGCTTTAGATATAGCTAAGTTGTTGACGAAGGATTTCTGGAAAACTAATGTTTCATGATAA
CTTTATTCCTGATTTATTCGTATTGAAATTTGTAGTTGGTAATAATAATATTACGAGTAGCTTACCTTCAA
ACGTTTTGAAAATTACTGGAATCTGTTAGGTATATTCTTCACTACCTATTTGGGGTATATTGATAGGAAAT
AACTTCAACTGTTGATTATTGATAGCAAAATAACTTATAAAAAAGTGAAACACAACTTTTTGTAAACTA
TTTCCATCCAGTAGTCGTCCCCAGTAAAATTTACACTTTTGGAAATTCTAAATCTTTGTTACATATTTTTG
GTAACGGTTTCGTAAGTATTAGATCTCATAAATTATTTTTATTACTGTAGGTAGGTAGGTAGCCTACCTCA
TGTTTTTGTTAGATTGTTTTTTGTAATCAAAATTTTTATAAGTAACTATATCATATTTACTTACTTTAAAAA
ACCAGTGGAATTCATAGAATTGAATGCCTAGTCAAATATAAAACGATACCATCTTTTTAATAATAGTAAA
TCCCAGTCTTAGACAATCATAAATAACATCATTTAATAAAAATGTACCTACATATTTACTATGAGAAATAA
AAAGTTATAACCACTTTTAGATCTCAGCTTCCACTTTGCTTGACCAAATGACCACAGTGACGTCACACC
AAGATACCAACCTGTGTTAACCATAATAATATATGGCAGGGGGTTAATATCATAAACCCCTCTTGAGTCT
AAGTGCCAATTTCTCCATAGTTAGAGCATAAACCAGGAAGTAGTGGTTGACTTTTGACACATCTGTCAT
GAATTTTATATGGAGATGACGTATAATTTATAAACCCTGTCGGTTAGATAACCGACTATGGAGAAATTGG
CACTAATGGTTAATGTTAAATAACATAATTGGTTAATGAAGGCGATTTTGTAGGCGTACATTGTACCTAA
TTTTAGTATTGTCAACAAAAAATACGAACCACGCAGTCGTCTTTTAAGTTGAATTTCGTGCAAATTATA
AAACGCTTTGACACTGCTAAATTTAAAATTTGTGCCTTCAAAATCGTTACCAATGACATAATAGGTTAA
AATGAATGACGAATAATATTATGTGTACGATGGCCATCACATATTTAACTTAACTTTTGACGCCAGTACC
TAAGCTGAGGTCTGTTGTACTTATGGTTATGAAGATATGAATTATAAATGAAATCATTGTGCATTTTTGA
AATGATTAAACAAAATATTATCCGCCCATCACCATTGGCGTCCTTATATAAATAGTTAGTTTCTCTGTACG
CATCGGTAATCCTTTATATGTATTATGCACTGTGACATATTATTATATAATGGCAGATATTAAATATATCATC
CTATTTTCTTTTTAACTCTGTACTAAATTGCAAAATAGATCTCTTTCTTTGGACCAAGCCTCAAAAACAT
ACACACATAAAAATACTAATATATATATTGTTTGTCTAACCCAACATCTACATGTATAATATATAATACCGAA
GTTAAGTGTAAATAGTTAGACATCACATTTTGCGTTAGTGGTTAAACTGGGAATACTTACCAACTATTTT
TTTTATTTAAATTTAATATTAAGATTTTAATTTTAGTTTTTAATGATTCTAGTTGTAAATGTAAATTGTGATT
GGATCCTAGTAATTTTAAGATTTTCTCGTAATCTAGGTTTAAGGTGAATTTAACATTGTCATTTTATTTATA
ATTAGTGTTTGTGAATATAACAAATAAATTATAGTTATAATATCCGCCCAGTCGTGTTTGGAATACGTTGG
TGGTGAGAATTGTCTGTGTCTGTGAATGAACCGATATTCGTCCCTGATTGTGCAAGATGGTCGGTCTCG
GGCGTGAAGCATGTAGGACATTGCTATACCACAGGCCAAGCCGTCGACGATGGCTATCCTTGTAAGTAT
ACATACATACATAGCCTGTCCATTTTCATATACTTTAGAAATAAGTCCTACATTTTGCGATAACAAAATTC
TTTTAGTCTGTCCAATCGGGCCTTTACTATTTGAAGTCCTCTAAAATTTGTCCATTTTTTGTCCACTAGAT
AAGACGTAGTGCTAGTTGATACAGTTTTGATATTGGCATAGCTTTTGAAAATATAGATTGTTCTATTCTAT
TAAAATGATTTGTGAAAATAGCCCATGTAGCGCCTGAATAAGGACAGTTTGTAAGAAATTTCTTATTTAT

TAAAATTGTACATCATACAAGCTTTTCTCCTTAGAACCATCCTATTATTCTTAGATAATTTTGTAAATATTA
TGCGTATTTTCCACCTACCATCTCATCACAGTGATAAATGTTTCTGAATAAAATCATTT
>TCONS_00002083 geneID=XLOC_000403 scaffold_100:234165-257708(-) Unclassified
NNGCTCTTGTGATTATAAATTTAAATTAAAAAACAAAATTTAAATTAATATCTAAACACCGCGTCTAAT
CGAAATATTTTTAGACTAAACCCCCTTGATCCTCCCTTTTTGATCTTAACTGTTGATGGTGAACAAAAA
GTTTTTTGCAAAATTTTTAATATTTATGTCGGTTACATTTGTGGAGAAATTTGGAAATTTGAAACGATTG
ATCGTTATTGAGAATATTTTTATTTTAAAGTATTTTTTTGGACGGACGTGACAGTGAGCGAACACATATA
ACGTTAGAGTTTAAAAACATTTAGAGTTTAGTTATTGTAAGGACTAACTATTTCATTGTAATGGACGTTA
ATTATTTACTGCCATAAGTTTTAATTGTAATGTTAATAGGACATTTGGCTTTAGTGTAAAAACTGGACTG
TTTTGTCAGCGAGAAGAGTTTTCTTGGACCAAGACGACCCACAACACCAGACCGTCTGCTTCGAGCA
CTTTTCTTTCTTTAACAAAACCCTCAAAACTACGGGAATAGGATTCTACACCGGATAAGAAAATAAAAT
AACATTCATGGTGAGTTTTTTAATTGTATTAATTACAAAGGATTTTGTCTATACTTAACGTAAAAAGGGG
TGTTATAAGTTTAACGTATCTGTGTATGAGAGTGTGTATCTGTCTGTGGTAGTGTAACTTACAAACTGCT
GAATCTGTGTATGAGAGTGTGTATCTGTCTGTGGTAGTGTAATTTACAAACGGCTGAACCGATTTTCTAA
TTGTTTCTTGTTACCCTGATGTGTTTTTCGCCATATTTTACTAAAATTGGTTCAGCCGTTAAAAAAGTTAT
ACTACTTTTAGTGTTAAGTTTCAGGGGTTTTTAATGTTTGTTAGGTTAAGTTATTACTATATAGAAG
>TCONS_00002375 geneID=XLOC_000403 scaffold_100:234165-260396(-) Intergenic
CCCGTAACCTCCGAATGACCTCATTTTTTTTTCTTCAATGTAAATATTCGGCTCGTATTGCCGCATAGTCT
ATAAGTGGTACTAAGTTCCTTAACCGCGTTATTTTTTGTTTAAATAATTGTGTTTCGTAGTGGACTTTTGC
TGATAAATTGACTTTTGTGTGGACTTTGGATTTTACAGAAAATAGTTTAAGTGACACGAACTGTTGGTA
AAAATTAGATTCGTCTTAAAATATTTTTAATAATTGTAGGTTTAAAATTATCGTAAGGACTGCCAATTGTT
ATTTTTGTGGTGCTTGAAAATTCGGAGGAGCAACGTTTGTAAATTTCACCATCAGTGTGCCGGAAAGA
ACTTGGATTTTTCGACTGTTGCAGACGACCCACAACACCAGACCGTCTGCTTCGAGCACTTTTCTTTCT
TTAACAAAACCCTCAAAACTACGGGAATAGGATTCTACACCGGATAAGAAAATAAAATAACATTCATG
GTGAGTTTTTTAATTGTATTAATTACAAAGGATTTTGTCTATACTTAACGTAAAAAGGGGTGTTATAAGT
TTAACGTATCTGTGTATGAGAGTGTGTATCTGTCTGTGGTAGTGTAACTTACAAACTGCTGAATCTGTGT
ATGAGAGTGTGTATCTGTCTGTGGTAGTGTAATTTACAAACGGCTGAACCGATTTTCTAATTGTTTCTTG
TTACCCTGATGTGTTTTTCGCCATATTTTACTAAAATTGGTTCAGCCGTTAAAAAAGTTATACTACTTTTA
GTGTTAAGTTTCAGGGGTTTTTAATGTTTGTTAGGTTAAGTTATTACTATATAGAAG
>TCONS_00002236 geneID=XLOC_000405 scaffold_100:321331-325106(-) Splice junction overlap
GTGTTTTCAGGTAACCTCGGTTTATCGAACGACTGTACATTGATTTAATCCCACCCCAGTCCCAGGGTC
AGTGCCTCGCGTGCTCCTCAGACGGCTGCCGGCGTCCGCGCGTCGCCTCGTGTCCGGCCTGCAACAA
ACCTGGGACTCCCCGGCCGACTCCAGCTCCTCATCATCATCCATCATGTCCACAGGATCCTCAGTCACC
GCCGCCGGCGTCTGGGCTCCGCGTACCACTAACTCATCATCCTCAACCAATGGAAACTCCTCGTCCAG
CGAACACGACCGCACGCAGTCCTACAATATGGACATAGACTTTGACTAATGTTTAATGCAACCTCCTTC
TGTCGTCTGCTAGCCTGGTACTTCTCGGCGCGTGATTGAGCGGTGAACGGATGAGTTTTTAGAAATAGC
TCGTTGTGGGCGGCGTTTGGAATTGGCATGTGATTGTTGTGTAAGAGTGAGGCCTTTTGGTGCGTTTCG
TCGTTGCTGCCATACATTCTTTAATTAAACCGTTTCTTTGGGACGACACGACGCAACGGGCCAATGGGG
CCTCACCCTAAGAGCACGTACTCAAAATGGCGAGCTAACAAGCGCGCGACGTCACGCTATCACCTATG
GAACACTTGTGAGTGGGATCTCCCACATATCTATGTTGTATAGTGTCAAGCCCTGAGATGCATTGATATC
CGCTGCCGAAAAATGTAGTTTGGAAGTTCAATTCTAGGCAAAAAAAAACTATGTACAGTTGTGAGACT
CGCGAGATTCTTGATGTACAAGGAGTGTGTGTTTCAAGTACGCAGTTTTGAAGACGAAATAAAATTATT
ATAAAATACTATAGTCACCGCTCAAGCCCGCGCCGGGAAGTACAAGCTTGTCTCGTATTGTGGCTGTGT
ATTGTAGGACTAGCGTTTAACTGAGGACGCTCCTTGAGTACATACGATACGTAATCAGAGTGATTTCCA

CTTATAAGTGCACGCTGCCTTTTTAATATATGTAGGTATATGAGAAAACATGGGATAGCTTAACAAACCT
TATTTTAATTGTAATTCAAACAGGAATGTCCCGAGTTAAACGCTAACTGGACCTATAGTTTAAATAAATA
AAACGCGTGCATAGTTCCAATATGGCGAATAGTCTAACAGATAGTTACTTGTAGGAAGCTAAGGTAAGG
TTTAGGTTTGTTTGGAGTCGCATTGGTTGTATGTGAGGGTGTGGCGATTGTAAATTTGTTATTTTTATCTT
AAGTTAACGGCAAATGCATAAAAATATGTCTTTTATTTTTTCAATTTAAACAGCAAATTTGAAATGTGTT
TGTGTGTGAGTTTTATTATTTAATGTTTAGTGAAGGGAGCTAGTTGGTAGAGCGACGGCACGGCTGAAT
ACAAGTTTCTACAATTAATTCCTTGTAAATTTTGTAAATATTAATCATAATTTTAGGCATTTGTTGCTGGT
ATGTTATTTACCGATCTTCTCTTCTGCTAATATCTAAAAGAGTAATAATGTAAATAAATATAGAAATTGCG
TAAAAAAGTAATATGTTGCTCATTATTTTTTTACAATATTAGAAACGCA
>TCONS_00002445 geneID=XLOC_000412 scaffold_100:533043-535042(-) Intronic
CTGAAACCGTATACCTAATGTAATTACCAGGAAAATGTATATCAGTACACAATAAGTTACACATACATAG
CCTACAATGTAAAAATTCTCCTGCTGAGGGGGGAGAATGTAACGAGCCGTTACCAAACCATCCACCGC
ACCTACCCACATACCGAAGCCACAAGAGCGCCCGCGAGTCAAGGCGGGGCACCACACAGGCGACTC
CGGTGAATACAAGGGGCGCGGGGCGACCCGCCTATAAATAACCGCGAGCCAGCCGTCCGACCACCAT
TCGTCACCTCCTGCTCCGGCGAGTGACCAGTCCGTTCCCGAGATCCTTCCCCGCACCTATATCTATCTA
AGTATCTATTCACGCATACTAAGCTCACAACACCGTCCTCCCTACGCTGTGGGTCGCCTACTAGCGGAT
CGAGGTTCCCCGGAATACACGGACGCCTGGGTCGCCTAGTTTAAAGCGGCCGTGTACCGAACTATACA
ATCGCTGAACACACACCGGCCCTCAGCCACACGTTCTTATCAACACAAGTACACTCCACAAATCCTGG
AACAAATCCACACGTACCACCGGTATTCGCCCGACCTCCTGTACCCTGTGTTCCTTCCCTTTATTAAGAT
AGAAACATCCGCGTAGGCCCCTTCAGGGCCCGACCAATATAAATATCAATAACCGTGATAAGTATTTGT
GCATTTGTGTAGTGTGTTAATATTAAGTACATAAGCTGTAAGTACCCTAGATATATAAGTTAACCTGGTTG
TAAATAAAGCGGGTCCAGTGCCGCCTGAATTGTGTTACACTAATTAATCGGCATTATTAATTCTCACCCC
GTACACCTACCGAGGTTATTTCCCTCCAGGGGCCTCAGGACCAAGATCAGGGTAGTTAGGACCGCTTC
ACGGTCATTGTAGATCCCCCAGGCGACCAGGCACTCCTTCAGAGGCCGGCGTCAAGCTGAGCTAGTCC
AAGACCGCTTCCCGGTAGATATCAATTGAGAGGTATTAGGTAGGTACCAGCCCTAAGCACTAGGGACG
TTAGTCATAAGGGTAAGTATCACATAACAATATAGCACGCAACCCCTACTCTCGGGGGAGGCCAGGCA
GGTCGATTCGCCCAGGGGTCGCTCCCCGGTGATCCAGACCAGCTCCTGCTGAGCTGCTACATTACACT
ATTTTTCCGGCGCATCTAGAATCCAACTTCCTACCACTGACTAATCCTTCAACCACAATCACAAAGCCT
CTTCAACACCACTTCAAAGACTATACCACTCACGTCATTGCTATTCTGCAGTCCGTACGGGCAGGTTCC
GTTGGCGACACAGTATGTGAGATCCAGTGGGTCTCCGCTGGCGTCGCGTAGCACGGTCCAGCCCGCCA
CCAACGCTATCACCATGTACGAGATTGTAGACAGATATAGTATAGCTATGTATACAATTCACAGCATTAC
GCACCTAGATGGGAACCTAAAAAGTGTCTTTCACGTATTGGATCATAGAGATTGCAATCACCAACTTCC
TAACGCTGACTCATTCTTTCATCACTATCACAAAGCCTCTTAGACACCTCTAAAACACTATAGGCCTTAC
CACAAAAACTTAGACAGTGTTTAAAAGGTGTTTATAGCTGTCAAACGCCTACAACATCTGTCAAATTTC
GTTTTAAACACTA
>TCONS_00002073 geneID=XLOC_000395 scaffold_100:570034-592678(+) Unclassified
CGGGCAAGCGGACTCAGTCTGTGAGCGCCTTTCTAGACGCACGCACGTTCCCGCCAAACGAACTGTC
AAATAACGCTCTCGAAAAAAGTTGCGTACCGAACTACAAAAACAAAAAAATACCAGTGATTTTATACT
GAACTTTGTGCAGTTTATTACAGTGTTTTTAAGTGGTATCGTGGATTTTTTGTGATTTGAACGAGATTCG
AGCCGGATATAAGGGACAGCAGTCGAGGAAATGGGAGTGCTGGTGTGATGCTGTC
>TCONS_00002540 geneID=XLOC_000503 scaffold_1008:1747-5928(+) Unclassified
GCACGCTGCTGTAAAAGTGGCGTTAAGCACACAGATAAGGCTCGGTCCCTCCAGCCAGTGTAAAAAG
CATAGCTTCCCCCCGCGCACCGACCACCTTCCATCGAGAGGATTACACGAAAATGTGTGCTGTAAAATA
TGGACTGCAATATAAAAAGCGCATACATAGATGTTAATCTTACTGTTAATAAGCATTAATCTATGTGCATT

TGTGTGAGTGAACTCTGTTCTACATTCGTGTATGTATTCTACGCTTCTTAAAACGTATATCTGGGTGCTT
CATCAATCTTTGCTTGTTTAAAAAGTTACATTCCTATTGTTGCGAAATAGTTGTCAAAATCATTCGATTA
CTTCTTACTTGTTTTTATCGATTATTTTATCCGATATTCGTTCGAAAATGGAATTGTTGAAATTTATGTTAG
GCAGAGATTATATTTTTCGATTTTTATTATCCTAATCGTAATGTTTATTTCTGATTGTTGTATTTCAATGTA
GTCCTCAATAAGTAGGTTACGCAATTTGTTTAAAATGCAATTATTCGTAATTATATAACACATATAAATAA
ACCTCCGATGTTAATTCTAGCTTGAATGTAGTGTCAATTTGTTATTGTGGTTATAAACACATGTAAACCT
GCTTATTACTTATAGACGCTTTATGTTACTTCAACTTGCAGGTTAAGTGCCAATTTCGCCATAGTGGGTT
AGAGTATAACCAGGGATTATTGCTTGACTTTTGACACATCTGTCAAGAATGTTATATGGAGATGACGTAT
AATTAATAAATCAATCCCTGTCGGTTAGATAACCAACTATGCAGAAATTGGCACTAAAGGTTGTGGCTC
ACCTTCCAGTTGTTTAAAAAAAACACGCTAAACTGTTTTATGCCATGTACAAGTGCCGATAGGTGTGCC
ACTATCATTATATTTCCTACAAAACTATGCGGCGATGCAATAAAATATGAGACGTGCATCTTATGCATTGT
TTGGCCTTTCACTTGACACTTTACAAATATGTACAGAGTAAGTAAGAGGCAAAGAAGAGTCCTGAATA
TGGACTGCTTCTTCATACTTCAATCGCTTTATACTTGCGTCATTCATTGTCATTGTAATTTAAGCACCCCT
TGAATGATTGGTAGGTATACGTTGATATACATATCTGCTGGAGTAAATTGTTAAGCTGGTAGATTTAATAC
GAGGATAATCTTTGACTTCCGTATAGCGATAAATAATTACCATAATGGCCACTTGTCTCAAATCTAGGAA
AATTAGAGTTATGTGAGGTGGGCTACAGTCACGTCTTATAAAGGTATAGACTCAGTAACTCGAAATACA
GGGCTAAAAGATATGTTGTATGGTTTTTTTAATAGAGATCCTAATCCTAAAAATATTTGTTTCGGGGTCC
TTAAAGACATGTCAGTAGCCCTCCACTCTCTGGTGCCTAGTTTGTCACACCACTATTGTAATAGTTTTTA
ATCCAATGTCCGCTTGTTTTTAATTAAAATATACATTAAGACTAATCAGTTCTTTTTTATGTAGTATCTAA
GTGACTAGTTATTGTTTTTCGCTAAACTAGTGTCGAATTGCCATCTTATACAGCAGAAGGTTGGCTTGG
AATGTTTCAGTCTATTACTAATAATGTACTGTGAAATACCGCACAACTTCTAGGATTAATTTGGTAAGCC
TGCCGAGTTGAGGAGGTGTGACTTCTCACCGAGGCCGTATATCCAGATAGCTTTTTTACAAGCCTTTTG
AATTTACGTTGGCGTTGTCGCTCAACAACTTTCAACAAAAGACTTGTAAAGGAAACATTTTAGTGTCG
GGAGATAGTTTTCACTTAAAAGGTGATATGGGTTTAGGCATTAATTGACATTTATTTGATAGCGTGTTTT
TGTTTTGTTATAAGTATTCTATTCAATTGTTTTGATGTGTTGAGTTATGTTGCCCTAAGCTGTAAGCATAC
GGTTACATTTGATGTTAAAATCTCTCAGTCCTTATTGATTTAAAATAGCTAGTGCGAAATGTTTCACAAC
AAACTTGTGTTAGATGACTATGATTTATAGAAAATTTGTGACTAAAAACTTTACTCCCGCACCAATGGC
ACTAAGTTAATTACTGTCTATACCCACTCCCGAGATATAAGCGTAAAACAAGTAAGATTAAGAATAGTA
CACATAAGATTGTATAAGTTATTAATGCAATTGTTGGTAAAATTTACAGTTAAATAACCTAAATTCTACA
AGAATGTAGCTTTGCCCTATAGTTGACAAGGGTATCATGTTCACTCGCAAGACCTACAAATTCAATTCA
ATCATTTATTGATTGACGTAAATATTTTAAGTGAATTGAAAATAAAACACTCTTCTTTCGTACCATACTAC
CTATAAATAAATAACTACTGTGTTATTCTAAAACAAAACTGATAGTTGTACTGACAATTGCATTCAATTTT
AGTAGTAAGATTTTAATGTATGTATTGTTCAGATGCAAATATTTAGAAGAATCAGCATGTAAATAATAAA
AATATACTAAAACTAAATTACTACGCCGCCTAAGCGGTGCATTTTTTTATATAAATTCATTTATTAGACGG
GGGTTTAAAATGTATATTTTAAAACATTGGGCTTTTTAGAATAATTTAGTGTTTTAGAATTTATGTATGCC
TTTAGAGAAATGTATTAACTCGAAAATATTGGTTCGCCTTATAATTATTACATATAATAATGTACAAAACA
GTAAATAATTATCCACCGATATGAGAATTAGTTGTACCTAGCTAATACTTTTGACAACAATGTATATTTAC
GTTTTATCGTCTCCGTACGCTAGCATTGTTTGAAATTGTAATGCGAACGCATAACTAATTAAAGTCAAGT
AAAGAATAGACTTTAATTGCAAATTGTCTGATGCCATTCGTCTAATACATCTTGCATAGCTTTATTGTATC
TTACGGAAGACATCAATATACGGTAATATTTGGTATATTCAGTTGACGAATATCGAATACTGTGTAAATA
GGCAGCCGCTGCGTTTCATGGTGTAATAAAAATGCCAAAATTCATTAATGTGTTGCGATATCTAAATATG
TATAACTAATTAAATGTTTCTTGTGTAACAATTCAATGTATAACTTGGTGTTTTCTAAATGTCTTTAGCTT
ATGTCGTAATCCGTTTCAAAATTTAGTTTACAAGATAATTTAGACTGAGTTGGGAATACGGCCTTTTTTA
TATCGATAGATTTTAGTTATCTGTCTCTGTCTACTATTTCATACCGACTTCAGTATAAAGTGTCTTGTAAA

GTATGAACATTAGCATGCATGTTATTTTTATACAGAAGAATTGAATTAGAAAAAACGGATAAGAGCCGG
TGTGTGTCGTATAGAAAAAAACATAATTGAATAAAATTAAGATTTATGCGCTGTCGAGTAATTCTTCCAA
ATATTGTAAAGCCTCTGAATAATTCAAAATATATTTATATTAACT
>TCONS_00002561    geneID=XLOC_000503    scaffold_1008:1882-2737(+)    Intergenic
GGCGATAAGCACACAGATAAGGCTCGGTCCCTCCAGCCAGTGTAAAAAGCATAGCTTCCCCCCGCGCA
CCGACCACCTTCCATCGAGAGGATTACACGAAAATGTGTGCTGTAAAATATGGACTGCAATATAAAAA
GCGCATACATAGATGTTAATCTTACTGTTAATAAGCATTAATCTATGTGCATTTGTGTGAGTGAACTCTGT
TCTACATTCGTGTATGTATTCTACGCTTCTTAAAACGTATATCTGGGTGCTTCATCAATCTT
>TCONS_00002600    geneID=XLOC_000507    scaffold_1009:27456-28637(+)    Intergenic
CGATTACCTACATATGGTAGTCCTGGTTAAATGTGGATATTTACCCCTCAATAGCAAAAAAAGGCGTTAT
TGTTTGTATTTCTGAATGAAACTAGGCAAATAAATATATGTATAAAAAAATATGTGTAACCTACCTATAA
GCCTACCCATTATTATTTCAGGTGAAAACACAGCCTATCGATGTAATGCGCCGATAAACTATCATGACTG
CATTGATGTAATGCGCTAAAAAACTGCAATGCCTCCGAATACATGATTACCAGATAACAAATCCTGGCG
CAGCTTACGTGTGTTCTTCAATAACGTCGCAAGCTACAGTGGCTGTTAATAATTTTTGCAAGTTCTACA
CCTACAGGATTCAGTTATCTGGGCATTGTAGATACCCTATTATCACCTACGAAGTTATTTAGGTATATTTA
TTAAAACCATTATACAAGGTGTTGCAAAAAGGGTATACTAAGCCGAAAGCTACATGTGCAGCATGGTAT
ATCTAAGCCCGAAACTGAAATCAGAATTTAAATATTCGCAAAAAAATATATCATTTTTCATGGAAACTTA
GTTGGTCACGTGACTTTTTACTATGGAC
>TCONS_00002650    geneID=XLOC_000532    scaffold_101:161327-170573(-)    Intergenic
GGCAGATCGCGTATCCGCGCTCACGCGAACCTCTCACAACTTTCACCTCCGCGATATCTATATCTGTGAT
AAATGCACTATAACCGAGTGAAATTAATTGTGGACCTTCAACTAAACCTCCCTCGACAGCCAACGCCG
GCTGACAGTGGCCAACATCAAGCATTACGGCAAACTAATTTCTCCAGTGCACAGCCGATTCTACGAAA
CTGCTTATAAAGAGTTTACCGAGAAACGAAACGGCTGCAAAGTGGATTTGAGGAAATATTGTAATTGC
TAGTGGGCTTGTGTGTTTCCGTGTCTTTGTGTTCAGTAAGGCGTCTACATTGGATCCCAATCGACCGAA
AGGACCTCCAATGGTGGTGAAACTACCAGACTCATGTGTGAAGGTGGCGG
>TCONS_00002929    geneID=XLOC_000535    scaffold_101:288059-289123(-)    Intergenic
TCACAGTAGAGAAAACTATTAACTATATCTAAATTGTGTTAATTTATCAGAGATACTCCCGTCGACATGT
CAATGAATCCTATATAAACAAATATGTGTTTAAGAATGGGAAACTACTTTTTACGATTTCAGGGTTGGAT
CAAAAATAAATTTAATGTTGTCACGATATCAGCACATTGATCTAACAGGTCACTAATAAGCTTTCATCCT
GTAGGCTTTATCCCTATGACCATTTATAACCAACTTTTTAGTAACACCCTGTAGAGCACCACACACCAAA
TTGATTAAGTACCTAAACAACTAAGAAATCAATTTCTCCTACTACATTTTTATAGCCGTTAAGCAAAAGA
ATATGGTAAAACAAGAATGATGTTAGAAGTGTACCTTTTAACTTTGACAACAATAATAATTTCTTTTGTT
GAAAGTGCTCCGGCTCCGGTTCCTGAATATGATGCACCTGTGACACCCTATGTAAGTTCATCATCATCA
TCAGCCAAGACAATCGACTGTTGATGAACCGTTCAGATTCTGTCCATTAAGTATCTGAGATTAAAAAAC
AGACTTTAACCAATGC
>TCONS_00002662    geneID=XLOC_000544    scaffold_101:519973-522773(-)    Unclassified
TTCAAGTTTAAATTTTGTTTGTTTTGATTTGATTTAGTTGTGTAATAAAGTGTTTTGAATCAATTAAAATT
ATAAACAACAGTGAATTTATAACCTTTACCATGTCCTCAGTGGTAGCAAATCGTGATCGGGATTGTTTCT
CTTGTCGCATGGTGAGCGGCACCGGTTTACTCGGAATTGGGGGTTATCTCGCATATCATGCACGAAAAA
ACCCTACAAAAGCTGGTAGACTGACTATTTCATCTTTCGCGTTGGCTTTTATTGGACTTGGAGTAGCGA
GATGTGGACAGCTGTATCCTTTCGACAGGCACACCAGAGCTACTTAGATCCAACAATTCGGTGGAAAA
GGTAAAATGACTTTAAATTCTATGATGTAATAGTACAATTTGCTGTACTTTAAATTCTAAGTCTGTAATGT
GAAGCATTTTAATGAACAATATTGTTGATAGATTCTTGAAGTAGGATAGTCTAAGTGATGGTTATGTTATA
TTAAGATTTAGAATGGAAAAACTTGTAATGGAAGCTGTGGCATCATGCATTTATATATGAACAATAACA

ACTAGTTTTTTTAAGTTATTGATTGTTATTTTTATAGATGAGTAAGAAAATAAATTGTATTTAAACATAC
ATTTTCAAGGTTTTAATAATATAGAAAATTACTTATAATGCTAAATGATATATCTAGCATTTCCTGTCTCCT
CATTTCAGCATTAATCTCAGCATCACAACACAATGTTTAGTTTTCAGATCCTAAAATAAATTTTATTGCA
CCTAAAGCTTAATGAGTGAAACAACATTTAATTTGGTTCAACAATTTTAATAAACATTCTTATATTTAAC
ACTTACACATTATCACAATTTTCTATTTTTCTATTAGGTACCTACCTACATTTACCTAGATATTTGAATGGC
GTCTAAAACTGGTAAGCCTAATGTAGGTACACAAACTAATAATCCATATTGAAAATCTTTTTTATAAGTT
ACATTTAAATTACGTTAATTTCATTAAAAGTTGACCATATTCATAAATACAACATTTCATATATTATATATT
TTTAGGTTTAAATGATTTTTAAATCATAATTTTAAAAGTTATTGTCATAGTTGCTGCATAAAAGATTAAGT
ATTGTAAGGGTGGGTTAGATTTCGGAAATGTAAGATAAAAATGTGCCTAAATACCTGGTACTGCACTCT
TTGCACTACAGTAAAGTATAAAGCTGTCAGTATCTTGCTCAAACTATACATATAGTATAAATACATATAAT
ACATACTATTTATAAAAATGTACTGCATACTGTTTCTACATAATTACTACAGTTACAATAGTAGTAAAAAA
ATAAGATAATTAGAAATAAACCTTGTTAAAAATTATTTCAACTTCATTTTAATTAAAAAAAATAGCACTT
TTTTAATGTGCTATATTATGTATAAATATTAATAAAATTTTTAGTACCTACTTTTATCTACAATCCCCATTGT
ATAAACATGAAGTAACTTTGCCAAACAAAATTAAAAACCTAACACTCTGTAAAATGAAGTAAAATATAC
TTATTTCTATTATTATAGAATCTTTGGGATAATCATTTCAACTGACTCAACCACATTATAAAAGGCTTAAC
ATCACCCATCCTTCAATCTTTCTTTAACCAGTATAACAATAAGCAATCTACTTAACTACATTATTTTGTAC
TAAAACTAAAACAGGTATTATCTATAATACCCTATAATTAATATATTGCAGTGTTGCTGATATAAATAAGT
AAAAACATAATATATTAAGTTAAATTGGTGTCGATTGTGATTATAACTGCTTATTGCTACGACAACACAC
CCCCCCTTTGGACGTCAACCTAGTATAGACAGAGATTTAAATTGCCTCAATGTCCTGCACAGTAAGGGC
CAGTTTTCTTGATCCGTAGCAAACCATTGGTAGAAAGTCACAACCATTACTATACCAATAAAATTTATAT
TCAGCTGTCATGCTTGAAATAGACTTTGACCAATGGTTAAGTTAACTTAGGGACCAAAAAACTGGCCC
TGAGTAATAACTAGTCCTAAAAGGTTTTCCCTCCTATAGCGAGCATGATGTTCTCGAAAGCTATCCAGT
TGATGAGCGCCCAGGGCCCCAGCCGCATCCACAGCGGCA
>TCONS_00002862 geneID=XLOC_000548 scaffold_101:630133-639240(-) Unclassified
CCGACGCGCCGTACATACTGGGGATGGAGCACAGGAGGCGGAGCTGCGTGCAAATTATCCTGAGCCC
AGGTGCTACGGGGCACCTGTGCGATGGGCGAGAGACTCCACTAGGATACGGCATTGGGCCCTTTCAAC
TTTATATCTAAACTAGCTGTTCCTGCGAGCTTCGCTTCGCCATAAAAAAAATTCCCTTGAGAAATCCGG
GATAAAAAGTAGCTTATGTTCTTTTTCAGGATCTACACCATATGTATACCAAATTTCATACAAATCTGTTG
AGTAGTTTTGGCGTGAAAGAGTAACAGACAGACACAGTTACTTTCGCATTTATAATATTAGTTAGGATA
CAGCAGAGGGTTCGTGTCTTGGACCATGCGAATGCATCGTCTATTCCACCTTAAATGCAGTGTTATTCT
TTAAAACCATTTTAGGCTCCAATAATCACGTAAATACTTTCTACTTTGATACTCTATTAAATTATTTGTAG
AACAAAAAACTAAGTCTCTCACTACACAAATTTATAACACAAGTTAGTAATTTATTACTAACAGTATAAT
TTTTCGCAAAACTTTCTTGCGTCGAATTCGCTGGTAATGATTCGCAGTAGTGTTTATTGAAATTTGATCT
CTAAGCCAATAGGCGCAAGTGCTAAATTTCTGGTTATTTAGCTGTCGGTTTGCAGTAAGCCAGTGAGTA
ATGTTGTCCACTCCATCATGACGAATTAGTTTGTTTGATGTAAATAAATGGCCGTTTTAACTACACATTG
TAACCTAGTTCAATTAGATTTATAGTGCCCCATGTTAACTAGTCATTTGTTAGTACGATAGTTTGTTTGTT
AGTTCCGAGTATTTTTATAAGTATAATTAAATCGAATCGATCTCAAAACGGAGAGTAAAATTACTCATAC
GTGATAGTGATATTGTTAGAAAATTCAATTAGGCTAATTTCTTCATTTGATATGTATATTCACACATTACA
ATAATTACAGTAAAGACACTACAATTATAATATAAAATTCAAATAGACTTATCACGTAACATAACACGGG
TATTATTCGTGAGTTTTTAGTAGCTACTTATCGTTAGAAAAATATTAGGCAAAGCCATTACTTGCACATA
AACACAGCCCAATATTGTGGTCTGTTTATTTGCAAGCCACGGCGAGCCGTCTACCAGGAAGACATAAA
TTAAAACACAAACACAGCTCCCATTTCTGAGCAAACACACAGTCATTGCCGTTCTGTTTGCATTGCCA
ACTATGCTACGAGCCCCGACTTCGTAGCGCTACGCCGTAGCGCGTAGCCCGTAGCCCGTAGGTCGTAG
CGTCTCGTAGAGTTTGTGTTAGGTAATAATGATAGCTTCGTCTTTAATCTTGGAAATATAGCTGTTACTAT

TGTTAGTAATGAGTCCATCTGCTATAAAGTAGCTAGAAATATTGTAATGTATGTTATAATAGTTATGAATT
ATTTATTAAGATATCTACCATAGTATGTAAAGTGGAGCATATTGCTTAGGATATATTACTTTACTATTTAGA
ATAGTTTTTTAAATAATTAAAAATTTATAACGTACACAGTAGGTATATTTGTATACTCATCCAGATATAAA
CGCAATGGAGATTTCCAAATTCAATAATAATAATAATAAATTCAGTTTCGGCTATTTTCGGCCACCATCC
ACATGGTTCACCTCTACCGCTCGGCTATTCGCAGCACGCGACCAATAACTCACAGCCTTCTGCCACTG
ACTCCGAGGAAAAGGCGCTTTTCCGGGGGGAAATATTAAAAGAACGTTAAGTAATTAACACCGCAAAT
AAGATGAGTAGTGCAGCGTCGACTTCATGTGTTAACAGACGGTTAGTTATACGTACATAATTAATTGTA
AGCTTGGGTATAAACCTATGCTCTCTTGTTAAAAACGAAGACTTAAGATAGTTCTAGTTTGAACCCATT
TGAGACTACTGATTTAGATAAAGTTCAGAAATGAAATCATGTTTATTACGGAGAAATTACTTAAACGTG
ATTTATGTGGGTGTGTATATAGGTTCCTGTACCTGGATCACTTGAATGAAAACTTTATGTACTACAAGCT
ATGTACATTGTACGAGTACATACGTACTATTTTGCCACCGATCATAAAATATGGATATTTTGTTTTAGAGC
AAAAGATTTCAGCAACCGTATATGTGTATAAGTACAGCATAAGTCCAAATACCCCTCCAGATCAGGTGA
TTAGTGAATCTTAGGTTAACAATAACAAGATAATATACTTAAAGAGATTTTGTAAAATCAGACTTTGTAT
TTAAGATGAGTCTCTGTTCATTAAAACCACTAAGTATTATAGTTATGAGAATAAGAAAAAAGATCAACC
ATAAGAATCTCCGTTTGAAGATAAAAGGAATAGATCAGAAAGAAAAATAAGGCTTTGACGAAATAAAT
CTGACCCTTCAGTAGTACTCGTATTCATTCCGTTTTTATGTCAAATCACTTTTTAGGGCGTCCGCTACGT
TCAGGGTTCTCGGCCGCGGATAACTGATTTTTATAAATGTTCTTATTGATCGATCGGTGGACGCTTTTATT
ATTTGTCTGCTGCCGTGGGGTGCTACACTAAACTCAGCGGAAGTCCTTAGTTCTAGAATGTACAGTTCG
TTGCAAAAGTTGTATAGTAAGCCAAAGGTTGATTTGGGTGGTGATTCTAAGCAACTAATGTTCTGATTT
CGGAAATTAGTGAATCAAAAAACTGTAAAACTTTACGTAACAACTAAAGTTTAAACTAACATTTACTAT
ATTACAAGACCCCTGTGACTCTCCTAATATAGCTTAAGTAGGTATATTTCTCCTCTCCAGAGATCTCAAT
TCGTGAAAAATCTACACATTTTTTAATGTAAAAAAATGATAAACATGTAAGTATTTACTTTGGCGGGAC
AACCAACATTTTTACACTTAGATTTAATCTAAAACAAGTTTTTTGGGAGCTGTCCTAGTTTTCTTTTAGT
AAATGCTGATATATTTTTCAGAGCGCGAGGCCTTATTTCCTACTGAATTATTTACGTTATCTCGTAAGCAA
TATCCTAGTTATAATATGTATGACTTGTGACTTCTGAAAAATGACATATCATTCGTTTTTATTTCTCAC
>TCONS_00002736 geneID=XLOC_000550 scaffold_101:675696-677463(-) Unclassified
TGAATTCAGTGAAAGTCGGGTGTGAATAAGTGCGGCCAACTGGCCAATGCGCTTTTTAGCCATGGCGG
GCATAGCAACGGTGGCGATCCTCATGCTCCTGGCCTCCGCGTCATCAGCCCCCGTGCCCCGCACCCTC
GGCTCGCTGGTCTCCGGAGACACGTTGGGGCTGGAGCTGCCCACGGGGCTGCTGGCGGGGGCGGGG
GCGGGGGCGGTGCAGGTCGTGGTCAACGTCTACACCGACAGCAGCGGAAATATCCAGGTCGACCCGG
CGGTTTTTTAACCTGTTACCCTTGGACCCTTTCAATTGTGTCTGATTTCCCGGCAAACACGTGATGGCA
AGGGAGTTTTAGACGATGAAACTTTGATCTACCCAACGAGAGAGCATTATAGTTTTATATCGATATTTTA
TGAGCGTATAAGATACTGAGATGTCGTTAAGAAATCTTCATAAAGCCAGAGTGAATAACGTAGAGATAT
AAAAGTGATAAAGCCGAGTAAAGTGAACACAGATGCAAGCATGATTCGATGATGCGAATGCAGTGCCT
TGACTGTGGACTTCACCAGAACTGTTGTGAGTCGAAGGATTCAGATCAACATGTCTTCGACTAAAGAA
GAGGAGTTATATGCTGAAGGATTACAGTAGATATATTATGTCCTGAAGGATGTAGTATAAGTACCTATAAT
TATGTTTCTGTGAAGGAAGAACTACTTACATCAATCACAGGGAGAGGACGCCCGTGTGATTGGTTTTT
CTTTTAACTTACTTAAGCATGATTATATCTGTACTTAATATAAGTTGTTTATAAATTGCCTAAAACACTGA
CTTATTTTCTAAAACATAATTACAAGTTGCATTTTTTTTCAAAGAAATAATGTTTCAGGGAATATCTTTCT
GAAATAATGAGAGCGCGCATCTGGCCGTCTAGACTTCATTATCCATGAAACTAAAACCAACCGCAAATT
ACAGATCAACCTAACTAATAAAACAACGTAACTGAAGATAAAAACTATAATTTCTGAACTAAACTGGG
AACCGTCTTGCAAGAGGAACATGTGGTTGCTAGGTACTCTAGGCACCTACCATAATATTAAACCCAATC
GAACTGCTACACACACAAACCACGCTTTTTCGTGCAGATTAATAACAAAACAGCTACGTAACTGGTAA
CGTGAACGTAACAAGTAGGAACTTAATAAAACCCAAAACGATCGAAACTCCTACCTATCCATTACGGG

TCCAATAAGTCTCGCGAAGAATGGGGGAGCACTGTTCTATTTCCTGAATCATGAAACTTACCCATCTCA
AAAAGATAAGTTTTATCGGCCATCCCCCCGGATTCTACCGGTGACGTTTTTTTAACCGGTTCCGAGGCA
ACTAATGCGACTCGGCGACCCCTGACGGCTGGAGGAAGGAG
>TCONS_00002814 geneID=XLOC_000525 scaffold_101:693741-731735(+) Splice junction overlap
CTCGTTGAACGTAGCGATCGTGTGAGCTTATTCTCTAGAACTTTTACGTGATTTTATTGTCTCGCGCGCG
TATTTAGTTTGAATTTAAAAAAGTGTCTTATTTGATTATGACGTTTTATAAGTGCCATTTCCGGTTAAATT
AACTCGATTATTTATAATATTAGTTTATAATTATAAAAGAAAAGAGTTTTGTGCAAAGAAATAAAATGTG
GAAATAATGTTTCATTAAAACAGCATTTTAACTGAACTTAGTTATAATTTTTTTTTTGCATCTAACTTCAA
AATATACTTAATAAAAAGTGTTCAATTGACTTTTTCTAAATCAGCTGTGAATTTAGTTTTTCGCTAAGGT
GCCATCTTGCAGAACTAAAAAGTTTACTTTTGAAGTCTTGTTGTTGTTTGTTTGTGTCTTGTGTTTTTAC
CAACGATTTATGAACGTATTAGTGTTTGTGATGGTGTGAGCCGACTGCCCGAGGATGAGGTGGTGGCT
GGTGCTGCTGCTGGTGGCTGCAGTGGCTGCGAAGGGGAAGAAACATAAACACCAGAAACCTGGTCG
AGCGGTCCTGGACCGGCTGGCTAGCAC
>TCONS_00002846 geneID=XLOC_000525 scaffold_101:694017-702269(+) Splice junction overlap
CTTCAAAATATACTTAATAAAAAGTGTTCAATTGACTTTTTCTAAATCAGCTGTGAATTTAGTTTTTCGC
TAAGGTGCCATCTTGCAGAACTAAAAAGTTTACTTTTGAAGTCTTGTTGTTGTTTGTTTGTGTCTTGTG
TTTTTACCAACGATTTATGAACGTATTAGTGTTTGTGATGGTGTGAGCCGACTGCCCGAGGATGAGGTG
GTGGCTGGTGCTGCTGCTGGTGGCTGCAGTGGCTGCGAAGGGGAAGAAACATAAACACCAGAAACCT
GTGCGCGAGCTCAAAGCGGACGACTGGCGAGAACTTGCACAGAAC
>TCONS_00003801 geneID=XLOC_000660 scaffold_102:57919-58923(-) Intergenic
CCTACCCTCTCCTATCGGAGGTCGGCAATCAAACAAGGATGTGCTAAAGCGGCTGCTTTGATGTCAGG
AGCTAATTCCTATAGTATGAATGCTCCTGCTGATATGACAACATCTACTGAAGAAGTTAAAATATCTGAA
AAACCTAAGGAAGAAGCTCCTAAAGATCCTCCTTGTGAAGAGGATAAAGAAGATATACCAAGCGGAG
AAGAATACTCAGATGAGG
>TCONS_00003325 geneID=XLOC_000670 scaffold_102:676850-688011(-) Intergenic
ATTACAGTATGGAATACATTATTGAAATGTGAATGTTCAAATAAAAAAAAAACAAATGAAGCCAGCTCT
CAGCTACAACAAATAAGATAACAAATTGGCACTTCAAATCATGGAGAAAACCGTGCATGCCCCTGAGG
GGATGGTGGTGACGCCCCACCACCTGGCGACTCAGTCTGCCATCTCCGTGCTGAGAGACGGCGGCAC
CGCGGTGG
>TCONS_00003266 geneID=XLOC_000671 scaffold_102:703871-704836(-) Intergenic
GTTTTACTTGATTTATTTTTAACACAAAAAATATACAATATCAATAAATATAGTAGTCACATTGGATCTATA
TCACTCGTCGTTAACTCCTATATTTTATTTTACAATGTTGTTATACAACAAAATTATTAACATTTAATAGTT
ACTTATTATATTTATACCACATTCGCGTTAAAATTGTGCATCTGTAAATTACATTTAAAGTGTTCAATCAAT
AGTATTGAGCAAAATTAATATTGCTTGAAAATAAATAAAAATTGCTTTTAGAAAATTCACAATTACTTTG
ACCCTGCAAGAGTCACCAAGATATGTTTTGGCCGATTTCTTGAAGAAGCTCGCTCATGTTATATTTTGA
ATACTGGACAAAAATATAGATATCATCTGATTACGTCTCATAGTAAGCAGCTTAAAATACAATAAAATAT
GGCCTAAAGTACATAACCTATGTTTTACGCAAATCTTTTATTTATTAAGAAAAATAATCTCAATGTAAACT
ATAAAAATGTATGAGATACGACAATACCTGTTAATAGTTCTAGATTTTAACAACACATATTCTTATTTTCC
TTGTTTTTGAAACAGGAAGAATATATTTAGTACTCTTAAATTGTTCAATAATCATATTATTGAATGCCTGA
TATCAAACCATGTGATAGGCCGAGATTGTAGCGAAACAAACTGCAAAAGCGTAATAAAAACCCATAAA
AACTTTCCTACGTGATTTGGTACATAAAAAAAATGCTAAAAATGGCAGTTTCTTACAAAAG

>TCONS_00003653 geneID=XLOC_000675 scaffold_102:829985-831473(-) Splice junction overlap
AATAGTTTTGCCATGATACTTCTTTTTGTAATAAATATTATAATAAAACTGAACATTTATTACTTAGAATGC

GAACACGCCACGCGCCGTCACCCACACTAAGTACCTATTTAGAAGTCTCAACACCACACCATACCACA
GTGGGTCGCGCACTCGGCTCGTCGATCCTGGACATGACGGCGCAGCACCCCAACTCGCGGGTGCCCA
ACTCCGAGTACCGCTCCGTGGCCGGGCTCCGGGACTGGCTGCGGAACAACGTGAAGAATATGAGGCC
ACAGAATGACCCCGTCATCGAGGTGTACTGTAACTGACTGATCAGATCTGAATCATTTAAACCAAAAA
AAATGTAAACGTTATCAAAATATGATCTTAAAACTACCTTTATTGGCTTTATTTATAATTTATACCAAGTT
AAATTTTATATAACAAGGG
>TCONS_00003107 geneID=XLOC_000649 scaffold_102:895515-901437(+) Splice junction overlap
AACACTTAGCCAAGGAGGTGGCGAAGTCCCCCTCCCCGCACGGGCCCACCCCGCCGCCCTCCCCGCG
CGCGCCCTCCGCCTCCCGCGATGAGAGGAGCAAGGGCAAGTCGAAGGCCAAGATGAGCAAGGAGAA
GCTGAATTTGTCAAGCGTGGGCGTCCGCGCCGTCCCGGGCGGTTCAGAATCCAGTGGCTGGTGCAGC
GGCGGCGAGGGCTCGCTGGAGGAGCCGGAGGACGCGCTGGCGGCCGTGCTGGACGCGCTGGCGGCC
GAGAACCAGCAGCTGAAGCGGCAGCTGGCGGACGCGGGGGAGCGCGGCGCCAAGACCAGCAAGTT
GGAAGAAGAG
>TCONS_00003176 geneID=XLOC_000686 scaffold_102:1484394-1486415(-) Intergenic
GCGGGCGCCCGCGTGATAACGGCCGCCCGTGGCCGTCTCTACGCCTCTACGCTCGCCAGTCCTACCCT
GGAGCGCACCAGCAACGCTCGTGAGTTCGTAAATACGCATTCAGAAGAAACAAGTGAAGTGCCGCAA
TCATTCGATTCGTTTGTTCCCCCGGAAATTTGAATTTGGAATCTCGAAGTGGATTTTGTTTTTGTGTCGT
GCCAGCCAGGAAGTGTTTTGTGTTTTGAGTTCGCTGTGTGGAGTTTGAGATGCAAAAAGTTTGATGTA
TAACCCTGCCAAGCGTGGTG
>TCONS_00003729 geneID=XLOC_000690 scaffold_102:1567803-1569266(-) Splice junction overlap
TAGGAATCCGTAGACGAAAGCCTGGACTCACTTCTGACATACATTCAACAGTACCTTCTTCGGCCGAG
GACGCCTTTTTACCGGAAGTGCGTGAGTTCCAACCGGATGGGCACGCCCTGCGCCCGCGCACCACTTC
TGGTGGCACCAGAAGAACCGCTGCCACTAGACGGTAAGTTTCTTTTTAATTCCGTAATTTTCAAATTTC
TTGGCTGAAAATCAATTTAATCTAGATATCATTAACTTTTCTTTAATCTCAGGGATTTGGTTAAGGGATCT
ATAACTTACATTAGGTAAAGTTTGCCGAGCATTCGGATGAATATTAACTGCTTCTAAAACCAAATGAAC
ATCATTACTATGCACTGTTTTTAGTTATTGTTCTATTCACATGAGATTTATTTTATCTTCAAATCGATCTTTA
TGATGGTGTATTTCTTCCAATTTAGTTAATTTAGCATGTAATCAAGCTATCTCATCAACCATTGGGCCACT
TCGTATCATGATCACTTCTTATAACAGTATTATTTGAAAAGATTTTGTTTATTTGAACACAAAATTGGCA
GGGGAATGTGTTTTTACTTTGTTAATCAAACAAAAAAATTGTCATCTTCATGTTTTAATTTTAATTTCTGT
TACTTTCTTGCATCTGTTAGGAGACGGTTGTGGAAACAGTAAGTCTGGCTCCGAATCATTTGGCAACTG
CTTGGGCTTGTATCTAGCGGTGTAGTTAGTCGTAGTATTTTTTAATGTTTTAACAGCATTTTGTACACAA
ACCAAAACTTCACACCGCGCACCAAAGTGACTATTTAATGGCATCACACACGATTTATTAAACTATGAA
AAGATTTGATTGTTTTAACGACTATTATTGTATCACCAGTAATCAGAAAACAATCTCTGACATTCTTTTAT
TCAATTCGTAATAACAGTCATGTTTTTCCTAAAAATTAAGCTTGCATTATTTTCAGTGACTGTACAGTTT
GTTTCGGTATTGTAACCTTGTAACCTTTGCTAATTTATACACAAATGCATAACTATTGTATGTTTTTAGCT
GTCTCTTCTATATTTAGGTACCTACTAACCTTAAATTTTGCAAACTACTCCAGTTCCTTTTTACAACAAA
ACTTCATTCAGTACCTATTCAATTAGTTAAATAGAAGGAGATCAGAGG
>TCONS_00003867 geneID=XLOC_000776 scaffold_1021:7962-9169(+) Intronic
CAAGGTACAAATTTAATCGTGTCCCAAGGTACAAAAAAGCAATATTTAACCAAAATTTAAATATCTTTAT
TTTTAATTAATAGGAATCTTTCAGATAATTGCCATGTCATAAAACTAAATAACTGAAAAGTTATACGTGA
CATCCATGTCTTTATTTTGAGCATGCCTCAATGAGATAAAGCAACAGAAAAAACTATACAAAAGCTACT
TTTGACTCCAAAAAACTTGATACTGTCTTCACCACACACTAGATGCTGGTATGATCACGAGACTCACTA
GTTTTGCCAAGCGAGTAAAATACTATGCCTAGGGTAGAATTTTAATGTGTTTATTTAGCCGGTTTTTAAA
ATACAATCAATGTCCCGAGGTACAAGCGTCCCAAGGTACGTTACGTTACCCTAGTACTGCTGCTAGATG

AATCTGTAAAAAAACATTGTACCTAAATGCATCGTTGTTGAGTGTCACATTTTCATAATATTTTTACATTC
ACATTCAGGTGATGCAACGGTGAAACTACGGTTAACTTCTTACAAAGTCATCTATAGTTTTTAATATACA
TATCTGTTTTACAGTTGCAGACAAAGCCACAAGACGAGCGTTTTACATTGGTCTATTTGTGGTATCCTT
GCTTGACTTTGGAGGCAACTTCGTCATATCCATATACGCATCTGTTATCATCAAGAACTCTGGAGTCAC
AGTTAGACCAGAGCTGCAAGCGCTGTCGCTTCCAGCGATAATGATTATAGGGTCTTTGGTGTCTACCGT
CACTGTTGAGAAATATGGACGGAAGCCGTTGCTTATATCAAGTCTTATTATCAACGGGTTACCAATGGC
AATACTCGGCATACTCCTAATATTGCCCACATATGGGTTTGAGATTGCCCAATGG
>TCONS_00004116 geneID=XLOC_000817 scaffold_103:357098-469848(-) Intergenic
CTCTCGTTGTGAGAGCTTGTGTCTCTTTTCTCCCGATCGCCTCCGCGTATATCCCAACAAAACATCGTTA
ATAGTTAAAATAAACTGTGACTGGTTTTAGTGCTTCGGTTCACGTGTCCCGCGGTTCCACCGGGAACA
GCAGTCTGTTAACTGGATCCGATCTGGTTTTTGGTTCTTTTTGTTCCGTTCGCAAGTGCAAAATGTGAT
GTTATGTGTGTTCAGCGAGCGTGCCGACCTGTGTAACGATAAAAGCCTCAAAGTGAACTCAATGCATT
TGATGTGTGAATGATATTTTGGAAAGAGATCTCCTTTGAGGTGGATTCACAACATTTTGAGATTACAGA
TATGCCCGAAACTTTAAGAATACACCAATCCTGATCTGGTGCAGCCATGTCGCCCAGCCCGCGCCTACG
TGAGGACAATTTGATGGACGGATCAGCTCTCACGTGTAACTACAGGACTACAATGGTAATACCTACATA
CATCCGTATAGCTTCAAACTCAAAACAGTTACAACCGAA
>TCONS_00004014 geneID=XLOC_000819 scaffold_103:713765-715753(-) Splice junction overlap
CAAATCCATAAATTTATAGCAAGAAGATAAAAAGACAAAATAAAAAATGAAGTTGTTTTTAAGGTTATA
AATAGTAACTTTTGCTTGGTTATAAAAGTGAGTTAAACAAAATGGAATCGCCAGAGGAAACAAGACAA
AGAAAAGCGATGTTTCGGCGCGTGTACATCTTGCTCATAGTGAACATGTTGGGGATCGCTGCTGTATCA
GTGTTGTTCACTGCCCTGGTTCTGTATATTCAAAGTATTTCGACCAAACAGCCACTGTGGCTTGATTCA
AACTTGGATTATTTAAAATTAAAACCGATTGATGCGCTCAGCAAAGACCAAAACTATAGTTTATTTAATT
ATTAGAGTGTTCTCTCAGTATTCCCGAGTAAGGTCGGCGGCAGGTGTGATGTTCGCACGAGCGGACAC
TCCTGAGTCGGATGATACCGCTCCGCCGAGCGAGTTATGAACAATTTCTGGAAACATTTCAAACAGAG
CTAGTCTGTTATATTTAAATAAAATTTAATATTTCAATTGCTATGTAAGTAAAAGAAAGATTAAATAAATT
ATTAAGTGAAGGAAATCCCTACCCC
>TCONS_00004068 geneID=XLOC_000808 scaffold_103:781568-782055(+) Splice junction overlap
GCCAGGAAGGAGACGGAGAGACGAGAGAGGGATAAGAGGGAGAGAGAGGAGCGGGCTGAGAGGA
AGAGACAGGAGCGTGAGAAGCAAGACCGCGAGCGTAAAGAACGCAAAGCGCAAGAGGACAAGGAG
GCCGGCGTAGTTCCCGAGGAACAGAAGGAGACAGAAGATGGGGAGGGAATCAAGAAAGAGGGAGA
CGACGCTGAGGGAATCAAAAAGGAGGCAGAAGATGCGGAGAAGAAGGAGGGAGATGTGGAGATGC
AGGCCGCTGATGATGCTAAGGTA
>TCONS_00004199 geneID=XLOC_000844 scaffold_1030:1507-5166(-) Unclassified
TTGATATTAATATTTAATATGTATTTGTGTGCATATATCAGCTGTCGATACCCATATTACAGGCTCTACCTA
GTTTCGTGTGTGAGATGACCACACTTATTATTATATTGCTATTATATTCATCGTAATAATGTGCGCGAAAC
ATTACCACACACATACTGTTACTTTTATCAAACACCCCTCTAACCCCCCCACCTCTATCTCCCCAGGAGG
ACCTGGACAAGGAGATGCTGGGCATCAACGTGCCCACGGACCAGCTGCCCGACGTGCCGTCCGCCGA
GCCCGCCGCGCCGCGCCCCGCCAAGGCGAAGCCTGCTAGCGAAGACGACGACGATCTGGCTAAACTG
CAATCGTGGGCGACATAAATGTAAGTGTCGGAAGCGAAACCGAATACATATGTATATTGTAAAGTCTTC
GTTGCACATCCAAATACTTAGCTATTCTAAGGCTGCAACCCTTTAGGCAAACAGTATTCTCGTTACTAG
GCTATGGTAATTTGCGTAGAGTGAAAGAAAAACTGGCTAATAAAAATAAGGAAGGTCGAAGACAGAG
TGTTGTGACGCTCCTTGGGATTGGGCTATGATCAATTACAATTACAGCCTCAGTATTAGGTACTGTCTAT
GGTAGATCTATTTCCCTGCGCATCACTTTGCCCTTTCAGGGCTTTCCCGGGGGAATACCGGGATAAAAA
GTACATAAAACCTTCCTCTGAAATCACCATAACAAATAAAATCCGTTGCGTAGTTTGCTTTATACTATGT

AGTGATTTGAAAAGCTGAGTGTTTGGATGTGTAACCGTTGTATGGTTTGTGATAATTTGTACGATTGTGT
AATATTTGAATATGCAATTATGCACATTTATTTATTGGACTAAAATTAAGATTTTTGTTGGAGGTTTATTT
TGTTGGCTGAATATTCAAATATTGCGTAATCTTACCGTCTTTCGGATATTGTAATGTATAGGAACAGAATT
GAAATCTAAATCTGAATAGAAAATGTTATTTTCCATTTCTCACAAATTAGAACCATAACTTTTTGTTACT
TTCGCTGAGTTGCACAAATGAACTTTAATCTAACGTCGACCTTAAATGTATTGCTGCCTTGCTTTGATAG
TATACGGAAAGAAAGGCAGATATAGATTTAATTCCCATATTAGCTTCTTTTTTGCAACTCAGCCTTAGAA
TCTCACAGACTGTTCATGCAATGTAAAGTTACCATACAATATTCTTTACCAAATGTTCATTCAATGGAAA
ATATAATGTTAGTAATTTCAGTAAGCTTAAGCTTAATTTCAGTTAGCTTTTCTTTTGATTTACACTATTCA
GATTTAGATTTATAAAAGATTGTCCATTTGGTGCAGTCGGTCGCGATAGTAATATTATCAAAAATTGTTC
ATCAATCACACTTATGTATTCAGTAAGTACCTAACGTAGCTTTTGCCGACTGTTAGGAGGTTTGGTTTAA
AAACTGGTGTAGGATAGGAATTCACATATTGGCGTTTAAAAACACCAACTGTGCTACCACAAAAAACT
TTAAACATTGTTTAACTTGAACTTGTTTAAAACGAAATTTGACAGATTTTGTTGGCGTTTGACAGCGCT
AAACACCTGTTAAATACTGTCTAAGTTTTTGTGGTAAGGCCCCAAAAGTCTTGCAACTTTTAAACCATC
TTCAAAATGAGGGAAGTTACGTATGTTTCCCTTTTTCACATCAGTTTTAAGTCAAACCTTCGTAGGATA
ATGTATTCATGTTAAGATTGTAATCGGAAGTCTTTTTTCTGCCTTAATAATCACAATTATACAATTTGTAG
TCAAAATATTGATGGTGTTCTAAGAGGCAGGGGAAAATTGTTTATATGTTGTATATATCTTGCTATTATTT
ATATTTTTCTTATAATTCTAATAGTTTATGCAGGTATGTTTACAATAAATCAATTTATCAAAGTGTTGTGTC
CCGATAATTTGTTGGGTAAAACTTTAAATAGGTTAAGTGCCTGTTTTACAATGTCTGTATAATGGTTAAA
CCTGTTGGATAAAATACATGCTGTCACTCTCGGTAATTATGTTTTAGACAATGACAGTATGTCTTCTATC
CCACAGATAGTCCTTACCCAGACATTGTGAAACAGGCCCTAAACATCATACATAAAATATTTGAATGTC
AAAAAGATTTTATTTTTTTATCAGTTTCGTTACGTTTGATTGTAGCGTGTCTGACAAAGTTTAGTAAAAT
GCATACTTCTTCCGAAAATTCTTATTAGTATTTAGTTGTCGTCTATGGAGCACCATATACATCTGTTTCAC
TATACACATATACAGAATGTTGCAAAAAAGGTTTAGGGGCCCAGGCTATATTAATAATATACAAACTTAT
ATCAGGGGCTCATTGTAAATATTTTATGATTTATGGTCTGGAAACTATCACGTTGTCAGATATGCTACAA
CCTCTTTTTGTTGGTAAGGTTAGGATAAGCTCAAAAAGAAAGTTTTTTGTGCAAATTATTAATATTATTC
TAGATAGATTCGCTTTAGCGCCTATCCTAATGTAAACGTAATTTTCTAAGCGGTAATATTACTTGTG
>TCONS_00004216 geneID=XLOC_000847 scaffold_1031:16293-24558(-) Unclassified
TCCCTGCTGCTGATCGTGGTGGCCATCGTGTTAGTTCGCGCGTTGTCGCGGCACAAGGGAGAATACCT
CACTCAGGAGGAGCGCGGCGCGGACGGCGNNNNNNNNNNNNNNNNNNNNNNNNNNNNNNNNNNN
NNNNNCGGACACGGCCGCCTTCGCCCCCGCCACCGGCCCGCGCGTCACCAAGCGACGCGAGTTCTTC
ATATAGACACACACACTATAGCAAACACATACACACTCTGTATCTTACATTCATCATAGCAGTATAGTGG
CAGCTGACTTATCTGACGCATTATTCAGGAAGTCGACATAAACAAAGGCACGTCTGTAACGTCATTTTC
TTCACCGATACAATTAACAAGACATAAGCAGCGTCGCTCACCTGTCAAACATCTGTCAGATTCATACAA
GGAACGTCATATTTGACAAGCGAGCTGGTGAGCGAGCTAATGTTGGGTGGGGTTACCCCACAGTCCAT
TTTCGGTCACTTCTAACTTCATGACGGTATAAAGAAAAACAGGACGTCAAAATTTAGGCGATCTGGAA
CATTGTACAACTACATTCTTGGTCCGGTGACAGCTGAGCTATACATTCTGGCTTCTGTTTAGCCATTTGA
CGCGGTCAAGATAAGTTGGCTTCACTGTAACCTTCTGTTTGGGAATAGTTGTCGTAGCAATTTATGACA
CTTTAAATAGCGGATAAAGACATCCCGATTCTTTAAATATACATCACCACACCTATATGCTAATTCAGAA
CATACCACAGGCCAAAATCGACAGATTGCCATAAAATAAAGATGAAGTTAAACGGCGTTTAACTCATTA
GACTTAGGTACCACGTTTTTCCACTGCCTAATAAACAAAAAGGCGAGTGTCCATCGTACTATTCAGTGG
ACATACGCCGGAGGAAAGAATCTATCGCACATTTACAAAATTATAATACTAGGTATATAACTATTTAATT
GATATTAGTCGAGAAATAGTACAAAACGCATCATTCATATCACATCTATCACTGTCAAACTCTCAAATGT
TCCAAGCGTTTATAAAATACGTTGCTTCATAATGTCAACCATTTTGAAGTAATTGTTATATTGTAAACCAT
TACCTAAATTCAATCTCTCCATAGCTTTTCTTCTTCTAACCTATTTCTCCCATAAAGTGCCATTTGGCTAC

TGCTTTCTCTTTCTCTCTCATGGATATTGTATGAAACGTATATGCGATAGCTATATTTTTACACACCGATTA
TGCCATAAACCAAAATGCCTTCTTGTAAAGTTTATTCGACATCGTATGTATCTCGTTTTATATTTTTATAC
AGATCTGATAAGTTTAAAGTTGCTGTTTTACTATATCTATACTATACTGTATGGTATATCTAAAAGAGCAT
TTTTAAAATACAAGCAAACTGCTTTACTATACAACTGACGTATATTAGTTATAAGTAATTACTTAGTTATG
TTTCTTGAAGTTACGAATAAAAACGAAATATTAATAAAATTAAAGTTAGTCAGATTATAAAACGTCTGA
AGAGTGAATGTAGTAGGCCTGTCGAGAACTGTGCCTTTCAGAGTTAAGTGAAACTTAGATTAAGGGAT
TGATATTTGTACAGGGTGCTGTTTTATCATTGGTCCAGCAAAAGTCTTATTCACCATATAAAAATTGAGA
AATGACTTTATATTGTTAAAACACGGAGGATAGTATCTGTTAAATTACCTATTTGAAATAAAATAATATTT
ACAAAGATAAAAAAAAATTGCCAGCTAGGCGCTACTGTTACAACAGCACTCTGCATTCGTAACTTCATGT
AGACTAGTATTTTGGGTGTATTAAGTTCTCTCTGAATTTTCTATTATTTAAGAATCTACTTAAAACGCGAC
ATTATAAATTAGACTGCTAATATTAGTGCGTAGATTTGTATTTTATGCAAAACAGTATTTTATTTTTCCGTC
CAAAATATTTTTTTTAACTTATAATGATGTAATCATTTATATTTTATTTGCGTGACATTATGTGGGATTAATT
TTGTATTAAACAAATTATAGTTTTGTATTTTATGTTGTGACTTAACAGTTTTTTATTCGATCGTGAGATTAT
GTGCCTTAATCTCTTCCAGACAAATTAAGAGGCACCGAAGTAGTAACCGCATACATTGAGACTCATTGT
GTAGATGTAGAGTTTTATTTTAGTACTTTTAAATCACGAATAAATTTTTCACAAGAT
>TCONS_00004250 geneID=XLOC_000859 scaffold_1038:21280-24157(-) Exonic overlap (-)
ATTTAGCTACTGTGTCCCACGCATTTTTGCAGATATTGTCTTTGGAAGCTCTGAAAAACCTATTTTCGTC
TACATGACGCGTGTAAATAAGATGCTTACTAAATTTACTTACGGAATATTTTGAGATATACTCACTTGTTT
ACGCGTTTTTGAGGTGGCCTGAGGGTATGATACAAGAGTAATACATTGTGGATCCGATTTTGTATTGTAA
TTTTCAAATTATAGATACCTACCTAGCCAGCGAATCGTGAGAAGATGATGTCGAGATCCGCAGCAGTCC
TCGCGGCCGTTGGCCTCGGCCTGTCCACCTTCAGCGTCCGCCAGATGGTCATCAGCCAGACCAGAAG
GATCTAATATCCTCATTAGCAAACGCTACAATCAATTGTCACCAACACCCGACAGTATGAACATCACCT
GTCAAAGTTAGCTGTCAGTTAAAGCTCTGTTCCCATTGGTAGATGGGTTTTAAGGTTGTTCCACTGTAT
AGCGGAAAGAGCTGAGCTGTTTAATAGTTGAAGCGAGTAAAGTGTTATCTGTTATCCGCTGTTGTTTTC
GGCAGACGCTTTATGTACGCTTGACGCTATCGCTCGCTCGTTTCAGAGGATGCAACGCTAGTATATCTT
CGCTACCAGTGGAGGCATGACTTCTTCAAGTAGTGAAATGTTAGGGAACTGTTGTATATGTTTGTCTGC
GATCGCCTCTCAAATTCGATGTTTCTTTGTGACTTACGTGACGCATCGAATGTAATATGTTAAATTTTGT
GTGCCGACCGCTCTAATGTTGAATTTTTCAGGCGATTTATTTAAATTCTTATCTTTAATGTGTGTTTCTGT
CTGACTTTGAATGTGAAATGTACATGTGTTTTGAATAATGATGGGAAAGCTTTGATTTGTATAATAATGA
TAGTTGAAATCCGAATGTTTTGGTCTATTTTTTTAATTATATAGTGAATTAAGTTAAGTAGTCACCATTTT
CATGAAACTAAGGACTTAACTATTATAATTTAGAATGGAAGATCGCGCGTGGTCGATTCGTGGATGTAC
TTATTGTGATGGCAACCAAATACAAGTAGATCCATACCGTAATCCAAG
>TCONS_00004252 geneID=XLOC_000859 scaffold_1038:21286-24214(-) Exonic overlap (-)
TGGCAAGAGAACAAACATAACTCTTTCCGTCTCCATTGCACGAAGAAAAACCTGCAAATTTAGCTACT
GTGTCCCACGCATTTTTGCAGATATTGTCTTTGGAAGCTCTGAAAAACCTATTTTCGTCTACATGACGC
GTGTAAATAAGATGCTTACTAAATTTACTTACGGAATATTTTGAGATATACTCACTTGTTTACGCGTTTTT
GAGGTGGCCTGAGGGTATGATACAAGAGTAATACATTGTGGATCCGATTTTGTATTGTAATTTTCAAATT
ATAGATACCTACCTAGGTATGTGCTTAGCCAGCGAATCGTGAGAAGATGATGTCGAGATCCGCAGCAGT
CCTCGCGGCCGTTGGCCTCGGCCTGTCCACCTTCAGCGTCCGCCAGATGGTCATCAGCCAGACCAGAA
GGATCTAATATCCTCATTAGCAAACGCTACAATCAATTGTCACCAACACCCGACAGTATGAACATCACC
TGTCAAAGTTAGCTGTCAGTTAAAGCTCTGTTCCCATTGGTAGATGGGTTTTAAGGTTGTTCCACTGTAT
AGCGGAAAGAGCTGAGCTGTTTAATAGTTGAAGCGAGTAAAGTGTTATCTGTTATCCGCTGTTGTTTTC
GGCAGACGCTTTATGTACGCTTGACGCTATCGCTCGCTCGTTTCAGAGGATGCAACGCTAGTATATCTT
CGCTACCAGTGGAGGCATGACTTCTTCAAGTAGTGAAATGTTAGGGAACTGTTGTATATGTTTGTCTGC

GATCGCCTCTCAAATTCGATGTTTCTTTGTGACTTACGTGACGCATCGAATGTAATATGTTAAATTTTGT
GTGCCGACCGCTCTAATGTTGAATTTTTCAGGCGATTTATTTAAATTCTTATCTTTAATGTGTGTTTCTGT
CTGACTTTGAATGTGAAATGTACATGTGTTTTGAATAATGATGGGAAAGCTTTGATTTGTATAATAATGA
TAGTTGAAATCCGAATGTTTTGGTCTATTTTTTTAATTATATAGTGAATTAAGTTAAGTAGTCACCATTTT
CATGAAACTAAGGACTTAACTATTATAATTTAGAATGGAAGATCGCGCGTGGTCGATTCGTGGATGTAC
TTATTGTGATGGCAACCAAATACAAGTAGATCCATACCGTAA
>TCONS_00004304 geneID=XLOC_000868 scaffold_104:45884-52221(+) Unclassified
GGCGGCACCTCGCGCGCCTATCACAACTACGAGTCGTCGAAGCCTCCGTCACTGGGCGAGGGCTCGT
CGCGGCCGCCCGACGACGCGTCGCCGCACTGATGCTCGGGACACACACGTATTGCATATCAATATCATA
ATCTTATCGTGTAGCGCCGAGGAGCGACCAGCGACAAACTCGGTTTTGGAACGCACCCGCCCGCCATC
TTGCCAGCGCTTCTGTGCACAAAATGGCGGGCTGTCAAAAAATGATTGACGAAATTGTAAATGGATTT
AATTTGTGACAAGGAAAGGTGTTTTTTGTCGTTCCATTTTAAACCGCAGCCGCTAGTCGCCTCTTACAC
AGCTCCAACATCTACGTTCAGTGAATCCATGCCAAAACATGTTTAAGGATATTATATCCCCCTTATATTAT
CTAGACGTAAATTGTAGCAATACAATTTGGATATATTTTACATGTACAATTTCTTAGAGTATAACTAACGT
GACCATACGTCCCGCTTTGGGCGGGACAGTCCCGCTTTCAAGCTGTGCGTCCCGCTGTCCCCCGCGAG
GGCAAAAATGTCCCGCTTTCATAAACAGACCAAACAATAATTTAAATTACCTACATTTTGTAACTTCATC
CCATATTTCCTTTCTTTCTTTTAATCACTTGAGACAACTATTTATCTTCTTAACTATCACTTACTCATGTAG
ACTATAGGGAGTAATATGAGTCCGAGTTCCACCGAGACTTCGCGAGTAACTTCGTTCAGTGACGATCAT
TGACTCGCTCTTGCTGTATCGTGTACATATAAATTTATCTCGCTCACCTACCGATTCCTAACAAATTAGA
AATGCGTGTTTATATGTTTTCCAAAAAAGTAATCTGTATTAACTTTTATGAATGTGAAAATAAGATAATAT
GCACTATTGATAAGTACTTTAAATTAAACTACTCAAATTATAAAAAAATCAAAGTATGATGTTTAAGAAT
TTTGTAGCGTCTGGTATGAGAACACCCAAAAATTCCGAAAAACTTTTTCCGAATTTGAAACACCGAAT
ATGTACCTAATTTACAAAATATTGCAATAATGATTTTTTAAATATTGATTTATAAAAACGAATAGATATAAT
AATCACGAAGGTTTGTTATTACGATTGTTAAATAATACGATACGAACGTAAAAAAATACGATACTAAAGT
AAATATACGAAAAATAAAATACAGATTTATTATAATCACAAAAATATCAAATCCGGACCAAAAATACGAT
AATTCGTGATTTTGTCAAAAATAAAATGAACGTTTGAAAAACTTTAGAACCAAAACCTAAAGATAGGT
GTGACGACGATCAAAGCGAGGAGGAGCGTGTTAGGTGCACATAGATATCGAAAAACAAAGCGGAGCG
TTTCAAATATAGAGTAAAAGTATTTTTAAATAGAAAAGTATTTGTAGTAAAGCAAAACAATTGATCGGA
TTTTTACGGTCTTTAATCTTAAAAACCTTTGGACCTAAACCTAAAAAAAATTACAAATTGTTAACTAAG
TTACATTCGGGGATTTGTGTTTTCGGAATATTAAAACTTCGGGATTCCTAATTCAAACAATTCGATTTAAT
AAAAAATTGTACATTTAAAACATTGAAATTATAACATTTGAATAGGCTGGCTGAGCTGAAATCAAGGGG
ATATGTACAAAGGTTCCACTCGAGAGACCCACGTACAGGAACTTCACCCCCTATGAGAATAGAATAGA
ACATTCGAAAAATTTACTTTGGTCATTTTAATACATCTGTTGTTTGAAATTTCGTTTATCAAATTTTCGTA
ATTTTCTTTATTCGGAAACTTAAAATTTACTAGAACTATGAAATTCATCATTTAAAATATCGTTATTTTCAT
ATTCGTAAAAAAATAATTAAATAATAATAAAATAAAATAAAATACCAACTGTACAAGATTATTAGATAAA
TATAAAAAAATGGTCATTTACCATTCATGCATCTCTTTCACTTTTCACTCTTTCATCGTAGGGATTGAATT
TCAATTGATGCTCTGAGCGAACTGAGCGAAAAGATAAGAAGATAAGAAATAAATATAATGTATCTTTGA
GGAATATAAAATTCCGCAGGTGTCCCGCTTTGACAAAAAAAAATAAATGGTCACGTTAAGTATAACTGA
AATAGATTTGTCCTCGATCAATGCAAGTAATGCAATCGTAAATTAGGTCAAATATTCCGATCTCTAATTAT
ATTACATACATATGTATACCTAACTTTAGATATGAAGAGCCTGTAAACAGTATGGCATGGGTGCACTGGA
CCGCTGTGGGCGCGGCCGGCTGAGGTCTATACTCGAACAAACACTCACTAGTTATAAATTTTCTTAAAA
ACAGTAACGATCAAAAACTCCTTTCGAATGTACAGAAAGATTAATATTTATTTCTATCGCGCGATATTGA
CGAAGGACAGTGGTGACACTCCCTTGTAAAAAAATTATATTTATTTAGGTGATATTTTTCTATTGGCTAC
TGATAAGATTTTATCTGTCGTTATGCGCCGCCGTGCGTCGTTGTAAGTAAAGTAGGTCCGCGCGGGGCC

GATATTCTCTAGCAATAATTTATCAGGTTCCTAAGACAACTCTTGTAGTTAGGTTCTATTTTGCACGAATT
TTGTAAAACACTGCCGCTTTGATTCTAAAATTTATTTTATAAAAATATTAGTCGACCCATTGCTTCCTTTA
TTTTATAAAAAAAAGTCATTTTGTTTGTTAATCAAATTTTTAATAATATATGTATATGTGAGGCATTTACGT
GCTGTATGAGGGACCGTTGCAATTGCAAAGTAAATATATTGAAATGGCATAATTTTTAGAACTAAAATA
GGAATATTATGTAGTCATTTCTTTTTATTACTATAAGTGTAAATGGGAAAGAATTTATTTTGGAGAGTATC
AGGAATGGATATTAAAGAGTCTTTGGAGTGCTGTGATCAAGATGGATATAATGCAGTCATATTTTTAAAT
AATACGTAAGAATTATTAAGTAATGATAAATAAATAACCTTGTGTACAGTAAGGTAGCGGTCTCCGCCGT
CAATTGCGCCGCGGGTCACGACGCCATGCTCCGTGTGTTGGTCATATCAAAAAAGCAATAACGTTAATA
TAAAAATCGAATTATTTTTAAAAAAGTACTATTTTAACGAGTTGTTATCACTAGTAAATTAACTAAACTA
ATAAAAAAACTAGAGAAATCCACCCCGGTGGTAGTTGTTTAAAAACAATAATTAAATAAATAAAATATAT
CCTCGATCCCGTATATGACTCACGAGAAACTGTAATGATCATATCATTTTGCAATATTCCTGTTTCAAAA
GATTTTATATAAAAATTGTATATAAGCGTACATATACGTAAGACGTATGTGCGGTCCGCACTAGGAGGCA
CGCCAGTAGCGGTTATTTACACTACATCGAATTGAAATTGGTGTACCTACGGATAAGTTACAGTTACCA
GAATTAGGATACTGTATGAAGTAGTCAAGCACTACAAACTAAATTTTTACGATGCCAACTAACTTAATG
CTCAATTTTGCCAAATTTTACTTGCCGAAAATATCTTGTCTAATGATGAATGATACCCGCGAGTGTCGAT
CTCTCCTCGCCGTAGCCGTGAGGAGGGCCTCTTAAATTTGCTCAATCATGTTTCTTTACTAGCCAGCTC
TGAACATTTCCAAGCTATTTTTGGCCCAAATAAAATGTATATCATTAGAAAACAATTGAAAGGTAATTTA
AAACGTAAATTTAGAGGAATGGGAGACATTAGAATTATATCGAAAATATTGCAAGTAGAATAAAAAAA
ACTTGTGTGAAGAATGCTCGAGATGCAATATGTTAATTTAGTGCCAAATGATAATTTAACTCGTAGCAAT
ATATCGTACCCTAATGTCCTCGTGTAACTTCTCAACTAGTGTTAATGACCTGTAAGGTATCAAACATTAA
GTTTCTTTTTACAAGACTATATCGTTTTTACAGGGAAGTGTTTAATGTGTATTGTTGGACGGTTGAGGGG
TGTTGAGATATAATACTCAGGTTACAGAGTGTAAAAGTAAAGGTTTCTAAAATAGACTTTTAAGTAAAA
AGTCTCGATTTTCTCAGTGATACGGGACTCTAAATTGTTTGTTTCTGCCGGCGGGCGGCGACGCTCGCC
ACTAAAGGCGGTTTTTCTAAACCTAAATCTAAATAATACCTATTTAAATTGGTTAACTAATTTGTCATAGT
CATAGATTTGATAGTTATAAATTACTGTTATGATTTCATTTTTACACTCTGTACAAATGCAAAGAAATGAT
GAAGCAAAGGAGCAAGAGAATTTATTTACTCAAATAGGTTTTTTAATAGTTTATAATTAATATTTTACTG
AATTTTAATATTTACATTATTTTGCATTTTATTATACTTAGAGGCAGCTGTGTGAACAAGGGTGAATGATG
AAACAATTTCAATTGATCTGGTTACAGAATTTTAATGAAAACGATACATCGAACATGACATATTGGGCA
CAAAATAATAGTTATCTAATGTTGTTGCTTTGCTATTACCAGTTTGATTATCAGATGTTAACATAACCCAG
CGCACTACACTCCGCTTGGAGTGGTGCCTTCATTCTGGCAGCTTGTGAGACGGTGGTTACTGGGCCAG
CTCGGCCAATCACCGTCGAATATACTCAGTACTATAACCCTGTATGTTAAATGACCCCGCAGGTCTGCC
CGCCACCCCTAGCGGGCGGGCGGCCGCGGGCCTCCTACAATATTCGCCAACCCTCAAGTTTCCGAGGC
GACAAAGCGCCGCATGTCTATATGTGAGCATAAAAACTGTGTAAAACAGTGTTGTGCCAAATTGTGTG
CTAGTCTTTATAAACAGAAATGGTTTGTTATGATTGTTTCAAATTTTATTAAGTCCAGAATGCGTTTGTTT
TTGTGCTGTGAAAGTTTTTTTTATATTTCGATCTAATCAATCAACAATGTTGATCTCTTCCGTATTTTAAG
GCACTGTGTGGTCATGTTGTTACTGATATTCTTACTCTTGATACTGTTACACTGTAGTTCTCCCCAACGT
GTGGTGTGTTTCTGCCAGAACTAACTAATTTATTCATGTCATACTAATGTTGTTCTTACTCATGCATTTTT
CTTGAATTTCCTTTAATTTCAGATCAAACTATATGACCATATTGTGGCTCCTATTAGAAAATGACTATTTA
CTTTGATAATTTGTTTACTTACAGGCATTATAAGTAGCAAAAAACATTAACTATGCACATAATCCATGTA
GGGGTTTAAGAAATGGGGAATGTTTGCAATGTACTTTTCAAATAAATGTTATATAATTCTATAAAATTGT
TGTATTGAAAATGCTAATTTTGCCTATTTGTCTCTTTCAAAGCCATTTGATATGTTATTTACATTTATAGGT
AAAACTAAATGCACTCATTTTAATGCTTTGCCGCTTGTGCAATAATAATACTAAATTTCGTTTTGTTGCAT
CATGAAATGAAATTTATAATCATATTATTTACTACTTTTAAAAGACAAGGCAGTAAATCCTAGGTTTTGTT
GTGCATTTATTTTTTATTAAAAATATGAAAAGTCTACCCACAAACTCATTGTGGACTGTTATGTGTATGC

GACATGAACACAGGAAGTCATCGTCGGTTTATTTTGTATAAATGATGTGAGTCAATTCTAGTAATATTCT
CCTTTACTTTAGAATTAATTTATAAACAAAATTATTATAAAATTGCTCAACCAGATGCAATATATGAGTTA
TGATCAATTTGTATGTTTTTATATGTTAAATCATTTACTACATATTTCATTTATATTATATTACTTTGCAAGT
AAAGGCAGCGCATGTTAGTCTATGTTAATTATTAAGAGAGGCCAAATGTTGACTTGCTAATGTGAGACT
GTTTTGCCAAGAATCAGAAAGTATATTATTACCTACATTGTTTTAAAATAAATGCTTTTATATAA
>TCONS_00004345 geneID=XLOC_000886 scaffold_104:133640-134501(-) Splice junction overlap
GGCGTCACCAGTAAAAACAAAAAAAAAACATGTTTCCATTGTACAACACCGTCCACACCGCACGAGT
CAGTGAGTCGACGTTCGTTTCAATGCGTTTACGCAAAATAAATGGTTACGTGGCGGAAAGTTTTACTCT
TTTCCGTGTTCATTCGTGAAATAATATGTTATGATGACTCGGTATTGTACCTCACTGAGAGCACTCTGTC
GCACTCCTTCGAGCAGTTCATGAACAATCTGGACGACTCCGTGTACCGACAGAAGAAGCAGCGGCGG
CGCCTGGACAACC
>TCONS_00004359 geneID=XLOC_000892 scaffold_104:234029-236046(-) Unclassified
AAAATATAAAAATGAATTTATTCTTTGCAAATAAAGTTGATTGAACTTTGATGACAGGAACTGTCAAGC
CTAAAAAAAATTACAAGCCTTCCTTTTGCAGTGAAATGCATTCAAGTTTTAAATCTATTTTCCTTTTTTAT
ATTGAAACATCATAGTTTTGCAGTTTTAATTCAGTTTACTAAATGCACAAGTTTATAAGTAACCTCATAT
GTCTAAAAACATCGTCCATGTGTAAAGTTTCATGAAGGTTTACGCCCTTGAAAATCAGTGTTACATACA
AAGAAGGCTAATGTCAGCATAGCAACAATACTTCGGATATACCTCTATTCCTATATTATGGAATAATTATT
GTACTTATTCGGTGCCTTTATGAGATGAGCGAGTATAACGTGCATGGTGCTGTTCACAAAGAAAGAATA
TTAATTTCATAGTTTATCTTATGGTAAGTGACCGGTGCTCGTTAGAGTAAAATGATCACTAGAAGATGGT
TCCACCCATCGCTGAACGGGGTGGAGGCAGAAAAGCTGCTTTTGGAGTGCGGACG
>TCONS_00004467 geneID=XLOC_000895 scaffold_104:419720-482957(-) Unclassified
GCCACATCTCCTGAGGATTCCGCGCCGGGTCAAACCGAGTGCACCCACCCTGACGTTGTGCTGTGAGT
GAGAACGCAGTTCCTGTGATTTGTTTTGAGTGTGACGTCACGTTAGTGGATGTTGTGAACGTAACGAA
ATTGTGTTTGAGTGCACTATGCGGTTGTCCGCACCGTTATGAATGGTCTTGATCGCTGCTTCTTCGATCA
ACGTGAGGGACAACAGCATGGGGATGATTGCAGTTATAGTTATATAAGCCTGGGCCGCCTGCACCCGT
ACGGCTCGGGCAGCGGCAGCGGGTCGAGCGGGCGCGCGCGCAGCGGCCGGTCCTCC
>TCONS_00004868 geneID=XLOC_000970 scaffold_1046:1053-2082(-) Unclassified
GTGCCACTTGCTGCTGGAGATCTACGGCGACAGCATCATCCACCTGATCGAGGATTTCGGCACGAAGG
GCGTCTGCCAGAAGATCGGGCTGTGCGCCGCGCGCGCCGGCACCTATGTCGACCTGATGAGGGGCGG
CCGTTGATTCTACAATCAACAACATCCTAAAAATAAGATTGTAGCTGTAATTGGAATCTCCTTCTTTACT
TAAGCCATGATTTTTACCAAATCTTAGGTAGTTTTAAGCTTTTTTGTGTTAACAATCAAATATTGTGACTA
TTCCTTATCGCGACGGAGGAAGGGCGAGATCGGAGATTAATTTTTGCTTTTATGTCATTCCACAATGGA
GATTTTTAATTATTTTTAGGAAAGTGTAAAGTGTACTTATAGATGTAAAGAATAGTCATAGCATTGATGC
GTCCTTAGTTTCTGGCTATCGTAGAAGTACGTAAAATGTTTTATGTAATCTCTTAAATATGTATTCTATATC
CAGTAATTGTGTCGCTATTTCTTTCCGCAACGAAGCAATATCTTCTAGTACGTCAGCCTGTGATAATGTT
ATCATTCATTTTGTAAACAGTTTCTATACTTAATAAATAAATTGGGAAA
>TCONS_00005040 geneID=XLOC_000998 scaffold_105:102916-109578(+) Unclassified
ATGCAATGTTCAATCTGAATATGAATGCAAGGAAATGCCTTGTGTCTTGATTTTATGAGCATGCGCATGT
AATGACTGAGCATTCGCTTGCAGCATGCAAAAGCTTTACTTGTTGCAGCTAAGGTGAGTATAGTTAGCA
GTACCTTTACCTTTCAATATCGCAGAAACAAGCTTACGGTCACACGGTTAGGCGGGCCTAATGAGAAA
GTTTATCGAGCGTTTATTGCGTAGTTAGTTCAGCGGGAAGCTCTGCCGCGAGCGAGCGCTCCGCAGGT
GTGCCCCTCGCCCCGCGCCCGCCCCGCGCCCGCCCGCGCCGCACTACCGCACTCCGCAGCTTTCAGTC
GACAGCCAGCCTTACAAGCGAGCGCACGCTCCGCTCACTCTCTCGGAAACGCAACGCGCAAACTGTA
AACACGACGCATTATCTTCCACGCTTCCGTTCCCGACTTAACCTGAATAGAGTGTGCATATCCTGTGAC

TAGTGTCGTGTTGTGCCAAAACAGTTGGACTTTTGTGTGACGGATGATCGAGTAACTTCCTATACATAC
CGCTGAATGCCTGTGATAACCCCTGTGATGTGCGAGCAAACCTGTGGATATTTTTCTATGTGATTTACCA
GTGCCCAAGTACAAACCGAGTACAAAAAAAACGGACAGTTTAGTGTGTAAGAGTAAAGATGAAGGA
GGA
>TCONS_00005054 geneID=XLOC_000998 scaffold_105:115801-135400(+) Intergenic
TGTTTGGCCACATGGCTACTACCAGCCGGCGGGGCCGCTGATCAAGGAGGCCGGCGCGGACGCGGAG
CGCGCGCTCGCACGGTACCACCCGCCGCACTACCACCCACCGCATCCACCGGTGCTTCCGCAGTATGG
CCCGTACTACCCGTCGGAGCTGTGCTACGGGCGCTACTACCGGCCGCCCGCGCCCGCCTACCACGTGC
CGGCGCCGCAGCCTGACGCCCCGGTTAGTATATAATATACTATAGTAACTGCCGCAGTATGGCCCGTACT
ACCCGTCGGAGCTGTGCTACGGGCGCTACTACCGGCCGCCCGCGCCCGCCTACCAC
>TCONS_00005093 geneID=XLOC_000998 scaffold_105:120045-135406(+) Intergenic
ACATTTCGCACACATCTACTGTTTACGCTAATTTTTGCGAAGGTCAGCGGTAAGGCCTTACGAAGACCT
CGCAATTTTTATCATCATCATTGTAAGCGTATATAACATTACAGATATCATGATTTTGCGCATATATTTAAT
GTAGTTTGAAAGCTTATAGCTTAGTTCTGTTGTTCCGCGATGCTAGTGCGCTTCGGTGAAATAAATCTCT
GATAGTGATGTGGAAGAAAGTGTTAGCCAGCTACTACCAGCCGGCGGGGCCGCTGATCAAGGAGGCC
GGCGCGGACGCGGAGCGCGCGCTCGCACGGTACCACCCGCCGCACTACCACCCACCGCATCCACCGG
TGCTTCCGCAGTATGGCCCGTACTACCCGTCGGAGCTGTGCTACGGGCGCTACTACCGGCCGCCCGCG
CCCGCCTACCACGTGCCGGCGCCGCAGCCTGACGCCCCGGTTAGTATATAATATACTATAGTAACTGCC
GCAGTATGGCCCGTACTACCCGTCGGAGCTGTGCTACGGGCGCTACTACCGGCCGCCCGCGCCCGCCT
ACCACGTGCCG
>TCONS_00005024 geneID=XLOC_001019 scaffold_105:333906-354263(-) Splice junction overlap
CGGCTGCGCCTGCGCACGCGACGGACGCGTCGTGTCCTCCTCCCGCTCTTACCGAACCTATTGTTTTC
GCCTCCAACTTACACGGCTTACACCCTTCGGACTTAAAAACATTCCGGCTCTTAGACCGGGAGTGCGA
TGGACGCGGCGGTCGCTCGGTGAAGTGCAGTGGACAGTGAGTGTGTGATGATGAAGTGAAACAGTTA
GTGGTGGTGATCAAGATGCCGTCGGACAGCGGCGCGGCCTGGGCGCCGCCGGCCTCCAATGGGGAGA
GGAGGCTGAGCAACTCTTCGGATGGGAGCGCCGGGAGTGGATACTCTAAAGCTCTATCATCAGACAGC
GCGGAC
>TCONS_00005152 geneID=XLOC_001019 scaffold_105:333908-393154(-) Splice junction overlap
CGTCGAGGCCGAAGCACGTGCGTCGTCGCCGCGCCACTCGTTGTGAACGTTCATCTTACTCGTCTTCA
ACTTACTCCCTAATTAAACCCTGTGACTAAAAACCAACTACCTGGACCCAAACAGTGCTGGACCTAAT
CACGGTGCCAAATACAGTGATAATATCTCCTTCTAACATCTCTCGGAGCCGGCGGAACGCTGCGTTTTT
CGCGGTAATTTGCCGACGCATTTGTTCGAAAGGGCTCTTAGACCGGGAGTGCGATGGACGCGGCGGTC
GCTCGGTGAAGTGCAGTGGACAGTGAGTGTGTGATGATGAAGTGAAACAGTTAGTGGTGGTGATCAA
GATGCCGTCGGACAGCGGCGCGGCCTGGGCGCCGCCGGCCTCCAATGGGGAGAGGAGGCTGAGCAA
CTCTTCGGATGGGAGCGCCGGGAGTGGATACTCTAAAGCTCTATCATCAGACAGCGCGG
>TCONS_00005153 geneID=XLOC_001019 scaffold_105:334331-354263(-) Splice junction overlap
CGGCTGCGCCTGCGCACGCGACGGACGCGTCGTGTCCTCCTCCCGCTCTTACCGAACCTATTGTTTTC
GCCTCCAACTTACACGGCTTACACCCTTCGGACTTAAAAACATTCCGGCTCTTAGACCGGGAGTGCGA
TGGACGCGGCGGTCGCTCGGTGAAGTGCAGTGGACAGTGAGTGTGTGATGATGAAGTGAAACAGTTA
GTGGTGGTGATCAAGATGCCGTCGGACAGCGGCGCGGCCTGGGCGCCGCCGGCCTCCAATGGGGAGA
GGAGGCTGAGCAACTCTTCGGATGGGAGCGCCGGGAGTGGATACTCTAAAGCTCTATCATCAGACAGC
GCGGAC
>TCONS_00005154 geneID=XLOC_001019 scaffold_105:334333-393154(-) Splice junction overlap
CGTCGAGGCCGAAGCACGTGCGTCGTCGCCGCGCCACTCGTTGTGAACGTTCATCTTACTCGTCTTCA

ACTTACTCCCTAATTAAACCCTGTGACTAAAAACCAACTACCTGGACCCAAACAGTGCTGGACCTAAT
CACGGTGCCAAATACAGTGATAATATCTCCTTCTAACATCTCTCGGAGCCGGCGGAACGCTGCGTTTTT
CGCGGTAATTTGCCGACGCATTTGTTCGAAAGGGCTCTTAGACCGGGAGTGCGATGGACGCGGCGGTC
GCTCGGTGAAGTGCAGTGGACAGTGAGTGTGTGATGATGAAGTGAAACAGTTAGTGGTGGTGATCAA
GATGCCGTCGGACAGCGGCGCGGCCTGGGCGCCGCCGGCCTCCAATGGGGAGAGGAGGCTGAGCAA
CTCTTCGGATGGGAGCGCCGGGAGTGGATACTCTAAAGCTCTATCATCAGACAGCGCGG
>TCONS_00005166 geneID=XLOC_001006 scaffold_105:439207-488471(+) Intergenic
CGCGACGGTCGCAACCCTTTATGCGGAAAATTCATTATAATAATTAGTTTCTTAACTGAAAATAAAAGT
GACCGATTTTCTCCAGCCGGTTGACACGCTGCACTCAAGTTATTAGCCATGGCGGTGTGTCGCATGGC
GTACAGCGTGGCGGCGCTGGCGGCGCTGTGCTGCGCGCTGTCGCACACGGTGCGAGCGCTCGACCTC
AAC
>TCONS_00005887 geneID=XLOC_001086 scaffold_106:34887-41393(+) Exonic overlap (-)
GGACGGCAAGCGAAGTACGCCCTAAAAACAACAAAGCATCGGCATTCACCCATGCCCTGCCTAAATG
CACCCGGATACTGGCAATGTGTTTACATACCTACTCTAGGATAGTGATAGCCACTCGGTTGGAAGAACC
TAGGTCTTGCGAGTGATAGGTTAGCTTTCTGAATGTTTGTGTATATACGCGTGAGTATGGCGTAGGCTTT
TAGATTCTTAGATGTTATGTTTTTATACGTATACAGCGTATCCAGAAACCAGTGCCAAAATCTTCAGCAA
ACAACGAGATAACCAACCATTGTGTTCGTTCTTAATTTAAAATTTAAAACCAGCAAATAAAAGTGACA
GTCGCAACTCAAGATGGTCGCCGTTTCAAACTTAAGCAAATTGTGAAAATAACAACTGAGACTAAATT
GTACTGAAAATCGCTTATATGGCTTTAAAATTATAATAAACTAATATATCGAAAAATATGAATGACTGAAA
TATCACGATAAAATTTCATAACATAAAAAGTTACAGTTATTACAAGCCATCGATTTAAGTGAAGTGATTT
GTTGTTCTATCTATCTCCAAGTAAATAATTATGTGTACTGAACTAGCACAAGAGTTTGTTTGTTAACTTA
AATAATATAAGTAGACGAGTGCCTAAAAGAGACTCTAGAGACCTACAAGTATAACTGAAGAATTTAAA
ACGTACCGAGTAACTGAAAAACCATAATGTCGCGCGACAACCAGTGATAAAAACCCCGTAGTGACAG
TTCAAACTTTTAAAAACTGACCGTTACTCTCCATCCAAAATGGCGGCAACGGCGCCAATCTAAGTGCC
AGTGAACTTTTGACGTGTCGAGTGACAGCTGTCAGTTTGAAATCCAAGATGGCGTCGTCGGCGGCGG
TGGCGCGTCGCGGCGAGTGAGGGCGGCG
>TCONS_00005418 geneID=XLOC_001093 scaffold_106:197640-199196(+) Unclassified
AAAAAATATTCCTGTAATCATGCAAGTCACTCGACTTTCACTTATAAAAGCATATTTTTTATATAAGTATA
CTTATTTTTAACTGTAATTGCTCTTTATAATTAATCTAAGCTTAACTAATAAAATTTCTTCTTCAGCAAAAT
TTATAAGCAGAGTAATCTAGGTGTAGAATGGAATTGCGCCTTGTGAAAGTCATGCTTCTAGACGTCACG
TGATGCGAATCCTAATTAATTCGTTTTTTATCTATCTTGATGATGGGTTGTGTGTGCTATCAATGATTAATA
AAAGAGTCATAGCTAAAACTACTGTATCTTCTCTATATATACTTATTGTAGACATTTCAATACAAAAAAA
CACAGCAATACGATAAATACTCAAAAGTACAACGTAAAGATAGCTTTGTTGTTAGTATTAAAGAATATA
ATCTAAGATCCCACTGCATTATTAAAACTACGAACTAATTCCACGACTAAGCTGATTCCAATAGTGCACT
TATAAGAATTTACCATCGCTTTATTTAGCGTAAGTCAATTTTGTTGTAAGGTTATATGAGCTGTAAAAAAT
ATACATAACATGTTTTATTTTTACATGATTTACGTGTCAAAGTTTTGGTGTAATTTAATTTAGTGATAATTT
TGGTTGTAAACGGTATGCCAAAATGTTGGTTGACATACTGTGAAGCCATAATTTCCCCTCGTCAATTATT
TTGGCATATCGTGGCATATTGTGCTATAATAGTTATCTAATAAGTGAGAACGAATCCCAAGAGTATTGTG
GAGTCACATAATGTAAAGTTTATTACTGTTATTGCACTATTTTAATGTAGTTTTTATGGACCAAATTTACT
CTAGTTATATTTAGTGCAAAGTTTGCGAGATGTTCAAGCACTAGTGCAATGGTTAGACCTTTAGGTTACT
CAGAAGGCCTAAGATGATCACGTGATTTCACTGTTACATTTTACCTTACGTTATTAATACGATGCGTAGA
TTTATATAGACTTGCCATAATGACGACAAATTTTAGAAATAGATACCCAGGTAAATCAGTTTAAAGGCTG
CCTTATTTATTATTCAGAATATCAAGTTGTTGCGGTACCGTTGAGATTGAGGAGGTGGTCAGTATATTAA
ATGAACTAGATAACTTAAAACACGGCACACGCTAACTCAGTCGGCAGCGAACAATTAGCTTACTAAGA

ACGCAGCATAAAATATTGCCTATTTGGAGATTGTTGAATATTTTATCTATAGTTCGACTATAATACGAATA
GAATTCTTTTGAACCATAAACATAAGGTAGCGTCTAATAAAGGAGCGTTGTTTTGGACAGTTGAGATGC
TTTAGCGATGATATCGAAATTTCATATTTTCTTTATACTTACAAGTATCAAATATACAATTCGGAAAACGT
ACCTAGTTACAATTTATGGACTCACATCACATTTAGTATATGTACTTAAGTATTGAAATAATTAACATTTT
CAACTGAACGTGACAGCC
>TCONS_00005855 geneID=XLOC_001099 scaffold_106:512852-513643(+) Intergenic
TGACGATTTATTAAACAAAATAAGGTGTTTATGTTTTTTTTAAGTCTGTAACAACATACACAATGTAAAG
TTTTTAGGTTGCCTATACCCACCAGAAATTTTCTAGAACTATTCTGCCATGATGTGAAAACTATTCGTTT
CTACCAGTGCTATGCCATGTGGACCAATGAATTTGATTGGTGGATATTAAACGCATCCGTAGCATCATTT
CATAGCACAGCTCTGGAGCATAGGCACCCTTAAGCCTGTTGGTTAAAACTATAGAAAATTACCAGTGAA
TAACATTGTAAAGTTATAAAATACTCAGATACAGATCAAACATAGTATTAAATACACAGTAGGTAGTAAC
AACAGCATAACGTATTTTTGTCAGGCGCGAGAGAGTCGCCACCTAGTGACGCTGGCTCTGTACTAGCT
GCGCTAAAAATAATGACGTTTACTGTTCACTGTTCAACGGTGAGAAAAACTCGCACTTATTACTCGATT
CATTCATATACCTATCTGGCTACATAAAAAACGCATACTTATTTAGAGCTCAAATTAAGTTGGAAAAGTA
GCAAGTGATTGATTAGACCCTTAAAAATGTACCTATAAGTTCCCACAGCATTGTATAAACTCCTTTTATT
TAAATTACAATAAATATTTCATTTTACAAGATG
>TCONS_00005752 geneID=XLOC_001122 scaffold_106:763860-886903(-) Splice junction overlap
CAGCGGGCGGTGCGGACGCGTCCCTACGGCTCTCTCCTGAAACTTTTACGAATTTTACGAAACCGTCT
CGTCGTCACGGTTCAAGTGTCGGCGCCTGTGATGTTGAGTGTGAGTGACGCGGTGTGGCGCGACTGA
AATTACGCTCGTGTGCTTGTGACATTTCGTGAGTTTTGTGATTAACGGAAGTTGACAAGATTGCCGCCG
CCACTCACCCGCAGTGTAACCGTCCAAGTGTCAGTGCAGTGAAGTGCAGTGCAGTGCAGTGATGTAA
GATGAAGCTGTGGAAGAGCCTGAAGGTGTCGATAGGGAGCCGCGGCGACGCGCGCTCCTGCCAGGAC
TTGCCGCAGTGGGGGGCGAAGGGCTTCGAGCTAGATGTGTTCAAATCGCACACGTCGCTTTCGACGG
ACTCGGGGCTGTGCACAGAGCGCGGGTTGCGGCGCACCCGGGTCGACGTGCGCCGCCCGCCCGGGC
AGCCCTTCGGACTCAAGCTGGCAGGTAAGCTAAAGTTTTGATGTCATTTTAACTGTAAGTATTTTG
>TCONS_00005690 geneID=XLOC_001125 scaffold_106:962357-981698(-) Exonic overlap (+)
GTCAATGTCAATTTGAGAGTTTATTTCATAGATTCCCAAAATCAAAATTGATTTAATTTCAAAAGTGTTT
CAAAATTATTTGTGTTTAGTTTGGAATTTAATTGCTTATTCTATTATCCGAGACCAATTTTAATATGTTTCC
CGGCAATTTTTCTTCAGTTAATATGCACACAGAAATACGCACTGTTTAAGTAAATAAATAAAATCAAGC
ATAATCGTGCCCATCCCGCTTCCATAATAAGGCTTCGGAGCGGGTCACAAAGGACTAGTATATCTGAGC
TGATCACC
>TCONS_00005795 geneID=XLOC_001129 scaffold_106:1033344-1034801(-) Exonic overlap (+)
TCTAGAACATAAAAAATCACTTAGGTATTTTCTTCTCGCATAAATTAGCGCCAAAAAAGTTTCAATTGG
TTTTTTTCTATGTACTGCCAGTCTAGGTATAACTTTAAAAGGACAGGTATGCTACCCCTAACTTCTCTAC
ACGTGACAGTGAATAACCGTGAAATTATCTCTCTTAAAGAGCTAGCTAAGAAAAAATCACCCTCTCTCT
ATTCTCTCATCTCCTAAATAGTAGTAAAATTATCTCTCTCCATAATCTTTATAGAACAACGCAGACTCAG
GTACCTACCTATCTCTCTTCACACCTCAAAGGGTTACGTGTGATCGTGTAAGTGATGATGTCCTCCTTG
GCTGGTGAAAAAACCAAATAGGCCATGGATGAAAGTACCTTAAACCTTTAGATAGCTTATTTTTCAGCA
GATCAGCTTGTCATAGTTTAGCATCCCTGACGCAGTTCAGGCGGGGTCAGTCAACCCTCGCGGCCGCT
TCCCGGTTCCACTAGGCCCCCGCAGTCTCAGGCCACTCCTCACACAGACACTACACTACTCCTGGGAA
AGATGCAGTGCTACCTCTGCATGACCTACGTTGTGTTTTTCTGCAACGCACTTCCAACTAAAGACAAG
GAACCGACCAATCAGCCCGAGCGGAGGTCCCTATTTCGAGATGTGATCGAGTCCCTCCGCATCCGCGC
GCAGCTCCCAGACGACTTGAACCA
>TCONS_00005501 geneID=XLOC_001132 scaffold_106:1063677-1064572(-) Unclassified

GACCCCCGAGGCCCTTGAGGCCCCCGCGGCCCCCGCGCAGCGCCCGGCTGGCAGGCGCGGCTGACG
CCCCTCAGCCAGGCTTGAGGGATGGCCAGCGGAACCGCCACACTATGTTTATTTATCACCTTCGTGTAA
TTGACCAATCCGGCAGTTTTGTGGACGCGCGGCGGCGTCACGAGCCGCCAGCTCAAGACCTCCGGGA
ATCATAACCACCATATCGACAGATTTTATGTATATTAGTTTGTAGTTTATCTTTAAATGTAAGTAGATATGT
AAAACGTTCACCAATTCGCATACAACATAAATCAAACACTAGGTTAAGTATATAACTCTCACTTTTCCG
TTTTATTTTTCCACGAAAAAGCTTTTCCATGTTATTTTCATTGTCTATGCACGTTACAAGCGTATTAATTA
AGGTTTACTGCTACGAAAATGTTGTAAATAATATGTACTTTTCCATTCTTTCAACATTTTTCATTAGATTT
AAGTAAGTTATGCTCACAATTTGTTGTAACAGTCAGCAATAAAGTGCGTATCTTTCTCATCGAGC
>TCONS_00005912 geneID=XLOC_001133 scaffold_106:1064821-1068534(-) Intergenic
ACTACATCGCCTTTACAATAAGGTTCCGATTCTACTTATGTTTTTGATAAGTACGAGTAGCTTCATTATTA
CACCTAACAAAACAAATTAAAATTAAAATCTCACATTCGTCGTTTTGGCTGTGGGCGTGATTGGTTCAT
GGTATAAAAGCTAGGATGCGCGGGCGCTCCTCGCACTTGGGCGATGTCTCGGGACGTGAGTATACTCT
CTCTTTAACTTCCATTTTGAATTGCCTTCCGCCATCTTGTTCAGACCGCGTGGTGCCGTGCATTTTTAAT
TGATTCCAGTGATATCAAAATTCATAACTTTTCCCCCCGTAAAGAATGAAAAATTGTAATAAAGCTTTTA
TTCGTGTGGTCTGTTACGTAATTTCAGTGAGCTGAGGTATAGTGTAAGAAACGTGGTCTAGCTTTGAGG
TTACTGAAGTGTGGTTTGTTTGTCCGCAGTGAGCCAGAGTTCCTTGACTTTCGCGGCCTGTAGCGGTAT
GGTAAGTTATCTTTTTATTTTAATACATTTTAAGCTATCTGCCGTATCGTTTAAACATATACCTACACGAG
CTTTTACGTCACTGAAATTATTGTTATGTCGGCCATCAAAGCTATTGTGCCAAATATATAGTATTGCTCTC
GTTCTATTTACGCTCTGCTAAGCTGTGAAATTCTATTTGCAACCTGCGATATACCTACAATTTATGTAGCA
TAAGGCAACTTGTTTATCACTCATCCTCATACTACATGCTATAATAACCTCAATACTTTGCAAAGGTTAC
CTCATCACCACCAACAACGTGTCTTGATTAACCTTCCATGCCATCTTCCGCTGCTGTTATTTGCCACCAC
CCCGCT
>TCONS_00005913 geneID=XLOC_001134 scaffold_106:1066028-1068385(-) Intergenic
GTTTAATTTGGGTGTGACGTATGCATATGAAATCTCCACGTGGTGACATTACCTAGCGTATTTAATAATA
CACTCTCGAGATATATAAAAGTTCCACTTGGCATTGCCGTTCATTGTGAGTTTATTTATTAACTTGGTCTA
CTTATAGCAACCTTACCTATATCTATCTACAATGATACCAACATTCAGCAAAATCTAAACCACCTAGACA
TTCGTTTAACTTTCATTTACTGTAGTCTCGCAGACCTGATGGCTTTTTAAAAATGTTTTCTGTAAAATATA
ATTTAACGTCCGTCAACATTTCGCTAACATCCATTAAAGCAGGTACTTGTAAAATTCTGGAAACATCTTT
GAGGCGAACACTCGCGCTTCATGCAAATGAACGTAACTTATAAATATGAAAATTAGAAATGCGAATCA
GAAAGATAAATTTAAGAACAAGAAATAGTCTGTTGACTTCGTTTTCCAGGCTCTGTGTAGGTAAACTG
AATTAATTTCCGTACATTACGTTGAATATTGCTTATTTTTCGTGACTGTAATCTTTCATGGTATGAACAAC
AGCTACCTACAGATTAGAATTTAGATTTAGTTACATATAGTATACATAATACACTCACGGGCAATGAAAA
GGTTCCATTGACAAAAGAACCAAATTACTCCTAAACGGAAAAAGCTAGCTTAATGACGCCTTCTGCAA
C
>TCONS_00005755 geneID=XLOC_001135 scaffold_106:1168657-1169250(-) Unclassified
CTATCACTTAACAAAAATACAACGCAGTGCCAAACGATAGATAGATCTCGTGCCAAGTGATCGTAGAC
AATCTTAGTTATTTGCGCATCTTTAAATATTCTCTGCTAGATTGGCTTCGTTGAATAATTTAACAACCTAA
TTTTTGTCCATTAACATATACTGCTTTATCAATTATGTACGAGAACACCGAAACCCCAATATCAGTATTAA
CACACGCTTCATAATGTCAAGAAAACTTGTCTTACAAGTTTGGATTTCTTAGAGCATGGGGCCGACTTC
GAGGCTGCTATTTCGGCCACCGGTAAGTTTCACACTTCTATTTTATTGTTTCTTTAGTATATGTATACATA
CCACACATACGTATTATTAAAGTAATGTACCTATCTATTTTATTGAATAACTGCCAATTTTTGCACTATTCA
TTACGCATAAACATGAACAGTAGCAAGGGCGTACCCAGGATTTCAGCTAGGGGGGGGCAGCTCTTGA
GATGATGGTCGGTCGGG
>TCONS_00005936 geneID=XLOC_001191 scaffold_1060:11782-14543(+) Unclassified

CTCAAGAGTTGTGCGTTGATTTTGAAAGCAATTTCGTGTCTGTTATTAAATTTTATTTTACTAAAAACTC
AAGATGCCTTGTAAAGGTTGTGGTGACGGCTGTAAATGCTCCGGCTCAGCTTGCTGTACGGCGTGCAA
GTGCGACGCGGGCTGCCACTGCGCGGGCAAGAAGGAGACCACGCCCACCACCACCGTCAAGAAGTA
GACAGAAGATGACAGAGAGCAGGAGATGAGGTGGAGACGTGTTTACGCGGATTGATGGTTCTAAAGC
AATATAAATGTTATGTTTCTTTCTTGAAATTTTAATGTATTATGTATGATTTTCCTATGTAAAGTAGGTTTT
GATGGCTGTAAATGCTGAATTTTGTTGCTTAGAATGATTGATTACATCACAATGTTAGGTATCTTGAAAA
ACTGTAATTTTCAGGGATTTTAAATAAAGC
>TCONS_00005942 geneID=XLOC_001194 scaffold_1060:18307-19481(-) Unclassified
CACAGCCACCAACCAACTATACAAGATGCCTAGCCCCTGTGAACCCTGCAAAGACACTTGCGCGGAC
TCCTGCAAGGATTCGTGCGCGTGCGCCGACGACAAGTGCTGTGATGACGGCTCCAAAGGTGGCTGTT
GCTAAACTCAAGACTATCACATCTTCACCAGCCTTCTCTTCCACCTTCTACTGCCTTCGAAGTTCTTCT
GACCAGAATGTTAGTCAAACTAATGGCTAACGTGTGTTTTGCTGTGGCCATAAGCCTTACCTTTCTTAC
CATTATGGCTGCATACTGTATTCTATTGTAATTCCTGTAATAAATTATGTTTGGTGAAGGAT
>TCONS_00005937 geneID=XLOC_001192 scaffold_1060:23425-25453(+) Unclassified
CCCGCCCCAGTGCGCCTCGGTCTAGCATCTAGACCACTTCCAGCCGCTGAACTACCAACATGCCCGCC
CCGTGTGAATCTTGCAAGGACAGCTGCGGCGGCGGCAAGGCGGCGTGCGGCGCGGGCTGCACGTGC
GGCCCGAGCTGCGCCTGCGGGGACGGGAAGGCCTCAGGGGGAAAGCCCTGCTGCCAAGGATCTAAG
AAGTAATTGTCTCAATGAGGCTGTAGAATGACGAAGATGGAGTGAAGGATGGCTATGCTTAGAGTTAA
AGTACAGTATTTTTATTATTTAGGTATCAGATAAATTATGTTTAGATTGGCTAATTTGATATTGCAAAGAA
ATTTATGAAGTAGGCAATTAAATCAAGAATTATTTTGCTATTTAGAATTTATAAAATTTATACATTACTTAT
CTTATAAATTGTTATGACATATTTTATGGAGAAATTTAATAGTCTTGCAATTGAAGTCTCATGGAATAATT
ATATATTGCAGAAGACGGCTCTGTAGTTTATAAAATAATTTAACTCTTATTTAAGTATTTATTATATTACGT
TAACTGGAGAGAATACTCCTCCAGAAGTACTGGCAATACTCATTAGATGTAATAAAAATGCGACATCGC
>TCONS_00006002 geneID=XLOC_001201 scaffold_1063:22405-22880(+) Unclassified
ATCGATTCTTTGTAGGAATGATTGGAAACGAAGTCCTTGTTCTAAGTGTAAGACTAAATCAAAACACCG
GCAGCCATCGGAAAATTCTTATCTTATCATTTACATAAATTTGCACAACATTACGTAGTTCTATTCGCTTT
GTGTGCCGTCTCAACCTGCGTTTGAATAAGTTCATAAGTTCAAGTTTTTCAAAGTGACTTTCAAAACTC
ACATAACAAAATAACCTTAAAAATGGCTACAAAGATTAAACTGAATAATGGGAGAGAAATGCCCATGG
TTGGTCTGGGTACCTATTCGCGCCAAGCAGCCCCTGGGCAGTGTCGGCAGGCGGTGGAGTGGG
>TCONS_00006623 geneID=XLOC_001232 scaffold_107:422253-422678(+) Intergenic
GTCCAAATGTCATCTGACGTTCGACGTCAATGTCACGGTCGTTGACTTTCATTTTTGGTTTTATGTGCTA
AAAATATTAATTTTAAGGGAAATGCGACATAAATCGCTCAAAATAACTCAAGGATAAGTCAGTGTTAGA
CAATACACAAAGGAAGTGAATGTACATTTTCAAGATGTTGAAAGGAGTGGCAATATTTAGTGCAGGAG
TGTACACTGGGCTGTACATTGCTCAGAATTACCAGG
>TCONS_00006430 geneID=XLOC_001235 scaffold_107:508781-518404(+) Splice junction overlap
ACAATATTATGCTCACGACTGTAATCCCCGAAGGGGTAGTCAGAGGTGGCTTGCATAGCAATTGATATA
TCTCGAGCTAATTTAGGTACGGCGTATGTAACTGACTTGAATATTGACATTCAGTTGTTAAAAAATTGTC
GATTGTTTTTAGTTTATTACTTATGTTAACATTATCATTTGTCTTTTTGAATTGACTCGTCATGCCGTATTA
GACATGATGACTGCCAAAAATGAAGGCGTAATGACGGTCACCGACTACTAATGATGAATTGACATCATA
ATTCACAACAAATAGGTATCAAATTGATAACAATGACTTATCTTATTTAATTCTGATTCAACAGACGTCA
GTAGCGCGGGCCGACTTTGAGACTAAATAAGACACAATCTTTGACTTTCTGATTTATATTATGTTTGTTA
CTACCTACTTTCTTTATTTGTATCATGGCGTCTTTTAATAATATGTACTATAAATTTACTGGCCATTGCTGT
AAATTAATTATACATACGCTTCTTCCCTTATCTATCATTCTCATGAAACTTGCTCTAATGGCCTTGTAAAA
CACTTATAAAACAAAAACTTCATAGTGTCAGTCCTACCCTATTTCGTTTGAATATTAGAAACATAACCCC

TATAAATTATTTTTAATATAGTCCAGTCTGATCCAACAACTATTCTTGTTTATCCAACGATATGCTTTTACA
CTAAACTTTATATTGAGGTAGAAGATAGAGTCTAATGCGTTTTATTTAAAACGAAGCATGTCCTTCCTAT
TCTTCAGCATGAGATTAGAGTTACCGATTAAAAACTTAGAATTTGTATCTATTTGAGATCCTAGTCCTCG
AGAACTAATTGCATGTTTAGATTTTGGCTTCGTTACGAGAGTAGAGTTTTGATTGAAGGAGCGAATAAG
CGGAGAGTGGACCACGGTCCGTTAGTTGGTCACAGGCGGCGCGCTCGGCGTCCGCGACGTATAGCGG
CGTCTCGCTCGCACTCTTACACCCGTCTCTTTCGCGAACGCCGAGCGCGGCTGCCGGCTAGGTAGGTG
GGGGTGAGACGGTTGATAGAGCGGAGTTGCTAATCAGTGGGCGGCTCCTACGGGGACGGCGGCTACG
GCTGCGAGCGGTTCGCGCGGCGCCCGCCCCCCCTCCCCGCGCACCCCGCGCTGCCGCCGCGGCACGA
CACCCTCGCCTGTAATGGGGCGAGTGGGTCCGGCGGCGGTACGTCAGAGTACGTGAGGAACGAGCTG
AGAGCCGTGGTTGGAGCTCGCAGCTCCCGGCCCGATTTGCACCAGCAGATGCAGCCCGAGCTAGACC
CGCTACTCAATTTCGACATGCCTACACCCGAATACTACGGCGGAGGGCTCGGCGGCGGCGGCGGCGGC
GGCGGGGGCGGGGGCAGGTGCGCAAGATTCCTGTTTCTGCGGGTTTACTCTATCCTAACTAATATTACA
AATGCGAAAGTAACTGTGTCTGGCTGTCTGTTACTCTTTCACGCCACANNNNNNNNNNNNNNNNNNN
NNNNNNNNNNNNNTGCCCGCGAGCCTCCTCGGTGATGCGTCTACGCGGTACCTCTCTGGCTGTAAAC
TCACCTCGATGAGCATAACATATAGTACACGTATTTATAACGACGTCTTTAACGTTAGCCGTAGTCCTTT
AGTGATATTGTCGAGTTGTAAAAATCTTTTTTATTATTATTGTTAGGTCGTAATATATTTTTGTCTTTAGTT
CCCCGTGAACCCGATAAGTTACATTTTGTATGTATAAATTTATTTTTTGTAATATTTAATTGTTTCTATGCA
GTTTTCGATCAAATGTAATCTTCATTCATCGATATGAAACCAAATGAAGTTCTTTTGTGTCATGTACAAT
TTCTCAACAGTATACCTAGTATACTGGAAAGCTATGTGTGCGTAGAAATGGCCCCTGAAATATTAAGAAT
ATCGCTTCGAAATATGTTGGTCGATAAGATCGCCACAAGACCTATTAGGCCGGATCTAGTAGATACGATT
GAACACCGTATTGAGCGCAGGGAGCCCAATGATGCAAAGTTTACAATAGACATTTAATTTGTGCAATAT
TGTATAGAAATACATATTTTGGCGTTGTGAACGTTTTGTATTATAAATGAAGTCATATGTTACCTGTTATG
GCAAAGTAATATGTATTATAGTAGAGTTAATTTTGACGTGAATCAGTTTCTTTTAGTGAAGAAATTGTAT
TTTTTCTGTATATAATATGTTGTTAAATTAAACTATTGCTTTATTGTAAATATTATTTGTTATAATTTCTTTC
GTTCTCACATTTTCCTGTAATATTCAAATCGTTTATGGATATTTAATCATTAATAAGATTTTCACTATTTTA
CAAATTATAATATGAACTCAAAAGTATTGCATAAATAACGCAACGGTCGGTTGAAGACCACAAGTCGA
CTGCCGGGAGCCGCAACTTCTCCAATGTAATCTATTACTCGTTCTCTCAGTGTAATCGTTAGCTATGATT
ATTACGTGTTCGGATAAAGATGTTAGATCGCCTCCACCGGTGCGTAGTTTAGTGTGAGCTTAGTGTAAC
CGTGTACGTACTAGCCCAGCTCCAGTGGCGTCTCTGTGTGACCCACAGGCGATAGTGTTTAAACGGAA
TGTGAACATAATTCAATGTCATTGTTAGGACATTTCTTTAACTCTAGGCACAAAAACATTAGCTGTTGA
CGTCGCGGCTTTAGCTGTAGATTCGGTCGTGTGACATCACGCCTTCATACTGTCTAAAGCCGCGCTACG
TCTTGACGTTATGCGTAAGAAATTAGAATCGCACTATACAATGTGTACTGTTTATTAACATTTGTCAACT
TTTAACTTAACTAATTGTAAGAAATCTAAAATTGTGTGATGATGATGAATGTGAATTAATGTATTTTCATA
TTTCCGTTCGATATAATCGGTAAAACGTTTAAGCTTAAAGTAAATGAAACACTATAATGTATCCCTAAAA
ACGCAAACCCGGGGACCATAAAGGAAATGTCTGCAGGAATTGGGAACACCGGCGACGACGGCCGTGT
GTCGCGTCGCGGGGCGTCGCGGCGACACGAGGCGACGTTGGACCGAGCAATGTGTCGTCATGATTCG
CTTGTGGCGGTCTCTCGGTCCAACAGTCGTCATCATTCGCACTAGGACTGGCTAAAGCGGCTTTATTTA
ATCAATCTCGGCTCAATCGATATACTAATTAGCATATTGTAAATACAAAGAGATAGAGTAAATGAACAAC
AGTGGTAAGAAGTTAGCTGTAGCCGTGTGTAAAGTACTTAAACCTTTTTTGATGCTCTTCATCTATTGTA
GAGATATTTTGTCTCCTTATTGTAAATTATCCCACGTAACACCAACTTACCCCGTAGTAAGTAGACCTAC
ACCTAATTAAACTGCCATTGTGATGTGTTGAATGCCTACCTATAAATGAACGTACAAATAAACTGATACA
TATGTAGAGATTACCTACCCGTAAAACAAAGGAAACCTAAGCTGTCTAAGAAATATTTAAATATTTTTAT
ACATAATGAAATAGTTGCGAGTTTTAGATTCGACCAAACCCTTTGTCGCGGTGACGTCTTCATTCAAGT
AGCTACGAGATAGTAGAGCCGCGACCGAGCATTCCACGTAGTTGTTGACATACACGCTCATCTCTGTG

AGGGAAAATATTACGATTTAGTACAAGTGAGGGCCTTCGTTATGAGACGATGTTAAAACAGATAATATG
GAGTCTATTTAGCAATTGGAGATGATATAATTCAAAATAAATTAATATACCGTCACATGGTGTCAGAAAG
ATTCAAACATAAAAATATATTTTTGCTATTGCGATTGATATGGACTCGCGACAGAGTTAAAAAACGTCC
AACTTAATTGAGTTATGAAAATCATGTATTGTCTGAATAAGTGTAAAGGTGCCTGGAATATAATAAATAA
AAGATATTTTTCCAATACCGCCATGTTGAAAAAGTTATTTTTTACAGTATATTAGACGGAAGTGATGGAT
ATAATAGAGAAAGTAGAATAGAGATGTAATAGAATTCATACAAAATAACCAAACTTGTTGTTAAGAGAA
GGAACCAGTTTGTCGTGAGCAAGTTGATCCGACGTGCCAGGTTTGATGCTTGATAAAATTCGATTCGC
AGATGTTTTATGTCACACAGATTCCAAACAAATTATGTATTTGGCAGCATACTGACAAAACCTACTTAC
AAATATTGTTACACAAAAATTATTAGCGTTATATGGCGACTCAAGTCTTCCAATGTAACTGACCATACAA
TTCGAAGGACAACATGTCACCTACTCAGTATTCTACACCACCATCGAAAAGTTTCCTTTAAAGAAAGA
GTAATATTTTGATACCACGACAAACTAAAATGGGCTTTATGTAGTTCTCAGTGTAAGTGTTTGATGATAT
GAGACATGTGTCTGTTAATGGATAAATGATACCTCATAACATAATATTATACCTATGACCTAATGTAGTAT
ATCGTTACAAATTGTATGAACTTCCCTCCATTTATTTGAAATGGAGAATAAACACTTCCTTAGCTGTTTT
GTAGAATTTGTAACGATTCAATTATTCTTAAGCATCTAAGCATTAAAATTAAAAAAATATAAATGGCGAG
GTTAATTGAAAAATACGGTCTATATTTGAATTGTTATAACGTGTCTCGAGTTTTATGAAAGATAACATATT
TGATTAGGTACATTACCGAACTAAATAACAACATTGATTATTCAAGAAGTTGCATCAATATTCCGCAGAG
TATGTTTTCGAAATTAATAGGGTCCAAGGTTTCCATCCAAAAATACTGAAGAAATTAAATTTGGACAGT
TTTAAAGATTGTATGAAATACCTAATATTAGTAATATACTATGTACAACGGACTCTGTGGAAAAGTAACG
ATCAGCTGAATGAATAATTAATGTACCTATAAAGAAAGAAACATGGATGTGTACGGGGGATCTTTGTTT
CAAATGGAAAACATAATTAGTTTTAGTTGATGCAATTCGGAACAAATTATACGAAAGTTAATGATGATAT
GAAGTACATAATTATAATAACGTTTATTTAAGTGTCGCAATGGGAAGCCGGCCTTTCGGCGCCTGCGCA
CCATTTACATTAATTAATATTCTCTAAATTAACATTATTTTGTGCACGTTACAACGAAAGGATCAGTGGAT
TACAATAAAGCATAAACGTAGGGAATCGATGTGTGATATAATAAAGGTAACATAACTAGCGGGTGGACG
ACAGTCGCTAACACTCCGAGATAGCTCCCGTACATTAGAGATTTTATTTTTTGTATAAAATCATTATCTCT
GTCTTGGCCTGTCGTCTACGAGTAGTATGTACATTTTTCTAATTTCTAAGATTAATTTGTAGAGTTGGGC
ATGAATATTGTTGTAAAAACTAATTGTGTTTTTCTCAGTTAATAAAAAAAATCGTATTGGTCAGTCGCAG
AAAGGATTTTTGCGAGTATGCTTTATTCTAGCTAGTAATGAGAGTTAAATGTTAGCACATCTCCAGTGAT
TAGTTACGGGTCGCCAGGCTCTGGCAGCAGCCTCCTGCTACAACATATGATGAGACTAGTCCTCGTTAG
GCAACTATCTTTTTTGCGGGTGACCGTTCAGTCTGTGTTATTATGTTCTTCATTCAGATGTTAGAAATAA
GGCCTTTTGTGTTGCTGTGCATTTCTACACGTTAAATGTAGGTATGTTTATATATTGTAAATAGTTTAATA
CAGTATATTTTTACGACTAGGATACACAACCAAAGTTGTTATAAAGTGATGGTTGATTAAATAATAAATT
TAATATAGGTCGTTATGTTTATTTTTAAGAAATGATACAAATTAAATGTACATTTATATATCGAACATCCAA
TGTTTTGTCGAAGCCTTTTACACAACCGCCCTGATATTAAAAAACGAATTTATTATTTTGGTAAAAAAG
ACATAAAGAAAAAGAAGATATAAAATATAACGGTTTCTCTACGGCTTACTTACCTCTTTTGTTGTTCCTA
GTAGCTTATTCAGGTTCATCTTCCGTCGGTCAATTACTGAAAAAATATAATTGATGCATTGTGTATGTAA
ATGAAATCCTGAAATTTAATATTGAATTAATTTTAATTATAAAATTCAGACACGAGTAC
>TCONS_00006328 geneID=XLOC_001235 scaffold_107:509990-518404(+) Exonic overlap (+)
GCGCCACCCCGCCACGATACTCATCGACCGCACACGCACCTCTACTGCTCAACATGGACATAGATACG
CATAGATTTACTTTATTTATAACAATTCTAAGCTTTTTCGGCTTGACTTACTCGACGGAACTGTCTTTTGT
CTAAGTACGAAGTTGTCGTTGCTATGACGTCGTATGATAAGAAGTAAATTGCAAAATTATAAAAATGGG
GTAACAAATTGACATTTATTACATATTTTACAATCAGAGCAAAGCAGAGTCAGTAACCTGTACCTGAGG
TGCAATATACGCGATAAGCAGTGCATTGAACTTCATATTTCGTTATCGCGTTGTTCTCACATTGTGCAAC
CGATGCGTTGTCGTGACTTGCAATAGTCGAATCAACTTCTTGTCAGATGATTGATGTCACCAATTTGTG
ATTTTCCATTTCGTTGTTTGATAAAAGATTAATTAGCGAAAATTCGTAACAATATGCTTTATGGTAACATT

CGTAGATAAAATCTGCACTCAAGTAACAATTCATTCAAATATACAGTCGATGGAACAGTATTTATGTAAT
AAATATTAAAAATTAACAGGCTTAACAAAGGTGGCAGTTTCAAAACTGGGTAAATGGTAGCGGTAGAC
CGAACTATACGAACATTTCTATTATAAACAGCTTTGCATGGTATAACTACAATACGCATTTTATTAATGCC
GCTTCTAAATATCTATCGTAGCCTTTTACGTAGCTGACTTGGATCAGTCTGTCTTTACGCACCATGAATG
TTGTTGGATATTGTATTGTGTATATTGACTTACATTTTGTTGTGTAGGTAATGGGGCGAGTGGGTCCGGC
GGCGGTACGTCAGAGTACGTGAGGAACGAGCTGAGAGCCGTGGTTGGAGCTCGCAGCTCCCGGCCCG
ATTTGCACCAGCAGATGCAGCCCGAGCTAGACCCGCTACTCAATTTCGACATGCCTACACCCGAATACT
ACGGCGGAGGGCTCGGCGGCGGCGGCGGCGGCGGCGGGGGCGGGGGCAGGTGCGCAAGATTCCTGT
TTCTGCGGGTTTACTCTATCCTAACTAATATTACAAATGCGAAAGTAACTGTGTCTGGCTGTCTGTTACT
CTTTCACGCCACANNNNNNNNNNNNNNNNNNNNNNNNNNNNNNNNNNNNTGCCCGCGAGCCTCCTCGGTG
ATGCGTCTACGCGGTACCTCTCTGGCTGTAAACTCACCTCGATGAGCATAACATATAGTACACGTATTTA
TAACGACGTCTTTAACGTTAGCCGTAGTCCTTTAGTGATATTGTCGAGTTGTAAAAATCTTTTTTATTATT
ATTGTTAGGTCGTAATATATTTTTGTCTTTAGTTCCCCGTGAACCCGATAAGTTACATTTTGTATGTATAA
ATTTATTTTTTGTAATATTTAATTGTTTCTATGCAGTTTTCGATCAAATGTAATCTTCATTCATCGATATGA
AACCAAATGAAGTTCTTTTGTGTCATGTACAATTTCTCAACAGTATACCTAGTATACTGGAAAGCTATGT
GTGCGTAGAAATGGCCCCTGAAATATTAAGAATATCGCTTCGAAATATGTTGGTCGATAAGATCGCCAC
AAGACCTATTAGGCCGGATCTAGTAGATACGATTGAACACCGTATTGAGCGCAGGGAGCCCAATGATG
CAAAGTTTACAATAGACATTTAATTTGTGCAATATTGTATAGAAATACATATTTTGGCGTTGTGAACGTTT
TGTATTATAAATGAAGTCATATGTTACCTGTTATGGCAAAGTAATATGTATTATAGTAGAGTTAATTTTGA
CGTGAATCAGTTTCTTTTAGTGAAGAAATTGTATTTTTTCTGTATATAATATGTTGTTAAATTAAACTATT
GCTTTATTGTAAATATTATTTGTTATAATTTCTTTCGTTCTCACATTTTCCTGTAATATTCAAATCGTTTATG
GATATTTAATCATTAATAAGATTTTCACTATTTTACAAATTATAATATGAACTCAAAAGTATTGCATAAATA
ACGCAACGGTCGGTTGAAGACCACAAGTCGACTGCCGGGAGCCGCAACTTCTCCAATGTAATCTATTA
CTCGTTCTCTCAGTGTAATCGTTAGCTATGATTATTACGTGTTCGGATAAAGATGTTAGATCGCCTCCAC
CGGTGCGTAGTTTAGTGTGAGCTTAGTGTAACCGTGTACGTACTAGCCCAGCTCCAGTGGCGTCTCTGT
GTGACCCACAGGCGATAGTGTTTAAACGGAATGTGAACATAATTCAATGTCATTGTTAGGACATTTCTT
TAACTCTAGGCACAAAAACATTAGCTGTTGACGTCGCGGCTTTAGCTGTAGATTCGGTCGTGTGACATC
ACGCCTTCATACTGTCTAAAGCCGCGCTACGTCTTGACGTTATGCGTAAGAAATTAGAATCGCACTATA
CAATGTGTACTGTTTATTAACATTTGTCAACTTTTAACTTAACTAATTGTAAGAAATCTAAAATTGTGTG
ATGATGATGAATGTGAATTAATGTATTTTCATATTTCCGTTCGATATAATCGGTAAAACGTTTAAGCTTAA
AGTAAATGAAACACTATAATGTATCCCTAAAAACGCAAACCCGGGGACCATAAAGGAAATGTCTGCAG
GAATTGGGAACACCGGCGACGACGGCCGTGTGTCGCGTCGCGGGGCGTCGCGGCGACACGAGGCGA
CGTTGGACCGAGCAATGTGTCGTCATGATTCGCTTGTGGCGGTCTCTCGGTCCAACAGTCGTCATCATT
CGCACTAGGACTGGCTAAAGCGGCTTTATTTAATCAATCTCGGCTCAATCGATATACTAATTAGCATATT
GTAAATACAAAGAGATAGAGTAAATGAACAACAGTGGTAAGAAGTTAGCTGTAGCCGTGTGTAAAGTA
CTTAAACCTTTTTTGATGCTCTTCATCTATTGTAGAGATATTTTGTCTCCTTATTGTAAATTATCCCACGTA
ACACCAACTTACCCCGTAGTAAGTAGACCTACACCTAATTAAACTGCCATTGTGATGTGTTGAATGCCT
ACCTATAAATGAACGTACAAATAAACTGATACATATGTAGAGATTACCTACCCGTAAAACAAAGGAAAC
CTAAGCTGTCTAAGAAATATTTAAATATTTTTATACATAATGAAATAGTTGCGAGTTTTAGATTCGACCAA
ACCCTTTGTCGCGGTGACGTCTTCATTCAAGTAGCTACGAGATAGTAGAGCCGCGACCGAGCATTCCA
CGTAGTTGTTGACATACACGCTCATCTCTGTGAGGGAAAATATTACGATTTAGTACAAGTGAGGGCCTT
CGTTATGAGACGATGTTAAAACAGATAATATGGAGTCTATTTAGCAATTGGAGATGATATAATTCAAAAT
AAATTAATATACCGTCACATGGTGTCAGAAAGATTCAAACATAAAAATATATTTTTGCTATTGCGATTGAT
ATGGGACTCGCGACAGAGTTAAAAAACGTCCAACTTAATTGAGTTATGAAAATCATGTATTGTCTGAAT

AAGTGTAAAGGTGCCTGGAATATAATAAATAAAAGATATTTTTCCAATACCGCCATGTTGAAAAAGTTAT
TTTTTACAGTATATTAGACGGAAGTGATGGATATAATAGAGAAAGTAGAATAGAGATGTAATAGAATTCA
TACAAAATAACCAAACTTGTTGTTAAGAGAAGGAACCAGTTTGTCGTGAGCAAGTTGATCCGACGTGC
CAGGTTTGATGCTTGATAAAATTCGATTCGCAGATGTTTTATGTCACACAGATTCCAAACAAATTATGTA
TTTGGCAGCATACTGACAAAACCTACTTACAAATATTGTTACACAAAAATTATTAGCGTTATATGGCGAC
TCAAGTCTTCCAATGTAACTGACCATACAATTCGAAGGACAACATGTCACCTACTCAGTATTCTACACC
ACCATCGAAAAGTTTCCTTTAAAGAAAGAGTAATATTTTGATACCACGACAAACTAAAATGGGCTTTAT
GTAGTTCTCAGTGTAAGTGTTTGATGATATGAGACATGTGTCTGTTAATGGATAAATGATACCTCATAAC
ATAATATTATACCTATGACCTAATGTAGTATATCGTTACAAATTGTATGAACTTCCCTCCATTTATTTGAAA
TGGAGAATAAACACTTCCTTAGCTGTTTTGTAGAATTTGTAACGATTCAATTATTCTTAAGCATCTAAGC
ATTAAAATTAAAAAAATATAAATGGCGAGGTTAATTGAAAAATACGGTCTATATTTGAATTGTTATAACG
TGTCTCGAGTTTTATGAAAGATAACATATTTGATTAGGTACATTACCGAACTAAATAACAACATTGATTA
TTCAAGAAGTTGCATCAATATTCCGCAGAGTATGTTTTCGAAATTAATAGGGTCCAAGGTTTCCATCCA
AAAATACTGAAGAAATTAAATTTGGACAGTTTTAAAGATTGTATGAAATACCTAATATTAGTAATATACT
ATGTACAACGGACTCTGTGGAAAAGTAACGATCAGCTGAATGAATAATTAATGTACCTATAAAGAAAG
AAACATGGATGTGTACGGGGGATCTTTGTTTCAAATGGAAAACATAATTAGTTTTAGTTGATGCAATTC
GGAACAAATTATACGAAAGTTAATGATGATATGAAGTACATAATTATAATAACGTTTATTTAAGTGTCGC
AATGGGAAGCCGGCCTTTCGGCGCCTGCGCACCATTTACATTAATTAATATTCTCTAAATTAACATTATT
TTGTGCACGTTACAACGAAAGGATCAGTGGATTACAATAAAGCATAAACGTAGGGAATCGATGTGTGA
TATAATAAAGGTAACATAACTAGCGGGTGGACGACAGTCGCTAACACTCCGAGATAGCTCCCGTACATT
AGAGATTTTATTTTTTGTATAAAATCATTATCTCTGTCTTGGCCTGTCGTCTACGAGTAGTATGTACATTT
TTCTAATTTCTAAGATTAATTTGTAGAGTTGGGCATGAATATTGTTGTAAAAACTAATTGTGTTTTTCTCA
GTTAATAAAAAAAATCGTATTGGTCAGTCGCAGAAAGGATTTTTGCGAGTATGCTTTATTCTAGCTAGTA
ATGAGAGTTAAATGTTAGCACATCTCCAGTGATTAGTTACGGGTCGCCAGGCTCTGGCAGCAGCCTCCT
GCTACAACATATGATGAGACTAGTCCTCGTTAGGCAACTATCTTTTTTGCGGGTGACCGTTCAGTCTGT
GTTATTATGTTCTTCATTCAGATGTTAGAAATAAGGCCTTTTGTGTTGCTGTGCATTTCTACACGTTAAAT
GTAGGTATGTTTATATATTGTAAATAGTTTAATACAGTATATTTTTACGACTAGGATACACAACCAAAGTT
GTTATAAAGTGATGGTTGATTAAATAATAAATTTAATATAGGTCGTTATGTTTATTTTTAAGAAATGATAC
AAATTAAATGTACATTTATATATCGAACATCCAATGTTTTGTCGAAGCCTTTTACACAACCGCCCTGATAT
TAAAAAACGAATTTATTATTTTGGTAAAAAAGACATAAAGAAAAAGAAGATATAAAATATAACGGTTTC
TCTACGGCTTACTTACCTCTTTTGTTGTTCCTAGTAGCTTATTCAGGTTCATCTTCCGTCGGTCAATTACT
GAAAAAATATAATTGATGCATTGTGTATGTAAATGAAATCCTGAAATTTAATATTGAATTAATTTTAATTAT
AAAATTCAGACACGAGTAC
>TCONS_00006347 geneID=XLOC_001262 scaffold_107:513755-518834(-) Intergenic
GAAGTCGACATTTATTGCACTTATCATCTCACTCGCATCTGCATTGTGCCCGCCCAAGTGCCCAGTTATC
TCGAAGACTGTAAAGCTACTGGTAATTGACCGACGGAAGATGAACCTGAATAAGCTACTAGGAACAAC
AAAAGAGGTAAGTAAGCCGTAGAGAAACCGTTATATTTTATATCTTCTTTTTCTTTATGTCTTTTTTACCA
AAATAATAAATTCGTTTTTTAATATCAGGGCGGTTGTGTAAAAGGCTTCGACAAAACATTGGATGTTCG
ATATATAAATGTACATTTAATTTGTATCATTTCTTAAAAATAAACATAACGACCTATATTAAATTTATTATTT
AATCAACCATCACTTTATAACAACTTTGGTTGTGTATCCTAGTCGTAAAAATATACTGTATTAAACTATTT
ACAATATATAAACATACCTACATTTAACGTGTAGAAATGCACAGCAACACAAAAGGCCTTATTTCTAAC
ATCTGAATGAAGAACATAATAACACAGACTGAACGGTCACCCGCAAAAAAGATAGTTGCCTAACGAG
GACTAGTCTCATCATATGTTGTAGCAGGAGGCTGCTGCCAGAGCCTGGCGACCCGTAACTAATCACTG
GAGATGTGCTAACATTTAACTCTCATTACTAGCTAGAATAAAGCATACTCGCAAAAATCCTTTCTGCGA

CTGACCAATACGATTTTTTTATTAACTGAGAAAAACACAATTAGTTTTACAACAATATTCATGCCCAA
CTCTACAAATTAATCTTAGAAATTAGAAAAATGTACATACTACTCGTAGACGACAGGCCAAGACAGAGA
TAATGATTTTATACAAAAAATAAAATCTCTAATGTACGGGAGCTATCTCGGAGTGTTAGCGACTGTCGTC
CACCCGCTAGTTATGTTACCTTTATTATATCACACATCGATTCCCTACGTTTATGCTTTATTGTAATCCACT
GATCCTTTCGTTGTAACGTGCACAAAATAATGTTAATTTAGAGAATATTAATTAATGTAAATGGTGCGCA
GGCGCCGAAAGGCCGGCTTCCCATTGCGACACTTAAATAAACGTTATTATAATTATGTACTTCATATCAT
CATTAACTTTCGTATAATTTGTTCCGAATTGCATCAACTAAAACTAATTATGTTTTCCATTTGAAACAAA
GATCCCCCGTACACATCCATGTTTCTTTCTTTATAGGTACATTAATTATTCATTCAGCTGATCGTTACTTTT
CCACAGAGTCCGTTGTACATAGTATATTACTAATATTAGGTATTTCATACAATCTTTAAAACTGTCCAAAT
TTAATTTCTTCAGTATTTTTGGATGGAAACCTTGGACCCTATTAATTTCGAAAACATACTCTGCGGAATA
TTGATGCAACTTCTTGAATAATCAATGTTGTTATTTAGTTCGGTAATGTACCTAATCAAATATGTTATCTT
TCATAAAACTCGAGACACGTTATAACAATTCAAATATAGACCGTATTTTTCAATTAACCTCGCCATTTATA
TTTTTTTAATTTTAATGCTTAGATGCTTAAGAATAATTGAATCGTTACAAATTCTACAAAACAGCTAAGG
AAGTGTTTATTCTCCATTTCAAATAAATGGAGGGAAGTTCATACAATTTGTAACGATATACTACATTAGG
TCATAGGTATAATATTATGTTATGAGGTATCATTTATCCATTAACAGACACATGTCTCATATCATCAAACAC
TTACACTGAGAACTACATAAAGCCCATTTTAGTTTGTCGTGGTATCAAAATATTACTCTTTCTTTAAAGG
AAACTTTTCGATGGTGGTGTAGAATACTGAGTAGGTGACATGTTGTCCTTCGAATTGTATGGTCAGTTA
CATTGGAAGACTTGAGTCGCCATATAACGCTAATAATTTTTGTGTAACAATATTTGTAAGTAGGTTTTGT
CAGTATGCTGCCAAATACATAATTTGTTTGGAATCTGTGTGACATAAAACATCTGCGAATCGAATTTTAT
CAAGCATCAAACCTGGCACGTCGGATCAACTTGCTCACGACAAACTGGTTCCTTCTCTTAACAACAAG
TTTGGTTATTTTGTATGAATTCTATTACATCTCTATTCTACTTTCTCTATTATATCCATCACTTCCGTCTAAT
ATACTGTAAAAAATAACTTTTTCAACATGGCGGTATTGGAAAAATATCTTTTATTTATTATATTCCAGGCA
CCTTTACACTTATTCAGACAATACATGATTTTCATAACTCAATTAAGTTGGACGTTTTTTAACTCTGTCG
CGAGTCCCATATCAATCGCAATAGCAAAAATATATTTTTATGTTTGAATCTTTCTGACACCATGTGACGG
TATATTAATTTATTTTGAATTATATCATCTCCAATTGCTAAATAGACTCCATATTATCTGTTTTAACATCGTC
TCATAACGAAGGCCCTCACTTGTACTAAATCGTAATATTTTCCCTCACAGAGATGAGCGTGTATGTCAA
CAACTACGTGGAATGCTCGGTCGCGGCTCTACTATCTCGTAGCTACTTGAATGAAGACGTCACCGCGA
CAAAGGGTTTGGTCGAATCTAAAACTCGCAACTATTTCATTATGTATAAAAATATTTAAATATTTCTTAG
ACAGCTTAGGTTTCCTTTGTTTTACGGGTAGGTAATCTCTACATATGTATCAGTTTATTTGTACGTTCATT
TATAGGTAGGCATTCAACACATCACAATGGCAGTTTAATTAGGTGTAGGTCTACTTACTACGGGGTAAG
TTGGTGTTACGTGGGATAATTTACAATAAGGAGACAAAATATCTCTACAATAGATGAAGAGCATCAAAA
AAGGTTTAAGTACTTTACACACGGCTACAGCTAACTTCTTACCACTGTTGTTCATTTACTCTATCTCTTT
GTATTTACAATATGCTAATTAGTATATCGATTGAGCCGAGATTGATTAAATAAAGCCGCTTTAGCCAGTCC
TAGTGCGAATGATGACGACTGTTGGACCGAGAGACCGCCACAAGCGAATCATGACGACACATTGCTC
GGTCCAACGTCGCCTCGTGTCGCCGCGACGCCCCGCGACGCGACACACGGCCGTCGTCGCCGGTGTT
CCCAATTCCTGCAGACATTTCCTTTATGGTCCCCGGGTTTGCGTTTTTAGGGATACATTATAGTGTTTCAT
TTACTTTAAGCTTAAACGTTTTACCGATTATATCGAACGGAAATATGAAAATACATTAATTCACATTCATC
ATCATCACACAATTTTAGATTTCTTACAATTAGTTAAGTTAAAAGTTGACAAATGTTAATAAACAGTACA
CATTGTATAGTGCGATTCTAATTTCTTACGCATAACGTCAAGACGTAGCGCGGCTTTAGACAGTATGAA
GGCGTGATGTCACACGACCGAATCTACAGCTAAAGCCGCGACGTCAACAGCTAATGTTTTTGTGCCTA
GAGTTAAAGAAATGTCCTAACAATGACATTGAATTATGTTCACATTCCGTTTAAACACTATCGCCTGTG
GGTCACACAGAGACGCCACTGGAGCTGGGCTAGTACGTACACGGTTACACTAAGCTCACACTAAACT
ACGCACCGGTGGAGGCGATCTAACATCTTTATCCGAACACGTAATAATCATAGCTAACGATTACACTGA
GAGAACGAGTAATAGATTACATTGGAGAAGTTGCGGCTCCCGGCAGTCGACTTGTGGTCTTCAACCGA

CCGTTGCGTTATTTATGCAATACTTTTGAGTTCATATTATAATTTGTAAAATAGTGAAAATCTTATTAATGA
TTAAATATCCATAAACGATTTGAATATTACAGGAAAATGTGAGAACGAAAGAAATTATAACAAATAATAT
TTACAATAAAGCAATAGTTTAATTTAACAACATATTATATACAGAAAAAATACAATTTCTTCACTAAAAG
AAACTGATTCACGTCAAAATTAACTCTACTATAATACATATTACTTTGCCATAACAGGTAACATATGACTT
CATTTATAATACAAAACGTTCACAACGCCAAAATATGTATTTCTATACAATATTGCACAAATTAAATGTCT
ATTGTAAACTTTGCATCATTGGGCTCCCTGCGCTCAATACGGTGTTCAATCGTATCTACTAGATCCGGCC
TAATAGGTCTTGTGGCGATCTTATCGACCAACATATTTCGAAGCGATATTCTTAATATTTCAGGGGCCATT
TCTACGCACACATAGCTTTCCAGTATACTAGGTATACTGTTGAGAAATTGTACATGACACAAAAGAACT
TCATTTGGTTTCATATCGATGAATGAAGATTACATTTGATCGAAAACTGCATAGAAACAATTAAATATTA
CAAAAAATAAATTTATACATACAAAATGTAACTTATCGGGTTCACGGGGAACTAAAGACAAAAATATAT
TACGACCTAACAATAATAATAAAAAAGATTTTTACAACTCGACAATATCACTAAAGGACTACGGCTAAC
GTTAAAGACGTCGTTATAAATACGTGTACTATATGTTATGCTCATCGAGGTGAGTTTACAGCCAGAGAG
GTACCGCGTAGACGCATCACCGAGGAGGCTCGCGGGCA
>TCONS_00006512 geneID=XLOC_001236 scaffold_107:545164-567661(+) Splice junction overlap
CGCGCCGCTCGCCAGGCCCACGCGCGCGCGCAACACGCATTCGCGATAAACATTCGCCAAAATTCAG
CGCCAATCTAAGCAGTTTCCTTTTATAGCCGCCGGACGGCGACCGCTCTATAAATATCCCCGTGCCCTA
CCGTGGGTAGTGCTTTAGGCAATATCGGTGTAATCTGTGCGAATATCCTCATATTTGGCATGCAAAGCTC
CGAGATATGGGACTTGAGAACTTTGAATTGAATAAGTTTAGCGGCATGTCGTAAAATCCGAGAAGCTTT
TACAGACGCGTCGGCTTTGGATTTTTCATGAGTGTTCTGTTTACGTGACCGTAGAGTTGCCGGTTTTGT
TGAGGCAGGGTAAAAACAACAACTTAGTCCAGCATATCGATATTTACGATTCTGTTATTCTCCAATAAG
ATTGTTGTGAACAAGACTTTTGGAGCTATTAAAATCAAATAGGTACAAGGTGACATGCTTCTTATCTCA
AAAAGGATATCTTTAGATTATGTATGCGTAATCTCCCTATGACGATTGCAAACGATAACAGTGTTTTCAA
TTCTTTATCGATAAACCTTCTTGAGTCACGTAAGCCATTACTCAATAGTAAAACAGGTGAATGAGTTATA
GATAGACAATTAAAATTGAATTATTGATCTACACCTAAATTAGCAGAAGTTGGGAATGTTGGCAGTAAA
ACGTGGTAAAACTACGCTAATGCGACGTGGAGACAATAAGTGCTAATTGAAGAAAGCTGTTCACTACA
ACTACAAAACCTTTTGTGTGTGTCAATATATGACGAGTGAAGTGCAGTGCAGTAGGTACATAACTAGAC
ATAATAGACATAGAGGACATCAGACTGCGGCATAAGCAAGAAAGTAACATGATGGGAGTACTCACATG
GTTGAAGGGTCTTTAACTGACGTCTGGATCCATCTCTCTCATGATGTACCGGAACGGAAGGCTCGGCA
GCTCCAGCAGTGGCTCCACATCATCAGGCCGCTCCGACCTGCAGGTGTCGGAGAGCCTGGAGCCGCC
GGAGCCCCCGCAGCTGAAGCAGCCGCTCGCCGCACAGCCTTCCGATGCGGCGCGGTTCACGGACACG
AGCTGGCAGATCCACCGCAGCTGGTCCCGGG
>TCONS_00006545 geneID=XLOC_001236 scaffold_107:545170-546127(+) Intronic
GCTCGCCAGGCCCACGCGCGCGCGCAACACGCATTCGCGATAAACATTCGCCAAAATTCAGCGCCAAT
CTAAGCAGTTTCCTTTTATAGCCGCCGGACGGCGACCGCTCTATAAATATCCCCGTGCCCTACCGTGGG
TAGTGCTTTAGGCAATATCGGTGTAATCTGTGCGAATATCCTCATATTTGGCATGCAAAGCTCCGAGATA
TGGGACTTGAGAACTTTGAATTGAATAAGTTTAGCGGCATGTCGTAAAATCCGAGAAGCTTTTACAGA
CGCGTCGGCTTTGGATTTTTCATGAGTGTTCTGTTTACGTGACCGTAGAGTTGCCGGTTTTGTTGAGGT
GAATGAGTTATAGATAGACAATTAAAATTGAATTATTGATCTACACCTAAATTAGCAGAAGTTGGGAATG
TTGGCAGTAAAACGTGGTAAAACTACGCTAATGCGACGTGGAGACAATAAGTGCTAATTGAAGAAAG
CTGTTCACTACAACTACAAAACCTTTTGTGTGTGTCAATATATGACGAGTGAAGTGCAGTGCAGTAGGT
ACATAACTAGACATAATAGACATAGAGGACATCAGACTGCGGCATAAGCAAGAAAGTAACATGATGGG
AGTACTCACATGGTTGAAGGGTCTTTAACTGACGTCTGGATCCATCTCTCTCATGATGTACCGGAACGG
AAGGCTCGGCA
>TCONS_00006360 geneID=XLOC_001236 scaffold_107:545848-560491(+) Splice junction overlap

CAGTAAAACGTGGTAAAACTACGCTAATGCGACGTGGAGACAATAAGTGCTAATTGAAGAAAGCTGTT
CACTACAACTACAAAACCTTTTGTGTGTGTCAATATATGACGAGTGAAGTGCAGTGCAGTAGGTACATA
ACTAGACATAATAGACATAGAGGACATCAGACTGCGGCATAAGCAAGAAAGTAACATGATGGGAGTAC
TCACATGGTTGAAGGGTCTTTAACTGACGTCTGGATCCATCTCTCTCATGATGTACCGGAACGGAAGGC
TCGGCAGCTCCAGCAGTGGCTCCACATCATCAGGCCGCTCCGACCTGCAGGTGTCGGAGAGCCTGGA
GCCGCCGGAGCCCCCGCAGCTGAAGCAGCCGCTCGCCGCACAGCCTTCCG
>TCONS_00006472 geneID=XLOC_001240 scaffold_107:686171-691981(+) Intergenic
GTTAACTTTGGTCTATAAGTAGTGAGGCATTGTGAATGTTTTGTCACAGTGGATGGTTTGATCAGTGGT
TTGTCATGGGGTTTAGTCCAAACAAGGAGTGTTGTTGGTTTTGCATTGTTATGTTTAGTCCTGAAATAA
AACAAATAATTTTATAGGCATAATTCAAATAAAATTACGTAGGTAAATGTAGAAGTTGAAGAGCAAATG
ATTAGGATACTTATTTAAAAGGTGAGGGGTTGACAATAAGTTAAAATATGTTGGTTAGTCTTGTCAGTAT
TCATTCAAAACTGTATGGGGAATAATTAACTGAGTTAGATTTTTATAACAATAGATCTATTATAAACACA
AGATCCGTAAAACAGAGCTATTGTACGTATGAATAACTAACAATTCAGGTGTTGTTTATTTTGAATAACT
ATAATTTGAGGTCAAGAATTTAGTTTAACAGCATGCATACACGGGTTTTCAATAAAATAATGACACTTAC
CTATTAGATTCACTCATTATTTAAGAGTTTCATAAGTTTAATCGGGTAATCTTTAGATTTCTTCATGCGAT
CGATATTGTTGAACACAACGAAGCATGCTTTTGTTTTGTTTAGTACCGGTAAAAAAAAAAGAAAAGGT
TAAGGTTATGTCAGGTTGACATTGACAGTTTAGTTTGACAGTTAGACAGGTAAGGCACATTTACACAA
ACAGCCTAGAATAGGAAAGAATAAAGGATATGGAGACCGTGTTTTTCAAATAGGGGTTGTGAGGGAG
AGATGAGATATATCTCAGAAATATTTTTAGTTACCTAGTAACATTATTTTCGACTTAGGTAGTTACGGAG
GGTAGGAAAATAGTTGAAGTAGAAGTAGTAACTTTCAGTTGAATCTTGAGCTTCCTTTTGGCGGTTTTC
GAAGATCTTTGATA
>TCONS_00006654 geneID=XLOC_001308 scaffold_1071:19770-22914(+) Intergenic
TTACTAGCTGATGAAAAAGACGACCCACCAACTACTAGTATGTCTCTTCCATCATAAAGGACATGCAAA
CAGCTGCTGAATTTATATTTTTTTTGACTAGAAGGTTATTTACATCCTGGAAGTAACATGCCATTCAATG
GTGGAATACCACAAATTCAAGAATGGAAGGTGCTAAACATGCCATCAGACCTAACATGATATTCTTGAC
TGTGGACACTACAAGGACCAAGTGATTGAGACAGAGTGGTGGCAACACTGACTACACCACCGGGTTG
AAATGAAATAGAACAGATATCTGCAGCACACATTTTTTGCAGGACAGGACCGAATGAAATGATAAAAA
TGCTGCAACAGAGACACGGGATTGCTGAATTTCCATATAAAATCCAGAATAAACGTCTCTATCTTCGAT
CGATCTACTTATCTGTAAAGCTGCTGATACGAGAAAAAGGGCGTGGGACATCAATGAATAAACTCAAA
AACATCCAATAAGTTGATCTGGAGTTGTCTTGGACTTGACGAACATCGTACATCCCGTTGAACAAAGT
AACATGATCTGCAGTATTCTTGGTCACCATTGGATTAAATACAATGAAGATTGTTGTAATATCTCCTGAT
TTCTTGGTGAGCTGCAGGTACTTATTCAACTCAACAAAATGCGATCCTCATTACGAAAAAAGCGTGTTT
CTTGGTTTTGAAAAACAACATGGAATGCAACAACGGTCTGAAAAGCAGCTGCTACACAGATGTTGAA
ACTGGTATTATCAACACATAGACTACAATGCAAATAGATGGACATTTTTTTT
>TCONS_00006655 geneID=XLOC_001308 scaffold_1071:19770-22914(+) Intergenic
TTACTAGCTGATGAAAAAGACGACCCACCAACTACTAGTATGTCTCTTCCATCATAAAGGACATGCAAA
CAGCTGCTGAATTTATATTTTTTTTGACTAGAAGGTTATTTACATCCTGGAAGTAACATGCCATTCAATG
GTGGAATACCACAAATTCAAGAATGGAAGGTGCTAAACATGCCATCAGACCTAACATGATATTCTTGAC
TGTGGACACTACAAGGACCAAGTGATTGAGACAGAGTGGTGGCAACACTGACTACACCACCGGGTTG
AAATGAAATAGAACAGATATCTGCAGCACACATTTTTTGCAGGACAGGACCGAATGAAATGATAAAAA
TGCTGCAACAGAGACACGGGATTGCTGAATTTCCATAATAAACGTCTCTATCTTCGATCGATCTACTTAT
CTGTAAAGCTGCTGATACGAGAAAAAGGGCGTGGGACATCAATGAATAAACTCAAAAACATCCAATAA
GTTGATCTGGAGTTGTCTTGGACTTGACGAACATCGTACATCCCGTTGAACAAAGTAACATGATCTGCA
GTATTCTTGGTCACCATTGGATTAAATACAATGAAGATTGTTGTAATATCTCCTGATTTCTTGGTACTTAT

TCAACTCAACAAAATGCGATCCTCATTACGAAAAAAGCGTGTTTCTTGGTTTTGAAAAACAACATGGA
ATGCAACAACGGTCTGAAAAGCAGCTGCTACACAGATGTTGAAACTGGTATTATCAACACATAGACTA
CAATGCAAATAGATGGACATTTTTTTT
>TCONS_00006670 geneID=XLOC_001308 scaffold_1071:20177-21036(+) Intergenic
AACATACCACTTTACTGAACAAATATTTGTACTTATAACTTATGAGCTTAATCTATAAATCATCTCTAACA
AGATTTTTCATTACAGGTTATTTACATCCTGGAAGTAACATGCCATTCAATGGTGGAATACCACAAATTC
AAGAATGGAAGGTGCTAAACATGCCATCAGACCTAACATGATATTCTTGACTGTGGACACTACAAGGA
CCAAGTGATTGAGACAGAGTGGTGGCAACACTGACTACACCACCGGGTTGAAATGAAATAGAACAGA
TATCTGCAGCACACATTTTTTGCAGGACAGGACCGAATGAAATGATAAAAATGCTGCAACAGAGACAC
GGGAT
>TCONS_00006662 geneID=XLOC_001308 scaffold_1071:20186-21330(+) Unclassified
CTTTACTGAACAAATATTTGTACTTATAACTTATGAGCTTAATCTATAAATCATCTCTAACAAGATTTTTC
ATTACAGGTTATTTACATCCTGGAAGTAACATGCCATTCAATGGTGGAATACCACAAATTCAAGAATGG
AAGGTGCTAAACATGCCATCAGACCTAACATGATATTCTTGACTGTGGACACTACAAGGACCAAGTGA
TTGAGACAGAGTGGTGGCAACACTGACTACACCACCGGGTTGAAATGAAATAGAACAGATATCTGCA
GCACACATTTTTTGCAGGACAGGACCGAATGAAATGATAAAAATGCTGCAACAGAGACACGGGATTG
CTGAATTTCCATATAAAATCCAGAATAAACGTCTCTATCTTCGATCGATCTACTTATCTGTAAAGCTGCT
GATACGAGAAAAAGGT
>TCONS_00006650 geneID=XLOC_001308 scaffold_1071:20302-23171(+) Intergenic
GGAATACCACAAATTCAAGAATGGAAGGTGCTAAACATGCCATCAGACCTAACATGATATTCTTGACTG
TGGACACTACAAGGACCAAGTGATTGAGACAGAGTGGTGGCAACACTGACTACACCACCGGGTTGAA
ATGAAATAGAACAGATATCTGCAGCACACATTTTTTGCAGGACAGGACCGAATGAAATGATAAAAATG
CTGCAACAGAGACACGGGATTGCTGAATTTCCATATAAAATCCAGAATAAACGTCTCTATCTTCGATCG
ATCTACTTATCTGTAAAGCTGCTGATACGAGAAAAAGGGCGTGGGACATCAATGAATAAACTCAAAAA
CATCCAATAAGTTGATCTGGAGTTGTCTTGGACTTGACGAACATCGTACATCCCGTTGAACAAAGTAAC
ATGATCTGCAGTATTCTTGGTCACCATTGGATTAAATACAATGAAGATTGTTGTAATATCTCCTGATTTCT
TGGTGAGCTGCAGGTACTTATTCAACTCAACAAAATGCGATCCTCATTACGAAAAAAGCGTGTTTCTT
GGTTTTGAAAAACAACATGGAATGCAACAACGGTCTGAAAAGCAGCTGCTACACAGATGTTGAAACT
GGTATTATCAACACATAGACTACAATGCAAATAGATGGACATTTTTTTTTTGATGTATGCACATTGAACA
ATGAACAAAATGCAACCCAAATGATTGAAGAATAACTACCAGTCCTTTG
>TCONS_00006651 geneID=XLOC_001308 scaffold_1071:20302-23171(+) Intergenic
GGAATACCACAAATTCAAGAATGGAAGGTGCTAAACATGCCATCAGACCTAACATGATATTCTTGACTG
TGGACACTACAAGGACCAAGTGATTGAGACAGAGTGGTGGCAACACTGACTACACCACCGGGTTGAA
ATGAAATAGAACAGATATCTGCAGCACACATTTTTTGCAGGACCGAATGAAATGATAAAAATGCTGCA
ACAGAGACACGGGATTGCTGAATTTCCATAATAAACGTCTCTATCTTCGATCGATCTACTTATCTGTAAA
GCTGCTGATACGAGAAAAAGGGCGTGGGACATCAATGAATAAACTCAAAAACATCCAATAAGTTGATC
TGGAGTTGTCTTGGACTTGACGAACATCGTACATCCCGTTGAACAAAGTAACATGATCTGCAGTATTCT
TGGTCACCATTGGATTAAATACAATGAAGATTGTTGTAATATCTCCTGATTTCTTGGTACTTATTCAACTC
AACAAAATGCGATCCTCATTACGAAAAAAGCGTGTTTCTTGGTTTTGAAAAACAACATGGAATGCAAC
AACGGTCTGAAAAGCAGCTGCTACACAGATGTTGAAACTGGTATTATCAACACATAGACTACAATGCA
AATAGATGGACATTTTTTTTTTGATGTATGCACATTGAACAATGAACAAAATGCAACCCAAATGATTGA
AGAATAACTACCAGTCCTTTG
>TCONS_00006652 geneID=XLOC_001308 scaffold_1071:20302-23171(+) Intergenic
GGAATACCACAAATTCAAGAATGGAAGGTGCTAAACATGCCATCAGACCTAACATGATATTCTTGACTG

TGGACACTACAAGGACCAAGTGATTGAGACAGAGTGGTGGCAACACTGACTACACCACCGGGTTGAA
ATGAAATAGAACAGATATCTGCAGCACACATTTTTTGCAGGACAGGACCGAATGAAATGATAAAAATG
CTGCAACAGAGACACGGGATTGCTGAATTTCCATAATAAACGTCTCTATCTTCGATCGATCTACTTATCT
GTAAAGCTGCTGATACGAGAAAAAGGGCGTGGGACATCAATGAATAAACTCAAAAACATCCAATAAG
TTGATCTGGAGTTGTCTTGGACTTGACGAACATCGTACATCCCGTTGAACAAAGTAACATGATCTGCAG
TATTCTTGGTCACCATTGGATTAAATACAATGAAGATTGTTGTAATATCTCCTGATTTCTTGGTACTTATT
CAACTCAACAAAATGCGATCCTCATTACGAAAAAAGCGTGTTTCTTGGTTTTGAAAAACAACATGGAA
TGCAACAACGGTCTGAAAAGCAGCTGCTACACAGATGTTGAAACTGGTATTATCAACACATAGACTAC
AATGCAAATAGATGGACATTTTTTTTTTGATGTATGCACATTGAACAATGAACAAAATGCAACCCAAAT
GATTGAAGAATAACTACCAGTCCTTTG
>TCONS_00006647 geneID=XLOC_001308 scaffold_1071:20983-23905(+) Intergenic
ATACAGGACAGGACCGAATGAAATGATAAAAATGCTGCAACAGAGACACGGGATTGCTGAATTTCCAT
ATAAAATCCAGAATAAACGTCTCTATCTTCGATCGATCTACTTATCTGTAAAGCTGCTGATACGAGAAAA
AGGGCGTGGGACATCAATGAATAAACTCAAAAACATCCAATAAGTTGATCTGGAGTTGTCTTGGACTT
GACGAACATCGTACATCCCGTTGAACAAAGTAACATGATCTGCAGTATTCTTGGTCACCATTGGATTAA
ATACAATGAAGATTGTTGTAATATCTCCTGATTTCTTGGTTTTGAAAAACAACATGGAATGCAACAACG
GTCTGAAAAGCAGCTGCTACACAGATGTTGAAACTGGTATTATCAACACATAGACTACAATGCAAATA
GATGGACATTTTTTTTTTGATGTATGCACATTGAACAATGAACAAAATGCAACCCAAATGATTGAAGAA
TAACTACCAGTCCTTTGAATTTTGCTCCATCTACACCATGGAATTTCTCAACGATGTGAAAGTAAAGCT
GCATAATTGTATTAAACAGGACAGCACAACGATATGTATCTACAAAAAAAAATACTGTGCAACATTCTGC
TTTTTTCATGGATTTTGATCATCTTGAGACTCCAAATCAAAGAATAACTAAAC
>TCONS_00006648 geneID=XLOC_001308 scaffold_1071:20983-23905(+) Intergenic
ATACAGGACAGGACCGAATGAAATGATAAAAATGCTGCAACAGAGACACGGGATTGCTGAATTTCCAT
ATAAAATCCAGAATAAACGTCTCTATCTTCGATCGATCTACTTATCTGTAAAGCTGCTGATACGAGAAAA
AGGGCGTGGGACATCAATGAATAAACTCAAAAACATCCAATAAGTTGATCTGGAGTTGTCTTGGACTT
GACGAACATCGTACATCCCGTTGAACAAAGTAACATGATCTGCAGTATTCTTGGTCACCATTGGATTAA
ATACAATGAAGATTGTTGTAATATCTCCTGATTTCTTGGTACTTATTCAACTCAACAAAATGCGATCCTC
ATTACGAAAAAAGCGTGTTTCTTGGTTTTGAAAAACAACATGGAATGCAACAACGGTCTGAAAAGCA
GCTGCTACACAGATGTTGAAACTGGTATTATCAACACATAGACTACAATGCAAATAGATGGACATTTTT
TTTTTGATGTATGCACATTGAACAATGAACAAAATGCAACCCAAATGATTGAAGAATAACTACCAGTCC
TTTGAATTTTGCTCCATCTACACCATGGAATTTCTCAACGATGTGAAAGTAAAGCTGCATAATTGTATTA
AACAGGACAGCACAACGATATGTATCTACAAAAAAAAATACTGTGCAACATTCTGCTTTTTTCATGGATT
TTGATCATCTTGAGACTCCAAATCAAAGAATAACTAAAC
>TCONS_00006671 geneID=XLOC_001308 scaffold_1071:21139-23430(+) Intergenic
TTTTAATATCGCCCCAGTCTGAAATTGTCATGTTGATCGATTATTATGATTGTTTCCTTTGAAGAACCATA
CATAGCTTATTATTATTATTTTTCAACATTTATGTTATTTTTTTCAGATAAAATCCAGAATAAACGTCTCTA
TCTTCGATCGATCTACTTATCTGTAAAGCTGCTGATACGAGAAAAAGGGCGTGGGACATCAATGAATAA
ACTCAAAAACATCCAATAAGTTGATCTGGAGTTGTCTTGGACTTGACGAACATCGTACATCCCGTTGA
ACAAAGTAACATGATCTGCAGTATTCTTGGTAAGAAAATAATCTATTGTTTGTGTACCTGTTTTTTTTTT
CACATAACATGATGAGATCGAAACCCCAATGCCTGCAGAATATACAATGTTGATCAATGGATCTGATTAT
ATTGTTAATCCATAAATCCATTACCACTGATAATATTGTAGTCTAACCAAAAATATATCTTTACAGGTCAC
CATTGGATTAAATACAATGAAGATTGTTGTAATATCTCCTGATTTCTTGGTACTTATTCAACTCAACAAA
ATGCGATCCTCATTACGAAAAAAGCGTGTTTCTTGGTTTTGAAAAACAACATGGAATGCAACAACGGT
CTGAAAAGCAGCTGCTACACAGATGTTGAAACTGGTATTATCAACACATAGACTACAATGCAAATAGAT

GGACATTTTTTTTTGATGTATGCACATTGAACAATGAACAAAATGCAACCCAAATGATTGAAGAATAA
CTACCAGTCCTTTGAATTTTGCTCCATCTACACCATGGAATTTCTCAACGATGTGAAAGTAAAGCTGCA
TAATTGTATTAAACAGGTC
>TCONS_00006663 geneID=XLOC_001308 scaffold_1071:21435-23111(+) Unclassified
TGTTGATCGATTATTCTGATTGATTTGAAATAATTTGTTCTTGGATAATTGGATCTTGTTTTTCCTAATTCT
CATTCCAATAATGTTTTTTTTTCTCAGGGCGTGGGACATCAATGAATAAACTCAAAAACATCCAATAAG
TTGATCTGGAGTTGTCTTGGACTTGACGAACATCGTACATCCCGTTGAACAAAGTAACATGATCTGCAG
TATTCTTGGTCACCATTGGATTAAATACAATGAAGATTGTTGTAATATCTCCTGATTTCTTGGTACTTATT
CAACTCAACAAAATGCGATCCTCATTACGAAAAAAGCGTGTTTCTTGGTTTTGAAAAACAACATGGAA
TGCAACAACGGTCTGAAAAGCAGCTGCTACACAGATGTTGAAACTGGTATTATCAACACATAGACTAC
AATGCAAATAGATGGACATTTTTTTTTGATGTATG
>TCONS_00006667 geneID=XLOC_001308 scaffold_1071:21435-23111(+) Intergenic
TGTTGATCGATTATTCTGATTGATTTGAAATAATTTGTTCTTGGATAATTGGATCTTGTTTTTCCTAATTCT
CATTCCAATAATGTTTTTTTTTCTCAGGGCGTGGGACATCAATGAATAAACTCAAAAACATCCAATAAG
TTGATCTGGAGTTGTCTTGGACTTGACGAACATCGTACATCCCGTTGAACAAAGTAACATGATCTGCAG
TATTCTTGGTCACCATTGGATTAAATACAATGAAGATTGTTGTAATATCTCCTGATTTCTTGGTACTTATT
CAACTCAACAAAATGCGATCCTCATTACGAAAAAAGCGTGTTTCTTGGTAAGTAAAAAAACTAACGTG
TAATACAAGCACTCACACTTATAATCCCTCACGGGACACAGGTTTCATGATGAGATCGATATATCGCCCC
AATGCCACTGAACAAAAATGTAGATTGATGGTCCTGATTTTATATTTCCATACGTACTGGAGAAGTTTAC
ACATAATTAGAAAATAAAGCAAATTTGTGTTTCCCAGGTTTTGAAAAACAACATGGAATGCAACAACG
GTCTGAAAAGCAGCTGCTACACAGATGTTGAAACTGGTATTATCAACACATAGACTACAATGCAAATA
GATGGACATTTTTTTTTGATGTATG
>TCONS_00006672 geneID=XLOC_001308 scaffold_1071:22375-23430(+) Intergenic
GATCGATATATCGCCCCAATGCCACTGAACAAAAATGTAGATTGATGGTCCTGATTTTATATTTCCATAC
GTACTGGAGAAGTTTACACATAATTAGAAAATAAAGCAAATTTGTGTTTCCCAGGTTTTGAAAAACAA
CATGGAATGCAACAACGGTCTGAAAAGCAGCTGCTACACAGATGTTGAAACTGGTATTATCAACACAT
AGACTACAATGCAAATAGATGGACATTTTTTTTTGATGTATGCACATTGAACAATGAACAAAATGCAA
CCCAAATGATTGAAGAATAACTACCAGTCCTTTGAATTTTGCTCCATCTACACCATGGAATTTCTCAAC
GATGTGAAAGTAAAGCTGCATAATTGTATTAAACAGGTC
>TCONS_00006816 geneID=XLOC_001337 scaffold_1079:1465-2555(+) Intergenic
GTGCTCATTTTATACCTTGTTTCACGGGGAAAGACATCTGACAGAACCCTTGTTACATTCGAATAAGGG
TAGTTTTTGTTTGTTTCAGATGACTTTCCCCGTGAAATGGAATATAGGCTGTAGGAAAATTTATAGCAAT
GCTTGATAACATAAAGCTATAATTCTGCACACCCACCTTTTTTCAGAAAGAAATTGCGAGACTATTGAG
AAATAATACAGGTCGGGTGTCAGATAGTATTTTATTTATTGAAACATAGTAAACACGGGCGAGTATAAA
GGTTTGTGATTGTCTCTTAGTTGGACTATACACTGGACAAATTTAAAAAGCTATGAGCTGCCCAGACAC
TGCACTACCGCCCCTACCTCACCTTCCAGGTCTAAGCACGGCGAATACACTAGTAAATTAACAAAATCG
AGTGCCGGTACCTCCGATCTACGTCTGGAAACACGACGACTCTGCCGAAAAACTGTCCACACGCTGTA
ATGTGGGAAAGACACACAGCCACTGCACTTGTGTCGCCGCTTCACGTTTTATTTAAAGACGTGCTAAA
ATCTGCAGACTTCGGTCGTTCTAATGCAGTGCGAATGGCGCATAGCTGAATAAATAAGAGTAAAAACT
GAAATGACAGCACGTCAAGAACAAGACCTCATTAGAAGACGGAATTATGCGTTTTGTACCTCGTGAGC
AGCATCGTTGATTATGTGGACGTTTTTATGAACTCACAACCGTTACATTAGCCA
>TCONS_00007207 geneID=XLOC_001364 scaffold_108:5537-18588(-) Intergenic
CGGTGTTCAGATGATTTTGGTGTTTGTCTATGATTTTGTGTTGGTTGTGATTTTCGTTTTGTGTGTAAAG
TGATAATATCATAACGTCGCCGCGGCAAAATCAAATTTTAAATTTGGAAGCCTTCGAGAATCAAGACTA

GTGATGTACCACCGGCCATGTATCACAGATAATGATTGGATACGCCACGCCTACCGCATCAATCAAAAA
CGCGAGGGTGGCTGACCGAAACCTGTTGAGCTCTTCCTTCACGCTTCTTCACCCCTTCGTGCCCTAGT
ACTCCTTGAGGTTCCAGGGGGATGGGAGCTCAAGTGAGGGTGGCCAGGAAGGATATTTTGCGAAAGC
ATGGATGTCTTGTTAGACGCAATGTCGCGGGTGGCAAAAAATAAACATGTGAAACGAGGCAACCTGTG
AAGAAAACACAAAAAGATGGAAAGTTTGTAGTGTGCTGGCGTGACGGACTCATTTAAACCACTTGATT
AATGTTATAAAAGACAAAGCGGTCCAGACACTTCCGGTGAGCGTAGCTGTTTCCGGCTAAAACGACGC
CATCTTTGAGAAAAACACCGGAAATTCCAAGAGTTGATGCTGGTTTTGTTTATTGAGAAACATAATAAT
ATGTATTATTATACTTACCTACTTATACCTAAAATAATAAGTTCTACCAATAGCTTAAGAAACTTTAGGTAT
GTAAGCTATAAAATTTACTTTGAAATTATACAATCTCATGAATATTTGAGTAAAAAAAACCATTAACCGG
GAACCAGAGACAATTTTGTTTCTTATTTATATTAATGCTAAACGACCACACTTCCCAAAATTGGAAGGA
GGCTGCGCGAAAAAATAAATTCGAACATGGAACGGGAGTTTTTTTTTCATAACGCTACATTAGTGTTG
GTGCCACAAGTTATTTATGGTAGGCATTTCGTGAATTTATGTCTTAGCCGAAATGACGGGGGGCTGGCA
ACCCCGTAAGTAAAATGTCATTTGGTGTCCCTTCTCTAAGATATTAGTCGTCATTCGTATGTATTAACTGC
AGTGAGAATGGGTGAAAAAAGACGGGTATGTCTGTAATGTGGCATGGGATAGATAATTTGATTTCCTTA
CCATGCGGTAGCTTTACTTGGATGTTGTTTTACACTCACGAGCAATAGAAAAGTTCCAGGGACAATAGC
ACAAAAATACTTCTAAACGGAAAAGGCTAGCTTAATATTGAAGAATATTTAAGAGATCGCTCTCAGCGA
TAAGGCCGCCT
>TCONS_00007208 geneID=XLOC_001364 scaffold_108:5537-18588(-) Intergenic
CGGTGTTCAGATGATTTTGGTGTTTGTCTATGATTTTGTGTTGGTTGTGATTTTCGTTTTGTGTGTAAAG
TGATAATATCATAACGTCGCCGCGGCAAAATCAAATTTTAAATTTGGAAGCCTTCGAGAATCAAGACTA
GTGATGTACCACCGGCCATGTATCACAGATAATGATTGGATACGCCACGCCTACCGCATCAATCAAAAA
CGCGAGGGTGGCTGACCGAAACCTGTTGAGCTCTTCCTTCACGCTTCTTCACCCCTTCGTGCCCTAGT
ACTCCTTGAGGTTCCAGGGGGATGGGAGCTCAAGTGAGGGTGGCCAGGAAGGATATTTTGCGAAAGC
ATGGATGTCTTGTTAGACGCAATGTCGCGGGTGGCAAAACAAAGCGGTCCAGACACTTCCGGTGAGC
GTAGCTGTTTCCGGCTAAAACGACGCCATCTTTGAGAAAAACACCGGAAATTCCAAGAGTTGATGCTG
GTTTTGTTTATTGAGAAACATAATAATATGTATTATTATACTTACCTACTTATACCTAAAATAATAAGTTCT
ACCAATAGCTTAAGAAACTTTAGGTATGTAAGCTATAAAATTTACTTTGAAATTATACAATCTCATGAATA
TTTGAGTAAAAAAAACCATTAACCGGGAACCAGAGACAATTTTGTTTCTTATTTATATTAATGCTAAAC
GACCACACTTCCCAAAATTGGAAGGAGGCTGCGCGAAAAAATAAATTCGAACATGGAACGGGAGTTT
TTTTTTCATAACGCTACATTAGTGTTGGTGCCACAAGTTATTTATGGTAGGCATTTCGTGAATTTATGTC
TTAGCCGAAATGACGGGGGGCTGGCAACCCCGTAAGTAAAATGTCATTTGGTGTCCCTTCTCTAAGATA
TTAGTCGTCATTCGTATGTATTAACTGCAGTGAGAATGGGTGAAAAAAGACGGGTATGTCTGTAATGTG
GCATGGGATAGATAATTTGATTTCCTTACCATGCGGTAGCTTTACTTGGATGTTGTTTTACACTCACGAG
CAATAGAAAAGTTCCAGGGACAATAGCACAAAAATACTTCTAAACGGAAAAGGCTAGCTTAATATTGA
AGAATATTTAAGAGATCGCTCTCAGCGATAAGGCCGCCT
>TCONS_00007209 geneID=XLOC_001364 scaffold_108:9156-18588(-) Intergenic
CGGTGTTCAGATGATTTTGGTGTTTGTCTATGATTTTGTGTTGGTTGTGATTTTCGTTTTGTGTGTAAAG
TGATAATATCATAACGTCGCCGCGGCAAAATCAAATTTTAAATTTGGAAGCCTTCGAGAATCAAGACTA
GTGATGTACCACCGGCCATGTATCACAGATAATGATTGGATACGCCACGCCTACCGCATCAATCAAAAA
CGCGAGGGTGGCTGACCGAAACCTGTTGAGCTCTTCCTTCACGCTTCTTCACCCCTTCGTGCCCTAGT
ACTCCTTGAGGTTCCAGGGGGATGGGAGCTCAAGTGAGGGTGGCCAGGAAGGATATTTTGCGAAAGC
ATGGATGTCTTGTTAGACGCAATGTCGCGGGTGGCAAAGTAAGTAGAAATAAACATGTGAAACGAGGC
AACCTGTGAAGAAAACACAAAAAGATGGAAAGTTTGTAGTGTGCTGGCGTGACGGACTCATTTAAAC
CACTTGATTAATGTTATAAAAGGTAATAAATTCACTTTATCATGCCATTAAAAACATAGTATCAAACATTA

TCACCGAGTGTTTTAACATAACTAACCCA
>TCONS_00007243 geneID=XLOC_001342 scaffold_108:88463-145382(+) Exonic overlap (-)
GCGATAACCGTCGCCTGCGCACGCGACGCGACCGCGCGTTCCTAATTCAAAATACGAACGTTCCGAAG
TGAATTGTAACTGGCCAGTGTTTTTATACAATATTTTTCTTAATTTTCCAACTGCTGTGGTGTGTGTGTG
ACGGCTAGGGTTGACAGTAAACTTGTTATTCAGTGTTCTCGGATTGCGAATCTTCGTAGCACAGTGCAG
GTTGTATTATTGCTTTGTTGACTGCGGGCAGCTGTCAAAATATTTCTTGGAATATCAAGATTTACACTCT
GCTCTACTGGAAATCCAGGCATGATATAATAAGGT
>TCONS_00007195 geneID=XLOC_001342 scaffold_108:173034-174593(+) Intronic
TCCTACTCTCATGTAACAGACAAATCCACCCCAATATTCCATCGATACCAAAACATCAAAATACCGCCA
TCTAGTTTTTTTTTCTACAACCTAATACACTATTTAATTTATCAAATATATAGGTACCTAACTACTACTTCT
ACTTCAACTATTTTCCTACCCTCCGTAACTACCTAAGTCGAAAATAATGTTACTAGGTAACTAAAAATAT
TTCTGAGATATATCTCATCTCTCCCTCACAACCCCTATTTGAAAAACACGGTCTCCATATCCTTTATTCTT
TCCTATTCTAGACTGTTTGTGTAAATGTGCCTTACCTGTCTAACTGTCAAACTAAACTGTCAATGTCAAC
CTGACATAACCTTAACCTTTTCTTTTTTTACCGGCACTAAACAAAACAAAAGCATGCTTCGTTGTGTTC
AACAATATCGATCGCATGAAGAAATCTAAAGATTACCCGATTAAACTTATCAAACTCTTAAATAATGAGT
GAATCTAATAGGACCAAACATAACAATGCAAACCCATCTACACTCTTTGTTGAACTAAAACCCATGATA
AACCACTGATCAAACCATCCACTGTGACAAAACATTCACAATCCCTCACTACTTATATACCAAAGTTAC
TATTCTACAATAAGATATGACATACTTTTTGAATGCTGGTGATGATGATGATGACGACGATGTTCTATATT
TGACAACTAAACTCTGAGACTACCTATATGAAGTAAACATCCTGACTCAGGTGAAGAAGTGGATTGCA
ACGGCACTCAAAATAGGAACAACTAAACTACCCACATAATTCCTAACTTACAAAGAATATATTGATACT
AAATGAGGTCCTAGCCTACTTTACCTGGTCTAACACTTTATCAACAACAGTAACTTGCTCTACATTAATA
GTTTTCTAAATTTATTGTTATGTCTAACCGCT
>TCONS_00006878 geneID=XLOC_001368 scaffold_108:193230-195563(-) Exonic overlap (+)
AATACAACTACAGTGACGACAGCGATAGCGATTAGAGGAGCGGCGGTGGTTTGTGGTCCAAACTTGTA
AATATGAAAAATGATATTCGAGCAGCAAAATGAGAGTGAAAGCAGATCGAGGCAGCACCTTTAGGTG
AATTGATACTGACGTAATCTCTTTACTTTTACTGTCTACTTCTAAGCACACCTATGACTTACTGCTAGGTA
TTTATATGTGAAACTTAGTGATACGTAATTTTAAAGTAGAAAATATTTAGGTAATGTTCTTAACATTTTAC
TTAACCCTTCTATGGTAAGTTTGTACAAAATAAAAACATGTTTCTTTAACAGTGAAATAATTTAAGTATG
TACCTATCTGTTGTCTTTTAAGATAAAACTGTGGTTCACGATTTCGACTTGTAAACTATGTAATTAAATTA
CTTTGTATAAACTCACCAAGAAAAGGGATCCGGGATTTTCTCAGCTCAAAAATTCCCCCGTGACTGAA
TTGTGAATATTTTTGAGTGTTTACTCAATATTTTTGTGTACAAATGATTTGGTGGAATCCATTGAATTTAA
ATAACGTTTCTATATCTTGCTTTAAATAAATTTTAAAAGTCGACATACTATATTTATTTCTTATTGTTAATAT
CAGTATTCTTGTGTAATTGACATACTTACCATTATTTGTTTCACAGATTTCATACTTTACCTACACACTATT
GGGTGTTATAGTTATAGTGTACTATCTGAGAAATTGTATAATTTAAAATAAACAATTATTTTTGGAATCTA
AATATTTTACATGACATGCCTAAACTTTTCTAGAATTTTGTGATGCCATTTTTTAAAAGTTACCGTTTAAA
ATTTTATCTTTTTTCATTCCATTGTATTATGATTTGAGGATTTCTTTAGCTGGTTTAAATATAAATCCTATAT
TTATTACGATGTCAATTGTAATTATTGTTTTGTTTAGTTGTCGTTTCGTAGTATGTTCCATTCTTTATTATTT
TTCTATTGTATTTTTTCCTTACTTTTAAACTGTGACAAATGATTTTTGT
>TCONS_00006879 geneID=XLOC_001368 scaffold_108:193230-203184(-) Splice junction overlap
CCGGCCAAGGAGGACTCTCCCCCGCCCAAGAAGAAGCCGGCTGGCAAGAGAAAGCTGATGAATGTG
GACAAAGAGAAAGAGAAGGAGAAGCCCAAGAAGCGACCGGCCAAGGCGCTCGACAGTGACGAGGA
CGACAGCATATTTGACACGAGAAAGAACAAAAAACCCAAGAACCCCCCCAAAAAGTCTAAGAAGAA
GGCGAGCAGCGACTCCGAAAGCGATGCGCTAGTACTGAGCGACGACTCGCCGCCGCCCGCCAAGCCG
CCCGCGCGCCGCGGCAAGCAGACGAAATACAACTACAGTGACGACAGCGATAGCGATTAGAGGAGCG

GCGGTGGTTTGTGGTCCAAACTTGTAAATATGAAAAATGATATTCGAGCAGCAAAATGAGAGTGAAAG
CAGATCGAGGCAGCACCTTTAGGTGAATTGATACTGACGTAATCTCTTTACTTTTACTGTCTACTTCTAA
GCACACCTATGACTTACTGCTAGGTATTTATATGTGAAACTTAGTGATACGTAATTTTAAAGTAGAAAAT
ATTTAGGTAATGTTCTTAACATTTTACTTAACCCTTCTATGGTAAGTTTGTACAAAATAAAAACATGTTTC
TTTAACAGTGAAATAATTTAAGTATGTACCTATCTGTTGTCTTTTAAGATAAAACTGTGGTTCACGATTT
CGACTTGTAAACTATGTAATTAAATTACTTTGTATAAACTCACCAAGAAAAGGGATCCGGGATTTTCTC
AGCTCAAAAATTCCCCCGTGACTGAATTGTGAATATTTTTGAGTGTTTACTCAATATTTTTGTGTACAAA
TGATTTGGTGGAATCCATTGAATTTAAATAACGTTTCTATATCTTGCTTTAAATAAATTTTAAAAGTCGAC
ATACTATATTTATTTCTTATTGTTAATATCAGTATTCTTGTGTAATTGACATACTTACCATTATTTGTTTCAC
AGATTTCATACTTTACCTACACACTATTGGGTGTTATAGTTATAGTGTACTATCTGAGAAATTGTATAATTT
AAAATAAACAATTATTTTTGGAATCTAAATATTTTACATGACATGCCTAAACTTTTCTAGAATTTTGTGAT
GCCATTTTTTAAAAGTTACCGTTTAAAATTTTATCTTTTTTCATTCCATTGTATTATGATTTGAGGATTTCT
TTAGCTGGTTTAAATATAAATCCTATATTTATTACGATGTCAATTGTAATTATTGTTTTGTTTAGTTGTCGT
TTCGTAGTATGTTCCATTCTTTATTATTTTTCTATTGTATTTTTTCCTTACTTTTAAACTGTGACAAATGAT
TTTTGT
>TCONS_00006978 geneID=XLOC_001345 scaffold_108:226432-228684(+) Intronic
TCTGAGAAAATCTTTATTAAATTATTTAGTATTAGAGCAACGGAAAGTTATGTCTGTCTCAACATGGAAA
TAAACAAAAAGTAAAATGATTAACAAAAAATAGATTCGCTCTTCTATTTACATTGCTGACTTTGCGTCG
GTCAAACTAATAAAATACTAAATAAAACAAGTGTGAATACCATAGACGAAAGTGGTTTTTTACGATCGA
GAGAAAAGTTACAGTAAGTTTATCACTTATTTTACCTAACCAACTACCGACAATTTAATCAGAATAATTC
TACAAAATGCTTCGCTAGCTTGCTCCACTACAAGCTAAAGGGATTGGTGATGTTAGCTTCATAAAGTAC
CTAGTTAAAACAAAACATGGCATAAAATTGTCTCCATTACAGTCTTTTTTTTACTTTTTTGATATGTTTTG
AGGTGACAAACAAAGGAATTTGAAATCTAAATCTGCGTTTTCCTTTGACCATCAGTGGACCATCACCA
ATCCCTGTCTACAAATTAAGTACATATTTATTTCTATTTACATGTTTCATAAATATTAATTTTCTACCAATA
AGTTATGTTAAGGCGTCGTGAGGTCGCAAGCGGTGCGACTCCTAGGTAGGTATACTTATACTTGAAATA
TCAATTTGAAATTTTAAACTGTTTGTTTGATCACAGAAGGTACATTATATAAAAATAAAACGTGTTTTTT
GTATCTCTCACTGCATTGCAGTTACACTACATACATTTTTCATTATACCCATGGGTTTTCGCTGACGTCAT
CCAATGAAATGTATACAGATAAAATAAAACTTTCTTAGGCAATGATTGTACGCTTCATCAACATCAGATA
AGATAATATTTAGAAAAATATTCACAAAACTATCGTCTATATTGATTTTAAAATATTTAAGTTTCTCTTTA
GGTAATCACAAATGACTAAGTTAGGAAATACATTAAATAGATTTTCGATAAATATAAGTGTTACTTTAAG
TTTTTGTTTAACAGTTTTACCTTTTACAGAATTGGACACGGTCCAATGTCCACACATGTCGGTTCGTTGT
CAACACATTTATTCAGATATTTCCATAAAACGACATGAATTGGCACCCTCTCATCTCAGTCTCAAACCTA
TAACTCCTAGGTACAATATCCATAGACAATTAAATAAGTTCCTAAATTAAGAAAACATACTATTTCTTAAT
CCGAATAAGTTGAGAGATCTAAACACACATAAGTAATTATTTAAATAGTTAATAACTCCACAATATACAC
TGACTTTTTGTTACAATATTTTAATATTAACATTAAGACAATTAATTAACAATTTATATTACAAACTAGTC
GAATCCAGGTGTTTAAGTTACCTTGTTAAAGTCAAGTAACAAGTAAGGACTGCACAATATGCCAATGA
CATTTCTATTTGTAAGTATGAAGCAGATGCTTATTCTTTAATTTAGTTTGTATGTTTGTTTGTACCCGAAA
TGCGAATAAGATAATATAGCCAAGCGCGTAAAGTGATTGTTTTTAATTAAATGCTTGAGCCATGAATACT
TTAATTAATTTAGCATCTGCGACAACAGAAAGAATTAGTCCTATATCTATTTATTTAGTCACTGTCTACAA
TTACAGCCCCATTTTATTGGCAGCTAGTGGAGAAGGCAATCATACCACAAACAGGTTATAATCTTGGTC
ACCATAATCATCATCTATAGATAGACAACATCACAAACAAAGCCGCGAACCGTTGTCATCGTCAAACCA
TACAGTTTACAAACGTCAATTATCACCGTCCACCATTTTGTCAACATGCGCCATCTTGAAACTACAGTT
TTTTAACCTAAAACTTCCTTGCTTACTGTAGCGCTGCTATCTGTCAAATTCCTAAAAAATTAAGTCACTTT
GCAAGGCAGTTTTTCTTTTACAGCGTCTATTTTACAAATGGCGGTTATACTCATCATTTTGTCAAAGCAT

TGGTGTTGTGTTAGGTACTAGCGTTCGGGTCCCCGAGTGTTCCCGAAGGCACCCGAAGTTGTCCGAAG
GCACCCGAAGTTGTCCGAAGCGTCGTTTAGAACCGGTCGCGCCGGCGCGCTAGTCAGTCCGCGATGT
AACGCGCATGAGTTGTGTCGTCGTCATCGTCTCGTCCCGGCCCTATGCTCTCTATTTCGTCGACTTCTTC
CAGGTTCTTCGTGCTTGGGTTGCATAGCCT
>TCONS_00007060 geneID=XLOC_001369 scaffold_108:323602-356525(-) Unclassified
GCTCACCTGCGGGCCCGGTGGCTGTGTGAGTGCGCGCCGCTCAGTCCAGCCGTCAGATTGGCGCGCT
CAGGTCGCCCCGCAGTCCACGTGTCTGATAACTCTGTTGTTAAACAGGCTCGCCCGCGCTCGCAACTG
CGGAGGCCACGATTGTTGTTTTTTTTTAAATTTCGTGCTGACATTGATTGATCGGGTTAGAGGACAGTG
CTAGTGGACGTGTTAGTGGCGAAGTGACTTTTAGGACGTTGAATAGTCCTGCCTTATCAGCAAACTGA
GGAATATTTGGCCAGCGGAAACCTATTTTTGGAAATATCTCAAAGGCGATGCGGGGCTGGCTCCGATG
CTAGCGTCCCTGCCGCGCCGCGAGCTGGACGCCGAGGCG
>TCONS_00007391 geneID=XLOC_001369 scaffold_108:323602-356525(-) Intergenic
GCTCACCTGCGGGCCCGGTGGCTGTGTGAGTGCGCGCCGCTCAGTCCAGCCGTCAGATTGGCGCGCT
CAGGTCGCCCCGCAGTCCACGTGTCTGATAACTCTGTTGTTAAACAGGCTCGCCCGCGCTCGCAACTG
CGGAGGCCACGATTGTTGTTTTTTTTTAAATTTCGTGCTGACATTGATTGATCGGGTTAGAGGACAGTG
CTAGTGGACGTGTTAGTGGCGAAGTGACTTTTAGGACGTTGAATAGTCCTGCCTTATCAGCAAACTGA
GGAATATTTGGCCAGCGGAAACCTATTTTTGGAAATATCTCAAAGGGTGTCGCAGGCGATGCGGGGCT
GGCTCCGATGCTAGCGTCCCTGCCGCGCCGCGAGCTGGACGCCGAGGCG
>TCONS_00006932 geneID=XLOC_001378 scaffold_108:723969-725442(-) Intronic
ATTGCAACATCCTGTATAGTTACAAATATTTATGGCTTTCAAGATTTTTCTTCGTATATTTTAGTAGGTATG
AGTTGGTGACTAGAAATCTACCATTCTAGAGTCAGAAGTACATTTGGATAGCCTAATGTTTGGTCCTTC
ATCTTTTGTATATAAAAGTCTAGGTTACACCGTGTCTGTACATAGGTTTTTCGAAATTTTTGCCTTCAAG
GTCTTTCAAGTCAAGTTTGTCGTTTGCGTTTTTAACTGTAGGTCTTAGTTAGGTTTTAGATTTATATTTAT
CGGCTGTTGACTATCGGTAGGTTTATCGGTAAATCATCTATACTACCTTTGCCTTTAGCTACTGAAGTTTT
AGGTGTGTTTTCTTCTTTGGGGACTTTTATAAGCCCTCATTTTTGTCTCCTAGGTTCTTCTAAGATATGGA
TCGATAATCCTTCGCAAGGAAATTTACACAATTAGTGTTTTTTTTTAAATAATATTTGTAGCGTGATAACT
TAATGAAATCTTAAAAACAAATCTGTTTTTGTAATTTTATTATTAGAGGCTTAATTTTTGGGGTAGTATTT
TGTTTGTTATGAAATTCATATCTCGTATCACAACGCCATCTAGTAGTCACTAGAGTAAACTAAATTAGGT
TTATTTTTGTGAGTTTACACCTAACTAAATACATTTTACTATACATACCTACATACTTATTGATTTGTTTGG
GGTTTTTAATAATATTTTAAGTTGTTTTTGAAATTGCTGTGACGATTTCAATTGTAAAATATTATTTTTACG
CGCTCATGATTGTGAAAATGGGGTTTTTTTAACAGTGAGTGAGAATAGAATAGAAATTGATTAAAAAA
ACACTGTTAAAGTCTCCCGTTTTCTTGTTTCAATAAATTGCTATTGTTTTTTGTTTTTATTATCTAAGATAT
TTGTAAATTATCCAACTAGTTTTAGTTTAAATATGTATCTGACTTAAGTCTGTTGTAAAAAAAAATACTTCG
AAAAGATCTGTATACTACCGCGTGAAAAGAATAAAAAAATTACTGAGAGCGGTACACAAAATATATTTT
GAAATGTCAGTGCTTATTTTTTTAAAAGGCGGTTGCTCAAATTATCCAAGACAGAATTGTAACACATGA
AAAAAATAACTTGTGACCAACACTATGTTTTTGTAAAAAAGATGATTGTAAAAAAAGATGGCGACCGC
CAAATAAAAAAAAAATGGTTCGTTGTTTCTTGAACGCAAACGATTATGAATATTATGATGTAGCTTCCC
GCTACTC
>TCONS_00007322 geneID=XLOC_001379 scaffold_108:745700-747628(-) Intergenic
GCAGACTCTAGTCAAGGTTATGCCACTGACGGAATTATTGGAGGAGCCAGTCGACCAACCTCCCAGGA
GAAGAGATAGACGGGGAGATTCTCTAAGGGACATAATAGACCGCCGCCGCCGCCGCTCGCGGTCGTG
GGAGCGTCGCGGCAGACGACGACACGCGTCGCCCGACGACCCGAGGCGACATGATAAGAGACGTCC
TCCCTCCAGATCGCCGTCCCCGCGTCGGTATAGGAACAGGAGTCCGCCGCCGCCGGCCATGTTGGATC
GACCACCCAGCCGCTCCAGGTAACATCATCATCATCAGCCTTTAATCTTCCACCGTTAGATATAGGCAA

AATGAGCGTCACAACACTCAGTCCTCGGCGTTCCTCATTCAGCTAATACTGTTTAAGGGTGCTTACATA
AAGAATCGGGTTCCCTGGATCTACCCGTACCGGTAACTTGATTAGGTACTGCTCACTGAGAGATTTAAA
ACCGGGAAATGCTCTAGCGCACTCGCAGAAAGATAAACAGCAGGTTACCGGTACGGGTAGATACAGG
GAACCCAGTCTCTCTATGTAAGCA
>TCONS_00006936 geneID=XLOC_001380 scaffold_108:850376-887830(-) Exonic overlap (-)
AAGAGCTCGATATTTTAACAGCCAGTTTATTTCCATAATTTTTAAACATAATAAATAATTAAAAAATCAA
CAGACACCATTATTACAAGCATACTTTATAAACCACATTTTAACCATTTTTACTAACATTCGCTACAACTT
TACTGTTCTATTGTACATACATACAGAGTAGATAAAAACGATAAAACTTTGGTTGAGTTCATTGTCAGA
AATGTCGATTTTGGAGTGCACATTTTGTGCTATACTTTCTAAAGTATGTTAGCCACTCAAGCTAGGTTTA
AACGGCATTCTTGACATAGGATGTAATTCTTTTATAAAATCTAGACATTGATGTGTTCCATCCAAAAAAC
CTCTTTCAGATTTGTCGGATGACATGTTTTAAATAAAAAATACACGCATGGCCTAAGAATTATAAATTAG
ATAAACAAAACAACATTTGAGCAATAATTAAATACATTAACTAAACCCGACATTGCAAACTCTGTCATG
ACAATAAAATGGAAATCATTATGAAAACATACTTAATATTCGTAAAGGCAGTATACAAATAGTTTTGGCA
AATTCATTACTAAATAAACATAAGGAGTATTCAAGCGAGCCATGGTATGTGCATTTCGAATATAAAAATA
AAATAATAATTAATATGTTTTTTTTTGTCTTTGAGACTAATAAAAAATGCCCTTTTTTACAAACATATCCC
CAAACCACACTGTTTCAGATATACGAGACCCTGCATTATCACTCATTGCTTTTAAATAACTGAATAAAAT
GAAACTATTTTCATCAACATAAACTCATCAAGTGTCCTTATTATTACAATTTTGTCATACCAACATCGTAG
TCACTTCAAACTACGGTATATTTATATTGATTTCGGGACTGATAATGATAATAAAATGACATCATTGTACA
AAAGGCTCTCCCATAAATTCATATTCGTTGATATAAATAAGAAAATCGAAAGATCTGACTTTTAATGTTA
AAATGGCTACATTTTGTGACATAATTATGTTTTTAATTAAAAAATATAATATAGCCTAACTACAGTGGACC
CCAAAAATTATAAATAAATAATTATTCGACCCGCACCGGCCATGAGATTAAACCCACATATTTACTACAT
AATAAGTATATAACAGTATTTCTAATGACCGGTGCAAGCTATTAACTAAGGTATTCGCTATATACTAAATA
AATATAGGTACATATCCATGCCCAAAAGCGATATATTGAGGAATGTTTTAATTTACAACAGGTTTTGTAC
GTTTACAGAAATCTATACAATAGTATGTGGTACCTTTTTAGTAAATGTAGGACAATCTAAAGATATTCAG
ATATCTTAGAACCTTTTACAAACCGATTCGAAAAACAAAACCTATATTTTAACTGAAATACAGTTTAAA
AGTTTAAACTATCGGCTCATTTTTCTGTTTCATATTCTCGATTACAGGGCCTCGAGTGCGAGTTTTTTCA
CCTATCGAGAAGTGAAATCGAAACGCAAATTTGTATGGCGCGGGAGACACGTCGCCTAGCGGTAAGTA
GCTGTACTAGCTCCGCGCCATACATTTTCGATTTCACTTCCCGATAGGTAAAAAAACTCGCACTCGAGG
CACAGTTTATTTATATACTCACTTATTGCATAACAATTTTATCCAATAAATAGTGATTCGATCTACGATTAT
TCAGAAAATAATTCCCATATTTTTTTGGTTCTATGAAAAATAAATACAACTCTGATGTACATAGAGTTGG
TATATTTTCT
>TCONS_00007667 geneID=XLOC_001509 scaffold_109:131411-141868(-) Unclassified
CCGGCGGCGCTAGTAGGGCGGCGGGAGCGGCATCGCGCGCGCGACCACAGTCTCATCTAAGCCACAG
CTATGCGAACATCTCCCCCCTAATAACCGAATATTCCACACTCGACATTCAAACCCGCGAGCCGACGCG
AAACTTTCAAATAACGAATCCGCGTGCATTGAATTTTTTCTTTTTCGTAACCGGCCAGCTTCAGTGACT
GTTCAAAATTTGAAGTGGCAGTTTCCCGCTAAACGGTAAAGTTTGATGTGTTGGTGTCTGTAAAAGTG
CGTAAAGAATTTTCAGCGCAAACGTATTGTTTCAAGTGTTTCCTTTTTGTGTATCGTTTAAAACATTTTG
TTTGTGTGGTGTTGTTTGGTTGTTATTTTATTTGTGCCCTTTATGATTTGGATTTTTTGCCGCTGGACTTG
TTGGTGCAACACCATGGGAAAGGATTTCGCGCGCGTTCTGACATCAGTGAATTAGTTTTATTTTTAAAA
AGTCAAATTTAATTCTCCATAAAAACAAAGATGCTGGGGCGTGCTCTGCAGAGTGTATCCTCAGTGTCC
CACAGTTTTGCCGCGAACTGCATCAACTTCAGCTCGGCTGACGACAGCCGGTGCTTAGACAAGTAGA
CAACAAAAAACAACAACAACAAAATATACTTAAATAAAATAGTAGAACAAAACTAGTCAAAGCAACA
CTCAAGCATCAATTAGATTAACGGTTACCAAAACGCGTCGCGCGGTCATAGAGTAGCGAGTGCACAGA
CACGCCACTTCTGTGGTATGCTTTATATTGTATTTTGTATCGACTGTGCGCTGACTGGTCGCTGCGTCTC

GTGGCTTCCCGGAGCGCGACTGCGGTGCTGGATGCTACTAACTGTATACACGT
>TCONS_00007692 geneID=XLOC_001516 scaffold_109:436045-446555(-) Splice junction overlap
GTCGCCCAGGGCGGACGTCCCACGGACCTCAATAATATATACCGCGAAATAATAACAACGAAAATAATT
ACCGAAAAATATGTTATAGTGAAAACTTATCGATATAAATAACGAAATTAAATAGAAAAGAAAAATCCA
TCAGTTAAAAAAATAATTTTAATTGATCGTGATGATTTTATCGCACTCAGTCAAGGTTACGATCCGCTAC
ACATGTGTTTAATGTGACTTTAAGTTTGTTTTGAAGAATGCAACTGTTTGAACTGCATGCATAGAACAC
GGTCGGTTAGCTAGTGATAATGCAAGTGCATGCTTCCGCCGCCCTTGGCTAGGCCATGGCTGCATCTAG
GCGGTTTCTGAAATTTGGAATTCGGCATTCGAATTAAATTGAATTTGGAAGCTCACGCCAGTTATGAGT
TCGCCAGATACTCCGAAAACCGCTTTTCATTTGGACATCATTTGAATTCCGAACAATATTTGTTGCCAG
CCAGTTCCTCTGAACACAAGGATAAATGTTTTGTTTTTGAACGAAGCCCGCTCTTATCAGATGTTTATTT
GACTAAAATTTGTGGAATAATTTAGTTTTAATTTAAAACTTGGTGTTCGTATTTGAAATTACCTATGTTTG
TTTGTGTTTTGTGCTAGTGAATGACCATGGAATATGACAGATTATGTGTCAGTGATACTGATAAGAAAA
GTGTGTCGGTGGGCTGAAATCTAATTGGTCTATCAACTGTCAAAATGTCATTGCAAGAGGCCTGTTCCG
AGCAAGAGGCCCGCGTCACCGAGCTGCGGTCCCTAGCCGCTCGCGTGGCGGCGGAGACAGGCTCCC
GGGCGCTCCAAGCCGACGCGGACGCACTGGCCCGACGCCTGCAACTGGTGTCCGGGGCGGTGCAGG
CGCTGGCTGACCTCGGTGAGGCGCGACAGCTG
>TCONS_00008594 geneID=XLOC_001469 scaffold_109:538573-593665(+) Exonic overlap (+)
GCCGCGCTCTGCGTTCCCGAACGGATCGCTCTCGCCATCTTGATTTCTGAACGTACGAAATAATTCGAA
CGCGTCAATGCTATTTGAATTCCGAACGGTCGGGATGAATTCCAAAATTAAATATTAAAAACTGTTGAA
GTTTTTGTATGTGTGAAGTGTGGTTGAAAGTTTGTTGAGACTGTGTACAGGAGTTTATGGAGTGATTTA
ATTTAAGGGTCGATGAACCGAGGGATGGAGTGACGTCACGTCCAGCGTACGTGTGGTTTGACCGGCA
GTGTTTCAAAGGAATGCCTTAGAAACCGTGATGTGAAAAACTGACCAACATGTGGTTCCCGCTTTTAG
TGTGGTTATTTACATATTTAGGGATGTCAGTGGGCGGCGACGGCAACGTGTCCTCCACTACAGAGACTA
CCCCGGCGCTGGA
>TCONS_00008409 geneID=XLOC_001518 scaffold_109:716159-719002(-) Intronic
CAGCCTGCTGGTGTGAGGACAGCGGCCCCCGCCCCCCGACCCCTGTCCCTGCAACTCGCAACAGAAA
CAAGAATGGGACAGAAATAAGGACATCGGGCGAGCTGTGCTTATGAAAAACAGCTTTTAGAGTGTCG
AAATAAGGTTTCAAATGGATAAAAATAAATAAGCGGGAAAACAAATACAAAAGAAGAGACACAAGTA
ATGTGAGTCATAAATATGGACGAATCTAGTTTATAATCATAAAAAAATGCTTATGAAAATGCGTATATAA
AAAATGCATAAAAATTTAACTTAAGTTACTTGTAGACGTTAATTAAGTTAGGCTAAATTAACACGCGGG
CCAAATGGTTGGAGAAAATTAACGAAAAAAAAGAATTTTTCGGCTTAAACAAAAAGGCGGGCAGATT
TGATGATTGACACATCAGAAGCACAATGGTAGAATAAATAAGATTAGACTAAATTAGTATTTTGGAAAC
ATGTTTAGTAGAAAGTAACATAGTAAAAAAAACATAGAACGGTTCACATCTAAGAGTGAGAAAAGTTT
TTTAAACATTTTTTATCTATGAAGATTGTTAGGAACGCTATGTGATGTGTCATAGAATTAGAGATACAATA
AAAACTGTAGTGGACAAACTTTACAAAGAAGTCACTAGTCATTTCAGACTTTTTTTTGTTTTTTGCAAA
AAAAAAATGGACAATGCCA
>TCONS_00007856 geneID=XLOC_001476 scaffold_109:1065759-1066281(+) Splice junction overlap
GCGGGAGCCAGAGCGAGAGGCGGCGAGGCGCGCGGCCGCGGCCAGAGAGAGAGATGAGAGACGAG
CACAGATGGAGAGAAATGCGAGGGCTAGCAGTGTGCCACAGAACTCGGGCGGAGCGCCCTCCTCCCC
CCTCCTCACGTCGCTGCTCAAGTCCTCGCCCGTGGTCACCACGCCGCAGCACATCTCACACCCCGCCA
ACAGCGTCG
>TCONS_00007732 geneID=XLOC_001526 scaffold_109:1150100-1151854(-) Unclassified
ATTTTTTTCACTCATATTTAACTTGAAATCCCATTTTTACGTAACTGTCCTTTTTAGTATTATTCTTGCTAG

TGTAAAAAAGGTTTTTACTCGTATTACGGTGTTGCCAGAGTGAAAATAATATAAATTGTGAGGACAATA
TTGTTTGCAATATAATACCCTTGACTTTAATGAAAATCATGCTAGTATCGTACAGTCAGCAAAAGAGATA
TGTATACCTCCCAGCGGAAGATTTACTGACGGGTGATTCATCTACTTTCCACCTGTGAGTCTTAGTGCC
AATTTCGCCATAGTCGGTTAGAGCATAACCAGGTACTTATTAGTGGTTGATTTTTGACACATCTGTCAAG
AGTTTAATATGGAAGTGACGTATTATTGGTCAACCTTTCCCTGGTTACGATCTAATCTAAATTGGAGCTC
AGCCCCAGTTTCTTCATAGTCGGTTATCTAACCGATCAGGGATTGATTCATCAATTATACGTGTCCTCCAT
ATAAAAATCTTGACAGCTGTGTCAAAAGTTAACCACTAATACCTGGTAATGCTCTAACCGACTAACTAT
GCAGAAATTGGCACTTAGGGTGAAACGGTTTGCTCTGTGGTTGGTTCTTAATCATTTCTGTTGACTGTA
CCTTTGTATTTAAATATCTTGCCTTGTGTAAATTATGGTTTGAGAAAGATAATACTATAGTGTTGTAAAGC
TATAAGGTTGTTGTTTTTTAATAATTGCGTTATTTGCATGGCAACAATTTTTATTTAAAACTACAACTATTA
TTGTTTGAATCAGCCTGTCAAATTAAAACCTTGTTAAATTTAAGATTAGTCAATAATCGTTGTTTTTTTT
GTATTATTTTATTAACTGTTGATAAATTAAATAATTTTATTTATAGTTTTGCCAAATGTCACTCATTTTAGT
GTTATTGTCAGCAACTCATCACAAAAGGTTTTTTAACCAAAGATAATTCAAAAATAGTAATTATATTGCA
TAGATTAAACCGAATGAGGTATATAAAAAGTATATTATGTACTTACCAGTTATATTTATTTTGAAAGTCCC
TGAAGAGGGTTTGTGCCCGTGATAACCAACTATGTAATATATACGTAACTGA
>TCONS_00008420 geneID=XLOC_001527 scaffold_109:1167211-1174398(-) Splice junction overlap
TATGCCATATTTAAGTGCTGATGCATATCGACACCGATGTTATAAGTACATTATCTAGTTATTTGTATTGCT
GCAGTGCTATTCTAAAAAAAAATTGGTCTACATTGACAAAACATTCCTATTTTCAAGATCAACCCTTCT
GTCTTTCGATTCTCCATACGAAAATCTTCTCTGAAATGGATCAATCCAATTTCACCATAATTAAAGCAAA
ATTGTAAGAAGTAGTCCCAAATTCATCTCATAAAAACCCCAATCGAGTGAGCAATAAGGAGCCTATATT
CTACGACCATTTCACTTGTAAAGGAATCTCAAACCTGACATAACCTCATAAACCACCAAGCGCTAAGG
GTTGCCACATCAACCGCAAACAATTCCTAACCTAACCTCAAAAACATCTAAAAATCAAAACACAAAGT
AAAAAAAGCCTAACCTAAAAAAAAACAGGTGATAATCTAAACAAATCGTGAAAAAATCCGCCCAAAG
CGCTGAATTCTGAGCTAACCGACGACCATTCCCTACCTAACCTCAAAAACAAGCGCTCAAAACAAAA
CACGTCTCAAAATCGCTTGAAGCATCACCATTAACCTCAAAAAGACCTTAAAAAACAAACACGAACG
TCAGAAAATCCGCTGAACGCTGAATGCTGACCTAACCTCAAAAACAAGCGCTGCGAACAAAATACGA
ACGCCACAAAACCGCTAGACACGCTGGATGCTGAGCTACTACGAGGCGCTGGTGGTGGTAGCATGCG
TGGCGACGAGCGTGGCGGCGCTGCTGCATGAGGTGTGGCTGTCGGCGCTGCCTGGTGCCGCGCCGCG
GGGGGGGCCGCCCCGCGCGGTATGGCGGGAACAATTATCAGATATCTATATGTTTCGGGAAGATTATCT
GTTTTACTTTGGCCTTACCGTGCATGTATGAAATGTATTTCAAACGGGAGTATAACGAGAC
>TCONS_00008421 geneID=XLOC_001528 scaffold_109:1211894-1235902(-) Intergenic
CGAAAAATGCACTTACGGAGTTTCACTTCCAGCCAGTACTCACCCCCACACGCGCAGTCTGTGTGTGT
GTGTGTGTGTGTGTCTGTGTGCGCCGAGTGCACCGCGACGCTAGGTCAGTACTCGACGATGCATTCAG
AAGCATGCACTCGCCGCTCCCAGCGCCAGTCGGGGCAAAAAAACACCTCAAAAGTACAAAAAACGC
GGTAAAGACATAGTGTTATCCGAAACGGGGGGTCGATCGACTCGGGGGCACGGATTTCAGTTTTCGAA
CGTGGCTCTTTGTTCGCCGCCATGTTGTCTCCGCTGCAGCCGGTCGAGATCTGTCTTCTATTGGGCGAA
TTACAGGAATGCAGAATGTACGACGGAGGCTGGCCCGTTTCCCGGGCCACAACTGGGACTGTCCGGG
TCATCGAGCCCCACTAGACTCGGCCGGG
>TCONS_00008571 geneID=XLOC_001530 scaffold_109:1284668-1285068(-) Exonic overlap (+)
TTTGAAATTTGAATTTGAACGTGTTTCGCCGAACTGATTTTCCTGTTTTATCACTTTGTTTATTTATATTC
AATAAATTCAATAAGTAAGAGTTACTAAAGTACGCCTTATTGCAGCAGTTGTTTGTTTGTTTGCAGTATT
CAAGTTAGTTGTGAACCAACAACAACTACAACCACCCACCAAACCCTCGCAAAAATGCCGGCGGTAT
ACACATTCACTCGTAGCGAGAACGAGATTCTCCAAGACCTGGTGAGGGTGTTCAGCTCATCACGGG
>TCONS_00008646 geneID=XLOC_001531 scaffold_109:1335059-1336983(-) Intergenic

ATATTGTGGTGGAATAAACATGTTCAAGAAACTTCTGCTTTTGTGTGTGGTGATGATGCTGATCGGCAG
TAGCCTACAGCAGGGCGGAGGCGGCGGCGGCGGGGCTGGTGGTGGTGGGGAAGCTGGAGCCGGTTT
TAGCGTAGGGGGCGGCGCGAGTATGGGAGGGGGCGCCGGTGCCAACGGCGGCGGGGGCGGCAACGG
AGGCGGCGGCCGTAAATGAAGACGGACAATCGGCATACGCTTACTCATACTCCAACCCCAACAACCAG
ATGACATCCAGTTACTCCAAGTACGTTCCGGACTCCACGAACAACGGTCAAGATTATAAAAAGTATTAT
CAAAATTAATTCATTCTGTTATTGGTGCTTAATTGGTTGATTATGTCATATTAAATTTTGATTCACCAGC
>TCONS_00008606 geneID=XLOC_001487 scaffold_109:1381041-1381989(+) Intergenic
GGAAAATTGTAATGGTTGATCGTGCGTGATATTATTGTGTTGTTCGGCCGCTCGCGTGTAGCACAGCGG
GGAGTGTGCGTGTGTGCGTGTGTGCGTGTGCAGGACGCGCGAGTGTGCGGGCGCCTACTTCCTGCGC
CCGCGCAGGGTACAACGACTGCGTTAACCTAGAAACTGTCGGCTCCGGCCACAGTAATGTGGCAAAC
GCGACACTAAACTGAAACAGGGAAACGAAATGGCTTTAGAGTTTATAATATTTATGACACGCAGCCAA
GAGCGACCCGACGACGCAAACAACCGTGGATCCAGCCGAGTCAACCGACTCTATTGGATGTGCAAAT
AAGCCCGACGGTTCGTGAGGAGAGGAGACGATACAAACATCGCGAGTGCGTGATAACGAGC
>TCONS_00008116 geneID=XLOC_001539 scaffold_109:1633528-1638689(-) Intergenic
CAATAATGAGTTATTTAAACGCAGATAAAGCTAGTCAGGCAGTCTCTGTTTCGTCTGATAAGAGTAGTT
TGAACGTGTGAGTGAGGAAGTTAACACGTGTTAGTGAACTGTGGCACCTCCTGTATACTCCTGGTTGC
CTGGTACTTTTGGCAAGAATTGTTCACCAACCAGTTAAATATTTATAAACAAACGTGTGAATAGATTTTC
ATAAATTTAAGACAATGTAAATATTTTACTGCACAAATTCTGCCTAAGTATACTAACACATAGTGATTAA
AAGACATTGAAAAGAAGTGCAATAGTGATAAATCAAAATGGATGAAATTCAACATCAACACTCTCCTG
AATTGGAACCTTTAGAAGCGGCATCCCCCCTCCGCCTGCCCCCTCTCAGACACCGG
>TCONS_00008657 geneID=XLOC_001542 scaffold_109:1683324-1686474(-) Intergenic
CCCATATTCTACGCGAAAAAATCAAAACCGTCAGTGTTCGACTCTGTATTTCACTTATTATAAAAGATTT
TTAAAAAATCTTTTTTTGTTTTGGTAAATAATGCAATTTTGAAGAAGAATCTGCAATAATCCGAAAATCG
AAAAAAATCAGATCCGTCACTGTTCTACGACTGCGACGATATATTCCAGTTTACATTCTAAACAACGCA
TGTTCGGAATCCTTAAAACTGTGAATACCTTCCAAGTTTTGGGCAATAGTAGGTATCAGGTCTGAGCAA
AATATTGAGAATATTTTTTGGAATCAAGTCTACGTTCTTTGCCAGTCTGTCTAATATTTATTATAAGTATTT
TGAGGTGGACCGCCAACGTTGTTGTTTGACTGGTGCCCTCCTCTCCATTAGCACTCTTCTTACTACAGC
ACTTGCATGCAGCTTCTACACATGAACATTTAGGTCACGTGACTCTCTAACAGTGGCGAAGGGGGTGA
TTCCCGCCCCTTTCCCTTTTGTATTCTCTGAACCTCACGATAGTTCATGATAGGTAAGAATATACACTCA
CGAGCAATGAAAATGTCCCAGTGACATACTATAGAAAGTCAATAGCCGGTGGAACTTTTTCATTACTCG
TGAGTGGATTTTTCACAACGGCTTCCCCTACAAAGAAATATACCCTACGTCACTGCATTCTACTTTAAAT
AGTACGTTAAAAATTGTTCAGTATAAGCCCCCGATTCATCCCCGTTCGGGTGTTTTTACCATGGTGCGG
ATTTCATAGCCCTTTCGGGTGTTTTTACCATGTTGCGGATTTCATAAAGCATTCGGGATCGCACGCCATT
TACCCTCGTCCCGCGTGCGTATTCCACGCATTACAATTAATAAGTGAATGAAAATAACACTTGCCGGTG
TCACGCTTGCGGGGCAATCCCGCGTGCGTAATGAAATCCGCTAATACCATATTAAAAACATCCTTAGTAT
ACCACTTTTACAACATTCTGTACAATTAAAATTTTTGTTCAGGTGGGACGCCAAGGCAATTATGGCTGT
GGGCATGACTTTTGCCCTCCCCCCATAAATCCTCGCTTTTATATAAAGCTCAATGCTCAAACCTTAGAGG
ACGGTAAAGGTGGCCCTGGTCTGCCGTAGAAGAGAAAAGTCAAGCGAATTCCTTAGCTCAATTAATGG
CACACATTTCCAGGGACGAATGAAAAAAACCTTCAGTTTTGTCAATAAACACAAATTCATTTGAGTGG
TTTTGTTTTTCTGTTTTTCTTCATGTGAATATCTTGCAAAAACTGAGTATTTCGTTGCTAATCATAAATCA
TTACTCTCCACCTCTAAATGATTTCCGTTACAATAACTCAAAATCTGGCCTTTTATTAAGCCCCAATTAA
AGAGATATTTTTATTTTTTAATCTTATTCCATCTAAGGGTGGATTCGACCAAATTCGAGTAACCCTACCTA
TGTCAGCGTATTTTGTATTTAAACAAACCTCAAACCAAGATAAGTAGCTTATACCAAGAATTAAAAGTT
TTTTTTTATGTAGTCTGCATCGTGTCCCACTCCCACCCAATAAAAAGTAACACCACCAAAATTGACGGA

CAGCACATCAGCTGATTAGAAAATGTCATCATAAAATTAGCATTAAGCCCACTAGTGCAATAAAGAATT
AAATAAATAAATAATGATGTTTCATACTACTATTTCGTTCCTCGTTCATTCAATTATTCATTGTATAACTTG
GTAGAATGCAAATGTACCTTACTTATCCCAGATATTTGCTATCTTGTTAATTT
>TCONS_00008658 geneID=XLOC_001542 scaffold_109:1683324-1686474(-) Intergenic
CCCATATTCTACGCGAAAAAATCAAAACCGTCAGTGTTCGACTCTGTATTTCACTTATTATAAAAGATTT
TTAAAAAATCTTTTTTTGTTTTGGTAAATAATGCAATTTTGAAGAAGAATCTGCAATAATCCGAAAATCG
AAAAAAATCAGATCCGTCACTGTTCTACGACTGCGACGATATATTCCAGTTTACATTCTAAACAACGCA
TGTTCGGAATCCTTAAAACTGTGAATACCTTCCAAGTTTTGGGCAATAGTAGGTATCAGGTCTGAGCAA
AATATTGAGAATATTTTTTGGAATCAAGTCTACGTTCTTTGCCAGTCTGTCTAATATTTATTATAAGTATTT
TGAGGTGGACCGCCAACGTTGTTGTTTGACTGGTGCCCTCCTCTCCATTAGCACTCTTCTTACTACAGC
ACTTGCATGCAGCTTCTACACATGAACATTTAGGTCACGTGACTCTCTAACAGTGGCGAAGGGGGTGA
TTCCCGCCCCTTTCCCTTTTGTATTCTCTGAACCTCACGATAGTTCATGATAGGTAAGAATATACACTCA
CGAGCAATGAAAATGTCCCAGTGACATACTATAGAAAGTCAATAGCCGGTGGAACTTTTTCATTACTCG
TGAGTGGATTTTTCACAACGGCTTCCCCTACAAAGAAATATACCCTACGTCACTGCATTCTACTTTAAAT
AGTACGTTAAAAATTGTTCAGTATAAGCCCCCGATTCATCCCCGTTCGGGTGTTTTTACCATGGTGCGG
ATTTCATAGCCCTTTCGGGTGTTTTTACCATGTTGCGGATTTCATAAAGCATTCGGGATCGCACGCCATT
TACCCTCGTCCCGCGTGCGTATTCCACGCATTACAATTAATAAGTGAATGAAAATAACACTTGCCGGTG
TCACGCTTGCGGGGCAATCCCGCGTGCGTAATGAAATCCGCTAATACCATATTAAAAACATCCTTAGTAT
ACCACTTTTACAACATTCTGTACAATTAAAATTTTTGTTCAGGTGGGACGCCAAGGCAATTATGGCTGT
GGGCATGACTTTTGCCCTCCCCCCATAAATCCTCGCTTTTATATAAAGCTCAATGCTCAAACCTTAGAGG
ACGGTAAAGGTGGCCCTGGTCTGCCGTAGAAGAGAAAAGTCAAGGTTAATATCGCCAAGACTGTGTTT
TTGACTGGTGCCCTCCTCTCAACCAACGCTCCTCCGAATTCCTTAGCTCAATTAATGGCACACATTTCC
AGGGACGAATGAAAAAAACCTTCAGTTTTGTCAATAAACACAAATTCATTTGAGTGGTTTTGTTTTTCT
GTTTTTCTTCATGTGAATATCTTGCAAAAACTGAGTATTTCGTTGCTAATCATAAATCATTACTCTCCACC
TCTAAATGATTTCCGTTACAATAACTCAAAATCTGGCCTTTTATTAAGCCCCAATTAAAGAGATATTTTTA
TTTTTTAATCTTATTCCATCTAAGGGTGGATTCGACCAAATTCGAGTAACCCTACCTATGTCAGCGTATTT
TGTATTTAAACAAACCTCAAACCAAGATAAGTAGCTTATACCAAGAATTAAAAGTTTTTTTTTATGTAGT
CTGCATCGTGTCCCACTCCCACCCAATAAAAAGTAACACCACCAAAATTGACGGACAGCACATCAGCT
GATTAGAAAATGTCATCATAAAATTAGCATTAAGCCCACTAGTGCAATAAAGAATTAAATAAATAAATAA
TGATGTTTCATACTACTATTTCGTTCCTCGTTCATTCAATTATTCATTGTATAACTTGGTAGAATGCAAAT
GTACCTTACTTATCCCAGATATTTGCTATCTTGTTAATTT
>TCONS_00008621 geneID=XLOC_001497 scaffold_109:1700928-1702552(+) Intergenic
ATGAAATCCGCTAACACAATATTAAAAACATCCTTAGTATACCACTTTTACAACATTCTGTACAATTATAA
TTTTTGTTCAGGTGGGACGCCAAGGCAATTATGGCTGTGGGCATGACTTTTGCCCTCCCCCCATAAATC
CTCGCTTTTATATAAAGCTCAATGCTCAACAATTTGGCGGTGACAATAATCTTGACCATGAACCGGATTT
ACAAAACTAAGTTTATATCGCCAGGACTGTGTTTTTGACTGGTGCCCTCCTCTCAACCAGCACTCCTCC
GAATTCTTTAGCTCAATGAATGGCACACATTTCCAGG
>TCONS_00008622 geneID=XLOC_001498 scaffold_109:1716283-1716975(+) Intergenic
AAATAATATAAGCTAACGATAGTGCGTTGAGTTGATTATAATAGTAAATATTCGAAGCGCTACTCTGACG
CCCGCCATATATTTTTTATTGCCCATAGAGACGATGGAAAAAGTCCTGAGATGCCGGCATGTCTGAAAT
GTATTTTTTTTGGTTTTTCTATATAAACTGTAATTTTTGCATACAATGATAATCCACCAACCGCGGATGTG
CACGGTCAAGTGTGTGATCCGCCTAATGTATTTCATTTATGTTTCCAGCGTTTCCGCCGCAAGCTAGAC
GAAAGGCCGCCGAACACGTACCGCTTCGAAGACGCTACTCCATTTTACTAAACGGAAAAGTTGGTTTA
ACAGCGCATCAGAATATTATTTACCAAGTTAAAATGTAAAATTACCGCCTTTTTCAAATTTAAAATTTAC

TTAATGTTTGAAAAAGTTGGCATTTCGTGTGTGGAACTTTTTCATTGATCGTAAGTCTATTTAGCGCGTA
TTCAAACGCCACCTATATGTGCAACATTATAATTAAAAGAGTTACACGTGTGAGC
>TCONS_00008659 geneID=XLOC_001543 scaffold_109:1747149-1750691(-) Intergenic
GTCAACTGAAGAATTTCAATATGAAGCTGTTAGTGTTTGTTTTGTCTCTTTTCGCCGTCCTGGCAGTAG
TGCTTGGACAGGATCCGGGCTCTGAGGGGGGCATCCGTCTTGTGTACCGCGGGCTGCGCCGCCCCCCC
TACCGACAGCGTTATCCTAACTCGTGTAAGCGCGGTGGATTAATAATCTGTTGAAAAACTTCAGAATAG
AATCAACTCTTTATTTAAATAAATAATAAACGTCATAAAATATTGGAATTTTATTATAATTATGTGTATCTG
TTTCCATAAAGATAAAAAAAATATTACAAATACTTACAAAAGCAAAATGAAACGTTCATTATGTCTTGCT
ACGCGCCGTAGCAAGCGGTGCTACAAGACTGCTACGAGACTGCTACGAGTCTGCTACGAGACTGCTA
CGAGACTGCTACGAGAC
>TCONS_00007775 geneID=XLOC_001544 scaffold_109:1776911-1808562(-) Exonic overlap (-)
GTAAAAGCGGGGCAGGTCAGTGGCCGGGTGCCGCGCGGAGGAAAGTCGCTCTTGGCCGTCGCGCG
TTAGTTTCTTTTTTGCAACGGCGCATGCGCGCGCGGCTCGCTCGGAGGTGCGTCAGTGTTGCGTGCAG
ACCACGTCGACGGCGGACGCGCCACGCGCCGCGCCCGTGCCCCCAGTGCCCCGCGTGCCCCCAGTGA
GTGCAGTGAGTGATGCGGGACGCGGAGCGCGCGCCGCCGCTGCCCGCGCGGCTGTACGAGCGCGCCC
TCAAGCAGTACCAGAAGCTGCTGGACCTCACCAAAGACAAAGTCGCCGCACCTGGCAAGGAGGATG
AGAGGTGGCGCAAGGAGGGCGCTCTGTTCCGCCGCTGGGGCCGCGCCGCCCCGCAGGAGGCGCCCG
CCGCGCCCCCGGCCACCACCACCCCCTCCGATCTACTCAAGTTCCTAGCTATTGTAAGTACTACTTAAT
TTACAATGTTAGTTCATTTCATACAATCAGTTAGCTAACTAACAAAAACAACAGATTTTTAAACCAGTT
GGTTGAGTGGGTGTCGGAAATTTTCATTGAGTTTGTTA
>TCONS_00007940 geneID=XLOC_001544 scaffold_109:1776932-1853453(-) Exonic overlap (-)
AAAACGGAAAAAGAATTTTCCGAAGGGAAAGACTTGCTACGAAAACGAAGGTCGCCGCACCTGGCA
AGGAGGATGAGAGGTGGCGCAAGGAGGGCGCTCTGTTCCGCCGCTGGGGCCGCGCCGCCCCGCAGG
AGGCGCCCGCCGCGCCCCCGGCCACCACCACCCCCTCCGATCTACTCAAGTTCCTAGCTATTGTAAGT
ACTACTTAATTTACAATGTTAGTTCATTTCATACAATCAGTTAGCTAACTAACAAAAACAACAGATTTTT
AAACCAGTTGGTTGAGTGGGTGTCGG
>TCONS_00007777 geneID=XLOC_001545 scaffold_109:1854964-1858036(-) Unclassified
GCATTATTTAGAACAGCTGACAGCTACATTCATTGTCTTCACGTCAGTGGAAACTGCGAAATTGGTTCT
TTTTGAACACGACATATATTATTGACAGGCGGATATTGTTCACGAAACAACTCTACCCGCAACTTGTTG
TCGTATTTAATAAGTTAATAACAGTATGTCAAAATAAATGTTGCTTTCCTTGAGTGGATTCGCGAGACAT
ATCGAGTGGTCAGCGATAGCGCGACAGTCGCCGGCCGGGAGGCGTGTACCGAGGCGGCCAATACGGT
TCGTTCGGTGCTCAGTTTGCATTACTCATTATCGGTGTAAAGAGCGATAATCCTCGTGCGGGCGGCAGT
GTGTGCGTGTGGACGCGGGCGCCGGTCACGGCGTGGAGCGCGCCCGACCCCGCCAGCCATGGCGACC
GCCGCGCCGCGCTACTGCTTCCCTCCGGAGACCTACTCCGCCGTCTACCCCCCCGCCCCCGTGGTAAC
GTCAGCTGAAGATGGATGTGAGAGCCGGGAG
>TCONS_00008019 geneID=XLOC_001545 scaffold_109:1854986-1870357(-) Splice junction overlap
CCAAGTTATGACGGCTATAGCGCATGCCGCGCATCATCAAAGAAGCGTGTTTTTGCAGAGCCCGGAGC
TCCTAGAGTTACTCTCCGGCTCACTGATCGCGGGCGCCGCGGGCGAGGTGGCGGCGGCCGCGCGCGC
CGTGTGGGCGCTCGCAGCCAACAACCACCGGGCGAAGCTAGCGCTGCGGTCGGCGGGCGTGTCCTCC
GCGGCGCACACGGCCCTGGAGCGCCTGCACCGCGGCGGCGGCGGCGACCACGCGTTGCAACTACTC
ACCTATGTGAATACTGTGTTGACGTCCACGTGACTTGCGGTGGTAACGTCAGCTGA
>TCONS_00008437 geneID=XLOC_001545 scaffold_109:1858567-1860072(-) Splice junction overlap
GCCGCGCGCGCCGTGTGGGCGCTCGCAGCCAACAACCACCGGGCGAAGCTAGCGCTGCGGTCGGCG
GGCGTGTCCTCCGCGGCGCACACGGCCCTGGAGCGCCTGCACCGCGGCGGCGGCGGCGACCACGCG

TTGCAACTACTCACCTATGTGAATACTGTGTTGACGTCCACGTGACTTGCGGTTAGAGGTGTAAGAGAT
GCTACCCGATTTTTGAGCCTCCTTGATTGAGCCATTGTTAGCCTTCATGAATATTGTTGAAGCCTTCAGT
AACCACAGAGAGTGTCGGCGAATGATGGTCCCGGGACTGCGCGGCAGTTATAACTAAACAAAGATAG
ACGCTTCAATGGTTACGCTGCGAAATCTAAAGTCGATTGATGAGAAGGCCACGTTGTATGATTACAGTA
TTAATGATATTAAAAAGATCTTTGAACAAGATCTGTTTTAATTATATTTTTGTAGTTGTTTATTTGGTTTG
AGTGATGTGACAAGAACGTAATATAGTTTGCTAGTTAGTCGCAGGCGCTCATGTATAGTATTTCTCACCA
TTATCGAAAGGATAACTTTACTGTCCTAGGTATGTCGGGTGTATTTCGAGTGATACGTTATAGTATAGCTT
TAGCTTAATGAAAGCCAATAGAGGAAGCTTATTTCGTATACCTGATTTGTAATTAAGACGACCTTAGTAA
AATTAACTGTTAATAATTAATTTAATTACTTAACCAGGAATTGCAATGAAATAAATTTAATTAAATAATTC
CGTTCTACTCATAGAACGTGTGGATATGGAGATCTAATAAGTCTTGAGAGAACGTATTGCCTAGCGGAT
GTACAAACAACGTGGATTATTGTTATTTTTCTAGTGCCACACAATGGAGGTGGCATCTTGTGACGTGCA
AGCAGCAGTTCACACATTTATGTTCATTATGGAGGGCTCATTTACACAATAATAAAGTTTTTACTACTGT
TTACAAACTCAACGACGACGCCGTTTCGCCACGTAGTTGTTACGTTATTTATTATGATCTCAAATTAATT
TGAAAAATAAAAGCG
>TCONS_00008336 geneID=XLOC_001545 scaffold_109:1858567-1862413(-) Splice junction overlap
CGCCGCGGGCGAGGTGGCGGCGGCCGCGCGCGCCGTGTGGGCGCTCGCAGCCAACAACCACCGGGC
GAAGCTAGCGCTGCGGTCGGCGGGCGTGTCCTCCGCGGCGCACACGGCCCTGGAGCGCCTGCACCGC
GGCGGCGGCGGCGACCACGCGTTGCAACTACTCACCTATGTGAATACTGTGTTGACGTCCACGTGACT
TGCGGTTAGAGGTGTAAGAGATGCTACCCGATTTTTGAGCCTCCTTGATTGAGCCATTGTTAGCCTTCA
TGAATATTGTTGAAGCCTTCAGTAACCACAGAGAGTGTCGGCGAATGATGGTCCCGGGACTGCGCGGC
AGTTATAACTAAACAAAGATAGACGCTTCAATGGTTACGCTGCGAAATCTAAAGTCGATTGATGAGAA
GGCCACGTTGTATGATTACAGTATTAATGATATTAAAAAGATCTTTGAACAAGATCTGTTTTAATTATATT
TTTGTAGTTGTTTATTTGGTTTGAGTGATGTGACAAGAACGTAATATAGTTTGCTAGTTAGTCGCAGGCG
CTCATGTATAGTATTTCTCACCATTATCGAAAGGATAACTTTACTGTCCTAGGTATGTCGGGTGTATTTCG
AGTGATACGTTATAGTATAGCTTTAGCTTAATGAAAGCCAATAGAGGAAGCTTATTTCGTATACCTGATT
TGTAATTAAGACGACCTTAGTAAAATTAACTGTTAATAATTAATTTAATTACTTAACCAGGAATTGCAAT
GAAATAAATTTAATTAAATAATTCCGTTCTACTCATAGAACGTGTGGATATGGAGATCTAATAAGTCTTG
AGAGAACGTATTGCCTAGCGGATGTACAAACAACGTGGATTATTGTTATTTTTCTAGTGCCACACAATG
GAGGTGGCATCTTGTGACGTGCAAGCAGCAGTTCACACATTTATGTTCATTATGGAGGGCTCATTTACA
CAATAATAAAGTTTTTACTACTGTTTACAAACTCAACGACGACGCCGTTTCGCCACGTAGTTGTTACGT
TATTTATTATGATCTCAAATTAATTTGAAAAATAAAAGCG
>TCONS_00007780 geneID=XLOC_001545 scaffold_109:1881564-1892966(-) Exonic overlap (+)
ATTTAATCGTTCAAGTAACATTCGAACAAGTAATGGATTAAAGTAAGTTGCTCTGGTTATTGACCTTTAC
TTTTAATATCGAATTACTGCAAGTACCAACTTTGTACGCAATATTTTATGGTAATTTAAACTGAGGACTTT
ATCGTAAGAGGCGACCGCGCCGTGAAGCGTTTAGCAATCTTAATAACGCGGCTAGAGTTCTAAGGTTT
GATTCGTAGCCGGGCATAATATATGTTTTGTCACAAATATTTGCTCTTGAGTTGATCAGGGTTGAAAAAT
GCCATTACCCTATTTTATTGGCACTAACATGGCAAACGTTGGTGCAGTAGTGTGACTCTGTGTACTTCA
CTAATTTACTTCATTATTTATCATTAGTATAAGATGCAAATGTGCTGTTTATGTCTGTAACTGTATTGTATA
TCATTGTACCTACCGTAATTGTAATTTGTAATGTTACTTATAATGTACTTAAGATTTTAAAGCTTTAACGG
ACGTGATTAATTGTCTGTGTATTTACATAATCAGGCGTCATGAAAATTTTATTATTCACACGGTTAATCAT
TTGTAAG
>TCONS_00007661 geneID=XLOC_001502 scaffold_109:1905626-1906331(+) Exonic overlap (-)
CCAGAAAGCAATGCCAGTGCCTCATTGACTTGCAAATGGCTGGTAGCATTCTGCAGCATCAGCAAGGC
TCTAGAGCTAGCATACACCGGGCAGGCGGATTGCAACGTTGCCACATCGTGCGCGGTCGGACGGAGG

GACAGCGGGCCGGGCGGCGGCGGCGCGTCGCGGAGTATGCGGGTGCCGTTCCAGGCAAACGACCAG
TGAACCCTGATCGCTGAGCGGTAGTCTTCTTCTGATTGGAGC
>TCONS_00007791 geneID=XLOC_001548 scaffold_109:1979010-1979977(-) Exonic overlap (+)
AGCACAGTCACATCTACACACTAGCAAATCATCTTTGTCGCATTAGCTGCGGATTCGCGGGAGAGTATC
CACTTTTGCTGATCTTTTTGTTGGATGCCAGATGCAAGACTCCCGCAGAGTTCTGTCAGGTTATTGTGG
AGAGCTGTGCAGTGCTTGTAACGGTATAAATACTAGGTCCCTGGGAGCGACGGCGTAGTCGACGCGCC
GCGTCTAAATAAAGAACGCCTTCGCTCGGCCTCGCGATGCTGCGCGCACGCAGCAGTGTTCGTCCAAT
AAAAACCAAACGTAGTACATATAATATTTCCCCCCCGATTTTTGATCCGTTTCGCACGCGAATCACGTAA
CTATGAGTGACAATTGACGGTCGGAGGAACCCGAATGAAATGTCAGTGAAGTGAACGAGCCAAATTG
AAGTTGGGTAATTGGTCAATAGAAGAAACGACAAAATGAAGTTCACAATAGATGTTCTGGGGATCTTC
CTCAAAATCGTTGTTAGGGTAAGTGGTATTTAGATCAGTGGGAGCAACAGACGCCATTCATGCATCAGC
CATTCTTTGAGCCAGATATCACGCATGCAGAGCCGTCTGGTCATCTGCCATGCCGAGTGAAAATGTCAA
AAGTCTGGCAGAACCAGCAACCTTAATACCTGAGTCATAATGGTCAATTCTCGTGGCTGATCTCACCGC
GATGTTGCTGTAATGGCAGGAACACAACTCTAGCATAACGTAATTAGTTCGTCCTTCAATCGAATGGTT
CTTGTACAATTTGGCCTATTGTCAGCATTCAAATCAGTGATTAGGTAGGTGTATTGTATTGTTCTATTTCC
TATGTAAAATACAGGTGTGTGTGCCGTGTGTTGTGATTGTGG
>TCONS_00008188 geneID=XLOC_001506 scaffold_109:1994380-2028614(+) Exonic overlap (-)
GTTCAGTTTCGCTCCGACCGGCAGTGAGTACGGACGTGCGTTTTATCTGTCGATTCATTCCCCCAGTAC
CGGTAACCTAACAAGACCAAAAGGACGTTTGTTGTCGAAATTAATTGTAATTACCTGCATGTTTAATCT
TACGAGTGAAGTTTAAATTATGTGTGTCAGTTAAGTGTTTTAATTGGTGTTTTTTTGTGATCTGACGTTA
ATTAGTGTTTACGGGACATCGAGCTGCAAGTAAAGGTACTTATTTGTGGTTTTCTATATGAAAAGGAGG
TTCTATTTCAACATACAGGTGGGAATGTGTTCTGT
>TCONS_00008187 geneID=XLOC_001506 scaffold_109:1994385-2015804(+) Exonic overlap (-)
GTTTCGCTCCGACCGGCAGTGAGTACGGACGTGCGTTTTATCTGTCGATTCATTCCCCCAGTACCGGTA
ACCTAACAAGACCAAAAGGACGTTTGTTGTCGAAATTAATTGTAATTACCTGCATGTTTAATCTTACGA
GTGAAGTTTAAATTATGTGTGTCAGTTAAGTGTTTTAATTGGTGTTTTTTTGTGATCTGACGTTAATTAG
TGTTTACGGGACATCGAGCTGCAAGTAAAGGTAAATTTTAATAGAAGCATAGACAAGATAATAATATGG
TGACTAGTAAGTAGTTTGCGGAGTAAAAGTACATTTGACTGAACTATTACCTATGAAGAAACACACACT
AGAACCTTCTCGTTAGAGAAGTAACTACTTATTTCCTTGATGGAGAACTCGTTTAAGATTTGTCTTGGC
CAGACAGTAATTTTGTATGAAAATATGAACAGCGCCCCTTACGGTCGGTAGCAAAACTAAAATTTTACA
AAACAAAATACATCGAGTAATTTTACTTCACGTTCACGATGAAAATTCATGCCTAGGGCTCAGACAGCA
CTGACTGCCCA
>TCONS_00008720 geneID=XLOC_001717 scaffold_1090:6012-10382(+) Exonic overlap (+)
GGACCGCGACAAGTTGTGCCGGCTGATGGAGGACTCCCTGCGCGCGAGCTTCGGCCCCGCGCCCGCG
CGCCGCCCGCAGCTCGCCAGCTAGTAGCCGCGCGCGCGTCTGCGATCCTGACGGAGAAGGAAACCAC
CTACGAAGAGCCGAGCGGAACCGTGCGGGGGTCACCGACCTCCGCCGTCACCTTGCCGCCCTAGTAC
GAGCCTAGCGAGTTCGCAGACAAACCACGAACAAACGCCCACAGAAGTGCAAGCAGAGGCGCTCGA
ACGCTCCACAGACTCCTGTGAGCCTAGTACTAAAGCTCCATTCACGTTCGCAGACGGAGATACTTGGT
GTAGAAGTATGTAGTTTAATCTTGTCACTGTAATAAGTTGGAATCGGATATGGTAGTTCGGTAGTTCTAA
CTTATATTCACAGAGTGTCGATATAAGTTTCCGTGAGTATGATGATCAGAAAGCCATGTTAATAACTCAT
GGACAGCCTAAAACGTTTATTCTGAACACTAGGAACACTGTGGTGATATCGGCCGCCATATTGGATTTT
AATTTTAAAAGTATGATATGAATTAGTCACCTTTAGAGTAGATAAATATTTAATTAAGTAATTAATTACCA
TACATATTTCTTGCATGACCAAGTATGTCAAAATCATCAATTAAAACAGCTTGCTTGAATAAGTACTAGG
CTATACTTAGCGCGTAATGTTCATTGTATTTCTGAAGAAAAGATATGTTAGACATAAAATAAGAGCTAAG

TAACTTGTAGGTGTAAATGAGGCGTTGGCTGGGCGAATTACTGGGGTGAGTGAATGGGCCAATAAAAT
TATACGTTAGAAGAAATTAAATTTCGATTCGCATAATTAGCGTGATTAAATTGACCTTCCAATTATAATTA
CCATAAATTTTTGATCCGAAACATAAAAACAACCTATTCTTTGTCTGTTTCTAAAAAAATACTTGGCAGA
TATAAAGGTCCATAGTATCTAAAAATACTTCCGAAGCACTAAATAATAGTTATTATCAATTTAAACCGTC
GCTTTACTTTAGTCAAGCTTTATTTTTTACATTATGAGTCTACCATAGACATAAAATGTAGATAGGTAGGA
GAACTTACGAATTTTGTATGGCTGAAAAAGTGTATTTTTGCAGAAGGAACATATAAAATCAAAGAAAAT
CCAATTATACTGAATACAAGTATTATTATTTTAATTATAACTCGATTACTCGTTCGTTCGTTTCAGAAAAA
AGCACTTTCATATCTACAGAAAAGTAGGTTATGATTACGTGACATTATAAGCATAAATAAATATGTTTAG
TCCTAGTAATAAAACATGCATTTCACCCCCAAAAGTGTACCTACTAGTCTTTTGTGAATCTATTGCACTA
TCACGAATGTTATAAAGGGAAGATGTAAAAATATTGACACTTTAGGAAATTTTAATTAAATTGTAAATTA
AATTAGCCAAGTCAAAAGTTCGATCGTGTTAGACGGACATCGAGAATGCTTTAATGTAAATCCCGACG
AAGCCACAAAATGGTAATATTTATCTATTTATTCGCCATTGAATTCATATTGCAGCACCGGAGCTTTGATT
GAAAAGCTGTTGAAAGTGAAACTGAAAAAATATGATAAACACGTGTAAGCGGAATTTCCTATAGGCAG
AATTAAATATTATTCGCTATCAAACTATCATTATTATCAGTTATCAAACCTATTTTTAGAAATAACGAATAT
AATGTATGTTGTTGTTTGATAAATATTAATAAACCTAAAGTGTAAACTACACGTCTCACCGATATACTTAT
TGAGTCTTAACACAAAAAAAAACATTGGCTCAAAAGGTAAAATTCACTAACATGGCCTAAAATACTTGT
TAGTTTCAATTCATTAATTTTAATATGACCAAGTTGTGTCTTCGTTCGATGGATATATTAAATAACTAGGA
TTATTAGGCTTGTATGATTGTGAAAATGTAAGAAATGATATTTATATATTTAATTACTAAATACATTATTATT
TTAACTATTGTATAGCGAGGCTTCCTCTTAGAGCTTTATGGTGTGTGTTAAGTATACGCGTTGAACATAC
TCACTATCGGTATAGGTTTAATATCTGTCGAGGTTCAAACAGTCGTTCTGGTCAAGATAATGTGTATATC
GCGAGATTCTTTTTCTATGAAATTTCTATTTTTAAACAAAGTTGGTCAATTTGCGTAGGTAAGGAAGATA
TATTGCATTTAATACATGGACATTTGTTTTTAAATAATTTCTGATTTTGTACAGCGTTGTGGTGCTATTCT
CAGCGGTGTTTAGGAAACAGTTTGTATGTTATACAGTAAAAAAAAATGCTCCGGTAAGGCACCTTGTTCC
TAATTTTTGTTCGATATATTTTTTTGGTGCTGTGACCGAATAATTTTATGAATTAATGGAGTTAAGTTTTAT
TTATTTCGGTTAACGCTAGTGGTGCCAAAAATGCGAGGTGCCAAGTTATTTAACTTTAGACCTTGTGGA
CAAAACGATAAGAAACAATGTTACTTCCATGCCTGTTTTCTCTGTAGAGTTTGTGCAATTATTTTCTGAA
TTTTAAGCGTTTGGTACAGGGAGATCTGACGGACAGCTTTATATTATAATGAACACAGTTTAGACTTTTA
TACGAATAATAATTAATAAATACATTTAATAAAATCCTTAAAACAGTTCAATAATGTAAAAACAATTGAT
AAAATCAGATTCTAGGGTCGACAGTGGACACCACCCGTGAAATTTGGAGCAATATATTGTCGAATCGA
ATAAGACATAATATCTAGTACTGTTGAGTCCAATTCGTGTCAGTTTGAAGTCCGATGGCGTTGAACCAA
AGGTATATTTCCAACCTACATTGCAATTCCAAAAACATATTCCATCCAAGAACCGTCAGATCTCCAGAA
TGAAAGGGAAAGAAACATACTGTCTCGCTCGCGGCTAGAGCGTAGGCGTAGGATAGCGTAGTAAACG
AAAGACTGGCTAATGTAGGCGAACATAAGATTGTTGTTATGTGAAGAGTGGCTCGCTCTGGTGACTCT
TTGGCGAAACTACACTACAAAAATAGAGCAAAATAAGGAATCAGATAGTGGTGCCATCTATAATATTGT
TAAGGGCCAAATGGCGCAAATAATTTCTCATGATTAACGTAATATTGCGTTTTGATGTGAAGGAATAAA
ATTTCAATATCATAAACCAACTTGTAATGTTTTTGTTCATCTTAAAGTGAAGTTAACGCCTAAGAGCCAC
CTCAGCATATATCGGGAGGCAGGTATCTAGCTTGTAACGTGTTGTATCTTGTAAATATTCGCTTGTAGTAT
GTAATTTATATCCAATACCATGTATTTTAATATAAAAACAAGAGTATTTAGCCGCGTCTCCTGTACCCGAC
CCGCGTTCATTATTTATTATTCATGGTCACTTATCTTATTTACTTCACTTGTAAGGAGTATTTGGACAATTA
AAACAAGGTAATTAAAAGTGGTTATCCCAAAGTTGCAACTAAAAGTACCGCTTACTAAAATAATACTTT
ACCTGCAAGTATGTAGTACCGACATTTCAAATTCGTAGAGCAGAAGTTGTTCAGAATGATAAGTTTCAC
CTCTTTTCGGAATACGATGCCATTATTTTAACACCACTCCCAAGTATTCATTCATAGCACCTTTTAGTCTT
CTT

>TCONS_00008728 geneID=XLOC_001717 scaffold_1090:6305-10404(+) Unclassified

CCGCCCGCAGCTCGCCAGCTAGTAGCCGCGCGCGTCTGCGATCCTGACGGAGAAGGAAACCACCT
ACGAAGAGCCGAGCGGAACCGTGCGGGGGTCACCGACCTCCGCCGTCACCTTGCCGCCCTAGTACGA
GCCTAGCGAGTTCGCAGACAAACCACGAACAAACGCCCACAGAAGTGCAAGCAGAGGCGCTCGAAC
GCTCCACAGACTCCTGTGAGCCTAGTACTAAAGCTCCATTCACGTTCGCAGACGGAGATACTTGGTGT
AGAAGTATGTAGTTTAATCTTGTCACTGTAATAAGTTGGAATCGGATATGGTAGTTCGGTAGTTCTAACT
TATATTCACAGAGTGTCGATATAAGTTTCCGTGAGTATGATGATCAGAAAGCCATGTTAATAACTCATGG
ACAGCCTAAAACGTTTATTCTGAACACTAGGAACACTGTGGTGATATCGGCCGCCATATTGGATTTTAA
TTTTAAAAGTATGATATGAATTAGTCACCTTTAGAGTAGATAAATATTTAATTAAGTAATTAATTACCATA
CATATTTCTTGCATGACCAAGTATGTCAAAATCATCAATTAAAACAGCTTGCTTGAATAAGTACTAGGCT
ATACTTAGCGCGTAATGTTCATTGTATTTCTGAAGAAAAGATATGTTAGACATAAAATAAGAGCTAAGTA
ACTTGTAGGTGTAAATGAGGCGTTGGCTGGGCGAATTACTGGGGTGAGTGAATGGGCCAATAAAATTA
TACGTTAGAAGAAATTAAATTTCGATTCGCATAATTAGCGTGATTAAATTGACCTTCCAATTATAATTACC
ATAAATTTTTGATCCGAAACATAAAAACAACCTATTCTTTGTCTGTTTCTAAAAAAATACTTGGCAGATA
TAAAGGTCCATAGTATCTAAAAATACTTCCGAAGCACTAAATAATAGTTATTATCAATTTAAACCGTCGC
TTTACTTTAGTCAAGCTTTATTTTTTACATTATGAGTCTACCATAGACATAAAATGTAGATAGGTAGGAG
AACTTACGAATTTTGTATGGCTGAAAAAGTGTATTTTTGCAGAAGGAACATATAAAATCAAAGAAAATC
CAATTATACTGAATACAAGTATTATTATTTTAATTATAACTCGATTACTCGTTCGTTCGTTTCAGAAAAAA
GCACTTTCATATCTACAGAAAAGTAGGTTATGATTACGTGACATTATAAGCATAAATAAATATGTTTAGTC
CTAGTAATAAAACATGCATTTCACCCCCAAAAGTGTACCTACTAGTCTTTTGTGAATCTATTGCACTATC
ACGAATGTTATAAAGGGAAGATGTAAAAATATTGACACTTTAGGAAATTTTAATTAAATTGTAAATTAAA
TTAGCCAAGTCAAAAGTTCGATCGTGTTAGACGGACATCGAGAATGCTTTAATGTAAATCCCGACGAA
GCCACAAAATGGTAATATTTATCTATTTATTCGCCATTGAATTCATATTGCAGCACCGGAGCTTTGATTGA
AAAGCTGTTGAAAGTGAAACTGAAAAAATATGATAAACACGTGTAAGCGGAATTTCCTATAGGCAGAA
TTAAATATTATTCGCTATCAAACTATCATTATTATCAGTTATCAAACCTATTTTTAGAAATAACGAATATAA
TGTATGTTGTTGTTTGATAAATATTAATAAACCTAAAGTGTAAACTACACGTCTCACCGATATACTTATTG
AGTCTTAACACAAAAAAAAACATTGGCTCAAAAGGTAAAATTCACTAACATGGCCTAAAATACTTGTTA
GTTTCAATTCATTAATTTTAATATGACCAAGTTGTGTCTTCGTTCGATGGATATATTAAATAACTAGGATT
ATTAGGCTTGTATGATTGTGAAAATGTAAGAAATGATATTTATATATTTAATTACTAAATACATTATTATTTT
AACTATTGTATAGCGAGGCTTCCTCTTAGAGCTTTATGGTGTGTGTTAAGTATACGCGTTGAACATACTC
ACTATCGGTATAGGTTTAATATCTGTCGAGGTTCAAACAGTCGTTCTGGTCAAGATAATGTGTATATCGC
GAGATTCTTTTTCTATGAAATTTCTATTTTTAAACAAAGTTGGTCAATTTGCGTAGGTAAGGAAGATATA
TTGCATTTAATACATGGACATTTGTTTTTAAATAATTTCTGATTTTGTACAGCGTTGTGGTGCTATTCTCA
GCGGTGTTTAGGAAACAGTTTGTATGTTATACAGTAAAAAAAATGCTCCGGTAAGGCACCTTGTTCCTA
ATTTTTGTTCGATATATTTTTTTGGTGCTGTGACCGAATAATTTTATGAATTAATGGAGTTAAGTTTTATTT
ATTTCGGTTAACGCTAGTGGTGCCAAAAATGCGAGGTGCCAAGTTATTTAACTTTAGACCTTGTGGACA
AAACGATAAGAAACAATGTTACTTCCATGCCTGTTTTCTCTGTAGAGTTTGTGCAATTATTTTCTGAATT
TTAAGCGTTTGGTACAGGGAGATCTGACGGACAGCTTTATATTATAATGAACACAGTTTAGACTTTTATA
CGAATAATAATTAATAAATACATTTAATAAAATCCTTAAAACAGTTCAATAATGTAAAAACAATTGATAA
AATCAGATTCTAGGGTCGACAGTGGACACCACCCGTGAAATTTGGAGCAATATATTGTCGAATCGAATA
AGACATAATATCTAGTACTGTTGAGTCCAATTCGTGTCAGTTTGAAGTCCGATGGCGTTGAACCAAAGG
TATATTTCCAACCTACATTGCAATTCCAAAAACATATTCCATCCAAGAACCGTCAGATCTCCAGAATGA
AAGGGAAAGAAACATACTGTCTCGCTCGCGGCTAGAGCGTAGGCGTAGGATAGCGTAGTAAACGAAA
GACTGGCTAATGTAGGCGAACATAAGATTGTTGTTATGTGAAGAGTGGCTCGCTCTGGTGACTCTTTGG
CGAAACTACACTACAAAAATAGAGCAAAATAAGGAATCAGATAGTGGTGCCATCTATAATATTGTTAAG

GGCCAAATGGCGCAAATAATTTCTCATGATTAACGTAATATTGCGTTTTGATGTGAAGGAATAAAATTTC
AATATCATAAACCAACTTGTAATGTTTTTGTTCATCTTAAAGTGAAGTTAACGCCTAAGAGCCACCTCA
GCATATATCGGGAGGCAGGTATCTAGCTTGTAACGTGTTGTATCTTGTAAATATTCGCTTGTAGTATGTAA
TTTATATCCAATACCATGTATTTTAATATAAAAACAAGAGTATTTAGCCGCGTCTCCTGTACCCGACCCGC
GTTCATTATTTATTATTCATGGTCACTTATCTTATTTACTTCACTTGTAAGGAGTATTTGGACAATTAAAA
CAAGGTAATTAAAAGTGGTTATCCCAAAGTTGCAACTAAAAGTACCGCTTACTAAAATAATACTTTACC
TGCAAGTATGTAGTACCGACATTTCAAATTCGTAGAGCAGAAGTTGTTCAGAATGATAAGTTTCACCTC
TTTTCGGAATACGATGCCATTATTTTAACACCACTCCCAAGTATTCATTCATAGCACCTTTTAGTCTTCTT
ATTCCTCTTTACAATTTTAAAA
>TCONS_00008848 geneID=XLOC_001741 scaffold_11:597365-597801(+) Intergenic
TTTATGAGTCCGTGGTAACTCGAAATAAATGAATTATTATTATATTATTATTAAATCTCAAAATCTCAGTAA
TGTTGCTTACAATATTAATACGGCATTCCTTGAAGGAAAAAAATAAAGTCAGTTGCCATCATAAAATTG
CTTCACAGACAGTTAAAATGGTTCATACGAAAAGTTATTGCGCTGGGGTCAGGCAGAAATACATTTTG
GTTCTCTCTTTCCTCAGAAATCGAGAAATCACTAAATCTTGAACTGAAAATATTTTTATGGCAAAGAAA
ATATATTTTTCCTTGGTTTAGTGGCTGCGACGCATCATAACCTTTTCAGTTAGTACAAAATGTAATTCCTA
ATATTACAGTAAATAAATATAAGATTAAGTAAAAAAT
>TCONS_00008851 geneID=XLOC_001744 scaffold_11:782877-787585(+) Unclassified
GTGCTTTATTGATTAAAAATTAGTATTATACATAATTTCATTAAAATTAAGAAATGTTGGGTTAATAAAGT
AAACCACTTGTTTCTTACTGAAACTGGAAGCTGCTCCGAAAAATATTTACAAAATAACAAAAAAAAAC
AAACTTTAACATTTTGGGGCAGTGAGTCAATTTTACCCCTTCGTCCAAGTACGCAACAAAATTTCGCA
ACTGAAACTGAAACACTGAAAAAAATAAATATTTGCAAACAATACCAATTATTTTTATTTGAACGTGGA
CAAAAGATAATTTTAATCATATTTTATGCAGTACGATACCGATCACAATCATGCAAAGAAAATTATACAA
AGTTTTGTATCGAACAACATTAGAAGAGTAGCACTTGTTTGTGATAATATCAAGAGGCAAATATACCTA
CTGCCCCATTTAAAATAAAACGTGTCGTATGAAACAAACAAAACCTTAGTTGTATTATATACTAAACTAA
TTCCATCGCAGTACGCACTATAGTGATTTGTTATTGTAAAAGATGTTTAAAATGAAAACATACCTTATTC
AGAGAAATTAAGTAATAGTGAAATTAAAGGTAAATATCAGCATCTGAAGTGGATTTTTCAGGATATTTC
AAAGGCGGATTAAATATATAGATAGAACAAGAGATCAAAGCCATCGGTATCTATACCTACCTTAGCTTTA
CCCAGAAGAAAGGCTACCAGTAAAAAAAGTCATGTTAAGGGTTAATTTCTCTAAATAAGGAAATAAAA
TAAAGTAACACAATAGTAGTCCAGTGCGGAAAGTGCCAGGTATTCAGGACTTGTTTTTAAATACCTGAT
CATTAGTATAATAATCGAGAGTTATTTACATAAAATCGCCATATTGCACATTTAACATTATGTATACGTACA
ATATACATGATCGCTGCCGTCACTTTAGAGAATGGTGTCGATATAAGTTATGAACTTTATAGGAAAATGG
TTTATTGTTCAAGAATAAGTAAGCAGTTGGAATATTTTGGGAAGGCTAGAAGGAATTTCTTCCACTTCC
TGTATTAGCAGCTTCATGTATTTACCTACACAAGTAAGTAGCTAAACTATATTTTAAACACTGGACTACA
TAATTTACCGCGGTACTTAAAACACTTTAAGTTTAATGAACTTTACAAATAATAATTTAGTTTTAACACTA
TTATTTGAAAAGGCAAACAAATTGTCTATGCACTATTTATAGCGTTTAACGGTATTCTAGGTCACTAGTA
AAAGTAACAACTTTAGTGTTATACATCAGAATTTTACTTTTGGAAATAAATTGTTGGATGGGTATGATAT
TCCATATTTATGAAAGAAAGATGAGTCTATGAAATAAAAATATATAGTTCACGATATGATAAATAGAAAA
TATAGTTTACAAATGAATGAAACTGGGAAAGTCATTGCTTTTAATAGTGACTCAAATCCTAGCACTTATT
TTTACAACTATTACATCTAACTAAAGAATTATCACTTTGCAACCCAAAAATCTTTTTATATAATATTTACA
ATGTTGTACAACCAAAAATATTTCATATATTGTATTTATATAATAAATATTTTAGAACCTTAAACTCTCTGT
ACAGTTTTTTCTCCATAAATGTTTTTTTTAAATATATAAGTAGTTTTGTTTTTTGACAATTTTGTTTTTGGT
ATCACTCGCACTGAGTGGTCGAAAAATATAATATTCCACTTTTGTGTTAATTGCATTAAGTTTATTATTGC
GTTTTTGTTTATTTATATAAGTGTTTATTATGAATATAATGAGATACAAGGCTGAGATTAGATTTCCTATAT
ATAAACAAGAATTGCTATATAGTAAGGCGGTGCCTACTTAAATATTAAATTCTAGAAAGCCCCAATGCTT

TGTTATAACTATAGCTTTTTATAAAAATGGATCGATGCCATTTTCATCTCAGCCCTTAACGAAACTTAGTG
ATATTAAAACAATCCCAATTTTACTATCCCCACTAAAGATTATAAAATAATTAGATGATGGAAAGAAAAA
CAAGACAGTAAAAGTGAATGAATATAATTAAGTTCTTTGCTATTTTATTCACAAACACCGAGTAGTGAA
TAGTTAACTAAAAAGCATGTTGTATTTATAATTTGAACACTTTTCTTCTTTAAATAATAAATAAAATAAAC
TATAAGTACCATTCAGCTTTCCTGTACATATTAGATTAGATTACCGTTGTACTATCTAAAGTTATGAAGAG
TAATCAATTAAAATATATTACTCAATGTAAGTGAGATATTTGCTGTTCGGCTTGCCAGCAATGTCGTATTT
AAAACCACCGACAGAAAAAGAGGGGTGTTATAAGTATTTACTTGATACACATAACGGCGAGTGTACCT
ACCTACAATTTCAAATTGTGTACATAAGCCATCTTTGGCACTAAATATATAATTGAACGATATTTTAAGCG
GATGGTTAGCAAATAAAGTAAGCCTCTTATGATGAATTTTTCAATCCGGTATCTTAAGAGTTCTAATATC
AGTTTTTAATCCAAAAAAACATTCCATCATCATTTAAATTGGTGTTACTAGCGTAATAAGTGTAGATATT
ACACGGTCAAACTAACTAATTTAGTATTTAGTTTTCTATTAAAGACATTATATTATCATCACTTTTGAATT
GCATTAGGCCCTAGTCTAATGGTGAAGTCCATAGGTTATAATAAGTAACTTTACAATTCGCAAGCTTAGT
ATACTTATTCTCAGTGTGAGCGAGAATAATCACGATGTTATACAGGATGTTACAAAAAGGAAGCTAAAA
GTCAGTACATAAAAAATTCGTTTCTTAGAATGAATGACCTTTACAGGGGTTGACCCCTTTTCTACTTTTT
CTAACACCCTGTACATATACTAAGCGTATCCGTATATTCACAGTGTATTGTATGTTTTAAAACCGTGTTTT
ATTTGTTAATGAATCCAACAGTATTGTGCTATAAGCGGTGTCATAGTAGGGTCTAATTTTATGATGCACT
CATTATATTTGGCAGTGTTCAAAGTGATTGGCACCTTACGGCTATCACTTTAGAGTACATAATGCTATTA
ACTAGCGGTGTGTAAGAACCCTTGAATTATAAGCCAGCATTATATAAATGTCCTTGGGTAGATAATCCAG
AAATTACAGTCATAATAATTTAAGTTTGCAGAGGTGTTTTGCAAAGTGTCAGATGTATGTTTTAACTCCG
ATAATTTCGAGGATTAATTTGTCGTAATTAAGTATACCTAAACTTTTAGGTTTTAATTAAGTATATGCAGT
GAAATGACATTTCATGGGTTTCCAATAAGAATAATAAATAGGCTTGTAGCTAATTGTGAGTATTCTACAA
CAAATGAATGCCAAGTTTGGCTATACAGAATGTTGCAAAAAAGGGTGACTCAAGACTCTTCTCTCAAG
ACTCACTCGTAGAACGAAAGTTGTTAAGAATGACCCCCTGAATCACCCTCTTTCGGCTTAGTATACCCT
TTTTGCAACACGATGTTTTATAATTTGTGATTATTATGAGTTTGTAATCGAATCAGATCCCTACTGAGACT
ATTTTACGAAAAAACATTTTCAAACTTCATTTCACTGCAGCTAAATAAATAAAAGATGCACTACCTATAT
GGCTGCTTTTCCACCAGAGATGTGCTACGTGAAAGAAACTCCTTCCCACCAGTGCTAAGGTTAAAGGT
TTAAGTTTATTTCCTATAAGAATATTACTTAAAACGTTTATTTAAAACGGGAGCAAAATTTTCTAATTATA
TTTAGTTTAGGTTCAATGGATTGGAGTGTTAGTGACTGGCAGGTTAAGCTATGTGCACATCTCTGCTGC
CGCTACACGGGTTAGTTATCGACGGAGATAAACGTCACTATATCGCGATTCGGCATAAATACGACTGAT
TGGTGGAACGCGCATTAAACGAGTTTATCAACGGCGATAACAGACCCGTGCCGCGCTCACTGGTGGA
AAGCTATAGAATCTTCTGTCAGTTTACTGCCAGTTTCAATACTCTATCTATGAATAGCTGACACTGGCGG
TTATTTTCATAGAATGGACAACATTTCAAAAAACTAAAGCTGCTATAAATCGAAAACCATTTTGGGATTT
AGCTCTAAAGGCCACAAAAAAAAATGTTTTTGTATTTTTTGTATGTATCATTTTCGAGAAAATCATAAAAT
AGTAAAAACGTGCTGATGACGTCATGCATCAGGTGCGATGCATTTTGATGATCCAAGTCTATAGATTTA
AAAATAAAGTAACTGTCACTTTAATTTTTAATTGACGTTGACGTTTTATCGTGACGTTGTTGTTTATTTG
GGTCATTTTCATAAATTCTGTTCTGACACTTTCTGACAATTTTTTTTATTTATCTTTCAGCTATCGATAGAT
AGAGTATTGAAACTGGCAGTTAGGGTCTACTCATGGTGTGTATCTTTGTATGTGAATGTCAGTATGTGTG
TGTGTATGTATTATGTTTGTGTGTGTTACTCGTGACGTCACTCCTCGTCGGAATCGGGGTCGCTGGAGA
GCTCGGCGGCGGCGGCGGCGTGCAGCCGCGGC
>TCONS_00009261 geneID=XLOC_001756 scaffold_11:1267775-1272851(+) Intergenic
CCGTGTGAGTTGCCTGTAAGGTGGTGTTTCGAACATTACGTAAACATTTTCTATACAGTTCAAGCAGTT
TTAATACTGAAACATAGTGATTTAACAGTGGATAGCAGTTATTACCTCGAATACTAAAGCCTCCTTATTA
TTTATAGTGGGCTATTTCAGATGTTTATGTAATTTGAGAAGAGTAGATTAATCAGTGTCGCCGTGAGCTT
AGTTAAGTTGGGGGGAAATATAACTCTTATGATACGAGTGACATAGATATTGTATGTTGTGTTTAGAGAG

CGGCAAGGGATCGGGGATGTGGTCTTCACTCAGACGATGACAGCAGGGATGCGGTCGCCGCCCGCGC
CAGCTTGAGATGACCGGGGCGACCAACTACTCGTACGATGAGACCATCCGG
>TCONS_00009372 geneID=XLOC_001786 scaffold_11:1362170-1373741(-) Intronic
GGCCGGATTACTCTAGGAGTTGTTCTTTAGTTCATTTAGTTTCTATATTGTCTGTGATTCGCATTGACTGG
CCGGTCACTCGATTCATTCATTCATTCATTCAAGATGGCAGAAAAAAACAAGAATAACTTTTAAGGATT
GCTGTTTGTTATTAGATTCCTTTTTGAATAGTTTGTGTTCCTTATAAAAGTATTTTACTTCAAACTTCCTC
AGTGTCTTATTTTCAGCATCTTCTATAGTACCAAGCTAACATTTCAGAATTGAGATGAGTAAGTCTACGC
CCACCGGTAAGTGACTTTATTTCTGTGCTTAGTTATGAAAATTGTTATCTATATAACAATATGCAGTTTCT
ATGATCACTAAAAATTAATTTACGTATCATTAACCACTATCTTAAGAAATTAATATCACGTAAAGTCCAAA
TAAGGTTATAAAAGGTAAAAACGCGATCATCACGCTATATTCGGCGAAAATTTTATCATTGTGATTAATT
TCAATATCGAAAAGTGCAAAACACAGCTTTATAACTAAGGTAGTTATGTATTAAAATCGTTGTCTTGCA
AACAGCAACAGTGAATGCTGCAGTACTTTATTTGAAATATCGCGTGCGTATTAGGCCTAAATGCAAAAT
GGAGCATTCGCAATTCCTCCCATTATTGTGAATTAGCAGTAGCAACTGTAGCAAGTCAATCAATAATTAA
TAATCAAAAAGTTTATTTGCGTCTTTGATTCAATTGATCAAGTAATAACTGAATTTTAGCATGAACTGAC
GAAACCGTTTTATAGACACTGACTGGGCTATGTAAAGAACCAAAAATAAAAAAAAAACTTAATATTGC
GATGTAATATCCACCATGGAATGCCAACCGTCCTTTAGAGTATTGTACTCTATTACTCTATATCATTTAAG
CGTGCTCTATAATCTATCTATCTATAACACTAATAGAGTACGCTAAAATACCCTTTTTTTTACACATTTTTA
CTATTCATATAATAAGGCAAAAACACATAATCAAAACCAAAAAAAAAAAAAAAGTTGACTATTTACTTT
GGACGATCTTGAACGCAGCCACTAATGTCATTACAAACGTCAAATGTCACATGTGACAAGTTATTTTGA
GAAGTTTTGAGGTTTGTTTTGTTTGTGACTTTGCTTTTGTTTGTTTGGAATACTTTTATCCATTCGTCATG
ACGTGATTGATAAACATGATAACGCCGATCAAGCATCCTGTACTGATAATAATAACGCAAGCACCCCAT
CTAAAAATTGAAAAGTTTTGACGAAGAAAAAGGACACAGAAGTACACACAACTTTGGGAAAATGAGT
TTTCATGGCTAAGAAAAGACTCAGAAAGTGATACAAATGCAAAATCCATAATCTGTGGCGAAGTTTTC
TCTCTATTCCATCAGCTGGAGTTGGACACATTCTGAGGAACAATGAACATACAGTGTAGCCGACCAAA
AATTCACCTAGCAACGTTGCAGCGTAGCAGACCAGCGTCAGACCAACATTAAGTACATCAATACGAAC
AGCCGGAATATGGTTTACTAATTACTTATAGTCTCTTTGAGTTTTGTTTTTAATTATGATTGCTAAGTGCA
TTTAATATCACCGCATAGATATAATAACTTTATTAATTTTGCTAATTCATTCAGTTTGTTAACATAAATTTA
GAACTTGTTAGTTAGCGTTCGAATGGCCGACAGGGAATGGGGACATTCCCAATAACAGGACGGCCGA
CTGTTAGCGTTTAAATTTACCAATGCTATTCTTTACTAATATCAAGCTACTTATTTCATCATAGGTCTGGC
CGGATTACTCTAGGAGTTGTTCTTTAGTTCATTTAGTTTCTATATTGTCTGTGATTCGCATTGACTGGCCG
GTCACTCGATTCATTCATTCATTCATTCAAGATGGCAGAAAAAAACAAGAATAACTTTTAAGGATTGCT
GTTTGTTATTAGATTCCTTTTTGAATAGTTTGTGTTCCTTATAAAAGTATTTTACTTCAAACTTCCTCAGT
GTCTTATTTTCAGCATCTTCTATAGTACCAAGCTAACAATCCATACTCTACGATAAATAATTCAGAACCAT
TAAGACTTGAAAACATATTGTATTGTATTGTAATTTATAACACTGAAATGTTAAGTAAATATACCTTTTTA
GGATTTGACATAAATTTTATTAATTTATTTGAGTACCTAGGTACTTAGATAATCTTTTAAATAATGTAAAA
AGCCTTCTCATTTGATTCTCACAGCAGCAAACAAAATATTTACCGTAGCAATCTTTGCTTAGCCAACTT
AGAGGTATTCAAGCCAGTTAATGGCGTTTTAGGCTTTTTAAACACACAATGACGACTACGCTTTCATCT
CGGCGACATTATAAGGCAATCATACGACAGCTTGGTGGCCACGGTGCACTGGGGCGCCTTAAAAGGTG
TCTCCAGGTATGGAAACCAGGCCCATTGTGACCGGTTGTTCACCTAAGAAAAACTTACGGAATTCTAA
ACGAGCGTTTATTTGCCCGCGCTTCGGTGCAGGGTTGCTGTTGAAAGTTTTAGT
>TCONS_00009422 geneID=XLOC_001787 scaffold_11:1521622-1522428(-) Exonic overlap (+)
ATTTTATGTATAGTTTACTTGATTTTACAGGCTCTACGTTACCCACTTCAAATGTTATTCTATGCGTGTAA
ACTTTAGTAGTAGATAGATGAAAACATTACTTGCGAAAAGTTAATTTCAAGGTTTGTGGTGTTGGTGAC
CCTCGTGAAGTGATAATTTGTTCTTAATTTTATGAACCATAGTAAAGATTTACCACAATAAAAAACTGTG

TGAAGTGTCAAAAACTTAATGATAAACATTGTGCGTTAACGTGAACTGTTAACCGGTTAAATTTCTATT
GGTGGTCTACAATGAGGTTGTGTGGTGTGTGGATGATGTAGCCTACTAGTGGCTAACCCAGAACGCCG
GGCGGAGCCCAGAACCCTTTAAAATATTGTAATTGGCTGTTCCCGCTCATTTGTAAACATGACAGACAC
GCTGGTCAAGGTGAAGACCGAG
>TCONS_00009295 geneID=XLOC_001789 scaffold_11:1554647-1557736(-) Exonic overlap (+)
GCCCGACCTGCGCCCGCCAGCGCACTACATGCATGACCCTGAGCGAGACAGCTGCGTGGCGTGCGTG
GCGCCACTGCCGCTGGCCCCGCCCCCCGCGCCCGCGCCGCACTAGTGCAGCCAGCGATCAGCAGTTG
CAGCACAAAGATATAGCTTTCCAATAGTTGAAAATGTTTAATTATTCATTAGGTACTGTAACAGCTCAGC
CGTTAGATAAATTCCACTCCACCTGTTCTTGGTTTCACTAGTTTAAAATAATAACAAGACTAAAAAATTA
TTACAATAAGTAGGTACTTACCTTATATGATGCTCCCTGTGCTTCACTATTTGGCACTGCTTACCTGTTTG
TCACTGCCCAACTCATGAATGAACGTAGGCTTAGGAAGGCACTAAACTTATCCTAGATGCAATCTTGAA
GTAGATATGAAATGTGCAATATCTGTGTAAATTATATCCACCAACTGCGAATGGGGAGTGAAATCTCAA
AACACTTCAGATTTGTTCATGTAAAGTATCTGAAAAAGGCTTATTAACTCGAAATGCTGTAATATGAAAT
ATCCTTGCACTTCATTGATTGTGAAGTGGTATGGTGATGAAGTCGCCATTGACGCTATATGATATAAGGA
CAGTAAATCGTCTTGGTTTTAATGTGTAAATAATTTTATCTTCTTCCTAACATACCATTAATGTAGATATAA
CTTAGAAATAAGGATAAAACATTTTAATTTAGAAACAATTAAGCAATCAGTCTAGTCAAGTATAAACCT
AGTCAGGTATACCATATCTTACACTCTGTATTTGTAAGATAGTAAGGTATTCTTGTATGTACAGTATTCAC
ATAATGTGCTACCTAATCAACTAAAATAGCGATGCTAAGCGCCTTCTTATAATCTTGTACAGCTTACCTA
ATGTTACTCGTTTCTGAAAGTATATTACTTAGCTATTTGTTTCCATAAAAACACCATTATAATATGACTTA
AATTAAATACATATCATAATGTTTACTATGCAAATGTAGATAATCTTTCAATAAGTAAGCGCCAATTCATT
ATAGAAATAATTGTTAGAGGCACA
>TCONS_00009698 geneID=XLOC_001923 scaffold_110:643227-681906(-) Unclassified
GTTGGACATAGTTGGACGATATCGCACGGGCGCGATGCGTCGAAAGCGCGGCGGCGGCGGCGGCGGT
TCCACAGAGTAACTTATTCAGTTCGTTGGAGAGCGCAGACGCGGGCACGCACGCTGCGCGCGCAACG
CTCTGTTCCAAGTTTGAAAACTTGCCGCGAAATAAAAACAAATTTCAAAACTGTGCTCTATGATAGTGA
TGTGCCCTCTTGTTCTGTGATTACGTGCCAAGGGAAGTGACTGCACCACCCGGTGTTCATGACTGAAG
ACAGCAGCATTTTTCTTTAGCCGAGTGTCGATCAGAAGCCGCCATGCTGCAGCAGATCTTGCGCGACC
TGTGGGTGGACCCGGAGA
>TCONS_00009996 geneID=XLOC_002002 scaffold_1104:14175-16316(+) Splice junction overlap
AACCTATTATCGTGTCACATATTTACATAGACATTAAAGTGTTTTAGATGTAAACAATACAATATTATTTC
GTTGTTTGTTTTAGGCAAATACAAAATCGAAAGTCTGACAGCAGCAACGTTTCCCGGAGCTATAAATGT
AATAAAAGTATCCTTCTGCCAAGACGAGGCAGTGAGCATAGGGGCGGAAGTGGACAAGGATCCCCTG
GCT
>TCONS_00010013 geneID=XLOC_002005 scaffold_1105:10690-11760(+) Exonic overlap (+)
GATGTTCAGTAGAGAGTGGTTACATCGTGTCGTGATGTCTGCGGGTTATCGCTAACGTTCATATTTACTT
TATGTACATAATTATCATATTATTAAGTAACTAACAGTATTGCAGTTACAAATAGGAATTAAATTAATTTTA
TAATTTTAGTTAGAATTGTGATAATGTATTACCGTACAAAAAGCTGACATTGGGTCCTGAATAAAGTTTG
CACGGAGTTCCTGTGAATTCTATGAAGAATGGTACAAGTTTAAAGGAAAGACCGCATCCTACCGACAC
CACAAGTAGTTAGCATCAATGGAATAACTGTCTACAAATACACGAAGTATTAACAGAGAGAAGAGTAC
CTAAGGTACCAAGCTACATCACAGATTTATCTCTAAATAAATTCAAGATGCAGGAAACAGCAAAACAA
AACCAGCAAACAGTATTTACAGTTGAAGATAAAGAGCAAACACAAGTTACGAAATATGAAAACAATG
AAGTGCCGTTTGAGGAAGCATTAAATATGACAGGTGA
>TCONS_00010066 geneID=XLOC_002011 scaffold_1108:14972-15975(+) Splice junction overlap
GACAAGAAGCTAGCAACACGCGTGCTAAGCCCGCCCAGCTACGCCGCGCCCTCCTCCGCGCCTCGCG

CCGCGTCGCGCTCCCCGTCGCGATCGCCCTCGCCCGACCGCCCCGACAAGATACAGTTCATCACCGAG
TTCGGAGGCGACGAGGAGACTACGCCATCAAAGCCATTATATTCCGAGAAACTAAAACAGAATCTAAA
GAAAACAGCACTCTACTCTGAAGTAGCTCGGAAACCTAAAGCTAGGT
>TCONS_00010061 geneID=XLOC_002011 scaffold_1108:16253-16775(+) Splice junction overlap
CGCGTCGGCGATCTCGATCGTTCTCGCGATCTCGATCCAAGTCCAGCTCGAGATCGAGATCCAAGTCG
AGATCGTCGAGCCCCCGGAGGGGTAGTAATAGAGGACGACGCGACAGCACTGAAAATAATTCTAGTG
GCAAGCGGTATGACTCAAGATCAACACAAGAGAGGTAAGCTGGTATATTTATTATTGATATGATCTCGAT
ATCATCATTGATATTGGAAACCAATATACAGATTGGTAAGTAAAACACACACTAAATTGCC
>TCONS_00010081 geneID=XLOC_002011 scaffold_1108:19237-19916(+) Splice junction overlap
TAAATTATTATTCTCTTGCAGGGCATGTCGAGAGAGGCTATATCACTAAAGGAGAAACTCAAGAAGAA
AATGCAGGCACAACTATCAAGGCAACTTCGCGCAGACAAACGGGCTGAAGCGGAGCGGTTAGAGCG
AGAGTCGCGGCGACAAGCGAGGCGAGACGAGGAGATGAGAGAACTGGCCATCAAACTGCGCCGCAA
GTCTGTATACTAGTGCCGTCTCTGTCTTACACTGTGGGGGGAT
>TCONS_00010263 geneID=XLOC_002025 scaffold_111:413112-427260(+) Intergenic
GGCCCACACTCGCATGGAAGGGTTAATAGCAACGTATAGCCAGTCGACCTTTGACCATGCCACTGTAT
GGTCTGTGAAATTAAAAATTTGAATCAAGAAAGTTGTCTGCCAAGGAGACCAAGACTCAACAACAAT
GGCATGGCAGACCAAAACTACACACCTTCGCAAAATGAAGATTCTAAATAAACCAAAACCAACTCAC
CGCCACCGCGGTCTCAATCTCCCAGATGACGATATACCTTGAATACCTGACTTCAAAATGGATACTAAA
TCAGAGATCTAAACTGGCATATACAATACAATATAACTTTATTGAACAAAACACACAACATACATTAAAT
AACAAAAGGAGTAAGCACAATAGGCGGCCTTATCGCTAAAAGCGATCTCTTCCAGGCAACCTTTGGGT
GAATGAGAGACTTATTGAGTAAAAGAGGAAGGTGTACACGTATTGCATATACTTAGTAGAAAAAGCTG
AGTGGATGTGCCTTTTTGACACGCAGCATATTGTCCAATGTTTTGTACTGGTACATATATTTTTTTTGTTT
GATTTAATTATTACTTAAGTTCTTTATATATAAGTACTCAGCTGTTTTTGAAAGTTACTGTATTTTTGTTTT
TTAATTTTTTTTTGCTGTGTGTCAAATAAATGATTTCTATTCTATTCTATTCTATTCTAAAAAAGAATAGTT
ACCTCCTATCCCGCAAAAAGGACAATAAAGGATAAAGGTTCGACTATAAATGCTAAACATGCATAGAA
CAACGTGGACTTGGGTGTACTCCGGAAGCATGAAAACTACGATGCGTTACGGGTCTTTGATTGCCATG
CCTCCGCTGTCCTGTTTCAAGATGGACTTAAGAAGCAAAAAGGAAAGAGTCCAAGGGTGGCCCCCAC
CCCCGCGGCAATGGAATACAGAATAATAATCGCCAAAAGAAACGAGGGGGGGGAAGGAAAAATATAA
ATAGCCTTAGGAACCTACCTACCGACTTTCCCGGCGAGATATTAAAATTTGTACAGAATTGCTCCAGCA
GACCGTTTCCAATTTAGTTTTAAAATATAAATTGTGTTCCTCAAAAGAAGCATTTGCTTCTGTCTTGTCT
CTGGACGGGCGGGCATCTTGTTTTTGTGTCGTTTGGAATTAAAATGGCCGC
>TCONS_00010264 geneID=XLOC_002025 scaffold_111:413112-427260(+) Intergenic
GGCCCACACTCGCATGGAAGGGTTAATAGCAACGTATAGCCAGTCGACCTTTGACCATGCCACTGTAT
GGTCTGTGAAATTAAAAATTTGAATCAAGAAAGTTGTCTGCCAAGGAGACCAAGACTCAACAACAAT
GGCATGGCAGACCAAAACTACACACCTTCGCAAAATGAAGATTCTAAATAAACCAAAACCAACTCAC
CGCCACCGCGGTCTCAATCTCCCAGATGACGATATACCTTGAATACCTGACTTCAAAATGGATACTAAA
TCAGAGATCTAAACTGGCATATACAATACAATATAACTTTATTGAACAAAACACACAACATACATTAAAT
AACAAAAGGAGTAAGCACAATAGGCGGCCTTATCGCTAAAAGCGATCTCTTCCAGGCAACCTTTGGGT
GAATGAGAGACTTATTGAGTAAAAGAGGAAGGTGTACACGTATTGCATATACTTAGTAGAAAAAGCTG
AGTGGATGTGCCTTTTTGACACGCAGCATATTGTCCAATGTTTTGTACTGGTACATATATTTTTTTTGTTT
GATTTAATTATTACTTAAGTTCTTTATATATAAGTACTCAGCTGTTTTTGAAAGTTACTGTATTTTTGTTTT
TTAATTTTTTTTTGCTGTGTGTCAAATAAATGATTTCTATTCTATTCTATTCTATTCTAAAAAAGAATAGTT
ACCTCCTATCCCGCAAAAAGGACAATAAAGGATAAAGGTTCGACTATAAATGCTAAACATGCATAGAA
CAACGTGGACTTGGGTGTACTCCGGAAGCATGAAAACTACGATGCGTTACGGTGCCTCCGCTGTCCTG

TTTCAAGATGGACTTAAGAAGCAAAAAGGAAAGAGTCCAAGGGTGGCCCCCACCCCCGCGGCAATGG
AATACAGAATAATAATCGCCAAAAGAAACGAGGGGGGGGAAGGAAAAATATAAATAGCCTTAGGAAC
CTACCTACCGACTTTCCCGGCGAGATATTAAAATTTGTACAGAATTGCTCCAGCAGACCGTTTCCAATTT
AGTTTTAAAATATAAATTGTGTTCCTCAAAAGAAGCATTTGCTTCTGTCTTGTCTCTGGACGGGCGGGC
ATCTTGTTTTTGTGTCGTTTGGAATTAAAATGGCCGC
>TCONS_00010157 geneID=XLOC_002040 scaffold_111:519774-536676(-) Unclassified
GCCCGCCCAGTCGCCACCAGACGACTAACGAAAGCTTCCACATTCAAAAAACCTCACCAAAAATTAA
AAACCTGTCACCCACCAGCCGAGTTCCAAATTCAACATTTTCCAAAATGGCGCGAGTGATAACGACCC
AGTGACCGGTGAGCGCGCAGGCTTGTCGGGAGAATGGACGGAACTTAATTTATAAAACGTTGTGTAAA
TCGATGTACATAATATTATATACGTGAGAGTGCTGTGCTGTGTTGTTGTTTAAACCGATTCCTCAGACCC
TATGGGACCAACCTTCAGGATATGCCACGTCGTATACAAAAGCAAAACCTTACTAATTTGAAACAAG
>TCONS_00010400 geneID=XLOC_002076 scaffold_1114:5429-6112(+) Intronic overlap (-)
GTCTTCAGCCCCTCAAAGTAGTCCCGGCTCCACTTCTCCCGGGCGATGGATCGTATGACGGCGGGCGC
GTGCAGCTCGGTGTCGAAGTCGAAGTGCCTGGTCGTCGCCGTCCACTCGCCCTGGAAGTCTACCTCGA
CCGTCTTTGAGGGGTCGTTCTGATACCACATAGGTCCCTTGTACTGCAGGGTCCACTTCTTCATGGGCT
CGACAGGCGTCATCTTGAGACCCTCAGCACCAAACTCTCCCACTTCTGCTCCAAATAACACTGTATCC
GGAATCTTCTTACTGCATAGCAGGCCTTTACCTGGT
>TCONS_00010434 geneID=XLOC_002080 scaffold_1115:20092-22747(-) Unclassified
GTATAGTACAAATTACAAATGTCCAAAGTTCTACAGTGACGGTGATAAGACTGAAGAGTCCAACAATC
ATGCGGCGAAGGAGGCCGGCGGCAAGTGAGGCCACGAGCGAGTAACAACATCTTCATAACGATCTGT
TGCGTGAAAGCAGAATGACAAAACTAAGGTATTGAATTTCAACAAGTGTTCATCCGTAAATATTAATAT
TTTTAATGAAAAAGTTTGTTAATTTATCCATGTTTTGCATCGATCAAAATGTAAAAAATATGTCTTATTAA
ATACCGAGGATAAATATTCTGTAATTCTCACGGAAAACCTTATAAATCAAAGTTAATAAATCGAGGTAA
AGCATGTCAATTAATCATCTTATGTCATCTGCTTTTATTCAACAGATCCTATGTTAGTTCTGGCAGATTGT
CTACGGTTTCCCATATCAATGATAAAATTTTCATTATTTGTAAATGGTAAAATTCCATTCCATTTTTGACG
AAAGTTCTATTCTGATAATCTTCACGACGTTTTTTATAACTGATAATGTTTTTTCAAACCAAAGTACTGC
CTTTATACAAATGGTCGATTTTCAAAATAAATCTTATCTTTGAGGCCTTGGGAAATAACTGGATCATTGA
AAATACTATATTTACCAAACCAGTAAAATATGTATGTAGATAACGAAGACACTACCACGATTGCCTTACG
TTTTATAAAGTAACTTGTAATTAGGAACAATATTTCAATTTAAAAGCCTTTAAAATTATATTTCTTACAAA
ATTAGGTTCATAGTTTTAAATTGGTATTAATTTGGTTAAGTTACTTAAATAAAGCCTAATATTTAAAGCCT
TTTTGAAACGTCTGAAGCGGTTTTAGTGTTTTAATACAGCTCAATCGTAAAATACAAAATATTAAAGTG
ATTTTTCTTAACTTAATTAATCGCATAAGGGTTGTAAATATCATGAGAAGTGTCAAATATAGATTGAGTG
CCTGTTTCACAATGTCTGGCTACAGTGCCGACGCATCATATTATAAAGTATTCTTGAGATCTAACTTCTG
TCAAGTGGCCCCTCAAAGATGGCGTGTATACAATAACGAACATGGCCTTTCGTAGTACTATGATTAGTA
ACTTGAATGTCGACGTTGTGGTCATCATATAACCACAAATAAACTATTCAGGACTTATGCAAGTATACAT
TGTGAAACAGAACCTTAGTGCAGTTATTTTTAGATTGAACTGAGTGTGAAACATTTTATGAATTCTATTT
ATTGAAGGCCTACTAACAAATTGATACCTAAAAACGGTACTAACTTTTAAATATTAACCTTAGTTTTACA
ATCGTAAATTTTATGTCAACATAATATTTAATATTTACATGAGTTTATTATAAACTGTCATAGTTTATATTTT
GCTATTTTAATTGTTAAACTCTGGTGATTAGTTTACGGATGACATTTCATAGTTAGTATGTAAACTAACAC
CGGTTGTATGTATATTAGTTCCGTAGGCTATAGGAAGGTGCATTGTGTAGCATGTAAAAGTTAAAAATAT
ATTTTATATTACGGGCTAGCTTATTTCCAAATATGTTAAAAGTATTAATTAACTGGAATGCATCTTATTTGC
TTGGTTATTTAACGGCGCTTTATTTCAACATACAATTTGTACTTCTTCTTTTTCCCTGGATATTTTGTCTCT
ACTCCATGATTGGCATCAAAATGTCTGCATAAATATGCTACATTTACAGCCCTTTCAGTACAGTTGGCAT
AAAGTTCAGAGCTTTGTATCATAATTATAAAACTTGGAATAACTTATTCCATATTCACATCTATTGCTTTT

CGATTCAATTCGGCTGCTGCTGTGCCAATCATGACAAAATGGTAATATATTCATTCGTCTTATCTGATGG
ATTTCCTTATTATATCTTTTTTCCTTTCTAATGGTTTAAACCAGCAATGGTTTAGCAGTTACTATAGCTAA
AATAACGATAAGCCTAAATGTTAACAAGTTACAATGATTAGCGTTTCTGTTATATTTCCAAAATATTGCTA
GGCAAATTAATTAGGTTTAAGATATTGTTTAATTTCAG
>TCONS_00010593 geneID=XLOC_002114 scaffold_112:168609-171256(-) Splice junction overlap
GTGTATTGTATATCAGACCTTTATAGCGACTTCTTGACAAGCTCAGAACAATGCCTTTGTTCTTGATTTT
ATGTGATCAATGGGTCGTTCCCCTCAGTGAAATATGCTGCCTCACCTCAACAGTTTCATGTTTTCTATGT
ATTACAAATCTTGCTGGTCCTTAGGGGAACCCTCCTAACTGCAGACGAACCCATTCTCCCTATCCCACC
AGCTAGAAATTCACAATGGCGCCACAACACATCCCTTCATATAAACACCTATTACTTCCACAGGCGCTG
CTAACCGCCGACTTCGAGATCGGCCACTACATCCGCGAGCGCGTGGTGTCCCGCGCCGTGCTGCTGTA
CACGGGCGAGGGGCTCGACGACGACGATGATGATGACTACGAGGAGGAGGAGGACTCGTTCTCTGAC
GAAGATTCTGGCACCGAGGAAGTGTCGGACGACTGAGGTCGGCGACGGTCGCCCTCTGCCCTCGCCT
TCTGTTAGGCCAGCTGTCGCGGGACTAATGTCAACAACACTCTCATGTATCCTCACTGTTGACACTAGT
CCCTTCGCACATATAAATGGTTGTGCTTCAGTCTAACCGCGGTCATATCTACTGCATATCTAAGTCTAGG
CCTTTTGGCATTTTACCAAATAAACTAACGAAATTACTTCGCACAGCGAATAGAGAATACTCCCTCAGC
GTTATTTTATTCTGTTAAATTACGATAGTTGTTACGCTAGATGTTTAATGGAGGATTTCCAGCTCGCATTT
AGACTTTTCTATGCATGGTTTATCCGCTGACTAGATAAATATGTTTTAGAATTTCACCGTGAATACTTAGT
TCTGCCGTTGAGCAGTTTTATTTTTTACTACTTAGATTAGAAGCGAGAGAAGATGGTGCCTTCTTAATGT
ATATGCATGTTAGAGTTATTGGATATGTTATAACTTCATATACTATGGATATTGCATTTCTATTTTAGCTGTA
CATTTTCTCGTTTTATTTTTTCATTATTTATTGTATAACTATAGAATGTATTTTGTGTACGATTTCATGTACT
TGAAAATTTCATGTGATATTTCTCTAAGGCTATATTTTTGTTTTGTTTCATACTTAAATAAAATTTAAGTTT
CATTTTGTCATTCCATGTTACAGATAGTATTGTTATCCAAGCAGAAACTGCCGTTTAGTTTAATTAAGGG
ATAAATTGGCTATGGAATTGATAAATTGAATCTCTTGTTTAGTGCCAAACAATGCATTTTAACGATTTTAT
TATCACTAATACACTGTGATCCATCCTTTGATAGGTTTTGTAATGTTGACAATAGCTAGGTCGGATTATAA
TGTATATTTATTTTTATTTACGTAACTTTTATATTTGAATAGCATAATATGGTCAATGTAGCAGGCAACTCA
TGAGAACCTTATTTCTAAACATAGTTACGGCTTTATTGTGTTGGCATGTCGTTATGATGACATTAAATTG
AATGCTTTTTATTCAAAGTGTGTTTTATTTTTTTGCTTGGACCTTTTAATTTTGCATGCGTTTCGATTCGA
ACAATGTGGAAATTGCGGTGGCTGAAGAAT
>TCONS_00010765 geneID=XLOC_002115 scaffold_112:187073-194859(-) Exonic overlap (+)
TCTGTAGTAGGTATAATATGACTACCTTTATCCGCATTTACTCTTAACGACAACAGGCAAATATAATTCTA
AGCGCTTATGAACTATAATTTTGAGCGGGTCTTATACATCCTAAGCGCATTGATTTAATAACCATAAAAT
GTATTGAACCTTTTCGGTAATTTATTCCCTTTTTACTCTGTAAGTTTATCTTTTGATTACTTCCTTCCTTAC
TCATATTATTACTATGCCTTAGGTCTTGGCTGTGTGTGATAGATATAGCAATATTTAACAGTGTGAATGAA
AAAGCAATATTTTAAAATT
>TCONS_00010901 geneID=XLOC_002104 scaffold_112:338667-339134(+) Intronic
GCATAGTGTACTCGTAGGTCAGGCAGAATGAAGTATCAAATATTATTTCACACTTTGTTAATCTTAAGTT
TTTCAACAGCCGATGAAACCTGCGAACCCCAAGACCCAGCCGCTACTGTCCAACCATGCCCCGACCTC
ACGGAGTCTTGTGTCCAGGAATACTTTCAGCAACGAGTTCAATGCACTCCAGTCTTTCAGGAGTCTGG
AGCTGTCAAATTTGATACCATCACCA
>TCONS_00010681 geneID=XLOC_002122 scaffold_112:359459-371407(-) Splice junction overlap
GGCACCGTGCGCACCAACATCAACTTCAACAAGATCTCGCAGGGCACAAGCAACTGTCCAGATATGTA
CCGAAACATGCATTACACAGCGACTCAATGGCCGAGGGAACACAGCATGCGATACCGGAGCATCGAA
GATCTAGAAATGTTATCACCAGCTTAAAGCCTCATCTGTGGGGTTACCACCACCGAACATTAGCAATTA
>TCONS_00010621 geneID=XLOC_002126 scaffold_112:633883-635178(-) Splice junction overlap

CAGACGATGAGGACAGCGACAGTGACAACGACTCCGTGACCAGCGAGGACTTCGAGCAGTACCTGG
ACTCTGTCACCGGGACTGCTGCACAAGAAGACTCGGACGAGGAGCTGGACTTCCTGACGGAGATGGA
GAAGGCGAAACAGAACAAGAAGGCGAATAAGAAGAGCAAAGACGAGGATGAACAGAGCGACGATC
AGTTGGGAGGTGA
>TCONS_00010992 geneID=XLOC_002178 scaffold_1123:8715-12289(-) Exonic overlap (+)
TATGTACACGGCCTGCACCACAGGTTACTCGGCCTCAGATGCCGCTGTCAGTCTTTGAAGCCACTTAA
AAGAGGGGGTCATAAAGATAGTTACGATGGCTATTTGGCAGGACCAAAGCTAGCAACGGGTGTCTCTT
GCAAGAAGAAGACGACATGATGACCGTCTCTTTCATACCCAAGAGGCCGACGAGACAAGAACCCAGT
TTAAATGGATAAAAAAATATTCTAAACATATTTACTGTTCAACAGATTTCGAGCGCTCAAGCGCTGTATA
CAAATAACAGGCATCTAAAAATGATTTTATGATGAGGGTGCTTTTTCCATCGGATAAGCGCTATGCTACG
TTGCTTTGTATGCATTTGATATCCACCAATCATATTCATAGCTTCACATAGCACGTATATGGCGGAAAGGC
GCCCTAAAGAATAACGATTATTCATTCAAAATCAAACTGATCAAAACCCTATTGGATTATTGTATCGAGA
TTTGATCGCTTCTAGTCTCTAAAATGCGGTTGGCTGACTTCAGGCGGTCTCAAGATAATGATGTTTTAA
CGTTTGTGAAGATTTTCTCGAGCAAATAATTTAATTGCTTTGATTACTCAACATTCTGACGCTATGATATC
TACGCGCTGCAAATTATTACATTTATATTAGCGTCGTAGGTAAGACTCAGTTTGCATTTCAAATAACACT
CAACTTTTATGTATACATAATTATAAACTACTAAATAAGATATATTTCTCCCAACCATGTAGTCACTTGATA
AGTTAAATTAATTTCAGTGTATGCGTTAGAAAGCCATGTTTTCTGATATTTTAAGTAACCAGTGCTTGTC
CTACAATAATCCAGTCAGAAAAATATAGAAATACCTAACCTACAATAGTTTTTAGCAAAAGGAAAAATG
TGTAATGTGCATTGGTGATTTATAAACTGTATTCTATATGATAATTTACCTGTGCTCTAAACGTTCAGTGT
TGCCTTCTTGTACTTTTGTAAAAGTAGAAATACATTTCTAGCGAAATTTCTTAAACCGTACCTATAAATT
AACCGTAATACCTGATGTAGCCATAATTTGTATATACAGTTACCATTTTATATACCTAAATTAATACAGTTT
GTATTTGTAAAATGTACCTACCTACTTTAGGACTTATAACATAATACAATTTAAATGTTCAGTATGTCTAC
CCTATCCATTAAAGGGGAGAAATATGGGTGATATAGATAGATAGATAGATAGATAGAGTATTCTTTATTTG
TACACACACATAAAAGAAACTTAAGGCTTAAACTAAAGGTATCATGTATTTTTAAACCATAAAAAACAG
GCTAACTTAATTTTAAGACTCATATTATGCTAAAAGTAGACTTCTGTAAATATAACATAATATTGTTAAGC
GTGAATGGGATTAGTATCAAAAGGATTTATTTAGCCTGTGACGAAATATCACAAAATTTTTAATAACAG
>TCONS_00010966 geneID=XLOC_002178 scaffold_1123:8738-11795(-) Unclassified
CTTTATTTGAGGGGTATTTTTATATTAAGGAATATGTGTTGAAGGGGGGTATAAATTTATGGGCATAAGA
AATGGGGCATTCTTGCATTAAATTATATTAGCAGTGAATATAAAACTGTAGAGGCGAACTAAGGAGCTA
ACGGAACGTGCTATTAATAAGCATAGTATATCAAGCGTAATCTATTCCTCATATTCATATCAACATACCTA
CATATCCCTACCTATTTAATTTACCTTACATATTTACTTTAATTTTGTTTATTTATTTCAACCAAATTTTCTT
TCTTTTTCGACGCCGTTTTAGCAAGAAGCGTGATACAGGGAAATCTGGATTCTTGGTGTAGCCAGATCG
GCTATTTGGCAGGACCAAAGCTAGCAACGGGTGTCTCTTGCAAGAAGAAGACGACATGATGACCGTC
TCTTTCATACCCAAGAGGCCGACGAGACAAGAACCCAGTTTAAATGGATAAAAAAATATTCTAAACATA
TTTACTGTTCAACAGATTTCGAGCGCTCAAGCGCTGTATACAAATAACAGGCATCTAAAAATGATTTTAT
GATGAGGGTGCTTTTTCCATCGGATAAGCGCTATGCTACGTTGCTTTGTATGCATTTGATATCCACCAAT
CATATTCATAGCTTCACATAGCACGTATATGGCGGAAAGGCGCCCTAAAGAATAACGATTATTCATTCAA
AATCAAACTGATCAAAACCCTATTGGATTATTGTATCGAGATTTGATCGCTTCTAGTCTCTAAAATGCGG
TTGGCTGACTTCAGGCGGTCTCAAGATAATGATGTTTTAACGTTTGTGAAGATTTTCTCGAGCAAATAA
TTTAATTGCTTTGATTACTCAACATTCTGACGCTATGATATCTACGCGCTGCAAATTATTACATTTATATTA
GCGTCGTAGGTAAGACTCAGTTTGCATTTCAAATAACACTCAACTTTTATGTATACATAATTATAAACTA
CTAAATAAGATATATTTCTCCCAACCATGTAGTCACTTGATAAGTTAAATTAATTTCAGTGTATGCGTTAG
AAAGCCATGTTTTCTGATATTTTAAGTAACCAGTGCTTGTCCTACAATAATCCAGTCAGAAAAATATAGA
AATACCTAACCTACAATAGTTTTTAGCAAAAGGAAAAATGTGTAATGTGCATTGGTGATTTATAAACTGT

ATTCTATATGATAATTTACCTGTGCTCTAAACGTTCAGTGTTGCCTTCTTGTACTTTTGTAAAAGTAGAAA
TACATTTCTAGCGAAATTTCTTAAACCGTACCTATAAATTAACCGTAATACCTGATGTAGCCATAATTTGT
ATATACAGTTACCATTTTATATACCTAAATTAATACAGTTTGTATTTGTAAAATGTACCTACCTACTTTAGG
ACTTATAACATAATACAATTTAAATGTTCAGTATGTCTACCCTATCCATTAAAGGGGAGAAATATGGGTG
ATATAGATAGATAGATAGATAGATAGATAGAGTATTCTTTATTTGTACACACACATAAAAGAAACTTAAGGCTT
AAACTAAAGGTATCATGTATTTTTAAACCATAAAAAACAGGCTAACTTAATTTTAAGACTCATATTATGC
TAAAAGTAGACTTCTGTAAATATAACATAATATTGTTAAGCGTGAATGGGATTAGTATCAAAAGGATTTA
TTTTAGCCTGTGACGAAA
>TCONS_00011021 geneID=XLOC_002187 scaffold_1125:11186-12222(+) Intergenic
CACACAAACAACACCGCGAAACACAAATCGACCGTTCAAAACAAAACAACCCTTTCTTTCAGACAAG
TGTTTTAAATACTGGTTAGTGATGGGGTTTAGGAACCCGAAACTAGTGAATCTGTTGATGTGAATCGTG
AACTGTGTGGTTCCTAGGAAATGTTGTGGTTGTGATGTGTTGATTGCTACACGCATGAACCATCCAGTG
GCACACACCCGTGACCAGTGGAGCTCGGAGTGCCTGGGATGTGAGCAGTAGGAGGGCATATGGGGCA
ATGCGTGTCGCGGGGTCACCCCGGCTCCTACGCCTTCTCCCGCCACTACGACTCCGCCAGTCTAGGCA
GCCTCAGCTCGGGACCTCAT
>TCONS_00011385 geneID=XLOC_002221 scaffold_113:41568-42225(-) Intronic
AGCAAAATGAATGACAGACTTGTTCCACGCAATGCTGCTAGAAATTGAGAGATCCGATGGAAATATTC
AAGAGAAAAATGGATGTGTTGTCTCATAAAGGAACCAGGTAGATAAGTTCACTATTTAGCTTAAATGAA
TAGATCTAATTTTGTAATGGTGATAACAATAATTTGCACAACATCTTTCATAGCATTATCTTGCAATGAAC
AAACCACATTTTAGAATACGCCAATTGGTTTTGTAGATAAGTAATAAAAATATATAGCTTGTATATACTTA
TAGATATTCACCCAAATATTTTAATGAATGAAACTACTTAACAACAAAAAAACATGTATTGTGTTCACTT
AAGATTTATAAAGCACCTTAGACATTACAAATTCATATAAATAAGTGAGGAGTTAACTGAACGAAGTCA
GGACAGTTTACAATGAACCAGTAAGGGAGGTGTGTTTGTATATGTTTAAATATGTCTCAGTGAATTCTTT
GGATACCTATATTTCATAAAACTGAGTAGGTACTGTTTATTTATGCGAAAAAAACATTTAATTTGCACTCT
AAAAATCATGCA
>TCONS_00011344 geneID=XLOC_002205 scaffold_113:201919-202976(+) Unclassified
TAAAAAAAAAACGACTTTATGTTCCTCCTATAGAAAACGCCAATTTCATATGTAAGGACCTAATATGTTT
TGCGAGGAAACATCACAAAAGTAAAAGCTAACTATTGCACACATAACTTTAAGGCACTAACGCTATAA
ATTATTATGCAATATAACTTATCAGAAGTACCTACTCGTACATAAAGTAATATTTCAGTTACATATTTACA
AAGTTTATACAAAAACGGAAATAATGACTATCAGATTTCATTTATGATTTGATCAATGTTGTTAACGTTG
AATGTCTACTAAATAGTAAATTCGTAAAATCTTATTGTATGCTAGTTCAATATTTACTAAAACAAATATAT
TTTTTAATTTAATAGAGTTATTTGTTGCTTGTTTGTGGTGGTACGTGTTTCGGTTGCAAAGTTAGTTTTAT
TACTTTTATTAGACTCTAGGTTAACCCATCGCACCGAGCAAAATATATTATTTGGGCCCTGTTTCACAAA
TGTCTGTCAAAAAAATAATAACAGAGAGTGATAGCATGTATTTTATCTCACAGGTAGCCACTTACCAGA
CATTGTGAAACAGGCCCTTAGTAACTTGTTACTTGGCGTTAAAACACTGATAACCATGATACATACCCC
AGACCAGGACAACACACACCGTAGAGAGATGGACAGTGGCTGGGACATGAGTTATTGCTATAACGTAT
GGACAGTGGCTGTGACATGAGTTATTGCTGTAACCTGTGACATGAGTTATTGCTACAAACTGTGACATG
AGTTATTGCTATAACCGGTTGTGCGAGAGTGTGCCGATGATGTTGTTCGCCATCGGGACGTCATCGTTG
CTCCACGATGACGCGCCGCCTTGTGAGCTCG
>TCONS_00011345 geneID=XLOC_002205 scaffold_113:201919-202976(+) Unclassified
TAAAAAAAAAACGACTTTATGTTCCTCCTATAGAAAACGCCAATTTCATATGTAAGGACCTAATATGTTT
TGCGAGGAAACATCACAAAAGTAAAAGCTAACTATTGCACACATAACTTTAAGGCACTAACGCTATAA
ATTATTATGCAATATAACTTATCAGAAGTACCTACTCGTACATAAAGTAATATTTCAGTTACATATTTACA
AAGTTTATACAAAAACGGAAATAATGACTATCAGATTTCATTTATGATTTGATCAATGTTGTTAACGTTG

AATGTCTACTAAATAGTAAATTCGTAAAATCTTATTGTATGCTAGTTCAATATTTACTAAAACAAATATAT
TTTTTAATTTAATAGAGTTATTTGTTGCTTGTTTGTGGTGGTACGTGTTTCGGTTGCAAAGTTAGTTTTAT
TACTTTTATTAGACTCTAGGTTAACCCATCGCACCGAGCAAAATATATTATTTGGGCCCTGTTTCACAAA
TGTCTGTCAAAAAAATAATAACAGAGAGTGATAGCATGTATTTTATCTCACAGGTAGCCACTTACCAGA
CATTGTGAAACAGGCCCTTAGTAACTTGTTACTTGGCGTTAAAACACTGATAACCATGATACATACCCC
AGACCAGGACAACACACACCGTAGAGAGATGGACAGTGGCTGGGACATGAGTTATTGCTATAACGTAT
GGACAGTGGCTGTGACATGAGTTATTGCTATAACGTGTCACATGAGGTATTGCTATAACCTGTGAAATG
AGTTATTGCTATAACCGGTTGTGCGAGAGTGTGCCGATGATGTTGTTCGCCATCGGGACGTCATCGTTG
CTCCACGATGACGCGCCGCCTTGTGAGCTCG
>TCONS_00011359 geneID=XLOC_002225 scaffold_113:201919-202976(-) Intergenic
CGAGCTCACAAGGCGGCGCGTCATCGTGGAGCAACGATGACGTCCCGATGGCGAACAACATCATCGG
CACACTCTCGCACAACCGGTTATAGCAATAACTCATGTCACAGTTTGTAGCAATAACTCATGTCACAGG
TTATAGCAATACCTCATATCACAGTCGCAATAACCGATTTCACAGGTTATAGCAATACCTCATGTGACAC
GTTATAGCAATAACTCATGTCACAGCCACTGTCCATACGTTATAGCAATAACTCATGTCCCAGCCACTGT
CCATCTCTCTACGGTGTGTGTTGTCCTGGTCTGGGGTATGTATCATGGTTATCAGTGTTTTAACGCCAAG
TAACAAGTTACTAAGGGCCTGTTTCACAATGTCTGGTAAGTGGCTACCTGTGAGATAAAATACATGCTA
TCACTCTCTGTTATTATTTTTTTGACAGACATTTGTGAAACAGGGCCCAAATAATATATTTTGCTCGGTG
CGATGGGTTAACCTAGAGTCTAATAAAAGTAATAAAACTAACTTTGCAACCGAAACACGTACCACCAC
AAACAAGCAACAAATAACTCTATTAAATTAAAAAATATATTTGTTTTAGTAAATATTGAACTAGCATACA
ATAAGATTTTACGAATTTACTATTTAGTAGACATTCAACGTTAACAACATTGATCAAATCATAAATGAAA
TCTGATAGTCATTATTTCCGTTTTTGTATAAACTTTGTAAATATGTAACTGAAATATTACTTTATGTACGAG
TAGGTACTTCTGATAAGTTATATTGCATAATAATTTATAGCGTTAGTGCCTTAAAGTTATGTGTGCAATAG
TTAGCTTTTACTTTTGTGATGTTTCCTCGCAAAACATATTAGGTCCTTACATATGAAATTGGCGTTTTCTA
TAGGAGGAACATAAAGTCGTTTTTTTTTA
>TCONS_00011478 geneID=XLOC_002227 scaffold_113:230291-251756(-) Exonic overlap (+)
TTACCTGATTATTTTCCTTGACAGAGCTTGCGGCAGCACTTGTGAACAACAGTTCAGAAGTGCCGTTA
CTTGAGCTGTGCTCCGCATCAGCATCGCCATTGTTCACATTCTCCTTCATCACCACTCGCTCGCTCAGG
TTCTGTCCAAACACAAACCCAGTGGAAGACGTAGCAGCTTGAGCAGGGGCTGGCACAGGGTTCGCAC
TTCTCGGGGTTGCATTTGAAGAACCTAGTGGCATAAACTTGGGAGCCTCAGCAGCATCCTCCTTTACTT
TTTCTTCTACATCATCAGTATTTTTTTCTTCTTCATTCTTTTTCTCTTCTACTTTTTCTTCTGTCACTTTTAG
GAATGGATTGCTAGTAGGCTTTAGCTGTGGAGGCCGTAGTACTGACCCTGAAAAACATGAAAAGTTTC
ATTGAACAGAATCTTTGCATTTTAGTGTGGAAACTGTAGAAGTCAAGGACAGCAAGTATGCGCCTTAG
AATTAGAAGATAGTTAATGAGAAATTAATTGATGAGATAACAGAAAAACTCACCGAATGCATTACTGCT
TTTGTTTGAACTAGATGCTCCAAATCCTGAAGAGCCAAACCCTCCCAGTCTAGGTTTGGCAAGAACCA
CTCGGGACGAGTGAGGTGGCCCATTATTGACGTCT
>TCONS_00011414 geneID=XLOC_002229 scaffold_113:324465-334316(-) Intergenic
CTTCAAACTAACAAAGTCTGTAAAAAGCTTTCTCAGAATAGGCTAGCCGGCACAAAATACTTTTATGAT
AGTATAAGTTACTATGCAACGCCCTGTATTCCTACTATCTTTGTAATACTGAATATCCAAAAAAAACATG
AAAGCAATCAAATCGTGCGCTATCTATAATAATAAATAAACCAAAAATATAAATGCAATTTAATATTTATG
AAATGCATTCTTCTATAAAAAATATTTTGAATAACAAAACTAATGCTAATAATAATAAGGCACTTAACTT
AGTTTTATTATGCAATTACATATTTTTATAATGTGGTTACACTTGACCCTATAGTGAGTCACAGGAAGAA
ACCCGCTCCCAAAGCGCGCCTCTAGACCAGCCCAAAAAGGACCCCT
>TCONS_00011509 geneID=XLOC_002214 scaffold_113:427321-428558(+) Unclassified
CTTAAATATATACACAATAATACAGAACTTACGTATAAATTTGCTGTCCGCCAAAAAGGCGTCCACTCA

GCTATTGCAGCGCCCTTGGCCACAGCCAAGGACACTGCGCTGGTGTTTCAGTGGAGGCCTCTTTATTA
GTGACGCACAATAACATTTAATAAACAACAAAAAAACGAAAGACTTACAAACACAAGTGAAACAAAG
TGATAAGTGCGCGAGCGACGACATTGAGGCGGAGCGCGTTCGCCCTGAAACCCGTTTTCAGGCAAGA
ACGGGACACCCAGCCGACCACACGCGTCGCGCGCGCACCGAAACAGACTAAAGAATATTCAGAACAC
GGAATAATATCAAAGGAAAGTCTACATAAACATCATACTTATGGAAAGAAAGCAAAAATGGAAGGTTT
ATTACTTATAATAAATACATATGCCTACGTAAATGAGATGCTTCCACTTGTTCATGGATGAGCAAATCCG
GGAGTTAGCGGTTATACCTACTTAACGGAAGACCCACCCTGCGCGTCGTGAATAACTTCGAGATCACG
TATAGGCGGTGGGATGTTATTAAAGATATGACTCGCAAGATATTGGAAGCAATGGCGATACATAGCAGT
TTTGTGTTTGGGCGTAAGCATGAGATATGCTGATGCTCTCGTGCTAATGGAATGGTGATTGCGAGCCCA
GTCCAGCTTGTCATACAGGTAATCAGGTGATTGCTGCTTCACTACACCGAAGAGAAGTGTTGCCAGGT
GCAGCTTCCATCGCGCTGCAATTTTCAAAAGACCTCCCTTGTTCAAGAACGGTGTGACATGTGAACGA
GGAGGAATCGTGAAGCAGTATCTTGCGCATGCGTTCTGAACCCTCTGTAAGATCTTTTCTGTTTTACGT
AATAAGCAAGGTCCGTATACAGTG

>TCONS_00011549 geneID=XLOC_002214 scaffold_113:427713-428276(+) Intergenic

AAAATGGAAGGTTTATTACTTATAATAAATACATATGCCTACGTAAATGAGATGCTTCCACTTGTTCATG
GATGAGCAAATCCGGGAGTTAGCGGTTATACCTACTTAACGGAAGACCCACCCTGCGCGTCGTGAATA
ACTTCGAGGTAATTGCCGTTTAATTACTGGTTTTTTGTAGATGTAGTAGGTATTGTTTGTACCGTAACTTA
AAAGAGCTTATGGTTTTCAGATCACGTATAGGCGGTGGGATGTTATTAAAGATATGACTCGCAAGATATT
GGAAGCAATGGCGATACATAGCAGTTTTGTGTTTGGGCGTA

>TCONS_00011313 geneID=XLOC_002214 scaffold_113:428007-435163(+) Intergenic

GATGCTTCCACTTGTTCATGGATGAGCAAATCCGGGAGTTAGCGGTTATACCTACTTAACGGAAGACCC
ACCCTGCGCGTCGTGAATAACTTCGAGGTAATTGCCGTTTAATTACTGGTTTTTTGTAGATGTAGTAGGT
ATTGTTTGTACCGTAACTTAAAAGAGCTTATGGTTTTCAGATCACGTATAGGCGGTGGGATGTTATTAAA
GATATGACTCGCAAGATATTGGAAGCAATGGCGATACATAGCAGTTTTGTGTTTGGGCGTAAGCATGAG
ATATGCTGATGCTCTCGTGCTAATGGAATGGTGATTGCGAGCCCAGTCCAGCTTGTCATACAGGGGTAA
CTGAACGTCTTCACACAAACAGTTCGCTCTGGAGCATGGAGATCGAATAGAGTGATGACGTACTCAGT
GAACTCAGATACCATTCGGTTGACATCATTAAA

>TCONS_00011614 geneID=XLOC_002215 scaffold_113:552325-552760(+) Intergenic

ATTTGTTTTTGTGTATTCTACGCTAAATTTGACGTGATTTTGTGCGTGATGTCCGATGACGTTGACGGAG
ATATCCCACAGCTGTTGCAGCAGTGGAGTTTACCTCAGTATATTGATACGTTTCAAACGATGAAAATTA
CGAGGAAATGGAGTCAATATAATAATGGTTTCTGGGAAACATGCAGGACGAAAAAACAATTATGGCGA
CCATCCAAAGCTAAGAAAAAAGGACAGAAACTATAGCACTGTTAATGCTTTCATATTATAACTGATCTG
GATATTGATCTTCAACA

>TCONS_00011550 geneID=XLOC_002219 scaffold_113:929259-931401(+) Unclassified

GATCTATGGCGGGAATACACATTGGGATCTCGCTATCTTGGGGAATTTACACCGATGTTGTCAAGATCG
ACTTTCGATACGTTTATGTCGCGTTTTGAACTTGAATCCGTCAAGACAAAGTGACAAATACTAGAGAGT
GATCAGGATTTCTGACCGCGGGGAAGCAGTACAGTGCAGCAAACTTATCTGTGCCGGCGGAAGTGGA
CTAGATATATTCTTTGATCATTGCTTACTTATAAATAATGTAATTTATTTATACAAAACATTATGTAACATA
GTTCGGTTTTTTAAAATTAACGTGCCATTAAACCTGAAGGGTTTGTCTTGACACTAGAAAATCTTACCT
TATGTATATCTTATGTAGTTTATTAAAACCGTCAGGGTGAATTACTGCTATGGGCAAGAAATGAAAGAAA
CATTTACGTGATATCTCGTGTCATATTTGTTATTGTCAGCTGCTGATCGCCCTGGGTGTAAGTACCTGAC
GTGAATTTAAATAAAATAGCTTTTCAAATATTTCATGCTAAAACTTGGTACAGGTTTGCTTATCTTCCTGT
ACCATGTGTGTGTGTGGTTGTACCCTTATATGGCTAGCCAGAGCTTAGCCGAATGAAACAGAATAGAGC
CACTCTTGCAACTAACGAACATTGTCATCCGACTTCGCAGTTCACAATAACAAGTTGGTGCAAATAGGT

AGTTTGCATTGAACAATGTAGTTGTAATATGAAGAGGTTGAATAATATTCTAATTTTTCATTCCTATGTTC
CCACGGCCGAGCGTTGAGATCTACAGAGAACAAGATGTTGAGTGTGCCTGACCGCAACGCTGCTTCTT
ACAGTGACTCGTTCGCTGTGAAGGCGGCGTGTTTGTGGAACACCTTGCCCGTAGACGTGCGACTTTCA
CCTTCACTTGCTACCTTTAAAAAGCGCGTAAAGGATCACTTTTATAAAACCTTAGGTTGGTAAATAATAT
GTATTATATTATATAGTTATATATGTATTTATATAAATATAGCGGATATATATTAAGTCTATTTTGTCATTTAG
GTATTATGTGTATTTGTATTATTATTATTTATGTAAGTTAAATAGGTACTTAGTTAGTTTGTATTTTTAGTTT
GTAGTTTGTTTTAGTTTAGGTACTTTCCACATTGTTTTTTCTTTGCTTAAATTGATTCAAGTAGGTACTTA
TAGCTTTTTATTGTATTAAGGGAGTTTAAGAACTTTAGTTTTCCACCAAACAAAGGTTGGTTGGAAGAA
ATTGCTTCTTGGCAATAAGCCCGCCTCTGTATATACTTACTAGTTTTAAGTCCATTTTTGTAACTGTGTCT
ATCATTATACAATAAAGTGTTTATAAAT
>TCONS_00011650 geneID=XLOC_002288 scaffold_1131:10175-13708(+) Exonic overlap (-)
ATATTATGAAATTCATGAGCCATCAAATAATTTTATACTTATGCAGTTTTTCATAGAAAGAAACGATAGCA
TACTATCTCTGTTTCGAATATATATGTTTCTCTCTATCACCCATTTCCTCCTTCGAGTCATTTCTATACTTAT
TATTTTCTATAAAAGATTAATAATAATTTCATTTTATAGTATTTAATCGTAATAAAATAATTAAATAAATAG
GAGAATTTTTCACTATTTAAAAATCACGCAGTGTTTTAAGAAATTTCTGACGCAACTTTCGTTGAGATG
AGATGAGATCACAATACATCAAGGAACTTCTCTAGAAGAATGTCATGGTCTGACTAGTGGTTAGGAAG
GTAATTTCTTATGGGTCTGAGTCTGACCCTTGTCTGACCTCAGAAAGTCTGACAAAAGTGACAAAGGA
TGAGATAAATGGATCTTTGAATATTTTTATATGAGATTTCCCAAGAATGAAGATAAGAAAAGAATATGGC
CAGCCATCCAGATGTTGTTCCGGTATCACAATAGATCATCATCATCCAGTTCAATGTAATGAAGAACCA
CACATGAGTTCCAGTTGTGAAGGCATTAGCTAATTCAAATTTAAATGTTAACATACTTATTCCATAATTAA
TGGAAAATCTAATTCCTTAAAGAATTTAGGCAGTAAGATTGGCCTCCGGCAGAATTGTATAAAACAATA
TTGT
>TCONS_00011729 geneID=XLOC_002308 scaffold_1137:400-3987(+) Exonic overlap (+)
CTGTGATCTTCCCCACTTATAACCTTCCCTTTCCCACTTCTCCAGCTATGCATGGTGCGCGCGGCGCGGT
GCGCGGCGGCGGCGCGGGCGCTGGGCGCGGAGTCCACCACCCTGAGGTTCCTGGACGACGTGACCC
TGAGGTTCTTGGACGACGTGGTGGCGCTGCTGAGCGGCAACGTGGCCGGCGCTGTGCGCGCCCGGCA
GGGGCCCCTCGGCATAGGGGGTGCGCGCCCGGCAGTGGCCCTTCGTCATAGGCGACCGGGACGATAG
TAGGAACTATGTTGTTTGAGGACAGAGATCACCATTATGACCGATAACTGCAGTGAACCGGCAGTGTA
CAGTCGTCGGGCAAACAACAAATAATACATAGATTTAACATAATAAATACAACTAGATTTTATTGGATGA
TATTGGTTTGAAAAAATACAAATGATATCTATGTAAAGACGTAGTAGGTACTTACAAAAAATTAGGTAGT
ACTTTATTACTTAGTAACATGCTCTAGCAACTGCTATATTTTTAGTTATAAATATAAATTGAAGCTGTTTC
CTCAAGTACAGAAATTCTAATCGAAGTTAATCCATAATAGTATGCGCTATAGTTGACATCGAAACATTAT
AAGAATCTCTATCAGACTTATGAGACTGAATGGTTAGTGATGTACATACTTCACTATAATGTTATATAACT
TATACTAATTTAGAGATTAAAGGAGAGTCAATAATGTAATTTAGGTCTAAGAATAACAACTACGAAGTC
TCTAGAAAATATAGAATTCAGATCTCTTATCTTCTCGTTTAATTTCTTCCGGAAAATAATTACGTCAAATA
TTGGCACAGTGATTGATAGCAACAATAGTTCATTACGTCGCCGCTCGCATCTAAAC
>TCONS_00011738 geneID=XLOC_002310 scaffold_1138:15890-19144(-) Unclassified
TCCCTGTCGAAGCAGAGTTCGCTGGCGGAGTCCAGGTTCGACTCCTCTGATGAGGAGCATACACCCGT
TGAGGAGCAAGAGCACATGGTTGAATGAATACGAAGACGTCAACAACGAGTATATTAATTTGTTATAAA
TTTTACTAATTATTTTCTAATATTCGTTTAAGGAGCATCTATTATATTTACTGATAAAAGGTTTGCAGCGA
AAACATTATCTATGATATTTTAATGATTTATGAAGCTACTGTATTTTTGAGTGACTAAGCTCCGCTCCGGC
TTAATTAATTTGAAAGAAAGAAAAAAAAACATTTATTTGTGAAGTTTGTAGTTTTAAGAAAAGTAATAA
TAGAGTGAATCTTATGAGCGACTTTAGATATCTTCCCTCGGACTGTGAATCTTCTATCTTTGTATTTAGG
GCTTAAAATGCTAAATCCCTGATATTTTATCTACACTGAGTATACCTAAGTATGTTTAGTGTAAGAAAAC

CTTTTTAATAATAGTTAAGTAGTTTTGTCACAAGAATATAATTATCGTACGGAAGTGTTTGTCAGATCAA
AAAATAACTGTCAATCTTCATGTAAAGCTCCCAATAAATGAAACTGTAAGGTCAACTCATCAAACTTCA
TCATCCTCATCAGACTTAATTTAAACTGATAAATAAGAAGTTCAATTTGATAATGTGATTTCATTAGTCG
GGAGCATTTGATTTACACCCTGTTTATATGAAGCTTGATAGTTCTAATCGTGCTATTAACAGTAATCGGT
ACCTACTTCTGTTGGATGAACACGGTGGCAATTTGGTGGCGGACAACACGGGTTGGCAGCACTAGCA
GCAGACGAAACGTGTCGCTACGATTCCACTGGTTCCTATGGTTTTCATCCACTTTTCGGTATACCTAGTA
CGCAACTGCCACATCGCATTCAGTATTCTTCGTATAGAACATTACAGAAGATTCACACAGGTGCGACCA
TTTCATTTGCATTGCCGACACCGTTTCTCCATAAAGTGTTGTTTGGAAATCTGTTTGGTCCTATTCGTAC
GATACCGATAGGTGGACGCTTACCTTACCAGTCAGCGACATGTCGCTCAGCGACGTCCATTTAAGTACA
TAGAAACCAGTGATGCCGTAGTCTGCAACCTCATGTCGTTGGCTATCAATTTCCACGTATATTATTTAGG
ATTGTGTTCCCAGTAGGCAAAATAATCACTCGTTAATAGGTTTACATAACGCCGCAAATTTTGCATAGTT
AGCACAACAATATCAAACTGTTAACACTCGCTCTACTAAACGATCCGATACACCTGCTAATTCAATCGA
CAGCAAGCATTTTGCGTACCTGGGAATACAGTCTTGTACTACCTACAGTTAAACTTTGTAGCGGTTTAA
TATTCGGTGTCATTTTAACTTGTAGTGGTTCTATTCTATGTGGTAGAGCTCGCCTCGACGCTATGTGATCT
TTACAGTAAAGTAGTGTAGATTTATACGTCGTTTCGTGAAGTTTTTAAAATAGTTTTTAGTTCTTGTTTG
TCACAAATGATTTTCAAGACGTTTTCCCAAAATGTATATAGGTGCCTATGGATCACAATGATTCTGGATT
CACTTATAGGTACATATGTGCTACAAACGAACTTAAAATATTTATTTTGTTTGCTTAAGTGTGTAGCAAG
GATATTTACTTATGCTATATTGTAATTATATATAGCCAGTGAGCCTTTCATCCCTGCCTCGACCAGGAATT
AAACATGGCACCTTCAGTACTTTATTCTGTACTAACTGTGGTACAGTTAGTACAGAATAAAGTACTGAA
GGTGCCATTCGGTCATCTCTAAAAGAAGTAACATATCGACACACAGATACCATACTTTTGTACCTAGTC
ACTGGACTATTGTACCTAGGTACTTATTGGGTCTAGTAAAGTTGTACTCAATGATTACCCTGGTAAACTT
ATAACGGGTATAATTAATAATTAAAAATATATTTCTGATTTGCAACGCTTTTTTCTGAAAGTTATTTTGGT
TTTAAATTGTTACGAACTAATAAATTTTTACACAAAGTTATGAAAAATATAAAGCCA
>TCONS_00012034 geneID=XLOC_002317 scaffold_114:73095-74637(+) Splice junction overlap
CGCTTTAGTAATCAAACTGTTTTCTATTTTCACCCTCTGACAGAGAACAATTTGTTCTTTGACAAAGAG
GGATAAAACTGTTCAAAAGCTAAAGTGGTCTATAACTGTTTTATGAATAAGGGGGTAAGGTTATGGGTA
TATCAACATAATTAAAAAATTAGGAAATGCTGTTTGTGTGGTGTAATTTTCGCGTCATAGGTACCCGAGA
TGAGCGAAGGATGACAGGAAAATAACATTTGTTATAATAGCTCTAACTAAACTAATTTACTTCCCAGGC
TACGCGGTAGCGGCGCCGGACGCTCGTGAGTTCTACCGGCGGCAGGAAGTCGCTTGGATACCGGAGC
TATGTAACAAATACCCGAAGAGCCTTCTCGAACGGACTGACCTGTCTCCAGCCGCTAGGGAATGCATA
ACGGAGCTGCACCTCAACTCGCCGGTGCTGCCGCCGGCCGCCCGGCG
>TCONS_00012015 geneID=XLOC_002339 scaffold_114:94283-99271(-) Unclassified
GGCCCAACGAACGCCAACGAACGGGTTTCGTTGACCTTCGTTGCTGCAATCTGCCCTGTATTATGTATG
ATAAATATCCATCGTTGGGATAACCGTTGGCGTTCGTTGGCCCGGTCTGTACGCAGCTTAAGACGACAC
AAAGGTCTCCTATTTTATGATGGTGATGAATTAAAATGCAAACTCAGATTATTTTTAAATAGGTTTCTTTT
ATTTATTTTTCTATCTACTATTTATGTACAGGGGGTGTGAATATTAAATATCTAAAGAAAAACATTATTATT
AGGACAACAAACTAATCGCCAGCTTTAACTAATCATCTTGGTGCTTCATCCTATGTCTACTGTAAATTAG
AGAACTTAAAACAAATAAAATAGTCTACATTCGTCATGTTTACACGCATTCAGTGTTGGTCTGTCTTTA
ACTTGGATGAAATCGACTTTAGACAAGTGGTTAGTTAGTAGTATTTTTTGTAAACAAATATTTAACAAA
GAGGTAGATCTCCTCAATAAAGGATTTAAACCGCTTTGTGATGCTTTGATCATGCAAATACGTATTGATA
AGCAAAATATATCGGTTATTTTAGAGTAGCCAGCGATATTGATCAGCTCATGTTTAGATTTACATCATCGT
ATAACTTGCAATGAGTTTGTATGAGCTTAGTTAATCGGATACACGATTTTCACGCCGATCTACAAACCTT
CTATAAAATCTTTGTAAGTTCTATCAATACCTACGGGGGTTAAGTAATATTTAAAGCAGACCAACAACTC
GCAAACACAATCATAACCGAAATTAAGGAGGAATTACTGCATATTTAGCACGCTTTACAACTACAGGTC

GATTATGTTTAATGCAGCGATTAACGAGGCATGATCAATCGGGACTTAAGCCGGGTCCAGACGAGTGTA
ACGTGTAATGTAACACGAATTCTACGTGTGGACGTCACAACGAGCATTACATGTAACGCTCGCAGCTG
ATCCATCGGCTGTCGGTTTTTCGCGCGAACACAAAAGCGCGCGTAGTGCTCGTTTGGTTGAGTTCGCG
TGACGTCCACACTGTTGACGCTAAATTACACGTTATGTTACATTTCACGTTACACTCGTCTGGACCCGG
CTTTACGAATACCATAGATCGAAGCGTTGCCACGCCATTACCATATTTAATTGAACGTGATAGGGTTGTG
ATTTAGCTTGGCATCGCTACGCTATTCTTTGATTCTACAAAATCATTGTGGCTTCAACCAAGTTACCAAT
ACATTTTTCAAAAAGTATTAATCCAGTTCTTCTAGATATTTACGAATGTTATGTTGTTATTATTATATAAAT
CTTCATAGTTCATGTTGTCAAATTGTAATGTTTTCCAACAATTTAACACAAAAGTTTCCATCTGATGATA
ACACTAATAGTACTGCAGTTTCAACTTTTAACCACTTTTGTATCTCATGGCTATAACAACAACGTTGGCA
TAGTTCTATTATATTCTCTGTGGGGTGTAAGTATTGCACATGGCTCTCTCGAATGGAACCTTTGTGCATAT
CCCCAAAGTTAAAACTCTTTTCCTAAGCTTGGATCCTTTTCCCACCACACTGGTCCAGTGCGGGTTGGT
GGGTTCACACATCTAGGTGTGCTAAATCTAGGTTTCCTTGCGATCTATCCCCATCGCACTATGAGTACTA
GGCCATGAGATGGAATCTTTCAGGAGTGTTTCTTTCTAGGTTGCATTATGTAGAGAAGACATAGTAATT
GAAGTTTTTAGTAATGCATGTATTTTTTTTCTTATTACCATACATAATATATTATTGTATAACAACTATTGGT
CGCAATTTTACATCTAAAGCATATCCAATTTACACACCATACGTAAATAAAGCCACTTATTCTTATTGACA
TAATATTGTAATTTAACAAAGCACACGGTTAACGTAAATACCTAGGTTGACCTGAAACCTAGTAGATTTT
TATAGTAGGTTCAGTAATAATAATAGTCACATTTCATAGGATGTCCCAACGATACATATAATATAGACGTA
TACATATCAAATGTGACCAATGATATTATTGATTCTACTTCAAGTAGATGCAAGACACTATACATTTCAA
AGATGCCAGAGAAACTATAATTTTCAATAAGTATAGGTAATTTATATTGAAATGTGTAGTGATGCAAGTT
GCATTATTACATTAAATTATTCAAGTAAGTATTTCTCCCTATCGCACCGCACGCTTTAACAAAAAAATATA
TTTTATTTAATGGATGTGAAATGATTTCCTGACATTGGCCAATAGAAGATGGGCTATTAAACCAGTCATA
TAAATGAAAGAAACATATAATGCATTGAACGATAACATATTAAAATTGCCGCCGGTACATACATAGGTG
GCAGTAGGCAGAGAAGGAGCATATTACTGTTGTCTGCCATTTACATATTGTTTCTTTATCCGAACGGGTT
ATTGAATCTGAATACTTAACTGTATATTATGTATGTATTATTTGTAGGAAGATTAAAAAATCTTTTTTTCGG
TAACTGCCTTTATAGTAGATGCTCTGCGGATTTATAGTACCTATTATCAAATCCATAAGTAGCTGTAACTA
CAATATTATAATCTAGCGAGGTTCGGTTACTACATAATATTGGTGTGTAATAATAAGGCTTTTATATGATGT
ATACTGTATAAAATTTCACATATGTATAATTCAAGGCAATGGGGATATATTATAATACATAATAAATTTAGA
TCCGATAGACTCAGCGTCCTCATATAATTAATTAGTAATTATCTTTTCACGCTATTGCAAATAACACTGTA
ATGCATTGTGTTATTAAGGAACAGAGCTAAATGATATTTTAGTTAAAATATATGAAACAAGACAATGGAT
TTTTCATAAACTTATGTCCTTACGTTAAAATACTCATTAGAAAGTTTTGGCACAAATGCAAAATAATCAT
TAGCATTTTTTATGAACAGACACTCAATGAAAGTATTGCAAAGTATTCATGAGAATACATAAACAATTA
GGTGAACACCAAAGTATCCATTGAGATCATTGCTATTAACGAAACATAGGATTGTTAAGTTCAAAACTA
TAGCGACACTGTTACTGAAAATTATTATCGGAAAAGAAAGAAGAATAGAAGACAACTAGGACTTAACA
CAAGGTATTATGTATAAAAAAATATTTCCACAACTTGCTGCAACCAAAATGGCGCATAACAAAATACTC
GACGTCACAATACAGACCTTAAACTCTTACGCAAACGCTTCAATATCGAAAGCTTTACAAAACTATTAG
ATAATATATCGCTACCTACTAAACAAAACGCAACCTCTCGGATAAAAACTAACCAAAAACGTACGACG
AAAAACAACAACTTTTATACTACTCAAATAGGCCGTTATGCGATCTACGCCGCGATTGGTCGGCCGCCT
CAACTCACGCTCTGATTGGCTATCGACACGACCGGTTGCTGGTTATTGGCGGAGATGACTGTGGACCC
TGGGG
>TCONS_00011830 geneID=XLOC_002319 scaffold_114:95668-96367(+) Unclassified
TAGTTTCTCTGGCATCTTTGAAATGTATAGTGTCTTGCATCTACTTGAAGTAGAATCAATAATATCATTGG
TCACATTTGATATGTATACGTCTATATTATATGTATCGTTGGGACATCCTATGAAATGTGACTATTATTATTA
CTGAACCTACTATAAAAATCTACTAGGTTTCAGGTCAACCTAGGTATTTACGTTAACCGTGTGCTTTGTT
AAATTACAATATTATGTCAATAAGAATAAGTGGCTTTATTTACGTATGGTGTGTAAATTGGATATGCTTTA

GATGTAAAATTGCGACCAATAGTTGTTATACAATAATATATTATGTATGGTAATAAGAAAAAAAATACAT
GCATTACTAAAAACTTCAATTACTATGTCTTCTCTACATAATGCAACCTAGAAAGAAACACTCCTGAAA
GATTCCATCTCCCGCACTGGACCAGTGTGGTGGGAAAAGGATCCAAGCTTAGGAAAAGAGTTTTAACT
TTGGGGATATGCACAAAGGTTCCATTCGAGAGAGCCATGTGCAATACTTACACCCCACAGAGAATATAA
TAGAACTATGCCAACGTTGTTGTTATAGCCATGAGATACAAAAGTGGTTAAAAGTTG
>TCONS_00012090 geneID=XLOC_002320 scaffold_114:126854-148903(+) Exonic overlap (+)
GGCCGGCGTCGCCGCTGTCACCGCTCGCGGCAGATAACCTCGCGCCTCCACCAGACACCGCCGTGCG
CGCCGGCGCGTATACTTACTTACGAACTACCGTGATTGTGACTTGCTATATTATAGTGCTGTTTACCTTGT
GATTTGGATTGCCACATCGCTAGTTCATCGGCTTATCAGTTCAACTTATCAGGTTATCGGACATTGGAGA
AATACATTAGGACTTAGGGTGTAAGTGCAGTGCGAGACAGCCACGTGTGATACAGTGTCATAACCAAG
TGTAGTGTCAGCCGCAGTGGACGGCCGGTGCCGCAGTGTAGTGCAGTGAGTGAGTCGGTGACTCGTG
CGTGAGTGTGTGAGTGAGGCGCCGGCGTCGGCGTCGATCAATGCGGGTGCGCCGATGGAGCGGTGAT
CGCAGCTTCACTACTTCACAAGGGCAGCGCTGGTAATATGGGCCAGTGCGAGCTAAGACTCAGCCCTT
GAGGCACTCCGGCTAAATGAGACAAAACAAACAATTGTGACGCGCTGCGGTGTAGTAGTTAAAGCGG
CTCTGCCGCTTTAAATGGACAAACGGAAGATGCTGCCAAAGAGAGCGCGTATGGATAGCATGCGGGG
CCCCATCAACAATGGGCCCCTACAGTCGAG
>TCONS_00011799 geneID=XLOC_002346 scaffold_114:572127-664295(-) Exonic overlap (+)
CGGCGTTGGACACGCTCTACGCGCTTCGCATTGCGAAAACGATAAATACGCTACGAATTAACCCAATA
AATCAACCTCTTCTCATGAAATCTCCCATTTAAAACAGCCCACGGACCACCCGTGAACAGAACTCTTC
GTTATCTACTCACGTTATTTTGTTTTCGGTTTGTTTTTGTTGTTTTCACTCGTTTATTTTGCCGGCAAAAC
GCCGGTTGCATGTTAAGTTGTGATAATACGCAGTGACGTTCAGTGTTTTGGTTCAGTGACGGTCGCAG
ACGTCTGAAATATTGTTTGAGTGACGATTTTTGTGTGTTTTTGTTTTGTTTCTGTTGGTTTTTTCCCGGC
GCCGGTCGAGAGGTGTGGTTGTCAGGTGAGCACGTGTTTTATCGATCGCCGCGGCTGTCTGGGGAACG
CAGAAATCAATTGATGCCTGCAACACTCGATCTTCAGACATACTTACTCCGTGATCGATATACAGCCCG
GGGCGAACTGCAAACAGAATTGACAAATAAGGTAAGTTTTCATTTGTTTTTCGCCAATATTAATTTTGA
TGAATATTCAGATGTATTGGTTTTTTAAAGCATTCACTAGTTGCATCTATTGACCCCACTTGTGTGGGTAT
ATTTTATAGTATCGCAAACTCGGACAAAGTAATTTTAATGTGCCAGGACTGAAATCGAGAAGTAATCTA
GCCTATCATACTAGTAGGTATCTGATATTTCGTTCAAAGATAAAATAATACGTTGAAAACAGCGCCGCTG
CTATCGAACTTCAGTGATGGCGCGCACTCTATTACTTCTTCTACAATGTGACTTACAATAAATACATATAT
TTAAATTA
>TCONS_00012120 geneID=XLOC_002328 scaffold_114:685209-685802(+) Intergenic
AAGCGTTTTACGGTTTTCTTTTATAACATTGTACCGATGTAGCAAACAATTTCACATACGGTATTCAAAT
CCCGCAGCTGTGGCTTGCTCTCTGCTATTATCGTTGCTATAAAACGACACAAGAGCTAGACAAGGGTTC
GTCACCATTTTGAAAGGATTGGCCAGTCGGATGAGCGTCAGTCGCTGACTGGCCAGGGTTTGTCGGAT
GCGATACTGGACGTCATTCAAAGAAGCTCCTTTCGTACACCCCTTACATAATACACTCACGGGCAATGA
AATGGTTCCACTGAGAAAAGCACCAAATTACTCCGTAATGGAAAACCCTCTGCAACATTAAAGTACAT
TTAAT
>TCONS_00012095 geneID=XLOC_002330 scaffold_114:709416-709971(+) Intergenic
CCGCGCCGCACGCTCAGTGGAACGAATGTATGGGCTTCAAAGATAGTGTGGGATGCATTGCTGTCCGC
TGGAACGATGGCCTTTGACAGGTTCGATGAGGAAGACCGAGGACCACTCTGTCCTTGTGATTTCAAGA
GGGGTATGTCCAGCAGTGGACGCTTACAAGCTAATTATTCATTTTAATACCGGAAGTTGTAAAAGCTCA
TTGGCAAAATCTATTACTTATATTAAAAGCTACACTGATTAAATTTTAATTATTTAAAACTTTATACGTTAC
TTGTATGATTTCTTATGACAACCAACACACATGCCAACACAGTATTTTTATAACATCTATATGACTTCCTG
TCTACCGCATTTTAGCTCTATGGTCCAAATGATAAAAGTTTAAAACGATACGACGAAGTATGGACTGCG

ATTAGAATAGAACAAAGTAAGGTGGAGATTGGCCCCTAAAGCTCTTCGCACTGATTGG
>TCONS_00012181 geneID=XLOC_002410 scaffold_1140:8484-11297(-) Splice junction overlap
ATAAAGACAAGCTGACGAAGTTCTTTAAACAAAATGGATGGATGTAGTTCGTCAGAGGTAGACGTAGC
CTAAAAGCAGTCATCCACGATGAAAAGTTGTATGTATTGTATTCATAAAAAATGTATGAACGGTTGTTAA
TTGAGAGTATTTATTCCTATTCCCATTTGAAAATATTAATGATAATTTAACATAATGAAATGTAAAAAGAT
AGTGGCATTGCCGATTTGTTATATTATTAGTAATTATTACACACTAATTGAGCTAGTTTTTTCTTTTAACTT
TTTTTACATTTGCAAGTGATTGCATAGATGGAATATTACTTTTAAAAGGCAGCAAAAGCCTAAGAATTG
CCACTAAGGATGTGGCCCCATTGATGCGTTGAGTCGCGTCGTCGCCATAAAAATATATGCAGCGATGAC
GCAACGCACCAATGGGGCCTCGCCCTAAGCTAGCGACCCTTTTGGTAGAATTATTTCATCTTTGCGTAG
AGTAGGGATTTAACGTGTTTGGCTCAAACCCGTAAAAAAATGTACGCTTTATATGAACATGTTTTGCAA
AATGTATATTAGATTGGATGTCTCAATTTTTTGAGACCCAACGGCGTATGTGAGAAAAAAATGGCGGAT
GTAAATTGATAAATAATTGCTTGATAGTTTTGTATAGAATGCTTGTGTTTTGTAAAGAGTTTTAGTTTGTA
TCGGTGTCTGCGGTGTAAGTTTCGATAATTTACGTAAACGCAGTTTTAAATGTGTTTTTAACTTTTCATG
TCTCATCTGTGGTCATATTTGTCTTTTGTTTCAGTAGTGACCGCTAGAGGCGCTAGTTTGTATGTAAGAA
CGCAAACTTAAAAATAAACTCGCATGCGTAAAATGTCCCAATTTACACAACAGATATACAGTAATTTTG
TATTTGAACAAAACTAAACTAACTGCTCCACTGTAGTCTTTTAATCACTGAAAAATGCGACAAAATATT
CCACGTATCC
>TCONS_00012375 geneID=XLOC_002430 scaffold_115:49830-50948(+) Exonic overlap (+)
CAAAATTGCACCTTAATACTCATAAACATACGGTGGTTATTGCAGGACGTGTGCTCCGAGGCGATGGAG
CTGTGGGGCTACCGGCCCGCGCACAACCGCAGCCACATGCTGAGCGAACACTTCGTGCCCATCGGCC
CATACCAAGTCGCACTCTAACTCCACAGCCACTGAGTCGAATCTCCAACTGCACTGTGAAAGATAAAT
TTACGCCGTGACGGGTTCTCTTGAACTCTTTATGGAAAACTACCAATTCACCTTACATTAAGGTATGAA
CATTTAAAATTTAATTTGCTCCCACTGATGTTTGATTTATAAAAGCAATGTCAATTTAAATTACATATATTC
GGAAAGGTAGGAGGGTACATAAAACATAAGGAAACCTACATACTCATTTTACACGAGGTTGCATAAAT
GAACCTAAATGTCTCACATCGAAATATTAGTGACGATTCATATTATATTTACCTAGATGTTATACGTGAAT
ATTGTTTATTTGTATATTTTTTTACTTTACTTTGTTGTTTCATGTTTTTGAAAGACTGAATGTGTTTCCAGC
AGCTTCAATATACTCTTCCAATACTCTATTCCTTAATTAGATGCGGAGATTATTTTTGTAAAGGTTAACTC
CGGCACATTTCACTATAAGTTTAAAACTATCGAATAGTTTAGCATTTCTGTCGCACAAAAATACTCTTAG
AATTAGCGCCGCCATCTTTAAAATATTTTTCAAATTTAACATTTAACTACTTG
>TCONS_00012327 geneID=XLOC_002458 scaffold_115:204121-204969(-) Splice junction overlap
TTCCTGCAGGTTTTGGCACCAGCGGTGGATTTGGCACAAGTACCTTTGGTACTGCAGCACCATTCGGT
GGAACATCAACTTTTGGCAGCACTGCACCAGCATTCGGTGGAACTAACACACTTGGTGCCAACACACT
GGGAGGCAACACACTTGGGGCCAACACATTTGGTGGGGGATCAACATTTGGTGCAGGATCTGCTTTTG
GGTCATCATTCGGTGCCAAGCCAGCGGCCACCGGCTTTGGTGGATTTGGCGCAGGTCTTGGCACTACT
AACACTTTTGGCCAACAACCTCAGCAGCAGCAGCAGCAGCAGCC
>TCONS_00012413 geneID=XLOC_002452 scaffold_115:681102-685988(+) Unclassified
GTGGATCATGTAATGTAAATACCTGGTAATCTTTTAAAAATAAAAGGATTGTAATAAAGATTTTAGTTAA
AACATTTTTGAATTTAACCAATTAATACGAAACTTACGAACAAAAAAATACGACTGAATTACTTAAATAT
TTATGAAGGCTGTGCACTGTACAGAAATTTTGCTTAAGAAATTTAAACCACCCCCATACTTAAACCTTT
TTACCAGTAGTGGTCGAAATATGGCCACCATATTTTAACTATGTATATTCTATTGACATAACGTCTAAAAC
CAATGTAATATTTTAGCTTTTCTATCAGCGAGGCAGGTGATTCAATCCGTACGTTTTCAGGCTCGCCGAT
GAACAAACAGAAGCTGCACAACCAGATGCTGCGGGGGAAGGAGATGGGCGTCGCGGAAACGTCGTT
GACTCGAGCATAACAAGAGGAGGGCGGCGACGGCGACTGGACTTCCGAGGCCCTGCCTACGGCAGTC
TAGACGCGACCGAGCCCCTCGCCACCACCAGTGGAACCATCAATGATGGCGTATAGTGCCGGCAGAG

ACGGTTAGTGACTAGCGCCTGAATGAAGAAGTCACTGTGGACTTGTGGAGTGAATATTTTGACGATTC
CGTTTTCAAGAGTGTGTGTGGTTGGAGCTGTGTTTAGTTACTTAATGTTTTTAGGTTGATGATGCAGCT
GAGTGTTAGTGGTATAGTGGATTATGTAAATATCTATGTTAAACTTAAGTATATCACAGGATATAATATATT
ATCTACTAATATCTTGGGGAATAGCGAAGGGTATGGACTTGATCTGTATTTTGTAGAGGCTGGTTTATCT
CACTGTACCTATACACAATAGGTATAACTTCACATAATTTTGCAAGGTTTGTAAGAATTTTAAAGCTTAA
TTTTAAGCAAAATTGTGTATTATAAAAATAGTTAGAGAATGGCTCCTAATCATAATTAACAAATGTTGAT
GGTTTTGAACCGATGATACCTATAAGTAATGTTATTACGAACCAAGGTTCGCTTTCTTAAACATAAAAAA
TACCTTTCTTTAACTATAATCAATGTATTTTATAGAAGCAGTTTATAATTTAAGGCCTTATATTTGTAAATA
TTATTTGATGTAAATACTTATTAAAAAATTATAAATTCTTACGCAATTGACATAATTACAATTATTAAATAT
GAATAATTATGTACCTATGTATTAAAGTAGTGTCAATGGTTTGATGATTGTTAAGTGCATATGTGTGCTCT
CAGATAGCCGTAATCGAATGAAATTACTATGTCTTAAATAAACGACTACCTAGTTAAAGTACTTGAGTA
AAATATTACCAATACATAATCGACTGATTGAGGTCTCGTATGTAGCATATCTCTTTGTATAACAAAGAAA
ATAGATTTAGAAAATTTAGCTATTTGATACGCTAGCAGATATTTTTTGGGGCTCGATTATGATATTATAGTT
TTTTTTATATAGCTTTTACACGTGCCTTAAATTACAAAAAAATACGTTTTAATTGAAA
>TCONS_00013085 geneID=XLOC_002543 scaffold_116:449669-451464(+) Intergenic
TATATATCCTCTTTTATGTGTAGTAAGTAAGAATAAATGGCTATTAATTATGGCTCTCTCAAAACTATTTG
GTAAGAGTAAATCATTATAGCAAATGGACAGTCGTATTGTAAAAAATACTCAAGTATCAAATCATTTTTA
TACTACACCTACCTTCAATAGACAGTGATACCACATATAATTTTTTTGAATTATCTGTGCTTTCTTCAGGA
CACGCATTGGGTAGCCAGGCTCTAGCTCTGTTTCGTGCTTATAAGCGTACGTAAAACTTTAAATACTTA
CCCAGTTCGAAAGATGTCCTCATCATACTTATGACACGTCCCTCGAGCTATAATCCTCCAAAATTGAAA
CTCGAAATAAAAGTGAAATAACACAATAAATCACACAAAAATGGGATCAGAAAATTTAAGTTTCGCAT
TCGCGAATTGGCGGCAAAACGAATTGTGTGGGTGCAAAAGATACCATTATAAGGACACACGGTGCTTC
TAGAGTTTGTTTTCCTCGTCGACTCACTAGCAGGCGGACAAACATGGGTGAAAAACTTTTACTGATAG
CACCTCTGGTGCTCTTAGCTGTTTTGGATTCCACTGTGGATGG
>TCONS_00013211 geneID=XLOC_002616 scaffold_1165:2719-8578(-) Unclassified
AGATTGCAAACGGCGCGGCGATAGAGGCCCCGTGCGCGCTACACTGTCCGCCCAACGACCTCTGTCA
CAACGCCACGTGTTGCGGACCCAACCAACAAGAAGAGAGTACGACCCCGTCCCCAGCCGTCACTCCA
TACCTCTGCCGCCAGCGCAGCATGGGCCAACGGACCGCTTCGACGGGATCCAATTTAGACCAGGGAC
CAGCGCCCAGCCACCACCATCTAGACGCCCTGGAGTGTGGCTCCTTACTCCCAGCGACCGTGCAAGA
AGACTCAGTGAGACTGAGCGTCTGACCGGCACCCAGCATCGTGGTGACAAATGCGTAAGATTGTGCC
GCCCTGAAATCGCCACGTTTGCCTTTGTCCAGGTATGTGAGGCCAGTTTGTGTTGACGCGTAAGTGCG
TGTCACAGTGACTGTGGTGATGAAAGTTTAGTGTCATAGTGATGTGGTGGTTTTAGAATGGCTGTTTTA
TACGAACATTTGATTCGGAACGGTTTATACGGGAGTTGTTCGGGATAAGTCTCGTTTGTTGTTGCGTGT
TAGAGACTAGTGAGCTAGAATGTATTCGATATGAACACCAATGAAATTTTATTTACATAGAATCTTGTAG
TTTAGGATATGTTATAGTTAGGATAGTAATTGTAATCGGGTAGAATGTGTGCGACTGGAATGAATGAAAA
TTAGTGAAATGAAATAAGATATACAGATTCTTTTTTATTTCAATCGGCTATGGTAAATAGTAACGTAACTT
TAAATATTTAAAACTTTTAGATAATACTATTATTACGGTTGAAACCCAGATTGTACTGTAGACATTTGGAC
TTTCTATTTACTTGTATGCAATTCTACACCCAAAGTTAGTGAATTGAACTTTGATAATTATAGGGTCACA
GAACTGACAAAGCCAAATTGTTAGAAAAATCATATCCGATATATCACATTGTAGCCTTATATCCCGTTTA
CCATGATTTACGATAACTTCAGTAGGTCGTCATCAGATAAGTACAAGCCCTTGTAGAAATAATAAACAT
GCTATAATTCAGTTTTTGTAAAACCTACAATACGTCTTTTCGTCTAAGTTTATTGAAATTGAAGTTTTAAT
TTCAGCCCATTTAACTAATTAAATACATTTCAATTTGTTAGAAACAACCTGAGAAATTAATAATAATTAA
CTATATCGTCTAACGGTATATTGCATAATGCATAGTTTTATCATTCTGTGTATATGTACTCAGAATCAAATG
TGTTCATAATAAGTGAGAGAAAATACCTCTTCAGAAAAAACGCAATAAAATAAAATACGTGTTTACAGT

GTAGTTTAAGTCGCATATATATGTAGATTTGTAATATATTTGCCGAATTATTGCATTAAATAAACCTACAAC
AACAAATTTTGCGTGAGCATTATTACTGGAGACAGTATAATAGGTATATCGGCTTACCACAATTTTATATT
TCGATTCGATACTGGATACAGCTTTTGACAATGTAGAAGATATATAAAATGTACCTATTTTCTTCAATAAT
AAAAAATCTTCGGGGAACCAATAAATATTTCAGTATCAAAGGCCAAGTTATTTCTTACTCGAATGTCTG
AATATTTGCAAAACGAATCGAATATTTTTTTAATTTATTTTAATTTATTTACATACATTAACACATTGAATG
CCGACTACGCAATATTGCGTAGGTACCAGTGATACGAGGTACCTTTGGCACTTAATGTTAAAAAACCTT
GTATCCCTGAAGTTTCTTTACATAATGTGGTATGGCTGATAGAAACCTCATAACAAGATCATCTAAGTTC
AACATTAGCAGACATCCTGTATCTTATACTTAAAAATGTGATCGTATATTTTATAGTTTTAAGTAGACTAA
AGCCTATGTCTGGCTAGCATTTTTAGTATATGAATCTAGGTAAGAATTATATGCGGTATATTTCATAGATA
GATAATAATAAACACAATTTTATAGTATGCTTTAGGATCGGTGCACTTCTAATCTAAGTAGATGATGACTT
TTGTTAATGCTTTTAACATTTTATGCATAAATTAGTGCTCAATTTATACTTACGTAGCGTTTGCAGTTACA
AAATACAGCTTTCACGTTATTTCCCATCTACAAACAGAGTTAAAGAAAATATTATTGGGAAAAACTATC
TCATCTCTATGAGCTCAAATACCATACCTATTTTGACTTAGCATTTAGATTCTCATTTTCATAACTGCATAA
TTATCTTTTGCATATTCTTCAGATTGCCTCATAATTTAAAGTTTACTCTAACAGTAGATTTAACTGTAGAA
ATCTGGTAAATCCAATCCTGTGATGAGAAATATACGGCTATTATAAATACCAATACTGCGGCCACCTTCA
CCTTTTACAAAATGTTTTCTTGTCTGCTAAAACCCTTTGATTTACTTATATATTTTTATATCTGATTTCGCA
AACTAGGCCAATTGGAAACAGTATTCCAGTGTGATTATATGTAATTATCGATTTAAAGTTTGGGAGCTTC
CCTTCTCGGCTATTTATTGAATTATGATCGTAGCAACAACACTTCTGTAACTTGTAATTTTATGAATTTAA
AATCATAGTTAATTTGTATTTACTATAACAATATGAATTTTATATATCAAATATATTTACAAAAGGAGTATT
GTTGACGAGAACTGATTGTATCACGTGAGGTCATCGACACAGCTCAATATTGCAATGTATTATATTGTAT
TTATGTACAAAATGTATAATATTGTACATTATATTATGAAGGCAAAGCCTTTGTAAAGTATGTTGAAGTCT
CGCTTTATTATGCTTTGTTTTTGACTGGTTAATTCGAATTTAGATTTTTTAAATGATTTTTAATTTACTTTG
CGAAAAGTATCGATTTTTAACGTATTAATTATGGCG
>TCONS_00013233 geneID=XLOC_002624 scaffold_1167:5390-7813(-) Intergenic
TAAAAAGTCAGTGATGGGAACTGCGTATTCAGTGTCGTGCAATTTTAATCACAAAACTTTTCTAATTAT
CGTGAATTACATAAATAACTTATAAATTGTGATAAGTGTGGACCGTAACATGTATTACACATAAAATTCC
AAGTTTAATTTGAAGTTTTCCGCAACAAAATGAACTTTTTGTGAATGACTTCAAAAGTACCGTTACTGA
CTAATAAATGCATTTTAAAGTGCCTCGAATATAAATAAACGAAACTATCATCACACCCAGCTGCTTCAG
ACATGGTAGTTCGCTGGCCGAACCACCACTTGTGCTGCAGCTCCAGCTAATCCACCCCCACCATGGGA
CAGCAGACCTCCAGGAAAAGCAGCAGCAGCG
>TCONS_00013387 geneID=XLOC_002631 scaffold_117:74236-81992(+) Splice junction overlap
CGGCCGCGATGAATGTGATCGATTGCCGTGTTGTTATCGTTATCATTATCCACCAGCGCCGCTTCGCGA
ACGGTTTCATGTTTCAAGACAAAAACATTACCACTATCCAGCGATTGTTTCTGTAGATATTCTGTTTATA
TTATAGTGTTACATACAAGTGTAGTGGCTGTTTCATGGGAGCTAAAGCGAAGGCGCGCAGAGTACGGA
GAAATATCAGTGATGATATCAAAGCGCAGCTATCGGAGCAGACGCGAGTCTTGGAGGCGCGCGCCGA
GGCGGCGGGCCGCGGCGCCCGGAGGCCCGCGCCGAGGCGGCGGCCGGCGTCGCCACCGAGTTGCAC
GACTACTGCAAGCGACGCGCCGACCTCGAGCTCGACTACTCGCGCTCGCTGGACAAGCTCGCGCGGT
ACGCGACCCAGCGGCATCGCGACCAGAGGCAC
>TCONS_00013472 geneID=XLOC_002636 scaffold_117:488255-490490(+) Intergenic
TTCTGCCCCGCGTTGCGTCGACCGCTTCGTTCTCTCCACGTCACGAGCACACGTCTCTAGCGGCCTAAT
ATTGACGTAGCGTCGATGGATTGTTTGTGTATTGATAGAAACGAAACTTTAAGTTGACCTGAGTGTGAT
TAGTTGTGTTTGCGCGGTGAAATGGTCAAATTCTTTGGTTGAAATTGTTTGTGCTTCGTAACTTTTGTG
CCTGTGTGAGCGTTGAAAGATTTTATATTGTTTTGTTATTACTGAAAAATGGAGACAATGGAGCCAACT
TGCCTAACACCGGTAAGTTACTTATTTTAATTAGAACTACACCGAGCATATCGACCGACCGCGGGCGCG

CATTTTTCATCGAATTCCATCACTGAACTTGCGCAAAGTTGAGAAACATGCACGTAGTCAACACACAA
GTTTGAATCGAATATATGGATGCCGGCTGGAAATATGAGGTCGATCGTACCGTTTCACGTGTGAGCACG
TCTTAGGTACGTATTTTGAATTTGGAATCGGCGACCGCTACTCGGCCCTTCGCCGACGATGTCCACCGC
CCATCGGCGACACCTCGGCGACACCGATACCGAGTCCCCGGGGCCCGCAGTCAGCGGGCCCGAGGTG
ACGATCGCAGTTGTCATCCATTGTGTCCGCATCAGTTATCGCATTCGCAAATGACGAGCAGTCGCTATC
GAGCCGTTCGATTCTGTTAACTACTCATTTAATAAAACCAACGAGCGATGCAGATGGACTATTTTCGCG
TTCGAATTCCGTCCATACCGTTGCACGGTTGACTATTTTCGTTTCGCACAAAGGGCCTATAAGGCGTGC
TGAAGTTTTATTGCCGAGTGGAAATAGAGTTTCGCGGTACTTTCTCTTAATCATCGGGCCGTTTCTGGTA
TCTCATTTTGTTTGACCCGTTATCGTTTTCAGCACTTGTAATCGGTGGAGTCATCCGCGTACTCCCGGCT
CTCCGGATGTGATTTGACGTCGGAAATATGTGGTATCGCTTTGGGCGAGCAGTACTGTAACTGTCTGTA
GTTAACAGAGTTGTGGACAGTGAGTTTCCTTGTTGCAACGTGTGCACTTGTTTTCATGGATTTGGTGAA
TATTGGTTGAAGTTTACGTGATGGCATCATCAGTGTCGGA
>TCONS_00013301 geneID=XLOC_002654 scaffold_117:730466-731170(-) Unclassified
CGCCTCTTTAGCATAGACAAAAATGTCGTAACTTTTTTATTCTTACATGCCAGTGATACACGCGTAATTA
ACATTATAATGACTAAACAAATTATTAATTTTACAGCAATCTGCAGCGTAACAAAGGGATCGAGGCCAG
TCGCGTACGAGCGAAGCGTTCGTTGTAATTCGAGGTTTGCTCTGGTACCATAATTCCTACATCGCCAGC
ACAGAAGAATAGCGGGAGAAAATACTGAGGAAATAAATTCGTAAACAGTAAAAAAATAGTTTTTCTCT
GACCTATATACCTACACGCTTTGCCAATATCTTACGTAGGGGATATTAAACATCTTCTATATAATTTTAATA
GAGTTAATAAACCTACTTAATAGGCCACATTTGAGAGCAATAGCAACGATAAATTCTAAATTAAATATAA
GAGTAACAAGA
>TCONS_00013303 geneID=XLOC_002654 scaffold_117:730466-733392(-) Unclassified
GAAATTTCAACAGATTGGTATTACATTTTCCAAAAGAAAATTTTAAGATTAAAAAAACTTTCAAATTGG
CATAGGTACTTATATCAACCAAAAGTTGCTGAAAAAACATTAAAAACCTTGTTTTCAGGACCCTATCAG
TCAAGTCCGAACCTAAGTCCTAAATCATTTCCTTGTGGGACGTAGAAATCAGAATCGGGAAGTCGGAT
CATAATCGCTGTTTCGCTCGCCTATGAATCGCTCCGCTTATGCGTCTGTGGCCGTCGGTTTTTCGCTGCG
CGCGTTTCATGGCATACCCCGTGGCGTCAAGCGCCATTGCAAACCTCGCTGCCGAGCCTGATAGACGT
GTTCTAGTATTAGACTAACAAAAAATACATCATTGTGCATTTCCTGGTCTATTCCAAGTAATATAACTTC
ACAAAAATTCAATTCTAAACATAACCAGTGAAAACTATTGCATTTCCTCAAGTGGTTTGCGGGTATTCC
GGGTAAATATAGTCGCAGTAATATTTCATTACACTGAATCATCGACCCGTGTGCTTTGTTGAAGTAATTG
ATTTGCCAACTATTTCCGTGTCAAAACGACCCGAAAACCATGGGCGTGGTACAGGAATGTGTCTTTCC
GTCGGCGGTTAGTGCAGAGCCTTTGTCTACTGCCCGACGCGAGGTTGACCTAGATAAATAAAAAAATG
AGGTCATGAAGAATTAGGTCATCGCTTGGTGGCAAGATGCGCAAAGCGCGCCCCTGGATCATGAGACG
GCACGATGTCCACTCGCCTCCGCCGTGAACGTAGTCGCGGGTCGCCAGCTAGCACGCGGAATAGATAC
GTCGCAGTTCCAGCTAAATCGATGACAGCGGCAGCGCGCGCCTAATGCAATCTGCAGCGTAACAAAG
GGATCGAGGCCAGTCGCGTACGAGCGAAGCGTTCGTTGTAATTCGAGGTTTGCTCTGGTACCATAATTC
CTACATCGCCAGCACAGAAGAATAGCGGGAGAAAATACTGAGGAAATAAATTCGTAAACAGTAAAAA
AATAGTTTTTCTCTGACCTATATACCTACACGCTTTGCCAATATCTTACGTAGGGGATATTAAACATCTTC
TATATAATTTTAATAGAGTTAATAAACCTACTTAATAGGCCACATTTGAGAGCAATAGCAACGATAAATT
CTAAATTAAATATAAGAGTAACAAGA
>TCONS_00013302 geneID=XLOC_002654 scaffold_117:730636-732390(-) Unclassified
AGATTATAGCCTGAAGATAAGGACCGTTGGCGGAGTTTGAAAATTTCAGTTTTATTTCCAATCTGTGAG
TGGCGCGTTTGACGTTCGGTAGCCTACAATTTTACGTGTGCTATTACGCAATCTGCAGCGTAACAAAGG
GATCGAGGCCAGTCGCGTACGAGCGAAGCGTTCGTTGTAATTCGAGGTTTGCTCTGGTACCATAATTCC
TACATCGCCAGCACAGAAGAATAGCGGGAGAAAATACTGAGGAAATAAATTCGTAAACAGTAA

>TCONS_00013516 geneID=XLOC_002654 scaffold_117:730644-743674(-) Exonic overlap (+)
CAGGTACGTCCAGAAGAAGTCCAAAGACGACGGCGAGCCGGCACGCTCGCATTCGCCGGCTCGACCC
GCGCAGTAACAGTCTTCCCGCACCGCTGACAACCACAATCTGCAGCGTAACAAAGGGATCGAGGCCA
GTCGCGTACGAGCGAAGCGTTCGTTGTAATTCGAGGTTTGCTCTGGTACCATAATTCCTACATCGCCAG
CACAGAAGAATAGCGGGAGAAAATACTGAGGAAATAAATTCGTA
>TCONS_00013572 geneID=XLOC_002654 scaffold_117:730668-743662(-) Exonic overlap (+)
GAAGAAGTCCAAAGACGACGGCGAGCCGGCACGCTCGCATTCGCCGGCTCGACCCGCGCAGTAACA
GTCTTCCCGCACCGCTGACAACCAGTATGTATTATAATAGTTATGTTATTCAGAGTATGGCTATGTAGGTC
TTACGTTGTTTTGGATGTTATCCCGTTGAGTAATGGCCTACGCACACTGGGACGGCATGGCAAGGGATT
TTTCAATGTCACAGTTTCATTTTAAAGCATAGCTATTTATGTCGTATTTCTGTAACAAAACGGTCGAATG
TCCATTAGTTAAAATTAAAATCCCTAGCTATGCCGTCTCGGTGTGCGTAGGCCATTAAGAGCGAAGAGG
AATGGAATATTTTAAGGTTCCCATGCCATCCCTGTCGGCCATCGTAGATGCTCAGTGATGAAGAGTGAA
GGGGTTTTTTGGTACGTGGCGTGAGGAATTATAGTTGATGGCACGTTGAGGGTTAGAAGGTCGAGATC
CTATTGTTTGAGGTTCGTCGTTGTAGGGTTTTGTTTTACTGCCAATTTAAAACTATCTACCTTGCAGTTA
CTTCTATACGGTTTCAAGTATTAACCTTGTAAATATATCTAAATCATTATCGTAAAGACAGCTAAATTGTG
TGTTCCTTTTAACAATCGTAACTTGGCGGAATTTGCTGTGTTAGTCACAGGCATAAAGCCACAACCAAA
AGAGTTTTGAAAAACTGTATGAAACTAGACCATTCATATTATGACTCAGTATGATTTCAGCTGCACAATA
TCATTGAGTCCATAAATCCTATATTAATACAGCCGTAAGCTAAAATAGCGACAAACCTATTGGCTACTGC
CCAATGGCAACTAGATGGCAGCACCATTGATTCTTACACAGACACTAGCGCCACCTGTCAATGTTAGCC
TCGTGCCAAAAGACCCCTTTCACATTGTAGACTGCATTTCCTGTGAGACAAGCAATAAACTGCCTTATG
TCAAGCAAGTTAAGGTTTTGTTTGCATGTCTTCTTTTCTTATTTATTACAAATTATGTAAATTTTCAGGAA
TGTACCCATAACCGAGGTAAAATTCCAAAGTAGGTACATTAAAGAAATAAATAAATTAAATATCATTTTA
ATATTAACCAATACGAATTATCCATGTCTGAATTTCAGTGAATAAAAAAGTGAGAATGAAATGGTTGTA
AAACGAATAAGTAACTAAATATTGAAATGAAAAATTCTTTCACGAAAGGTGAAAATTGGTTGCCGACG
ACATGAGGTTGGAAAAAGGTAAGCGTCCACCTATCGACATCGTACGTATCGGACCAAACGGATCATCA
TAAATGTATGGAGAAACGGCGTCGGCAATGCCGATGAAGTGGACGCACTTGTGAGTTTCTATACAAAT
AATAGTGAATGTGATGCGTCTGTTGCCAAGTGGGCGCTTACCAAAAGACAACGTTATCGTTATTACTGG
TTTCTATGTATTCATCGCTTGTCAACATGTGCAATATACATACTTCCAAAAAATTATAAAAAAAAAAACA
AAAAAGACATTCTTAACCCACTGACGGACACTGACTGCATTTGACGTCCTCCACACTTTTAGAAAAAA
ATCACAACGTCGAATGCAGTCCCCGCTCTTTTTCTAATTATTTCAACAACGGTCAATGCAGTCCTGTTTT
ACCTAGAGTTTGTATGAAGACCATCTTGTTGTGCTTTATTTGGAAGGATTTCAAAGTTATATGTGCACCC
AGAGTACCTTTTTTTCACACAGCAATAATTTTTCATACAGAAAAAAGTGTGAACTCACACGCACACAA
CGGTCATCGACGTCGTGATTTCTTTACTGCGTCTAGGCAGTGTGCCGGCATGTGCACCAGCAGATCATC
ACACAACTCCCATACAAAAATGTTGTGCGTTTTTATGAGTTTCACCGTTAATCTAGTGTCCATCAATGTG
TTAAAATCGGTCCATAACTGGCGAAGATATCTTTGAAAATATACGCAAAGAATTGAGAACCTCCTCCTT
TTTTACTAAATCGGTTAAAAACTAGTACTGCCGATGCGGCATTAAAAAAAATTAAAATTAAAAATTATTT
ATTGACATCATGTAACACAAGTATGTATACAAGAAATTAACAGTAGGTAGGTTGAATTATTAATTAATCT
ACCAAGTTCAATGCTACAACAACCTAATGTCGTCGGCTATCAATACATGTAATTTATAAAAGGGCTAAG
AAGTGCCAATGAACTGGAACATAAACATAATATATAAAATAAAATAATAGTAATTAAAAATTAATATGTA
TGATAGATTGGCTTATAAAGTTCCTTAGGAAATCTCCTAAATTCACGAATAGTTTATAGTTGTTATCTTAA
ATTTCCCGTTGTAATTATAATTTTCATTGATCCAGATTGTAATTTCATTCACGCACATATTTTAGATAACGT
AAGTACTTACTTGTTTTTAAACGATAAAAGTTATAAGCTTATAAAGAATAATAGGCAAAACAACAAAAA
ACTACACGCTTTGTTGTGTTATTAACCGCTTATAACTTTTATAATTACAGGATGTAGAATCTAAACGCCAT
TTTATTATAATGTAACAGTATTTATAACTAATTAAGCACGATACAAACAGAGGCTACCAACCACTTTGCG

ATTGGTCCGTGGTCCATCCAATCACGAGGCTGTAAACTGCAGCCTATTTTCCCAGTTCCACAGAATAAT
AACTAGTACCCCAGGTCATTAATATGCAGGAAATGAAAATTACTGCTGTCCACTAACAATATGAAACCA
TTTTAAAAGTATAAAACTGGCTAGTGAGGCTCACGTTCCTTGTCACGGGAATTTAAAATCTACCAAGTT
TGCAACAGAGTCTAAAGTCGAGTAAAGTGCGATAGCAGCGAGATGATTTATCTAGGACGCTAAAGGAA
GGCCGTACAAGGAAGATGGGTCAGGGCTTTACACATACAAACGTCACTCTATAACTAGCGGCGCAATC
GATAAACGCCTTACACGCCCTTGCATTAGAGCTCACGTTTTCCGCAAGGTTTTCCGCCGCCGTTCTTGA
CTTTTCTTTGTTAACACAAACTAGTTACTAATGAGTTGGTTACGTGTTTACGCCATGAGCTATGGCTTTA
TTTTACATGCATTTTGTTAGTAATAATGTCTATAAAACAGTACGTATATCTACTGCATTTTTTCATACTTGC
ATTAAAAGGTAAATAATCTTAGTGTGTGTTTTTATTAGAATTTATTTCAAAACGAGTAGTAATTTTTTAAA
ATAATAATTTGATTAAAAGTCTCGCTAAAATCGTTTATATTTTATGCCAAGTTCTTTTGAAAAAATGCAGT
AAGTATAGGGTAAGTACTTATGTATTTTATAGACATCGCGTTTGATGTATAAATAGCAATGAAACCAACT
TCATGAGGAAATAGTTTTAATTAGCTGCCATTTACGACTAAGGCTTTTTTTTTAATAAAACCAACATTGT
AGTTTATTTATAAAGTTAACACTAGTAGCGATTATGAAGGCACCAAATTTAAATTTACCACTGACTTAAA
AGTCTTTGTTCAAAAATCGACTATGTGAGTGTTTACGTAAATCTCATTTACCAGTGTAATTTTTTTGGTT
TGGTGGATAAAATTTATTTTTGTGCTTTTAAAATCGACATATTTGTAAATGTAATATAATTTGACAAATGG
ATCACAAAAATACGTAAAAGAACTGAAGGAATGACCCACATTCCAGTTAAAATATAAATACCTTATAAG
TATTTCGGAAAGGTGCATTATTATTATTACTATATAATATCATAATGAATTATCAGTTAGGAAATACACTTG
TGAGTTTGGCGTTTTAGATTACGTAGCGGAAAATAATTTAGGCCATTAAAGTAATCTACATTAATAATATT
ATAACTTTATTTATCACACCAGTTTTGGAGTGTTTGTCTTACAACTTAAGCATTTTTATTCGTGTACAGA
ACCCTATAAACATTTCCCACTCCATTCGGAATTGAATAAAAAATACACACGTAAGCGCGGAAAATGGGA
TCAGACAAAGCATTTGCACGGCGCCGTACTGAAGCACAAAGAAAATAATTGTTTTCTAAATAAAAACC
AATTGATGAATCTAACCGCCCATAGGAAGGTACCTAATTATGCTAAAAATTAAACGCTAATCGGCTTATA
CTCGTAACTGTGAAACGGCTGAGCCGATTTTGATAAATCACAAGACCAACGGCAATAAGTAAGCGATA
GAAAACACAAGGAATAACAGCGCCTTCGAATGCATCCATCTATTTTCCACCCACTACAGAACAGAGAC
ACACAGATAGATACGTATAACACCCCTCGGGGTTGGTCCGGGGTATGATTGAAGGCTGCACAGGGCGT
CCAGATCAGACAAAGCATTATTCCACGGCGCCGTACAGTAGCTCAAAGAAAATATATTTTCCTACCTAA
CCAGGGTAGGTTATACCCAAGCTAAAATTGAAAAACCTATCAGTTAAAATACTCGTAACGTAAAACGG
CTGAACGGATTTTGTTAAATACCTACCTATAAACAACAAACTAATGCTTATAAACCGAAACTAACAAAA
GAAAAACTAATCAGATCCAATCCATCCAGTCTCCACTCTCCACCAACAACAGACAGACAGACACACA
GATACCTCGACCCTACAACACCCGGGGTGGCTCCGGGGCAGGCTGGGGCGTCGCCCGGCCGCGGCCC
CGCGCCCCAGTTCCGCGTGACATGGTTATTTCAATTATTTCGTTTACGTCCACACTTCTGGCGCACCCA
CTGTGACAACATGATGATTCTAGAGCACTCATGTTTTGTGGGCTTTTGAGGCTGCTTCGCTTTTGGTTG
GAATAAATTAAAGATTTAAATGAACTCGGAAACATGCATAGATTTAAATTATACATAACGTAATTAGTTT
TTATTGAATTTTATGGTAGGTATACTAGGTGTTTCCTGTAAAAAGAACAATGAATCTACATGATTTTCACT
ATGAACGAAATACGATCATGAAATTCAGATGAGTGAAATTTAACCTAACATGTTACCAGATTACATTAA
AAATAAAGGAGTCCTACACTGCAATAATATATAAGCAATACAAACCTCAATTCAGTAAAAGTAAAAACT
AAATATTTTCCAGATAAATCTGAAGAAAATTACCTACCTCCTTTGTCTTCCCGATCTAGACAAAGGCAT
GGCAAGGAACAGACGTCCGGTCGTTATTTCAAATCACTATTTCCGTCCATATACATAATATAATTTCGAG
TCTATTTCCACGAACCACTCAAAGGGGCCGATATCTAAATTTGCTGAAAACAAACAATCGAGGCCAAT
TTCTCTGCATCTATCAGAGCGGAGGCCGCCTGCAATGCCGACCCCATTCGGTGACCTGATTCCTAACAC
CAGAAGTAGGTTATTGCATGAATATATATTTATACATGGGTAAATGCTATTCCCCTAAGTTTGTGTTCTGC
CAGCCATAAATTGAGAATATTTTTCAGTTAAAAAGGGCAACGGGGAATCATTGCAATTTGAAAAGAAG
ATGTGGGGGTTGAAAGCTACAAAGAAAGATAATATATAAAATCAGTAAAAAATATATATTCTTTGTTTCA
CAAGTTAAGTAGCTACCACATTTGAGGTGACGCATTTTACAGGAATGACGTAATATAAACGACGTTCAA

TGAATATCTAATAATAAAATAATATTAGCGGTTACATGATTCGGTGAGGAAATAGTGGGGAAATTTCGTG
GAAGCCTTACGACTGTTATTATTGGGTGCCTACGCAAGTGAAATCCCTTCACTAATCCACAGCTCAAAA
TAGGGTCAGAACATAACAGTTTGGTTTATACATTAATGTTTATAATTACTAAAATAGTAAATTATAAACTA
CTCATCCTGCTCACAAGTTTACGAATAAATGTAAAAAAATGCGCGTTTCCATATAATAAACTATTTTATCT
ATGAAATTTCGAAACGAAAAAGTAGGTCACGACACGCCTGCCGATAAAGAATTGAAGATCTGAATCG
GCCTTGATTAGGTAGTTCGGATCCCATATATCTTTTAGGTATATAAGAACTAGCTTGATCCCGTGGGTTTC
GCTTGGCGTTAAAATTATCTCTTGGGAAAACTGGCATAAAATTTGCCTAAGTGTATCCTGCGTTATAAGC
ATGCTGCATACATAAATTTAATCAAAATCCGTTTATATATTTTTAAATAAGTACATATAATATATGTAGGTA
GATGTCAATACATTCAAACTTTCGCCTTTTTAACATTGGTAGGACTACTTACATGTTTTATTCTAAATAAA
AGAAGCTAAAAGCATCACAACCTTTCTGATTGACTGCATAATATCCTTTATATATACTTACGTAGATAAGT
ATTGACAAAACATGTCAGAACGATGATATTTTTATGTATGTTTTGGTTTCACATAATTATAATGCGCATGC
CTTCTAAAGACACAATAAATACGCTTTATGTTTACAACAATTAATAGTTCAAAGTGTGTAAGTATTATAA
TAAACACCCCCTTATTCATAGAGAAGTTACAAAACGTTTTAACTAATAAACTGTTTTGTCCCTCTCCAA
CAAAGAACAATTTGTTATTTGACAGAGAGGGACAAAACAGTTTATTAGTTAAAACGTATTGTAACTTTT
CTATGAATAAGGGGGCCAGTTTCTATGCAACGTTTAATTTTCTCAAACTATGTCATACAACTTTAGGATT
CAATGCATCGTAGATTGCTATCAATGGATATGTTTTATAGACTGCCTTTTTATAGGTAGATACTACGTATG
TACTTCGGCTTTTATATAACAAGTTCGAAAGAACATGATACTTACCTAATGAAATGCAGGAATTTTTGCT
GCTCAATTATAAATTTAAGAGAGTTCGAACGCACAAGTGTATCTTCCTGACATGAAATAAACTATTATAA
TTACAATAATTGACTTATAATTATAATAGCTACTTATTTTTGGTACAAAGATTATCATATAATTGTTTAAAA
TTAGTGGTATTTATTTATAATTTGCTTTCAAGTATTTCAGTAAGTTATTTATCCAAAGTGTCGGATTCTGT
CTGTGAGTGCAATTTTAATAAAACCGATTTTTACTATCATCATGACTATGTAAAAATATTACAGTGCAAA
TTTTTGTTGAAATTGCGTTCACACATAGCATAAATTTTAATACTAATTGCCCTAGAAAATTGTATAAATTG
AAAATTATTTTAAGTTATTGAATGATTATAAAAATAAAGTTATACAATGTTCTTATCTTGTATGTTTTTATT
AGTAAATACGACTGAAGGGAATATGTACTGGCCGAGCTGAATTAGTATTCAGTAATAAACGAAATATTT
TGCCTGGTGATAATGATTATATTTTCATGAGTTTCAGTTTCACAATATTGTATTAAAAGCAAAAAAGTATA
TGAAATATTTCGTGAAAGTTTTTTCAAGTCTACATGAAAATACTTTTCAGTAAACTTTACGATTACTTAC
GTACTGCCAGCGACTTGTGTAATGTAATTAAACAGTTTTCAGGGACCAATGATTTTTCCAACCCTGAAA
CTTTGTATACAAGCTCTGGCCAACAGCCTGGGAGCTATACTACTGGAGGAGAGGATATAGGCGCGAAA
TCCTTGTATAATATGGATTGTACGTCGCATTAGCCATCGTATCTCCGTTGGAACATGCGAACTGCGTATTA
GACGTGGCGCGTCAGGAACAAAGAGCGCAATATCGACTAAGCTTGAACTAAATTTCGTTGTCGGCGG
CGCCCTGCCTTTGCCCTAATTCGCCAGCGCCGCGATAAAACTAGCGAACAGTCGCCACTGTGCGCGCA
ATCGGGTCACGCACACATCTGACGGCCACGTGCTGGCGATAACGACTCGCTCGATTCACTCCAATTTA
GAACGGAATCACGACGCTNNNNNNNNNNNNNNNNNNNNNNNNNNNNNNNNNNNNNNNNNNNACGCTCTTT
GTCACTTCTAGCATATTCCGCGCCCAATGTCTGCAGTCGCCATCGCATTGTGCCAATCCGCTCCCATAA
AGGGGTCTCAATGCTCAATGGCGATTTTTGATTTGAATAGAACAAAGCGAAACTGGTTTGAATAAAAA
TCTTAAGACAGCTGTCCACTGTTACAACATTCCCTATGCGTAAATCTATAAATAACACCTTTACATCTAG
TAGGTGATGGAGCAGCGTATGCTGGGCGGCGGTTGCAAGCGGTAGCAGACCGATGTAGCGCGAGCTC
CGTAGCCGCAGAGCGCTGTGTAACGTGGATCGATACCGCGACCCGCCCCGAAATACCGATGTCTGTGT
GACCTGCAAACTTTCAATACAGTGACATCACTGTTTACCAGTTACGAGCTAGGACCCTATCAGTCAAGT
CCGAACCTAAGTCCTAAATCATTTCCTTGTGGGACGTAGAAATCAGAATCGGGAAGTCGGATCATAATC
GCTGTTTCGCTCGCCTATGAATCGCTCCGCTTATGCGTCTGTGGCCGTCGGTTTTTCGCTGCGCGCGTTT
CATGGCATACCCCGTGGCGTCAAGCGCCATTGCAAACCTCGCTGCCGAGCCTGATAGACGTGTTCTAG
TATTAGACTAACAAAAAATACATCATTGTGCATTTCCTGGTCTATTCCAAGTAATATAACTTCACAAAAA
TTCAATTCTAAACATAACCAGTGAAAACTATTGCATTTCCTCAAGTGGTTTGCGGGTATTCCGGGTAAAT

ATAGTCGCAGTAATATTTCATTACACTGAATCATCGACCCGTGTGCTTTGTTGAAGTAATTGATTTGCCA
ACTATTTCCGTGTCAAAACGACCCGAAAACCATGGGCGTGGTACAGGAATGTGTCTTTCCGTCGGCGG
TTAGTGCAGAGCCTTTGTCTACTGCCCGACGCGAGGTTGACCTAGATAAATAAAAAAATGAGGTCATG
AAGAATTAGGTCATCGCTTGGTGGCAAGATGCGCAAAGCGCGCCCCTGGATCATGAGACGGCACGATG
TCCACTCGCCTCCGCCGTGAACGTAGTCGCGGGTCGCCAGCTAGCACGCGGAATAGATACGTCGCAGT
TCCAGCTAAATCGATGACAGCGGCAGCGCGCGCCTAATGCAATCTGCAGCGTAACAAAGGGATCGAG
GCCAGTCGCGTACGAGCGAAGCGTTCGTTGTAATTCGAGGTTTGCTCTGGTACCATAATTCCTACATCG
CCAGCACAGAAGAATAGCGGGAGA
>TCONS_00013603 geneID=XLOC_002694 scaffold_1173:546-2584(+) Splice junction overlap
CAGATTCCGTCCCAACTGCGCTGCCTCCTGCCGCGGCTGCTGCTGCTCGCACGCGGCGCACGCGGCGC
ACGGCCACCACGGCGCGCACGCGAGACACGCTCATCATCATCACGCGGAGGCGCGGCGGCGGGACCC
GGTGGTCGCTCCTCCCTACCTCGTGCACGAGAGATTGTGGCAGCGGCAACATCACATGCTGGAAGCCC
AGCGACGCAGTATGATGGCGTCGCCCGGCTACGGAGTGGTGTACGTGGC
>TCONS_00013645 geneID=XLOC_002710 scaffold_1179:2281-3429(+) Splice junction overlap
GAGCATGGGAGGAGGTGCCGGCGCCAACGGCGGCGGTGGCGGGGGCGGCAACGGAGGCGGCGGCC
GTAAATGAAGACGGACAATCGGCATACGCTTACTCATACTCCAACCTCAACAACCAGATGACATCCAG
TTACTCCAATTATGTTCCAGACTCCGTGAACAACGGTCAAGATTATAAAAAGTATTATCAAAATTAATTC
ATTCTGTTATTGGTGCTTAATTGGTTGATTATGTCATATTAAATTTTGATTCACCAGC
>TCONS_00013668 geneID=XLOC_002722 scaffold_118:291671-296443(+) Unclassified
TTATAATCTAAAACAGGTGTGTATTGGAATTCAAAGTTCAATTTCAAAGAATTATAATTATAACGGTGAA
ATCACAAATTGACTACAAGAAATTTGCTGACACGACAATGTATTTTATTAAATACAAACATCAATGAAG
TAGTTTGCTTTCTGACATACAACATCATGTTTAGATTTCATTATCTAAATACTATGTCAATTCTTAACTGT
GTTTCATTTCAATCCTTAAAACAGTTGTTCAGGCCTGCGTAACTAAAATAAATACGTAGCTATTTTAGAA
TGACGCTTAAGTTTGAGTCTGCACCGTCAGAATTTATTGATAGGGCAGGGTGCTAAGAGGTCAATATTC
AATGGTGACGTTAAATTTTAAAGCCAAGCAGACTAAATTATCCCTTGATAGGGCAACACCGATACACAT
AAGTGACATCAAGCGAGTTTGATATGTAATACACAACATTTAGTGATTATTATCGAATAGTAAAAGTAGT
CCCACATGCTCGAATTGGTCCATCATTGGGGCCTATGTCCAGTTAGCATAGCGTCACTCAGTGGGTTTT
TTTATTAATATAGCATTGCGTGTATTAAAAAAATACTTAACATACAGTCAGCTGCATAAATAGCTATACAC
TTTTGTACCTTGTCTAACTCACCACCTGACAATTCATTCATTCCAACATTGACAGTTTCTTAATAAGGTA
GTATGACATGGTACAAAAGTGTATAGCGACTTATGCAGCTGACCGTACCTACACATCTCAATATTAGATT
GACTTTAGATTTGTTACACGAAACGTAAAGTATTTTTTTTTAATTGCTCATGCTAAATTAAAATACTGTAT
TACATAATTATACTATTATGTTTGCATTATATATCCATAGGTTCCCACTGAAGCGATATTCTCGTAGCACCC
TGAATATTGGTATCTACAGACCTTCTCTGAGACAAGCTATAGCGCGCTCGTGCGCAGCACACAATGCTA
CACTTCCAATAAACGTAGCCCTTTCATTGGCTTTTTATATAACGTAACATTATTACGTAACATTATTTCCT
TTTTTCGTATTAAATCGCGCAAAATGCCCGCTATGTCTTGTCTCCATTTTCGACATTTAAACGTTAACATT
ACAAATTACAACTACGCATGAATCTTAGGACATTTTATTTTTATTGATACTTACAAGAAACATTTAATAAT
AATAATAACCAAATTATTTATTAGCTAATTATCCTTTAATCTCATAGGACATCATATGCACCAAAACTTTA
AATAGTTATAAGTATTTATTGTTTATAGTTAGTAGGTACGTGAAGAAGATTAGAGGAAGTAGGTAAGTAG
GCTATAAGCATTAGGAGAGAAGTTAGCGTGACTTATTTTAGTACCTAGGTACCTAATTGAGAGAATTTT
GTTCGGTCGTTAAAGTGATAGAAATCTCGCTATGTGTTTATTGGACTTAGACAAAACAGCAATGTTTTAT
ATGTAAATTAAAGTAGGTATATTTAGATTGTGATTGATTAGTTTTCGATACTTTTTATGTAGCTACGTAACT
ATCTATATTATTTTGTTCGATGATTATTCTCTTAAGCATTTAAGTAGTTATTTAAAGTTGCCATTAAAGTAA
CATGTGGCGTAGTACAAATAGAGGGTAATAGTAAAAGTGAGGGATAATTGTGGGAAATATTGCTATGGA
TAGTAAGTTAAGAAAGAGTAGGATTGTGATGTCGTGCAGTGGTGCTGTGGGAATGTCCCACGTTCATT

GGCTTGCGTTGACTACAAGCAACATGTGATTGGATGATCCTCATCCCGACATCTACATAGTGATTTCTTC
TCAATAAGTTTAAATAAATAGATCAATCGTTCTACATTTTGTAATTACTTATTAGCACCCACATAATGCTA
GCAATCAACTAGATAAAATAAGTTTTCATAAATCATCTAACTCTTTATTATATTTTCTTTCCATAATGGTGT
CTTTTAAATACGTCTATTCAAAAGTCAAATTACATAGTATTTAATTTGTCGTCTACGGCTTAATTTATAGT
GGAAGACCGTAACAAAATTACTCACTGTCGATCAAAGCTTGTAAAATAACTAGATAAGGGGCTAAAGC
GAATGACTCAAGGGTTTCTTAAAACTAAATTTAATAAGTTGTTTGTCTGATCTGATACTTTTCTGCAATA
ATTGATACACAAAAACAAATCGGAATTGCGTTTTTTTCACATGAAACTGTTGAATTATAGTTGGCGATA
ATTATCCAAAAGTTATTTGGTAAATATGATTGGTTTGGTTACGATAACTACGGCCTTTAGGAGCATCTTT
GTAAAGAGGCGACCTATTCAAAGGGATCCGCCAACATATGGACTAATAAACTGCCATTAATTAAAAACT
GTGTGACAAACACAATATTTTCCTAGAAATCAGCATGCC
>TCONS_00013911 geneID=XLOC_002723 scaffold_118:466313-467230(+) Intronic
TTAAAAGGAAGGTGTTCAGATTAGTATGTGTATAAGTGTGATTGTCTGTAGATGAGTAGTACAAATAAA
AATTAAGGGTAAGAGTAGATATTACTAGAGATTAGCGTGTGTTGTCTATTTGTGTCGTAGTCAGTAGATC
AGTAGAGTTTGAAAGTGTATATGCGTCGATTGAAATGTGAAATTATGTCCTGTGAGAATGCTGAAGTAA
AAAGAAACCTGTGTCATGTGACTGTCTGGAATAAGAAACAACAACATATATACACAAAAAACAGT
>TCONS_00013966 geneID=XLOC_002728 scaffold_118:679027-690193(+) Exonic overlap (+)
CCCGCACCGCTCAGGTGGACAGGCACCCTCGCTCTCTGAATTAATCTGACAGGCTATCTTTTGACTGG
CTTTTCATGCTACGAAAAAGCTACACAGGTACAAAGGAAAAATAATAAACGACAAATTTCTACGGCCT
TTTTCAGGCGGCACCGAATATTCGGCCGAAACTGTGCGCTATCCCGGCTCGTGTACCCTCCTCGCGCAC
CGCAGTCCGCCCCGGACGCTCCACCGCATCACACTCTTAACATGCTCTCCATTTAAAGAGAACAAGAT
AAAAATTATCTCCGTCCACCCATCCAACAATCTTCAACCCCATGATTCTATCGTCCCGTTTCGACGGCA
ACATTCCAAGTAAACAATGTGGTTGAAAGTTGTTTTTCTTTCTCCTGGATGATTTTGTTGAGAAGATGT
CGAGCACCTCATGCGGCTGTAAGAGGTGCGAAATCGCGTAAGCTGTAAGATGCTGACAGTCCACCAC
CACCATGGACTACGGATAGAACGACATCACGCCTGTCAATTTTTTCTTCTGCAACACGGCCTCATCTAC
AAAACCGACGGATCCATCCGCCGTCCTTTATTTGTTTCCCGCTTGATTTCATCATACTACATCCTGCATC
AAACCTGTACTGAACTGC
>TCONS_00014003 geneID=XLOC_002787 scaffold_1182:4496-6413(-) Intergenic
ATAAATTTGTATTCGTCGAGTTGAATGGAGAACCACATTTTTCGTGTTTTTCATGAATAAATTTGTCTA
AAATCTTTCAATTTTTCATTGAGTGTTGCTGGCGAGTTGGAGAAATGCACACGGCCAGTACTGATGCG
AACACAAACGGCGCCAATCCCGCACGGGATAAGGTGGCCATCCTTGATGCCGGATCACAGTATGGAAA
GGT
>TCONS_00014403 geneID=XLOC_002807 scaffold_119:340754-343387(+) Intergenic
CACCTTTATTTAAGTACAAGATGTATACCTACATTTCTCAAGATCACATTACTTGTCGGCAAGTGTCACA
CATACCCTCCACTGTACTCACCGACAAACAAATAGGTAGTTCCAAACATGTCTGCGTATTGAATGGGGC
CGAGTCCTCGGATCCTCCGTATTGTGTGCCGGCAGGACGCACTACCTGCCTTTTGTGTGTTCTGCTGAA
TGCAGACAAGAGACGGGGGCAGATGGCCGGGTCTCGGCTACGGCGGAACGAATGTCGAATAGCCCAT
GTGATTGAAACCAGTGCTTTATGTTGTAGTTACACCGAGTAGGTACCTACGGCAGCGGCATAGATAAAT
AGGTAGCAAAATATGTGGCTTAAAGGAGAGTTTAGATTTTTGGCGTCCACGAAAGGAGTCCCCGAGTC
TCCGATGGAGATATCAACTGCTCGGGTGCACCTTGTCTGGACAGTCAGCAAATGACTATTCGGACTGG
CTAATCCATTCACCACCGTTGTAGTTATACGCTAAGAAGAATATTTATTTTATCGATTAATCGAAAATATT
CTCTATTGGATTCATAGGTATGTACTACTCTAGGTACCGTTTGGGTTGACTTTTTTCTGATTTCGACACCC
GTAAGTAGGTGGGCCATAAAATTGTGCGCCGGCTACGTTTTCCAATTTCACTAAGGCATCGTAGGAATT
CCGTTTCAGCTCCGGGGGAATGTAGATAAATAATACTAAAGTGTCTGCATGAAATAGCAATTTTTGCAA
GGTAAAAGGAAATTACAGAGCGATAGTTTTCGCTCTAATATTCTCTATAAGTATCTTTAATCGATTTTATA

TGTTTCAAAACATTAAAACCCGTATTTTTTTAAATGATAGGATATTATGATGATGCAACCATATCAAAAT
ACGTGTGATTTGTGTGTTGATTACGTGGAAGCATCTCATTTGTATGCTGATTTATTTATTCAATTTGAAAT
AATGTTTCCGGACTGAAAACAACAAGGAATAAAGAGAGAAATGGACTCAACTCAAGTGTAGTAGGTC
AACAGGGTATTTCGTTACTCTTTGTCATCAATTAAAAGTAATCTCCATTTCATGGACACTTATTGGAGGC
AAGGCACTCGTAAAATAAAACTCCCGTCTTAAAAATATTCTGCAAAAATGTTGCTTAATTTTGTTTGCT
CTACAAAAGACATAAACTGGCAAGGTTGTAAAAAGCTCTTTTACCATCATCGTCTTGAGAATTTATTTA
GCAACATTTACATAGTCTACATAATTTATAGACTTTATAGTGTTAGACTGATATTACCTAGGGTCATGGAA
GTCTTGGAATATTTGATGAAACGTTATTTTAAAGAAACTTGCTTCACCATGTAACATTATTATTAGCCTA
GGTTTTAAAAACGATAACGAAAATTAATCATTTTTTTAAACCTAGGCTAATAATAATCTTTGATGCTAAA
CTTGAAGGAAGACGCACATCTTCACAGTTACCTATTTCTGTGCAACGCAACACCCGTACTACCCGACG
AATGAAAAAAAATACATCTGCCAACTAGAATCACGTCTTGTACGCAGGCAGGTGTTGAGAAAAAAACA
ACCAAAAAGTTACTGTATCTTGCTCAACTTTAGCACCATATTGC
>TCONS_00014345 geneID=XLOC_002808 scaffold_119:365828-394730(+) Unclassified
TCGCGAGCGAATGGCCGACGAAGGAACAATACGCACGCAAATGGTGGTGTGGAATTTGGGTGGACTG
TTCCCTTGTGCTTGCTTATAGTGTAAAGCTTATTCATATCTGTAAATATTGTACTCCGCAGTGTTAGGCTT
CCTTGTTTTCTTCCTGCATACCTGAAGTTTTTGCGCGAAATAGTGAAGTTACTAGTCTTGTGTATTTCAA
GTGTATTCAGCAACTATTGTGGACTTCTGGCTTTTATTGTAAATAAAGGAACTCGTTCTTCAACATCGTT
ATCTGTTAATGAGGGAGGTCACCCCGGGACCGCGGCCGGACCGCGCCGCCGCAGCCTTCCAACAAGT
GAGTCCACATCACTACATACATAAAGCCCCATTGCTATTGGAAAACAAGCACAACTTCAAACTGGAAA
AATAAACTCCAAAAAATATGCATAAGCCGATCAAACTCCGTGCAAAAGCCGAGCTTTAATCTCACGGC
CACTACTCGCCACGAGTCTCGCTACAACAGCAGACAGCATTAAACTTTCAGCGGCGGCGACCCGCTCC
CCACTTCCACTTTACACTAACACCTATTAAATTGGTCACACGAGATACCAACGCGACGAAAACAGAGC
TCTTACGATCATCTACCATTCCATAGCATCATCCATAATTTCTCTCCGAC
>TCONS_00014266 geneID=XLOC_002808 scaffold_119:365832-430503(+) Exonic overlap (+)
GAGCGAATGGCCGACGAAGGAACAATACGCACGCAAATGGTGGTGTGGAATTTGGGTGGACTGTTCC
CTTGTGCTTGCTTATAGTGTAAAGCTTATTCATATCTGTAAATATTGTACTCCGCAGTGTTAGGCTTCCTT
GTTTTCTTCCTGCATACCTGAAGTTTTTGCGCGAAATAGTGAAGTTACTAGTCTTGTGTATTTCAAGTGT
ATTCAGCAACTATTGTGGACTTCTGGCTTTTATTGTAAATAAAGGAACTCGTTCTTCAACATCGTTATCT
GTTAATGAGGGAGGTCACCCCGGGACCGCGGCCGGACCGCGCCGCCGCAGCCTTCCAACAAGGCGG
CGCGGCAGGCGGGTGCTAGTGCCACGCGCACCCTGGAGCCGGCATCAGGGACATCCGTGAAGCGGTC
GTGGCGGCGCCATGGACGACACGGGCAGCAACAAGCAGCGCCAGGCCACCAGGGTCTTCAAGAAGA
GCTCGCCCAATGGGAAGGTAAGTTATATGTGACTTTACACCTCAGAATAATAACGCGCTCTCGTTTAGC
TCTATCAGCTGCAGCTACAC
>TCONS_00014088 geneID=XLOC_002809 scaffold_119:453138-453900(+) Intergenic
GCCTCGTAGCCATAATGCGCGGGCTGGGCGCCGCCGCGGCCGCCCTCCCCGCGCGCCTACAAGCCGA
GGGGCTCCGCGCGCGCTGCACCCGCGTGCTGCAGGCCTGGGCCGACTGGGCCGTGTACCCGCCGCCG
CTGCTGCTGCTGCTGCACCGCGCGCTGCGCCGCCCCGCGCAGGAGGCTGAGGACGAGGCGCCGGCG
GCGGCGGAGGGCGGCGCGTCCCCCGACAGCGCGTCGTCGGGCTCGTGGGACGGCGCGCTGGACGGC
GCCGCGCTGCGCAGACTCGCCGCCACTAGACTGCCAGCTAGTGATA
>TCONS_00014478 geneID=XLOC_002825 scaffold_119:467134-470144(-) Splice junction overlap
GCGGTCGCCGCGGCTCGGTGCGTCTGCGCGACCTCGGGGTCTACGGTCGCGAGGACGCGCCGGACCC
CATGACGCAGTCCATGATAGAGACCACGCCCGCGCCCGCGCCGCTGTCCCGTGTGCCTAGCGCTCCCG
GTAGTCTGAGGGGTCTGATGCCGCTGGGCAGTACCCTTCCGTCCAACCTGCCGTCTCCGCTGTCTGCT
AGAAGGATTCCCCCCGAAAGCCCGCGGCCTCTACGCAGACAACACAGCATCGCCAAGCCGGCCTCCT

TCCTCCTGACAGAGCTCTACATAGGCGAGGACTGAAGACCGCACGCAAAGAAGAACACAAGCTTTCA
CAAACAACACTTTAGCTAAATGTATATAACGTCTAAAATAGCATGCGAGGCTGTCTCGCAAACGAGATG
GTCATACGCATGAGTATACTAATCAAGTAATGTCACGGGAGCAATTAAATGCATTTCTGCTTTTTTTAATT
AGATGTAAGCGCCATTTTGGTTGTCACTGACCAATGGGGAATGAGTATAGTTAAAAAAAGATGGCGCC
ACTTGCAACTTGCTTACAGCTCTGTTGTGATTGGTAGTTGGATATGCCTTTTTATTTATAAATTATCTGAT
TGGCTGAGAGGTTGCGATAGTGCTGCCAAAAGTTTTCCAACTCTCGTCTCCATTGTAATTTAATTAGTC
AGTAGCCCATTAGGCAGTATTTATGGCTTATAATATTATACTTAAGAGTAATATATTATGAAATATTATCAC
TTTAGTTAGTAATGTAACAAAAAGCTTTAACACAGACTTATGGTAATGTAGGAGTAAGCATAATTATGTA
GAATGTTTTTGTACTTAAGCACTTATTGCTGAATAAATAAAGTTGAAAGTATCTTGTGTTTGTTTGTCTG
TAGTTGCATGGCACCTCCTTTGTTTTAGAGCACGCAGCATGATCATGATTGTTTGTAATTAACACATTAT
TTTAGCTATCTGGCCAAAGTATCTGGCAGACCTAACAATATAGTATACTTCTGAGACGTATACTACAGTA
TAACAATTTGTATATTGCGGGTCACTATTGTATATTACTATGTATAACATTATGCTATATTTATTCCCTATTA
TTACTGATCAACATTTGAAATATGTATTAGATGTTTATTTCGTTGTGGTTTGACATAATCATACTAAAGTA
TTTGTTGTGGCGCAATCCCCATTAATATGAACCATTTTAATTCGATTCGACGCGAATCGCTCAAACTCAT
TGCAGGACAAATATTCCATTTGGTCTCGGTAATTCGATGAACATTATTGCATACTTACATCATTCGATATA
CATAATTTTTATCAAAGTATTTCCATTCATTTTAGATCAGTTCTACACCCTGTAAAAGGATTTAATACTTA
ATAAGATTTACATTTTGATTTGGCTTTTGATACATATCTCACAATACACAACTACAATCCTTACGGTCCGT
TTCCACATGAAATCCAACATGAACATTATCAAGTATACGGAGTTACTAAACCCCGGTATTTTGACGAAA
GATTGTGCCAAAGTTCAATACCATGCGCACGCGCATAGACGATCAGATTTTCATGATTTCAAAACCATT
AGCCACAACCATCATTACTCCTCTTCTTCAACCACACATAGGTAACACTCAATCAGCCACAACCATTCA
TTCTAGTCTTCTACCACACATAACACTCAGCTCTCCCCTCCCCCCGCCTCGCTTGCTAACCCC
>TCONS_00014090 geneID=XLOC_002810 scaffold_119:536787-541879(+) Intronic overlap (-)
GTGGGGAAGTAAGGTCCGCAAGCTCTGGACTGGCACTGCCTGCGCAGGGTGAATTTACAATCTTTCTT
CAGGTACTCGTCAACTGGGATCGGATTCTTCACCCATTCGATGACATCGTGTTCGTTCACCACAAATAC
ATCGTACAGCTGAGTGACAATGTCCAAGAATTTTTTGAGGGCGGACTTGGAGGCAGGATGTGAAGTG
AAGTGAGCTTCGTGGATGTAATAGCCGAAGGGAGCACGGTTGCCAGT
>TCONS_00014091 geneID=XLOC_002810 scaffold_119:541305-541940(+) Intronic overlap (-)
TATGTTGTGGGTAGGGTAGTAAGGCCCGCATGCCCTTGACTGGCACTGTCTACGCAGAGTAAATTTACA
GTCTTTTTTCAGGTACTCATCGACTGGGATCGGATTCTTCACCCATTCAATGACATCGTGTTCGTTCACC
ACAAATACATCGTACAGCTGAGTGACAATGTCCAAGAATTTTTTGAGGGCGGACTTGGAGGCAGGATG
TGAAGTGAAGTGAGCTTCGTGGATGTAATAGCCGAAGGGAGCACGGTTGCCAGTGTAGTGTCTTTCGA
AGTTGCGAATGTAGAATTCCAGCCAACCATCTTCGTCGTTAGGTTCA
>TCONS_00014094 geneID=XLOC_002812 scaffold_119:548824-556218(+) Intergenic
CGACACTATCCCAGTTTTGAGATAACACTCAGTTTAAATGGTTTAGCTTTGAGTTGCGTTTTGTCAAAA
TGCAACTAGTTTTGCTATGTTTGGGTTTGTGTGTTTTGGTAATGGATTTGGCGGCTGCAAATTATACATT
CACGTATGAAATGGCGTCCCGCTGCAAAGAGCACACCTGCCGCCACGGGTACAAGTACGAGACGTAC
TACCTGGCGGACGCCTCCAGCAGGCAGGCGGCGTTGCGCGA
>TCONS_00014095 geneID=XLOC_002812 scaffold_119:548824-556218(+) Intergenic
CGACACTATCCCAGTTTTGAGATAACACTCAGTTTAAATGGTTTAGCTTTGAGTTGCGTTTTGTCAAAA
TGCAACTAGTTTTGCTATGTTTGGGTTTGTGTGTTTTGGTAATGGATTTGGCGGCTGCAAATTATACATT
CACGTATGAAATGGCGTCCCGCTGCAAAGAGCACACCTGCCGCCACGGGTACAAGTACGAGACGTAC
TACCTGGCGGACGCCTCTAGCAGGCAGGCGGCGTTGCGCGA
>TCONS_00014277 geneID=XLOC_002816 scaffold_119:733163-757198(+) Exonic overlap (+)
TGGCACCTATAGGTACCTACAGGGTGGAATTTTATAAGTGATGTTTCCATTGGTAGGTAAATGTGCCTGC

CTGTCATACCAAACTCATTACGGGACCACTGAAACATTTTCGTCGAGTAGGGAGGTAAACAGCTGTTT
TACAATAAAATTATTACCTAATGATAGAATCATATTGTTTTCATGCTTGTCAATATTATTACTTAGTCAAAT
TATTCTACATATAATATAAGTAATACAATAATTTGGACAGTGTTGCGCCCGCGCAAATATACTTAATTTAA
ACTGAGAAACTCCTACTTTTTTGAATTCGGTTAAATTATAAATGAATCTAAGTAATTCAGTCAGCTTCCC
AAACCGCATTTTTTCATTCAATCACAATCACAACATAGACGGTAGATACTTATAGGTATATATTAGTTTGA
CAAAGTTCAAAAGAGTAGAACTACTTATGAAGCTGAATGTAGGTACGGTAGTCTACAGTTTTTACCTTG
TCAAACTCACAAACCATCGAAATAAAATCGTTGACTCTGATTAACTTTCCATTCAGGATTTTAACAATA
AGATTATGTAAGTGACAAGCTGCTCGTTATTTGAAAACTGTTACAAAAGTTTACAAAAATATCATATTTT
ATCTATCTACTTCCTTACTAGACTATTAATTCATGACATGATACAATAATAAATACTGTAA
>TCONS_00014290 geneID=XLOC_002830 scaffold_119:755964-757048(-) Unclassified
GGAAAGTTAATCAGAGTCAACGATTTTATTTCGATGGTTTGTGAGTTTGACAAGGTAAAAACTGTAGA
CTACCGTACCTACATTCAGCTTCATAAGTAGTTCTACTCTTTTGAACTTTGTCAAACTAATATATACCTAT
AAGTATCTACCGTCTATGTTGTGATTGTGATTGAATGAAAAAATGCGGTTTGGGAAGCTGACTGAATTA
CTTAGATTCATTTATAATTTAACCGAATTCAAAAAAGTAGGAGTTTCTCAGTTTAAATTAAGTATATTTGC
GCGGGCGCAACACTGTCCAAATTATTGTATTACTTATATTATATGTAGAATAATTTGACTAAGTAATAATA
TTGACAAGCATGAAAACAATATGATTCTATCATTAGGTAATAATTTTATTGTAAAACAGCTGTTTACCTC
CCTACTCGACGAAAATGTTTCAGTGGTCCCGTAATGAGTTTGGTATGACAGGCAGGCACATTTACCTAC
CAATGGAAACATCACTTATAAAATTCCACCCTGTAGGTACCTATAGGTGCCACAGCACCCTGCCAATGA
AACATCACAATTACTTTTTTTATGTGACCCAATCATGGAAGTAATAAGTAGGTACTTACGTACGTACGTA
AGTACCTACGTATTACTTCCATGGACCCAATATAAGTTTATTTCACAAGACTTGCCAATATCAAAATATA
AAAAAATACTTCATGAGCTTTGCTGAGGATATGTGATATTATTAAAATAAGATAACAATAAAATTTACCC
TCTATAAGTAGGTACAGTGTAAATCTAAATCAGTGAATCATTTCAGGCGTCCTGTTAAATAAAGATACTT
ACCTAATAAGATGCTTATGAATTTGATTACTGATGTGGGTCCTCACTGCTTGTTTTTGTATAAATTTGGAT
AGTATAACTAGAAGTAAGTAAAGTACAG
>TCONS_00014440 geneID=XLOC_002831 scaffold_119:947253-950849(-) Intergenic
GCTCTCACCGCATTCGTACAGCAGTGTGAACAAACTAAACTCAGGATTAATTCCGGCTAATTACGTGAT
ACCTATTGAAGTTCTCATTGTTAATTGCGTTCATAACATTTCCCGTATACCTATAACCTATTAAGTGCATG
TTTGAACGGAGTATAATGATCGTGATTTTCCTATCATTTCTATTTGAAATTGTGTGTTTGTGTGTCGGTGC
GATATGTGTGTGTTGTGTGCTAATTTAGAGTAAAAACCTCTGGATGACTTGTCAAGGTATTGCACCATG
TTTTGTGGTTA
>TCONS_00014655 geneID=XLOC_002916 scaffold_1195:5116-7633(-) Intergenic
CAGAATCATAAAGCTAGGAAACACTAGTTTTTGACATAGTTTGACAACCAAATGGTGTAGTGGTTAGT
GACCCTGCTGACTGCTATGCTGAAGCTCTCGGGTTTGATCCCGGCCGGGGCAGGTATTTGTTTAAAGA
CAGATATTTTGCACTCGGGGCTTCGGTGTTACAGTTGGGTCTAAACAACTTGATAGTTTTGAAGTTGGC
TGCGATTACACTTTTGTATGTTTTCTTTCATAGTGGTTGATTGCGCCTCCTTTACTATCAGACTGGCCGA
AAATAATATTATGTCACAGCACAACGCTTACCTGTACTCGTATGTGCCTAGCATGCTGCTCTTCATCCTA
ACTTCCTAACTTAATATTATAAATGCGAAAGTAACTGTGTCTGTCTGTCTGTCTGTTACTCTTTCACGCC
AAAACTACTGAAAAGATTTGGATGAAATTTGGAATACATATGGTCTAGACCCTGAGAAAGAACACAGG
CTACTTTTAATGCCGGAATTCCAAGGGGAAAACTTTTTAAGGCGAAGCGAAGCTTGCGGGAACAGCTA
GTATAGAATTTACCTACTTCTCTGTCGTCGTAAAGTCTGACTGTCTATAATAGGTGGACATAGCACCTGA
TTAGAATATTAATTTTGTATTAATTTATTAATCAGCAAAACGAATGCACATAGCAATAATTCACTGCCTCC
ACATATGAGTATTATACTAAAGTTAGTTCGGTGAGCAAAGGCAAAGCTTTTATAATCGAGGCGTCAGGA
ACATAAGTGCCAAGCTTTTAAAACAATTACTTACTATTGTTTCCCAACAATATCGGCAAAAGCGCTGAA
AATAACAAACTGAATATGAATGTCGACCAAGTTATTAGGTCGCAGTTTGTGTTCCGTAGGCACTCAGTA

CTGAGCCCACAGTACTTATCATTATCATCATGTTTTGGTAGGGACACTATATTATGAGTGTTATTGATTCC
TACTATATTAAGAGTGTTTTAGTTAGGTATATATTTCTGCCATTCCAACATAAAATTTTCAGGGCAGTGCT
GAAATACCCAATATATGATAAAGTAAATTAATGCTGCAAAAAACCTAAATTTTCAAAAGTTGTTAAAAT
AAAATTACGTCAAACTTTTTGCCTTTCGTCTCTACTGTTGCAACATCCTGTATAGACTGTTCTCATTCTT
GAGCACCGCGCTGGCACGCCGCGGACGCTTCACACTCGTGTCGCTGATGTACCTCGGAGCGGAACAA
GGGAAAAACAACGAGGAGTATTTATATTTACCTCCCGCTTTCAATCACGTTAGCGCAGTGCCAGTCCAC
TCCAGTAGGCGCGCGGGGGAGGCGCATAGTTCCAGCTACACCCGGCGAGTCGGCACTTTGAAGCGGC
GCCGTGTCCCGGAAGGGGTAATTAGGTGAAAGATCCACACGGCCGAGGGTCACATCATCGCGTTACAC
AAATTAGAGCATATTTATTGAACGGAAAATCTCGTAAGCGCCTCTTTGTGCCGTGTGCAGTGAGCCCCG
GTGGGGGCGGGGGCGGCAACACGGCGGGGGCGGCGAGGCAGGGCGGCAGGCAAGGGCTCTAAGTA
ACAACACCCATTAAAGACACCATTATATGTATAAAAAAAACATTACAGGTACACAAATAACATAGGTGT
AAATTCAGATATTTGACAACGGTATTTTGTTTCCCCAAAATGCGTTCACCGCGGACCGCACTCGCCAT
ATATCTGCTGAACTAATCTTTTGCGCCACATTTGTTACACAAATACCTTTTACTAGTTGCAAGATTTATGT
TGTTTGCAGTTTTTTTATACTTATTTTAATGCTGTATTTTTTCTTTCATGACATCATCGTGAAGAATGACA
ATAAAAATACCATGGACCAAGAAACACCATGACTGTGCTCATCTTGTATACTCATCCCACACCAGTATA
CGGCTCGCTTGTCTTTACCAGAGTACAATGTACGGGACAGTGGGTTGCCAAGCAGCAAGTTACCTCTG
AGTACCTTACCTACCCCTCACACACACAGGAAACGAGCTCAGGCGCTTCGACAGGAGGAA
>TCONS_00015444 geneID=XLOC_002963 scaffold_12:232163-232900(-) Intronic
AAAACATCAGACAGAATGATGTCGTTTTTGAAACAGGCAGCAATAGGTGTGCTTTGTGGTTTACTGTT
TGTTTCAGGGTTCGGTATGGAAAAGATGGTGGGTGAAGGCAAGTGGTCAGTCCTGGACGTGCGCGCG
TGCGACAACCCCGGGCAGTACCGCGACCGCGTCACCGTGCAGAGGCGGAAGCTGAACCGAACTCAT
GACGTGTTTGATGGGAGTTTTGATCTGAAGGAGCTCATTGATAATGATACTGCTGTGAGTACCTATTAA
GTAACCTCTACAGGGTAAAGGGAATGGGG
>TCONS_00015048 geneID=XLOC_002928 scaffold_12:351436-358000(+) Intergenic
GGATGGTTGTGTTACAGTGGTAAGCCCAGAGGCAACTCTACAATTATTTTTTATTGGTCACGGTATTTTG
AAATGTTAACTGAAGCGGAATGGGTGGGTTGAGCTGGTCTTTTATATTATAATCATTAATCACGAATTCA
CGGGCAATAATTTATTACTTAATCATAACATCATGTGTATATTTTCCCGCCGTAAATTAATTTGGAATTAA
CAGTAAAAGGACGTGAGCAACCAACGTTTCCTCTGAAACTTATCAGTCGAATTTGGACATAATCTCGA
ATTTCATGAACCCAACTCGTTAAACTAACGAAATAACACACAACCGCAAGAACAATAATAATGACGCC
AACCTTTATAGAATTTTATAACCTAAAGTTAACAATTTCAACTAAACTATGATAAATTTCGTACGGTGTA
AGTAATAACTGAGGATTACAATATTCGTTAGCCGATACGGTAACGGCGTTCACAATAATTATGACACTG
AATTTGTTATGTAAGAATTTGAACACACAAGTCGCTTGAACTAATGCTATAAATTGGTAAGCGCGCCGG
AAGTTGGAAGAGCGCACCGGATGTAGAAAAATCAAGATGGCGGCGATGACAGCGACTTGTAAACCAA
CCGATAGATGTTACGTGTTGCGTAG
>TCONS_00015304 geneID=XLOC_002969 scaffold_12:541683-546109(-) Splice junction overlap
GTTTTTCCATTGGTAATGGACGGCAACTCTATTTTAATCTTTGAACTTAAAATTAATAAAATACAGTCCA
CTAAACCCACTTCGCATTTTATTTATACATGCGAAAGTGGTCCTTCGATATAACAAATAATAAATTGCAG
TTAAAAAGTGCAAAAGTTGTGCAGTTGTGAGCAAAGCGGCACGTGCATGCACGCTCAACAGTTTGGA
TTGCAGTGTATTTCAGAGTATTCAGAGATAAGTTTGATCTAAGGTGATGATACACCTTAAACTCCACTAC
AACATATTGCAGCGGTGGCGGCAAGCTTACGGACATCGGGACTTCGGCAAAAGTGTGGAGCTTATAAA
ACAAAATATACAAAGCTTGGAATACCACTACGGATCATTTAGTGTTGAGGTTGGTTGGACATAAAAAAT
ATCAATAGTTAAATAAAAACATGTAATTTTAAATACTTGGCGTGCATCTTCAGTCCCATTGTATTCCTTTC
AAAAACTGACGTCGCCTTTCG
>TCONS_00015303 geneID=XLOC_002969 scaffold_12:541783-545635(-) Splice junction overlap

CAGTTTTGTTTCTGAGGTTGGCAGCACTGCGTATCTGTCACAGTTGACAGATGACGGACGACACTTCG
TAAACAAAACATCGAGTTCAGTGTTTGTGTGATAAAAAACCTATAAAAGTGCATTAAACAGTAAGTGA
AGTGAATTTAAACAAATTAAACGATGGAAAAGGAACTAAAGTCATTAATAAAAGTATTCCGCACGCAA
TTAAAAGGGACATTTACGCGAATGCAATCTTTTGTAAACAATCCTAGTTTTTCCAATGCAAATATTGAGA
TGTCACAGGCCAGCAAGGATAAATTAAACAGTACACTTAAAGGCTATGAAAAGGGGACTTCGGCAAA
AGTGTGGAGCTTATAAAACAAAATATACAAAGCTTGGAATACCACTACGGATCATTTAGTGTTGAGGTT
GGTTGGACA
>TCONS_00014699 geneID=XLOC_002936 scaffold_12:1118530-1119349(+) Unclassified
ATCACATTGACAATTTGACATGACAAGTCTCTTCTCATTCTCAACTTCTCTGTCTGCTGTCTGTTTTGTT
TACTATTATTTTCCAATTTCTTTCCTTGATTATTCTACCAACCAAGTCATTCATCATAAATTTAAACAGATA
GAAATCTTCAAAAATACAAGATGTTTAGAAATCTATGTAGTGTATCAAAGACCACAGTTTATTGTGCAT
CATATCGAAGAAATTTCAAGAATTTCGGACACAAAAAACAACCAACTCCCCCAATTACGATGTGGTGG
ACCGGATTCCTAACATTCACCTTTATAGGAATGGCAATAGAATGGAAAAGAGTTGCAAAGTTTTTTCTA
GGAGATGCAGCCGAGGAGGAGAGTGTACCAATAACACCAAGCACCCTCTATGGCTCCCGAGGGACAG
ATTTGTGAAGTTATGAACAAGTGTATAGTTGTTATTCAAAATAAATTATTGTCAAGTTTTAGATATATTAG
GTTAATTATTTATTGTAAATGCTAAGAGATTATGTTGTTTAATTATTTATTGTAAGTGCTAAGAGATTATGT
TGTTTAGTAGCCAAAGTGTAAGTCTACTTAGTAAATAAATTCAATTTTTACACACCAT
>TCONS_00014928 geneID=XLOC_002977 scaffold_12:1170655-1171772(-) Exonic overlap (+)
TGAAAATGTTACTTTTATAATCGTGTGATCCATGTTTTATCTTGGAGATCGAAATGCTAATATAATGGGAT
GTTATTTAAAAGTAAATAATGATGGTGTGTCATCGAAAAACTATGCTCAGTTTGTCAGTTGTAAAATATA
ATATTGAATAAGCAACTAAATAAGTACCTACCTATGTTATATTTCATACAATTTTAAATAGTCATAAGACC
TATGTTATTTTTATTTGACGTTCACAATAAATGTTAAAACTATGATTTTAAGCTACATAAACCTATCTTTGT
TTGCTCCATCACTTTACATTAGCGAAGATCAAAACCCAAAATAATTTATAACTCCTAAAAGTCAGTGAA
TGAATCTCTAAAGAAATGGGCACAACTTTGTATAAGCCAGCCGCGAAACGTCGGCGTAACTTTGAAAG
ACGAATAGCTTCGGAGCAACGAACCGCGGTTCCTCCAGACAGATCGAATCTAATACAATTTGGTCCCA
GATACAAGAATCTCCGCCGGCCGCTGGTTGTAAATCACGGCGCTACGTCGATCCGCGTCTCAGCGCGA
AAAATAGAATATTTGATGCTGGGAATAATTCCCAGATAATAGAAGTCACTGGTAGTGAGTCGCCGCTCA
GGACACGACGCCCCCTGTTAGCGTGAAGATAGTGCAACATGCCCGGATACCAACGCCCGCCGTTCTGG
AGAGTTCCTCATCAGAGGAGGGACCCTGAGGACGAGAGACAGATCATCTTCGGAAACAGGAACAAG
AATAAGGTAAAAATTTTGATTTGTAATTTAATCTGAATGCGTTATGATAATCTTGCAATATCTCGGTCGGC
GTCACGATAGGTACGCAGACTTGGCAAACGGAATGCTTTGAAGTCGTATGCAGTGTGTTTTTCACTCAT
ATTTGTTCGCTA
>TCONS_00015088 geneID=XLOC_002939 scaffold_12:1207029-1209176(+) Intergenic
AAACTGTTCGGCAAAGCTGAGCCGCCCGCGCCCCAAACCGAAACAGAGACTGAGCTAGTACCGCGC
ACCATTAGCTACAACGGGCGCGCGACGGCCGCCTCGCCCGCCACCGGCTCCAAGGCCGCTCACAACA
AGGAACTCCGAAACCTCCTCGAAGAGTTCAGCATGCTCTAAGCACTCACCACCACATCTCACATCCCC
TATTGTGAAGCAACCCTACTCTAAAACCGTGCCACTAACTTTGTCGTAAAAAAGTATACCATTGAAACC
TCAGAAGCAGACACAGTCGGTAATGTGTCAGAATGACAGTTAATTGAGTGTTGCCATTTAAAAAAATA
TTTCAGGGTTGCCATTTTTTGCTTTCGCAAAGTTGGCTTGCACGGTTGTGAATAATATCATTCGGAATTT
ATAGCTTACCGATTTTTGACGTATAATACCTGTGTTTCAGTATAAAAATAAGTTGAATGAATGGTATGTTT
TAGAGCAGAGTTGTGAAGTGATAGGTTCACCCTACGACGGTACCTGAACCGTAGCCAGCACCGGACG
ATTCAGTGTCACACTCGAAACGAACTAACTCGCGGAGTTTTTACATTAAATGTTCATTTTATTTTGTCTT
TTGTCAGTATTTAACTTAATACTATTATAGGTAGACTAGTTAGATAAATCCTATTTATTTATTTAACGTAGC
ATTAAACGTGAGTGTAGTTGGTTTATTTTTATTGTTGTCGCAACGTCGCATTCTCACGTGTCTCCACTCT

TTTGTTATGAACAATTTCTTTTTTTTTCAAATAATTTATGATATAATACAGTAAAATCATAATAAATTCAC
ACTAAATGCAGGACGGTAATTTAATTTACTTAGTAGAAAATGATAAAATTACTTATTTTTTTTATTAATA
TTAATTAACAATATTCTCGGTACAATGTTGTAGTTAGCGCATCGCCTTCGCGTTTCATTTGCTAGTCAAA
GACGTCGAACGTTGTCGGTGATATTGTTTAACTTTTTCCTGCACTCGTATAACGATATTCTATTTCAGATA
TTATTAGCTAGTATACTAAGCACTGGTAAAGTACACTGTAGGTCAGGTACATAATGTACTGTACATAGCT
CGCTTGTAGGTTCCGTAGTTACCACTACCGTTGAGAACGGAAAACTTTCGGTAACAATAGTAACACTA
ACAGCTTTGTAAATTATCTCGCGTGGACTTACGTACCTAACTCTCCTTTCTGTAGTAAATCTGTGAGAAT
AAGCTCTATAATATTTTAAAATCACTCTCCTATGATTAATTTATTGTCACATTTCACTATTTTGGTTATCAT
TTTATTTGCTATAACGATTTTATTAACTTATTTGATTGTAATACGCAGGCTTAATTGTATTTGAAGTTGTTA
TTTTACCGTTAAATTTGATAATTTGGATGTTTTAAACTGTATTTTGGTCGTAGTGGCCGCCTTGCTCAGG
TCGCATGGAGAAATGGAAGGGAATCATGGATGAAGTAGCAGAAGGGTTCGGCAGGTTTACCTGAGCC
AATTTATTGCTAAAATTAGTTGGACGAGTTAATTTACAAATAAATATATTCAAATTTAAATGATTGTGTTT
TTTT
>TCONS_00015132 geneID=XLOC_002941 scaffold_12:1316584-1318529(+) Exonic overlap (+)
CGCGTGGCGCCCGCGTGGCGCGAGGGCAGCGAGGCCGTCGTGGGCAGCCAGATCAGTGACAGCAAA
GAGGCTATGCTGTTAGTGCGCAGTGCTGGAGCCCCCCTCCCCCCGAGTGCCGCCGCCGTCTGCGCCGC
CGTGCTATTTCTCACCTCAACAGCATTATCTTCGACGTGATAAATTATATACAGACTTTTATATCGGCAAC
GTGATTAACGCTGGCATTTTGTACAGTGGCTAAGATTTATCTCTTGTACTTGGTTGAAAATGAAATGTG
GCTATGTTGGGGTTTAGTGGTTCTTAGTTGTGTAATAAGTGTTTTTATTTTTATGTTTATTGTTTAGTATAC
AAAGAATGCTGTTCAATTCTACTACTCAGACAATTAAAAAAAACTTTAATAGAACCAAATTTCTTTTCA
ATGTGTTATAACTGTTTTTTCATATTTTTTTGTCTATTTATACAATAGTTATAGGTTGTTTCTAAGTATACTT
AGATCAAGTTATTAGAATAAGTGGCTATAGTCAGTATAAACGTATAAGAAAAGTGCATATCATAATTTAA
CTGAGCTAAGAGTATAATTATTTTATTACTTAGTCTTTCGCAACTTTAGTAGGTACAGTAACAACCGAAC
TCATAAAACTGGCTTCTGAATTTTAAATCGATGTACCTAGCTCAAAAATACCAAAACACATATTTTTTTT
ATTTAATTAGATACTGTACTAAAGGTGTTATATGTACCGCCAAACGTAATACATTAATCTTTCGTGGGACA
TATCGGTATGTATGTTATGTACCCTTTGTTACCTATTTACTCAATCTAAATTTTGATTTCTAATAATAAATAA
TTTATAAAAATTGTAATTGCTTTCTATAAAAAACAGTACTGATATACCCAA
>TCONS_00014713 geneID=XLOC_002942 scaffold_12:1369545-1370436(+) Intergenic
TCTTGAACACATGTCTATTCTGTTTAAATTTTCAGTTGTGTAAATAAAAAGTTCAGTATGAAAATACCGA
TGTTAATAATATTTATTTGTGCGTTATTGGAATTTGGTGCCCTCTCTCAAACATCGAATACGATGAAAGA
AACAAAGACATTTTCCCGTTCAACAAGGAGCGTTGATCACTTGCCGGGTAGCACGAACTACGGCCGC
ACGACCACT
>TCONS_00015295 geneID=XLOC_002945 scaffold_12:1598249-1637568(+) Intergenic
TGCATTCAGAAGCATGCACTCGCCGCTCCCAGCGCCAGTCGGGGCAAAAAAACACCTCAAAAGTACA
AAAAACGCGGTAAAGACATAGTGTTATCCGAAACGGGGGGTCGATCGACTCGGGGGCACGGATTTCA
GTTTTCGAACGTGGCTCTTTGTTCGCCGCCATGTTGTCTCCGCTGCAGCCGGTCGAGATCTATCTCCTAT
TGGGCGAATTACAGGAATGCAGAATGTACGACGGAGGCTGGCCCGTTTTCCCGGACCACAACTGTCC
GGGACAGTCAGCCCTACAAGACTCGGCCGGGGCGGAAAAAGAATATTTTAATTCAAATGAATAAGTGC
CGAGGACCATTGCAGGATCATGTGGATGAAAACTGAAGAGCTGATATTACAAGATTGCTGGAAAGGAG
AGCAGTGCCCGAG
>TCONS_00015441 geneID=XLOC_002945 scaffold_12:1598255-1642401(+) Intergenic
CAGAAGCATGCACTCGCCGCTCCCAGCGCCAGTCGGGGCAAAAAAACACCTCAAAAGTACAAAAAA
CGCGGTAAAGACATAGTGTTATCCGAAACGGGGGGTCGATCGACTCGGGGGCACGGATTTCAGTTTTC
GAACGTGGCTCTTTGTTCGCCGCCATGTTGTCTCCGCTGCAGCCGGTCGAGATCTATCTCCTATTGGGC

GAATTACAGGAATGCAGAATGTACGACGGAGGCTGGCCCGTTTTCCCGGACCACAACTGTCCGGGAC
AGTCAGCCCTACAAGACTCGGCCGGGGCGGAAAAAGAATATTTAATTCAAATGAATAAGTGCCGAGG
ACCATTGCAGGATCATGTGGATGAAAACTGAAGAGCTGATATTACAAGATTGCTGGAAAGGAGAGCAG
TGCCCGAGGACCATGCACCATCAAGTTGGACCATCAGGTCTTGGACCATCACGTCTTGGACCATCCAA
GGAGAAGACTGAGCGACAGTTTCCAGAAGATATGGATAACATTGCCGGGCACCACGATTCGGCTTTCA
TCATTTTGCACCTTCTGAGGTTGGA
>TCONS_00015442 geneID=XLOC_002947 scaffold_12:1654307-1656284(+) Intergenic
GTTTTTCGATGTGTCAGAATTGTTTTGAATGTTTTGTTTGAATTTCTCGCTATGTTTTAGATAATTTAGCC
GTTTTGTGTTGTTTTTGGACGATAACGGGTGTTAATGAAGCTGGTTTCTGTAATGTAGAAGGTGATAAC
TGAAACTGAACCAGCTTCTTAACATTCGGTGTTGATGCAAAAACAAATGTGGACGAGTTGTAGTATAC
AGATTTTAAATGTTGTTAGTGTTTTGGAGTCGACTACGTGATAGCTACTGTGATAATAGTTTTAAACAGT
AAATAAGAAAGTGATTGACTTAAAGTTTGCCTGTAAACAGACAAACTAACCTTTAGCTTCTGATGTGTA
ATCATGGTCAATGCAGTAGAAGAAACATTTGATTTGCCTTGACCTAATGAAGTGAAATCAAATCGAAA
GTGAATTAGTGAACTTTAAATTGAAGTGTTTGGATCGATCGTCGAGTGATAAACGAGTGATTTGATGAC
TCAGTGCATTGAGAAACATAAACGTTTATAGCAGACAATTAATAAAATTATAAGGGAACTGGGTTGTAC
AGAGCTCGTGAATCTTGAGAGATACTCTCGCGATGTCTTCAGTCTCTCCCTATGTCATATTTATGTGCTG
ATGCATATCGACATCGATGTTATAAGTACATTATCTAGTTATTTGTATTGCTGCTGTGCTATTCTAAAAAA
AATTGGTCTCCATTGACAAAACATTCCTATTTTCAAGCTCAACCCTTCTGTCTTTCGATTCTCCATACGA
AAATCTTCTCTGAAATGGATCAATCCAATTTCACCATACTTAAAGCAAAATTGTAAGAAGAAGTAGTCC
CAAATTCATCTCATAAAAACCCCAATCGAGTGAGCAATATATTCTACGACCATTTCACTTGTAAAGGAA
TCTCAAACCTGACATAACCTCAAAAACCACCAAGCGTTTAGGGTTGCCATATCAACCGCAAACAATTC
CTAACCTTACCTTAAAAACATCTAAAAAACAAAACACAAAAGTAAAAAAAGCCTAACCTCAAAAAAA
AACAGGTGATAATCTAAACAAATCGTGAAAAAATCCGCCCAAAGCGCTGAATTCTGAGCTAACCGACG
ACCATTCCCTACCTAACCTCAAAAACAAGCGCTCAAAACAAAACACGTCTCAAAATCGCTTGAAGCAT
CGCTGAATGCTGACCATTAACCTAAAAAAAGACCTTAAAAAACAAACACGAACGTCAGAAAATCCGC
TGAACGCTGAATGCTGACCTAACCTCAAAAACTTTCAAACGCTAAAACAATCACTTGAAGCGCCACAT
CGCTGGATGCTGAGCTACTACGAGGCGCTGGTGGTGGTAGCATGCGTGGCGACGAGCGTGGCGGCGC
TGCTGCATGAGGTGTGGCTGTCGGCGCTGCCTGGTGCCGCGCCGCGGGGGG
>TCONS_00014897 geneID=XLOC_002948 scaffold_12:1754653-1755447(+) Intronic
CAATATCATACAAACAACTCGATCAAATCAAGATCAATCTCACATCCTCATCAACCCGAGTATTTGACC
TTCTGCTTGATCTAAGTCATTGAATCCTTCAGCAATGGGAGACAGTAAAAACAAGGGAAGAAAAATAA
GTCTATACTGGAATCATTTTACGGAATTAGAAGTGGAAGACATGTCTAGACGAAGAGTCAGGTGTGAA
CACTGTCATAAAGTGTTAGGCCTTGGGGCTTGCGGCACAGGCAACATGGGACGCCACTTAAAGAAAA
TGCATCCTGCCGTAATGATGATGATTGGAGAGGCTGAAGGTTTGGACGTGTCTGAAACACAAATAAAT
TCAGAAACAGAAATAGAG
>TCONS_00014803 geneID=XLOC_002989 scaffold_12:1759329-1760525(-) Intronic
GTCAACGTGAAGTAGAATTCATCGAGTAATTTTGTATGAAAATATGAACAGCGCCCCTGGCGGTCGGTT
GCAGAACTAAAATTTGCCTACCAAATTCATCGAGTAATTTCATGTCACGTTCACGATGAAAATTCATGC
TAAGGGGTCAGTATCGTCAACTGTCACTGTTACAATGTAAGGCAAATTTGTTTGTTCCAAAAGTGACAT
GTTTTTTCATGTTTGTGTAGTTTCGAAAATAGTATCGAATCCTGTGATCACTGCACTGGTACTGACTGGT
ACTGGTATTTGCTGTATTTGTCACCGTGGTGAAAGGATTAGGCCGCCCCTACATACCTATTAGATACTTG
TTATTACTAGGATAAGCAAAAATGAAAATAAGGTATTACTTATTATAAATACTTAAGTATTTTATTTATTTG
GTAATTAACTGTACAGATTTACTTTACACCTAACTATATTTACATTACAACAATGTGGTAAATGCGTACTA
GCGTCAACCACAGAAAAAAGTTTGTAAAAACCTATACAGTAGGCCTTCAGAGCCTCTAGAACGAGTTA

CAATTTACGAAAAAGAAAAAAGTTTACTTTTTCTTTAGCCTACATTACTTACCTATGTGTCAAAAGTTTC
ATAGTTTCCGTTAGAGGCGCCAATTTGTATGCGAGGAAACAAAAACTTTAATATTTTTCAACAAACTA
TCAAACCCGTACTTAGTCCCACGATAGGTCTTTAGTAGTCTACTCTGAATCCTCCAGTGGATTGGTTAG
ATCCTCTACTTTGTGCACAACAGTAGACTGCCAACTGTTCATTTCAGCTTCGTGTAGCGCATCATTTATC
ACTTTCTCAACTAAAATCCGTTGCCGGGTGTCAAGAGCTCTCATTTTCATCCCTACTAATTTAGTAAAC
ACATCTATTCTATCATCCTCTTGCTTCGTAAGAAGGGGGAGAGAATCTAGTAATATGTTTGGGTCTTCTA
TCCTTAAGTGAGTGTTGTCTTTTTGTGAATGTTTCTGGGTTGAATTTCGTTTGCTTCGCTTCGCTTTTGG
CTTCGCAAGTTCTTCGTCAGATTGCACAATGTAGAACT
>TCONS_00015143 geneID=XLOC_002951 scaffold_12:1847050-1858571(+) Unclassified
GGCCGTTATCATTTCATAAACAATACGGAGATTGGCGATTCAAATAAGGAACACTTCAAACGTGCGAA
GTTCCATATTGAAACGGACGCTCGGGCACCGCGCGCATCTCAGGATCGACTCCTGCGCAACACATT
TAGCAAAACCTGCTCTCTGATTGGCGGACAGTTTCAGTCCATTTGTGGAAAGTGTTTTGTGGTTGGTG
GACTAAGAAAACCTTCAGGTGGTGGACAATGAGTGGTGGAAACGGTGCGCTTGGAATTTTTCAGTTC
GCCAGAAGTGCACCGGCCATATCTATAATATTAGATATAACATTTATAACAATCATAAACTCTATCGAGA
AAAGTGTCTGAATACAACAAAACATATTCCAAAACCAGTACCGCTCAAGAGAATATATAAATAGCTAAA
TAAATACCCTAGTGATATACCTAAATACCTACTTAGTGTTTTTTTTCAAATACTTTGAAAGAGATTAACTA
ATTTCGTTAAATAAAATACGTGAAATAGTACTTTACAAATAGTGGTTGTG
>TCONS_00015840 geneID=XLOC_003193 scaffold_121:168066-168909(+) Unclassified
ATCTCAAGTTATCTCAAGTGTGTTCCGAAATGTACCTATAAAGTGAAATATTTGGAATTGCGAGCAAAT
GAATATAAATATCTGGACTCCAGAATCCAAGAAAGAGAAAATAAAGAACAAAGTTTCATTAAAAAAAA
GAACAAATCCATCCCAAAGTTGGCAAAATAGATCGAATATTACACAAAAGCATAAACATTTTGGAGTTT
AAGAAGTCTTAGGTATTTTCAAAAATCTTTGATAATATTGATTGAATGTGGAAAATACTAATTACTAACA
TGATTTTGCTACAGATTAGATACAAATATTTTTTCTTGTTAGGTTAAGTCAATGAATTTGCTAGTACAAAT
TTTACAACGCACCAATATCTTCGTGAGCATAAAAAGTATTTGTGTTTCTAGGTCTCGCGTTTGAGTCATT
AATCTTTGCCGAACCGGTTCACGTTTTCTAGTCGTATTAATATTTACATTGACGAAGGTCAAGATGTTCA
GGTTTATGGTCATTGCTGAGACTATTTTCGAAAACTATTGTAACAAAAGATAAGATTCAAATTTAACACT
TGTAGAGATATAAGTGAATTATAATTATAATCAATTAAAATGTGCCTAATTATTTAACAATTATTATTGTTC
AATTACTTATAGGATAACAATGTAAATTCTAATGATTTTATTAACTAATCAAGAC
>TCONS_00015874 geneID=XLOC_003200 scaffold_121:169101-170713(-) Intergenic
ATTTTTTTAATTTTATACACACGGAGGGTATCGCTAAACATAACCCTTTGGTGGTTCAATTAAATTCGATA
CCATCTGTCATATTTAGGGTGTCTTCTACATCAAATTATTTTTACTTTGTTTATCCCGAGTCCTGGTCAAG
CGGCTTCGAAACAATGTGGCCAAAAATTCGTTCAATTTATTATTCCCAATTTTGGTCCCGGTTTGATCCC
TTTTAGTTGCCATGTGCAAGGCAGCCAAGTCCAGACAGCTCCACCCTAAAAAACCGTTTGTACCAGAC
GAGATTCCTATTCCGTCACTCCCGTTGTGTTTTGTATGCTTTGTCTCTTTCCCGTTAGCTGTGTAGCAGG
ATACATCTTCCGTCTCTTTTTCGCTTGAGGCCGGCGCACATTGTTCCGACTCGTGCTAATTCGTATGTTT
CCGCTTCCCACACGAGGAGCCGCCATATTTGGCGGGACTGTCAAGTCAACGCCAGTTTGGCGCGTATC
AAGCTAACGAGGAACAAGGTCAATTTCCATCGATGAGAGCTTCCTCACCACATTTTTCATCCTTGAGTG
TATTGATCGTAGAATCGCAGCTGTAACAGACTCTTTCCATTTTATTTCAGATGGAGACAGGTTGGGCGG
CTCTTCCTATGGACCATCTATGGCGAAAAATTCTATGGCATTTTCATCTTGTCCTGCATCA
>TCONS_00015945 geneID=XLOC_003202 scaffold_121:226390-227264(-) Intergenic
CAACAACATGGCGTCCTTACGTTGTTGTTATTTTGCTGAATTTTTGTGTGAAATTTGTAGGAAATCCTCA
TCAAAATCATAGTTGATTATTCAAGAAGTGTTCAATAGCGGGTCAGTGGAGTCAGACTCGGTGGAATTT
TGAACGATCTTTCCCGCTTATACATAAAAAAACTTTATTGGACTGTCTAATCCTTGCGACAAACCCAAA
AGTGTATCTCGAGTGTTTGCTATGACACGCTAAGAAGATTGTTTCCATATCTGGTCTAATCTGGCAGGGT

ATTCTTTAATAATTTAGCAGAGGCTCAATACAAGGAAAATATGGGGATAACATTCAAAAATCTTCGCCTA
TAAAACCTCTGCAGCATATTTATGGTCAATAGCTACCGGT
>TCONS_00015890 geneID=XLOC_003194 scaffold_121:266199-278912(+) Intergenic
CTTTCGATTTCACCTGGACTGGCCAGTTGGTGTAGCGCTCTGAGCTGTGTTTGGTGTTAGTTGTTGCTG
GATACTTTTAATTTCAACGTCTTAGTTGATGAAAAGTTTCAAATTGGTGGAAGATGTTGAGGTGCAAAG
GCATCCGTCAACGAGGGACCAAGAAGCCCAGCAGACATATTGTGATCCAAAATGCTCAAATGCCACAA
TACAGATCAATAAGATTTGAATCTGGAATGCTACATGCGGGTGAACATTTGCATAGAATGGACAGATGA
GGAGAGGCTTACACTCATTTATCATCAGGTGATAAAATCTGCACATCCACGGCAGCGGCAGCGGCATC
CTGCAACAATGAAAAATAAATATTTTTTAAGAATTACAACTTATGATAAGGACATAAAACTCTCCGAC
AAACAAAACCATACATCGCTGGTCTTAATTAACCAAACATCCTGCCAACAGTAAGGGGATTGGTGAAT
ACGCAGAATGCATCACTGTTTGAGTGAGGAGATGGTGAATAGACCATTGTTGTCATATCTCTAGATAAA
CTACAGCATTAATCAATAACAAATGACAATAGGGCCGCTTACCTCAAACGATGAGCTCTAGGACCAAAT
ATAGTTGAGCTCCGAGGTGACTCTGGTTTACCAGTCCGTAGTGCTATTATCAGGAAATGGAAGGGAGC
GTGGAAGGTCACGACATTTTGGTGGTTGAGTGATACTCGGTGGTGTA
>TCONS_00015925 geneID=XLOC_003204 scaffold_121:307904-345763(-) Unclassified
AGCGATTAAAAAAACGCGAAACTGAATAAGTTTGACAAATCCTGCTCCTACCTGGACCATTACATAGA
CGGACGCAGTGCAACCCTATTTTTTTAGTACAAATTGTATCATATATCTTAAGATTAAAATCTAGGTAACA
TTAATATATAGTGTTTATTTATTAATAGTACAAACGGAAATAGCTGGTAGTTGTTAGAGTAAATAATACAG
GGTCACTTTTGTATGAAGTCAATGTGATTGTAGTTTTGTAAGTTCGATCGCGAGTTTGAAACCTGCTCG
CTCAAGCTGCTTACGGACCACCACCGAGGGTAAATATTTACAGATTCGTCGAGGATCCGATGCAGAGA
CACCGACCGTCCAGACCGCGTGACCCGCTCGTGAGTCCGCCCGCTGCCGCGTAGTGAATGGAATCCG
AAGTGTCTGTGTGGTGATAGAACGGTGTTAGTGTGGCCCCCAGGCCAGGTCGTGCCACGGACGGGAC
GGACGCGAGCC
>TCONS_00015847 geneID=XLOC_003205 scaffold_121:391934-392942(-) Unclassified
ACGCTAAATTGTACCGGCAACTAAATCGTACCGCTACCTTACTTTCTACCAGTTGGTTTATTTTAATAGT
CTGGCAACACTAATTCATTGCTGGCAACACTAAAAAACCACTGACAATATGGCGCTTGCCTGTGTCAA
CTGTGTCGTACGTAAAAACCAAAAAATGATATTTCATTTGTAATAAGCTATTTTCTCGTGAAAAAACTTA
AGGATTCTTGAGGAAACGATGTCCGAGAATAAAAGAAAATACTATGCATGGACACAAGATGATTTAGA
GGCAGCACTGAGGGCATTAAAAGCTAAAACTGTTGGACTGAATGAATGCGCACGCCGATATTGTTTGA
ATTCTTATGTCACAAATACTAAATAGTTAATTACCTAACTATATTAAGTTTTACTTATTCGTACAATTAGGT
AAGTTAGTTACCATTCTTTAAAAGCATATATATTTTAATACTGTTTTTCTTTGCTATGTTTATTGCTGCCAT
TTGAATAAAAATGATTAAAAAAACATAGGATTTTATTTTATTAAATATTACATTGATCAATTCATTCAAAA
TGTTAGGCAGGCAGGTACAATTTAGGAATCACAGATTTTCTTACTTGCAATTCGTACTTCTAGGTTTTAC
AGAAATATATTTTTTATGTTGTTATATGGATTTACAGCTTAATTAATACCAGTAGTTTTAATCTTA
>TCONS_00015842 geneID=XLOC_003196 scaffold_121:612227-683914(+) Unclassified
ACACCTGATGTGTTTTTTAAAAGAAAGTCTTTCACCTTTATTGCAAAATTTTACGGAATTAATAACGATT
ATTTATGACTGTTTTGTCCAGATATTTCGCAATGACAACAATATGATGACGCTTGTCACAACTTAACAAA
GAAATATTATTGTAATAGGTTATTTTAGTTCAGTTTGTGTTTAACAGTTTAAGAATTAGCACTGTAGTTAG
TGTGAGTGCTTTAAAATACCTCTACATCGACATACACGTGTTTTTTACGTGATATTTTGCGGTAAATGTT
TCAGCAGGTGCTTCCATCTGTGAGTGCGAGCCGGAACTGTTGCGCGGTGGAGGCTTCGCGCTAACGA
CCTGTTTTCACGCAACAAAGCCTTTGGCGCTGCTGGTTGTAAGCCTTCAGAATTGCCGCTAAAACGCT
GCGTCCGGGCCGAAGGCTGCGCGGCAAAAACATTCATCTTCACCGAAGCGCCGCCCACGACGCTTGC
GGCCGTCCCATTGGCTGCGCACTTCCCGCCCTCTAACGGCTACAATTATACACCTAGACCAGAACAGCT
AGGCATTTCGAAAATACATTTCCCAAAAAAACCTTCCTCTTCTATACCTTACACTATAAGACCTCAAATCC

AACGACTGTTTCAGCTTTCAGAGGCCATGAGTAGCTGGCGAAGGCAGTATTTATAACGAGCTTACTGA
AACATTTTACATTTGAGGTGAATTTGCGAGATGCTGGCGTAATTGAAAATTTGTTGTATTGACCTGAAC
GGCCTTTTCAATGGCTGGAGGAATGCAACGTTATTACGGTCTACACGTTTTGGTTNNNCAGGAATGCTA
AGTAATTACGTTTATACAGTCTTGTTTGATAGACTCGCCCGTTCGGGTAGCTTGTTCATTAAATGTGCCC
TGTTTTTGGGTACAGTAATATGGAATTTAAAAATTGTTTACTAGGCA
>TCONS_00015978 geneID=XLOC_003258 scaffold_1215:456-2034(-) Unclassified
GATGGACGAGGAGAAGCTGCGGAGAGCCATCGAGGCTCAACAGCAGTTAGAACGCGAAGAAGCCGA
GCGCAAAGCCAGAGAAGAGCAGGACAAGCTTACCCGCCTCAAGGAGGAGGAAGAGAAACGGAAAG
CTTTAGAAGAGGAGCTCCGGCTGAAGAGAGAAGAGGCGGAGAGAGAGGAGAAGGAGAGGATCGCG
AGGAAACAGAGGGTTGAAGCTATTATGGCGAGGACGAGGCTTCGGAAGGCGGAAGAAGACAAGAAG
CAGCAAGAGAGCA
>TCONS_00015974 geneID=XLOC_003259 scaffold_1215:6576-9935(-) Intergenic
CCAGGTAGAGCCTCCTCCATGACGCGCCTCGACCGCGTCGGCGCCTCCAGCCGGCAGAGCCTGCTGC
AGACCAAGAGCGCGTCGGGCGCCAGCACGCCGGCGTCGCGGCCCGGCTCGGCCATGTCCAACTGCA
ACTCCAACTCGACGGTGGTGCGACGGACCACCCCGCGGCGGCCGCGGCCGGCTTCCATCGCGGGGAC
TGGTGTGAATACGCCCAGAGGCGCAGGCGAC
>TCONS_00015975 geneID=XLOC_003259 scaffold_1215:6576-10337(-) Intergenic
CTCCATGACGCGCCTCGACCGCGTCGGCGCCTCCAGCCGGCAGAGCCTGCTGCAGACCAAGAGCGCG
TCGGGCGCCAGCACGCCGGCGTCGCGGCCCGGCTCGGCCATGTCCAACTGCAACTCCAACTCGACGG
TGGTGCGACGGACCACCCCGCGGCGGCCGCGGCCGGCTTCCATCGCGGGGACTGGTGTGAATACGCC
CAGAGGCGCAGGCGAC
>TCONS_00015967 geneID=XLOC_003259 scaffold_1215:7539-11165(-) Intergenic
AGAGCCTCCTCCAGGACGCGCCTCGCCCGCGTCGGCGCCTCCAGCCGGCAGAGCCTGCTGCAGACCA
AGAGCGCGTCGGGCGCCAGCACGCCGGCGTCGCGGCCCGGCTCGGCCATGTCCAACTGCAACTCCAA
CTCGACGGTGGTGCGACGGACCACCCCGCGGCGGCCGCGGCCGGCTTCCATCGCGGGGACTGGTGTG
AA
>TCONS_00015998 geneID=XLOC_003260 scaffold_1216:2380-5999(+) Unclassified
GCATCGTTCAACGAAGCGAATTGCTTCAAGCGCTGCCAGCTAGCGACAGAAAGTCACAACACTGGCT
GGTCACACCACACTAGTCGAGTGCGTCGCGCGCCGCGGACGCATTTTTAGTTTTCGCGGTTTTCGTCG
CGCGCTCTTTTGTTTACAACCGTTCTATTCACATCACTCCGAACTGTGACTTCATCATTTTCCGCTTGTT
TAGATTACAGACTAATAAAAATACCACACGGTAATTTGAAAACATTATTGCGTTAACAGTGTACAGTGC
TAGATCACAGTGATAAAAGATAATCAAAAGCTACGGGGTCTTCAGTGAATAAAAAACTTAAAAGTGAT
GTATTAGTGAAAGTATTAAGGACATTTTTAACAATGAGTGTTTCTCTTACTTCTATGGAGACTCAAAATG
AGGAGATCGGTAAGTTTATTATTGCTACAGTATAGAGTTACTTTTATTTAATACACATCAGTTTACCTTTA
TATGTTTTCTTTAAACCTATTTTTTAATGCATCATCCCAGTTTGGTTTCTAGCTGCAAGTAAATTTAATATT
GTTAGAATATCTGCAGTACCCCAGGTTTTCCGCACTTGATAAAGTACGGCGTAGAAACATAAATAAACA
TAGACACATTGCGGGAACAGTGTTCTTGACCTATTCGCGTCCAGTTCGCCTACGGGAGGCCCACGCCT
GGCATTATTACCATCTCATTTTACCTGCAGGTACTGTGCCTGTGCTAATTGCAAATTCTCGCAACATTAC
TAAACATTCAAGTTAATTTTCATACCTGAAGCCAGTTTTCCCACCTACGTTTACATAGTTAACAGTCTTA
TCTACATTCTCCGTTGTGTGTTTAAACATTATGGGTACGTCTGAGTGGCGTTTCACTGCATTCAACAGGT
TTCTACATTTATTTAAATGCAAAAGTTGTCAGTCATTTACAAGTCAGGTTATCCTTTCATCAGCTCTACT
CGTGTGTACATAAAGATACCGTAAAAATATCTCTTCGGGAAATGTAAACATAATAAGAGATAAATGCGA
TTCTCTTCCTAACAATACTAGTAACGTAAAACTTCTTGTTTTCTATTCCATGATCAAACGGTCAAAGACA
AAATTGTTGAAAATATGCCAAGTCGGTCATCGTTACATTACTTAATCGCGGATATGATTGATCCAAAGGT

TAGCTATCATTACCGTTTTAGGTACGGTGCGGATATCTGTGCTTAATGGATTTCCGGACGCAAACTGAA
ACTGATCATGCCTTCAGCATTTGTTGACTCCGAAA
>TCONS_00016000 geneID=XLOC_003260 scaffold_1216:2475-5659(+) Intergenic
CGCGCCGCGGACGCATTTTTAGTTTTCGCGGTTTTCGTCGCGCGCTCTTTTGTTTACAACCGTTCTATTC
ACATCACTCCGAACTGTGACTTCATCATTTTCCGCTTGTTTAGATTACAGACTAATAAAAATACCACAC
GGTAATTTGAAAACATTATTGCGTTAACAGTGTACAGTGTTTCTCATTTCTCTCAAGCTAGATCACAGT
GATAAAAGATAATCAAAAGCTACGGGGTCTTCAGTGAATAAAAAACTTAAAAGTGATGTATTAGTGAA
AGTATTAAGGACATTTTTAACAATGAGTGTTTCTCTTACTTCTATGGAGACTCAAAATGAGGAGATCGG
TAAGTTTATTATTGCTACAGTATAGAGTTACTTTTATTTAATACACATCAGTTTACCTTTATATGTTTTCTTT
AAACCTATTTTTTAATGCATCATCCCAGTTTGGTTTCTAGCTGCAAGTAAATTTAATATTGTTAGAATATC
TGCAGTACCCCAGGTTTTCCGCACTTGATAAAGTACGGCGTAGAAACATAAATAAACATAGACACATTG
CGGGAACAGTGTTCTTGACCTATTCGCGTCCAGTTCGCCTACGGGAGGCCCACGCCTGGCATTATTACC
ATCTCATTTTACCTGCAGGTACTGTGCCTGTGCTAATTGCAAATTCTCGCAACATTACTAAACATTCAAG
TTAATTTTCATACCTGAAGCCAGTTTTCCCACCTACGTTTACATAGTTAACAGTCTTATCTACATTCTCCG
TTGTGTGTTTAAACATTATGGGTACGTCTGAGTGGCGTTTCACTGCATTCAACAGGTTTCTACATTTATT
TAAATGCAAAAGTTGTCAGTCATTTACAAG
>TCONS_00016025 geneID=XLOC_003271 scaffold_1219:1395-2161(-) Unclassified
GTAGAATAGACAAGCTAAAATTACCACTTACTATCAAAATCGGTTAAACTAACCTTAAACCTATATGAAT
CTATGAATGCATTGGATATTGGCCATTCCTAAACAAAAATAGTAAGGAAAAAAGATTTTATACAAAAATA
TGAATTTGATTTCCAAACAGTATCGTTCAAAATATACATTTCATTTGACAATATGATGAATTTAATTCAGA
AATATTCACTATCTTTTTCAAAAAATACATAAGGCACTTTGTAGATATTCTAAAAACATTTATTATGAAAC
CACAGTTAGATTAAGAATAAAAGAACATTCGAATAATAACAACAACAGTTTAGCCCCTAACTATATTAC
CAGAAAAAGGTTTAGACCAGGTTTAAATCGTGTTTAACACGGTATTTGACAGGTGCATAAGTATTCGAC
AATGCTAGACATGCCTTTTACATGACGTCTCTATCTAGTTTTTCTAGGGTAAGTGCCAATTTCACCATTC
TCGGTTATCTAACCGACAGGGATTGATTTATAAATTAAACGTCATCTCCATATAAAATTCATGACAGATG
TGTCAAAAGTTAACCATTACTCCCTGGTTATGCTCTAACAGAGAATGGTGAAATTGGCACTAA
>TCONS_00016534 geneID=XLOC_003304 scaffold_122:67454-68714(-) Intronic
ACCAGTTTTATAGTGTCACATGTGGTCTTTCCAGTACTTTTAACAATATCCAACTGTTTTAACCTATGATA
AAACATACGAAAACTTACGTGAAACTGAAAAAAATATCAATTACTGGGAATTAAGTTGCCATGGAATA
AACTATTGTACCTACTTAGTATTATGTATACAATGTTGTTATCAGAAATGGGACAGTTAAACGAGAAACC
ATTCAAGAATTTACGTTTACAAATGGCAGAAGAGGTGTGTTTTGTTTGCTCAGCGATAATGTGAACGG
AGGTAGGTAATTCTGTGAGCTGCAAGAATGGGCTTCATAAGCGGCGCAGAGACCAGCAGTTCGCCCTC
GCCGTATAACACACG
>TCONS_00016363 geneID=XLOC_003304 scaffold_122:67455-75877(-) Exonic overlap (+)
GGCTAAGTGCCTAATGTTTAAGAAAAATTGTCATTATGTTGGTAATACGCAACGAGAGCGCTATTCTCT
CCCACAAGTGTAGCCATTCACAGCCTTATCTCCGTCAGACGACATGAATATTGTGAATAATCCGAGTGC
TTGTCGGTCTGTCAGAATATTTTGTTAATTATTTTTCTCCAGTTCAATATGTGGGTACGCGCCAGCGCAT
AAGGACAGTTGTATGAATTTGTGTGTATTTGACTGCTTGCCCGGTGCTGTGATTAGTCAGGCATTTGTT
GAGTGTGGTGACGAGTTTTGTAACTACGGTAGGTAATTCTGTGAGCTGCAAGAATGGGCTTCATAAGC
GGCGCAGAGACCAGCAGTTCGCCCTCGCCGTATAACACAC
>TCONS_00016098 geneID=XLOC_003304 scaffold_122:67477-75819(-) Exonic overlap (+)
CGCTATTCTCTCCCACAAGTGTAGCCATTCACAGCCTTATCTCCGTCAGACGACATGAATATTGTGAATA
ATCCGAGTGCTTGTCGGTCTGTCAGAATATTTTGTTAATTATTTTTCTCCAGTTCAATATGTGGGTACGC
GCCAGCGCATAAGGACAGTTGTATGAATTTGTGTGTATTTGACTGCTTGCCCGGTGCTGTGATTAGTCA

GGCATTTGTTGAGTGTGGTGACGAGTTTTGTAACTACGGTAATTCTGTGAGCTGCAAGAATGGGCTTCA
TAAGCGGCGCAGAGACCAGCAGT
>TCONS_00016257 geneID=XLOC_003307 scaffold_122:178469-182657(-) Intergenic
ATTTGAGTGGTTTTTAACTAATGGTCGCCATTTTTGACCAATGGTTGAGTTAACTAGGGATCAAAAAAC
TGGCCCTAAGTCATTTTAAAGTTTTGGCTTCACACATCATTTATATTTAAATGATTTTTAGACCTTTACCT
TCATCTTTATAGGTACATTATGTTTTAATTAAATATAAGAATGTAGGATAGTTTAGGTAACGACAAATTGT
ACCATCCCCCCACTACTCCGACCTTATTTTACTAAGCGATTTAAATTAAATTATATTTATAATACGTACCTA
CTTACCCCACAGAAAATAGAATAGAATAGTAACGTGTAAATAA
>TCONS_00016536 geneID=XLOC_003309 scaffold_122:239481-245226(-) Splice junction overlap
CTGAGGTCACAGTAAGCGCACGCGCAATAAACAGTTTAATTTTTTAACTGACGGTTATGTGTGTTTAAC
GGATAGTTAAGTTTGTTTAACTGTCAGTTGCGTGTAATTAGTGAGTGTTTAGATGTGTTTGTTGGATTTC
GTGCATGTCTACTGTCATGGGGCAGCTGAAGCGCTTGCTGCCGACCCTGTCTCGCAAGGAGAAGGGG
CCCCGCACCAGGACATCATGGTATGCGGAATGTGGAGACGAGACGCCCCCGTCGCAGCCCTCGCCGC
GGCCGAGCACCACCTCTTGGTACGCAGAAGCGGGCCTATACCAAGCCGGCAGTGTGACGTCATCAAG
CGGCGCCTCCTCCGCCTCCCCCCCCGCCTCGCCGCCC
>TCONS_00016422 geneID=XLOC_003309 scaffold_122:305030-322783(-) Exonic overlap (-)
ACTGTCATCAGCAGCAGCAGTAATTTTTTGTGTTGTGTTGGTGTTCCTGTTGGATAAACGGGCTGAACT
TTTTGAAAACTTTGTTACTTACTGCTAAAGAGATTTTATTTAGCTTTCCATGGTGGTTAGTGATATGCTG
ATGTGTTGTGTTTTCCTTTGTGGTTAAGGCGAACGGTTGTACTGCAGAATGATCATATTACTGTGGGTC
GTACTCGTTCAATTCGAGAAACGGTGAGTCTGGCACGTTTCATTTTTCAGAGAATCAAGTATAAATATA
AAACGTGAAAACCGTTATTATTACCTACAATTTTATGGCTAATGTTTAGTTTACAACCAATCTAATAAGA
AGATACTATCGATCGATCGATCTACGAAATTAATGTGTTGAATCTAGATTTTTCGTCTCCGTAAGCCAAT
CTATCCACAGCTGGAATTAGTTTTTCTAAATGTGTTTTTAACAAACTTAATCATAATTAAATATAGCTATT
TTTAGCAGTACGTTGGTAGCTATCATAAAGTAGAAATACGTTATAAATACCATCCATATCACTTTCCTTGG
TTTCCTAATAATGCATTGACTAATTCCAATAATGAGAATAAAGATAATAAGTAATAATTACGATCTTTCAC
AATTTCCCCAATCACTCAGACACGGAGATAGGCCCAGCCAGTTTCCGAAACGTGATGTTTTAATTTGTT
TTTTATCATGTATCTCTGAGCACAGCCTTGTTGCCAAACCGAAGGCAAATGTTATTAGTGTAAGGTGTG
GTTTATTATTATGAGTAATATTATAATTTTGGTGTATGTTATTATGGACGTTAAATACCTTGAATCGTCGTA
AAAATATGAAATGGCGAAAAAAAAAACAAAGTTAATGAGGTAGACAGGTACCTAATTAACTTAGGGCTG
GTCCAACAGCTGCATTAATTGTTACTTAACGATAGCTTTATGTTTAGACAGTGTTCTCTCATAATAGTATC
TCAATTATAGTAGAGTATAAC
>TCONS_00016286 geneID=XLOC_003311 scaffold_122:418250-432179(-) Exonic overlap (-)
CGGCCTTTCGTCTGTTAGTCCAATAAAACAACAAAGCTGAAGTCACACTAGTTGAGAAAACTATCAAA
GTTAGTCCAACATATTATTTCTTGTGGTATCAGTTTGTATGAAGACCCACGTTTGATGTTCATTCTACAC
AACGGTTGCGAGATCACTAGACCACCGAATTATGTGTGGCTACAGCTTTATTTCTCAGTTATATTCATAA
AATTAACCATAATATGTTTCTGATTACAACAGTGTTTAGCGGTAGTCTAGATGTAGCCTAGACATCAATC
CAGCGCACTTTCTATCTGAAGTTAAACACCAAAACATTATTAAATGAATACACAATTTAGAGTACAGTT
ATACTATACTATTTCACAATAAATCTACAATAATATATTCACAAAGCAACACTTCAAGCGAAGGAAGCTA
GTTAAAACATAGATTAATTAGCTGTCTATTCAAAGTCAATACAATTCGTTGCTCTAAAAGAGTCTGTTTT
CCGGGGTGTTAGCACGGTTTTACGAAATAAATCACAACATATCGGCACACTACTCGCGTTCTGTGCTTA
GGGTTACATAAAGCAAAAGTGTGATACTGTTATATAATTTTGAAAAACCAAGAGATGTCGTTTATTGGC
CGTCTGTAAATTTGTTTTTAAGTTGAGTTCAATATTGGCACATTCTTTTATAGCATATTAATTCAATTTAC
GTAGAAATAAAAACATATAAGTGTTTCTTTATGACGGAAACTTGCGTTTTATTTTTTTGTCGGCAACATC
TATGGTTTGAGTGAGAAAGAGAATAGTGCACACGGCGATTGCGAATCTATTTGTAGTTGAT

>TCONS_00016899 geneID=XLOC_003379 scaffold_123:119575-120789(+) Exonic overlap (-)
GGTAGGTAGGTAATATAAAATGAACATGGAAATAAAATACTTTGTTACAAATTCTTCTAACTATCATTAT
AACAAAAATGTGGTTGACCTAAAATTTTACTAACTATAATATGTATAGGTACTGTATCAAATCATTATGGC
ACATCTAAAAGTTATCTATAATAATGACAAATGAGAGGTAACATAAGTAACTGTGAATGGTCTGTAGGT
ACTTCCTATGTCTCCTTTTTTTAGTCACATAATTTTTAGCTGTATAGTGGAATGCACAGAAAGATCATAG
AATATTTTCAAGTATAAATAAAATCTTTCCATGATCTTTCAATCTTTGTCGACTGTGGAATCACGAGTAC
ATAAATAACAGATAAATAAGAATATTTGTACTTAATTGATTAGAAATAATATAATTATGTTACAGATAGAC
TCTACTGTTACTATAAACCACTTTATTTTCATTTAGTTTGGCCGTAAATACCTATTTATATCCTCCTCTGAC
CAATGTTAAAGCTCCGACATTGACCCACAAAAACCAACTTTAACCCTCCAAACCCAACTTTGACCCAC
AAATTCTAACTTTGACCTCCAAACCCAACATGTGAGACTTGAGGATGTGGATGCGCTGTGAGTGGTTG
GAAAGGGTCCTAAGGGACTGTACGGTTAGTTACTATAAACCACTTTATTTTCTTTTAGTTTGGCCGTAA
CTATCTATTTCCTCCTCTGACCAACATTTGACCCGAAAACTCAAAATTTGATCCACAATATCCCTCTTTG
ACCCGCAAAACCCAACTTTGACCTCCAACCCCAACTTTGACCCTTTATCTTCAGACAGCATCTTTCTTC
CTCAACCCCTTCCGCGTAGTCTTGAGGTGTATAAGCTTGTTGTGCGTCCACAGGCTGGCGGACTGCGC
GAAGGCGGCGGGGCA
>TCONS_00016629 geneID=XLOC_003381 scaffold_123:145911-160635(+) Splice junction overlap
GTTGCCGCGCATGCGCCGCGCGCGCTGCAGACAAACTGACGTTTCCCCTCTCAGTGCTCGAGGAATAT
TCATTGAGGAACCTGTAAATATTAAACTGTAAATACGCTTTCCGAAATTCGAATGCACGTGACTTGTTC
CGGTTTTGAAAACCCTAGCTGAAGACGGCCGTATGGGTGTTTACGCGGATCGCTGCTGAGATTAGAGA
AGCAGCGCCATGATCGACCCGAGCTCCTCAGAGGAGGACACCGACGATGAGCACAAGAAGCAGCCC
TCCAGCAAGCTGCCCGCGGCGCCTCCGGGGTCGCTGGCGGCGGCACGGCCCTCCGTGCGCGGCAAGC
CCGACCTGCCGCGGGGCTCCACCGCCGGCCTGCCGGGGAAAGGAGCCAGTCCACTGACGACGGCGC
CGCTGGCCGCGCCGCTGCACCCCGCCACGAGGCAGAGGGAGGCAGCACTAGCGCCGCCACCGCATG
CGTAAGTGAGACAGATAATTACATGAAGA
>TCONS_00016646 geneID=XLOC_003383 scaffold_123:241935-264268(+) Unclassified
ATTGTTGTTTATTTTATTCGGCGCTATGCTCCGACACGGCGAGCCTTTCGACTCAGTTTTAACGCGCACG
TTTTATGAAACGACAACTATGCGGCGATGGTCGCTCGCCGGTGATAATTATTCATATCGGAACCGCTTCT
AGCGACAGAAATAGCACGGTAAACAAATTCGCGAGTTTTATTGGCAGGCCCGTTGTTGATTATTATTAC
CTTTTTTAACTTACGAGTTTTTTCGCGAACTACCACATGTCGGTGTCGCCTCTACCGGCTGAAGTTGTC
TCCGCGAATCTTAATGGTACTTAATCATAGTTTATTTTTGCCGCGCAACGTGTGTTTTAATAATTGTGTTA
GTGCCAGTTGTCGCGTTCTTATTGCTATCGCTCTTAATTAGATCCTTGCGTGTTGCGGAAGTGTTCTCCA
AAATTTAATGTTATTTGCAAAGATTTTAAATTAAATATTTTTGGACAGTGCGGTGCGCCAAATTGTTTTG
TTGTGACTTTATTTCGACAATTTTGTGCCAATTTAATTTACAATTTGTGATATCTCCAAGTGTTAAAAAA
AACTTTCCGAAGTTTGGCGGGAAGTGCGCTAGCGAGGTGTTAATGAAGAAGTAGGCTGTACACAGTAT
ACAGAAAGTTTGGAAAAGTGTTGAACATACATTGGATGCTGACTGCGAGTGTTTTTTCAACGCGTTTT
CGAGGCTAAGCGCTAGTTGTTTGAACAAGTGTGTTTTCTAATGGATTTATTTTGAGATTGTTTAGAAAA
CATTCGTCGGTGCGACGGCGCAGTTCAGCCGCGTCGCGCTAGTGGAGTGCCACAGGGCGCGCGACTG
ACACCCACATTGTTCTTTGTCGACAGAAATAAAAAAAACAGTATTATAGCTCAATGAAGTGGCGACAA
GTGTGACAGAGAAGGCCGCAGTGTACCTGGATGTGTTTGCAGAGGGATGAAGAGGATGGTTGTTACTT
AGATGAGTCGACACCAATGGTGCAG
>TCONS_00016959 geneID=XLOC_003383 scaffold_123:241963-264262(+) Intergenic
CTCCGACACGGCGAGCCTTTCGACTCAGTTTTAACGCGCACGTTTTATGAAACGACAACTATGCGGCG
ATGGTCGCTCGCCGGTGATAATTATTCATATCGGAACCGCTTCTAGCGACAGAAATAGCACGGTAAACA
AATTCGCGAGTTTTATTGGCAGGCCCGTTGTTGATTATTATTACCTTTTTTAACTTACGAGTTTTTTCGCG

AACTACCACATGTCGGTGTCGCCTCTACCGGCTGAAGTTGTCTCCGCGAATCTTAATGGTACTTAATCAT
AGTTTATTTTTGCCGCGCAACGTGTGTTTTAATAATTGTGTTAGTGCCAGTTGTCGCGTTCTTATTGCTAT
CGCTCTTAATTAGATCCTTGCGTGTTGCGGAAGTGTTCTCCAAAATTTAATGTTATTTGCAAAGATTTTA
AATTAAATATTTTTGGACAGTGCGGTGCGCCAAATTGTTTTGTTGTGACTTTATTTCGACAATTTTGTGC
CAATTTAATTTACAATTTGTGATATCTCCAAGTGTTAAAAAAAACTTTCCGAAGTTTGGCGGGAAGTGC
GCTAGCGAGGTGTTAATGAAGAAGTAGGCTGTACACAGTATACAGAAAGTTTGGAAAAGTGTTGAACA
TACATTGGATGCTGACTGCGAGTGTTTTTTCAACGCGTTTTCGAGGCTAAGCGCTAGTTGTTTGAACAA
GTGTGTTTTCTAATGGATTTATTTTGAGAATCTGCAAAGTGCGTACTCTGAAGATTGTTTAGAAAACATT
CGTCGGTGCGACGGCGCAGTTCAGCCGCGTCGCGCTAGTGGAGTGCCACAGGGCGCGCGACTGACAC
CCACATTGTTCTTTGTCGACAGAAATAAAAAAAACAGTATTATAGCTCAATGAAGTGGCGACAAGTGT
GACAGAGAAGGCCGCAGTGTACCTGGATGTGTTTGCAGAGGGATGAAGAGGATGGTTGTTACTTAGAT
GAGTCGACACCAATG
>TCONS_00016863 geneID=XLOC_003383 scaffold_123:242673-280535(+) Exonic overlap (+)
GATTTATTTTGAGAATCTGCAAAGTGCGTACTCTGAAGATTGTTTAGAAAACATTCGTCGGTGCGACGG
CGCAGTTCAGCCGCGTCGCGCTAGTGGAGTGCCACAGGGCGCGCGACTGACACCCACATTGTTCTTTG
TCGACAGAAATAAAAAAAACAGTATTATAGCTCAATGAAGTGGCGACAAGTGTGACAGAGAAGGCCG
CAGTGTACCTGGATGTGTTTGCAGAGGGATGAAGAGGATGGTTGTTACTTAGATGAGTCGACACCAAT
GGTGCAGCTACCTATACTGGGTCCGGAGCCG
>TCONS_00016852 geneID=XLOC_003401 scaffold_123:288146-289367(-) Intergenic
GTGATGTGTAATTGTGTATGTGGAGGTGAGTAATTTTAATCAATTCTTTACGAGTGTGTGTCTTCCAGGT
AAATCGTGTTATGGTGGGACAGGTTTTTGGTGGTTTACAGTGTTAATTTATTGACAGTAAAAATGTTGT
AGTTTTTTGTAAGTAAGCATTTATGTGATGTATGTGATGTGATGTATGTTTTGTAACCGTCTTATTTAAAT
TAAATAATGTACTTAGGTATCTGTGTAGCAGTGAAACAAACAAGAAGAACCTTGTATTTTTCACCTGTG
CAAATGTAATAAGTAAATTTGGCTGAACGTTACGTGTGTTTGAAGATTAAATTGATGAGAATCCTGAAA
AGCTGATCTGTAGGTATCCTATGAATGCCAATTCTATACTTTGTGTTTTTTGAAATGAGTTTTATTGCATT
GTGCAATGCAGAGTCAGAAGGTTGTGTGTTGTAATTTTAAGTATAGTCTACATTGTATCTTGTGAGTAA
CAGTGTCTGTGTATGAAATTAGTGTATTTTATGTCGTGTTATCGTGTTAAAGACAGAGTCAGTGTGAAAT
GTGTAGGTATTTAATGTACTGTACTCGTTTTAAAGACATCGTGTAGCGTGTGGTTCTGTGTTGGTATTTA
ATGTAGTGTAATCGTGATAAATACAGTCTGGATGTGAAATCTGTAGTTATGTGTAGGAGTTTAATGTTGT
GTACTCGTGTTAAAGACAGCGTCAGTGTGATCTTTAGTTGTTATGTGTAGGTATTTAGTGTAGTGTACTC
ATGTTAAAGACAGTATCTATGTAAAATGTGTAGTTCTGTGTAGGTATTTAATGTAGCGTACTCGTGTTAA
AGACAGCGTGAGTGTAAAATGTGTAGTTCTGTGTAGGTGTTTAATGTATTGTACTCGTGTTAAAAACGG
GTGCTCTCGTGAAATGTGTAGGTATTTAATGTAGTGTACTCGTGTTAAAGACAGTTGTTTTTGTGTGATA
TGATTAATTATGTGTAGGATTTAATGTAGTGTACTCATGTTAAAGACAGCGTCGATGTGGAATGTGTAGT
TATGTGTAGGTTATTAATGTGGCGTACTCGTGTTAAAGACAGAGTCAGTGTGAAATGTGTAGTTCTGTG
TACATATTTAATGTAGTGTACTCGTTTTAAAGACAGAGTCAGTGGGAAACGTG
>TCONS_00016660 geneID=XLOC_003388 scaffold_123:421621-422952(+) Unclassified
ATTACGTGTCTACATTCCAAATTTAACTCTACGCCGTTGAAATAAGGAAGAACCTTTGCTGTGCTAATTA
GTCGCAAAGAGACGGATGGAAGACGCGTGCCGTGCGAAATGACTGTGAAATAGTGTAGTTTTTCGTG
TTCGCGTAAACCTTATGACGATGTATTAAGTGTGATATTGAAATAGTCGTTGTTTGGTTAGCATAACTGA
GATGGTTGTTAGGAAGTGAATGACATGATTCAGTCTAGTTCCACACATTCAATTGATATCACCTATAAAA
GTTGGCTACTTTCATAACGCGTAAAATAACAAAGTGTGAGTGTTTCAGTGCAGTTTTCAGAGGATTGTC
AGTGAGAAAGGTGTCTTGAAAAAACTGTGTACGTCGTAAACTCATCGCCGTGAAAGCGCGCAGTAAG
TGAAACAAAACTGACAATAGCATCACTCATTTATAATGATCGTTATCATTAGAGTCACACAATTATATTT

CATAATATTCACCATCTTTTCTTCTGTTAATGGGTCTTATAAATTCTCCCGCCCGAATCACACTTTGTACC
CTTTACCGTTTAAAATTACAATATTCCTGAAATTCCATTTCTCGAAGACATTTCGTTTCAATATATCATGC
TTAATAGTATCGTTGAGAGGCTCGCTTTAGCATAAATATTTGCAATTAAAATTAGAGGCACCCCTATCCT
TGCCATTATTCAAATTCAGCCCACAACGGGAGACCGCAGAAAGTTCCAAATTAATTAAGAAAGG
>TCONS_00017019 geneID=XLOC_003388 scaffold_123:421798-442983(+) Exonic overlap (+)
AAATAGTCGTTGTTTGGTTAGCATAACTGAGATGGTTGTTAGGAAGTGAATGACATGATTCAGTCTAGT
TCCACACATTCAATTGATATCACCTATAAAAGTTGGCTACTTTCATAACGCGTAAAATAACAAAGTGTG
AGTGTTTCAGTGCAGTTTTCAGAGGATTGTCAGTGAGAAAGGTGTCTTGAAAAAACTGTGTACGTCGT
AAACTCATCGCCGTGAAAGCGCGCAACATGCGTCGTTCCTGGAGCCGCACCTGCAAGGCGTGAGGCG
TCGGGACAGCCGCAGCTCGGACTCGGACCGCGATGAGAGGTCAACTGAAGATGACGAGCGCTCCCTC
CTGCGCAGCTCGCCC
>TCONS_00016875 geneID=XLOC_003390 scaffold_123:473926-475181(+) Intergenic
GAGTAAACAATATGCAAATCATAAATAAATGGAAATCATTTATTTTTTATTGATAATTGGGGTGATAGCAT
TAGTCCTATACCTAATATCGCCTTATTGGATATTTATTCTTACATTCGCAATTCTTTACGCGGTCTACAGTC
GAATTACTATGATATATTAAATAAATATCTGTATTAATAATCTGTTACGTGGTAGAAGTCATAACGTGTGA
CGGAAAAATATGATTGAAAACAGATAGAAGACTATTGTGCTAATAATTGGCTAAAAAGAAGGAAGTGG
TCATGGATTTCCAGGCAGTTTACATGTGATAGTATTCTACTCAAACGAC
>TCONS_00017113 geneID=XLOC_003413 scaffold_123:682795-685511(-) Intergenic
GTTGTGTTCTTTGTCAGGTTCTAGGTTGCCTAGGTCCTCTGTTGCCGACCTTCTGGGACAGTATATATTG
TTCGGAGCCGCCCTCATCGATATAGTTTGCACTGTGAAGGCTCCAGAGCAGTTCTAGTGATATCTATAC
ACACTTAACCCATCAAAATGAAATACGTTGTGTTCTTCTTCCTTGTCTGCGTGGCGGTGGCGGCGGCG
GCCCCGCAGTGGGTGGCGGGCTGGCCCGGCTACCTCGGCCACGGCCTGGTGGCTCCACTCGGCGCCA
GGACTCTGGTGGCCGGGCCAACCGTAGTCAACGGCTACCCGGGAGTAGTCGGCGGCCGCCTGATCGC
CCCCGGCTACGTGGGCCACGTCTGGTAACTCCCCCCTCCCCCCGCGCGCACCAAGCCTCACCC
>TCONS_00016686 geneID=XLOC_003394 scaffold_123:726759-727352(+) Unclassified
CTTCCGTACAAAACTCATACAATCCGTAAAATGGCAACATAAAAAAAGTATAAATAGTTGCCATAACAA
AAATATTAATTTCCGCTTTCAAAAATTATCGTCTTACTCCTTTTAACACATGTAAAATGCATATTTATCAC
TCTTTTTACAAGCTAAAGAGCTTCAGTGGTGCGATCTATGGGAATCTAGCAGTTTACCAACACGACAAT
GGCCGAACAGCCCGTTTGAGTTATTTTTTATAGGCCATTACAGAAAGACCTGCCATGTCTTTGGTTTAA
AAATGGCGTATACAAGCTTTTTGTTTGCCGGTTTACGCTGTTGATGAGTTGACGAGACCCCGCGCGTTG
ATTTACGAGGCGTATACTTCACGAATATGAAAATAAAATACATGTATATGACAGCATCGAAAAATAGTAG
ATAATGGAGAGTTCGGAAGACGATGCGGGCG
>TCONS_00017413 geneID=XLOC_003480 scaffold_124:170826-173915(-) Intergenic
GCGAGGCGCGCTGCGGCGCCGCGGGCTGCGCCGGCGCACAGCTGTGCTGCCCCACGCAGTGCGGCG
GCGCCATGTGCGTGGACGCTATGACTACGAGACACTTTGTTAACTTAGGTAAGCTGTTAATTTAGGTAA
GAATCTATTTAGTTACAGCTTTTTAGACATGTATAAACAGGCATGCAAAAACATACACAGACAAAAATA
CAAGGTAATAAAAAGTTAGTACGCAAAGGCGGGCTTATTACCAAAAAGCTATTTATTTCGACCCTTATA
TCATTAAGCAAGTTAAATGGTATGGTAAGAATATAGAATTCAAGTAGCCTTTAAAAAGTAGAATACAAG
TGGACAGATCAACCCAGCTTCTACCTAAGAGCGCCGAACTACGCTCTGTCTTCAGTCTAAGATAAAAA
TACAGAGTAAGAAAAAAATAGTAATTTCTTCCAGCCAACCCATACATCATTGAGGTATAATGTCAGTAC
AATGGTAAGACCACAGAATTCAAGAGCTGGAAATCGCCTTAAAAACGTAGAATACCAGCGGTAACGT
CAATCCAGCTTCGAGCTAAGAGCGCAACAACGCTCTATCCTCAGTCTAAGGTGGTCGGTCCCTATCCCT
CTCCTCGCCCGGCTCCTGCCCCCCTCCCCGTGGACGCGTGCGAGGCGCGCTGCGGCGCCGCGGGCTG
CGCCGGCGCACAGCTGTGCTGCCCCACGCAGTGCGGCGGCGCCATGTGCGTGGACGCTATGACTCCG

AGGCACTTTGTTAACTTAGTGTAGTGTTCACTATTAAACTGATATAAACAGACACGTAACCGAGATATC
GGCTCGCAACAATCGCACACGTCTTTCCTCCACCGCGGCCGGTAGGCGACTGCCCCCCGCGCCCCGC
GACATGCGTCTGCGATTCTCAAGACGACAGAATGGTCATACACAGATGTAAACACTACATCCCTCCCTT
GTCATTTAAATTTTGATTTGATTTGCTCAATCATTACCAGATTTTTCCAATCCATATAAAAACAAAAAGC
TCAGTAAACCCTTGCGTAATCCTCATTCTATCGAGAATAATGAAATTCAAACAGATTGCTTCTTCGACTG
TCCTTCAACTATCATTTTGAAATGTTAGCATTATATTATTCTTTGTGCCTAACCTTACCTAACCCTTTCATC
CTTTCCTTTTGCACATAACTTCTTTTCCTCTTACTTCCCTGTTTCGTACTCTCTGCATTAGCTTCTCTCTC
AGAACATTGTTAATATATTCCCTGCCTTGTTTATCAAAATTGTCGAAAATCATATAACCAGGTGAACACC
TCTGTACATAGCTGCCTAAGCTCTGCCTGGCCTGAACCTCTCAGCTGTAGGTATAACTCCAGTTAGATC
AAACAACGCTAACTCAGCTATTCTACCACCAACTTCAAACCACTCAACCAACATTTTCAGTGGTGTCG
CAATAGTCTCACTGTTTATCTAGAGTTAACCTTTCACACACACACTTCACACCACGCAATGTAATGCTA
AATCTAATTTAATTCTCATCGCAATTTAATGCTCACCGCAATTTAATGCCCATCGCAATTTAATGCCCATC
GCAATTTAATGCCCATCGCAATTTAATGCCCATCGCAATTTAATGCTCATCGCAATTTAATGCTCATCGCA
ATTTAATGCTCATCGCAATTTAATGCCCATCGCAATTTAATGCTCATCGCAATTTAATGCCCATCGCAATT
TAATGCTCGTCGCAATTTAATGCTCATCGCAATTTAATGCTAATCGCAATTTAATGCTCATCGCAATTTAA
TGCTCATCGCCAGCGAAACTTCGCCAGGCTACGCAATTTAATGCCGCCATTTAAGGCCCCTCGCCATTT
AAGGCTACGCAATTTAATGCCGCCATTTAAGGCCCATCGCCATTTAAGGCTGAGCGCAATTTAATGCCA
CTCTTACAGTCACAATCTAATTTTGCCATGCAAGGCCAATTGCCAGTCGAGGCTGAGTGCAATTAAATG
CCACTCTTACAATCGCAATTTAATGCTGCCATTTAAGGCTGAGCGCATTTTAATGCCACTCTTACAGTCA
TAATCTAATGTTGCCATGCAAGGCCAATTGCCAGTCGAGGCTGAGTGCAATTTAATGCCAATCTTACAA
TCAAGTGTAAAAATAAAAGTGATTATTAACCAACAAAGGAGAATGGCCACTTCTCCACGGCGCCACGA
CTTGCCAGAGCAGCCATCGATGAAACACACACCAATGTGCAGTCCATGACCACAGCACATCCCGGCG
CAAACTTCACCACTATGAGGCACGGGAGAACGAAGACACAGGTGTCGAAAGATCAAGTCCCCGTACT
GTACAAGACGCCTCCCTCCCTCAAGCAAAGTATCCCTTTTACAAGCGGTCTAGATTCTTTCAAACATCT
TCCCTTATCCAGAACACCCTTAGCACTGAATAAAAACAGGTCCCGCTAGCAGCGGCAAAGCGGGTCA
GACTTGGCCATTCTCTTTTGTTTACATTTTGTGATGTGGTTGCAAATTCCACACTAGGCGATACAGTTCG
CCAACGTGAATATTTCGTGGAACTTAGACTTACGGTATCTAATGTTTTTTTAAACAAATTT
>TCONS_00017371 geneID=XLOC_003484 scaffold_124:318348-321978(-) Splice junction overlap
CGAGTACGACATGACGATAAACGGTCTGCATTACTTCCAGCCGGACACCAACGCGACGTAAGGGCCG
CGCCGCTCTTGTTATAGATATTAAGTTACGTTACTATATTGTAGGCTAAGCGTTGTTGAACCCCACTGGG
AACTGTTATGTATGTAAGAATGAAAATGCCAGGATAATTGTTAACGAAAACACACCATTCATATACTTAC
TAAAGTGTTGTATCAGTGATAAGTCTACCTGAGAAGCCTAATTGATAGATACTTAAAAGAGTTTAGGTAT
ATTTAGATGTTCAACTGTTAATCCTTTACTATCAACTGGTTAGTGACGTGTGAACAGTTTAAAGTCTAAA
TTTTCTATAACTAGGTATGTTTTTTAAAGAAGTATCTATCTATGTTTAATAAAATTAACTGTTGAATAAAG
TACTTATGGTTAGTTCGTTAAAATATGAAATGCATAATTTGATTAAAATGGCCTTAACTGTGTAAGTTCG
AACTTAACAACTGATTTAGTCATCACTATGATGTTAAGACATAAGTTACATAAAAATGTGAGTAATGAA
ACACTTTTGAGAGAGAGAGCTAGTCAATCAGCAAGAAGCTAAGTTTGTACCTTACACAAGTTATACGG
GTACTGTATAGTCTGCGGTAGAGAAGCCTTACCCCTCTCAAGTAGCATTATTACTCTGAGGGGTCACGT
TTCTTTGCCCCAGAATCTACGTGTATAGTGTTATGTATTCGCGAAATGAATTAAACTAATCTCTTCATTCA
TTTTGTTTCGGGTTTTCGAAAATTATTAAAATGGTTGATGGTTATGGAATTCTAAAAGTAATTAAATTTT
GTTAGCGTAAATAGATATGATAATTTTGTAACCATCAACTATGTATGTAACCAGGTATAGGTAGGTCATAC
ACTTGTAAAATTTGTAGTGGGTAGTAAAACAAAATCCTAACTACTTTAATTGTAATTCTCATCGTTATTT
ACTGTAAAGTTCGTTTATCTGCTACAATATGTCTACCTATTTGTACTTGTATTGAATCTTAGACAGATGAG
CACTTTAATTTTATCTGAAGACTAGCAACATGTACATGGCATATCTATACAATATCTGGAGCTAATTAATT

AATCTTCTTATGACACCTTCACCCCTTTTGAATTTGATAATATTAATATTTTTAAATTACAATTTATTTTTTT
GTTACTTAGCTACTACTCTTATCCCTTTGAAATTGTTAATCCTGCAAACAGGGTATTCATTTAGCGCCTA
GTTTTTGCTTTATTTTTTATGATCATATAATACTTAATTGTAACCTGTAGCTTTTGTATTATTAGTTTTAAGT
GAGGCTACCTATTTATATCATGCGTTTGGACCCCGTTACGCGGTTATAGGTAATGATGTAACAAGTAGGC
CTTCATATTTTAAAAATATAAATTTCATAAAGGTGCTTGTGTTACAGTTAAATTCCACGTTTATCACAGTT
TCTTTGAGTAAATATAAATATACTTACATGTCAAAATATAGACTTGATTATACTACACCAAATAATCTTAG
GCAAATAACACATTGTTTAAAAAATACCTACTACATTATCATAATGGAAGCTATAATTTTTATACCGTGTG
TGATAGTGACAGGTAGATTTCGCATATCTGAAGGTAGTAGGTACGGTTCCATATATGTAAGTGCGTAGGT
GTTAGTAAAGCGTAATGTGTCAATGTTGCAATATGTACACTGCCACGGTCCACACAGTGTAGGTCCACA
CAGTGTAGGTATATTGCAAGCCATAGTGAGATTATACTTAGTAAGAGTTTAGAATTATCTTTTAGAGACC
TATCATTTTTAACCACATATTTTCTACTATATACTACTGTTATGAACACTCATTTCATAAAGCTTACATTGT
GAAGCCAATGCAGATTGCAATAAATACTAGCTTTGTACCGACAGGGTTTGCGGGTAGGTATTTAGCAAT
ACTTTAAAACATGTAGGTACTTTATGAGTACCTATTCCTAAATACCATTCTGTAAACACAAGATATTCCTA
TGGCGTGGAAAAAATCATAACATTTATAAAATTAGTTTTTAAGTTAAATTTATGCAATAATTAATAAGTG
GTCTTAGTGATAGAGCTAAATAAAAGTGAGATAAATGGTTAAATAAATCACAAATTTCCATTATTCAAAA
TATACATAGGTCGGTATATGTGCCACATTATTTTTTCTAAATTAAAACCTATATATAGTTAAATTGAGGCAA
TATTATTAATGTTTGTGGATAGTTCATATTCATAACATACTTAACTACTTAACATAAGTACCTAGTATAAAT
ATTTTTATCGCAATTTGTTGTTGTGTAAATACATAGGTTATTTAAGCATTTTTTATAGATGCCAATTTAGAT
GTTTAGTAGCATTACTCCATAAAATCTGTTTCTATTTGATGTCTCCACATTTAAATCTTGAAATAGGTAGT
GCGAGTGAGTCCTTTTATTATGAAATTCGATCTTAAAAATCTATTGTAAATAATAGTGGCTTTGACTTTTA
CAAAAACTTACTATTCTTAAAATTATCTTAGAGGTGACCTAAGTACCTACCTGTTCACTATTTTCTTTTTT
ATCTTTATCTCAGTAGGTACCTAAATCAACCTAAATTCCTATACAACCTAACACTTAGGGCTGCTTTTTC
AGTAGTGAAATAAAAGTTACCTACCTGAGGAATACTTTGATTGAGTAGGGACTAGGGATACATTTTAAT
TTTTGTGGTATAAAATGAAATTATTTCTCTGTTGAAAAATCAGTATACTATGTATTCTAGAGTAAAAATAT
GCTACTTAGTTAGGTACTTACTGTTCGGCATAGGTATCGGAACCATGCATGGCAAGGGAAGAATGATGT
AAAAAAATGGCGGAATTAACTTGTATTATCATCATCACTGACAACTGTACCCAAATAGATATATTTTTGG
AAAATAGTGTTGTTAAAGAATATATTTGCGTATGTTGAACACGGCAGAGTTTTAATATGTTGAAGTATGA
AATAAATTGGCAAGTAGGTGCCCGATAATTATCTATAGTTGTGCATTATTATAAATAAATAACATTTGGAA
ACCATGTATAATTGAACAGAGTGGTTGTTAAGTTTCGTATTTTATAATAAAAAATAGTTATGTATTTATGA
TATATAATTATGATAAAAACAGATTAAAGTTATTAATTTAC
>TCONS_00017372 geneID=XLOC_003484 scaffold_124:318348-322187(-) Splice junction overlap
CGAGTACGACATGACGATAAACGGTCTGCATTACTTCCAGCCGGACACCAACGCGACGTAAGGGCCG
CGCCGCTCTTGTTATAGATATTAAGTTACGTTACTATATTGTAGGCTAAGCGTTGTTGAACCCCACTGGG
AACTGTTATGTATGTAAGAATGAAAATGCCAGGATAATTGTTAACGAAAACACACCATTCATATACTTAC
TAAAGTGTTGTATCAGTGATAAGTCTACCTGAGAAGCCTAATTGATAGATACTTAAAAGAGTTTAGGTAT
ATTTAGATGTTCAACTGTTAATCCTTTACTATCAACTGGTTAGTGACGTGTGAACAGTTTAAAGTCTAAA
TTTTCTATAACTAGGTATGTTTTTTAAAGAAGTATCTATCTATGTTTAATAAAATTAACTGTTGAATAAAG
TACTTATGGTTAGTTCGTTAAAATATGAAATGCATAATTTGATTAAAATGGCCTTAACTGTGTAAGTTCG
AACTTAACAACTGATTTAGTCATCACTATGATGTTAAGACATAAGTTACATAAAAATGTGAGTAATGAA
ACACTTTTGAGAGAGAGAGCTAGTCAATCAGCAAGAAGCTAAGTTTGTACCTTACACAAGTTATACGG
GTACTGTATAGTCTGCGGTAGAGAAGCCTTACCCCTCTCAAGTAGCATTATTACTCTGAGGGGTCACGT
TTCTTTGCCCCAGAATCTACGTGTATAGTGTTATGTATTCGCGAAATGAATTAAACTAATCTCTTCATTCA
TTTTGTTTCGGGTTTTCGAAAATTATTAAAATGGTTGATGGTTATGGAATTCTAAAAGTAATTAAATTTT
GTTAGCGTAAATAGATATGATAATTTTGTAACCATCAACTATGTATGTAACCAGGTATAGGTAGGTCATAC

ACTTGTAAAATTTGTAGTGGGTAGTAAAACAAAATCCTAACTACTTTAATTGTAATTCTCATCGTTATTT
ACTGTAAAGTTCGTTTATCTGCTACAATATGTCTACCTATTTGTACTTGTATTGAATCTTAGACAGATGAG
CACTTTAATTTTATCTGAAGACTAGCAACATGTACATGGCATATCTATACAATATCTGGAGCTAATTAATT
AATCTTCTTATGACACCTTCACCCCTTTTGAATTTGATAATATTAATATTTTTAAATTACAATTTATTTTTTT
GTTACTTAGCTACTACTCTTATCCCTTTGAAATTGTTAATCCTGCAAACAGGGTATTCATTTAGCGCCTA
GTTTTTGCTTTATTTTTTATGATCATATAATACTTAATTGTAACCTGTAGCTTTTGTATTATTAGTTTTAAGT
GAGGCTACCTATTTATATCATGCGTTTGGACCCCGTTACGCGGTTATAGGTAATGATGTAACAAGTAGGC
CTTCATATTTTAAAAATATAAATTTCATAAAGGTGCTTGTGTTACAGTTAAATTCCACGTTTATCACAGTT
TCTTTGAGTAAATATAAATATACTTACATGTCAAAATATAGACTTGATTATACTACACCAAATAATCTTAG
GCAAATAACACATTGTTTAAAAAATACCTACTACATTATCATAATGGAAGCTATAATTTTTATACCGTGTG
TGATAGTGACAGGTAGATTTCGCATATCTGAAGGTAGTAGGTACGGTTCCATATATGTAAGTGCGTAGGT
GTTAGTAAAGCGTAATGTGTCAATGTTGCAATATGTACACTGCCACGGTCCACACAGTGTAGGTCCACA
CAGTGTAGGTATATTGCAAGCCATAGTGAGATTATACTTAGTAAGAGTTTAGAATTATCTTTTAGAGACC
TATCATTTTTAACCACATATTTTCTACTATATACTACTGTTATGAACACTCATTTCATAAAGCTTACATTGT
GAAGCCAATGCAGATTGCAATAAATACTAGCTTTGTACCGACAGGGTTTGCGGGTAGGTATTTAGCAAT
ACTTTAAAACATGTAGGTACTTTATGAGTACCTATTCCTAAATACCATTCTGTAAACACAAGATATTCCTA
TGGCGTGGAAAAAAATCATAACATTTATAAAATTAGTTTTTAAGTTAAATTTATGCAATAATTAATAAGTG
GTCTTAGTGATAGAGCTAAATAAAAGTGAGATAAATGGTTAAATAAATCACAAATTTCCATTATTCAAAA
TATACATAGGTCGGTATATGTGCCACATTATTTTTTCTAAATTAAAACCTATATATAGTTAAATTGAGGCAA
TATTATTAATGTTTGTGGATAGTTCATATTCATAACATACTTAACTACTTAACATAAGTACCTAGTATAAAT
ATTTTTATCGCAATTTGTTGTTGTGTAAATACATAGGTTATTTAAGCATTTTTTATAGATGCCAATTTAGAT
GTTTAGTAGCATTACTCCATAAAATCTGTTTCTATTTGATGTCTCCACATTTAAATCTTGAAATAGGTAGT
GCGAGTGAGTCCTTTTATTATGAAATTCGATCTTAAAAATCTATTGTAAATAATAGTGGCTTTGACTTTTA
CAAAAACTTACTATTCTTAAAATTATCTTAGAGGTGACCTAAGTACCTACCTGTTCACTATTTTCTTTTTT
ATCTTTATCTCAGTAGGTACCTAAATCAACCTAAATTCCTATACAACCTAACACTTAGGGCTGCTTTTTC
AGTAGTGAAATAAAAGTTACCTACCTGAGGAATACTTTGATTGAGTAGGGACTAGGGATACATTTTAAT
TTTTGTGGTATAAAATGAAATTATTTCTCTGTTGAAAAATCAGTATACTATGTATTCTAGAGTAAAAATAT
GCTACTTAGTTAGGTACTTACTGTTCGGCATAGGTATCGGAACCATGCATGGCAAGGGAAGAATGATGT
AAAAAAATGGCGGAATTAACTTGTATTATCATCATCACTGACAACTGTACCCAAATAGATATATTTTTGG
AAAATAGTGTTGTTAAAGAATATATTTGCGTATGTTGAACACGGCAGAGTTTTAATATGTTGAAGTATGA
AATAAATTGGCAAGTAGGTGCCCGATAATTATCTATAGTTGTGCATTATTATAAATAAATAACATTTGGAA
ACCATGTATAATTGAACAGAGTGGTTGTTAAGTTTCGTATTTTATAATAAAAAATAGTTATGTATTTATGA
TATATAATTATGATAAAAACAGATTAAAGTTATTAATTTAC
>TCONS_00017214 geneID=XLOC_003484 scaffold_124:331463-333102(-) Exonic overlap (+)
CAAGGCTCATTGATTATTATTTCAACTAAAAGTAAACGGAAGTACGAGAATAGCCAACAAGTGGGGAA
AATTACCCCGAAACGGAGCACATTCTGCCGCTCTCTGGCTTTCAGGCGGACTTCAGTCAGTGTTGTCG
AGACCATTGCGCAGGGCACACGTGCCGCGAGCTATCGACCGCGACCCAACCGAATAATAATATAACTC
CTCTACTCGGTACAACATCAGCGTTACATTGCAGTGATTGTGAAAAACAAAACTCTTATAGCAATTCAA
ATATTCTATGGTGGTTTGTGGGAGCATTTTGTAAAGGCGCCACTCTATTAATAGCGAGATCTCTGCACTG
AAAGCAGCCTCTGGAAGTATTTCGATATAATAAAAAGTGTATTTATAAAAAAGTGACAACATGGCGATG
GACACTATGGCCGTTGAATTGCCGGATTTGAATACGATAACACCAGAAGCGCCTGTTGAAGAAACTGC
GGAGGAGA
>TCONS_00017182 geneID=XLOC_003471 scaffold_124:373409-373864(+) Unclassified
TGCAAAGAGTACTATATCATATCAGATGGTAAACTTTTGGTAAAACATTAATCTAACCTACCTAATCTAC

CAATTACAAAATGAGTTCTACTTTAATAGTCGCTGTAGCATGTGCTATGACCTCAGGCTTATTCGCTGCG
TTCCTATACCTGATAACAGCGGTGGACCCAGCGCTCCTGGAGGTTGGACAAAAGGCTGTGTTGATGAT
AAGAGCTTTGCGTAATGAGTCTCAGAGAAATCGCTATGATTTACTGGTAAATGGGTTTCAATAAAGATT
ATGGATGAAACTTAGCGGGTTAGAAAAACTGCCAAAATGGTAATATTAGTAGCCAGTAAA
>TCONS_00017224 geneID=XLOC_003489 scaffold_124:526049-527304(-) Exonic overlap (+)
CCATACAGGTGATCGCCGCGCCGAACCAAGACCGCCTATGTTTAGCTGTCGCGAAGAAATTAGAAGAA
GCTTTCGGCGGATGGCAACCAGTGCCGAATGACCCGTGAATTCATTCATGTATGATTAGATTAGATATAG
CCTTAAGAATATCTAAACTTTTATGGGATAGACTTTGAAGAGGGTATAGTAGGTACTAGGTACCTAACGT
TACTGATATTATTTGTATGTAACTTTCCTATGTACTCTAAGACGTAAAAAATCTGTTACTGATTGGTAAAG
TGAGTATAAATTATTTTTATGAGTAAATTAAAATCCTAATCCTTATCACATCATAACATGTCATAAAGTAA
AAAATACATGAATACTAAATTGTTATAATACATTTATTAAAATCAATATTTATGGCATTGTATGGATTATGT
ATTACTTATTTGTAACTAATGTTATACTGTGATGAAGCATTAAAACATAACTGGGGATGAAATGTGCAAT
ATTAGGTAAATTCGGTATTTTAATATCGTAAGAATGTATATTATTATATACTAAAATTATGTATTTGGTAAA
ACTACAGATTTTGTATAAATAAAATAAGCTGACTTTATGTATTTTTGTAGATCAATTTTTTTTACATTGAT
GAAATGAGACTTTACCGCTACCGAAACTTTAAAATAAAACTTATTTGGACAACTATAAAGTATTGTGAG
TAAAGAACTAATTAATAATAAATATTAATGTACTTATTAGTGTAACTCAAACCACTGGTATTGCGTGGAAT
ATTTTTTTGATCATAACTACTATTTATAAATCCTGACATAATTACTTGAATGTTGTTTGTGTCTTGGCATAC
TTTGTAAGGTCGAGTGTTACGAATATTGCTACACGCATTTTAGGAATGTTATGCAAATAAAGTATAAATA
AATGTATTCTGGGA
>TCONS_00017225 geneID=XLOC_003489 scaffold_124:526049-527763(-) Exonic overlap (+)
CCGTGGCTGTTCCACGCCGGAGTGGGGTTTTGCCCACAGCTATACAGGTGATCGCCGCGCCGAACCAA
GACCGCCTATGTTTAGCTGTCGCGAAGAAATTAGAAGAAGCTTTCGGCGGATGGCAACCAGTGCCGAA
TGACCCGTGAATTCATTCATGTATGATTAGATTAGATATAGCCTTAAGAATATCTAAACTTTTATGGGATA
GACTTTGAAGAGGGTATAGTAGGTACTAGGTACCTAACGTTACTGATATTATTTGTATGTAACTTTCCTAT
GTACTCTAAGACGTAAAAAATCTGTTACTGATTGGTAAAGTGAGTATAAATTATTTTTATGAGTAAATTA
AAATCCTAATCCTTATCACATCATAACATGTCATAAAGTAAAAAATACATGAATACTAAATTGTTATAATA
CATTTATTAAAATCAATATTTATGGCATTGTATGGATTATGTATTACTTATTTGTAACTAATGTTATACTGTG
ATGAAGCATTAAAACATAACTGGGGATGAAATGTGCAATATTAGGTAAATTCGGTATTTTAATATCGTAA
GAATGTATATTATTATATACTAAAATTATGTATTTGGTAAAACTACAGATTTTGTATAAATAAAATAAGCTG
ACTTTATGTATTTTTGTAGATCAATTTTTTTTACATTGATGAAATGAGACTTTACCGCTACCGAAACTTT
AAAATAAAACTTATTTGGACAACTATAAAGTATTGTGAGTAAAGAACTAATTAATAATAAATATTAATGT
ACTTATTAGTGTAACTCAAACCACTGGTATTGCGTGGAATATTTTTTTGATCATAACTACTATTTATAAAT
CCTGACATAATTACTTGAATGTTGTTTGTGTCTTGGCATACTTTGTAAGGTCGAGTGTTACGAATATTGC
TACACGCATTTTAGGAATGTTATGCAAATAAAGTATAAATAAATGTATTCTGGGA
>TCONS_00017636 geneID=XLOC_003540 scaffold_1247:6654-9444(-) Unclassified
CTTTATACATATATAATACCTAGATATATAAAAATGTTACTTATCATAGAATCTTTAATACGAAAGTAAAGT
TTTTAATAAGGATTGGGATTAATATAATATGGGGCCTTTATTTACTTAGTACATATTTGACGATACGAAAT
TTATCAAGTCATTTCAAATTTACATTCCATATGTTGAAGATATTATAGGTAATAATTAAAAGTATCTCCAA
AATTACTTTGTACTTAGATTCTCTGTTTAATTCGTATTTTACATTTTATTTGTGGAATATTATGTAAAAGCA
TGAATTAATTAAACGAAAAGCCGCTTTTCAGTCTGACTAGGGTTATGAAAAAGTCAAACTTCGAATTA
AGAACATTCGATTGGAAAAGACAACTTGAAGAAATAGGTTACTTTTTAAACTCCCTTTGACCGTGTAA
GGAGTTTGATCTTACATAGGTGCATTAGTTAAAATAGTATTCAAATGTTATAGCTATTTCCGTGTTACGAG
TTATGACACGACTTGACCAAAAATAGTCACTGGACCTTTTCAATTATAGCTTTTGTAATTATTAGCATTA
ACACGTTTCTGTTACTTACCAAGTTCTTCAATATTAACTTAGGTTAAGTAAATTAGTGTGATACTGTGGT

ACTTAGTCAATGTAAGGATTTATACGAGATAATATTATGTGAATGTGATACAAATATTGATGAAAATACAT
TATCTGAATACCTACGTACTTAGTTAACCGTTCACCCACCAAACTTCAAAGCAACATTATAAGTGTACA
AAGATTAGTTATTTTTGTAAATCTGACAGTGCAGTCCTTAAACCTGTGTTTCCTAACACTTGGTGCTTTT
ATAAGGAAATAGACAAATTTTTGATCGATTTAGTAAATCATTTATGACGTTACATTTGCCTGTTTCTTTAT
AACTGCATCCATCCATCTGTCTATATCATAGTACCCATGATTAATGCATAAGACATAAGGGCGACATCGT
GTTTTTATGTTCTGTACATTGAACAATAAAGTACTTATACTTAACTATTGAGGATAAGTACTTCATCCTTT
ATAGAAAACACATTCAAATTTAAATGTATGTACATAACTAAATTTAATATTTTAGCTTGCCACATTAACTA
TGTTACTTTATAGTTTAAAAAAATATGTTACCTCTTTCATGTAGTGATTAACTTATTTTATAATGCTAATAAA
ACGAGGAAATTTAGTTTTATTATTGCTCAATTTTTTGGCTCAATTTTTGTAATAAGTAATTATATTTTGGTC
TTCCTTACCTATGGAAAATGTGATAAAGATACTAGCATCTTGGGTACAAAACTAACTAACTACCCAGTG
GTTTTAGAATCTATGTTATGAGAATCGAAAATGAGCCTTACATATATCCATATCCATTGTGGTGCTATAAA
ATTGGATATAACAAAATATATAGGTATGGAATTTTAGACTAAGGTGGGTAATTTCATTTACCTATAAAATA
TTAGTATAAACCCTGAGCAATAAAGGAATTTAAGAATTGTTATCATTAGAGGAGTATGAAAAGTTTTTTT
TTATATAACGCCGTAATTAAAAACGTGAAATTATAATGTGTCATTTTATTATTTACACCTAAGTACACATA
TTTATTTGATAAGAACTTACATACAGTAGGTAGGTACATTTTGTACGATTATGCTTAATTATTAGGTACAT
AATTAAGTACATAAGTAGTTACATCCTTAAATTTTTAATAGGAACTTAACTGTACAAGGTGAAAGAGTT
AAAAAATCCTTAGAAAATAGAAGAAGAGACTAGTGCAACTGCACGGCACAACCCAAACAAACTACAT
ACAGCGAACAACAAAGTATGTGTAAACCAATTTAAAGGCAAGAACGTCAAATGAAAAAATCATTAAG
TACACTAAATGCGCTTATGGGAACACACAGAAGCCGTTGTATAAGTAATTTTGCCAAACGATAAGGAC
GCATACCAAAAGGTAAACTCGTAAAAATCCATCCATTCCACCCAACCACTAAATCAATCATTCATATTC
CTCGGATAATCTTCAGGTAGCTTGAACTCCGATATCGGAGACTTATACAGGGGGTTCTGTTGCCGCTGG
TTCATCATCATCATGCTCCGCTCCGTCTCTGCAGCAAACTTGGCGTATGCCCTCCTATCTGCATACACTT
GAGACACTTTTACTGCTATAATCATAATGATGCCGATGGCTATGACGCAGCCCATGATGACCAGTCCTGT
GGATAGGATCTGCGCTGTGATTGGCTTAGAGCACTCTCTGCTCATGAGAGCTATGTCCATGTGGACCAT
GGACTTGGACCCGGCGGTGTAGGTGTACTGGTACTCGCATTGGAGCCCGCTTTCTTCCCGGCGGAGTA
TGCAGCGGGCGGTGCCAATTGGG
>TCONS_00017644 geneID=XLOC_003541 scaffold_1248:220-10867(+) Unclassified
TAGAGTGACCCCATTAGTCAAACGAGTACAAAGCAGCGATAGCGCCACAGTGCTCGTCGCTCATTGGT
CGGGGACGGACGCACGCCATTGGGCCCTCGATCTCCAATTCGATTTTTAAATCCGAAAATCTTTTTGGC
CAGTGCTTTGTGCGTTTGAGTGCATTTTTTGTGACAGTGTGGTTCTTTGTGGATTATTTTTGTCACGAAA
CATGGACGACCGAACACGCAGTGCCAACTTAACCAACGCAATGTCGATCAATTTTTTATAATTTAAATA
TTTTTACAAAACACTTTCAGAGTGTAAAAGCAAACAACCAGTAAAATATCTGTGATTTTTATACAAATA
AGAATTTAAAGTGATCTCTATACATTTTTAACTAAACCAGCCAAAAGGTGAAATTATATCTGCAGTATAT
GTGAAGAATCGAGATTAATCAACCCTCAATCGTGGTATACGCAAAAAGGGCGTAACTCATAAATTTTCA
TATTTTATTTTTTATTAAAAAAATATTATAAAAGAAAATGCCAGCAATACTGTCAAAGATCAGGGAGCAG
GCGTCGCGGGTGCAGCCGACGGTGGCCCTGGGGGTGGGGGTCGGGGTGGCGCTGGCCGCGTGCGCC
CTCTACGGGACACGGGAGAAGAAACAACCTCCGCCCTGTCAGAACAACAATAACTATAAGGTA
>TCONS_00017668 geneID=XLOC_003546 scaffold_125:59879-60548(+) Splice junction overlap
GTTTTATTCTCATTTCAGACGTTTCGACTGAACAAAGTCTTACAAATAGGGATTTATTACGCAAGACGT
CCAAAAATACAGAAACGTTCGCTGACCGCATTTTCCCTCACAAAGCTTACACTCTCCCTCCGCTCTCG
CATCTGAAACGTAAGCTGAATAAGCAATTTAAGGAGGCAACAGAAACATTAAAACTGAACATCAAGA
AGCCGATTTCGCCTTTGAACTTGAACAAAAAGTTTCAAATCGACCTCCCGTCGAAACCGAAGAAGTTT
ATATTTCCGAAATCACCACTTCAGCTAAATAGGCAGTCGCTGAAACGAGAAACTCTCAATCATGGCAA
GTTCAATGAGAAGTATGCGCCCCTCTTTCAAGATATTGGCTCTTTGTTCGATGATCTGCTGGGCGTCCA

CGAACGCGAATACATCGAGCGCGAGACGAAGTCCCGACAAATCCATCGCAACGTCATTG
>TCONS_00017818 geneID=XLOC_003557 scaffold_125:439449-448731(+) Unclassified
GAAATTGGGACGTCTCGCGCGCGCTCCCGCCGCTGGGTGTTTGTTACGTGTTCGGAAATCAACGTCGG
GTCTTTTGACTGAAATTTCAGTGACAAAAATATCGCGATTGTTGTGTGTTTGTGGTGCAGTTAAATGCT
GGTTTTGTAAGCGTTTCCGCAGCCAGTAGGAATAGTTTGGTGTTGAGATTTTGTTTCAAACTTGTACGC
TGACAAGCCGGGGGAAATGTAAACTTTCGTTATCTAGGATGTAGTTATTTTTAAAGTGATTGGACGGGA
GTAAAACGAAAGACTTTCTGTCTGTCTGTCTCTTTTCCTCGCATGTTGCATTGCAGTGACAAAGTGTGT
CAGTTTGTGATGTTGCCGTTTTCGGCTAGGGCTATAAAGGGCGAGAGCCGCCGTAAAGATGAGAGAGG
GCCGTAAACGTGACTTTTTCGGCAACAAGATGCTACTGACTTTGTACGCCGCCTTGCTTCTCTGGAGG
TTCGACTGTAGCGCCGTGGATAATGTTAAAGGTAAGCCCTTGACGTAATAAATAACTCTGTAGAAGTGA
GTTGTTTAATAAAAT
>TCONS_00017983 geneID=XLOC_003606 scaffold_126:6694-18890(+) Unclassified
CTCAGATATTCATACAGCCCAAAACTCATTAGTTTTTTACTTTTATAACTATGTTTGTTTCGATAATGCT
TTTTTCACTGATGAAAATATGACAGTTTAATATGACTCTGACTTTCATCTAATCTAACAGCCATCACTAG
TTATTATCTGTGATTTTGTCATTGTCGATGGTCGTCGTCTCGCTGTCGCTGTTGTTTGTTGGTGTTTTGTG
CTTTGTAAATTATTGGTTTTCATCGAAGTTTTACAAGCTTTCTACATGTAGTGTGTTTAATAGTTAGTATT
TTATTATTTTGTTTTCCTGAAAGTTCCCTTGTGTATTGTAGGATCAGAAGAGCAATCAAAATGGCATCAA
CTACAATGATAGAGACAGTCACCATAACTCGACCACTGAAGGTGATAGCC
>TCONS_00018021 geneID=XLOC_003622 scaffold_126:70120-73503(-) Exonic overlap (+)
TCGCCAGTTAGTGTCTGACCGTGCGCGCGCGCACTTGTGTCAACTGTCAACATGCTGAGTGTCTGCAC
GGGGCGCGCGGCGCGGGTGGGCGGCGCGCTGTTGGGGCTCGCGCTGGTCGGCGCGCTGGTTGCCGTG
CTGGTGCTGCAGCCCTGGGCGCCGCCGCCACAGCAGCGAGTGCGCTACCAGAGTGCTGCGGTTGCTG
CCAATGGATATGAATGTGCTAGTGTTGGCAG
>TCONS_00018186 geneID=XLOC_003609 scaffold_126:85493-90204(+) Intergenic
AAAACAGATAACAATGGTTACAAAATTTAAAAAATACAACTAAACATCTGTTCTACATTGATACCCAAA
ACATGCATGCACAACATTAATTATTTAGGATGGAACATTACTCATTACATGTGTTCTATGCTATAAGTAGT
CTCAGTGGAACAGCCGCTGCAATTCATCTTTAATGGCAGAATTCCCCGCTTTAGTGATTGCAAGTGAAC
AAGCTAGCTATAATGTTCCAAAGCACGTTAGAAAGCTTGCGTGCAAATGAGTGTAGGTACTCACTGGG
CGCCCGAGGCAGGAGGGAAGAGGGAGCGAGACGTCCGACACAAACCAATATGGCCGCTGTGCCTTAT
GGGAGACTCCCCAAGATGAAACGAGAGCCACCGATGTTGTACAACACGTCACCAAAAACTTGAATAT
TTAAAATGTATTATAAAATATATCGACAGAGCCAACAGAACACACGTCCGTCCGATCCGAACTGTACTT
GAGGAAAAAA
>TCONS_00018200 geneID=XLOC_003654 scaffold_1260:2073-4339(+) Unclassified
CGTGGTGTCGTGGCGCGCGCCGCCGCCGCCCGCCTGTCGCCAGCACTACGAGCCGCCGCGACGCCCG
GCAACACACCTGGCGACGACTATAGATGCGCTGAGGAGTCTGTTGGAGACCAATGGGAACTGTTAGTA
TGGATACCCATCTTGCGACCACGATTGATGCGCTGAGGAGCCTGTTGGAGACCAATGGGAACTGTTAG
TACGGATATGATTTTGGTAAATTGTGAAGACGCGTTTGAAGAACCGTACCGGTTCGCCCGCTCTAATTA
GACGTGCATTGATTTACCATATAAGCCACTCTCCATTTACCGGTTATACGCCGGGAAATGAGACACAATT
AAAACTGTATCGTATTTTGGTTAAAGAACCAATGGCAAGCTTTACGGAGTTTTGTGCCTCGAAACATCC
ATACAAATCAGTGTGGTTAAAAGGTTAGTGGCGTCACTTAGCTAATCGCGGAAGAGATTCGCGGTTAC
AAAATTTTATCGATCTAGCTATAATAATTTTCTATAGAATAGCGCCACCTATCGGAGCTAGCGTTAGCTAA
ACGCAAATTTATCCTAAAAGCATCAATCCCCCGCCAGAGATTAGTTGCACATTGCAGTTCAATATTTTGA
TTTATTATTATTGCCCCTAAACAACGTGAGGGTAAATCAGAAATACAAAATGATTGTATTACTATGTCTAT
ACTCCTATTAATCACGAAACCATAAAGATTTAAATGACTCGAATAGTGAATTTTTGCTTATATCAGAAAG

CACGTTTTTTACATGATAAGGTATTAGTGAGTAATTATTATGTACTTGGTACATTCCTTAACAAAGACTG
GGTCAGTAGATCTGCATTTATATGCATTATATAATTGTATGAACGGATTAACATTTTGTAGAGTAAGTTTA
TTGATACGGTGATATGAAGGAGTTGTAGAACGCCGAGTCTTCCTTAAATCTGCAATCAAATATCACAAA
AGTTGATTATAGCGTCTTCCTAATTATTATTTACAAATTACGTGATAATTATAAGAGTTAAACTGTATGGG
TAAAGCATGACAAATTATTTACTTTAGTACTTAGTGGTACATATTTAGTTTATAATATATTACTATGTATTG
AAGGGGCCAATTCAGCATCGTCTACCAAATATATTTTAGTAAATTGCAGAATTGACCCCTCCGTCGCGT
AGTTATATTTTAATTTGTTCATTGAGGCTTTTTTAGACTTGTGCTTTTTCATTGTTTATGTAATTTGTATTG
GAAAACAATAAAATAAACTTATTCAAGTATTCATAAAAATATAGAATACGTGACTCCAATATTGGTCGCT
TTCCATCCTTTGACAGTGGAGCGTATGTGACAGTGCTTTATGAGGCGCTATGCCAATATGCCATAGACCT
ACTACATGCGCTATGCATAATATTAATAATCTACGTAAGATCTCGATTCAAAATGACATTTATAAAGGTAG
CTAGAGTTTTGTATATAACACAGACTGAGTACCGAATGTATTATACAACTGTTTAAACAAGTATTATATTT
ATTGTTTACTTTATACAGTTATTTTAGAGTAATTAATTAATAGTATCTCTTGTTGTGTCACAAAGGTTATGT
TAATATAGTGAAGGTTGCTGGGGAGTAGCCCACATTTGTGCCAAAAGGGAAGGTGTGATGTTCGTGGA
GTAATCCCCAGCTAACCACGAGTATATCTTGCCAGTACTAATTCTAAATTATACTGTTCATATTATTATTAT
GTAGTTGTATTTAGATAGTTTAATTCTGAAGATGGCATAGAGACGATTGAATTGTCTGCCTGTCTAATCT
AATCTGATTGAATCTATTGAGTGCCGAATGTGGACACCTCAGTTCCTCATTCATCGTTGTCTTTGTGTCT
CATCTTCAGATCTCTAGATTAGTTTAGTTATATGTAAATATATTATTATAGTTAAATATTATCATGTGCCTTA
CTATAAGTGGTCTTCCAACGAGTAAAATGAACTTCCATTTGTTAGTTGTATAAGTAGAGGT
>TCONS_00018236 geneID=XLOC_003661 scaffold_1264:11077-11712(+) Exonic overlap (+)
ACACAGTGACTCTGACGGCCAGTAGCAGCTCCATGCAAGAAGCGGGCTCGCAGTCCTCGGAGTTCAA
CACTTTAGTAGACACGGCCTGCTCCATCATCGAAGCGCCGCACCAGCAGACGCTCGGCGCCGCGCCGT
CGCCAGGCCCCAGCGGTTCGGGCGCGGCAGAAGCGCGCGCGGCCGCCCGCAGTCCGGCCAAGACTG
TTATTGTACGTGCTGTA
>TCONS_00018267 geneID=XLOC_003666 scaffold_1266:10854-11596(-) Intergenic
CGTTTAGCGCAGCCGTGGCTGAGGCCGAGCTGCACGGGGTGCTTGGCGTCGCACGCGCAGGTGCCCG
TGCTGGGGTCGCAGAACACGTGCTGGAGCTGCGGGCTGCACGTGAACTCGCACGGGGAGCCCAGGTA
TATGCCCTTCTCTGGAAGTGAAGGAATATGGGAGTGGTTATATGGGGATCTTTTTTTAGCGTATCGATGA
CAAATACTAGAACTATACTATAGCAAATACTATAGGGTTGGTTACCGATGCGAAAGATTAGCCAGTCCG
ATCAAATGTCAGTCAATTTGCCAGGGTGATGTTGGACGTATATGGGGATTTTGTTCAGTAGGGATATGT
GTGTTGTTCTTCCTCTGTTGCAGTGGGGTGCATCCCCTGTTTTGGGTCAACCCCTAATTAATTGTGATCA
AAACCACTGGCAAAATGGGAGCAGCAACAAAGAAAATTAGTAGAAAGCATGAGAATATGATTATTTAT
CTCTATGTCTATGTTACAATAAGGACGAAAAAGATTGTTGTATAGGTACAGTACAAGCGCATAGCTAAG
GCGATGTATTATTTCTACCCCTCCACATAAAACGATCGGACCGATGTTTTGGATCAATTATATAAAAGAA
AGAATTGGGTCACCCTCGGCCTGTCGTC
>TCONS_00018500 geneID=XLOC_003688 scaffold_127:640898-642082(+) Unclassified
ATTTTTAATAATACCTCCTAATAATTTTAAACCTCCTGACAATAATTTGAACAGGTCTTGTTGGTACTTAA
ATAACGAAGGAGTAAATATATTTCCCAAAAATACATGTTAGGTTACAGGACGAAAACATTACTATTTAA
AAATACTACAATCCTGATCTTGGTGCTTCTATCTGTACGGTGCTTTGTACTAATACTATATCACTGAAGAT
TTTCATAGTTACTATCTGGTTACGTACTGGCAAAAGAAACATGATCTCAGATACATAAATAAGAAACAC
CCGGTGTGGTGAAAATCTATTTTAACCCAACCGGATTATTAGGGTTACTGCCACATTAATTTATTACCAT
AGATTCCGTATGTTATGAATTATGATATCGTCTACTAGGGACGCAATGTAGGCAGTTTAAAAAGGAACT
GTTGGTCGAAAAACAGTTTGTAAGCTCCTCACTTTACTACCCTCATGGTCTCATGCCACTTCCCCACTC
CTCATCCATAGTTACAAACCCAAAAAGTATCATCCTCCCTTTCCTAATCAATTCCAATAACCAAAGATCG
CTCGACAACCTTTAAAAAAGTTAAGTACACATAACAGCCGCTCTATATACCCAATGCTTTATCATGTTTT

AAGTTTGAATACCGACTCGACTTCCACAGTCCTACTATCGCTTTATACGGCATGGCATGCACACTTTTAA
TCAAACTCAACGTATTAACCTTCAAATCATTCCAACTTAAATTCCACCCGGTCAATTCATCTCATTTACA
GATTAGGCGTGGGTACAGGCGTCAAGAAGGGGTTAGTAGTGGAATGGCCGGCATACGATGCGGCGCG
GCCGGCGGCCCCCAGGTTGGGCGCGCGGGCTATCCTGCCCAGCCACTGGTCCGGGTGAGGCAGGGCG
GGAGTCACGGGGAGAGGGGCTATGGGCACTGGCTCCTCGGTTAATGCTCGTAAACCTGAGGAGAGCT
GCTGACACATGGCCGCTACTGGGTCTGAAGGTTCTGCTGTGCCGTTCTCCT
>TCONS_00018558 geneID=XLOC_003715 scaffold_127:859757-861652(-) Splice junction overlap
GCCACTGCGCCACTGTATGTGTCTACACTGTGTTGTATATATACACTAGGGGCATCAACCCGGCTGCGG
GGTGCGAGTGGCGCGACCGCCGCCACTGCCGCCGCTGCTGTACCGCAACATGATGGCCGAGCTCGCC
GTGGCCGAGTGCCGCCACTGCGCCACTCTAATGTACATGGAAGACATGGAGATCCAGGAGGCTACAA
AGGGCCACTGTCCGTTCTGCCGCACCCCCCGCGCGCCGCCCGCCCCCTCCAGCCCCGGTTCGCCTGCC
AGCGACGATGCTTCGCCTTAAGAGATATGAAACGTGTGGATGATGCTTCGCCTTAAGAGATATGAAACA
TGTGGACGATACTTCGCCTTAAGAGATATGAAGCTTGTGACTTGGTCGCGTTAAAGCCGAATAACACA
CTGTTTAGTGCGAGTGTTAAGTAGACGTACGTACATTGCTGGGTTTGTAGGAGATATGAGCCTTGTGAA
TTTTTAGCAATCGTTTAACCCTTTTACCAGTGTTGTACGAAATTTGGCCGCCATTTGATCTTTTATTTATA
CTTTTCGCAGAAAGAATCTGAAAATTTTACAGATACTTAATTATGTAGGTACCTATGTTTGCAAATGCAT
TTAAAATATTATTATAGTTAGTGAGAATAATCATATTTTATATTATAATGTATAAAATTTATCATCACAGAG
GTCGTTATGTACTATCGAACAAAAAAGTGTGGACCCCCTTAAGGATCATTTTCCTTTGCAGGCATCAGC
CATCAATGACTCGCTCTTGACCAATGGGGTACAAGTTGACCAAAAGTTACTGTCTCTATTGCCACCAGA
TGTGTAAACACAAAACAACATTGCATTTTCTTAAAGGTGCGAATGTCCGAAAGCAATAAATAATACAA
AGGGAGAAATTTAAAATAATGGGGTTGGTTAAAAAGACAGGCAAAGGCAAATTTGGGATTGCAAAGG
ATTGGTTTTAAAAGAGGCAACTCACTTTTTTGTTCGATAGTACCTTTTGGAAACTAGCATTTCACTTGC
ACAAATTATTTTAGGTAGCAATTTTTGGAAATACCTTATAGTTAAGTAAGTATATAAATTATATTTTACTAG
GGAATGTAAAAACTACGCGTTTGTTCTTCCTGTGTTCCTCATTATAAATATTATGTTCATTTTACCCCACC
TGCCTGACCAGTGTTTTACATTTACGATTTTTTCCATCATAATTTTGACACAAAGAGAATCTTCTCAATA
ATTTGATAACGAATCCGTCCA
>TCONS_00018557 geneID=XLOC_003715 scaffold_127:859759-861485(-) Unclassified
GTGTCTACACTGTGTTGTATATATACACTAGGGGCATCAACCCGGCTGCGTGGTGCGAGTGGCGCAACC
GCCGCCACTGCCGCCGCTGCTGTACCGCAACATGATGGCCGAGCTCGCCGTGGCCGAGTGCCGCCAC
TGCGCCACTCTAATGTACATGGAAGACATGGAGATCCAGGAGGCTACAAAGGGCCACTGTCCGTTCTG
CCGCACCCCCCGCGCGCCGCCCGCCCCCTCCAGCCCCGGTTCGCCTGCCAGCGACGATGCTTCGCCTT
AAGAGATATGAAACGTGTGGATGATGCTTCGCCTTAAGAGATATGAAACATGTGGACGATACTTCGCCT
TAAGAGATATGAAGCTTGTGACTTGGTCGCGTTAAAGCCGAATAACACACTGTTTAGTGCGAGTGTTA
AGTAGACGTACGTACATTGCTGGGTTTGTAGGAGATATGAGCCTTGTGAATTTTTAGCAATCGTTTAAC
CCTTTTACCAGTGTTGTACGAAATTTGGCCGCCATTTGATCTTTTATTTATACTTTTCGCAGAAAGAATC
TGAAAATTTTACAGATACTTAATTATGTAGGTACCTATGTTTGCAAATGCATTTAAAATATTATTATAGTTA
GTGAGAATAATCATATTTTATATTATAATGTATAAAATTTATCATCACAGAGGTCGTTATGTACTATCGAAC
AAAAAAGTGTGGACCCCCTTAAGGATCATTTTCCTTTGCAGGCATCAGCCATCAATGACTCGCTCTTGA
CCAATGGGGTACAAGTTGACCAAAAGTTACTGTCTCTATTGCCACCAGATGTGTAAACACAAAACAAC
ATTGCATTTTCTTAAAGGTGCGAATGTCCGAAAGCAATAAATAATACAAAGGGAGAAATTTAAAATAAT
GGGGTTGGTTAAAAAGACAGGCAAAGGCAAATTTGGGATTGCAAAGGATTGGTTTTAAAAGAGGCAA
CTCACTTTTTTGTTCGATAGTACCTTTTGGAAACTAGCATTTCACTTGCACAAATTATTTTAGGTAGCAA
TTTTTGGAAATACCTTATAGTTAAGTAAGTATATAAATTATATTTTACTAGGGAATGTAAAAACTACGCGT
TTGTTCTTCCTGTGTTCCTCATTATAAATATTATGTTCATTTTACCCCACCTGCCTGACCAGTGTTTTACA

TTTACGATTTTTTCCATCATAATTTTGACACAAAGAGAATCTTCTCAATAATTTGATAACGAATCCGTC

>TCONS_00018590 geneID=XLOC_003716 scaffold_127:861816-863213(-) Splice junction overlap

CGCGCCCGCGCTCGCGCTGCCCTCGTACGAGGTCCTCCCACTAGTTCTCTTCCGGCCGGAACCGGAAA
TATCCCCCGAGGAAGCGGATGAACTGCTGGAGCGGACAGAGATAGAGCCTGACTCCACGGACCATCT
CACGATAGGCGAGGACAGCGATGCGCTGTTTCAAGATGTCGGGTTTGAGGGCGAAGGCGAGCCAGAC
GGCGCAGTGATATGCAGCCGGGCGGCGCTACTGCGCCTCAACCCGGGCTGCGTGGTGCGAGTGGCGC
GACCGCCGCCACTGCCGCCGCCGCTGGACCGCAACA

>TCONS_00018817 geneID=XLOC_003791 scaffold_1276:5942-12570(+) Intergenic

CATTCTGTGACCGGCTTACAGCCATTTCCGTTTATCTTCTCTCTGGTTTGGTTATCTTCCTTTCTGTGCAG
CCAGTTACTTTGTGATTGTGGAGAATTAATCATTAATAGTGACTACCAAAATAAAACCGAATCTTCAATT
TGTGAAACTATCACTTATTCATCAGTGTTTGTCAATGAATCAAAATGATAGACGATAATTGAACTAGCG
ACTAAGTGCCCTAATTTATCAAAATTATTCAGTGTGCAAGTGAAAGTGCTAAGAAAATGGAAAAGTTTA
ATTGAGAGACTGAGCTAAAGCTCCACAACCCTCACCTTCACCCATCAATGCTAAACTACAGACTCGTG
TCAACACATTTCTTGAAGCAGCCGTAAATCCATTACGAGACATTAGAATAAGGACTCAAGTAGGCTAAA
TTATGTGCCGCGACCGAGATATGATCCTCCTTCGCAGTGGAATAGTGAAATAAGAATTCTGAACGCATC
AGACTCTATCCAGTCTATGGTTTTAAAGTGTCAAGAATCGCGCCAAAACGGCGACCGATTCCGCCTTAC
CGCTTCGTTTTAGTTCATTCAATTTGTTTTATATAAAGGGATAAGCAATATTGTGGGTTCACTTATGGATT
TATTTATTGGACGAGCAGGGTCAGCGGGCCACTGAGGTCTCTCTCGACCCTGGCAAGCCGTCGGCGTA
ACATTTCTAGGATATTCGACTCCATTGTTGGCGCAGCAAGATATTGCATCGGTTACGGGCGCGAGTCAG
ACCCGTCAGACCGCCGCGTGTCGACCGGCAGCCGGCGCGACAACATGGCCGCGCCGGAGGGAGAGC
GCGAGGTGCGCAAGCGCAGGGAGAGACGGGACTCGGGGTGCGCGCATGAGAGAAAGGAACGCCGC
CCGCACAGCGAGGAGCGCGCGCCGCCGCCGCCGCCGCC

>TCONS_00018818 geneID=XLOC_003791 scaffold_1276:10435-12570(+) Unclassified

CAAAATAAAACCGAATCTTCAATTTGTGAAACTATCACTTATTCATCAGTGTTTGTCAATGAATCAAAAT
GATAGACGATAATTGAACTAGCGACTAAGTGCCCTAATTTATCAAAATTATTCAGTGTGCAAGTGAAAG
TGCTAAGAAAATGGAAAAGTTTAATTGAGAGACTGAGCTAAAGCTCCACAACCCTCACCTTCACCCAT
CAATGCTAAACTACAGACTCGTGTCAACACATTTCTTGAAGCAGCCGTAAATCCATTACGAGACATTAG
AATAAGGACTCAAGTAGGCTAAATTATGTGCCGCGACCGAGATATGATCCTCCTTCGCAGTGGAATAGT
GAAATAAGAATTCTGAACGCATCAGACTCTATCCAGTCTATGGTTTTAAAGTGTCAAGAATCGCGCCAA
AACGGCGACCGATTCCGCCTTACCGCTTCGTTTTAGTTCATTCAATTTGTTTTATATAAAGGGATAAGCA
ATATTGTGGGTTCACTTATGGATTTATTTATTGGACGAGCAGGGTCAGCGGGCCACTGAGGTCTCTCTC
GACCCTGGCAAGCCGTCGGCGTAACATTTCTAGGATATTCGACTCCATTGTTGGCGCAGCAAGATATTG
CATCAACGGCCACATTACAGACCGGTACAGAACCAACGTCTGTCCAATTGGGGGTCTTTTAGAAGCAA
TCATGGTACAATTGGAAATTGAGAAGGGTTACGGGCGCGAGTCAGACCCGTCAGACCGCCGCGTGTC
GACCGGCAGCCGGCGCGACAACATGGCCGCGCCGGAGGGAGAGCGCGAGGTGCGCAAGCGCAGGG
AGAGACGGGACTCGGGGTGCGCGCATGAGAGAAAGGAACGCCGCCCGCACAGCGAGGAGCGCGCG
CCGCCGCCGCCGCCGCC

>TCONS_00018829 geneID=XLOC_003796 scaffold_128:15757-46319(+) Exonic overlap (+)

ATAATGACATTCAGTCACTTTTGGTTCTGTAAGTACTAAGGTAAATTCATAAAAAATAAAGTATACATAG
GAGAGGCTGTAAGATTATTTTGAAATAGCTGTCAGTTTCAAGAAGGACAAGCTCCTACTCATATCGCAC
TCGTTTCTTCATATTTGTTATTTCGATATTACGTTAGGGTCAATTCAGACGTACCACAACCACAATCACA
CCACAACCACGCCGTGTGGTCGGGCGTATATTTTGTATTGCAAATGAATAATCCAATTCACACGTGCCA
CATTTTGCCGTTGTCATCGACCACCTGTGTAATACGACCACATCGCAACCACATCGCAACCACGTCGCG
TCGGCAACGTGCAACGTGCTATTAATTGCATACGACCACGTGGTTACCACATCGCAACGACAGCGACA

ACACAACCACATCGACATTTTGTCGCTGCCACAACGCAACTGCAAAAAGCCGCTGTCACACTATTCAC
ACGTAACCACAGCTTGACCACATTATGTCGCTGCGACGTGGTGTGGTTGTGGTACGTGTGAATTGACG
CTTATGTTATGCTATGCACCGTACTTACAATATCAATCTACCTCAGTATTGATAAACCATAGCTCATATCA
GTTCAATCTCAATCTATTTTATTTGAAATAGAATTTCTTGCAAACTAAATACGGATACTCACAAACGGTC
TCACTCTCATTCGTAAATGTTTAAGTGGTGATAAACGCCAACTTAAGTCCCAGTAATTCAAAATGGCGT
CGTTTTTTCTGCAGATAATCTCTAGTGGATCGGGCGCGGGCCCTCGAGCCTCGTGACACTTGCAAAGC
CTCCTCGCCACACCCGCCACCATTCCACAAGAAGGGCTGAGCCCGGGCGCCGCGAGGATGCGGCGGC
GGCGCGTGCAGCGATGAGCTAG
>TCONS_00018836 geneID=XLOC_003796 scaffold_128:56921-58043(+) Splice junction overlap
AGAACGGGAGCAAGCGAGAGGGCGAGTCCCGCGCGCGCTCCATCCTCAAAAACAAGGAGCGCGGCA
CCGACAGCGAGCTGCTCATACAGGAGACCACCTCGCAGGGAGCTAATGGAGATTCCGGCAGCGAGTC
CTCTCCGATCCGGCTGAAGGACTTCAACAGGAACCCGGGACAGAACCACAGGGGGGGCGGCGGCGA
GCGGGACCTGGTCGCCTAGCCGCCCCCGCGCCGCGTGGCGTCCGCGCCCCCGTGACGTCCACGCCCC
CGTGACGTCACTACACACTAGGAACCTGGTGGCCTAGCCGCCCCCGCGCCGAGTAGTGTCCGCGCCCC
CGTGATTTCAGCGACCCCGAGACGTCAGCACCCCCGTGACGTCTCTACACTCTAGGAACCTGGTCGCC
TAGCCGCCCTCGTGACGTCACTACACACTAGGGACCTGGTTGCCTAAGCGAACCTGCCTCGTGCCGTC
AACGCCCCCACACCGTGTGGCGTCAGCGCCCCCGTGACGTCACCACACACTAGCTATTACTTCTAGCG
CGCAATAATAACATTAATTAAATAATTTTAACATATCATTGGGCGATTGTGAAAGCTTGTTGTTGAATATA
GACGATTTTCATTTCGTAGGATTAAAAATACAACTATAAATAATGTAAAAAAATCAATTACATCATAAATT
ATAATTTATAATGTAATAATCGTT
>TCONS_00018912 geneID=XLOC_003796 scaffold_128:56931-58003(+) Splice junction overlap
CAAGCGAGAGGGCGAGTCCCGCGCGCGCTCCATCCTCAAAAACAAGGAGCGCGGCACCGACAGCGA
GCTGCTCATACAGGAGACCACCTCGCAGGGAGCTAATGGAGATTCCGGCAGCGAGTCCTCTCCGATCC
GGCTGAAGGACTTCAACAGGAACCCGGGACAGAACCACAGGGGGGGCGGCGGCGAGCGGGACCTG
GTCGCCTAGCCGCCCCCGCGCCGCGTGGCGTCCCCGCCCTCGTGACGTCACTACACACTAGGGACCTG
GTTGCCTAAGCGAACCTGCCTCGTGCCGTCAACGCCCCCACACCGTGTGGCGTCAGCGCCCCCGTGA
CGTCACCACACACTAGCTATTACTTCTAGCGCGCAATAATAACATTAATTAAATAATTTTAACATATCATT
GGGCGATTGTGAAAGCTTGTTGTTGAATATAGACGATTTTCATTTCGTAGGATTAAAAATACAACTATAA
ATAATGTAAAAAAATC
>TCONS_00018999 geneID=XLOC_003804 scaffold_128:488600-490122(+) Intergenic
GGAGACCGTGCGCCTCAACGCGGCCTGGCTGCGCCGCGACCTCGCGTCCACCACGGCGTATCTACAG
GCCTACCACTGAGCCACACGATGCACGGACCATTTGCAAGACTGAAACGACAACGAATTCCACAATTT
CGTTTCATTCGAAGATGATAGCCATTATAATTCTAGCTAGCTTTATTTATTTTCTGTGCATTGCTATCAAG
ACCAAAATACCTTCACTTATTTAATCGGCCAATCCATTTACTTTTCAATTTATAAACCAAGCATAGTGCC
ATGGGTTTTGAAGTAGTTATTAACAAAGGTGACCTGAACTTTTTTTGCATTTCTTATTTGCTTTTAATAA
ATGTTGATTATAGTTTATTCTTAATGAAATACGAGTTTATTCGGATACATATTAGAGCATACTATCTAAACT
CCATTTTTACCCAAAGACGGAGTTAATATAATTTACAGAAATGGCTATTTAATGTTATTTACCTGCGCGTT
TATCAGCAATGTTGTATTTATATTATGTTACTTAAAGATATACAATTGTACGTATCTGTAAAATATTCTGCC
ATTTATTTACCTACTGAAGAAGCTAAAGTTTACTGGCCTATTACGTGTAAATGTGTACAAAATACTTATA
ATTTAATTTATGTTTCATAATGTTACGATTTGTAACTTTGGTGCTGAATGCATTTTTTATTTCGTTTCATTT
TGTTTTTTTATCAGATAACCTGGCTACATTTACCCTAGTTCTGAAAGTTGAAAGATAGTAAAATCTCCCA
TTTTGCCCTGCAAAATCCCATAAGTTGTTACCATATCGCTTTTTAACTATTGAGATAAACAGATTCTTTTT
TTAAATATAATGCAAAGTGGACTCGATTCGGAATTAAGAGAAGTTGGCGTCTGTGAGTTGTTGTAATGT
TCGAAGTATAAAATTGAAGTTACCATCACCTAGTGTCTACTTGTTGTCTATCACTCACTTGTTTTGTAAG

ACTGTGTTATCGCAATAAAAT
>TCONS_00019000 geneID=XLOC_003804 scaffold_128:488730-490122(+) Intergenic
GCGGCCTGGCTGCGCCGCGACCTCGCCTCCACCACGGCCTATCTACAGGCCTACCACTGAGCCACACG
ATGCACGGACCATTTGCAAGACTGAAACGACAACGAATTCCACAATTTCGTTTCATTCGAAGATGATA
GCCATTATAATTCTAGCTAGCTTTATTTATTTTCTGTGCATTGCTATCAAGACCAAAATACCTTCACTTATT
TAATCGGCCAATCCATTTACTTTTCAATTTATAAACCAAGCATAGTGCCATGGGTTTTGAAGTAGTTATT
AACAAAGGTGACCTGAACTTTTTTTGCATTTCTTATTTGCTTTTAATAAATGTTGATTATAGTTTATTCTT
AATGAAATACGAGTTTATTCGGATACATATTAGAGCATACTATCTAAACTCCATTTTTACCCAAAGACGG
AGTTAATATAATTTACAGAAATGGCTATTTAATGTTATTTACCTGCGCGTTTATCAGCAATGTTGTATTTAT
ATTATGTTACTTAAAGATATACAATTGTACGTATCTGTAAAATATTCTGCCATTTATTTACCTACTGAAGA
AGCTAAAGTTTACTGGCCTATTACGTGTAAATGTGTACAAAATACTTATAATTTAATTTATGTTTCATAAT
GTTACGATTTGTAACTTTGGTGCTGAATGCATTTTTTATTTCGTTTCATTTTGTTTTTTTATCAGATAACCT
GGCTACATTTACCCTAGTTCTGAAAGTTGAAAGATAGTAAAATCTCCCATTTTGCCCTGCAAAATCCCAT
AAGTTGTTACCATATCGCTTTTTAACTATTGAGATAAACAGATTCTTTTTTTAAATATAATGCAAAGTGG
ACTCGATTCGGAATTAAGAGAAGTTGGCGTCTGTGAGTTGTTGTAATGTTCGAAGTATAAAATTGAAGT
TACCATCACCTAGTGTCTACTTGTTGTCTATCACTCACTTGTTTTGTAAGACTGTGTTATCGCAATAAAA
T
>TCONS_00019001 geneID=XLOC_003804 scaffold_128:488841-490122(+) Intergenic
GCGGCCTGGCTGCGCCGCGACCTCGCCTCCACCACGGCCTATCTACAGGCCTACCACTGAGCCACACG
ATGCACGGACCATTTGCAAGACTGAAACGACAACGAATTCCACAATTTCGTTTCATTCGAAGATGATA
GCCATTATAATTCTAGCTAGCTTTATTTATTTTCTGTGCATTGCTATCAAGACCAAAATACCTTCACTTATT
TAATCGGCCAATCCATTTACTTTTCAATTTATAAACCAAGCATAGTGCCATGGGTTTTGAAGTAGTTATT
AACAAAGGTGACCTGAACTTTTTTTGCATTTCTTATTTGCTTTTAATAAATGTTGATTATAGTTTATTCTT
AATGAAATACGAGTTTATTCGGATACATATTAGAGCATACTATCTAAACTCCATTTTTACCCAAAGACGG
AGTTAATATAATTTACAGAAATGGCTATTTAATGTTATTTACCTGCGCGTTTATCAGCAATGTTGTATTTAT
ATTATGTTACTTAAAGATATACAATTGTACGTATCTGTAAAATATTCTGCCATTTATTTACCTACTGAAGA
AGCTAAAGTTTACTGGCCTATTACGTGTAAATGTGTACAAAATACTTATAATTTAATTTATGTTTCATAAT
GTTACGATTTGTAACTTTGGTGCTGAATGCATTTTTTATTTCGTTTCATTTTGTTTTTTTATCAGATAACCT
GGCTACATTTACCCTAGTTCTGAAAGTTGAAAGATAGTAAAATCTCCCATTTTGCCCTGCAAAATCCCAT
AAGTTGTTACCATATCGCTTTTTAACTATTGAGATAAACAGATTCTTTTTTTAAATATAATGCAAAGTGG
ACTCGATTCGGAATTAAGAGAAGTTGGCGTCTGTGAGTTGTTGTAATGTTCGAAGTATAAAATTGAAGT
TACCATCACCTAGTGTCTACTTGTTGTCTATCACTCACTTGTTTTGTAAGACTGTGTTATCGCAATAAAA
T
>TCONS_00018931 geneID=XLOC_003806 scaffold_128:506331-508052(+) Intronic
ACCCGACGCAGTATGCCGCCTGCAGTCAGTCCACTACGTTTTGCGGCCAACACATGCCGAGATAAATG
GAAATGTTAGGATAGGAGCAGCTATAATTTATTTTTCTAATGGTGCGAATTGTGAGCTGTGGAATTGTGG
ACGAAAATTGTATATTTGTTTAAGGATTCGCCTCCGCAGTAACGCTTGCTGCACGCGCGGAGGCGTCGT
AGTCAATCATGTTAGATATTATGTTATCATTATCATTTTATGTACAGACAATTCAGAGGACATTATTCACT
CATTGAAACATAAAATGCTACGTTTACGAACTTTATAGTTAAGATTAATCAAGTTACATGTGTAGTGTGT
ATGTACAGTTTCAGTAATATTTCCGAATAGGAGTATAATTAATACTGATACTTCATCAAACTTTCTATTCA
AATTTGTACCTACCTATTTAGGTATACTTACATAATATAACGGTTTGTAATCACTTGTTATTAATTATTTTAT
AAGTGTTAATGAGCCATGTTCTTCAAGGTATTATCAAAAATCATAAGAAGATATTAATGTAAATAGGTGT
AGCGAAATTTAGCATAAAAGACAATAGAGAGAATATAAAGTGATTATGTATCGTGGTAATAAAATTATTT
TTTGAAGTTATCTCTAACGACGAGCGTATTTGAAATTAGAAATGGCAAATCAACATTGCATGTTCTAGT

GTTGTGCGCAATACAGAGGACGCGGGCGTTTCAGTGTATTGCTTGTAACACAATTATTATATTACGATTA
TTATAGATTATTTGCCCTAGTTTGCGTTTAATTATTTCAGATATTGTTGGTAACAGATATTCGGAAATGTTT
GGTAGGTAAATGTTAATGTCTTGCTCTCTTTATATTTCATGTAAGAGGGAGACAAATTGATGTTTTCCAG
CTTCCATTCATAAAGGTTTTAATGTTAACAATTTTGAATAAACTAGGTTTAAATGGGTAATTGTAATGTG
GATGTCATCCTCTCTTTAATAGTATTCGTATGCGATGCGACCATGTAAGTTAATCATTACAATAGTGTAGA
CGAGTATAATACAAAAAAATCCCGTTTTTTGTTTGACCATGTCCTATTTGAAAAACTGTTGATTAGTTTC
TCACGACGCAAACTATCATATAAACGTTGATGTCTATTGTATTCATTATTTAGTACATACGCTGCTAAAAC
AGCTGCTTAAATTAGCATTAATAAGAATTTCTGAGAAGAAAACGAGTACCTGTAACGTTTACATTTTCAT
ACTCAATAGATTATTAATGGTTTTCTCATAACACCGCATCAACTATTATATGAATTTGTACAGCATAAAT
>TCONS_00019110 geneID=XLOC_003811 scaffold_128:565617-627506(+) Intergenic
AACGGAGCTCCGCCGCGGCACGCGACGATCAGAAAAAAAAACAAACTGCGACAAACTTTTCGAACG
CATTTGAAATTATTCGCGCGGCAAGAATTTTGAATATCGAACTAATCGAACGTTCTGTGGATTTTGAAA
CTTACGCCGGTTTGCTTTTTATTGTTGAAAGAAGTTTTTGATCTGTGAGTGAAATGGAGTGAGTGGTTT
TTGTTTCAAGTTAATAACAAATTAAAGTATAATTAAAAGTGGTTTTGTGGAGTTCAAGCGTCAAACTAC
AACTGGATCGTTAAAGAGCGCGACAATGCAGGGCGCCCCCCCAC
>TCONS_00019204 geneID=XLOC_003885 scaffold_1286:840-1804(-) Intergenic
CAGAGCTGCACGGTCGCATGATCTCGGTGGAGAAGGCCAAGTCGGAGTCGGAGTCGGCGTCGCGGCG
CGCGCCCGCCCGCCACGAGCGCCGCGGCAGCAAGGAACGGAAGGACGACAAGGATGAGGACGGCA
AAGAGAACGCGGACAAGTCTGGCGAGTCGAAGGGCAAGAAGGAGGGCGACGCGTCCGGAGAGGAG
GGCCGCGCCGCCTCGCACGCGCGCGATAAGAACGACAAGGTAAG
>TCONS_00019407 geneID=XLOC_003902 scaffold_129:332840-334696(+) Splice junction overlap
CCAGGATAGCTCCCGTCACCGAACAGACTCGGACTCGGACCTGTGCATCGACGAGCAGTCGCCCGCG
CCCTCCACCATCGACCCCCAGCTTTCAATTAATATACGGAACGGATATGGCCATGTTTAACCAAGTAGC
CGTAGGCACCTGTCGACTGTGATATTGATATGTTATAGTCTGAGAAGAAACCGGTTCGCATAAGGTATAT
TGCTGGTGACATTATTAAGATCTCAATGTTAGTATTGATGTTGCATAGTAAAGACTTGAAGTAACCCAGT
TTAAAAACTTTGTGAGCCTATACATAAAAGCTGATACAAAAACAAAAATTAAGGTAGGTATTTATGATT
GTTATCGTTACGGTTTATGAAAAAAAACATTCACGTTCATATAGCCGCAATATAAAAAATATTGACAGAC
TAGCTTTTAAAGACAAGCTATAAATAATTGTTTCGGCTTGAATGATTCAGTATCGGCATGGCATCAGCCG
CTACGCAGAAGACTGTCCCGCCTAAAAATGCGAACCGATTTCTCCGGCAGACACTGTAGTAAAATATAT
AGGAAAATTATTTTTTAATTATTGCACGCAGCAAATGTGGCCTGGAGTCGTGGACACTCGCATCTCTGT
CGAAGGGATCACAGGTAGTAATATATGAAACTATATCGACAACCCACCTTACGGGATCAATGTTATAAA
GCCGGAAAGTGCTATGATGTAACCTGGCGCTTAAGACATAATGTTATTGTGACGGTGGATTGTAAGCCG
ATGTTTATGTAAGGGTGAGGCCCCATTGTTACGTTGCGTGGCATGCGTCGTTGCAAAGGAAATGGTCAT
AAAAAATGGGGCCTCGCCCTAACTTGTTGTTAATGACGGATAACGCAGCGGAAATGGAAACTGTATTG
TGTATTTTAGGTATAAGGCAGTATAACAGTCGTGTGTGTACAATACCATACTTGTGCATACGTACATCTC
CTATAGATCACCCTTGGCAGAGAAGCTATACATAGAGTCTGATATTGGTCACTAGTTTATCTTTCCGGGT
GTTCTTCTTACCATTTCTGGCACATTTTTGAGATATAGAGCTAATATTATTTTGTGTTTTTAGCTAAATGT
GTGCTTAGTATTAGTTTAAGTACCTATATACCTACTAGGTAAAATTAATT
>TCONS_00019582 geneID=XLOC_003921 scaffold_129:376959-377597(-) Splice junction overlap
GTGTGACCAGCTGAATGGCAAATCACGGAGTAAAGTTCATTGGAAGCTCTTGCCCTGACACTGTCAAG
CTCTGCAACTGCATCATCTGAATTTTCTCCAAAACATTCCACATGGGTGATGCGACGGCGACGCCGAC
GACGTTGCGACGCAAAAACGGCAATAGCCCCGAAATGAACCTCTGTAGCCAGCCTTATATCACCATCT
GTCAATGTCAAAAACAGAGTGACTGGCTTGTAAGCTGATTTTGAAACATAGCCAGCACAACGTCGTCG
TATCTTTCGCTTTTGTGAAGGCTTTTTGTTCTTTAAATTTCTGTTTCCTTGGATTAGTTAGAAAATTTAGT

TGGCAAACAGTTTTTGCTTGTGCTGTGACGAAAGCATGTAACCAACTACTACACAGTTTGACTAATATT
TACTTGGATACTTCTAAAAACCAATACAATGAGTTATCAAGATGAACCTGGTGGCTACGATGTTGGCTC
ATTCCCTAAGGACTTCAGCTACGGCCCATTTGAGCAGAATGGGGATGGGGACAACTCGTCACTGCAAG
ACG
>TCONS_00019369 geneID=XLOC_003924 scaffold_129:474546-475485(-) Exonic overlap (-)
TGGATTTTAGGTTCTTTTTTGAGTGCCATACATTAAAATTTTATTGTTTTACATCTACATGCAGGTGAATA
TGTACATCTAAGTACCCAGGCAGGTTCAATAGCTATGCATGAATGACTGCCACCTACAGTTGAGATTAC
GAGCAGTAACTCAGTCGAGATCGTCTAACTTGACATGTATAGAGTCGCCAAGTTAACATCTATAAGGCA
CTTCAACACCTATTAAATAATGTGGATACAACGCAATTCTTATCACAAAGCATTTGAATATTGCACCGTA
CTTCTATCTCATAGAGATGACTAATTTTCAAACGCCCTGCTTTTAAAAACGCTACAAATAAAAATGCGTT
TGAAATCAAGACCGCCCTGTCACATAAATAATATCTATGAGGTTACAATCTCCTATAACTACGTTCAATT
TACGTCGCGTTTAAAACATGCCAGACACCAGCTGCCACGAAAGAAATCCCTCTAATCTTCAACAAGAT
CAGTCTTTCGAGTGTTCAATCGGATACGAGGTTAGTAATACCCTGTATTATGCGGTTGATCGCCGACAG
ATCCCAAACGGGGGAACTACATCGCATTAGCTACCACAAACACACACGAGCAGAACTAAGTTTTTAAA
CAAGTGATGCGAACTCCTTGGCTTGGACCTGCTTAGTAGCTGCCTTAATCCGAGGTCTTCCACTAAGG
AGGTCGCATGTCTGTGGCTGTTGGTGGCTAGTTCCCGTGCGTGTAGTCGCGCAGGTCGCGGTTGCCGA
GCGGCAGCGTGGGCCGGTAGTTGTGAAGTAGCTCCATGGCCTCCATGGCGGTCGCCTCGCCGCAGCGT
AGCTTGCGCATCGTCG
>TCONS_00019701 geneID=XLOC_003964 scaffold_1292:8965-10751(+) Unclassified
CGAGCCCAGCCGACGCCGGCGGTTACTTCTCCTCAAGAAGACAAGAAGTTCTACTCGAGGCCCAACA
GCAGCGGCTCCCGGTCGTCGTCCGGATCCGGTTCATCATCAGAGGAGAGCAGCTCCGAAGAATCTTCC
GATGAAGAAGCCAGTGACGACGAGGAGGAAAAGAAACCAGAAAAGAAAAAAGAGACAAACAATAT
CAAATCAACCACCACCTCGTCTGCGAGTGACTCTGAGAGCGACAGTTCAGACTCGGAGGAGAGCTAC
GAGAGCTCAGACAACAGCGATGATCCGAAAAGCAAAGGAGATAAGACTGACTCTAAGCCCACCACA
ACCACAACGACCACCAAAAAGAACGAGAAGTCGAACTTGGAGCTCCTTCTAGAGTTGGACTCGGTGG
CTTCTTCCCTGCCCACTATGACGCCCACTTGCGGGGGACTGCTGTCGCCCAGTGCCGGCCCTAGTGGA
GACGGTATGCAGTAAATTAGAGTCATAGACTAACATTAGAATAAT
>TCONS_00019702 geneID=XLOC_003964 scaffold_1292:9440-10793(+) Unclassified
GTCCGGATCCGGTTCATCATCAGAGGAGAGCAGCTCCGAAGAATCTTCCGATGAAGAAGCCAGTGAC
GACGAGGAGGAAAAGAAACCAGAAAAGAAAAAAGAGACAAACAATATCAAATCAACCACCACCTCG
TCTGCGAGTGACTCTGAGAGCGACAGTTCAGACTCGGAGGAGAGCTACGAGAGCTCAGACAACAGC
GATGATCCGAAAAGCAAAGGAGATAAGACTGACTCTAAGCCCACCACAACCACAACGACCACCAAAA
AGAACGAGAAGTCGAACTTGGAGCTCCTTCTAGAGTTGGACTCGGTGGCTTCTTCCCTGCCCACTATG
ACGCCCACTTGCGGGGGACTGCTGTCGCCCAGTGCCGGCCCTAGTGGAGACGGTATGCAGTAAATTAG
AGTCATAGACTAACATTAGAATAATAAGGTATATGGTAAATAACACGTTGGATACTTTCCCAAATAA
>TCONS_00019712 geneID=XLOC_003965 scaffold_1293:8218-9570(+) Intergenic
TTAATTTTATACCGTGAGTTTAGAGTTCACATATTTACAAATATGTTTAAGAAAATACGAACATTGTGAT
GCATACTAAATTTGATTGGGTTATTGTGTGAACGGCGACATAAGCACGGCAACAAACTTAAAACAAAC
AGGTAAAGAGAGCAACATTCCTACAATATCGAGCGTTATCAAGGCAATTTGCACAACCAGTTTAAATAT
TTCATAATTCAACTTTTTAATTCGACATGTTTCAGTGGCGTAGACCTTGGACACTAAAATGTTTGCAAG
CTTAAAGAGTAAAATTAAGGAGGAAACTGGAAATGATATCAGCAAACTTACAAGCAGCTGGCGGGCG
GGCAACTTGTTGGGAAGAATATCGTTTAGGGATGAATCGACGGCAACAACAACACCAAGCAGCTCCG
GGGGAATGGGAGACACATCCACATCAGAATCAATATCACCTCAACTGGAGGCCTACCTGTCCGAC
>TCONS_00019740 geneID=XLOC_003970 scaffold_1296:422-828(-) Exonic overlap (+)

ACGAGCATGATATTCATATAACATATTCGTCCTATACGCTCACGACGGAGCGTTCATCCGCGTGGCGTCC
CAAGGAGGGCGCGGCGCCGCGCGAGCGCGAGGGAGGGTTCCGCGACCGCGAGCGCTACCCGCCGCG
CGACGGACCCCGGGATGGACCACGTGATGGACCCCGGGACGGACCACGAGACGGACCGCGCGACCG
TGACGGACCTAGAGACAGGGACGGACCAAGAGACGCTAGGAGGGACGATAGGGATGGACCGAGGAG
GGATGATCGGTG
>TCONS_00019735 geneID=XLOC_003971 scaffold_1296:1637-2491(-) Unclassified
CCCGGACCGCCGCCCCGCCGCCAGGTCGCCCGACCGCCGCGCGCCCGCCCGGTCGCCTGATAGGAGA
GGACCGCCCAGGTCGCCCGACCGCAGGGGACCACCTAGAGATGACAGACGCGACGACCGCGGGCCG
CGCGACGACCGCCCGCGCGACGACGGCGTGTGGCGCCGCAACACCGACTGGGAGCGAGACAGGGCT
CCAGCAGCCTACGGAGGTCGCTCC
>TCONS_00019745 geneID=XLOC_003971 scaffold_1296:1984-2529(-) Intergenic
CCCGCCCGGTCACCGGACCGCAGGCCGCCGGCCAGGTCCCCGGACCGCCGCCCCGCCGCCAGGTCGC
CCGACCGCCGCGCGCCCGCCCGGTCGCCTGATAGGAGAGGACCGCCCAGGTCGCCCGACCGCAGGG
GACCACCTAGAGATGACAGACGCGACGACCGCGGGCCGCGCGACGACCGCCCGCGCGACGACGGCG
TGTGGCGCCGCAACACCGACTGGGAGCGAGACAG
>TCONS_00019765 geneID=XLOC_003973 scaffold_1297:3492-6656(+) Intergenic
GAACCTATGTCAATATAATAAAGACGTCCTTTTCTTACTACACTCTTCTTGTCAATGTGAATGAGCACAA
ATAGGTACATAATATAAAAGTTTCTCGTTTTTAGAAGTACGCAGAAGAAGCACATACTTTATAGATAAAT
GACCAATCAAATCATTATACAATTTGTAACAAACCAATAAAGCACAAAACATATTGAAATAAAAGATGA
TAAAGTTCTGAAAAGTATTTTATTTACGAAATTAATATACAATAATTCTTGTTCATACATCAATTACTTTT
GATAGCTCACTCAGCCGTCGTTCAACCGACACTTACATAAATAAAACCAGATTATTATCATTTAAACGT
GGACAATTCAAGTGACGTCATATTTGGTCCAAATCTCTATGAAACTGTAGTCCAAATAGAGAAAGAAAT
ATCCACCGAAGGCATATTGATAAGTTCTTCCACTAGAAGAGACACGTTTCAGAGAAACTTGGACGTTA
AAACACGCATCTTACAGTAGAATACAGATTGTTTAGTAAGTACAGACAAATAAGCTTACGCCTCCGCGA
TCGATGGGCGATCACGCACGTCACATAGAACTACGCTTGCATCTAACGGGTACATAACAGTTTGAACTA
ATCTCGGCCCGACCTGAATATATAGGGGACGGTGTTACATATTTTGTTCATCGTAACCAACTTCTACGTA
ATGTTTATTAAAATGTATGCTTACTACTTAAGAGAGTTATCCATTACTTCCAATATTATTACAATTTATGTC
ATTTGCTTTGCGCACGGCCCCAATGCCAAAGAGTATAAGCTACGATTATTAATTCACTAGAGTGTGGAG
TAAGCACGCTGGTTGCTACGAGTGGCCGTAGTCTGTCACTGCGCCGGCGGCCGCCGCGCCCGTGGTCT
TTACATAAAACTGGCAAAAATCTTAACGCTAATTTGGGACCGCTGCTGCCGAGAGTCGCCCTGAAGGC
ATCGAGAGTACTTTCACCCTAGTTACTTACACCCAACTGGATATAATTGGTGTTTCATTGATTCAGTCAT
TACATTTCATATGCATGCATAGCAAAACTGGCTTCAACAAGTCAAACTTACATTAAACGGACCCCCGAT
GCCTTCAAGCCGACCAGTCAACGGCTGAACTATTGAATGCATACAAGCAGACACTCACACATACATAC
AGACATTATAGAAACGTTCGCGCTAGACACTCCCGGACCTAACGTTAATAAATTACATGGCTTCTCGAA
AACTTACTATAAAAATGTGATTAATGAAGATGGTACTGAACAATCGTCCAGCAATTGTCTAAGCCAATG
TTCAGCCAACTAGTTTTATTATATTTATAACTTAATAAAAATCCTTAAAAAGCGAGACGCAAATGTAACC
AGAGTGGCGGTGCCGCGCCGCCGAAAATTAAATTATAATAATTTACAATTAAAGTTACATAAATCCATTG
GGAAAGTATTGTTTTTAAAAGAAACCTATTAACTATTAATGTATGTAAATTATCCTCCGCGTGTCCAGTC
TGTAAACAATGACAAAAATGTTCATTCTAGCGTCGAATAGAATAGAAGAAACAAAAAAATCCTCCGAG
AAAAAGATACCGATAAAACGAAACGTTTTATCGACACGTAATAGCGATAAACAGAGCCAAATTTAACA
AATTATTGCAAATAACAAGTAGCACTTTTGCAACGGACACTGTTGCGTATTGAGAATCATTTGATGTGT
TTGGTGATTGACACCGCATTTAAATTTAATACTTTTGCAAGTTTCGTAGGAACGATATCTCTGTAGTTTA
GTGCATTTAAATTTACGGACAATCACCAGATACCACAAATGAACATTGATATTACCCTCGAATTGTTATA
GTGAGCTATGAAAACAAACGTACTTCATACGACAGTAAACGGAAATACAAAACTAAAATGCATAAACT

CTTCCATTTGCGGCCCACTGTAGATCCAATCCGCTTTTAGTTCTTATAAATGGAGCTGTTAAAACTAAGA
ATGGATTGAGATCCTGCAGTCTTCACAAAACGATAACAAATAAGGTGGAAGAACAATGAAATAGGCCA
CTAATTACATGATGTGAAAGATTCGATTTCATATGACGGCATCGTACGCAACGTATGTGTATGAAATTAA
TGTCATCGATTCGATTGATTTTGTTTTAAAAGTTCGCTGGACTCGCTTTGTACGATCCGTCATATAAACA
CTTTCCATAACGCTATTCATGGTGAAAAACATGCCGACACTCAACCATAAAACAGTTCATTCTTAGAAT
TAGGCAAAACTTATACAATTCTACATACTGCATCGTATGTTTTCACTGTGAAAGCGTTATAGGGAATGGA
CATAGATTCAGCTCACAAATACAACTCAAGGGCTACAACTGATAATGCTAAATACAGAGAAATAACCTT
TAAACCTTCCCAACTACCGTCTCTTATAGCGTTTCAGTCCTGTAGATAGCCTTATAAGACGCCACTGTAT
TGAACAATATTAAATTTATCAACTTTGAGTATTCTTTCCACATTGGCATTTCATGTTACAAGTTTATTGTT
AGTTTCAAGCAGTGGCGCCATCTACAACATCATCCAACGTCCCGCAATCCTAACGACTTTAGACAGGTA
TCTTGGTATAACAGGCTTAGAAAGGCCCTCTTCAGAAGGCCTAAGTCCGGCTCTAAAACGCTATAAGC
TGACGGCAGTAGAGGTTCTCGGGTAAAGGAGACACAACTGAAGGAGCTCGCGGCCGCATC
>TCONS_00019802 geneID=XLOC_003984 scaffold_13:151060-153919(+) Unclassified
TATTTGTCGAGTCAACGACGCGACGCGTCGAAGCACCGTTTTTACTGGCGATCATGGGTAACGTTGCA
GTCGCGTCGGTTGAGAGTCGCGTAGTTGGAGAAGGGCTATGTTTGTATGCCAGAGTTTGAATTTCGATT
GAACTGTAATACTGGGAGGGCTTATTGAATGCTGTCCAATACTGCTGTTAAATGAAATTGTTTTAAAAC
GGTGGAAAATGTTTCGCCTCTGGTAAGGCATTATATATCTGCAAATTCGATATTGCTGGGCACGGTAATA
CGGTTTGATTCGGATATAATCTCATTAGTTCCTGATGTTTATGTTTTATGTGACCTAATATGAAGTCATACT
GTATGTCCGATGTTATGTTGAATGTCTAAGGTTGCCCTTCCAATGAAAACTCCTATCAATTAGAATTAGA
AACCTTGAAAAAAGTGTGCTAGTTTTCAATCGGGCATAGATTTATAAAATTCATACTAAATAAAAATAAT
ATTAAACCTTTCTTACCTAAAAGAGCTTGAATTTTTAAATGAATTTATGATATAATTTCCATGTTACCTTT
ATCTGACAAAATTATTATCACATTTGATAAATAATTCTCGCTGATTACTAAGCAACAAAATTACCTCTTTC
AAAAAGTAATTTTCCGGTTAAAAAATAATAAATGATCACAGCTGTATATTTAATGTTGAGCTGCTCGAG
AATAAGCCCATTGTCCTTTTAATTTTAATCGTTAATTATAAATTAATATTAGTAAAAACTTAATATGGAATG
CACTGGATATGCACTGTAGTGATAGTTAAACATGACTAACCCAATGAGTATAAAATGTGCCCCAAATGG
ACAAATACAATCTTGGTTGATTCCAGTGATTCTTACAGTTAATAAAAAGGTAATGAAAACAAAAAGGAT
CCAAGAATATGTCTTTTGAAAAGCGACATAATCGCATGTATTTTACTAAATATCACAATAATCTTTATACT
TACTGTAATACTAATAATGTAGGAAATATGCCAATCCCATGTATAAGACAGTAGGTTTAGACCATAGATAT
TTTAGGCTGTCGACATCAACGCGTCATTAGATTTGTAACAATGTCTTTTGATAGTGTAAGTATCCCTTGA
CATAAAATGAGCAATAATGTGATTAAAATGCAATAAAACATGGTAACAATATCTACAGCATAGAAACTT
TTTAGCGATCAAGCTGAAGGCAAATTGTGGTAGATGGATCTTCCACTTGATCACTCATAATGTTGCTCCT
ACGATTTGTTTATACAGGACAGCCAAAGTTGCATGTATACAGCCTTCTGACATTTCATTCAATTAATTTA
ATTTAGCAATGTAGTCTTCATTCTAGCTGAAAACTAACAATTGCTTGAATATTATATCGCGTTTTGTTTTA
AACTATAACTTTCACATGTAACGTTTTTGTTTGTGAATTTGTTAGATGTATGTAACTAGGTTTATGTTTGA
GTTTAAAGACTGTTGAGCGAGGTTGCTATGTTTGTTCTCTATTGAAGCAATACTCATTCATTATTGAAGT
TTGCGTGACAAAACTATGTTAGCAGTAAGCGCAGGCCACACCGCCAGCTTACGATGCGAGCGAGCTA
GGCGAGTGGATATCGTTTCACTTCTTTACATACTCTTCACACCGCGAGCTACCGACTAAACCCGCAACC
GCGGCTAGTTTAGCGTGCCTAGCTGGCTTTGTAGCCTACGCTTTAGTCATTGGACTATTTCTGGAAAAA
TTTGCTACTACATATTTAAAATCAATAGATTTCACCTTTATAAAATACAAAATTACTTCAAATCAAATCAT
ATATTTTATTGGTTGGGAGTGTGTTGTACTGGCAGTATGGATGTATTTAGATTATTTTACTCAGCTAAGGA
AACCCTCGACAGTCCCATCCAGTAGGTTTTAGAAACTAAAACTTGATAGCTGTGATGAGGCTTTTATTA
TAATTTAATTTTGTAGTCAAATTCATACAACTTAAATTCAACATTCCCTCGATAAGATTCAGTATTCTTTC
AATGTACAAAGTATTCGTTGTTTAAAATCCTCAAGGTGTATCTTTAAATATAAATTACTTGTGACATCACT
AATCATTAAAATGTATGTGCGCT

>TCONS_00020486 geneID=XLOC_003985 scaffold_13:156265-156782(+) Intronic
CTCTATGGTAATTAAAAGACAACAATGGTGGATTTGAAAGTCCGCCATAACTAAATCTTATTTCTAAAAT
AGTGACGTTCGTATTATTTTCTAATTTGTGTTAGATATCTCTGTTGTGTAGTTATTCTTGTTTAATTTAATT
ATTTTTAGTTTACCCAATTCACGAGCCGTGCAGGCCGTGGACACCATGGAGTCTCCAAGAAAATTTAA
CGACTGCTATTTTTACTACTATTCCACGTGCACGAAGG
>TCONS_00020240 geneID=XLOC_003985 scaffold_13:156284-156782(+) Intronic
CAACAATGGTGGATTTGAAAGTCCGCCATAACTAAATCTTATTTCTAAAATAGTGACGTTCGTATTATTT
TCTAATTTGTGTTAGATATCTCTGTTGTGTAGTTATTCTTGTTTAATTTAATTATTTTTAGTTTACCCAATTC
ACGGTGAGTGTATGATTACATGCGGTTTATTGATTTGACACATGTGTGTTTTTTATTTTGGAACTTTTCG
CAGAGACATGCCTTGCAAATTGTCAAAATGAAAAATTCATAATTTTGCTGTTCGTCAAATTAAATCCCA
CGTGTAATTTTAGTGATCCCATAAACAAGTGATAGGGGCGATAAGCCGTGCAGGCCGTGGACACCATG
GAGTCTCCAAGAAAATTTAACGACTGCTATTTTTACTACTATTCCACGTGCACGAAGG
>TCONS_00020318 geneID=XLOC_003986 scaffold_13:173519-175953(+) Exonic overlap (-)
ATTTTTTAAATGTATGTAAAAGTAAGTAAATAACTGACAAAGTGACAAACATAAAAAGTCTTAACAAAA
TCGCACTCTTTATTGTTAGGAATTTATAGTTCGACTGTAAAGTAGGCCAAGTAAGGGGTAGGTTAGCTA
AATATATTATACCTTCTTAGAAATTAGTTTACCTACTCAATATCTAAAAGTTACAAACACAGACGAAATG
AAAAAGGCGATTAGTACCGAAAAAATCATTTATTATATTATACTGTAATTAAAACTTCCAACATGACACA
ATCATTTCATCGGTATTATATCTACTTAGCATAAGATAATAAATTCGAAATGAATACCACTAACCATCCAC
GGGCTAGCAGATTTTATATAACTTATGTTTGTGCGTTTGTAGCACAGTGGAAAATGAATCACATCTTCTA
AAATTAGAAAAACATATTTACATTAACATTTGGAACGATTTCAGTTACATAAAATTGAGATGAGACATTC
AAAAGTAGGGTAGGTACATCACTAGTAATTATAGGTACCTAAGTAGGTAAGTATATAAAAATTACTAAAC
ATTTACAATTTAGTTACCCAAAGGTGGTAATAATTTACATCTTACACATAGTTGCCCTGTTTACTTATAGA
TTTTTTACAGACAGTAGAGATTCTTCATCTACACAAGTATTTACATAGTTTTTAAGTAGGTACTGTAGGG
AATCAATTGGTACCTATTTATTAAAATGAACACTAGGTACCTAAACATTAAACACATCCGTTTTGTGATC
AGCACTTACCCAGGTATGTGCATAAGGAGAAATCCACATACAGATTGTGAGTTTAGTTAGTTCCGCAAT
TTACGCCATAAAAAAAATTCACAATAAAATCTTAATATTATTATCATTTATGTACTTACTACATACATACATT
GTAGTATATATAGTAAACATACAAGTTCTTCAGGACTCATGGCGAATTGCTTTAGCTATTGTGCTAAATA
AATAATTTCATACAAATCCGGATAACCTGTGACTGATTCTCCGAATCAGTTCTGACCAAAAATATAAATT
AAATACTTACTGTACATACATAATAGGTATGAGGTACAAGTACATCATACACAAATTTTAATAAATTCTTA
TTCTATCTAGAAGTAAGTACATTATTATACGTTCTTATGTCTAACTATCTACTGATGAAACGCCTTTCGCC
CACTCAAATTCATCACAAAAGACTGCATAAAATATAAGTAATATTTTGTACAATCAATTTATATTAAAAC
AAATATCATGATACCCAAAATAGGTTTGAAAAATAAAATTTGAGCCTAACTTTTGTGCTCTTCTATGATT
TCAGTTTGTGGCATCTAATAGATATGTTGATTCTTTGAGACAAACAATAATTTATAATAATAAGATATACA
TAACTATAATGGTAGTTCACACAAAGTTGAGCACCTATAGGTAGGTACTAGTACTATAGGTGTTGTGGG
GCACAACACTAATTACCTACAGGATTTAGGACGTATGCTCCTGAAAAATACATAAGTACATACATACATA
ATATGCTCATGACTGAAATATCCTCAGAACCTTTTATGATAGATCGAAAAAGTTTTAATTTGACCAGTAG
CTCATATAAAATTGTCAGGAGTATTATTTACACCCTGGATACTATGTGTAAGTACCTATTGAGTTTCCTCC
AACAACTACCCAACTAAGTAGGTAAATTTCATTTTGCATCAGAGAAAAACCAAAGCGAATCTGCATAA
CATATACCTAAGAATTGGTAGCGTGAACCTTTGCGGTCAACATGTTTGAATGAAGTTCTAATAAAATGA
CGTGTAACTTATCTATACCTCGTTTCAGCTAGAGATGCTTCCTGTGTCGTTTTGAGTTTTACGGTTTAAA
GAGATCCTTTGTTTAAATTCTAACGATTTTGTCTATAATCCATTCCACGGGGAAAGGCATCAGATGCAA
ACAAAAACTACCCTTATTCGAATGTAATAAGGGTTCTGTCAGATCATTTACATAAGTATCGACTTAACTA
TTGTATGAGTCGCATATCCAATGAAATGTTATTAATAAACACAGAATTAGCAATAATATTTCTGCGTAATT
TGGAGCTGTTATAGCGAAGATCTTGGCTGAAACGAGGTACATTGAAGAGTAGAAGCGAGCTTAGGTCC

ACCAAGGTCACTGCAGTCGCGACCAGTACTGCTGGGACTCCTGGCCCGGCACCGCCAGTGGGACCGA
CACCCCCG
>TCONS_00019897 geneID=XLOC_004029 scaffold_13:286405-289346(-) Unclassified
GTCTTTAAATAACATAAAACGGAATACCAGATACCAATAGATATAAAATAGCAAAATAATTACATAGGAT
ATAATTGTACCAGCTATAATAGGAGGCCATTTTAGCACGATAGTGAGAACTTAGACTTTCTTAAACTAAT
ATAAAAATTAAATATAATTTGGCACTTGTGTGGCCGAAAGTCGTTGGTTATAGACAATATAGTATATTGTA
TGTAAAAGAAAAAATTGATGATAGCTTGTAATAACAAATAATTAAAATGCAAAAATATAGTAAATATATA
GTATAGTATCTTACGCAACACATTGACATAGTTCATGTAGAGAAGCTCTTACTCTAAAAATAACTTAAAC
CTACATAAAAGACGTAATAGTTAGTAGTATACTTCCTAACATGCATTAACACTGATATTCAAAACATTGA
GATCAATATTTGTCTTATCCTATTTGTATAACGTTATATTAAATTGTAATTAAGAAAAGTGTAAAGCAAAG
TTATTCAATTAACTTTCACATACGATTTTACAAATATAAGATACAAACTTATGTGTTTACATACATACGGT
ATAGTTTCGTATATGTAGGTATATTAGGATAGAAAAGTTCGATCAATAGATATATAGAAATATATCAATAC
AAATAAATAAATACCATCACATTTTTCCTTTGCCTAGCTACCATTTTATCAAAATATATAAATATATTTTCG
ATTCTCATTTGTCTAAGACCATTTTACAAACATGATATTTTAAATATGAGTATTTTAGTTACCATAGGACC
CTGTAATTAAGTCAGGGTTGGGTTTAGATATACATAATCTAAAATTAGAAGATTAATATTGTATAAGTACA
TAAATACAGAATTTTATAACTACAATGTACTTAGAGAAATCACAAAAGAATGCTTGTGAACCTACGAGT
TCAACACAAAATTATTGAATATTTTGTCTAAAAATTGTGCCTTCTACCGTAAATATTTGTGCCATAATTGG
CGCCATCTTAGATTTTGGATCTACAATAACAGACATCAATAAACATTTAACATCAATATTTTTCTCCCCAA
TACACATACTGTTGTAAGCTTACTCTTACTACACCAATACTTTTGTTAATCTTTTTAACTTTTCGGTTAGT
AAATCTCTTCACGTCAAGTATAATTCGAACCATGACGAACCCTGCGACTCACGGGCTTATACACTTCTT
CTGTAAGTTAACTACATTGCCTACAAAAATATATTACAAAAGTATTTTTTATGAAATTATGTGCAGGGTG
TGGCAGGAAATGTATTATTTTTTATATTTTATATTACAATACCAAAGGGGGATTTTTCAGTTTACCATACC
CTTAGACGATTTAAGTAGATAAGATATATTTTTTCACACCGGCTACAATGAAATCCACCCACTGGAGGG
TTACTCCATACTGACGAAAAACGAACGAACGAGTGCTGTCGTGCAATCGGCACGTTTAATACGACGAG
ATCGAGTCGTGGCTCAGCAGCGCTCCAAAACTTAGTTTTCCGTCATATCTAGTGACACCCCTGCACCCT
ACACACATTCCATACAAAAAATACTTTTTTTATAATAAGTCTTAACCTAAGAAATTGCCGTTCGGCTTT
GTCATCTAAATTCAAATTAATTTTGTTTTAAAAACATGTAATTTTTTCTAGAAACTCAATCGTTTGGTTTT
CCCGACTCCGTTCTGGAAATTTTGACTTTTTATGTTATATGAAAAATAAATACTTTCACTTATACATACTA
TACAAAACGGCAACTTCATAGGTAAAGACCTAATACACTCACGGGCAATGAAAAAGTTCAAGTGAGA
AAAGCACTCAAACTCCGATCGGAAAAATTTGATTATCGGCTCTCCGAGTCTATAAACTTGTATTTTCAA
AACTTATCACTGTATGAAGCCAAAATATAAATAAATACTTAAATGAAACAGAGAAATCTACATAACATAA
TGACGCCATCTGTAATATTTACGTACATTTAATGAAGCATCAAAACATTACCAATTAAAACGTAAGTATT
TTGTTAAAATTTCCTTTTGTACACTTATATTTCATATTTGTATGAAATAAATGAATAAATAATACATGTTTA
AAAAAACTAGGGCCATTTCGTGTCAGCATTTTGTAAGTCGACCTTTCTCATTCCCCGTGAGTGTTTAGG
CAACGTCCAACTTACATGACGAGGTGTACAAACGGCCTCGACCATCAGTGTATCTCGTAGGTGCTGGT
CACTCGCGCGCCCGCGCACTGCTCGCGGCCTATCTTCTTGCCGTTGACGAGGAGTTCCATGGGGTTGT
CCACGCGGATGGATTTGTAGGTCTCCT
>TCONS_00020422 geneID=XLOC_004030 scaffold_13:304440-327534(-) Exonic overlap (+)
AACCGGCCAAGTTGCGAGTCGGTTCGCGCGCTGCTTTGAAATAACTGGCCGCCTAGACGTGCCGATAA
AAAAAAACATCTATGAAAACAAAAGAATAAAAGCAAACGTCCTTCCCAATTCAAATTTTTGGTGTCCT
GTGTGTTTTTAGCAGTTTTGAAAGTTTTGCCGTTTAGTCAAATTTCCGGACTGTTCCATTGTTAGTGAAA
TTTGAAAATTATTATCAATGTTGTTGTTATGAAAGGATGTTATCAATGAACCAACCGCTGCTTCTGGGAA
AACAGTCACTTTGAATTTTCATGGGAATAAAACGCTGGTGGATACCCCAGGTGTGATGGTGCAGGTGC
TGACAGTGCTGCTGCTGGCGGTAGCACTGACTCGCGCCGCCTCCACCTTAGATTCAGAACTAGATGTC

GGCAAAGCTATTAATATTTTTATGAGGTGAGTTGGTCAATGTTTTTTTCTTTATATTTCCTGATATCGGGA
CATCTAATTTGAAAATTTAATATTAATAACTAGGTAGGGATAGAATGTTCGAAAAAAATACCGG
>TCONS_00019815 geneID=XLOC_003993 scaffold_13:457292-464557(+) Splice junction overlap
CCGCCGACCGCGCCGCCACTCGCACGCGCCATGCCTCGCGCACCCACACCTACACGCATTTACTATGT
ACGCCACTGAGACGCAGAAATATGTGGAGTCATCGCTGGGCTGGGCCGGGGAGCGGGGGGAGGACTC
GAGCTACGACTCGGAGTGCGACGACGAGGGGGGGCACCGCGCCGCCTCCCGCACCCCCTCGGACAC
CCTCGCTGCCGAGTTCGCCGAGTACGTGAC
>TCONS_00020199 geneID=XLOC_004039 scaffold_13:512526-512902(-) Intronic
GATAAAATGGCGTCTGTGTCTGTATCTACGTTAGTGACTGAGTTAAATTAATAATTTCTTAGAATAGTTC
AGTGTTTAGTTCAAATAGTGAGATTTAAAAAGATTCTAGAAGTGTACTTAATATTTTACGACTTCGAAA
GGGCCTCTTAACGTTTTTATAAGAGTGCGATGTCCGAGGAGACAGAACGCCTTGGCGGGAAGTCACC
GCCGGCAGTCGCTGAGAATGGCAGCCCGAGCGGTGGAAGCG
>TCONS_00020290 geneID=XLOC_004040 scaffold_13:595626-596723(-) Unclassified
GTGCATACATGCAATCAAGGCTTTATGTACATATCTTAGTAGATACGAAAATATGTGGGTATCATCGATTA
CCTACATATGGTAGTCCTGGTTAAATGTGGAGATTTACCCCTCAATAGCAAAAAAAGGCGTTATTGTTT
GTATTTCTGAATGAAACTAGGCAAATAAATATATGTATAAAAATAAATTATGTGTAACCTACCTATAAGCC
TACCCATTATTATTTCAGGTGAAAACCCAGCCTATCGATGTAATGTGCCGATAAACTATCATGACTGCAT
TGATGTAATGCGCTAAAAAACTGCAATGGCTCCGAATACATGATTACCAGATAACAAATCCTAGCTCAG
CTTACATGTGTTCTTCAATAACGTCGCAAGCTACAGTGGCTGTTCATAATTTTTGCAAGTTCTACACCTA
CAGGATTCAGTTATCTGGGCATTATAGATACCCTATTATCACCTACCTTCCTCACCTACCTTATTAAGTTG
CATGAAATGTCAATG
>TCONS_00019836 geneID=XLOC_004004 scaffold_13:825777-826432(+) Exonic overlap (+)
GTAGACTTCCTTGACAACATTTTATATGAATCAAGGAATTTAAGTATTAAAACTCCTAAATGCTAACATG
AGTAAAAAGTTTAACAATACATTTATAAGTGAAAACACATCAGAGATCCCCAGCAGTAGCCGTGTCGG
CGCGGAAGCCGCCTGCCCCAGGGATGGCCAGCCCGCTCCAAGTGCTGTCCGAAGAATTGAGAGGAAA
GACGCGGAGGTACAATGTTCGATGAGCGACCCGGGACTCCATAGGGAACCGATGCTAGTCATCGTCGA
ATCACCAGGCGTCAGTAGCCCCGGCTCCACAGAAATTGTCGTTGACGGCAAACAGAACCATAATGCCC
>TCONS_00020510 geneID=XLOC_004044 scaffold_13:869453-872374(-) Unclassified
GGACCAGCTAGTCGACTGTGCACACACACGAAATATACAACGATAGACCACAACAGTCTTCCCCTCTA
ATTAGATAACAGATTTTATTCCTTATTCCTATATCTGAGATTTTATATCTATTTCATACGTGAGATCCTGTG
AATTGTTGACAATACTTTATTGCTAATATAATTGTTTGTATTATTCCATAAAATGTTGCTCAACGTGTAAG
AGTATTGAATTGAGTGAGTTTTGATGTATTTAGGATATTGTAGTTTGCGTTATTTAGTGGTATTGTATGCA
ATATTAAGTAATCAGATTTAAATCGTGATTGTTTATGAAACCGAGGGTTATGTTAGAGAGTGGCAGCTAG
CGTTACATGGGGGTTAAGATAAAACATTAGATGCCTACGCATGCAGACGAATTTAATCCTATCGGTTGC
CATGTTCACTTAGGATTTTGACACTATCAATTTCTGGCAGCTGATTGGTTGTTAAATGGGAGCATGTGCT
GCCATCTAGTCTTGATATAGACGTGTGCAATTTGGTTGGTTGTAAGGAAGTTCACCTTTGTAAGATGTA
AGTTGTATTAATACTCGCACCCATGACTCGAAGTAACCTTCTTTCAGAATGCTAGGGCACCTTGTTTGT
AAGTAATTATGATTCTACTCTTATGTCGCTTACATGAATATCTTTTGGATGCGTAGAACATAACAAGACC
AATTTTAGCAAGACATCGTACCGATAGGGACTTTAAATAGAACACCTATATATTGGGAGTGGATGATCAT
AGATAGAGTCACGCAATGATAGAATGTATGGGTTAACCGTGTGCATAAAACTAGGTATATGGCCAACGA
AATTACCTCGATGAAGTGTGTACATTTTAAATTTTGCTGTACCCTTTGGTAGCCCTGTATTGTCCTAACT
AGAGAATAGTATTGAAATTCTCAGGGTGCTAAAATCATTGCCACTAGGAATTACTTATTCATTTTTTTAA
AATAAATGAATAGCTGATTATGATTACTTACACAATATTTTAGCGGCACTCATGTGCCATAAATTTGCCA
GGCTGCCTTGTATTTGCCACATGTTTGAATATACATACGTTTCTAGGTTTTCTTTTAGTTAAAAAAACCTA

TGTCCTACTTAATTTTATTGTATTTCTGTACCTACATTATTCTTAAAAGGTTTTTTTCCTACAGCTTCGACT
TTTAGTTTTGTGTATTTATGCTATTTTTATATATTCTTGTGTGGTAAGATAGTTTCTAAAGCCATGATAATA
ATATTTGTTGTGTGAATTATATTGATAGGTTCATTTTTATGCTTATATTTTTATAGCACCATGAGTTGTGTG
AATCAACACTGCAATTTAGTTTTCATTGTCTGAGATTATTATAGCTAAGGCGGTTTATATGTATATTTTATT
TATATATTTGTTGATTAGATGATTTTGTAGATAACTGTTACTCTAAAACATGCCTTATGACGATAATTAATA
GGTAGTTGAGGTCTAAATGGTTTTGTGTTCATGTGATTTTTATTACTCAGCAAACTTATGAGAGAATTGC
AATACTTACATAGTTAGTAAAAATTTAAGACTTCTATTTTCTTCCTCTATATATATCTCGGTATTCAATATT
AATAAGATTTCTAAGTTTAGAAATGTGATAATTAGGATGTTTTGATGCATTTAGTAACTTTCAGAGTTTG
CTGGAATATATAAAATTACATTTTCTCTTTAAATTACTGTACAGAATTAGACATACACGCATATCCAACAC
AAATAAATTAGGGCTTGATGAAATTATTTTCTTATAGAAATAGTAATTTTGGAATTTATTATTTGAGCTAA
GAGCTCTCCTTACTGTTTCCAATTTAGTTTAAAGCAGTTTTAGACTAAAATAACTCGTCCAAGTTAATCT
TATAATTTGTAAACGAATTTATTATGTTTATGTTGGTAAAATGACACTTATGGAGTGATGTAAAATACCAT
ATCAGGCATTTATACTGTAGTTAAGTATATACATGTATTAAATCTTTAATTTATTGGTTAATGAAAATATAG
ATAATGTCATCACATTTCATGGCTTTTCCTCGCTAAAGTCACAAAATGCATTATTTTGTTAAGTGAATTTT
TAGGCCGGATTTTTATCAGTCACATAATTTATCTGGGGAATAATAAAATTGTGAGATTGTCATTAGAGGA
ACGTAAGTTTCGACAATGGCAGCATGCTCTTTATTCATCACACAACGTTCTTTTGTTAAATTAAAACCT
GCCCTTAATTTACAAAATGTAGAGAGAAGTGACACAACTGTATTGTTTGTATATAGGTTAGCTTTTGCA
GATTCGTCCATTAATAATTACAAGTAAGATTTTATTTCACATTATAATTTAGATTTTGGTTAGGT
>TCONS_00020104 geneID=XLOC_004044 scaffold_13:869521-872458(-) Unclassified
GGGCCAGCTAGTCGACTGTGCACCCACACGAAATATACAACGATAGACCACAACAGTCTTCCCCTCTA
ATTAGATAACAGATTTTATTCCTTATTCCTATATCTGAGATTTTATATCTATTTCATACGTGAGATCCTGTG
AATTGTTGACAATACTTTATTGCTAATATAATTGTTTGTATTATTCCATAAAATGTTGCTCAACGTGTAAG
AGTATTGAATTGAGTGAGTTTTGATGTATTTAGGATATTGTAGTTTGCGTTATTTAGTGGTATTGTATGCA
ATATTAAGTAATCAGATTTAAATCGTGATTGTTTATGAAACCGAGGGTTATGTTAGAGAGTGGCAGCTAG
CGTTACATGGGGGTTAAGATAAAACATTAGATGCCTACGCATGCAGACGAATTTAATCCTATCGGTTGC
CATGTTCACTTAGGATTTTGACACTATCAATTTCTGGCAGCTGATTGGTTGTTAAATGGGAGCATGTGCT
GCCATCTAGTCTTGATATAGACGTGTGCAATTTGGTTGGTTGTAAGGAAGTTCACCTTTGTAAGATGTA
AGTTGTATTAATACTCGCACCCATGACTCGAAGTAACCTTCTTTCAGAATGCTAGGGCACCTTGTTTGT
AAGTAATTATGATTCTACTCTTATGTCGCTTACATGAATATCTTTTGGATGCGTAGAACATAACAAGACC
AATTTTAGCAAGACATCGTACCGATAGGGACTTTAAATAGAACACCTATATATTGGGAGTGGATGATCAT
AGATAGAGTCACGCAATGATAGAATGTATGGGTTAACCGTGTGCATAAAACTAGGTATATGGCCAACGA
AATTACCTCGATGAAGTGTGTACATTTTAAATTTTGCTGTACCCTTTGGTAGCCCTGTATTGTCCTAACT
AGAGAATAGTATTGAAATTCTCAGGGTGCTAAAATCATTGCCACTAGGAATTACTTATTCATTTTTTTAA
AATAAATGAATAGCTGATTATGATTACTTACACAATATTTTAGCGGCACTCATGTGCCATAAATTTGCCA
GGCTGCCTTGTATTTGCCACATGTTTGAATATACATACGTTTCTAGGTTTTCTTTTAGTTAAAAAAACCTA
TGTCCTACTTAATTTTATTGTATTTCTGTACCTACATTATTCTTAAAAGGTTTTTTTCCTACAGCTTCGACT
TTTAGTTTTGTGTATTTATGCTATTTTTATATATTCTTGTGTGGTAAGATAGTTTCTAAAGCCATGATAATA
ATATTTGTTGTGTGAATTATATTGATAGGTTCATTTTTATGCTTATATTTTTATAGCACCATGAGTTGTGTG
AATCAACACTGCAATTTAGTTTTCATTGTCTGAGATTATTATAGCTAAGGCGGTTTATATGTATATTTTATT
TATATATTTGTTGATTAGATGATTTTGTAGATAACTGTTACTCTAAAACATGCCTTATGACGATAATTAATA
GGTAGTTGAGGTCTAAATGGTTTTGTGTTCATGTGATTTTTATTACTCAGCAAACTTATGAGAGAATTGC
AATACTTACATAGTTAGTAAAAATTTAAGACTTCTATTTTCTTCCTCTATATATATCTCGGTATTCAATATT
AATAAGATTTCTAAGTTTAGAAATGTGATAATTAGGATGTTTTGATGCATTTAGTAACTTTCAGAGTTTG
CTGGAATATATAAAATTACATTTTCTCTTTAAATTACTGTACAGAATTAGACATACACGCATATCCAACAC

AAATAAATTAGGGCTTGATGAAATTATTTTCTTATAGAAATAGTAATTTTGGAATTTATTATTTGAGCTAA
GAGCTCTCCTTACTGTTTCCAATTTAGTTTAAAGCAGTTTTAGACTAAAATAACTCGTCCAAGTTAATCT
TATAATTTGTAAACGAATTTATTATGTTTATGTTGGTAAAATGACACTTATGGAGTGATGTAAAATACCAT
ATCAGGCATTTATACTGTAGTTAAGTATATACATGTATTAAATCTTTAATTTATTGGTTAATGAAAATATAG
ATAATGTCATCACATTTCATGGCTTTTCCTCGCTAAAGTCACAAAATGCATTATTTTGTTAAGTGAATTTT
TAGGCCGGATTTTTATCAGTCACATAATTTATCTGGGGAATAATAAAATTGTGAGATTGTCATTAGAGGA
ACGTAAGTTTCGACAATGGCAGCATGCTCTTTATTCATCACACAACGTTCTTTTGTTAAATTAAAACCT
GCCCTTAATTTACAAAATGTAGAGAGAAGTGACACAACTGTATTGTTTGTATATAGGTTAGCTTTT
>TCONS_00020037 geneID=XLOC_004044 scaffold_13:870302-873040(-) Exonic overlap (+)
CGAGTTCAAGTGGCAGGCGCGGGCCAGCTAGTCGACTGTGCACCCACACGAAATATACAACGATAGA
CCACAACAGTCTTCCCCTCTAATTAGATAACAGATTTTATTCCTTATTCCTATATCTGAGATTTTATATCTA
TTTCATACGTGAGATCCTGTGAATTGTTGACAATACTTTATTGCTAATATAATTGTTTGTATTATTCCATAA
AATGTTGCTCAACGTGTAAGAGTATTGAATTGAGTGAGTTTTGATGTATTTAGGATATTGTAGTTTGCGT
TATTTAGTGGTATTGTATGCAATATTAAGTAATCAGATTTAAATCGTGATTGTTTATGAAACCGAGGGTTA
TGTTAGAGAGTGGCAGCTAGCGTTACATGGGGGTTAAGATAAAACATTAGATGCCTACGCATGCAGAC
GAATTTAATCCTATCGGTTGCCATGTTCACTTAGGATTTTGACACTATCAATTTCTGGCAGCTGATTGGT
TGTTAAATGGGAGCATGTGCTGCCATCTAGTCTTGATATAGACGTGTGCAATTTGGTTGGTTGTAAGGA
AGTTCACCTTTGTAAGATGTAAGTTGTATTAATACTCGCACCCATGACTCGAAGTAACCTTCTTTCAGAA
TGCTAGGGCACCTTGTTTGTAAGTAATTATGATTCTACTCTTATGTCGCTTACATGAATATCTTTTGGATG
CGTAGAACATAACAAGACCAATTTTAGCAAGACATCGTACCGATAGGGACTTTAAATAGAACACCTATA
TATTGGGAGTGGATGATCATAGATAGAGTCACGCAATGATAGAATGTATGGGTTAACCGTGTGCATAAA
ACTAGGTATATGGCCAACGAAATTACCTCGATGAAGTGTGTACATTTTAAATTTTGCTGTACCCTTTGGT
AGCCCTGTATTGTCCTAACTAGAGAATAGTATTGAAATTCTCAGGGTGCTAAAATCATTGCCACTAGGA
ATTACTTATTCATTTTTTTAAAATAAATGAATAGCTGATTATGATTACTTACACAATATTTTAGCGGCACTC
ATGTGCCATAAATTTGCCAGGCTGCCTTGTATTTGCCACATGTTTGAATATACATACGTTTCTAGGTTTTC
TTTTAGTTAAAAAAACCTATGTCCTACTTAATTTTATTGTATTTCTGTACCTACATTATTCTTAAAAGGTTT
TTTTCCTACAGCTTCGACTTTTAGTTTTGTGTATTTATGCTATTTTTATATATTCTTGTGTGGTAAGATAGT
TTCTAAAGCCATGATAATAATATTTGTTGTGTGAATTATATTGATAGGTTCATTTTTATGCTTATATTTTTAT
AGCACCATGAGTTGTGTGAATCAACACTGCAATTTAGTTTTCATTGTCTGAGATTATTATAGCTAAGGCG
GTTTATATGTATATTTTATTTATATATTTGTTGATTAGATGATTTTGTAGATAACTGTTACTCTAAAACATGC
CTTATGACGATAATTAATAGGTAGTTGAGGTCTAAATGGTTTTGTGTTCATGTGATTTTTATTACTCAGCA
AACTTATGAG
>TCONS_00020035 geneID=XLOC_004044 scaffold_13:870435-872546(-) Intergenic
GCGCGGGCCAGCTAGTCGACTGTGCACCCACGCGAAATATACAACGATAGACCACAACAGTCTTCCCC
TCTAATTAGATAACAGATTTTATTCCTTATTCCTATATCTGAGATTTTATATCTATTTCATACGTGAGATCCT
GTGAATTGTTGACAATACTTTATTGCTAATATAATTGTTTGTATTATTCCATAAAATGTTGCTCAACGTGT
AAGAGTATTGAATTGAGTGAGTTTTGATGTATTTAGGATATTGTAGTTTGCGTTATTTAGTGGTATTGTAT
GCAATATTAAGTAATCAGATTTAAATCGTGATTGTTTATGAAACCGAGGGTTATGTTAGAGAGTGGCAG
CTAGCGTTACATGGGGGTTAAGATAAAACATTAGATGCCTACGCATGCAGACGAATTTAATCCTATCGG
TTGCCATGTTCACTTAGGATTTTGACACTATCAATTTCTGGCAGCTGATTGGTTGTTAAATGGGAGCATG
TGCTGCCATCTAGTCTTGATATAGACGTGTGCAATTTGGTTGGTTGTAAGGAAGTTCACCTTTGTAAGA
TGTAAGTTGTATTAATACTCGCACCCATGACTCGAAGTAACCTTCTTTCAGAATGCTAGGGCACCTTGT
TTGTAAGTAATTATGATTCTACTCTTATGTCGCTTACATGAATATCTTTTGGATGCGTAGAACATAACAAG
ACCAATTTTAGCAAGACATCGTACCGATAGGGACTTTAAATAGAACACCTATATATTGGGAGTGGATGA

TCATAGATAGAGTCACGCAATGATAGAATGTATGGGTTAACCGTGTGCATAAAACTAGGTATATGGCCA
ACGAAATTACCTCGATGAAGTGTGTACATTTTAAATTTTGCTGTACCCTTTGGTAGCCCTGTATTGTCCT
AACTAGAGAATAGTATTGAAATTCTCAGGGTGCTAAAATCATTGCCACTAGGAATTACTTATTCATTTTT
TTAAAATAAATGAATAGCTGATTATGATTACTTACACAATATTTTAGCGGCACTCATGTGCCATAAATTTG
CCAGGCTGCCTTGTATTTGCCACATGTTTGAATATACATACGTTTCTAGGTTTTCTTTTAGTTAAAAAAA
CCTATGTCCTACTTAATTTTATTGTATTTCTGTACCTACATTATTCTTAAAAGGTTTTTTTCCTACAGCTTC
GACTTTTAGTTTTGTGTATTTATGCTATTTTTATATATTCTTGTGTGGTAAGATAGTTTCTAAAGCCATGAT
AATAATATTTGTTGTGTGAATTATATTGATAGGTTCATTTTTATGCTTATATTTTTATAGCACCATGAGTTG
TGTGAATCAACACTGCAATTTAGTTTTCATTGTCTGAGATTATTATAGCTAAGGCGGTTTATATGTATATT
TTATTT
>TCONS_00020036 geneID=XLOC_004044 scaffold_13:870435-873047(-) Exonic overlap (+)
TGCAGACCGAGTTCAAGTGGCAGGCGCGGGCCAGCTAGTCGACTGTGCACACAAGCGAAATATACAA
CGATAGACCACAACAGTCTTCCCCTCTAATTAGATAACAGATTTTATTCCTTATTCCTATATCTGAGATTT
TATATCTATTTCATACGTGAGATCCTGTGAATTGTTGACAATACTTTATTGCTAATATAATTGTTTGTATTA
TTCCATAAAATGTTGCTCAACGTGTAAGAGTATTGAATTGAGTGAGTTTTGATGTATTTAGGATATTGTA
GTTTGCGTTATTTAGTGGTATTGTATGCAATATTAAGTAATCAGATTTAAATCGTGATTGTTTATGAAACC
GAGGGTTATGTTAGAGAGTGGCAGCTAGCGTTACATGGGGGTTAAGATAAAACATTAGATGCCTACGCA
TGCAGACGAATTTAATCCTATCGGTTGCCATGTTCACTTAGGATTTTGACACTATCAATTTCTGGCAGCT
GATTGGTTGTTAAATGGGAGCATGTGCTGCCATCTAGTCTTGATATAGACGTGTGCAATTTGGTTGGTTG
TAAGGAAGTTCACCTTTGTAAGATGTAAGTTGTATTAATACTCGCACCCATGACTCGAAGTAACCTTCT
TTCAGAATGCTAGGGCACCTTGTTTGTAAGTAATTATGATTCTACTCTTATGTCGCTTACATGAATATCTT
TTGGATGCGTAGAACATAACAAGACCAATTTTAGCAAGACATCGTACCGATAGGGACTTTAAATAGAA
CACCTATATATTGGGAGTGGATGATCATAGATAGAGTCACGCAATGATAGAATGTATGGGTTAACCGTGT
GCATAAAACTAGGTATATGGCCAACGAAATTACCTCGATGAAGTGTGTACATTTTAAATTTTGCTGTACC
CTTTGGTAGCCCTGTATTGTCCTAACTAGAGAATAGTATTGAAATTCTCAGGGTGCTAAAATCATTGCCA
CTAGGAATTACTTATTCATTTTTTTAAAATAAATGAATAGCTGATTATGATTACTTACACAATATTTTAGCG
GCACTCATGTGCCATAAATTTGCCAGGCTGCCTTGTATTTGCCACATGTTTGAATATACATACGTTTCTA
GGTTTTCTTTTAGTTAAAAAAACCTATGTCCTACTTAATTTTATTGTATTTCTGTACCTACATTATTCTTAA
AAGGTTTTTTTCCTACAGCTTCGACTTTTAGTTTTGTGTATTTATGCTATTTTTATATATTCTTGTGTGGTA
AGATAGTTTCTAAAGCCATGATAATAATATTTGTTGTGTGAATTATATTGATAGGTTCATTTTTATGCTTAT
ATTTTTATAGCACCATGAGTTGTGTGAATCAACACTGCAATTTAGTTTTCATTGTCTGAGATTATTATAGC
TAAGGCGGTTTATATGTATATTTTATTT
>TCONS_00020038 geneID=XLOC_004044 scaffold_13:870435-873409(-) Exonic overlap (+)
GAGTTCAAGTGGCAGGCGCGGGCCAGCTAGTCGACTGTGCACACAAGCGAAATATACAACGATAGAC
CACAACAGTCTTCCCCTCTAATTAGATAACAGATTTTATTCCTTATTCCTATATCTGAGATTTTATATCTAT
TTCATACGTGAGATCCTGTGAATTGTTGACAATACTTTATTGCTAATATAATTGTTTGTATTATTCCATAAA
ATGTTGCTCAACGTGTAAGAGTATTGAATTGAGTGAGTTTTGATGTATTTAGGATATTGTAGTTTGCGTT
ATTTAGTGGTATTGTATGCAATATTAAGTAATCAGATTTAAATCGTGATTGTTTATGAAACCGAGGGTTAT
GTTAGAGAGTGGCAGCTAGCGTTACATGGGGGTTAAGATAAAACATTAGATGCCTACGCATGCAGACG
AATTTAATCCTATCGGTTGCCATGTTCACTTAGGATTTTGACACTATCAATTTCTGGCAGCTGATTGGTT
GTTAAATGGGAGCATGTGCTGCCATCTAGTCTTGATATAGACGTGTGCAATTTGGTTGGTTGTAAGGAA
GTTCACCTTTGTAAGATGTAAGTTGTATTAATACTCGCACCCATGACTCGAAGTAACCTTCTTTCAGAAT
GCTAGGGCACCTTGTTTGTAAGTAATTATGATTCTACTCTTATGTCGCTTACATGAATATCTTTTGGATGC
GTAGAACATAACAAGACCAATTTTAGCAAGACATCGTACCGATAGGGACTTTAAATAGAACACCTATAT

ATTGGGAGTGGATGATCATAGATAGAGTCACGCAATGATAGAATGTATGGGTTAACCGTGTGCATAAAA
CTAGGTATATGGCCAACGAAATTACCTCGATGAAGTGTGTACATTTTAAATTTTGCTGTACCCTTTGGTA
GCCCTGTATTGTCCTAACTAGAGAATAGTATTGAAATTCTCAGGGTGCTAAAATCATTGCCACTAGGAA
TTACTTATTCATTTTTTTAAAATAAATGAATAGCTGATTATGATTACTTACACAATATTTTAGCGGCACTCA
TGTGCCATAAATTTGCCAGGCTGCCTTGTATTTGCCACATGTTTGAATATACATACGTTTCTAGGTTTTCT
TTTAGTTAAAAAAACCTATGTCCTACTTAATTTTATTGTATTTCTGTACCTACATTATTCTTAAAAGGTTTT
TTTCCTACAGCTTCGACTTTTAGTTTTGTGTATTTATGCTATTTTTATATATTCTTGTGTGGTAAGATAGTT
TCTAAAGCCATGATAATAATATTTGTTGTGTGAATTATATTGATAGGTTCATTTTTATGCTTATATTTTTATA
GCACCATGAGTTGTGTGAATCAACACTGCAATTTAGTTTTCATTGTCTGAGATTATTATAGCTAAGGCGG
TTTATATGTATATTTTATTT
>TCONS_00020105 geneID=XLOC_004044 scaffold_13:870440-872906(-) Intergenic
CCAGCTAGTCGACTGTGCACCCACACGAAATATACAACGATAGACCACAACAGTCTTCCCCTCTAATTA
GATAACAGATTTTATTCCTTATTCCTATATCTGAGATTTTATATCTATTTCATACGTGAGATCCTGTGAATT
GTTGACAATACTTTATTGCTAATATAATTGTTTGTATTATTCCATAAAATGTTGCTCAACGTGTAAGAGTA
TTGAATTGAGTGAGTTTTGATGTATTTAGGATATTGTAGTTTGCGTTATTTAGTGGTATTGTATGCAATATT
AAGTAATCAGATTTAAATCGTGATTGTTTATGAAACCGAGGGTTATGTTAGAGAGTGGCAGCTAGCGTT
ACATGGGGGTTAAGATAAAACATTAGATGCCTACGCATGCAGACGAATTTAATCCTATCGGTTGCCATG
TTCACTTAGGATTTTGACACTATCAATTTCTGGCAGCTGATTGGTTGTTAAATGGGAGCATGTGCTGCCA
TCTAGTCTTGATATAGACGTGTGCAATTTGGTTGGTTGTAAGGAAGTTCACCTTTGTAAGATGTAAGTT
GTATTAATACTCGCACCCATGACTCGAAGTAACCTTCTTTCAGAATGCTAGGGCACCTTGTTTGTAAGT
AATTATGATTCTACTCTTATGTCGCTTACATGAATATCTTTTGGATGCGTAGAACATAACAAGACCAATTT
TAGCAAGACATCGTACCGATAGGGACTTTAAATAGAACACCTATATATTGGGAGTGGATGATCATAGATA
GAGTCACGCAATGATAGAATGTATGGGTTAACCGTGTGCATAAAACTAGGTATATGGCCAACGAAATTA
CCTCGATGAAGTGTGTACATTTTAAATTTTGCTGTACCCTTTGGTAGCCCTGTATTGTCCTAACTAGAGA
ATAGTATTGAAATTCTCAGGGTGCTAAAATCATTGCCACTAGGAATTACTTATTCATTTTTTTAAAATAAA
TGAATAGCTGATTATGATTACTTACACAATATTTTAGCGGCACTCATGTGCCATAAATTTGCCAGGCTGC
CTTGTATTTGCCACATGTTTGAATATACATACGTTTCTAGGTTTTCTTTTAGTTAAAAAAACCTATGTCCT
ACTTAATTTTATTGTATTTCTGTACCTACATTATTCTTAAAAGGTTTTTTTCCTACAGCTTCGACTTTTAGT
TTTGTGTATTTATGCTATTTTTATATATTCTTGTGTGGTAAGATAGTTTCTAAAGCCATGATAATAATATTTG
TTGTGTGAATTATATTGATAGGTTCATTTTTATGCTTATATTTTTATAGCACCATGAGTTGTGTGAATCAAC
ACTGCAATTTAGTTTTCATTGTCTGAGATTATTATAGCTAAGGCGGTTTATATGTATATTT
>TCONS_00020154 geneID=XLOC_004044 scaffold_13:870454-872714(-) Unclassified
CGAGTGAGTAACAAGAGCTATATTCGATTGTAGCTGTGAGTGAATGATGCAGACAGTTTATTAGGCAGG
CGCGGGCCAGCTAGTCGACTGTGCACCCACACGAAATATACAACGATAGACCACAACAGTCTTCCCCT
CTAATTAGATAACAGATTTTATTCCTTATTCCTATATCTGAGATTTTATATCTATTTCATACGTGAGATCCT
GTGAATTGTTGACAATACTTTATTGCTAATATAATTGTTTGTATTATTCCATAAAATGTTGCTCAACGTGT
AAGAGTATTGAATTGAGTGAGTTTTGATGTATTTAGGATATTGTAGTTTGCGTTATTTAGTGGTATTGTAT
GCAATATTAAGTAATCAGATTTAAATCGTGATTGTTTATGAAACCGAGGGTTATGTTAGAGAGTGGCAG
CTAGCGTTACATGGGGGTTAAGATAAAACATTAGATGCCTACGCATGCAGACGAATTTAATCCTATCGG
TTGCCATGTTCACTTAGGATTTTGACACTATCAATTTCTGGCAGCTGATTGGTTGTTAAATGGGAGCATG
TGCTGCCATCTAGTCTTGATATAGACGTGTGCAATTTGGTTGGTTGTAAGGAAGTTCACCTTTGTAAGA
TGTAAGTTGTATTAATACTCGCACCCATGACTCGAAGTAACCTTCTTTCAGAATGCTAGGGCACCTTGT
TTGTAAGTAATTATGATTCTACTCTTATGTCGCTTACATGAATATCTTTTGGATGCGTAGAACATAACAAG
ACCAATTTTAGCAAGACATCGTACCGATAGGGACTTTAAATAGAACACCTATATATTGGGAGTGGATGA

TCATAGATAGAGTCACGCAATGATAGAATGTATGGGTTAACCGTGTGCATAAAACTAGGTATATGGCCA
ACGAAATTACCTCGATGAAGTGTGTACATTTTAAATTTTGCTGTACCCTTTGGTAGCCCTGTATTGTCCT
AACTAGAGAATAGTATTGAAATTCTCAGGGTGCTAAAATCATTGCCACTAGGAATTACTTATTCATTTTT
TTAAAATAAATGAATAGCTGATTATGATTACTTACACAATATTTTAGCGGCACTCATGTGCCATAAATTTG
CCAGGCTGCCTTGTATTTGCCACATGTTTGAATATACATACGTTTCTAGGTTTTCTTTTAGTTAAAAAAA
CCTATGTCCTACTTAATTTTATTGTATTTCTGTACCTACATTATTCTTAAAAGGTTTTTTTCCTACAGCTTC
GACTTTTAGTTTTGTGTATTTATGCTATTTTTATATATTCTTGTGTGGTAAGATAGTTTCTAAAGCCATGAT
AATAATATTTGTTGTGTGAATTATATTGATAGGTTCATTTTTATGCTTATATTTTTATAGCACCATGAGTTG
TGTGAATCAACACTGCAATTTAGTTTTCATTGTCTGAGATTATTATAGCTAAGGCGGT

>TCONS_00020200 geneID=XLOC_004044 scaffold_13:870454-873044(-) Exonic overlap (+)

AGACCGAGTTCAAGTGGCAGGCGCGGGCCAGCTAGTCGACTGTGCACCCCCACGAAATATACAACGA
TAGACCACAACAGTCTTCCCCTCTAATTAGATAACAGATTTTATTCCTTATTCCTATATCTGAGATTTTATA
TCTATTTCATACGTGAGATCCTGTGAATTGTTGACAATACTTTATTGCTAATATAATTGTTTGTATTATTCC
ATAAAATGTTGCTCAACGTGTAAGAGTATTGAATTGAGTGAGTTTTGATGTATTTAGGATATTGTAGTTT
GCGTTATTTAGTGGTATTGTATGCAATATTAAGTAATCAGATTTAAATCGTGATTGTTTATGAAACCGAGG
GTTATGTTAGAGAGTGGCAGCTAGCGTTACATGGGGGTTAAGATAAAACATTAGATGCCTACGCATGCA
GACGAATTTAATCCTATCGGTTGCCATGTTCACTTAGGATTTTGACACTATCAATTTCTGGCAGCTGATT
GGTTGTTAAATGGGAGCATGTGCTGCCATCTAGTCTTGATATAGACGTGTGCAATTTGGTTGGTTGTAA
GGAAGTTCACCTTTGTAAGATGTAAGTTGTATTAATACTCGCACCCATGACTCGAAGTAACCTTCTTTC
AGAATGCTAGGGCACCTTGTTTGTAAGTAATTATGATTCTACTCTTATGTCGCTTACATGAATATCTTTTG
GATGCGTAGAACATAACAAGACCAATTTTAGCAAGACATCGTACCGATAGGGACTTTAAATAGAACAC
CTATATATTGGGAGTGGATGATCATAGATAGAGTCACGCAATGATAGAATGTATGGGTTAACCGTGTGCA
TAAAACTAGGTATATGGCCAACGAAATTACCTCGATGAAGTGTGTACATTTTAAATTTTGCTGTACCCTT
TGGTAGCCCTGTATTGTCCTAACTAGAGAATAGTATTGAAATTCTCAGGGTGCTAAAATCATTGCCACTA
GGAATTACTTATTCATTTTTTTAAAATAAATGAATAGCTGATTATGATTACTTACACAATATTTTAGCGGC
ACTCATGTGCCATAAATTTGCCAGGCTGCCTTGTATTTGCCACATGTTTGAATATACATACGTTTCTAGG
TTTTCTTTTAGTTAAAAAAACCTATGTCCTACTTAATTTTATTGTATTTCTGTACCTACATTATTCTTAAAA
GGTTTTTTTCCTACAGCTTCGACTTTTAGTTTTGTGTATTTATGCTATTTTTATATATTCTTGTGTGGTAAG
ATAGTTTCTAAAGCCATGATAATAATATTTGTTGTGTGAATTATATTGATAGGTTCATTTTTATGCTTATATT
TTTATAGCACCATGAGTTGTGTGAATCAACACTGCAATTTAGTTTTCATTGTCTGAGATTATTATAGCTA
AGGCGGT

>TCONS_00020153 geneID=XLOC_004044 scaffold_13:871065-872129(-) Intergenic

GGCAGGCGCGGGCCATCTAGTCGACTGTGCACACACACGAAATATACAACGATAGACCACAACAGTCT
TCCCCTCTAATTAGATAACAGATTTTATTCCTTATTCCTATATCTGAGATTTTATATCTATTTCATACGTGA
GATCCTGTGAATTGTTGACAATACTTTATTGCTAATATAATTGTTTGTATTATTCCATAAAATGTTGCTCA
ACGTGTAAGAGTATTGAATTGAGTGAGTTTTGATGTATTTAGGATATTGTAGTTTGCGTTATTTAGTGGTA
TTGTATGCAATATTAAGTAATCAGATTTAAATCGTGATTGTTTATGAAACCGAGGGTTATGTTAGAGAGT
GGCAGCTAGCGTTACATGGGGGTTAAGATAAAACATTAGATGCCTACGCATGCAGACGAATTTAATCCT
ATCGGTTGCCATGTTCACTTAGGATTTTGACACTATCAATTTCTGGCAGCTGATTGGTTGTTAAATGGGA
GCATGTGCTGCCATCTAGTCTTGATATAGACGTGTGCAATTTGGTTGGTTGTAAGGAAGTTCACCTTTGT
AAGATGTAAGTTGTATTAATACTCGCACCCATGACTCGAAGTAACCTTCTTTCAGAATGCTAGGGCACC
TTGTTTGTAAGTAATTATGATTCTACTCTTATGTCGCTTACATGAATATCTTTTGGATGCGTAGAACATAA
CAAGACCAATTTTAGCAAGACATCGTACCGATAGGGACTTTAAATAGAACACCTATATATTGGGAGTGG
ATGATCATAGATAGAGT

>TCONS_00020004 geneID=XLOC_004014 scaffold_13:1065617-1066425(+) Intronic
AGGCGAGGGAAAATCTTCACATATTCCTTCCCCAAACATTCAGTATAAAGAGTTAGATAAGTCGGTAGA
TTGGCAGGAGCTGTTGCAACCGAGGGGGCGGCTAAAGTCTGCGGGCAGTGCTAGTACTAGTGCTAAA
GATTGGGCGGACGTCCTGGCTTTACCCGTCGATGGATTTGTTGGTGTAAGCATCCCAACAACACTGGA
AGTGACCTCACTGTCAGACAGTTGGTTGACC
>TCONS_00020299 geneID=XLOC_004049 scaffold_13:1078175-1124627(-) Splice junction overlap
AGCAACTTGGATTGGCTGTTCGAGTGCACGCATGCGCGTCGTATTTTTTCTTCTCTTTTTTTTATTGGTT
TGTTTTATGTCATGTCCGGACCCTCGCTTGCAATACCCCGCTTGTGTATTTTTAGTTCTAAATAAAGTTTA
GGTGATAATCTTTCGATGGTAGAAATAGTTAACAAATTATTTATAGTATTGTGATGATAAGTGCTGTGAAT
AACTGTAATCAGCGTTGGGAAACTAAGGCGGCACCGTGGAGTCACGTAAACGAAGGTACAAGAGCGG
GGTGCGCGGCGTGAGAGGCGGCGCGGAGGAGGCGCGGCGCGGCGGCCTCAAGGCGCTGCCGGCGA
AGCGCGCGCGAGCCCGCTGCCGGGGCATGAACATGGGCC
>TCONS_00020160 geneID=XLOC_004054 scaffold_13:1538440-1563944(-) Exonic overlap (+)
GTCGGTGTGTGCGGCGTGTGTGTGCGTGTCGCTCGCTCGTCTGTTTACGCTCGGAGTCGGCGGCGCGC
GGGCCTCACGTGGTCGCGGCAGTCCAGCGCAGCCCCCGGAGACGCCGGTACAGTCCGCAGCGAAGC
CGACATCGACACCCGCGCCGACGTTACCGCGGCTCATAAAAATTCAGTGTTTTCAGCGTATGCGATGA
ATAAATTTTGCGAATCGTGTCTGTGTTACAAAAAGGTTTTCGGTTGCTATGTTTTTGGGCTTCCACAAG
TGTTAGTGAGGGACGGCTCTGATGCACAGCGACTTTTGGAGGTGATCCCCAGTTGTGTGAGGCACCCT
GAGGCACCGGGATACGGCTTCTGTAAGGATTCCGTGTAGACCGGCGGAAAAACTGATAGTATAAGATA
ACGCTCCGACCGTCGTCGCCGCGAAGACTCGTGAGGAGGACCAAGTGCCATTTGAAACCAGTTTACC
AGAGCAGTGATATTTAGCGTTAGCGTAGTGTTAAGTGTAGTGTAGCAAACAAGCGTAGACTTGTGGTG
TTTAGTGAGAAAAGTTACCAAAAAACAACCACGCTGGCTCCGTACAAGGATATAATGAATTCGTCGGG
CATTAGCGCGGGTGCTGTGCCGATGATCAAGTGTGAGGACACGTCTGCGGGTGTGGACGGGG
>TCONS_00021231 geneID=XLOC_004119 scaffold_130:435540-436263(+) Intergenic
TAAGTCCAACGTTATTCGAGTACCTACTCATGCTAAGCTAAGATTATGATATTATTGAGTCACCAACTTG
GTGACCAATATACGGAAAACTGGAACAAGATCAGTTTGAAAGCCTCGAAGAAGATGAAGATGATGGA
TCAGAAGAAGAAATCTCCACACAGACAGAAACAGATTCAAATGCTGGAGGACTTCCCGAAAACCGAA
TAACATCTCAAGAACCAATAGTTCTTGAAGATGTGCCTTCCCCGGATTCCGTCGCATCACTTGCAAAGT
CCGAAGGCTTTAGTCCTGTAGACTCAAACACATCCAGCAATAAACTATCTAATCAACTAATTAATAAAA
CCTCAACAGATTCACTTTCTCCCAAATCTTTCAAAGATAATGTACAAGAAAAAGAAAAAGACC
>TCONS_00020618 geneID=XLOC_004120 scaffold_130:439860-440754(+) Unclassified
AAAAAAATAAATATAAAATTCACAAAAATATTTATATGTTTGGTAAAAAAATTAAATGAAATTTTCTGTA
AATTAAGAAAGAATTTAACTAAAAATACACACACAATGCCGCGGTGGATACCGAAAAGCATTAGCTTT
TCCGTAATCGTGATGTATTTCACTTCAGATGTTTTAGGATAAACCACCACCATCTTCCACCATACCACTT
ACTATACTACTATGCCAAACCTCACAGCCCATTTCCGCCAGAATCAAATCTATGTCGCCTTGAAGTTCA
CGTTCTTTTTATCGTTTTTGAGCTTCATAATATTGTTGTTTTACAGCTGGTGGCTGTGTCTCAAGTTGGAT
CGGCCAAGTACATAGATCCAGATGTTAAAAAACGAG
>TCONS_00020619 geneID=XLOC_004120 scaffold_130:439860-440754(+) Unclassified
AAAAAAATAAATATAAAATTCACAAAAATATTTATATGTTTGGTAAAAAAATTAAATGAAATTTTCTGTA
AATTAAGAAAGAATTTAACTAAAAATACACACACAATGCCGCGGTGGATACCGAAAAGCATTAGCTTT
TCCGTAATCGTGATGTATTTCACTTCAGATGTTTTAGGATAAACCACCACCATCTTCCACCATACCACTT
ACTATACTACTATGCCAAACCTCACAGCCCATTTCCGCCAGAATCAAATCTATGTCGCCTTGAAGTTCA
CGTTCTTTTTATCGTTTTTGAGCTTCATAATACTGGTGGCTGTGTCTCAAGTTGGATCGGCCAAGTACAT
AGATCCAGATGTTAAAAAACGAG

>TCONS_00020877 geneID=XLOC_004120 scaffold_130:439883-440787(+) Unclassified
AAAAATATTTATATGTTTGGTAAAAAAATTAAATGAAATTTTCTGTAAATTAAGAAAGAATTTAACTAAA
AATACACACACAATGCCGCGGTGGATACCGAAAAGCATTAGCTTTTCCGTAATCGTGATGTATTTCACT
TCAGATGTTTTAGGATAAACCACCACCATCTTCCACCATACCACTTACTATACTACTATGCCAAACCTCA
CAGCCCATTTCCGCCAGAATCAAATCTATGTCGCCTTGAAGTTCACGTTCTTTTTATCGTTTTTGAGCTT
CATAATAGTAAGTACTCTTTGCAGTATATTTTCTGTTACAGTTCGAATGAACTTACTCAATAAAGTTGTC
ACAAGTTGTTGTTTTACAGCTGGTGGCTGTGTCTCAAGTTGGATCGGCCAAGTACATAGATCCAGATGT
TAAAAAACGAGGACATTTCAGTTGCGTTATTGAATTCATTCTCA
>TCONS_00020949 geneID=XLOC_004121 scaffold_130:626606-627530(+) Exonic overlap (-)
GTGCTCAATATGAATATCAGTAAAAAATCCTAGACTTAAGAAGTACAAATAAACCTAAAAATAATCAAT
CCTCCCCTTTAACTTTATATTTACATACCAAAAATATAAACAGCTGTTAACATAAATAAAAAGTTTCTCTT
TAAGTTATCCAGTAGTGGCAGCGTGAGTAACTGTAATAGGTATGCTACTATGAGGCAGACATACTACAA
CAAACACTAATGATTTATACTGAATTTCAACACACATTTAGCTATCTATCAATCTTAAATTTGTTATCTATA
CAGTAAACGATGTATGCCCATGTAAAATACGCTGGTAGACGACACTAATCATTACAAATTAAGCGATAA
GACCCCCACACGTACCTATGTCTTAAAATAAGTTACGAAACTGTTAAACAAATCCACATTTCTTTCACA
AATCCACTATCCTCATGTGTGTAAAAAGTAAAAACTAGAAAAGTATCTAAAGTTCTGTTTGTACGTTTC
ACAAACGGGCAAGCCCGAGTCGTGGGCTTCGCCCAAGGTTTGTATCTTGGCCCAGGCGCTGGCGATG
GTGGCCGGGGTCATGCTGTGCATGGAGGCGAAGTCCCAGTAGGCGCAGGCCAGCGGGACCACGGGCT
GGAAAGGGCTCAGCCGGTCGTCGTGAGACAGTGGCAACCCTAGCGCCTTGACAGCAACTAAGTATGT
GAAGTAAACATCTTCTATGTTTATAAGCGGAACTTTGTATGAGGCCTCGACGATCTCTGGCAGGTATTCT
>TCONS_00021139 geneID=XLOC_004150 scaffold_130:654979-669912(-) Exonic overlap (+)
AAACCACGACGAAGGACTATGACCTCAGTACAAAAATACAAAGAATTTCCTCCATCCACCTCAACTGT
GGAGAATGAAGCAACAAAAGTGGAAAATAATAAATAAAACAAAAGTTAACAAAAGTGTCATATCATTA
CGGAGCGTCGCTCGGAACGAATGCGCGCGCTGCATCGAAAATTGCAGCGCAGGAGCAATAAGATGGC
GTTTATTTCGATATAAAATATCGACGTCTTGAGACTTATATTATACCAAAACATATAACTACAAAGAGCCT
AAGAACCTACCATGGCTTCCAAGCAAGTGGAACATCTTCACCTGGAGGGCCTGGCGAGGGTACTAGC
TGATGCCGAGCTGGAGTGCCACCGGGCTCGCGAGGAGTCCAGC
>TCONS_00021084 geneID=XLOC_004152 scaffold_130:797427-798716(-) Intergenic
GAAAATTTCAATAAACAATTTATTCCAACAACTTATTTTGTGGGATTTAAAATCGATATTAATCTTATCAA
GTATGGAGGAACGACTTTTTATTCAACGTTGAAGAAAAAAACCGTGTATGTGTGTAAGTCTTGTCACTT
TTGACATTTACCATATTTTTTTTCTGTTGAAAACTTTAGCCGAAATTGTCTGAACTGTTTGAAAAGAAGT
TTCTTTTAAGTATCCAAAATGCTTCCCCTTGGTGACGAGAATGCCGCACCGCAAGCAACAGCCACTGG
GACTAGTGCTGGTGCTGTTGCTACAAATAGTGCTTTGCCCGACAGAATTGAAATTATGTGAACTCCCAG
AGGCGCAGAGGCTGAAGCCAAAGGAGACTTGATGGTTTTGCAGTGCGAGCAGATACAATGCGATAGC
GAGAAGGCGGAAGCCATGAAAATGGTAGCTGCAGCAGTAAATACACAAACTGCTGTAAATGAGGGAC
ACAATGCCAGATTAATGGCATTCATAGAAGCAGAAAAAG
>TCONS_00020790 geneID=XLOC_004123 scaffold_130:839542-840493(+) Splice junction overlap
CGTCAAGGTTGCAGAGCGTGCGAGCAGAAAGCGCGAGGGTGGCCCAGCCGTCGCCGGGTGCCCACC
GAAACTCTCCGCCGCACACCACCAGTACACCACACATGCCCAATAGGGTCGTCGATTACTTGGCGCCG
GAATCTCCTATGGACGCCAACGAAGATTTGATTTTGGAGCTCCTATTCAAAATGTAAAACGGAAGCTGT
CAGCCCTTCAACAACAAAGAATGACGAAAAAAAAGAAAGTAGTTTAGTGAACTAGCTGGCCACAAAA
TTTATAAGCAATTATTGCAAATTGACGTGGCATAAACGATCAAAAGGCTATATAGCACAGATACGTCAC
CAAAAGGTTCATGATTAAATAGATTTTGAAAAATATGTTTAACCAGGTTTGACCTAAAAGACCTACGAG
TAATAACAGCACTTCAAGCCTTGTCTGTTTTAAAATATTTCATTTGACTTTCTTAAGTCTTCAAACAAAA

TGGATTATTTCTTTAAGTCTTAAAGGATATTAGTAATTTATTGAACACCTATTAGTAAGTAGCTTAGCATC
TATTGTAATTA
>TCONS_00020803 geneID=XLOC_004126 scaffold_130:916403-917106(+) Intergenic
CACGGACGAGGGCGAAGAACACGATAAAGATAGTGAGAAAAATAAACTGGTCGAACCGGAATTCTCT
AATGTGATAGTGAATCAGCCACAAAGTATTAACCAGCCCCCTGAGACTGGCCGCATTCTTGACACTTTA
GACGATCAAAACCAGGAGAAAGAGTTGAATGAGAAAAGAAAAAACCTTCGTGATAATGATGATAAGA
ACAAATACAAGCTCTACAGGTGTGAAAGAGAAATTTATTATTTCTGAAGATAGATATAAGCTTGCAC
>TCONS_00020649 geneID=XLOC_004130 scaffold_130:1081057-1082056(+) Intergenic
GCCTGTTTCACAATGTCTTGATAATGGCTACCTCTGGCATAATAGACATGCTGTCACTGTCTAAAACATA
ATTTCAGAGAGTGACAGCATGTCTTTTATCCCACAGGTAGCCATTATTCATACATTGTGATAAAGGCTAT
AAATTGTGCAGATTCTTGCAATTTGTCATCAGGAGAATTTTTATTGGATGGAAAAAAATAATTTTACAC
AAGTATCAATAACATCACTGTTTGATTGACATATTTATAATATATTTATGTTGTGTGGTGTCTATGACTGAT
TGTAAATAGACGCGTATACTTTGTGTACAATGATGTGTACAATTAAATGTTAAGTAGACAATTCAAATAG
TTGATTATACGATTACTAAGTGGCACAGTGTAATATTAGACTAAAATATTATTCTGTAGTATATTTATTATA
AATTATTGCAATCTCACCACAACATTTCCTCACCCAATAGAAAGAAGAAAGTATGATGTTTTTTTTCTTA
CAGGATGTAAACTATGGCATATTCTAAAATATCAGTTCCTATTGTAATTTCATGAAAGTACTTACTTATTT
CGAATAGTAATTATTCTACAGTGTTATGAAAATATAATGAAATGTTTTTTTTTATATATTTAAAAAAATGTA
GCCAAATCGGTATGTTTTGGTCGCACTTTATCTTTTTTTACCTTTCATATTATCTTGCGCTTATTTTACTGA
AACTTTTAATGGTTTTACTCATCTTACTGTACTGCCCTAAATGTAATTTATCTTTTTTATTAAAGTCCTATG
TTGACCGTTTTAAGAATGGCAGCTTATGGTTGTACAAGAAATAACATGTTTGGTTTAATTATACTTGTTA
GCATGATCACGTGTTTTATTAAAGTGTTT
>TCONS_00021091 geneID=XLOC_004162 scaffold_130:1110313-1132634(-) Exonic overlap (+)
CGAGCGTGCTGTGCGCGCGCCGCTGCCTCTGACACTCTGGGTAACTCCGGGTCTGGTTCTAACAAAAA
CAAACAGAATCTCGAGGTGCCCATGATGATAAAGAACGGCCGCGTGCGCCCGCGCATGGGGGTCCGG
TTCGCGGAGACGTCGCTGACGGCCGTGCCGTCGCGGCCGGAGCCCCCGCGCCCCCGCGGCGTGGCCT
GACAGCCCCCCGCGCGGCTGGTGTGCGCGCAACATGAGAGATGTCGAGTATTGCATCAGTTCTACACC
GAACGAATGGCTGCGACAGCTAGTTTGGTTTAGACCAATAAGAGCAAAGCTACGTTACCAATGGACCA
ATGGTAGCCTGGAGGTTGTGATTCGTGTATGAGTTGGAATGGTGATGGTGCGGATTTAAAGTAAGAGG
ACCAATGAAAGCGTTGTTATACATTTACCAGTGAGTGTAGTGCTGCAAGATTCATTAATGATATCTACCT
GTTTTGTGTACCTATTATTAAAGTGGTGTAAATATTTTTGTACGAAATTAGGAGGAGATAATATTTTAAGT
GAGTAACTGAAGTTTGTATTATTATTATTATTGCACTTGACTCAAACTTATGTAAGTGCTTGACAATAAG
GGACATTTGAAAGAAAACTATAGACAGTTACCAAATTCAATACTTTTATGCTCACTTTAGTGCTAAAAT
GAATGTGTTTGAATATAGTGACTACCTACCTATTTTTGTAACATACCACCTATGCCGTTAAGAATTAGGT
ACTATTGCAGAAAAAAATCTAATCGAATCTAAAACGAATAAAATAAGTAATCGCGATTGATCCGCCGCAA
AATCCGATCCGCATAATTCAATAAATACCTAATAATAATAATAAATTATATTTTATTATATGTGTCAATTAA
GTACCTACCTATTTTAACCTATTTTAAGTTTAAAAATATATTGCCTACCATAGTTTTCTTTATTC
>TCONS_00021092 geneID=XLOC_004162 scaffold_130:1110313-1132634(-) Exonic overlap (+)
CGAGCGTGCTGTGCGCGCGCCGCTGCCTCTGACACTCTGGGTAACTCCGGGTCTGGTTCTAACAAAAA
CAAACAGAATCTCGAGGTGCCCATGATGATAAAGATGTCGAGTATTGCATCAGTTCTACACCGAACGA
ATGGCTGCGACAGCTAGTTTGGTTTAGACCAATAAGAGCAAAGCTACGTTACCAATGGACCAATGGTA
GCCTGGAGGTTGTGATTCGTGTATGAGTTGGAATGGTGATGGTGCGGATTTAAAGTAAGAGGACCAAT
GAAAGCGTTGTTATACATTTACCAGTGAGTGTAGTGCTGCAAGATTCATTAATGATATCTACCTGTTTTG
TGTACCTATTATTAAAGTGGTGTAAATATTTTTGTACGAAATTAGGAGGAGATAATATTTTAAGTGAGTA
ACTGAAGTTTGTATTATTATTATTATTGCACTTGACTCAAACTTATGTAAGTGCTTGACAATAAGGGACA

TTTGAAAGAAAACTATAGACAGTTACCAAATTCAATACTTTTATGCTCACTTTAGTGCTAAAATGAATGT
GTTTGAATATAGTGACTACCTACCTATTTTTGTAACATACCACCTATGCCGTTAAGAATTAGGTACTATTG
CAGAAAAAATCTAATCGAATCTAAAACGAATAAAATAAGTAATCGCGATTGATCCGCCGCAAAATCCGA
TCCGCATAATTCAATAAATACCTAATAATAATAATAAATTATATTTTATTATATGTGTCAATTAAGTACCTAC
CTATTTTAACCTATTTTAAGTTTAAAAATATATTGCCTACCATAGTTTTCTTTATTC
>TCONS_00020956 geneID=XLOC_004131 scaffold_130:1232705-1235041(+) Exonic overlap (-)
CCGGGATTTGTCTGAAGGATCACATCATAATAAAATGAGGTACTGATGTACAGTCAACCAAAAATATCA
AGAGGCATAGTGCATTTGAGGCACGGTAAAGTCGTCACCTGTACAGTCTAGTTATTATTCTGTGGTACA
ATTTACACACACTGAACTAGCTCCAACAATTTTGCGTTAAGTCTCACTACTTGACTGGTATTGAAAAAC
CTACACTAAGCACTCAGTACTAATATGAAGTACCTAACAAATCCCGTATCCAGTTGTGGACCCCTCGAT
TTAGTCCGTATCCGTATCTTCACAATAGACCGATATAGCTCAATGGATTTGGATTATTAAATTAGATGAGT
AAGTACCTACTTATACATATAAATAGATTTGGATTATTTCCAGAGGCCTTTTCCTGTGCCATTATGTCCGG
TTGTTGACTGTCTGGTATATATGAGGCCACTATGTACATAGTTATGTATGTACTATTTAGGGTCAGTTATT
GATAGTTTAGGCTACCTCTCTGAAATTAAAGATTCAGTCCATTTGTCCGGATGTCGTTAAAGTAAATGA
AACTTTGAAACACTCCTTTTGCGTTGGGTGGATGCCGGTTGATGGATGACTTCATAATAATGACTTTAA
TTTTGCTTTTATGATAGTATAGCCTATAAATAAACCTATAACCTAACCAACGACGATAGAAACTTCCAAA
ATTCAACTCTAAAAGTCGCATAGTTTTTCATTATTTTTCTTTGAGTATTAATTTTAACAATCGCAAAATAA
CAATATACTTAGCTATGCTATGCTATTACATTATTAATTCATTTATTTCAGGCAATATCACAAGAAAACCCA
TATAAATTCAGTCATACATAATTATTCTTATATTATATGCTATTTTCTATTCTACAACGAATCATTTTTGATT
TAGGACATTAGTGGACTCGGGTGCACTAAATGTTATCTTCTAATAAACTTGACATGTATGGGGTGGGCA
GGTCTTAGCACCAGGTCAGCTAGCAG
>TCONS_00020906 geneID=XLOC_004165 scaffold_130:1260117-1264171(-) Unclassified
GAGAAGGCAGTGACGACGAAGAGGAAAAGAAACCGGAAAAGAAGAAAGAGACAAACAATATCAAA
TCAACCACTACCTCGTCTGCGAGTGACTCTGAGAGCGACAGTTCAGACTCTGAGGAGAGCTACGAGA
GTTCTGACAACAGCGACGATCCCAAGAGCAAAGGAGACAAGACTGACTCTAAGCCCACCACAACCA
CAACTGCCTCCAAAAAGAACGAGAAGTCGAACCTGGAGTTACTCCTAGAGCTGGACTCCGTGGCTTC
TTCCCTGCCCACCATGACGCCCACTTGCGGGGGGCTGCTGTCCCCTAGCGCAGGACCAAGTGGAGAC
GGTATGCAGTGACATAGACAAATACAGGGTGTTGCAAAAAGGGTATACTAAGCCGAA
>TCONS_00020724 geneID=XLOC_004165 scaffold_130:1296887-1298034(-) Unclassified
TCAATATTATATTTTAAACATGTAAAATTTTGTATTTAATATTTATATGCCCAAAAAATATTAATTTATTTCA
CATTTTCCTGAAAATAACATCACAAAATTTCAAGTAAAACTTTCAGTTATCAAAATGGGTGATTTTAAAT
ACGCTATGCGCATTTTTAGAATCATAGTTGGTTTAATTATTATTACTATACCATTTTTAACGCTAATTGTTC
TTGTGATGATTGCCCCTCTGTGTTGGGAAAAAATTGTGCTCTACATGTCCAAGTCATATAATCGGGTTTT
ATTGGCAGCAGATAAGATGTCACAAACATTATGAAGCCACAGGGAGTTCAAGTGCACAACCCATTTGG
CGCCTACATTTTGAAAAGGTTTTTTCTATGAAAAGCTAATGTAATTTTATGAATTTTAT
>TCONS_00020726 geneID=XLOC_004167 scaffold_130:1299208-1300000(-) Intergenic
TTCACAACAACATGAGAACAACAACTAAATAAAATAATTATAAATAGTTATAAATTAAAAGTGTACACA
ACAATGTTGAATCCAACATGTGCATCAAAAGCCCGTCATTCAAAGAGTACTCAGCGACCGTATGGAAC
AACTGCAAATGGATGGGCAGAGCAATCGCTAGCCAAAAGTGGACAATAATGATGATTACTTGTGTATTT
ATATCCTTTGGTCTCATAGTATTGTTTATGTTGCTGATGATTGAACCACCTTCGGAGATACAGAAGACTA
ACGTCCCTCCAAACAGTCGACGGAGGGCTATACACTATTTGTTCAAATTTACCCAACCTTGTGTTAGAG
CAAGTGCCTGATGGTGATGGTCCT
>TCONS_00021303 geneID=XLOC_004265 scaffold_131:353011-354315(+) Unclassified
AAAAGCCTACCTATCTAACTGCAAACATAAATAAATAACTCCAAACAAGCCGATATGTTCTGATTTGCA

GATGCTGTAGGTATCTCAAGATGGTGTTATTTAGTTGCTTGCCCTCTTGATAAGGATGACCTGTTGATAG
CCGGTTGGAAGCGTCTGGTCGCGCTGGCTCATACTGAATCTGGACACAGAGGGAACCACACCTCGGG
CTCGCAGCGGACCACACTCCTCAGATCCCTTCAATATGTTCATTGGTGTAACTTCTAGTGGAGACAACG
TAAACTCACACTACGATTATCTCATTTGAAGCACGGATAGGTACCTACTGAAACATAATATTTACTGTGA
TCATGGCGTTAGATTTTATAGCAGGATGCATCGGAGGATGTGCCGGTATCCTGGCAGGCCACCCGCTGG
ACACCCTAAAAGTCCACGTCCAGTCGGGGCGAGGCAGCGCGCTAGAATGTGCTAGAACTCTCCTGAN
N
>TCONS_00021492 geneID=XLOC_004265 scaffold_131:353448-354315(+) Intergenic
CTCGATGTATGCAAATAATGGAAATCTATGAGATGAAAGGTCTTTATGGTAAATTCATTTCCTGATTGAC
TCGAGTGCGATTGTGTGGTTACGGGCGGTCAGAGAATCACTTATGTTCCTGGCTGGGAGTTGAAATGC
TTACGACATAGTCACATCTTTGAAAGGACCTTTGAGATGGGAGTGCTATTCTGTAAAAATATAATGGTG
TGGATGAAGATAAGAATGTAACTAAGTCGTTATAAGACTGTAACCATGAAACAATATAACATAGCTAGG
TACATTTAAACAGCAGGTAAATACGATACAATTTAAATAACAACTGAAGTAAGAACGGATCTTAAAGTA
GGCGTCGTCGATAGTGATACAGCTACGACTCTTTGTTATATTTTAGATTAGCTATCTCAGGTCTCGGATA
GAGCCAATATTGATGTTGACCTTTAACGTCGATCACTCAGCGCTCATTAACTCAATCTCTAGTTGACATT
TGCCTCGACGACTTCATTACGAATCGAAAACTAATCTTAGCAATAAAATGTGGAAATTTAATACAATTTC
AAATAGAAGGGTTATTTATATAATCCTTTTACTTATACAAACATAATTGCTATTTTTTTCAGGTACCTACT
GAAACATAATATTTACTGTGATCATGGCGTTAGATTTTATAGCAGGATGCATCGGAGGATGTGCCGGTAT
CCTGGCAGGCCACCCGCTGGACACCCTAAAAGTCCACGTCCAGTCGGGGCGAGGCAGCGCGCTAGAA
TGTGCTAGAACTCTCCTGANN
>TCONS_00021324 geneID=XLOC_004280 scaffold_131:362303-363553(-) Unclassified
TTTTCACAATCGCATGGCAACTTCGGCAAGGGCAGAAACAAAATGAAAATATTGCTGCCGCGCTATGT
CACTCGGAAAAAAAAGGTTTGACATGTTTGACATTCTTACATAATTTTTTTGCTAACCTAAAAATATGTT
TTATCAAATGACTTGATCGGCGATTTTGAGTTCTTTTTTATGATTATTTTTTATAAAACTTGGCTAAATCT
CGATTGTGTACTATCGCGCTGTATATCCGTAAAGGATTTTTTGCGTACATTTAGCGATGATGTCTTTACGC
TAAAGTGATTCTTTCGAAATGGACACCGACCCGAATAACACTACGGCGGGTCGCGAGACA
>TCONS_00021475 geneID=XLOC_004282 scaffold_131:387152-389144(-) Intergenic
TGAATAAACAAGAATGTGGCCTCGAGTAAGAAATAAACATCGCTGTGACGTCTGCCACTCATTTATTTG
GACAGTATGGTACATTAACATTATGTTTTCCTGCTAGTATATAAGGTGTTGATGTGCCAGCGCACAGATC
ACTGTGAACACAGTTGTACTGAAAACATGAAAATGTATACAGCAATCATTGCCATGGTGGCCCTGATAT
GCGTCGCTGCTGCGTCACCGGAACCCAAGAAATTACATGCTCTATTTCAAATTACAGCTACTTAAGTGC
AATGGAAATCATTTTCGAAGGACTAATGCATAGTTTCAGCCTCAGAGCATGGACGAAACATGCATG
>TCONS_00021331 geneID=XLOC_004282 scaffold_131:388276-388997(-) Unclassified
CACAGTTGTACTGAAAACATGAAAATGTATACAGCAATCATTGCCATGGTGGCCCTGATATGCGTCGCT
GCTGCGTCACCGGAACCCAAGAAATTACATGCTCTATTTCAAATTACAGCTACTTAAGTGCAATGGAAA
TCATTTTCGAAGGACTAATGCATAGGTAATAATACGAAACATGGCTATTTGAATAAATTATTTTAATATAA
AAAATATATAAACAATACTTGTGTATCTTTTATACAGTGTATATTTTATTAAATACGCTTGAAATGCTTAAG
GAACGTCATAGATTTATTTTATATTACCACCTATAATCTTCTTTTTCACAAATATAAAATATTGTGT
>TCONS_00021530 geneID=XLOC_004266 scaffold_131:390564-403911(+) Exonic overlap (-)
CTAGTGTGTAAACTCTGCTCTGGCTATTTACCTCGTCAAAACTGTTTGTTTTACTTCCAACTTTGACACT
TATTACGAATTTCTATAATCTACCATATACGAGTACCTACGCATACAGCTGATGTTGCAATTTATACCCAT
GGAAGTAATAAGTACTTAGTACTTATTATAACCATGTTTATACCTAAAATACGTAAGTACATATACTAAAT
AACTTGAAGTACACACAATTAGGTACGTACATAGAG
>TCONS_00021777 geneID=XLOC_004329 scaffold_132:34333-40867(+) Exonic overlap (-)

CTCGTAACTAGTATACATAGAGTAACTTTGATAAGCCACACAGCATTAACGATAACATAGGGCTTACCTC
AAAGCAGGCAAAGGCTAGGCGGTCTTAAGGCCTTCGTATACCCCAGGCATCATTAGCGCAACCTTGGG
AGCCACGTATGTATGTCGCTTAGCGCTTCTCATCGATACAAATTGCACTACTTTCATTATTTAAGTAAACT
ATTCATATTGTTAAATGTTAAGGCATCGCAAATGCGAACTGGTGTTCGGTGAAATAGGGCAGTGTCATG
TTACTCGACTAATAGTTACTGTGATTATTACAAGACGCACTGGGACTATTGAGACTATCATGGGTACTCT
CTCTCTCTTTTGTTTATTTATTAATAAATGTGCGTTGCTTACCATATGGGTATGCAGGATAATCAACAGTT
TCTGTTAGAATATGCAAGTAATTTGACACTACTCTCTATTTACAGGCAGCATAATTATACGTATCATTGTA
AATGTAATACTATTTCATTCATAAATTATTGGTGTTTAGTTTCACTCACTATTGCTTCAGTTGCTTAAATCT
AGTGTAGATATAATATGCAGACGGGGAGATTTCCAAAACACATTGCCTCGTATTAATTATAGTCAAAGTA
CATAAGCTAGCTGAACTAGGTACCTACTAAGTATCTATTTGCGAGACGGCTTTAATATAATTTAAATAATT
TGTTTAGTCCTACCTACTCTAACTACAGGCTATGTATTGTTGTCCTTGCTATATGCAGGTCTGAGGTCAC
GAGAACACACCACGTTATATCAACAACAACATTTAACAATCATAAGATGTCGGACTGCTGTCGCTTCAG
CGCTGAAACACCAACATCACTCTTATCTTCGACGGACTTGCTTTGTGAAAACACAACCAAGCTATTGT
TGGTTCTCTATTCGTCAATTT
>TCONS_00021907 geneID=XLOC_004330 scaffold_132:162002-164857(+) Exonic overlap (-)
CTGTTGACATAATCATTAATAGTTAAAACAAGATATTTAATTCTAATTAATTACCTGTTGTGAATGGAGA
AGGAAATTGAGGAATGTAATGTTTATTATTTATATTATAATTTAGGTAGGAATTTATTTAAATTATTCATTC
TAAGGGCCTGTTTCACAATATCTGGATATACCTGTGGGATAACAGACATTTTGTCACTCTCTGTAATTAT
GTTTAAGACAGTGACAGCATGTCTTTTATTCCACAGCGAGCCATTATCCAGACATTGTGAAACAGGCCC
TTAATGATTGAACGGTACGTGTAGATTTGTAAATGTTTTCCATGTACTATGTTTTTCAAGTAGCGTCATCT
AAGTTTATTGATATGTACATGCGCCCGCTCTTTATTTTTTTTTGTATTTCCACTTTCTGCGAATGATGAAT
TCATCCTCAAAACATAAGGTTAAGGCGGTGCCTACACTGCCCGCATACCGCCGCGTGTTGCGGTAATG
CGTGTTTATACCATGCTCGTAAATCTCCGTTTGTATATAAAAGACACGCACAGTCTATCTTCGGTGTATT
CATGCATTGGTACAATAAAAACGAGGCGAGAACCGGGGCAGTGTGAGAATCACCTTAAATTATAGAAC
ATTATAAAATGGTAACCATTATATAATGGGTTCAAAACTACCGTGATTGACTTGTCCTCATAAATAACAA
ATAAATCTATGACACAGTTGACATCGTCATGCCATTTGTATTGGTTACAAGACTACAAGTTAGATTTAAG
TGATAGGTAAATGACAAGTGATAACAATCATCAATATTTCAAATCGAGCAAATTCTATAGTGTGAAATAC
GTGCGAATAAATTTACGCTTTTGACAGTTAGATACAATACGGTTGAAACAAAACTGCGGCTAATACACA
GAAGAAACAGGTAGGTACATAACCACTTTAGGATTTGCTCCAGTCCGAAAATAACCTTAAAAATTAAG
TGCATTGGTCAACAATTAAACTATTCTATCATATGAAACAAAGTGTTTTACAACCGAATTCTTATTATCCT
AATATATTAATCACTCGTAACGTAAGTAATGTATTATTTAGTCACCAAATTTATTATGAATCTTATATATAA
ATATGCAGGTTACGACTGTACCCCGTTGAACTACGGGTAGCGAGGGTGAGCGACCTCGCAGTCTCGTA
TTCTAGAACGAATCTTAGGTCTAATCTTAAATCTGAGATCTACCTACTCAAAAGTAATCTTGTTATGAAA
ATTTTTGTTAGCTGTCACGTATCAATTTAAAAGTTTCATGCAGTGTGCAACAAATATATTGATTTTGTTTG
ACTATATATTATTATACTGTTTTGTATTTGTAAAGTTTAGGTTACTGATTCAAGTGACCGGTTGAACTAGT
GTAGGTATAATTCTTGGGAATAATAAGTCTTGTATTGTGATGTCATAATAACAAAAGCTGAAGATTAGTC
AACATATTTTAGAGTCTTCATAATGAAATAAGTACCTACTTACGATTTTTGATGCGTTTTCCAACATCATT
TACAATACAGTGAGTCACTACAGGTCTCCGTGTTTTTATTTTATTTGATATCTGCCTATTTATTAATCAGA
ATATTATTTTCTGTCTCTAATTCAGTGATGTCATACATTTGTTGATGTTGAATAAAAGCATGTAGTTATCT
CTACATGAGATGTCAGACTCTCATCATCTTCTTGCACTTAAAAACAGTTGAACTCTATATTTACGCGTTT
TGACGTGCGTGCGTTATACCTTCTCTTCATCTATCGTGTGGTCAGTTTCAGATGATGTAATGTAAAATAG
AAAAAGCGACAGTGATATCTCATATTATGATGGATCAATTGTGGATCTGTGGCCAAGGGCGCTGCAATA
GCTGAGTGGACGCCTTTTTGGCGGACAGCAAATTTATACGTGAGTTTATATATTATGGTGTATAATTAAG
TATGATTGCTGTCATTGTCAAATAAAGTTTTTTTTTTTATCTTTCAATTTTATACGACGATTACATAGACG

AAATTTGCAGCGTTTCAGCTCAATTGGGCGAATATCTTAACACTGTTGTATCCGCTCAGAAGACATCCA
TAAATACATAATTTGGTAAAATCTGCTCCCTATTTTGAATTTTATTTTAAAGGTTAGGAAATTTGAATCAA
TCTAAGCTATCATTAATCTAGTATAGTATTCAAAATTAGGTCCGTGGATTATCAGTAGTGTAATAAGATAA
TTAGTTGTCGTATTAATTATCGTTTCGATTCAGATGCAAAATTACACGAGTATCACTCTAATGGCACCTAT
TACTAACCTATCCGTGGATTGATCGATCGTATTTTCTTTAATTACTCAGAAACCGGCCAAAATTTCCATC
ATACCAGTCCTAAGTTTTCAAAATTATTAATACCAGACATCCATTATTAAAAACTTACACAATGAAATCT
CGAAATTTGAAAAATGCATGACCAAAAACATTACCATCAACGATCTCAAACAGGATGCCTGTTCAACT
AGTATTTTTACACGCCCACCAAAAATTGTCGACTAAGACCGTAGTACAGTTAGGTGTAGGTTAAATTTG
AAAAATGCACGACGAAAAAATTACCATCATCGATAATTCTTCACACGCCCACGCAAAGACTCGTTGTA
GAAGACACCGTGCGACAGTTGCGTGTAGGTGACCGTATCGCCGCGCTAGTCGGAGTTGCAACCCGTG
TCGTGCAGCAGCGGCTGCGCG
>TCONS_00021754 geneID=XLOC_004331 scaffold_132:199630-207222(+) Exonic overlap (-)
CGTCTGTCAAGCGGAGTTGTCGCTTGACTTCGGTTCGCGGAAACATTGCCTGTTTTAAAGAATCCTACT
CATTCGCAGTTATTTTACGTTAGTTCGCTTCAGTGCTAATATAAATTAAATATAATTTAGCCGATTGGTCT
ACTTCGACCAATTGCGACTTTCACCATGCGGCAAATATTGCAATGTTTGTTGTCCCCACGTCTGTACAA
GATCTACTATGATGGAACTCCGTATTACACCAAAAACAAGTTGGAGCGTTGGGGAAGCAATGTCATTAT
CTCGGT
>TCONS_00021757 geneID=XLOC_004332 scaffold_132:258343-278532(+) Intergenic
CAGAGACTCACCGGCCCTCCCCATGAGGGCGTGATCGGGGGGTCGCACTTGTGCTTCACCACGGACA
GCTACGTCTTTCGACTTTACGCCCGTATGCTGCTGCCACCGGTCTTTGGGCCGGTTCACCGGCACGCAC
GGTTCAGTATGATGCTATTCTTAATGAATCCGGCGAATTGGGTAAGCATACCTAACATGTTCATTTTACAT
AAACCTGTATTAAAC
>TCONS_00021850 geneID=XLOC_004347 scaffold_132:374946-378472(-) Splice junction overlap
CGCCCCCCGCCAAGCCCGGCCTCGGGGACCACGCGGCGCTCATCAACAAGTTTGACATGGCCTCGTT
GAACCTGAACAATGGATTCAAAGCCTCTTCGTGTCCAAACTGCGATGTATTGAAAAATAGGTTGCTGC
ACACCCAGAGTAAGGCAGAGACTATTAAGAACAGCATCAAGGACATGAATCAGAACCTCGAAGATCT
AGAGAAGAAAGAAAGGCGGCTAGACGCCGTAATAATATACTTACTGAAACAACAAAACAGGACAGGC
CGGCGACCGCAAATAGGAAATTAATAGTAAATAATTAGGTTATGTTATTTACATTTATGAGCAAGACAAT
GCATTTTTCGCCGTTACCATGTCATCATGAAATATTTAATATTCATTATTTCAGAGTTTCCCTTATGGAAA
CCCATTCCACTCGTATTGTTTTTTTTTAAATTGTTTAGGTAAAACCCCACTTGGACGACGGCACTTGCTG
CTGATATAAACAAAAAATATTTTTTGACATAATTATTGTTACTGATGCATTGTAGTCATAAACTAGAAATG
TGTGTATAATAGTGTTAGTAATGTGTAACTATGTGTAATGTATAGTGTGCTTCGAAACGTATAGTAGGTAC
AATCAGCTACATAAGTAGCTACCAACTTAGCAAACCGTCAATGTTTGAATGAATGAATAGGCAGTTAGT
GAATTTGACAAGGTACAAAAGTGTATAGCGACTTATGCAGCATACTGTACATCTAAGATCTCAAACACT
TTTTTAAAATAATTTCGTAAGTAAACAAATCTATTTAAGTCAACACAGTGCTAACAAAGCCCGTGTGGC
ATAGCTCTAACGTAGGTAAGTTTAGACAAGTATAACACTTATGCGTATAGTGTATTTTTGACGAAATCCT
AATATTGTTTAAGATGGCATAATAGAGGATGATTGGTGGCCTAGTCATTGATGACTGTGATTTACGTCCG
TTACATAATATTATGTACGATGTATCTAGAGATATTATATGTAAGTAGTAAGATTTAATTAGAATGAATGTA
ACTTACGAAAATGGTATTACAGAACTGATACTAGAAACTAGATAAGACAGTAATAATAATAAATATTTTA
GTAGTTGGCTACCAAATGAGATACGTAGAGAGGATAAATATGTGACAAGAAAATGGTGACCGAAAAAG
CATGAGTCACATATTTTTTCTTTATCCACTCAGCAGGCAGGGATGGAAATATTATTAATAATTTGGTTTTT
AATATAATAAATAAAATACTGTAAACTTTTCTATTCGAATTTATTATCTTGGAAAACGCAGACTGGGGTG
GGTGTACCCGCGGGAAGCGTCATAGCGATCTGAAAAAAGTCTGGAAATTTTTAAATGAGTTTAATTATG
AATTTACTCTCTACGACTAAAATGTTTTTGCTGATCCATAGAATGTTGATAGGTCACGTGATTTTTACTAT

GGCTAGTCAAGAACATTTTTAAATATTTCGACTTTCCTGAAGTTGATGAACAAAAGTGGTTCAGAATCA
GAATGTCCCATTAGTCAACCTTTAAGGCTAACGTCACCACTGTAGAGTGGGTTATAATACTAATTTGTTA
ACTTAAGCCTTATAATAAAGACACTATGTTACAAACTAATAATAAAAATATATGATTATATTATACCAACT
GACTTTTGTATATCAAAGAAATATTTTGCAAAATAAAATAAATTGTGACA
>TCONS_00021853 geneID=XLOC_004347 scaffold_132:381640-384261(-) Splice junction overlap
ATTTAATTTATGTTTCGATTGATAATTTTATAGTTTATTTCATAAATTTGCTGTAAATATCGGTCAACGGTT
TTGATTTCGGTTATTTTATTTTGTGTTTAATTTCTTTAATTTGGATTTTATGCACCTTGCGTTCTCAATACC
ATTCGTTCGTGTTGTTCTGCAACGGTTCCAGTAAATGCAGGATTCAGATCTGGACCAAGGCAGACGGG
TTGGAGTTGATGAAAAATATATTCACTTCCGACCCTCACCTAATTTCAAGAAAATGAAAAAGGTATGTA
CGGTGGCCTGCGCCTAAAAGTATACAGGCGGAGTTTTTAAAATAGCAATCCCTGATGATGAAGGGACC
AGCTACATCTGTTATAAATCCGGGGACGTGTTGTGTGATGTGTGTAGCAGTGGTGTTTATGTGGATGTG
CTTGATCAGCATGTGAGCTTGAGATCGCTATTTTAAAAACTC
>TCONS_00021735 geneID=XLOC_004337 scaffold_132:392619-400619(+) Intronic
CGTGGCTTCGTTGAACTTGGACAATGGAGTCAAAGCCTCTTCATGTTCAAACTGCGATGTTTTGAAAA
ATAGGTTGCTGCACACCCAGAGTAAGGCAGAGACTATTAACAACAGCATCAAGGACATGAACCAGAA
GCTCGAAGATCTAGAGAAGAAGGAAAAGCGCCTAAACGCCGTGCTAATATTCCTACTGAATCAACAAA
GCGCGCCGACGGGCCGGCGACCGCAAATAGGTAATGAATAGTAAATAAATAAGTTAAGTTATTTACATT
TTGGAGCAAATCAATGCATTTTTCGCCGTGTCAACATAAAATATTTAATATTAATTATTTCAGAGTTCCCC
TTATGGGAACCCATTGCTTTTACGTTGGTTTTTTTTTAATTGTAAGGTAAAGCCACAATATATAAACATTA
CATATTATTTGTGACAGATGCATTGTAATCATAAACTGGAAATGTGTGTTGCAATCTTTGTATAATGTGTA
GTATGCTTCGAAACGTATCCTTAGTACATAGGTATATCTAATGTCTCAATCACATAATAATTATGTAACGA
TAATCAAGTTATATAAAAATATCTTAAGTAAATAAATATTAAGTCATAGCTCTAACGTAAGTTTAGACAA
GTAGGTAGAAAACTTAGCTCCCGTTTCGCCATAGTCGGTTAAATCGTAACCAGGCATTGGTTCATCACC
GTTCATCAATTATACGTCATCTCCATATTAAATTTTTGACAGATGTGTCAAAAGTCAACCAATAATCCCT
GGTTATTGGCGAAATTGGCACTTAGGTATATAATTTATTTTTGACAAAGATGGCATGATATAGGATGATTG
TCGGCCTAGTTTAGTCATTGATGTGATTTACGTCCGTTACATAATGTTATGTACGATGTATCTAGATATTAT
AATATGTAAGTAGTAAGATTTAATTAGAATGAATGTATCTTAAAAAACTTTTATTACTGACTGGTAACTA
GAAACTAGATAATAGGAATAAATATTTTAATAGTTGACTACCAGAGAAGGTATAACTCGGGTGGATAAA
GATGGGACAAGAAAATGGTGGCCGTTACATATTTATTTTTCATTGTGCAAAAGCATGAGTCAGATTATTT
ATCGTTATCCACACGCGAGTTTCCTATTTGTAGGGATGAAAACATTATTAATAATTTAGTTTATAAATAAA
TTAAACACTGTAAACTTTTGCTATTATTATTTCAATGTCATCATACTCATCATTCGTTTTAATACATATCTT
AGTAAG
>TCONS_00021689 geneID=XLOC_004350 scaffold_132:545495-610955(-) Intergenic
CAGAGCCGCGCCACACGTGTCTCCACCGCGCGACCGCCGCCGCCGACCAATCGCGACCACCTCACCC
CCCACCCTACCCCACCGGTGATGTCTCACGGTTGTCGAGCCGGTTCAAAAACATCGCGTCTGCATTTC
TTTCATTTACCGGTCTCCTTAACATTCAGTGCATAGTTCATAATGACTAATCATACCACCTTCCTTCATAG
TAACATTAAGTGTAGTGACATTCTTGTAGGCTTAATCGTATCGTTAGTGACACAAGTGTAAATAAGTGA
CTGTTCGGTGTAAGTTAGTTTAAGGACAATGACAGCTGCTGCTGTTTAGGTTTAGGTTTAGTAAAGTTA
GTGGTAAAGTGGCTGTTTGAGCGTTTGTTCAAGATGTCTCAGCCGATCAAACGTTCGCTCAGCAGTGA
GGGCGGAGCCTCCAAGCGGGACTGGAAGATGAGGCTCAGCCTGAGGAGCAGGAGTGGCCACAGTGT
GG
>TCONS_00021669 geneID=XLOC_004339 scaffold_132:690204-708310(+) Exonic overlap (+)
GTATAAAGTGGAACCTTGAAGTCGCTCGCGCAGTATCCACAGTCACTTTCGAGCGAGGACGTACCTGA
ATAATTTTCTAATCACTTTTCATGTGTTTTTTTTTGTAAAATGATCCGTTTCTTGGTTTTTCTTGTTTTTGT

GAGCTTCGGAGGTGTGCTGAGTGAGAAGGACATAGGCCGCCGTCGTGTCGTCCCTGTCATACACGTG
GTGGGCTCGCACTACGATGTGGGATTCGATG
>TCONS_00021981 geneID=XLOC_004397 scaffold_1328:5616-6106(-) Intergenic
GGTGGCGTGGTGGTACACACACACAGTTAGTTGAGCGGCGTGGTGGTACACACACACACACACACAG
TTAGTTGAGCGGCGTGGTGGTACACACAAAGTTAGTTGAGCGGCGTGGTGGTACACACACACACAGT
TAGTTGAGCGGCGTGGTGGTACACACACACAGTTAGTTGTGCGGCGTGGTGGTACACACACACAGTT
AGTTGAGCGGCGTGGTGGTCTCGCCAGGGTGCCCACACACAGTTAGTTGAGCTGCGTGGTGCTACAC
ACACACAGTTAGTTGAGCTGCGTGGTGGTACACACACACAGTTAGTTGAGCGGCGTGGTGGTCTCGC
CAGGGTGCCCACACACAGTTAGTTGAGCGGCGTGGTGGTACACACAC
>TCONS_00022253 geneID=XLOC_004405 scaffold_133:33268-34845(+) Intronic
CACAATAACAACCTAACCTAACCTACTAACGAAAACGGTAAAAGAGCATTTGATTTACTTATCCATTTA
ACTTTAAGAAGTATTCTGTAATAAATGGGAAATATTGTGTGGTCTTTAATATGGCTTTTGATTTTAATTTT
CATTGGTTGGTGGATAGCTCTCCTCTTGTCGGTATTCTATGTTATATTTCTACCTTTTGGAGTTTGCATTC
CAGCATGCGCGCCGATCACTGATTTATTACTTGCTATAGTTCAATTTCCTGGATTCTGTGCGGAAAAAAT
GAAAGCTGGCGAAGGATTCTAAAATGTACACTGAAGATGGCACGATGTGCATAATTATAATGTATAAGT
ACCTTGTGAAACATACC
>TCONS_00021991 geneID=XLOC_004406 scaffold_133:37508-38545(+) Exonic overlap (+)
TTACAACACACGTAAGAAATGCAGAGGAAGTAGAAATTGTAAATATCTATATAAATGAAAAACAGTAA
CTAAAAACATAGCAAATGGTAAAAATATAAAATCATCAGTGCTTCAGTATCTAAATCGATTGTGATGGC
CGAAAACAGTTTTCTTTGGTCAATAATTTGGTTTGCGATCCTGGTTTTAGTTTCATTCTGGGTGGCTGG
GTTTGCTGCCCCAATATACATTATTCTGCTACCTTTCACAGTTTTCATAACATCTTTAACGAGTTCAACG
GACTTCTTCCTAAAGGCAATGCAATTTCCTAGATTCTGCGCATATAATATGAAAGAAGGGCTACCATACT
CATAAGTTCATAACAAACATCTCCAAATGATGAAATACCTACACACAAAGAGTTACTGAAACAGACGT
GTATGTAGGCATAGGTACGAAATACGTGAAAATCTGTAATGGCTTCGACGTTATAGTGCTTGGCATGTG
AATTGTATTGAAATTACAGTATTATAACAAAAAATACCCGTGATAGTAAAGCACCCATAAAAATTGTAAA
AGTAACTGTAATAATTTTAATTAAAATTTGGTAACGTATAAGTGACACTGTCAATGTATG
>TCONS_00022077 geneID=XLOC_004407 scaffold_133:39250-40037(+) Unclassified
CAAAAACTTTCTAAATCATTTTATAACCTACTCATACAAAATTATTCAAAATTACCAAAAAAAATATTTT
CTAAATTATGAAAGTAATCTGATTTCCTCTACAAATGAACCATATGAACCCTTAACCCTTAACATTGAAT
GAATAGGTATTAACCTAAAGAGATGGCATTGGTGGTGGGCACCCCCTTCAGCATCATCTGGGCAACCTT
CTGGTTCCTGATGCTAATTCTGGTCGCGTTCTGGGTCGCACTGATAGGTGGAGTTATGTATGTCCTCATA
GTGCCGTACTCAGCATTTGCACCTGATGCCCTAGCGACATGTTTAAATTTTCTCTTCAAATGTATGCAAT
TTCCACTGTATTGCACGGTTATGATGGCAGATGGAAAGCCCTGGGATGGGCCCAGCTGATGTAATAACA
AACGAATAGTAGGTCGGTCTCCACAGAATTAAATAAACGTACGTT
>TCONS_00022099 geneID=XLOC_004408 scaffold_133:48922-50098(+) Unclassified
ATTCAGTTTATTCGAGTTACACTGACTAGGTACACGAGTTAAATCATTCGGAAAGTTATATTTTATAAAA
TATTATGGAATCTATACTTTGGTCGTTCCTTTGGTTTCTCATACTGATATTAATTGGGTTTTGGATTGGGAT
TGTTGCTGCTATCGTTTATGTGGTACTGTTACCTTATACAGCTTTTATACCATCCTTATCGCCTGTCGTGT
TTGTCTTGTTTAAGGGTGTCCAGTTTCCACTACATTGTACTAATATGATGATAGCTGGTAAACAGAGTTA
ATGGGAATGTGTACTTGTGTACGTATACCTTGTTTCACGGGGAAAGACATCTGACAGGACCCTTATTAC
ATTCGAATAAGGGAAGTTTTTGTTTGTATCAGCTGTCTTTCCTCGTGAAAAAAGGTATAGAGAAACCCT
AAAAATCCAAAGTGCTATTGAAAATCAAATTACCTTTTTCCCTAAATCATATACCTAGTACCTAATTGTC
GAAAAACTTCCCAATAAAATGCAAAACTTGAAAATATTTCATTTATTTGCTTTTAAGCACAGGTATTATG
CTTTCATTATTATTGACTAGTACCTTTGGTACCTACGAGTAAATGAACGGACATTGGAAGGCTGCTTCA

GAAGCAGCTCGTGTCGCTATGACAATGATTTGCCGGCTTTCATCTGTTGGGCACAATATTTTGGAAATT
CCATTGCTTTTTTCAAGAAG
>TCONS_00022011 geneID=XLOC_004416 scaffold_133:445613-449037(+) Unclassified
CGTTGGACGACTTTTCGTACAAATTTTAATTTATTTTATTTTGTTGTGGTGTTTCGTTGTGTTGTTATTTG
TAGATTTTTATTCGTTATTTTTGTACTTGTGTCCTTACAAGTTTGGTTTTTTGGGGTTTTCTTACAGCTATT
TTAAACGTCATAAATCGTTAGCATTCTCTCCCTGGTTGTTGTTAAGGGTTGAAAAATGAATAAAACAGT
AACAGGGAGGAACTCGTGTCTATTATTGAATTATAGTCAAGCTCCTTGTCCTTTTAAATCCGTGGGCATT
GCTCTGGTGGTGGCATCAAGGCCTCTGCCTCTGACGCCGATTCATTCTCCATCTTGAATTTAACTAGAT
CGAAATTACTTTTAAAACTTCACTGGTTAAATTATGTTTTCCTTGAATATTAAGGCTACCAGTTAATTCA
CTACAGGGTACATGCCTGGGTGAGAGTACAACAGGTATTTTATTTTATCCCCATTGCTATAAGTTAAGGA
GTTTTGACAATTTTTTGACTATGATGAGTTTTTCTTGGACCATTATCGTTTATAGGTTCCCAGGATATAGA
GCAAGTCAATAAGCTGTAAACATCCAGTTCATCAATAAGCAAGATTGTCAGTCAACTGTATTTTTTTTT
GTGTTTTGCTTCCTGATGGTCAAGCAGGATGTTTGTACATATTTTGGAAGACACTTCAGTCTCCCTCCC
AACACAGAGTATTATCACTTGGACTATTTGCTGTGGTTTTATAAAGTAAAGTAAATCATCCTATCCATCG
CTAGGTTGGCTCTTGTATTAATTTTTCTGTTGGTTAGCCGGGATTCTCATAGGCCAATTCACACTGAATA
TTTGCTTGATGTGGGCATGTTTCAGGTCAACTAGCATGAGGGAGCTGAGCAGTTCAATACAGCCTTAC
AATTCAATTGGCCAAACCAAATATTCATATCATGCAAATGTTCAATGTCTTTCACAATGCTCTGCACATA
CATGCTACAAGAGATTGTTACTATAGCACAGTTTAAACAGTACCAACTAATTGTTACATGTATGTTATAA
CTCTGTACAGTTTAATTAACAAACATCTAGTGATTTGTCACTACAGTGGGGCTACCAAACATTGGCAAT
TAACAATACTAAAGCCCGTCACAGACTTATCTATAATATATATTAATACACCATAGAATTGGTGACTACAC
AGAAATATATATTATAGCTGAGTGTGTGGTCACTGCGAAAATGTATAGAAAATATATAGTAATATGCCTAG
CTATAAACATATTAATATATATTATAGCTAGGTCTGTGACGGGCTTTATCCAGATCTGGCTGACGTTTGAC
AGGGAGCTGCACTGATGGATTGTTAATTGCTAATGTGTCGATGGTGGTATGGCAGTCCATGCTTTTCCA
TCATTTGTTGAGTATGAGGTAGTCTCATCCACTGCTCATTCTACCCATTATATTGGTCCTTATGCAGATGC
TCAAATCAAATCAAATGTTTAATTGCCACCAAGTGTACATGAATGATCTTATAATTAATAAACAGCACTT
AAAACTTGATACCCATACAGGCACTGCAATTTAGCTTAAAACTATGAGAAGTAGAAACAAATCTTAAA
AATAACAAGTACCTACTTAACCCTTATTCATAATAATAACTACAATTATTAAATCAACATTCATTTTACTC
CAACTAATCAATAATAATAAAAACAAGCTTAACATTTTCTATTTATTAAATAGCTATTTAACTTATACTTAA
TATTAATACTCATCTTTATCTGAGTATTAGCTTGTCTCATGGAATTTAATTTTGATTTTACTTTTGGTTGTG
AATAGTTTGGTTGGTAAATATGGTTTCTGAAAGAAAGTGTAGACAATTTTAAGTTAAACTGTCCCAAGT
CAACTTAAAATTGTCTACATCAGTATCAAACTTTATACAATCTCATTTACTAAATTGCATTGTACATTATT
TTATTTAGATTTCATGTCACATAATTGCTTTATATTTATTGTCAGGCAATGTGAAACAGTAAGGAAATGC
ACAAAAAAAAATGTTTTTGGAGTAATGTCTAATATAATTCAGTTTATGTCAGAATTCTTCGCTACTTGGAT
TTTCGGGAGTGGTATTAAGTTCTTCGCACATTATATTAACTCACCGGCGACTCGACTCTATGCTAGACAT
GGATATTTAAGCCCTGTGTCATATGCTCAAGGTTTAATTTCAAAAAATGATTATAGTGTACAACGAGTGG
CCTACGCATCGTACACTAAATGTCTAAAAAAACTAGGTAGTGACTAGGTTGTGGCTAGTCGTTTCACAC
TTTGGTAGTGTATGGGTTGTGACTAGTCGTG
>TCONS_00022398 geneID=XLOC_004490 scaffold_1338:7119-8073(-) Unclassified
ATAACAAGTAAATATTTACTTACTAATCGCAAAGGCAAACAACAAAACTTAATGTTTTTTCGAGACAAA
CATAAAAGAACAGAAAACTAGCTGCCTACACCTAAAATGAAGAGATGAAATATCCTGGTCTTTCGGTA
CCAAATTGTAACGTTTCGCCCTACCGCCTGCCTACTTAACTCCTACCTAATGGATTTACTAAGAAGATAT
TTGAAACTACAACAAAACTTACTTGAGTGAAATAAAAGTGAAACAAATACTGCTTTGAATTGGCCATG
AAACATCCAGTGAACCCCTACGTTGTGATGTAACCCAATAAACGAAAGATAGTGAATTAAATAAAACTA
TGACCTAGAACTGATTACATACTTCCTACCTGCCTATAATTTTTGACCTATATACTACAACTTTAATGTAT

AACTTTTATTTGTATGATAAATACATATTTACAGGTAAGTACATAAAGATAATAATATAGATAATAATGTTT
TTTGGTGTTGGTTAATAAGGGAAGCCCCTCCATACGCTTTGTAGGCCTTGATCACTACACCCGATTGAA
TCAATATATAAGACTCTAAAATAGATAAATAATAGCAAATATTCAATCGGAGCCCTCGTGGAACACAAC
CATTGACTTGGACATTCCTCCGACCAACTTATTAATCACTTCCCTTGCTCCTAACCTTATATATTTACATG
GTTTGTCTTTTTTGTTCATGTAAATAAATCAACATGAATACATGTTATCAAAGCTAAGGGGACAATTTTA
TTTATAATCATAAATTAACTGAAACTAGAATAGTTCAATAATTTATAGTATAGTAACTTTATTAAATCATGA
ATTTCATAATAGATAAGTATTT
>TCONS_00022785 geneID=XLOC_004525 scaffold_134:368725-370653(-) Intergenic
ATTTTAGTTTAAAAAATATTTACGACAAACAAAATGTGATATGTGATAGACGTCGACAAATAATCTAGCA
ATAAAATAAAATGAAATTTAAGCGGTCCAAATTTAATTCAGTATTCATATTATAGTGTAGTTTAAGTGTAT
GTGTTAGGTTACCTAGTTTTAAAGTAGTTTTAAAAGAGTTTTATATGAAAATGCGGGCTTGGTCGGGTG
ATCGCCCAGAAAGTGAAGACGTAATGGGCGCATCTGTGACGAGAACCGGAAGTGGCAAGGAGG
>TCONS_00022476 geneID=XLOC_004534 scaffold_134:510570-511185(-) Unclassified
AAAAATGATAGGCCTGATTACTTCTTAAACATATTATAAATACTGTAGCTTTATATTTTTTAGTTCAATAG
CATCTTGTTTTTTACAGATGGGCAACTGTTCTTTGCTGCTTCGGTTGGTAACTATCAGTTTGCCTGGTCC
TACAACTGAAGATTTATGTACTTGATGGCTGTACAGTAAATAACAGATGCTGGGAAGGCAACTATTTAA
GATTCAGAGGATG
>TCONS_00022581 geneID=XLOC_004534 scaffold_134:510571-510975(-) Unclassified
GTGTTATATTTGATAACTACATGATATTAAATGATAGGGCTGATATAAACAGACATTCTTTTAGTAACTAT
AAGTAGTCAATTTCTATTACAAAACATAATTTTCTTTGTTACAGTTTGCCTGGTCCTACAACTGAAGATT
TATGTACTTGATGGCTGTACAGTAAATAACAGATGCTGGGAAGGCAACTATTTAAGATTCAGAGGAT
>TCONS_00022738 geneID=XLOC_004534 scaffold_134:510704-512108(-) Intergenic
GTTTGGTCGTTTTGCAATTTCTGCAATTTCTCTAAATTTAATATGAATAAGAACTGTGCGAACCAATTTT
CCGGCAGTTTTGTGCCTAATAACGCAGCATCACAACTTCCTATAAAGATTCTTCCACACTTACAGACTG
TAATATTCTTAAATCGCGAATGATAATGTGGATTGTCAATACATGAATATTCCACCTAAAAGTGGTTATAA
TGCCACCTGAAGACCAGGTCATTGATGATACAGCCATGGTTATCGACTAAGGGAATAAAGTAGCGGGT
GCAGTATGAGAGGATAGAGCACCAACCACCATCACATAAGAAGTGATGGGCAACTGTTCTTTGCTGCT
TCGGTTGGTAACTATCAGTTTGCCTGGTCCTACAACTGAAGATTTATGTACTTGATGGCTGTACAGTAAA
TAACAGGTAATAAATCAATATTTTTATAACTGTTATAATGTTTAAAAAAATATTTACCATGATGACATCAA
CACGAGCCGAAAATGTGTTATATCTGATAACGAC
>TCONS_00022578 geneID=XLOC_004517 scaffold_134:713216-714829(+) Unclassified
TGAGCCGCAGCGGCGGCGGAGCGGTGACAGGACTCGGCTCCACCTGGAATTACGTAACACCATCAAC
AATCTCACCACCACTATCACCTAATCTTATCACAACACTATCACTCGGACATTTTCAACACTTTCCCTCA
CTAGAACTTTTCAAAGGGTCAACATTTCTACAGCTTTTAGATTGCAGATACGAACCATCAACACGAAC
ACTATTATCGCCACGTTCCGATACATCTTATCTGTTTTATGTGCTCATTTTAGAACGATTGCCACCGATAT
GTGAAGAATCACGTAACTAAGAAATCGCTTCCTTACAATTGCTTGTGTTTTGCTGATTCATCAAAACCG
TGGTTCGATTGTCGCGACGCTTGCCGCTGCGCTTGCAAGGTCACGCGCTTGACCATTGCAACAGTCAG
CCAGTGGCCGCAGGCAGTTGCACTAATCGATGTTACGTGTTAAGATCCGGTGTGGTAATCTCTAACTGA
TAAAACTATAGTATCCCCTAACTATGCAGTGGTTAGTTAGCGGCTGTTATGCCGGAAGTATAGTTTTGTA
TTTATTCGTTAAAGCGTACTACAAGAATGAACTTGAGGTATTTGATGCGGCTGGCAGTTTTTGTCACGA
TTGCTCTAATAACAGCAGAGGTTTGATGTTTTCTTTTATTATGTCTGCCTTTGTTTGTAGGCTACACGCG
CGGCGCACTTCTATGCACAATTTCTTGCAGTCTAATGTCTATTTCTTTCCTACGTTACTGTCTATCGTCTC
TAACCTACCTACTCCCTATAAATGTCCTAAGGTGTCCTCATAACATAACTTCTCCTGTTCTCATAGGCGA
GTCTGCTGCTCCGGCACTCGCTGCTGCGGCCACAGCTCGAGGAGAGCTGCTTCACGTCGCGCCCCGA

GGAGCGCCCGCTCATGTACCCCGTGTTCAAGGTCTTCGGCCAGTTCAAGGACATGCTCGTCAGGATGG
TCAACGTGCTCGCGGCTCATGCTAAGGAAGCCCGGCTCACTTAGATGTGGTGTAGAGTTTTATAAAGTA
CAAAAAGTTCTGCCTTGCAGGCTCTTTTGAGTAGTTGTTTAGTAGAATTGAATAAGCCGTATTTGACAG
CAGATTCAGCGATGCGTTTAAAAATCTGTCACATTTATGGCCTATTATTTTGACTCTAGTGTAGGTACTAT
CTTGATTATTAACTTTACAAACGAGTTCTTTCGACCTACCTAGTGTATCGGTCTAACGATCTTTAATATAA
TATTATTATACCTACATACTGGAGGTATAAACGGTGCATGCTAAAGCTTGTCCTGAAAGAACACGTTTAT
AAATATTTATATAGTTATAAGATTTTATAATGTTTTTTACCTACACACATTGTACTGTAATGTTAGTCATAA
AGTATGAATTAAATTGATTTTAAAC
>TCONS_00022707 geneID=XLOC_004517 scaffold_134:713634-714829(+) Intergenic
GGCCGCAGGCAGTTGCACTAATCGATGTTACGTGTTAAGATCCGGCGAGTCTGCTGCTCCGGCACTCG
CTGCTGCGGCCACAGCTCGAGGAGAGCTGCTTCACGTCGCGCCCCGAGGAGCGCCCGCTCATGTACC
CCGTGTTCAAGGTCTTCGGCCAGTTCAAGGACATGCTCGTCAGGATGGTCAACGTGCTCGCGGCTCAT
GCTAAGGAAGCCCGGCTCACTTAGATGTGGTGTAGAGTTTTATAAAGTACAAAAAGTTCTGCCTTGCA
GGCTCTTTTGAGTAGTTGTTTAGTAGAATTGAATAAGCCGTATTTGACAGCAGATTCAGCGATGCGTTT
AAAAATCTGTCACATTTATGGCCTATTATTTTGACTCTAGTGTAGGTACTATCTTGATTATTAACTTTACA
AACGAGTTCTTTCGACCTACCTAGTGTATCGGTCTAACGATCTTTAATATAATATTATTATACCTACATACT
GGAGGTATAAACGGTGCATGCTAAAGCTTGTCCTGAAAGAACACGTTTATAAATATTTATATAGTTATAA
GATTTTATAATGTTTTTTACCTACACACATTGTACTGTAATGTTAGTCATAAAGTATGAATTAAATTGATTT
TAAAC
>TCONS_00022780 geneID=XLOC_004517 scaffold_134:713640-714725(+) Intergenic
AGGCAGTTGCACTAATCGATGTTACGTGTTAAGATCCGGTGTGGTAATCTCTAACTGATAAAACTATAGT
ATCCCCTAACTATGCAGTGGTTAGTTAGCGGCTGTTATGCCGGAAGTATAGTTTTGTATTTATTCGTTAA
AGCGTACTACAAGAATGAACTTGAGGTATTTGATGCGGCTGGCAGTTTTTGTCACGATTGCTCTAATAA
CAGCAGAGGCGAGTCTGCTGCTCCGGCACTCGCTGCTGCGGCCACAGCTCGAGGAGAGCTGCTTCAC
GTCGCGCCCCGAGGAGCGCCCGCTCATGTACCCCGTGTTCAAGGTCTTCGGCCAGTTCAAGGACATGC
TCGTCAGGATGGTCAACGTGCTCGCGGCTCATGCTAAGGAAGCCCGGCTCACTTAGATGTGGTGTAGA
GTTTTATAAAGTACAAAAAGTTCTGCCTTGCAGGCTCTTTTGAGTAGTTGTTTAGTAGAATTGAATAAG
CCGTATTTGACAGCAGATTCAGCGATGCGTTTAAAAATCTGTCACATTTATGGCCTATTATTTTGACTCT
AGTGTAGGTACTATCTTGATTATTAACTTTACAAACGAGTTCTTTCGACCTACCTAGTGTATCGGTCTAA
CGATCTTTAATATAATATTATTATACCTACATACTGGAGGTATAAACGGTGCATGCTAAAGCTTGTCCTGA
AAGAACA
>TCONS_00022879 geneID=XLOC_004604 scaffold_1349:766-2189(-) Intergenic
CGTCGGAGTCGTCGGAGGGGCGGTCGCGCAGCTCCTCGTCCGCGTCCGCCGGCGCCGCCTCCGCGCA
CGACCTGCACAAGTCCAGCTCTGACTCGGTGGTGTCGAGTGAGAAGCGGCCGCACGCCTCCTCGCAG
TCCATCGGACCCACTCCCAGCAACAACAGCAGATCGCACAGCCACACGTCGCCCATCAACATCCCGG
G
>TCONS_00022885 geneID=XLOC_004609 scaffold_135:108499-112209(+) Exonic overlap (+)
CAGACGGCTCTCCCCTCTTTCAGGTCGTGGAATGCAAATGTATTGTTTAGCTCCACACCTTCAATAATA
ATTCGGCTCGAAATCTTCGAGATAACAATAGATGGTCGTGGATAATTAGTAAACACCAATTGTTGGTCA
GTGCGGGCGGAGGCTCAGTGGAAAATAACAAATTGGGTGCACAAAATTAATCTTGGAAGTACGTTACG
TGATTCATGCTCAGGTGGAAAGCTGGTGAAATGTGCCCTTGTGTGGAACTGTCCTCCGTGCAGGATTG
TGACGGATCAAGGCATTAAGTTGGTAGTTCTAGTGTTGTGTTCTAG
>TCONS_00023111 geneID=XLOC_004609 scaffold_135:111965-113011(+) Intronic
TTCGGCTCGAAATCTTCGAGATAACAATAGATGGTCGTGGATAATTAGTAAACACCAATTGTTGGTCAG

TGCGGGCGTAGGCTCAGTGGAAAATAACAAATTGGGTGCACAAAATTAATCTTGGAAGTACGTGATTC
ATGCTCAAGTGGAAAGCTGGTGAAATGTGCCCTTGTGTGGAACTGTCCTCCATGCAGGATTGTGACGG
ATCAAGGCATTAAGTTGGTAGTTCTAGTGTTGTGTTCTAGTGCCCTTAACGGCCAGTGACGGTCGTGAT
GATGCCATCAGTGAGGGTGGTGGTATGGGGGCTGGTCTGTTGCTGTCTATTATCCCAGATTGGACTGTT
TGTATCAGGATACGAGGACT
>TCONS_00022972 geneID=XLOC_004610 scaffold_135:145337-150960(+) Unclassified
GTAAATAGTTTATTAAAACTGTGTCAGTTCATTTGGTAGTTCACTATAATGCACTGTCGAGCAGATTATT
AGGGTCACACGACCTTTGCATCTTTTATATCGTCACAAACACACTAATATTAATTACGATGACAAAAGTT
TTTGAGCGTGTAAGCAACCATGTTTTTTACTCTTTCACGGAAAACTTTGATCAATAGAAAACTTATGAC
ATGGATTAGGACCGAGTCGAAATCACCACGGGATTAGGTAAGTACACCTACTTTTTAGCGACCATTTAC
CAGTCTATAAATGTATGTTAGTCAGCAGGGCGATCACGAACAATATAGAATCGAATGCGAGTGCGACTA
GTGGCAACTTGACAGCACTTTCGAACAATTTTTACCTATAGAATTGACAGTTGTCGCACTCGCATTCGA
TTCTATACGTTAGCCCTGTTTTTCGTGATCGCCCTGCAGGTTAATGTAAGTAGCTTTACACAAAAAAAAT
ACATTCGAAATAAAAAGCTTTTTTTACGACGTATCTAGTTTCACCGAATAATACGCCTGTCAAACCCGT
CATTTTTTTCTCGTATTCGTGGTATAATTATGTGAAACTCTGTCTAATAACCTGCAAGCCACTGTACTATT
ATTATCGAGTGAGTACTCTTGAATACGCGTATCGGTCGCCGCTACACCCAGCTAAGTGTGACGTAATCG
CTACTCATTTTATGTAAAAAAAAAAACTGTCAGTCTTGTATGCCAGTACCGGTTTATATTCGAAGCGGTAC
GCACGCTTCTTTCTGAATTCAAATATAATCCGAAATTATAAACCAGCCAAAACAAAATGGCATCATCAT
CTCAAACGCAATTGCCTTCAACCGAAGAGCAATTCGACGATGAAAAGTTCAAACCACATCTTGAAGA
GTTGGAAAAAGCCAAGACAGACCAAGAGTTGGATGAGAAACTTAGTGGCATGTCGAAAATCGGTCTT
TCGATTTCCGGCTGGATGAAGTATGTGTTCAATAGAGATACTAGTCGTGAAGAGTTTAATGCTGTTTGG
TTGACGGTGAGTAAAGTAATCTATCTATGATTAAAATAATGGTCTACGGCTAATTTCATTCAGATCAGCA
CTCTACTATAATAATAGTGTCCAAGTGAGTTATGACTAGCAACAAAAAAGTAGAAGGTTAGTATACAGT
AAAACTACGAAGTCCTCAAAACGGAAGTCGATCCTAACTAAATTGTTAAAGACCGTATTTAATCAATCT
ATTATATTTATTTAAGGACAAATTCGTTACAAGTTATTATCAATCGGAATTTTATTTTTATAATATACCTATG
CAAATGTAAGAAAATAACTGACAATGACAAAAAGTTCATTATTGTCAGGACTTTATAGTTTGACTGTAA
GGGTGTGAACAGTTCTTGCCATACTAACTTGTGCAATATTTTCACATTATATACCTACACAGTCATGTATA
TTAAATTTCAATCTGCGTTATTAATTATTCCAGATTACATAATTTACTTTTATTCATGAGAAGGTTTTTTTT
CTACTAAAACGTATGAAATGCAAGTCTAGACAACATCTTTAGTGTGACA
>TCONS_00023268 geneID=XLOC_004610 scaffold_135:146079-150424(+) Intergenic
CCGGTTTATATTCGAAGCGCCAAAACAAAATGGCATCATCATCTCAAACGCAATTGCCTTCAACCGAA
GAGCAATTCGACGATGAAAAGTTCAAACCACATCTTGAAGAGTTGGAAAAAGCCAAGACAGACCAA
GAGTTGGATGAGAAACTTAGTGGCATGTCGAAAATCGGTCTTTCGATTTCCGGCTGGATGAAGTATGT
GTTCAATAGAGATACTAGTCGTGAAGAGTTTAATGCTGTTTGGTTGACG
>TCONS_00023057 geneID=XLOC_004610 scaffold_135:146081-150426(+) Intergenic
GGTTTATATTCGAAGCGGTACGCACGCTTCTTTCTGAATTCAAATATAATCCCAAAACAAAATGGCATCA
TCATCTCAAACGCAATTGCCTTCAACCGAAGAGCAATTCGACGATGAAAAGTTCAAACCACATCTTGA
AGAGTTGGAAAAAGCCAAGACAGACCAAGAGTTGGATGAGAAACTTAGTGGCATGTCGAAAATCGGT
CTTTCGATTTCCGGCTGGATGAAGTATGTGTTCAATAGAGATACTAGTCGTGAAGAGTTTAATGCTGTTT
GGTTGACGGT
>TCONS_00023216 geneID=XLOC_004610 scaffold_135:146091-150654(+) Intergenic
CGAAGCGGTACGCACGCTTCTTTCTGAATTCAAATATAATCGTTTGTATTTTGTTTATGATTGTGATTTGA
ATCGCGTTTTCTTTTTAAAAAGTGTCTTATTGCTGGTATAAGTATACAGGGGTTGCAAAAGTACTGGTG
GTTTTTCTTAGTTTAATACCATTTTTGTAAAACCCTGTATATTACCTGCATGTGCGGGTCTATACTACCCT

CAAAGCAATCCTTCAATTTATTTTCCATTATCATTATCACAGCGAAATTATAAACCAGCCAAAACAAAAT
GGCATCATCATCTCAAACGCAATTGCCTTCAACCGAAGAGCAATTCGACGATGAAAAGTTCAAACCAC
ATCTTGAAGAGTTGGAAAAAGCCAAGACAGACCAAGAGTTGGATGAGAAACTTAGTGGCATGTCGAA
AATCGGTCTTTCGATTTCCGGCTGGATGAAGTATGTGTTCAATAGAGATACTAGTCGTGAAGAGTTTAA
TGCTGTTTGGTTGACGGTGAGTAAAGTAATCTATCTATGATTAAAATAATGGTCTACGGCTAATTTCATT
CAGATCAGCACTCTACTATAATAATAGTGTCCAAGTGAGTTATGACTAGCAACAAAAAAGTAGAAGGTT
AGTATACAGTAAAACTACGAAGTCCTCAAAACGGAAGTCGATCCTAACTAAATTGTTAAAGACCGTATT
TAATCAATCTATTATATTTATTTAAGGACAAATTCGTT

>TCONS_00023258 geneID=XLOC_004636 scaffold_135:216885-217436(-) Exonic overlap (+)
ATCGCATTTTGCGTTAGTAACTCATTGCGTGGTTAGAAAGGCTACCATTAACATACTTTTATTCCTTTTT
ACAATTAAATTATCTCAAAATGGTTAACTCTACGTCTATATGGATATTGGCCTTGCTAAGCTTCAATGTTG
AAACCCGCAAGCGCCAGCAAAAAAACCTAAAACGCAATGAGATCGGGGCAGGAAAGCGGTTCGGCA
TCACATCGGGATTATAAAGTGCAAGGTCGTTTGTTCCCCAGCGGCGG

>TCONS_00023031 geneID=XLOC_004624 scaffold_135:445843-457353(+) Exonic overlap (+)
GCCGACAGTCCACGACACTGGCTGTACGCTTTCAAACTCTTTGCCTCAAACCTATAACGTGAATTAAA
AACAAAACTTATCGAAACATATCGGCGATAAAATTCGAAATTCAAACCGATGTTTTTATTGTTAATCGA
AATGTTACAATAAAGAAGCGGGTGTGGTGTCAAGTGCGTGGTTGCGACAGGCGGACGCGTGGCGGAA
CATAAAAACAAACTTTTCGTCTCGTGGTGAGAATGGTCCTCGACAACAATTAAGTAGTTGGTAAAAGT
GAGTGTTGTGTTGTGCGACCGAGCGTCTGAATGTACCTGCGGCCTGTCCAACTGGGATTCATTTAGGAT
TTACTATGTTGAAATTGGAGTCCCCGGCGGTGCGGCTGGGGGGGGGGGGCCGCGGTGTCCACAGGA
GTCCCCGGGGGTGCGGCTGGCGGTGGCGGCGCCGCCCGACGCCGGCGTGCTCGCGGTGTACTGGGCC
GAGTACGACCTGCAGGGCAGTGCCGACGACGACAACCA

>TCONS_00022950 geneID=XLOC_004645 scaffold_135:549540-567635(-) Splice junction overlap
CAAACTTTTACACTCATTAACTTGAAAAAAATACATTGACGTAGGAAAAACCCAAGAAAAGTAATGCA
AACAGTAAAAAAAATATAAAAATGTGACAAAATATGAAGAAGTGATCAAAATTGCCAATGTGAACCCT
TCGAACTTCCGCGAATCTTGTGATTTATTTAAATGTGACAAAGTTACAACCGGCACCGCTGACCTATTC
TGTTATTGGGAGTCTCCAAGATGCCAGACACCTTGTCTCTCACGACCGGAGTCAGCCTGGGCCTGCTG
GCTGGCATCGTTTCTGGAGTGTGCTACATCTTCGCCAATGGACTTAATAAGCCGAGGGAGTCTC

>TCONS_00023261 geneID=XLOC_004645 scaffold_135:567305-579757(-) Exonic overlap (-)
CAAGTTATTCAACCGTTATTTTAATAGATTCTCCTGAGAAACGATCCGTTATCAGTTCACCATCTCGGCT
AATACCCACCCTACTGGCTTGATAACGCAACGCACGGCGTGTCATCTCATCTCACCTCACTAGTCTAGT
GACAACTGTCAGTCAGCCACGCAGCCTGCGCAGGCGCCGCTTCAGCGCAAACCAACCAATAACAACC
TTATTACTAAACAGCAAAGTTGACTTGTGATTGGTGCAACCAGGTTATGCTCTTTGAACAAACTGAATT
TCAAGTGGTTCTAAGACAAAAGTGATATAAAGTGTGGAAAGTGTATTAATTGTTTCAAAGTTAATGAGT
GTAGAAGTTTTGATGTTATGTTGGTTGAATAAAAGGAAAAACCCAAGAAAAGTAATGCAAACAGTAAA
AAAAATATAAAAATGTGACAAAATATGAAGAAGTGATCAAAATTGCCAATGTGAACCCTTCGAACTTC
CGCGAATCTTGTGATTTATTTAAATGTGACAAAGTTACAACCGGCACCGCTGACCTATTCTGTTATTGG
GAGTCTCCAAGATGCCAGACACCTTGTCTCTCACGACCGGAGTCAGCCTGGGCCTGCTGGCTGGCATC
GTTTCTGGAGTGTGCTACATCTTCGCCAATGGACTTAATAAGCCGAGGT

>TCONS_00023262 geneID=XLOC_004645 scaffold_135:567305-579757(-) Exonic overlap (-)
CAAGTTATTCAACCGTTATTTTAATAGATTCTCCTGAGAAACGATCCGTTATCAGTTCACCATCTCGGCT
AATACCCACCCTACTGGCTTGATAACGCAACGCACGGCGTGTCATCTCATCTCACCTCACTAGTCTAGT
GACAACTGTCAGTCAGCCACGCAGCCTGCGCAGGCGCCGCTTCAGCGCAAACCAACCAATAACAACC

TTATTACTAAACAGCAAAGTTGACTTGTGATTGGTGCAACCAGGTTATGCTCTTTGAACAAACTGAATT
TCAAGTGGTTCTAAGACAAAAGTGATATAAAGTGTGGAAAGTGTATTAATTGTTTCAAAGTTAATGAGT
GTAGAAGTTTTGATGTTATGTTGGTTGAATAAAAGCAGGTATTTTATGATCATGAACAAAATTAGGAAAT
GTGTGTGAGGTTAAATCATTCAATAGGAAATCGAAACTACACAAACTTTTACACTCATTAACTTGAAAA
AAATACATTGACGTAGGAAAAACCCAAGAAAAGTAATGCAAACAGTAAAAAAAATATAAAAATGTGA
CAAAATATGAAGAAGTGATCAAAATTGCCAATGTGAACCCTTCGAACTTCCGCGAATCTTGTGATTTAT
TTAAATGTGACAAAGTTACAACCGGCACCGCTGACCTATTCTGTTATTGGGAGTCTCCAAGATGCCAG
ACACCTTGTCTCTCACGACCGGAGTCAGCCTGGGCCTGCTGGCTGGCATCGTTTCTGGAGTGTGCTAC
ATCTTCGCCAATGGACTTAATAAGCCGAGGT
>TCONS_00023578 geneID=XLOC_004715 scaffold_136:46826-47634(-) Unclassified
GGTCCGCGGGGAGCGCCGGGGCCGTCCGGGCCGCCGGGCGTGGCGGGCCCGCCCGGGATCAAGGGC
GACAAGGGCGAGCCCGGGCTCCGGGGCAAGGCGGGAGATCGCGGAGAGAAAGGAGACGCGGGGCC
CATGGGATTACCGGGTCCGGTCGGTCTCCCGGGCGAGGCGGGGCGGCCCGGCGACGGCGGCGCCCGA
GTGAGTACCTCCCTATACCTCCACATAGATATACACTCACCCTGTATACCC
>TCONS_00023607 geneID=XLOC_004693 scaffold_136:102913-103458(+) Unclassified
GAAAAAAAAATCTCTTACCTATTCTACAATACAAAACAATTTCTTTTAGCATTTAATAATTGCTATATCTT
TCCCGTTAATTCAAGAAAAACATCACTGTTCTCAAAACTGCTGAGTTGATGCTAAGTTATGGGTCAATA
AACTGCGGAAACAAGTGACACTCACTCACGCAGTTATTTCTAGCGTCTGTTTCTGCAAGAGAGCAGTA
TAAGATGGAGGACGACATAGACATAGAGGAGCATGATCTGTTGAACAACCCAGCCTTGGGGGGCGTG
TTTCCAGAT
>TCONS_00023309 geneID=XLOC_004704 scaffold_136:481180-485379(+) Unclassified
CGTTGGGCGTGTTTATGAAGCAGATCGCGGTCAAAGTACACATGGGCTGCACATGTTTCAAATAAACG
TATTGAATACTTTCGGCGAATATTTTCTGCGAGAATTCACCTACCAACTGCGCTGACAAGTCATGTTGG
ATGTTGGACTGGGAAAAAAAATCGCGAAAATTAACTCTTGTTTGTTTTGGAGGAGAAAACTCCGACTG
TCAATTTTCCAGTGATGTGTTTATAATGTGAAGGATTTGTGATCTGTGAATGACATGTGAAGGCGGGAG
ATTCGATTAGTTTGTGCGAAGTTCCAGCTGATCGCGGGATTTCTGAAATGTTAATAGTTGAGTGAAAGT
GTGTTGTGAGTGCGATGCGGCCGGGTCACAGTCGCCAAGCCAACATTCTGGCTTGCCCTTGTAAACCA
CATAACTTTTATACAACTCGTATGCCAGTGTAGTTTTGAGTGTAGTAAAATAACTGTACCTATCTATACA
GCCATGCCGTTCCCAATGCGCGA
>TCONS_00023318 geneID=XLOC_004706 scaffold_136:569073-594536(+) Unclassified
GTCGCGATCCACACGTTGCGCGGGGAAGTGCCGCCGTACCGCGTTCGCATTAACACTTTTTTCGAAGT
TCTTTGTACAAAATTTCTTTTAAAAGTTGTAATAAGTCGGTTTTGTGTCAAGATATAATTTAATACGAAC
CCTGTGAGTTATAGACTGCAGTTCACGGTGTTACTTGTTGAGTTGTTACGTTAAATTGTGTTTTTTTCTG
TTATTGTTGAGTTTTTTTTTGTTTGGTGCATCGTGCCGGGTCTGAGACCGGTGGGCCCGCGAGGAGCCG
GCTAGCAGACCCTAACACACAAAGCCTTTAGGCGTGCAGTAGTGGGGGTCGCTCGGCGGCCCCGCAG
CCGGCAAGCTGGCCGCCAGCGCGCTCAGCATGAACCCGCATTACCACGGGTATGCGGAGCTCAGCTC
GCCGCACCCGCCCTCGCCCGAGGCCGCCTTCGCCGCCAGCAACGGCCACGAGTACCCGC
>TCONS_00023574 geneID=XLOC_004707 scaffold_136:623148-623473(+) Intergenic
GTTTAATTTCGCGTAGCTTGTCTAAACCACTTGCTAAAACTATTTTATCTGCCTAATAAAATGCGATTACT
AAAAGCCACTTACAACCATTTCATTGGTTAATTCCTTCTCTGAAACTTTAATGCATGTTACAGAATTTAG
CGTTTTAGCTAACAATTTTAAGTTTATGATTTAACAGGGTAAATTTGATCAACGAACCACGAGAATATAG
GCCACATAATAATAATAGGTCAATTGATATAAGCGATGATCTGCTTACAAC
>TCONS_00023676 geneID=XLOC_004732 scaffold_136:727863-728512(-) Intergenic
GCTTCTATCTCGTAAAAATTGGACGTGATCTTCGTATAAAATAGGCCGTTTATGACTCATGGTGACTTTG

ATTGATCATGTAGCCAAAATTATCCATTGCAATCTTAGTCTTCATTAAAATTTTGTACGAAATTGACTTGC
GGAAGATATTTATAAGTAGTGAAGTTTGTTTATACATTGCATGGGAATATTTTGTGAGATCTATTAATTGT
AATCAGTATTTTGTTAAAGTAAAAAATGGCAGTGTTTTTTAACCGCCTTGGACAAGTTGGCTTAGGCCT
TGCGTTTGTTGGAGGTGCAGTCAATTCGACACTTTACAACGTTGATGGCGGTGAACGAGC
>TCONS_00023774 geneID=XLOC_004794 scaffold_1365:578-2633(-) Unclassified
TTTTTGGCACGATTTTTAGCTCGCGCTGTTGCTGATATGCATATCGCCGCGAACTCATAATTGCGCGCAC
CGACAAATAAACTTATATGTTACATTTCTGAGACAAAAAATAATGATATGTAGTGCGCGCACTCTCTAAC
TAAGGCATGGCAAATTTGTTCGATCGTTTGTATAAAAAATGCTTTACTTTCCCCGAGCGTTTATATACCAT
ACCTACGTTTTTTTAACCATTGGGTACCTCAAAACCTTGAGAGAAATTTAATGTGTTTCCCTGCTGATGT
TGTCCCCCTGCGCAGGCGCACGTGGCCCGGCTGCCGCGCGAGGAGGTGCCGGCCGCCGTGTGGGCCG
AGCTGGCCGCCGCGCCCGCCGCGCCCGCAACCCACCCGGTGGAAGGCGGGTACGCGGGAGCCCCCG
TGGAGCCGCTGCCGCCCGGCATGGAGTGAGCGTGGGACGCCGCCGCTGCATCGCCTACCACACTAAA
CACTTAGCTAAGTTATCCCTTACTTATTTTTACGGCCTGTATTATACCAGACTGTGGAATTTAAAAGCATA
AAACGGTTATCGACATCAGAAGAAAGTTGCTATCTGCCACCAAAGTATGTTTGTGGTTAATACAAATAT
TTTGAGATCCATAATGCAAAAACATTTCCAAAGGAAAGAAGGCAAAGTAATTATTAAGGTCACCCCAA
AAACATTATTTTTTTTACTATTAATTAAGAAAGAAAACAAAAATACCTATAGTGCCTCATTACAGATTTAT
AATATACATAATTGAAATGATTAATAATATACTGCCATAATAATTCAATTCACTACTATACCATTTACTTAA
GTAAATTATATTATGTTATGGTTTGTATTTTTTGTATGTTGTCATATCTTGTGTAGTAAAAGAGGTATATATT
TTTCTTTCATGAAGTATTTATTGCCAGTAATAGACGACCGAATGGCGTAGTGGTTAGTGACCCTGACTA
CTGAGCCGATGGTCCCGGGGTCGATTCCCGGCTGGGGCAGATATTTGTTTAAACACAGATATTTGTTCT
CAGGTCTTGGATGTGCCCGTAAAATGGCAATAGGCCCGCCCCCTATTACATTGGGACTAACATAACACT
CTGGCGAAAAGTGGGTGCAGCAATGCACCTCTGCCTACCCCGCAAGGGAGTACATTAGTACAAGGCG
TGAGTGCGTGTGTGTGTAATAAACATACCGTAGTTTTTATGATTTTTTTGTTAAATATAAATTAATTGAAC
ATCTGTTTTAGTTAGTATCTTTGACGAGAGGCAGCAAGTTAATGGTAAATATTGTTTAGTTTACTTGTAA
ATAATAGAACGATAGTGTGTAAACCCAC
>TCONS_00024137 geneID=XLOC_004828 scaffold_137:405577-407618(-) Intergenic
CTAATTGCCACTAGTTAAGCTGTAAACATATTTTTACAAATATTAAATTAAAGCAAAAACTGCATTATATA
AAAGCTGAAAATATTGCTCATACAATTTTGAGTAAAAATATATTTTCAGACTTATTTGTTGTAAAAAAGT
AATTAATTCAATTGAAATTTATAAAGGTATCATTGGTGGTACTGCGTAGATATCAGAAGTTGGACCACAT
TTTTATAATGTGCTATGTGAGCGGATTCAGTGTTAAGAATCTTAGAATACCCGATCAAAAATGAATGATA
TATCTCTTCATTTTATATATAATAGTGTCAGTATAATTTCTCGTGTAGCTAGATCATCTAGAAACAGCCAA
ACTAAGCATCACGTATTATAAAATAGTATTTTATTATCATTAGATATTAAATATCCCATAAAAGACAGCGAT
ATACGTAGTGGTAGGTGGTTTACTCGTAAAGTATAAATCGTCTAATATTAGTGTAGTGATCCATTTTACGT
AGATAGTAGGTATTTTCAACTTGTTTTCATTATTGTCCTGGTCAGACAGGCAGTTGCAAGGTTTTCTCA
AATAATATCCCGTATACTATCACACTCAATGTGAACGTTGAATTAAGAACCTCCTCCTTTTTTTGAAGTC
GGTTAAAAATGGTCCTATATCAGTTCAACAATGGACGATTAAACACTCGGTCCTCGGCCTTCCTCGGCA
ATAACCATAATACATCTTAACAACTTGTCCTTCTGAATAACTGCATTTAAAAAAAAATATATTTCTTTAAA
CATCTCAGATTAATTATGTTGTTGAGGCAAGAGTCACTATAATAATGTTCTCTTTAATACTGTGCATATTT
GCATTAATCAAACCTAAAATTAAGTACCCATACATAATACATCAAGTAAACCTTGCGACTGCTATTTGTC
TATCTTAAGATACGCCGTTTTACCATTCCTAGATATTTGTAATCTGTAATTATAGTATGACATACTTACATT
ATATTAGATATTGATTTTACTTTCTACCCTAGATAGGTAATATTGTCTTTTGTGTTTTTACGAAGGGCTGG
TGGTCCCTTTTATAAAAATTAGGCATGTGTGCAGCCATAGATAATTTGGTTGTGTACCTACTTTAATTTGT
TTAAAATAATGCAATCTTGACAATATTCAGTTTTTATGAATCTCTACGGTATTTTAAAGATTGTATAAATT
AGATAGAGTTATTTTATATTAAGTTTAATTTGCTGCCATTGTAGACTTAGCTCAGTTGTTTTTGTTTGTTA

TTTATGAGTTATCAAAATATATTTTTAAATAAATCTTTAGCAGGGATTTCAAATTTAAAGCTTGAAACA
AGGACTTGTTTCATTTCAAAGGCATAGTAGTACATTGGCCGTCTGTGACTTTCGTCTGTGAATAGACAT
ATAATACAGGATGTTGCAAAAGTGGGGTGACTCAGCTCATCAATCTAAACAACTGTGGTTCCGCGACA
TAAAAAATCTTGAAATATGATATAGTTGTTGTACTTCTCTTACGTATAAAAAGGCAAGTGAAAAATAAA
CTTAGTATTTCTCTAAAATTTTCGAAAGTCGCTGAATAATGATTGCTGTTTAGAATAGCCTCCCAAGTCA
CACCACCCAAGCACACCTTGTGAGATTTAAAAAAAACTAATTTAAACAATTTAAGACTTTCTAACCTAA
ATTTAACCTATTATTGTGATCGCAACCCCTTTACTTTGCGCTTTAAAAATAAGGTTGTTACACTTACTTTT
GACCGTTGGATTTTTACACTCAGATTTAAGTTGTTTATCGCACGAATTTATAACTCAACCCTCTAAACTT
AAGTGTCTTCGAACAAGAAACATTTGTATTTTTTAAATTGTAAAGCACACTTTTGCCTAACATGTCGTG
AATGTACAC
>TCONS_00024110 geneID=XLOC_004834 scaffold_137:587653-588690(-) Intergenic
GATGGGTTTCTGACCATCTTTTGGCATCGGCTATCTCGGTCATGATCACTAAACGAATAGTGGGTATGA
GATTTATCGATACACTAGAGGTTGCAAAATTTTTGTGAAAATATTCTGTGATTTGGTTAGCCGACTAAAT
GAAGACTTCATTTCACTTCAAACTACGACTACTGCATTTATCAAATCTTTAATTTGCCGCATTATTTAGA
AAAATGGCGATACTGGAAGCTATTGTCAAATGATTGTCAAAACAAACTGCAAGGAACCATCCAAAACA
GAGATTGATTTTGGTGTGTGTTTTTTATAATTACCTGAATCCTGTGTTTTAATTGTTCATTATATCTAAGGA
TGGCGCCTTTAAGAAAAGTCTCAGATCGACAAATGGACATTTTGTTGGAGTTTGTTGAAAGACATAAC
TAAAGAGCTTGCTGTTGGCCGGTACCCTCCCGGCCCACTAGGCATACAATCAGCACGTCGGCTATGGG
ACAGTGCAGCATTACAATTATATAAATTATATTCAATTGGGTCTGGAGTTACAAAAACTGGTCAAAAATT
CTGACGGGCATTCACATGTAAAGTGGCAATGTGCAATCATAACTTAAAGGTTAAAGTAGAAAAATTATT
TTAATAATGATTATAGTAAGACTGGTAGAAGCAAAATGTGATAGGTAGTTATGTGATTTAAAATCAAAAA
TAAAAGCC
>TCONS_00023830 geneID=XLOC_004819 scaffold_137:612979-617126(+) Unclassified
NNTTAAGCTTTCACTACTGTGAATGAGTAAAATAAATTCATAACAGTATTATCATCAAATGATAAATATG
ATCTAGTCTACGTGTCCACTGGTTTCGTGAGATATTTAAGATAGTCTATCATTCCATTTCAAATTATATGT
AAGTACCTAAAAGCCGTGAATGAGAATATGATTGAAAAATTAGCAAAATTATTTACAAATCTGAGTCC
CACAATCCAACTTACTCTAGATAAGTAAATTTAGGTGAATAATGTTTATATCTTCCTTGTTAATTCGAGA
GAGCTAAGAGATGGATGGTTCATTTTATAAATATCTATGATTGTTTGTATTACAGGCAGCTGGTGATCTC
TTTGACTATAAATGGAAATACAATTTACACCCATAAGCATCAGAGTTGAAATTACAGTAATGGCATCAAT
TTCTCAAGAAAATATTACTAAACAATGAACTATGAAATGATCTTTTAGCAGTTAGGAAATAGTTCACAG
AAAGTGACTACTTAATTTTAAGTTTATAAAAACATGACATTGGTCGATTTGATTTATATTTATTGACAGCA
TCACTAAGTACTAAGCAGTATTTTACATTATGTATTACTTATGTATTTTGTCAGTTGCACAGTTATATGAA
ATGACTAAGATTATATTTGTAATAGTAATAATATCACTCCAGTGGCTGAAGTTTTAGTTAATATAAAAGGT
GAATACTGAAACATTCAACATGCGATACATACATAAGTATTAGGGTCATGTAACAATGGCAACAAGCCA
TTCGATGTTTTTTAGACTAAGAACTAATTGGTAATAAAATTGTAAACGAAAAGTCACTATTTTTATTTTT
TCTCAGAGACTACGATCTTCTAGGAATCCTATTTGCACACAAGTACCATCAACAAAACAGTATAAAAAC
CTTTTCAAACCAATAGGAGAGCATCATATTTGAAATATATGAAAAGGTTGTACCAATAGTTATTTTAATT
TGAAAAATACATATATTTAAATTATGATGGTGCCTTCTATAGAACTATGAATCAAGTTAAGCTCTGAAAG
TTTTAACGAAATGAAGATCTAAAGTAATGAGAGAAGTATAGTGAATAGAAAGGGTTGAAACTAATATTT
GATTTTTTTAAGTTACCAAGCCATTCAGGTAGCATTGCGCATTGGAGTGAGCAGTTTTGCGAATGTATT
GATAGACTCGTAATATTTACGACATGACAAGGTCTTGGATGAGACGGTATGATTAAAAATCTGCATCTAA
TCTATATCCAAGACTGAGAACTTGAAACTCTCACAAGCGAAGAATTATGGAGCAGCAGACGAGGACAT
CATAAGATATACTTACCTGACCTGACGCTTCTGATTCAAGACCATGGCGGACATCTAAAAAATGATAAA
CAATTACATGTTCTAGAGCGAATAAACATTTTAAAGGTTGACTCAAGGTTTTCCTCTATTCTTTCTCTTT

ATCCTAAGATTATTTATACCGTATTAGGTATTCTGCGATACATGTAACTTGGCCAAAAGTTATGACAGACA
TTGTTAATAGCAGCTAATAAGACGAAAATTGAAATGTAACCTTTGATTCAGTCACTGTCTCAGTAAATA
GAACAAGTTACGTTAAAA
>TCONS_00023831 geneID=XLOC_004819 scaffold_137:612979-617126(+) Intergenic
NNTTAAGCTTTCACTACTGTGAATGAGTAAAATAAATTCATAACAGTATTATCATCAAATGATAAATATG
ATCTAGTCTACGTGTCCACTGGTTTCGTGAGATATTTAAGATAGTCTATCATTCCATTTCAAATTATATGT
AAGTACCTAAAAGCCGTGAATGAGAATATGATTGAAAAATTAGCAAAATTATTTTACAAATCTGAGTCC
CACAATCCAACTTACTCTAGATAAGTAAATTTAGGTGAATAATGTTTATATCTTCCTTGTTAATTCGAGA
GAGCTAAGAGATGGATGGTTCATTTTATAAATATCTATGATTGTTTGTATTACAGGCAGCTGGTGATCTC
TTTGACTATAAATGGAAATACAATTTACACCCATAAGCATCAGAGTTGAAATTACAGTAATGGCATCAAT
TTCTCAAGAAAATATTACTAAACAATGAACTATGAAATGATCTTTTAGCAGTTAGGAAATAGTTCACAG
AAAGTGACTACTTAATTTTAAGTTTATAAAAACATGACATTGGTCGATTTGATTTATATTTATTGACAGCA
TCACTAAGTACTAAGCAGTATTTTACATTATGTATTACTTATGTATTTTGTCAGTTGCACAGTTATATGAA
ATGACTAAGATTATATTTGTAATAGTAATAATATCACTCCAGTGGCTGAAGTTTTAGTTAATATAAAAGGT
GAATACTGAAACATTCAACATGCGATACATACATAAGTATTAGGGTCATGTAACAATGGCAACAAGCCA
TTCGATGTTTTTTAGACTAAGAACTAATTGGTAATAAAATTGTAAACGAAAAGTCACTATTTTTATTTTT
TCTCAGAGACTACGATCTTCTAGGAATCCTATTTGCACACAAGTACCATCAACAAAACAGTATAAAAAC
CTTTTCAAACCAATAGGAGAGCATCATATTTGAAATATATGAAAAGGTTGTACCAATAGTTATTTTAATT
TGAAAAATACATATATTTAAATTATGATGGTGCCTTCTATAGAACTATGAATCAAGTTAAGCTCTGAAAG
TTTTAACGAAATGAAGATCTAAAGTAATGAGAGAAGTATAGTGAATAGAAAGGGTTGAAACTAATATTT
GATTTTTTTAAGTTACCAAGCCATTCAGGTAGCATTGCGCATTGGAGTGAGCAGTTTTGCGAATGTATT
GATAGACTCGTAATATTTACGACATGACAAGGTGGGTTACTCAAGGTAAAATTTATATTATAATTTTATTA
TTTAGTAGTATAGTTAAGTTTAATTAAGTAAGAATAAGAACAATGAGCACCAAATATGACAAAAAACCT
AGAGAAATGAATTTCTAACTATGAATTTAGCTACCTAGCTTGTAAGAAAATGGGATGTGAACCAATGGC
CAGTTGTTGAAACCTGCCTGCAAACAGGCTCATATCGATACAAACAATTAACATTATTAGGGAGATAAA
ATTTAATTAAGGCTGTACACAGCTATAGTTTACGATTATACAATTCATCTAATGAATGTTTTTCATGCCCT
AGATTATCACGTAAAGTATACGGGCAAAAAAAGGACATTGGCCGTATTTTTAAGTACTTACTAGTAGGT
CTTACTCAAAAGTTTCGTCACTGTATAAGTACAAGTTTACTGTTAAGCTAACTTTAAATGTAAACACTAC
AAATTAGAAGAATTTCCAAATACAAAGAAATATACAGAGGGCACATGAGATATGGTTAGCCATTTACGT
AATCTCATGTGTATGTGTACCTAGACCTACTCACGAACACTAGCTTGGTAAGATACATGAAATAACGTTT
GATAGAAAATATCTATCTATAATATACCTACCTATAAGCTAAATATGTTAAGACTATGGAAAATATAACTA
GGTAACATTTATTTGAGGCCTCTAACATAAAGCATAATGCTAGAAACATTAATGAAATCATCACGATTAG
GTCGATCGAAAGTTTGTGGCATTTTGATAAAAGCCATATTTAAAATAACGCATATTTTGAGGGCATAACA
GCCATTATTAAAGTAGAATAAGCATATTTAAATATATAATATTATATAGGTATAGTCTCAGTGTACAAAAG
AATAGGCTAAGGAGAAAACTCAACTCTCAATTTCTATATAGGTAATCAGTAAATTTAAGACGTTCGATG
CAAGGGTCGTGTCAATCGTTATACCTATAAAATGATCATATTTATATAGAAGCATAACATGATGCATGATA
AATAAGCCATCTCTAGTACACTGGCATTGTCCCTATGTAAGTAAAAATTAGATTCTATTGCAGTTACTATA
ACATTTATCTAACCATTTTCAGGTCTTGGATGAGACGGTATGATTAAAAATCTGCATCTAATCTATATCCA
AGACTGAGAACTTGAAACTCTCACAAGCGAAGAATTATGGAGCAGCAGACGAGGACATCATAAGATAT
ACTTACCTGACCTGACGCTTCTGATTCAAGACCATGGCGGACATCTAAAAAATGATAAACAATTACATG
TTCTAGAGCGAATAAACATTTTAAAGGTTGACTCAAGGTTTTCCTCTATTCTTTCTCTTTATCCTAAGATT
ATTTATACCGTATTAGGTATTCTGCGATACATGTAACTTGGCCAAAAGTTATGACAGACATTGTTAATAG
CAGCTAATAAGACGAAAATTGAAATGTAACCTTTGATTCAGTCACTGTCTCAGTAAATAGAACAAGTTA
CGTTAAAA

>TCONS_00024128 geneID=XLOC_004819 scaffold_137:612980-616873(+) Unclassified
NTTAAGCTTTCACTACTGTGAATGAGTAAAATAAATTCATAACAGTATTATCATCAAATGATAAATATGAT
CTAGTCTACGTGTCCACTGGTTTCGTGAGATATTTAAGATAGTCTATCATTCCATTTCAAATTATATGTAA
GTACCTAAAAGCCGTGAATGAGAATATGATTGAAAAATTAGCAAAATTATTTTACAAATCTGAGTCCCA
CAATCCAACTTACTCTAGATAAGTAAATTTAGGTGAATAATGTTTATATCTTCCTTGTTAATTCGAGAGA
GCTAAGAGATGGATGGTTCATTTTATAAATATCTATGATTGTTTGTATTACAGGCAGCTGGTGATCTCTTT
GACTATAAATGGAAATACAATTTACACCCATAAGCATCAGAGTTGAAATTACAGTAATGGCATCAATTTC
TCAAGAAAATATTACTAAACAATGAACTATGAAATGATCTTTTAGCAGTTAGGAAATAGTTCACAGAAA
GTGACTACTTAATTTTAAGTTTATAAAAACATGACATTGGTCGATTTGATTTATATTTATTGACAGCATCA
CTAAGTACTAAGCAGTATTTTACATTATGTATTACTTATGTATTTTGTCAGTTGCACAGTTATATGAAATG
ACTAAGATTATATTTGTAATAGTAATAATATCACTCCAGTGGCTGAAGTTTTAGTTAATATAAAAGGTGAA
TACTGAAACATTCAACATGCGATACATACATAAGTATTAGGGTCATGTAACAATGGCAACAAGCCATTC
GATGTTTTTTAGACTAAGAACTAATTGGTAATAAAATTGTAAACGAAAAGTCACTATTTTTATTTTTTCT
CAGAGACTACGATCTTCTAGGAATCCTATTTGCACACAAGTACCATCAACAAAACAGTATAAAAACCTT
TTCAAACCAATAGGAGAGCATCATATTTGAAATATATGAAAAGGTTGTACCAATAGTTATTTTAATTTGA
AAAATACATATATTTAAATTATGATGGTGCCTTCTATAGAACTATGAATCAAGTTAAGCTCTGAAAGTTTT
AACGAAATGAAGATCTAAAGTAATGAGAGAAGTATAGTGAATAGAAAGGGTTGAAACTAATATTTGAT
TTTTTTAAGTTACCAAGCCATTCAGGTAGCATTGCGCATTGGAGTGAGCAGTTTTGCGAATGTATTGAT
AGACTCGTAATATTTACGACATGACAAGGTGGGTTACTCAAGGTCTTGGATGAGACGGTATGATTAAAA
ATCTGCATCTAATCTATATCCAAGACTGAGAACTTGAAACTCTCACAAGCGAAGAATTATGGAGCAGCA
GACGAGGACATCATAAGATATACTTACCTGACCTGACGCTTCTGATTCAAGACCA
>TCONS_00024666 geneID=XLOC_004890 scaffold_138:43687-45337(+) Splice junction overlap
CTGGAGCGGTTACTATACAAGGCGGCTGGAGGAGACAGGGTGACCAGGGTGCTGGTGCTGGTGCCTA
CTAGGGAGCTTGGAGCACAGGTGCACGCAGTGACCAGACAGAACTAAGCGTTTAGCTCCTTATCACC
ACTAGTAGGACGGTCCCACACTTCATAAATTTGAGATCTGACACGATTAAATCAATCCATTTGGTTAGTA
TGTGTTCGTTTTGTATTCAATTCGCGTTTATCACTGTGGAGCGAGTCCATTGCTCGGGCGCGCGGCGGC
GGCGGCGGCGGCGGGCGGTGTGTGCGGCGCGCCCCTACATCTACACTTGAGCACCGTGGAAGGAATA
AGTGCGCATAAGAACAGATTCGCAGTGACGTGGAGTGGGGAAATTGAAAAGACAGTGATTTACATTG
TTTTAATCATTGCCATTGTATGGATTTTGATGGTTTTAGCTGATACACAAGGAAGTTTTAAGTTCGCTGC
GCGCATTGTTTGGAACAACATACCTCCCCCACTCCACTACACTGCAGTGAAATACTTCCAAACAGCAG
AGCTCTTAGACAATTATAAATCTTAAAATATAAATAAACATCTTCTCAAAATGTCTCCCAAATTATGAGC
ATATATCCAGCATAAAAATCTTTTAAAATAACGCGTCATATTCTCTTTTTTCTTTTCATTTTTTTTTATAGA
TATACAACATGGCACACTGTTATGTAATAATAAATTAGTACGCCGGTACCTAGTTACCTTGTCACCATAG
AATAATTGAATAATATGGATGGATATAAGAAGCTTAACAAGCGCATTATATAACATTCAAAACAACGGTT
GGACAAATCATAACAGGATTCCACACTCGGGGGCTTATACAGACGTCTCTCTCTAAGCCCCTAAGTGT
GAAAAAGACAACAGGATAGCGCATCAGATGCGTTAAGACAACGCGGTCGTAGTTGGTCCTCTGCGTT
GTGAATTAAGACTAATTGTGAGAGTCTCGGGGACGCCTCATTTGATGGCCGCGTGGCGATTCTCCGCG
TGTAGGCA
>TCONS_00024431 geneID=XLOC_004904 scaffold_138:44410-45876(-) Splice junction overlap
ACGGCTACTTCGCGCATCTGCCGAGGAGGCTGGAGGAGCTGCATGATGGTGAGTTTGCCGAGGTGTC
AATATTCACAGTGGAGGGCGTCTGCCTACACGCGGAGAATCGCCACGCGGCCATCAAATGAGGCGTCC
CCGAGACTCTCACAATTAGTCTTAATTCACAACGCAGAGGACCAACTACGACCGCGTTGTCTTAACGC
ATCTGATGCGCTATCCTGTTGTCTTTTTCACACTTAGGGGCTTAGAGAGAGACGTCTGTATAAGCCCCC
GAGTGTGGAATCCTGTTATGATTTGTCCAACCGTTGTTTTGAATGTTATATAATGCGCTTGTTAAGCTTC

TTATATCCATCCATATTATTCAATTATTCTATGGTGACAAGGTAACTAGGTACCGGCGTACTAATTTATTAT
TACATAACAGTGTGCCATGTTGTATATCTATAAAAAAAAATGAAAAGAAAAAAGAGAATATGACGCGTT
ATTTTAAAAGATTTTTATGCTGGATATATGCTCATAATTTGGGAGACATTTTGAGAAGATGTTTATTTATAT
TTTAAGATTTATAATTGTCTAAGAGCTCTGCTGTTTGGAAGTATTTCACTGCAGTGTAGTGGAGTGGGG
GAGGTATGTTGTTCCAAACAATGCGCGCAGCGAACTTAAAACTTCCTTGTGTATCAGCTAAAACCATC
AAAATCCATACAATGGCAATGATTAAAACAATGTAAATCACTGTCTTTTCAATTTCCCCACTCCACGTC
ACTGCGAATCTGTTCTTATGCGCACTTATTCCTTCCACGGTGCTCAAGTGTAGATGTAGGGGCGCGCCG
CACACACCGCCCGCCGCCGCCGCCGCCGCCGCGCGCCCGAGCAATGGACTCGCTCCACAGTGATAAA
CGCGAATTGAATACAAAACGAACACATACTAACCAAATGGATTGATTTAATCGTGTCAGATCTCAAATT
TATGAAGTGTGGGACCGTCCTACTAGTGGTGATAAGGAGCTAAACGCTTAG
>TCONS_00024482 geneID=XLOC_004892 scaffold_138:166019-168022(+) Unclassified
TGCTGCAGGTCGCTCAACTGGTCGCCGACGCATGACAGCCCGATCTACACACTCAAGAAAACTTAGA
CTAGTACTCCTTTCCTCTCTTCAAAACGTATGTATTACTCTTTTTGTATACTAATGTTACCCCTTTCCCTTC
CCTCTAATGAACTTAGAACAATAACTAGGATAAGTCGATTATAATATGTCTCATTTGGCACTTTTATGTCG
ATAATATTTATTGCCTCGATTGTAAGCTTTGAAATCGCCATGAAGAGAAATATAAGCGTTTTTATAAGAA
TTGTGTTATATATCGTCGGTCATTTGTTTCGTCTTAGTGACCGCTAGAGGCGCTGCTTTGTATGTAAGAAT
ACGAAACGTTTTTCATATGCTTCAAACATCAAAACTTACACTCGAGTTACTGGTCGTTGCAAAAGTGAT
TTTATAAGCCGGTTTTACAAGAGTTTTCATAAGTCGTAGAGTAAAAAATAAACAAGCTGAAGCCCTGTT
TTCATACTTAAGGAATGCAGCGCTCTGATTGGCTGAATTAACGTTCAGCAAGATTTATGTTCAGGCTGA
AATAGTTTGTCAAATGAGAAAATTAATATATCCCTTTTGCATCACCTAATGTATCAGAACAATTTAAGTA
CACTACAATTTAAGGTAATGCTATTGCTTGAGATTATTAACACATTGATGGACACTAGATTAACGGTGAA
ATTCATAAAAACGCACAACATTTTTGTATGGGAGTTGAGTGTTGATCTGCTGGTGCACATGCCGGCACA
CTGCCTAGGCGCAGTAAAGAAAACACGACGTCAATGACCGTTGTGTGCGTGTGAGTTCACACTTTTTT
CTGTATGAAAACTTATTGCTGTGTGAAAAAAAGGTACTCTGGGTGCACATATAACTTTGAAATCCTTCC
AAATAAAGCACAACAAGATGGTCTTCATACAAACTCTAGGTAAAACAGGACTGCATTGACCGTTGTTG
AAATAATTAGAAAAAGAGCGAGGACTGCATTCGACGTTGTGATTTTTTTCTAAAAGTGTGGTGGACGT
CAAATGCAGTCAGTGTCCGTCAGTGGGTTAAGATAGTGAATGACACAAAATAGTGTATGCTGTCGTAA
ATAAAATGGCGGTTAAATGTATGTTGTTTAATGAAATTTATGTATGAAGTGTTTTATCAGGTAACGAACG
TATCCGTTACGTATTTAAAAACCATAAGTACATTACCTTTATCGTGGTTATTTTAATTTATCCGCGGAAAC
ATTTCTCAGTAGGCA
>TCONS_00024673 geneID=XLOC_004896 scaffold_138:396464-400260(+) Intergenic
ATTTTGCAGTGTTTACATCTGTTTTAACTGTGAAACGAAACTATAAAAACAAACATTCTACAAATCAGA
AAAACCTACAATAATTATCACACAACCTCGAGAATCATATTTATATATTTTTACTGCCCCGCGTAGTCTTC
ACATCAGACACTAAACAAATTATCCCATTTCACCCCCGAAAAAATACCCGACTGAAACTCAAACAAAA
GCCTGAAAGTCGACAAACCCAAGAGCCGCGGGTGCGTTTACCCCGTCCGCCCTAGCCGCCCGCCTCA
GTCGCTCCCCAGCGCCCCTGACGGCTGGGCGCGGAACTAACGAGAGTAGCGAGACGTAGGCTCTGTG
AGAATACTGCGTTGTTGTGTTGTGTTTGGCGGGTGGTTCGATACGGTGCGTATTTTTGGATTTTTGTGGT
TTAGTAAAAATAAGAGGTAGGTACGTTTAAAAAGAAACAGTCAGCCGATTATACTTTTAATTGAATATT
AAAAATAATAAGAATTAATAAAAAAAATAAAAAAAATACGATCAATTCATTATATATTCAGGAGATTTGA
AAATAATAAATGCTAAATAGTATGCAAAAAATAAAGCTTATTTTTGTGTGCAAATAAAGAATATCTTATC
TTATCTTATCTTATTTGAAGGTATATCTTTTGTCACAACAAAATTACACAAATGCTCAATGTCAATAACA
AAAGCTCTGTTATCTGTTAACTTAACGTGTTATCGGAAGCTGTTGTTGCCACTTATGAACTTTAATTACT
TTGGTCATTAGCGATGCCATGTGTTGTCTTTTGTTAAAAGGATAACCATATTTAGCAGAAAATAGAATTT
AACTGTGTAATTTTTTAACACCGTCTGAGATTAATTGCTTTTTCATTTAAAAACTTTCAACGTTTTTAAA

GTTAAAAATTGTGTTTCCTAATATGGTACACGGGACTGGTGCAATATGGTTTTAGCATTATGTCCATCTC
TTGTACAATTAGTTATACCAAAAAATATATGAGTTATTTTTTGTTTCATTAGTCATATCCTCACTATCTCCA
TTTATTTAAATACCAAAGGGACAAGAATTAACTTTAGACTTATGAAGACGGATTAATAGATCCAAATGT
CCCGAATATCTGAGTATGATTGTCACCCTCTCTGCCCAAATAGGATAAGTTATGTCCCTTTCAATGTCAA
TAACTCTTTGGACAGTAATATGGTAGTCTATGATGTGATATGGAATCGAATGCGGCCACAGAATATTGTC
TCCAGTTTATAGTGAGGGTAAAACCTTCATACTTTTGTCGATAACAGTCAGCAATTTTGTTTTGAACTGT
GAAAATTGAAAATTATGCATCCACTGTAGTTTTAAAGGTTTGCAAGACTCGTAAATAAATTTAATTATGA
ATAAAAATAGTAGGGACTGGTCACTGGTTCACTAGGGAGAAATAACATTTATCATTCAGTTCCTATTGA
GGGTTTATTAAATATGTTCATAATTATCTTTTGAAAAATAGCTTTTCCGCTTGAGCTTCGTATGGCATTTA
AAAGTATACAACCTGTGGGAATTTCGGGATAAAAACTTATCTACTGTATAGCATTCAGGAAAAATGTAG
CTTTATAATAGTTAAATCATTTTTGAAATGGGCCGAGCAGTTTTGAAGCAATTCTAATCAATACTAAGAA
AACAAAAGATATAATGCTTACTGGTTTCAGAACAATAGCGTGGTTTCACATGATAGTTAATGATTGTATG
ACTCAGTCACCCGAACGTTTATCGATGCCCGTTTTGTACAACACTAGTGCTACTACAAAGCTTTTGATA
ATGTAATGATGTACTGTCACTGCCGACACATCTCTCGCTGTCGACAGACGTGACAGTTAATATTAACCT
TTTCTATTATATTTATTTATCATTAATTCATGATTTTAC
>TCONS_00024516 geneID=XLOC_004895 scaffold_138:400276-401108(+) Intergenic
GTTCAATGTTGAGTTAAAGAGAATTTTGATAAATCAGTGTTTACGAATGTTTGAGATTAGTTTTTATCTT
TGTGGTATTTTGGCGAGTGTTTGTTTATAAAAATGTTATCAATGCAGCCAGTGAACCAGCGGTTGTTCA
GTTTTAAGACGTTGAAAGAGAGATTCGAACATGCAAGGCCGGCGGGCGGAGTGCAGCGGAGCGCCA
CCAATGCAGACATGCCCAACCGCGGCGGCACC
>TCONS_00024629 geneID=XLOC_004897 scaffold_138:539749-542801(+) Unclassified
ATTTGAATGATGTGTCAATTACATATTAAATAACATGTTTTTTCACATTTAATAATAATATAGGAATCACA
CATTCTGTGCAAAGTCAAACGGAGGAGGAGGAGGTGGAGGCTAACGTAGCACACCCTCCGGCTAGAC
TTGCGAAGATTGGAGAAAATTTACAGCAAACTGATCTCTCTAAGCAAGGGAGTATTCTATTCTACTATAT
AATAGAAATAGGGAGTAAAAACAACATACATTTTCCCTTTATCAACTCCGCCACGTTGCTCGACAGCTT
TAATCATATATCCGACCCATTTGTATGATTATGTGTCCCCTCTGCTACGAGGGATGGGGAAGTTATAGGA
AGAGAGAGTCTACTCCAGCAATGCGAATATCTTTAAAATGCGCCTGACAACTAGAGAACCGTTGTAGA
GTTGTTGTTGGGCGTTTCAATGAAGAATCAATAAGTCTTCTCTCAGTCTGTTGACTTCACTCTGCGATC
ACGCAGGATCGGATAAATATTTATGATACGATATTGGAAATTCTAAGGTGAGATACGAATGAACGAAAA
GAAAGCTTTTGTATTTCAGCTGGTGATTCTGTAATAAATTCATGGTTGGC
>TCONS_00024545 geneID=XLOC_004913 scaffold_138:590965-594445(-) Unclassified
TGCTATCAATATGACGCAGTGTTTATTTACAAACAGAGTGAGCTACACAAGAATAACAATAATTGTTATT
TAAAAACAAAACATTGTGCAGCATTTGTTTTTGTAACTCGTGTACAAAGACATAGCAATAAAAAAAATA
TATAAAATTTACAATAAAATATTTTTTTTGTTACATTTTATTGTGAGTGTGGAAGTGAAATATAATAATGG
CTGTTTGGAATCAGGACGATGGCTCCACTACGAGAAGACCGACAGCAGAAAGGATCAAGAAACTGCT
TCTCCTGTCAGTCTTATCGCATATCAACGTGACAACGACGTTAACAACACACAACGCATAACACAAAA
CTGTCAACGGTTAAATACAACGTTAGATGCGTCGCGTGCTTACTCCGACTACGTTTTATTAAAACTTAAT
ACTGAAAATGGAATTAGAAGAATTTTGATCAAATATACTAAATCTAAGCTGTAACTTTTGAGTTTTAATT
GACCCGAAAACTTAAAAGTAAAAATTACTAACAGG
>TCONS_00024564 geneID=XLOC_004900 scaffold_138:685938-697484(+) Exonic overlap (+)
GCCCAGGGTGCGCCAGACGTATACAGAAATTTGTTGCAAAAAGTAGGTATACTAAGCGGAAAAGGGAT
TACTTTTTTCGTTAATTTTCCAAACTCGTAGGTCAAAGTTGTTTGGAATGACCTCCTGGGTCACCCCCT
TTCGGCTTAGTATAAGGTAAGTTTGATACATAATCCGCGAAGGCTGTTGTTAACTTTACATTAGCTATCT
ACACGAGACTACACATAAACCAAAAAACTATATTTTAGCAGCGGTGACTGTAGCATCAGCAAAAATAC

CCAATGTTTTGTTTTCCTGATGATGTAAGCTCATAAAGTTATGACATACGGCATTTCCTTAGCAGTCTCT
ACATACGTAGAGACATCGGAACTTGGCAGTGTAACATAAACAAAATTATGACCGAACTGCCTTGAAAA
ACTTAGTGCCACTTTATCTTCAAAGCAACCGTACGTATGCGCGTTATAAAAAAAATGTTTTATAAATTTT
GAGCGCGGAAAAAAAACGGCGGGAAAATTTATTTTTAGGTAATTTTTTAAGTAGTGTTTTTAAGTTTAA
TAATAAAATTAGTCGAAATGGAATACTCGATTGTGATTTTAGTTTTATTAAGTTTATGTGTTGAAAGTGTT
TGGCTTCAAGGTGTGGGCTCAGGAGACGATGGAAACCTGCGAGTGTGCGTGGTGGAAGGCCGAGGG
CTGTACAAGCGCGCCGGCCGGTTCTGTCCGAT
>TCONS_00024612 geneID=XLOC_004917 scaffold_138:705931-732158(-) Exonic overlap (+)
CGACCACTTCTACAGCGTGTTCAGAAATCATAAGTACAGCGTGTTCAAGATTCATGACTTCAGCGTGC
GCTACATGCCTCGCTCGTTCGACACCTGACAGGCGGCCGCCGGCGCCGCCGAGCTCGGCGCGCTCCG
GAAGCAACTCGACGAGAGCGTTCTAGGAATAACAGGATTTCAAATCAGTAAGATGTCTATGATGAACG
ACAACATGACTCGGTTTTACGCCAAAACTAAAGCTGAATAGAAAAGATGGATTCCATTCAACAGGATC
TCATTCATGAAACAACTATTTACCTAATATCAGCATTAACTCACTGTTAGATCTCCGTTAATAACTAGTTT
TAAGCTTAAATACATAACTCAAAGTTACACGAATTTTCATAATACAGGGTGTTGCAAAAAGGGTATACT
AAGCCGAAACCAGCATGTGCACCATCTGGAACCAGCCCAAATCTGGAATCAGAATTTCAAAATTCGCG
AAAAAAAAACATTCTCCATAGAAAAAAGTCATGTGACCAACTAAGTTTCTATGGAAATTCTGATTTCA
GTTTCGGGCTTAGATATAACATGCTGCACATGCTGGTTTCGGCTTAGTATACCCTTTTTCAACACCCTGA
TAAATCATAGTCTTGGAATTTCGTCTGTGGATTAATCATCAAAATAATTTAGCACATTCTTTTCAACTTT
ACCCTTAGTTAGGTAGTTTGTAAAAGTTAAGTATAATGTTATGTATGTGATTCAGTAATTATTATTGTAATT
ATAAGTAAGATATGTGTGAACCTAGAACCCTAAAATATGATTTACTAGCTGTTCCCGCGCGCTTCGCTTC
GCCTTAAAAAGTTTTCCCGTGGGAATTCCGGGATAAAAAGTAGCCTATGTTCTTTCTCAGGGTCTAGAC
CATATGTATACGAAATTTCATTCAAATCCGTTCAGTAGTTTTGGCGTGAAAGAGTAACAGACAGACAGA
CAGACAGACACAGTTACTTCCGCATTTATAATATTAGTTAGGATTTATTTAATGCCCGTACGGGGCACTA
TATTATTATGTACCCTCTATATTATTATTTCCAATTTTATTATACCTGGGTAACAAAACTTATACCAAATCG
CTTAAAAAGCGTAAGTAGGTGTAATTTCTTGTAGATAGAAATTCATTTTGTAGTTAGTACCTAGTAGTTT
ATACTTATATTATACTTAGGTACCTATTTTATCAGTAGTGGGATCTACCTATCACACAAGCAAATTTCCAC
TATCGAATGAAATTGTTTAAGTAAGTACCTACCTACTTATATGGAAATAATCAGTTTAATTCAATTAACTA
AATGTACAGTTTTTTTTATACACTTTGCCGTTTATTTAATTAGGTATTGGGCTTAGTATAAACTATAAAGTA
TAGAAGAAAAACTAACTTTGTG
>TCONS_00024613 geneID=XLOC_004917 scaffold_138:705931-732158(-) Exonic overlap (+)
CGACCACTTCTACAGCGTGTTCAGAAATCATAAGTACAGCGTGTTCAAGATTCATGACTTCAGCGTGC
GCTACATGCCTCGCTCGTTCGACACCTGACAGGCGGCCGCCGGCGCCGCCGAGCTCGGCGCGCTCCG
GAAGCAACTCGACGAGAGCGTTCTAGGAATAACAGGATTTCAAATCAGTAAGATGTCTATGATGAACG
ACAACATGACTCGGTTTTACGCCAAAACCAAAGCTGAATAGAACAGATGGATTCCATTCAACAGGATC
TCATTCATGAAACAACTATTTACCTAATATCAGCATTAACTCACTGTTAGATCTCCGTTAATAACTAGTTT
TAAGCTTAAATACATAACTCAAAGTTACACGAATTTTCATAATACAGGGTGTTGCAAAAAGGGTATACT
AAGCCGAAACCAGCATGTGCACCATCTGGAACCAGCCCAAATCTGGAATCAGAATTTCAAAATTCGCG
AAAAAAAAACATTCTCCATAGAAAAAAGTCATGTGACCAACTAAGTTTCTATGGAAATTCTGATTTCA
GTTTCGGGCTTAGATATAACATGCTGCACATGCTGGTTTCGGCTTAGTATACCCTTTTTCAACACCCTGA
TAAATCATAGTCTTGGAATTTCGTCTGTGGATTAATCATCAAAATAATTTAGCACATTCTTTTCAACTTT
ACCCTTAGTTAGGTAGTTTGTAAAAGTTAAGTATAATGTTATGTATGTGATTCAGTAATTATTATTGTAATT
ATAAGTAAGATATGTGTGAACCTAGAACCCTAAAATATGATTTACTAGCTGTTCCCGCGCGCTTCGCTTC
GCCTTAAAAAGTTTTCCCGTGGGAATTCCGGGATAAAAAGTAGCCTATGTTCTTTCTCAGGGTCTAGAC
CATATGTATACGAAATTTCATTCAAATCCGTTCAGTAGTTTTGGCGTGAAAGAGTAACAGACAGACAGA

CAGACAGACACAGTTACTTCCGCATTTATAATATTAGTTAGGATTTATTTAATGCCCGTACGGGGCACTA
TATTATTATGTACCCTCTATATTATTATTTCCAATTTTATTATACCTGGGTAACAAAACTTATACCAAATCG
CTTAAAAAGCGTAAGTAGGTGTAATTTCTTGTAGATAGAAATTCATTTTGTAGTTAGTACCTAGTAGTTT
ATACTTATATTATACTTAGGTACCTATTTTATCAGTAGTGGGATCTACCTATCACACAAGCAAATTTCCAC
TATCGAATGAAATTGTTTAAGTAAGTACCTACCTACTTATATGGAAATAATCAGTTTAATTCAATTAACTA
AATGTACAGTTTTTTTTATACACTTTGCCGTTTATTTAATTAGGTATTGGGCTTAGTATAAACTATAAAGTA
TAGAAGAAAAACTAACTTTGTG
>TCONS_00024616 geneID=XLOC_004917 scaffold_138:729070-732158(-) Intronic
CGACCACTTCTACAGCGTGTTCAGAAATCATAAGTACAGCGTGTTCAAGATTCATGACTTCAGCGTGC
GCTACATGCCTCGCTCGTTCGACACCTGACAGGCGGCCGCCGGCGCCGCCGAGCTCGGCGCGCTCCG
GAAGCAACTCGACGAGAGCGTTCTAGGAATAACAGGATTTCAAATCAGTAAGATGTCTATGATGAACG
ACAACATGACTCGGTTTTACGCCAAAACTAAAGCTGAATAGAAAAGATGGATTCCATTCAACAGGATC
TCATTCATGAAACAACTATTTACCTAATATCAGCATTAACTCACTGTTAGATCTCCGTTAATAACTAGTTT
TAAGCTTAAATACATAACTCAAAGTTACACGAATTTTCATAATACAGGGTGTTGCAAAAAGGGTATACT
AAGCCTAAACCAGCATGTGCACCATCTGGAACCAGCCCAAATCTGGAATCAGAATTTCAAAATTCGCG
AAAAAAAAACATTCTCCATAGAAAAAAGTCACGTGACCAACTAAGTTTCTATGGAAATTCTGATTTCA
GTTTCGGGCTTAGATATAACATGCTGCACATGCTGGTTTCGGCTTAGTATACCCTTTTTACAACACCCTG
ATAAATCATAGTCTTGGAATTTCGTCTGTGGAATAATCATCAAAATAATTTTAGCACATTCTTTTCAACTT
TACCCTTAGTTAGGTAGTTTGTAAAAGTTAAGTATAATGTTATGTATGTGATTCAGTAATTATTATTGTAAT
TATAAGTAAGATATGTGTGAACCTAGAACCCTAAAATATGATTTATTTATTTATTGCCCGTACGGGGCACT
ATATTATTATGTACCCTCTATATTATTATTTCCAATTTTATTATACCTGGGTAACAAAACTTATACCAAATCG
CTTAAAAAGCGTAAGTAGATGTAATTTCTTGTAGATAGAAATTCATTTATAGTTAGTACCTAGTTTAGGTA
TACTAATATTATACTTATGTAAGTACCTATTTTATCAGTAGTAGGATCTACCTATCACAACACAAGCAAAT
TTCCACTGTCGAATAATAATAATAATGAAATTGGCAAGTACCTAGGTACTTATATGGAAATAATCAGTTT
AATTCAATAAAATGTATAGTTGTTTATACACTTTGCCGTTTATTTAATTAGGTTTTGGGCTTAGTAACTAA
ATAACGATCTAACTATACCTACTAATAACTATAGTATCTATAGCTGAATTTAAACAAAATTATGAAAAGCC
CACTTTGTGCTCAACTATTTTT
>TCONS_00025082 geneID=XLOC_005004 scaffold_139:153020-154760(-) Intergenic
GAATATTAATTCACATACTGCGAGCGATACATAAAATTGACTCAATATCTGTAGAAACATTTGTGTTTTT
CCTATTGCTATGTTCGTTTGGTCAGCTATTAGTTTGTATTTTTGATTCACCTTCAGGCAAATCTTACAATA
CACCCCCTATGGACCCAAGGGGAGATGGATCAGACAAAGATGCTTTTTATGGAGATATCGGGGAGATG
ATAGATCTCTTGAAAGAGACCAAAACAATACGACAAAAAACTACGAAAAATTCAAAGAAGATTATTAG
CAGGCTGAAGAAAACATCTCACACAAGAGAGTGAACTAAGAATCAACAACCAGTTTTCATTTTACTCA
GTACGTGCGTAGTTTTAGCATAATATAATGAAGTCATCAGAAGCCCAATTCGAACGATTCCAATTTTATT
ATACCGTTAGGGTATCTCGTACGGCCGTTTCATTATTATTGGTGCTCATGACAGGAGCCTTCTGAAGTGA
CGTCATCACATAGTATCAGAGAACCCTGTGCCGTCAGAGACTGAAGCTAATCCAAGGAACATTTAAGT
ACTTTTCTAAAGCTGGCTTTGATTTGCACATCCTCTAGTGGTCTGCCAATCGCCACCGACATACACGAA
AGGAGAAGAATGCGTTATGGAGACATTTGCCACTTGGCGATCAAGATGGAGGTTCTTCTATGACATTA
ACATGATTTACC
>TCONS_00025005 geneID=XLOC_004994 scaffold_139:435694-470950(+) Splice junction overlap
GATTTAACCATTTTACTAGTTTATGTTTATATTTATGTGAGTTTCACGTGCTTGTGCAATTTTTACAAGTTA
TTAGTGCTGTTAGATATCATAGTGCACTTTGTTGGATCGCCTTTGGATGTGTTCAGTGTTCAATTCTGTG
ATTATTTACTCAGTGACAGTGCTTTTCGGGCTGTGATAATGCTATTAATTGGATGATGCTGGCGTGCTGA
CGAACGGAGAAGCATGGAGAAAGCGAAGGCGTACATCAAGAACCGCTCGCCGCCGAGCGACAAGCC

GCCGCCGCCCGACGACCGCGTGTGCTTCGTGTGCGGCGGCGCCGGCTTCGCGGACCAGTACCGGATA
CGGGTCAAGCCGGACCCTCAGTCGTCGGAGCCGTACTTCCCGTTCCTGGGCGCGCACCCGCCGCC
>TCONS_00025070 geneID=XLOC_004994 scaffold_139:486615-492954(+) Splice junction overlap
TCTAGCTCTTGTGCTACCTGACGGTTACTTGTGAATGTATTCTTCATAAAACCAACATTTCGTTGTCATT
AACTCAAAGTAGTATTCAAAAATCATCCGTATCATATCACTTAAAAACCCATTACAATGATACGATATCA
ACCATCACATGAGGCGCAAAGCATCACCACACCCCCGAACCAGGATCGTTAAAGTCCAATGATACTTG
AGCACTATCACAACACGGGACTGATAAGAATTAGAACCCGCTACACCGCAGTCTTGGTCTGACCGTCG
CGGAGAGCAGACGTCTTTTACGCATAAGATTGTGAAGTCTTACGAAACTTTCTAAACTAAAACAGTGT
TTTTGTAGACTACAAAGTAATGATGGGTCCATAATAGCCAGGCAGTGTTGTGTGCAACTGAGTGACGTA
TAAAGTCCTGTGTAAAGTGTCAAGTGAAGTGTGAGTGACGGACGATGTTGGATCGCAGTGGAGTGAA
GGAGGAGCGACTGTCGCCGGTGGATTATAGGATGCGC
>TCONS_00026329 geneID=XLOC_005067 scaffold_14:125255-127077(+) Exonic overlap (-)
TGTTTCTGTATCAGTTTTATTGTATCTATTCACACACCTTGACAGCGGCACGAATATTTTTATGCTTAGC
AAGTAAAAATAAATTACCAGTATCCAGATTTATAAGGAAAAATATAATTTAAGAATTAGGGAATTATGGT
AATGGGAGTGCTCACGAAGTGCTATTAAAATGATTTGTCACTATGATTTTAATGAAATTCCGCTGCGGC
CTTAGCTCAGCGTTATTACATAAAACTAGGTAAATTAGACATTAAACAGTTCGTATATACTCCCCTGGTC
GTTTAATTCGTTGTTTATATATATATAGTTATGTAAGCATAAAAGTGTTCATGTCTTTCTTCAGTATAATTTTT
ATTTACTATAATACAGTTATTAAGTTATTGCGTTTAGTTCTCTTAAATATTCGCGTTAAAATTACAATATAT
ACTTACAACAGCGACGTATGTACAAAACTATAGACTCAATTTCGGACTTTCAGTCTCTACAATATTGGA
AAGTATTTTACAATAAAACAAACCGGACAGATACCTATACAATGTTAAGTAACGTAATCTATTCTAGTAA
CTATACAAGGCCTTGTCACACCGAGATAAACAAATCACATTATCGAAATCAACAAAACTTGACTGGTTT
CAATGCCAGCATGAGACAAAACTTTGATCATAAAATGATGTTTTAATTTCACAAGTTGTAAAATAGACA
ATCATAGTCTTCTGACTAGAAATTAATTACACAGATACATATTTTAAAATCGTTAACGAATCTATTGTAGA
TCAAACTTATGTTAAACTGGTATTAAAAACTAGGTACTGATCATTTTAAGTAGATAAGTATTAATAAATAT
ATGAGAACCTAATTATTTTTTTATTTAAGTATCTTTTAAAAACAATGGACATAAATGGACGTCATTAGGC
ACTAAATATAAAGGGAACGATTTTTGAAAAATAAAATACAATTACAATACTACATAGGTATACAAATAAA
ATAAGTTGTTCTAAAAATCAGTTTCGTTCACCTAGACAAAACGTAACACATATTATAATACCTATTTTGA
AATCACGTTCGTTCAACCAACTCCAACAATAACTATAGCAAGTTTTCGCAAGGCCCAACGTGTATTAGC
ACCTAGTATTGTCGGTCGGTCGGACGCGGCGCCCGACGCTTCTGTCGTGGCCATCGCGATGTGTCCAC
TATCACATCTACGCTTTCGCTACCGGTGCATACCTTTATTTACTATCGTGTATACATGTATACAATACATAT
TTATAACATTAAATCTAGAAGTCAATATTTACAGTTCGGTTGTCTGGGCACCGTGAGGGCGCGGTTATT
GTCTGGCGCATGGGGCGGGCGCCGGCGTGCTGCCCGGGGGCGCGCGCGACGGGAGATGCTGGTACGC
CGGGTGCAGCGGCCGCTCGTACGGCTCCGGCGACTCGGGGTCGCTGTCTGCTCGTGATTCAGACCATT
CCGATTCTGTTCTTTGCCCCAGCAAGTCTAGTCTTTGTTGTGG
>TCONS_00025388 geneID=XLOC_005142 scaffold_14:147209-164744(-) Unclassified
CGGACGACGCGCGAGCGGGACCGAAGCGCCAATTACTATCACCTGGCGGGCAGCTACCGCATTACCC
ACTGCCGCCGCGCGCGCGACCACGCGCTCGCTGCACCACGCGGTCCGCTCCTGCCATACTCCCGCCGT
GCACTACAATCGATATCGGCAGCGGCCCGATCGCCTCAGTGCACCCCTGCCTTACGCCGCTACCCCGCC
GTCGACTGTGCGATGCCCCGCACCCAACTTTGCCGATCTACGTTCCCGTAGAGTGCTACGTTTAGAGA
CGAATTTATGCAACTGTGAAGTGTGTGCGGACAGTGACTTGGACATTTACCTGGTTTTGTACAATTGTT
ATACTGTCGAACGCAAAAGTGACTAAAAATGACATCCCAGTTTGCCTTCAGAGGATCACCTCCAAACA
CGAGCACCATATCGACAGCATCCAACAGTCCGCCGCCGGCGCAAGCGGGGATGAGC
>TCONS_00026464 geneID=XLOC_005144 scaffold_14:365523-369594(-) Exonic overlap (-)
GGTAAGTACGACAGTAATCTTTTAAATGTTATCTATTTGTGTGAAAAATAGCATCAATGCCAAATAGGGT

CGAAAATTGCTTTCCACGGCCGCTGTTTCCTTTAAAACTTTAAACGTGCATCATTTATACACTACTTTGC
TACTTTTTACTTTATCGGGCAAAAAACATAAGTGGCTATAATTTTATAGAGGACTCGGAGATAATTTGGA
TTAAGAAGGCTGCGTTCGTGTAGCGAGGTGTTTGTATAATCACAAAGCTCTGAGGCGGCGGCGGCGAG
CAGACTAGACCGGCTTTTGGTTCTACAGCCGACATGGACGACTGACGCAGAGAACCTTGTCGCAGTG
AATAATAGCATTATGGACTCTCCTAATGTCCATTTGTAGAGTTTAGTATGTTCGAACAAGCTTAATTATCT
AACATTGAGTGTCTGTGAATGTCAGCCAGTGTAACAAATACTAAGAAAAATAAGAAAAATCACAGTTT
AGGCGCCGTGTTTAGAAAAGCGACACCCTAAAAGTGCCCCAATTTGATCGCAATGAAATGAAGCGTA
GACTTTACAGTTTTAAGATGGGTCCGCCAATGTTAATGGTCTGTTCTGTGAAGTAATGGCGGGTCTGAA
GGGTAATAAAACACTGGATGTGACGGTTTTGTTCCCAATGTTGGATCGAACACGATCAGCTTGTCTTGG
TTCAACGGAAAAGACTTCCCTATCTGCAAGTATAAGTACATACATAGATCTATTTATTAAGTTAATTCATT
GTTACAATAATAAAAAATATGCAGTGTACAATTATAAAGTTCTTTGGTTTGTTTACGTTTAGCTTTTTAAA
GTTATGACATTTTTAATAATTCGTTTTACCTTCTCTATTTACGCTAAGAAGAAAGTTTGAGACACCTACT
TTTGCTTCATTTTTCTACGAACACATTTCCGTAACACTATATTTTCTGCCTTTTTTAGCATTTCAAAGTGC
AAATACAGAGTAAGTCGTGCTCAACAACAAGTCAGGCAATTACAGCGTTTCATTTAATACTTAAGTTTT
GCTTAACTTGATCTTCTCAAATGACAATCCGAGGGTAAAACAGGTGAATTTATAGCTGAAGTAGCTTAC
CGATCTCGCATTGACGATGTTCCTTACAGCACCCGCAAGCAGCTTTACCAGGTCTTCGTGGCCTCGTTT
GCAGGCCCAGTGCATCGCGGTCTAAACCTGTAGTTTCAGCACGAAGAGGACGGGAGCATTGCCGCAT
TTACCAGAAAGAAAACATATGGGTAATTAATTGCTTTGCCATCATCATGATATCATATAAATATTCGCCAA
TAAGGTATTTT
>TCONS_00025215 geneID=XLOC_005068 scaffold_14:366360-368477(+) Splice junction overlap
CAGAGCTTTTACCATTTTATTATTCGTATTTAGTAGATTCAGCCCATAATATACGTAGGCCGCTCTGCCCT
CTACAGATTACAGAGAGTGTAATTCTATCTTCCTTATCCTTTTTATACATCCATAGTTAAATTGTTTTAGTC
ACTTGTTCAAGAGCGACTCGAGATTTATTTGGGGGAATTTAAAAGCCTTGCTCTGCGCAAGGACCAAG
TAAGCCCTTCACGATATTTATTAGCTCATCCGGCGATAAATTAGTTAATTCCATGTCCGGCTGACCCCAA
AATGTTTCCTGGATAAGATGTTTTAAATAAGGCCTTTGAGGTTGAATCTTTGCTTTGTCTTATTTGACTA
AAAAAGTGGGCGAATGAATGAAACACCCGAGATTATTGGGTGGAACCGGAATATAACTTTTAACGAGC
TTTATTTAGAAATTGTCCTCTTACATTTTTATACTTTTTCTTGTTTTGTTTTTATTAAAATACACCGGATTA
CGAAATTAGACACGGCTAGTAGGTGTTAGGCGATTATACAGACCATCTCATATTTATGCCACATGAAGG
GTGACAATCTAACCCACAAGCTAGTAATTGGAGTGCATTCGCTTCAGATATTTGTAATGTTATGTTAAGT
GCTTGCAAAATCTTTTTCATTAATTAATTATTAACAAAAGTTATTTTCTTTTCCATATTATCTTCTCTGACT
GGATCATATGCAGTATACCGCGATGCACTGGGCCTGCAAACGAGGCCACGAAGACCTGGTAAAGCTGC
TTGCGGGTGCTGTAAGGAACATCGTCAATGC
>TCONS_00026467 geneID=XLOC_005146 scaffold_14:482340-483885(-) Exonic overlap (+)
GACAAATTGAACAAATTCTCAAATCGAAACTAAACGATTTTTTGCCTTTTGCAAGCGTTTACGATGATT
ATCAATATATTTAATTCTTAAAGTGCTCAAATGTAAATAATAATCATAAATGCCTATTCATAAGTCTTAGAT
TTACTGTGTAAAATGAGTTATTAAATGAGAAACTAAGCAGTGAATCCCGTCAAAGATGTCGACGCATCT
CGAAACATACAAATTCAAACACAAACATGAGAAGGTAAGCTTAAATTCAAAAAGTAATGCACACAGG
CAAGGTGCAGCATATTTTTTCGTAAACCAATTACCGGTTTTACCTTTGTTATGTAGGAGGTAGAAAGAA
ATTATTGTTTAATAGGACTACTATTGGGGGTATGCCAATTAAAATGTGCGTTTGCTTCTTCCTCTTGCGTA
TAAATGTCGTGAAAGGTTATTTTTTTCCCTGAACGAAACAAGCAACTTTTATACCTAATTGTGCATTTTC
TGGTATAAGGCCACACGGGACTTTTATTAATTCGAACACAACCATGAGAAGGTAAGCATAAATTCAAG
AAGCAATGTGCTTTCACCACCACAAGCACATGTGGGAACTTGTAATTGGCTATTTGTAACTTGATTATAT
CATATTGTGTTATAGCTGAAAGTTCTCCAAATATTATAAAATCTAATTAAAATAAAATACAGTTAGATTTA
TAGAGACTTTAATAAAAATGTTGAGAAGCCTTACCTAAAAAAAATTCCAAGACAAGCAACTTTATTTTA

TTACTATATGCTCTGGTTATTGAATGCTCCATGGTCTGAGGGAGGGCTATAACAAAGTGTTCAAAAAAC
TGTACATTAATAGCCGTCGATTTGAAGGGTGTGGGGGTGGGGGGGTGAGGGAGGGGGGCAGAGGGCA
TCCTCTCGTACCCTCTGCCACCCGCGGAGTCTCTTGGTTTCCAAGATATTTGTGGTTGGAGTTAGGTTA
GGTTTGTTTTACTCTATATTTGAAATGCTCCGCTCGCTTTGTTTTTCGATATCTATGTGAACCTAACATGC
TCCTCCTCGCTTTGCTTGCCGTTGCACCTATCTTTAGGTTTTGGCACAGAATAATAACTAGTACCAGTAC
AGAAGTCCACCTTCGCAATGGCTTTCGAAGAAATATGACTCCATCCCATAATCAATAAGATCTAATTTA
GATCGCTTTCCAGCCGCACACGTTTTTATTTACTTACCTACCAATTTATTTTTGATGGAATATGCGCCTTA
TTTCACTGGACTACGGTGCATAATAAGTTATACGTGGTTATACGCATTGAAAAAGAAGCCACCTTAGGG
TTGCCTCGGCACCACAGACTATAACGTTTACTAAGCAACGGACTGAGTTTGTAAAAAGAATGTCATGA
TTTTAAAACAAAATTAATTTGAATTTAGAATACAAAGCTATTCCAAACTTATTTTCTATTGGAAATGACT
AGTAAAAGCTACGAC
>TCONS_00025714 geneID=XLOC_005149 scaffold_14:558548-562814(-) Unclassified
TGATTATAAAAGTATTGTATTAAAAAGAGTCGTTTTATTATCATTGTCGTTATGCTTACGCGTTCATTAAT
CATTTTAAGGAATTGATTTCTGTTGGTTTCATATATTGAAAGCTCTAAAGTAACGCACATAAGTACAAAT
AGATCTTGCCAATATTAAGTAATGATTAGCTATTAATATCGCAGTATAGAAGAATTACAATGTATGTATGA
GGATTTTAGACGTGTTGTTTGTGCTGTAATTAGAATATGATGTTATGATCGGCTTGAGTAGCACTGGCCA
GTTCTAGTGCCAGCTACCACTTATGGCACTATGTAGGGAGTGGGCAGCTGTCACCAGGACTGGCTGGC
GGACCAACAGTAGGTACATAGTATCGGTCTGTCGATCAAAAAAATAACGTAAAACGGTCCTCGTGCTG
TTTGAATACATTACACTCGAGCCCCGTCTGAGCAGTGCTGTGGACAGGCCGGTACTAGAGAGATAACG
GATTGAATTATTCTAAAACTTAGACTGGTGATCTTCTAAGCTTGTGATCGGAAATAATACAAAACATAGT
AATACTTAAGTATAAATAAAATACTTTGTAACTACATTTGTAAACTGTTTTGTAATGTTAATATTATAATGC
AATACCATAAAGCTTCTAAACTAAGTGGATATCAATTATTACCCAGTACAGACACATAAGTACATACATA
TTTTATATAACGTCGTATACTTGCGTAAAGTTATATTTGCATGGGCCTATAGTGATGGGAGTGCCCGAGAT
TGACCCCGTACAGCGATCCTTGATACCCTGCGCTTGCGCCACCTATTTATTGACGTTGCCTTCAGTGTGT
CAAACTTCACAATCGCTATTGGTGTATTCCATATTTTACTATTTATATGCGAAAGACTGTGTTTGTTAATT
TGTCCACATTGTACGTTGCACCGGAGCGACAGCAGACCCGTGGTTTGCAAGCAGAATGTATAAACAAT
AAAACTGTAAACTCACCCCTCATTATTTTATTTGTTATAATTATGGATATTAATTGGAATTATATTTGCAAG
AGAAGGTGCACTCAACATTATGCAATAAAATCATTATATCACACTAGAGATAATATGAAAATAACATAAA
AGCATAATGGCGTTTCCATTTTCGCCAAGACTGATTAGATTTTTTTATATATTATAAGCGTGTCTGTAAT
ATCCTAGTGTAACACGTACAGTAAAGATATTTTATTACAAGCTGTCGGAAATCTTTGTGTTATGGATGTC
ATTTCAGCTAAGTACGGTGATATGCGTCGGTACGTACGTACGGTAACAACGTAATGGACCTCTGTTAGG
TATATTAGAATATCATTGGTAATAGTATAAACTTATCCTGAGAAAATGTACACTAAAAGATGAATCTATCT
CCCGGAGAGGATAACAGGTACAAGGCTAACAGGTAAGTACCTTTATCTACGAATTCGAATATCATATTAT
TACTATATATTACAAAAGAGTTGATCTAGCTAGCGCTCCGAACGTTATACTAGCCGTAACATGAACATTT
CCGACGTGTTTAATGAAATAGAAGCACTGTTATAAACAATCTCATTATTTTGAAATTATTATAAACTTTAA
TACTTAAATTCCATCATTTAGTTTAGGTTGAGGCTCCGAATTTAAAATAAACATTATGTTGTTGTATTCAT
GTATAAATGTTGTAACAATGTTTTATTATTTATTAAATAGCATTGAGATTTATTTTGAAGATGCAGTTGTAT
TTGTTTCATATAACATGACTGTTTTCTGAATCAACTGAATTTATTTAATACATAGGTAGTGTGGCAAACTT
TTAGATACTCTGTTAGCGTTGTGATTCATGATATCATAAATAAAATTATTTAAACAACTACCAGCCTTTTT
AATCCGTTATTTAAATTTTTCAGTCATACCACCACGCCAGTTCAGTCTGGGCGGGCGAATAAATAAACT
GCAAAGAACTGCCTCTAAATTTATAGAATTGCCTTACAATTTACAGAATTGCGATAGTACCTCATTATTA
TTGTAAAATTTTAAACAAACACGGTCTCTATTGAGTCCAATCAGGCTAGCATATGATTAGGGCCGAGCG
ATTGCTTTATCAGAAGTTGATATTTTATTTATTTGTAGGTACATGGTGTGCCCTCGTCAGAGGGCAACTG
TGCACTATGAGTACTAGTCGGCAATAATATGTATTATGATATTTGTCATATCTATGACATTCTATTTCTGGG

GTGTCTAGTGGTTTACCTACCGCTAGGTGGTAGTTGCACTTAAATTCACACATACCGCCCACGCTCAGT
TATGAAACTGTGAAAACTTACAGCCATGCAAAGGCTACACGCGACAACGAAAGCTTGGCGACTAGAC
GCGACGACGGCTACGACCGCGAATTTTCACATATTTATTATGCAATGTTTTTCATAGTTTGCGTCAGAGT
CGCGATGGCAAAAAAAAATGAGAAAATTCGCGGCCGTAGCCTGTTGTCGCGTCTAGTCGCCGGGCTGT
GTCGCTGTCGCACGTCATCTAGTGTGCGCCTAATATAGCTTTGGCAATATTTCTACATTGCTTTAAAATA
AATCTCACTGTTACTGCTGATATGGTATTATTTAAGTTTCCCTGAAGTGTGTGTTTCATTAGGAGCTGTAT
TGAAGTAGTTGTAATGATTTTATTAAAAATAAGAATTATATGTTGTAGGTATTCCTGTGAATGATTATGTG
CAATATGTGTGTAATTAATTTCAATATCTTATCTTGTTATTAATGTTATTAAATAAATAATTAATGCTTTATA
TTTTATTATCATTAATTTGTTACTCCCAAAGGATAAATTATAGTTTTTAAATAAATGTTAAGCGTATAGTTG
GTATCTATTAATAATTTACTCAAGTGGAAGTAATAAGTACTTTTTATATAAAATTATATTAAGACTGTAGCT
TCACTAAATGCAAATATCAACTATGGTGAGTTCCACTGCGATGCGACATGAAAAAGAAGTGCCTTAAA
ACCCGAACATAAGGTTTTTTACTGAAATTATAATTTAAACTATAATATAACTGAAACTAAAACTGTTTTC
ATTAAATTTCACTAAAATATAAAAATCTGTGAAGGTTACTTCCCTTTCATGTGGCATCGCCGTGGGTCAG
CCTGTACACGGGAGCCGATGGCGTGGAGGTTGACAAAGCTGTCGTGAGATGTGCTCTCGGGTTTGATG
ACCGCTGCTCCACCGTTGCTCTCTTATTTCCACATGTTTTGCCTATAGAAACATGAGAAACCAAATACTT
GCAGTATTCTTTTCATTTAAAATTACCGTGGGATGTTATCAGCGATTGACGTGTAGTTAATCGGTAAACT
ATAAATACGGTATGGTATCTATACTCTCTTACTATACATTGTGCTACATACTGGCTAAAAATACTTATAAA
CATATTATTATACACAACCCATAGTAACGCGTGAAATTTGATGTCGCCAACCCCTCCGATGATGATTGAC
CCACACGATTTTCAAAATACCCTACGCTTAATCTTGACTTAATACATGTGTAAGTACCAAAATTTATTAC
TTAGTTACTAAGTATTATTTTTATTTATTGTATTGTATATTTTCTAAATGATGCTATCTCATGTCTCTATAGG
CAATTCATACGCTATACTTATAAAAAATTGTCGTAATATTAATAATGATCCATGAAGCTTATTGACCACAT
TGTTGACAAATTGTCTAAATCACCATGACCCCAGCTGGTTCTCGAGATGCGAGAGCAAATGAAAAAGC
ATCGGTATTTAGTCCTTTACCATGTATTTATGCAAACAATAAAAGTATACTGAGCTCCA
>TCONS_00026469 geneID=XLOC_005151 scaffold_14:627216-631864(-) Unclassified
TATTGGTATACATACTTTAATGGCGATAGATTAATATTACATTGAATCTTAAACTAACACTGCATGCATGC
ACACAAGCTCGGTTGTCGGTACTAGTCGGTAGTCGGTACAATACGGCGAGCCATTTCAATCTCTATACT
ATTACTTAAGATTATATTCATATTGACACAATAATTGAAAGTACAAGAAAATGAGATACCATCTACAGAC
AAAGAGTAATAATCTTAAAATTGTTCACTACTTCAATTTATTTAAATTTAAATTATATTATAAACATACTTT
CAAAGTACCTATAGTACGTAATTAACGTTTAGACAATTATGAATAATTTGTATAGTTATAGTACTTACTTA
CAACAATACAACTGCTTCAGATGTAATGTTAAGTTAAATCTTATTTTATAAATCTGAAGTTATATAAAATG
CAGTGGGGTTGTCCCACAGACTCAAAACTTTACTTTAATTTTTATTATTAGATATTAGTTAAGTAGTACTT
ATTGAATATCGATACTTGGGGCTGTCATCTAATAGAGATTCCGAACAGGGGCCATCAGAAAGCCAAGC
AAAGTCAGTTAATATCCGTAGTATCGGGACTCACAGCCCCAGAACAATAATTTACTGTGTTATCATTCGA
TAAGATACAAAATTACGTTAAGTGAGCCTTTCAAGTGTGATAGTTATATTGGTTAAAATATGCTTATTCTA
ATCTTACTGCTTTAGGTATATTATAGTATGTAATGTATTTATTTAAATGGTAGGTATGCTATACTACTCGGG
AGGTTTCGCCGTGGGGTGCTGATGACAAGACCCGTGCCTGCCCCGCTATCTTCTACTCCTAACACTGA
AACAGGCTCCGCGGTGGTATGTTGAGCACAACTATCTGTTTATATCATGGTATATGAATCACTCGACGTA
TGATTTAAAAATGGTGGTGCAATGAAATGGAATCTACCTACTAACTATGATGATCTCTAAATGTAGGAA
GCAGATGGTTTGATTTTTGATGATGTTTATGAATTCGCAGTGATGGTGGTGACAATGGCGGGTGGATGA
CGTAATGCGGGCGCGAGGCCGGCGACAAGGAGCGCGGCCCGGGGGGCTCTGCTGCTCCTACTCACTA
CTTAACTATCAACTAATACCATGCGGGAATGTTTTGATCAACAAACGCTTTGCAATACTTATCCTATACA
TTTCTGTCTTACATAGTTTCAATTACTTCTCTAATTCCTTAAGTTTTCTCTAGTGGCAGTGATGGTCAGCG
GTTATACGGGAGGGACTTATGTCAACCTTGCCCCAAAAAACCAAACAAAAAACACTTATAATTTTGATA
AATACCTAAAATTATGTACACTGTTAAGTGTTTACTATTTAACAAACTACTGTTTACAACCTTCTCCCAC

AAGCCTCCTTAACCTACAGTAAATTCAATACATTATGTGAATTTACCATGGCCTACTACGGCAATTAAAA
TTATCTCTAATGTTACATCTCAAACATGTTATACTCAATCTATTGGCCTTACACAGGAATATTTATCCTAA
CACAAGAAAGATAATATTTATTAAAACACTACTTTGCGTTAAACTTGAAAGATTCAAAAAGCTTTATTTA
TTTTTAAAATTTATGCAACACTGCTGAGCTTAACCAGAAACTAGGAAAAGTCTACGCCTAAGAAACTT
AATTTAAGCCTGTTTCAGTGAAAACCTATACACAGTAATTCAAACACTTCATAGAGCCTATCTTCCAGG
AGATTCATCTTTATAGTCTAGAAGTTAAACCTGTTAGCTTGCGCGCAGTACTCAAGTAAAAATACAATG
CTTTAGATATTCCCCTCCTCTACTCATACGCAAACACCCCCTGAACTACAGGACATTACTTTACATATTC
ACCGACATTCAATAATTACAGGGTAAAAATGACACCCACCCGAAAATAAAAGCACGAGATCAAATACC
AATTTTCTCGTATAAACGGACCATCTGAGTTTTATCATTCTCCTTTTAACATTAGTCGCCTTTTACGACAC
CCTGAGTGTAAGGGGTTGGTGCTATTCTATTCTGCCGGCCACCACACGGCTTATGGATGGTTAAATTATT
TCGTACATTTCGCGAGGGTATCATTTAGATACACCCTGTATAGATATTTGCATTGCTATTCTCGTCAGATG
TCATACTAATATGTAAAGTAATTGCGTACAGCAGTGGCCAGGGTTGGTGAAAATGTTTAATGAATGAAA
TGAAGCATGAAGTGATGACGAGACTTGTGAAGTAATGATGTGATATAACATGTTATGATTGTAATGACA
AGTGAATGATAATGATCTTGGTGAAGTCTTTTGATGAGTCCACAGCTGTACGCCAACGGTGATGAAATA
GATGATCGATAGATACATAAAGCAGTAAGTGTGAACTTATTTTAAATGAGAACAAAGAAAACTTTGAGT
CTTCTGACATGAAAATGCGTTGTTCTAAGTTTGAGTATGTTTGAGCTGCAATATTGTGCCTAAAACCTAT
ATGATAAGTTGGTAAGAGAGCCAAGTTATACTAATACGATGGTGGCACGTAGGATTGCCCTATGAATGC
GGAATGTTGATGATATTATGAGTTCATACATTCACGTGCAATGAAGTGCAAATTAGTCGCTGCAGTGAA
CTACCAATTCCAGGATAATTACTAATATGTCCTATGTGCAATGCAATAGGTAATTTATGCGATATGATGGC
CTAATAATTTCAATGTCATAATGTGCCGGCGAAATGTGGGTTGAAATACAGTGAAAGCTTTATTGTAGC
ACTATTTTGTGAATATAGTGATTTTAATACGAAATTTCGTGAATTATTTCAATTTAAAGCGGTATTCTCTG
CAAGTCGTGAAGAAATATCACTCGACCATTGCACTTCATGGCCATGACTAAATCTTAAAAATACATTAT
CAAAATTTTTATGGACTATGAATCACACAATAACATGAAAAACAGTCTTGCAGGCTTTTTCGTTTATCA
AAAATTTGTAAAATGTAAATTTTCTTTTTATAAACCCATAAAGAGGATTCCCAAAACAGTCTACAAGCA
CAACTCTTGGGTCTAAATCTAGACTTGTGCTTTAATCTTTTATCAAAAACTCCACCAAGTTAGCTCTACA
GGTTTTCCGTCAGGGTTCCGTCTGCAAGAACAGTTCAAGTCCGAAACTTCAGTTTTTGGCATAAATTTT
CGTCGTGCTTCATTTTCATTCGTGATTTCGAAAGCATGGTCTCTAAATTATTTTTTTCAATTTAGTATTAA
GTTTTGCATTTCTGACATTGTAATTAATTGAATTTGAGAAGTGAGGGAACCCTAACTTCGCGCCTGACA
TTCTACTAATTTCCAATTTAGTTTGACAAATACTTCTAGTTTTAATAATGCCTATTCCATAGGAGAATAGC
AAAGTGTGCTTAAAATTTAACTATAATTTTAATATTTAATTGTATCTACTATGGTTTATTGAATATTGGGGG
AAAAATATAGTATCAATTGATGTTAAATTCTGCGGGTTATAAATGTTCCCAGGACTAATTGATTGTCAGT
GTCGTTTAACTAACACTGGTACAATTATGTTTATAGTGTATATTTTATTATAATTATAACTAAACATACAAT
TGCTCCTAAACATCAACTACGCTAATGGACATACACTTTTAAGACATTACTATGCTGCTTGAATATTACG
CTATACTAAAATCGTTGCCTCTTTCCATAAAATCGTAGAATCCAAAGATAAAATCTCATAAAGCTACTAA
ATGCCGTAAGTAGTATGTCCACGTTGAGAGAATCGCCCCTTTCTTTCCTTATATTCCTAACGAGTAAAAT
TATGATTATACCGGTTGAGTACATATTCATATTATATATTAATTATTGAATATAAATGTTTACTATTTAATTCC
TTAACTACGTATATTAGATGCAATGGATCAAAATAAAATTAATGTTTAAAAATGAATTTAAATGAAATGA
ATTGAATATAAAATGTAATAAAATCAACTAACTACAATAATAAGTCTCAACGTTTTTTTAAGTAAAGCAT
TAGAAATCATTAACAATAATGTCACACGCGGACTTACACGATAATGTCACAATGTAATATTAATGTTAAA
ACAATCAACGTATAGCGGGACGCACATACCGCCTGCGACAACTCAAATTAAGGGAAAGACAAATGGAT
TTACACATTTATGCTTAGGTATAAATATGTTGGTACTGTCTGCATACCAACTCGACTACATGTGAATAATT
CCTCATTGAGTAGATGTATTGATTAGACTACGTTACATTAGCGGTTACATGGTGAGAGTGTCGACGCAC
GCCTCGTATCTCTGTTCATTTACTTCTCTTTCTCCTTCTCCTTGTCGGCCTCCTTCTCCTTCTCGTTCTCC
TTCTCGTTTTCCTTCTCCTTCTCCTTCACCGGCTCCTTCTCGGCCTCTTTCTCCTTTTCCTTCTCTTCGGC

>TCONS_00025946 geneID=XLOC_005077 scaffold_14:702512-706141(+) Exonic overlap (+)
GCGCGGTCTGCGAGCGTTCCTTCGCCAGGAGCGACCACCTGGCGCTGCACATGAAGCGGCACCTCCC
GAAGACCAGCAAATGACACGCGAGACGGGGCGGCGACCAGCCGCCCGCGCAGGACTCAGCTCAGGA
GGAATGCAATGAAAGCATTTTTCTGGAGTGCGACCTGGATGCTAACCTGATGGACACCCTGTACAGCG
TGCTGTGATGCTGTGACCGCCACTCCGAGAAGAATGCCCCAACTTGCCCAAGCACTGGGCAATGTGA
CCCGAAACGTGCCTTCACCTCCCCCGCCCTCACTGTGAATTAAAATGCCTTCGTAATTTATAATGTTGTG
CCTTTTTAGACATTACTTATTACAAAAATAGCACCTCCTCTAAGCAATCATCATCACATCGTATCTCCCC
GTAAGTTAGATAGAAGCAGCATCTGTTGTGCCTTATATTGCCCACTCGTATAATAGTAATAGACTTGTGT
ACTGCCTTATTTTATAGCCTGAGACTAGTCGTCTATACTTTAGTGCCTTCACCATAATGCCGTGGCCTTA
GCCGTAAATTTATATTCAATTTAGTCGACTATATTTTATGAACATTGAGAGGATAAATATTAAGTAAATAG
AAACTTTTTTCGGATTTTATGATGAAGACTTTTGCTGTGAAGTAGCTTAATAAGTTTCTGGTGAGAGCT
TCGACAACTCGAGGAGTTATTTTCATATACTTAGGTACTTACATAAAACTTAAAATAAATGCTAGACTTC
TTGAATCTTCAACTACCATATAAAAATGCAGATAATTAACGAATCAATCCGATAATCCGTTGAATTTAATT
AATTCTCGAGTGAGGTTCAAAAATATCAGTTAGCCATAATTTAAATTATTTATTGTAATATAAATAAGATA
AGTATTGTAAAAATCGCATCACTTATATCAGCCTTACCAAGGAGATCGCTTTACCTAGGTACCTACCTAC
TTTTATATTTTATTAATAAATTAACATGGCGTCGCCATATTTACGGACATAAGTCCAATTTAAATCTTGTGT
AAGATTAAAATCCGATTAGTGGACGGGTGATAGCAATGCAATTGCGTTACTTAACTTAAGTAATTACTTA
CTATGCAATTTGCAGTTCTTCCGTTACAATACATAGTAACTAACTATGTAGGTATGTATAATTAAAGAAA
CGTTTTCAGTCATGCACAATTTACTACCATTAGAGTTGCATAATTGAAAGTTTCCAAGTGACATGAATTT
AGACATTGTAAGTAAACTCCTCACACCGAAAAGGATTCAGTGTATGTGTACCGTCGTATATCAGTTTTT
TTAAATACATATACACCTAAGTTTTATAACTTACTTAACCTAATTATACTTTTCGAATACATTAATCAGTAT
TTGCTCACCGTGTCACCCGTCGCCACGAAGATCTTAGTATTCTGTAGATATTATATAATTTTATTTATATGA
TAATGATAAGAGAGCAAGGAATATCCAAGATATTATAGTTATCCTTACAATCTATATAGCGATTTCTAGAA
AATATCATTTAGCGCCTACCTAGTTATTAATTTAAATGTTACTGGGCCCCTAAGGCTCACACTTGACTAC
CTGATATATTTATATTGGTATAGAGCCAGATATTTTTATAAAAACCATATTTTATGTTTTTAAAACAATTAT
GCCACAGCCAGCATTCTAGTCTCGATTTAATACTAAAAAAGGAAAATTCTCATTTCAAATATTAAGAAA
ATCTCTAAAACTAGTATAAAGGGTCCAACTAAAAAAAAATCTTTTTGTAACTTTTAATTAATTTCCGTGTA
AATTAAATGGTAGTAGGTACATAATATCTTAACAACGGACTTTATGTTGCCATACGAAAACCTGCCTGCA
TAATATATTATGTTATGTTTAAAATTTTTGTGAATTTTATATTTTTTTAAATAATATGTGATATTCTTCGTAA
ATCACTGTGTACCTACAATCTTTTGGATAGTAATAACTATGATAAACTCTACAGAAATAATCGTAAATATT
TTGTAAATGAGCATTCTCCTTTTTTATAAACTTCAAATGTATGTACTTACTTACAATTTAGATTAAGCTTT
TTATCGTGTGCCTTATTTAATTATAAATTAAATTATAAATCACATATCTTTAACATAAGTGCGTCTATGCCA
TATTTCTTATTTAAATAATCTACCATAAATAAGTATTAAGTACGCACAGTAAATCTAAAAAGAGACGCAA
GTTTGTTCTAACCATTTAAAATACCCTATGAACCACCATTGCGATTCCCAGTTACACTACTTTTCAGCGT
GTCTTTTTGATTTTTAAGTACATATTTATATTCTTATTATAAATGAATAAGGTGGTCTTAGATTCTTACCAT
GTATACGTAACAACTGTATTTAATAGGTACTTTATAAATTCAATGAAACTGCCGCCGCACCGCAGTTAAA
TATAATTAATTTTGGTGTAATTTACAATTTTAATTTAATGCAAGTATGGCGACTGAAAATCTTTGTTTTTAT
TTCAAAATAAATTATGATAACTAAGGGCGTGTGCTAAAATGCTAAAACAATATTTTAACTAGTCTAGCGG
CGCGGTTGGTAAGGATTTTTTTGTATTTATATTGATATAGGTATATATATATGTATATATTTATCTCCAAAAC
GCCTAGCCAGTCTGGAGATAATTGTCAAAACCTCTCCATTAAAGATGAGGCCCATGCCCTGCAGTCAA
CTGCTTTTACGGTCTGTGAAAGATTCAGAACCTTTACCGTTGTGAAAATGTTGTTTAAAACATATTACT
CGTACATAATAGTCGGTTATAAGTTCTTATGACCGATTTAAATACCGTATCTCATTACCAATATTTTATACC
TATTATTAACTTAATAAAATTTGTATCTAAAATCAACTATTCATATCGGTTACCACCCCCCGAGTAACAGT
TTGATGCTCGCAGTCACAGGTAAAACTATAAAGTAGATAAGCTAATGTTCGGTTGTACCTATGACAGGT

AATCATAATGAGGCACACTATTCGGGGACTTCAGTAATGGCCATAGAAGATTATATACCTAATGCATCTA
AATTCAGGAAGGATCCGCGCTTCTGCCCACAGCGCTCGCGCTGGCGTTTCAAAGATTAATCACAGAAG
TTGGCGTTTCAAAGATTGTCCTATCAATGTCCTGCAACTCTTGGAATCAACTGTTTTACTCTGGGTGGT
AAGCAATATGATTTGTTAACTGTACATTGGGTGTTTAGATATCCATATTGTTACGATATTTATATTATCATT
GAAGAATTTTGG
>TCONS_00026000 geneID=XLOC_005156 scaffold_14:793061-794236(-) Intergenic
AATATTGACAAATTAGTAACTAAATGTACTTATTCATATTACTACTTCTGACTCCTATGTACTTACATAGA
ATATGTATACTACGTTAATGTAACCAAGTCAAGTCACAGGTTACAGGTTTTTAAATGTTATGACTAATAA
CTTAAAGGAAGAATGCAGGGTGGCTTTTGACGAAGTTTTATTAGCGTACCTTTTACATACTACTACCAG
TAACTATCGCCGCAAAAAAATGCGATGGCTCAATATCACTGTCATTCATTAACAACGATTAAATAATTAC
TAACCTTTGTCTATCAGTCAGTAGGTACCTATTGTGATTCTTACGTTGATGCGTAAATTAAGTTACGTATT
TGTGAATTTCATAACTTACCTAATCATTAATTGGTGACAACTTCCATGAAAATTGTGTTTGAGTGTACTT
ACAGAAACATCTCTATGTTGTGTGTGTATTTAATACAATTGCACGCTATATAGCTTATAATAGGTATAATA
CGTACTTTAACGCACATAAATGTTTACATAATACGGAATGGGCACTTTGACGAATCGGCACAATTTCATC
ACTGAATGATTTATTTGTATGTACCTATGCACGAGGCCGATTCGTGAAATGTGTCAACCTAGATACTCAA
CGCCAAAACTACAGAAAGTAAAGAAGGGTTAATGAACACTACATAGGCATCACACTTAAATCGTTAAG
TATAACATAATTACCTAGAAGAAAACAAATCTACTTACTTAACTATTGTAGAACTCGAATAAGTAGATAG
TATGCACATTGTTTATTATGTTTAGTAAATTGAAAATCGGTCCAAATTATTATGGCTAAACAGCAATTGTT
CTTAAAAATGATGAAAAATCTGCATCTTGCTATAATGAAATGTGATAAACTATTAAATTGTAAAACAACA
CGAAGTTTCAGG
>TCONS_00025258 geneID=XLOC_005083 scaffold_14:876409-880464(+) Unclassified
GGAGTTCCCATGAAACAATTCATATTTTATACATTTTATCAGCTGATAATACCAACTTTTTATATGAATAC
TGAAGGCCAAGCGCGGGGCGCAAGACGAGGCCTTCTGGAATTGTATTGATATTAGAACCCCACCGTAA
CTGATGTTTTATCGAATTAGGAGTAGGTTTTCTATGCAATTCGAATGACAAGACTGCTCATGTTTACTTT
AAGCCAATCTCACAGGGATAGCATTTTTATAAGTATTTATCCAGTAATGCGGAGTGCTGAAAGCTAATA
AATTGGCTTTTTTTATTAATATATGAATTATAATGATTTGGTTGATAATAGTGAGCAAATATCAATGCTTTT
TTTTAGGTTTAAGTACTTCATTTTTACTATTTTTCATAATACAAACCTGTGTCAAAAATATCTTGTCTTGT
GCACGACCATTATATTTTAATTTTTACAGAAGCTTTGACACCATGTGGCCAGCTCACTACGGGTGAATTA
CTTTGGAGACGTTTTGGATATGTTGTGGTATTTCTGTACGTTTGACATTTCTGCATAATACGAAGTGGTA
ATTTACGGTAAAATTAATTCAAATGTGATTTTTTGAATCAAAGTTCCCTGTAAATACACAATACCACTAC
ATGATAAGCAATAACGTCTTCAAAGTAATTTACCTGTACACTGTTTACTTTTGAATGAATTTATTATTACA
ACTACGTGATACTATTGTTTTTAATCTTTTTTAAATATGATTTCACATTGCATTTACTTTTCCGTTTTTAGC
TTTATTTGAGTGACGTCATGTAAATCTCCCATACAATTTAACATAGCAAACAGTTTTATATCTCGATTTTT
GGATCATCCAATTTATCATCCTTTACATCTCATCCTTTTATAGTGTAAGTTACATATTTAAGATATTAGAAT
AATTTATGTTTATTATATCGTTGTTCAATAACAACTTAAACATTCATTACACTTTACTTATAAAAATAAATG
GTTTAGGATAGTTTTATTGATAAAGTGGTTCATAAGGTTTTCAGTATTTATGAAATTTGTAGTACGAGTA
AATTTTGAAGTCGCGTTTCACGGAATAGATATAATTTCCTATTAAATTATCTTACTACAAGAAATATTTAA
GCTATTTACATGCCAAATACAAAGATTTCTTAATAATCTGGCATCCCTAGCTTACATTGGCTCGATTTGGT
GTTATTTTGGATAACAAAGCTTGCATAAGCATCAAATATGTAGCAAGATACCTACATTTACTTCCATTCC
AAAATAGTTGTTCAAACTGCCAGTTATTATTATTATTATAAAGTTAATCTTAAACTGGATCACTTTACAAT
ATCTAGCTTTAAGACGTAAACCATTTTGCCGTTTTCATGTCGGTATACGATTTAGATGTTAATCTCTTCAT
AAGTATACCTAATCTAGATAATTTAAGTATTACTAGTAACTACTTGTTCAACTTCGTTTGTAGATCTGTAT
GGCTCTTATTCAGGGATTATTTTGTACAGTGAAATCGATTCTTCTTTTTAACCGACTTAAACAAAACATA
ACGAGGAGGTTCTCAATTTGTCATGATGTATTTATGAAACATATCCCTAACGTTTAGTTACATCATTTGA

CACTAAACTTTTTATTAAAAAAATTAATCGTTTTTATTGAATCCTATGCAGGCTATAAAATAGCTGCTATA
AAATCTAGAATAAGAGTCTGCAACCATCTTAATATGACTGCAGAGTTTACTAAAGTTTTATCACTTTTAT
ATTTAAAAATGTAGCTGCACCTGAGAATCTGCCGTGTTATTATTGCAAATTTTCCCCGAATTCGTAGACC
TTTTTGACACTTAAAAAGGGTTAAAAATTCACGAAATACACGAATTTCATCATTTTGACCGACGCCGAC
ATAAAGAGTCGAAACTCGTGTTTCAAAGTAAATTTTAACCCTGTTTTAAATTTGCCATACGGTCTATGAA
TTTGAGGCTTAAAATATAAGATAAATATTGTTACAACATCACTGCTTTCCTCTAAGTTAAACATGCCTAT
GTATCTGAGTTAGTGTATCAGACTGTGTAGCTACCAAACGTCTTAGACAGTTACATAGATACACATACG
CGTGTACACCTTAAGTATTGACTTGGACGATCATTTATTAAATATGCATTTAGTTAAAGCCCAACTACATA
TTTAACTGTCAGTTTACAAGAGGTACTTTTATAATATTGGTCATATTTATGACTGTATTTAGATTTTTTATT
TAAATATTACCATAATTTGTACTTATTAGACTATTATTCGGATAAATTTAACAGTCTCAATTAATTTTTCAC
GTTTTAAATATTTGAATTACTGTTAACTAGATATCGGCAAGAATATAGTTGCTTGTTGCTCATCTTAACAT
GTAGTATGAGTTGTTTTTGACATGTATTTTAAAGTATGTCATGTAATTTTTATAGTATATATTTTAATTTATG
TCCGTTCACTGATTAAATTTAATTCTGTATAATTACGATGGAATGATTCCTGAGCGAAGATTGATTTTGTA
CCTACATTCCCTATTTTATAAAATTTGTGATAGAATGATACAAAAATAATACGTAGTTTAACATGTAGGAA
TTTTTATCAGTTTTATGTTTTTTGTTTATTTATGTGTATTACCAAACAAGTCTCATAAAATAAATGTCTGCC
GTCAGTTCAATCCAACGTTAGGGTACTATGTACATACATAACTTACTTTTGGTGAGACGGATAAGTTGTT
TCAATGTAGGTAATTTTTAAAGCGAACAAATTTTTAATATTGAAATATAAAATACTTTACGTTTCACATCT
TATGACTCGTGTGAAGGGGCCATATTCCTAAGGATGTAGAATTTTGTACACTCATTTTCTATGCTACTGG
CACTAAAGTTAAAGTTATTCTTATGTATATTATGTCTACTCTACTCCATTTGTATTGTTTACTTTAAAACAC
ATAATAAATTAATGACGGGTATTATACTTATTTTTTGTCGAACAATTAAATATTTATTATAATTTTCCTTGG
CGATA
>TCONS_00026478 geneID=XLOC_005158 scaffold_14:906382-919769(-) Splice junction overlap
TGCCATTCGTATAATTGGTCGCATTTGCAGCGTTCACTCACACCTTGTTTTTAATTTAATAAATAATGGCT
ATTCATACATTTTCCATACATTCAAAAATAAGCAATTGTGTTCTTAATTTGAAAATCCAGTGTTATACCAG
CCACCTACTTGCGGAGATTCATTGTGATTTCGTGTAACTTTTGGAAAACAATAATATTTTCGTGACAAG
GATATCGATCGTTCCAATCACACTGACAAGTCGAGATGATTAGTAAGTAGGAAGGCTAGATAGTGTGAG
AATACGTCGGACAGGTGTTGTGGTGATACGGTGCGATATGGGCCGGGGTGGCGGCGCGGGCGCAGGT
CTCGTTCAGTTTCAGTCAGCTGCGCCCGCGCGACCGTCGCGCCGCAGGAGCCATGAGTAGCGGGAAG
AGGAAGCTCTCCTTTCTCGGATACGCGCCGCCGAGCTTCGGCGGCTCCGAAGATGAGGTCCGCTTGGA
CGAGGAGATGGAGCATGCAGTGTGGGGC
>TCONS_00026059 geneID=XLOC_005086 scaffold_14:1050303-1053587(+) Exonic overlap (-)
TTGGTGGTATAAAATATAAAGAAGGTACAAATAACCCTTTTCACAACTTCAATATTTGTGATTTAGTTCC
TTGTTAATGTTAATTATATAATAAAGTAACGAGATAATCTGACGTAGTTGGCACTCATGAGGTGGCGGCT
TAGTACAACCCAGAGAAGAGAAGTTGACCAAAACTCTTCCAGTATAACAAATGTAGTAAGGAG
>TCONS_00026270 geneID=XLOC_005164 scaffold_14:1160449-1164927(-) Intergenic
GTTTGCGTTGCGATCCTGTTCTGCTTCACAAACCACGACGTCTTGACCGCCGCCCGCACCCACTTCCA
GCGCCGGTTCAACACCAACGAAGCTCTGCCCATGACTGGTTTGACAGCCGGAGAGAGCGTAGCGAAC
ACACGAGACAACGCAGCCTGAAACTGAACTGACTGAATCCGAATTTGTATCTTGTAACCATTCTCAAA
ATAATAATATCAATTATACTTACTCACGTAGAAAGCTTGCACTGATATGACGCATCATACACACTCCGTA
CTATTGTGTGGAAGGATCTGCCGGTCATTTTAAACATACATTAGCACCTAGAATAATAAGTCTCTCTATA
CTATACCTACCCAAATGGGTTGGCTATTGCCTCCTTATCATGATCAATATAACTTAGTATCTTAAACTATG
TTTAAAATTTTATTGGCAAACGTAAGTTAATTAAATATTGTTGCTAAGTTAGGTATATATTTATAATAAGTT
AAATATATTTGGTTGTATATTTTTCTTAAATTATTTATTACAATTCGTTCGTTTATGCGTCAAAATGTCTTC
ACCATACACTCCCTGGTACACGCATGACAGTAACTGCTGCGATTAGGAGAGGTCAACCTCCTCCCCCA

TCAATGAAATACCTACTGTCCTAAAGTTTCAAATATTATATTTAAAATAATATTTTTATCAATATATTTTTC
TCTGCTCCGTCCGCTGACTTCACTAAATTTTTCAGTACCGAAGTATTATTTTGATATTATTTTTTGCAATA
AATAGTAGGTACCTATCATTTAATTACATCAATATTCATTTCCTAGATACGAATTTAATATATCTAATCCTTA
TCAGATTTATTTAAAATATTTATGCCTAATCTTTACGGAGCAGCCACAAACACTGCACAGTAGGACATAG
GCCTCTTCCAAGACTGGAAGGTTTTCGCATAATCCCCATGTTTAACATCCAGGTTACCGTACTACCACC
GACTCATTAGCAAATCAGAAATCCTTCAGTAGTTTCGCTTATCTGTCAAACATGTCTGATCCATGCAAG
TCCGATTCGATTTTAGGGTCTGTTAAAGATTGTCGATTGCTAATGTTCGATGGTGGTAATCCCGCTGGTT
GAATATATATTATCATACAACACAAACATTTATCACCCTATATACGTACCTACATGATAATGAATATCTGTT
TTTAACTAATTTAATATCTGGTCCCGGTCGGGGATCGACTTCTGTCAAACTAGCAACAGCAATTTATCGT
AAAACACTAGTGTCCGTCTAAAATTATAGTTTCTGAAAATGTACATTTCTGTGCGTCCTTTCAGCAGATA
TTTTTATTACGAATATGATAATTACTTAAATAAAAGCTGGAAGGTTACCCTAGGTTGACAAAAAAACGAA
GGAAAGCTGTTATGAAATCGGTATGTTTGATTCAACGCGATAGAGTAGTGGAGTGGAG
>TCONS_00026271 geneID=XLOC_005164 scaffold_14:1160449-1164927(-) Intergenic
GTTTGCGTTGCGATCCTGTTCTGCTTCACAAACCACGACGTCTTGACCGCCGCCCGCACCCACTTCCA
GCGCCGGTTCAACACCAACGAAGCTCTGCCCATGACTGGTCTCCTCGCTAAATAAGTGAAAGGATCAT
CGCGTTTGACAGCCGGAGAGAGCGTAGCGAACACACGAGACAACGCAGCCTGAAACTGAACTGACT
GAATCCGAATTTGTATCTTGTAACCATTCTCAAAATAATAATATCAATTATACTTACTCACGTAGAAAGCT
TGCACTGATATGACGCATCATACACACTCCGTACTATTGTGTGGAAGGATCTGCCGGTCATTTTAAACAT
ACATTAGCACCTAGAATAATAAGTCTCTCTATACTATACCTACCCAAATGGGTTGGCTATTGCCTCCTTAT
CATGATCAATATAACTTAGTATCTTAAACTATGTTTAAAATTTTATTGGCAAACGTAAGTTAATTAAATAT
TGTTGCTAAGTTAGGTATATATTTATAATAAGTTAAATATATTTGGTTGTATATTTTTCTTAAATTATTTATT
ACAATTCGTTCGTTTATGCGTCAAAATGTCTTCACCATACACTCCCTGGTACACGCATGACAGTAACTG
CTGCGATTAGGAGAGGTCAACCTCCTCCCCCATCAATGAAATACCTACTGTCCTAAAGTTTCAAATATTA
TATTTAAAATAATATTTTTATCAATATATTTTTCTCTGCTCCGTCCGCTGACTTCACTAAATTTTTCAGTAC
CGAAGTATTATTTTGATATTATTTTTTGCAATAAATAGTAGGTACCTATCATTTAATTACATCAATATTCATT
TCCTAGATACGAATTTAATATATCTAATCCTTATCAGATTTATTTAAAATATTTATGCCTAATCTTTACGGA
GCAGCCACAAACACTGCACAGTAGGACATAGGCCTCTTCCAAGACTGGAAGGTTTTCGCATAATCCCC
ATGTTTAACATCCAGGTTACCGTACTACCACCGACTCATTAGCAAATCAGAAATCCTTCAGTAGTTTCG
CTTATCTGTCAAACATGTCTGATCCATGCAAGTCCGATTCGATTTTAGGGTCTGTTAAAGATTGTCGATT
GCTAATGTTCGATGGTGGTAATCCCGCTGGTTGAATATATATTATCATACAACACAAACATTTATCACCCT
ATATACGTACCTACATGATAATGAATATCTGTTTTTAACTAATTTAATATCTGGTCCCGGTCGGGGATCGA
CTTCTGTCAAACTAGCAACAGCAATTTATCGTAAAACACTAGTGTCCGTCTAAAATTATAGTTTCTGAA
AATGTACATTTCTGTGCGTCCTTTCAGCAGATATTTTTATTACGAATATGATAATTACTTAAATAAAAGCT
GGAAGGTTACCCTAGGTTGACAAAAAAACGAAGGAAAGCTGTTATGAAATCGGTATGTTTGATTCAACG
CGATAGAGTAGTGGAGTGGAG
>TCONS_00026709 geneID=XLOC_005090 scaffold_14:1358912-1362228(+) Exonic overlap (+)
CAGCAGCTCAGCAGCGGAGTGTACCAGCTGTTTTCGGGGAAATAAACACAATATCGTTAGAGGGTAGA
GTATTCCGGAGCTGGGGCTGCGCCTGCGCCGCCACGCCGCTCCACGCGCTCCACGCAATGCACGCCG
CGACTTACACTACTCTAATTCAATGTCTACATCTAAATTTTATCCCCCTTTCATCTAAATTTTATCCACCTA
TGTCAAACCCATATTATAATGTATTGTACTAAGCATATCAGGACGTGTTAGCTCCTTAAATTGGCTATGTA
AACAAAACCGATGACCCGTTGGATTTCCGTGTTATTCAAAATTGTAGAACTGAAGATAATGCAGCGGC
CTAAACGATGCGTCTGCCGAGAGTGAGGAGGATTGAGAGAGCAAGTTGGTTTCCTATCACGATTGCAT
GCAAAGAGCCGCGTCTGTGCCCTCACCGGATGCTGTTTGCACTTTCCTAACCTTGTAGCGATACCGAC
ACTTCCCACCGAAACGAAGTTACCACCCCGAGTCGTTGCATGAATGACCCAG

>TCONS_00025445 geneID=XLOC_005169 scaffold_14:1518175-1519671(-) Intergenic
GTTTCATTGAGTGCGTGAATACTTTCTTGCGTAGGCCTTTTCAAGAAAATAATAAAATTGGCAGATATG
GCTACTATGGCTGATTGTCCCTGATTACAGTCCATAAACGAATAAAAATGATACCCAGATATGTCACTGC
AGTGACATCTGTCACTTTATCTTCATTGTTGAAAACGCTCGCTGGTCGTCTGCTGTGTCGTGAATTTTTA
TAGTTTATTTTTGTTTACCCGACTATTCTAATTTATTATTTAAAGCGGATTTAAAAGGTGTGTGGTGAAAA
TGGAGAAGGGTGGTAATCCTCCTCCGTATGCTTGGAGTAACAACATGCAGGCGCCGCCGGCCGCGCCG
CCGAGCTACTCGCAGGCCGTGGGCGGCGTGGGCCCCTCCAGCCCCTACACCCCGCAGTACCCACCATC
TGCGGGACCGCAAATAGTGACCACAGTAGT
>TCONS_00026711 geneID=XLOC_005093 scaffold_14:1519853-1530192(+) Intergenic
GTCGTTCGTTGGTGGTGTTATTGCTCTCTACAATTTTCTTGTTCTTGTTCTTGTGGATTTTAATGATACAC
TCGAATGAAATCAACGAAATCTTTACGGTCCATTTAAAATTCAATATGTATTAGTGAAAAATTGTGCGAA
TGTGTTGGAAATTGGCTTAGGGTCCACAGCCGAATATTTAATAGGCAAACCTGGCAACACGGGCACTT
GCACTGGAGTCCAGCAATGTTCGTGACAATACAGTGAAGGACAGAGAGGTCAACATGAGAGCTGGTT
CTACTCTACAAGTGAGTCAAGCCAAAACAAC
>TCONS_00025744 geneID=XLOC_005174 scaffold_14:1687957-1691906(-) Unclassified
CTTTTCTAATTGCAACAATCAACAAGCTCTTTATAGACCTACCAGCAAATGAATAGGTAGTAGAGCGTT
TTATCTACTTTTTGCATTGCATTGCAGCAAATATTCTTCTCAACCCAATTGTCTATATTCTCATGAAATTAT
AAAAAACTGTATTGAATTTCCCAAGCTATCAAATTATACCGTATTAAATACACTAGAAAAATGCACAAC
TTAGCCAATGTTTACGCAAACTCAATGTCACGCGAACCGGTTGTTGATCATTAATAAACCGGCGCGGGC
GCAGGCCATACTACGAAGAAGGAAAATTTACATTCTGTACAACTCTTTGTCCTAAGGAGACGTGTTTCT
GAACCTAAATGTGCTTTAGGAAGTGAATATCTAAAGTATACACAATGTTTACCAGACCTTTAATAGTTG
GTCAATAAATTCCTAATAGTGACGATAAAAATATTTCTTAATAGACATCGCATCGTGTGTCTGTTTAAATA
TTAGTGTATTTTTAAAATGTGGCGTAGTTAAAGCCACGCACCGCAAGCAGCAGCAGTGCACGACAGTG
CTGCAACTTCGGCCAACGCGCTGCAGCGATGACCTCTAGCCACCTTGCACTCTAGAGCAGTCGCCGTG
ACGTCACGATGCTGGAGCACGTCGTGTTTGCCCCCACCTGGCTGCTGATGATGTGGTTGGGAGCCTGC
ATACTGTTCAGCCCTTTTGCAATATTTTTTGTCATCTGCCTGCCCCAGCTACCGCTGTTGGTGGTAAGAC
GCGCTTGCCACATTCCTTTTGAGTACATCTGACGAACGTTCTAGAGTCTGTTTGGAAGGTTCGAGAATA
ATGTGGCTGTTTTTGTCTTTGATGGATAAGTTAAGTAGGGTTAAGTAATGTGATTGTGATGTTTTCCGTC
TTTAATGGTTAAGTTATTTGTGTCTGATTTCATGTTAGGTGATTGATAGTCATTGTTAATAAAATTGTAAA
CATTAGAACGTGTAACGTAGAATTTCATAGATCAAAGCCATTTAAATAAGGTTAGTTTTTGCTTATTTTA
GAGGCATTCGTAAAACGCTCTCTCATTTATTTCTTTATAGTTATAAGCAGGGAGCACTAAACAATAGATA
CAAAATGAAATGATTGTGCCTTTCAAGCAGACTCCAAAGTCACTAAAGTGATAAATAATTATAATTTCG
ATGAGTAGAAAGTGCCCGCAAACAATGTATTAGGTATTTCAGATTCAATGTAAATATTATGTAAGTACCT
ACCGACTTAGGTGTAATAAAAAAAGCAAATAAAATAACTCAATATGTTATTAAGCTGAAG
>TCONS_00025745 geneID=XLOC_005174 scaffold_14:1687957-1696998(-) Unclassified
ATTTGAATACGAGCTAACCACTTTCAATGGGCATTCGTCAGTTTCCGTTATCCGTCGGGCGCGTTCGGC
TCAAAACAAAACATAATCTGCGTCTGCGCTTGTTTTGAAACACAACTGAGTCACCCGCCGAGCTCCTC
ACTACCGGCGCGCGGCATCGCACTTTTACTTTAGTTTTTTAATTGAAATAATACTGGCTGAAGATGGAC
TCGGAGAAGAAACTGAAGCCAAAGCCAGGTGAGGGGTACCTGGCCGGGTACAACCCCTGGAGCCTG
TTGTTCAAGCCTCGTGCGCGCACTGAGAGCGAGACCAGTGCCACTTCTAGCACCAGCGGACAGACCT
ACCAAGCAGGGCCCCCCCGCGACGCGCTGGCGAGGCATTCCAGCAAGGATGAGGAGTATCTGTGGAT
GATGTGGCGTAGTTAAAGCCACGCACCGCAAGCAGCAGCAGTGCACGACAGTGCTGCAACTTCGGCC
AACGCGCTGCAGCGATGACCTCTAGCCACCTTGCACTCTAGAGCAGTCGCCGTGACGTCACGATGCTG
GAGCACGTCGTGTTTGCCCCCACCTGGCTGCTGATGATGTGGTTGGGAGCCTGCATACTGTTCAGCCC

TTTTGCAATATTTTTTGTCATCTGCCTGCCCCAGCTACCGCTGTTGGTGGTAAGACGCGCTTGCCACATT
CCTTTTGAGTACATCTGACGAACGTTCTAGAGTCTGTTTGGAAGGTTCGAGAATAATGTGGCTGTTTTT
GTCTTTGATGGATAAGTTAAGTAGGGTTAAGTAATGTGATTGTGATGTTTTCCGTCTTTAATGGTTAAGT
TATTTGTGTCTGATTTCATGTTAGGTGATTGATAGTCATTGTTAATAAAATTGTAAACATTAGAACGTGTA
ACGTAGAATTTCATAGATCAAAGCCATTTAAATAAGGTTAGTTTTTGCTTATTTTAGAGGCATTCGTAAA
ACGCTCTCTCATTTATTTCTTTATAGTTATAAGCAGGGAGCACTAAACAATAGATACAAAATGAAATGAT
TGTGCCTTTCAAGCAGACTCCAAAGTCACTAAAGTGATAAATAATTATAATTTCGATGAGTAGAAAGTG
CCCGCAAACAATGTATTAGGTATTTCAGATTCAATGTAAATATTATGTAAGTACCTACCGACTTAGGTGT
AATAAAAAAAGCAAATAAAATAACTCAATATGTTATTAAGCTGAAG
>TCONS_00025450 geneID=XLOC_005174 scaffold_14:1694130-1696950(-) Intergenic
TATCCGTCGGGCGCGTTCGGCTCAAAACAAAACATAATCTGCGTCTGCGCTTGTTTTGAAACACAACT
GAGTCACCCGCCGAGCTCCTCACTACCGGCGCGCGGCATCGCACTTTTACTTTAGTTTTTTAATTGAAA
TAATACTGGCTGAAGATGGACTCGGAGAAGAAACTGAAGCCAAAGCCAGGTGAGGGGTACCTGGCCG
GGTACAACCCCTGGAGCCTGTTGTTCAAGCCTCGTGCGCGCACTGAGAGCGAGACCAGTGCCACTTC
TAGCACCAGCGGACAGACCTACCAAGCAGGGCCCCCCCGCGACGCGCTGGCGAGGCATTCCAGCAAG
GATGAGGAGTATCTGTGGATGATGTAAGTACATTCGGAATACCTCATACAACCTCTCCC
>TCONS_00025671 geneID=XLOC_005106 scaffold_14:1809016-1813336(+) Exonic overlap (-)
TCAAAGTGAAATAATCTCAATTTGTTTAGCTAATACAAATACATAAAAAATATTTTGTTTTCATCTAATTG
TAAAAGTAAGTGAAATCTTCTAACAATTTAAAATAATTGGTTTTGTGCTGCTGATAAGACATGATAATCA
TGTAATAAGGAAAAGGCTAGGAAGAAGAAGAATCATCTATCAAGGCAATTTTTATTAATACATAGTAAC
CTACATAAGACCAGTTAGACCATAGAACCTGAATCAAATGGTGACCGTCTGATGAACTAATGTGATAAC
TAAAAACCTGAGAGCAAGATTGAGTGTTCCTGGGAATCCCTTGAACTAATGTGATGATTATAAATCTGA
GGTCAAGATTATGAGTGTTGTTGGGAATCTCTTTAAAAATGTGAAGATTAAGTATAGTTTCCTATTAATA
ATTACTTATCAACCAGTATTCAAATGAAGAAAAGTAATAATTGCTGAATAAATTTATCATTTTCATTAATC
AGCTGATAAATTTATGGTAGAATGTTTTTTTTATACCTATTATGTTTCTGCATAATTAGCAAATTTAATTGT
TATTACCTAACTTTACAATAAACATGGACATATTATACAAAAGTTACATGGGAAGTTAAACATATCATTG
GGAAGTGCACGGAAATGGATGGGAGGCTTAGAATATTTTTTTATTTTTATGCATCCATTAAATTTAAAGT
TTATTGTAAATACTGATTTGAAGTTTGGTTCGTTTTAAATGTTGTCAATAAATAATTTTTAATAATTTTGAT
TTTGTTATTAGGTGCAATACATATACTGTAAACAAAACATGAGTAACGGCCCGGGTTTATGATATCACCA
GAAATTGAATTTTAAGTAATCATTATATGTATTGCAA
>TCONS_00025305 geneID=XLOC_005106 scaffold_14:1809024-1809608(+) Intronic
AAATAATCTCAATTTGTTTAGCTAATACAAATAAGTAAATAAAAGATATTTTGTTTTCATCTATTTTAAAA
GTAAGTGAAATCTTCTAACAGTTTGAAATAATTGGTTTTATGCTGCCGATAAGACATGATAATCATCTAA
TAAGGAAAAGGTTAGGAAGAAGAAGAATCATCTATCAAGGGAATGTTTATTTTAATACTTAGCAACCTA
CATAAGACCAGTTGGACCATAGAACCTGCATTAAAATGGTGAACGTCTGATGAACTAATGTGATGACTA
AAAACCTGAGAGCAAGATTGAGTGTTCTGGGAATCCCTTGAACTAATGTGTTGATTATAAACCTAAGG
TCAAAATTATGAGTGTTGTTGGGAATTTTCTTTAAAAATCATAATTAGCTAAGTACTTGTTATTAACTTCA
CAATAAACATGGACT
>TCONS_00026371 geneID=XLOC_005179 scaffold_14:1835973-1836491(-) Unclassified
GTTTGCACATCTAACGGTTGTAATATACGTTGTGTTGTTGAAACACATTTTATTTTATTAATTTGACATGG
AATAAATTGATTTATTCCTCTTTTTAATGTAATCTTAAGAATTAAGGTAATAGGCGTAAATGTTTGAATCA
GTCATTAAACTAATAACAGTGAAAGTGTTTAATAATAAGTGACTTATTGACGATTGCTGGTTGGAGTTTA
TTTGGAAATTGTGAGCTGCCGACA
>TCONS_00026018 geneID=XLOC_005180 scaffold_14:1844140-1847232(-) Splice junction overlap

CTTTCCTTTACTAGATAACTCGTTGCGCGGAGTGCCGGGCGAGGTGCGTCGCTGCGATAACAAGGTCG
GCGAAGTGAGGCGAACTACTTGGTACTGCGAATGAGGAGGGTTACTTTATACTGACGAAAAACTAACA
AACGTTTTATACAACGAGATAAGCGTAGCTCGCGTAGACGTCGCTTCGAAGCTTCGTTTTTCGTCATAT
AGAGTGACCCCCAAGGTTCGCACTTTGAATTTCATGCAACCTCCTTGATGAGGACATCTGACGCGATG
TTTTCTTCTTCATCCTGGCCACTTCACTGGCAAGGAGTCTTATGTGCAGGATTAGGACATATTTTGTTAT
AGCTGACCAACGCAACGCAACCGCCATGATACGTAGTATATTTTGAATTCAACAATAATAAGTAAAACA
AATGAATAGAAGTTTGGATTTGGAAATGATGCTTAGTCTAAAATAAGGTTAACTAACGAACAAACAAA
AGTATTTTTATATTGCAGCCATTATTACCATCTTAGCAAAACTTAAGATATTATATTAAAGGTCCGGGCTA
AAAAGCTGAACATTGAGTATTAACTTGTCAATCATTTGTGTATACACTGCCGGACAATATATGTTCCACT
TACAAAATTTCGACACTAAACGACCCCAATTTTTTCTAAGATTTAATTTGAAATTGGAACAAAATACTT
CCGTTTTTAGTTTTTTATATCTTCATTCTCTGTTAAATATACTTTAATGTTGCAGAAGTCGTCATTAAGGT
AGCCTTTTCCGTTTAGAAGTAATTTGAAACTTTTCTCAGTGGAACTTTTCATTGCCAGTGAGTGTAATAT
GGCTATAGCTTCAATTACACAGTCTGGTATTAATATTTAACATTTTAAATAATAAAAAAGAAATTAACTA
AAGCTAAAACTATTTTTAAATGTAAAACCTTAGTACTTATAACTTGTTCGTTTTGTTTAATGAAAAAAAT
CAATGAGATTAGAATCTAATTAATAATAATTAAGAACTTTTCAAATTATTAGGTAACCAACATTGGAAAG
GAAAGCAAATAACCAAAGTAATATCTTATGAAAATGTACAAGGACTTAGTTAATGTGATAATTATAAAA
GAGTGTATTATCTAAGAATGTGAAGTATTCGTATCTAGGAACTGAAAATAAATTAAATTTTGTATATAAA
ATATAAATGTTATTTATTGTGTAAGTTTGTATCAAC
>TCONS_00025903 geneID=XLOC_005189 scaffold_14:1996312-1997806(-) Unclassified
CCTATACTGGCAACTCTAAAATACAAATCGTTCCTTTCATTTGGAAAATGTCGCACAGTGCGCTGTGTT
TTTAAATCGTCATTTATTAATGTAATCTCTTGACTGAGAGATACATTGATCAGTTGAAGTTTTAACAACA
CCTTCTTAAAACATGCGTGCCAAGTGGCGTAAGAAGCGTATGCGAAGGCTAAAGCGTAAAAGGAGGA
AGATGCGTGCGAGGTCCAAATAAACTAATTGGTACTCAAGTGGAAAAGAACATGTTTCTGATTGGTAG
CTTGTGTATCAAATAAAAAATACAAAAAATGCATTTGTATC
>TCONS_00025471 geneID=XLOC_005189 scaffold_14:1996821-1997855(-) Unclassified
ATCGATTCTCTAATCGATAAATTAAATATAAAAAAGAATCGACTAGAAACCTATACTGGCAACTCTAAAA
TACAAATCGTTCCTTTCATTTGGAAAATGTCGCACAGTGCGCTGTGTTTTTAAATCGTCATTTATTAATG
TAATCTCTTGACTGAGAGATACATTGATCAGTTGAAGTTTTAACAACACCTTCTTAAAACATGCGTGCC
AAGTGGCGTAAGAAGCGTATGCGAAGGCTAAAGCGTAAAAGGAGGAAGATGCGTGCGAGGTCCAAAT
AAACTAATTGGTGAGTAAATTCTTTCCACTTACTTCTCCATGCATACTTCTTCAGATTCCTCATGTCTTCT
AACATAATAACCTTAATGTCCAATAGGTTCTGGTATTATTCAGATGTTATTTAAAATACAATTTGTTTGAA
CTACCTAACTATTTCAATATATTTGTAAAGATCTTAGGATCTGCATTTTGAGGTTATGTAGTCTTGAGCAT
GGTTTTTATTTAATGCACTGCCACTGCATAACAACACTCCTTGGTCTATCTTACACAGGACAAAAGTTA
GCCTCATGTCTGTTATATCTTTGGTTAGTAATCAAATTTAAAGTAGGTATGTTTACTGGTGAAAGAGTAG
TAACTTGCTTGCTCAAAAGAGGAATCACCAAGCAATACCATACAATTTATCGTTTCAGAAATACAATTAT
ACCCATGTGCTCCACAGTTAAACAAAGACAAAGTCCCCAATATCATTAGAATAGTTGTTAAGCTATAGA
TATTATCTATTAGATAGTACCTACTCTTAGGAACCATTTGGGTGCTTTAATAGTTATGTTGGTCAAAAGGT
CTTTATTTCCTATGTTGTTAGGGACTATCAAACCATGGGGAGTAATGTACAAGATCCAAACAACTTTATA
GATTTCTG
>TCONS_00025522 geneID=XLOC_005208 scaffold_14:2838134-2839329(-) Unclassified
ATTTGTATGACTCTGGTTTAAAGTCATAGAAACGAAAAATATACATAATAAACATAAAACTCATAAATCC
ACCCTTCATATAGGTACTTAATGAATTCTTGGTGTGAACATTTTGAGGAACTGGAACCAGAACCAATAA
TAATAAGCATTCGGACTCATTTCAATGATTGACCTGCTAGCAAAGAAAAGAACAACAAAATTATTCGA
AAATTGACAAGTGGAGGGACTGTCACGCTTTGATCCTAATTTTTGACAAAAAACCAAATTTTGTGTAA

AAAATGAATTAAGAAAAATAGTTGTGAAATTATGTGTAATTCAAAATTGACACCGAAATTTCGTAAGCT
ATAAAGGCTTGCCTCGGTGTCCTTACGATTACGATTGAGGATTGGACTGACAAAATACAAGAGCGGAA
CCCTCGGGACTCTGGTTTCCCGATCGTAGTCAAAAAGAGAAAAAATAACAGACCAAACCAACATGATT
TGTATGTCCATGTGTACGATGATTGCTGCGGCTTCGGTACTGGCGTGTTTCCCCGTCATCAACAAACAC
TTGAAGCTACAAAAGATATTGAATTGTCTGAAGGGCAAATCGTAGTTTCACCGATACGAGATGATCGTT
TGTTATATACCAATTATTATAAGCCCAACGGTTATCCTCGTTATAAGCTGCAGTTGTATCCTCTTGACGAC
AGTGCTATATAACTGCTGGCCATGTATTAAGATCTCTATGATCGAGAACAAGCCGCAAGAGCCTGAAAA
ACCTAAGACAATGCC
>TCONS_00026240 geneID=XLOC_005132 scaffold_14:3011648-3012755(+) Unclassified
AGCGCTCATGCTTCAATATCGTATTAATCGTGCTGATTGCACGTCAGCGCTCGTTCGTTCATTTTTCGTC
AGTATAGAGTAACCCTCCAGATTGGCAGTGTTGTGTACGGGCCCTATGACGTTTTTCTTCTCTACGTATA
GCCGTATTTTCCGCTCATGATTCGAAAAGCGAAGTATTTTGCGCGCATGCGGTCTTGCGGAGGAAGTTT
GTTCTTAACTTACTGACACATTTATGCCTAATGGTGTGTCTTCGAAACTCATGATAATAAAAGCCCTAGG
CTTGGTGATTTAGCGCAGTTAATACAATTCGATTTCTATTAAGTATTGTGGTTAAAATATTTGCCATCCTG
ATCCTTATTCGGATTTTAAATAAATTCAGTATTGCTATAAATTTTGGGGAGGGTATGAACATTTTATAATAT
TTGGGCGGGTTGCTAAGCTTGAGATTTAAAAGTAATAATTCGAAATTTGTAAAATAAAACGTCTCTATT
TTCTCAATTTGAGCTAAGCCGGAGTTATTATAATAGGATAATAATAAATAGTTATGGTTACGACGCAAAA
TTAGTTAGTGTTCAAATTATAAATACCATTCTTTTTCGATGCTGTCGAATAATGTCCTTAACAAAGCACA
ATTGAGCCGCAAAAAGTTGTGTTCGCTAGAATTTAATAAGAATAAATAATAGTTTAAGGTTTGTATTATG
AAAGCTTACACTGAATTTGTTATAAAGCTGATATTTTTAAAGATCAAATTATAATGCCACAAGATTACAT
GTAAGTTTATTTTTGTTTTACATTAAGTATTTATGAAGTTCACTAGGATTTAGTTGCTTTTGAAGATAGTT
TACTTACCTAGTTTTCTTTCGTAGAATCAAAGATTTTTTCTATACTTATTAACTTTTATTTAACTGTGAGA
CTTTATACATATTATTATTAAATCATTTACATTAAAGGTGAGGTGACTGATAAGGTGTAGGAATATAAGAG
GCTATTAGGAAAAAAATATAGATATCTGTCCTTTAAAATATAAAATTGCTTTTAAGCATTTTTTTGTTTATTT
AA
>TCONS_00025865 geneID=XLOC_005133 scaffold_14:3019959-3025004(+) Exonic overlap (+)
CTGACGTCGCGGTAAACAACTTAGTGATCCAAAAACGCTGTCCTGACTTTTGAAATTAGAACCCATTA
GTGTCACTCATATGCAGATATATTAAGGAATAGGCAGAATTACCACCGTGATGCAGCTCAGCTGAGACT
AGCGCTATGGTCGCTAATAGAGCCGCTTCGCCAACATGGCCGATGAAGAGAAACTCCCGATCCCGCCA
ACTCTTCTTGAGAAGCTCCTCTTCTACACGACAGCTTCCTTCGTCTTACTCCT
>TCONS_00026768 geneID=XLOC_005212 scaffold_14:3043901-3045161(-) Intronic
TGACATTCGGCACAAATTCAACTTCAACATGATCGAGTGAATAAGCAAAAGTTCCAGTTATTTTAGTAT
CAAAATGATAAGTGGCAGTACCTGCATGACCATTAATGCAGCAATATTGCTATGTCTTATTGTGGATGGT
GCTTTGGGTATAGGCTTCTTTGCAAACCTACGGAAACGCTGTGAAGATGGGAAGAAGAGGAAAAATA
GAAAGGTTAAGTGTTCTGTCCCAGAAGGCTGCAAAAAAATCTAAATGCACGATATTATGAAGAAAAGTA
TTGATGGTGTAAAGTGAAAGTGTTGTCTACGTTTGCAATAATAATCTAAATTTCTGGTCACTCTATATTT
GCGTTTATTTGATATAAACCAACTCTCCATTCACTCAGTAAATACAAAAATATAATACTTAATAAACTCA
AATTAATAAAGGAAATAAAGGTGATATGAAATGTTGTTATTGGCAATGCTTGGCTTGAATCAAATTTGG
AGCACAAAACAATAGAAAGAAGTATTTACCTTGCTAAAGAAAATGAAATTGTAAATAAAAATAAAAAC
CTTGATGTCTATTCCAAACTGTCAAAAGGACCTTCTGTGATTATAAAAATGACGTTTCGTAACATAAAAT
AAACATAAACTTTATTCGTTTGAAAACAGCACTAAAATCTCATCATGTGAATCTTCAGGCTAAATAATTT
GGAAAACATCCATGAGGGATTACATTGAAACACCACAATGTTCTGTGGGATACCGATTAGTTGTGTGA
CCTTAAATTGCTTTCTGATATTGTTTTGCATCTTGGACGGAGCTCTCGACTGTGGGATCATCAGGAAATT
CCAGTACATCATGAAGTGTGTCCTACACAAGACACAGGAGAGGAAGAAATGCGCATGTGGTGCTTGTT

CAATACTATAAAACTCTATTTAAAAATACA
>TCONS_00026308 geneID=XLOC_005214 scaffold_14:3073532-3074795(-) Unclassified
ATTCGGCACAAATTCAACTTCAACATGATCGAGTGAATAAGCAAAAGTTCCAGTTATTTTAGTATCAAA
ATGATAAGTGGCAGTACCTGCATGACCATTAATGCAGCAATATTGCTATGTCTTATTGTGGATGGTGCTT
TGGGTATAGGCTTCTTTGCAAACATACGGAAACGCTGTGAAGATGGGAAGAAGAGGAAAAATAGAAA
GGTTAAGTGTTCTGTCCCAGAAGGCTGCAAAAAATCTAAATGCACGATATTATGAAGAAAAGTATTGAT
GGTGTAAAGTGAAAGTGTTGTCTACGTTTGCAATAATAATCGAAATTTCTGGTCACTCTATATTTGCGTT
TATTTGATATAAACCAACTCTCCATTCACTCAGTAAATACAAAAATATTATACTTAATTAACTCAAATTAA
TAAAGGAAATAAAGGTGATATGAAATGTTGTTATTGGCAATGCTTGGCTTGAATCAAATTTGGAGCACA
AAACAATAGAAAGAAGTATTAACCTTGCTAAAGAAAATGAAATTGTAAAGTTAAATAAAAATAAAAAC
CTTGATGTCTATTCCAAACTGTCAAAAGGACCTTATGTGATTATCAAAATGACGTTTCGTAACATAAAAT
AAACATAAACTTTATTCGTTTGAAAACAGCACTAAAATCTCATCATGTGAATCTTCAGGCTAAATAATTT
GGAAAACATCCATGAGGGATTACATTGAAACACAATGTTCTGTGGGATACCGATTAGTTGTGTGACCTT
AAATTGCTTTCTGATATTGTTTTGCATCTTGGACGGAGCTCTCGACTGTGGGATCATCAGGAAATTCCA
GTACATCATGAAGTGTGTCCTACACAAGACACAGGAGAGGAAGAAATGCGCATGTGGTGCTTGTTCAA
TACTATAAAACTCTATTTAAAAATACAG
>TCONS_00025379 geneID=XLOC_005137 scaffold_14:3352663-3353975(+) Splice junction overlap
GCCGCCGCCGCCGCCGCTGACGCCATGCTTCGGGAGTCACTGGTGCAGGACTGCACGTGCAGTTATGT
CAACCCGTTTGTGGCGCGGTCTGTTTGTGTTAATGAGGAGAGGAAGGAGGCTGCCCGCAGACGAAAA
TGAGCTGTATACCAACGCAACAAAACTGTGATTCGTCGAGCGATACCAAATACTCTTAGTTTTACACGG
CCCGTTGTAATAATACCTAAAACTTACACATTTGTACCAAAGCCTATTTAATTGTTCGGCTGACTTTCAC
CAACTTCATTTAACTCACGGTTAAGTTAACGAGGGATACTCTATCTATCTGATTCTATTTAACATTAAATT
AGAAAGTGTCAGATTGATTATTTTCTACAGATTTACTAGAACCTAAGCTAAATGATGTTGGTGTCAGTC
ACCCTCTAATGTGATAATTTTAAGTCATAATTTTATTACCATATAAGCGAAATAATTTTGTTAAAATATCAC
CTCTAGTCTTCTACTAAGACTTAGTAAAATCACTTTAGGGGTGTATTTAATGTTTTATATAATTATATTTTT
TGAGTAATCTGTATTTTAGATAGCATTTACCAATGTCTAGTTTCGCATAGATTTGTTTAAGTATATTATTAT
GATCTATGGTTGTTTTGTTTTAGGTGTTATTAGAGCAGGTTTATGTTCGTCCTAGTTTGTAAATGTATCTC
GTCTGCTTGTAATTAACTCTATTTATATTCCATTACTACAATATGCCGGTCGTACCGAAAGGTATTATGAG
TAATCATGCGCATTGATTATTAATAATTGAAGACAATAAATATGTTATTAAATTTTATATGAATTTTCTTTTA
TAACCTTAAAAATTAGAAACCCACAAAACGCAATAAGGAATGAAATGCC
>TCONS_00027151 geneID=XLOC_005434 scaffold_140:95875-100006(-) Exonic overlap (+)
GGATTTTCCTCAGTGCTCGCATGAAGACAGAAGTTTATTTGTAAATATTGTAATTTGTAAATACCACTGA
AACCATTTATTTTCTCAACGTTTAAGTGATTTATTGGAAAGTGGCAGTGTTTTATGGATAAAAATATAAG
CGGAAACCTTACTTGAACCCTCAGAGGGCAAGAGCGAGCCGCTATGGTGATGGAAGCCAATCCCATTA
CTGACCAGCACGCTGAAGGTAAACAGATTTTTACATACGTATTAATTCTTAGGTAACTAGGTATATTTTA
AAAAGTTACAAGATACTTATTTTTGTCTCTCG
>TCONS_00026878 geneID=XLOC_005399 scaffold_140:100539-113935(+) Unclassified
CTCCGCCCGCCCCGGGTTTCACAAAAATTTAACTTGAACGAAGACAGGACGTGGGCGGCATAACAAC
GGTGGATTGTTTTGGAACGTTCAATAACAACGGATGTTAGAACTTAACACAAATTACTAATGTTGACGT
AAATAAAGCTTTTACATAATTAATTTGAGTGTTAGTGAAAAGTTGCCGTGAATTTTTTTGAAACTGTTGG
GAATTAATTTGTGATAATGACAGCGTCTACAATGATTGAACTGGAATCCTGGATAATATACCAGTGTGTC
CTGAATGAATGCCATGTCTCAAGTAGTTGAACTGCCCTACGGCTTTTTAGTTGGACTTTGTTTTTGAGT
TGCAGCCATGGTTTCTACTTGTAAGTTTCTTTTTTTTATTTTGTTTGTTTTTTTTTATTCTAATTATTTTGT
GTTCGTGTATCACTATTATCTATTACTGGTACAGGCTATACACCTTCATATTGACATTAAATTTTTAATACT

GAAAGCTGGATTAGGTAGTAGGTAGTACTTAATTACTTATAATGAAGTACTTATACCCAATTAACATATTT
ATAACATGACAGAGACGACGTATTAAAAATAGTTAAATACAAAACCAACGTGAAAACGCATTTATTACT
TATAAACTTTATTCATAAAGCTGCAGAATAAGGTTCTGTTATCGCGGTGCAAGGAGGCTGCGTGGCCGT
TGCCATGGCAACCTCTCGGAACTTCCGCTACACAAGGTCGGCTCGAGGATACCACGCAATCGATAGGA
AACAACCTCCAAACGTATTTCATATTAAAATTTATTATTTACCACGAGAAAGTGAGAAAAATCTTTCTTT
TGTAGTAAAAAATGTTTATGC
>TCONS_00027535 geneID=XLOC_005436 scaffold_140:204733-205872(-) Intergenic
GTCTTAACGGAATGTTATCAGTGCTCATACTTGCATGTACATGGAAGTAGAAGTGAGCTGGCAAACCC
AATTTTGGGTTTATGCTGAAATTTTCGTATTTAATTAGTTCTGAAAAACGATATAAGTTGACTGTGATTC
CTGCTACGATGCTAGGACTCAGGAGTAAAAGTTCATGCATAAATGATTCCATTTTCAAAACACAAATGC
CCCCAACCCTGTGACCAGCCAGCAACCGGCGCGGGTGCGGACTGCGCCTCCTTGGTCCTCATCTCCTT
TCGCGGCGGGAAGGCGACACCGAGAGATCTCGGCCGGCGCTCGCCAGTCGCACCACAGCACGCGTC
GACTCGACATGTAAGTACGACCCACGCTCCTAACACCCCAGTGCCAGTGATCAGTGAAAATATAAAGT
GCATTTTTTTCAATCAGTGAGCTTTGATCTTAAGTTGATTTTAAATTTATTTCGTTGCGTTT
>TCONS_00026929 geneID=XLOC_005422 scaffold_140:1057357-1060155(+) Exonic overlap (+)
CAAGACCATCGACAAGACGAAGAAATGTCAGATGTCATGTCAACAACAAACGTCAAAACAAGATTCA
CGATTTTTTGTTTTCCTGTTTCATTATTTCCGAGATTCCTCAAAAATTTCTTCTTGTAACCGTGGTATCTT
GATTGTAGTTATTTATTTGTTAAATCCAAGCACCCTGTCGTGAAGCATTTCGAAATTGCTAATTATATTGA
ATACGTTGTTATTTTTGGGTGTACTATTTTTGTCCGCTCTGTGCTCCACATTCGGGACATGAATATAATTT
GACAAGCAGGAAAAGAATGTGCTTCCTGGATGACCTCACCCAGCAGTGAGCCCCTGTGATGGTGATG
ACTGCGAGCGGCGCCTCCGACGCGCTCCGGGAGCGGCTGCCGGGGCGGGGGCGGGGCCCGCGGGGG
GGGCGCCTGCCGGTGCTGTGGCGGGCCATCGAGGATGCCCACTACAGCTACCTCGAAACCGATGACA
GCCCGGAGAAGATACAGCAGCGGACCAAGCTGGAGG
>TCONS_00027279 geneID=XLOC_005424 scaffold_140:1078868-1079285(+) Intronic
GATAATCATATTGATTTCTATCTTTTCAGGTTTCGGCCAAGGCGGTCATGGTATCCACCACGGAGGCAGC
GAGGGCGGTTTCGGCCACGGTGGCAGCCAGGGCAGTTTCGGCCACGGCTCTCACGAGGGAAGCTTCG
GCCAAGGCGGACACGAGGGCAGTTTCGGCCACGGTGGCAGTGGAGGCCACGGACACGGCAGCCATG
GATCCTTCGACGGAGGCTACGGAGGCGGCAGCCATCACGGTGGATACCAAACTCACGGCTACTAATTC
CTAGTATAAGTTCTATTTAAATAAAAGTATTTCTAAGTTTTTCCTCTGGTTTCTTTTAATATTTAATAATCT
TCTAAAGAGTTGTGATGGGCT
>TCONS_00026939 geneID=XLOC_005425 scaffold_140:1081503-1083537(+) Unclassified
ATCGGCTGAGACAGTCGGATTGATGTGACATGCGTAATTACTGTGAGACAATTTCCCAGCAGATCTCAA
TTATTATCTAATTTCCAGCACGCATAACAATATAATTTTCTCATTAACAACTAAAGCCGGTCATAGATATA
AAAGGTGGCTGCCAGGTACATCCTGAAGTTGTTTCTACACTCTGGACACAGTCAGTAGACGAGGGATT
TTCGTGAAAGAAAATGGCGAAAACTGTGCTGATCTTCACACTTCTCCTGCTAGCCGTCCTGGCCGTGG
CTGCAGAGGAGACGACAACCGTCGCTGCTGACGCTACCACGACCCCGGCGGCCGCCAACGCCACGG
CTGATGCTGCGGACGCTACCGCTGACAATGGCACGCCTAAAGACAAGAGCGATAGGTGAAGAGAGTA
AGGGTAGAAGGTGG
>TCONS_00026989 geneID=XLOC_005459 scaffold_140:1085664-1087330(-) Exonic overlap (-)
ACGTGAAAAGAAAAACTGGGCATGTTTGAGCCTAATCGTCGCATTGTTCACTTTTTTCCTGTCGTTTAA
ATGGGCCAATTATACTATCAATTGATTGATGTGTCTTAGAATCATAGCAGAATTATAGTGTACACCCTGTA
TATAAAGACGGTCTTGAACTTGGCACTTCATTCTGGAGAGCAAACTCTAAGGAACTTCTTTGAAACCA
TGGCCCGGTTAATTCTATTCATCGCGCTCCTGCTGATCGCAGCGGCAGTAGTCATGGCCGACCTGCCCG
GGGGCCTGAAGGTCCCTGACCTCCCGAAGACGGACAAGCTGCCTGATGCTGCAGGGAAACTCGGAG

ACGCCACCAAGAACATCCCTGGTCTGGGCGGGCTGATATGAACCGATGACTACGAAGACATCCGACCG
TGACCTGAGAGATTTTACCCTAGTTTTAAGTCAAGC
>TCONS_00027551 geneID=XLOC_005460 scaffold_140:1105975-1106755(-) Splice junction overlap
GTCCAAGTCGCTGAGAACTCCGGCTACAATCAGAACGGTGGCTTCCAAGTTGATCCCGGCTTTGGTAA
ACCATCATTTGGACAGGGGCAAGGTAAATAAAATTATTACTATTACTGAACAAAATACTGGCAAGTTAT
TTGGTGATAAAAGGGCGTAAACTAAAACCCGTGTATGCACTTGAGGAGATTTATTGTAGAAGAACAAA
AGGAAACGGATACAGATTAACCATAGAC
>TCONS_00027803 geneID=XLOC_005557 scaffold_141:142330-144516(+) Unclassified
AGGTGAACCCCCCCTCCCTGCTGTCCACGATCGAGCTGGTGCAAGCGCTCGGGGGCCCCGCCCTGGC
CGGCGAGGCGCTCTGCTGGTGGACGCACTTCTGCGCCGCCGTCGCCTACATCAAGACCATGGACTACC
CGCGCACCTAGCGCCGCCCGAACCACGCCCGGTGTGCCGCGCGGTCTAGCGAACCTGTGGCTCGCTT
CACGCGCGCGCGCCCCCGGCCCCACTCCGTGTGAAGTGCCTGGCCGTTGCCATGGTAACCGGACATG
ACGGACTATAACATTCGTTACTAGCATAATAGTGGCTGAGCTGCAGAAGGTATGACGCAAACTGGTGTC
ACAGATGCTTTAGCATTAAATCCCTGTAAATAGATTTAGTGATGGAGTGTCAGGAAAAGATACCAGTTT
GTATTGCCACTTGCAGCTTATGATATACATAATATTGCATACCTACCAATATGGTAAAATATATTTTGATCA
TTACTAACTACATATAGTAGTCAAAAGAAGTGGAGCAGCACTGTCGATTGTGTCATTAAAATAGGAATT
GAAATTGAAGCAACGAATCACGTAGAGCTGTCGGGCACAATCGCCAGTGGCGACATCTCTTACTCGTT
TTCAAGCATTGTCTCACTCACACCCGTGGCTCTTATAGCCAAAGTGTGAGTGAGATGACAATGTATGGC
CTTTTTCGCCGTGATATCCTCTCAAAGCTGTAAAATTTTAACACGACGATATTAAAATTTAAATTTACATA
TTTATAAAAATATATAGGAGCGGTTTTTTGTGTAAATATTTTGGCTTGAAATTTATGTAATAGCCTCTTATA
TTTGTTGCTTTAGAATCAGAACCATGCCCTATCTATAGGTAGGCGCATGTAAATGTAATACTTACATCATA
ATAATTTAATGTAGATAATTTAATATGTTTAAGGATCTGAATAAAGAAAATAAATCGAATGTAAAATGTTC
CACCCAAAATTTTATCTTAGAATAAAAAAAATCATTACATTTTGGGTGCAACTGTATCTAGAGTGGTCTA
GCCTTAGCTCCTCTGTGTGTGATTCTTAAGACATTATAATAACTAAAGTTGAACTTTTGTTTAAAGCCGA
TTTTGTCACATTATACACGACTGTGATCTAGGGTTGGGTACTATATAGGAAATTAGGAACATGAGACTCG
TAAATTAAGTAAAAAAATATAATATGAAAGGATCCAAATGCCAGATATTTTTTGATAAATTGATATTCTAT
GGATATGAGTAAAGACCGGTGTATGTAAAATAAACGTGTAAAGAATCTATTAGGGCGGTACTTCTCATT
ATTGTATGTCTAATGTCTTGTTATATGTATATTTGTATAGCTGGATGGAATAGTTTGCTTGTTATTGATTTAT
TTGTACCACCTTTAATATTTTTTATCATTGCTTTTTAACTGAAATATTGATATTAACCCATTGTTTAATTTTA
CATAACTTTATTTAGCTAAGCAAATAACCAAATGAAACTATTTATCTTAGTTACTAGAAAATAAGCAAAA
ATGTGTATGTAGAGATTTCTGGTATATCTTCCACATTTAGAGTAAGCTTTAATAATAT
>TCONS_00027868 geneID=XLOC_005566 scaffold_141:437411-440133(+) Splice junction overlap
TGAAAAGTAAGATATCAACATATTTTACAGAATTATTTCAGTTTTTATTATTATTATTACGTAAGTACTTAC
ATAAATTAATGATGTCAGCATCATCACCAGTTATTTAATCACTGCTTTTTAAATTCAGTCCCAATGACGA
GGAGGAATCTGAAGATGCAGAAGAAGATATATCCAAGTCTTCTCGGTAAGAAGGCCTAGTCAGAAATC
TAGATCGAAGGAAAGGAAGAGGGAGAAAGAAGCAATAGACTACGTTTCCAGTAAAGAAGATAATGTT
AACGAACCTGACGATTGAGATATATATAAGGTTGGTCTTTTACAGTAAATATAAATAAATAGTATCGATG
TTTTTTTGTCCTCA
>TCONS_00027869 geneID=XLOC_005566 scaffold_141:437440-440133(+) Splice junction overlap
GAATTATTTCAGTTTTTATTATTATTATTACGTAAGTACTTACATAAATTAATGATGTCAGCATCATCACCA
GTTATTTAATCACTGCTTTTTAAATTCAGTCCCAATGACGAGGAGGAATCTGAAGATGCAGAAGAAGAT
ATATCCAAGTCTTCTCGGCCTAGTCAGAAATCTAGATCGAAGGAAAGGAAGAGGGAGAAAGAAGCAA
TAGACTACGTTTCCAGTAAAGAAGATAATGTTAACGAACCTGACGATTGAGATATATATAAGGTTGGTC
TTTTACAGTAAATATAAATAAATAGTATCGATGTTTTTTTGTCCTCA

>TCONS_00028273    geneID=XLOC_005668        scaffold_142:432314-448319(-)    Exonic overlap (+)
GCTTTCTTCTTATTTTATTTTTTTCTTTCTCTTGCTTTAATTTATATAATAAAAATATGTCAACCCGAAAGT
AATAGCTCGTCGTCCGCCGTGTTTGCCGGTTCGGAATGTTATGAGCGTTCGCCGAGCATGTGGATGGCT
GTTTGTGTTCGGTGATTGTGGTTGGTGTTTGTGACTTTGTTTGCTGTTTGCCGTGTTTGTGACAAACAG
CAAGCGTATGGATGGGGCTTAAAACAAATTGCCCATACAAAAATGCTCACAAGAGTGCTAAGCTGTCT
CAGGCGGGCCCTATGCCAAGCGCCAAGCCGCGTTATGCCAAAAAACAGGCAGTGTTGTGCCAACCAC
CGCCGGGGAAACACTCCTGTCACCCGCCATTTCCCGCGAATAAATCCGCGGGAACCGTTCGAAAACC
CCCATATGAAGGAAGCAACACTGGTTACAAGTGCCCAAACCCTCACGATCACCCTGAGGTGTTACCAC
AATACTAACTTCTACAGAATACAGGAGGGTAGTCGAGAATGAAACGCGATGAGTCGGCCAAGAGTCT
GACTCGTTACCAAGG
>TCONS_00028100    geneID=XLOC_005669        scaffold_142:464287-464767(-)    Unclassified
TGCATCTTTTCGGTACTTAGGATTCAAGGACTTTTTAAAAGTCCTTGGTAGGATTAGAAGCTATGAAAT
CGTATTCTACGGTTTGACATAAATATTGGCCACAGAGTAAAACTTAATCTAACTTGTCTGTGGCACAAA
AAAATGTCGTCGTCTTTCTGAATAATATTGGAAAGGAAAACTAAAATTGATTGTTGTTTTTGTAAGAAA
CAGAAGAATGTGGATGCGAAATTAAAAAGAAAATACAACTGCATAATACGTGATTGAAATTGTATAACT
TCCACTCTAACCGGAACAGTATATTATCCACCATGACCTTAAATATTAATAACCTCCGTAACGAGGGCGG
CTTCGGTCCCCAACTGCGAGTTATATTGCCGCCTCAAGATGGACA
>TCONS_00028229    geneID=XLOC_005653        scaffold_142:601601-607798(+)    Intergenic
AGAAGGGCTAGTACGGGGCGCATTTAAGACGTGACAAGAGTTTTGCATCGCGCTCACTCACATCGCGC
AGCCTCAAGAGCGAGCGCGACGGAGAACTTTTGTCACGTCTTAAATGCGCCCCATGCTAGCCATTCTG
ATATGGCTAAATTACAAAACAGTTTTTAAATCCCTTTCCCGATTTCAGCGTTTAAAAATTATGTAAATTC
TCATGCAATACCCGTCATCAAAGGAAAATCAGGAAGCCCACCATCACCATCTAGCGACAAGAAACAG
AAACCACTTCTTCTCTTCCACATTCATAAAATAACGAACTTGGATGGAACTCAACACTGATTCACGACG
TCACACATTGCCAAATCTATTTTTACCACACCACTGTGTGTGAACTGTGAAATAAAAATGAGTGTTATT
TTTTTATCTTTTGCGTGGATGCTGGACGATCACCCATGGACCTTTTCTCATTCCTGTGAGTAAATCAAAG
AGGCGCGCACGCCGGCGTCTGCGCAGCGAAATAAACATAATTTATTATGAGTGCGTGCTGTCATGTCAT
GTGTTTGTTTACATCGTGTTTAGTGTAAACAGTGGCTAGTGTGTTATGTGCACAATAAATTTATTTGTGT
TTATTGATTCGTCATGTATTCTCTTCGGGTGATGCTGCTCATCATCAGCCTGGCGCTGTGCTGGCTCTCA
GCAGCTGACGGGCACCCCCTGGGCCCCCTGGGAGCCCTGTGGAGCCCCCCCGACCCAGAGACCACA
AACACCAAAGACACAAAACCGGAGAGGTGGACCCACGTTTCCGATGACTACAAAAAATATAACTACC
TGATTACCACGTATTTGTAAATAGGGTTTAAGTAAGGGTATGAAATTGTGATTTATTTTTATGAATAAAA
GTGAGATTTAATTTAATATTAC
>TCONS_00028257    geneID=XLOC_005653        scaffold_142:603467-608519(+)    Exonic overlap (+)
CACCACTGTGTGTGAACTGTGAAATAAAAATGAGTGTTATTTTTTTATCTTTTGCGTGGATGCTGGACG
ATCACCCATGGACCTTTTCTCATTCCTGTGATGCTGCTCATCATCAGCCTGGCGCTGTGCTGGCTCTCA
GCAGCTGACGGGCACCCCCTGGGCCCCCTGGGAGCCCTGTGGAGCCCCCCCGACCCAGAGACCACA
AACACCAAAGACACAAAACCGGAGAGGTGGACCCACGTTTCCGATGACTACAAAAAATATAACTACC
TGATTACCACGTATTTGTAAATAGGGTTTAAGTAAGGGTATGAAATTGTGATTTATTTTTATGAATAAAA
GTGAGATTTAATTTAATATTACAGCTAATCATATTTGTTACTATAGAAGTTAGTGTATTAGAAATCCTTAG
GGTTTCCCGCAAGATTTTTTCACTGAAAGTTATGTGTAGTCACTGACTATATACGTGTAGAGTTTGAGTC
TGTTGACTATGTTACACCGAATAAAAATAAATGTATCGTTTAGCCGATATTAGCAAATACAGATGCAATG
GCATAGTTATAATATGTAGGGAACTAATTAGATTATGCAACTATACCTGTAAGAAATAATTAGTACCTACT
TAACAACAAAACGTTGATACTTTTGATAAAAAAAGCACTGAGTCACAGGCTGAAAGTATTACACCTAA
TTCGCACATTACTTAATCCAGGCTCATTAGACTAAATACTAAAAATAGATATTTAAAAAGAGCACATTGC

ATTTGGATATCATTTGCAAACCCATCGGGAATAGTGCAACTACTGCAACTGTCCTAATATTTATAACAAA
GTCCTAGAACGCAGGTACTTACAGTTCTGTTAATTTATTAAGTGTCTCAGTGAAAATTAAATAGACATCA
TGAACACAGCAGCGGTAAGTTTTTTTAAAATTACATTTACCTATTTAACTCTAACTAATAGTTAAGTATAT
CCTTTGTATGTATATGTACAGACCTAGAGGTAAGGGCAGTTGGATTCCTACTATTTCATATTTATACAGAA
CTTTGCTGATCCTGTGTTTTTCATTCAAAATGTTCCCGAAG
>TCONS_00028345 geneID=XLOC_005746 scaffold_143:162586-165168(-) Unclassified
ACTAGGTAGTACTCGTTATTCTATGATTATACTGACGAAAATTCAGGCAATGACGCGCGATACGCAATAT
ACCGGTGGGTAGTGCCTGGATGAGGATGGCCGAGGACCGAGCGTTGTGGCGCTCCTTGGGAGAGGCC
TATGTCCAGCAGTGGATGATTATTGGCTGATGATGATGATTAATATAACATTTTAAATATAAGGATAAAGG
GCTCTTTGTCTTTGGAGAAGAACCGTGACATAACAAGGAATGTCAATGGCAGGTAGAACTTTTTATTG
CACGTGAGTGTAAGACTAAACAGCCGCGTTCCGCTTTTTTTGCATTGGTACGTGTCTCTGACGTGAGA
AGCAGGCGCTTACCCGACTGAACTACCACCACTCTGAAGTATTGTTAATTTAACTATTTTCCAGGAGAC
GACCGAGTGGAGCGGGCCCGTGTGACTGATAGCGCTCAGTATGACCGGGTGGCTGTGGCCGGGGCCT
ATCTCCCGCTCCTATGAGACCACAGTGGATCCCCGATGGCAGGGGCCTACCTCCGGCTCCGATGAGGC
CACAATGGAGCCCCGACCGCCAGCTTATGTGTAAACTTTGTTGTCCACATGCCAAACATTATATTACATA
GACATTAATTGTTGTCACCTTGCTAATAATTAAGATTGAAAATGTTCGCAATAGTTATGATCTATCCTGGC
TGCAGAGATCACAGAAAAAATACTGATGTGAATACTGAAGTCTTTCTTATGCTGTATTATAAGCCAGGG
AAAGGCCAGCTTTTGTTTGTTAATACTGTAGTTCAAATACTTATAATTACAATAGAATAATATACTAGTCT
CATTCCAAAGATGTGGTAGGTAGATAAGTATAAATTGCAGTTTTTCACAGTATTAGTTAGTGTGGTAATG
TTTAAGCCGCCAAAATGCTTTGGTTCAGGTCTGATAGGATAAAAGTAACTCAGTTGAAATCCCGTGGTT
ATGTTAGGATTTCTAGTTTTTCTTAAACGGGATTTCATCGTATCCTAAACTAAACTATTGAGTGTAATTTT
TTTGTTAATCTTACTTCTTAAATTGTATATTTATTTCCATTGTTAACAATAAAATTATTGTTACATTAGAAT
CTAATTTAGAGAACTACTCGTACATATATGAGTTGTCTTGTCCTCAGTTAACCAAGCCAAAGACATAAA
ATCCTAGTCTGTTGTTGTTTATATTATTGCTATACTTTTTGAGTAAAATTATATGTACCTATACTTTAGAGT
GATTTAATCAATTTGAAACTTTGAACATTTCAGTGTTCGAAAGTAGTACGGACAATAAAAGTACTGCAG
GATTTAGGTATCTCTAGTGTAAGATCTATAAAAATAATAGCAAAAAAATATATTTTTTGCTTTGTTTTTGA
GCAAACCTTTTACATTTTTCTTGGAATAGTATAAAGTAGTAACTATTATAATAAATGTAACTTTCATTTAA
AGTTAAAAAGATAAGTCTGTGTATTCTGAGTAAGGATTGTTGTTTCAAGAAATAAAGACTTGTAACTTT
CTATGTAAGTATTAACTTAAGGATTCATTAGATGTAAAGGCTAAATGGTGTACATAATAGTCAATCATGT
GAAGCATTCAATTCTAAAGGTGGTGTACATAAATAATTGTTAGTACTCACAGCATTTTATTTTGACTGTG
ATTAGTAAATTAAATACTCAAATATACACTGCTATAATAGTTGTTACATCTGTAATCAAATATCTTTTATTC
TCAGTTTGTTTTTAGACTTCCAAAGATCTACTATGCCTCAAAACCAAATATGTATAGGGAAAATATCTAC
ATATTGTTACTAGAATAGAGCCAAATTGTGTATTATTTAGATGTTTTTATTACTCATTTATAGCAGTGATTA
GTAACCAAATACCTGTTAACCTGTGGTAGAGCAAAGCTCGAAGTTAATATTGTAGATTATTCAATCCGAT
AAATGTGATATGTAATAAGTAGTTACCAAGCATATTCAAAGTATTTGTAAATTTTTAAAATTCTAGATTGT
ACCTACTTATTTATTTTGTAAGTGTTGTAAAATGTATTTATAAGTATTTATTTTATCTCTGAATAAAT
>TCONS_00028530 geneID=XLOC_005730 scaffold_143:269302-270654(+) Splice junction overlap
CAAGAAGAGTGCTACTTGTGGGTGCGGCGCCGCCATGGGAGGGGGGTATAAAGGCTGCGGGCACGGG
CACGCGGGGCACCCCGGCGCGCGGCACTGGTCCTGCTGCGGGAGCACGTCCAAGAGGAGCATCTGCA
TGAGGCCGCAGTCGCAGACTTACCAGTTCTCATTGTGAAGAGTAGGATTGTTGATCGATGCAAATTTAT
TTATTACTCTGTTTTTTTAATCTCAGATTTTTATTGTGAATTTTATTGTACTGTCGGCAGTGGGTGGTGCT
TGTCGTATTACTACTAAAAATATTAAGGTTTGAGGTGAGACATTTTACTTTGAATGCATAACAATATTGA
GGTGTAACCACTTAGTTATTACTCACTTAATCCGCTGGCTCAGCGACCCGAAGTAGATCTTGGCCTCAG
ACTGTAACAACTTATGTGAAAAATATTATTTATATGACAAACATAAGATTATTTAGCGTTGTCAAATGCCT

AATGCTTCTATCAATCTCCCGCTAAACCTGTTAGTTAGTTAGTGTTAGTCAACCTTTAAATTTGAATAGG
CTAGAATGCGCCGGCAAGACGGTTTCCGTTGACCTGTCGGATAGGTAATTAATTTGATACCAATCACAA
TCACAAAGCAGTTTACGCCAATCAACATCAACGGTGGAGCTTTGAGCATTTATTTTTAGGTTCTTATTTA
TATTGTGCAATGACTCTGCAATGAATGTAATTTTTTCAAAATTTATTTAGAGATAAGTAGATAAGGTTGT
AATTTTTAAGAAAACTTGGAC
>TCONS_00028572 geneID=XLOC_005734 scaffold_143:504872-507539(+) Intergenic
CTAACTACTACTTCTACTTCAACCATTTTCTACCCTCAGTAACTACCTAAGTCGAAAATAATGTTACTAG
GTAACTAAAAATATTTCTGAGATATTACAACTCTCCTTCACAACCCCTATTTGAAAAACACGGTCTCCAT
TATCCTTATTCTAGGCTGTTTGTGTAAATGTGCCTTACCTGTCTAACTGTCAAATTTATTAAAACTAAACT
GTCAATGTCAACCTGACATAACCTTAACCTGTTTGGCTGTTTTTTTTTCCGCACTAATCAAAACAAAAG
CATGCGTCGTTGTGTTCAACAACATCGATCGCACGAAGAAATCTAATGTTTACCCGATTAAACTTACGA
AACTCTAAAATAATGAGTGAATCTTACAGGTAAGTGTCATTATTCTTTCAAATACCCTGTATGCATGCTA
CTAAACTAAGTCCTCATGCCCAAATTATCGTGATTCAAAATAAACAACCTCTGAATTGTTAGTTATTTAT
ACATACAATAGCTCTGTTTTACGGATCTTGTGTTTATAATAGACCTATTGTTATAAAAATCTAACTCAATT
AAATATCTCCCATACAGTTTTTAATTGATACTGACAAGACTAACCAACATATTTTAACTTATTGTCAACC
CCTCACCTTTTAAATAAGTACCTATCCTAATCATTTGGTCTCCAACTCCTATATTTACATAATTTTATTTGA
ATTATACCTATATCATTATTTGTTTTATTTCAGGACCAAACATAACAATGCAAACCCAACTACACTCTTTG
TTGAACTAAAACCCATGATAAACCACTGATCAAACCATCCACTGTGACAAAACATTCACAATCCCTCA
CTAATTATATACCAAAGTTACTATTCTACAATAAGATATGACATACTTTGTGAATGCTGATGATGATGATG
ATGTTCCTTATTTGACAACCAAACTCTGAGATTATATACTATGAAGTAAACATCCTGACTCAGATGAAGA
AGTGAATTGCAACCGCGCTCAAAATAGGAACAACTAAACTACCCACATAGTTCTTAACTTATAAACAAT
ATATTGATACTAAATGAGGTCCTAGCCTACTTCACCTGGTCTAACACTTAATCAACAACAGTAACTTGCT
ACACATTAATTGTTTTCTAAATTTATTGTTATGTCGGACC
>TCONS_00028328 geneID=XLOC_005735 scaffold_143:536282-616650(+) Exonic overlap (+)
GCGTTTTGGTGAGCGCGCGCGCCGGCCGTAAGACTCGGGCGTCAGTGCTGCATCGACTGTGGCCGAG
GTAGCCTCCACTTAGCAACGTGCTTAGATCACCGGTGACTTATCGATCTTTTGTGTTTTTTTTTCTTCCT
CGACGCATGGACTTTTAGACTCTTTGGTGTGTGAAAGTGCTATCAGGATATAAGACCGGGGTACAGGCT
ATTGCGGTGTTCACGGATTCATTGCTGTTTCTGAAAGAGGTCACCTACAAGAAACTTATAACTAAGGTT
GGACGAAGC
>TCONS_00028885 geneID=XLOC_005846 scaffold_144:6515-7155(-) Exonic overlap (-)
AATGATAAATAATTATGTTATGTTGAACTTACCTTCAAAGTCATGTTAGTTGAATGACTGCCACGTTTAT
CTCACGGCACTGATGTAACAGAAGTAAGCCCTTGTTTACAGCGGCGGCCGCAGTCAGCTTCATTATCTA
TTCAAATTATGGAGCCGGTTTGTTTATACAGAAATTACTGGAATTTTATTCGTGGATCAACTTGTTTTTA
CGATCGGCCAACCATTTTATTGAAAGGGGAAAACCACAGAAAAATCTAAGTAATAAGGGGAAAAACA
AATCGATATAGCCGGTAATCAACGGCTTCGATAACAGTGGCAATAAGGGGGTGCAAGTAAATAACCTTT
GTTCAAAGCTATCACTATCACTGCCTGGGCACCTAATTGATATAAA
>TCONS_00028942 geneID=XLOC_005832 scaffold_144:258184-258803(+) Exonic overlap (-)
TGCGGCTAGCCTTTGGATCCTCACTCTCTTCCTCGTCTTTCTTGAACACATAGTACACCACAAACGCTG
TGACTGTGGCTAGAGACAGCCCCAGGGCCACTGGCACAGCTAACCTCGCAAACGACATGGTGTTACC
TTTTATTTAAAGCCGTCAAGTGTTGGTAGAATACTGCACAGTTTTACATAAATACACAAACAAATCTTTA
CAAAAAATAATAATGAAATGAAATCGCTGAAAAATTCGTTCGGC
>TCONS_00028682 geneID=XLOC_005857 scaffold_144:357817-382339(-) Intergenic
ATTTGATCATTCGATTATCAAAATTTGTGGTTCACTGGTTTGCAAATTCGATTATTGATATTAGTGGTATC
AAACCATTCGATTGTTGCTGTCATTCATAATCGCCCATCATTAGCCTAGACCTAGGCTACCACCCCATGC

AGTTTGTCGTAGGTACTTACTACTTACTGGACACCCTGTACTAATGAGGAAATATGCTGGCCCGATCAA
AATTTTCTAAGCTATACTCATCTATGGTTGAATGGTG
>TCONS_00028627 geneID=XLOC_005837 scaffold_144:384745-386443(+) Intergenic
AGCAAAAAAAAAACCGGCCAAGTGCGAGTCGGGCTCGCGCACAAAGGGTTCCGTAGCAGCAAATAT
AATATTACAGTTAAATCAACCTATCTCAAAAACTATAAGAGATACTTTGATCAAACCAAAAATCGTTGA
AAGAGTTAATTAGCATGCATCACCTCTATTTTTTTTAGAATTTTATACCCCGTAGTTATAAAAATAGAGGG
GGGGGGACATACTTTTTACGACTTTGAGAGCTGATATCTCAAAAACCGTTCACTTTAAGGAAAATGTTT
TTTAGAAAACTTTATATCATTTTAAAAGACCTTTCCATTGATACCCCACACGGGTATGTACATCGAAAAA
AAAATTTCATCCCTCAGTTACATGTATGGGGGGCCCCACCCCCAATTCTTTTTTTTACTATTTAGTGTCAT
ATTTTTGTAGCGGTTCATACAACACATATTCCCATCAAATTTCATCACTGTAGTACTTATAGTTTCCGAGT
AAATCGGCTGTGACAGACGGACAGACGGACAGACGGACAGACGGACATGACGAAACTATAAGGGTT
CCGTTTTTGCCATTTTGGCTACGGAACCCTAAAAACGGTCCTTAAAAAATGACACTGCAAGAATCGAA
TATGACATTTCGTTCCCCTCCTCGTACTTCACCCCCTCCAATATTCGGGCGCCATTGAGAATTTGCATGA
CAGTGACAGCCAGCACGCCAGGCCGCCATTGCTTATTGTTGTTGAGAAGTGCTTGCGTCGATTAATTTC
CGCCTCTTTAAATCCACAATTTGTGCAATATCGTGTAAATTTTCGGCAATGTTTCGAAAGTGTTGTGTGC
CGTTTGCAATAATAAACGACAGGACGTTAAATTGCATAAATTACCCGCTGCAGAGGACAGCAAAAACC
GGTGGTTAAAGTGCATAGGTTACGAAAATTTTGGTGAAATAATCACCACGAACATGTTTGTGTGCCATA
AACATTTTGAACAGAGATGCCAAGAAGACAACAGATCATAGTTATTCCAAGAAGCGCCACATTGACCA
TTCATATGCACAACCACCAAATAAAATGGCATGCCTTGAAGTTAAGGGTATGTAGTAGTATTTTAGTAA
AATAAAAAACTATTTAAATAATCATTTTGTTTTAATTATATCCCCCGTAATGTATTTATTTTTGAATGGGCC
GTATTTTCAATCGCCGGACAGCACATTTATTCCTAGGAACAAATTAATGTCTGGCGATTGGAAATATTGG
CCATAAGGAAAATGCCCGATTTCAAACCGTAGGGTGCTCGGTTATATATTTACCCCCTTATTCATAGAGA
AGTTACAGAACGTTTTAACTAATAAACTGTTTTGTCCCTCTCCGACAAAGAACAATTTGTTCTTTGACA
GAGAGGGACAAAACAGTTTATTAGTTAAAACGTATTGTAACTTTTCTATGAATAAGGGGGTA
>TCONS_00028882 geneID=XLOC_005844 scaffold_144:753617-754424(+) Splice junction overlap
AGGAACTAAGCGATGCAACCCTACCTCATGAAACGGTTGATACTGAAACCATAGAGTGTGACGAATGC
ACTGAATATGAACCCTATAAGGAGGGCTTGCCAGAAAGTCGGCCTGTGATAGTTCAGGCTGAAGTTCC
TAAGGTGGTGATCTGTGAGCCCTGCGCAGAAACTGACTGAACTTTACCCCTGCGCAGTTTTGCACTGA
CGGGATTTTATTTTATAATTTGCT
>TCONS_00029188 geneID=XLOC_005961 scaffold_145:520658-525389(-) Intronic
CGCCGCGCCCGCGCAGCCACCAGGGACCACCCAGTTCATAGCGCAAGCGCAGACGCTAAACACCCAC
CAAGTGCGGCGGAAATCCAACCAATCGGACGGCTCATAGTGAACGTGCGAACTCTAGCTCACTGCCC
AAAACTAAAGTCTGTGTACATACGTCAATATACCTTATTACCTAGGACTTAGTTAATTTATTCATACGTAA
CTCCGGACGGTCGAACAGTGTGCAATGAACTCTAGTGAAATATATTCTCTGAAGTTGGGATTCCACTTC
GTGTTGGGACAATGCAGCCTTTGCCTCGGTCTGAGCGCATATTTGCCTCGGGCGAGGCAAACTCCAGA
GTCGTACTGTCGCATCAAGGAATCTCTACTTTATGTTGCGCCGCCGTGTTGTGTTCACTTGATATTAAAA
ATATTTATTTCGTAATTATTTACTCGTACTCTTGGTTCGTTTTAAATGTGACTTGTTGTTTTAAACGAATTT
GGACTTTCTCTTGGAAAGTCCAAATTCCAACTAATTATCGGGGATTATTACTGGCCGATGTTTTAATACA
TTTTGATGAATGTTATCAATTAAAATCATAAAATGGCCTCTGATATGAATTTCAAATACAATGCGAGAGG
ATCACGATATGTTCATGTTCAAATGTTCCTATTAGCCGCATTAGACTATTTTATTAATAAATTTATTTAGTA
TTTATTAATAAATCAGCCAAAAACACAGCAATTACTGTCTGGAATAGCTCAGAAAAATTTGTAGAAGTA
TAATTTAGTTGGTTATCATGCATTGTGTTTGAGTCTCATAAAAACAAGTTACCTACTTTGCATTTCGATTG
CAATGAGTATACTTTGATGTGTAATCCAAGATTTTATTGATATTTAAGATTATATCGTAAATGAACAGGGT
GTTGCTAAAACGGTATCGTCATTCGAAAGAACTTCATAATACTCGTGGTTCTGAGCAAGATTTGTTACC

AATGTACCGATTAGTCGGGGCATGCCTGGGATTTCTAATTTAGTTAGCCAATGCTCGGTCTGTATAGTCG
TCCCCTAGGGCAGAAATATATAGTCCCAGCATTTAAAAACCCAAAGCATGCCTCGCCGAGGCGACGTC
GATGTTAGCACGTTATAAGTCGAATATTTGGCATGTACCTACTCACTGAAGTGGCATGCCGAGTATTCG
ACTCGTAACGTGCTGCCATCTAGCGAACATTCATTGTCAGTTAGCAGTTACCGTCCTTTACGAAACACC
ATAGGTATGCATTTAGTAGCACTGGTCGCTTCGAATCATATTGGTTTTACATAATAAGTTCTACGTATTAT
GCTAAGTACATCAGTTTTTTTAAAATAAAACTCGTAAATCAAAAGCGCGGTTTAGAATGACGAGTAGTG
TGATGCGTTTACTGATTTTCATACGATTTTAGCAACTTCCTCTTCATTAGCCGAACAATTATAAAAATGA
ACTTCAATTCATCTTTAATCATGTACACTGCATGCATGAATTATTTGTTATATTTATATGTATACGAATCAA
TCATGATCATTTTAAATGTTTCGATGTCACAAAGTACTTATTTTATTTTTTATATGTTTTAAAATGTATGTA
TTTATAGTTTAGAACCACCGAATGTTAACATAGCAAGTTCCAAAAACATTATCGCATTAAGAAATCATAA
CAATCATTACGTATATTTCAACTACCTAACCACATTACGTTATTGTATTATATTAGTTAGCCGCAGTAGATA
GTTTATTTTGGTAATACTCAAAATTTTGTTGCTCTAAAAAATATATTTGATGCTTTTCCAATTTCGTTGGT
ATCTTCAACCAGTTTAGAAATTGATCTCGAATGTGCATGTAATTTAATGTTAATGTTTCGGCCTTGTGTT
GTTTTAGGTTTACGTTGCGCATTCCTATCAAAAGGTATGACCGATATTAAAAGTGTGAATGGTACTTAAT
AATTTACTAACACAAAATTACCTTAGACTTAGAAGGAGTTATATGGGAGAGTCTTAATCAGTATTAAATA
CTAAAAGTTTTTTAATGCCGTGTATTGATTAACTGTGTGTTGGCATGCGATTTGCATCTATTAAAGTACA
ACACTTTGTTGTGATTATGATAGAATCATTCTTGTAAATAAGTTGTAAATAAATAAAATTGTATAAAATGT
ATGCGATATTCAGAAATGGGAGTGTAATAGGATTGTATAAATATCTCGAAAAACAATAAGGTTGAAAGC
GATTTATAGCAATAAAATAATAATTAGACTAAATTGCCAATGATGTTAGCGATTTTGAAGGCATCAATTTT
AAATTTAGCACTGTCATAGGCTATGTGTAATAATGCAAATAAGATTGTCAGTGCTTAACATTGGTGCCTT
TGGAATCCGTTCCAATATATTGTTAAAATGCTCTGACTAAATCAATTAAGGGCTGATTCTGAAGTAACAA
ATTTAAAATTTGGTGCTTTTAAACCAGCTCATGCATTAATATTATCCAAAAACAGACAGGTTTGCCACAT
GTGAAGTCTAGTTATAACTGCTAGTCAAAACTTGTTATTTCAGAATTGTCCAAATTGCCTATAAATCTAC
AGGGTTACCACAAATCAACGAAAGTCGTCATTTTTTTTCGGTTAAAGGTCTTTTAGTTCTGAAAATGCA
TGAGAGTTTGAAAGCGAGCAGCGTTGCTCAACTCCCTTAGTTTTACTTCTAGATCTGAATTAGTTCTTA
ACACTAGGGCTTTCCTAATCAATGTAAACAAACCGAAATTGTGCTATGATTGGCTTTTGGATCAAGATA
AAATTGTAAGTCGTTTGGAATTCTTTTTAAGAATCACTCATAAAGCCTACTGTTAAATTGATGACTATAA
TGAGATTAAATGTTACAGCCCTTGGTGTGTTCAAAATGCTTATGTTAAGCTTAAATGGCATAATAATATG
ATGTTAGATGGGTATTATTTTTATGGCATACGAGTAGATAAACCGACGCAATATTATAATCACTTAAGGTT
GTAAATGCACAAATGAACATTAAGTAATATAGATATTTTCACGTACTGACTAGATAGTACCAGATTTAAT
GTACTGTATCTGCCAAAAGTGGATAACTGGAAAATTTAGTGTGCTATTTAATCAACTAGCGATGAATTA
AATCAAAATGTGACACATAATACGATAAGGCTTCGATTCCTAATAGTCTTTGTTATAAAAATATACCGAT
AGAAAATGAATTAGTAATGTATCTACTCTCTTAAGATGCATCTACATCTTAATACTCTCTAAAAGGGACT
CTTTTCATCGCCTTTTGATAAACAATGTCTGATAAATTCTTACTACAATCCCTGCCGGCAATCTTATCCCC
TGTAACTTTATTAGTATTAAATTTTGGATTCACATGATTTTACTAGAAATATATTTTTAGAATTCAACAATC
GGAACCTTTTGCAGTTTTTAACATAAGTTTTGTTTATCACTAAGCCATGAAGTGAGTCTCAATAAATATA
TAGACTGTTTTTTCGATACATCACCTGATTGAAAATTTAATATGGAGGGATTTTCAGAACCATCCGTTTA
GTACAAGCCAAGATGTAAGACTGCCTTTCTTACTCGGCAATAAATATAATTAATTTATTATTATAGTTGTG
TTGGTTTGTGTGTCTGGGCCTCGTATAGTAGCGTGAATCAATGATCTCATTTGTTTAATGGACAATAGCT
CAATGTGTAGATGTTTTATAGTAAATCACATAACTTTGACCTGCACATGTAAATGACTATTTTTAAGAGA
ATCTACTATGATTGTATATATTTTTTGTAATGAAGATTTTATACGAATTATGGCCCAATTCTATGCGATGGT
TGCTTGAAATTTCCAGTGTCAACTAAGTATGTGTATAATATTATGATATGGTAGTGTAAATATATTTTATGT
AATGAAATGAGATGTGATATCCATTCTGATGTTTATAGTTTGTAGGTGTATTATAAAATGTATATTATTGCG
TTGACATTGATTTTTGTGTATATTGATATTGTAAAAACTAGGACGTAAGGATGTTGTTCAGAGATTGACG

TTTCGAACATAAATAAATAATTTTTACATG
>TCONS_00029027 geneID=XLOC_005950 scaffold_145:534148-534791(+) Intronic overlap (-)
CTTTCACCTTCCACATGATCCCTATCCACGTACAACTCTCCCAAAAACTCCTGATCCGAAGGCCTCTGC
AGTATCGACAGTTTAATATGGAAGAAGCCGGCACCAGCGTGCGGTGAGGTTTTCTCAGTCTCCAGAAT
GTACTCTTCGACCAATTGCGGTTGGCAGTCGGGCGATGGGCGTGGCCTTTCGTACGCTCTGGCCAATC
GCAACGCGGCTGCTGGTTCGGAGGGCAGGGCTAAGGGTTGTGGCTCCATCTTCGGTGGTTCTATTGGT
TTGAATACCTCCGGCGGTTTCTTGGGGAACTTGGAGGTGAGCCTGGTGTGCGGCAGCGGCTGGCGGC
TGTTCCTGGCTCGCTGCCGGTGGATCAGCTCCAGTAGCTCCAGGCCGTGGTGGTGTGTGAACTGGTCT
AATTTGC
>TCONS_00029288 geneID=XLOC_005985 scaffold_1455:4575-5599(+) Unclassified
CGTCAACCAAACGTCAAAATCATCGAAAATAAAACTTGCTTTCATCTTGTGCGTGCATTATTTTCAATTA
TTAGCTAAACTTAATTATTTGTGGTCTTTGTAGGATTACTGACAACACTACACCAACTGAGGTATCTGGA
TATTTGTCGGAGCGCGTCATATCTTTAGCAGAGTGGGCGCGGGCGGACGGCGGACTTCCTCATGATGT
CGTTAAAGCCGAGGAATGTGGACTTCCAGGAGACCTGGGCCGGGCTGAAGGAGACGGTGAGTTCATC
ATTCATACATATCCTCCTCAAGAATGTTGATGTAA
>TCONS_00029564 geneID=XLOC_005989 scaffold_146:109276-123726(+) Intergenic
TCCATGTTAACTGTTTTCAGTTGAGTAAAATAAAAAAAAAAATACTGTAAAACCTTAAAAAATTTATTG
AACTGAAAACCGTTAATTGCTATAACTGTCGATATATTATGACTACATTCAAATACTGGCAGGCAGTCAC
AGTTTATAGTAGTCTATAGTCGATACATACATTTCGTATATGGTGTAAATATGGTACTAAAAGAATAAACA
CATAGGTTGCAATGATCAAAGGCCACAATACTTTTAAATTTCACGATTATACATATTTTCTTTTGCTGATA
CATTGAAAGGGCTATTTTCTAAGTCACTTTAAATTACTGTCAGACATAGCTCATCTAGTCAGTGCGGCG
AAGTGGCAGTAGACTGGGTTCACTCCCTAATCGACGAAACGAAACGGTGTGATCAAATTGCAAGGTC
AAGGAAATTGGAATATGTGGAAATTTCAAATGACAGTTCTGCTAAAGGGACCTGTACAGGTGTATGGT
ACTAACAGTAGGAATTTGTGATATAATTAATAGGTTTTTATTTAAAGTAGTGCCTACAACTTCTAGTGCC
TACAGAAATAGCGTGATTTGCTTTTAGCATTAAGTCCACCATTTGTACACTTATTTATATATTTGTGCAAT
AAAGGATTAAATAAAATAAATAAATAAATAAAATAAATTGTGATCTAGTGCCTTTGTTTACCACATTCGA
CTTCTTAAGATAACCACTGTATATATGTATATGGTTAAAAAAAACGATTTATATTAGCAAATTAAATTATT
CTTCAATGGAAGAAATTATTGGCAGATTTCCTACCTATGTACAAGGCCAAAAAAATAGCATAGCAATAC
TAAACTAAGCCAGAAAAAACTTCTTCTCGTCTAGGTTCTTAGGATTATTACGCAGAACAAAATCCATAA
AAAAATATTTTCAGGCCTTGTGAACGCACCATTTTCGTATTTGTATATTTATAAAATGTCACAAGATTTTA
TTTGGATCTATTCGATCGAAGGGTGTTTTTAATAATAACCCAACAGTTTTGCATACCTATAGGAGATTAG
CAATTGACGGAGAAATAAAATTCTTTAAATATCAGTGGAAACTGACGGACGTACTACTAATCGACGCGT
AAGCGGCTTAGGAATGACCTACAACCCTCGTCTCAACCAGGTAAGAGAATAATAGTAAACCCCGACCT
CGCGTCTGCTAGCATACAGACTATTTACACGCATCCGAGTCTCGAAGCCGCCAAGCTGTAAGAATACTC
CACGTAAGAAAAGAAAGGTCTTCGGAAATTCCAGCTTAGGGCTATCAAGTTTGGACAGTTCCTATATA
AACAAAAAGTGTCTAGCATCACCAGAGAAGGTTTAAGTCTACAAAGGCAGCCAATGATTTCATCAACC
ACGCAGCACTGACATCAAACAAATTCAAGGCTATGATCCTAACATACCATATGCTTCCTCAGATAAAAC
TTGTCCTAAACAAACCGGGAAAAAAATCGATCAAAGTCATAATGTCA
>TCONS_00029336 geneID=XLOC_006001 scaffold_146:110242-124810(-) Intergenic
TCTCCGCAGCTGTCCGCAATGAATATCCTGCTTTTACCTCTTCATAAGCGTTTTTGTACTGCAACAAGTC
GATCTGCGCCCTCAAAGTCTTCCTTACACGTACTCTCGGCATCTAAAAAATAAGAAAATAATATGAACT
CATTGTTTCAACCGTACCCACAAATATCGTTTCAACCGTACCCAACCACTCATTTTTTACTAACAAACTT
GACATAAAGTCTAGCAACAAAGTATTAAAACACTTAAAACACAAATGCTAATATCAACGAAACAATTG
GAAATAGAAAACAAAACAAAATACTTACTTTTGTCGAAAAAAAATACAATGAGGCCACCTACATACGTG

CACGGCGCGCAAAATGCGTCGAAGCACTGTGATTCATGTCTGGACTCGCAAGCCGCGTCCCTCTCCCC
TTGGACCCCTCGTCGTACCGCCCATAGTTTAGGAATTATTAGAGTTGTTTCAACCGTACCCAGGCTACC
CTACTTGAACGTTTTCAGTATACCTATTTCTGAATAGTGTCAGCCGATTTCCTTAGTCAATTTCCGATCAT
ATAATGAATGGGCTATTAGTCATATGGAGAAAACAATTAAATATATAAACAAGCGCGCATGCAAACATTT
TTCAATTTCATAATACTGGTGAATGTTATGACCCAGCATTTCCAAACATTTTTCCATTTCATAGTAAATAC
CACTTTCCACAGGATTTTGTCACTGACTTTCTGGTCATTAATACTCATACATACACATACAATACCCACT
CATAATAGAATGAGCCGTTTGTCATGTAGGAGGTTCTTCTGTAAATCCCAGAAAATTCCCTGACTTGGT
ATGACATAGTCATGAGGTTGCTTGAACGTCTTCAGTATGCCTATTTCTGAATTATGTCCGCCATTTCGGG
GCAGCGGGGGAGTGAACACTGTCTTTTTGTAAGATTTGTTAAGAGAAGAGCCATGAAGTTGGTTCAGC
AAATGATTTCTTGCTTTTTGGAAGTCATTGAAATGCTGTCCTGTGACATTATGACTTTGATGGATTTCCC
CTGGCTCGTTAGCATCAGCCTCAGCAGTTTAAATGCTGTCCTGTGACATTATGACTTTGATGTATTTTCC
TTGGCACGTAAGCATCAACCTCAGCAGTTGAAATGCTTTCCTGTGACATTATGACTTTTATCGATTTTTT
CCTGGTTTGTTCAGGACAATTTTTATCTGTGGAAGCATATGGTATTTTAGGATAATAGCCTCTAATTTGTT
TGATGTCAGTGCTGCGTGGTTGATAAAATCGTTGGCTGCCTTTGCAGACTTAAATAATTCTACTGTAATT
CGATTACGGCCTGTCATTTTGACACCATCTCTGGTGATGCCAGACACTTTTTGTTTATACAGGAACTGT
CTAAACTTGATAGCCCTAAGCTGGACTTCCCGAAGAGCTTTCTTTTCTTACGTGCTCAATGTCCCGTAC
ATGGAGTAATCTTCCAGCTTGGCGGCGTCGAGACTCGGATGCGTGTAAATAGTCTGTATGCTAGCAGAC
GCGAGGTCGGGGTTTACAATTGTTCTCTTACCTGGTTGAGACGAGGGTTGTAGTTCATTCCTAAGCCGC
TTACGCGTCGATTAGTAGTACGTCCGTCAGTTTCCACTGATATTTAAAGAATTTTATTTCTCCGTCAATT
GCTAATCTCCTATAGGTATGCAAAACTGTTGGGTTATTATTAAAAACACCCTTCGATCGAATAGATCCAA
ATAAAATCTTG
>TCONS_00029546 geneID=XLOC_005989 scaffold_146:117980-123719(+) Intergenic
GTAAGAGAACAATTGTAAACCCCGACCTCGCGTCTGCTAGCATACATACTATTTACACGCACCCGAGTC
TCGACGCCGCCAAGCTGTAAGATAACTCCATGTATAGGACATTGTGCACGCAAGAAAAGAAAGCTCTT
CGGGAAGACCAGCTTGGGGCTATCAAGTTTGGACAATTCCTGTAAAATCAAAAAGTGTCTGGCATCAA
CAGAGATGGTATCAAAATGACAGGGCGTAATTGAAATGCAGTAGAATTATTTAAATCTGCAAAGACAG
CCAACGATTTCATCAACCACGCATCACTGACATGAAATTCAAGGCTATAATCCTAACATACCCTATGCTT
CCACAGATAAAACATGTCCTAAACAAACCAGGAAAAAATCGATCAAAGTCATAATATCACAGGACAGC
ATTCAACTGCTGAGGCTGATGATAACGAGCCAAGGAAAATCTATCAAAGTCATAATGTCACAGGACAG
CATTTCAACTGCTGAGGCTGATGCTAACGAGCCAGGGGAAATCTATCAAAGTCAT
>TCONS_00029380 geneID=XLOC_005989 scaffold_146:118291-119647(+) Unclassified
TCAAGGCTATAATCCTAACATACCCTATGCTTCCACAGATAAAACATGTCCTAAACAAACCAGGAAAAA
ATCGATCAAAGTCATAATATCACAGGACAGCATTCAACTGCTGAGGCTGATGATAACGAGCCAAGGAA
AATCTATCAAAGTCATAATGTCACAGGACAGCATTTCAACTGCTGAGGCTGATGCTAACGAGCCAGGG
GAAATCTATCGTCGTTGTTATCATCGTTGAGCCGCGACGAGTGTTCCACCACCACACACACCTGCCGCC
GAATAATCCCGCCCCCTCCTTACGAGCCGCCCGCCTCCATACACTACGTCGTGACCACCTGCGGCCGC
GTAGTGCCCGTCATCGACCTCGGCAACTTGTTCATCTCACAGGCTACATGTCAGATGTGCTTTCTCTATT
ACTTGCACACTGAACTAGAACAATAAACATTATTCTCCCTAAGATGTGCACAGGTCTTCTTGTGGATGT
GGACGTCTTGGTCAGTAAGAAAATACCTGATTAAGTATACATTAAGTATTCTATGGGAAAGACACTTGC
TGTATACCGGCGCGTAACTACAAGGCGGCGAC
>TCONS_00029304 geneID=XLOC_005989 scaffold_146:123062-124810(+) Intergenic
CAAGATTTTATTTGGATCTATTCGATCAAAGGGTGTTTTTAATAAGAACCCAACAGTTTTGCATACCTAT
AGGAGATTAGCAATTGACGGAGAAATAAAATTATTTAAAAATCAGTGGAAACTGACGGACGTACTACT
AATCGACGCGTAAGCGGCTTAGGAATGACCTACAACCCTCGTCTCAACCAGGTAAGAGAATAATAGTA

AACCCCGACCTCGCGTCTGCTAGCATACAGACTATTTACACGCATCCGAGTCTCGACGCCGCCAAGCT
GGAAGATTACTCCATGTACGGGACATTGAGCACGTAAGAAAAGAAAGCTCTTCGGGAAGTCCAGCTT
AGGGCTATCAAGTTTAGACAGTTCCTGTATAAACAAAAAGTGTCTGGCATCACCAGAGATGGTGTCAA
AATGACAGGCCGTAATCGAATTACAGTAGAATTATTTAAGTCTGCAAAGGCAGCCAACGATTTTATCAA
CCACGCAGCACTGACATCAAACAAATTAGAGGCTATTATCCTAAAATACCATATGCTTCCACAGATAAA
AATTGTCCTGAACAAACCAGGAAAAAATCGATAAAAGTCATAATGTCACAGGAAAGCATTTCAACTGC
TGAGGTTGATGCTTACGTGCCAAGGAAAATACATCAAAGTCATAATGTCACAGGACAGCATTTAAACT
GCTGAGGCTGATGCTAACGAGCCAGGGGAAATCCATCAAAGTCATAATGTCACAGGACAGCATTTCAA
TGACTTCCAAAAAGCAAGAAATCATTTGCTGAACCAACTTCATGGCTCTTCTCTTAACAAATCTTACAA
AAAGACAGTGTTCACTCCCCCGCTGCCCCGAAATGGCGGACATAATTCAGAAATAGGCATACTGAAGA
CGTTCAAGCAACCTCATGACTATGTCATACCAAGTCAGGGAATTTTCTGGGATTTACAGAAGAACCTCC
TACATGACAAACGGCTCATTCTATTATGAGTGGGTATTGTATGTGTATGTATGAGTATTAATGACCAGAA
AGTCAGTGACAAAATCCTGTGGAAAGTGGTATTTACTATGAAATGGAAAAATGTTTGGAAATGCTGGG
TCATAACATTCACCAGTATTATGAAATTGAAAAATGTTTGCATGCGCGCTTGTTTATATATTTAATTGTTT
TCTCCATATGACTAATAGCCCATTCATTATATGATCGGAAATTGACTAAGGAAATCGGCTGACACTATTC
AGAAATAGGTATACTGAAAACGTTCAAGTAGGGTAGCCTGGGTACGGTTGAAACAACTCTAATAATTC
CTAAACTATGGGCGGTACGACGAGGGGTCCAAGGGGAGAGGGACGCGGCTTGCGAGTCCAGACATG
AATCACAGTGCTTCGACGCATTTTGCGCGCCGTGCACGTATGTAGGTGGCCTCATTGTATTTTTTTCGA
CAAAAATGCCGAGAGTACGTGTAAGGAAGACTTTGAGGGCGCAGATCGACTTGTTGCAGTACAAAAA
CGCTTATGAAGAGGTAAAAGCAGGATATTCATTGCGGACAGCTGCGGAGA
>TCONS_00029384 geneID=XLOC_005989 scaffold_146:123384-132218(+) Intergenic
TCTTCGGGAAGTCCAGCTTAGGGCTATCAAGTTTAGACAGTTCCTGTATAAACAAAAAGTGTCTGGCA
TCACCAGAGATGGTGTCAAAATGACAGGCCGTAATCGAATTACAGTAGAATTATTTAAGTCTGCAAAG
GCAGCCAACGATTTTATCAACCACGCAGCACTGACATCAAACAAATTAGAGGCTATTATCCTAAAATAC
CATATGCTTCCACAGATAAAAATTGTCCTGAACAAACCAGGAAAAAATCGATAAAAGTCATAATGTCA
CAGGAAAGCATTTCAACTGCTGAGGTTGATGCTTACGTGCCAAGGAAAATACATCAAAGTCATAATGT
CACAGGACAGCATTTAAACTGCTGAGGCTGATGCTAACGAGCCAGGGGAAATCCATCAAAGTCATAAT
GTCACAGGACAGCATTTCAATGACTTCCAAAAAGCAAGAAATCATTTGCTGAACCAACTTCATGGCTC
TTCTCTTAACAAATCTTACAAAAAGACAGTGTTCACTCCCCCGCTGCCCCGAAATGGCGGACATAATTC
AGAAATAGGCATACTGAAGACGTTCAAGCAACCTCATGACTATGTCATACCAAGTCAGGGAATTTTCT
GGGATTTACAGAAGAACCTCCTACATGACAAACGGCTCATTCTATTATGAGTGGGTATTGTATGTGTATG
TATGATTATTAATGACCAGAAGGTCAGTGAGAAA
>TCONS_00029452 geneID=XLOC_005989 scaffold_146:131621-133418(+) Intergenic
CTTGTGAAAATGGCTGACATTATTCAGAAATAGGTATACTAAAGAAGTACAAGTACCCTCATGAATATC
CCATGCCTATTCAGGGGATCGTCAGGGTATTTACAGAAGAACCTCCTACATGACAAAGGGCTCATTCCA
TTATGGGGGGTTTATTCATAACCAGAAGGTCAGCGAGAAATGCAGTAAATAATGGAACTCCTGAGTGA
TCTTCCAGTATCTGGACCTAGGGGATGACGATTTATATTATTGTCTGCATTTGTCTCCATATGACCAAGG
GCCCACTCCGCTATGGAGAGCCGATATTGAGGGATGCCTCAAGTAGGACCTAATGGAATCAGATCACTT
TCGTCGTGTAAAAATTTAAAAAGAGATTGTTTTTTATCCATATAACTCCGGCTACGTTTCGCCTGGGAAT
GAAACTTGTGAAAATGGCTGACATTATTCAGAAATAGGTATACTGAAAACGTTCAAGTACCCTCATGAA
TATGCCACACCTATATCAGGGAATCGTCAGGGATTTACAGAAGAACCTCCTACATGACAAAGGGCTCAT
TCCATTGAGATTGTCGGATACGGCCGCTACAGTAGGAATCGTAGGGCAAATGTACCAATTTCGTGATAA
ATCTTCACTGCAGAAACCAGTGGAAGGGTAAAATATGTTTAACCTGACAAACTACCGAATGATCAACA
GTAGGTATCATCGTCAGAAACGGGAAGGGGAATGATTTTCGACTAAGTACAATCGCGATGAAATTTCGT

ATAAAAATCGTCGATAAATACGACATGACCTCAGCGGCGACATCTAGTGCTGAGGGGAATATTAGTACC
TATATTAATTATTCTCAAAATGGTCGGTATGGGAATGACCAGGGAAATTGGAACTACATCAAATTCGAG
CTTAGTTCGAAATGCAAACAAATTGAAAATCGGAACTGGGAATAAATATAAATTCGGTCAGGTGACCTA
TTAGGACAAAATTCAGAGTCGTATATCAGCAATTGCTTGCTTGTCTTTGAATATTTTTCCATATGACTAA
GGGCCCATTCCGTATAGGGATATAAATGGAATTTAATAATTGTTCAAATATGCGTACTTAGAATACATTAA
GAAATTGTTACTTAGTAAGTACTTCAAGGAATCGTAAAGACAATTTCGGTTATGAAATGTCCATGTGGG
GAATTATGCTGAAATTAATAAATAAAAACTAGTTACCTGTTCAAGGACAAAATAATATCTTGGCCAAAG
AGTCAAACTTTCGCCGTAAGTCAGACCAAGCATTGCAAATTTGCAATACGAGTGGCAGAGACGAGCA
GACGTAATACCCACAAGAAGATCCGTGTATACTGTATACCTCCCAGAAAGGTTGGTCCTCATATTCCAC
ATTTATTAAAAGGAATCAGAAACTACTTACTAACCAAAAACTGAAAATACAAAATGAATGGAGAGGAA
AATGGAGCATTTTTTGCACCGTAAAGACAGTAAAGAGCTAAGAAAGGCAGTAACAACACAAAC
>TCONS_00029436 geneID=XLOC_005991 scaffold_146:177262-194436(+) Exonic overlap (+)
CTTTTCTTTTAGTTACCTATTGTATATACCTACCAACACAAACATCAATTTCGCATTTCTCATGTTATTATG
CATTGTTTAATAGCGTTTGTGATTGAGCTAATAAAAACCCAACCATCTATCGTTCAATCTACCTACTTCA
ACAAATGGTCCAACCTCCGAAACCCTGTCTTAACTTTCTTTTTGACCTTTGTGGATCAAAAGATTGATT
ACTTTCTCCACACATTTTACATGCACATAAACAATACGTAAAACGATACGTATAAAGAAACAATATTAA
AATGGCGTCTATAACAAAATGAAACTAAGTAGGAGCGAGATAGCATGATTTTTTA
>TCONS_00029474 geneID=XLOC_005993 scaffold_146:251831-252689(+) Intergenic
CTAGAAGCTTGTATAAGCTGATGCCATCATGTTGGGCTAGAAGCTTGTATAAGCTGATGCCATCGTGTA
GATCTGGCTAGAAGTTTGTATAAACTAATGCCTAGGCAAGAATCCTGTATTACCTATACCGAGTCGTCA
GTGTTTCCCGGTTTGAAAAGGTTCTGTTCTGATCTGATATTATATGTAATGTCATGCATTGTGTGATGTAT
TTTTATGTGTAAGCTAACTCCCTATGTGTTTTAAGCTAGATACCTACCTACTAAGATAGTTATAGACAAAT
TGATAATAAAAACTATTGTTTCCTTTTGTAAACTGTGTTACTAACGTTCCTAAGGTAGCTAATAATATGTT
CCCAATACTAGGATAGCTATTATTCAATAAATAAATAAAAAGTTGACTGTGTTGTCTAATATTAATGATAG
TTATTATGTTCTAGTTTTATTAAGATAAGATTGTAAGCAAGTTGTAAAATTTTATGCGTTGCCTACCCAGT
TGTTGATTAGTTGATTGTTGTTAATTTTGTGTTTCTAATAAAACTTCATCGAATTATTATAATGTTTAATAT
TTGAAATGTAATTAAGTAAAAGAGGGCGCAGAGAGCGCGCCGAATCAGAATGGCCGTGTGGGCGTTT
TCTGTATTTGCTCAGAATAATAACTTTATTAATTTCTAAACGTAAATTACTATACTAGTTATGTTCCGAATT
TTCTGAACGCATCCTAACCTATGACACTCTATCTAGACTATGGTTCAATAGTTCATTTGTAGTCAAGTTC
AAAACTAAC
>TCONS_00029503 geneID=XLOC_005994 scaffold_146:256914-393692(+) Exonic overlap (+)
CGCCGAGACGCCGCGCGGCCGCGAGCGCGAAAATTGCGGGAAAAACATAAACAAGTGTCAGAAAAC
CGGCAACGCTCGTGAACGGTTCGAAATGCAGTTCTAAATTTGAAAAGGAATAGCTTTTTTAAAAGTGT
GCAGCCAGCCAGTGCTATTTGATTTTGTTTACAGTGAATGTTTTTCTTCTAAGTGAGTGTGTGTTGTTTT
TTGGACTGCCTCAGCAGCGGTGACCAGTTTTTCAGGTCACGCTATCAGTGAGTTCCGTCGAAAAGCAG
CGGAGTGAGTTGAAAGTTAAAGTGAAAATTCTGTGGACTGTAGGTGTGATTGTGATAGACATTTTTTGT
CATGTGACGTGTGTGAGACAATGTTGAAGATACAGATGCTAGATTCTATCGTGAGCAACGACGCTGAC
GATGGTCAGGGGCAGCCAGCTAATGGGCGACAACACGTGTCGGCGCCATGGAGCTCATCGACGACGA
CAGGGGGCGCTCGAGCGCAGCACGAGTTGTTCGAGTTGTTGGAAAGGGTACAATGTTCCCGGCTAGA
TGACCAAAGAGCCGTACTACCGCCG
>TCONS_00029317 geneID=XLOC_005994 scaffold_146:256979-324973(+) Intergenic
CCGGCAACGCTCGTGAACGGTTCGAAATGCAGTTCTAAATTTGAAAAGGAATAGCTTTTTTAAAAGTG
TGCAGCCAGCCAGTGCTATTTGATTTTGTTTACAGTGAATGTTTTTCTTCTAAGTGAGTGTGTGTTGTTT
TTTGGACTGCCTCAGCAGCGGTGACCAGTTTTTCAGGTCACGCTATCAGTGAGTTCCGTCGAAAAGCA

GCGGAGTGAGTTGAAAGTTAAAGTGAAAATTCTGTGGACTGTAGGTGTGATTGTGATAGACATTTTTT
GTCATGTGACGTGTGTGAGACAATGTTGAAGATACAGATGCTAGATTCTATCGTGAGCAACGACGCTG
ACGATGGTCAGGGGCAGCCAGCTAATGGGTA
>TCONS_00029588 geneID=XLOC_005994 scaffold_146:422854-452992(+) Splice junction overlap
GTCGATAGTGTCAGTGCATTCGCCGGATTGTTTACGCGTGTGGTTTAAAAATAAATGTTAATAAAAGTT
CTTATCGAGTACAGAAACAAGCTGTTATTAAAGGTTTTCCTTGTCGATGCTAATACCGCTATCAGTCGA
GTATGGGACGTTTTACGTCGCATTTTCCCCACAAATTTCAGTTTGGGCACACGTATTTTATGAACTTTGT
TTTTTTACACATTTTTTACATATTTAACAGTGTGGTGTTGTTTTAGTGCGATTGTTATTTTACCATGAAAC
GGTGAAATTTGTAAAATATATTATTTGAACTAAAATGTCGCCCGGTGGCAGAAGGTTTTCGTTCCCTGT
GATTGTGGAAAAGTTGATTCACAGGAAGAACTCTTCGTCCCGCACATCCTCCGTCAACTCCGATGATG
TATTTTGGCCCATAGACGAACAAGGGGTCTCCCGGGTCGAAACTCG
>TCONS_00029459 geneID=XLOC_005994 scaffold_146:422855-436020(+) Intergenic
TCGATAGTGTCAGTGCATTCGCCGGATTGTTTACGCGTGTGGTTTAAAAATAAATGTTAATAAAAGTTCT
TATCGAGTACAGAAACAAGCGTAATTGACCATTATTGAATTGTTTTACCGTTTTCCTTGTCGATGCTAAT
ACCGCTATCAGTCGAGTATGGGACGTTTTACGTCGCATTTTCCCCACAAATTTCAGTTTGGGCACACGT
ATTTTATGAACTTTGTTTTTTTACACATTTTTTACATATTTAACAGTGTGGTGTTGTTTTAGTGCGATTGTT
ATTTTACCATGAAACGGTGAAATTTGTAAAATATATTATTTGAACTAAAATGTCGCCCGGTGGCAGAAG
GTTTTCGTTCCCTGTGATTGTGGAAAAGTTGATTCACAGGAAGAACTCTTCGTCCCGCACATCCTCCGT
CAACTCCGATGATGTATTTTGGCCCATAGACGAACAAGGGGT
>TCONS_00029530 geneID=XLOC_005994 scaffold_146:423281-436030(+) Intergenic
CGCTTAGACAATATCGTTGTTTGGCCTTTTACACATTAAAAACCTAAATGCGAATAGGTATGACCTCAAA
TCCATTACCATAAAACTAGTCAATTAACTAAAACCTTATTTTGTATGTGTTAAGTGTTATTAAAGGTTTTC
CTTGTCGATGCTAATACCGCTATCAGTCGAGTATGGGACGTTTTACGTCGCATTTTCCCCACAAATTTCA
GTTTGGGCACACGTATTTTATGAACTTTGTTTTTTTACACATTTTTTACATATTTAACAGTGTGGTGTTGT
TTTAGTGCGATTGTTATTTTACCATGAAACGGTGAAATTTGTAAAATATATTATTTGAACTAAAATGTCGC
CCGGTGGCAGAAGGTTTTCGTTCCCTGTGATTGTGGAAAAGTTGATTCACAGGAAGAACTCTTCGTCC
CGCACATCCTCCGTCAACTCCGATGATGTATTTTGGCCCATAGACGAACAAGGGGTAAGAAAAAGG
>TCONS_00029325 geneID=XLOC_005997 scaffold_146:662876-674985(+) Exonic overlap (-)
CGCGAGCTTTCGCGTGAGTAGACGTGAGGGAGGGGTGGCGCGCTCTGGTGGCGAAAAGCGGAAAAC
TTTCTGAATTATTTCGTATTACAGTTTTCAGTGTCTGTTTCGAGTCAGTGGTTTCGCGCGGTAATTTAAA
GTGATTTGAAGTTTCAGTGGCGTTTTCCGTTGCAGGGAGGAGTATTTTTCGGATCCTACAATACCAGCT
AGGTTTGCCTTCTTTCTGGCTAACATCAAT
>TCONS_00029782 geneID=XLOC_006079 scaffold_147:140977-159951(-) Exonic overlap (+)
GCGCGCGTATGACGTGTCTTTGCTTCTAGTTCATTTATTTTATTCGCAATAACGCCGATCGTAAAATTTTC
AGTGTTCAGCTTATATTTGCCAATCAGTCATATTGCTGAAGTAAAATTAAAAAGTTCAATATAGTTTCAT
TGTGTTGTGAAGTTATTGATTTGGACTTTAGTGTGGGAAAATCCTACTCGGAATTGCCTGTTCACAGTG
AAAAATTTGCTTACGTAATCATCGTTAAGTTATAAAACTGAACTATTCTGAAATAGAGATTAAACATAGT
TGCATGTTGCATGAAAAAAGAAACTTACTTTTCTTTCCCGCGCTCTCAAACGTCACCTCAAAAACCAA
ACGTCAAAAACCGCTTACAACCAAAGAGGTAGCGGTTAGCGTCTGTGAGTGCGGACTCCTTGGTGTC
GCGTTGCGGAACTAGTGAAGTGCAGTGATAGGGCTCCGGTCGCGACGCCAATGCGCCGCCAGCGCCG
CCATGTCGCCGCCGCGGGCGCTGCTGGCGGCTCTGCTGCTGTGTCAGCTGGTCGCCTACGGCGGGCAT
CAGCCGCTTACCAAGATATTCTACA
>TCONS_00029776 geneID=XLOC_006064 scaffold_147:361310-361986(+) Splice junction overlap
CCAGATCATCTGCCTCGCGGCTCTCACCGCGCTCGCCGCCGCTCAGTATGGCCACGGCGGCCACGGGC

ACGCGTTCTCGTCCCAGCACATCTACCGCCACGACGGGCCCGCGCACGTGGTGCACTACAGCCACGA
CGGCCACGACGGCCACGCCGGCCACTACGGCAACAACGGCCAAGACGGCTACGCACATGGCCACGG
TGACCACCATGACTACTATGCGCACCCCAAGTTCGAGTTCGAGT
>TCONS_00029693 geneID=XLOC_006086 scaffold_147:495737-496387(-) Exonic overlap (+)
TATACAATTAACAACCTTAATGATGAAAAGCGTCCTGTGGGAACGGCTACAAAGAACTTAAATCTGAG
CGAAAATTTCAATGTATTTGTAGCCGAAATCAGTTGATTTTCTAGCTAATTTCTGTGTGAATGTGTAGTT
ACTTCAGTGTTTTAGTTAACAACTATTGTAAAAGCTTCAGTTATCCGGTAAATACAGTGTTGACTCTTGC
TGTTGTTTCGTGAACATGAGAGTGATTCGAAGATAGAGCCTCCGCGCAGCAGTCAGTTTGTGCGATGG
ACAATGCTGGCGTGAAGAAGCTGGTGACCAGTATGAAGGTGTCGGAGGGGATGAAGTCTGAAGAGTC
TGATGATGAGGTGTGGGACAGCGACGACGATGACGACACCATCGCGAGGATCCAGCGCGCGCACTAC
GCGGCCAC
>TCONS_00029875 geneID=XLOC_006069 scaffold_147:508493-511892(+) Intergenic
CCCTTTCGGCTTAGTATACCCTTTTTGCAACACCCTGTAGAAAAACAAACGAATCAGAGTTGACATGCT
ATCGGCACGTTTAATGCAGTAGAGTCGTGGTTATCTCCTTAACTTTGTGTTCCCTAAGCTTGCGAGTGC
CTAACCCTTGTATTTGTGTCCCGATTGCATGACAGCTCTCGTTCGATTGTTCTGTAGTACCGCCATGCCT
CCATAGGTACGGTCACCTTCATAAGTAGGTATACACTTTTTTACCTTACCAAACGAACAAAACGTCAAT
GTTTCGATGATTCAATTTAACAAAATATTAAAATAGTTGGTGAGTTTGAAAAGGTACAAAAGTGTACAG
CTACTTATGAAGCTGACTGTTCAAATTTCGTCGAGATATTTAAAGTGCTAAAAATAAGTTTTATGTTTTA
TTTATGTAAAATACTGTTGTGGCGTTTAGGTGTTCCTATTATTTTTATCATCATGTTTTCAGTCTGCCTAAT
TTATGTCAGTGCTTTTTTGTTTTCAAAACTTATCTTAAATGTAAAATAGGTTATAGTTATGTGGGGTGAG
AGCAACATACATGAGAAATAAATAGAGACCATAACCAATAAACCACATAGTCTAATTATAGTCTGATATC
AAGTTTCCGGTGTTCCTCTTACCCCACATGACCTTAATTATTATAGCCGAGAAATTCCATGTATTTAAATT
ATACTCTTGTGTAGTTTGAAATTATGAATTATGAAATAATATTATGAATCTACTACTTATGTACCTACATAC
TCTTCTATATTATGCGTAATTATTCGGCCTAGGTTGTTCGATGGTAGATAGCTGATAGAATTTACATGATT
CTCCCTGCTAGTTGAACCTGAGTGGTTTATCAAGGTTGTCATGATAAGACCCGTATGCTTTATTCAACTC
ACATACATTTCAGGAGATAAATTAATGCAAGAATTGTCTTATTTGATTATGAATAGAATATTCATTATTTAT
AGAATACCTATCTTGTAAATTTATAGTGTGTGTTTTCCTTATTTTGCCAAATTTATTGTACTGTACTGTGA
ACCCCAGTGGGATATTTTGTGTGTTTTTATTCGATAAGACCAGTGAAGTTTTCTGTAGCATCTGGTTAAA
TAAGACAAGATTGTAGATTAGCTAGGTATTTTAATTTCTGTGATACTTTGCCATTGTGGTATAAGGTATTA
TTATCATTTATTTATTTTGTTTAGTTACTTAATACAATACCCCTCGATTTAGATTGCTGATTTGTGTGTTAA
TTATTTACTTATTCATTATATTTTACGTAAAATTGGTTTTGATCCGTTAATCTGACGAGTGAACTATAGCTG
ATACGAACATGGTTTTTATGAGAATATACCTTAATTTATTATTAAAATAGCAATATTATATTCTGGTAGTGA
CCTAAAACGTGTGGTTAAAGTAGGATAGCTAAGATATTTGTATACTTTCATCTAGTCACTGGCTGACTTA
AGATTTGATGTTACAACAGAATCATAACAGCGAAATATGGCCTTAAAATATGTAGGTTATTCTTCAATGT
AATTATGAAACCACATTCCGGGTTATATTATAGTTTAAACTAGACTTGTTTAGATAACGGAAAGTGAGAT
TAACTTGACTTTGAGTAATGGGTAAGGAGCAACTGGCTTGACTTCAAAATACAGTCTTGTATACTATAA
TGTATGGTATGGGCACACTGTTTACTTAAGTAAGGGATGCAGTTAAAATTACACAGGCATGCTAGGTAC
CTGCTAGAATAGATTACACACATAATGCTTATACAGAGTGTTGACTCCGGTGGTCGTTCGTACAAATTTT
GTTCTACAAATTTTGATAATTCGTGAAATTGTGTAGAACATAAGTTTTTGACCTCTTGAGTCACGCAAC
ATCTGTATAATAATAATATTTCAGTATGTACTAAATATAGTACTAACTAGCGCACTTTACAATGAACTATTA
AAACTCCACAGCTTTGAAGGTCTGGATTTTCGATATGCATTTGTTTTACGTTACATATTCGAATAGGTAT
TTCTCATTTCAGTGGGTTACAAATACTTTTTGGTCTTGAATGCCTAATGTAGAATCTAGTACAGGTATCG
TTATGGCTTCATACATAAATAATACAGTCATATTAGTACTTACTCCTAGTTGCAGAAAGCTTTCAATAGGC
CTTGAAAATGTCGTCACCAAATGTAATAATAATGGATTCATTGTGAATGTCATAAGTTTCTGCATTTTTT

GTCACGAGCAAAGACAGTGCTTAGGTACGTGAGATAGTTTCAGGCTACAGCTACTTAAAATAATTTCA
AATATGTTTGCCTTTTGATATTATATCTAATAGGAAATGATACAGTTATGTAGACAGTCAGAGAAGCAAT
GTTCAAATTACTGTGTATCCTTTTAGGCGCAGGCCACCGTACAACGTACATCACCCGTAACAAATGAAT
CAAACTTACTTAATTACGTTGTGGACATGAGCACCATCGTCTATTCAAAAAATAAATAAATTTTATGTCA
AATAAAATTAAAAAAATATTTTCGAATTTCAATAAGTTTCATTCAATTTATACGGAATGCCTTTATAAATG
TATGACATTCTATGTATATTTATCGTAGGCTTATGGTTTATCTAGGAGAGCTTTCTTAAAAATCTGTATATT
AGCAGAAGCAATATTATACTTACTCTTGTCCTTATAACCTTTGAGCTGTTGTATTTAGGATCCTATAACTT
GTAAATTACAATAAGGCCTTACAATGTTTATTAACTCCATAAGCCATGCTATTTAATTTACCATGAATAAG
GGTACTTATGTCAGGATTGAAGGACATGTTATTGGATTGTGATAGTCTACACTACGTGGCAATCGTAGA
ATTCTCATTAAACAGGTAATGTTTCACAATGAATATGATATTTTTACTAGGATTTGAGATAAATTGATAGA
GCTAATCGAGAACCCAGTTAACCATCAAAACTTGTTTGAGAGTCGATAGCTTAGATAGTACTTATGGTA
AAATAGTGAATCTTTAGACTAGAAAATATATAGGGAGTTTACAAAATAGAATATGAATAAAAATTGAAAT
GACTTCAGCTGTTATCCAATTTTTATACATTTTTACGGCATTATGTCAACAGTGTAAATTTTTGTAATGTT
AAAATATGTAGGCGTTTTACTCCATATAGGAAATGTAGCATTTTGC
>TCONS_00029945 geneID=XLOC_006089 scaffold_147:508864-511886(-) Intergenic
TGCTACATTTCCTATATGGAGTAAAACGCCTACATATTTTAACATTACAAAAATTTACACTGTTGACATAA
TGCCGTAAAAATGTATAAAAATTGGATAACAGCTGAAGTCATTTCAATTTTTATTCATATTCTATTTTGTA
AACTCCCTATATATTTTCTAGTCTAAAGATTCACTATTTTACCATAAGTACTATCTAAGCTATCGACTCTC
AAACAAGTTTTGATGGTTAACTGGGTTCTCGATTAGCTCTATCAATTTATCTCAAATCCTAGTAAAAATA
TCATATTCATTGTGAAACATTACCTGTTTAATGAGAATTCTACGATTGCCACGTAGTGTAGACTATCACA
ATCCAATAACATGTCCTTCAATCCTGACATAAGTACCCTTATTCATGGTAAATTAAATAGCATGGCTTATG
GAGTTAATAAACATTGTAAGGCCTTATTGTAATTTACAAGTTATAGGATCCTAAATACAACAGCTCAAAG
GTTATAAGGACAAGAGTAAGTATAATATTGCTTCTGCTAATATACAGATTTTTAAGAAAGCTCTCCTAGA
TAAACCATAAGCCTACGATAAATATACATAGAATGTCATACATTTATAAAGGCATTCCGTATAAATTGAAT
GAAACTTATTGAAATTCGAAAATATTTTTTTAATTTTATTTGACATAAAATTTATTTATTTTTTGAATAGAC
GATGGTGCTCATGTCCACAACGTAATTAAGTAAGTTTGATTCATTTGTTACGGGTGATGTACGTTGTACG
GTGGCCTGCGCCTAAAAGTATACAGGCGGAGTTTTTAAAATAGCGATCCCTGATGATGAAGGGACCAG
CTACATCTGTTATAAATCCGGAAACGTGTGATGTGTGTAGCAGTGGTGTTTATGTGGATGTGCTTGAGC
AGCATGTGAGCTTGAGGTCGCTATTTTAAAAACTCCGCCTGTATACTTGTAGGCGCAGGCCACCGTACA
TGGATACACAGTAATTTGAACATTGCTTCTCTGACTGTCTACATAACTGTATCATTTCCTATTAGATATAA
TATCAAAAGGCAAACATATTTGAAATTATTTTAAGTAGCTGTAGCCTGAAACTATCTCACGTACCTAAGC
ACTGTCTTTGCTCGTGACAAAAAATGCAGAAACTTATGACATTCACAATGAATCCATTATTATTACATTT
GGTGACGACATTTTCAAGGCCTATTGAAAGCTTTCTGCAACTAGGAGTAAGTACTAATATGACTGTATT
ATTTATGTATGAAGCCATAACGATACCTGTACTAGATTCTACATTAGGCATTCAAGACCAAAAAGTATTT
GTAACCCACTGAAATGAGAAATACCTATTCGAATATGTAACGTAAAACAAATGCATATCGAAAATCCAG
ACCTTCAAAGCTGTGGAGTTTTAATAGTTCATTGTAAAGTGCGCTAGTTAGTACTATATTTAGTACATAC
TGAAATATTATTATTATACAGATGTTGCGTGACTCAAGAGGTCAAAAACTTATGTTCTACACAATTTCAC
GAATTATCAAAATTTGTAGAACAAAATTTGTACGAACGACCACCGGAGTCAACACTCTGTATAAGCATT
ATGTGTGTAATCTATTCTAGCAGGTACCTAGCATGCCTGTGTAATTTTAACTGCATCCCTTACTTAAGTAA
ACAGTGTGCCCATACCATACATTATAGTATACAAGACTGTATTTTGAAGTCAAGCCAGTTGCTCCTTACC
CATTACTCAAAGTCAAGTTAATCTCACTTTCCGTTATCTAAACAAGTCTAGTTTAAACTATAATATAACC
CGGAATGTGGTTTCATAATTACATTGAAGAATAACCTACATATTTTAAGGCCATATTTCGCTGTTATGATT
CTGTTGTAACATCAAATCTTAAGTCAGCCAGTGACTAGATGAAAGTATACAAATATCTTAGCTATCCTAC
TTTAACCACACGTTTTAGGTCACTACCAGAATATAATATTGCTATTTTAATAATAAATTAAGGTATATTCTC

ATAAAAACCATGTTCGTATCAGCTATAGTTCACTCGTCAGATTAACGGATCAAAACCAATTTTACGTAA
AATATAATGAATAAGTAAATAATTAACACACAAATCAGCAATCTAAATCGAGGGGTATTGTATTAAGTAA
CTAAACAAAATAAATAAATGATAATAATACCTTATACCACAATGGCAAAGTATCACAGAAATTAAAATAC
CTAGCTAATCTACAATCTTGTCTTATTTAACCAGATGCTACAGAAAACTTCACTGGTCTTATCGAATAAA
AACACACAAAATATCCCACTGGGGTTCACAGTACAGTACAATAAATTTGGCAAAATAAGGAAAACACA
CACTATAAATTTACAAGATAGGTATTCTATAAATAATGAATATTCTATTCATAATCAAATAAGACAATTCTT
GCATTAATTTATCTCCTGAAATGTATGTGAGTTGAATAAAGCATACGGGTCTTATCATGACAACCTTGAT
AAACCACTCAGGTTCAACTAGCAGGGAGAATCATGTAAATTCTATCAGCTATCTACCATCGAACAACCT
AGGCCGAATAATTACGCATAATATAGAAGAGTATGTAGGTACATAAGTAGTAGATTCATAATATTATTTCA
TAATTCATAATTTCAAACTACACAAGAGTATAATTTAAATACATGGAATTTCTCGGCTATAATAATTAAGG
TCATGTGGGGTAAGAGGAACACCGGAAACTTGATATCAGACTATAATTAGACTATGTGGTTTATTGGTTA
TGGTCTCTATTTATTTCTCATGTATGTTGCTCTCACCCCACATAACTATAACCTATTTTACATAAATAAAAC
ATAAAACTTATTTTTAGCACTTTAAATATCTCGACGAAA
>TCONS_00029832 geneID=XLOC_006071 scaffold_147:662283-672613(+) Unclassified
CGGCCGCCGCGTCACCTCGCCCCGCGCCCCGCGCCCCGCGCCCGACGGCCACCATGCTGGCCTGAAC
CAGCCCTCGCGAGCACGGCACCCTTCAGCCGACAGTACTCAAAGTGCAGACTGAAAACAGTGCATAT
CTCAAACGTCGCCATGAAACAAAACCAATGCCGTTACTAATCCAAGCCGACGGCCTCACGCATTAGGA
ACTCAAGTGAACGTAATTTCAGACAACCTTTACCACACCTGCCCCTAACCTTTTGGAGCTAATAGCCG
ACTATTAATAAACCCGCCGACGCATCAAACCCACAGTGTCTAAACAGTGAAAAAAGGAAAGAAACAA
GTGCCGAGTGATAGTGAGCGCGGACCATGAGTGAAGTGCAAAGTGTGTGTCTTGTGTTGTGAATGCGT
GTGTCCGCATGTGGTGGGAGGCGAGGGCGGGCGGCTAGGGCGGGCGGGCGGCACCGGCTCCA
>TCONS_00029834 geneID=XLOC_006074 scaffold_147:765019-787984(+) Exonic overlap (-)
TAATTTCTCCTCCCGCACCTACCGTTAAGTTATTCATAGAAATTGAAATTCCAAGTTGGGCGAGCAACAT
AGTGATGCATCAGCACGGTCGAGCGACGGGCGCTCCGCTCGCTTGCCCGCGCCGCTCGTGGCGACTG
ACGATCCACGCTCGCTCGCTCCAGTCTCCAACCAGCCTCGACCGCGGGCGCGCCTCCCGCCGGCCAC
ACCAAGACAAACCCGCGAAAAAACGCGATGTTTACCTATACTGTGGAATGTGGTGTTAAAGAAAAAA
AAGAAAATTAGTGACAGTGATACGAGTTCGGGATCAGCTCAATCGTGATTTATAGCCGATTAAAGCAG
CCGAGAAAAACAGAATTAAAGAAAAAATCAATCTCAACTTATGTTAAAGTGGAACTGCACCGTAGGC
AGAGCCAGACGCGTCCGTCAACAGTGCCAATTGTATTATCTGAAGCAGCAGTGTCAAAAGTGTTGTAC
CATGAGTGTGTGGTGTCAGTGATGGGTTGGTGAGTGATGTGAGTGGAGTGAGTGGGGATGAGTCGGC
ACCAGCTCCGGGTGCCGGAGTTGCGGGTGTCGGGGGCGCCGCCGGAGCCGCAGCCCGAGGATGACA
CGCCGCGCGCGCTGCCGCCCTTCGCGCTCACGCTCGCGTCTATACCGCGGCGGAGGCATTCGTGGATC
TG
>TCONS_00029985 geneID=XLOC_006074 scaffold_147:765320-787838(+) Exonic overlap (-)
AAAAATTAACCGTGCTCGCGTTAGATACGAGTTCGGGATCAGCTCAATCGTGATTTATAGCCGATTAAA
GCAGCCGAGAAAAACAGAATTAAAGAAAAAATCAATCTCAACTTATGTTAAAGTGGAACTGCACCGT
AGGCAGAGCCAGACGCGTCCGTCAACAGTGCCAATTGTATTATCTGAAGCAGCAGTGTCAAAAGTGTT
GTACCATGAGTGTGTGGTGTCAGTGATGGGTTGGTGAGTGATGTGAGTGGAGTGAGTGGGG
>TCONS_00029986 geneID=XLOC_006075 scaffold_147:793018-794091(+) Intergenic
AATTACTACATAATTATTATACATCTACGATCTGCGTCCAATTTGCGGATGACTAAAAAAATTCCTTCAAC
AACCTCATTCTCTTGGCTAACAAAAGATGATTGACAGTGAGATATCATGGAGCATGGAGAATAATTTCC
GTAGAATAGAGGATGCTGGCTGAAAGAGCGATGAGACGTCACGAAAGAAAAAGAAAATTTCACGGTG
TGAGTTGCGCGGCACGTCGCCTGCGCAGTACTCGCCACGCCGCCCGCGCTCATATCGTTGATTGAATAT
TTGAATTAAGAACACTTTTGAATTACGAACGATTTTCCATTGACAGCTGATGAAAAAGTTGGCGCGGA

ACTCCGTGTGTGGATCGGTTTTGTGTGAAGTGTAGAGTTGTTTTGTTTCGTCATTAGTTCATTACGTATG
TTGAAAAGTGTGGAGGTGTGACATAGTTTTTCAGGAGAAGATTCGTGGACTGAAACTCGGTGCTCTTT
TCTTTTCAGTGTACTGAATGTGTAAATTCCGTGCGTTAGTAGTGATTAGAATGAAATGTGATAAAATATT
AGTTTTTGCACTGATTTAATAACCATAGTACTAATGCTTGTGTTTTTAGGAAAGATTCGACCCTGACCTG
AAATAACGTTCTAATAACCCATAACTTCAAATATTAGT
>TCONS_00031200 geneID=XLOC_006165 scaffold_148:83565-84258(+) Exonic overlap (+)
GGTGGCGCAGGTGCGCCTGGAGCAGGCGGTTCCGGGGCTGGGGCAGGTACTGGAAGTGGCGCAGGT
AGTGGAAGTGGGGCAGGAAATGGGAGTGGGGCAGCAGGTACTGGAAATGGGGCAGGTTATGGAAGT
GGGGCAGGGGTTGGACCTGGCACCGGAGCAGGTGATGGTAATTTCGGAAGAGACGGAAGTAAACTTG
GCAAGGACGGAACTCCAGGAGATGATGACGACATGCTGGGAAAGGGTCTTGGGGATGGCACTCAGG
GGGTGGGTTTGGGAGACCGCGGGGTGACGGGCGCGGGCG
>TCONS_00030523 geneID=XLOC_006165 scaffold_148:83705-84263(+) Exonic overlap (+)
TGGAAGTGGGGCAGGTGCTGGTAGTGGGGCAGGTGCTGGAAGTGGGGCAGATGCTGGAAGTGGGGC
AGGTGCTGGAAGTGGGGCAGGTACTGGAAGTGGGGCAGGTGCTGGTAGTGGGGCAGGTACTGGAAG
TGGGGCAGGTTATGGAAGTGGGGCAGGTACTGGAAGTGGGGGAGGTTATGGAAGTGGGGCAGGGGTT
GGACCTGGCACCGGAGCAGGTGATGGTAATTTCGGAAGAGACGGAAGTAAACTTGGCAAGGACGGA
ACTCCAGGAGATGATGACGACATGCTGGGAAAGGGTCTTGGGGATGGCACTCAGGGGGTGGGTTTGG
GAGACCGCGGGGTGACGGGCGCGGGCGTGGCG
>TCONS_00030940 geneID=XLOC_006184 scaffold_148:709899-717142(+) Intronic
GTGGGGCGGCAGTGTCGCGCCGAGGCCCGCGCCGGCTCCACTTGATCAATACACATTACCTAATAGGT
AATCTAAAATACTGAAAAAATACGTACAAAATTATGCATTTATAAACAAGCTAGTGAAAGTTTTGAAGA
AGCAGTTTAAGTTAATAGTTCTCATTTAAAGTATGTGTAAGCACGTGGTACTAATTCCCATGAAGACAG
TCGATACAGCGATGCAGAAGA
>TCONS_00030255 geneID=XLOC_006230 scaffold_148:820875-822653(-) Splice junction overlap
CTGTAGGCAGGCGGCAGGACACTTATCGTTCTCAGACTCAGAATAATAACACATCTTGCTAGAATGTGT
TCTGTGGCTCAAATGACTGTCAATGTGACAACTGACAACGACAGATTACCAGTTTTCAATGTTCGTGG
AAAATTCGTAATTTCGTAAACTTACAATATTTTCATTCATTACATTTACGAAAAAGAAATATTACTTCTGC
CTCTTATAACTATTATAGGATTCCACCCTCATATAGCAAAATGACGAGTGTGACTAAAAGAGTGCAGAA
TTCAATCAAAGGATGGTACCTTAGATATTTGCTTGCCACTGATCTGTACATAGTGGAGCCTTGGGAGAA
AGTTGTTATACATGTGTTGTTTGCACTACTATTTGCTGCCTTCTGGTACTTCAACTATGTGATGGTGGCAT
CAGGTTCATCGGAGCTGAAGCAAGGACATGCAATAGTGGATCCAGTATTATGAAATATCTAGCTATTTT
CAAGTTTTCATCAATTAATATTTTAGCAGCCATTAGGTTTGGTTTGTAGCATCATCATGTTACCTATTTGT
CAATTTTATGATGCTACAAAGGAAATCAGCTGGTGTGGTTAAGTTTTGTATTTATGCTTCTTTCAAACAC
TAAAGTAATTTTATAATTGATGTTATTAATTAGAAATTCCTAAGCTGTTACATTGTGTAATTTTGTCAATA
ACCCCACCTATCTATACAGGTATTGCAAAAAGGGCACAAATTTTAAAATTCTGATTTCATTTTTGGGCTT
AGATATACCATACTGTACATGTAGGTTTCGGCTTAGTATACCCTTTTTGTAACAGCCTGTATATTAGTTAA
GACATTCCAGTGATGAACTATTCTCTAAAAATACTTCATTCTTGTTAATACATTCACCGTGCCTTTATTCA
CATTCACATAGTTAGGTATATGACTATACTTTTATTCATACCAAAAATGTTAAATAATCCTAAGAAAATAA
TTATAAATTATG
>TCONS_00030269 geneID=XLOC_006233 scaffold_148:948564-955968(-) Exonic overlap (+)
GTTTCGGTGAGGCCGCCGTGACCCAGTTTCGCGTTATTCGCATCTTGGACCGCCAACGATGCACATGTC
TCGGCTCTGACGGACCTCAGTCGCGATATTCAGCGAAAATCGGGATATAAACGCGAATTCAGTTACAG
TTCTAGTGACGGTGTATAAAAATTCGTGAGGACTGAAAAAAGTACCGACGTGAAAGCCCTAAAACCTA
TTCGTGACATACATTTTTGAAAAAACCGCGTTTGGGTTAAAATTTTCAATCGAGCCACTTTATTGGTGG

CAGGGCGGTGTTCTGCGCAGTGATTGGTCGCAATCTGACAGGTGGCGCGCTCGACGAATCAGAAAGC
CGTATTTGGAGGTTGTAGACTTTTGTAGAGCCACTGCAGCGCCACCTGCACGATGAACTTGCTAAACT
TGCGCCTTTCATTGAATAACCCGGAGTGTTTTTCTTACGCATGCGCTGTGAGGCCCGTCGAAGGGCCG
AACAAAAACGGTTCGAAAGTAAATCTGATGTATTTATGTCAGAAAAATGGTGAAATCTGTGAAGTGGC
TACTAGGAATTAAGATATTCTTCATTTCCAATCATGACCGAGGGATATGAGGAGACATGGTTGGTGCGG
GTGCGGGCACAAGTGATGTGCCGGGACGAGGCGACGGGCGGCTGGCTGGCGCTGGGCGGCGGCGGG
CTCGCTAACGTGTGCGTCGGGAAGAGGAAGGTGGACCGCGCCGCGCAGGGGGC
>TCONS_00030270 geneID=XLOC_006233 scaffold_148:950384-955550(-) Intergenic
CATTGAATAACCCGGAGTGTTTTTCTTACGCATGCGCTGTGAGGCCCGTCGAAGGGCCGAACAAAAAC
GGTTCGAAAGTAAATCTGATGTATTTATGTCAGAAAAATGGTGAAATCTGTGAAGTGGCTACTAGGAAT
TAAGATATTCTTCATTTCCAATCATGACCGAGGGATATGAGGAGACATGGTTGGTGCGGGTGCGGGT
>TCONS_00030843 geneID=XLOC_006194 scaffold_148:981650-992602(+) Unclassified
GTACCGGTAAATTTAATACATTTTAAATTCATCGATGCCCATTTAACCGTAATCATGACATCACTAAGCC
GCAGCACGCACACCAACGCACCCGTATCGCGACGCGCTAGAGATGCGATAGCGTACACAAGCGGCAT
AGGTGTGTACCCCCGAGACAAATGTACGGAAATTTTAGCCGCCAGTCGAGTTTCTGAACCGAAAACG
GGACGACGCACGCTCCTTGCCGGTCCCAGCGTTTGATAAGTGCCTGAAAAAAGTGCCTTTAGTGTCAG
TGTGGGAGTGTCTTACAAAGAAGTTTTCTTTTTTATAAAAACCAGTGTTAACAGCCAGTGTCGTTTTGT
GAATTCAACTGTTTCGTGAAACTACGAGATGGAATTGAGGAGATTGTCTCTGGTTCTGGCGTTCTTCGC
GATCGCAGCCGTCTCACGATGCA
>TCONS_00030290 geneID=XLOC_006246 scaffold_148:1500329-1503592(-) Unclassified
GCCGGCCACGCATATATAAGGGTGCAGGACGAATAACGGACCACTTCCGAATTTGAACTGCTACAGTT
AACATCCCCGGGGGTCCCTGGCCCCCGGTGGAACATTACTTTAATAAATCAGCGTTATCAAGAAAACTT
AGTGTCGCCTAAATTGAAAAATAAGTTGAAACTTATTTGAGTTAATAAGTTGAATACGGCTGCGATGAC
AACTCGAGAAATGAAGCATCAGCTATGCAGACTGAGAAACAAAATTCACCTAAATTATCGGTTAAAAT
GAAAAATTACCATCACCGTATTTTTCAAGAAATAATTCGTTTCCGGATCCAATAATGATCATGGCACAAT
TTACCAGAACCTGAAACAGAGCACCATCGTCACATCACGGCCTTCGATTTTCAACCAATCTTCAACAG
CTGTTTCATTCAAGGAAACTGCTCCGTTGTACCCTGAAAATGAAGACACAAGACGACTTAACAGATAC
TACAGCTCAGGTGATTTACTCTATTTAACATCGTTTAGGTACATCTTCAAAGTATTATATAGACCCCCAA
AGAATTACCACTGCTCCCCAAAGATACATAGGATAAGCTTACTTAGGTTTAGAACTTGTTTCACGATAA
CTGGATGAAAATTATCTGCGAGATAATAATCACACTGTCACGGTCTAAAACATAATTTTAGAGAGTGTC
AGCGTGACAGTATCTCACAGTTAACGGTAGTTCAGAAAACGTGAAACAGGCGCTTAGTGTTTAGGGTT
TCAATAAGCATTAGGTTTAAGTGTTTCAGTCTGTACGTTTCTAAATCAGTTTGTAAACAAGTGTAGGTC
TTAAGCAACTGGTTGTCGGTGACAGCTACTACTCCTCGGCACACAAATCGTACGCTATCGATGCGATGT
CGAGACCCTTAATGTTCGGCATTTGTTGTTAAAAAGTGTTTCACAAGTTACCTCTATCACACTTCGTGC
CTTTGAGTTTCTGTTATTTCAACTTTGTTTACTTGTTGTTTGTTTGCTTTTATCGTTGTGTTTAAATTTCG
AATCAAGATCAAACAATCTCGAACTTTTGTTTCAACATTAATTGAGTTACAATAATATGATAAATTATTC
GGAGCATATAAAACAATATGCTACGGAGGCAAAAAAGTTAAAATCATTTTTGAACAATGCGATTTTGTT
AGCATTTCTGTTACGTTTGTGTTACGTCACTGTTAGTTATTGTTGTCACTGTTAAACGTTAATATTCTGTT
AAGTTTTTAACCTAATCAATAGAATCAACTTATTAAAAAAATATTACATACCTAAATACAATAGAGTTCT
CACGCTGAGTCTAATCGATTCAAATTCAAAATACATTCGGTACAAATACTGTCGAAAAAACTCCTATCT
CTTTTTATTAAGTTTCTGTTAAGTTTTCACTTTGTTACAACCCCGGGTTAAGTTTTTGTTTGAGACAGCT
CTAGGAATGGCTGTAATGACCTAAAAATTGCATTGTTTCTTGTTCTTGTTATGACATCGTTTCGTTGAAT
TTTTGTTTTCCGGAAAGTAAAATGGCCCGAGTTCAAAACCATGTCTATAATACCTTTCTGCAACTTTCT
GCTTCAGTTATTTCTCCCATGGTTCGTTAGCAGTTACTTAATTTAAAAGGTTGAATTTTGGAATTACTGT

TGTATACAAAAATACAGTTAAGTTTTTCGTTTGAAATATTTCATTTCACTGTTATTTTCTAAAGTTAACAA
AAAATGTAAATACTAACTTGTTTTGAATCTCGTTGAGTTTTTGTTAATTAATTAGGTATTAAGTTTCTTGT
TAACGTTGTTTGAATCCCCAGCGGGACTGGTGGTCTTAACTGTTAATTGTTGCTCAAAACTGTTAACCG
TTGAATTTTTGTTTTGATATGATTGTTTTTGTTGATTTGCCTGTTGAACTGTTTTGTTTGTTCCGGTTATTA
TTATTAGTTATACAAAAACACTACGTACCTTATCAAAATCAGAAAAAAAACTTACACAAAACTAAATTA
CATGTTAAAGTTGAAAACAATCAGCAAAATTAACTAAATAAACTAAAAGATCTTCTCTTTAAAACAATA
TGCAATAAATGCATTACATTTCAAGCTTGTTGCTCTATTCATCAACACTTCTTCAATCTGTTGTTCTATTG
TGCCTTAGTGAAGTGCCTCAACTTGTTATTTTTGTTATGTTTACAATTTCCTTAATATCGCCTCGTGGTAG
GGCTCTCAATGGAGTCCTGCCATAATGCGATTTTACGATCGGGTACGGAAACATTTTTATGACCAAAAC
AGTATAGTGTCTTTTGCATCTTTATACCTATACTAATCATGTCTTGAAATAGCTACTCTACAGTGTACAAT
CAGGATATGAAGATTTCAATTTTTGTTTTTTTACCTCCATTTGAATGACACATTGTTTCCTTACACGATCT
GATTACTTTTTGTTTCTTTCTGTTTCATTTTCGTTCCTGCCTACTGATTATTTTAACCCCCAATTCTTATTG
GATTTTCCTCTAAAAATCCGCAATAGCTTACATATAATTAACTTCCAGTTTCACACATTATTTCACATTTC
CGTTGGCTGCACACTCCAGTTAACACTCAATGGGGTTAAAATTGTCATTATGCGTAATTGTTATCGTTAT
CACTGCTGCGTTATCGTTAGTTGATATTGTTAACTAATTGTAATTGCTGTTTTGTTATCTAATTGTAAGTG
TTGAATTTATGTTAAGTTTGAACTGTGATTGTTAGTATCCGATGGCTGTGTTGTTTGTTAATTTTATGTTA
AGTTTTTGTTTTAACTGTTCATGTTCATTGCCAACATTAGGGTTCCGATGTTTTGATTCAAATTAAACAA
ATGAGATCATTTCAATTGTAAGGTTCTAGGTTAGTGGTTCCGTCTGTTCCGGTTTACCCCGTGAACGAC
ATTGTTGATAAATAATCAACTTCACCTAGTAATGTAGTAATAATTTTAATATATTTTTAAGTAATACTTAAT
TGCTTTTGTTTAGATCGGTGTATGTTAAAACTGAAGCTTTGTGTAAAATTAACAAATAGTTTAGGAAGCT
AATATATAACGAATTTATTTATTAGTGACGTGTGAAAGTGTGAAATAAAAAGAAAACACGAA
>TCONS_00031391 geneID=XLOC_006360 scaffold_1487:2043-4737(+) Intergenic
CAAACAGCTGCAAGTGAAGTAAATCGAGTCGGATTTTTTAACCAAATATTAGTGAAAACATTCGTGAA
AGTAATAAATTCCATTATCTGAGTGAAGCAGTGATGTCTATCAATGTGATGCGAGTGTAGTTTTAATGAA
AACCTCAGGCATTAAGATCTACAACCAACGCTCCCACGGATGGAATGCCACTCGACTGTACCTAAGCG
GCGGCATGCTGGTGGTGCGCG
>TCONS_00031575 geneID=XLOC_006419 scaffold_1492:3500-4841(+) Splice junction overlap
CCCTACTGCAGACATGGCCGCCCTCGTCGCCCGCGCACTCACGCTCGTGCTGCCCATCATCCTTCTTCT
TGCTGCGACGGGAGGAGATGGGAAGCCGTTCTTTGGATTGAGCGTGCTGGATGAAGCGTTGACCAAT
TCGACGTCGCTGTTCGCGGCGCTGTCGGCGGCGCACGCGGCGCACTCGCTGCAGACGCTGGCCGGCT
TCATGGACGTGCTCGCGCTCGCACGCAACGTCACCGTCACAGCACTGACCGGGTGAGGTGCGGCGCG
TCCACGGACACATGACTCACTCCAAAAAGACTAACTACCTAATTACCACAACGAAATTGGACCACCAA
GCCACAAGGATAGGCGAACGACAAGGATATACCAAAAAGCCCAAGTGCCATGTACTGTAATAGTAGGT
TATGTAGTAGATAGCTATTTAAAATTAAATGTAGCTAAATAATTAGATTAGATAGTCAAGTAGTATAAAAT
GGTTAAAATAATAACCTACCTGTAGATAAGGGT
>TCONS_00031592 geneID=XLOC_006425 scaffold_1495:3936-4399(-) Splice junction overlap
GAAAACTAAAAAGTCCAAAAGTAGAGAAAATTCAAAGGAAAGTGTGAATGGTGACGACAGAAAATA
CAATAGTCCAAGCCATAATCCGAGTTTAGATGGGTCACCTAGCAAAAGTAAAGTAAGTTTTTCTAACTT
GAAATAAGTGTAAAGTGCCACAAACTTATCTGTTCCGGTGAGAGCTCACGTAAATGTCTAATATTTCTT
GATTCTATAAGATTTTAAGTTAGCCTGTAGTGGTAATTCACCCTTGCGGTTTTAATAAACCCATCTAATCT
ATATGAATGTATAATTCATTGGTAAGTTATGAGATATGGATCAATTTAGTTCGCTGTCACCGGAACAGAT
AAGTTTGTCGCACTATA
>TCONS_00032442 geneID=XLOC_006439 scaffold_15:476623-478172(+) Intergenic
CTTGAAAACTAGTAGAGATCATTATTGGCAAAGTTCAGCTCTACATAAACATTACATTGGATTATTTACT

GTGACTTTTACAAACGAATTTATAAGCAATCCAAACAATTGTGATTTTACATAATATTATTGTAACACCG
ACCACACTTTAAATATAGCAGTTTCTATTCTTGGCTTCAACCCGAAGACTGCCGAAATTATTCGGTGAA
ACGGCACTCAGAGCTGTTTATGAACTATGTTATATCATTATATTTCACACTTCGACCATTTACTTTTTCAC
ACTCCTATTTAGTGCTTTTACATTTTAATTTGTTATCAACTATTTACCATTTGACCAACATCCTGTTGTTG
TTGACAGTTGACAGTAGATCTACGTCGACACGACATTTTTTTTTTTTTTTTTTTAAAGGGTTTTGGCT
TGGCCACTGGGCCTTTAAGCCATGTCTTTATTTTCACATTTCACAGCACAGTTGCACTTTCACTTCTTCA
CTATATTTTATTGAAGGTATTTTTAATATAATAGTATATAGCATTAAAGCACACTTTTGATTTTAGTAGCAT
AATGAGGCGGCGGGAGGGATTATCTTCATTTGTTATAAATTCACTCACTTCACTTGCAAAAACTAAACG
TTCCATGTTGTGGGTAGGGCATTCAAAAAACATATGTTCTAGCGTTCCAACAGCATTTTGACATGATAA
ACATGTGCTATCTTCTATAATGTTAAATCTTTTTAAATGCGATGGTGCTTTTGTGTGACCAAATCTCAGTC
TATTCATTGTTGTGATAAAATCTCGACTGGCTATTTTCAAATCATTATACCAGGGCTTAGAAGGAGAATC
TGAG
>TCONS_00032063 geneID=XLOC_006504 scaffold_15:738285-741898(-) Splice junction overlap
ATTTCTTTGTGTTACATCATTTGACGCTATTCCCCCGATTCCTACAGGAATAATGCTTCTCTTGTCAACA
GAGATGCTCGATATCGCTGCGTAATTCATTATTTTCGTGTAATCAAAGTACAAAACGCGAGTGTTTCGA
GTCTTTCTTTCTGTAGTGATTTGATAAGTCGCCTTCTGCGCCAGTGAGTGTAAAGGTTAGACTTGAACA
AGTATTCGTACGCTACGGAACTATTTTTTTGAGTTTAACTTCGTCAAAATATCTGTGATTTAATTGTAGA
AAAACAAAATGAAGGCTATCCTGTTTTGCTTCGTCGTAGTCCTGGCCGCGGTCGTCCCGGCTTTCGGT
ACACAATGCCACGTGAACCCAGTCCCCATCCGCAAAGAATGGATCACGGTGGTGCGCGGCTGCCGCG
ACGCGATGCGGAAGCAGATCCAGACGGAGGTGGGCGCCTCGCTGCAGTACCTGGCCATGGGCGCA
>TCONS_00031753 geneID=XLOC_006504 scaffold_15:738287-741957(-) Splice junction overlap
GACTCGTTCTGACGCATACATTTAGATCAGATGTTGGCTGAGGTTTTGTGTTTCGAGAAATTTCTTTGT
GTTACATCATTTGACGCTATTCCCCCGATTCCTACAGGAATAATGCTGTAAAAACATATCTACATTGCTAT
TATCCCTATCCAACCCCGTTTCGAAGCGCAAAAACTAGTAATAAAAGCGAGTAGGGCGTACAAAAATT
CCATGGAAACATTTGAAACACAAAACCTCGACTGGGGCTTTTGTGACACCATTCGCTGTCCCACTCAT
TCATTGGCAGTCTCTTGTCAACAGAGATGCTCGATATCGCTGCGTAATTCATTATTTTCGTGTAATCAAA
GTACAAAACGCGAGTGTTTCGAGTCTTTCTTTCTGTAGTGATTTGATAAGTCGCCTTCTGCGCCAGTGA
GTGTAAAGGTTAGACTTGAACAAGTATTCGTACGCTACGGAACTATTTTTTTGAGTTTAACTTCGTCAA
AATATCTGTGATTTAATTGTAGAAAAACAAAATGAAGGCTATCCTGTTTTGCTTCGTCGTAGTCCTGGC
CGCGGTCGTCCCGGCTTTCGGTACACAATGCCACGTGAACCCAGTCCCCATCCGCAAAGAATGGATCA
CGGTGGTGCGCGGCTGCCGCGACGCGATGCGGAAGCAGATCCAGACGGAGGTGGGCGCCTCGCTGC
AGTACCTGGCCATGGGCG
>TCONS_00032712 geneID=XLOC_006505 scaffold_15:753431-754935(-) Intergenic
GGCCGACCTGCAGGGGCACACGGTGTACAACAAGTGCCAGCAGGCGGCCGCGCCGGCGATCCAGCA
GGCCGCCTCGCCCATGCCCTGTGCTGTGCCGCAGACTGTACAGGCGCAACAGATGGCGGTGGCGGCG
CAGCAAGCGCTGCAGCAGCAGGCCATACAGCAGCAACAGGTACCGCCGCCCGCCTACCCCAACCGAC
GGTAGCCTC
>TCONS_00032631 geneID=XLOC_006450 scaffold_15:1157004-1159558(+) Intergenic
CGCGCGACCGAGCGGCAACACCGCGCCGCGCCTCGCCTCCATCTCACGACAACTACACCAACCAGAC
CTCCACTCCGCCTCCTCACCTCCCCACATGCCAACAAACTCATAAGCACCACCACTCCCACAATCATAA
ACAAGCAAACGTGATATTCAAGTGGTGTTCGACACAGGATCTCTCCTAAATCACATAACAAGGAAACA
AATGTCGATCCGTCGCAGCGACGCAGTCGAAATCCAATTAAACTCTTAGTGGAATCTGGCTGGACAGG
TGGAAGTCTTCGGCCGGAGTGTGGCGATCGATCGCGGGGGCATGCCGGCGCCCCGGCTGTGAAGGAC
CAGGTCACAGGACGTGTTTGTGCAGGGGCCACACACACGTAAACAACCGACCACGATCAATGCCAAT

GGACGCTTCTCGGAGGTGAGGCAGATGATGTCGAGGGTCAGCGGCGGGTGACCGGCGGAGGCA
>TCONS_00031679 geneID=XLOC_006464 scaffold_15:1528786-1535183(+) Splice junction overlap
TCACTTCTCATAGAATCCCGCTCGGTTTACGGCAAAACAGACGTGTTTTGATTTTGTTTTAGCGTAACT
CTTTACAAAATCGGCAAAAGTGAAGTTTAAATTGTACTTTTCAGTGTTTAATACGTGAGTTAAGTAATG
TCCATTTGGATGGGTGTGTTTAATCTCGGAATAACGGTTGGTGCTACGGTGGCTGTTGTTACAAGAAAT
GCGTACACCAAGAAGGGGGTGCAGCGCGCGCGCAGACGCAGAGAGGAGGAGGAGAACCGGAGCCC
GCTGAGCGACAAGCCGCTCGGGAGGTGACGGCCGGG
>TCONS_00031680 geneID=XLOC_006464 scaffold_15:1533140-1535182(+) Splice junction overlap
GAAAAAAATAAGTAGGTTACTGTAAGGTAATATCAATCTTATGGCCTTGAGTTAAGCAAGCCTATTTAAT
TGAATTTCTCTGAAAAATATGCTTGGTCTCACTTTTGATTTGGACCTTGTGTCGCACAGTTTTGCGCAA
ACATTGATAAAATTGACATGGATTTTATAAAAATTGCTTTGGTTCCAATAACACTGACTGTGGGATTGAC
GTATTTAAATGTTAAAAGAAATGCGTACACCAAGAAGGGGGTGCAGCGCGCGCGCAGACGCAGAGAG
GAGGAGGAGAACCGGAGCCCGCTGAGCGACAAGCCGCTCGGGAGGTGACGGCCGG
>TCONS_00031979 geneID=XLOC_006516 scaffold_15:1556879-1557726(-) Splice junction overlap
CCCGCCTATTGGAGCTACGTCGCCGGACAGCTCGCGCTCGGTGTCGGAGGAGCCGATGGAGCAGGAC
GAGGGCAAGTGAGCGGCGCGCGCGCCGGCCCGACCGCCCCCGCCCGCCCCGCCCCGCCCGCCCCCG
CGGCGAGACACTCGATACATTCCCCACTGCTAGTGGCTCTACTGTAAAATACTGTACATTATATTCATTT
TGAATCGAATGGAGGCAAGCCCTCCACTGTTAGGTACCTCGAATCTTTGTTCGTTTATTTTTGTTACGTC
GTGTTCGGTGGCTGTATGTCTGTTTTTTTGTTGTATAGACAATGTTGTTGTTGTAAAAGATTTTACTCGT
AAAGTTGTTCGAACGTGCAGGTTATTTATTAAGGATGTTTATGTACTGCGAGGTGGTTGCAGATGTAGA
GTTAAGCGGGAAGTTTTATGTATATTGTGGTAGGCAGGGGGGGGAGGTGCAACTGGACAACGCAAGA
AGTGACCTTGCTCTCAGCTTTACTCCGTGGCTAGGGCTGTTTTTCGTCATTTTGACACTTTTATGTTGGA
GTTTTAGCGGCCGTTTGTGGCCGCGTGTCCCCCCCGCCCCGGGGTAGCGGGGAGCGGGGGGGAGCTG
GGCGCGGACATTCCACAGTGACAGCCTGTCTGTAGCCGGTTGCTAG
>TCONS_00031951 geneID=XLOC_006476 scaffold_15:2180859-2189028(+) Exonic overlap (+)
CGAAGGTGTAATTTTCTTTGCCTTATCCACGTATTATGTTCTGAAAATCTTGAAAAAATACTTAAACTTC
GCGGCCAACCACCTTTTTCGATAGAATAGAAATGTAGCTATCATAAAAATTATAAGAGTTCACCAAAAA
GCTAAAGTTATTTTTTTCAAATTCGGGATAATTTTTTATCCATTATGTAGGTTAAAAAGAAGAACTGCCC
AGGTACATTAGGCACTATAAATTACATATAAGTACATATTCTATATTATATTTAAAAATAAATATTTACATA
GGTATTTAACATAGGTTTTGAAAAGATAGTCAAAGTTACCAACTATAATTACCATTATATTTTAGTTGTCG
ACACATTTTAATTTTTCCTCTAATATCGTTTTCGCAACCGGTGGCATAAATACCAATATTCCTTCTTCAAC
TTCACTTCTCCGATAGTATAATCCTGTTATTCTGACTCGCAGTTGCATCCTTCTGTGATTGTTCTATTAAC
TTGTTACTATCGTACATATTTCGTATGACTTTCAGTAGCCAGTCTAGACCGACTCCAGTTTTCGCCGATAT
TCCTAGGATATCTAAGTTTTGTTCTTTAGTTAGTCTCTTCAGGTTTTCCTTTCCTTCTGGCAGGTCTATTT
TGTTTAGTATTAT
>TCONS_00032678 geneID=XLOC_006542 scaffold_15:2302674-2303490(-) Splice junction overlap
CGAGGTTCATAAAATAGTATGAATTTTCCAAAAGCTTTTGTATTGAGGATAACTCTATATTTATACCCCG
GGGCAACACCTCGACCTAACTGTAAAGGTGGTCACAACGATTCACAAAGTACCGTATGGTATTAAAAG
ATTGAATATCAAACAATATTTTAACTTTTTGGTGACATGTAATTGCGAGACTCGTGACTACTAAAAATAA
CTAATAAATCACTGTAACTTTAAGACAATATCTCACCTTTTTTACCTGTCACGGCGCAGCAAATGTTGA
GAACCACAGAACTGATGAATACTCAAATGTCGATCCACACATAGATAATGAGACAAACTGGACAAGTG
AATCAACTTTATTGAAGAAGATAAAAAAGGTATAAATACGATTAGGATTGTGAAATGAGACATTCATAC
AGTTAACCCTGTTTGAAACCGACGAAAATGCGGGGCTTTTTGATTCTCTCGGTTACCCTGGCGGTCGC
CTTGGCGGCAGAGCCGTAAGTATTGAACGAAACTCTTATAAGTACACCATAAAAATCATTAAGATGGTT

TACAAAACCATTATTAAGAGAGAAATCCTAAGTGTTCTGTGAAAATCTAAATATCTATCTTGAGTATATA
TGTTCCAAAAGAGTGATACAAAAATATCAGCGAGTTATTTTTGAAATGTACTTCCTAAAG
>TCONS_00032812 geneID=XLOC_006690 scaffold_150:145047-146155(+) Splice junction overlap
GGGGTGAAATTTCCATGTGAATAAAAGTGAAGTGTAATTTGAAATGGCCTGTTCATTAAAAGAACCTA
CAGTGACGTGTTGTGGTGATTTAAACTATAATTTTAAAAAGAATTGGAGTACTAAACGATTAAGAAAAA
AAATATTAAGAAAAGATAGTTTACGTAGAGGCGCTGGAGTTAACGTCCAAGATGACAACGGCTACACA
CCACTACATCATGCCTGCCTACGGGGACACAAGTATGTTTAGGGCGGATTCGGCCAACGTCGAGTAAC
TGTTTACTCACGAGTAAAGTACCAGTTACTCGCGAGTAAGCAGATTTTGCATTCTACCAAACCTGAAAC
CAAGATAACTAGCTTATCCCAAGAATAAAATGTTATACCGCCAAAATTTACCGTGTAACGAGCTTATCC
GAGAGTAATTTTGTAATGGCGGCGGCACATCAGCTGATTAGAAAACCGTCATAATGACATATAAACACA
TCTGTCAAAACATAAACAAAAGTACGTTAAAAGGCAAAGTCTCAGAATAACAACAGCGAATAAAATAT
TAAAACATAAACAATTTCATACTTTTATAATCCATTCAAACTA
>TCONS_00032911 geneID=XLOC_006719 scaffold_1500:2393-3775(-) Unclassified
GTACGCTTTTGTCAAGAATAATATCACTCAAAATTTTGTAACCCATTTATCAATAACATGGCAGTACAGA
AATATACTGTATATTATGTAATATGAGCTATAGTATACACTTACTGTAAAATCTATTTATATTTACATATTATA
CTGTCTCACTTTTAAAAGCCTTTAACAAAGGAATGCAAAATTACACTACTATAACAATTAAAGCAACAA
TTTCTAACAAAAACCCTCTTTAGGCATTTTATTCATGACTTCAAGCTTCAACCATAAATTTAAATTACAG
TATTTGGGAATGTCATAAAAAATTGGACACTGAATGCAAATTATTTGGTAAGTTTTGAGTGTTAAAAGATA
TAGGAATCCTTTTGATCGTATCTATTCGTATAGGCCTCATATGTCCTAAGATACAAAATATCTTTGTACCT
ACCAATTTCTCAAATTATTTGAAAAAGACAGGCCATTATTTCAATGCACATAAAGTGAAATCTTTACTGT
TGATCTACACACTGGTATCCAATTGTGACTTACTCTGCAAACGTTTCTATCAGCCTTGCTTCATCGCATT
ACTTTTAAACCATTTACAAAAATATTTATATATCACTATCCAGGCGCAATTTGTTCAGTTGTCAATATCAC
AAGCAATACCTTCTTCACCTACAAACCTACCAGTTGTTTCCAATATCCAACTAACCCGTAACAACAAAT
CAAAAAATATCTCTGTCCACTCACCATAACCCTGTAGCTTCACAAACAGACTAGTCCTTAACTGCACAA
GTCACATCATCACGACCCATCAAGTCCCCACTAACAGGGCACGGGTCTTTAGGCCTGGTCCACCACTG
GCCTAGTTACTGGCCAAGTCACATGACACTGCACATTTTCTTCAAATCGCTATCCTCTGGATCCAGCAA
CTCTGGGTCACTCACCGGCTGTTTCCC
>TCONS_00033837 geneID=XLOC_006771 scaffold_151:176943-181214(-) Intergenic
GAATGAGAGCAGATACCTTATCCGTTACGTCACCATTACTATATATCATAAAGAAATACATGTCAAAGAG
ACATGTCGAAGTACTTTTTTTTAGAAAAAACTTTTTTTTGTGAAATAGGTATAATTAAGGGTAAGTATTC
ATTTAATTAAGTAAGTACAACAAAGTTTCCTAAGTAACAAAAATCAAACCTTATATTAAGGTGTAGTCG
GGTAAGAACCCGAAATGCTCGTTTGGATCGCTCTTAAGTTTTTTTTTTTATTTTTCTCACTATATTATTTT
TACCACTTTTTCAGATTTTTTGAAACGGTATAACGGTACGGTTATTCAGACACAAGAAAAGCAAACGA
ATGGCAAAATTAAATATTTTTCGCCACAGGATGTTATCCCAACACAAGAAACGAATGACAAAATTAAAT
TTGAAAGCATGGTTGACGCATGGAAAAAAGTAGGTAAATATATTAATTTTAGTAAAACATAAAATAGCA
GCATATAAAATAAGATATTAGGCTTCAGTTGCCCACGAGCTCTGTGGCCGCGTAAGTAAAAATAATTCC
AGCC
>TCONS_00033597 geneID=XLOC_006773 scaffold_151:230447-232741(-) Unclassified
CCATATTGTACTTTCAAGCGTATGTAATAGTTAATTTTTATATGACAGCATCAACACCTCACCGGCCGCC
CACGCTGTACTGCCACGGCCCCGACAACACCTAGCAAGCATCCTCCCCAAGGACAAGCAGTCTGTTA
GCGCCTGTGTGCAGCACAGACGAAGCAGACTTCTGATGTCCGCGCCGGGACTCGAACTAGAGACTAC
TCCACTAGCAACCACTGTGCCATTCGAGAAAAACATCATTTAGAAGATTTTTTTTTACTTTTGCTGCGA
CTATGTGGTGCCGTGTCCTGTATGTGAACAACATAACTGTAAGTACAACCAGTTGTTTTTCACATATAG
AATACGGCGCCATATTGAATCTACGGAGGATTTAATTTTTATTTATTTATTTTATTCTTTTATTGCACAATAT

ACTAATGTAATTATGTACAATCAGGTGGACTTCATGTATATTATGTACCTATTTCGCACAACCTGGGACTA
AATAAGTACGGTATATCGTAGGAACAACAATAACGAGGTTTGTATTGTAGTAATATGGAAGAAAACCAA
GTGAAACTTTGGCAGCACAATCGACTGTGTCTTTCAGGTTCTGACCAATTTCATTACTGGGGAGAAAA
AATGGCGACTGAACCAACCAATTAAAACTGGGGAATCGGTCCTACTCGACAGTGGCGACACTGGCGA
CATCTGGTATTAGCTTTGTGTTTTCCTGCCCATTGATCGATATGCCAAGCTTCTTAGACGATGTTTTTTTT
TTAATTTACCTAGATTAAGGCTTTAATAGTGAGATAATTTAAGGTTTAAGCGTAAACCTTTGGGCACCTC
CATGATTTTACAAAATAGATTTAAGGAAAACCACTCAGAGATAATAGTCATAGAATATTATAAATGTTGT
AAAATTGATGTATATTTATACCCCCTTATTCATAGAAAAGTTATAGGACGTTTTAGCTATTGAACAGTTTT
GTCCCTCTCTGTCAAAGAACAGTTTAAGTATTCTTTGACAGAGAGGAACAAAACAGTTCAATAGCTAA
AACGTCCTATAACTTTTCTATGAATAAGGGGGATAGAATTTATCTGTAGCTGTTTTAAAGTCACATAGGT
AAGTTTATTTAATAACAGCGCCCGCTTCACGGGTCTGTTATCGACGTCAATAAAATTGATCAACACGCG
TTCCATCAATCACAGCGTCATATGTATGGCGAATCGCGATATAGTGACGTTTATCTACATCGATAATGAA
ATCGTACAGCGGCAGCCAGTATCTAAAAATTTATATTAGCATGATTCACTTTTTATATATTTAAAACCGCT
TTTGAAAATATAAATATATAAATGTTTTGTTTTACACAAAGTTTTACGTACAAAATCAGAAAGTTGTGAA
TTTTATCCCTGTGATTTAAAAACATTTTTGATAGATTTTATTTGAGTGACGATGTTTTTTTATTATTCCACG
ATTAACGGCCATACAGTTTTATACAATGTCAAATTTACAACACATAACCCTTATTTTAGCTTCGCCGAAG
TCTGTTAGAATAGAATAGAACTGTTAAAATAAGGATGGCAAAAGTAAATTAATTACATTGTATATGTTGT
ATTTTGAATCGATGTCTATCTTTACTATTGTGACCTCTTCTATACAGTGGGTTACTGTCCAAATTTACGAA
CGAATAAACGAGAATTTTCACTCAATCGGCACGGTTAATACAACGAGATACTTCATCATGATCATCATC
AGCCAATAATGATCCACTGCAGGTCATAGGCCTCTCCCACGAGATACTTATTATTATTATTAAATTATTTA
TTGCACAAAGTAAATTGGTACAAAGGCGAACTTAATGCTAGCAGCATTTTTTACCAGTTAACCTTTGGT
GATGTTGAAAAAGTTATTGGTGGTGCTTAAAGGCTTTGACGATATAAGATGGTCTATCTATATTGACCAT
CTTATATATTATATACTTAACGTTCTATTAAGCGTGACAATCCTTGTTAGTTCGGCCCG
>TCONS_00033758 geneID=XLOC_006780 scaffold_151:394962-395544(-) Intergenic
ATATTTAGTATACGATTTCCATCCAAAACTAACCCAGTGGCCATACCCCGTACCGATTGTTACCTAGGTA
ACAACGTGGGTCGCCGCAGCCGGTACAAGAAACTAACCTAAAGATTGTGATAACTTGATAACGATAAC
AAAACAACGTGTGAGTGAACACCAATATGGAGAATGGAACATGCACAGCTGTCGTGTGGAAGGCCGG
AGAAAATTGTGAA
>TCONS_00033447 geneID=XLOC_006800 scaffold_151:1008864-1019703(-) Exonic overlap (-)
TTCTCGAGTATAGACCACGAATAGGCAAACGCAGCGTGGGAGGCCTTCCTGTCTGGATCAAAGACGA
CGACCACAAGTATATTTTTTTTTAATTGGATGCCTGTTTAAATTTTATTTACAGAAAATGTATTGTACAAC
ATGACGTCCCTACCCCAATGATGACCACGAATCACAACACAATGTTTTACACTGATTAATATTAAATTTT
GTCTTAGCTTAAAATTTGTTGAATTTGATCAGTTTGTTCCCAAATAAGTCTTACAATTGGGGCAGTAATG
GGCACTTTGCTTACAGCACGGGACCACATAAGGAATAGCGGCCAAACAGCAGAGCATGCACCAACTG
GAACAAACGAAACATGTCATTGTAATTCATGATACCATACTTACAAAATAATCAATTACAATCCTCTTTA
TGTCCTAATGCAAATGCAGCTAAACAGAAAAAACAGTACAAAAGGCAGCCAGACAATCATTAGGTATG
CTACTTGTATCCTCCTTATAATCTTGGCTCAAGCATATTATCTGATTCTCCTTTATATGTATGCACAGTAAT
TGGCAGCTAGCTGAGCTTAAAAATTATAATCACAAATCCTACCATATCTGTTGCAGATAAAATATGAAC
>TCONS_00033380 geneID=XLOC_006801 scaffold_151:1028401-1029781(-) Splice junction overlap
ATATTTATAAGTTAGAACTGCAAACTGATGCATGATTATTTATTTACAGGAGAGACTACTAGGAGGTTTA
TCAGACAAACATAAACTGAATTCAAGCAATGGACCTTTACAGGTGGACTTAGAAGAAAAATAACAGTT
AAATAAGGTAGGAGGGTTTATAGAAGTGGCGAAACGTCATTCCAGTGTGCGGCCGCTAGCCACCCGG
AGGTAGCGCGCGGCACAGAGCAGTGCAAACTACATTATATACGGGATAGTTCACGGGCTGCCATTGCC
TGCGACCGCCTTGTGTAAAACAATTCTAAATAGACTGTACCGTTGGCAAGTTCATACAAATTTTGTCTT

TGACAGCTTCATAGTTGAATCTGGACTTGGTGGAATGTAATATGTAGTAAGTAGCAAAGAATTGAATTG
TTGTCGAACTAACGGTAGTGCAACCAATATCTATGAACATAGATCCTGTGTCACGTAAGTCCGCACGTG
TCAATGCCAACGTTAATATTTAGGTAATACATATTTATTGCACTTACACAAATTGTGTTTGTAATACATGA
TACAGTGCATTCTTTCGTGAGACAGGACCTTATAGTGTTCAGTTTACTTAAACCTAATTATTTACAGTAC
CTACGTAAATGAATGGCAGCCTGATGTCTAAGTGACTATGAATGTTAACATCCTCGAATTAATTGTTAAT
TAATTACAAAGTATACTTTAATTGTTTACAATAGACTTAATTCTTATTTCACTATTTTAGGAATGTGTAAGT
ACGCATACGCATTATTTTTTTATTATCACACAAATCCATTTTAAGTATGTATACCATCATCGATCTATGAA
TTATTTTATGATTTTATTGTATAAAACAAAACTAACTATTTCGGTATTTTCACTATAAAAATATCATGAAA
AATGAGTTTATTTTAATGTTTTATCATAATCATAACTGTATAAGCGGTACATAGTTAATGAATTGACTTTG
CAAGTAAAAATGCTTATTTTTAGATAATTTGTGT
>TCONS_00033678 geneID=XLOC_006763 scaffold_151:1154466-1155785(+)Exonic overlap (-)
GGATGACTACCAGGGTAGGAGGAGGTTCTCTTTGGGCTGAATCCACTTCTCTCAAATGCATTACACATG
TATCTCCAAGATACAGGTGAGGGATTTATTTGTAGATATATACCAATCTTACCAAATATAGGAGATTCCTT
CCGCCTTTTTTTCTACTTGGATGAATGTTATGATAGGATTTGAAAATACAAGACATAGACAATGATATTTT
AAAAAGGGCATTTTATTTGCAAAAATAGATTTGATCTTATCATAACTCGATCGATCGACTCGGAACCTTT
AGTTTATTAGTAGGTATCCCTGCTCCAGCCCCAACGTTTAGGCATCTCATCTGTACTGTTTCGTTGCTAA
AAAGACCAGCTTATTTAAAATATCCACCCCTTTAATAATTTCACTTATGTTTATTATACTGTCTTATGTCTT
AAATTAAATAGTCCTAGCGTTAATTACTCAGGACAACAAACTAACAAGTTGAAGAGACAACCTATGAA
TAAAAAGATAAATTAAAGATTATCTCTTTGTAGTCCGAATTGCACTTCATTATTTTGTACATAAAAGTCC
GGCCATAGCCGCCTAGTTACTTAAACTTCAGTCCATTAAAAATGATAGCATTAGAAGTATTAGAACTGC
AGTCTTTTCGCCAGCGAGCCTCCGTCGATGTTCTCTAGAAGCTTCCTCTTGAGTGAGGGTTTCTCCGTC
TCCACCTTGGACAGGTTGGAGGGCATCGGCAGCTTGTTGCCGACCGACGGCGCGGGCTTATTGCTGAA
ACAACATTTCATTATCAAACTACTTTCCATATGATTGTATGAAAAAATGCACGCGCCGCGGTGCAGTCG
GCGCGGCGCGGCGGGAACAGCGCGCGCAGCCGCTCCAGCAGCAGAATGAGGTCGTCCGGCG
>TCONS_00033997 geneID=XLOC_006921 scaffold_152:1802-4825(-) Exonic overlap (-)
GATTGGTTCACGCTAACTCCGCGAATTAGGTGACCCAAGGGCTCTGCGCAATGTTTGTGCTACGTTTTT
TTTGTATTCTCTGTTACAATATTGTATACATGACAAGATAATTTATCAAGAAAAACACATTTAGAGTAGG
CAAGGTCCAAGTCATCATGGTATATTTTCACAAGAGCGGACTAGAACCAAGCATACTCTTAAAGTGTAT
ACGCCGTTTTAACGAGGAAATCATTCAGCCATACACAGTAAAGGTTGACTAATAAAATTGCTAACAGT
GGCCGACAGGGCGGCGCTGCCATCACTCGGGCGGGTTGTCGAAGTAGTCTATCTTGGGTTCGGCAGC
GGCAGGGGCGGGCTTGGGCTGGCTGCGCGCCGCCCAC
>TCONS_00034004 geneID=XLOC_006926 scaffold_152:91940-93845(-) Splice junction overlap
GATTTGTCGAAAATGGAGTACAAAAATCTATTTTCGCATCTATTTTAGATAAAATATCTGAGAAATCTTG
CCAACAACTAGATATGTGTGATAGAGCATTTGCGAAAAACAAGTATAGAATCACCTCAGCGAATCAAT
GCCTCTTGACTGATGTCCAGACCTCGTTCAACGCTCAAGTCTTACAACAAAGCATTGTTTTGATGGATA
GTGATGAAACGATCAGCATTAAAGCGGAACCTGATATCCTCGAGGAGGGCAGCGTGAGCGTGCACCC
GGCGCGGTGGATCAGCGAGGTGGTTGTGGAGCCTACGTTCAGCTCTCTGTCATTCACCACCCTCATCA
CCATTAGCCTGTAATCATCATCTACTGGACATAGGCCTTCATCATCCACTGCTGGACATAGGCCTTCGTC
ACCCACTACTGGACTTAGGCCTTCATTCGAGCACTCTGTCCTCAGCCTTCATCATCAAGCTAATCATCA
CTACTGGCATAGGTAATTGTTCATCCTTCTTGGCATGTTCAC
>TCONS_00034005 geneID=XLOC_006926 scaffold_152:91973-93968(-) Splice junction overlap
ACCGTATCGCCGTCGTATTATTGAGCTTTTTTATTGTAGCAACACTAAGAATTGAATGAACGAACTGAA
CTTTGAATGAATGAATGGGCTTCTGTCATGTCAATAAAAAAAAGTAAGATTTGAGATTTGTCGAAAATG
GAGTACAAAAATCTATTTTCGCATCTATTTTAGATAAAATATCTGAGAAATCTTGCCAACAACTAGATAT

GTGTGATAGAGCATTTGCGAAAAACAAGTATAGAATCACCTCAGCGAATCAATGCCTCTTGACTGATGT
CCAGACCTCGTTCAACGCTCAAGTCTTACAACAAAGCATTGTTTTGATGGATAGTGATGAAACGATCA
GCATTAAAGCGGAACCTGATATCCTCGAGGAGGGCAGCGTGAGCGTGCACCCGGCGCGGTGGATCAG
CGAGGTGGTTGTGGAGCCTACGTTCAGATAGCTCTCTGTCATTCACCACCCTCATCACCATTAGCCTGT
AATCATCATCTACTGGACATAGGCCTTCATCATCCACTGCTGGACATAGGCCTTCGTCACCCACTACTG
GACTTAGGCCTTCATTCGAGCACTCTGTCCTCAGCCTTCATCATCAAGCTAATCATCACTACTGGCA
>TCONS_00034197 geneID=XLOC_006926 scaffold_152:92863-93840(-) Unclassified
GTCGAAAATGGAGTACAAAAATCTATTTTCGCATCTATTTTAGATAAAATATCTGAGAAATCTTGCCAAC
AACTAGATATGTGTGATAGAGCATTTGCGAAAAACAAGTATAGAATCACCTCAGCGAATCAATGCCTCT
TGACTGATGTCCAGACCTCGTTCAACGCTCAAGTCTTACAACAAAGCATTGTTTTGATGGATAGTGATG
AAACGATCAGCATTAAAGCGGAACCTGATATCCTCGAGGAGGGCAGCGTGAGCGTGCACCCGGCGCG
GTGGATCAGCGAGGTGGTTGTGGAGCCTACGTTCAGGTGAATTCACTCCCTTGACTAACCCTCGGTTT
ATTGGCTTGTAGGTGTCGATTCTGAAGGCACCAATTCTCATTTTGAAGCGGT
>TCONS_00034220 geneID=XLOC_006902 scaffold_152:125463-128561(+) Unclassified
ACGCTATTCAGTTGTATGACTAAAGGACAACTCGTCAAGTCGTTGATTGTAACGTTCGACGTCTTGACT
GAACGTCATTCAGCGAAGTCGTTGAATGGGATATAGTATAGCAACTTTGTAATGTCTGGTTAGGGTGAA
CATCATCAGACAAGAGTCAACAAAAAGACTATGTTACAAAGCTCTTATCGCAAAAATTAACTAAACAA
TAACAATAAACGTACCTTCATATTGTTGTTCATAATTATCCAGTTTCGCCACTTTTTTTCTTTGAAACTAT
CCGCCCGCGGCGTAATAATAGGTAAATCCATGTTGTCATACTATTTTAACACAAATTACGCACTTTAAAG
CAAATTTATTTGCAAAAAACTAACAATATAGGTGCTTTTTGTGTCAGCTGTTAGCTGTTAGACGCCATAT
TTGTTTTATTCACGACCTGACTGATTTTAAGTCAGCTAACTAGTTGGACGTTTTGTGACGCTTGTACAAT
CAACGTTCGGCCTAGTCATACAACTGAACAGCGCCATCCAATGAACGGGCTAGTGGTTCAATCAAACA
GGAGATTAAACCAGTTGCCTGCGACATATCCATCGTTAGGTTAGGCGTTCGTTGGGCCGTTCTGTACGC
ACCATTATAACAACTTCTAATGAGAATTCCGTTACTGGGTAGGCAGAGGTGAATTGATGTCTGACTTTG
AGATTCTTCCATTTTCCTAGATAAAAAATACTTAGGTAACTGGACTTTCATCAAAATGGTTAAACGTATT
TAGTCAAACATCTGTGAGTAGTGTTAGTACAAAATGTATTTAAAATTTGAATTCAACACAATTTTAAATA
CGTTTAGCGTTTGGTAAAAGTCGCCCTGAACAATAAAAACGGAAGAAGCAATATAACGAAGACAACTT
AGAATTTTTGAGAATTTTTGATATACTAAAACACTAAATTATTAGGAAGGCAAATAAATTAGAATAACAA
GCGTAAACACCAAATATGAGGTCTTGTAAAGTATAGATATAGAAGGGGGAATTTATAGTAAATATTATTC
GATGTATAATGTTAGTAGTGTATACAAGATAGGCAAAAGAGAGATACTTTTTACTGTATAGTAAATTAAA
AAAATATTAAAATATTTTATCCAATGACGAAAGAAAAAGAGGACCCGGCATAGACATCGATACTTAAAA
ATATATCGTCGTCTCTTTTCAACTTATTAGTGTTATCGTACGTAAAAAAACTAATCGTAATTTTTCTTTCG
ACATTGCTTTTATCCCAAAAATGATAGGCGAACTGTCAACGTTTAATATTATGAATGTTTTTTTTAATTGG
CTGCGCAAAGTTTATATTTTGGCGAATCATGCACATACACTATTCCAATTTCAGAGGGGCTAGTGCGGG
GCGCATTTAAGACGTGACAAGAGTTTTGCATCGCGCTCACTCAAGTCACTCACATCGCGCAGCCTCAA
GAGCGAGCGCGACGGAGAACTTTTGTCACGTCTTAATAGCGACCCGTGCTACAGGGCCTGGATACAAA
TCATACAATAAGTCAAGTCAAATCAAGATATGAATTAAATACTATGGGATATCTGTTTGTGGTAGATCTG
TGGATATCTCTAAAACCGTGAAATTTAAAACGTCAGCATAATGTCACAGGAATCATATTAGCTAAAAAG
AACTAAAAGTATGCGTTTAATAAGAATCTTATGTAAATTGTGAATAATAAATTAATAAACAAAAACTATG
CTTATGTACATACCTGCCATAGAATCTTAAATAGTTAAAAGAAGTGTTTGAAATAATATATTTAAATAAAT
CCTGTACGAAGAATAATTATATTTAGTATCTATCTATCTATCTATGTATTTGTGTAGATATATCAGCTGTCC
GACTCCCATAACACAGGTTCTACCTAGCTTGAGGTCGGATGGCCGTGTGTGAGATGTCCCCACATATTT
AAAAAAAAAATTATTGATCTCATGCTAATAAAGTTTGCATAAAAATTACTTTGATGCCCATTAAGGGTGC
CTAGGTATCATTTAGTTTTTTTTTACCAGGATTAACGATATTTATAACGAACTAGATGGAGTGTCAGTGTA

AAAAGAAACGTCTTGACAAAATAACACCCCACTTCTACACTGAGCTCTTATTTTGTATTAGTTCGTTTA
GAATATTTGTTACTTTAATCATTGGTATAATGGTTGTCATTAGGTTGAACTTAAAACGGTTTAACAGTAA
ACGAACTGCGGTCATTTTTTATATAAGACTAGCTGTTCCCGCGCGCTTCGCTTCGCCTTTAAAAGTTTTC
CCGTGGGAATTCCGGGATAAAAAGTAGCCTATGTTCTTTCCCAAGGTCTAGACCATATGTATACCAAAT
TTCATTCAAATCCGTTCAGTAGTTTTGGCGTGAAAGAGTAACAGACAGACAGACAGACAGACACAGT
TACTTTCGCATTTATAATATTAAGTTAGGATACTGTACCTATCTAGTTCGTATATCGAAAACAATCGATGG
TTAGATTTTAAAAATATATATATATGTGTTAGTGTATAGAAATATGGGATTGAAAATGTTTAATTACTAATA
CTTTTAACAACGTTTACTGCAAAGTTTTTAAATATTGTTGAGAAACAAATATTATGTGTGTATTGCCGAA
ACGAAAATTTAAATTTTGTTTTTTTATTAATTAAACGTTACGGCCTATATATTTTTTGTTTTATATTTAAAA
CTTTGCAGCAAAACAATGACTATATACATACATATTGTTAGTCTGTTTGTGTGCCTCGTTTTGCGCATTTT
TTATACAATTAAGTATACATTATTAGGCAATTATACAAAAGTTTTATTAAATTATATATTATTGCTGATTTTG
TAAAAAAAAAAAATTAAACAAATCGCGTTAAGTCTTA
>TCONS_00034355 geneID=XLOC_006931 scaffold_152:372537-375762(-) Intergenic
GCGACACACTGCTCCCGACGACGCGGATTGAATAATTTAATTCGCTGGAACTGATAAGGAAAACCCAA
TTTCTCCGAGTTATCTTAAGCACTTCTGCGCAATTAAATTTAAAATTTTCTTGTCCTCCCGGATCGCTAA
GTGTGTTTTTCTTTGAGGAAATGTTGTTTTACCGTGGAAGGTTGCCATGAACTTTGCACAAGGGCGGG
CGATAGGGCGCCGCCCGCCCAACGCTCAGTGTGAGACCGGCCGCCCAGCGAGCGCCCGCGCCCGCGT
GACGTCACGCGCCCGGACACTTCTACGACCCACCACTACGTTCGTGTTGCAAAAAACTATCTCAGTGC
GCCTGAAAGTGTTGGTTTCCATAACTTTTGAACACTTTTGAAGCTGGACAGGACTTTGTTAAGTTAAA
CTAAGTTCAGTGTTGTTACTAGCCGGCAGTGTGGATCCCGAGAACCAGGTGGGTTCGCTAGCATTGTT
GTTGTTACTAACTTTTTAAATTTGATCTTATCACGAGAATCATGGCGAACAATGACAAGCTTATTTTCAT
TTGTATTTAGGCCTGCTGAAACTTTTATAATTACGGTCAAATAAAT
>TCONS_00034078 geneID=XLOC_006908 scaffold_152:508831-509880(+) Unclassified
CAAACACGTCGGCAGATACGTCCAAATGGAATCTTCAAACTTTGCGACTTCGCACCTATCTTCGTAGG
CTGAGCCAGCCAATGGCAGAGTGAGGTAATTTTTGCAAATCTGTCTGCAAACACTCAGTCAGATATCG
TCAGCACAAACAGCCACAGTGCCTGAACGCACGCCGGTAGTGGAATATTTCAATAACTGAACTCTAAT
AAACAGTAAATTGTCATAAACTCATCACAGACGCATCAGTTCCTTGGAAGATGTAGTTGATCAAACGG
TGGTCGATGAACAAGAAGTGTTGGTGTAAGACGATCGTGCAAGGCTTCTCCGACATGTGCTAATAAGG
AATGAATCGTGAATGTGGATGATGTGAAAAGGTTATAATTGAAAAGATTACCAAAGATGCCAGTCCGC
CGCTAC
>TCONS_00034023 geneID=XLOC_006937 scaffold_152:611208-615460(-) Intergenic
ACCACAAGGATGAAAAACCTATTTTTCCTACAGAATCTCTGTGTTTTCGTAGCAGTCGGGCCGGGCGAT
AGGAACCCGTTTTTATACGCAATAAATCATTTTCTACCAAAGAGTTTTCGATAATATCCTGTATTTTGTAA
TAGTTTTCCTGATAACCTTTTTAGCCAGTAGTTTTGTGGCATATTTTAAGAATAATTGTAGGATTTATTGA
ATAGTGACTTCAGTGTTAGATGTGATTTTACGATTGTGAGTGGCTCTTGATATCGAATCGATCAAATCAA
GTACACTACAATAGCGTTGCGAGTTTTTAATTGAGATTTCTTAACTTTTTTGAGCCGGCCATGGCTCCCG
GCAAGTTCGGGCTGTTCAAGACTCGAAGCCGGGACCAGCCCGAGCAGCCCAGCACCGAGAACCTGC
TGCAGCACAGCAGCAGCGGGGATA
>TCONS_00034443 geneID=XLOC_007026 scaffold_153:218141-219275(-) Exonic overlap (-)
GGGTCATTAGGTGAGATGATATTCTAGTTCATTGACTGGTTCATTATCATCATTAGATTGTCTGTGTCATT
AGATCACACTTTAATTAGAGCTTCGAACCCGATCGAACCCGCTTATTGTACTTGGTTTGCTTGTACGTAT
TTGAATAACTCTGTGTCTAGGGTATCAGGGAGTAATTTTAATAAAATAATTACAAATCAATATTTTAAATT
ATACATTTTAATTGATTATATATCGACGGAAATTCATGACATTAAACAAAGAAAATAGTAGACTTGAAAG
ATAGGTAGATCACTGTACATTATTCAATTTCAATCCATACTTTATGCAAAAAATGGGTAAGTAGATCCCA

CTGTAGGTACTTTGTGCAATGTTTTTTCAAAATAACTGGATCTAGGTGAGGTACATACTACATACAATAT
GGAAGAGACAATAAAGTATTTTGTAAATAAGTATACAAAATAGGCAAGATAATAAATGAAATCATTTGG
TATACAATCACAAATAAACTGTATTTATTCTACACAATAATTCACAATCTGTTTCTAAAGTGATATGTAAA
ATTATTGAAAGGTACAAAATCTAAATGCCTACTTAGTATTCTATCTATGTGTAGGTAAAACTATATGAAAT
GCACCAAGTGTATAATTTTCACTTAACATATTCATATAATTACAAATTCAGTAATAATTACTATAATAAGTT
ACCTGTTTGTATTGCGTACATGGATTTTGAGATTAATTACAATGTTTAATTTTGCATTATATTGCTGAAAA
GTATTATCATATTCCATCTAACATATTTCCCCATGATATAAACAGTTGGTTCAGCAATTGTATAATTTCCC
TTTCAGTAACAATCACATTTGTTCACTTCACAGTGAAACTCATATCAAAGACACACACAATGTCTCCGG
CTCCACATTCACAGTCAACCACTTAAAACACTGAGCACCTTGTATTGTCCATTAGTGAACACAGTCTTG
TATTAAATGGGTAGCGTGCTGGACACAGATGACT
>TCONS_00034444 geneID=XLOC_007027 scaffold_153:225195-228279(-) Splice junction overlap
GGCGGCCGTCGGCCCGAGTCGGACGCGGAGGAGCGCGGCGGGCGCGGCGAGGCGGGCTACGTGCCC
TTCCGCGCGCGGGGCAAGGGGCCGCCGGCCAACAGATCGCGTGATCGCGGCGGCGACGGCCCGCGC
CGCGGCGGCGGTGGCGGCGGTGGGGGCGAGGACAGACGCCCCCCGTTAGAGAATGGACGAACTCAT
CCCAGCGGGCGGCGACGCGACGACCGGCGCCGGCAGACGGACGACGAGGGCTCCGCGCTCGACTCA
CACGACGTGTCCAGCGCGGACCGAGAGTCGGCGTCGTCGGACGAGCGCGGTGGCGGCGAGGCGCGC
AGGCGGCGGCGGCGCGCAGGTAACACACACACACACACACCTACACAGACAC
>TCONS_00034446 geneID=XLOC_007027 scaffold_153:225195-228279(-) Splice junction overlap
GGCGGCCGTCGGCCCGAGTCGGACGCGGAGGAGCGCGGCGGGCGCGGCGAGGCGGGCTACGTGCCC
TTCCGCGCGCGGGGCAAGGGGCCGCCGGCCAACAGATCGCGTGATCGCGGCGGCGACGGCCCGCGC
CGCGGCGGCGGTGGCGGCGGTGGGGGCGAGGACAGACGCCCCCCGTTAGAGAATGGACGAACTCAT
CCCAGCGGGCGGCGACGCGACGACCGGCGCCGGCAGACGGACGACGAGGGCTCCGCGCTCGACTCA
CACGACGTGTCCAGCGCGGACCGAGATGTGCCGGCCGTAGAAGATTCTCTTTCTGCCAAGTCGGCGTC
GTCGGACGAGCGCGGTGGCGGCGAGGCGCGCAGGCGGCGGCGGCGCGCAGGTAACACACACACACA
CACACCTACACAGACAC
>TCONS_00034445 geneID=XLOC_007027 scaffold_153:225231-228192(-) Splice junction overlap
CCGCCGGCCAACAGATCGCGTGATCGCGGCGGCGACGGCCCGCGCCGCGGCGGCGGTGGCGGCGGT
GGGGGCGAGGACAGACGCCCCCCGTTAGAGAATGGACGAACTCATCCCAGCGGGCGGCGACGCGAC
GACCGGCGCCGGCAGACGGACGACGAGGGCTCCGCGCTCGACTCACACGACGTGTCCAGCGCGGAC
CGAGATGTGCCGGCCGTAGAAGATTCTCTTTCTGCCAGTATGTTGCCAGCCTAAAGAGTCGGCGTCGT
CGGACGAGCGCGGTGGCGGCGAGGCGCGCAGGCGGCGGCGGCGCG
>TCONS_00034725 geneID=XLOC_007037 scaffold_153:412602-413928(-) Exonic overlap (-)
AAACAATAAGTCGGCTGTGCTGCAGGAGGTTAGATACCTTGCAAAAATAAGAAATACAATAATACTTTT
ATAGCTAGGATAAAATAGTTAGAATCATGTTACAATATTGGCAAAGCATCGTTATTTTAATTGCAATTTTA
GTTGTTCTGATTTTTGGTTTCGCAATAATCCCGCGATGTATCCGCAAGCGCAGCTCCGGCTACAAGCCT
CACGAGACGTGCCGCGGGCTGGAGGCGTCCGCGCTGAGCGACAGCCTGTCCGTGTCCGTGCCGCCCG
TGCCCATCTACTTCTTCGTCACGGAAGCACGCTGCGACAGTTATTCTGTTTAGCGCCCCAGTGGTTAAT
CAAGTGTTAAGTTCTTATAATAACGGTTTGTACAGTTGTTGCTACAGATATCTGCAAGATTATATCTGAT
GGCACATAGGGAAAACTGAAGGCCAAATATGTTAAGTATTTTCTGTTAGTTGATTCCTACTCTTTATTAA
ATTATTGTTATTAGATGATTTTAAATATTTTTAGAATGTTAAATATTCTATATTCATAATGGTTCTGTAAGTA
AAACGAATTGTTAATGTATTAAAATAAAAAGTTTTTATTTATTTACTTCTTCACTGTTTGGCTTATTTAAA
TTACTGCTAGGGTCTTCTGTTTGCGTTGAAACTTGGCTGTTGGTATCTTCATTTGATTCAGATTGTTGTT
CATCTTCACTGCTGCTGTCTGATTGTGAGGATGTTGGAGCAGCATTTTCAACTTTAGGCTTTTTACCCTT
CATTTTAGCTAGCTTCTTTTTCTTAAGACGTTTAGCTCTTTTCTTCGCTGTTTTCTCATCAGCGGCTCTTT

TATTTGCTT
>TCONS_00035093 geneID=XLOC_007096 scaffold_154:89656-92086(-) Exonic overlap (+)
CGGTGTAATGCGTTCTTGACACGAAAGACTGTGAGTAATTGCCTAAAATGACCAGTGTGAAGTGCCCC
TTATGATAGTGATGTGAGGACTTTACTGCGATGACTTTTAATCGGATGTTGCTGAAATTATCAACATGAA
ATACAATGTGCCTTACAATCTTCCTCCTCGTAGAAATACTTACTTAAAACTTTTGTAGTTTTTGTACTAAT
GTCCAAACTTTTCAAAAAAATCTATCAATGAAGATCTATCTTCAATCCATCTAAATATATTGTATATCTAT
CAGAAATTACAACGAATTCAGTCCTCAATCTCTGTCCAAGGATCACATTATGGAAACCTCCAATGTGTA
TCTGCATAATGTGTAACAGAAGACTGAAATTGAATGTCCATAATAAATAAATAAATCAATAAGCACTGG
CAGCCGAGACTTTACTCTTCTACCACAAAAAAAAGTATGAGTAAAACTTCAACCAGCCAGCACCACC
GCGACGCTCAGAACAAGTATTCAGCCGCACTCACAGGCTCGCGTGGATGGTAATGCGATGTCGGTCTG
GGTGGGAAGCGGCCGCGGCGGGGGCGCGCAGGCCTCCGCCGCCACCGCCGCCGCCGCCCTGGTCTC
CACCAACACCTCCATGGAGTCTGGCATCGAGGCTGGAGTACTACCAACTGTCACCGGGTCACTGGCCT
CTGCTGGAGACTCCCTGGGCTCGCTGTCCAGAGCGGCCGAGCCGATGTATGATACTGACCACACGGAT
TTGGGCTCGGAGTTGGA
>TCONS_00034852 geneID=XLOC_007085 scaffold_154:127648-129657(+) Unclassified
TCATAGTGTAGTACGGAGAGTCTCGCCCGGCCGCCGCTCAGTCAGGCATTGCCCGCGGCATCGGCGCT
ACGCCCGACGCCGGCCCGATCACCCGGGTCGCGGGTGTGTGTCCGCGCCCGCCGCCGACCGTATTCCC
CGGTTCGCGAGTTTGTGTCCCCAACATCGCCCGGGATCAAAAAACAAGTGACAGTTTAAAAAAACAA
AAGTGAATCTAGTGGTTTTCCATCGATTAGTGAGGCTCACTAGTTGTGTACGAGCTGCCGAGTGACAGT
AAAGATTGTGATGTGCGCGCGTCGCTGGTGAAGTAATGTTTATAAAGAATGTGGCGGGAGG
>TCONS_00035066 geneID=XLOC_007101 scaffold_154:477991-480763(-) Exonic overlap (+)
CGGAGGTGGACAGTGAGGAGAAGCTGGTGCTCATCCGGCCCGGCAAGCACGACGCGCAGACCAGCG
GGGTGGATAGCTAGGACGAAGACAGTGACGCCGACGGGGACCAGTAAGTCCTGACGGGTCACTGTCC
AAGCTGAGGGGCCAAGCACACGGTCCGACGAGCCACTGCTGAAGCCGGACCGAATGACGGGGTCAA
ACGGGTCACTGTGCTAAGAGACTGGAGTAAGGTTCAGATCATTCCCGGTGCAATTAAAGTGGAGACTA
AGGTTTTGTGCAAGTGAGTGCTTACGAATATTAAAAGCTACATAGTAGCATATGAACTGTGGGACAGTA
TTTTGAATTTTTCTTACTGTTGGAAAAAATACGTTTCTGTTGAAAAAGAAAAGTCAATTTTGGAACACC
CTGTAGTTAATGAATTACTGATGAGTGATTTGGCGGTATAGTAGCGACTAGCGACTGTGTAAATAAGAA
GCGGTAATGTGTATAGTCAGCTCTGATAATATCGCAATCAGTAAACATGAAAAATTATATTTTTTCTCAAT
CGGCATATACAACATTACAATAGATTTACAGAATAAGCATTAATTGAAGTTATTACTATAGCTGACCGTA
CATTTATACTTGTATGTAGTGGAAAGCGGTAAAGACAGTATATCATGATAATGTCGGGAAGAGATGATAC
TATTTTGTAAATACAAACTGGTTTTGTCGATGAAATGAACTCAGTTTTTATGAGTAAAAATAAAAATATT
TGTATAAATAATTAAGTGTAATAATCATTATTTTGATGTTATGGTTGTAAATGTTGTGTATTTTTGTTAAAT
AATTTGATGGCTAGAATATGAAATCAATTACATTATTGTATATTATACATTTATTTAGTAATTTGAATGTCT
CTCTGTAAGTACGAAGTGTTGCATAAATTATTTGGATTTAAATTTGTAGGCAGCTTTTTACCTATTTATAT
ACAAGTGGGCGATATGTGGGTAAGTTGCGATAAAGGGACCACTCGCAACAAAATGACACCAATTTATA
GTGAGTGACAATTTATACAAGATAAATGTTTTAAAATTAGATCGGCTTAAAATAAGTTTACCTACGTAAC
TACATGTCATATACTACATATACAATACATAATAATATAATCACACCATGGAATAATGACTCAAAATAAAA
TGTATGTGCCTATAGTTATTAACAATTTTGCATGTTAAATACCATACCATAGATAAGCGAAGGTTTTTTAT
ACAATGAAACATGAATATATTGCGATTTTCATGACCGATTTTACATTTTAACTCTTATCACTATGTTTTTA
ACTTTTGACATTTTAATTGCTAGATTTTAACAAACTATATTTACCTACATACGTAGTTGGTGAATTATATTT
TAAAGCTCGGTTAGCTTCAGTAGGTATACACATGTATCTTTTCAAACTTACAAACAGATGACTTTGAAA
ATGTACAATAGTGAACTAATTAAAGTCTGTTTTTTAATCTCAGATACTAAATTGACAGATTCTGAACGGT
TCAACAAGAGTCGATTGTTCCGCCAATAGGACGATACGTCACTTACTCGCGGGACAATCGACTGTTGAT

AAACCGTTCAGAATCTGTCAATTTAGTATCTGAGACTAAAAAACAGAAATTAATGTGGCTGATTGAACA
TGTACAATGTAATATTGTTATATTAACCCATTATTAACCCGATTATGCCATAGTTTCTGATGGGTGTGTCAT
CAATATTTTAGTTTACTTATACGAATGTGATCCCGAGCAATGAAAAAGTTACAGTGGCATATGGAACGG
CAATGGCTTGGGACTTTTTCATTGCTCCTGAGTATATCAGTCATCAACTTATAAAAATTATGTTATTAAAA
TACAATCTAAATGTTACCTAAATACATTTGCGTTTATATCAAACCATTTCACATTGTGCTTTGAGAAGATT
TTTCAAAACCCTAATTCTCAATCGCACGCCTTTGGAGGCGTGTGCTAATATTATGGCCAAAACATATTAC
TCCCTTGGCTAGCTCTTGGGCAGCGGTCACAGTTCATCAGTTCATTCATTCTTAATCATTATCATTATAGT
GAGTACAAAATCAATAATTTCTCTAACATCGACGCACAAATGGGTCCAACCGGCTTGCAACATACTCCT
GTAGTCCGTTATTTTACTAAATAACACGTTTAAATTTAATTGTATAGTGTAAATACACGACTGTATATAAT
TTTATTGTTGAACCAGTTG
>TCONS_00035101 geneID=XLOC_007102 scaffold_154:500279-501371(-) Exonic overlap (+)
TGCTTTGCTTACTTTAAACTAATTCGCTTCTAACACACTAACATAACTGCACTTATAAACCTTTACGTTT
TGTCAAAATGGTTTGGTTGACCAAGTGTTTATCATACATTTACTTAGAATACCTTAATCGATTCTGAAGG
CACTAATTTTAAATTTAGCACTGTTAACATTCTACTTTACGATTAGCTGAGACCCTAAATCTCATCCTCC
ACAAACATTTCACAGTGCTACAACAAAAACAATTGCCTTCACAATCCGCTTCACAAACACTTCCAACA
ACCTATTCCTAACCCTCCTCCCACACAGATGATGTCGGCCATCAAGTCGGGCCACGTGGCGTACTCCCG
CTTCGCCCGCATGGCCGAGATGGAGGAGGAGCACCACGAACAGGAGGTGCTGAAGAAGAAGATCAC
GGTGGAACGCAGCGGCGGCGGCCTGTAGGCGGTCACGGAACTACTAGTGTTATCCGGATACCTCATTG
GCTACATAGATCGTGGCGCCCCGACTCATCAAGTGCACCCTCAAGTATTATATCGTTAATCATCTTTATA
CATTTGAATGTAGATAAAAGTCGATATGCACATCCGATATTTCAATATTTATAAGATTTTTCAATATTGTAA
TAAGCAAACAAACTAACGTAAGGATTTGAGTTGAAGCAATACTTAAATACTATTGTGATGTATAATTATG
TTTTTTTTTCGAAAGTTTATTTATTCAGACGTTTTTCGTCTTTAGACATCGTTGTATTATTTAAATCAAAAT
AACA
>TCONS_00035140 geneID=XLOC_007107 scaffold_154:633342-653303(-) Unclassified
AAGGCCGCGGCGATCGGTTAGCGGCCTACATCCTTTCTCTGAAATAATAACAACAAACTCCGATAACG
AATTGAAAATAACGATAACGACTCTCTGTTAATAAAAAACGAACTTGTTTTTTTGAGAAAAAAAAGAA
AAGAAACCGGCCGAATAAAAATGTAGCCTGTATTTAGGAAACTGGACCGTAATGTTGTGGATATTGAA
ATGTGAAGCGTAATGTAAAAAGTTTTGACAGTTCAGTTTTTTTTTCTATTGCCTTTTGCTTTGCTTGTGT
TTTTCGTTCATGAAAAGTTAGAAACTTGAAATGCAAGTTTTTCTTCGTAAATCCTACAAGTTTTCATGA
GTTCTTCAGTGTGAGAATAAATGTTACTAATTGGGAGATGCTCCTAAAAGCATCCGCTCAGGAATAGCT
ACAGATGGATGCTAAATACAGATTCAGAATGCCGCTTCCCAAGACAATAATAATATTATACAGTATACAA
GTACTTGTACATGTATAGCCATGTTCAATTAAAAATACATATTTGATAGTAACAATCAAATTATGTGATTT
TTTTTATTAATATTTACAATAATCGAGGCCAGTAAACCGCATTATTGATAATAAAAACATGTAATTAGTAT
CTTAATTTAATTAAAATAAGGCCAGTACTAGTTTATAACAGTTTTATATGAAAAGAGCAGCATGTTTAAG
TCTACACACATATTAATGGAAGTTTTAAGCCTTTAAATTGTGCCCAATGACCCATCCCAAGGACAAGTG
ACATTCGGGGGTAGAAAGCTTCATGGTTTACATGGATGATCGCGAGGGCG
>TCONS_00035060 geneID=XLOC_007093 scaffold_154:707179-708292(+) Splice junction overlap
ATGGTGCAGAAGATGTACCAGTGTCATCGATATTTCCATTGCTGGAGGACCACCCTGCTGTGCCATCTC
CTTCCTGTTCCTCAGTGGTGGGTTCACTGGAACCTTCAGCCACTACGTCAGCTCCTGCCACTCAGGAT
AATTATGAAGTTGAGGAAGCTGTAACTACTCCAGTTCATACAAATAAAGAGGCAGTGCAGGATCCCCA
TCATCCTACAGAGGACGTGCAACGGCCTGATGAAAGAGAGTCCCGTGCGAATAGGACTCCTAGACCAT
CTGTCGGAAGGGGTTGGTTAGGCGGCACGCTGACTGCTCGGCTCGTGGACGTGGAGGAGCGTCGGCT
TCTCCAGACGGATCGTCGCATCGACGTTGAAGAGCGGCGGCTGGCTGTCGAGACGCAACGACTCGCC
GTGGAGACGCAACGACTCGCCGTGGAGGAGCGACGACTCGCCGTGGAGGAGCAACGACTCGCCCTT

GAGCAGGAGCGCCAACTCGCC
>TCONS_00035570 geneID=XLOC_007187 scaffold_156:440531-441725(+) Intergenic
ATCAGTATTAAAATATAGCCGCCAAAATTTGGTCTTGATCGTAGGTGTACAATGCTGTAAATAGTCCCAT
TTTAATAAATTAATTTTGTAATTTACCTCTGCTTAATCGTTATATATAGTATATTACGGTATTACTGCTGAG
AATTTGTTTTTCATGCATTTACGAGCGAGGGCTATTAGGCCCAAGAACCACTTAACACTCCTGGGACAA
ATGAATTCGTATAGTGCGAGAGCCGGCAGTAAATATATTAGTATGTCAGTAAATGTTAAAAGAAAAAGA
AGGACATGTGATACTTAAACATAGTTTAAACCAGTGAATGGACCTAAAGCTGACAATTTTGTAGTAAAA
ATGTTAGTGCTAGTGCAAGTGTAACGTGTCAAGATAATCGGCTAAATCCGGACGCCCGCGCCGTTGGA
AACTCGACCGTATTTTGACGATTATAAGAGTCAAGATCACTCGGTTGAAGTGCGTCTCCAGCTAAGATG
GAGTAAGCTATTTGCCTCTCTTTCTGTTATGTAAGAGCGTAATATTATGTTTGTTTCTTTGGAACGCATCT
TGACTAGGACAGCTATTTTAAACACACTTACATCTTTCATACAAAACTGAAGACTCGAATAAATATTAAT
TTAATGTGTGTTCTACAGTTACGTTAAATTGATATGTAATTTCTTTGCGCGCGTATCACAAAGTTTGACG
TTATCTGTGGGATAAATATTGTGCTGTCGCTCTGAAATTATGTTTTAGACAGTGACAGCATGATGTTTGA
TTTTAACTTTTACGCAGACATTGTGGAACAGGCTCTTAGTCTCTCGTCTTGTATTGAGAGGTGTTTATTA
GCTCTGCAGTGCTAGTGTCGGTTAAATATTCAGTAATGCCTTACAATTTTATGAGATTTTTGGTCGGTAA
AGGTTTTTTGTATCACAATAAAAAGGA
>TCONS_00035572 geneID=XLOC_007189 scaffold_156:617833-619778(+) Intergenic
CGCAGGGCCGCCGCGTCAACTCCTCGCTGCAGCGGCTGGAGCAGACCCACGACGACCTGTTCCAGTT
GTAGCGACCGAACCGGCGCAGATATGCGCGCTTCCACTGAGCTGTGTGTGACATGGTTCTATCTACGCT
GCGCTATGTGACATGGTTCTATCGACGCTGCGACTCTGACGTCCAATGACGAAATCTTCGAACTTTCGA
GATCATACGAGACATAAAACATAGTATCTACGAGTGATAAGTACTGAATTTCTCATAACGTTAAATATCC
ACATCCACTATTCCGGTACATGATAATAATAGTCTGTAAACACACTATTTGGCTTATTTAGAGGATTACAT
TGTACCAATATACATGATACTACAGTAATAGTCGGATCACTAAAGTTGGGCCTATAAAATGGCGCATATG
TATTTGCCTCTCGCTCTTATCATCGAAAATAGGGCCGTGATTAGATTCTCAACTCTAGTGACTTACCATA
ATACCTAATTTATTATTTATAGATACATTATCGGAAACTAATGTAATGCATAGATAACAATTATCTCTGTCT
GTGAAATTTGGTTCATCATTGATGGGATTTAAATGTTGTAAAAGAAACTTTGAATATACGATTATGAATT
TAGTGAAAATGTTCACGTGATGATATCTATATTGTACCTACACAGTTGATAAGAGACATGGATATTTTTG
AGGCAGTTCTGTAACATAAAGTCAAACGTCAGTATCATACGTATGGCACCAAATGAATTTTCATAAATG
TATGGAGAAACGGTGTCGGCAATGCCGATGAAGTGACACTTGTACGAATTTCTATACAAAGAATACTG
AATGCGATCTGCCCGTTGTGTACAATGCCGAATGGCATAAAAGCATCCTTACCTTATTTGCACCTTAATG
TCTGTTATTCTTACTTACAAGTTGTAACATTTGTGTGTGTGTTTTTTGAAAATATATAACTACTATACAGT
TATAATCGCAATGAAAGATTTCGATTTTCAGCGGACTGCACTAGAGGCGCCACTTTGTTTGGAAGAAA
CGGATCGAAAACTTACATAGGTAGTTCACGCAAAACAAAAAAATGTACTCTAGAATGTTTCCTGTATAT
ACTTTTACGTGTATTTTTATGTCGTAAAAGATTGTTGTTATTAAAACCCATATATTGGTAATCTTGTAGAA
ACCTAC
>TCONS_00035773 geneID=XLOC_007235 scaffold_157:158119-163055(+) Splice junction overlap
CCGCCCTACCCACCCTACGCGCATTATGATGGTACGACAACACTAGCCAGTGAATACGCGTACGCAGC
ACCGTACTCGCAGTACTCGGCATATGGGGGGCCGTATGCTGCTCATTCCGCCGCCACGGGGCTGTTGTC
CAAGTGATATCAAAAAACGCCGTGCGCTGAGCAATGACAGACGGCGGAAAACAACGAACAAAAATAA
TCAAGTGATAAACAAAAAGACACTCTTATCAAAACCAATGTGGCATTGTGAATCTTTGTAAATTTTCTA
AATAATAAAATGTCTTCTCTTAAATTAGATTTTGCTTTGGTCAGTTACTATTATTAATGTACAAATGTTTG
AAACATGTAGTTTTTGTTAAATTGATTTGATAAAGGCCCAGTTTTTCATTTTATTTCGGTACTTTATAGTG
CATACTTGAAATATTTTACGGCCAATATTCTCAAATAATA
>TCONS_00035822 geneID=XLOC_007252 scaffold_157:288164-290859(-) Exonic overlap (+)

AGGGAAACCCGCCAGCCAACCCACCTCAGGTACCAGGCAAGCAGCCGGACGCAGGAGCTTCTACCG
ACAAGAGCAACGAAAAACCAGACAGCATCGACGACAAGAAGGCGAGAAAGGACGGGGCCGCGCC
TCCAGCTCATCTTCTAGCGAGAAGTGAAAGAGACAAACTCGGAACTCACTCTTACCCGTTAATCTAAA
CTATGGAATGGAACCTTACGCCATGTCATTTGTGTAACATTTTATTTAAATTAATTTTATGCATGGTAAA
ACAGGGTAACATGTAATTTTACCCAAGGTAAAATATTATTTTAACGCGATTCGCCTCAGCGAAACAAAA
ATTTAATTATGATTTTGATTACAATTCACCAGACCAAAACGTTACAATGAATTTTACGTAGATATATTTTG
TGCATAATACTTACTAAAATTATTTTCGGGACCATGGCTTTAAAAGTTTGTGTATAATTTGGCTAACTCGA
TCGTTTTGTCACGATGAATTTGTAGTATTATTTCTGTTTTTCTTGCGCCCGCCATACTAACTAACTGCAGA
TTGTAATGTAATGTAGCTTAGATTTAAAACCATAAATGTGATTTATAAATAGAATTAAAACTGTCTATACT
AGTGAGTGGGC
>TCONS_00035875 geneID=XLOC_007252 scaffold_157:296709-298151(-) Splice junction overlap
GCCACTTTTTATGTGTCAAAATCGTATCTGTCATCTATCGATAGATAGAGTATCGAAACTGGCAGTAAAC
CTTCCGTCACAAGTCCATCTCCCCTCACACATCATGTCACCCCCAGGCCGTCATCCCTCCACGGCCTCA
AACACAAGCTGGTGGCGGGCTCGGGCTCCACGGAGGTCAGACGGGCGTCGCTACAACACATGCCGCT
GTCGCCGCTGGCTCGGACGCCGTCGCCGCAGCCGCATAGTCCTACTGGGTCAAAACTACCACCATTAT
TTTCCGGGACCATTTCTTCAGTATAAAAGATAAAGTAACTTTTAGACCCCTTTTTTAAGTACAGAGATTT
ATCCTTTGGAATGTATGTACAAAGTTTTTGAGTTTGTCCCCAAAATTTATAAAACAACCGAATTAAAAAT
AACCCCAATTTTACGCGTTCTTTCTCTATGGCATAACATCTGTGTTAACAAGACATTTTGAACAGTATTT
TTACTAACAAATGATTTTTGTAATAATCATCACGATAAGATAGACCTGCAAAGCAAAATGTAGCTATGAA
GTGATTAAAAAAGTAATACTTTGCAAGTTTATTTTGGGATTCATAATTCTAC
>TCONS_00035628 geneID=XLOC_007240 scaffold_157:343609-344300(+) Unclassified
TAAATTTTATAAAAATATTCCTGTATAAGTACATGTTTAAGAGGGCAAGTGTCATAAACAATACATACTT
TTGATAAGAGAAAGCAAACAAATTGCCCTATAAATAGAAGCCAGATTCATATGTATGCAAGTATTTAAT
GTTGTATTGTTTGGAAAACAACTGAATTTGTGAAAATGAAGACGGTGTTTCTCCTCGCCCTGGTGTTGT
GTGTGGCGGTGGTGCACTGCCGCCCGTCCGTGGATGAATCATCCGTAAGTAACATTAAACAAAACATG
ATGCGCTATTTTCACATTATGTATCATCATCATCACGACCCATTACGTCCCCACTGCTGGGGCACGGGTC
TCCTTCCAATGAAGGAAGGGTTTAGGCCTAGTCCACCACGCATTATGTATGACTAACTGAAATCTATTT
CAGGGTCACN
>TCONS_00035776 geneID=XLOC_007240 scaffold_157:343620-344202(+) Unclassified
AAAATATTCCTGTATAAGTACATGTTTAAGAGGGCAAGTGTCATAAACAATACATACTTTTGATAAGAG
AAAGCAAACAAATTGCCCTATAAATAGAAGCCAGATTCATATGTATGCAAGTATTTAATGTTGTATTGTT
TGGAAAACAACTGAATTTGTGAAAATGAAGACGGTGTTTCTCCTCGCCCTGGTGTTGTGTGTGGCGGT
GGTGCACTGCCGCCCGTGAGTACCTACTACTTACTAAACATCTAGTAACTACTAGAAAGAGTAGAAATA
AGAATCCAATTATTTATAAATTAATATTTTTCTGAGGTACTTAATTAATTTTTTATTTCCACAGGTCCGTGG
ATGAATCATCCGTAAGTAACATTAAACAAAACATGATGCGCTATTTTCACATTATGTATCATCATCATCAC
GACCCATTACGTCCC
>TCONS_00035694 geneID=XLOC_007241 scaffold_157:344546-351048(+) Unclassified
CCCCTATCACTATAACATAACAGGCACCGGTCATCTGAAATTTAAATAGTCAAAAATTATTATAATAAATT
GTTTCTTTTGTTTATTTCAGGAACACAAACACGGTGATGAACACAAACACGGTGATCGTCATGATGGG
CACCATCACCACCACAACAGAGGACACGGCTCATCGNNNNNNNNNNNNNNNNNNNNNNNNNNNNN
NNNNNNNNNNNNNNNNNNNNNNNNNNNNNNNNNNNTTGTTCGCCTCTTCATCATCAACATAAATAATTAT
ATCATAATCACTCACCATCTAATGAAAAAGTTCCATCTGCTATCGACGACGTTCCATAATTA
>TCONS_00035629 geneID=XLOC_007241 scaffold_157:344558-350567(+) Intergenic
TAACATAACAGGCACCGGTCATCTGAAATTTAAATAGTCAAAAATTATTATAATAAATTGTTTCTTTTGTT

TATTTCAGGAACACAAACACGGTGATGTAAGTACTTGCATAAGTATATAATAATAGGTTAGGTTAGTTGC
AGCAAATGCAGATCAATCAAGAGCGAGAACATTTTCTCACTAAAATAATAAAAACATTTATTAGTATTT
C
>TCONS_00035777 geneID=XLOC_007241 scaffold_157:344622-352607(+) Intergenic
TTTTGTTTATTTCAGGAACACAAACACGGTGATGAACACAAACACGGTGATCGTCATGATGGGCACCA
TCACCACCACAACAGAGGACACGGCTCATCGNNNNNNNNNNNNNNNNNNNNNNNNNNNNNNNNNN
NNNNNNNNNNNNNNNNNNNNNNNNNNNNNNNTTGTTCGCCTCTTCATCATCAACATAAATAATTATATCAT
AATCACTCACCATCTAATGAAAAAGTTCCATCTGCTATCGACGACGTTCCATAATTATGTCTTTGGAACT
TTTTCATTCGGACTCGCTGCCGTGCCCCCAAGGTCCACGTGAACTAAAGTGTTAGAGTGCAGAATATG
CACCCGTGTTTGCTATTTTCCTCAAATTGATATATTGTTATTAACAAGTACATCGTATTTAATATAATTAAT
GTATTCCCTTTCTTGCATTTCAGAAGCACACCCATGAAGAAGGTCACGAGGGCCATCACCACCACCAC
AACAAGGGACATTCGTCTTCAGAAGAGAAACACACCCATGAACACGAAGCTAAGACTGACGACAAA
GCTTAAATCCAGGAGATCGAAGAAGATTACGAAGAGTTTGAACAGTCAATTGGAAGAAAAATTCAAA
ATTTAAAAATTTTAAGACCAACATGCAGATTTTTGGTTATCTGAGAAATACATTCCAAAATTAAACGCG
ACAGACTGAATTAAATTAGTTATACAAAGG
>TCONS_00035631 geneID=XLOC_007241 scaffold_157:350230-350890(+) Intergenic
TTCAAAAGTAAATATTATTTTAACATTCAAAAACTTATTTAAGTATAGTTATTTAACCTGCAGTAACGAA
AAGGGTAATATGCTCGTTTCTCCTTAACTATCAAAACTCCAACTCAGTATACCTATCTATAAACGTTAGG
TTAATTTCTGAATTCCTGAATAAATATTATAACACTTAAATTCTTTATTTTCTTTATTTCAGGAACACAAA
CACGGTGATCGTCATGATGGGCACCATCACCACCACAACAGAGGACACGGCTCATCGN
>TCONS_00035632 geneID=XLOC_007241 scaffold_157:351104-352580(+) Unclassified
ACTAAAGTGTTAGAGTGCAGAATATGCACCCGTGTTTGCTATTTTCCTCAAATTGATATATTGTTATTAAC
AAGTACATCGTATTTAATATAATTAATGTATTCCCTTTCTTGCATTTCAGAAGCACACCCATGAAGAAGT
AAGTACCTACCCATAAAATAGTATTTATAGGTAGTTAGTAATAAGATTGAAAATATGATTACATATTTTTAT
TTGATAAACGGACATTATTATTATTATTCTTTTAATTTCTGATAAAAAAATATGGTATAGGTATAAACATAAT
ACACTCACGGGCAATGAAAAGGTTCCACTGAGAAGAGCACCAAATTACTCATAAACGGAAAAAGCTA
GCCTAATGGATCCTTCAGCAACATTGAAGCACATTTTAATCAGGATATTACCACTTAAAACGGTAATATT
TTTTCCAAATATAAAATTACATGTTTCAAAAAATTGGGGTTGTTTGGTGTCGGCATTTTGTGAGTGGAA
CGTTTTCATTGCCCGTGAGTGTAATAATATAGCCTGAGCTACCGGTGATTTTTGTTTTTGTATCGCTGTA
AGTGGCTTGCCTAATAAACTGCATTAATTTCAGGGTCACGAGGGCCATCACCACCACCACAACAAGG
GACATTCGTCTTCAGAAGAGAAACACACCCATGAACACGAAGCTAAGACTGACGACAAAGCTTAAAT
CCAGGAGATCGAAGAAGATTACGAAGAGTTTGAACAGTCAATTGGAAGAAAAATTCAAAATTTAAAA
ATTTTAAGACCAACATGCAGATTTTTGGTTATCTGAGAAATACATTCCAAAATTAAACGCGACA
>TCONS_00035695 geneID=XLOC_007241 scaffold_157:351223-352810(+) Unclassified
AGAAGCACACCCATGAAGAAGGTCACGAGGGCCATCACCACCACCACAACAAGGGACATTCGTCTTC
AGAAGAGAAACACACCCATGAACACGAAGCTAAGACTGACGACAAAGCTTAAATCCAGGAGATCGA
AGAAGATTACGAAGAGTTTGAACAGTCAATTGGAAGAAAAATTCAAAATTTAAAAATTTTAAGACCAA
CATGCAGATTTTTGGTTATCTGAGAAATACATTCCAAAATTAAACGCGACAGACTGAATTAAATTAGTTA
TACAAAGGGTAGATTTCGTTTTATTATTACCCGATTATTTTACTCTACCTTACCTTATTAACACCATGTAGT
ATGTAGGTTTAAATATTTTTATGCAGGTAGGTATATTGTATCTAGTCACAATGTAGAGACGTTTAGAGAC
GTCTTCCTTAGTACAAATGACACAATCGATTATTTATTAATTGATAATCAAATGTATTGTAATAATCTA
>TCONS_00035696 geneID=XLOC_007242 scaffold_157:358561-360468(+) Intergenic
CAGGAAAAGAATAACGCCAGTTCGTCATCATCATCATCTTCTTCCTCTTCTTCATCGTCATCATCATCAT
CATCATCGGAAGAAAACTGTATTGATGACGACAACAGCTCGGCAGACCACCACAAAAAGGGTCGTCA

CCACAGGGTCCGCAGATGCAAACACTAAAGGCCTATGACACTGGAGACAAAATGGAGTTGTGAAAAT
GTGCATGCAGCATTTAAAATGGAATCATTTATAACATACTTACGGCAAACATTGTAGTTAAGTCGAACT
GTAATGATAACTTATATATGTCGCAGGCATCTGGTTTAATCTCCTTTTTGATTGAACCACTAGCCCGTTCA
TTGGAAGGCGCTATTCAGTTGTATGACTAAAGGACAACTCGTCAAGTTGTTGATTGTGACTGAACGTC
ATTCAGCGAAGTCGTTGAATGGTATATAGTATAGCAACTTTGTAATGTCTGGTTAGGGTGAACATGACC
AGACAAGAGTCAACAAATGACTATGTTACAAAGCTCTTATCTCACAAATTAACTAAACAATAACAATAA
ACGTACCTTCATAGTGTTGTTCATAATTATCCAGTTTCGCCACAATTTTTTCTTTGAAACTATCCGTCCGC
AATAATAGGTTAATCCATGATGTCACACTATTTTAACACAAAAAACGCACTTTAAAGCTAATTTATTTGC
AAAAAACTAACAATATGTATAGGTGCTTTTTATGTGAGGTGTTAGCTGTTAGACGCCATATTTG
>TCONS_00036169 geneID=XLOC_007289 scaffold_158:140939-146253(+) Intergenic
GCCTATGGAGGATGTTGGGTTAGGGGTAGGGGTTTATCTTTTCCAAATGAGGAATATTCAAAGCTGGAC
CAATGGATGTTCAAGAATATATGTTACAGTCACAGCCACATTTTTCACAAGCATTTCTACAACATTCAG
GACACAATGAAGAGTTAAACAGCAATTTGCAACAGTTTTCTAAAATACAGCACATTTTTGTATGTCTCA
GTTGGTACCGATGTAAGTACCTATACAGTTTCTCATGATGCAGAGATGTTTAACTGCTGAGTCAGCGGC
AAATTATTGGTCGATGCGAATAAGAAAATGTTCCTTGGTTGACACTCTCGCTGGATAATAATATAATTTT
TTTATAGGAGCATTTGAATCAACTTCGTTTCAGTACTTTACTTGGTTTATCGTCCTTTATTATTATTATAGC
ATTAAATAATGTAAGTATAGCTTTAAAGATCGGGCTAGAGCTTACGTAAACCGGCTTTTAGCGTACGGAT
CTTTCATTTTCGTTTATTCAATTTAATATATGCCATGATATAAAGCACAAATTATGGCTTTTGTAAAATATA
TGTAAAAACGAGGTAAAAACACTT
>TCONS_00036150 geneID=XLOC_007302 scaffold_158:300003-311262(-) Exonic overlap (+)
CTATAATGAAGTTTATACGTTACTGTGTTTTTATTTCTTGCGGCGATTGGTCGTGGCAATGTTCAAACTC
CGGTAAAAAATATTGATATGTAAACGTGATAGTGCCAAAAAAATACAGCATTCTTATTTCGAGGTGTGT
AAGAGTTATAAAACAATTTGGTGATTTTTGTGTGAACTGGACTTTGAAATCATTTAGAAGACCGTGTCT
GTTAAAATTCTGCAGATCCCTGCTATGGTGAGGTAACATGGCTACCACCACTACCATGGTAGTCTCGCC
TCTGGCTACTCAGTCGTCGCCCAAGAATAAGAGGAAGCTGTTTGCGGAGGCCAGGCAGAACAGTCTT
CAGAAGTTGAGCATCATTTTCGACCATGGGAATAAGGGTAGGTAACCACTAGTAGGTAGGAAAATAAT
AAAATTCGATTTAAAAAAGACTACGACATGAGTTCTACTGAAATTTCGGGTAACATACTGAAACCTACC
GGATCAAACATGACTTTATCTTCAGCCATTTTTGAATAAATTAAAAATCAGTGAACCTGAATGAAACTT
GTGAAAAAGAAACACTTTGTTTGTGAACAAACTTCAACAAACAGAACCAACAAAGCATGGATGATAA
CATTTATTTCAAACTCAGAATAATAATATTGTGGTCATTCCGAAGATCAGCGTCGCGTCGATAGGAAATA
CTAGTTAGAAATTTCATAGATCAACGCGATATATACCCCCTTACCTCAGCACCCACACTTCCAGTTCGAG
AGAAACATATTTAATAATAATAAGCAAAAGTTATAAGTAACCCCAAAAAGCGAG
>TCONS_00035927 geneID=XLOC_007294 scaffold_158:390789-392129(+) Exonic overlap (-)
TCGGGGAGTAGGACGTAAGATACGACGTTTGTGCTCATTAGGGCTGTCGCTTTCGCTGCGTCATAGAC
GAGATGTCTCGCAATAGCCTCCACCATCATCTTGTGCTCCGTGCTCACATCTGCCCCGTACAGACTGGA
GTGGCTGTGGTTGAACAGGATCTGGCGGTCCAGGTGGGTCGCGAGCGACGCGTTGTTGTTGGCCGGA
GAGAGCGAGAG
>TCONS_00036279 geneID=XLOC_007377 scaffold_159:20487-31973(-) Exonic overlap (+)
CCTCAATGGTGCGACCGCGTGGTCACAGTCCTTGTGAGTCGCGGACGACTCACAGAAGTCACAGACA
CAGCTTGAGCTAGCGGGTCGCAGTCGGGCGACAGTCGTGGCGAGTGACGTGCGTGAAGCGTGCAGCC
GTGAATACTGCGAGTGTGTGCACTAGTGTGGAGTGAGCTTTTGTGGTTTCAGTTGTTTGTAGCATCATC
AGCAGCATGGTGGCGCGTGGGCTGTCATCGGAGTCCGAGGGCTCCGCGGGGGCGGGCGCGGGGGCG
GAGNN
>TCONS_00036278 geneID=XLOC_007377 scaffold_159:20518-31935(-) Exonic overlap (+)

TCGCGGACGACTCACAGAAGTCACAGACACAGCTTGAGCTAGCGGGTCGCAGTCGGGCGACAGTCGT
GGCGAGTGACGTGCGTGAAGCGTGCAGCCGTGAATACTGCGAGTGTGTGCACTAGTGTGGAGTGAGC
TTTTGTGGTTTCAGTTGTTTGCATCATCAGCAGCATGGTGGCGCGTGGGCTGTCATCGGAGTCCGAGG
>TCONS_00036525 geneID=XLOC_007364 scaffold_159:157155-295194(+) Exonic overlap (+)
AAATAATATTACATTGATTAGTTCGCTCGTTCCGCTCGTGTGGGGAGGCCGCGTCGCGCGCGTCCAGAA
ATAACGAAACTACTTTTTCGCAGACAGATCCGTTGAAGAAACCGGTTTGAAAGTGACTAAGGTGTGGC
GTGGGCGATTATTGTGGCTCGTTTTAACCATATTTTATTCCTTAACCGTGAAAAATCGGGGTTTAGTACG
ACTTTTTAACTTTGAAAGTGGTAAAAGTGCTACATGACTCACTTACTATTGGATTTCTGAACGTTATCAA
GATTATTGAGATTGAGCGGGAGGGAAGATATACCGCGCAGGAGTGAGCGGGACAGCCTCTAGTCCAGT
TCAGAACCGGTTGAAGAAGGATACAAGTTTATTTTTTTTAGTGGTCGTTTTCGAACGCTCTCTCTAGTT
TTAGAGTTTGGTTTTGGAATGCTGTGAGTGTGGTTCGTGGATTTGAGCAGAATCAAGTGTTCCGTTTTT
GAAAGTGGCGCGAATTTGTCTCAAGATGGCCGTGCTTCCCGCGCTTTTCGGATTGCTCCTGGCTGCTTT
TGTATCAGGTCAAAACGTGGACATCACCCCCCGCGAGGCGGTGCGGAGGGTGGGGGACGAGGTCACC
GTGCTCTGCAAGGTGGCCTACCCGATTGACTCGTGTCGGTAA
>TCONS_00036585 geneID=XLOC_007383 scaffold_159:325539-327769(-) Intergenic
ATGGAGTACAAGAATCTATTTTCGCATCTATTTTAGATAAAATATCTGAGAAATCTTGCCAACAACTAGA
TATGTGTGATAGAGCATTTGCTAAAAACAAGTATAGAATCACCTCAGCGAATCAATGCCTCTTGACTGA
TGTCCAGACCTCGTTCAACGCTCAAGTCTTACAACAAAGCATTGTTTTGATGGATAGTGATGAAACGAT
CAGCATTAAAGCGGAACCCGATATCCTCGAGGAGGGGAGCGTGAGCGTGCACCCGGCGCGGTGGATC
AGCGAGGTGGTTGTGGAGCCTACGTTCAGATAGCTCTCTGTCATTCACCACCCTCATCACCATTAGCCT
GTAATCATCATCTACTGGACAATAGGCCTTCATCATCCACTGCTGGACATAGGCCTTTGTCATCCACTAC
TGGGCTTAGGCCTTCATTCGAGCACTCTGTCCTCAGCCTTCATCATCAAGCTAATCATCACTACTGGCAT
AGGT
>TCONS_00036550 geneID=XLOC_007388 scaffold_159:452165-453586(-) Splice junction overlap
CGGACGCCTGGCGCTCGGCTTCCCGAGACGCGGTGAGAGGTCATTCAGCAGCGTGGCCTCGCTGCTG
CGGGTACACCGGCGGCGGCGCGCGCTGCTGGCGGCTCCGGGGGGCGTGGTCGCCGCGCGGCTCACG
GAGCCCCCCATCCACTATCAGACCCCAAGCAGCCTGCCTGTAGTTGTGCAAGGGGGGGTTGAGTAGG
ACAACAACAATCATGAATCCAGAGAATTCAAAAAATTGTCAGGGGAAAGTTGTCAGTGAGCCACCTG
ATATAATGAGTGCCGTTCCACCAGCGACACATTAGCAATTGACAATCCTGAATCCAGATACTTGATAGA
GTAAACAGGGGAAATTTGTCAGTGAGACACCTTATGTAATGAGCCAAGTCCAAAAAGAGCCCTCTACC
AGTCTACTACCAGACCCCGAGCGGCCTGCCTGTGGTTGTTGTGCAAGGGGGGTTGGGTAGGACTGCTT
AGGGAGCTGATGAAGAAGGAAAGCAGACGAATTGTCGATACGCTGATGTGTATCATGCACTGATTACG
TGACAGCTCTCCCAAACTTCCTATTTCATCAAGCAAGAGTAGCCCCCTGTACTTTATAGACATCCGGCG
CAGCCTAGTTCTAGCAAGCAAGCTCTAGTTTCACAATAGTCTGCTAGATCATTAATACCCTGTTACATTG
TTAAG
>TCONS_00036601 geneID=XLOC_007374 scaffold_159:589710-590155(+) Intergenic
CGAGATTACTATGTTTAGGTGTGGACGTACACATTCAGAAACTTGAGTAAAGCAATACGGCATTATTAA
CATCGGGAGCCATTATTCGCGTCAACGGGTTTGGCTCAACTGTTGCCGGCGATAAATTGAGGAAAACG
ACGTTTAAATGTAACTACAGATATACAATTTGAGCCACCGACTGTGTGTGGGTTAAAACTGCGAGCGA
>TCONS_00036644 geneID=XLOC_007436 scaffold_16:6436-47391(+) Exonic overlap (-)
TTTCCATTTCACATTATCGGTTCACATGTATGCTTAACATTTATGTACAAGAAAAAAAGGAACAAATTCC
ATTCAACAAAAATGTGTGGTTTGGACTGAAACGGGATTGAACTAACACAAGCGCAAAATATATATTTTA
AATAATCATAGTTGGATGATAATAGCAAGGTCAAAAACTATTTCAAATCAATGGAATTTGATACTACTTA
TATGAAAAAAAACATTACTTATTACATATTTGGAAATAAATATTTTGTGCGGCATGTCTTGATTGCTACA

AAACATTATTTGAATATATATCAACATAGTTAACTCATGAATGGCCCAGTAGATTACATACTTATTGAGA
AACAGAAAATTCATCCGGTCACCCCAACAACAGTAATCAGTATTTAAAACTACCGCCATCAAACACGC
AGTATTAATAAAACTAGATTAAAAACGTAAATATTCTTAACATAAGAATAACTACAACAGAACATAGTAT
AAATAGCTCACTGGAATTAAGAAATAAAAACACGTTCGTCGTATAGTTACTTGGTATTGTACAATCGTC
AGGTTCCATTTAAGTTATATAGTGGTAACAGGTTGAGGTATACATTTTACTACTGTTCCATTAAATACTCT
ATTTAATATTACATGATAAAATTTATATATCTACACATGTAGTAGTAGTATAATTACATTTAGGTACCGATT
ATCATCTTTCTTTACTCACATTCCCAAAAATATATATATTGTTATAAAATTGTACAAAATACTCCATGCAA
GGAACAGACTTTTTCATAAATATTGCTCTGAGTTTCTTTTAAAACAACTTTTTTAACATTTACAATGCAA
CTGGGATATTATCTGAATATAATGTCGATAAATAACAGTGTATTTAATAAACGCACCGAATACGGTTTCTG
CATCGCAATAATTTGTACAATAGGGAAAAACTTTTTACAGCTTTTCAGAAAAAAGCTATCGACGCCATT
TTGTTCGATCGTACAACATAATGTCAAACCTTCCGCGCCTTAAAACTAGATAAAATTTTAATAGTTTACA
AAAATAGTAACAAGTTTTGTACTAAATCCGTCCAGTTTTGTACATTTACGGCGCGGGGACGCCAAAATT
TTCAAATAGATTAATTTTTCATATATAAAATCAAACTTTATTTATTTTCAGACAATTTTGCTATACATATCAT
ACTAATAATAAAAAAAACAATTTAAAATTTTGCACGTAATATTACTCTTTCACAGCCATCATTCCACAGA
CTAGAGTCTGTCAATTCATTAAAATAATATAGACAATTTTGATTTTTTACACATTTCTAAAGTGTTGTTAT
TTTATTTAAACAGTGATCTACAAGTGCTACTATAATAAATCAAATAAATTCTTCCCACAATTTCCACTTTA
TCCATTACCGAAGTAAAAATTAAATAATTAAATGCAGACTAAACAAAATATGGCAAAAGATCTTATTCC
AAACTTCTTGATATGTTACAACTTTCCCGTGGTGTTTCCAACACTATGGCACAATCAAAATGCATCCGA
ATCATACCAATAAAGTTTCAAATAAAGGCTAGCTAAGCTAGATTATTTTATGTCAATAAAAGCTAAGTAA
TTCCAGCGACCCCACGCCTTTCGTTTCTACTAAAGAGTTGTAGATATTGCAGGTAATATTACACTAGTAA
CTTACCGTGGCAAGGGCAGGCTACACAGCCAAGCCGACTTGGTCGTAGATTGGCAGTGTGTAGCGCA
GCCCCAACCAACACTGTGTAAAGTAACGCACGACTAACAATAGTAATACTAACATTATTACTAAAACAT
CACGTCGAAACATTACAATTCTAGTAGGCATCACTTTGTGATGTGTCCCGCACATATTGCGCCTACACA
CATAAATATTTACGTTTAACACTAAGGTACTCAATTCAACACTCGCATTCAAAACCAACTGTGTTACAC
ACAAGAAGTCTTTTTACAAACAAAAAAGTTTTCAATTTTCATCGCCACTCCTTGCCGCCAAAACTTGA
CAGTGACATTGACAGCTGTCAGTTTGTTTTCTTTTCGCGTCCGCGCGGCTGTACTGCCAGTATTATTCC
CGCCGTGCACGGAGGTCGTCCGTTTATCGGAGACAGGCCTCGAGCATTTCTACGAAGAGTTTGTTCAT
GGGGGCGCGGCGGTCGCGGTTCACTCCGGACCAGAAGCGGCGCACGGCGAAGTCCGCGTGTCTCAG
GGCTGGCAACACCAGCAGCAGCTGTTGCAGCGCCGCCAT
>TCONS_00037692 geneID=XLOC_007522 scaffold_16:68950-70410(-) Unclassified
CTTTCTCTTTCTTTCTCTGTGTATTGAATGCATTTTTGTGTAAATAAATGCAAAATTCATCATAAATAAAG
TGATTTTGATATTCCAGATATATTTCAGAAATACAAGACAATAGAAGGAACTCAGATTTGCCGAGAGAT
AATCGAAAATAGCTGTCAACAAATCTGTTTAATTTACATCATAATTTCTTTTTCTGCGTTCTTATTTTCTC
GACCAATAGTCATCTTTTCAGTATTAGCAGCTTTCATAGGCTAAAACCACTTTCAGCAACTTTATTACCA
GTCACTGGTAAAAAAAAAATCTGCATCGCATCAGGGCTTTATCAGTTCAAGAATATTTTAACGACCTGA
AGATTTGCTGCGTAAAATGTAGCTGCCGTTGCGCTGTCCCGATTTATTTAAGG
>TCONS_00037217 geneID=XLOC_007527 scaffold_16:216838-218117(-) Splice junction overlap
GGGCCACCAGCCCAGCCGCGTATGTGCCGCATGGGAAGGTGCGACAAATGTTCGAGGAGCGCCGTAC
AAAGGGCATAGACAAGAGCTACCCGCTACAGCCGATCCACAACACGCGGCAGCCCCTCGCCAACGGG
CACAGCAAGTCCAGCACCGGCACCAGCGTGAGACCGGAGAAGAAGCGCCCGGGAGTCGTGGGCAGG
AAGCCGGTAAATGTCAATCATAAGAGTGAGAATCGGTTGGTGAACGGCTCCGGCGACGTGAACCACA
ACCACGAGGCGGATCTGCACACACACAAACACAACAA
>TCONS_00037922 geneID=XLOC_007448 scaffold_16:691900-713114(+) Unclassified
CCGCTATCTTTTATCAGGGGCGCTATGACGCCACGGCCGGCGCGCTTCGCGTAGGGACGAACAGTTGA

TGGCGGCGCGCGCTCTGCACGACACATCTGCCGGCACTGGCTCGCGGACGCGGTAGTGAAAAAAAAA
ACACGAGTGTTCCATATTTGAAAATTTGAATTTTTCGAACGAGAACCGCCCCTGTTTGGATTGAGGATA
AATAACTGCGCTGAGCGTGACACAGTGATCCTGTAGACCAAGGATTTTATTATTATAATCATGAAATTAT
CGACAAACTTACAGGTAAGAATATTTTTTGATAAAATAAATAACTCTACCTATAAATTCACTTTTTATAAC
AATAATTTAAAAATATTTTTCGTGTGAAGATTCACTTCACTTGCTTGGGTTATTATTATCAACTAACTTAA
TTTACCAAGTACAGCCTTATCTAGATACTATATAACTATTATTATTAGGCAGAGGGTACAGCGACTTGCTC
AAATTGCTTTAAGTTTGCTCGACCTAAATCTATGTTAACCGTGTAAATATACTACAGCAAACAAAGCTAA
TAATTTCCCAACTAAACTAAGCAGTTAACTGTACACCAGCAAAGTGCTTAGTTACCGTCGATAAAGGTT
CTACCACCAGTTGCCTCACTAAATCAATTCACCAGAACATAAAACAAAATAGCCGTATATTAAACGTTT
AGTTCTGTTAATAAAAACGTCAAGATGCCACGATAAAGATTATCTTCAGTGGAAACGAAAGAAGGTCT
TTCAAAAGTAGTTTGCTAACATCACATATAAACAATAGACTGGATAGTGCACTTCGCAACTGAAGCCAT
TTAACAAGTAAATTCAGCAAAAACAGAAATAAAGAGATTCACATAATAAATTTAGCCGTTTTGCGTCAT
AGTGCCAAGGTCTACAGATGTTCT
>TCONS_00037923 geneID=XLOC_007450 scaffold_16:745478-746587(+) Splice junction overlap
GTGAGAGGTGGCAAGCCTGTGTCGTCTTCGCCTAGCCACAACCTTGGCAATGTGCAGTTGCGTCCTTA
AACTTGAGGTTTAAAAAAAGCAAATTAAAAAGTTAAATTCTCTATGGTGTAAAATACGAGTGTATTCCA
CATTCAAAGGGAAATATGGCGCCTTTGCTAAAAATTCTGTGGCTATCTGCTTATCTGATGGTGTCCAACT
GCCAAGAAGATAGCAAGTCTCAAGAGGACGCACTGTATTCTAGAACCTATCAAACGGAGACAGTAAG
TAC
>TCONS_00037367 geneID=XLOC_007543 scaffold_16:940653-941702(-) Unclassified
GCAAAATTAATTTTACCAATCTATTGTTTCAGACACTCCTGCAGTCAAAGGCCAACAATGTAGAACTAA
ACACATGCGTTGCGAATGTGCAAACACACTGACAAACACTATTATATACAGAGTGTTGCAAAAAGGGT
AGAGTTAACCTGAAACAGGTCGTAGTGTAACCAAAAATGTGTTAAATATAAATTTATTAATGAGCAAAA
AATTATAAAAATATAAATTCGCTAACAGCCTCTAAAGACAGCTTTGCAAATCTTGATGTAATTTTTTTGC
AATTATAAAAAATATAAAAATGCGAGTGCGAAAGTTCAATGGGGCTTATTTTTGCTTGCCAAACCCTTT
TTGCATCCTATAGCATAGATATAATATTACTTCGCATATAATATCATATCGACCATCTCCCACTTTGGGGTA
AACCAAAAAATATTGAAAGAAAATGGTCTGTAAAATGCTTAGCAATATTATATTATGGTGTATACTTTGT
GAATACAAAATGTACAAATATTCTGACAGTGTCACAGAAAATTTTGAAGGCACTTAACCTACCTACGTC
ATTTTATTTCTCCAAACTTATTAAAATTTTAATAAAAATTGTAACCTATTTTGCCATCATCATCGCTTAGCA
TTGCCACTGTAGTAGTTATCTTTACTACTAAACTTATTAATTTAAAACGGTAACTGTGGTTATAATTCGTA
GATAATTTGTTCTTATACAGAGGATATTTTCAGTAAAAAAAAATACACTTTCAAACGTCACAGACGTTTTA
ATTCGTAGTATATTTAACTGAAGCAGTCCCCGCTACACCGAATGAATGAATCGATTTCTTGTACGATTAT
TGATAATGTAAAGCATCTCGGTTAGTTTTTAATCGTCCGG
>TCONS_00037534 geneID=XLOC_007543 scaffold_16:940653-947131(-) Splice junction overlap
TAGACGCTGATGACATAAAGGCGATCATGTCCGGATCCACCATACGACCAGACAAAGTTGGCAAGGAG
CCAGTGCGAGAGAGCAAGGACAGCAAGGAGCCCTCCCCCCCGGGCCTCATGCCGCACCCTGCGCCCG
CCTAGCCGTAGGTACTCCTGCAGTCAAAGGCCAACAATGTAGAACTAAACACATGCGTTGCGAATGTG
CAAACACACTGACAAACACTATTATATACAGAGTGTTGCAAAAAGGGTAGAGTTAACCTGAAACAGGT
CGTAGTGTAACCAAAAATGTGTTAAATATAAATTTATTAATGAGCAAAAAATTATAAAAATATAAATTCG
CTAACAGCCTCTAAAGACAGCTTTGCAAATCTTGATGTAATTTTTTTGCAATTATAAAAAATATAAAAAT
GCGAGTGCGAAAGTTCAATGGGGCTTATTTTTGCTTGCCAAACCCTTTTTGCATCCTATAGCATAGATAT
AATATTACTTCGCATATAATATCATATCGACCATCTCCCACTTTGGGGTAAACCAAAAAATATTGAAAGA
AAATGGTCTGTAAAATGCTTAGCAATATTATATTATGGTGTATACTTTGTGAATACAAAATGTACAAATAT
TCTGACAGTGTCACAGAAAATTTTGAAGGCACTTAACCTACCTACGTCATTTTATTTCTCCAAACTTATT

AAAATTTTAATAAAAATTGTAACCTATTTTGCCATCATCATCGCTTAGCATTGCCACTGTAGTAGTTATCT
TTACTACTAAACTTATTAATTTAAAACGGTAACTGTGGTTATAATTCGTAGATAATTTGTTCTTATACAGA
GGATATTTTCAGTAAAAAAAATACACTTTCAAACGTCACAGACGTTTTAATTCGTAGTATATTTAACTGA
AGCAGTCCCCGCTACACCGAATGAATGAATCGATTTCTTGTACGATTATTGATAATGTAAAGCATCTCGG
TTAGTTTTTAATCGTCCGG
>TCONS_00036924 geneID=XLOC_007546 scaffold_16:1125624-1129969(-) Intergenic
TGAATTGATTTTGTGATCAAGCTATAAAGGATTTGTGGTTACCTTATATGAAATGTTGGAAGAAGGACCT
AATTTCAAGAAGATGATAAAATAGAACAGTGACGGTTCAATAAAAGATTTTCTAAATCCAAGATGGCG
GAAAACGAAGGTCCTGTCACGATAGACGATCTGCCCATCGCGTTCCAAGTAAGCTTTTTCTCTAGATAA
TATACAGGGTGTTGTAAAAATACACTCATGAGAAATGA
>TCONS_00036707 geneID=XLOC_007469 scaffold_16:1449827-1466134(+) Exonic overlap (+)
CGGAGGGGAGCACACGCATTTTCCAACAAAATAACCGACTTATTTATTCGCGCCAATAATTTTAACAAG
AACTTTCCATATTCAAAAGTTTTTTGTAAATAAAATTTGGTAGTGTTCAATTTTTTTCCCGATATTCTCTT
GAAATCGGGAATAGTAACGAAATTGCTTATAAGAATTTCCAGTGTCCAGTTTCATCAGAAGATAATCAT
CGATGACTGCGTTGAGGTGAAGCCAACCAGCCAGCAGCCATGGGTCGCAACTCGCCGGCAGTTGACT
CGAATGGCGAGTTGAGCATCCTGCTCGTGATCACAGTCGCGATAGGCCTGCTACTCATGCTGTCTCTGC
TCATGTGCTACGCATGCGTCCTGCGCAGGCTCTGCTGTGGCACCGCCAGAGAGAGGAAGCAGAAGGG
CGGGAGAAGGACGGAGAGCTACCCGATGGGAGACGTCACATTGCTGTCTCAGCCGACGGAGAGGGG
AAGAGTGTAGTACATAATGGATGATGAGGAGTGAGGTGTTGACTTGAGTGATGATGATGATGGTGATGA
TATACTTGGGTGTTAAAAGTGACAAAGTTTAATTTTTAAGGCGTGATGTGTTTGGTCCAATGAAACGGA
GAAAGATGTGATGATGGAGCCTAATTTTTTTCGAAAATTACAAGTTTAAGTGCCAATTTCTCCATTCTC
GGTTAGAGCATAACCAGGAAGTAGTGGTTAACTTTTGACACATCTGTTATGAATTTTATATGGAGATGA
CCGACAATGGAGAAATTGGCACTTAGTTATACTTAATTAACTATGAAAATCAATCATCATTATTTATAGCT
TAAAACTAGTAGAAGGAAGTAATTTTTGAGTTTGTTCTAAACGCGGCCATCATATAAATGTGGGGTATT
TGGTACATTTATTAACATTACAACTATCTATGTAAGTATATGTCTGATACCTACATTAGATTGGAAAACAA
AAGCTGCCTGGTAAAATAAGTAGATAGGTTAGTGTTAAGTAGTTAAGGTACATTTATATTAAATCTTTTT
TCTAGGTACTTAATAAATATTATGGGCAGATTTATTATAATACAATTACTAAAAAAAGGGTAAGTATGTAA
TCCAAAAGGAGTCCCTTATCACATCAATCTAATTAGCTGTAGTACCTAATGAACTACTTACCATACTTAT
TGTAAAAATAGCCTTTTGTAATTACCTACCCTTTCATAAAATTATTAATTATCAATTCAAGATATACCTAC
CAATATTTTAAAAAATCGGATACAGTTTTTCAGTTGTATTGTGGTGGTGGGAGATTGTAGGTAGGTAGAT
CTGTATCGATTTGACAGTGCCAGTGGGCTGGCCATACAGGTACGCCGTAGAACCAATGACCGGTGGGG
TAAACGAGTTCCCGAGTGGATACGCACGAACAGGCGAGGCTAGCGTGGGACGCCCTCCGGCCCGTTG
TATGGCTGGATATGGTATGGATAAGGAAGGCCGAGGAAGAGTGTTGCGGTGCTCCTTGAAAGAGGCTG
GAGAATTGGAGAGATATCCAGTAGTGGACAATTAGGGGCTGATGATGATTATGCTGACAGATTCGACTG
TAATCTATTATTATTTAAGTAAGTAGTGAATAGTTTTGTTAACTGACTTTACCAACGTATTTTTTCCTCATC
GAAAGATGGAAAGTATCTATGTACCTAGGTATTATTAATTGCGGTAAAACGCATAATGATATCATTTACC
CTTTTTAATTATAAATACCTAGATAAGCC
>TCONS_00036717 geneID=XLOC_007475 scaffold_16:1707110-1708258(+) Unclassified
AGATCTAAAAGTGCATATCTTCTACATATTGGGCCAGTTACGTGCTTCCCATTATGAGTATGATTAACTA
GTCTTTATAGAGACATACCTACATAATTGACGCTCTTATTTATAATGGTATAATTTAATTCCAGCGGTGGG
CGAGATGGGCGTGTGCCTCGGGTGCGGCGTCATGTTCGCCCTGAGGTCCTGCATCTACGCGTCTCGGT
ACGACTCGGGTTATGAACACACTTGGATCAATATAGATATACCATATGACACTGAGTCAATACTCAAGG
CGTTGGACGTTAAAGAAGAGGAATTTATCCTTAATCAGAGGGGGTAAACGTACTTAATAATAAGATTCA
TGTATTTTTTTGTCTATGTAAGCCCTACTTTTTTATTACGTAGTTTGTCTAGAGACCTAATATAAAAACAT

ATATTTTATCAATCCTATTATATATATCCAGCTCATCATATATTTCAATCAAACTTCAAATAGCTGAATCCA
CAATCACATAAATGTATAATTTATTGTATCCATCGCCACCAAGACAAATTTCTAATATTATGGCAGAATTT
TATTGTACCATCAAAATTTTATAATTGTTAATTTATATTAAACTCAAGTTAAAATATTTATTGGATCAGTTA
CATTTTACTACGTATTCTGATACAAACCGAAGTTCAACATCCTTACAAATTAAATTATGAAACGTGTTGC
CATTATGATCACACAATTTGTAAAAATAAACATGATTGGATTATATTATAAAATTTAAAGATCATTAATCA
TGTTCGCAGATTTGGAGCTATTTGAATAAAAATATTAAATTAATATAATAATTAATTATGCGTCTACTTTGA
TTTAAAACGTTCTTTTATAAGTTGTACTACTAGTTCCTATATCCACGTTTGATAGCGATACACAGACACA
CGTAAGTAGGT
>TCONS_00036949 geneID=XLOC_007558 scaffold_16:1837862-1868544(-) Exonic overlap (+)
CGAGCGCCGCGCCGGCCGCCCGTCCCGCCTTCTGACACCCTGTATCAGTGACTGCGCGTGTGTGTGTC
CGAACCCGAGTGTCATACACCCCCGGACCACTGCTGTCGAGGTCTCCTGTACCTGTTGCGTTTGTTTAT
ATATGTGAAAACAACAATATTCGCGAGCGAGTGAGAAGAGAAATGTGTTTCAACAAGTGTCTGATCAA
ACTGGCGGATTATTTCCGATTACACGCACAGTCGGACTAGAGAGCCATTGAACAATTACAAGCTCGAA
ATGATCTACAACAGCAAAATTGTGTTTACAAAACTGTTATTGTGCATTTGTGTGTGCGGAGCTCGGGGC
CGGTCGTGGGCGAACCACCAC
>TCONS_00038295 geneID=XLOC_007479 scaffold_16:1979004-1981994(+) Intergenic
GAAGTACGGAAATATTTTGTAATATTTAATGTTTTAGGTACATAATTCTTTTATAATAATTACAGGCACCC
GAAGACAGCAGCTAGTGCATAAACATAGGCTATGCATCTGAAGCCTCGTGCGTTCGGTGATTTCATTTA
TTGTATGTTGTTAGCTCTGTGGTTACGTGGACCAATGGCATACAAGTAAAGTCGGTAATCTGGACATAAT
ACGTTATGACTATGGTCATTTATCACAAATTTAGCACATAACTACTGAATCGATTTCGATAAAACACCGT
TATAAATATATATCAATAACACAAGTGGTATCAACTTGTGTCGGACTGTACCACAGGGACCCGCTCGGG
AGGCGAGCGTTGTATCAACGAGACTAATGCTGTTTGTGGACATCGCATCTCGCACGAGGACAGATAGT
GAGATATATAGGTTCATACGAGACAAGAATATATATACAGGGTATTCCCAAAACGGGGGTCGCTGAACT
CTTAATTCTTAACAGTGGGGTTAACATCACCGAATATCGGCAATAAAGAATCCCAAAGCAACGTCGGTC
GCTTGTCAAAACGGACGTTACTTGTATGGATGGTGGTGGTACGATAGCAGCTTTAAAAAAGGGACATA
AGAGGAGGAGCTTTTTTTAAGAAATTCAAAGTTTCTTCACACGAATTTCGCACTGATTTACAAAACTCT
TACAATAATATCGTTAGTTCTATGAGTAACTACGAGGGC
>TCONS_00037885 geneID=XLOC_007573 scaffold_16:2603523-2607605(-) Exonic overlap (-)
AGAAAACTCAAGCTAGCATGTAACAAACCTCTTAAAATAAAGATTTTAATAACCTTAGGTATAATTATCT
CACCTAGAATACAGGGCCAACATCTTATCCCCAACATAAAGCAGCAGATGATAGTGTGGGTAGGGCGT
CTTTATCTCCTTGGCAGCACTCTTCAAGGCACTGAGGGCGGCCGCGGTGACCCCACTCTCCCCGATGA
TGCCACTGGCTCCGGGTATGGAGTCCCCTTTGTTGCGACAGTCGAGGAGCGCTGATAACCACCTCTGG
TATACTTGGGAGTGAAGACTGAAAGATATGCTGTACCAGGGTCTTGCAGTCCAGCAGCTCCCGGTGAG
CATCATCTACATCTTGCTCACTTATTATGAGGAACACATGGTCCAACCACTGCAAATGGTTAATTTTGTC
ATGCTTTTAATGTAGTGAAAATAAAAGGATATAAAACACTGGAGGTGAGGGAGGATAAAAGTATTAAAT
ACAATTTATAGTATGAACCTAATAAAGATATAAATATTTTACATAATGTAATTTATGTTTGCTACACGATTT
GGTCTACATATTCTGATGATGAAATTAGCTAATAAATAGACAACGAACAATATTTCTTATTACCTCATCAA
ATACTATTGAAGTTCCATCTTTGCATTGCATGGATGAATAAGTATTTCCAAATTGGGCAGCCATGACCCT
TTGAGATGTAATAATAGGTGATAGTAGCTGAGAGACATTGTAGCTGTCTGTCACACCTTCATTATTATCA
TTATCCTGAAAACAAGCAGTACTACTTATAAGAAAATTAAGAGTAAATTAAAATTACACAATATATTAAG
TACAGTTAAAGTACATTTACATTTCTTGTATTGTAATCGATATTAAGAGATTCATACCTGGCCACTAAATG
CCTTCATGCGATCTAGAAATGTATCATCCCACTTGGAAAACAGTAAATCGTTCACATTATCGAATATTAG
CACTGCCTTCATGTCGGTCAATCAGAAATTATCGTCCTGCAAATGTGAAGTTCCTCTGGTCAATTTTTAA
TCGCTACTCATATTACAATAACAGGCGATACTCAGTGAAGGGATATCTTCTCTGGGGGTTGCAGACTCC

CTAATTTCCCAAATTATCATAAAACTCCCAAAATCCAAACAAGACATGAATGAAGACAACAGTTAGCA
CTAGGGTTCGAACGATACTTTCGATACCGAGTAAGTATCGATACTTTCGTTCGCGATACCCGATACTGAG
TACTCGATACTTTTGGGCCGATACTTTTTTGGTATCGATACCCTCGTCTCGATACACAAATCGCTGGTAT
CGAGTACTTTGTCGTCACATTTTAATATGATAGGCCTTCATAAAAGATCTTAAGACTAAACAATCCGTTA
AAACTTCAGTCTTGCATTTTTTTTGTGTTTATTATTTGCTATTATCACCAGAACTTTTTTTTAAATGCAA
TGGAAAAGTAGATTTTTATGCTGGTGAAGGTACAAAAATTAACTATTTCGCAAGTACTCGATACTGAGT
AGTATCGATACTTTTGGACCGATACTTTTTCGGTATCGATACCCTCGTCTCGATACCACATGAGTACTCG
GTATCGATACCGGACGCCGATACTTCGAACCCTAGTTAGCACTGACACTGACAATGACAACAAACACA
AATATAACATCTGACATCTGGTTGTCGGTATTATTTTGAGGATTTACCTACTGTACTTGAATATTGTAGGG
ATACAAGACTCATCATCATCACAGACTGTAAATTAAAGGCTCTGTGGTCTTTGGCAGTAAATGTCAATT
TGACGGGAGTTAATTTTATTGATGTTTGCATCATAGAGAAAAGAACACTACGTTTTAAGAGATGGGAGT
ACAAGGGAAAAAAAAGAAAAAAAATACTTGTATTAAACAGAGGGTACCAACAAAACAAAAATTATGAC
GTACAAAACTGCCAACATAGCCCCGCTGAAATAGTGATGTGCTTGTTATGGATATTTTTTATCGATAACT
AGTAAAAAAAAAAAAGTGCGAGTGTATGTATTACTTTAATTTCAGTTTTACACAATGACACCAACGTTAA
GTCTCGGTTCAACGTTATGTTATGTTCCTACTTGAACACAAAAATATAATCCATAAAATAAACTCGAGAT
TTTCCGTTGACGTAAGTGCTATGATATAATGTTGAAATAAAATAAAAGTGGAAAAATATAATTATATTTAT
TTATTATAAAATAAAGTGTCTGCAGTAAGTCCCACACATGGAATTGTTTTTTTTTTTCAATTGTGTTCTTA
AAATGTTGTGGTAGAAAATGATTGCCACAAATTCGTTTAAGTTGATGTAGCTTTTCAATAGGCACATAA
ACCAGATCCTCCTTACCGGTCATCTTAACCCACTGTTTACATCTGCAAACAACATTTTTATAATTATTATT
AAACAATTAAATATTATTTAAGTAGGTATAATAAAAGTCACTGGTAATTTTTGTATAGAGATCCGAATTTG
ATTTTTGCTAATTTCCAAAACTAGTAGACAAAAGTTGTTCTGATTGATGAGCTGTGCGTACTAATGATAA
ATAAAAATATCGATATCGATAAAAAAAAACATTCATGCACTATTGTTGGTTGTGCTGACCCTCCAGTTATT
TTATTAGACTTTAACTTTTTATACATATAAATAATCTAAATCCTATGCTTTAGCCTTTAATTGTAGCTTACC
TTTCCTCATCCAGTGGAAATTTGGCCATGAAAATTCTCTTTTGGCCATCTATACGACCGGCTAGCGGCT
CTTAAACCACATATTTCACAAGTTTTATAAAACATAATTATTATCAATAATATCAAGCAACACTAAGG
>TCONS_00038259 geneID=XLOC_007575 scaffold_16:2631078-2632544(-) Exonic overlap (-)
AGCATTTAAAGTGGTTGTTTCTGGGCGCATCCGTGCTTCCCCGGACGGCAAATTATGAAAATGTTTTTA
TTAAAATATAAAGTTTAACTTACATAACAACATACTGTGATCCTCACACTAGGTAACACCTGTATCGTGA
GAGATCAGGTTCGAACTTATACAAAACAAATCATAAAGCCCGGTTTGTCACGCAGGACGTAAAATGAA
ACATTTTTGAAACTATTAAAAATAATGAAGGCATGCATATAACAAAAAATATATTGTAATTACGTATGCC
AAATTAAAACCGTAAACAAGCAAATATAGAACATATATATAGTATTGCAAATATAGAACATATATATTAAG
CCTCCCAGTTGCTGTTTCCGCAGCAGTCAGCGGCCTCGGGCTCGCGGGCGGTGGAGACCACAGTAGA
TAATAAATTGTCTGTCCTGCTCTCGCACACGCCTATAGACTATGTCTTGTCTGCTCATCTGCGTCCAAGC
ATACGCCTAGCCTTAATCTTGTCAAATATTACAGTTTACAGGAGTGGCGGTAGCCAGCTTCTCACGCCA
GAGATACTGGTTCAAATCCCGGCGCTGACATACCATGTATACCATAGCTCTTACGGTGAAAGAAAACAT
CGTGAGGAAACCAATACATCTAGATTTCGGCGAAACATACACCGAAAAGCGGGTGAGTCGCTTGCGA
GTCATCTCAGACTATCCTTTCGGGGATTACAGTCGTGAGATAACTATGTATGTTCTTACATTAGCTGCCA
AACTGTTGGCGGCTCGTCCGTCAACAGTTGAAGCCGCGGCGCGGGGCGAGGGCGCGCGGGGGCGAC
GCTCCGGGGTCAGTGACCTCGGGCTGGCGGGGGGAGAGGCGCGGCTGTGAGGCCGGGACTCCCAGG
CGGGTTGCGAGCTGCGGGGGAAATTGGTGTAGGTATAAGAGTATATTTAAGGCTGACAGAATATTGCA
GTTCAAGAGCGAATTCACGTCTCTCGAAATTAACGATTAATAGCTGATGATGATACACCGCAGACGCGG
TCGCCCCGTGCACACATGGCGGCGCACAGTGGACGGCGAGCTGCGAGCGCAAGGCCTGAGCTGGAC
GGAAGCCAGAGCGGTTGCTGAGGACAGAACGGCGTGGCGCACGCTTGTAAAGGCCCTCTGTACCACC
GGGGTACCATAGGACAGCAACAACAACATGATACAATTAAACCGAAGGAATCATCCCATCAGTTTTGA

ATAACTAGATATGTACGGATGGTACTCATCAGTTTCATAGTTCTCTTGCATAAAGAGCAGAGCATGTTGT
CGATCAGTGGCTCCCTTGCATTAAGTTGTCCTGAACATGTATTTCTGGTCTATTACAC
>TCONS_00036814 geneID=XLOC_007503 scaffold_16:2843493-2845644(+) Unclassified
CCAGCCGGTACCCTTCGTCTACTATACACTCTGCGAGGTCCGTATCAGATAACAGCGACCACTACGCCA
TAGTGCAGTCGCGGCCCGGCAGCCGGCACTCTGGCATGCATCGCAGCAGACACTAGGTATACCTTCGA
CCTTCTAGTACAGGTTTCATAGCTGGTTGAAAACTATAAAAGTTTACGTTTTCTCGTATACATATATTGTA
ATATTTATATAATACAGAATTAAATAAATATACAAAGTTGCACCTCTAGCGGAATCTTTTGACAGATAATG
TATGCAAATGACGGAGGAAAACGTAAACTTTTTCGTTTTCGTAAATTGCAAAACCTGTAGTGAAACCA
ATTTGTTCGAAGAACTACACAATAAAGCCTTTGTTTTTTACCAACTACCAGAAGGAAACATTCCATATA
GGTACATAATCTTATGAAACAATAACTGTATGTATACTTAGCAAGTTACCTACCTAAAGTGTAGTGGAGT
GTCACATACCTTTATAAAATCAATCATGTGCCATCTACCTATTGTACCTAAAACTGTAAACTTGTTAGCAT
TATTTAAAAATATGTACCTAATTGGTGAATTTATTATTTGCAACGAATCCGTCCATTACTTTGGTTATGATT
AGATAATTAAAATAATTATTATAAATACATTAACGTAATAATCTAGGTTTGTATTTATGTAGCATATTAATTA
GGAACTCTTTATATTATGAAATAAATAAAGATTCAACCACCATCAATAAATATAAATTGCATATTGGTGGA
TAAGTTAGTGCAGATTTTTCGTTATTTAGATTTCAGAAATGTTTCACGTTTTCAAACGCTATATTTATACA
AATAAGAGTACATTCACAACGAATTAGTATATGATAGTTGAGCATTTTTGCGGCATTATATCCTTTTGTTA
TATGACCCAAATGTGGAATACGGTTAGGTATACATTTCATAAAAGTTTATTTATGCCATTTCTGCTGCAC
AACAAGGATAATTTCTTATAGGTAATTGGGAATGGTTGGAATTGTAATAAATCCTATTTATTTAAGTAGGT
ACCTACCCAAGCATAAAATGTGTTCTAAAAAATCATTCCAAGGAAATTCAGCCAGGGAATACAAATTA
AACATAATACATAATATGATATTTAGCCATCAGTTTCTGACCTATTATGCGCTTATCAAATGTAGTAAATA
AGTTAAATTAACTACTTAGTATTAAAAAACCATGGAATACCACATCAACACTGTGAATATAACTATTAGC
CTAGTATGTAAGTTATAAATAAATGTTTATTTTTGTATAAAAGTAACGTTTTGGTGTTTGTGTGTGATTTT
GGAATTAAGTTACTATACTTAAGTATTTTGTAGTAAATTAAATAATACACTTAGGTACCTAGTAAATGAAC
ATAATTTTTAGATCTTTAAATAAATACGTAATTATGCACTTCAAGCTCGTTTTATAGCGAGTGCTCAAATG
TAGGTTGATGATTGTACTTATATGAATCTACAGCTTTTAGTCATGCGATCATTAAAAATAAACTGACAAA
TGTTATTTAACAGACTGTGTACTTAATAAAAAAAACTTAACAGAGCCGACTATTTTACTTCAACCTCCTT
CCTTCCTTCGACTATGGCCATGTGTGTATTAAATGGCATTAGGTAGTTGTGATTTTGAATCTAGATAAAT
GAAAGAATGTGGTATAAATTAATATAATAATTGAATAAAACTTGACGATATCAATAAGATCAATGATATCT
GTATACCTATTTCCTCGAAAAGATTGCCTTAATCAATGGGTTCAGAATTAATAAAACAGTATTTTGCACA
CATATCG
>TCONS_00036999 geneID=XLOC_007581 scaffold_16:2899359-2902284(-) Intergenic
CTGATTTTTGAGGCTCAATGCATTGTTTTCACATCAGTGCAAGCATAATATTTTCCTATCTTTTTAAGTTT
CTATGTGCTGTGTTAAACTGGCTCTATTTGTAAAGGCTCTAGTCAATATATGAAGCCGTAACTACAAATC
AGCTTATTGATCATTATAAATATAATGAATAAATCGATAAATATGGTGGATAGTGTCATGGAAAGCGCTGA
TTCATAAAAATGAATTTTGGTTGAGTTTGTGGAGCGTTTGCAATGTTAGCAGATTTGGGAGGCTTAAAA
GTTTGTATAATTACTGAGATTATAAGTTATTTACACTAGTTATTAACGACTGCGTTGGCGTTAAAGACATA
CAAGTTAAATCTTGAAACGTGATCCTTATTGTTAGACGTGAAAATACAACGAAAAGAAGTCTATAGGA
CTAATGGTTAAACAGTTTTACGCCGTTACTATTAAATAGTTTGTATTATTATATTGTTTAAAATGCCTTTTT
GAATAAAGTTGCGATGACTTTTCTGACTGAACTAACGGTTTCTTATAGTAGAAGACCCACGATGACAC
AACTTGTTTTTAATGTCTTATTTGTTATTACTATGATAATGTCATAGTGAGAAATATGATCAGTGCCAAAT
CGTAGACATCATGAAATGGCAAAATAATAATAAATAAAAACGTAGTGCGTCCACGGTAGGTAGAAAAG
TAGAAAATGTAGTCTCGGCTGAAAAAAAATCAATGACCTAACAATTTGTATATTAATACTAAGTAAGTA
ATACCTATCTCACCATGGCATTATCAATAAGATATTAAATATAAATTTAATCTCGTCTGTAGCGATTTGTAA
TCTGAATAATAATTATGGTTCATTAATTATAACCTTGTAAAGTAGCTTTCGTTAGTACGTAAATGCACTTT

ATTTGATATTGATCACATTCATACATATCCAATTTAGTAAAAAAACTAAAGTTTTATTGTAGATGAATCAC
TATTTTCAAGTAAACTGCTAACCGGTATGAAATCTTATGTAGTTTCGAACGGATATCCCTACATAACATA
ATAAGTTATGAGTTATGATGCGCCACGTAGATTAGCTTGCTTCGTGTATAATAATGAAATGAGGTAGAGA
ATGCATGAAAGAGAAGTGCAATAACAATTTAACCATATTTATGTAATTTCATTTTAACAATATGAAATTG
ACAATTGTTTAATTTTTTTCCTTGTTATCAGCACCGAATATTATTAATAAAATTGTTAACATTGAATTGTTA
TATCTGGACCAATGTTGTTTAATGTTGAACTTTGTGGAATTAACTATAGTATACTTATTTAATTTGATGTAT
GTTATCGGCTATCTTCTATTGCATTATCATTGTCACCTTTATGTAACTATATAACGGGTTTCCTACTTTAAA
TCGAATGCCTATTGATCGAATAACACATCCACGAACACTATAGGTATACGTAAAACATTCATGAAATCGA
TGCATCAGTCTGGCTCGAATCGTCAAAAATCGAATAAATCCAGCTCGAATGCTCCATCCCTAACGATTT
TTCTAGAACGGTCATGGGACTGAGTTCGGGGGTTTTCGATTCGATCAGTAGACATAGACATTAGATATG
AAGTATCTTTATCATAATAATTTCATTTATACGGCATGGATTTTAGAGACTGTTTGAAGCATCTACAATTC
CACAGATCGATATTATAAGAGTAGAGAACCGGAATAAGTACCTATTCAAAATAAACCGAATAAAATAAC
ACTCTACTCTTTTACATCAGGTTCCCAAAATGTGTTTTACTAAATCAGATTCAGAAAACGCATTAATATT
TATTTTCCGACTATGGATACTTATAGCTCACCCTATCCCGAAAATGCATTGACTGTAAGCTAATAAGTAG
GTACAATATTATTTTTAGTTAAATGGTTGTATGTAGGTAGGTTCATAAAGATGGAATGGAGGTTCAAAAA
GCTTTTTAAGTATTTCTGCTTAGAACCACGTCATAAATGTCAATACCACCGCATGTGCACGACTTACTTT
CATTATAATTAATTTAATATTGCAATAATTCCCATAATCTAGTATGTAATTAAGAGCTTAATGAAATAGTTC
ACCCAATTTATAATAATTCTAGATGTTAATCTGATTTCAAAGATTACCTCTTACCAATTTATACATAACGT
AGGTATTTCAAATGAATGAGTTCAAATAGTAGGAAATAGTTTATCTTACATCGAAGTAGGTATGTCATTG
TTAGATATTTAATTTTGAATTTTTTGATAGTTTTTTTATAACTGCAAATTTTGATTTGAATATTTGCATCAT
TTTCTTGGGTAACTTTGTCATATTTATATGATATATTTTAGGTTCTTTAAATACACCTACCTACTTGAATTA
AATGGGGTGACTAAGTCCACATTTAAACAACATCACATAGGCACTATTTCAGTACAATCGACTCATGCC
ATTTCATAAATCTCATAATGTTATAGCCATGTCTGAATGAACGATCTCTATTTGTACGTATAATAATTAATA
TAATTTAATATTTTGATGTTATTTTAATTATACGAGGAAGATTACAAGCGTTTGTTGACCAATACGAGGGT
AATGTTAGGATTTTAACTACAATAATGTAGAATTTGCTTCAAACAGGGAATATTTTATGTATTTTACTGAT
AAATGCTGCTTTGAACTGAATATCTCCATTGTTATTTATAACTATTTTAATTGATCTTTTGACACTTCTCA
CTAAGCATCACGAAAATAAAACTTAAATTATACT
>TCONS_00037272 geneID=XLOC_007582 scaffold_16:2916109-2918435(-) Splice junction overlap
CCCGTCATTGAGGAGCTGGAGAACGAGGCGTGGGACAACGGCATGGACGCCGAGATGCAGGCGGGC
TTCCTCGAGTTCCTCAAGATGTCCAACCAGATCAAGCGATAACCGCCCCTCCCCCCAACGCACGACAG
ACCAAAATGTCTCTACCGATATATTAGGACAATGAAAACAATAAAAAAATATATAAAAACGATATAAAC
CCTAAAAAGATATTATTAGATATCAAGAGATGTATTGCTCGTTTATGTAGTGGTGGATTACAACTGTGTA
GGAAATTATGATGGCATCTCGGGCCCCGTCTTGTATTATAAGGATTATAAGTTGCAGACTAAGTTGAAAT
GCTTATATTTGGTGTAAGTATTCCTTTACTTTTAATTGATTTTTTATTATCTTAATAATTATAATGTTAATAA
TGCCTGCAGTTTGGTCTGTCGTCTTAATGGTAATAGATGTTTACGTATAAATAATATTGATTTATAGGGTA
CAAATCGCGATCGCATCCCTGTCGTTAGTGTGTGTGGTAGTGTGCATGAGTATCACTGGTTCGAGATCA
TTTAGACGTAAACATAATGCCGGATGAATCATTTTTAAAAAGTTTGTAACTCTGGACTAAATGTAAACA
AGTCTGTAGATATACTTATGTAAGAACTTTGCTTTCGTCTATTTGATTTTCATCTGTGCCAGATGCATTTA
AATCTATTTGTTTACTTGTTAACTTTACACACGATGAAATGAATTTAAATTAGGTATTAGTTGTTATTCGA
ATTTGATTTCTTATTTGATTTTAGAGTCTTTAGGCCATATGTTGTAGCCATCGTCTCTTGTTTACATTTCGT
CTATTAATTTACTGAGACTAATAATATTTTGATGATCTCGTAATTTCTCCATTTATTTGTTTCTCTCGTGCT
CGTGACATCGATTGCAAATGTTGTGCCTATTTGCAGCTGCTTCCACGTATTTAGCGTAAAGGCAAATAG
AAAATATTATTAGAGTTTTGCGTGCTAATTAAGCGCACACTTATCAGTCTTGTAGATTTTAGCAGGAGTT
TAATATTTTCATTTGTCTTTCCGTATTAAGATGCTAACAATATATTTTATCACCATTCTCTCTAAACCTAGG

CAACTGATAGATGCAAGACACAGAAATGCAACTTTATTTGGCGGCTCGGACCTAAGAGCAATTAGTTA
AGTATACAAATCAATATTGCAATATCTCCACTGTGGTATTTGGCAGTATGGAACCATTATACATGCATAAT
AAAAACATTAATATAGCGTTACCGAAGGTTGCCTTTGAACTGCCCAGCGGATGTAACATTACGCGTTCA
TAGTTATGACAATACTTAAATAAGCCAGTTCCAATGACGACCCCGTCAAATGCCCTAAGTCCTTAATGC
AATCCTACATCCTCTTGTTCCACAATTTCTGAGGCATACAGTTTTCCTTCCAGAACTTTCGTTTGAGCAC
AATACTTACAAAGTGCAAAAACCAAAGTGTTTCTGGTAGTCCAATAAATGTTAAGACTGCCTTATCGAA
TATCTTAAATGTTGTACGCGGTTTATGATGCCTTATATCAAATCTATTAAATTCATGGAAAATTATTGTGAT
TGAAAGTTTATTGAGAATGCCTCCTGAATGAGTGGGTTGGATTGTAGATTAAATAAATGACCTAATTGC
ATACGTTCTTTGCCTAAAATATATATACATATATTACAATAAAATGAAGCCGGAAATAAATCTCAATTTTG
CTATATTAAAGATACCAGAACTTTTTGAAATATATTTATGCAATACAATAATTTATTTCAGCAATTAGGAG
AATGTTTGTAATATTCCATTGTATTTTTGTTGCAGTGATATTATGAATGTTATAAGAATAAATGGATATTTT
AATAAAAGTCATTTAAAT
>TCONS_00037986 geneID=XLOC_007508 scaffold_16:3024917-3025271(+) Intergenic
CAATTTTGTTTACGGTAAAAAGGTTTTTGTTGACCTTTTCAATTACTTATTTAATTTAATTTTGAGCTTTC
TTTAGTTACTCTCAGCCATAATATTAAGAAGTAAAAACCATATTTGGTGCTATATATTTAATCCGGGGATT
CAGTTGATTTATCGAGGTTTCATTTCATAAACGTTCTGTGAGAGTTTAGTGAGGAAAAATGCCAGGGT
>TCONS_00037821 geneID=XLOC_007510 scaffold_16:3039538-3040582(+) Unclassified
CAACTACGCCACAGTCACTATGACGAAATTGTAAGAAATTAACTACCTAATATACACAGTAGTAAAAAC
AGCGAATACAAACAATATCGTACTGCTCAAACACTCTTACTTATAGAACAACAACATGCACTTATCAAA
ACACACGTCCAATACGTTGTAGTTACAAGGTTTTTACTAGATGGCATCACTGTGCGGACCGTGCCTGCA
TACTTTTAGGCGCAGGCCACTGTACATACCCGTGTGGGGTATCAATGGAAAGGTCTTTTAAAATGATAT
AAAGTTTTCTAAAAAACATTTTTCTTAAAGTGAACGGTTTTTGAGATATCAGCTCTCAAAGTCGTAAAA
AGTATGTCCCCCCCCCTCTATTTTTATAACTACGGGGTATAAAATTCTAAAAAAAATAGAGGTGATGCAT
GCTAATTAACTCTTTCAACGATTTTTGGTTTGATCAAAGTATCTCTTATAGTTTTTGAGATAGGTTGATTT
AACTGTAATTTTGCTGCTACGGAACCCTTTGTGCGCGAGCCCGACTCGCACTTGGCCGGTTTTTTCGTT
TTCCCTTTAGGTAGGTTTTGGTTTTCCCTTGTTACAAAAAACCTCAGCATAAAACGCAGAAAATAAAAT
GTGTGCATTTGACAGCTGTCATAAGGCTCTCATGCTATTTAATTAGTCTGTGATTATACTGGCTTTGCTTT
GGCTTGGCTTGACTTGACATTGAAAATAACCAAAATATTTTGCTTTGCTTTGCTAAAATAAATTTAAAAT
GTATTTCTTGGATTGTGTGTCCAATAACTAGAACTCCTGAATCAGACCATCAAATAATCAGTTTCTTGCT
ACAATGGAAGAGACTAGTGCGGGTCCTCCGCAGCCGAAGCAGCCTCTTAATGATGAAACTATTGAAG
GAGCATGGATAGTGAGCCAGTCGAAGAAAACCGGCCGTAGTTACTATTTCA
>TCONS_00037201 geneID=XLOC_007511 scaffold_16:3070122-3071351(+) Unclassified
CTCATACAAAATTATTCAAAATTACCAAAAAAAATATTTTCTAAATTATGAAAGTAATCTGATTTCCTCTA
CAAATGAACCATATGAACCCTTAACCCTTAACATTGAATGAATAGGTATTAACCTAAAGAGATGGCATT
GGTGGTGGGCACCCCCTTCAGCATCATCTGGGCAACCTTCTGGTTCCTGATGCTAATTCTGGTCGCGTT
CTGGGTCGCACTGATAGGTGGAGTTATGTATGTCCTCATAGTGCCGTACTCAGCATTTGCACCTGATGC
TCTAGCGACATGTTTAAATTTTCTCTTCAAATGTATGCAATTTCCACTGTATTGCACGGTTATGATGGCA
GATGGAAAGCCCTGGGATGGGCCCAGCTGATGTAATAACAAGGCACATAGTAGGTCGGTCTCCACAGA
ATTAAATAAACGTACGTTGTCTGAAAATTATGAAAACTTTTCACTACTTACGTACCTATTCCGATCGGTA
CCTAATATTTATAAGCATTTCATTCATATTTTCATGACGATTTTTAATACGTATTTTGGACGAAATCACATA
TAAGGTACCTCAACTTACTAAATATTACCACTGCTATGTGTACACTGAATCTAGGACACATAGAAAGTA
AAAAATTAAAACATAGTTAGGTAAGCAAGTAGAAACAACCAGTGGATCAAAAAAAACTTTAAACTTTA
ATGTAAAACAACACAAAACACTCACTTAAAACTAGTTAAATAAATCAAAATTTACAGGCCACGCACCA
TAACCATATTGACTGAATGAAACAGAAAGACAGACGGTGAGACGCGTCTTGCTGTTCGATTCAGTCAA

AGAACGGTTATTCAATTGATTTTCCTGCCTTCATCATCTTTGCGCAGTAGAAAGGAAATTCTACTGCTTT
TAGTAAGAATTTTG
>TCONS_00037990 geneID=XLOC_007512 scaffold_16:3072625-3073505(+) Unclassified
TAGGTACTAAACTATGAAACAATAAATATGACGTAAAACTGAATTGAAAATTATAAAGAAATTATAAAC
TAAGTATACATTCAGTTTATTCGAGTTACACTGACGAGGTACACGAGTTAAATCATTCAGAAAGTTATAT
TTTATAAATATTATGGAATCTATACTTTGGTCGTTCCTTTGGTTTCTCATACTGATATTAATTGGGTTTTGG
ATTGGGATTGTTGCTGCTATCGTTTATGTGGCACTGTTACCTTATACAGCTTTTATACCATCCTTATCGCC
TGTCGTGTTTGTCTTGTTTAAGGGTGTCCAGTTTCCACTACATTGTACTAATATGATGATAGCTGGTAAA
CAGAGTTAATGGAAATCCAAAGTGCTATTGAAAGTCAAATTAAGTACCTTTTTCCCTAAATCATTTAAAT
TCCTAGTACCTACTTGTCGAAAAACTTCCCAATAAAATGC
>TCONS_00038322 geneID=XLOC_007519 scaffold_16:3487270-3488549(+) Intergenic
CGGCATGCGCATACTGTACCCCGCGCAGAACAATGTCACGCTCCGAATGCTGCTGCTCGAGAAGCACA
AGACGGGCTACCGGCCCACGCCGCTCGACGTCAAGTACAAGCTGTGCGAGCTGCGCACCAAGGACA
AGTACTTCCTGCCGGCCATACAGAGGTTCTGCCCGCAGGATCTAAGCAAGTGCCCGTTTCTGGGCGTA
ATAGAAATAAAGGAGATGACCTTCGAGCCCAAGATGCTCCCCCTCAAGTACTTCCCGTACCAGCACGT
GCGGCTGGACCTGACCTGGACCG
>TCONS_00038639 geneID=XLOC_007783 scaffold_160:80629-83936(+) Exonic overlap (+)
CTGGTTTCCTGGTACTTTTGGCAAGAATTGTTAACCAACCAGTTAAATATTTATAAACAGACGTGTGAA
TAGATTTTCATAAATTTAAGACAATGTAAATATTTTACTGCACAAACTCTGCCTAAGTATACTAACACAT
AGTGAGTAAAAAGACGTTGAAAATAAGTGCAATAGTGATATCAAAATGGATGAAATTCAACATCAACA
CTCTCCTGAATTGGAACCTTTAGAAGGTAAGCATCATAAAATATAATATACCCAACTGTTTGTAAAGTTT
GTTTTGAAACTTATCGACATCGATATTGTTTTCAGTTTTTATTCGGAGTTTAATGTACTAGTTATATACAAT
ATTTATTATCCTTCAAACAACTATTTATTTTAACGCCGTTAGTACACGGCAGGGCTGAAGGCCCCGGATA
CTCCTGGATTTGATGCCCAGCCGGCCACAATTACTTGCAGTAACATAGCTCACCCAGAGAGGGTAAAC
AAGGGAG
>TCONS_00038561 geneID=XLOC_007785 scaffold_160:127499-128088(+) Intronic
AAGAGGAGAGGCATCCCGCTCAGCTTTAACGAAATCTGGCAGTACCGAGCCAAGCAGGTCAAAGGAG
TGAAGTGCAATCTTTAAATATACTTAATTATATAACTTTTTTATTCCTATTCCCACACTTGGAAACGTGAA
ATTTATAAATTATCGCTATAAGTTTTGGATAAAACTATAAAAACTTACTTTATGGAGATGGAATAACGAAT
TTGTACATGACATTGTGTATG
>TCONS_00038489 geneID=XLOC_007787 scaffold_160:160134-162130(+) Exonic overlap (-)
CTCGGGATGAGGCTCTGGCTCTGACTTGGGTTCGGGGGAATGTTCTGATTTTGGCTCAGGACTAGGTT
CGGGTTTGGAATTGGGTTCAGAAGTAGGTTCAGGCTCAGATTTAGGTTCCGCGCTAGGTTCAGGCTCA
GATTTAGGCTCGGCACTAGGCTCTGGCTCTGCGCTGGGCTCTGGTTTGGGTTCAGCTGAGGGTTCTGG
TTCTGCGTTGGGCTCTGGGTGTGGCTCTGGCTCGGCCTTAGGTTCTGGCTCTGGCAGTGGCTCCGG
>TCONS_00038698 geneID=XLOC_007815 scaffold_160:591230-597828(-) Splice junction overlap
AACTTCGGCTGATAATGTTCGCCAAATTTATCGCTTAGAACCCGGTGACGATTCGTTATACGTTCGCGA
GAAATTTGTTATCAACTAATTATTTTTGTGAGCGAAATCGTTTTTTAAAACAATTTCAATAAATTGACAT
AACTTTTAAGTGAAATACGAAAGTTTAATTAACTATAAATAACCAGTTATTTAGTAGGTAACTAACTTTT
GTAACAGCAACTACTCGAACCCAAGTGCTCAAAACAAAGACGGTACATAAAGTTAAAAGACAGTGTA
CATAAAGTGGTACCAGCCATGCTAGTACCTATCAGACTGTAGTGCGGTGCTGGTGTGGACTCAAGGTA
CCAGGCGCGCAGAGCCGCCAGGAGATGTCGGAGCCGGCGCGGCGCGGCGCCGCCTACACCATCGAC
AACATCCTCGGACGACACTCCCCGGACGAGCCATCGTCCCGGGGTGCGACTCCGGAGTCGGAGTGCG
GGCGCTCGGCCTCGCCCCCCTCGCCCCGCGCGCCGCCCCCGCGCTCGCCCTCCCCGCAGCACGATGAC

GTCACCAGCCGCCCGCGCAAGGTTGTATGTACATCCTGGATGGCCATGGTTGCCCGATACTTTTGTAG
>TCONS_00038774    geneID=XLOC_007796    scaffold_160:618359-619244(+)    Intergenic
GCCCCCTCCCGCCCCCTGTCTCTGCCTGGTGAAAGCCACTTGAAGCTGAACAAGAAGCAAGGAGGCC
AGACCAGCGAGAAGTTCTCCCTACTACAGCATCCGAGGCCGCTGAGACGACGACAAGACGACAGCGT
GAGATTGGTCGCAGATTGTGACAAGTAGCAAGGAGGATGCTCAGAAGAAGGAGAAAAGAGGTTATAG
TGTTGTCTGTGGACTGACAGTTGTGTTTGACAGCTGAGGTATTGTGGGTAGACTTGAGATTATTGATTA
TTGCAGGTTATGTGAGCTGTTGATTGGTGTATGTATGGAGTGGATTGGTCGGTTTTTGTTTACCATCCAT
GTATCTTGTATTGGAATTCGATTAGGCCTGGCGACCAGCGTCACGCCGTAGGCCGCGTCACGATGCTCA
CTACGTCTACGCGCCAACGTCGGCGTGTGAGGGAGACCGCATCAGTACATTTCTATAGTGA
>TCONS_00038775    geneID=XLOC_007796    scaffold_160:618461-619244(+)    Intergenic
GTCTCTGCCTGGTGAAAGCCACTTGAAGCTGAACAAGAAGCAAGGAGGCCAGACCAGCGAGAAGTT
CTCCCTACTACAGCATCCGAGGCCGCTGAGACGACGACAAGACGACAGCGTGAGATTGGTCGCAGAT
TGTGACAAGTAGCAAGGAGGATGCTCAGAAGAAGGAGAAAAGAGGTTATAGTGTTGTCTGTGGACTG
ACAGTTGTGTTTGACAGCTGAGGTATTGTGGGTAGACTTGAGATTATTGATTATTGCAGGTTATGTGAG
CTGTTGATTGGTGTATGTATGGAGTGGATTGGTCGGTTTTTGTTTACCATCCATGTATCTTGTATTGGAAT
TCGATTAGGCCTGGCGACCAGCGTCACGCCGTAGGCCGCGTCACGATGCTCACTACGTCTACGCGCCA
ACGTCGGCGTGTGAGGGAGACCGCATCAGTACATTTCTATAGTGA
>TCONS_00038533    geneID=XLOC_007816    scaffold_160:627577-630487(-)    Intergenic
CACGCTGCTCGTCTAGAAGACTGTGCGTGCACGGCCGGCGCATGGGGGTGTAGCGCGAAAACTGTAG
AACATTTTGTTGATATATGTACTAAGGCAAATGTTGTTTCTTGTGACTGGTGATACCTAACTATTCTTCG
GTGAACGTTCTTAGTAGTGTCGAGATCGCTGTAGAGGCTGACGGGACCTCGCCGGGATCCCGCGGGA
ACTGGGAACTCCGTGATTAGAGAACTCCGGGATTCGGGAACTACGGGATCCGGGAAATACCGCGGGC
CGCCGAATGCGCTCCGCCACTAAAGTAATGCCCTTTTGTCAAGTGTCTGGGTGATGTCGGAAATTGTAT
TGTAATATTTTTTTGTATTGGGTAATTTATTCGGTAAAAGTACATTGGTTTTCTTCTGATGACCTGCATTT
GTTTAAAATATTTTTTATTATTTTTGTAATGTTCTGGTATTTTTTTATATAAAATTTGTAAGTACAATTTCCA
ACTGAACCGGGAACTATTATTTTATTCGACCACCGCCGACAGACCAACACATTACACTAAAACTCCAAT
TTTACCATCTCTGAGTACAAAATTGCCAAAAAAATATATTCAAAGATTATTTTTCCTTTTTCTTACTTATTTA
TTTAAAAAAAACAACTGACATGCTAAATCAATCTTTTCCACCTGCATCTTAACTTTCAGCTCTTACTTTT
TGTCTTTTCATTCCCTTCACTATGTTATGAACAAAAAAAGCTTGTAAAAAAGATTATTTTCTGTATAACA
GATTCTATATTTTTTATTATACTTTTACTTTAAGAAATCTTTATTATTCAATATTAATTTAGTGCTAATATATT
TTCCTCCAAAGATGAATTAACATAATATATTTCATGTCATATATTATGTGAAACGATCGACAAAATGGCCC
CTACATTAATGTTCATTTATTTAATCATATTTTTATTTTATCCCCTGACTCACTATAATGGACTATAGAATGT
ACGGTAAATGGGAAGCGTTCTAGAAAACTCTAGTAGGTACTTATTGTTATCATTTTTCACAAAAGTTGC
TATTTCTAGCTCATGTTGTATAGATAAATATCAATTTTATATCTGTATATATAATTATTTTGTTGTATAAATTA
ATCAAATAGAGTTAGATCTGTAATTTTATTTAGTGTGTTCTGAATGCTGTTGCGTCAGAATCACTTTTTAA
TTACTTTTTTTTTATATTCGCAAGGGTTTATAAGAGCGAAACCCACCAAAAAAATTCATTAAACATTAATT
TAAGTTTTATTCTTCAGTCCGTCAAATTAACTTATTGTAACTTATTTTCGGCTATGATCCTTAAAAAAATG
AAATTTAGCTTTTGTTGTAAATCAATTAATTTTACAGCAAACATTTTATTCCACATTACAGGCCGCTTAAT
TCCATGGAATTATTTTCTTATACAAATTATCAACATCACCAACTAACTTTTGTATTCTTAAGTATTAATTTG
AATATTAATGATCTCACGTCTTTGTCACACAGCACCAATGTAGTAATTTGTCATTCGTGTAATTGGTCCT
TTTATATTCCTCTATTTATTCGCATATTTTTCTAAAAAAATATTATTTATTCATATAGAATTTATCATATATGT
AGAGTAGTTTTGAACGACCAACCAGAATTTAGCGATTTTGTCTGGACGAATCAGAGCGATCATGCTCG
ACATTCAAAACTAGTCTAGATTTTTAATAAATTTGCAAGTCCAATGACATGATTACTCTATTGGAAAATT
TATTCATGTGGACACTCATTTTACCAAAAATATAATAAATATAATGTACCTATGGAAAGGAAAGTCGGAC

AATATCGCGACGAGCACAGTCGTCGCGACACCTCTCTAGACAAATACAACTAGTTGAGTACAGACAGA
TATATTTTTGTTACAACTACTATTGTATCTTTATTAAAAATAAAGAAATTGAAATTTTGTACAATTACGCA
GTTCATTTCTAAAGATACTAGGCTTTTCATTTGTTGAATTACTTTCCACCTCTGTTATTAAATTTGATCAA
ATTCGTGTACAGTGCCGCCAACTAAGCAGACCCTCTGACGCACGATCGCTTTTTATAAGTCAGCTGTCA
GGAGGTCAGT
>TCONS_00038534 geneID=XLOC_007816 scaffold_160:627577-639008(-) Unclassified
GGCGTCTCCACGGCTGATCGTTCGCCCTCCTCCGTGTCGGAAAGCGACGAGGTGGACGTGATAGGCTA
CACGTCGAACCAGAGCGACAGCGAGTCGCTGGTGTCGGGGCGCAGCGGCAGCGGCGACAGCGGCCT
GGGCGTGCGAGCCGTGCGCCGCCGCCGCGCGCCCACAGACACCACGCTGCTCGTCTAGAAGACTGTG
CGTGCACGGCCGGCGCATGGGGGTGTAGCGCGAAAACTGTAGAACATTTTGTTGATATATGTACTAAG
GCAAATGTTGTTTCTTGTGACTGGTGATACCTAACTATTCTTCGGTGAACGTTCTTAGTAGTGTCGAGAT
CGCTGTAGAGGCTGACGGGACCTCGCCGGGATCCCGCGGGAACTGGGAACTCCGTGATTAGAGAACT
CCGGGATTCGGGAACTACGGGATCCGGGAAATACCGCGGGCCGCCGAATGCGCTCCGCCACTAAAGT
AATGCCCTTTTGTCAAGTGTCTGGGTGATGTCGGAAATTGTATTGTAATATTTTTTTGTATTGGGTAATTT
ATTCGGTAAAAGTACATTGGTTTTCTTCTGATGACCTGCATTTGTTTAAAATATTTTTTATTATTTTTGTAA
TGTTCTGGTATTTTTTTATATAAAATTTGTAAGTACAATTTCCAACTGAACCGGGAACTATTATTTTATTC
GACCACCGCCGACAGACCAACACATTACACTAAAACTCCAATTTTACCATCTCTGAGTACAAAATTGC
CAAAAAATATATTCAAAGATTATTTTTCCTTTTTCTTACTTATTTATTTAAAAAAAACAACTGACATGCTA
AATCAATCTTTTCCACCTGCATCTTAACTTTCAGCTCTTACTTTTTGTCTTTTCATTCCCTTCACTATGTTA
TGAACAAAAAAAGCTTGTAAAAAAGATTATTTTCTGTATAACAGATTCTATATTTTTTATTATACTTTTAC
TTTAAGAAATCTTTATTATTCAATATTAATTTAGTGCTAATATATTTTCCTCCAAAGATGAATTAACATAAT
ATATTTCATGTCATATATTATGTGAAACGATCGACAAAATGGCCCCTACATTAATGTTCATTTATTTAATCA
TATTTTTATTTTATCCCCTGACTCACTATAATGGACTATAGAATGTACGGTAAATGGGAAGCGTTCTAGA
AAACTCTAGTAGGTACTTATTGTTATCATTTTTCACAAAAGTTGCTATTTCTAGCTCATGTTGTATAGATA
AATATCAATTTTATATCTGTATATATAATTATTTTGTTGTATAAATTAATCAAATAGAGTTAGATCTGTAATT
TTATTTAGTGTGTTCTGAATGCTGTTGCGTCAGAATCACTTTTTAATTACTTTTTTTTTATATTCGCAAGG
GTTTATAAGAGCGAAACCCACCAAAAAAATTCATTAAACATTAATTTAAGTTTTATTCTTCAGTCCGTCAA
ATTAACTTATTGTAACTTATTTTCGGCTATGATCCTTAAAAAAAATGAAATTTAGCTTTTGTTGTAAATCAA
TTAATTTTACAGCAAACATTTTATTCCACATTACAGGCCGCTTAATTCCATGGAATTATTTTCTTATACAA
ATTATCAACATCACCAACTAACTTTTGTATTCTTAAGTATTAATTTGAATATTAATGATCTCACGTCTTTGT
CACACAGCACCAATGTAGTAATTTGTCATTCGTGTAATTGGTCCTTTTATATTCCTCTATTTATTCGCATAT
TTTTCTAAAAAAATATTATTTATTCATATAGAATTTATCATATATGTAGAGTAGTTTTGAACGACCAACCA
GAATTTAGCGATTTTGTCTGGACGAATCAGAGCGATCATGCTCGACATTCAAAACTAGTCTAGATTTTT
AATAAATTTGCAAGTCCAATGACATGATTACTCTATTGGAAAATTTATTCATGTGGACACTCATTTTACC
AAAAATATAATAAATATAATGTACCTATGGAAAGGAAAGTCGGACAATATCGCGACGAGCACAGTCGTC
GCGACACCTCTCTAGACAAATACAACTAGTTGAGTACAGACAGATATATTTTTGTTACAACTACTATTGT
ATCTTTATTAAAAATAAAGAAATTGAAATTTTGTACAATTACGCAGTTCATTTCTAAAGATACTAGGCTT
TTCATTTGTTGAATTACTTTCCACCTCTGTTATTAAATTTGATCAAATTCGTGTACAGTGCCGCCAACTA
AGCAGACCCTCTGACGCACGATCGCTTTTTATAAGTCAGCTGTCAGGAGGTCAGT
>TCONS_00038786 geneID=XLOC_007816 scaffold_160:629014-630742(-) Intergenic
GGACGTGATAGGCTACACGTCGAACCAGAGCGACAGCGAGTCGCTGGTGTCGGGGCGCAGCGGCAG
CGGCGACAGCGGCCTGGGCGTGCGAGCCGTGCGCCGCCGCCGCGCGCCCACAGACACCACGCTGCT
CGTCTAGACTGTGCGTGCACGGCCGGCGCATGGGGGTGTAGCGCGAAAACTGTAGAACATTTTGTTGA
TATATGTACTAAGGCAAATGTTGTTTCTTGTGACTGGTGATACCTAACTATTCTTCGGTGAACGTTCTTA

GTAGTGTCGAGATCGCTGTAGAGGCTGACGGGACCTCGCCGGGATCCCGCGGGAACTGGGAACTCCG
TGATTAGAGAACTCCGGGATTCGGGAACTACGGGATCCGGGAAATACCGCGGGCCGCCGAATGCGCTC
CGCCACTAAAGTAATGCCCTTTTGTCAAGTGTCTGGGTGATGTCGGAAATTGTATTGTAATATTTTTTG
TATTGGGTAATTTATTCGGTAAAAGTACATTGGTTTTCTTCTGATGACCTGCATTTGTTTAAAATATTTTT
TATTATTTTTGTAATGTTCTGGTATTTTTTTATATAAAATTTGTAAGTACAATTTCCAACTGAACCGGGAA
CTATTATTTTATTCGACCACCGCCGACAGACCAACACATTACACTAAAACTCCAATTTTACCATCTCTGA
GTACAAAATTGCCAAAAAATATATTCAAAGATTATTTTTCCTTTTTCTTACTTATTTATTTAAAAAAAACA
ACTGACATGCTAAATCAATCTTTTCCACCTGCATCTTAACTTTCAGC
>TCONS_00038879 geneID=XLOC_007859 scaffold_161:123640-124652(-) Splice junction overlap
CATGACAGTCCTGTGGATGAGGTTCAAGTGGCAGAGTTCGGCGGAGACGACTGGATGGACTGAGAAG
ACTAGGATCAACCAACAAGTTGCCTTACACGCTAGTTGATAAGTATCAAATTCAATTTATGCGTAGTTA
GATATTTGTGAATACAAAAACTAGACATTGGCCTTGTTCACTTAATTCACGTTCGCACGTCTGTTTTGTG
TTCATGAATATGATCAAATTATTTATTAGAGTATTGAAAGAGTATTTACTATAATATATTATTATTATGAGG
CTAGCTTGCCAACGATGATATAAAGTGATGAGTATAATAATTATTAATAATGTTAATGTTGTGCAAATATT
GCAAAT
>TCONS_00038880 geneID=XLOC_007859 scaffold_161:128737-130272(-) Intronic
AAAATAACATCTGCTAATATTTTTTACTGTATTTTTACTTTTACATCGGATTCGGCTACCAAATTTCAATAT
TAGGATTAATATACAGTGTCTTTAAAAAACTGTGATACGTGGATTGGTATTTTCTGCATATTATGGTTCAA
CAAGGCTACTACGGCGCACCTCAACAGCCAGATTTTATAAGAAATTAATCGATAAATTGTAAGGAGCGT
CTTTACAATGCATGGTATGTCTTTAAACGCTTTTTTTTCGTAGAAAATTGGGTAGTAACATGTAAAAA
>TCONS_00038984 geneID=XLOC_007860 scaffold_161:170741-173990(-) Splice junction overlap
CTGTGACAAAACTTGGACAACTCTTGGGTTTTTCACCGACACTGCGCGTAACGGCACCCAACTCTCGC
ACGTGTAGCAAAAACTGAATACGAAGTTTGTATAAAAAAGTATTTAGACATGTAATAAATTATTACCTTA
CGTATTCACAATTAACCAAAGACGATGACTTTATAAAGCCACTTCCAATTTAAAAAAAAAAAACATTCG
AAGATAATTTTGAATACGTACGAGTAAAATTGTAAAAAATGTGTTCCTGAAATTTAAATAACGACTGATA
TTAAATATTGTAAATATTATTATTCAATTCGAATGATAGCAGTTAAGTTATTGTAAGAGAAACGAATGTTA
TACTTAATTATAATATTGTAAGTAGGTACTTGGCATTATTTTGTGTTTTTTTAATCTTAAGTGATCTGATAC
TAGAACCTTTTTCACAGTGTAATATAAAAAAAATTTAAATTGAGGCATTTCAGTATGCATGTAGGTATAT
TAAATTGAAACAAACTTATAAGAGCAAAAAATTATAAAATTCAAAAGTACTTATACCTACACTTTTGTC
GCCGGTCCCTGGGTCAGATAAGTTTATGTCATTTTACTCTGTTGATATTAAGTTAGTACCTATATCACGTT
TAAATGAGCCGTCAACCTCAAAATTATCTTTAAACCTGCGATAAAAATACATGATGTTCAAAAAAACCT
TTAAAAGTCCGTGCCTCGTTGAGAACCTATCCGATAAGGTTGTCGGCAAAGCAATTTCGCTATCTGTAC
ACATCACATCATTCTAAATCTCATTTGCAATTAAATCTTAGGAGAATCATACATATCCCCTTTCATAATCG
TATTTTTAACACAGCATCCTGATATAAAATGAAATATTTACAATATTAATTTTACCTCAGAAGCCAGAGA
GAAACCGCAACGCATTAAAAAGCTAGATTGTATAAAAACAGGACGCATATCTCTCGTCTCTCTCTCGCG
CCACGCGACTGTTGCCCTCCTTGTCTGGGCGCCTGTCTTCAGCACTGGCCGTCCCTGTCTCTCGCCAC
GCTTATACAGCTGTCTCTGTCGGCGGGTGAACGGTCAGCGTATCCGTTCGGACTGGTCCGACGAGGTG
ACAGGTGTTAAGAAGCTGGATACAGAAGGCTCAATGTACTTACATTCCTTTGCCGGTTGCGCGTCTCG
AAAAAAAAACAAAAGGGATTGTCTTTGTCCGAAGTTTCAAAGTTAAAAATTGATTTTTTTTTGCTACCT
TTGCTGTAATTTATAAATATGTGGATTTGAGGATTTTATTAAATAAAAACATTACCCTCCTCCTTCGGCAG
TCGGGTAAAAATAGTCCAATAATAAAAATAGTAGCAACAAATGACTCGCATAGCCAGTTTAATTGTACT
TAAGTATTATTGTTTTGGCGCGAAAGTTTTTTTTTTTGCTTATCTCAAACATGCATTCGACACACTTACA
GAAACAGCCACAATTATTATAATTGCACCAGATGCTGGCCGGTAATGTTGCGTGATTAAAAAGAAAATA
CTCTTGTCTTGAAAAAATATGGCCTTCTTATTTTGAATAAATAGCAGAAATTGTCATATTGTGTATTTCAC

CAAGCAGTTTTTTTTTATTTTATTAAGTAATTGTTTCAACTTGGAATGAATAAACTACTGTCAATTAAAG
CTAAACATATCAACATGTAGATTTTGTGATAAAATTACTTAGTAAAATTTGTTTTTATAGTCCCTACTCGC
GAAAGGAATCTTTATAAATGATTATACCTACTTGATACTAACACAGTTATATCTACCAAAAATCTTGATAC
GCGTTTTAATACTGTTAAATAATGCCACAATCCAAAAATGTACGCA
>TCONS_00038964 geneID=XLOC_007860 scaffold_161:170741-174095(-) Splice junction overlap
CGCCGCCTTACTCCGCCGTCAGGAGCTCCGTCGTCATTCCTCAAGGTAACGGCACCCAACTCTCGCAC
GTGTAGCAAAAACTGAATACGAAGTTTGTATAAAAAAGTATTTAGACATGTAATAAATTATTACCTTACG
TATTCACAATTAACCAAAGACGATGACTTTATAAAGCCACTTCCAATTTAAAAAAAAAAAACATTCGA
AGATAATTTTGAATACGTACGAGTAAAATTGTAAAAAAATGTGTTCCTGAAATTTAAATAACGACTGATAT
TAAATATTGTAAATATTATTATTCAATTCGAATGATAGCAGTTAAGTTATTGTAAGAGAAACGAATGTTAT
ACTTAATTATAATATTGTAAGTAGGTACTTGGCATTATTTTGTGTTTTTTTAATCTTAAGTGATCTGATACT
AGAACCTTTTTCACAGTGTAATATAAAAAAAATTTAAATTGAGGCATTTCAGTATGCATGTAGGTATATT
AAATTGAAACAAACTTATAAGAGCAAAAAATTATAAAATTCAAAAGTACTTATACCTACACTTTTGTCG
CCGGTCCCTGGGTCAGATAAGTTTATGTCATTTTACTCTGTTGATATTAAGTTAGTACCTATATCACGTTT
AAATGAGCCGTCAACCTCAAAATTATCTTTAAACCTGCGATAAAAATACATGATGTTCAAAAAAACCTT
TAAAAGTCCGTGCCTCGTTGAGAACCTATCCGATAAGGTTGTCGGCAAAGCAATTTCGCTATCTGTACA
CATCACATCATTCTAAATCTCATTTGCAATTAAATCTTAGGAGAATCATACATATCCCCTTTCATAATCGT
ATTTTTAACACAGCATCCTGATATAAAATGAAATATTTACAATATTAATTTTACCTCAGAAGCCAGAGAG
AAACCGCAACGCATTAAAAAGCTAGATTGTATAAAAACAGGACGCATATCTCTCGTCTCTCTCTCGCGC
CACGCGACTGTTGCCCTCCTTGTCTGGGCGCCTGTCTTCAGCACTGGCCGTCCCTGTCTCTCGCCACG
CTTATACAGCTGTCTCTGTCGGCGGGTGAACGGTCAGCGTATCCGTTCGGACTGGTCCGACGAGGTGA
CAGGTGTTAAGAAGCTGGATACAGAAGGCTCAATGTACTTACATTCCTTTGCCGGTTGCGCGTCTCGA
AAAAAAAACAAAAGGGATTGTCTTTGTCCGAAGTTTCAAAGTTAAAAATTGATTTTTTTTTGCTACCTT
TGCTGTAATTTATAAATATGTGGATTTGAGGATTTTATTAAATAAAAACATTACCCTCCTCCTTCGGCAGT
CGGGTAAAAATAGTCCAATAATAAAAATAGTAGCAACAAATGACTCGCATAGCCAGTTTAATTGTACTT
AAGTATTATTGTTTTGGCGCGAAAGTTTTTTTTTTTGCTTATCTCAAACATGCATTCGACACACTTACAG
AAACAGCCACAATTATTATAATTGCACCAGATGCTGGCCGGTAATGTTGCGTGATTAAAAAGAAAATAC
TCTTGTCTTGAAAAAAATATGGCCTTCTTATTTTGAATAAATAGCAGAAATTGTCATATTGTGTATTTCACC
AAGCAGTTTTTTTTTATTTTATTAAGTAATTGTTTCAACTTGGAATGAATAAACTACTGTCAATTAAAGCT
AAACATATCAACATGTAGATTTTGTGATAAAATTACTTAGTAAAATTTGTTTTTATAGTCCCTACTCGCGA
AAGGAATCTTTATAAATGATTATACCTACTTGATACTAACACAGTTATATCTACCAAAAATCTTGATACGC
GTTTTAATACTGTTAAATAATGCCACAATCCAAAAATGTACGCA
>TCONS_00038988 geneID=XLOC_007865 scaffold_161:388923-391978(-) Intergenic
GTCGATGGCTGGTCAGCCGCTGGCGAGGCCCTGTAAACCCATATATCGCAAGTGGTTAGCCGGTGATTT
GCTGCGTGATTGTGAAATTGTTTGTTTTAAAGAGCGTGCCTCCGCTCCGCGGTTAAAGTTAGTACAGTA
CACACCTAGCCGCGAACGGCGGCGCCACCTCCGTCGGCCACGGCGCATACGGCGCCGGCAGCGGCAC
CGCAAATGGTGGCAAAGGCCAGGCCGGGTACGGCGCCGGTGCGGGCACCGAAACATACCGGTATCTG
AAATTTAATAAAAAAAATATGATTATAATTAATTATGATACGGATTCATTTGACATAATTATATCCACTATA
CTTGTCTTGCCACTGCCAGTATAAAACTTTATTATCTACCGATGACTGGTAGTCACATTTAGCCACCTCT
TCAACCGAACTACCTACTTAACTTAAAGAGTCGGCCCATAAATGTAGTGAGTCACTACATTTACTAGCC
GACAGGGGCTAAGTTAGCAAACAATATCGTAGAAAAACAAATGAACAGAAATTAACCTTGCTATTTAG
CCAAATCACACATTACACCATCATATTCTTTCTTCATGCTCCCTACTGATAAAAGTACTGCGGACAGTGC
ACTGATCTTGACAACTTGATACCCTTTGAACACATCTGTAATCTCAGATTTTCATCTGGAATATTTCCAA
AGATCTGGAAAATTGCAGCTGTTACACCAATACATAAAGCCAGTGATAAAGAGAACCCTTCTAACTATA

GCTATAGGCCTAAGTCTTCGCCCACTTTCCTATCAAAACTACTCGGCAAAAACTTACTTGTAAAACCAA
CGCGGACGTGGGAAAATGGCGTACCAGCCCGCATGCTTGATGAAATCTGCACCATATTTAAAAAAACA
CCCTCTGTGCTAGTCACTTATCGACGTATCATTTTAATTATGGGCATTACACGTAATTTTCCATAATGGCC
AGGCCATTTTGAAATTCAACTATTACGAATACGTACGAATAAACTGTGATCCTGGCCGACAAGAACCA
GATCCTACCGGCTACTACTACTCAAATTAACTAAGTACAATAATAAAAGAATGAAAGAAACTTACTCAT
CCAAGTCATTAACAATAAATTAAATTTAAAGAGAGAAATAAACTTTTCAACTTACTTTTCCATAGTAGTA
GTAGTCACTTATGGCTTCACAGCATAGACTAAATAGAGATACTATTTCGTTTTTGGTAGGTACTATTTCC
AATTCCATGCTTCCCGTTTATAATATTCTCTATGATTTTCACCGCGAAGTACTTTTAGTTTAGGCCGTCTC
CGCACCTCGATTATTTAACAATAAA
>TCONS_00038889 geneID=XLOC_007866 scaffold_161:402194-403452(-) Splice junction overlap
TGACGCTTCAAGAGGACCAAGAGGTAAAGATATTGGAGAAAATCAAGGTGAAGATGGAGAGAATCAA
GGCGAACCAGAAAAAGATACAAGGCGCTCTCGCTAAGGACCCCATACACCACGACCGAGGAAGTGG
CTCTGAAACTGAGTCTGAGTCGGTTAGGCGCCGCGTTTTTGCGAAGAGAGCCCAAGGTATTTGAATTA
GTGCGTTTTAAACAAGTTTGGTGTTGGACCATTACGCTTATGTTCGTCCATCCTATACATTTCATGAATT
GTGGTGTCCCTGTACATGTTATAATGTATATTTATTTTCCAGCTAAAACTTTGTCCCAATCTTCAACGGGC
ATGGTATTGTTATGAGCACG
>TCONS_00038840 geneID=XLOC_007867 scaffold_161:456303-457950(-) Intergenic
TAATAAAAAAATCTAAAATATAAAAGTACCTATATTCAATTCGAACCTATGAACGAAAAACGTTACAATT
AAGTACTTAAATTCTAAGCTGCCCGTAAAATTTACACATCAAGGAACTTTAACAATTTAAATATACAGA
GTATAAATTTAATATTAAAAACTATATTAAAGAAAAATATACATCATTAATATAAATTTGGTATTTGTATAA
GGTGTCGTTAATCTTGTAATGCGAATAAGCAATGAGTATTCAACCGAATTACAAGAGATTGTCACAGTA
ACCTCCCAGTATTATACAAGGTCAAAGGTAAAGTAACTTAAAAGTTCAAGAAAAAGGTTTACGTCCTA
CGTTACCTATGACTATATTTACACCCTGTCTCAATTATTTATTAAATGACTAAACATTGAATAAACGTTTT
CTTTCTTTCTTTCTTTCTTTCAAAATGTACTCTAATCTACGTATATATTTAACCATTAGGAATGTACTGTCA
TTAGGTGACTGAATTTATAAATCTTCCACAAAATAATAAGCCTGCGTAGGTTATTAACCGCTATTTTGTT
CCCAGACGTTTGATGTATCCGAGAGAAATAAAATAACCATACCTGTTGGCCATATACCTACTACACACTA
TTTAAGTAGCATAGGTCCTATTACAAAAATAACGATAACGTGTAGGTAAGCTTTTCTGTAAATATGTTTT
GACCTGTGTTCTTGGTCTATTTTATTAAAATAATAAGTTCATATGCCTATACATATTATATGAGCCATAACA
AACTAAGACCACTGAAAAACACGATATGTGGTTTATGTTTTTACATAAATATAC
>TCONS_00039047 geneID=XLOC_007920 scaffold_1610:958-2272(-) Splice junction overlap
CAGTCTTGGCAAGCAAGTCAGGCGAGCTCCCTATCTGGTTCCAAGCGCCTAGCACCCTGGGTTCGATT
CCCCGACGATGACGATGAAGAGGCCGATGAGGCGGCCGACACGGAGCAGTGGCAGGGGCTCGCGCC
GCTCGTCACCTTCGCATAGTTATACTAAATACTTTAAGATTACGATATAGGAATCGTAGCAGCCATTTTA
AGTTTATATGAACGTTCCGCTCTTAGGTCTTTTTAGATTAAATTGCATTCAATCTAGATAATAGCTATTTA
GTTAATAATGCTCCGTTTGCATTGAAAATCAAATTTGATTTCAACGCAACGGTTTTTTTGTAAATTTTAT
CAAAGCAAGAAGTCCGCTAACTTTGTCGATGGCACAGTTATAAATTTAAAAGTTAATTGCTTTCAATTC
ATTTTTTTCGAACATATAAAATTTACCTACCTCTAAATCTATAATATAACATACTTATGTAAATTTTATTGA
GGCTAATAATTAAATTAAGACCCTTAAGGTACAATCTATAAGTACCCCTTATTCATACAAAAGTAGTTCT
TTGACAGAGTCGGACAAAGTTGTTCAATAATATCTAAAACGTACTCCAACTTTTCTATGAATAAGGGGG
TTACTTTTCTATCTATGGTTTATGAGATGTAAGTGGATAGGCGTAATTATTAATTAAGTTAATTTTAAGTTA
TAAAAATGTAATGAATTTATAAAGTGCCATTTTTATATAAAAAAATAAATAATTGACTGATTTGAAAAGT
TGGTTCACGGGTGATAATTATACGGACAAAACGGTGCTATAAAATTCTAAAATTAATAAACAGTAAAGT
TAAGGGATAATAGGTCTTAAGCTAACAATAATTTGCTTATAAAGATATGGAATGGTGATAATTTGCTCTTT
TCCTTAATCATAAGTAAGTAGGTAGGTACTTGATTACTTAGTTAGGTTAGGTACATTGCAACACTTGATA

TAATAAAAAATATAATTGTATTTTTTCTTACATTTCCGTG
>TCONS_00039375 geneID=XLOC_007967 scaffold_162:101238-103052(-) Intronic
GTATTTACAGTTTTTTTAATATAATATAGTAGTTATGTGTAATACAAATTTTACGCATTAACAATTTGTGTT
GTTGTAAAGTGCAGTCGTTCTTCCAAGGGCACGTATAAAAAACTAGTCTGTGGTTTAAAAAAATCAGA
AATGAAATGACGTATGAAACGTCATAATCGTCACATTGAATGTTGTGTTGATGGTGAATATTTATTTTTG
GACAATGTTTATTACAGAATTTTCATTTAAATACCTATTAAGATTAACGCCCTTCGTTAGTTAATTTGTTA
GTGTAGGCTGTACAATAATTTAAACATAGGTACGACCCAATTATATCATGTTCGTGCAATTTTCCTGCTG
TATATCATGTTCGTGCAATTTGCCTGCTGAAAAATCAACTCCGTCTCAATAAGTTCAAATAAAGTGAATT
AATACAGTAAAAAATAGTTTTATTTTACACGTTTTCTTAGTCAAGCCTCCAAATTATATCTCCAGCAGAC
CAAAACCAAGTCTTCTACCTTCCAACTCATAATGGCTGCTATCAACTGGTACACAAGAACTTCAAGTAC
TACCTTCACAACCGTCTCAAAGCCAAGCTCAGTTGGACTTGCGTTCTGAGGAAAAAGACAGGGTGCT
TGAGCCGGATATCCACCACTCTGATTGGCCGCTTCCTGGGCATAATCGAGAGTCACAACCACAACCCT
ACCTTGACTATCATGGATCTAAAACGGAAGAAAACACAATTTATTGTCTCTCCCCATGAAATGATTCCA
ATGTACTTTATGTAAATGTCTAAAATCTCATTACATTTGTATTTGAGCATACAATAACTTTATAGGTAGTAT
TTTTGAAATTGATAATGTGTGAACAATATACATATGTTTTTAAAACACACATTAAATCAGTAAATTAATCT
TAAATGTTGTTTTAAATGTTAATCTACAGTGTACTGACCATCATTTTCATATCTGAGCACTGCTATTCTGC
TCCGTGTAATGTTGTAGTACATAGTTAGGCATGTGGCAGTATTCATATACAGAAATAGTCAGGATGCAGT
AGATCTCACCTTTAGATGTTATATGAATAAAAACTATAGTATTTCTATGTATTATTCCAGTCAATCTTTTTA
A
>TCONS_00040067 geneID=XLOC_007967 scaffold_162:124073-125057(-) Intergenic
CACGAGTAACGATAGTGAAGTTTCTACCAGAATAAGTGATGAATATACATTACTACCATCACAAAGAGG
AGGTCAGCTTCTATTGTGCAATGGATATCGTTATAATTTCAAACGCAGAAGCTCTCAAGGAAACGGAG
AATGGAAATGCTCAATAAAAAAGTGTAACGCCATTTTAGTAATAAACACTATCGGCATGATAATCAAGA
CAGGACAACATAAATGTTCTCAGGAAACAGCTACTACGAGTAATGAAATAAAATGTATGATAAATAGAT
GCATTGAACGAGCTAAATCTGAAAGTACCCCAGTCTCGACTATTTATACGGAGGTTTTGGAAGAGATG
AAAGATGCTGGGATGGACATTATGTCACAGAACTTACCAAAATTTAGTAGTATAAAGAAAACGATCTAC
AGGCATAGAAAAAAGGCTTTACAAGAAGATTATAGTCCGTTAACTTAAACAGATGTCATCATGTCAATA
CGAAACAAACCTTTGCTTCTGTACAGAAACTATACATATGCCAAAAAATATGGCCACAAGGGACCAAA
GCATAAGTTAAACAGATGGTGTTGTTCATCTAGAAATTCTAAGCAGTGCAAGGCTTTTGTGGTTGCTAG
TGACGAGGGGAGGGTAATGCTGACACTCAGCTTCCCTCAACATACTCATAAGGAG
>TCONS_00040126 geneID=XLOC_007969 scaffold_162:127109-129226(-) Intergenic
TGCCGCTTCTATTTTACCAAAAATACACTTATACCAAGCAAAATCATCGACCTTTGAAGAATCTGAAGC
AAATTGTCAAGTGGTGCTGCTCGTCTGCTCGGTCCAAGGCCTGCAAAGCCTTCGTGATGGCATCTGAA
GAAGGCATGGTTGCGGAAGGTTACACGAGTCACAATCACTCAGAACCATCCTTTTATTTTTCTTCCTCT
GGTGGTGTAGTTAGAATATGAATTGCAGAAAGATTTTGTGAAAATATTCTGAATTTTTTGTACAGCTTTG
TGATAAAACTGTAGATACCAACATGTTCGTATGGAGGTAAGACTGTTAATAACCTTGTGGGCAACGTTG
GTTTATTCATGCATAATTATAGGTTTCATAAATGCATTACGTGCGGCGAAAACGTATGATTTTCACTAGTA
TCCCGGTGATGGTTTTAAACTCTGGTACCCTACTTTCAGCACGCTTTGTATGTTTTACCTTATAAACTTT
GAAAAAGGCTGAATGTAAAAAAAATCATATTTGAAATTAAGCGAGCGCTATCATATCTTAACCATTAAG
GTGGCGAATCACTCTGGAAAGGAATACTAATAAGTTAAATACATTTTATGTTTAAAACTACTAAAATTCT
TCTATAAATCAAATTATCTGCGTCGGAATTTATATAATATTATGATAATAATGTTAATCATTACGGGAACGC
GTCCCGTTAGTCCCGTAGTGTTGTTGCCTGAAATACTTAAATCTCAACATGTATGCTTTTAGCATTTTCC
TTAATTTTAGAGTTAATTAAAAATGGAAATACTAATCATTTCAGCAACCATGAAATCAAATCATAATGAA
AACAAAAAGAAGAGAGCTAGGAGACAAAAGAAAATAAAAGATATTGCGTATTTACGCCACAGATTCTT

AGCAAATGCAAGAAAAAGAAAGTATTTAGATAATTTGACTGCCGAACAAAGAGAAAAAAAACGAGCT
AAGGACAGGGAGTATTACTACAGAAAAAAGGCGCAAAAATGGGATATTTAAGTTACTGGTATGATATG
ACACAACGTGAAAATAAGGAAGGCATGTTAGGAAAGATCAAGGAAAATACAAAAAGAATTACATATC
GAAATTTAATTAAAAAAAGCTATTTAGTGACATATTTAGTGATTTGTTATTTTTTGTGAAAAATGCCCAG
TTATACTGGTTAAGCAAGAGTAATTACATCAAGGAGAGACAAACCACTTCTTTACTACAGAAATTTCAC
GTACGTTCTCCGTAATCCTATAAACACATTAAGAACAGCTCCCGTCAGATGGACATGTTCTTCAAGGGT
CTCCAAATTGTGCAAGGCGTACCTCATGGCCACACTAGATGGCGTCGTCGCAGATAGCTACGAACATC
ACAGCCATCCACACCCAAATATGTATTTCAGTGAAAATGGAACTGTAATCAGAGTTTAAGAAAAAGAT
CCGATTTCGGTGGCATATTTTTTAAACTTTTTACACTGTCACTATCAGTAGTGTCAGTTTTAAAATTTAA
ACTTAGTAGCTAAAATAATTTCGTCAAAAAGGGGTAGACCACTTCTCTACTACCGATGTTTCACTTACA
CGAGAAATACGTCGTTAAAATCTAAGCATCCGGCTGCAAAATGGATATGCTCGTCGAGAGTGTCCAAG
TCCTGCAGAGCCTACCTGATGGCCACTAAAGAAGGCTTCGTCACAGGGAGCTTCGAGGCACATAGCCA
TATGCCCCCTAAAATTTTCTTTACAGAAACCGGACATACTATCAGAATTTAATTTATGATGTATTATTGTT
ACTTCTCTGTATAGTAGACGATGATGGTGACGTATGATAACTAGCGTTACATTAATAATCATGATTATTCA
GTATTCACTCCAGC
>TCONS_00040068 geneID=XLOC_007969 scaffold_162:127640-128577(-) Intergenic
GGAATTTATATAATATTATGATAATAATGTTAATCATTACGGGAACGCGTCCCGTTAGTCCCGTAGTGTTG
TTGCCTGAAATACTTAAATCTCAACATGTATGCTTTTAGCATTTTCCTTAATTTTAGAGTTAATTAAAAAT
GGAAATACTAATCATTTCAGCAACCATGAAATCAAATCATAATGAAAACAAAAAGAAGAGAGCTAGGA
GACAAAAGAAAATAAAAGATATTGCGTATTTACGCCACAGATTCTTAGCAAATGCAAGAAAAAGAAAG
TATTTAGATAATTTGACTGCCGAACAAAGAGAAAAAAAACGAGCTAAGGACAGGGAGTATTACTACAG
AAAAAAGGCGCAAAAATGGGATATTTAAGTTACTGCAAGAGTAATTACATCAAGGAGAGACAAACCA
CTTCTTTACTACAGAAATTTCACGTACGTTCTCCGTAATCCTATAAACACATTAAGAACAGCTCCCGTCA
GATGGACATGTTCTTCAAGGGTCTCCAAATTGTGCAAGGCGTACCTCATGGCCACACTAGATGGCGTC
GTCGCAGATAGCTACGAACATCACAGCCATCCACACCCAAATATGTATTTCAGTGAAAATGGAACTGTA
ATCAGAGTTTAAGAAAAAGATCCGATTTCGGTGGCATATTTTTTAAACTTTTTACACTGTCACTATCAGT
AGTGTCAGTTTTAAAATTTAAACTTAG
>TCONS_00040038 geneID=XLOC_007969 scaffold_162:134573-138168(-) Intronic
GTCATTTATTGCACAAAAAAATATTCCAACTTAAATCAGGATTAATAAAGTGTAGTGAGTGTTATTTCCC
CGTAGTGATATATAAGATTTGTGTGAAAGTGACAATGTGTCCATAACTGCTTAAATACATAATCATAAAT
TGATTATTTGTGATGAAATAGTGATGATGTTGATACAGAAGGAACTTCAGGCATGCATTATGATGGTGTG
AAATTTTTGCATCCAGCCTTCAGGTAAGTCCAGTAGATGTGCCAGTTATTTATAAGGATTTTTTTTCAA
GACACTATATTGGATGAAGTTATACCTATGTTATATAAATATACTTAATAATGAATCCTGAATACAACTGG
GTAAGTTTAAAATAAAACATATTTTATTTTTGAATAAAAACAACAGAGCCATACCTGTGGGTTCGAGTC
CCATTAAAAGTCAATGAGTCTAAATTTGCTTCTAGCTTTTTTATGGACAAATAAAATTTGGTGCTAGACA
AACTTAACATTGACAAAAAACAAAGTGCAATTCACTACTAGTCCTGGGTTCGATACCTGGTTAACAAT
GTATGGAATGGAATTTTTTAAATAGGCTGGACTGCTATTACATTGCATTGTAAGTACTTTGTCACATAGA
GACATGCTGTGCATAGCCCTTTCCATAGCTTGCTGAGTTAATCTAAGCTTATTCAAGAGGCCTAAAGTTT
GAGACCATGTCTCGAGTTGTAACCAGCATCTGTGAACACACGCTGTTTTTTACACCTCCCACTAATAAA
GGGAGAGCTTTGGGTTCGATTCCCATACTTAAGTGATTTTTAATATTTGATTGATAAATATTTGTGGGTTC
GACTCCCATATTGTACTAAATAAAATTAACAAATCAGGGTCCAAAAGAATTATGTGATTGAGTTCAAAT
CCCATTGGTCAGTAGTGATGCTGCCTACTTTTATTATTTTGTAATATACTAAAATCCCTTATGGGAGCTTC
TTAGTTGAAAATGTAACAGGGTTCGAATCCCAGTAAGTACATTAATATGTGGTCTGGATGACTGATGGT
GATGGCTGCTGGGGCAAAGAGTCTGGTTATTTATTTAATGATGTGAGTATCCAAGTGAAAATTTGGTAT

TTACCTACTAATGTAACCTTACTTACATCTTGAACATGAATTGTTATGAACAGTGTTCATTACAACTCTTT
ATAATAGAGATAATGTTTTGGGTTTGAATCCCATCATTGCCCCTACACATCTACTACACAATTTCAATTTT
AAATTAATTGTGTGACCAAGTGAGAATTGGAGATACTATTGTTCCAGGAATGGGTTCGAATCCCACTAT
CACCCAACTACCTACTTGATTTAGGTGTGAATTCGATCACTTTGTACAAGGATAAATTGAATAGACCAA
AGAAACCACAAATAAGGGTAAGGGTAAAGGCTGGATCATAATAATACGAAGCTAGGAGAGAGTATTGC
TATGTTCCTTGAGAGAGGCTTATGTCAATTGATAAATGTTTCTAGACTGATGAGTGAAAGGTAAATACA
ACATATAGAGTTTGCGGCTTATTATGGGTTCGAATCCTATAATAACTTATTATTATTAATCTTCGTCAATTT
GCCTCTTTGTGGGTTTAACTCACAAGAAACTTCAACGAAGTTGGAGCTACATAAAGAAACATTATAGT
TTTTTGATTTATGAGTGTCAATTACTTACAAATATATGATGTGGGCTCGACTCCTATACTTTTTAAACGAT
ATTGGGTCTAGTCTCAGCAAAGATTGTTTGTTGGCACCTACAATAGCCTGTTGCTGTGGGCATTGGATA
ATGTAGTGTATGAGATATTTCTATGGGACCATCAAACTTAATAAAGGTTGGTATTTTTTTATCTATTTAAG
TACTGCCATCATAAATTTCTGATGATTACTG
>TCONS_00039077 geneID=XLOC_007930 scaffold_162:155013-156612(+) Unclassified
ACCATCTGCAGATTCTTCAGCACACGAGTACAGGTTTATTTCCACAAGTCGAGCTCAGCTTCTTATGTT
AAACAATTACACATTTTCGGAAAGAGGAAAGAAATCTGGAAGGTTTCATTGTTCCAAAAAAGATGCTG
GTTGCAAGGCAAACTTGAAATTGGATCAATATGGGAACATTAAACGGATAGCTAACATTCACAACCAC
GACCCACCAAAATTCATTGTATCTCAAAATGGGCATTATATTCCAGTATAATAACCATAGTTATAGGATTT
GTGTTCATATTTATGTAAAGGAGTAGTTCCAGTAGGTACATACCTACTTCCCGCTTATTTAATACAATCAA
CAAAAAAAATCAATTTTTAATCTTGAGATACCGCTGCCGCTGGAAGGACGAAGTCGCCAAAGACCTGA
AAGAGCTGGAAGTGTCCGACTGGTCAGAATTGGCACAGAATAGAGAAGATGGGCGCACTTTAATGTC
GGAGGCCAAGATCCACTTCGGGTCGCTGAGCCAGCGGAGTAAGTAAGTAAGTTTAATCTTGCAGATCA
CCCAGAGTATAAGTTTATTTCTACTACCCAATCTCGCCTTCTTATAATGAATGGTTATAGTTTTTCGTTAC
GAAATAAAAATAAGTGAAAATTCTTTTGTTCCAAGAAAAGTAACGGATGCAAGGCAAATGTGGCGCTC
GACAAACTAGGTAACACTAGACGGTTTACTGACACTCATAATCATGATCCACCTAAATTCATTTTAACTA
ACGACGGG

>TCONS_00040100 geneID=XLOC_007932 scaffold_162:164973-166774(+) Intergenic
CAGCTTTTCCCTACGATTTCATACTTGACCGAGACAGTGGGAGGCAACTACTGCTGTTCAAAAATTACA
CCTATTTCACTATGAGAAATAATGGACATTATGGACACTATTATTGCTCCAGTTACATAGGTTCCAGATGT
ATGGCGCATCTTAAATTGGACAATCTGGGCAACATTGAAAATGTAACCGGTGTCAGAATATCGGTTTAT
ACCATCAAGAAGAGGAAACCCATTGCTGTTGTTAAACGAGTATACTTTTGCCCGCAACGGACGTCTCT
CCAGACTTTATTACTGTTCTACTAACAAACTGACGTGTAAAGCATTTGTAAAACTTAGTCCTGATGGGG
TAATCGTACAAAGTAATCTACATCATGATCACCAACCGCCGAAATCTTTAATGTCTCAGAACGGGGATT
ATGTCATTAGAATCACCCAAAAATACAATTGACAGACATTGTAAAATATTATATAAAAGAGAGATATGAC
GTTTCATTTACAGATGCTTATGCCTGATGAATAAAGTTCATTTTCCTTTGAAAACGGAGAAATGTTAGAT
ACACTGTATGGATTTGACAGCTGTCATAAGGCTCATGCTATATTTTGAAAAATATACTTAGTAAATTAAA
TTTTTACGCATCGGTATGGTTAATTTCTATAGAAAGGCCAATTAGAATATTTAAATAATATTATGTTCTTAC
GCTAGTGCCTACTTTAAGATTATTATACACATCTTCTGTTTTTTTCTTTTTTTCTTAAAGAATCAACTAAA
AACAAATTAATAACTTCCTAAATTTATATAAAACAGATTCAACATTCCATGTAGCATAACGATCTTGACA
CCCGTCAGCTGATGCTATTTACCTATTAATTGGTAATAAAATAAATTTCCTCAAAAAAAATACAAAAATATA
TTTGACACATGCATATGCTCACGACTGTAATCTCCGAA
>TCONS_00039677 geneID=XLOC_007932 scaffold_162:168597-175274(+) Exonic overlap (+)
TGCAAAAAGTTCTACTGCTCCAAGAGACAAACTATAGTGTGTAAGGCTCGAGTCACGCTGGACGACG
GTGGCGCCATCGCGGAGGCCGATGTGCACCACACGCACCCCCCCGATGAGTTCTTTATTACTAAATCC

GGAGAGTACATCAGACTCAACAAATAACACACCTAACAGCTGCATTTTATTTTTAATGTTTGCATTACG
AGCCAACTTACCAATTGTTCCCAACGAAAAGAGGGAAGACGTTACTCTTGATGAATCATTACACGTAT
AACGAGAAACGTAAACTGATATACTACTGTTCGAAGGCTCCCACGGGATGTAGGGCGTGCATAAAGCT
CGACAACGACGGAAGCATACGAGAAGCTAACACGAACCACACGCACGAACCTATGAAATATGTCATC
ACTAAACAAGGCAATTTGTGCAAATTATAGTCAATGCTTATATTGACTGATACGGGGGTATTGTTATTGT
TAGGTACGACCCCTCTCAGACGCATTGTTACTATGAGAGGGGGTCAGATTTATCTGCCCCTGACCGTAC
GTATCAAAAATATCACATATTAATATTACTAGCTGTTCCCGCAAGCTTCGCGATATAAAGTAGCCTGTATA
ATGTGTTAATTTCGTTAAAATCGGTTTAGCCATTTTGATGTGAAGAAGTAACAAACATACTCTCATACAT
ACACACATACTCACAAACTCTCGCATTTATAATATTAGTAGGATTCAGTGTTAAGTTTTGGATTAGTTAA
ACAGAGTTTTGCTGAAATGCACGGAGGAATACTAATGTCGATTAAATTTATTCTATTGTAGCCTTGCTTT
GTCATTATATCTAAAGTGGCAGATATTTAGTTTAAAGCTCCTTTGTGCAACTCAGCATTAGTATAAAGTAT
TTAATGTTAGTCGTTATTTTAACGGATATTTCATATACCTACAGTAATTGCGTGTTGGCGATAACTATTGA
TTTTATAATAATATATAAATGTACATCACTGCACTTGAGAGAGAATAGAATAGTGTTTCACTGTAATTATT
TTATTAGCAAGCAC
>TCONS_00039678 geneID=XLOC_007933 scaffold_162:177208-178337(+) Exonic overlap (+)
AAAAAAATTGCAGTTTAATAAAGTCAAACTTTTACATCAAGAAATAATATATTTTTGGCGATCTCACTAG
GGTTCACCTAGGTGCAATCCTGTGCAATTATATATGCGAAATTCAATAATTACGTATTACAGGGTACCAA
TACAGGATGTTCCCTACCGGCAAAGGAAAAAGCTTACTCTTAATGAATGAGCACACATACACTGAATA
CCGCAAAACAACATTTTATTGCTCGAAAAAGATGTCCACTGGATGTAGAGCGAAGGTTCACTTCAATA
AAGACGGAAGTATTCGTGAGGATGTGATCCTAATTGCAAATTGCAATGTGCGACCAAATACTCACAAG
ACGCCCGAGAAGAGTTAAGAAAACAATTCCACAGCTTCAATGATTCACAGAAATATCATTTTTATAGCG
AGTACACTGAGCGGAAGGATAAACTAAGGGAAAGAACAAAAAACGAGTGTAGTAGGAGGAAATATAC
TTTCATATACTACTTCCCTCATATAGGGACTAAAGAAAAGGTGTGCCGCAGTTTTTTCAGTAAAACTTT
GGATATATCTGTTAAAAGAGTATACAATTATTATAAAAAAATGGATGTGTTTCCGATGCCCATTAAAATAC
TCTACCCAAAAGAAGAGCCTTATGATACCTATGATGAATAATAGCGAACAACCAAAACAAAAAAAGGA
GAATTACTGCTCTTTTGAAATAAATAAAATATTAAATAAATAAAACACATGCATTTAGATCGTTAAAATC
GCCGGCAGAATTTTAATTTAATATTTTTTTTATATATTTGTACATTATAGTGTAATACGATAAAGTTTATTAT
TATTACCTATACATACAATTTACG
>TCONS_00039085 geneID=XLOC_007934 scaffold_162:178679-180196(+) Unclassified
GAGACCCAGTCCGGAATTCAGTTCATTAACTTAAAAAATAATAAAAGAATTTTATTAGTTGACGGGTAC
TCCTTTGCACAAACCACTCTAAGACATTGGTATTGTTCGAAGAAAGCGAAGAATTGCAAAGCTAGAGT
ATTTTTCTCTACTGATGAAGAATCTGTCACATTTTGCCATAACGAACACAACCATCCACCTCCAGTGTAT
AAGTTAACATCAAGTGGATATTACGTTAAAATATGCTAATGATCATTTTTATTCAGCTTTGGCGTATACGG
ATTTCAGTATTATAAAAACAAGGAAAAACAAACGGGCCATCAATATGGGGGGCTATAAGTACAACCAA
ATGGTCAGCAAATATATATTTTATTGCATTAAGAGACAATCGTATAAATGTCCCGCAAGACTGCGTCTAA
ATGCTAAAAATCAAATAGTGCATAACATCCCTGGCCACAATCACAATCCCGAATACGCCATGTTGCCCT
ATTTTTGTAAATAGGTTTATGTGAATCACGCTAACTCTGCACATGTTATAAACCGGTCACACTGAAAAAT
TTCTGTTTTTTTCAAAAGTTGTCAGTTTTCCTTGCATAGTTAAGTATAAGTATATTAGTTCGATTATAAATA
TTTTATTATTAGTGACATGGTAATTTTAACGTCTAAATATTAAAATTACGTAAAATATACCTAAAAAATTC
TGTACTTCCTTAGCTATTAATACTTTTTCTGTAAATGAGACCACCCTGTGAATTAGACCACAATAGACTT
TGGTCCATGGTCCACGGCATACACTGAGCTTGGTTTTTAGCCATTTTGACTTTCTGGGCAGCAACTTCC
TGCGGACTTCTGCTATCTCGCCCTATTTGTATATTTCTCAACTTTTACATAAATGGAGGTCCTTGACCTCG
ATCGTGATCCGAGCCCTTATAGACTTAAAAGTGAAAAAAAAAAAAAAAATATTTTCTAACCCGAATAATT
AAGACACTTCTCGATGTTCGTTATGCGGTCCACCGGGGTTTGTTTGAGATCTTGAGAAGCGCCTCTATA

GGTAACGTTTGTAACTAATAAAGTTCCATAAAAATCCCCGGTGGACTTATCGAATAACGATACTATCAA
CAATATCAACATGACTGTGTCTAAATTATTCGTGATTGCACTTTTGAAATTATAGTGAGTGCAAGATTCT
TCTCACGAGAGCCATGCTTTTATTATTATACGAAAT
>TCONS_00040102 geneID=XLOC_007934 scaffold_162:178679-181347(+) Intergenic
GAGACCCAGTCCGGAATTCAGTTCATTAACTTAAAAAATAATAAAAGAATTTTATTAGTTGACGGGTAC
TCCTTTGCACAAACCACTCTAAGACATTGGTATTGTTCGAAGAAAGCGAAGAATTGCAAAGCTAGAGT
ATTTTTCTCTACTGATGAAGAATCTGTCACATTTTGCCATAACGAACACAACCATCCACCTCCAGTGTAT
AAGTTAACATCAAGTGGATATTACGTTAAAATATGCTAATGATCATTTTTATTCAGCTTTGGCGTATACGG
ATTTCAGTATTATAAAAACAAGGAAAAACAAACGGGCCATCAATATGGGGGCTATAAGTACAACCAA
ATGAACGATATAATTATAGACTGATTCCGACGAAGAGTGGAAAAAAAACGTTAGTTCTAGGAGGGCAC
AAATATTACCAAATGAACAGCAAGTACATATTTTACTGTATCAAAAAGCCGAGCGGCTGCAAGGCTCG
GACGAGGCTGAACGAAGATGGATCCATCGCCTTCAGTGAAGCTCACGACCACCAGCCCCAACAGTAC
CATTTTACAGAGATGGGGGAATGCATCAAAATTGGATGAATAAGAATTTAATTAATGATAATTAAGGAA
GATTTAGTTATGGTTATCATATGCATATAAATATATGTTAACCACCGGCGATCTAGCTCAAGCCTTAGCTT
CCTAGATCATTACAGACAATACAGAGTATCCAATCCGCAGCGTTTGGTCCACCAGCGAGCCTCCGGAG
ACAGGTCCATGGGCGATGAAGATGATAAGGAGATCAGCAACCCGGCAGGGAGAGTCAGTGACATTTT
GAATAAAATTGTACAATTCCGTAAGAAATAG
>TCONS_00039586 geneID=XLOC_007934 scaffold_162:179235-180833(+) Unclassified
TCAGCTTTGGCGTATACGGATTTCAGTATTATAAAAACAAGGAAAAACAAACGGGCCATCAATATGGG
GGGCTATAAGTACAACCAAATGAACGATATAATTATAGACTGATTCCGACGAAGAGTGGAAAAAAAAC
GTTAGTTCTAGGAGGGCACAAATATTACCAAATGAACAGCAAGTACATATTTTACTGTATCAAAAAGCC
GAGCGGCTGCAAGGCTCGGACGAGGCTGAACGAAGATGGATCCATCGCCTTCAGTGAAGCTCACGAC
CACCAGCCCCAACAGTACCATTTTACAGAGATGGGGGAATGCATCAAAATTGGATGAATAAGAATTTAA
TTAATGATAATTAAGGAAGATTTAGTTATGGTTATCATATGCATATAAATATATGTTAGTAAGTAGAATATA
TGTATATATATAAAATAATATGTAAATATTAGGTACCCACATAGATTAATTGATGTTGCATTATTTTATTATG
ATCATAATTATTTAAAATTTACATGTAAATAACTGTATTTATGTAACTGTATCATAACTAATAAACTCATAT
CATAAGAC
>TCONS_00039941 geneID=XLOC_007971 scaffold_162:185954-189155(-) Unclassified
CCCGGTATTACGCAATACCGGTATTGCGGTATTGCATGCCCTAGTTCCTACCATGACCATGGTAGTAATT
AAGCTTCATTATTTGTCAAAAAAAAAAACGGTAAAATATGCTATTTATTATTTTATATAACTTTTTACAAAT
ATCACATTTATCATAGACAGATAGGTATGTATCTGTCCAGGTACTTAAGTACTGCATAATTTCTGGAATCA
TATTTTACTATAAAGCGTTACTTTAAGAAAACGGGACCCACACCTACACTACACATCCAATGTCAATCA
AGGTCATAACTATATGTTTCACATCTAGATTTGATAGCGATTATGAAACTGAGTTGCAAAGAGGAACTTT
AATCTATGGTCAACCTTCAACGATTGGCAACTTCAACTTTAACTTCAGCTTTATGATTGGCAGCATATTA
AATTTTACCTTACCTATTTCGAATAAGTACCCTTTCTATTTAGATAACCTTTAAGTACCTATATTGTAACTA
GTACCAGTTTTGACTATACCTAATAAAATTTAAAATTGTTCCTTCAGAATCGCAACCATTACACTTACCT
ACTTATAAACCAACAATCTACAATTTATAAAGTTCACCATGGGGTCCAATATGCAGCGGGGGCGGCTGG
TGATTGTGCTCCAGAGAGTACACGATCTGTCCCTCATTCGTCAGGTACAGCGTGGACGGGCACTTGTT
CTTGCGGGTCGACGAGCAGTACCACCGTCGGTTCGTGCGTCTCCAGAATGTGTATCCTTTGTAGAGGA
TCAGCCCACTCCCCTTTCGGTTCCTGATGATCTCGTAATGTTGGTTCCGCAAGTAAGCTGCGAAAGGG
GTTTCATGCTTTAAAAGGTGTGTTAGCGCGTGAAATAGTTTAATGATTATTGTCTGAAGTGAAGTGAGA
CTTTTAAATTATTTAGCTGGAATGTGATTTGAAAAATGGGAAATATAAAGCAACGGAAATAGGTCGTAT
ACCAACCGTTGATTCACCGTTATTCACAAAGGAAATATGTTTATGTGTGTGTTTGATATATTATATGGCGC
ATGGTGCGATTGAAGCGGAGATTTTAGCTGTCATGAGCTATGCCTTGACTAACGAATTTGTGCTGTTAA

CTCTACATTTCTCCGTACAATACATCTATTGTGGATTCACGAGAGTTGAAAAACATCTCTCCTCGTCTCC
TATTTGCATATCTCCGCATTGGTGGAAGGGCCTACTTGGTACTTAGCTTGTCTTCGGCCACAGTATACCT
CAGTGCATTATGTTCGTGAAACGTGATGTTTTTTACTATCATTGTCGTTACTTTACCAACTGTAAATAATT
CGTGTAGTGCAGCTAAAAGTTAGAAGCTTTGGTTGTTAATACCTAAGTTGAGTACAGCCGGATGGAAC
CTATAATTTTTGTAACGTTAGGCCGTGTCCCTAGTGGACAGACTGTTAGCGGCGAGATACTGAGATAGT
CAGCGCGCGTAACGCTACAAACTGCGTACTAAACTAGCAGACTGTGGACACTCCATTTCAAATATATG
GATGAGCGTACCTACTAAATCGATACGCCGCAAACTCAGTCGCTGACGGTTTGCCTGCTGGGTATGGA
GTTGGGACACGGCCTTAATATTATGTAGGTATATTCATTACTCATCATCGTCATCATCAGCCAATACATGA
CTGGCTAACTCCAACTTTTATGACTTACGTTACGTATCACTGTCTACTTTTGTGAACCATTAGGTATCTCT
AAACAAAAGCTATTATGCTCATCATAAATAAAACTTTATTATTCAAATACCTAGGTAGGGTAGGTACCGT
TTAGTATATTTAAAAAATCGTGATACAAAACTATTAACTATATTTTCATGTAAATTCCTTCAGATGTTATAT
GGTATTTAGGCGGGGCGTGGTTGTGCCCTCCCTCCACTGCAATCAAAGCCCCGTCCTGGTCTAAGCGC
AGCTTAGCACTGCATTTGTGGCTGAGCCGGCTAGAGCAGTATCTATTCAAATCTTTGGACAACAAACTC
ATCCTGCTAAACGTGTATCCTTGGTACATAATGAGAGTCCGTCCATTTTTGTTCACGCATATGGTTTTGT
AATCTGTAAATAAGGAGGGTAAATGAAGCTATAGTAATAAATATAATATTGATAAAGGAAAAGTTTCAA
GCCACTCGTAAATCTGCAAATGGCGTTGACTTATGGATTATTGATAGACCCATTATTTAACATTAATGTTT
TATTAATATAATAATGGCATTCTTCTCAGCTCTAGCAACTATTACAACACAGCCAAAGTGCTGACATAGT
TTCCAAGAGATGAGAGCAAAGATGCTGCTTCAATTTACACAGGAGCTAGGCTATCTCAGCGGAATATG
TAGGTACAAAGGTTCCACGTACAATAATCGATACATGGATTCGACAGCAAGGCTCATAAACTATATTTT
GAAGTAAATCCCTTCAGGTGTTTGATGGTATCTAGGGGGGCCGTGGTTGTGCCTTCCTTCCACTGCAAT
CAACGCGCCGCCCTTGTCCAATCGGAGCTTAGCACTACATTTGAGGCTTTTTCGACTAGAGCAATATCT
ACTCAAATCTTTGGACAACGCACTCATCTTACTAAACGTGTATCCTTGGTACATAATGAGAGTCCCGCC
TCTTTTGTTTACGCATATAGTTTGGTAATCTGTAAATAAATAGAATACATTTTAAATTATC
>TCONS_00039714 geneID=XLOC_007971 scaffold_162:186034-190802(-) Exonic overlap (-)
CGTGTAACGTACCTTGGGTCAAAACAAGCATTTTTACTTTTATCTTAATTTAATAAAATCTGGCCACACT
AAAGCTTAAGCACAAATACTAGTGATAATAGATTATATATCCTACCGAATAAAGAAAATTTCACATTAAT
ATCTTAGTTGTACGCTGCGATAGCTTCATTCAAAGTTGGGGTAGTCGAAAATGTCCCTAGGTACGTTAC
GTTACCCTACTTAATTTAAAATACTTTTACAATATTTTGATCTGATTTCACTTTTTAGGTTGTTTTCTTATT
TTGTGTTGTCATGTCAACTTGTCATTTTCATGTCGCTCCTGTCAGGCTGACAGCTGAGCGGTAAGCGTA
CCACAGATTATAAAATGAAATGCGACTTTGAGATTGCGTCCCAATTTTCTCAGAATAAGTATACATTTTC
AAACAGTAAAGAAAAACTGAGTGAGAGTGGAAATTGAAACGGCCGACCTACTTTTAATAAAGCGTTA
TTGTATTCCTAAATGCATTAAACTACAAGAAGATACCTACTTCCTATCTACCAAAATTTTTCTTTTGGTAA
ATAGGTAGTTAATTAAGTTATTTAAGGACATAATAACTACTTTTGATATATTTTTGTATTGTAGAGTAGTA
AAAAGGGTGTAAGTATACTATAGTGCCACAAACTTATCTGTTCCGGTGTTAAGCCGAAATAGGGCGACT
CATACCTATGACGTAATTTTAAAAAACTTTAGCCTGTCTTTAGTTTTAGGAATTTTAAAGTAGGTAAGAT
ACCTATACGCTCCAGGAATAGCAATGTATTTTTCCACTTTGGTAAGCATTTAAGTATTTAACCTAATTAAT
CTCATTAACTTTACGAGTATACATATAGAGTACTTAATCTTTGATCAGCAAGAAATGAGTAAAATAACTA
CTTACTACTAACTATGATGCATATCCATTCATGAAAAATAACTAGAACACGTCCTATACAGACCTATATTT
TTTTTGTTAAAAAATATATAGGTGTAGGTTCCTACTAGGGCATGCAATACCGGTAATATTTTCAATACCG
GTATTGAGTTCCGGTATTAGAACTCAATACCGGGATCCCGGTATTTATACCGGTATTAGAATTCCTTGTTA
TAAATGACATATATTTTGTTTAAAGGCCGTATAGGGCAAAATACAACAAGAAAAAGCAATCAAATTCTG
TATTATGCAATATTTTTTACGAGAATTGACTATTAGAGTTAAAATTTTAGAACGCATAGCAAACAAGCAG
GTTTCCAAAGGCCGAAAACCGCATGTAACGGTTGAAAACAAGTGCACTTTCGTAGTATATAGGAGCAC
AAGGCTAACTATATCTTAATCGCTTGATTTATAGCCTAAAAAATTTGCGATTCCGGTATTACTTCAATACC

GGTATTAACTTTCAATACCGGTATTCAAAAAAGCGTGGATTTAGTCCGAGATCCCGGTATTACGCAATAC
CGGTATTGCGGTATTGCATGCCCTAGTTCCTACCATGACCATGGTAGTAATTAAGCTTCATTATTTGTCAA
AAAAAAAACGGTAAAATATGCTATTTATTATTTTATATAACTTTTTACAAATATCACATTTATCATAGACA
GATAGGTATGTATCTGTCCAGGTACTTAAGTACTGCATAATTTCTGGAATCATATTTTACTATAAAGCGTT
ACTTTAAGAAAACGGGACCCACACCTACACTACACATCCAATGTCAATCAAGGTCATAACTATATGTTT
CACATCTAGATTTGATAGCGATTATGAAACTGAGTTGCAAAGAGGAACTTTAATCTATGGTCAACCTTC
AACGATTGGCAACTTCAACTTTAACTTCAGCTTTATGATTGGCAGCATATTAAATTTTACCTTACCTATTT
CGAATAAGTACCCTTTCTATTTAGATAACCTTTAAGTACCTATATTGTAACTAGTACCAGTTTTGACTATA
CCTAATAAAATTTAAAATTGTTCCTTCAGAATCGCAACCATTACACTTACCTACTTATAAACCAACAATC
TACAATTTATAAAGTTCACCATGGGGTCCAATATGCAGCGGGGGCGGCTGGTGATTGTGCTCCAGAGA
GTACACGATCTGTCCCTCATTCGTCAGGTACAGCGTGGACGGGCACTTGTTCTTGCGGGTCGACGAGC
AGTACCACCGTCGGTTCGTGCGTCTCCAGAATGTGTATCCTTTGTAGAGGATCAGCCCACTCCCCTTTC
GGTTCCTGATGATCTCGTAATGTTGGTTCCGCAAGTAAGCTGCGAAAGGGGTTTCATGCTTTAAAAGGT
GTGTTAGCGCGTGAAATAGTTTAATGATTATTGTCTGAAGTGAAGTGAGACTTTTAAATTATTTAGCTGG
AATGTGATTTGAAAAATGGGAAATATAAAGCAACGGAAATAGGTCGTATACCAACCGTTGATTCACCGT
TATTCACAAAGGAAATATGTTTATGTGTGTGTTTGATATATTATATGGCGCATGGTGCGATTGAAGCGGA
GATTTTAGCTGTCATGAGCTATGCCTTGACTAACGAATTTGTGCTGTTAACTCTACATTTCTCCGTACAA
TACATCTATTGTGGATTCACGAGAGTTGAAAAACATCTCTCCTCGTCTCCTATTTGCATATCTCCGCATT
GGTGGAAGGGCCTACTTGGTACTTAGCTTGTCTTCGGCCACAGTATACCTCAGTGCATTATGTTCGTGA
AACGTGATGTTTTTTACTATCATTGTCGTTACTTTACCAACTGTAAATAATTCGTGTAGTGCAGCTAAAA
GTTAGAAGCTTTGGTTGTTAATACCTAAGTTGAGTACAGCCGGATGGAACCTATAATTTTTGTAACGTTA
GGCCGTGTCCCTAGTGGACAGACTGTTAGCGGCGAGATACTGAGATAGTCAGCGCGCGTAACGCTACA
AACTGCGTACTAAACTAGCAGACTGTGGACACTCCATTTCAAATATATGGATGAGCGTACCTACTAAAT
CGATACGCCGCAAACTCAGTCGCTGACGGTTTGCCTGCTGGGTATGGAGTTGGGACACGGCCTTAATA
TTATGTAGGTATATTCATTACTCATCATCGTCATCATCAGCCAATACATGACTGGCTAACTCCAACTTTTA
TGACTTACGTTACGTATCACTGTCTACTTTTGTGAACCATTAGGTATCTCTAAACAAAAGCTATTATGCT
CATCATAAATAAAACTTTATTATTCAAATACCTAGGTAGGGTAGGTACCGTTTAGTATATTTAAAAAATCG
TGATACAAAACTATTAACTATATTTTCATGTAAATTCCTTCAGATGTTATATGGTATTTAGGCGGGGCGTG
GTTGTGCCCTCCCTCCACTGCAATCAAAGCCCCGTCCTGGTCTAAGCGCAGCTTAGCACTGCATTTGTG
GCTGAGCCGGCTAGAGCAGTATCTATTCAAATCTTTGGACAACAAACTCATCCTGCTAAACGTGTATCC
TTGGTACATAATGAGAGTCCGTCCATTTTTGTTCACGCATATGGTTTTGTAATCTGTAAATAAGGAGGGT
AAATGAAGCTATAGTAATAAATATAATATTGATAAAGGAAAAGTTTCAAGCCACTCGTAAATCTGCAAA
TGGCGTTGACTTATGGATTATTGATAGACCCATTATTTAACATTAATGTTTTATTAATATAATAATGGCATT
CTTCTCAGCTCTAGCAACTATTACAACACAGCCAAAGTGCTGACATAGTTTCCAAGAGATGAGAGCAA
AGATGCTGCTTCAATTTACACAGGAGCTAGGCTATCTCAGCGGAATATGTAGGTACAAAGGTTCCACGT
ACAATAATCGATACATGGATTCGACAGCAAGGCTCATAAACTATATTTTGAAGTAAATCCCTTCAGGTGT
TTGATGGTATCTAGGGGGGCCGTGGTTGTGCCTTCCTTCCACTGCAATCAACGCGCCGCCCTTGTCCAA
TCGGAGCTTAGCACTACATTTGAGGCTTTTTCGACTAGAGCAATATCTACTCAAATCTTTGGACAACGC
ACTCATCTTACTAAACGTGTATCCTTGGT
>TCONS_00040127 geneID=XLOC_007972 scaffold_162:204281-205253(-) Intergenic
ATACATTCTGCCGCAGAAGTGCATCTGGTGTGAAACAACGATGGTTCTGCTCTACTCATCACCGCAAG
AGGTGCCGGGCGCTGATACACACGTATGCTGAGTTCGTAACTTCACAAAAGGGCAAAACATTGATACG
AGTTGGCAACTACACGTTCTGCGTACAAAATACATCTGGAGCGAAGACGCGGTGGTTCTGCTCTACCC
ACAACGGCAAGAGATGCCGAGCGGCACTACACACATACAACAACGAGATCATCAAAATAAGCAATGT

TCATAATCATTAAATCTATGTAAAAATTTACGCGCTTTTTTATTCACTTCGTCTTAGATTGGTACTACACA
CACG
>TCONS_00040128 geneID=XLOC_007973 scaffold_162:209274-210004(-) Intergenic
CACGTTCAGCTTGCAAAGTTATGTGGGTTTAAAAAAACGATGGTACTGCTCCACACACAACCGCAAGA
AGTGTAGAGCGCTTCTACATACATACGACAACGAGATCATCAAGATACATAATATCCATAATCATTAAGT
GTTTTGCTTCCTACCTTAAAGACTCTGTATAATTTTGAGAAGAAATTGAGGTTGTGTACACGACATCAA
AGAAGGGCAATACATTGATAAGAATTGGCTTCTACACTTTCAGTCTACAGAGCTCGGCCGGGTTGAAG
AAGCGGTGGGTTTGCTCCACTCACGCGCACAAGAAATGTCGGGCAGTTCTACACACATTTTCCAACCA
GATCATTAAAATACATAATGTCCACAATCATTAAATCAATATGCAAC
>TCONS_00039942 geneID=XLOC_007974 scaffold_162:210130-211771(-) Unclassified
TTAGTTCTCAAGAGGGCCTTAACCCCAGCAGAACGTGCGAGAAGATATAGAGCTAAATTGAAGAAAA
ATCCACAGAAATACGAAGAATGGAAGAAAAAACAACTAGTAAGAAGTAAAAGAATAAAGTTTTCCGA
TCTATCAGAGCATGAAAAAGAAAAAAAGCGTAAACGATGGAGGGAATACAATCGAAAACGAAGCAAT
ACGAGTAAATATAAGACCCTGAGTACGTTAATTCTCGAAAAGGTAATCGATTAATAAAATATGGCAACT
ACACCTTCCGAAGAAAAAAGTATTCAGGTCTAAAGAAGAGATGGGTTTGTTCCACTCACCACAACAG
GAAATGCCGAGCTGAGTTATACACATTGGAAGACGAGATCATTAAAATTAGCCACATTCATAATCATTA
GGTACAAAATATATTATGTGAATAAAGTGGGAGATGCTTGTGCCAGACAAACGGTGACTTCAGCATCA
CCCCCTTTTGGCAACATTGTATAACTAGCAGTCTAGCAGTATTTCAAAATACATTCTGTGTAAAAAGTAT
TCTGTGATAGAAAACAAAAATGAAAATTCGCCGTGAGTACTAAACACAACTCAGGAAACAGCGTTTA
AAAAAAGTTGTGATGGTTGGATAATTCGTTGGCGTATGTGTTTCGTTTCTGAAGGAGAATACTTTGAAG
GTGGTAAAATAAATTTGGATGAATAAAAAACATTTTGTGTTTTATTGATCCAGTCCCGATACTTTTTACA
CAGAGTAGTA
>TCONS_00039165 geneID=XLOC_007976 scaffold_162:229879-230750(-) Exonic overlap (+)
CGAGTACTTTACTTCACGTATTAGTGGTAAAAGATTAATACGCATTGGCCAATACACATTTTATAATAAA
AGTACTAGTGGCTTGAAGAAACTATGGGTGTGCTCTACCCACCACTGCAAGAGATAGGATATCGAGTA
CATAACTTCGCAAAAAGGAAATCAAATGATACGAGTGGGCAGATATAAATTTTATCTGAAATCTAGAGC
AGGTGCCAAGAAGCTGTGGGTCTGCTCGACGCACCACCCCAAGCAATGCCGCGCTAACATATCCACAA
TTGAAGACGAGATCATTAAAATTAATAATATTCACAATCATTAAATAAAAGGGATGATTTGTCAGTTTAT
TCCCGCGAGTTTCTCTTGGCTTAAAACGTGTTGAATATACCGGATGTTTTAAAATTCCCCCTTAGTCACC
TCCTGTCAGCTTGCTATACCACTTTGGCAACATCTGCATACTACTCGCATTTTTGTTTCTTTATAGGTAT
>TCONS_00039719 geneID=XLOC_007979 scaffold_162:256140-259647(-) Exonic overlap (+)
CAAGTCCCCCAATAAAGAAAGATGGCAATGCGGCACGCACCAGAAATACGGATAAGTCGAATACATAA
CTTCACAAAGAGGTAAGCGCATGATACGAGTCGGCCACTTCACTTTCTGCCTACAAAGTTCTACTGGG
GTGAAGAAGCGGTGGGTGTGCTCCACACACATCAACAAAAAATGCCGAGCCATGCTGCACACATTTG
AAGACGAGATCATCAAAATATACAATGTACATCACCATAAAAAATAACGATACAAAATACACTCACGAG
TAATGTGAGTAGTGACATATGGAACGGCAATGGCAGGTGGAACTATTTAAATTCTCGAAAAAAAATATT
CATTTTCCATTGAAACTTAGTTGGTCACGTGACTTTTTACTATGGAGAATGAATATTTTTTTTCGCGAAT
TTTGAAATTCTGATTTCAGTTTCGGGCCTACTTATACCATGCTGCACATGCTGGTTTCGGCTTAGTATAC
CCTTTTTGCAACACCCTGTATAAGTCCCTACAAACTAAGTCTATAGACTTAATGTCTAATATCTTACATGT
AATTATACTTATATATCTAAAATAATGATATAGGTATATTTTATATGTTTACATAATAAAGCCTGTACAACAT
GACGTAGGTAGTAATGAATAGAATTATAAATCATGAGGTTTTATTTAGGATCGATGACAGATAATTAAAA
AAAGATTTTTTTTGCTACATACAGTTTTTTTTTATTTGAGATCCCTAGGTAAAGACCTGTCCCGACTATAT
AGAAAATATAATAGCATAGAAGTACAGCTTTTTTTATTACAATTACAGAAGAATTCGAGTACATTACTTC
ACGAAAAGGCAAGCCAATGATACGAATAGGCGCCTTCACTTTCTGTCAACATACGTATTCTGGATCGA

AGCACCTGCAGCGGCAGCGGTGGATGTGTTCTACTCACAAGAACAAGAAGTGTCGAGCCGTGCTATA
CACCTGTGGGGATGACATAATTAAAATATACAATGTCCATGATCATACCTAAAACTGTAAATAATATACAT
AGTGATAATATAGTGATACTATCTTACAAATAGTGGTGGTGGTGGTGTTTCTTATTTAAATAAATAAATAA
ATATAATGAGCCGTTTGTTAAAAAAAAAAAATTTCATTTTTGGAGAAACAGGTGGCGTTGTCTGAGTTGT
ACTAACTGTCAAACACTCAGAACACAGATTTTGTGTCAGCTCACATCTTATTTCCACAGAGGAAATTCT
ATATCTGCCCTCAGGCGTTCTCTACAGCAACGACGCTTGAAAAATACACATTCTGAATTTGTAAATTAG
ACTAAATAGAAGTACTATTAAAAGTAGTGAGTTCTTATTCTGAGTTTAATACTTCAACCTCTTTGCTCTC
TGTGTGCAATATTTGACGTTTGGTACAACTCAGATGCACTATGTTGTCACGACATCTATCGGCTTGAAC
AAAAACTCAACTGACAGCTGTCAAGGAAAACGGCTCATTATGCCTTGTATGTGAATCATTTAAGTCTGT
TCGTAAACCTTTGTTATGTAATTTGTCTTTTTTGTGTACAATAAAGTGTAAATAAATAATAACCACATAGT
TATGTAAATGAGGGCAACCTTCTCAGGGATACAAATCTCAAATATGTAAAGATTAAACCCGGGATTTAC
TAATTTATCCAATTAACCTTTTTTATTAAATAAACAGTTCAAATTTTAAATATTTTTTAAAAATAAATAAA
GGGAATATTTTAATAATAATATATAAATTAAATATACCGTTTTCGTGAAATTTGCTAAACGCGATTTTACC
TGGAACGCATCTCTCGCGTAAAGTGGAGTATTACATAATATTAAGGTAATAAATAAC
>TCONS_00039720 geneID=XLOC_007979 scaffold_162:259350-260157(-) Intergenic
CTGTATTTATTGAAAAGAGAAAGTTTGGCGGTCGTGCCATATATCTCTCCGGCCACGTGTACCAGTTCA
ACAGAACCTCGGGCGTGAACAAGTGCAGGTGGTTGTGCCGCCGCCAGCGCTGGCACAGGTCCAATTA
TAATGACGTCTAGACGTGGAGCTCGGGTACTACTGCTGAACAACTACAGATTCTTGATACAACGCAAG
TCCCCCAATAAAGAAAGATGGCAATGCGGCACGCACCAGAAATACGGATGTAAAGCTTACATTTATACT
ATAAATAGCCAAATGGTAAAGTGCAATAATAATCACTCTCATCCACCGCCTCCGAGTAATTTTGATTCTT
AGTTATGTTTATTCGCAGTATTTTTTGGACATAATGCATATTTGTATTACTTAGGTTTAGCATTAACACGC
CGGGATCTTTTTAAATCAGTATAGATTATAAATAAGTAACGTTTAACTAAGTATGAATTAACTATACAATA
ACTAACCTGTACC
>TCONS_00039723 geneID=XLOC_007981 scaffold_162:261847-263796(-) Splice junction overlap
CTTAAAGCCTACTTTTTACCCGACTGCCGAAGGAGGATATGTTTTTTATAATTATTTTTCTTTCTAAACAA
CCAACAGTTGACATTTAAATTTAAATTACGAAACCACGCCACCTTCCTACTTTTAAATATCTTCTTATTT
GCTTTATCTACCTGCTTAACCTCATATTTCTGTAGCTGCAGTTTCAACGGGCACAAACAAGTTATTATAC
TGTATATAAGTAGTTGAGTATTTTCCTGTTTTCCATCTTTATTTGTATTTTTGAGTGCATTAATGAGTTGTC
GTATTTGTAAAATGAAATTTGCTACAGTTACGAAATTTCCTTTCGTTATTAATGTTTATTAATTTAAAATT
CTTATAATAATGTATCATCATTAACTGATTTTAATTCTACAGGGTGTATGGTAAAGACGCGCAGTGGTCA
GATGTCGATACGCATGAACGATGGGCACGTATTCTTTGTACACAGAATATTCAGCAAAAAAACTAGATG
GCACTGCGAAGGCAGAGACAAGAGGTCCTGCAAAGCCTGCGTTTTTACCATTGGGGACAACATAATC
AGTTACAACGATAAGCATAACCATGCTGCTTCAATAATATAAAAATTACTTATAGGTTTTCTTTAAAACA
CTGAGTTTACCTAATACGTCATAAAGTAAAGATACAGGCGACCGAGCGTGGCGTCCGCAATATCATTTT
GGAAGATGGCCACCGCTTCCTGGTGCACAGCCGCGCGGGCCGCCGCGTGCGTTGGTTCTGCACCGGC
CGCCACGCACACAAGTGCAAGGCCAGCATCATGACCATCGACGATCGCTTGCTGAGCTACAGATGCAT
ACACAACCACAAGCCGCCTGTCGTGCTAGTAGATTAATAAATTTAAAATGTTTAGGTTACGGTTGTTAA
TGTTAAAACGAAAGAAAAAAACGCTAAAAATAAAGGACGGTTTCCTATAAGTAAAATTTGTTTCAACT
TGAAGATTTTTAGTTTGTAGTTTTAATTTCCCTCCAGAGCCTGTAACACCTTTTATATCACAATGATTACA
TAAGTTAAAGTGGGCGTGACGGTTGTCTATTTTTAACTTTTTTTAAATATTTTAAATTATTTTTTGGCTCT
GAATTTGTTTTTTAATATTATAAAGCGATGTTAAATGTCTAGATTACAATTGTTAATTTCAAAACTACCGA
AGACTTAAAAAATAAAAGAATGTTTCTTATATTTAACACTTTTAAGTCTAATGATTGTGATCTGTTTTGT
GTGCCTTAAATAAATAAATAAAATATGAGTAAACTTTCAACTTGAAGATTTGTAGTTTGTACCTAGTTTC
CCGTTAGAGCCTGTAACATTTTATGCTCTTAGCCGGCTTAAAACAGATCTATCTTTATGCCTAGTTTTATA

ACACCTCGTTTACATTGTAACATCATCAATTATACGTCATCTCCATATTAAATTCTTGACAGATGTGTCAG
AAGTCAACAAC
>TCONS_00040132 geneID=XLOC_007981 scaffold_162:262184-263912(-) Splice junction overlap
GTCTGACTTCTTCCCCGCTACATTGAAGACTCCTGAAAGTTATTAAAGGTGTATGGTAAAGACGCGCA
GTGGTCAGATGTCGATACGCATGAACGATGGGCACGTATTCTTTGTACACAGAATATTCAGCAAAAAA
ACTAGATGGCACTGCGAAGGCAGAGACAAGAGGTCCTGCAAAGCCTGCGTTTTTACCATTGGGGACA
ACATAATCAGTTACAACGATAAGCATAACCATGCTGCTTCAATAATATAAAAATTACTTATAGGTTTTCTT
TAAAACACTGAGTTTACCTAATACGTCATAAAGTAAAGATACAGGCGACCGAGCGTGGCGTCCGCAAT
ATCATTTTGGAAGATGGCCACCGCTTCCTGGTGCACAGCCGCGCGGGCCGCCGCGTGCGTTGGTTCTG
CACCGGCCGCCACGCACACAAGTGCAAGGCCAGCATCATGACCATCGACGATCGCTTGCTGAGCTAC
AGATGCATACACAACCACAAGCCGCCTGTCGTGCTAGTAGATTAATAAATTTAAAATGTTTAGGTTACG
GTTGTTAATGTTAAAACGAAAGAAAAAAACGCTAAAAATAAAGGACGGTTTCCTATAAGTAAAATTTG
TTTCAACTTGAAGATTTTTAGTTTGTAGTTTTAATTTCCCTCCAGAGCCTGTAACACCTTTTATATCACAA
TGATTACATAAGTTAAAGTGGGCGTGACGGTTGTCTATTTTTAACTTTTTTTAAATATTTTAAATTATTTT
TTGGCTCTGAATTTGTTTTTTAATATTATAAAGCG
>TCONS_00040070 geneID=XLOC_007981 scaffold_162:263182-264251(-) Intronic
CAAATACTACAATGTCGCATGTAAGTACATTTAAAATGTGATTATTCATAGTTTCGGCCTGATTTAGGAG
TCCATTATTCCATCTTTACCGTCCATCATTTTCATTCTACCATCCAAGCTTCCGCCCTACTGTCTGACTTC
TTCCCCGCTACATTGAAGACTCCTGAAAGTTATTAAAGGTGTATGGTAAAGACGCGCAGTGGTCAGAT
GTCGATACGCATGAACGATGGGCACGTATTCTTTGTACACAGAATATTCAGCAAAAAAACTAGATGGC
ACTGCGAAGGCAGAGACAAGAGGTCCTGCAAAGCCTGCGTTTTTACCATTGGGGACAACATAATCAG
TTACAACGATAAGCATAACCATGCTGCTTCAATAATATAAAAATTACTTATAG
>TCONS_00039459 geneID=XLOC_007982 scaffold_162:265234-266156(-) Intronic
ATAGGGCACAAATTCGGGGTAACGAGTCTGGGCGCGCGGACTGTGGTTACCGAGGATGGGTACAGATT
CAGGGTGCATAGCGTCAACGGAATTAAGACCAGGTGGTACTGCGTCGGAAGAAATTTATCCAAGTGCA
AGGCCTGCCTGTTCAGCATCGAGGACCAAGTAGTTTCATACAAAGCGGGTCACAACCACCCGCCCCC
GGGGTCGCTTTATTAGTGATATTATAATTTGATGTCGGAAATTGACTTTACTGATTAAATATTTTGCGTAA
AGGCGTGCGAATGTACATATCAAATCGAGGGGCGCGAACCATAATAGACAATCGCGGCCACAGGTTCG
TGGTGCACCGTCGCAACGGCCCCCGCACTAGATGGCGCTGTGCGTGTTACAACAGCCATTTCTGCAAA
GCGTCCTTCACTACCTTTGGCTTTGACGACGGGAACGCCGGGATAGTGAAGCGTAATGATGTGCATAC
GCATTTTGCACCAAATTAGTGACGAATATTAGTCCGCCTGACGAGAAGTGCTTACCAAAAATTACTTAG
TTAATTACGAAGCAAGTAAGTAAGTAGGTACGCTCTTATATGGCAGCGTTCATAAAGAAAACAACCAA
CAAACACATCATGTTATAATATCTAGTCTTATAATTATTTAAGTATTCATTTTATAATTTGACCATGTCAATA
TAATATAATGTAACACGTATTGTTGTTCATATGTACTAAATAAATAAAATGTATTTTATTCACG
>TCONS_00039724 geneID=XLOC_007982 scaffold_162:265234-266156(-) Intronic
ATAGGGCACAAATTCGGGGTAACGAGTCTGGGCGCGCGGACTGTGGTTACCGAGGATGGGTACAGATT
CAGGGTGCATAGCGTCAACGGAATTAAGACCAGGTGGTACTGCGTCGGAAGAAATTTATCCAAGTGCA
AGGCCTGCCTGTTCAGCATCGAGGACCAAGTAGTTTCATACAAAGCGGGTCACAACCACCCGCCCCC
GGGGTCGCTTTATTAGTGATATTATAATTTGATGTCGGAAATTGACTTTACTGATTAAATATTTTGCGCAG
AGGCGTGCGAATGTACATATCAAATCGAGGGGCGCGAACCATAATAGACAATCGCGGCCACAGGTTCG
TGGTGCACCGTCGCAACGGCCCCCGCACTAGATGGCGCTGTGCGTGTTACAACAGCCATTTCTGCAAA
GCGTCCTTCACTACCTTTGGCTTTGACGACGGGAACGCCGGGATAGTGAAGCGTAATGATGTGCATAC
GCATTTTGCACCAAATTAGTGACGAATATTAGTCCGCCTGACGAGAAGTGCTTACCAAAAATTACTTAG
TTAATTACGAAGCAAGTAAGTAAGTAGGTACGCTCTTATATGGCAGCGTTCATAAAGAAAACAACCAA

CAAACACATCATGTTATAATATCTAGTCTTATAATTATTTAAGTATTCATTTTATAATTTGACCATGTCAATA
TAATATAATGTAACACGTATTGTTGTTCATATGTACTAAATAAATAAAATGTATTTTATTCACG

>TCONS_00039946 geneID=XLOC_007983 scaffold_162:267222-269194(-) Intronic

TCGAAACAAAGAAGCACAGTCGGAGGAAGAATTGTTTGGCGTTGTTGTCAACATAGTAGCCGGAACT
GTAAAGTGTTAATATCTACAGTAGATGTTAAAATATTGGTTACGAATCGCGGCGCACGTACTTTAGTTTT
TAAGGACGGCTACAGATTCATACATCACAGACGCAGCGCGATGAAGTCTAGGTGGCGCTGCGTGGGCG
TCGACAAGTGGGCCTGCAAAGCCTCCGTGTTCACCATCGAAGATGAAATTATCAGCTACAACAACAAC
CACAATCATGATCCTCCGTATGTTAACATGTATTACTGTAGATAAAGTAATCCTGCCTTGTGGTGATTCA
CCTTATACTTATAAATACAGTTTGTTGCAAAAGGGGTATGCTAACCTGAAAGCCTTCCGGCTTAGTATAC
CCTTTTTGCAACACCCTGTATATATTAAAGTATTAACAGATTGATGGACACTAGATTAACGGTGAAACTC
ATAAAAAAGCACTGAAAATTTGTATGGGACACATGGGAAGCTGGTGCACATGCCGGCACACTGCCTAG
GCGTAGTAAAGAAAACGCGACGTCGGTGACGGTTGTGTGCGTGTGTGAGAGTGAGTTGTATGAAAAC
TTATTGCTGTGTGAAAAAAAGGTACTCTGGGTGCACATATAACTTTGAAATCCTGCCAAATAAAGCACA
ACAAGATGGTCTTCATACAAACTCTAGGTAAAACAGAACTGCATTGACCGTTGTTGAAATAATTAGAA
AAAGAACGGGGACTGCATTTGACGTTGTGAATTTTTTCTAAAAGTGTTGAGGACGTCAAATGCAGTCA
GTGTCCATCAGTGGC

>TCONS_00039726 geneID=XLOC_007981 scaffold_162:269666-271592(-) Exonic overlap (+)

CTACCAGTACGGCTACCACCGCACGGCCGGCGTGAAGACTCGCTGGCAGTGCGTCAAGCAACGACGG
TACCTGTGCAAGGCCTGTCTCTACACGGTAGATGGCGCTATTATCAAAACCAACGCCGACCACAATCA
CCCCCCCACTACTGATAAAGAATAGTCAAATCAATGGTAATATCACTCTCGTGTCGAGGCAAACCAATT
TTATACATGGAGGGCTACAGATATTCTAGGCAAACAGTTGTAGGATTGAAAACTAGGTGGCGCTGCTGT
TCACATAATCGAAAGAAGTGTCATGCTGCCGTGTACACTATTGAGGACGAAGTTGTCAGTACAAGATC
AGAGCATACACATCCGCCAGTGACGCAGAAATGAGTAGATAACTAAGGGCTCCGCCACATAAACATGT
CTTGTGCCTCTCGTGGGTTATCAAACACATAACACGATACGTGTAAACGAGTCACGTCGTAAAAACGT
CTCCTTCCCACTCCCAGGTGGTTCAGTGGGAGTCAGCGAGAGAGGGTCACGACGCGACGTCCAATTG
TTTTGTTTATGTGTGGGAGGCTTAAAGTGTTTATTTTTATGATAAATACAAATATATATAATATGATTTGTT
TCATGTTAAATAAAATGGTTTTTGTTATTTGAATGTATACCTACATCAAAATAATTTTTAG

>TCONS_00039617 geneID=XLOC_007986 scaffold_162:283610-287117(-) Splice junction overlap

TGAATCCCATTCCCGAGTTCATTCATAACAAGGACGGCAGACGGATCCTGGTTCTGAACGGATACAGAT
ACGGAGTACACAACCACCGCCTCGGCCTGACGAACTGGCGATGCACCAAGCACCGCACCCGCTCCTG
CAGGTCCATCGTGAAGACATTCAGAGATGAAATACTCAAGCCGTGAAAATATGTAAGCTGAAGCGTGG
TAGCGGCCTGCGGCTGATAGCCCACGGGTTCACCTACGGCATGCACAAGACCACAAAAAAATACATCC
TGTGGCGATGCACCAAGAACAGATCCAAGAGGTGCCAGTGCAAGGTCAAGACCACCGACGGCAAAAT
AATAGCTATCCTCAACTTCCAC

>TCONS_00039733 geneID=XLOC_007986 scaffold_162:288230-290149(-) Splice junction overlap

AGGTAATCAGCCATCCTTCATCATGAGTAAGGGCAGAAAAGTTATGATTTTAAACGGATATAAGTACGG
GATCCATATTAACAAATACCCGATAGTAACTTGGCGATGTACGAAACATACCTCCGAGGCATGCAAATC
TATAGTAAAAATGTATCATGATAGCATTATTAAAGTAACCAGGCACTCACATTAAAAAGGCAAACACAC
AAAAACTGAAATTTATAAATGGAAATCATCTAATTGGCCAATCTCATACGGTGGAAAATTCTTGAAGAT
AAAGTTAGAGTTCAATCCTTGTGTCTAAAAAACAACAGACTTTTAGGTTAAATAAGTGGTCTTTTCTGG
GAATATTTTATTTTGGCATGCGCCTGGATCATAAGAGAAGTAGCATATTCCTTGACCTGGATCCTGCCTC
GTTCAGAGGGTGGCCCCGGCTATTCAGCGCGGCAATGCTGCGAGTGTCATGGACACCATCAAACGTGG
TTCGGCGGCGTCCTTTGTCATGAGCAAAGCCAGACAAGTCATGATTTTAAACGGATATAAATTCGGGGT
TCACATAAACAAATACCCCATAGTCACTTGGAGATGCACGAAACATAACTCCAAGTCATGCAAAGCTT

GCCGTGCTCGTAGAAAACGACGGGCGGAAGAGACTAAAATACGCCGGCTACAAATACGGGCTCTACT
ACAAGGCGGGGAAGGTGACTAGCTGGCGGTGCACCAAGCACAGCAACATGAAGTGCAAGTCCGTGC
TGAAGACAATTGACAGCCGAGTGGTGTTCGCGGCCGGACACACGCACCCGCCTCCTGAGCACGATGA
TCCGTGAATAACTCACGGAAGCTAAAGGAACGCCTCTTTTGTTTCATGTTATCGCGTTAACATGTCCTT
GTGAACGCTTTTTTGTTT
>TCONS_00039734 geneID=XLOC_007986 scaffold_162:288671-290149(-) Intronic
AGGTAATCAGCCATCCTTCATCATGAGTAAGGGCAGAAAAGTTATGATTTTAAACGGATATAAGTACGG
GATCCATATTAACAAATACCCGATAGTAACTTGGCGATGTACGAAACATACCTCCGAGGCATGCAAATC
TATAGTAAAAATGTATCATGATAGCATTATTAAAGTAACCAGGCACTCACATTAAAAAGGCAAACACAC
AAAAACTGAAATTTATAAATGGAAATCATCTAATTGGCCAATCTCATACGGTGGAAAATTCTTGAAGAT
AAAGTTAGAGTTCAATCCTTGTGTCTAAAAAACAACAGACTTTTAGGTTAAATAAGTGGTCTTTTCTGG
GAATATTTTATTTTGGCATGCGCCTGGATCATAAGAGAAGTAGCATATTCCTTGACCTGGATCCTGCCTC
GTTCAGAGGGTGGCCCCGGCTATTCAGCGCGGCAATGCTGCGAGTGTCATGGACACCATCAAACGTGG
TTCGGCGGCGTCCTTTGTCATGAGCAAAGCCAGACAAGTCATGATTTTAAACGGATATAAATTCGGGGT
TCACATAAACAAATACCCCATAGTCACTTGGAGATGCACGAAACATAACTCCAAGTCATGCAAAGCGT
ACGTGAGAACGTTTTATAATTCAATTGTAAAAGTGGCTTATAGCCACTCACATTAGAGTAACAGCTTGT
AAAAATTGTGCATAGTGTATGAGTGCGGAAAGTTAGTCATTAAAGTCATTAGGGAAAGTAGTGCAGTA
AGCAGTGGAGTCAAATTACACTAAGAGTTTTTAGATGATGAGGTTTTTATGTCCATAAATTTTTCAATAA
ATTTC
>TCONS_00040134 geneID=XLOC_007986 scaffold_162:289513-292063(-) Intronic
ACAGAGTTTACATTTATACCATCTCAAAAAGGAAAGAAGCAACTGCTGATGTATAAGCAGTACACATTT
TCCCAGGATTCCTTCTCGAACCGTTACTACTGCTCTAAGAAGGACCGCGCGTGCAAAGCGAATGTTCG
ACTAATCGGAAATCGGGTTAAACACGACTTGGACATGTGGCACAATCACCCGCCACCTACTTACATGAT
TACTAAAGATGGCCGATATGTAAAGCTCTAAAATGTAATCAGCCATCCTTCATCATGAGTAAGGGCAGA
AAAGTTATGATTTTAAACGGATATAAGTACGGGATCCATATTAACAAATACCCGATAGTAACTTGGCGAT
GTACGAAACATACCTCCGAGGCATGCAAATCTATAGTAAAAATGTATCATGATAGCATTATTAAAGTAAC
CAGGCACTCACATTAAAAAGGCAAACACACAAAAACTGAAATTTATAAATGGAAATCATCTAATTGGC
CAATCTCATACGGTGGAAAATTCTTGAAGATAAAGTTAGAGTTCAATCCTTGTGTCTAAAAAACAACA
GACTTTTAGGTTAAATAAGTGGTCTTTTCTGGGAATATTTTATTTTGGCATGCGCCTGGATCATAAGAGA
AGTAGCATATTCCTTGACCTGGATCCTGCCTCGTTCAGAGGGTGGCCCCGGCTATTCAGCGCGGCAATG
CTGCGAGTGTCATGGACACCATCAAACGTGGTACGATGCAGAACGATCCATTCTTTTAAATTAATAATT
ATGTTTTCGTTATTATTTTAAGTTTTATTTGCATATTGTAAATTACCATTTATATTAATACATAGATAGGGTT
CTTTTCCAATAATTAAAGTAGCATTTTTTTGCATGATGTGGTT
>TCONS_00039949 geneID=XLOC_007986 scaffold_162:293657-294590(-) Intronic
GTTTACATTCATACCGTCTCGCCGTGGACAGAACGATCTACTCATGTATAAAGGACACACGTTCTCCAA
GGTCCCGTCTTCCACGAACCGGTACCAGTGCTCGCAGAAATACCGCGGATGTAAGGCGGACGTCCGCA
TCATCGGCCCCCGGGAGATACGCGTGACCGTCGCGGAGCACGACCACCCGCCTCCGATATATACTGTC
ACTAAAGATGGCCGCTACGTCAAAATTTAATTTGCGAGTTAAGTTTAAAATTCATACGATCTCAGAGTG
GAAAAAGTAACCTGCTCATGTATAAGGACTACACATTCTCCCGAAATGTGCGTTACATCAACAACTACT
ACTGTACTAGGAAAAACTACGGATGCAGAGCAAATGTATCTTTTAAAACTGTAATAATATGTATGCTGTT
GGTGTATCTACCTATAATAAATAAATAAATAAATAAATGTGAAAGTGCAGAGTAATCGAGTGACAGAGT
CTGGTGCACATAATCATTCTCCTCCTGCCTTTATAGTCAGAAATGGCATTTATATTAGAATCTAATATTGC
CTTTTGCTAGATGGTACATTGAGTATTTTGTCGAGTCTTACGAATATCAATATGCGTATTATTTTTCGTAA
ATGCCAT

>TCONS_00039739 geneID=XLOC_007987 scaffold_162:302081-303572(-) Exonic overlap (+)
CATTGAACACGTTGTGTGTGGAATTGTACCTCACTCCGCGTTTCATATCATCGAGACGTGGAATGACGC
ATATCCAAATAGGAGGCTATAAGTTTGGTGTCAGCAAAGATAAGGGAATGAAGAAACGGTGGTCCTGC
ACTTCTCATAACAGCAAGTGCTGCAAAGCATCCCTCATGACTGTGCTTGACAAGATTGTTACCGGTTAT
TCGCGACCAAGAAAGGAAAGAAATTGCTTCTAATGGGCGACTACGAGTATAACCAACTCGGAAAATA
CATGTTTTATTGCACTGATAGGCTAAAGCATGGATGCAAGGCAAATATTCTTCTGGACGCCGACGGGAA
CGTCGTCAGGCGTTACGAGACCCACAACCATGCAACGTCTAAACACGTGATTACCAGTGATGGCAAGT
TGCGTAAACTTTAATGTTGTTTAATAACAGAGCGTGTTTCACTATGTCTCGATAAAGGGTAAAACTCTC
TGAAATTATGTCGCTGTAATCGCCAA
>TCONS_00039169 geneID=XLOC_007987 scaffold_162:302747-303687(-) Exonic overlap (+)
CCGGCAGGCGGGCCCCAAGGCGCGCTGGTGGTGCACCACGCACAAGTGCAAGGGCTGCCGGGCCAC
CATCATGACCTATGATGACGAAATCATCAAGATTAATAATGTTCATAACCATTGAACACGTTGTGTGTGG
AATTGTACCTCACTCCGCGTTTCATATCATCGAGACGTGGAATGACGCATATCCAAATAGGAGGCTATA
AGTTTGGTGTCAGCAAAGATAAGGGAATGAAGAAACGGTGGTCCTGCACTTCTCATAACAGCAAGTG
CTGCAAAGCATCCCTCATGACTGTGCTTGACAAGATTGTCAGTATAAACAATGTACATACGCACTAAAT
AAGTTTTAAGATAACTTATTGTACGGTCAGGGCCAGAGAAACATGACCCCCTTCTCATAGTAACAATGC
TTCTGAGAGGGGTCAGGTTTCTCTTTACCTATAGGCTGTAATTTACATAAGACGAAATGTATTATAAAGT
GGTGGCGGTG
>TCONS_00040104 geneID=XLOC_007938 scaffold_162:307554-308031(+) Intergenic
CCTTAATCTAAGTTCTTAAAACTAAAATAGAACAGCTTATGTTAATAAAATAGTATAATATTTATAGACAA
GTTTTGGTCTAGAACATTTACAGATACAGAAGAGTACCATCTTTAGTAATCATGTATTTTACAGGTCCAT
GTGTATGATTATCGTCGACTTTGCGAATAGACCCATCTCTGTCTATGTGCACGCTCGCTTTACAAGAAGT
CGAAAACTTCTTTGCGCAGTAAAATAATGATTTATATATAAGTGACTTGACTTCATTATAAGTGTACCCA
TTCAAAAGAAGTAATCTTTTTCCTTTCCTGGTCGGGAATATCCGGTATTCTTGGTATTCTG
>TCONS_00039743 geneID=XLOC_007989 scaffold_162:307557-309272(-) Intergenic
CTAACGGAGATGTGGTCTGCTACTATTCATTGGTTATTAATTTAACTGTTTTATTACAGAATTCCCGTACC
GGCTATTTCCAACCAAAAAAGGAAAAAGTTTACTTTTACTAAACGATTACACATACAATCAAAACTCTT
CATTCAGTTATCATTGTTCCAAAAGGATTTCATTAGGCTGCGGCGCGAGCGTAGGTCTCCACAAGGAC
GGGAGGATTCGAAAATTAGATGAAAACCACTCACATGGACCTGTTAAATATGTTATTGGAAATGACGG
GAGTTTGATCAAATTGTAATATTATTTAATTTAGGTTATATACATTTAATATTATAAATTTATTATTTTTCATA
TGGACAATTATTAATATGCTATACTTATTGTAACTGTCTCGACATGCATTTTTTTTACCTATCTCATAATTC
CTGTTGCCAGGAATCTAGAAGTTAATTTAAAAACGCTTGCCCGAAACGGAAACAACTTCTCAAGTACA
AAATCTCTCCTCTATTTTCAGATGTCCCTCGGTTCACATTATTATTACATCGAAAAAAGCAAATAAAAAT
CGGTGGATGCAAATTTGTTTTTAAATAAATTCAGTGACTGGACTGGCTCCTGCAATTACTAAAGTTGTT
GCATATAATTGTATATGAAGGCAGGAAACAAAAATAAATAAAAATTTAATAATCTTGAATTCCACAATTC
ATCGGTATTACTCTTAAAACGGCAAACTATTGAATGTTGTGATAATTAGAGAGGATCTTGTTAATATACT
AATAGGTAATGGCTCATCTGAATTGGATGTTGTTCGGATGCCTTTATTAAAAATGTTTCGCTAATATCAG
GTATGTTCTTTTCGTTTACTATATGGTCAGATAAATAAAGTGCATTTAATGATAACCAAACCATTGTATAT
TTTTTCAAGTGTTTCAAAGTTTTATAGATTAACCCCCTTATTCATAGAGAAGTTACAGAACGTTTTAATA
AACTGTTTTGTCCCTCTCTGTCAAAGAACAAATTGTTCTTTGTCGGAGAGGGACAAAACAGTGTATTA
GTTAAAACGTCTTGTAACTTTTCTATGAATAAGGCGGTAAGACTCTGATTACCCTTCCTCCCTTTCAGCT
AACAATATCACTTCTGCAATTGTTCAGTTATGAACAAAATTGTTATTGGAAAGTGTGAATACCAAGAAT
ACCGGATATTCCCGACCAGGAAAGGAAAAAGATTACTTCTTTTGAATGGGTACACTTATAATGAAGTCA
AGTCACTTATATATAAATCATTATTTTACTGCGCAAAGAAGTTTTCGACTTCTTGTAAAGCGAGCGTGCA

CATAGACAGAGATGGGTCTATTCGCAAAGTCGACGATAATCATACACATGGACCTGTAAAATACATGAT
TACTAAAGATGGTACTCTTCTGTATCTGTAAATGTTCTAGACCTGAATTTTTTACCAAAACTTGTCTATA
AATACTATTTTATTAACATAAGCTGTTCTTTTTAGTTTTAAGAATTTAGATTAAGGAATATATGATTACTAA
AGATGGTACTCTTCTGTATCTGTAAATGTTCTAGACCAAAACTTGTCTATAAATATTATACTATTTTATTA
ACATAAGCTGTTCTATTTTAGTTTTAAGAACTTAGATTA
>TCONS_00039951 geneID=XLOC_007989 scaffold_162:307716-309217(-) Intergenic
ACAGAATTCCCGTACCGGCTATTTCCAACCAAAAAAGGAAAAAGTTTACTTTTACTAAACGATTACACA
TACAATCAAAACTCTTCATTCAGTTATCATTGTTCCAAAAGGATTTCATTAGGCTGCGGCGCGAGCGTA
GGTCTCCACAAGGACGGGAGGATTCGAAAATTAGATGAAAACCACTCACATGGACCTGTTAAATATGT
TATTGGAAATGACGGGAGTTTGATCAAATTGTAATATTATTTAATTTAGGTTATATACATTTAATATTATAA
ATTTATTATTTTTCATATGGACAATTATTAATATGCTATACTTATTGTAACTGTCTCGACATGCATTTTTTTT
ACCTATCTCATAATTCCTGTTGCCAGGAATCTAGAAGTTAATTTAAAAACGCTTGCCCGAAACGGAAAC
AACTTCTCAAGTACAAAATCTCTCCTCTATTTTCAGATGTCCCTCGGTTCACATTATTATTACATCGAAA
AAAGCAAATAAAAATCGGTGGATGCAAATTTGTTTTTAAATAAATTCAGTGACTGGACTGGCTCCTGC
AATTACTAAAGTTGTTGCATATAATTGTATATGAAGGCAGGAAACAAAAATAAATAAAAATTTAATAATC
TTGAATTCCACAATTCATCGGTATTACTCTTAAAACGGCAAACTATTGAATGTTGTGATAATTAGAGAGG
ATCTTGTTAATATACTAATAGGTAATGGCTCATCTGAATTGGATGTTGTTCGGATGCCTTTATTAAAAATG
TTTCGCTAATATCAGAATACCAAGAATACCGGATATTCCCGACCAGGAAAGGAAAAAGATTACTTCTTT
TGAATGGGTACACTTATAATGAAGTCAAGTCACTTATATATAAATCATTATTTTACTGCGCAAAGAAGTT
TTCGACTTCTTGTAAAGCGAGCGTGCACATAGACAGAGATGGGTCTATTCGCAAAGTCGACGATAATC
ATACACATGGACCTGTAAAATACATGATTACTAAAGATGGTACTCTTCTGTATCTGTAAATGTTCTAGAC
CTGAATTTTTTACCAAAACTTGTCTATAAATACTATTTTATTAACATAAGC
>TCONS_00039173 geneID=XLOC_007990 scaffold_162:319843-321694(-) Splice junction overlap
CACAAGCATTCCAAGGTACCCCCCAGTTCGTGACATCAAATCGCGGCAACAAGCACATACTGCTAGGT
GGCTATAAGTTCGGCATCCACAAGACTAAAGGCCTGAAGACGCGCTGGCTCTGCACGGCCCACAACA
GCAAAGCCTGCTATGCCTCCCTAATGACCATTGAAAACCAAGTCATCAGTATGACTAATTATCACACCC
ACTGAATTCAAAGTAATTGTTGCAAAGTGCTGTTTATTCTACTGATAAAAAGGAAACTTATGCTAGAGC
GCATTTTTCAAAGTTGTAGACTCGAATTAGAAACGGCACAAAAATAAAAATCAATTTTAAAATACAATA
AATTAGTCTGTATAAATGCTTTGAATCATCTAATGTCTCATTGTAATTATGTATCTCACTTAGCAAATCAT
GTACTCCACGTTTATGAATTATAACAGTATCTTTCATTGGCCCCTGACTGTTATTACAACTTCGATCCAAA
TCAAATTTAAAATGTAGTCCTCCAGTGTCCCACTATTCCAGTACTGTACACACTGCAATACCTAAATATT
AAAAAATGCCATATTAATAAAATATTATAGATCATCATTTATGTTCTTTCAGACGGGCTGAAGTTTACCTA
CATAACATCGAGGCGCGGCATGAGGCAGATAAAGATCGGCAACTATAAGTTTGGCATCCGAAGCACAT
CCATGACGGAATGCGGTGTGAAATCTCGGTGGGTTTGCACCGCCCACCGCAAGAACTGCCCGGCCAC
CATTATGCTCCTCAACGACCAGATAGTCAGCATACACAACCCACACACGCACTAGTAAATACTGGTTTC
ATACCTGAGACTGCTCTGGTTTAGTTAAGCCTCTGTTAGTATGGAATATGGAAAGAATTGTTTGGCTAA
GTTGACACGGTCTATATGAGGATTTTTGAGACAGCATCTAGCAGATAAGTTGTGTGTATTGATTTCGGCT
ATTTTAATGTTATTTTTAGAATTTATAAAGTTACTGAACTCTATCGCGAATCATTTCAAAGCTCAAATTGT
AAATCTGGACGATTAGTCCACACATGTAGCTACCTAAGCATCACTGACTCTTCCTCGGGTACCTGGAGG
CTAGCTGGAAGAGATAATGTAAAGATGTGGTATGTTACAACACCCTCTATTTTTTAATTAACAATTTTTT
AACATAGCATGGTAAAATATGAAAGGAAAAGAAACAGAAAAGGTGTTTTATTTTAAACGTTTTGCGTT
TGTTTCTTGGTGACCTCAAACTAAATATATGTCGTTATTTAAAACTTTAAATTAACATAAGTAGCTCATAA
TTATAACTATAGGTATATATTAAGATAAATTATGTTAGATATAGAGTTGTGGAGAG
>TCONS_00039746 geneID=XLOC_007990 scaffold_162:319917-321228(-) Splice junction overlap

CTAGCGTTATTACTTCCAGCATTCCAAGGTACCCCCCAGTTCGTGACATCAAATCGCGGCAACAAGCAC
ATACTGCTAGGTGGCTATAAGTTCGGCATCCACAAGACTAAAGGCCTGAAGACGCGCTGGCTCTGCAC
GGCCCACAACAGCAAAGCCTGCTATGCCTCCCTAATGACCATTGAAAACCAAGTCATCAGTATGACTA
ATTATCACACCCACTGAATTCAAAACGGGCTGAAGTTTACCTACATAACATCGAGGCGCGGCATGAGG
CAGATAAAGATCGGCAACTATAAGTTTGGCATCCGAAGCACATCCATGACGGAATGCGGTGTGAAATC
TCGGTGGGTTTGCACCGCCCACCGCAAGAACTGCCCGGCCACCATTATGCTCCTCAACGACCAGATAG
TCAGCATACACAACCCACACACGCACTAGTAAATACTGGTTTCATACCTGAGACTGCTCTGGTTTAGTT
AAGCCTCTGTTAGTATGGAATATGGAAAGAATTGTTTGGCTAAGTTGACACGGTCTATATGAGGATTTTT
GAGACAGCATCTAGCAGATAAGTTGTGTGTATTGATTTCGGCTATTTTAATGTTATTTTTAGAATTTATAA
AGTTACTGAACTCTATCGCGAATCATTTCAAAGCTCAAATTGTAAATCTGGACGATTAGTCCACACATG
TAGCTACCTAAGCATCACTGACTCTTCCTCGGGTACCTGGAGGCTAGCTGGAAGAGATAATGTAAAGAT
GTGGTATGTTACAACACCCTCTATTTTTTAATTAACAATTTTTTAACATAGCATGGTAAAATATGAAAGG
AAAAGAAACAGAAAAGGTGTTTTATTTTAAACGTTTTGCGTTTGTTTCTTGGTGACCTCAAACTAAATA
TATGTCGTTATTTAAAACTTTAAATT
>TCONS_00039748 geneID=XLOC_007991 scaffold_162:321826-322691(-) Intronic
GTTGGTTGGTTGGTTGTTGGGGTAAGTTAATTAAAGTATTCTAAATATTTTCAGGTCTCATCACGTTCGT
GACTTCAAAGCATGGACGATACTTAGTGTACGGCAACTATAAGTTTGGCGTCCACAAGGCACATTGCA
CAGCACGCAAGACTCGGTGGCGATGCACCACTCATCACTCGAAGGGCTGCAAGGCTGCTATAATCACC
AACGGAGATGAAATAGTTAAAGTTCACGGTTTTCATTATCACTGAAATGAAGATCGTAACATCAGGTTC
GTGACTTCAAATCGTGGAAACAAACAGATAATATACCACAACCATAAATTTGGCGTCCACAAGGCGTG
TTGCACGGCGACCAAGACTCGCTGGCGATGTACCACGCACCACTCTAAAGGCTGCAAGGCCTTCATCG
TCACGAGCGGGGATAAAATCACTAGTGTTCACAATTTTCATTATCACGGAGATTTAAAGTACAAAAATG
TGTTTCTTAACAGTGTCTAAGGCGTAATATACTGAGACTTTTTTATCAAATTGATTTATCTGTTATTACGG
AGAGAGTATAATGTAGTCTGCGTTGGCATCACAGATCAGATATTTGTTTACGTCTTGTTTAAAAAAGAA
CAAAAGTAACCCTTGGTAACCCTTATTTCATAGAGC
>TCONS_00039751 geneID=XLOC_007992 scaffold_162:324332-327119(-) Intronic
TAAGGTTATAATATAGTATTTATTTCAGAGTACCCAGAGTTTATCACGTCACAAAGAGGAAAACAACTG
ATAAAGATTGGAAAATTTAGGTTTGGTCTCCAACATCGCAACCCACATACAAAAAAATGTCGATGGAA
TTGTTCTACCCATAATTCTAAAGGTTGTAAGGCCACCATCTACACCGTCGAAGATGAAATTGTGTTTATA
AAAAATGATCATACCCATTCATAGAGCAACGAAGCAGGCATATTATTACCAGAGCATGGTTATTCCCAA
CCAATTCAGTTTGTGACAACCAACCGGGGCAAGCAACAGATCCACATCGGTGGCTATCGCTTCGGGTT
CCACTTCTGTACGAAGAGCGACCGCGTCCGCTGGAGCTGTTCCCTGCACAGCTCAAGACTCTGTAAA
GCATCTATAATCACTTATAAAGACAGTATAATTAAAATGAACAATTGTCATAATCATTAAATATGTTAAAC
TCTCCATTAGCTTATTCGAATGTAAAAATACAATTTTATTATTGATGACAATGTCCTCTGATCTGGTAAAT
GTTTAAATATAAGAATAACCCTTGAGCCCATTCATTTTTCGCATGTCTATATTGTAATAAAAATGAATAGC
AAAAGGTAACTCAACATAGCTCTACCTCTATAACCAGTTTTACCAATAGGGTACCAATTCTTCATGGTCC
GGAGAACATGGGAGACAGCTAGGTACTTTTTTTAAACATTCATACTACACAAAGCTCCTCTACCATTTA
ACTGTCCCAACATAACATCCAATTAATTTCAGGCAACACAATCGTGTTCCTGACCTCCGAACGCGGCA
AGCGGCAGATCATAGTGGGGGATTATAAGTTCGGCATCCGGAAACGGAACCCGTCGAACGGGCGCAC
GCGCTGGCGCTGCACCACGCACCACTCGCGCGGCTGCCCCGCCTACCTC
>TCONS_00039753 geneID=XLOC_007992 scaffold_162:325653-327086(-) Intronic
CCAGAGTTTATCACGTCACAAAGAGGAAAACAACTGATAAAGATTGGAAAATTTAGGTTTGGTCTCCA
ACATCGCAACCCACATACAAAAAAATGTCGATGGAATTGTTCTACCCATAATTCTAAAGGTTGTAAGGC
CACCATCTACACCGTCGAAGATGAAATTGTGTTTATAAAAAATGATCATACCCATTCATAGAGCAACGA

AGCAGGCATATTATTACCAGAGCATGACTACCCAGAGTTTATCACGTCACAAAAAGGGAAACAACTGA
TAAAGATTGGGAAATTTAGGTTTGGTCTCCACCATTGCAACCCACATACAAAAAAATCTCGATGGAATT
GTTCGACCCATAATTCGAAAGGCTGTAAGGCCACCATCTATACCATTGAAGATGAAATTGTGTCAATCA
AAAACGATCATACCCATTCTTAAAGTAACGTTGCAGACATAATTATATAATGCACTGTACTTATACAATG
CAATAATTATATATAACGGCACGATGTTTTTTTTTTAGTTATCATATTTGTTACCCGAAGGAACCTCAATA
ATACATTCTTATACTTAAATAAATAAACCTTTGGAGTACGGAGTAAAAACTCTCACAACGCTTTTATTGA
CGACTTATGAATATAG
>TCONS_00039756 geneID=XLOC_007994 scaffold_162:338038-340100(-) Exonic overlap (+)
CGCAGTGGAAATGTTCACAAACAAGAACGGCAAGAAGATGCTGCGCATAGACGCGTACCGGTTCACG
ATCAACAAGCGCGTGCGCAACAAGTACTGGTGGCGGCACCAAGAGCTGGTGGCGGTGCTCGTCGCGC
GGCGCGCGCGGCTGCCGCGCCACCGTCATCACGCTCGACGACCTCGTCTACAAGATCGCTAATGAACA
TAATCACTGATTTGCTACCCATGTTAGTTAATTGCCGGTGTCCTTCTCGATGACCAAGCGGAATCGCCCC
CGGCTGGTGGTCTCAGGGTTCTCCTTCTTCAAGAAGCGAGCCGGCGTCATGAGGGACCACTGGGAGT
GTTCCACGCACCACAGCAAGGGCTGTCGGGCCTACGTCTGCACCGTCAACGACGAGATCATCAAGAT
GCACAACCAACACACGCACGGATGCGGCGCTAGTTCCTTGCAAGAATATTTGGTCTCGAAGCAGAACT
GTGTTTGATGCCATATCAATGATATGATATCCATATACAATTGTTTGTTTTTTAATAACATAGTAGGGTATT
ATTAGGTATAACGTAATTTATAAGTAACACCAATGTAATCTGATGTGCAGATAAACTTTTACTAGGTTTA
AGTCACATTATTTAGTAGAACAATAATGTAAACTATTAAATGAATAAAATAAATAAATAAATAAAATAATT
ATACAGGGTGTTGAAAAAGGGTGCACTAAGCCGAAAGGGGTTGACTCAAGGGGTCATTCTGAACAAT
GACTTAGTAACCTTTTTGCAACAGTCTGTAGATTAATCTTGTAGGCATTTAATTGAGCAATGAGTAGCTT
TCTTTAGGGTTTCAGTAAGTTATAATAATTACGTATTCAGTTAAATAAAAGTAAAGTACATGCTAAATGT
AGAGCATGCCTATTGCATAGTCGAACGCATTTATAAGACATAGTTGCATTTGTATTTTAAGGTTTCGAAT
ATCAGTATTATTATTTTGTACTAGTAGGCA
>TCONS_00039758 geneID=XLOC_007995 scaffold_162:342831-344751(-) Intergenic
TCAGCAGTGGTGCATTACACTTTTTCCAAAAAGGGGAATAAACTTATAAACTACGATGGTCACCGATTC
TTCAGAAAGAGAGTGATGGGCCACAAGGAGTATTGGCAGTGCACCATGCACTCCCAGAGAAACTGCC
GAGCAACCATGTCCGTCTACCAAGACACCATCATACGCATTAATAATGTACACTCTCATTAATAAACTCT
GAACAATGATATTTAAACTACCCTATTAAGGGGAAGTGTATTTATTATTTATTTATTTATTTAATTATTAAG
CTTACATATTAGTTGTAGTTTTACAATGAGTCCCACAAAACTGTTTGTATACAATTTGCCTGTGGGATCG
GAGTCTCTTATACTAAACAAAATACTTTTTATGTTTTAGGTATTGTTATACATAATTGGTAATATCACAAC
AAATAATAAAATTATAATCTGAGGTGAACTTGTATCAAGAATACATGATTACAACTTCACAACGGGGCA
AGCCGATCCTATGCGTTGGCGTCTATAGGTTTTGTAAGAAAAGTAAATCCGGCTTGAAAACCAGATGG
GTTTGCTCCACGCACAACCACCGCGGCTGCAACGCCATCGTTCACACGTTCGAAGATCAGCTCATTAA
GATTAAAAATGTTCACAATCACTGACACATACGATCCAGTGGGTAATCTCCTTAGTATCTACGACTAATC
AA
>TCONS_00039688 geneID=XLOC_007940 scaffold_162:350947-351470(+) Intergenic
AGCTTATACACTAACACAAAGGCACAAAAAGGGCGGCGTTATCGCTTGAAGTGATTTTTTAAAGACAA
CCTTTTGGGTGGAAGGATATACATTATATACATATATCATCAAATAAATCTGTAGTGAATTCGTTAAGAAT
GAATGTAGAAGATGTTATGTTTTCCTTCATTTTGAACATTGCCGACCAAACACGAAGCTACGTAGGATA
GGTCTAAAGGCAGTAAAATAATAAAATAAAAAACAAGTAAATAATAAATACGTCAGAATATACAAGGC
ACTTTTTTCCTCTAAATTTATTCAAACTAAGTGGCAGGTGGATGGTTATGCACATTGTTGACCTTGATGA
TCTCGTCAAGGTAGGTGAGCAGGGCGGCCCCGCACTTCCTGTTGTTGTGCGTCGAGCACGACCAGCG
CCGGCGCGCGCCCGACTCCGACTGCTTGCAGAAAGTG
>TCONS_00039767 geneID=XLOC_008003 scaffold_162:417568-418898(-) Unclassified

GTTCGGCAACCCCGTGATCGAGTACTCGGGGTTCCGCTACAACCTGCACAAGACCAGCCGCCTGCCC
AAGGGGCTGTGGGTGTGCTCACGCGTGTCCAGCGGGTGCAAGGCCAAGTTGGTCACGCTGCTCAACC
AGATCGTGAAGGCACAGACGGAGCACAACCACGCGCCGGTCAAGAAGAGACTTCAGTAGTTTTAAGT
GTAAAGGCATTCAGATCTTAGCGAGTTACCTGCATGTGCTCGACAGCGATTGCTTTGGTCTAAATAGAG
CTTTTTATCCAGATGCTTAGGTCGTTTATCCACCGCACGACGTCAAGGTCACGTCACGAGAACAAGATT
ATTTCAGGCCGAGTTTAGTATGACTTGACGGCGACGTGACGGATGTGCGGTGGACACGCGGCTAAGTA
TAATGTATATGTCAGCCTAGTCATACAATCAAAAAGGAAATTCAACCAGATGCCTGCGACATATATACTT
ACCGCTACTTCGGCGACGTCATCTCGATCTTATGATGTGACGTTGACGGCTTGCGGTGGACAAACGAA
CTTACTTTCAAGAATTTAATTTAGTTATTTATGTACGGTTGTTTATTATGACTTTCTGTGATAAGGAATAT
ATTACTGTTTCATGTCGTTTATAGTTTTTTGACTAAAGTTTAAGTATGGATAGATAGAAGTAAAAAGCCT
AGTTTAATAATTTAGTAATTATGGCCGAAGTAGGTATTTCCGCAACTAACGGTCTATGCTTTTCAGATAC
CCAGCAGATTATGTTCTCAGTGACTCGCTTCGGCAACCCTGTCATTATAATGGGCAGCTATCGTTATAAC
CGCAACAACAGATATCTTTCTGGCCCCAAAGTATTTTGGACGTGTAGTAAAGCCTACGTTGGATGCAA
AGCTAACGTCACCACCGTTGATAACGTCATATTGAGACACAATATGGAGCACAATCATTGAACTTTGAA
ATCACTCTTGCATACCAAGTTATTATCAGGATATGTTCATTACGTTAGAAATTTACGAACTAATTAATGAT
TAGTTACTTATTTTTTTAACTTCATATTCTTTAAGTAACCTTTAAATATTTTTCATAATTTTAACCCTAATAA
CAGTATTTAAACCAGATTGGCGA
>TCONS_00039376 geneID=XLOC_008007 scaffold_162:429449-434258(-) Unclassified
ATTTTAATTATTTAAAATCGTATGCTTTTTTTCAGAAGCAATATTCTTGAAAACGAGGCAAGGCCACGCA
AGATTGATACTAGATGGGAATTCATTCTCACTGCACGGTGACTACCGCTACAGAAGCCGCGCTAAACT
GCTGTGGGTGTGCGCGCAGTACCGCCGCGGGTGCCGGATGTCCCTCACCACGGTCGGAGAACAAATT
ATTAAACGGAAGAATGAACATAATCATTGAAAAACACAGGGCTAAAATTCACGAAAACGAGAAACGA
CAATCCAAGATTGCATCTAGATGATTATTCATACTTCCTGCACGGTGACTCTAAGGGCCGCCCCAAGAA
GCTGTGGCTGTGCAACTGGTACAGCCGCGGCTGCCGCGCCTCGCTCACCACCATCGGAGACGAAGTT
GTCAAAAAAAAGAATGCGCATACGCATTGAGAACGAATGCGCTGCGCGTTATCTGTTAATAAATGAAA
G
>TCONS_00039956 geneID=XLOC_008007 scaffold_162:429449-434258(-) Intergenic
ATTTTAATTATTTAAAATCGTATGCTTTTTTTCAGAAGCAATATTCTTGAAAACGAGGCAAGGCCACGCA
AGATTGATACTAGATGGGAATTCATTCTCACTGCACGGTGACTACCGCTACAGAAGCCGCGCTAAACT
GCTGTGGGTGTGCGCGCAGTACCGCCGCGGGTGCCGGATGTCCCTCACCACGGTCGGAGAACAAATT
ATTAAACGGAAGAATGAACATAATCATTGAAAAACACAGCGTCCGCCTACATGCCTGGCGATGGAGAT
CCTCCACCCGGTGAGTTGGCGGACCTGGCGCATCACTGCGAGTCCAACTCCCTAGAGCTGATCATCGC
TGCGGATACCAACGCGCACCATCCGCTCTGGGGCAGTGAAACTCCCAACAGAAGGGGTGAAAACCTG
GGCTAAAATTCACGAAAACGAGAAACGACAATCCAAGATTGCATCTAGATGATTATTCATACTTCCTGC
ACGGTGACTCTAAGGGCCGCCCCAAGAAGCTGTGGCTGTGCAACTGGTACAGCCGCGGCTGCCGCGC
CTCGCTCACCACCATCGGAGACGAAGTTGTCAAAAAAAAGAATGCGCATACGCATTGAGAACGAATG
CGCTGCGCGTTATCTGTTAATAAATGAAAG
>TCONS_00040145 geneID=XLOC_008007 scaffold_162:429647-435273(-) Exonic overlap (+)
ATATGTCGTTTAAATGACTTATTTTACCTTACCCTGTCAGTACTTGTATTTTTGTATCCATATATCAACCTA
CGTACCATTTAAAATTATGACAAAGCAATGTTTCTTTTTCAGTACCAATACCAATATTCATGACAACTCC
GCGTGGCACTGCAAGGATGATAGTAGATGGATATTCATTTGCTCTACACAAACCTTTTAAAGGGCAATT
CAAGTTGCTATGGACATGCAGCCGCTACCGGCGCGGATGCCGAGTGTCGATTATGACTGTGGAAGATA
AAATAGTTAAACAAAAAAATCAACATAATCATTGAAAAAAAAAGAAGCAATATTCTTGAAAACGAGGC
AAGGCCACGCAAGATTGATACTAGATGGGAATTCATTCTCACTGCACGGTGACTACCGCTACAGAAGC

CGCGCTAAACTGCTGTGGGTGTGCGCGCAGTACCGCCGCGGGTGCCGGATGTCCCTCACCACGGTCG
GAGAACAAATTATTAAACGGAAGAATGAACATAATCATTGAAAAACACAGGGCTAAAATTCACGAAA
ACGAGAAACGACAATCCA
>TCONS_00039775 geneID=XLOC_008009 scaffold_162:440381-441421(-) Unclassified
TAAAATTTCAGTGGCAATTTTCACTAAATCCTGCCTAGGGAAGCCGTCGATTGTGATAGGCAGCCACCG
ATTCTGCATACAAAACGTGAAGAGGTCTACTCAAAGGCTGGTGCGCTGGGTGTGCACCAAGCGGACC
TCCCAGCAGTGCCGCGCGGCGCTGCACACCGTCGACAACGCGGTGGTGCGAATCATTAATCAACACA
ATCATGGATAACGTGGCCGTGTGAAGTGATATTCACCACATCAGTCCACGGGAACCCGTTCCTCAAAG
TAGGAGAGTTCACATACAGCATGTTCACGCCGTCGCGCACCTCGGCGTGCCTCAAGAAGCGCTGGGT
GTGCTCCAAGTGGCGCTCCGGCTGCCGCGCCACCCTGTATACTGTGGACCACCACATCATCTCAATGA
GGAATGAACACATGCATACGAGCAGACGGAATTGACAATAACTTAGTGTTTATTATATTTGTATGATAGA
ATTTTCTTTGGGTACTTACCTATAATTAAATGCGGTTTATTTAACCATAGCAATGTCTACGGTATATCTGT
GCAATGTTATCTTGAAC
>TCONS_00039464 geneID=XLOC_008013 scaffold_162:449326-449834(-) Intronic
TGACACTGCGCTATGACGCTGAGGTTTTACCTTTACAACATCGAAATTTGGCAAGCCGGCGATAGCAAT
GGGCAAGTATAAGTTCAACAAATCTTCAAAATGTCGCGGACCCAAAGTCATCTGGAACTGTAACTGGA
CCAGCCGGGGCTGTAAAGCCTTACTTGTTACTATTGATAACGAGATCGTCACCTGTAGAAATGTACACA
GCCACTAACTCATTGCGTGCCAGCAGTATTTCTATTGTAATTAGTACATAATATGTGTGTGTAACTTCATT
ATGAACGCAATTATTTCAATATGTAAATAGTATGATGTTTATAAT
>TCONS_00039782 geneID=XLOC_008016 scaffold_162:499752-501273(-) Exonic overlap (+)
GCAATATCAGTTTGTAACGTAATTATTTAAGTACATGTTTTTTGCAGACTGTACAGATGAACCACCACTT
TTCTACAACACCCCGAAAGGCGCCCGCGGTATATTGTACAAAGGGAGCAGGTTCAACTACGACGTGTA
CTCTCCGTACACGTACTCCCGGTGGCGATGTGTGAAGAGGAACCACGGCTGCAAGGCGATGCTACAC
ACACGCGATGACCGCTTCATTAAAATATACAACGTTCACACGCACACGGACAGGCAGAGACCGTGTCG
AATGGCGGCGAACACTCAACGCTAATTAATGTCTTTGAGTTTGAGCTAACCGCTGAGCTGCTGCCAGA
CGTAAACTACAGACCGCAGAAGTAGTGACGGCTGCATGTGCATAGTATCTTTATCTATGCAGATAAACG
TTACTATATCCTGATTCGCGGGACAGTCGATCGGTGGGACGTGCCACATATTTCTTCCATACGCGCCAG
AAGCACCGCGGGCTGGTGGTGGGGCGGCACCGGTACAACCTGGTGGTGAAGCGCGGGAACTACGTG
AAGGACCACTGGCGCTGCGTGAAGCGCAACACCGGCTGCCGCGCCGTCGTGCACACGCTGCAAGAC
GTCGTCGTCATGATCAAGGAAAACCATAACCATCCATAGGTTGAACAAAGAACGCACTGGGAGGTCA
CTCAGCAGTATGACGAAAAACTAAGTTTCAGAGCGCTGCTGCGCGAGCCACGACGCTATCTCGTTGTG
TTAAACGTGCCGATTGCACGGCACTCGTTCGTTAGTTTTTCGTCAATATAGATTAATCCTCCTGTTCCGG
AACGTTCGACAGTGCTGCCTCTTGAGAACCATGAAAAAAGAATCTCGTGTCCGAGTTCATAAACGGGT
TACCATTTAGATGTACCTATAAATGTTGTGTAGTATTTCTTATTCCATTAACATTAATATCTAAGGAATGTT
ACTAGAATATTGTGATTACTATGTACCTTCTTACTGCTACATCTATCAAATCAATACATACAACATAATATA
TTATTT
>TCONS_00039101 geneID=XLOC_007943 scaffold_162:519986-520568(+) Exonic overlap (-)
CTCATTAAGCGTTCGCTGATGAACGATCCAAATGGACGTCTTCGATATATCAAAATCCTTCATGTCGGAT
TATGACTCTATTTTTAATGCTACACCAGGGTGACGTATATTTGGAGCGTCCGTATTAGCAACAATGTACC
TAATACCTAAAATATACATGTATCTGGGCGATAACGCGACAAGATGTACCTACCTACACTGCTTCAAAAT
TTAGCTTTGTTTTTTTAAACGAATTTCCTTTTTTCGAAAATCATAAAAGTTTTCACGTAAAATCGTAATT
AAATGCCAGTATTTTTAAGGGGACAGTCACTACACAACCAAATAGCGATTTTTTTAAACTTGAATTTTC
ATAATTCAATATAGTTTAATGCGCAATTTTTTTACATT
>TCONS_00039958 geneID=XLOC_008019 scaffold_162:541148-543390(-) Unclassified

GGGCGGGTCTGCTCATCTATCATTGTTCCTAAACAAAACTAGGTACTAAAATATTAAATGAATCATCCGC
AAGCGTTTTGTTGAGCGGAGAGTAAGATGAAGATGAGTTTAATGGCATGGCGTAGACAGGAACCACT
CATTGAACAATTTGACCAGCGAACATAACTCGTGCCCACTTCAAGACAAAAGGACGAAGGAACCAGC
TATTCAATTTGAATATCGTCGAGGAAACAATCAATACGACTGATGGTAATGACCAGCGCACTTCGGATG
CACTGAGCCTCCTAAGCTCTCCACTCTGGCATAATAATTCAAGGATCCTATAGAAAGGTAATCATTTAAT
TGACTTGAAAATATATGTATTAATAAAAAAGCGTTTAAAATTTGCATTCGTTATTTATTATTCAGTCAAAT
AATACAAAACTTATTCAAACTATTATACACTTTGGTAATCGCGATTTGACTAGATAGGTACAGTACAATC
ATACATGTTACATGTAGGTTAAGTAAATATTTTTCACCGTGATGAATGAAAAGAATGTGATAAACATCAC
CAATCCTAGTTTATATCAGTATGTATATTCTATATTCACTCTTCCAACTACGTATGAACAATTAACAAAATA
TTTTATAAAAATAATGGAGATACATCTCGCTGTAGTTATATTGGAAAACTTGGTAATAAGTGTAGTCAAT
ATTTCACTTCTGAGAATTCGCAACGAAATTACATATAACATTGAAATCGCAAATAATTTATGACTCATTG
AAAAGAACATTCTACCGATTAATAGCTGCTATAAATATATAAGTACATTAAAATTATGAAGGTGCCAACC
ATATCGAAGGTATGAATTTGATTTCCTTACATTATGATCACGACAAAACACTAAACATAGGCATACTCGT
TTATTAATATACCAATTAGTATGTTAGCTCTTAATCCAAAAAATCTAACATTGATAGCTTACTTTTTACTGT
AATATTTTATTATAATTCAATAAAAAGAACTTGAAGGCAGTAGGAATGCAATAGTAAATAGGATATTAGG
CCTTACATGATATGACTAGACCAGGAATGTCAGTATACTGTAGACTACACTAGCTGAGAGCCACGAGAC
GAGTGGTCCATGCTAATAAAATTGTTGCACTTGTTGATAGGAACTTAACCAATAAGATACAAATAAGAA
ATATGGCTGAATTTATATTCAAGACGTAACTGAGAATAAAAATACTAACTCGCACTAGCAATTTTGACA
AAGCCGCAAAGGTAGATTATAGTAATACAGCGTCACAAACAAACCTGGTACAATTTTGGGATATTTTAC
TCATTTTGAATAAAGCCAAAATTCGGAAGAGTTATCCATAAATTATATCAGATTTGCAGTAGGTATAGAG
>TCONS_00040149 geneID=XLOC_008019 scaffold_162:541148-544262(-) Intergenic
AATGTTCGCTCGCATGGCACCAGATTCACTTCACAACGATTTGTCTGTCTGGTGACGTCACACGCTAAC
GTCATCCACCCCGCGGTCAAGGACTCTCCAGTTAGGGTTTTAGTGTTCCTGAATCTCGATGGCCTAGAT
CTATACGTCAGCATTTTGCGTAACAGAGCGGTAGGTAGGTACTAGGTAAAAGCTACAGTCCGTAAGCTA
TGAGAAAAGACTGTAGGCTTTATTAACACTTTTTTTTTTCATTTATAAGAATATATTTTGGATTGTAACAC
CACCCTCGACTGTATAGCACGCGGCGCCAAGTTTAACTGTCTAATGGAGAAAATTATCACAAAAGCTTT
AATTAAAATCTAGGAACTTACATTTGCACAAGACACAATCCAGAAAAGTGCACACAAGATTTTTTCCA
GTAAAAAAAAACCCGGTCACTTTCAACACACCAAGTTTTCCGCACCTGTGTCGGCGACAGGACTGCC
GCGCTGCGCCAGGAGCCAGTTGCCCAACACTTGATGAGCAGCAATCAGCTGCTTCCGGCTGAGCAAT
CCGCATTGTTCCACCGCCTACTTGGATTAGCTGAGCCACGCTCGCGGACGAATTTAGTTGATGTAATGA
CCAGCGCACTTCGGATGCACTGAGCCTCCTAAGCTCTCCACTCTGGCATAATAATTCAAGGATCCTATA
GAAAGGTAATCATTTAATTGACTTGAAAATATATGTATTAATAAAAAAGCGTTTAAAATTTGCATTCGTT
ATTTATTATTCAGTCAAATAATACAAAACTTATTCAAACTATTATACACTTTGGTAATCGCGATTTGACTA
GATAGGTACAGTACAATCATACATGTTACATGTAGGTTAAGTAAATATTTTTCACCGTGATGAATGAAAA
GAATGTGATAAACATCACCAATCCTAGTTTATATCAGTATGTATATTCTATATTCACTCTTCCAACTACGTA
TGAACAATTAACAAAATATTTTATAAAAATAATGGAGATACATCTCGCTGTAGTTATATTGGAAAACTTG
GTAATAAGTGTAGTCAATATTTCACTTCTGAGAATTCGCAACGAAATTACATATAACATTGAAATCGCAA
ATAATTTATGACTCATTGAAAAGAACATTCTACCGATTAATAGCTGCTATAAATATATAAGTACATTAAAA
TTATGAAGGTGCCAACCATATCGAAGGTATGAATTTGATTTCCTTACATTATGATCACGACAAAACACTA
AACATAGGCATACTCGTTTATTAATATACCAATTAGTATGTTAGCTCTTAATCCAAAAAATCTAACATTGA
TAGCTTACTTTTTACTGTAATATTTTATTATAATTCAATAAAAAGAACTTGAAGGCAGTAGGAATGCAAT
AGTAAATAGGATATTAGGCCTTACATGATATGACTAGACCAGGAATGTCAGTATACTGTAGACTACACTA
GCTGAGAGCCACGAGACGAGTGGTCCATGCTAATAAAATTGTTGCACTTGTTGATAGGAACTTAACCA
ATAAGATACAAATAAGAAATATGGCTGAATTTATATTCAAGACGTAACTGAGAATAAAAATACTAACTC

GCACTAGCAATTTTGACAAAGCCGCAAAGGTAGATTATAGTAATACAGCGTCACAAACAAACCTGGTA
CAATTTTGGGATATTTTACTCATTTTGAATAAAGCCAAAATTCGGAAGAGTTATCCATAAATTATATCAGA
TTTGCAGTAGGTATAGAG
>TCONS_00039191 geneID=XLOC_008021 scaffold_162:622070-627179(-) Intergenic
TTATAAGCAGCGTTCGATAGATTTGTCGAGTTTTTGTGTTCGTTATAGGTGTTTATGTCAGTGTGAATAA
ATGTGAAATTTATGGTGAAACAGTTATTTATAGAGATTTGTTTTCACCTATGTTGTCTACAAAATTCAGAT
ATTTGTATACAGGAATATAATTGACTTTCAAAAACACCTCCAAAACACCTAGCTAGCCTACTCAGTACT
CAAGTTCAAGCTACAAATAACAAAACTCAACAACCAAAACATATCCATCATATCAAACAGACCAAAAG
TGTCCAAGTGTTTGTTCGCATCGCGTTGTTTCCTCTCCATCGCTGCGTGAGTGGGTGCAGTGTGCGTCG
TCAGCGATGGTGGTGCAGTGATGAGCGGTGATGGCGTGGCCGGGGGGGACGCCCGACTCCGGGGACC
TGCCGCTGCGGCCGGTCGCCAGCGAAGACACCTTGCTGAGCGCCGGGCCGCCCACCGACGAGGCCA
ACACTCC
>TCONS_00039517 geneID=XLOC_008033 scaffold_162:1027525-1033168(-) Exonic overlap (-)
AAAGAAACAATATGAAATTTACATGAGTATTGACAGACCTAAGGTTAGAGCGAGATAGCATGAGTCTG
TCCGCCATTTTTATATTATTTCTTTATTTCTCGGCGTTATACTTCTGTCGCTCACTGCGAGCGCGAGATAA
ACTTATTACAGGTTCTTGTGAGACCCATGCTACAGCCCCAGATGATAAAACTCCAGTCGCAGTTGATGC
TATACAGTATACAGAATATTTACTATTGCTTAAGCTTACTTAAGGGAGGCCGCCCTTTACGCCTAATATAC
GATATATTTTGACATCAAATATTGCACAAACGCCTACGCCTGTGGTTGGTCTTTGGCAAGATTTTGTAAC
CCCAGCCGGTTTCTAGAATGACAATCAATGCGACGCGGTGTAAGATATGTTGAAGAACAAAGTTCTCC
CTCAGCGAATGGTTTAATTGTAGATTTTATTTGATGGACTTTACCAAAACCAGAAGGAAGTTAAGTAGG
TATTTCTTGTTCATATTGATTATATACACTTCTAGAAACCGGCCCCCTGAACTAACTCACTGTGTGCCAA
TCGGATATAATTAGTAGTTTTAGTTTGATGTTGGATTTAGATAATTGGTTCGTTCAAATTGATTTTAAAAC
TGTTTTAACGTTAAATATCTACTAACTTACTACATACATACCTACCTAGATAGTTCTATTAGATTTCAAAC
ACAAACTTGCTCATAAAGTAAGAGCCATTGTGGGATTCGAATGTTTTGACAAGAAAAATGCTTGTCGT
ACGTGTAAAAGTAGGTTCCCATATTTATTTCAGTAGCTAGTCGTTAGGATAAACTATATTTTCTGTATGAT
TACTGATAACCGAGTTAATACTAGATTGGCGCACAGGTGATGTTTTCATTTCAAAGATAATTGTGCCCTA
TAAATATCTATAACTAATTATTCCGTTCATGTGTAATTAAATAACGGATTTAATTTAATCAAGTGGTGATT
GTAATTTAGTCACATTTTAATAATTTACATGCACAGATTTGCTCATTCATGATAGCAGTAGATATGCATTC
TATTTAGGTGAACTAGATATCTAGTTCACCTGCTATTAAATAAATGTATGGTTATTATTGTTTTGTAAACTA
AACATAATGGTACTAATGAAGTTAACGAAACGTTAACAATTTGTCCAAAAATATGTTGGAGTTTACCCA
AAAGCTATAATGATAATAATACCTATGTAGTTCGTTTTCAAATACATATTTACTTTTTATATCATCGATCGG
GTTTTGAACATTTATTTTAAACTGAATACCTACTGGTAATGTATTGCTTTTACAGTGAGGTACAAGTAAG
TGTATGGGAGATAGGCTCATCCTATAGAATTGAAACATTTTACATCCTTAAATTTTTCTAAGAAATAATC
GACATAACTTTCATCTAAAAGTGTCATTTTTATAATTATTAGTTGTTTCATCCATTCCTCCTTGTTTTAGGT
TTAATACGAAATTGTTGTTCATTTTCATAAAAAAAGCATTTGTAATTTAAGAAAAGGGTATAGTAAAAAT
TTTGTTAATTTTACTATAACACTATTTTGTATAGAAATATATATTTCTATAGGTTACTTTTAAATTAATAAAA
GCACTTGTCTGCATATTTGCAAAATCTTGCGTAATTATTACGTTAAATACTGTAATTTGTGTAAATATTTTT
ACTTACAATTTAAATATTTTGTCACTTTTAATACACTTGATTTTGTAATAGTTTCTTAAATAGTACATGTTA
TTCGTCACATCAACTCATAAACGATTTGTTATGCCATAGGGAATTTGTAGGAAAATAACATACTAATTTA
AACCATCATCGATTTTTCTAACAATATTTAGTCAAGTGCTATCCTATTCAGAGTTGTGTGACCTGGAAAA
TAAATTATTTCTCAGTCAAATGGTGAAACAAATCGGGGCGACCACAAGTCCGCACTCGCCAAAATATA
CCTACATACTATGTAAAAGAAAACAGGTGACAGTAGATCGGGAAGTTGACTGAATTTCTTTTAGCTCTT
AGACAGAAGGTCAAATAGAGACATGATAACATCGTACGGTAAAAATTCTAGCAAATAAGCAACTATAAT
ATAGTTATGAAAATAGCAAATGGACTAATGATGTCTCGTCTGTCAATACAAATTCAGGCCATTTTTATTC

CTATGTTACCCGATCAACATCTGCATCTATGGCCTTGGTGACCTTTGTGTAATGCTAGAAAATATAGTTA
CAAACACAATATTTAACAGATAACTGTTGGTTGACCTTATTTATTTACGAGTTTTTTTGTGGTGGTTATA
AAACAAGAATTTAGCTTTTTTGTAGCTTTATAATATTATTGTAGTGGTTTATAGTTAATTAAATTTAAGTT
GTATAAAGTATGAAAGGATTACAGCTTGGTTTATTAAACAGATGTGACGTTTTTGAGTAGGATGGAAAG
TTTCCTTCATTATGGTATGAAACGAATGACATTGTAAACACAGTTTTTTGTTACATCCTCAAATAATTTTA
TGTATTAGGCAGTATGATATTTAATATACAACAATCACACATAACACAAGTCACATCTTAATCAGAGATA
AAAACACCTTCATTATAAGACAGTAATCAATGTCCTGTCCTCTTCTTTAACCAAAAAGTTTTCGGATATG
ATAATTATAATGTACTTGGTAGATGTACAACTTGAATATTTATTAAATTCACAGGCCAAATTAAAATAGAA
CATGGTTAAATTTTTGTTTGGTGCAAATTTCAATACAAGTATTTTTTAAAACTAGTATGCACACTTACCT
ACTATATTTTAATATAAAATGTGTGTACTCCTGTGATGTATTTTAGACTGCTAGGATTGTCTAAAATTGAA
AGAATCTGGGTGTAACAATATTATTATGTGACAGAATGCAATTAATAAAAATATAAAAGAGAT
>TCONS_00039642 geneID=XLOC_008036 scaffold_162:1210961-1216582(-) Splice junction overlap
TATGCGCCTGCCCTTGTGAGGTGTCACCACGCGCGATGTAGCAGAGATGTGAAAAGTGTAACAATAAA
AGTGAAATCGAGCAAAGACTATAAGGTACCCAGTGCATTGACATTTGAATCTACCAAATAATTGTGCTT
GAATATTTCGTTCCTGTGTCTTATGTATGCATGAGACAGTATTTCGTGGGCAAAGCTCTGTTATGTTAAG
TATTAAAGTTTGAATGTGAATCTTCAATAGAGACCTATGTCTTGTTGTTTTGTCAATTTGAATTGTGTTAA
TAAGTTAAGAATATTATTTTGAATATATGACATGAATATTTTGAAATTATCTATATATGTTTATTTTTAACTT
TTCTTCATAACGTAAGCTTTAAGAGCTCAGAAGAGTAGGTTCAGTTATCAGAAAGCAAATAAAAAAAC
CTGCACAGATTGGTATTTTCTACAGTAAGAAAAGGGCAAACGAAAAATGAAACACAGTTTTAAAAAC
AATAAAATAGATTTTCATTTATTGTGAGACGAAAAATACAACAATGGGACTTATGACCTAGAATGGTTA
GTGGCAGACTGTCGTGTCAAAACAATGTTGTTAGGTCAGACATGCCGCGGTCGCAGCATTCCTAATTTA
TGTCTTTCATTGGGGAACTGTGTCAACGAATATCAACAAAACATAGTTATATAATACACAAACCGATAC
CATGACCTCTCTAATAATCATTGATTCCACAACTAATATTATTTAAACATGTGAAGTTTCAATAGTTTCAT
TTTAACTAATTTTTAAAAAGGCTTTTATTGATTCGAAGCTAATACTTACTTACAAGATTGTATAATAGTTT
GTTTATAGTAGAGCGGCGGGGCGCAGTATCGATGGCAGTGAGCCCCGCGCGATCCAACCATCTCATTC
AATACTACTATGAAACGTTCTATACAAAATTGAACATGCCGATTCACTAAATCGTTAGTTCTAAAGTCAC
GTGGGCGGGAGCGGCGCTCGCAATCTTCACTAACTCGAATAAGTACATGTCTTACTTTATAACTTGAAA
ATGCCACTGCTGCGATTCTACGTCGCCTAGAGGTGCGGGGCGCGGCCCGGGCGGTGGGGGCACGTGA
CCCGCGGGGGCACGGGGACCCACGGGGCACGGTGCCCCCGCGCCCCGGGCCCCGCCCGCGCACCGT
GTATTGCTCATAGAGAAAATTTCCAAAATCAATTCGTTACACCGTAGACTATATTTGGTTTAATAAACTT
GAAGTATTTATCAATAATCAATAAGTACATTATAATAATTGAATAATTAGTTATTTTGTTTTTTCATAAGAG
ACTAGCATTACAAATAAATACCTTCAGCTATACAATTTATAGTTTATTTCTATTTTAAACATTTCGAAGAT
GTTTAAGTGGACTACGTCGGTTAATACACAAGTGGTCGTGGGTCTTGCATACAACTGTCTACGCCGAGA
TATGACTTACATGTACACTTACACTTAGGGGAAAGGTCGTAAAAACAAAAAGAAAGTTATTGAAATTTA
TAACAAACACCTTAACTAATACTTCAATGTAATATGTGTGCGGGCGCGGCGGCGCAACCAAACAACTTT
ATACAAGGCCCCGCGCGCCCGGCCCCGGCAGCGAGGCGCGGATCTTACGCATTTTATTTATTTAACCAA
GTATTTTTTGTAAATGTATCCGTAATTATTTGTTTCAAAAAACACACTCCCATTCGCTCACGCTTGCGCTT
AAATCTAAAATTAATAATTAGACAATATTTTTTATTTGTTCAACAGACGCTCGCGTCGCGCGCGTATAAT
AATAATAATAAAATATTTTCAAAACAATCGAATAATTAAACACACTAGTTAACAATAATCGAGCGGCGA
GTGCGTTCGCGGCGGTTAGACGGCGGTCTCGGTGTGGCTGTGCGAGGACAGACGCTCGACGTCGAGG
TCAGCGGGCGCCAGCGTCTCCCGGGGCTCGTACTCGACGGATGACGAGTGCGAGAACCGCTTGGCCA
GCGCCGTCTGCTTGCGGAGCGCCTCCGCCAGCGCCGCGTCGGCCAGCGAGCCGCGCGAGTCGAGCCC
CTGCCGGAACGCGTTCACCACGCGGATCTACGCACCAACAACATGATATTAGAATGAGGTGACGGTGA
CAGCGGCGCCGCAATGCTCTGATTCTTGTGTGATTGGAAACTACGGTTCTCAAGTTTAGGATGTCAGGT

ATTTACATGATCAAGGTGGCTAATAGAGAAAAGGTTTGACTTGGCTAATCGGTATAGAGACTAAGGAA
GGTGACGTTAAATTGCAAGTTTGCACTGAAGCTAGGGTGGCAGTGTTAAAAATCAGAGCATTGCGTTA
GTAAGTCCCGTCTCGTCTCGCGCCAACGGGCCATAGACACCCATGAAGCACCTCGAGCCATATAGAGG
AGGCGGAAAAGCGTTAGGTCGTTCAATCCCAGTCACAGATCCAGTGGACATGCAGTGCGCTGTGTCC
ACGGTAGGGTTATAACTCGTTTGTTCTTCCATTGGTGCCAATTGGTGTGGTGTGGTCATGCATATCTGAG
TCGTCTGTGACTGTATTCGATCCTGGGACTACGACTAGGCTACGCACATCAGGGAAGCAACATTTACTG
TCAATATTCAACGCATGTCTCGGAAAGCGTTTTACAAGCCCATAGCAGTTATATTCACCAATGTATCGCC
AATATAAGACAAGATCTTCCACCATGGCCCATGGTCCTAAGCCTTACAAAAATGGAATAATGAGTATTT
TTTTCAACCTAAGAAGTGAAACTATATGTTTAGATGACGCCATACTTTTTTTTTGCTAAGTTTTTTTTAA
AGCTATGATTGCTAGAAGAGATGAACCCCTGGATTACTCGAGTTGGGAAATTCACTAATTTTGTCGTTG
ATAGAGTGCAAAATGTGTGAAGCAAATATATTACTATTACTGCAATGATGTATGACGCATAAATAAATAA
AAAGCTAGGGTATATCTAATTCTAATATTCAAACGGTTAACTTTATATATTTTATATCAAATTTATTTAATTA
TTAACCCCATGTAAAACTTTACAGGACACATATTTCTTGTGTGCAAAAATCCTGTCATTATGTGGTAGGT
ACTTTGGTGCACAGACAAACTCACTCTTTATATGAATAGTTGTTACTGTTAGAGACAGGCAATATATGTA
CAGAAAATACACACAAAGTGTCACAAAACATCAATATCAAATGCTCACATCAATATTTAAAACACCCAT
ATATGATTATTTAGATTTTCATATTTATAAACATATAAAAATATGATATAGGCACATGCGATAGGATCCATG
CGGATGATGTCATGCATTTTTATATATTTTACTGACCATTTGTTTCATATAAAAATCAGTTAATAAATTCCG
TTTACAAAACAAATGCTCAGCAAACGGAGAAATAGAAAGAGACGGATGGAAAAGACAAGATAGTCA
GTACAGTATTAAAAAAGAGAGGTTTAATAATAAAGGACGGAACGAAACAAACAAAAAAAAAAAACG
GTACAAAGGTGTGAGCGAGCGAGACACCACGTCTGCCCAAAAAAGGTTGTTTAAATCTTACAGCTTG
GTCTGTCGATGTCTTGCTATAGGGCACTGGTATGAGGCGTTCCTGTAACTCGCCACCTATGACCTTCCG
TGTGCAATGGGGGTTTCATGGAAAAGAAAATAAAAACGAGAATATGAAATAGAGGTATACACGGCACG
CAAGAGAGGAAGAGATAGGTAGTCACAGTCATCCTGCTGTGAGGGCGCTAAGGTATACAATATACATA
GATAACATCGCCAGTGCTACTGGACAAACACGATTCTGTTTTGCCATCATTTTTATATACTCTACAAAAT
ATATATAAAATTATATGTATATTTTTCGTGGACATGAAAGAATCGTGTTTGGCCCTTGCAGTGAAAGGTAT
TAAAGACAAACATAGTGTATTTGAAAGTTACAATACAGAGGTATCAACTGGAGTCCTCCGTAGCAGTG
CTCCTCACGAAGCGGGCTCGAAAATATAGAGTAAACAAAATATAGATAACGCAGGGTATTCTCCTAGAC
CGTAACAAGCACGTTCATGATATGTGCAATATATCCTAACTCATTTACACATTATTATTGGCCAGGGCTCC
TTGAAATCCGTTAAAAATAAATAAACAAAAATTCAACTGAAAAACATCGAACAGTAAAAGGTTTTACA
CATTTTTTGATAAGTGTTCTGTGAGGGAAACCGTCTTAGTGCCTGTGTGTATGGGTGTGTGGAGACCCA
TTACTGTAGTGTGTTTTACTGTAGTCTTCTCCGCATGCCAGCTATGTTACAATTCTATATATAAAAGCACA
CGTGATCGTGTTTAAGTATATAAAAACGGTCGCTCACATACCAGATCTCCTGTCCCTATAGTAAAACTAG
TCAAATTTAACAAAAAATCGGGAAAAGCTGCTGTGATTTGCAAACAAATTTAATGGAATCGGGATAAA
ACCAAACCCTAAAGTCTCTTTTATACACATAGTTTACACATATTCTATATACAGCGAGACGTTAAGCACT
ATATTATTATTATACATCTTTCTAACATGCTTAGAGTTGGAAAAATGGTTCTTACTAAGACGAACCATGA
AACACTGTTCTATAACTTTTTATATATGCAGTATATTTATATGAAAGTCAGAGAACAGCGGTTACCCTAGT
ATTCCTATGGTAGGGTGAAAGCTACTCTACTCTAAGCTTAAACAGTGTAAGCGCTAAGCGTTTCTAACA
TCAAGACTAGACCGCACGACAACCAGTGTTGGCCCCTGTTGGCCACAAACCACAAACATTTGGACCA
ACATTTACACAACACCTCCGTAGAGTTACCTTAGTCAGTCAGCTCCGCAATGGTTGGCGAAATGAAAA
TAACTTTGGAGTATAGATACTTTATTACTAAAGTTTAATAGAGACAAGAAGCATAGAGTAACGGATAGA
CAGAACCAGTTTAGTCAGTATATTAGCAAGAGACATGCTGCGTAATAATTTTAAAGTGGATCCTTAAGA
TTTATTTTTTCACTAATCGCCGTGACTTTATCCACTATAAGCGTAAGTATATTAAAATTTTTGGAAAATTT
TACTCTTAGTTGATTTTTTTAAATAACTAATTGGTGTACAC
>TCONS_00039477 geneID=XLOC_008041 scaffold_162:1291503-1292048(-) Splice junction overlap

CTAATACTTGAAGGTGTCCTGAAAGTCCTGATCCACGACAAAACGAGAGTGAATAAGACACGTATGTC
GCACATTTATTGAATGTTGACGGCGTACAGAGTAACGTATAATACTCAAAGCGTTGTGTGTATAGTATAC
CTGAACTAAATGTTGAGTATCCAATATAGTCATGCGAATTGGAACAGTATGCCAATGATATTAACGCTGC
TTATGTGTTTTACGAAGTCTTTTCTGAATAATTGTTGATAGAAGAGGCAGCTAAAGAGTGTTGATTGAA
GGGCCTTCGTGAGTCGCACAAGTTATACTCGTTAGTTAGTTGAAGCGGCGTTTTATCAATATTGTCGTC
AAATGGAAATAGACTATATGCTACGGGCG
>TCONS_00040496 geneID=XLOC_008275 scaffold_163:21271-22018(-) Intergenic
ATATTAATGAGAGTCATGATTGCACTGGGCTTGGATCATTGTCTTTTTTTGCTGTCCGTACCGAGCTCGA
GCGTGCAACACTGCAACATTGTGGCACTAGCGGAGCCGCAGCCATTCAGAATATGCTGTCCCCTAGCG
TTGTGTGACACGCAGTGATTGCTAAACTGAAGTGGACTCTATTGGCGATGGTGAAGGGTGATGCTGAA
GATGTGCTGGCGGGTCACAGCGGGCGGCTGGTGGCCACGTGTGACCTTGACCCTCGAGGCGGAGGAT
ACAAGACCGACGCATTGTGTGAAACCAGCGCGAGTCGCTCGCCTGACTCCGGCATCAAACTTGGAAA
TATAAAAATGCGAATAAACATTTGGTTAGCGGAAACTTTTCCAATAGAACGGCGCTCATGCATATTGCA
TCACACAGCT
>TCONS_00040238 geneID=XLOC_008266 scaffold_163:332961-334626(+) Unclassified
CTCGTTCACGCGCGCTCGAGCAGAGTGGATGTGCCGTCGCACCGAATAACGAAACACGAAATAAACA
TCGCGAGTGTAGTTCAAGTGCAGTGAAGTGAATGTGATAGTTTCCTATTATAACAGGATTGCTTTGGAA
CATTATAATGGTGGTGGTGGGCTGGCGTGAGGCGCGCGCCCGGCCATGAGGAAGTTCATGCGCACGCG
CGCGCCCGCGCCGCCGCCGCTCGCCCGCCCGCTCGATCGCCCGCTCACAAACGG
>TCONS_00040487 geneID=XLOC_008281 scaffold_163:396097-398108(-) Splice junction overlap
GTTTGTGCCAGGAGAAGACAACAAAGAAGGCCCAACGCTAGACCTGCAGCTGTCGCCGCCGCCACC
GCCCGTCGAGCGGACGGGCTGCTTCATGTTCGCGAGACGAATGCTCTGCCCCCAGAAGAAGGTCCCG
CCCCGCCTGCGCCCCGTGCCAGTGCCGGAGCACCTGCTGCCCGCGACCAGCTGACCGCCTCGCGACC
AATCACAGAACGACGAGGAATAACGAACAGCTATGAAGATTAGAAGAGAGAAAAACAAGTGAAAAA
AGTGTCGCTGTCAAAAGTTTCCTCGTAGTGACCGCTAGATGGCGCCTGAAAGTTAAATTTAGGAATACT
ATGTAAAATATACCTACTGGGGCCGTCACACGTCGACACTAACTATATTACAGCCAATGAGGATTAACG
AACAGCTGTAAAGATCAGAAGAAGGGGAAATAAGTGTGGTGGTATGATTCGCGACCACTCACATAAC
GACAATCATTATGTTAATGTTAATACAGATCAATAGAAGAGAAAAACCAGGGAGAAACGTGTCGCTATC
AAAAGTTTCCTGGTAGTGACCGCTAGATGGCGCCACTTTGTAGGTATGTTGGAAGTTAACTTTTATAGT
TTATTCAAACACAAATACATATACTAGGATTACAGCGGGTCTAGTAAGTACACTATAGCAGTTTTTGATA
AAGTAAAAAAAAAAAGTTTGCGTGAGCATAAAGTCATAGTAATTACATACATACAATGCTCAACAACAG
CTCTATATCCCGTTCCACGGGGAAAGACATCTCTGACAGAACCTTTATAACATTTGAATAAGGGTAGTT
TTTGTCTGTATCAGATGTCTTTCCCCGTGGAATAATGGGATAAAGTGGTCGATATGATGAAACAACTGA
CAGGAGGTATATGCAAAGAAAACAAAATGCTGACATAATACGGAGATCAGAAGATCCGGTAAATGACA
GGGTCTCCCGTTATACAGCCATAAGAAAGAAAACATTAATCACACCATTTAATATTTATATGAATGATTG
AAATAATGTAAAGATAATGTGAACTTCAATCATGTTACAAATCTCTGAG
>TCONS_00040488 geneID=XLOC_008281 scaffold_163:396097-398259(-) Splice junction overlap
GTTTGTGCCAGGAGAAGACAACAAAGAAGGCCCAACGCTAGACCTGCAGCTGTCGCCGCCGCCACC
GCCCGTCGAGCGGACGGGCTGCTTCATGTTCGCGAGACGAATGCTCTGCCCCCAGAAGAAGGTCCCG
CCCCGCCTGCGCCCCGTGCCAGTGCCGGAGCACCTGCTGCCCGCGACCAGCTGACCGCCTCGCGACC
AATCACAGAACGACGAGGAATAACGAACAGCTATGAAGATTAGAAGAGAGAAAAACAAGTGAAAAA
AGTGTCGCTGTCAAAAGTTTCCTCGTAGTGACCGCTAGATGGCGCCTGAAAGTTAAATTTAGGAATACT
ATGTAAAATATACCTACTGGGGCCGTCACACGTCGACACTAACTATATTACAGCCAATGAGGATTAACG
AACAGCTGTAAAGATCAGAAGAAGGGGAAATAAGTGTGGTGGTATGATTCGCGACCACTCACATAAC

GACAATCATTATGTTAATGTTAATACAGATCAATAGAAGAGAAAAACCAGGGAGAAACGTGTCGCTATC
AAAAGTTTCCTGGTAGTGACCGCTAGATGGCGCCACTTTGTAGGTATGTTGGAAGTTAACTTTTATAGT
TTATTCAAACACAAATACATATACTAGGATTACAGCGGGTCTAGTAAGTACACTATAGCAGTTTTTGATA
AAGTAAAAAAAAAAGTTTGCGTGAGCATAAAGTCATAGTAATTACATACATACAATGCTCAACAACAG
CTCTATATCCCGTTCCACGGGGAAAGACATCTCTGACAGAACCTTTATAACATTTGAATAAGGGTAGTT
TTTGTCTGTATCAGATGTCTTTCCCCGTGGAATAATGGGATAAAGTGGTCGATATGATGAAACAACTGA
CAGGAGGTATATGCAAAGAAAACAAAATGCTGACATAATACGGAGATCAGAAGATCCGGTAAATGACA
GGGTCTCCCGTTATACAGCCATAAGAAAGAAAACATTAATCACACCATTTAATATTTATATGAATGATTG
AAATAATGTAAAGATAATGTGAACTTCAATCATGTTACAAATCTCTGAG
>TCONS_00040317 geneID=XLOC_008269 scaffold_163:447544-448980(+) Splice junction overlap
ATTAGAACACTATGACATCACTATGGGCATAAACAATACGCTGTCAATGTCAGTCCGCCATATTTGTTTA
CATGATTGTAGGATTCTATTGCAAACGACTATAGTGTAATGCAACTCTGTCTACATAGGGTTAGGTTAGT
AGTTAATAAGGAGGAGCACATTTGTGGATCGTTAATGATCGTGAGTTACTTTATGAAGTATAGCTTTTAT
CATTTTATCTTCCAGATCATTGGCCACGGCCTGCTAGCCACCAATAATCTTATCTAGGTACATTATCTCTT
CAGCTTTCCTTCAACTGTATCTGCCGGAATCGGAAATATATTCTCAAAATAATAACGACTTCGTGCTTTA
TAGTACGTACCTCAGAATAATAACTTGCATAATATACAAACCTTTTCCTCCTTCCATCCACAAGTTTAGA
ATTTTTACTGTCATTCAGTAATCATCGAGTCCATAGACTAATATTGTGATCGAGTCTTCAAATAAATAAA
GAAATAGGTACACAAGAAGACATAGCAAAAGGAATGTTTCTGAAGAGCGTGTTGGCTCCATTGCTGC
GGCGGCCCCCGATGGTGGGGCGAGTGGTGGTGCCGGCTCGCAGCTGGACCCAAGTGGGCAACGTCAT
CAGCACTCCACCCAGGGTTCCCTGCAGCTTTGCCGAAAAGATGGTGCACGGCTTTATTATGATGTTCAT
TTGGTTATCTCCAATATGTTTCTTCCTGAGCCAGCTTAAAGTGTGGAGACGAACGTACGTGGAAGACTG
AATCAGAATTTAGAAAATAAATAAACTGTCGGCGATGGAATAAATAAGCGTTTCGTTTTCTATGTAAAA
GTCCCATTAAGATCTTCGCACAGTACCTCGACGGTGG
>TCONS_00040250 geneID=XLOC_008270 scaffold_163:463122-468622(+) Intergenic
TCTCTCTTATTTGGCGCGCTTCAGTGTGGCGGGTAACGTTCGCGGGCCAAGCCGCTCGCAAAAAACGC
GGGAAAATCATCTGTGAAGTGAAAAGCTCCGTGAAAAATAGTTTTTTTGTACCTAAATTACAATATTA
CTGGAATAAATCAAAATGTCACCAAAAAGAAAAGCGAGCGAAGAAGAGTTCGACACAGACGCTCTG
ACTGCCGACCCTGAACAGAGCCC
>TCONS_00040390 geneID=XLOC_008284 scaffold_163:505647-510180(-) Intergenic
TGAACCCCACTGTTTCTGTTCAGAGCCCCTATACCAGAATGGAATTGGCAACTAGAATGGCTACCAGA
AATTACACTTCTGAGCAGTGGAAGAAGTGCAGGAGCAGAAAGAAGAAGAAACAGTAGAAGAGGAAC
CAGTGAAGGAGACTCCTCCATCGCCAGTAGCGACGCAGGCGAAACCAGAGACGAAGCCGAAAACAC
CACGCGGACGCGGCCGGGGCCGGCGCGGCCGCTATTAAAAAAACAAACTAAGGATGCGTTCAGAAA
AAATAAACTAATTCATGGCTTCGTTCCGAATAATTAAATAGTACTGTAGGTTTTAAAGAGTTGCCTTGGT
ATGTGTAGGAATGGGAATGTAAGATTTTTCTTATGATCTTATTGTAACTGCTAGTTTTTATTACGGTATTC
GATATTAGACGCGTCGTATACAGCTTTTGTAAAATTCCTAGTTTGCTTTTAGGAGCCTAGGTTAGATGCA
TTATAAAGTTCATAGATTTTGGTTTCATTAGAAGTGGTAGCAGCTATGATATCTTTGTATCTAAGTGCATC
TATTTCGTAATTTTCTAATGTCGACTGTAGGCAATTAAGCCCTTTGTAGTTATTTACATTCTTACATTGTG
TGCTTGAGTAGGTACTATAAAATTAGCTTATGTTTAAGCTTTCACTATTTCTAATTACTTAATCTTATCTTA
ATAGATATTATGGTTTTTGCTTTTTAAATCCTAGGTGTAGTGCTAATGGGATAGGATAACTTTAAAACACC
CAAATTAGAGAGTTGCACATAAATTTAACTATGGGAG
>TCONS_00040369 geneID=XLOC_008272 scaffold_163:531298-531968(+) Splice junction overlap
TGCTTATTGACAAGTGCAAGGAGGAAGAAAGAGAGCGCCTCCGCCAGCAAATAATAATCGAAGAGCA
ATCGCCCAAAATGGCGAACCCTTACGAGCCTATCAAAATGAACTACGAACCGCTGCGCAGGAAACTTG

ACAGTTCAGAACCTAAAGAGCACCAATCACGAAGCAGGAGACGACGACGGGAAAGAAAGAACACA
GACAAGACAAATTATCAAATAGACACACAGAGTAATGTGGACAAAAACGCGATACAAGTGCGCAAAG
AAAACAACGTATACCCATCCTGGCACGAGCCGAATAGAG
>TCONS_00040518 geneID=XLOC_008329 scaffold_164:18774-34271(+) Exonic overlap (-)
TTTGAGTGCTTTGACAGAGAATGACGGCTACAATGTACTCTGTGACGGCTTCAGTCCGACAGCTAGAT
TTGGTATTGCCTTACATAAAAACGTTTCCCACAATAACTTTGGGGAAAATTATGTCTCAAACGATTAGC
AAACCTAATAACATTAACAATGACCGACGATTTGATGGTGCAACACGTTGCAGTAAAATTTTAACTGTA
ACGACTCTGTCATTGCTAAGTTTAACCTTAACTATTACGATAACCGGGATGTTGATGCAACCCACCCTA
AGAGTTGGATTCACAACCCTTAATCCCTCATACTGTTTTTTTTCATAAGGCAGACTCACAACAAAAGCC
CATAAACTCACTGAAAGTGGGGTCCTGCAGCTCATTGCTGGAGCTCTCCGGCGACGAGTCGAGATCGA
GCGACGAGCGCCGCGACGCGTCCAGCGAGCCCGACGCCGAGCGCGCGGCCGAGGCGGCTGAGGACG
AGTCTGCTGAGTGGTCCATGGCCA
>TCONS_00040777 geneID=XLOC_008330 scaffold_164:22103-36780(+) Exonic overlap (-)
CTCAGCCAAGAACTCGCTGGGTGCGGACAGAGTCTCATTCCCATCGGAGCCATCATCAGCGCTGGCCG
ACGACTGCGCCGGCGGGCAGGCGGCCATCTTGCACTTCACACACTTCCCCTTACGGCGACCACCTTTG
AGCTTCTTCCTCGCACGTTTAGGCGGGGACTCACACCGATGAATCGGAGAAGGACACTGGTTTGGGG
ACCGCGACTGTCTTTGGGCCGCTAGCTCTAAAGCAACACTAGACGCGGCTTTGAGCGCGTCTGTGTGG
CGAATTGGTTGCCAGGAGATGACAGGGGAAGCGGGGGGTGAGGGGCGAGGGACGTGCCGCGCGGGG
GGCTGGTACAGGCGCGCGTTGGGCACGAGCCGCCGTCGCAGGCAGCGCTTGCGGTGGCGAGGCTGGT
GGCAGTCTTCGCGGGACTTGGGGGCACGCCGCGGCTTTGGGCCTGGTTACAAGCGAGGGTTAGGATG
GTAGCACTTGGGTAGCGAGCGACGCTTGAAGAAAGGCGCCGGCCCACGTTGCACAGGACTCGGGTTG
ACTTCGATCACGTCTTCCGGCTCCGAGAC
>TCONS_00040563 geneID=XLOC_008344 scaffold_164:39430-47832(-) Splice junction overlap
CTTGTTTTCCTTTTTTCAATTTATTTGACAGAAAGATCATTTGGTAATCAAAATCAATGAATAAAATTAAT
TAAATTTAAAGTTAAAACTCTCATAAAATTGTATTTTAATGTAGTAGTTGTACATACAGTGAAATATCACA
TAATTGTATAATGCTTTATAAAGACCAAAAATCGGCATCGTCACCGTAAGGTCCGCATCAAGCCAGTGT
CC
>TCONS_00040657 geneID=XLOC_008348 scaffold_164:112494-118184(-) Unclassified
NNAGGAGGTAATTCGGCCCCGTGGCAACCCAACTACGGCTTTCTTACCTTCAAGAGCGAGGCCAGCG
TCAAAAATGCTGTGCGGGCGTAGGACTGTCTCAACAATGCTGTGAGTATTGCTACAAATCCTAGACAC
ACGCATATATTAACAAGTCAAGGCACAGATCCCGCCGACAGATGGCACTAGCTCACTCGCTTCGGCCC
CGTGGCGGAGCTGCGCGTGCACAGCAAGCCGGCCGCGCCGCACGGCCCGAGGCACCCCAACTACGG
GTTCCTCACGTTTGAGAGCGAGGCCAGTGCGCAGGAGTGCCTGAATAATGTTCCGTTGTACTTCCCGG
AGAACAGCCCCGACGGCGTGAAGCTGAACGTGGAGGAGAAGAAGCGCCGGGCGGAGGCGCCGCGC
CGCCGCCCGCCCTCGGGCTCGCGCGCGCCCTTCCCGCGCCCATACCGCCGCTAGGGTGGTCTGATCGC
GCTACGGGTCCCCTTACATTAAGTTACAGTTCTTAACATCCGCCGACTGCCGCCATCTCGCGATAACGA
CCTCTACTCCATACACCCAAGATCTGTATTGCAAAAACAGTCGTTATTGCGTATTTTTATTTATATTAGAC
GGCGCTAGCATAGTCACTCCGCACACAGCGCTCCCCGCAGTGTTTGCCAAACGTTGGCTATTTATAATG
CACTCTGGTAGTCGCTCTGTTGGGCCCAACGTTGGAGCAAAGCTTGGCTGCGGGAAGCGCTGTGATTG
GTCGGTTAGTGTAACGCGTAGAGCTAAGTCTTTCTCGCGTCTATTGCGAATTGTGCTCACGATCTGTTTA
TACGAACCTGGATCTTATGTACTTATTACTGAACTCAGTTTCTCATGTGTAGATTCAGACGGTTCAACGT
GTTGCTAACATCCAAGTTGTATAACAGATCACATTATTCCATTCTCCTTTGTAATTAACACGCTTTGCATT
TCGCTTCACATCGTCTAATAATTTGGTTTAAGTAGTTAGATAGTAAATTAAATAATTATTTGCGTTCAGAG
CGCGTGCGGGTCAAGCTTATATTGTTTATAATAAATTGATAACAGAAGGGATGTCATGCAATTTTAATTT

TTGCGAGCATTATCGCCAATTTACCGATAATCCAATGTTTAATTTGTCAGCAAATTAATTTCTGAACAAA
ATGTGATTAACATGGCCGCCGCACGCGCCTCTGACACTCCAAAACTTACCACTCGCTCTATGGACATGT
CTACTGAACTACGACCGACAGTTTTTTCACTGATCAACACTACCATTTTTATACGCCATCATCAATCCAT
CCTTTTGTTATCATCTTCATCATCAATCATCCACGTTTTTCTTGTATGTCAGTGAAAAAACTGCTCAAAA
TATATTTTTAAGGCGTCACAAAAGACGTAAACAGCATTTGGTATACGGGCTGACGCGGCAGCGCGGCC
ACTGCTGCTTCCTGTGCTCTGATTGGTCGGAGCGTTGCTGACGTCACGCCCCTTTATTTGTGCTAGACT
TGTCGAAGGCTTAGGACTGAACTCTAGTGTGATTTCGGGGTGAAAAGTAAATGAGTAGCGGACTGGCC
GTAAGAACAGTAAAGATGAACACTATAGTGACGATGTATCGCTTTAACCCGCTGTTTTCAAACTACAAC
GTTTTCATTAGTCATCGTAACTGTTAGACTGATCTACGCTTGTAAAACGTAGCTCATTGGTCCGCTACGC
AGTTACTCTGTCTCTCCTCTCGATGGTTGTTTTTTATACTGCGCGTGCGAATGATAAGTTCGGTTCCTTA
ACTTCGGGTTAGTTCGTTAGTTATGCGATGAAGATCCTCTCCTGTGACCCTAGTTGTGTCTAGTTAATTT
TTAAGTATTCATATTCTAGTTGAAGCGATCTCGTCTAGAAGCGGGTCAGTTTAAAACATAATAATAATAT
TCACAGGACAGGATCACTCGCCACGGATACGACTCGTGTGTAAACATTCATTATGTATTAGTTTACCTTT
GTATGTATTTCTCCGATTTTCATTTGGGAATACACTTTCTGTTTGTTAATAATTGGGAGTTGGACATTAGG
GTGTGCGCAACCTGCTGTGTGTCGACAATACACGGAGACGCAGCAGTGTGGGCAGCCCTACACCGAT
ACGTTTAATTTAGATCGAAAACTCAATACTGTTGAGTTATTTCGAGAAGAACTTGTCGTAATTTTTGCTG
AGATTTAGACCCTGGTATTTTGTTTGGTAAACCTTACGGCAAGTTGACCGGGGGCCTTATTGACCAATA
TAGTTATAAGTCTGACAGGAGCATTTTAATTAATGGCGAGTGTACCAATCACGATTGCAATAAGTGACA
GCGCCTGTCAGATTTATAGATCATTAGCCAATGAGGCCCACAGGCCCGCCCGGCGCCGGCAAACATTT
GATCATGACGGTTTTGTGATTTTCTATCTGTTTTGTATATTTTATTTTGTATAACTTAGCATATACTTCTCC
ACTCACAATATACATTATATTTTCAACAATGTATTTATTTATTTTTGTCCAAATTACTCATCTGTTATTAGTT
TTACCCTGTAAATGTGAACATTATACCATCAACCGTGTTGCTCTTGAATATCCACATCTTACTTGTAATAA
GTAGTCTACACTAGTGATATAATGATGGTTTCAGGAGTAACATTAACAGTGTAAATACCCTGTTCTCAAA
ATGGTAAATATCATTGTCATTTAATCATTTTTGTATAATTGGTAAAAGTATTCTACATTCGGGTTGAGGCG
ACGGCTTCCATTTAGAAAAATGGTCGATACTTTTATAAAATTTCTAACCGAGTCCTAATTTAGCTACACA
TTTGTCATCATTATTATGATTTAGCCTCGCCGATAGCCGTGATAATAAATTTATGTTGTAAATACGTTCTG
GTTGATTCTTTTACGTAGAGAAATTAAACTTCAATAATATAATAATTACTATTATTAATATACTAACTTGCT
ATAGTTCTAGAATCTTAAAGGCTATTTGGGATTTAATGTACTTTTTTAAATTTGCTTATAAGCACTTTAAC
TTGTCTCCTTAAAGGGCAGAGATAAATAAGTTCGTCAAGTTAACTTCTTATGAGCTGCACGATATTACGT
AAAAATTATATTCAAAAATAGATGACAGTTGATAAAGTAAATAGTTAATTAAGTCAGAGCGAGGCAGTG
TGATTCTGTACTTGACGGCTACAAGCGCCACCTACCGACTATTTTCAAATAAATGTATAGTTCTATAAAT
AGCTACGAAAAATAATGTCCTCAATTTATAAAATATACTTTTATATTATTTTAAAGGCCTTTCTAGACATA
CGCGATATTCGATATGGATATGGGAAACGGTATTTGTACTTCTTTCGTCGCGTCGCTCAGCACGCGTAAC
CGTGTGACACTACGTGCTACCTGCGGTAACGAATATCGAATATGTCTAACAGACCCTAAGTCATTGCTG
TGCCAAGCTATCAAGAGTAGCTTGTACCTTGTATAACCACATGTTTCACTTTGTCAACCCACATACATAC
AACTGCAGACACTGAAATACTAATTTTATGTGTAGGTATTTCTTACAATGGTTAATTTAATAGATACGAG
AATAAGTTGGCAGTGACCAGTTTTGAAGGCTAAATTACAAAAAACCAACAAATTTTGTTTTCATTGAA
ACGATTTGTTGAGCAAAATTTCATGTACTTATTTATTGTATATAATACTTCCAACTCATAATAATTTCCCTA
AAGTATGGACGGACATCGTGTTTTATAGTATAGTGTATTTATGTAAAATGGCGGCCTTTGCTAGTAACAG
TTAATCAAAGTATTAATTTCTGCTCAAGAAAGTATTTGTTCACAAAAAAATGTCCGCAAATATGTTACAT
GTACGGTGAAACTTCCTGAACAAGACTGCTTCTGTATAAATAAACTGGCTACTAATGCAGACTTAGCGC
GTCACTGCCACACTAGGTAAAATGTTATTTAACATACAGGGTGTTACATTGCAAGTCATCATGTTACAA
GTTATAAATTTTAATTCACTAAAATTAACCACATAATATTATAGGTACTTGTCAAAGCATCAGTTTATCAT
ACAGCGCCTTTTTGTTATTTTCATGGTAGCCATTTTGTACAATAAGGTTGTAACGCCCTGTGTGGATGCC

AACAAAGCGATGTCGGTCCCAGAATGGCCGGACCACTCTGTTTTCAGGCGATTTGTCTAATTGTGTTTT
TTTTCTTTGATCATTTAAAACGGTAATAGTTACAAAACACACAGCTAACAGGAACAGTCATACAGATTG
TAATAATTTGAATACAATTGACAAATCGCCTACCCATTCCCATCAATTCGGTATTGTTTGTGAACTAACT
TTAGACACCATTGTTTTTTTACGAATCCCATTTTGTTAAAAATAGTGTTTAATTGTATTTCGTGGGGTCTC
AAGTTAGTGAACATATTATTTTGAAATTGTCTTGCGTGGTGACACCAGCGGTGCGTCGCCGCCGCGAC
AGTTGCATATAGACACACGCACATACACGCCTGCTTTGTGATACTCGGTGTATCGCGTGTGAAACACAC
TGTATCGCTTGTGACACATAGTGTATCGCATGAGACGCAAAATGTATCACCGTACCGTGTCAGCTGTGC
GTGCGTGTGTCTGTATGGGTAGCGGCGACAGTGAGATGAACTGCCCTCGGTAATAGAGATACTTTTTGT
AACACT
>TCONS_00040572 geneID=XLOC_008348 scaffold_164:114098-117522(-) Unclassified
CGAGGCCAGTGCGCAGGAGTGCCTGAATAATGTTCCGTTGTACTTCCCGGAGAACAGCCCCGACGGC
GTGAAGCTGAACGTGGAGGAGAAGAAGCGCCGGGCGGAGGCGCCGCGCCGCCGCCCGCCCTCGGGC
TCGCGCGCGCCCTTCCCGCGCCCATACCGCCGCTAGGGTGGTCTGATCGCGCTACGGGTCCCCTTACAT
TAAGTTACAGTTCTTAACATCCGCCGACTGCCGCCATCTCGCGATAACGACCTCTACTCCATACACCCA
AGATCTGTATTGCAAAAACAGTCGTTATTGCGTATTTTTATTTATATTAGACGGCGCTAGCATAGTCACTC
CGCACACAGCGCTCCCCGCAGTGTTTGCCAAACGTTGGCTATTTATAATGCACTCTGGTAGTCGCTCTG
TTGGGCCCAACGTTGGAGCAAAGCTTGGCTGCGGGAAGCGCTGTGATTGGTCGGTTAGTGTAACGCG
TAGAGCTAAGTCTTTCTCGCGTCTATTGCGAATTGTGCTCACGATCTGTTTATACGAACCTGGATCTTAT
GTACTTATTACTGAACTCAGTTTCTCATGTGTAGATTCAGACGGTTCAACGTGTTGCTAACATCCAAGT
TGTATAACAGATCACATTATTCCATTCTCCTTTGTAATTAACACGCTTTGCATTTCGCTTCACATCGTCTA
ATAATTTGGTTTAAGTAGTTAGATAGTAAATTAAATAATTATTTGCGTTCAGAGCGCGTGCGGGTCAAGC
TTATATTGTTTATAATAAATTGATAACAGAAGGGATGTCATGCAATTTTAATTTTTGCGAGCATTATCGCC
AATTTACCGATAATCCAATGTTTAATTTGTCAGCAAATTAATTTCTGAACAAAATGTGATTAACATGGCC
GCCGCACGCGCCTCTGACACTCCAAAACTTACCACTCGCTCTATGGACATGTCTACTGAACTACGACC
GACAGTTTTTTCACTGATCAACACTACCATTTTTATACGCCATCATCAATCCATCCTTTTGTTATCATCTT
CATCATCAATCATCCACGTTTTTCTTGTATGTCAGTGAAAAAACTGCTCAAAATATATTTTTAAGGCGTC
ACAAAAGACGTAAACAGCATTTGGTATACGGGCTGACGCGGCAGCGCGGCCACTGCTGCTTCCTGTG
CTCTGATTGGTCGGAGCGTTGCTGACGTCACGCCCCTTTATTTGTGCTAGACTTGTCGAAGGCTTAGGA
CTGAACTCTAGTGTGATTTCGGGGTGAAAAGTAAATGAGTAGCGGACTGGCCGTAAGAACAGTAAAG
ATGAACACTATAGTGACGATGTATCGCTTTAACCCGCTGTTTTCAAACTACAACGTTTTCATTAGTCATC
GTAACTGTTAGACTGATCTACGCTTGTAAAACGTAGCTCATTGGTCCGCTACGCAGTTACTCTGTCTCT
CCTCTCGATGGTTGTTTTTTATACTGCGCGTGCGAATGATAAGTTCGGTTCCTTAACTTCGGGTTAGTTC
GTTAGTTATGCGATGAAGATCCTCTCCTGTGACCCTAGTTGTGTCTAGTTAATTTTTAAGTATTCATATTC
TAGTTGAAGCGATCTCGTCTAGAAGCGGGTCAGTTTAAAACATAATAATAATATTCACAGGACAGGATC
ACTCGCCACGGATACGACTCGTGTGTAAACATTCATTATGTATTAGTTTACCTTTGTATGTATTTCTCCGA
TTTTCATTTGGGAATACACTTTCTGTTTGTTAATAATTGGGAGTTGGACATTAGGGTGTGCGCAACCTGC
TGTGTGTCGACAATACACGGAGACGCAGCAGTGTGGGCAGCCCTACACCGATACGTTTAATTTAGATC
GAAAACTCAATACTGTTGAGTTATTTCGAGAAGAACTTGTCGTAATTTTTGCTGAGATTTAGACCCTGG
TATTTTGTTTGGTAAACCTTACGGCAAGTTGACCGGGGGCCTTATTGACCAATATAGTTATAAGTCTGAC
AGGAGCATTTTAATTAATGGCGAGTGTACCAATCACGATTGCAATAAGTGACAGCGCCTGTCAGATTTA
TAGATCATTAGCCAATGAGGCCCACAGGCCCGCCCGGCGCCGGCAAACATTTGATCATGACGGTTTTG
TGATTTTCTATCTGTTTTGTATATTTTATTTTGTATAACTTAGCATATACTTCTCCACTCACAATATACATTA
TATTTTCAACAATGTATTTATTTATTTTTGTCCAAATTACTCATCTGTTATTAGTTTTACCCTGTAAATGTG
AACATTATACCATCAACCGTGTTGCTCTTGAATATCCACATCTTACTTGTAATAAGTAGTCTACACTAGT

GATATAATGATGGTTTCAGGAGTAACATTAACAGTGTAAATACCCTGTTCTCAAAATGGTAAATATCATT
GTCATTTAATCATTTTTGTATAATTGGTAAAAGTATTCTACATTCGGGTTGAGGCGACGGCTTCCATTTA
GAAAAATGGTCGATACTTTTATAAAATTTCTAACCGAGTCCTAATTTAGCTACACATTTGTCATCATTATT
ATGATTTAGCCTCGCCGATAGCCGTGATAATAAATTTATGTTGTAAATACGTTCTGGTTGATTCTTTTACG
TAGAGAAATTAAACTTCAATAATATAATAATTACTATTATTAATATACTAACTTGCTATAGTTCTAGAATCT
TAAAGGCTATTTGGGATTTAATGTACTTTTTTAAATTTGCTTATAAGCACTTTAACTTGTCTCCTTAAAGG
GCAGAGATAAATAAGTTCGTCAAGTTAACTTCTTATGAGCTGCACGATATTACGTAAAA
>TCONS_00040525 geneID=XLOC_008334 scaffold_164:140832-143925(+) Exonic overlap (+)
NNGCGGTTACGATTACTCCGGCTACCCGAATTACGCGGGCTACCAAGGTGGGAAGCAGAGGGGAGGC
GGTGGGGGTCGCACCAACCAAAGGCACCAGCCTTACTGAGCACCGTCCCTCAAGTACCTGCCGATAG
TCATCTCACTCCAATAGTAAGGTTAAGTCACCGGTCATGAACTCTTCCAACGCAGCGTCTTGCGTCGG
GAGCGTCATTCCATTCCACATTACGTTAAGCGAGTAATCTTTCTAAGATTTTTAAGATTTAAATTATCGTA
TGTAACGTCGCACTTGGTGGCGATTGCATATTTATTAATATAGATATGGAATAGATTCACTTGAGTATTAT
TGCCGATTGATTTGATTCAATTAATTTTTGGTATTAATCTTACCTTTTCATTTAAAACTGAATTAATTCTAG
ATAATACTAAACAGAGCTTCAAAGTAACAAGAGTTTGTATGTAGTAGATGAAAGTTGAAGATGTAGATC
ACCGAAGAGGTGTTGATGTATGCGTGGTAGTGTGAGTGTTGTATCGAGTCGCCCTCCACTCGACAGTT
TTGTCAGTCATTTGTTTGTTCAGGATCTCATTGCAGAAATGTTGTAAGTTTTATATTTTTTAATTTGGTAA
TAAAAAGGAATCAGAATCATGTAATTTTATACAAAGTAGATGTTTCTTCCAAATGAGTAGGGATGAAAT
TTAAATTCTAGAAATAAGTGATCGGACATGAACAAACTGAAGATGTACGAATGTATATCTAAATAAATA
AAAGAAATGTGTAAATAAACTATTGACTTTCAGTCTGCATGTTATAATTATGTTCTCAAGAATCTTAAGT
TGCCACTCACCTATAGGTAGACACGGGGTACGTCAGACTATTTTAAAAAACATACACTTCGAACTAGGC
CGGTGCTAAAACAGAAT
>TCONS_00040690 geneID=XLOC_008334 scaffold_164:140833-143790(+) Splice junction overlap
NGCGGTTACGATTACTCCGGCTACCCGAATTACGTGAAGGGCACCGAAGATCAGGCTGATCAATGCGA
TGACTTTACAGCGGGCTACCAAGGTGGGAAGCAGAGGGGAGGCGGTGGGGGTCGCACCAACCAAAG
GCACCAGCCTTACTGAGCACCGTCCCTCAAGTACCTGCCGATAGTCATCTCACTCCAATAGTTAAGTCA
CCGGTCATGAACTCTTCCAACGCAGCGTCTTGCGTCGGGAGCGTCATTCCATTCCACATTACGTTAAGC
GAGTAATCTTTCTAAGATTTTTAAGATTTAAATTATCGTATGTAACGTCGCACTTGGTGGCGATTGCATAT
TTATTAATATAGATATGGAATAGATTCACTTGAGTATTATTGCCGATTGATTTGATTCAATTAATTTTTGGT
ATTAATCTTACCTTTTCATTTAAAACTGAATTAATTCTAGATAATACTAAACAGAGCTTCAAAGTAACAA
GAGTTTGTATGTAGTAGATGAAAGTTGAAGATGTAGATCACCGAAGAGGTGTTGATGTATGCGTGGTAG
TGTGAGTGTTGTATCGAGTCGCCCTCCACTCGACAGTTTTGTCAGTCATTTGTTTGTTCAGGATCTCATT
GCAGAAATGTTGTAAGTTTTATATTTTTTAATTTGGTAATAAAAAGGAATCAGAATCATGTAATTTTATAC
AAAGTAGATGTTTCTTCCAAATGAGTAGGGATGAAATTTAAATTCTAGAAATAAGTGATCGGACATGAA
CAAACTGAAGATGTACGAATGTATATCTAAATAAATAAAAGAAATGTGTAAATAAACT
>TCONS_00040634 geneID=XLOC_008334 scaffold_164:140833-143925(+) Splice junction overlap
NGCGGTTACGATTACTCCGGCTACCCGAATTACGTGAAGGGCACCGAAGATCAGGCTGATCAATGCGA
TGACTTTACAGCGGGCTACCAAGGTGGGAAGCAGAGGGGAGGCGGTGGGGGTCGCACCAACCAAAG
GCACCAGCCTTACTGAGCACCGTCCCTCAAGTACCTGCCGATAGTCATCTCACTCCAATAGTAAGGTTA
AGTCACCGGTCATGAACTCTTCCAACGCAGCGTCTTGCGTCGGGAGCGTCATTCCATTCCACATTACGT
TAAGCGAGTAATCTTTCTAAGATTTTTAAGATTTAAATTATCGTATGTAACGTCGCACTTGGTGGCGATT
GCATATTTATTAATATAGATATGGAATAGATTCACTTGAGTATTATTGCCGATTGATTTGATTCAATTAATT
TTTGGTATTAATCTTACCTTTTCATTTAAAACTGAATTAATTCTAGATAATACTAAACAGAGCTTCAAAGT
AACAAGAGTTTGTATGTAGTAGATGAAAGTTGAAGATGTAGATCACCGAAGAGGTGTTGATGTATGCG

TGGTAGTGTGAGTGTTGTATCGAGTCGCCCTCCACTCGACAGTTTTGTCAGTCATTTGTTTGTTCAGGA
TCTCATTGCAGAAATGTTGTAAGTTTTATATTTTTTAATTTGGTAATAAAAAGGAATCAGAATCATGTAAT
TTTATACAAAGTAGATGTTTCTTCCAAATGAGTAGGGATGAAATTTAAATTCTAGAAATAAGTGATCGG
ACATGAACAAACTGAAGATGTACGAATGTATATCTAAATAAATAAAAGAAATGTGTAAATAAACTATTG
ACTTTCAGTCTGCATGTTATAATTATGTTCTCAAGAATCTTAAGTTGCCACTCACCTATAGGTAGACACG
GGGTACGTCAGACTATTTTAAAAAACATACACTTCGAACTAGGCCGGTGCTAAAACAGAAT
>TCONS_00040747 geneID=XLOC_008334 scaffold_164:140841-143781(+) Exonic overlap (+)
CGATTACTCCGGCTACCCGAATTACGCGGGCTACCAAGGTGGGAAGCAGAGGGGAGGCGGTGGGGGT
CGCACCAACCAAAGGCACCAGCCTTACTGAGCACCGTCCCTCAAGTACCTGCCGATAGTCATCTCACT
CCAATAGTTAAGTCACCGGTCATGAACTCTTCCAACGCAGCGTCTTGCGTCGGGAGCGTCATTCCATTC
CACATTACGTTAAGCGAGTAATCTTTCTAAGATTTTTAAGATTTAAATTATCGTATGTAACGTCGCACTT
GGTGGCGATTGCATATTTATTAATATAGATATGGAATAGATTCACTTGAGTATTATTGCCGATTGATTTGAT
TCAATTAATTTTTGGTATTAATCTTACCTTTTCATTTAAAACTGAATTAATTCTAGATAATACTAAACAGA
GCTTCAAAGTAACAAGAGTTTGTATGTAGTAGATGAAAGTTGAAGATGTAGATCACCGAAGAGGTGTT
GATGTATGCGTGGTAGTGTGAGTGTTGTATCGAGTCGCCCTCCACTCGACAGTTTTGTCAGTCATTTGT
TTGTTCAGGATCTCATTGCAGAAATGTTGTAAGTTTTATATTTTTTAATTTGGTAATAAAAAGGAATCAG
AATCATGTAATTTTATACAAAGTAGATGTTTCTTCCAAATGAGTAGGGATGAAATTTAAATTCTAGAAAT
AAGTGATCGGACATGAACAAACTGAAGATGTACGAATGTATATCTAAATAAATAAAAGAAATGTGT
>TCONS_00040729 geneID=XLOC_008334 scaffold_164:142681-143729(+) Exonic overlap (+)
ACTTGTATAAAACATTTCTATCTAATTCCTATGTGTTTAATATTAAGTATGCACGATTCTTGATAAGTAAC
GATTAGGTGTTCACTTATAGTGAAGGGCACCGAAGATCAGGCTGATCAATGCGATGACTTTACAGCGG
GCTACCAAGGTGGGAAGCAGAGGGGAGGCGGTGGGGGTCGCACCAACCAAAGGCACCAGCCTTACT
GAGCACCGTCCCTCAAGTACCTGCCGATAGTCATCTCACTCCAATAGTTAAGTCACCGGTCATGAACTC
TTCCAACGCAGCGTCTTGCGTCGGGAGCGTCATTCCATTCCACATTACGTTAAGCGAGTAATCTTTCTA
AGATTTTTAAGATTTAAATTATCGTATGTAACGTCGCACTTGGTGGCGATTGCATATTTATTAATATAGATA
TGGAATAGATTCACTTGAGTATTATTGCCGATTGATTTGATTCAATTAATTTTTGGTATTAATCTTACCTTT
TCATTTAAAACTGAATTAATTCTAGATAATACTAAACAGAGCTTCAAAGTAACAAGAGTTTGTATGTAGT
AGATGAAAGTTGAAGATGTAGATCACCGAAGAGGTGTTGATGTATGCGTGGTAGTGTGAGTGTTGTAT
CGAGTCGCCCTCCACTCGACAGTTTTGTCAGTCATTTGTTTGTTCAGGATCTCATTGCAGAAATGTTGT
AAGTTTTATATTTTTTAATTTGGTAATAAAAAGGAATCAGAATCATGTAATTTTATACAAAGTAGATGTTT
CTTCCAAATGAGTAGGGATGAAATTTAAATTCTAGAAATAAGTGATCGGACAT
>TCONS_00041011 geneID=XLOC_008351 scaffold_164:154478-155881(-) Intergenic
CTGTCTTTAATATTTGAAATAAGTAAACTATACATAGGGCATGCATGCCTACATACTTACAAAAATAACA
AGGATATATAGTCAATATTTATTAAATAAATACATATTTACTCATGTTTTATAATATTACGCTACTGTCCTGT
AGCATTTGCTTTAGGACAGCTCTACAATGCATACGACCGTTCGTATATGCTTTATTATGTTCTTGTTACGA
ATCTGCATAAGTATAGGTATATAATCAGTTGAGTTTTGACGTAGTTACTTCTCTTATTGTTTTGGTGGCGT
TAAAGTTGGGATGTGGTTTAACCAAATATTTTATATGTATGATGTATGTTTGTTTAGTTATACGGAGT
>TCONS_00040528 geneID=XLOC_008335 scaffold_164:255697-268151(+) Unclassified
GCCAACGCACGCACAGCAGGGGACAGCGTTCCAAATTCAAACTTTTGACAGTTCAAAGTTGCCAGTT
AGTGAAGTTTTTAATTCACGTTGGTGTTTTCCCCTTAACTCTTGGATTGCTGCCCTCCCGCAGTTCATCA
TCAACTCTACACGATGGCTTTCTTCAAGAGTTTGATGTTGACTGCGCTGCTGTGCGCCGCGCTGACCA
CCGCCGGCGCGCGCCCCCCCC
>TCONS_00040662 geneID=XLOC_008355 scaffold_164:315868-317076(-) Splice junction overlap
CCACCGCGAGATCCACAAGGCAGTGAGCAGCTCCGTGTCCCGCGGTCGGCCCCGCGTCATGCGAGCT

CAGAGCACCACCGACGACGTCGACCCACTGTGCGACACTACGCAGACACAGATCCCGTCATCCATCG
AGCTCCTCCTCTCCAACAGCGAGCCTTCCCCGCCCAGCGTCAGCGCCGCCGCCCTGGCGCAGGCGCA
GGAGCAGTGGGCGCGGCGCGCGGCGCGCGGGGTCGTGCAGTTCGCCATGTGCTGGATGAAGTTTG
>TCONS_00040901 geneID=XLOC_008359 scaffold_164:367586-369991(-) Exonic overlap (+)
CACGCCGCACGCCTCGCTAACCAGTTCTATATAGTGTCACATGTGTTCTTTACAACCTTATACAATATCC
AACTGTTTTAAACCAACGATAAAATATGCGAAAACTTATGTGAAACTGATAAAAATATCAATTACTGGG
AAAAGTTGCCATGGAATAAACTATTGTACCTACTTAGTATTATGTATACAATGTTGTTATCAGAAATGGG
ACAGTTAAACGAGAAACCATTCAAGAATTTACGTTTACAAATGGCAGAAGAGGTGTGTTTTGTTTGCT
CAGCGATAATGTGAACGGAGTCAACGGAGGTAATTCTGTAAACTGCAAGAATGGGCTTCATAAGCGGC
GGAGAGACCAGCAGTCCGCCCTCGCCGTATAACACACGTAAGTTTCATTCCCGAAACAAAAACAAGA
GCATATATACGTAAGCTAAGTGTGTCTGTCTGTATGTCTGTTTGTTACCAGTAAAATGTGCATTCAGTAC
TGAACAAAATGATACAGAAGAAAATGGACACAAACATCATAGTAGGA
>TCONS_00040790 geneID=XLOC_008340 scaffold_164:543663-544644(+) Intergenic
CAAAATACGACCTACTGTTTAACTCCCTTGGGGATAACAGATGGTCTTATAAGTTTGGTGCGTGTGAAC
TTTCATAGATCTGTGTCTGTGAATTTTAACTATCTGTCGCATATAGATACTATCTAATATGGTCAGACTGTA
TGTATAAATTTATGAAGGATGTCAGTTGAATAAGTATAGTAGTAGTTTTTGAATTAAGAATACATGGTAG
AATTTTATCAGTGTAGTATGGTAAACACTTCCCATTTGCAGGTATCCAACACCCTGTATACTTACTGAGT
TATTTTACCATGATACCAACTGTGACTTGACTTTGACTTTGAAATACTTAGTTATCTGCCTTCTCAAGTG
AAATGAAGATATACCTAGGTAGATAATCTATAACTTCCGTATTTTTTTTACATATTGGTCCCGCAATGTTC
AGTTTATTTTGTAGGATTCTAGGATGTGATGTCTATTCACTACTGTTAAACCGTATTGAACATTTCGATCC
GGCTCGAAGGACCATGAAAAGGAAGTGTAGTGATAATTTCACAGTCGCCTTGATCGTTGGTAGACGCA
ATGTTTATTAAAATTAGTAGTAGTAGTAAGGAAAGCACAAAACACGGGTTTATAGTGAAATCTATTGAT
ACATATTATTATTATGTAATAACTGAAAGTTAGTATAATATACGGTATTTCAGGTACATTGACCTACCGAC
GGCTAATAAGTACCTATTGATAAAGTTCCACTCTGTATAATCTTAATATGCCTTCTTTTTAATTGATTACA
AAATAATGCTGGCTATCTATAATTAACTTTATTGATTAATAAATGGC
>TCONS_00041049 geneID=XLOC_008340 scaffold_164:544945-545814(+) Exonic overlap (+)
TCTTCAGAATGCTGCTAATACTACTTATGCTTCTCTCGGCCCAGCCAGCCTTCCCTAGTGGGGGTTTACA
CCTAGGGGGGGTGGTGAAACATGTGGACCAGGACTGCCTGACAGGAACTATGACGGACGTACAGTCC
TGGGTCGGTCCTGACGGTCACCTGTTGATGGACGTCGGTATGTCGATTTTTATTTTAAGATTTGGTTCGC
TGTACTCCCTACTAGATGGCGTTGCTAGCCTTGGTTTACTTCGCGGTGTGTTTAGGATGCCCTCCGGCTA
GATGGAGAGAAGACTAGTTTGCGAAAGGTAATGATTGGATGCGGAAAGC
>TCONS_00041076 geneID=XLOC_008406 scaffold_1642:106-794(-) Unclassified
CAGGAGACAGCGGCGGACACGTGGTCGGGCGTGGGGAGCCCCGCGCACGACCCCTGGGCGCCCGCC
GCGCCGCCGCCCGACCCCTGGGCGCCGCTAGCACATATGAACAGCAAGTCCCCGCTGAGCGCGATGT
CGTCCCCATCCTCGGAGCTGGAGCAGTTCGACGCGCTGTCGCAGCGGCCGCGCAGCAACGGCACCGC
CGCGCCCGAGCCCGACCCCTTCGACCTCACGCCCCTCGCAC
>TCONS_00041224 geneID=XLOC_008427 scaffold_165:608395-641588(+) Exonic overlap (+)
CCGTGTATGAAGGGGCCTAAAACTAATTAATATTTGTGATTATTAGAGGATAATACTTAAACGAGGTATC
TAAAATTTGGGGAAATGCGAAGTATCGCAGTTTTATATTATTTCCGTTACAGTTATAAAAACTGCCATCT
TTAACATACATATTTACATTTATTGATGTACACAACAGCTGCTAAAGAATTGATATTCAGAATTCATAATG
AACATTATGATTACGTTAAAACTATGCATTTTAATAGTTCCACAATCAAAATTACATTTGATTATTTATACA
TGCAATCCAGTATGAAACCATATCATACAGATTTCATTCGAAGTTGCTGTAGTAATAATATTATATAACCA
GAGTGTTTTTACCCGCATTAAAACATATCCACTTATCCAGTATCGAATGTGATTATCGTTTATTATGAAAT
CCATTTGATTATTAAGACTTGTTAAGATTTTGGATGATGCTGGAGCAATTTTGTACCCGTAAAAGGTAAG

CGTCTACTTTGCGGCATCGTACGCAACGGACGCATCGCATTCAGTATTCTTGTATAGAAAGTCACACA
AGTGCGTCCACTTCATCGGCATATCTGACCCTGTTTCTCAATACATTTATAAAAATCTGTTTGGTTCGAT
ACGTACGATACCAATAAATGGACACTTACCTTAAATGGGACACAAATGCCATTTCAAGACATACAATAC
CATCAGATATATAATCCCATCTTCAACATCCCAGATCAATAATGCACTCTATTACTAACCACGTAAAATCG
GTCTTTCAAGTCTTTTCCCCAGCATACCATAGAGTAAGAAGAGTTAGATGTTCCATTTTCTCCCTCAAA
GCCTATTATCTGAAGGCTTTGACCACCCACACGATGAATATTATGGCCAGGATGATTCCTATGATGCTTA
GAATGAGCATTTTTCGTCGATACCTCCGTTGATGTTCTTCTGCCTTTGCTAGTTCGTCGACTCCAGATTC
TACGTTGGCTGCCGTCGCTTCGATGTGACTCTCAACTGTG

>TCONS_00041141 geneID=XLOC_008438 scaffold_165:609114-641590(-) Exonic overlap (+)
GACACAGTTGAGAGTCACATCGAAGCGACGGCAGCCAACGTAGAATCTGGAGTCGACGAACTAGCAA
AGGCAGAAGAACATCAACGGAGGTATCGACGAAAAATGTTCATTCTAAGCATCATAGGAATCATCCTG
GCCATAATATTCATCGTGTGGGTCGTCAAAGCCTTCAGATAATAGGCTTTGAGGGAGAAAATGGAACAT
CTAACTCTTCTTACTCTATGGTATGCTGGGGAAAAGACTTGAAAGGTTTGCGAAAGCCGATGTTACGTG
GTTAGTAATAGAGTGCATTATTGATCTGGGATGTTGAAGCGGCGAT
>TCONS_00041453 geneID=XLOC_008502 scaffold_166:23296-31769(-) Unclassified
CGCGTCCCGCTCGCTCGCCAGTCGTTCCGAGTCGGAAGCCCGTCAGTGTCTCGCGAGTCGTGGTGTCG
TGTGCACGTGCCGCTGCCTACTCTATCGCGACCGACGCAGTGCCGGTAACACGGTCATCGGAAAAGTG
CATGTGCGTTTTCCGTCGAGAGCATTGTGTGTGCTTGTGAGATGTGCTATTGTTTTCTAGATGTCGTTCA
TATCCTGAATACCCACGAATCCGTATGAGGAAGTAAATGTGCCGGGCGGCCCTCAGGAGTAGCTGTAC
TGACTGTCCCCCCGCGTCGGCGCCACAGACCCCGGTGTGTGTGTGTGAGTGAGCCGCGCCCCGCGCC
CCGCGCCCCTCGCCCCTGTCGGT
>TCONS_00041950 geneID=XLOC_008502 scaffold_166:23298-71808(-) Exonic overlap (+)
CAAACTGCCCGCCTCGCGCATGCGCGGCGAACTCACAACACCACGGCTCCAAGGCACGCCAAAACCA
AAGATAAGCAATAAATAAAACATCAAATATTCACTCTAATTACATTAACCGACCCACCTTCTTTCGTGTT
TCAGTATAAGTCTATGTTTGTGCAATGTGACGTAAAAACTGAGCGATTCGATAACGTTTCCCGAAGTGG
TTGGACGAGGAAGGACATCCGCCATAAATGCCTAAATAACCGGGAAATTAAGTGAAAATGAACTAATT
TTGAGTGTTAACGCATTATGTGTCATCGTGAAAGCTCTGCCGGACAAAGTGACAGTAAAGTGGTGGTC
GGACAATACAGAAATGACGAGAATGTGAAAGGTCACCGTAACACCATCTTGCTGAGAGCCGAATAATA
AATGAACCCAAGAAAATCGTTAAAATTATGACTTAGACATTAAATAATCTTATCAAAATCAAATACATAT
CAGTATTGTGCCTGTAGGTTTAAAAATATATCAATTAAATTCAGTAAGACTTAATATAAATATTCAACGTT
AACGCGATATTTTACGTAGCATATTTTATGATATTCATGTTGAATTTACAGCTAAAATTCATTGTATAAGG
ACAATTAACTTTTACCGTGTTATCACCTGTAAAGAGAAGACTGAACATAAGGATTCAAAGCAACTCCT
GACTCTGATGTCGTTCATATCCTGAATACCCACGAATCCGTATGAGGAAGTAAATGTGCCGGGCGGCCC
TCAGGAGTAGCTGTACTGACTGTCCCCCCGCGTCGGCGCCACAGACCCCGGTGTGTGTGTGTGAGTG
AGCCGCGCCCCGCGCCCCGCGCCCCTCGCCCCTGTCG
>TCONS_00041801 geneID=XLOC_008482 scaffold_166:296582-313287(+) Unclassified
GGCCTTACCTCGGTCCGCACACGAGCGCTCGCCGCTGACATTTACGTAGTGCGCCCCGTGCCGCCTTC
GAAGCGCGCGCCTTTCGTGAAAAAAAAACCAATTCCATATTTGAAATTGTGAAAAAAACATCCGACTT
CAATGTGTCAAAACTCAAACCTATTTTAATCTTCTTATTATTTTTCTTCTGTAACTGGCCAGTGTTTTTTT
TTTGTTTTTAAAAGTGAGTTATTCTGTGTGTTAGTTTTGGGGGAGAAAGCTCATTTTTCAGAGCATCTCT
ATAGCAACGCTGACGTCAAGTTCAGGGGCGGTTGGTGCATTTTGGTTCGATAGCTGTATTTTGGGGGT
GTTTATGCGTTGTTTTGAATCTCGCAAGCTCTGTGGAACTGCTGCTCAAGAGTGTTGTGTGAAGCAGT
GACCGTGCAGTGAGACACAAACATCGCCAGTGTTCAACGCAGTGTGTGTAGTGTAGTAACTGACGATT

CTGCAATAATCGAGTTCTGCATCGATTCAATGTGTGAATGACCCAAATGCCAAGAGTTTATGGACAAGT
GCACTTATATCAGGAATCATGGTAAGTGCTAACGATAATGTTTCTCACAGTTCTGAAAAAGGG
>TCONS_00041803 geneID=XLOC_008483 scaffold_166:357138-369010(+) Exonic overlap (+)
GTCTGTGACCAGCTGTCAGTGTGACACTGGAAGCGCGGGTTACAAGCAAGTGGGCGTGTACCGACGA
GCCAAGGTGCCCCTGACGTCAGTGGGCGTCAGCAGCGGCGGCAGCAGCGAGCCGGAGGGCGGGGTC
ACCCCGCAGGGCCGGGAGAAGAGCCCACAGCCTCCGCCTGAGCCCACCTGAACCCAGGGCAGGAGG
GCTAGCACGGGGCGCAATTAAGACGTGACATAAGTTTTGCATCGCGCTCACTGACATCGCGCAGCCTC
AAGAACGAGCGCGACGGAGAACTTTGCGCCCCGTGCTACTAGCCCAGGAGGGAGTGTAGTGATAATT
AAGTGGATGTGGACCGAATATATGTGACTTGTAAGTTGTAAGTGATGTGAGATACGAAATTTTAATAGG
AT
>TCONS_00041747 geneID=XLOC_008484 scaffold_166:450385-451520(+) Exonic overlap (+)
GTCGCTGCAGTCGGTAAATGGACGTTTGATGTCGTGTTTGTCAAGATTGTGCTATTGTGTGTTTACAGC
TATTATTTCTCATTTACTATCATCCCAATCAAAGTTAAAGGAATTAAGGGGTAGATGAAGGTGACAGTAT
GTCTCGCAACAAAGACAAGCATGAGGGCGCTAACAATCGCTCGTACCACATCATGTCAGAGGCGGAG
CGGGCGTCTGGCCGGCGAGGGGG
>TCONS_00041690 geneID=XLOC_008485 scaffold_166:612094-614931(+) Exonic overlap (-)
CATGTGCCCAGGCCGAATGCCGGGATAGTGTGTCCATTGTACAACTTTATCTTTGGCACTTCACCCATAT
TTTTTAATTATTAAGTCTAAGTACTTATTTAGGTATTAAGTAAGTATACAAAGTGTTTTGCTCGCGTTCTC
ACTTCAAACTGACTAGATGAATAAGATAGTGCCTTGTTTATAGGTTCATATTTATGCTTTATACAGGGTG
GATAGAATTGAGTTTGAGTTAGACTTGAGTTTTCATATATTATATGTAGACCACGTAATGCTGCTAATTTG
TCGGGGTTTATAATATGGATCATATAACATCTAAAATGATTTATATATGCAAGTCCATTATTTAAAATTAAC
AATACTTATAAAATAATCCACAACATCTTCTGTCATAAAAATATAAAATGATTTCATTGACGACATGCTTC
GTCCATTTACAAAATAAATAAGTCAGCAAACAAGCTTCGTCCTCCAGTTAAAAAGTTAAAATAGCCCG
CCCCAGGACGCACCCGACCCAAAGGTTCCCATGACGCTGGCAGCATTACGGCGCTGCACTGCGAGGG
AGATCCTCTGCATCAAGTACGCCCCAGAGCAATTGTCATGGCTCTGCATAGATTGATATGGATCTACTTA
GGTACTTATCACATGGTTACTACTGAAATCCTAACTAATATTATAAATGCGAAAGTAACTGTGTCTGTCT
GTCTGTCTGTCTGTCTGTTACTCTTTCACGCCAAAACTACTGAACGGATTTGAATGAAATTTGGTATAC
AGGTAGTCTAGACCTTGAGAAAAAACATAGGCTACTTTTTATCCCATAATTCCCACGGGAAAACTTTTT
AAGGCGAAGTGAAGCTCGCGGGAACAGCTAGTATTTAATATTTTTTATAGGTAGTTAGGTACTCCAAAC
GAAAAGGACAGGTGTTAACTATACGATTAACGTATCTGTGTATCTGTCTGTGGAGGCGTAGTTCCCAAA
TGGTCGAACCGATTTCGATCGACAGATACACACACGGATACACAGACACGAACTTATGCATTGGCCCT
TTCTTTTTATCGGGGTATAAAAAGGAAGTGTCATTATTTTCTCAGTAACTATGTAGGTATTAGGTACACC
ATATTATACGAAATATAACTAAGTGCCAAGTCACCGACTGGCCTAATTCAATGAGGCATCCTGTATATCA
TAGTTTTCAGCGTGATACGAATATGAATCACAGATTTCATTATCACTGTTAGTTTAACTATGACGGAATC
TGCCACCACATAAACTTTGGTGGAAAATTGTTTTACCTACTAGTGTTGTTGCTCTTGAACACTATCGATA
GTGTAGGTTGCCTGTAAGAGATCGCCCGAAGCGATAAGGCCGCCTATTGTTCATTTGTTGGTAGTTTAT
AAGTATTTTCGGCATGTTTTAATATTCGTGATAAGTACGTCATAAATTCATATTCTATTCTACAGTCACTC
ATAAGTATATACCTAGGTACATAATAATATATT
>TCONS_00041750 geneID=XLOC_008486 scaffold_166:628212-629577(+) Intergenic
ATTTCTCAATGCAATCTATCTAATCTATCATCCCAATCAACTAAAACTGAATGCATAAAGAGTATACTTAA
TTTTTTGCTAATATACAGTTTAATTGTTTCAGGTACCAGGGAATCAGTAGTGAAGTGGAGTGTTCGCGA
GTGTGAGATGTAGCTACTCGAGTGCGCGTGTGTGTGTGAGCTAGTGATGGTGTCAGTGTGTGGGTGAG
ATGGCGGACGATACCACCATCATCGAGGGAACTGTCAAGTTCCGCGATGGAAAAAAGTGGAAGCCGC
GC

>TCONS_00041635 geneID=XLOC_008514 scaffold_166:742672-744157(-) Splice junction overlap
TTTAGAAGAGTTCCAGAAGCGAGCCGCCGAGAAGCAAGCGGAGAACAAGCAGCAGCAGCAGGAGA
GTGCTCCAGAGAAGATGGATGTTTCCGAACCTAATAAATCTGAAGTGGTAGACGTAGAAGCGCACGCC
GAAGCGGCCGCGCAGGCGTCCCGCTCTCGCATAGAGAGACAACAGGCGGACGAGAGACAGAGACAG
GAGAAGGAGAAGCAGGAGAAAGAGGAG
>TCONS_00041622 geneID=XLOC_008491 scaffold_166:845627-846529(+) Intronic
CAATACTATTATTAGCCATATTAAAAAATAAACTAAATCTATAAAAAAAAAAACAAAAAAGGAATTTTG
CAAGTTCACAGGGATTTGAACCACCATACCGATTATTGTAAGTCTTGTATCATACCAACTGAGCCATTC
AATCTATTACAATTCATTGCAAAATTTTGCTTCATATCATAAAAACAATGAATCATTGTCACTGGCACAA
CTACATGCTTACAATATCCGTGTGTGGGTTGCCCAGAACTAAATAATAACGTCGACGTCGCTGACACAC
ACATACACTACCTACGTTAGTGTGCCCTGCCAACCATTTAACGTTGCCGTGTGTACCTACTTAATACCTA
ATTGACTAACTACCTCATGCAATAAAATACCTAGTTACCTGCTACTAAAGGTATGGGGTCGTGTTGTTCA
TCTTTGGAGGAGCCGTCACCACCGTACAATGCGACAGCGACACCCAAGAGAGATTACTATACGGTATG
TTGTTTTAGGGTTCCGTTTCTGAAATGAAAAAACGCCACCCGTCGGA
>TCONS_00041957 geneID=XLOC_008518 scaffold_166:865307-867166(-) Exonic overlap (-)
CTCGAGTGTGAAATTGGGTAGATAGGAAGTTTAAACATTGGTTGAAGAATGCTAGTTCACTTACTTTAC
AATAGACACAGTTAACAGTACCTATATGTATTGTATTTAGAAATGAATTTGTATCTCATAATTTTCCATGT
GATATTAATTTCTTCTATATTTTTTACTTCCATTTCCGGTCAGCCAGTAGCGCCATCGCGGGAAGCGATA
GTGGCGCCATCTAGACGCGGCATGGTGGTGAAGAGGTCCGAAGTGGCCCTCATCAGGTCTGTGCTCG
GAAAACGGAAGAAAATCAACTTCAAACACTACGACGGGCCGATGCAGCTCAAAACTAACTAAATACT
ACATAATTATTATTATTCCACATACGTTTGTACCTTATTTTCGCCGAAGTCTAAAATCTGAGTAGGGAGTT
GTTGATCCGTTGAAAAAACTAATGGGTATAAGGTATCTGACCCTAGGGGCCGTACATAACCCACTTTTT
ATCCCGAAATTCTCACTGAAAAACATGTTAAGGTAAAGCAAGTACTGCAATAAAATAGAGACATTTGT
TTTTTTACATTATTTATTAAAAGAAAACTTAAAATAGTTTAACCAAACAAAATTTACCGCTTCGAATCAG
ATATAAAAACAAAGTCATCTTATAAATAATTATAAACTCAAGTTATTATACGTTTCTGTACAGTCTATACT
TCAAAATAATGATTATGCTTTACGATACGATTTTTGTAAAGTCATCGTTTTAGTTGAAAAAAGGTAACAC
AAGAGGTATTATGCTGCGTACAGACCGGCCAACGAACGGGTTTCGTTGACCTTCATTGCTGCAGTCTG
CCCTGTATTATGAATGATAAATATCCATCGTTGGGCGTTCGATGGGCCGGTCTGTACACGGCTTTTTTAG
AATGTACACAAGGCGAAATCTTACCACTGTTTCTCTCATAGAGAACGTTGCTAAGTATGATAAAATTCC
ACGTTTTTTATTTTATTTTAACAAGCTTTATAGTTTGGGACCGCTACTCAGCTGAGACACGAGAGCCCTC
GATCATACTGTACCGAGCAGTTTTTAACCGCCGAAGAAAAATAGGGGTTTTATAAGTGTGACGTGTCTG
TGTATCTGCTGTGGGTGTGCCTTGGCGAATTTTGACCGTATATCAAATAGCTTTAGGGTTGAACAGTCG
TCGAGACTTCGATGGCTTCTCTTCTGGTTCTGGCGGCGTGGGGTCACGAGATAACTGCAATGATATAAA
TAAATAATCAATAAATATGTGGGACATATCACACACGGCCATCCGACCCCAAGCTAGGCAGAACCTGTG
TTATGGGAGTCGGACAGCTGATATGTCTACACAAATACATAGATAGATATATTGAATATATAAACGACAA
CCAGACACAAGGCAAGCACAAATGCTCATCACACAAATTTTTACCCTGAGCGGGATTCGAACCCGCG
GCCCCCAGCTTCTGAAGCAGCTTCACTACTATCTACGATGCCGATACTTTGAATTGTTTCGGAGTAAAA
CAATCTACGCTTAACTCTCTCCAATGGCGGCGGTCATCCGTCCCATTGGGTTCCGAAGGTGGTATAACA
GATTACTATTAGACTAGTGCAGCTTATACAGGGTCTAACAAAAGTCGTATACTA
>TCONS_00042009 geneID=XLOC_008596 scaffold_1662:480-2554(-) Unclassified
CACCCAGAGTAAGGCAGAGACTATTAACAACAGCATCAAGGACATGAACCAGAAGCTCGAAGATCTA
GAGAAGAAGGAAAAGCGCCTGAACGCCGTGCTAATATTCCTACTGAATCAACAAAGCGCGCCGACAG
GCCGGCGACCGCAAATAGGTAATGAATAGTAAATAAATAAGTTAAGTTATTTATATTTTGGAGCGAATCA
ATGCATTTTTCGCCGTGTCAACATAAAATATTTAATATTAATTATTTCAGAGTTCCCCTTATGGGAACCCA

TTCCTTTTAAGTTGGTTTTTTTTAATTGTAAGGTAAAGCCACAATATATAAACATTATATATTATTTGTGA
CAGATGCATTGTAATCATAAACTGGAAATGTGTGTTGCAATCTTTGTATAATATGTAGTATGCTTCGAAA
CGTATCCTTAGTACATAGGTATATCTAATGTCTCAATCACATAATAATTATGTAACGATAATCAAGTTATAT
AAAAATATCTTAAGTAAATAAATATATATTTAAGTCATAGCTCTAACGTAAGTTTAGACAAGTAGGTAGA
AAACTTAGCTCCAGTTTCGCCATAGTCAGTTAAATCGTAACCAGGCATTGGTTCATCACCGTTCATCAA
TTATACGTCATCTCCATATTAAATTTTTGACAGATGTGTCAAAAGTCAACCAATAATCCCTGGTTATGCT
AACCCACAATGGCGAAATTGGCACTTAGGCATATAATTTATTTTTGACAAAGATGGCATGATATAGGATG
ATTGTCGGCCTAGTTTAGTCATTGATGTGATTTACGTCCGTTACATAATATTATGTACGATGTATCTAGATA
TTATAATATGTAAGTAGTAAGATTTAATTAGAACGAATGTATCTTAAAAAACTTTTATTACTGACTGGTAA
CTAGAAACTAGATAATAGGAATAAATATTTTAATAGTTGACTACCAGAGAAGGTATAACTCGGGTGGAT
AAAGATGGGACAAGAAAATGGTGGCCGTAACATATTTATTTTTCACAGTGAAAAAGCATGAGTTAGAT
TATTTTTCTTTATCCACACGCGGGTTTCCTATTTGCAGGGATGAAAACATTATTAATAATTTAGTTTATAA
ATAAATTAAAC
>TCONS_00042304 geneID=XLOC_008614 scaffold_167:642-6302(-) Intergenic
CTAGAACAATCGAAATAAATTATTTAAATATTGGTGGTCATCGTCGACGTTTCAGATTGCTTAAGCGGCT
GCCAAAACCAAAACCCAAAATACCTATCATACCAAAAAAACCCATACCACCAATAACTATAAAACCAC
CACTATCAGTACTTCCAATAATAACAAGAAAACCAATAATAACCAGAAGAACAATACTCACAAAAAAA
ACAAGGCCTACTAGAGGAACTACAAAACAAACTACCAAAGGGTCTACGAAAGCGCATACTAAAGCAA
GTACTAAACAGCCAACTAAAGAAACAACTAAACGGCCTACGAAAGAAACTACTAAACGGCTTACAAA
AAAAACTACTAAAAGACCTACTAGAGGAACTACAAAACAAACTACCAAAAGGTTTACGAAAGAGCAT
ACGACA
>TCONS_00042305 geneID=XLOC_008615 scaffold_167:28544-29394(-) Exonic overlap (-)
ATATTATTTTTATAGTTATTATTTATAAGTAAGTAGGTATATCTCTGTGTCTTGATTTTAATATTTATAAACT
TCTCTCCTCTCAGCCTAACGCACTTCCAATCACTTTCTCGACATCACCAAAGGTTAACTGGTAGACAAT
GCTACTAGCATTAGGTTCGTCTTTGTAAATTATTTTTTATGTGCTATAAATAATTTATAATAATAATAATAA
ATGTGATTCACAGATTCGTCTACGGAAGAGTGTTGTTCTTGCGAGGAACAAGATGATAACGATGACGA
CGACGAAAGAGGAGTCACAGACGGAGAAGAGAAGGAAGAAGAAGATGATCAAATCATCACGACCGA
ATACGTCCCCACTACTGAGATATGGGTCTCCAACCAACGAAGGAAGGGTTTTAGGCCTAGTCCTAGAA
GAAGAAGAC
>TCONS_00042306 geneID=XLOC_008615 scaffold_167:28544-37623(-) Exonic overlap (-)
CACGTATATCGGTATCGCACGTATCGGACCAAACGGATAGTCATATAGATTCGTCTACGGAAGAGTGTT
GTTCTTGCGAGGAACAAGATGATAACGATGACGACGACGAAAGAGGAGTCACAGACGACGAAAGAG
GAGTCACAGACGGAGAAGAGAAGGAAGAAGAAGATGATCAAATCATCACGACCGAATACGTCCCCAC
TACTGAGATATGGGTCTCCAACCAACGAAGGAAGGGTTTTAGGCCTAGTCCTAGAAGAAGAAGAC
>TCONS_00042257 geneID=XLOC_008607 scaffold_167:88720-90883(+) Intergenic
AAGATGGTCAAGGAGATCAACATCGCGCTGCTCACCAGCTAAGGACTTTCCATGGGGACATTTGCCGA
CTTCAAGTGTAAAGCAACGGTATCCCAATTACCCACTATTCGTCGTCGCTTCATAACCGTTCTTGGTTTA
CAGTTTTACATAACAGTTTCGTTAGCGCTGCTATCCTCGATTGTTGTAAAGATAATTACATAGAACCGTA
AATGTAAACAGTGTATATTTTTTCTACTTTGTATATCTGTCGACAGTGAACGGTTGATATTAATTTTAAAG
AATAGAGTGTAATGGATCACTTTTTTACAAAGTTGCAATCGTTTTGGATATAAGTTTCTGTGCTTACGTA
GCTTTTATGTACAGATATTACGGTTCAAGAGTAACTAACAATATTTTTAACGATATAATTTTATTACAATAT
AACTTTATTTTATTTGGTAGCACAGGAAATGAATTATTAGGGAGTCGTCAATCGGAATTGTACATATTAA
AATATGTATTGTGTATCTTTTTTCCAAATTATTGTGGTATGTAGATTATTAAATTGATATTAATAGCGTTCC
TGAATTTAAGGAAAGATTGACTACAAATTGCTAGCATGTAAGTGATAAGTTACAATAAAAGAGCGCAC

TTTCATTATTGTATATCGACAACGTTGGTTTGTTAACTGTAATGGTGAATATTCTCTCAAATAGAATTATT
AAAAACTAATGTAAACTTAGTGAAAGTCAGATTCGTATTATATTTTAAGTATAATGGTATAAACCGAGTA
CGGTTTAGGTAACAGCTGTGTAAAAAGGAAATACAGATTCTCTTAAAATAAGACTGTTTTATTTACTTA
CCGATTTTAACATTTGATTTGCATGCAGTTAAATTCGTAAATTTAAAGCGAATCTAAACCTAATATTGAC
TTTAGGGATAAAATTAAATGCACAAATAAGTTTACTTTTTTAATAAAGTATTATCTCAGGAATCTGAACT
GTCCAGGAGGGTCGGGTATAATTATCACTAAAATTAAATATTTACTTAACACGTGGGATTGTAGCTACAG
CAAAATTGGACAACAGTTAAAAATGTATGACAATACTGCAAATTTTGAACAGTTTCTTTGGCCATTTCA
GTGATTCTGAATGAAATGCTGAGTATTACAATGGCATATGACATCATAGCTGGCTTTCAAAGCATGTATA
AATTTAAAATAGTTAAAAATTAAATACCTACTAGAAAAACATTGTCAAAGGATAAAAAATATAATACAG
C
>TCONS_00042071 geneID=XLOC_008621 scaffold_167:365044-446833(-) Splice junction overlap
AGGATATGCAAAACAATACAAAAACAATCGACATTATGCAAGCCTACCCCGCCCTTAGACCAAAGGAC
TAAACACTACAAAACGTCAAACCTTATTAACCGACCATTTTCAAACTAACCTATTTAAGCGTGCGTTCC
AAAGCCACAGAGGATATCGAAATAGGTTGTGTATGACAATATTATCATTCTCAGTGAAAGTAGAAGGAC
GCTGGCTGCCAGCAGTGCGTCCCGAGCGCGCGCAACGCGTGAACGTTTCCGAACGGCGACAAAAAG
GCGCGAACGACGCGCCTTGAAAATAAAAAAGTTTGACAATCCGAAACGGACGCGTTCAGGATTTGTG
CGTGAAAATGCTGTTTTAAGTTTTCATGGAACTTAAACTCAGTGGAGTTTCGAGATAAACGACTTTAGT
TAGTTCTGAAATGTGTAAATGTGAGTGAGTGTCCCGTGTGTGAGTGAACTGTGACCGGACAAGGAGAT
TTCAATTGATTTCCTAATGACTGAAACTGAAAGATAAACGGTCGGCATGGAGAGCCGCGTGGGGCTGG
ACTACATCGTGGAGCACGCGGAGTACGCGGGCAAGCTGGCGGCCGCGCTCGTTTCGCCCGCGCAGCC
CGTCAAGAAGCAG
>TCONS_00042070 geneID=XLOC_008621 scaffold_167:365045-446616(-) Splice junction overlap
GCAGTGCGTCCCGAGCGCGCGCAACGCGTGAACGTTTCCGAACGGCGACAAAAAGGCGCGAACGAC
GCGCCTTGAAAATAAAAAAGTTTGACAATCCGAAACGGACGCGTTCAGGATTTGTGCGTGAAAATGCT
GTTTTAAGTTTTCATGGAACTTAAACTCAGTGGAGTTTCGAGATAAACGACTTTAGTTAGTTCTGAAAT
GTGTAAATGTGAGTGAGTGTCCCGTGTGTGAGTGAACTGTGACCGGACAAGGAGATTTCAATTGATGC
CAGTTTTGGATGATCCGGATTTCCTAATGACTGAAACTGAAAGATAAACGGTCGGCATGGAGAGCCGC
GTGGGGCTGGACTACATCGTGGAGCACGCGGAGTACGCGGGCAAGCTGGCGGCCGCGCTCGTTTCGC
CCGCGCAGCCCGTCAAGAAGCA
>TCONS_00042314 geneID=XLOC_008621 scaffold_167:418333-446833(-) Intergenic
AGGATATGCAAAACAATACAAAAACAATCGACATTATGCAAGCCTACCCCGCCCTTAGACCAAAGGAC
TAAACACTACAAAACGTCAAACCTTATTAACCGACCATTTTCAAACTAACCTATTTAAGCGTGCGTTCC
AAAGCCACAGAGGATATCGAAATAGGTTGTGTATGACAATATTATCATTCTCAGTGAAAGTAGAAGGAC
GCTGGCTGCCAGCAGTGCGTCCCGAGCGCGCGCAACGCGTGAACGTTTCCGAACGGCGACAAAAAG
GCGCGAACGACGCGCCTTGAAAATAAAAAAGTTTGACAATCCGAAACGGACGCGTTCAGGATTTGTG
CGTGAAAATGCTGTTTTAAGTTTTCATGGAACTTAAACTCAGTGGAGTTTCGAGATAAACGACTTTAGT
TAGTTCTGAAATGTGTAAATGTGAGTGAGTGTCCCGTGTGTGAGTGAACTGTGACCGGACAAGGAGAT
TTCAATTGATGCCAGTTTTGGATGATCCGGGTAAGTTACCTTTACAAATGTCGTTACATTTTG
>TCONS_00042111 geneID=XLOC_008621 scaffold_167:438091-446605(-) Unclassified
CGAGCGCGCGCAACGCGTGAACGTTTCCGAACGGCGACAAAAAGGCGCGAACGACGCGCCTTGAAA
ATAAAAAAGTTTGACAATCCGAAACGGACGCGTTCAGGATTTGTGCGTGAAAATGCTGTTTTAAGTTT
TCATGGAACTTAAACTCAGTGGAGTTTCGAGATAAACGACTTTAGTTAGTTCTGAAATGTGTAAATGTG
AGTGAGTGTCCCGTGTGTGAGTGAACTGTGACCGGACAAGGAGATTTCAATTGGT
>TCONS_00042165 geneID=XLOC_008626 scaffold_167:548126-571458(-) Exonic overlap (-)

CTTACATTTATAATTTATTTTTGTTTTTTATATAGTTAAGGTAGCAATATGTACAATTTCTGTCAATAGTTA
AAACAGATTTATGTTTCTGATGCACAAGAACTTAGCTTTGCCAATACAACTGTAGAAATATGTCTTTACT
TAATTACAAATATAGCAAAACAACCAATTTTGCTTCATGTACTATGACTAATTAGCAAAACGTCCAACC
AATGGTGGTCAGAGAAGAATATCTTGGACATTAAATTTTTATTTATGGACACTCTGAACAGAAAGTTAA
AATTTGTAGTTATAGGGTCAAAGGTCATATTTTTAGACTTGGTTTCAATCAATGAAATGGCACAGAATTA
TGGAAACTCCGTAGTTTTAGGCACTAACATTCGTTATCTGTTTACATAATGTACCTACATTTAATTTAAAC
ATTGTGATGAATGTATCACTCACGAGGTTCTGATTGGACCGATGTGAAATGTGTAAATTTAAATTCTCTA
TACTATGGCTGCATCAGCCTTCGAAGACATACAGTGAGAAACATTAATTTATACACATACGTAGGTGCA
GATATCCGCATATCAAGTGCTTTTTAAACCGACTTCGCAAAAAAGGTGGTTCTCAGTTCGTATGTACGG
TCAGCTGCATAAGTAGCTATACTTCTGTACCTTGTCAAACTGACGAACCGTCAATGTTTCAATGAATTG
ACAGGCGGTTTCAG
>TCONS_00042213 geneID=XLOC_008613 scaffold_167:598383-614784(+) Exonic overlap (+)
TTAAGAACAAACAGAATTTCGCATCCATAACAAGTTGAATTCTGTGTTTTTCTCGTCCACTTCTAAGAT
TCGATAGTTATTCTTCTAGATTACAGGCTAACATTGGGAACATTGTCCACAGCAGCAACAGAACGGCA
AGGAGAACGAGCGCGCGGTGCAGTCCGGCAAGAAGCCGTCCAAGCTGCCGGCGCCCAGCTCCGCCA
ACAAGCCGCTCAGCGAACGGAACACCACGCGGTGATGCCTGGAGATTTTCATGACATTTTCCGCTAGA
GGCGCCACATTGTGTCTGTATAATTTTGACTGTACAGTGCGACAAACTTATCTGTTCCTGTGAGAGCGA
ACTCAATTGTTCCATATCTCATTACTTACTAATGAATTATGTATTGATATAGATTAGGTTGGTTTATTAAAA
CCGCAAGGGCGAATCACAACTACGAGCAAACCTAAAATCTTATAGAATTAAGAAATATTAGACATTTAC
GTGAGCTCTCACCGGAACAGATAAGTTTGTGGCACTGTATGTTATTATAAGTATTAATTATTACAATAGG
GAATATGCGAGTATGTCAAATAAAGGTCAAATATTATTTATAATAAACTTTATAACTACGACTCCATCAAT
ATTTTCGAGTATAATTTATTTTTGAGCAAGTAAATGAAATTGAAAAGTACAAAAACAAATATATCCCGAC
GAATTGAGAACCTCCTCCTTTTTTTGAAGTCGGTTAAAAAAACTTCGGTAAATTCTAACTTCAGAGAA
ACGTCACCCATTTTCTTAGGGCGGATGTGACGTCAAAATTCTTTATATATCAATCTCAGAATAATAACTT
ATTTGCGTTTACGGCGGAAGTTAAATAAATGTCAAGTGTTAACATAGAACAACGCGGACTCGGGTGTA
CCCTCGGGAAGCGTCATAGCGATCTTTCTTGATACAAAAAAGCCTGCCAATTTTTGCGGGGGGAAATT
TTACCGTTTACCATGATTATGTCTCATGGAACATATTATGAGTTTTAATTCCCAGCAATAAGGGTTACGTG
AAGTGACATTTAATAATGAACACAGTGTCTTCATTTTTCTTGCCAATGGATGTAACAATTATCGATAAGT
GTTATCGATGAATGATACTAATTTCAAATTAATTATGTATAGTTAAATTGAATTAGGAATGATTAATAAAC
GTAGTCACCCGCTTTTCGCTGTACATTTATACTCAGGTGATAGGTCGCGAGCCGTATCGCCGTTTTTGAA
TGAGTACAATGCACTGAAGTTGTCGGGTAAGTGGGCAACCCGACGGTCAGATGCTTTCTAGCCGCCCC
TTCGGGCGGCCTCTGACTAGGCTTAACGACTGCTGCCGAAGCAGCAACCGGGACCCACGGCTTAACG
TGCCGTCCGAAGCACGGAAGCCTATTTCGTCTCCAGACCATTCATACTCAATGAGTGTTAACAAAGAA
ATGTTAATGTCACCGAGTGTTTGTGATGCTCGTTGTGATCAGATACGATGGATCACATAAAAATTCTTCC
AATTCTCTCTTGATATGCTTGTTTGTTTGCAAAGTTTAGGACTTTTTATATTTTTTGTTGGTAGTGTATGG
GCTGCCACTAGTTGTTCTATTGTAATGAGGCGTAAACAGCCATTCGCTAGTCAACGAACCGCTCAAATA
TGCTCCATATTGCAACACAATAAAATTAAATTTGTATTGTAAGTATTATGACATGAACATGACACAAGTT
GCTCTTTCTACTTTTAGGTTATAATGGCAGTCGTTAGTATATTTCAAAAGTTGATATTTTTTTCTAAATTA
AGTTCTGGAAGAATTCTCGTAATCAGAAGAACTGGACACATTAAATTTATTACGCAGTACCTATGAACG
AGTAAACTGTGGTCAGCTGCGGAAAAGGACAGCAAAGACTATTTAAAAGTCATTGAGGTTTTTTTTAA
ATTGGTAATTTTGACAAATTGTATTAAGTATACTTATTATTTTAATGACTTTTTCAAATTATATTAATCGTA
AAAAGTTGATTGCATATTGTAAAGTATAGGTTGGATACCACAAAACTTTATTAAATTGTGTAACTACCTA
GGTTAAGTTAGTTTTAAAAAAATTCACACACAATGTATATTGTTTATGAATAAATAACTATGAACTATGAG
CATCTGTCAATGTACAAACTTACACCTTCTCTCAGTAGCCAAGTCCCACCAAGAGGGGCCAGTTTTCT

CTGCAGGCCACTGTACCTACGACGGTTGTAAATCAGAAATAAAAACGTATGTATATAGTTTTCAATTGT
TTATTTAGCAAACAGACGAAGTACATTTATAATACAGTACTGCAGTAGCGTTTTCACTCTTGCTCATCGA
TGTATCCGCGTGACCGTAAAGGTACTCTTGTCAGGCAGATAAATTAATAAGAAAGTAGAAAAACGTCC
AGATA
>TCONS_00042363 geneID=XLOC_008669 scaffold_168:549-1594(-) Unclassified
ATTTTATCGCTCACACTCTCATTTCTCAATACACAATTTATTAAAAACTAGAAATTGTGCGATATGATTTT
AGACTTCAGTAATAATTTTGGGCCCAATAGCGTGGTTACATAAAATTTTATTCTGCCTAACTTTTATCCTC
AGAATACTAACAGCATTGCAGAAATTTCGCTGTATTTGACGTATTTATGACATTGACATTTCACTTTCAT
CGACTGATTCACTCTCTGTGATTCATTTTGATTGTTGTAGTTGTTGGTTGGTCCTGCGTGCAGCTTATTT
TTTAGATACAAATAAAATAGATTATCAGTTTGTTTTGTAACTGGTTTTCTGTTATAAATTAAGGTTTTCCA
CGAATTTATTGCTCTTTAAGTCCAAAATATATTGTGTTTCTTTAGTAAGGATATGAGATACATGATTCGGT
AATGGGCACATGTTGGTGCAAGGGAAAAGTCCGTGATGATGGTGAGCAGTATGCACCAGCGACATCG
GCAGCGTCCCAGGCCTCCCCGGCCGACAACCGGATACGGGGACCCAAGTGTGTGGATACACACGTTG
TTGACCAACTCGTGCTGGAAATGCTTGGACTTATAGCCTCATTTATTGACA
>TCONS_00042364 geneID=XLOC_008670 scaffold_168:3429-29440(-) Unclassified
CGTCAGCGGTGGCTGCAAATCACTTGAAGGAAGATGCCCGTTGGTGCCTGTTTGCAGGTACTTACTTG
GAAGATTCAGAAAAGATGCAGTTCATCCAAATACAATCTCTAAGATAATGTCGGGTTCAAAACAAACT
AGGCAGACATCTCTTATCCAAGAAGTCCCTAACGGATGAAGTCGTGGTTTTCTACCTACCCGCAAACC
CTTCAAGCGACAGCAGCGCCTGGCCTGAGGGTGTGGACTGGAGGGGTCACGGCACGCTGGTGATGCA
TCTCCAACTGTCAGATCATGGGTCAGCCAGGAGCGATGTCGCAGGAAACAATCAACAATGATCTCAGA
ATGCAAGAGATAAATTATAT
>TCONS_00042428 geneID=XLOC_008670 scaffold_168:3429-29440(-) Unclassified
CGTCAGCGGTGGCTGCAAATCACTTGAAGGAAGATGCCCGTTGGTGCCTGTTTGCAGGTACTTACTTG
GAAGATTCAGAAAAGATGCAGTTCATCCAAATACAATCTCTAAGATAATGTCGGGTTCAAAACAAACT
AGGCAGACATCTCTTATCCAAGAAGTCCCTAACGGATGAAGTCGTGGTTTTCTACCTACCCGCAAACC
CTTCAAGCGACAGCGCCTGGCCTGAGGGTGTGGACTGGAGGGGTCACGGCACGCTGGTGATGCATCT
CCAACTGTCAGATCATGGGTCAGCCAGGAGCGATGTCGCAGGAAACAATCAACAATGATCTCAGAATG
CAAGAGATAAATTATAT
>TCONS_00042501 geneID=XLOC_008670 scaffold_168:3440-29826(-) Intergenic
GCATTGGTTAGGGCTCAGGTAGACGCATTGAAGCGTGCTTGTTCCATGTATCCGTGTGTGTGTAGGTTC
CTGAATGTGTTATTGTGTCTGTGTGTGTGTGAAGCTTGGAGGGTGACTCGCCACATGGTGTTACTGTGT
CGACGACCTATCCAAGGACAGAGCTGAAGTTACTTTCTAAATGATTATCTGCTAACTTCACAAAGGAG
CTTTATTGCACCGACGGACCGGTGCTTGCGAGGGGGATACGGCAGACGCTGATTAATCCGTTGGTTGG
CGGCGTGGCGTCAGCGGTGGCTGCAAATCACTTGAAGGAAGATGCCCGTTGGTGCCTGTTTGCAGGT
ACTTACTTGGAAGATTCAGAAAAGATGCAGTTCATCCAAATACAATCTCTAAGATAATGTCGGGTTCAA
AACAAACTAGGCAGACATCTCTTATCCAAGAAGTCCCTAACGGATGAAGTCGTGGTTTTCTACCTACC
CGCAAACCCTTCAAGCGACAGGTAATGACCGATCATTTATTATCAATCTTAATGAAACATAAATTCTTAT
TTTAAGTTAATTTTAGTTAAATAGATTTATTTTTAAGTTGTCCATGACTGCTTGTGTCTTATGCTGTTGCG
AAGTGTGGGAACCCAAGATGTGCTTGCGTGTATTTATTTTCTAGATGTTTATTTGTTTATTGCTCGCGAT
GTCAGTGAATGAGGTTATTTGAATAGTTTTTGTGGTGCTCTCCAGCAGCGCCTGGCCTGAGGGTGTGG
ACTGGAGGGGTCACGGCACGCTGGTGATGCATCTCCAACTGTCAGATCATGGGTCAGCCAGGAGCGA
TGTCGCAGGAAACAATCAACAATGATCTCAGAATGCAAGAG
>TCONS_00042365 geneID=XLOC_008670 scaffold_168:3442-29448(-) Unclassified
CGGCGTGGCGTCAGCGGTGGCTGCAAATCACTTGAAGGAAGATGCCCGTTGGTGCCTGTTTGCAGGT

ACTTACTTGGAAGATTCAGAAAAGATGCAGTTCATCCAAATACAATCTCTAAGATAATGTCGGGTTCAA
AACAAACTAGGCAGACATCTCTTATCCAAGAAGTCCCTAACGGATGAAGTCGTGGTTTTCTACCTACC
CGCAAACCCTTCAAGCGACAGATCATGGGTCAGCCAGGAGCGATGTCGCAGGAAACAATCAACAATG
ATCTCAGAATGCAAG
>TCONS_00042407 geneID=XLOC_008670 scaffold_168:17301-29463(-) Intergenic
GAATCCGTTGGTTGGCGGCGTGGCGTCAGCGGTGGCTGCAAATCACTTGAAGGAAGATGCCCGTTGG
TGCCTGTTTGCAGGTACTTACTTGGAAGATTCAGAAAAGATGCAGTTCATCCAAATACAATCTCTAAGA
TAATGTCGGGTTCAAAACAAACTAGGCAGACATCTCTTATCCAAGAAGTCCCTAACGGATGAAGTCGT
GGTTTTCTACCTACCCGCAAACCCTTCAAGCGACAGCAGCGCCTGGCCTGAGGGTGTGGACTGGAGG
GGTCACGGCACGCTGGT
>TCONS_00042561 geneID=XLOC_008670 scaffold_168:17585-22284(-) Intergenic
CAAAATGTGCAGTTATGAAACGACGTCATGTTTTATCACGTACTTCCCTGGTACTTACTTGGAAGATTC
AGAAAAGATGCAGTTCATCCAAATACAATCTCTAAGATAATGTCGGGTTCAAAACAAACTAGGCAGAC
ATCTCTTATCCAAGAAGTCCCTAACGGATGAAGTCGTGGTTTTCTACCTACCCGCAAACCCTTCAAGCG
ACA
>TCONS_00042562 geneID=XLOC_008670 scaffold_168:17585-32676(-) Intergenic
CAATTTAAGAGACTCGAATTTCAGAAGAAAGTATATCATAAGAACAACTGGATAATTTTGTATTGGGGA
TGATGTGTGCGGGTCTCATTAGGCGCGCTGGCGCAGGCCTCGTGCGAAGTGGACTGAGACGAAGACG
TCGCGCACGCAACTAGCGAATCCGTTGGTTGGCGGCGTGGCGTCAGCGGTGGCTGCAAATCACTTGA
AGGAAGATGCCCGTTGGTGCCTGTTTGCAGGTACTTACTTGGAAGATTCAGAAAAGATGCAGTTCATC
CAAATACAATCTCTAAGATAATGTCGGGTTCAAAACAAACTAGGCAGACATCTCTTATCCAAGAAGTCC
CTAACGGATGAAGTCGTGGTTTTCTACCTACCCGCAAACCCTTCAAGCGACA
>TCONS_00042408 geneID=XLOC_008671 scaffold_168:102087-108543(-) Exonic overlap (-)
TAGAAGTGCTATTAATTATTTTAAATAGAGATTTTATTGTTACTTGTTTTGTTATAATTATTATCACTGTTA
AGTATTTAATATTTATATGTTGTAGTGATGAATGATTGTGTAGAAACTAATTTGTGATTATTTTTTCTTTTC
TGTCATGACAGCGCAGTCCTTCGCTACTGGTTAACTGCCGGATTATGAGTAGGATGAGGTACATGCAGG
GCTAGAATAAGCAGCCGGATCACTCCTTTAGAACAAGATAGTTGGTCAATTAAGTTTAGGCGCTATAAA
CCAGTTAGGGCTTGTGTTTAAGTAAATTACACTTTAAAATATGTATACTGGAGCGTCCTATCATAAGCAA
TAAACCAACAAGAAAGAGTATCACAATAAAACACAAAGCTAAACTGCGCATGAATAAGTAAGTACCTT
TTAGTTAGCTTAGTCTAGGTCCTCTAGGACACTTAAAGCCCAGCTTATGCCTCTCGGGATAGCAACCAT
TTCTTTGGCACCAACCGACACTCTATCTTGTTATTTTATTGTTGATCAAAGGAAGCGTAGCTATTTACAA
ACTGAACTATAATGTCGCACTATATTTGCCTTATTTGGGTTGTTTAATTATTATAATGTTAGATTACCAAAA
TAGTGTGAAGGTGCATACGTCAAACTTTCCTTCAGCCCATTTATAAATATATACCTAGGATACTAATATTA
ACCGTAACCGTAACTTACCTACAAATACCTAATACGCTTACCTTACACGCATGCACGAAACACATATCA
CTTTACCATTTGTAAATTTTATAAAATACATATTATACAAAGCAATATTGAATCGAGAAATCTGGATACCC
ATATTGATGGTGGCTGACCTATGTACCTACACATACATGTATAATATAATAAATAAGGACTCGGATAAGAT
ATGTGATCAGTAGTTAACTACCTACATTTTATTAAATAATTCGGTACCTTGAGAATTAAATGTTATCGTTA
TGATGTCGATTGTCATATTGTGTACAACCTTATAATTAACTGTAACCCTCTTATGACCTGTATTTATTGTAT
GCTGTTTATTATAATTGTCTATGTATCCATATAGTTACCTACGTAAAGTAATTCATAAGATACCTAATTACC
TACCATTATCTACCGCTTTTCGTTTTTTTACAATTTTGGTGGTTCGTTGTAGATAGTTTACCAGTGTTTTT
GGACTGTTGCGATGATCTGAATTTATACTTACATAATATTGTGAAGACATAAAATAAGTGACACTAGTGA
GAAATATTTATTAAAATGTAAAAGTTTCTTATCTTAATTTTTATATAAAAATATGGTTACCTCTAATTATTAT
GTCAGTACCTAATAAATGAGCGTAAGAAATAAGTATATATTTTTTATAGCATGATTTTGACATAGGTATGC
CTTAGATACACATGGTAGCTAGTAGGTATAATAATGGTGATATTTTATATACTGCCTCCGTTGGATGTTAT

GTCTCTCATGCTTATTTATAAAGAAAAAACTGCGATCATACATAAAAGCCATGTCTATTGTATCATACAA
GTACCCAGTAACGTTTGTCCGTACCTAGTAATCTTTAAGTAGGTAGAGACAGCCTAAAAAATAATTTTG
TAACTAATTATGCTTTGTATTTATGTATAATTTCACCATAGAAATTCTCATAATTGTTTAATATTTTTTTGT
ATACCTTGGTAGGTACGGTGGCACGCACCCCTTTAATAAGGTTGGTATTTTATAATTTTCAATGACTTAT
CGATTTAAATTGACAACAGTAATGATGACTTTTCTTTATTATTTTGTTAGGATGGTTTTTAAAAGTATTTT
TGTATAAATAATGCTAACATATTGATAAGATATCTCATGTGAAGATATTGTTTCGTAAACATTCCTTTGTA
AAATCCAATAAACATTTCTG
>TCONS_00042498 geneID=XLOC_008667 scaffold_168:573579-597290(+) Exonic overlap (+)
ATTAAAAATAATATTTAGTTTTAATTGTGAACAATTATAGCGGGCTATGAAGATAGGTAGCCTGCTAAAA
CAGACAGCTGTTATATCACACAAACCGGAGCGTCTCCCTACATAGCGCAAACTGGTGCCTTGTATTGTT
TCCGCTGTTTGGAAAACGCCCCCCCCCCCCCCCCCCCTCGCCGCCCCCCGCGAGGACTCCGCTCCGAT
CCGAACCAGCCGGGCGGCCATCTTGCATCGTTTCGTTTCGTTTCGTCTCAGTTCAGTATTACGGCGCGT
GTTGTTGTGGTTCTGCGTTTTCCGCACAAATAATTCTGCTTCGCGATTTAACTCTCTCTTCGTTTGTGTT
TTGGTGCATATTCTATTTTATTACTTACGCCAAATTCCCAAATCAAATATCAGGAAACGAGATAAAATGT
CTCGGATTAATGCTAGTCCTCTGTGGGAGTTCTATCGAAAGGTGGATCATTGCGACAAGAAAGCTCAAT
GCAACACTTGTGGTGAAGTTTTGAGCTACAAATCTACTACTGCATGTTTGAAAGCTCATTTAAGGCGA
AAACACATCGAAGTGTATAACGCGGTGACGGCGGCGGCGGCGGAGGGCGCGGGGCGCGGGGCGGGA
GGCACGCAGGTACGCACACTACTTCATTTATTCATATTTCGGTAGGTCTAGGTATCGCTTCTTACATACT
AATAATTTATTTCAGACCACATTGACCGAGATTTTATCTGTTACCTACGTCACTGGCACTGATGTATCAA
ATATAGGGTTTCGTTCCCGTGTTCGGAAATTTGTCCTTCCCGAAACCGGAAATAAAATAGCATTCCCGG
GTTTGATTTTTAATCGATTCTATGCTATATTTTCATCATTATTTATCTGTTTCTCCTACCTTAAACCAAAAA
AAATAATCGGCAGGCTTATCGTCTACGATCAGAAACTCGGTTTTGTTGTAAATTATTGGAAAATCATACG
TGTTATTGTTTTCGCGCAGATGCATTGCAAAGATAAATGACTTATCTACATCACCTTGTTTCACCTCTTC
CTGGTTCAATTGTTTCATCTTTATTTAAAGTGCTATTACTTGAATGAACGAAAAACGCTCTATGGTTTGC
GCGAATAACGTCTCCAAAGTAATTAAGCTGAGGGCCTATCACAGGCTAACGTGGCAATTCGCTAGCGT
TGTACGATACTAAACAAACATTGAGTAGCCACAAAAATTTAATGGCTCCTCTTCACAATGGGCCATGAT
GTCCATCAAATGATAGTCTATAATCGCCGCCATGATCGGGCCACCGCACCGTCCATACTGTCGTGACCG
TTAATTTGCTTGACTGTCTACTGTGAAAATAACACGTCTCGAAGATAGACATCGAAACGATCGCGGCAA
TGGCCGGCAGTACTAAAAATCACCCGTCCCGCTGTCCCCGTAATTCTTGTCCCCGTTAAGCGGGGACA
ACGGTACTTTCAGAAAATAAATAAAATCATACCCGTTGTCCCTGTTGGACGTGAGCTGCTCAACGGGG
ACAGCGGCACCTAATAAAAAATAAAAAATCAGACCCGTTGTCCCCGTTCTCAAAATAAACTTTAAAAA
ACACAAGTATATTTTTTATTTCATGAATTTTATATTATGTGTATCTTCACATCAAATCGCTTTTTTAGCTTA
GATCAATTCATAACTAACCTGGGTGATAATTTGGCAAATTTGATGAACGGTGTAACTTTTAAAGGATGC
GCTAACGGCACGACGAAATACTGCCGCGAAACAAAAGCACAGATAAGATTGTTGTTTGCAAAACTCT
CATTGGCTTATTCTAAATCAGTCAACCAATGAGAGATTCCGTAACTACACTACACTTTTGAACAAAAGG
AGCTGCTTACTGGAGTTATTTAGACGTGTGTGATGCATAGATAAACGGTGTCTTTGGTGAAATCGTAAA
AAGTGCATGTAAGTACATTTATGAAACTACTAAATACCTACCTATATTCGTTATTATGTATATTATTTCATAT
ATGTATGATGATTATATAAAAATGTTGTTTTTTCATAGGTATCAACAATGTCACAACCGGGCACTTCGGG
AGGAAGTGGTGATTTAAAGTACATAAACAGAAAGGGCGGTACAATACTTCTCCATGCCGGACATCAAT
TTACAAAAAAATCTTTGTATAAAAGTGGAGCAGTCGTATGGGAATGCCACTTACGAAAGAAAAACAAG
TGTACTGGTACATTAAAGATTAAAGACGAGCCGGTCGCCAAGAAGCCGCGCCCGGACTGCTTCTCCCA
GGT
>TCONS_00042545 geneID=XLOC_008667 scaffold_168:588071-592738(+) Exonic overlap (+)
TACTTTGTTGGAAATTACAACTTGCATAAGGACCTTAGGATTCCGACCATCAGGAAATTTCTTAAGGAT

ACCTCAGCCCGTTATTTCGACAAAGCATCGCTAGATTCCAATCCGTTAATCTCTTCCTCGTGCGATTATA
CTGTTAGGGTTTAATAAATATTTAATAATATACCTCATTAATGCAGATTTTATTACTTACGCCAAATTCCCA
AATCAAATATCAGGAAACGAGATAAAATGTCTCGGATTAATGCTAGTCCTCTGTGGGAGTTCTATCGAA
AGGTGGATCATTGCGACAAGAAAGCTCAATGCAACACTTGTGGTGAAGTTTTGAGCTACAAATCTACT
ACTGCATGTTTGAAAGCTCATTTAAGGCGAAAACACATCGAAGTGTATAACGCGGTGACGGCGGCGGC
GGCGGAGGGCGCGGGGCGCGGGGCGGGAGGCACGCAGGTACGCACACTACTTCATTTATTCATATTTC
GGTAGGTCTAGGTATCGCTTCTTACATACTAATAATTTATTTCAGACCACATTGACCGAGATTTTATCTGT
TACCTACGTCACTGGCACTGATGTATCAAATAT
>TCONS_00042406 geneID=XLOC_008668 scaffold_168:599051-599920(+) Intronic
AGGTCGATCACGCTGTTCGACGACGCGGCGTTCCGCGCGCTGCTGCAGCTGATCCCGCTGCCGGCGG
CGCAGCGCGCGCGCATCTCCTCGCAGTTCGTGCAGGCGCTGCTCGACTCCGACGACGCCAGCGACGC
CCCCCACGATGACACACACGACGACGACGACGACACCAGGGACATGGACACGACCACCGCTGACGA
CCAGGACGACGACGAACAGCCGCTGCGGATCAAGAGT
>TCONS_00042580 geneID=XLOC_008719 scaffold_1686:1331-1951(+) Splice junction overlap
TGATCTTCACGTGCGCGCTCAGCGGGGTCATCATCATCGCCAACCTGTACACGTGGGGCGTGGTGCTA
GGGGCGCTGCTGTTAGGCCCGCGTGCCAGATTGAACGCCGCCGCGCGCCGCGGGGCGCCCGCGCTGG
CGTTGAGGCCTGAGGTGCATGCTACTACCAGTGTGGTGTCCTGCCTAGACGCGTTCACCGGGCAACAG
ACCCG
>TCONS_00042771 geneID=XLOC_008737 scaffold_169:77912-85517(-) Intergenic
TAAACAGGAATGTGAGAGCGTGAACACTTACCAAAGTCCAGGAGAAACAAGAACGCTCACATCAGA
GTTGCAGAGATCCATCAACATGCCAGAAGAAATTGCTTTTACGCAGGGATGGCGTATGGCGAAGCTTT
CATTTCGTGTGTCATTAAAAAAGGGGGAAGCGCCAGTCTTAATGATCCGAGTGTGTTATCAAGATCCAT
AAATCAAGCTCAATCGCTATCTTACAGTCTAATGAAATAAAAACCCGACACGGCACAAATCTTCTTCAA
CATGAAGAATAATTAGTGCCTGTTGAAGATTTATGCTTTCTTAATACTATACACCGACACCATACGACTG
TGATCGTATCTTACAGATCGACATAAGTCTCAGAGGCGAGAGAGTAATATTTCAAACGACGGAGATTGT
GCAAACGAAAATTCACTTTATGCAGAGGAAGATGGAGAAGGTAACTCCATAGAGTATATCACTGGAAA
GACGCTTGCATCAAAGCAGGTAGATCCAAATTAAAGATTTGCCGGACCGCCTTGGATGAATTGAAATG
AATTATAGTTTTCAGTCAGACAACAAATGAATGAGC
>TCONS_00042764 geneID=XLOC_008724 scaffold_169:129048-130463(+) Splice junction overlap
GGGGTTTTGGTTTTTAAGTGCGTACTAAAGCCGAAGAAGTACGGCCTCCCCGGCGGCGCCTGGAGCG
AGCCCGCGCAGCCCGAGATGCTGCCGCGGCCGGCGCTCCGGTGACCCTCAGGACACTCGGCTACATT
ACACCCGGAGTCCACTTCACACGCGGCTACGTAGATAAGCGTCACTACTTATAAAGAAAAGAAATGAA
AATTTATTTGACAAACTGACAAAAAAAAACAGAAAATAATTCAAAACAAACATAACACATCAGATTAC
TGTTCAGAGCGTCAAAAAGGCACCTGCATGTGAGATTGGTCATTTATATAAGTTTATCGACGTCTATAA
CGGACCCGTAGTGTAAGGTTTGCTATGTATACTACCTTCACCGCACATTAGTAAACAGCAATCCTAAAG
AACCATATCTGTTAAAACTGACATAATGTATAAATTGTATGGATCTGAGCCATTATAATATTAGGTTGCTT
AGATAAAGAATCGGGTTGACAGGTTCCCTGTATCTACCCGTACCGGTAACTTGATTGGGTATCTCACTG
AGAGATTTAAAAGCGGGAAATGCTCTAGCGCACTCGCAGAAAGATAAACAGCAGGTTACCGGTACGG
GTAGATATAGGGAACCCAAATCTCTCTATATTAGCACCCTTAGGTATAAGCAAGGATTTTGATTGCTAAT
GTGTCGGTTGGTATAGTGATAATGATAAGGAAGTACGTTTAAAATACTATATTCCAGTGGCATCCCAAGT
TAACTTTTGGGTTTTAACTTTCTCGGTACACACCGCAAGAGTTTATTTGAGGGCTCAAATAAAAAACTT
GCAGTGATCCTGTGATATCACGGATTTAAAAACAAATTGTAGGTACCTAGTCAGCTTTATGGCTGCTTA
AATTAAAAGTTACCAAATGAATGACATACGATTTATAATATTTATATGTAGAGTGTAAGTATTAAATGAGA
TTAAAGTCATGTACGTAAGATTTTGAAAAATAATACCTATATGTAACTGACTGTTGTAGTTTTTGTGTATT

TAGTATAAATGAC
>TCONS_00042786 geneID=XLOC_008738 scaffold_169:144331-145094(-) Intronic
TGAAACGAAAAGTTCAAACAGCCTTCGGCGGCGGCGTTTCAGCGTGCGAGCGAGACCGGTCGCCATC
GCTGACTTGCTTCAGCGCTCAGACCAGTCCGTGCCGTCCGGCTGAACACAACTGTGAAACAGTTTCAT
AATAAAATATACACTACTGTGAGTGAAAAGTGGACACAATGCACACTACATAAGATCCATTTTGCAATG
GCGGACTATGCAAAGAAAATGAAATAAAGTAACATGCAAGATTTTCACTTCATGAGAACAACAACACA
TGAGAACGATAGAAAGTTAAGAGTAAACTAGTGGAACATCAACACAAAGACTGATGGCCTGATCTCTC
GGGATTGAGTGAGAAAATAGCACCCCCACGTGTCGTGACGAGAATGCCAGTTACGCAAGCTTGGGT
>TCONS_00042773 geneID=XLOC_008742 scaffold_169:317086-348254(-) Intergenic
TCCGAAGGTCGAAGTTGTTGATACCGACGCGCGGAACCCGATTTTCATAAACAACGGAATTTCAAACG
TATACCTACGCGAAATCGGCTCAAAATCAAATAAACGCATAATTACTGCATGCAGTCTCGAAAAATTAA
GTTCCATTTTTGAATTTCGCGTTCGAAGGATTTTTTCCTTTTTTGTTTTTTAATTGTGTGGTGCGGGAAA
AAGTTTCGTTCAAAAAGTTAGTGCCGCATTTTTCGTGTCTTGTTAGTGGTTTTTGGGAATAATCATGAG
TTTGAAAAAGATAGACCAGTCATCAGGCTCTGACGGTGGAGGGGAGAAGAAGTCTAGACTCAACGGG
TTCCTGAATCTGAAAACCCCTCTCATAAGCACCAAAACTCTTGG
>TCONS_00042793 geneID=XLOC_008781 scaffold_1693:1742-2776(+) Unclassified
CGGAAAATACGCAAGCAGATGAAACCTGTCTTGTCTGTATCAAATGTCATTATGTCAGTGTCATTCAAA
CTATTAAAAAAAATGCGTTAGCAAGCAAATATTCAAGGGAAGCGAACGTATTTTATTCATAATACTCATA
ATGTCTTGTTATTTGAGTATTTAGTTGCATTTTCAAACAAATAAAGCGTAGAGTTGTATTTATAATACATT
AAATTTGTATTCAAAAGTGTTTATGGCCATTTAGCAACATGCATCGTCGCAGAGGTCCCAACTTCACTT
ATGTCAGTAGTTGTGTGAAATACATGATTTTTGTGTTAAATTTCATATTTTGGTTATTTGGTGGTCTACTG
ATTG
>TCONS_00043595 geneID=XLOC_008842 scaffold_17:152997-159279(-) Intergenic
GACCGTCAACAGTGAAACGTCGTGCCCGTCGCGCAACCACCAACTCGCACGATCCACAAACTCACAC
CAACAACACAACTTAGTTCCTCATTATCAGTTCCCGCGGGTGTTTTGGTGTTATAAGATTTTTTTGTGAT
TTGACTGACCGCTTCTGAAGTGCAGTTTGAGTTTACGTTTGTTTGGATATTGGACAAGTGATTGAAGTG
ACTGTGCGTGCTATGCCGCATCGTGTTTAGCAGCGTTTCGCAGGCGATGCTCAAAGGTCAAGATAAGC
TGTGCGTGTGGGGTCTACACTCGCATGATCGTGAATCACTT
>TCONS_00042816 geneID=XLOC_008799 scaffold_17:694527-694977(+) Exonic overlap (+)
GATTACAAAACGCGAGTGTTTCGAGTCTTTCTTTCTGTAGTGATTTGATAAGTCGCCTTCTGCGCCAGT
GAGTGTAAAGGTTAGACTTCAACAAGTATTCGTACGCTACGGAACTATTTTTTTGAGTTTAACTTCGTC
AAAATATCTGTGATTTAATTGTAGAAAAACAAAATGAAGGCTGTCCTGTTATGCTTCGTCGTTGTCCTG
GCCGCGGTCGTCCC
>TCONS_00043086 geneID=XLOC_008847 scaffold_17:717610-729247(-) Exonic overlap (-)
TATAATTAGGTGGTAGTTAGGTAAGTAAGTATAATTTAACCTAACCCAGTAACAACACCTTAAAATATCA
TTTGGTGCAAGTACAATGAAAGTAAAATTTAGGAGCTTCGTTATCACTTGTATGAGTATCCAAAGATTC
ACGTTTTTCCTCTTTATTTGCTTCATCACCATTACTTTTGTCTTTACTTGCATTAGAATTATTTGAAACGA
ATCCTCGTTTCATGGTGAGCTCATTGGGAATAGGTACTTGGCCTATATGGTCAACCCCTGGCCCAGTCC
TCAGGGTGAAACGGTGGTCAGATTTGCGGTACTGAGACTCTTCAGGTTTGTATTGAGTGCGGCTGTTA
TGGAAATGGTAGTCTCTG
>TCONS_00043093 geneID=XLOC_008850 scaffold_17:791450-797883(-) Intergenic
TTTACACCCATTACCATTACCCGAAAAATTATAATAATAATAGCGTGAGTTGTGACGGTTTGTTTCGTTT
CTTTTTTTTTCTAGACGAGGAGCCACTCCGCATGGCGAGAAGCAACGCTATTGCGATAGTGAAATACA
CTACAGCCTATCCCTTTAGACTCCCAGCCCATGAGATGATAAGACGAGCAAATCAACATCGCGAGAGA

GAACGCTATTGCGATAGTGAAATATGCTACAGCCTATCCCTTTAGACTCCCGGTCCATGAGATGAAGTG
CGTTTTCTGTGGCCAACAATTTGAAGACCCCGCCGAATATCGACAGCACATGAAAACTGAGCATAAAA
AATTCAACGTAGTAAGATCCTTAAGCCATGTGAAGGGAACGGATCTCTATCCCAAAGTTGACTGTAGT
GAATTGGCTT
>TCONS_00042836 geneID=XLOC_008807 scaffold_17:895794-896323(+) Intergenic
AACGCGTCTAACCTAAAAAAATATTCAAAATTTATCAACAAAAGACAAAGTCAAGGTTTTAAAAATGT
TATTTATTTATATTTTAAGCTATTTAGCTATGCTCCTGCAGGCTGTTTTTCTTACACTAGCTACTGCCACA
GGACTTTATTACCTCGCCGAGTTGGTAGAAGAATACACAGTAATAGCTAAACATGTCATAACATGGCTT
ATTGTGGTAA
>TCONS_00042854 geneID=XLOC_008813 scaffold_17:1048970-1050554(+) Intronic
ATGGGAACTGCGTATTCAGTGTCGTGCAATTTTAATCACAAAACTTTTCTAATTATCGTGAATTACATAA
ATAACTTATAAATTGTGATAAGTGCAGACCGTACCATGTGTTACACATAAAATTCGAAGTTTAATTTGAA
GTTTTCCGCAACAAAATGAACTTTTTGTGAATGACTTCAAAAGTACCGTTACTGACTAATAAATGCATT
TTAAAGTGCCTCGAATATAAATAAACGAAACTATCATCACACCCAGCTGCTTCAGACATGGTAGTTCGC
TGGCCGAACCATCACTTGTGCTGCAGCTCCAGCTAATCCACCCCACCATGGGACAGCAGACCTCCAGG
AAAAG
>TCONS_00043416 geneID=XLOC_008816 scaffold_17:1190507-1211453(+) Intergenic
CTTGTGTAAAGAACAGTGCAATATTTTCATAGATACTGAGTAATTTTCATACCTCAGTGATGTAACCTAG
TGACATTAGTATACCAACTGTTAGCAATACCTATGTACTAAAATACAGTTATTTACTTACATACATAGCGT
GGTGTATATTCTTCGTTGTGTATTAGTGATATAACAGACTAGTCTTTATGAAGGAAATCAATTGGGATTTA
TTTATATTACCTATGAAATTACCTACTCAATTTGCTACTAGGTATCTAAATCTGGAAGAATTACAAAGGGT
CACTATGGAATGCGTCAGCGTGCACCTTTGTTTGCCCCGACATCGTATCTCACTGACCAGCCCTGGACT
AACAACGAACAACGCTGTTGCGTCAGTAAACAACTACTTCAGAAAAAAACAACTGCCGTAGGACACG
GAAGAGAAAACTCCAGCCCCTCGCTACCTCCGTCTTACATATTTTATTGACCTCCCGAGATCACACACG
GCACGGCGAAACACAAGATTTGTAAGCAAATGTAACAACAAATGATGAATTTTGTATAGATGTTTATGC
TTCGTTGAGCCTTTGTTTCTTTTTTAAGGAATTGTGCTGTAGCTTTATTTTTATTTTAGCATATTGGTGAC
TTGTATTTATTACTATTAAAGAAGATAGGATCAATAATTGCTCATCAACTTTTCTATATTAAGCCACACAT
TCCATTTTCGACATTATGCGGCTGATATGTGTTAGATTGGCCAGAATGTGATTGTGAGTGATATGAAGTG
AACAAGTGGCCCCACACGGCGTTGGCTACTGTGTAATGGTGTATATGGCAGTAACGAATGATTATAATC
AACAATAAATGTTATGAAATGATTACCCAAAGAGTGTGGAGGGGTCATATTCGGCTATGCGTTGTTTTG
TGTACTATTGTGAAAATGTAAACTTGCTGGCTACCATTAATAAATTGTTGCCTACGTAATTATAATACCTA
CCTACCTATTTAGGGTAAATTAAGTTGTGCATTAATGTCCATTTTGGTTGGGCACACGCCATGCACCGAT
CAATATGTACCTATGTATTCAATATAGTTGTGTGGACATAGGTCTTTTCTTTCAATCAAATTAAATCTCAT
CGTCATAAAATTCTTTGACAAAGTTGAATAAAGTTTAATGGTGTATTTGTCACATACAGTTATAGTTTTAT
GACCCATACAAGTCCGAGGTCGATTAATATCAGTGACTCTATCAAGGAAAACCTAAAAAAGGAAGAGG
ATCCCAATGCAAACATTAATTTCTTTAATTTATTTCGTTAATACCTTGTAATTAAGCTGACTTGGCAAATA
AAACCATTCTTTTAGTTGCCAGTATGACTACTTAACATATATATTTAATTAAGAAGTAAACGACCGGTGA
AGTCTTTATCTTTTTATAAAGAAGGCACTTTTAATTTCTCAACTATGTTGATGGTTGTGATAAACAGAAT
AAATACAAATATGTGCATATCTTCGGATGAATCTGCGGGTCGAGTTGCCGTGTGAGTCGTGAACTCTGT
AGTTTACTGTATCAGAAGGTGCGCTATTCCCAAATCGCACTTTAGAGAGACCCCACGGGCAAAGTATT
CTGACTACTACATAGATATCTACGTACTACGAAAGCGCTTGGTTTACGCAGCAAACGAATATTGTATTCG
AAACACGTACTATATGTACGATAGAACTTGTAGGTACCTATGTAGTTCTATTCTTTTAGTTTGTAGGTATT
TTTATTTTTAAATATTAAGGCATCTTCTCGTTTTTTGTCGGGAAAGGTAAAGTGTTCACCAAATTGCTTA
TCGCAGTGACAAAGATGTTTCTGATCACAAAATTGAGGCAGTACGGTGAGCACGTTAAAGTAGCTAAG

TAGTTGTTGCAAATAAAATAAAGTGCGCCTGTTAAACAAGATTACGTAAAATGTTCCACTGCAATTGCT
TTTTTAACACTTAAATGTGTCTTACTCGTTTTTAATCTTAATTTAAAAGTCATTTTTCCTATTACAAATA
TGAATGCGCATAAAAATGAACGTTTACTTTTCTCAATGTAGTTAATTGCAGTATTCGATGCTTGCCAAAA
GTCGATGGTAAATCGTTCAATTTACGATTATTGCCCACTTCGATACTCCGATTGTAGGTTTCTTTAAAAG
CGAACTTGGCTTTACGCACGCGAGCCACAAGTTGCGATTCCACCCTCGAAAAATAACCTTACTGCGAC
CATTTTAGAGTTGTATATCGAATAACAAACCATCTAGCACTATGTAAAGTAAGTTTACACGATAATACGG
ATATTTTATGGTTATAGGATCGAAAATTTGCGTGGAAATCGACATGTCGATGTCTATCAAGTACCTATTTT
TACTGCCCGTCTATCGGCCATGCGATACTTGTCCATTCTATTATTGCTTATGAACCTATGTAACTCCTATA
AAGGCTGAAGCGAGGGTGTCATTTCATGGAGGGTACTGGACTTAAAACAGGAACTCAGAGGCTAACC
CTATGATAAGATAGATAGATAGATAGGTACCTACCTACCTAATTTTTTCACTTACTAGAATATGTTTAATTA
CTTATTCTTACTTTCTGTTAAAGTTTTTCACTAGATCAATATTCTATATTTTAAACAGAACACTAATTTACC
GGCTGTTGCAAAAGTGCCCAAATAAAGTAATTCAGGGGTCATTCAGAAAAAAATATTTGAGTCTCTATA
GAAATTAGAAACTTTGTTGATCATGTGAGTTTTCACTGCCTATGGGAAACTGTTTTTCACGAATTTCGT
CAAAAAATCGTCCTACAACTTTTGTGTGATAAAGTATTTTTATATTTATTTAGTTTTAATTTTCTTACATAA
CAATGTAATAATTAATTATATGCACC
>TCONS_00043417 geneID=XLOC_008817 scaffold_17:1221295-1242491(+) Intergenic
AACTTGGTCTAGCTCATTGTGATAATGTGTCATATTGTCTTCGACGAAAGGCTCTAATAGCTTTCTTTTAT
CTCCATCATTGATTTATTGCTGTGTTCCATCGTCTGCGGTGACAACAATGGCAGTTCTGAAAACTGCGG
TGGGGACAGAAGCAGAGAACCATTGCTGTTTACGTGCCAGCTCCACACGCTGGCAAGACTCGCATAA
CTTGAATAACGAAACGAACACCGGGGCCAAATGGAAGTGCACATACCGACCTAAGGATCCACTTCTG
AAGAATTGCCTTAAAATTGCAAAAAAAAATAGATTTTCATGAAACAAGGATCATGTTAAGATCATTATTA
ATCACGGACAGATTCATATTACTTATCTCCTGATTGATACATAGACGAGTAAAATTATTAAGTATACCCGA
AAAAATCTGTAAAATAAAAAAGTACCTAGGTACCAAATTTTACCGTATATAAGTAGGTATATTTTTGTAT
ACGTAACAGAATCAAAAACATCGCATACCTAGGTATATATATCTACGAGACGAGTACCCAAGGTCTATT
CCACAGAACTCTTCTATTGAAATTGACAAAACAAAACGACATCACTCAGGCACGTCACACAATCGTGA
CGTGTGTCGTATAGTATTTCCCCG
>TCONS_00043462 geneID=XLOC_008864 scaffold_17:1324347-1331142(-) Intergenic
GTTCGAGAGCTGACATTACTTGCGGTCGACCTTACTTAACAGGTGAGGCCGCGGCGTCCACAAGAACA
TGCTGCTAAGATTGCCTTGCCCCTTCACGCAGTCCTCACTGGCCAGTCTAAGACAAGGTGAATATAATA
ATTTAGTAACTTTACTGTTCCCCCTCCCGTAAACATCAGCCCTGCAAGGCACAAGAATTTTATTAAGTT
GTAGGCTGAAACGACTGCCTGTTTAACCGCAAGGCAGCGGTGATCAAAATGCATAAATATGACATTTAT
TAAAACTGCAGTTATTATAAAATGTAGATGTTAACGGAGATATTAAAAACATATTTTTATTGATTATTTTG
TTGCGAGAATACCCTGAGCAGTGTAATAATAAAACCTCTATGTTTGAGAACATTATAACCTATTATATTTC
GCATCTTACAGTCACTGATCTTTACTTCCCGTTAAAAATGAGGTTTTTCAACCGCCGTACGCGTGGGAA
AAGCTCGCGGAATCAGACGCAATCCAATTTAAATGCAAATAAAAATCCTGTCCTTATTCGCGAGAGAC
ATAACTACTAAATTACGGCGTTTAATACAATAATCACTGACAGTTCTTTCGGTTAATGGCCGGCTCCAAC
CTCTTTACAAAGCATAAAAAAGTTACCCGAGGCGAAAAGAAAGTTCGGCTTCGATATTTAAATTTTCA
GGTCGGTGCGGAAACTTGCTCCTTACCAATTACACTTGGCTCAAAATGGCAGCCGCTGCGAGTAGGTA
GCCCCATTCCTGATCGTTCATTTGCGCAACACCGCCGCTGGCGGGAGATAAGAAAGCGTTCTTATTTAC
GTGCGGGCGGCATGGCGGACGAATGTCGGAGAGGATGTTAATGTAAATATTTATAGACGATCGCGGCA
CAGGCGCAGCTTGTTCCTCTGTTTGGTTCTGTCATTCAACGAGGCTGAGGCCCGTCTGTGCCTTGTTTG
TGCTATGTGGAAATAGATAACATGACTGTCGTGTCATGCTTTTAA
>TCONS_00043538 geneID=XLOC_008864 scaffold_17:1324498-1351657(-) Intergenic
ATCTCAGCAAAATGGTGTGAGCGTCAGAGAGACTTTTCTGAATAAATCCTCAGATTTTGCTATTTGCTT

ACGGCCACAGCCTAGTAAGGTTCGAGAGCTGACATTACTTGCGGTCGACCTTACTTAACAGGTGAGGC
CGCGGCGTCCACAAGAACATGCTGCTAAGATTGCCTTGCCCCTTCACGCAGTCCTCACTGGCCAGTCT
AAGACAAGGTGAATATAATAATTTAGTAACTTTACTGTTCCCCTCCCGTAAACATCAGCCCTGCAAGG
CACAAGAATTTTATTAAGTTGTAGGCTGAAACGACTGCCTGTTTAACCGCAAGGCAGCGGTGATCAAA
ATGCATAAATATGACATTTATTAAAACTGCAGTTATTATAAAATGTAGATGTTAACGGAGATATTAAAAA
CATATTTTTATTGATTATTTTGTTGCGAGAATACCCTGAGCAGTGTAATAATAAAACCTCTATGTTTGAGA
ACATTATAACCTATTATATTTCGCATCTTACAGTCACTGATCTTTACTTCCCGTTAAAAATGAGGTTTTTC
AACCGCCGTACGCGTGGGAAAAGCTCGCGGAATCAGACGCAATCCAATTTAAATGCAAATAAAAATCC
TGTCCTTATTCGCGAGAGACATAACTACTAAATTACGGCGTTTAATACAATAATCACTGACAGTTCTTTC
GGTTAATGGCCGGCTCCAACCTCTTTACAAAGCATAAAAAAGTTACCCGAGGCGAAAAGAAAGTTCG
GCTTCGATATTTAAATTTTCAGGTCGGTGCGGAAACTTGCTCCTTACCAATTACACTTGGCTCAAAATG
GCAGCCGCTGCGAGTAGGTAGCCCCATTCCTGATCGTTCATTTGCGCAACACCGCCGCTGGCGGGAGA
TAAGAAAGCGTTCTTATTTACGTGCGGGCGGCATGGCGGACGAATGTCGGAG
>TCONS_00042960 geneID=XLOC_008864 scaffold_17:1325248-1365644(-) Intergenic
CGCGCCGCGCAGGCGCGTACGCACGCCCGACCAAGATGGCAGCTATTTTCCAACTTTAAATTCAAATT
TCAAATACCTGAAATACTTTTGTTTTTAAACGCTTAAAGATCCAATGACTTGCAAATAGTTCGGTTTACG
TGTTCGCGAACGGTGTCCTAAAGAAATCGGAGTGCCTTTATTGTGACTCAATTTTCCTAGTAGTATCAG
TTGTTATGACTCAATGACTTTAGTGCCATATGGTTGTGTCTTTATTCTTGTGATTCATCTCAGCAAAATGG
TGTGAGCGTCAGAGAGACTTTTCTGAATAAATCCTCAGATTTTGCTATTTGCTTACGGCCACAGCCTAG
TAAGGTTCGAGAGCTGACATTACTTGCGGTCGACCTTACTTAACAGGTGAGGCCGCGGCGTCCACAAG
AACATGCTGCTAAGATTGCCTTGCCCCTTCACGCAGTCCTCACTGG
>TCONS_00043463 geneID=XLOC_008864 scaffold_17:1351585-1393024(-) Intergenic
CGCTCGCGTCACTACGTGCATCGCCTCCGCCGCGGCCGGCCGTGCCGCCATCAGTGCCCCCAATTCATT
ACTTTACTACCAAACAACACCACTAAAGTGATCATACTCCTTTATCGACTATAAATCTGTGCTGTGTTGT
GTCTCGGATCGCGTGTCGCGGGATAAATCTGTGCTGTGTTGTGTCTCGGATCGCGTGTCGCGGGTACA
GGTTACAATGGAAAACCAACGAGATTAATCCGTTCAATTAATCAAGCAGAGCCTTCGCTAAGATCTCA
GCAAAATGGTGTGAGCGTCAGAGAGACTTTTCTGAATAAATCCTCAGATTTTGCTATTTGCTTACGG
>TCONS_00043418 geneID=XLOC_008818 scaffold_17:1361028-1380148(+) Intergenic
CTTTCGCTTAAGTAACAGTCAGTTATATTTCAAATGCTATTTTCGCTTTTACGTTCGTTGGCGCGTGAAC
ATGTTAAACAAACCCTCGTTGCTGTATCAGTTTTGATAAATATGTAGCTGTTATAGTTGTGATTTATATTC
AGGTGGGTTCGTGTCACGTTATAGTTAAATTTTTACGATGCTGATATGGGGTGGGTAGAGTAAAGCCGA
CGATTTGTTTATCGTGCAAGAGGAGGCGACGACAGGGCCAGGCAACATGCGATGACACAGAGATATC
AAGTGTATGCCTGAATATACCGCTTTATGAACAGCGCAAGAAACGCAAAGGAAATTAAGGAGGGGGCA
TCAAATATTCGCATTACGAAGCGGCGACGTTGCCAAAAACTTTTTTCATCACCAAAAAGATGGAGCGC
TATAATGAGGGTTCGCTCCGCAAAGTCATAAAAACACCTACAACTTAATAAATTAAACGGGATCCTGAA
AGATTTATCACATTGCCCTTATTGCGACTCGTGACTTCAAAGAATAAAAAAT
>TCONS_00043419 geneID=XLOC_008819 scaffold_17:1409953-1416439(+) Intergenic
GGCAACAAAGTTACGCGGAGCTGTTGGTCTTTGAAAAGAAGAAATAAAGCTTCTCCACATACGAATTT
GCAGCTTGGGTTGTGTGGAATATAAGGCGGGTGCACACATAGGTACAAAGTTGCGTGTAATTTATGGC
GGCGTGGTTTATGGGCCGCGTTCGCACATGCGCCGTACTGTCGTGGAGCGAAGCGTTGTAAAGCTGCC
GGGGTCAGGTGACATACAGAAAGTGGTGTTTAGGAACAGCCCGCGGACCCGTGAAGAGTTGCGCATT
ACCGAACGCTCGGGCTTAATAGAACCCCACTTCTGCCTCCACTATAATTATAATTACAGCCCAAGTTTA
AGTCGATTACCTACTTAACGTGTTCGTACTTTAATTTAAATGAATGCAAGCTTTAAACTAATGTGATATG
AAAACAGTTTAGTTTTATCCAGGATTGGCTTATTTTTTTTAGGTTCACGATCATAATTATAATATCTAGTC

AACTAATGTAATGTAACCTACCTATACAACAACATAATAATTTAATCTAATAAAAGGTAGAGATTTCATTA
AGCAAATGAGGACGCCTTTTTGTACTATTTCGTTTTTAGTTTATAACTTGTTTTTTGTATGATTTCTTTTT
CGTTAAAATAAGAGTATGGTATAGTAAAAGCCTGTGTAATCTTTTATAATTAGTTAATCAGAAGCCCGCA
TAGGAAAAGGCAATTATTTCTAGTATAACGTTTTTATTAAAGACACGTGCGTCGTATTGAGGAATGATAA
ACTATGTAATAAGTATTAATTTATGATATATACCTACTTACCTTACCATATTATACTATATTATGTAACAATG
AATGTTAAATGTAAATTTTGATGTTTAAATGTATATATTATGTATAACTATGGGACCTGTGCCTG
>TCONS_00042867 geneID=XLOC_008821 scaffold_17:1445297-1447316(+) Unclassified
GCGCGCGGCGCCACACTCGCGCACGACGACCTGGTCGTGGCGCTGGCCCGCCTGCACCTCGCGCATG
AACGGTACCGCAGCCTGGAAACGAAGTTGAAATCAAATCCACTCTCCCTAGAAGAGCTGATTCTGATG
ACGATCTACTCGTGAGGCGGCGACGTCTCTGCTTCTACTACATACTTGCTACTCGTACTACTACTTTACT
ACCTAAAGTATACAGTCCACGCAACGCCATCTGTGTTTAAAGTGACATACTTATTTATTGATAGATTTTG
TCACGATAAGAGATGATGGTATTAGCGCCCAGGGCGGGCGGCAGGGCGGAAATGAATACTTACATAGA
ATCTTTAAAAAGAGTAGGTAAAGTGGACTTACTTAAAATCTACCATTGTTTCAGATTACCTATGTAGTTA
ATCAGGTTAAATTTTTCAGGTATCATTTCCAAAAGCCGTACTTAAGATCTTTTTTAACACTGCTGTGTTT
AAAACCTATTATTCTGGCGGGCGCTAAATTGTAGTAGGTACGTAGGTAGTATTTAATTGTAATGGAGGTC
CAATAGTGTTAAGTCTTGTTGCTTGTTTCATCGCGGTTGTTGTCGGTGCTCTAATGTTATATTTCTGTTTG
TGTAGGTAGTTAGAGTGTGCTAATAAGGAACATGGTTTAGAGTGACCAGTTCTTACGCTTATTGAATGA
ATTATGTGTAATATTTGCTATTTATAACAATGTGTAGGTAAAAACATACGACTTGCACTATGTAGCTACAA
AAATCCAAGGGCTCTTCTTGTACCAAAATTTGGGGCAAGGTCTCTTTCTTAGTGGTTTACTCAACATGT
TTTTACACCTTAATGGTCCAAGGCATAGTGCAGCCAATCCCTTACTAAGTGAATTTTAATATCCTATAGTT
ACTTATTCCATTTACATATCGTTTTGTAAATATTAGAATAGAAACTTATCCTCTTTTAAGTTAAGTATAACA
TAGCTGAAGAAGTCAGATAGGGTTACGCGGCTCCTTATATTCTATGGCTTGTGAGCAGCTTGTGAGCGT
AGGTGCAATCACCAAAGAAACTACATTCCAAATCACTCCAGATTAGAGTACAAAATATGTGTAATATAT
AATTTTGTCGGTTTCTATTTCATTCACAGGCAGTTGCAAAAAAATACAGCTAGTTACAGAAAATTATTTT
TGCTATATTTTTCGAGAATACCTAGTTAAGTACTTCTTACAAATATTCTTGGAA
>TCONS_00043424 geneID=XLOC_008824 scaffold_17:1525231-1526167(+) Unclassified
CTTTCCACTTTCACCATTATCCAGCTCTACAAAGACTCGATCTTCAACCTACCACTTCAACTCCCCCTTG
TGTAAATGACATTAAACTGCCTTGTTCAGGTCCGATCCTGGGCGGGCTGGTCTCGTATCTGCTGAGCTT
CGAGACGCTGCTGCGGGCCATGGGCGTCGCCAACCTTCTGTACGCCGTGTTCCTCTACCGGGCGCTGA
AGAAGGATCCGCTCACACAGTGTTCTGATGATGACGAGGAAGACGGCGAGGACCTGCAGTTGTCTAC
AGTCGCGGTGGGGGACTACGACACCCTGCGCTAGTGGCTCCATCTACGACGGACGCCTCCACGATCG
GATTAGGGGCAGCAGGATGGTCACTTTAGTTTTACGAACACGATTAAAATAGGAGCGTTACTGCGCTA
ACTACCACTCTATCTCATTGTGTATTAGATGTACCGATTGCATGACA
>TCONS_00042878 geneID=XLOC_008825 scaffold_17:1529774-1530757(+) Splice junction overlap
GCAGGCCCCCGACGTACTCGCTAGGCGCGCGGCTGGCAGGGCTCCGCCGGTCACAGGCCTCGCCCGG
GCCCGCCGCCTACAAGCCCTGCACGGATAACAACAAGAAGAGTTCCCTGGCGTATTCGTTCGGCGTGC
GGCACTCGCCGTGGGCGCCGCCGCTCGTCACCCCCGCCGACAACATGGACTGTCTGTAGCTGACTGTA
GCCGACTGTAGCCGACAACATGGACTGTCTGTAGCCGACTGTAGCTGACAACCTGATGTCCTCCTCAT
CGTGATCCGATGACGGCGACCTCAGGGGCTAGTAGGACGTCTGCGGCGGTGCGCGCGGATGTGACTC
GCGAAACCCAGCTTCGTGGCAAAGGAACCCTTCGTCTGAAATCCGGGACAGTAGAGCAAGGAAGGA
AGGAAAATTAATATAGGCTGGAAGTTTTTCCCATTGTTACAAGAATGTAAATTTTTGGTCAA
>TCONS_00043522 geneID=XLOC_008827 scaffold_17:1538654-1540006(+) Splice junction overlap
CTTTTGTCTGTGGTCTTTGTTTTGCCGATATCGCAAATTAAATTAATATTTTAAATTTAATTATCAAACAA
ATTTAAGGCACAAGGTCACTGTTGTTTTGTGCATCTTAACGTATACTTCCTATAATTATCTAGCTAAGCC

ACACGATACGCACTGTCCAGAACTGAAAACCGGTTCTTTTGGCTTGCATTTTTCCTTATCACAACTGAG
AGCACTTGTGAATCGTTCGGTAAAACCCCTCGCTCGGGCTTCGCCGATGACTCACGCCTTTAAAATTA
ACAAACACGGTGTTTTCTGCATTAGACAGTCGAGTGTAGTGTGCCAAGTGATGTGCATGTGCCAGTGA
TCACGATGTTTCACCGCGATCATAGTGACGAGGATTTCGATTTCGACGACTTTAAAAATCACAAATTCG
ACGACTTCCACAAGTGCTGAGTGAGGAGGTCTCGGTGACATCAGGAGTCGTCGTTCCTGGGCATGGG
TCTGATGTCGATCCTGTACATCCTCATCGGGATCGCGGTGGCGGCCGCGCTGCTGGCGTG
>TCONS_00043346 geneID=XLOC_008827 scaffold_17:1538668-1540018(+) Splice junction overlap
CTTTGTTTTGCCGATATCGCAAATTAAATTAATATTTTAAATTTAATTATCAAACAAATTTAAGGCACAAG
GTCACTGTTGTTTTGTGCATCTTAACGTATACTTCCTATAATTATCTAGCTAAGCCACACGATACGCACT
GTCCAGAACTGAAAACCGGTTCTTTTGGCTTGCATTTTTCCTTATCACAACTGAGAGCACTTGTGAATC
GTTCGGTAAAACCCCTCGCTCGGGCTTCGCCGATGACTCACGCCTTTAAAATTAACAAACACGGTGTT
TTCTGCATTAGACAGTCGAGTGTAGTGTGCCAAGTGATGTGCATGTGCCAGTGATCACGATGTTTCACC
GCGATCATAGTGACGAGGATTTCGATTTCGACGACTTTAAAAATCACAAATTCGACGACTTCCACAAG
GTAAGCGGAGCCTTGGACTGGATGTGTTCTGTTATGGTCAGTGCGTTACCGGGTCGCCATTAGACGCG
CGCCGTGAATGCTGATGTGAACTACTAACTAATAATAAACAATTGTTTTTTGTTTGTGCTGTCTCTGGGC
AGTGCTGAGTGAGGAGGTCTCGGTGACATCAGGAGTCGTCGTTCCTGGGCATGGGTCTGATGTCGATC
CTGTACATCCTCATCGGGATCGCGGTGGCGGCCGCGCTGCTGGCGTGCTGCTGCCGCCG
>TCONS_00043426 geneID=XLOC_008828 scaffold_17:1572394-1575328(+) Intergenic
CCAGACACCAACATCACTCCTGTTTATTTACCACTGCTCTTTTCTGACTAAACCACCAATCCAACACCG
AAATGAACCAATAAATTACAATATAATTACACAGTTTTAAGACTAAAACACTGTTGAAAGTGCTTTCAA
GATGTAGTTTAAGTGAAGTAGTGAAGTGTGGTGAGGTATCGATCGCGGTGTCCGGAGTTGTAGCTTGA
AGTTAACAATTTATTGTTGCCTTGCACCCGCAGCCATGCAGCGGCAGGCGGGCCCCCC
>TCONS_00043466 geneID=XLOC_008867 scaffold_17:1596749-1598810(-) Unclassified
CGGACAGATGATATATCTACACATATAGACTCGAGTATAAACATCTTTCTTGAAACAAATATCTGCACCG
GCCGGGGATCGAACGCGGGACCAGAAGGGTCACTTTTATATTATATTGAAGAAAATTACAGGAGCGTT
GTTAATTTTGTATTTACAAGAGGAAACAAAAAAATAATCGTATACAAATACATAGATAACAGGTTTTATT
CAATGTAGCTATCATATAGGTATACAGAACTTCAGTATACGCATTTACACATAATTATCTCTATTAGGTATT
CGACCCTATGTGTAACTACATAAACATCACACAAATTTTATTACAGAATCTATATAAATTATATACTTATA
AGTACCCATATTTAGATTTAAGGCCGACCACACATTTTTTTACATACAACACTTATAAAGAGCCATCTAA
AAGTTAAATAGTTACTGCTTATTGCAACACTCGTGCAATAATTCAATTGCAAATTAATAATAAGGTACAG
TTCCGAAGAAACAACTACAGTTCCCCAAAATTGATAATGCACATAGAAATCTTCGTCATTGTTCGGCAA
CGGTCATATTCCCGAGCGTTAGAAAACTATTATGTATTTAGAGTGGTTAAGTGCATTTTCTTCATGAAAT
TTTAGTAGAATTATGTAATTGGTGCTAAAAGAGATAATTGATTTATCAGTAACGAAATTTGATTCGATAAT
TCGTAATATTCCATAATATTAAAATTTACTTCGAAAATGTTACAGTAGGTACTTTTCAAGTGTCTTACTGA
AGCTTATGTAAAGATGGATGAAAGAAGATTTGAATAACACAAGGTTTATATTATAAGGAGAAATGGATT
TCAATCAAAC
>TCONS_00043726 geneID=XLOC_009034 scaffold_170:35697-41943(-) Exonic overlap (-)
CGGCTACTCGCGGAGCGCGGCCCTCCTCGCCGTCACGTAACCACGTCTTATTTATCGCCACCACGTCTG
CTAAAGAGTTGGTAATAGTCACAGTCAGTTCGTCGTGTCCTGTACCAAGAGAACCTGCATTAAGCAAA
CCAACCTTAAAGTATTTAGTGTTAGTCATAGTTATGTATGATTAGAAGTGCCAATTAAAGGATAACAAAT
TGGATGTTGTGAAGGGAAAAAGGCAACACAATGAGTACACAACCGGATTTGCAGCGAGCACGCTGAA
CCCGAGGTCATTCCGCCCGCCGCACCGATGCCCCACCGCCTCTTCATGCCTCCTACTAGAAAAATGATC
GCACACAGAAGTTAAGCACTATTTCGACAGCATTTATACACATTAATATCATTACTAATACAATATTCAA
CATATCCTAAACTATGGTACACTCGCAAATAAATACAAATAATACAGTCTATAAGTTTATCAATAAGTACA

TCATTATTGTTTTTATTTTGCAGATTATCCACAACGAAGCAACAGAACATGGCCAGACAGGACATACAC
ACACAGAAGAGAATATGACATAATACACATTTACAAAGTGGAGCGAACTGGTTCCTCTCCAAAAATATT
AGTTAGGTCATTGACGCATCTTATTCTATACGACGGCTTGCCTTCAGCTTGGCGTACAAACACACGCCC
ATCCTTAGTCCAAACGAAACGCCATGAAAACCGCTTACCTAGATCTCTTGCTTTGCCAAACAGTTGCCT
ATTGAGTTTAGTGAGCCGCTCGTTTATGTAAAACCTCTTCGGTTCTCCTTCGAGGCCCATGTCGGCTGT
GGTGAGGCCGCGGCGCACGCGCATGGCGCGCAGCAGCTCGTCCCGGG
>TCONS_00043845 geneID=XLOC_009035 scaffold_170:52430-53537(-) Intergenic
TAAACTTATTCTATCAAAATGAAAATATCTTCAGCAATCGTTGTCCTCGTTGCTCTGGTTTGCAGCGTTG
CCGTTGCCGCTGCTGTGTCTGCCCGCGAGAGAAATGCTCACATTCTCGGCGCGTTAGAACGCTAAAGT
CGTCTATCACTAAGCTCTTATTTTTATTTTAGTTTTTTGCGGAAACTTACTGGCTTCTGTGTGTGTAATAT
TGTTGGCTGTATGTTTTCCCAATAAAGAAAATTAAAATTAAAATTAGAAGATTTGTGACTTTATTCATTG
GTAC
>TCONS_00043929 geneID=XLOC_009094 scaffold_1703:146-1096(-) Unclassified
CTACGACTTGGTTTCAGAAATGTGTCACAATGACTCAATTATAATGAATTTTAAATAGTCCTGCCCCCGC
GCCCCACATCCGGTACACCCGCGCCCTCGGCATCGCCACCGACACCGCAGCTGCCATTTATACACGCC
TGTGAGACGCACAAGTTTCGACGAATAATCAAGGTATATTCGGACTACATTCCGCTATCTCCGAGTAGA
CATTAGGTGATGAGTTGATGACTGATGATGATGAGGCCACTGAAAAAAAGTGTTCAGTTTTACAGGTT
CTTGTCACACGTTACGTAATTAACATTTTTATTAATGTACTTATTACAAAATGAAGAGGTGTCGTAGGCC
GATCGGGCAGAAGAAAGCGTGTTGTAGAGCTCCGAGGGAGGGTCCACTGTCCAACAGCGGGTGATGT
TGGCTAATGAACAATGATGATGATCTACACATTGCGAAGTACAAAATAATATATGAGTTCTTATCCACAC
TCGCACATAAAAAT
>TCONS_00043985 geneID=XLOC_009114 scaffold_171:393151-395371(-) Unclassified
AAACCGTACAGTTATACCGGTCCCCTGTTTAAAGCATGATTCACGCTACACCGTGTCTCGACCGGGCCG
TGCCATTAACCTTTAATTAAATGGCACCCTGTTTTATAAAACCCAACAAGTACTAAAACCAACAAAATA
TAGTGGTTGGCTGTTTAGACCCTGTGGTAGATAAGTCAGAAATTTAGTATAATCTTCTTAGTTTATGGAC
AAAGGATAAGTTTACACCATGTAAGGTTTTGATTCCTCTAAGCCTTGCATGGTGTAGAGAGGTACAGCA
AACGAATTATTCAGAACTGTTTTAAGTTTTATCTGGTAAATGTAGGTGCTGTAGATTGTTTGAAGTTTGG
GACCATGTCATGTACCTACACACATATGTACGAATTATGGTATGTGTTTATAACAATGATGCATTGATGAA
AGTGGTTTTCGATTTGTGCTATAGTTAAAAGACAAATACAGTATCAGGATGCCATGCTGAGAATGTTTG
TAATTATGTATTATGAAAGTTATCTGGCTACGGTCTGCTTTATAAGTATCCTTGTACTTTGTCAAACTAAC
AAAAAAAAAAACAAATGAATAAGCGGTTAGGGAGTTTGACGAGGTACAAAATTGAGGGTAACAAAG
CTGATAGTACTCACCACTGAATGTAGTTAGCTTCGTTATTACAATATAACTCGTTATG
>TCONS_00044211 geneID=XLOC_009104 scaffold_171:460580-462726(+) Intergenic
CTGTTCAGTCGAATAGCCGGCCGGTACTCCGCTAACCTGCCTGCTGATGTAGACTACACCGACCCTGA
CAGCATGAAACGCGCAAACTCCCTGTCCGCGCTCAGTAGCTCTCCTCCTTCGAAGCCTCCGGAGGGG
CGGTAGCTGGCACCCTCCCCCCTCACTCTGAGGAACCTCCTCCTCCGGTACATAGTCTTCATGTTCATA
CGGATGCTGGCTGTCAAGTCATGATAGGAGGTTGAAAAAGTTTGGACCTATTTTGTGACACGTATGTG
GATACGAACTTTAGAACTTTGTCACAGTTGCTTTTAATAACTCCACAGAGTCGGTACTTAATTTATTGAA
AAATATTCTATCAAAAGCAAAATAGACAATGTAATAAAGTTCGTAGATACATACGCTTGCTAATTGACTA
GGCTGTAGGACAGACTTTATTGAAATAAAATGTTAAATTAAGGCTTTTTCATGAAGGTTGCTTTTGTAG
GTACCTCTTTTACTATTTTTATTTGGATAGATAGTGTCACAAAGTTTTAAAATCACGGCTAACATACATTA
AATGTTATCATAACTCGATAGAGGTGCTACTTTACCCAAATGTGTTATGATCCCACTTGACTCTCATAAA
CTCTGATCCCCGTATTAGACACCTACACTACACTAGGGCCACGTAACTTGGCCAAAATGGTTAAATAAT
TTGTTTTGTTCGACACATTCCGTGGCCAATGTGGCCATCCTCGCCTATGGAAAATGAGGTTTACAGAAA

CTCTCACAGGGACACAGATTTTTACGGCACTAAAAATAGTATTTTATCTGTGCCGGCACCTACAGAGGG
CCTAAAATAAATTGTGAGCGGTGGTCAAGTTACGTAATTAGCACTGTACTGTAGGTACCTTCTGATAAT
CACTTTTATACCTTCAGTTTAAAACACTTTATAGTCTTTATACATACTATTACCTACACTTATTTCTTGTAT
ATTTTTAACTTCACAAAATTCAATGCTTTGACAGAGCCCCACTGTAATCATAAGTAGGTGAAAAGTAGA
CGTTGTTTGTAGGCATACTGCAAATCTTATGAACAAAATCATGAATTTTCTAACTGTGTTTATTTAGTTTA
AGTTAATAAAATACTTATACGAAGAGATTTAATTAATGATGACCGGCTCTTTTAAAGTTGAATTAAACAC
GTAACTAGG
>TCONS_00044051 geneID=XLOC_009117 scaffold_171:497351-500909(-) Unclassified
GGCGGTCGAGCTGTGGGCCAGCGACGTGGACTGCCCGCGCCGCCGACGGAGAAGACAGGTGGCGGT
GTCCCCCCCCTACGTGGTGTACATGCTGCCCGACGCCGACATCATGGACGACTGGCGACTCATCCGGA
AACTCATCGACTGATAGTCCACCAAGTTCACCAACACGCTATAAAGGCTCGATCATAGGGTGTTTTTAA
TGTTGCGCCATTCTCCCGCGTGTATATACACGCATTACAATAAATACTTGTATGAAAAGAACACTTGCGG
GTGTCACGCGTGCAGGGCATTCCCGCGTGCGTGATGGAATCTGCACGATATTTAAAACACCCTTAAAC
ACGATTTGACGTCACGAGGTCGTATCGTGTTTATGTGCAAGCCTTAACGTGTTCAGAAGTTTACGTTAG
GCTAGCCACACATCTTACCTCTCGGCTAAATAATCGAGAGCCAAGAATATCCCGCTCACCTGCATTATAT
GTTTTTCAGATGGAAAATTTTATAAAATATTTATTAATATACATATCTATGTAAATATTACTAAGGGTGCAA
AATATAAAATAAACATTGATAATTGCTGTGCCGATATGTAAGATGTGTTTTAGCCAGATTATGAACACTA
AGCAACTCCGTAAGGGTAGGTTTAATAGTTAAGAGCCTTGATTGTAAAGAAATAATTATAATATTATGTT
GGTATAAAATAGGTGGGTAACATACTTAATAATATATATGTATATTTCTTCTGAAACTGGCCAATGGATAG
ATTCTGAAGGCCAATTTTTTTACTATAATACCTACCAACATGAAAAATTACAATTACGCAGCCATTCAAA
AAGTAGTATAACGTACAAATAATTTTGCGTTTTAACATAGCTAAATTTAAATTTGTAAGTACCTTCAGAA
TCGTTATCAATATCCAAATATGTTTGTAATGTGCTTTACAAACCTCCCCTCGCTATTTCTACTGAGCATTA
TTTTTAAACTCGCCGTGAAATAAGAGCCGATACCCTTCTGTAATGTTGGTATTGATTTGTCTCCTCCCCG
CTGTAGATAGCTCGGAAAATAACTATTATTATTGTATTCCGACAACTATTAAGTTTACACATGCGAGGGA
CATAACTTTAAGCTAATGTAATAGTTAAAAAAACAGTTAAGCGGTAGTGGCGGCGTTTTTACGATAAGT
TACTGATGCGCTATACAAGCGATCTACGCAACGGCAGCTAAATAGCGGAATCCGATTGTGCTCTATTTT
CATAGTTTCATAAAACGGCGATATGGTTCGCAACCTATCACGTGAGTACCAATGTGCTGCGAAAAGTGG
GTGGCTCGCTTGTGAGCCACTGACTACCACTTCGGGGATTACAGTCGTGAGTGCATATGTATGTGTATG
TGTTTCATACGAGCAGGCTAGGACCGAGCATGGACCAAGAATATCGTGTAAACGCCGCTTTAAGGTAA
GCGTCCAACTTTCGGCATCTTACGTAACGCAAGGGACGCTTCAGCATTATTTGCATATAAATTCATACAT
TACAGGTACTAATCATTGTCCTGTGACATAAGTGCCGTTTCTCCATTCATTTATGATAATCTGTTTGATGC
GATACGTATACCGATAGATACAGACGCTTGCTTACCTTTACCTGAAAACTATTGTTAATTCAAAATTATC
GTAAGTAATCCTCGTCCTAATATCGTAATTATTCCAACATATATTTTTCTCTAATGTGTGTAGATATTGGTG
TAATAATTATGTATTCGTATTGTTATAGCAGGTGGGGAGATATATATTATTATGTTTATAATAATATTAATCA
TTTTATGTGTTAATTTTGTTACAATTCGGGCTTTTTGTAAGCTTTCAGTAACGCTATGTAAGTTCAGTACT
GTATTTTTCAAAAAATATGGAGATCGTAAGCGAAAGCGGATCCATAGATTTTTTTATCTAATACATGAC
CTTGTCTAGGTCTTAATTTTGTGTTGTTTGTAATCTTAGTATAAATTAATTAATAGTAATGCTTTTACATTA
TTTTGCTTTTAAAATTCTTCACTTTTACAGTCTTGAAATTGGCGGCGGACTGGTTTATTTTAAATCATATA
ATATAAAATAATATGTGGGTACATTTTACACACTGCCATCCGACCCCAAGCTAGGCAGAGCCTGTAATAT
GGGTATCGGATAGCAGATATAACGACACAGATACAAAGATCCATCCACATATTAACAACCAAGATCCAA
GTAAGTACAAATATCACATCTGTCTTTAAACAAATGGCCAGGATTTGAACCCTTCCGGGGACTGCAGCA
TAGCAGTCAGGGTCATTAACCACTACACTATTCGGTCGTCACCATGCCGTATTGACGTTGCGACATGCC
GCCGCCGCCGTCTGCTACAACCTTATATATCATCCTCCTCCGCCAAACGGCACCTTTATGTTTTCGACTG
ATTAATTTAAATTAGCACTGATTTGATATTCGCGTTTTAGATTTTTTAACTATTTATTTAGAGTAGGTTACC

TAGTTTATTAAATTAAATAAAGTCTAAACGATACACCAACCACTTATTTCATTTAGGTCCATTTTTGCGGT
CCATCACTTAAAGAAAGGTGGACGTAAAGTTGTTTTCTTATTATATAAACACCCACGAGACCCATGGAA
TGTAAAAATAGAATTACTTATAACTTTTAAATTAATAAATAAGAATGATGGGTACCTACGTACTTTATGCT
GAATACTTATACAGGGTGTTGCAAAAAGGACATACTAAGCCGAAACCTTAAAAACATTTTCCATGGAA
ACTTTGTTGGTCACAGGACTTTTTTGCTATGGAGATAATTTTTTTCGCGAATTTTATACATTTTAATTTTA
TTTCCGGGCTTATATTAGAACATGCTGCACACGTAGGTTTCGGCTTATATCCTTTTTGCATCATGCTGTAT
AGTTGATAAAAATTATGTATTTCTATTGTAAAGATTTAAGATTACATTAAATTGTGTAAGGAATAAGGAC
ACTGAAAATACCGTCAGTTTTTGTGATTTTTAATACAAGTTAATAAACGTTTCTCAGT
>TCONS_00044165 geneID=XLOC_009119 scaffold_171:601589-608787(-) Intergenic
TGAAGCTCGCTCCTTTGCCTCAGCGAGACGTAGCGCGCGATCGCGCAAGAGAAAAAAAAAACAAAA
CCACAGAATACCCATTTTCTCGTTTGGCGCGCTTTTCGTTCGTGTGTTTTCTGTGCTAAAATAAATGGAT
GGCTATGTGCATCGAATTCAAAAAACTATTTCGATATTTGCGATACAGAATTTGGCTCGTATATTCTGAA
TTCTGATATAAGTAGTAGAAATGGCGTTAAATAATGAATGGTGTCGTAAAAAACCGGTGCTCCACGG
>TCONS_00044477 geneID=XLOC_009150 scaffold_172:300078-303125(+) Exonic overlap (-)
ATTAAAATTAAACTAAAAAGAAGATGCTGGCCAGATCGCTGCCACTGTCGGGTAGGGTACCCATGACG
CTGGCCGCGTTACCTCGCTATACCTCATTATAAATTGTTTCAGCTAAGATGATTCCTGTGACAGTTTTAA
ATTTTCCGAATTATACCATTGCGCAATCTGTGTCAATTAATTATAATACATTGAAGACTCATACAAGATAG
ACAAAATCTAGATAATTTCATTATGGGATATGTCTTAAACTGCAAAATTCAAAACGTCTCCATAACATCA
CAAGAATCATCTTAGCAGAAACAAGGTATAATACACACTTACACATGTAAGGGGTTGCTTAGGAATGTT
AAATAAAATAATACACTGAGACAAAGAGTTTATTTAATTAGAAACTAGGGTGTAAATTAATTTACTATTA
TTACAAATGGAATTTGCATGAAATCAAAACTGTTATGACGAGACACTAAACATTACATTGTTAGGTCTAT
ACATATTATAAGATCTAATAAAATGCTTCTTTTAAAACTAATTTTCAACTGAAAGTTATTCTCTACACAAT
TATGCAAGCACATGTATTATGTAAAACTAATATTCGTGTGTAAAATAAATTATTAGTAAGACACATCATAG
TACACTGCTAACTATTAATAGTAGATATAACTTTAAATGAAAATTAAATTAGCTAGCCCATACTTTTCTCT
ATTGCATTTTCGATTTGAGTCTCAAATCCTAATTATAAAAACATAAATACAATGTATATTATCGAATTCGA
TAACGTAAAGCAAACAAAATCGGTCACACTACCCAACCATCTAAACCAATACATAATGTGTATATCTTA
CAAACGTTAAGAATAACTAAATATACATCCAAAAATCACAGTGATCTTTAATGTATGGAAACAACACCA
ACACACAGGGATGAAAGAAAAGGACCTTGAAGAACTGAAGGTTTCACTGACAGAAAAAAGGATACA
GTTATAACAAAAATGTATACAGTATACACCCAAAATTTGTTCAGCAAATATGCCGAGTAAAACAGTATA
GTTAGTCTAATCATAAGCATATTTTATCAACTTCAAATCGTATTATAATATTTTGTATATTATGTTGTATTGT
GTAGGTATGCTAGGTTCGTAATCATTACGTAAGTGTACAAAAATCATAATTATTTTAAAAGGTTTTGTAA
CATACATTCTATCTATTTAACCTTCTAATCTAGCAAATATTCAAAATGATATAATGATAGCCCAGCATATAA
GCGAAAGCTTATCAGTTAGTAGTATTTTTTTAAACCTTGCCTTATTAATTTTAGGGTAGTAAGTTGATATT
GTAGAGCAACACCAATCACTAAATGTCCATATATTTTTCCATATCAAGGCCACTGGCTATTGCGCTTTAA
GTATAACATTATGAACACATCCAGGCCTAATTTAATGCAGACCTTAGGAAAATAATACGTATACAATCTA
CTTATGTATGTAGGCTGTATATTGCGCAGAATTTCTCGTATTATACGCCAATCGAATTCGGTTCAAGGTCC
TTTGATTGCTTTAGAATAGGCTTGTGGTGAAGAAACAAAATTTACTAATTATAATATACGCCTATAAATG
ATGAAACCAGCATTATTCTGTAGTTGAAGCTCTTAACCTCGTCAACTTCTTTAGCCCAAATTCAGGTAG
AAAGGATAATACAAGAGTTTTCAATGTTCAACTTCAACTTTATTTTGTGGCAATCTGTCGGTTTTTCCA
ACAATTTCTGCCAGTTAGGTAATCTGCAATTTACTACGCAGTTCACACTATATTCCATTCCACAGGGAAA
GACATCTGACAGAACCCTTATTACATTCGAATAAGGGTAGTTTTGTTTGTATCAGATGTCTTTCCCCGTG
GAATGGGATATAAGCATATAAAATGACCGGACCCAGTCTACCTAAACCCGGGCTAACTCCCAACCTCCC
AGCTCCTAGCTCCTATCCTACTCTGTGTGGTTCACTGGCTTCCCACAGCCCCCCTAACCAGTGGCGCAT
TGCACGCGGTTCATGCCGGGGTGGTCCTCGTCCTCCTCGTAGGCCGGCCCGTTGGCGGCGCGGCGCCG

GCGGGACTCCTGCTCGGGGTCTAGTTCCATGAGGGTGCATTCCTCAGCTAGCTCGGGGATCTCGACCT
G
>TCONS_00044390 geneID=XLOC_009164 scaffold_172:483319-485096(-) Exonic overlap (+)
CTTGCACGTACTTACATCGGTTTCACGTAAATTTCAGCGTTAAACTTGCAATCTCGTGAACATGATCTAT
TGTGAATTAATCCGGACTAACATTGCCTGTGATATCAATTCACAGATCGTCTGAAGTCGGGAGGGAACA
CCACCATGGACACATGCAGTTTCCGCTTCACTGCCCCGGAAGGGGGAGCGCGGCTCGCCACCACGGC
CGGCGCGACCAACACCATCGCCACCGACCACACCGCGTTCATAGCTACAAGGTTGCTTCATGAGGACG
CTTATAAGATACGGGGG
>TCONS_00044434 geneID=XLOC_009156 scaffold_172:580455-581921(+) Intergenic
ATTTTTAAATATGTTACAATTATTTTGTGCATTTTTGTCGCTTACAACACATGATTAAAGACGATACTGGC
AACACAGGATTAAAAAAAGTAGGGTTCAGAAAAAATATGAATAGGGAATGGCGGTTCGAAGTTACAA
TTGTTACAAAGGTGCATGTGAATTACTGATAAATAAGCTTTATTACTGTTTAAAAAGTTTAAATAAAGTG
TAAGTGAGTAACTAAGGTGTTTAAATTAATAATTCAGAAGTGAAAAGACCAGCTCACCGATCGCAACA
TGTCTGATGATAGTAATTGTACTCCACCTATTACGGAAGAAAGAAGAAAAAGGAAAAGTTACGATCCA
CTGGAATCCCCCTTTACTAAGCGGCGTTGTAGTAGGTCACGACCATCCCGCTCGAGATCTAGGTCTCGC
CACAACAGACCGCATAGGCGACGCAGACACACCCGTTCGAGCTCCGTGCATACTCCGAGTAGGCGCC
GAGGAAGGAGTCGACCCCGCGCTCGTGGCTCTCACAAGCAGCACTGGGAACACAAATACGAGGCGA
GACATTCTACGAGTCCGAGTACCAAGAG
>TCONS_00044511 geneID=XLOC_009203 scaffold_1724:280-1332(-) Splice junction overlap
CACGAAACTGCAAAACAAATAAAAATTCCAGATATTTTGTTTAACTAATAATTACAATCACACAAACCA
CTACAACATCAAGATGTAAATGATAGAGAACCTTGTGTTCATCGCTGTAGGCACCATATTACTCTGTGC
GTTGCTGTACATAGTTATCTTCCATCTCGACCGGCGGTGGAATCACAATGGCATGACGTCGTCGCGCTA
CGTGTCGTGGCTGCCCAGCTTCAGCGTGGAGGTGACGCGCGCGCCGCCGCCGCCGCCGCGCGCGCAG
ATCGACGCCATGGCTAGACAGGTG
>TCONS_00044529 geneID=XLOC_009211 scaffold_173:71552-72486(+) Intergenic
CCCAAACAAACATTTATTAATAAACATATTTAACAATAAAATGTAATCCTAATAAACGATTTCTCTAACA
GGTACATAATTTTAAAATATGAAAATTACAGAATTATCTTATTTTAGCTTACATATATAGGTAGTTCATATC
ATATTATGTCCTATTTCATAGTATGGTCAGGCTACGCTAGAGTAGACTAAATAATAAATACAAGGTAGAA
TTTTGTTAAGTTTCCGTGAGTCAACAGTAGGTACATATCGGGTACCCTACCATAAATGATCATCACTGAT
AAAAATGTACCTTGTACCTACTTATTGATATTTTTGTGACGTGTACATTTATACATAATTATAGTTACAAAT
ATTCAGCTCTTGCTGAGTCTGTGCCATTTTGCTGGGAGGGCTGGATTTTATCAGTGTGCTTTCCGTCAG
CTGGTCAACTGAGGTCTGTACGCTGCTAAGGGTAAGCGCATTGATAAAAAATCACCCAGTATTTGCAG
TTTTACATGATTATTTATTTACTTACTGTGATGTGAAATTTGAGAGAAAATTAATTTAATGATATCTAATAC
AGATGAAATTTATTTACAAATATATCAATTAATCCACTACATAAAGAAGAAAGTATTAACAAGGATTTTA
CATAAAATGTATTCTAAAACAAAACAAAAATAATATTTAAAAGTAGTTAAAAGTTACGTTGTACCTATGC
CTAAACACCTAAATAGGAAGATATTTAATTTTTACAATAATGCATTT
>TCONS_00044589 geneID=XLOC_009238 scaffold_173:71552-72486(-) Intergenic
AAATGCATTATTGTAAAAATTAAATATCTTCCTATTTAGGTGTTTAGGCATAGGTACAACGTAACTTTTAA
CTACTTTTAAATATTATTTTTGTTTTGTTTTAGAATACATTTTATGTAAAATCCTTGTTAATACTTTCTTCTT
TATGTAGTGGATTAATTGATATATTTGTAAATAAATTTCATCTGTATTAGATATCATTAAATTAATTTTCTCT
CAAATTTCACATCACAGTAAGTAAATAAATAATCATGTAAAACTGCAAATACTGGGTGATTTTTTATCAA
TGCGCTTACCCTTAGCAGCGTACAGACCTCAGTTGACCAGCTGACGGAAAGCACACTGATAAAATCCA
GCCCTCCCAGCAAAATGGCACAGACTCAGCAAGAGCTGAATATTTGTAACTATAATTATGTATAAATGT
ACACGTCACAAAAATATCAATAAGAAGGTACAAGGTACATTTTTATCAGTGATGATCATTTATGGGTAC

CCGATATGTACCTACTGTTGACTCACGGAAACTTAACAAAATTCTACCTTGTATTTATTATTTAGTCTACT
CTAGCGTAGCCTGACCATACTATGAAATAGGACATAATATGATATGAACTACCTATATATGTAAGCTAAAA
TAAGATAATTCTGTAATTTTCATATTTTAAAATTATGTACCTGTTAGAGAAATCGTTTATTAGGATTACATT
TTATTGTTAAATATGTTTATTAATAAATGTTTGTTTGGG
>TCONS_00044799 geneID=XLOC_009213 scaffold_173:101213-102145(+) Intronic
ACAGTTTGTCTACTGCGAACTCAGCCTTACTTGCTCTTTATAAAAGCACCAGTGATTTATGATTTGACA
AAAAAGTGATTTGTTTATCTGTATGTATGATATGGAATTTCGTTTAGAGAATGCTCTCTAAAACCTTAAAT
ACAGCTCCAGACCCTTTAGCATGAATTTTCATCGTGAACGCGACGTGAAATTACTTGATGAATTTTTAT
CGTGAACGTGACGTGAAATTACTTGATGAATTTTGTAGGGAAATTTTAGTTCTGCTACCGATATTGTTTG
TTAAGGTGATATCTCATTGCTGCGGGGCGTTGTTATCCATATATTTGTCAGGGTCCTATCATAAAAAATAC
ATGAAAAAACGCACCACAGCAATGGAG
>TCONS_00044934 geneID=XLOC_009241 scaffold_173:231718-240487(-) Splice junction overlap
GTTCTATTCCGGTTGTAAAATCGATTGCCTTCGAGCCAGCGGACCGTGAAACAACGAGTTTCAAGTTT
CAACTGTTCTATTGCAAACAAAAATTCACCCTCTCAGTGCCCGTGCTGTTAATTAGTGCCAATGCCAAT
TGAAGTGCTTAAACTGATCGTGTCCTAAAAAAACTGTGCCAAAAATTTACCTCAATAATTTGGATGCTC
TAACTTTATTTTTCTATGGTGCATTTTGTATCCAGCCAGCATTGACTGTCCAATAGTTCCAAAACAAAAG
AGTTCTAATTTTAAATAACTGGCTGGTGTAGAAAAAAGTTTACCCACATGGAGGATGTAGCAAAAGTG
TTTGTTCCAAGCTTAGTTTGAGGATGAGACGGGTGTTTGGGGTGTGCACCGACTCCTGGCTGCTCTGC
GGACAGAATGTGAGGCGTAGCTGTCACTAGATGTTGTCTGGCTCCCCGCACGGGTCGGTGATGTCCTG
CCAGTCGCTGGCCGCGCGCGCCTACTCCGACCCCGCCGCGGACATGACGCACTGCGCTGGCTTCCAC
AGATCGGTTCGCTTCAGCTCAGAGTCGCGCAGCTCCACGAGGGCGTCCCACAATGACGCGCACACAA
GCGACAAGAGGGAGAACTCAGGTGAGGCTCCTTTTATGGTACTTAGAAAGCAAACAATATATCAAATT
TTCTGTTAATTTGTTCCTCCGTTTTTGTTTGTGTTCTATGTGTAAATTGTATTTTTAATGTGTAAATGTAAT
TTTGATGTTTTA
>TCONS_00044936 geneID=XLOC_009241 scaffold_173:232833-240487(-) Exonic overlap (+)
GTTCTATTCCGGTTGTAAAATCGATTGCCTTCGAGCCAGCGGACCGTGAAACAACGAGTTTCAAGTTT
CAACTGTTCTATTGCAAACAAAAATTCACCCTCTCAGTGCCCGTGCTGTTAATTAGTGCCAATGCCAAT
TGAAGTGCTTAAACTGATCGTGTCCTAAAAAAACTGTGCCAAAAATTTACCTCAATAATTTGGATGCTC
TAACTTTATTTTTCTATGGTGCATTTTGTATCCAGCCAGCATTGACTGTCCAATAGTTCCAAAACAAAAG
AGTTCTAATTTTAAATAACTGGCTGGTGTAGAAAAAAGTTTACCCACATGGAGGATGTAGCAAAAGTG
TTTGTTCCAAGCTTAGTTTGAGGATGAGACGGGTGTTTGGGGTGTGCACCGACTCCTGGCTGCTCTGC
GGACAGAATGTGAGGCGTAGCTGTCACTAGATGTTGTCTGGCTC
>TCONS_00044607 geneID=XLOC_009241 scaffold_173:232833-267339(-) Exonic overlap (+)
CGGATGGCGTTCGGTGCGTACATTCGTAGCGTTTGTCGAGTTATTTTTTAATAATTTCAGTGAAAACTGC
AAAGTTATAGCTTAAAATGTGTGTGAAACCGAAATCGTGCTGTGCGGATTCTTCATCCGAGGGAACAA
TGAGCATATAGGTCGATAATTGTTGAAAACACAAACATTCATTCGAAGTGATCACTCAATAATTGTTTGT
TTTCTTTTTACATCCTGTTTTATTGAATTTCGTTTCAATTTGTGGCGTTAACATCCTGTCCTGTGCTTTGT
TAGTGCAACGATGACCCAAGTATGCTAGTTGAGTGATTTAGTTCAAGCTTATGGTGGTGAAAACAAGT
GAACATCTGGATTACCCTTGTACACCACAGGAAAGCTGAAGGGCTC
>TCONS_00044937 geneID=XLOC_009245 scaffold_173:316427-317344(-) Unclassified
TGAGTAGTCACTTGAATTAATGTTAGAAATATTATGTGAATGAAAATGTAGCTAAGTGGATTGTTCTGAG
GTTGTGAGTAACTAAAATAATGAGGGTAGTAGGGTTAGGTTTTTTTTAAATACTACCAACATACCTACA
CATTAATTCATAGGTTACCTTGCTCTTGGGAATTCAAAGGTTATGTTACTGGGTGCAGTTGCAAGTTGT
GTAACTGAAAATACTTCATAGGTAGCAGGCTCTCTAACTGTGATGGCTGCCGACGCCCCCGCGCCCTC

CGCGCCGCCCTCGCTGCAGGCCATCAACCAGGAGATGGACAAGCTGATGCAGAAGTACAATGTGGTG
AGGTTCACCGAGCCG
>TCONS_00045101 geneID=XLOC_009226 scaffold_173:445283-451186(+) Exonic overlap (+)
GTTGAGAGCAACGATCTTATTCGAGTCTCCGCTTATCGGATTCGCCACTCGGGGCGCGAGCGACCGGC
GGACGAACAACTAAACGCGTGATTTAAACTAGTTACGGGAAGTTTGGAAAGAAGTGATGTGTGTGAC
AGTGACGGTGACATAGCACCCCGCGCCCTGCGCCAGCTCTCTGCCACATCATTATCACCACAAGATCA
GCCGCCATTCACACGTTACGGGAGGCGCAATATTAATAAAG
>TCONS_00044684 geneID=XLOC_009227 scaffold_173:492249-493453(+) Splice junction overlap
CGGCGTCGTTCCCCCGCGTCCCGCGCGGAGCGGCGGCGGCGGCGCGACGGCAGGGACCGCGAGCGG
GACTCGCAGCTCACGCGCTCGCTGGAGCGCATCCACCGCAAGAGACGGGCTGAGCGCCGGGCGGAG
CGGGAGCGCCTGTCTGATGCCGAGCTCAAGAGGACTTATACGGGGTTGGACAGAGCTTACGCGGAGC
AGTTCATAGCGGTGTGCGACGAGAGCCGCTCAGCCGCCGCGGACCGCAGCGACTCGCTCACCAGCAC
CGCGTCCAGCGACCTCACCAACCACACCAGACACCTCGACTTCTGATGCTGACGCGCTAAGGGTTAA
GGTTCATAGTTGAACGGGAAGACTGCAGCGGTTTAGACCTCACCGCGCACACCAG
>TCONS_00044942 geneID=XLOC_009252 scaffold_173:495172-496174(-) Unclassified
GACACGGCGTCCCGCTGGACGCGCGCGGGCCGCCGCCCCCCGCGCCCCGCCACCTGCACCATCCCCA
TAGACGACGACGATGAGGACATTCTAGAAGGCATGGAGGCGGGCTCAGCGGGCGACTCGCTGGGCAG
CTCGTCGGAGAGCGAGCTGCGCGTGGGCAGCGCCGCGCGCCGGCCCAGCGCCGCGCCGGGCCGCGC
CTCCGCGCTCAGCGATAATGAGTTCTAGTGTGTAGGGGATAAAGGGGGTTTTTGTGTGGTGGGGGGTG
GTTAGCTCGTGTAAGGGTTGCCAAATCTGCAAGCACCGATGCTTTGCCGCAGTACGAAGACGAAGTGC
CTCCACACTCTTGTATTGCTCTTCGCGATTGATGTAGCGGAGCCATTACAAGTTTACACGCTGTATAAAT
AAATTATACTTATTTTAAGTTTCAAAAGTTACTCGGCAAGAGTTTAGGCAGATGAAATGGTTTATAAAAA
TTAAACGATGC
>TCONS_00044631 geneID=XLOC_009256 scaffold_173:667505-673189(-) Intergenic
CTATTCTATGTTTTTGTCATTCGCGAAGTCCGTCGTTCAAGTAAAGGTTGTGCGTGTAAAAATATATTTT
ATTTTGTAAGAATAACTGTAGAAATTCAGCAAGTTAATGAATATTATGTCTGTGCATACAATTCCTCTGG
TTACACCTTTTATTCGAGGCCCGAGGTTTCCCTACCGAAAGAGCAAGTCCCCATTCTTCCCGGGCGTG
GAGGAGGCGGCGGTGGCGGCGGCGGGCGCGGGCTCGCCCCCCGGCCCCGACCAGACGCAGGACGTG
GTCAACAGGCTGAGTGCTGCAGGTCTGTCGCTGTG
>TCONS_00044998 geneID=XLOC_009233 scaffold_173:960802-1028340(+) Exonic overlap (+)
GTAAATTAATATACAATAAGATATTTTAACATTAAATATTATTGTAAATAGTATAACTGATGGACGTCACAT
AGAATGCAGAAATGTTTTAAACAATTTGTACATTTAAGTGTTTTTATAAATAAACAATATTTGTTTAAAA
AAACTGACCAAGAGTATAAATGGAAATATATACCCAATGGGGGATGATACATAAACTATACGAACACAC
TAGGGCTAGGGTATCACTATCTATCCATAAAAAAGGCACTACATTATTAAATTTTAAATGTAATCTTCAGT
GAAACACTTGGTCAGTTTTTTCAAAACAAATATTTTGTCTTCGTACATTTTATAAGAATACGAATTAATT
AAATGAATGAGATGGAATGTAGAAATAATTTCATTATTAATCGTATAAGACTAACTAACCAACATGTATA
CGATACGGTCTAGCCATTTCGACATTTCTACCTACCACCTATATCTTGAC
>TCONS_00044959 geneID=XLOC_009263 scaffold_173:963682-968848(-) Splice junction overlap
GTCACAGATGTGTGGACCAGCAAGGGACCGTCTGAAGAATCAAGTTCGTGTAACGCGGGTCCCAGCA
CGCCGCGTGCCGCTTCACTCACTCCTCACGAGCCGCCTAAACGTCAGAACTCTGACAGCGTCGCCGA
AAGAGCCCGGGGACTCCGCGCGTGGCTCAAAACTGCACCCTAAAACAACTGAGTAAGAACTAAAATA
CAAGCAACAACTAACAACACCTGGCTGGTAAGAAATAGATGAATATGATTTGTTATGCAAAAAGTATA
GAAAAACTGTTTTGAAACTACTTATTGGAGCCGATGATAGGTTGGGTAAATTTATACCTACAATTTTGTG
ATTCCCTTGAGTTGTCTGTGGTGTGTTATGTCAGATGAGTTGATTTTACTAATTAATTAACTACAAATAC

CATTATACAACACAAAATTATTACAAAAACGATTAAAAAATAATTGAAATTGCGTTAAGAAACAGTGTC
TTTTAGTAAATCGCGTGTAGTTTTGCCGATAACTTCCATATATTTGATCGACATGACAATATGTAAAGGTT
CTATGTCGGTTATTCTGACTACAGATGTATTTCAATTACTCGTACTGATAAGAGTAACGTTGTAGGTAAG
AGTAGCACAAATGAAAATAACGATGCCTAAATTGACGCACAAAGACATAGTCATTTCAATGACATGTC
CAGTCATTTTACAGTTGTTTCAAATTTTAATAAAAAAATGTTTCTTAAAGCTACTGTATTCTTTGTAAAA
ACTTCAAATTTCATTTTAAACTATTTCTACGTGTTAAAAATAAAGCTCAATAAAACCAAAAAAATATTGT
ATGTTGTAATAGCACAAATTTTATAAAAATATAATTAGCACAAGTTATGAACATTCCTTTATGTACAAACC
TAGTCGTTTTGCTTTAAAATAATAATGACAAAGTCACTCTACTTAACTTTGTTAAAAACAATAAAAATAC
GTAAGTAGTTAAGTACATAAGATTATACCTTATGTATAGAATACCATGAACATATACAATGAAATGAGCC
AGTTGCATTCCAGTACACATGGAGTCCAAAATTTAAAGAGTATCTATCTACCTCCGCCTATTTTTGCTGT
GTTGAAAGATCAATCATGTTGAAAGTCATTGTTATTTCTAGTCAATTATACATTTTAGTTATTTATATACCT
AATACGCGGACAAATATGTGGGATGCCCTCCAGCTTACTAAACCGGAGACCTTTGGAAAGTCGGTATAT
AGTATGCTGCATGAAGAAGTTAGATCATTTATCTAACAGTAGTTATTTTTTTATTGCAACTGGCTACTTCT
ACTAACTATGAACTTGAATGTAATCTTTAGACTTTTGAATGTGACTCCAATTTATG
>TCONS_00044960 geneID=XLOC_009263 scaffold_173:963745-969072(-) Splice junction overlap
TAATTAATACCTACTTCTATTTTAATGCTTGAATTTTGCCTGCGTTTATAATTTTGTTCATAATATTTATACG
CCGCTTATAATGGTATTGACAAAACTGGTAATTATTGCGCTTGCAACACATTAACATCGCCTCTGTCTAA
CAGTATCATTCCCACACACAGGACAGCTGGAGTGGCTCGGACTCGGGCTCGGAGGCGGCGCGGCGCC
CGCGAGACCGCGATGTCACAGATGTGTGGACCAGCAAGGGACGTACGTATCATCAATATCATCATCATC
AGTAAGGGCCACTTGCACCAAAATATTAAATCGAGATTTAGTGTTAAATAGTGTGCCATAAATTTATATG
TACTGACGTTTCTTGACTAGAATCTAGATCTAATCTTTCACTGCTATGCAATGTAAAGGTCCTGGAGGGT
TACTCTATCCCGATAAAGAACGAGCGGAAAAAAGCCAAAGTATGTTTAATACTACAGAATTATCGTTTC
ATTGATATATTAAAATTCGCATGTGTATAAATTCGCAGCGTCTGAAGAATCAAGTTCGTGTAACGCGGGT
CCCAGCACGCCGCGTGCCGCTTCACTCACTCCTCACGAGCCGCCTAAACGTCAGAACTCTGACAGCG
TCGCCGAAAGAGCCCGGGGACTCCGCGCGTGGCTCAAAACTGCACCCTAAAACAACTGAGTAAGAA
CTAAAATACAAGCAACAACTAACAACACCTGGCTGGTAAGAAATAGATGAATATGATTTGTTATGCAAA
AAGTATAGAAAAACTGTTTTGAAACTACTTATTGGAGCCGATGATAGGTTGGGTAAATTTATACCTACA
ATTTTGTGATTCCCTTGAGTTGTCTGTGGTGTGTTATGTCAGATGAGTTGATTTTACTAATTAATTAACTA
CAAATACCATTATACAACACAAAATTATTACAAAAACGATTAAAAAATAATTGAAATTGCGTTAAGAAA
CAGTGTCTTTTAGTAAATCGCGTGTAGTTTTGCCGATAACTTCCATATATTTGATCGACATGACAATATGT
AAAGGTTCTATGTCGGTTATTCTGACTACAGATGTATTTCAATTACTCGTACTGATAAGAGTAACGTTGT
AGGTAAGAGTAGCACAAATGAAAATAACGATGCCTAAATTGACGCACAAAGACATAGTCATTTCAATG
ACATGTCCAGTCATTTTACAGTTGTTTCAAATTTTAATAAAAAAATGTTTCTTAAAGCTACTGTATTCTTT
GTAAAAACTTCAAATTTCATTTTAAACTATTTCTACGTGTTAAAAATAAAGCTCAATAAAACCAAAAAA
ATATTGTATGTTGTAATAGCACAAATTTTATAAAAATATAATTAGCACAAGTTATGAACATTCCTTTATGT
ACAAACCTAGTCGTTTTGCTTTAAAATAATAATGACAAAGTCACTCTACTTAACTTTGTTAAAAACAAT
AAAAATACGTAAGTAGTTAAGTACATAAGATTATACCTTATGTATAGAATACCATGAACATATACAATGA
AATGAGCCAGTTGCATTCCAGTACACATGGAGTCCAAAATTTAAAGAGTATCTATCTACCTCCGCCTATT
TTTGCTGTGTTGAAAGATCAATCATGTTGAAAGTCATTGTTATTTCTAGTCAATTATACATTTTAGTTATT
TATATACCTAATACGCGGACAAATATGTGGGATGCCCTCCAGCTTACTAAACCGGAGACCTTTGGAAAG
TCGGTATATAGTATGCTGCATGAAGAAGTTAGATCATTTATCTAACAGTAGTTATTTTTTTATTGCAACTG
GC
>TCONS_00044577 geneID=XLOC_009235 scaffold_173:1094337-1095613(+) Intergenic
AGTCAACATACTGTTACAGGTGTAATAACCATATTTTTGTTAGTTCGATATGAACAAATAATGGGTGAAA

GATTCGTTTAACAATAATTAACATAATTTTATTATACAAAAATAGCTTTGCGCTTTTTACACGTTATTCAC
ATTTAACTACTATCTTAAAGCTACGGTTCCGCAGTTTTCAACACGCTCAGCGTAGGTTTTGACCATTAA
AAGTTATAATATAAAACTAGGTTACTAATACAAGAAAAAGTAAGTAGGTAATAGAATTATTTCAAATTAT
AATATTAAATTAATACTTAATAATTCCTAAGCCAGCGTACAATAATAGTTGCTGACTAAACTGACTTGAT
AAAAACTGTGGTTTGTATTATTAATTTAAACATAATCAATTCAATCCAACATCACTGACTTCACCTATCT
AGACTCCAATACTTTACTAAAATGTTATGCCATCTACTTTTGTCAGTCGTTTCCCTAATATTAAATTCTAT
ACAAGATATACACATATTGGTAGGTATAGTTAGAAATTGAAATTATTTTATTGGAATTGATAAGTGCTATT
AAATACCTATAAAGTGGGTACTTATAATGAAATTATATTGCTTTGACATACACGTTTCTGGTTATTATTTCT
AATTTACATGTTCAAGTCTATCAAGTTGCGTTAGTTCCAAATAAAGTAAGATCTAAAATCGTTAGCTTGC
TTTTATACATTTACTTATAAATGCTGTGTTAAAAATTATGTC
>TCONS_00045262 geneID=XLOC_009348 scaffold_174:277350-277993(+) Exonic overlap (-)
ATATTGGCTGATTATGATGATGAGATGTGACTGGAACTTATCCATTGCTCGTAACTATAGTTCCGTTTACC
AAGTCCTTCTTCTTAAAGACGCAAGCCACGAGGCACCTGGCGCCGCTGCCGTCGGGTATGACGTGCTG
GTGCATGAGCTGGATCTCCTCCGGAGTGACCTGGTGCTGCTTGGCGCACTCCATGCCGAGCTGAATGA
ACTCCTCGCGGATCTCCTTCTCAGTGCGGGCCTGGTG
>TCONS_00045191 geneID=XLOC_009353 scaffold_174:347145-370180(+) Exonic overlap (-)
TAGATCATACCACTTAGAAGATAGGGGCTTAATTGATCTGTAATTATGGCAAACAGTGGTTAACTGCGC
TCCTTACTTCACTTCTCTAACCAAACACCGTATGATCGGTCCCTATTGATGACGTTTGTGGAGTTTGTTA
ACAAAACAATCAGTGATCGTGTTTTGTCAATAGAAATAGCTTGTTTTGGTAAATGTGGACTTTTAAGAT
TTTATTATCTTAATGTGAAAGTGATTAGAGTTAGTGGATTTATTTATTGTATTACTTTGAAACAGTTAATG
CCGGTGTAATGCAAGTGTGACAGTATTGAGGATGATAAACCATATGTTTTGAGAAATAAATCAGCCCAT
TGTGCTACAAGAAGTGTCACCGACTTATTCAAGTGTTGCTGTGACTTAAGTGGATTGAAGCAAAAGAG
AACAATATGTCTAACATTTGTCACCGCTACATCAGACGACACTGTGAAGAGAAAAATGGAGGATCCTA
CATGAAGTTGCTGAACACACTATCGACGGATCTGGAGGAGATGCTCAGCGAACTATACGAGGGGAAA
CATGTTGTCAG
>TCONS_00045471 geneID=XLOC_009354 scaffold_174:403065-406955(+) Intergenic
AAACACTGCTGTTTTTGTGTCTTGTGACTTTGCCTTTTTTCAAGTGTCCAATAACAAAAATAGTAATGC
AGTTGGCATCGACAGTAGCGGAGATTGCAAAGGGTTATGCACCTCGAATGGGCGTCTGCGCTTGCAGC
GAAAAAGAAAAAAATAAGCATCACGTCCCGTTGGAGTTCCAAGTTCAAATTAAAACCGTTTCTCGTCC
TGCAGGGCTGCCCACGTATTATTGTAGCCGCCCGGCCAGTGCGCCGGCGCAGACGATTGTGATCGGAC
GATCCGATCGAGGAATCGCGCGCGCATTAAAATAAATATCGCGAATAAAATACAGATGCATTGAAACGC
GACAGTGGCGTGGTTACAGTGCGTGAGGTGTCGTCTTGTGGCATACTTTTGTTGTAATGTAATGTTTTG
AATTGGATCTAATTGGATTGAGCTTTGTATGACCTTTCATTTTTGCAGTCTTGGTTTTTAAGGCCCTTAA
CTCTTCCACCAAGCAAACTCAGTGATTTGTAAGATTGTAAGTGATGTGTGAAGTGTAGTGAGTGACAT
AATGCTGAGTATAACAAAGTTCACGGCGGTGTTGCTGGAAAGTGCGAGGACTTCAGAAGCGAGGAAC
AGGTCGAGACGAGATCCATGGTCTTCAGAGAGTGTTCCAAG
>TCONS_00045362 geneID=XLOC_009356 scaffold_174:467680-468162(+) Exonic overlap (-)
CCCATTCTAGACCTTCTGGGGTGGCATACAGATCTATTTTGGGTTCTTACCTCCTCCCCCTGTCCCGTGT
GTATGAGCGAGCCCAGGCGGCGCGCCGCGGCGGCCGACGTGAGCGCCGTGTCGCCGTCTGCCGGGAT
CAGCTCCACCGGCACGCTGTGCAACATAAACGTGCCTGATGTGGCGCCGTCGACATTATTAATCAGATT
GTTCAGGTTGACAGCAGCTCG
>TCONS_00045554 geneID=XLOC_009356 scaffold_174:467730-468463(+) Exonic overlap (-)
CTCCTCCCCCTGTCCCGTGTGTATGAGCGAGCCCAGGCGGCGCGCCGCGGCGGCCGACGTGAGCGCC
GTGTCGCCGTCTGCCGGGATCAGCTCCACCGGCACGCTGTGCAACATAAACGTGCCTGATGTGGCGCC

GTCGACATTATTAATCAGATTGTTCAGGTTGACAGCAGCTCGTGGAGCCGTCACACGGCAGCGCAGCC
CGCAGCCCGGCACGGCCGTGAAGCCCGAGCATCGGCCGGCTGAAGACCGACCCACCACTGTTGTGCA
GAAGGTTACTAC
>TCONS_00045352 geneID=XLOC_009372 scaffold_174:490729-504426(-) Exonic overlap (+)
CCTGACGTATGTTACTCGGCGCACTTTCGAGTGAAAGTTATCGCGGCTAGAAAAATGTGCGCGGCAAT
TTTTGTTTCTTAATTTCTGTTCTAACCGGCCAGTGTTGCGATCAAAAATTTGTTTTTATCTTGGAATATTT
AGTTTTTTGCTGGAGGACCAAGGAGTGGACATGATACCATGTAGGTTAGTGATACCCTCCGTGCGGAC
CCAGGTGTGTGTAGTGTGATGTTAGAAGGAGACAGATATAGTGCAAGTGCGTCAGAGTGAACAGGAC
AGCAATATGAAGAACTTCAAGTACCAAAAGTTGGATAATGTGGAGATTGACGACCTCCAAGAGCAGCT
GGTGGCGGTCCGCGTGCCGATCCACGGCATGACCTGCCAGTCCTGCGTGAGGAGCATAGAGGGCTCA
GTCAGGGAGCTACCAGG
>TCONS_00046114 geneID=XLOC_009415 scaffold_175:180334-182106(+) Unclassified
ATTTTCTAAGTATTTGTCAAGCTTCATGAAATGTAAATAAGTTGAAAAAAGAGACCTAATTTCATGCCTT
CCTCTTGTATTTCATTCGATAGCTTGACTAACTCTTTTCAATATTATTCGGTCTAAGACAAGTAATTCTAG
GTCTAAATATTTTATCTAGATGGTGTCTGCAGTTAATTAAATGTCAGCCATCCTGTGTGAGACGCTTGAT
GTTTATGTTGGGGAGTTAGTAGATAAAATTGTAGATTCAAATTAAAATTGTTATTTTTTATAAGAAAGAA
AAGGATTTTCTACATTTCCAATCGCTTGATTGGTTGGGTTAGATTTTTCCTTCTCAAACTTTTCATCTAA
ACGCAAGGTTTTTATAGTATGTATCGTTTAATCCTTATCTTTCAACCTAGACGGCATTGCCTGATAAATTT
TGGTAAAATTTTATGAAACAGTCGTACCCATAACATTTGTATCTCTACACTTATAACAAATCGCGAGTGT
ATTAATTGATGCGATATCAGTTGGTTCTAACAGGTCCGACCTTTCTCCTCTATCGCTTCAATAAAACAGG
TTTTTAACATATCTGGGTAAGAATCCAATACGAAGACTCTACAAGGGAGGGCCATCTCATCAGTCAAAG
TTGTGATTGTGTTGCGGCGTTGATTGTCGACGGCCGCGGCAGGTCCGCACTCGCTTCGGTTCCAACAT
GGCGTCCATTGGTCTGTCAATATTTGCGAAGATCTCATTGAAAGTTACTGGGTAAGTTGCGTCCGCGAA
CACTGCATTTTGATCACTGTTGCATGGGGCTTTTGGTTCTATTTGATGTATCAAACGAGTGGCTGCGATT
TGTGGCGAATTCTGCTTTGTGGGTGCCCCTCGTCAGTACGCCGTAAGCAGCGGTCGGCTGTGTTATGAT
AATATTCTTTGTCAGTCGCATAATGGATGATCCGTCACTCCTAGAAGAGATTGTTAACACTTTTTTATTT
GTCTCAACAGAATTTCTTCGTCGGCGGTTTGGTGACATCTTTCTTTTGAAATATTGAAAAGTTTTATTCT
ATTATTGACTGGTTATTGCGGTACCTAATGCACATTTATATATTTTCAGGTGTACCCAATGGATTGATCTA
AAGTCTCTGAAAATGTAAACTATGAGACAGTCAAATGATATTGACAAACACAACTGGCTAATTTTGGA
AAGATTGCCAGAATTAATTTAAAGTTGGTACCATGCTGGATAGATGCAATGATCCTTTATTCGAGATGAC
AACGATCTGACGGCGAAGTGGTTGATGAAAACACAGAATAGAAAACGATAGATGACGGCAGCTCGAG
AAGACCTAAGCAATAATGGACACGATAATAGAATAAAAAATGAGG
>TCONS_00045836 geneID=XLOC_009418 scaffold_175:241339-242579(+) Splice junction overlap
GCCACTCAGACTTACGAATATGTTCGATCGCATCAAGCAATGTAAACACAGAATCATGATTCGGCAACA
CAATTTTTTATTTGTTTTTAATTTGCGACAAGGGACCATCCTCATAGCGGTCCACCAAATAGCGCTGGCG
CTGTTCGTGCTGCTGGTGCTGGCGGCGGGCGCGGCGCACGCGGCGGGCGCGGCGGGCGCGCTGCAG
GCCGACATGGCGGACGCGGAGGAGCGGCGCGAGCTAGACCTGCTGCTGCGCG
>TCONS_00046053 geneID=XLOC_009447 scaffold_175:270267-270982(-) Intronic
CCTCATTCCGCCACCGACCTTGGTCCCGTGAGCATCTCCCGTGTCGTGTCTCAGTGTGTGTTGTGTACC
CTATTGACAAGATGATCATCTGCCTGTCTGGATTTGTTATTGCCGGAACCTCCCCGGCCCCGCTATCCGT
CTCCAGAAATGATATTGTAAGTACCTACATATTTGTTAAATTTTATTAGTCATTAATTTATCCTACTATGTA
CCTATGTACATTAATTTACTTTTATATTCTTTTACGTACATAACAATCTCGTCATCATTAATGTAATTTTCC
GTTACGGTTTCAACTCTAAGTTTATACCATTTACTGATCCACTGTAAGACACCTCATCATCAGCCACCCT
TCATCCATTATTGAGACTACCTAGGAACGACAACACAGCCCTCTATCCTGTATTCATATTAGCATACAGG

TGTGAAAACTGCCGAACTAAAAACACCCTTTCCTTCTTATCTAAC
>TCONS_00046122 geneID=XLOC_009431 scaffold_175:766589-769950(+) Splice junction overlap
TGGACTTCTGTACCTGGTACTAGTTATTATTCTGTGTCCGTACGTAAGCCGCGTCCACTAGAGTGTCGG
CTCCCTTATGTCTAATTCTGTCCTATTCTGAACCTTAGTCTGTGAAATAAAAAACTAACCTCTCTGCACT
TGACAAGTGACAGTTGACAGATACTACAAGAAAATAGAAAAAAAAATCTCTTACCTATTCTGCAATAC
AAAACAATTTCTTTTAGCATTTAATAATTGCTATATCTTTCCCGTTAATTCAAGAAAAACATCACTGTTCT
CAAAACTGCTGAGTTGATGCTAAGTTATGGGTCAATAAACTGCGGAAACAAGTGACACTCACTCACGC
AGCAAGAGAGTATAAGATGGAGGACGACATAGACATAGAGGAGC
>TCONS_00045695 geneID=XLOC_009462 scaffold_175:799935-804471(-) Exonic overlap (+)
GAAAGTAATCTTAGATTTATCTTATATGTTGCATTTCAGCTAGCATTGACCGTGAATTAAACATATTTTTT
GTAGCTTAGGCCAAATATGACGGATAAATAGTGATTAAAATAAGAAAGTTAGAAATTTTTGTGCTAAAA
TCTTGGAAAAATAGTGAAATGCACAATTTACAGCCATGGATGTTACAATGCCTTTTTGTGGGCATAGCA
AACCTTGGCATGGTATGTGTTGGCATCAGCACGGGGTGGACATCGCCGATTCTCGTGAAACTGTCAGA
CGCTG
>TCONS_00046069 geneID=XLOC_009464 scaffold_175:891757-901123(-) Intergenic
CGCAAGCCGCCGCCGCGCGCCTGTCTCCCGCGCCCACCGCGATCGCTACAAACACATGTTCATTCAGA
AAATCTAACAGACTTCTTCGTTTGCATCCTGCAAACAGATTTATAGTGCCATAAGAGACTTAGTGAGGT
GCGTTCAGAGACTCGCAGCGCCAGTGCTAGTGTGCCGGCCGACCTGTGGATCCCGGGTCGACGCCATT
ATTGCATTTCATTCAACCAGGATCTTCATAAGAGTGAGAGATGCGGGACGAGCGCGCCTCGCTGCCGC
GCGTGCGCATCGCCGTCATCGGCAGCTCCCGAGTGGG
>TCONS_00046157 geneID=XLOC_009464 scaffold_175:891794-901121(-) Intergenic
CAAGCCGCCGCCGCGCGCCTGTCTCCCGCGCCCACCGCGATCGCTACAAACACATGTTCATTCAGAAA
ATCTAACAGACTTCTTCGTTTGCATCCTGCAAACAGATTTATAGTGCCATAAGAGACTTAGTGAGGTGC
GTTCAGAGACTCGCAGCGCCAGTGCTAGTGTGCCGGCCGACCTGTGGATCCCGGGTCGACGCCATTAT
TGCATTTCATTCAACCAGAGGATCTTCATAAGAGTGAGAGATGCGGGACGAGCGCGCCTCGCTGCCGC
>TCONS_00046181 geneID=XLOC_009465 scaffold_175:941403-951956(-) Exonic overlap (+)
CGAATCCTTGAAAGCGTTCGTCCAAAACTGGTTTAAAGCCAGCGCAGTGTGTACGAAGTTAAGTAAAT
GCTTAGTATAGCGTGTGGCGAAGCTTGAGCGGTGTTCCCTGATTGGCGTACTTGCTATAGTTGGTGAAT
TCGCGGGCGTAGTGCGACTGCGCAGTTGCTGTCTGGCGGTGCGAGCGCGCGCGTCCCGACCAACCGA
CCGACCGCAGAATATATTGTTCCAAATTCAAATTGTAAACACGTGCTGCAGAGATGCCGAGTCCAGACT
ACAGCGTGGACCTGGACCTCGGGGAGCCGCCGCCGGAGCTGCTGCAGTACGCGAGCGAGCACTGCG
GGGAGCTGCCCGACACGCGCCTGCAGGCCGTCGAGGACCTCCGGACTATCATC
>TCONS_00046075 geneID=XLOC_009472 scaffold_175:1192343-1193658(-) Unclassified
GTCAGACCCAGCATTGTGTTATTGTGTGTCACTTTCAATCTTAATAATACTTAATAATGTTTTTGTTACCA
ACTTAATTTACGAGTTTTCAATGAAATTATTTGCAGTAGTATCACAAATTATTTGACCAATAACAGGAAA
AAGGCACACATCACAATGTGCGACCCAGGACGCTGCAACTTATGACACGCAAGATCCAGCCGAGCTC
CCTGAATGGCCAGCGCATTTCCCGAAGGTCGTAAAACTACCTAAAGAGCGGCTACGGAAGAATACCA
GTCATACCAACAACTCAACACTAAACTCAAGCGTTATACTCTGTTGTCGTGAAAGTCCAGTGTTTCGTG
ATCGCCACCGGAAGCCTACTAGATGGGACCGAATTTGGGCTGTTTTAAACACGACATTCATTGGCAGA
ATCCATGAATATTTGAAGAACATTGTTCCTCATCTCGCTCAAGATTCTTTGGAGTACTTATTATAAGAGT
TTATAATCGGAATTAGCTGAAAAATGTATGTGTATTGGGAAAAAAATTGACTGTTTATGTTTATTTTGCT
GATTACTCACAACACAACAATGATACTCGATCGTGATATGTGGTGTATTAGTTGTTCTGGTTACAGTAGC
GTGTTCTACTGAGAGGGGGAGGAATTGCTTTCCAAAGGCATGGAACGACATTTTGAAGATATACCATA
GTTTTGGCGTAAAAATTTAATGTTTATGCTTACAAAAATCGGCAGTTTATTTTTAATTTGCTGATTAGTCA

CAATCATGATCGCATTATTAATAATAGTTCTGGTAGTACTCTCGGGGTACTTGTACTGCAACCAGCAACA
GTCGATCAGGGATCAGCTGGATCCGTCAAGGAACCTTCAGAACAACTTCCCGTGCAACTTCTTCAGCA
GGGTGAGTCAATGGTTCTCAGACACGTGGAACAGTATCTTCAAATGATTAACTTGGGATAAGGAGTATT
CATAGGCGCAAAGCTCTCAATAATGTACTAAATTTATAAGGGTTCTTATACTTATACATTTACAGGGTTCT
TTTATACTTATAAAAAGTAGTATTTTTATAATAAAAAAATAACCCTAGGTATATTATATTTATACAAACTTT
ATATTTTCTTGGCAAGACGGTGT
>TCONS_00046272 geneID=XLOC_009563 scaffold_176:317053-320196(-) Intergenic
CACGCCATCTATAATGATATTCATATAGATTTTTTTTTAAGTATAAAATTCAATAGATAGGACTTTTCAATA
TAGATCCAAACGTTTTCTAATGCCTAAGAAAATAAATGGGTAATTATTTTTAACAGCAGAAATGGACTTT
ACATTAAAATTTATTTTGGACAAACATGTTACGATATAGTTAAATAAATAATAATAATTAGTTATTACAAT
AATTAATGTGAACCAAACTCATATAATATAACAATACTGGATCTTGTGCAGAATTTATTATGACCAATCTA
GTATAATAGTCTAGTAACATGTCACTGGTGAAACATAGGTTCAAAAATTTTAATGTTCATGAGGTGGAG
TGGTGTAGGTAATGGTAAGTATTACTATAAAATTTTATATTATAATAAAGTACAATGCGTTGTTGGCCATG
AAAGATGAAGATACAAGAAGCTTCAGGTATAAAATACATTAGACGTGAAAAACAAAAGGCACAACAT
TCATTTTAACCTATTAACAAAGGCACATTAAATCTAAATTCATAGTATTTAATCTGAGGGATGATTCCGGA
TCTATAGCGCTCTGGGTACTGATGGATGAGAATATAAGGGTGCTTACGGCATTTTAACTATGCTGGGTCA
TACAGGTAAGATGTATACACACTCTATTATAATATCCCCCTTCGCCATTTTTACGTATGTATAGTAGTTACA
TCAATGTTTAATCATCTGCTTTTTATTTAATTCAATTTCAATTAATGTTTACAAAAGTGTCCGCTAGAGGC
GCTGCTAGTTAATGTATGAAAAACCGAAATATCAAACAAAGTTTTATAAACTACAACAGTAGGTAAGTA
GTACACCAACTCTGGTGCCCTAGGTTTAGTAGGTTAGGTATAGTTTGGTCCAAACAAGTAGGTAGATAG
ATAGACCTAAAGTTGGCAGCCTTTGTACCTTGCCTCTGCCAACCTTTTAAAGTAGGTAGTTTGACCGTG
GTTTGACCATTGACGAAAGATTTGTCATTGGGTTTGACAAGGTACAAAAGTGAGGTGTCAACTTCAGG
CCTATAAACCATAGACTAAAATATTACTTGTTTGGATAGAAGGACAGAAGCACACTGCACATTACAGAT
ATACACTGCTTTTATAATTTATTTGCCTTATTTTGTGACTTTATAAACACCCAATGCTCTGAATACAGTTA
CTAACAGATACAATTGTTACTAAATATAGGAATACACAATTAATTACTCATGTAATATTAACTATGTGACA
ACTTTTATAATTAAAATTATTTGGAGTTGGTTGTCATCATTGTTGGTAGCTGTATTTAGAACAATACTTAA
AATATTTTTTCTAATTGAACGATATTACTAAGTTGTGAGATTTTGTAAGTGACGATAGTGAAAATTTAGA
TGAGATAACTTCACAATTAGAAATGATTAAATACAATTATTATGATAAGTGTATTGCGAAATATTTACATT
ATACTATACATATTCTTTATAAGTATAGATACTTTTAGTAATTTTGCAAAAGTTAGGTGCAATATACTATAA
GTAGAGAGGAGCCACACTAGTTTTCAGTATATAGCATGTATGGGCTTGATTTATTTGAGCAGCTATTTTT
TTTTATGATCTCCATATTTGTGAAATTTGTATGAGAATATATATTTATTAAGTACATTATCAGCAGTAATATT
CAATATTGATTATTTATTACAGATATGAAATCTAACATGTATATAAATATATAATTAATTAAATATTCATCTA
CCACATTTATTTCCTACGAAAAGCTCTTTTACACTGGCGTTATTACCAGTTTTTTATTATAAATTTTACAC
GTTTTTTTATGTTTCATATTTTAGCTAGCTAAACACATAACCTAAGTTTAGGCACACATTTAATTAATGTC
TATTATCTATAATAATATATTTTAATAGTGCATACAGTTCCTACACGATGCGTTCGTTAGTGAAACATAAA
AAAGTGAAAAGGCAGTGGACGAATGTTATTGTCTACTTATTTAAAATAAATGTTCTAAATGCGTTATTAG
AAAATTTACAATTTAAATATTTTTTGCCATATGATTTTAAAAAATCCAGAGAAGTGTAATATTTTTTCGGT
TAAACCAGTAGATAAGTTACCCCCGTGTTATATTATTATAAAACATTGTGTGAGGTTTGTAGTTAATTCGT
GTAATTATTAATTAATAAATTTATTCAATTGGCAGTTTGTTACTATAACGTTCATTTAATTAACGACTACG
GTTACTAATTACAGTATAAAATAAAGTAAGTGTTCAAAACTATAAAAAATAGTAAGTCTACGACCCATG
ATAAATGAATGAAAAATGTTAACTACTTATTTATCTATTCACATTGTCAATACGTAATACTGAGCCGGCAT
AACACATGGCATCAAACAGATCTTATAACTAAAATTGTTAAGTCTTTGACCAAAAGTTGTTCTCGACTT
TTATTCAAAGACTTTTCGTAGGACTACGTAGGATGTATAGAATGACGAGTTGTTTAGCTTTCGGCTTTTA
AGTACCCTTTTTGAAAAGCTTTATGTGACACAAAACATATGATATGAAAGCTTACTCAAATACAAAAAT

AAGTACAGTATTTTACATAAAATTGCGAAACGAAAATGACTAGGATATTTAACTAATACAATACCTTACA
ATTAGTTATGTAAACTACCACACTTTATCAATATATCTAATTCAAAATAATACTATTACAAACAATTTGTA
CCAAATATATTAAAGTAG
>TCONS_00046199 geneID=XLOC_009553 scaffold_176:345014-412250(+) Unclassified
GTTATGCTTTGCGAATGTGCGACGTCGCGAGAGTTATCTGTATGAGTGGTTTTTGCTTTGTATCTTTTTA
TTTTAACTAATAATGGATGTAAATGAAGTGATTATAAACCAAACGTGATAATATTTGTGATATAATAACGC
TATGGCCCTCGAAATGCCCTGTGTTTTGGTGTACTATGTGATCCCGTGCCATCGCTCGTCACTTCGGTTG
TTTGTCAATGACGTCTGCGTTTAGCGCAGCAAAAAGTGGATTTTTTATTTGATTGTGACAAGTAGTGCA
AACGTAAATGTTATATTTGGTGCAAATGGGTGTGTGTTCTTAAAATGGATGAATATTTTCATCGTTGGAT
CCTTCTTTTATCAACAGGCGACATCAGAACAGGCTCATGAAGAAAGGGCCGCACAAAATGATGCTAGA
GTCGAGCCGGTGAGTACATACATATTATCATTATGTAAATATATGTACATAGTAAGTAAATACGTATAATT
AGGTATATTTGCAGATATAATACGATTAGTCGATTAGTCCTAGTTATTAGGCAAAGCTATGACTACCATAT
GTTGAGAATAGGAGTCTTATTGTTTTTCTTTTCTATCTATAGTGATCGTTTTGAGTTTCCAG
>TCONS_00046201 geneID=XLOC_009554 scaffold_176:428791-436627(+) Unclassified
GGGATCTCGGCCGACGATGCAACTCGCGACAGTAGGGCCCAGCGTTTACGACATAATAGAGCATTAGT
TGGATCCACTTTTGACATCATTATCTGTGGGCTCGAGTGACGATAACCTGGCGGAGGCGCCCCGCGCC
GCAGTCTCGCGCCAACCTCCGCACCGAGTGGACGTCTTGAGCCACGCTTCACCGCCCAAACCCCTCC
CCCCTCAATGAAAGAGGCGCGGAATTTTGGCGCGCTCATGCAACTTGTATGCCGCCGCGACTGTGTGT
GAACGCACTTGGATTGCAGTGTTGGATTTGGATTTTTATACCTACATCATAGAGAGACCTAAATTTAAAC
TGCACGAACTCAGATACAAACCAGCCTATACAAGCAATTGCCATAACCCTCACGACAGCGCTACTCAA
TAACGCAACAAAAACAAAAGCTAACTTCCCAGTGAACCCACAACCATGTTCTCGCGTGGTGTGGATTA
AGTGATACACGAGTGAGACCCGAGTGAAGTGAGATCAAAGTGAGTTTTACGGTGAATCCGCCGGGTG
GTCGGGGCTCGTCGGGCCGCGAGGCTCCACGCGCGGGCATGCATATGTGCTACGAGCGGCCAGGAGT
CGTGACTCCCCTTAACGGGGCTGTTGG
>TCONS_00046268 geneID=XLOC_009554 scaffold_176:428829-436593(+) Unclassified
CCAGCGTTTACGACATAATAGAGCATTAGTTGGATCCACTTTTGACATCATTATCTGTGGGCTCGAGTG
ACGATAACCTGGCGGAGGCGCCCCGCGCCGCAGTCTCGCGCCAACCTCCGCACCGAGTGGACGTCTT
GAGCCACGCTTCACCGCCCAAACCCCTCCCCCCTCAATGAAAGAGGCGCGGAATTTTGGCGCGCTCAT
GCAACTTGTATGCCGCCGCGACTGTGTGTGAACGCACTTGGATTGCAGTGTTGGATTTGGATTTCGCTA
CTCAATAACGCAACAAAAACAAAAGCTAACTTCCCAGTGAACCCACAACCATGTTCTCGCGTGGTGT
GGATTAAGTGATACACGAGTGAGACCCGAGTGAAGTGAGATCAAAGTGAGTTTTACGGTGAATCCGC
CGGGTGGTCGGGGCTCGTCGGGCCGCGAGGCTCCACGCGCGGGCATGCATATGTGCTACGAGCGGC
>TCONS_00046200 geneID=XLOC_009554 scaffold_176:428940-436606(+) Intergenic
AACCTCCGCACCGAGTGGACGTCTTGAGCCACGCTTCACCGCCCAAACCCCTCCCCCCTCAATGAAA
GAGGCGCGGAATTTTGGCGCGCTCATGCAACTTGTATGCCGCCGCGACTGTGTGTGAACGCACTTGGA
TTGCAGTGTTGGATTTGGATTTGTTAGTTTTTTTTTCGCGGTTCTAGTGTCTCCTGCGCACGGACTTATA
CCTACATCATAGAGAGACCTAAATTTAAACTGCACGAACTCAGATACAAACCAGCCTATACAAGCAATT
GCCATAACCCTCACGACAGCGCTACTCAATAACGCAACAAAAACAAAAGCTAACTTCCCAGTGAACC
CACAACCATGTTCTCGCGTGGTGTGGATTAAGTGATACACGAGTGAGACCCGAGTGAAGTGAGATCAA
AGTGAGTTTTACGGTGAATCCGCCGGGTGGTCGGGGCTCGTCGGGCCGCGAGGCTCCACGCGCGGGC
ATGCATATGTGCTACGAGCGGCCAGGAGTCGTGAC

>TCONS_00046325 geneID=XLOC_009590 scaffold_1762:1-840(-)Unclassified
GCGGCGGCGGCTCGGGCCCACACTGCGCGGGCCGAGTGGGGGCTGCCCCCCTGTGCTTGTGAGGCGA

GGGTCGAGCCGGATAGGGCGAGACGGCAGGCGGCGAAGGCTCTGTTGCATGCTGCGCTTAGCAAGAA
CATCAAAAAGGAGGAAAGCAAAGAGGAGAACGGGCCCACAAACGGCACCTCTCCTGTCAAGTCCGA
AAACGGCAAGACCACACCCGTAGTCAGCTGGCTGTCCG
>TCONS_00046422 geneID=XLOC_009597 scaffold_177:5323-6089(+) Unclassified
CAAGACTGAAGCCGTCACCCGTGTGACCTGTCAAACTGACAGATAATCTGTCATCATATAACCTCAAAT
TTTAGGAAATTCTGCCTAAAATCGATAAAATAATGAATTGAGATGCTATATATTCGTAACAGAAATACAT
TTCGGGTTTGCATTTAGCCTGTTAATTTTTTAAGTGTGTGCAAGATGTTGAGGGCGGTGTCAAGATGTT
ACCTGGAAGGGCCGGGCTTGGACCTGTTAGTTTTCCGCAGGGACGCCCTCAAAGGATTCCGGTCTGGT
TTACATTCCACAGCAGTTCATGCCAAGCAATCTCGGAAACCTAAGGCCAAGAAATACAG
>TCONS_00046460 geneID=XLOC_009602 scaffold_177:117952-118968(+) Intronic
GTTTACAACAATCCCGTTAGTTAATTAAATCAATTGTTTGTTTGTTCGTCTATTAGTGACGCATCTCTATT
CTGAATAAAAAGCCGTTTCAGTGGAATCTGTTCCATCATTCAGCAGCATCATGAAACTGTTCACAGCAC
TACTCTTGGTCCTTGCGGTGTGCACTGGCAGCGCCCTCGCCGGTGGTGGCGGCGGCGGCGGCGGCGG
CAGTGGTAACGGAAACAGGTACTACGGTTATAATTATACCTACAACCGGGGCGGCGGCGGTGGTGGCG
GCGGCGGTGGCGGCAAATGAACCCAAAAATAGAAAACACGCACACTCTTGCAGATTCTTAATCATGTT
TATACTTAAGCATAATGTGTAAGTGTTTTAAATAAATGTTTTTGATTTTAATATACCTACTTAGTTTATTTT
TTG
>TCONS_00046387 geneID=XLOC_009622 scaffold_177:191509-192442(-) Unclassified
CCGCGCTCCAACATGTCGCCAGCTACAGCCTTCGTCATCGCCGCCCTAGCTATGGCTTTTGCAGTTGCC
GCTTATCATGAAAGTAATCGCAAACACATAATGGTACTTGATGATCAATGATTTCAAAAGGAAGGATAC
GCCCTTCAGAACATTTTACTGAAACCTTCTTCATACAAACAAGATAACTGTCATCTGTCTGCGTGGTTT
TTGTTGCAAACTTATGTACCTATTGTAATATGCTTTTGAAAAATAAACGAAATAAATAAAATAAATTTTA
ACTATGCCAGTACCGATCTATACTTATCCTGACGACGCAACCTTGTACGTGCGTTCAAATTTTACCGTCA
TCGTCACGTCACCGGAAAGTACTTAATAATAATATGAGTGAGTTGCATGCGGTTATACCTTTGCGATGGT
AATCAACAAGTGACAAACAAAAATGGACTAAGTACGTCTACGTACATTCATTCATAATATACATACCTG
TGAAAACATTATCCTCCGTGCAGGTCGGGTAAAATTATAACTAAT
>TCONS_00046563 geneID=XLOC_009603 scaffold_177:312633-316352(+) Splice junction overlap
CGGTCCGTGCACCCAGAAGCGGCAAACCTGGCGTTGACGGCTGCAGATCTAGAAAGAGAGCGCGGG
CGCCCTCTGTCGAAGTACGAGCGAAACATGATGATCTTCAACTGGCTCCACACGCTGGACGAAGCTCA
GCCCTTCTAGAACATTCTAGACACTTCTGAAACGTTCTGCAGGTTTGTAGAACGTTCTACGGGCCTCTA
GAAAGTTATGCAGGCTTCTAGAATGTTCTACAGGCTTCTATAACGATCTATTGGCCTCTAGAACGATCTA
TATTCTTCTAGAACGTTCTAGACACTTCTCAACATGTTTCTAGAACATTCTACAGGCTTCTAGAATATTT
ACTGCTTCTAGTCATTCCATGGTATGCGGTGTGAGTTGTGACCGTGTGAGAGTGTAAGACCCCGTTAGA
TGTGAGAAATAGCCAATGTAAATTGTACAGCCTGGCAAACAGAAATGGATAGGTACTCAATAATCATTT
GAACTAGTGTTGGCTTAATTTTTTACCCATATTTTATTAAGTATAGCACAAATGTATTAAATCGGTATCTT
TTTTCTACATTATTATCTTTAAGAACAGGTAAAAATCAGATGATATTATATAATAAAAAAAGTCATTTTAT
ATTTCCCATTCTTATTTGCCAGCCTGTAGTAGAAATACTCAGAGAACTAACACCGTACCTACAGTCAAA
CTATAAAGTCCTGACATCAAAGAGTACGATTTTGCTAAGACTTTTTCTTTTTGTGATCAGCAGCTATTTA
CTTACTTTTACATATACATTTTAAAAATAAAATACCGGTTGATTATGGATTTTTAAAGGATTTTTCCTTTAA
TAAATATAACAGATCGATTTAATACGATCTTTAATAATTAGTTAGAATCCACTTTCATTTTCACGACTTTAT
AGTTGGACTGTAAGTTTTAATGCATTTTGCGTATATGCGTTAACTGCCTCACATTCCACACCCATTCACA
CTCCTACAGAAAAATTATATTCGTATGTTGATAGTTGTTATTAAGCTAACTAATTTTGTTGGATAATGTAA
GTAACTAAGTACCTACTTATACCTATTTGTAAGTAAATTGTAAACAGAGGCTGGTCACTTGCAGTTGCA
ATTTAACCCTTATTGTCTACGAATTAAGAACCTGTACCTATAAATCATACAAATTATATCACATAAAGCAC

ATGTTTGCATAAATTACGCTGGATATCTAGATTAGTAGGTAGATAGGTCCCGATAAAGCTAACTTTGCTA
CTGCAACACGTGCCGACACAAAAAAGAGTTACAAACTTTTTTTGACACTACAGTCGTTGAAATATAAT
AGGCCCGTTTGGACAGCTACACATATTTGCTATTTACTCTTGATTTACTTGATGATATACATAAAAAGAC
ACATAATCTGGCTTATTATTTAACGGTCGATAGGTAAAAGAGCTTTTGATCCACCAAAAGTTACTTACTG
TGCTACCACAAAAAACTGTAAACACTGTTTAACAAGTGTTTAAAACGAAATTTGACAGATTTTGTAGG
CGTTTGTCAGCGCTAAACACCTGTTAAATACTTTCTAAGATTTTGTGGTAAGGCCCTTATTGTTTAGATT
TTTAATCACCAGCTGTCATTCCATACATGTTGTAGCTGTCAAAACGGGCCTCTTATATTTCGACTACTGT
AATGTGGGTGTGCGCTGGAGTGGCTCACATTATCTCTTTTGTTGACTGTACATTTTACTCTTGTTCATTA
ATTTAGGGCTACTACTGAAAAAGAAAAAAAATACCTCTAAGAGACAAATAGTACCTGTTGCAAAGTTA
GCTTTGCCAGATTCTACTTGTTTTAAATATACCTAAGCTGCCACCATCACGACATAGACCGATTGACAAT
ATATTAAACCATTTTAATAGATTAAAATGTTTTTAGGGCTGATTTTTCGATAGTCAGCTAAAGGTGGAAT
GGACACTGGACACTGTGAAGTCTGAATTTTAACAGTTTTATTCCTAAGATAACATAACATTTATCTGACT
ATCGAAAAATCAGCTCTAAAAGATACACTACTTAAGCTAATAGATAGTAGGTACATATTATTAAGATTATA
TTAAATAAGCGGATATTATTGTGTTGAAAGGTTGAGACCGTATAAGTAAATATACTGAATGGTTTTAAGC
CATGGCTTGCGGCCGCGGGTTCGAATCCCAAGCAAAACATTTATGTGTGATGAGCATTTGTGTTTGCCA
TGTGTCTGGTGTCATTACCTATTTAATATGTATCTATCTATGTATTTGTGTCGATATATCAGCTGTCCGACA
CCCATATCATTATTTTCTGACATAGAATAATAAGCACTAGTACTACTGTACTTGTTATTCTATAGTTCTGCC
TAGTTTGGGGTCGGATGGCCGTGTGTGAGTTCCCCACATATGTATTATATTATGTTATTATTTCTGTGGAA
GCAGAAAGAGGCACGAATCCGATGGCATAAAGTTACTGCTAAATAGTAGGTAGGTATGTTAGGAACTG
GTTATACGTCA
>TCONS_00046626 geneID=XLOC_009623 scaffold_177:315255-321495(-) Intergenic
GTGGTAAACTAATTGGTAGGAACATAATATTTAAGTACTTACTAACATTACAATTTATATTTATACAACA
TACATAATAATTAATTATGTAAATCAACTCTTACAATGCAAGCTTAAGATGGTTTCACGTAGTCTGATTTT
ATTTTTGTTTTTCATAAAATACACCCAATTTATTTGAGATCCTATATATCTCCAGTCATCATCATATAATCA
TCATCATCATCATCATCAGGGGAACATGACTGGAGCCTCCTAAGTGACGTATAACCAGTTCCTAACATA
CCTACCTACTATTTAGCAGTAACTTTATGCCATCGGATTCGTGCCTCTTTCTGCTTCCACAGAAATAATA
ACATAATATAATACATATGTGGGGAACTCACACACGGCCATCCGACCCCAAACTAGGCAGAACTATAGA
ATAACAAGTACAGTAGTACTAGTGCTTATTATTCTATGTCAGAAAATAATGATATGGGTGTCGGACAGCT
GATATATCGACACAAATACATAGATAGATACATATTAAATAGGTAATGACACCAGACACATGGCAAACA
CAAATGCTCATCACACATAAATGTTTTGCTTGGGATTCGAACCCGCGGCCGCAAGCCATGGCTTAAAA
CCATTCAGTATATTTACTTATACGGTCTCAACCTTTCAACACAATAATATCCGCTTATTTAATATAATCTTA
ATAATATGTACCTACTATCTATTAGCTTAAGTAGTGTATCTTTTAGAGCTGATTTTTCGATAGTCAGATAAA
TGTTATGTTATCTTAGGAATAAAACTGTTAAAATTCAGACTTCACAGTGTCCAGTGTCCATTCCACCTTT
AGCTGACTATCGAAAAATCAGCCCTAAAAACATTTTAATCTATTAAAATGGTTTAATATATTGTCAATCG
GTCTATGTCGTGATGGTGGCAGCTTAGGTATATTTAAAACAAGTAGAATCTGGCAAAGCTAACTTTGCA
ACAGGTACTATTTGTCTCTTAGAGGTATTTTTTTTCTTTTTCAGTAGTAGCCCTAAATTAATGAACAAGA
GTAAAATGTACAGTCAACAAAAGAGATAATGTGAGCCACTCCAGCGCACACCCACATTACAGTAGTCG
AAATATAAGAGGCCCGTTTTGACAGCTACAACATGTATGGAATGACAGCTGGTGATTAAAAATCTAAAC
AATAAGGGCCTTACCACAAAATCTTAGAAAGTATTTAACAGGTGTTTAGCGCTGACAAACGCCTACAA
AATCTGTCAAATTTCGTTTTAAACACTTGTTAAACAGTGTTTACAGTTTTTTGTGGTAGCACAGTAAGT
AACTTTTGGTGGATCAAAAGCTCTTTTACC
>TCONS_00046745 geneID=XLOC_009687 scaffold_178:11806-12353(-) Intergenic
TGGAACGACACAATGGATCAATGAATATTAGGTATGTTAAAAGTTAAGTAAGTTATATTTTTACCGCTTT
AACATGGAATTATTTTAGCAAATTCACAGCAAACTTTTTAAAAATTATGATCTTAACCTAATTATTTTTAA

ATTACATTCATATTAATAACTTAATGCACAAAAGATGACTGTATCAGTATAGGTGTCGGTAAAAGTTGAT
ACTGAATTAATTCATAATTATGGTAACTATTTGATATAACTACATAATATGTATTGTTTTTTTACATAGTA
AGCACAAAATATTATAAAACCATTGTGTACGTATTTATAATAGTGCGGTATCGTGAATGGTTTTTAAAATC
GACATTATCGATTATGATTATCTGCAATTCATATTTTCATTACAGTAATAATGTTATTCATATTTTTAAAA
>TCONS_00047128 geneID=XLOC_009690 scaffold_178:104891-105353(-) Intergenic
CTATTCCACTCAGTTAGTACAACTAGACTTTCAGGGCTTCACATGTTTGGCATTATACTTACACCACTTC
ATTCCTGGGAGACTTCAGTTACGCACAAAAATATTAGTAGAATGGGACACTTGCTGGTCATATACAGAC
GAAGAGTTACCGCTAGATGACACTAGAAGATGGTTACAGATAGGGGAAGCCCCTTACCCTGGCTACTG
CAGGGCCCCAGGCAGTGGGAAATGCATCCCTAGTTTATGTAACTGTAAATCTTTAACAGAAATATGGAA
GAAGAGAGCAGCGCAGTTTACTACCAAAGATAGGTTCAGCAAAATAAGAACTGATCATTTAAAAAAAT
GTCAAGGCGATAATATAGTGTGTTGAGTACCTAGTGGATCGGAGT
>TCONS_00046902 geneID=XLOC_009684 scaffold_178:565647-566106(+) Splice junction overlap
GTTTGACTTGAGCTTGTGTTGTGTTTTGTATTTTTGCCCTGAATTTAATTATTTCATCAAGTTTTGTGAGT
GTAATAAGCGTTTTTGAATTCGAATAGCCATGAATTATTTCTATCCTTGACAGGGTTCACATTCATGCCAT
AATTGTAGAGATTGCATTCTATAACGACATTAAAATGGCTACAATACCATGTGTTCTTATTCCATTGTTAT
TAGTATCATCTAGCGTTTTACTTGTTCATGGCAATCTGGCATACGACGTGCATTGGCTTGGAGGGGCAG
CTACTATAGACCGGGACATACCTTCAAATAAACAGACTGTTGATAT
>TCONS_00047381 geneID=XLOC_009734 scaffold_179:4468-7200(+) Intergenic
CGGCAAGATGCCTGCGCCGCGCCTTCGCCTTACGCGTTTTCTGTGTAATTCGCGCCTAACGATAAGGAA
TTGATCAATTGCGAATAAGAATAAAATAGCAACACCCAAGTTCATATAATGTCAATTATCTAATTATAGTA
AGTTTGTTTGTATCGTTTGAATCTGCTGATTGAGGTACGTATGGTGAGATATGTGAGTGAGGGCCAACA
ACAGAGTGACATGAGCATGGGTTTCAGGGGAGTTAAACAATTAAACATGTATTATCAAGTTTCGACTAC
CGTGTCTAATAAGCAACGATATATTTTCAGTGATATCTTCTCCAGTAACAATAAGCATTAGCGATGAATC
ATTTACAACCCGCTTCTACTTTTGTTGCACTGTTTTTTCAGATATTACTTATATTTAATCTTACTTAAATCA
GGGTCTAATCACTTCATGTGATTATACCATCGATGACCAAATTGACCCTGAAAAGGGGGAAACTAAAC
GAATTATTATTATTAATGACTTATTAAACACTTTAATCTAAGTTGAATATAGTGTATTTTTATTGCAATATT
GGCAATACAATAAACGTCATTGGGTGAAAATCAACTATCCTAGAAGAAAGACAACTCCAGAATCAAAC
CATGTGGTGGGGTGAGATAGGCATCCTGAGCATTGCGCTGCCACCTATCTAGGAGAAGGAAAACTCCA
AAATTAAACCAGGGGTTGTGGTGGGGTGAGATACGCATCCTGAGCATTGCGCTGCCACCTATCTAGGA
GAAGGACAACTCCAAAATTAAACCATGGGTTGTGGTGGGGTGAGATGGGGTTGTGATGGGGTGAGAT
AGGCATCCTGAGCATTGCGCTGCCACCTATCTAGGAGAAGGAAAACTCCAAAATTAAACCAGGGGTTT
TGGTGGGGTGAGATGGGGTTGTGGTGGGGTGAGATAGGCATCCTGAGCATTGCGCTGCCACCTATCTA
GGAGAAGGACAACTCCAAAATTAAACCAAGGGTTGTGGTGGGGTGAGATAGGCATCCAGAGCATTGT
GCTGCCACCTATCTAGGAGAAGGACCACTCCAAAATCAAACCAGGAAAGTCCACGCGTGACGAAATT
GGCCCATGGACTAACCAGTCCTAGCCGGTTCAAAGAGAGAAACTGAAGACAGCCCGTGTTGTAAAAA
CAAAGCGATGAAGTCGGCTACCTGAATGGTAGGTCGGACTAGAAGATGGCTAGTATCGGGATACGAAT
CACTCGGAGAGACCACTGGTCTTTTAATAACGGAAAGGCAAATATTTTTTTGTCTTGTAGTGATGGTTG
CCACAGCATTATAAAAACAATAGAAATAAACAGTATATTATACTGGTGTTGCAAATGTGATAGATTAGTC
GTAGAATAAGATGTTTAGAATACCCCTTTAGTCATAGTCACCCCCTTTCCGCTTATTGTACCCTTTTTGC
AACACCTAGTATAGTATATTCTCAAATCATACTATCGCATATTATAACTACGCTCGACAATACATAACAAA
TATATTGTAAAAACTTTTTTTTCTATTGGTTTTTTTTTGTACAAAGGACTGTGGCATTTTAACGAAACGT
TTCGAGAACAAAGTGAGTCGTACGTCTTATTACTAGTAATAGTTTAACAGCCCAGAGAGATTTTTGGCC
GCTTCGTCACTCCTAGTCATATGGGCGCCTAGTGAAGCGGTGGTAGATCCATAGTATGTGCCAACATAC
TGGGTGGTATCCGGTGATATTTTGACCCGTTTTCCCCCATAGGGTGAGATAGGCACCCTAAGCATTGTG

CTGCCACCTATCTAGGAGAAGGAAAACTCCAAAATTAAACCAGGAGTTTTGGTGGGGTGAGAAGGGG
TTATGGTGGGGTGAGATAGGCATCCTGAGCATTGCGCTGCCACCTATCTAGGAGAAGGACAACTCCAA
AATTAAACCAAGGCTTGTGGTGGGGTGAGATGGGGTTGTGGTGGGGTGAGATAGGCACCCTAAGCATT
GTGCTGCCACCTATCTAGGAGAAGGAAAACTCCAAAATTAAACCAGGTCCGGCCAGTCCCTGATTCAC
GTAGTACCAAATTAACAACAACAACTCAGGACTAGTCTTGTCAAGTGCTTACCATCGAGCGAATATTGC
TGTCAATCTGCACCTGATTGTGACAGTTTTTGGAAAATAAAAGTCGTTGTTGGTAAAGAAAATATGAG
AACGGATCAAGCCGTCTGCCTGTAAGGTAGGAGCGGCTAGCTGCTGGACAGTATCGAGATACGAGTCA
CTCGGGAGACCACTCTGGTCTTAGACGAATTGGTAATCGAATGTGAAATAGACAACAACAAAAGTACA
TACAATCAAAACAATCAATAGATCGTGAAAAATAAAGAAACATTTTTTTCCTAGTGAAAGTCAAACAA
ACAACTTTACCTATTCGGAAAAGCTCACCCGGATATAAATAATGATAGATGACAAGATGTTCTGATGAG
GCGAAAGCGTCGGCTTGAGAGCGGGAAGAGGATATGCGGACTCGTAACATATTCTAAATAGTAGTAAT
TAGGTGGCCAGAGAGTTTGTTAGACGTTTCTTCTCTCTTAGTAGAATGGCCTAGCGAAACGGAGGTAG
ATAAGTAAGGTAAGGGGGGT
>TCONS_00047304 geneID=XLOC_009742 scaffold_179:55730-58093(-) Exonic overlap (+)
GTCATGTCTGACTGAATGAAATTGAAGTTCAAAAATCCGAAAAGCAAATTTGTTGCAAATTGTAACAA
AAGCGTATTATTCAGTATGATATTGAAAAATACTTGATTTTAGGTATTTAGTTAGAGAAAACGTGAATGC
AATATAATTAGTTCCTTTCTTTGTGGTTTTAATAGTGTATTACGGATGAAATGAAGAAGAATTTGAAGAG
ACCATGTGAACAACTTTAAAAAGAGATTAACACGCATCATCAAAAATGATAACATCTGAGGAGTCAGA
TTCCATGAGCACCGTGCCCTCGGAGGGACATCCAGAAGACATGTCAG
>TCONS_00047307 geneID=XLOC_009744 scaffold_179:169688-171784(-) Unclassified
TCAAAATTTAAGGAATGCTAAATTTGCAAAATATTCATAATTTACTTACTAGTTAGACAAGACAAAATAT
TCAGCACCTTTGGTAGTCAGTTATAACTATAATACATCACTTAGATACTCGTTTAAGATGTTGTTGTTACA
ACAATCTACGATATATTGCTTTAATGATATTTTCATATTAAAATAGCAATTAGTTTTCAACGAATGGAGCG
GAATATATTATAATATACTTATATACTTAATATTATTTCAGGACATCTTATCGGTATTTTCTGAATTACATAC
AACACTACTTCAAATTCAGGACATATATCGGTTAGCTATATTCTAAATTATATACAAATAGCATAATTATGT
GTAATTTTACTTATCAGTTATAATAAATATACAGAGTCAAATGATAGGAGTTAGGTACGTACCTGCTTACT
AACTATTTATAAATTATTGCTAATTTCATAATTTTTGACAGAACATTGATTAATACATTACATAGAATGCA
AAAATAAATTATTATATACTCGATAAAGTATTTATTATTTGTTAGTTTCTATAAGACACAAGATAAGTCCAT
CGTTTTTGTTACTATCAAATATATTGTACAAATATGTACCTACAATTGTACCTAAAATCGGCAACAAGTAT
ACAAGTCAAAGCAGATAACTAACGACAACTTACTTAATATTCACTTCAATCACGTTCAAATGAGACAG
AGACAAATCTTTACGACCAGCGAAAGACATAGACAATAGCTATAAATAAATTACGATTGCCTGTACGAA
TTTATACTTTTTCTTACTATAGTATAATTATGTATGTATACTAAATGTACTACTAAGCAACAGCCACAACC
GCAATTTGATTCTGTAAAATCACAGTCACAGGGTAGAACAAATAGACGAATATCGGTCACAGAAGTAC
CTACCTACTAGTCATTCTAAATAACTGCACATTGTAAAATTCATACATTTTGGGTATCTCTTAAAATTTTG
TTGGTTGTGTAACAAAAGGTGTACAGAATGTCTAGTTGTGTGACCTCCTACCAGCATGACGTTGAGTA
AAGGGACACCTGTAGAGTGTATACCCACGAAACTTTTAATTATTATTTAATAAGTATTCCCATTTACTTG
AAGCAAGTAGAGACTGAGCTGAATTAATATCGGTAGACAAAAAATATGAAACCGAGAGGAAATCTACT
ATGCATATCACCGCGACGCTATGACGTTACAGAATCGTTTACATATTTACATTATGTATTACTGCTCGATC
CCGATTATATTATGTACAAATAATGGCCAAGCAAGGCACTCCGATTTGTACTTACGCTTCTCTAGAGATA
TTTTTCAAATATGCTCAGAAACTCGCCTGCTACAGTTTTCGAAATATAATAGGTAAGAGTTCATACATTC
ATTCACTTCTCAGAATAGACTAGAAGCACAGCTTTACCGTAGAAATACCAACAGTATATCTATGTCTATA
TTTAAGCATTAATGAAACAACACATAATATAATAATATGTATAAAGTAACTAGTCAATTGTTGTTAAATAT
GTAATATTTTTTGTGATTTATGTACTTAATTCGTAGGTAGTAAGCTATATTTGTTCTATATGTCTACTTGCA
ATAAAATACAAAACACATGATTGCGTCAAGGCTAGATATGCAATTAAAAGGAATTGTCTTTGTTGGACT

TGGAAGTAGGCGGTTTTATGACTGAATAGCTATTTCTAATAGAAAACGTTATGTAATAATCTGAATAGTA
TCTACTAATTTAATAGATCTGAATTAATTTAACAGTTTAATATACACGATGGAATCCATCCATACTATGTA
AAACTATGACGATGATATTTTACAATAACATTAT
>TCONS_00047379 geneID=XLOC_009746 scaffold_179:228372-228833(-) Unclassified
TAAAGACAGTAGTGCAAACTAATTATAATGTTGTGGACATTATATAACAATATAAAATAAAAATAAATTG
TCGCTCTTTACGCTTAACTTTAATAGTAGAAATATATTAAGTATGTTAATATGATAATTATGTGGTAAAGTA
AGTACTTTACCGATAAGGTATATTTGTACTTCTGCCTTACAATCACCCTGCACGGGTCACGATGTACCCC
ACTCACACTCTGTCACATGAAAGGTCACACACACACACACATACAAACGCGTCCCATGTCTTGTAGTC
CACGCATAAAATATAACAACCTATTTATGAAGGATACAATCGCATTAGTAAAGTTACACCAATAAAGCTG
AGGCCCCTCATGCTTGATGCAGCCTAACCC
>TCONS_00047388 geneID=XLOC_009740 scaffold_179:252373-254726(+) Exonic overlap (-)
CCGGCGCAGTGCCCACGAGCCCGCGCCCGCGCCGCGCCGCGTCCTACACCGCCACAAGGACGATCCA
TCCCTTACGGCCTAGTAACTACCCTCTCGGTACTCTTTATTTACTGCGCTGTGTCACGCGTTCGACGACT
TACTCAATAGATTATACACATACATGTACATTATGACATCAGCTGTGCGTTTCCGTAACTAGACCTCCGC
AATAAGGATTGTTTGGGTGCAGCAGTAATTTTTCTTTAGCACAAGTTATCCACATAATTATCTTACTAGTA
TATAATAAGGACACCGACCTGGGGCTGCCGTTGCAAAATACAAAATTGAAATATTTTCTCTATCGTCCTT
AGGCAAGTACATAATAATATATAGTAGCAAAGTTGTGGAGATTGTAAAGATAAGTTGTGATTGCTATGAC
CCCTGTCCGGTGTCCGTCGGTGAATAGTCCAGCTCTTCGTACAATGTCAAGTTACATAATAAAAAGCAT
AAGTTTGCAGAATCCAGTCCGCAGTGCAATGCAGCTCGTTAGATAATGCAGTCCCACCGACCCACAAC
ACTACATAGAGTAAATAATCTATACACATAGTTACCAACCAACTGACGGAAGTGTTACATAAAGCTGAG
CTCGATGGTTCAGATAAAATATATTGAAGATTTACACTTGGAATACCAAACTGGATCCTTAAATTCAAGT
TAAGCCTTATTTGCAGAAAGGTAATTTATATTTCATAATTATTTGTAGAGCATAGCCTACAAGTACTTGCC
TCTACTCTCTCGTGCGCCATTTACATCGTAAACACTAAACACACTCGACATTTCCAGAATCTAGATCAG
ATGACAGATCTATTCATTATTATAGAAGCAACTATTGGAAACGTATTCCGTATTGTGATGTTTAATTCATA
ATTTGGAGTATGTGTTTGGTATGGATGTTCTGCATCACTATTACTACTACTAGCGGATACCAATTAATATT
TCAATGTAAACAATACCAAGAAGTTCCTTGTAAGTCGTGCGAAGTACCTGTAGACCGGGGGTGGGTCG
TCAAAGGGGCGACAAGTGGTCGACGTCAGTCGCAGTTTGGCAGCGGCGCGTTACACACGTGCTTGCA
CCATGGCTCGCCACTTCCACCGTCAGGACTCGCTGCCGTACATGCAGCTGACTGAGCTGCACCCTCCG
CCGCACTACCTCCCGCCGCAACCCTGCACTCTGCACCAACTGTGAGTGAATGCAGCTGTACCATTTTT
ACTCCCAGCCGAAAATACGCGAATCAATCTACAGGATGTTGCAAAAGTGGTGGGTGTAATAAGCCGAA
TGGGATCACTCTTAACAATTTCTATTCAACAATTACTGAAAAACATATGCATAGTTACCATACTAAGGCC
TCCATACCAAATTCGAGGGGAAGAAAAGGC
>TCONS_00047295 geneID=XLOC_009741 scaffold_179:567024-587581(+) Exonic overlap (+)
CGGCAGCGCCAGAGTCGGTCGCGACGCGAGCGACACGCGACTTCGCACGATCACCAGTTTTCCACAG
AGAAAACTCGGCGAAATAAAAACTTGTTTAATAGTTTGTCCAGTGAGTGAAAATTTTAACGGATACTTA
AACTAGCTGCGCCTGCGTCGTTGATCCATAGCACACCAGAGATTTCTACACTACAGGAACACGGTACC
AACTGAACTGACCTGACAAAGATAACAAAGAAACCGATTGTGGTACTGTCCTGATCCCAACAAGTGC
ACATTGCAGCCGTCTTAGTGCAGGAGCCAGGAAGGCGGTACAGACTAGACTGTACTGAAAAAGACTA
TAAAGAAACAGATTGTGGTAGTGTCCTGGTCCAAACAAGTGCACAGTGTACTGGAGCCATCACACAG
GATCTGTGACAGCCGTCACATACAGGGTAATGCGTGGAGCGCGCGGCCCCGGCTGCGGCGCGGCGCT
ACCATGAACCGGGAGACGGTGACCACCAAGCTCCAGGCCGGCATGGACCGCTGGCAGAACACCTTCG
AAGACATGATGCATCTGCGCTCTTCCAACACGCACACGTTTAAGGATAACTATTGGGTCAG
>TCONS_00047278 geneID=XLOC_009741 scaffold_179:567027-589948(+) Splice junction overlap
CAGCGCCAGAGTCGGTCGCGACGCGAGCGACACGCGACTTCGCACGATCACCAGTTTTCCACAGAGA

AAACTCGGCGAAATAAAAACTTGTTTAATAGTTTGTCCAGTGAGTGAAAATTTTAACGGATACTTAAAC
TAGCTGCGCCTGCGTCGTTGATCCATAGCACACCAGAGATTTCTACACTACAGGAACACGGTACCAAC
TGAACTGACCTGACAAAGATAACAAAGAAACCGATTGTGGTACTGTCCTGATCCCAACAAGTGCACAT
TGCAGCCGTCTTAGTGCAGGAGCCAGGAAGGCGGTACAGACTAGACTGTACTGAAAAAGACTATAAA
GAAACAGATTGTGGTAGTGTCCTGGTCCAAACAAGTGCACAGTGTACTGGAGCCATCACACAGGATCT
GTGACAGCCGTCACATACAGGGTAATGCGTGGAGCGCGCGGCCCCGGCTGCGGCGCGGCGCTACCAT
GAACCGGGAGACGGTGACCACCAAGCTCCAGGCCGGCATGGACCGCTGGCAGAACACCTTCGAAGA
CATGATGCATCTGCGCTCTTCCAACACGCACACGTTTAAGGATAACTATTGGTCTCTTCACGCACATCT
CTTGAACT
>TCONS_00048097 geneID=XLOC_009825 scaffold_18:16056-16839(-) Intergenic
ATGCAGTGTCTTTGCAACTAGATATAAGTACAGTTAGCAAATATATTATATCAATTTTTAAACATAGATAT
ACTTACCTATGTTTTTGCTAACTATTCTTAACAATTTTGATGAGATTATGGTGGCATTATTAGCAACAATG
AGCAAAATTTTTCCTAAAAAGCTTTGAATCATCCCTACACCTTCAGTTAATATTACATAATTTGTAGCAC
TATATATTATTACTATGGTTATGTATTTGTATTAAGGCACATTCTGGTTCGACATGTCATCTTAATAAAGGC
GTTTTATCACAATACATTTATAGTGATTTGAGAATGGCACGGCATAATAGGTAAGTAGTTGTAGAAAAAT
ACATTCAATTATAGTGTAAATTTAGTAGTTTACAAATGCATAGGGATTGCATAAAATTCACAAAGGTACT
GTAAAATATATTTCCACTAGATAGCCTTACATTATTTGTATCGATATTATTGTCAAAATATAAAACCCTGCC
GATGTATAAACCCTAAATAATAAAAACCTGTACATCACACCCAAATAGCGCCCGATACTTGCCAAAAAA
AATAATTGGTGATCTCTCTCTACAACATAGAAAAAACATATTGTTTTGAATACTTATGAAGTAGGTACTA
CATAATAATATTATAAGTAGGTATATAACACAGAAAATGCTTAAGTTTTGT
>TCONS_00048098 geneID=XLOC_009826 scaffold_18:79826-81411(-) Splice junction overlap
GTTTTGGTTAGTCACACAATATGCCGTGTTAGGACGGCTTATTTCCGACATGTTTTCCTTCTAAAACCAC
GTTTAAAATGCCATTTTGTGTCTTCCCATTGGTTTTAGTGACAGTACAGAGAGTGATGGATGCAATGCT
AGTGGATTCAGTGTTAAAATAGTGATTTATATAGTTTTAGTGATAAAATAGGCAGTGAATAGTGATGTAT
ATTTTGAAAAGGGCGAAGACGTGGTTTCGGGATGAGGAGACGTTCCAAGAGCCGCTCAAGGTGTCGT
CAGTCCGCCAGTACGACCGCCTGCCCGAGTGGAGCGCCGAGCGCTATGACGTGGAGTACAGCAAGAG
CTCCG
>TCONS_00047426 geneID=XLOC_009789 scaffold_18:135724-152673(+) Exonic overlap (-)
AATATACAAAACCAAAACAGTTTCGCTTTTAAACTAATTACAAATTATCAAGTTCTGAATCCAAGCCAA
TCCTTTCGGTAGATTAGGTTCACAGAGTAGGTTATTGAAGTGTTTACCTGTGGGAACCTGTCCTCCTCA
GCATTGAGGAGATCCCCGACGGAGGATGCCTTGCCCTGGCTGGACAGGTGCCGGCGGCTGCTCAGCA
CCGACGACAGCTCATCCAGGTACCGCAGCGTCAGACAGAGCCTTTGGAAGCCGA
>TCONS_00048284 geneID=XLOC_009831 scaffold_18:235361-236807(-) Intronic
GGGCCGTAAATGAAGACGGACAATCGGCATACGCTTACTCATACTCCAACCCCAACAACCAGATGACA
TCCAGTTACTCCAAGTATGTTCCGGACTCCATGAACAATGGTCAAGATTATAAAAAGTATTATCAAAATT
AATTCATTCTGTTATTGGTGCTTAATTGGTTGATTATGTCATATTAAATTTAGATTCACCAGCATTC
>TCONS_00048101 geneID=XLOC_009831 scaffold_18:235365-236339(-) Intronic
GAGGCGGCGGCGGCGGCCGTAAATGAAGACGGACAATCGGCATACGCTTACTCATACTCCAACCCCA
ACAACCAGATGACATCCAGTTACTCCAAGTATGTTCCGGACTCCATGAACAATGGTCAAGATTATAAAA
AGTATTATCAAAATTAATTCATTCTGTTATTGGTGCTTAATTGGTTGATTATGTCATATTAAATTTAGATTC
ACCAGC
>TCONS_00048285 geneID=XLOC_009832 scaffold_18:238988-240437(-) Intronic
ATTTACTTGCTGAATAAACCTTCAAAATGTTCAAAAATCTTTTCGTTTTGTGTCTGGTGTTGCTGATGGT
TGGTAGTAGCCTACAACAGGGTGGTGGTGGCGGACAGGGAGGAGGCGGCGCTGGCGGGCAGGCCGG

CGGCGGTGCTAATGCCGGGTTCGGCTTCGGTATGAACGCGGGGGCGGGGATGGGCGGCGGCGCGGGC
GCAGGCGGGCAAGGGGGAGCGAATGGAGGTGGTCAAGGTCGGAAGTGAAACTTTTAGTAGCTAATGC
CCTATCATATACTTACTACTCAACACAATCATTTTAGCTGAAGTAAAAGTACCTAGTATAATGTAGTAAA
GATTAATAGGAATAAATGATACTTTCGT
>TCONS_00047886 geneID=XLOC_009797 scaffold_18:505668-508221(+) Intergenic
AGTGAATCAGTGAATGAATTTAGCGTTTATTTCTTTAAAAAATTAACAGTTGATCTAAGTTAATATTTCT
AAATTACCAAATAAAGACAATTCGTTGGTATGATGTGCAAGTGGTAGAGAAATTGTAAAATAGGATTTT
CAACTTGGGATGAAAATTGTCGCCATTTTCGCACTGATTGACGTGAGCTAGCGTGAAGTGATAAACTC
ATTTTATTATTTTATTTGAAATTATTATGATTGGTGACTAAAACTATGTTTGCACTGAAACGAGTGATCAG
ATATTGATAATTATAGGATTGAAGCTAATATCTCATTACGACTTCAATGGGGAGCTACTTCGATCCGCAG
GCATGACGGAGAAGATCTTATATCCAGG
>TCONS_00047672 geneID=XLOC_009803 scaffold_18:860819-863439(+) Splice junction overlap
CCGAGCGGGGTCGACGGTGAGCCAGGGCCCGCGCCCTCAAAACTACTACCGCCCGTCGCATCGATCA
CAGCAAATAATGGAGTCGGACGTATTGTACTAGAAGATGGCAGCGTCATACATTTCCCGTTTTACCATT
AGCTTCGAAGTAATTACTAAGTACTTATTATTATTAGTTAGTTATTAGTACCTATATACCTAGTAATTTGCT
AATGAACCAAACATTGGGGAGACATATCCAAGCCTATCTGAGTACCATGACTATGAGCCAAGCCTGGA
GTTGGATGAGTCAGAGATCATACAGATCAGTTTCTTAACTTACCCAGACGTAAGTTAAGAAACATCTTT
GCCAGAGTAGGGTTGTCGAATTACAATCCAACATTGGCAGAATCCAAAAATTATAATTTAACAAGTTAA
CCCAATAGCCACATATGGTACTTACTACAATGGTATTCAAAAATTGAATTCTGATATTTCGAGAAACTTA
TACAAAAATTATTTATGGCAGATATATATTTGGAAAGACGGGTGCATTCCCCTAGTATAGGAGTTCGCTC
AAATGGCTCAATGCTACATCCCTGCTACGATACAGTTCTCATTGAATACAAAATTAATGATATATTATAAT
ATGTTATAAAAAAACTCAATATTACGGCTTTTTTGTATTGGTCTTAATTCAATAATATGCAAGTCACTTGC
TTTCGTGTTCGTGACTGAGATCAACTCAAGGGCCAGCTGAAAACCAGCGCTGTGGCCTCACTGTCCAC
AGCATATGCTGAGCTGGTGCCGTTTTGACACTCTGGACAGCATCCTTTATTTGTTTGTTCTGTATGAAGT
ATTCTCGTTATTTTGTTATTGTTTCTTATTATTCTGTCCCAGTGTGTCAAATAAATGTATCTTTCTTTCTTA
CTTTCTTTCTTTACAATGGTAATTTAAATTTATAAAGTTACCCCCTTATTCATAGAGGAGTTACAGAACGT
TTTAACTAATAAACAATTTGTTCTTTGACTAACACTGAGTTGCAGAAAGGCACTTTAAGCTAATGTTGG
TATAAAATCTATGTCTGTCTCTTTCTGTCCGTATACTGTCAAAGCAAGGCAGGTATACATTTAAGGTCAT
CATTAGCTTAAAGTTCCTTTCTGCAACTCAGCGAGAGAGGGACAAAACAATTTATTAGTTAAAACGTAT
TGTAACTTTTCTATGAAAAAGGGGGTAAATTAGCAGGCAGCTTACCTAACCGCCGTCGTAGCAAGCCT
GGCGTAGACGCGCCGCTACGAATTTACTACGCGGTGTCGTAGCAGGGACGTAGCAGAGCGGGCGAAC
TTCAATTCAGGGTGAATGATTGAAATTTATTAATTTGTATAGGTAACTCTTGTTATGTCAGCATTCAAACT
TTTGATGCCAAAATAGATTTATTTAAGCTTATTGTTTCTCAAAGTATGATTATAAGATACTATTCATAAGAT
GAATATTATAAGATGGTCTAATCCTTATACTCAATCGAGGACGTTGAGTGTTATGGATTATCAAGGACCC
TATCTAAAAATTCAATATTGCTGAGCTAGCTATAAAAATTATAGCTGTCTTAAAGAATGTTATTGGTCAAT
CTATTTCAAAAAAATGCAGCCCTATTCTTGAATAAATTCATGATTGTCCTTTGCCAGATATTTTTTGAAG
CATTTAATCATGTTAAGTTATTTTAAATATATTTTTCGTATTTTTGTCGTTAACATTTCTACTTTTATAATGA
TTAGACTAGTAAATGAGGTACACCGGGTATCTCAATATTGGATTAGGGTGTTTTTAATAATAATAATTGA
GAATATGATTAAGTTTAACTAAATGTTA
>TCONS_00048042 geneID=XLOC_009845 scaffold_18:1175195-1177779(-) Splice junction overlap
TTGCTGCGGAAGGAGCGGGACCGCCTGCAGCAGCAGTGGTCGTGAGACAAGCGCACGGAGGCACCA
GCTGTCCTGTCACCGCACCATCTTGGGAAGAAGAAGAAGAGATCCAACAAGGAACCCCTTAACTAGG
GCTACAGATTACATGAACGATAAATGCAAACAATATTGCGTGATTAACATGTAGACAGAGGATAAAAAC
TCTTTGCTTCTTTATGAACTGAATATTAGTTCTGTTGATGTATAAATAATGACGATCGCATACACATTTTA

AATTCTACAATTAATAATAATGTTATCATTAATGATCCATAGGGAAAAAATAATGTAAATTTACTTTTAATA
TGATGGAGAGAGGACAAGTAGGTTTACTTGAGTACTTACTTAATTAATAATACTACTCAATTTGTAGTTT
TTCACCCTATGTTACATGGGAATCTACCACAGAAAACTTAATGAATCTACTCTAGCAAATATGTTAGAGT
GTTATTCCATCTGATGGATGTTGTAAATTAGAGTTAATAATATAATAGATTAGAGCTATGCACAAATTTAC
TATACAATGTACGTGCATTTTTGACTTTTGTATGTAAAAACCCTAATTAGTGGTAGTTATGTATGATGTTA
GATTATGTTTAGGACAGAGTCCAGTGATTTATTCTTATGCGTAGGTATTATAGAAGTTATACTTAATTTAT
TTGTATAAATTTCTGCATAGTTTATTAATAAAACATTTCTCCCATCCGACATATTGCTGCTCAGTGTCTTC
AAAACATTATAACTAATTAAATTTATCAAAGCAAACCACGAAAGATCAAATATCAGATCACGATAACAA
TAGCAACCTACATGAGGGAACATTGTGATGGTTTAAAATACAGAGCCAGCTAGAATAGGTATGAGTAA
GTCTCGCTATCCAGCATTCATTATTACCGGTCGCCCCGGCCGGTCGCCGCCGGTCGCGCCCCGGGCGA
AGCGAGCGAGGGGCCGGCATAATCACCGGTTCTTGCCCAAATGCCCCGGGCAACCGGCACCGTCGCC
TGTGGAGGGTTACTCTACATTCACAAAAAAAAAGAAAGAAACGATAGCTGTTGCGCAAATGAGTTAG
AGTCGTTATTAGCGCAGCAGTGATAGAAAAATTACGTTTTTGGGAAGTTAGAATAGAGGTAGAGTTACT
GAGCCGCCGGCTATGGAGATAACGTCTATGTGAATATAAATCCGTTGACTGTGTTATTTTATTTCGATGG
GGATTTATTTGTTTGTTACATAGTTGTGTCCTAAATTATATTAACTAAGTATATTTATATTAGGTACTAGCC
TTAGGAACCTTAATGGATTTTGATGAAAGCTCTCGAGCGTCTACATATAATATAGTTTGCGTAATATTGA
AACAAAAAAAAAAACAATCAGTGTGCTCACTCTCATTTTTGATCGGTAGATACTAGATAATAGAGGTG
CGTTCCAAATTGGTAGGTACCTATAATTAGTTAGACGTGTATTATAAAGCCAAAAGAGGGTGACAAAGC
TTTTGTATATTATTTTAAACAACATATTTATTTCTAAGAATATTTAAAAGTAGTGAAAAAAAAAAAGATTT
CTGAGTCACTCTCTTTTTGGCTTTTTTTATCCGACTTCGAAAAAGGAAGGGGTTCTCAATATGTCTCCAT
GTATGTTTATTTTCTATATCTTTTTTCGAAAAATCATGGCTATCCGATCGAGGAGTCGGTTGTGTTGTGA
GTATTATGTTTAGAGGCATGCATGTAGGTATGGAAGTAAGTAGCTACATATGTATTAAACTATTAACATTT
AATTTTATTGTACCTAGTACGTAACTAGCACTATGGTTAGGTACTAAGCTAGTACTTAAGTCTAAACTTT
AACTTTTGTGAAAATATCAGTTTACTTAAACTTTATTAGGTACTAGGTATAAATACTGCAGATAATCTAC
AGTGTAGATTATAGGTAATTTTAAGTTATAAGCAGGTATGTTCGAAATAAACTGAACTTATAGGTATTTT
GGTTGTAGTCAAGTCAATAACTTCATTATTACTTATATTGTTTAAGAATTTAAGATGCAATTTATTATATTG
AGCTAGCATT
>TCONS_00048043 geneID=XLOC_009845 scaffold_18:1175195-1178236(-) Splice junction overlap
TTGCTGCGGAAGGAGCGGGACCGCCTGCAGCAGCAGTGGTCGTGAGACAAGCGCACGGAGGCACCA
GCTGTCCTGTCACCGCACCATCTTGGGAAGAAGAAGAAGAGATCCAACAAGGAACCCCTTAACTAGG
GCTACAGATTACATGAACGATAAATGCAAACAATATTGCGTGATTAACATGTAGACAGAGGATAAAAAC
TCTTTGCTTCTTTATGAACTGAATATTAGTTCTGTTGATGTATAAATAATGACGATCGCATACACATTTTA
AATTCTACAATTAATAATAATGTTATCATTAATGATCCATAGGGAAAAAATAATGTAAATTTACTTTTAATA
TGATGGAGAGAGGACAAGTAGGTTTACTTGAGTACTTACTTAATTAATAATACTACTCAATTTGTAGTTT
TTCACCCTATGTTACATGGGAATCTACCACAGAAAACTTAATGAATCTACTCTAGCAAATATGTTAGAGT
GTTATTCCATCTGATGGATGTTGTAAATTAGAGTTAATAATATAATAGATTAGAGCTATGCACAAATTTAC
TATACAATGTACGTGCATTTTTGACTTTTGTATGTAAAAACCCTAATTAGTGGTAGTTATGTATGATGTTA
GATTATGTTTAGGACAGAGTCCAGTGATTTATTCTTATGCGTAGGTATTATAGAAGTTATACTTAATTTAT
TTGTATAAATTTCTGCATAGTTTATTAATAAAACATTTCTCCCATCCGACATATTGCTGCTCAGTGTCTTC
AAAACATTATAACTAATTAAATTTATCAAAGCAAACCACGAAAGATCAAATATCAGATCACGATAACAA
TAGCAACCTACATGAGGGAACATTGTGATGGTTTAAAATACAGAGCCAGCTAGAATAGGTATGAGTAA
GTCTCGCTATCCAGCATTCATTATTACCGGTCGCCCCGGCCGGTCGCCGCCGGTCGCGCCCCGGGCGA
AGCGAGCGAGGGGCCGGCATAATCACCGGTTCTTGCCCAAATGCCCCGGGCAACCGGCACCGTCGCC
TGTGGAGGGTTACTCTACATTCACAAAAAAAAAGAAAGAAACGATAGCTGTTGCGCAAATGAGTTAG

AGTCGTTATTAGCGCAGCAGTGATAGAAAAATTACGTTTTTGGGAAGTTAGAATAGAGGTAGAGTTACT
GAGCCGCCGGCTATGGAGATAACGTCTATGTGAATATAAATCCGTTGACTGTGTTATTTTATTTCGATGG
GGATTTATTTGTTTGTTACATAGTTGTGTCCTAAATTATATTAACTAAGTATATTTATATTAGGTACTAGCC
TTAGGAACCTTAATGGATTTTGATGAAAGCTCTCGAGCGTCTACATATAATATAGTTTGCGTAATATTGA
AACAAAAAAAAAAACAATCAGTGTGCTCACTCTCATTTTTGATCGGTAGATACTAGATAATAGAGGTG
CGTTCCAAATTGGTAGGTACCTATAATTAGTTAGACGTGTATTATAAAGCCAAAAGAGGGTGACAAAGC
TTTTGTATATTATTTTAAACAACATATTTATTTCTAAGAATATTTAAAAGTAGTGAAAAAAAAAAAGATTT
CTGAGTCACTCTCTTTTTGGCTTTTTTTATCCGACTTCGAAAAAGGAAGGGGTTCTCAATATGTCTCCAT
GTATGTTTATTTTCTATATCTTTTTTCGAAAAATCATGGCTATCCGATCGAGGAGTCGGTTGTGTTGTGA
GTATTATGTTTAGAGGCATGCATGTAGGTATGGAAGTAAGTAGCTACATATGTATTAAACTATTAACATTT
AATTTTATTGTACCTAGTACGTAACTAGCACTATGGTTAGGTACTAAGCTAGTACTTAAGTCTAAACTTT
AACTTTTGTGAAAATATCAGTTTACTTAAACTTTATTAGGTACTAGGTATAAATACTGCAGATAATCTAC
AGTGTAGATTATAGGTAATTTTAAGTTATAAGCAGGTATGTTCGAAATAAACTGAACTTATAGGTATTTT
GGTTGTAGTCAAGTCAATAACTTCATTATTACTTATATTGTTTAAGAATTTAAGATGCAATTTATTATATTG
AGCTAGCATT
>TCONS_00047585 geneID=XLOC_009846 scaffold_18:1197349-1198790(-) Unclassified
AGTGCTAAAACTATAAAATTTGTAATGAGGTTTTAAAAACCATACTTTACTTATGTGTATTTGTTTGTTAA
TTCTTATTTATTTTTAAAATAAGTATGTACCTACTATAATTTGGCAAATCTATTATTTGAATTACTCCAGAA
TTATTTAATGTAATATTTTAAAAACCAAATGAATAAATACCATACATAAAATCAAGGCATAAAGTAAACA
AGAGTAAACATTAGAAAAAAATAATCCGTTTTTAAAGGTACCTTTATAATATACCTACACGCTATAAAAA
ATATATCCTACTCTAATATGTAAGTAATATTCATTTTTTTTTACATGTGTATTTTGTATTATATACTCACGTG
CAAAGAAACAGTTCCAATTAAATATCCGACACTAAATGGTCTGAATTTTTTATACATTTAATAAGAAATT
TTAACAAAATGTTGACGTATTTTCTTGGTATTATTCTGGTGATCTTTTAAAACTTTAATATTTCAGACGGC
GTAATTAAGCTAGTCCTTTCCACTTAGGACTAACTTGGTGCTTTTCTGACTGGAACTTTTTCATTGCCCG
TGAGTGTAGTTTTCAGAAAATTGCTAGCATTTTATAGTCTCTAATTTCAATATTAAAAACTCAACTTAAT
ACGAATTTAAGATTTCTAAATATAAAAAAATATATTATTTATTATATAAATATAGTATTATTATTAATATTGA
AATACTATAATATGAACTTTTAATGATAAATACAGGATGTAACTGGCTGTGTAACCTGCGCGGTAGTGGT
ACAGTAGACCAAACCACGTAGCTCTCCAAAAAAATTACATAAATCTGTTAAATTGGCCTACTTCTTTCT
CATCAACCACCGTAAAAATTAAGCTTTAAAACTCTAATAACTAATAAAACAGATGTTTACATACATACGT
GTACAATTATATATAGGATATAACGCACGTTAGGAAAAATTAAGACTTTATAAATTGATCTACTTAGGTAT
TAGAATTAAGTACATACCTACCTATAATATAAATGGTTAAAATAAATTTTATACCGAAACAGCTTACATTG
CCTTTTATTAACGAGATGCTTTTCTTCCTTGGCTATAAGTAAGTATTGCATATAAATAAAAATATGATAAA
AGATGTCTCGGGATTGTTTAAACAGTCTTGTTATGAATAGATCATGAATAGGCAATACCTAATTACCAAG
CTCACATGTTAATTCAAAAATCTATTCACATCATTAAGTGCGTAGTTAGAATGGGCAAGTACAAAAACA
TCTAAGTACTTACATGCAA
>TCONS_00047503 geneID=XLOC_009810 scaffold_18:1208657-1214254(+) Unclassified
CGAGTACATTAGAAATAAATAACACAGGTATTTGAGAACAATCACACTTTATTTAATGTGAATAGGTAC
ACAGCAATTTTACGTATAATTTAATTATAGTAATAATTACAGAACTTAAAAATATGTAGGTATACAAGTAT
ACCGGAGGTATTTTTGTTTATTACCGAAAGAAAGAAGAATTCACAGAATTTAATGTAGCCCTGAAAAA
ACAGTGACATAATAATCTAACTTTTTATTCTGAAATATTGATCGTGCAAAAGTACTGGCCACATATTACTT
AATAAAATTTTCAAAGAAAAATATTCCTATTAAGATTTTGTATTTAATTTTTGTTTGTAATTATTTTTACAA
GTACAATTATACTTTCTTAAAATGAGCAAATTTTTGCTCTTGATTCTGAAGGGCAGATTTTAATTTTAGG
ACTGAGTTATTTAATTTGAAAAAGATCTAAAATATTTTTTTATTTGATACCTTCAAAACATTTTGGATACG
CTACCAACGTCATTTACCGTTAGGGCCAATCCACAGGTACCATAACACAACCACACCACGTGGTCGGG

CGTATAATATGTTTTATTGAAAATGAATAATTCTATTTACATGTACCCATGTACCACATTTAGCAGTCATTA
TCGACCGCTTGTGTGATACTGACGAAATAGTAACACAACGCAACTGTCAGCGACACCTATAACTTGAC
ATAGAGATGACATAGACAGTCTTGTCGTAAACATAACGCATAACTACAAAAACATGTGATACTATTCAC
TCGAAGACACTGTTTGACTATACGCAAGATTTCGATTTCGTGTGGTTGTGGTTCCTACTACGTGTGGGT
TGGCCCTTAGGGTTGCATGTTAAAGAACCAAGGTAGTGCTGTTTTGTAACCTTTACTCTTGATCTGTCT
GAAACTTCATAATTATTTCGTCATCACGACATAAGTAATTAAGCTTTATACCATGTTTCACTAACTTATCA
ATTATACCTGTCGCAGTAGTTATTGTATTGATTATTGCGATTGATTTGTGGCTGTGCAATGGATTGTTCAG
TTTTGTTTTGGCGAAATATTTAGACTATTAAATTATCAATTAGCTGACATCTTAGGGTAAAATCATATAAA
ATACGTCTTACGGTATTTTCATATTATTGCGAAGGCACGAGCTAGACAGTGTCGCGGTAGATCGTATAGT
TGTTTTAAAAAAACATAATTAACTATTAACACGTAATTATAGGAATATATTTTTTAAATGCGACTACTCGC
TGAAGTAGTTTTGCCCTAAATATTACACTGTATATAAAGAATCTTAATAAAACGATGTCTATCGACTTAC
ACATACTTACTTTGAATACAGAAATGAGTGCAATACAATTTAAGTAATATCATACACTAACCCCCATTCA
TAGGACAATACACCAATACAATTAATGGTTTGAAATTGGTGCATAGTACACGATTTTCATATTTTCAAGT
GACACAAAGGGTAAAACTCGGTTTCTCGATTTATGTAAGTTCTATAAATACGGGGTTAAATTTTAATACT
TCATCAAAGCTTGTAATTTATTTTAATTTGATTTAAAACCCTTGAAAAAGTATCAAGGTTTACGTGTAAG
TGGTTGTATATTAATGTTATTAAGTCACATAAGACCAAAATAAAATGTTGTACATAGTCACACAGTAGTA
AACTACTAGTTAACATAAACAAGTAACGTATTTATAGGAACATTTAGTGATCTACTTTAAACTCTATAAA
TATGGGAATTAAATCATTCATAATATAATTATCATGAAAATTTAACATAGTAAACAGTCCTTAAAACATAA
AGGGAAAATATAGGTAATACCTATTTAAGTATATAATATTACGTACGTTTAATTCGACTCATAACGATAAA
ACAGATTTTAAACTATTTGCCGTAAGTTCATGCCACAATATGATCAATAATTTCTTTGGAATTTAAGTAC
AATTTATAAGATTTCTCCGACGTTGATGTAAACCACCGAGAGAAAACCTCCACATATACATTCTACATTT
GTACCCACTTATTTTACATTGTACTCGTTCTAAAACGGCGATACGGCTTACAACCTATCCCGTGGTACAA
TAAGATCTCGTTCCGATTAGCGCTCACTATGTGCTGCTGACTGTGAGACTCCTTAGTCACAGAAACTCT
GCTCACTCAGTTGAGACCCTTTGTACAGATGATAAATATTTGATAGTAACTTGAAAGTTTTCGCTGAAA
GCGCCACTTTGTATGCCAGAAAAACGTAAACCTTTCATAGTTTACGGAATCTATCAAAACCTGTGTTAG
ATATACAAGACGTCGAAGTTATGGGACCAAAAACCATCGTCAGTGACAAAATGTGAGCCAAATCGAG
ACGAATCTTAAAATTTAAGCAAATAGTGTGAAGGTGGCCTGGAAACACAGACCATATAGCCTAAAAGA
CAACGCATAGATTCAGTTCAGTGTGAAAATCTAATCCCTAAAAGTAATAAGTTCCGGGAGTTTATTTTAT
CTATTCTTATTAGGTAGGTATCTGCAAGGGTGTTTAGTGCCTGACTGCACCTGGCTCTATCTATTGGAAC
GTTTTTGCAAACCCTAAGGTCTAAACTGCATTCCTAAGCTTGGAACATTTACCACCACGCTGGTCCATT
GCGGGTAGGTAGGTTCACACATCTAGATGGGCAGGTTTCCTCACGATTGTTTCCTTCACCGTAAAAAC
ACATGCTATAATTTTACTTGGACTACGGGGAGAGGATATTTTGTTTTCATTTGAACGTTCAATGGCATAT
TTTTTTTTAAAGCATAATTTTGGCGAACGCAGTTCTCTGCCAAAGTGCCGCCTATCAGGTTATATAGTCT
GTGTCTGAAAGTCAGCTATGACGTAGGCCACCCTCCCAAGGGAACAGTTAGAAACATCTACAACTGTA
TACAACAATGAAATCTAAATGCACATCATTTATATACCTAGCTATAACAAATACATTGATACTAGAAGCA
CGATATAAAATTAAGAACATAATAACGCTTATAGTACTTAATGTACCGAGAGTAAGTACGTTAATAATAA
GAATACTATTTTGATTATAAAGTAACGGTGTACTACTTCATAATACCGAAATGGTTAATTCTGTAGCACC
ACATTAGGAGTAAAATCATGTATATTTTAATAGACTAAGCTAAGCATTAGGACAAATACAGTAAGGGGC
ACGGATTAAGGGCACCCATTGTACTATAAAGTGTACATGTTTGTCTAAAGTCAACCACTCACATTTTAA
GGTATTTCTTTGTCTTTTCATGGTATCCAGCCAAACAGTGGTTGACTTTACTTCACATACTTTAAGATAC
AATCAACATAATTAATATGTAAAAAATATAAGTACAATTTGTATAAACAATTTCCTAATTCGGCCATTTCC
TCAATACGCTACGAACTTTTCACACCAGATGAGATTCATTTATTGTCACTTTAATACATGCCTCTATTAAA
TTAAAATAAATACACTGTACAAAATTACTAGGTTTAAAATTGCGCTACCAACCGGCGATAGCTAATGGT
CACTGCTGACGCATCAATTCGGAAATCGGTACATTCTAAGGTAAGTATAAAATAATAATAAAAAACCAT

GCTTGATTACCTGAAAAGTTTACTAAATCCTGAATAGAATAAGTTAAAAGAGAGAGCGTTGTCTCTTT
>TCONS_00047771 geneID=XLOC_009824 scaffold_18:1548512-1570423(+) Unclassified
CGAACATCGGGCGGCCGACAGCCGTATTCAATCGCCACGCGCGCCGCGGGCCGGAGGTCGCCAGTGG
ATGAGTGAGCGCTCGGGGGCCGGCGCCTCCCGTCGTCAGTGTCAGTACTATCGGAGCCGGTGTTTTAA
GTGACTATTATGAGCGACAGGGACAGGTCTTCGCCCTCCCTCACGGACACCCCGCTGAAGACGTACAT
CGGCCGGCACGACGACGACTATCCTCTCATAAACGGAAACGGTGAGAGCAAATTTTACTCCTGTATAAT
AGCATCGGGGCGG
>TCONS_00048543 geneID=XLOC_009960 scaffold_180:135575-137046(-) Exonic overlap (+)
CAGAAACTCATCGACTCCACGGTTGTGCCGCTGGTGGTTAGCGCGAGGAACTGGGTGATGTCGCCGTC
TGCCAGCGTCGACGTCGAATGAGCGAGAAGGTTCGAGAACAGTCGAGAAGCAGCGGCGATCGTCTAT
TATTACGTTCTGCGTTATGTTCGCGTCTATATTTTTTTATACAAAAAAACGAGGAAAATCACAAAAAAAT
AAGCACAAATAAGTATGTCTTGTCTATTAAGTATCGTTTCGCTTCTTTTTTTACTGAAATCTATTTAAGGT
TTGTTTATATATTTTGGCTGAACAGCTTGTGTTAATACTGTCTTAACAACTGAGATTACATCGATCCGAG
TCGTTTGTTATTTGTTGTGGTAAAATCGAATTTATCTATTGTCTGCAATTATACTATTTATCGATTTTAATT
TTCGCTTTGGCTGTTGTATTAGTCTTTTAGGTGTTATCGATTTACCTAGAGCGATTCACACGAATATGCCT
TATATAACATATCTAAGTCAATTTCAAGGGTATGTAATTTATGTTGTACATGTATTTGAAGATATATTTTAC
AACATTATGTAAAGTGCTATAAAACCTAGTGTACGTCTGTTCGCCGAGGGTGTCAGACAGATAGGCGT
GTTCATGTTTTATTGTTCTCACCCCAGGGTGATAAGTGCTTCAAATGTTTAAACTGCATAACATAGGCTT
AAGAAAATTATTGACAGATTAATTAAGTATTTATTACAAGGAGTACGCGACATTTATTAATTTTATCAAG
GAATGTCTATGGTTTATCAGTGTGTGTCACATTTATGTAGGTCTGTGAGATTTTAAAGATTAAATTAGATT
TAAACGCGTTGCCATAATACTCCACTGGCAGGCCGCTTCCTAACAATGTTAAGTGACATTCAGTCATAG
TTTTTACTAATAAAATATAATTATTAAATATTTTTGTTCTCGAATTAAGAATCACTCTCGTTACAATAATGT
AGTTGAAAGTTATCGCTATTTTTACAAGATAATAATTAATTTAATATATTAAGTGACATCATAACTATATTT
ATAAGAGAATAACATCTGTGTTAAGTACGGGATCGCTCCACGCTTTCTGACCACATACTGTTTGGGGAT
CACTACATAGGGGATAATGCTTAGAGTAAATTATATTATTGAATGTTAAAATTTATAATATATGTATTATAC
CTATATTTACTTTTTTTGAATCGAGTAGCTGTGTGATCTGATCTAAAACTTTATACCTATAGAATGTATAA
ACAATGAAACTTAATAAATGATACAAAAGTTATTGTTTGTTTTTATTTTGGTCCTCCCTCACTATTCTCAT
ATTTAAAAGTACTATAGCGACATCTGTTGAG
>TCONS_00048674 geneID=XLOC_009955 scaffold_180:561694-562181(+) Exonic overlap (+)
GTGTAAATAATTTGAAAATAATTTTTCAATAACTAAATTATTTTAAATCAAATGAATAGTAGCATTTTGTG
AGTAAAATCCATGTGAAGCAATGGATGCGATGCGTCAGTCGGGAGTTTTCAACATGGTTTCGTGGCTG
TTTGGAAATTATTTATTTTAAATCGCAAGCCATAATAATTAATACAGTTTTATCCTCAAAACAGCGCTAAA
TAAAGTACAAGGCCCTAATTTTTGTCTCTGTAGTGCTTATAATTCTATCCCTGTTCCGATTACAATCACG
AAATGAGTTCCGCTGAGGATGGAGAGGTGGCTGCCGCGGCGCCAGAAGAGGTGCCTGTTGGGAATCC
TGATGAACTCACAAAAAATCCAGAGGCACTG
>TCONS_00048908 geneID=XLOC_010019 scaffold_181:168603-220786(-) Intergenic
CTGGACCGGAGCGGTCGTGTCGCGCGCGTCGCTGCCGCCGACATACGAATGTCTTCCGATAAAGTTGG
CCAAACTATTGGACGATTAGGCACACTTGGATAACTTCCTGTCGTTCAACTATTGTGAGTCACTAGATT
AGTGGATGGACAAGTTCGGAATGTGAAAAGATAAATATTATGATTCTCCGAGAAGCTCCACATTTTGAG
TACAAGAAAAATATCAAAGCTTTAATAAA
>TCONS_00048749 geneID=XLOC_010003 scaffold_181:242151-243737(+) Exonic overlap (+)
GCTGTTTTATTCGAAAACTTTAAATTCGGAATTGTGAACTACCTACCTATAAAATTCTTAATTTTAGAAA
TTGGTATCAGTAGCCGGGAATCGATGGTCCCGGGTTCGATTCCCGGCTGGGGCAGATATTTGTTCTCGG
GTCTTCGATGTGCCCGTAAAATGGCAATAGGCCCGCCCCCTATTACATTGGGACTAACATAACACTCTG

GCGAAAAGTGGGTGCAGCAATGCACCTCTGCCTACCCCGCAAGGGAGTACATTAGTACAAGGCGTGA
GTGCGTGTTTTTTTTTTTTTGGTATTATCATTTAAATAAAAACAGATTATAGTTGTAGGTACCCTGTCTAG
CTAGCCCCGCATGTTCTTTTTTCGTGAACATGTTATGATTAAATTATATTACCAATACTTATATGTACACCC
CGTATCTTTATTTCATGAGGAGCTGAGTTTCAGAACTTTACTAAAACATGGAAAACTATCTGCATACATT
AAGTAAAAAACTGATTTATATTCTTTTAAATATTCGTTTATTCCTAAACAGTTAAATAACAATAGTTTTAA
CTAATCTTAGAAATAGTTACTGTTACATTACTAAGTAATTTAACTAACTATAGATTCATAACAAAGAAGT
TCAAATACCTATTATAGGTATCTAATAAAGTCCTTGGGTCACTTCAAAAGTAACTCGACATTATTGAGAG
GATAATTGGATATTTACTTACCGTAACAAAATCCAGAATGACTAGTCAGAGATAATTATGTAGTCGCATA
CATTTAGGAATCAAGATTAATAAACTTGCTACAACATAATACTGCGGTAGGTATGTTTTGTTATCTTCTG
GAAATCACAATCGTAAGGAACGATTGTTCTCATAAGGTTTAAGATCTAGACGTCGACGTATCATCACTA
AAGCAGTTTAGTTTACCGTTTACCCGTATCAGAAGATTCAAATATTAATCAACGAGGCTACGATACGGC
TATTTAAAACAGAGAAAATGCACTCCAGTCATTTTAAACATACCACATCGCACGTATCTCGTAAGAGCA
ATTTATGCTGAATTTTTCAACTCTGAAACCTTGTGCTACTACCATGGTGCTCGAAACTTCGGGACTTCA
GTGGTGGCGCGGAATCCATCGCACTTCGCGTCTAATGTGACGCAACTGTCTATGACGTCTCATATGGGA
TTAGATTTTTTACAACAAAGGCGACAAGTTGCCGCTAGCGTCACGTCACGTCGCCGTCCCGCGCCGCC
GGATGTGTGAACGGACCTTTAATTTTCACCACTGCTTTCCTATTCGTCCTTCAAGACATCTCCAATTAAA
GTAGTTTCCATTTCTAGTGATATTTGATTGCCTGTGTCTTTCTTGGTATTTTTCTTTTGCCGCTT
>TCONS_00048927 geneID=XLOC_010005 scaffold_181:387984-400528(+) Intergenic
AAGAAATTTTGGTGGGAAGTGAGTGGGGTGCGCCGGCGCAGCGCCAGTGCCCACCACGCGCGGCCC
GCGCCCGGACTGTCAGTGCAGTGAATGTGAACCATAGACTACCACCGAAATATCCACTATTATTCCTCG
CGCGCGCCCAACAGTGACCCAGAACCTCAATACAAAGTGTACGTTTGAAAAATTATAAAAAAAAATAC
ACGACGGGATTTTGCTCTATTTAAGAATTTCCCCGTTGTTAAATAGTGCAAAAAAACTTTGTGTGTTTT
GGGATAAATGTAATTGGCATTCGCAATGGAGTGACGTCCGCCCCCCCCGCGCTCGTTCCCGGGAACAG
TTGACGATCGGGATATATCGCTCGCTCTGGACTGATCATGTGCTATTTGTTTCGGATGTGACACTGACCG
AGCGTCTGCTCTTTTTATTGGAACTTGACTCTTCTTGGCAGGTGGTGTGTGAATGGAGCACAGTTAGA
GGACCACTGGTGTACAATAACA
>TCONS_00048928 geneID=XLOC_010005 scaffold_181:387984-400528(+) Intergenic
AAGAAATTTTGGTGGGAAGTGAGTGGGGTGCGCCGGCGCAGCGCCAGTGCCCACCACGCGCGGCCC
GCGCCCGGACTGTCAGTGCAGTGAATGTGAACCATAGACTACCACCGAAATATCCACTATTATTCCTCG
CGCGCGCCCAACAGTGACCCAGAACCTCAATACAAAGTGTACGTTTGAAAAATTATAAAAAAAAATAC
ACGACGGGATTTTGCTCTATTTAAGAATTTCCCCGTTGTTAAATAGTGCAAAAAAACTTTGTGTGTTTT
GGGATAAATGTAATTGGCATTCGCAATGGAGTGACGTGAGTTCGGACCGACAGTGACGAGTGCGGCA
GGACGCCCCGTGGACGCCGAGCCTTATATATTTTTTCTTTTATATTTTTTCTCTATCCTCCTTGTAAATAAT
CGAGTTATGTTTGTATAATTCAATTATTTATGCCAATCACATTAGTTCCTTATTTTTGCTAATCCAACGCTT
TTTATTTTTTAACTAACAGCATGTCTATATTTTATTGGCATTTTTTCATAACCTGTAGTCAGAATACCGATA
TTTGAGTAAAGTAGGGCTTATTTAATGAGCGGCCTAACGAGGCTCGGTTTGGGCCGTGACAGGTTTTTT
TACAGTTTTAACGGCGGCGGCCGCGGCGCGACACCAATATTTAAATACCTTTGTTTTTTCACGTTCAGA
GAAAAACTGAACATCAATTGGGAAAATTTTGATGAACTAAAAAGTTTTTTTGGTGTTGTAGCATAAAAT
AATTTAAAAAACAGCTCGGTGAATGTTGTAAATTCTTTTACATTTTATTGTCAGTAATTTATTTTAAGAC
AGTATTTTAGCTATCTTATTTTATTATTTTTATCTCTACTTACAATAATTAAACTCTTGGATCAGGTTTTACA
TGAGGCCGACTAACCAAAACGAATGTAGGTCACACAGAGTACCTATTCAAATTGTGTCCCACTTTCCG
CGTCTCTAACAAATTCCATGCGAAGTTGAATTTGACTAACAATCTAGAGTCTTCACTTGTTAAACAGAT
CCCGGTCGCGACTGAGCCGCTGTTTGTACATTGTTTCTAGTGTCCCTCCTTGCCGACTTGACCTCTTCT
TTCCGTGTCCGTGTCGAAGGTCCGCCCCCCCCGCGCTCGTTCCCGGGAACAGTTGACGATCGGGATAT

ATCGCTCGCTCTGGACTGATCATGTGCTATTTGTTTCGGATGTGACACTGACCGAGCGTCTGCTCTTTTT
ATTGGAACTTGACTCTTCTTGGCAGGTGGTGTGTGAATGGAGCACAGTTAGAGGACCACTGGTGTACA
ATAACA
>TCONS_00048787 geneID=XLOC_010023 scaffold_181:512304-514027(-) Intergenic
ATCCCTTTCGGCTTTTAAAACATCTGGTAGAGTTTACATTGTAATTATGTAATTTATTTATTGATGATAAC
GAAGCTTCTTTTCAATTTTCAGACCAAAGAGTTACTGTAACGCCCCCACATCGCATAGCCGCCAAAA
GTGCATTCATGCGCAGCGTGCATCGCCTCAATCTGAATGATGAAGAATCTTCTAAAGCCACTGACGGA
CAATGTAAACTCATGAAATATCACACCTACAACAAGTTAGAAAAGCATAATGAAGCACAATCCAGAAA
AAAGTTAAAATGTCTAATAAATAACTATTTAATGCTCAAAAAAATTGTTTTCATTGTATAAATATATAACT
TTTAGCCTAATTATGGATCTACTCAGAGTTGCTCAATCATTCGTTAGCTTATGAAAAGTTATAAAGAACA
ACAATCTTAAATCCATACCTACTCGAAGAAGAATTACTTTAAAAAACTAATGAATGATTAAGCGACTCG
ACGTAGAGAAGAAATAG
>TCONS_00048962 geneID=XLOC_010023 scaffold_181:512531-513901(-) Unclassified
ATAGCCGCCAAAAGGAATGCACATTCCTGCAGCGTGCATCGCCTCAACCTGAATGATGAAGAATCTTC
TAAAACCACGAGCAATACCAGAGAGTTACTGTACCGCCCCTCACATCGCATAGCCGCCAAAAGTGCAT
TCATGCGCAGCGTGCATCGCCTCAATCTGAATGATGAAGAATCTTCTAAAGCCACTGACGGACAATGT
AAACTCATGAAATATCACACCTACAACAAGTTAGAAAAGCATAATGAAGCACAATCCAGAAA
>TCONS_00048847 geneID=XLOC_010007 scaffold_181:528207-533162(+) Exonic overlap (-)
ATTTGCACTTTATCTAGGAACGGAAAAGGTTCCTGGAGTTGATGAGATGTAGAGAAATTTCACTGTTCC
CACTATGTCTATTTTAGGTAAATAGTAACATAAATATATATTTTTATTGCCCATTAGACTCAATTGATACTT
CTATATCATTAAATCATAATTAAATAAACATTAAGGGACTTTCAAGAAAATTTACAAATATTCACATGCA
CTCACCTCTGAAACTTAAGCAAAAAGTGACAACAAAATTTTTCACTTTAACTTTTTTCTTGCTCTTTTC
TAATAAATTGGCGGCAAAGTTTTGTTTCTTGTAAAGATTAACTTAAAAAAATGCCCTTCTGCGGTGGCT
ATAACAAGACAACATTACTTGACACTCTTTATTTGACATAGACACCAGCGCCCTCGTTAAACTGGGTAA
AAAAACAGTGGTTGTTCATGAAACACTTATATTTTTTTTATGCAAAAACCATAGACAATGTTTTAAAAG
AAAGATTTTTTTAAATTCATTCCACACTCTCGTCTAACATTATTAAAAAGAACTATTTAGGTATCTTTCAT
ATCGTGTTAGTAAAGTTAGTAATGAAATACGATTCTAATTTGAAATCCCACGAGCTATCTTTTAAATAAG
AATAATTTTATCGTATTGGTTTCGCTTTGTGCATGGTGTGCTTTAAGCTGCATACAGACCGGCCCAACGA
ACGCCCAACGATGGATATTTATCATACATAATAATACAGGGCAGGTCTGAGGTAAAAAACAGTGGTTGT
TCATGAAACACTTATATTTTTTAATGCAAAAACCATAGACAATGATTTAAAAGAAATACTTTTTAAGGTC
ATTCATTCCAGACTCTCGCTCGTCTAACATTATAATGATTTATTATTAAAAAGAACTATTTAGGCATCTTT
CATATCGTGTTAGTAAAGTTAGTAGTTAATGAAATACGATCCTAATTTGAAATCCTTAAAGAAGCGCAA
GAATCTGCCACGAGCTATCTTTTAAATAAGAACAATTTTATCGTATTGGTTTCGCTTAGTGCGTGGTTTG
CTTTAAGCTGCGTACAGACCGGCCCAACGAACGCCCAACGATGGATATTTATCATACATAATACAGGGC
AGATTGCAGCACCGAAGGTCAACGAAACCGCCGGTCTGTACGCAGCTTTTAGCAAACAAGTGTACCT
AACTAATATTACCATGCCACTTGGCTATTCTTATTTGCTTGTTATCAAAAAAATACCATATCGTGTGTGGTC
AGTATACATGTTATATTCCTCAAAATGATTGCCGAATTCGCCTATATTTTTGGTATTTAGCAAATACATCA
AACATCCAAAACAAATGCGAATAGCCAAGTGTGTGATTACGCGTATCAATAGCGACGCGCATTAAACG
TGATTACGCGTATTGAAACGTATAAATGCACATTTACGCGTATCGAATCGCGTTTCTTTAATACAACATA
ATTTTATTTCCCTTCATCATGATACACACTCATTGATGTATGTAATAGTGTGTTTATTATAATAATCCACTAT
ATAGCCACACTTATACTGAAACGGTTATACGTGATTACCTACATGCTTATGCGGATTGAAAAGTGTGTGA
TTACGCGTATCGAAGATTTGCCGTACAATAACTTTTTAAATAAAGAACATAG
>TCONS_00049100 geneID=XLOC_010064 scaffold_182:5939-7937(+) Unclassified
ACAGACCTTAATTTGTTATATTATTTAACAGTAAGTACCTACTAGATATTTGCCAAATTTTATAGCTGTTT

GTATTTGGAAATACATTTCATTGTAAGTAAATTATATTTCAGCTAGAAATAGCGTTTTTTTTCTGAATGGT
ACTGGTGAAACCATCCATGCCGTGTCGATGCTTTTGGTCTTTTACCAATTCAATAATAGTAGCTACAATG
TAACTTGTGAGAAAAATAATTATTAAGTATTACTTACCTACTTATTTGAATTTGACTCATTTCAACTAAGG
CGACTAGTTATAACTACTAGTGTTTAAGTAGTGTATTAAATACTCTTGCACCACAACTACCTCTAATACTA
TTTTTTTAATTTATAAAACTCTTCCATCCAAAGGTTGTCTGGTAGAGATTGCTATAAGCAATAACGCCTT
TTTGTACTGTTCTTATTTTTATTTTGTATAGTTTCTGTTTTTTTTGTGCAAATAAAGAGGATTGTATTGTAT
TGTATACTGTCACTAGTTCGAGATTTGGCTGTGGAGGTTGCTACAAATCGGGAAGCTTGGCGTGAGGT
CTGCCGGAGCCGTGCAACTGAGCTTCTCAAAATGAAGCAATTTCATCACGATTCACGTGTGAATCAAC
ACTAACGATCTCTAAAGATGTTTAAAAAAACATTATTTTTTTTGCTATTTGTGATCAAGATTTCACAGAT
CACGGGGACTTGAGACTCCAGGGCAGTCCAGGGTGAGGGGTTCAAACAAAATCGTGACATTCTTACA
GAATGACAGATACCAGCTGAAGTTATCTGATGCCCGAGAGAAGACCTCTCAGGCCGTGGCCCCTTACA
CGGTGGTTTCGAAGAGAATGAAGGAAATGGTGTCCTGAAACATGTTCAGCCTTTTTCAATGTGAGAAC
AATAACGACGATGCGCCGGCAATGGCAGGACCGTCCAGAGAACCACTTACAAGCCAATAAGAAGATC
ACACCCACTAAAAAGCCAATAAGAAGATCCAACATGGAATCCCATCACTTATAGCAGTCAGGAGAAGC
CATATTAGTTCTTAATATGTTTCTTTCTTTAGATTATTTTGCCTTATTGTTTCCTTTTGTGAATAATTTGTTT
CATATTATTATAGGACAGTGCGTTGGTAGTTTTAGTACCTAGTTTATTAAATGTGTAATAGAAATAAATAA
TTAGTAAG
>TCONS_00049136 geneID=XLOC_010076 scaffold_182:412472-414055(-) Exonic overlap (-)
GTATCGAGAGCCATTAGTCTTGCCGGAGTTTCCTTTATGCGTTCAGATGCCATAAAGATAGACCCTGGA
GCGCTCGGACTTGGCTGGAGGCAGTACTGTAACTGTGAACTTGGGAGAGTACTTGATTGTTGTGGGTC
CCCGCAACCTTGCTGACCATGTAGCAGGTCTGCCAACACACCTTTCTGGCGGTACATAAGCCACCATG
CTGTGACCGTCTTCGTCCAACTCCCGCCCCCCGACTACCCAAGTGGCCCACTCCTACATCAGTTCCGGT
ACCCGCTCTGGCATATTAAAAACTTTAATCTTTCGGCGGATCCCAACATTGGGAATACGGTCTCTTAGC
GAAATATCTCCATGGCTTTCCTTGTAACTCTGATTCGGTGCACATTCTCCCTGCTCAGCGTCCATCATAT
TTCGACAGCGTCGGTTAGTGTGAGCAAGGCATTTACGAAATATTATCGAATTACCTACACGATAAACTA
ACAAGCAACTTTCCGGTTAAATTACAAATTTTGAGATTCTATGCCGCAAACATCACAATCACAACGCTC
ATGATGTGGCATTTTTA
>TCONS_00049408 geneID=XLOC_010127 scaffold_183:114742-116169(-) Intergenic
ATTTATCACCCTCTTAGACTGTGAGCAACAGATCATCAATATTACTCAGGGATATTTATTTATGTTTCATA
TTTTACCAGTATTATAAATGAAACGTGAATCGAAATTTGAAAAATAAACTTCGTGAGGAAACCTAGATT
GCAGACGTGTATCATAAGTGCACTGTACCTACTCATTCAATAACGGCGATACGGCTCGCAACCTATAAC
GTGTCTACAAATGTACAGCGAAAAGCGGTTGCAGTTTTTTTGATATTTTCTGGTGGTATCACATGCATTT
ATATTATTTTTTCTACCTTAATTCTATCCAGATTATCTGAAAGTAAAAAAAAAATACTTACTAATCCTCTAT
CTACTGAAACCTTTAATAAAACTGAAACACATCAACAGCCGCTACACGGGTACGTAATCGACATGGAT
AAACTCGTTTAATGCACATTCCACCAATTATTTATGGCAAATCTGGATATAGTGACAACCCATGTGTGTC
GTGGTAGGTACGTTTATCTTCGTCGATGACGAACCCATGTACGAACAGCAGTTACTATGTCAAACAGAA
CTACAGACAAAGAACCTCCAAGAAAGATCCCCAAGGAGCGAATCATAATTCCCGGGCAAAAGAGAAT
GAACTATAAATGAAAGATTACGCGGAGCAAACGGTTAACCCCATCCTATATATTTAAAACGCTTCAATT
CTCTTGGGAGATTCCAGTTAACGCAGGTTAATGCTTCCTAGACGGTTCACTCTTGCTGGTTTATACCTG
GCCTGGCAAAATGCTAACGTGTATTCTACGCTGGATCTTTTGTAATGTTACGTAAACGGAACGGAACAG
ATATATGAATGGCAAGAAAACATACTATAAATATCGTTGTAAGCGACGTTTATATTTCAAAAACTCTTCA
CGAATAAAGACTTCATAATATACTTATGACTTTTTTATATGTTTTTGTTAGTATTTTCATTTTTTTCAATTTA
CCAAGCAATATACATAGTTAACACCCAGTTGCAGAAACATCCTTTAAGTTTTAAAAACATCATTAAACC
TTAAAGTCCAATTAAGTCGTTGCAGAAAAGCATTTTAATGTGACGTTTCATGAGACTTTTAAATTTATCT

AATGAAATGTCAAATTTACCCTAATGAAATGTCAATGTCGTTTTTTCTTTAATTTTGCGTTGCACAAAGC
CACATTAGTCACTAAAGTGACTTTATTTTGACGTTTGGTCCCTTAAAAAATACTTCAAATCTAATGACCC
AAAAAGCATCCATTATAACCAATTTCAAATTTATTTTCTTATAATAATTTGTGTATTTGAAACTATTTTAAT
CAGTGTAGTGTCTTAAACAGAATTGTAA
>TCONS_00049169 geneID=XLOC_010111 scaffold_183:167611-170660(+) Intergenic
ATGAGTTCTTGTTGTATAAAGCCGATTTTGAAGTGCTGAGTTTTGGATTGCCTAGAGCGTGGTGTTGAA
GACTTTGAGAAGACGTTGCGGTCGAGGACGTCACGCCGTCAGGAAAGGCCGTGTTACAAATATAACT
CCGAACCTCAGAATAATAACAGGAAGCAATCGTATTTACACCGCGACGACGTACTCTGTGGTCGGCAG
ATAAGCCATGGCGGGGAACGTCAAAAAGGAGATGTCAATGTCATTGTCAAGGAAGGAGGGAGTTGAT
TTGAATGGAGTCGTGCTCTTGTTGTATTTTCTGATTTAATTTGTTTGTTATTGATATAAATCCTCATGTTTT
CAAGTTGTATTAATGATTTATGGTAAAACGAACCTATTTCACGAATGAGTTTACTTTTTGTGTCACCTTT
TCCTTCTGGTTTTAAC
>TCONS_00049174 geneID=XLOC_010115 scaffold_183:487521-503067(+) Exonic overlap (+)
TGGAGGAACAGTTTTCTTCTGAGTGTGTTAGTGATTGATAAACACTACTAGACTAGGCTAGACTAGGCT
TTGTCACTGCGGTGGAAATATTCAAGCAAAGCACTGACATCCCAGGGTTCACCCGACGTAAGAATATT
TAAAAAGTCACTACGGCGATACGGCCCGCGACCTATCACGTGAGTACTAAATTTACAGCGAAAAGCGG
GTGGGTCGCTTGCGAGTCACCTCTGACTACTCCTTCGGGGACTACAGTCGAGTCGTGAGCATGTGTGT
GCATGTATAAAGTCACTTGGAAAGGCTCCTTGCGTAGATTATTCAGAGCTGCAACAATGTAATGTATGA
AGCCGTGGTTTTTGCACACGTCAGGTTGCGCACGCTCCGCTCGCCTACCGTCCACCGCCGCCGCCGGC
GCCGCCGCCGGCAGACACCGCCGCGGACTGGCGCGGGGATTACCAGCTGGGAGTCCCGTGTTCGAAT
AAAATGTGATTAATCTTAGTGAAAAACCTAAAGTGTATTTTAAATGTGTAAACGACTGACAAATGAAGA
GTTGTGGAAATGTTTTCGTGGCTTTGTGGAATATTGTGTGCAGTGTAAAATGCTTAAAATCCAATGTTAA
TACCTAATTGTGAAGTACTTATTGACATGATGCTAAAGATAACCTTGCCCGTCAAGTTGGGATTTGGGAT
GTCAGCAGACGTGTACGTGTATTACGAGTCAATAGAAAAGATAACTAATATTCAGAGATGGAAGCCCA
CCACGACCACGGTAGCCACCAAGCCCACCACGACCACGCGCCCACGGTAGCCACCACCCGCCCGCGG
CTGACGGCGAGTGCGCGCCCGGGCACCTCATGACGTTCCACCTGGGCGTGTGCCAGGAGATCCTGTT
CTCCGGCTGGGTGACCACC
>TCONS_00049234 geneID=XLOC_010115 scaffold_183:487521-503067(+) Exonic overlap (+)
TGGAGGAACAGTTTTCTTCTGAGTGTGTTAGTGATTGATAAACACTACTAGACTAGGCTAGACTAGGCT
TTGTCACTGCGGTGGAAATATTCAAGCAAAGCACTGACATCCCAGGGTTCACCCGACGTAAGAATATT
TAAAAAGTCACTACGGCGATACGGCCCGCGACCTATCACGTGAGTACTAAATTTACAGCGAAAAGCGG
GTGGGTCGCTTGCGAGTCACCTCTGACTACTCCTTCGGGGACTACAGTCGAGTCGTGAGCATGTGTGT
GCATGTATAAAGTCACTTGGAAAGGCTCCTTGCGTAGATTATTCAGAGCTGCAACAATGTAATGTATGA
AGCCGTGGTTTTTGCACACGTCAGGTTGCGCACGCTCCGCTCGCCTACCGTCCACCGCCGCCGCCGGC
GCCGCCGCCGGCAGACACCGCCGCGGACTGGCGCGGGGATTACCAGCTGGGAGTCCCGTGTTCGAAT
AAAATGTGATTAATCTTAGTGAAAAACCTAAAGTGTATTTTAAATGTGTAAACGACTGACAAATGAAGA
GTTGTGGAAATGTTTTCGTGGCTTTGTGGAATATTGTGTGCAGTGTAAAATGCTTAAAATCCAATGTTAA
TACCTAATTGTGAAGTACTTATTGACATGATGCTAAAGATAACCTTGCCCGTCAAGTTGGGATTTGGGAT
GTCAGCAGACGTGTACGTGTATTACGAGTCAATAGAAAAGATTTCAGCATTTGTGTATAAACGTCTCTT
GACTCTACAAAAGGCTCATTACAACTGATTCACGTACACAATCGATTATTATTTATAAGATTAATTTTATC
GTACCAATAATCATAGGATATTACTCTTATACGTCTCATTAATTGATAATCGATCGCATTGATCCGTGAAA
CTTCGCTGAAGCTATATCTTCGGCTGAAGTATCGGCCACTCATTCAGTAGCGAGCAGTGCGTGGATATA
TCAGCGTGGGACGCCCTCCGACAGCTCACAGATAACTAATATTCAGAGATGGAAGCCCACCACGACCA
CGGTAGCCACCAAGCCCACCACGACCACGCGCCCACGGTAGCCACCACCCGCCCGCGGCTGACGGCG

AGTGCGCGCCCGGGCACCTCATGACGTTCCACCTGGGCGTGTGCCAGGAGATCCTGTTCTCCGGCTGG
GTGACCACC
>TCONS_00049333 geneID=XLOC_010115 scaffold_183:490528-500741(+) Intergenic
CGGCCGCTGCGCGCGCACGTTGTTCCCGCGCTGTTTGCCTGACGTTTCACAACGCATTTACGAAGTTTA
TTACGTGCGCGACTGTTGAAATCATTTGTTTACAAAACAAACAACAGACAGAAAATCTTCAACATTATT
AACATAATCGGTGGTTTTCTTTTTGTTTTTAAAACTTGTGTGCTGTGTACATCTGTTCCTTTGCTGTGATA
TTTTTGAATAATTCGATAACTAATATTCAGAGATGGAAGCCCACCACGACCACGGTAGC
>TCONS_00049340 geneID=XLOC_010116 scaffold_183:561679-565091(+) Exonic overlap (+)
GGGCGACGCCGCCGCCGCCGCCGACCCGGATACCTGATTGACAGAGCCCTGCAAGGAGCCTCCGGAC
AAGGGCGCCCCCTCCCCGCGCCCCGCGCGCCCGCTCCCGCGCCCCGCGCTGCGCTGGCGTCGCCTGC
TCGTCAAGGTCAAGTAGACTCTACACTCTAGATCATAAGGTATTCAATTATAAGTCTTCAGAAAGTATA
GGTTTCCTCCTTCATGTATCGGTATCATATTCAGTTATTGGGCATATTATGCGAGTACATCACAAAATTGA
AAACAGGTTTTTCTTTTACCTTTGTTTTCAGTTATGGTTAGGTGTCTCCGACACTGCCAATTACAGTGCC
ACAATCTTATCTGTTCCGGTGACAGCTCACATAAATGTCTAATATTTCTTAATTTTATAACATTTTATAAG
TTTGCTCGTATTGTTAATTCGCTCTTGCGGTGTTAATAAACCCATCTAATCTTTTACAATATACAATTCATT
AGTAAGTAATGAGATATGGATCAATTTAGTTCGCTCTCACCGGAACAGATAAGTTTGTCGCACTGTACT
GTCATCAAGAAACAAAAGCACAGAAGTCACATTTTTTGTATTGTCAAAACTTTAATTTCTAATAGAAAA
AAATATTTACACCACCAAGCATACAACTCATTAGTGAATAATTAAAGAATTCCTTCTCTGAGATTGCCTA
ATGATGATTGGTGCCTTCAGAATCCTCTAAAATATGCGCCTAAATAGAAAGTAATGTTGTAATAATATGG
ATATAGTACTTAGTTATTCTATGATGGATATGCTCTCATATTAAGAACCACTCGCTTCCTAACTCTACACT
GCTACTCTATGCCCAGCCTAATAATAGTTAAATGATACCGATTGTAGTTGACACAGAAACATGCGATGCT
GCGATGTCTAATGTTTAGTTTATGACAAACATAGCTGTGAGTTTGGGAACAAATTAGGCTTACAGTAAT
TTTAACAGAGCCTGTAGGTCACCTTACAGTTACAATAATGTCATTTCAGCAAAATATATTATTTGTACCT
AATAGGAATTTAAAATATGAATATTAAAGTTTTCCTTAGGTTGTACAATGTTTACATTCCTAATAATAGTT
TCAGTGATTTCGTTCGAGGGTAGGAGAATATTATTTATTTTTATTACATTTTGTAATTTATTTTCCATTAAG
TTTTCGAATTTATATTGTAAGGGCGAGACGTCTATAATCATAAGTGTATTAAATTAGCCTACAGATTTTAT
GTCTAATTAAGTTAAAGAATAACAAATCTTATGATTCGTTTAATAACAAATGGAGTGGGTATTTAAATCA
ATGCAGTTTTATTCTAAGTTTATTATTTACTAATATGTAGTACCGAGTATGCTTGTGAAATGATGTTTGAG
TAATCTTCCTGTACTTCGATACTAGGTTCATAACCCTTGGCTTTAAAAGCATTTTTGAAAAGCATCGCAG
TGCTTGTAACCGTGGGACAAAATGTGCACAAAATTAGGTATAAAACTAAGTTCTAGTACGTAGGTATGT
ATGTCATTATTTAAACATTAATGTTCGATGTTATATTTAAGCTAATCATACGAGTATAATGATGTTGATTCT
GAAGGCAGAAATTCTAATTTTAACGCTGTCAAACTAAAAAATTTGCTAGCATAAAATGACATTTACGGA
AACAATCATAGAGTCGAATTTTAAACAAAGAAATTAAGGTTTTGACATCGCTAAATTTAAAATTTCTGC
CTTCAGAATCGATATCACTAACCAAACAAATTCTAACATGACAGTGCAAAATACATAAGAATTAAGAAT
TTAAACCATTCCTTTGAAACTTTTTGAGAGACAAGTGGTCGCTAGTAACTTATATATGCTTTTAATAATC
AACTAGATTCTAGATCATTCCAACGTAGCCTTTTCTACTAGTCTTTCTAGATTGTTAAAGCTCCCAGTAG
TTAGAATAACATCTGTTATTTATTTTCGCACTGGCAAAGAATTGTTTTATGTTTAAGTATAAACGTTATTT
TAATATGTAATCGTCACAATTAATAATTATTTATATTACATGCTTAAAAAGATTTCAAATTTTTAAAATTTA
TTAAGTATACCTATCTTGATTTTTTAATGAGTAGATGTTTTTTGCCAACTCATTTTAAACTGAATGTTTTG
TAGGAACCTATCGGGTCTATGTAGTAAATTAGGTAAAAGAAATAGTTTTTTTATGAGCTACGTACTTTTA
CGTAATATTAAACTATTTTTTTATTGAAATCAGTGCTTACAGACAAATAATACATGATATTATAGAACAGA
CACCTAGCGTATTCTTGAATGTATGTATATGAATGGCGATAAAATGAGCTACGTATCGGCCTAAAGCAAC
TCTAGTAGATAGACACAATATAATATATTTAAATCAGACTGACAAATAAAACAACTTGCTATTTTACCGA
TATTAGTAAAGTTTTTATTGTATGTATACAAAAATCACAAACTTTAACTAAGCTTCTTCATAAAATAGACT

TAATTATTATGATTTCGTGAAGTAACACGCTGACAAACTCTTTGTGCCAACATATTGTACCTATTTTGAA
CTATTTTCCACAACAACATATTATAATTGATTTTTTATTAATTTTAACTGCTAATCATATACATTTTACATAA
TTATAAACTGTGATGTCTATTGGTTTTACTGTTAATATTAAAACTTACAATGCAAGCTTCCATTTTCTGTG
ATCTTTTATATATTTTCTATACTTTATTTTTTATTTTTAAGTTTTTTC
>TCONS_00049240 geneID=XLOC_010119 scaffold_183:588502-590640(+) Exonic overlap (-)
GGCGGGGGCAGGAACAGGTCGATCTCGCGCGGCTCCGCGATGGGTAGGCAGAAACGGGATACCGGA
AGCCATTCGTTCAACATCGGGCAGTGTTGCTAGTATATGCAGAAAAATGAAACGACCACTAAGGAGCG
TTACAACATTATCTGATGCATTAAAATCGGTTCAGTCGTTGCAATTACCTAAACGATCACTATCCGTAGT
AGAAACAAAAGACACATGCGTTACTCGAATGTACCCATACTACGTAAGAAATTAGCTCTCGATAAAAA
ATGTTAAAACCCCGAAATTTATTAGACAATCAACTTAAGTTCAAGTGAACCTCCTCCAGGCGCTCGGC
GAGTACTGAGCGTCGGAGCTGAATGTAAACACGCACTGCCGCTCGCGCTGCCGGTCCTTTGACTTCCG
CACAAAAAACCGCCATTTAGGGGAGCGCCGGGCAGCGCGAGTGGCAATGGCAGCGGCATGAGAAGTA
TCGCGTTTACACACTGCCCTGCCCACTTCCCTGCCGCCTTCCCTGCCCACTTCGGCTGAATGTAAACGA
GGTATTACAGTTCTCCCATATTAATAATGCGCATGCAAATCTTCGCAAACTGTCGTATTCCCGGAAACAC
CCGAATCATTGAATCGTAAGAGCACTTGCATTTTGATCCTTGTTCGAAAGAAATAGTAGTCAATATCATG
TGACACATAATCGAAAACAATTTGGGCGGTCGGTGGCTGTCACCGATCAGTCAAGGGTCTCAAGGTCA
AGATCAATATTACTTTTGTGGAATTATAAAGAGCTGCAATTACACATCGTTCTTGTTATTTTTTTCAAATG
TGAATTTAATTTCAACTTTAATTATTTTTGACGATGCCATATTATGAGGCACATTTGAGAATAAAATATAC
GTCACATTGATAGACTTCACTTTGCCAATAAAGCCACACCCAAAAGACCAAGTTTGTAATTGTAATATT
ATTGTGAATGATCAGCGCTGTAAATACTTTTGAACTGAGATTTTAATGTAAACATTTTTATTTATGTGAAA
TAATCAGGGGTGTAAATACTTTTGAACTGAGATTTAAAAATTTTAATATAAACCTGTGTTTAAAATCCAA
TTCTCCTTATAATATAAACCTTGTGTTATTCAAACCTTCTTTCATCCATCTTTACATCAGCTTCAGTAAGA
CACTTGAAAAGTACCTACTGTAACATTTTCGAAGTAAATTTTAATATTATGGAATATTATGAATTATCGAA
TCAAATTTCGTTACTGATAAATCAATTATCTCTTTTAGCACCAATTACATAATTCTACTAAAATTTCATGA
AGAAAATGCACTTAACCACTCTAAATACATAATAGTTTTCTAACGCTCGGGAATACGACCGTTGCCGAA
CAATGACGAAGATTTCTATGTGCATTATCAATTTTGGGGAACTGTACATTACTAATTAGCGCAATATCATT
TTTTTAATGTAAACTTTTTGTAAGTTTGACACATCTTTCACCTAGCTGCCTACAAGTGTGGCCATATTTC
ACCATAATTCTAATTTGTGTACCTATCCAAAATAAAAAAATAACTCGAATATACTAAAAAAATCGAATTG
GTTAAAACGCTTCGTGCTACAAGCCAGCGGCCACTACCATAATCATCTGCCAGTGATCGCTAATCCTAA
TTAAACTTGAACCTAGTTAGAGATGGAGCCACTGGCGATCTCATCTAAAGAAATGTGAATTATACGATT
AGCCAATAAACTGTTTTGCGATACTAATGCCATCAAGTAGAGTTTCCTCTTTATCACCTTTTCATCCAAT
AGCCCTACCAATCAGTTGATCAAGTTCATGCGACGATCTCACCAACGCCATCTATTGCTTCGCCTCGGC
CCCACTAGCGCCATCTAGTACAGATTATCCGCGACGGGCGGCGTGGAACAAGCTCGCTCGGCCGTGGG
GGAGCCACTGTCGTTGACGAGGCGCTGGCGCTCGCGGAACATGGATACTCGGATGTTCTCCAACTGGT
GGTAGCGACACAGCTTGAGGACTGAGAAGACGGCTAGTCCCACCCATGTGGCGAACGCTCCCCACGC
GCCGAACTGTGG
>TCONS_00049609 geneID=XLOC_010176 scaffold_184:14329-15094(-) Splice junction overlap
CAGTTCCACAAACTTATCTGTTCCGGTGAGAGCGAACTAAATTGATCCATATCTCATTACGTACTAATGA
ATTATATATTGTTAAGGATCAGATGGGTTTATTAATACCGCAAGGGCGAATTACTACTATGGGCAATCTTA
AAATTTTATAGAATGAAGAAATATTACACATTTACGTGAACTGACACCGGAACAGATAAGTTTGTGGCA
CTATACACCCCAACCCTATAAATGCCAAAAAAAATCGAACCGCCACCAGTGAAAAGTTTGTTATCATTA
TCAAAATAACAGCGAGTTTAAATTACATTACATTTTGCAGTGTTTTACGCCTGATATCCTAAAAAAATGA
ATATGAGTGAACGCTCGTTGCATCCTTCTCCGGCTCAACTAAATGCTTTGATAGACTTCCTCGAGAGAC
ACCCACACTTGGCCAAGGGTATCGCGAAGACCCAACATGCCAGAGAGAAAAATAGACGCCAGTGGGA

TGATTTAAGCTTAACTTTAAACAGTATGGGTGGCACTTATAAATCTCCGAAGCAGTGGACCAAAGTTAG
TACATAATGTCTGTTATACTTGTCAGAATCACTTAACATTCCTGATGATGCCAAG
>TCONS_00049463 geneID=XLOC_010177 scaffold_184:31730-59443(-) Exonic overlap (+)
TTGATAGCCTTTGATCATCTTGCACTCAAATCTTCCATAGGTAATCAATAAAATCACCAAATGTACACAA
TGTACAGAGTTGATTAAAATCACGTTCAGTCAAATTCCTTGACAAACTCCCATTAAACAAGGTTTACTT
AAACTTGTTTGAGGTAACAGAGTGAATCTTGTGTCAACAACAAGAGAGCCTGGACTAGGCGGCGGCC
GCCAGGTCCTTCAACAGCCTGTCCACTATCTGGTTCTGATACTTGATGCACCTCTTCCTGGGCAGCAGT
TTCAGCAACGAGGCCACGTAGAGGCCGAATTGGTCGAGACTCTTGAACTCTTGATGTGGATGGTCTAT
GCCCTTGAATCTGTCCAATGAGGAAGCGTCGTGCTGCGTCGTGATTGGTTCGTTGTTGACAGGTGGTT
CGATCTTGCGCTTCTTATTGGCTGGCGGGGTTTCGGCTTGTGGAT
>TCONS_00049681 geneID=XLOC_010168 scaffold_184:284883-286372(+) Intergenic
TGCCGCAGTACTCCTGGTACCAGGCCGCTGACGCCAACCCTGGACTTTTGACGGTGTGGCCGGCAGT
GTAACAGCCTATGGACAAATACAGAGAAAAACTATACTAAAAATGTATGTTGACGATGTTATAACGTCA
AAATTAAAAGGCGCAAAGTTGAATTAAAAAAACTTATATGGCGTCTGATTTAACTTGTAATGGCACAAC
CATCTGTAATTTAAGAAACTATTTAATAAAAAAAACATTGTAAATTTTTATTGATTAAAATTATAAAGAAT
AGTGAATAATTGTGTATTATAGACGAAAATGGTATCTGAAATCGATAATGTGCTTAGTGAAATGATTTTA
TTTTATGCTAAATATTTTTATTCAATATTTTAGCTTTTACACGTAGTTTGCAAAAATATTGAAAAAAATATT
TGATGTTTGTATTTGTTTAAAAAATGTGAGGAAATTGTTACTAAATGAATAAACCAATCTTTTAGGCATA
TTGGTACTGTGCTCAAACGTAGTGCTTATTTTTGAAGCCTTTGGTTCCTTACGTTTTTGTGTATGTAGTT
TTATTAACTTAGGTAATTTAATTTCAACAAGAAGGGTGGTTTATTCATATTGGTATTAAAAGTCCTTGGT
CCTATTTACGAAACGGGCATAACTTCTGTTTTGTCAAAATAATTGTATCCTTTTTGTAAGTCTTAAGTCA
CGAAATTTTGTTTCAAATCCTA
>TCONS_00049473 geneID=XLOC_010186 scaffold_184:425087-426154(-) Intergenic
CTTCGGTCAGTGTTTTACTGTAGCTGTAAGCAAATTAAACGAGCCCGTGCCCTGTGATAGTCCTTTAAT
ACCATCAATGGATCTAAATAGGTTTAGAGAAGTGTAAAATGTAGCACCTAAGATGAAACCCATTCTGCA
GTACCTGAAGTCATAATATTTTGTATATCTCAATATTTTAGGCCCCTTGCTCAAAGCTAAAGAAAGGGAC
AATGCATAATGATAAGTTTATATTACGTGTCTGTGATACATCAGTTTCATAAATAAAATTTTAGATAGAGG
CTGATTTAAAATGTTTTATTTATACTTTTTTCATGTTAGTCAGAAAGTTTACCATTTGTTTAATGATCTGTA
CCGTTTACTAAATTAATAAATATGGAAAGATTATCGTTTCAGTTTTAGCACCGTGTGTGATTTTGAAACT
CATTTCATAGTTTTTCAGGTTCAAATTATAGTGGTTTTTTCTCCTTTCGGGACGGTTATGTTTTGTTTTGT
GCCTATGTTTGTGAATTAACTTATTGAGGGAACTCAATCTCTATACTTGTCATTTATTTTTATTTGCTTAC
ACTATATTTATTTACTACCTGCTGTTTTCTGTCTATTAAATTAATAACATTTCCATTAAATGTCTAAGAACA
CCTGATTAATGTTTTGCTTATCGAAGCATCTACTACAAACATTTGATTGTGTCGCAAAAGTGATTCTCTG
TTACTTTAGTGATTTTGCATGATTACCTTACCTTTTTGCGACATAATGAATTGCCGATTGTATAAAAAACT
AGCTAACTAAAGTAGGATAGTTGTTAATATAGAACGTAGATCATCAAAAAACTTACTGATTATTTAAATT
AAAAATAAACTTTTCATTGCTTGGTTTTGATTTATAAATAATATTTCAATTTTGA
>TCONS_00049521 geneID=XLOC_010173 scaffold_184:495584-496591(+) Intergenic
CAGGAAATACTACTGGAAGTAATGTTACCGGTGTAAAAAAAACTGCTGGCGGAAATGTAATCGGGAAT
ACATCAATTGAACGTGCCCAAGCAAACAAATCCGGATCTGAAATTACTATGGAAATAAAACAAGAGCC
ACCTGAAGATGCCTGTACCGAGGAGGTTGATTCTACGGGTTTTATAGTCTTCGGCTCACATCGATCTAT
CGAAGGCAAAAACAAAAAAACTTCGTAATGACAAAATAGTTTGTCTTCTCCATTTCCTTTTACTTTAAT
TACCATTTCACTCAATTAAGTAAAAGGTACTTTACCTTCAAGAATAAGGTAAAATTACTTAAGTTTGTTA
AGTTATACTTTGTAAAATTATATAGCAAGAGCCCTATGGCGTTTGATTTAGGCTTGGGAAATATAGTTAAT
TTTTCCTTGAATACAAAGAAATGACACTTAAAAGGTATGGATTTGACAGCTGTCTATATTTCGTTAATTT

AATAAACATACCTACCTAGCATAGAACAACGCGGACTCGGGTGTACCCTCGGGAAGCGTCATAGCGAT
CTTTCTTGATACAAAAAAGTCTGCCAA
>TCONS_00049745 geneID=XLOC_010219 scaffold_185:3760-44953(+) Exonic overlap (+)
TAGCGCTGCACCGAAACGCGTCGCGCGGAAAAACCGTTAAAAAAACTAGTGCTAAAAATAGTTGCTT
TTTTCCCAACCGTTTTTTCGAAACTGCTATGTCTAGTTCGATTACCGTTTTTCAATTTAGACACCGATATT
CGATAATGATAGCGATAATGCCGTTGGAATGTGATAGTGTAGGCGTTACTTGCATCTCTTTCGCACGGTA
GATTTTTTTTCGGAGTTTTTTTTTCGCTGAAGCCGAAAGTGCAGTGTTTTGTGCTAAGTGGTTGGTGAT
TTGGTTTTAATGAGTGTTTAAGATAAAAGCACCATGAAAGCGCCAGACAAACTACAAAACTGAATAAT
TAGTCAAATCTTAAAAACGATTTCAAGTGCAATACGTAAATATTACTTTTAAGCATAATTTATTGAGATTA
AAAATAAAATATCGTTACAAACGTTGTTAACTAGTGAAACTGGTGCTAATTAGTAAAAAATAAAAACA
AATCAAGTGCAAACGAAAAAACAATGGAAACCCGCAAACACGCCAAAATGTTAAACATTCCAAATTT
CAAAAACGAGTGAACAACCAAATTTAAAAACTTTGAGTTCCAAATTCAAAACGTCAGTCCTCGGAAC
ATGCGTTCAAAAGTGACTGCAAAACCCCAAAATGCGTGACACTCCAAACCAGTGAACGTTCAACTTA
AAAACATTCCGTTCCAAATTCCAAATTCAAACTCTCAGTCCTCAGAACATGCGCTCGCATGAGTGACG
TCTACGAGCACGGGTGTGAGATGTATACCGCGTGGTACTCCCGCGGGGAGGGGGGCATGCACCATCAC
CAGATGGGCCAGTTGTACCACCACGGACCGCCGATGATCAAGACTGAGGCCGGGGTGAATAGCGACT
GTATGATGGCAGTGGACTACGCGCCGCCTATTAAGGCGGGTGCGTCATCAGCGTTCGTGTGGAGCGGC
GGGGG
>TCONS_00049885 geneID=XLOC_010219 scaffold_185:10039-44950(+) Exonic overlap (+)
AGATAAAAGCACCATGAAAGCGCCAGACAAACTACAAAACTGAATAATTAGTCAAATCTTAAAAACGA
TTTCAAGTGCAATACGTAAATATTACTTTTAAGCATAATTTATTGAGATTAAAAATAAAATATCGTTACAA
ACGTTGTTAACTAGTGAAACTGGTGCTAATTAGTAAAAAATAAAAACAAATCAAGTGCAAACGAAAA
AACAATGGAAACCCGCAAACACGCCAAAATGTTAAACATTCCAAATTTCAAAAACGAGTGAACAACC
AAATTTAAAAACTTTGAGTTCCAAATTCAAAACGTCAGTCCTCGGAACATGCGTTCAAAAGTGACTGC
AAAACCCCAAAATGCGTGACACTCCAAACCAGTGAACGTTCAACTTAAAAACATTCCGTTCCAAATTC
CAAATTCAAACTCTCAGTCCTCAGAACATGCGCTCGCATGAGTGACGTCTACGAGCACGGGTGTGAGA
TGTATACCGCGTGGTACTCCCGCGGGGAGGGGGGCATGCACCATCACCAGATGGGCCAGTTGTACCAC
CACGGACCGCCGATGATCAAGACTGAGGCCGGGGTGAATAGCGACTGTATGATGGCAGTGGACTACG
CGCCGCCTATTAAGGCGGGTGCGTCATCAGCGTTCGTGTGGAGCGGCGG
>TCONS_00050044 geneID=XLOC_010225 scaffold_185:577705-583193(+) Splice junction overlap
TCAGATAGCGGAACAGATGGAGAGTGCGATAGCGAGCGCGGCGCTGGGCGCGGGGCTGGACGCGGC
CCGCGTGCGCCGCGCCATCGTGCGCCGCCTTAGGTACAGCGGAATCCCGTTCACGTCATCCGAAGCGC
TAATAGACGCGGTGTTGGATGAACAACTGAACGAGGAGGCCTGGAGCGTGACCCCGCCCGCGACGTT
GTATGCGAGGGATTTACTCATTGATACCATCCGACGGTACAA
>TCONS_00049903 geneID=XLOC_010238 scaffold_185:596475-631582(-) Exonic overlap (-)
TACAGAAATTCATTGGTACCTTTACGCTGCTGCTTCCCAAACTGTAAAGGGTCGTATAGATGGACTTCG
ACGAGGACAAGATAGTCTGCAATTTTCCTGCTTCATCATCAGCGGGGATGGATATTGACAAAACTTGAC
GAGGAATGTGAATGGAAGCGTCAGAATCGATCCAGTACACTGATGAACATGAAGGATATGTGGTGAGA
TCGCATTACCCAGATACAGGACGACTACACATTGCTGTCAATCTATTTAGTACGAACCTATGTACATAA
CTATTAGGACCTTTTTTTCGAGAGATCGATAAATGTTGCAAATCGGCCGAACTACTTACAATATCCAGTT
GTTATTTTACGTCGGG
>TCONS_00050015 geneID=XLOC_010238 scaffold_185:596509-635155(-) Exonic overlap (-)
CTTTGTGAATACGTTAATTTGATAAGTAAACAATCGATAGTGGCTTATCACACCACTACAGATTTTAGTG
CTACTGCCAAAGGAAAAAGTCGTAGTCGAGGCAATCAAAATTATTTGGCAAAGCTACCTTAACTAAGT

ATTAGCATAAAATAATCCAAATAAGAAAATGAAAATGGAAACTTATGAATTTATTGAATGAAGAAAAGT
GAAGAAAAGTTTAAATAGACAAAGACAAGACAGATGCACAATGTACGGCCATAGAGAAATTCATTGGT
ACCTTTACGCTGCTGCTTCCCAAACTGTAAAGGGTCGTATAGATGGACTTCGACGAGGACAAGATAGT
CTGCAATTTTCCTGCTTCATCATCAGCGGGGATGGATATTGACAAAACTTGACGAGGAATGTGAATGGA
AGCGTCAGAATCGATCCAGTACACTGATGAACATGAAGGATATGTGGTGAGATCGCATTACCCAGATAC
AGGACGACTACACATTGCTGTCAATCTATTTTAGTACGAACCTATGTACATAACTATTAGGACCTTTTTT
TCGAGAGATCGATAAATGTTGCAAATCGGCCGAAC
>TCONS_00049866 geneID=XLOC_010238 scaffold_185:596675-634753(-) Exonic overlap (-)
TTTTGAATAGATATCAGTTAGTAGGTAGATAGTTATTATGCACTTACCATTCCCTGCCTTGGCTGCTTCAT
TCCTCTCTTTTTCAGCAGTAGCATTTTCAGAAACTTTGCTTGTGTTTGCTTCCATAACCCGCAGTTATAA
ATTTCAATTTCAGGTTAGGTATGATTTAACCTGTTTTCCAACAATGCCGTTGTCAATACATGCTGAAAGT
TGGTTTTGCGATGATTTATGCCATTTACAATATTCACATATTAATATAATCATTTTCACTTTATCATTATTAT
TTTTATTTCAGAAATAAGAAAATGAAAATGGAAACTTATGAATTTATTGAATGAAGAAAAGTGAAGAA
AAGTTTAAATAGACAAAGACAAGACAGATGCACAATGTACGGCCATAGAGAAATTCATTGGTACCTTT
ACGCTGCTGCTTCCCAAACTGTAAAGGGTCGTATAGATGGACTTCGACGAGGACAAGATAGTCTGCAA
TTTTCCTGCTTCATCATCAGCGGGGATGGATATTGACAAAACTTGACGAGGAATGTGAATGGAAGCGT
CAG
>TCONS_00049778 geneID=XLOC_010227 scaffold_185:632712-636321(+) Intergenic
CCAAAGCACCGCGCACGGTCACTCACTAGTCGCGCGTCTACGTTCTACAGCCTGGCAAAAAATTGTAG
AAAACAAAAAAGGACGAATTTGGAAATTTAGTTCATCATTGCCTTGTACCTACCTACTTTTTAAGTACT
TATGGTTAAAATAATATGTCGATTAAGAATCAATAGTCATAATACTTATATGAAAAATTAAGGTATTAATAT
TTTTTATTGAGTTTCCATTTCAGTTTTCTAATCTTTTTTGCCAGGCTGTACGTTCTTTAAGTGTTCTATAAT
AAAGGAAACTAAGTTCTGAGTGCTTGGAAACATTGTGAAGCATGTATTGACAACGGCATTGTTGGAAA
ACAGGTTAAATCATACCTAACCTGAAATTGAAATTTATAACTGCGGGTTATGGAAGCAAACACAAGCA
AAGTTTCTGAAAATGCTACTGCTGAAAAAGAGAGGAATGAAGCAGCCAAGGCAGGGAATGAAAGAG
ATAGCGAGCTCTACTCGCGGTCGTCCTCACGCGCTGTCACTTCACATTGCGATTGCGCTGGGCGGCGC
GGGGCGCGTTCCCGAATCGTCGAGTCGAGAAGTCACACAGACTGCGCTAGTGGCTGCGAGTTTCGCG
CTCTCGACGGAAGTGTGTGGTGCGTGCTTGTTCTGAGTGTCAGTGAGAGTGAGAACAGCTGATATTGT
TATCAATTTGGATGTGGCCGTATAATATTACACAGTGAAGCTCTTTATGAATTCCTTGGATTGCTCTTTTC
GTGCTGAGGACAAATTGAAAGGTAATTTTCCTACCTATTTGGAAAGAAACGATCACACATACCTTTATG
ACGTGTATGTATCTAATTTGGAACGAGGGCGATTTCGATTTAGTCAAGAGCCTTGAGCTTATTGTCTTTG
TATTTATATGGATGTTAATCCTAGTACATAATAGTTAAAGCCAATGTAATTAAATTAACACGTACTTATCTC
CAATAAACCATCGTTTTACAACTAACCTCCTGTTATTCTATTTCAAACATAACCTCAAAATGTCTAATTTA
TTGACTGTATTCGGGAAGCATTTTACTTTTTGTTTAC
>TCONS_00049837 geneID=XLOC_010227 scaffold_185:632720-634706(+) Intergenic
CCGCGCACGGTCACTCACTAGTCGCGCGTCTACGTTCTACAGCCTGGCAAAAAATTGTAGAAAACAAA
AAAGGACGAATTTGGAAATTTAGTTCATCATTGCCTTTTTTCTAATCTTTTTTGCCAGGCTGTACGTTCT
TTAAGTGTTCTATAATAAAGGAAACTAAGTTCTGAGTGCTTGGAAACATTGTGAAGCATGTATTGACAA
CGGCATTGTTGGAAAACAGGTTAAATCATACCTAACCTGAAATTGAAATTTATAACTGCGGGTTATGGA
AGCAAACACAAGCAAAGTTTCTGAAAATGCTACTGCTGAAAAAGAGAGGAATGAAGCAGCCAAGGC
AGGGAATG
>TCONS_00049922 geneID=XLOC_010227 scaffold_185:633918-634707(+) Intergenic
CAAAAAAGGACGAATTTGGAAATTTAGTTCATCATTGCCTTTTTCCATTTCAGTTTTCTAATCTTTTTTG
CCAGGCTGTACGTTCTTTAAGTGTTCTATAATAAAGGAAACTAAGTTCTGAGTGCTTGGAAACATTGTG

AAGCATGTATTGACAACGGCATTGTTGGAAAACAGGTTAAATCATACCTAACCTGAAATTGAAATTTAT
AACTGCGGGTTATGGAAGCAAACACAAGCAAAGTTTCTGAAAATGCTACTGCTGAAAAAGAGAGGAA
TGAAGCAGCCAAGGCAGGGAATGG

>TCONS_00049894 geneID=XLOC_010227 scaffold_185:633925-634707(+) Unclassified
GGACGAATTTGGAAATTTAGTTCATCATTGCCTTGTACCTACCTACTTTTTAAGTACTTATGGTTAAAAT
AATATGTCGATTAAGAATCAATAGTCATAATACTTATATGAAAAATTAAGGTATTAATATTTTTTATTGAGT
TTCCATTTCAGTTTTCTAATCTTTTTTGCCAGGCTGTACGTTCTTTAAGTGTTCTATAATAAAGGAAACT
AAGTTCTGAGTGCTTGGAAACATTGTGAAGCATGTATTGACAACGGCATTGTTGGAAAACAGGTTAAA
TCATACCTAACCTGAAATTGAAATTTATAACTGCGGGTTATGGAAGCAAACACAAGCAAAGTTTCTGA
AAATGCTACTGCTGAAAAAGAGAGGAATGAAGCAGCCAAGGCAGGGAATGG

>TCONS_00049957 geneID=XLOC_010227 scaffold_185:633928-634708(+) Intergenic
CGAATTTGGAAATTTAGTTCATCATTGCCTTGCTGTACGTTCTTTAAGTGTTCTATAATAAAGGAAACTA
AGTTCTGAGTGCTTGGAAACATTGTGAAGTGCTTTGGTTTTATATCTAGTTCTAATAACTGATTCAATAG
ATAATTATTTTAAGTATTTCAGTGAGTAAACAAAAACTTACTGACCTCTATGGCCGTACATTGTGCATCT
GTCTTGTCTTTGTCTATTTAAACTTTTCTTCACTTTTCTTCATTCAATAAATTCATAAGTTTCCATTTTCAT
TTTCTTATTTCTGAAATAAAAATAATAATGATAAAGTGAAAATGATTATATTAATATGTGAATATTGTAAAT
GGCATAAATCATCGCAAAACCAACTTTCAGCATGTATTGACAACGGCATTGTTGGAAAACAGGTTAAA
TCATACCTAACCTGAAATTGAAATTTATAACTGCGGGTTATGGAAGCAAACACAAGCAAAGTTTCTGA
AAATGCTACTGCTGAAAAAGAGAGGAATGAAGCAGCCAAGGCAGGGAATGGT

>TCONS_00049779 geneID=XLOC_010227 scaffold_185:634430-636321(+) Intergenic
CATAAGTTTCCATTTTCATTTTCTTATTTCTGAAATAAAAATAATAATGATAAAGTGAAAATGATTATATT
AATATGTGAATATTGTAAATGGCATAAATCATCGCAAAACCAACTTTCAGCATGTATTGACAACGGCATT
GTTGGAAAACAGGTTAAATCATACCTAACCTGAAATTGAAATTTATAACTGCGGGTTATGGAAGCAAAC
ACAAGCAAAGTTTCTGAAAATGCTACTGCTGAAAAAGAGAGGAATGAAGCAGCCAAGGCAGGGAAT
GAAAGAGATAGCGAGCTCTACTCGCGGTCGTCCTCACGCGCTGTCACTTCACATTGCGATTGCGCTGG
GCGGCGCGGGGCGCGTTCCCGAATCGTCGAGTCGAGAAGTCACACAGACTGCGCTAGTGGCTGCGAG
TTTCGCGCTCTCGACGGAAGTGTGTGGTGCGTGCTTGTTCTGAGTGTCAGTGAGAGTGAGAACAGCT
GATATTGTTATCAATTTGGATGTGGCCGTATAATATTACACAGTGAAGCTCTTTATGAATTCCTTGGATTG
CTCTTTTCGTGCTGAGGACAAATTGAAAGGTAATTTTCCTACCTATTTGGAAAGAAACGATCACACATA
CCTTTATGACGTGTATGTATCTAATTTGGAACGAGGGCGATTTCGATTTAGTCAAGAGCCTTGAGCTTAT
TGTCTTTGTATTTATATGGATGTTAATCCTAGTACATAATAGTTAAAGCCAATGTAATTAAATTAACACGT
ACTTATCTCCAATAAACCATCGTTTTACAACTAACCTCCTGTTATTCTATTTCAAACATAACCTCAAAAT
GTCTAATTTATTGACTGTATTCGGGAAGCATTTTACTTTTTGTTTAC

>TCONS_00049923 geneID=XLOC_010227 scaffold_185:635365-639957(+) Intergenic
ACCTACTTACTTACCTACCTACTACCTTTCATCACAATGATAAACCCTAGTGTTAGGTGACAAAGTGAC
ACGCTAAGGAGAAACCGTGACACCCATAATACCCAGTATTACAGATGGGGTTGCATAATAAAAAATATT
ATTTACTTATTGTAGATAAACAACCACCAAATTCATACAAGACATGAATACACATGAATAGGTATAAATA
TGATGGCTTGTCATAATCTAATCACCAAACCATACCAACGGAGTGCCATATAAAAAAGTCTACTTATTTA
GTAGAATAATTTAAAAAATCTACCACACTTGGCACTGCGAACCGGAGCTACTATAGCAATATGCATTTTT
AACGTGCTAGAAAGAGATAGCGAGCTCTACTCGCGGTCGTCCTCACGCGCTGTCACTTCACATTGCGA
TTGCGCTGGGCGGCGCGGGGCGCGTTCCCGAATCGTCGAGTCGAGAAGTCACACAGACTGCGCTAGT
GGCTGCGAGTTTCGCGCTCTCGACGGAAGTGTGTGGTGCGTGCTTGTTCTGAGTGTCAGTGAGAGTG
AGAACAGCTGATATTGTTATCAATTTGGATGTGGCCGTATAATATTACACAGTGAAGCTCTTTATGAATT
CCTTGGATTGCTCTTTTCGTGCTGAGGACAAATTGAAAGTTTTTCATCAATTTGTGCATAGAACAATGT

GTATTAGCAGTGTGACGTGAACTTTAGTGTTAGTGATTGTAGTGAAGTCAAAATGCCTCTCCTAATCGA
CAGAAACCACTTAAATGGTAAATATCGATCGGTGTATTGTTTGGCAGTGCGGCCATTGCCTTGATGCGT
TCAGTGTTTTGTTACAATTTCATACAGTTAGGTTCGATTTTGGTTAAATTCCAGCAGTTACTGATTATCA
ACATGAAGAAATCTTACAAAAATCATAAACACACGTATCAAGATTAGCTATGTACTTACATACCTAACT
GTAAGAAATATTAAGTCGTACCTATTACTACATCATTCGTATAATACTTATTATTGCAGTCACGTTATTCA
AAGTATCTATACTTTTGGATCCCTTAAAGGAATGTTATATTTTTGTTGTAAAAAAAATTGATTGCTCAGTA
CGTTGGGTGCATGCGCACTAGTCCCCGAGTGAAGGGAGGCGCGGGGCGCGGGGCG
>TCONS_00049958 geneID=XLOC_010227 scaffold_185:635758-637118(+) Intergenic
CGCGCTGTCACTTCACATTGCGATTGCGCTGGGCGGCGCGGGGCGCGTTCCCGAATCGTCGAGTCGAG
AAGTCACACAGACTGCGCTAGTGGCTGCGAGTTTCGCGCTCTCGACGGAAGTGTGTGGTGCGTGCTT
GTTCTGAGTGTCAGTGAGAGTGAGAACAGCTGATATTGTTATCAATTTGGATGTGGCCGTATAATATTAC
ACAGTGAAGCTCTTTATGAATTCCTTGGATTGCTCTTTTCGTGCTGAGGACAAATTGAAAGAAACATGT
TTGGCAGTCGGCCTTGTCAACCTACTGGGGCCTCTTGCTACACAATGTTATGGCATGACCTCACTATTT
GGCACTAGTACTTACTATGGATTCTGTAACACCATGTTATTACCATGGTAAGTATATTTTCATACTTAAAT
ATGACTGTAATGTAAATAACACTTCTACTAAAAAGAGTTTCTATAAAGTCTTAAACCATTTTTAAATCAT
AAAATGATACTTGTGGCGCCATCTATATGAATGTTTTCGCAGCTGCAGCTCTG
>TCONS_00049970 geneID=XLOC_010240 scaffold_185:660697-661695(-) Intergenic
TGAGAATGTACCCATGTTTGATTTAAAAAAATCCTGTACCTCCTGCGGGTTGACTGGCAGAAAATAGCT
TTTAGCATTAAGTCCACCCTTTGTACTTTTATTTGTAATATTTGAACAATAAAGAATTAAATAATAATAAT
AAATAAAGGCCCTCCACCTGCTTACGGTAGACATGCTTAAATCCTACATTTTTATATCACAGCATATTATT
GGCAGGCATTCATAGTAGGTAA
>TCONS_00049789 geneID=XLOC_010229 scaffold_185:668030-669139(+) Intergenic
CGCCTGCTCATACAGGAGTCATGAAGATGTTAAGTGCCTAATACAAGAACCTCAATGTGCTACGTATTG
GAATGAAATTCCTTGTTGTTCCAATGTTAATATAAGTGTTAAAGAAGACGGGCTAGACCTGCCCACAGG
TGTAGAGAAACGCAACCAACTCTACTTCAGGCGGTGCGATGTTTTGCGCAGTTCTTAGAGAACATAGT
AATACCTCAAAGGCAACTGTAGTATTTTACCTAATTTAATTACCTATAATTGTGATTAATAAGTATTCCTA
ATAACGTATTTTTGGTGCATATTCAATTATTATGTAAATTACTGTCGGGGATTTAGTTTGTGGTTGTGAAT
AATTATGGTACTTACTTTCTTGTAGTGGTCTAAGTAAATCTAATTTACTGTTTACATACTTTTAGTCATGT
AGTTCATAGTAATTTTGAAAGAATAATATGTTTAGCTTATACAGTTGAATACTGACTATGTACTAAATATG
TATCTATCTACCTATGTTATTAATTCCATGTAGTCACACAGAAACTAAATTCCAATATATACGTCTATTGTA
TACTCAATTATAATTATCTAGTATCTTCCTACATTACCTACATGCCTATGTTATATTACCTATAC
>TCONS_00049897 geneID=XLOC_010231 scaffold_185:771349-774154(+) Unclassified
GACCTGGACAAGGAGATGCTGGGCATCAACGTGCCGACGGACCAGCTGCCAGACGTGCCGTCCGCCG
AGCCCGCCGCGCCGCGCCCCGCCAAGGCGAAACCTGCTAGCGAAGACGACGATGATCTGGCTAAACT
GCAATCGTGGGCGACATAAATGTAAGTGTTGGAAGCGAAACCGAATACATTTGTATAGTCTTCGTTGCA
CATCCAACCACTTAGCTATTCTAAGGCTGCATCCCTTTAGGCAAACAGTATTTTCCTTAATAGTCTATGG
TAATTTGCGTAGAGTGAAAGAACAACTAGCTAATAAAAATAAGGAAGGCCGAAGACAGTGTTGTGGC
GCTTCTTGGGATTGGTCTATGTCCAACATTGGACGATAACAGCTTTATGACTTTAATTTATGAGGTACTG
GCTATGGTAGACCTATTTACGGGCGCATCGCTTTGCGCTTTTAGGAGTTTCCCTCGGGAATTCCGGGAT
GAAAAGTACATAAAAACCTTCCTTTGAAATCACCATAAAAAACTAACTCCGTTGCGTAGTTTGCTTTAT
ACTATGTAGTGATTTGAAAAGCTCAGTGTTTGGATGTGTAACCGTTGTATGGTTTGTGATAATTTGTACG
ATTGTGTAATATTTGAATATGCAATTATGTACATTGATTTTATTGGACTAAAATTAAGATTTTTGTTGGAG
GTTTATTTTGTTGGCTGAATATTCAAATATTGCGTAATCTTACCATCTTTCGGATATTGTAATGTATAGGA
ACAGAATTGAAATCTAAATCTGAATAAAAAATATTATTTTCCATATTTAAAAAATCAGAACCATAACTTT

TTGTTACTTTCGCTGAGTTGCACAAAGGAACTTTAATCTAACGTCGACCTTAAATGTACTGCTGCCTTG
CTTTGTAGTATACGGATAGAAAGAGGTATACATAGATTTAAAACTTCTTTTTGGCAACTCAGCATTAGAA
TCTCACAGACTGTTACCATACAATATTCTTCTCCAAATGTTCATTCAACGGGAAATATAATGTTAGTAAT
TTTAATAAGGTTTTTTATGTTTTCATTTACACTATTCAGATTTAGATTTATAAAATATTGTCCATTTGGTGC
AGTCGGTCGCGATAGTAATATTATCAAAAATTGTTCATCAATCACACTTATGTATTCAGTAAGTACCTAA
CGTAGCTTTTGCCGACTGTTAGGAGGTTTGGTTTAAAAACTGGTGTAGGATAGGAATTCACATATTGGC
GTTTAAAAGCACCAAAAGTCTTGCAACTTTTAAACCATCTTCAAAATGAAGTTACGGGAAGGGAAGTT
ACGTATGTTTCCCTTTTTCACATCAGTTTTAAGTCAAACCTTCGTAGGATAATGTATTCATGTTAAGATTG
TAATCGGAAGTCTTTTTTCTGCCTTAATAATCACAATTATACAATTTGTAGTCAAAATATTGATGGTGTTC
TAAGAGGCAGGGGAAAATTGTTTATATGTTGTATATATCTTGCTATTATTTATATTTTTCTTATAATTCTAAT
AGTTTATGCAGGTATGTTTACAATAAATCAATTTATCAAAGTGTTGTGTCCCGATAATTTGTTGTGTAAA
ACTTTAAATAGGTTAAGTGCCTGTTTTACAATGTCTGTATAATGGTTAAACCTGTTGGATAAAATACATG
CTGTCACTCTCGGTAATTATGTTTTAGACAGTGACAGTATGTCTTCTATCCCACAGTTAGTCCTTACCCA
GACATTGTGAAACAGGCCCTAAACATCATACATAAAATATTTGAATGTCAAAAAGATTTTATTTTTTTAT
CAGTTTCGTTACGTTTGATTGTAGCGTGTCTGACAAAGTTTAGTAAAATGCATACTTCTTCCGAAAATT
CTTAATAGTATATAGTTGTCGTCTATGGAGCACCATATAAATCTGTTTCACTATACACATATACAGAATGT
TGCAAAAAAGGTTTAGGGGCCCAGGCTACATTAATAATATACAAACTTATATCAGGGGCTCATTGTAAAT
ATTTTATGATTTATGGTCTGGAAACTATCACGTTGTCAGATATGCTACAACCTCTTTTTGTTGGTAAGGTT
AGGATAAGCTCAAAAAGAAAGTTTTTTGTGCAAATTATTAATATTATTCTAGATAGATTCGCTTTAGCGC
CTATCCTAATGTAAACTAATAACATTTTCTAAGCGGTAA
>TCONS_00050496 geneID=XLOC_010295 scaffold_186:211530-216661(+) Unclassified
GGCGTTTGTTAAAAAAAAAAAAAAAGTTCCTTTTTGGAGAAATAGATGGCGTTGTCTGGGGTTGTACCA
ACTTTCAAACCCTCAGAACACACTCAGATCACAATATGTTATTCTGTGAGGCTAACGAAATGTGAAAG
TGACGTAGGTAGGTAGAACATTGACAAAAGAATAAAGAGGACTTGGCATAAAAATCGGTACTTAAAAA
TATATCGTCGTCTCTTTTTAACTTATTGGTGTTATCGTACGTAAAAAAGGACAACAGTAGTTTTGTCTTT
GCCTAAAATTACAACATTGCGATCGGTCGCCATGTTGATTGACCAAGATTGACATCCAAACACAAGGT
ACTTCAGCTGTCAAACAATTTGTTTGTTTACATTTTTCAAGAAAAAAGTCGCCATTTTAATTGACACCA
AAGTTTAGGTAAGCGCATTTTTTTCGTTGATATGGCATGTCCTCTCTTTATTCTTTTGTCAATAATAATAA
AAATAATACTACAATTCTACCTGAATTGTAGTATTATTTTCTCTATGATGTCGATGTCACTGTTGCTTCAG
CGTTCAGTGCGATTGTGCGTACAGCCGTTCTTTTCAAGTTTTCTCATCAGTTCTCGCACGCAGCACCCG
TTCAAACGGCGCGCATAGAGAAAAGAACACTACGTGACGGCGCGGGTAGAACTATTCACAGAATACTT
AGCACTATCCCAAGCCACATGCAGTCTGAGCCTCGGAAGGATGGCTCGCGCTACCACCCGACATTTAA
TCACAACTAGTGAGTTCTTATTCTGAGTTTAGTACTCTTTGCTCTCTATGTAATTTTTGACAGTTGGTAC
ACTGCGTTTTCACGCCATCTATCGGCTTACCCAAAAGCTCAACAGACAGCTGTCAAGGAAAACGGCTC
ATTTAGTAAATGTATTGAGCTTGGTATTGAGCTATTCAAACTATTTCAGAGAATCGAGAACTTTTTCAGT
GTCCATTACTATTTGATGAAATTTGGGATCCAAAAGACACAATTTCACAAAGTTGGTAAAATCTGTTGG
CATCGTAGACGTGTTTTTATGATCTGCAACAAATAAAATACAGGGCCATATTGGACAAGCTGGTGCATT
TCAGCTGTAGACAGAAAAAAAATTACAGGATTTTCCACATGATTCGAGGACGAGGGTGGGGGACAAAA
CAAATCACAATGTTACCCTAAAACAAGTTTTATTAACTTACACTTACAAAGCTATAAATTATAAATATTTA
CAGAGATTTCAAAAATATACTAAAATAACATTTGAATAATGAAATTGATGAACATACACAAAAGGCAAA
TGTTTCAGGCTGGTCAAGTTGTACTGCAAACGAAATAAAATGTTTCACTGAATAAGCAACATTTTGACT
CTTTCATCTTATGTTAATTTAATAATACTCAATATACATAGGATCATCATCATCTAATTGCTCCAAGCTACA
CATATCGCATATTTCTTACATATAAAATGAACCCTATATCACCAGGAATAAGGCACTGTAGTTTACAGTCT
GATAGTTGATGGTGTTTTTCAGAAGGTATCAGTATTATTTACATAAATAACTACTTGGGAAAGATTATTAA

TGTCTGTGACCAAACTGATCCTTAAATTACACTGTAATTAATAATTGATAATCATCCAGATTTTAACTTAG
TTAAGAAAGAGTTGATGCCTCGCTGATTCTTCTTGGCCTCTGGCGTTTTGGCTTCAACAACTTTCGCTT
GTTTAGTCTCTTTCTTCTCAGGTTTAGCTTTCCTCTTTTTCTCGTTTTTGGGACTCTTTTCTTCCCCCTCT
GGGGCTTTCTTTTCCGGTTTTTTCTTTTTGTTCTTTTTCTTTTCTGTTTCCTCGGCTACCGGAGCAGGTT
GCAACAAAGGAGTTACGTCATCCACGATCATTGCTAGTTTCTGGAAGTAGTTCCAGAGAGCATCCACA
CACTTTTCAGGGCGGCCG
>TCONS_00050548 geneID=XLOC_010295 scaffold_186:212414-216006(+) Intergenic
GGAAAACGGCTCATTTAGTAAATGTATTGAGCTTGGTATTGAGCTATTCAAACTATTTCAGAGAATCGA
GAACTTTTTCAGTGTCCATTACTATTTGATGAAATTTGGGATCCAAAAGACACAATTTCACAAAGTTGG
TAAAATCTGTTGGCATCGTAGACGTGTTTTTATGATCTGCAACAAATAAAATACAGGGCCATATTGGAC
AAGCTGGTGCATTTCAGCTGTAGACAGAAAAAAATTACAGGATTTTCCACATGATTCGAGGACGAGGA
GATTTCAAAAATATACTAAAATAACATTTGAATAATGAAATTGATGAACATACACAAAAG
>TCONS_00050497 geneID=XLOC_010295 scaffold_186:214399-216661(+) Exonic overlap (-)
GTAAAGTTCTTGGATCATTTAGATTGTGACTCCGAACAGGACAAGCTGGTGCATTTCAGCTGTAGACA
GAAAAAAATTACAGGATTTTCCACATGATTCGAGGACGAGGGTGGGGGACAAAACAAATCACAATGT
TACCCTAAAACAAGTTTTATTAACTTACACTTACAAAGCTATAAATTATAAATATTTACAGAGATTTCAA
AAATATACTAAAATAACATTTGAATAATGAAATTGATGAACATACACAAAAGGCAAATGTTTCAGGCTG
GTCAAGTTGTACTGCAAACGAAATAAAATGTTTCACTGAATAAGCAACATTTTGACTCTTTCATCTTAT
GTTAATTTAATAATACTCAATATACATAGGATCATCATCATCTAATTGCTCCAAGCTACACATATCGCATAT
TTCTTACATATAAAATGAACCCTATATCACCAGGAATAAGGCACTGTAGTTTACAGTCTGATAGTTGATG
GTGTTTTTCAGAAGGTATCAGTATTATTTACATAAATAACTACTTGGGAAAGATTATTAATGTCTGTGAC
CAAACTGATCCTTAAATTACACTGTAATTAATAATTGATAATCATCCAGATTTTAACTTAGTTAAGAAAG
AGTTGATGCCTCGCTGATTCTTCTTGGCCTCTGGCGTTTTGGCTTCAACAACTTTCGCTTGTTTAGTCTC
TTTCTTCTCAGGTTTAGCTTTCCTCTTTTTCTCGTTTTTGGGACTCTTTTCTTCCCCCTCTGGGGCTTTCT
TTTCCGGTTTTTTCTTTTTGTTCTTTTTCTTTTCTGTTTCCTCGGCTACCGGAGCAGGTTGCAACAAAGG
AGTTACGTCATCCACGATCATTGCTAGTTTCTGGAAGTAGTTCCAGAGAGCATCCACACACTTTTCAGG
GCGGCCG
>TCONS_00050416 geneID=XLOC_010316 scaffold_186:410277-424178(-) Exonic overlap (+)
CTTCGATAGCGGTTGGACGCGTTGCGTGCTCTTTGCTCGAAATAAAAGCGCGCGGAAAAAGTTGCTAG
GCGGGAAAGTTTTTTTTTTTTTAAATCACTGTAAGGTCTGAAAGGCCAAGGACTATATTGATTCCTTTG
ATTGACCTCAAAAAACTATTTGGTGGAAAGTGCTTAAATTTTTAATACTTTAGCCGGCTTTTAAGTGCTC
GTGTTTAAACTCTTGTGATAAGGATTAAACTAAACATTATAGTGGAGGTGTGAACTCATAGGTTTAAGA
AATTCTTTGTGTGACTGTTTCGTGTTGTTGGCCTGCTGAAACCATTTGGATTATCTAAACTTTTGATAAC
CCCTTGAAATGTCCCGGCATAGTTACAAAAACAAAATGGCGGGGCGGTGAAACAAGATGGCTGCCAC
GATGGCGTCGCCCTCTGGCGAGCTGCGGCCGCAACACTCGAGGGACAACTCCAGGGACTCTGGCATC
TTCCACACCACCAAGGGACTGTGGAATGCTCCGGGACTCAATAACTGCTTCTTGAACAGCGC
>TCONS_00050373 geneID=XLOC_010318 scaffold_186:512545-513372(-) Intergenic
CGGCAACAGTCATATATTATTGAGGGAATATCATGTAAAATTGAACACTAATATTTATGTAAAACATCTAC
TATGCTAGTATGTACTTATTATGCTACTGTAAAGTGAATATTATTGTGATGTATGTATTACTATTTAAATAGT
AAAAGTATTTTAAAAACACTGGAAGAGACTTTATGGTTCTGCGGTTCCAGAAAGTAAGTGTAGTGTATA
TTTTTTCATATAATATTATATTGTTAGAGAAGTTTATTGTACGGTCGACGTTCGCGGAAGCAATATGGTGG
GTGGCTAACTAACACCCTTCTATTGGTTGTTTAAGGCATCTACATTCTACAATAGTAGTTTTAGTCCATG
GCGTGTACTGTGGTAGTTGTTGTCAGTTACTGTTATATTTTCTGCCACGAAGTAGTTTGTCAGGAGTGG
TTGTTTAAGATAAGCGTCCCTCTTTCGGCATCGTACCCAACCAACGGCATCTCTTTCAGTACTCTTTGTA

TAAATTCACACAAGTGCGTCCACTTCATGGGCATTGTCGACGCGGGTTTTCCATGAAAATTCATTTGGT
CCGATACGTACGGTGGACGCTCACCTTTATGTTAGCTTTTCGC
>TCONS_00050421 geneID=XLOC_010319 scaffold_186:518248-521227(-) Exonic overlap (+)
AGCCGGTGGCGGCGCTACACGCGCGCCTCAACGTCGCCTCCACACACTTGCAGCAGTGTGCACAGAT
CTGCGCCTCATGCTGCGGGCACTCAGCCGGCGCTCGCTGCGTGAACACCCACTGCCCCGTGCTGTGGC
GGCGCGTCGCGGCGCAAGGACACCTCACGCAAGCACAAGATGTCGTGCGGGAACTGCCGCACCCAC
TGTGCCGCGAGGAGTTGCAGTTTTAGTTTAGTCATAGCTTTTGGGAGCAGAAAAGGAGACTTACTCAC
GAGATGTTGCAAAAGTTTCAATAGTAGCCGAATGGGGGTAACCCAGGTCGTCATTCTGAACAACATAT
GCTCTACAACTTTAGGAAATAAGTCAAGGCATAATGCGGTGGCTAACTGACCGGCGCTGTGGCGACGC
TTCGCGGCGCAAGGACACCTCACGCAGGCGCCAGATGTAGTGGGGGAACTGCCGCACCCACTGTGCA
GGGAGGAGTTGCAGTTTTAGTCACAGCTTTTGAAGAGAGCTTTTGGAAGCAGTAAAGGAGACAAACA
CACATACATACTAACGAGGTGTTGCAAAAGTGTCAAGTTGTTGCATAGTAGCCGAATAGGGGTGAGTC
AGTACAGAATGACGGACAATTTATGCTATACGACTTTCGTAAATTAGTCATAATGCGGTGGCTAACTGA
CCGGCGCTGTGGCGGCGCTTCGCGGCGCAAGGACACCTCACTCAGGCCACAGATGTCGTGGGGGAAC
TGCCGCACCCACTGTGCCGGGAGGATTTGCAGTTTTAGTGAAAGCTGTTGGAAACAGAAAAGGAGAC
ACATATACCTACAAAGCTACTCACGAGGTGTTGCATAAGTGCCAAGGTGTTGCATAGTAGCCGAATGG
GGGTAACTCTGTACAGAATGACGAAACAACTTACGATCGTAGATATAATATTTTAGAAATAACTCTTAAT
GCCATGGCTAACTGTCCGGCGCTGTGGCGGCGCGTCGCGGCGCAAGGACAACTCACGCAGGCACAAG
ATGTAGTCAAGGAACTGCCGCACCCACTATGCAGCGAGGAGTTACAGTTTTAGTCACGGATATTGTTG
AAGAAAGAGGACACATACACACAAGGTGTTGCAAAAGTGGTTATAGTAGCCGAATGGGGCAGACTTA
GCTCGTCATTCTGAACTACTTACTTATGTTCTACGACTTTTGTAAATTATTATTATAAAATTTTATTAAGTA
ACACCCTGTGTACCTAATCCGCTGCAGGATGCTCTTGGGACCA
>TCONS_00050569 geneID=XLOC_010319 scaffold_186:545169-545730(-) Exonic overlap (+)
TGCGACTTTTGTCATCATGCTCTACCAATACGCGTTGATAACGCACTGTTTTCCTGCGCTCTGATTGGCC
GGCGCGAGGGCGTGCGCGCGCCGCCCGCCATTTATATAAAAAGCGCAAACGTCAGAGATCGCCAGGC
ATTCCCGCTTGAAGCGTCTCGCGGCGCGTTCGCGTCACTCCGCATAATCTATACCTGTTCACGTGTACC
TTATCAAATAACTGGATCTCATTATTTTTAGAAACATAAAGACTGAATAATTTAAGGTTTTATTTTAAATA
ACTTAACTACTTAAAATGGCGTACCTGCAATCCATGCCGAAGGCTGAAACCATCAAGCTGCGTGAAAA
GCACGTCGGGTAAGTTTCATACCTTTTTCCATCCTTCAGTCCCACTACGATTTCGAAATTCAAACATCG
AATATCAATACATCCTGAGATGTCATACCCTAATCATATTATAG
>TCONS_00050136 geneID=XLOC_010320 scaffold_186:559385-560055(-) Splice junction overlap
AAAATTAGGCTAGGAATCTGTAGGTCGGGCTAAAGAGGACATCGACCCCGAGGAGCCGCCGTACCGC
GCGCCCGTGCCCGTGCCGGTGCCGTACCAGCTGTACCCGTTCCCGTACCCGTACCCGCCGCCTATATAC
CAGCTGCAGTACGCGCCGCCGGCGGGCCCCCCGCACCCCGCGCCCCCTTACGCGCTGCCGCAGTACTT
CGTGCCGGGGCTGCTGCACGGCCCGCGGCCCGCGCTGCCGCCGCACCAGCACGCGCTGCCGC
>TCONS_00050307 geneID=XLOC_010321 scaffold_186:599139-626003(-) Exonic overlap (+)
GATACTCAAAAATGATTAGGTACTAAATGCAACAAAAATTCAACAAATATAATATTTGAAACATTTAAAT
GAGTGCTTTGGGGGTTGTAACAAGTAAGAATTGAAAACTAAAAGCGAATTTGTATGGCGCTGCAGATT
CGCCACCTACAGCAGCATAAGGCTGTATTTATTGATAATAACGGCAAGGCACTAGACGTGTCCAGTTTC
TGTACACGAGATAACGAACTAACTATTCATAAAATTTCAATCCAATGATTTGAT
>TCONS_00050518 geneID=XLOC_010324 scaffold_186:800212-801561(-) Unclassified
CACGTCTGCTGCAACCTCGTCAACCAATTTTTACCATAGTTTTCTACACGCTGCCTGGCTTTGGAATTC
ACAAAACTGCGTGAAATATGTTATCGCTAAAGAATGTTTACCAACAGTTCAGTCTGGCCTGCGGAGCC
GATGAATGGCATCGCTCCCATGACTGAGATCACTATTTGATTGTGGTATTATCCTCCGATTGTTGACAAA

ACGAGACTGCACAGCAGTAGGCACTTTGAACTTGATCATTGCGCCGCGTGAAATAAGATCTGAGATGT
GAATATCCGTAGCGCAGGATAAATAAAAAAATATATATAAGTATTTTAAATCGTTCATATGCCTACCTTGG
TAGTAGGTACGTAACTTGTGGGAATACTAAAGAAGTAAATAAATTAAACTTTATGGCGAATACTTTGTG
CTAACAGGAAATGTGCTGTGATTTTTGTGATGTGATTGCTTTTTTAGTTAATCAATGTGAAAGGGAATC
AAAACAAAGCATCTAACCAACCCGGGAACTATGCTCGACCGATTCAACTGCAGTCCCCGCCGGCTGG
CCAGCCGGGCGCTCCCCAAGCTACCCATCTCCCCATCCTGCGCTGGGCTCGCGGGTACTCCGCGGCGG
CGGCGGGGGACCTGGTGGCCGGCGCCACCGTCGCCA
>TCONS_00050664 geneID=XLOC_010383 scaffold_187:115175-117093(-) Unclassified
CACTTTTACCAAACGTTAAACGTATTTAAATCAAATAATGTTAAATACATTTTGTACTAACTGACAGACG
TATGACAGGCTACTAAATACGTTTAGCGTTTGGTGAAATTCCACCTAACATATGGAAAAAACAAATGTC
ACTTTTGGAACTAACTTTGCCTTACATTTTTACAGTGACATTTGACGGCAACGTTAACGGAGGTTTCGA
AACCTGTGCTCGCGGTACTGATTATTTTATCACTCTGGAAGGTTATCATGAAAATGAAAGGCCAGTTTA
AACTAAAAATCTCATTTATTCCTCTTTATATACACAAAAATATAAATTTGGCCCAAGCAGCAAGGGTTTA
GTACTTAATTTAATTATAATAGATACATTGCGAATAAAGGAAGTATATTATAAATAGTTTTAAGAAAGGAG
ACGGAAACATATGATACTATAAAAGTCAAGATTAGAAAAGGAAATTGAAAGTAGAATACATAATAATTT
CAAGTAAGTATAGGAAGATAAAAAAAACTTATTAGAAAGTGAATCACAGTTTAGAAAAATATTATAGAA
TAATAGCATTCTTGTAATACAGTTTGCATGTCTTTACATAATAATATATCATCTAGTCTTTGCTAAAAGGT
AACCAATTTGAGTTGAGTATATGGACCTGAAGGGCTAGCACGGGGCGCAAGATTCGCACGGCAAGAA
GACGTGACAAAAGTTATCCGTCACGCTCGCTTTTGAGACCGCGTAATGTGAGAGCGCGTTGATAAAAC
TTTTTTTACATCTTGACGTGCGCTACTTGCGCCCCGTGCTAGCCCTACAGAGCTTTGAAACACATTTAA
ATATTCAAAGTTATAAGTATTCATGTCCTATAGTTTACTTTTAGGAAAATATAAATTAATAATCAGTCACA
TCTTACGTTTGATTAGCACTGAATAATAGAATTGTATTCGTCCAAAAATTCACTTTATATTCCTCATTATTA
TTTTAAAATCGGCACTAAAAATATACAGAATACCTATTTTTTATTAAGTCAGACAAATTGGTAATCGAGT
CCTTCTATTGGCGTCGTCGCTAATACATTCTACATACAGTTACATATGCCAGAGTCAATAACAAATAATAA
TTATTGACAAAAAGCCAACTCTTTTTGTGAATGTAGAATCTTAAAAGTGAGTCAAAAAATTTGTGACAT
TGAATACTACTGAACAATTTTTTTGTGTTAATAAATATGCATATGCATGCAAACAGGTGCATAAATATGC
ACCTGTTTGCATGAAATAAATACAGGGTAGAAAATGGGTACAAGAGAAAACAGTGATTTCTGTTTTTTT
TTGTATACAAGACTCAATAAATTATAAAAACATTGCATTAGAACGAACGTTTTTTTATATTTACTCTTGCA
GAACCATTTATGTTTTCTTTATATACCTACTACCGACTGCGCCAAAATTAAGTGCATGTTCCTATATATTAT
ATAAAATCCCTATTCTTTTCCAAGTCTAAAGCATTGAAGAAATATTTAAATATAATATTATCCTTGGAAGG
ACACATTTTTGAGACATTGTAAGCGATATAGATAGCTTTGTTGCGAATACGGAATAGAATCTAAAAGAC
GTTTGCCAAACAAGCATATTATTATGCATCACCTTGGAAATTACGCAATAAAAAGGATTTCAATTTTACG
TACCTACTTTTAAAATTTCTATGAATGTCCAATCCAGTCATTTCCCGAATAAGAATTTAATTTTGGAAAC
ATGTTTTAAAAGCCTAAATATTAACTTAC
>TCONS_00050607 geneID=XLOC_010367 scaffold_187:122708-130309(+) Unclassified
CCTCCGCCATCAACTGTCTCACTAGCGAAATGGCTGAAATAACGCATCTTGTATCAGAAACTTCAAGAA
TCGAAATAACATCTTATGACATCCAACAAGCGGAAACTGAAGGGGTATATACTTTAAATAAATAAAAAT
AAGCTTTAGTGCCCGTTTCACCATGTCTGGATAAAGTTATAGGTGTGATAAAAGTCCTATGGTCAAAGA
ATATCATTGTGTCATTGCCTAAAACACATAACCTAGTATCCAGACATCGTGTCACATACTGTATATAGCGT
ATTGCAAAAGTTGTATACTTAGCAGAAAGGGAATAACAAAAGTTGTTCAGAATGACATCCTGAGTCAC
CCCCTTTCGGCTTAGTAGACCCTTTTTGCCACACCCTGTATAAGATGGTGTGGGTGAAGTCTGTCAGAT
ATGAACATTTTAATTTTACTATTCAACCAAGCTGTAATGTTCCTAGCTAACAGTGCATTTTGTACTAACT
GACAGACGTATGACAGGCTACTTAATACGTTTAGCGTTTGGTGAAATTCCACCTAACATATAGAAAAAA
CAAATGTCACTTTTGGAACTAACTTTGCCTTACATTTTGACAGTGACAGTTGACGATACGCAACGTTAA

CGGAGGATCGAAACTTGTGCTCACGACTCTGTTCTTTTTCCATATATTTGTGTAGTTTCGAAAATAGTAT
CGAATCCTGTGATCGCTGCACAACGAGGCGACGTCACCACCCAACAGAGCGAATTATTATACTAATAA
GGGTAAACGTTATTGTTATAAAATGTAATCTTGCTAATTAGATACTACTCAAGAATTATTCGATATGATGA
AGTTGGATTATACCAATAATTGTCAAATACAATCACGAGCAATAAAAAGTCCCACTTACAAAATGCTGA
CACCAAATTACCCTATTTTTATAAACATTCAATTTAGAATTTGATCAAAATCCTAATCCTAACTAATATTAT
AAATGCGAAAGTAACTGTGCCTGTGTGTCTGTGACTCTTTCACGCCAAAATTTGGTATATATATGGTCTA
GACCCTGAGAAAGAACATAGGCTACTTTTTATCCCGGAATTCTCAAGCGAAAACTTTTTAAGGCGAAG
CGAAGTTCGCAGGAACAGCTATATTTAATACTTTTTTAGTTGGAAATATTCTTACACGCTGTTAAATGTA
CATCAATATTGCAGATGGGGGGTCACTAAGCTAACCTTTTCCCTTTAGGAGCTGTTCCCGCGCGCTTCG
CTTCGCCTTAAAAAGTTTTCCCGTGGGNNNNNNNNNNNNNNNNNNNNNNNNNNNNNNNNNNNNNNNNNNNN
NNNNNNNNNNNNNNNNNNNNNNNNNNNNNNNNNNNNNNNNNNNNNNNNNNNNNNNNNNNNNNNNNTGTAT
ACCAAATTTCATCCAAATCCGTTCAGTAGTTTTGGCTTGAAAGAGTAACAGACAGACAGACAGACAG
ACAGACAGACACAGTTACTTTCGCATTTATAATATTATTAGGATTAGGATATTAGGATTAGGATTTGGTGC
TATTCTCACTGGAACTTTTTTGTAAAGTTACAGTTTCATTCATACTGAAAAACAATGAGAACTTCTGCA
ATTTTGTATTCGTGTGTAATGACATAAAATCTTTTTGGTAACATCCAACAACATCATTGTATATTTCCAAT
TGTTATTATGTGAGTTAATGTGTGTATAAAAATGATGTATTTATATTTCAGTGTAATTAATAATAAAAGAAT
GTTTTTGATATTTACTGCCACACGAAAAATTACTGTAAATATTCAGAAATAATTTTAATGGTTTTTAAATT
GTCTTTTTTAACTGGAACCTTTACATTTTTCGGTCGCTTTTTCAGTATGATTAGGTGATTAGTCCTCAATT
GATTTTAAAAACTAGATCTGATTATTTTAAATAGTATAAAATATTTAAGAAATGTTTAAATAGCTCTGATT
TTAAGTTTATATATATTTTTAGAAATAAGTAGCTGAATTTTTTAATTATTTTAAATGCATTTTTATTCGTTAT
TTCAATAGCCGTAAATTATGTTAGTTTACAACTTTATGCATTTTTAGTGTGTCTTAGTTAGGTATATTTTTA
TACTTTCATCACAACTGTAAATCACATAAATATTTACCTACTTCAATTTATGCATTTTATAAAACAATGTC
GCCAGTTTGCTATAAATAAGTATGATTGATATCAAGTGTATTTAACACTTACGAGAGTAAATATATTGAA
AAACCTCTTCTTAGTTACTTTTTTCGAATTCCAATCAGTTTCCTATAGTGAACGCATAAGTTTCAATAATT
TTATAAATGGAATGAAATCTCAGAATATCAATATTATTCAAATATTTTCAGTTCAGAAAATGCATCCTTAT
ATTTTTTCCAACAAGTACCTAAAGAGATATACAGAGTGTTGCTAAAGTGGTGACTCAGGTTCATCATTA
TAAACAACTTTAGCATATTTCATAATTGTTGAGAATAACCCCATCCCCAAAAACTCTTTCGGCTTACTAA
AGTCTTTTAGGGTATTCTATGCCGGAACCTACCTGTGCAGCATGTTATATCTAAGCTCTAAAATAAAATC
AAATGAATGACAAAATTCGCGAAAAAAAACCTTATCCATAGAAACTTTGTTAGTCACGTGACTTTTTAC
TATGGAGATATTTTTTTGGGGTCTTTCGACATTCTGATTTAATTTTCAGGCTAACATGCTCTACACGTAG
GTTTCGGCTTAGTATACCATTTTTGCTTCACCCTGTAGATAAGGAACGCTAGAGGAGAAAGAAATCAAG
AGCCTCAATAGGTGTCTAAATATTCATGTTTGGATGTTTGGCCATATGTCTATGAAAAAATCACTACGAG
TCTACAATTTAAGTTTTGTCAAACTGCAAAGCTCACATAAATTGTCGTGAACATTGATTGCATGTACTTA
GTTATTTGTATATTGAGAGTCAATACTCAAAATAAAATAAAATTGTATGTATAAAAAGGTTTTTGTGTTAT
TTTCTAGCCCCGTACCATCTTAAGAGACTCACTAGATACTTAGTTGTAGATAACTACCTACAATTATTTTA
AATGTTGATGCTATGCTTGATCCTGAACAAATAAAACTAACAAGGTACCTGCTTCAATAATTTGTATTAA
AAATACATATAGAACATTAACTTTCCTTCAGGAATAAGTACCTACCTACACCAAAAAGTGTATTACTTAG
GGTAAAAATACTATTTGCAATTTATATTTAATAGCAGTTCCCGCGAGCTTCGCTTCGCCTTATAAAGTTTT
CCCGTGGGAATTCCGGGATAAAAAGTAGCCTATGTTCTATCTCAAGGTCTAGATCTAGACCATATGTATA
CCACATTTCATTCAAATCCGTTCAGTAGTTTTTGTAGTAGACATACAGACAGACAGAC
>TCONS_00050635 geneID=XLOC_010375 scaffold_187:393732-414107(+) Exonic overlap (+)
GCCGGCCCCGTGGATACTCGTGTATGACTCCTCTGATTCAGCAGTGTGTAGTCAGGCAGTGGTGCGCG
GTGAAGTGGAATGCTGTGAGATCGGATGTGGTGAGCCGATGCTGAGCCGCGGGGCACCCTGGGGCAC
CAGATCGACCAAGTGTTGGGTGTGAAGGAGGAGATGAAGACGGAGCACCGCGTGTGCTGCGCGTGC

GGCGTGGGCATCGCGGACCGCTTCGTGCTGGAGGTGGGC
>TCONS_00051400 geneID=XLOC_010436 scaffold_188:285672-286835(+) Intergenic
TAAAAGTATCCAGTTCCATTGAGGTCGAAGCATTCATTAAGGTGTCGCTCTACCCTATCTGATGAACATT
GCAGTTATACCTGTCTGTCGTCTGTGATACGAGATAACTAATATGGTAAGCCTCAAATATTAATCTTTCC
GTATGGTTTGGGTGTTCTGCCAGTTTGTTAATAGTTAAACACTGTTAGATGATGATAAAACTCCCCCTGA
TGCCACCATGACGGTTTTTACAAGCTCAACAGGACTGAATAATAAAGCAACTAAAATTAATAGGTACTG
GAATAAAATGTTGTAGTATAAAATAACTAAGTTTAAACTTTTACAGGTCGCATCTGCCAAACAAATTGA
AGACACAACTTACTGTGAAGCTAAAAGCGGTCCAAGTTTTAATCCCTTCCTGGGTTCAAGAGTCGGTC
CAAACATCACAATTTTGCACATGGTAAGAAATTTTACTAATTTATTACATTAATTAACTAACATTCTTATG
ATGATCAACACAGACCTGTTCTGAAATATTATTGAGGACA
>TCONS_00051401 geneID=XLOC_010436 scaffold_188:285737-286946(+) Intergenic
CTATCACAGACCTGTTCTGAATTTCTTTGAGGACAGCTTTAAAATATTACCTGATTATTTTTTAACAATTC
AAATATATTCATACATATTTTAAAGATTTATCAATCACTAATACTGAGTTTATTTTCGTTTACAGTATCCAG
TTCCATTGAGGTCGAAGCATTCATTAAGGTGTCGCTCTACCCTATCTGATGAACATTGCAGTTATACCTG
TCTGTCGTCTGTGATACGAGATAACTAATATGGTCGCATCTGCCAAACAAATTGAAGACACAACTTACT
GTGAAGCTAAAAGCGGTCCAAGTTTTAATCCCTTCCTGGGTTCAAGAGTCGGTCCAAACATCACAATT
TTGCACATGATTCCGCTGTCATCATGTGAG
>TCONS_00051402 geneID=XLOC_010436 scaffold_188:285998-286835(+) Intergenic
GACGGTTTTTACAAGCTCAACAGGACTGACAAATTATTACAGTCAACTTAATCCTAAACCAATGTAACA
TCATCTACATGTTATTAATATTAAATAACTCTTCCAGGTGTCGCTCTACCCTATCTGATGAACATTGCAGT
TATACCTGTCTGTCGTCTGTGATACGAGATAACTAATATGGTCGCATCTGCCAAACAAATTGAAGACAC
AACTTACTGTGAAGCTAAAAGCGGTCCAAGTTTTAATCCCTTCCTGGGTTCAAGAGTCGGTCCAAACA
TCACAATTTTGCACATGGTAAGAAATTTTACTAATTTATTACATTAATTAACTAACATTCTTATGATGATC
AACACAGACCTGTTCTGAAATATTATTGAGGACA
>TCONS_00051041 geneID=XLOC_010440 scaffold_188:436139-437125(+) Intergenic
CGTAAGCAAATATTTTTTAATCTTTTATGGCTTTTCTCCTGTACCTACATGTTAAAACTTCAGGTAGGTAA
TAGGTAGCTGCTATTTACGCTATAATGTAGTCCAGCAAAAAAAGGTTAATGTAAAATGACCAAAGTTTT
GTTTTCCTATTCTTACTTTTTTACTGAATTGAATGTTTCAGTGAAGTTGTAGGTAACTCGTTCTACTGATT
GTAATGCTCTCTTCCCTTATGAAATAAGAATATTTATTCAAAGTTAACATTTATTAATTTGTTATTATTGTA
ACTGTACTTACTTTGTAGGCATTAAGAGCGTCGAGTCAAAGATCTGTATTATTTTCTATTTTTTAATACTT
TTATTGTATAAAATAAATTTATGTTTCAGAATCACTACCTATACTGTAATATTTATACTGTCTAATACGAGG
TATTCCGATTTTCAGTATGAATTTTTAGTCGTTTACGTAGAACCGCTTCCATAACGTATCAATGTACATTA
TAATTATATTACACACGATAGTATTGTGGACTGAGATTATTTTATGTGCATTTAGAGTTCGTAAAAATTAG
GACATTTCCGTGGATTAGCCATCTGTTGGTGAGTAGAAGAACTAATCCGTAGTAGCGACATCTGTAGTT
GAGTACCGGAATCTTTTGGCATCACAAAATAATATTATAGTCGTATTTCTATAAAACAAAATGTATAGTGT
ACTTATTATTAAACAGTGCCATGATACACACAGTCAAATAAACTTGTGAAATACATTCATAATATATTTTA
AAGCAATAAATTAACTTAATTGTAATTCAGGGCAATTATATACCTACCTACCTTATTCCTATGTAAATAGA
CACAATCCCTCAAATTTTGTTACATCACAACACACAAAAACCATTGTTGAGACAAATTACAAATAAAA
AACTGG
>TCONS_00051174 geneID=XLOC_010459 scaffold_188:452256-454466(-) Splice junction overlap
GTCGTGCGCGAGCTGCGCGCGCAGCGGGCACAGTCACTGGCTAATAGACACCACTATATCTTCGTTTAT
AAGGTACTAAGCGAGTATGGTAACAAGCTGATGGGTGGAGGCAACGACACTATCTGAAGGAAGCTTC
ACTCTACTCGTGTGTAAATTGTAAAAATCTTCATAAGACAATAATTCCATGACACTACAAGGAAATGTAT
AGTTTTAATCCCTATTTGGAAATATACCAAAGGCTATGTTTTAAGATCTCTTGATCGCTCAAAAAGGATT

TATCAATCGACTTATTCGATATACCATTATGACGAATAAACTAACTTTATTTATAGCTAACTCTTAGATTCC
TACTTATTTTAGATGATGTGCATAATACTTAGTTGAGAGTTATTGTAGGCTTTTTTTATATTTACCTATACA
TGTATATTTGTCAATTTGTAATAATGTAATGCTGGGTTTAGACGAGCAATAAATGTTGCAGAAGTCAACG
AGTGCGAGGGTGTAAACTACATAATTTTCAAATAATTTATTTACTTGCAGAAGGCGACTTTTGCAACTT
TTATGGTCAATAAAGTACCCACTTAGTTAATGTTATCAGATAATATATGAGGATGTTTACATAACTAGATA
TGTCATTACCACATTCGAAGGTGCTTTTATTACCTAAGTAGGATCACTTATTACACTATGTTATTATCCCA
TACCAACAATAAAGTATGTCTCTGAATAATAGTAAAAAAAACGTTTGTCGTACTCCTTATAAAAAAGTT
TAAATGTCAATGCTTACTACTTAAGTAAAGAAGAGAAATCATTACCTAAAATTACAATAACGGTTACTCT
AGTCTAGAATCAGATGTATTTTTGTGTTGGGCATAACATAACATAACATAACATAAATAAATTAAACCTT
ACCTTAAAACGTGCGTACATGAACCAATAAATTATGTATGTTATTGCTTTTTCAATCAAAGTAATTGTTT
TGATAGTTATTTTATGTATATTCATTAATTTGTTAAGCACTGTTGTTATTAGCATAGGTATAGTAATTAAAT
TTTCAATGCAGCTAAGATGAATTGACGA
>TCONS_00051369 geneID=XLOC_010459 scaffold_188:470053-499901(-) Exonic overlap (+)
GCGCCTTTCTAGACGCACGCACGTTCCCGCCAAACGAACTGTCAAATAACGCTCTCGAAAAAAGTTG
CGTGCCGAACTACAAAAACAAAAAAAATACCAGTGGTCTTATACTGAACTTTGTGCAGTTTATTTCAG
TGTTTTTAAGTGGTATCGTGGATTTTTTGTGATTTGAACGAGATTCGAGCCGGATATAAGGGACAGCAG
TCGAGGGAATGGGAGTGCTGGAGTGATGCTGTCCACAACGGATGAAGCTCGGTAGAGGATGAGATAC
GATTTTCGAGAATGACTCTAAACCGATCATGAAGTGATCATGGAGTCACCAGTCAGAATATGTTTAGG
>TCONS_00051224 geneID=XLOC_010460 scaffold_188:500444-501524(-) Intergenic
AAATCGAATACTTTACACGATTTTTGTAGTAATGATAAGATAAAATTAGGTGCGTAGTTAATTTATTGTAA
TCGGTTTTAAATTGTAGTCAAATGTACACGCGAATATAATATCTATTTATGTAGTTTCATGTAATTTAGTTT
TATACAATTTTATTTGTACATGTTGTTTTTTGTTGTTGAATACCTGTCAGTCTACTAATTTATTGGATTATG
ATTAAGTGAGCATTAAAAATGCATTTAAGTTATTTTTTATCGTGCTGATTCAGTAGCAACAAAAAATATA
CCCACGTGAAAATAACGTTAACTTTCTTAAGACCATTGAAAAACAATGACTCTAAGCATGGATAAAATA
AAATTTTGTTGTTGCTTCGGAATCGAGCTCAGTATGTATATCCAAAGTTACCAGTGAATCAATCATATGA
CATTTTATTTTAG
>TCONS_00051832 geneID=XLOC_010518 scaffold_189:119611-135640(-) Exonic overlap (-)
AGATCTCTTTCTTCAACATTGGGGTGCAGTTAAACCAGAACTGTACAACGGAATATCAGATTCTACAAC
AACCGAAATAAATTATTTAAATTTGGGTCATCGACGTCAACGTTTAAGATGGCGGCCAAGATATCCTTT
TGGTCGCAGACCCAGACGACCTAAACGACCCAGACGACCTAAACGACCCAGACGACCTAAACGACC
CAGGCCACCTAAACGACCCAGACCAAGACCCAGCCGAACTAAAAAACCAAAAACAAAACGAACTAC
TAAAAGGACCCCTAAAACCAAAAAAACAACTAAGGGGACTACTAAACGGCCTACTAGAAGAACTACA
AAACAAACTACCAAAGAGTCTACGAAAGAGCCTACTAAAGGAAGTACTAATCAGCCAACTAAAGAAA
CAACTAAAAGGCCTACGAAAAAAACTACAACAAGACCTCGTAAAGGTAAAGTACTGAAACA
>TCONS_00051741 geneID=XLOC_010519 scaffold_189:129821-131329(-) Unclassified
CGCCTTCGCACATTTTTTCCAATCAAACAGTTTTTTTCCATAAATTGTGATATCTCTGAGTAAAATGGGA
AGAAAAAGAAAAAACGAAACGAACGAGGAACGGATTCGTCGAAAAATTAAGAAATACGAAGACCGA
TTAGCTTACGAACATCGACGCCGCCAAGATCGTAGTGGTGCGCCCACGCCCAGTGATTTGAATTCTCA
AAATGGCGAAAACTACATGGTTCCGGAACTCAGAAATGAGCCGCACATGGACAGCCCCACATTTTATG
ACTGGGAGGATGCCAGCACTGGATTCCCACATCAAGAGTTAACCCCGGGAGCCCCAAACGAGGAGCC
CGCCTCCGCCATGGGCGCGG
>TCONS_00051785 geneID=XLOC_010502 scaffold_189:129916-131223(+) Intergenic
GTGGGGCTGTCCATGTGCGGCTCATTTCTGAGTTCCGGAACCACTATAAACAATAAAAGGTAGGTGTC
AGATTTGTCCCGATGACATTTCGCAACACATTTTCCTTAAACGAAAGAGAATACCCCGCTGGTAGCTCG

TATCGTGAAAACTTACAGTAAATACGTAGGTACTGTAGTTTTCGCCATTTTGAGAATTCAAATCACTGG
GCGTGGGCGCACCACTACGATCTTGGCGGCGTCGATGTTCGTAAGCTAATCGGTCTTCGTATTTCTTAA
TTTTTCGACG
>TCONS_00051833 geneID=XLOC_010520 scaffold_189:136042-136656(-) Intergenic
AAAATTTCACGGTGTGACAATCTTCTAGATAATGGTTTTATACCTTCGAAGTCGTTTGCATTCAGCCTTT
TGACATCCATGTCGTAACTATGCATAGAACACGATAAATCATCAACGGTTATCGGTTCTTCGACAGATGT
GGTCGGCTTATTGCCATATTTGTTTCCTACTATGGATTTGTCCTATGTACAGCCATGGATATTCA
>TCONS_00051838 geneID=XLOC_010522 scaffold_189:179800-189528(-) Exonic overlap (+)
TCGAAATAAATTATGTAAATATTGGTGGTCATCGTCGACGTTTCAGATTGCTTAAGCGGCTGCCAAAAC
CAAAACCCAATATACCTACCATACCAAAAAAACCCATACCACCAATAACTATAAAACCACCACTATCAG
TACTTCCAATAATAACAAGAAAACCAATAATAACCAGAAGAACAATACTCACAAAAAAAACAAGGCCT
ACTAGAGGAACTACAAAACAAACTACCAAAGGGTCTACGAAAGCGCATACTAAAGCAAGAACTAAAC
AGCCAACTAAAGAAACAACTAAAAGGCCTACGAAAGAAACTACTAAACGGCTTACAAAAAAAACTAC
TAAAAGACCTACTAGAGGAACTACAAAACAAACTACCAAATGGTCTACGAAAGAGCATACGACAGCG
CAAACTGAAAGATCTACCGAACC
>TCONS_00051528 geneID=XLOC_010525 scaffold_189:313490-314312(-) Exonic overlap (+)
TGCCGTTTTGTTTTTCGTTTGGCCCGGTTGGTGGTGTCGTCGTTTTTTTAATCACTCTTCTTATTTTATTC
CACCCACATTCATTTGGTCGTTGTTGGACGAGTCTCGGCAGCGGGTCGTATCAATTTTAAAACTATTGT
TATCAGGCTTTCTTCATAAGATACAATATGGCTGGAAACAACACAAGAAGGCTGATGCAGTATGTCACT
GACCACACTAGGATATCCACGCGTGCCTGTGAGTATACCGGCTGAGCAGAACCCTTCAACCTATTCAA
CAGGGGAATAAAGGGAGCCTACCTGAGTTACATATCAACTGATCCTTATA
>TCONS_00051529 geneID=XLOC_010526 scaffold_189:315077-315592(-) Intergenic
CTCTTCTCCGCCCTGCGGTCTCAGAAATGTTTAAATATTTAATTGGAAGTTCAAATGTTGTCCTTATCTA
AAGTTAAAATATTATGAATTTATTTTAAACAACACGCAGTTATAACTTTAACAAAGACGCCGAATAAAA
AAAAAGTAAACACCACTTGTCGCGTTGCCACCTAGCCGGCCGGCGATTATGAACTGAAAATATTTGAC
ACTTTTAAGAACCACACACATCTGATTCAAGGTTTTAGCAAAATTACTATCGTAGGGCAAATTTATCTTT
ATATTAGTAAAATACTTATGAAAGAAAGAGATAAAGAAAAACTTCGATATTTTGATAGAATCTAGAGCG
AAC
>TCONS_00051665 geneID=XLOC_010529 scaffold_189:416788-438568(-) Intergenic
AATACTTATTTTATTCAATTCAAACTAAATCAAAATCGCGGCCCAGGCGGGTGGACGCAGAGACGATCT
CATTTTTTTTACTTGCGATCCAAGTGTTCACTAGTGCATTTGTTTACCAACAAATAGTTTAGTTTTCGTG
AAATCTTATTTAATTTTTGTCATGGGCGAGTTCGGCAACCGGCTGGGGCTCGACGAGCTGGCGGGGGG
CGCCGCCGCCATCAGCAAGGCTCTGCTGGCTGAGTTCATTGGCAACTTGCTGCTAAACCTGTTTGGA
>TCONS_00051717 geneID=XLOC_010529 scaffold_189:417220-438639(-) Unclassified
GATGTCGCTAGCAGCGCTGTATCCGCTTTATTTCCCGCCACCGCCACTACAATCGCGCGGCGGCTTGTT
CAAATACTTATTTTATTCAATTCAAACTAAATCAAAATCGCGGCCCAGGCGGGTGGACGCAGAGACGAT
CTCATTTTTTTTACTTGCGATCCAAGTGTTCACTAGTGCATTTGTTTACCAACAAATAGTTTAGTTTTCG
TGAAATCTTATTTAATTTTTGAGTCATGGGCGAGTTCGGCAACCGGCTGGGGCTCGACGAGCTGGCGG
GGGGCGCCGCCGCCATCAGCAAGGCTCTGCTGGCTGAGTTCATTGGTAACA
>TCONS_00051778 geneID=XLOC_010535 scaffold_189:532012-577430(-) Exonic overlap (-)
GTCTGTGCGGCAATGCTCGAAATACGTGTCTCGAAATTTTGCTAGTGGCGACACACACGCGCGCGAGT
TTGAGCCGTAATACTACCCACATTACGTAGTTCGCTCCGCATAGCGACCGGCCACCGTAACCTGACCCG
CCGTGCCACTCCGTAGCCCGTGCTGCGTCGAGAACCGGCGGTGTCACGCGAAAGTGGTCCGATACCG
CTCAGTGTTTGTGTCGTAGTTGTTTTGTGTTTGTGTTAGTGCGCACATAGCACAAACGGACTATAGTGC

AACCCAGGGTTTTTATTGAAAAACTCTTGGGTTCACGACCTCAGGAGCTACCTACTCTTGTTGCTGCC
GACTCTGTGAGCCTCCGCTCGCGGGCGCCTGTCCTGGGCTTCTCACCAGCGAGATCAACCCGGGTCG
GCTGCACGCGCCGAGGAGTGCTATCCAGGGTCCTGCGCACCTAGGTAGCTACCTGCACCATCGAGGAT
GGAGGAGGACGAAGGTGGAG
>TCONS_00052442 geneID=XLOC_010636 scaffold_19:145630-218052(-) Exonic overlap (+)
CAGAGGCCTCGCGACACCGATCCGTTATCGGCATCGATAGATAGGTTTAACGTGCGTTACACCAATCAC
AGCGCCGTTTTTATCGCGATTCGTGATATTGTAACGTTTATCTACGTCGATAACGGACCCGTGCAGCGGT
GACAGGCATCGAAAACTGTCCGCGGATCGAGTGCATCATGATAAAATGATACAGTTGCTAATGTGGTAT
AGTAAGCCAAATAAACACAGCACGTCATACTTAACAACTTTTGGAAATTCGGGAAAAATTATAGTACTT
GACTTTTAACTATGGGATTAAAAGCCACGTAACTTCAAGCTTCTATGGAGAAGCAAATGTATTTTCTTT
CGCGATTTCACAAAACTTGTTCAGAATGACAATGCGTCATCCCTTCGCACTATACCTTTTTAACACGC
TGTATGTTATTATTTTTTGTGAGAATAGAGAGAATAACTTGTTCAATATAAATTTCGGTAATCTGTGTTAA
TTAACTGCGAGTTTCTATAACTCTATCTATCAATTACTGGTAATTACTTTCATACGATTTTGACAGATTGT
TACTTTTCATTACGTCAAAATCGTATGAAAGTAACTACCAGTTATAGATAGATAGAGATATAGAAACTGG
CAGTTAGATACTGGTAAAAATGTACATATTTTGATACCAACAGCTGCCAAGTTAGCACATTACATGGAA
GCGCTTACCCAGCTACAATGCAAGTAAAAATAACAGAGGAAAAACTAATTAAACTAAACGTAACTAAA
ATTAATAATACAACTAATAATTACAAGTTAATCAATTATTATAATAATAGTAACAAAGAAACATAATTTTAT
GTGATAATACGCCGCTCGCTCTATTTGACAAGAATTTTATAATCATTTGTAGAATTAACTAAAGAGTTTG
AAACAATACTTAAAATCTGCCCGATGACGTGGCAGTGGGCCCAAATAATATACCTGCAAATGCCCTTTT
TTAAGATGGCTTGTTAGTGTGACTGGCTGTTGTTTGATATCTTTTATCAAATATAGCTTAGCAGTTCAGTT
AAAGGAAACTTCAAAAAAGGAATCAATTTCTATGATTCTGTTTTTTAGTTATTATTTTGTCATTATTTCTT
TATGGTGGTGTTCCAAAAAGCAATGCAGCAAATTATTATTTTACTGTTGATGTTTTCAAACCGACTTAA
AAAAAGGAAGTTCTAAAGTCATTCGTAAATTTGTATGTAGGTTTTTTAAATATCAGTCAGGTTGTTAGA
AGGAAATTACAAAAAGTGACCTTTCCACTGAAGAAGGAAGAATTCGTGGCAATGCTACTCTCTGGAA
GTGAGGTGGCGAGCGGCTATCATACATACAGTTTAAAGGTATTATACATTAAAATTAACATCTAACGATA
CTTATTCACACGGTATTGGTACGGGTGAGGACATGGCACGCGCTCCGCCTCGGCAGGGTATGGACACA
AAAAAACAAAAGTTTTCCGTAAAAGGTAGGTACGGTCAGCTGCATAAGTTGCTATACACTTCTGTACC
TTGTCAAACTGACGAACCGTCAATGTTGCAATGAATTGATAGGCGGTTTCAGATTGACAAGGTACAAA
AGAGTATAGCTAAGTATGCAGCTAACTATACTTTCATATTTTAGGGTCTCAGCACATATAAGCGTAACGT
AAACGGATCGCCTTTTTTACTTCTGTCAACCAGGCGTTCGCGAGCTCGCGTTAAAAGGCTGAGGCCAC
ACGAGCGTAATTTTGTGAGTTGTGAGCGGCGCAGAAAACAAAGAAAAACAACGATCCCTTTACGTTA
AGCTTGTATGTGCGGTGACCTTTAACTTTTAAATGCTCATCATTCATTTTTATACTCTATATTAGAATGGC
TAAAACATAATTGAAGATATAATATTGAAGTACTCTCTAGTATAATAAATGTCCATTATCCACTGCCAAAT
ACGGAGCTTATTATTTTATTACAAAAATAATAACATTTGCTATTTTGTATATTAATAATATTATGATAAGGG
TTATTTATTGCATTCCACTAATTCAGCATAACACAAACTGCAGGGAAGAAACTAAGTTACTGTAACATT
AACAAAACAGACATTTCGAACATTTGAGAAAGAAAATACAGGAGTTGCGCAGAATAACAAAGAAAA
ACATACGATGCAAAATCAAATAACAATTCAGAACAAAATAAAAAAATCCCAATAATAGAAAACAGGGT
GAAGAATACACAATATTGAATTTTAACATACTAATATTGTCTAGCAGACATGCGTCACTACAGTCGTCGA
AAAATAGGCCCGTTTGGACAGCTACACAAATTTGCTATTTACTCTTGATTTACTTGATGAAATTCATAAA
AAGACACATATTCTGGCTTATTATTTAACGGCCGAAAGAAAAAAGAACTTTTGAAGCACCAAAACTTA
CTTATTGTTTAGATTTTTAATGATTTAAATTTGACACCAGCTGTTTTGTTTAAACCTGTCACCTCCCATAC
ATTTTGGAGCTGTCCAAATGGGTCTATTATATTTCGACGACTGTAGGTTCCAGATGTTTTCGATACAGAG
GCGTTATCATTCAATACTTTAGTGGCTAAAACAGTTACGAGCGTGATTGAAATTGATATTTTTACTAATT
AACTGCACAACGATACATAAGAAAACGATAAAAAAACAACATAATAAACGAATTTTTAAAAGAACTTT

TGTAAAGAGAAAAGATATGTAAAATTTTATTTACTTGTGAAGTTTAGTTTTAACGTTATAGTTACTTATTG
CACAAGCGGTCACCATTGCGTATCCTAAATTCCGACTTTTACCGTAACATCTCTACACGAGTCCTAATC
ACTATTTTTTCCAACGCATACGGACGTACAGACATACAAGTTAATAAAATACAAACTCCCTAAAACAGA
AAGTGAAGAAACGTAACCTCAAGAAAACACCTTACATACGTGGTTCTGTTAAGAAAAACTGGCCTAC
ATTATATTATTAAAAATAGCTCACTCTACACATAGCGACGGCTCACTCACACATGTACATTATATATTTAC
ACACACAAAGCTAAGGCATCGACGTCACAAATCAAAACAAAGTTATTATAAAAAACTCGTGTGTTCAA
ATTCTTAACGTGACGATATCTAGAACCGTGGGAAATATCGGTAACTTTCACAAAAAAAAACAGTACTAC
ATCTATACGTTTTTTTTTTTATATATATAAAATTCTGTGACAATTCCGTGCGCTTCCCGCCTTTAATACAAT
GGGATAGAGTCGGGAATGCGCACGAAACATTTTCAGATACTGGAATCACCTGAGGACAAAAACACAT
GATATTTTGGCAAATCGCACTAAGCGTCCTTTTAGTATGGTGTGTAATACCACACGCCCTTGCACGCACT
ACACATCCTACCCCATTTCACATATATTGCGCTTCCCTTACAGTTACTTATCATCAATCAAGTGATTTAAA
CTGATCATAGATTCTATATTTCGGATCTTAGTAGAGGCAACACCATCTTATTCGATATTGTATAACAGGCC
CTCATAGCCTATTTTCTTTATATTTATAAGATCTTATCTTCAGTTATCTTAACGCTATATCTTCTATAATCAA
TTTAAATCACCGCCATCAATGTTTACCAACACCAAATAAAATGTTCACTCTCGACAAAAAAAAGAAAC
ACTCCAAAATTCACACCCCCGAACTAGCGCAATATATGTCGAAATAAACCTTATTTTAGTAACTCTTTCA
CATCACAACACGAATCTTCTGGAATCCTGGACCTTTTCTCTCAGCCCTCCCACTGGCAAAAAACAGGG
>TCONS_00052021 geneID=XLOC_010636 scaffold_19:148527-218065(-) Exonic overlap (+)
AGACAGATTTACACAGAGGCCTCGCGACACCGATCCGTTATCGGCATCGATAGATAGGTTTAACGTGC
GTTACACCAATCACAGCGCCGTTTTTATCGCGATTCGTGATATTGTAACGTTTATCTACGTCGATAACGG
ACCCGTGCAGCGGTGACAGGCATCGAAAACTGTCCGCGGATCGAGTGCATCATGATAAAATGATACAG
TTGCTAATGTGGTATAGTAAGCCAAATAAACACAGCACGTCATACTTAACAACTTTTGGAAATTCGGGA
AAAATTATAGTACTTGACTTTTAACTATGGGATTAAAAGCCACGTAACTTCAAGCTTCTATGGAGAAGC
AAATGTATTTTCTTTCGCGATTTCACAAAACTTGTTCAGAATGACAATGCGTCATCCCTTCGCACTATAC
CTTTTTTAACACGCTGTATGTTATTATTTTTTGTGAGAATAGAGAGAATAACTTGTTCAATATAAATTTCG
GTAATCTGTGTTAATTAACTGCGAGTTTCTATAACTCTATCTATCAATTACTGGTAATTACTTTCATACGAT
TTTGACAGATTGTTACTTTTCATTACGTCAAAATCGTATGAAAGTAACTACCAGTTATAGATAGATAGAG
ATATAGAAACTGGCAGTTAGATACTGGTAAAAATGTACATATTTTGATACCGACAGCTGCCAAGTTAGC
ACATTACATGGAAGCGCTTACCCAGCTACAATGCAAGTAAAAATAACAGAGGAAAAACTAATTAAACT
AAACGTAACTAAAATTAATAATACAACTAATAATAATTACACGTTAATCAATTATTATAATAATAGTAACA
AAGAAACATAATTTTATGTGATAATACGCCGCTCGCTCGTTTTTGAGAAGAATTTAATAATCATTTGTAG
AATTAACTAAAGAGTTTGAAAC
>TCONS_00052191 geneID=XLOC_010637 scaffold_19:148917-218775(-) Exonic overlap (+)
ATTTAATTAATATTTAAAATGAGCGAATACAGGCGGACAGGCGAGACGGAGAATACACCTCCTCTCTTC
ACACACAGGTAATAACGCAACCAAAATTATCATTGTCAAGATTACTTTAAAATTTTGGTCAATAAACTA
AAGTTTTTCATGAAAAAATTAATAAAAACTGAAAGAAATATTTGTTTTATAAATGGGTAAAAAAATTGT
GGAACTTCAATGTAGGTATTTAGCTCCATTTTCTCCATAGTCGGTTATATCGAAACCAGGGAAAGGTTG
ATCAATTATACGTCATCTCCATAATTCTTGACAGATGTGTCAAAATTCAAGCAATAATCCCTGGTTATGC
TCTAACCCACTATGGCGAAATTGGCAGTTAAGGTTATAAAATATGGATTTGGTCAAACACACACTTATG
TTATAAAAGCAGTTTATTTTGAGCAACCATTTTGTACCGACGACGGTCGGGTTCTGTTTTTTTTTCATAA
AAATCAGTTTTCTACAATCATAGACATTTTGAATACAAAAATTAACAGTTTTTAATAAAGAAAACACAA
TCGATAGACCGAACCGCGTGATTAGCTATAGCGATCGCCCAAAATGACAGCTGTCAGATATCAAATATAT
ATATAACCCATTACTGAAGAACCAGACAGATTTACACGGAGGCCTCACGACACCGATCCGTTATCGGCA
TCGATAGATAGGTTTAACGTGCGTTACACCAATCACAGCGCCGTTTTAATCGCGATTCAAGATATAGTAA
CGTTTATCTACGTCGATAACGGACCCGTGCAGCGGTGACAGGCATCGAAAACTGTCCGCGGATCGAGT

GCATCATGATAAAATGATACAGTTGCTAATGTAAGCCAAAAGAGACACAGCTCGTCATACTTAACAACT
TTCGGAAAATAGGGAAAAAAATATAGTACTTGACTTTTAACTATGGGATTAAAAGCCACGTAACTTCAA
GCTTCTATGGAGAAGCAAATGTATTTTCTTTCGCGATTTCACAAAACTTGTTCAGAATGACAATGCGTC
ATCCCTTCGCA
>TCONS_00052192 geneID=XLOC_010636 scaffold_19:217628-218137(-) Intergenic
ATTGAAGAACCAAACAGATTTAACAAAGGCCTCGCGGCACCGATCCGTTATCGGCATCGATAGATAGG
TTTAACGTGCGTTACACCAATCACAGCGCCGTTTTTATCGCGATTCGTGATATTGTAACGTTTATCTACG
TCGATAACGGACCCGTGCAGCGGTGACAGGCATCGAAAACTGTCCGCGGATCGAGTGCATCATGATAA
AATGATACAGTTGCTAATGTGGTATAGTAAGCCAAATAAACACAGCACGTCATACTTAACAACTTTTGG
AAATTCGGGAAAAATTATAGTACTTGACTTTTAACTATGGGATTAAAAGCCACGTAACTTCAAGCTTCT
ATGGAGAAGCAAATGTATTTTCTTTCGCGATTTCACAAAACTTGTTCAGAATGACAATGCGTCATCCCT
TCGCACTATACCTTTTTTAACACGCTGTATGTTAT
>TCONS_00052356 geneID=XLOC_010642 scaffold_19:289159-292936(-) Intergenic
GTATATAGAAAAATACTTATCACCAAGCAAAGATGTCATTTGTTTGACTATTAACCACTTTAGGATAATAT
GATTGATTTTAAAAGTTTTGGTGTTATTGTTTTGAAACCATTTTTAACAATAGTCTAGTAATATTATATTA
AGAATATTGTACAGACAAGAGCAAAAGTTCCTCTGCTTAGTCACAAAATGAAGTGTTCTTTACCATTAA
TGCGGAACATTATTGTAATCGTTTGTTTTAGGTTTCCGCAATGCCCTGAAAGAAAAAGCAAATGGATAG
ATGCAGCACATCGTGGCGACCTTTGGTTCC
>TCONS_00052308 geneID=XLOC_010644 scaffold_19:332221-332719(-) Unclassified
TATCGCTCCTTGGGAGAGGCATACCTATGTCCAACAGTGGATGATCACGGGCTGATGAGATACATTGGT
CATAGAAGAGCATCTTTAGATTTCAAGTGATATTCCCAGGACAAGTTAACCCCAACACACTTAGCAATT
TTCTTTAAGGGACGAACTCTGAAATACAGAAAAAAAATATTTTTGTTGTATTTATATTTCTCAGCTGTTT
TGTTCAAAATCACAGTTGCTCCCTTCAAAGTAGTAGTACTGTCATGTACTTAAACACAATAGACTTGCC
AACAGGAACATCACTGATAAACCCCCACCCTATCTCTATAAATTGTCAGATTCTGAACGGTTCATCACC
AGTCGATTGCCCTGCTAATAGAATGATTATGTCGCCTAATCGCGGGACAATCGACTGTTGATGAACCAT
TCAGAATCTGTCAATTTAGTATCTGAG
>TCONS_00052758 geneID=XLOC_010595 scaffold_19:402601-403253(+) Intergenic
AAGGTGCGTTCTCTTCATCGGCATTGCCGACACCGTTTCTCCATACATTTATGACAATCCGTTTGGTGCG
ATACGTACGATACCGATAGGTAGACCCTTACCTTTAAGGTACATCCAATTGACATGTTATTGAAATCCAT
TACATTTATCATTATATTTACGTACTAAGTACTTATTATTTCCATGCATTTATCAGACTTGCGGGGCAGGA
CTTTATGTTAATGCAGAGAGGTTGTCTAAATAGGTATAAAGCATTTAGAATTTATCATGATTAACTTCTTG
TCTAGTGGGCTATGACGTTGCACACATTAATAGGTATTTAATAACAATTAAAAATAGTTCTAGTGTGGAT
TATGAAACCAGTGTAAAGCATCCATCTTAGATTTATCATAGTTCGTTCTTGATGATTATTATTAATGGTGT
TAATTACCTAATACAGACCTATTGTTTTTCTTTTAACAAACCGCATCAAAATCCGTTAGTTACACAGGTT
TAAAAGTCAAGGCACAAAGCACA
>TCONS_00052404 geneID=XLOC_010602 scaffold_19:666640-668282(+) Unclassified
CTCACGCTGATACAAAAGATATTTTTTTGTGATTTTTTGCCTATTTTATTTCAAAATCGTGAATTGGCTTC
AGGAAGCTAATATTTATCGTTTAAAAGCAATTACATTCTTGTTACGTAGTGGCTGGCCCTTCAATATTAC
TTATCAAATAATAGATTTGAGACACTTTGAATGAGACTCAGGTCTTGCATTCAAAATTATAAACTGTGA
AAAAATTATTAAGGATCCTGCTAACCTTACAAGTGCCAAGCTACCTCCCTCTGCTGACACAATGGTCTC
AACATGTGTTCTGCGGACCTCTGCTGTAGATGTCTGATCGGGTTCTCGTGTTGCGCCTGGTGTATCGCA
TACTGCGCACTGTGTGGTACTTGCATTATGACGGCGAAGAATAACGATCCACTGTCGTGATTGGTTTAT
GACGTGTTTGGTTCGTGACGTGTTGTTTGGTTAATGACGTGTTGTTTGGTTAATGACGCGTTATTTGGTT
AATGACTTGTTATTTAACGAAAAAGTATTTTTTTTGTGAATTGTTAAGTTTGTGTTGTTATACTGGTGAA

CGAGTAATTTTCAGAGTGTTATTTATGTGAAGTCGTCAAAGATGTGTTGTGGATTTTTAGAGTGCTGTTC
CATGTTTTGTGGTTTTATTTTGGGGGTTTACAGTTTTTGCTGTAACATAGAGAAGGATGACCTCCACATA
ACGATTCCGCAAGCCAATTAGGATCGCCGCATACTTTTTAGTAACGGCCGTAGTTTATACGACCGTTACT
AAAAAGTGTGCGGCTATCTTTAGAGTTTAGTTTATAATGCGGTATCAAAGAGAGTAAAATTTCAGTTGA
ATCTGTTAAAAATATACTTATTTATTTATTTCAAAGTAATTTTCGTGATCTAGCAAATGTCTGTAGCTATG
AAAGTCCATGTTATGTTTCGCAACTGGTTTTGTAAGTCCTATATGTTTTCAAAGATCAGGTTATTTACTTT
GTATTAAAATAAAAAGTGAAGTAAAAGTTAGAAATTTTCTTTTTATTTGTCATTTAAAAATAAATAGCTT
ATCGTTGGTATAAAATATTTTTTACAGATAAAATCTTACCAGTATTAATGTAACGAGAAACTATATTGATG
ATACAT
>TCONS_00052551 geneID=XLOC_010665 scaffold_19:1073254-1073858(-) Intronic
CCATATATTAATAGTGCTATTTTTCAAAAATCCGTCAGTGCTATATCAACCGTTGCAGAGTGCAAATTAC
CTACTTATTGGTTGTTACATTACAATTTGGAGTTCTCATCACGCCGAAATAGTAAGAACTCTTCAAGAA
GTATCTACCTAATTGATGAAATACGTCTATGAAGCCTAAAATAATACTCCAGTATGACACCTGCACGATA
CCAGCCTCCTTGCCTCGACATGTCCGTACCACCTCATCTACCGA
>TCONS_00052453 geneID=XLOC_010668 scaffold_19:1208854-1215806(-) Splice junction overlap
TGATCATCCTAGAGAGGTGGCATACCACGAGCCCTCATCCCCCGGTGGTTCCTTCTGGTCTCGCGTGCT
GTGTCTCCCGCTGTACACAGTCCTAGCGGCAGGCGGCGCTCNNNNNNNNNNNNNNNNNNNNNNNN
NNNNNNNNNNNNNNNNCGCCGCCGCTCTCAGGGCGGACTACCTGGCGCTGGGCGGGGCCGCCGCGC
CGCCCGCGTTGAGGGCGCTGCCCGCCCAGGGGACTAGAGTTAAGGTGATAGAGTACCTCTCTACCGAC
CCCGCTCTCCGCAACCAGACGGCGGCGGCGCTGCTGTCGCGCAACTACACATTGTTGGCCGATCGAG
CTGACGGGATCATGTATGTACTGCAAGAGGGGGGGGACTAGACCCTAGCGGGGAGAGGGGTGGAGGT
GGACTAGCGTGGCGGTTGTGAAGGGAAACATGCTCATAATATACCTACCTACTTTGTACAACATAAAGA
GTATTGACTACAACATTTACTGGTAACAGAACAACGAGCTCTCTGGGCCTCTACGGGTTTTGCAATTTA
CGTAAACGAAAAAAGTTTTGGTTTTCTTCTGTCACCTGCAATACATTTTCTGTCAAAAGTTTCATGACA
GTTTCCGCTAGAGGCGCCACTTTGTATGCGAGAAAACTGAAACTTGTATAGTTTTCGACAATCTATCAA
ACCTGTACTAGACGTAATGATAATTATGTTCTAGTATCATGCTTTTACCCGTATTGTACCATTCGTAAGAA
CGTTACATTACACAGAAATGCTCGACAAAATGTTGTAATCTATACTCACCTTAACGTGAGCTCTGTGAC
AGCTGACAAATCCATAAATTTTATGTGTCATTTGTTGGTTTTCAAGGGAATATTAGAATTCGATAGAGGC
GCCACTTTGTATGCGAGAAAACGTAAATATTTTTTTGTAAATTATAAAAACTTATTTCTGCCTACATTTTC
TGTTCAATGTTGAAGTTCTTTATGTGTAAAATATACTGTAAATTTGACAGTTGGTCATATACTCTCAGCA
ATCTCCGTATACTTATTTTTGTGTTTGGGTTTAAAATAATATACCTTATCTGTTGCATAATAGTCCACTCAG
GAATCTCTAAAACAACAGGAAGCATTATTATGTCAACTCCAAAATTATTCGATTTTATACTTATATATTCC
GAAACATCATTAGTTACACAGAATGAGTCTGTGAAATGTATCAGTATTTGTGTTGTGCAGTAACAATAC
AGTCGTATGGAATTCTACATTTGATATATTCCTCGCAAAAACTAGTACCTAATGCTCTTTTTAAACTCGA
ATCTATTCTTTATACCTCTCCTTACAAATAGTAAGAAGACTTCTACAAAACAGTACCTAATAAGTATTTAA
TCTCAAGATAATTTAATTAAACGGTTTTTTCAATAGCCAGATAAAAGTTATCTGATGGATAAATAAACGT
GTCTGAGCTTTTATCTGACCGATGAATCATCACGACCCATCACGTCCCCACTGCTGGGGCACGGGTCTC
CTTCCAATGAAGGAAGGGTTCTAGGCCTGGTCCACCACGCTGGCCTAGTGCGGGTTGGTGGACTGATG
AATAATCAGGCCTAATTGAATAAACGAACAATTGAGTGGATGAATATCAAACTGCCATATGTCCACCTA
CCCGAAAAATTTACTTTTTGATTTGGTCGTGTTCTCCGTTAAAATGTCAGTTTGCCTATGCCCTTCAAGT
TCCAATAAGTGCAAGGCATCCATGAGAAAAAAGCCGTTAAAAAAGTAAGTTTTTTTCAAATCCTTTTA
AGTCTAACCGTTTTGCATTACAAATTTTCACAAGTCCATTCCGGCCAATCACAGAGCGTAATTTTTTCA
ACATGGCGTCAGTCTTGGTGAGAATTTCGAGGCTGTGGTGTGTGTTCTCAGTGTTTTATAACGAATATG
AATGAAAATGTTGAGGAAATTGTGATTGTGGACCCTGATAAAGCTCGGAAAAGGAACAGATTGAAGA

GTGTAGCAAAAAAACGACAGAAAACTATGGCGAAGTAAGTTTAAAACCTCTTTTTTTCCAACCCACGT
GTCAATCACAAAGCATTTTCGTAGTAAACACAGGGCCGTATCGGGTAGAATATAATACTGTAGGTAGGT
ACTTTTCTACCGAAAATTTTTGGTTGATTAACTTCATTGTATTATATAGAATATAGACTATATTAGTCATAC
TGAGTCCGGCCCATGATATTAGTATTTTTCAAACAACTATAAGTCCTTATGACTCATATCAAAACAGCAT
AAGTCTTGTATGGGCATATCAAACTGCTATAAGTCCAAACTGTTTATTAACTTTTACGCCTTTCTATGAG
TGTTATAATGGATTTGTTTATTAAAATACATTCTTAAGGTCACAAGTCAACTGATTAAATCACGGGGCAA
GTTCTATGACCATTAAATAATAGAGAAAACAAAAACCTCCATTTCCCAAATTTTGCGTGGACGTGACAT
ATGGCAGTTTGATATTCATCCACTCAATTTAGATGTAATATTAATTTTAATTTCTATTTACAAAAGAAGTG
CCTTAAAATTGTAATTTTTATTTGCAGTTTTAAATCATTTTTCAAAAAAGTATTATTATACAGTTTAACAA
TGTCTGACTAAGTACTAAGTACACCCGTGATATAAAAAAAACTATCAAAATCAAAACAGCTTAGTGGA
AACGAAAAAGGTGACTTCACATTTAATCAGACATTGAAAAACATGCCCTTAGAATAATGGTATACCCAC
ACATAGTTATGTCACTAAGGATACAATTAGCTTCACTATTCCAATAATTATAATGAAGAGAAAGATTAAA
CGATGCCATAAGACTGCCTTTATCTTAGTGTGGGGTTGACACTTGTGTCATTGCAGTTTAAACCCTGAA
GAAGTGGTCTTACGGTATTTATTGCTCGGACAATAATAAGGTTTGTGCTATAAGATCGGTAATGTAATAA
TTAAGATAGATAGGTTTTAAATTATAATTCTCACTAGTTTAATTCTGATGATCTACACCAGGGGGCGCCTT
ACGCTAACGTTGAATAAAAATATAATCTAGCTTTCTAGTTATACCTAGGCCTTTACTCAGCACTCCATGA
CACTTTACAAACTTGTATTTTTGTCCACATTTTCTACAAAATGTAGAGTTTAATTTCTGAAATACATATTT
TCTACAGAACATGTATAAGTAGGTACGTACGTACAGAAAGTTTACACTTTCATTACAGCCAATTTGATG
ATTTATGATGAAATGAATGTATCAGCACTAGTTGGAAATATTCAGAACACATTGTTAACGATATTCTTATA
AGTATTGAATTAATCTCGCACATTTTGTGGTTACTTTCTTTCTAAAGGTTTCTGAATCAGCACTCCATTTT
GATATTATGATAGTTATTTCCCTGAAAATATAAACTAATGGAGTGTAAGCTCGTATTTATCCGATTAAGAT
TTTCTTACCAGTTCTTTTCTCATATTCTACTTTGACTTATTAGCCATTGCTTACAATTTTTCAACGACCTG
CTCACAATCTTTAGAGCAATGGCATGTCTAGCTAATGCCATCACAGTGACGTGATTTATATGATTTTGGC
AGCTTTCCAGTTTTGAAACCTACGATCGTAGAGTTCCCTGATTCGCATTTGGTAATATTATGTCAAACTC
TCTAATGACAAAGAAAACCTATTTCTTTTAACCCTGTGTTTGTCCCTGTCTAATTCATTCCGATTGCCTG
ATTTTAATTTTAAGTACTTTAGTTGTAAATCTTTTAATCTTAACGCATTTTAAGCACAAATTATTTAAATG
CGCATTCCAATTTTATAATTTGATTTTGAAGACTGAATGTAATTTTACTCGAATCTAAATGTCTTAATTAT
TTATTGTATAAGAATTGTCCGTATAATTATTTTATTTTTGGCAATTTTTACAGAAATATTTTTATTTTGAATC
GCTTTAATGACAAAATTGTAACAAATGATACAAAATGAATTTGAAATACTTTTTAATACGATTGATAATA
AACAGTTTATGTGATTTCTTTCAGCGAGTATTGTGTAAGATTACCTAAGTTAAGCTTAGTTATCTATGCG
CTCAATTTTTCACGAATAAATGGCTTTAAATTTAGTCCCTGTGTTTAATTTTGGTACCTACTTACTG
>TCONS_00052552 geneID=XLOC_010668 scaffold_19:1208904-1214416(-) Splice junction overlap
TCATCCTAGAGAGGTGGCATACCACGAGCCCTCATCCCCCGGTGGTTCCTTCTGGTCTCGCGTGCTGTG
TCTCCCGCTGTACACAGTCCTAGCGGCAGGCGGCGCTCNNNNNNNNNNNNNNNNNNNNNNNNNNNNN
NNNNNNNNNNNNNCGCCGCCGCTCTCAGGGCGGACTACCTGGCGCTGGGCGGGGCCGCCGCGCCGC
CCGCGTTGAGGGCGCTGCCCGCCCAGGGGACTAGAGTTAAGGTGATAGAGTACCTCTCTACCGACCCC
GCTCTCCGCAACCAGACGGCGGCGGCGCTGCTGTCGCGCAACTACACATTGTTGGCCGATCGAGCTG
ACGGGATCATGTATGTACTGCAAGAGGGGGGGGACTAGACCCTAGCGGGGAGAGGGGTGGAGGTGGA
CTAGCGTGGCGGTTGTGAAGGGAAACATGCTCATAATATACCTACCTACTTTGTACAACATAAAGAGTA
TTGACTACAACATTTACTGGTAACAGAACAACGAGCTCTCTGGGCCTCTACGGGTTTTGCAATTTACGT
AAACGAAAAAAGTTTTGGTTTTCTTCTGTCACCTGCAATACATTTTCTGTCAAAAGTTTCATGACAGTT
TCCGCTAGAGGCGCCACTTTGTATGCGAGAAAACTGAAACTTGTATAGTTTTCGACAATCTATCAAACC
TGTACTAGACGTAATGATAATTATGTTCTAGTATCATGCTTTTACCCGTATTGTACCATTCGTAAGAACGT
TACATTACACAGAAATGCTCGACAAAATGTTGTAATCTATACTCACCTTAACGTGAGCTCTGTGACAGC

TGACAAATCCATAAATTTTATGTGTCATTTGTTGGTTTTCAAGGGAATATTAGAATTCGATAGAGGCGCC
ACTTTGTATGCGAGAAAACGTAAATATTTTTTTGTAAATTATAAAAACTTATTTCTGCCTACATTTTCTGT
TCAATGTTGAAGTTCTTTATGTGTAAAATATACTGTAAATTTGACAGTTGGTCATATACTCTCAGCAATC
TCCGTATACTTATTTTTGTGTTTGGGTTTAAAATAATATACCTTATCTGTTGCATAATAGTCCACTCAGGA
ATCTCTAAAACAACAGGAAGCATTATTATGTCAACTCCAAAATTATTCGATTTTATACTTATATATTCCGA
AACATCATTAGTTACACAGAATGAGTCTGTGAAATGTATCAGTATTTGTGTTGTGCAGTAACAATACAG
TCGTATGGAATTCTACATTTGATATATTCCTCGCAAAAACTAGTACCTAATGCTCTTTTTAAACTCGAATC
TATTCTTTATACCTCTCCTTACAAATAGTAAGAAGACTTCTACAAAACAGTACCTAATAAGTATTTAATCT
CAAGATAATTTAATTAAACGGTTTTTTCAATAGCCAGATAAAAGTTATCTGATGGATAAATAAACGTGTC
TGAGCTTTTATCTGACCGATGAATCATCACGACCCATCACGTCCCCACTGCTGGGGCACGGGTCTCCTT
CCAATGAAGGAAGGGTTCTAGGCCTGGTCCACCACGCTGGCCTAGTGCGGGTTGGTGGACTGATGAAT
AATCAGGCCTAATTGAATAAACGAACAATTGAGTGGATGAATATCAAACTGCCATATGTCCACCTACCC
GAAAAATTTACTTTTTGATTTGGTCGTGTTCTCCGTTAAAATGTCAGTTTGCCTATGCCCTTCAAGTTCC
AATAAGTGCAAGGCATCCATGAGAAAAAAGCCGTTAAAAAAGTAAGTTTTTTTCAAATCCTTTTAAGT
CTAACCGTTTTGCATTACAAATTTTCACAAGTCCATTCCGGCCAATCACAGAGCGTAATTTTTTCAACAT
GGCGTCAGTCTTGGTGAGAATTTCGAGGCTGTGGTGTGTGTTCTCAGTGTTTTATAACGAATATGAATG
AAAATGTTGAGGAAATTGTGATTGTGGACCCTGATAAAGCTCGGAAAAGGAACAGATTGAAGAGTGT
AGCAAAAAAACGACAGAAAACTATGGCGAAGTAAGTTTAAAACCTCTTTTTTTCCAACCCACGTGTC
AATCACAAAGCATTTTCGTAGTAAACACAGGGCCGTATCGGGTAGAATATAATACTGTAGGTAGGTACT
TTTCTACCGAAAATTTTTGGTTGATTAACTTCATTGTATTATATAGAATATAGACTATATTAGTCATACTGA
GTCCGGCCCATGATATTAGTATTTTTCAAACAACTATAAGTCCTTATGACTCATATCAAAACAGCATAAG
TCTTGTATGGGCATATCAAACTGCTATAAGTCCAAACTGTTTATTAACTTTTACGCCTTTCTATGAGTGTT
ATAATGGATTTGTTTATTAAAATACATTCTTAAGGTCACAAGTCAACTGATTAAATCACGGGGCAAGTTC
TATGACCATTAAATAATAGAGAAAACAAAAACCTCCATTTCCCAAATTTTGCGTGGACGTGACATATGG
CAGTTTGATATTCATCCACTCAATTTAGATGTAATATTAATTTTAATTTCTATTTACAAAAGAAGTGCCTT
AAAATTGTAATTTTTATTTGCAGTTTTAAATCATTTTTCAAAAAAGTATTATTATACAGTTTAACAATGTC
TGACTAAGTACTAAGTACACCCGTGATATAAAAAAAACTATCAAAATCAAAACAGCTTAGTGGAAACG
AAAAAGGTGACTTCACATTTAATCAGACATTGAAAAACATGCCCTTAGAATAATGGTATACCCACACAT
AGTTATGTCACTAAGGATACAATTAGCTTCACTATTCCAATAATTATAATGAAGAGAAAGATTAAACGAT
GCCATAAGACTGCCTTTATCTTAGTGTGGGGTTGACACTTGTGTCATTGCAGTTTAAACCCTGAAGAAG
TGGTCTTACGGTATTTATTGCTCGGACAATAATAAGGTTTGTGCTATAAGATCGGTAATGTAATAATTAAG
ATAGATAGGTTTTAAATTATAATTCTCACTAGTTTAATTCTGATGATCTACACCAGGGGGCGCCTTACGCT
AACGTTGAATAAAAATATAATCTAGCTTTCTAGTTATACCTAGGCCTTTACTCAGCACTCCATGACACTT
TACAAACTTGTATTTTTGTCCACATTTTCTACAAAATGTAGAGTTTAATTTCTGAAATACATATTTTCTAC
AGAACATGTATAAGTAGGTACGTACGTACAGAAAGTTTACACTTTCATTACAGCCAATTTGATGATTTAT
GATGAAATGAATGTATCAGCACTAGTTGGAAATATTCAGAACACATTGTTAACGATATTCTTATAAGTAT
TGAATTAATCTCGCACATTTTGTGGTTACTTTCTTTCTAAAGGTTTCTGAATCAGCACTCCATTTTGATAT
TATGATAGTTATTTCCCTGAAAATATAAACTAATGGAGTGTAAGCTCGTATTTATCCGATTAAGATTTTCT
TACCAGTTCTTTTCTCATATTCTACTTTGACTTATTAGCCATTGCTTACAATTTTTCAACGACCTGCTCAC
AATCTTTAGAGCAATGGCATGTCTAGCTAATGCCATCACAGTGACGTGATTTATATGATTTTGGCAGCTT
TCCAGTTTTGAAACCTACGATCGTAGAGTTCCCTGATTCGCATTTGGTAATATTATGTCAAACTCTCTAA
TGACAAAGAAAACCTATTTCTTTTAACCCTGTGTTTGTCCCTGTCTAATTCATTCCGATTGCCTGATTTT
AATTTTAAGTACTTTAGTTGTAAATCTTTTAATCTTAACGCATTTTAAGCACAAATTATTTAAATGCGCAT
TCCAATTTTATAATTTGATTTTGAAGACTGAATGTAATTTTACTCGAATCTAAATGTCTTAATTATTTATTG

TATAAGAATTGTCCGTATAATTATTTTATTTTTGGCAATTTTTACAGAAATATTTTTATTTTGAATCGCTTT
AATGACAAAATTGTAACAAATGATACAAAATGAATTTGAAATACTTTTTAATACGATTGATAATAAACAG
TTTATGTGATTTCTTTCAGCGAGTATTGTGTAAGATTACCTAAGTTAAGCTTAGTTATCTATGCGCTCAAT
TTTTCACGAAT
>TCONS_00052216 geneID=XLOC_010670 scaffold_19:1296229-1299686(-) Unclassified
CACGTCACTTGTCGCCAGCGAAAAATCCGCCGATTGGTACGCTCATGAATGTGGGCCTAACGTACGCG
ACGACGACGACGTACCTTTAAGCAGTGGTTGGTGTTTGCATTTTCATGAAGTCCGCCCACAGTCCAGT
CGGCCGCGGGCACTCGCTTGGATCGCTCGTGTAACCGTGATTTTATTTCCTTCACTCCACCACTTCTATC
CTACCGTGATGCGAGCAGCTTGGCTCGCGCTGGTGGCATGTGCCGCCGCCGCCGCCGCCGCCGCCCCC
GAGGAGACCGCGGGGGGCGCGGGGGGCGCGGGGGCTCCGGGGTACCTGCTGCCGGGAGACGTCAGG
CCCTCGCACTATGACGTCATGCTGGCGTTCGATGTCAACGCTACTAACAAGTTCCCCTTCTTCGGAGTT
>TCONS_00052076 geneID=XLOC_010672 scaffold_19:1425231-1439681(-) Exonic overlap (+)
CTAGTTGGCAGGTGTAAACAACTCTTCCATTTCCACAGAGTCCATAGATGGTTCAGCCACTGTCGGCA
CCGCTCGTGGCGAACCCGGATTGATGCATATAACTAAATAGCGGTATTTGACGAGAATAAATCGCAAGA
TTCAACTGAGAAAGTGATCGGTTTAGGCGCGGATTACGCGCCAATACAGTGCCAGTGTTCCAATATTCA
AATTCACACCTCAACGTTCTATTTTATCTTTTCTTTTTTCCGTGTGTGTGCCAGTGGTGACTTTTCAGTT
TGTTGTTTTTGGATATTTGGACATCAGTCTACAGTTGGACGCCATGGCCACCCAACCGGCCTTGTTCAG
TTGGACCACGGGG
>TCONS_00052820 geneID=XLOC_010624 scaffold_19:1487085-1489117(+) Intergenic
GCTCAAATGCCAGAGAATAAGTTTAAGTGTTACATTAAAGAGAAACTCAGTAAAAAAGCTTATTATAA
AATTGATGATTACTTAGAGGACGTTAATGCATGGCTGTGATAAGTAGTTAGCTCTGCTCATATTATTATAA
TAATAATTATTAAGCTTATATGTATTGTATACCAACTAGTTTTAAGTCTTTTTTTGAAAAGATGGCTCAGG
AGTTTCTTGCCTCCGTTCTTCTCCTTAGACAGAAAGAGCCCCCCCTTATCCGAACGGAGAGTAATTTGT
AAAAATTGACGTTCATCAGAAAATTTTATATTTGTACGATGAATAAAAAAATTTGAGTTTGAGTTTGAG
TTTAATTTCATTACGTGATGAAGGCTGCGTTGACCAAAATAAAAGGGTCAATTTAAGCAGCAGAGTAA
CCGGAATAAAAAACAGCAACACCAGATGTTATGATGATAAGTTTGGTGATGATCTACTGCGTGCAGCTT
GCAGCTAGGCAGTCTGGAACCGTGTCACAACCATGGAACACCGAGTCCTTGCTGTACGTGAATACCTA
CGAAGTCACGTAGGGGTGGCCTCAGCGTGTTCCATATGAAGCTGGCTGCATAACGGAACGAGTGGCG
AAACAAAGCGGTTTTGTGCTTCGGAGTGACCATAAGATAAGTGGACGCCCTAGTATTAATTGTATGACA
GTCGCGTGACCCTTCAAGTTTGTTGTATAGGTACTGGGGTGATTTATACTTTATAACACCAAACAGAAG
CG
>TCONS_00052438 geneID=XLOC_010631 scaffold_19:1659509-1660938(+) Unclassified
CCTGAATTTCAGCCAGTCTTCTCGGCACCTACTGCTCTGAAAGTCCTAAACCTAACCGCGGCAGAGAA
CCAAATTTTCTCTCTCAGTATAATTCTAACATCTTCATATATGAAAAACATATATAAAACATCCAAACTAC
GAGATCACTTACGGTTCATATATAATTGTATGAACGATCCCGTAAACCACAAAGAGATCTGTTTTTCGAC
GAAAAATATATCTTACAAGTAGTGCCAGACTATAATTTAGTCTTTAAAATTGTAGCGATAAAGTTGTAGT
TTAAATAAAAGTATATATAAAAGTTTCAAGTTCAACGCAAAAGACTAAGACCACGATAAAGATATTAGT
TAGCGAGCTATGTCAAAGATGAATCTGGACGGCTTTTTTTCCTACTGCAAGCAAATAGCTGAACACATA
CAATTGGGAGAAAGAATGGCGGCGCTCTCCGGACAAGGCACCCCGAAACTGACGTCATCCGTGG
>TCONS_00052009 geneID=XLOC_010631 scaffold_19:1659540-1660919(+) Unclassified
CTGCTCTGAAAGTCCTAAACCTAACCGCGGCAGAGAACCAAATTTTCTCTCTCAGTATAATTCTAACAT
CTTCATATATGAAAAACATATATAAAACATCCAAACTACGAGATCACTTACGGTTCATATATAATTGTATG
AACGATCCCGTAAACCACAAAGAGATCTGTTTTTCGACGAAAAATATATCTTACAAGTAGTGCCAGACT
ATAATTTAGTCTTTAAAATTGTAGCGATAAAGTTGTAGTTTAAATAAAAGTATATATAAAAGTTTCAAGTT

CAACGCAAAAGACTAAGGGAGAAAGAATGGCGGCGCTCTCCGGACAAGGCACCCCG
>TCONS_00053042 geneID=XLOC_010792 scaffold_190:124337-175159(-) Exonic overlap (-)
CTCAAAACGATAACCGCGGAAGGAGATGTGTCTCATGCTATAGGGTCTATTTTCCAAGGCAAGGTTAA
AATTTTCCGGTCGTAAACTTACAGAGTCGTTTATCGCACATTACAGTCGTCATAGAAATGATTGCTGAAT
TATGTTCTTGTCACTAAATTTTTCTTTGAACGTTTTCCTGTTTGCCACTTTACATTGTCATTACCATAAAC
CGAAATTGTACAAAAAAAGCTCAGTTGGCAGAAACTGTTTGTTTACTGAATAAGTGCGACTTCTTACT
AATTTGTCATCTTTCATTAACATCTTAGTAATTCTATTAAAAAATATCATCGACGAATCTCATCAAATTTC
AGTGCCAAAATATCTAAAAAAAACATGTATTGACACTGAAATTTATTAATGCGTCTACGATGAATTTTAT
CGAATTACCCAGCAGTTCCACAAGAAGATACAACGAATCAGCGAACGACATCACTGCCGAAATAAAA
AAGAAAACAATCGACCAATCAAGTAGATCGGANNNNNNNNNNNNNNNNNAAAAAAAAAACAACCC
AACAAACAAGTAGACCGGAAGAGGAAAACCTTCCAAATTCAAACTGAATGAAGCGACTCCGTCACG
GCAGTTTGAAATAAGAACTTTTTTATTCTTCTAATTTCGGGACTAGTGATGTTTGTGAGTGTGCGTAGTG
TAGTGGTGTGCTGTAGTCGCGGCAGTGACTAGTGTTGTGTGTAACATTAGAAAAACTGGAGGTCGACG
CGAATGAGACGTCCGTTGTTGGCTCGCTGACGACAGATGTCACCCCAGACTCTGCAATGTCGGCCGAT
GGGTCGACGACGGACATCAATGGTCTACTCAACGATACTGAATCCGATCTCACCGGCCTATCGCATGTT
G
>TCONS_00052993 geneID=XLOC_010792 scaffold_190:124337-176096(-) Exonic overlap (-)
CGACCGCCGCCGCCGCCGCACGCGCGGACAAACAGCAGTTCCACAAGAAGATACAACGAATCAGCG
AACGACATCACTGCCGAAATAAAAAAGAAAACAATCGACCAATCAAGTAGATCGGANNNNNNNNNN
NNNNNNNAAAAAAAAAACAACCCAACAAACAAGTAGACCGGAAGAGGAAAACCTTCCAAATTCAA
ACTGAATGAAGCGACTCCGTCACGGCAGTTTGAAATAAGAACTTTTTTATTCTTCTAATTTCGGGACTA
GTGATGTTTGTGAGTGTGCGTAGTGTAGTGGTGTGCTGTAGTCGCGGCAGTGACTAGTGTTGTGTGTA
ACATTAGAAAAACTGGAGGTCGACGCGAATGAGACGTCCGTTGTTGGCTCGCTGACGACAGATGTCA
CCCCAGACTCTGCAATGTCGGCCGATGGGTCGACGACGGACATCAATGGTCTACTCAACGATACTGAA
TCCGATCTCACCGGCCTATCGCATGTTG
>TCONS_00052992 geneID=XLOC_010792 scaffold_190:124337-176098(-) Exonic overlap (-)
CGCGACCGCCGCCGCCGCCGCACGCGCGGACAAACAGTTCCACAAGAAGATACAACGAATCAGCGA
ACGACATCACTGCCGAAATAAAAAAGAAAACAATCGACCAATCAAGTAGATCGGANNNNNNNNNNN
NNNNNNAAAAAAAAAACAACCCAACAAACAAGTAGACCGGAAGAGGAAAACCTTCCAAATTCAAAC
TGAATGAAGCGACTCCGTCACGGCAGTTTGAAATAAGAACTTTTTTATTCTTCTAATTTCGGGACTAGT
GATGTTTGTGAGTGTGCGTAGTGTAGTGGTGTGCTGTAGTCGCGGCAGTGACTAGTGTTGTGTGTAAC
ATTAGAAAAACTGGAGGTCGACGCGAATGAGACGTCCGTTGTTGGCTCGCTGACGACAGATGTCACC
CCAGACTCTGCAATGTCGGCCGATGGGTCGACGACGGACATCAATGGTCTACTCAACGATACTGAATC
CGATCTCACCGGCCTATCGCATGTTG
>TCONS_00053077 geneID=XLOC_010792 scaffold_190:124349-176096(-) Exonic overlap (-)
CGACCGCCGCCGCCGCCGCACGCGCGGACAAACAGTGTAGTGGTGTGCTGTAGTCGCGGCAGTGACT
AGTGTTGTGTGTAACATTAGAAAAACTGGAGGTCGACGCGAATGAGACGTCCGTTGTTGGCTCGCTGA
CGACAGATGTCACCCCAGACTCTGCAATGTCGGCCGATGGGTCGACGACGGACATCAATGGTCTACTC
AACGATACTGAATCCGATCTCACCGGCC
>TCONS_00052841 geneID=XLOC_010786 scaffold_190:209180-212021(+) Unclassified
TACATATTTCATACATGAACAACAGGACATATCTATTTGAAGCGAAACAATGCTGATGATACGATGATAT
GTATTAAACAGATATATTATTGTACATACGGTCTGATTTAATGAAAAGGTTGCCAAGTGGTTGCTAATTTA
TAAACGATTCCTCAAACAATATATCTCAATTATATGTAAGTAATTATGAGATTTGAGTAACCTTCGGCTTC
TGCTATAATTGATTGCAGGATTGTCCTTCATCAGGACCTAAGTTTGCGCTAAGGAATATTTACTCAAATG

AGAAACTGGACCATTTTTTACTTAAAATGCAAACCGTATGTAACTATGCTTTATAAATGATCAGCTATAG
TTATAATATGACATGAATAGTTTTGGCTCGAGACCTAAAAATAATTTTGTTATAATTTTCTCTACAATGAA
TAATTGAGCAACCATACTGATTGTCTTTTATAGAAATAAATTTACAACTTTTGCGCACTACTTAAAAATA
GGAAAGCGGCTATTCAAACTTGCGTATTGGCCACTGCCATATGTCTTGTATAGTTCCTTACATAGGTATAT
AGTGCAGAAGTAAATTGACACTTCATTGTTATCAGTGGCCAAATTATTAAACTATAAAGTAGCCATATAA
GACTGCGTGAGCGTCCCAGTAAGTACCTACACTACAGCTACTAACATTATGACTGGCAGTCTGTTCGA
A
>TCONS_00052959 geneID=XLOC_010794 scaffold_190:234542-325840(-) Splice junction overlap
ATTATGAAGCTGCTCATTTTGTATTTTAAGTAACTCAAATTAATGTTTGTAAGTTGCTGTTCTGTTGATTT
CTCGTCACTCGCAGTCAGTCGCTCACTTAGTAATTCTATAGCCCAAATTAATTAATTTTGACACTTAGAA
CTGTAATTTACAGTCACTCGTTTTATTGCTACCCCTAAGGTTTGACAGACTACTCTGTGATCTACTGTCG
ATCTATCTAGTCTATGATCAAAAGGGTGTCAAGAAGTCATCAATATTAAAGTTTTTGTTGTTTTCGTGTC
TTTATCTGTGATTTTTGTCCATAAACTGAATAAGTGCACCTTTAAAAGTTAACACACCGTCTGGGTGAA
GCCATTGGCGACAGCCGCTGATCTTCACTTGCCATGAGCCGGAGGGCGGCGGCGGCGGGCGCTGCGG
GGGCGAGGGGCTAGCGCGAGCGATGCAGTCCGCGCCCCCTGGTGGCTCGCCCAAGAGGCAACCCAA
CGCGCAGGGCGCCTCCACAACAACCGAC

>TCONS_00052989 geneID=XLOC_010791 scaffold_190:533775-534169(+) Intronic
GAGCATATTTTTTAGGTTATATTTTTGTTTACACTACTTTACACTAAAAGCTAGTAAATATTTTCATAAAA
TGATATTCTATTAGTGATTAAAGAGCTGAAAACCTACAATACCTATGTTAAATTATACTCCTTAATAATGT
CCTGTACTATAACAGAGAGTCTCAATATTTTATACGAGGAATCATCAGCGTTATATTTGGGGTCAGAGAT
AGCCGAGGTGACATCGATAGATCCCCTTCAGTTTCATCGTGAATACGTATCCAAGAATATACCAGTCGTT
ATTAGAGGTGGCTGTGCTTCCTGGCCCGCAT
>TCONS_00053004 geneID=XLOC_010800 scaffold_190:568497-574175(-) Unclassified
CAGAGCATGGCACCTAGAGTTAAGATGCAATCTTAAGTTTTTGCACATTTTGCTTGAGGTTATTTAAGG
ACACGCCCCCTCCCCTGCCGTCGCGCGGCGCGTACTTCGCGCGGCCCTAGTGAGCGCGCTGGCGCCG
CCCGCTGCTGGCCGCGCTGCGCTCCGCCGCGCTGCTGCTGCTCATCTACCTCGCCGCCGAGATGTTGC
AACACTAGCCCCACGGACAGACCTTCACAAAGGTACAAGAATCACGTTTCTACGACTTAGGATAGCTG
AGATAAACCCGTGTTTGTAAAATTAAAAAATTCGAAATTAACTTAAACTTATAATTTCTGGTTTGGTGTT
ATTGTTATTTCTGTCTCAACGTTGAAATGTGGCAACACTAGCCCCACGGACAGACCTGTCTACAGGTAC
AAGAATCACGTTTCTACGACTTAGGATAGCTGAGATAAACCCGTGTTTGTAAAATTAGTAAATAGCATA
ATTGGAAAATTTCAAATTTCTCGTTAGGTTATATTATGCCTATTGTTGGTAGTATCTCAATGTTGAAATGT
GGCAACACTAGCCCCACGGATACATCTTGCCGAAGGTACAAGAATCACGTTTCTACAACTTAGGATAG
CTGAGATAAACCCGTGTTTGAAAAATTACTTAATTCGAAAATTTCATGTATCTGGTTTGGTCTAATTGTT
GTTAGTATTTTAATGTTGAGATGTGGCAAACACTAGTGCCACGGACAGAACACGTTTGTATCTTTCCAA
AGGTACAAGAATCATGTTTCTACGACTTAGGATAGCTGAGATAAAACCTTGTTTGTAAAATGTAGAGTT
TCTAAATTTACCTTTAAGGCTGCGTTACTGAAATGTTTCAACGCTAAGATGTTTTATCATCACTGAACTG
ACCGACCTTTCCAAAGGAACAAGAGTCACGTTTCTTAAACTTAGCATAGTTGAGATTCATCTAATTTAC
TTACTAAAGTTTTTGTAAAACTTTAGAAAAACAATCATTTCCGCCATTTGTTTCTCATTTACATCTCCAC
CGCGAAGTAGATTAATGATTATTATGAGTAATCGCATACATAGGTACTGACGAAGGATAGCCGGACTATC
CTAAGGAATAATAGCTATGCTTGTATCCACAGTAGCCCATCCATACAACACGTATTCAAATGTTTATTCG
GTTCCTAGAAAATGATAGCAGATCTATGTTATTTATGATACCTAATTAATCTATGGTAGGTAGATAGAGAA
ATAAAATCGGCGAAATATGTTTCCGCGGCGGTGGCGTATATTTCCCGAACTAAGTCTGAAGTTATTTTTC
TCGTAACTAACGTATATTTCAAAATACAAAAATAAGCCCTGTCTAGTTTTAGTCTAGCTAGACTTAGTGC

CAATTTCGCCATAGTTGGTTAGAGCATAACCAGGGAGTAGCAGTTGATTTTTGACACATCTGTCATGAA
TTTTATATGGAGATGACGTACTTATAATGAATAAATCAATCCCTGTCGGTTAGATAACCGACTATGGAGA
AATTGGCACTTAAACAATTAATTTTAACGGGTCATTCATTTCACAAGTTCTTTCCAAATTACTCTATAAG
TCAGTCACATAATATCATTGAACTATCCTAAGTCTATAAGTAAGTAATAACTCGACGCAATGTAATTACAT
TTGTACCAATGCAAACAGAAGTAGATTTTTGTATGGCTATAAAACATCTTTAAAAAAAGTTAAAACAGG
GTTTATACTTTGAGGGTATTGCTTTTAGAACGAAGTAGAAACTGCAGCAACATTTTTGTGGATTAATGA
GGATATAAATGAAGATCTTTACGAATATATAGAATTTAAATTATATACAAGGTTGTGCAAGAGTGGTATA
GTAAGCCAAAACGGGGTCATTTTTTGTGAATTGTTTAAAGTCTTACAACATATGAAAAAATATGATTAG
AATGTCCACCTGAGTGACTTCCCTTTCGTTATTCGTAGAGCTTACTATACACTTTTTGCAACATCCTGTA
TACTTTTGCTACTAATAAAAGTATTACTTTTAAATAGCTTGACTACAGGCTTCCGTCTTTTCTGTTATCAC
AACAATAACAAATTTCTTAAATCAGTAAGTTGTTAAAAAAGACATCGCTAAAAGTTCATTTCTGCGTTA
TACAGACTCTGCTTCATATTAAATCTAGAAATATCACATCCAGTTTTTTTTAAGTCAAGTTTACATCAAG
TGAACATTATTTGGTCTTGCAGAATCTCCATAAAGTGAAAACGTGTCGATACTTACATGTGTTAATTATT
AGCTTTTAACAAGAGTTAAATATAAAATGTGATTTTTAAAATTACTTTATTAAACCGAAAGCAATTGTAA
GCATACTTATAAAGTTATACTGAATATCATAATAAAAAAAAAACTTTAAGTAAATGAACTACGTGAGATGT
GTAAAAAGTATGTATATTTTAGTCCTTAAATTATGTAATTTTAAAGGTCAAACATAACAGTAATGCAACT
TTATACCATGTCGATTTCAGTTGGTGGTAAATATTGATTATTGCAAGAAATCATATCATAAACAAACATCC
ATTATGCCTATAAGTTATATTCAACTGTTTAGGTATATTTATTATGTGTAAGAATATCTCATCGGTTTTTGC
TTAATGTGTCTAGTCCCTTAGCTTGTCCATGTAGTGTAGATAGATATTTTATATATGTTTACTTGTATTACT
TATGTATCTTACTTGATACTTCATATATATTTCAAACATTACCTATGAAGGCTCTAGCAAGCACACTTTTTA
TCACCTGTCTATCTACATTTTCCGTATGTTCCGTTTCCGTCTTCCACTCGTCAATTCCCTTCCAAATGTAC
AAAAATGTCAATTGACTTCCTGTTTTTTGGTTTTTATTCATATTATTCTTGTTTTCCCGCTAAATGGACAA
TCTTGTTTGTGGCCATATGCTATTTATTCAAAGAATAAAATCTAGTTTTAATTATTATCATAAAACCTATTG
TTGCTGCTTCATAAACCTATGCCAATATTTTAACCGACTTCAAAAAGGAGGAGGTTCTCAATTCGTCAA
AATGTATGTTTTTTATGAAATTACTAGGCGGAGTAGACAAGACACAGAATTAGTTAAGTAGGTGTCCCT
CGACGGCTCTTGTATCGATTTTGCATTGATTTTTTAAGTTTTATTTTGCTCAGCATGGGGAGTGCATGTTT
TTTACTCCGTTTGAGTAGTGGTGAGTAAGCGCAATAGGGAATATGCATAAAAAAATTTAATACTTTTATT
GGATAGTTTTACATCACTTTATTATAGTAAAAAAAAAATTCAACGCCAAAATAAGTAATTTAAGGTATTTT
AAAGAAAGTTAAAAAATTACAACCATCACGACCACTTTGAAATTCCCGTCGGGATGGCGGGCTGGCGT
GACGTCATAATCTTTTAATTTCACGGCTCAGAATAATGAGTCCCGTCAGTCGGCATAGATTTTTTACCTA
ATCAAGTAATCACAGAGTACTTTTGTATTTTCAACAAACCAATGTTGTCATGGTAAAAAACGAAAAAG
GAAGTGTATAAAGTGTGATCAGTGGCTGTTTTGTAATGGTAATAAAGTGTGACCAGTGGCTGTTTTGTA
ATGGGAGCGCGCAAACCAGAGGACCTTTGTACACAATATTACGCTATTATGTCAATATGAATATAAAGT
AATATTTGTATTGTATGTACTTACTGTAACTTTGGTTCTCAGTAAGAGAGGCTAGGGTTAGTGGGTCATT
CTATTCTATTATCTGTGGGGGTGTAAGAACCTGCACCTGGCTCTCTTGAATGGAACCATTGTGCATATCT
CAAAGGTCTTAACCCCCTTCCTAAGCTTGGACCATTTCCCACCACGCTGGTCCACTGCGGATTGGTGG
GTTCACATAAATATCTAAATGTGCTTTATCTAAATATTGAGATTTCCTCACGATGTTTTCTTTCACATGCT
ACATGATTATCCCCGAATTTAATTCTACAAAAATAATAACGTATAACATACAACTTATCTTTTATTTATAAT
AAAAACTTTTCCGCAGCTGGCCACAGTTTAATCGTTCATACTGCGTAATAAATTTAATGTGTCCAGTTCT
TCTGATTACGAGAATTCTTCCATAACTTAATTTAGAAAAAAATAACAACTTTTGAAGTATAAATAAATAA
ATAAATAAATAAATAAATAAAATAAATTGTGTTGCAATATGGAACATATTTGAGTGTCTCGTTGACTAGC
GAATGGCTGTTTACGCCTCATTACAAAAGAACAACTAGTGGCAGCCCATACACTACCAACAAAAAATA
TAAAAAGTCCTAAACTTTGCAAACAAACAAGCATATCAAACAAGAAGTGGAGAGGTTATAGGAGGCT
AGGATCTATTGGAAGAATTTTTATGTGATCCATCGTATCTGATCACAACGAGCATCACAAACACTCGGT

GACATTAACATTTCTTTGTTTACACTTGACATTTATTTAACTTCCGCCGTAAACGCAAAGAAGTTATTAT
TCTGAGATTGATATATAAAGAATTTTGACGTCACATCCGCCCTAAGAAAATGGGTGACGTTTCTCTGAA
GTTAGAATTTACCGAAGTTTTTTTGTACTTTCCAACTTCATTTACTTCCTCAAAAATAAATTATAATCAA
AAATATTGATGGAGGCGTAGTTATGAAATAATAAAGTTTATTATAAATAATATTTGACCTTTATTTGAACC
TCTTGCATATTCCCTATTTTTTATGGCGTGGGAGAGTAGCCACCAAGCGATATACGGGCTTCACTAGCTC
CGACCATACATATTTGGGTCGATTTTCACTACTCGATCGCTTAAGAAAAACCTACACTCGGCCCTCAGA
TCACTTTTTAAGACTGATTCGACTAAAGAAACCACTCATGTGTGGAAATGGCCAAATTTATTTAAACCA
AAACATTTGCTAGTTTTATCTTTGTTTAGTATCATTATTATTTCGTTACAAATATAGGTTAGTATCATAGTC
ATAAAATTGCTGATAGAGTTTCATTTCTTATTTCCATGTTCGTTCTGTAAGATTTAATGATAATAGCATAC
CCAATGGGTGCCTACTGGCAGTTATTCTCACTGTGCGAACAGTGAAACGCAAAATATGTAAGTATTATA
GCAAGTGTACTTACACTTTTACTTTACATAAAAAGTATTATACTTAAGTGTTCAAATATAAATTTCAGGTA
TCAGTATCATATATGGCAATTATTTAATTCTTACCAACCATCAATTGCATATTTTACTTTAAATTTAATAATT
ATACATTTCTTTGAAATTATTTGCC
>TCONS_00053443 geneID=XLOC_010859 scaffold_191:368390-376044(+) Intergenic
GGAGAAGCAAGTCTGGGGAAGTCATCAACAAATATATTAATCAATCAAGTATTTACTTAGCCTTCGATC
ATCGATGGATATTAATTTTATTATGACGTTGAAGTTCCTGAATGACTAGCATGGGCCGGAGCAAGGAGC
CGGAGGTGCCGGTCAGTGACCCCCTGGAGGCCAAGAAGCACCGGGACGAGGCCACGGGGCATCTCA
AGAACGGGAACTATGATAGAGCACTGCACTCACTTGAGAAGGT
>TCONS_00053153 geneID=XLOC_010873 scaffold_191:676032-677308(+) Exonic overlap (-)
AACACAGATTTCTCGGGAAAAAAGTCACGCACTTCGCGAGTCGACCGCGCCAGCCGGCAGCCGCCTC
AAGACGCTTCGTTGCGTCTCAAGCAGCCTCGCCGCGTCCGCAGCCCCCGCGCTGAAGCTGCAGCGAG
CTTATGGAGTCCATACCGAACACATAACATGCTTACCGCACTTGCAAGAACTAAAACTGGAGCAAATAT
CGCGTAGTGTGAAGCCGCTCCCCGCTGCTCTCCTCACCCCCGCGCCTTACTCCTCGCCCCCCCCGCCC
CTGGCCTGGTGGTGATGGACACGGTGTTCCCGGCGCGGCTGCCGCGGCGGCGCGGGGCGCGCGCCCG
CTACCGCACGCAGCCCGTCACCTTCGCTGAGATCAAGGT
>TCONS_00053380 geneID=XLOC_010874 scaffold_191:713369-714114(+) Exonic overlap (+)
TATTTATAGACACTTTATTGTATACACAAAACAAAGACAAAAACATGAAGACAGAAAAAGTAAGCAGT
ACACAAAGGCGGGCTTATTGCCAAGAAGCAATTTCTTCCAGCCAACCCTTGGTTGGTGGAAAACTAA
AGTGATATTCACACGAAACTCTTTTATANNNNNNNNNNNNNNNNNNNNNNNNNTCTATTTTATTCTCAGA
CATGCATGATTTCACATTAATAATATGTTTGCACGCCCTACAAGGGTAGAATGTAGCAGGTCATCTCTGT
TTTTCTGTAACATCTTTGTGTACTTACCTACGTTCATGCAAATAAATGATTTTGAATTTTTTTATCTAACA
TCGAGGAATGGCTTTAAATATTGCTTAGGGAGTAATAATGGATTTCTTGCTTTTNNNNNNNNNNNNNNN
NNNNNNNNNNNNTTTCTTGCTTTTTATCAATGTGTATTAAAACATATTTAATGTAGAACATCAGTAGTGA
AACTGCAAGCTGCTGGGTGATCCTACCTACCCTGACTCCCGGCGGCACCTGCACCACGAGAGGGCCT
CACCGCGGTCTATGCACTTTTCCCTGACTCCCGGCGGCACCTTACCTCACTCTGTTCTATGAACTTCAT
CTTCATTGACAAAGTAATGACATGCTATATTACCTGGTCGCAGTGAGCAGCTCCGGGCT
>TCONS_00053740 geneID=XLOC_010923 scaffold_192:240391-241571(-) Splice junction overlap
CACAGTAGGTAATTATAGAAACTTGATTGATTTAGCCATTAAGGTATTGTGAAATTTTATTGTGATTTCTT
AGTTTTTAAATATATAACCATTTACAAGTAAGTTTTTATCATAGAAATATATTTGGTCAATCTGACGAATT
TTATTGTGGATTAGTTTTATCTTGTGGAATCGTAAAGATAAAGATTAGTGTGTCACACATCACATGACGG
CACCTTTTCATTATTCTTAACATAGTTATCAGTACAATATTTGCAAAGAAGCAGCTTGTTACTACAAAAG
AGAAATATATTATGTTTATTCAGTTGCAATTATTTTCCTAACATAGTGAATAGTGATACGTGTTTTGTGCT
CTTATTATTGTGATCTTAAACTGCGACATTCCAAAAAAGGACAAATCGATGGCGCTGCAACGGGACCTA
>TCONS_00053769 geneID=XLOC_010923 scaffold_192:247726-248155(-) Exonic overlap (+)

GTATCAGTAAATTTTGGTTTTCTACTCGGAAAATATATTTTTAATTTTCAGTTTTTCAGTGTTTTTAATTTT
ATACCGTGAGTTTAGACTTCACATATTTACAAATATGTTTAAGAAAATACTGCATACAAAATTTGATTGG
GTTATTGTGTGAACGGCGACATAAGCCCAGCAACAAACTTAAAACAAGCAGTGGCGTAGACCTTGGA
CACCAAAATGTTTGCGAGCTTAAAGAGTAAAATTAAGGAGGAAACTGGAAATGATATCAGCAAACTTA
CAAGCAGCTGGAGGGCGGGCAACTTGTTGGGAAGAATATCGTTTAGGGATG
>TCONS_00053872 geneID=XLOC_010960 scaffold_193:304732-306990(+) Unclassified
AACTATTATATGAAATCGTTTAGTTGTGATTATTACAAAACATTCCATGAAAAGACGTTTGCTAACATTT
TTTATAGTCTGATGATGCAATTATGATCGTGGCAGTTTAAACATTAATCTATGTACCTAGTACTTAATTTTT
TTACTGATTAGTTTTTGAGTACAATGCACTTAAGTTTAGGTACACGTTGTTTTTTTGTTTTGTCTCGTGT
CACGTTTTTTTACAAACAATGTTTATTTAGAAATCATTTAAGGAATTGCATTTTGATGCTCACTTTACGTT
AACGTTTATTTTTAGAAGATTTACATAATCTTAGGTTAGTAGGTTGTTAAGCTTTGATTATTATAACAAAT
TAGGATTATATTACGTTTAGTTTTTTGGCGTTTGCGTGCTGCTATCCCTTTCTCTTTCCCTCCATCAGTTT
TTCCTTTTCCCTCCCATGATAAAGACAACGTATATTTACTACTTTAACCACATAACCTTATGCATATAATT
CTCCACTTAGCTTTATAATCATGGTACTACCTCACACCACATCTATTGTAGTCATGTCAAATATTAATTAC
CTATTAAGTTACTTTTGGGTAATATTTCAGAACTATTTCCTGGCAACATTATCTTAAAATATATACCATAG
ACAAAAGAACTTACTCTTGATTACGTTGTGTATGAACTTTTTAAATGACACAAATTAGTTCTGAAATATG
CCCAATTGGGTGATATGTTAAAGATAAATTTTATAGTTATCTTTCGGTGGTTTAACGTTTATGTTAGTGGT
ATCACCGTTGGTCTGTATATCACAGGATATAGGAATGGGGCTTTCACATACCAACACGGTGAAATGGTT
AATTATTTGTCTATGTCATCCTTAAAATTTATAACCTTTTGTTTCATTGACACATTATTAAGAAAGGTACG
TAACTCCTGTACTTACCAGCTTTTAAAAGAAATTTTTTGCTGATCCGGCAGGCACAAATCTAAATTTATA
GATATATTTTAATGTAATATTAACGATAATACTAAATTTTGATTTGGGCATTAATAAAATTGGCAGTGTCA
ATGTAACAAAAGCAAAGTCTAAGTTGAAATCAAATTATGTATTTAATTAGTAAATCGCTGGTATATTCTA
TTTAATAGCACTTTATTTGGTACATATTTGTTTTGCTAAAGAATAAGAAGAATACTTAATAAAATATGGGT
AAGGCAACCGTACGACGCAGTTTCAATCTATAAGTATGGCAACACAAATTTATATCTACAATTTTTACCG
CGTAAGCAGGGACGTATTCATTTTTCATATTCTCTTTCGTATATTCCGTTGTGTTTTTTCTGGTATTTGTCT
ATGGTTGTATCTGCTTCCTACGATTGGCTGCCCTGAAGTGTAGTAACATGCTGTATCCGTATGGTTTCTG
TGCTGGAAAGCATGCGACATCGGCTGAGCGGTAAGTGTTTTTATATGTTTAACTGAAACATGGGTTGAA
TACTAAAATAAGTACGGGGTAGCATGGAAAGCTTAAACTAGCAAATTGTTTATACAAAGGGTCTGTTTG
CATGACTGATTCGTTGGTACGAGATTTCCTGAAGTTCTTTCGCTTGATACGTTTAAACCTATTTATGGCG
ACTCCATTTCGCTTCGTTTTCCACAGCACTATGCGGATACAATGTGTTTCGTAAGCCAGTGTACACAGA
CTTTCCCTTAAAAGTGTATTTTCCAAAGAAGTTTCGTGTACGTCTCTCCATTTTACCTTACTTACATTTTC
ATTAAACTTTCACAGTAATTAGTACACCTAACTACTTTAAATGCTTTCTGGTTTAGCGGTAAGCAGTCTC
CCAGTCACTAAATAAAAAATAACTCAGCTGACTTAACCATGCTTTAATGTAATTTTCTCTCGTGTCTTAT
CGCGTTTTTTTCTTCACTGATTTTATATATGGTATTATTGCCATCTAAAAGTTAAAATATATAAAAAAGTC
AATGAAAAAAAAAATGGAACGTTAACTATTATTATTTTTAAACTATAGTGACTGGGGGAAGTGGTGTGAG
GCTGAGTTATCATTTTTATGTTTTACTAGTTAGGTATACAGCTAAGTTTGGTGTTTTGAGTATAAAAAAC
CACTCAACCATATTTGAAATATTATTTACAAAAAATCTTATTGCCGATTTAATTTTTAAAAATTCAAAGA
AAGTTTATTATTACTTATTTTTTTA
>TCONS_00054010 geneID=XLOC_010960 scaffold_193:305759-307874(+) Intergenic
AAATCTAAATTTATAGATATATTTTAATGTAATATTAACGATAATACTAAATTTTGATTTGGGCATTAATAA
AATTGGCAGTGTCAATGTAACAAAAGCAAAGTCTAAGTTGAAATCAAATTATGTATTTAATTAGTAAAT
CGCTGGTATATTCTATTTAATAGCACTTTATTTGGTACATATTTGTTTTGCTAAAGAATAAGAAGAATACT
TAATAAAATATGGGTAAGGCAACCGTACGACGCAGTTTCAATCTATAAGTATGGCAACACAAATTTATAT
CTACAATTTTTACCGCGTAAGCAGGGACGTATTCATTTTTCATATTCTCTTTCGTATATTCCGTTGTGTTT

TTTCTGGTATTTGTCTATGGTTGTATCTGCTTCCTACGATTGGCTGCCCTGAAGTGTAGTAACATGCTGTA
TCCGTATGGTTTCTGTGCTGGAAAGCATGCGACATCGGCTGAGCGGTAAGTGTTTTTATATGTTTAACT
GAAACATGGGTTGAATACTAAAATAAGTACGGGGTAGCATGGAAAGCTTAAACTAGCAAATTGTTTATA
CAAAGGGTCTGTTTGCATGACTGATTCGTTGGTACGAGATTTCCTGAAGTTCTTTCGCTTGATACGTTT
AAACCTATTTATGGCGACTCCATTTCGCTTCGTTTTCCACAGCACTATGCGGATACAATGTGTTTCGTAA
GCCAGTGTACACAGACTTTCCCTTAAAAGTGTATTTTCCAAAGAAGTTTCGTGTACGTCTCTCCATTTT
ACCTTACTTACATTTTCATTAAACTTTCACAGTAATTAGTACACCTAACTACTTTAAATGCTTTCTGGTTT
AGCGGTAAGCAGTCTCCCAGTCACTAAATAAAAAATAACTCAGCTGACTTAACCATGCTTTAATGTAAT
TTTCTCTCGTGTCTTATCGCGTTTTTTTCTTCACTGATTTTATATATGGTATTATTGCCATCTAAAAGTATAT
TCAATGTCTCAAAACCATGGGGCTCTACTTAACCTGCTAGAGGCTAGAGCGTTTATAAAAATTGGGATT
TTGTCATTTAAAAGTAAAATTTCAG
>TCONS_00054334 geneID=XLOC_011026 scaffold_194:37275-42154(+) Splice junction overlap
TCGTGCAGGCGAAGAATAGATACGGGTGGAATGAGGTGAGCGATATCTTCCAATTCCACACACTGGGC
GGGCCGCACTCCGTCCACGCGGACGAGTTGTCCCCGATGTTCTCTACAGCGTCCCAGCGAGAACTGA
CAGCGTGCCTCACGACGCTNNNNNNNNNNNNNNNNNNNNNNNNNNNNNNNNNNNNNNNNNNNCGCTC
GCCTTATATCCCCTCCTACTCCTCGTGCAGTGAGCGCGTCCGCCAATCAGAGCGTCCACGATAGTGTTG
ACTAATCACGGCGTTGCGTTGATTGTCAGTGTTTTATTTTGTTATCGGAGTTACGGACTTAATGAGCCAT
TTGTGCTTTTTGGTGGATTTTTTGCAGCTTTTATGGGAAAATGGCAATTTGTGTAGAATATTCTTCTCAA
ATGAAAAGCCTTAAAAAACAGCATCAGAAGCAACTACCGACTTAAGGGCCTATTTAGGTATAACTTCA
AATGGCTGGGTAGGGAAGGGAATGCATGAATTGTGAAAATATTCCAGATATTGAAGGCAAATAAGAAA
TCTCACTTTTTTATTTATGTAAAAAATTATGACACCAAAATACGAGTACGATATAACAAGAAAAGTTTTG
TAAGCATTTAAAGCGCATAAGAAGTGTTGTGAATTTTTTAAATTTAATTAAAAATACTTATTCATAGACT
GTGTCTCTTTAATAACATAATGTTTGATAAGTACATTTTTTATTTAGTAATTTTATAATAGTGTATAATCTC
GATTTAGATTTAGATTATTATATCATTGTAAATACCTAGTCACAATATTTTGTTAGATTTAAAATAACCCCA
GAATTAAAAAGTAACCCCAGTTTCCGTAAAACCCTGGATTAGAAGTCGTGCATTTACACGATTGAAGA
AGAAGGATTAGAAATAATCTGCCAGTACTTTTAATCACTGTTTCGGTAACTTACCAGTAACCAGTGATAT
CAGGGCCTCGAGTGCGAGTTTTTTCACCTATCGAGAAGTGAAATCGAAACACAAATTTGTATGGCGCG
GGAGACACCAC
>TCONS_00054344 geneID=XLOC_011045 scaffold_194:41856-47474(-) Intergenic
CTGGACTATTATCTATAGTATTTGAATAAAAATAAAATGTCTTTAGACTTGTTATTGGTAAAAATAGATTT
TCTTGAAGAAACGGAGGAAAACATTGGATGTTTATTTGGAAAATATTTCTTTGTTTTATAACTTACTTCG
TACACAGATCGCATTAAAACAGTATAATATAGCTAAGCATACCTCCCCATAGCTCCCCAAAAGAGAAGT
GAAATCGAAAACGCAGATTTGTATGGCGTGGGGCTAGTACAGCTACTTACCGCTAGGTGTGGTGTCTC
CCGCGCCATACAAATTTGTGTTTCGATTTCACTTCTCGATAGGTGAAAAAACTCGCACTCGAGGCCCTG
ATATCACTGGTTACTGGTAAGTTACCGAAACAGTGATTAAAAGTACTGGCAGATTATTTCTAATCCTTCT
TCTTCAATCGTGTAAATGCACGACTTCTAATCCAGGGTTTTACGGAAACTGGGGTTACTTTTTAATTCTG
GGGTTATTTTAAATCTAACAAAATATTGTGACTAGGTATTTACAATGATATAATAATCTAAATCTAAATCG
AGATTATAC
>TCONS_00054391 geneID=XLOC_011048 scaffold_194:164429-166061(-) Intergenic
GTCAGCCCGTCAGTGATAGTTCAGCTGACGAGGCTAGACCAACTAGGTTGGTCTAAGATAATACGATTT
TATAATGTTACCCAAGTGCTGCAACGCGTTTATTTAGTCCCTATTCAGGGTAAGTGCGCCTAAGCGAAG
CCCGAGGGACGAATTAATTGGTTCACCCGAGGTAAACACTGTTTTTCATTAGATTTTCTCACCCGAAAA
GAATATTAACAACAAAAAGAATTGCTCACCCTGATTACCATCCTCAGGATTGTTACTTGAACTTCATCAT
CATTCATTTGCATTTCAGATGCAGGAAAGCAATTTTACCTTTGGAATAAAAAAAACGATTTACTTACCT

ACTTTTATAGCATCAGTGGATGATGGAGATTTTGATAATATTTTTTGGTGAATGGAATTCACCGGTGGGC
CTTTTTTATTTTCAATATCATTTGTTAGCCAGTTAGATTAGATTTTTTACCGAATTCAAATAACTGAATCC
GAGGCATGGTACCACCTTCAAATAAAAAACATACCTGTCAGATGAATTGAGAACCCCCTCCTGTTTTG
GAAGTCAGTAAAAAAATTATAGTACCTAATAAGTAGTACCTAATATTATAGTACTGCATTCATTCTTACTT
TACACAAAAGGTAAACAGTCTTTGCATGTCTTTTTATTTCCATTTATTTAAAAACCAGATTTTACATATAA
CATAACGACCAATGAAAATTCTAATTACTGCATACGATTCATATTTGGCTTAAGAAATTATAACCATGTAT
CTGTATTCCGACAAAAACTCAAGTGGCTCCCAATTCGCCTTCGACGGAATACTCACATACTTTCATTAC
CTACTTTATTCGGTACTGTTCAATCCTTGTTCCCCAACATACCTTACGTTTTGAGTTGAGATGCTCAACA
CATAGCCGCTCACTACGTTCATCTCATAACCTTCGCCTTCATGTTCCCTCTTGCACAACTGTGTTTTATG
AGGGTTCATTCACATTTCAGGCTGGATATGACAGAGGATAGAGTAAAATGGCGTGACATGACGCACAA
AGCCGACCCTAAATAAGGAAAAGGCTAGGAAGAAGAAGAAGATTCACATTTCAGGCTGTAATTATGG
AACGGTCTGCCCATTGAAATCAGACTCGCACTCTCTCTACCATCCTTCAAAAACAAGCTCAAACAGCA
CTACTTAAATTTATCTTATCCTGATTAGAATGCACCAGCAATTATTTATTTATTTTTAATAATATTTATGTAT
TATAGGTATAAATTTGTTATTAACTATCTAGGTTTCATGTATTATTATAGGTAGGTATTTACATATCGTTTAT
ATTTTATATTAATGTAGTTTATAAATATCTCTTGTTTTCACACGATACCCA
>TCONS_00054408 geneID=XLOC_011048 scaffold_194:164550-166170(-) Intergenic
TTTTTCGAAGTAATTTGGAGATAACAAACTGATAAAAACAAATTTGTTACAGCGGATGTGATTAATGAA
GCAGGAAAATTATGAATCTTTTAATTTCAAGCCAAGCTCTGTCAGCCCGTCAGTGATAGTTCAGCTGAC
GAGGCTAGACCAACTAGGTTGGTCTAAGATAATACGATTTTATAATGTTACCCAAGTGCTGCAACGCGT
TTATTTAGTCCCTATTCAGGGTAAGTGCGCCTAAGCGAAGCCCGAGGGACGAATTAATTGGTTCACCCG
AGATGCAGGAAAGCAATTTTACCTTTGGAATAAAAAAAACGATTTACTTACCTACTTTTATAGCATCAG
TGGATGATGGAGATTTTGATAATATTTTTTGGTGAATGGAATTCACCGGTGGGCCTTTTTTATTTTCAATA
TCATTTGTTAGCCAGTTAGATTAGATTTTTTACCGAATTCAAATAACTGAATCCGAGGCATGGTACCACC
TTCAAATAAAAAACATACCTGTCAGATGAATTGAGAACCCCCTCCTGTTTTGGAAGTCAGTAAAAAAA
TTATAGTACCTAATAAGTAGTACCTAATATTATAGTACTGCATTCATTCTTACTTTACACAAAAGGTAAAC
AGTCTTTGCATGTCTTTTTATTTCCATTTATTTAAAAACCAGATTTTACATATAACATAACGACCAATGAA
AATTCTAATTACTGCATACGATTCATATTTGGCTTAAGAAATTATAACCATGTATCTGTATTCCGACAAAA
ACTCAAGTGGCTCCCAATTCGCCTTCGACGGAATACTCACATACTTTCATTACCTACTTTATTCGGTACT
GTTCAATCCTTGTTCCCCAACATACCTTACGTTTTGAGTTGAGATGCTCAACACATAGCCGCTCACTAC
GTTCATCTCATAACCTTCGCCTTCATGTTCCCTCTTGCACAACTGTGTTTTATGAGGGTTCATTCACATT
TCAGGCTGGATATGACAGAGGATAGAGTAAAATGGCGTGACATGACGCACAAAGCCGACCCTAAATAA
GGAAAAGGCTAGGAAGAAGAAGAAGATTCACATTTCAGGCTGTAATTATGGAACGGTCTGCCCATTGA
AATCAGACTCGCACTCTCTCTACCATCCTTCAAAAACAAGCTCAAACAGCACTACTTAAATTTATCTTA
TCCTGATTAGAATGCACCAGCAATTATTTATTTATTTTTAATAATATTTATGTATTAT
>TCONS_00054417 geneID=XLOC_011035 scaffold_194:385645-422776(+) Intergenic
CGAGCGTCGCGGTCGAACGCCATCAACCGAGGCCGAGTTTATTCAAGTAGTCTCGCATCAGAAAGTTA
CATAATTCGGTTGCTCCACACACAGAAGCAACACTTTTGTGTGATAAAAGGTTTCAATAGGTTTTGGA
GGGTTTTAAGCGCCATTCGAGGTGGAGTCTTGTAGTAACCTTCAGGGGGGGTTTGAGAGGCGACGCC
AGCGCCCCCGCGCCAGTGCGCCCGCCGCCGCCGAGCCAAGCATAGCCGTGCTGAGCCCGCGACTAAA
ATACACACTTGTGACTAAAACCAATCGTTATGGAAATGTGTCAGAGAAATATAGTGACAGCAACTGTTT
AGTGAACATACCTAATAAGGACGTATTGACGACATGTCTCCTATACTGCAGTCGCTGATGCGGTGGCGG
CGCGGAGC
>TCONS_00054223 geneID=XLOC_011035 scaffold_194:416784-422777(+) Unclassified
AGAAGCAACACTTTTGTGTGATAAAAGGTTTCAATAGGTTTTGGAGGGTTTTAAGCGCCATTCGAGGT

GGAGTCTTGTAGTAACCTTCAGGGGGGGTTTGAGAGGCGACGCCAGCGCCCCCGCGCCAGTGCGCCC
GCCGCCGCCGAGCCAAGCATAGCCGTGCTGAGCCCGCGACTAAAATACACACTTGTGACTAAAACCA
ATCGTTATGGAAATGTGTCAGAGAAATATAGTGACAGCAACTGTTTAGTGAACATACCTAATAAGGACG
TATTGACGACATGTCTCCTATACTGCAGTCGCTGATGCGGTGGCGGCGCGGAGCC
>TCONS_00054358 geneID=XLOC_011035 scaffold_194:420472-422776(+) Unclassified
CTCCACGGTGGCTCCTCACCAAATCTTCCAACAGAAATGATCCACAGTCGCGAATCTAGTTACAGTAC
CAAACAATAAGCAAGAGGTGATCTAGTGTGCAAGCTGTAGAATAAGGTCAGTGGTCACGTCCAACGAT
CGATTTCAGCTTCAGGCTGCCCACAGGCCTTCAGTTTCATGCGGCAGATTAAAGTGCCCACATGTCTCC
TATACTGCAGTCGCTGATGCGGTGGCGGCGCGGAGC
>TCONS_00054424 geneID=XLOC_011039 scaffold_194:476176-487292(+) Intergenic
GTTAATAAATTCATTGTTCCCATTACCTTATCTGCTAACTGTTCATAATAACACCACTAACACCTACTGTG
AAGAGACTGGATTTGTAATTTTATCACTTCCATAGTGAATTGATGATACTTAAAGGTTCTAATACAATCA
CATGATAAAGATTTTAATCATTTAAATGTCTTTAGTTACCTACTTTGATGTCAGTAACTTAGGTACTTAGA
TACGATAAATAAATATAAATCTTATTTTTCTCTCTAATGTACTTAAAATGTAGCTACCTATATTCTGTTGTC
ATAGATGATAGAACATATAGTAGTGCATACATACAATATAAGTATAGGTACTTGAGTACCAAACCAATCC
AATCTACCTGATGCTCTCCTAATCTACCTTACACCATAGAATAACAAGTACCTACTAACTTAATATTAGTC
TTATTACCTAACTACAATATTTATGTACCTGGACATTTACAAATAAAATAGATAGATACCCTAACTACCTT
CTTACGTTATTTTGGTGATCAAGTGCGACAAATTCTTACTTTCTAAAAATAAATACTTAGTTATAATAAAC
AAC
>TCONS_00054247 geneID=XLOC_011053 scaffold_194:523501-523922(-) Splice junction overlap
CACACACTTGATAATAAGAATAAGGAAAAACATGAACGTAGTGACCAGTGAATAAACTTTGATCACAA
TAATCTCAGTCTCACACTGGGCAGGCGAAGCGCCGGTCCCACGTCACAAGTCCGCGACCGGTCGAGG
TCCAAGATAATAATATTAATAATATCACATTCACCAGACCAGTTTCTGAACGTGTCATTATCACTACTGTT
TGTTTTCGCCAAAATATGCAGAGGTTAAGTTTGGTGTGTGTGTTGGTGTCGTGTGTCGGTGTCGTGGTG
GTGGCGTTGACCACTTCGTCTCCTGACGATGAGCTGGACCTCTCCC
>TCONS_00054701 geneID=XLOC_011083 scaffold_195:23624-26699(+) Exonic overlap (-)
TCGCTTCTAATAAAAACTTGTAACTAATTACCCTGATGGGCTGGTTGTTGGGGTTGCTCCAGGAGACGG
TCTTCTCCCGCACGGTGAGGGGAATCCAGGCGAGCTGCACGTGCTCGGAGTAGGCCACCCGGCCGCG
GTTTCCCACGCTCAGGTCGTAGGCCTGCGCCGCCATCGCCGCCAGGGAGATCAGGAGTAGGGCACGG
AACAACATGGTGGAGGTAGTTATGGAGTAGGGAAGTGTGGTGAACTGGTGAAGCTATTGACT
>TCONS_00054683 geneID=XLOC_011101 scaffold_195:48628-51108(-) Intronic
CGATGGAAGTGCTTGGAGGAAGAAAACAACGTGACGTATAAAAACTGTGCAACACCACACCTACAAT
AGTGCCATACATTCTCGTTGTCTACAATAGGTGGCCACATTTTCTCTGGTTACCCACAGCGAGATATTAC
AATTTGCCGCCCTTTTTCTTGTGCTGGCGCGCCAACGTAAATCAGATTCCGATATTCAACATACGATCG
AACATTAAGAAACAAATGCAACTAGTCTATGATGGTCAACTCAGCGAGCCAATCCGGTGCGAGAACTT
CGTTTCCTGACGTCGCGGTCGAGCCGAACGAAGCCGAAGCAACCCCGGACGCAGCCGAATGACGTAT
TTTTTTTAGTAATCTCAACTTCAACTCAACAAGTCAACAACGCCGCCATTACTGTTTCTTTGCTTCATTT
CGATGTGTATGTAACGCCGGTCTGATTTAAAAGTGTATTTCTGATTATACAGTTACATAATATTACAATTT
TGTGTTGTCTACATCAAGTAACTAATAACTGATTACTGCATCGTGACTGTTGTGTCCACATTATTGTAAA
TCAAAGCTAAGCGTCACCGTCACCATAATGGACAACGAAGAAAGGCGTGCCTCAGCTCTCGAGAACT
CCGAGCAGCCTCACTTAGAGAGGACAAAGATGACATGTGGCATCGGGGTAGCAGCCAGCGAGGAGCC
ACCAGATTCCAGCAAGCCTTCCAGCAAGTCTCCAGCGGCATGCCAGATATGTGCTCCTGCCCCTTGAG
TCCACGAGCTCCGCTACAGTTTCACCACACAATGCACACCATGGGAGTCTGCCGGCACCAACTATAGT
TAACACCAAATTTGGACACATCATAGCTGGTAACACATACAACCATTCATACTCTCTGCCCTTGGAAAA

GCACTGCCTGTTCTGTAAAACCTGTGATTCAGACATCACTGAAACTATTTCAAACTTCTGGCAAGCTG
AAAGCGTCCCTGAAGTCTTCACTGAAAAGCTTTCCGAACACAACACTTGTGAGCAAATATTCAAG
>TCONS_00054630 geneID=XLOC_011109 scaffold_195:315822-350862(-) Intergenic
CTCGGTTCGCAAGGACGTCTCGCGTTTCCGCCGCGCCGAAGCGAGACAGACATAGTACAGCCCTCGG
GCTAGAGCGGGACGAACGATAACTTCGTCCATTTTCAACAGGTCGTTATTGTTACATCCACTGAATGGT
GATTTCGTGAAATAATTCCTAACATCGCCGGACTTTTCTTTTATCAATGAGTACGCTTTAACTTTTACGAT
TACGAGTAGGCACTACATGGCTGTGTGGGATGGGTTATTGCCACTCTAGTGTTGTGAGCTTTTAAGACT
TGTGTCAACTTCGAGAAATGTTATTATAACTTCGGGTCGTTGGCTGACAAAATGTGCTACTTCCGCATA
ATAGTGATAATATTTCAGTAGCAAGTTGCAGTGAGTGCAGTGATTGTGCACGATGCCGAGTCGCCGCGC
GCTCGTCCGCACCTACCCGCACTTGTTGCTAATACAGATTAGCTTGGGGATCGCGGCGGGCATGTCGG
ACGTGTGGG
>TCONS_00054735 geneID=XLOC_011160 scaffold_196:25658-67800(-) Exonic overlap (-)
CGCCGCCCCGAGCCCGCGCCGCCAGGTCGCGCTCACGCAACTCGCCTCACACACACACACACACACC
GAGTGCATTCATCCCAAACGTTCCGAATTCAAAACTCGAGTGCAATAAGTGAATCCAAAAGTGCATGT
GCCCAAGTGACTAGTGTCTCTCTGATTGTAACTGGCCAGTGTTTGTGCTTGTGTTCTCAGTTCAAACTT
GGAATTTTAACGGAGTTGCCTTAAAAGAGATTGATATTGGATTGAAGTGCATCACACATACTTTGG
>TCONS_00054715 geneID=XLOC_011148 scaffold_196:207730-209220(+) Splice junction overlap
GCCGAGACGCAGCCGCCGCGCGCGCAGCCCGCGCCGCTCGCGCAGCAACACCAGGCTCTAATGTGGC
GCGACGGCCCCGCCGGCTACGAGCCCTACACTCGCGCCTCCTGGTTCTACCTCCGCGTGGCTCTACTG
CTAGTCCTAGTGGCGATATCCCTGGTTCTAGCCAGTGCGCTGACGCTGCTGGTGCCGGTGGCGATAGG
CCGCCGGCTGATGGCGCTGTG
>TCONS_00054921 geneID=XLOC_011152 scaffold_196:361405-364084(+) Intronic
GGAATCCCCGGATCACAAGGGGACAGTGGGCCGCGCGGTTACCCCGGTCCCGTAGGGGAGGCTGGGC
CGGCCGGGCCCGCCGGGGAGCGCGGGCCTAAGGGGGAGAAGGGAGAAAGGGGCGTGGAGGGGGTA
GGGGTACCAGGACCGGAAGGGCCGAGAGGTTTGCCTGGTGCTGTTGGACTGCCCGGACCGCCCGGCA
AGACCGGACCTAGAGGCGAATCCGGTCGGGAAGGATCGCTAGGCCCCCGCGGGGCGCCCGGGCCGA
GAGGCGCCTGCGACTGCCCCGCGCCCGCCTACTACGCGTATGCACCGCAGGGGAACAGCAAAGGACC
ATAGTTAGGACAACTAGTGATGACTAGCAAAAGGTTGATGATTGGTGAATCACCGAGCATCAGAGATA
TGACAGACATTTGAATAGCCCATCATAAGAGCCCGGCTGAAACAGGTGCAAGTTTTAAAGAATCAAAT
CTCATAGGTATTTGGGTCTTGTCAAAATAATGGTACAGATATTGAACTTATTGAGAAATCAATACATAATA
TTTTTTCAGGATCTTTCAGGGTGATGTTGGTTCTAAGTACCTATTCATGTTCATGGGCAGAAATTGTGCT
ATGCATTCTGAGATTTGGGTATACGAAATAAGATTAGTCATTGTAATATAAGTGCATTTATTGTAAAGTTT
TATATAACCTTGTAAAATATATAAGTAACTAAGTAAAATACGTTCTAAAACATAAACAATATAAATATTTC
ATTTGTCTCCATCATCTTGACTCACTATAAGTGGTAAGCCATTTATTCCAAAATGGACCCCCCTTAACCA
CCAACCTTTCCCAAGAATCTAAACTATCCCATCACATCAATTGATTCCAGGTAAGTATATCTATCTAATGA
TAATATTATTACTTCACTTCAGTTATTTGGTTCGATTTCTTTGACAGAGTCGATTTCAGCCTTGCAAAAG
AAACACTGCTTGCTGTTCAATAGGTGCTGCAAGATACACGATCTGAAATAATTAAGAGAAAATGTTATT
AGTATAGGATCTTCATCATAAATTATGAAGAAGCGCTAAAAATGGATTTCGCTAGATAACGCAACGGTG
CTTAGTGACACCCGATGTTTTATGACTGGTCTAAATAAAAGAGCCTGAATTTCAGCAGTGGACTGTACC
ATTTATTTTACTCCCTATTAGGTAAGTACTTTAGTTTTAGCTTATACGCAAGCTCACCCGTTAGACCTTAA
CATACAAAATATTTTTAGCAAAAACCTCATGAAAT
>TCONS_00055061 geneID=XLOC_011209 scaffold_197:477874-478422(+) Unclassified
GTTAGGTAGGTACTACGACACATTAACTTTGGAGCCTGAGTATATAAAGTGAGCAATATTTTTCACAGA
AGAAACATTATTTCCTCCTATGAAATATTGAATAAGTACCTATAATTTTATTATGTTAGCAAATTCAGTGC

ATTTGACTTTAGAGTGCTTAATTTCTTTTAACAGTGCGAATGTATAGTATGAATGAATTCACGATCGGAC
ACCTACAAATTAATGTAAGCCTCACTTAGGTTATTTCTAACTAGGAAATTTATTAGGTTGGCACTATAAG
CCTAGATCTACCCAAAATACTTTTATTTATCACGACCTCTTTCTAAACATGCCTAAAATAAATAAATAAA
ACTTGTGCTAGTCTTGACTAAAACGTATTTTGGGTGTTGCTTGTAATTTTCTCCAGTTTTACTACTTTTTA
TACTGACACCAACAAACAAGACACAACATTTCATGGGG
>TCONS_00055220 geneID=XLOC_011220 scaffold_197:544442-564565(-) Exonic overlap (+)
GAAGCACGTGTGTCGAAGCGCGTTTAAATTGGCGCGAATTTTCTTTTAAATGTCACAGTATTTACTGTG
ATGAAGTATTAATAGTAATTTTTTACGTTATTGAAAATAAATTGTGATGTCCGATCGTATAAAATACGTGG
TGAGGATCGTAGTTATTACAGTTCTGTATTATACATTGATAAGTGTTGTAAAAGAAATCAAACAATATTG
GAGTGGATCCGGTGAAACACAGCACACTCGCTAAATATTTGGATTTCGTTACGTCAATAGAATTTTTAC
CGGCACCCAATATGGAGCACTTGGCGAATTTCACCGATGACATCAACTCTAGTGTGCAGCCTAGTGAA
TTCTCCAAG
>TCONS_00055412 geneID=XLOC_011244 scaffold_198:259950-262503(+) Splice junction overlap
AGTCGATTTTCCAGAACTTGAGACCTCTTACTTTAAAAATGTCGAACCAAATTCTAGTGTTTTTACTTTT
GACTACATTGATAGCTACAGCGATTGCTAGACCTGCTGATGATGTCGAAAAAGACGCAGAAAAACTTA
AGGACAAAGTTCCAAAAGACCCAAAAGGCGAAGTAAAACCTGAAGACATGAAAAAACCAGGGGGTC
CAGATGCTAGTAAAAATAATATGCTAACTGACGGCCTGCAAAAGATCCCTATATTTGGCAGTTTTGTGA
GTACGGGCACAAAACTAGCCCAGCCG
>TCONS_00055349 geneID=XLOC_011244 scaffold_198:259953-263059(+) Splice junction overlap
CGATTTTCCAGAACTTGAGACCTCTTACTTTAAAAATGTCGAACCAAATTCTAGTGTTTTTACTTTTGAC
TACATTGATAGCTACAGCGATTGCTAGACCTGCTGATGATGTCGAAAAAGACGCAGAAAAACTTAAGG
ACAAAGTTCCAAAAGACCCAAAAGGCGAAGTAAAACCTGAAGACATGAAAAAACCAGGGGGTCCAG
ATGCTAGTAAAAATAATATGCTAACTGACGGCCTGCAAAAGATCCCTATATTTGGCAGTTTTGTGAGTAC
GGGCACAAAACTAGCCCAGCCGTTATTAGGTGGTCATTTCTTCTGAGATAAGTCGATGACGACGGCTC
CAGAATTACAAGTATAAAGGGTCTGTAACTCTGTATACTTATACTTACTGA
>TCONS_00055446 geneID=XLOC_011244 scaffold_198:271936-275466(+) Exonic overlap (+)
CAGGATTATAATACATACTTACTACGGTTGTCTTGAAAAATCGCTTTAAGCGATAAGACCGCCATTTTTG
TACATATGTGTTAGTATTTAAGTTAAGTAGTTAAGTTTATTTTATGTGTGTGTAAAAATAAAGAATATTCT
ATCTATCTATCTATTTATATATATATACTTACTTAATTTGTTTTCAGTTTCAACATGGATGCAAGGGGGGAA
GAAAATGGTTGATGATATGGATAAAGTGGCAGAAGATACAGCGAACAAATGGTTGGCACTCTGGGGAT
AATTAGTAATTAACGACAAAAGGAAGTAACGAGTTCGTCTGTATTTGAAAGTATTAGTAATAAAAATAA
ATAATAATTACATACTTAATCTATAACTACCTACCTATTCTATCCCGTGTGACCAGACTTTTCGAATGGAA
CCTTTGTGCATATCCCCATAAGATCTAAACTGTAAACTGCCTTCCCTAGCTTGGACCATTTCCCACCACG
TAGTACACTGCGGGTTGATGGACCAGAAGGGCTAGCACAGGGTGCATTTCAGACGTGACAAAAGTTC
TCTGTTCTCCGTCAATCTCGCTCTTGAGGCTGCGCGATGTGATGTGAGTGAACGCGATGCAAAACTTTT
GTCTTAAATGCGCCCCGTGCTAGCCCTTCAGTGCGTGGACTATTTAATTGTTGCAAAAAGGGTATACTA
AGCCGAAACCTACATGTGCAGCATGTAAATTCAAAATTCGCGAAATAAAGTATTCATTTTCCATAGAAA
CTTTGTTGGTCACGTGACTTTTTTACTATGGAGAAAGTTTTTTTTTCGCGAATTTTGAAATTCTGATTTC
AGTTTCTTGCTTAGATATACCATGCACCATGCTTCACATGCAGGTTTCGGCTTATTATATCTTTTTTGCAA
CACCCTGTATAATATTATACCTATACATAAACATTTAGTTAAATAACTATACATAATGAATAAAAAGAATA
AGTAATATGCAAATTTGTAACTAATTTAAAATCTGATGTATACCTACTTAAGTGTAATAAATGTCTTATAT
CACAGTGCGTCTATATCATTTTTTTCTTGTTAGGCCCTGTCCGCACAGCGAATATTTCGCCGCCATACAA
ATTTGCGTTTTTGATTTCACTTCTCGATAGGCGAAAAAACTCGCACTCGAGGCCGAAGCCTTGCAGAA
GGCGTCAATAATTGTCTCTTCCATCCAAAGGTTGTCTGGTACAGATTGCTTTAAGCAATAAGACCGCCT

TTTTTGTACTATTTTATATATCATTAATCATCACGACCCATTACGTCCCCACTGCTGGGGCACGGGTCTCC
TTCCAATGAAGGAAGGGTTTAGGCCTAGTCCACCACGCTGGCCAAGTGCGGGTTGGTGGACCCCAAC
ACAAGCAAGCTTGTGCTGAGAGAGTTGTTGGGTAAGTGGGCAACCCGACTGTCAGATGTTTTCAAGC
CGCCCGAAGGCCTCTGACTAGGCTTAACGACTGCTGCCGAAGCAGCAACCGGGACCCACGGCTTAAC
GTGCCGTCCGAAGCACGGAAGCGTCCAGAAAAGCACCACTTGAAATTGGTCACCCATCCAATGG
>TCONS_00055439 geneID=XLOC_011256 scaffold_198:276024-278166(-) Splice junction overlap
GCCATCGACACCGCCGTGGCCCAAATGATGAAAGACAAGCGTGTGTCCACACGCGACGGAGACCACG
CCCTATCTAAGCAGGTTGTGAGCAAACCCAGCAACAGTAGCGCTAGTGTCGCACCCGCCAGCAGTGT
GAACGCACCCTTTAATATGCGCGGTGACAGGAAAGGAGTCCTTATTTAGCTCTTATATTTTTAACGAG
TTTCTCGAAATCTGGGGGTTTAGTGTGAGTTTTTCAATACCAATCGAGCTGTGAAACCTAGCGCAAAAT
TATAGGCGCGGAGCTATTGCACCTATTAACCGATAAGTGATAAGTGGCATCTCTCACGCCCTATACCAAT
TCATGTTTACTCGCCGCTTCTCAAACGGTGAAAAAACTCGCACTCAGGGCCCCGTTTGCACAAGTTTT
AACGCACATCATTAAAATTTTGTGACGCATGATGTAGAAACATACCAGTTAGTGCCTTTTTTATAACTAT
AAATTATACGTTGATACATTGCCATTGCCACTGTATAATATTTTCTAATCGCTACCCAAC
>TCONS_00055335 geneID=XLOC_011256 scaffold_198:295943-309601(-) Exonic overlap (+)
GTGAAATAAAATAAACAACACCGCTGCGGAATGTTTTCAGTTTTTACTAAAAGAAAGCTTTTATAAAGT
TTTAATCAATTAGGTATTGAACTGAACGATTATAATCTTTAAGATTTTGTTGAATTCAGTGAATGGAGTG
AACCTAGTTTCAGAGTCCGTAGAATTTGAACACTCCAGCCATGGGTCGCAGGGTGACGGTGGCGGTGT
GCACGCTGAACCCGTGGGCG
>TCONS_00055432 geneID=XLOC_011248 scaffold_198:352136-367180(+) Unclassified
AGCGCGGCCGAGCGGAGCGCACGTGCCACCAACCTAGTCCGGACCGGCGCGCCATACATCGCGGAAA
AACTTTTCAGTGCCGTCAAAATATTTCACCGATCACTTTCCTACACCATATATCTGTTGACAGATATGAA
CTTTCCATACTTTTTGTGTTGTGTTTTTGTTTGATGGCTCAGTGCAGAGACTCGTTTGAAAGTGTTTGA
AGGGGCTAATTAGGAGACGCAAATTGTTGGGGCCATCGTTTCATGAAGGTGAGTCAGGAGAGAGTCA
CTTACAAGCCATAAATCTTATCTGCTAATAATTACG
>TCONS_00055353 geneID=XLOC_011248 scaffold_198:352147-389824(+) Unclassified
GCGGAGCGCACGTGCCACCAACCTAGTCCGGACCGGCGCGCCATACATCGCGGAAAAACTTTTCAGT
GCCGTCAAAATATTTCACCGATCACTTTCCTACACCATATATCTGTTGACAGATATGAACTTTCCATACTT
TTTGTGTTGTGTTTTTGTTTGATGGCTCAGTGCAGAGACTCGTTTGAAAGTGTTTGAAGGGGCTAATTA
GGAGACGCAAATTGTTGGGGCCATCGTTTCATGAAGTGCGAGAGGCGCGGGCCTGCGCTGGGTCGCG
GCAGCCCCCTGCCGGGCGGGATGCTGGCCATGGACGTGACCAAGGAGGCGCGCGCGTCGCCGCTGCC
GGCCGGCGCCCAGCCCCCGGCCGCCGGTGCGCCCGCGCAGCCGCCGCAGCAGCCCCAGGTACACAC
GGCACCTCCAAATGGTTGGCAGGGCACACAAACGTAGTGTATGTGTGCACACTAACGTGGTCTATGTG
TGCAATAAAGCGGAAATCGGTAAATACACCAGTAACAAACCACACACACACACACACAACAGCACAAAC
GAGTTTGCGCGTAGGCAAACGCCCTTACACCAGCTGTGACATAAAAATATCAACCGGTCGAGTAAGTC
AAATAGTTATAAAATTGACTTGAAGCAAAATTTCACAACTTGTAGCTATACTCTTCGTAGCTCATTGGTA
GTATGTTAGACTTATGAAACATAGGTCGTGAGTTCGAGTCCACTGATGGGTATTTTTATTTTACTTTATTT
AAATTTAGCGTTAATTTAAACGCTGTTGAGTATAAAATAAATTTATTTGAAATTGACAAATGACTACAAG
CTGCATAGCTGTCGACTATTTCTCTTCGCTAAAAATATATGATTCGTTCTTCATTTGAGAAGTAAAACCA
TTCTACCCTCATACAATATGAATATTATGAATAAACGAAATTAGTTTTTAGTTTTAGTGACAATTCACCTC
TGCGTCTGCATAGTGATTCACTTTGTGATTTGTCAATTTATA
>TCONS_00055679 geneID=XLOC_011309 scaffold_199:72882-73995(-) Unclassified
CTCCTTGCGTCTGATGTTCTCCTAATAATTTGTTTATTCGGAGTGAAATATCTGATGGAAGCGATCCACG
CCGGCAACATCTACAGCAGACCTGGAGAGGTCCTCTACAGATATGAATACTAATAATACAAGTTGTATA

ATCTATATACTAAGTTAATTATGTACTAACTTTAAAAGTTAAGTACAAATGAGACCAATGATTTATAAGTT
CGAAGATTGTTAACACTTTATGCTAGTAATTGTAAGGTCATCGACTGTTACAATTCAATTTTGTATTTATT
ATTGTTATAAGTACTTATAAATAAGCTATAATAATACGTGATATTGTACTCGAAATAACAATAAATATACA
AACTTAGACCATGTAAATAAAGTTCAATAAATCATCTATGAAAGAAAACTCTGTTTCCATCATACCACC
CATTAACAGCACACGTTATAAATAGTAAGTATACACAATCACAGTCAAAACTAATACTCTAAAATAAATA
TTAC
>TCONS_00055892 geneID=XLOC_011279 scaffold_199:118935-119592(+) Intergenic
TTTCATTGCGTTCACAGACTAACTAAAACTCTTGTATAATATTTGGCAGTGCAGCATTAAATTACGTTCA
TGACAAAATCACTGCAAGTTATAATTTTTCACTTTACGAATAATCTCAATATGTTTACGTTATAATAAATC
TTTTGCGTTTTGTTCGTAATGATTTGTTACATACTAGGGGTAGGAAAGTGATTATATCCTTGATATAATCA
CAATTTGTTGCCGTGGAGTGATCGAATTTATAAACGAAAGCAATTTAATAGGAAGCGCAAGAGGCTAG
GTTTTGTTATTACGGAAGCATTTGTGAAGATAGATAGGTAGATACTAGGTAGTTGATAAAGTTAGGATGA
GGAAATGAATAATGGAAATAATCCATTTATTAAAGTTTACTTAATAAGTAGGGGTTGTAGTAATGTTATG
TAGCTCGTTAAAGAATTACAAAAGATTAGGGGTTGTATAACATAATGCTTGTAGTGGAGTTAGAGAATA
TAATCATCATCATCATCAGGAGTTGTTTAATATAATGGATTCTTATTCAGGACATTGTC
>TCONS_00055893 geneID=XLOC_011279 scaffold_199:118935-119698(+) Intergenic
TTTCATTGCGTTCACAGACTAACTAAAACTCTTGTATAATATTTGGCAGTGCAGCATTAAATTACGTTCA
TGACAAAATCACTGCAAGTTATAATTTTTCACTTTACGAATAATCTCAATATGTTTACGTTATAATAAATC
TTTTGCGTTTTGTTCGTAATGATTTGTTACATACTAGGGGTAGGAAAGTGATTATATCCTTGATATAATCA
CAATTTGTTGCCGTGGAGTGATCGAATTTATAAACGAAAGCAATTTAATAGGAAGCGCAAGAGGCTAG
GTTTTGTTATTACGGAAGCATTTGTGAAGATAGATAGGTAGATACTAGGTAGTTGATAAAGTTAGGATGA
GGAAATGAATAATGGAAATAATCCATTTATTAAAGTTTACTTAATAAGTAGGGGTTGTAGTAATGTTATG
TAGCTCGTTAAAGAATTACAAAAGATTAGGGGTTGTATAACATAATGCTTGTAGTGGAGTTAGAGAATA
TAATCATCATCATCATCAGGAGTTGTTTAATATAATGGATTCTTATTCAGGACATTGTC
>TCONS_00055524 geneID=XLOC_011285 scaffold_199:408341-409842(+) Unclassified
CCCTAAACACTGTGTCGCGCGCATGTCAAAAATCACCCCCGCCCGCCCCCTACGTCACATCCGTCACC
CATTGAACTGTCAAATTTTCCTTCTGCTTCCTTCCCGAAAGAAAATAAGATAAGAAAAATGTTTTTCAG
TAAATTATAATATTTGCTTCTATTTTCTCTCGATACCGATTGAGAAACACCAAATTATTTTTCCAGGATTTT
ACAAAATGAGCTCAGCGAATACAGGTGCAGTGTTTTTTCTTGGAAGTCGCGCTCATTATCAAGTCCTA
ACCCCCCCTTCGTCACACTCGTACGACCTGGAGAACGGGAACACATCGACGGAGACAATCTACCAGA
CCACCTCGACCGAGAACATTTATAACAACCCGTCTACCCTGGAGTGGCCTAACACCTTGAACAGGT
>TCONS_00055851 geneID=XLOC_011286 scaffold_199:507356-507733(+) Exonic overlap (+)
GTTGTTTGTTAAAAGTAAAAGTGTTATTATTTCATCGTTTTGGCAATGGCAAGTCATGAACAACCATCTT
GTGGTCCCTCTGAGGAAAGACAGAGGCGGAAAAGGACCTCGCGAGCATCTGCAGGTCAGATAGAGG
AGCTGCTGGAAAAATTAGAGGCACGACCATTTTTAGCCTATAAAAGGTTTACGGGCTTGCAAGGCCGT
GAAAATTATTTAAAAGCGTGGCAGCGGAACTAAACAACATGAACGGCTCGACAAAAACGCCGGAACA
GTTGATGACGGTACATAATAGTAC
>TCONS_00056023 geneID=XLOC_011320 scaffold_199:798440-800817(-) Intergenic
AGGAGTTGTTCTAAATGGAATCAACTTATTACTGCCAACGGAACCAATAATGCAAACAGTCGATGGAC
AATGAATAAATACATCGCCGACGAGCCGATCCCAAACAAATTGGATACATTCAGTATTACGATTGGAAA
AATGACATATTCTGCTTATGCCAGCATTTTAAGAAGAACTATTACAGATTGGATTAGTATCAACACTATG
ATAAGTATAAATTTTACGAGTAAAAGATATAATAGGTGTGCACTATGTGTAAGGGCAAATGAAAATAAAT
AAATTGGGCTAACTCATTGACCACTGAAGACC
>TCONS_00055591 geneID=XLOC_011323 scaffold_199:879078-879657(-) Splice junction overlap

TCTAGAGTTCGATGAAGATAAAAAACGAATGGAGGTATTAGTAGAAGATTTCAAGAAAAGGATGGAGC
TTCAACTAGCCAGCTTGAACAGCAATATGGAGATTGTATGAAGGTGAACGACTTTAGTACTTATAGTTT
AGAATATAAGGCATATTTTTATAAGCCACACAAATAATGCTTTTGCATGCTAGTTATTTATCCTATATTAA
GTATGTAGTTATAGTATTATTTAAAAAGTATATTACATCATGTTTATCTGTAAATAAGGAATCAAGTAACG
ATAAGAATATAATTTATTTTGTAATAAATATC
>TCONS_00055551 geneID=XLOC_011296 scaffold_199:920819-926770(+) Unclassified
CGCCGCGCCGCCCGCGCGCCGCGCCCGCCCCGCCGGCCCCGCTGCTGCATACGAATAGAACACAACT
CAATCCGCTCCAATAACATTCAGTTCCGAACCGACTCTCTCAGCGAGTGCACGCCTCGACGCGATAAA
CAATAATAATCCCGATTTGCAACTCATTGCATAAAAACTGAAGTGTTTACGGAAAGTTAAGAAGAAAA
CTCGACATTGCGTCAAATATTTAAATCTTGACATTCCCTGTCATTGCATTGATAAAATATAAACAAAACC
AAGTTTCTCTACATCATATTTACACGAAAATATTTTTGGATACAGGAAAGTAAAACTTGGGAAACATTTT
TACAGCAGTATGAAAATCATATGTTTCGACATAGTTTAGTGTTGTGTTTTAACTATAAACAATTTGCTAAT
AACTGAAAAGTTTATCAATAAAAATGTCTGCGTTAACTGTGACCACGACGCTGACCAACGGACACGTG
AAAGGGAAATTGGACACGACTCTCAACGGATACAGGAAGAATGGATACGTCAAACCAGAAACAGACT
TGCCGTCGCTCCGGCTGGCGGCGCCCTACAGCACGGA
>TCONS_00056598 geneID=XLOC_011420 scaffold_2:386933-387988(+) Unclassified
GGACTCGCGATTTCCAACCGTATAAATCTATGATGGGTTTCTGACCATCTTTTGGCATCGGCTATCTCGG
TCATGATCACTAAACGAATAGTGGGTATGAGATTTATCGATACAATAGAGGTTGCAACATTTTTGTGAA
AATATTCTGTGATTTGGTTAGCCGACTAAATGAAAACTTCATTTCACTTCAAACTACGACTACTGCATTT
ATCAAATCTTTAATTTGCCGCATTATTTAGAAAAATGGTGATACGAAGCTATTGTCAAATGATTGTCAAA
ACAAACTGCAAGGAACCATCCAAAACAGAGATTGATTTTGGTGTGTGTTTTTTTATAATTACCTGAATC
CTGTATTTTATTTGTTCATTATATCTAAGGATGGCGCCTTTAAGAAAAGTCTCAGATCGACAAATGGACA
ATTTGTTGGAGTTCGTTGAAAGACATAAAGAGCTTGCTGTTGGCCGGTTCCCTCCCGGCCCACTAGGC
ATACAATCAGCACGTTAGCTATGGGACAGTGCAGCATTACAATTATATTAATTGGGTCTGGAGTTACAA
AAACTGGTCAAAAATTCTGACATGTGACAAAGTGGCAATGTGCAATCATAACTTAAAGGTTAAAGTAG
AAAAAATATTTAATAATGATTATAGTAAGACTGGTAGAAGCAAAATGTGATGGAAAGTTATGTGATTTA
AAATCAAAAATAAAAGCC
>TCONS_00056089 geneID=XLOC_011421 scaffold_2:393656-396603(+) Unclassified
CATCAAAATACACATTTATTTAATTAATTCTTTCTTTAAAAATATAAACATCTATACACAACACAAGTATA
CATTTAATAAACACTTTGATTTAAGTAAAGAGTAGGTTCAAAATTGTTGGTTCGTTCACATATAGGTATT
GGTAACTCAGGGTAAGTACTTACTTAATATTATTAAGTATAATAATTGATGTACTTACCTATAGACAGGTA
CCTACTTACAGACGACGGAAATATTAAGTAGGTACAGAAACAGAAAATATAATTTATTTATAGGTATTTC
AGTGGTACGGTCACTCTTACAAGTCTTAGGAGTCCAGTTTCTTTTTTTTCAAATTCAGAATATATAAATA
CCGATTGTCTGTAGTTATAAAAATAAAATCATTAGGCTATGACTATACTTAATATAGTTAGGTAATTTCTC
AATATAAAACTCTAACAAAATAAACTAAACAAAATTTAAATAGCTCTATCTACATAACTACTGATCACAA
TTCACAACATCTTCTTTTTGTATATTTATATTCCTCAATAAAATGCATTTACTTACTATAATCATTTAAAAA
CCCTTCCTTCAATGGAAGGAGACCCGTGCCTCAGCAATGGGGACGTGATGGGTCGTGATGAATAATTT
AAGAATTTCTTTACAATAATTGTAATGTAGCGATTGACTTAAAGTACTTAACTAATTTATTTATGAGATTT
TGAATATATAAATAATAAAAAATACTTAATACTTATTTATGATTATTATGTATTTCAACTTAATTGACTTGAT
TTGAAAAGTGTCTACTTAATTGATAATAAAATATAAAATAAAATTACCTAGTTACTTTACAACCATTTAC
GGGTATAAGGGAAGACAAAACATAAGATATTTCAGAATTTTTTTATCGGTAGGTAGGTACCTATAGTAGA
TAGTTATCAGAGTTAAGAACGAGTAGGTAATTAAAACTTTTTGTTTAAATTAATTGAACTGTCTACAGTA
TTATTATACGTAGGTAAATAGACATAGAGTAGTCACCCCAAAAAAACTGGAAAAAAATCACACTGCATA
TTTTTTTAACAAATAAGTACCTACCTAAACTAAATTCATTTAAGTTATACAGGGTAACTTCACTCTATAAT

AATTTACCTGGGTGTCGTATCCATACCATGTTACCCACACTAAGGTCCAACCAAATGTAGGTAACTATAA
TCTTAAGTAAGGCATCGCAAAAACTAAGAAGAAATTAAATAAAAGAAGGTAAGTAGCTAAGTAGGTAC
TTAACTATTAATATCCAAATAGTACACAAACATACATTATTATTAAAATGACGTGTCTTACCTAGTACATT
TTAATTGATAAGGTGTACCTCCCTAGAGCCATTATAATAGATTAATTAAAGTTTTTGGCAAAATACACTG
TAGGTAAGTAGCAATGGTGACATGTCATAGGTACTATTAATTATAAACGGAAGTAACTACAGAGATGGT
TTAGGATTAATTCGTTCGTTGACTAAGGTTAGGCAAAGAAGGTTGGGAATGTAGTAGTCGCCCAACCG
ATTTCAAACAGTTCTTTCTACTTCTAACACTTAAAAAAATACATTCATTTATTTATCTACCAACAAGAAC
TACATATCAAGGTATCATATTTATATTAGGTATGCATTTAATGTCCATAAGTACACTATAGAGGTTGTCATA
AAAATGTATAACCACGTATTTAACTGTAAACTATACCTAATCATGACATGACAACCTAGTTACATGACGG
TTTGAGTTTTAAGTACAACAACGTCAACAACCCTGTAGTTTACACTAAAATCTTTTTTTTTGTTTTTTGT
TTTGCTTCCACTACTTTTAAGTAATAAATATAATAAGAGTGAGACAAAACCCTGACTTCCATCCGTAATT
AAACACCACTTATTAACCATCAGTTCTTCCAATTAGCCACCTACCCTACCTATACCTCACCTACACAACT
TTCCTAATCACGACCATTCTACCTTGTCTATGTCTCCCGTAAGTTTGCCAAAGTCACCATTGAATAATGT
GAACCCCTTTACCCTTGCTTCGTTGGTGGTGGTTGGGAAAGGTGACATAAAGAAGGTCACAATATTCA
ATATTTTTGCAGTGCTATTCTGTAACATTATCGTCAAAAAAACGTGGAAGGAATCGATGCTATGTACACA
TTTCGCAATTATTTATTTATGCATAAGTCAAACTGAACAATTTTGTCCACGATCGCTCGAAAATAAAGAT
TTATGAATCTTTAAAAAAATACCAACCATTAGACAATTTTGTATGAATTATTTTTTTCGGAATCACCAATT
TATCAATTTCAGCACTTGGTAAAAAAAACAGGAATATCTCTTTGAACGTAAAGTTTTTTGCTACTTCTA
CCTACTAATTCCATCGACTTTCAAGGGGCAAAAATTACCTAATAAATAAATGCAAAAACCAACAAACGT
TTCGACGCTAAAGTTACAGAACAGCACAGCTGATAGCCACCGCCTCAGCGCTCAGTGCCTGCGGTAGC
GCAGCGGGCGCCGGATGGTCCTCCGCTGTCACAACCCACGGCGAGGCTGCTGGGGCGACCTCCCGAC
ATCCACATTGTTGTTGTTTTGGTTACGAAGTATCAGCATCCGCATGATGTCTTCAGGGAGATCGTCGTC
GCTGTCGTTGAACCTGAA
>TCONS_00056265 geneID=XLOC_011422 scaffold_2:419415-420699(+) Splice junction overlap
GGGTACTTGACGGTTTCACTTGTTTAATGTAGATAGGAAAGTATTAAATTAAAAACCAAATCAAAGTAT
TTTAAATCGGACTCAGTCCCACCTCCCTCCGTCCGTCTATCGCCGAGTCCATCGCGTCAGGGCAGACC
AACCCTCGCCCCAGCGGGCTGCCGCAGGTCATGATCTACCGCTCCATCCTGAGGCTCAGCCAGGGGA
GCTCACAGTACCAGAAGAAGAGCAGTTGGAGACGCATCTTCGGACGGTCGAGCCAGCAGACTGTGCA
AGTGCAGTCGTGAGG
>TCONS_00056539 geneID=XLOC_011425 scaffold_2:671903-676066(+) Intergenic
CTGTGAAGTTATTATTCCGAGCGGCAACTAGTCTGTGAATCCTCTATTTCAATATAAATTGTCTATGGTCA
TATTTTCCTTCTTGCCGCCATTGCGGTTTGTCAACAACACTACTTGTGCAACTTGGATAGATAATCTTCA
CGGATTAAATTAACAGCTTAGTGGTTCTTTCCTTTGATTATTGTGCATTGTATCATGGTTGATTGTTTTCT
TTATTATTTCTTTGTATAATTTCGATTCCACAGTACAATTTGGTTTATAACTTAGATTCTAAATATTTCAAT
TGTAATAGAAACTCAAGTGCTTATTCTGACGATTGTGTTTTTCTTTCTCCCGCCGCTTCTGGTTTCTAAA
ATCAGAAGTGGGATTCGCTTACAGCCCACAAGTGTTTAATTTCCATCAAGAAACAATCAGCCATGGTA
GAAATCTAAAAATTCGAAATGTCAGACGACTCAACAAGTCAACATAACCTCAAAAAGTGTTTACGTTT
TTCGAGAGCAAATGCGCCATAAATCTACGGGATTTCGTTCTGGTGGAAACCTCTTTACCCACGTAAAAT
ATTCGGTCTCGTGTAACAAAACAAATTCACGCGTACTTATTCATTTGTTCATATCTACTTACGGTCTCAA
TGCTCGCGCCGGTTATCTCATCACGAGCTATGTAAACAGTGTTGTTGTCGCGGAAGTTCTGTTGTATAC
AGTGTTGCATGACCCATACGATCATCTCACGTTAAAGTGCCATCGAATAAAGTAAAGTTCAATTTCAGA
CTGCATCTATTGTTTTGTAAGGAGCTTGAGCTGAAGCAAGCAGATTGTTTAGCCAAGCAACGGCAGTG
TAGACACACAAAATCAAAGGAAAGTTAACCTAAGTCCGTACGGGACTCGCAGATAGTTCCAATGATGT
CAAGTAGATTATAATTTGGTTGTAAACGAACACTCTCTGTCAGAGTTTGATTTCTTGAAGTTAAACCCA

AACTAAAGTTTTTCTAGTGGCAATATTAGCTTGTTGGTGACAATGAATCTGGTCTGGGTTTATCACCGT
GGCGGTGAAGACACTGGGGAGTCAAGTTGGTGTCAGTTACTTACTGTCCACCTGTGGCGCAGCTGGA
TGTGTGTTCGAAACTAAACTTCAAAGACGGTAAACAAATTTACAAGCCTGGTGCAAAATCTCCAGGA
ACCATGCAAGTGAGTTCTTTATTATTTGTCCACATCAGCTAGATTCTTCTGTGAATCGCTTCTTATGTGG
AATCCCATAATAATTTATTAATAATGATAATATCATAATGATTTTCTAGTTTTACATAAGTAATTTACTACA
AATTGGTGTTCACTCATAGAGCACAGGAAAGTTGAATTTCAAGATAAGACAACTGTAGGAAAACACCT
AAGATCCTGTCAAGTAACTTACAAGCTGTAGCTCCTCCTCATCATGATCAGGTCCTGGCATTCCCAATT
GTATTTTAGCCACAGGGCTCGAAGAGTAGACCTCAACTTTGTCTGATCATGTGCCACACCTTTGACTTC
ATATCAGTTTATGCCGAGGCTGCTGCATACTGCACTATAGTGTGACCATGCGTGGTTATGGTTACTGTTT
ACTGTCCCTTTCGCTAAACATTATATATCAGCACATTTAGCACAAAAAGGCATCAGTTTTATCTTGGCCC
AAAATTTTATACCTTATAAAATTAATTTTGTGTTCGACAAACCTACATTTGAAGCTAAAAAGAAAGTGT
CTACACTACTAAAAACCTTTACATATGATGAAACAGAGCTTTTTATTAAGTAATACGTACTTATTCAGTTT
ACGTACTCACACTCATTCTGACTCACATACAAACACACACACACACACTCTTTCTTACTCACACACAA
ACACACACACACACACACTCACACACACACACACATTATTTATTTTATGTCTTTATTTATATCCTTCCATC
TGAGGGAACATAAAAATATTGTTTGAACATTGAAAAACAGGTCACCGCCGATCACTGTTACGATCCTC
ATAAGAGGTGCGGGCCTACTTAATCATAAATACAGGCTACATGTGAGCCTAGTTATGATAGTAGTCATTT
TTTGATTTTATGAATAAAGATTTTTGATATTAGTAAGTGAACATTTTGGATGAATGGGCTGCAGGTCGGC
AATATATGGCTATGCCACTCCTTGACCTGGATTTCCAAGTCAAATCTCCTCTTGTTGTTAACTGGTACTC
AGGACCAAACTACTCATACCCTCACAATGATGCTGCTGTGGTCCAGCTATTATGTAAAATTTCCAATGA
AAATTAATATTTTCGTTATTTGACTGAAAGACTAAGATGTTCTTTAGACATTACACTTACATCCATAAATG
TTGTTATGGGAGAGTTCAATAAATATAGCCTTTTTCGAACCTGAATTAAAGTTATTTATTGTTTCAGAGA
TTACTCTGAATGACAAGAGATGGCTGGCCATTTTGGTCAGACTGGCCGAGCTGGATGGCAACCAGATG
GTGCCGATGCAGTGCCGCCG
>TCONS_00056101 geneID=XLOC_011427 scaffold_2:895067-897552(+) Splice junction overlap
GCCGCCGCCCGTGCCCGAGGCTATTCAAGCGGCCTTAGACCAAGCAGCTAAGGAAGAAGCCGAGAAG
AACAAGCAGCAGCAGCAGCAGAATTACTACCCTAGCTCCACACCAGCTCCCTACTACCCGACTACCCA
AGCGGCCTACTACCCGACTACCCAAGCAGCTTACTACCCACAACAACAACAAAACTACTACCCGCAAC
AGCAAACTTACTACCCGCAACAGAACCAACAAAACTACTACCAAGGGTAGAGTCTAGTCTAGATTAAA
CAGCGAATTTATATTGATAGTTAAAATTAACGATAGAGATTCGAATTACCATAACTGTGTTAAAGAGGAA
TTAATTTAAGTCTCATAAAAAGATACTCATTTTAATTTATTGGTGAATGTCGAACAGTGTAACGGTTAGC
CTATTAGTAAGTATGTTTGAGGAAACAAAATGCTTTGTAAATGTTACGCCCGTCCGTAAATATTTGCAAT
AGCATTGCCAGTTATCTTTTTCACGAAGATTTGTTTGTGAAATTGATTACTGCTCATACGATATACGCTG
CCAGTGATACTTGCTATTATGAAATAAACCTTTTTATAAGAAACAGTTTTACCTTGTTTGCATTTTATCTG
TAGCACC
>TCONS_00056800 geneID=XLOC_011429 scaffold_2:976883-985183(+) Splice junction overlap
CGCCTGACCACCAGAGCATAGAGCAACGCATAGTGGAGGAGTACCGTCGCGTGCGCGGCGACGCCAC
GTACCCGCAGCAGNNNNNNNNNNNNNNNNNNNNNNNNNNNNNNNNNNNNNNCACAGTGGCGGAGTCC
CGTCGAGTTCCTGCACCGCAAGCTCACGCACATGAAGCAGGCCGTGCACCACTACGACCACACGCGT
AACCTGAAGCCCGAGCGCGTGTCGAACGCGAGCACGACGCAGGCATACTGACACGCGCCCCGCCGC
CCGCCGCCCGCCCGCTGCCTCTAACCACCACCGCCGCAACCGGAGTACACCCCCCGACGCTCCCGGC
AACCAATCAGCAACGCCGCGCTGACTGTCTGCCAACCAATTACCGGATTTCCGAACTGATAAGTGAAA
CCGCGTAACGCTTTACTACAGTGCCTCGTAAACGGTTATTTTGTGTGTGTGTATTTATTACTACTGCAAAAG
TTTATTTATTGAACGCAAACTTGAGGATCCATCTGTGTCGCCTGCCAGTTGGCAATAGTATATATTGGCA
CCGTTTTTTCGGATGTAACGGAGACGTTTATAATAATTGTTATGGTTAAGGTGATTCGCGTGTAACGAAC

ATGTTTAATGAACATATAGATTAGCGTCCAGAAATATTTTTATTATTGACGTATGTTTTTTCGAGATACAA
ATGATCTTCCATTAGTTTTTATTGTTCTGTGTCATCTTAACCATGAATTTGTAATAATTGTGTAAGCAAAG
ATTGGACTCTTTGCATCTCTGATTGGAACTCATACTGAACTAACATAGAACGGTGTATACATACTGTTAA
CTCCACATCGTATTTTATACTATATGTAACTCCATACTTACTCTTCTGATTTTATACATCTTAGATCATCTAT
TTATAAATTGAATAGCATGTATATATTTTAGCCATAACACCAAAACTATTTATTTCATGGACCAATATATAC
CAATAAGCAATAGGAAATTATAGATGCAGAATGTAGTAGTTCATTAGCCCCTATTACCCTCGCAATGTCG
CATAGGAACGATGACTGCGATATGTCATTGGTGTCATTTGATTACTGTAGTATAGTGATCAATGGATTTG
CGACACTAATCGATAAATATTTGCAGCCACAAAACATATTATGTTGAACGAAGTATTCTGAAGCATCAA
GGGCAAACCTACATACGGCAGCTAGAATAGAATCAAAGTGGCCTATATTTTAACTGGTTGCATGCCATT
TAAAAGTTTGATGTTTGCAATTTAAATCAGTATAACTAGTTAGTGCTGATACATATTTATGCAGCGGTTAC
GAAAAGAGCAATAAAATGTTTTTAATTACAAATATTTATCTTCAGTTCAAATGACCTCAATGTATCTGGT
TGATTATTACATTGTAAATAAAATTGTGTATAGTAAAAGTCGAAATCTTAGTAAATAATAGTTCAATAGTA
GATATTGTTTCGTCCACGTTTAATTCCATTCGAAAGTAATTTCCGTCTAACGTTCATTGGTGTCCAGTTA
AAGTTTGCTTATACTCAATTATTTTACATATTATCCACAGTTTTTAAACTCTGAGGTGTAAACGGAAAATA
TACACCAAGGCCCGCGATCTCGGACGAAAAATAATGAAACTTCAACAATTCGAGAGTCAATTTTAACA
GATTAATGGCCTTTTAATGTAAATTAACCCCATATATCTATACATTTTAACCCTTACGTCGTCCCTTTTAAA
ACTGCTTCTAAAATCCATCCATATAATATTGGAGAAGACCGAAATTTGATTTTAATTCTTCTTGCCCTGC
TGTATATAGTAATTTATGAAAATATAATACATTATTGTTATGTAATGTGTAGTAATACTATAGTTCAGTCAG
TATAAATTGTTACATCAAACTACAGATGTACGTACACGCAGGCGTGTCTTGCAACTTTCACCGGCAAGG
CTGTAAATATCGGGATAGAGGCTCTAACTTTTCGTGTCACAAATATTAAACACTTTATGACGGCAATATA
ATCTGAAAATTAGTTAAGGAAAAAGTGTCGACGCGAAATTGCAACACATGCCTGCCTACCAAACGTAG
GAATTGCCAATTGTTTATTATTAAAAATATGATTTACTTGAATTGCCAAAAAGAAAAACTTTGCGTGTCC
TAAAAAATGCAACAACCGGCCAGTTTAAAACATCCACTAGGAATAAGAAATTATCTGGTCCTCTTCGG
AAGAACCCTCCATTACTACAATAAAATCGTAGTAATTTTAAACTACATCCGGGCCATGCGTCTGACGGA
AGTATAACTATGTCCAAAACAGTACTTGTGACCCACGATATGGTGCCTTACTACGTTGTATTACGACTTA
TTGCGCTTTGTGTTGTCTTCACAGAGCCAAATTAGTATCACATGTTTACTAAACCTTCCAGTGTGTCACA
TGAAAGATATTACACGATTTTCCCGTCACAAATTATGCGTCTTACTAAAGAAATCCGGAATATCGAATGC
ATGATAGTCCGAAAGCGATGAATTTCTATCAAACATTCAGCTTCACTTTAGTACCGTCATGAACTGGTC
AGATGTAAAAAGCCGTCTTGCATATATTTGTCCTGATTGTAACGTCCATATTTGAAAATGATTTAGCGTT
AAGTAATTCTGATTTTAATCGCAGTAATTAAAACAATCTCTTTCATGTCCAAGAATCCAAATGAAACTTA
CCATTTTCTTATTAGTGGCAAGCAAACGTGAATAGGATTATTCAAATAAATTAAACTTATTTCCGCTAATA
TATAAATGTAAATACCTGTTTGTTATTAAATGCATATGTATAGTAGAAGACATATGACAGGTTAACGTCA
AGTTGTTGAAGATTGCGTTTTATTTAGTTCGTTTTCTGTCAATTATTATATGTCAAAATGGCATTTTTTATT
TCATTAAAATCGTCAATTGTTTTTTTGTATATAAAGAAAGTTTTCATCAAGTCAAGTTAGGCAGTCAATT
TATATTTGGAAGTGCTGAAGTGCCCTTTTGTTGCTCATAATAATAAATAATATGTATAATAAAGTAATTAT
GTTATGGTTAAATTGCTTTTTTACTCATTAGAATGCTCAAGAACGGTCATGATATTATATCATTTGAAATT
AGCCTCAAGCATTAATTTCACTTTTATCACTATTATTTTTAATCTATTACTAACTTTTATTATTTTTTCAAA
GAATTGAAGTACCTATTCATATTATCATCATTATGCCACAATTACTTCTTTTAAGCTTCATAGTTAACAAA
GTGCCGTAAGCAGAGACAATAATACTGTGAGAGATCTTTGTTATATAAAAATACTTATGTTTTACTTGCG
ACCTTATCTTTGTTAAATTCGAAGCGAAAAGAAATATATATTTCTTGCCTTACATACATCATGAATGAACT
ATATTGAGTATACATAATATAAACATTTACAGAGCTCGATACACCTATTAACTCGATAATTTGAACAAACC
GTTGAAAATGTTATATTGTTACCACTAGTCTAAGCTCTATCAATTGTTTTTGTGAATTAATAATTGATTATT
GTAAATTTATTTGTTTTTTCGAAAGGAGATTGTAATTTCTTACGTTATATATTATATTTTGGTGGATGTTAT
CTGATGACTGTTGCGTTCGTTTTGTGAATAATGCATTCCATTCAAAGATCCGTTGACTTCGCACTGTTCA

TGATGCACTCACTAATTGAATAGTGTAATAATAGATACATATTATATATTTTTACAGGCCTTAATATCCAGT
ACAGGTCGATCCAATGTGCTTCCTGTTGTCGATAAGGTTACTTGATAACCTCGACGAGGCTAACATTGG
ATCCACTTGCACATACCAGACCACGGTTAGAGCACTAATTTTGTAATAAATTGCAGTATCCCTTGATAGC
GTAATAAATGTATTGTCACAAAGGGAGTGAATCGTCAAATATTGCTCGATTGTTACCGGATAAGTAATTA
GGACGGCAAGGTTGTTGTCTGTATTTTGTCATGTTTATACAAAGGAAAAAATATCTCAATAAATATATCA
TAGTCCATTATAATGAGTAGGCGATATTTAGTCTGTATAATAGTGACATCATTTTTCTTAAATCAACTTTA
GTAATAAACTAGGAGTACTAATTTCTGAACTTGGACCGAGACACGACGGTGGCGCCCCCAATTTTGTG
GTTACCACAGAATTTTAATTATTATTAGTCAAATTATATCAACCCCATATGTTTAACGCAACAATGAATTA
GGCCTATTTTGTTTGAATGTGCCCACGTTATTGTTTGATCGTTATATCTGGCGGCGCGTCGTGTCTCGTC
CAATCACGCTATTATACTTAACGTAGCTGATATTAGCGTCTAAGGCGGGCGTTACGTATACAAGATCCTT
ACTTTACAATTACTTTAGATCATAGAACCTTTGAGTTTGTGTATTCTGATCAAATGTTATAATGTAAATGT
GGTGTACAGTAATATGCGGGCGGGCGGGCGCGGCCGGGGGCTGACATCGATCAAAGACTGTCGCGGC
GCCGAACAATTTTGAATACAATGTAAATAGAGTACGAATTAGGAATTAGACACGTTAAGGGATGTAATC
GTAGTGTTGTATTTTTATTACTATATTACAAAATATATATATTATGGGGAATTATGAAGTTTAAAATAGTTG
CGGTGCCCCCCGGCGCGCCTTGCCGCCCGCCTACCTGAGTCAAGTGTAGGACGATGCGGCAGTGTTAT
GTGTGTACAGAGTTATACCAGCTCGAGTCGGGCACGCGGGCCCTTGCCAGCCGGTTCGAGCGAACGA
CGACAAGCTATGAATACTGACATCGGCTCACGTTAATTAGCTTTTAAATAATTTCATCAAAATATAAAAT
ACACAAAAAAAATGGATATAAATTTCTCGACCAAGTACTGGTTGTTTTTAAATTATTAAAATGTAAAAA
AAAATATAGTATATAAACAAAGTATAGGTACAATTTATGAATTATACTTTTCGGCATTTCGCTGAGTGTGT
GTGGGAGCGCTTCATGCACTGATATCACGAGCACTCGCGCCAGCAACCTACACAGTGTCGAATGTTTC
TATTTATTTTCTCATTTCTTTTGCTCATGTCCTCTTCCATCGCTTAATTTTTATTTTTCATCTGTCTATGTGT
ATTATGGTTTTTGTTAAGGCAGGGTAATGCAGAAATGTGAAGAGGTACTTTTTTAATTTGAAAACGATA
ATTTGTTTGAATGTTAAAATTGAGTTGATTTTATCCATTCAATGGACTTTGTGTTGTTAAGAGAAGTGAT
CGAGTTTGTTATTTTTAATTAAGTCAATTTGAGGTTCGATGAACACTTTTGTGGCAGCATAATTAGTATAT
TTTAACTATCAAGTTACCACAGAATCAATAGTCAAAGTAGCTCTCTTTGGCGTTGCCTCTTAGTGATTCG
AGAGAGGTGGGAGCGCGCTAGCCCCCGCCACGCATCCGAGACACATGAGCTAAAGAGACGATGAGTA
TCATTATAAAAGGTACCTATTCAAAGTCCGGGAACAGACTAACTTGATTGTATACAACATAAGTATAGTG
TTGCTGCAATATGCATTTATGCCATCTTCATTTTAATTTGAATTGTGGGATCAGTGCCAAACTTTCTTCTT
CTTGTTTAGCTCAAAACGATGCAATTGTTAGCATGCTTGGTGAAACAGCTATGTAATTGTTTAGTTAGTC
TGCCATTTTGTGAATATTTTGTTTTGATAGTAATATGTAAATCTTTTATAATATATTTGTTTACAATAAATAA
ATGACAACATTATAAATAAATAAATTGTATTTTCCATTTAAACTGATGAAAAAAATATAAAAAGAATAAT
ATGCA
>TCONS_00056103 geneID=XLOC_011429 scaffold_2:977900-985189(+) Exonic overlap (+)
GTCCCGTCGAGTTCCTGCACCGCAAGCTCACGCACATGAAGCAGGCCGTGCACCACTACGACCACAC
GCGTAACCTGAAGCCCGAGCGCGTGTCGAACGCGAGCACGACGCAGGCATACTGACACGCGCCCCGC
CGCCCGCCGCCCGCCCGCTGCCTCTAACCACCACCGCCGCAACCGGAGTACACCCCCCGACGCTCCC
GGCAACCAATCAGCAACGCCGCGCTGACTGTCTGCCAACCAATTACCGGATTTCCGAACTGATAAGTG
AAACCGCGTAACGCTTTACTACAGTGCCTCGTAAACGGTTATTTTGTGTGTGTATTTATTACTACTGCAA
AAGTTTATTTATTGAACGCAAACTTGAGGATCCATCTGTGTCGCCTGCCAGTTGGCAATAGTATATATTG
GCACCGTTTTTTCGGATGTAACGGAGACGTTTATAATAATTGTTATGGTTAAGGTGATTCGCGTGTAACG
AACATGTTTAATGAACATATAGATTAGCGTCCAGAAATATTTTTATTATTGACGTATGTTTTTTCGAGATA
CAAATGATCTTCCATTAGTTTTTATTGTTCTGTGTCATCTTAACCATGAATTTGTAATAATTGTGTAAGCA
AAGATTGGACTCTTTGCATCTCTGATTGGAACTCATACTGAACTAACATAGAACGGTGTATACATACTGT
TAACTCCACATCGTATTTTATACTATATGTAACTCCATACTTACTCTTCTGATTTTATACATCTTAGATCAT

CTATTTATAAATTGAATAGCATGTATATATTTTAGCCATAACACCAAAACTATTTATTTCATGGACCAATAT
ATACCAATAAGCAATAGGAAATTATAGATGCAGAATGTAGTAGTTCATTAGCCCCTATTACCCTCGCAAT
GTCGCATAGGAACGATGACTGCGATATGTCATTGGTGTCATTTGATTACTGTAGTATAGTGATCAATGGA
TTTGCGACACTAATCGATAAATATTTGCAGCCACAAAACATATTATGTTGAACGAAGTATTCTGAAGCAT
CAAGGGCAAACCTACATACGGCAGCTAGAATAGAATCAAAGTGGCCTATATTTTAACTGGTTGCATGCC
ATTTAAAAGTTTGATGTTTGCAATTTAAATCAGTATAACTAGTTAGTGCTGATACATATTTATGCAGCGGT
TACGAAAAGAGCAATAAAATGTTTTTAATTACAAATATTTATCTTCAGTTCAAATGACCTCAATGTATCT
GGTTGATTATTACATTGTAAATAAAATTGTGTATAGTAAAAGTCGAAATCTTAGTAAATAATAGTTCAATA
GTAGATATTGTTTCGTCCACGTTTAATTCCATTCGAAAGTAATTTCCGTCTAACGTTCATTGGTGTCCAG
TTAAAGTTTGCTTATACTCAATTATTTTACATATTATCCACAGTTTTTAAACTCTGAGGTGTAAACGGAA
AATATACACCAAGGCCCGCGATCTCGGACGAAAAATAATGAAACTTCAACAATTCGAGAGTCAATTTT
AACAGATTAATGGCCTTTTAATGTAAATTAACCCCATATATCTATACATTTTAACCCTTACGTCGTCCCTT
TTAAAACTGCTTCTAAAATCCATCCATATAATATTGGAGAAGACCGAAATTTGATTTTAATTCTTCTTGC
CCTGCTGTATATAGTAATTTATGAAAATATAATACATTATTGTTATGTAATGTGTAGTAATACTATAGTTCA
GTCAGTATAAATTGTTACATCAAACTACAGATGTACGTACACGCAGGCGTGTCTTGCAACTTTCACCGG
CAAGGCTGTAAATATCGGGATAGAGGCTCTAACTTTTCGTGTCACAAATATTAAACACTTTATGACGGC
AATATAATCTGAAAATTAGTTAAGGAAAAAGTGTCGACGCGAAATTGCAACACATGCCTGCCTACCAA
ACGTAGGAATTGCCAATTGTTTATTATTAAAAATATGATTTACTTGAATTGCCAAAAAGAAAAACTTTGC
GTGTCCTAAAAAATGCAACAACCGGCCAGTTTAAAACATCCACTAGGAATAAGAAATTATCTGGTCCT
CTTCGGAAGAACCCTCCATTACTACAATAAAATCGTAGTAATTTTAAACTACATCCGGGCCATGCGTCT
GACGGAAGTATAACTATGTCCAAAACAGTACTTGTGACCCACGATATGGTGCCTTACTACGTTGTATTA
CGACTTATTGCGCTTTGTGTTGTCTTCACAGAGCCAAATTAGTATCACATGTTTACTAAACCTTCCAGTG
TGTCACATGAAAGATATTACACGATTTTCCCGTCACAAATTATGCGTCTTACTAAAGAAATCCGGAATAT
CGAATGCATGATAGTCCGAAAGCGATGAATTTCTATCAAACATTCAGCTTCACTTTAGTACCGTCATGA
ACTGGTCAGATGTAAAAAGCCGTCTTGCATATATTTGTCCTGATTGTAACGTCCATATTTGAAAATGATT
TAGCGTTAAGTAATTCTGATTTTAATCGCAGTAATTAAAACAATCTCTTTCATGTCCAAGAATCCAAATG
AAACTTACCATTTTCTTATTAGTGGCAAGCAAACGTGAATAGGATTATTCAAATAAATTAAACTTATTTC
CGCTAATATATAAATGTAAATACCTGTTTGTTATTAAATGCATATGTATAGTAGAAGACATATGACAGGTT
AACGTCAAGTTGTTGAAGATTGCGTTTTATTTAGTTCGTTTTCTGTCAATTATTATATGTCAAAATGGCAT
TTTTTATTTCATTAAAATCGTCAATTGTTTTTTTGTATATAAAGAAAGTTTTCATCAAGTCAAGTTAGGCA
GTCAATTTATATTTGGAAGTGCTGAAGTGCCCTTTTGTTGCTCATAATAATAAATAATATGTATAATAAAG
TAATTATGTTATGGTTAAATTGCTTTTTTACTCATTAGAATGCTCAAGAACGGTCATGATATTATATCATTT
GAAATTAGCCTCAAGCATTAATTTCACTTTTATCACTATTATTTTTAATCTATTACTAACTTTTATTATTTTT
TCAAAGAATTGAAGTACCTATTCATATTATCATCATTATGCCACAATTACTTCTTTTAAGCTTCATAGTTA
ACAAAGTGCCGTAAGCAGAGACAATAATACTGTGAGAGATCTTTGTTATATAAAAATACTTATGTTTTAC
TTGCGACCTTATCTTTGTTAAATTCGAAGCGAAAAGAAATATATATTTCTTGCCTTACATACATCATGAAT
GAACTATATTGAGTATACATAATATAAACATTTACAGAGCTCGATACACCTATTAACTCGATAATTTGAAC
AAACCGTTGAAAATGTTATATTGTTACCACTAGTCTAAGCTCTATCAATTGTTTTTGTGAATTAATAATTG
ATTATTGTAAATTTATTTGTTTTTTCGAAAGGAGATTGTAATTTCTTACGTTATATATTATATTTTGGTGGA
TGTTATCTGATGACTGTTGCGTTCGTTTTGTGAATAATGCATTCCATTCAAAGATCCGTTGACTTCGCAC
TGTTCATGATGCACTCACTAATTGAATAGTGTAATAATAGATACATATTATATATTTTTACAGGCCTTAATA
TCCAGTACAGGTCGATCCAATGTGCTTCCTGTTGTCGATAAGGTTACTTGATAACCTCGACGAGGCTAA
CATTGGATCCACTTGCACATACCAGACCACGGTTAGAGCACTAATTTTGTAATAAATTGCAGTATCCCTT
GATAGCGTAATAAATGTATTGTCACAAAGGGAGTGAATCGTCAAATATTGCTCGATTGTTACCGGATAA

GTAATTAGGACGGCAAGGTTGTTGTCTGTATTTTGTCATGTTTATACAAAGGAAAAAATATCTCAATAAA
TATATCATAGTCCATTATAATGAGTAGGCGATATTTAGTCTGTATAATAGTGACATCATTTTTCTTAAATCA
ACTTTAGTAATAAACTAGGAGTACTAATTTCTGAACTTGGACCGAGACACGACGGTGGCGCCCCCAAT
TTTGTGGTTACCACAGAATTTTAATTATTATTAGTCAAATTATATCAACCCCATATGTTTAACGCAACAAT
GAATTAGGCCTATTTTGTTTGAATGTGCCCACGTTATTGTTTGATCGTTATATCTGGCGGCGCGTCGTGT
CTCGTCCAATCACGCTATTATACTTAACGTAGCTGATATTAGCGTCTAAGGCGGGCGTTACGTATACAAG
ATCCTTACTTTACAATTACTTTAGATCATAGAACCTTTGAGTTTGTGTATTCTGATCAAATGTTATAATGT
AAATGTGGTGTACAGTAATATGCGGGCGGGCGGGCGCGGCCGGGGGCTGACATCGATCAAAGACTGT
CGCGGCGCCGAACAATTTTGAATACAATGTAAATAGAGTACGAATTAGGAATTAGACACGTTAAGGGA
TGTAATCGTAGTGTTGTATTTTTATTACTATATTACAAAATATATATATTATGGGGAATTATGAAGTTTAAA
ATAGTTGCGGTGCCCCCCGGCGCGCCTTGCCGCCCGCCTACCTGAGTCAAGTGTAGGACGATGCGGCA
GTGTTATGTGTGTACAGAGTTATACCAGCTCGAGTCGGGCACGCGGGCCCTTGCCAGCCGGTTCGAGC
GAACGACGACAAGCTATGAATACTGACATCGGCTCACGTTAATTAGCTTTTAAATAATTTCATCAAAATA
TAAAATACACAAAAAAAATGGATATAAATTTCTCGACCAAGTACTGGTTGTTTTTAAATTATTAAAATGT
AAAAAAAAATATAGTATATAAACAAAGTATAGGTACAATTTATGAATTATACTTTTCGGCATTTCGCTGA
GTGTGTGTGGGAGCGCTTCATGCACTGATATCACGAGCACTCGCGCCAGCAACCTACACAGTGTCGAA
TGTTTCTATTTATTTTCTCATTTCTTTTGCTCATGTCCTCTTCCATCGCTTAATTTTTATTTTTCATCTGTCT
ATGTGTATTATGGTTTTTGTTAAGGCAGGGTAATGCAGAAATGTGAAGAGGTACTTTTTTAATTTGAAA
ACGATAATTTGTTTGAATGTTAAAATTGAGTTGATTTTATCCATTCAATGGACTTTGTGTTGTTAAGAGA
AGTGATCGAGTTTGTTATTTTTAATTAAGTCAATTTGAGGTTCGATGAACACTTTTGTGGCAGCATAATT
AGTATATTTTAACTATCAAGTTACCACAGAATCAATAGTCAAAGTAGCTCTCTTTGGCGTTGCCTCTTAG
TGATTCGAGAGAGGTGGGAGCGCGCTAGCCCCCGCCACGCATCCGAGACACATGAGCTAAAGAGACG
ATGAGTATCATTATAAAAGGTACCTATTCAAAGTCCGGGAACAGACTAACTTGATTGTATACAACATAA
GTATAGTGTTGCTGCAATATGCATTTATGCCATCTTCATTTTAATTTGAATTGTGGGATCAGTGCCAAACT
TTCTTCTTCTTGTTTAGCTCAAAACGATGCAATTGTTAGCATGCTTGGTGAAACAGCTATGTAATTGTTT
AGTTAGTCTGCCATTTTGTGAATATTTTGTTTTGATAGTAATATGTAAATCTTTTATAATATATTTGTTTAC
AATAAATAAATGACAACATTATAAATAAATAAATTGTATTTTCCATTTAAACTGATGAAAAAAATATAAA
AAGAATAATATGCAATGATA
>TCONS_00056802 geneID=XLOC_011431 scaffold_2:1002710-1004817(+) Intronic
GTATGAGAATCTTACACTGAAGTGAATTTTAAATCCAGATTCTAACATAACAATCGGTGACCTAATGATC
TGTCCAGTCTGATACGTAATGAAGTGTTGAGTGATCGAATCAAATACGGTTGGTGTTAAATATTAAAAT
AAATATATGTACAAAGGAAGTTTTCCCTTACTCTATCTAAGGCGCTACCAAACCATCATACTAATATCGA
CTAACACCGCCAAAGATCAGTATAATAATGCGAAGTACGAGAAATGTGGAAAGTTACCAAAAAGTGTG
AAGTGTGAATTCAAAG
>TCONS_00056850 geneID=XLOC_011430 scaffold_2:1026919-1028168(+) Exonic overlap (+)
TTTGTATCGTAAACAGTGTTGCTTGGTAGACCGAGCGTTGCGGTTTTAAATACAGTGTCTGTGTTTGAA
CAGTTCTCGTTAATATATTTGCAATTATTGCATTAAAATTAACAACTAAAGTCAGTGATGTATAATGTTAT
AAAATTAAACAGTGCTAAATAAAAAATGACGGACAACGCTTCCTTCGATTATTCAGACGAATCGAGCG
GAGAACTTACGCCAGACCTGAATA
>TCONS_00056114 geneID=XLOC_011430 scaffold_2:1039728-1041745(+) Splice junction overlap
ACCAACCTGCTGCTGCTCACTAAGGTGCGGCAGTCCCTAGAAGCGCTCTCTGGCGTGATCCTCACAGA
CATGGCGCAAGGAAACAACATGAACCTAGAGATACCCCCAAACGAGACCGCGTTCAGTTAGCGAAAT
GTCTCCGGTGAACCGTGTGGACTATGAACCCGTGAGTTTGCTATAAGTCGGTTAAACACAGAATACAA
AACCATAGAGTTTCGTTACGCTTTTAAACAATATCAATGATTGCGACAGGTCAGATCGCTTTTTGTTATA

CCTACCTATTTATTTATCTAATTGAGTACTGAACTACTGACATTGTGTTGTTAGTAGTTATGTATTTTTGTG
TTCTCTTACTAATAGATTAGGTCTTCTGTTTTATAAGCGTAGCAAAACAAGCATTGGTGTGATATAAGGT
ATTTATTTTCGCATTTATCTATTCGGTCTTTGGGTGTGTGATGAAATGTTTTTTCAGTAGAAGACAATACT
TAATGTATACTACATTATACATTACATGTTTTGTGTGGCTAGGCAAAATGGAGTGACCAAAACCTTACTT
AAAACCATTGAGATATTATTGTTTTTAATTTATTTAGAGTTTAATCATGTTTCTTTTTATTTTCGGTTATGA
ATTCAATGTTATTTATACATCATTGTTAAATCTGTTACGTTATAATTTGATTCAACAAATTACAGTTAAGCT
AAATCATGAGACCACGTTTATTTGAAGGTTGTAACTGCTATTTGTTGATTAATACATTGAGACAAACCAT
AGGAATGGGTAATTTATATTGTGTTAAACACAGTGCATTACTGTTTTTTCAAAATTTTTAGTTATTCGTGC
TTACGTACACGAGAAAGAATAACATGAAAAAAATGATGAATATTTTATTGACAGTCACGTCTTTAAAAT
TACTGGTGTGTAAATACTTGTGAAATACGGAATCTGACAAAGCAACTCCTATCCTGGTCAGATTGTGAA
ACACTACTATAGTCAGTTAAAACAAATAAATGGGATTAGATATGGGTTAGTTAGGGTGGTGCTAGTAAA
GTAAGCATGGTTGTACATTAAGGTACGGTCAAACTAACAACATTCCTTTATGTTATTTGAAGTTAGTACC
GTTAAATGTTTTGCCTAAGAAATGTGGACACATAGGACAGCTGAAGAAAAAAAATACATCATTTATCAGT
ATCTTCTTAGCTTGTCAGTAACAAACACCACATCCGGTCACCGTGGTAAAAAGGCGTATGCAGGCTGT
TGCAAAAGCGGTTTAGTAAGCTAAAAAGGGGTGACTCTGCTCCCTTTTTTAAACACCCTGTGTAAGGT
ACAGTTGCTTCGTCTCTCAGGGACGCTTATAACAATGACAAGTATCTAGATACTGTGTTTGTAATGTATA
CTTGTTTCTTGCCATTATCTTAAACTTTTATAACGCATTTTCATAACATACCTACATTTGTATCAAATCCAA
TCTTTTGTAATGCATGTTTATATGTATTCTAATTTTCAAAAAAATACTATTAGTCATATTGAATCCGGAT

>TCONS_00056665 geneID=XLOC_011440 scaffold_2:1293736-1295497(+) Unclassified
TATTTTATTAGAAATTGTTTTCAGGAAACACACTCTTCAGCATATCTCCTAATTCCTGTTATGGGCCTGTG
TTGAACTTAGTAACATTTGGTTATTTTATTAAGTCTACCTAATAGCTTATTGGAAAACCTTACTCACCCGA
TATTTACGAAAGCGAATAAATAAGTATTTCGGTAAAATTATGATGAGTTTAAAAGCAAGAGCAAAAAAA
GGTTCGGTTTGTGAAACAAACTTGTTCCAGGCAAAATGTGCGTAGGCTTTCAGCTTCAGCAAATATAAT
AAATATTCACCCCGAGGCTCTTAGCCCCGCAAATAAACAGAGAATATCATGCATTAGCCACTTTCAGCA
GCGCTGCCGAATGAAATTATTAATTTCAAATTAGAGGAGGGAGTTCCGCTGAAAACTGAAACAAAGGA
AAACTTAAAGTTGAGAGCATCGGGATTGGGAATAATGAAAATTTCATTATTCATACTAAGCTAGGTTAG
GTATGCGAAACATAAGCTGAGGCTAAGAGCACTTTTTTCAGCCTTTTGTTAATCTTCCCGATCAGCTGC
TTTAAGTTTTAAAGGTCGTTGAAATCTAATACTTGTGCGTCTTGTTCAGTTTTGTGGAAATGGCGGGAG
ACACAAGATGGGCAATTTTCCCGTTTTCAGTCGGCGCTGGGTGTCGGCCGGGCTCGGCTGCGCTCGTG
GAAATGTATCTCGGTCTCGCTCTAGCCGCGCCGCGCCGCTCTGTCGCTCGCAGTACAGTCGGGGCTGG
CGACCGACTATCGCGTCTACCGGTTACTCACAGCGAGACACACTGCCGCCTTATCGCTAAAAAACAAA
CGAATTACACTTTCTGACATAAACAAAGCCGGATATTAATTAAAACCATCCTGAAAACACTTTTACAGC
TATTTCAGGACGGTTGGGCCGTCAAACAGTTTGCCATCTAGACGACCGGCGTCTTAGATCGTGTTGGC
CAGGTGAAGTGCATTAGTGTCGTTTACTCGCTGAATTTCGGTGAAAGGAGGCAGCGTTTGTGGTTGCA
ATGTGCCGTTTAGTGGGTTCTGTAGTTAACTGTGCCTCGCTGAGTGAGATCTGAGTGAAAGTGTTGAG
CCGAGCCAGTCAAGGCGGACGGCCTGCATCACCGACTCCCATACATGCACATTACACTTTAAACAGGA
CAAGACAACACAATGAAGGTAGGGAAGTACCTAGTAAACTAGGAACTAGGTACGAAGCTCACGCGGA
AAACTTAATGATACTTAATAATAAGTAGGTTACGAGTAGTTGGTAATGGGATATTCTATACCTAGGTATCT
ATGTACTTACTTACCTACTTTATTTAAATCAAATTAAAGGAAGA
>TCONS_00056414 geneID=XLOC_011441 scaffold_2:1357773-1359406(+) Exonic overlap (-)
GGGGCATTCCACGGGTAGTGGGAAAATGAGATATTTTTTTATTAATCTAATCAAGATTATTTTATTGTTAA
TTCAATATTAGATATACATATTTACATGTTCTGTTATTTTTTTTCTGAAAAAAGCAGGAAAGTATTTTTTT
ACATACGTTTCATACGTTGTTTTTTTGTTCTGAACATGTGGTACGATTTTTTGGGTCATGTTCATGTCCCA

CCAGTCACAATTTATTTGGTCTATATTTCTTGCAGAAACAAAGCTTGCTCATAGTTTATAGTTCACATCTT
AATATATTTAATCATTTAATAGCACATTCAAATACATTTTGCTATGTTAAGTATTTGGACTAAAATCTCGTT
TGACGGAGCAGAGGTCGGGGCTGAGGTATTTTTGGCTGTCCCACCAGTCACACTCCTTAAACCGTTAA
TATATCACCAATAAATGTACATGGGTTATTAATTTGGTAATCATTACATTACTTGGTTCATTATCTACTATA
CAGTATATATTTAATCATATTATATGATTAAAATAAAAAGTTACAGGTCTAAACAATAGCAATTCTGGAGT
AAACAGTCCCACCAGTCACTATGGAATGCCCCTAAAGTGATTCTCCTAACCCCTCTAAAAATATGAAAT
ACCTAGAATAAGTAAAATATAACCATTGAACTTGGGCTCGCGGTATGGCATCTTTATCTACGTATGTATG
AATCACGATCTGCGTAAAACACAGCGATGTGATACTCGTAGGTGAGACATACATTACACGACTTGTTAT
AGTATATAGTTAGATAAAAATGCCATGTCGCGGGCGTTGGATTGAAATTGAAGAAGACAATAAATATAA
AAATGTATGAATATAGCGATGGTTTAATTATAGAGCTTCCACTATGTTATTGTATTTTAGACGTTTCCTTAT
GTCTGGTAAATAAAGTTGGTTTTCCTTTGCAGTCGTAAAATATGTATGGGATTTCGAATGTCAAAAAAC
CAATAATATAATGGACAGAGTATGGTTAACATTTCCGTGTTTGTCACCTGCACAGTGCTCTCAGCTCGG
TCATGGCTGCTTGAGACGCAGCCGTCGTTTTCCTTTCAGTTTTG
>TCONS_00056461 geneID=XLOC_011442 scaffold_2:1372671-1373730(+) Intergenic
ATAAGGTGTAACGGTTTTTGCACATCCCTACATTTTAATTCATTCATTCATTCGGTTGTTCATTTCATTCA
TTCATTTGTTCGAAGTGTGGTGATGGTGAAATAAAACTGAAAAATGTTAAGAAAATAGCAATACAGAA
ATTTATAAATTGGCCTGAAAGTTGTGAAGTGAACAATAATAATACAAAAAGGAAAGAATTCAGTGATTT
TCAACATATACAGATTTGAGAAATTCTGATTCAGCAAGGTCAAAACACACCGATGATGTAAAATGCTTA
TTATGAACACCGCAGTCATCAAAGTACGCTAAACTGGAACTGTAAGTAATAGAAATTCATTATAAATGA
AATGTTGTATTACGCTACCTACATAATAATTTTCTCTTAACATGCCTACCTGATTTCTGATTTGTGAACTG
TTAGAAATAAATAGATATTGGAGTAAATGAGTTATTGAGTGCATACCACAACTACTGGGCAAACAGATT
GTGGGACATCCCACCTTAGATATAATAACCAGTAGTGGTGAGATGAGGACAACCAAGGACAGTGTTGT
GGCACTCCTAGGTTTCCTCCTGCATCTAGTGGTACATACATGATTTTTGGCTGATAATGATTAGCCTTATT
AGATTTCCCTTAACACAGCTTTTTACTGAAAGTGTCATGTCAC
>TCONS_00056807 geneID=XLOC_011442 scaffold_2:1393000-1395275(+) Intronic
GTCAGTTCGGACGGAAGCGCACGTTTGCCTGCGCCCGCGCACGGCCCCTAATCGCGTGTCTAGTGCCG
GACACATTATTACTACTTCATCGTGAAATCTCAGAACTAACCTCAAATCTGGAGCAGCTGATGTCATAG
TGCTCCCAGTTGAATTCCATTGAAATACACAGTGCAATTAGTGGAGACGCGAGGGATAAATGAGTAGT
GAAGTGTCGTCGTGAGTGGTGGTGTTTAATTGGGGCGGGGGTGGTGGTGGGTGAGGCGGATGCGAGC
GGGAGGCGCGGCGCGACACGGCGG
>TCONS_00056137 geneID=XLOC_011445 scaffold_2:1445981-1482727(+) Unclassified
GCGCGCCGCGCCGAGTCGCGCCACTCGACACGCGAGCCGCGAGGTGACCGACCCGCCCCCACGCAA
CACGCGTGACTACTACTACTCCATATCGGATGCACCCAGCCTTCGCCGGCACCGCGGGAACCCCCCAC
ATTGAAACGGAAGCAATAAACGGAAAGATGATCGACAGAGCGCTGAGCTACTTCTTAGACAAATACG
ACACAAGCACGATCGCGGCGATTCGGTGAGAGAGGATGGAGGGCGCGGACGCGCGCGACGAGTGGG
AGGCG
>TCONS_00056203 geneID=XLOC_011478 scaffold_2:1535573-1537500(-) Exonic overlap (+)
CGCGCACGCGGCTATCTAAACCGCGATAAACCATCGGAACAATAACATAAACACATTACTTAAAACGTT
TCATGCCAGCCAGCCACTCAAAGTTGCACTGACCTCGAAGCAGGCACATCCTGTGCGCGATTCACAAT
TTAAATTTATAAAAAAAACAAAAGGCGCGCGCGCGGCTGCGGCTGTCCAATCCGGTTCTGTGAAAAAT
AAACAAAAGTTTTAAAATTAGTTTTAAGTTATTTTAAGGACTTTGCAACTTCAATGTTATTGTGCGTGCG
TGACAAAAAAGAGACATTTTGTGTGAAGTAGGTAGAAAGTAGACCTTTGCGAGTTGTTGTGAAACTTT
CTCATTTGAATAGAGCTGCTTTTATATTACTGGTGTGTAAGATAGGCGACAATGGCGGTGCGATGGACC
GCGCTGGTGCTCGCGCTGGCAGCGGGGGCGGTGAACAGTATGGACACCAGCTTCAACACCACACAGG

ACAATCTTAG
>TCONS_00056142 geneID=XLOC_011447 scaffold_2:1565421-1566458(+) Exonic overlap (+)
CGCGCGGCTCGAGCTGGACGCGGAACTGGCGCGCAAGATGGCGGCCGAGCGTGACGCCACGCAAGC
CGTGCTATTGGCCGAGATAAGAAAATGCAATCGTCTTTATTTGGAAAATATGCTACTGAAAAGTAACAG
TTACGACTGGGATATACGACCATCTCGGGTGAAGGGTGAATCTGAACATAGCACTTAAAGAGAGCGTA
ATTTTAAGCGTTATTTAAT
>TCONS_00056714 geneID=XLOC_011489 scaffold_2:1952145-1953970(-) Unclassified
ATTTCCAGGGTAAATCGACGTTGGCGCTAAGCTGGCTGGTCCTATCGACAGTGTGGGTGCTCCTCAAC
ATACTGCTGCTCATCATCATCAGCTGCACGCACCGGGTGGCAATGTCACTAGCTTGCGTGATCCTGGCT
TCCATATTCAGTGTCGTGTATATTATTCTGCTGCTCTACGGACTCCTCGTTGGCTTCAGCTACTGGGTTG
CGTTGAAGAATTCTCGCCGAATAATTCCACA
>TCONS_00056995 geneID=XLOC_011611 scaffold_20:180639-189723(-) Intronic
GTAGGAATTAAACGCGTTTAGCACGATTTTTTATGAAAAATTAAGTGTAAATTACCCTAAATTCAGTGCT
AAGTGGCTGAAGGATTATGTGCGTAAAACCGTGGTGACAAAGTGTGAATTGGATTGTGATAGTTTTCA
CGACGTTGGTGCTTAAATTTACGCTATAACTGAAAACCTGTGCTAAAACTAAAAACATGTTTTTTATCC
CACAGATAAGTTTTATCCAGGTATTGTGAAAAAATAGTTAATATCGAAAAAAAAATCTAGCCACGTAGC
ACACAGTTCAGTAACAAATAATACTACGAACTTAAGTTTCAAAATGCGTACAAAAACTAAAAAACTAT
GTCAAAAACAGTGAAAACTTAAAAGAAACAGTGAACGAAGATATGAATAGTGAACCAAAACATGACC
AGTGCAGACGACATGTGAAATGAACAGTGACAGTCATCAGTGATGTTGACGAGTGTGATGATTTAGCG
CAGAACTAAGTGAGAGTCATCAGTGATGGTGATGAGTGTGATGATTTAGCACAGAACTAAGTGAGAGT
TATCAGTGATGGTGATGAGTGTGATGATTTAGCACAGAACTATGACGGTGATGACGGAGGCTCGTTGG
CAGGGGACCGATCATCATTTCAAGCCGACAATCATCCTGAGATCAATATGGAAACGCACTTTGTCATTC
AAGAAGAGGAAGGTGAG
>TCONS_00057588 geneID=XLOC_011611 scaffold_20:180639-189723(-) Intronic
GTAGGAATTAAACGCGTTTAGCACGATTTTTTATGAAAAATTAAGTGTAAATTACCCTAAATTCAGTGCT
AAGTGGCTGAAGGATTATATAAGTTTTATCCAGGTATTGTGAAAAAATAGTTAATATCGAAAAAAAAAT
CTAGCCACGTAGCACACAGTTCAGTAACAAATAATACTACGAACTTAAGTTTCAAAATGCGTACAAAA
ACTAAAAAACTATGTCAAAAACAGTGAAAACTTAAAAGAAACAGTGAACGAAGATATGAATAGTGAA
CCAAAACATGACCAGTGCAGACGACATGTGAAATGAACAGTGACAGTCATCAGTGATGTTGACGAGT
GTGATGATTTAGCGCAGAACTAAGTGAGAGTCATCAGTGATGGTGATGAGTGTGATGATTTAGCACAG
AACTAAGTGAGAGTTATCAGTGATGGTGATGAGTGTGATGATTTAGCACAGAACTATGACGGTGATGA
CGGAGGCTCGTTGGCAGGGGACCGATCATCATTTCAAGCCGACAATCATCCTGAGATCAATATGGAAA
CGCACTTTGTCATTCAAGAAGAGGAAGGTGAG
>TCONS_00057589 geneID=XLOC_011611 scaffold_20:180639-189723(-) Intronic
GTAGGAATTAAACGCGTTTAGCACGATTTTTTATGAAAAATTAAGTGTAAATTACCCTAAATTCAGTGCT
AAGTGGCTGAAGGATTATCCACGTAGCACACAGTTCAGTAACAAATAATACTACGAACTTAAGTTTCA
AAATGCGTACAAAAACTAAAAAACTATGTCAAAAACAGTGAAAACTTAAAAGAAACAGTGAACGAA
GATATGAATAGTGAACCAAAACATGACCAGTGCAGACGACATGTGAAATGAACAGTGACAGTCATCA
GTGATGTTGACGAGTGTGATGATTTAGCGCAGAACTAAGTGAGAGTCATCAGTGATGGTGATGAGTGT
GATGATTTAGCACAGAACTAAGTGAGAGTTATCAGTGATGGTGATGAGTGTGATGATTTAGCACAGAA
CTATGACGGTGATGACGGAGGCTCGTTGGCAGGGGACCGATCATCATTTCAAGCCGACAATCATCCTG
AGATCAATATGGAAACGCACTTTGTCATTCAAGAAGAGGAAGGTGAG
>TCONS_00057590 geneID=XLOC_011611 scaffold_20:180639-189723(-) Intronic
GTAGGAATTAAACGCGTTTAGCACGATTTTTTATGAAAAATTAAGTGTAAATTACCCTAAATTCAGTGCT

AAGTGGCTGAAGGATTATGTGCGTAAAACCGTGGTGACAAAGTGTGAATTGGATTGTGATAGTTTTCA
CGACGTTGGTGCTTAAATTTACGCTATAACTGAAAACCTGTGCTAAAACTAAAAAATAAGTTTTATCCA
GGTATTGTGAAAAAATAGTTAATATCGAAAAAAAAATCTAGCCACGTAGCACACAGTTCAGTAACAAA
TAATACTACGAACTTAAGTTTCAAAATGCGTACAAAAACTAAAAAACTATGTCAAAAACAGTGAAAAC
TTAAAAGAAACAGTGAACGAAGATATGAATAGTGAACCAAAACATGACCAGTGCAGACGACATGTGA
AATGAACAGTGACAGTCATCAGTGATGTTGACGAGTGTGATGATTTAGCGCAGAACTAAGTGAGAGTC
ATCAGTGATGGTGATGAGTGTGATGATTTAGCACAGAACTAAGTGAGAGTTATCAGTGATGGTGATGA
GTGTGATGATTTAGCACAGAACTATGACGGTGATGACGGAGGCTCGTTGGCAGGGGACCGATCATCAT
TTCAAGCCGACAATCATCCTGAGATCAATATGGAAACGCACTTTGTCATTCAAGAAGAGGAAGGTGAG
>TCONS_00057306 geneID=XLOC_011578 scaffold_20:688136-706792(+) Exonic overlap (+)
TCTAGTATTTAGTACGTGTTTTTATTATGGTTTGTTTCTATTATTATATTTAATTCTGCATTAAATTATACAA
TTTCCTAACTAACATTAAATAAACCATTTCATGAGATTTGAGTAATGCGAGATTAGGTTGTTTGGAATGT
AGGTATTTAATCAATGAAGGCTAGAAACTGTACATTCTATGAAACTTATGTTATTTACACCGCTATATTAT
ACCGAATTGTGTAAACCAACCTAATTTCACATACAACTAAAGGGATAAATGGTTTACATTTTAGTATTAT
ACAACAACATAGCGCCGACATAAAAACTGCCTCATATTTTTTTATTTAATAATACATTTTTTTTTATTTGA
TTTACATT
>TCONS_00057465 geneID=XLOC_011579 scaffold_20:696633-701392(+) Exonic overlap (+)
CGCGCGACAGTACCAGCTGCTGTGTGTCTCCGGGACACTGAGCACCGAGCGGGAGTATGAGGCCAGC
AAAGTGCTCCAATCAGTTCGCATCCCATAGGACAGGTGAGCCGGGGCTGATCCCTGTAAATGTAAACA
CTATCAGAAACACACCACAGAAACTACAAACTTATGTCTAACGAGATAAGGCAATTCTAATGGCAACG
TTAATGTAGGTGAGCCCTAGTCTTATGAAGCTGACAAATAATGTGTGCGTAAGTAATGTAATAAGAAAG
TGAAAAATTATGTATATTTAATTTTGTCGTGTATCTATGCAGTTATGTTGTCTTGGAATTGTGTATTTGCTG
TTGGAATTGTTTCTTCTCCTGCTATTTAAGTGCAAAAAATCTAATTCTTCTTTATTGAAAATGTCACATA
AGATAAGAACATTTTGTTAGTCGTATACCTCGAAATCAAAAAATTCTTGTAGCTTTTCACCTATTCGCAT
TTCACCTTGATAGCGTTTTTTGTTGTAGCGTATAATATCAGTAGTATATTTTTTTACTAGCATATATTTTTA
ACTGTTTTTTACAACAGTAGCAAATTTTTATGTAGCATGTATTATCAATAGCTTATTTATTAAGCTGCATAT
ATTTTGAATAGCAAGTAAGTATTTTATGTCGCATCTTATTTGGGTCGCATATTGTTGTAGTGTAAAAGAA
ATATCGCGATATGTACCTAATAATGCATTACACACAAATATCTATCAAGGTAAAAAGCGACTACAGTGAA
GGCAGGCAGGAAAGTAAATCTTTAGACAATTGTCAGTAGCGATCAAAAATTATACTACAAGAATAAAA
ATCTATTCAGAAATTTTGATATCGCGGCGAAATGCGAATAGGTGAAAAGCTACAAGGATTTTTTGATTT
CGAGGTATACGACTAACAAATGGATAAGAAATACTTAGGGTGGCTTGCACCAAAATGTTAATCAAAATT
AAATCTATGATCTCTGGCTCTGACATTATACTGTCAAAGCAAGACAGCAATAGATTTAATTTCATTGAAA
TGTTTGGTGCAAGTCACACTTAAACTACTCAAAACTATTTGGTGATACTTTTGCTGTCAATTATTTTACT
TCTTAATCTTATGGTTCGAATATCACTATCAACATATTTTACTTGCAACGATATAAAAACGCTCAGCTCCT
AACCACAACTTGTA
>TCONS_00057787 geneID=XLOC_011588 scaffold_20:972561-974360(+) Splice junction overlap
CGCTCTCTCAACCCGACATGGGGAAGTTAGCGCAGGCGCTATGGTCGGCGTATGTCCTTTGTTCGAAA
CAAGTCAACATTATACTTTTGTGTTTGGATTTTCTAGTTTCAGCTCAGTTACTTAATTTTGTGTAAAGAC
ATGTCTCACATTGAAATTGCTATCACAAGCTCAATAATTTGACAAAAGTGTTTTCTGTGCAGTGTTTAC
GAATAGCTTTCGGTGAGAACGAGCTTCGAATCCCAAGGATTGTACGCATGAAACCTTTGGGTGACTTT
TTTTTCGAAACCGATGTTAACGTTATGGGCCCCGACATGATTGCGCCATC
>TCONS_00057435 geneID=XLOC_011630 scaffold_20:987966-989097(-) Unclassified
CTTAGGTACTATATCATTGTCAACTTTGTAAGAAGATATTTATTTTGCTCGTACCTACGAGAGTCAACTT
GAGACCCGCTACCTATACCCGTCTCTTTCACTCTCACGTGATAATAGAATGGAGTGAAAGGGATAGGAC

CATTTTTCAGGTGTCAAGTTAACTAGCGTAAGTATACTTTGCTCGCTTATGTGCGCGGTCGCCGCCCTC
GCACCATCGAGTCAATATTGTGTCATAGAACGAGTAGGTACCTACTTAATGTATTTTGGTAAATTAAGTA
TTTAGTTATCATCAACATCAACAGCCAACGCACGTCCATTGCTGACCATAGGCCTCACCAGTAGGTATAT
ACTGCCGGAGCCGGATTTCATGCAAAAACTGTCGGAAGTGGTTGGTTAGGCGGTACGTGCTCGGCTCG
TGGAGCTGGAGGAGCGTTGGCTCCACCAGACGGAACGTCGCACCGACGTTGAGGAGACTCGCCTTTG
AGCAGGAACTAGGAACGCCAACTCGCCCCCTAAAACATGAAAATATACCTATAATATACTTACTGTGAT
TTTTATTATTTAATTGTAATATTGCTATAATATACTGTACTGTGATTTTTATGTAGATCCATGGTAAATTATT
ATAATGACTGTTTAGATAATAAATGCAACAAATTAATACATACTTAACTGTGATTTTTATGTAGATCTAAG
GTAAATTATTAAAATTGCCATGTTTAGGTAATAAATGCAACAAATGAATACATAATATTATAGGTACTTTT
TAATGCGAGAGGCCTATATAGTTAGGTGATACTAGGCCAC
>TCONS_00057573 geneID=XLOC_011589 scaffold_20:1027781-1028545(+) Intergenic
CATTACAGTAAATAGTCTCACATTATTTAATGTTTTCGCTGAGATCGGCAATCACCTTGAGGTGAGTGTG
AATCACCACCCTCACCGGTGTGTTGGCAGTTCAAGAATAGCGTGCGGTCTATTTAGATGGCTGTGCGTT
TGTTTGCCAGCGAAATGTGTCATTGTTAACTAATTTTAAGTAACAGCCATTGCCAGCGGTTACGACACG
GGCCGGCGCGCAGAGGTGGTCGCCGCCGCGAGAGCTGGACTCCGATACGCTCATTAATTTGATGCCTC
CGATCACTTGTCATCACCATCTACAACGTCCGGCATGTACCAGTTCAGAATAAAAACTTACATACCTAC
CAAAAACACTAGGGAGTTTCTATTACTCTTCTGGTAGTAGATTACGAGCGGATTGATTCATGAGCTCCT
GAAACGGTAAAATTCGCGTGCTTCGTACAGTTTATAGCGCTGTATTCTTTGTATGTGCGCTTGTGGGAAT
ACTGGAGATAGGCCAGGTTTACTGAAATGAAGACTATAAAATACCCCCCTGTCTGATGTGAGCCAGTG
CTCACTTTTAAATGCATGAAATTATATATTAATTAGTGTAGGGCGAGCGGAGCACCCTGATAGGCCGTGA
CATTATTGGTGGAGAGCAACGCAAACATCACAATGTAAAACACGATACCGTTTAGCCGATTAATTAATT
GCAGTGGCCCGAGCC
>TCONS_00057402 geneID=XLOC_011603 scaffold_20:1259616-1267146(+) Unclassified
TGATATTTTGTTTTAATGGTTTTATTTAGTTTAGTTTGTTACATTAATTCACATTCGCACTGTTTAATTCGG
TTCATTTAGTACCTATATTGTGTATTATAAATTGTAAAGTAACTTGCATCACATTTAAACGATTCATTACC
AATACAAACCAGTGTAACCTATTTAAAATCGGAGACTTTGAAGTCAGATGTAGCGTGGGTATTCCCAGT
GGGTGCCGCAACGATCTGAACAGCAGAATCTGACCTGAAGAGACTACGATTGCGACTGTATTGATCGC
TTTTTAAGGTATGTGAAGACATACACTCAGTCTTCGGCGTTCCTTATCCAACTAAAGAAGAAAGATAAA
ACTTTATTTGACATAGACAGCAATACTGATAAAATCAACCACACAAATCACATTTATACAAAAAAAAAT
TGCTGTCATAACTACTTGTTAACTGACTTCGAAAAAAGGAGGAGGTTCTCAATTCGTCTGTATGTATGT
ATATATTTTTTCTAATGGTCTATGGTGGTAGAACGTCCCACACTGAGTCTCCACTCGAGG
>TCONS_00057583 geneID=XLOC_011603 scaffold_20:1329096-1331420(+) Splice junction overlap
GGGGGTGGCTGCAAGAGTTTGTACATATTCGGCGTTTTGAAGCGCGCCTTTAGGGTATAGCAATCTCGC
GAAGGCGTAGATAAAATATGTAAGGCCTGGGTCTCCTATTTTATGCTATATGCGGCGTCCGACTTTGGCG
TAAACGTTTCGATCAACGTCCTACGGCCTACCTACGGCGTCTACGCGTCAACGTCCGCGTGTGAGGGA
GACCGCATCAGTACATTTCTATAGTGAGCATCGTGACGCGGCCTACGGCTTGATGCTGGACGCGACCTA
CGCCGTAAATAGGAGACCCGGGCCTAAGTGCGTCAGAATCGCACGCTTGCATGTGCACTCCTTCGATA
GACCCTAAAGCTCGCGATTCAAAACGCCCGATGTGTACGCTCTAAAGGCCATGTTACGGCATGCCTCTT
CTGCAAGGCCTGGGTTGTCTTCTCGGTGAGTGCTTGTGTATGTAGTGGTAGCAGGAGATATGTTATACG
ATGCCTCATTTACATGCTGGCCTTGGTTGGGGCGAACCCGGCCGTCGTGCCGTGGATGGAGCGCGCCG
TCAAGCTGCCCCTGTTCCGCCAGCTCACGCCGCGCGACATCGCCGTGTGCGACGCCTGGGCCGAGTA
CTCGATGGACCCGAAGCTGTGGGGCAGCATGTCGTTCGTGCAAGTACG
>TCONS_00056985 geneID=XLOC_011605 scaffold_20:1470255-1478086(+) Unclassified
CTCGGAGCTGCGCGTGCGCGGGCGCGTCTCACACGCACCCGTTTGTAATTCGAACTCACTCGGATTCC

TAATTCAAATCTGTTTTAGCAACTTCTAAACATGATATTGTAACAACGGTGTCCATTAGTGTTTACTAAA
GTTTAGTTTCAGTGTACAAGTAATACAGTGTTTTTGATTGTATGATATAAAGTTGTGTTTGATTGATATTT
TTTACACCACCACATCCCACTGGGCGCTGTAATAGCGGTGTTCCGACCCAAGAAATGAGCTTATAATTG
GCCTATATCTAAGCCCAAATGATTCGACTAAAGGAGCCTTAGCTGAGCCAACATGTCACAAAGAGAC
>TCONS_00058035 geneID=XLOC_011736 scaffold_200:544755-547140(+) Splice junction overlap
GTCATTTTTCCCTGACTTTCCAGGTGGCCCATCAATTTCACTGACTTTCCCTGACCACCTTGAGCTTCC
CTGACTTTTCCCTGACTATCCCTGACTTTCCAGAAAGTGGACACCCTGATAATATTATGTTATGCTAAAT
TCATACTTCATGTTTTTTAAGAATCTGGCTGGTTTTCTGGTGAACCCTACTAACTGCAGACAAACCAAT
CCTCTTCATATCCCACCAGTTAGAAATTCAAAATGGCGTCATAAACTCATAACCTCTGGGTCCGTCTGTT
GAAAAATCTAATAAATAAACATCAACCCTTACTTCCTCAGGCGCTCCTAACCGCCGACTTCGAGATCGG
CCACTACATCCGCGAGCGCGTGGTGTCGCGCGCCGTGCTGCTGTACACGGGCGAGGGGCTCGACGAC
GACGATGATGATGACTACGAGGAGGAGGAGGACTCGTTCTCTGACGAAGATTCTGGCACCGAGGAAG
TGTCGGACGACTGAGGTCGGCGGCGGTCGCCCTCGCCCTCGCCTTCTGTTAGGCCAGCTGTCGCGGG
ACTAATGTCAACAACACTCTCATGTATCCTCACTGTTGACACTAGTCCCTTCGCACATATAAATGGTTGT
GCTTCAGTCTAACCGCGGTCATATCTACTGCATATCTAAGTCTAGGCCTTTTGGCATTTTACCAAATAAA
CTAACGAAATTACTTCGCACAGCGAATAGAGAATACTCCCTCAGCGTTATTTTATTCTGTTAAATTACGA
TAGTTGTTACGCTAGATGTTTAATGGAGGATTTCCAGCTCGCATTTAGACTTTTCTATGCATGGTTTATCC
GCTGACTAGATAAATATGTTTTAGAATTTTACCGTGAATACTTAGTTCTGCCGTTGAGCAGTTTTATTTTT
TACTACTTAGATTAGAAGCGAGAGAAGATGGTGCCTTCTTAATGTATATGCATGTTAGAGTTATTGGATA
TGTTATAACTTCATATACTATGGATATTGCATTTCTATTTTAGCTGTACATTTTCTCGTTTTATTTTTCATTA
TTTATTGTATAACTATAGAATGTATTTTGTGTACGATTTCATGTACTTGAAAATTTCATGTGATATTTCTCT
AAGGCTATATTTTTGTTTTGTTTCATACTTAAATAAAATTTAAGTTTCATTTTGTCATTCCATGTTACAGAT
AGTATTGTTATCCAAGCAGAAACTGCCGTTTAGTTTAATTAAGGGATAAATTGGCTATGGAATTGATAAA
TTGAATCTCTCGTTTAGTGCCAAACAATGCATTTTAACGATTTTATTATCACTAATACACTGTGATCCATC
CTTTGATAGGTTTTGTAATGTTGACAATAGCTAGGTCGGATTATAATGTATATTTATTTTTATTTACG
>TCONS_00058341 geneID=XLOC_011790 scaffold_201:36281-38673(+) Intronic
GTCCTATATGTATATTACGGTGCCCAATCTTAAATCGACCTAGGTTCACCTTAGACAAGTAAAGGTTCAC
CCTATAATCACACTACATAATATAATTATTATTATATTTTTATCCTAGTCAGTTGATATTAGGATTATTACCT
ATTTCTAAGCCTTTCATTGAGTTCATACGCAGACATAGATTGGTGTAACTTAAATACCTATCAGCATTTC
TCTGTATTTCCTTTAGTCATGAAAAAAACGATGTTTCTATA
>TCONS_00058463 geneID=XLOC_011803 scaffold_201:39585-43220(-) Intronic
TTCTATATTTTATATTAACTGTTAACAATTTGTATTTTAGCCGCGAGGGAAGCGAAGAATCGAATAAAAA
ATCAGCCTAAAATCTTCGGTCTTTTCCCTGTGGATTGTACGCCAACAACGGAGGAGCCAGAACAGCGC
CTAGATCCATGAGCACGCTTTTACGGTGGTCGTGTGCGCCGCATGACGCGGTTCACGCGCAGCCGCAG
CTACGTGGTCGGCTGGATCTAGCGCGCGCGCGGCGCCGAGCGACTGTGTGCGGATGAGTGCGTCCTAT
AAGCCTGCGAGCGACGAAGCATTTCTTTACATGTCCCATGTTGGGCGCCAAACGCCATTATGTGTGTCC
CACGGTGGGCGCCAAACGCCATTATGCATATCCCACGGTGGGCGCCAAGCTTACGCCGCTGTGTTTCT
GTAATTTAAGTTGCAGATGTTTGTGGTATGTAGGAAATTGTATAAGGTGGTTTATTGGTTCACAGGTTCA
CACCTGTTTTCTTGCTGTATGGAAATGCTTCGTTGTTGGAACAGCTACGGCTACGCGTGTGCGGGGTTG
CGTTGTCGTATGTTTATGTAATGGACTTACATTATTATGAATGATAGATATATTTGAAACTTGACCAGTTG
TGATAAACCAAGCTGTCTAAGCGCACATCAATACAAATGACTTCCAAAGTTGTTTTTCCCCTGTCCTTT
TTATTACTTTATCTATAATGATAATGACGCATTTTTTCAACTAAACTATTACTGCCATTTTGTATTTTGACA
AAAAAAAGTGAGCCTAACAGAGCATATACGACAACGCAACTCCGTCACAACTTATTTAAGGACAGTAT
ATTTGACGATAGAGTGTGTGCTACACAAGAGTTGTGCTTTAAATTTTACTTCTTATTTATTAAGAAAATA

TTTTATACAACAACAATTTTCATAGTGGCAGGACCATCACAGAAATTCAATGTATTCGACTATGAAATTA
ACGGTTTCAAATCATGATTCTTGTTGCTGAATCTTTTAGCATAAACTTTAATATTAATTTTTACTCTAGTG
TGCTAATAATCGTATTAATTAATTTTTATATTGATAAATAAATGTAATATATTATACCTTACATGTGCACATT
CCATTAATGCAAAACATGGTTTTACATTGTGACCTTCCCAGAGTTAGCGTTAGTCCCTTACGATTATTCT
GATCTTGTAATCCACATAAGTGATTATTTTCGTCTTCGACAGACGTATATATAATCCAGAGTCCTGTATTT
GGGCACTAGGTTCGAGCTGAGTCTAGTTAACTATTAACTCCACCATGTTCTGCGTCAATGGGTGTTTAA
AGTCTTAAATTTTCAAATAAGCACTAGGTTTGCTTGCAGTCAGCGTAGCAAAGTGGATTTCATATCTAA
GCTTCAGGATTGAGCTAAAGCAAGCTTTCAGTGTCGCCGTATGGATACGATAATGTAACCTTTTGCTTC
TTTGAAACAACTATTTTATAAAGTAAATTGCTTAACTTTAAAAACGTAACTTTATGTATATTTGTTAGAG
AACTGGGGGAACTATGTGTCCATAATATTGTGATAAAACGTCAAGTTTTTCCATTAAAAACTGCTTTGA
TTTTTTGTCTTTAGTTTCAAATGAAGAGAGATTATTTGTTAGATTTTTAATGTGTATCTTTATTACGAAATA
TATTTTAAAAATTAATTTAAAAATGAGTTAATATGAATAGATTTATTATTATATATGTTTTGGTATTACGGT
CCAGGAACATTTGAAATTCAACGAAAACCTGACTAGTAGAGTCGACCAGTTTGTAGTTTGTGACAAAT
GTAAAAAAAACAAATAAACATTACGTA
>TCONS_00058462 geneID=XLOC_011803 scaffold_201:39615-42253(-) Intronic
CGCGTGTTTCAGTACGCCAACAACGGAGGAGCCAGAACAGCGCCTAGATCCATGAGCACGCTTTTAC
GGTGGTCGTGTGCGCCGCATGACGCGGTTCACGCGCAGCCGCAGCTACGTGGTCGGCTGGATCTAGC
GCGCGCGCGGCGCCGAGCGACTGTGTGCGGATGAGTGCGTCCTATAAGCCTGCGAGCGACGAAGCAT
TTCTTTACATGTCCCATGTTGGGCGCCAAACGCCATTATGTGTGTCCCACGGTGGGCGCCAAACGCCAT
TATGCATATCCCACGGTGGGCGCCAAGCTTACGCCGCTGTGTTTCTGTAATTTAAGTTGCAGATGTTTGT
GGTATGTAGGAAATTGTATAAGGTGGTTTATTGGTTCACAGGTTCACACCTGTTTTCTTGCTGTATGGAA
ATGCTTCGTTGTTGGAACAGCTACGGCTACGCGTGTGCGGGGTTGCGTTGTCGTATGTTTATGTAATGG
ACTTACATTATTATGAATGATAGATATATTTGAAACTTGACCAGTTGTGATAAACCAAGCTGTCTAAGCG
CACATCAATACAAATGACTTCCAAAGTTGTTTTTCCCCTGTCCTTTTTATTACTTTATCTATAATGATAAT
GACGCATTTTTTCAACTAAACTATTACTGCCATTTTGTATTTTGACAAAAAAAAGTGAGCCTAACAGAG
CATATACGACAACGCAACTCCGTCACAACTTATTTAAGGACAGTATATTTGACGATAGAGTGTGTGCTA
CACAAGAGTTGTGCTTTAAATTTTACTTCTTATTTATTAAGAAAATATTTTATACAACAACAATTTTCATA
GTGGCAGGACCATCACAGAAATTCAATGTATTCGACTATGAAATTAACGGTTTCAAATCATGATTCTTG
TTGCTGAATCTTTTAGCATAAACTTTAATATTAATTTTTACTCTAGTGTGCTAATAATCGTATTAATTAATT
TTTATATTGATAAATAAATGTAATATATTATACCTTACATGTGCACATTCCATTAATGCAAAACATGGTTTT
ACATTGTGACCTTCCCAGAGTTAGCGTTAGTCCCTTACGATTATTCTGATCTTGTAATCCACATAAGTGA
TTATTTTCGTCTTCGACAGACGTATATATAATCCAGAGTCCTGTATTTGGGCACTAGGTTCGAGCTGAGT
CTAGTTAACTATTAACTCCACCATGTTCTGCGTCAATGGGTGTTTAAAGTCTTAAATTTTCAAATAAGCA
CTAGGTTTGCTTGCAGTCAGCGTAGCAAAGTGGATTTCATATCTAAGCTTCAGGATTGAGCTAAAGCAA
GCTTTCAGTGTCGCCGTATGGATACGATAATGTAACCTTTTGCTTCTTTGAAACAACTATTTTATAAAGT
AAATTGCTTAACTTTAAAAACGTAACTTTATGTATATTTGTTAGAGAACTGGGGGAACTATGTGTCCATA
ATATTGTGATAAAACGTCAAGTTTTTCCATTAAAAACTGCTTTGATTTTTTGTCTTTAGTTTCAAATGAA
GAGAGATTATTTGTTAGATTTTTAATGTGTATCTTTATTACGAAATATATTTTAAAAATTAATTTAAAAATG
AGTTAATATGAATAGATTTATTATTATATATGTTTTGGTATTACGGTCCAGGAACATTTGAAATTCAACGA
AAACCTGACTAGTAGAGTCGACCAGTTTGTAGTTTGTGACA
>TCONS_00058147 geneID=XLOC_011792 scaffold_201:143808-144362(+) Intronic overlap (-)
TTCTGGGTGCTTTTCGCACGCAAGAAACTCCTCGTGTAAGCAATGTGCCGTCCAATACCTTTCAAACTC
CTCTGCATTTTCAAACACTAAACTGGCATTATCAATTTTCAAGCTGCGGTCTACGGGGAAGGTCTCTCT
CAATATCTTCTCATTTATGAGCATAAACTCTAGTTTCTTTGTCCTGATAATGTCATAGTCTATGCCCAGGT

ACATTAGTAAGTACAGCTTCCCCAGGGTGTCTCGAGTTAGGTCACTCAGTTGGTAGCAAGGGCCCGCT
TTATCTCTCATACTGAAAAACAAGAAATAGATTTGATGATTTCTAAGAC
>TCONS_00058520 geneID=XLOC_011807 scaffold_201:149742-153253(-) Exonic overlap (+)
TATTTTTCAAATTTTCACTCGTGTTCACATTAATTTGCACAATACCGTAGATATGTAGGTATGTAATGAAT
AATATATGGATCTTGTCGTAGGCAAAGGTTGAATTTGAATTTGCCTACAATTTTTATGACTAAAAAATAT
AAAGTAGGTATTCAGTGAAATCATTAGGTATGTTAAAGTAAAGAAACATGTTTTACTACGAGTATCTTAT
CTGTTTATAATCACGTGTTGCTACAGTTATTTACAGATAAAATAAATTAGGAGCTAAGCAAACATAATTA
ATTAAGTAGTAAACTTGCGTCACTTTATAACTAAGCTGTATAGACAAGTACACAACAATAATGGGCATG
GATTGCACAATAATTGGTTTAAAATTTCTCGAACGCACGTTGCTTGAACTGGGGTCAATGATTTGCATT
GCATAGTAAAACAGCTAAGATTAGGTTCAGTTGTGCGTCAATCCGCTTGCATCCACTTTCGGTTCATGT
AGATTTGTGTTATTGACGTCAATGCAATGCAATGACCCTAATTATCTAATATTATATGTACCTATATAATTT
AGAAAGTGTTTTGTTTGTAAATGTTTGAATTAACCCATACCTTAAACCAATTTACACGACTTTTGAGATT
GAATTTTCTGATGGAAATTATATCCTAACTAATATTATAAATGCGAAAGTAACTGTGTCTGTCTGTCTGTT
ACTCTTTCACTCCAAAACTACTGAACGGATTTGAATGAAATTTGGTATACATACGGTCTAGACCTTGGG
AAAGAACATAGGCTTTTATTCCGAAATTCCCACAGGAAAACTTTTTAAGGCGTAGCGAAGCGCGCGGG
AACAGCTAGTATTATATAAATCTTAAGTGAAATTATATCTTCTATCCGACCTTAGTTCTCCAACAATGGTA
ACTTTGCAACCCTGTAACTTACACATTAATGGCTAACATTGCAGGCGCGTCCGCAGAGGCCACGACGG
AAGCGTCGACGGAGATCCCCGCGGAGTCCGCGTCCCCTGCCTCGTCCGTCGCCTCCCCGCCGCTCGTC
CCGCAGCTGCCATTGGCCGACGCCTTCCTAGACCACGCCTCCTTCAAGCAGGAGCCCAATATGAGTCC
TGAACATTCATCGCAAATATTGAAGACTGCTGATATTATATTGTTATAACATTTTTATAATCTAAAAGTTT
AATTAGCGAAAGAAACAAAAATGGCAACTATGTAAACGTTATTTAATAAAGTAAATTTAAATTTTACTA
CAAAGAAATCCTATTGTATAAATTGGTTATGGAAAATATTTACAAAAATACTGCAATCAATATATTTTTTA
CCTAGTGTAATATAAAATATATTTTTTGTTTCTATTGTAGTAAGTTATAATTATCTATGTCTATGAGTGGTG
GGTGAACTCGAAATCAAAGACACTGTACTATCAGAATTAAAATTAAATATTTACCTGAATATTAGAATGT
TTATAATATATACTTATAAGTAAATCGAGTGCCTTCTTGTGACAAAGTTTTATAAAATGTATGTATATTCAA
AAACATATATTTGAATAATATATGTAAATAACATAATATACCAATTCTGGTATAGTACCCTGTTTACCTATG
TTATATAGACTTTAAAATATATTACATATTATTTTATATAAATAGAATAGCGTGAGGATGTTTTCGAACTTT
TGAGTCGACCGATTTTGGCTGTGTGTCGTTTAAAATGTGTGAAGGTGATGAAACAGGGTTGAAACAGT
AGGTGTATTTACATTTTTAAAACGGTATGAAAGTTTTAATTTTAACGTATGTTTTAGTATGAGTAATGACT
TTTGTGTTTTTGTAAAATTATACTGCTTTGGTATTTGCGAAATGGAATCCTTATTATAATGTGGAATTAAA
TAGTCTACTAAATGGAATCCCTTTATTACTGGATATCTGAGAGGCTCTTCAAAGTTACCTATTTATTCATA
ACAAATGATATTTGTACCAAAAAACTAAAAAAAACTAAGCTTACAGAGTTTTAACGCTCGGTGTAAGA
TTGAGTAGATATCATAGTTTAACTTTGAATTGCGTCTGAAAACGACAATAAGCCAGTAACATTTATTACA
CAAAGGGTCGGTATTAGGTACAATTAGGGCGAGGCCCTGTTGTTATGTTGCGACAGCGATGCAACGCA
GCGCACCAATGGGGCCTCATCTTAATTGATTTTAATTTATGTTTTCGCACATGTTTTATGTTCCTTGCTTT
GGAATCCGTTAGTAGATATATTTTATATAAATTCTGTGTTGAATGCAGATATTGTATTTTGCGTTTTCTACG
CACGTATTGGTGATGGTGGTTGATTGTCAATTTTTGTATGGGCTGAGTCAAATTTTAATTATAAATATTTT
AAGATTTTTTGCTCGGGGTTTATTTGAAGGATTGTATTACAGATGAAAAATCATTCGTCTCGTTACAATA
TAAGGTCATAAGTCTTATAATATGTACGCTGATTGTTGGCTGATCATGATATAAATTTTCTGACTTGGGAT
GTTGAAACAGCCAGTAACACAGTATGGAGACTATATTAAAACCTATGATAGTTTTGTAAAAATATATGCA
TATATTTTGTTAGGTATTGACAAAGTTCTTCCTAAAGTGACCGCACTATACTGTGATCATACATTGATGTA
GACTGCATGTATTATGAATTTATGATAATCTAAATATATGTATTTAGTTACGTAGTTACTTTAACAAAGTGT
TGGAATGCTCAATAATAGTTACCTTGTTAGTAAGTTTATTCACTTGCTCAGTTTTTTTAATTTACTATTTT
CACATGCA

>TCONS_00058165 geneID=XLOC_011801 scaffold_201:518186-518921(+) Exonic overlap (+)
ATTTAAAAAAATCGAATATAAGTTTTTTTCTGAAAATAAGTGAAAATATTTTTCATCAATAAATCGAGTT
TTTATGAAAAATAGCTGATAATTACTGTTCTTATAAACCGACGTAGACATTAGTATTACGTATGGTTTAAA
AAATACTATTAATTCACAGTATTTATTCATTTGCACACAAAACACTTCTTCCCGTTATCCCGAAGAAATT
GCAGTCTGCCACGTTCAGACTGTGTTTGTATGTTAATTTCTTGAAGATGCTGTTTATTTATTAGGTATCC
GGTTAAATTTAAGTAGCTAGGAACAACACTATGTCCAGCATGTCGGACCGCAAGGCAGAGCTGGAGC
GCAAGAAGGCGAAGCTGCAGATGCTTCGTGAAGAGAAGGACCGCCGCCGCCGCGAGAAGGAACAAA
AGGATGCTGAGGAAGCCCT
>TCONS_00058832 geneID=XLOC_011874 scaffold_202:303199-339149(-) Exonic overlap (-)
GCCAAACGCACATGTGTGCTCGACTTTAAATTAGTGTATTTTTTTATAAAGTGCAGTATACATTATACAC
TGTGTGTTGTGAGCAATGCTGTGTGGGACGGCCGGAGCGGTGGCCGCAATGGACCGCGCCTACATGTC
TTTGAGGGCACGCAAGCCGAATAAAACTACGAGCGAGCAGTCGTCTCCGTTCCGCCGGTGGACGACG
GGCAGCGGCGCGGGCAGCTCGTACCGGCATCACGACCACAGGACTAATATTTATAACAAGGTGAGACA
TTTACACTGCTGCATAAAAAACCAATGGACAGCCAGGGACTGTCAGTAACTGGCCTCGGAG
>TCONS_00058833 geneID=XLOC_011874 scaffold_202:303263-362016(-) Exonic overlap (-)
CTCAGAAACGTTAGTTGAACGTATTAAATATTATTTACCTACATGTATGTATTTCGTTAACACCATGCAGT
ACCTACACACATTATGTACACATAACGCGTGCGAATGTGGATGGAAACTGTCGCTATCGTAATCAGTCT
ATCACGAGCGGTCCACCGTTGCGGACCGACAGACGCCTTTTATTGCCTTGTTTACCGCACCCGCCATTT
TGGAAAACGTTACCGAACTCATTTTGAAAATGGAACGGCCTATGGAACACGCTCAGAATGCCAAGATT
TAAATCCAAATTAAATCAGCTGTTTTTTGTTTAGTTCGCGTGTTACAGTGTTGTTGTATTCAGTGTTCTA
ATGCAAAGCAATAGTGGTGGTTTCGGTTCGTGTGTTCGCTTTTATTGTTATGGTAGTGTGTGAAAATTGT
GAGAAATAACCGTCTTAAGGGAATTAAGTGATAAGTGTCGGCTTGTTTCATGATTCTCCTTCAAGATGG
CGAATCGTGTGATGGGATACATGATCTGTGGATACAACTCCGTCGATAATTTATTGAGCGAGCAGTCGT
CTCCGTTCCGCCGGTGGACGACGGGCAGCGGCGCGGGCAGCTCGTACCGGCATCACGACCACAGGAC
TAATATTTATAACAAGGTGAG
>TCONS_00058823 geneID=XLOC_011866 scaffold_202:617475-619120(+) Exonic overlap (-)
TGACTAAGTAGTTTACCTAATAGTAAGTATTGACTACAAGGCAAATCATGGCTTCATATTTCATATTTACA
TAGTATTTATAATTATAAGCAATACATATCTAAGTATCTAAAATTAAATACAATATTTAATGGAATGCCTTA
GTAGCACTGTAGTACAGTCGGGGCAAACAAAGCTGACCCCTCTTAGATGAGTCAGGTTTCTTTGACCC
AGAATAAATAACTGGTCCTCTAAGAATTCTCAAAAATATTTGTTACTTTGATTTGATAATTATCCGGTTG
ACTGAAATGAGGATTGGGCACTGTATGGAGTATCAGATGATGATGGTGATGATAAGGATTATTAAGTTAT
TAAGATGGCACAAAAACAAATAAGTTGTAATCATGATGTTGCTTTAGAGCTAATTTCTCACCGATGCTTT
AAGTACCTATGGATGAAATTTTTGGATGGTGAAACCTACATTTCTAATTAGATTATATAGACCTTATTTTA
TTACTCATCCATTATTATTATTATGACGGATATATGAAACTAGTGAATTTTATTTTCATGATACAAGAAAAT
ATTAACAATAATTTTAGTAAACAATCTGTCATTTGAATGTTCATATTTTTTATCACAATTATCATCACCTAT
ATCCAAAGCACTTTTGACATCAAACTTTACTTGTATTTTAAATTTTGCTGTGTTTTACTTTATTAGTAGGT
ATGTTATATGTGTTATGATGATACTCAACTAGTCCCTTATGTAGGACATGTAAGGAAGTAGATGAAACGG
TTGAACATGTTCTGTTAGCTTGTCCACCTACTACAGAAACAAGATTATCTTTTCTTGGACCAACAGAGC
GCCTATCTCAGCTATGACAACATCCAGGCTAGCTGCTCCAGTTTACTCGGTAGCTAGGCTGGCTGAGCT
GAAATCAAGGGGATATGTACAAAGGTTCCACTCGAGAGAGCCACGTACAGGAACTTCACCCCCTATCA
GAATAGAATAGAATATATCTGTTATGTGTTATACAAGTTATCTTCATCATCACGGCAGTTGGAATAGCAC
AGACCGGGACACCTGACAACTTTATCAATGTAGTCAAAGTCTACACGTCCCATCAATCATGAAATCGC
CGTGGATATCCCCTGGTATATCCTCTTCATCATCAGGTCAGTTGGAGTGGTTCCTGGCGGGGTGCGGCG
CGGCGGGCTGCAGGGCTGGGTTGGCGACGGCTA

>TCONS_00058919 geneID=XLOC_011938 scaffold_203:110626-113490(+) Exonic overlap (-)
CCTCGCTCTGGATGCGTGCCAGCCAGATAGAACCAGAGACTCAATCATTCCATACATGAGCACGACTT
GGTCGAACCTCCCCCGCGTGTGCACGTGTGTCGCGGTGCGGTGCACGTCCTCGTCTGCCACGGTGTGC
CCGTGGATCGCCATCTCCACCATGCGCATGCCCACGTCGATCTTGGGCGGCACGCGCCGGTAGATGGT
CT
>TCONS_00059029 geneID=XLOC_011948 scaffold_203:328146-359202(-) Exonic overlap (+)
CACGCTCGCGCCAGTCGGCAGCTCGGCAGTTCTTCTTGTCGCGCCTTCGTATGAGCCGTGTGTCGCGC
TCGGATACCCCCCGCGCCCCGCACACTCCCGGCCGCCTCGCATTCTAGTTGTGGGTGTCGCGCCGTGC
AGTCCTCGCGTGTGCTACCGAACTTGTGCGCCGTCTGTGAGTGCTTGAGTTCCCGCGCGAGTGTCAGT
GTGCCGCAGGATCCCCAGCCCGCACCCGGAGGATGTTAACAGCGCCCGATAGCTGTTGACGTCCACAC
CAGAAGCCAGCTCCAGGCAGTTTGCTGTTGCCCCCGCTCCCCACACCCTCTCCAAGATTGCTGCCACC
ATGGGTCGGAAGAAAATACAAATATCCCGGATTACGGATGAACGGAATCGGCAGG
>TCONS_00059079 geneID=XLOC_011948 scaffold_203:344599-359202(-) Intergenic
CACGCTCGCGCCAGTCGGCAGCTCGGCAGTTCTTCTTGTCGCGCCTTCGTATGAGCCGTGTGTCGCGC
TCGGATACCCCCCGCGCCCCGCACACTCCCGGCCGCCTCGCATTCTAGTTGTGGGTGTCGCGCCGTGC
AGTCCTCGCGTGTGCTACCGAACTTGTGCGCCGTCTGTGAGTGCTTGAGTTCCCGCGCGAGTGTCAGT
GTGCCGCAGGATCCCCAGCCCGCACCCGGAGGATGTTAACAGCGCCCGGTATGTACCTACTGCCAAGC
CTTCTAATGTCCGTAGCGGCTCCAATCTATTAAGAAAAAATACACAAAC
>TCONS_00059302 geneID=XLOC_011990 scaffold_204:44503-89866(-) Intergenic
CGTAAATAGAGGCCGAGCACGCGCCGCGCCGCATTCGTGTGAACGTACCGGCACGCGAAACTTTTTGT
TCCATTATCTTTTTTGTTTTTAATTTGAATTCCGAAAATAAGGCTTTCCAAGTTTGTAAATAGCCTGTGTG
TGTGCCAGTGGTAATTAGTGCAGTTTAGGAAGTAGTTCGGTTGTGAACTTAGTCGGTGGCCTTTAAGTA
TACGAATATTTTAAGGTTAGATTTGTAAGTGCATTTTCTTTATGTCAGTAAACAGTGTGTTTGTGCATAAT
GTGGTGAATATTCAATTCCTTGCTTGCCCCAAGAATATTCTAACGAAGTCTAAAGCCTGAAG
>TCONS_00059194 geneID=XLOC_011991 scaffold_204:189029-190232(-) Exonic overlap (+)
TGACGGCGGACGTGCACACCACCGACTGTCCAAGCGACGACGATAGCCGGTCCACTGCCCTCACCTC
TGAAGCAGAAATCAGCCAACATGACCTGCAGTAGACTTGTTGACAAGATATTATAAAATTATACTTTATT
ACTTACCAACTGAAACTGAAAGTGCCTTTCATACTTTTATTACCTGCAGGTACTTACTTATATTGTTGCT
TTTGCAGCTACTGTAAATACGATATAAGAAATTTAATATTGGTTCGATTTTATGTTCCCCCTTAAAATAAT
TAGAGGGAAAATTACAAGTAACCTTTTTGATACTAATTTTTTTTTGTTTTGTTAAGAACTAGATGTTCGT
TTTTTTGAGATTTTGTTTCTTATTTGAATTGTTCTGTCATGGTAACTATAGTGTGACAAACTTATCTGTTC
CGGTGAGAGCGAACTAAATTGATCCATATCTCATTACTTACTAATGAATTGTATATTTTAAATGATTAGAT
GGGTTTATTAACACCGCAAGAGCGAATTAACAATACGAGCAAACTTAAAATCTTATAGAATTAAGAAAT
ATTAGTCATTTATGTGAGCTGTCACCGGAACAGATAAGTTTGTGGCACTGT
>TCONS_00059167 geneID=XLOC_011987 scaffold_204:228542-271894(+) Exonic overlap (-)
GTCATTGTCAACAAGTCAATCGTAACGTAAACAAATAAAAGTGATTTTTCATTTATTTTTATTATTTATTA
TATTTTTACTGTTACAATAGAGTTAGTTATGTTATTGTGGTAGTCAACATATTAAACTGCACCTCTCCTGC
ATCTAATGACTGATGATCCTCAACGCAGACCCAGTTATGATTTCATGGTGTTATCAGTCGGCACATGGC
CACGATCGATTGAAGACTAT
>TCONS_00059795 geneID=XLOC_012041 scaffold_205:3238-15652(-) Exonic overlap (+)
GCTGCTGCAGTGCGGAGCGCTCAAATTCAAATTAAATAATAAAGAAATGATTTAGACGAAACATTCAG
AAAATAATTCCGTTAGTGACGTAGCAAGTGACGTTCAGGATTGCACGCCGTTGCGTTTTTAGGAGACC
CTCGGCTCTGCGCCCGCTCCCCCGCCCGCGCGCCCCGCGGCCCCCCGCGCGCCCCTCGACGTAGACC
AGTGCTGGGACGAGCGGCTGCTGCGACAGC

>TCONS_00059405 geneID=XLOC_012046 scaffold_205:183454-200522(-) Intronic
GTTTAATTTCTTGCGGGTTAGGTAATTGATAACTTCACTTTATTTTGTTTGACACGTTTATTCACTTTTGC
AGTAGGTAACAATTAAAATACATTCAACTCTTTTACGACATATCCAAAACCAAACCATACCGAGATGTA
GAATTGGTTAGACAGCGCCATCTCGGGAATAAATTAGAAACTAAAAACTTATACAATTACAC
>TCONS_00059407 geneID=XLOC_012048 scaffold_205:341888-348770(-) Exonic overlap (+)
AAACTAAGCTGGCAACACTGGTTTCGCGCGCTAAATCGATTTTCTACTCCGCTTTTCCCAAACCCACG
AGTGCCGTCAGTGTGTATGAAAAATTGTGGAAATACAGTGTTTTATCCGTGCTTTTCGCTAAAAAACTG
TTCCAGAGATAGTTTGAAGTGGTTTTCTGTGTGATCCTAATTAGATTGACTGGTGTTTGAGTCCTTATTT
GGAGGATTGGGGTCCGAAGAGGATAAGCGGACGTCATGCCGGCGCGGTGGTGGTGAGATGGACGCGC
TCGAGATGGAACCCATTGGCAAATATAAGGCGGACAGGAACGGATCCGCTAAAGCGGCAGCCTGGAG
GAACATGGTGGACGCTGATCTCGATGACGTCGCGTTCTTGGCCGACTCGGACAAGGCG
>TCONS_00059701 geneID=XLOC_012051 scaffold_205:410206-421189(-) Exonic overlap (+)
GCTCCTTTAACGCGCTGTCTAAATCGTCCACTTTTAGTCGAAATATGTAGTAGATGTATTTTGTTTTGAC
AGCAATGACAGCACCAATCATCGATAACGTCAATAAAATTATTTATACTTTTAATACTATACTATTGTATA
CTATGATTACTATGTATATTGTATACCCATCACTAGTAAATGATAAGCAGTTTTGACGTTTAGTCTTCGTC
AGTGAAACTTATCGGATTTTGAGACTTTGTGAGGATTTCTGTTGGTCCGCCATAGGCGATAGGTTTGCC
GATGCTCGTCTAGTGAGGAGATCGTTGAATATTCG
>TCONS_00059521 geneID=XLOC_012052 scaffold_205:414002-419481(-) Exonic overlap (+)
CGGAAACATAGAAAGAGAAATAGTGCCAAACAACGACTCATGCGGGGCGTCATTCAATGCAGACAGC
CAGATCAGCGGCATGGAGTTCCAGGGCAGCTTCAGCGGCTACGGAGACAACTCCAACTCCATCGCTC
CCATGGAATGCTGAGATCAGCAACAGAAGTATTTAGTGTGCTGAAACATGACTGAAGATGAAAGTTGA
AGACAAAACTCGATATGAGTGGTGACTAACAGACAAAGTGCTATTGAATATGTTGTGAAAATTTTGTAT
ATATACATTTGTAGGTTTGTACTTTAGGTTTGGCTGTACCATGCTGTAAAATGATAAGTTTTAGTTAAGAT
GTTCACTGGCTACGATATTTGTACTGTAATTGGCTGATTTTTTAAGGTTTTAAGTTGTTAGAATTTTAGCC
AAAACCTAGACTGACTAATCTGACACTAAAATGGAGCCCAGTACAAATATCATAGCCATTTATTTGTAA
ACATTAAGTAAGGACGATTTTACATAACAAAGATTATTTTTACAAATTGCATGTATAATTTTTGTGTAAA
CTTGAAAAAAGACAATAAGTAATTAAAACATGTCAAATGAAGTGTACAGCGAAATATAGTAGGTATGG
GATGGGTATGTCATCAATCATACTTATCACATGGATATCTATAACCCAATAACCTATCATAGGCTTCCCAC
GGTGCGATGATTTTGCGGACGAACCCGATAGACCGAAGTATGCTCTGGTCTGGTATGCTGCGCGAACC
ACGTCATCTCTATCTCTTAACTCCGTTTTTTTTTCCACTTTAGTTGTTTTAAGCGTGCCGATTGCGTGAC
AATTCTTGTTCGTTCGTTCGTTGATTTTTATTTTATTTATTTATTAACTACTTATATTATCTTACAGGATAAA
CCTAACACAATAATAAAGAAAAATAACAAACACAACATTTAGAGAGTAACTGGGCAACCTACCACAA
AAAACTTTAAACACTGTTTAACAAGTGTTTAAAACGGAATTTGACAGATTTTGTAGGCGTTTGACAGC
GCTAAACATCTGTTAAATACTGTCTAAGTTTTTGTGGTAAGGCCCTAAGGAACTAAATCTGCGTCGATT
TGGAGAGTGACCCCACAGGAACTAAACTACAGTGAGCGACGCCATCGTCCATTGTGCGTGAGGGTCC
GTAGAATCGCGAACCGTATTGCTACTCCACTTGGCTATTCTTATTTGCAAAAACTGCCATGTATGCATTG
TGTGGTCATAGTATTTTCGCCAAAATAAAACTCCTGTTTAGCAAATATAGCCAATGGAAGTTTTGGCAA
ATACGCAAATATCAGCCAAGTAGCAGCATAATTATGACTTAATAATAATTTCCCTGTACACTTCAAGCGT
GACTTGTATCTGATCCGAGTGATCTCATTATGTTGGTTACTTGACAGATGTGGTGTTAAAAAAGCATGG
ATGATATTCGTAACTATTATTGATTTGATAATGGGTGGGTTTTTTGTTAGATAACAAATTAAGTGTGTAAA
CTCATTAAGTTTTCTTCTGTATTTATAAGACTAAATTAATGTGTATTAGAGATGCCACGAATATTCGGCAA
CTATTCGGTATTCGGCCTATTCGGCCAGTTTTTTCAATATTCGGTATTCGGCCGAATAGTAAGTAGTATTC
GGCCGAATACCGAATACTTAAAAAAGTAGCAAATATCTCACAAAAAATAATAAAAAAACAATTTAATCG
AAACATTTAACTATCAAACAATAATTAATTGCGAAAACCTGAATACAACATCGCGCTTGCTACGGTTGC

GCTTTTCTTCATAAAATATACAATACCTGCTACTGAGACACAGGTATTTTTAACAAAATATATAAGGTTT
GGCTACTGATACGAGTTGGTACAACTGGTGGTCAACCAGTTATACGGATCTGCCCTTAAGATTAAGAAG
AACACTCTTTAAATAAAACTTTAACTTATTGAATTATACTGCATCACCTGCCGAATATTCGGTGGCCGAA
TATTCGGTATTCGGCTGAGAGCGCCAGCCGAATATTCGGTATTCGGTATTCGGCCAAATCACTATTCGTG
GCAACACTAATGTGTATTATTGTGTATAAGTTTAACATGTTTTTTTCATAAGGTTATAAACTAAGTAACAG
AATGTTCTCCATGCCTTTTTTTCTACCCTTTTTGCATTTCTTGCTTTAGTTAATTTATTACACACTTGCTG
GTCTGGAT
>TCONS_00059927 geneID=XLOC_012055 scaffold_205:505334-506043(-) Splice junction overlap
CTTTTCAGAGCATTGGGTTATGAATAACCATGATTACTTTACACGTTGACACTTTCTGATTATACTTACTC
CTATTGTTAGAACCACTCGGCAATATTCACACACACAATATTTAATGCCGCCATTTTGTTTCACAGATCA
AGCTGCCCGCTAGCGACCCCGCTGTCAAGGCGGAGAAGCTGCGAGTGAACCGCGCCAAGTCCGTTAA
GCTGGCCTTAGCGAAGAAGCCCAAGAAGGCGGTGAAGAAGACCCCCCCGCCCCCCCCTAAGAAGAA
GGCCGCCGGCAAGGTCGCCAAGGTCGCCAAAAAACCGGTGGCCAAGAAGTAGAAGGGACAATATATT
TTATAAAGGACATG
>TCONS_00059384 geneID=XLOC_012034 scaffold_205:594719-600987(+) Exonic overlap (+)
GCGCGCAGCGTCGTCGCCACCACGCGCCCTAGAGCGCCGCCAAAATCGAAACCAGACTCCGTACCAG
CCGTCGCCACAAAAACACAGTGATAATAACAACAACAACAAAAAAAACACAACAACTTGTAAATAAC
AAACAAAAAAAACAACAAGCTGTGAGAAAAACTTGTCTGTGTGTCTACTATAAGTTACTTAAACAAA
ACATAAAAATGCATTTTTTTAAATAAAATATAAATAATAACATAAAAACTGAAGTGTGTTAAAGGTTAGT
TAAATGTAATATGGCTAATACATAGCTCGGTTAGGTGTTATATATGATATAAAAAAGTTCAGTTTTTATAA
TTATATATGATATGAAAAGTGGTGTGATGTGGATTGAAGGCCGGATCGGATGCGGTGCCGGTCCGGTTC
GCCGTACGGACTCTGGATTTGCAGGCGTTTATTATGTGGAGCCTAGCTTGTATGCATCGACAATAAGCG
ACGCCGCGCGAAGTCATCGTAAATATAATTACTATAATAACATATTGACTATAAACTCGTACACAGAATA
CGCTCTAAGCTTAAGCACCATTAAACGTGTATAACTATAAAATATATATATTTTTGACAAAACATGTAACA
TATTATATTATATCTATTATAAAAAAAAAATGTGGTAACGAACACAAAAAATAAACCAGTCACACGCGAG
GTAAAAAAACTAGTGATTTTCTTTTGTTTAATTAATTATAATATATAAATTTGCAACAAGTGAAATTTTAG
CCCGTAAGTTTTTTTTTCCTAATTATAATTATGCTTGTGTAGTTAAGATTTTTCGGAGGTGGAAACACAG
TTTTTTTTTAATGATTATTATTTCGTGGTGCTGCGAATTTAGCTGGCATGTTTTTATTTTAATGTTTTCTTC
ACAAAAGATTATAGAAACATGTCACTTAGCTTGTCGTCAATCAGCCAATTTGCAAAAACGCGAGTGAG
TTATTATTCTGAGCCCTTCAGTAGTGAGGGCGGGATTTGTATATTCTGTGTATAATAATTGTTGTTGATAA
TACCGAACTACTAAAGTGAAAGTTGATATTAGTATCTTGCTGTATAACTAACGAGTAATTCTAAAGCAA
ACATTGTTTTACTGTCTCCAAAAAGACTGCAATGCGTATGATTGTTGAATTTATTGAATCATAAAAGATT
ATTATCGGGTGTCATTAAGTTAGGTATACTTAATAATAATGTTTTGACTAAACTCGTATAGTTTTCCTGTT
CACGAACCATGCGGGCATACAAAGGATAAATTATTGAATCCATTCCTATTTTTGGCAAATTTTGAGGTTA
AAACATGTGTATTTTTTTAAGGTCTTTACCTACACTTTCTTTACTTTATGTTGACCGTCTAGCTAGGCCGA
TTACATTAGCTTAGTAAAACATGTAGTTTAAATCCAAATTCAGTAAATATTGCACAAAAACGGTCTCCTT
TGCATGCCCGCGTGCTATATAACTGCCACTATGTACATAGTATATGACACAATTGAGCACATACTAAGTT
TAAATGTTGATAAACGAATCTTCTGGTTTTAGCGTGATTGTGAACCGAAATTTTGTATAAACTATCAGTA
ACAAGTATTATTTTTAAGTCACTTATGTATAACAATATTTCAATTTGTTTAACAATTTGAGCGTACATGCA
TTTTTATTAATTATGTTTAAAATTAATCGCCGTAACCGTTTACTCTCCCGCTAAAAGTTCACAGATTAGA
AGTTCAATGTTCACAGATAATTTTTACTAATTTGCTCAATGTTTTTTTTTTAACTTTTCGTTTTCTCTAAG
AGTGTGTACGGTTTAAGAAGTATGTCTTTTCATTTGTTTGTCTGTTGCCCTTATGCCTTAAATTAATTTAG
GTTCGAGCATTTAGTCCCAGAGTTTTCCAGTGACAAACAGACAGATGAAGGGATAACGTGGTGTGTTG
TTAATAAAAGTCGGATTACTATAAACTCGGGGTAAAATAATAAATTTAATTTAAGCTAAAATCATTTTTCA

TTTAAAGTTTTGTTGTTGCAAAAAAATATATCAACATTTGGGAGCAAATAAGTTTAGCCTCTGCTTTTCA
GAATTACAATCTGTGTGTTCGCGGTGTTACATTAATGTGATTAGGAAATAATTGATAATATTATAAATATT
TACGTGGTAAATACTTTGATATGCACTAATTACTAAGGAAAGCGACAAAGCAAGAATACAGGTTGTTTT
AAGGCGAAAAGCAGTGTCTTTACGACTTTTGTAAAATCCTGTATTAAATAGTCCCCCTTATTCATAGAG
AAGTTACAGAACGTTTTAACTAATAAACTGTTTTGTCCCTCTCCGACAACGAACAATCTGTGCTTTGAC
AGAGAGGGACAAAACAGTTTATTAGTTAAAATGTCTTGTAACTTTTCTATGAATAAGGGGGTTAGTCTT
TGTTTATTTGCTCGGAAGTGTTACTGTAACATTTAAACTTCAAATAATAAAAACTTAGACAAGCGCATAT
TCTTCATCTAGGATATGGTTTTAGCATTTTGTTGTTAGCAAATTTCTTTGAGTCCTAAACATACTTTTTTT
TAATAAATGTTTTAGGAATAACCAATTAGACTCACAGAGTCTATTCGGTACTTACCCTTAGACTAAATTG
AACTTTTCTAAGGCAATAAAATATCGCCATTTTGCGCAATTAATTAAAAGTGATTCGCGTTTAAAAAAGT
AGGGAATCATTCGTTTTGAGGTTATGTCAACTATTTTTCTTTCTTTATTTTTTTTTAATTTTAAGTCGATAG
TGTGCTTGACCAGAGCATTTGTGGTGTACTCTAAGAATATGCATAAATATAATATACACAATACAAGTCT
AAAGCGATAAATAACGTATATAAGTCACGCAGTTTATATACGCGATAAAATTCATAACACGACAGAGGC
AAAATATATAGGTCCAAAATTCATGCAAGCTATTGGTTGTTGTATATGTATACCCTTAGTCAAAACTATTC
GTAACCCATTAATATTCGTCTTTGTATGCCTTTCTAAATTTAAAACTGCCACTAGTTATTTGGCGAATTTC
AATATTTTAAGCAATATTAATAAATGTTATGTAGTATCATGTGCATCGGTGGTCAATTTGACTGTATGTTG
ATCGTGAGTTTTAATAGTTCACGCAAACGGTAAACTTTGCGTACTCTGGCATACAATATGGCGGAAAGT
ATCATAAAACTTATGACATAAGCATTCGCACTAAAGAGGTGTAAACGCAAATTTTAGAGTTTTCGTAAA
TTATGAAAAACTTACGCTAAACGTACACTCACCGTAATGTTAAAGAAATGTCGTAATGTTTATCTTTAGG
TAGTAAAATTTACTAATGAAATTAATGAAGAAATGAATTTAAATTCCACTGTCTCCTCATACAAAATGTA
TAACTTATTGTAGACGTTTATTAGTAGGTTTAGAGTATCACTCGCAAATGTTCTGTTAAATTAAATTTAGA
GACTAACTTGTTTAATAAATAAATACAAAAAAAACTAAAATAAAAATGTTGAAGACATTAGCTAGGTCG
CTTCGGGTTTTTTTCTAAGTTCCTATTTCACAAAAGCCATAACATTTTAATCCTATATTATAATTTTAGTCG
CACAAGTTACAGAAATTGCAAATGCAAATATTACTCAAAAATATATACTCAAGTATCAGAAATCGAAAG
TGTAACTTGCAATTTCTGCAACTGTAAAAAAAAACCGTTGATTTTTTTATACCGTCCCCAAAATAATATTT
AGATCCTAGATTGCATAGCCATTTCAAAATAGTACGGTTATATGATTAGAAATAAATAAAATATTGCCTAT
AAGTGATATGAAGCGATCTAATGCTTTAAGTTATGACATGACATTGTATTTTTATCTGTTTTTATAAATAA
AAAAAACATCGAAACCTGTAATACAATGATGAGGGCTTGCTTATAGAACCGAATGCAAAAAATAAGAA
ACAAGTATCTTTTTCATAATGTTTCTAAAATACGTTGAGTTGAGAATTCACTTTTTTATTCATAAAAACA
AATGAAAATCTCAGTGTCAAAATAAAAAATCTTGGCTTTTGATGCACTATTATAATTGTTAAGTTTTATTA
TTATTATTTCAACTGTTGCTATTTTTTTCTTTTCTAAGTACGCCAAAGGGGAGGATGTGCTAAAAAGCGT
AGGTATACTGTATAAATGAAAAAATATACAAAAAAAGTCTTGCTATTGTTTCTAATTCTAAGTGTAGATG
TTAAGTTAGTGTATACGGCTCGTGCATATTATTTTTTCAGTGGAAAAATAACGAAAAATATATGGCAATT
TGGTTGTTGTAATATAGAATGTGTAGTACTGCATCGGTTAGTCATTACTTAGTTAGGTTTCAGTTTGAAA
GTTATAAATTAAGGTAATTTTGTAAGGACGCTTGCTGGCGGACGTACGGGCGGTGGCAAAAAATTGTG
TATAAAATAACTGTAAATTCCTAATTTGATGATTATAGGCAAAGGTGTACGTTTTCTTACTAATTTTACTA
TGCATGAAATGTAAAAAAATCTCCAATTGTTTTTTTTTTTGTACAAAATTAAGAATGTCTTTTCGGTTGT
ACTACTGTCGCCACTGTCCCGCGTCCGCCCTTACTGGCTGTAAGTTATACATTATATACATATTATTATATT
GATAAAATAAATTAATAATAAAAGCAAGT
>TCONS_00060405 geneID=XLOC_012172 scaffold_207:128387-129023(+) Exonic overlap (+)
CCGCCGGCGCAGCGCCCGCGAGCACCCCCGCCGCGAGAAGTACCTGCAGAAGCTCATGGCCCGCTCC
ATCAGTGCGCAACAGGCAGCGGGGAGTTGTTAGATAATGACTTGCTGCTAGAAGAACTCATAGCCCAG
TCCATTGCACAGAGGAAAGAAGAACAGAAATCTAAGGAAGACATCACACAAGACACTACACGATAAA
ACAAAAAGACATTTTGTAAAGACAACTTAGAATATAAGCACAAAAGATTCAGTATTAACGCATTTTGTG

ATCAAATTTGTACATTGATAAGTACAGCTAAGAAATCAGAGATCATGAAGATTTGATGTATTTTTTACTT
ATACAGTGACTAGAGACCATGCTGATAATTTATTTTTAAACGCTAGTTAGAAATACAGGAATTTACAAC
>TCONS_00060131 geneID=XLOC_012174 scaffold_207:231318-258495(+) Exonic overlap (+)
CCGCGAAGCGCGATTAACACGTAAAAACGCGCTCCACTCCGCGGCCGAATAGTTTAATATGTAGTCTTA
GTGCCACCGTGAGGACGAAACTTTAAATAAGGTTTTATAAATAGCAAGTGAACAATTATGATCGATATA
TCGGTGCGGCATGGATCGTTTGCTGCGCTGTGATTGGTCGGCATGGACATACAGAAGACACACCTGTG
CCCCCGATTGGTGGATTACCTAACCATCGTCGGGGCACGACC
>TCONS_00060376 geneID=XLOC_012197 scaffold_207:364484-365099(-) Intergenic
CCTTGATTAGAATACACTTTAATGCTGCTACTTCACTATTCGCATTTGCGTATTTGCCAAAACTTACATTT
GATATGTTTACCAAATAGTTAACTTATATGCAAATACGGCAATAAGTTTTGGAAAATACGAATAGCCAAG
TTAGGTAGCAGCATACCTCCCACCACTATCAAAAAGATAAATGGGGCAGCAAGCCATTTTTTCAATATC
AATCAAATCATAAAAAATATTCAGCCTTAACATAACCTAACCTAACCTATGTAAAAGTAACATCAAGTCA
TCATCCAAGTACCTAATAAATTCACACAGATGACATTGTGATTGGCTCAAAGTTAGTTTCACTCTATCAC
AGTTAGTACTTGATCTTGTACATACAGCAAACATATAGAAGCTTAATTCTTTTTAAGAATACATCATTTAA
TTTTCTCATATTGAGAGGCACTCTGGCGCAGCATACCTTTAATTCATTAACACATTGGAAAACACATGAT
TTACAACTTATTATGATTTATTGCAAGCAAAATATCATTGCTAAATTAGTGATTTACAG
>TCONS_00060327 geneID=XLOC_012181 scaffold_207:588622-591386(+) Unclassified
CCCGCAGTCCCCAAGCGGCGGCTACAACGCAGCGTGCGCGGGCGGCGCGGCGGCGGCGGACAAGGA
CGGCGGCGGGTGCGCGGGCGCAGACGACGCGTCCAGCGGCTACGGCAGCCCGCCCGACCCCGACGT
GTGCGACGCCGCGCGATGACCGACACTGCCGCGCCGACTTCTCGCTTCGGTAAAGCCTCACTAGAAG
GGCCGCGGCCCGCAGGCGGTCGGGAGTTGACGGGCGCCACAATACTTCATCACACGCATTCACCATCT
CCACCATAACGATAGCTTCAATTTAAACCACAAATCTACACCGAATTTATTTACGGCTAGCCAAGATTAT
CTTATTCATGATTCGCTCTAAACACTTCTGATGCATGTCTAACCACCGAATCCTGAAGATTAAGAATAAT
GAATAAACAGCTTTCAAGACTTAAAATCCCACGTCAAGTATGACCTTGTCTTGACTATCAGCCCGTAAT
CATCCACTTTTAGACATAGGCCTATCCCATAGTTATCAAAGTTTATTATGTTTTTTTCAAGTTTCAATATCT
TAATGCTATAAATCTCTATGGCGTGGTTACGTGGCTGAGGCTATTTCCGAGTCGGTGGTTAGATGTGTAT
CAGGCTTTATTTTAGCTCTGGGTATAATGTGGGCACTTTAGAATGTCCGGACGGATTGCGATACCGGTG
TCAGAACATGATATTTCCATTATGTATACGATTTGAAAACTTGTTTACATTTTGAGATAGCGCGCCGGCA
TACACTCCAGCTGCTATCTGCAAATAGTCTCACTCTATCTTTACTTCTGCAGTTTAAATTAAAGTGATAA
TGATAACACTAATGTAAATTTATATTTCCTTTTTAAGTTGTTGTTTACAAATCCGTTGCCTAAATTCTCTC
ATGCAACGACTCAAGTCCAGACACTAAATTCTATTGTTTATTGTAAGTGAATTCGATCTCATTGTTGTGA
AAAATGTACAGTTAGTGAAGATAATTATTGTTATCATACAAGGTATGAACTTTGAGCGCTGTTTTTTTTG
GATCGGTTAGTGTTTTTTTTATGTTATTTGTACCAAAGATGATTTTTTCGTTGAATTGTTCAGTTGTGGTT
ACACAGACGCCGTGTTTTGTTCAGTTGGCGGCGTGAGTGTCGCCATCTAGTGTTGTCTGTCACGCCCA
GTTTGTAGATAGTGGGTAGTTAGTAATATTGGTACATGGATGAGGTGCAATGGTTAGTTAATAACATGAT
AGAGACATAAACTTGTTAACTCCGCGTATATTTATGTTTCTCGGCCATAGCTTGGATCACTTGGATATGG
ATAGTATGGATACGACCACAACTTCTATCCGATCTTAATTCAAACTCGTGCTAAATACGTTATTTCTATTA
AAAGATAATAAAATTAAATCTAGTTGTAAGTATAAGAAGATGCTATAATTCTCCTACTTTTAAGA
>TCONS_00060287 geneID=XLOC_012185 scaffold_207:702442-703483(+) Intergenic
TAAAGATGCTTTTTCTTGACACACCATGTTTATTTTTATTTGTAGGTATATAAAATTTATAAATAACGATAT
ACCGAGAGTATAGGCTTTTATTACAAAAATGTTATTTGCGAGACATTGTAAGACAACTGAACTGACACT
GACACGCAATTTCTTCATAAAAGCCATAGATCATGACAATGTTAACATTCAAATACTACTTATCTATGTG
TCTTGTTATTGTTCTATGGTCTTGTTGATAACGCCATCTTACATCTTTTTTGGTAGTAAGAGTAATATAAA
GAGATGGCACTACCTTCTACTAAAAGTTCAGCCATTAAGGCACTGACTCCCCCCTGCTCAGTGCGCGA

GCAAGCGTGATACGGAATGAGCGTGTGGTGCGTTTTGCGCGAGCGTTGACGCTGCGATATATTTTTAT
TATTTTGCGATTTATACCGCCAGACTATCAAAGTTTTGGAGTATCAAGTCGAGAAGACAAGAAAAAGTA
TTTATTCAAAAAGTTACTTTCATTTAGTTTGCGCAGAATTTGACCAAAAACTGTGAATTTCCGTATAGAC
GCGAAAAAGTGAAACATTTCGAAACAAAGGACTGTTTTGTACATTTTCGCAGAAGTGCGAAATTGTGT
TCAAAAATTATTTAAAAAGTGAGTTCGTCGACCCTTTAAATTCGTGCTTCAGTGGTTTACTTCAAGATT
TTCATCAAGAAATCCATAACCATAACCCTGGAATAAGTGTCGAGTTGGTTTTTTACATGATGTCTACTGC
CATAATGCATCAGTCTGCGCTGTACGACTTTGGTGATCTGTTCTTGAAGGTAAG
>TCONS_00060651 geneID=XLOC_012185 scaffold_207:702501-730662(+) Intergenic
TAAATAACGATATACCGAGAGTATAGGCTTTTATTACAAAAATGTTATTTGCGAGACATTGTAAGACAAC
TGAACTGACACTGACACGCAATTTCTTCATAAAAGCCATAGATCATGACAATGTTAACATTCAAATACT
ACTTATCTATGTGTCTTGTTATTGTTCTATGGTCTTGTTGATAACGCCATCTTACATCTTTTTTGGTAGTAA
GAGTAATATAAAGAGATGGCACTACCTTCTACTAAAAGTTCAGCCATTAAGGCACTGACTCCCCCCTGC
TCAGTGCGCGAGCAAGCGTGATACGGAATGAGCGTGTGGTGCGTTTTGCGCGAGCGTTGACGCTGCG
ATATATTTTTTATTATTTTGCGATTTATACCGCCAGACTATCAAAGTTTTGGAGTATCAAGTCGAGAAGAC
AAGAAAAAGTATTTATTCAAAAAGTTACTTTCATTTAGTTTGCGCAGAATTTGACCAAAAACTGTGAAT
TTCCGTATAGACGCGAAAAAGTGAAACATTTCGAAACAAAGGACTGTTTTGTACATTTTCGCAGAAGT
GCGAAATTGTGTTCAAAAATTATTTAAAAAGTGAGTTCGTCGACCCTTTAAATTCGTGCTTCAGTGGTT
TACTTCAAGATTTTCATCAAGAAATCCATAACCATAACCCTGGAATAAGTGTCGAGTTGGTTTTTTACAT
GATGTCTACTGCCATAATGCATCAGTCTGCGCTGTACGACTTTGGTGATCTGTTCTTGAAGGTGAGCCC
TGAGTTTGAGGTCGACG
>TCONS_00060155 geneID=XLOC_012185 scaffold_207:702789-736516(+) Unclassified
CGAGCAAGCGTGATACGGAATGAGCGTGTGGTGCGTTTTGCGCGAGCGTTGACGCTGCGATATATTTT
TTATTATTTTGCGATTTATACCGCCAGACTATCAAAGTTTTGGAGTATCAAGTCGAGAAGACAAGAAAA
AGTATTTATTCAAAAAGTTACTTTCATTTAGTTTGCGCAGAATTTGACCAAAAACTGTGAATTTCCGTAT
AGACGCGAAAAAGTGAAACATTTCGAAACAAAGGACTGTTTTGTACATTTTCGCAGAAGTGCGAAAT
TGTGTTCAAAAATTATTTAAAAAGGTATCGTTTTGACAGTGAGTCGTTGCTCTGTTTATATTTAAAAAAA
AAAACCACCACGGCCAACTTGTTGGACGTAATATTTTTGTACTTGAAGAAGGGTAAAAAAAACAATTA
AGCATCTATTTTATTATAAAATTATTGTTCTATAAATCCATTCGCTTAGCTATCATAAATTCTAGTTATTTAT
TTCGTGGACATTAAAAACATAACCTAAAAATTGCAGTGAGTTCGTCGACCCTTTAAATTCGTGCTTCAG
TGGTTTACTTCAAGATTTTCATCAAGAAATCCATAACCATAACCCTGGAATAAGTGTCGAGTTGGTTTTT
TACATGATGTCTACTGCCATAATGCATCAGTCTGCGCTGTACGACTTTGGTGATCTGTTCTTGAAGGTGA
GCCCTGAGTTTGAGGTCGACGTCGCTGGTCTCACTTCCGTAAGTTGTCGAGACGAACTCAATACGGTT
TCCTACATAAAACAACCTGGCCTTGCCGATAAAGTATAACTTATTTGAAAAAGGGCCCTGTTCGTTATG
ATTACTTTAAACATCGGAAAGATCGTGAGAATCTCCGCGAGCCGAGTAATAAAAGGTTGTTGTAGTTGT
TGAGCAAAATACACATTAGTCATAGCGTAATAGGCTCCACCATTATTTCTTTGTGCGGGACACGTCATCC
AGCAGGGAAAGCGTGCGTGACGTAACGAGATATCTGACTGTAAAAATTACACGGACAAAGGAATTGC
AGCGGCTAGATGTCCCTCACGTAAGGGCCCTCTTGCCCGCTAATGACCAGCCACTACACTGTTGTACA
ACAATGGCCCACTGTCATGATGACTTTCATAACCGCATGGATTTCGAGCATCCCATGTAATGTGTCGCAT
TATGCGACTGTCACAAACGGGCATATTCATGCACTGAACCATTTACAAGTTGTTATGGCGGCGGCAACA
TTGTTTACTTGCGAGTTTCTGCAAATGAGGCTCGCAGCGGCGAGTGGTGTTGCATCTATAAATAAGGAG
GTGCTAAAAGCGGCCCGGCCGGGGGACGTGCTTCCTCCGCTTTGAGCCGGCCCGGGTAAACACGCCC
GCCCGCCCTTTATGGCCTTACACCTCCGCTCTACTCCGCACCCTACTCACGACCATGCTGTATACTAAAC
CCAATAAAACAAAGAGGAAATTACTATGATACAGCCTCATATAACAACATCAACAATAGATAGCCGTCC
ATCGAAGAAAACCTCAGTAATTTTCGAGTGATGTCAACCGAGGCAATGAACTCTGACGACAATTAGTT

TGCTGTTTAGAGTCACGGTGTTAATCACATGGAAAATCACGAGTTGCCGTCAAAAAGATAATTTTCTTG
TTTTGTATCTTATATACCTATTGAGTACTACAATACAGTGATAAAGCTATGTGGATGCGGTGCACGGTGC
GACGGCGGTGGTGTCATCTGCATGCAGGCCGCGCACGGGGCGTCCGGGCTTACTCATCTAACGCTCGC
CATCTAAACAAATACCATCACTAGACTTACGTGCCATCTTTACATTACATATTCTTAACGTCTAACTATTC
AAATTTGTTATCCTTACAACATTCGGTCTAATATTCAAATGAATTGCGTCAGTTTTCGGCAATCTGACTG
CAATTGTCTTCTGGTTTGTTAGTTGAATTTCTTCAGCTTAAATTAACCGAATGAGTCGTTCACTGTTCTT
TATTGGATGGCTCTCCGCGGTTATGACTTCTTTTGGAACAAACCATGTGGTCGTAAATGCTAGACTTAG
ATTAATATTAGCTTTAATGTGTTCCAATCATAGTTACCTCCTACAGTATAAAGTAGTTCATTCAAAACATG
TGTGTGTGTATGTATGTGTGTGTCGCCGCGATAGCGCCGCTGCGGGAGAGCAATGCACTGCTCTAATTG
TCACTGCACCGTCTCACACTTTACCAACTATTTTAAATACGTCATGTTGAAATGTTGTACACCTCACCGC
AACTAAGCACAATGTTTAATTTAAAAGTTGGGCAACGGCTTAGTTTTGTTACTCACTGTAAGTAGATTA
CTTCACCTCACTGCCTGTCGGGCTGTCCATAGCCACAAATGCCAGTACTAAAGTAATTAATTAGAATTG
TATCCGTGTTTGTCTCTTAACTGATTGTTATATAGATAACATGGCTGTAACCTGGGGAAACTACAACTAA
ATAATATAAGTTCGAAACGCAATAGCAGTTTCTTTGTTGAACTCGATGTAATTGTTGTTAATGTGTCATA
AATCGTAATTAATATTAATTGTTTACGGTTAACATGGGCCATGACCAGCAAGCAGGGCACAGTACATGA
CTCACTCGCGCGCTTCCCAAAACAACGGTGCATTGTGGAAAATCAAACTTTGCATTTCTGTGAAATATC
GACACGCAAATGACGTTGATTCTCAGACAAAGACGAAAGGTTGAGGAATAACCTTTAACAAGTATAA
ACAGTCAACGATTGTATAGGTTGTTGCCATTAGCGGAAGGTTGCAAGTCGTACCCACTAGGCGGCTGA
GGGGCCGCAGCGTCTCCAAGCTTGTAATTTTCGCGCAATCATTCAATAAACGTGATGGGGACAATCGA
CAAGACCCTCGGCCACTCTTCGCTGAGAGAAATGGACCAATTTCGATGACACTTATACCCATTACTCAC
TTGTGCCGCTCGCTCGTTTGTTTTGCTGAACTCGCGAATCGCGTCTTTGCACTCGTAATTGTGAATTTG
CGAAAATTCATTTGATGACTGGCGTAAAACCGTAAACAATAATAAAGATTGTATGTTTATGGGAGCCGG
CCAGCAACTTTCCGCTGTCTACACCAGGCGTGTTATAAGTTCATTTAAATATAGAACGCATTCGATTAAA
GGCAATTAACGTTTAATTAAAGCAGTTCTCTTGTTTGAATTGCGCGGCTGGCTAATGAGAGTCAGTGTT
GGGACCCGGTGGTGGCAGCGGGCCTGACGCTGGCGTCATTCGTCAGATCAAACGACAGACTCTGAAC
CGATGGAGATGGAGAGCAATTGTATTTTAATTGTAACCAGCCGTGATGGCAACTACCTACTTGCTATTTT
ATTTTCCTAGTCTCGTTGAGCCACTTTATGAAGTGTAATTTAAGATACATAACATAACTCGTACTAGAGG
ACTACACGTTAAATTGTTATAAAAATACCCCTCTATTACATAATTCCGCCATGAGGAAAATGAGTCGGCT
CGTTACAGACAAGTTCTACTCCGACCAGTGGTTAATTAAATATTCTGATTCTGCAACGCAAACAGGCAT
ACGGCAACGATGACCGCCATCTTTGTGCCGTACAATTTCCCATGACTTTGTAATGTTAGCCTCTGACGT
GTGAATGCGATGAAAAATAGTAAACCAAATTCAGTAGCATTGTACTCGCGACCGCATGAACTGGGGGT
TATTCGTCAAATCATTGACGTGTATTTGTATCACAAAGAGGAAACGGTAACTGAAATTATGTTGATTTGT
TGGAATGCGTGCAACCATGCAGTTATAACTATTGTTGTTTATTGAGGTTTTCCTCAAAAGTGTAAAGAT
GAGACTAGAGGGTTCAATGTTCATGCAGTCTTGCAGTGTTGAACGCGAACTTCCAAACAGTTACTAAG
CGGCTGAAACGTTAGTGAGTTCACGTGTTATTGTTTACGCAAACAAAGATTGCTGACGAAGGATGCTC
GCTAGGTATGAGAGTTTGTTCACCTCGGGTTAACGCAGTTATTTCTGTATCACTGTATGTTTAATGATTG
CTATCTATTGGGAAGTAGGATTCGGAAAGGTTAAAGTCTGAACGGTAATACACGATATTTCAGATCATA
GGTGCCTTTCGTCTGTGTTTGCACTTCATGGCTTTCTATGTGGTATCATATAGTATGCAGTTTTTTTTTA
GTTTATTGAATACATAGACATTCTGTATTTGTATAACATCTTGCCCGTATGACACTGCTTATGCGAATTAC
GTCCCATGCGAACTTTATTTACAAAAGCTTTCATTCCTAATGCAATCTTGCGTTTCCAAAATATATCATGT
CTTCCTCGCTTAAAATAGAACTGTCGAGTCGAGTTAGAATAGAATCCCCACTATCATTGATGTATTGAAT
CAATTGCGACAGTCATTGTTTGCTTATATGTCGTGCCCCTGAGAAGTATTTAAAAATCAATTTATTGACC
CATTGTATTTCCATTTCTTTATTGGACTCGTAATTAGGAAGTTCCCGTCAAGATACATGTTTGTCGAGTC
CCTTGTTATGACATTAAATTTGTCTTCTTAATTAACGTCCGTACGTTTTAAGATATACAAATAGCGCGCA

GCAGCAAGATCATCAATCAACAGAGTATGGGTTCACGGCCGGCCTCGGCCAACTGTTGTGTTGTATGT
AGACGCACATTGTGCTGCAGCAAACTTGTTCAGCTTTAGACTGGCTCGTATGTGATCTGTGGGGCTAAT
GAGCTATTCAAACAAACAAGCCATCGTGTCCTGCTCGTTGGACTGAGCGTGCTAACACTCCGGCGCAC
ACGTGACCCGAGTTGCACCACCATCTTTGTCATATTCAGAGAAGCTTTCAAACCTCCTAAAATTATAAG
CGTATTTATCAACAAACACGGTCACAAGGCACGCCGTACAATGGTGATGGAGCAGTTTGTTGCCCTGA
ACAACGTAAACTGTTGACGTGGACTCGCTTAAACATATGGTATTATAATAAACACTGCAATCGCGGCGG
TGCGCTGTACGCGTTGCAGTTTCGAAGGCAACAGCCGCAGCCATTTGAAATCGACAGTGATTACAATT
TTCCCTGCGGCATGATTGCGCGGGCGCAACGTCCGATGGAAAGCAGCGTTTAACCAATGTTCCGCTCT
GATAACTCTCTAATTACGCGGCGGCCGTTTTCATTCGACACTTGCCCACCACCGAGTTAACCTCTGCGC
TTTTCCCTGCAACATTTCATCCACCGCCGCCCGCTGCCGCCGCCGCTCGTCCACCGCTCATTTGTTTGC
TATACTTTCGCCTTTGACGCTTGAGTTCCATATTGTTCCGCTGGACACAAATATATGGTTCCCTATAAATT
CCCTACAACGTTTCGCTTCTCGACGTTTCTGGAGTAGGACAATGGTAACTTTCCCAAGTCGTTTATTAAT
AGCCGTTTTCCAACGCCTGGTTTATGTTATAGGTTCCAATGTTGCCTGTGAATTTGTCTTCGCGTTTAAA
TATAGCTGAGCTTCTCGGCTGCTATTTAATAACTGCATTTGCATCCCTTGCGACATGGACTGTTGCGGAT
TTAGCAAATCACGAGTGGTTCCGCTCAGTCACGTAAACGATGTTTGCTATTAGCGTCTTGCGCCGGCAG
AGGAACAATCTCCAGCGAAGGACAGCGGGGCGACCTTTACTCACAATCGTTCTGCCATTACCTGCAGA
ACAATACACCCATATCATGCCCATACGTACAACTAGAAGCGATGTAATTATTTATTATTTTGTTGCTAACT
GTGGCTATTCACACACCAGCCAATCCATTGTTTAGTTTTCATGGCAAAAAATGCTCCGTCTAAATAAAT
GGTGCTGTGTATCGGTTTGTACTTATTGTTTTGTTTCTTACAGGTAGTTAGTTACTTAGGTTGAACACCA
AGGTTCACTATAGTACCTGGTACAGCCACTTCTAGTAAAAAATACGACACAAGCGTAAATGTAGATACT
AGTTCTCTATACGGCTTCGGTGGCTGCTTATAATAATTTGTCTGTTGTATCTAAAACACTAGTGTTTGCG
TGTTTACGATCTCCGGACGAGGCGGAGGCGAATAGAATATACAATGATGTGTCATTCACATCGATTGGG
GCCGCATACAAGATGGAGTGTGTCATACATAATGTATAGTGCTATGTTCCGCGTCTCTTCGACTCGGCCG
ACCTTCGAATTCACTCACGGCTCCAGCGGCGGCGGCGGAATGCCGCCGCACCGGGCGACTTGCGAAA
CCAGCGTGGGCCACATTGTCTCAGCTGAGTTATACCCTCCTGCCTACCCTTTACTCACCCGCCAGTCGC
AACCACGTAGGAGGTAATAGCCACGGTCGGCACAATGGCTCCGTGTGTAAATTTCACCTAATAAGCGT
AACTGAACGTCGCCGCGTGATCGCAGAGCTCGTACAGTACACATAATGGAATACTGTTAGCTAACACG
ACACGCGGCGACCGTTGGCCCGCGCCGGCGAGAGGTGTCGCGATGGCACGATGACAATAACTCAGGC
>TCONS_00060331 geneID=XLOC_012187 scaffold_207:752124-753369(+) Unclassified
TATCCTACAGTCCTCCTATCGCCGTTTCCGTTTTCTTTGACGATATTATTAGTCACCTATTTAACCTGAGC
TGAAACCCTCTACAGTTTACTATTATTAAAACGTTTTATAGTGTTTAGCTCTATTGTGTTGGAGTGCTGG
GGCGCATGCGTGCGTTCGATAAACTTTGTTTACAACATTACCTATAGGCGATATGGCGGTCGTTGACAC
CGTTTGAAATTAAAAAATCTTAAGTTAATTTTTTGCGGAAATAAATTTTATATAAATACTTACCTATCTAC
AAAAAAATAAATAGGTATTCTGCTCCACATTATCCACATAATATGCTCCACCGCTGTGCTGATGGTGATT
GCAATAGGTATATTTTGTGCAATAGGTACTTCCTTGATATACTAACACACCATCCTTTTTAGATGTTCTTA
AGTACTTGTATCGCACGCTGCCGCTGGCGATGATCTTCAAAACACTTTTAAGAGGATCCTGTGACTGTG
GCCAGATTTGCTAGTTTTTTGCTAATGTCGATTATTACTAGTATAACCACCTACTATACTAGAGATATGTC
AACCACTGTGGTTTACTTGTCTAATATAGTAGTAAGTACCACTCATACCAACTCCTTAAATAGGGCCTAT
ATTCAAAGTCAGTCTATAAAGTTCTGAAACATGTGTCGGTCTTATTCCCCAGACGTAGTTATTTGTACAC
AGCCTGTGTCGTTTTAGTTGAGTATTCTACATAGTTTAGTTTTCTTGGTGAACCCATCCCTATTCCTATAT
TTTGATATCTGTTTGATACTGAACAACACAGGTGCATATAGGCAGAATCATACAGTTGTATTAGGGAGG
TTAAGACTAAGGCGTTAATTGAAGGACTGCTAGTGCTATCAGCTGTTCTTAAAATAGAGAGACATTAGT
AAACCATCATATAAGTATTATCTCGAGTGCCTAGCTCATTGACGTCCAAACGAAAGTGTTTACTATCTGT
GCTTGTAATTGTACGTCGTAATAAAACTATTATTCTAGAAAATGGAATGTTTATCATTATCTCACAGCTGG

GTATGATCGTGCGGTATGATTGTGTATCAACTGTTTTACTTACCATATTATAAGATCTGGACTGCCAAGTT
GTAAATGTGAACAATATACTTAGGTACTTAAATATAAC
>TCONS_00060871 geneID=XLOC_012295 scaffold_208:290625-291848(-) Unclassified
TTCACCGGTAACATTGTTTATGTCGAGGCTCCGTTATTAAAATAGATAAAACATAGTTATCTCGCAAAAC
TAAAGTACATAACTTATTAACTTAAAATCACCAACAGTAAAGCTAAGTGAATTAAGCTTCAGTTTAATA
AAAATCGTGTTTAAAGTGATTAATTGATCAAAATAACATCTAACTGCGGGTGTGCAAATAAATTCTGTG
TGATCGCAAGCAACTCTCGACGCTACGCGCAGCACGCAGTGACTACATTCGGTAAAGCAGCCCTCCA
GCCGATGAGTCAGTGACGTCGCATCGGTCTTGTCACAGAGTAAGCCGCGCAGAAGTGTTTACCACTTT
GCACAGTCGATCCTTG
>TCONS_00060910 geneID=XLOC_012296 scaffold_208:314154-316868(-) Intergenic
GCCACTAACTGTGCGCGTGTGTTGCCATGCAGTATAGTCCGACAACGCTAACTTTGCAACGGCTTAAAT
GGGTTGTTACTTAAGATGTTGAGATGCATCACGAGTAAAAACAATTATTTCCTAATACTTATTGTATTTTT
TTGTGTTCCCATTTGTTTCTGTCGCAAAGTTAGCTTTGAAGGACTCATATCAGACTTTATAGTGATGTAA
CGAACATGACAGTGGACAGTTTTTCAATATTGATTGATTGTTTTCAATAAGATCTGTTGTTATATGATGAT
GTCCGTGGACACCAGCACAGAAGCCCTACTGTCGAAGGCCCGCGGTCGGTTCTCGGCGCGGTTCGGG
CGCGAC
>TCONS_00060912 geneID=XLOC_012298 scaffold_208:372371-373180(-) Splice junction overlap
CCAACATGTACAAACATAAACAATTAAGTTTTCGAGTTTTCGTTAATTTTCGTGTTTTCTTTTCTCTGAA
GTGTTTTCTACGTGTTTTCCTTTTTGTTTGAAATATATACGAGTATATACTGCCGGGGCCGGATTTTATGC
AAAAACTGTCGGAAGGGGTTGGTTAGGCGATACGCTGACTGCTCTGGAGCTGCTGAGGAGCGTCAGC
TCCACCAGACGGATCGTCGCATCGACGTTGAGGAGCCTCGGCTGGCTGTGGGGACGCAACGAGTCGC
CGTGGAGAGGCAACGACTTGCCGTGGAGACGCAACGACTCGCCGTGGAGACGCAACGACTCGCCGT
GGAGGAGCGACGACTCGCCTTTGAGCAGGAACGCCAACTCGCCCCCTAAATATACTGTGATTTTTATTA
TAATTGTAATATTGCTATAATATACTGTGATTTTTATGTAGATCCAAG
>TCONS_00060877 geneID=XLOC_012302 scaffold_208:445310-445854(-) Splice junction overlap
GGTATGCGCTGAAGTGTCAACTGCAGGAGGCGCAAGTGGCCCTGGCATACGGACTTGCTGCAATACA
GGCGCTGCCCGAGATGTTCTGCGCACGCGCCGAGCGACACGCCGAGGCCGCGCGCCTGTGGGCCGAT
ATACACGCGACTCTTGACCCAAGCCACAGTAGACAGTAATATACACACGGCTCTCAATGCAAGCAGTA
CTAACGCGACTCTCGATGCAAGTTACAGTAGACAGTAGTATACATGCCGCCCTAGACTAGTGCAAGTAC
AACAGAACTCAGATCTCCCGCGC
>TCONS_00060975 geneID=XLOC_012289 scaffold_208:529032-573232(+) Exonic overlap (-)
CGAAGATAAATAATATCAAACAAATGAATGCTGTTTAGTATCTGTATAAATACCTTTGGTAGGAAGAAA
GTCTTCATAGTATTTTTACAACCTAGAACAAACATCATGAATTATGCAGTAAAATCTTTATTGTTAATTAA
TTTGTCAATATTGGCTTATGGGAGCTATGGTCTAGCCATCCCCGTGAACACAGAGTCTAATGTCAACCG
CCTCTTCCGGTCTACCCGCATTCATGGAGGTGTGACAGCTCCCGAAGGCTCTGCCCCGTATTTACTGGC
TTTGGTGATAGGAGTTAACATCGGGCA
>TCONS_00061629 geneID=XLOC_012379 scaffold_21:8007-8446(+) Intergenic
AAACAGGCACACATGCAAAAGCTAATTTTGTTGCAATTACTCAAGGTTCTTTATCCAACATGATGAAAC
TCTGCTCTTTTATCAGAGCCGGTCAGTGTGAATGCACCCTTATACACAAACTGATACCTGACAGTTGGA
TGATGAAGGTAGACTACAGGAGATGATGAGACTATTGATCCTTGGTATAGCCTATCGGTAATATAGGAC
AAAAATATGTCTAGAAGTTGCCTGATGATGTTAATGAAATATCCAAGATCTGAGCACAAACATTCCCGC
CA
>TCONS_00061345 geneID=XLOC_012436 scaffold_21:103267-104819(-) Unclassified
AGGCAGCTGTTTACACTGTATAAACATACAAAGACACATCCTTGTTGAGGAGAGGTGACCGGTCACTG

CAGCCACAGGCCCCGAACCAACGCACGCGCAGAGTCGTTCACGCCGCCACACATACCGGACCTCCTT
GCGCCTCGCTCAAGCGCGCCAAATTAAAAAAAAAAAACAAAAACTTTTTAAACAAAGAGGAAACAG
ACGACGAGGATGGATTAAAGTGATAGTGAAATAAAGAAAAACTTTTAACAAAGTTAGTTTGAGTTATC
AAGTTCCAGTTTAGTCACGGATCCCATTGGCAAGATACACCTTTTCTGATCTACATGACGTGAAATGGC
GTATCTCAAGATACTATCAGCGATCACCATCATAACATTAGGTAAGTCATTAAGAAAAATATCCTCATCT
CAGGTCTCAGACTTCTTCTTGTTATACGTTGCCGGTAACAAATAATGCATTTAGTCGTTTATCACCGTGG
AATGGATTAGCCAGTCTGATTGTTGTCAGATGCTGACTGCTAAGGCTTCCCCATCTGAGAATAAATAAC
CTTACCTAATCAGTAATTTTCAGGGGTAGTTAATATAATCTTTTAGTTCCTACAGAACTTACGCTACACTC
ATACAATACTTACTTACAACTTATAACTACAATGCTCTCAGATGTTTCTAGGAAAAATAAAGCTAAATAA
ATATAGAGATTCACTCATGTGGGAAATAATAGTAAACTTGTGATGTCACAGATGATTAACAAATAGGTG
TGTACTTACCTACTACTGTGCCCGTGCCTACAACCAACTGTTGAGATACCTGCCTAGTTATATGTATATT
GTATAATCAACAGATTTAATAGATTTTTTATGAGTCACGTTTCCCTACGTGTGCTGCTGGTGTACTGGTA
GCAATATGTACAATTCGAGGTCGGAGTAAAACCAAAGACCCATACTCATAATCGTACATATATATACACC
TACATTATTGGGGATACTGAAGCTCTAGCGGGAGGGAACCGTGAGTTATTTTACTACTTCAAAAAGAAA
TTTTAAAAAAAACATAAAGAGCTTAATTGCACATGTACCTAATTGCTAAATCC
>TCONS_00062111 geneID=XLOC_012436 scaffold_21:103995-104821(-) Unclassified
CTAGGCAGCTGTTTACACTGTATAAACATACAAAGACACATCCTTGTTGAGGAGAGGTGACCGGTCAC
TGCAGCCACAGGCCCCGAACCAACGCACGCGCAGAGTCGTTCACGCCGCCACACATACCGGACCTCC
TTGCGCCTCGCTCAAGCGCGCCAAATTAAAAAAAAAAAACAAAAACTTTTTAAACAAAGAGGAAACA
GACGACGAGGATGGATTAAAGTGATAGTGAAATAAAGAAAAACTTTTAACAAAGTTAGTTTGAGTTAT
CAAGTTCCAGTTTAGTCACGGATCCCATTGATACACCTTTTCTGATCTACATGACGTGAAATGGCGTATC
TCAAGATACTATCAGCGA
>TCONS_00061887 geneID=XLOC_012385 scaffold_21:275441-277425(+) Intergenic
TTGTGGATCCACTGCATCCCGGACCGTGTGACAGAAATATAACAGACAATAGTGTGACTCGAGTGCGG
AAGGGAGAGAGAGGTATGAACTAAGCGAAGAGCTGAGGGGGAAAGAACATATTCAGGGACTGTCTG
GTTGTTAAACTCATATTTTAAGTTATACTCTTTGACTTAATTCTCATATTATGATGAACGAAGCGGCTTGT
AATCTAATTCCTATTTCGTTAAGTATTAAATTTGATTTTGAATGACAAGCTGGCCAATAAATACGTGTCTA
GAACTTAAACCAAAATGTATAAGTCAAACCATTTAAAGATGTTGATGTCAGTTTTCTAAGTTGCGTTTA
GAAAGTAGATTAATTTATCGTTATTTTTGTAATGAGTTAATTAATTTAATTTAAGACTGAACTTGTAACCA
GTATGGTCTCTGCGAGTCGAGTGTTGTCTTCAACAAGTCAAGTAAATAGTGATTAGGTGTACTAAATTC
AGTAAATAATTGGTAGCAGCCTAAACCCTAAACTATTTTTTAACTTATTACACACTCATTGTAGATAAAT
AATTCTATAGTTTTTACTGATTAACACTTTAAACTTAATAAATTTTGAGCACCAAATCGTACTTACTATTT
AGTCAAAATATTATAGGACTGTAGTTAGCACAATGTTCTGAAAATAGGTACTTATATTTAGAGAAATTTC
CATGTTTCTTGTTATAACTAACTATAAATACAGGATGTTGAAAAATAGGAAATTTTAAGCAGGGAAGGC
AATACTTTTGGCATTCTATAGAACTTTTGTTTTTTAGAATGTCAGATTATTTATATTACTGAGTAGGCCTTT
ACTGAAAAGGTACTTAATTAATGTAATTTCAATCAATATGGCTTAGTTAGAACCCATTTTGCAACATTCT
GTATAAGCTTGTAGGATGAATTGTTTCATCGGAGAACTGACTGACATAATTGTTCTAAAATTCTAGTGTT
TTAAGATTAATAAATATAATTTTACTTCAGGAGCATGGTGTGTGACGCTGGCGCAATATTTATTGTTAAG
TTATATGATGTATGGAGTTTTAGTGCAATATGTTTTGATGCAAATGATATAAAATTTACGTATGAACGTTT
TGTTTATTTTTTTTATGATTTGACGATTCATTCTATCCGAGGTTTCGCGATGACAAATTATGTTGTTCCGA
CATGTTATTGAGT
>TCONS_00062115 geneID=XLOC_012440 scaffold_21:323563-333200(-) Exonic overlap (+)
CAGGTCCAAGAGTTCCCTGAACCAACGCCGTCCATCACGGCTTTAAATTAAACAAATAGCGAGCATGA
GTTTATGAGGGTAGCGACAGGCGGCGACATGGCGCGGGGCGCGTGCGCGGTACTGGCGCTCGCGCTG

CTCTGTCCCCTCGCGCTCGGCCGGCACGACGACGACTTCGCCTTCGACAACAACGACGACTCGCAGG
TGG
>TCONS_00061821 geneID=XLOC_012440 scaffold_21:323593-333198(-) Exonic overlap (+)
GGTCCAAGAGTTCCCTGAACCAACGCCGTCCATCACGGCTTTAAATTAAACAAATAGCGAGCATGAGT
TTATGAGGGTAGCGACAGGCGGCGACATGGCGCGGCGGGGCGCGTGCGCGGTACTGGCGCTCGCGCT
GCTCTGCCCACTCGCGCTAGGCCGGCACGACGACGACTTCGCCTTCGACAACAACGATGACTCGCAG
GTGGGTTGAGTCCACGTGTAGCGAGCATGAGTTTATGAGGGTAGCGTTTGGTGGCGACATGGCGCGGG
GCGCGTGCGCGGTACTGGCGCTCGCGCTGCTCTGTCCCCTCGCGCTCGGCCGGCACGACGACGACTT
CG
>TCONS_00061779 geneID=XLOC_012388 scaffold_21:646474-647222(+) Intronic
CTGGGATCTTCGTTGCTTATAAGTTAGAGTCGCGATGGTGTAAGGGATAAGGTCTCGGCTCTCATTTGT
GATGCTGTGGGTTCGAATCCAGCCATACAATGAATTCTTAAGAGTTAGGTACCTAGCAAATGTGGGAAC
TAACTACACATGACATAGCACAATGTTGACACAAAAATGACAAACCCTCTGACACGTTACGCAGCTAT
TTGTATGTCATGAAGGCGTGCCTCCAGCGACGTTATTTATGACATTAATTCATCACATCTCGTCATATTAG
TCAGT
>TCONS_00061982 geneID=XLOC_012391 scaffold_21:773890-784114(+) Unclassified
CCTACATACAAAATGTCAAAAAAATAAACAAAAATACAAAATTTAAGACCAAGAACGGAAAAGTGCC
GGTTTTAATAGTATTTATCCTATCTTCTCCATTTCTGTGCTTTGATTAAAGTGAACTAGCAGCGGTAGTGG
CTGCAGTAATACATCACACATCAAATGTACTACATCAAAGCTTACTGCTCAATTTCCCCGCGCGTTTTCA
TCACAACTCCATAGTCAAAACTCGGCGTCCGTCGAAAAAAAGAGGTAAGGGTTTCTTTGTCTCAAAAT
AGTTGTGATTACGTCACTGCTGGGATAATTAGAAGTTATAGCTATTGGCGGTACATGCGGCGTGTCTCC
CGCTGGGGTAGACGGTGCCAAAGTTTCATAGATGGATGCTATTTCCGTGCAAAGTGAAATCCAC
>TCONS_00062025 geneID=XLOC_012445 scaffold_21:838124-839049(-) Intronic
CATCAGTCTTCAATTTATTTATTCAAATAAATTTACAAATTACATGCAAGCTATAAAGTAAGCTACGTGC
AGACAGTCATGTTAATATAAGCATTTTGGCAAGAAAAGGGAAATCAGACTCATTCTCAGCAGATCGGG
GCACTGGAGCGCCGGCGACCGCAACACATCACATGTATACTTACATACGGCTCAATCAGACCCAGACT
ATTTATCATTTAATGTATATTTACTATTGGAATCAATAACTAGATTGTCATAGTTGCTCAACTAATACTGAA
TATTGAGTTTCTATCCCTATCAATGATTATTTTAAATTGGGGCTTTCATAATCGATGTCCGTATCGTCTGA
GACGCGTACAGAGTTGTACTAGAACTAAACAAAATTACTTAGTTTATGAGTATGATATGGTCAACAATA
CAGTATGACTTATCTTAATCGGTAGGTATAAAATCCTGTTTCAACATCTGTACACAGTACAAATTAGAGA
TCATTGTGTAATACTTGTGGTATGATTCGACGTGAACAATCATATTTATCAAATTGGCAGTCTTAAATCA
GTATTTTGGTCACTGAGATCGACCTAATGAACTAGATTTGCATTAGGTACTGATAAAAACCTTCAAAAA
CTTTTGTACTTTCCTAAAATAATATTAGTAAATGGCAGATCTACTTAGTTCAAAAGTGAACATTACAAAA
ACTTACA
>TCONS_00061514 geneID=XLOC_012401 scaffold_21:922172-923983(+) Splice junction overlap
ACACCAGTAGATATAGGTTACATGATCTAGATCTAAATCAAGAGACATTAGTGCCAATTTGCTAATGAGT
TTCAGTTATTAGCGACGTTCTAGTCACGGTGGCGGGAGCGCTAAGCTAGGTAGTTTCACGGTGAGAAT
TTCCACGTGATATATGAGTTGGGAAAATTTGATCGCAGGGTGCCGGAGGGGCGCGCGGCGGGGCGCG
GGGGCGGCGGCGGAGGCTTGATATTGACCTAGATGTTCATTCAGAGTTCGGTCGATGCGAAACTTGAC
ATCTGCGCGTTCCTGCGTTTTATCATTTTCGCTTCAATGTATGTTTAACTGGTCTGCTTCTTGATTTTGTT
TTATTATTCAATTAAAACTTAAGTAAGCTTTACAAAGACGAGGGTTTTCTCGTTTGTTTGCGTCGGTTCA
GTCAGCTTGCGCGAAGTCAGTCGATAGGCGGTGTGTTATGTGTTGTGTGGTGCCGAGCGGGTGCCGCG
CCGTCGCCTGCCGGAGCTCGCCTGGAGCCTCCTGCAGGGGCAGATAGGAGCTCATCTCTCTAAACCAA
CATGTCGTCCTCCAACAACCTCATCCCAGCTGTCACAAAAGGTGGTGCTAAGGATTTAAACCATGCAG

AAAACGGCGACGAGGATGCGGGCACCGCGGCGGCCGAGGAGCCGGAGCGCGGCAACTGGACCGGC
CGTTTCGACTTCTTGCTATCTCTGCT
>TCONS_00061939 geneID=XLOC_012453 scaffold_21:1005307-1006187(-) Exonic overlap (+)
CAAATTGAAACGGCACGACAACGCAACGCATTGTTATTTTCCTGTGTTTTAAACATAGTATTTTCACATT
ACACCTATTTTCTGCGAATTCACAATGCATAACTCCTGTCATAAGTGTTAGCCGAATAATGGAATGATTC
GCAAAAAACAAGAAGTTCTTATAAATTATTCCAAGTGTCATTCAATTTTGTATTTTTTCTCGAACACTTC
TCGCTTGAATCTAATCAATCAAAAAGCCTGTACCTGAACTGCATTTTATTTCGTGTTTCGTGTATACTCT
CAATTTGTAGACTGGACATCCTCTTCACTTGACTATTTGATTTGAAGTATCATCTAGTGTAACTCTGTTT
GATGCCTTGCATCCATCACGGCTTCTAGAAGAACCGAAGAATTTGGCCATCAAGAATTAGAAAAACGA
TACAGGATGCCAGCTGCAAGTTTTGGATCAGTGGCACCGATACCAGCTAATGAAGAGTGTGGCATCAA
AGATGTGTGGAACCACAATCTTTATGAAGAATTTCAAATTATAAGACAGGT
>TCONS_00062162 geneID=XLOC_012407 scaffold_21:1178049-1180769(+) Exonic overlap (-)
CAGTTTTATTTTACCAACATTTATTAAATTTGTACAATAACAAAATATCATCGAACTGGTGTCAGCCACG
CAACGGAATGCAATACCCTCATACTTTTACACACAAAACAATGAATTAATCAATTTATTTACGTGAATTA
ACTTAAGTCACAGAATTATTATAAAACTTATATTTGTCTTTTCTTCAGTCATTACTTTTGGATGTTTTCAC
GATTCCAGCAGCAGATTGTTTATACTATATTATTATACTTTCGTTGCATGGAAAAGTGGGAAAAATTACA
CATTTTACAGTCATAAAAATAGGAGGGCATTTCACGCGACATTCAAAATCTGTTGTTGCAAACATTGCA
CTTATCAAAGTATAAATTACTTATCGTCTCCCATTGCGTGGCATTGTTTTATATACATTGCATTCATTTATA
CATATACTGTGCATATTCATAAGATTCATCAGTTGCAAACATGGTAGGTATATAATTATAATAATCACAAC
ATTCATTTTAGCATACATAGCATTGTGCCTATCAAAAGTAATGGCATTAGACAGTTTAATATTGGAGGTAT
GTATTTACAACGCATTTAGGAATAATTCAGTTACATATTTCACAACATTAACTAAAAAATAACACACAGC
TAAAGTAATACTATTACACGTAATTTCAGTTTTAAATACAGTGTCTTGTTTATAAATATTTTAACCAAATT
GTGCATATTCTTAACGATATAAGTAATTAGATACCTATTTGTGTTCCATAAATATAAGTTACATCCATTTCG
TGTAGTATAAGCCATTTTTTATTGCCTCTTTTCAATTTTATGTCATTTCTATCTACTGTTATAGTCTTTTTAA
GGTTTGGGCTGTTGAAAGTCTTCGCGTAGGCAAAAAATATTTTTGTAAACCAACCCGAGCACATAAATA
TTTGTAAGTTAACTTATTATACATTCAAAATAGTGACCACAGCCAGTTTTTTACGTATAAGTAAAAGGTT
AAAAAAAACTCATAATTTCTGTCAAGGTCGTGTCAATTCCATAAAAAAAATATGCAAGGTGAATTTTTTT
AATACTCAATAAATAAATTGAAAAGTTTTATTTATCAAACCACAGAACCACAGCAAACGAACCACCT
GCTTTGGCCCTTGATTTGTATAGTCCCCTACATTCTTGTGCTAAGTAAACTACTTAAAACATAGAAAACA
AAATGAAAAAAGTGAATAAACGTAGCCACATTAAAGCAATAAAACAAATAAATACAGTATTTAATCAAT
TCACTAAATAATTACATTCAAAAACAATGACCACGAATGATGAAAAAATTTGGAATCCTAAAAATCTTG
AAATCTAGTAAGGAAGAGGTTTCATTCCTGCACTTGTGTGATAAAAATATAATAATATCATCTTTCTTAC
GGTTTATGTCAAGTTTCACACAAAATTTAAATAGTTCAATATAATTAAGTAAGTATATAAAACTTCCGTG
GAAACACATTTTGCATGACTCTCATACAAATATTATATGTCTTTTACTAAATTTACATGAGTCGCACACA
AGTTTTATATTGATTCTTTCACAAAAACCTTCCTTTGTTCGAGTCCCACACATTTTTTGCATGAGTCTCT
CATGTTGATACCACACTTGGCTATTCGTATTTGATGTTTTCTATATCTGCCAAATACCAAAAACTTTGGC
AAATTCAGCAATAATTTTGGCAAATACACTGACCACACATGGCAGTTTATGCAAATACGAGTTGCCAGC
ATCAGTAATGTTACATAGAGGATGATGTATGTGTCTACTTGACATTGATCCTGTTCATGATGGTGGTGTA
GGCGAGACCGAGCGCCCAGGACACCTCGTGGCCGTTCACCTTCTTGTATAGCTGTTGAGAGAGAAAC
ATTGTGGTGAGTAGGTATAGTAAGGATGTTGGGTAAGTGAATCAATGTATGTAGACATGACAGATTACA
GAACCCTGACTATTGTGAAACTTATTTATTTATACCACATACATTAATTGCATATACTGATAGCTGATGTT
GCTGATTGGTCTGCTTACGTAAATCTATCCACTCACGTTGTACACACAAGAGCACGCCAAGATGGCGTC
TTTACAAAATGGCGTATTTTTCTTTGCTTGTAATAATGAACGCTATTTATCAGCAGATTAAACACTGACTT
ACCGATATAGGATGGTTCAGAGACA

>TCONS_00061377 geneID=XLOC_012454 scaffold_21:1190609-1196632(-) Splice junction overlap
CGACAACATAAAAAATCGTGAAAATATGTATAGTATTTTTAACTACGTTTTTACAGTATTAACCATGTGT
AACTAAAGTTGTGCCTCATTTATTTACCGGTTGTGATGTTTATATCTGGCCGTTATGGGCAAATTCAGTA
CTTACAGGTATTTAGAGTTGAAAGATGATAGTGCGCCGGCGGCCGCCGCGCCACGGGCCCGCAGCCTC
GCGCAGCCGCGCCGCCTGCGGAAGGTGTTTGTCGTGCTCATCATAGCCGCCGTCTGCGCCACATACTT
CACCGGTCTATTCGCGGGCCAGAGTCGTCTGCTGGACGGGCTGGCGCGCTCGC
>TCONS_00062349 geneID=XLOC_012454 scaffold_21:1194316-1196647(-) Splice junction overlap
TTTCAATTCAAATCACGACAACATAAAAAATCGTGAAAATATGTATAGTATTTTTAACTACGTTTTTACA
GTATTAACCATGTGTAACTAAAGTTGTGCCTCATTTATTTACCGGTTGTGATGTTTATATCTGGCCGTTAT
GGGCAAATTCAGTACTTACAGGTATTTAGAGTTGAAAGATGATAGTGCGCCGGCGGCCGCCGCGCCAC
GGGCCCGCAGCCTCGCGCAGCCGCGCCGCCTGCGGAAGGTGTTTGTCGTGCTCATCATAGCCGCCGTC
TGCGCCAC
>TCONS_00061943 geneID=XLOC_012458 scaffold_21:1246022-1268442(-) Exonic overlap (+)
TAGAAGAGCAGGTTCATGACTAAGAAAAAAAACCGCATACAGCCTACAAGACACGATAAAAAAGTCA
TTACCAAGGACCTACGGCGAGGCTCTCTGAAGTCTTCGTGCAAGACTCCGGGCTCCGGGCATCATAGT
CGCTGCGGTGCCGCAGCCACGCGCACGCGTCAACTACACGCAGTTTTAACCTGATTGGTGGCTGCATT
AGTCAGGGTAAAGAACGCGTGAATTTAAAAAAATAAAATTAAAAACCTAACTGGCCGCACGGATTTTC
AATCGAAGTCAACGGGCGCCATTTTGGAAGTTTTTCCCCCGTGTATTTTGACAGCTTTGAATTGTGTTC
GGAAATTAACTGGTTCTATTGTGTGATTGATTGATCGATTTTTGTTCAGTGCACTATTTTTATTGGATTTG
ATCCACCATGTTTCAAAGCGGCGTGTGGCAGCGCCTGTCGGGCTACTACAACTGGTACGTCCTGGGGC
TGGTCTCCGTGGGGTACATCCTGGGAGAGCTCGGAC
>TCONS_00061398 geneID=XLOC_012463 scaffold_21:1595147-1596355(-) Unclassified
CTTAGTTACTACACGCGCGTTCTGTAGCCCGGACGCTTTGCCGCCGTTTTCAGTGTCAATAATAGTCGT
TTCCACCGGTTCTCGTACGATTTGTGGCCAAACTGGATTTGTACGGAGCATTTTTAGAAGAATCGAAGC
ATTTTAGCTGTTTGTAAAGCTATATCGAGCGGGTGTTTGTAAACTGGTAGCTCGCTCTAGCGGCGAGCG
CGGTGAAACAGCCGGCGGGGCGCGCGGGGCGGGAGCTTCCTCCCCCTGCTTCAGTCTCTGCGCAGCA
CTCGAAGAGGCAGGACGTGTGTCGAGCGACAAGCCAACACACCACTCGCGCGGCAACTACGATCAG
TGACAGTGTCGTAAAAAAGTGAATTGAACAGAAAATGCGTATCGGATTGCAGTGCCCACATTATTGGA
TGCAGTGAACGGATCACACACATTGGATTGCAGTGTTTTTGGATTGTACTTTTTAATCAGCAAAACTGG
GGATGCATAAGATGGAACTTGGAGACAGCGTGTACGCCGCTGAGCGTATAATGAAGAAGAGA
>TCONS_00061599 geneID=XLOC_012466 scaffold_21:1876915-1893694(-) Exonic overlap (+)
CATACACCGATCCAGTTTGCGCCGGGCCGGACTGTCGGCATACCGCGTCCGTAACCTCTCAACTGCCA
GTGATGTGATCAAAACTATTATTAAATCGTTTCAAAGCGTTATAATCGATAAACAAAGAATTAAACTATG
TGGTGGAAGTGTTTGTGAGCCGTTTGTCGAGGATTCCTGCGCAGGAGTGGCTTGGAAACGGAACATC
AAAATGGCCGCTGCGTGGTATGTCCCGATGCTGCTGGCTCTAGTCTCGACAACACTGGCTGGTGAGAA
AAAAACTAAATTCAAATATAGAAAAAGAAACTCGTTCTTCAACAATTTGACGGTAATTTATAGAAGTGT
ACGGGCGTTTAACAAGTGGAGGCGTCGGCCGGTGTTGCTACAAATCAACAGTCTTTTATAAAGCCCCG
GTAAGCTCAGAACTATGCGTTGAAATAATCCCGTGTGTAGGTTTAGGGACCCGTGGTGGGGTAATAGG
TTATAATATAAAGGTATGTAAGAGGTTTGTAACGTTGCCCTCCGTTAATGAAATTAATTTTGATAAGTTG
GAGCTTTTTGTCCAATATTATTTTAGGCATGGAAATGGTATCATAAATTCAGATATCGCACACCAGAGTAT
ATATGAATATTTTAAAAGTGTGTTAGTACATACAGCTAGG
>TCONS_00062103 geneID=XLOC_012425 scaffold_21:1969265-1976561(+) Intergenic
AACGGTTGGACGCGGTCGGTTTAGACGTTGCGTTTGAAAAAAGAACGCTTTATTTTCTTCTTTCAAAA
GAGTTTTAAGTTCTTTTCTTTTTTAAATGTGGAATTCTGTGAACATGGTGTTTAGATGTTGAGGTTGCAG

CATTTTTGCTCCTAAAGTGCTCGATAACCGAAGTGGATTTTCAAAACGAGTGTACCTGTGTGTTTTGAT
TTAAGGGCATAAGATCTCAGCCATTCGTCGTGGAAAGTCATAAACAACACAAGAACCCCGGCCAAGG
GATATTAAAGTTCCACACTGCTTTCTACCACATATAATTGCCGTACAAAGTCACTCGAGATTATAAAGAT
AAGGCAGGATTACAGCCGGATTGTAAGTCAGAAAGAAAAAGTTGAATTTATTGCAAATGTTGCATCCG
CCATTGTTGCAAACGACTTTAATGATGTCGGATTAGAAGAGAGTGACTTGTTACGAGTCCATGGTGTCA
TTATCTCTTACGCAATGCATTTGATGCAAAGGTCTCAGATAACTTTTATCTGACATTTACGGAGTAGTAA
TGGAAGCTCACGAGGAGCGTTTGAGGTAATTGCCAATCGTGAGCATAAAAATATGCTATTACAGAAAG
TGAATCTAATTTGGCGTAAGAGAGAACATTTTTAGCTTGTAACACGAGCAATTTAACTTACTCCTTATAC
ATTCAGAAAGAGATACAGTTCTACATAGGAGTAATCGCAGGATTTCCAACAACTAACAACCAGGAGCA
CAGGTTATTCAGGAGTACAACATGATAATATCATCAAAATAAACTAACATAAGGACTTCATGAATGAAC
AAGAGACAAACTACATGATATCAACAAAAGTGGTTACTACCAGGAAGACATGCGTCGAAAGAGGTCG
GAGCTCAACGTGCCAGCGGCAGCAGAACCAAAGTACTAGGGTTCCAGGAAACTCAAGGCGGTCTGT
GCTGACCCGATTTGCGCCTTAGGGCCGGGGAGCAAAGACCAATGAGTTTATC
>TCONS_00061804 geneID=XLOC_012428 scaffold_21:2288602-2291079(+) Exonic overlap (-)
AACAAGTGCCAATCGCATTATCGCTATATTACAACCCTTCAGTCCATCAAACATTATCACATCACTTTTT
TCCCATATTTCTATTTGCTTATAAACTTATACCTAATAAAAAATTAAGATAAACATTCTAATTTGTCATATA
AAATTATATTCTAATGGATGTTTGTATTTAGCTACGTAATACTAAAGACACGCGCAGTCACAAACAAACC
TCACAAATACTAAGAGGCAATGCTAGTCAATCATAAATAAAACGCCATTAAATACTAAAGGAACATAAC
TATCAACTTACTTATAATAAATCAGGGGATAAAGTGCCAAGTAGTGTGGCTACATACAGTTATAAATCGA
AATAAAAATACTGCGAAAACTAAAAAAGTA
>TCONS_00062300 geneID=XLOC_012474 scaffold_21:2411819-2413630(-) Intergenic
GTAAATATATCGTTTCAGATCACTTCAAATGATAGATTAGATAGATGTAGATTTGTATTTTGTAGATAGTT
GTCCACTTCAACGCTCAGTTTACTATAAGACACAGTGTGTAATTCATAGATATTATTCCTGGAAAATATG
TAATAAAAATCAAGTAAACTGTTTTTCAGTTCAATAAATTCGGAGACAAGAACATACCTTCTCAATCTT
TTCATATCTCATACAAGTACAGTGCCACAAACTTATCTGTTCCGGTGAGAGCGAAATAATTTGATCCATA
TCTCATTACTTACTAATGAATTGTATATTGTAAAAGATTAGATGGGTTTATTAACATCGCAAGAGCGAATT
AACAATACGAGCAAACTTAAAATCTTATAGAATTAAGAAATATTACACATTTTTGTGAGCTGTCACCGG
AACAGATAAGTTTGTGGCACTATAACAGAACTCAAAGTAAATAACCATAACCAATCGAATACGATTTAC
CGTCAACAATATAAAATTTTGGTCAGTCTGAAAACACGCGTCTTTATCCTAAGTACCTACGTGCTTGAC
AATTATTATACGGAGTTCCTTCAATGGCTGCGAACATCATCAGTCTTCACAACTGAAACGAAACATGCA
AACGTATCGGCATTTATTGGTTTTCAGTATGTGTGTAATTAAATGCTCGACTGATAGTGCGGCTCACGAC
ATTCACAGTGCAATTAAAATAGGCAAGAGAAACC
>TCONS_00062400 geneID=XLOC_012616 scaffold_210:111588-114881(-) Exonic overlap (-)
AAAAGCAGTGTTTATTCGATACGTTAGTATATAAAGAAATTACTTACCTAATTATCAGAAACAGTACAGC
GAACAACGTACAACATTCAGCTCCGTCGAAGTCATGAAAGTTGTTATATTTTCATTGAGTTTTGGCTCT
GTCGTGTATCTGTGTTTTGCAGATCACCGCTGCCGTCTAAACGAAGTGTACAGATGTAAGGACATTTGC
CCGCCACTCAAAGAGACCTGCTTGGGCGTTGGGAAAGCTTTGGAATGTTCAAACAGAACGTGCCTGT
ACAAATGCTACTGTCGGGACGGCTACTTGAGAGAAAACATCGACACCGCATGCATGCCCATGTTCAAA
TGCCCGCAGTTCAAAAATACCCTAGACTTGATATAAAATAGCCATAGCCCGAGTTTTAAGTACATACTG
GCTAAGTTTAATGTAGAAATGGAACCATATTTTTTGGGCTAGCAGCCCTAAATTATCAGCCGTTGCAGC
ATAATGTCTAC
>TCONS_00062473 geneID=XLOC_012614 scaffold_210:531028-547763(+) Exonic overlap (+)
CGACACTAGACCAGATACATCGACACTAGGGCAGAACAACACTAGGCAAGATAGGGCGACACTAGAC
CAGATACATCGACACAACAGCGTTTAAAGTGACACTAGACCATACCTATGCCACGCTATGGCAGATGG

AGCAAAACTAGACCATATAGAGTGATACTAGACCAGATAGGGCGATACTAGAAAAGATAGAGCTACAC
TAGACCAGATAGAGCGATACTAGGGCAGGAAAAACAAAACTAG
>TCONS_00062483 geneID=XLOC_012614 scaffold_210:531030-547395(+) Exonic overlap (+)
ACACTAGACCAGATACATCGACACTAGGGCAGAACAACACTAGGCAAGATAGGGCGACACTAGACCA
GATACAGCGACACTAAGGCAGAACAACACTAGGCAAGATAGGGCGACACTAGACCAGATACAGCGAC
ACTAGGGCAGAACAACGCTAGACAAGATAATGCGACACTAGGGCAGATAGAGCGACACTAAGGCAGA
ACAACAATATAGGCAAGATAGGGCGACACTAGGGCAGAACAACACTAGGCAAGATAGGGCGACACTA
GACCAG
>TCONS_00062474 geneID=XLOC_012614 scaffold_210:531035-547392(+) Exonic overlap (+)
AGACCAGATACAGCGACACTAAGGCAGAACAACACTAGGCAAGATAGGGCGACACTAGACCAGATAC
AGCGACACTAGGGCAGAACAACGCTAGACAAGATAATGCGACACTAGGGCAGATAGAGCGACACTAA
GGCAGAACAACAATATAGGCAAGATAGGGCGACACTAGGGCAGAACAACACTAGGCAAGATAGGGCG
ACACTAGAC
>TCONS_00062435 geneID=XLOC_012614 scaffold_210:547272-547679(+) Intergenic
GCTAGACAAGATAATGCGACACTAGGGCAGATAGAGCGACACTAAGGCAGAACAACAATATAGGCAA
GATAGGGCGACACTAGACCAGATACAGCAACACTAGGGCAGATACATCGACACTAAGGCAGAACAAC
ACTAGACAAGATAGGGTGACACTAGACCAGATACATCGACACAACAGCGTTTAAAGTGACACTAGAC
CATACCTATGCCACGCTATGGCAGATGGAGCAAAACTAGACCATATAGAGCTATACTAGACCAGATAGA
GCGATACTAGACCAGATAGAGCTACA
>TCONS_00062436 geneID=XLOC_012614 scaffold_210:547369-547679(+) Intergenic
AGGCAAGATAGGGCGACACTAGACCAGATACAGCAACACTAGGGCAGAACAACACTAGACAAGATAG
GGTGACACTAGACCAGATACATCGACACAACAGCGTTTAAAGTGACACTAGACCATACCTATGCCACG
CTATGGCAGATGGAGCAAAACTAGACCATATAGAGCTATACTAGACCAGATAGAGCGATACTAGACCA
GATAGAGCTACA
>TCONS_00062829 geneID=XLOC_012669 scaffold_211:222365-223071(-) Exonic overlap (-)
GGGCCGGCGTGGTGGAATAAAGCACCCTTCCACTATTGGAAGGAGACGAGACAAGACGAGATGGGTG
ATGATGAATAGCTAATATAGAGCTACAAAACACCAACCATCATAGTTGACCATAAGCAGTTGTCCAGTG
GTATTATTGTTCGGGTCAGCGATGGCGACTGCAGACCCGTTGTAATGACTCGCAGTGCGGTTCTGGATC
CAGCTGATGGACACGCTGGTGGTGTTGCTGCTGCCGTGCTGGAACTGGACTGTGGCACATTCCCCTTG
CAGCTGCAGGTCTGATGCGGCCGCGGATAAGAACCAGGTGCCGTC
>TCONS_00062836 geneID=XLOC_012677 scaffold_211:582937-583677(-) Splice junction overlap
CTCTTCTTTAAAAGTTTCAAATCATATAATTTTGTAACTATTTTTGTTACATCTTTTACATTATCTTCAACC
TTAGGCTGACTGTTATAAAGCTAAGAGTCTGTTGTGAAGATGGGCCCTATCAAGCCACCTCCGGAGCA
GAAGCCTCCTCCTGACCCTGAGGCTGAGCTCAGCAATGCCGTTAGGATGTTCAAGAAGCTAGCGCCCC
GTTGTAGCGTGGCTCAGCGCGCGAGCGGCGCG
>TCONS_00062732 geneID=XLOC_012681 scaffold_211:697102-698127(-) Splice junction overlap
NNGGTGACGTCACTCCTCTCTCTGTGCGTCCCTTCCACTGCCCCCGTGCCCTGGGACCAGGTGGGCAA
GGCGCGCTGGGGCGACGGGCAACTTGATACAAACGTGGAAAAGAAATCCGACTTCAAGCCGGAGCC
GCTTCTGCTATCGGCCGTGGCGCGCGCTCGTCGCTCGACTAGCTACGTCATGATCGCCAGCAGTGTCGT
CGCTAGGGGCATACCTGTCAGGGTGGTGGAGGCTACTGGTACTGGAGGGTCATAGTGGGGGATGATAG
TGGTATTGTAAGGGTGGATTCCCACTAGAGGCCTAATGACAGTGAATCGACCCTAACGCGTCGCCCTA
GCGTTTGTTAGGCCCCGGGTCTCCTATTTACGCCGTAGGTCGCGTCCAGCGTCACGCCGTAGGCCGCGT
CACGATGCTCACTACATCTACGCGCCAACGTCGGCGTGTGAGGGAGACCGCATCAGTACATTTCCATA
GTGAGCATCGTGACGCGGCCTACGGGAGACCCGGGCGACCCTATTTTAGATGTTTACATGCAAATTATG

GATAGTAATAATTACAACTTACTATTGGCTGATGATGATGATGATGATGAATAATTACAAATAATGAC
>TCONS_00062820 geneID=XLOC_012660 scaffold_211:996005-998146(+) Splice junction overlap
GGAGTGATAATGATATTACAATGATAACGATAAAGTGGTGGTCAAATAGAATCTAGACATTGCTTCTTAC
TACATCACCAGCTGTGACATATATTTTCTGTTTTCTGTTAATTCTTTGGATAGCAAATTTTGCTATGGCAT
CGCCGTTTGATACCCTATATATTGTATATACCTGTTATGGACGAACGTTATCTACCTATTCTGCTAACATTA
TCATTTAAATGAATATACTTCTAAAGTATCTGTATCACATTACATTTTTATCAGTATTAGAACTACTGTGTT
TGAAGTAAAAGTGTCTTACCCACCAAGTGAAATAGTTTATTTATAATCTTAAACCGTTAGTACTTACCAC
AAAAAAATTTAATAGTTTATTTATAATTTTAAACCAGCAATAAGATGGATATGTCAAAGAACATTTTAGA
AGCACCCGATGTAAAGCCCTCATACGCATCCACACTATCCACTAAGAAATCGTTTGTGCAATCAATGAA
CGCGACAGTGTTCCAGCGAGCATCGAACCTGCTACCTGAAGAGGACCGCCCCCGATCACCAACCCCT
CCACCTAAGGAGACTAAGGATGAAGAAAATAGCGACATGTCTCGTCAGTAGTTTAAAAATATAATAATT
ATATTTTTGGTTCAGTGACTTTTCATACTAGCAATGAGTGGGGGCAGTTTTCTTTGACCCGGACTGTAAT
TAAATTCATCAGCTCTGTTACACATTCAAATGGATTGTCTCAAGAATATTTGTCGGGGAACGAAGAAAA
AACCTCAAGATCCACGACTCCCAAACGATGGGTTAGTGATCCTAGGGTCTGCTACTGCTTGGGGTCAT
GAAGCAATAGTCTACGAGAAAGCATCGAACC
>TCONS_00062821 geneID=XLOC_012660 scaffold_211:996005-998146(+) Splice junction overlap
GGAGTGATAATGATATTACAATGATAACGATAAAGTGGTGGTCAAATAGAATCTAGACATTGCTTCTTAC
TACATCACCAGCTGTGACATATATTTTCTGTTTTCTGTTAATTCTTTGGATAGCAAATTTTGCTATGGCAT
CGCCGTTTGATACCCTATATATTGTATATACCTGTTATGGACGAACGTTATCTACCTATTCTGCTAACATTA
TCATTTAAATGAATATACTTCTAAAGTATCTGTATCACATTACATTTTTATCAGTATTAGAACTACTGTGTT
TGAAGTAAAAGTGTCTTACCCACCAAGTGAAATAGTTTATTTATAATCTTAAACCGTTAGTACTTACCAC
AAAAAAATTTAATAGTTTATTTATAATTTTAAACCAGCAATAAGATGGATATGTCAAAGAACATTTTAGA
AGCACCCGATGTAAAGCCCTCATACGCATCCACACTATCCACTAAGAAATCGTTTGTGCAATCAATGAA
CGCGACAGTGTTCCAGCGAGCATCGAACCTGCTACCTGAAGAGGACCGCCCCCGATCACCAACCCCT
CCACCTAAGGAGACTAAGGATGAAGAAAATAGCGACATGTCTCGTTACACATTCAAATGGATTGTCTC
AAGAATATTTGTCGGGGAACGAAGAAAAAACCTCAAGATCCACGACTCCCAAACGATGGGTTAGTGA
TCCTAGGGTCTGCTACTGCTTGGGGTCATGAAGCAATAGTCTACGAGAAAGCATCGAACC
>TCONS_00062772 geneID=XLOC_012660 scaffold_211:997438-998658(+) Splice junction overlap
CATACTTAGTGCTTTCATTGGAGTGCAGGTGCAGTTTTATGCCCATTTCCTTATATGGAGGACGTAAGAT
TATTTAGATTAACGTTTAATTAATTTAAACTACAGGTTACACATTCAAATGGATTGTCTCAAGAATATTTG
TCGGGGAACGAAGAAAAAACCTCAAGATCCACGACTCCCAAACGATGGGTTAGTGATCCTAGGGTCT
GCTACTGCTTGGGGTCATGAAGCAATAGTCTACGAGAAAGCATCGAACCTGCTACCCGAAGAGGAGG
AACCCAAACCTCCGACCCCTCCACCTAAGGAGCCAAATGTTGAAGAAAAAGGCAACGCGTCTAGTTC
TAAATAAATATCATTTTTTTATGTGTTAAAAAAAAGTTTTGTTTAAAAAAAGTTAATAATAATTAAAAAA
AAACTTTGTACCGACAGATGTTTGACAGGGGCGACTAAATTTTAACTTTTTTTTTAAGTCCACCTAAGT
ATATAATATAAGTAAATAACAGTATTTCCCAAACTAAAATTAAATACATAAAGTACCAGGACTGCATAGA
AACGTAGTAGGTATTAGATAGAATCACCATTGTCAATGAAACTACTAATCGAATGACAGTGACAATTATT
CGTCTCTTTCGCTCTTATGAGAAACGTTGTAGCCCTCTCGCTCCTTCGCAATCAGTAGCGGTTTTGTTAT
CAGTAAAAAGTAGCGGTCAAGGGCGAACCGTTATTGCGATGTTTGTGGTTCGATCGTGTTTTTTTTCTT
TAGTTTGTG
>TCONS_00062901 geneID=XLOC_012660 scaffold_211:998971-1012584(+) Unclassified
AAAAGACGCCACTGACCTCAATTTCGTCGTCAACAAAACCGACAAAGTTTCATTGACAAGCAATACTC
AGAACTTGGGTTTTAACGCTAATGAGTTAAAGTCGGAAACTACTTGATTGGTTTCGCTGAACAACTTT
GTCACAGTAAGCTGTGGTCGCTGTCAATGAATCTTTGCAAACCAAAATGGCCACAGTTGCCATCTGTG

CTTGAAGCGGGAAACAGGAAAACATAGTTCGAATTTTAAAATTTAAATTTTGTGTGTTGTTTGATAATG
ATTTATTTTTAATTTTGGCGTTATTTGTGTTTTGTGCCAGTATTTTTGGATTTCTTGCGGATTTATTTGAAG
TCCTCCTTCCACAAAAAACCGGCCATGCCGCCTACCAAGGACCAGGGTAATAAGTACAACTACACGAG
GAGTTTTTCTAATAGTAAGTAACTACAAATATTTTAAAGATTTTGATACACACATAATTATTTCTGCCTAA
AGTAATGCAGAAATCATGCACCCCTTTACCTATAAATAATAAATAAATACCTAGTTGGTTGCGTAAACTC
TAATACCTCAGATATTGCACTTATAATTATAATGTATTATACAACGTAGACTGAATAGTTGCAATATCAGT
TTTAGTCCCGAGATTTTTGAGAGATCATTACAGTCTGGTATATCACTGCCCACAGTTGCTAAAACCGCA
TGAATGTGTGTAATACCGGTGTTGAATGCCTAAATCCATTTAAGATTTAGACTCGAAGCGTCCGTAGTC
GAGCGGGCCTCAGTGATCGTAACTGATCGCTGAGGTTAAGCAACAACTGACACGGTCAGCCATTGGAT
GGGTGACCAATTTCAAGTGGTGCTTTTCTGGACGCTTCCGTGCTTCGGACGGCACGTTAAGCCGTGGG
TCCCGGTTGCTGCTTCGGCAGCAGTCGTTAAGCCTAGTCAGAGGCCTTCGGGCGGCTTGAAAACCTCT
GACAGTCGGATTGCCCACTTACCCGACAACTCTCTCAGCACAAGCTTGCTTGTGTTGGGGTCCACCAA
CCCGCACTTGGCCAGCGTGGTGGACTAGGACCTTCATTGGAAGGAGACCCGTGCCCCAGCAGTGGGG
ACGTAATGGGTCGTGATGAAATTTTATCAGCTATGTTAAAACTACCACTCTGCAATTATCTACCTACATG
GTTTAACTGAATATTTCTCTCTACTTCCAGACGAGATGTCAATGTCGGCACGGGTCTACCATCCAGCCA
CGAACCTGATGCCGCCATCGCCCCCCGTTTCCCCCACCGGCATAGCTCCCAGCTTCAACACCAAATACT
CACCCACCAGCACCTTCGGAAGACAAAACAGCCAATCAAATTCGAAATACTCCTCATGACAGAGAAAT
AAAACATGGCGGAGCAGAATCGAATAAAAACAAAAGACGATCGCGCGGGAAAGTTGAAAATGGAAT
CTGTTCGGTCCAGCTGCGTTCAAGATTCTGAATGGTGGTTTTAACGTATCGTTCAAAT
>TCONS_00063129 geneID=XLOC_012738 scaffold_212:166613-217515(-) Exonic overlap (+)
GACGTTGCTAAAGTATTTAGACCGTAAAATATCTGTGTATTTAAAAGTAAATTGAGAAGTCACCGTAAA
GTTTTTAGGTACTGAAGTTTGCAATGTGAGTGAGCTAGTGTTTAAAGTTTTTGTTGTTACGACGTTTGG
AAGACACTGACAAAAGGCACAGCTTTCGTTCGTAAACCGGAACTGTCACCCAGAGGAATGAACTCCA
TTGTGTCCACTCGCGGACAATGTGCTTTATAAAACGGAAAAGTTACAAATATCAGTTCAAAGGGCCAT
GATACGAATCTCTTGCTCTAACTTTGAAACATTGTGATTCTAACTGTGAAAACTAGCGCATAGTTCAGT
GAAAACCATGACGTTTTGAAAACCAGTGATCGTGTGATGTAGTTATTAGCTTCTTCAAAGTTTAAATTG
CTAGGATAATTATATTTCCCAAAAAACAAACGTACAAATACAATCACTAACGTTCCAAAGATATTATACC
ATAAAAACTTTCTACTAGTGCGAACGTCAGTGAATCTTGACAAATTAGCTGCATGTATCTCTCGGTGTT
GCACCTAGCAGTGATAGGTCAGCTGTGCACACACCGTGGTAGTGTCGCGCCGTGATTGGT
>TCONS_00063134 geneID=XLOC_012742 scaffold_212:330327-331001(-) Exonic overlap (+)
CCACCAGCCTGCTGGCTCTGGCCACTGCCACTGAACCGGATTACTCTGATGTGCTATCCAGTGCTGGT
GATTTCAACCACAGAGATGAGTCGCTAATAATACTGTGAGGTCTGTGAATCAAACATCACATATACATC
GTACATAGTGCGGATACGACGGAGATTCGAATCATGTCATGCAAATGCATGAACACCGACTGTGTATCG
GCCGAGAGTGAGAAAAAGAACAAAATAAGTGGAGCCATTGAATAGACCCGATAAGAATAAAAAGGAA
TCGGCCGTTTGGTGATGAAATGAAAACAAAGGAAGTGTTCAGAGAGAGAGATTGTAATAAGCGTCCA
AATGAAATAGGCAGCTGAAAATACGAACAAAGTATGTGTGCATTTTGCACTTACATGTACCTATTACGT
TAGATGCATATTAGTTTTTAATTAGATATACACCTACCTGTACTGTATATACTTAATAATGAAGAAATGTG
CTGCCCCCTATCTTATGTTGAC
>TCONS_00063159 geneID=XLOC_012746 scaffold_212:402534-402893(-) Intergenic
GCGCTCACTGATTATAAATTATTTGTTTTTGTGTATTTTACGCTAAATTTGACGTGATTTTGTGCGTGATG
TCCGATGACGTTGACGGAGATATCCCACAGCTGTTGCAGCAGTGGGGTTTGTGGGTTTACCTCAGTATA
TTGATACGTTTCAAACGATGAAAATTACGAGGAAATGGAGTCAATATAGGTAATAATGGTTTCTGGGAA
ACATGCAGG
>TCONS_00063789 geneID=XLOC_012840 scaffold_214:72434-75515(-) Intergenic

ACTCTGCAGCTGGGAGAGTTCACGAACGACATTCTCCTGGACGACGTGCAGTCTCTGATCAACTCGAC
GCCGAGCAAGCCGGACAACGTGCTGACCTGGCTGTAGGGCCGGCCCAGCACCCCCATGGCCCACTGT
GCCTGGTACAGCCGGCCCCGCCGTGGCTCACAGACTATTGTAGGTTACGTTAAGTTAGAAGAGTACTT
ACCGCGCCAGAGCGAGACGACGCGCCCGGAAACGGTAATGCTGATGTAAATTTCGCTACAAATGACA
ATAAGCTTAGTGTGTGCGTGAAACGGACTCAAGTGAATGAATATCTCGTGTTTAGTTTAGTGATACTGT
TCATGTTACAACGGTGGCCTTTATAAGTGATTTATTATTCAGAATTGGATTTTAACGTCTCTGCATTTATT
TAGACGAGATAGAAAAACTCAATTCTGTGTAAGTGATATGAAACCCCTTATCGGAGTATGTATGAAGAT
TAACTGTAAGTGTAAGGTGCCACTTTTATGGACTCACTTGCATATAATTATATGTGTTTTGATATATACTA
GTTAATTCAAGAAGAAAGCGGTGGAGCTGTCTCATATCATGTAAATCATTGATTGTGCCAAAATCAAAT
AACGGCTCTATTGTTTTCTTCAGCAAATTTATTTCAAAACGGGCATTGTAGTATGAAACTTTAAGCCTTG
TAAGCCAATATCTACATACGTTTGTGAATAACTAGATAATAACAAACTATTAGTCTATTTCTACAACACG
TTCGTGTATCTGTATGTAGTAACTGTATGTTTCGAAGTGTTTGAGCCATGACTATCATTATTGGCTAGTCA
TATTAGGAATATTGGTATAGGGACTGCTCTGGAGGAGGCGGCAGGGGCAGTCGCCGCAACGCTTCTTT
CGTCACAGAAAGTTGCGGGTTGCGGGACAATTTATAAACAAAAAGATGACAGGTTCTCTATAGTCTTC
ACGTAAGACTATAAGGAATCCTTAACTTCTAAAAACCTAGATAAATCTAATTGGCTAATTTCGAATGAAT
TTCATGATTGCCTTAATGTGCCTTAAATAACTTTATTTATTGTTTTTTATAATCTAGTAAATGTTGAAATTT
TCTACAAACGTTTTATAAGTTTCTACAATTGTTTCTAAAACGGAAATCGTTGTGCGTATTGGATAATCGA
GATAATTAAGAATACTTACCTTTTTGTAACGTGTTTGTCTACTTACACCGTCCTTGTGTATTTTCAATGAG
AGATAATTAGACGTTAAGTTTTTATATATTACTTATTCATAAAATAAATATGTTGGCAACTACTATTTACTT
AGTTATCATTATGTTTTTTTTAAATATATGAAATGATGCTTGTATATGGGTGCAGCTAGTTAATGGCATAA
CAAGTATAGAGATAAAGTAAGACTGTGTTGGGATCGAGTGTTTGTAGCGAGTGTGAGATGTCAGTGAG
ATGAGATGAGATGAGATGTTACAGAGAGTGCAATATCGCAACTGAGCCGTGCCTCACAAGTGATCACC
ACTTCCAGTTTAGGTGCCAATTAAACAGCTAAATATATATAAAATTATTTCATAATTGTTCTGAACCTCTA
TCCGGACTGGACTATCGATAGCGCACTCGTATTAAAACTTTGCCAAACTAATAACTACTCTCTCAGAGA
TGGCAGAGATGCGATAGTGAGGTCCGGATAGTAACCTATCCAGTGGATGGAGTCGCATTGGCACAAGT
GTTGATTGTTATCTGTAGGCTATCTGCTTCTTAAAGAATGTGCTTTATGTAGTGAAATGGTGTGAGAGGG
TGCAATAATATTTGTATGCTGTCAAATGAGAGTAAAATCGATTTAATAATGTATGTTCTCTATCCTCTCCG
CACCTATGCACTACTTATCGTGCATACTCAGAAGGTCAAGCTATAGCTAACGAAATGTCATACAAAATG
GTATTTGTATTATGTAGCCCACCTAGTCTTCATTTGTTTGTGTGAACGTCCCCATTTGACAAATTAGATTA
TTGGAGCAATGCGATTTTTTAAATTAAGCTACATTGAAAACTTTGCCATATTAGTGAAAATGCTAAGTAA
ATCTTCAGTTTTACTGTTCTGTAATTATGTAACTAGACTTAAAACGGTGCTGGGTTTATGAGACATGTAA
CGTTACTATATTCTATGCACTTTGTGTTGTACTTTACACTGCATCAGTACTACACATATTGTTCGGTTTATT
ATTCTTATTGATTGATGGAAAGCTTATGAGTGCCTGTTAACTTTAAACCTAAAAGCCAGATGGTAAATTT
TCCTACTTATCATTTAAGGTATATCGGCAACTTCAATAGTTTATTCAAGATCTTATTTTTCCTTGTTCTTTT
ACTATAATTAAAAGCCGAAAGATTTTATAAAACATTCCATGTCGTAAAAAAAACTGCTGTTAGCCGCAT
ACTTTGTACTGAACTGCGCGACGGCTAAGACAGGTCTAATACTTTATCTTGTTATGTGAATCGTGACTA
AAAAGGTGTATTTTCAGAATAATTTTCGGAACAATATAAATTTCAGTAAATTGACATTTAATTATTTCTAT
TTAGCTTTTACGAACACATACATATCAACACATTCTGATATGGAAGTTTTCTCTGTAGTATATAAAGTAAC
ATTATTTGTATCAAATTATTAATATAAGCAATGAAATTTAATGTCAGAGTATTTTAGGTACTGTAATTTCAT
TGTAAATAATGCGATATTCTTTATCATTTAAATAAATCTTTGTTTTAATATCATCCTTGTGTTTCACTCACT
TCGCCATTTTAGAGCTCGCTTTTTATTTTCGTAATCTGTGAATATATTATTTAATACATTAATAATAGTAAT
TGGACTTCTTCCATATGACGATATACGACAAAACATGAATAGAAAATAATAGATAAGTGGATTTTAAATT
TTCCAATTTGACTGGGATTATACCAATCTTG
>TCONS_00063427 geneID=XLOC_012844 scaffold_214:185681-193148(-) Exonic overlap (+)

GAGACCGTGGAGCGACGCGGCCGCCCCGCGCCCCGCGACTGCATTCCGAATTCAAAATTGCTCACAC
AAACATTTGAAAATCGAACACTTCCCCGGACGTCTGTGGATTTAAGAAAAATATTGTGTTTTGTGCCTG
TACTTTTAATGAAATTGTTGAATGAACACAGTTTTGCGAACATTGCGGTGCTTTGTGTGCACGATTGGG
GTCAAATAAAAAGCTCACCTCTATGGTCCAGCGCGTGAGGCGCCGGCACCATGCACTCGTTCGACACC
ATGTCCTACGGGCACAAGAAGTACCGGCCGCCGCTGTCGCCCGCCAGTTTCTATCAGCAGGACCTGTA
CTCCCGTCCGAACACGCTGCGACCGCAGGTCAGTGTCATAGACTAGCATAGCAGAAGACCCGGTGCG
CTTTTAAAAGTTCTCGGTCACGCTCGCTCTTGAGGATGTACGATGTGCAGCGCGATG
>TCONS_00063777 geneID=XLOC_012833 scaffold_214:331341-332273(+) Intergenic
ATTTAACTGACAGCGCTAATTTTTGCTATTCTGAAGGCAACGATTTTAACGGCCAACTAAATTTAATAGG
CTGATTTGACGTCTTTAAAATGAGTGTTCCCATACTTAGGACAAATACCAAATGATGGCAGCGAAATAT
AGATATCATGGAAATATAGTCGTTATTTTATTTTTTAAATCTTAATTTACTTTAAATACATAAAAACATGTT
AAAAATATATTTAAAAATATACTAAATGCATTTACAGCCTATTATTATCGTGAAAAGCATAACTATTAGTG
TTTCCAATGTGACTCATTATAGGTAATTTAGAAATAAATTACTGGCAACCCCAAAAAAATACATTGACTT
TTTCATTGTTTTCTTTCTCCGATTGCCGGGAAAAAATTCGATGGTGATAAATACGATACGGAAGATCTTA
AATTATCCTTGATAGCTACTTGCTTACAAAGGTTTTCATTACACAATTTTTCCTATAAGATGCAAACATAA
CCTTAAAACATCAGCCAGCAAGTTATATGAAACACAACACCTGCATCAACAAGTATGAAAGGATCCAG
TCGCATTCCCGTTCAGACTATCTAAGTTACCTAAACAGAAGGTTAGTATTTATACTTACTATGTAATAGA
GTGAAAAAAAAAGAAAAACCCATGTAAGTACTTACTTACAAAATTTACAAAATTAAATGATTGAGTGT
TAAAGATACTTAAGTACTTCAATCTCTCTTAGCGGTGAAACGATTCATCATCAG
>TCONS_00063578 geneID=XLOC_012854 scaffold_214:349453-367364(-) Unclassified
CAACTTCACTTTATCTTCAGTCAGTGACATTTTGTTGTGCGAGCTGCTGTGTTGACATTTTAATCAGAC
GGAATTTTGTTGACAAATAGTCTTGTGTTTGATAAATATCTAGTGGTTTCAAAATTTAATGTTCTAATTTG
GTTGCTGTGGACATAAACTGCAATATTTTAGTAACGTTGTGAACAAAACTGTGTGTGCAAGCGTACAAT
TACACCTTTCCTTCCTTTCACAAAGGATCACTAGACGGGGCCCTCATTGTTGTCTCTCTGCGCATTTCA
CAATAACTGCTTCAGCAGTGCATAACGTGGAGAGGCTCGGTGCGTTGTGTGATGGGCGCGCGGTGAG
CGCTAGCAGCCTCCGGGATGACGGAGGGCGACAAGCAGGGCGCGGGCGGCACCAACGCCGCCCCGC
AACACAGGAAAGGAC
>TCONS_00063778 geneID=XLOC_012834 scaffold_214:375703-378226(+) Splice junction overlap
CGCCCTGGCCGCCAAGAAGAGCCACCCGCCCAAGGCCAAGATCGTCTCGCTGCCCTACAACAGCTAC
GGCCACAGCAGTCACGGACCATCATGGAACGTAGAAGTACATAGCTGGCACTAAGTTTGGTGGCTCCA
GTTTTCTGACGTCGCGTCTCGCGCACTGCAGTATAACTTCGAGCATCGTTCAATAGATGTCTTTTAAGG
AATTGTTATGTTTATGTACTAACGTCTGTAGTCACCGGTGTGTTTTAGTAGTCACAACTTTGTAAATATT
GCTGTTAGAAGCCTACAGGCGGCAGATTCCGTGACTCCCTGACTATTTCTGGAAGGTGTGATACTTGCT
AAGCCGTCTAAGGTAGTATAAAGTTCCCCGAACAAGTTGTATTTTCGTGTCGATGTTGCGTGCGACACG
CTCGCCGACCCAATCTGCGAAATGATACAACTTGTAATGTTATTACAGTGGATGCGCTTGATATAATCTC
TAGTTATATGACCGGGACCGGCCCAGTTTGTTCTACACCATCTTGATAACTGCTTTTTATTTAGTCAATAA
TTATTGTGAGACATAGAGGTTATTATTGTAGCAAGTATGACATGTTTTGGAAAGTATGTATGTATGTAGCT
CCTCGTCTTTTAGTGATGTACTTGCAAATATGCTCATATATGTGATCTATTATTCACGAATCTGTCGATTGT
AAGTGACACCTAGGTTACAAAATATACTAAGACTTAGATCTACATGACGTCACGTAGCATTTAAGTTTA
GTTCTGATTGTTATAATATTTATAACAAGTCGTGCC
>TCONS_00063392 geneID=XLOC_012835 scaffold_214:384560-388861(+) Splice junction overlap
CAGGCTTCTACCGGACATTGTGAGACGCGAATTCGATTGTGACAGTGCTATTTAGGCAGAAAATATAAG
AACCATGAGGGTATCTCCAGTAGTGTTGGGGCTGGTGGCGGCGTGCGCGCTGGTGTACGCGGGCGAG
GCGCCGGCGGCTCCGGGCCCGGTCGTGGCGCTGGTGCAGGCGCAGCAGCCCCAGGTGGACGGTCAG

CTCGCCCAGGCGCCGAGGCAGAAGAGAGGACTGCTGCTTGGTCTGGGAGCGGCGGGCGCGCTGGGC
GCCGGGGCGCTCGGCGTGGGCGCGCTGGGCGTGCTCGGGGCGAAGGCCATCGGCGCCGGCCTCATCG
GCGGGGCGCTGGCCTCCAAGTTCCACGGC
>TCONS_00063696 geneID=XLOC_012836 scaffold_214:491886-522401(+) Exonic overlap (+)
GGGACCCACGCCGCCTCTCGCGACTACATACTTACGTAAGTCGCCGATCACGCGCTGACCAGTTCCTAT
AAACTCTGCTACAAACTTGATTTGCCAACATTTTACCTTGTGTTAAATGCAATCAAAACACACCATAAG
AGCAGAAACCAAGTGTAGTTGATGACTTTCGTAAGTCTTCAGTGGCAATCATAAGTGGGTGAATTTTAT
TAGACGTGCTTTAAGACTTTTTGTGTTTCTAGAGGACTCTTTCAGGATTTGAGTTGTGGGATTGTTGAG
ACGCTGACGTTGTTAGCAGAATCTCTCATCGCTGTACTTCCGGTGTGGGTGTAAATCACACACAATCAC
ACACACACACCAGTGCAACAGCCAGTGGCATAGGATGGGAAGGGCGGGCGGGGTCGCTGATTGGTCA
GGTTTGCGGATGCGAGTACCAGGAGCGCAGTGAGTCGTGGGAGGTGTGAGGGGATGGGCGGGGCGG
GGTGCGGGCGCGCGGGGTGGCGGTGACTCGCGCGCACGATGCCGCCGGGCCTGGTGCTGGCTGTCGC
CGCCTTCTGCGCAGTCGTCAGCGCGCAGATCGCCTGGACACCTATCTTCCTCGATGGCAGCA
>TCONS_00063896 geneID=XLOC_012892 scaffold_215:233407-235393(+) Splice junction overlap
CAGCGCCAACTACACTTGCGAGCTATGAGAAAGTTCCTCCTGCCATTACCGTTCCATATGTCACTGGAA
CTTTTTCATTGCTCGTGAGTATACTATCAAAGATTAGCTATTCAACATAACGGAATATATATTTTTATAGCA
TTAATAAAATGATTATTTCAGAGACTACAACCTAATGGGTTCGCGCAACCGCAACAATAGAAGACCGAT
AACTCTACCTGAACTGTCGGCCACCGGCCATCAGATCCACAGATCTACCGTGTATGCCAATCCTCATAC
ACGGCCTAGAGCTAATGGATACGAGTCTAAGAAGAAAGTCATGAATAATGAAAATGTCAACACAGGG
AGGACCCCCGAAGAACAAGCAGCAAACACCGGGCCAGCGGAGGGGCGGCCACGGCCGAAGCGGCA
ACACCGCAAGCAGCACGCCGCCACCGACACATAACTACTCCTCGCGCCCGCCACGCCACTCAGACGC
AACAGGTTTCGGAAAATACTGGACAGAATCCTACCCGGTCGACAACGGAGGAGTGTATACGATTCAGA
AGATTTTGAAGACCAAAATTTACTAGACCTAACACATAACTACTCCTCGCGCCCGCCACGCCACTCAG
ACGCAACAGGTTTCGGAAAATACTGGACAGAATACTACCCGGTCGACAACGGAGGAGTGTATACGATT
CAGAAGATTTTGAAGACCAAAATTTACTAGACTAACTGCTTTACAGAAAGGCGTAGAAGCTGTCGTGT
CCATAAACGTGCTTTTAACGGAACTTGAGTGGTGTCTGAGCCTAAATAAATTTACACGACTGTAGTACT
CTCAGCGAAAGCGTAAGCTCGTATTCTATTATCTAAAAGTAGCTAGCATAATTATTGAAGGACGAGATTT
TTTTCACATAATATAAATATGGTATGGTACAATTCGGATTTTTACAAATCGTCTAATAACGCGTATAGTTT
AAAAGCGTATTTTGGTGTCATTTTTGTCTGTAATGCTATGGTTTTCTTGATAAGGGCAATTTAAAAAATA
AAATAAATACTCTTATCGAGAAAACCTGCCTTACAATCAAGTCCATGTAATTATCCAGTGTTTGATCTCT
GTATTTCTATGTTTTAATTATTGTAATACATACCTAGTAATATAGCTATCCATATTCGAATAATCACCTTAGC
TCTAGTCTTAGGTGAATTTTAATATTCAAATTCTTGCCGAATATTTCATGTATATGTGACGCTTTCTGTAA
GTTATGAAAACCGGTATTATACCGGTATAAGCCTGGTCTATGCACATCACTTTTACGATATTTCGACACG
AAAATTTATAATTGTATCAAGTTATCGACCAGCTTATTGACTAGCTGTACTTACAGAAAATAATTCGTTTT
ACTATATCCGTGTATGATTATAGGGATGTATCTTAATAACATAGATTTTAGAAGCAAGGCGGTTTTGATTG
AGAGTACTGCGGTCTTAAAAACAATTTGTAGCCTTATCAATATTAATCTATGAAATACCATTATGATTATT
TACTATTGTATTGATTATTATATTATAATATATTTATTGCCAC
>TCONS_00063997 geneID=XLOC_012896 scaffold_215:395030-397427(+) Splice junction overlap
CACATTATCAGATAAGACAAACTGTAGCAGCCAGTCTCCCTTTACCGAAGCTATTGTGTAGTCTTTTAG
AGATTAAAGATATTCTGCGTTGCTCTGTTCAGAGCCTCTGAGAGATATCTTATCTGTGATTCTGTTATCTT
GGTCGAGTTTCTTCACAGGTGAAGAGAACTATGTGATACTGCATGTTTTCAGTGAATTTTTGGTTATTTT
TAACCTGTAATTGTGTCAGTGGAATGTGTTGTGTGAGTGCGAGTGTGTAGGTTTGGATTCGGGATTACA
GGGTCGTAACTGGAGAGAGCGGATGGTGGGGGATGTCGGCCCCCATGCTGCGGGCCCTGTGGCGCCA
GGTCATGCGCCGCCGCGACTTCGGAGGACAGGAGCAGCTGGACGCAACGCCGCTTAACAGATGTCTA

AACACATGGG
>TCONS_00063860 geneID=XLOC_012908 scaffold_215:446084-447092(-) Splice junction overlap
AACAAAACAAACGCCAAATTATATTTCGGTGGCAAAAGGATTTCAAAGATTTTAACTCACGTCTGCT
GATTAATTAATTTGAGAACATTTTACATATTTACCTAGGTAGTTTAGTGAAGTTGAAGATGGAAGCCTGT
GTAAGAGGTTTCTCATTCATGCCGGATAGTCACGATGCGACGGCAGTGGATTTGCAGTACATCGAGGG
CAGTGAGTTGGAGCGCGCGTGGAAGGCGACGCGACGGGAGAGAGTCAAGGCACAGGAGAAGAGGA
TATGGAGC
>TCONS_00063861 geneID=XLOC_012908 scaffold_215:446394-447092(-) Splice junction overlap
AACAAAACAAACGCCAAATTATATTTCGGTGGCAAAAGGATTTCAAAGATTTTAACTCACGTCTGCT
GATTAATTAATTTGAGAACATTTTACATATTTACCTAGGTAGTTTAGTGAAGTTGAAGATGGAAGCCTGT
GTAAGAGGTTTCTCATTCATGCCGGATAGTCACGATGCGACGGCAGTGGATTTGCAGTACATCGAGGG
CAGTGAGTTGGAGCGCGCGTGGAAGGCGACGCGACGGGAGAGAGTCAAGGCACAGGAGAAGAG
>TCONS_00064001 geneID=XLOC_012898 scaffold_215:451543-452793(+) Intergenic
AACTCACTACTTTTAATAGTACTTCTATTTAGTCAAATTTTCAAGTTCTGAATGTTTATTTTTCAAGCGTC
GTCGTTGCTGTAGAGAACGTATGAAGGCAGATATAGAATTTCTTCTGTGGAAATAAGATGTGAGCTGAC
ACAAAATCTGTGTTCTGAGGGTTTGACAGTTAGTACAACTCAGACAACGCCATCTGTTTCTCCAATAAT
GTAAACTTTTTTTTTTAACAAACGCCTCATTCTTTGAAACGTGACGGATTTTCTTAGAGCGGATGTGCG
ACATGTCAATCTCAGGATAATCTATGCCCATAGAACTGAGCGTCGGCGCGACTAGCGAGAGTCATTATT
CTGAGCTGTGAAACGAACGTGATGACGTCACACCTCTTCATACAAAGTTTTGGGCGTCTATACGAGTC
GGTTATAAATTTTTAACTCTGTTTAAAATTACTTTTTTTGGCTTTGATTTTTTTTAACTATAATAAAGCGAT
GTACAACTATCTAATAAGAATATGTGCATATTCCCTATTATATTGTTATTTAATAGTTATTTTGCCAAGTTA
CCGTCCTTGTGGTGAGCAAATATATGTCTTCAGTTCATTTCGAATAGGTATTTACTGCTGACGTAATGTC
ACTGCCCCCCGC
>TCONS_00064214 geneID=XLOC_012973 scaffold_216:500850-508713(+) Exonic overlap (+)
CATTCTAACACCACATCACGATAATGTACAAGTGAAATAAAACATTAATCCTAGAGTAAAGTGTAATTA
AGAAACAATAGCATCATGTTTTGTACAAGAAGAACTTTGAGGTTGTGTTCATTTGATTAAACAAGTGAT
ATTGAACTTTCGGTTAAATATTCATTGTAACGTGTTGCTTGATAAGGAAGATCCTGGAAACAAGTGACA
ATGGATCCTGACAAGCTGTCGACGGTATCCTCGACGACGGAGCGCCGGCCCCTGATGGCGGCCCTGGT
GCTGGAGAGACGCATCCTCTTCGGCTGTACCATCCTCGTGGGGCTCAGCACCTTCAT
>TCONS_00064235 geneID=XLOC_012977 scaffold_216:526450-527433(-) Exonic overlap (+)
ATACTATTATCATCGAAAAGAAAACACCATACGATCAGTCCTTATTAACATTAGCATAACAGCATATTTT
GTTTAATTTTTTACGATCACATAATGAACCCATCATGAAAAAACACCGTGAAAGTCTCAAAAGTCAATA
GAAAAAAACATGGTGCAATTTTCAAAAGTCAATACATACAATAATAAAATACAGTGCAAATCTATAGAC
GATCAAACGGTCCGCAAAAATTAGACGTCAACGGACTATACGCAAGTTGTGGTGGCTGTTAAAAAAAT
ATAAGCAGGTAAATCTACCTAGTGATTAAAGGACACTGTTCATCAAGTTAGAAACACAATTCGATATGG
ATGAGACAAGCGAGGCCCAGGCGGTGGCGCATACCTTGCTGGAGCATCAACCGCAGACACCGCAGAT
ACAAGATACGAAACTAATAAGGACATTGAGATCATG
>TCONS_00064466 geneID=XLOC_013001 scaffold_217:121834-123082(+) Intergenic
GCTGCTAAATACCCACAGGAATTCAAGACAATGATGCAGCAATCAGGCGAGCTCCGCCTCAAACTCGA
GGCGGCGGTGAAGGCGAGCCAGCAGCACAGCAGCAGACAGCGCAGCGCGCCGCAGAGACCCGTGTT
CGACACCCGGCCGGCACGCCCCACTATTGAACTGAAGACTGACTTCAGCGACTTTAGGTAAACTGGCT
AATGGATCTATGTACAGCCTGCTTGCACAGATTTAGGGTGAGGCCAGACGAGCGTAATTTTATAAGTTG
TGAGTCGCGTATTCTGATATTTCTGCCGGAATCGCGACTCAAAACTCACAAAATGACGCTCGCCTGGCC
TCACCCTTACAGGCGTGAGTGAATTTAGACGACGACGAATGACGAAATTAAAAGTTTGTTTTTTTGTA

AAAGAGAAGTGCCAATTACAGTAA
>TCONS_00064492 geneID=XLOC_013001 scaffold_217:122312-125863(+) Exonic overlap (-)
CACAGGAATTCAAGACAATGATGCAGCAATCAGGCGAGCTCCGCCTCAAACTCGAGGCGGCGGTGAA
GGCGAGCCAGCAGCACAGCAGCAGACAGCGCAGCGCGCCGCAGAGACCCGTGTTCGACACCCGGCC
GGCACGCCCCACTATTGAACTGAAGACTGACTTCAGCGACTTTAGGTAAACTGGCTAATGGATCTATGT
ACAGCCTGCTTGCACAGATTTAGGGTGAGGCCAGACGAGCGTAATTTTATAAGTTGTGAGTCGCGTATT
CTGATATTTCTGCCGGAATCGCGACTCAAAACTCACAAAATGACGCTCGCCTGGCCTCACCCTTACAG
GCGTGAGTGAATTTAGACGACGACGAATGACGAAATTAAAAGTTTGTTTTTTTGTAAAAGAGAAGTGC
CAATTACAGTAAATTTATTCCAAGCACTGTATTTCTTAGAAAAAATATATCAGATTATAATAGTTAGCATG
CTATAAATATTAATTAATAAAGATCTCTGAATATATGATATTCTGACAGATCACATCGACTGTAATGATATT
GTATGGAAAAACTATAGATGAAATATACGAAATTAGATGCCTGCCTAAGTAAAAAAATTGAGTTTACAA
ATACTTATTTATATTTATAATATAGTAACCTACGCTGACGGGTTGCATTTATAGTATTAAAGTAAGAATTTT
AAGTACGAATGATCTTTGATTTAGTTATAGTTCGTATTTTAGGACAGATTTTGCAATAGGTATGCATTTAT
GTTGCGTTGACAGAAACAGGCAATACGGAGATTTAATTTTTCTGACTATACTTACCTACCTTTTAAAAAT
ATAGCATGAGTTCTCAGAGCTATGACTTTAGATTGAAAGAGGATTATGTCCGGTTAACTAACAAATTTA
GTGCCAATTTCTCCATTCTCGGTTAGAGCATAACCAGGGAACACAGTGGCGGCTGGTGCCTCAGATAC
CCGGTGGTGCACTTTATGGGTGTAGTAAATTTTGGAAGAAAAATAGATTATTACAATTAGACCTAAAAA
AAATATAAGCATATTTTACCTTATTTATATTTTAAATCAATTCTGCGTTCCCTTTTTGACGCGAAATTGCTT
CTCCGCCGAAACGAAAACAACACACGGAAAGCAAAATAAAGCATTTAGTCTCTCACATCCACATAAC
CATTTCTTTATATTGTAGGTACATTTCACTTTTGAAAATACACTTATAAGTTTTATTTGCTGTTTTGCACTC
CTGTTTTAAGATGAGAAAAGGCCGAGGCGGCCCAGCAGATTTAATGTCCAGTCTATTTTGAAATGACCT
ATTGCTTTTTATGAGGTCAATATTGTATTTTGCACTCATTTTCACTCACACACACATACACACACACACA
CACGCACTCACGCCTTGTACTAATGTACTCCCTTGCGGGGTAGGCAGAGGTGCATTGCTGCACCCACT
TTTCGCCAGAGTGTTATGTTAGTCCCAATGTAATAGGGGGCGGGCCTATTGCCATTTAACGGGCACATC
CAAGACCCGAGAACAAATATCTGTGTTTAAACAAATATCTGCCCCAGCCGGGAATCGAACCCGGGACC
ATCGGCTCAGTAGTCAGGGTCACTAACCACTACGCCATTCGGTCGTCCCACACACATACACACTCACG
AAATCTTTACTACTTTCTTAGTTAGTAAATAATTAGAGTTACAAATTAATTACCTACGTATTCGCGCGCGA
AAGGTGAAACTGTGACAATGAGATGAGATGACAAAAGTGCATCGTTCGTAGGTCACGCCACCGGTCA
AGGCCGCCGCCCGCGCCTCCCGTGGCGTCATTCGTATTATTTCGGCAATTTCCGGCCGCGTGCGGCGCA
CGAATTTATCCTGGAACTGCATACAGTTTGTACTGAGCATCTTCCGTCTCACGCGCCGGGCGATTTCCA
TCCTGTTTCATACTAAATGTAGGAGCTCGCTCCGTGCGACAATCCGAGCGCACGAGGCTGTTTTTGTTC
AAGCAAAAATAAGATCTAATTCTTTCGCAGTAGAAATCAAAATAGGCCAGCTCGATTTAATTTAACTCG
TAACGATAATCAGATTTATGTAATTTCTGTTTAGGGTAGGTATGTTGTTATATTGGTATTTTTTGTGAAGTT
TTTTTTTAGAGGTACGGTACCTAGTATGGTATAGGAAGAAGGAAGGTACATATTTAGAAGGTTTTAAAT
GGTAGTGCGTTGCACGGGCGCACTTAAGGTGCAGCCGCCACTGGTAGTGGTTAACTTTTGACTAGTGT
TGCCCAAATGCAAGAACAAGACGAGACTTAGCCAGTCTTGGTCTTGGTCTTGCGCCAATACACCTGGT
CTTGGTCTTGGTCTTGGTCTTGCGCCAATACACCTGGTCTTGGTCTTGGTCTTGGTCTTGCGCTCCCAG
TCTTGGTCTTGGTCTTGGTCTTGCAGCAAGAGTCTTGCAAGTCTTGCAATTAGACTTGCAGTTTTGACA
CATCTGTCATGAATTTTATATGGAGATGAGACGTTTAATAACCGACAATGGAGAATTTGGCACTAAGATA
ATCAAAATTATCTTAAGAGAATTTAGCACAAAGATAATCAAAATTAAATCTATATATAATTAAATTTTGAT
TATTTTTGCTGTGCAAGACGCCGTTAATGACAAATAAAATGGATTTTACAGATGTCATTGGGCTCCTGCT
ATATTTTACAAAGTATAATGTAAAATTTACCAACATGATCAACCAATTTATTTCCGAAGTGTATTTATAGA
ATTAAGTTGTGTAATCTTTATTTTACTATAACATGCATGAATGTCGAATATTTATTCTTAATTATTTACTAAT
ACGTACTATGTTTTGCACAAGACCCTAGATAGTGGCTTTTAGAGTAAAACTATATTTAAGAATTAAAATT

TTAAAAGTATTATGAAATTATGATGAAGATAAACAATGGTATGTGGTATGTGCGACTGTTGTAATAAATA
TTAAATATACATTATATCCAAATAAAAAAACAGTTGTATGAGTTAAAGATCGGTAAAAAGTAAACAATA
AAATCATCTTCCATACTTTATGCGGTAAAATATTAATTAAACCATTATTTATTCACTCTGTAAAATCTAAAT
GATTCAGAAATTCATCCTAGCTACGTCAATTAAAGCCATTATCCACAGTGTATACAGTATAGCTACTACT
GACC
>TCONS_00064558 geneID=XLOC_013014 scaffold_217:163923-165028(-) Unclassified
AATAAATAAAACCTAAAACCTAATTTTAATAAACCTAATAAATAAACATACTTTAATTAAGTGATCTAGT
TCTATTACGATAAGTGATAATAAAAGTGTAGGTTCTAAAGTACGTTCTCACCGACAAACCGTTTCCAATT
ATAAGTAGCGAATAGTTTCGTCCACTCGCGACGTCACCCTTTGTTCACCTTAGATCTAGCGATAAGCGA
CCGGCCGACTACGGGAGGGGCGCTTTGATTTGCACGGCGATCAGTCTCTGCTCACCGCTCTGGCAACT
AGCCGCTCATAATAAATATCATACGTACCTTGTCTGCGAGTACCTAACTCGTCAAATCTAGACGTTAATA
ATTTTTATAATTCCTCAAATAAGTGTTTATACGTTGTGCCCATCTGGGAACAACTACCGGACCGATTAAA
TCGGCCAATCTAGTTCCGGACCACGGAAGACTCACCCTGGGCGACTGTCCCGGGCTTCCCCTCCCGCT
ACCTGAGTAGCTGACCTCGTCGGATCCCTCGACCCCGACGTGGACCTAGACCTGGAGCTGCACCTGG
ACCTGGACCTGCACTTCGACAGGAAGCCAAGGAGAGTGCGGTATTTCTAGACCGAACCAGTGCGCGT
GTGACACCCTTGACCTTGGCCTAACCGACGAC
>TCONS_00064601 geneID=XLOC_013003 scaffold_217:377385-378445(+) Intergenic
GCGCGCGCCGACACCTGCTTCCACGACACGATCGCCGTGTGCGGCACGATGGCCGGCGAGACGCGCT
CGTTCCTCGACGAGTGCGACATGTTCGAGTACTCGTGTGATAACAACTTGTTTTACACCCACATAGATG
ATACTGATGACTGTCCGGCACCCAAACCTGGATAAAGAAGTATGATACAGTTGAATAATGAAGGAATTA
TTGTGTTTTGGGGAGTTCTTTAATAAATGGGTCATTTAAAAAAAATACCATAGCATTAACAATTATGAAT
TTCTATTAATTTAATTGTACTCGTACATTGTAAATAGAAGAAAATATTGATTTTGAGGTTTCATGGTATAA
AATTAACCAGA
>TCONS_00064723 geneID=XLOC_013047 scaffold_218:54633-56737(+) Exonic overlap (+)
CAAATAGACTGTCAATGTGACAACTGACAACGACAGATTACCAGTTTTCAATGTTCGTGGAAAATTCG
TAATTTCGTAAACTTACAATATTTTCATTCATTACATTTACGAAAAAGAAATATTACTTCTGCCTCTTATA
ACTATTATAGGATTCCACCCTCATATAGCAAAATGACGAGTGTGACTAAAAGAGTGCAGAATTCAATCA
AAGGATGGTACCTTAGATATTTGCTTGCCACTGATCTGTACATAGTGGAGCCTTGGGAGAAAGTTGTTA
TACATGTGCTGTTTGCGCTATTATTTGCCGCATTCTGGTACTTCAACTACGTGATGGTGGCATCAGACTC
ATCAGAACTGAAGCATGGACATGCACTAGTGGATCCAGTATTATGAAATATCTAGCTGTTTTCAACTTTT
CATCAATTAATATTTTAGCAGCCATTAGGTTTGGTTTGTAGCATCATCATGTTACCTATTTGTCAATTTTAT
GATGCTACAAAGGAAATCAGCTGGTGTGGTTAAGTTTTGTATTTATGCTTCTTTCAGACACTAAAGTAA
TTTTATAATTGATGTTATTAATTAGAAATTCCTAAGCTGTTACATTGTGTAATTTGGTCAATAACCCCACC
TATCTATATAGGTGTTGCAAAAAGGGTATACTAAGCCGAAACCAACCTTTCACAAATTTTGAAATTCTG
ATTTCATTTTTGGGCTTCGATATACCTTACTGTACATGTAGGTTTCGGCTTAGTATACCCTTTTTGTAACA
GCCTGTATATTAGTTAAGACATTCCAGTGATGAACTATTCTCTAACAATACTTAATTCTTGTTAATACATT
CACCGTGCCTTTATTCACATTCACATAGTTAGGTATATGACTATACTTTTATTCATACCAAAAATGTTAAA
TAATCC
>TCONS_00064846 geneID=XLOC_013047 scaffold_218:54658-56719(+) Exonic overlap (+)
GACAACGACAGATTACCAGTTTTCAATGTTCGTGGAAAATTCGTAATTTCGTAAACTTACAATATTTTCA
TTCATTACATTTACGAAAAAGAAATATTACTTCTGCCTCTTATAACTATTATAGGATTCCACCCTCATGTA
AGCACCTACAGTTTGAGTTTAGCTCACTAAATTGATATTATCACTACACTACAGAAACAATTTCTCTTTT
TTCAGATAGCAAAATGACGAGTGTGACTAAAAGAGTGCAGAATTCAATCAAAGGATGGTACCTTAGAT
ATTTGCTTGCCACTGATCTGTACATAGTGGAGCCTTGGGAGAAAGTTGTTATACATGTGCTGTTTGCGC

TATTATTTGCCGCATTCTGGTACTTCAACTACGTGATGGTGGCATCAGACTCATCAGAACTGAAGCATG
GACATGCACTAGTGGATCCAGTATTATGAAATATCTAGCTGTTTTCAACTTTTCATCAATTAATATTTTAG
CAGCCATTAGGTTTGGTTTGTAGCATCATCATGTTACCTATTTGTCAATTTTATGATGCTACAAAGGAAA
TCAGCTGGTGTGGTTAAGTTTTGTATTTATGCTTCTTTCAGACACTAAAGTAATTTTATAATTGATGTTAT
TAATTAGAAATTCCTAAGCTGTTACATTGTGTAATTTGGTCAATAACCCCACCTATCTATATAGGTGTTGC
AAAAAGGGTATACTAAGCCGAAACCAACCTTTCACAAATTTTGAAATTCTGATTTCATTTTTGGGCTTC
GATATACCTTACTGTACATGTAGGTTTCGGCTTAGTATACCCTTTTTGTAACAGCCTGTATATTAGTTAAG
ACATTCCAGTGATGAACTATTCTCTAACAATACTTAATTCTTGTTAATACATTCACCGTGCCTTTATTCAC
ATTCACATAGTTAGGTATATGACTATACTTTTATTCATACC
>TCONS_00064867 geneID=XLOC_013068 scaffold_218:465374-466631(-) Exonic overlap (+)
TTGGTGTTTTTCTCAGTGGAACGTTTTCATTGCCCGTGAGTGTATTATCAAAATCGGCCTAGACGTTCG
AAGTTATGCGTCTTTTAGTATCGAAGTTATTTTGTTTCAGGAAGTAACATTTACACACAGATGTCGTTGG
CACTACTACCGGCCGTGGCCTACATTGTATTATCGTAATGGGAGAACTAGATAAACAACAGCAACTTAC
CAAAAAAACAACAAAGAAAGTATCTACATTCCAAAGCGTGTATTTATCATCACATCTCTAGTCAATTTA
TTTAATTTTGTAAAAAGTTTTCCTTCTGCACAGAGGTAAAGGCTTCAGGTAAAACTTATTTTATTGAGA
CAAAATTTGGTAACTTAAATTAAGTTTGACAGTTCTGACCGACAATCGGTTTATTTACGCGATACCTGCT
ATCAAACGCATATTATTTTACGCACGCGTTTAGCAGGGAATGCCAAAAGTACGGTCAACTGCATAAGTA
GCTACATAAGTAGGTACAAAAGTGTACAGCTACTTATGCAGCTGACTGTACAAGCTTAAAAGCAGGTT
TTGAAGATTATATCCAATGTTAAAAATACACTCACGAGCAATGAAAACGTTCTAGTATCAAAGCACTTC
ACTTACTTCTAAACGGAAAAATCTAGATTAATTACGCCGTCTTCAATGTTGAAGTATATTTAACAGCACG
GGATCCATTTGGGTGGAACTTTTTCCATGCTCGTGAGTGTAATAAGCGGGCGCGTCCAAATAATGTCTC
CATCAGAACAAATCTATGTAAAAGAACTAAAGTACGAGAAACCAACAGTAAATATGAAATATAAATTAT
TAGATATTGTTTTTAATCCCTACAGAATATACCTAATAGGTATTTATACGTTTACAGCCTGGCTAATAAGC
AAAGAAAACGATAATAGACACTCAA
>TCONS_00064798 geneID=XLOC_013071 scaffold_218:683767-685879(-) Intronic
GTCTGAAGTTCTCACTGTGCTTCGCTATTCGGGTATTTGCCAGCGCGCTCTCAGCAACTATCCACTGCC
GGCACCTGATGATATGGGGAGTGTTCACACCGAAACTGCTGTTTGAGAGCGGCGCGTGTGCGGCCGC
CCTTATAGGAGCCATCTTAGGGGCCGCACTTACAGCCTGGCACACACCCAGCCAACAGAAAAGCAGC
TGACAAGACAAGCGGACTAGGTAAACTAATGTAAAACGAACACTTAAATATGGAATATCGACCAAGAA
TGATGAGGTGACGACGAAAGATATAGTGAGGAAATCTTAGAAGACTATAGGTCTGTGGAGACAATGAT
AAGGTACAGTACAAGTCACAAGAGTTGACCTTTTTCTAAATGGCGGTAGTGAGCCCATGTTGACTGTA
GCACTCGGACCGTCGCTCGGTCTGTGACGCCCTCTGGCCGCAACGTACCAAAAATTACTTATGTGAAA
TTGTCACCTCATGTGAACTGAACTGTATCTTCAGCAATAAATATGAACACGATTGGAGTGGAGAATGAA
AACCACAAATATACAGTGGAAAAAACAGTTGAGAACATTAGTGAAAACTGTCGATTTGGATTTTTACA
GGATTGTTTTTAGATAAATGGTATATTTTTTTCTACAAATTGAGTGCATTTTTTCGCTTTTGGTGAATTTT
GTTTATGTTGTGATTTGATCGATAAAAAATTATTTAACTTTTAATGACATTTATATGATATAATTAAAATTG
ATCGGAATATAGTAAGTAATAGGAAAAAGGACCGGATTGTACCTTTGTAATACTTATTGCATTATTATTAA
TACTGTTACATTTATAGCAATGACGTCTGTGCTGAAAGATTATTCAGCCTGAAACAATTTCAACTTAGA
AAATCTTGTAACTTCTATGTTATCTGTTAAATAATGTTTTAATTAAGAAGAGTTAAAATATTAAAGTACAT
TCACTGCCATAAGGGGCTGTTATTAAATGATACTTAGGTATGTTAAATATTGAAATTAGGAACAAGTATT
TAAAATAATGATGTAGGTATTATTTTACAAGACCTAAGCCAATGCTAGTGTTATGTTAACATAAGTAGATT
AGAGCTAAGCTATCGAACAAGTCCAAATCTATAAGCTCTTACTTAGTGCAGGGCCAATCAGAACTGTG
CATACTTAATGCCTTTAGTGGGCACATATGGCATGTGTAGATTTTAGCCACGTTTCATCGTTTTAATATGA
ATATAGAGGAATGCCCTGTAAGGGGTGCAATAAAATATTGTAGATAAGCCAAGTACTTCTTAAGACCCT

TGGAGTTCTCGAAATATGGCAGTAGAATAGCACATTTTACATTTTGAGTAGGTATTTGTTATGTGTAAGT
GTAGGTACGGGGACACTGGCAACTTATTAATCTTATGAATTGTCCTGGATATGTGTAACATTCCTACTGT
TTAAGGGCACAAACATCTATTGTAAGGACACAAGTTCTGTACTGTATAGCATTTACTTATGTATACCAGC
AAACATTTTTTTTATTTAGTTAGGTACCGCAATGCCAAAAACACAGTGTATATTTATTTGAAAAAATATT
ATACACGCAGTTTTTAAATACCTACCTGATTTTCCACGCATATTATAGATTTGATAACCGACTTTGGATA
GTCAGAACAATATTCAAAATCTAGTTGTGAAAAGTATTATTAAACAATAGTTTACTTGTAAATGCTAGTC
AATATGCCTTATTTATTTATGTAAATTAAATGCATTTCTTGTATGTGCATAGTGAAGAGAAG
>TCONS_00065055 geneID=XLOC_013113 scaffold_219:116976-118621(+) Splice junction overlap
GAAGGACCGGCGCAAGTTCGGCCTCCGCAAGAAGAAGTCCAGCTCCAAGTGTGCTTCCATCGACCTA
CAGCCGCAGCACTACATCGACCCCTCCAACTCGCATATGGCCTTGTCAGCGCTGACAGCGCCCCCCTC
GCCCGGTGGCGGCGCGTCTTCGGTACCAAACTCCGCCGCTTCGTTACCTATAGACTTTCCTTAAACATT
CCCGACTCCCTAAGACCCTGTTCTAATACGAAGAATTAAGAAATAACATTTTGTACTTTTGTTTATAATA
TTTCGTTAATTTAATCGTGTG
>TCONS_00065205 geneID=XLOC_013122 scaffold_219:155815-165032(-) Splice junction overlap
GTAGGTAGGCGTTTGTAGGTATAGATAGTAGCCGGATTCATAAGTAGAGAGTGCGAGGGAGGCTGCGG
GCGTATAGTATCGGTCGGATAACCTTCGTCCCGCCCGGCAGTATAGTATTATCATAATAATATAAAATCAA
GTAATCCACTTAGTGGTCAGTGAGTTTTCGCTAACAACAATTTAGTGTTACATTCACGTTATCCAAACG
CTCAATCCGCGATCGCCTAAACGAAGAAATCGTCGCATTATTGTGTAAAAAAATCGAATAAGAATTTAT
ACAAGCAAGACTGAAGTTTTAAGTGTTCATTAGTAATATTAATAAAACAAAATGTCCATGGAAGGCTTG
GAATACGAGACCATTGTCCAAAATGGGCCTAAAAAGGTAACCATGACAACCGGGCTGGACCGCGACA
CGATACGATCCGCATA
>TCONS_00065119 geneID=XLOC_013121 scaffold_219:469950-470549(+) Intergenic
CACGACATCGCCACGATATCGTCACGACATCGCCACGATGTTGCTGCGATATCGTCACGACATTGTCAC
GATGTGGCTACGATATCGTCACGCTGTTGTAACAATATCGGGATAGTATTGCGACCAAGGGCCTACTCC
GTTTCGGCGAATACGAAAGTTGCTCGCAGGAATAACCATCGATCCTTGGATCCTTTGATTGTCATGTTA
GAAAGAGACATCGTGACATTTCAGGGAACTTTACCATCGACTTCGTATTCGTTATTCGGGAGTAGTTCT
TCTACTTCGTAAATACATCGCAGTGGTAACGTTAGAAGTAAGCACATGTGATGGGTTGTGGGCACTCGA
CGCTCTGAGGCTAACCGGGGTAACTCTATATGACGAAAAACTAAATATTTTAGAGCGCTGCTGCGCGA
GCCACGACACTATCTCGTTGTGTTATTAAATGTTCATTCGTTTTTCGTCAGTATAGAGTAACCCTCCTGT
AACTTTACACGTCACACCGGTAGATGTCAGCGCTGCCGTGATTAATTTCAG
>TCONS_00065758 geneID=XLOC_013189 scaffold_22:102916-105229(-) Splice junction overlap
CGACTTTACCGACACGTGTTCCAGAACCTCCGCGACTCGGCGGGGCGCCCTCTATCCTCCAGCCAGTA
CTCGCTGCAACAAGCCGCCGACGGGAACGTCGTGCTCGTGCCCAAGATGCCGCACCACACAGATCAG
CTGATGAACCGCACTTCCGACGATGAAATGGACAGGAAACCCAAAAGCTACGACCAGTGACATCCAC
CCAACTATTTCTATTCGAGATGGTAACACCACGGATATAAATAAAACCTGTACAAACTTATTTACGTGTG
ATATGCGAATATAACTGTTATTTTTGTCCTGTCAAAGCTCAAAATGTCTTTGACATTTGTCTGTTCAACG
TGTCGTTTTGTTTTTGTTTCGTTCTATCATTCATTTTTGTCGATAGTGGCGCCAACGGCCATCTTGCGGC
GTTGTGCGGAACTAAAAATATTTTCTTTCATTATGTGGCATGAGGTTATAAGCTATTCATATAGCAATGAT
TTTTGTTGCTTGTATACTATTTCCAGTAATTCTAGAATGATTGGAAAGATAGCTTTGGCCTTCGGCAATTT
TCCACGCCATTTTATTTAAGATCACACTTTATGTCTAGTTTTAAATAAAGTGGAGTGGAATTTACTGTAC
TTTTTTAAGGAATGGTTGTGGTTTGACTATCGATGCTGTTAAACCTAAATCATTTCTGGACCGCCTCATA
ACAGTTTTTACATTGAGTCTATTATAAATATTAGTTACGTATGAATAGTTCTATCTAACAGAATAAATTTAT
AGGTATCTTGTTGATTGTATGTGTGCTCTCGCAAAATTATCATCTCGAAATTGAAGAAGTAAAAAAAAA
CAATAAAAAATGTGATATTAAAGCAAAATTCCATTTTTAATACGTAGTTTTGATTTAGAACAAATTGTTT

GAATGGTGTCTCTGTTTTTTTGCCAGTAATTGTAATTTTATGCCGTCTATGTGAAAGGCTATGTCGTGGC
AAGGATCATTGCAAACTGTTACAGTGCAGTGCCAGATAATTAGGTCAATTATAGTTTGTGTGTTTTCCAT
AATTATTTGGCACGCATTGTATAGAAAAAAACTAAAGAATACACAATGCAGATTTTCTTGCTTTTAGTGT
TTAAATAATTTCTGACAGTTAATAGATACTAGCGCCCTATTTCTTTTGAAAAATGATCCTTGTGGCAATAT
AAAGGCACGCTTTTTTACTAATCTGTGACTGAGTATGTATAACCCATAGATTTAATATAAAAATCGATGC
CCTGCATCGATATTGCAATAAAAATAAATCGTAGATTCGATTTTTAAAGTTTACTTTGTATAGTGTTTCTT
GTAAGTTTTTATTTATGTATATTATTATTATAAATAGGTTATGTTTATACTACGAATAGTTTGTTAGATCTAG
ATGTGCTATTTTGTAAAGAAAATATCTAGAAATTTATTTTAAAATGCCGTTTCTGGATTTGAGTACAAAA
TGAAGTTTAAATGTACCTACTTGTGTTTAAATTAAGAAATACACACAGATAGGAATTCAAAAGATCTTTT
ATTCATTTTTGATTCCTGAAAAAAATATATATA
>TCONS_00066284 geneID=XLOC_013189 scaffold_22:114635-115999(-) Splice junction overlap
CGGAGCTAGTACAGCTACTTACCGCTAGGTGACGTGTCTCCCGCGCCATCCAAATTCACGTTTACTCAC
CGCTTCTCGAACGGCGAAAAAACTCGCACTTGGGGACCTGGTATTGGAATAAGGGTGTGTACAAATCT
AAAAAAAGACCGATCTTATCTCAAAAAGCGATCTCTTCTAGCTATACTCGCTCTCGCGTGTTATTGTATT
TAACCTCTTCTGTTTTTTGCAGGGATGTATACGATGCGAGTGTTGGACGGGGGCGCGGCGTCGCACCAT
CATGCTCCCCCGCATAATCACACGCATATGATGATGACCAGCGCAGAGCGTGACTCGGCGTCCGATAGC
GCCGTCTCGTCCATGGGCTCAGAGCGGGTTCCCTCTCTGTCGGACGGCGAGTGGTGCGACGCGGGCA
GCGACTCCGCCGTCGACTACCACAGGTGGG
>TCONS_00065865 geneID=XLOC_013189 scaffold_22:191861-197894(-) Intergenic
CGCTTCTCATTGGCTGCTAACGTGCGTTTTCCCGCGTATAAACCGTCCAAGTTGAAAATCCCGCGTTTG
CTCCCCGCCTCGTGCGTTCGATCGCGTTTCAATTGCGTCGGTTAATACCAGTGTTTACTTTTTAAATTTT
TGAGTGTAAAGGCTTGTGATATTTAAGTTATATTTTCGGATTGTTTGCAGTTTTTTGTGCCAGTGAGTGA
GTTTTGGATTGTTTCACAATATTACATAGTTGCCGAGATGTTGGAAGTGGACCTGCCACTCATGAGGAG
CCTCAGCCCAGACCCCGCCGACCTGCTGGGGCCGCCGCAGACCATCTTAGAGGATAAAGGTCCATGTC
TTACCGAGCTGGACCTGCCAATGATGAGGAGCCTCAGCCCAGACCCGGAAGTAGCGGCTTCTGTCAA
CCGGAAGTTGATTGACCAGG
>TCONS_00065997 geneID=XLOC_013157 scaffold_22:392358-393696(+) Splice junction overlap
CGAGCATGCTATCTCGACTAAGTTACACTCGTCAAGTTCACACCACATGAGCATCGCACAATACGCAA
GTAACAGATCTGTCAGGACCGTCATCGCCTCCAGAGCTGATCACGACGGACTGTGACTCCATCATCGA
CCCCGACTCCGAAGATGAAGCGGAAAGAAAGGACTCACTCGACGTCCACGAAGAAGACGAATTATTC
GAGTGCTGATCGTATGGAGACGCTTTCATATCCTCTGGACACATACACGAGAC
>TCONS_00066203 geneID=XLOC_013157 scaffold_22:393114-393844(+) Splice junction overlap
CCTCCAGAGCTGATCACGACGGACTGTGACTCCATCATCGACCCCGACTCCGAAGATGAAGCGGAAA
GAAAGGACTCACTCGACGTCCACGAAGAAGACGAATTATTCGAGTGCTGATCGTATGGAGACGCTTTC
ATATCCTCTGGACACATACACGAGACCCGTAGGTTTTAATAATAATTTTCCTAATAAGACAAAATATACC
TAAGTAAATGTACCTAAAACTATACTTACCTACATTAGTACATATCGTAAAAGGATTTTCCTTCCCAACT
GTTATCTAGTAGTTAGTGTGTTGGTACAGTAAGG
>TCONS_00065827 geneID=XLOC_013159 scaffold_22:403078-409090(+) Intergenic
TGTTAATGCGCCATCTATGTGTCAAGCTGCGAACCATGTGGAACGAGGCCTGACGACCACTCAACACA
ATCACACAACTGGATCGATCGAGTACTACAAAGTTCCTGAAGAAAACGCTAGATGGCAGCACTAGAA
GATTTATACTTATGAATCGTCTATAAAATCCCGTGCGCATCGAGGCGCGCTCTCTTTACCACGATATATTT
CAACCGTCATATACCTACCTGCTTGTGTATATTAAGTTTATATTTTATATTGAAGAGCATCCTAATAGCTAG
ACCCTTCAGAAACTAGAAGAACTGCTGAGCTGTAGACGATTAGACATTAACATATGGACCTTCAACGC
CGGATACCATAACCAAGAAGAAGCAGCCACGTGAAGCGGTTAAAGGCAAAGACGGCATCTCCAGGGT

GGCTGACATACGCACTAAAACTGGAATCACACGACGGGCTGTCTCGAAGATATGTCCACTGCCGGTGG
AGCCATCCAGCCCTGAGACGAAGGGCACCACAGATGACTGATGATGATGCGGCTCCTTGAAAGCTATC
GGGCAGCTTTCAAGGCGGGCGGCATGTTAATGCGCCATCTATGTGTCAAGCTGCGAACCATGTGGAAC
GAGGCCTGACGACCACTCAACACAATCACACAACTGGATCGATCAAGTACTACGAAGTTCCTGAAGA
AAACGCTAGATGGCAGCACTAGAAGATTTATACTTATGAATCGTCTATAAAATCCCGTGCGCACCGAGG
CGCGCTCTCTTTACCACGATATATTTCAACCGTCATATACCTACCTGCTTGTGTATATTAAGTTTATATTTA
TACTTAAATAAATAATTTTAAATCAAGAAATCAACATAAATATTCA
>TCONS_00065292 geneID=XLOC_013158 scaffold_22:415933-416390(+) Intergenic
GGGTACTAAGTTCAAGGGACCCGTACTCTATACTCTACCTACCCGTATACTAATTCATTGATACGAAATT
GAATTGAAGACCACCCAATTCTGTACATAAAAATAATATATTTTGCAATAATAATTATAAGTACTTAATAA
AATATAGTGGTTGACCCCTTATCGGCTTACAACATAAGATTTGCAACACGCTAAAATAATTTTAAACCAA
GTTAAAAGGAAAAGTCTAGCATACTAAATTAAAAACGTAAACTTATAGTGAGATCTAATCTATAACATC
GAATCTAGCTAAATATTACATAATATCTTACATTTAGATATAGTCAAGGAATAGGTAGCTTGCTTTCGGTA
TTAAAACTTGTTACT
>TCONS_00065413 geneID=XLOC_013194 scaffold_22:483505-486545(-) Intergenic
TGTCAAGATATTTTAATGGAACAACAATAAGAGGCAGAGCTAATGTAGCCAAACTTACTTATGCTGTTT
TTGCTGCTATAATAGTTTACAATATTTTTAAACCAAAAAAGAAACACACTGTTGAGAATGAAAATTAAA
CGAAGAAATACTGCAAATTAACATAAAAACATAAAGAATGGCCGGAGACAGTGCAGCAGATGCCGCG
GCAGAGGCCCAGCTGAAGGGTCTGGCGAAGCACTTCAACAGTGTGACCAACAGAGGACGAGCAAAT
GTAGCCAAGGCAACATATGCATTCTTTGGAGTGGTCATCCTATACAATGTTCTGAAGCCCAAATCCAAG
AAGTAAAATATACCTTTAGTTAAGTTATAGCACCATATTTATCTGCAAATCCTTAATTTGTAAAGTTACTT
GATGATTTAGCAATATAATCACATCAGCATACATCTCTTTTATTTAATTAAATCAATAGTGAGTACATGAT
TGAAATACAACAGCTTCAGTTTGTAAACATTTTTATTGATAAAAGAATACAGGTATACAGAAATAAATA
GGTTCTTAATTTTAAAATGGACAAATCATTAAAAATATAGCTTACAATATTTGTATCAGATCTGATCATAG
AGGCAGTCTTGAAAAATACATTTTGCAATTTGTGGTCAAAATACTCTTTGGAATAATATACTCAGAGTT
GAGCTATACTGTAGTGTAGAGAGTAGGGTGAAGAGGAGCACTGTCCAAAATAGTTTTCCAATAGGGTG
TAGAAGCTACTATGCCATATGGTATTTTGTGCAATATTATGGCTCTCAGTATAAAATTTAATTCTAAATAAT
ATGAAGCATTAGCACCTACAGAGCTGCAGCAATGAGATCTAACATTTAGTGAATACCTACTTGTAACAT
CAAAATCTATTAATCGCTTTCATAATCATAAAACAATACAACCTAGTGAATACATGAATTTTAATATCATA
CATATCCCATAATCAACATTCAATGACTAGCTTCTTCATATTTTGTTCTTGGTAAAATGCACCTTTCTTTA
AAAAGTCTATCTAAAGCGAAGCAATATGTAGATAATTCCATAGCCTCGTAATAATCACAGTTATTTCAAG
CTCCGACCCAATATAACAATTATCCAACACTACATAACTCCAGCATGGTGTATAAAACTTTAACATCCTA
CTGTTAATACATTATTGGAATGTCGGCAGTGTACATACCTAATCGTTGTATACTCGTGATTGCCTAAACA
ATCGAGCCTAAGGCACTAGCCTGGCCAAACAAATGAACATCTACGCTACGGATGGGATGCTACATGGC
GCGGGAATAAGCGTCTAGCCTGTCTAATTTTACAGCTACTACGCCAACAACACTGCGCAGTCTGCGCAT
TTGTCGTGTTTAAAATATTACTAGTAAAGAGTATAGATTTATTGCGTCACTTTATATTAATTTAAAATACTT
TATCGCCAATTTCACTATCATGGACTGGCGTTGTTTGGTAAAAAATAATATACCTATACTGAGTGTAAGC
GTCTTTATACATTAACCATAACTCCAAAAATTAGACCCTCATGCCAAGGATTAGCTGTTTATTTATTTATA
CCATATTCATCATCATGCCTCAGGCTATGACACGAAATG
>TCONS_00065414 geneID=XLOC_013194 scaffold_22:483505-486584(-) Intergenic
CGGGCGACGAGTTAGAAGATGACCCAAAATTAAAAGGTAGCCAAACTTACTTATGCTGTTTTTGCTGC
TATAATAGTTTACAATATTTTTAAACCAAAAAAGAAACACACTGTTGAGAATGAAAATTAAACGAAGA
AATACTGCAAATTAACATAAAAACATAAAGAATGGCCGGAGACAGTGCAGCAGATGCCGCGGCAGAG
GCCCAGCTGAAGGGTCTGGCGAAGCACTTCAACAGTGTGACCAACAGAGGACGAGCAAATGTAGCC

AAGGCAACATATGCATTCTTTGGAGTGGTCATCCTATACAATGTTCTGAAGCCCAAATCCAAGAAGTAA
AATATACCTTTAGTTAAGTTATAGCACCATATTTATCTGCAAATCCTTAATTTGTAAAGTTACTTGATGATT
TAGCAATATAATCACATCAGCATACATCTCTTTTATTTAATTAAATCAATAGTGAGTACATGATTGAAATA
CAACAGCTTCAGTTTGTAAACATTTTTATTGATAAAAGAATACAGGTATACAGAAATAAATAGGTTCTTA
ATTTTAAAATGGACAAATCATTAAAAATATAGCTTACAATATTTGTATCAGATCTGATCATAGAGGCAGT
CTTGAAAAATACATTTTGCAATTTGTGGTCAAAATACTCTTTGGAATAATATACTCAGAGTTGAGCTATA
CTGTAGTGTAGAGAGTAGGGTGAAGAGGAGCACTGTCCAAAATAGTTTTCCAATAGGGTGTAGAAGCT
ACTATGCCATATGGTATTTTGTGCAATATTATGGCTCTCAGTATAAAATTTAATTCTAAATAATATGAAGCA
TTAGCACCTACAGAGCTGCAGCAATGAGATCTAACATTTAGTGAATACCTACTTGTAACATCAAAATCT
ATTAATCGCTTTCATAATCATAAAACAATACAACCTAGTGAATACATGAATTTTAATATCATACATATCCC
ATAATCAACATTCAATGACTAGCTTCTTCATATTTTGTTCTTGGTAAAATGCACCTTTCTTTAAAAAGTCT
ATCTAAAGCGAAGCAATATGTAGATAATTCCATAGCCTCGTAATAATCACAGTTATTTCAAGCTCCGACC
CAATATAACAATTATCCAACACTACATAACTCCAGCATGGTGTATAAAACTTTAACATCCTACTGTTAAT
ACATTATTGGAATGTCGGCAGTGTACATACCTAATCGTTGTATACTCGTGATTGCCTAAACAATCGAGCC
TAAGGCACTAGCCTGGCCAAACAAATGAACATCTACGCTACGGATGGGATGCTACATGGCGCGGGAAT
AAGCGTCTAGCCTGTCTAATTTTACAGCTACTACGCCAACAACACTGCGCAGTCTGCGCATTTGTCGTG
TTTAAAATATTACTAGTAAAGAGTATAGATTTATTGCGTCACTTTATATTAATTTAAAATACTTTATCGCCA
ATTTCACTATCATGGACTGGCGTTGTTTGGTAAAAAATAATATACCTATACTGAGTGTAAGCGTCTTTATA
CATTAACCATAACTCCAAAAATTAGACCCTCATGCCAAGGATTAGCTGTTTATTTATTTATACCATATTCA
TCATCATGCCTCAGGCTATGACACGAAATG
>TCONS_00065575 geneID=XLOC_013194 scaffold_22:483652-485354(-) Intergenic
GGAATAATTGCAAACATCCCGCATATCTATTTCGTTAGATAAAGTCATGTTCACAATTTCAGACCATTAA
ATACTATGACAAAGTCTTGAAATACATTTTCTCATCTACGCATTTACAGAATGGCCGGAGACAGTGCAG
CAGATGCCGCGGCAGAGGCCCAGCTGAAGGGTCTGGCGAAGCACTTCAACAGTGTGACCAACAGAG
GACGAGCAAATGTAGCCAAGGCAACATATGCATTCTTTGGAGTGGTCATCCTATACAATGTTCTGAAGC
CCAAATCCAAGAAGTAAAATATACCTTTAGTTAAGTTATAGCACCATATTTATCTGCAAATCCTTAATTTG
TAAAGTTACTTGATGATTTAGCAATATAATCACATCAGCATACATCTCTTTTATTTAATTAAATCAATAGT
GAGTACATGATTGAAATACAACAGCTTCAGTTTGTAAACATTTTTATTGATAAAAGAATACAGGTATACA
GAAATAAATAGGTTCTTAATTTTAAAATGGACAAATCATTAAAAATATAGCTTACAATATTTGTATCAGAT
CTGATCATAGAGGCAGTCTTGAAAAATACATTTTGCAATTTGTGGTCAAAATACTCTTTGGAATAATATA
CTCAGAGTTGAGCTATACTGTAGTGTAGAGAGTAGGGTGAAGAGGAGCACTGTCCAAAATAGTTTTCC
AATAGGGTGTAGAAGCTACTATGCCATATGGTATTTTGTGCAATATTATGGCTCTCAGTATAAAATTTAAT
TCTAAATAATATGAAGCATTAGCACCTACAGAGCTGCAGCAATGAGATCTAACATTTAGTGAATACCTA
CTTGTAACATCAAAATCTATTAATCGCTTTCATAATCATAAAACAATACAACCTAGTGAATACATGAATTT
TAATATCATACATATCCCATAATCAACATTCAATGACTAGCTTCTTCATATTTTGTTCTTGGTAAAATGCA
CCTTTCTTTAAAAAGTCTATCTAAAGCGAAGCAATATGTAGATAATTCCATAGCCTCGTAATAATCACAG
TTATTTCAAGCTCCGACCCAATATAACAATTATCCAACACTACATAACTCCAGCATGGTGTATAAAACTT
TAACATCCTACTGTTAATACATTATTGGAATGTCGGCAGTGTACATACCTAATCGTTGTATACTCGTGATT
GCCTAAACAATCGAGCCTAAGGCACTAGCCTGGCCAAACAAATGAACATCTACGCTACGGATGGGATG
CTACATGGCGCGGGAATAAGCGTCTAGCCTGTCTAATTTTACAGCTACTACGCCAACAACACTGCGCA
GTCTGCGCATTTGTCGTGTTTAAAATATTACTAGTAAAGAGTATAGATTTATTGCGTCACTTTATATTAAT
TTAAAATACTTTATCGCCAATTTCACTATCATGGACTGGCGTTG
>TCONS_00066237 geneID=XLOC_013197 scaffold_22:545429-546150(-) Intergenic
CGAAATTTCTAAAATAGTTCTATATATTTTTTCTTGTATTCCTCATTTGACCTCGTTCGGATAGCCATAAG

GATTTATGAGTATCGGCCATCATTCATAAAATGACATTTATGAACTAAAAAAATATCAGAATCTCGGAAA
ATCAGTCTCATTCATCTCAATGACAGAACTCGAAGTTTAAATAACCAGCCAAAATTGTTAATCTCTTTTT
AAGCGAGCCTTAAAAATTATGAAAGTGGTTTTAAAAGAAAACGGCCAAGTGCGAGTTTAACTCGTGC
AAGCAGTGATTATTAGTTTCGGAAAAAAGTGGCTGTGACATACGGATGGACAGACAGAAAAACTGAC
ATGACGAATTGGCAAAAACAAACCACTAAAAATAGAATTTCCCAAAAGATACTATCCGCGTATACATGG
AGGCATGTCACGGGAGCGGCGTTCGGTGTTCATTTATTATCTGTGGCTCGGGATCGTAAATGTAAAGTG
CCCTATATATGAATACAATTTAGATAACATTCGGTTTTTTATTTATTTTACCATCAACCATTGTTCCACATC
ATAGTTTAATCGATAAGGCAGGCGTATATGTATCCCAGTCTCCAAAAAGTTTAGTTACATGTCCAAATAA
AAC
>TCONS_00066211 geneID=XLOC_013163 scaffold_22:556466-557757(+) Intergenic
AAGAAAAGCTATTCCTTTGATCGTAACTTCTGTTCTCCACAATGTCTGCTTCTTTTTTGGACAATAATTC
CAGAAACGGTGTAACTGCATATAAAAGTTATAAAAAAGGTTATTCGGGTTAGTAAAACATGCTACGCCG
CTACGAAAGTGCTACGAGCTTATTACGTAGACAAGAAGCAACGCTACAAACTGGCTACGCAGCTACGA
TGCTGTACGAGGAAGGTATTATTAAATTATTGTAACAATTGACTAACGATGTACCCAAGTTATTTACTTTA
TGTAAACTTTGTATGGTTATTTAAC
>TCONS_00065433 geneID=XLOC_013202 scaffold_22:833592-836509(-) Exonic overlap (+)
CCGCGCGCGGCCCCTCACGCGCACACTCCGCGCGCCTCCGTCGCGACACGAACCGAATCGAAATTCG
AAAACTGACGGTTGGTGGACATGAAGTTTGAAAAGTGCTGACTGGAATAGTGCTCGGAATCGCATGTA
CGAATGCAGGCATGACATAAGATTTCTACGAGGAAATCAATTTATAGTCGTCTTAAGCTCCAATGGCAT
GAGGACAGTGTGGTCGGAGTAAGTAGAGTCAAGTGATGCGGCAGACGGGTCGCCATGCCGGACTGGC
GACACAACGTGCTGTCGACGAGCGAGGCGTACCTGCCGCCGGCGCGCGAG
>TCONS_00065434 geneID=XLOC_013202 scaffold_22:838330-856324(-) Intergenic
GTAGTGGATGTGCGTGAAAGTTTAGACGGGCTAACGAGCTAATGTTTACGTGCGTTTACTGAAGGTTT
AGGTACTGTGTTTACGTTGTAGTTACCGGTCTACGTGTAAAGTTTTACTGTTTTTTTTTAATAAAAACTG
TTTATGACTTGTCAATAAAACATTACAAATTGAATGTTTTATGGTCTACAGCTCAAAATAAAAATTGGTG
AAAGCGTGGTTCGTCGCTCGGAGGAGCCAGTAGCCTCGGCAGTGTCCACCAGTGTGTACGACCGCAG
TGCAGTGTTGTGAATGGGCCATGGAGTAATTAGTGATCAGTGCAGTGAGCTAGTGCCAACATGAGGAA
GGTGAAGTGTGAGGGTCCCGTGGTGATCCCGTACCCGTGCCGCGCTGAGGGTTGGATAGATCGATGTG
GTTAAATATGTGAAAAATAACAACATACTTATAAAAATCGAAAAGAAAGATGTTGTGATAGTCTGCAAA
GGG
>TCONS_00065964 geneID=XLOC_013202 scaffold_22:852239-856508(-) Unclassified
TAACGAAAGACATACATAGGTACCACAATGTCTATCACGATTCGCGATAGCGGTAGGTGGGCAGGTCA
ATACCGAAGAGAGCGAGATATCCGATATTCTTGTTCCGCTCGACTCACAGATTGGACTGAGTTGAGCA
ACATAGAATAGACGTGCTACTTCTATAGTTTTTTATAAGCTTCGGAGGGTAGTGGATGTGCGTGAAAGT
TTAGACGGGCTAACGAGCTAATGTTTACGTGCGTTTACTGAAGGTTTAGGTACTGTGTTTACGTTGTAG
TTACCGGTCTACGTGTAAAGTTTTACTGTTTTTTTTTAATAAAAACTGTTTATGACTTGTCAATAAAACA
TTACAAATTGAATGTTTTATGGTCTACAGCTCAAAATAAAAATTGGTGAAAGCGTGGTTCGTCGCTCGG
AGGAGCCAGTAGCCTCGGCAGTGTCCACCAGTGTGTACGACCGCAGTGCAGTGTTGTGAATGGGCCA
TGGAGTAATTAGTGATCAGTGCAGTGAGCTAGTGCCAACATGAGGAAGGTGAAGTGTGAGGGTCCCG
TGGTGATCCCGTACCCGTGCCGCGCTGAGGGTTGGATAGAT
>TCONS_00065337 geneID=XLOC_013179 scaffold_22:1238830-1240149(+) Exonic overlap (-)
GCATATTTTGTGATGATAACGAAAAAATATCACATTTTTAGGTAAAAAAAGAATTATTTGAGACTCTTAT
AAAATATAAATACAATTTGTAAATGTGTATAGAATGTATAAAATAATTTTGAATTGCATGAACTGCTTACA
TAAGTATCCAGTAAACATAGGTAGTAATTTTATGATATTTTACCGAAGTTGACAATCATTTGCCGGGATT

CGAACCTGAGACAGTTGAGTCATTACAATTTAAAATACACAAAGTAAAAATGGCAGTGATAAGCTGAA
AGTTTGAGTAGTTAAGTAAACGGGTATTTACTTTGGCATACATTTTACTTTTACAGCACCTTAATACTTA
GTTAATATTATTATTTTAAAATAGGTAACCCTGTATAATACATATCAGTTAACGTCTTGAATACTTATAAAA
AATAAGGTGATGGGTAGAATCGATTTCGCTAACTTTTCAACGGGTAAAAGATGAAAGATTATTTTTTCT
GATAGCACTAATGATTGAATATGAGTAAAACGTACAAAGTCAGTGTCAGAAATGACAAATAATAATGAT
TTCCCTAATAAACTTAAATTTTATTGTTGTAGGCATTTGTTTTCTTTTGCAAAGTTAGCTTTGTAGTCCTC
TAGTATTTTTTAACACAGCAGTCTCGAAAAAGAGGCTTCTAATAATCAAAGATCAAAATCAGCCAACCT
CCTGAAGCATACGCCTAAAAATATGCACAAAGTAATCAATATAGAATCGCTTACCAACCCGAGAACACT
GCTTTTGGTGAAAAACCATCCGTACTTAACTTTTTTCTTTGTCAACCTCCTTTTGTCTACGCTCAACTTT
TTCGGCCAGCTGTCAAAATCGGTCGGGACCTTTTTGCACTGATTAGGAAACCATAGTCTCGCGTCACC
CATAGAGTTTTTCGCTTGGATGCAAATGTCAAAATTGCCGCCTTTTTCCAGTGACGTTTGGAACTCCTT
GTCTATGGCGATGGTGATGGAACGCAGGTTGTACGCCACGTCGTTTGAGTAGAATAGTTTGTTTTTGTC
GTCTTTTATTGTTATGAAGGTGTCGGCTACGTCTTCTGTTGTTAAAACGAACCAGTGAACGTAGATTGA
GGTTTGGG
>TCONS_00065844 geneID=XLOC_013181 scaffold_22:1286428-1299283(+) Splice junction overlap
GTTTTCCGCCCGCGGCCCCCTCCTATCAACCGACCTAAACTAAACCAACAGTTCGCATCCGTGCCGTTT
GCGTGGTGGACGTTTGCTTTATGGAATGTATAATTTTATTGTGCATATCAAATCGTTTAAAATCCTTAAAA
TACCTTAATTTCCTTGTTTATCTCTTCGAGAAGTGAAGTTCCCGTGAATATCGTGAAGAAACAACCAGA
TTTCAAGTAAAAATCGAGTGGTTCGTGTGATGGGCGTGGCCTGAGCTCGCCATGATGAAGCGGACGGT
GGTGGCGCGCGCCGGCGCCGACGAGCTGGCGCAGATGGCGCAGGCGCAGCCCACGATACAACAGCA
AGGTGCAAGCAACATGCATACATTAGCCCAGACGGCACAACCACAGCTGCTAGG
>TCONS_00065845 geneID=XLOC_013181 scaffold_22:1291199-1299283(+) Splice junction overlap
GTCGCAACCTCGCCCACCTGACCGTGTTGATGCCGCGCTCGAAAAAGTTGCGCCGTTGCGCTATTTGG
AGGCGCACGACTGTTCGTGCGGAGCGTGTGCCGTGACGCGTTATTATCTCTATCAGTTGCCTAAAAATA
CTAAAGAAAGTAGTTTTTCAAAGCTAACCGAACTTATCAGTGTGGAAATCGACGCATATTCATAAAGGA
TTCATCGTGGTTCGTGTGATGGGCGTGGCCTGAGCTCGCCATGATGAAGCGGACGGTGGTGGCGCGCG
CCGGCGCCGACGAGCTGGCGCAGATGGCGCAGGCGCAGCCCACGATACAACAGCAAGGTGCAAGCA
ACATGCATACATTAGCCCAGACGGCACAACCACAGCTGCTAGG
>TCONS_00065354 geneID=XLOC_013181 scaffold_22:1330967-1332680(+) Unclassified
GCGGCGCGGCGCACTCGCATCATGCGAGGCATAAGAGGAAACCGACGGTCGCGGATTTGCATGATTG
CTGCTTGAGCAATCGGTTGAGGTTACTAGCGGCCCGCCCCCACCACCTGTACCGGCGCGGCTGTCTGA
AGGCTGCTCGAGACTCCCAAGAACGCTTACAAGCACGCCGCCGCCGCCGCTTCCACGCCTGGCTGCG
GCGACGGTCTACCGACACGTAGACATAACTTAGACTTGGGATGTGTCGCCAGATGAAGGATGCAGAAG
TGAAGCGGAAAGGGGCTTTGAAAAACGGCTTGTTTCGGGTTTAAATTTTCATTTTTTCGAGCGGTTAA
AAGTACCGGAAAAAGTGGTTTTGAAAAGTCGTTTCGGAGGATTTGATGGCGACATTGAAAAAGTGTA
ATGTTAGTTTTGATGGTCGCGCCTCGTGTGCGAGTCGTGTGTGAAAAGTGATGTTATAAGTTTGGATGG
CAGACGGTCTAAATGCTGTATTTTCATTGGTTGCTTAGAAAGCCTTTGGATTCAAACTCTTACAGCGGA
TTTCGGACGCGAATCGCTTAGGAGGTGCGGCCATAAGGCTCAATGTAGGTTGTAACCTATGTGTAAAG
GGGATATGGACTGCATTCGCTGACATTATTAAAAATATATTGGGCTGGGGCTTTGTGAATATAGTCTTATT
TCGGATAGCATCGGTAACATTAGTTTACTTGTGATTGATGTCACAGCCACTAAATAAGAAAGAGATTTG
TAAAGTTTGTCAAGAAGCTAAGACAGTATTGTAAATGTAATCAAAGCTTCAGCCCAATCGCCGTCGTTT
CTAAAAGTAGTTTATTTAATTTATTGTGCGTACACGCCCCGGCGTTAAGGCAACAGGGTGATGCATTTTA
ACGTTCTCGAATAGCAAACGACGGCTTTTGATTATGATATGAAAGATGAGTATTTTTCGGTGACATCAC
GTTTTAGTGGTGCACTTTTAACTGTAGTTGAAGATGATGATGATGATGAGTCCATGTCCCTGAAACGAG

GGACTAAAACGATCTCTTCTGTTTTAAAGTACAGTGTAATTATTTGTTGTACATATTAGTAAGGCGAGT
GCAAGTGTTTTTTGTAGTATAAAGAAGGAAATGTTGAAGAACCAATGTTTGTTAATTTGTAAAAAAAAT
GACTTGTCAAGAGGAATA
>TCONS_00065730 geneID=XLOC_013215 scaffold_22:1531756-1534261(-) Splice junction overlap
CCAAGCCCAACACTAGCAGTCGGATAGCGTCCTTCACATCCTTCCTAATCCAAATCCTAACCTCTTTAG
TCATCGAACCTACCACCTTAGTCTACACCTGGGTGCGAGCCCGCCTCTTCCCCCCTCCTCCCCCCAACC
CCCGTCGTCCCACCCCACCCCCCGCGACGTCTGCGCCCGGCCTTCGCTACAGACACAGAAGCAATGT
GCACAGATTAAATGGCGATGCTGATGATGATGAGAATAATACTTGGAATGGGAATTCTACGCAGCAGAT
GTAAGGTTTTTGGAGGATATAGTGGGAGATCGAGCTGTAAAAACTTAAAGGTGAGTATAGACTATAACT
ATAACATTTTGTCGAGCATTTCTGTGTAATTTAACATTCTTACGAATGCAGGCAAATGCATGATACAAAA
TTCGTGATACACTAGGACATATCAGAGATTAGCTCGTGGTTGTGTTACAAGTAAATGTTGTAGTCTATAC
TCACCTTAACCCAAATTTGGTGTAGCGCCATCTAGCGATACACGCATTGTACTAAAGTAGCGCCGTTAA
AAATTGCGTTTAGTTTTCACTGCTCGGAAGGTTTAGGAACTTCTTACGGCGCATGCGGGGTACTGTTTG
GTATACGGTGTGAAGAAGTTAGTTGGTGGATTGAAGTTTGTTGTTTAAACATTGTTTAGATCATGTACCT
AAGTAATTTATTGCCTCAATTTAATTGTATCCAACAGGCAAGAAACTTGAGTCGTGTAAATCGCGCTTA
ATGTTTCAAGTTCTTCGTTATAATTTGGCGTTTAGCAGCTGTAAATGTAGGTGAAACTTTATTTAAATTC
ACTTGCTTCAAAGTAAATTGCAGGCAAATACAAGCACAGAGAATGTTACCCTTCATAACAAAAATACG
CTCAAACACATCAAAATGGCACACCCTTAAAATAGTTAGTTTATTACAAACGCCAAAACGTCTGTTGA
GATTGGACTTAAATTTTACTGTGTTCTTATTTGGCTAGTAAACTTATTATTAACTGCTCTTCTGGACATAA
GCCTCTCCCAAGGAGCGCCAGAACACTCTATCCTCGGCCTTCCTCATCCAGCCACTACCGGCAACCTG
TCATAGGGTGCTTACATAGAG
>TCONS_00065378 geneID=XLOC_013187 scaffold_22:1689231-1706776(+) Splice junction overlap
AAATATCTTGACTCGATTTCAACTTTAACTCAGTCTCTAAAGTTACGCTGCGTTGACTAGCGCATCTTAT
TAGTAGTGGCGTGGTTTATATTTAGTTTAGTTTGATCTAAATGATCAGGATTAGCGAAGTCGAATGCCTA
AAGGCGTAAGCTAAGATACGACGTAAAGTTTGATTCAGTGGCTCTCGCGCAGTGTCCGTGTAAAGTTG
ACAGAAAGCTCGCACAAAGTTTTACAGTGAATCAAAAACATGTTTCTGAACTGATTTGTGTCCATTTG
TGGGAGGATTTTCAGAATTCTGTAACGAAGCGCGGATGAGCTGAAGCATGTCTCTGGCGGATGTGACG
TCAGCGCGCTCATCGCCCGGACCGGAAGCGTGTCCCGAGTGCGACACCAGCGCCTCCGTCACTTCCG
ACTTGCATAAGTACCAAGTAGCGTGCGTGTGCAAGCCGTCAGCTCAGTACGCATGCGCGGACGACCCC
GGCCN
>TCONS_00066459 geneID=XLOC_013318 scaffold_220:493351-500898(+) Splice junction overlap
GGTGGAATAAACATGTTCAAGAAACTTCTGCTTTTGTGCGTGGTGATGATGCTGATCGGCAGTAGCCTA
CAGCAGGGCGGAGGCGGCGGCGGCGGGGCTGGTGGTGGTGGGGAAGCTGGAGCCGGTTTTAGCGTA
GGGGGCGGCGCGAGCATGGGTGGGGGCGCCGGCGCCAACGGGGGCGGCAACGGAGGCGGCGGCGG
CGGCCGTAAATGAAGATGGACAATCGGCATACGCTTACTCATACTCCAACCCCAACAACCAGATGACC
TCCAAGTATGTTCCAGACTCCATGAACAACGGTCAAGATTATAAAAAGTATTTTCAAAATTAATTCATTT
TGTTATTGGTGCTTAATTGGTTGATTATGTCATATTAAATTTTGATTCACCAGC
>TCONS_00066477 geneID=XLOC_013349 scaffold_221:16318-48082(+) Exonic overlap (+)
TATTTGACACTGTTGACGTTGAAGTGGCGGACGACCGCTGCGGTCCATCTCACTCTTTTCAATTTCATT
TCATTCTGTTGGTTTCGTTTTTTTGACTTGCGTGTTGGGTTTTTGGTGGAGTTCCAGTTTCCAGTTTATA
TTTTCTTTTTGAAACGGCACTTTATTGAGGAAATCTGCAGCTGGAGAAGTGAAATAATTGGCCCAGAAT
AACTTCTGCCAGTCGCTAGACGAGTGTTTAGTGATCGACACAGAAAGTTACCATTACTCAGCTCAGCA
TCAACACAATGGATGCTCAATAAGATCTTCCCCACCAAAAGTCTGCATCAGAGAACGGGGCTCGGGCC
GCGGGCACGTACGATCCGCGGAGTTATCGGCACTTTCTCTCGCCGATGCGTTGCGCCTCCACTATTACT

CAACAGACAGTTGTGCAAAGCGAACCAGTCGGACCTGCCGTCTCGATGTTTCCTAACGGTAGTGCAG
CGTGTGTTTAAGGACAGTTGAGTGTGTAATGAACTAATGAACAGTTGATAGGTCAGTGGTGTTGCTGT
GAGTGAGTGTTATTGAAGATGCGACGAGGCAGCGGCGGCGGCGGTGGCGGCGGCTGCGGCTGGGGC
GGCGGCGTGCTGGGCCGCTGGTCGCTGCGCCGCCTGCTGCTCGACAGCCCCCTGGTGGGCAGGAGGC
TCGCACACACAGTGCTGGAGCAGGGCGAGGCGCGCGACCGGCAGGTGAAGCTGACGGCAGTGGAGG
CGCCGCGCCAGCACACCTCCGCCGAGTACTATGAGGACTACGAGGAGCCGGGCACGCTGGAGTGCCC
GCTCTGCTTGGCCCCGCTGCCGGCGTGCAGCCTC
>TCONS_00066729 geneID=XLOC_013364 scaffold_221:67169-69054(-) Intergenic
TTGTTTAATTTGTATAATACGATACCCATACCCTTTTTTCCTAAATTCCCGTTTTTCCTACAATTGCGTTCA
GACATCATTTATTTCAAAAATGATGAATGGATTTCAATAATAATGATATCAAATTAAAGCTGACATCTTAA
GCTTTTAAATGACATATCATTTTTATTATTTCTATTCATAATTTTACTTGTATTAATGTATGTTTGCGCCGTC
AAGGCTTGAGTCGATTTGGTTGCTCGCGAGTGTACATACATTATCAGGCTATCAGCGGTCTACCTATAG
CCTGGCTGTATGCAAAGCTTACTTATGAGACAATGTCCCAATCTCAACCAAAGCTTTGTTCATATAAATT
TCGATTAAAAGGGATGTTAGGTATATTTAAAACGGCTTGCTTGTATTTTAAAGCACGTATAGTTATAGAT
TGTTGCTGACTACTTATGTATAGCATATATTATTCTAATGTTAGCTCAATAATTTATGTGGATAGTACCTAC
ATACATACCTATGTTACAAACTAATTAATGCATAGGTTATGTCAATGTAAGTGTAGTATGTGTTTAAAACT
TTCTGCAAAGAACTTTGTAAAAATTATAGTGGTATATCTTCCAAATGAGCTGAAATATTATGTTAAAGTA
GTGTAGTTGTTATGTAACCTTAGTGCGAGTTTTCTGTCTTCGGTGTCACTGAAAACGACTGTTTATGGC
GCCGAAGTGTGGTTTAGCTTTAGGCACTCGCACCTAAAGTACCTTAAATACAAACAACAATACAGATG
GATAACTTGCGAATGATGAGAATCTCGCACTAACAGAAGAATAGGCCTCAATGTTAAATGATGAAACT
ACACTAGGTCCCATAATGTTAACATAGCTATGTAAGATGAATGTGATACAATATCCAAGCCAATATATTTT
TGTATAAATTGTAAATATGTATATTGTTATATTACTTTTTGAGGATTTGCTTATTTGAACATTACCTTCCTTT
TTTGCGAAAGTATGATTTGGAATAGGTTTAAAATATTGTAGATTATTGGAATCATTACTTTACACATTATG
ACTAAAATGGAAAAAATAGGACAGTTAATTTAATTATGCACAAATTCTAATTAAAAATTTGTTGATATTT
ACTTAATAATATTACACACACCCCTGTACTAACACCATTACGGGAAGGGGTAAGCGTTATGTAAATAAG
TTACTAAAAAATAGTACCTACCCACTAGACAATATAATCCTGTGTTCCCCATGAGATACACCTAGTATTA
CTATTTACTGCAACAACTATACATAGACGGATGTAAATTTATTTCAGAAGAAATTAGAATAAGAATCTAT
CTAGTTAGATACTTATGTAGGTACTGAAATTGAAAAAGAAATATGTAATGTCTATTGAAATAGATACTCA
AAAAATTGAGGACAAGGTAGGTTCTCTTAGATTTTGATAAATAAAATCATCGTCAACGTTTCATAAAGT
GCAGTATGTGTATGAAAAGTATTGTCTTTTTTGTTTTTATGTATCGTCCATTCCATGACGCCCTTAACCGG
CTTGAATTACTGTACCAAACGATTCGAGTCTTGTTCATTAAAAGTAAATACATATAATTTAAGGAATTTA
TTTCACTTCACAACTATAGCTACAATAAGTGTTAAGTTTAAAATTTTATGTCCTATTTGTAGAATAAAGAT
CTTGCATTTGCGAATTGTAATTATTCAATTTTATTGTTGAAATACGTGGTATTCATGGTGAATACTAAATA
AC
>TCONS_00066561 geneID=XLOC_013371 scaffold_221:149119-156401(-) Exonic overlap (-)
AAATAAAATACTAAGCAGGTACACTATATAACCACGTACAGTCAGCTTCATTAGTAGCTATATACTTACC
TACTTATACTTTTGTACCGTGTCAAACTAACAAACTTCCTATTTATTCCATTCAGTTTGTCAGTTTGACA
AGGATTACCTACTAGCGATCACAACACTGCATCTGTCACGGTATTCATAGTTTTTCAGTGCAAATGCTT
GATTACTGTCGCAGATTTTTGCTTTTTGCAAGTGTTGCGACGTCAAATCGCAGTAGTGTAGGTGCGTTT
AGAGTAACTTAAATAAACTATTGTGACGTATCTGCAGTCTACTTGGCTGCGGCCTTCTTCGCCTTGAAC
TCCTTGATGAAGGCGCGGAACTTGCCCAGCCCGGCCAGGTTCTCCTCCTCGTACCCCTCCACCGAGCT
CTTCAGGTGCTCGTACCAT
>TCONS_00067069 geneID=XLOC_013411 scaffold_222:104502-125938(-) Splice junction overlap
CTTAAACACGACGCCGTAGTAGCGCCAAGCCAATCTGTTTCAACTGTTTGGGCGGGAAACGTAACGTA

AACGTAAATAGTTCCGATTTTCAACGGCCAGAGTAAAAAACTTTTATTTTATTTATAAAAATAACTCGTG
ATTTATCGCCTCTTTTGAACTAATAATGAGTGTTCTGTTTTGTAAATTTACTAAGCGTTAAGGATGAATA
AGTGTGTTTAAAATAAAAGTGTCTGTACACAGTGAAACTTTTGTTATGTTTTCTATAATATTTGTGGAAG
TCTTTTGGATTTTTGTAACTGTTTCCATTTACTGAAATGGCCTCTACTGATGTCGCGGATAACCTTGACG
GAAAAAATGCAAATGGCAATAATGAAGAGGAGGGCACAGAGCTGGAGCTGGAGGCTCTATACAGAAG
ATACTGCGTCAGGTTAAAACACGCCCTATTCGTATCAGCACTATGTGTCACATTGGCGTGCAGCGTCTC
CCTCCTGTGTGCAGTCTGCGTGCTGAATCCACC
>TCONS_00066878 geneID=XLOC_013419 scaffold_222:498457-539059(-) Unclassified
TCCGGAGTTCAGCGTCCGATAAAACTAAGACGATAACGAAATGACTCCTCGATGAGTCTACAATCATCT
AAGAACATCTTATCAGCGGTACATCACGCCCAACAAACACAAAGTGGCTGTCTCCTACTTCAAACATC
GTCGTCGCAGTTCACCAAAAATCGATTTATTATATCTGCTCTTACGCCACAATGGGAATTCTTAGTAATT
TTTCCTACTTAACTAAAATAATGGAACAAAATAAAATTTGCAATAATGCATATCACTCGGTTTCATATATC
TGCTTGTCAAACATCTTGTTGTAACGTGATATTGTTTACTGCAGTTTTGTTTCCACCTCGTTCTGTAATTT
CCGTTGCGGCGAAGCAGAGGCGTCCACGTGTCTTACCTAGTTTTAATTATCGAATTTGATTCAAGAGGA
AAACGCAAAAATGCCTCCCTAGTCTTTAAGTTTATTGACTTTATGATGTTCTTTGATCTTTACAACATTT
TTCTGTTTTCCTTCTGGATCCTATAACTGACGGTATTTATGAATATTTTATTTATGTAAATTTATGATATTAT
ATACAATAAATGTTAAACTGTGTTGTCTCAAGTTTTGAATTACATTGACTAACATTTACTAAAATTTCTA
ACGCATGACTTCAAAATGTTTTACTGTATCAACTCGCGAGTGTGCATGACTATTATAAAGTATTTAAATA
GTTTTATTATTATGTTATTTGATAAATTGCCTCTCCACTGAATTCATAAACAATATAGCACCAGCATATTTC
AACACTTTCTCAGTGGCATTTTACTTTCGATGCTAGAAAAGACGAATATTCGTTAGTGTATTCGGTGGA
CGGACAATACAATACCATGACATGTTATTGAATAAGTCTGATTATTTTATATTTTAATTAATTGTTTTGGGT
TTAGTTTCGGTATAGAAATGTTTACTTCAACAATTTGTTATTGTTTAATTGCGATTATTAATGTAAATATTG
AATATATAATGTGAGAGAATCGTCGCCTTTGCTCTAAAAGCCAAGGTTTTAGCACCAAATAGTAAAATG
TTGAACTTGGTAGTGCATGGCAGTACCAGAGTTGCATTTAAGTTTTTTTCCAAGAAAAATTTGATTATG
CAATCAGATGATTTTTAATTTAATCGATTTATAATTTTAAACTATTTTATACATTACGAGTAATTTTAATAA
ACTATTTCGGCATGAA
>TCONS_00066822 geneID=XLOC_013419 scaffold_222:498458-578374(-) Unclassified
GTGCAGTAATGAAACTGCACTTCAATTTTCGATCGATCATATGTTTTCTTTGTGTAGTTTTTCACCTAGT
TTATGTGATGGGGTTTAAGTTTCACGTGTTTTAACAGTAATTAGTATAGCCTTGAATATTCGCATTCAGTC
CGGTTTAAACTAGAGGTTAAAGTAGTTTCCTAATAAGGTTTTAAAGAAAGTGCAAGTCGGTGCATATAG
TAAGTGCACGTGGTTCAAAATTTTCTAATAAAATTAGATGATTTCAAGGTTTTAAACGGCCTAGGGGGT
TAATTGAATAAAAATCAACTCTCACTCTATTGTTAACCGAGATTTCAAGTGAAAATATTGTATAGTACTT
GTAAAAACGTGCTTAGACTAGAGTGTGAAAGATTTGATCGACGTGTGACTTTGGTTAGTTTTGTGGTG
GAAATCTCTGCAGCCGCGTGGAGTTTCGATTACAGCCTCCTCGGATGAAGTGCTAGTGATTTTACATTA
CCTACAAAATGTCTAAGTGCAAGTGCAATCAAATGGCGATAAAATTTTATAGTGCTCAACGAAATAATA
TGAAGTGTGAAAATAAGAAAATGACGCAAAATAACGTAAATAAATATTATTGTGAGTGTCGATGGAGA
ATGTGAAGTAGTGTACCTTGGATTGCAGTGTTAACTCGCAAGATGGCGCTGTTGGAACGATGACGTAG
TTCCGCTATCCGCCGCTAGATGCCTCTGATCCGGAGTTCAGCGTCCGATAAAACTAAGACGATAACGAA
ATGACTCCTCGATGAGTCTACAATCATCTAAGAACATCTTATCAGCGGTACATCACGCCCAACAAACAC
AAAGTGGCTGTCTCCTACTTCAAACATCGTCGTCGCAGTTCACCAAAAATCGATTTATTATATCTGCTCT
TACGCCACAATGGGAATTCTTAGTAATTTTTCCTACTTAACTAAAATAATGGAACAAAATAAAATTTGCA
ATAATGCATATCACTCGGTTTCATATATCTGCTTGTCAAACATCTTGTTGTAACGTGATATTGTTTACTGC
AGTTTTGTTTCCACCTCGTTCTGTAATTTCCGTTGCGGCGAAGCAGAGGCGTCCACGTGTCTTACCTAG
TTTTAATTATCGAATTTGATTCAAGAGGAAAACGCAAAAATGCCTCCCTAGTCTTTAAGTTTATTGACTT

TATGATGTTCTTTGATCTTTACAACATTTTTCTGTTTTCCTTCTGGATCCTATAACTGACGGTATTTATGAA
TATTTTATTTATGTAAATTTATGATATTATATACAATAAATGTTAAACTGTGTTGTCTCAAGTTTTGAATTA
CATTGACTAACATTTACTAAAATTTCTAACGCATGACTTCAAAATGTTTTACTGTATCAACTCGCGAGTG
TGCATGACTATTATAAAGTATTTAAATAGTTTTATTATTATGTTATTTGATAAATTGCCTCTCCACTGAATT
CATAAACAATATAGCACCAGCATATTTCAACACTTTCTCAGTGGCATTTTACTTTCGATGCTAGAAAAGA
CGAATATTCGTTAGTGTATTCGGTGGACGGACAATACAATACCATGACATGTTATTGAATAAGTCTGATT
ATTTTATATTTTAATTAATTGTTTTGGGTTTAGTTTCGGTATAGAAATGTTTACTTCAACAATTTGTTATTG
TTTAATTGCGATTATTAATGTAAATATTGAATATATAATGTGAGAGAATCGTCGCCTTTGCTCTAAAAGCC
AAGGTTTTAGCACCAAATAGTAAAATGTTGAACTTGGTAGTGCATGGCAGTACCAGAGTTGCATTTAA
GTTTTTTTCCAAGAAAAATTTGATTATGCAATCAGATGATTTTTAATTTAATCGATTTATAATTTTAAACTA
TTTTATACATTACGAGTAATTTTAATAAACTATTTCGGCATGA
>TCONS_00066961 geneID=XLOC_013419 scaffold_222:498462-576038(-) Intergenic
CAATTTGTCCCTCTGTATGTACAAGATCCGGAGTTCAGCGTCCGATAAAACTAAGACGATAACGAAATG
ACTCCTCGATGAGTCTACAATCATCTAAGAACATCTTATCAGCGGTACATCACGCCCAACAAACACAAA
GTGGCTGTCTCCTACTTCAAACATCGTCGTCGCAGTTCACCAAAAATCGATTTATTATATCTGCTCTTAC
GCCACAATGGGAATTCTTAGTAATTTTTCCTACTTAACTAAAATAATGGAACAAAATAAAATTTGCAATA
ATGCATATCACTCGGTTTCATATATCTGCTTGTCAAACATCTTGTTGTAACGTGATATTGTTTACTGCAGT
TTTGTTTCCACCTCGTTCTGTAATTTCCGTTGCGGCGAAGCAGAGGCGTCCACGTGTCTTACCTAGTTT
TAATTATCGAATTTGATTCAAGAGGAAAACGCAAAAATGCCTCCCTAGTCTTTAAGTTTATTGACTTTAT
GATGTTCTTTGATCTTTACAACATTTTTCTGTTTTCCTTCTGGATCCTATAACTGACGGTATTTATGAATAT
TTTATTTATGTAAATTTATGATATTATATACAATAAATGTTAAACTGTGTTGTCTCAAGTTTTGAATTACAT
TGACTAACATTTACTAAAATTTCTAACGCATGACTTCAAAATGTTTTACTGTATCAACTCGCGAGTGTGC
ATGACTATTATAAAGTATTTAAATAGTTTTATTATTATGTTATTTGATAAATTGCCTCTCCACTGAATTCAT
AAACAATATAGCACCAGCATATTTCAACACTTTCTCAGTGGCATTTTACTTTCGATGCTAGAAAAGACG
AATATTCGTTAGTGTATTCGGTGGACGGACAATACAATACCATGACATGTTATTGAATAAGTCTGATTATT
TTATATTTTAATTAATTGTTTTGGGTTTAGTTTCGGTATAGAAATGTTTACTTCAACAATTTGTTATTGTTT
AATTGCGATTATTAATGTAAATATTGAATATATAATGTGAGAGAATCGTCGCCTTTGCTCTAAAAGCCAA
GGTTTTAGCACCAAATAGTAAAATGTTGAACTTGGTAGTGCATGGCAGTACCAGAGTTGCATTTAAGTT
TTTTTCCAAGAAAAATTTGATTATGCAATCAGATGATTTTTAATTTAATCGATTTATAATTTTAAACTATTT
TATACATTACGAGTAATTTTAATAAACTATTTCGGC
>TCONS_00067047 geneID=XLOC_013419 scaffold_222:576557-578340(-) Intergenic
GATCATATGTTTTCTTTGTGTAGTTTTTCACCTAGTTTATGTGATGGGGTTTAAGTTTCACGTGTTTTAAC
AATGATTTCAAGGTTTTAAACGGCCTAGGGGGTTAATTGAATAAAAATCAACTCTCACTCTATTGTTAA
CCGAGATTTCAAGTGAAAATATTGTATAGTACTTGTAAAAACGTGCTTAGACTAGAGTGTGAAAGATTT
GATCGACGTGTGACTTTGGTTAGTTTTGTGGTGGAAATCTCTGCAGCCGCGTGGAGTTTCGATTACAGC
CTCCTCGGATGAAGTGCTAGTGATTTTACATTACCTACAAAATGTCTAAGTGCAAGTGCAATCAAATGG
CGATAAAATTTTATAGTGCTCAACGAAATAATATGAAGTGTGAAAATAAGAAAATGACGCAAAATAACG
TAAATAAATATTATTGTGAGTGTCGATGG
>TCONS_00066897 geneID=XLOC_013409 scaffold_222:615645-616329(+) Splice junction overlap
CTCAGTGCTGCTTGTATTTGTTGCTAAAAATAATCAAAGCCCGTACCGTCGTCACAAGCCTCATCGGAA
GCATCCTCTCACTAGCGCGGGGGCTGACGGACGCGGCGTGCACCCGCGCGCTGGACGCCGCGCTGCG
GGGACTGCTGGGCGAGCGCACGCTGGCGCACCTCATACGACTGGGCCACGGTGAGTCAAACTCTCAA
ACTCAATTTTTTTTATTCATCGTAAAAATATAAAATTTTCTGATGAACGTCAATTTTTACAAACTACTCTC
CGTTCGGATAAGGG

>TCONS_00066938 geneID=XLOC_013410 scaffold_222:646740-647063(+) Intergenic
CACCACTATGAAAATCTAAGGAGTATTCCCGTGGAAGAACAGTTCCAAACCCCAAGAGGACCAGCTC
AAGAGCTCTGGGGTGACGTGGCGTATATTCGGCAGCAAGTCGAGCGGGAACCTGTCGAAGGCGCTCG
GCTCTGAGGGGCCCAACTCCCTGAGCCCGACGTCCTCGGCCGCCAACACCCCGCATCACCACAAGCG
GCAGGGATC
>TCONS_00067144 geneID=XLOC_013470 scaffold_223:178922-179605(-) Splice junction overlap
CGCCGTGTAGGAAGGTTCTTATATTCGAAGCTAAGAAATTCAATCACCTAATAAGACTCATCAAAGAAG
AAGTGGACAGTCAGCAGGCGTAATCTATAAGAATAATATACATCATTTCAATCGATTATCAACCCTTTTG
TATACTCGCTTCAGTCGCTAGCTCCCATGTATAACAAAGGCAGCAAATATGCAAAAAATATTACTGTGT
GTTTATTGTAATAACACTGGGGTGTAGAAATACAGCAAAACAAAAATGAGAATCAATGGGCACTGTAT
GTACGCCCAAAAGGCTGATAATCGAAATATTGCTGCTTATTCGCAAAAGTCACGTGATATAATCTTGTGA
TGTGTATGCCATTTGTATCGTATGATAAAAACTATTTAACTATAATATTAAAA
>TCONS_00067251 geneID=XLOC_013480 scaffold_223:439440-441788(-) Intronic
ACTAAAAAAATTGCTAGCATAAAATGACATTTACGAAAACAATCATAGAGTCGAATTTTAAACAAAGA
AATTAAGGTTTTGACGTCTCTAAATTTAAAATTTCCGCCTTCAGAATCGATATCATTGTCAACAGTTGCC
TGCGCCGCGCTGACATCATAAAGGTGTTTGATGGCAGAGACACCAACGCACCCACCATCGCGATGCTA
TGCAACGAGCTTACAGGCTACGAGGTGCTATCAACA
>TCONS_00067296 geneID=XLOC_013512 scaffold_224:14354-15783(+) Unclassified
AGGAAGTGATTATAGTGGAGTGGGTGCCGTAAAAGATGATGCCAGATTACACGAGCTCGTAGTATCTA
CCAGTGTCAATATCATCAAGTTGTAAAACGTACGTAGGTACAATAGTCGTCATAGTTAGGATCTCACTC
GCAGTTAACAATCTCGGGACAGCTCATTCACACTTGCCAGTAACGGTGGTATGCGCGGTGGGTGTGGT
CCACTGAGGTGACTCTGACTCGCGGACACACTGCGCCATGGCTTTGACACATAAGGACTCCAGTAGG
AAGCGCAAGATGCAGTCAGACTCGGTGTGGGCGGCGCTGAGCGCGGAGC
>TCONS_00067475 geneID=XLOC_013516 scaffold_224:176110-182800(+) Intergenic
TCGGCAGTGCGCCGCTCGCGTCCGCGCCGCGAACGCATCCGAACGTTTTAAACGCGCGAAATATACGT
TTTCAAATACGAAAGCGTGACATTTTCTTGACAGAGCGCTCTTATCGATAAATCTGAATTAACGTGACA
GTTAAGTTTGGCTGGTGAAATGAACATTTTTGTGCAAAATGCTCAAGTATCATCGCCGTTCACACAGGC
ACAAGTCGCCAGTGCAGTGTTTAAGATGGAGATCATCGGTGGACTCGGACGCGGCGAGCACGACCGC
GGTGGAGGCTACTCTCGAGCGGCTGCGCGGCACTAGGGGCGCGCTGGAGGAGCTGTGGGCGGGCCG
GGAGCGTGTGTGTGTGTGTGTGAGAGAGAGAGACAGAGAGAGA
>TCONS_00067314 geneID=XLOC_013516 scaffold_224:258210-264072(+) Intronic
ATTTGTTCAAAACTGTAATTGTTGAAAATCCTTTTACAAATCAGCTGTCATTAGATACGCACTAAGTTTA
ATTAATTGGGTAGTAAACAATATGTGATTAAAATGATTGTCCAGTTTAGGCTATATTTGAAATCGGAATT
GATAAATGTATGTTTTAATGCTTGTATAAGGTAGCATCGTAGGATATTATATATTAACCTGTGTACTTTATT
GACTTGTACATTTCCAATGTGCTGATTAATGACTGTTAAATGAAGCAGCTATGTGACAAAGTGATCGCA
CATGATTTAAAGCCAATACATCTCAAATTACTAACGCAATTGCCCAAATTCACAAACATATCGTAGAAA
AATTATCTTACACTATAGTCTACCACCCAGGGTCTGTAATAAATTAACCATTTGCTAATTAAATAACTTAG
ATATGACTGTAATAACTAATAAACAATTAATAGAACATACGATCTGAGCTACGTGTACTGTCAACATGTT
TTAAAAGAAACAAAATATTGTTTTTTTCGCAGATGCAATAATTTAATTAATTTTAAAATTGAAATGTGTG
GTACAAACCTGCTGAAACACCTTGCGGTGACCTTGATAGCGGCTGAAGTTAAGAAAATTATATCTAAA
AGGTGTACGAATGCTTATGAAAATAGGAAGCGAACACAACTGAGATGCTGCTGTCAATGCCACACCCT
TTACCATAAGTTTTCATTAGATTTTAAATGAAGACATGTCATTCCAAATACTTACATTTAAAGGTATATTT
ATTTGTAATTTTACGTATGTATGATGTAGGTTTTATTCCGTGTCATGAGACTAATTTCAAGGTTTATAATTA
TGGTCCAACCACCTATTGTTGGTGGATTATGGTTGTTATAGTGGTCTGTTGCATGCACTAATCACTAATC

TTGTCTATATAATATTTTGAACTACCATTGCTTCCATGGCCAAGGACCATTTCACAATCTAATTAAGTCAA
AACGCAAAACTAGCAGCAAGAATGTCTAAATAACTCTCTATCAATCAACTCAGTTTATCATACCTTTAG
CATGCAATATTTTATTAGTATTAAGGATTACATACATTCATACTTGGTAGTTTTAAATATGAATCGTGTTAA
AAGACTGCAAGGATAGTAATTAACGGTATGTTGCGAGTGATGGACTAGATATAACGGATCGGTTAAGGT
AGGCTACCCTATATTTATTCCATAACATTAATCCTATATGATATGTAGCGCCATTTCACGTCACTTTATTAT
AGACGAGCACATGGCAAAGTAACATTTGCAATAAAATTATTAGTGAGATTTTTGAAACCAATTACATTT
AAAATAAACATTATTCTTAATGGTATGTAGGATCTTAAATACCTACCACTACTTAATGCTTTAAAATTGTA
AACTCAAACCTCTTACTGGTATACTGTTCAGTAATGAATTAATGATACTTACTAAAATAAGCCACAGGC
GGAAGAGACGGCTCATGAAAGGCACTTCGCATGTTTCTCTGCGAATATGATTTAAAACTCTTAATTTTC
GGAGATATTTTATCTTCTCTGTACAGTACGTGAAGGCAATAGTTTTCTACTTAGGTATGGTTTTTACATTA
CATCAGCTGTATAATGTTCATGATGGCATCCTTTCAATGCGTTCATAAAAACATCATACTAGACGTACCT
ACAGACCGTGTTTAGAAGTAAGATTTTATTATGGACTACGCTGAAATGAGTTGGCTTTGGCTATATATAT
TTTTTTATCTGGGGCATTTATAAAACTAAGTTTTGTTTTAATATTGGGGCCAAGTTTTTACTTTTAGCAAT
TTGCAGGTGGCTGCGTCCGCGCCACTGCCTCTACTATTTATTCAACGCAATCACTCCGTTATATAATTATA
TTCTTTATACGCAACTGTAGGCTAGGCTCAGTTTTGTACTCACTCATGAAAATATACTTATACTTAAAGA
GTTTGATGGGTTGTCTGTAAAATGTAGCGATCTAACCCCTTATGGAACTCTAACCACTAAAAGTTTTGT
AATGGTGTATAAATCATTAATAAGTGAACCACTGAAAAGTAATTATTTATCAATGTAATGCTTGTAACTC
AGTTGGTAATTCGTCTATGCTTATTAAGTTTATTTTTATAAAATTTGTAAGGAAAAGTTGATAAAATTTGT
GGATCATCGACCAGCGTAATAACGATAGAATGCCGTCGTTTATATATTATTATAATGTATTACAGTTAAGA
GTAACATTTAAATTGTCATGTAATCAAGGTGAAAGTGTAACTTTGTCCTCAAAACAATATTTAGAACAT
CTTTTAGGGCTTATCAATTTAATCTAGTCAAACTATTCTTATACGTGTTGTAGAGAAATCTTGCCTCCTAT
GTCGAAATATACCTACCTAACATAAATATGTTTAAATTTGATATTATAATTAACTCTAAATCAGATCTATGA
TCAGGAAGGTTAAGTTAAGGGCAATGCACTTTTTAACTGTCACTATGCATCTTAGCTATTACCTTAATGC
TAACTAATGTTATATGTACATTTAGAGCTGGTGTCTGTATATACATCTCTTACAACAAATATCAATGTAAC
GTTACAGGAGTAACTAATAAATGTGAATAAAAAGGCTTGTGGTTTTCTGCTTTCAGTCCCGCATGCTTT
ATATATTTTTTTGTTTTCTGTTATTTATAATTTGGGAGCCAGCTTGTAGCCTCACCGCAGTGCGGGCCTGC
AGTAGCCGGGCAGCACCCGTGTA
>TCONS_00067420 geneID=XLOC_013527 scaffold_224:264865-284901(-) Intergenic
TTATATATAATGGTTTAATATCATAATTCCATTAGATACCTACATAGTGGTGAGAGTGGTACATTTCTAAAC
GGTGACTACTCCAATACATCCTGTGTTTGTGATGGCTGTAAAATGTTTCAGGGGTCCAATTTTTTACAA
CTAGAGTGCTGGTTCTACACATTTGAATCTTGAATGACACATACATTCTTTACTTTACTTTTTAATATTGA
AATGAATTACAATAATTAAAAGAGGCTCGTTTTAAATAATTAAGATACTTCCATATCCAAACAGCCATAG
GTTACTTCAACATTGCATAGCACGCGTTATGAATCAAACTATAAAGTGTAAATTGGCCCCTGTTCTGTCT
TGGACAGTTCATTAGACCGGGACTTGACAATGCGTGAGTCCTGAACTGCATACAATATGAGGCAAGAA
TGCTGTGTTTTTAAATTATACATTGTTCATAATACCTACCTCTAGTAAAAGCCAGCTTTACACATATTTTA
AAACATAATGCTCTTAATTATTTCATTCATTGTGTTGGTTCCTCGTAGAGTTATACATTCTGACTAAATTA
ATATCTAAACTATACAAGTCTTTATAATATTATTTTTAGTAACATTGTGTAAGGCAGTCGTTATGAAACTT
ATACCTACAATATAATCCGAAATTGATAAGATATAATCGTGTAGCCAAGGGCAGCGTGCGGCACACTGA
ATCATTATTGCAGCTACCGCTAACACTATACGCGTTATACTTACACATTATTGTAATTTGATCACAAAATA
GAACAAAATTACAAGCTAAAACTTACTACTATATACAATAAAATAAAAATAGATTTAAACAAGTCATAC
GTTAAATTAGGAATAAGCATGAAATTATCGTAATACAATTATTTAGCATAAATCACAGAGACTTCCCCTA
CATCCACTCGTAACTTTATATAGATTTATAATCACAAGTTATGTAGGTAAGTAAGTTACTAAAGCAAGCG
GGGGTCAGTGACCCCGAGCAGCGTCGCTGCCGTGGCTACAGTAGGGGACTGAGCAGAACTTCTAATT
TCATATTTACTATCTTAAAATTTTGGTTTGATTACAGATAAATACCTTAATTTATTTTTAATAATGTAAAGT

TCCGCAATCCGCATACATGCTCTGAAAATTATCTATAGGTATAGAAAAACCGAAATGACTTTGAATGGG
CACTAATTTTGATCGCTATCTCATCAATACTTTAAAGCAAATTCATTTAAAACTCATTTCGTAGCATGACT
AGCATTCCTCTAATGCTCACAACACGATTTCTACATTTTGATACGAGTCCAGAATATTTTACATTTATTTA
TTTATAAAGCAAGAATTCAATACAATCTTACCATAATCATTCAGAACAATTTCAAAGGTGGGAAATATG
ACTACAGAAATATTACGCTAAACATTTGGATCCTTTTGAGTACATAACAAAGAATATTACAAGATCTAAA
AATACTGCCGTTCGCCTGCAAAATGTAGCTAAACCCAACATTTAAAAACATGTTAAGCCATCTTGGTCG
CTTTGATAAGCGTGTCATTCTTGTGATGTGCTACAGTGGAAGTTTTGAGAATTGATAATGCGCTGGAAC
CTTGTCCAAATGAAGTCATAAGTAAGGTCGATACGAGTAGGCACTCGTTGGTCACTTTTACGTTAGAAC
GAAGAAAG
>TCONS_00067492 geneID=XLOC_013528 scaffold_224:354271-375959(-) Splice junction overlap
CACGACGCGCCTCCTTCCTATACAACATTTCTAGTAGTTCCCGACCGCGGCTCCTCACGGAAAACCTAT
GAATCCGTACGGCTTATTATGAAACACTGTCATCTCTAACCTATACAAATATTTTAAAAACTCAAAAATG
GTTATCAAGTAGCTTTTATCAGTGTTAATTCTTAGATTTTTATAGTAAATAGTGATGAGCGGCACTGTTCT
TGTGGAAGCGACGAGTTCGTCCGGCCTAGATGAGGAAGAAGACTTCTATACATTCAAGGAAGAGCCG
TGTTCACCGTCGTCTGGTGGTTCCCCGGAGCAGAGCTACCAGGCGGCGGATAGCGCCAGCGGGGCGG
CGGGCAAGGCGGCGTCGCCGTGCCGAGTGTGCGGCGACAAGGCCTCCGGGTACCACTACGGAG
>TCONS_00067493 geneID=XLOC_013529 scaffold_224:354855-367010(-) Exonic overlap (+)
CCCCCCGCCTCCTACCCCACACTGACCACTGAAGCACGAGACTTCGACTTGTCCTTCTTCGACGACGG
CAAAGACAAACCGAGGCAGCCGCCACCGCTGCCGCCTCCGCCGAAACAAGATCTCTGCAATGTGGGC
GGAAATACAATCTGAAGTTCATTAATAAACTTGGACGTAGCATGCACCCTCCTCCCAAAACTATAGAAC
AAAGCAATATCCGGCCAGCCTGCACATGGATAGGCAAATTTTACGCACAATATATCTACCTAAATTGTTT
CCTATTTATTTACTTTCAGTCATTGAATAAATTATTTTGTAATAGGTACTTAACTAACATCCTACTTTTATT
GTACATATTGATTATTTCTGAAAAGGTTTCTACAGTGATTTCATACTCAGTAGTACATTCAGCGATTACA
GC
>TCONS_00067463 geneID=XLOC_013523 scaffold_224:480907-488334(+) Unclassified
CTTCTTTGTGAAAAATGTCTATGTAAATAAAACGTAACTATGACTATTATGGCCCTGTGTAATAAAATATA
GGTTTCAGTGATATTTTGTACTTAAAAATCTCAGTTTTTACTTTTTGAATCTCAGTTTCTTGTTTAGTATT
ATTGGTGTAGTATTATTATAGGTTACATAGTATTTATATTGAGTGCAGGATGCACCCCAGTGGACGTCGTC
GTTAGATAATAATGAGACAAGTGGCTTCAACAAGAGTTCATCAAATAGGATTAAGGAATATAGGTTTTT
GCGCACTTTTATGCAAACGTTATGATAATATGCTGCCATTTTACACAGTTAAAAACTCGGAAAATAACTT
TATATTTAGTATTATATTTTAGCAGTTAGCTTAGTCATAAGCAATTTTTTGTGATTACTAAACCTGCCTGAA
TGATTCTAAGTTATTTATTACGTTCATAATTATTTATGCAAAATAAGATAGCAAGTCTAAATTGGGGGAAA
TAAAGATATACTAAAATTCTAATGTATAATTATGACGATGAAAATGGAGACATATGAACCACGATATCTAT
GCATAAGTAACGTTAATTATTGTCACATAAATATTTAAATATGTTGCATTCAAATTTAAAAATTCTGTGTA
CGGTTCCAAATGACGTACATGGGATAGTGCGCTGTATGGCTGAACAAAAATGTGACCTCAACATTAATG
CATAAAGTACCTAGTTTATTTCCATAAAATTGTTGATTTGTACAGGAGCGGTTTTGTAGGTCATTAGTTAT
TTAAGCGCTTTGCTTAAATGTATGCTCTTAATCCATCCAGTTTTTTTTTAACAAAGTACGTTTGTGTTTTG
GCTGCAAATATAAATAGTAGCAAAATATTTTCCATGAATGCATATTTATCGTGGTCCGATTCTATGGATTG
GAAATTCCACGTACAAACATAAAATCAAGTAAGGAATAATAATAAAATTATGAACAACTAAAAGTATTA
ATAATAAAAGATGGTTTCAGCTAAAGACGTGCTAATGTGCCTATGCGTGCAAAAATACCTCAAAATTGA
AAACAAACAGTTCATGTTGGTTTCTAATAGTAATAATGGAATATTTAGGACATAATTATACACCGATACC
TACATGAAATGCAGTCGATATAATTATGTTTAACGTAATGAGAAGTTTTCTGCAGGTCTAGTACGGTTAA
TGATAGCTTATGCTATTTTACGTTTTATTATTTAAAAAAACCCAGTAATCTATCATAATAACATAAAATCC
GGAAAATGCATGCAGATATTGCACATGGCAGAAGAAAATAGTTTTTTTTTGTTTAAAACTATCAAACTA

TAAAACCGGGACTTAAGCGTGCTGATAGATATTCATAGCGCCTTTGAGTTTTAACATTATCTTTTTGAAT
ACAGTATCGTTATCAACAGTATAATACTCGTATTATGTATGTTAAGTATCCTTCTTTTATATACTCTGACAG
AAAACTTCTCATCTTTTTTTAATGTCGGTAGTTGCTTGGTATAACATTGAAACATTTGTAAAATCAATAA
AATTAATCAGTTATTTAAAGTGAAAAAGAAATATTATAATTATATAATTGTTGTAACGTTTATAACATTTA
AGAATAGTAACGGCTATTGATTTCAAGAACAAAACTTAACATTTATTATACTTAAGGTGCACTGAAAGC
ATTCGTGTGTAATGTTAGAACTCAGATTTCGAACACAATATGAACATGACTATTCAGGATAATTATGAAT
AGTCATATTTAACGCAAAAAAACGCACGAAAAAATCCACTGCTGGATATGAGCCTCCTCGAAGACTGA
AGGGTTATTGCCATAATCGCTGGAAAGGCGGGTTGCAGATACGTTCTTTTGAAGATATAGATATTAATTA
TTCTGATGGATGTGTAAAATGAGTCCAGTGTACCTATCGCGAACCTTGACAATGATTTTTGGATTCAAA
TGGCCACTGATTACTTCGTAAGTGTTACATATTTCGTTTTGACTTCGCGATAGGGCCGCTGGTCATTGTG
GCACTGCAAACGAATGCTATCAGTGATGAACACCTTTAAACAACGGGTAAAGATTTGTACCTTTTTTAC
ACTAATTTACATTCCATGTTTAGATTTACATATCTGTTTTTGGACTAAATCACATTTAAAACATATTTTTAT
TATTATTTATTTTTGGTTCGGAAATAGTAAGGGCTTATTTTATAAATTTATAAGTAGATAAGACAAAAGCT
CAAGGTAACTAAAGAGTAAACATCCATATTGCAGCAAGAGAACTGGATTTATTAATTTAATAGCCAAGT
CAAGAGTGCCAAATGTTACAGATTTTCAACATGTGCGGGCAACTCTTTTAAGTATACATATTTATTAAAA
TAAAGGAAACAATAATCATGATTCTAAATAGTATTATAGGTAAATATTATGATAAATTGGTATTCTAAATG
TGCAGGTAATTTTTAAGCTTATTAAATATTGTCAGAGGGAATATAAATAAATAAAATATATCAAGAAACT
TCTGTAACGAGAACCTATCAACAAATGAGTACTTAATTTCTTTCAGATCCCAATTTTTTTGTGTCTCTGT
GCTGTTATAATACTTATTATAGTATCCTACCGTCAGTCATCCCTACTTCGAAGTTTATTCAAATTGACGAA
AGAACTGTTAATAAAATCAGAACATGATTATAATTCCTCCAGGAAATTAATATACCCTGTATTGTATCATG
TTATTATTCTTGCCGTAATAATAGTTTATGCCGTAACAGAATACCTGCCTAGGTTGACTAAGTACTTACAT
GTTTTGGGAAACTGCCACTTAGCTTCTGCACAAAAAAATAATGTCCACTGTTCTAGTTTTAATATTGTAC
AATTATTTAAAGTTTTAATATAGGTTTCATAAATAACAGTCACTTCTTTAAATCCTGTAATCCGTTCAGCG
AGGTAGGCAGAGTTATATTTCAGCCCAACTTTAACCCTGTTTACAAGTTACTAAAATATTTTTAAGCCTT
GATTTTTCGGTTTTGTTTTGTGCCATTGCTAGGTGCCATAGGTCGATGGCTGTGCCATCATTGTAATAAA
ACTAATAAATGTAATGTTGTCTTAATGTCATTCCTTTGAACTTACTTGAGTAGCTAAGCTATTTCTATGTT
TATAAGATGTATTTTACTTTACACAACACTTTGTTCGTATATGTTACGCACATCTTGTCTCATGAAATCGT
GTACTTATCTAATGTCAATAAATCGTACCGAA
>TCONS_00067825 geneID=XLOC_013568 scaffold_225:78995-84640(-) Intronic
CTCACTGGCCTCGCCGCTACGCCGCCGCGCAAACCCGTTACTTTCAAATTATCCACGAACGACTGCTGT
ACTTCAGTGTAGAGATTTACTACATTATTATAAATACAGGATATCCAAAAAAGTTTTAGTTTCAGTGTGT
TTTTGTAAGTGCTATTTCCAGCTGAAAAGCGTGGATTTTTTTCGGTGAACTACCTACTGAAACTTAGCG
TGCGGGGGGCGGCGCTGCTGGTGCGGGGATGGTGGTGCGGCCCAAGCGCTAGCCATGGGGCTGACCA
CGCGGGGGGCCGCGCNNNNNNNNNNNNNNNNNNNNNNNNNNNNNNNNNNNNNNNNNNNNNNNNNN
NNNNNCACGCCGCGGCGCAAGGTGAGCCTCCACCACGCAATGGCACGGGGATGCGGACCACTTCTCA
AAACCTATTCATAGCCTCTATTGTCAAGTGCATGCGATCTCTAATACACTGACCTCCGCGTGATCGAAGT
ATCGAGGGAAACAGTTCACGTGCAAACTGTAATAGCCTGAAGTGAATGGGTGTGTAAACCCGCTCTCG
GTGATTGATGGCGGCGAGGCGAGTGCGTATACGTTTCATCACCGGCTCTGAAGTAAGGAAGAACCAAT
CAAGTGCACGATATCCCGGTTAACTACTGCTGTTACAGCGGTATCCGTGTCTGGCGGCAATCATAGACG
TAATAGGTATCAATAACAGTGATATTTCGGAAACTACAGCATTTAATCGTGTCCGCTTGTCCGCGCCGTC
TTAAGTTGATCGTGATAATTCTACTTCGAGTACTTAGATTTGTGCAATTTTTTTGTCCGACGTGAAGGTT
CTTGCATGTTTCCTAATGCATTTTAACGATTACTGAGTATCATGCAACACAAGCCCATTGTAGGTACATT
GTTCTGTCCGGTGCACAAGTCAACAGATCCTTCGCACGCCACTATTAGGTTCCAGCTAAGACAATAAA
CTTGCACGGAATTATACTTTTATAATGCCTAATAATTCTTCACACGAAAAGCTCCT

>TCONS_00067815 geneID=XLOC_013557 scaffold_225:88935-90671(+) Splice junction overlap
CGCCGCGCCCGCGCAACACCATGAGCAAGCTGCTCAAGGCGCAGAGACGGTGAAGCAGAGCGACTC
GCGGCCCGCGTCGCCGCCGCCGCCCGACAAGATCAACATACAGCTCGGCGCGGGCACGGTGGTGGGC
GGCGCGTGGGCGAGCTTCCCGCCCGAGTACCCGCGCGCCGCGGACTTCACGCACTGACCGTAGACAC
ACACGCACACACACACACACACACACACACACACACACACACACACACACACACACACACACACAC
ACACACACACATTGACATCATCACGGCTGCGGAAACCATGTCGGTAGTACTGATGGCGATGTCTTAAA
GTGAGGTAGGTACAACACACTTCAATACGTCGTCGCCATAGCGTGCTAAGGAGGCAGGGCTATGAGGC
CGCGTCCACGGCACCGACTAGTGTTATTTACTCAACGGCAGATATTTGTACGTAATTCGCCGCCGTAGA
GTAAATAATACCAGTGCGTTCCGCCGCTCCGCCCGCTGGCGTTGTGGACGCGGCCTTCGGTACAAATA
ACGTGCTGTAAATCTTTTATTATATTTTCAATTGATTGACAAGTGTAAATATCGATAAAACTAGTTTGTGT
TAAGTTACTGTTAAAACTAAAACCGTTTACTTACATGATTTCAACTTTTATTATTTAGTCAATGTATTATT
TTATTACTGTTACAGCAACTTTATTGTGTTGAAATTGATTACATTTATTCTAGTTGGTTCTAACAAAATAT
AATCATATTGCACTATCATGTTGTGAATATCTCGACCGCCCCTAGTACAAACTTTTTGTTTTACATAGGTA
CTTTGAAATAAAGGTGCTCAAAGAATTTTTTCACTGGACTGCTAATTCTCATCATACCGAGATTTCCTCA
TCAAAATTAACTGAAATAAATCGGATGTCTTCAATAAAGGGGGTCTGGATTTCTGAACTATGAGATACA
TAATTATTATAAGGTATTATTAATTATTTTAAAGATATTAAACTATCTTAAATGTACATAGGCTTAGATTGTA
TCTATTAGCAAAGTCATTTGTATATTTAGTAGGTAAATATATAAAAATCTATACAGTA
>TCONS_00067767 geneID=XLOC_013558 scaffold_225:94855-96775(+) Splice junction overlap
GGGCGAGGAGCCTGCGCGCCCCCCGGCAGTAGGGGCACCCCCGCGCCCCCGGGAGTAGTGACCCCCC
CACCCCCGCCACCCCCGCAGCAGTGACGCCCCCGCTCCTTGCCACCCCATCGCCGCACCCTCCCAAGC
TCATGAAATGATACAATCGCAAGATTCCCAAGGAGCCGAGAACCTGACGGCCAAGGAGCAGTGGTGC
TACTACCGGCTGAGCGGAGCCGCCCTCGCCGCCGCCGCGGGGGCGCCGGGAGTGGGGGCGCCGGGA
CTCCACGCGTGACCGACTGGTCAGTTCATCCTTCACACACCTCAAGGAATGTAAAATAAAAACCCTTA
GTCAGACAGTGCTCAAATTTATTTTATAATATTGTCTTACAGGTAATATTTGAACAAATTTTGAGAGTTG
CATTTGATACTGCTGTGATAAATCATACAAGTTTTATAACATTGTTGATAAACATAATGATTTTAATTTATT
CTATTACATATTTACAGCGTTAAGTAAATGCCTTTTATTTTACATTATCCTATTACTTGAGTATAATCTGAG
TTTCATGTTAGTCATTGTTGATTATCAATGATGAGTTTATTGAGAAAATAGTTAACTACAAGTGGGAACT
GATTCATAGCGGATACGGACAAGTATTTACACAATATTCTATCTGGCATAACAATAGGAACATTTCTAAC
CTAGTGTAATTTAACTTACGTAAGTGTACGTACTCATTATTAGTACAGACCTATGTACACAATATTCAGAT
CCACGCTGTGCCTGTGAGTAGGTAGGTACATAACGACTTTCATTATGAAATATACACAACCAAAAATTT
CTAAACTTACTCTCATTACCTACACGCTTTGTGTTTTGTCTATAAACCTACCCATGTTTAAGTTATTATAA
TATTAAAACGTAGCTTAATTACATTACATAGGTTTCTAATTAATATTTACACATGTAATAATACTACATTGA
ATTTGTTCTCATGATTGCCTCCTGCCCCACAGAGCCCCGCCCGGCCGCGGGAGGTACAGGAGGCTACG
AGATCTCCACATTAGATTCACATACCTATAAGTGGAGATCTCCACATTAGATTCACATACCTATAAGTGT
GTAAAATCGTTTCAGTCACGGCGTAACGACATCTATTCAGTCAGATAATCATTACATGAATTACCTAATA
ATATCTACAAGTCAATAAACCTATGTACAAACTAGTGAACGACTTCGTAATGGTGTGAAACCACATCGA
AAGATTATAAAGCATCGAATTGGTTGGTGATTGGTCAACCCTTTCGACAAGGTTATACTTGGTCCTTTCA
TTTTTATTGATAAGCGACATAGGTTATAGACGCAACCTCCTGTTTACTAATGAGTTGGGAGTGCGTGCTA
CGCCGCTAGTGGCGCATGTTCGCTGCGTGTTCGCGCTCGTGGCTTATGTTCGCTGCGTGCTACGGCCCT
AGTGGGGCATGTTCGCTGCATGTTACGCCCCCAAAGACGCATGTTCTTGCGCGCTCGCTACGTGCTCG
CCATGAGCTTGGCGTGCGCGGTGACGTTGCGCTGGTGCAGGAACCCGCTGCCCTCTTCCTCTTCACCC
GTGTTCAGCCCGTACTGC
>TCONS_00067597 geneID=XLOC_013569 scaffold_225:95654-97203(-) Splice junction overlap
CGCACGACGCCTATGCGCATGCGCACGCGCATTACCCGCAGCAACACTACCTACATGCGCTACAGTAC

GGGCTGAACACGGGTGAAGAGGAAGAGGGCAGCGGGTTCCTGCACCAGCGCAACGTCACCGCGCAC
GCCAAGCTCATGGCGAGCACGTAGCGAGCGCGCAAGAACATGCGTCTTTGGGGGCGTAACATGCAGC
GAACATGCCCCACTAGGGCCGTAGCACGCAGCGAACATAAGCCACGAGCGCGAACACGCAGCGAACA
TGCGCCACTAGCGGCGTAGCACGCACTCCCAACTCATTAGTAAACAGGAGGTTGCGTCTATAACCTAT
GTCGCTTATCAATAAAAATGAAAGGACCAAGTATAACCTTGTCGAAAGGGTTGACCAATCACCAACCA
ATTCGATGCTTTATAATCTTTCGATGTGGTTTCACACCATTACGAAGTCGTTCACTAGTTTGTACATAGG
TTTATTGACTTGTAGATATTATTAGGTAATTCATGTAATGATTATCTGACTGAATAGATGTCGTTACGCCG
TGACTGAAACGATTTTACACACTTATAGGTATGTGAATCTAATGTGGAGATCTCCACTTATAGGTATGTG
AATCTAATGTGGAGATCTCGTAGCCTCCTGTACCTCCCGCGGCCGGGCGGGGCTCTGTGGGGCAGGAG
GCAATCATGAGAACAAATTCAATGTAGTATTATTACATGTGTAAATATTAATTAGAAACCTATGTAATGTA
ATTAAGCTACGTTTTAATATTATAATAACTTAAACATGGGTAGGTTTATAGACAAAACACAAAGCGTGTA
GGTAATGAGAGTAAGTTTAGAAATTTTTGGTTGTGTATATTTCATAATGAAAGTCGTTATGTACCTACCT
ACTCACAGGCACAGCGTGGATCTGAATATTGTGTACATAGGTCTGTACTAATAATGAGTACGTACACTT
ACGTAAGTTAAATTACACTAGGTTAGAAATGTTCCTATTGTTATGCCAGATAGAATATTGTGTAAATACT
TGTCCGTATCCGCTATGAATCAGTTCCCACTTGTAGTTAACTATTTTCTCAATAAACTCATCATTGATAAT
CAACAATGACTAACATGAAACTCAGATTATACTCAAGTAATAGGATAATGTAAAATAAAAGGCATTTAC
TTAACGCTGT
>TCONS_00067957 geneID=XLOC_013570 scaffold_225:126241-127302(-) Intergenic
GTTTGGCGCGCGTTTTATTTGGTTGGGCGGCGGCGTTTGTTTATAGATTTCTAATTGAATAACAAAGAA
AAGTGTTACAAATAACTGAAATTATATAAGTTTATTGTTTCAATTCATTTGAGAATAATATTTGTGTACCA
GAACTATATAAAACTTGTGTGAACTGAACTACCGTTGTATTGAAAACGGTTATATTTGGTATGATGATCA
TCTATTATTAAGTGTTTGTACTTCATTTATGAAGTGGTACTCATAGTTCACGCTGATTTGTTTAGCAGGAC
TGGTCAAAATACAGTTTCAGTCAATTGCTGAGTTGGGGACACCATGGCTGAGACGGTAGATATTAATTG
GGCAAAAGAAGTAGAAGCAGATGAA
>TCONS_00067611 geneID=XLOC_013574 scaffold_225:199847-201540(-) Unclassified
AACTAAGATCGGATACTACGACGTTACCGGAGCTCTTTATGTGTAGAATACGCACCATAGAGTAGCGGT
TAAACTGGGCTACAGGCACCCAGTGGGCACCCAGCTATATTTATAACTCCCTTGCTTTGTATGACCCGG
GGACACCATCATTACCATTGTGCCCGCGCCTCCAACGTCTGTGAACTGTCGCGAGATAAGCTAGGATTT
CCAAGAAAGGCTGATATGATTATGTGGTTGAAAGTTGTATAGGATAATCCACCTTATACTTACTTAGATA
GAAATTGTAAATAACAGTAATAGTTGACAGGATAAGTAGATTGCTTTGTTTCAATGACGAATTTAGATAT
ACCGTTAGTTATTCGTAGGTAACATTGTTTACTTTGCCTGATGTGGCTCGTTTTGGATTAGAACATGCCC
TGTCCTCCGAATGATGATAGGTGTACGTAGTTAGAATAGGTACATCATCAGTTTCCAATCAAAGTTTGC
AGTGAACATGATCATCGCTTTACCAAATTGATCTCATTATAGCATTAGTAGAAACTTATTTTTAGTTTTTT
CCCCGTTTTTGAACTTCTTTGTTTAGAGGTTAATTGATACAAATTAACGATATCTGTTTCGTTTTAATGTT
ATATTGTGAAAATATATTATATCTATTATGTAATAAAATTATAAATTAAATTAGTTTCCTATTTTTGCATTGC
TACGAAACATTGTTTGAAATATTGTGAGCTCTTTTTTCAGGAGCGTATTACATTAAATGCCGT
>TCONS_00067954 geneID=XLOC_013566 scaffold_225:520909-525983(+) Intergenic
CGCAGGTACGCCGCGAGAGGCGCCGGCGCGGGGGGCGCATGCGGCGCAGCTGAGGGCACGCCGCCC
CCGCCGCCGTGCTCTGCGTGGACCTGGCGGGGGCGTCTCGCGTCTGAACGTGGCGCCACACTGAGTG
GGGACCATAGGTAAACCGACCTATAGTCACGTTCGCAAGAGATGTGAGACCTCTGTGCTTATTATGGAC
AAAGTGTTAGTGTTTAGTTTTAAATATAATTTAGGTGCAGTTCATTTTTGTGTGCAGGATTTTTACTATAA
TACCTAGGTATTGTTGTTTTATTTAACGTAGAGTCATTGTGTAAATTCAATATCTTTTCTTATCGGGAAAC
ATGGAAATGTTTGCGACAGAGCGATAAGGAATTTTCCGTTTTCCATGTATTCTTATAAGTTAAATAGATT
TGGCTCAACTGGAAAAACTAACAGAAAGCTTTGTATTTTTTCTCTAATTAGGTAAGAAATAAAGTGTTT

AAATGGACAACATTTCAAAAAACTAAAGCTGCTATAAATCGAAAACCATTTCGAGATTTAGCTCTAAA
GGCCACAAAAAAAAATGTTTTTGTATTTTTTGGATGTATCATTTTCGAGAAAATAATAAAATAGTAAAAA
AGTGTTGATGACGTCATGCATCAGGTGCGATGCATTTTGATGATCAAAGCCTATAGATTTAAAAACAAA
GTAACTGTCTCTTTTATTTTTGATTGACGTTGACGTTTTATCGTGACGTTGTTGTTTATTTGGGTCATTTT
CATTAATTCTGTTCTGACACTTTCTGACTATTTTTTTATTTATTATTAAGAGATGACCTCTATATAATATGT
CCCAGAGCATGCCACCGCCTACACAGCTAAAAAAATGCTTCTGCTTATTTTTTTACATGATAAGTACCT
ACTTTCCATCATCAGAAATATCAAGGTCATTTGAAAATGACCCATTTGTTGGTTGGCATGTAAACAAAG
TTTAAATGTCACTTGTCAGTGTCATTTATGTTTTTTTTCGAGACTCAGGCAATAGACAAAAATAAAAATT
GAGCCTAATATGTGACGTCACTTCCGGTTTTAAAATAATTAACTTTAAAGTTAAAAATAACATGCAAAA
ATTTATGTATATACAAAATAAAAAAATACGGGTCCACTAATTTTCTATAAACTTTCCGAATAACTAATAAA
AATATAGGGTAAAGAAAATGAAATGTTGTCCATTGTTGGTGAGCCATATCCGTAAGATTTTGTTGTAGA
ACGCAACTATTTATTAGATTGTTTTGTCATATGTTAGATTAGTTTTGTAGTTGTGAGTGTTTTAGTAGCTC
TGTATAAATCTTTCTTCCTATAAATAATGGCGCATAGCTGATGATAATCTCATACATGTCATTTAAAAAAA
AATGGAAAGGGGTATTCGTGAGAATTTATGACCCAGTTAATACACTTATTTACCGTCTCACAACTAACA
CTAGAAATATGCTTTATGCTTCTTGTAACATACTGTGTCAAACTCCCGTTAAACTTGCCTAGTTTAAACT
CCTTTTGTGGTAATACAGTTAGTAACATAATAAAATAGAACATGTTCGTGCCTTGCTATTCAGTTATTAA
AGAATGAATAGGCCTTTTTGCCTATTCATTCTTTGTAGCTACTGCCATTATAATGAACACCAATAGAGTA
ATACCCACTTATAAATAGATGATCAGTTGCCTACATAAAATTTTCAGTAAATTTCTACCTGATAACTAGCA
GTTCTCATACGATTTTGACAGACCTAGAATAGAAAATAGAATAGGACAGGCGAGCGAGACAGCATGCG
TTTATCCGCCATTTTCACTTTGTTTCTTTATGCACATACGTTTTAGGTGTTTGTACGGTCAATTGGTCAGA
TTCATTAGTAGCTATAAACTTTTGTTCCTTGTCAACTCACTAACCTTCACTGTTCTACCACAAAAAACTA
TAAACACTGTTTAACAAGTGTTTAAAACCAAATTTGACAGATTTTGTAGGCGTTTGACAGCGCTAAAC
ACCTTTTAAATACTGTCTAAGTTTTTGTGGTAAGGCCCTAACTGTGCTACCACAAAAAACTTTAAACAC
TGTTTAAGAAGTGTTTAAAACGAAATTTGACAGATTTTGTAGGCGTTTGACAGCGCTAAACATCTGTTA
AATACTGTCTAAGTTTTTGTGGTAATGCCCTAAATGTCTGGCGGTTTCCTAGTTTGACAAAGTACATAA
GTATAGGTACTTACTTATGAAGCTTAGTTGTACTAACTATATTTCCTCGCCTTTTTCTGCATTATCTTCCTG
TAGATGTAGTCTTTATTGTTTATAGAAGGATAAAGATAAATTATTAAAATTGTGACTTCCCACTTTAGACT
AAATCGTAT
>TCONS_00067783 geneID=XLOC_013566 scaffold_225:522712-524212(+) Intergenic
CCATAGGTAAACCGACCTATAGTCACGTTCGCAAGAGATGTGAGACCTCTGTGCTTATTATGGACAAAG
TGTTAGTGTTTAGTTTTAAATATAATTTAGGTGCAGTTCATTTTTGTGTGCAGGATTTTTACTATAATACCT
AGGTATTGTTGTTTTATTTAACGTAGAGTCATTGTGTAAATTCAATATCTTTTCTTATCGGGAAACATGGA
AATGTTTGCGACAGAGCGATAAGGAATTTTCCGTTTTCCATGTATTCTTATAAGTTAAATAGATTTGGCT
CAACTGGAAAAACTAACAGAAAGCTTTGTATTTTTTCTCTAATTAGGTAAGAAATAAAGTGTTTAAATG
GACAACATTTCAAAAAACTAAAGCTGCTATAAATCGAAAACCATTTCGAGATTTAGCTCTAAAGGCCA
CAAAAAAAAATGTTTTTGTATTTTTTGGATGTATCATTTTCGAGAAAATAAT
>TCONS_00068203 geneID=XLOC_013634 scaffold_226:58164-77983(-) Unclassified
TGGCGGTAGCTGTGTTATTTGTGCTCGGGTGATAAAATAGGAAATGATACACTTTTCACACAAAATATC
GGATTTTTGGACGTTTTATGAAATACTTTGTGTGTGATTCAGTTATTGGTGTCGTTTTTATTTGTTGCAAT
CGTTATTTGTTTGTGAAAATTGTGTTTTATTTGGCGTAATCTTTGGTTCAGTGCAGTGGTCTGAAGAAG
GATTTGTACAGCTTGCAGCTTTTATTTCCTGCAACATTCTGCATTATTTACGAATCTATGAGGTACGCTCT
ACGACCCGGCGGTATTAGCTGACTCACCGGCC
>TCONS_00068148 geneID=XLOC_013624 scaffold_226:166569-168844(+) Splice junction overlap
CATAGTTCCTATAGCGAAAGTGGTATTTATGTACGTGATCAAAGTGTTGAGAGAATACGAAGAGAGAG

CTATAACATGCGCGGCCGCCCGCCCCCAATCCCTGCCAGGGGCGGAGGCGCTGGGCGGCGGCGGGCC
CGCCTCGCTGTCCAGCCTCACTCCTGACCACCAGCTCAGGGAGGACTTCACTGTGCTGGCTAAAGATC
TGGTGCATAGCTTGAAGGGGATTGTTG
>TCONS_00068394 geneID=XLOC_013699 scaffold_228:85896-93629(-) Unclassified
CAGACGCCGCACCGCAAGCACGTGTGCCCGACCCCGCGCGCCCTCAGCCATGTGGTGAAGTAAAACT
GTCAGTGATACACGTACCGGTCTCGCGGCGGCTACCCCACTCTGAACGGTGATATTTGTGATTCCGAAG
TGGTTCCCCCGATTTGGAAAATTGGATTTTACGAGCGAATAATGGCCTTAGCCAGGTATAACCAACAAA
CTGCCAGAACGCCTTGGAAGTACGTAACCCTTTTTGTTTTACTCTTTAGTTTCTTAACTCGCGCT
>TCONS_00068425 geneID=XLOC_013699 scaffold_228:100405-116518(-) Intergenic
TATCTTATGTCAAATCCGTTTTTGTTCTGTCGGTTCGGTTCTTTCTTTCTCTTGCTAGGTTTTGTAAATTA
ATTTCCATTTATCTTTATTATTGTGACTAACAGTGCTATAAAAATTACGGAACGCCTTTATTTTTATCGTTT
TGGTGACAAGAAAAAATCCAGTGAGCAATATTTGAAGACTGATTGGTGTTTCCGTACAAGGATCCGGT
GCA
>TCONS_00068447 geneID=XLOC_013703 scaffold_228:348357-348889(-) Intronic
AAAGGCACTACTCATCACTCTTTTAGTTTCTAAAGAAGTCAACAGTTGTGGGCTAGATTTCTTGGCTTA
CAAAGTTATCGATGGAGTCACTGGTGCCTATAACTACGCTGTCAACGGCTTCAAAACAAGTTTAGACA
AACTTAAGGAGATCCATCATGATGTTTCAGAAGAAAAAGTAGTGGAGAAAAGCACACCACCTCCTTAC
TCAGAGGCATTAGTTGAATATTTCAACTCAAGACAAAATGCAGAAAACCAAAACGGAATGGAAGGGA
ATACTATACCACCTGATTTTCCAGAGAATAATAATATTCCTGATGTTCAGAACGCGTTTTTATATAAACAT
CGACCCCCAAAAGTGTTACCAAGAACTACTACTGTCAAAACACCAAAACCCAAGCCAAAAAAGTGG
GAAGACTGGTTCATCTCAACCACAGAATATTCACG
>TCONS_00068726 geneID=XLOC_013734 scaffold_229:23943-26317(+) Intergenic
CCCCCCCCCGCCAGGCCCACTCGGCGACCCCAATCGACCACTCTTCGCTCCCAAAAGACCAGGACGA
CGCCTGGTCGACGCAGCACGAAAGGGGGCAAGGGCAAAGGCAAAGGCAAAGGGAAACGCAAACGG
CGACAGCGCCGACGCAAGCGACGCCGCTGTTACAGACACATGGAGTGTATGAAATTTGGTATCACGA
GCTACTTAATGGAAGTAATAAGTACACCAGATAAAAATGACGAGGAGGTCAAAGGAGTAACTGTAGCA
GGTTTATGGAATAGTGTTATGGAAAGTTGTATGCAAGGT
>TCONS_00068517 geneID=XLOC_013762 scaffold_229:406161-408963(-) Splice junction overlap
CGGTTGCCGGCGGCAGTGTATCGATTCTCGCCAGTGCAGTGGTGTGCCTAATTGTTTGTTTATTCACTAT
ACAGTCTTAGCACACTGTTTTCTTAGTAAATAATAGAACATGGACGTTTTGGTGCTTTAACTTGGTCTA
GTTTTTTGTTGTTGAGTTAGTGATTAAAGTTGTTTTGATCCTACAAAATGAAAAGCAGACCCCAGACCC
GAAGCCCTCAGAAGCGTCGCCAAA
>TCONS_00068652 geneID=XLOC_013765 scaffold_229:428451-431124(-) Intronic
GGCAGGTATTAAGTTAATAAAATTAACCCTATAACCCTTGTCCCTAGTGGATAAAGAACCAGCTCATACT
TATAAAATTCGTATCTATACGTCTATCTTCTACCACTTATTTACCAACATTTGCCTGTTCCATTTATAATCA
CACTATCTACTTCATTATTTCCTCTTTCTTCACACCTCAAAAACGTCATGTGTATTGCCTTATGCCTATAT
GCCTTAATTACCCTTGCGGATAAAGAACCAGCTCATTGTTATTAAACTCGTATGTCTAACTTTTCCCATTT
ATTTACCACCATTTGCCTATTAAATTTTTACCCACACAAAATCTACTTCATTCCTATATCCTCTTTCTTTAC
ACCTCATAAACTTCAGCAAACTAACTCTAAATTTTTGTACATATGTGTTAGTATTTAAGTCATATAAGTTT
ATTTTATGCCTGTCTACAAATAGAGAATATTTTATCTATCTATCCACAGGAGCACCAACCCGAGATCCTC
TCATTCTCTAGCACGGAATGGCACGTGCCCTTCACTGTCGGCGCCGTGACGTCCCGCGTGATACGCCG
TGAGGCACCGCCGTCGACACACTAGCTGTAAAACTATCCGATTTGGACCTGCTACCTAACAGTGCCAA
TGTGGACCTTATCGGCTCTCAACAAGTATCAGATTCCAACAGAGCCGTTTGTGGTCACCCTCAGTTGGA
TGATATCCATATAACAGTTTTTGTTAAGATTTAAAATCGTTTTTTTATGTAAAAGAAGATTTTTGTTATTTA

TTTTAATAAGACTTATCGAAGATGTTAAAAAATATTTGATTCCTTAGCTAGTGTAATAAGTGATGTGATAA
TTATTGCAGATCTCATTTTAATGTTACTTGTAACTAACATTTACTAGTTTACTTGTTTGTTGATCTTTAAAT
CTGCATGCACTATGTTGAAACCTTAGGAAATAAATAAATAGCTATTTTTATTGTACTTACGGAAAATAGG
AATATAAATATGTACTCTTACATTGCATTATTATAACAAAGACATAAGAGTTAAAGATGTTATAAAATGGT
AAGATTATATTTTTAAATATCCTACAAAGTGTATCAAAATTATGTTTGTAGAGGT
>TCONS_00068756 geneID=XLOC_013806 scaffold_23:291-3664(+) Unclassified
GAAGCCGAATCAATGGACACAGCCAGTGATTTATTTAGTAGCAAACAAAACAAACGTGAACTTTTTAC
GAAATTACCCTTTTGCCTTTACAGAACTTTGAGAACACCGTGAACCCGATCCTCAACAAGCCGAAGCC
TAAAGAGAAGACCCCCCCTCCCGCGGCCGACCCCCCCGCGGAGGGCGCGCCCGCGCCGGCGCCCGC
CGAGAACATGGACGTGGAGTAGACCCCCCGCCAGCTCCCGGTGCTTTCAGAGTGCAGCCTCTTCTCA
CGGACTGTCGCTTACTATTTGATAAGTCATTTACAGCCGCGGGTTTACAGTCCCAATTTTGATTTGTGTT
CGTGCGATTGATGCGAATTTATTCTAGAGACTTGTAATTTTCATTTTAATGATCTACTTCCATAACAAATG
AACTATCATTCGACTTTTTGTATAAATGAGCTGCGGCCGATACATGAGTTCGTTATCGACGTCGTTAAAC
TTGTTTAATACGATTTGTACTAATCGTATTTATGGTGAATCGCGATATAGTGACGTTTGTCCACGTCGAAA
TCGAACCCATGTAGCAACAGCTATACCTACTTTCAGTTTTATTAGGTTTTTTGCAACGCTGCACGCTGA
AAACACTCCTCGCACCACCCTCCACTGTAGATCCACTGTAGACCTCAAACTTTACAGTACAGAGCCTT
CATACCATTACTTATCTGCTTTATTGTGATAAGTATTGACTTCACTCTTGACTGACTAGTACGTTTAAAGT
ATACATTTTCATGTATAATTTTCGTCAGCTTCTTGTTTTATTTGGCCATGTTTGAGGTCGGAACTTAACGG
ATCATATTTGTGCAATGTGCAATACAGCTTGTCCGGTATGAGTGATCTACTAAACGATAGGAGGAATGT
GTTGTGTGCACATTATACACAGTATATAATTTATGATATACTTATGTAGTTTAATTTATACACACTCGCCTG
GCTCGAGTCCCGTTTGTTTCGAATTCCACTGTGGACATTTTGTATTTACATTAAAGAAAAATATTAGTTA
TGATGTGATTGATAGACAAATTATTGTAATAATTAATTCATTGCAAATGTTTGGAGGCCCGTTTTTTATTT
AACGAACCGTGGAGAGATAGTAATTTATTGTTTAAGAGGCTTGCAGATAGCTTTTTGACATTGTAATTTT
GTTGGTCCTTACTATAATACTCTATCATCTCTTTGCGGTCCAACTGTACTTAGTTTTTAACTATGCATTTTA
TTTTAATAATTCTAATATGATGGCAGGTGCTGCCGGTGTTATAAATTGCCACTGAATGAGCAATTTGGGA
AGATTCTGGGCAATTTAGCACTTTTCATTTTTACTCGAATCGCACATAAACATTCCAATTACTAAACCAT
TGAAAAAAGTAGTTAAAATTTCACAATACAAATACCTAAAATAATAGTTTTCCAATATATGTACGTTTCA
CTTCTGTACTTACACTTTAGTGTGGCTGTGGCCAATGTTGCTCATTCAGTGTAGAACTTAATGCATCTTA
CTCTAAAAATAATATCACCGTTTAACCTGAGTGATATGACATTGCATTGTATACGTAGCTACGACCAGTA
TAGTTCTCAAAATTGAATCAAATTATGCAACATATTATGTTTAAAAAAGTTTTTATTTAAACAATCTCTTT
TTGATTTCTAATAGTATTAACGCCTAAACTTGAATTCCACCATCGCACAGGTCTCCGGGCCTATGTTGTA
TATTTTACAAATAAATGGTTCAACCATTACTGTGTTATTTGTGCATTTTAAATGTTAAAGCTTAGTAAAAT
AATAATATTTAACAAATCCACGTTCTGTTCTTTTATACCTCCTGCTTAGGTTTCTTGGGTTTGCAAATAGT
TTTCTACTTAAGTGTATTATAAGACATCGGGCGATTTGCCCCGCTTTTTTGAAAATTCACGAACATAAAC
GAAAATACTTTTAAATTATAGTGTTGCCAACCAGATTTAATATTCTGTAATTATTTTACTAATTTACCTACT
ACGAAAAAGTCACCATAAAATATTATCTATCTTTTCTCTAAATTGCCATACAAAATGCCCAATGTCCTTT
CAGATTTTATTGGGACAAGGTACAAAAGTGTAGGTACTTATACATAATGATGCCCATGACTAGGTTCGTT
CAGCAAAAGGAATGTATGTAAGTATACCATTGAACTAATATTACTCTAGTACACACCCTTTTATAATAAC
AAAGTTGACTGTAGGCGTAGTGTTTTTCATCCTAAGCCCCACTCAGGGTTACACGGAAAAGAAAAGTC
GTCATCATCCATCCTTTTCAGGCTGGGGGGTAGAGCGTATTTGTGGAATGCCACATTGTATTTCCAGGG
GAACAGACTGGTTTCACCATCAGCATTGTAGCTGACGGCGAGCCCGGGTGGCCGCAGCCGCTGCAGC
TCCTGGTACAGACCCTCGGGCAGTGGCTGGGGGACGCTTCCGCTATTCG
>TCONS_00069054 geneID=XLOC_013850 scaffold_23:113262-117791(-) Intergenic
AGATAATCTACTTCAAGCAGCTCCAGAACGAGGAAGACCGCATCAGCAACCAGCGCATAGCGTTGTTC

CTGCAGCAGAGACAGGAGCGGGAGGCGCGCGCGCGGGCCGAGCGCGCCGCCGCCACGGCTGTGAAG
CAGAAGGGGATCGAACACATCTCCAGGTCTCAGAAGTAACATAACAGCCATATCATCAAAAATTCGTC
AAAAAAATAAAGTTACACGCACATAATAACACATCATTCTCAGTCATTCTCACATATTCCATGAACACTA
AAGATGGAATATAAGGTCAAAA
>TCONS_00069349 geneID=XLOC_013868 scaffold_23:1029153-1032960(-) Splice junction overlap
GCAGACCACTGCTGGACGGTAGGCCTCTCCCAAAGCACGCCACTGGATGCGATGGATAGATTTCATAT
CATCAATTAGAGGAAGTTTGGCAGAACGGTTCATGGGCATCGGCAGACATTTATGACCAGGCCACTGC
CTCTTTAGGTTGCATGTTTTAAAGGTTGCTGTGCTGTGGTCATATCTGGTGGGGCTATAAAGGTCAATTC
ACACATCAGCGAAGTACATTAAAAACGTTAGGTACATATTTCACTTTGGCAACCAATAGTCGAGGTGAC
AAATGTGTTATTTTATATATTTTTATAATCTATTCGTTTTTTTTGTTCTTCTATTTTATGTTCTTTTGTTTGAT
TTTATTTTTTTCTTTGTTACAGAATATTTAAGATGTTAAAAGTTAAGGGCGCAATGTAGATGTCGACATA
GTGTATACCTACTAGAGTTACAAGGAAAATAGCTATATATTATTACAATAATAATATATTTTAAAAATTAAT
TAATTTGCAATCAATATGTGAAATGGAAATCGCCTGCTTATTCGAAAAGCTCATATCATCGTGAGCAGTG
ACCATTCGGCCGCCATCACGTTCATAGTCAACTAAGCTCCACATTTAAATAAAACTTAGCATAAACTTG
CACCGCGCTGGCTACGTCACGAGATCGGATACTTTCGCTTTTTAATAACAAAAATAATACACTACTTTC
GGACACTCGCAGTTTGTTATCCTCTTTTAAGTTTTAAGTTTGTTTCGTCCGATCTCGGAGCTCAGCGGC
GCGGTAAAAGAATATATAATTTAATGTTGTAGGAATAATTTCCCTAGGATAAGAATAATTCCGTAAGACA
GTCACGTAAGGTTTGTTTTTATTGAGGACATTATATTTTGTATTTTGTGTCTACTACTGACTTAATTTTTTA
TATATTGATGTGATAGCGAATATCACAGCCTTTAGTTGAGTGACTAAGCAAATTCGGGCTTGTGTGTCCA
CCAATCAAATGAAGTTACTTATTTTTCCTAAAAATTATATAGTTTGAACGGGACAGATCGGGACCATTTT
GTTTGACAAGAGCGCAGCACGAGTCTGTGAAATTTGCGAATCAGTCACCTACTTATAGTACATAATACT
ATGTATGAATTATTTGACATCGGAAGGGTATCATTGTACAAAAGTCTTTCTATATTTCGGCTAAAATACTA
AGATTTAATTGTTATAATATTGGTTTTAAAAAATATTTCGTAAAATTTTATTTTTTTATAGTAGTCTGCAAA
TTGCAGTTCAAAAATTTGGCGTAAACCTACTTATTTTAAGTGTATTTTAAGGTGTGTAAATAATACACTC
TGTGTCTAAGTTTGTATAATTTTGGGATGATTCAAAAGTAGTTTACACCAAATAATTACAAAAAATAATT
AGCCTACATAAATAATTTACACCCTAAATACAAATGCGTCCCGCCATGAATTTCATAATGATTATTTTTTA
TACCCAATGGTAAATATTATCAAACTTTAGGCAGCAAATTGTGGATTATTTTAATCTTCAAAAATGTTTA
GTTCAAGATAAGACATCACTATCACAGCTACTATGTCATAATATGCGTATAACATTGCGAATGTTGAAAT
AATCGACAATGATTCTAGATGTACCTATTGTTATTGTATTTGTTATTATTTACCAAGCTATGTTCCAGTGTC
TGATTCACTCAGGCTCTTTATTGTGTTCATTCGTTATGATTGGTTGGCTTATTTATGATACGCATAGTAATA
GTACAATTTGGAAGCGCGTAGATACAGTGACATTGTGAAATGACGAAATACCTAACCCATATTTAAATG
GCAATCCATATCAACACTATTGAAATACCGCAGCTCAGATCACAATTTAGATTAAGTTACATATTCGCTT
GACGATTTGTGGGTGCTTCTTGGCATTTGTAGCGTTCAATATGTTGTCATGTAACAGTAACCTGTGTTGT
TGGCCGATGCGTCAGTCCGTGGGTGTAGCTAGGCTAGGGGTTGGGGCGCGCCGCGACGCTCGAATGC
GTTCACTAATATAGATCCGCTATTTTTCGATATCTATAAACGCACGTCTCTATTTTTGAATGCATTTACTAC
TAACCTGAGTCTTCATTATGTTTTTATAGACTAAGGTTCTTCAAATGACCTACAATTTTAGTTATATCTTT
GTTACTGCTTCAACACTTCGCTTGCCTCTCGGTGCGGTCCAACTCTTCGATTATAAGATAGACATTAAAT
GTGTATTTGTGAATTTTAATCTTATTTTGGTTGACGTTTCCTTATTTACGTGTAAATGGCCGAGATCTGTA
GCTTAGTAGCGACGGAGTGATAGCGTGTGATTGGTTCCCTTTTACAATTGGATAAAATAGATAATGAAG
TAAAAAGGTAAATAAAATATTAATCGCTTTTTTATTTTACTCCCTGTCCTTTTGTACATATTATAAAATATA
CAGTATCATACATTAATTAATAGATTTACAAAATATTACAATAAGTAGAAATAGTTGTTATGAATTCAATT
AAGTACATTACAGTACACGAAAGAAATATCTTAAATTATTATGTGATTTGTATAATCAGAAATTTGAGTA
ATTCAAAATTTTATACAGTGAGATCCAATAATTTAAGTCTGTTCTTGGACGGATTAGTTCAGTTTAACGT
TCTATAAAATATTAACTAGTCTCTAATATCAGACTTTAAACTAAGGGCGACCTCATAATCTGTCCCTTTCA

GTAGGTATACTTATTGTTACAGCAAGGCAGCAATAAATTCAGGTCGACCTTAGTTCCATCATGCAACCT
CAGCGTAACTGCCTTGCCTTATTCTAAATCTAGTCAAATGTCTTTACAGCGGTCACCCTAAATGGAATGT
TCATTAAAGCTGAGAAACGTTGTTATTTAAGTATACAAGTAATAATTACATACCATCCATGTAAATGTAA
CAAATGGAGGAACTATATGAAAAAACTTTTAGAAATTAAATAAGATTTTTTACGCCTGCCCTTGACCAG
AAATATAATGTACTTGGTGCCCTTTGTGGTCAGCCCTAAAGTGGGGTTTACACTATATGAGTATGCTAAC
TCAGTCTGAGCCCACTCGTATGACTATATAGCATTATCATCAGATTCCAAAGATGACTTTCGTCCGAGAT
TAATAGGGTGCGCTTTTCATATGGTTTTCCGCGATTTTTATCATAATATGCGCCGACACACGTACAGTAC
CTACACACTCATACATGTTAAAAAGTATTGCGCTACTTAAAAAACGCGTACTCTCATATGCTGTAAATAC
CTACTGTCAAAAGGCCATAAGTACATCTCTAACGAGAAGGACCATCATTATATCTCATTATGCCTATAAC
AAAGACAGTCATCAAAGTTTGGTCTCCGGTTTGATGCACAGTCGGCGCGCGGGTCGCGGCTCGCGGT
GGTTCTGTCGCTGCGACGAGGAACTGTCGCTGT
>TCONS_00069430 geneID=XLOC_013869 scaffold_23:1068624-1070101(-) Intergenic
CGGAACTCCAAAAAAGTTCAATGTAGGTACAGCAATTAAACTTGATGTCTATCTAAATAAGGAAGTCA
CTTATCACCAAAATCATAATTAGATGGCTATTCCAAACAAATCTGGATTGAATATTACCAATATATCATTAT
ATTTAAAGAATGTTTAAAATTTACAATAAGTAAATATAGGTACTTAAAACTTAATTTAACGGGTCTGGAT
GTGTTTGAATTAGCACTACAGGTCATATTTTTTGCATTGTAAAAGAACTTGAATTTTATACAAGTTTGTA
GAAAAGCAGTCATTTGTTTCAAACATTTATGGTATTCAAACAAAAAGCGATCAAGTTTTTATATCATCTA
AAATGACGTCATCTTCATATTACACACGAATACTAGTAAATACTAGTCGCATGAGGTTTGAATTTAAATT
GTAATAAATAGGCTAATCAATAGACACATTTAATATTTCTTGAATAATACAGAGGATAATCGCGGCTGCA
GAAACCTAACTACGAAACCGTAAAAACGCATTAAGTCTCCATAAAAAACATAATCCAAAAAACAGATT
TAGCTCCTTTTTTTCTTATGTCCATAATTTTCCGATAATAGTTTTCAGCTTATCTATGTTAATACTATCGAA
TGACGAGTTGTTATTAAAATATTTAACAGTCAGCACTTACCAAACATCATACTACCTCTTGTCTGTATCC
TTTGGGTCTCTGCTTTCTCATCATAGTAAAATCTCATGTCCGATGGTGGTTTTAATATTATTTTCCCATATG
ATCGTTCAAATTGAGTAGGCATACCCCACCTTTAACAGATCGACATTGAAGAAGTGATAAAATCACACA
GTGAATAAAAGTATTATAAAATACAATGCATACAAACATCAATATGTCACAGATACAACCAACACAATG
TAAAAATGGTGTAAAATCAGGGGGAAATATGATGAGTGAAGACGAGGGCCAATATGATACAAC
>TCONS_00068900 geneID=XLOC_013869 scaffold_23:1071656-1072156(-) Splice junction overlap
GCAGGGCCTCCAGGGCGCGAAGGGCGCGGTCGGCATCCCGTCGCGCGCCTCCACCGCCGCCGCACCT
TTACGTACCAGCCAGGTCCATGTATTTGGTGGCGTCAGAGAGCAGTGCAGTAGAGAGCGATGCTCCCT
GGGATGACCAGCCCAGCTCGGGAAATAACTCAACATTAAACGTGTGCCCAGAAGACCTCTCGCGCAT
GCGCGCTGCGCGCCC
>TCONS_00069416 geneID=XLOC_013831 scaffold_23:1133616-1135107(+) Intergenic
GTCCACCGGGACTGATTGGTCCGTTGGGGGCCCCCGGACAGCCTGGTATCCGGGGCCCACCCGGGCC
GGCGGGACCGCAGGGCGAGAGGGGGAGCGAAGGACCACGGGGTCTCACCGGGCCGCAAGGGGAGC
CCGGTCCGCCGGGGCCCCCGGGGTCGTCGGCACCAGCGGGTCCCACAGCCAGCCTTCCCGGACCACC
GGGGCCTAAAGGAGATGCAGGACAGAAGGGTGAAGCCGGCTCCCCCCCCGCNNNNNNNNNNNNNNN
NNNNNNNNNNNNNNNNNNNNNNNNNNNNNNNNNNNNNNNNNNNNNNNNNNACCCCGGGGCGGCCGG
GTCCTCCCGGGCCGATGTGGGCCAGCGCTGCGCCGCTCTTGATGGGATCACAAATGACGGAATCCATG
GTGCGAAATATCTGCGAAGACTTATTTAGAGATAAGCTGCATGAGCTGACAGCCGGGATGGCGGGCCC
GCCCGGACCGCCCGGGGCGGGGCGGCGGGGCCCTCC
>TCONS_00069185 geneID=XLOC_013831 scaffold_23:1135649-1136941(+) Unclassified
AGGGGGTAGGGGTACCAGGACCAGAAGGGCCGAGAGGTTTGCCGGGTGCTGTTGGACTGCCCGGAC
CGCCCGGTAAGACCGGACCTAGAGGCGAATCCGGTCGGGAAGGATCGCTAGGCCCCCGCGGGGCGCC
CGGGCCCCGGGGCGCCTGCGACTGCCCCGCGCCCGCCTACTACGCCTACGCACCGCAGGGGAACAGC

AAAGGACCATAGTTAGGACAATTAGTGATGACTAGCAAAAGGTTGATGATTGGTAAATCACCGAGCAT
CAGGGATATGACAGACATTTGAATAGCCCATCATAAAGAGCCCGGCTGAAACAGGTGCGAGTTTTAAA
GAATCAAATCTCATAGGTATTTGGGCCTTGTCAAAATAAGGGTACAGATATTGAATTTATTGAGAAATCA
ATACTTAATATTTTGTCAGGATCTTTCAGGGTGATGTTGGTTCTAAGTACCTATGTTCATGGGCAGAAAT
TGTGCTATGCATTCTGAGATTTGGGTATACGAAATAAGATTAGTCATTGTAATATAAGTGCATTTATTGTA
AAGTTTTATATAACCTTGTAAAATATATAAGTAACTAAGTAAAATACGTTCTAAAACATAAACAATATAAA
TATTTCATTTGTCTCCATCATCTTGACTCACTATAAATGGTAAGCCATTTATTTCAAAATAGACCCCCCTT
AACCCTTGGTAGAACAAAAGGGGCCCTTTCCCAAGAATCTAATCTAAACTATCCCATCACATCAATTGA
TTCCAGGTAAGTATATCTATCTAATGATAATATTATTACTTCACTTCAGTTATTTGGTTCGATTTCTTTGAC
AGAGTCGATTTCAGCCTTGCAGAAGAAACACTGCTTGCTGTTCAATAGGTGCTGCAAGATACACGATC
TGAAATAATTAAGAGAAAATGTTATTAGTATAGGATATTCATCATAAATTATGTAGAAGCGCTAAAAATG
GATTTTGCTAGACAACGCAACGGTGCTTAGTG
>TCONS_00068919 geneID=XLOC_013873 scaffold_23:1225024-1225746(-) Exonic overlap (+)
GGGTGAATCACGAAAATAATAAACTTTGTTCTCTAACAACTTTTAGTAAAGAAACGTACTAATTCTGTG
GAAAATAACAGCTAAACATACTATAACAATACAAATCACTAACGTAACTGACAGAGCTAGCACGTGAAT
ACCTGAAAACAATATTCAGCAACAGTTCTTAAATACGGCATGCCGATAATGATTGCCACACATTGTTCG
ATCTCCGAAATATTACGAGCCAATTAGCATTATTAAAGACACCAGACGCGTACAATGTACTTAAACAAA
TACTCCTGGCACGAGTCGTTTAGTCGGAATGTTGAAGATAGTGTTGTTGGTTGTGTGCGTGGCGCTGC
CGCTGGGAGCCCCCAAGGAGCACCGGCACCGATTGCGAGAGTACAAGCGGAGCCGCGACGTGGACA
AGGCTCTAAAGGAGAAGATTAAGAGAGACGCGGCAGAACTGATGAGGGAGAAGGAGGAGCAGTTTG
AGGCCGAGAGGGATTACTGGATGGAGAAGTACCGACACAGGCAGCC
>TCONS_00069072 geneID=XLOC_013873 scaffold_23:1225025-1225752(-) Exonic overlap (+)
CTTTCTGGGTGAATCACGAAAATAATAAACTTTGTTCTCTAACAACTTTTAGTAAAGAAACGTACTAAT
TCTGTGGAAAATAACAGCTAAACATACTATAACAATACAAATCACTAACGTAACTGACAGAGCTAGCA
CGTGAATACCTGAAAACAATATTCAGCAACAGTTCTTAAATACGGCATGCCGATAATGATTGCCACACA
TTGTTCGATCTCCGAAATATTACGAGCCAATTAGCATTATTAAAGACACCAGACGCGTACAATGTACTTA
AACAAATACTCCTGGCACGAGTCGTTTAGTCGGAATGTTGAAGATAGTGTTGTTGGTTGTGTGCGTGG
CGCTGCCGCTGGGAGCCCCCAAGGAGCACCGGCACCGATTGCGAGAGTACAAGCGGAGCCGCGACG
TGGACAAGGTCCCCTCCCCAGCCCCCAACCTCTACAGGACCCTGCCGAGGTTCCGCGAGGCCCACGA
CCCCAAGATCAAGGCGGCCACCGAACACTTCCAACAGCGAATGAGAAAGCTCAAGTCCGGCTCTAAA
GGAGAAGATTAAGAGAGACGCGGCAGAACTGATGAGGGAGAAGGAGGAGCAGTTTGAGGCCGAGA
GGGATTACTGGATGGAGAAGTACCGACACAGGCAGC
>TCONS_00068922 geneID=XLOC_013875 scaffold_23:1245185-1246487(-) Unclassified
GGGGATAGCTTAGCAATTCTACATAACCAATAATTAACTAGGTATCGGCCATGCCATGAGTTAGTTCATA
GCAGATTTTAGAATAAACATAAGTAGGTCTATGGTACAGCTAATTATCAGATAAGGTTTTGTGGCTTTCG
TTCAACTTAGACTGTGTCTACTAACACCATTGAAAAAAACACTTAACATAGTAAGTCGCGTCTTCTTAA
TTATTTTTTGGGTAGGTAACTACTTCTTGCAAAGTGTGCACTGCCTGACTTTACCTAATTTTAAACAGGC
CATCCAGTATATATAATATTGGAGGTTTTGTACCTTTGAAATAATGAAGACACTAGCACGGTGGCGACCA
TTTTTCTATGGTGAATGGATGAAGGGACTTGGGTTCACAATTCACATCACATAGGGTTCACTATGTAGAT
ACTTACTTACTTATAAATATTATGTGGTTATAAAGAATCTTTCGCCTGTGCATAACAATGAATACGTTTTT
TTTATATTAATGATATATAGGGTATCTGATAAAGCTCACTATAACATCATCACAACAATTTCCACAACAGC
CATCTTATTACCTAACACAGATTTTAGACCATAATAAAGATTATAACTACCAACATAATACCTAATAACAA
TATACCTGCTTTAACATACTCATTATATTGTAATCTTTCCTCTTTATAATAATTAGGTACTTGCCTATATTAG
TGTTGATATTTACTTCTCTCTCTTAAATTATTTCTTTTTCAGGTAACATTCAGAAATTCTAGCGATAAAGT

AGGTATATATGTATCTGTCTTTTCCCGTTATCGTTGGGTACTTGCAGTTTAATATTTTAAGTAAATGATTGC
CATTGATTTAAACGATCATACCTAACTATTTTAAATACAACGTATACCTACTTCAAAACAAAGTCCTTGC
TTCAAAATATAAATTACACAATCATTTCTATCCTTATAAACAACATTTAGATACTTGCTACAAACACTTTT
CTTGATACAAAACATTTTAAGCACTTAATTAGTTATCATGTTTTTTTTTAAATTCTATAAGTACTTACAAA
AAGTTGCAAAACCATTTTGCAGATAGGGTTGCATAATTACCTAATAAGCTCACCTTTTAATTCATAAGTG
TAATATTTTTACTTAAGGTACACCTACA
>TCONS_00069520 geneID=XLOC_013951 scaffold_230:8750-14343(+) Exonic overlap (-)
ATTCCAGAGAAGAAGGTGGTAGAGTTCATGGACAAGAAGTGAGATGGCGCCACTGGACACGCGAGC
AGACTGACCAACACGAAATATTCAACATAAATAATATGGAGTCCTTCACAGTAGACACGAGGAAGGCC
GAGAAGGGATGCTGGCCATGGGTTGGATATACAGGTTGTTGCAAAAATCATCATTCTATCTAGACTAAT
CTTTGGTTATCCAAAATAAATTGTATCCAAAATAATATGACCCCCTGAATCACCCCTATTTGGCTTACATA
ACCACTTCTGCATATTGTATATTTACATCCTGTATATTTATAAGTATGGAGCCCCTTCAAAGAAGCTTGTG
TCCAGCAGTGGACTATTCTTCTTTCGTGTCAGTGAACATGCGAATTAAATCTGTCCAGACCAAAATATA
GCAACTTTGATAGGATATTCTGGTTGGCCTGGAGTAAGTCTTCCCGAGTGCAGGTGGAAGGGCAAAGG
GGACAGGAGAGTAAATGCTACATAGTCTGGGGGGCGGTACCGCAGTCGCAAAGGTTGGTACCGTGGA
CTATTAGGGGA
>TCONS_00069582 geneID=XLOC_013970 scaffold_230:262668-263365(-) Unclassified
GTCTTCTTCATAGACAAGAGTACTTTTTCAGTGATAAACAGACTTTTATAATAAAATATGACTTTATGAA
ATTTTTGACAGTAGGTTTAGCATGTAATAATTTTATTACCTATACTTAATAAATAATGCGTAATGTAAAAG
ATAATTTTAGTGAATCTGCTAAAGTTGATGCACCTTCTAAAGAAACACCAACTCATCGGAGTAAACCGA
AACATGTACCAG
>TCONS_00069547 geneID=XLOC_013971 scaffold_230:281994-282806(-) Splice junction overlap
CCCGGTTGCTGTTTCGGAAGTCGTAAAGATATTTCAGTGTCATTATGAAAATCTTACATACTTAATATAAT
ATATTTTTTTTTGTTTTTCATTACTTTATAAAGATTTACCTACCTGTTATCGTTTTTCTAGTTTACTTACATC
AATATTATGTTTTTTTCTTTTATTTATTTTGTTATTTTCTTTTCTTTTTAGAAGGAGAACCGCTNNNNNNN
NNNNNNNNNNNNNNNNNNNNNNNNNNNNNNNNNACCGCTCTCAATCCAGGGTGCTTCAGTGTACCGA
GGTGCGAGCACGGACGGCGCATTCTCGCTTCGACCAGAATCAGTCTTATTATCAACAGTAATTATGTAC
CTTCTTGTGCGGCTTTTCTAAAAAAAAAAACTACTACTTATGTATAATTTATTGTTCTAGTTCGGAGCAGA
TTATTATGTACCTTTACCAATGTTGAATCATTATTAAAAATATAAGTACATAGGTACCTACCTACCTAATTA
AATATATTGTAGCCATTTTTGTGTGTAGAAAAATGTGTTAGCTTGGTACAAGTTTAGTGATGTCACAACC
ATTCTGAATCCAGAAAAATAC
>TCONS_00070153 geneID=XLOC_014038 scaffold_232:28073-49917(-) Intergenic
GGTCGCTCTCCAGTGGACGGTTGTATTGCAGCTCTACGCCTTGGGAATAAGCTCTACTTTCCAACAAG
AATCAGAAATTAATAGCAAGCAGAAGCTGTGACGATGCAGACTGCCTACCACTCCTTTGCCAGCGTGT
GCGTCAAAGATGCCGCCAGAATGACTGAAGATGGAAATTATAAAGATATCAAATACAATTTTTTGCAGT
TAAAAAACACATAGATTCTTACATAAACTCTCGCGCTACACTTTCATCTCAATTAATATTTTCATAATTAT
ACGTATACTTAAAGGTAAACAATCTTAGTGTGTCTTTTAAATTGAATGTTAGTAAAACAAAC
>TCONS_00070152 geneID=XLOC_014038 scaffold_232:28075-45217(-) Unclassified
CGGCTCGCCACACGGTTAGACGCGACTCAGCTCTTCCTGACAACGACACTTATTTTGTAGAATCAGTT
CCCAATTTTTAAAATTTCAAACACCTGCTGAGCTTTTAAGAAGCTGTGACGATGCAGACTGCCTACCA
CTCCTTTGCCAGCGTGTGCGTCAAAGATGCCGCCAGAATGACTGAAGATGGAAATTATAAAGATATCA
AATACAATTTTTTGCAGTTAAAAAACACATAGATTCTTACATAAACTCTCGCGCTACACTTTCATCTCAA
TTAATATTTTCATAATTATACGTATACTTAAAGGTAAACAATCTTAGTGTGTCTTTTAAATTGAATGTTAGT
AAAACAA

>TCONS_00070154 geneID=XLOC_014039 scaffold_232:53408-60374(-) Intergenic
GTTGAGGGAGAGTTGGAAAGGGATGGATGTGGAAAGCGTGTGTTTTGAAGCTGGCAGAGGATGGTGA
TTCAGTCGGAGCTGGTGTCGGTTACTGAGTGTCAACAGTAGCGTCTGCACTCGGCCCCAGTTCGTTGT
AGTGATTGTGTCCTAGCCGTGAGAGAAGATAGTGAACTAGTGATTGTACTATAATTTTGATACTAAGAA
GTCGGTTAAATACAAAGTGAAAAACTACAAGCAAATAAGAAATTATTCCGTGAAGCAAGTGAATTTAG
AAAAATTATAAATATGAAAACAAACGAATTGTGTTACAAAAACGTATAGTTACATATACTTACATCCTTA
CGAGGCTTTAGTTACTTTTACAATGCCATCTTCAGATCAGTGCGGCTTAGGGGCGTACCATCTGCAAAA
CTGGAGTAGTAACGTCGATTGAGGCGGGCTGAAACAGCCTGTAATAATGGAGTGTGGGAAGGCTGCT
GAAATCATGAAAAATCCAACAGATGGCTCCACCAGCTGAAACATACAAGTAATGGAACTTTTTCAGGC
CTAGTTCGTGTGACGCCGAGCGAAAACGCTACGCAACAAAATGGCGGGTGAAAGATCAATATTACCTA
CCGACAGGTATGCCCATCTCACTCACACTCAATAATATTCTGGTCCTGTCTCACTCTGTCGCGACGCTT
GCGTCCCAGCCTCAATATGACGTAAGAGAAGAAAAACCCTAGTGGGATTTTTTCACTTCCCGTATAGA
AATGTGTGGTAATAAAGTTTTCAAATAGATCTTGTTCACCGACATGGTTAGCGATTAGTGGCGAATGTC
ATTGCCGCG
>TCONS_00070186 geneID=XLOC_014051 scaffold_232:308160-310418(-) Exonic overlap (+)
GTACGGCCGGCGGCCAGGGCTACGGCGGCATGGGCGGCGCCCCCGACGCCGCGCCCGCCGGGCCCGC
GCGCCGCTACTAGCCGCGCCACCGGGGACCTCTTGTGTGAAGTTGCGCTTATTTGAAGCTAGGCAGGC
AGACGATAGAATACTTAGGAGAGGAGGTAGGCCACTACATCTAGAAGTTAGTCAGGGAGGGCCAACA
CAAGAGGGACTTAGCAACCGGCAAATCCTACACTCTATAGTGTAACATTTATTATTCATGTCGATGCGAT
GTAAGGTTTGTTGAAGGTAATTGTGTAGGTACATAATTTTTTTATCTACTACTTCATACATTTTAGCACTG
ATCACAGTTTTTACATTGATATCAATACACTTCATGCACGGCCCATTCACTTATCAGGTTTGAGCTATCCT
ATGTCCTGTTTAAATATAATTGATTGTTTTCATATTGTTTATTCCAAAAAGTTATGTTAGTGTCTCTGTAA
AAGCTGTGTTTATAAACAATATATATAAGGTTCCTAGTTATAAGTAAGAAAGATTTTATGTTTTTTATAAT
AACCTATTATAATGTTGAGTATTGACTGTTATCGTCAAAAATATTAAGGAAACATTCCAAAAATAAGAAA
GTAATTATATAATTTGCTATAATAGGAACAATATCTGTGCTCAATGACAAAATTTAAAACACGATATAAA
AACATTATTGTTAGACATAGGGTAGATAAATGACTCAAACTTTCTTTTGTTCTTTGACTGATTCGTGAAT
TTTGCCTCTAATCTAAGAATACTGTATTGTTTGCGTTATAATTCTATTTTTATGTTCTTGCCTTTTTCTTTT
AAATTTTCTTAAATATGGAGTGTTGACATAGATATAATTGAAGTATTTTGTTGTGTGCAATAACTGTATGA
AATAATCCGTATTTTCCATGGTTTGTTGACATGGCCTCTGGGACGAGCTCACCCATGGACACGGCCATG
GTAAGTTATGCCTCAATAAAACTATAAACATTTTAAACAATCTTTTATTTCTATTCATTTAATATTTACAAC
GCCTATTAACATTTATATAGTATACATTTAAGTAAAATTAAAATAGTACCTATACATTTTGGGATAGTAGG
CATGCTTAATGACTCTCTAATATTAATACAGATTTTAAAACCAAGTATAATATGTAATAGGTAGAGTAGGT
AAAGTCTACACGATGATGTAAAAATATGATTAAAATGCAGATGGGGTTCTGGGGAAAAAAATGAAACT
CAAACACTGTTGTTGTTTTTTTCGGAAATTCGCAGGAAAAATATTTCGTTCTACACACCATAATATAGTT
GATGGTCTCATTCAAATATTATTTCCCATGTACCTATAAGAATAATAATTTTTACATATAATAATAAATACT
TATATGAACTTGCTAAAAAAAATAAAGCTATGTACTCGGGTACATTTTTATCTGTTTTATATAATTAATCCT
ATACCCTAAACCTAACCTATCTTCCGTAGGTACACAGTTATAGCATTGCGGTAACAGGATCTTATCTCTA
GCTAGAAGAATCTGAAGATGGTGGTGCAAGTCGCAATCTGTTTTAAATTATCTTAGATTAAGGTTCTATT
AAAATTAGTCGTTTGGAGGCGGCAAAGGCTGAACTGGTATGTAGGGGCAGGAGGTCTGGCAGTACGG
GGCGGCGAGCAGGCCCGACGACGACGGGAACAACCCCGACGCGGCTGCTAGGTGCCTGGAAACAAA
G
>TCONS_00070240 geneID=XLOC_014054 scaffold_232:435883-461423(-) Exonic overlap (+)
GTTGTTATTATTAAATACAATACTATTTACACTAACAATACCTACTAACATTAATACTAAGTTCACATACAT
CACACACATCATACAACACCAAACGTATCGTGCCCGACGGATAAATTGTCGTGATACATCGAGAACGC

ACAAAATATTTTATCCGTAGGTTATTTGTTGGTCAAGTCGAGCGGCCGCCGATACCGGTCCGGAGGCAG
GTTCCCTGCCCCGCGCGCCCGCGCAACCTCGCGCGCCCTCCGCGGCCCGTGCGCCGCCAGTCGAAAT
CTCAACTCCGACGCGAAGCCTGTGCCGCTTTACGCTAGCCCGCTATTGCTTATGTTTACGCGATCTTTC
CGCTGTATACCTAGCTTGCTTATTTACTAGCCTATGCTTTATACCTAAGTGCTAAATAGTATAAACTCTGC
TGTGTTATTGGCTTGTGCATTCTCCTTCTAAATTCTATAGACTTTGTGTGTGCTTTGCTTGTTCAATATAC
AAACCTGGACTGAACTGTAAGTGTTTGCCCCGAGGAAAACACACCAAGCACAATGTCCAA
>TCONS_00070228 geneID=XLOC_014034 scaffold_232:507400-510057(+) Intergenic
CAAGTCTCGCATGGGATGATTCATTTTGTAGCATTATCTAAATTTGTGTTTGTGTTACAAGACGCATTGA
TAGAATAAAGAGTTCTCTTATATCACTATGGATGAAGATTCTCAGGCGGTTTCAAGATTCAATAATCAAT
ATAATACTCTGAAGTTTCTTTGGGATTACATAACCATTTTTCCAACACCCTGTACGGAACGGCTTATGGT
CTCAGACGTATGTTACTACCAACCTCATTCTACTATCTATGAATATTATATCCGATCCTGCCGAAACATGA
ATGCATTTACATAAGGTATTTAGATACATCTCTAACCCGCTTACCAAACGTGGAGATTACAAATCCCTCC
AGACGTAGATGAGGGCCATGTTCATCGGTGGAATGCTAACATTTGAACAGAGAATCATCAAGAATGCC
GGCCAAGATTAATATCTCACAGAAGCTCCAGGATAGGAAGGGGGAATGAGATGCTTTCGTTCCATTTTC
AAAAATAAACTAACGGAAAATAAAGCTTCTTGTTAAGCATCTGTTAAAGTAGAGAAAGGCAAAAATGC
GGACTTCGTGCGACGCCAGTAGATAAACGTCCGACTAAAAGTAGCAGCTTAACATTTTTGTTTTTTTGT
TCGCCTTCGAGATTTAGCTCTTCCATCGTTTAATTACTTTAAAACTGTCTAGTCAAACATAAAAATGCTT
TTCAATATATTTTGTTATCTGCATAGTTTATGGAAAAATATTATAATAAGGGGTTGAATTGCAGAATTACA
TACACGTAGGTATATTTTGTGGACTTACCTTTAGGTAACATTTATCGTATTAGAAACATAAATAATCATAT
ATGCATATTTAACAAATAATGATAATTACTACGACTATGTTTATTTTGAGTAAATTATGTACTAGGTATGAC
TTTGATAATCAAAACCAGCACCCACCAAGACAATAATTGATCATTTGCGTTTATTTGAAAAGCAAAATA
GATGCCGATATCTATCATTATATAGCTACACATTTCCAGAAAATGAATGTGGTTAATTTTAACAGTAGCAA
TAAACTAATTGCGCAGGTATCGGGATAACGGACATAATTCCTAGAACCTGTCGTATTGATAGTGTTAAC
GTTCCTTGGATGCCGTGAAGAATAGG
>TCONS_00069966 geneID=XLOC_014055 scaffold_232:616271-639302(-) Intergenic
CAACAACTGGAAATCGCTCGCAGTGCACGGGCGTAAATCCTCAAAATAAATCAGAGTCGAGAAGCCA
TAGTGCCGCCGTCAGCCTTGTGAAATATGCTGCCTGCCCGCAAATAAGTTATTGAGGCAATTAATAAAT
ACTACGGGAGCAGAGGCGGGTGGTACTGTGACAAGAGGGAGTTTGTTACCATTTCACCAGAAACAGT
CACTCTAGTTTAGGCACCCCTAGAGAAGGGGGGAAGGGGACGTCTGACGATGAAAGATGACCTCACC
GCACCGTGATTAACGACCGAGCCATTCATCATGCAGCCTACTAAGTACACAAGCAAACGTTTCAGACC
ATACTTGTCTGAAATAGAGTACGTTTTCTGTTGAACGAAAGTGGCGCGGTAACATGTACTAATTTATAA
AATCGACTGTGGTAGTTTTTATTGTGATGGAATAAAGTGTTATATG
>TCONS_00069945 geneID=XLOC_014035 scaffold_232:789950-806953(+) Intergenic
TACTAGGAGAGCATAGCTAGCAAACTGTGGACACACGGCATATTAAACCGATACGCCTCTAAGGGCTG
ATTTTTCAATGGTCAGATAAACGCTATCTGACGAATAAAATTGTTGCTGCCCCTGTCAAATACAAACAT
CAGTCGCGATAGCATCATAATTATTCCCCAGATAACCCTTATCGGACCATTGAAAAATTAGCCCTAACTC
AGTCAGCCGTGAATAGTTTGTTTACTGGGAACGCAGTATAAACATTCCTTCGAAACGCTATGAAATCGT
ATACCTATATAATAATTATGAATAAATCATAACTAAAAACTACAAATCACAGCAGTCTTTTAAAAGATCC
AAATTAGTCATAGCACAGTAAAATTTTCTCATCTAGTTTATACATAAATAGTGTGTGTATATTATGAGTTC
ACAACGTGCCTGCTCGCAATATTTTGTTCTTATCTCTTAATTAACTATCCACTTTAATCTTTAGCATAGTT
TTACTTTGTATTAATTTAGTGTGGGGTTTTATACTGTGTATGTAATAGTTACGTATGTACGGTTTATTGCGT
AAATTTTATGAATTCACGTGAATAAATTTGTTGAATTATAATACCG
>TCONS_00069971 geneID=XLOC_014059 scaffold_232:789950-806953(-) Intergenic
CGGTATTATAATTCAACAAATTTATTCACGTGAATTCATAAAATTTACGCAATAAACCGTACATACGTAA

CTATTACATACACAGTATAAAACCCCACACTAAATTAATACAAAGTAAAACTATGCTAAAGATTAAAGT
GGATAGTTAATTAAGAGATAAGAACAAAATATTGCGAGCAGGCACGTTGTGAACACATAATATACACAC
ACTATTTATATAAACTAGATGAGAAAAATTTACTGTGCTATGACTAAATTGGATCTAATCTTTTTAAAGAC
TGCTGTGATTTGTAGTTTTTAGTGATGATTTATTCATAATTATTATATAGTTATACGATTTCATAGCGTTTC
GAAGGAATGTTTATACTGCGTTCCCAGTAAACAAACTATTCACGGCTGACTGAGTTAGGGCTAATTTTT
CAATGGTCCGATAAGGGTTATCTGGGGAATAATTATGATGCTATCGCGACTGATGTTTGTATTTGACAGG
GGCAGCAACAATTTTATTCGTCAGATAGCGTTTATCTGACCATTGAAAAATCAGCCCTTAGAGGCGTAT
CGGTTTAATATGCCGTGTGTCCACAGTTTGCTAGCTATGCTCTCCTAGTA
>TCONS_00069983 geneID=XLOC_014061 scaffold_232:843508-873037(-) Splice junction overlap
CTGCAAGCGGTTGTGGGTGGGAGTTTTGTTGTGAATTTTGTTTATTTTCAACAAGAAATCGTAAAACCG
GCGTTGGATTTTCTCAAAATGGCGGGGGCAGATGGCGCTGGTGTGGCGCGCTGAGCGCGGCCCCCTC
CCCGCCCCCTCCCACTCTGTTGACTACTCTTTGACGGAGGTTTCGTTTGTGCTACACGACCTGTCCTGG
ACCGCCGATGGCCCCCCCGCTGGTCATCATTCTCAGTTGGAGAATGAAGTGATGGTGGACACGTGGAG
CAGCATGGACTCGTCCAACTCTCCGCTGACCGACTCCGGCGCGT
>TCONS_00069987 geneID=XLOC_014062 scaffold_232:901690-902663(-) Intronic
GAGCGCCGCGCGGCCGCCCGCCGCTGACGCTGACACCGCGACAACAATAAAAAAAGAAAAAAACAA
CACCCCGATAACAAAACTTATGAATCAAAATAAAAATAGTGCCGCTACTAAATTTTCTTTAGTCACAAA
ACGCGCGGTTCACGAACCGGAGTTAGTTTTAGGTTTTTTTGTGTAATTGTTTTCTAGTCGGACTCCGAT
TTAAATAAATAAGTTCAAAATGTTTGCATTTATAGTGTTACCTGTGATTTTATCTTCATTTATAGATCTGA
CTCACCAAGCACGCACAT
>TCONS_00070416 geneID=XLOC_014132 scaffold_233:274859-276842(-) Unclassified
TTATGTTATGGAATACGTCGTACCTTAGCACATTTAATCTAGATTGTAGAACACACTCGTATAGTAAGCA
AGAAAAGTAATCATAGTTGCAAATAAGATTTTATTATTTTATTATTATTGATAAGAAATGCTAAAATCGAA
AAACTTACAAAATCGTCTTAAATTGAAGATTAATTATATCATCGTTTTTATTTCATTTGGCTAGATTTTATG
AGTTAGTCGTAGTAAATGCTCGCAAAAATAACGGTTGAGCACCGCTACTTAAGATTAGCG
>TCONS_00070279 geneID=XLOC_014110 scaffold_233:281606-282045(+) Exonic overlap (+)
CAATTTGACAACTATCTGTCAGTACCACATAAACAAAACGGAACGTTATTATTTACACCACAGCAAAGC
AAGTGTCGATAATTTCGACAAAATCTTTTAAATTATAAGAACAAATATACAGAAACTAATCAATTCGTAA
TTTTAAACCTACGGGACAGATAAATTTATATTCCAGCCAAGTGGGCGCTATTAGCTTTTAAGAAAAACC
AAGATGAGGATGAGAAACCTCGACGTGAGCAACAGCAGCACGGACAGCAGCTCGGGGTCGTCGTCG
GACTCGAGCTCGAGCGGCTCGTCGTCGTCCACGTCGGGCTCGGCCTCCAGCAGCTCCGACTCCGACT
CCTCGGCCTCCGACCAC
>TCONS_00070539 geneID=XLOC_014117 scaffold_233:382529-383689(+) Intronic
CTGTTTATTAGTAGAGTGAGTAGTGTAGTGACTAAACGATACATGATTTTTCTTTGCGGCCAGTGTATTA
TTGTAATTTTACGAGAATGCGGGGCTGCTTGATGGCATTTTGAGCGGGAAAGTGGTAGAAAACCCGAG
TGTAGTGCAGTGAAATGGTTTGCAGTAAAAGATGTGGTTGTGGTGCATTTTGCCGAAGATTTTTCTGTG
GGGATAAGGATAAAGATAGTGATGAAAAAAACTGCGAAGAAGAAATAGATGAAGATGGTGTTCCTGTT
ACCTTGGTTTGTTTTTTTGTCTGGTGTCTTAAGACCTACCTGATTTATTGTACATAATATATTGCATACAA
ACAGTTGAATCGAATGTTGTCATTAAGCACTCTTTTTTATAAATAAACAGTCGTCAGTGTTTATTTTTGT
ATTTGCGGGTAGTTGCCCATTTTGAAGAGGGTAGTTTACAAAGGACTTATATTATGTTGATGCTAGACAA
TCAATCAATCAATTCAATCTTACTGAAAATATAATTTTGTGATTTATAAGTGTTTCCTTATAATAATGCTAT
TCAAACATCTACGAAATCGAAATGACACACTGCTGACCACACCCATGAAATACGCGCAGTGAAGGCG
AGTGCAACTTAGAGGCCACGAGTGTACTACTCAACTCTATTGTTTTGTTTTTAATTGCAGCCAATACAG
TTGTAAC

>TCONS_00070781 geneID=XLOC_014191 scaffold_234:446979-447500(-) Unclassified
CGTGAGACTAATGTAAAATGTAGGAAAAAAGTAAAGCATTAGACAAAAATCTAATTAGCCAAACGAAA
ATGAAGTGATAGGCGAATAGAAAACATTAGATTTAACAATGTGAATAGGAACTAGAGTGCTGTGGCAA
AGATTTACAATGAAATTACTGATGAGTGATACTTGTTTGCTACTGTACTTATATTACTACAAAAGTTGCTA
TGAATTGGGCCAATGATGCTGCACTGAGAACTGGGAAACAAATATGCCCGGTCCAGAGTAGGCCGAG
ATGCCATGTCTGCTGGGGCCTGCATGGGCGCCGGTATCGCATCGAGGTCCTGCCTGGAGTTTATGCGTA
TAAGTATATATGAGACATTCTGAAAATATTTCATTTAATTGAAAATTATTTTACCGTAGGTACAGTATCTAT
TTAAACAAACTAATTAATAATTGCG
>TCONS_00070866 geneID=XLOC_014254 scaffold_235:127933-129394(-) Exonic overlap (-)
CTTCCCATGTATCCTGTGCCGCCTTGCGTTCCGGTACACGTGTCCGCCAGTGGACACGAGTGTGCCGA
GCGAGCTGGCGGGGCTGAGCGCCGTGTGGAGAGAGAGCTGCGAGCCTGACGAGTCGAGGGGGCGGT
GGTGGAAGTCCAGGTCTTCGTTTGTTGGGGTGCGTTCGCCGTCGCTGCCCTGCGGCGTGCGTCGCGTG
AGCTCGGGGCTGCAGCTCCAGGGCGACAGCGCGCCCAGCGGGCTGTTGAGCCCCGCCGTCACTAGCG
TGTCG
>TCONS_00071123 geneID=XLOC_014255 scaffold_235:191445-192971(-) Exonic overlap (+)
GCTATGTCCTCGCGCATTCCATGCGGCCAGCACTCGGCCAGTCTCGCTCCGCGCTCGCTGGCACGAGA
ACGCATGCTCGCGGCCGAAACTAAATTAAAAAGTTAGTGTTCCCGCCTCTTCGCATTTCAAATTTGACG
TTTCATAAATCGAGTGGGGTTGTGTTTTGTTCGATTTCTTCAAACAATGGCTTCAGTAGAGAGTAGCAG
TAAAAATGGCGCAGCAACGAATCTTATCAATAAAGCACCATGG
>TCONS_00070871 geneID=XLOC_014257 scaffold_235:211526-212146(-) Unclassified
CTCCTAACCTATAACACCGCTTCTTTTCAGTTAAGTTTCAAGATTAATAAACTTGCCTAACTAGGTCTTA
CATGTAGCTATTTAATAGGAAAATATCCTCCTCGATTGGGTCGAGCCCCGTCTTCTATAAAATACCTCGA
GTTAGTAATGTTAGTAATCGATTTGAAGAGTAAGCATTGAAATTTGCTTGTAGCGTAGTCACTGTAGTGT
ACGAACCAAAGTTTCAACCCTGTGAAAGCCAAGTTTCAGCCGCGTCGCGCCGCGAACGGACGTCTTT
CACGGAATGACAATACGTGCCAGTTTTGCGTGTTGATTTCTTTACGCGTTGTACCTTGGAACTATTTAAA
TAAATATGAAAATTATGCAAGTATAATGCCTCATAGAAAACTGAAGGAGAAAAGCCTAGACGCCCTGC
AAAAGAGCACGAATTTCAGCAAGTGAGTTTCACGTGTGTTGTGAGTTTTCGTATCAACTACGTACCGA
TAAAAATAATATACAGTA
>TCONS_00070932 geneID=XLOC_014258 scaffold_235:297093-297497(-) Exonic overlap (-)
CAAGTGCTGGGTGACCGGCACTGGCCAGGTCGGAAGTTGTTAGCCCCATTCTGTCCAGACCGCTGAG
ATCCTGGCGTTTGAGATCCGTTCGCACGAGATCCTGGCACATGGGATCCCGGCGCATGGGATCCCGGT
GTTTGAGATCCAGGCGCATGAGATCCTGGCGCATGGGATCCCGGTGTTTGAGATGCAGGCGCATGAGA
TCCTGGCGCATGAGATCCGGGCGCATGAGATCCTTGTGCTTGAGATCCCCGATGTTGCGCTCCGGGA
>TCONS_00071146 geneID=XLOC_014299 scaffold_236:135873-153137(+) Unclassified
GCTCAGATGCTCTGTGTGCGCCGGGCGGGCCGCGCGCGTTTCCGAACGACACTCGCGGATTACGGTAC
AACAATTACCGAACGCAAAATATTCTTTTTTCGAACACCGTTTTTAAAAACTCTCATCATCTGTGCCGG
GGTCGTCCCATCCCTCGTTCGAATTCCGAAAACTGTTCAGAAACTCAATCGGTAAAGTACGACTAGAA
AGTTTGCATTGCATTTACAGTATTTTCTATACAAATAGCAGTGGTTGCATGTGTAATAGAGTTTTATTTTG
TGCTTTTGTGCCCCCAGTGTAGTTCTGGACGAGTTTTGAGTGTTGCCGAGTTGTTGGTACTTACTGTCA
ATATCTACTACAAAGCCATTAGGTATGTTGAACCAGAAATATTTTAAATACTTTTATAATCATTTGTAGGT
TAGGTTAGTTACAAATTAACGAAATGTGAAAAAGAATTTAGGAAACTTTGCTTTGGAAGTAACAAAGC
AAGTCTTCTTATCGGGCCGTAAAAGTGCTAACGTGTGATTAACAAACAAACCCTTAACTTAATTACAAA
AATTCTCCGAAAGGTCCTATTATTAGTCCAAAACCAACAACACATAAAAAGAACAAACAACCATGACC
CATGTATACAAACTGATAGTACGGCCACTTAATATTATCGCAATTGCATGTACTTAGGTACATATTATGAC

ATAAATACTTACATAGGCATGTAACATAACATAGACTTGGTCTCAAAATTGAATTATTTCTTACTTAAGTT
AGTGTTTTTTTGGTGTGAAAAATTATTCGG
>TCONS_00071307 geneID=XLOC_014308 scaffold_236:264654-267346(-) Unclassified
CAGGAGGACCTCGACAAGGAGATGCTGGGCATCAACGTGCCGACGGACCAGCTGCCCGACGTGCCGT
CCGCCGAGCCCGCCGCGCCGCGCCCCGCCAAGGCCAAGCCTGCTAGCGAAGATGACGATGATCTGGC
TAAACTGCAATCGTGGGCGACATAAATGTAAGTGTTGGATGCGAAACCGAATACATATGTATAGTCTTC
GTTGCACATCCAAATACTAAGCAATTCTAAGGCTGCGTCCCTGTAGGCAAACAGTATTCTCGTTACTAG
TCCATGGTAATTTGCGTAGAGTGAAAGACAAACTTGTTAATAAAAATAAGGAAGGCTGAAGACAGAGT
GTTGTGGCGCTCCTTGGGATTGGATCGTCCAACAGTGGACGATTACAGCCTAATGACTTAAATTTATTA
GGTACTATCTATGGTAGGACTGTTTCCCTGAGCACCTAAACTAAAGGGAATACTGGATAAAAATTACAT
AAAAACCTTCCTCTGAAATAACCATAAAAAACAAAATCCTTTGCGTAGTTTGCTTTACTATGAAGTTAT
TTGAAAAGCTGAGTGTTTGGATGTGCAACCGTTGTATGGTTTGTGATAATTTGTACGATTGTGTAATATT
TGAATATGCAATTATGCACATTTATTTTATTGGACTAAAATTAAGATTTTTGTTGGAGGTTTATTTTGTTG
GCCGAATATTCAAATATTGCGTGATCTTACCGTCTTTCGGATATTGTAATGTATAGGAACAGAAATGAAA
TCTAAATCTGAATAGAAAATAATTATTATTTTCCCTCTCTAACAAATCAGAATTAAACTTTTTGTTACTTT
CGCTGAGTTGCACAAAGCAACTTTAATCAAACGTCGACCTTAAATGTATTGCTGCCTTGCTTTGATAGT
ATACGGAAAGAAAGGCAGATATAGATTTAATTCCCATATTAGCTTCTTTTTTGCAACTCAGCATTAGAAT
CTCACAGACTGTTCATGCAATGTAAGTTACCATACAATATTCTTTACCAAATGTTCATTCAATGGAAAAT
ATAATGTTAGTAATTTCAGTAAGCTTATTTTCTTTTCATTTACGCTATTCAGATTTAGAATGATAAAAGAT
TGTCCATTTGGTGCAGTCGGTCGCGATAGTAATATTATCAAAAATTGTTCATCAATCACACTTATGTATTC
AGTAAGTACCTAACGTAGCTTTTGCCGACTGTTAGGAGGTTTGGTTTAAAAACTGGTGTAGGATAGGA
ATTCACATATTGGCGTTTAAAAACACCAAAAGTCTTGCAACTTTTAAACCATCTTCAAAATGAGGGAA
GTTACGTATGTTTCCCTTTTTCACATCAGTTTTAAGTCAAACCTTCGTAGGATAATGTATTCATGTTAAGA
TTGTAATCGGAAGTCTTTTTTCTGCCTTAATAATCACAATTATACAGTTTGTAGTCAAAATATTGATGGTG
TTCTAAGAGGCAGGGGAAAATTGTTTATATGTTGTATATATCTTGCTATTATTTATATTTTTCTTATAATTCT
AATAGTTTATGCAGGTATGTTTACAATAAATCAATTTATCAAAGTGTGTCCCGATAATTTGTTGTGTAAA
ACTTTAAATAGGTTAAGTGCCTGTTTTACAATGTCTGTATAATGGTTAAACCTGTTGGATAAAATACATG
CTGTCACTCTCGGTAATTATGTTTTAGACAGTGACAGTATGTCTTTTTTCCCTAGTTAGTCCTTACCCAG
ACATTGTGAAACAGGCCCTAAACATCATACATAAAATATTTGAATGTCAAAAAGATTTTATTTTTTTATC
AGTTTCGTTACGTTTGATTGTAGCGTGTCTGACAAAGTTTAGTAAAATGCATACTTCTTCCGAAAATTCT
TATTAGTATTTAGTTGTCGTCTATGGAGCAACATATAAATCTGTTTCACTATACACATACTACAGGATGTT
GCAAAAAAAGGCATATGGAAAAGGATTTATGGTCTGGAAACTATAACTTTTTCAGATATGCTACAACCT
TTATTTGTTAGTTAGGATAAGCTCAAAAAGAAAGTTTTTTGTGCAAATTATTAATATTATTCTAGATAGAT
TCGCTTTAGCGCCTATCATAATGTAAACTAATAACATTTTCTAAGCGGTAATATTACTTGTGC
>TCONS_00071338 geneID=XLOC_014310 scaffold_236:305680-326653(-) Exonic overlap (+)
TAAAATTGTTACTCTATATTTCATACAAGTCACAAGATTAATACGCCCTTTATCATAGACTATATAGTCTAT
GCCTATATTTTAGAGGCTCTAGAATTTTAACCTATATTTTTCGTGTCCAAAGAGATATTTTGTTACTTCAA
TATATATAGTGCCACAAACTTATCTGTTCCGGTGAGAGCGAACTAAATTGGTCCATACCTCATTACTTAC
TAAATGAATTTCATATTGACATAGATTATATGGACCAATTTAGTTCGCTCTCACCGGAACAGATAAGTTT
GTGACACTATACCTACAATCTAAATGTTTCCATAACCTCTACCAATGTTGCTACACAAAAAATACGTAAA
ATTTCGGTAAGAATACTGAAAAAGTAGTCAAGGTAACCGTCACCTTTCGGTATCGTATATCGCACGTAT
CGGACCAAACGGATTGTCATAGATGTATGGAGAAACGGCGTCGGCAATGCCGATGAAGTGGACGCAC
TTGTGTGAATTTCTACACAAAGAATACTGAATGCGATGCGTCCGTTGGGTACGATGCCGAAAGGTAGA
CACTTACCTTTAGGTGGCATCGCAGGCGAGTCACCCAGTTTAAGACATCACATGCATAATAATATTACA

GTGCTCCAAGATTGATAATGCGCATAGAAATCTTTGACAGATCGGTTGTTTTCGATTATGTGACACATGA
TATTGACTCCTATTTCTTTCGAACAAGGTGCAAAATGCAAGTGTTTTTTTAAGGCTCTTACGATTTAATG
ATTCGGGTGTGAAGATCTGCATGTGCATTATTAATTTTGAACAAGTTTACGTCAGCCTATTGATAAAGGA
AGATTAGAGCTACATTTTCGAATTATTTCAGAAGTATAGATTTGTCTTGTTAAAGTGAGGCATTAACACT
TATTCCATGCCAGTCTTAGTGCCAATTTCTCCATAGTTGGTTATCTAACCGACAGGGATTGATTTACTAAT
TATACGTCAGGTCCATATAAAATTCATGACAGATGTGTCAATGATGTCGATTCTGAAGGCGGAAATTCTA
ATTTTAATGCTGTCAAAACCTTAAATTCTTTGTTTAAAATTCGACTCTATGACTGGTCCCTGAAATCTCA
TTTTATACTAGCAATTTTTTTTTGACAGCGTTAATATTAGAATTTCTGCCTTCAGAATCAACATCAATATC
CCTGGTTATGCTATAACCGACTATGGAGAAATTGGCACTGAAACTATCGTCTTAAACTATGATAGAATCC
ATATTTATTTGTTGTAAAAGTCGATTCTTTCTTATAAAATACACAAATGTAGAACCGACCGAACAAAATG
TTTTTAATTTAAAAAAAAACGCTTGCCTCTTCGTATTTATCGCTTTTTTACGATTGTGTATTTTGTCTATTT
ACTTTTTAAATGTACTAAAAAGTTCTTTTAAGAATTCGGTATCGTCTCGAGAAATTATGCATTTTTCTAAT
GAAATAAAAAGCATTTAATTTATTTAATTCATTATTAAATTATTCTTATAATCATGCCATCACAATGTTTAT
ATACGAATGGTTATAAAAGGCCTAGTTTTAGGGTATTATTACAGTAGTTTTTGGGTTGTGTGTAGTCAAG
CAAATAATTTTGATATTTCAGTGTGTAAATGTTACGTTTATAGTTAACGTATTATGTGATTTTTTTACCAAT
TTCATTAGCTGTTATCTGTTGAATCGTTTTATATAAAAGTTTTTGGGATGTGAGTCCGTTAGCGTGATGCA
TTTGAAACAACCAGAAGATATGTATTTATTTTAACTATTTTGTCTGCGGTGAGTGTGTTTTGAACGCACT
TGCATAGACAAAGATCCTAATATCTCAAACTCCTTATTCTGAGGAATTTCGGGACTTTCAATAAACGTAT
ATTTGTGGCATCTACAAGCAACATAAGTACTCATTATTAGATAAAATATATTATCCAATTCAGTCTTTAGT
TTAGTTCTAATCCCGTAGATAAATACTAATATTGTCACATTTTTCTATAACTTTTGGAAAACTTTTTAAAA
ATTGTTTCATGTCATTTTGGATGCCGTATTATAAAAACAATGTTTGTAGTAATCAATAAATAATTATGTTG
GCAACATTTATTTTCAAATATACAGGTTGTTGGAAAAATTGTATATAGTAAGATTAAAGGGGTGGGTGAT
TCAGGGTGTCACACTAAATAATGTTGATACATGTTGACAAAATCTATTCGCTATGTATGGTAAATATTCTA
AAGTTGTTGAAAATGACCCCTTGAGTTTCCACTTTTGGTCTACTATACAATTTTCCCAACACCCTGTATA
CCAACGTTCAGTTGCGTTAAATAAATGGTGTTATACCATTGCCATGTATGTAGGTTATGTAAGTAGAAGC
CTGCATAGGGCTATAAAATATTGTAACAAATTGAGAAAGCGAAATGGATACGTTGGCTTATTTACCGAT
GATGATGATGATGATTTTTAGTAAACATTTTAACATACGTACACAAATCTGGGCCTCTAGTACGGGTTAC
GCAGTTTACGTAAACGAAAAAAGTTTTGGTTTTCTTCTATTCTATCATCTGCCTACATTATCTGTCAAAA
GTTTCATGATAGTTTCCGCTAGCGGCCCACTTTGTATGCGAGAAAACTGAAACTTTTATAGTTTTTGAC
AATCTGTCAAAACATGTACAGTCCTCCCATATTAATAATGCGCATGCAAATCTTCGCAAACTGTCGTATT
CCCGGAAACACCCGAATCAGTGCCTTAAACAAAGCACTTGCATTTTGATCCTTGTTCGAAAGAAATAG
TAGTCAATATCATGTGACACATAATCGAAAACAATTTGGGCGGTCGGTGGCTGTCACCGATCAGTCAA
GGGTCTCAAGGTCAAGATCAATATTACTTTTGTGGAATTATAAAGAGCTGCAATTACACATCGTTCTTGT
TATTTTTTTCAAATGTGAATTTAATTTCAACTTTAATTATTTTTGACGATGCCATATTATGAGGCACGTTTA
AGAATAAAATATGCGTCACATTGATAGACTTCACTTTGCCAATAAAGCCACACCCAAAAGACCAAGTT
TGTAATTGTAATATTATTGTGAATGATCAGCGCTGTAAATACTTTTGAACTGAGATTTTAATGTAAACATT
TTTATTAATGTGAAATAATCAGTGCTGTAAATACTTTTGAACTGAGATTTCATTTTTTTTTATATAAACTT
GTGTTTGAAATCCATTTCTCCTTATAATATAAACCTTGTGTTATTCAAACCTTCTTTCATCCATCTTTACAT
CAGCTTCAGTAAGACACTTGAAAAGTACCTACTGTAATATTTTCGAAGTAAATTTTAATATTATGGAATA
TTATGAATTATCGAATCAAATTTCGTTATTGATAAATCAATTATCTCTTTTAGCACCAATTACATAATTCTA
CTAAAATTTCATGAAGAAAATGCACTTAACCACTCTAAATACATAATAGTTTTCTAACGCTCGGGAATAC
GACCGTTGCCGAACAATGACGAAGATTTCTATGTGCATTATCAATTTTGGGGAACTGTACTAGACCTAC
TGGCTTATTTAAAAGTACGATTTTTTACCCTCCATTTGTTAAAAATCACGTAGGTGCATTGCATGCAGTT
ATTCAAATATATCCTCGTGGTACGGGTCTTACGACATCGATAAACTATTTATCGCGAATCTTGACATATTG

ACGTTTATCCACGTCGATAACGGACCTATACCAAGATTGAAATCGTACTTTAACGCCTGAACGTGAGTA
ACTATAAAATCACTGATTTTAAATGATTGGCGTAACAGTTTACAACTATTTATTTATTTCTCACGCTTTTA
ACACAAGTGATATTTAGATAGAAGGTGGCATTCAATGCAGAAGGAAAGTAGGAAAACGAACAGAGCA
ATTGAAGTCTGTTTTTTAATCTCAGATACTAAATAACAGATTTTGAACGGTTTATCAACAGTCGATTGTT
CCGCGATTAAGTGACGTATCGTTCTATTGGCGGGACAATCGACTATTGATGAACCGTGCAGAATCTGTC
AATTTAGTATCTGAGATAAAAAATCAGACTTTCGAATACAGGAAATTAGAACCTTTTGCCCGAGCACAG
GATTCGAAACCTCCGTTTACGTTGCGTATCGTCAAATGTCACTGTCAAAATGTAAGGCAAATTTCTTAG
TTCCAAAAATGGCATTTGTTTTTTCCATGTTAGATGGAATTTCACCAAACGCTAAACGTATTTAGTAGCC
TGTCATACGTCTGTCAGTTAGTACAAAATGTATTTAAAATTTGATTTAAATACGTTTAGCGTTTGGTAAA
AGCGACCCTTTGTGTAGATTCGAAAATAGTATTGAATCCTATGCTCGCGCTACTTGTCGAATACTTGATA
GCTTATAAAAAGCAGTTAAGGCCGAATCCTATTTTTGAGATATCATGGTATTTTGATAAAGTTCTAAATC
AAACCTGTCATTGCTCGTGCGTGACTGTACCTAAATATGGTAACACCAAGAATGTGATTGAATATTGTAT
GTTTTAGTGAAAAATAACGCTGATTTAGCTCACACTTATTGTTCAGTTCCCGCTGCACGGGTCAGTTAT
CAATGAAGATAAACGTCACTATATCTCGATTTACGATAAATATGGCGCATTAAACTTAGTTACCAACGTC
GATAACAGACCCGTGCCACGGGTTCAGCTATAAAATCGACCTTGGGTCGCTATGGCAAAATAAAATAA
AATGCAACTCGGCGCTGTTTTTCAAAATGAAGGTGCCAATTGCGACACAATGCTCGATCTCTAACGATA
TATCTACGTCGATAAAGAGCCCGTGTAGCGGCAGCTTGCAGTCCGCGGACAATTGGCACCTTTAACGT
AAAACTTTTGATATGAATGGAAGCACTTTTAAGCTTGTTTTTAGTCCATTATTGTAAATACATTACTTTTT
TACACAATATGGTTGTATATATG
>TCONS_00071368 geneID=XLOC_014310 scaffold_236:305680-327577(-) Exonic overlap (+)
CTTTTAGTCATTAAAAACTTTTTAAACATGTGATAGACAGTCTGAACTGTTAATAAGTTAATTTTCGCAC
AAAACTACAAAATTAAGTTAAAATCTTAATCTTGTAAACGATCATACGTTAGTTGGTGAGCGAATTTAA
CGATGTTTGTGAAGTAATTATAATAAAAAGGATTTAGGAAAGCTGTCGTTTATAGGGTGACATTTCATAA
GTGGCCGTACCCGTACCAGGTTAACGTACCTGACGTACACTGAAGAGTAATAAAAACACATCTTCAAA
ATTGAGTTTTCAATTAAGGATTAAGTTCAATAATACCACGCGCTCTTACGGTGATGGAAAACATCGCGA
GGAAAACTGTACATCTAGATTCTAGATGTGTATCCTAAGTGCATTATTTATTTCATTACTTATCACAAAAC
GGCGATATGGTTCGCAACCTATCACGTGAGTACAAATGTGCTACGAAAAGTGGGTGGCTCGCTTGTGA
GCCACCTCTGACTACCCCTTCGGGGATTACAGTCGTGTATATCTTCAAAATTTCGTCGCTGGAAAAGCA
AAAATTACAAAGTATATTGTTTTTCAAAAATAAGTTCTTTACACTGATAAAATGTCACTCTGTACATAAT
ATTATATAATTACGAGCCTTAATAGTCTGTACTTACAGTCAAACTATTAAGTCCTGACATCAAAGAGTGC
GATTTTGCTAAGACTTTTATGATCATCAGTTATTTACTTACTTTTACATACATTTTAAAATAAAATACCGAT
TGATTATGATTTTTTTACGCATTTGTTCTTTAATAAATATAACAGACCGATTAAATACGATCTTAAACAATT
AGTTAGGTTAGGATCCACTTCCGTTTTCACGACTTTATAGTTGGACTGAACATTAGTATTTAATTTTGTG
ACATTAGTTTTCAGTATAAAATTGTTACTCTATATTTCATACAAGTCACAAGATTAATACGCCCTTTATCA
TAGACTATATAGTCTATGCCTATATTTTAGAGGCTCTAGAATTTTAACCTATATTTTTCGTGTCCAAAGAG
ATATTTTGTTACTTCAATATATATAGTGCCACAAACTTATCTGTTCCGGTGAGAGCGAACTAAATTGGTC
CATACCTCATTACTTACTAAATGAATTTCATATTGACATAGATTATATGGACCAATTTAGTTCGCTCTCAC
CGGAACAGATAAGTTTGTGACACTATACCTACAATCTAAATGTTTCCATAACCTCTACCAATGTTGCTAC
ACAAAAAATACGTAAAATTTCGGTAAGAATACTGAAAAAGTAGTCAAGGTAACCGTCACCTTTCGGTA
TCGTATATCGCACGTATCGGACCAAACGGATTGTCATAGATGTATGGAGAAACGGCGTCGGCAATGCCG
ATGAAGTGGACGCACTTGTGTGAATTTCTACACAAAGAATACTGAATGCGATGCGTCCGTTGGGTACG
ATGCCGAAAGGTAGACACTTACCTTTAGGTGGCATCGCAGGCGAGTCACCCAGTTTAAGACATCACAT
GCATAATAATATTACAGTGCTCCAAGATTGATAATGCGCATAGAAATCTTTGACAGATCGGTTGTTTTCG
ATTATGTGACACATGATATTGACTCCTATTTCTTTCGAACAAGGTGCAAAATGCAAGTGTTTTTTTAAGG

CTCTTACGATTTAATGATTCGGGTGTGAAGATCTGCATGTGCATTATTAATTTTGAACAAGTTTACGTCA
GCCTATTGATAAAGGAAGATTAGAGCTACATTTTCGAATTATTTCAGAAGTATAGATTTGTCTTGTTAAA
GTGAGGCATTAACACTTATTCCATGCCAGTCTTAGTGCCAATTTCTCCATAGTTGGTTATCTAACCGACA
GGGATTGATTTACTAATTATACGTCAGGTCCATATAAAATTCATGACAGATGTGTCAATGATGTCGATTCT
GAAGGCGGAAATTCTAATTTTAATGCTGTCAAAACCTTAAATTCTTTGTTTAAAATTCGACTCTATGACT
GGTCCCTGAAATCTCATTTTATACTAGCAATTTTTTTTTGACAGCGTTAATATTAGAATTTCTGCCTTCAG
AATCAACATCAATATCCCTGGTTATGCTATAACCGACTATGGAGAAATTGGCACTGAAACTATCGTCTTA
AACTATGATAGAATCCATATTTATTTGTTGTAAAAGTCGATTCTTTCTTATAAAATACACAAATGTAGAAC
CGACCGAACAAAATGTTTTTAATTTAAAAAAAAACGCTTGCCTCTTCGTATTTATCGCTTTTTTACGATT
GTGTATTTTGTCTATTTACTTTTTAAATGTACTAAAAAGTTCTTTTAAGAATTCGGTATCGTCTCGAGAAA
TTATGCATTTTTCTAATGAAATAAAAAGCATTTAATTTATTTAATTCATTATTAAATTATTCTTATAATCATG
CCATCACAATGTTTATATACGAATGGTTATAAAAGGCCTAGTTTTAGGGTATTATTACAGTAGTTTTTGGG
TTGTGTGTAGTCAAGCAAATAATTTTGATATTTCAGTGTGTAAATGTTACGTTTATAGTTAACGTATTATG
TGATTTTTTTACCAATTTCATTAGCTGTTATCTGTTGAATCGTTTTATATAAAAGTTTTTGGGATGTGAGT
CCGTTAGCGTGATGCATTTGAAACAACCAGAAGATATGTATTTATTTTAACTATTTTGTCTGCGGTGAGT
GTGTTTTGAACGCACTTGCATAGACAAAGATCCTAATATCTCAAACTCCTTATTCTGAGGAATTTCGGG
ACTTTCAATAAACGTATATTTGTGGCATCTACAAGCAACATAAGTACTCATTATTAGATAAAATATATTAT
CCAATTCAGTCTTTAGTTTAGTTCTAATCCCGTAGATAAATACTAATATTGTCACATTTTTCTATAACTTTT
GGAAAACTTTTTAAAAATTGTTTCATGTCATTTTGGATGCCGTATTATAAAAACAATGTTTGTAGTAATC
AATAAATAATTATGTTGGCAACATTTATTTTCAAATATACAGGTTGTTGGAAAAATTGTATATAGTAAGAT
TAAAGGGGTGGGTGATTCAGGGTGTCACACTAAATAATGTTGATACATGTTGACAAAATCTATTCGCTA
TGTATGGTAAATATTCTAAAGTTGTTGAAAATGACCCCTTGAGTTTCCACTTTTGGTCTACTATACAATT
TTCCCAACACCCTGTATACCAACGTTCAGTTGCGTTAAATAAATGGTGTTATACCATTGCCATGTATGTA
GGTTATGTAAGTAGAAGCCTGCATAGGGCTATAAAATATTGTAACAAATTGAGAAAGCGAAATGGATAC
GTTGGCTTATTTACCGATGATGATGATGATGATTTTTAGTAAACATTTTAACATACGTACACAAATCTGG
GCCTCTAGTACGGGTTACGCAGTTTACGTAAACGAAAAAAGTTTTGGTTTTCTTCTATTCTATCATCTGC
CTACATTATCTGTCAAAAGTTTCATGATAGTTTCCGCTAGCGGCCCACTTTGTATGCGAGAAAACTGAA
ACTTTTATAGTTTTTGACAATCTGTCAAAACTGTACTAGACCTACTGGCTTATTTAAAAGTACGATTTTT
TACCCTCCATTTGTTAAAAATCACGTAGGTGCATTGCATGCAGTTATTCAAATATATCCTCGTGGTACGG
GTCTTACGACATCGATAAACTATTTATCGCGAATCTTGACATATTGACGTTTATCCACGTCGATAACGGA
CCTATACCAAGATTGAAATCGTACTTTAACGCCTGAACGTGAGTAACTATAAAATCACTGATTTTAAATG
ATTGGCGTAACAGTTTACAACTATTTATTTATTTCTCACGCTTTTAACACAAGTGATATTTAGATAGAAG
GTGGCATTCAATGCAGAAGGAAAGTAGGAAAACGAACAGAGCAATTGAAGTCTGTTTTTTAATCTCAG
ATACTAAATAACAGATTTTGAACGGTTTATCAACAGTCGATTGTTCCGCGATTAAGTGACGTATCGTTCT
ATTGGCGGGACAATCGACTATTGATGAACCGTGCAGAATCTGTCAATTTAGTATCTGAGATAAAAAATC
AGACTTTCGAATACAGGAAATTAGAACCTTTTGCCCGAGCACAGGATTCGAAACCTCCGTTTACGTTG
CGTATCGTCAAATGTCACTGTCAAAATGTAAGGCAAATTTCTTAGTTCCAAAAATGGCATTTGTTTTTTC
CATGTTAGATGGAATTTCACCAAACGCTAAACGTATTTAGTAGCCTGTCATACGTCTGTCAGTTAGTACA
AAATGTATTTAAAATTTGATTTAAATACGTTTAGCGTTTGGTAAAAGCGACCCTTTGTGTAGATTCGAAA
ATAGTATTGAATCCTATGCTCGCGCTACTTGTCGAATACTTGATAGCTTATAAAAAGCAGTTAAGGCCGA
ATCCTATTTTTGAGATATCATGGTATTTTGATAAAGTTCTAAATCAAACCTGTCATTGCTCGTGCGTGACT
GTACCTAAATATGGTAACACCAAGAATGTGATTGAATATTGTATGTTTTAGTGAAAAATAACGCTGATTT
AGCTCACACTTATTGTTCAGTTCCCGCTGCACGGGTCAGTTATCAATGAAGATAAACGTCACTATATCT
CGATTTACGATAAATATGGCGCATTAAACTTAGTTACCAACGTCGATAACAGACCCGTGCCACGGGTTC

AGCTATAAAATCGACCTTGGGTCGCTATGGCAAAATAAAATAAAATGCAACTCGGCGCTGTTTTTCAAA
ATGAAGGTGCCAATTGCGACACAATGCTCGATCTCTAACGATATATCTACGTCGATAAAGAGCCCGTGT
AGCGGCAGCTTGCAGTCCGCGGACAATTGGCACCTTTAACGTAAAACTTTTGATATGAATGGAAGCAC
TTTTAAGCTTGTTTTTAGTCCATTATTGTAAATACATTACTTTTTTACACAATATGGTTGTATATATG
>TCONS_00071413 geneID=XLOC_014358 scaffold_237:56406-57008(+) Intergenic
TTTAGAGACTTACGCCCTGCCCCGGCCCAATGATGTCGTGGCGGCGGTGGAGGCTGTGTTGACCAACA
AAATCAGCAATTCGGCAGCGCAATAGTGGTAGGAATCTCTAGCAAAAAAAAACGTACACGAATTCCAG
AGATTTTTTAAATTATATTGTAAATATTTTTTATTTCCATGTTAGTTTGGGTGAATAATTCTCACTGCCTGT
TCATTACACATGATATTGGATATATCAAGCTGTATTATGATTGTGAATGGAAATTTATACTAGTGAAATCG
TTATTTAAATAAATGTTATTTATTAGTAAAACTTTCAC
>TCONS_00071821 geneID=XLOC_014399 scaffold_238:5780-7876(-) Intergenic
GCTGTCACTACCCGGGCTCGTTATCGACGGCTCGAGCTGTGGCTGATAGTACCGGGACTCACAAGTTT
TTCATCACCATCTGATGAATCCATCTTGTATCGGCCACGGAATTATGTATACTTCATCATCAGTCTATAGC
AGTTCAATGCTCTCCCAAAGCACGCCACTGGATGCGATCTTTAGCTTTCCGCATCCAATCATATCCAGC
CACCTTTCGCAAGTCGTCACCCCATCTAGCGAGAGCCGTATGTATACTTAGCAAAGTAATACATTCAATT
ATTTTCATTATTGCCGAAATTGGTTTTCTTTTTCTTATCGTGTAGGTGAAAAACACAAGAGATAGTTATT
CTAAGCTTAGTTTGGAAAGTAAGGTATTATTTTCAAGCGAGACATTTCTTTGGTACCGTCACTCTATGTT
GTAATTTTTTTCAATAATTCAAAGGGATATTTTATAAGATTAAATAATTACCCATTTTTTATTAGTTAACCA
ATGTCCAAGCTACAGCGTCAGCAAAAACTCTGTGTCGGTCAGCTTTTATATAAAGCTTTATCTACACCG
TCCCGGTTTCATAGTTCATAACTGCTGTAAACAGAACATAAAACTTTACGAGATGCGTAATCATTGTGG
CTGCGGTTACTCATCACCTGCTGGCTATTCTTCTCTATAAAATTTAAAAACTTTGTATATTTAAAAACTTT
AGTTCTCCACCAAACAAAGGTCGGTTGGAAGAAATTTCTTCTTGGCAATAAGTCCGCCTTTTTATACAC
TACTTTTTAAGTCACTTTTTGTAAATGTGTTTTTAATATATATACATATATGTAGTAAGTGTTTAAATAAAT
AAATAAAAGTCAGTCGAAAACAAATTACTAATTGTCCTTTTTGTATTTAGACCTCAAACCATCGGGACG
TTTTCGAGGGTCGGTTAAAAACGTTTCTTTTACAAAATTAATGCATTCAGTAGTTTATGTTCGGAATTCG
CCTTTAACAAAACGCTAAAGCGTATTGTACCTACATATTAAAATGCCAATGGAAAGGTAACTTATAAAA
TCCCACCCTGTATATCTTCATATCAAATTCAGCAAAGCCGTAACAGTGTCTAGCAGGCGCTTAATCCAG
AGAACTCGTAACATATTTGGCGAAGACATAAAATTTTGTAATAGCCTCTCTAGTGTTCCCTAGAAACGT
CCCTTACACATTTGAACACAGATAAAAGAGAAATGATAGTCGGCACAACTTCAATTATTACGATGATTA
TTATCATCATTTTAGTAAATGTTAGGTATATATAGGTATTGATAACCAAACCAACGTTAAAAACCCTCGA
CGCTAACAGTCGCATAACTTTTGAACTGCTGAGCCAATTTTGATAAAAAATGGCTAAGAACACACACA
CCCCATAAAACATAACGAAAATCCGTTAGGGAGCTACGCAACCACAGACAGATACACAGTCACACAG
ACACGTTAATTGTATACCACCCTCTTTACCAGCTTACTGTTGAGGGTAGCCTCCCCTTTTTCTTATTAGC
GTGACT
>TCONS_00071601 geneID=XLOC_014390 scaffold_238:162008-166715(+) Intergenic
TACGCAGAAACAAAGGAACCGGATCTAAAGAATCTTAGTTATGGCATTGAAGAAAACCATTATTTACC
CGGCATGGTGGCGGGCGCCGTGGCGGTGCTGACGGAGCAGGGCGACGCCGAGTCCGCCGCCAGCTG
CCTGGCCGGCGCCACCGTCAACCCGATAGTAAGTACAATACAACATTATTAAATATATACTTACCTACTA
CCTGACCCGTCACGAGCTTCGCTTCCCCTTAGATTGTTTTCCTTCCCATGAAGGAAGTTCTCTCGTCCC
TAAGGTTTTTTTTAACCTGGACCTGATCCTTTCAGGAGGTGATACAGGAAGGCATGTCCAGTCGAT
>TCONS_00071605 geneID=XLOC_014390 scaffold_238:168641-173954(+) Intergenic
ACCCCGTATAATCCCATACTGTTCTTATTTTATTTTAGTTTATTGTATGGAAAACCAACACAGCTTTATAC
TAGTAAAAAAACCAGCTAACCTAATTAAAGATATACAGGGTGGCCCAAAGAGTGACGTCCAAAGGAAA
AATGCCAAGGGGTAGCCAAATCACCCCCATATATTATGTTCAGCAATTCTTAGTATTTTAGGAGTTATGA

TTCTTTTAAATAATTTTCTAAGAAAATTGACCTTAGTGGATTAATTCATTATGTTTTATTATTTTTTTGGAC
AACTTTTTTTATTTTTTTTTATTTTCGTGTCGTCCTCCTAAATTGATCTTTTAACCATAAAAGTTTCAGCT
TCCTAGCTGTAATATAACATAGTTATTTTTTAATCGAATGTCGAATTTTACGATCGAGCTAGTATGCTCTG
AATTTTCAACTTGAAATTAAAATTGAAAAAGATAATCTCATGGTCCCTTATTAATTTTAAAAACTTAGTA
ACTCCTAAACTACTAAGAATTGCTGAACATACATGGGGGTGATTTGGTTACCCCTTGACCTGAAGAATC
ACTCTTTGGGCCACCCTGTATATACAAAAATCTAATTATAGGTTTTCGCATATGAAGACGATTTAACAAT
TTATTTTATTTTAATATTGACGGATCACTAACAACTATTTGTTACTTAGTAGTTACTATTATACAGTTGTAG
TAACAGCGAGTCATTTAGAAGTGACCACTGTTAGGATAACAACATATTAGAGTACCAGTAGTAAGTATA
AGTAAAGTAAAATAGTTAAGTAGGTAGGTTGATAATTAGTTAGTTAGTAGTACTTATAAAGTTCTATTTC
GATGGATAGATACATCCACCATCGTCTACGACGAGAATTCTTTTTTATATTCGATATCTTTTTAAACATCG
AACATAAATTATGTATTGCAAAAAGATACAGCGCGGCCGCTGTAATTATTTCGACGTCTGCCATTGCCG
ACACTTGAAAGCGAAGTGAGTGGTAGGGCAGTATGTAAACACACACTCCTCTCGATCCTGCTGCAGC
AGTATTTAGGGACTTATCGCCGGCAGCGCGAGGGGCAGCGGCAGGGGCTGAATGTAAATGGGGTAATA
GACGTTCATCGTATTCACATCAAAAAACGAAAAGGTAAATTTCAAATGCGTTTTTCTCAATAAGGGGCC
GTCCATTAATCACGTGAGGCTCAAAGGGGGGGAGGGGTATGGAAAACCTCACGAAACATCACGAAGG
AGGAGGGGGGGTCTCGTTAGATCACATGCACCACGACCCATTACGTCCCCACTGCTGGGGCACGCGTC
TCCTTCCAATGAAGGAAGGGTTTAGGCCTAGTCCACTACGCTGGCCTAGTGCGGGTTGGTGGACCCCA
ACACAAGCAAGCTTGTGCTGAGAGAGTGGTCGGGTAAGTGGGCAACCCGACTGTCAGATGTTTTCAA
GCCGACTGCTGCCGAAGCAGCAACCGGTACCCACGGCTTAACGTGCCGTCCGAAGCACGGAAGCGTC
CATGCAGAAAAGAACCACTTGAAATCGGTCACCCATCCAATGGCTGACCGTGCCAGTTGTTGCTTAAC
CTCAGTGATCAGTTACGATCACTGAAGCCCGCTCGACTACGGACGCTTCACGTGTACATACAGACATC
ACGTGTATTATTTTTTTGTCAAAAAGCGCTAGGTATTTCTGAAACGAAATTTTGAAAGGCGCAAAAATT
TGAACGATTTGTAGTATGACCTTGATTGACTCGCCCGCCGTTGCAGCAACGAGCTCCCGGCGCGCCGC
GAAAGACTTGATCTTTCAGCATCTACAGGGTGTTGCAAAAAGGGTTTACTAAGCGGAAACTTACATGT
GCAGCATGGTATATTCTATTATATTCTATTCTCTGTGGGGGTGTAAGTACCTG
>TCONS_00071825 geneID=XLOC_014402 scaffold_238:187711-188934(-) Intergenic
CCTACTGCTACTGCGATTGCGACGCGACATTGCAATGACGCATAAATTTGTATTTTATAAAAATGAAAAT
AGGTTAATTTACTAATTAGTTTATTATTACTGTAGTTAGGTACATACTTTGTTACTGTACAATTCTACATAA
GTACTACCTACTACATATATTTACTTACATTGTAACTAAATAATAATTATGTACCTACTTAAAAGTGCCTAC
TTAAATTAATAATGAGCCCCGATTTACGGCGTTCGTGATTCCAACTACCATCCTGCCTTCCATCTGTCCT
CTACCTGCCTACTTCACTGCTACCCTGAACACTACTTCCTCCTGAA
>TCONS_00071627 geneID=XLOC_014403 scaffold_238:206330-219526(-) Unclassified
AGTAGAAGTAGGTAAATCTTAAGCCTGTTGATCTTAATAGTCCAATAAGTACAACAAAGTGTGGTTTAT
CAATTCCAAAATTGCTTTATCATGGTGTTCAAACTATTTGATAACATAATTCTTACGAAACTGGGCGGAT
ATATCATTTAAGTAAATTAAAAAGTTTATCTATTACGTGTAGTGACGGCGGTTATGCGTATCAGCGAATAT
CGGACTAGATTGGTAATCTGTGTTGTGGACAATTCTTTTCCAAATCAGAAATAGGTACCACGCCGTTTG
TCGCGGACTACAGTTCGCCTGGTGTGATACAGTACTTACTAACAGTTAAAATATACGAGACATCTATTTC
AAAACTTATACAGTCAAACTTGGTGGCGACCCCAGGCTCCCCCATACCGTCGACGAGCCGCACGACG
CCTGAAGAACTGGCGGAGATGGAGATGGCGGCCTTCAGGGGGCTACAGGGAGATGATCAGGTCGTTT
GGGTAGGAATTGAAGCTTTGTGTAAGTAACTATGTGGTATTATGGTCTAAACTGCCATCTGATAGTTGG
ACTACTCGTATTTGTAATACTGTGTCACAAGGGGGAAAACTGTTCGTCGCCGACTGAGTTAGCGGCGA
CGACGGTTTGAGGTAAGCGTCCATGTTTGTATAGGTATCGTACGTATCGCACCAAACGGATCGTCATAA
ACGTATAGAGAAACGGCGTAGGCAATGCCGATGTAGTGGACACACTTGTAAGGTTGTACCTAAGATAG
GATATACCGATAGGTG

>TCONS_00071630 geneID=XLOC_014405 scaffold_238:270922-272993(-) Unclassified
TGGCATTACTGTTACATATTATGTAAATTTATTAAAAAGCTCAACCTAATCTGCCCACAGCGATAGAAGA
CGACGCCTCCACACTTCTAATAGACGTGTTGGTGTGCGGCGCGTGCGGGTTCGAGTCCTCGTCGCGCC
GCGTGTTCCGCGCGCACATCGACGAGGCGCACCCGCCGCCGAGCACAGAGGAACAAAACGAGGAGG
AGGAGTGAAGCAGAGAACAAGGAACCTCTATTCAACATCACACAAGTGCAACAGGGTGGGCTTTTAT
GCAGAGTTGATTGTGAAAATTGTGCCTACATTACTGTATGATTTTTTGACTATTTAGTTTAGATTAAGTAC
TTCAATTAGCCATATGGGTATCATTATAGAAAATTAGGAATAGCTCGAGCAGATAAAGAAGCAAAATGT
AAAGGAAGGTATTATTGTTTCTTTATTTGTGCGCGTTATAACAACTATTGCGTACGACTGAATCGTCTTT
AGATATGAAAATTGTGTAAATGTCAATACCATTCGAAATTATAACGAGACTTGTTAATAATTATGTGAAC
CTTACGTTTTTTTATAACTACTTACTAAGTTACTACAAAATTACCTCCATTACAATTATGAATTTTAAAAA
CTTTTATTTTTAAATTATGATTACATTAGTTACAGAGACCAAGTTTATCAGTCGTATTATCAGCAGCTAGC
TTGCTTGTGTGGGGTCCACCAACCCGCACTAGGCCAGTGTGGTGGACAAGGACTAAAACCTTCCTCCA
TTGGAAGGCGACCCGTGCCCCAGTAGTGAGGACGTGATGGGTTGTGATGATTATCAGTATATCACACAT
AAGACATTTTATCTAGACATGGTGTAAGGGGCAGTAAATCGTGTATGTATACTTTTTACTTTCAACCCAA
TGAACTGATGATCTTGTTCTAAAGATTATGTAAAGTAATATTAGTTAAATCTAAAGGTAAGCTGTTAATA
AAGCATTTATTCTGTTTATAACTGTTGTGTATATTTTATTCATCCGTCATTTACATGATATAATTATATATAC
ATTCTGAAACCGGGTGTTAGTGGTGACAACACTGACAACCCACCATACTCAGCAAAATTTATTATAGAA
TTTGGGAAGTTATTAGTTTTAACCAAGTTTTAACTATGTTAACGAAACAACTTGATGATAGGAATGCATC
TTCGCTTACCCTTCTA
>TCONS_00071796 geneID=XLOC_014394 scaffold_238:303820-305434(+) Splice junction overlap
TTCGGTGGACGATGAAGAGGAGGAGCAAGTAATGCTAGAGCTGGCGGCGGGCGCGCCGCTGCTGCTG
GACCACCTCATGCGCACGCCGCTCACCTGCGCCGTCATGTAGACCACCAGCTCCTCATGAGAACTTCA
CCAGAAAAGTACCAGCTCACCACCAGAGAGGCACCAGGTCATCACTAGGGAAGTACATCTCATCATC
AGAAACTGACATAACAAGTGGATGGATCTGATATATGACTGCAGCGGTCAACGGTACTTCATGCTTTCT
AAGCACCAGGATACCAACTCTCAGCATCAGGATCTGACAGAACAAGTATGAATGTAACAAATGACTGT
AGCAATCAGCAGTACTTATGGGTATCGTATATTTATAGCTGGCTACTGCCAGTGCACTAGCTTGTCACCA
GGTCACCACTTTCATTTTTAAAGCAACTAAGTTTCTGTGAAATTTGTCATAGGCTCAAGGACTCAAGCT
TGAGGCAAAAGCCAGAATCTGTTCTGCTGGTCCATATATCCACAATCAGCATCAACAATCCTTTTAAGC
AGCAATGTATAACACATATTATGCCGATTTGCAGCTTACTTATAGGTCGATTTGATTTATATGCCGTTCAC
TGTGCCCCGGCATACGATAGCAGCTAGGTGATGCTTTGTGAAATTGAGCGAAGACTCATGGGTGAGTC
AACGAGCTTTTGTGTTTCCAAACCGTACTAAAATTAGGTATTGATAAAATAAATAGTACAGAGATAAGT
GTCCCTCCACGCTTTACTTTCAAAATACCTAAATGTTAATAAAATATGAGGTGACTATAAAATGCCGTCG
TTTTTGTGTGGCGGTAGTCATCGGTTCATTAGATATTAAGAGTGAATACCTATTGAAGGATTGAAATATA
ATAATAATTGCATCTAGTCATTTAATAA
>TCONS_00071615 geneID=XLOC_014394 scaffold_238:304520-305905(+) Unclassified
CAACTCTCAGCATCAGGATCTGACAGAACAAGTATGAATGTAACAAATGACTGTAGCAATCAGCAGTA
CTTATGGGTATCGTATATTTATAGCTGGCTACTGCCAGTGCACTAGCTTGTCACCAGGTCACCACTTTCA
TTTTTAAAGCAACTAAGTTTCTGTGAAATTTGTCATAGGCTCAAGGACTCAAGCTTGAGGCAAAAGCC
AGAATCTGTTCTGCTGGTCCATATATCCACAATCAGCATCAACAATCCTTTTAAGCAGCAATGTATAACA
CATATTATGCCGATTTGCAGCTTACTTATAGGTCGATTTGATTTATATGCCGTTCACTGTGCCCCGGCATA
CGATAGCAGCTAGGTGATGCTTTGTGAAATTGAGCGAAGACTCATGGGTGAGTCAACGAGCTTTTGTG
TTTCCAAACCGTACTAAAATTAGGTATTGATAAAATAAATAGTACAGAGATAAGTGTCCCTCCACGCTTT
ACTTTCAAAATACCTAAATGTTAATAAAATATGAGGTGACTATAAAATGCCGTCGTTTTTGTGTGGCGGT
AGTCATCGGTTCATTAGATATTAAGAGTGAATACCTATTGAAGGATTGAAATATAATAATAATTGCATCTA

GTCATTTAATAATAGTCAATGCCTAAAATTACTTAGGTACCTACTTACTTATTTATTACAAATTGAATTTTA
TGTTCGTACTAGCTGTTCCCCCGCGCTTCGCTTCGCCATAAAAACTTTTCCCGTGGGAATTCCGGGATA
AAAAGTAGCCTATGTTCTTTCCCAGGGTCTAGACCATATGTATACCAAATTTCATCCAAATCCGTTCAGT
AGTTTTGGCGTGAAAGAGTAACAGACAGACAGACAGACAGACAGACAGACAGACAGACACAGTTAC
TTTCGCATTTATAATATTAGTTAGGATAGTTAGGATTAGTTAGGATTGAATTTTATGTTCGTATTAACGTTA
TATTACAGCACATGCGCTGTTAGTTTTTACACATACAAATAAACAATGCAAACTATTGCAGTAGATTTTA
CCTAATGTCATACAAACATTGTACAACATCGACCATTTCGGAAGGAATTGTATTTTAATAGGCA
>TCONS_00071715 geneID=XLOC_014406 scaffold_238:325832-335067(-) Unclassified
ACCGTTCGCGCCAAACCACGGAACGTATTTAAAATCCGCGTTCCAAATACAAATCGAAAAAAGAAAA
TTACTTTTGCGCGGTTTTGTTTTTTTTTTCTGCGGCCGGCCAGTACTGTTGTAGTGATGTGACATAACAT
AACATAGCGTGCATTATTGATTTAGTCCAAGTTGAAAATACTATCTACGGCTTCAATATTGTTTTCTTGGT
TTCTCGGAAAGTGCATTTATGTTTCGCTACGATATTGATTCGTAACGGATGACACGTCTATTAAGATTGT
GGTCGCCGAACAGTGTGGTGGTGTACACCAGTGCCAATGAAAACCAAACTCAAAGTAAACTTTACAA
TCAATAGTGCTAAGACTCCTTCCAATAACC
>TCONS_00071905 geneID=XLOC_014407 scaffold_238:351686-352537(-) Intergenic
CGCCTGGACGAAAAAAAACATGTCTGGTTATTTTCAAACTGAAAGATTATGCTAGAACATTAGAAAATA
TTCTACAAAAAACTTGCATACGCATATGCATCTAGCAAATCCTTATAAAATCTACTTTAGTTCAGGTAAT
TTGAATAGCAGTTTGCGCCATAAGGAATTTTGGGAATTCGTGTATCATATTGACTTTCTAACTGTTACAA
AGAGTAGAAACTACAGTGTGACAAACTTATCTGTTCCGGTGAGAGGGAACTCAATTGATCCATATCTCA
TTATTTACTAACGAATTATATATTCACATAGATTAGATAGGTTTATTAAAACCGCAAAGGCGAATTACCAC
TAAGGGCTAACATAAAAACTTATAGAATAGAGAAATATTAGACATTTACATAAGCTCTCACCGGAACAG
ATAAGTTTGTGGCACTGTACATAATATATGTAAGGGCAAGGTAACATTTTGACTCAATTTATAAGGGTAA
TAAAAATCACGGACATTATGGCGCAATCCAATTTGAAGACTAAATTTATAAACGCTTCCTCACTTCTTTA
GTGCCTGGGTCGAAACATCACGAGTCTTCAGATAGTATGCGATCTAAGCGTGGGACGCCCTACGGCCT
AATGGATGTTGGAAAAGCCCAGGCAACTTAAATCATTAGCCTTCCCCACTTGGGATTGCATTCTCAGTA
GGCGAACAGGTTGCCGACTTAGGGTGCTTACATAGAGAATCG
>TCONS_00071897 geneID=XLOC_014409 scaffold_238:398983-399599(-) Intronic
GTCTAAATTAATATTACCTATTCTTTTTATATTTATTTACAAACAGCGTTTTTGCTCTTATTATTGATGTGG
AAATCACCTTAAACATTTTCAAGTAATGCCATCTCCTTATTCCGAGGGCTGGCAAGACTGTTACCAGCC
GAACCTGTGGTTCTTCTCCTTAATACGTCTGTTCAGGATGTTTGTTAGTCTATTCCCAAGATATTAAACC
TTTTGAGTAAGCCTTCA
>TCONS_00072006 geneID=XLOC_014460 scaffold_239:407759-433381(+) Splice junction overlap
GTCGAGCAGAGAGGACGTGCCGCAGGCTTAGCGCTGGTGTGATCGTGTGTGGAACGCTTTCGCTTCA
AAATATTCATAAATCGAACACTCGTAGTTACAATAAAAAGTGTAAATAAAAGTGATCTTGTATCCGGAC
TGTATTTTTGATCCGAGGTCAATGAACTTGTTGTTTACAAACATTACGTGTGAACTTGCAGTGGTTTGC
TCAACGAGTATTTGTTGCTGAGTGCGAAGTAAAATTGTGTTTCTGAAGTTAAAAGGACTTTCGTGTTG
GTTAAATAATCGCTTGTGTTAATTTTGTTGGTTGACGAGAACAGAAAAAGGAGTAAAAATTATTTTGTA
TCAGTTTTTCAAGCTCTTCCAATCTGATAATACCCCAATGACTGATTAAAAGTGTTACTTAGACTATAGA
CAATGTGCTCAAATTAGACTGTTCTTAATGTGGTAATGCAAACTGTCCATTTTTGATCCTTTAATAGTGT
CGAAACCATATGTGTGTAAGACTAACGTAATATGTGCTAAAACTGTTTTTGGTGAACTCTGAACATTAT
GAACTGATGAGTAAAGTTGGAATTCGGCTAGAGGTTGGAGAGCAGCGGCATTGATGGCTACGGATGG
GCACCAACTTATCTAAATTGAACAAGACCAGAGTCTCCGGTGTTAATCATCGCGAGGGAGCGGCCCGC
AACGATGCGGAGCGCGAGCGGCGCGCGCGCGGCGACTGGGA
>TCONS_00072063 geneID=XLOC_014492 scaffold_24:241272-242327(+) Splice junction overlap

ATTAGCTGTAATTAGGTGCTGCGCAAGTCGCACCCGCCGTCCGTGCTGGCGCTGTGCAAGAAGTTCCA
GCCCGACTACGAGAAGCCGTCGCGGCTGCACGGCGGCGGGGAGTGCGCGTGCGCGCCGCCGCCGCC
GCTGCCGCCCGCCCGCGCCGTGCTGTGCTAGCCCCCCCGCCCCGGGGACAGACTCTGCCATCTCTGTC
TATGACACGCGGCCCCCGCCAAGGCGCGCCGCACGTGCAAAAGCAACTCTTATAAGCTCGGAATACA
GTCGACCCTGTGTCCAACATCTAAGAACACGTTGAGTAAGCACGGCGCGCCCCCGGCCGGCCCCCCT
GTCCCCCACTAACACTTGTGCCACAAGAAATTATTGCTATACCTGTTGAATGTAATAGGCAGTCGCGTC
CCGTCAGTGATGTAAATAAAATAAGTAGTTAAGTACTTTAGAGTTACTCGAGTTGTATATTTTTATTGGT
AGGGCCCCGATTACGCGAACTCGCCGTTCCGTCTCCGCGAGTCCCGCACTCGAGCGAACACGTGAGT
TCGTGAAGTAATCGCGCTCCTACATCGATGGTACAAAAAGCATAATTGATTACGCCAACAATAAATGTA
GCGATCGCTTGCGACGTTTGTAAATGTGTCTAAGATTG
>TCONS_00072322 geneID=XLOC_014495 scaffold_24:320072-323192(+) Splice junction overlap
CGGGTGAAGGTCTCGCCATGGGGGCTCGCGACACTCACTCAGTGCTCACCAGTTTTTGTTTTAAAATG
TGAATGGTGCAAACGGCATGATGATATGCCACGTTCCTCTCCTGGTGACGTCCATGATCATGATCTCGG
CCACTCTGTCCATTATTTGTGTCTTCTTGTTTAAGCCGGAAGCGATCCGTCTGCCCGACGCGCCGTGCG
ACGAGACGAACCCGTGTCCCGACAACAAGCGCTGCTGCGAGGGCGGCTGCTGCCGGCTCGACCTGCC
CCGAGCCATGACCTGGATGT
>TCONS_00072069 geneID=XLOC_014495 scaffold_24:320075-323828(+) Splice junction overlap
GTGAAGGTCTCGCCATGGGGGCTCGCGACACTCACTCAGTGCTCACCAGTTTTTGTTTTAAAATGTGA
ATGGTGCAAACGGCATGATGATATGCCACGTTCCTCTCCTGGTGACGTCCATGATCATGATCTCGGCCA
CTCTGTCCATTATTTGTGTCTTCTTGTTTAAGCCGGAAGCGATCCGTCTGCCCGACGCGCCGTGCGACG
AGACGAACCCGTGTCCCGACAACAAGCGCTGCTGCGAGGGCGGCTGCTGCCGGCTCGACCTGCCCCG
AGCCATGACCTGGATGTCGGACTACACCTGGCTGGATAACTGGCGCCTATTCCTGATCTTCGCGTCAGC
CGGCATAGTAGCAGCATGCGGCGCGN
>TCONS_00072070 geneID=XLOC_014495 scaffold_24:320075-323828(+) Splice junction overlap
GTGAAGGTCTCGCCATGGGGGCTCGCGACACTCACTCAGTGCTCACCAGTTTTTGTTTTAAAATGTGA
ATGGTGCAAACGGCATGATGATATGCCACGTTCCTCTCCTGGTGACGTCCATGATCATGATCTCGGCCA
CTCTGTCCATTATTTGTGTCTTCTTGTTTAAGCCGGAAGCGCGGTAACGTCCACAGATCCGTCTGCCCG
ACGCGCCGTGCGACGAGACGAACCCGTGTCCCGACAACAAGCGCTGCTGCGAGGGCGGCTGCTGCC
GGCTCGACCTGCCCCGAGCCATGACCTGGATGTCGGACTACACCTGGCTGGATAACTGGCGCCTATTC
CTGATCTTCGCGTCAGCCGGCATAGTAGCAGCATGCGGCGCGN
>TCONS_00072769 geneID=XLOC_014498 scaffold_24:413099-415062(+) Exonic overlap (-)
CCTTTACTAGGCAGTTCAATCAAAGCGGATAAAGTAAATATGTAAATGATAATAATAAATTAGTCATAAT
TTATAACTATAACACAGCCACGACAGAAAAAATATCACAAATGCTCTTACAAATAATAAAATGTGGTAA
TGACAAGCTACAAAATTTATTTTACGCTGAGTTTCAGAAATGGACTTTAAGCTAATGACGGCCTTAAAT
GTATAGCTGCCTTGTTTTGACAGTATACGGATAGAAAGAGACAGACATAGATTTAATGCCAACATTAGC
TTAATGGCACTTTCTGCAACTAAGCGTTAAAAAATGGCACTTAAGACACATAAATCAGTTAAACTTTAA
CTAGCAAATTGAAAACATAAATATCTATGTACTGTACTGTAATTTATTTTAACCAATAATTAAATAGCTAC
ATGTAAAAGCTACACTATAACCATAAATATTTTAATAATAACAACGCTTATCAACCATTCAACGTCAATCT
ACATAATTATTATTATAGTTTGGTAAGTATTCCCATACGATATTTAAGCAAATGCAAAATCGCACATTATA
CATTTCCAAGTATGTAGATAGATACATAGCTACGTCTATGTATTCTAGTGTCAATTTGGATTTAGTACTTA
CTTACATAAAATTATTAATATTTTGACTAGTAGACCGAGAACGGTACCCAGGTACGCTATTTCATTTTATG
AACTTTTTCGTTTGGAGCTTATATTAATCAAAATCTAGATTACGGACCTAATTTTATGCTAATAGGCCTTT
TGGGATTTCTATGAATACCTAAGGCAATGTAGATAGGAAAGGAAAATAATTAAATTCATTGATTAATTAT
TATCTCGATTTCAATATCAATCCCACGTAAATATTTCTGTTTATCAATGTCACTGCTGCAAATATATTTTTC

GTTTAAAATCTAAATTTTGTTTCCCATTTCTTAAGTTTCCTTTTATTGGCCAATCTTCATTTTTTTTCTATA
ATACTGAATTCCATGCAGCCAACGCTTAAAATGATCTGAGCAATGCAGTTCAAATTACCATACATAATG
AAAACTATACCTACTAAAATATGTAGTGGTTTTACTTCACACGTGAACAAATCAATCATTTTTATACATTA
TAGCATGAGCATGAAATAAATTAGTAGGTAATCTCTTCCCGTAACGGTACTTTTAGTACTAATTTCCTTT
AAAATATTTTGTAACATGCCTTTCATGGAATAGGTAGTGTCTTCTGGAAATCAGACGAAAAAATGCGAT
GCAGCATCGCACACTACAATGTCATCATGCAGGTAGCCATTGTGAAACAGGCCCTAAGTAAAACAAAC
GAAGCCACACGCTTACGTTCGCATAATCATGCCCGTCCAAACATTTATTCTATGAGCATTAAATTACCCA
ACTCTTACTGTATTTAGTACCGAATGTACATTCTACCAATTCCGTTCCCCAACTCAAATGATACAAAAAA
CCCTTTATTCGTATCCTCACTGCGAACTGCAACCTAGCCATTCACTATCCATTAAATAACTCAAATGATAT
AAAACAAACTCATTTGTTCGTCCTCACTGCCAAACCTAGCCATCAACTAACCAACCTAACCTTAAAATT
AAAAGCTAAACTTTCCTCTGCACTTGAAGAAGCCCATGCCGGTCTTGTTGTGCAGCAGCGAGGTGACC
CCCACGTAGCTCTGGATCACGATCTCCGCGTGCTGGATGCAGCACGAGGATGACTGCATGGCTTCGAT
GGCGCGCTGGAGTTTCACGGCGAATGTCTCCATGTCGTATGTGGCGCGCTCTTCTAGGGTCGCGTCGAT
TGCTGGAAGGAAATTTTGTGGTGATTGAGGAATGTAGGGCA
>TCONS_00072711 geneID=XLOC_014528 scaffold_24:431642-447557(-) Splice junction overlap
CCAGCGCACTGTTACCACCACTCCCAAAAAAACAAATTAGTAATTTGGTGTTCGAAATTTAAATTAGTG
ATTCGTCTCACGCGGCTCCTAGCTAGACCTCGCCTCTTCGCGCCACTGACACATTTCGCGGTTGGCGCG
CGGTTTGAATATAGAGCGTTCGGTTTTTCTTTGCTCCTGTTTGTTTTTTAATTTGGTGTATTTCTTTGGTG
CCTGCATTGAGTAATTTTGGGGACCTTAAAATTGAGACTGTGATTATCTGCATTTGGACTACTTAAAGCT
GTTTTGAAAAGATGGAGCTGCGGCGCTACTCCACCCCCCGCACCTCGTTCCTCACCTGCTGGATCTTC
CTGCTACAAGTATGTATGTTGCTCACCACTGTAAGATTCATAATTAGGGCCTTATTTACTTACTATTGTGT
AGGAGTTCTTGAGTGGAGACCACGAACAG
>TCONS_00072106 geneID=XLOC_014506 scaffold_24:906586-911388(+) Splice junction overlap
TGACGACAAAATGCGAAGCTTGTCAAATCAAGTTTACTAAAAAAAGATAAAATTCCCACTACTACACA
AGCCTAGCACAGTAGTTATCTTGTGCTGAAATACTACAATTTCCATTCGTGTGGCGACCAAATTCGAAA
TCACTTTGTGACTTCGATTAAGTTTCGCTTTTTACACACCAACACGAAGGATTCAATTTGACACGCTGG
AAAGATATGGCAGCAGCTACATCTTTTGCTTCTGATTATCATTGGATCAGCACAGGAAGCAGACGCAAC
TCAATCGAAAGTGTCCTCAGCATTCAAGGGAGAGAAACAGATGGTGGACTCCTGTCGATCCTTCAGG
GAGTCCCTGCTTCATCAGACTGAGGAATTGGAACCCGACAATTAACTAACAAAC
>TCONS_00072478 geneID=XLOC_014537 scaffold_24:908663-911388(-) Intronic
GTTTGTTAGTTAATTGTCGGGTTCCAATTCCTCAGTCTGATGAAGCAGGGACTCCCTGAAGGATCGACA
GGAGTCCACCATCTGACAATGTTATTAAATAGCCACATTAATAACTTGACAGAAAAAAGGCTTAAATTA
AAAACTGTATTTTCTAAGTTAAGTGGAGTGGGTAAGGGTATGGCTAAAATTGTGGTAATGATAAACACT
GTAAGTAGGTAGGTATAGGGACATTTAGTTATTAATTGAGGAATCTAATGTTAAATCAAAAAGAAATGG
GACATATAAAAGAAAGTAGCAATGAAAAGGCTTACAAAGAGGTGTTCAGACTCAAAATGATAATAAAA
CAGTGAGTCACCGCCGCCGAGTAGGGAGGATAATGGCTCGGAATATTTACGCGCCAACAAGTTCCAAC
TCGAATGCACAAACTACGTCTAATGCAATGACTCAAACGGTATGCAGTTAATGTTACAAAATATTTTCG
GAAAGTGTGACATTTCCACTCGGATTACCGAGAATTTACAGCACAATAGTCTTTGAAATTGGCTGTTTG
CGGACACTGTCGTGGTCTCTGATAAGAAAAATGTTTTAAGCGGAAGTTATTCGCGAGTACCGCGACTT
CCGTTCGTATCTCGATAGGATCTTTACAGTCATCTTGAGATAGATAACTATTTCATTTGGTTACAATTAAA
TAGCGACATACCTACTTGAAAAGAAATAAATACAGAAAATTGGTATGCCGCATTACATAACAATTATAAT
AACAAAGTAGGTAGGTATCTACTTAGGCTACAACTTGTAAATGCGCTGACTGGGCAATAGTCGGTGTTT
ATTTGGGACAACATAATCTAAGAGCACTTGCGATTTATCTAAGCAGCCTCTATCTTCGAGAATGTTGAG
ACCACTACATAATCATAAGTATGCAATTATGAAAAAAATCTGTAGGTACCTACTTATAAAATTTCAATAAT

AAATAAAAAGTCAAAAACAACACACAACAACCTTAAACATGCATTCAACACACATTCATAAGAATACC
TAGTAAAATAACTAATAATAGACATAAAATTAGTTTGAAGTAAATTCAATAGTAGGTATAATACCTGTTT
CTCTCCCTTGAATGCTGAGGACACTTTCGATTGAGTTGCGTCTGCTTCCTGTGCTGATCCAATGATAATC
TGAAACATTTTATTGGAAAAA
>TCONS_00072774 geneID=XLOC_014510 scaffold_24:960121-961061(+) Intronic
AGACTGTGTCTCCTTTATCAAAGTTTATTTGGAAAAACCAATAACAATAAACTGTTATAAAATACTATGA
ATTTTGTGAAGACTATCGCGTTGTGTTTGTCCGACGTTCACAAACAGATTAACAACTTTTAAGGCTTCT
TTGCTTTAAATTAGTTACGCGCCATTAAAACTAAATAAACTAGGCTTCAATAATAACAGGAAACACCGA
TTCTTTAATTGTTTTAGAACAACCTCGATATATCTATTTGGTAAATAAGTAGAATTTGTTGGTAAACAAC
GTGTTTTTCCGAATGGAACATAAATCTTTTCGATACCTAATGGTTTTTTAAAACCAACAAAATTATACTA
AGATACCTCACGATAAAAATCTGAT
>TCONS_00072665 geneID=XLOC_014543 scaffold_24:1202323-1204723(-) Intergenic
AGCTTTTTTAATTAAATTCTATTTTTCTTTTCAGGATATGACACACTTTTTGAATGCTGATGATGATGATG
TTGATGTTCTATATTTGACAACTAAACTCTGAGACTATATACTATGAAGTAAACATCCTGACTCAGGTGA
AGAAGCGGATTGCAACGGCACTCAAAATACGAACAACTAAACTACCCGCATAATTCTTAACTTACAAA
GAATATATTGATACTAAATGAGGTCCTAGCCTACTTTACCTGGTCTAACAGTTTGTGAATAAAAGTAACT
TGCTCTACATTAATTGTTTTCTAAATTTATTGTTATGTCTGTCCGCTACCCTTTCATCACATGATATCAATG
TTATATTTTCTTTTCTTTATTAGCTGTAACTCATACTTGTATTTGTTTAGGGAAATGTCTGATAACATCTGT
TACTAATTGGCTCTCTAGAATCTGATAATTAGACATTTGGACTTTTCCGGTTACTCTAAGTTAGGATGTT
GGCATAGATAGTGTTACAGTTCTATCCCAACTCCTATGATCAATGTTGTAATATATCTACTTTTTCAAACA
TTCAAGTATAATAATTTAAGAGCCTAGTTTTTAAGCACCTAGTCGATGACAAGCAAAGATCCGATCCAG
AAGCACAATCAGCCACATCAACAAACCAAAGAACCCGCACAACAAAACTCATACAACCAATACCAAA
CCGAACCAACCCAGCCAAAAGGATAATTAATAATTATAGACTAGTTGCTATAAGTACCGATTCCCCCGC
AACCAGGCCAGTCCAATCCAGCACCAATCATACAAGCCCCGCCACGCAAACTCCCCGATCCACCGATG
CAGCCGACCGCACCTCCAGACCAAAATCCCCACCCCTCATCTACCTAGGCCAGAATTTCTTTATCTATT
CTAATCATTTCAGGTTGTTCATAAGGACTAAAATGCACCGTCTTTTCGCTACATTTGTTTCTTTTTATAAA
TTTTTATTTTAATAAATTTACTGCTTTTACCTAAATTAAGACCATATCATTATTTTACCTACAACCCCTATT
CACTTATAATTCCCTAATCAGCTACATAATCCGAGGAACCGTTCATAGTGATTCAGACCAACACTAATAT
GACCTACCTACAGATAAATCAGTCTCTATACAACTATGTTTTATGTTATTCTCTTTAATACCTTTACCTAC
CAATTAAATTTTATAAATAATGTCCTGAACTAGAATCCATTATATATCCAACCCCTGATAATGATGACGAT
TATTTCCTCTTACTCAACGAGGAGCATAACCACATACATATACAATTCCTGATGATGATGATTATTATTTC
CTTTAACTCCACGAAGAGCATAGCTTAGCCTTATACAACCCCTAATCTTTTGTAATTCATTAATGAGCTA
CATAACATTACTACAACCCCTACTTATTAAGTAAATTTTAATAAATGGATTAAGTTTTTCCAAATCCGCAT
TCTAACTTTATCAACTACCTACCTATCTATCTTCACAAATGCTTCCCTAATTACGTTCCGTTCCAATACAA
AACTTAGCCTCTTGCGCTTCCTATTTCATGATAAATAATTCTTTCGTTTATAAGTTCGATCACTCCACGGC
AACAAATTGTGATTATATCAAGGATATAATCACTTTCCTACCCCTAGTATGTAACAAATCATTACGAACA
AAATGCAAGAGACTTACCTATTATAACGTAAACATATTGAAAGTATAAGTAAATTTAAAAAATATAACTT
GCAGTGATTTTGTGCCGACATGAATGTAAAATAATGCACACAGCACACTGCCAAATATTATACTTGTCT
GTGACGCAATAAAAGTTACTCTGTTAAAAAAAATAACCTTTTGACTTTTATTGTTTGTGGTGCAATGCT
GCATAAAGTTTTTCCAACAAAAAAAACTAACTTTTGAAACTTTCTTCCAGCCATAACATTAAAAGAAAT
GCCTTTATGTGAAAACTGCCATATTGAGTAAATATTTTCTTACTAATCGCAAAGGCAAACAACAAACCT
GAATGTTTTTTCGAGACAAACATAAAAGAACAGAAAACGTGCTGCCTGACACCTAACAAGAAGAGAT
GAAGCAAAATCTAATATCCTGGTCTTTCGGTACCAAATTGTAACGTTTCGCCCTGCCTCCTGCCTACTTA
ACTCCTACCTAATGGATTTACTAAGAAGATATTTGAAAACACCCAACAAAACTAACCTGAGTGAAATA

AGAGTGAATCAAATACTGCTTTGAATTGGCCATGAAACATCCAGTGAACCCCTACGTTGTGATGTAACC
C
>TCONS_00072720 geneID=XLOC_014543 scaffold_24:1204462-1206366(-) Intergenic
TTTTTTTCGGCACTAAACAAAACAAAAGCATGCTTCGTTGTGTTCAACAATATCGATCGCATGAAGAAA
TCTAAAGATTACCCGATTAAACTTACAAAGCTCTTAGATAATGAGTGAATCTAATAGGTAGGTGTCATTA
TTCTATCGAATACCCTGTATGCATGCTATTAAACTAAGTCCTTATCCTCAAATTATGGTGATTCAAAATAA
ACAATCTCTGAATTGTTAGTTATTCATACGTACAATAGCTCTGTTTCATGGATCTTGTGTTTATAATAGAT
CTATTGTTATAAAAATCTAACTCAGTTAATTATCCCCCATACAGTTTTGAATTAATACTGACAAAACTAAC
CAACTTATTTCAACTTATTGTCAACCCCTCACCTTTTAAATAAGTATCCTAATCATTTGCTCTTCAACTCC
TATATTTAAATAATTTTATTTGAATTATACCTATATCATTATTTGTTTTATTTCAGGACCAAACATAACAATG
CAAACCCAACTACACTCTTTGTTGAACTAAAACCCATGATAAACCACTGATCAAACCATCCACTGTGA
CAAAACGTTCACAATCCCTCACTACTTATATACCAAAGTTACTATTCTACAATAAGATATGACACACTTT
TTGAATGCTGATGATGATGATGTTGATGTTCTATATTTGACAACTAAACTCTGAGACTATATACTATGAA
GTAAACATCCTGACTCAGGTGAAGAAGCGGATTGCAACGGCACTCAAAATACGAACAACTAAACTAC
CCGCATAATTCTTAACTTACAAAGAATATATTGATACTAAATGAGGTCCTAGCCTACTTTACCTGGTCTA
ACAGTT
>TCONS_00072192 geneID=XLOC_014543 scaffold_24:1204998-1206365(-) Unclassified
TTTTTTCGGCACTAAACAAAACAAAAGCATGCTTCGTTGTGTTCAACAATATCGATCGCATGAAGAAAT
CTAAAGATTACCCGATTAAACTTACAAAGCTCTTAGATAATGAGTGAATCTAATAGGACCAAACATAAC
AATGCAAACCCAACTACACTCTTTGTTGAACTAAAACCCATGATAAACCACTGATCAAACCATCCACT
GTGACAAAACGTTCACAATCCCTCACTACTTATATACCAAAGTTACTATTCTACAATAAGATATGACATA
CTTTTTGAATGCTGATGATGATGATGATGTTCTCTATTTGACAACCAAACTCCGAGATTATATACTATGA
AGTAAACATCCTGACTCAGATGAAGAAGTGAATTGCAACCGCGCTCAAAATAGTAACAACTAAACTAC
CCACATAGTTCCTAACTTACAAACAATATATTGATACCAAACAATATTGGACAAAACATTACAATCAAA
GTTCTAACCTACTTTACCTAGTCTAACACTTTATCAACAACAATAACTTGCTACACATTAATTGTTTTCTA
AATTTATTGTTATGTCTGACCGCTACGCTTTCAACACTTTTTTTTTCAAATGATATTATTGTTATATTTTCT
TTTCTTTATTAACTGTAACTCATACTTGTATTTGTTTGGGAAAATGTCTGATAACATCTGCTACTATTTGG
CTCTCTGGAATCTGATAATTAGACATTTGGGCTTTTCCAGTTAGGCAGCGCTCCC
>TCONS_00072930 geneID=XLOC_014633 scaffold_240:190565-191348(+) Exonic overlap (+)
GCCCAAGGTGCTCAAAGTGCCCTAGGTGCCCAAAGTGCCCAAGGTGCCCTAGGTGCCCTATGTGCCC
AAGGTGCTCAAGGTGCCTTAGGTGCCTGAGGTGCCCCAGGGTGCCCGAGGTGCCCAAGGAGCCCAAG
GTGCCCAAGATGCCCAAAGTCCCCAGGTCCCCAAGGTTCCCAAGGTCCTTAAGGTCCCCAACGTGCCT
AAGGTCGCCAAGATCGCCAAGGTCTCCTAGATCACCAAGGTCTCTTAGGTAACCCTAGGTCTCCAAGG
TCGCCAAGGTCCCCAATATGCCCAAAGTCCCTCAGGTCCCCAAGGTGCCTAAGGTCCCCAAGGTTGCC
AAGGTACCCAAGGTGCCTAAGGTCCCCAAGGTCCCCAAGGTCCCCGTCCCCGTACATAATAATATTCCC
ATCTTTATCTTAATTCGCATCCATATCCCCACCCTTATTTGAAGATATTATAAAGATATATTATAGTAGGTA
GGTACCTACTACCTACTAATTATTTAATTCTAAAA
>TCONS_00072983 geneID=XLOC_014641 scaffold_240:467464-471985(+) Unclassified
GGGGGCGCAGCGGCGGCGCGGCGCCGGGCCCGAGCGATCAATACGCGCCGACCGCGGCCGAGGCCC
CACGGAAACACCCGAAGCCCCCCGAAGATAGGCGGAAAAACCCGAATGCGCGCCGCACATGACAAA
TGTCACTGCGAAACCGCTCAGCAAGTCTCAGCCCTCGCTGAAAACCGTGAAAAACAGACAAAAGTG
CAACCCCAACAACTGTATGGTGATTGTAGTGTGTTCATTGTGCCTCTAGGATTCGTTCAGTGGTGAAAT
AGAACTGAGATTCGCGAAGATCTGTGTAAAAAGTGTGCCGGGCGGCGGGTGGCATGCTGGCGGTGCG
CTGAGTGATGAGCGCGGCGCGCGCGTCCGCCCCCCGCGACCCCGCGCGCGCCTCTCACCGCACCAGG

CA
>TCONS_00073116 geneID=XLOC_014693 scaffold_241:8180-11442(-) Unclassified
TTTTTATTAAAAGTATTATCAAAATGTTAATGCACGATGATAGTTTTGTTGAGAATACCATGAGCAATCAT
ACAATTACAATTGCAATTTAGTATTTTACTCAGTAACATTCAATTTATTCCAATGATTTATATACAGTGAA
CTGAAGAACTGTCTGTGTAACTATTATGTAAATAATATCAAGTAACAAGTTTTAGAATAAAATTCTGTAA
TCTATGCACTCGTCGAATAGTTTTAGGATTTATTGTTTTTTGTGATGAAACACGTTGCCTAGCCAAATGT
AAAATGTACTTTTTAGTTTATAATAATGTTTAGTACTTAATTTAAATAATTTTAATAAGTACATTATAATTA
AGTTCGTAACCTTATGGATCGTTACGTTTATCGACATTCGTGTCGTTCGTGCAGTGCCGTGCGCCGTGC
GTGACGTATATTCTGGACTATACATAAAAATGTGAAACTTATAATACCTAACTGACTAGACTGAGAGTGT
TACTTTTAAGCAGTGAAAGTGTAAAAGACGATCGTAAGTGTATTAAATTAAGTTATACCTAGTTTACAG
TTTCCAAAATGTGAGGATTTTGTATGGTTCTTATGGTGGTAGTGGTTTTAATACAATACGTAGGTACTAA
ATACTTATGAGGATGATAATATTATACTTTCCTATTAAATTTAATTTGGCCCGTTATTTACATTCGATCACT
TAGCCTTTCATTTGCAGTATTGTAAAGAATAGATCGTGACCAGATACAAAAGATATTTCGTATGTTATTT
AAAAAAAACTGCGTTTGGTATGTACAGGGCGGAAAAAGTCGTGTGACATAATCATTTTTCATTACCATG
TTTCGTTTGGACCACGAATAACAGGGCCACCCTGTATAAAACTCAATGTTGAAAAATAGCTCAATGCG
GAATTTGTTAAAAACAAGTTTATATTCTTGAAGAAATTAATGACGTTGTCACAGTTCAATAAAACTATTA
AACCTCAGAACATAGATTTTGTGTTGAATCAGTCACACCAGCTTACACCTTATATTCAAAGGCTAATGC
AGTCTCACAGTCTGTAGTCCTTGTTCTGCGTAGTGCTGCGCTTAATGGTCACACCTGCATTCTGCACTA
GACTAATGATAATAATGTAAAATATAAATATCTAAAAAATATTTATTATATTGCTATCTTCAGCGGACAAC
CAAGTAAGTAAATTAGGTTGCGTACCTAATTTCCTTGCTATCTGATGGATTGGTGGTGCCTAGTTATTAC
AAGAAAATCTATGTAAACCTTTGCTTGAGCTTCTTCTTATTCACCTTCCTATGCGTTGCGCGATCATTCT
CCTTTGTAAAAATATATAGGTATGAATGTTTTCCATGTCATTTAGAAATCAGAAATATAAAATCCCAATAA
AAAGTTAAAAAAAGTACTTAGGTACATGAAAAAATATTACGATGTATATTCTGCAAGCAACAGGGGAA
AATCATATAAAATCATATCTCTATACATATAGGCCAAGAAAAAAAAGGTGTTTCGAATTCCCCGAAAAC
GACCCGTATCTCCAGGTTTCCGTTGGTCAAATAGTTATGGGATATCCTGTATATTATCACAAAATAAATAT
GATATTATTTGAATATATTATGATATAACTACCTACAATTAAAATAACAATTACCATGCTTTAAAGAAAATT
GTTTTCATAATTTATTGCAAATTACTGTACCTAACACAATTATTACAAAAAATACTTGCCATTATTATTGTT
TAGAACTTTCCTTTCAATGTTCAGTGTTTCTTATTTATTGTGACTGTAATAAATAATTTCTGCTCCTGCCT
AATCTCAAAGTTATTTAAATGTAAAATGTATATAGGTATCTATGTAAAAAATATGGTAATTTTTAATCTCA
AACAAAGAAAACTGGTATATTGAATTATTAAAATCACAACCGTGCCTTAGGATAAATAAGTGTGTATGT
ATGTGTATATTACAATAACGGCCAATATGCCTTGAATTTCTATCAATATCTGGCAGCCGGCGCTTAGCGC
TGGGTCAGAATGTAAAATCATAGTGTATGGTCAGATTTCATATACTTAGTGAAATATCTTATTTCTTTATC
AATTTCTTATGTGACGACCCAATGGTGTAGTAGCTGATAGTTACCGTCACTGCTATACTTCGATCCCCGA
TCGGTGCTGATATTTGTTTAAAGACAGATGATTGTACTCGGGTCTTGGGTGTTACCTATATAATATTTATC
TGTGTCTACGAATGACATAGATCTGTGTAGATACCTATACCAGCTATCCGATACCCATAACATAGAAGGG
CTCTGCCTAGCTTATGGTTGGATGGCCGTGTGTAAGATATCCCCGCATATTACATATTTATTGTACACTAT
TCTAATTCACTATAATAAATTCATTAGATTATGATAATTGCTTGTAGGAAAAAAAATGTAAACAAAATTT
GAAAGTAGGTAAGAAAAATTGGGTATCAGGCGCTTAGCCCAGCCCAGCTACTGAGCTACCATCGCTAC
CTATAACAGTTTTTTTCGTAATAACAATGTTTATGAAATTAGAGTTTATTGTGTTTGTGTGTAACACAGTA
TCTTAA
>TCONS_00073118 geneID=XLOC_014695 scaffold_241:25501-26795(-) Unclassified
CTTTATTTGAATCACATGCATATTCCCCATTAGCTTTATAGAATCACACAGCAGGTCTAATAGAAGAAAA
TACTTCCCATGACCTCCCCTATAACAGAAAAATATTTGTCGGTGCATGTGCCGAGCAGCCTGTATATTAT
ACTAATGTTGTACTGAATAAAATAAGTACAAATTTCAATTATTATGATTGTCATTTATTGATTAAGGAGTC

TGCCCACTACCCTCAATTGAAGGTATAGCACGGGGCACATTTAAGACGTGACAAAAGTTCTCCGTCGC
CATCGCTCTTGAGGCTGCGCGTTGTGCGCGATGTAAAACTTTTGTCACGTCTTAATTGCGATCCGCCCT
TCTGATTCATCGAGGGAATGACTTTTGGGATATGAACTGGGTAATAATAATAATGTTTCTCTTTCTACTG
TGGGACGCTAATATCCGAGTCCGCGTTGTTCTATGGTGTGTTCAAATTCGCACAATCACCCATAGCATTC
ACCAGTGAAAGCGGGTGCTTCTGCGGAGTCAATTGAAACTGAAAATCCCCAAATATTTAAATCTGCTC
GGAACTTAAATTTTGTCTCTCTGACCATCGACAACACGGGCTTACGGGGTAAGCAGGGAAAAACCTTT
GTAAGAGAAATAGGGAGAATACCTGTTATGTTTTGTTTATGACTTCAACAAGGGAAACCAATCACTATA
CTAGAAATGTAGACGCTATAGAAAAGAACTCAGCAGACTGCAAACAACCAGAAAAAAACCGGCCAA
GTGCGAGTCGGGCTCGCGCACAAAGGGTTCCGTAGCAGCAAATATAAACCAAAATTACAGTTAAATCA
ACCTATCTCAAAAACTATAAGAGATACTTTGATCAAACCAAAAATCGTTGAAAGAGTTAATTAGCATGC
ATCACCTCTATTTTTTTTAGAATTTTATACCCCGTA
>TCONS_00073325 geneID=XLOC_014708 scaffold_241:280010-285432(-) Splice junction overlap
ACAAATTCATGACTTTATTATCGCGTCACTAAAGAACTATGTATTACTAGGTAGCCACTATTAGGAACAT
CCAGCCATTTATAAGTGACCACTGTTCTATTCTATTCTACTATTTCTGATCTTCTGTATAATCTGTAACTTC
AATAAACTCCCTAAGATTAATCTTGTGTGGATAACGGTTGTCACATAAGCTTGCTGTCCCATCTATCTTT
TCATATAAACGCTTATACAGCATGTCCAAAGCCATACCCTTCCTGATTCTCCAACAAATAACAACAACAT
TTATCTTTTCAGGTCTGACCCTGGGCGACTACGTGAAGTGGGAGGAGTGGTGGTACAAGTACCAGGAG
TGGCTGAAGCGCGAGCGCGCCTACGAGCGCTGGGCCGAGGGCGAGCAGGCTCCGGTACGCCGCGAG
AGGCGCCGGCGCGGGGGGCGCATGCGGCGCAGCTGAGGGCACGCCGCCCCCGCCGCCGTGCTCTGC
GTGGACCTGGCGGGGGCGTCTCGCGTCTGAACGTGGCGCCACACTGAGTGGGGACCATAGGTAAACC
GACCTATAGTCACGTTCGCAAGAGATGTGAGACCTCTGTGCTTATTATGGACAAAGTGTTAGTGTTTAG
TTTTAAATATAATTTAGGTGCAGTTCATTTTTGTGTGCAGGATTTTTACTATAATACCTAGGTATTGTTGTT
TTATTTAGCGTAGAGTCATTGTGTAAATTCAAAATCTTTTCTTATCGGGAAACATGGAAATGTTTGCGAC
AGTGCAAAAAGGATATTTGCGTTTTCCATGTATTCTTATAAGTTAAATAGATTTGGCTCAACTGGAAAA
ACTAACACAAAGCTTTGTATTTTTTCTCTAATTAGGTAAGAAATAAAGTGTTTAAATTGTTGGTGAGCCA
TATCCGTAAGATTTTGTTGTAGAACGCAACTATTTATTAGATTGTTTTGTCATAAGTTAGATTAGTTTTGT
AGTTGTGAGTGTTTTAGTAGCTCTGTATAAATCTTTCTTCCTATAAATAATGGCGCATAGCTGATGATAAT
CTCATACATGTCATTTATAAAAAAAAATGGAAAGGGGTATTCGTGAGAATTTATGACCCAGTTAATACA
CTTATTTACCGTCTCACAACTAACACTAGAAATATGCTTTATGCTTCTTGTAACATACTGTGTCAAACTC
CCGTTAAACTTGCCTAGTTTAAACTCCTTTTGTGGTAATACAGTTAGTAACATAATAAAATAGAACATGT
TCGTGCCTTGCTATTCAGTAATCAAAGAATGAATAGGCCTTTTTGCCTATTCATTCTTTGTAGCTACTGC
CATTATAATGAACACCAATAGAGTAATACCTACTTATAAATAGATGATCAGTTGCCTACATACAATTTTCA
GTAAATTTCTACCTGATAACTAGCAGTTCTCATACGATTTTGACAGACCTAGAATAGAGAATAGAATAG
GACAGGCGAGACAGCATGCGTTTATCCGCCATTTTCACTTTGTTTCTGCACATACGTTTTAGGTGTTTGT
ACGGTCAATTGGTCAGATTCATTAGTAGCTATAAACTTTTGTTCCTTGTCAACTCACAAACCTTCAGGG
CCTTACCACAAAAACTTAGACAGTATTTAACAAGTGTTTAGCGCTATCAAACGCATACAAAATCTGTCA
AATTTTGTTTTAAACACTAGTTAAACAGTGTTTAAAGTTTTTTGTGGCAGCACAGTCAATGTCTGGCGG
TTTCCTAGTTTGACAAAGTACATAAGTATAGGTACTACATACTTATGAAGCTTAGTTGTACTAACTATATT
TCCTCGCCTTTTTCTGCATTATCTTCCTGTAGATGTAGTCTTTATTGTTTATAGAAGGATAAAGATAAATT
ATTAAAATTGTGACTTCCCATTTTAGACTAAATCGTATGCTTATAGTAAATCTTAAATATTTCATTGACCT
TTTCTAAAGGGTCATTTTAATGTGTAAACTAGGTTAGTGCATTAGTTAAGAATAATTTAAAGAATATATTT
GAAATTGAACAAATAACGTTAGATTGTAATGTTAAAAGTACACTGTCTTTAGACTTATTTAATGATCAAA
GAAGGGAGATATCTGTAAAGAAGTTAGAATTACAAGATTAACATTTTCCTACCCCTAAGTAACTAGGGG
TGTGTTTGATGTATTAGTTCGAATAAAATACAATAAGTATTTTAAAATGCTTAATTTTTCTCCTATTGTAC

ACGAAAGTACAGAAAGTTTGATATATTTTAACTAGATAACAATTTATATTTTAACAAATCATGATGATCT
GTAGTCGCTATTTAATGTAAAGGAGATTGAAAATTATTATAAATAAAATAAAATATTTAATAAAATTACAA
TGTGGTTCTATATTGAGTATCCTTTAGCAAGAGAGGGCCGTTTTCAGTACATCTATTTAAAAATACTTATA
AATAATTTAGATTCTCTATCACCAATTGATTTAATTATTGGAATCACAGTGAATTTAATAAGCGATAATAC
AGGGTGTTGCAAAATTGGGGGGGTGTTGGAAAAAAAATCCATAGTGAAAGTCACGTGACCAACAAAG
TTTCTATGGAAAATGAAAATTTTTTTTCGCGAATTTTCAAATTCTGATTTCAGTTTCGGGCTTAGATATA
ACATGCTGCACATGTAGGTTTCGGCTTAGTATACCCTTTTTGCAACACCCTGTATATTACGTAGAATATTT
GCTACATTTTTATTGGTTCTGGAACATAGCATGATAACTACATGCATAAATAAGGTTTCTTCCGCCATTAT
TTTGTTCGCTCTAGCTGGTAATTCATCAGTCAAGTGGAGAGATGACTAAATCACGGCTTAAGGCACGTT
TACTCATTAGTAACTACCCTCGACTGA
>TCONS_00073186 geneID=XLOC_014684 scaffold_241:280456-282358(+) Unclassified
TATAAGTATTTTTAAATAGATGTACTGAAAACGGCCCTCTCTTGCTAAAGGATACTCAATATAGAACCAC
ATTGTAATTTTATTAAATATTTTATTTTATTTATAATAATTTTCAATCTCCTTTACATTAAATAGCGACTACA
GATCATCATGATTTGTTAAAATATAAATTGTTATCTAGTTAAAATATATCAAACTTTCTGTACTTTCGTGTA
CAATAGGAGAAAAATTAAGCATTTTAAAATACTTATTGTATTTTATTCGAACTAATACATCAAACACACC
CCTAGTTACTTAGGGGTAGGAAAATGTTAATCTTGTAATTCTAACTTCTTTACAGATATCTCCCTTCTTTG
ATCATTAAATAAGTCTAAAGACAGTGTACTTTTAACATTACAATCTAACGTTATTTGTTCAATTTCAAATA
TATTCTTTAAATTATTCTTAACTAATGCACTAACCTAGTTTACACATTAAAATGACCCTTTAGAAAAGGT
CAATGAAATATTTAAGATTTACTATAAGCATACGATTTAGTCTAAAATGGGAAGTCACAATTTTAATAATT
TATCTTTATCCTTCTATAAACAATAAAGACTACATCTACAGGAAGATAATGCAGAAAAAGGCGAGGAAA
TATAGTTAGTACAACTAAGCTTCATAAGTATGTAGTACCTATACTTATGTACTTTGTCAAACTAGGAAAC
CGCCAGACATTGACTGTTTGTGAGTTGACAAGGAACAAAAGTTTATAGCTACTAATGAATCTGACCAA
TTGACCGTACAAACACCTAAAACGTATGTGCAGAAACAAAGTGAAAATGGCGGATAAACGCATGCTG
TCTCGCCTGTCCTATTCTATTCTCTATTCTAGGTCTGTCAAAATCGTATGAGAACTGCTAGTTATCAGGTA
GAAATTTACTGAAAATTGTATGTAGGCAACTGATCATCTATTTATAAGTAGGTATTACTCTATTGGTGTTC
ATTATAATGGCAGTAGCTACAAAGAATGAATAGGCAAAAAGGCCTATTCATTCTTTGATTACTGAATAGC
AAGGCACGAACATGTTCTATTTTATTATGTTACTAACTGTATTACCACAAAAGGAGTTTAAACTAGGCA
AGTTTAACGGGAGTTTGACACAGTATGTTACAAGAAGCATAAAGCATATTTCTAGTGTTAGTTGTGAGA
CGGTAAATAAGTGTATTAACTGGGTCATAAATTCTCACGAATACCCCTTTCCATTTTTTTTTATAAATGAC
ATGTATGAGATTATCATCAGCTATGCGCCATTATTTATAGGAAGAAAGATTTATACAGAGCTACTAAAAC
ACTCACAACTACAAAACTAATCTAACTTATGACAAAACAATCTAATAAATAGTTGCGTTCTACAACAAA
ATCTTACGGATATGGCTCACCAACAATTTAAACACTTTATTTCTTACCTAATTAGAGAAAAAATACAAAG
CTTTGTGTTAGTTTTTCCAGTTGAGCCAAATCTATTTAACTTATAAGAATACATGGAAAACGCAAATATC
CTTTTTGCACTGTCGCAAACATTTCCATGTTTCCCGATAAGAAAAGATTTTGAATTTACACAATGACTCT
ACGCTAAATAAAACAACAATACCTAGGTATTATAGTAAAAATCCTGCACACAAAAATGAACTGCACCTA
AATTATATTTAAAACTAAACACTAACACTTTGTCCATAATAAGCACAGAGGTCTCACATCTCTTGCGAA
CGTGACTATAGGTCGGTTTACCT
>TCONS_00073140 geneID=XLOC_014709 scaffold_241:287673-288851(-) Splice junction overlap
GACCAACTTTGAAATAGTGAAAGCAGCAATTTTTTTGCGACATCAGCTTTAATCGCATACGTGATGTAC
CTATATTGTCACTCATAAAATTCCTCCCTATATTGACTCTGATGAACTAACAATATAACGGATAAACTAAT
ACCGAAGGCACTCTGTACCGTCTTTCTACATTCTGCTATAAGTTTATAAAAAACCACAGTAAATAATCTA
TAAACCTGTCTTCCTCACCAGCCCGCGTTCATCCGGCACAGCGCCGCCGCCGCGCCCGGAGCCGCGC
GCCTCCGCCGTGGTGCAGCCCACGCGGGAGCTCCTACGAGCTCGTGATCACGCCAAGCGCACCCGAG
AGGAGAAGGTCCTCGAATGGCAGCGCTCCCAACTAGCAGTGAACCGGCCGCCGCCCGGA

>TCONS_00073405 geneID=XLOC_014741 scaffold_242:331542-332598(+) Intergenic
ATGTCTCAGCTGTTTATGTGCTACGTACTGGTTGTATTTACGGATGAGTGAGATAAGTGTTGCAGTGAGT
AAATGCAATTTCGTGTTATGTTATGTTTGTGTAGTTAAAGTCTAAAGTCGAAAACAAAATGTTGTTAACT
AATTGAAGACTATTTTTTGTGAATTAGCTAGTTATTCGTGATGTGCCATGAGTTAAATTTAGATGAATCG
GAAAAAAACCTATGAATTTACAGATGTAACAAGGACCTACAGGGAGTAGAAATTTATTTTACAAATGAT
GAAAGAAATCTATCAATCTGCCGGATAACTTCTCAATAAGGAGGCCCCAATGCAACTGGAACCTATCTG
ATATATACATAGGTACGCACTAACTGCC
>TCONS_00073419 geneID=XLOC_014747 scaffold_242:343081-343873(-) Exonic overlap (-)
CTCATTGACGGTCGCATACGGGATGCCGAAGTACTGGTAGAAGCGTCCATTCTCACTGACGCGGCCCC
GCACCAGGCCGCTGCTGGTCCTCACTAGCGGCGCGGGCTGCTGCACCAGGTTGGCAGCGATCAGCGA
GAACAGAGCCACCCACTTGTTCATCCTACGCTGGCTGAAAGAGCGCCGCTCCGCACGCACCAACTTTT
AAAAACAAAACATTTCCGCGCGCTTTTATATTGTCACTTTTGTTTTTTTTTCGAACACTCGTTTTAATTTT
AATTTTGACACATTACATAATCTGTGGTTTGTAAAGTTTGGTTGTGTGTTTTTTTTGGTTGAAAAAAGA
AAATTAACAATGCAACGTTTGAGCGCGGCAAACGATGGATTGGTAATGATATGGTTGTGTTTATTAAGG
CGGCTGCTCTTGTCAGTGGTCAGTAGTTTTCCTTCGTTAATGTATTGGGCATGATTAACGTCATAACATC
TTTCGGAAATTGGAAACAAC
>TCONS_00073428 geneID=XLOC_014750 scaffold_242:451657-452885(-) Unclassified
CGTGAGTCTTACCAACTGTTATGAGTTTTTTGTGCTTTGAAGAGTGTAGTCACGAAAATCGTCTCTTAG
ACAATTTAGACAATAACAAATTACACATTGTACGTATACCAAAAGTATACAAAGATTAAATCCCGCTTTT
GGAAATCTTGACTGGATAAGCTTTCGTTTAAAAAATTGTATTATGTATATCCATTTGAGAAAAATATATCC
CAGAATAATATCTTTAAAACAATTTTCCCTCGTCACGCTGACCTCATTGCATCTCATTGACTCATATTCC
GAGGCCTGCGGCCTGCTGAAGTGCTCAGTTGTCAGATGGTTGTCAATCACTGTTGGTCACTGTGTCAC
TGTCAAAAACACCACAAAACTGCAAAACAAATAAAAATTCCTGATATTTTGTTAAACTAATAATTACAA
TCACACAACAACATCAAGATGTAAATGATAGAGAACCTTGTGTTCATCGCTGTAGGCTCCATATTACTC
TGTGTGTTGCTGTACATAGTTATCTTCCATCTCGACCGACGGTGGAATCACAATGGCATGACGTCGTCG
CGCTACGTGTCGTGG
>TCONS_00073783 geneID=XLOC_014772 scaffold_243:32894-80883(+) Intergenic
CGGGAACGCGCCGAGACGCTTGTCTATGAAGCGGCGGGAAACTCGCGCGAACTTTGACATTCGAAAT
TTGAAACTATTTTCTTTTTAAAAGTGTAGAGCTAACCGTTTTAGTGAAGTGAAGTTCTGTACAGCCAGT
AGTATTTTGCTACTGATTTACAGTGTTTTTTCAGTAAAAACCTCCGTGTTATTAATAATTTTATGCAATCA
GAGATTGTATGTGAATGTGCGTATTTAGTTTTCTTTCGTAATTTGTGTGGGATTCAGTGTTTATTGTTTGT
GTTTGCAGATACTTCAGCTATATGCTTTAAAAAAGCAGCGACATCTGAAGTGCAGCCAACAC
>TCONS_00073650 geneID=XLOC_014782 scaffold_243:505206-506050(+) Exonic overlap (+)
CGTAAAAAGTCTCCTGTGTTTTGGTAATTTTTTCAGCTCATCACTTCATCAATTTCTTATTGATTTATTTT
ATTTAAAGCGTTACCTTATTAGTTATGTGCATAACGAGATGAAGATGTGAAATACTTTGAGTTAATCGTA
GAACACGAGAGCTATGAGCATTCTAGTTTCCGTTGAGATTAAATAAATAGGTATTTCATTCGCTGTTGTG
ATCGCAAATTTTGCGAAGTTTTCAAGAAAATGGGCAATACAAGTAAGAAATTGGCAGAGAAATGCACA
ATCCTGACCAAGGATGAGCAGAAGATGCTCGCGGTGACATTCAGGCCGGTGAGCCGGAACTCGCCGA
GGGTCCGCGAGGACGACCTGGTCGTGAGTGGCACACTTGACGCACT
>TCONS_00074276 geneID=XLOC_014864 scaffold_245:178884-180231(-) Intronic
CGCTCTTCATTCTACAATTAGATCATTTTCGTACTTATTTTTAGATCTAGGAGTTTATTATTTATTTTATTAG
TGAAAACTAAACGCGAATCGTATATGACGCGGAGCTAGTGTAGCGTATATACCGCTAGATGGCGACACT
AATACGCCATACAAATTAGCGTTACTTTTCAATTTTAGAACTGTGAGTTCTTAGACATGAGAGTCTTTGC
AAAATTGACAAATTCTCAATTTCTTATTTTCTCCTCTTTTTTGACAGAATGCACGCCCAAAGGCGAAAA

TAACCTACAAATTGGTATTGGCTCTTTTAATATTTAAAACTTGACATTTGTCTATTTAGTATCTGTGATAA
AGAAAACTTTAATTTAGACTAGCATTCCCGAAGCTAGTAAGGTTTGGTCCTTCGGTAGGTGGAAACGG
ATACACAGCATCACTATCTTCTGTCTCCCTCTCCTTCAAGCGCCGTCTATCTGTCTGTCCCTCTCTGTTC
CTGTTTCGCTTGGGGTAAGGCTTGCGTATTGGACATAATTATTATGGATCGTGAATAGTCAAGTCACACA
CACTCTATAAATATTTAGAACTTGACATTTGCCTATTTAGTATCTTTGATAAAAGACTTTAATTCAGACTA
GCATTCCCGAAGCTAGTAAGGTTTGGTCCGTCATTAGGTGGGAACGGATACACAGCATCACCATCTTCT
GTCTCCCTCTCCTTCAAGCGCCGTCTATCTGTCTGTCCCTCTCTGTCCCTGTTTCGCTTGGGGTAAGGTT
TGCGTAAGTGGACATGGCTGGTATGTAATTTGCTTGAGAGTCTTTACAAAATTGCGAAATTTACACTTT
CTTATTTTCTACTCTTGACCTACAAATTAGTATTCACAAAACTAGCCGAAGTATCGGGTGGCGGAAACG
GATACACAACATCACTATCTTCTGTCTCCCTCTCCTTCAAGCGCCGTCTGTCTGTCTGTCCCTCTCTGTT
CCTGTTTCGCTTGGGGTAAGGTTTCCTCAGGTGGACATGGCTGGTATGTAGTTTGCGCGCGCCGTCGTT
CTTGAAGCATTTACCGCAGAATTCGCATTTGAAGTCTTTCGTTTCTG
>TCONS_00074433 geneID=XLOC_014917 scaffold_246:337757-338676(-) Intronic
CAGAACAGTAAAGTAAAACCCATCAAAGCTACAACCACGACTAGTAAATACCACATTGAATATCAAAA
GCCAATCATGCACGACCATAACGACGCCATAGTAAACCTCACACGTGCGATCACCAATGGACTTAAGA
TAGCTAATCACTCGCCCAAGTCTGAACCGATTCGAGGTGATAAATTTGCTAGGCTGCCTGTCAGCTAGA
GACCTGCCCAGGTTTTCTGGTGATGCACTTGAATGGCTGCGCTTCAAACACGCCTTCGAGTCGACGAC
GAGAATAGGCCAGCAGGGCGATCACGAAAAATCATCAACAATAATGTTATTAAAGTGAAAATTAAATT
CACATTTGAAAACAAAACCAAGAACGATGTGTAATAACAGCTCTAGATACATAATATAATTCCACAACT
GATTGACAATTGTCCTCCTTTTCTTTCCGAACATAACGATCTTGTACCCGTATCATGAAAATTCGAGCTA
ACGAATAGAATCGAATGCGAGTGCGACTACTGTCAACTTGACAGCACTTACGAACACTTTTTACCTTTC
GAATGAGTTTCGAAAAGAATGACCACAGAGTACATAATATTATTCTGACAATGACCTATGGAATGATAG
CTGTCAAACTTTCGCAAATCTATACACCGCCATTTTGTTGTTGACAGGTTGACAGCACTTTCGAATAGA
CTATCGAATAGAATATGCTGCGGTTTTTCG
>TCONS_00074600 geneID=XLOC_014985 scaffold_247:360363-366680(-) Unclassified
TTTCGATCTGTTTGCTAGCGAAAAAGGTTATCAAGTTACGTGAGCGGGCGCCGGGCGGCGGCGGCGG
CAGTGGCGGCGACGGTGGACTCTGCTGTTGCTCACGCGCAGTATCGTATTGCTGCGCCAGCGAATCGC
GCGCACTTTCGCTCTCAGGCGCTTCCGACTTCCTCCTTCTACTCCAGACGCACCCCACGGGATTTACAC
CGGTTCGTGAAAAATAAAAGACAATCGGACTTCGACCGGTTCGTGAAAAACAAAAGACTATCGGCAC
CGCTTCGCTTTTCAAAATTCGCGCCTTTTTTTACGTCTTCTGTTTATACACGCGGGACTGGTGTAAATAG
TGTTGTGTTTTTGTTGTTTGTTTGTTTTGATTCGGGACAAGCTGACTATGTTGGTGATCCTAGCCTTCAT
CGGAGTGAGGTTCGCACTGAATCTGTCCCGACAGAACGGCAGGAATCTAGCGGATGAGTGCTACACT
AGAAAATGCACGACGGCCCCAGTCGACATGTCGGTACCGGAGTGGCTGCACTACGCCGCCGCAGCGC
TGTTCCTACTCTGGGTGCTGCTGGGCCGCAGGAGGAGCCTCAGCAG
>TCONS_00075021 geneID=XLOC_014986 scaffold_247:414549-426915(-) Exonic overlap (+)
TGGAAGTGTTCAGTGCATATTGATTAACATCTTTATGCCTGCCTTAGCTCTGACAAAGTATTAATCGTGC
CGATCTGCAGTGTATCACTCATGCGACTCATTGTGCAGGGACATTCAGCATGGTTCGATCGTTCGGTTG
AGACTTAACTTTGACCCCAGAAAATGTCAGATGTGGCGGACAAACCACGTGAGAACGCTGTGACCGG
GGAGTAGAACATGTTCCAAGTGCAGGGGTCGTTCGAGCAGTCGCTGCGGGGCGGGGGGGAGGCGTC
CCCCGGGGGCAAGACCCTCGCGGCGCTGGCCAAGATCCACCGCCACTGCAGCGGCAAGGCGCACGC
CAATGGCTGCGTCGATCCCCGCCACAACGCAC
>TCONS_00074610 geneID=XLOC_014990 scaffold_247:643436-646199(-) Exonic overlap (-)
CGCGCGCACTATACCATCACTATTATGAATTTGAAGCAGAAATAATACAAAATATTCATGAGAATTTAAC
AAAATTTAATACCAGTCGAGTATTGTCCTAAAATAATCTAGGTAAATTAAGCTACTACTAGGAACATAAT

TTAACTGACCCACTGATAGATAGAAGTTATTCAATGCTAGTTCCGCAGTTGATTGGGAGTGACAGCCTT
AACAGATTATTGCACACTAAATTATTCTAGAAATGTATCAATAAAAATCAATATCGGTGTAATAACTGAT
AAGAAGAAAATAGAGTACGTATATTCATTAGGTAGATTAGGATTTAATCTTGTAACACCGTCTAGTACCT
ACCTAACTCACTTTCACGGACATCTAATCTACTGGTGGTTGAGATACATAAAGTAATGTTTATTTAATTT
AAAATGAAATAAAAAAATAATACTTCTTTGAATGTGTTAGGGCTGGTCACACCGGCTGTGTAATTTAAT
ACTTTTAAACAGTCAGTTAATTTATATCGCCAACATGTCATCTCAATAAATAAAATTAATGTTTTAAATAT
ATCTGGACAACATTTCAATAAGTATACCTATAAATTAAAGGTAAATAAATACATTTTACGCAATCACACG
ATATATTATAGACATATTTTAACGATCAGAGTACTTACCTAACTGATTTAGGTAAACACCTAAGTTTATAG
AATAATTAGACAATAAATAAAATTATTTATTTTAATATAAAAAGAGAAATGTGATCTATTCTATGGCTAGC
CGCCATATTTTACTTTTATCTACCGTCAGAGGGCGCTCATTAAACCGTCCTATGAAAAGCTATAAAAAAC
GGTTTCTGGTTTAATTACGTATACAATTTTCACACATCGATTTGTCCGTCATAATTTTATTTCAAAAGATA
TGATAAAACATGAAATCTCTCATGTAAAATTTCCATTGTTTGGTTGATTATCCCTCTGCCTTTTATGGTGA
CCATATATACACTCCAGTCTATCGGAGAGCTCTGCTAGTGCAGGTATACATAGGTACACGGGAACATGTA
TATAGTGGGGATACTGTGCAGTTTTTAGACCTATGCAGACGACCGGAGCAAAATCTAAAAACAATTATC
TCTGGTGAACCCTTCCGGAGTTCCCGACACACACTACGACACACGGGACACAGACAGAGCTCTCCGT
TTCTTTACATTTACTTACAGATAAGGTCCGCAAAAAAACAGAGATATAGGGTTATTTATTGCCGGTGTTT
GTTTTAAATTACCAAAACAAGATCCCGACGAATTGAGAACCTCCTCCTTTTTATGAAGTCGGTCCACAA
TATGTTTTATTTGAGACGCATATTAATTAGATTAACGGTAAATACGTTCACACAGTCATCTCCGCTGGGT
GTTAGAAAAATTAAGTAGTTATAACATAAAAAAATCAATATATTCATGAAAAGGTAGAAAAACAGAAAT
AGACAGGTGAATAAATAACAAATTGTGTTGTTAAAAAATACCCAGCGAAAAATACTCTTAACTCATTCG
TAATTTGTTAACACTTAAATAATACTTTAGGACTGCAATAAAACATTAAGTATATTAATTTTAATATAAAAT
ATATCTAATTAGATACTTATTTTATGACTTGGTATTTATCTGTCGCTGCTGTGACAGTATAAAGTAGGCTT
TTACAATGAAGACGCTATCATAATTCAACTTGTTGTCTCTACAGGGAGAGACTTGGCGACTATAGGATT
TGACACTTTTAACTTTCCCCTTTCGCTTTCCCGTGGGAAGATCGGGATAAAGATAGGCTAATGTAACTG
AGTTGCATCGCAACAAGTTGGGTCGTTCCATTGCAACATGGATCTTAGGTGTAACCCCTGGAATATAAT
AGAATTGCCCCAAGTCTCACCCACCATAGATAGAATATTATAACGCACATATCAAGTTGTTTATAGCGCC
ATCTAGCGACAAATTTGAGTTCTCATCATTACGATTCATGTGGCCAAGCTTTATGACACTAACGCCATCT
ATATTTTCTTAATATCAATTTTTCTGCGTTGCAATATTTTATCTAAGCTTTTAGGACGTTACATAATTTACC
TAGACTTATGGACTAAAGCGATTTCCACTA
>TCONS_00074753 geneID=XLOC_014969 scaffold_247:643757-645283(+) Exonic overlap (+)
AGCCTATCTTTATCCCGATCTTCCCACGGGAAAGCGAAAGGGGAAAGTTAAAAGTGTCAAATCCTATA
GTCGCCAAGTCTCTCCCTGTAGAGACAACAAGTTGAATTATGATAGCGTCTTCATTGTAAAAGCCTACT
TTATACTGTCACAGCAGCGACAGATAAATACCAAGTCATAAAATAAGTATCTAATTAGATATATTTTATAT
TAAAATTAATATACTTAATGTTTTATTGCAGTCCTAAAGTATTATTTAAGTGTTAACAAATTACGAATGAG
TTAAGAGTATTTTTCGCTGGGTATTTTTTAACAACACAATTTGTTATTTATTCACCTGTCTATTTCTGTTTT
TCTACCTTTTCATGAATATATTGATTTTTTTATGTTATAACTACTTAATTTTTCTAACACCCAGCGGAGATG
ACTGTGTGAACGTATTTACCGTTAATCTAATTAATATGCGTCTCAAATAAAACATATTGTGGACCGACTT
CATAAAAAGGAGGAGGTTCTCAATTCGTCGGGATCTTGTTTTGGTAATTTAAAACAAACACCGGCAAT
AAATAACCCTATATCTCTGTTTTTTTGCGGACCTTATCTGTAAGTAAATGTAAAGAAACGGAGAGCTCT
GTCTGTGTCCCGTGTGTCGTAGTGTGTGTCGGGAACTCCGGAAGGGTTCACCAGAGATAATTGTTTTT
AGATTTTGCTCCGGTCGTCTGCATAGGTCTAAAAACTGCACAGTATCCCCACTATATACATGTTCCCGTG
TACCTATGTATACCTGCACTAGCAGAGCTCTCCGATAGACTGGAGTGTATATATATGGTCACCATAAAAG
GCAGAGGGATAATCAACCAAACAATGGAAATTTTACATGAGAGATTTCATGTTTT
>TCONS_00074558 geneID=XLOC_014970 scaffold_247:650771-658969(+) Unclassified

TTGAAACTAGTTTGTTGCTACATGAACACCGTCAGCCAAAATATTAATTTACTGCAGCAACCCCAAAGA
ACTGTCACTTTTGACAATCACCTTAAATCCTGTGTCCTTGTTTTTCATTAACCTTGCCGATAGCGGCATC
TCTTTCTCTCAAATTAATGTTGATGTGCCTTCGCTTGTAAATTTGTAATGCCAAATGCCATGTCAGTTAAT
TATTTTGTGAGGTATGCGGTTGTGATTGTGAATGATTTAGTCGTGAAAAATCCTCGTTTATTGAGTTTT
GTGAAGTTTGTTTTGTGACCAAAATACCTGTGTAGTGCTATAGAAACTTGTGTCCTTTACGGAGGCCAT
GAAGACCAAAACGGATAAGAGTAAAAACGGAGACAGTGACTCGGAGAGTCGCCCAGAGAAGCACAC
GTCAGCGCTGGCAAGCGGCCCCCCGCCCCCG
>TCONS_00074755 geneID=XLOC_014970 scaffold_247:654586-658976(+) Unclassified
TGTTTATCAAAATAAAAGTGTAAAAAACAGTGTATATTGTTGTGTTTTCGTGTAAAAGATGTTGCTCTC
GGCGGCCTCCACCGCTAACATTACTAAGAGCTCTCTCAGTACAATATATCGGATTCACAAGAGCCAGAC
GGTCTTTCATGTGAGTCGCCCAGAGAAGCACACGTCAGCGCTGGCAAGCGGCCCCCCGCCCCCGGCC
CCCA
>TCONS_00074951 geneID=XLOC_014971 scaffold_247:687555-688820(+) Splice junction overlap
CTGTGCTGTCAACACTTTGTCATTTTTGTCATGTTTACTCAGTTTGCTTTATTTTTATTGTTATAAATCTGT
ATGATATTAGATGTTTCTTCAGTTATATACCTACATCAATTTCCAAATTTTTATTGAATGAATCGTCATCCT
TGTCCATACGGTACCTACACAATAAAAGTGGCAGTCCATAAACCGCTGTCTGCTAATGAGTGACTTATT
GGCAAACGAGTTGAAGAGCTTTTGACTAATCAATATAAAATAGAAGCTGTGAATAAAATATCTTGCTCA
AAAGGCTCGTGCGGTGTCCCCCCCGTCCCCCCCGGCCCCCCCGCAGATGTCGGGGTCGTCGTCGCGC
GGCGCGGACATCATCGACCTGGACCGGTACATGCGCGGCGTCGCGCCCGCAGACACCGCCAGAAATC
GTGGCGCGCTCATAGTGGATGCGGGAGGCAGGCGGGCGCGGGGCGCGTGCGAGCGGGACGGCAGCA
CGTGCTACTCTCTCGCTCGCTCCAAGGCTCTCTCGCATGACTCGGGCGTGTCCGACGCCAGCTACCGG
CGCCGCGGGCGCAGGTACTCCAAACCGAG
>TCONS_00074950 geneID=XLOC_014971 scaffold_247:687567-688822(+) Splice junction overlap
CACTTTGTCATTTTTGTCATGTTTACTCAGTTTGCTTTATTTTTATTGTTATAAATCTGTATGATATTAGAT
GTTTCTTCAGTTATATACCTACATCAATTTCCAAATTTTTATTGAATGAATCGTCATCCTTGTCCATACGG
TACCTACACAATAAAAGTGGCAGTCCATAAACCGCTGTCTGCTAATGAGTGACTTATTGGCAAACGAG
TTGAAGAGCTTTTGACTAATCAATATAAAATAGAAGCTGTGAATAAAATATCTTGCTCAAAAGCAGGCT
CGTGCGGTGTCCCCCCCGTCCCCCCCGGCCCCCCCGCAGATGTCGGGGTCGTCGTCGCGCGGCGCGG
ACATCATCGACCTGGACCGGTACATGCGCGGCGTCGCGCCCGCAGACACCGCCAGAAATCGTGGCGC
GCTCATAGTGGATGCGGGAGGCAGGCGGGCGCGGGGCGCGTGCGAGCGGGACGGCAGCACGTGCTA
CTCTCTCGCTCGCTCCAAGGCTCTCTCGCATGACTCGGGCGTGTCCGACGCCAGCTACCGGCGCCGCG
GGCGCAGGTACTCCAAACCGAGGT
>TCONS_00074722 geneID=XLOC_014972 scaffold_247:694404-709878(+) Exonic overlap (-)
GAAATACCTACATGGTGACTAGCTGTTAAACCACCCAAGATAATGAGATCACTGGTCATAGAAAATTAA
AAAAATGCAGGTCACTCTTTCACTAATTACAGTTCATAACAATTTTAGGTAGACTGACAGGGGCAGAC
TGACAGGGGCAGACATGATAAAATTAAAACCACAAATTAGGTTAACAAATTTAAAAGACAACACTCGA
ACATCACAGTAGGTATACGCATACAGGGTGACCGCAAGTCTAGTGCACGCTGTGAGATACTGGAGAGA
GGAACCATCATTGCACAAATATACAACAGTTTCATTTGATTCATATTATTAATTAATAGCACTTAGTACAT
ACTTCAGTATTCACGAGGTCACTATTGAACACACATTCATTATTATTATTATAAGCGAAGCACCTACATA
CATACACAAAGGACATTTCCACTAATCCATATTTTACGTAAGTACTTACTTAACTTTAAGTTTGAAAATT
TCAATCAATCGAATATTTAAGATAAATAATGAATCACATTACAACATTACAAATACAGATATAAAATTTAA
AATAAATATCACAATGGAACACTGTTTGTAAAAAAAGCTACCTTATGTTTTAGTTAATAAGGTCCCTTAG
ACCCTTATACCTATTTAACCCATATTATACCATATGCAGGCAGTGGCTAACCGTCATTCGAAAATAAAGA
GGTTAGTTACAAGGTAAAAAAAATTGATTATCTAAAAAAAACTTAACAATTTATGATGAAAACAAACG

ATGGAAGAACCACATAAAACAACAAATATCGGACCGCGACTTATGTTTTTCTCTCCGCGTACAGCCATA
GACACCTGACCACTCTCACAGTGACTTTGGTACTGAGAGGGGTTATGATTCTTTGCAACAAATTGTAA
GTACAAACATATTGTATTCTTGTACATTGTCGCCCTGTATACCTGCATGCCCTGTCACGGTAAACTATGAT
GTTTGTATACAACTAATGGACGAATGGCTGACGAGTATAATGAACCTACTTATTGCACATATTAAGCTAC
CGTGACAAGGTTCTAACTTGCCTCATATTTGAACGTAATTATACCAGCTACAGTTCCCATCAATACATTC
TTATTTTCAAATTAAAGTTACAGTTTAACCAATTAATTTTTCATCCTACGCTCCATAGATTTCACTTCATA
CCATTTTAACAATATCCATTTATCCATACGCTTATATACATATAACTCTTAGTGTTACATTTCATTTAATTGT
ATAATTTAATCGGCCAATTTTATATTGCACATAATTTAATTACACATCCTCACCATTATCACATAAAAGAA
ATAATAATCTAATTCCATTCTATCTGTGCGGCATACTTATGCAAGGTCTTCGACCAATCAATCAGTAACC
AATCAGAAGACAGCTCACTAAATTGCAACCAATCATCGTGCCGTATTCTAGTTACTTGCTGGCAAGTTC
AATATTTACACCGCACTGGATTATAGTGCTTATTCTTGGGGCGGTAAGGTCGAGGGTGGCTCGCTCGCC
GCCGCGCAAGGTCACTGACTGTTCGCTAGTTCATCCATTCGGCA
>TCONS_00075105 geneID=XLOC_014991 scaffold_247:695120-709674(-) Exonic overlap (+)
GACCTTGCATAAGTATGCCGCACAGATAGAATGGAATTAGATTATTATTTCTTTTATGTGATAATGGTGA
GGATGTGTAATTAAATTATGTGCAATATAAAATTGGCCGATTAAATTATACAATTAAATGAAATGTAACAC
TAAGAGTTATATGTATATAAGCGTATGGATAAATGGATATTGTTAAAATGGTATGAAGTGAAATCTATGGA
GCGTAGGATGAAAAATTAATTGGTTAAACTGTAACTTTAATTTGAAAATAAGAATGTATTGATGGGAAC
TGTAGCTGGTATAATTACGTTCAAATATGAGGCAAGTTAGAACCTTGTCACGGTAGCTTAATATGTGCAA
TAAGTAGGTTCATTATACTCGTCAGCCATTCGTCCATTAGTTGTATACAAACATCATAGTTTACCGTGAC
AGGGCATGCAGGTATACAGGGCGACAATGTACAAGAATACAATATGTTTGTACTTACAATTTGTTGCAA
AGAATCATAACCCCTCTCAGTACCAAAGTCACTGTGAGAGTGGTCAGGTGTCTATGGCGGCCACTGTA
CGTGGAGAGAAAAACATAAGTCGCGGTCCGATATTTGTTGTTTTATGTGGTTCTTCCATCGTTTGTTTTC
ATCATAAATTGTTAAGTTTTTTTTAGATAATCCATTTTTTTTACCT
>TCONS_00075068 geneID=XLOC_014974 scaffold_247:981445-985144(+) Exonic overlap (+)
CCTGAGCGCGCTGACCTCCGACACAGCGACTCTGTCGCCGGCGCTGGACGAGTCCGACAGCGGAGAC
GCGAGTGAGCCCGCGCGCTGACCGCGGTACGGACCTGGTCATCGTAGGTCGCCTTGAAAATGCCGTCT
TCATACGTTCAACCAATGATGACAACATATTGTTAGAATTCCAAGTGCTCAAATTATTAGGGACCAAATT
TAGATAGAATATAATATTATTATTCAGTATATTGACTGCTATAAGATAAGACAAGTATATAAGTAATAAAA
AATAGTTGCATTTATTAAACCGAGTGCCAAATATGTAGCATTTTATAGCATTTACTGTGACAACGCTTCA
TCAGCTCATAATTTTACGGTGTGAATCCAAATAATTTACAAAATCTATTTTGTAAACTGCATAATATATTA
TCTATTATCAACGTATCAACATCTGTCCTTTGTTTCTTCATAGTGACCGCTAGAAGCATATTTTTCATGAA
TAAGCATACTTTAACGGTTTGGTGCTACTGTTGAATAGTGGAAAGATGAAATGTACGTACACTTTATAG
GCAATATTTTGATGTTACATAATAATAAGTGAGTACCTAGTTTTTCTTCGTATACTGAAGCACTTACTAAA
TATAAGACTGCAAACTTGCTCAAATATGAAGCCAGTATTCGTTGTATGTAGTTTGCATGGATGCCAAAC
CATACCATCATGACTTATGCTTAAACTTTTGTATGTAAGTACCAGTGGCGGATTTACAAATTTGCCGCCC
GTAGGCCATCAAATTTTTGCCGCCCCTGAAGATCCTGTGTTACGAGTTTTTTATTTTATTTTTTCCGAAA
CGCTATTGAAATATTTTTAAATTCATGATCACATTTATGTATCTAAGTATACAGGGTGTCCCAAAAGTCAA
CGTCATACCTTAAAGGGCTGATAAGCTAGCTCATGATTGCAATATTGCTCAGAATATAGTAATATCACCTT
AATAAAAATTCGATCGTTTTCGTGATATTGACACTTTTATAAATTTTCTGAAAATGTACACCTCGAATCA
GTTTATTGTTTTTTTATCTTGCATATTGATCACAGTCCAAAAAAATACGAGTCGAGTTTTCATATTACGGT
AATCATATTTTTTATAAGTAACTTTGACTTTAGTTACATAATTACTGTAAAAAAAAATTACACTGTCCTGG
CACCTTATTACAAAATATGCTCATTTAATGTAGTTTTTAAAACGGTATTAGCCGTTGCTACCTAGGTATGT
TTTATTTGGAAAAAAGTTATTGTCATTTTAGTACAAAAGGAGCTTGATGTAAAATTGTTCAAAGAAAAT
TTAGTTAAAGTAAATAAGTTGTGGACCCGGGCAAATAGATGACGCTGTGACTTAGAAAACCGTGCTTT

TTTAAAAACATACATCATTTTTTGGACTGTAATCAATAGGAGGACTATACACCCTGTATAGTCCTCCTATT
CAAGATAAGCTCCCTCTAAAAACAAACTTAATACGATATACGTCATGGTCACTTCTGCAACTTTAAATGT
AAGTTTGTAATCACAATATTGACTATCACTCGTAAACTTGTTAAGTCTACTTATCTGAGATAGTTTTCCTT
TTAATTTCGTCATTATGTACTACAGCACTTTGAAGCTTAGTATTTCGTAAACGAATCACTAAATCAAAAA
ATGGTTTGGCATACTTTATTTTTAAAAGACCTATTCAACGATACCTTACACAATCGAAACAGCGGCAGC
GAGGAAAAAAAATCACCCCCACTTTATTTGAACGAGAGGTAAGTACCTACTTACAGTAGGTACAAACC
CCAAAAAATATTTTTTTTATTTTATTTTATTTTACTACTTACTTTATCGGACGGACAACAAAGTGATCCTAT
AAGAGTTCCTTTTTTCCTTTTGAGGTACGGAACTCTAATAACAATAAGGAATTTTGTACCGGTGGCCCG
CAAAAAAGTGATAAGAGGTGTCGATTTTCAGCAAATGCATCAAAGTGTCAATATTTCGAAATCTATTGT
ACCTATTTTTATTAAGGTGTTATTATGATATTCTGGCCACTCCTGAGCTGGCCTATCAGCCCTTCAAGGCT
TGACATTTACTTTTGGTTTATTTATATGGATTGTATATAATTATAAAAAAATACTCTAATTTTTTTTTTCAA
AATTTGCCGCCCCCCTAAATCTGCCGCCCTAGGCACTGGCCTTTTTGGCCTATAGGTAAATCCGCCACT
GGTAAGTACATTATCAAAAGCAACAAAATTTGCTGGAAATAAGTAGCAGTTCCTGTATTTTTATTGGCG
TGTACCAATTATACATTTGGCGGTATGTACTCAATACTCAATACTCAATCTTTTATTGCATCCATAAGTTTT
ACAGGTGTTAAATACATGAGGAGTATTTCAGGAGTACCTAGACACCTAAGCTAGACAGTAATTTTCTCT
AGACAAAGCATAATATTTCATTTAGTTATGATGTAAGAAAGAAGTTAGCTCCATACTTTATTATTGATGG
TTTATTATTTTAAGTTTCATTTACTTATGTAAGGAGTCGAGATGTTGCTCACTACCTATGTTGTACCTAAT
TTGTAAGTGCAAAGATTTAAGAGAGATTATTGTGTATAATATGTAGGTACCTTGTAATACTTTTGCCTAAT
AATTATAATTAGGTAAGTATATGACTTCTGCGTGATATTATTTAACTTATAGAGGAAGTGCCTTATGTTTAT
TGAAGAATAAAGGT
>TCONS_00074898 geneID=XLOC_014974 scaffold_247:981534-985120(+) Intergenic
CGCGGTACGGACCTGGTCATCGTCGCCTTGAAAATGCCGTCTTCATACGTTCAACCAATGATGACAACA
TATTGTTAGAATTCCAAGTGCTCAAATTATTAGGGACCAAATTTAGATAGAATATAATATTATTATTCAGT
ATATTGACTGCTATAAGATAAGACAAGTATATAAGTAATAAAAAATAGTTGCATTTATTAAACCGAGTGC
CAAATATGTAGCATTTTATAGCATTTACTGTGACAACGCTTCATCAGCTCATAATTTTACGGTGTGAATC
CAAATAATTTACAAAATCTATTTTGTAAACTGCATAATATATTATCTATTATCAACGTATCAACATCTGTCC
TTTGTTTCTTCATAGTGACCGCTAGAAGCATATTTTTCATGAATAAGCATACTTTAACGGTTTGGTGCTA
CTGTTGAATAGTGGAAAGATGAAATGTACGTACACTTTATAGGCAATATTTTGATGTTACATAATAATAA
GTGAGTACCTAGTTTTTCTTCGTATACTGAAGCACTTACTAAATATAAGACTGCAAACTTGCTCAAATAT
GAAGCCAGTATTCGTTGTATGTAGTTTGCATGGATGCCAAACCATACCATCATGACTTATGCTTAAACTT
TTGTATGTAAGTACCAGTGGCGGATTTACAAATTTGCCGCCCGTAGGCCATCAAATTTTTGCCGCCCCT
GAAGATCCTGTGTTACGAGTTTTTTATTTTATTTTTTCCGAAACGCTATTGAAATATTTTTAAATTCATGA
TCACATTTATGTATCTAAGTATACAGGGTGTCCCAAAAGTCAACGTCATACCTTAAAGGGCTGATAAGC
TAGCTCATGATTGCAATATTGCTCAGAATATAGTAATATCACCTTAATAAAAATTCGATCGTTTTCGTGAT
ATTGACACTTTTATAAATTTTCTGAAAATGTACACCTCGAATCAGTTTATTGTTTTTTTATCTTGCATATT
GATCACAGTCCAAAAAAAATACGAGTCGAGTTTTCATATTACGGTAATCATATTTTTTATAAGTAACTTTG
ACTTTAGTTACATAATTACTGTAAAAAAAAAATTACACTGTCCTGGCACCTTATTACAAAATATGCTCATT
TAATGTAGTTTTTAAAACGGTATTAGCCGTTGCTACCTAGGTATGTTTTATTTGGAAAAAAGTTATTGTC
ATTTTAGTACAAAAGGAGCTTGATGTAAAATTGTTCAAAGAAAATTTAGTTAAAGTAAATAAGTTGTGG
ACCCGGGCAAATAGATGACGCTGTGACTTAGAAAACCGTGCTTTTTTAAAAACATACATCATTTTTTGG
ACTGTAATCAATAGGAGGACTATACACCCTGTATAGTCCTCCTATTCAAGATAAGCTCCCTCTAAAAAC
AAACTTAATACGATATACGTCATGGTCACTTCTGCAACTTTAAATGTAAGTTTGTAATCACAATATTGAC
TATCACTCGTAAACTTGTTAAGTCTACTTATCTGAGATAGTTTTCCTTTTAATTTCGTCATTATGTACTAC
AGCACTTTGAAGCTTAGTATTTCGTAAACGAATCACTAAATCAAAAAATGGTTTGGCATACTTTATTTTT

AAAAGACCTATTCAACGATACCTTACACAATCGAAACAGCGGCAGCGAGGAAAAAAAATCACCCCCA
CTTTATTTGAACGAGAGGTAAGTACCTACTTACAGTAGGTACAAACCCCAAAAAATATTTTTTTTATTTT
ATTTTATTTTACTACTTACTTTATCGGACGGACAACAAAGTGATCCTATAAGAGTTCCTTTTTTCCTTTTG
AGGTACGGAACTCTAATAACAATAAGGAATTTTGTACCGGTGGCCCGCAAAAAAGTGATAAGAGGTGT
CGATTTTCAGCAAATGCATCAAAGTGTCAATATTTCGAAATCTATTGTACCTATTTTTATTAAGGTGTTAT
TATGATATTCTGGCCACTCCTGAGCTGGCCTATCAGCCCTTCAAGGCTTGACATTTACTTTTGGTTTATTT
ATATGGATTGTATATAATTATAAAAAAATACTCTAATTTTTTTTTTCAAAATTTGCCGCCCCCCTAAATCT
GCCGCCCTAGGCACTGGCCTTTTTGGCCTATAGGTAAATCCGCCACTGGTAAGTACATTATCAAAAGCA
ACAAAATTTGCTGGAAATAAGTAGCAGTTCCTGTATTTTTATTGGCGTGTACCAATTATACATTTGGCGG
TATGTACTCAATACTCAATACTCAATCTTTTATTGCATCCATAAGTTTTACAGGTGTTAAATACATGAGGA
GTATTTCAGGAGTACCTAGACACCTAAGCTAGACAGTAATTTTCTCTAGACAAAGCATAATATTTCATTT
AGTTATGATGTAAGAAAGAAGTTAGCTCCATACTTTATTATTGATGGTTTATTATTTTAAGTTTCATTTAC
TTATGTAAGGAGTCGAGATGTTGCTCACTACCTATGTTGTACCTAATTTGTAAGTGCAAAGATTTAAGA
GAGATTATTGTGTATAATATGTAGGTACCTTGTAATACTTTTGCCTAATAATTATAATTAGGTAAGTATATG
ACTTCTGCGTGATATTATTTAACTTATAGAGGAAGTGCC
>TCONS_00075345 geneID=XLOC_015054 scaffold_248:151466-152554(+) Unclassified
GAGCGAACCCGCGACGAACGTGTTTTCATGAACTTGGCAAAGTTTTTTTCATAAATTTACAACGAATG
AAGTGATATTTTTCCTGAAAACTATGATTATGTGATTGTTTTTCTCGAAACTACCCATTTTAGTTGAAATA
TCTTCATAAAATAGAAGTAAACAGTTTATTGTTGCGGCGTAGTGCGTGGACGTTACTATTTGGTGCATTT
TGTTTATTTATAGACGGTCGCAGTTTGCAGCGATGTAATATTGTAATTATACCGGTGGCGCTAACATGTC
TCAGTTGGATACAGCGATACATCTCGTGGCCGGAGGAGTGGCAGGCACAGCAGGTGCCGTGGTGACA
TGCCCGCTAGAAGTTGTGAAGACTCGTCTGCAGTCGTCCAAGGGGCTGTGTGCGCCCCCCTCCAGCC
CGCCCCCGTCTGACAGCACTGCGTCCAAG
>TCONS_00075278 geneID=XLOC_015055 scaffold_248:182646-183062(+) Intergenic
TATTTTTTTGCATCGGTATTCGTAATTTTGTTTTGCTATTCAAGATAAATAAATTCTGAAAGTTGCAACAA
CACAAGAAAAATGTCTACTAACCAAGTTTTCAACCAACTTCGGCAAGATTTGCAGAATGAACTTACTA
CAACTAATCAACTTCTTTATTTTATAGCTACAGAATTAAGGCAAATTAAGGAATTCACCAGTCCAGGGC
GTGCATTTGAAACAAATATTAAAAAAG
>TCONS_00075466 geneID=XLOC_015057 scaffold_248:219800-220725(+) Splice junction overlap
TTCAGATGCAGCACGGGCGGCGCACGGCGGCGCTGAAGGCGTCCTACGAGGCGGTGTCGGGCGAGC
CGCTGCACGCCCGGCCCGCGCACCACGACCACGGTCACGGGCTAGCCCCGAGCCACAGTCAGTCGGC
GCACGCGCACCACACCACCGCACACGCCGCGCACTCCGCCTCATCCTCCTCGTCCAACAAGCGACAC
AACAAACAGAAG
>TCONS_00075448 geneID=XLOC_015078 scaffold_248:235826-238292(-) Intergenic
GTAAATAGGAATGGCGGGGTAAATTGGAATAAAAATCTGCTTTTCGAAATCGATCTGTTATCGTCATTCT
ATTTATGAGAGATCAATACTTTTTTTATTTTTGAACGAGCTTATATTTTTTTGCTACTGGCAACACTGAAA
ATTTAAGCCAGCCAATCAGGATTCACTAAAAAAAATACGTTGAAAGTGAGGTCGTCGTGGAGAGGCAAT
TATTGCAGTTTTAACCCTTAATCAAATATAGGGAAATTATTTCCCACTTGGTCTGAATCGAAAAAAAATAT
TTTTATTTTTTTACTGGTTACTCTGATCAATATCAACATTAGAAACAAAATAATCTTGATTAAATTTTAAA
TTTTATTAATGCCAACACTTTAAAAAAAAACCGCAAGCTGTCATCGTACGTCAATGTCAAAATCACGATT
TCAATTTTTCTGTGTCAAAAACCGGTTCTCGTGACGATATTTCAAAATTTTTGGAGAAAAAAATCTGCT
AATTACCTAACCACAATACTATTGTAAACTTAACTTGTTTTACAAATGGATTTTATGGTGATTGGCACGG
TAAAACTGGTGTTTCTTGCTTTTTCGTAGGTTCAAATAAGTGGGAAATTATTTCCCACTGTTTGTTCCTG
CAGTAAAAATATACTAAATTTATCTATACATAAAATATAATGTTACTTTCTTGAACGAACTAATATGTATG

GTTTTTGATAAGTATAAGCGGTTTATTACTCCAGATTCTTCTTATTTATTTGCAGCATTTTGAAACAATTA
TTAAAAAGAAACGTAATATCGCTTACTACGAAGAATTTAGAAGAGACTGTCGAAAACATAGCTGAAAT
TTCTGATTTATCAGACACAGATTCGGAAGACGACTCATGCTATGTCAACAGTATTAATTCTCCTTCCGAT
AGTTACCTCAAACAACTTAACGACTGCATATGTCCTTATTAAAATATTGTTCTTAATACTATTGTATCATT
TTATAGTTTTTTTTTCTAAAACAAAAAAATATTTATAAAAAAACAAGCAAAGTAGCGGCAACTAGCTTG
TATAGAAACTGTAGTAAATATTCATTTAAATTTGTGATCTGTACCGGATAGTGGGAAATAGTTTCCCACT
TGATATTATTTTGCTTTGCAATCACGAATAGTGGGAAATTATTTCCCATAGCAAAACCCCCCTTTCCCCC
CACTAAAAAAAAATAAAACATTTTTTTCGTAATCTACGGACCCAACTTACCTAAAAACCATACATGGTT
TACATTTTTATCAAAAAAAATCATCCATTTGATTAAGGGTTAAACTTTATGTTTTATATGTGATAAAAAAA
GAAAAAAGGTTTAATTCGAAGTAATTTGGATTATTAGGTGTGTATTGTGGCTGGAAGAAATTTGAAAAA
TGCCTCGCAACTACAACACTTAACTTTAACACGGACGAGGAAGAAATATTGAAAATTTTAGAAGATTA
ATTTTTTTTTGTTGATTTGAAAGAAGTTTTTTTTTTGTTAACTAGGTAATTTTGTTATAATGCAAAACAA
AAATATGATCTCAGACAGTTGTTTTTTACCTACTTTTTATTAGTATCTTTGTTATAAGGTAAAATAAAAAA
TTTATCTCACAAAGTTTTTTTTGTAACTATTAAACACATGCCAAAAGGGAGTACCTAGTATCATTTTTAG
TAGGTAGGTTCAAATACTAGAGTTTTTTTTTTCAAATTAGTAAAGTACCTTCATATATTAGATAAAGTGA
CAGCCCTAGTTAAAATTACGAGTAACCCCTCTCTCGAACCTATTAACCCCGTCAGGGGTTAATTGGAAT
CGAGTAGGGTTTTTATAAAATTTCAGAAAATATAAAAATAATTTGGGAATCTAAATCTACTGAACATCTA
AAAGAGCATACCATACATACTTAAGTATTCCATGAAATACCCTATATGTTACGGAACAAGCTTTAAATTC
AAGTTGAAAGTGAAGATTGGACCAATTTACCCCGTTTTACGGTATA
>TCONS_00075467 geneID=XLOC_015059 scaffold_248:235826-238293(+) Intergenic
TATACCGTAAAACGGGGTAAATTGGTCCAATCTTCACTTTCAACTTGAATTTAAAGCTTGTTCCGTAAC
ATATAGGGTATTTCATGGAATACTTAAGTATGTATGGTATGCTCTTTTAGATGTTCAGTAGATTTAGATTC
CCAAATTATTTTTATATTTTCTGAAATTTTATAAAAACCCTACTCGATTCCAATTAACCCCTGACGGGGTT
AATAGGTTCGAGAGAGGGGTTACTCGTAATTTTAACTAGGGCTGTCACTTTATCTAATATATGAAGGTAC
TTTACTAATTTGAAAAAAAAAACTCTAGTATTTGAACCTACCTACTAAAAATGATACTAGGTACTCCCTT
TTGGCATGTGTTTAATAGTTACAAAAAAAACTTTGTGAGATAAATTTTTTATTTTACCTTATAACAAAGA
TACTAATAAAAAGTAGGTAAAAAACAACTGTCTGAGATCATATTTTTGTTTTGCATTATAACAAAATTAC
CTAGTTAACAAAAAAAAAACTTCTTTCAAATCAACAAAAAAAAATTAATCTTCTAAAATTTTCAATATT
TCTTCCTCGTCCGTGTTAAAGTTAAGTGTTGTAGTTGCGAGGCATTCTGAAAAACATGGAAAAATATCA
GTATTTTATAATAAACGAAGATAAAAGGAAATAAAACATTCTTTTTTAAAATAATATACCTACCTAACTA
ACATAAAAAAAATCTAGTTTGATTTAAAATAAATTCTTAATGTAATTTTTATTTAAGCGACTACAAAAGA
ATGATCAGTTCATTTATAAGGGTTAATTGCATTATTCATAGAATAGGAACCGTTTTTTTGGAAATTTAATA
CATACTTTATTTAGGCTTTGGTTTTTGACAACCCTATTTAGGAATTCCAATTTACCTCTTTACCAAACCA
AATAACCCCCGAGTTGTTCCAATTATACCTGTTCATGTGCCAATTATACCTGTTTGTGTGCCAATTTCCC
CCCTTCACGTTCCATTTATCTCCAAAATAACCTAAAAACGACTGTTCGACTAAAACTAAGATTTCCATA
AAAATACGCAATTATTTACTTACTTTCAAATTTCTTCCAGCCACAATACACACCTAATAATCCAAATTAC
TTCGAATTAAACCTTTTTTCTTTTTTTATCACATATAAAACATAAAGGTTAAAACTGCAATAATTGCCTCT
CCACGACGACCTCACTTTCAACGTATTTTTTTAGTGAATCCTGATTGGCTGGCTTAAATTTTCAGTGTTG
CCAGTAGCAAAAAAATATAAGCTCGTTCAAAAATAAAAAAAGTATTGATCTCTCATAAATAGAATGACG
ATAACAGATCGATTTCGAAAAGCAGATTTTTATTCCAATTTACCCCGCCATTCCTATTTACC
>TCONS_00075472 geneID=XLOC_015063 scaffold_248:268111-270160(+) Intergenic
CTAGCTGTTCCTGCGAGCTTCACTTCGCTATAAAAAGTTTTCCCGTTGGTTTTCCGCGATAAAAAGTAG
CCTATGTGTTAATCCAGGGTGTGGCTTCTGGTAAATGACACACACAAGTTAAAATTACTTATAACACCC
TTGTTTTTGTCATTGATAAACAGTTATTCAATGAAAATACAATGGAAAATTTATTAGTCACTATAATTTTT

ATTTTCTACTGTTGCTGCCTGGAAGAAATTACCAGGAATATTCCAGTCCAGAGATAATTGTAGACTCGT
GGGTAGGGCATGCAATACCGCAATACCGGTATTCGTAATACCGGTATTAGGGACAAAATTCACGCTTTT
TTCAATACCGGTATTGAAAGTTAATACCGGTATTGAAGTAATACCGGAATCGGACATTTTTTAGGCTATA
AATCAAGCGATTAAGATATAGTTAGCCTTGTGCTCCTATATACTATGAAAGTGCACTTGTTTTCAACCGT
TACATGCAGTTTTTGGCCTTTGGAAACCGACTTATTGTCCATGCGTTCTAAAAATTTAACTCTAAAACTC
AATTCTCGTAAAAAATATTACATATTACAGAATTTGATTGCTTTTTCTTGTTGTATTTTGCCATATACGAC
CTTTAAACAAAATATATGCCATTTATTACAAGGAAATCTAATACCGGTATTAATACCGGGATCCCGGTATT
GAGTTCTAATACCGGAGCTCAATACCGGTATTGAAAATATTACCGGTATTGCATGCCCTACTCGTGGGTC
CAGCTATGCCTGAAGTGGTGTAGGCGGATTGTGACGTCACGAGGTCTATTTGTGGTACCTATTGCAATG
CAATGTTACATTTTGGGTTACAGGATAGCTGTGTTGAATATTTCCATTGGGCTACTGCGACTACTAGCCC
ATAAAGAATGTAGGTGTAGATGAAAAGTGTTGTTTCAAAGATGCTCAGTATGTTGGTTTCCCTGAAGA
GGATTCCGGGAACCAGGAAGATTGTTGCAGAATAACAACATCATAATTGAAGATTCTTTTACACCCACA
TGAGGTTTAATTCGGATAAGTATAAATTCATAAGAGCCCAAGGCATTTTTTTAAAGGTTATATTTTGTATA
TTATGTGTGAACTACACTTTACCATCACAATTAAGTCCTTACCGACCGACAGACCGTATCCCGTTGTAGT
AAGTAGTGGTTAATATAGATGAGGAAGGTCTAGGAGGCTGTGGCGCTCATTGGGAGATAACACAATGC
TTGAATTTTCTTCATTATCATTATTGGCTGATGATGATGATGATAGGTCATCTGGGTGGTGGGTTCTTTTG
GAGTATTATTCTGAGATCGTTTTGTCACCTTAATACCTAAACGGGTCCCTCTATCTAACGAAAAAGGCA
GTTTAGGAGCGCTGATCCGCTAAGCACGACTATTGCATGAAAAATCCGATGAGTTAGTCGAGTAGCCC
TCCTATTCGCATCGAAAACGTGACGGATGCTGTGAATAAACATCACTATTGAAATGGAGATTACGTTTA
TGTTTATTTAAACCTTAGCTCGTGTAAACGCTGCGAAATGTTTTAATGCCTATTTGTTGTTTGATAATAAT
TATGATGCCGTAGAAAAACATTTCAGTATGAAATAACTTTTATGTAAAGAAAGT
>TCONS_00075583 geneID=XLOC_015144 scaffold_249:399187-403013(-) Splice junction overlap
CTGGACCCGGCAGACGAAGAAGATGAGACGGAAGACTCGCGTCCTGACGATGAAGAGGACCCGGAC
TGGGAGTGGGACGACGAACAAGACGACTCGGACGCCGTCGTGTGCAAGAAACACAAGATACAGTAG
CCTGTGGGTGCGAGGGAGACGGGAGATTGATACTAGATGCTGTAGTCTGTAAGTTTGAGGGAGACGG
GAGATAAACACAAGATATAGAAGCCTGTAGGTGTGAAGGCGACGGGAGATTAACACTAGATGCAGTA
GGCGATAAGTGCGAAAGAGATGCGGGTATTGGCGTTAGAGGAAGATTGAAGAGCTAAAGATGCCTCG
TCAACTGCAATTTTACTTTCTCTTTCTCCTAAAGTGTCGATGTCAAATATGGGCTGATATTTATGTACATA
CACTAAAGTTTATTATTTTATCTCAGACATGTGCAAATTGTAAAGTTGAATAAAATGAATTCAACCTGAC
GTTTGCACTTGTTAGTTTCATATCTGAGATAAAAGACAAGTGGTGAAAAAAAGTGTTAGTCAGATGAG
CGTCGCCATTTTTATTGTAGTAAAATTACCAAAAAATACGTAACGTCGGTTTAAATATTTAATTGATGGT
TGGGGATTGCAAACAAAAAAGGTAAACAAGGTTTTGCAACAGTGGTCTAATAAGCCGAAAGAGGTCA
CACTGGTAGCTTTTGTTACTCCCTTTCAGCTTAGTATACCTCTAATAGAACACCCTGTATTTGCGTTTTAC
AAGTTGGCGGCCTTAGTACTCATTTTGGCCACGTGATTAATTATCCTGCTGGCGCTGTATTCGTTTGTTA
CGTATGCATGTAAACACGCTTTTGAGTTTATATAGGTAGGACGGTGCATTCGGTTAAGAACTGAACCTT
TAAATAAGAACAACAGACCATAGCCGAATGATCATTTCTTAACTGAATGCTATGTAATCTGCCGTATATG
TATAAATTAGTTAGTTCAATTTGCATAACATACAGGATACCCTGCATCGGATTTAATTGATAAGATAAATG
CATTTTTTCCAATCTCTACAGGGCCGAAAGCTTAGAGAAATCCTCCCATTAATAATTGTATTATATTTGTA
TTGTGTATTTATATCTGAACGCAACATTCATAAATATAATTAAGTATATCTTCAGTGTACCTATCTGAAAA
ATATTACCATTGCAAATTAAGTATGATCCATTCTATGCGGAGCACCCTGTATATTGTGTTTGAAGTAAATA
AATAAATAAATATTTATAAGATAATCGGTTTAATTTTGTAAATAGTAAATATATGTATTTTTGTTTAAGCAT
ACTCGTATACAGGTGTTGTAGAAGTGGTTTAGTAACATCCTGTATACAGTATGCAACGCAGTCATGTTTA
GTGTTGTACCAAAGACAGGATTATAGAGCTCTATTTTATTATATTGTGGATCACTTACATTTTATTTTATTA
ATAGCACAGATTTTGCGTTCTATAATTTATTGTAAGTACATTGTAATTTGCAATGATTATAAAATCATTATA

TACTAGTATGTAAATAGAAATGTATTTTACTATATGAATTTATTACGTTATTATTAGGTTCTGTATTGCAGC
TATATATTTACACATAATTATGTTACAGTATGGCCGTTTCGTTACCTACTTATAAATCGGAAGGGTGGAAA
AGTATACAACATAGAAATTATCTCTGTTCCACTGAGCCATCTCGTATATTTTATTTCCAGGGCATTGTAAT
CCGAAAGGACCATCACACAACTTATCATTCTGATCATTTCTTTCAAACTGTATATACCTACTTCTCTGAA
CACAGCGACGCTTCATTCCGTAGTTTGCTACCGTAAATATAACTTCTATAGGTTATTAGATCATTAACTGC
CTGTTTCACAATGTCTGCATAAAGGTTATATGTGAGATAAAGTTAAATTGGCAACATTTATTTATTTTTAT
TTATTTATTTATTAACTACTTATATTATCTTACAGGATAAACCTAACACAATAATAAAGAAAAATAACAAA
CACAACAAAAAACTTTCTTAAAAAGACACAAACAACTAAAAATAAAAAAACAATAAACTAATCAACA
CATAACATATGAAACATAACCTTTGCGAACTCACCCAAAGAACATGTGAGAATGTCAGTCCTCTCAGAT
GCCTTGTTGATTAGCCTAATAGCCCTAGTGAGTGGAG
>TCONS_00075556 geneID=XLOC_015144 scaffold_249:399187-403669(-) Splice junction overlap
GCACCACGCACTGGACCCGGCAGACGAAGAAGATGAGACGGAAGACTCGCGTCCTGACGATGAAGA
GGACCCGGACTGGGAGTGGGACGACGAACAAGACGACTCGGACGCCGTCGTGTGCAAGAAACACAA
GATACAGTAGCCTGTGGGTGCGAGGGAGACGGGAGATTGATACTAGATGCTGTAGTCTGTAAGTTTGA
GGGAGACGGGAGATAAACACAAGATATAGAAGCCTGTAGGTGTGAAGGCGACGGGAGATTAACACTA
GATGCAGTAGGCGATAAGTGCGAAAGAGATGCGGGTATTGGCGTTAGAGGAAGATTGAAGAGCTAAA
GATGCCTCGTCAACTGCAATTTTACTTTCTCTTTCTCCTAAAGTGTCGATGTCAAATATGGGCTGATATT
TATGTACATACACTAAAGTTTATTATTTTATCTCAGACATGTGCAAATTGTAAAGTTGAATAAAATGAATT
CAACCTGACGTTTGCACTTGTTAGTTTCATATCTGAGATAAAAGACAAGTGGTGAAAAAAAGTGTTAG
TCAGATGAGCGTCGCCATTTTTATTGTAGTAAAATTACCAAAAAATACGTAACGTCGGTTTAAATATTTA
ATTGATGGTTGGGGATTGCAAACAAAAAAGGTAAACAAGGTTTTGCAACAGTGGTCTAATAAGCCGA
AAGAGGTCACACTGGTAGCTTTTGTTACTCCCTTTCAGCTTAGTATACCTCTAATAGAACACCCTGTATT
TGCGTTTTACAAGTTGGCGGCCTTAGTACTCATTTTGGCCACGTGATTAATTATCCTGCTGGCGCTGTAT
TCGTTTGTTACGTATGCATGTAAACACGCTTTTGAGTTTATATAGGTAGGACGGTGCATTCGGTTAAGAA
CTGAACCTTTAAATAAGAACAACAGACCATAGCCGAATGATCATTTCTTAACTGAATGCTATGTAATCT
GCCGTATATGTATAAATTAGTTAGTTCAATTTGCATAACATACAGGATACCCTGCATCGGATTTAATTGAT
AAGATAAATGCATTTTTTCCAATCTCTACAGGGCCGAAAGCTTAGAGAAATCCTCCCATTAATAATTGTA
TTATATTTGTATTGTGTATTTATATCTGAACGCAACATTCATAAATATAATTAAGTATATCTTCAGTGTACCT
ATCTGAAAAATATTACCATTGCAAATTAAGTATGATCCATTCTATGCGGAGCACCCTGTATATTGTGTTTG
AAGTAAATAAATAAATAAATATTTATAAGATAATCGGTTTAATTTTGTAAATAGTAAATATATGTATTTTTG
TTTAAGCATACTCGTATACAGGTGTTGTAGAAGTGGTTTAGTAACATCCTGTATACAGTATGCAACGCAG
TCATGTTTAGTGTTGTACCAAAGACAGGATTATAGAGCTCTATTTTATTATATTGTGGATCACTTACATTT
TATTTTATTAATAGCACAGATTTTGCGTTCTATAATTTATTGTAAGTACATTGTAATTTGCAATGATTATAA
AATCATTATATACTAGTATGTAAATAGAAATGTATTTTACTATATGAATTTATTACGTTATTATTAGGTTCTG
TATTGCAGCTATATATTTACACATAATTATGTTACAGTATGGCCGTTTCGTTACCTACTTATAAATCGGAA
GGGTGGAAAAGTATACAACATAGAAATTATCTCTGTTCCACTGAGCCATCTCGTATATTTTATTTCCAGG
GCATTGTAATCCGAAAGGACCATCACACAACTTATCATTCTGATCATTTCTTTCAAACTGTATATACCTA
CTTCTCTGAACACAGCGACGCTTCATTCCGTAGTTTGCTACCGTAAATATAACTTCTATAGGTTATTAGA
TCATTAACTGCCTGTTTCACAATGTCTGCATAAAGGTTATATGTGAGATAAAGTTAAATTGGCAACATTT
ATTTATTTTTATTTATTTATTTATTAACTACTTATATTATCTTACAGGATAAACCTAACACAATAATAAAGA
AAAATAACAAACACAACAAAAAACTTTCTTAAAAAGACACAAACAACTAAAAATAAAAAAACAATAA
ACTAATCAACACATAACATATGAAACATAACCTTTGCGAACTCACCCAAAGAACATGTGAGAATGTCA
GTCCTCTCAGATGCCTTGTTGATTAGCCTAATAGCCCTAGTGAGTGGAG
>TCONS_00075608 geneID=XLOC_015160 scaffold_25:135270-137944(+) Intergenic

CCCGCCTATAGGTACGCTGATAGTGCAGCCGACAAGAAGCCGTTGAGCCGGTCGATGAGCCGCCCAGA
TGTCAGGAAGAAGAAGTCCGAAGATTTTGATGACGATCTGCCACTGCCACCGGAGTAAATCCATATAC
CTACATCTACACGTTCCTAAAATATTAAGCACTGAACAATGAAAGGTATTTATTTAATTTTATACTAACTA
TGTGTATGAAACTGTACTTTTTGATAATGACGTAATTTAATATTGTATGTAGACTATTCAGATACGTAATA
AGGTCATGAATAAATGTGTTGCAATAATAACCACTTTGTATTGTGTCGTGCGTCGTCGTAGGTCAGTTTT
ACGAAATAACAGGGAAATGGAATTAAAGAAAAATCGAGTGTGTAAAAGGTATTCTATATTTATAAATAT
TCAATGTTGAACAGACTCATTGATACAAGACTAGAAACATATATTACATTGCAACAAAATATCACACATT
CAAATGAATGTTGCTAAACCATCTATTTTGATCCGTCTGTGTATAAATATATTTTTTGTTAGGAAATTATA
AAAAGTGCTCAGATAAAGGAATAACAATTATGCAGATAAATGGCAGACGGAAACATGCTATCTCGCCC
TATTCGTAGGTCTGCCAATACTCCTTCTTCTCTTCGTTAATACTTCTACGTTAAGTAACAGCCACCATTTT
GATTTTGTTTCTTTACATGAGCGCTTTTTAAGTAATAAGACTCTATTGTTACATAACGCTCACACGAGCA
CATTATACTTGAAGTATAACTGTTAATAGTTCTTATTTCTTTAGTGTGACGATGACGGTAGCAAACACAA
GAGTTGGACTAGCGTTTGTCATTGTTATAAGCTGAAGACATGGCGCGAAAGGATATTAACAGAATCGAT
CGATTCACGCGTAAGCGTCTTTCTTTCATGGGATTGCCTTTTGTACCATTAAATACTTACCACCATCAAG
GAAACAAATTGGCTCAATGGGATTTTCAATTCGTAAATCGAAGTAAGCGAGAAGAAACCATAC
>TCONS_00075727 geneID=XLOC_015212 scaffold_25:293326-295802(-) Intergenic
CAGCCGGCCGCATCGGTTTCTTTCGACGGAACAATTAAGTACTATCGCTGAAAGTGCAACTGAAAACC
GCCAAATGGACGGAGCGCGGTCGCGTGATTCTGACTTCAAAAAGTTACCAAGTTCAAGAGCAGCGTG
TAAAAGTTGGCCGCGTTCGCAGCGGCTGTGACTGGACTGCATTTATTTGTGCCGCATTCAGCCATTGTC
GCTATGGATCATCGCGAATTTCATTAAGACGGTATCATAAGGGTGTGTCCACAAG
>TCONS_00076399 geneID=XLOC_015175 scaffold_25:542632-543787(+) Exonic overlap (+)
TTTAGATATTATAACTGGGGATCCTTGTTCAGCAAACATGCAACCTGGAAAGGATACATTAGCCTAAAA
CTATAAATCCTAGGTGGTGTGTCGCCCCACAGTCATTATCTAGATCGCTAGTAACGACAGTTTTGTTCCA
GTTTAGCATAACAACGTTTTTATGACTTTGGAGTAACTGCATGCTAAGTGTATTGAGTGATGGAGGCCC
AACATAGTAAAGCACGCGACCAGCCTAGAGTGATGGGAGGCGCCATGGATAGCTGTGGTCGGTGTATG
AAGTTCTCCTTAATATGCATAAATCTAGTCACATTTATCGGCGGTCTGCTAGCGCTCGCACTAGGCGCGT
G
>TCONS_00076357 geneID=XLOC_015178 scaffold_25:650382-651536(+) Splice junction overlap
AAACGAGTGAGAAGTTAAACTTAGATGCATGGAGTGAATCGCACACAAAATAAGATAATCTTTCTTTC
TTTCAGGATGTTATAATATTTATAATACTCTTCCCACAAACAAGTACACGTTTGAAGTTTTTCTTGCACAT
TGGGGTGTCCACTTAGTTCAAGTTGTCAACTTATCTGTAATCTTCACCAAAACTTTAAACTATAACAATT
CAAACACGCTCTATTCTAGAATCATATAACATTTTTCGTATCAATGTTTTCCCATGGGAATTACGGGATA
AGCAGGGTTATGCAGGGTGTCAGATTACTACCTATACATGCCGGATATCAACTATTGTAATAATAGTAAG
TCCAGCCGTTTTCCCGTGAAAGACTAACAAACATCCATCCATCCGACTTACGTTTATAATATCAGTAGGA
TAAACAGCCATCGTGATCATAATCTTCCACACACAGCCATTGCTGGCCCCTCCCAAAGAGCGTTTCAA
AACTCTATTCCCTTCATTCTGTTTCATTAACAAACTCACACTTGTAAACCTGCCAATTCCAGACGGTGAT
CCACGTGCTAGCCCTGGTGGGGTCGCTTCTGCTATTCTTCGTGTTCACGTTGGTGTTCCAGACGCTATG
CGTCGTCTGCTTTGGACTCCTGTCTACGTACCACACCATACATATGGCCATGAAGGACCCTAACTACTG
GCTGTGTATCATACTGGCCACTGTAGCTGCTCTGTCGCCTAGATTAGCATG
>TCONS_00075861 geneID=XLOC_015200 scaffold_25:1432655-1442115(+) Unclassified
CGCACACGGGCCGCCGCTACGCGCACGTCTGCCCGCTGTGCGCGCGCCGCTACGACAAGCCGAACAA
AGTGCGCACGCACTTGCGCACTGCGCACGGCCTGGCCGAGCCGCCGGGTAGCATTGTGCGCATCGAG
CTGTCCAAGTGCGGATATTAGTGCTGTTGTAGTACCGAGCTGTTTAGATAGATGGCGTTGTTACGGGTT
TTTGTTAAAATGATTTTCGCATTGTTTTGCCGATAGATGGCAGTGTTACAGTATTGATGTTCTTTGCATTT

CTAGAAATTGAAATCTATTTCGCTATACTATAACAGTATTTACATCCGCACTTGGTTCAAGCTTCGGACA
TATGAAACAAAGTAAGTGCGAGCAGTGCAACAAAAAGTTCCACTCCAAGAAGTTCCTACGCAGCCAC
AAGTTCAAGTCTCACCAGACGCGGCGGTACACCTACGTATGCCACCTCTGTGGCTCCCAGTTCACATC
CACTGACTCAGCCAGGAAACATCTGACGCGAGCCCATGGCATTCCCAGGGACGCCCAACCCCCTGTG
TCTAGGATACCTAATGCTAGCATCCATATGTGATGAAGAAGTTGGTGGTTGGCGGTATAGATGGCGTTG
TTTATGGTTTTTGTGATTGATAGAGTGGATGGTTTGCCGCTAGATGGCGGTATGTGTCAACTGTGCATTA
AAAAGTGTGTAGAAACTTTAAGATTTATTTATGAAAAATTAAGTACTTACATTGTAATATTTTATGTAGTG
ATTTTATCTATAGTATTATACACCTAGTTATTAAG
>TCONS_00075782 geneID=XLOC_015242 scaffold_25:1815291-1816550(-) Intronic
CTGTGATCTTATTTCAAACTCCTTATTCTGAGTATTGACGGAAAATTCGGAAAATCTTTTTGGGAAAAG
TGAAACGCTGAAACTAGGTTGTGAGGTGTTGTGTTATTCAATTTTACAGTTTTTACTTGCTCCTGGGGG
CTTTAGGCGAATAATCATGGTCTGAGAACTTGCTTAGGAAGTCCCATAGATGGGCAAAACCTTAAAAC
ATGACGGACAAAACATGTAGGGCAAAGTAAGATCATGGAGGAAAAAGGGGTATTTTTCCGTAAAAAT
GACTGAAAGTGGTTAGATTGGCTGAAGCAACCGAGGGACATGGAAGACCCGGAAATAATTGCACGTG
AGCTGAGCTGTTCGCCGCTGCTCAACGATGAAGAAGAAGAACTTTTATCGCCGATAGGCCTAGACCCC
GAGGACCTCACCTTCGATGGCGCGTCGCA
>TCONS_00076657 geneID=XLOC_015310 scaffold_250:17892-19961(+) Intronic overlap (-)
CCTCCAGCTGCCTTGTAGATCCTCGCAGCCTTTTTTTTTGAATTATTTTTAAAATCGCTCCTTACTTTCTT
CCATTTTTCTGGAGTTTTTGTATCGCCTGTGCCATCACTGTTTAAGACGTTCGCTAGTTCATCCCATAATT
GTATGGCTCTTATTCGTCCTTTAGCGGTATTCGACGGCTTCGAGAGGTCCCCGTGCCTGCAAACATAAG
AGAAATCAGATATTTTTTAACAACACGTGTTTATTTCGTAAATAAATGAAATCGTGTAGGTAACTTACTG
CTCCATAAATGTGACCAATAAGTCGAACTGCTGTGGGCTAGTCCTCATAATTATGCTTGATTCTTGCGTT
TGATTAGCTTTTTATAGTAAATTAACACTTCAACTTCAACTTTTTGTTTTCCAAACTTGAGGTTTCACCG
CAAAAATTCAAGTGAGCTAGACTCGCTACAAATTATAGTATTGTCACGGTTTGTTTTCGAATCGAGCTG
TCAAATTATTGACTCACACTCCCACAGACAACAAAAACCGAGTGGTCTATGCCCGTTTTCGAAAAGGT
AACGCTTTCGGTATCGATAAATCAAATTGGAAGTAACTTACACTTCGAACTAAAGGTTCAGCCAGTGA
ACACCAGATGGCGCTGTTTGATTCCTATACAAATTTGCGTTTTCGACTTCTCTACTCGATAGGTGAAAA
AACTCACACTCAGCATACAGGCCATGTAGTTCACAATTAACTAAATGTAAGAATACAAAAATGAAATAA
GCACATTTGTGTAACCTAATTAGATAATTTCTAATATTTACAAGTACAATTAATTAAATCGAAGAATTTGG
TTACGAAGTTAGGAATGACTGATGTTATTAAAATATTCTCTGTACCCTATATCTTACATTATAATTTATTAC
AATAATTTATACACTTTAAAAGGTACATACCACACACTCCTTATCACCTAAGAATTAATTTATCACTTGAT
AAACATAATCTCACCATAAAACTTTGTGTCTCTAAGCTAAAATTATACAACTTTAAATTCACGAGACATT
AAATTGGCCTCGTTGACTAATTTTAACTATTGCAGTGAATACTTATGAATGCACATTGTGCATATTATGTG
GGATTGGTAACAACGAACATCGCACTCGTAACGAGCATAGCACATTGGGGATAAATACTTGTGCTTTTC
AATCCTCACAAACATTCTTTGACTGACTAAGAAGTCTCAAACAGAGCAATATCAACAGCTAGGCCGTT
AGGGTTCAGTATTGGTTGGTCACAGGTACTGGCAGGATCGCTTCATGACTGGCTTCTTGCGCAGGCCG
GCGCGCACGGCCTCCTCGAACACCTCGTTGAGGTTCACCCTCT
>TCONS_00076510 geneID=XLOC_015335 scaffold_250:180878-181265(-) Exonic overlap (-)
ATCAGGCGGTCCACGCTAAGTTACGAGTTAGGGGGCGCCGTAGAAATCTCGCCCAGGGCGCTGGTAGT
ACTAAAACCGGCACTGTATACAATGTTTAGCGGAACATGAAGCTGGACACATTGTGTCGAATTTGGAG
GATCTTTACGCTCGTACAGCGTCTCTGACGGGTATGTAGTTAACATTGTACAAATTGTAATAATTCATTT
TTTATCAGTACCAATGCTTCATTTATTGATAAACCTAGGTTTAGAGCCCAAGTCGAACATTGTTTACATA
CCGCTGGTTGTAGGTTTGAG
>TCONS_00076805 geneID=XLOC_015316 scaffold_250:194880-195914(+) Intergenic

ACTTAATATGTCTCGGTAACTTTACATTAGGTTTTATATTTGTAAGTCACAAAAAGAGAAGATATTCTTAT
TTTTTTCTTGAATGAGAATTTAAGCAAGCTTGGGAGGAGAATATGAGATCTAATGTGTTCTCACTACAC
TATACTAGGTAGGGTGCATAAGGTCGTGGGATTCTGGGAAAACTACTAACTACTGGAGAATCAAGAAT
CGAGTACTAAAGCTCCGAAACTCCGAATCGGTTGAAGAAGGTTGGGGGGTTACTCTTTAAATCAATTA
CTTACAGTGCCACAAACTTATCTGTTCCGGTGAGAGCTCACGTAAATGTCTAATAACTATTTCTTCATTC
TTTAAGATGTTAAGTTTTCCCGAAATGAGAATTTACCCTTGCGGTTTTAATAAACCCATCTAATCTATATC
AATATATAATTTATTAGTAAATAATGAGATATGGACCAATTTAGTTAAACCTCACCGGAACAGATAAGTT
TGTCGCCACGCTTATAATATCGAGATAGAGCCGAGGTTAGCGCTGCAGTTTTATTTTTCCGAAAACGCA
AACTACGAATCTATCTCGTTTTATTAAGCGTGCCGATTGCATTAAAATGTTGGTTCGTTCGTTCAATGTT
TTAGTTAGAGAGTCCTCTGAACCGCAGTTCACTACAAACACAAACAAAATATGACCCCTGCCTTCCTC
TTCGAGGATCACACATCATATGCATGCATGTACTGTATATATGCATGTTATACCTATTTAGAAAAAGTAAA
TATTACCTACGTGGGCTGAGCTCAAAGGTTTTATAGTACAGTAGGAAGGTAGAGAAAAGAAAAGCTTC
GTAAAAACATTACGAGACCCTAAGGGAACGCAGCTTTTAGACGGG
>TCONS_00076806 geneID=XLOC_015317 scaffold_250:207391-208880(+) Intergenic
GAACAAAGTTATTGTTTTATTCTGTTAAATTTCACTTGAGTGCCAGTAGAGTTATGGTTTCTATTTATGTT
TGAATAGATTCGCGTATTTAAATAAAATGATAATTTTCTATTTCCGCTACTGTCTTTTATAGAAAAAATAA
CACTGTTAGGTACTGTTTTTCGACTGGCATCTTGCACTAACTACTTACTAATGCATAAATATATGTATTAA
GAAGAAATCCAAAATTATAATATAGAAATGTCCGCTCTCAACTTGGTGGCAGCACTGTGTCTCATCGCG
CACCTCCACACATCTCACGGCTTCGACCGCTCACTGATATGCGAGACGCCTCCCACCCCGCCGCCTGG
ACCACCCCCCATTATGCCACTGCTGGCCCCACCGATGTATTACTATCCACCACCACCAGTGATCCCAGC
TGTAGCAGTGATACCAATGATACCAGCTGTACAAGTGATACCAGCTGTACCAATGGTACCTCAATGTTG
CTGACGGCGTCATTGAGCTAGCCTTTTCCGTTTAGGAGTAATTAGGTGCTATTAACACGGGAACTTAAT
TGCTTGCGAGTGTATTTTACTCATGCAGAGTGTTCACAGTGTAGTACTGAGGCGCGAGCATAGGATTCG
ATACTATTTTCGAATCTACACGAAGGGTCGCTTTTACCAAACGCTAAACGTATTTAAATAATTTTAAATA
CATTTTGTACTAACTGACAGACGTATGACGGGCTACTAAATACGTTTAGCGTTTGGTGAAATTCCACCT
AACATGGAAAAAACAAATGCCACTTTTGGAACTAACTAATTTGCCTTACATTTTGACAGTGACATTTGA
CGATACGCAACGTAACGGAGGTTTCGAATCCTGTGCTCGGGCAACTGATAGTTATAATAATATGTGAGA
ACATCGCAAACACGACCATCCGACCCCAAGCTAGG
>TCONS_00076486 geneID=XLOC_015321 scaffold_250:334330-337039(+) Exonic overlap (-)
CCTCAGATAAAAACTTGTAACTTCGACAAAAGAGTGCACATAAATATATATTTATTCCATATGATTTAGT
TGATCTTCACTTTTAACTTTACTAATAATAAATACATGTTTTCATGATTTCCCAAACTAATGAATTAATTC
GGTCACATACTTTTTACAGCTAAAAATCAGTTCTGTTATCTACTTACGCCATAGAAAATATTCATGTATAC
ATACTTATGTTTTTTCACAATAATTTTAAAATACATAGTTAATACCCGCATACCTAGCACAAGACGTGGC
AAAAGATTTGTGTATCACTCGTCATAAGAGCATATCATGCGCCTTCGAAAGCAAATGAGACAGAAAAC
TTTTGCCACGTCTTATCGTGCGCGTATTGCGCCCCGTGCTAGGTTTGCAGATTAATTAAAATTTCCATTT
CTTAAAATGACCATTCCATAGAACATCCATCAATAGCAGAGCTCGATACAAATTCACAAATGCACTAAC
CAATTTATCATTAATATTATTTATTACACAAACACAACAATTCATACAAAACACATACATTTACCATTCAA
AACGTAACAAAGTTTCATACACATAAAAACAACACTCATTACTGTGCTAAGGGCCACTACCAGGGTTA
AATTAATATATCGACGGTGGAAATTAAAAGGCAAAAATTACTAAGGGATATTTTTTTCAACAATGAGTG
AGCTATATAACCTGATTCCCTTTATATGTCCGAAAAATTACGCTTAGTTATTTTTGACTTTCCAGCGTCGA
TATGTTGCTATTTCGTGCAGAATAAGTACAAATAAGACTGCACTTCATGTAAAAATATGATGTGTCTTCC
ACCACACCGCTAGCTTTCATAATAATTGTATGGAGGAGAATAACACATACTTTGCCCAATCTGAATCTAA
AGTAAGTATGTAAACTAAAGAAGGTAAGGCCCATGAATTTTGCATCAGAAATTGACATTCAATGTATGA
GCAAGCGATATTATTTTGAAAAATAAATTCCTATTTTCGAATATTAACACTTTTAGCTATAATAAGAGTAT

CGTGGTTACTTATTTTTACAAAAGCTTTACACTATCTATAGCGATATAAAATACTTGCAGGGGTTCCAATT
TACAATAAATGTCTTATATTTATATGCCTGAATTTAATTATTTATATATAATTTTTAAGCCTTTGTAATTGGT
TTATTAGTAAGCCGAGTAAGCAGAGACAAATAATGTTAAATAGACATTTATAAAGATATACATGTTTATTT
AACACCGTTTAAATGTAGACTAAAAGCATATTTCGAGGTGCGACTAAAACAGATCTTTTACTTTGTTCA
TAAATATATTGCATTAAATTTACTAAAATTAATGTCAGTTATTGTCTTCAAATTAAAACAAATTGAGCATT
ACATTAAGAAAATGAAAATGGTTAAAATACGATGATATTGATGACTATATTTCAACGAAACATAAATTTC
ACTGTCTCTAAAGTAATAACTAAAGTGAATTACTTATTAATAATTATTATGATAAGTTATAAATATGTCAAT
CAATCATTTACAATTTTAATAAGTATGTTTTTTATGAACTTAGTAGAATTTAAATTTTTCGAATATATATAG
TAATTTAATGATTAAAACAAAATAACTTAAATGCAAAAGGTCGTCATATTGTTCAAAATAAAAATGTGTA
CATTTTTTGAGGCCATACAATGTATTTACAAAAAGAAATAAAAAATCATCAAGTTAATAAAATGTTTAGT
ACAAAATTACTACCCGATTATCATGAGTTTAATTATTTACTGCTATGTTTATTACTAAATTAGACGTTATTC
GATGCAGTTGCTGTAGATTGACAAACTAATAATATTATTGTACAGTAAAAACTAGTTTCTTTATTGGGGT
TACATAGTTTTTGTGAAAGCATAATTTAGAACCTGTTAAACCAACTTATAAGGATATTTTCCTCATTTAG
GCCCAGTAGAGTAAATATACAGACGGATTGGTTGCTACTTACCTAGATTTATTTAGTTACAATACAATAC
GATATAAAAGCCACACCGTTTATATTGTAAAGTGGTGTCAAATACTAAAAAGATAATCATACCAAAATC
ACTTCTAAGATCGCAATCATAAATTTATTTATGTATTGGCCAAATTTACTGTCAACGGCTATAAATTTAAA
ATATGTAGCGTCAGAACCGACTGCTATACCTTGTTCCACGAAAAAGGTCCCCTTATTGCAAAGGCGTG
GACAAACTACAAGCAAAAACCCTCGGATCTCCGAGGTCTCTCCGGGAAGTCATGAGCAACCTAGGTC
GTCTACTGGCTTTCTGGCGTGACCTTGGGTGGCTTAATAGCTAGTCACCCCATAGGTATTTCACGCATGA
TGGCTAGATATATCCTGGACCCTTATTATTTTGAATATAAGATCTAAAATCGCTTGTCATATGAGACTTCT
GAAATGGAACAAGGTATAATACCTTTTTTTTGTAAGTAGCAGAAGTGTGGCTATAACTTTAGTAATTTTT
TACAGGATATAGTTTCAGAATCAGCTCTCACTGACGGCCGAGGAGTCGTCAGACTGGTTGGTGCTGGT
GCAGTCGCCTTTCGGAGCTATTTTAGACGGCTTGGCGCCCTTGCCTACGTAGATTTCTACTTCCTG
>TCONS_00076808 geneID=XLOC_015323 scaffold_250:380417-383066(+) Exonic overlap (-)
ACTGGACTACTTCATAGCCTGGCTGTGGGAGTATACGGGTGCCATCTTGCACGAGGCTTATGACGCTGC
CTGGTGGATCTATAACAAGCCTGGCCAGCCCATGGCCGATGTTGACGAGTATAAGAAGTGTATGGGCG
ATGTGTTCTGTTGGCCCCGTAACAAGATGAGATGGCACCTAATGTACCCCTGGACATCGCGACCAGAAT
ATGGATATAGAAGAAATGAACAACAAACGGAAAACCCGAAACAACTGGATTACTGAAAAACAATACA
TTATATTATTATTAAATTATTTATTGCACATAATTTTTGTACATAGGCGAAATGATGCTAATACCATGTCAT
CCAGTTAACCTTGATGGCTTTACGAAAAAATTAAAATAGTAAACTGATTTTTCCATCGTGCAACTTCAG
CGATTGTGCATGGTCGTGGTGCCTACGACAGTAATGTTTTTTTCGTCTGAATAGTGTGTGAAACTGTCA
AATTCTAAAAGTAAACATCATAAAAATATAATTGCGAACATTACTAAACAGAGCGTAGGTATAAGACGC
ACCTTAATACTGTCCTTTTAGATTAGTCGCCATATAATTTAATCAATTCCAATACCGCTAATAATCACCCT
CAAAGTTTCTAACACTAGCACGGTCGGGCTCTAACTCCAGCACGACAAGGCTCTAACACAGCACGGTT
AGGCTCTAACATTAGAGCACCAGGTCATTATAACACTAGTAGTCTAGTACGGATGGCTCTTTAGGACTG
TCGGGGTCTAAAAATAGCATGGTCGGGCTCTAATACTAGCACACCAGGTCTCTAACTGCGCTCACACG
AGCACACTTGGTCTCTTACGCACGATCGGGTTCTAGCACGGTCGGGCTCTATCACTAGCACACCAGGT
CTCTAACGGCTCTGGCGCTGCACTCGCTGGCCCAGTAGATGGTGCCGCAGCTGCTCACGAGGCTCCA
>TCONS_00076836 geneID=XLOC_015365 scaffold_251:8347-36798(+) Exonic overlap (-)
CGCTATTTCTCTGCACATTTTAATCGCGATCGCAAAACTTACGAACGAACTTTGTAAAAAACGATACAA
ATCGAATGTATTAAAGCTCAATTGTGAATGAATTAAAGTGATTGGTGGACTGTGTGACAAAGTTTCCCT
GTTTAGGAGTTTAACAATATTTTACAGTTTACAATACTTGGAGGAGTTCGCGACATGCTCTAGATAATAG
>TCONS_00077189 geneID=XLOC_015421 scaffold_252:152020-157566(-) Exonic overlap (+)
CGGGCGAGCGACACCGGTCACACCGGTCGCCTCGCTTGTTTCAGCGCTCAGAACAGCCCGTGCCGTC

GGCTGAACACTACGGTGAAACAGTTTTACAAACACAATAGATACAGTGTGAGTGAAAAAGGAACATA
ATTAGCACCACACAAGATCTATTTTTCAATAGCAGATTGAAAAATAAAAAAAGGTTAACGTTTCGTGAG
AACAAAAGTGTATTCCTGATCATATGTAAAGTATAGACGGTGAAGAGATAGACAAGTGGAAGATAAAC
GCATAGACTGGCGGACTGATCCCGTGGGATTGAGTGGGAAACTAGTACCCTCCACGTGTCGTGACGAG
AATGCCAACGGATCTGGAGACTCTGTCGGAGGTACCCGACGAGGCGTTCGGCGAGCGCGAGCAGTTC
GAGCAGCTGTACTACC
>TCONS_00077081 geneID=XLOC_015424 scaffold_252:305760-322383(-) Exonic overlap (+)
TACTACAATACTTACAATATTTTTAACTGCCAACACACATTGCTACACAGAGTACAGTGCCACAAACTT
ATTTGTTCTGGTGAGAGCTCACGTAAATGTCTAATATTTCTTTATTCTTTAAGATTTAAAGTTTCTCCGTA
GTGGTAATACATCCTTGCGGTTTTAATAAACCCATCTAATCTACTATATCAATATATAATTCATTAGTAAAT
CAACTTAGTTCGCTCTCACCGGAACAGATAAGTTTGTCGTACTGTAGATAGATTGAGAGTACAAGACAT
TGACAGTAAATTTCAGACAAATTGTTTTTTTCTTGCTTGCTTGTATCAAAGTGAACTTAAAGTTCATAAA
TCTGTTTCTGAATCATTAACTTATAACGAGTTCCCGTTTTATTAATAATAATATTCAATTAGTTTATTAGAA
AAAGAGATTCATCCATGACACAAAGTCAAGATGTTGCCGTATCGTGCCTTTCAACTTAAGACTTTGAAT
TATTTACCTCTCTAAAGTATCGTATAGGGCGTAGGTTACATCTTATATCATATTATTATTTATAGATTTTATA
CTAAACATTTTACTTTCGGTAAGCACTATGTTTTCAAAGAAAATGCCAAGATTGCCACAAACACTTTGA
TAGGTAATGCTTCTTTTAGTAAACTTTAATGAATACTTCATATATTCCCCTGTTGAATAGTGTTGTAACTA
ATTACTACGTGAAACGGTCATCAGAGCCCCTAAAAGGTTTTTACAATTTACGAAAAAGATTATTTTTTTT
GGTTTTTCTTATATTATCTGTCAAAAGTTTCATGATAGTTACTGCTAGAGGCGCCACTTTGTATGCGAGA
AAATGGAAACTTTTATATTTTTCGACAAACTATAAAATCTGTACGAGATGTACAGGCCCTTAATGAAGTA
CTTACTTAACTTTCGTTTTTTCAATTTGTATTCAGAATCAGTTAATTCAAAATGTTCTAGGTACTTTCTAA
TAAAAAAGTGGTTTATTAAAAAAAAACTGATATCTGGATTGGTCTATCCACTCTGATTGTCCGCATAATGT
TATCACTCCTATTAATATTTATGTAAAGTAGGTATATTTTTCATGAATTAATTTTTTCAAGACCACTGAGCT
TATTAATGTTTTCAATTTTGTCTGTGATATAAAATATTTTTGTAATATCTAGATAGAAGGAG
>TCONS_00077172 geneID=XLOC_015414 scaffold_252:385984-389772(+) Splice junction overlap
GCTATGCGCCGGATCACGGAGGCCATGCTGCGGACTCAGCATGTGGACAGATGTGAGAGAATGCGTCT
GACCCAGGTAAGACGAGAGAGACTACGGCCGAAAGTGCAGCCGAAAAAGTTAACTAAATTCGGGAA
AAGTCTAGCGATCCTATAACGCCGCACTGTGGGGTTACCACCACCGAACATTCGCAATTAACGATCCTT
AAGCAGAGCCGCTCGCCTGTCAAATCTCCTGATGATGGACATCTGACAGATGTTTGACAGGCGAGCGA
CGTTGCTTATGGATGCTTAGTTGCTAATGTGTCGGTGGTGGTACGGTAACTGTACTAACAAAACGCCTT
AAACCCATATTTATTCTAAGCCAGGGTTACGCCAAAGTTACACTAATCTTGACTTAACCCTGGGGTGCT
CATGTCAAAATAGGTACATTGCAGTGGGCTTAAAAACAGGCTAGCTAACAGTAGCCCTTTAGACTAGG
TAAACAAGCCATGACATCACTGATATTAATCACTTTTAAAAATGATATTATTTATTTATAATTATTATCTTA
AGCCTATCATTTCAATTGAGTTTTGACTTTTGAAAATGTACCCGTCCGAAAATTTAAATTTTTTAATGAA
ATTCCGGTGTAGAATCCCTGAAGGAAGGATATTTTTTTTACGAATGCGTTCCAAGTTATAAAAATACTGT
TATTAATGATGAATCTCGTATAAACTAAGTTTAATGCAAATATTTATCTATTGCTTTCCGCAAATAAGTTAT
TTTATATCAGTCGCAAGGTTAGTAGAATATCAATTAATCCACCAGAACACTTCAAGATCGTTATGAATAG
AAATTTGTGTATTGACACACGTATATATATTGAATGCATTAATTATAGTACAGCTTGGGCAAAAAATCTTG
ACCCCTTTCAGAGTGATAATATAGGTCAGTGGTGCCACTCTTGAAAGGCAATGTATTGCGTCTGTCTTG
CGTTCCCAACATTTAAAAAATCATAACTTCTCATCAGAAGCGTTTCTCCAAAAAGTAAAAATACGAATA
TGATCAGCTAGGTAATGCCTGTTTGGAGAACCTAGAATCAATAATATAATGCCTACAATTTAGAAGTTAA
CTTGCAAAAAGGTCAAAAATGACTTTTTCAGCCTAAATCTAAAATAAATTGTACATTGTAGGTATTTGG
TTCTTACTGCTAGGTTCTAAGAAAAGGCCGTATCTTGATGAACATTTACGTATTTTTAGTTTTTGTAAAA
TCGATTTTGAAGAGAAGTTATGATTTTTTTAATGTTGAGAACGCAAGACAGACGCAATACATTGCCTTT

CAAGAGTAGGGGGGCAGGTTTATTTGTCCTGGTTTGTACCTTCATACAATAGTTACCAGTAAACGTTGG
CTTGTAATTGTAACTAAATAATTATAATATAATTATTGCCTTAATATAGTGCAAAGCGG
>TCONS_00076996 geneID=XLOC_015414 scaffold_252:385990-391871(+) Splice junction overlap
CGCCGGATCACGGAGGCCATGCTGCGGACTCAGCATGTGGACAGATGTGAGAGAATGCGTCTGACCC
AGATGTCGTCCCGAGAGTCCTGGGTAAAAGCTTTGAATAAAAAAGTATTTTCTTTTTGGCGTTAAAAG
CTTTTAAAGCTGTTTTTTATAACGATAATATTTTTTTATAAAAAAAGTGCCTGACGATTTGGCCCAGCGA
TTACGCAATAGCAAAAATACATTTATTTTATTACACTTTTATATAATTTTCATAGAAATTTGAAAAATATTA
TACGTTAATGAATTTTATTAATAGTTTATTAAATCACTCAATTATATTTTAAAGGCTCGAACAAAATGTAG
ATACATAGACATGTAGAAAGAATAGGTTAACAAATTTATATGATGAAGTCATTTATAATACATTATGTTTT
ATTTACATTTTACAACATTTTGAAATAATTTAAATTTACTATAATTTTTATACTTTGAAACTTTTTATTGGT
TGTGTTTGGACTATGTATGACACGTATGATGTCAGATATATTAATTACTATTATTACTTTTTGTAAAAGCA
CTATTCATTCTATGGTGAATGACCTTGGAGTCCTTGCTCGACGACACAGACACACACACAGATACACTG
AAACGTTAAACTCATAACATCCCTCTTTTTCGTCGGGGCTAAAAATTGATTAACATAACATGATGGTAC
AGACTCACAGAGACTCTGGCAGTTTGCTTACTATTTGTCACGCGCCTAAGTAAAAATTGCATATTTCTT
TGTTGTTGCGAAACTAACTAATAAAAAATAACTAAATTGTAACCATTAACACAGCACTCCTTTTAGCGT
AAATTCTTGCGTATAAATTTATAGTATTTCACTCCAATTATAAAAAAAAAAATATCATCGTGAAATACTTA
AATTTAGAGGGATAATTAAAACCATTAAAAAGATTTTAGTAATAACAAAAATTATAATCATGAAAGTTAG
TTGTAAAATAAAGGTTTCCAAAGTAAAATGCCTGCCCCATTATAATTATTTTTTTTCGGATTTTATGCATG
TAAAATTTGGTTGTGTAATTTTATTTATGATTTGTATTTCTTAAATATTAATTGGGTCCAAGATATTAATCG
TATTAAACTTGAGTGCAAGCAGCTAGGAGTGTTAGTAATAGAAGAATCATTAAACTTTTTTAAATGGTG
GATTTTGATAATATTCGTACAATCATTGTGTATAATATTTAGCACTTTAAAATAAAATATACTTTGTGACGT
AAGCACACTAAATTCCATTTTCTCACTGGAGTAAATCTACCTAACATGAGATTAAGTCCTACGCCAGCG
TATTGATTTTCACACTCTCTAATCAGATGGGAAAATTAAATATTCCATCATGGTAGAAACAAGCAGCCTG
CGTGCTAAAAGCGATCTCTTCCAGGCATATTATTATTTTCTTTAATGATTCTACTAGTATAACTGAAAATG
TAAAGACTAAAATGTTTCTAATATGCTCATTTAGATATTGTAAAAAAAATAGAAATTCGCTAGAACAAG
CTTGCTAAATTACAAATTTGAAGCTCTTTTTAAATTCCATGTAATTTGTTTAATGAAGGCAATTTATATAA
ATATTTATGTAAATTCTGGCG
>TCONS_00077115 geneID=XLOC_015426 scaffold_252:388630-389887(-) Exonic overlap (-)
GGAGCATGAAAAAGAGATATGTTTACGTATTAAGACTTAAATATAAATATTATTTGAAATAAAACACGTC
CAACAATTCATTTTATACCTAACTACTGCTAATATCTTATTATTACCGCTTTGCACTATATTAAGGCAATAA
TTATATTATAATTATTTAGTTACAATTACAAGCCAACGTTTACTGGTAACTATTGTATGAAGGTACAAACC
AGGACAAATAAACCTGCCCCCCTACTCTTGAAAGGGGTCAAGATTTTTTGCCCAAGCTGTACTATAATT
AATGCATTCAATATATATACGTGTGTCAATACACAAATTTCTATTCATAACGATCTTGAAGTGTTCTGGTG
GATTAATTGATATTCTACTAACCTTGCGACTGATATAAAATAACTTATTTGCGGAAAGCAATAGATAAATA
TTTGCATTAAACTTAGTTTATACGAGATTCATCATTAATAACAGTATTTTTATAACTTGGAACGCATTCGT
AAAAAAAATATCCTTCCTTCAGGGATTCTACACCGGAATTTCATTAAAAAATTTAAATTTTCGGACGGG
TACATTTTCAAAAGTCAAAACTCAATTGAAATGATAGGCTTAAGATAATAATTATAAATAAATAATATCAT
TTTTAAAAGTGATTAATATCAGTGATGTCATGGCTTGTTTACCTAGTCTAAAGGGCTACTGTTAGCTAGC
CTGTTTTTAAGCCCACTGCAATGTACCTATTTTGACATGAGCACCCCAGGGTTAAGTCAAGATTAGTGT
AACTTTGGCGTAACCCTGGCTTAGAATAAATATGGGTTTAAGGCGTTTTGTTAGTACAGTTACCGTACC
ACCACCG
>TCONS_00077001 geneID=XLOC_015415 scaffold_252:396497-398462(+) Exonic overlap (+)
ATATGATAGCCACTTGTACCTACTTTAACTCTAGCGCAAGTAATAAAACAACAATATTTTCTGTTATTTAA
TATGTCAAAGCTCGGTTTTATTTTTATTAATGGCTACGGGTTCTTAAATAATTACATTCATTCATAGCAGT

AGTAGTGGTTGAAGTATGAGTGTCGCTCGTATGTTTGTGGTAGCTGCGTGTTTGTGGATGGCGGCGGC
GGCCACGGAGGCTCCGAAATGGAAGCTCAACAACGGACTGGAAATACCAGCTCTTGCCCTCGGCACT
TACCTGGGGTTTGA
>TCONS_00077216 geneID=XLOC_015466 scaffold_253:11108-22208(+) Exonic overlap (-)
TTCCCTACTAATCTTTGAACGAAATTACTTCTTTCATTCACGTGAAGTTCATTATTGTTTTGTGCATTCAG
CTTTGTTATTGGTCGTCATCAGTTGTGTCGAGGAAAAACATGAACGGCCAGCTGTTTTTGTTCTTCTCA
GTGTTTAAAATTCACCATGAATCTAAATTGAATAGTGTCAATTCATCTTGAAACCGTGACATTTTGTGGA
TTGTGAGTGATTATTAATTTGAAGATACAAAATTGTTTCGCCAAAGGCAGCACACTCAGATACAGATAG
CTGCGCGGTGACTGTGCGGGACTATCAGTGTAGTGTTGTGGTGGTGATGGG
>TCONS_00077218 geneID=XLOC_015466 scaffold_253:21850-22209(+) Unclassified
ATTTCTGTCGCGTAGCACGGCAACATTATTCTGTGACATGACAGCCAGCTGTGTGGAATAGATTGCATT
GGAAGAAAAATAACTCGAAAAGCGTAGCCGTCCTTGATGTGTCATCAAATTCAATAATTTCTACAAAA
CGTGGTGCCTTTCCGTGCGTTTCTAGACCATCCGCGTGGCTCGCTCACCTGGCAGCTTCAGAATCTCAT
AGCTGCGCGGTGACTGTGCGGGACTATCAGTGTAGTGTTGTGGTGGTGATGGGG
>TCONS_00077391 geneID=XLOC_015467 scaffold_253:24544-27283(+) Splice junction overlap
GGGCCGCCCACCGCCACCGTCACCAGGGATCACTCAGCGAGCCCGGCGCGCCACGACGCGAGCCAC
GAGCGACCAGACTCCACCACCCCGCCGCCTAAACATTCACAAGAAGCAAGTTCGTCGAAGGCGCACA
GTTCGGGCAGCAAGCTGCTCATAGTTCCCCGAGATCTCCCGTATTTGAGACAGACTAACGCTCGAAGG
AGTATCGCAGCTGCGACGGGTGCGACGCTGGGCGCGTGCCTGACGCGGGGAGCGGGCTCGTCACAGC
GCCAGCGGCAGGACACTGCGACCAAGCGACAGGTGTCGGGCAGCGAGGGCGCTGCGAGCGCGGCGG
GCGGGCGGCGGGCGCGGCCTCGAGATATGCCGCAGCCGCAGCCGGCGTCCACGCGTAGGGACAAAC
GAGGT
>TCONS_00077335 geneID=XLOC_015467 scaffold_253:37892-44968(+) Splice junction overlap
TGCTTTTGTATGATCCAACGTGTAGCAGCTACCCTTTCATGTCATCAGTCCACTGCAAATAACGAAATA
AATACATAAATACATAACACATAATAACACTATCCACACCCTCCACAGACATCTCCGACGGCCGCCAGC
TCGCGCTCCCCCGGCGGCTCGTCCCCGCCCGCGTCCACGTCCAACACGTCGCTCGAGAACGCGAACG
CGAACCTGTCGCTCGCGTTCTCCGCGTCCGGCTCCTTCGTGGCCACCACCTTGCGTGAGTTGTTTATAG
CGACTGGCAACGAGGCCAGCGGCACCGAGCCCGGCCCCGCCTCCGCCTCCACGCATCTACACACACC
CACTTCTACTCACAGCAGATCAGGCAGTCCGCTCCAAGCGGCAGAGCTACTGAAGC
>TCONS_00077440 geneID=XLOC_015467 scaffold_253:37892-44968(+) Splice junction overlap
TGCTTTTGTATGATCCAACGTGTAGCAGCTACCCTTTCATGTCATCAGTCCACTGCAAATAACGAAATA
AATACATAAATACATAACACATAATAACACTATCCACACCCTCCACAGACATCTCCGACGGCCGCCAGC
TCGCGCTCCCCCGGCGGCTCGTCCCCGCCCGCGTCCACGTCCAACACGTCGCTCGAGAACGCGAACG
CGAACCTGTCGCTCGCGTTCTCCGCGTCCGGCTCCTTCGTGGCCACCACCTTGCCGACTGGCAACGAG
GCCAGCGGCACCGAGCCCGGCCCCGCCTCCGCCTCCACGCATCTACACACACCCACTTCTACTCACAG
CAGATCAGGCAGTCCGCTCCAAGCGGCAGAGCTACTGAAGC
>TCONS_00077244 geneID=XLOC_015482 scaffold_253:83691-84533(-) Intergenic
TATAGTGAATAAATTTATCCGGACCCATATTAAATTATATATATATCTATCCTAAATTTATCACATTTTAAAA
TTAAATTTCAGATGTTTATTGGTGCCGGCGCTATATCCAGTAAACAGTCGCCCATAGAGCAAGAAATCG
CATTGCGATAATGGAGATGGATCGGTCACACTATAAGAAGGGGAGCCGATAACATGGCAAGCATCGCT
TTCGAGTGGAAACTGCTAGGAGGCCATCGGAGACGCGGTCGCCCTGTGCACACATGGCGGCGCACAG
TGGACGGCGAGCTGCGAGCGCAAGGCCTGAGCTGGACGGAGGCCAGAGCGGTTGCTGAGGACAGGA
CGGCGTGGCGCACTCTCGTGAAGGCCCTCTGTACCACTGGGGTACCTTAGGACGACAACACCATAATG
CAGATCACCTGGGTGAAGATGACGATGCAACATGTGACGACAGGAGTTCCTGTCAAAATCTTTGATAG

TGCGCGTCTTCTTCTTCTTGGCGTCCTATGACCCCCCGTGGGGGAAAGGGCAGACACAACGGTTCTCC
ATCTCAGTCTGTCGACGGCAGCGAGCTTCACGTGGTCCGAGCCACGTCATCTCCG
>TCONS_00077356 geneID=XLOC_015482 scaffold_253:83913-86532(-) Unclassified
CATGAATCCGCGCGAATTGTCTTGCGAATTGACAGGCAGGAACGAAGAAAAATCTTTATTAAACAGTT
TGGGCATTTCAAATCTCAATGTAACCAAAAAGTTATCCAAATAAACAGATCAATCAGCAGCAAGTGAA
GAAAAACAATGGCTTTTCCGTTGTTTTCTGGAGTGGGAATTACAGTTATTACAGTTAACAAGACTTGGA
TAGCGGCGCAAGCGCCCAAATGAGAACAGATGTAGCTTGGCTATTTTTTCGGGAATGCCACCGCTGCC
CCCGGGCCATGCCTCTATAATGAGAAGATGTTTATTGGTGCCGGCGCTATATCCAGTAAACAGTCGCCC
ATAGAGCAAGAAATCGCATTGCGATAATGGAGATGGATCGGTCACACTATAAGAAGGGGAGCCGATAA
CATGGCAAGCATCGCTTTCGAGTGGAAACTGCTAGGAGGCCATCGGAGACGCGGTCGCCCTGTGCAC
ACATGGCGGCGCACAGTGGACGGCGAGCTGCGAGCGCAAGGCCTGAGCTGGACGGAGGCCAGAGCG
GTTGCTGAGGACAGGACGGCGTGGCGCACTCTCGTGAAGGCCCTCTGTACCACTGGGGTACCTTAGG
ACGACAACACCATAATGCAGGTAAGATATTGAGTATTGATACTAATGACTGTCTAATCTTTTCACCATAC
GTGCAGACATGAGGTGCAGCAATGTATTACTATATTATATACTATATTATCTGTCAAAAGTTTCATGATAG
TTTCCGCTAGAGGCGCCACTTTGTATGCGAGAAAACTGAAATTTTTATAGTTTTTATTTCACACAGAAA
TGTTATTTATTTGGCAC
>TCONS_00077245 geneID=XLOC_015482 scaffold_253:85112-86575(-) Intergenic
GTCCGAAATGGTTCAATGGGACTGGCAACACTGTAAAAATCATCATGAATCCGCGCGAATTGTCTTGC
GAATTGACAGGCAGGAACGAAGAAAAATCTTTATTAAACAGTTTGGGCATTTCAAATCTCAATGTAAC
CAAAAAGTTATCCAAATAAACAGATCAATCAGCAGCAAGTGAAGAAAAACAATGGCTTTTCCGTTGTT
TTCTGGAGTGGGAATTACAGTTATTACAGTTAACAAGACTTGGATAGCGGCGCAAGCGCCCAAATGAG
AACAGATGTAGCTTGGCTATTTTTTCGGGAATGCCACCGCTGCCCCCGGGCCATGCCTCTATAATGAGA
AGGTAAGTGTAGAAATACATTATTTTAAAACAATAAAGGGTAAAAATTTCCATTAGCGTCA
>TCONS_00077246 geneID=XLOC_015482 scaffold_253:85187-86783(-) Unclassified
CGCGCCAACTGAACTTTTTCGATCGCTCACTATCCATCTGTTGGCGGCTGGCAAAATCTTTTGTATCAG
TAATGCTCTCAATGAACCACAAAATAGACTGGATGTTCACTAGATGGCCCTGCTTGGCTACATTTCTTAT
TTTTTGGAGTTGAAGAGGAAGGTGGTATGTTACTGGCACATGGTCAGTTACTGGGTGTTTTGCCCTACC
GTCCGAAATGGTTCAATGGGACTGGCAACACTGTAAAAATCATCATGAATCCGCGCGAATTGTCTTGC
GAATTGACAGGCAGGAACGAAGAAAAATCTTTATTAAACAGTTTGGGCATTTCAAATCTCAATGTAAC
CAAAAAGTTATCCAAATAAACAGATCAATCAGCAGCAAGTGAAGAAAAACAATGGCTTTTCCGTTGTT
TTCTGGAGTGGGAATTACAGTTATTACAGTTAACAAGACTTGGATAGCGGCGCAAGCGCCCAAATGAG
AACAGATGTAGGTAAATATGATTGAAAATAATCTCATCCAACGAGCATCAAGGAGATTATGGCAGCAAA
CCAAAGTGCCATGCCAGTATTGTATTCGGGTGATCTTGGCTATTTTTTCGGGAATGCCACCGCTGCCCC
CGGGCCATG
>TCONS_00077327 geneID=XLOC_015483 scaffold_253:147631-148050(-) Unclassified
GCAGCCGTCGACGTCGACAGTCTTTCGACAGTCGACATTTTCAATTTCATTCAATTCAGTTTTCGTATTC
GGCGGCTGTGACTCGTTTACATTCGATCGGATAACTATTTATTTTTAAACGTTTCACTAAACATTTGTGG
TGTGTTTATATAAATAAGAGTGACAAAAGTTAAGTTGAAAATGAATCCCTACCAGCAAGGCCAGTCGG
GATATCCACAACAGCCTTACATGCACAATATTGGGTTCAGTAACAGCGCCCCACAGTCTTTTGGGAACC
CGATGCCTATGG
>TCONS_00077450 geneID=XLOC_015484 scaffold_253:174396-175526(-) Splice junction overlap
TTGTTACAAATGACAAAGTAGATTAAGATCAAGAAAATGCGAAATAAGGAATTCATTCTGAAATAATCC
CATTACGTTTTCCAATCTCACTGGAGATTTTCCCGCCTCGTAAATGCTAAGTCACAGATGTTCGTTTCGA
AACCGCTGGCTGTTGACTTGATGAGTCATTCTACTGTGAACAACGTTCAGAAAACATTATCTTGTAAAC

TAAGTTTTAAGACTGTGATTTATTTTATACTGGAGGCATTAATTTAAATTTTTATATTCACCCGACATTAG
GAAGTGGAAAGCGATGGCGAGAGTGTATTTAATTGAGTGGCCTTTTTAGGTCCTCTTCGACAGCTTCA
CCCTCGGCCGGTTCACCTCCTTCACCGAGGACGGCTGTCCCGACGGCTACATGCAGATCCAGGAAGCC
AGCAGGCCACAGGTTATCATAATAATATTTCGTCGCAGTCGTAGAACAATGACGGATCTGCAAAATAGC
AACTTTACAGGAGAGCCAGTTAAAGTTTTTAAACAACGTTTTGTTCGATAAAATGAAAACGGCTGAAG
CAATTCATTTGAAATTTGGTCACATTATTATAC
>TCONS_00077415 geneID=XLOC_015475 scaffold_253:264760-266569(+) Intronic
ATACCGTCTATGATCCTACCAGGTTCATGTCTCTTCATTGCTTCCTATTTCCGAATGAAAAGTTTTGTCC
AAGATAGCACTCCTTAAGGAAATAGTTCTGGTAACAAGTGAAACCTATGAGATACTCATCTCAAGTAAA
TGTCCTTGTAAGATCTCATTGAGCGACATGACTGTATTAATTGTAAAGAAATTATTTGGTTTGTTCTTATA
GTGATTTAAATCAACTTATAGAACATAAGGAACATCTGAATTTCTGAGCTGTAAACAAAAATGTTGATC
CTGTTTTATCATTAACAAATCAATTTGCAATGAGTACATCATGACATGTAATCGTCAATACACCATATTTT
GAAAGAATGATTTATGTATACATAGAACAACAACATTAAACTATAAGTTTTATAAATTCTGTTTGTTAAG
CTAGTAGGTATACAAAAATACTGTGATATAGTTTCTAATTGTATAGTGGCACTAATGTCATATTAAAACGT
TAAGTTTAAGTAAGAAATTAAAGGTTAATTATTATAACTTCATTTAAAAGTGGTGCACAAACATATTATT
TATATTTATAATATATGAAATCATGGAACAAAGAAAGTCATGTATGACCATAATTATATTTGTATTATCCTA
TTTATGTAGCCTGAATTTTGGATGAAAAGATTCATCAAATATAGGATTTACCTTTTATTAACTTTTTTCAA
GATATTGTTTTATCCAGATGATAATTTATGTAGATTTATCTAATACTGAGACTAAAATATAATACAGCAATG
TATCTTTGCCTTCCCCGTAGGATCTATAACATTGCATGGTTTTTTTAATTGGTGTGCTCAACTGCACATTG
AGTTATGTACATGATTCATAGAAAATATTCTATGTTTTTGTAGGTTATTTATATTTTTATATGACAGTCAAT
TGTTTTGTATTTTTGTTTTTATAAATATATTTATTTATCCATAAAC
>TCONS_00077604 geneID=XLOC_015527 scaffold_254:116336-146836(-) Unclassified
ATGAATTTGTATAGCTACTTATACCGTAGTTTACGCACGACCAAGCTTAGCCATGCAAACGGCAATATTT
CACTAATCCGTGCATTTCGCAGCAGGCCGGCAGTATTAATCGGAAGGCCGCGGCGATCGGTTAGCGGC
CTACATCCTCTCTCTGAAATAATAACAACAAACTCCGATAACGAATTGAAAATAACGATAACGACTCTC
TGTTAATAAAAAAACGAACTTGTTTTTTTTTGAGAAAAAAAAAGAAAGAAACCGGCCAAATAAAAATGT
AGCCTGTATTTACGAAACTGAACCGTAATGTTGTGGATATTGAAATGTGAAGCGTAATGTAAAAAGTTT
TGACAGTTCAGTTTTTTTTTCTATTGCCTTTTGCTAATTTGCTTTGCTTGTGTTTTTCGTCCATGAAAAGT
TAGAAACTTGAAATGCAAGTTTTTCTTCGTAAATCCTACAAGTTTTCATGAGTTCTTCAGTGTGAGAAT
AAATGTTATTAATTGGGAGATGTTCCTAAAAGCATCCGCTCAGGAATAGCTACAGATGGATGCTAAATA
CAGATTCAGAATGCAGCTTCCCAAGACAATAATAATATTATACAGTATACAAGTAGTTGTGCATGTATAG
CCATGTTCAATTAAAAATACATATTTGATAGTAACAATCAAATTATGTGATTTTTTTATTAATATTTACAAT
AATCGAGGCCAGTAAACCGCATTATTGATAATAAAAACATGTAATTAGTATTTTAATATAATTAAAATAA
GGCCAGTACTAGTTTATAACAGTTTTATATGAAAAGAGCAGCATGTTTAAGTCTACACACCTATTAATGG
AGGTTTCAAGCCCTTAAATTGCACCCAATGACCCATCCCAAGGACAAGTGACATCCAGGGGTAGAAA
GCTTCATGGTTTACATGGATGATCGCGAGGGCGCGGCGCGCCGCTCCGAGCCTGCTAGTATGGAGGCG
TGC
>TCONS_00077626 geneID=XLOC_015527 scaffold_254:130232-146836(-) Unclassified
ATGAATTTGTATAGCTACTTATACCGTAGTTTACGCACGACCAAGCTTAGCCATGCAAACGGCAATATTT
CACTAATCCGTGCATTTCGCAGCAGGCCGGCAGTATTAATCGGAAGGCCGCGGCGATCGGTTAGCGGC
CTACATCCTCTCTCTGAAATAATAACAACAAACTCCGATAACGAATTGAAAATAACGATAACGACTCTC
TGTTAATAAAAAAACGAACTTGTTTTTTTTTGAGAAAAAAAAAGAAAGAAACCGGCCAAATAAAAATGT
AGCCTGTATTTACGAAACTGAACCGTAATGTTGTGGATATTGAAATGTGAAGCGTAATGTAAAAAGTTT
TGACAGTTCAGTTTTTTTTTCTATTGCCTTTTGCTAATTTGCTTTGCTTGTGTTTTTCGTCCATGAAAAGT

TAGAAACTTGAAATGCAAGTTTTTCTTCGTAAATCCTACAAGTTTTCATGAGTTCTTCAGTGTGAGAAT
AAATGTTATTAATTGGGAGATGTTCCTAAAAGCATCCGCTCAGGAATAGCTACAGATGGATGCTAAATA
CAGATTCAGAATGCAGCTTCCCAAGACAATAATAATATTATACAGTATACAAGTAGTTGTGCATGTATAG
CCATGTTCAATTAAAAATACATATTTGATAGTAACAATCAAATTATGTGATTTTTTTATTAATATTTACAAT
AATCGAGGCCAGTAAACCGCATTATTGATAATAAAAACATGTAATTAGTATTTTAATATAATTAAAATAA
GGCCAGTACTAGTTTATAACAGTTTTATATGAAAAGAGCAGCATGTTTAAGTCTACACACCTATTAATGG
AGGTTTCAAGCCCTTAAATTGCACCCAATGACCCATCCCAAGGACAAGTGACATCCAGGGGTAGAAA
GCTTCATGGTTTACATGGATGATCGCGAGGGCGCGGCGCGCCGCTCCGAGCCTGCTAGTATGGAGGTA
AGACAGACAGATACACACAGACACACATGTACTGGGTAACTCGCTTTTCTCAGTGTATAATTAAGTTCT
TAGAAAAACTGCCTAAGAAAATTTCGGGATAGAAAAAAATCATCAAAATTGGTATAGCCGCTTTTCTC
GTGATGAGTAACAAATTTAGTGACCATCTCAGAAATTTTCGCCTTTATTATATTAGTGTAGTTTTAACTG
GTACATTGAACATCCAAGATAGTCGGTTAAAGCCCGACACTACCTGGGTCCTCTTCCCAGGTGTCCATG
TGGATTTTCCCCCAGCAGTGAAAAAAAAAAAAAAAAAAGAGAAAATAGAAAAAGAAAAAAAAAGAAT
AATATATATATATAAAAGATAGAATATATATAAATGAAACTTCCTTACCGAGTAGAAAGTAGATAAAAGAA
TTAAAAACTATCTCATAAAAATTACCACTCTTCAAAAGGCACGGACATAAAGTCTGTGGAGTGATAATA
ATTTATTAAAAAAAAAAAAAAAAAAAAAAACATCCAAGATCCAGGGACAAAAATGTAGGTACATACATA
TATCTGAACGGCTATGACAAAGGTTCTCCGTCGCGCTCGCTCTTGAACCTGCCCGATGTGATGTGAGTG
AGCGCGATGCAAAACTTTTATCATGTCTTAAATGCGCCCCGTGCTAGCCCTTCTGCAAACAAATGTAGA
CCTCGGCCGGAATTCAAACCCGGACCTTCGATTATCCCAACCAACCAGCG
>TCONS_00077473 geneID=XLOC_015516 scaffold_254:227722-347447(+) Exonic overlap (+)
GCTGCGCAATACGAGCGAGCGAGACAGAAGACGTGACCAACGCCACTTGTGCGACAGAGAGAGCAC
ATCACCTCTATATGCGTCGCCACCGCATAGCCCGCTGTGCAGTCCTGTAGTACATGACAATGGATCTCA
GCCAGCCAGCCTGACGCCTCACCCGGCGGTCACAGACCAACACTTCAACCGGGACACACACATGAGG
ATCATACTGGGGCGGGATAGCAAAC
>TCONS_00077605 geneID=XLOC_015529 scaffold_254:232557-352802(-) Exonic overlap (-)
CCCTGGTACAATAGAATATAATCTGACACTGTTCACTTGAAAATCGAAGCCGCACAAAGTTATATCATC
AAATCTTTTTGGATAAATAATGAATGTAGGTAACATGACAAAATATGTGAATGTTAAAACATTTTGTAGG
TAGGAGTAAATTCAAAATAATCTGTCTTAGCAAAGTCTCTACTATAATTCTAGAGACTACTAAAGCAGTT
TTTTTTTAATTACTGAACGTACGTGTTATCCCCAGGGCAGTTATTGGTGACTCCCAAATGAGTACAATCG
ACTCCTTGAACATCTCATAAACTGTTGTAGTTGTACTATAACTGTTGGTAGTTTGTACTCATTTTATTATT
TAAATGATTATATTGACTCACATCGAGCTTGTTATCGTTATTTTATAATGGTACTTTTTTAAACTCAATAAA
ATTTATCTCTATGTATACGTGTTTAGGTAAAAATGCGTCATTATTTATATTTTTTGTTCATTTTTTTTTCAGT
TTAGTAGTCTATATTCTAACAGAATTTTAAAATATCCTATTTTTAATATCGAATGAACTTATATTCCTCGTT
TATTGCTCATATCCAATCTTTTATATATCCAACTTCCCTTATAAATACGGAGTATTGCAAAAAGGCTATATT
ATAAGCCGAGCCAGGGGTTGATTCAGGGGGTCATTCTGAACAACTTTCGTTACCACTTTTGCAACACT
GTATAATTTCTGTCCTACACAGTAGCTGCGTCATTCTTTTATTGCACCTTGAAAGCTTGAGTTTTGTGAT
TTTTTAATATAAATCTAATTTTTCATCCACGAACTAACATAAATATCATCTTTGTGCCTGCAAAAATTTAC
GTAGTCGTGTCTTTGTCTTTCCATTGCAAAACGTCTTTCATCGTATAGATTTGTCTGCCACACAAGTTTC
ACATTTACACTACAGTACTGTAGTAAGTAACGTCATTTGTCCTCTATCCTAGAGTCTTACCCCCCTTATTC
ATAGAAAACTATTTTGTCCCACTCTATCAAATAACAATTTGCTTATTGATAGAGAGGGATAAAGCAGTTC
AATAGCTAAAACGTCCTATAACTTTTCTATGAATAAGGGGGTTAATAGAGTTGCATCCTTTCCCCCCCAT
CATTTATCTTATAATGTCATGCCATCTATCCTCCTCTGCGCCATCCTGCTCTCTTCTATCCTATAGTTTCAT
TTTCATACTGGTGACCCCGACATGATCAATTATCTCTATTGTTATCTATTGCATCTGTCTCTATCCTATAGT
GTCTTTATCTTCCGTCCTACCCGTCTACCCTATAGCGTCGCCTAGCGTCACTTCTCCCTCTCTCTCTCCTA

TAGCGGCATCTCTCTCCGCCGCTGGAACATTGCGCCACTATCAACCATATTCTGTCTATTCTGTGTTGTC
TTTTCTATCATCATATTACAGCCGCATCTATATTACAGTAGCGTCGTCTAGCGTCACCTCTCCCTCTCTCT
CCTATAGCGGCGTCTCTCTCTGCCACTGGAACTGAAAGGCGTTGCTCTTAACCGCGAGGATGAAGTTT
TAGCGGCATTTTGCATCATACTGGTGACCCCGACATGATCAATTATCTCTATTGTTTCTATTGCATCGGCA
GCATCTGTTTCTATCCTACAGTGTCTCTATGTTCCATCCTCTACCCTACATCGTCACCTCCCTCTCTCTCC
TATAGCGGCGTCTCTCTCCGCCACTGGAACTGGAACGCGTTGCTCTTGACCGCCGGGTTGTTGGAGCC
CGCACTCGCCGCTA
>TCONS_00077598 geneID=XLOC_015516 scaffold_254:232575-352802(+) Exonic overlap (+)
CCAACAACCCGGCGGTCAAGAGCAACGCGTTCCAGTTCCAGTGGCGGAGAGAGACGCCGCTATAGGA
GAGAGAGGGAGAGGTGACGCTAGACGACGCTACTGTAATATAGATGCGGCTGTAATATGATGATAGAA
AAGACAACACAGAATAGACAGAATATGGTTGATAGTGGCGCAATGTTCCAGCGGCGGAGAGAGATGC
CGCTATAGGAGAGAGAGAGGGAGAAGTGACGCTAGGCGACGCTATAGGGTAGACGGGTAGGACGGA
AGATAAAGACACTATAGGATAGAGACAGATGCAATAGATAACAATAGAGATAATTGATCATGTCGGGGT
CACCAGTATGAAAATGAAACTATAGGATAGAAGAGAGCAGGATGGCGCAGAGGAGGATAGATGGCAT
GACATTATAAGATAAATGATGGGGGGGAAAGGATGCAACTCTATTAACCCCCTTATTCATAGAAAAGTT
ATAGGACGTTTTAGCTATTGAACTGCTTTATCCCTCTCTATCAATAAGCAAATTGTTATTTGATAGAGTGG
GACAAAATAGTTTTCTATGAATAAGGGGGGTAAGACTCTAGGATAGAGGACAAATGACGTTACTTACTA
CAGTACTGTAGTGTAAATGTGAAACTTGTGTGGCAGACAAATCTATACGATGAAAGACGTTTTGCAAT
GGAAAGACAAAGACACGACTACGTAAATTTTTGCAGGCACAAAGATGATATTTATGTTAGTTCGTGGA
TGAAAAATTAGATTTATATTAAAAAATCACAAAACTCAAGCTTTCAAGGTGCAATAAAAGAATGACGC
AGCTACTGTGTAGGACAGAAATTATACAGTGTTGCAAAAGTGGTAACGAAAGTTGTTCAGAATGACCC
CCTGAATCAACCCCTGGCTCGGCTTATAATATAGCCTTTTTGCAATACTCCGTATTTATAAGGGAAGTTG
GATATATAAAAGATTGGATATGAGCAATAAACGAGGAATATAAGTTCATTCGATATTAAAAATAGGATAT
TTTAAAATTCTGTTAGAATATAGACTACTAAACTGAAAAAAAAATGAACAAAAAATATAAATAATGACG
CATTTTTACCTAAACACGTATACATAGAGATAAATTTTATTGAGTTTAAAAAAGTACCATTATAAAATAA
CGATAACAAGCTCGATGTGAGTCAATATAATCATTTAAATAATAAAATGAGTACAAACTACCAACAGTTA
TAGTACAACTACAACAGTTTATGAGATGTTCAAGGAGTCGATTGTACTCATTTGGGAGTCACCAATAAC
TGCCCTGGGGATAACACGTACGTTCAGTAATTAAAAAAAAACTGCTTTAGTAGTCTCTAGAATTATAGT
AGAGACTTTGCTAAGACAGATTATTTTGAATTTACTCCTACCTACAAAATGTTTTAACATTCACATATTT
TGTCATGTTACCTACATTCATTATTTATCCAAAAAGATTTGATGATATAACTTTGTGCGGCTTCGATTTTC
AAGTGAACAGTGTCAGATTATATTCTATTGTACCAGGG
>TCONS_00077621 geneID=XLOC_015519 scaffold_254:382929-388826(+) Intergenic
CGACAATATATTTCCTCACATTACTTAATAATAAATACTAATTAATTAAAACATGAAGATAAAATAATTTA
AACATAGAAATACAAATACAGACTGTGCTTAAATATTGGTAAAATCCTCCTAAATAACCCATATAAACCT
TCTGTAAACCGAGTTTCAAGTTTAGGTCCTCAGTTCTGGGTCAAAAGGGTACGAGCCTGAAGTTTTGT
TATATTGTGAATTAATTTCGCAGTAGCCCCGCAGTAACTCTGGGGTTGTTGGGGATTCCCCCGGAAGCT
GTAAGCTTTGTGCAGATTCCTTCGCCACCCACCCAGTTAATTCCACAACACTCAAGAAATCTGAGCAG
TTATTATTGTGAGCGGAACAATTGCCAGTTCCCACTTCGATACAGTTGTTAACTGGAGATACAGTTGTT
AACTGAACACAAATAGGAAACTCTCAAGAAGAAACGGAGAGATTAAAAGTAAGACGTATCCAGCGGC
AGGGTTACCGATCTTATTAAATTAAAATTTCTCGTGACCTATCTACTTCTGAGTACTAATATTGTATAGGC
GAAAATTTGTGTGTGTGTTTGTTACTTCTACACGTCAAAACGGCTGAACCGATTTTAACGAAATTTGGT
ATCGAGTTAGCTGGCACACTGGATTAACACATGGCTACTTTTTATCGCGGAATTCCCACGGTTTTTTTAA
GGCGAAGCGAAGTTCGCGGCTACAGCCAATAACGTACCTATAAATGTACAGCAAAAAGCGGGTTGCTC
ACTTGCGAGTCACCACTGACTACCCATTCGGGGATAACAGTTGTGAAACTCATGAACATGATGGAATT

CTTATGTAAAACTAGCTGTTCCCGCGAGCTTCGCTTCGCATTAAAAAGTTTTCCCGGATGAGAATTCCG
GGATAAAAAGTAGCCTATGTTCTTTCTCAGGGCTTAGACCATATGTATACCAAATTTCATTCAAATCCGT
TCAGTAGTTTTGGCGTGAAAGAGTAACAGACTGACAGACACAGTTACTTTCGCATTTATAATATTAGCC
AAGTTAGGGTTATTAGCTTAAAACAACGTGCTCTCGAATCTACTACTTTGTTTTCTACGGAGACATTACT
GTGTAGCAATAAGACCTCCGGTCACTGGACCAACCACGTCACATAGGAGGCGGTGGCTGGATGAGGA
AGACCGAGGACAGGGTTTTTGGGCGCTTCTTATGAAAGACGTGTGTCCAGTATAGTGGACGATTACCG
GCTGATGATGATTAAAAGGACCGATGTATGTACATAATAATAATATATTATGATCTGGATGAAGATACTGG
TTTTCTCCCTGTTTTCCTTCACCGTGGTATTATTTATGGGACTTAGGCTTATAACCTTATCAGTTAACAGA
CTCCGTTTCCTAAAATTTCAATGAAGAACCCTAGAAAATGTTAATATAAATACCCTACTAATAATAAATG
CAAATAAATTTGGAGGAAGTTGGAGCATGGAGGAAGCTGTCCCCCTGTGATAAATACATAGGCTTATTT
TAATCCCGGTATTCCCACGGGAAACCTTTTAAAGGCAAAGTGAAGCTAGTAAATGCTAGTCGGAAAAT
AAACAAAGTTTTAGAACAGAGCAGCCCTATACCACCTACGCTACGCTTCGAGGAGCCATGTAGACACT
GCCTAGACACCGCCTAGACACCGCCTCTAGACACTACCAGACAGAGACACTGCGGACGTCTCAGCAA
TCCCAGTTTACACTGAGATCATTAAATATGACAAATAAAAATACCACACAATTTTTTTCTGAAGCGAGC
ATTAGTTATAACACTGGACTACAATGGTAGTTTAACAGTGATATTTTTGATGTTCTTACATACAAACCGG
CACTAAGCTGAAACATCATCATCATCATCAGCCAATAATCATTCACTGCTGGACATAGGCCTCTCCTAG
GGAGCGCCACAACACTCGGTCCTCGGCCTTCCTCATCCAACCACTACCGGCTACCCGCCTAAGGTCAT
CAGCTATGCTATGCTGAAACAAATGATAGAAAATTTACAACACGAATTAAGCGTAAATTACCGCAATTT
ACAACCCAGAACAGTTTTTATTAATATAGGTAGTTTATGTATTAATACCAAGCTGAGTACAAACAGCTAA
TCGTTATAATTATACCATAATGTATGTATTTAGCTCAAAGCAGTTACTGCGCAAATGTTTGACACTTTATT
GTAACTTTCAGGAAGACAAAGGGCCTTTGTCTGATTCATATTTCTTTCAGGAGATAGTACTTCATGTTG
GAAAATTTACATTTAATCAGCGTGAAATCTGGAACGATGCACTGAAGGACATGTGAGAATATGCAAAT
CATTATGTACAAGGAAGTGTATTATGTACTTATAGGTATTATTATGTATTCTATTTATTGTTAGGTACAATA
ATTATTGAGT
>TCONS_00077623 geneID=XLOC_015520 scaffold_254:696418-701165(+) Intergenic
CTGGATTATTTATTTTTACCATATAATAATATATATTCATTATTGAAACAAAGTACGATACAAAATGTACAC
GTTGTCTTAAATTCATACTCTACAGTAGTTGTTACATCATACTTAACAATTTTTAGATCTACGTACATATG
TATATCAAGTTATTGTAGATAGGTAGGTATACCCTATATATAATTACGACATAGTATGTACAAGTTGAGAG
CCCACTGGGACCTCTCGCCCCTCTCAATACGTCAAACTTATAATTACAATTAAACACCTTAAAATATACT
GTTGGACAAAAAGACAATATTACAAAATACACCCTTTGACGAAGGTCATAAAAATCTTTGTCGTTTGA
GAAAACCGTGCCTTAACAGTTTCAAAAACATCTTAAAAATTAACTGTTCATTTCTAATATCTACAAGTTT
TAGAACTGATTATTACAGCGAAAACAAATGTGTTTCTTATTTGGTGAGCCGATCTACATTCATCGGGCG
GAGATTTTGGTAATTTCAAACTTCTGTTTTCAATTCATCACATTTCTTACTGCACAGAGTATTACGATTTT
TCGAAATTTTCAAAACCACCTCCACTTTAAACTGCATAGAGCTACTTACAAGAGAGATCCATCGGTAAC
AGTTTGTAAGAGCCTTGTTTTTTTGGTATAATTTATATTTAAAAAATCTATCCTCAAAAAGCATTTACAA
AGTTAGCTTGATTAATTACAAATTACAGAAACTGGAAACGTTTATCCGGCTTTTATACAAGAAAATATAT
TAATTAGACCGTTAAAAACTGCTCAAAAACCTCCAAAGACTAGATTGGTTCGGAACACAATGAGATTC
CTAGCCAACATTGTATCATTAATTACACATACTAACATACCTTCTTATACCAACTCGACCAAAACGTATC
CTTCGAACCAACACATTCTCGAAAGTATCGCACTCCGAAATTCTTTCAACGACAATATTGATTAGCTTC
CAATAATAAAATTATAATTCGTGATCGTTATCACCTACTAGTGCGTCAAAGTATAAAGTGAGCTTGCGCG
CCGGACGCAAGACAACGTGACTATAGCCTAGTCTTATGTTACTATTACTCGGTTACATAGTTCATCTAAA
ACATCTCAAAGTAAAGTACGAGTCATTCGCTCCTATAAGTACATGATTAATCAAGTATTACGAAGTGAG
GTTGAATTGCAAACCTATAAATTGCACTAGAAGCCAAATCGAATAATGTACATGTGCGGAATACGGGCG
CGACATCGGAGTGACGGCG

>TCONS_00077630 geneID=XLOC_015533 scaffold_254:766332-768616(-) Intergenic
ACTGGTACTATTATTTGTTAAGGTGAGATTGACTGTAGTAAGCAGCACTCACTGGTACTATTATCTGTTA
AGGTGAGATGGACTGTGGTAAGCAGCACTCACTGGTACTATTATCTGTTAAGGTGAGATTGACTGTAGT
AAGCAGCACTCACTGGTACTATTATTTGTTAAGGTGAGATTGACTGTGATAAGCAGCACTCACTGGTAC
TATTATCCTGTTAAGGTGACATTGTGTTTAGCAATGCTAACTGGTACTGAATTAGACTCATGGATTCATAT
TCCTACAACCAACGATAAGTTATTGATTAACTATTATACATTTAATCATAGATTTTGTACTTTCGCAGGAG
TCCTCAAGATTCTACGAATAACAGGGAAGATGCAAGTGCTCTCACTCAACCAGCATGGGATTCTGAAA
AGAAAACGGAGAAAAGATCCACGGAAGATGCTTCTCTATACTAATTGTATTAGTAAAATTATTCATTATA
ATATTTTAACTACAGCCTTAATTTATGCGTGGTGAGCCTGTTGTGTCGTTGTAATTTAAGAATTGAAACA
TGTATGAAAGTTTTTTTTTTTTTTTTTTTTGTTATTAATTTGATTTATTTACCTACTATGAATGTCGACTCC
GTATGTTGGTTAATATTCTTACCTCAGCAATTAATTGTGTTAGCTTGTGTATTATGTTTTTTTTAGGTAGGT
AATGCTCACTAATTAAATTCTCTTTATGATATTCAGTATGGCCGATGTGGAATAGGGGCATTCCAATAAC
AGGACGGCCGTCTGTTAGCTAAACTATTCCTTTGCTAAAAATTGTTGTTATTTTCCAATATTTTTAAATAT
GTTATAATTATTTTGTGGACCTTACAATCCACAAAACCCCAAAAATTATGCAAAATCCGATTTGTTTATA
CATTTTTGTCGCTTACAACACATGATTAAAAACGATACTGGCAACACAGGATTAAAAAAGGAGGGTTC
AGAAAAAATATGAATAGGGAATAGGGCATGCAATACCGGAACTATTTTCAATACCGGTATTGAGTTCCG
GTATTGCAACTCAATACCGGGATCCCGGTATTAATACCGGTATTAGATTTCCTTGTAATAAATGGCATATA
TTGTGATTAAAGGTCGTATAGGTCAAAATAAAACGAGAAAAAGCAATGAAATTCAGTTTTTTGTAATAG
ATATTTACGAGAATTGAGTATTAGTTTAAATTTTACAATAATTTTTTTTTTTTACATCCAGTGTCCGTTTAC
GCATGGACAACAGTACATATCAAACGGCCGAAAACTGCATCAAACGGTTGGAAACAAGTGCGCTTTC
ACAGTACATAGGAATACTATATCTTAATCGCTTTATTATAGGCTAAAAAAGTCCGATTCCGGTATTACTTC
AATACCGGTATTAACTTTCAATACCGGTATTGAAAAAAGCGTGAATTTTGCTCCTAATACCGGTATTACG
AATACCGGTATTGCGGTATTGCATGCCCTAATAGGGAATGGCGGTTCGAAGTTACAATTGTTACAAGTG
TGCATGCGAATTACTGATAAATAAGCTTTGTTACTGTTTAAAAAGTTTAAATAAAGTGTAAGTGAGTAA
CTAAGGTGTTTAAATTAATACATGGCTGTGATAAGTAGTTAGCTCTGCTCATATTATTATAATAATAATTAT
TAAGCTTATATGTATTGTATACCAACTAGTTTTAAGTCTTTTTTTGAAAAGATGGCTCAGGAGTTTCTTG
CCTCCGTTCTTCTCCTTAGACAGAAAGAGCCCCCCCTTATCCGAACGGAGAGTAATTTGTAAAAATTGA
CGTTCATCAGAAAATTTTATATTTGTACGATGAATAAAAAAAATTTGAGTTTGAGTTT
>TCONS_00077631 geneID=XLOC_015533 scaffold_254:766332-775007(-) Intergenic
CAGAGAATAAGTTTAAGTGTTACATTAAAGAGAAACTCAGTAAAAAAGCTTATTATAAAATTGATGATT
ACTTAGAGGACGTTAATGCATGTGTTAGCTAAACTATTCCTTTGCTCAAAATTGTTGTTATTTTCCAATAT
TTTTAAATATGTTATAATTATTTTGTGCAGTTTTGTCGCTTACAACACATGATTAAAAACGATACTGTCAA
CACAGGATTAAAAAAGGAGGGTTCAGAAAAAATATGAATAGGGAATGGCGGGTCGAAGTTACAATTG
TTACAAATGTGCATGCGAATTACTGATAAATAAGCTTTGTTACTGTTTAAAAAGTTTAAATAAAGTGTAA
GTGAGTAACTAAGGTGTTTAAATTAATAATTCAGAAGTGAAAAGACCAGCTCACCGATCGGAGTCCTC
AAGATTCTACGAATAACAGGGAAGATGCAAGTGCTCTCACTCAACCAGCATGGGATTCTGAAAAGAA
AACGGAGAAAAGATCCACGGAAGATGCTTCTCTATACTAATTGTATTAGTAAAATTATTCATTATAATATT
TTAACTACAGCCTTAATTTATGCGTGGTGAGCCTGTTGTGTCGTTGTAATTTAAGAATTGAAACATGTAT
GAAAGTTTTTTTTTTTTTTTTTTTTGTTATTAATTTGATTTATTTACCTACTATGAATGTCGACTCCGTATG
TTGGTTAATATTCTTACCTCAGCAATTAATTGTGTTAGCTTGTGTATTATGTTTTTTTTAGGTAGGTAATGC
TCACTAATTAAATTCTCTTTATGATATTCAGTATGGCCGATGTGGAATAGGGGCATTCCAATAACAGGAC
GGCCGTCTGTTAGCTAAACTATTCCTTTGCTAAAAATTGTTGTTATTTTCCAATATTTTTAAATATGTTATA
ATTATTTTGTGGACCTTACAATCCACAAAACCCCAAAAATTATGCAAAATCCGATTTGTTTATACATTTT
TGTCGCTTACAACACATGATTAAAAACGATACTGGCAACACAGGATTAAAAAAGGAGGGTTCAGAAA

AAATATGAATAGGGAATAGGGCATGCAATACCGGAACTATTTTCAATACCGGTATTGAGTTCCGGTATTG
CAACTCAATACCGGGATCCCGGTATTAATACCGGTATTAGATTTCCTTGTAATAAATGGCATATATTGTGA
TTAAAGGTCGTATAGGTCAAAATAAAACGAGAAAAAGCAATGAAATTCAGTTTTTTGTAATAGATATTT
ACGAGAATTGAGTATTAGTTTAAATTTTACAATAATTTTTTTTTTTTACATCCAGTGTCCGTTTACGCATG
GACAACAGTACATATCAAACGGCCGAAAACTGCATCAAACGGTTGGAAACAAGTGCGCTTTCACAGT
ACATAGGAATACTATATCTTAATCGCTTTATTATAGGCTAAAAAAGTCCGATTCCGGTATTACTTCAATAC
CGGTATTAACTTTCAATACCGGTATTGAAAAAAGCGTGAATTTTGCTCCTAATACCGGTATTACGAATAC
CGGTATTGCGGTATTGCATGCCCTAATAGGGAATGGCGGTTCGAAGTTACAATTGTTACAAGTGTGCAT
GCGAATTACTGATAAATAAGCTTTGTTACTGTTTAAAAAGTTTAAATAAAGTGTAAGTGAGTAACTAAG
GTGTTTAAATTAATACATGGCTGTGATAAGTAGTTAGCTCTGCTCATATTATTATAATAATAATTATTAAGC
TTATATGTATTGTATACCAACTAGTTTTAAGTCTTTTTTTGAAAAGATGGCTCAGGAGTTTCTTGCCTCC
GTTCTTCTCCTTAGACAGAAAGAGCCCCCCCTTATCCGAACGGAGAGTAATTTGTAAAAATTGACGTTC
ATCAGAAAATTTTATATTTGTACGATGAATAAAAAAATTTGAGTTTGAGTTT
>TCONS_00077632 geneID=XLOC_015533 scaffold_254:766332-775007(-) Intergenic
CAGAGAATAAGTTTAAGTGTTACATTAAAGAGAAACTCAGTAAAAAAGCTTATTATAAAATTGATGATT
ACTTAGAGGACGTTAATGCATGTGTTAGCTAAACTATTCCTTTGCTCAAAATTGTTGTTATTTTCCAATAT
TTTTAAATATGTTATAATTATTTTGTGCAGTTTTGTCGCTTACAACACATGATTAAAAACGATACTGTCAA
CACAGGATTAAAAAAGGAGGGTTCAGAAAAAATATGAATAGGGAATGGCGGGTCGAAGTTACAATTG
TTACAAATGTGCATGCGAATTACTGATAAATAAGCTTTGTTACTGTTTAAAAAGTTTAAATAAAGTGTAA
GTGAGTAACTAAGGTGTTTAAATTAATAATTCAGAAGTGAAAAGACCAGCTCACCGATCGAAAGACGA
CACTCATGTGATTAATGCAGCTGAGTAAAGCTGAAGAATGGATTATTTGTCGCTGAAGGGCTAAAATTA
GAAGGAGTCCTCAAGATTCTACGAATAACAGGGAAGATGCAAGTGCTCTCACTCAACCAGCATGGGAT
TCTGAAAAGAAAACGGAGAAAAGATCCACGGAAGATGCTTCTCTATACTAATTGTATTAGTAAAATTAT
TCATTATAATATTTTAACTACAGCCTTAATTTATGCGTGGTGAGCCTGTTGTGTCGTTGTAATTTAAGAAT
TGAAACATGTATGAAAGTTTTTTTTTTTTTTTTTTTGTTATTAATTTGATTTATTTACCTACTATGAATGT
CGACTCCGTATGTTGGTTAATATTCTTACCTCAGCAATTAATTGTGTTAGCTTGTGTATTATGTTTTTTTA
GGTAGGTAATGCTCACTAATTAAATTCTCTTTATGATATTCAGTATGGCCGATGTGGAATAGGGGCATTC
CAATAACAGGACGGCCGTCTGTTAGCTAAACTATTCCTTTGCTAAAAATTGTTGTTATTTTCCAATATTT
TTAAATATGTTATAATTATTTTGTGGACCTTACAATCCACAAAACCCCAAAAATTATGCAAAATCCGATT
TGTTTATACATTTTTGTCGCTTACAACACATGATTAAAAACGATACTGGCAACACAGGATTAAAAAAGG
AGGGTTCAGAAAAAATATGAATAGGGAATAGGGCATGCAATACCGGAACTATTTTCAATACCGGTATTG
AGTTCCGGTATTGCAACTCAATACCGGGATCCCGGTATTAATACCGGTATTAGATTTCCTTGTAATAAAT
GGCATATATTGTGATTAAAGGTCGTATAGGTCAAAATAAAACGAGAAAAAGCAATGAAATTCAGTTTTT
TGTAATAGATATTTACGAGAATTGAGTATTAGTTTAAATTTTACAATAATTTTTTTTTTTTACATCCAGTGT
CCGTTTACGCATGGACAACAGTACATATCAAACGGCCGAAAACTGCATCAAACGGTTGGAAACAAGT
GCGCTTTCACAGTACATAGGAATACTATATCTTAATCGCTTTATTATAGGCTAAAAAAGTCCGATTCCGG
TATTACTTCAATACCGGTATTAACTTTCAATACCGGTATTGAAAAAAGCGTGAATTTTGCTCCTAATACC
GGTATTACGAATACCGGTATTGCGGTATTGCATGCCCTAATAGGGAATGGCGGTTCGAAGTTACAATTGT
TACAAGTGTGCATGCGAATTACTGATAAATAAGCTTTGTTACTGTTTAAAAAGTTTAAATAAAGTGTAA
GTGAGTAACTAAGGTGTTTAAATTAATACATGGCTGTGATAAGTAGTTAGCTCTGCTCATATTATTATAAT
AATAATTATTAAGCTTATATGTATTGTATACCAACTAGTTTTAAGTCTTTTTTTGAAAAGATGGCTCAGGA
GTTTCTTGCCTCCGTTCTTCTCCTTAGACAGAAAGAGCCCCCCCTTATCCGAACGGAGAGTAATTTGTA
AAAATTGACGTTCATCAGAAAATTTTATATTTGTACGATGAATAAAAAAATTTGAGTTTGAGTTT
>TCONS_00077524 geneID=XLOC_015533 scaffold_254:767665-769046(-) Intergenic

AAGCGCTGTACTTAAAAACGATCGTTATCAAGTATCTAGCATAGACGGACATTCAAAACGTAAATACAA
GAGTGTCTACCCTGCTGATGCATTAAAAAAATGGATTACTTTTGCGACATCAGATGAAAGCGACAATG
ACGATGACTTAGAGACTACTGAAAATGAAGATGACTCGAACGATAATGTCGTTATGACTAACACTAATG
AACTACTACGAGTTGGACTAGCCTATTTACAAATCAATCCTACCATCATTTGAGTCCCAATCCCAGTTAA
CATTTTCAGAAAGACGACACTCATGTGATTAATGCAGCTGAGTAAAGCTGAAGAATGGATTATTTGTCG
CTGAAGGGCTAAAATTAGAAGGAGTCCTCAAGATTCTACGAATAACAGGGAAGATGCAAGTGCTCTCA
CTCAACCAGCATGGGATTCTGAAAAGAAAACGGAGAAAAGATCCACGGAAGATGCTTCTCTATACTAA
TTGTATTAGTAAAATTATTCATTATAATATTTTAACTACAGCCTTAATTTATGCGTGGTGAGCCTGTTGTGT
CGTTGTAAT
>TCONS_00077657 geneID=XLOC_015533 scaffold_254:767763-768618(-) Intergenic
TCACTGGTACTATTATTTGTTAAGGTGAGATTGACTGTAGTAAGCAGCACTCACTGGTACTATTATTTGT
TAAGGTGAGATTGACTGTGATAAGCAGCACTCACTGGTACTATTATCCTGTTAAGGTGACATTGTGTTT
AGCAATGCTAACTGGTACTGAATTAGACTCATGGATTCATATTCCTACAACCAACGATAAGTTATTGATT
AACTATTATACATTTAATCATAGATTTTGTACTTTCGCAGGAGTCCTCAAGATTCTACGAATAACAGGGA
AGATGCAAGTGCTCTCACTCAACCAGCATGGGATTCTGAAAAGAAAACGGAGAAAAGATCCACGGAA
G
>TCONS_00077606 geneID=XLOC_015534 scaffold_254:782562-785074(-) Intergenic
ATAATAATGAAAAATATGATGTCAGCTATATTACGGTTGGATAATGGAGGAGGTGATGACGCTTGCAGC
TGTCATCATTAGCTGGGAACAGCTATACCGGTCCTGGAGGGCAGGTATTCTAAAAATCTTTTGTATTTGT
TAACCCAGTTTATACATTCATGGTACCTAGGATTCCACACTTGTGAGGCGTGGTCGAGTATGCTCCGAA
CGTATGAGCAGAACTTGTATAAGAAAATTTTAATAAAAAAAAAAATAATAATTGTTTATTTCAAAAAACT
TTTCACTTATAACTAATACAAGGTAAAGACCTCCTATTAAGCATAAAAATACCTGTGCCAGGAGATCTC
GCTCTCCATATCAAATTATCATTTAAATTATTCAGTTTCAGAAATAAATGGAAGGTAACAATAATAACCTA
TACACAGGTCCCTTCTTGGACAAATTTCTCGTAGTACACTCGATTTCGGCATGAGTAATACACAACCTA
TCCCATCATTCGGTGTGCTGGTGGAAAATGTCTAGTTCTAAACTAAAACTACATCACATACAAATAAAA
AGAAAACCCTAATTAACACAAAACTAATAGGGCGACAAAGGCAGAAAAACAGCAACTTATGCACAAG
GACTTGTTCTGACGATGAGGGACAGAACTAGAAGCTCTTATCGCCGTACATAGATCTATTTAGGGTTGC
AAGAGTCTTAGGAAGAGAAGTAGCTCCTTTGCCTGGCATTTTCAGCACCCAAAACATTGTAATTTTGTA
AAAACAATTTTGGCATTATTATTGAAAGACTGCTTGCTAAAGATTGTATAATATGTTTATGAGTTTGGTTA
CTGATAAAGTTCAGTACATTGTTTATGATGATAATAATAATAATCCATTTATTTCAGATTGGTTACTGATAA
AGTTCAGTACAATGTTTATGATAATAATGATAATAATCCATTTATTTCAGATTATATGTTTGTGAGTTGGGT
TACTGATAAAGTTCGGTACCTATACTCAGTACTGATAAAGTTCGGTACATTAGGAAGATGATTGTGAGTT
GGGTTACTGATAAAGTTCGGTTCCTATACTCAGTACTGATAAAGTTCGGTACATTAGGCAGATGATTGTG
AGTTGGGTTACTGATAAAGTTCGGTTCCTATACTCAGTACTGATAAAGTTCGGTACATTAGGAAGATGA
TTGTGAGTTGGGTTACTGATAAAGTTCGGTTCCTATACTCAGTACTGATAAAGTTCGGTACATTAGGAA
GATGATTGTGAGTTGGGTTACTGATAAAGTTCGGTTCCTATACTTTATAGTACTGATAAAGTTCGATACAT
AAGGAATATGTTTGTGAGTTAGGCTACTGATAAAGTTCGGTACATTATGTATATGTTTGTTAGTTAGGTT
ACTGATAAAGTTCGGTTCCTAAAATCTATGTTACTGATTATGTTTGGTACATTATGAATATGTTTGTAAGT
TAGATTACTGATAAAATTAGATTCATATACTATTTATATTAGTGATAAAGTTTAATACATTATGAAAATGTT
TGTAAGATAGGTTACTGATAAAGTTAGATTCCTATACTCTATATTACTGATAAGGTTTCGGTAAATTATGA
ATATGGTTGTAAGTTAGGTTACTGCGAAAGTTCGTTTCCTATACTCCTTAGTTAATGATAAATGTTGGGTA
TATATTATTAAGTTGGATTGTTTTGTATAATTTGTGTTAACCGAAGAACATTCCCATTCATTTGTTATTGTT
AAATGCATTTATTGTTTCTTCCAAGTTGGGTTACTCTTGAACATTCAGTTTTTATAAACTTTAATTCATTT
AATTATGTTTAAATTTACTTTAAAAAAGCATTGTGATTACATTTTGTTTAAATATAATATTAGTAAACTTAG

CTATATTATTGTTAAACTTAATGATTATAAAACATATTGTTGATGTTAACTGTTTCTAAATTGTAGTTTATA
AAACATACTTTATTTCTGGTTTGATGATTGTAAGCTCTGTGCCATGGACCAATGTTCCCAGCCGATTGTT
AGGTTACGCACCGGCGTCGTGCGCGGGGTCGCACACGTTGTCAGGATGGACGATTGTTAGGTTCATTA
ACATTGTTGGAATAAGAATTGAATTTTAACTACAAAATGTAACGGAGGTTTTAAAAAAAAACAAGTGA
CGTTTGGGGAATTTTGGTCTATGGACTGGAGATGGATTGATTCTTGGATTTTGCGATTGTTGTTAAAAAT
TGTAAAAGTGCGAAAAGTACGAATATAAGCGAATGGTCGAAAGTTATACGGTGTCTCATTTCACTCAA
GGATAGAATACAAGCACACATAAGAAGGATTAGCGACTGTATGC
>TCONS_00077601 geneID=XLOC_015521 scaffold_254:783528-784804(+) Unclassified
AACCTTACTTACAAACATATTCATAATGTATTGAACTTTATCACTAATATATAGTATGTAAATCTAACCTTA
TCAGTAACCAACTTACAAACATATTCATCATGTACCAAACATAATCAGTAACATAGATTTTAGGAACCG
AACTTTATCAGTAACCTAACTAACAAACATATACATAATGTACCGAACTTTATCAGTAGCCTAACTCACA
AACATATTCCTTATGTATCGAACTTTATCAGTACTATAAAGTATAGGAACCGAACTTTATCAGTAACCCA
ACTCACAATCATCTTCCTAATGTACCGAACTTTATCAGTACTGAGTATAGGAACCGAACTTTATCAGTAA
CCCAACTCACAATCATCTTCCTAATGTACCGAACTTTATCAGTACTGAGTATAGGAACCGAACTTTATCA
GTAACCCAACTCACAATCATCTGCCTAATGTACCGAACTTTATCAGTACTGAGTATAGGAACCGAACTT
TATCAGTAACCCAACTCACAATCATCTTCCTAATGTACCGAACTTTATCAGTACTGAGTATAGGTACCGA
ACTTTATCAGTAACCCAACTCACAAACATATAATCTGAAATAAATGGATTATTATCATTATTATCATAAAC
ATTGTACTGAACTTTATCAGTAACCAATCTGAAATAAATGGATTATTATTATTATCATCATAAACAATGTA
CTGAACTTTATCAGTAACCAAACTCATAAACATATTATACAATCTTTAGCAAGCAGTCTTTCAATAATAA
TGCCAAAATTGTTTTTACAAAATTACAATGTTTTGGGTGCTGAAAATGCCAGGCAAAGGAGCTACTTCT
CTTCCTAAGACTCTTGCAACCCTAAATAGATCTATGTACGGCGATAAGAGCTTCTAGTTCTGTCCCTCAT
CGTCAGAACAAGTCCTTGTGCATAAGTTATTATTGTTACCTTCCATTTATTTCTGAAACTGAATAATTTA
AATGATAATTTGATATGGAGAGCGAGATCTCCTGGCACAGGTATTTTTATGCTTAATAGGAGGTCTTTAC
CTTGTATTAGTTATAAGTGAAAAGTTTTT
>TCONS_00077750 geneID=XLOC_015567 scaffold_255:135663-137671(+) Unclassified
CATTAGTTGATCATTTGGAAAATATTTGTGAAATAAAGGTAACTTTTGGAATAAATTTCTTTTTTTTTTT
TAGCTAAATAAGTACCTATATAATAGGTAACTACTCAAACAAGTTTTACAGAAACAATATACCAAAGTTT
CCCTTATAAATGTGTGTGTGTGTGTCTGAACGAAGTATCAATAAACCCTTCATTTCCCAGTTGTATTTGT
CATGATTATAAATCGCACTCAATATCAAATAATTAAAACTTCATTCTTCATCATTCTTTAATCAACTATGTA
AGACCTGCAGGTACCTTTAACATTATTATCATTCAGACTTTAAAATTACCTTTAAAGTGCTTTTCTTCCTT
CCACATTCTTTGCTCTTTCAGTTTTTCGAAACATTATAATCACGACATAATCCCCGGGAAATGGCTATGT
AAATAACCTACCCTTTTGTTTTCTCTATAAAAAAAAATTATTGCAGATAAATTTTACCAAAATTGAAAGT
TCCTACTCTCATGTAACAGACAAATCCACCCCAATATTCCATCGATACCAAAACATCAAAATACCGCCA
TCTAGTTTTTTTCTACAACCTAATACACTATTTATTTTATGAAATAATATGTCTATTTAACTAACTACTACT
TCTACTTCAACTATTTTCCTACCCTCCGTAACTACCTAAGTCGAAAATAATGTTACTAGGTAACTAAAAA
TATTTCTGAGATATTACAACTCTCCCTCACAACCCCTATTTGAAAAACACGGTCTCCATATCCTTTATTCC
TTTTCAGGCTGTTTGTGTAAATGTGCCTTACCTGTCTAACTGTCAAATTTATTAAAAAACTAAACTGTCA
ATGTCAACCTGACATAACCTAAACCTTTTTTTTTTCGGCACTAAACAAAACAAAAGCATGCTTCGTTGT
GTTTAACAATATCGATCGCATGAAGAAATCTAGAGTTTACCCAATTAAACTTACGAAACTCTTAAATAAT
GAGTGAATCTAATAGGTAAGTGTCATTATTTTATCGAAAACCCGTGTATGCATGCTGTTAAACTAAATTC
TCGACCTCAAATTATAGATATTCAAAATAAACAACACCTGAATATTTAGTTATTCATACGTACAATAGCTT
TGTTTTACGGATCTAGTGTTTATAATAGACCTATTGTTATAAAAATCTAACTCAATTAAATATCTCCCATA
CAGTTTTGAATTGATACTGACAAGACTAACCAACATTTAACTTATTGTCAACCCCTCACCTTTTAAATAA
GTACCTATCCTAATCATTTGGTCTTCAACTCCTATATTAATTTGAATTATGCCTATAGAATTATTTGTTTTAT

TTCAGGACCAAACATACAATGCAAAACCAACAACACTCCTTGTTATAAATTCCATGATAAACCACTGAT
CTAACCATCCACTGTGATAAAACATTCACAAAGCCTCACTACTTATAGACCAAAGTTACTATTCTTCAAT
AAGATATGACATACTTTTTGAATGCTGATGATGATGATGATGTTCTTTATTTGACAACCAAACTCTGAGA
TTATATACTATGAAGTAAACATCCTGACTCAGATGAAGAAGTGAATTGCAACCGCGCTCAAAATAGGAA
CAACTAAACTACCCACATAGTTCTTAACTTACAAACAATATATTGATACTAAATGAGGTCCTAGCCTACT
TCACCTGGTCTAACACTTAATCAACAACAGTAACTTGCTACACATTAATTATTTTCTAAATT
>TCONS_00077680 geneID=XLOC_015567 scaffold_255:136600-137656(+) Intergenic
AAGAAATCTAGAGTTTACCCAATTAAACTTACGAAACTCTTAAATAATGAGTGAATCTAATAGGACCAA
ACATACAATGCAAAACCAACAACACTCCTTGTTATAAATTCCATGATAAACCACTGATCTAACCATCCA
CTGTGATAAAACATTCACAAAGCCTCACTACTTATAGACCAAAGTTACTATTCTTCAATAAGATATGACA
TACTTTTTGAATGCTGATGATGATGATGATGTTCTTTATTTGACAACCAAACTCTGAGATTATATACTATG
AAGTAAACATCCTGACTCAGATGAAGAAGTGAATTGCAACCGCGCTCAAAATAGGAACAACTAAACT
ACCCACATAGTTCTTAACTTACAAACAATATATTGATACTAAATGAGGTCCTAGCCTACTTCACCTGGTC
TAACACTTAATCAACAACAGTAACTTGCTACACATTA
>TCONS_00077695 geneID=XLOC_015577 scaffold_255:150097-163524(-) Unclassified
CCTCAATCGAATCTCAAATTCAAATTTGGAACTTTTTATTCTTTTGTTGCGCTCGCGTCTTCAACGGATT
GTTTGGTTCGTGTGTATGCGATGTGTGAATGAATTTCAGTGACCTGTGATTTTTAAGGTACCCTTCAATC
AGAGCTGTCCATCACAGACCAAAATGGATGAAGACGGTGTAACATGAAGACAAAAATGATCCCTTTGC
TCCTAGCCAGTAGCAGTTGCCAAATACAAAACAGACTCGTTACTGTCATAGTACAATCAACTCCGAGG
GAACTTACAATCAGTTGTTCGCTGTTCAGTGCTAAACTATTACCGTGAATCAAATGTTTGTTTGAGAAA
AGTTTTTTTGTGAGTGAACTACGTGAGTGATATAATCGAAATGATGTACCGGACTTTGCTGTTGCATTTT
GCTATCGTGTA
>TCONS_00077734 geneID=XLOC_015579 scaffold_255:194590-196039(-) Intergenic
ATTTTTAATAATGTACTCGCTTCGAAAGGACGTAGAAAACACATTGGTTCAGAATGACATCGAAAACA
GGGACAAAGAAATAACTCGCTTTTAATTAAATTATAATAGAAAAAGAAATTCCTATGTTGATAAGTTATT
TTTAAGTTTAATTTAAAATACCACAAAATCTCAATAAATTTAAGACTTGTATTATATATAAATTTATGATAAA
ATTAACTCGGG
>TCONS_00077925 geneID=XLOC_015605 scaffold_256:5559-16979(+) Intergenic
AAGTGGAGACAGCAGCAGCAGGTGGTGGCGGCGCCGGCGGTGCTGCCCCCCGAGTTCGCGGTGGCC
GACGGGAAGGACTTCATCTACTTCACCGACGTCGCCATGCCGCAGATTGTTCAACAACAAACAGTAAC
GATTATGCAGGACATGACCGTGAAAAAGGATGATGTCAAACTTTACGAAACCCAGCAGGACATCACAT
ATTTTTAGTATAAACTGACCTTAGTAAATAATGAGTATCGGACTAGGTATTATAAAATGTACTTATAGGAG
TTAAGCGATATATGAATGTAAAAGCTGCCTAAAATGTTTGATTGTTTACTACTTATTTTAAATTAAATAAA
AAATGGATTTACAGTGTAGGAAACCGAAAAAGCAGCACTTATTACTACAGGTATTGTCTTAAAATTATA
GTATCATTTTCAACTTTGGAGACAAGCGATACCTAATCGGTAATTATGTGTGTGTATGTATAGTTTATTTA
GCTGTAAGTTGCTGACATAAAATAGTTATTCATATCGGCTCATAATCGTTAATGGACAACATTTCAAAAA
ACTAAAGCTGCTATAAATCGAAAACCATTTCGAGATTTAACTCTGAAGGCCACAAAAAAAATGTTTTT
GTATTTTTTGGATGTATCATTTTCGAGAAAATCATAAAATAGTAAAAAAAGTGCTGATGACGTCATGCAT
CAGGTGCGATGCATTTTGATGATCAAAGCCCATAGATTTAAAAACAAAGTAACTGTCACTTTCATTTTT
GATTGACGTTGACGTTTTATCGTGACGTTGTTGTTTATTTGGGTCATTTTCATTAATTCTGTTCTGACACT
TTCGTACTATTTTTTTTATTTTTTATTAAGAGATGACCTCTATATAATATGTTCCAGAGCATGCCACCGCCT
ACACAGCTGAAAAAATGCTTCTGCTTATTTTTTTACATGATAAGTACCTACTTTCCATCATCAGAAATAT
CAATGTCATTTAAAAATGACCCATTTGTTGGTTGGCATGTAAACAAAGTTTAAATGTCATTTGTCAGTG
TCATTTATGTTTTTTTTCGAAACTCAGGCAATAGACAAAAATAAAAATTGAGCCGAATATGTGACGCCA

CTTCCGGTTTTAAAATAATTAACTTTAAAGTTAAAAATAACATGCAAAAATTAATGTATATATATACAAAA
TAAAAAAATACGGGTCCACTAATTTTCTATAAACTTTCCGAATAGCTAATAAAAATATAGGGTAAAGAA
AATGAAATGTTGTCCATT

>TCONS_00077915 geneID=XLOC_015605 scaffold_256:15645-17002(+) Intergenic

GTTCAACAACAAACAGTAACGATTATGCAGGACATGACCGTGAAAAAGGATGATGTCAAACTTTACGA
AACCCAGCAGGACATCACATATTTTTAGTATAAACTGACCTTAGTAAATAATGAGTATCGGACTAGGTAT
TATAAAATGTACTTATAGGAGTTAAGCGATATATGAATGTAAAAGCTGCCTAAAATGTTTGATTGTTTACT
ACTTATTTTAAATTAAATAAAAAAATGGATTTACAGTGTAGGAAACCGAAAAAGCAGCACTTATTACTAC
AGGTATTGTCTTAAAATTATAGTATCATTTTCAACTTTGGAGACAAGCGATACCTAATCGGTAATTATGT
GTGTGTATGTATAGTTTATTTAGCTGTAAGTTGCTGACATAAAATAGTTATTCATATCGGCTCATAATCGT
TAATGGACAACATTTCAAAAAACTAAAGCTGCTATAAATCGAAAACCATTTCGAGATTTAACTCTGAAG
GCCACAAAAAAAATGTTTTTGTATTTTTTGGATGTATCATTTTCGAGAAAATCATAAAATAGTAAAAAA
AGTGCTGATGACGTCATGCATCAGGTGCGATGCATTTTGATGATCAAAGCCCATAGATTTAAAAACAAA
GTAACTGTCACTTTCATTTTTGATTGACGTTGACGTTTTATCGTGACGTTGTTGTTTATTTGGGTCATTTT
CATTAATTCTGTTCTGACACTTTCGTACTATTTTTTTTATTTTTTATTAAGAGATGACCTCTATATAATATGT
TCCAGAGCATGCCACCGCCTACACAGCTGAAAAAATGCTTCTGCTTATTTTTTTACATGATAAGTACCT
ACTTTCCATCATCAGAAATATCAATGTCATTTAAAAATGACCCATTTGTTGGTTGGCATGTAAACAAAGT
TTAAATGTCATTTGTCAGTGTCATTTATGTTTTTTTTCGAAACTCAGGCAATAGACAAAAATAAAAATTG
AGCCGAATATGTGACGCCACTTCCGGTTTTAAAATAATTAACTTTAAAGTTAAAAATAACATGCAAAAA
TTAATGTATATATATACAAAATAAAAAAATACGGGTCCACTAATTTTCTATAAACTTTCCGAATAGCTAAT
AAAAATATAGGGTAAAGAAAATGAAATGTTGTCCATTTATATGAGTACTATCACTGTCAG

>TCONS_00077832 geneID=XLOC_015611 scaffold_256:174572-175423(-) Intronic

GGATAAAATACATACTGTCACTGTCTAAAAAATAATGACAGAGAGTGACAGCATGTATTTTATCCGACA
GGTAGCCACTAACCAGACATTGTGGAACAGGCCCTTAATATTCTTATGTAACTTATAAGATTATTTAGTA
TTCTGTGAGAAGTAGGTAATTAATAGCATTACAACGGGTGCCCATGAATAAAACTATCATAACTAGATTA
TTACGTGTAGTGACAGCTCATAACTACTTCATTTCTCTGAATCACCATCAATTAAAGACACGAAATCTTA
AAAAAAACTATTATCATGCATTAAATGAGCTTACTTTGTGAGAACCTACTTATACAACATCTGTCTATTC
TATCTTTAAAAACACCTGAAATTATTTTACTTCAAAAAAGTTAAGTTTACTCAACCTTCATCTTTGCCAT
AGACTATTACTTGCTGATTAATATTAGGTATTTATTGTTGATAAAAATGAGGTATTTTTAAAG

>TCONS_00077845 geneID=XLOC_015614 scaffold_256:255413-257554(-) Splice junction overlap

TGCTGGCCGTCATGCAGCTCGCGCCCGACGCCTTCAGGGGGACAAGTCCGCGCGTGGACGAGCAAAT
CGACATACTAATGAAAGTCAACTGAGCACTTAGTTCTATCAACATAGTATAGTTGGGTCTTTCTGACTG
ACTTAGGTATAGCATCAATTTAACTGTTGTCATATCACCCACCACAGGCTAAAATATATACATAGTGCTG
CATTATTTTGTATTATATTTATCCTTATAAAATATAGGTATTTGTGATTAGTAAACTTAGCCGTTTTTAGTTT
GCTATGGAAGATATTGCTTCACTTTGTGAAACTGATGTTTAAAATACAAAATTACTAAGGGCTGATTTTC
AATATTCTTGTGAGGATTACAATAAAATTGTTGCTCTCACTGTCTAATGTAAGAAACATTCAGACGCGA
CAGCATCAGAATTTTTCCCTAGATAACTTTTATCTGATCATTGAAAAATCAACCTTTAGTCAACAAAATT
ACTATCTAGGTTCTTATCTGTCTTAATTAATGAGCATTTTTACTAGTACCTTTCGTAGTGATCGTAAAGCT
CTTATTTTATGTTTTGAACTCATATTATGTTGTTGGGGTTGATACCTGACCGATTGAAATGTAAATAAAAT
ATAGATAGCTTATAATGTATTGGTGTTATGAAATTTTATGTTTAAGTTATAAATATGTTTTATTGTACCGTA
ATGAAGTATGAAAAAATATTTAAATAAGATCGTTTGTTGATTATGTGTAGTTCTTTTCACTATAGCCCTGA
GTATTTTACTTTTGAGGCGCATTTAGTACAACATTACTCATTGAATCTTTTTATTTATTTTCATATTGTTAA
TAAACTTAGTTACACCTTTTTTACAGTGAC

>TCONS_00077910 geneID=XLOC_015614 scaffold_256:265749-266926(-) Splice junction overlap

GCGTGGTCGGCCTGACTTGTTCTCAAATCTTAGCCCGTGAAGTGTCGGTTGGTGCGCCCAAAAGTGAC
CTATTGTGCATCTCAATCGCATTGTAGCGAGGTCGCCACTGTTGCAACAGCGTCCATTCATAACAGAAG
TGATAATGGCTGAAGCGGAGGCGTCGGCGGGCCCCAGCGCGGCCAAGGACGTGGAGCCCGAGGAGC
GCCGCCCCGCCTTCCCGCCCATCGTCTACAAGGACATCGATG
>TCONS_00077912 geneID=XLOC_015614 scaffold_256:274796-275456(-) Exonic overlap (+)
GTCAAATGTCACAGAAACCGCAAAGTCAGCCAAAGAAATAGAAACTCTGAATCTGAAATAAATAATTA
TTTTCTCTCTTAAATACTGATTGTATCAATAGTTTTACCTTTATATTACATTGCACATAAAATTGCCTACCT
ACCATCTCCTATCGCAGTTCCAAAGTCAGTTAATTAAAAGCGAGTGTAGTGGCGATGTCTGAGCCAGTT
CTGCTGTATGTGTACGACCTGTCCCGAGGTCTTGCCTCTCTGCTGTCTCCTGCCATCCTC
>TCONS_00078225 geneID=XLOC_015691 scaffold_258:327549-329429(-) Exonic overlap (-)
CTGAACGCCGACGGCATCAGGTTGGGCCGGCTGTACGTGCCTGGACCTCTAGGCACCATGAGTATGCG
GAACGGGTCACAGTCTCCAGCCAGATGCGCCAGCAGCTCCCGCTTCTCTATCCCGGTGACGTGGTCCA
GGGGGTCGTGGAACTGACGCGCCAGGGACCCTCGAGTGGTGGACAGCCAGCGGGTGGCCCCGCGGA
GATAGTGGGCCATACTTATGATGGCTAACTGGGATGTTGAGGTAAGTTTCTGAATGTTTCAAGTGATTA
GAAAGGAATATTCACGTTCGCTTTGGTGTTTTAGCTCTAGTTATAATATTCTAGTCTAT
>TCONS_00078244 geneID=XLOC_015673 scaffold_258:367950-368940(+) Splice junction overlap
CACCGGCGCGCGGCAGTGCGGGCGGGAGCCGCGGGCGCGCGAGCTGCGCTGGTTCCTCGACACCTG
TGACATGACGGAGTACAACTGTCAGTATAGGCGATCATTTGAACCAGTACCGAGGACGAACTGTGTCC
ACATTCCCATAAAGCTGCAATAGTTTACAAAACAAGGAAATTTCCGATGATATCGCAGCCACAGGATGT
TTTGTTTCTTAGAAGTAGGCAATAGTACCTATCTAGTTAGTGTCTACAATGATGCACTTACCTACCTAGT
ACAGACTAAGTACCACATATCGTCATAGTAAAGGCTTGGAGCATGTCGTCTATGAAGAACATTTTCGGT
TTGTCTATTCAAATTTATAATTAATTAAATAAAGTACCTACTTACAGACATTATAGCACTATGCATGTGAT
GGGAAACCTATCCATAATAATAATGAACGTTTACTAATATTTTTATATTGCATCGAAGACATCCTAAGTTT
TCATCTTG
>TCONS_00078250 geneID=XLOC_015675 scaffold_258:401855-403786(+) Intergenic
AGCCAACTTTGTGTGAACAAGTTTCGATAGCAGCTGTTTAGTTTACCCGTTATGCGTTATTAGTTGAAAT
AACAGTTATTTTAATACGTTAATGTAGAAAATTGCGCCATTGCCGGTCTGTAAGCACCTCATAGAGGAC
AAGTTACATCAGCAACACAGCTTTCAGATGACACTTCTTCACAAGCAGAAAACCTACACATCACGAAA
AACGAATTTTACTGAAATATTATACTTAGATCAAATGAAGAAATATTTATTTTCGTTCCCGAATTTCATAA
AATTTTCGGAGTAAAAGCCAAAAATATGCAGTACTTACCTACTTATCAAACAAATATTTAGACTTGAAA
AAATCGTCCTATCCATTTAATGAAACTATTTGAAAAAGGAGGGGAGGGGAAGGTTTAGGTCGCTTCGA
TTTTGAAAAGATCCTAAAATTTTACATAATTAGCAGTCTACCTAATTCAAAAGAATGTATAGTATACCCA
AGTTACTATAGACCCAAAAATTAAAATATGTCAGAGGTTTCAAGAAAAATTGAGGAGCTCTAAATCTTA
CATGACGAAAAAATATGTTTAGGACTTCCTTTGTTTCTAGATCTTCCTAGTTTTTGAATAATATTTTTGGC
TTTTTTCCTGTTCATATAAAGTACCCGCCTTTAGTAAGTGAAAATAAATGATGTTTGTTAGAGACACTCG
CTGGTCCCTTCCTTCAGTATTTTGTAGGAATTTTAAAGCTGAAATTTCGTAAGACTATATTTTGTTGTTA
ACATACAATATTTTTAGATAATCTTTCTTTAATTTGGACAGTTTATCTAATGTACCTACCTAATAGTAGTG
AAATATCAATCCAAATCATATCTCGAAGCTTACCGATAATACTGTGTTCACAACAAATTTTAAAATGTTG
AAAATATTTATATAACCACTTACTCATAATTACTTCTTTATTTATATTTTGGACCAAAC
>TCONS_00078063 geneID=XLOC_015696 scaffold_258:404197-411100(-) Unclassified
TACAGATATACTAATTTTATATGGTTGTTGTCTATATTATTTTATAGTACATCGTATTTTTTAAACGTGTTTC
AAGATTCTAACATGGATATAACGCATTATTATTATGGTTTCCTTAGATTTTCGTTTGTTACCTACATACAGT
AAATAATGTTTCAGTACTGTGATAAGTAAGAACAACACCCACGTAGACAGTTATCCGTTAATTATATTCG
TTCGATAAAGATATTGTTCTATACATCTATAGGTATAATACTAAAAACACGACGTAATATACAAGTACGTA

CTTACGTGTTCTGTAACAATTTTTCAGATAAGTATTTATCATTTATAACATTTAATTTTAATATTCAAAAAT
TATTTATTACTTACCGTTAAAATATATATCAATAAAGATGGTAATCACGTGAAAGCCAAAACTTCCTTTAT
GGAATTCAACATAAGTAAGTAGGTAATTGCCAAAACATTGTCTTAGTGAAGACCGTCTTAGTGAAGAC
CGTCTCTGCAAAACTTTCAATCACAAAATAAATCAATGCCTTTTAACATAATACAATTTCCTACATAATG
TATATTATAGTAGAAGATAGGTAGGTACACGTACCTACGTATACATACATAGATATTTTCTTGAATCTGAA
ACTCAAAATGTGAAACCAGTGCTAATTTAATGCTTTATGTGCTATGCGAATATACCTACATACGTATTTAG
TTATACAAAATACTAAACACAAACTGAAATGCTATGCAATAATCATAATAATTTGAATCCACGATTGTGT
CTCAAAAAGGTACGCCACATATGCACAGATTATCGGTAGATAGCGGTAGGTACTTAATAAAAAACCAGT
TTGAAAAAAGTACAACATAAGTTAAAACCATAGAGTTATACACTGTAAACAAACAACAAAACATCATA
TTACAATAAACTACATACTTTTTAAGTAACCGTGTATAATTATTTTTGCTTATTATAACTCATAATAGGTCG
CTTTTATAATAAAAATCAGTCATAGGTATTGTTGGTTGTGTAGTAGCGACTAAGGCATATGTAATATGTAT
ACCTATTTAAAATTTTGCAAAAATTAAGTACAATGGTTTATTCGTAATTTGAATGTATGAGGTGTTGTCG
TACATCTCATTTTCCAAATTCAAAATTAAAAAGTATTAAAATTATAATTTGTTCCGTCTCAGCTACAATGT
TAATTAGTACTTCGTACCTTCCTAGTAAAAACTTGACCGACAGAATTATAAAAGCAACCTTATCTTACAC
TAAATTATTTTAACGGTTAACATATTATTTCTGGTGTATTTGTATTCTTACCTTACAGTGAATGGTTTTGGT
ACCTCTGTTTCGAAATACAAGAGCTCTTAGGTTTTTACAATTGCGTACCTTAACTTAATTAAATAGGTAA
TTTTCCTTTTCTGAAATTGATTTTAGTAGATATAATGAAAATCCGTATAATCTGTTTAAAGTGATCGTTTC
AGTGAGTATATTATTTATAACGGCCACACACACGTACAGGTCCAAGGAGTCGCATATCGGATCGGAGCA
AAATCGAAAATCAATTAATTCCAATGAACCCTTCCGGAAGACCGGAGTTTCCCACACACAGACTGCAC
AGACAAACCTCTCCGGGACTCCGGAGTGAGTATGGCCTTAGTTATTCAGCGTTGTTAAAAGGTGCAAC
CAAATGAGCAATCCATTACATTTGCGAAACAGAGGCTATATTTCTTAAAACTAGAAACTTATTATAATAA
CTTCTCCTTCATTCAAATTTTCAACAGTAACGGTACCTTTATATTTTAAACAATAAATCAATACATTTAGG
TAAGTAATTAAATTATTCACTTATAAGATATTTTATAATGATTAAACTACATTTGAAGTATTAATATAAGCA
CTGGCTTCTTTGTATGTTTTTCAGCAAATATGATTTTGAAAACTAGGTAAGTAATAGGTAAGTAGGTACT
TATACCTTAAAATTGATATTTTGAAGCCACAATTCAACTAAGATTTTTGAAACAATCTAACTAAGTAGTA
TTTATACAATTTCTCTATAAAAGCCTATAGTTTTTATTATAATAACAATGCACACGCAATTTAAATTACAAT
AAGTTGATAATGTTCATGCAACTTATTCAGAACAACTCCATAATTGTTTTCAGCAATATTATGCCACCAG
TGCGGATGAAAGGTAAATAAGTTAAGAAACTTATAAAATAAC
>TCONS_00079015 geneID=XLOC_015786 scaffold_26:126189-127215(-) Intronic
CTAAAATATTACAGTGTATATATTGTACCTAATTATTATTTAATTTATAAATGGATTGACTAATTCCCTAAA
TGGGAAACCTACTCACCTTCTGCCACTGTACCCAAGAGAAGCGTTGGTGTCAGTTTCTGCCCAAGCCG
AAGCCACGTCGCCGCTGCGGGTGTCTCGGCACCAAACCCAAGTGTCTGAGGATCAGACGGAAGAGTG
CTGGTAGGAGGAAGAAGAAGTGTGGGTTAGCTATGGTCAGTACTGGTGTCCAGGCGTGTCAATGTCAA
TGCTGTACCTCAAAAGCAAGAAGCAGAACAGGATCACCAGACAAAAACATCTGCCGATTCACCGTCG
ACGCCTATTTTGTATGATAGAGTTGTGGTTCTTACAATTTGTCGTCAAATAAATAGGGAAATACGATTCT
AATTTATTTTTTGAAGTATTTATTTGTATTTATAGATGTCGTCTTATACATAATGTACAGTATACATAAGATA
AAGGATTTAATTTCATCAGTAATCGAGCGAAC
>TCONS_00078577 geneID=XLOC_015786 scaffold_26:126194-126925(-) Intronic
CAGGAAACCTACTCACCTTCTGCCACTGTACCCAAGAGAAGCGTTGGTGTCAGTTTCTGCCCAAGCCG
AAGCCACGTCGCCGCTGCGGGTGTCTCGGCACCAAACCCAAGTGTCTGAGGATCAGACGGAAGAGTG
CTGGTAGGAGGAAGAAGAAGTGTGGGTTAGCTATGGTCAGTACTGGTGTCCAGGCGTGTCAATGTCAA
TGCTGTACCTCAAAAGCAAGAAGCAGAACAGGATCACCAGACAAAAACATCTGCCGATTCACCGTCG
ACGCCTATTTTGTATGATAGAGTTGTGGTTCTTACAATTTGTCGTCAAATAAATAGGGAAATACGATTCT
AATTTATTTTTTGAAGTATTTATTTGTATTTATAGATGTCGTCTTATACATAATGTACAGTATACATAAGATA

AAGGATTTAATTTCATCAGTAATCGAG
>TCONS_00079204 geneID=XLOC_015788 scaffold_26:306711-309150(-) Intergenic
GCTTAATATTCCAAGAGAGAAGTTGTGTAATTTATTTATTATCTCTTGTAGCTAAGCTTAATTATAATATTA
GTGATAACCGTTAACAGTTGTGATAACGTAAGTAAGGGCAAGACAGTATAAAGATGAAGCTGCGTAGC
TGCAACCGTGCCCTAGCGGCAGCAGGTATCCTGGGCGTGCTGGTGTCCACCTACGCCCTGTACGTGGA
GCTCTCCGCCGAGGCCCGGCCCGGGTACAAGGCGCTGTGTGACTTCTCCGAGGCCGCCAGCTGCTCC
CGGGTACTCACTTCGAA
>TCONS_00079205 geneID=XLOC_015788 scaffold_26:306711-309150(-) Intergenic
GCTTAATATTCCAAGAGAGAAGTTGTGTAATTTATTTATTATCTCTTGTAGCTAAGCTTAATTATAATATTA
GTGATAACCGTTAACAGTTGTGATAACACAGTATAAAGATGAAGCTGCGTAGCTGCAACCGTGCCCTA
GCGGCAGCAGGTATCCTGGGCGTGCTGGTGTCCACCTACGCCCTGTACGTGGAGCTCTCCGCCGAGGC
CCGGCCCGGGTACAAGGCGCTGTGTGACTTCTCCGAGGCCGCCAGCTGCTCCCGGGTACTCACTTCGA
A
>TCONS_00078506 geneID=XLOC_015755 scaffold_26:372912-373902(+) Splice junction overlap
GCGGCCGCCTCGCCACGGAACTCTGCTGATACGCGGACAAGCCTGTCGGCGTCGCCGCTGGTCGCCC
CGAAGACGCCCAAGGGCGTGCGTCCGCACACGCCCGGGTCCACGGGGGCCGCCGGCGCGCCGCACT
ACTCGCCCCGCGGACACAGCCCGAGCCGGGAGAGGGATAGCTTCAGTAGCATAAGCAGTTTATCGCG
GGCGAGCGTCACGACGCCGGTGTCGTCGGCGG
>TCONS_00078746 geneID=XLOC_015755 scaffold_26:372912-373913(+) Splice junction overlap
GCGGCCGCCTCGCCACGGAACTCTGCTGATACGCGGACAAGCCTGTCGGCGTCGCCGCTGGTCGCCC
CGAAGACGCCCAAGGGCGTGCGTCCGCACACGCCCGGGTCCACGGGGGCCGCCGGCGCGCCGCACT
ACTCGCCCCGCGGACACAGCCCGAGCCGGGAGAGGGATAGCTTCAGCAGTAGCATAAGCAGTTTATC
GCGGGCGAGCGTCACGACGCCGGTGTCGTCGGCGGCGGCGGGTGGC
>TCONS_00078715 geneID=XLOC_015791 scaffold_26:466860-468047(-) Unclassified
AAAAGTAATTTAGTTTTTGTTTAGAAGCTGAAACTCAGAATGCGGAGCCTTCCCACAGTAACGCGCAA
GAACACAGCACCAGTAGTCCTGAAGAGTGATGTGAATAATAATAATTACCATGAACTATTGTCTCGATC
CGGTTTACCATGTAAACCAGAGTGTGAACGAGTCGAGTATCGTGTGTTACACCTACTTACTACGCGTCC
CAGTCAATTTGTATGGAAATAATTTGATTGAGACTCTCGGAGAAGACTGATTCGTTAGTATACATGTATG
GGTATGTAGGTACAATAATGTTAGTATGTATTATAGCGATCGTGTGAGATGTATGGTAAATTGTAATGGAT
AGTAAGATCTTAGGTATCAAATAAAGAACTTACATACAATGAACAAGATAACGTGCCTCATTATCATAAT
TTTCCGCTAGTTTTACAGGAGTGATTACAACGAAATAAGGTTACTCTAAACTATGAATCAGTCAATGCA
TGGATTTATGTCGGTTTCTAAATCTGTTGTACGGCACTCGTTTTAATATGCAAATTAACTATTTATTATTTG
TTCTATTTATTGATGATTTAACAGTAAAATTTGATTTTGTGAGTACGAAAATTATGTGTATCATCTTGCCG
ACAAAGTACACAAATAAAATCTATAAATGATTAAATAATGTTATCAAGAAGCTTGCAGTGTGTTTATAAA
TGTATAAAACTAGCTGTTTTGAAGTTATCAAGACTGAAATAATTTAATGTTTGTTTAGGTTACGTTACAA
AATTACATTTGTAAATATGAAGGGATATTAATTATAAATTATTTTTCATACTACTTGTTTTCTTTTGAAATG
AAATACCTTTTTATGTTCTCTCATTTATTAACGATAAATGCTTTAGGAGCCCTATGCACTATACCAAACAT
GATATAAT
>TCONS_00078683 geneID=XLOC_015764 scaffold_26:516402-517492(+) Intergenic
TTGTTACGTTTGATCATGGTTTGACCATAAAAAAGGCACAAAAACATTTCCAATTAAAATTTAATGTCA
ACACAAATAAAATGTCCAATTAAACTAAAAGTAGGACTATTATATGATAATGTATATGAAATGACCTAGT
AAATGGATGGAATGCACGACTGTAACTTTAACTTCGTACTTAAATAAAACTTACAGTTAAAATACTTTT
GTTTATACGTCTTGTTATATTTTAAATCTGAACATGAGAAAGTTAGTAATCTGTTTATTGGAAAAATGTA
ACTTGAATGAGCACAATATATTATTACATTTAAAGCAAAGTTTTAAAAAGTAAAAGTAGAAGCCATCTG

TACTGATAGTTTGTAACACACAATTTTGTTATTACTGTTCTAGAGGGATTACTATATACTGTTACCAAATG
CTTTTACATACAAATAAATATGTGTAACTTGAATGTTATTTATGAATGATATATTATAT
>TCONS_00079023 geneID=XLOC_015794 scaffold_26:551248-555374(-) Intergenic
TGGATTCATACCAATCGCGCGGCAACTGTTTCTGTTCACACTCGCTGAGATAATTTACGTGATAAGACC
GCAGCTTATGATCGTCGTGGTTTTGATGGTGAATGAGTAAAATATGAAGGTGCAGTGATACAGTGATGT
GATGTTATTGTTATACACTGTGAGGAGTAGTGTAGCGATTCTGTGATTGTAAAGTTGTAGCTTAAAGGCT
TGGAGAGCCACCCACCGACATGAAGC
>TCONS_00078592 geneID=XLOC_015795 scaffold_26:557127-560235(-) Intergenic
CTTGAAGTTAAGGTCACTGATAAGTATAAGTAGGTATGTAAGTGATGTTGTCTGCCTATATCAATATCAA
TTATTTATGTATGTATGCCGTGTGTTTATATGCCCATCCATCCATATCAACTTATCATATCAGATAATTACA
AAATATTTACATTCGAGTGTGTATTTTTAAACTGAGTCTGTTTCAGATTAGATCTATAATTACGATACGAT
AGCATCGTTAACAAATAATTCAGTTTTTTTTACATTGTGATAAAAAAGGCAGTGTAGATGCCTGCGACA
TATCTATAAAATTCCTGTACTTATATGG
>TCONS_00078777 geneID=XLOC_015801 scaffold_26:836317-841429(-) Intergenic
CGCCAAGCGAGAGGTGCTCGTTTGTTGCGTGTCTTGTGTTTTTCAGAATTTGTTGAGCGATTGACGTTT
CTTTTTTGTATTTTACCTGTTTCAAATAATATTTCTGTTAATTACATACACGTGTGAACAATATTAGTGTTA
ATTTGTGAACCATACAACTTTGTAGACTTACGAACAGTGATGTTTTAACTGGACGCAGTGTTCCTTTCT
TGGGTTGTGGATATTCACCAGCAGTGCCTTTCGTCTGCCAGTTGATGGATAGTGCTTAGTTCTTCTATGA
AACAGTTGGATTGTGTGCCAGTACTGCCAGTGTGACTAGTGATTGTTGTCGCAGCAAGTTTCCGTAGA
GGAAACTTTCGAATCGTGCATAAACAGTGGGTAATGCACTCGTAGCGAATGAGGGGACGACGGCACG
CATTCCACTGGGAAGGCAAGTGCCGGCAACATAAGCATTTTACGGGAGGCGGGCGGGGCGGGGGCCG
CTGCGGCCGGCGGGGTGATGACCATGAACGCCACCCAGGTCCAGTGTGAGCCCCACGACTCCGTCGT
ACTTATCAAATGTGGGACGAA
>TCONS_00078557 geneID=XLOC_015774 scaffold_26:892554-895351(+) Unclassified
CCGTATTTTCGGGTATATGAAGGCGGGCGGCGCCTCGACGTGCACACTCGCTCGCAGCCGACAGCCGG
ACACCAACATGAACTTCTTCTACATCTTCACCGTCGCCGCTCTGGGCTTGGTAGCACTACTGGGAAAC
GCAAATGCTGATTACGTGAATCAGAATAGTGAAGATACCGATGGATTGTTGACTGGAAACCAAGTGAC
TGGTATTACTGCGTTGGGCAACCAGAAGCAATAGAACTTGGACCGAGGACACGCAGCCGCCTGCACC
TGCGGCATGAGGCGAGCCATTCCCGCGTCCCGTGCCTCACTATATCCACTATCCGCCAGCTTTATTGTTA
TTGTTTTTTGTATAATATATTCGTAAAATAATAAATTATGTGACTGCTTACTTTCTCTTTTCTATTATTGGA
GTAGGTTCCTACCGACTGACTTTTAGTTGATAAAACTACCTATAAGTACTTAATTTAACGAATATAATAA
GCCATTGTACTTGGGTTTTGTAGATTATAGTAAAGCCTTCGATAGCATATCACACGAGGCCATATTCTCT
GCACTACAAAACCAAAGTAAATATATAAATTATTTTAATATTTTAAAAAACATTTACCATTAGTAGGTACC
TACCTATAACATTTTATATTACAGCAGACATTACATAAGACAATGCAGGTTGTTGTGACCGCTGGTGCAA
TGAACACACAGAGATAAGTAAAGTGGACTGTATAATATACCTATTTAGGTTTTATTGAGCACAAACTAAT
ATTAACATACATAACGTACAAAGTACGGTGGTAAATGGAAGTCCTTTCCTAAGGTTTTTTTTCCTTTTTT
TATATTCAGTGATAAAGATGTTCAAAATGAGGGAAAAATATATAAATAAATCAGAACCACTTTAACAAA
AAAAATCTTTGCTTGAGGGTAACATTTAGTACAGTACAAAGACTTGGTCTTTCATTTTCATAATATATAT
GTTACTGTAAAAGCCCGAACAACGGTAAATCCTAAGATTTACGCGTAAAACCTAAGATTTACCAACTCT
TAATGGTTAAAGCCCGAAAGATTTTGCGATTCGCACTTTTACCACTATTCACTTGGTAAATCTTAGGATT
TACATTAAGGTACGGTAAATGTTGAAAAAGCCAGGTTATAACTTATAACCTCCGTAGTCGAACAAAGCG
TCCGTAGTCGAGCGGGCTTCAGTGATCGTAACTGATCACTGAGGTTTAGCAACAACCAGCAT
>TCONS_00079103 geneID=XLOC_015802 scaffold_26:949229-950666(-) Exonic overlap (-)
TTGATTCAAACAAGTAAAATATATATTACCTATTTTAGATTGATAAAAAATATTTATATACTTTTGCGTAAC

GCCTACCCGATTTTTCATTTTCCTTGCCCATTATTAAAAAAAAATGGAGGAAGTAAGTGAAGCTTTTATC
GTCCGAAACTTATTTGCTTTTCATTTCACCGTTAAAAGCTTTTTACTGTAGGTGTTTCCAAATTTTACTTT
ACAATACCTTACATCTGCTTGGTTGCAGTCAAATAACATTTCATAATGTTCCAACGTTTAATAAGGATAT
AGGCACACGACTATACAGGACATTTTGAGGGTTCTAAAAGTAGGGCTTTACTTTACACTGTGTACTTAT
TGTTATGCTTTGGTTGGAATGAGTACCGTTGCCACCCCACAATAATTATATTCCTTATTTATCATTACTTT
CCTAACCCAAAACTAAGTACCTACACTCACGAGCAATGAAAAAGTTCTACTTACAAAATGTTGACAAA
AACATTTAATTTAGAATTTAAACAAAATATATGTACTTCAATATTTGAGACTTTATGCGTTTATAATTCGA
GCAATTTTATGCTTTTATCACTGGCACTTTCTCATTGCTCATGAATAAATCAGAAAATCTTTCACGAAAT
TGCTCTCCTAGTTTCAACTCTGCACGATGTGGCAACAACACTCGGCATAATCGACTGTTGACCGGCGA
CCGCTGATTGAGTGTTCGGCTGAACTCATTAATTAATGCTCCGGACGGCCCAACTGACCGATGCAGGC
ATTTCGAGCTGGTTCGTCTCTTATTCCGTATGTTATATTGTTGGGTCTCATACCCTTAAATTTGTTGTTCG
TTTGGGTTTTCCTGCTTGCGTTGCGCAAGTTTCGGGTGTTTTGGGTGGCGGTTAAAAGTTAATAAAACA
ACTATATTTACTTACTATACCATTTTTTGTTATTATTTAGTAACACCACCCATAATACCTTTATTTTGAAAA
CACAAGCCCTTGAGATGCCTTCAGGAAACCAGTGTTCTTAGTCAATATCAAGTTTTCTAAAAGAACCT
TCCACTCCGAACACTTCGTCGAGTTTGGTCACACCTTCTCTCTTGCTTTCATCAGAACAAGCCTCGTCT
TGGTCGAGGAAGTATTGACGCCACTCTGGTGTTTGGAACGTTTTCATCCATGAT
>TCONS_00079013 geneID=XLOC_015782 scaffold_26:1256584-1258147(+) Intronic
GAATATGTGAATGACATAAAAAATATTGAGTTGTATCTTTTAATTGAAAATGTTATTAAGAAAATAGTTT
CTTTAAAGTATATAAATATAGTGAAAAACATCTTTTTATTAATGCATAATTTCAGTTGCATCTAAAGACTT
AATTATAAAATCTGTTAGTGTGGGTATAATATTATGATATAGGTTTATTCTACATTCATGTAGGACTTCTTA
AATTGGCCCAATTTATTTCATAAGTATTTAATAATTACGTTGTTCTACGGTAACTATCTATACTGTCTATTT
ACCTTATAGTACTATAATTCTTTATGTTTTGATTACTAAGCGACTATTTTACTATTATTCTATCATAATCGGG
AAGAAAACAAAACAAAGGTTTAGCAGCTCAAGTTATCAAAAATGCCGTGGTGATTTATTTAACCTGAC
TACCACACTGGAGTACCTACACAGTCATGTACTAGAGAAAAAATCAGAAATCGCATCGAAGCCAAGA
GCTAAACTCCGCCAGTAAGATTAAATGAATAGAAATGTAGTGGAAATGTATTTTTGCCTCTGATCTCATT
GATCTGGGTTCTAAAAGGCTATTAAACAGTATCTTATTGTACTTATGTACAGTGTGGTCCTACACGGTTA
TGAGGACGCTGCCGACCTCATCTGCAAAATGATTTGTGTTTCTTTTCTTCCCTATTATTTATTGTTTAAGT
AAATTGTTTCTATTATTAATATTAAAATGGGCCACGTTAAAAGTTTATTTTAGTTGTTATGTAACTAATAG
ATAGATAGATAGATAGATAGAATATTCTTTATTTGTACACACAGACATAAAAGAAACTAATAGGTATACA
TGTAGATCCACACATAACACTAGGATTTTATTGACACTTATTTACACATGGCAAGGTAAGCTAAATTTAT
AAAGATAATGGATTGTGAACGTTATTAACAAAATAGTAGTTTGTAACACTTGAGTTAATTAATGTAAATC
ACAGAACATGTCATATCTCACATTGTGAAATAAAATACCTACCTGTATGTACCATGAGTACTTTGAAACT
AAGAATAAGAAGCT
>TCONS_00079248 geneID=XLOC_015942 scaffold_260:3340-12090(-) Exonic overlap (+)
CCGCCCGCCCAGCCCAGTCGCGTCACACTCCCAGTGTTTGTTGTGATATCACCGCTAAAACTCCTGCA
GAGGACATCAGATTATTGCACAAGCATCAACATGACGTCGCCAGCCATCACCAAGTCGCCCGACAACC
AGAAGGGAGAGAAGAAGTCGCCGAGCCACTCCGCTGGGGCGCCAGCGGAGGCCGCGGCCACCGAG
CCCGCCGCGGCCAGTGCGCCCGCGCCGCCCGCAGAGACCTTCAGCTGGCTGCGGATCTTCAAGCTGG
CCGACCTCATGATGAAGCAGGGCTGCTAGATCACTAGACCCAAGCAGAAGAATGAAAGGAACATCGT
AAACCAACTTGCTATCGAAACTTTCGGCAACGATATTAATCGCATGATTGAAGGTTGGTCGGTCATATT
GTAGGTATCCATTGTTACAATATTAATATCTAGAGGTTTCTGTCGAGTCCTTTCTATACTAATTCACTACAT
AATATGGTATTAGTAGATTAGGTATTATATTTTTATTGTTAAAATACTTTAAGAATTTCTATCTGTTGTTAC
TTGTTGCTAGCATTCCTTTCCTTGGGATTTGTTTTCTATCTAGTCTTGTTATAGATTTCGATTTTCATAATG
TACCAAATGTTGGCATTTTATTTTTCACATATAACTGAGTCCCTGTTTATTATTCTTATTCGAAATCTATCT

TGATGTTCTACTGTTGTAGTTTAGATGTGGGACTTTTGAGTCCTATGAGAGGATCCAGTTGCGTTAGGTA
TAGTTAATTAGAAGTCAATTTTAGGATAATTAACACAGCATTTAAGTTACTTATATAGAAAAGTATAGATC
ATTTCAAAAGTGCATGTGATAATAATATTGAGTACCGTTGTTTGTAGTTTTTTTTGTGGAGGCTGATAAA
TGATAAATTACGCTCTTTGTTTCGTTTTTGGAAATCTGGACTTTACAAGTTATATTTTATTCCAATTATGT
AGACTGTTGAATGAATTTAAATAAATTCCTATTGATCTTACCAAAC
>TCONS_00079294 geneID=XLOC_015942 scaffold_260:3349-5817(-) Exonic overlap (+)
CAAGGTGTCAGGAAGGTGTCAGAGCATCAACATGACGTCGCCAGCCATCACCAAGTCGCCCGACAAC
CAGAAGGGAGAGAAGAAGTCGCCGAGCCACTCCGCTGGGGCGCCAGCGGAGGCCGCGGCCACCGA
GCCCGCCGCGGCCAGTGCGCCCGCGCCGCCCGCAGAGACCTTCAGCTGGCTGCGGATCTTCAAGCTG
GCCGACCTCATGATGAAGCAGGGCTGCTAGATCACTAGACCCAAGCAGAAGAATGAAAGGAACATCG
TAAACCAACTTGCTATCGAAACTTTCGGCAACGATATTAATCGCATGATTGAAGGTTGGTCGGTCATATT
GTAGGTATCCATTGTTACAATATTAATATCTAGAGGTTTCTGTCGAGTCCTTTCTATACTAATTCACTACAT
AATATGGTATTAGTAGATTAGGTATTATATTTTTATTGTTAAAATACTTTAAGAATTTCTATCTGTTGTTAC
TTGTTGCTAGCATTCCTTTCCTTGGGATTTGTTTTCTATCTAGTCTTGTTATAGATTTCGATTTTCATAATG
TACCAAATGTTGGCATTTTATTTTTCACATATAACTGAGTCCCTGTTTATTATTCTTATTCGAAATCTATCT
TGATGTTCTACTGTTGTAGTTTAGATGTGGGACTTTTGAGTCCTATGAGAGGATCCAGTTGCGTTAGGTA
TAGTTAATTAGAAGTCAATTTTAGGATAATTAACACAGCATTTAAGTTACTTATATAGAAAAGTATAGATC
ATTTCAAAAGTGCATGTGATAATAATATTGAGTACCGTTGTTTGTAGTTTTTTTTGTGGAGGCTGATAAA
TGATAAATTACGCTCTTTGTTTCGTTTTTGGAAATCTGGACTTTACAAGTTATATTTTATTCCAATTATGT
AGACTGTTGAATGAATTTAAATAAATTCCTATTGATC
>TCONS_00079247 geneID=XLOC_015942 scaffold_260:3384-8844(-) Exonic overlap (+)
TGACAAAGTTTAACTGTAACCGTTACTGTTACCTGTTACGTTTGTTTAGCATATCTACATTCCTGTTGCA
CCTTATATCATAGCATCAACATGACGTCGCCAGCCATCACCAAGTCGCCCGACAACCAGAAGGGAGAG
AAGAAGTCGCCGAGCCACTCCGCTGGGGCGCCAGCGGAGGCCGCGGCCACCGAGCCCGCCGCGGCC
AGTGCGCCCGCGCCGCCCGCAGAGACCTTCAGCTGGCTGCGGATCTTCAAGCTGGCCGACCTCATGAT
GAAGCAGGGCTGCTAGATCACTAGACCCAAGCAGAAGAATGAAAGGAACATCGTAAACCAACTTGCT
ATCGAAACTTTCGGCAACGATATTAATCGCATGATTGAAGGTTGGTCGGTCATATTGTAGGTATCCATTG
TTACAATATTAATATCTAGAGGTTTCTGTCGAGTCCTTTCTATACTAATTCACTACATAATATGGTATTAGT
AGATTAGGTATTATATTTTTATTGTTAAAATACTTTAAGAATTTCTATCTGTTGTTACTTGTTGCTAGCATT
CCTTTCCTTGGGATTTGTTTTCTATCTAGTCTTGTTATAGATTTCGATTTTCATAATGTACCAAATGTTGG
CATTTTATTTTTCACATATAACTGAGTCCCTGTTTATTATTCTTATTCGAAATCTATCTTGATGTTCTACTG
TTGTAGTTTAGATGTGGGACTTTTGAGTCCTATGAGAGGATCCAGTTGCGTTAGGTATAGTTAATTAGAA
GTCAATTTTAGGATAATTAACACAGCATTTAAGTTACTTATATAGAAAAGTATAGATCATTTCAAAAGTG
CATGTGATAATAATATTGAGTACCGTTGTTTGTAGTTTTTTTTGTGGAGGCTGATAAATGATAAATTACGC
TCTTTGTTTCGTTTTTGGAAATCTGGACTTTACAAGTTATATTTTATTCCAATTATGTAG
>TCONS_00079328 geneID=XLOC_015931 scaffold_260:89193-107521(+) Exonic overlap (-)
ACCTATTTAAAATGATTGCAAAAATATTGTGAACAATTTAGGTATATCTAATGGAAATAAGTAACGCAAA
TTTACTAATCACAGCAAGATAGAAAAATCGAGATGTTTACAATGGTTTTAAGTTTTGTATCAAATTTGAG
AACATATTTGTACAGAGTTTAGGAGAATGTAGTACACAATGCCAACTTCGTGTTTGTGCAAATTTTACT
TGTTAACATGGATAGTATTTAATTCTAGATTGGATAACCATATTTTGGAGGTTTTAAATGTTAATACTACC
TTTGTGCATATCCCAATGGCTCCATTAACACTGATAAACATCTTAATGACAAAATAGTTGTGTTTCAAAG
CTTGATAAAAATGTGGTCTCTCCGAGCTGGTATATGTATTATGAATGGACCCATTTCCCACCTCACTCAT
CCACTGCGGGTAAGTAAGTATTTTTATTTAAATTACAATATAACTTTTAACACCCTCCCAGAAGCTGTCT
TTAGTGCACTACATTCTCCATTTACAAGCTAGAACAAATAACTACCATTTACACATCCTTCCTAGCCAAT

GAGCATGGATTCCAGCTTGCCGCTCTCATGCAGAGCCTTCACGTCGGAGCCTCCCCCGACACAGTTGC
CATTGATGAAAACTTGGGGCAC
>TCONS_00079329 geneID=XLOC_015931 scaffold_260:99311-107493(+) Exonic overlap (-)
TTTTTTGTAGTTTAGGAGAATGTAGTACACAATGCCAACTTCGTGTTTGTGCAAATTTTACTTGTTAACA
TGGATAGTATTTAATTCTAGATTGGATAACCATATTTTGGAGGTTTTAAATGTTAATACTACCTTTGTGCA
TATCCCAATGGCTCCATTAACACTGATAAACATCTTAATGACAAAATAGTTGTGTTTCAAAGCTTGATAA
AAATGTGGTCTCTCCGAGCTGGTATATGTATTATGAATGGACCCATTTCCCACCTCACTCATCCACTGCG
GGTAAGTAAGTATTTTTATTTAAATTACAATATAACTTTTAACACCCTCCCAGAAGCTGTCTTTAGTGCA
CTACATTCTCCATTTACAAGCTAGAACAAATAACTACCATTTACACATCCTTCCTAGCCAATGAGCATGG
ATTCCAGCTTGCCGCTCTCATGCAGAGCCTTCACGTCGGAGCCTCCCCCGACAC
>TCONS_00079304 geneID=XLOC_015952 scaffold_260:316705-317132(-) Exonic overlap (-)
AACGCTATAAAAGAGTGCGCGGTCTTGAGGCCGCCGCTATACTGTTTCCGCACGGCCGGCCATCGCGT
CCTCACACACGGGACCAAGGACGCGGGCGGAACTCCCGCTCAGGACTCAGTGACCACTAGTTCAACA
CAAGATACGAGATGAGATCGTTATTTCTATTAGCGGCGGTGTGCTGCCTAGTGTTGTCGGCGTATGCCA
GCCCTGTCTCCCGGGTGGAGGCTGAGGAGACGAAGGAGGAGGCAGTGGCGCGCCAGGCGGCCCCGG
CGCCCGCGGCGTCTGACGATGATGATGACGACGACGACGACGATGATGACGATGATGATGATGACGA
>TCONS_00079818 geneID=XLOC_016008 scaffold_261:12645-14085(-) Exonic overlap (+)
GCTGTCCAGATCAGTGCGCCACCCGCAGCCAGCCCGAGCGCTGGGAGAGCGGCGAGCACACGCCGC
TGCTGCGCCCCTAACACCCAGTGTGGGTGTAAGCCTTGCGCGATTGATGATTTAATAGTCGTAACAAAG
AAGTGAACGGGAAAAGGAAAGAAAAAGGTAAAAAAAATGTTATTGAAAGGTGTAAAGGTAGGTTAT
GCTATCGTTGGCACACACTTGTCATAAAATAGCAGGCTATCATGCTAATAACAACCACATGTTGCTCGC
TTCATTATGTTATATTGAATATGCATGCTTGACTCATGTATTACCTTGCTATTCATTGTGCCAAGCAGCAG
GCGCTATTTATTAGTAAAATAGCTGCTATTTTATCACACATTGTGTGCCCTACGATAGCATATAGAATAGT
CGGATAGATTCATACTGGCTCTATTACTCAGTAGAATTGAAAAGAGCTACAAAGATAACGGTAGAGTAT
AGAGATGCGGATTTTAAAAGCTATTTTTGATCCAGAAACTGTAGATCAAAAATAACCTTTGATAGTGTT
GAATTAGAATGAGTATCGAATCGAACAAGGCATACCCCTATTCAAAATTGACAATGATCGAAAACCGCT
GTTTAATGTGATATTTTTTTGTGATTGTCTACTTGTTATTGTTTTTTTGAATAAGACGATGAAAACTTATG
AATATTTACAATGGTTGTTGAAATATTATAATTAGCTGCAGTGAGAGATGAGAAATGTGACGGCAGCGC
GTTCATCGTGGTAAATAAAAAAACGTGACATGCTAGAGATCTATGTTTTGTACAGATTTATTGGTATAAG
TACAGTTCGTTTTTATTGGGATGTAAAAATAGGAGTTAAGGTAGAAGATATATTGAGTTCATTTTTATATA
CGGACAAGACAGCTATTGAATTGTTAGAGATGGAT
>TCONS_00079775 geneID=XLOC_015992 scaffold_261:56193-60299(+) Exonic overlap (-)
GCGGCGGCGGCGGCGCGGCATTCACTTGGGATACTAGGGAAAGTTATTTCCGCTATCGAAGCCGCCTA
AGTCGCCACCGCCCGCCCCGCCGCCCGCATACGAAACCGCCCCGCCGCTCCATCGACCGCTGTTGCTC
CACTGACTGCTGTAGCCACCGCTGACTGCTGTAGCCACCGCTGACTGCTGTAGCTACTCTTCCGCCTAC
TTCGGACTCTTCCGCCTACGATCACACTCTACATTGAACACTGAAAGCTTAGTTTCCAAGAAAATTCAT
TCCAAGTATGAACTGGGATGCTAAACACAGTGTCTATAAAACCCGTCACAGACTTAGTGTTAAGTAAG
TTAGTGTTAGTGTTAAGTAAGTTAGTATTAGTGTTAAGTAAGTTAGTGTTAGTGTTAAGTTGTTAGTAAG
TTAAGTAAGTAAGTAAAATATGCTTTAAAATAGAGCTTGGAAATAGAAAGACTTATAAAACGATTTCAG
TTTGTATTTTTGTATTTAACAAATTTTCTTTTAAAAATAAAAAAAATAAAAATTCATTTTTAACAAATAGG
TAAGTACTTAGGCAAAAGTTTGCGGGGCATTAGACCGGAAACATCAGTCAATTTACGCAGTAGTCAAT
TACATGATCTTATGTGCTATCGCCCCACTTGGCTATTCGTATTAGCCAAAACTTCTATTGGCTATGTTTGG
GAAATAAGCATTTCATAGGCAAATTCGGCATTCGATTATGTACACATTATTATTATTAAATTCTTTATTGC
ACATAAATTTATTTGTACATAGGCGAACTTAATGCTAAAAGCATTCTCTTCCAGTTAACCTTGGGCGTTG

TGGAGAAAATGATAGGTAGTGCGAAAGACAATGAGAACAGTTTAATTCTGTTTAATATCTATTGTTTAA
CACTAGAATATACAGGGTGTTGCAAAAAGGGTATACTAAGCCGAAACCTACATGTGCAGCATGTTATAT
CTAAGCCCGAAACTGAAATCAAAATTTGAAAATTCGCGAAAAAAAAAATTCATTTTCCATAGAAACTT
TATTGGTCACGTGACTTTTACTATGGAAAAAAATATTTTTTTTTCGCGAATTTCCAAATTCTGATTTCAG
TTTCGGGCTTAGGTATAACATGCTGCACATGTAGGTTTCGGCTTAGTATACCAATTTTGCAACACCCTGT
ATACTTACTTATACATAATATCATAAAATAAACGTAAAAAATATACTTATAAATACATAATAATATATAAAT
ACCTACATACATAATTTAAATATATAGTTACTATAACAAAAAATAATAACATCATCGTCACACTTACAAAC
GATGATATTGACTCCTATTTCTTTCAATCAAGGTGCAAAATCCAAGTGCATTGTTTAAGGCTCTTACGAT
TCAATGATTCAGGTGTTTCCGGGAATACGACAGTTTGCGAAGATTTGCATGCGCATTATTAATATGGGA
GAACTCTGTATATCTCTGTGTTTCGTTTTAAGTAGAGATAAACTTCTCTCCTCTCAGCCTATCGCACTAC
CAACCACTTTCTCGACATCACCAAAGGTTAACTGGTAGAGAATGCTACTAGCATTGAGTTCGCCTTTGT
ACAATGTTTTTATGTGCAATAAATTATTTATAATAATAATAATAATAATAAGACTCCTATGACATTTGATTG
ACAGACAACGAACAAATGACAAATAATATGTACGAATTTTGACAGTTGTCATAGGGCTCATGGTATATT
TTACAAAGTAATTGTATAAATTTTGTATACCTATTTATATTTAGCAAAATATCTAAAGGAAATCTATTTTTG
TTAAATCTAGTCACCTAATACTTGTCAAGACAAACCCTTCAGGTTTAATGGCACGTTAATTTAAAAACC
TAACTATGTTACACAATGTTTTGTATAAATAAATAAAATATCACCTTGGG
>TCONS_00079889 geneID=XLOC_015992 scaffold_261:57923-59275(+) Intergenic
TATTTTTGCAGTTATTTCCGCTATCGAAGCCGCCTAAGTCGCCACCGCCCGCCCCGCCGCCCGCATACG
AAACCGCCCCGCCGCTCCATCGACCGCTGTTGCTCCACTGACTGCTGTAGCCACCGCTGACTGCTGTA
GCCACCGCTGACTGCTGTAGCTACTCTTCCGCCTACTTCGGACTCTTCCGCCTACGATCACACTCTACA
TTGAACACTGAAAGCTTAGTTTCCAAGAAAATTCATTCCAAGTATGAACTGGGATGCTAAACACAGTG
TCTATAAAACCCGTCACAGACTTAGTGTTAAGTAAGTTAGTGTTAGTGTTAAGTAAGTTAGTATTAGTGT
TAAGTAAGTTAGTGTTAGTGTTAAGTTGTTAGTAAGTTAAGTAAGTAAGTAAAATATGCTTTAAAATAG
AGCTTGGAAATAGAAAGACTTATAAAACGATTTCAGTTTGTATTTTTGTATTTAACAAATTTTCTTTTAA
AAATAAAAAAAATAAAAATTCATTTTTAACAAATAGGTAAGTACTTAGGCAAAAGTTTGCGGGGCATTA
GACCGGAAACATCAGTCAATTTACGCAGTAGTCAATTACATGATCTTATGTGCTATCGCCCCACTTGGCT
ATTCGTATTAGCCAAAACTTCTATTGGCTATGTTTGGGAAATAAGCATTTCATAGGCAAATTCGGCATTC
GATTATGTACACATTATTATTATTAAATTCTTTATTGCACATAAATTTATTTGTACATAGGCGAACTTAATG
CTAAAAGCATTCTCTTCCAGTTAACCTTGGGCGTTGTGGAGAAAATGATAGGTAGTGCGAAAGACAAT
GAGAACAGTTTAATTCTGTTTAATATCTATTGTTTAACACTAG
>TCONS_00079896 geneID=XLOC_016009 scaffold_261:79894-81172(-) Intergenic
CAACCGCCGCTTTCGATGAAACACCTTACTACTCATGGATCGGAGGAGGAAAAACGTGAGGAAACCT
GACAGGCTACAGGATCGAGGGAGTCTACATCCATATATTATACTGAAAAATAAAAGTAACGTAAAGTAA
AAAGCATTGTAATCCCCGAAGGGAGAAGTCAAAGGTGACTTGCAATTGCAAGCGAGCCACCCGCTTT
TCGCTGTACATTTTGTAATAACTGTTTTATATTTAAGTTTTGTGGCTTTTTCTGAGTCCCTAATGTATAAC
AATTATTGTGAAACTTCTAACAAATTTTACGGGACTAACGAGCACTATCTAGGTAGGGTAGGTAAGTAT
CTACCTTAACAGATATACTTATTAATAAAAAAAGCAGATAGGATGAAAGAATGCCAAGTCCCGGTTTTT
CAGAATCTTTGCACTGTGCAACGGAAGCAGATGAGAAGAGAAGCTTAGCAGTAACAGTCAATGGTTG
AGTCATCATGGCCGGATTCTGTCATGAAGTGTAGAGGTCGCCATGACGGCATGTATGTATCAGAATCCG
AAATCCATTCCCCTTTGTCTCGACTCACAACAAGAAAACATCCTTTACTCAAGGAATATTTAAAAAAGT
TTCCAAGCTCCATTGCTCATTGGTTGCACTGAAATAATTAAGTAGGTATTTCGGTTCGTCATCATCTTCA
TAACCCTATACGTCCCCACTGCTGGGGCACAGGTCTCCTTCCAATGAAGTAAGGGTTTAGGCCTAGTCC
ACCACGCTCCGAAGCACGGAAGCGTCCAGAAAAGAACCACTTAAAATCGGTCACCCATCCAATGGCT
GACCGTGCCAGTTGTTGCTTAACCTCAGTGATCAGTTACGATCACTGAAGCCCGCTCGACTACGGACG

CTTCCGGTTCGTGCTTCTCCCATTCCCTGGACATTCCAGTTTCCATTACCCAAGGATATACTATAAAAGG
ACTTGGGTGAATTGTTTTTCCAAGTAGGTAAGTAGTAACAGCAAGTCGCTGTTTATCTAAAAATCATTT
CTACCGAG
>TCONS_00079616 geneID=XLOC_015993 scaffold_261:103482-104372(+) Intergenic
TTTTATTAAAATTATTATAACAAATAATGTACTTACATACATGATTGGTTTCATTAGCTTTATGAATATAATA
TTTACTTATTTGTTTCGTCAAATTCAATCGCTAACTTAAACTACAAACTAAATTTTAATGTAACTAGGTAT
ATTATGTACAAATAAAAGATAAAACAAATAACAAAATGTGGACTGTACTGCATGCAGCATGCCTCAAGC
AGTGTTTCAATAAAATTTACTGGCACAGGCCTAAAACATGATTTTTATCATTCCCCATTGGCGCGGAGG
AGGCGCCATCTAGCGGTACACGCACCTCACTAGCTCTGCGACATACAGATTTGTTAAAACCTATACTAT
AGCCTGTTTTTTAATCTCCGTATCGTAGCCTCAGGGCAGTCCTCGTAGTCCACCAACCGGCACTAGGCC
AGCGTGGTGGACTAGGCCTAAACCCTTCCTTCATTGGGAGGAGACCCGTGCCCCAGCAGTGGGGACG
TCATGGGTCGTGATGATGATGTAGCCTCAGGGGTATCATGATCTTTCTCATAGGTAAATTTACGAAACGG
TGAAGTCAGTCAGTAATCTTCTATTATTGATATTGCTACGTGTGAAACTCATGTAATGATCTTATAGTAAC
CGTTTTTTGTATATTTACCTATAGCAGCTGGGATCCACAATATTTATGTATGTATTATATGTATATTTGGATA
ATATTACGTACAGTCCATCTACAAAGTCATGAAAACTTTTTGTCTGCGGCCACCGACCGGTAACTTTCT
>TCONS_00079787 geneID=XLOC_016013 scaffold_261:139997-143477(-) Unclassified
CGCGTGTGAATTTGCCGCTAGGGGCGCTGGTGTTGACTGAGGTTAAGTGACATTTTACTTTTCATATAA
TTTGTTTGGCGGGCTTCGTGATAAATTATATTTTCGTTCATTTCCCTGTTGAAAAGTGATCTCTTCGCGA
AATTATCGCAAATATGGATTATTTATCGATGAATGTGCAGTTGCGCATGCTTTGAAAGCTGAATTAAGGA
AAAGAGGCGCATAAATAAGCGGTCTCAAAGAGCAGCTAATTCAGAGGTTAGTTTGTTTACTGTTTACA
AACTTATTTTTTACGAAATGTATGCTTGTTTGCTTAGATAACAGCTATGTTTTTTCTTTTTACAGACACCA
AGACTATGACCGTAACAGTAACTTGGTTGGAAACGTAGAGGACAGCATCATACAACAAAATGATTCAG
TGTATACTGTTGAGTGGCCTTCAGAAACTTTGTATAGCAGTGTGAACACTAAAAATGAAACTCCAGATC
TAGATATTGTCACCAATGAGTAACTACTTTGGAATTTATGCACAGTGTACGTACATACCGTCGCTCTTAC
GGTGAATGAAAACATCGTGATAAAACCTGCATATCTAGATTTTCGAACCCACAAACCTGCAGAGGACC
AGCGTGGTAGGAAATGGTCCAAGCTTAGGAGTGGAGTTTAGACCTTAAGGCGGACATGGACAAAGGC
TACATTCGAGTGAGCCAGGTGCAGGTACTGACACCCCCACAAATAATAGAATACTTAGTATACTATACT
ACCATTATTTAGTTTCAACTAACAACTCAACCAGCATTCATTGTGTGGATTACTAATGTGTTATATTTTTT
GTATAGCAGTGTGTTTAAACGGAAAGGATAAATAAAAGTTAAATCAGTGTGTAACTCTTTTATTTTTATT
GTATTTATTAAATGCCTAATCCTTATTTACATTATGTTCTTTCTGAAATTAGTTATAATGATTTTCACAAAC
ACGGATAGACGGATAATAATTATTTCACAAGCCCTTTTTTAGAACAGTTTTATCACTTGGTAACATATGT
CCTCCTTTTCTTTCCGAACATAACGCATCTGCACAACATTGCGGGATAGTAACTCGATATGATGATAGTT
ATTTTATAATGAATAAGCTTATAAGATTCAAGATTTTAAAACTTTATTTCGCGCCAATTTGATGAAGTCGC
CTTTGATAAGTTTGACAGCTATGGTACCTCAACCTTAGTCGACAGTGGCGCCAACTGGTGAGCAAAAA
AACGGTAGTCCCCCTTCCATAGGGGGCAAAATGAAAACAGTAGGTATAGTTTACTCAAAGTTTTATTAC
ATTTTATCTGGTAAACTTATGGTAAGTATACTTAAATGTAGTTATAATAATTATATTGTACACAGTGGTATG
TAAGTATATTTAAAAGTTAAAGGATTGATCGGTACCAACTTGCTTAAATTCACATGTTGAAAGTAGTTG
GAAAGTAAGGTCTGGCGACATTCTTTGTACACAAAAACTTATTATTGATAGAATCACAGAATGCTTGCT
TTAGGGTGCTTACATATTCTTGATTACAAATTTGCTTAAACGTAACGTGTGAGATCGATGAAGCTGATTA
TTCAGCACTACAGGGTGATGCAAAAGTTGTGTAGCCAACTGAAAGGCCTGTGAAAAATAGTTAGTGA
ACATAATAATATTGGTATTTTCATTTAGATAGAAAGAAAGAAGATACAGCTTGTTGACGAACCTTTCGG
AATACTGCACCGCTAGTGAACACCCTGTATACATAGGCAATTGTACAATTTAAATTCTTTAATTATTAGAT
TCCACGCAAGCTTCAGTATTTGTATCGTGTATACTTAGGTATTTTTTACTTACTTACGTATTTTTTGCTTTA
AAGTAAAAAAAAAGGCATGAGGATGATCAGTGTAAGGTAGGTAGGTAGGTACTTATAATCTTTTTATAAT

ATCATAGTTTGGCACTTGGGCTGGGCTTGGGATGGGATACACCGAGGTATGTTATGGATACTGTAATATT
GAGGCGTGCGTGAAACCAGTCGAATTTAACACTATTTAGGCATATTGGGAAAAGTTCTAAGGAATTGA
TTGGATATTTAGAATTTGCTTAGCCATACGTTATCTAATCATTAAAATAATCTTACTGTATACTTAGTAGGT
ATTTTTGTCTAGGTATTATGAATATATTGAAGACTAGGAAGGAGTATATATTGCCTATTGCACCTTAAGCG
AAGACGCTTGGTCTATGGCCTAAGTTCCCGGCTCTATAAACATGCGCCAGATATTAGTTACACAAATAA
ATCTGCGCTAAATTACATTAGGTACAGTTCATGAGCAATTAGAATATATTTGACAGTTAATACAGTTGAT
TTTTTGTATGGATTTGAACTCTCAAGTATATTCTATATGCTAATGAACTGTAGATACTTATTAACTAAAAA
AATATGCACATTAAATTAAAATACCTAAATACATCCGCTGCACTGCAAAAAATAATAAAATAATGTTAGT
GCTAATAAGATAGCAGGGTTCGTAACATAGTTTGATACTTTGTGAAAGCTGAATTGTTCTTTGTATTAAG
TTGTGTGGATTCGTACGACGTGGGAGAGAAATTGTAAATGGTCACCAGTGTGCGAATTACGAATATTTT
TGACACTTGTCCATAGGTAATTCGATACGCGGTTCTCCTGGCTTTATTATTAGTTTCAAACTATACCAAC
ATGAGCGAGCACCCCGATCCGGAAAAAAACAGAGCTAACGTATAGAATCGAGTGAGAGTGTTTTAAC
TCGCATTCTATTCGAAAGTGCTGTCAAGTTGACAGCACTTTCTAAGCACTTTTTACCTATCGAATAGAAT
TACCTATGGAATGAGTGCTGTCAAACTTTTGCAAATCTATTCACCGCTATTTTGTTGTTGCCAGGTTGAC
AGCACTTTCGAATAGAACACTCGCACTCGATTCTATAATTTATCTGTATTTATCGTTTTTTCCGGATCGGG
GTGCTGTACAAACTGTCATAAAAATGATCGAATTACCTATCGACCAGTGTCAAGAATATCCGTACTTAG
CACACATTTATTTTGTTCATTCGAATTTCCGTAATTTCTACCCCCATCGACGAATCAAACTTACATAAATA
AACATTACACATCATTACTATTACTCAGAAGGTAATCGACAACAGACAAATCAAAATTTAAAACTAAAT
TATACAGCTAAAGTAAATAATAATTTATAGCTTCCATACATAAGTATAATGTATTTTTTCTTACTTGTCGTA
GATTTTGAGCAATCTTGGAGTGTCATTTTATTTTAATT
>TCONS_00079751 geneID=XLOC_016015 scaffold_261:269126-270493(-) Splice junction overlap
CAACAACCCACCACCACCAATACCTGGATTTGATGCCAGCCGGCCACCACCTATATTTCACAATATAGT
AGGCAAGAGAAAATCCTCTAATGCATATGAAGGTATGTATATCTGCCAACTCTTTTATTACGTAATACTAT
ATTTGAAGGCGCCAGTTATTTTATTCATAAACTAGACTGTTTCAATGAAATCGGATAGTTGCGACAACC
GAGCATGATGTGTTTACTCATAGATCTATCATCAACTTATTCATTTAATTGCTCGCGACAAGAAAAATAG
TTACATCGAGTCATGCTACACGCATAAGCGAATAAGGTGGAAATAGCGAGCAGACATGATTGATATATT
GTTATAAGTAAATAAGTTAATAACCCTTTCATTGCTGTGCCCTAGTCGGTCAATAAGGGACTAATACAAC
GCGGATGCATTACACACTTATAAAAGCGATTTCAAACACATTCGTTACTGATGGTACATCTTTCATTTAA
CTATTCTCAAATTAATATTTGAATAAATTATTTACGCAATTTAAATATACGATTCTAAGTGTATCGCCGTAC
CACAGCGCATATACATTACTTCGATAGTCAGTAAAATATCTTTATTAAAATGAGTACTCACGTTTTTGTC
ACTGCGAAAATATAATATTTACTAACTACCTACCTACACCATTTTTAAACAGAACGTTTAGAATATAAATA
TAAAAGTGTCTAGATTTAAAACATTATTAAGATGCTAGTGGATGATTTAAAATGAAGCTATGCTACCTCT
AATCTATGAGCTATTTTAAGGTTTTTGTTAAGCCAGCAAGCAAAGTTTTATTTCTAATGGTTAAAATTCT
TAAATGGTAAAACATGGAAATAATTTAAGGTCTCACAAGTTAACAGTTGTAGCAAATATTACCTACAAA
TATTATATGGAAAGATTGAAAAACCTGGGATCAAAATTAAAGGAAGGTGTGACAGCGGGCAAAGCGA
AGAGGAGCATATTAAGCACTTTATCCTACGCACCATTTGGAAGACGCTTTGCTGTACTCCTTTCTGTTTC
AGACACAGGCTGGCTTTGCTCGTCGTCGCACCTACTTTCAGTTTTATCGGCGTTTTCGTTTTCTATAATG
ATTTTCATCTCGATTTAGACATTTTTTTGCAAATTTTAGTACAACATACCTAC
>TCONS_00079532 geneID=XLOC_016019 scaffold_261:612175-613340(-) Intronic
CGCCATCTCATCGAGAAAGGTGTGTCGCGGAATTTAGGATTTCACCGAGGATTCTATACAATTTCAGTG
AAAAGTTTTGGTGTAATAATACTACTGGTTCGTAACTTTGTCTTGTGATATACATATAAGGTGAAAATTA
GTTGTTATGTTTGATGTTCAGCTGCTTGGAATGCACTTCGTTCATGGAGCCACAGGACAAAGGATTATG
GAAGCCGTGGAGCTGCTTCGTCATCACAGCCGTGTCTGGCCCGTCCCGTGAGGCCTCGTCGCGTCTCC
GCTAAGAGTTGATCTCGCGCTGGTCATTCTCGCGATCGAGTCGCGCGGTACCGTTATTCTGCCCGAGCG

CCGCCCTGCCGCTGGTCTAAGTATGTCGGTTGTCTTACAGCCGTGTTACCGGTCGCGAAGCTGCCCCG
GCGCGTGGCACCGCAGCCCGCGGCCCCCGGGCCCGTCCTTGTCCGTCGCGCGCGTCAGGAGCCCTCG
CTCCTAGCTCAGCTCGCTTCCGAGCCTCGTGCGAAGCCCTAGAGGATGTCGTCGTCACGACGCAATGG
CTC
>TCONS_00079972 geneID=XLOC_016079 scaffold_262:345452-350796(-) Unclassified
CTGGTGACAACATAACTAACCGTCTAACAAGTACAATGGTGTCGAGAAAAGTTGAATTGTGTGTTAAA
AGATAAATTTTAACGGGTTTGCAGCTACGAGGTATCTGCTAGCTGGCTTTTGACACTGGCCACATTCGC
ACCAGCCTTCACTAAGATACGAGAGGCTCGCCGCGCCATACCGGCCCGAACAACAATATTACCGTCCT
TGAAAACATTAAAATACATCAAACGTGGCGCGTTGCCAGTTTTCTGGACATCGACAGCGGTGTTGAAA
GCCTATCGCTGTCCCGCTCGCATAAAACTGGCAACGTAGCAAAACAGGGCGCTCTGTTAACAGTCATT
TCAAAGCCATCATCGTTTTTAGTCTAAGTTTTATTGTAAGTGCGACTAGTGTAGCTACTTATCAACTTTG
TACCCACGACGTTTATGTCCTGTTTAATTTTACTGTCTTCCTCTGAGACATCTGTTTTGTACAGTTCCAG
GCAAAGTATATTTTGTTTGAGTAAAGATTATTTATTTATACAGATTTTATATTTGAGAAAGGATATAATGA
CTATTCAAGTATTTATTGCAGATTCTTTATTAATGTTGCCTCTTATTAAGAAGTGATGGCAGTTGATGCGT
AATTAATTAGATAGAAATGGCGATTATTAGATGTTCTGACTGCCATCAAAATCTACAAGTTACCGAAATC
TTTTGTAGGTACAAACGTATAGATTTTATGAAATATACTTATTAATATTATTCATATATCCCTGTTTTTGCTA
ATAATTATAGATTTCTTACATACATGGTATACGCTTCATTTCGAAACGAAAAAATAAATAATAATTAACCC
CAAATTAAAACCTTTAAAATTATCTTGCGTACTATAGGTTCGTTTTAAACACGTAGTATGTAGTCTATATT
AGGAACTATATCACTCTTTTAATAACCCTTGCTTGTAACACAACTAATAAATATTATATTAACATAATTAC
ACCCATTTCTCAAACGTTTTTTTTTCCGATCAATAGAGTAATCTGTAGTATCTTGTTCATAAAAGGAAGA
ACCTACGCAACAGGTTCTTATACTAAGCATCTTGTTATGCGGTATCGGTAATACATATATAACTTATATGC
CCACCAGCATTGAAAAAAGCATTGAAATTTAAATATTTTAAAATTGCATTTAATATACTTACTTACTATTT
AATATTTAGTTTCATTCAGAATTTTTACCATAGGTTTATGGTATATTTTTAATTTTACTTTTATGCTTTCAAT
TTAAAACTTTTTTGTACAAGATACGTACAGACTATGAAATTATAAAAGTATTTCTTGTGTGAATAACGGA
TTGAGCTCATTTGTGTAGCAAATTAACATTCTAGAATATTATTTTCAAGACGAAATCATTCAGCAATTAC
AAGCTAAAGGGGACCAAATACGATTTGATTTATTTACTGGAAACATTCACACTGACACTCTTGATTTTT
TAACAATTCTGACATAAATAACAAGACTATTAAACTTAAAAATAACGCGGGGTAATACATCACTGTAGG
CAGTTTCCCGAATGTTTAATTCAAATACGTCGTTATTTATTAATTTATTAGCCATATATTTATAAACATACC
ATAGGTTAAGGTTATTATCTTTTAAAGATTTCTAGATAGGTTAGAAAAGAGTGAGCTTTATGGAAAATAT
ATACCCGTAATGTATTATGTAGATTTATTTGTATCGCAGTTATTGCTGTTAAGTCAATTTATATTAGAAAAA
AACTTTGAAGCCAGCCATTACTTGTACCTTGTTTGGGTTGCATTATACCCAGTAATGTTTTAATAACTTT
TTTATTATTTCTCCAAAGCAATATAATAAAAATAGATAATCTTTAGGTTAAGCAAATAAATATACTTATTAT
ATATACAAAAAGTTAAATATTACTGCCAGAACAAAAATATGATAAAAAATTAGTATTAAATTGATAGAAA
AATAATTTGGCAGTGATACGTAACGATTTGCGTTCGTGTAATCTTTAAGTAAATAAATAAGTAATAGTTA
AGGATAATGAGATTACATTACTTTTCAGCATTTAAATAGAGCTTGGCCCTTTGGATCTTGATCAATGTAA
CAGTCGTAAGATGTGAAAGTAAAATTAGATCCTCATGGCGCGGCTATGTTTGGGTGATGGTTTTAAACT
AAAGGTGTGTTATATGAGACACAGATGATAATGTACGAATGCCGATAGGTGTGGTGCGGAGGGCTGCC
GGTCGCTTAACGCAAGTGAGGGAACACCCTTACCAGCGCACGGCCTTGCCAGCGCCCTAAGCCCGCC
GAAATACCCTTACTGCCGCCATTTTGTTCACAAGGTGAGTGCTAACGACGAGGAGGAACTAACAGGA
GTCGCGGCCGCCCACGATCTCGACCAATCCTGGCCAATCCTGGCCAAACCCAGCCAACCTCGGCCAA
CCTCGGCCAAAGCCGCCGCGTAACACTCTCATCGGAACATCGACCACATGTAGCTATATCGCTGTTGGG
CCTTTCACGTTAACAACTACTATGTATTATAATAGTCTAACTATTAACATTTATTTGCCTGTTATGTTTATA
ACAAGATGAACTTATATCATTAACCATATTTTTCTCTAAATGGCACACGCCATCGACTATACTATTTACAT
TTTTGTTACTTTACTGTTTGTTGTTGCTTTGTCGTATTGTTGCGTGTCTACTGTTCCACGCAAGTGGTTG

TTAGGTTACCCGAAATTCCTTTTTAGCTTTGTATAGGGATTAAATTGATAAACTATACTTAATTATTTTGT
AGACAATATTATGTCGGTCATTTATAAATAATGATCTGAAAAAGTTCAAACAAATCCCTGTACAAAACTT
ATTATTATGAAAAGATAAAGAACTTACCAACTTTTTGGTACTGCAATCAGCATATCTAACACTTTTTTCA
AATAAAGAGAAATAGGGTTCTAGAACTTGAAAAAACAAATCAGTTTCAAATTTCAAAGTTGACTTTTT
TGTTGTTTTTCAATTCAATTTATCTCTTGATGTTAAAGTTACTAAGTATTTAGTAACTACTTACTTATTTCA
AAATATATTTTCT
>TCONS_00080280 geneID=XLOC_016102 scaffold_263:54799-56116(+) Unclassified
CTGTTTTTAGAAATTTTATTTGCTTAACCATCCCTTACAATGTTACAATACTATACTATGAGTGTATTTGTA
CTTATTATCTACTTACAATAATTTGAATGGGAAGGAAAACTACTAAAGATGACTCCATTGGTTAATGACT
AAACCTAATTAATCTCTTCTTGTTTTGTTGATTCAGGCGACATTTAAGTAATTTTGTCAGTTTTCTGATTA
GATTGCATTAGGTATTAGTTACACTTCTTGTCAGTGCTGCCAAAGTTTCGCCCATGTTTCGCTAGTTTTC
ATCCCCATTCCATTCATAGTGGGTAAGTGGCACATTAAAACATAGGTACTTAATATAAAGGTCACTAGGT
TACCTATTCCTAAGTAGCTAGTTATATTTTGTTTTCAACAGTCTTTTGTTCTTCATCCGATTCATCTGATG
GGATGATAGTGAATTTGTAGTACTTACAACCTGAAACATCCTAACTAATATTATAAATGCGAAAGTAACT
GTGTCTGTCTGTCTGTCTGTCTGTCTGTCTGTCTGTCTGTCTGTCTGTTACTCTTTCACGCCAAAACTACTGA
ACGGATTTGAATGAAATTTGGTATACATACGGTCTAGACCCTGGGAAAGAACATAGGCTACTTTTTATC
CCGGAATTCCCACGGGAAAACTTTTTAAGGCGAAGCGAAGCGCGCGGGGACAGCTAGTAGTTAATAA
TTTAAAGCATTTCTCGAAACAAAATCAGTTGAGTTAATCGAGGGGAGAGATGGGAATGTAGTGTACTT
GCCTGTAGGGTTTATCCGCACTTCAAAATCTTCCATCGATTTTAAAATGCGGTCATCTTCTAGGTGGTAC
AGGTCCTGAAAAAAACAATGAAAGCCTTTAAAAATATCTACGGATGGGCTTCATATATAGTTAGATTTA
ATTTTATGGGAAGGTATATATACCGACACGATAAGAAGATGGTATATTACATGACGCCATTAAGATATGC
CACAACTTCACTTTATAAAATGCACACACTATTTTGACTACCATTGTATTGTTGCAATCTCACCTGCACA
CGGTAAGAGTTCTGTGGCAGCTGCATCTGAGAGAAAGAGAAGGAGTACGACCTCGGGCTGCCGTCTT
CTCTTTCACTCACGAAGGCCAACGCATTGGAGCCGTCGTGGAGCTCACGAAAGCCAATGCGTTGGAG
CCGTCGTGGAGCTGGCGGTTCCACACTGAGATCTTCGTCTGAAAACAAACAGAATTCCGTTTAAACCT
CATGCTATTTCTTAATGGAG
>TCONS_00080148 geneID=XLOC_016103 scaffold_263:73486-74320(+) Exonic overlap (+)
GCCCTATGTGCCCAAGGTGCTCAAGGTGCCCTAGGTGCCTGAGATGCCCCAGGGTGCCCGAGGTGCCC
AAGGAGCCCAAGGTGCCCAAGGTGCCCAAGATGCCCAAAGTCCCCAAGGTCGCCAAGGTCCCCAATA
TGCCCAAAGTCCCCCAGGTCCCCAAGGTGCCTAAGGTCCCCAAGGTTGCCAACGTACCCAAGGTGCC
TAAGGTCCCCAAGGTCGCCAAGGTACCCAAAATCCCCAAGGCCACCAAGGTCCCAAGGTCCCCGTCC
CCGTACATAATAATATTCCCATCTTTATCTTAATTCGCATCCCTATCCCCACCCTTATTTGAAGATATTATA
AAGATATATTATGGTAGGTAGGTACCTACTACATACTAATTATTTAATTCTAAAAGTATTTTAACTAGTAA
CTTTGGTTTAGGGTACAATTTTTTTTTTGGCTTAGCAGGGTTACTAAAATGGTACTCATAAAATAATATAT
TGGTTACATTTACTTAGC
>TCONS_00080194 geneID=XLOC_016116 scaffold_263:147860-148396(-) Intergenic
GTTGAATTAACACTTGTCTGCTTCCAAACCTTTTGGTTAGGTGTGGCTGTGGTGCAGTGCAGATCTCCC
GGATGCCAGTGCTAGTGATGGTGACGGAGTAGTGGTTTACTCCATGGTGTTTGTGTAAATTGTGGTGTG
TATAAGTGCTGAATGGATTGTGTCACATTTGGATTGCACCAGCGGATCATCGGTCGAGCAAGTTGGGA
ACGGT
>TCONS_00080170 geneID=XLOC_016116 scaffold_263:147861-161480(-) Unclassified
CCAACGCCAAACAGCCCTTCATTATCCGACTGAGCGTACTCTGTCTCGCTCCCGCCCGAGCGGGGAAC
CGATAAGCGCGTCTCCCTCGCACCCGCCAGCCGGCGGGGACACGTCACGCCAGCGTCGCTCAGTTCC
CAGCTCCGCCGCCGGTAGCACCGCGGCGCTGACGTGCGTTACGCTACAAAGCTAAGCTTGTGTTTCAA

GATTCCTCTGTTATTTGTTTATCTATTGGATTTGATATTGCAGAACAGTGGCATTGACTAGCGCCCGTGT
GATGTATTGATGTGACTGAGTGTGTTCGCGGGATCGAGTGGAGCCCGACTCGGGAACGAGGTGGCTGT
GTTCGTTTGTGTGCTGAATGGTGATTAAAAATCTTCTTTTACGGGTGTTGAAGATCGGTCGTGGGGTAT
CGATCGTGTTTTGAGACAAAGCGTGGTGGATTGATGTGCCGGCACCGGAGGACAGCAGTTGAAGCCT
GAGGTGTGGCTGTGGTGCAGTGCAGATCTCCCGGATGCCAGTGCTAGTGATGGTGACGGAGTAGTGGT
TTACTCCATGGTGTTTGTGTAAATTGTGGTGTGTATAAGTGCTGAATGGATTGTGTCACATTTGGATTGC
ACCAGCGGATCATCGGTCGAGCAAGTTGGGAACGG
>TCONS_00080232 geneID=XLOC_016119 scaffold_263:210754-213310(-) Intergenic
NNGGGATCCAGCCCTAGACTGAGGCAAAAGTACTTTAGTCGAAGCTCGCTCTAGCTCTAGCTACTCTAT
AGATATGCTGATATTATTAGCGAATTATCTACAAATAACTGTTACCATTATTATTAGCAATGAAACTCCAT
CAGTACTCACAAATATTTTATAAGAAAAAGACATGTCAGCCTCGTTACCTACTTCAGATTAAAGAGGCA
ACGATGATGGTAAAAACTACTAACATAAAATGAAATAACTTGAAGATTTGTAACGTGAAATTTAGTTAC
AGCATGTTTGCTTACAGCCTAGGATATAAGTTTAAAACAATCTACTTAGGTATAGTTATAGCTGTGATTT
GTTTCATAACACGCATAGAACCTTGCTAGGTACTAGATACTTATAGAACAATATAAAACTGTCAAGGTG
TTTTAGGTAAATATTTTGACAAATGATGGCTTCTCAAGTGGCCACAATTAAATATAAGCACAATGTTGAT
CTACATAATATATTATTGTATGTACTTAGTTAAATCTATCATTAGGTAAACAATTTAATACAAATATGCCTC
TGATAGAGAAAGTTGTACCCGGAATTTAAGACGAAATTACATGAAACAAGAGGTAGTTACAGATATTA
GACAATAGACATAGTGTTTGTTGGTTAGAAACTTTGCAGTTGCTTGAGAATAACGATAATGGTCAGTAC
CTATATCATTATAGGTAACCTATGTAACATAACAAAGGTGGTATACCTTATATCGTATACCTATGATAGGTA
TAGGTACTTGTTGCACCAATCCATACTATCACATAGGGTACCATATCATCCTACATCATAATTTAAAATAT
TCCATGTGTCTCACTTATGGTCACTCAAATCCTGAAATTAATATTGTCACAAAAAAATACATGCCGTATA
ATTGAAAGCACTTATTTTACTTCTTCATTTCAATAGTGTTATATTACAATTTATAAACGCCAATGGTATTAT
TTTGAAAATAGATAATAACTTATTTATAAATCAATGAGATTAAAATCACAATAACGAAATCATCACCTATA
AGTAATAATAAATGGCCCATATTTGAAATTGTTATAAAATACTGTTTGCCAAATCACACCTTCCTAGTGT
CTATATACTTACCGATACTGATTTTCAGGCCCAGGTATATGTAGCACACATGTGGAGGTTGCATGTGTTT
TAGTTATCCGTCAG
>TCONS_00080173 geneID=XLOC_016120 scaffold_263:250384-277078(-) Exonic overlap (-)
GGTTTGGTTACTGAAGTAGGATATTTTTATCAGTGCGCGGCGCGCGCTTATAGTGCTGGTTTGTGTCGTT
TGGGCTCTGTGACTGTGACTTCTGTGTTGCCCGGGTGAACGAGTTAGTTACTACGCGCGTGAACGTAT
AAATCATAGTAAATCGCGGTTATTTATTTGGCAACACATAATATGGCTCATTATTTAAATAGAAATAGGTG
AACTAAACTTTATAAGTTTATTAGAAAAGGTGCCAACTTTTTACAAACTTTAAAATATCGAAAGTAACT
TTCGTAAATGCTTCAAAATAAATCCCAAGCTATTGAGCTGTCAAAGTCAATGACTGTCAGTCAAAAATC
TTAAATGTCAAATTTCTTGTTTTGATTTTACGTGATATTGTTTTGTGAGTATTTAATTTAAAATTCAAACA
AATGAAGATCTGTTAAATACTTTAATCGTAGATATTTAAGTGCCAGAGATTTGTGTATTGTGAAGTGTAA
CAATATTTGTGCTTCGAACATTGGTAAATAATATTTTTTCCCGTTGGCATTTACCCAGATTTCTTGATTTA
TTTGAGTGATTTGTGAATCAAAATCGTGATATCTGCAATTATCAGCACAACATACCGAGTGATGTTTGC
AGCGGACGGGCGTCTGAACACTGCCTTATAGTGAGATGGTGAGTGCCGAATACCAAAGTTGAACAGT
GTTTTCAACTCATCAAATAAGAGTTTTCTATGACGATATAATAAAAAACGTATGGTTTATTAAATCATCA
CCAAC
>TCONS_00080411 geneID=XLOC_016174 scaffold_264:9994-10862(-) Intergenic
GCTCGCGCTCTCCATCCAGCACCAGACACCAGCTACAAGGCAAAGACAATGATATGCTATCGAAGGTT
GCAGAGTGCATCGAAACAAGATTCGTGAGAGAAGTGTAAAACAATGGCATATACATCGTAATTGCTATT
ACCTAACTTTCGAACTGTCTCCGACGCGTTTAATGAGTGACAATTATTGTGCTTAATGTCTGCAGTCTG
TGAGTGCTCTGCAATGATCAGTCAGGTCTAGTTCTGAGTCAAGTGATCGGTGGTGATCAGTCGGGAAG

TGCTAGTGAGCTGTTTCAGAAGCCCTTTTTTCGGACAAGTTCAGAGGAAATCAACAGCA
>TCONS_00080511 geneID=XLOC_016169 scaffold_264:202131-204098(+) Exonic overlap (+)
CGCGCCGCTACGAGCGAGAGCGCTCCTACTCGCCGCGTATTGAAACAAGAAGTAAAGGATGAAGTTC
CTTGAAACTAACTGTGTTGAAGTTTTGAAAATGTTTACTTGAAAATTCCGATTCGAAAAGGCGTTTGCC
GAAACCCGCCGAGTTGGAAACGGCGTTAAAGAAACTCGGGGCCGGAGCGAAGTTTTCCGAAAACGT
TTATGTCCAAAGAAAGAAAGAAGCGCAAAGAATATGATCTGCGGATTAGGCGGCGGCGGCAGCGACA
CAGACCACTCGAAGATCATATTCTTTCTATCTTCTCACTCGTTTACTAAACGTTTCTCTCTGCAGTATCT
CTCCACACTTGCACACTGCTCACTCGTGATCTTCTCGTGCATTTGACGCAACAGTATATTATAGTATATTA
TATAGTAATTATGTAATGTATGTTGTTCAGCTGTAGTGACCGAC
>TCONS_00080670 geneID=XLOC_016231 scaffold_265:213169-214212(-) Unclassified
CCGCAAAACCACAAAACAAAAGATCAACAACGACTTCTAGTTCCAGTTTTATTAGCAAGAAGAAATCT
AAATTTTACCCGATTAAACTTACGAAACTCTTAAATAGTGAGTGAAACCTAATAGACAGAACCCTACAA
CTGAACATTAATACTACATTACTTTGAAATTGAAGTTACATTCTACATTTTGCAACAATCGCTGTGGTGG
TATACTATGATATAATTCAAGAAACATAGTACTGAAATTAAAATAAAACAAGAATGACACCTCTAATAAA
ACGGAATTAAATTACTTCCTTGATTTTTTATTTATATGTACCTTTAATGTATAAGTTTGTAGACTCTGAAAT
GCTATACTTCCCTTTTTACATTCTGAACTTATTTAGAATCAATACTAAAATTAAAATTCCTACTTTCTCATT
ATTTCCACTAAAATTTCACTCAATTTAATTTCGTTGTTCATA
>TCONS_00080778 geneID=XLOC_016231 scaffold_265:213176-214215(-) Intergenic
CAACCGCAAAACCACAAAACAAAAGATCAACAACGACTTCTAGTTCCAGTTTTATTAGCAAGAAGAA
ATCTAAATTTTACCCGATTAAACTTACGAAACTCTTAAATAGTGAGTGAAACCTAATAGACAGAACCCT
ACAACTGAACATTAATACTACATTACTTTGAAATTGAAGTTACATTCTACATTTTGCAACAAGTAAGTAA
ATTTATATTAGGTATTATTTTCCCTTCACGTTTCAGTATGATTGATAGTTCCAGTAATAGATAACCACTATG
TGGATTTGATTATGAAACTTACTATATGTTTGTTATAAAACTTTCTCCTAAATTTATTATTACTCTATTTGC
CTATGTGTATTCTGAACCATGTATTCTCTTCCAACTTTTATTTCTTTAAGAATGAAACATAGAACTTATTT
TATAATTAAGTCTCTCTCTCTCTTTACAGTCGCTGTGGTGGTATACTATGATATAATTCAAGAAACATAGT
ACTGAAATTAAAATAAAACAAGAATGACACCTCTAATAAAACGGAATTAAATTACTTCCTTGATTTTTTA
TTTATATGTACCTTTAATGTATAAGTTTGTAGACTCTGAAATGCTATACTTCCCTTTTTACATTCTGAACTT
ATTTAGAATCAATACTAAAATTAAAATTCCTACTTTCTCATTATTTCCACTAAAATTTCACTCAATTTAAT
TTCGTT
>TCONS_00080654 geneID=XLOC_016222 scaffold_265:373045-373974(+) Splice junction overlap
ATAACGCAGAGAATCGATAGCAGTTTATTGGTGGCGGCGCTATATCCGCCATGTTCCCCTCAGACAGAA
CCGTGTCCAGAACCACCAGGACAAGTTCGCTGGCTTGCTTGACACCGGCGTCTACTCGCCCTCCGTGC
AGCACATCCTGTACGGGTTTTGCAATTTACATCACCTGGGTGAAGATGACGATGCAACATCGACATGTC
GAAGTACCCAGCGAATGTGACGACAGGAGTTCCTGTCAAAATCTTTGATAGTGCGCGTCTTC
>TCONS_00080693 geneID=XLOC_016223 scaffold_265:417984-418837(+) Intergenic
CTTCTCTCAGTTTCGGACCAAAATCTAAATTCATTACATTTTTTAGGTTATCTAAATTATTTTGGTAAAAG
TTAACCTTGCTTGAAGTAAGAAAAAATGTGCGATCGTATGCGAGTAATCAACCAGCTTCAAAATGTTAT
GTCTGCTATCCAGGGATGCGACAGCTGCTGCCAACCGCTTCCTTGCTGCAACGCCTGCTGCGCCCGCC
CCTGCTGCCCTCCCTGCTGTCCCTCCCGCTGCCCCGGCTCGCTCGTCACAGTATACAATGCCATCCTGG
CGATCGGTCAGCCTCCAGTACCGGTAGCAGCCTTCGACAAAGCAACCATTGCTATAGGATGCCATCATT
GTAAACATTAGTCTTAAATCCGGTGTATTGTGGTTCAAGTGAAGTGAAGTAAATTATTGTAAAGTGGTG
TAACGTTATTTAAAAAATAGGAATAAAT
>TCONS_00080888 geneID=XLOC_016265 scaffold_266:117582-119454(-) Unclassified
CTAACCCCCTTATTCATAGAGAAGTTACAAAACGTTTTAACTAATAAACTGTTTTGTCCCTCTCCGACA

AAGAACAATTTGTTCTTTGACAGAGAGGGACAAAACAGTTTATTAGTTAAAAAGTATTGTAACTTTTCT
ATGAATAAGGGGGTAAGTCTATGGTTACTGACATGTTTTCACAGTATAACTACACTAACCAACAATAGAT
AAAGACCCAGTTTCCCATTTGATTTTGAGTTACATACAGTGGCTAAGGGCCAGTTTTTTTATTCGTAGGT
AAGTACCTACTACCTAGTTATCTCAAACATTAGTAAATAAACGCAAATAAAATATTTGATTTGATACTTA
CTAAATGATGATGGGCACAAAAATATGGAAGCTGGGACCATCACCAATAGAAATGAGTGATACGTCGT
TGAAAAGGTATTTTTTTGCAGAATATGAATAACGGAAGCGCTAGTCTTGATTTACTGTCCAATTAGAATA
ATCGTACCTTGAAAATTTTATATTTTATTTCAGTAATTTTAATGTTTTTGTCATTTTTTCACACTCATTTTT
AATTTTTATAACAAAATGATCATCATTCATTATAAGATTATATTTGTTTACTATCTCAGTAAATAAAACCAG
TTGAAAATAGTTTATCCCATTTTAATAATACGTGTTTACGTGTATTACGCCCTAACTGTCATCAGGAGAG
AGAGTGCAATGCCATAGAAAAATACTTCCTTCCGACGAATATATTTCAAATTGGACAACTTATACGGAT
GTGTCGTCATAATATTTTTTTGCTCACTTGAAAAGAGCTATCCAACGATGTATGGTATGACTCATCTTTAT
TGGACATAGGTCCCAAAACGCCCATTGTCATTTATAGATTCTAAATAGAACGATAAGTACGTCACTTAAT
CGCGGGACAATCGACTGATGATGAACCGTTCAGAATCTGTCAATTTAATATCTGAGATTAAACAGACTT
CAGTTAATAACAAACAAATTAAGTGCCAATTTCGCCATAGTGGGTTTGAGCATAACCAGGGATTACTGC
TTGACTTGTGACACATCTGTCAAGAATTTAATATGGAGATGACGTATAATAATACCTTACGATCTAACCG
ACGAATTTGAGTTTATATATTTAACTAACTACTTAAGTAACTATATAGATTTTTAATAACACAAAATGTCA
ATAATTAGGTTTAATTTGTTGTTAAACTAATAAAATGAAC
>TCONS_00081003 geneID=XLOC_016257 scaffold_266:120449-121222(+) Intergenic
GTGGAATTTCACCAAACGCTAAACGTATTTAGTAGCCTGTCATACGTCTGTCAGTACAAAATGTATTTA
AAATTTGATTTAAATACGTTTAGCGTTTGGTAAAAGTGACGCTTCGTGTAGATTCGAAAATAGTATCGA
ATCCGGTGCTCGCTGCACAGATGTCTTTCCCCGTGGAATGGGATATAGGAGTTATTTGGTGCTATTGTCA
CTGTAACTTTTTCATTTCTCGTGGGTAGGTATATAGCTATTTAAAATTTTGCCGTTTTTCCGATACTATATG
TACATAAAATACCTACTTATATAGTTTTTATTTATGTATAGGTACTTACTTTACTTGTTTACATAAGTATTTT
TTAATGAATGAAACACAATCAATAATAACAATTTACACCATACACGCTGAGATCCCGGGAGGGGTAGGT
AATTACATAATATCACTATTGTTACTGTACGGTAGGCGGTTAAGTTAAGCATGTACTTATAGCTGAATTTA
ATAATTTACACCACATACGCTGAGATCCCGGGGCGGGTAGGCAACTACATGTTACTATTGTTGCTGTAC
GGGTAGGCGGGTTCGCCGACTATGTCGATGTCGTCGTCGCTGTGGTCGGAGTCTGAGTCGGAGGTGTG
GCCCGAAGTCC
>TCONS_00080805 geneID=XLOC_016258 scaffold_266:181077-195477(+) Unclassified
CTCGGAGTCACCAGCATACGGCTTGGCGCTCTGTTTATTGTGTTTATTCGTGCAATTGCGGCGTGTGGG
CTGCACTGTGTTATGTCTGTTTATCTTTACTCCGGTCGCTTCTGTGTGTGAGTCTGAAGAAGGGGCTCC
GAGTATCAGCGGAGTGGCGGCGTGCGTGTGTGCGTGCGCGCGCGAGTGTGCGGCAGGCGCGGCGGC
GGCGGGCGCGACTCGCGAGGCGCCAGTTCACTGCGAAGCGCCGCGGGGCTGCCACGCTCGTGCTTCT
TACGTTCGTAACTCGTGATCCAGTAACTTTGTAAAGTGGAAACTTTTCAACGCAATTCCGGACGGATTC
CATGTTCGAGCACAGTTTTGAGTGTCGAGTGGAAGCCGCGACGCGAGCGCGTGCACGTGGTAGTGGC
ACGCATTATCGCCAGTGTTAGACTCGATCTATTTTTGAATATTCTCTAAACGTTTCACCGTGAAGTTTTC
GAAACAGGGACCGTGATAGTCGGGTGAACGGCCGCAGTGTGCACCCGCTGCATCACGTCCCAGGTTT
CTAGTGCTTTTGTTGACGGATAACATTACATTTGTGTTGTTAACCTCTGCCATCGAATCCAAGAGACGTT
ATCTTGACATTTGCGCGGCGCGGTGTCGCCAGTTTAATCTTTCCCTGGGAGGGTGATGGCTTCGTGTGA
TTAGTGGTCTTGTAGTGGCGCACGTAGTGTTGTTCCGTGAATAGTGGCTACTAGTGTTGTGCAGTGATG
TGACGTGGTGGTCAGTGGTCGGAATGTTCACGAAGCAGTCCCGCGCCCCCGAGCGGTGCCGCCGCGC
TCACTCGCACAGAGCCAGCAAGAAGAAGCCACTTCCGCACACGCCTTGGAAATTATTGATGAATTGCC
CACTCGTTAAAAAATGTTTTGGAAGCTGTGATTGAATGAATGTTCTGCATGAAACACCTAAACATACTT
ATGTTATTACGTTGTGTATACTCATCATTACCACATGACACAGACTTAGCATAAACGAACAGCTAGTACA

AATCGCTTAACCGCAAATCTCGACAGACAAACTATGTGAACTCAGAACTTGCGAATACCATTATCTAGT
GTTGCCAGTGACAGTGATATTGTTGTGTGAGAGACGTTTCGTTCCGAGGATGTAGTGGAGTGGCGCCC
CC
>TCONS_00080872 geneID=XLOC_016258 scaffold_266:181196-195565(+) Unclassified
AGTCTGAAGAAGGGGCTCCGAGTATCAGCGGAGTGGCGGCGTGCGTGTGTGCGTGCGCGCGCGAGTG
TGCGGCAGGCGCGGCGGCGGCGGGCGCGACTCGCGAGGCGCCAGTTCACTGCGAAGCGCCGCGGGG
CTGCCACGCTCGTGCTTCTTACGTTCGTAACTCGTGATCCAGTAACTTTGTAAAGTGGAAACTTTTCAA
CGCAATTCCGGACGGATTCCATGTTCGAGCACAGTTTTGAGTGTCGAGTGGAAGCCGCGACGCGAGC
GCGTGCACGTGGTAGTGGCACGCATTATCGCCAGTGTTAGACTCGATCTATTTTTGAATATTCTCTAAAC
GTTTCACCGTGAAGTTTTCGAAACAGGGACCGTGATAGTCGGGTGAACGGCCGCAGTGTGCACCCGC
TGCATCACGTCCCAGGTTTCTAGTGCTTTTGTTGACGGATAACATTACATTTGTGTTGTTAACCTCTGCC
ATCGAATCCAAGAGACGTTATCTACTTAGCATAAACGAACAGCTAGTACAAATCGCTTAACCGCAAATC
TCGACAGACAAACTATGTGAACTCAGAACTTGCGAATACCATTATCTAGTGTTGCCAGTGACAGTGATA
TTGTTGTGTGAGAGACGTTTCGTTCCGAGGATGTAGTGGAGTGGCGCCCCCTGGTGGTCGATGGAGTT
ACGGAGGAGCTAGCGCCCGCATGGGGCAAGCGGCGAGCATGTGTCGCCTCCACGGATCATGTCTGCA
CCAC
>TCONS_00080873 geneID=XLOC_016258 scaffold_266:181196-195565(+) Unclassified
AGTCTGAAGAAGGGGCTCCGAGTATCAGCGGAGTGGCGGCGTGCGTGTGTGCGTGCGCGCGCGAGTG
TGCGGCAGGCGCGGCGGCGGCGGGCGCGACTCGCGAGGCGCCAGTTCACTGCGAAGCGCCGCGGGG
CTGCCACGCTCGTGCTTCTTACGTTCGTAACTCGTGATCCAGTAACTTTGTAAAGTGGAAACTTTTCAA
CGCAATTCCGGACGGATTCCATGTTCGAGCACAGTTTTGAGTGTCGAGTGGAAGCCGCGACGCGAGC
GCGTGCACGTGGTAGTGGCACGCATTATCGCCAGTGTTAGACTCGATCTATTTTTGAATATTCTCTAAAC
GTTTCACCGTGAAGTTTTCGAAACAGGGACCGTGATAGTCGGGTGAACGGCCGCAGTGTGCACCCGC
TGCATCACGTCCCAGGTTTCTAGTGCTTTTGTTGACGGATAACATTACATTTGTGTTGTTAACCTCTGCC
ATCGAATCCAAGAGACGTTATCTGAAGCTGTGATTGAATGAATGTTCTGCATGAAACACCTAAACATAC
TTATGTTATTACGTTGTGTATACTCATCATTACCACATGACACAGACTTAGCATAAACGAACAGCTAGTA
CAAATCGCTTAACCGCAAATCTCGACAGACAAACTATGTGAACTCAGAACTTGCGAATACCATTATCTA
GTGTTGCCAGTGACAGTGATATTGTTGTGTGAGAGACGTTTCGTTCCGAGGATGTAGTGGAGTGGCGC
CCCCTGGTGGTCGATGGAGTTACGGAGGAGCTAGCGCCCGCATGGGGCAAGCGGCGAGCATGTGTCG
CCTCCACGGATCATGTCTGCACCAC
>TCONS_00080957 geneID=XLOC_016258 scaffold_266:181289-195568(+) Unclassified
CGACTCGCGAGGCGCCAGTTCACTGCGAAGCGCCGCGGGGCTGCCACGCTCGTGCTTCTTACGTTCGT
AACTCGTGATCCAGTAACTTTGTAAAGTGGAAACTTTTCAACGCAATTCCGGACGGATTCCATGTTCG
AGCACAGTTTTGAGTGTCGAGTGGAAGCCGCGACGCGAGCGCGTGCACGTGGTAGTGGCACGCATTA
TCGCCAGTGTTAGACTCGATCTATTTTTGAATATTCTCTAAACGTTTCACCGTGAAGTTTTCGAAACAG
GGACCGTGATAGTCGGGTGAACGGCCGCAGTGTGCACCCGCTGCATCACGTCCCAGGTTTCTAGTGCT
TTTGTTGACGGATAACATTACATTTGTGTTGTTAACCTCTGCCATCGAATCCAAGAGACGTTATCTACAA
ACTATGTGAACTCAGAACTTGCGAATACCATTATCTAGTGTTGCCAGTGACAGTGATATTGTTGTGTGA
GAGACGTTTCGTTCCGAGGATGTAGTGGAGTGGCGCCCCCTGGTGGTCGATGGAGTTACGGAGGAGC
TAGCGCCCGCATGGGGCAAGCGGCGAGCATGTGTCGCCTCCACGGATCATGTCTGCACCACAAG
>TCONS_00080967 geneID=XLOC_016271 scaffold_266:369597-371038(-) Unclassified
CTTGGAGGACGCCTTGTTGAAGAATATTACCCTTAAGGAAAATATGGATACGTTAGGAAAAGAAATAG
CGAGATTAAAAGGACAGCCGCCGCCGCAAGCGAGCAAGTAGCGAACATTGATGTACTTAAGAAGATT
CCCAAAAGGTTCGACGTTGTTACGTTAATTCAACTTATGCTATTATGGGTGTCCGCATAAAAAAATCGTT

TTAAGAATGGGGGAATTTTATTTTAATCAATGATTTCAAAAGGAAGGATACGCCCTTCAGAGCATTTTA
CTCAAACCTTCTTCATACAAGCAAAATAACTGCCATCTGTCATAATTTGTTGGAACTATGTCAGAGCCG
AGCTACTTATCCTGACGACGCAACCTTGTGCGTGCGTTCATATTTTACCGTCACCGGACAGTAGGTACA
TAATAATATGAGTGAGTTGCATGCGGACGTCCGTACAAGGTTACATCGTGCGGCCATTGTAGACTTTGG
TAGTAAAATAATTATCGCTTTAAAATGTGTTTATTTATTAATTAAGGATGATACGATATTCCTTTTTGACCT
TCGTATCATAGTTTTTGTAGCCTTTCACAACAAAAATAGGCATATTTATCACTAGAAACAAAG
>TCONS_00080818 geneID=XLOC_016262 scaffold_266:394165-395810(+) Splice junction overlap
CGGACCCGGAGGCGCGCCGGCGGAACCGGAGGCTCAGGCGGGAGGAGAAGGAGAAACAGAACCGG
GAGAAGGCGCATCAAGCTCGGAAAGTGCTGGAAGAGAAGAAAGCTGAACTACAGAAGACCAAGAA
GCAGCAAGAAGAGCTAAAAGACAAGCTGCAACGGCTCGAGTCCAAAGTCCTGGTCGGCGGAGAGAA
CCTTCTAGACAAGGCGGAGAGCCAGCGCGCGCTGCTGGAGCAGGCGAGCCGCGAGCTGGAGCGACG
GAG
>TCONS_00081048 geneID=XLOC_016314 scaffold_267:380662-443372(-) Exonic overlap (+)
CTCACGGCTAAATAATCAATTATAACGTAATGCAATTAAAATATAACGTTAATTATAGAAAGTAACGGTT
TAGATTATACGGTTTTATAATAGCTTTGTATTTAGATAATGTTTGAGTAGAGTGTAGCTTGGAAATGGAA
CATCGAGGCAATATAATATTCTGAATGTAGCCGAATTTATTTGGTATTGTTATTCTATATTTTGGTGTTGAA
ACATATTTCTGTTCATACAAAACATACATTTCTGTTACACATGACATTTCTATTATCAATGATATCATGCTT
GAGAGTCACGTTTTTTATGATGTGGTTCAAGCTACAGTTTTATTATATTTTTCTTGTAAAATTGGTTCATT
CCATGAGTTTGTATTGTATATTTGACACTTTTTGT
>TCONS_00081357 geneID=XLOC_016345 scaffold_268:73430-74558(-) Intergenic
ATAGGTAGCATAAGAGAAATTAAGTTTCGGAGTGCTGCCAGCTGCTGAGTGCGCCAACAACGACTCTC
GAACCTATTTCATGACAGCCCTCGTTTTTTTTCCAAGGTATAGTAACCCTCCAGGGACGTTGCAGTAGC
GCCATAATTTTTAACCAATCCTTAATGTTTTAAGTATATTTATTTCGTTATCCACTTATTGTAAAATTAATA
ATATATTGGGGGCTAAGCGTTATTTGAACTGGATGATTATAATAAACATAGTTTAATACCAAAAAAAAA
>TCONS_00081451 geneID=XLOC_016332 scaffold_268:146060-149625(+) Intergenic
GTGGTTTTTGCTGCCGGCTCGGTAAATAACGTTGGCCTGGCTAATTAGGAAAAAACTGTTTGTTTTTAT
TCAGGATCCTCTCGTTATATATTCTTGAGTGAAACCGTCGGCTCTTCGCACAGATTCATCTTTCTTTAAA
CAAACTTTCCTGACACAAAACTATAATGTATAACTTAGGTCAGGCCACGGCGGGGGTGCGGCAGGGCG
CCGGCGGGCGGGGAGAGGGGTACTTCATAAGTTCAATTGGTCGAAAAATTGGCACCGGCTAATCACGT
CCTTTGAATCTGAATGTTTAGTTCGAGAGTGAGACAGTAGATAAA
>TCONS_00081454 geneID=XLOC_016333 scaffold_268:232368-233735(+) Exonic overlap (+)
TTTTCTTGTGCGAAGGTCGTCTGTGAATCTTTCATTCTATTTTTCAGTGGACCTGAAAAACTCGTTTTAA
TGTTTCCGGATGGTGTATTTCCGCTTAACAATGGAAGTTGGGCTTTATTCATTTTCATGAACAGTACGTT
TCATTAAAAGTTTTAGTTTAGTTCTCATAATAGATTACAGTAACTTGTAGTATTTATAGTGGTTATAGTAA
TCAAATCATATTAATATTTATAAGGTACAATAATAACGTTAGATATTCATCAATAGATAGTAGTTAGTATTA
ATAACTTTACAATAAAGAAAACTATAATATGTGCTATGGCTATGGCATAATAAACTCCCAAAATTGTAAC
TTTTTCACGGTAGGGCTCGACCAATTTTCATCAAATAAGAACTTATCTCATCAAACTAAGAAATTAGAA
AGTATTTTAAAATGGAATTGCAATAGATTTTAAGCTACTATACAGGCAGTATACCGTATACCAAGAGTTG
TGGCTAATTTATGAGGCGATATTCTAATCTATTCTATTGACAATACGAGACGACATTTTTGGAGAGTGTT
TTAATTGACCCTAACTACTTTATCCCTCAACTTTTCAGACCTGGTTTGTAAATTTGTAATATTTTTTTATTT
TATCTTCCAGTGGCCAACTTGATTCCACATATGTTAAGAAGAAGAAACACTACTTCTTCCCCCAGGAAT
AAGAAAGCACCCGAACGATAAAGACTGAATGAAGGAACCAAGGGACCACTGACAAGACACCGAAAT
GAACAAATTATATTTTAATCATACTCCTATTTAGGTTATATTTTACGTTCGCAAAACCTTATTTACGTTTGC
TTCTATGGAATATATCCTATATGTATTAGTTATCGTTAGTTTTTCTTTCTTTGTCGTATATAGGTACTAAATT

AAGTTTTTTATGCATTGACGGTTTTATTAGATTATGTTTTTATGCACTTTGACTTGTATGCAACGGGTGCG
TGGGCCTGCTCGTTATAATTATGTGA
>TCONS_00081471 geneID=XLOC_016334 scaffold_268:269469-270469(+) Intergenic
ATATACGGGTGTGTACGGGATGCCTACTTGTAGGACACCCTGTATATACATATATACGGGTGTGTACGGG
ACGCCTACTTGTAGGACACCCTGTATATAGAACCGCTATAGGGGATGTTAAGTGGTCATCCTCTGTATAC
TGTATCTCTATACGGGATGCCTGCTTGTACGAGTAGGAGATCCTGTATATGCTGAACCTCTATACGGGAG
GCCTATACCAATACTTTCGCGGCAACATCTATACCAGTTTCAGATTTCGATAATCTGTCATGATTATAAGC
AGCAAAGGCTGTTGAAAGTAATCCTATATATTTTTTGTATATATATAAGTGAAAAACAGTGATGAAAGGA
CGTTACAAGTGAAAAAGACTTTAAATATTATGAACTATTCCGTGGTCATAAAATATTTATTATTTACCTAA
TTATTGCTTAATTTGTTAACTTGTAAGTACTTAATTGTTAGTGATTGAAACATACTGCCATGATGCCGAGT
GAGGTTTGTTCAAAGTATTTCAATATAATGTTAATATTATGGCGTTATAAAGGTGTTAAGGAATAATTCAA
TATAAAGGTACTTATGTATAAAATGTAGGTTAGATTTGTATATATTTCGAAATCCTCTTAAAAGTGAATTT
AACCTGTAGATGTCTACAAAAATGTATAATTTAATAATTTAGAATGATAAACCTAGCATTAAAGTAAGTC
TGTACAACATAATCGCATTGACCTATTTGTAAATTGATAAAATCTGTTCAATTGTTTTGATGATGCTATGA
GAATTAAACACGATTTGATTCGAGTAAAGATTTCGATATTCAAAAGGACTTCTTGTACAGCTACCTAAA
AGTACTTATAAATATTCAGGAAACTATGTACTTATAGCAAATGCCAGACTTGTTAACTGC
>TCONS_00081585 geneID=XLOC_016389 scaffold_269:64437-64857(-) Intergenic
GTCAGTGTCACTTGAGTTATCAGTAATTTTTGTTTGTATCAATAATTTTGAAAAAACATAGAAATTTGAA
TTTAACATAAAAACACTAATAGTAATAGTTTATTCGTTATAAATAAAATGAGTGACCCATTTATTTGGAC
ACCGAAGCTTACAAAGCAGCTATTTGAATTGCGATTTAAAAATGAATGGCTATTTAAAAATAAAAGGCA
ACCATGGCAGTAAGTTACTATGTTGTTTTATCATAATTGTGGGGCTAAAAAAGTCATATTGATAACAATA
GTTTCCATAGTAAGCTAGGCTAGTACCTTACAGTTAGGGTAAACAAAA
>TCONS_00081858 geneID=XLOC_016394 scaffold_269:290497-293034(-) Exonic overlap (-)
TTCGAAAATAATTAACAAAAAATAAAAAAAAACAAAAGATACCTAAATTAACAAAAGCATTCGATTGC
GAGAATCGTGTTTGCTAAGATCATTTTTGTCTAAAACACTAGTGAAAAAAAAAAGTTTACAGTACCAG
CCCTTCTTTTTATCACAGTATCCCATTTCTCTTTACTGCAACTTTAAATTTAGATTTCTAGTCTTTTTAAG
AAGAGGCGTTCCACAAGGCACGACTCTGGTTCCCCTGTTATACCTAATAGATATAAGACGCATGCGCCT
TATCACAATCCTTTAGTAAATGTTTCATTTAAGTACCAATTATAAAATTTAGATTTAAATGTAATTAGTGC
CAATTGGAAAATTTTGAATAAGTTTTGTTTTGTCTATCTAATATCTACCTTCACCCCAATTACAATACATT
CACTTTTCTATACTTCTTTCCTTCTTTATTCTAACAAAAGTTCCGTGAGAAGCTTCAAATTATAAATAAA
AAGTGGGTATTTTGATAGAAGACTAAAAAAATATATAGAACTCAGTATTTGAAATTCTACATTCATAGAA
AAATAGGTAGGTACAGAGATATCAAAAAGTGTACAGTAGATACAAGTTTATTTAACATCCAACATTTATA
TCGCTACTAAACATATAAAAAACCCCTTATACCTAAAATATAACAACAAAATTAAATCCAAACACGACA
CTGCAAGTGTAAAATATGACATAAAAGTGTTGCGTTATATGAAATAAAAATGAACCAATAAATAATAGA
TCTCTAATGACAAATTAATATGCTATTGCTATTGTAGATACACAACCAAGTATAATCGTTGCGGCAATCTC
ACCTAAACCATAAATAAATAATGTATAAGGGGTAGCTAAACATTCTTTGACGAACAAACATGTAAATATT
ATATTGACACATGTATTTTTTTTGCTTTTTTGCTACACACAATTCACACACAATAATTATCATATTCGATG
GTATAATATAATTGTAATAGACAGCAAAATAGTCGTCATAGTTAGAGTATTTAAGACAGTATTCAAATCT
CTATGGTTATCATCATAGTAATGTTACAATTGATTAAGTTAGTTTTCTCAATCTTAGGATCTCTATAGTGTT
AAAACTTGCCTGGTTGTTTAAAATATCATGTCATCCAAACACTCAACAATCAATCCGTCATTCAAAATA
ACTTTTGTTCCAGACCAGTATTTCCAGAAATTTGCTTTAGACGACAATATTTTTTTGACAACGATATTAG
AGATGTTTTAAAGTCAATTCAAATGGTATTGTTATAATACCATCTTACCAATCCGTGTTATTCCTAATCCG
GGTGTATGTCATAATCCGATGTTGAATCTAGAGATTCTAAATGCATAGACAATTCATGATGCTTCAATCA
AATAGGTTTATATTAAAAGTCCGTGTTTTTCCAATCCGTTCATTTTAAATTAAAATGCAGAAGACGTTGA

AGAAAAAGTTGTGTATTTTAAGAAAAAGAACCAAAATAAACACTACGAATAATGGAAAAATGTAACTA
AGACTAGAAATTCAAAGTGTTGAGTACACCAATCACGACTTGTCGAAGTTACCGAGGTCTATGACGCG
ACGATTTGTCACAGCGCTCTATGTGATTGGTGGAACTAAAACTGGTCCAAAAGTACGCCAACGTATAA
GCGATGCCCCAATGGTGCGTTGCGCGGCGTCGTCTCTGCGTAGTTTTACAAATATTTTTATGACATTGCC
CGTTGACATCGTGACACAACGTAGCGCAACGCACTAATAGGGCCTCACCCTTAGAGACCGTTTCACGG
AATAAAAACCTTTACGGAACCTGTAATACGATTGAATTTAGTTTGTGCTTGTTCCAGAGGACTTATTTAG
TGATGCAGTCTCTAACACGTTAAGAAAGCCCGATATATCCAAATGTGTTATTTATTATATTGACGTATAAA
TATTCGTCGTTAAACAAAGTAAACTCCTTAATACTGATACTACTTAAGTTAACTAAATCATAGTCCTGTT
TATTTCTGTATGTATATTTGCCATACAGCGATTCCATAATAATACTTTCAAGAAAGATAACTAAAACGGCT
TGTAACTAGATAAAAAGGATAAAAACAAAATTGAATAATTACTACAGTTCATGCGAAAGAGATTGGCTT
TCCTGATGCGCTGGTTCCGGTGAAGCGTCGGTCGAAGCTTGAAGATCATTAGTGGGAGGGTCGATGGC
CCCATTTTGTCCAACTGGGTCCACGTCCATAGTCCCATTTTGTGCCACTGGGTCCACAGAAGCACTGTT
TTGTGCCGCTGGGTCCACAGAGTCAATAGCCCCATTTTGTGCCACTGGGTCCACAGAAGTACTGTTTT
GTGCCCCGGGGTCCATAGAAGCACTGTTTTGTTCCCCTGTTTCCACAGAGACACTGTTTTGTGCCGCT
>TCONS_00081566 geneID=XLOC_016383 scaffold_269:424634-436555(+) Exonic overlap (-)
CGCTTATTAAAGACACAATAGGTAGCCAAGTACAAGTATAAAACTAACCTCAAACTCAAAGCTGACCA
GTTTCTTCTCAACCACCTCATTGTACGTATTCCTCCCGTAGTGTATGAAGTACAGAGGCCGGTCCTTGA
ACCGGTCGGCTGGTCGCACTTCCGACTCGAGGCTCCAGCTAGTCAGGTTTACTGAGGGGTATGGAGAT
AGGACTAGCGACATGTGGTGGGGA
>TCONS_00081567 geneID=XLOC_016384 scaffold_269:426171-438006(+) Exonic overlap (-)
ATAGGACAAACACAAGCAAAATTAAACAAAACACACCCATTTACAAAAATAACCTCACCCATAATCTA
TAGTCCTCGGAGTAATTACAGGCAGGTTACAATACGTGAACTCAGAACAATCACTCTTGAAGTCTATGG
TTTCTAGAGTGATGTTCTTCTGTGCCGCCAATGTTAAGACGTTAAGTGGGGTGTTCACTTCTGTTTTTCC
CATCCATATGCCTGACTTCTGGTCTATTAATTCACCAGCTCGGTTGTAGGTGAAGATTTCT
>TCONS_00081842 geneID=XLOC_016396 scaffold_269:461089-466505(-) Splice junction overlap
GTTTAAACACTCTAGATTACGATTCATGTTTATTTTTCGTTAATTTTATGAAATGAAATAAAAGTTTAGGC
GCTGTGAGTGAGCACGGTGGCAGTGGTTTGTGATCATGAAGTATAAACTAGATTTTTCAGAGGACCCC
ACGCACTCAACATACAAAAATGACGGGGAAAAAACATCTAAGCTACCATGCTATTACTTAGCAGCTCT
GTTTGGACTTCTCTTCCTGTTGGCGTATTTCGCATCGCTAATCGACGATGCTATGCCCACCGTTGTCACT
GACATTGATGCAG
>TCONS_00082431 geneID=XLOC_016413 scaffold_27:7989-9170(+) Intergenic
TCTTGTTTTGTATGGAACTTGTACTGTGCGTGACTAGCACAGTAAATTCCGCCAATATCATGTGTTGAAC
TAGGAAACAGTAGAGCAAGTTAAAATGTACGGTGGTATTTATGACTCTACATTCAAAATAATCGCGTAC
CTTAGCTACTGTTTCTTATGTCTATATAGTCTGTTTTTAATGACAGATACTAAATTGACAGATTCTGAACG
GTTCATCAACAGTCGATTATCAAACTGAACGATATGGCACAGAGATACATGACAAACCTATGTAGGTAC
TATAATAATATCAAGATTTAACCCTCTTTCCCTTTTTGCTTGACAGTGTTAAAATTAGAATTTCTGCCTTC
AGAATCGACATCAATATCGTTTTTTTAAATCTCAGATACTAAATTGACAGATTCTGACCGGTTCATCAAC
AGATGATTGTCCCGCGATTAACTGCCAGTTTCAATAACTCTATCTACCGATGACTGGTTGCTACTTTCAT
ACTATTTATTTTGACACATTAACTGCCGGTTTACCGGACATAATGAAAAGTCACAATCTGTCAAAATCGT
ATCTTAATCGTATTCTTTATTTGTACATACATGTACACACACACATAAAAGAAACTTACAAAACTTAAAT
ACTAACTAATATGTACAAAAATGGCGGTCTTATCGCTTAAAGCGATTTTTTCAAGACAATCTTAGGGTG
GAAGGATATTTTGATTAAAGAGGGGTCAAGTGTACTAAAGAATTCTAAAGGAAAAATTAAAATAATTTT
TGAGTTATAGAAACTGGCAGTAAAAGTTACAACCTGTCAAAATCGTATGAAAGTAACTATAAACCAGT
CATCGGTAGATAGAGTTAATGAAACTGGTAGTTAGGTACCGGGTGTCTTATGTCTAAGGGGC

>TCONS_00081983 geneID=XLOC_016458 scaffold_27:362348-367289(-) Unclassified
AATCGGTGTTGGGCGCCACGCTCGGGCTAAACGCGAATAATAATAAGTTTACCAAACTTTTATTCATTTA
TTAATAGCTTTTACGTTCATATAGTTTTTGTCTGGTGCTAAACGAATTTAGTTTATTTTGGCATTGCAGCA
TGCTGACGGAAATGCGAAGTGTGGTTTTGGAATACTGACTGTGCTCTACTCGTGTGCCGAATTGCGTT
CCTTGGTGATATTTTTAAAGCGCCACAATGGTAGTGGTTCTGAACGGCGTGCTCGCAGACGAGTGTCC
CACCTCCGTGCGCGCGCTGCTGGCCGCCCACCCCGGCTACCGCGACGCGGCGGCG
>TCONS_00082096 geneID=XLOC_016458 scaffold_27:362350-402121(-) Exonic overlap (-)
CCGCAACGCAGACCCAGTTATGATTTCATGGTGTTATCAGTCGGCACATGGCCACGATCGATTGAAGAC
TATAGACATAAATGTTTACGCCAACCTGAATGGTCTTCACATCGAAAGCTCGCATTAACTCGCCACAAT
GGTAGTGGTTCTGAACGGCGTGCTCGCAGACGAGTGTCCCACCTCCGTGCGCGCGCTGCTGGCCGCC
CACCCCGGCTACCGCGACGCGGCGG
>TCONS_00082459 geneID=XLOC_016467 scaffold_27:998578-1013596(-) Exonic overlap (+)
ATGGCGCCGACTGAAATAATAACAACTATAAACCTAAGCCAACCCCAAATCCTGTAATGGATTTGGAAG
ATTTGGATATATATATAAGTGTGTGGAAAATAAAGTAGTTCTTAACTCGGATTCCAATCAGGATGACTGAA
ACCCAAGGTATCATTGGGTAAAGAAAGTACTTACTAAGTCAAATTTAAGACTATAGTTCATGTCAGAGG
ACCTCCAGTGAAACTACGTTTGTGCATTCCTGATAGAAATATTTTTCTCCTCGGACTATCTCCCCAATTT
CAATGTGCGTCCAACCGCCCCTTGCGCGCACGCATATAAGCTCACACTCGCGACCAATCACATCATTTG
ATCTCGCGCCACCGCGAGACCGCCGAGTGAGAGTGCAAGTGTCAGTGAGCGGAAAT
>TCONS_00082460 geneID=XLOC_016468 scaffold_27:1013774-1014369(-) Intergenic
TTGATCAGCTGGCACCAGTCTTTGCTCTGAGTTCAACCGTGTGAAGAGATCGAGGAAATACCATGTGC
TTCATCATGCAAGTAATTTGTGGCGCGCTGCAGATCGCGGCTCTCGCCCTGTGTATTATTTAAAACTTCG
TCCTTCAGAACCTGTAGAATGTAATTAATTATCTTCCTGCAACTTTATTCCTTTTCTTCTTCGTCTTGAGT
TTTGATCACTATTTCACGGTGGATGCAAGGAAACTTTAAGG
>TCONS_00082446 geneID=XLOC_016440 scaffold_27:1065624-1065895(+) Intergenic
AGTGTAGAGGACTGTAGAGTGGAATAACCTAAGTGGTAATAGTGTCAGTGACAAGGTGGTAAGACGG
TGGGCGAAGTAACGGGTAACTCTTGTGAGGTTTGTGCAATGGTGAATGGTGGTAACAATAGGTTGATG
ATGGTTAGATAAGTGAGGTGAATAATCGAAGAACACCAAAATATGGCACCCGTTTACATAAATAAATGT
TAGGAAGCT
>TCONS_00081964 geneID=XLOC_016451 scaffold_27:1414532-1416312(+) Splice junction overlap
GTGATGTATTCAACAGTACACTAACACGTTATGGATTTACCTTTAACGCAATAGTTTAAAATAACCAGA
GTGCTATTTGTAAAAAAAAAACTACCTTCTGCTCACAGTTTATTCCACCTGGCAACACCGAGTTACGCA
GCCATTTTGTGGTGTCTGACTTGCAGTGAGGGATCGTCTCGAATTTTTGTCTATTTTACATGGGAGTATT
ACTTTTTGTTGTGATTAGCGAAGATAAATAGTTGGATTTGTTTTGGGAGTTGGTTTTTCGGTAATAAAAC
ATGGAACAAGACGTGGAAAGTGGTTTGGAGGGCACCCTGACTTCCTACCGACATATTTGCAGTATTTG
CCTGAGCACAGATCGTAACTTGGTGGTGATAAACGGAGACAATCAGCTCCAAGAAGTCTTTAGGCTTC
TCATGTATGATTTCGCGGGAGACCGGTTCTCCGACCCCCT
>TCONS_00081962 geneID=XLOC_016451 scaffold_27:1414635-1415869(+) Splice junction overlap
ACAGTTTATTCCACCTGGCAACACCGAGTTACGCAGCCATTTTGTGGTGTCTGACTTGCAGTGAGGGA
TCGTCTCGAATTTTTGTCTATTTTACATGGGAGTATTACTTTTTGTTGTGATTAGCGAAGATAAATAGTTG
GATTTGTTTTGGGAGTTGGTTTTTCGGTAATAAAACATGGAACAAGACGTGGAAAGTGGTTTGGAGGG
CACCCTGACTTCCTACCGACATATTTGCAGTATTTGCCTGAGCACAGATCGTAACTTGGTGGTGATAAA
CGGAGACAATCAGCTCCAAGAAGTCTTTAGGCTTCTCATGTATGATTTCGCGGGAGACCGGTTCTCTG
ACCCCCTGG
>TCONS_00081963 geneID=XLOC_016451 scaffold_27:1414635-1416032(+) Splice junction overlap

ACAGTTTATTCCACCTGGCAACACCGAGTTACGCAGCCATTTTGTGGTGTCTGACTTGCAGTGAGGGA
TCGTCTCGAATTTTTGTCTATTTTACATGGGAGTATTACTTTTTGTTGTGATTAGCGAAGATAAATAGTTG
GATTTGTTTTGGGAGTTGGTTTTTCGGTAATAAAACATGGAACAAGACGTGGAAAGTGGTTTGGAGGG
CACCCTGACTTCCTACCGACATATTTGCAGTATTTGCCTGAGCACAGATCGTAACTTGGTGGTGATAAA
CGGAGACAATCAGCTCCAAGAAGTCTTTAGGCTTCTCATGTATGATTTCGCGGGAGACCGGTTCTCCG
AC
>TCONS_00082230 geneID=XLOC_016451 scaffold_27:1419523-1420582(+) Unclassified
ACAGACGCTAGACCACACGTACATCACGCAATCCCTGTCCCCCCTCCAAGCCGTCATAAAGACTGACT
ACGACGGTATCTTCTTCGACTACTCCGACGTGCCGGAGCGCTGGGGCGTGGTGGAGGTGCCGGGGGC
GGGGGGAGGAGGGAGCCAGGCGGGGGGGGTGAAGGACGAGCAGTTTGATGTGGCGCAGGTGGTGC
GGAACAATATAGACCTGGATATGGACATGGATGGAGCACAGATACTGGAGGTGGGCGAGCTCGACGGC
GTGGACTCATCAGTGGACGCGAGCCTCTCCATGGCGCACATCGTGCTGCGAGCGCCCAGCCTGCCCAC
CCAGCTGCCACTACTTACTGTCAG
>TCONS_00082805 geneID=XLOC_016555 scaffold_270:27394-28264(+) Intronic
TACAAGGTAACACGACTAGGTATATAGTTATTTTCATATTACATGTCTATTAATAATTTGCATACTGATAAC
GCAGCCTTATAGCCCAATATTTAGGTATTATGTGTGCGACATAATAAGCGGGGCTTCCTTCATAGGGAAG
AACGGACTGAACACGCACAAGCTGAAGACTCATAAACATGATCAGGTAGGCGGACTTATGGAGTAAA
GTAATTTCTA
>TCONS_00082730 geneID=XLOC_016557 scaffold_270:67364-68987(+) Intergenic
GCTCCCCCCGCCGCGCGCGCCCCGACCGCGTCGACGAGTAGACAGCGAGACTAGTAGACAGTAGACA
GACAGACAGACCGCAGCCGTCGCAAGTTCTAGCGCCGCGCGCGCCCTGACCGCGTCGACGAGTAGAC
AGCGAGACTAGTAGACAGTAGACAGACAGACATACCGCAGCCGCCGCAAGTTCTACCGCCGCGCGCG
CCCCGACCGCGTCGACGAGTAGACAGCGATACAGTAGACACGCAGACTAGTAGACAGACAGACAGTA
CCGCAGCCGCCGCAAGGTCTACTAAAGTCTATCGGACTAGTAGACAGACAGACCGACAGACTAATAG
ACACACAGACGAGTAGACCGATAGACGAGTCGACAGATAGACATACATATACAGAACTAACTTAGGGA
CGAATGATAGAAATGATTATCAGCTGCAATATCGACGTAGATAAACATATACTATATCGCGATTCGCTATA
AATGGAATGAGTTTTTCGACGTCGATAACGAACCCGTGTAGCGGCCGCAGCGATGAGTATACATAATAT
AAGACAGGTATTAACTATATAGTTACAACCAGAACGATCGGTCGACAAGTTATATCACTGTCTGCCTGT
CTATATGGGTGTGAAGTCTGCACTACTACTTTTAAAATTACCACGTTTTAAGTGTCAAATCCTATGAAAC
TTACAGCGGTCATCGGTAGATAGAGGTATTGAAACTGAAAGTTATAGAAAATATTATAGATAGGTATATG
AAATGCATTTATTTCCATTCTCATCCTGTGTGTTGTAATATAAATAAGTTGTCTGTTGTCGATAAGGCAAG
GTATTTTATTGTTTCCCTGATCATCTAAGGACAGACGTTGCTTAAACATATAACAGCTCTCCTTTTATCAT
CAGGGGTAATAGGTTTGTTTATTGAATTGTATTGTTTGAGCAATGGAAATAAAATGGTATTTTTTAAGTT
ATTTTAAGTTGAAACTATACTTTTTTCTCCCAAATGTGTGTTTTAACTATAATTATGTACATAGTGTTTTG
GCAAATTGTGTGTGTGTGTTTTATTTTATGTAATAGATTCATAGTTTATGGCAAGAGCTATATACAAGGTGTT
GCAAAAATCGTTAATTATATGAATTGCGCAAAAATAAACGTAATATGTATTTAAGTACCAAAAAATCATG
TCAACTTTGGAATTAAATCTTGAAATCAGAGCCTTTAGTACAGGTTTTGCAATTTACGAAAGTTTACGT
TTTTTTTCTGTCAGCTGCGTACATTAACTGTCAAAAGTTTCACGATAATTTCCACTAGAGGCGCTACTTT
GTATGCGAGAAAACGTAAACTTTTATAGTTTTCGTCAAACTATTAAACCTGTACTAGACGCACATAAGT
TTTTTACAAACGTGGCCATTAGAGGGATATTTTTTTAATAATAATTTAAAATTGGTAGTCAAAGATATTTT
AATCCGAACTAGACCAAGTGTCAGTGTAACACCCCACATCTACCACTGAGCT
>TCONS_00082842 geneID=XLOC_016570 scaffold_270:372386-372789(+) Intergenic
GAAAACATTTCAAATACAAGATATTATGTATGACGAGTTCGGGCGAATGTCGGGGATGCACGACGCAA
GACATAAGACAGTACACTGATTATCCAACCAGATTCGAGCCTGAGAGTTTCGGAGATTTCAAGGTTTA

CATATCGTGTCGGTGAGAAACACTAGAGCCAGGGTGTGCTGGACGTATTTAGGTACAGTAAATGCGT
CAGGCTCCTTTAGTGCATTCCACTAATCAGATCATAAAATATTTTTTTCATCGGTCGGGCATCAACCGCT
GTAAAGTTTGAGTTAACTAATAAATAACTTAGTGTAAGTTTAAAAATATTATTATTTATTAGTAGGTAAGT
ACTTAAAAG
>TCONS_00083044 geneID=XLOC_016616 scaffold_271:324356-325821(+) Intergenic
GTAACACTTTGGATACCATTCATAAATCGATCTTTAAGGGTCAATTTCGAACTGAAAGTGTTGCATTTAT
TGTGTGTCCACTGTACCTATCGTTTATGTCCTGATTTCCAGTACGACTACTGATATGTTCATATGCTGGGG
GTATCATTGCCCTATTATAGTCTAGTAATGTTTTGAAATTCGCTTGTGTGATTCATGTCAAGCAGAATATC
AAGTAAATCATTAAAAGCTGATGCAATATTTCAAGCGATATGTTAAATTCTAAATAGTAGTGTAAGGAAC
TGATGATACATATGAAATTGACTTTCATTTAGTTTATGTTTTAACTTAGGATACATATTAATTAGTCTCGAC
TTGATATAACACAATTGTGAACTGATAGTCACATCTCATAGTATATTCGCAAGTTCATTAACTCTCCAGC
ACTAGATAGTTCAGTTTTATTAGAATACTTTTCACACGTGAAATGTACTTTTGTAACTGATAATGGTAAC
AATGTTATCTTTGGGCTTAACTAGATTTATTGTAAGTGCGGCTACGTGTGAGAACCATTGCCTTCATTTT
CGAATGTAGTCAAGTAATCAGATTGTTAATATTTAGGTATAGTCTGGAAACTCTTTGAGGTACTAAATTC
ATTCCTATTTTCTTTTGAACAAATAATAATAGCCACCTGGCTACAGCGAGATCCTCACTTCTGTACTGTA
TCTGTAATGAAATAATACTTTTTTACTATCCCAAACACTCTCAACAATTAGCGACTAATTTGAAGGCGCC
CTAAAAGCCGGCAGAAACGTTAGGCAAGTATCTAGATTTGGCAGTAGATAAAATAGGCATAAGCTATC
AAATTGTATGTTGAGAATGTTCTGAGTGCAACTTGTGCCTTTTAACATGTTGATACTAGGTCTATGTATG
GACTCTGATGCCTTAGTTAGGTACTGTGGCCAAGTTTTTTGTTTTTACACATGATTTTCTACTGGAGATC
CATGGTCAGAAGAAATTGAGAACTTATGGCTTTATAGCTCTTCTTTATCACTGTTAATAGGGATGGTGAA
TAAGTCCACAAACAGGAAGGTAAAGACATTAAAAAATGAGTTCTAGAGTCACTGTTATGTATTTATAAT
TGGTAAATTTTGTTAATATATCTGTATAACATGTAAGTGTATATATTAATTTTAGTATGTTAAAAAGGGAG
GGAAATTTGTTTTATGTAAATTATAAAAATAAAC
>TCONS_00082877 geneID=XLOC_016628 scaffold_271:325978-372552(-) Exonic overlap (-)
TACCTACCAAAAGGGCCACGGGATTTTGCAGACACTTTCGTAATCTTGGAAACGTGGGCTTCTTTCGA
ATGCCGAAGCGGTCTGGCAACGTAGCGCGCCTCACGCAAGCACTGCCGCACCGACCTCGCGTTATCG
CATCGCACCCGATGACGCGATTCTCCAAAAAGGTCACCACACCAGTCACCAGCTAGTCACCGCCGCAT
TCTCTATTTTGCTATCACCCATGTCTTCGTTTGGCCATCTAAACAATCGCTATTTCGATAATTCCAAGCCT
AAAGAAAAATAATATTATTTCCGAACGAAACTTGTAGCGGTGCTACCCTTCTCTTTCGCACCTAGCGCC
GCGCTTCGCAAGCCCCGCCCCCCTCTGATGGTGTCGCCACCTGGCGCCAACAGCGCGCAACAAACAA
AATCTACGCGTTGCCACTTCGTACATTTTCGCTCGACGTAAATTTCGAACGAAACAGTTCAATGGACTT
TCCGAAAATCCTAAACGCGTCAGTTATCGTAAAATGTCTTGTGTGATTGGAAAAAGTTGTAGTAAGTGA
TGTATTCCTTGTTATTGTTGTAGAAAAAGTGCAAATTGTGTTCAAGTTATAACGTGCCCGTGGCTTTCCG
TTGTGTGAGGGCTCCAACCAAGAAACGGGTAGCTTTCAAGTCTATCAGCGTAAAAAGTTCCCGCCGTG
TGGCCCGTGCGTGTGTGTGCGAGTGCGTGTGCGTGTGCAGTGCAGTGAGTGTGTGCGCGATGGGGCG
CAGCTAGCGGCGCGGACGCGGCATGCCCGCCTAGCCATGCCTGCCGCGCGCACGACGACATGGCGCA
GTATTCACAACGGACCCAGCCCATGCATAGCAGGCCCGGGCAGTCGGAGCACAGTCACGGCGCGCTG
GCGTACGGCGGCAAGCCGGG
>TCONS_00082876 geneID=XLOC_016628 scaffold_271:325986-346030(-) Unclassified
TGTCACTCGTCTTAAATCTATTATAATAGTTAAGTAATTTTAATATTCTAAAGTCTACATTACAACGTAAG
AACTGCTTGCCGTACCCGGAGCGACTGCCGCGAAAACCATTCAACAGGCTACCAGGATATGGGCGGG
GCGAGAGGGACGCGGTGTCATTGGACGAGCGCCCGAAGCACAATGATGGCTGCCGTCGCATTCGGCC
AATGAGCGGCGCGGTAGATGAACGCGCGTCCGGCCGGCGACGGGCGCGACGTTGCCAACTTTCTCGA
AAAAATATCAGATTCGACCTTCTTTGGACCCTGGTTTTTTTTTGTTTGCCCGCGTTATCATTCGTATTCC

ACGTTCACGTTTGAGGTCCTAATCCGTGTGTGAGATGCTGTGCAGGTGGTTTGAGTGTGTTGTGTTTTA
GTGTATTATGGTGTGGTCAACCCCATCAGGAGAGGTGGTAGTAGACTTCGCCACGAGGCCCGGGCAGT
CGGAGCACAGTCACGGCGCGCTGGCGTACGGCGGC
>TCONS_00082903 geneID=XLOC_016628 scaffold_271:326016-368855(-) Exonic overlap (-)
CTTGTTGCAGGTTCCCGCCGTGTGGCCCGTGCGTGTGTGTGCGAGTGCGTGTGCGTGTGCAGTGCAGT
GAGTGTGTGCGCGATGGGGCGCAGCTAGCGGCGCGGACGCGGCATGCCCGCCTAGCCATGCCTGCCG
CGCGCACGACGACATGGCGCAGTATTCACAACGGACCCAGCCCATGCATAGCAGGCCCGGGCAGTCG
GAG
>TCONS_00082935 geneID=XLOC_016630 scaffold_271:409071-411132(-) Splice junction overlap
CTACATTCTAGTTGGATACTTTATCACTATTGATTTTATGTCATTGTTCGCAGTCGAGGACATGATAAAAG
AAGTGGACTCGGATGGTGATGGACGCATTGATTTCTTGGAGTTCGTGCGAGCCCTCGGGGAGCCAGA
AGACGCGTGTGATGATGAAGACGACGAGGAGGTCACCGACCCGCCCGAGCTCATGTTGGGCAGACCC
GTCGCCGCCATGTAGACCACCACCTACGGCTAAGGGTGTTTTTAATAGCGTGCGGATTTAATAACTCAC
GCGGGATTGCCCAGCACGCGTTCTTTTCATACAAGTATTTATTGTAATGCGTGGAATTTGTACGCGGGT
CGCGGGATCAAATCCGTACGCTATTAAAACACCCTAAAGCAAACTCGACTATGCTGCTGCCTAAAAGAT
AAGATCTCAAATTTCTGGTACCGTCAGCTTCATAAGTAAAGTAGGTAAGTACACTTTTGTACGTTTTCA
AATCAAACTCACAAGTCACAACACAACTCACAACTCTCACGTGCACAAGCCGCATTTCATTCATTCAA
AAAACATTGACGGTGGTTTCTTTGACAAGGTACAAAAGTTTAAAAATACTTATGAAGCTGACCAATAA
GTTCATAAGTTCCCATGATGTTTATGGTAGAATTTGTGACGTGAAAATGTAAAATGGTACAGTTTTGTTT
AAATAACGTTAGAGTGCGTGGAACGGATGGGAATTTCAAGTAAAAAATTAGTCAGTTAAAATTTTTAC
CCGACCAACCATGGCTAAAGTAAAGGTACCTACTGCCTTACTTAGGTAAGTACTTTTACCTTACAAATA
TTCATCATGTCATGTGACAACTAGAGGATCTCAAGGTTCACTAAAATAATAATCATATACTTAACATAAT
TCCGAAAATAACGCCTAGCTATGTAATCTGACATAAGATTTTGTCTCCAAAGTAAACTTAAGCATTTTTC
TAATATGCTTTCATGTTATACCTATATTTATGTATTATCTATGAACGTAGATATTTTATTGTACATACTAAAT
AACGTTTTCAAACTATTTTCAATTGGGACTAAAATACTGGTTGTTACAATACTTAAGGCCATTCTCTTGA
GACCAGTTTTTACAATTTATTAATTAATCTTTAGAGAACAAAATTTAAGAAAATATGCTACTTAGAATTC
AGATAACCAAATTTATCTCATCTCTATCAAACCTATCATTAAATAAAATAATTCATAATCCTCTATATCCTA
CATATCCAATATTGAATTTATTTACAATTTATTACCTACCTAAAGCCTAAAAAAATATACGAAAGTGTTAG
TCCGCTTTAGATGTTGTGTGGCTGTGGAACTTTATTAATCACTACTAGATTTAATAATTATATTAAAATAC
TATAGCATGAAATAAAGTAAATTCCTAATAAAA
>TCONS_00083123 geneID=XLOC_016657 scaffold_272:13828-15342(+) Exonic overlap (-)
GCAGGGTCTTGTCGAAAGGTGTCGTTCAGCGTACAACTCGACATACCTAGCTAATCAACCAATCTACTA
CTAAGTAGATAGGTACTTGTTCTACATTGTTTGCCTCTTTAAATTCTAAACGGCCATCCTAACCTTGCCT
GGTCTCAGGACCACTATGGTTAGTACCAGTCTCTGTCGTGAGAAGGGCGGCTTACGTTAGGACAACAC
CAATACTCCTCCATTACCTCGATTACCTACCAAATTTGAAAAACTTTTTTTCCTAGGTACCTAACTACTC
AAAAAGCAGATGTGTTGATTTTTATGAAAATTTAAAGAGTCTATATTTAAAACTCTCTAATTTTTGGCAA
GTAGTCATTAATATAGCGATAAGCATTCGTTGCTTATGAGCTAGCCGATCAGCCCTCCAAGGCATGACGT
TGAGATTACCTACCTGTGTTTGCGGTATCAGAGTTATGCACGTTGATATATGAATTCAATGTCATTTTAAT
TATACCTACACCTACACTTATCGCTCAGATGCTAGGTATTAAACCTAGTCACATAATATGCTATGCGGGG
AATCAGATTTACCAAAGCACCGCGCACGGCCACTCACTAGTCGCGCGTCTACGTTCTACAGCCTGGCA
AAAAATTGTAGAAAACAAAAAAGGACGAATTTGGAAATTTAGTTCATCATTTCCTTGTACCTACCTACT
TTTTAAGTACTTATGGTTAAAATAATATGTCGATTAAGAATCAATAGTTATAATAATATATGAAAAATTAA
GGTATTAATATTTTTTATTGAGTTTCCATTTCAGTTTTCTAATCTTTTTTGCCAGGCTGTACGTTCTATAAG
TGTTCTATAATAACGGAAACTAAGTTCTGAGTGCTTGGAAACATTGTGAAGGTGAATAACAAAAGTGA

TAGAAATAAAAAGCATGTATTGACAACGGCATTGTTGGAAAACAGGTTAAATCATACCTAACCTGAAAT
TGAAATTTATAACTGCGGGTTATGGAAGCAAACACAAGCAAAGTTTCTGAAAATGCTACTGCTGAAAA
AGAGAGGAATGAAGCAGCCAAGGCAGGGAATGGTAAGTGCATAATAACTACCTACCTACTAACTAATA
TCTATTCAAAAGAAGTAGCTAAAATGTCGAGTGACTAAAAGCTAAACACCAGATATAAAACAAGTAGT
TACTCACCTAAATACTTAC
>TCONS_00083229 geneID=XLOC_016657 scaffold_272:13828-15342(+) Exonic overlap (-)
GCAGGGTCTTGTCGAAAGGTGTCGTTCAGCGTACAACTCGACATACCTAGCTAATCAACCAATCTACTA
CTAAGTAGATAGGTACTTGTTCTACATTGTTTGCCTCTTTAAATTCTAAACGGCCATCCTAACCTTGCCT
GGTCTCAGGACCACTATGGTTAGTACCAGTCTCTGTCGTGAGAAGGGCGGCTTACGTTAGGACAACAC
CAATACTCCTCCATTACCTCGATTACCTACCAAATTTGAAAAACTTTTTTTCCTAGGTACCTAACTACTC
AAAAAGCAGATGTGTTGATTTTTATGAAAATTTAAAGAGTCTATATTTAAAACTCTCTAATTTTTGGCAA
GTAGTCATTAATATAGCGATAAGCATTCGTTGCTTATGAGCTAGCCGATCAGCCCTCCAAGGCATGACGT
TGAGATTACCTACCTGTGTTTGCGGTATCAGAGTTATGCACGTTGATATATGAATTCAATGTCATTTTAAT
TATACCTACACCTACACTTATCGCTCAGATGCTAGGTATTAAACCTAGTCACATAATATGCTATGCGGGG
AATCAGATTTACCAAAGCACCGCGCACGGCCACTCACTAGTCGCGCGTCTACGTTCTACAGCCTGGCA
AAAAATTGTAGAAAACAAAAAAGGACGAATTTGGAAATTTAGTTCATCATTTCCTTTTTTCTAATCTTT
TTTGCCAGGCTGTACGTTCTATAAGTGTTCTATAATAACGGAAACTAAGTTCTGAGTGCTTGGAAACAT
TGTGAAGGTGAATAACAAAAGTGATAGAAATAAAAAGCATGTATTGACAACGGCATTGTTGGAAAACA
GGTTAAATCATACCTAACCTGAAATTGAAATTTATAACTGCGGGTTATGGAAGCAAACACAAGCAAAG
TTTCTGAAAATGCTACTGCTGAAAAAGAGAGGAATGAAGCAGCCAAGGCAGGGAATGGTAAGTGCAT
AATAACTACCTACCTACTAACTAATATCTATTCAAAAGAAGTAGCTAAAATGTCGAGTGACTAAAAGCT
AAACACCAGATATAAAACAAGTAGTTACTCACCTAAATACTTAC
>TCONS_00083250 geneID=XLOC_016657 scaffold_272:14402-15220(+) Exonic overlap (-)
GCACCGCGCACGGCCACTCACTAGTCGCGCGTCTACGTTCTACAGCCTGGCAAAAAATTGTAGAAAAC
AAAAAAGGACGAATTTGGAAATTTAGTTCATCATTTCCTTGTACCTACCTACTTTTTAAGTACTTATGGT
TAAAATAATATGTCGATTAAGAATCAATAGTTATAATAATATATGAAAAATTAAGGTATTAATATTTTTTAT
TGAGTTTCCATTTCAGTTTTCTAATCTTTTTTGCCAGGCTGTACGTTCTATAAGTGTTCTATAATAACGGA
AACTAAGTTCTGAGTGCTTGGAAACATTGTGAAGTATTTCAGTGAGTAAACAAAAACTTACTGACCTC
TATGGCCGTACATTGTGCATCTGTCTTGTCTTTGTCTATTTAAACTTTTTTTCATTCAATAAATTCATAATA
AGTTTCCATTTTCTTATTTCTGAAATAAGAATAATAATGATAAAGTGAAAATGATTATATTAATATGTGAA
TATTGTAAATGGCATAAATCATCGCAAAACCAACTTTCAGCATGTATTGACAACGGCATTGTTGGAAAA
CAGGTTAAATCATACCTAACCTGAAATTGAAATTTATAACTGCGGGTTATGGAAGCAAACACAAGCAA
AGTTTCTGAAAATGCTACTGCTGAAAAAGAGAGGAATGAAGCAGCCAAGGCAGGGAATGG
>TCONS_00083251 geneID=XLOC_016657 scaffold_272:14402-15220(+) Exonic overlap (-)
GCACCGCGCACGGCCACTCACTAGTCGCGCGTCTACGTTCTACAGCCTGGCAAAAAATTGTAGAAAAC
AAAAAAGGACGAATTTGGAAATTTAGTTCATCATTTCCTTTTTCCATTTCAGTTTTCTAATCTTTTTTGC
CAGGCTGTACGTTCTATAAGTGTTCTATAATAACGGAAACTAAGTTCTGAGTGCTTGGAAACATTGTGA
AGCATGTATTGACAACGGCATTGTTGGAAAACAGGTTAAATCATACCTAACCTGAAATTGAAATTTATA
ACTGCGGGTTATGGAAGCAAACACAAGCAAAGTTTCTGAAAATGCTACTGCTGAAAAAGAGAGGAAT
GAAGCAGCCAAGGCAGGGAATGG
>TCONS_00083252 geneID=XLOC_016657 scaffold_272:14434-15220(+) Exonic overlap (-)
CTACGTTCTACAGCCTGGCAAAAAATTTTTTCTAATCTTTTTTGCCAGGCTGTACGTTCTATAAGTGTTC
TATAATAACGGAAACTAAGTTCTGAGTGCTTGGAAACATTGTGAAGGTGAATAACAAAAGTGATAGAA
ATAAAAAGCATGTATTGACAACGGCATTGTTGGAAAACAGGTTAAATCATACCTAACCTGAAATTGAAA

TTTATAACTGCGGGTTATGGAAGCAAACACAAGCAAAGTTTCTGAAAATGCTACTGCTGAAAAAGAGA
GGAATGAAGCAGCCAAGGCAGGGAATGG
>TCONS_00083088 geneID=XLOC_016667 scaffold_272:50576-51630(-) Splice junction overlap
TAGCTGATGATCATAAAAAGTCTTAGCAAAATCGCACACTTTGATGTCGGGACTTTATAGTTTAACTGT
AATAAGTATACTTATAGGTACTTTATTTTTTTATTACTTCCATGGGAAGACTTCCATAAAACAGAATGTAT
GATAACTTTATTAAGCAGTTTAATTTTCATTTAACTCATCGCGGTGACTACTTACATAACGAGTTGCTAG
TGTAGAAGTAGCTTTAAAACAAACAAACGTTTCCCGCCAAATTGTATTTTTTTGAGTTTGACAGTAGAC
TGACAAATCTGACCTGACAATAACAAAATCAGCTGCTGTTTAATTTGAGTTACTTTCTTTGTTCATCGTT
TCTTTTAATTAAATAACTACTTCTTTAATGGTTTTCGACTTGTTTCAAAAGTGTACATAAAACAAAAACA
TGTCTACCTTAAAACCGATTCAAGCATTAAGCCTAGATTCAGTACAACAACAGAGTTTCATCAAATTTT
ATAAATCTTTGCCTGAG
>TCONS_00083150 geneID=XLOC_016659 scaffold_272:57651-58685(+) Intergenic
CCTGCTTCTGGACGACGGCTGCGAGTACAACGACGCGATAGTCTGCGAAGCTGACGGGCTGTTCCGA
GCTCACGAGCTACGCAACCACCGCCTGCTCATACAGGGTACCAACACTAGCAAGAAACACTCCTGAC
CCGAGGCCGAGCCTAAAAGAGGCTCAAAATACAAACACCACGCTAAGACCCATAATCTACACGATTAC
GGCAGCTTGGGCAAAACTAACACGAAACACAGACACAACGGTCGCCTTTCCCCAGACAGGAAAGCC
AACTTGGAAAACGACAGCGGAATGTCTTCGGAGGAAACAGACGAATCTCTCCAGGATAAAGTAAAGT
TTAATGTTAACGATAGTTTTAAGAATAACGAGCTAAATTGTACATTACAGAGTCATGAAGACGTTAAGT
GCCTAATACAAGAACCTCAATGTGCTACGTATTGGAATGAAATTCCTTGTTGTTCCAATGTTAATATAAG
TGTTAAAGAAGACGGGCTAGACCTGCCCACAGGTGTAGAGAAACGCAATCAACTCTACTTCAGGCGG
TGCGATGTTTTGCGCAGTTCTTAGAGAACATTGTAATACCTCAAAGGCAACTGTAGTATTTTACCTAATT
TAATTACCTATAATTGTGATTAATAAGTATTCCTAATAACGTATTTTTGGTGCATATTCAATTATTATGTAAA
TTACTGTCGGGGATTTAGTTTGTGGTTGTGAATAATTATGGTACTTACTTTCTTGTAGTGGTCTAAGTAA
ATCTGTTTACATACTTTTAGTCATGTAGTTCATAGTAATTTTGAAAGAATAATATGTTTAGCTTATACAGT
TGAATACT
>TCONS_00083303 geneID=XLOC_016672 scaffold_272:262151-264522(-) Intergenic
AGAGTGACCCCCTAGGGGCGGGTGCGAGGGGCGAGGATCGCGTGTGATGGATGGCCGCTGATTCATG
CTGATGTGAGCATTTTGAGCACAACGCACTATTGGGGAATCGGCCTTAACACTTAGACCTGCTTGAAG
TCAGCTCGCTAGTTCACTACAATCACACAAGCCGGGACGATGACTTGCGCACGGGATATTGATCTATCG
GGCC
>TCONS_00083172 geneID=XLOC_016674 scaffold_272:298684-303362(-) Unclassified
CGAAAATTATGATAATGATAACGGAAATCATTTGATACCAGTTGCTATAATAAGCCGACAATACGACCCG
TGGCTCCGCCCAGCAGTGGCCATTTTTAACTTCTCACCTTAACAATAGTTTCTTCCGTACCATTTTGACA
TATTTAAAAAAAAGCCTAAGAAATATCAGTAATATGTAGCCAGTGGTTGAAGTAGTGCACCTAGTTCGT
GGTACCAGTGAAGACAGATCGCGGAAGATAATGTCTTCAGCGAAGCGAGTGTTCCTGAGATGGGGCT
TCTTCTACCAACTGCATTGCCTGGGTCTGCCCATGTAATCGGCAGATTCCTAAAGATCTCTTCTAGTATT
CAG
>TCONS_00083386 geneID=XLOC_016708 scaffold_273:166590-188769(-) Intergenic
GTTTTTTGTAAAGTCTAGATATTTACTGTTGCGTATCTATCATTCTGATATTAATTTAAGTACTATTAATCT
AGCTATGCTTATTAGATTAATACTTATACCTGGTCTCTGATGGATCATGTCAAAAATGAGTTCATTATGTC
AATAAGTTTCTCAAAAAGCTGTAACTTTAGAGTTAAGTACGGACTGAATGACGGTGATGTTAACGTTTT
CATGATTTGATAATTAACATTTAGTTTTTCTTCTTTACAGATATGCCTCAAGTCCATCGTAATGTCTGCTT
CCACCGGTCTGAGTATGGGAGCCTTTACAGACATATTCATCAGTTTCTGATGATAACATTTGAGTGATCT
ATAAATCTGCGTTTGATAGATAATTCTCCAACCATTCCACATTGGATATGTTTCGATCAAGATAATCTTTA

GGTCCACATACAAAATTGAAGAATGGTTGCGGAGTTTACCTGCTGTTTGGAAGAGTCCATTGATGAAA
AATAACACGTCATTATAACCATGTACGTTTGTACCCAAGGAGGCATTGTATTTGGTCTCCTTAGTAATAG
ATAAAAAAAATGGGCAAACCTGTAAACCAGAAGCCTAAACGAAATATTTTTGTAACTTACAACGTATA
CTTAATCCCTATAGCCTAGGTGTAAAGCATGGCCAAGCATTTTTGAATCCACTGGAGTTGTTTATTATAA
CTTGCCTTTTTTAACCTACAAGTAATTAATGAATGTTCTTTACTTCTTCGGAAATCATGCACACCCTACA
CCTTACACAGCTAGGCAGAACTTTGGCGCTCGCGAGATTTTGACCAAATTACGAAGTGCCAAGTGTAA
AGATAAGCTAATGTGGCTTAGTCAACAGTGGGTAGGGGGTTCGAAAACTAATTTTGCATAGTAGAAAC
CCGTTGAATATACCAAGGTGTAAGTGCACTTGTTCTAATGTGTTTATAGAGGGATAGTAGGCAGGTGGA
ATGCGATTTGTTTGAAGTGTACTTACTTGGATGATGGATTGATTATTATTATGCTTGTTGATCTTGGCTTT
CCACGTCTACTCACTGTTTAGGTTAAATTTATAATTATAATTGTTTAATATAAATACAGGCTAAGTACTCT
ATAGTTACTGCATCTATGATAAAGTTTGAATCCAAAGGACAACGACCTGTTGTTTTTTTTTTTACATTGT
TTTTTAGGAATTAGAATCCACAAGATTCTCAATAAC
>TCONS_00083388 geneID=XLOC_016709 scaffold_273:183995-187618(-) Intergenic
CAGCCATATTGTTTTGGCGCGTGACGTCATCGGCCCACCGGATAACTGGCCATATCTTATTTCTCGTTAT
CAGTGGAAGCGCCTAAATATCCGGACCCGTACAGACTAGTACAAACGCATAGTAATGTTTCTTGTTAAA
CTTCTCTATATTATGCCGAAAGTATAGTATTGTTTGTAGCAGTAGGCATATTTCACAACAATATAAGTTTT
TGACCCTGAAAATTAACTCCAATTATGCTAAAAATTATGATAGGTAACTTCAAAAAATACTTGATAGCG
AATCATCTATGTTTCACAGTTCATAGTAGTTATTGGACGTTATGTTGTAGATTACTCTAGGTATCACCGTT
AAGTGCTAAAAACAATAAAATTATCTTTAGTTGAACCAAAAACATATAAATAAAATATATATACTTACTTA
TATTTCGAAAAACAGTACAAACAGCGGCCTTATTGCTTATACCAATCTCTACCAGAAAACCTTTGCTTA
CACAACACATGCAAGTAGCAAGTAAATCTAGACAGGTATGAATATTGCGGGAGTCTGGGGCTTCTAAC
GCCCAATTAGAGTGACGCGTAATTCTATTAGACAATCGCTGGCCTGCTCACTTCACGTCAATCGTATCG
TCGGGACGAAAAGTTTACGATCACAGATAAGTTTTCTGTGATTATTAACGACATAACGTAGTAGTAATT
AAGCTCTTGCTCTTCTACCTAAAAGCGTACCTGAGTAGACAGATTTCGTTGAAGGAAGTATTTTTTTAC
GAAATTGGTGTGAACTGTTGCTTATCACTATGGAAATTCAGGATTGGGATCTATGTTATACACGAATGAA
TTGGTATAAATACACTAATAAATTAAGAAAGAAGCAATTAATGTCTTGGATTAATTTTTTACTATCTTATG
TAGAGAGGCCTAAGTCTAACCACCATACTCGTATGTAATCCTTATCTTCACTCGCCAGTACCTATCTCGA
CCAGTGGCTCCCTCTTCCCTAATTAATCCTGTATCGAGCAAACCTCTGGAGATGACCCTGCCCCATAGA
GGTTGTATCTGAGAAATACGTAGACATCAGTAGGTATAGCAAAAAATATTAGTTTAAAAAGTAACTTTA
GGTAGCCAAATAGAATACGGGATGTTTTAAGTAGATAAATTCCGAGCCTGTATAATCGTATCCCATTATT
CATATAAAAGTTATAGGATGTTTTAGCTATTGAACTGTTTTATTAAAGAACGAAATTTGAGGGACAGAA
CACATAGCTAAAACTTCGTATAAGTATCCTATGAATAAGAGGGTGAGTATCTAAATAATTATGTAGCATA
ATTATTGGAATCGAACTATTCGACTTTCCACACTTTCCGCTTCGGTGACGTTCATCAAAATTACACAAA
ACACGGATTAGCTATGCCACCACAAAAATACCAGGTTTTATTAATATGCAGTCAGCCAGCTGTCACGTA
TCACCAGGCTAACATCCCTGGTCTCTAATGAGTTTAGCTGTGGATCCAAACGGTGGCCGATGTAGGCTG
GCTGTAGTTCCGGACAAATTGCGTTAGCCTGATCGAGGTGGTCGGAGGTGGGTAGATATATCTCTACTA
CGTCTGTGTCTATGCACTCCTGGTATCTATTAGTAGGTTTTGTGAATACAAAGCTATACTCACGGGCAAT
GAAAAAGTTTTAGTGACAATAGCACCGAATTACTCCTAAATGGAAAACACACAAGTGAACAGCATGAT
GATATCGTGTTTTCCAGAATATTACCAACTAAAAAGTAAATATTTTGATCAAATTCTAAATTGTTTTAAA
AATTAGTGGTTGGAGTGTATTTATTTGACCATGCAAAGTAATGTAGTGGTAAGAAAATAAGTTTGTAGG
TACCTACCGTAATTGTGCAAATTCCTAAAAAACAAGTTATTTTAAAAATTCAGCGGGCAAACAGTAACT
CAAAACTGTCATTAACAACACGACTTTCCTAGCTTACGAGTTTATTAAGCCGACTTGCGCGATCTAAAC
GAGGCATAAACCGACCCCTTTGTCCAGCTTTCTCCCTCGCTATGGGCAGTACACCCCACCAAATGTACT
CAGGAACAAAAGAGCTTAAAGCTCACATCCTGCTTTACCAATTTGATACCTCTAGCCGGTGGGTAAGG

TTCGTTTTTGATGAATAGGTGCTGGGATGCGGTGGGCAAGTTACTCGTAGGGGGATGGTCGAAGATCG
AAAGCGTAGTTTTTGCGAGTGTAGTGGTGACTTTTATTAGCATTGCAAGAAATAGTTTTAGCAAGCTGC
CTTTGTTAATTTCTTCTTGTTAGAAATTTATGTTGGCGAGCTTACGAATGGTGTCTAGTTAATAAGATGTT
TTATGCTGTTATTAAGGGCTTTCCTAATATGATTGTCGTGATTCCTCACTCATAATCTCGTTAAAAATAGT
GTCTTCTGTAGTGTGTGCCTTATAGAATCTTGAATTCCATTATGCCTAATAATTTACATATTACAAGCTAA
AGAACCTTTGTGTTAAAATGTCTCTTTTGATAGTATAAAATCTCTCAGTTTAAACCAAGTCAATGCTTTA
CTAACATATTTCCATCCAAAAATCATACTCATACAGAAAATCTCCAACTATGCCTGGTAGTAATAGAATA
ATTCTACAATTACCTACCTAAATAGAATCCTTTCCTAACATTATATGTATTTCGAGTAAAAAGTAAAACC
ACGTTTCTATTTACTAACACTCCTCCGCAGAATGTAGGTACAGGCAGCCAACACTAACATGGGTATTTT
TAAATTGGCCTGTTTCCGCAACATGTCCGCAACATAGTTTGTACAAGACCGGTCACAGAAAGTTAAAG
AAATGACCCTCTTTACGAGCCACACGTAACATTGTTAGATTTACGTGTGGTCCAAGTTACAAAAGTTAG
CTGGCTAATTAGATTCTTATTCGAGTATGCCACATTTGCCAACATAAGTATTATATGTATAAACTATACCTA
CATAGTTTTCTTATTGGGAAAACTAACAAGATTCGGTGGTTATCCAACTTGTTGTTCTAATAAGTCATTT
AGCTTTACTAGAGCTCTGCAGTACCTACACAAAGTTTATAAATATTTTTCTTTCTTACAATATTTCAAAG
AATAATAACAATCCAAGTAATATTATAAAATCGACAGTTTGAGAGTATGTGTGTGTGTGTAGGTTTGTTA
CTTCTTCACATCAAAACGGCTGAACTGATTTTAATGAAATTTGGTGCTAGCTGACACCCTGGACTAACA
CATATGCTACTTTTTATCACTGAATTCTCACGGGAAATCTTTTTAATGCGAAGCGAAGCTAGTAATAATT
TAAATAAAGGAAAGTGTTATTTTCTTGTTTTACACCACGCGGCCGCCCGTGTCAACTAAGCTGACGCG
GGTCCGGCGGCGGACGCTGCACAAACACAAATATTCCCCAGCTTTTCTGCCATTTGTGGCATTTCCAA
CGAGCTACCTACGTCC
>TCONS_00083390 geneID=XLOC_016710 scaffold_273:189539-210008(-) Intergenic
TTCCATTCCATTTTCAAATCTTAACCGGTGCGCTGCGTCTGCGCATAAAAATGCCGTCAATATCAAACT
GTGTTGTCGTTTGTGACATGAATTTGTGTTTCGAGTTGATTTATACTTATAAGTGATCAAATCATTGTCTT
TGATTTGAAATTAAGTTTTAAAGTGCATTGTTTTTATGCCGGCCGGCCAAATATAACCCTTCGAATGGCG
TACAGTCGGCGGG
>TCONS_00083309 geneID=XLOC_016705 scaffold_273:221783-223821(+) Unclassified
CTGACGTTAACTCAATGTCACAAAAGATTTTACATCAATAAAAACTTACTTACTTAGAGTATATATGATA
AAAACATATTTTTTGTACAGCTACTATATTTACTTTTACAGCTTTATTTGTAGCAGTTTAATAAGTGTACC
ATGTGCATTGTTAACGTAGCATTGGGCTTGCTGTTCGCGGTTGCGATCGTTTACTATTTAGTACTGGGAT
TTATCAGCCGCTATCAGAACGTATATGACATAACGATGGATATGAAGTCGCCATCACCAGTATATCTGGC
CACGTATACGGAACCCACGCCACGACACGAAGGCTCGAAGTTTCAATCACAACATGCGGAAAACCAG
GCTAAAGGCTAGCTTCAACATTTTACGGAACCCTAGTATAGGTCCAAAATCACAAGAATGAACTTTGAT
CAAGTACATTAGGTAATTAAATGATTTTAAAAGCACAAATTGATCAGTTGAATATCATAATTTATTATTTG
TGTAGTTGAGAAAATAACTTATGTCCAAAATTTACATAGAAGAATCGCGTGGACACTGATGAAAGTTTA
ACCAGGATCCCTACTTGAGGCACTTGTATTCAATGAAGCGATTAAGTATAAAAGCTGCATGACATTCTA
ATTACCTAACCTAACCTGGCAAGGATATTATAATCATGACCTATGGAGCAGTGAAACGTAACGCAAAAT
TGTATGGAGCGGAGTTAGTGCCGGTACTGACCACTAGGTGGCGTCTCTCCTTACTCGCCGCTTCTCGAT
CGGTGTACAAACTCGCAACTCTATACAGTCATGGCCGTTCGCGTATATTTTTTTATTTGTGAAGCAAACT
TATAGTGCCACAATCTTAACTGTTCCGGTGGTCAAAATGTGCAACTAACGAGACGTCATTGTCAAACGT
CATTTCGTACTCTGTGCGTTATTTGAGTTGTCAATTTTTGCGAAGAGGCTGACGCTACGTTATTTAATTT
GTTTTGTGATTTTCTGGGTGAAATAATGCCAAAAGCGCTGTAATGTGATGCAAGAAAAGTAGTACTATA
TATAATGGCTTTTATGCAGGAAGAATAACATATTCAGGCGCCTTTAATTCCGTTTGAAAAATTGAAAGA
ACGAGTTGCAGCGGCTACAGGTTAGTGTTGCCAAATATGACGAATAATTTTACCCATATACTCCATGTAA
TTTTATTAAATATTTTCCCGAGAGCGGAGAGGCCCGTGCCCCAGCAGTGGGGACGGGATGGGTCGTGA

ATAGTCATGATACTCGTGATGAAAAGTATATAATCTTTATTTTTCTTTGATTACAGATGTGAGGACATGAC
ATGATACTTTAAATCTTATGCAGTATACCAAGAAGAATTTCGCGCACCTGCAGCGATTTTAGACGAAAT
AATGATGATTTATGACATGTCATTGTTTTCCCAACCTATCAAGAGTTGGCTGAAAGAAATTGATT
>TCONS_00083313 geneID=XLOC_016711 scaffold_273:222601-224512(-) Unclassified
GTAGGATCGTAAAAAAAGTTATTGGGTGTACACAAACGAATATGTGGCCCCTATTGTCTCTATATTAGAT
TTAGGAACCTCTTAAAATCAGCATAAGCACTATTTAAGTATATTTGCTTCACAAATAAAAAAATATACGC
GAACGGCCATGACTGTATAGAGTTGCGAGTTTGTACACCGATCGAGAAGCGGCGAGTAAGGAGAGAC
GCCACCTAGTGGTCAGTACCGGCACTAACTCCGCTCCATACAATTTTGCGTTACGTTTCACTGCTCCAT
AGGTCATGATTATAATATCCTTGCCAGGTTAGGTTAGGTAATTAGAATGTCATGCAGCTTTTATACTTAAT
CGCTTCATTGAATACAAGTGCCTCAAGTAGGGATCCTGGTTAAACTTTCATCAGTGTCCACGCGATTCT
TCTATGTAAATTTTGGACATAAGTTATTTTCTCAACTACACAAATAATAAATTATGATATTCAACTGATCA
ATTTGTGCTTTTAAAATCATTTAATTACCTAATGTACTTGATCAAAGTTCATTCTTGTGATTTTGGACCTA
TACTAGGGTTCCGTAAAATGTTGAAGCTAGCCTTTAGCCTGGTTTTCCGCATGTTGTGATTGAAACTTC
GAGCCTTCGTGTCGTGGCGTGGGTTCCGTATACGTGGCCAGATATACTGGTGATGGCGACTTCATATCC
ATCGTTATGTCATAT
>TCONS_00083318 geneID=XLOC_016715 scaffold_273:342086-358399(-) Unclassified
GATCGGATTGGCCGTTGGCAGTCGTGGCTTGAGAGACGGTATTCTCAATTCAAATTGTTTTAAAAAATA
AAACGGTTTTTTATTTTTTTCTGTGAATGAAGTGGCAACGGTAACAGCTGTAGTGGACCGTATCGATCC
AGATTCTAAGGATCGTTGGCGATGCGTGGCACTGCAACTATTGAAAATTAGCTGATAATTTACCGGACA
CTTTACAAACAAAAGGCACGATGCCGCGCGTGGAGCAGTCCCCCGAGGACTTCCGCCAGCTCCTGCG
AGAGAAGGCGCTGAGCCACAGCCTGTTCTACCTGAAGCTGG
>TCONS_00083349 geneID=XLOC_016715 scaffold_273:343185-358492(-) Unclassified
TTATAAATGCCCGACTTACGGTATACCGCTTTTACTTGTATTATCTAGACTTACTATCTGAGATACGAAGC
GAGGCGTGTGGTGTAGTCAGTGGATCGGATTGGCCGTTGGCAGTCGTGGCTTGAGAGACGGTATTCTC
AATTCAAATTGTTTTAAAAAATAAAACGGTTTTTTATTTTTTTCTGTGAATGAAGTGGCAACGGTAACA
GCTGTAGTGGACCGTATCGATCCAGATTCTAAGGATCGTTGGCGATGCGTGGCACTGCAACTATTGAAA
ATTAGCTGATAATTTACCGGACACTTTACAAACAAAAGG
>TCONS_00083514 geneID=XLOC_016768 scaffold_274:446379-448044(-) Intergenic
AGGTAGTTACTCACATCTGAAGATTAAATTATTATCATAAAATATGAACGGGAAATGTCTGGACGGTCAT
GCATTCTGTACTAATAAGTAATGGAAATGGGACTTAGAGACGTATAGATCGATGAGATGCACCGAAACT
CCGGCTGAGTAACTTAGCTTAGAAATTACTACCTATATAGAAAAACTTCCTAGTGCTAGTACTTATGTAT
ACAGATAAGGAAATAATAGAAGTTGATGGAATCTTCGTTTCTTCACCATCACCAAAAAGAGAAAAGAG
TAAATAAAAATGTTAGCGGTTATCCCTACAGTGATTAGATACCAACAACAATCAGAGCTGAGGTATCAG
TATGAGGCAGGCAGGAGCAGTCACTGATGTCAAAATACTACTATAGTACTTTACAACATTTAGATCTAC
CTACTTATGATTGATAAATAAAACTAACACTATAATATAGATATTTTCTACAAAGGGTGAGAACATTCTGT
GTCGACCCGTCGTCGGATCTGCAATACAAGTGTAACTAATAGATGTATTTAGACATTATACCCTGGAGGT
GAATTGATAGCCAGCAGTAGGTACCCTTATCTATCAGGTAGATGATGATGATAACAGATAAATGAATGAT
TAATCGTATTTTAGTGTGAGTATAAACATCGCTAGCCGTATATTAGCTGTGTAGGTAAGGTCTAATACTAG
ATAATACCTAGGTAGGTGGGCACATAATGTAATAATTCTAAATATGTAGGTACTTTATACTATACAGGATG
CCGCATTTGTTAGACTCAAGTTTTCAAACTTTAATAAAGAGCTAAGGGAATTATGGGTTACTGACATAA
ATACTTGATAAATGATAATGTACCTACCTATCTAATGAGGTGATGAGCCATCCTGTATAGAATATTTATTT
CTCTCTACAGCAGAAGAGGGGTAGGATATATAGGAGCCATACCGTCATTGAATTTAATAAAATAGAATT
AGACAAGTAGGTAATAGTTAGATAAGTTAAAAGGGTTGTAATACGTGGTTTAATATACCTACGTATGTAC
ACTCAATGTAATGAAAAAAACCAGTGACATAGTTAAGGAATGAGTGCATTATGAAATGCTCTATACATA

TATCTCTATTACTTATAGTGTAAGTGTAGACACATCTTTATTTAGTGTTATTATATTTAATTTGACTTAGTA
AACGCACATTGACCCAAAATACTTTGTAGTAAGAGGCGAAAACAGTAGACAATACTTACACTTTATTG
GTCAGTTGAGAGTCTATAGTGTGAAATTAACTCGAAAAATGGTTTCCTTTTAT
>TCONS_00083505 geneID=XLOC_016768 scaffold_274:447736-449885(-) Intergenic
GGATACGGTAGTGTTATCCACAACATTTAGGTTTAGTGTTTAGTGTAAGGAGTAAATAATAATGTAGAGA
TGTAGAGTGTTACTTATAATGCTACAATTTAACTAAGCAATTCTAAGTTATTATATTATTTTGCCCAATAAA
TAACTCTAATGGATCACACATAAGATTAATAAAAAATGAGTGAAAAGTCACCCGGTTTCCTTCCGGTAG
AGCAGCAGATTTTTATGAATTTCAGGACTTTTTAAGCTAGGCATGACATTTAACTTTACAATACAAAATC
AAACTACACATGAGCAGATGTAGAGATGAAGATGACAATTATAACCCAGCCAGTGCCTACCGTGAAGA
TGAGACTGAGGCAGTAAAATCCAACTCTTTGATGCAACAAGAAGCCACCACCAGCAAGCGATCCTGG
ATAACTATGAAGCACAGTCTTGGTATTTTAATGTTACTCACATCTGAAGATTAAATTATTATCATAAAATA
TGAACGGGAAATGTCTGGACGGTCATGCATTCTGTACTAATAAGTAATGGAAATGGGACTTAGAGACG
TATAGATCGATGAGATGCACCGAAACTCCGGCTGAGTAACTTAGCTTAGAAATTACTACCTATATAGAA
AAACTTCCTAGTGCTAGTACTTATGTATACAGATAAGGAAATAATAGAAGTTGATGGAATCTTCGTTTCT
TCACCATCACCAAAAAGAGAAAAGAGTAAATAAAAATGTTAGCGGTTATCCCTACAGT
>TCONS_00083589 geneID=XLOC_016794 scaffold_275:66593-67672(+) Intergenic
CTGATATTCATTGAGTAGGTGCATTGTCTGGTCCACTTGACAAATTTGGACCAAACAACTATAGTTAAA
ATAGCCCTAGACACTCTATAAGCTCTAGGATTATAATTAGACATCTAACATTGTACCTAATTTAATCAGAT
AAATGGCGTGTTTAAGTAATAGGTACATATTTGACAAAGTAGGTACTTACCTACCTAGTTACATTAAGTT
CATTCTTTTAATATGGATAACATAATATGACTAAGTAAAGCTTTTACGTAAAGGACGATATACGACAGTA
ATGGTCAAAACAAAAATGAAAAAGCTCTTTCTCATGCTTTATGGAACTGAACTTGTCTTGGAAAGTAC
TAGTACTTATGTATTAGCAAGTAGCTATTGTCAAATAGGTACCTACTTGACGCATGCTTACAATGAAAGT
TATCTAACGTCTATAAAAGTTAAAAATTCGATAAATATTATGAGTAGGTACCTAAACAGTCCAGCTGTCA
TCGAAAGTATTTTTTGGACGGCAAATTATGGTGGTAAATAGAAATATGCTGATACCTGTAAATACATAGG
TAGTTGGCTTATAATTTAATATCTATGCTATTAAAGCTGTACGATGATATCTTTGAATAGGGCCAGTGATT
ACTTGAAATACGCTTGGTAATCTTGATAAACCATACATACTGCCAAGGCTAGCTCAATCTCGCTATTCTC
ATTTGTCAACAACTCAGCTATATTTGGAAATGTTTGCATCTTTCCGTTTCTTTGTAGATTATTGTTGGATT
TTGGAATGTTGGGTTGGA
>TCONS_00083928 geneID=XLOC_016821 scaffold_275:128861-161397(-) Intergenic
TTCGCCGTTCGCCGAGCGAATTCGTGTGTGGGTTCGTCGCTTTCGTCCGACGGACTGTGTGTGATGAA
ACGGGTGTGTGCGAAAGTATGAAGTGAAATCAGACCTAAAAGTGTGTGAAATGTTTAGTGTTAGTTTT
TAGTGTGTTAAGAATTCAAAATGAGTGCGAACGGATAATCTTTTTCCTCCTTCCCGTACCTTAAAACAC
TACGGTTTGGTGTACACGTAACATTTAGTAGGAACAGTTTCAAAATGGCTGTGTTTTGAAGGACTCTTT
GTCCACTTTCGTATGCTTCCTCCAGCATGATAGTGGGATCAGACCGTCACAATCTTGACTTCGAAAGGG
AATGCATGTCATGTACCCGGAGGCACCGGCCCAATACCGCACGAGTGACCCGCCCGCCCAGCCCGAG
CCCCCGGCGTCGCCGCGCCACCAGCCAGCCAAGAAGCGCCGCCTGAGCCCCGCCGACTACCGCCTGC
AACGGGACAACGCTGAGCTAAAGGTAACAACAACTCTATAGAAGGTTTTGGGAACTCGACATATTGA
>TCONS_00083760 geneID=XLOC_016824 scaffold_275:277623-333791(-) Exonic overlap (+)
CGAATTTAAGTATTTTGGACAGTGAAGATAAAATATGATGTAAATAATAATGATTATTATTGTAATCTTCA
AGTAGGCTGTCCATTTTTGTTTTCATAAAGTATAGTAAAGTAGTATGTATCTGCCTAGTTTGCCGTATACC
TATAAGCAGCAAACAACAAATAGTAGGTATATGTATCAGGTATTGAATGATATGAGACAAGTCAGGATAT
TGGAGTAAATTAACCATTTCCAACACAAACCAAACCAGGTAAACCAACCTTGAATAAAAATAATATAC
CGAAAGGTAACGAATGCCCTTTGACAATGTATTAAATTCTTCTGTCAGTATTTTGTGACATCCCACGCTA
TAAAAAGTTAAACTAACGAACGCGAGTAATCCTAAATGTTAGTTTCATGTGATTGAAGGAGTGGCGGT

AACTGTAGAAGCAAAAAATGTTTCTCAAAACTATTAAGGTTTTGTCCTAACAAAGAAAAGAAAC
>TCONS_00084001 geneID=XLOC_016801 scaffold_275:393385-394471(+) Intergenic
CTCTTCATTCCACTGGCTCTGTGTCATGCTTGTTGCGTTGAATGAGTGGATAGTACAACGAGTCCGCGT
TGTTCTATGGGTTCATGACATGAAAACAATCACAAAACTAATGGGCAATATTTATTAATCCATTCACCTG
TATTATAACAAAGCTATAGCCAAGATGACGCTGAAATATGAAGACAACACATAGATACCATGGGTCATG
GGTGTACTGCGTGCTATAGTTATTTTGTATATGGTGGTTTTTATGGTCAGTTACATCGCTTTAGTATCTGC
TTTGATTTATGTGAGAAGTCTTCTACTCAGGGGCAACACAATTCCATTTGAAGAACAATGTCAAGATTA
AAAGTTAAAAGACTGCTATAATTTCATCATATTTATTGCAAAATTGTAAAGGCACTCATTATGGACGGT
>TCONS_00083596 geneID=XLOC_016801 scaffold_275:394021-395225(+) Unclassified
ACAAAACTAATGGGCAATATTTATTAATCCATTCACCTGTATTATAACAAAGCTATAGCCAAGATGACGC
TGAAATATGAAGACAACACATAGATACCATGGGTCATGGGTGTACTGCGTGCTATAGTTATTTTGTATAT
GGTGGTTTTTATGGTCAGTTACATCGCTTTAGTATCTGCTTTGATTTATGTGAGAAGTCTTCTACTCAGG
GGCAACACAATTCCATTTGAAGAACAATGTCAAGATTAAAAGTTAAAAGACTGCTATAATTTCATCATA
TTTATTGCAAAATTGTAAAGGCACTCATTATGGACGGTACAGTTCATGCTAATTTAGCATAATCATTAATT
TTTACAATTACTGCGGAATGTTTGTAAAATCATGATAATAAAATAATATGAATTATATAAAATATTGTGGT
CAACTTGTACATAATTAATGTCACACTTATAATATAACTTAAGTAACTAAATAGATGCAAACTTGAGATT
CTTATAATATAACATATTTTCAGTCTATATAAATGGAAAGTAAGTATAAAGAAAATTATAGATGAGAGTAA
GTAGATTAAAAAAATAGAATGTAGATTTTAAAGTAAAAATTTAGATTTGTGTAAGTACTACTCGTATGAT
TAAGTATTTGATTTGGACGGCAGATTTATATAATTCACACGATAAGGCACTTAAAGATAACCACTACACT
ACAATTCGTTAGAAAAACATATAAAAAATTAAGATTTGAACGAGATACAGCGTAAAATCAGTTTACCCG
TAACTTTGGGTAATTTTTTGCGTGCGGCAACACCGGGCCGCCATTTTATCTTAGTTGGTACAATGCAAA
CAAGATGGCACCGAGCACAGTGAAGCTGCTCTGTATGGCCGCCGCTCCGGCCAGTCGGCACTTGAAC
ATGTCAATTCTGGTTTCTTTGTCGGCGATAATGTTCTGGAACTCGGCTTCGACTATCTGGCCGTCGTAGT
AGGTGATCAGGTCTTCGAATGGGTACTCGTAGCCTTGGAGACACTCACACTTGTAGCCGCCAGCCTCG
AAGCCACGGCCTTGAATCGGGACGCA
>TCONS_00083601 geneID=XLOC_016803 scaffold_275:414701-417901(+) Unclassified
TGCGGCCGCAGGACAGCCGCAAGCAGTGCTGCCAGTAGACAGAGTCCATTTAATCCTGGAAGGCGAA
AGAAGTAAGCGTGTATGGAACACTAAATGCAATCAACAGGAATCATTCCCATATAATGGTCAAAGCGA
CGTGAAAACATGAATATAACTAATGCTCAAACCTGTCTGCCCTTCTTTCAAGATTACTGACGTTAGAAG
TGATAATTCATCGATGGTATTTGTATGTAGAGTTATGCAGTGCGAATAGTTGCCTATTTTAACAGCAGTCT
CTCTAATAGACTCAAGACAAATATCTCAACTTAATTAACTGCCATTTAGGGTTTATGACTTTACTGTAATA
TTTTGTGAAGCAATCAATATAACATAATCATAGCTAAGTCTTAAACTATACAAGCCTAATAAGAATTCCT
TTAACATCTTTAATATTCTTCTAATTATGGTCTATAATATTTATGTGTATGATGTTAAAAGTAAGTTATACTT
GCATCGAACTATTTGTAATATTATAATCGCAAAGTTTGTTAAGTTGATGTTTGTGGAAGTATAATGTTGA
CTGTAAATGCGGGAGATAATTTTTGTAACAAAGGTATAAAGAACAGCGGTACAAAAATGGGAAAAGTG
GTCTTGAAATAATGGCAGATATTCTGAGTGGCAATAAATTCGTCTGCAGTAAGTGGCTCATGTAGATAG
CTTTGTGTATAATTATTGATATATTAGTGTCCGAACGAATAAAATTAACATACTAATAACGCGAACACCA
CGGTTTTCTAGTGCCCAGCTTATGTTCTAGACTAGCACCTAAACTTTATTTGGCGCTCCTAGGTCTTAAA
ATTAATACACACCGCTGTGTTAAATTATTCGTGGTAAATTTTATTATTTAGGGGAACAACTTGTTTCGTTT
TAGTAGATAATTCATATTATATCTATTTATTTAGTTAATTAAAAGTTATTTGAAGGCTAGACTACGGAAAC
TTTATTTTAATCGTTACAAGTTCATCTGGTAAAATGGATTGAATGGCTGACTACATAAGTATGCAATATCG
TTGTTTGGATATCAGAAAGGTAGCAATGTTGTTGGAATGGCAATTAACTTTCAGATATATATTTTTTGTG
TGCAAACTGTCATAGACGATGCTTACACCAGCCACTTAGCAAATGATGAACTTATATTTTCAATGCTAC
ACAATAGTATCTAAAGTGAACTAAAAACTAGTCACAAAACTCTGCTTCTATGGCCGTTTGCATCGCACC

TAGATGTTGCTAAATAACAAGATAGCTTTTAAAAAACATGTTTTCGTTTACTGATGTGCCTTCTCTGACG
TGTATTTCTTTTAAGCTTGTTTCTTTAGTCCGCTGTTAATATTAAAAATTGAAAATGTATATTTGGTGTTG
CAATAACGTGTTGGGGAAGGTTGTAGGGGGCGCTGTAGCAGCACTTCTCTGAATACTCATTCTAGACG
AGGGGTGTTCGTGCCATGAGGCCCCGGGGGTCACCGTGCCCCCGTGGGTCAGTCTGCCCCCGTGGGG
TCACCGTGCCCCGCCGGGGTGGCTAGCCTCCCCGGCCTGTTTCTCGCCGGACAGACAATCTGATGGTC
GGTACGTTTTGCCACGAGCCGGCATTATACGCTCGTTGTAACTAAGGATTATTATAATTGTATTATGGTCT
TACATATGCATGGTTATTTTTTTAGTGCCTATCTGTAATTACTTTATTAAAGACAAAGATAATAAAAA
>TCONS_00083602 geneID=XLOC_016803 scaffold_275:414786-417901(+) Unclassified
CGTGGACGTGCGGCCGCAGGACAGCCGCAAGCAGTGCTGCCAGTAGACAGAGTCCATTTAATCCTGG
AAGGCGAAAGAAGTAAGCGTGTATGGAACACTAAATGCAATCAACAGGAATCATTCCCATATAATGGT
CAAAGCGACGTGAAAACATGAATATAACTAATGCTCAAACCTGTCTGCCCTTCTTTCAAGATTACTGAC
GTTAGAAGTGATAATTCATCGATGGTATTTGTATGTAGAGTTATGCAGTGCGAATAGTTGCCTATTTTAA
CAGCAGTCTCTCTAATAGACTCAAGACAAATATCTCAACTTAATTAACTGCCATTTAGGGTTTATGACTT
TACTGTAATATTTTGTGAAGCAATCAATATAACATAATCATAGCTAAGTCTTAAACTATACAAGCCTAATA
AGAATTCCTTTAACATCTTTAATATTCTTCTAATTATGGTCTATAATATTTATGTGTATGATGTTAAAAGTA
AGTTATACTTGCATCGAACTATTTGTAATATTATAATCGCAAAGTTTGTTAAGTTGATGTTTGTGGAAGTA
TAATGTTGACTGTAAATGCGGGAGATAATTTTTGTAACAAAGGTATAAAGAACAGCGGTACAAAAATG
GGAAAAGTGGTCTTGAAATAATGGCAGATATTCTGAGTGGCAATAAATTCGTCTGCAGTAAGTGGCTCA
TGTAGATAGCTTTGTGTATAATTATTGATATATTAGTGTCCGAACGAATAAAATTAACATACTAATAACGC
GAACACCACGGTTTTCTAGTGCCCAGCTTATGTTCTAGACTAGCACCTAAACTTTATTTGGCGCTCCTA
GGTCTTAAAATTAATACACACCGCTGTGTTAAATTATTCGTGGTAAATTTTATTATTTAGGGGAACAACT
TGTTTCGTTTTAGTAGATAATTCATATTATATCTATTTATTTAGTTAATTAAAAGTTATTTGAAGGCTAGAC
TACGGAAACTTTATTTTAATCGTTACAAGTTCATCTGGTAAAATGGATTGAATGGCTGACTACATAAGTA
TGCAATATCGTTGTTTGGATATCAGAAAGGTAGCAATGTTGTTGGAATGGCAATTAACTTTCAGATATAT
ATTTTTTGTGTGCAAACTGTCATAGACGATGCTTACACCAGCCACTTAGCAAATGATGAACTTATATTTT
CAATGCTACACAATAGTATCTAAAGTGAACTAAAAACTAGTCACAAAACTCTGCTTCTATGGCCGTTTG
CATCGCACCTAGATGTTGCTAAATAACAAGATAGCTTTTAAAAAACATGTTTTCGTTTACTGATGTGCCT
TCTCTGACGTGTATTTCTTTTAAGCTTGTTTCTTTAGTCCGCTGTTAATATTAAAAATTGAAAATGTATAT
TTGGTGTTGCAATAACGTGTTGGGGAAGGTTGTAGGGGGCGCTGTAGCAGCACTTCTCTGAATACTCA
TTCTAGACGAGGGGTGTTCGTGCCATGAGGCCCCGGGGGTCACCGTGCCCCCGTGGGTCAGTCTGCCC
CCGTGGGGTCACCGTGCCCCGCCGGGGTGGCTAGCCTCCCCGGCCTGTTTCTCGCCGGACAGACAATC
TGATGGTCGGTACGTTTTGCCACGAGCCGGCATTATACGCTCGTTGTAACTAAGGATTATTATAATTGTA
TTATGGTCTTACATATGCATGGTTATTTTTTTAGTGCCTATCTGTAATTACTTTATTAAAGACAAAGATAAT
AAAAA
>TCONS_00084137 geneID=XLOC_016803 scaffold_275:415974-417676(+) Intergenic
GCAGTGCTGCCAGTAGACAGAGTCCATTTAATCCTGGAAGGCGAAAGAAGTAAGCGTGTATGGAACA
CTAAATGCAATCAACAGGAATCATTCCCATATAATGGTCAAAGCGACGTGAAAACATGAATATAACTAA
TGCTCAAACCTGTCTGCCCTTCTTTCAAGATTACTGACGTTAGAAGTGATAATTCATCGATGGTATTTGT
ATGTAGAGTTATGCAGTGCGAATAGTTGCCTATTTTAACAGCAGTCTCTCTAATAGACTCAAGACAAATA
TCTCAACTTAATTAACTGCCATTTAGGGTTTATGACTTTACTGTAATATTTTGTGAAGCAATCAATATAAC
ATAATCATAGCTAAGTCTTAAACTATACAAGCCTAATAAGAATTCCTTTAACATCTTTAATATTCTTCTAA
TTATGGTCTATAATATTTATGTGTATGATGTTAAAAGTAAGTTATACTTGCATCGAACTATTTGTAATATTAT
AATCGCAAAGTTTGTTAAGTTGATGTTTGTGGAAGTATAATGTTGACTGTAAATGCGGGAGATAATTTTT
TGTAACAAAGGTATAAAGAACAGCGGTACAAAAATGGGAAAAGTGGTCTTGAAATAATGGCAGATATT

CTGAGTGGCAATAAATTCGTCTGCAGTAAGTGGCTCATGTAGATAGCTTTGTGTATAATTATTGATATATT
AGTGTCCGAACGAATAAAATTAACATACTAATAACGCGAACACCACGGTTTTCTAGTGCCCAGCTTATG
TTCTAGACTAGCACCTAAACTTTATTTGGCGCTCCTAGGTCTTAAAATTAATACACACCGCTGTGTTAAA
TTATTCGTGGTAAATTTTATTATTTAGGGGAACAACTTGTTTCGTTTTAGTAGATAATTCATATTATATCTA
TTTATTTAGTTAATTAAAAGTTATTTGAAGGCTAGACTACGGAAACTTTATTTTAATCGTTACAAGTTCAT
CTGGTAAAATGGATTGAATGGCTGACTACATAAGTATGCAATATCGTTGTTTGGATATCAGAAAGGTAG
CAATGTTGTTGGAATGGCAATTAACTTTCAGATATATATTTTTTGTGTGCAAACTGTCATAGACGATGCT
TACACCAGCCACTTAGCAAATGATGAACTTATATTTTCAATGCTACACAATAGTATCTAAAGTGAACTAA
AAACTAGTCACAAAACTCTGCTTCTATGGCCGTTTGCATCGCACCTAGATGTTGCTAAATAACAAGATA
GCTTTTAAAAAACATGTTTTCGTTTACTGATGTGCCTTCTCTGACGTGTATTTCTTTTAAGCTTGTTTCTT
TAGTCCGCTGTTAATATTAAAAATTGAAAATGTATATTTGGTGTTGCAATAACGTGTTGGGGAAGGTTGT
AGGGGGCGCTGTAGCAGCACTTCTCTGAATACTCATTCTAGACGAGGGGTGTTCGTGCCATGAGGCCC
CGGGGGTCACCGTGCCCCCGTGGGT
>TCONS_00083963 geneID=XLOC_016805 scaffold_275:447722-451424(+) Intergenic
CTGTTGTTTTAGTTAATCACATCAGGTGATAATTTAATATTGTTATCTCTGGGAATCTCCAACTTCACAAT
ATAGTGATTAAACCCACTGTACCAATGTTGTTGCTTTCTGTGACTTCAAATAAGTCGGGCCATAAACTTT
ACTGCTCACTGTATAAAGAGAAAGATTATTTTAATGAAGTAATTATGCTTGACAGGAAGTTAAACTCCA
CATCAATTAATAATATTTCATGAATATTGCACATAGTAAATACATAATCATTGAATATCCACATTCAAAAA
CAATAGACAAACATGATCATAAAATATTGTCCCTTCGGCCGTTCGACACAATAAAAAGGGACCCAACC
TCAGCTATCGAAGGTTGGAAGCATCTTCTTGGCGTTTCGGAGACAAACACGGGATGAACTAGGGATTG
TCACCAGAATGTCATCATCATCGAGAGTCAGATCAATCGCTGACTGCCCTGGGTGCGCCTGATACATAT
ATGTATCACTCGGGGAGACTCCTTTCACTTACCCCTGGAAGTATTTACTTAGTAAAAAAGTATTGTCAA
GTATCAGTATGAATAACCCCAATAACCGCATTCGATCTACCAGGGGCACAGCGGTGGGCACGTTGCTGA
TGTTCGGGCGCGTGGGCTCCGTGGTGGGCGCCAACGTGGCGGGCGTGTCGCTGGCCGTCGCCTGCAG
TGCTACCTTCTACGGCTTCTCTGCGCTCCTGTTCCTGTGCGCCGCACTGAGCCTCCTGCTCCCGAAGGA
GGCCTCCACCACAGACGACACCGACAGCAGAAACAGCTGATATTCAAACACCTGTACCTGTACAGCT
GGGTGCTGACGTTACGAGGTGTAACGTAACATGTTACGCGGCGAACATTTGTGCAGGCAATACGCAAC
AAGCGGTGATATGGATTTTACAGCACTCTTTAGTTTTACACCAAAAATATTAACAACATTAACAAGTTGT
AACTGAATTAAAGGTGATCTTATCACTTAAAGCGATCTCTGTCAGACAATGTTTAGCATGAAAGTTCAC
TATGTAACATGTTACATTACATTCTCGTAACATTCGTCAGTACCTATCTAAATGCCTACCTGACCTGAGTA
CTTAGTAATAATAATAAACATAATTAATTATTATAATAATCTAGCTATTTGTAAATGCGGATATGTGTGATG
TTGTTATTTCACTTCGTCTTGAAGCGACAACGGTATGGTATGATTTATTAACTGTAACTTTGTGAAAAAT
CTTGTTAAATTCATATAAACAATATAAGTAAGAATAAGGTGTCTATATTATGTACTTAGCTATATGATAAAT
GATGTCTGTTTAAATTTATTCCTTAGGGATATGAAAACCCTAATTTTGTCATTGATTGATTTATGATATTGT
TTTGAATTCGGGTTAAACGGATTTTCACGATAATACCTTCATATTGTTCACATACTGACTGCTACATATTA
CTACGGATTGTAAAGTAAATAACTAAGGATTTATTTAAGTTTAACGAAATGTAAATGATAAAAGTAAAA
AATAAAGTAGGTGCTTTATTTGTATTTAATAGTCGTAGTTGAAACAACGTATTTAGTTGGGAAGAGTGTC
AATCAAGATATTAAGTTATTTTTACACATGACAATTACGAGCTTGTATACTGTATAAATGAGATTATATTT
TTATATAAATTTTGTTATATCTGATAAAGATGTTTTAATTACCGCACGTACGATGTACGTACACTATTTCCC
CACTACTTATCTGTATGTTTTTTACTTCTGCAAACAATAAGCATGGCGGACTTACCCAGTGGTATATTTG
AAAACGGCGTAGCTTAGGACAGAGTGTTGTGGCGCTCCTTGGGAAAGTCTCCTTGAGGATAATGATGA
TGAACAGATACCGTACCGCCGCCATCACCGACACATTTGCGATTAACAATCAATAAGATAATGTAATATC
AAACTGTCTGTCAGACGTCTCTATGCTGCGTCAGATTTAGTGTTGCCCAAATGCAAGAACAAGACGAG
ACTTAGCCAGTCTTGGTCTTGGTCTTGCGCCAATACACCTGGTCTTGGTCTTGGTCTTGGTCTTGCGCT

CCCAGTCTTGGTCTTGGTCTTGGTCTTGCAGCAAGAGTCTTGCAAGTCTTGCAATTACCTATTAGTCTAT
TACTATTTATTAAAGTTTACTTTTTGATTTTAATAGATATTTTTTTATTCGCTATGTTTATGGTATTAGTCGT
AATGCGCATAGGTCCAGTTTTTCATTTTCTTTGATACAGTTTTTTGCATCTCATAGAATTCCTAAGCGTAC
TTACCTATACACCGAACTGTTACACCTAACTACTCAGTGAAATAGCGCTCTGCAAGACGCAAGAGTCTT
GCAGGCTATGTCTTGTTCTTGCTCAAGTCTTGCACGGTCAGTCTTGGTCTTGGTCTTGCTAAAAATAAG
CGGTCTTGTTCTTGGTCTTGGTCTTGCAAAAACGCAAGAACAAGACCAAGACTGCAAGACCAAGACT
GAATTTGGGCAACACTAG
>TCONS_00083907 geneID=XLOC_016806 scaffold_275:513463-515398(+) Splice junction overlap
GGGGTCACTGTGCCGAGACCTACGGCGGAGAATAATGCTGAGGTATTGGAAGACTTTGCGGCCATGA
ACCTCTTCCCAAAGGAGCTGCTCCTGTGATCTAATCCAGAAGTAAAAGTACATAAATATGTAAGAAAA
AGTCAATCTCCCAGTGATACCGACAGTCGCGCTAGTTTCGTTCACTCATTCAGTCATTATTTTAGTTCAC
CTTGTTAGAACGCAAAGAATGAAAGGAACTCTGTTACAATATTTTTACTACTTGTAGTTTGAAAAGGCT
TTTATAGAATATTTTCTTCAAGCAAATTTCAAATTATGTGCAACTAATTGTATTGGTATTTATTTTTACTAT
TACGTTGACTATTATTTTTCTTAATCGAATGTTACGTTTTTTTTTTTATTGCAAAGTATATTTACTGTGATA
GCTCTAAGCTAGTATGAGTATGTCTCACTAAGAAAAATACTATCTTATGAAAGATTCCGCCGATTTTGAT
AGAATTAACCTATTTGGAGACTAATACGACAATAAAAAGATGTAGGATACATAAAATTAACTTTTTAAA
GAAAACTTAACTTTATATGTAAGGAAATCAAGACTGCAGAGACCAAAAAGCAATTCCTTCCTTGCTTTT
TTACTAAAATCTTCCAATATGTTGACTAAAAGTAATATCCAGACACATTCCTATAACAATACTTACCCTA
AGACTTATATTCCTGTCTTGCATTTACAACTTAAAATATATTACCATTTTAATAGTCAAATGTGACTAGTC
AACCCAACAACTGATAGGTTAGGTTGCAAACCGTATCGACGAGGTTATAAAAGTACTAAGTACATACT
GATGAAGAAATAGCTGCTTCTAAGCTACTTGCGATATAAATCCTATGAGATGTTCCAAAAGTTTTTTTTT
ACATATAAATTTTATTGAGATTATTATATTACTCATAGATTATGTTAAGAAACAAGGTTTCAGCTACGTATT
GTTCTTATTCGTTATTGTACTACGAAATAAGTTAATTAAGTCC
>TCONS_00083908 geneID=XLOC_016806 scaffold_275:513511-515398(+) Splice junction overlap
GTCTATATACTACATAGGTACATGATACATAGGTACATAGAGACAGCTGTGTCTCAGCGGCGCCGCGCT
GGCCGGGACGGGGTTTAGGGTCCCTGTGCCGAGACCTACGGCGGAGAATAATGCTGAGGTATTGGAA
GACTTTGCGGCCATGAACCTCTTCCCAAAGGAGCTGCTCCTGTGATCTAATCCAGAAGTAAAAGTACA
TAAATATGTAAGAAAAAGTCAATCTCCCAGTGATACCGACAGTCGCGCTAGTTTCGTTCACTCATTCAG
TCATTATTTTAGTTCACCTTGTTAGAACGCAAAGAATGAAAGGAACTCTGTTACAATATTTTTACTACTT
GTAGTTTGAAAAGGCTTTTATAGAATATTTTCTTCAAGCAAATTTCAAATTATGTGCAACTAATTGTATT
GGTATTTATTTTTACTATTACGTTGACTATTATTTTTCTTAATCGAATGTTACGTTTTTTTTTTTATTGCAAA
GTATATTTACTGTGATAGCTCTAAGCTAGTATGAGTATGTCTCACTAAGAAAAATACTATCTTATGAAAG
ATTCCGCCGATTTTGATAGAATTAACCTATTTGGAGACTAATACGACAATAAAAAGATGTAGGATACATA
AAATTAACTTTTTAAAGAAAACTTAACTTTATATGTAAGGAAATCAAGACTGCAGAGACCAAAAAGCA
ATTCCTTCCTTGCTTTTTTACTAAAATCTTCCAATATGTTGACTAAAAGTAATATCCAGACACATTCCTAT
AACAATACTTACCCTAAGACTTATATTCCTGTCTTGCATTTACAACTTAAAATATATTACCATTTTAATAG
TCAAATGTGACTAGTCAACCCAACAACTGATAGGTTAGGTTGCAAACCGTATCGACGAGGTTATAAAA
GTACTAAGTACATACTGATGAAGAAATAGCTGCTTCTAAGCTACTTGCGATATAAATCCTATGAGATGTT
CCAAAAGTTTTTTTTTACATATAAATTTTATTGAGATTATTATATTACTCATAGATTATGTTAAGAAACAAG
GTTTCAGCTACGTATTGTTCTTATTCGTTATTGTACTACGAAATAAGTTAATTAAGTCC
>TCONS_00083982 geneID=XLOC_016829 scaffold_275:514123-515348(-) Exonic overlap (-)
AGCTGAAACCTTGTTTCTTAACATAATCTATGAGTAATATAATAATCTCAATAAAATTTATATGTAAAAAA
AAACTTTTGGAACATCTCATAGGATTTATATCGCAAGTAGCTTAGAAGCAGCTATTTCTTCATCAGTATG
TACTTAGTACTTTTATAACCTCGTCGATACGGTTTGCAACCTAACCTATCAGTTGTTGGGTTGACTAGTC

ACATTTGACTATTAAAATGGTAATATATTTTAAGTTGTAAATGCAAGACAGGAATATAAGTCTTAGGGTA
AGTATTGTTATAGGAATGTGTCTGGATATTACTTTTAGTCAACATATTGGAAGATTTTAGTAAAAAAGCA
AGGAAGGAATTGCTTTTTGGTCTCTGCAGTCTTGATTTCCTTACATATAAAGTTAAGTTTTCTTTAAAAA
GTTAATTTTATGTATCCTACATCTTTTTATTGTCGTATTAGTCTCCAAATAGGTTAATTCTATCAAAATCGG
CGGAATCTTTCATAAGATAGTATTTTTCTTAGTGAGACATACTCATACTAGCTTAGAGCTATCACAGTAA
ATATACTTTGCAATAAAAAAAAAAAACGTAACATTCGATTAAGAAAAATAATAGTCAACGTAATAGTAAA
AATAAATACCAATACAATTAGTTGCACATAATTTGAAATTTGCTTGAAGAAAATATTCTATAAAAGCCTT
TTCAAACTACAAGTAGTAAAAATATTGTAACAGAGTTCCTTTCATTCTTTGCGTTCTAACAAGGTGAAC
TAAAATAATGACTGAATGAGTGAACGAAACTAGCGCGACTGTCGGTATCACTGGGAGATTGACTTTTT
CTTACATATTTATGTACTTTTACTTCTGGATTAGATCACAGGAGCAGCTCCTTTGGGAAGAGGTTCATGG
CCGCAAAGTCTTCCAATA
>TCONS_00083631 geneID=XLOC_016810 scaffold_275:629054-631134(+) Splice junction overlap
TTCGACAAGGAGGACCGCGAGGGCGAGGACAGCTCGGCGGAGGACGAGGACGACCTCAACGGGAA
CAACGACTCCGACGACGAACACGACGGGGACAGTTCCGACGGTGAAGACGACGACGATGTGTCACT
CAGTGCCGTGTACAATGATAACTTAGAGGAGGAGAGCTCAGCGAGCTCGCTGTACGAGGGCAGCGAC
GTGGAGGAGGAGGAGGACGACGAGGCCGAGGACAATGAGAACAACGCCGACAACCACAAGACCAC
AGGTCAGTTAATTAATAATATGTGAGGACATCTCACACACGGCCATCCAACCCCAAGCTAGGCAGAGC
CTGTAGTATGGATGGGTATCGGATGGCTGACATGTACCTAAGTCAATCGGAGGCGCTGCTTTCACCCAA
CCGTCCACTG
>TCONS_00083682 geneID=XLOC_016836 scaffold_275:987566-989958(-) Unclassified
TTTGAGTTTCTTTGACGAAACCGCAATAAAAATATATTTTTCTCTTTCACTCTTTGAATGAATCCCTAGG
ATTGAAAATGGATTTTCGGGATTGGACTGGATTGGCAAAATTCTCTGTAAACAGTTGTCAAAGTCTGA
GATTGTTTCTAATTTTCTATTATTTTTCTAGTTAATCGAATTAATCTAATTGTTCTAAAATGTTAAATACTT
GAAGTGTAATCATTCAAAGTTTATGTTGTAGTGTGTGCGACGTTCGCGACGGTCATCACTTTATTGATAT
AATGATAACCTACACGTTCTTCTCTGAGTATTTAAATGGATGCAACCTCGACATCCACCTATAGTTACCT
ACAACAAAACAATCGCTGCAATATCAAATAGCAAGTGCAGTATCACTGCGTCTAGTGACAGACACTTG
ATCCGGGTATCAAGTGACGGGAGTGCGCAGGTACCGTGGTGGTAGTGATGTGTAGTGTGGCGTGAGGT
GGTGGTGGCTAGTGATGCGCCGCGCGGTCATGCGGAGGCATGGTGGACGCGCCTGCGCAGCGACGGG
CCGGCGCCGACACCTGCGATGCTGCAGATCTGGC
>TCONS_00083854 geneID=XLOC_016837 scaffold_275:1001607-1002191(-) Unclassified
TTTAGGTGGAGAGGATCCTGATAACGCCAAAGAAGCAGCTGAGAAGGCCGAAAAGAAGCACAAGAA
AAAGAAGTCCAAAGACAAGGACCGCGGCGACTACAGCGACAAATCCGAAAGGAAAGACAAGAAGA
GCAAACACAAGAAGTCCAAGAGTGACAAGGACAGACAGAACCCGCAGCAAGCGCTGCTGTCTCCCA
ACGACACGTCAGTGTCTTATGACGACTACGATAGTATTTGAGTAAGACATCTTGACTTCAAAGTTTTGC
ACTACTGGGGGCGCCACTAACGACGTCTTTGGGCCTTAAATACCTACATATCATGTTTGTTTTTTGTAAG
CTAGCATTAGGTTTATTATTAGTTTTTATACGACG
>TCONS_00084272 geneID=XLOC_016894 scaffold_276:110628-113912(+) Exonic overlap (+)
CCCGCCCCCCTCGCCGCCGCCCCCGCGCCTAGAGACAGATCGCCGTCGCCGCCGACGGGCATGTCCC
GTCTGCGCGCCATGTTCTCCCGCCCGCGCCCGCGCCCGCCGCAGGTGCACCACCATCTACTAGTGCTG
CTCACGCTCTATCTCGCTCTCACGCCTGCCTCTGTGGGGTTGGAAGACGAGGATAGTGATAGGGGGA
CGAGGATAGCGATAGGGAGGAGTGAATCCACGCGTGGAGTTTCTATCTCGCTCTCACACGAAGATAGC
GATAGGGGGGATTACATTCATGTGTTGCAATATTCTATGAGCTGTTATCTAGCTCGCACAATGAGATCAG
GATAGAAGGAGCGATAGATCCATACGTTGCATCATTCTTTAGTTTGTCCAATCCTCCAAAATACCGAGG
ACGAAACATACCTATTTTACTCCCGTATATTTACTTTAATCCCCATTTTCTCCCCACTGATTTCACATCCA

CCTGTCACATTACTTTTACCCACTCCACATTGATGTCACTGCCCACCTTTTACATTATTTTTACCCAATAT
TTTTTTTTGTTTTTTTATTTACCCCCCTTCCCCTCTAGGAGGTCCCCCCGACGACGGCGGAGGAGGGCC
CGGCGTGGGCCGTGCACTATCCGCACCTGCAGTGCCCGCCCACCTTCGAACCCGAGACCTACAGCCTG
CAGCCAAGGCTTCTGCATAAAGAGGACAAGCTCTGACACCGGATTGGGGGATTAGTGGGAAGATTTG
TGAGGGAAAGGTTGCGGTTTCGGCGAACCACGTAACCTCGATAAAGATAAATACTCTTTATTCTGAGT
CTAGCACCAAACAAACACATAATATATGTACTTGGCCAGTTTCCTGTGACGCATTTACGTATATTTTTTTT
ATGTGCCTAAACTGTAATCGGCTTGTTTCTTTCAGTAAACAGTGGATGTGCATGTATAAATGCAACGTG
GCGTGCTGGACTAGAGAAAACATTTTTGTTTGTATAATTTTATTCATTCGATTATGTGATCATTTTAGGAA
GCTCTGTTCTATGTAATGAAAAGATGTAATTTATGCTAAATTATCAAATTGGGGACGTAAATTGTGATTC
TTGAGACAATTAAAATGCATGAACGTACATAATACTTGTATTGCACTATGCGATGAGATGTGAAAATTG
GCATAGCAGTAGATTTATTAACAAACATGACTTGATGATAATTATGAGACAAACACGTGCTGTAGATAG
CTAATTAGATAAGTCTATATAAATGTAAGTACATAATTCTATACTATCTGTTGAGAATCGCTGAATAAATA
AAATCTATATTAATCTATGCTTTCAAAAAACGAACCAGTTGGTTTAATATGAATTCAATTTAACTAAGTA
AATCTGAAACGTAGGTACCTAATTGAAATATTCTAAAAAGTAATTCTAATAAATATTGTAAAATATCAAG
CTAACTATAAAGTAGAGTTAAATGGAGGAAGACACACTGTACGACTGAAACGAGACGAAGAGACTTG
TCTAACATCCGACCTGAAAGGTTTTCTGTAAGAATAATCTATGTAAGTACATAGTAATAGAACATATTTG
TAGAACTAACTACATTTTAATGCTTTACATATACTTGAGCCATTACAAAAGTAGACATGCACTTATGTAA
AAGTAGACCCGACATGCCTGTAAGTTCGAGCGAAAGGTCTGATAAATGCTTCACACTTCAGTAATGTC
TCAAGTATAGCTGTTTCTATCTTTAAAATATAAAACGACGACTTTATAAATCTGATAACAGGGAAAAAG
AGTTAAGTTTTTAACTATGGTAGGTTTACTTATACATACAGATGTAAGTTATATAAAATATTTATAAGCAT
GTAGGTATGTATAATAGGTTGAGTCATGTTACTGTATGTGATTGCATACATTCCTATACTTACTGTATTATG
TTTTATACTGTGCAAGTGAGTCCCCAAATCTATGTACCAAATTCGTGGCTGGTGCATTGCGGGCATTTAT
GTCGATTCTGAAGGCACAAATTCTAATTTTAGTGCTGTCAAAGCTTTCAAATATTTGTTTGAAATTACGT
ACGTCGCCGTCGACTTATGACTATTTCCGTAAGGCCCGGGTCTCCTATTTACTCCGTAGGTCGCGTCAA
GTGTCACCGCCGTAGGCCGCGTCACAATGTTCATTATGACTACGCGCCAACGTTGGCGTGTGAGAGAG
AC
>TCONS_00084255 geneID=XLOC_016896 scaffold_276:281829-290299(+) Splice junction overlap
GCAAGAATTCACTCGGGGAGACCGAGGGCTCCATACGACTGTATGAAATACCCATGCCGTCAACCTCA
CCTAAAGCGACAGAAATGAAAAGTAATGCAAATAAAGAAAGCGGTCGTCAGTTGCGGTGCTGGCGAC
AACCGTTCCTCGTGGTTATACTCCTCGTGCACATGGACCTTATTGTCTGATCTGATATCACCTGAAATCT
TTAAGTTCAATAAACTTTGTAGCGAACCACAAAAACTATAGGCTGACAATGGACAACATTTCAAAAAA
CTAAAGCTGCTATAAATCGAAAACCATTTCGAGATTTAGCTCTAGAGGTCACAAAAAAAAATGTTTTTGT
ATTTTTTGGATGTATCATTTTCGAGAAAATCATAAAATAGTAAAAAAGTGCTGATGACGTCATGCATCAG
GTGCGATACATTTTGACGATCAAAGCCTATAGATTTAAAAACAAAGTAAATGTCACTTTCATTTTTGATT
GACGTTGACGTTTTATCGTGACGTTGTTGTTTATTTGGGTCATTTTCATTAATTCTGTTCTGACACTTCCT
GACTATTTTTTTTATTTATTATTAAGAGATGACCTCTATATAATATGTCCCAGAGCATACCACCGCCTACA
CAGCTGAAAAAATGCTTCTGCTTATTTTTTTACATGATAAGTACCTACTTTCCATCATCAGAAATATCAA
GGTCATTTGAAAATGACCCATTTGTTGGTTGGCATGTAAACAAAGTTTAAATGTCACTTGTCAGTGTCA
TTTATGTTTTTTTGCGAGACTCAGACAATAGACAAAAATAAAAATTGAGCCTAATATGTGACGGCACTT
CCGGTTTTAAAATGATTAACTTTAAAGTTAAAAATAACATGCAAAAATTTATGTATATACAAAATAAAAA
AATACGGGTCCCCTAATTTTCTATAAACTTTCCGAATAACTAATAAAAATATAGGGTAAAGAAAATGAAA
TGTTGTCCATTTCATAATTGACGTCGAACCTTTGACAATCCAGTGCAGGCCTAAATATTGTTAGCAACAT
TTTATTTCCCTAAATACTTTAATTGTTTGTAGACTAAAATGTATTGTGACCTACCTACATATCTTCCTACCC
GGTTAGTGACATATCAGCCCGAAACATGTGCCATATCATCGCTTATAATGTCAGAAATTAATATGCCGCC

CCTATTGACTACCTACCTTGAATCAAGCTATCCTGCTAATTCTGTAAAAATATAATCGTAGGCTCATCCCC
GAAGCAATGGCCGAATACCTAAGTATATGAAAAAGTCTCGTATTGATATTTACATTCATCGATATTTTTAC
AGAATCAAGTATACAACTATGCGTGTAGTAGTACTATATCATTTGAATTTTAACATTATTGTAAGAAATAA
AGTAAAAATTCAAACTCCATAGACCAAAAACGGATTGAGATAGTTTATTAATTATTGTGTATTTTTACGG
TCAATTTCCGTTAAACCTTCCAAAATATTAGCTAATAAGATTAAAAGTCTTACCCCAAGAAGTTTTGTCA
TTATAACTTCCAGATTGCTAAAGTGGCTTCCTTTTTGATAAATTGTTGTGTCTGAGTTAGAAATGCGCAA
AGTTTTAAATTATTTTGACGTAATACAAGTTTAGCGCAGACCACACGCGGCTTACGTACGGCTTTCACT
TATTCGCTGCCAAGTTCTCCCTTGTAGGCCTCCTGTGTCTGGATCAGGTTTTTATTCTTGAACAATGAGA
TTGAGTGTTATCAGTTAAAACAAAAGGACTTGCTCTTTGGCAGTGTAAGTGCAAACTCCTTTTTAGTTT
TCATATTAATTGCTGGCCAAATAGGAGTACATAGGATGTCGGACCGAAATTTGAGCAGACAAAATGTAT
GGTGTGACCACGTAGTATGAAAGATATGTTCATTATTTGTAAATGAATAGGTACTTTTTCTCTTAATTTAT
TTGAGTCTATCTTTAATTCTTGGGACGATACCTATACCAGAGCAACATAATGCTACTAGGTATCTGGATC
TGATATTTATTCACGAATACCTACATCATTATTTCGTGCTCTTTTCAGAGCTCTTCCAGAATTTCCGTATT
TTCCTTATAAACAAAATAAAATGATTACATATTTTATATTGTATGTATATGAACCATATATTATAACCACGT
GTAACTGAAAGTGTATTAACACGAAAAACCTGTGTAATTGGAAAATACCTATTTCCTTCCACAGTTAGT
GAATGAACCCTTCCGTCTTCTTTATATCACTGGCGCTATTACTTTCTCCTTTCCAAAAGTCCTATATAAAT
GTATTACTTCGTAATCCATTACAAATCTATTAGTGGTTCCCGTGACGAGATTGCTTACAACGTATTGGTT
AGGGCTACAATACACGGCCGTTAACCCATTAATAATTCCTGATAACCCGCAATTTTTGAGGTTGTCAGA
CGTCGTTATTATAGAAACTTTAAGTAGTAAGAAAATTACTTCCTGTAAAATGATGCAAAATTCATTTACC
TAGATTGTACTGGTAAGTATCCCTACCCTACACAGAACAGAATGAAAAACATTAAAATAAATCATCATC
ACAAGAAATTAAATTATTTATTGATATTATAATATTACCTATTAAAAATAGTTCCAAATTGTCCTAACTAAA
TGAACATGGCCAAGAGTGAGGGTTAGCTATCGGCACGTGTGTGGCAGCCCTTAGAGATGAAAGGTTGT
ATCCAGCAATGAGAAACACATTCAGTTCACATCAATGTCGCCAAGCTTCGCTAAATATCGTAAATACCT
ACATTACCGACCTAACTTAGGTATTCATTATACTATAGTCTATTTAGTAAATGTGCCAAAAATATATATATA
CAAACTAAGTTTTAATTTTAGCACTTTTGTTAATAATGTCTAGAGGAATCGTATTTTTTGTTACAAAAAG
GTGAGTATTTATTTAATATTGTTGGTAATATAACGTTATTGTGTAAGATTAAGATGTAATTAAGAATTTAGT
ATAACTTTAATATAACTATAACTATCCAAGCTTGCCCCCACGCTTGTTGAATTGTAAATATACAAAAGTC
TTGCACCAACTTTGTCACCTACTAATGATTTGTTACATTAACCACTAAATATGTTAGCTAAGTGTAAAAT
ATGTATCTTTATTGAGAATAAAGGAATATATCACTAGGCTTAATTATACTTTAGTAAAAATTCTGTACAGA
ATATTATGATGTGTGATTTTAACATTGTAAATGAAG
>TCONS_00084470 geneID=XLOC_016949 scaffold_277:84344-86133(-) Unclassified
CAAAATGAAGATTGTATCGGAAAAGTGTCGGGAAAGCTATTAAGAATTCAATAAGCACTAGGTAGCAC
AATCTCACAAAACATACGTAGCTATCTGTGCAGGTTCCTATATCGATTTGTCGCATTCGGTTCAATGGAC
GATATTTCTTCCCGACTGCTAGTGTTGGCACAACGAACGACTGAATCAAATTTGAATGCATCACAAAAC
CCACTATCATTTAAAGCTTGCTATTCTTTTTTACATGCAAATTCAACGTATTCCTATTGCAAAGCGACCC
GCTTCTCTTCTGTTGCATTAAAATGTAATCTTGAACACACACAGCCACAGTTTATGTAAGGGCTTTCAC
TGGATGTAAATACTGCTAGCGCCATCTAGCGTCGAGTAGCAGAACTAAATTAATACGCATCATTTTAATA
TTTTGGCTTCAAAAGTGACTTTTATTAAAACTTATAGGTTACCACTAGTTTATTATTTTAATTTAAATAGT
GTTTTAATGCTGTTCTTAATACCAGCAGCAGACTCTAATGAATTCAGTTGAAACATGTCCCTTGAAACT
CTAAACCCAAGTCATTCAACATTTTGGCTTACACAAATTATTCTTTTTATATTGTGATAATTGTATTCTTAT
GTGTACTTTTTGACAATTGGCTGATTTATAACACATATTTTTTGTCCCACAAGGTATCTATCAGTCAGAA
ACGAAGGACAGGGACAGCTACAATAACGCCATGGGACATTTTAATTTGTTCGGCATGGACTCCCCGCT
GGGGCTGTTCCCGCCCGGGATGGACCACAAGATGTACCCCATGATGGACCACGACCACCGCCCGCCG
CAGCTTGACACGCCACTGTTTACCAATGTTAAGAAGAAA

>TCONS_00084544 geneID=XLOC_016936 scaffold_277:388885-390853(+) Exonic overlap (+)
ATAAATTGGTGACTGTTGTAACAAAAAGGATATTATTGAGAAACATGGGTCAGGAGAAACCGAAAGAT
ACTCCGTATGAGGTTTGCTCACATCAAAGCGAGTTGAGCTTTTTCCAAAACTTTTACATTCAGAAGCTT
TTCCATAAAAATCATTATAACTACATCTTCCGACGAGTAGAAGAACTAATAATAACAAAAGCTATCACA
ACGCCATCTATTGTCGCGTAGCTATAACTAATACCCATCTCATGATTGCAAGATTAATGAAGTTTAAAAC
TGAAATGGAAAGATGTCGCTACACGTGGCAATTTTTGTTATTTTTGCTTGATATCCTGAAATAGACCATG
GGAGAGTCCAGTGGCCGGGTGTAAATCATTTTATAATTTGCGATAAACCGTTTAAAACATGACACGCTT
TATTGA
>TCONS_00084295 geneID=XLOC_016937 scaffold_277:426306-439427(+) Exonic overlap (-)
TAATGACTCGTTTTGTTTAAATAGAAGAAAAAGTATGAAAGTTTTAGATACTTAGGCATAAAAGTGAAA
AACATCTTCAATCCTTGTGTATGTAAAAAGTGTAGCCTGATTAAAAAGAACTACCTATAAAAATTGCAT
AAAATACTACCTTCCTTGTGCCACTTGCTGATTCTCTGCATACGATATACATAGATTTTTCTATCAAATCC
AAGTCCATTTTTCTTGTCCCCATTGAATTTCAAATGTTGTCACCTTATAAGCGTGGGTGTTTGTTATTTTT
GTGACTATCTCCCTTTGGATACAGAGTGAAGTGGTGTTGCTTATTTTTTAACTTGCGTCAAGTTTAAAA
CTCAGAATAATGAGGCACTTCCCCAT
>TCONS_00084321 geneID=XLOC_016953 scaffold_277:556479-573143(-) Splice junction overlap
CGGCCGCGGACGCCGCACGTTCCGTTCCCGCGCTCTTATCATTTGTTTTCGCGCCATATTCCACGATTTT
CTTTTTCAGCCGCTTTGCTCTGTGTGCCCTTGGTTTTAAAATTGGAACGCGTGAAACTTTTTTGTTTTG
GTTTGTGCGTGGATCGTGTTTTGTTTTGAGTGTGTTTGTGTTGTTGTGGTATTTTTTTGTGAAATAATCA
TGAAGTTCAAACTTTTCAAGAAGGCTAATCGGCAGGAAGGCCCGCACTCGACTCTACCGGACGATGA
GTTCTACGATGCCGTCGAGACAGGGTTCGACAAGATGGAGCGGCAGATCTCTCGCGTG
>TCONS_00084547 geneID=XLOC_016942 scaffold_277:597741-603201(+) Exonic overlap (+)
GCTTTGTGGAAGGTTGTGCGTCGCCTATATTTTAGTGAAGTTTTAACTGTGAAAACTGTTTATTTGCGT
GCCGTGTCGTTAGTGACTTGAGTCGCGAGTTATTTGTGATAGTGGTTCTTCAAACTGAGCTTACATGAT
TGGATTACCGCGTAGCGATAGAGATAGAGATCGAATAACGGTGAAAATTCGTAACATGAAGCGGAGCT
CGTCAAGGGACAGTATGCCGAGGTCGAAAAGAACGCGTAGTAGCATAGGCAGGTACTGACGACCCGA
CCTGAAACGACCACTCAAGTCTACTACGTGTCTTCGAAGTGTTACTAAAATCATCTATCTTACGTATAG
GTAAATTATGAGAAAAGAGGAATAAAATCCGTT
>TCONS_00084417 geneID=XLOC_016956 scaffold_277:601861-602836(-) Intergenic
GTACGGTAAAGCAAAATATTGTGAGGAAACCTGCACATCTGGATATTTGAAAACCCCACAGTGGACAG
CCAGACCTTAGAAGGGACTTTAGACCACTAAGGTTCCATTCGAGTGGACCAGGTGATGGCGGGTCCA
CCACACCCCCCACAAATATTACTAAGATTTGGGTCTTTTTAGAGTAGTACTTAACCATCGCCAACACGTA
TCTAGGAAATAGAATTTCATGGTGGTAAATTTAAATAAACATTCAAGCGAGTTAAGGTCGTCTAGGGTC
ATGTCATTATAAAGGTTTGGCAGTAGTACCCATACAGGTGTTCCCACGGCATATTTAAGTGAATTTTATG
TACTTACAGAGACTTACAGGTACAGTGAGTTTTCATCCTGAAGTGTGGTTGACGAGGGTGGCAGGTCC
TGGTGACTGAAACCTACCTATTGTTGGCACCTACATATACAGTGGGTAACGTGCTAAAAAGTCAGAATT
TGTATGAAGTTACGTTAATGTAGATAAAGACTTATGGTTTTTAGTTATGTGGCTATGAATAAAACTATAA
ACTTATGTACTTTGTATTGTATTCAACCATGAAAATGTGGATTTATGTAGCATAAATGTGAGTCCAATGC
CTCATTTTATTATTATTTTGCTATTTCCTCACTCAGCAATGATAACTTGTTAATTTCATTATGAG
>TCONS_00084562 geneID=XLOC_016958 scaffold_277:638954-639367(-) Exonic overlap (+)
ATCCATTTGAAATCACAAATACATTTACGTGGGTGACTGTGATTGTTCTAAATGCTTAAGTGCTTTATCT
AGCCCTCAGAGATAAGATGTGTACAACGACATTATAAAAGATTAGCGAGTGGCGTTGTATATCGTTGTT
ATTTTTGAAGTACTAACAAGGTAAGTCCAATATTATTGAATTTTTATTTGTTAATTTGTGGCCCCGCACCTA
CATATAAGTGTATTATAGTGACAAAATGAAACTAGTGATAGCTCTAGCCCTGCTGGCCGTGGCCTCGGC

TGACATC
>TCONS_00084566 geneID=XLOC_016987 scaffold_278:13681-15261(+) Intergenic
NCGTGATCCGGTGCCCTGTGTGTGAGCTGTGCGCTAAGATGTCGTACGATTCAATGGAATGTTGTGCTT
CATTTCTATTCTAGCTATAACATACTATAGTGTGATGTGCATGGTGTTAATTGTAGATGGAGATTTCACCG
ACGCTTCCTGTATTTTTATCAAATGTAGAGCTTTAAAACAGAGATAGGGTAGAGTGCGTATTAATAGAA
AAAAATATATATTAAAAATGTTAAGTTGGAAGGGCTCTCAGATGTCTGTATCTGCCTGGGCCGCGGGGC
CCCGCTAGATGAAACTAAATACAGAAATATGACTCAATCGTATCGTAACTCAAAAACGACCTTATTATC
GGATGAGGATGATATGTTTTAGGCGGTATAAAAGCTTTGGGGAGAGGAGCGCGGCGTGAGAGGGGCT
GATTCTGGATTCTGGGGCGTTATCGATTGATGGTGGCATCACCAGGCGGCGGCGCGCGTCGCGGGGCC
GTGGGTGGGAGCCACGCGCTGGGTTTATCGACCCTCGCGCACCCAGCGGCCCTAGAACCAGCCG
>TCONS_00084650 geneID=XLOC_016998 scaffold_278:73161-76169(-) Intronic
TGAAAAAGCTCGACGGCATTTTAGACACGGCAAGCGATGACATAGTGGTTCCATTTATAATTACGGTCA
CCGGCGACACTTCTGATAGTGATGATGACGAAGACGAAGATTTAAATTTGTTTTAATAAACACTTTTTT
ATATATAATCAGTTTTATTTTATAGTCTATGGCTTATATATTTAATCTAATTAGAACACTATGACATCACTAT
GGGCATAAACAATACGCTGTCAATGTCAGTCCGCCATATTTGTTTACATGATTGTTGGATTCTATTGCAA
ACGACTATACTTACCACTAGACCACAGAGGCAGTTATAAACCCCAAGCACAGAATAATAACTAGTACTT
ACCCAAAGTCAATAAGGAACGGTCGAGACGAGGGGGTACTTTGGCACCACGCATGACTAGGCTACGC
CCGCACAGGCTTAGCGAGTCACTAAGATAATAGCAAAGTAATACGAACGTTCTAGAATCTTCGTGAAT
GGCCGCATACTCTTTGATTATATGGTCGAAGCGCATACTATGCGCGCAATGTGATGTACTTGTGCTTGTG
ATTGATGTATGAAGGATGTATACATGGCATCATGACATAGAGGCATAGAGGGCATAGATCTTTGATTTCA
ATCAGAAAGTGATCAGAAATAACTGTTTAACAATACTGTTGTAAGTATATTATGATACAAAAAATATACC
TACAGCTACAGATTGTTGGCACACCCGACAGATTGCCATGAATAAAATTTCAATTCAATTCAGTGCTTA
AAAATCTTAATATTATGTCAACGCTTGTAAGTAATTTCATACATAACCTACAGTAAGTACTTTAAACCCAT
TCCCATTACGTATGAAAAGCATAAGTAAAACAAACACCTGTAATACAGTAATACAGTCCACTGCAGGG
CACTACATGGACCCTGGAGGGCATAATCTCCACTCTTGGCAGGCGGGTTTATGATTTCTGATAAAAGAT
GCTATGTTTGTTACATAGCAGCACAACACATATCAGTGATGGGATACGAACCCGGGACTTTCGGCAAGT
AGAGGGTTTTGAATCGGATGTCAGAGACGAGGGATCCCCATATAAAAGGTTTAGTATAAGTACCTACCA
TTTATGAAAATAATAGGAATCTATCCGCTCGATCCTTCCATCCTACTTTTAGTCTCAAAACTCTCAAAGC
CTCCAGTCCAGACCTTCACCGAGATAACGCTAGTTAGGTAAGTAAGTACTAGTTATTATTCTATGACCTA
GACTCCTCGCAATACATCGTGATAATCAAATATGTAAGTAAGTACTAGAGTGTCACTCAGCTCTAGAAT
CGCTTGCCTCACAAAACAATACCATAACCCTCCAGTGGATAAAAGGGCACAGAGCGGGT
>TCONS_00084884 geneID=XLOC_017044 scaffold_279:90838-91681(+) Intergenic
TGCGGCTGCGACCTCTGTGCGGAGGTTGGCGCGTGACCTGCGAACAAGGAGTTCGATACCACACCGC
TGATTACAGCAAAGAGGTTATCGCTTAATCATTGTGACTATTCATCAAAAACATAACAATGGCAAATTCT
AATACTAAAATTGAAAGTGATAAAAAAATTCGCTGATATTGACGATGAACTAATAAAAATCAAAAGACAT
GAAACTTTAGAGGAAACAAAAATAAAAGTAGAGACAGACGAACCTAAACTAGTGATAAACTTAGACG
GTGAAGTGAGAAGTGGTGATGAAAGTGAACTAGTGAATAAGAGTGGTGATGGTGATGCAGCTGATGA
AGGAAGAAGTGGGAACCAGAGAGAGAACAGACTCACCAGCATCATCGACCAGTTACGAGGAGCCAA
C
>TCONS_00084886 geneID=XLOC_017045 scaffold_279:117072-120345(+) Intergenic
CCGTAATTACACAATAGATTTTAATAACTATAGATTATAGCGGTGTTATTTCATTTACTCAAAATATATTAC
AACACCTAAAAATAAAAACATAACATTTTCATCTGTTGTTATAAATTTTTATAATTCCTCATGTTCCGTCA
GTCATGTGAGTCTCGGATCGAGCGAGTTTGTCGCTGTATTTTCTGCAATTTCTAAACGATAAAAAACGT
GTTAATTTCGAATTTAGAGTGCGGTTTAATGCGTGGTTTTTTAACTGTTGATTTCCGGTGTGTGACGTGC

GTTACTAGAACTGTTATCAATTAAATAGTTGTGTAAATAGTTAGGTTATCTGCGTAGTGACTGTTATCTG
CCCGCGGGCGTAATTAGAAATAGTGCCAGCTACGAATACGAAAACGAGAAATGAATAAGAAAGGCAA
GGGATTGTGTTGTTGGTGCTTTGCCAGCCAGTGCAGAATTTTATCAGTGATAGTAGATCAGTAGAAAGT
AGGTCAATGTAAACGGCATTCTGAACAATATTTGTACTTCCGCAGACGATACATACG
>TCONS_00085091 geneID=XLOC_017046 scaffold_279:156127-161695(+) Unclassified
AGCGCCGCCAAAATCGAAACCAGACTCCGTACCAGCCGTCGCCACAAAAACACAGTGATAATAACAA
CAACAACAAAAAAAACAACAACTTGTAAATAACAAACAAAAAAAAACAACAAGCTGTGAGAAAAAC
TTGTCTGTGTGTCTACTATAAGTTACTTAAACAAAACATAAAAATGCATTTTTTTAAATAAAATATAAATA
ATAACATAAAAACTGAAGTGTGTTAAAGGTTAGTTAAATGTAATATGGCTAATACATAGCTCGGTTAGGT
GTTATATATGATATAAAAAAGTTCAGTTTTTATAATTATATATGATATGAAAAGTGGTGTGATGTGGATTG
GAGGCCGGATCGGATGCGGTGCCGGTCCGGTTCGCCGTACGGACTCTGGATTTGCAGGCGTTTATTAT
GTGGAGCCTAGCTTGTATGCATCGACAATAAGCGACGCCGCGCGAAGTCATCATAAATATAATTATTATA
ATAAATAACATATTGACTATAAACTCGTACACAGAATACGCTCTAAGCTTAAGCACCATTAAACGTGTAT
AACTATAAAATATATATATTTTTGACAAAACATGTAACATATATTATATTATATCTATTATAAAAAAAAATG
TGGTAACGAACACAAAAAATAAACCAGTCACACGCGAGGTAAAAAAACTAGTGATTTTCTTTTGTTTA
ATTAATTATAATATATAAATTTGCAACAAGTGAAATTTTAGCGTGTAAGTTTTTTTTCCTAATTATAATTAT
GCTTGTGTAGTTAAGATTTTTCGGAGGTGGAAACACATTTTTTTTTAATGATTATTATTTCGTGGTGCTG
CGAATTTAGCTGGCATGTTTTTATTTTAATGTTTTCTTCACAAAAGATTATAGAAACATGTCACTTAGCTT
TTCGTCAATCAGCCAATTTGCAAAATCGCAAGGGAGTTATTATTCTGAGCCCTGCAGTAGTGAGGGCG
GGATGTGTATATTCTGTGTATAATAATTTTATTGTTGTTGATAATACCGAACTAGAGTGCCACAAACTTAT
CTGTTCCGGTGACGGCTCACATAAATGTCTAATATTCCTTCATTCTATAAGATTTTAAGTTTGCAAGTAG
TGGTAATTCGCTATTGTGGTTTCAATAAACCCATGTAATCTATATCAATATATAACTCATAAGTAAATAAT
GAGATATGGATCAATTTAGTTCGCTCTCACCGGAACAGATAAGTTTGTCAGACTGTACTAAAGTGAAA
GTTGATATTAGTGTCTTGCTGTATAACTAACGAGTAATTCTAAAGCAAACATTGTTTTACTGTCTCTAAA
AAGACTGCAATGCGTATGATTGTTGAATTTATTGAATCATAAAAGATTATTATCGGGTGTCATTAAGTTA
GGTATACTTAATAATAATGTTTTGACTAAACTCGTATAGTTTTCCTGTTCACGAACCATGCGGGCATACA
AAGGATTATTAATTCATCCATTCCTATTTTTGGCAAATTTTGAGGTTAAAACTTCTGTATTATTTTTTTAA
GGTCTGTACCTACACTTTCATTACTTTATGTTGATAAATCGTCTAGGCCGATTACATTAGCTTAGTAAAAC
ATGTAGTCAAAATCAAAAATTCAGTAAATATTGCACAAAAACGGTCTCCTTTGCATGCCCGCGTGCTAT
ATAACTGCCACTATGTACATAGTATATGACACAATTGAGCACATACTAAGTTTAAATGTTGATAAACGAA
TCTTCTGGTTTTAGCGTGATTGTGAACCGAAATTTTGTATAAACTATCAGTAACAAGTATTATTTTTAAG
TCACTTATGTATAACAATATTTCAATTTGTTTAACAATTTGAGCGTACATGCATTTTTATTAATTATGTTTA
AAATTAATCGCCGTAACCGTTTACTCTCCCGCTAAAAGTTCACAGATTAGAAGTTCAATGTTCACAAAT
AATTTTTACTAATTTGCTCAATGTTATTTTTTTTACTTTTCGTTTTCTCTAAGAGTGTGTACGGTTTAAGA
AGTATGTCTTTTCATTTGTTTGTCTGTTGCCCTTATGCCTTAAATTAATTTAGGTTGGAGCATTTAGTCCC
AGAGTTTTCCAGTGACAAACAGACAGATGAAGGGATAACGTGGTGTGTTGTTAATAAAAGTCGGATTA
CTATAAACTCGGGGTAAAATAATAAATTTAATTTAAGCTAAAATCATTTTTCATTTAAAGTTTTGTTGTTG
CAAAAAAATATATCAACATTTGGGAGCAAATAAGTTTAGCCTCTGCTTTTCAGAATTACAATCTGTATGT
TCGCGGTGTTACATTAATGTGATTGGGAAATAATTAATAATATTATAAATATTTACGTGGTAAATACTTTG
ATATGCACTAAATTACTAAGGAAAGCGACAAAGCAAGAATACAGGTTGTTCTAAGGCGAAAAGCAGT
GTCTTTACGCCTTTTTTAACACCCTGTATTAAATAGTGTCTTTGTTTATTTGCTCGGAAGTGTTACTGTAA
CATTTAAACTTCAAATAATAAAAACTTAGACAAGCGCATATTCGTCATCTAGGATATGGTTTTAGCATTT
TGTTCTTAGCAAATTTCTTTGAGTCCTAAACATACTTTTTTTTAATAAATGTTTTAGGAATAACCAATTAG
ACTCACAGAGTCTATTCGGTACTTACCCTTAGACTAAATTGATCTTTTCTAAGGCAATAAAATATCGCCA

TTTTGCGCAATTAATTAAAAGTGATTCGCGTTTAAAAAAGTAGGGAATCATTCGTTTTGAGGTTATGTC
AACTATTTTTCTTTCTTTATTTTTTTAATTTTAAGTCGATAGTGTGCTTGACCAGAGCATTTGTGGTGTAC
TCTAAGAATATGCATAAATATAATATACACAATACAAGTCTAAAGCGATAAATAACGTATAAGTCACGCA
GTTTATATACGCGATAAAATTCATAACACGACAGAGGCAAAATAGTCCAAAATTCATTCAAACTATTGG
TTATCGTATATGTATACCCTTAGTCAAAACTATTCGTAACCCATTAATATTCGTCTTTGTATGCCTTTCTAA
ATTTAAAACTGCCACTAGTTATTTGGCGAATTTCAATATTTTAAGCAATATTAATAAATGTTATGTAGTAT
CATGTGCATCGGTGGTCAATTTAACTGTATGTTGATCGTGAGTTTTAATAGTTTACGCAAACGGTAAACT
TTGCGTACTCTGGAAAGAGTCTGCGGAAAGAATCATAAAACTTATGACATAAGCATTCGCACTAAAGA
GGTGTAAACGCAAATTTTACAGTTTTCGTAAATTATGAAAAACTTACGCTAAACGTACACTCACCGTAA
TGTTAAAGAAATGTCGTAATGTTTATCTTTAGGTAGTAAAATTTACTAATGAAATTAATGAAGAAATGAA
TTTAAATTCCACTGTATCCTCAAACAAATGTATAACTTATTGTAGACGTTTATTAGTAGGTTTAGAGTATC
ACTCGCAAATGTTCTGTTAAATTAAATTTAGAGACTAACTTGTTTAATAAATAAATACAAAAAAAAACT
AAAATAAAAATGTTGAAGACATTAGCTAGGTCGCTTCGGGTTTTTTTCTAAGTTCCTATTTCACAAAAG
CCATAACATTTTAATCCTATATTATAATTTTAGTCGCACAAGTTACAGAAATTGCAAATGCAAATATTACT
CAAAAATATATACTCAAGTATCAGAAATCGAAAGTGTAACTTGCAATTTCTGCAACTGTAAAAAAAACCG
TTGATTTTTTATACCGTCCCCAAAATAATATTTAGATCCTAGATTGCATAGCCATTTCAAAATAGTACGGT
TATATGATTAGAAATAAATAAAATATTGCCTATAAGTGATATGAAGCGATCTAATGCTTTAAGTTATGACA
TGACATTGTGTTTTTATCTGTTTTTATAAATAAAAAAACATCGAAAACTGTAATACAATGATGAGGGCTT
GCTTATAGAACCGAATGCAAAAAATAAGAAACAAGTATCTTTTTCATAAAGTTTCTAAAATACGTTGAG
TTGAGAATTCACTTTTTTATTCATAAAAACAAATGAAAATCTCAGTGTCAAAATAAAAAATCTTGGCTT
TTGATGCACTATTATAATTGTTAAGTTTTATTATTATTATTTCAACTGTTGCTATTTTTTTCTTTTCTAAGTA
CGCCAAAGGGGAGGATGTGCTAAAAAGCGTAGGTATACTGTATAAATGAAAAAATATACAAAAAAAGT
CTTGCTATTGTTTCTAATTCTAAGTGTAGATGTTAAGTTAGTGTATACGGCTCGTGCATATTATTTTTTCA
GTGGAAAAATAACGAAAAATATATGGCAATTTGGTTGTTGTAATATAGAATGTGTAGTACTGCATCGGTT
AGTCATTACTTAGTTAGGTTTCAGTTTGAAAGTTATAAATTAAGGTAATTTTGTAAGGACGCTTGCTGGC
GGACGTACGGGCGGTGGCAAAAAATTGTGTATAAAATAACTGTAAAATCCTAATTTGATTATTATAGGC
AAAGGTGTACGTTTTCTTACTAATTTTACTATGCATGAAATGTAAAAAAATCTCCAATTGTTTTTTTTTG
TACAAAATTAAGAATGTCTTTTCGGTTGTACTACTGTCGCCACTGTCCCGCGTCCGCCCTTACTGGCTG
TAAGTTATACATTATATACATATTATTATATTGATAAAATAAATTAATAATAAAAGCAAGTTGTGCCGTTTT
ATTCTGTATCCTTCGGTTAAATTATATTAGAGACTTTCCTGAAAAATATTATCCAAAAGTTAGATATTACA
CATTGCGTGCAG
>TCONS_00084916 geneID=XLOC_017060 scaffold_279:162064-163238(-) Intergenic
GATGCGTCAACCAGTGGCGATGGGGTCTAATTAAAGAAGTACATATTGGATGCTTATATATAATATAAAT
AATTAATAACATAAAATCTTAAGTTTATGGTTGTAAAAATAAGTTATATTTTTTAAATTAATCAAAAATGC
TAAAGTAGGCATTAATGTAACCTAACTAATATAAAGCGATGAAGTTATTTATTGCAAGTTCAGTTTAAAA
TAAATTTTAGTTTTATATACATACAGTCTTAATTTAATACTTATTTTAAATATGTTTATTTGGGGGTCCAGT
AGTTTGCAAAATTCTGTTGTTCTTGCGCTGTTTTCGTTTACAAACTTATTTATTCATTTTGGAAATTCATT
AAAATGTTGTTGGAAATAAAGTTTATATATTTTAGTATACACCGCTGAGTTTTATTTAAATGTGATCTTAT
TTATGTAAGTAGCGAAGCAGAAGGTACACAATTTCAAAGTGAGGGTATTTTGAAAAACTACTTATAGA
CATTGTCAGC
>TCONS_00085033 geneID=XLOC_017061 scaffold_279:278148-279411(-) Intergenic
AGCTTGAATCTATTGTAAATATGTGTTTGACAGGGTGACATTTGAAACGGTATAATTATTAAAAAAAACT
AACAATGTGTGCGTATGACCTAATTCTTACAAGCTAGACTTTCTAACTATGAGATACAAAAATTCTAGCT
TAATTTAGATTTTAGTGTTTCTCTGTCAATGGACTGTTAATATGAAATTGGCATACTTACTGCCAATTTGC

TATATAAAACATAATGATATCCAAAATATCAGTCAAAATTAAGGCTCATAAGAACTCTGTTATATTTATGT
ACATTAACCTACAGAGAAACCTGACCCCTCTTAGATTGTCATTGCTACTGACGACTACTGGTCAGGTTT
CCTTGCAGACTGTACTACAGAAAGAAAAAAAACATTGTTATTTTAGTTTTTTTTACCCAGCTATGGTAA
AATACACTTAGGTATTTTCTTTGTTGACTAGCGTGGTCTTTAAACAACATTTTTTATATCAAAACCGTATT
ATTTATAATCTTAATTTGCTTATCAATTATTTAACTTGATAATTAAAAGTTAATAACCCCGATAATAATTAT
GACAAATAATTAAGGAATATTTATGTTGAAGGCAAGGTCCGATTCTGAGGGCCGAGTACGAATTTTTCT
TACCGTTTGAATAGTGAAAAGAAACTCTCAGCAAAATAAGGGTCAGAAAAATCCCTCACTCATTTGAA
TTTTACATCTATGTATAGTCTTTACATAAATATCTGACAACACAAATTGTTATGTCCACAGACGGACATA
GGCCACAAAATTATGTCCCCAGTCTTCGTCAGGCATAAAGTGTTTATTAATAATTATGCATCAGGCTACT
GTTATGTTGACTTAGCAATATTGCATTTATTACTTGAAAAAATCTTAACATTCCGGCTACTAAATTGAAA
ATTAAAGTTTACACAGTTAGTTTACAGTGAGTGACAATGCTACTGGTCAGGTTTCTCTGCACCGACCTG
TACTTAAGATTAAATTTTCGTTATTATACAGGGTGTTGCAGCAAGGGTATACTAAGCCGAAACCTACATA
TGCAGCATGGTATTG
>TCONS_00085045 geneID=XLOC_017049 scaffold_279:293587-294317(+) Intergenic
CAAACCATAGTCTAGCTATGATGTTTGTCACCGACACCCTATGGAGAACAATTTTAATCCCTCCATTAGT
GTTTAATACAGATGATAATCAATCAATCCGTAATATTTCAACACTGCACGGGATGAACCGTTCCGATTAC
CTTTCATCCATCATTTTTCCAAACGAAAAACCACGAGAAAGATATTCTTTTTACAGTCAAATTATACCAT
AAAGTATGAAACTTACTTGATAAGAGCGCCATCTAGCGCTTCTAAAACTCTTAAACATTTTACTCTGTG
CCAAACACAATATCGTACAACTTAACAACGCCATCTATCTATCTAGCCATAAAATATAACATCTCGGAAA
AGTTAAAAAGGCGCCATCTAGGCGTCAACAACGGCACTGCTTGGTGTTTTTAAATGACATTTCTTCAA
GAAGAAAATAACTTCTAATGCTTAACAACGCCATCTAGGCGTAAAATATAAAATCTCGGAAAAGTTAAA
AAGGCGCCATCTAGGCGTCGACGGCGGAACTAGTTGTTGTTTCTAACGGCATTTCTTCAAGAAGTTGA
GCACTTCCTGCGGGTAGTTCTGTCTGGTTGTCGACTTGGTGGCGCTGCGAGCGTATTCTAGCGGCGTC
ACCGAAC
>TCONS_00085655 geneID=XLOC_017099 scaffold_28:364430-366001(+) Splice junction overlap
AACAAACCTCAAGATGAATTTCTTCTATATACTGTCGCTGATAATGATAGTGATGTTGATAGCTAATATCG
GAAGTGTACACTGTCAGGGAGGGGGAGAGGGCGAGAAAGAGGGAGAAGAGGGACTGGGAGAGTCA
AAGGAAGATGCAATGGGAGCTGAAGGGGAACCGCAACAGGGAGACCAATCGGATTCTAAGGAATGA
GGAATTCTATATTTCTAAGATTCGCCGCAGTCTCTGTGACGTCAAAATAACACAACAATAGACCAAATG
AAAACCAGTG
>TCONS_00085493 geneID=XLOC_017133 scaffold_28:785712-790278(-) Exonic overlap (-)
GACCCTTCGTGTAGATTTGAAAATAGTATCGAATCCTATGCTCGCTGCACTGTAAGTTTTTTTATGAATA
AGGGGATTAAGCTCTAGTCTAAAGGCTGAAAGCTGGTACTTACATGATTAGATCAACCTAGTATGAAGT
AATTTAGTTTAGAGTTCGATTAATAAGGAAGCAAGTAATGATCTAAGGTAAGTAAATATGAAAAATAATT
CATAAATTAAGTCTTTTATTCAGAAAAAACGATACAATATAGCAGTTTAACAACAACACCATATGGAAA
TTTTTATAAAGTATTTTCTATTGTTAAAGTAAACTTAGTGGGATGAATAAACAGCCATTTGGGGGATAAT
AACCCCCAAATTTAAATGTCTAAAAAGCAGTTACAATTTAAACATAATATATAGTAATAATAAATATAATA
TGACAGTTGCAATAATCAATATGAAGCCCTCACTGACTTGAGGCGGGGGCTGCTCTGTCCTTGAATAAT
ACTAATTTATATACAACAGTCCATTCACCGCATTGAATTTACACCTTCATAGTAATAAAAAATAATAAAA
AAATGCACGTAACAAGAGCGATGACCTCGTCTAAATATACGGACACAAAACTACGACAAAAGTGCTAT
GAATCATGACCATGACTTCTAGTTTTACATAATAGCGAGCGGCGGCGTCGCAACAACTAACATCGCCCT
CCGCCAGAGGGCACCCTCACTAGTCCACTGCATCCTTCGCACAGTTCCTCGCTTATTCTACTACATTAC
AATAGTTGAAACCAAACCTAGTACATAACATTCCAATTAGGTATAAAAGAAACATAATGTAGGTAATCA
ACTTGTAGTGCACTCTCATGGACTTACAGAAAGACATCCCCCTAAGTTATCCATCCGAACATGCAATAG

CAGAGAGCTTCGCACAGTATGACGAATCTACCAAGATTATTCAACATGATTACATACAGCCCCTCTAGG
AGGTTAAGTTATGATCTAAAATAAGTTATGTGAATATCAATTTAGTCGATAGGTATATAATTATGATGTGC
TTAGTGCTTATAAAATGCTAGAGATCAAGTCATCCTTTGTAGAAAACAAAGCATACTTTAAGATCAAAG
TCGCACAGCGTGGCAGTAGTGAATATTTATGGTGTGTATTATAGTGATAATAATTGTGTTATAAATATGAA
GTCACCATCAAAATAATGCACATATATACGATAATTCAACAAGGTAATTACAGTGTATTTGTTATCCTCTA
TTTAAGTCTAAATGAGTCGATTTATTGTTAGTAAAAACTCGTAAATTGATGAACGTTTACAAAATATCTG
GAACTCAAAAACGAGACCATTCTATTTTACCAGGCTATTTTACGGGTTAATTATTCTTTTACAAATATTTT
AACCATAGGCACAGATAATATAGCCACAGGTAATATAACATACCGTGGAACCTATTATATTAAGAAAGTG
AGGTAATTAATATTGCGATTGCTTTAAAATGTACCTATATTCTTCTAGTGTCCTAGCTTAGAAAATGGTAT
AATTTTATTTGTTTTGATGGTGACTATGCTAATATAATATATAACTATAAAATGATATATTGACTATGTAATA
TGTATCTCTGTATTAATCTACATAAATATATTGCTTTTACGTGCTTGCTAGCGTGGTAGCGTCGCAATGCC
GGTAAAAGCAGTGCTGTAAATAACATTTTATGAAAGCACTAGTATAAGGCTGTCACTGATGTGTATTGA
ACTTCCCCAAAAAGCTACCGAGGTTATTGTCATGCGGCTAGTGAGAAAAATAAAACTTCCTTCAAATAT
TGTTAAGATCAATTTAGTAAAAAGAAAAAAAAATATCGTAACAAAAATATAGATGTAACTAATAAATTA
AATCAAATTCGATGTAGTTCAAAGTTGAACTTAAAATAGATATACATAAAATGGGTTAGCGTTCGCAGA
ATGCTACCGATTTTTTTTGATGTAGATTTATATGCAGCCACAAAACTAATAAAAAAACACATATTATGTA
ACCTACTTTAAACTGCCTGCAGTCTCAGTAGATAATAGAGAGATGAAACTCTTTTATGTTAGCAAGGCT
ATGGGCATAACATTATAAGACCGTGTCCTTTCCTACGGTTTATGTTTATTGATTAAAGATTAAAATATAAA
TGAAATGTGGCTGCAACTATTCAAATGACAATGTAAAATCTTAGCAGTTCATTATCAAAACCCTAGATC
TTGTCTACAAGTGATTGACTGTTTCTCCAGGTTACAAGTACCTAGTCAAATCGTTGTTAGAAATGTGAA
GGTGAACAAAAATAATGTTAAGAAAAATGAAGTATCATGTCTGAGTCGGCTTGTTGGTAGGAAGTTTT
GGGGATTATGTTATTTAAATACTGTCTTACAATACAAACACACAAAATTTGTACACAACAAAAATAATTA
CTGTGAACATAAAACACATAAACTGTTTGCTTACTGTAAAAGCTTAAATATTTTAGAATATTTTGTTATT
GTTATATTGCCCTCTATTTGAACTCTACATTTATTAACAAGTTTTTAATAAGTATAACATCCTCTTTCAATA
TTATATTTATAATAAGTATAATAAGATCTTATAGAAAAATTCCGTCAACAACTAGTGTTGTGACATTCTCC
CGCAAATAACTTGCTGAATATACAAATTGCTGCTCATTATGCCTGACTATCTGAAAGGACTTCATTTTAA
CGTGTAGATAATACTCTATACTAGCCAGGTACTCGGTTTCTCTCGCGCACAATCCTCAACATGACGGAC
ATTGTAAAGCTGTTGTCATGAGATATACAGCCTAGATGCCTCAACCACCGTCCCATGGCATATATCTCAT
TATATATATTTATTGAAGCAATATGAATTTACGCTTACAATAAAAATATTACAATGCCAATGTTGTTGGAG
TGAAATACCAGATATTTTGACATAAAGAGCACAAAGATTCAAAGACACATTGATACACAGCTACATTAA
GATCGTCCACACAAATTTTCAAATTCAATACATATTTAACAAGATTTCTACTAAAAATAAGATCAAGCCA
ATCCGTAAATATAATTATAATAATATTTCTGTGTAAACCAGATTTTTCTACTTAATCGAAGCATAAATCAG
TTGAAGCAAACCCATTTTCATAAGTATTATTTAACACTCAAAAACATCTAACCATGACGGGGAGGAAGT
CCACAAAAACTTTATACAATTTAACTTGATTAAATAATGTGATCTGTACATACACAGGGACTCTATAGCA
GGANNNNNNNNNNNNNNNNNNNNNNNNNNCAGGAATGCAAAATGTTTAAGCGAGAAAGTTAAGTTTA
CACTAAACATTTATTACAAAATGTAAAACACGAGTATACCGAGACACTACATACAATGTTAATAGTTTCG
TTTGTCTAACATAAGAGTTCACATACAGCCTGCACATTGCACAGGTGCCGAGGTGAAGTCAGTAGTCG
CATAGAGGGGTCGCCAGGACGACTGTGGGGGGTAACAGTTGGAAGGGGAGCGTTCACAAACCGGTG
TGCGAAGTAAAGTTCGGAGCTAGTAGAAAACTGTGAAGCTGGGTCGAGTGAGGAGTGTGTGGGATGC
GAGCGGCGAGCGGTCGCGCGTTCACATGAGGAACTTGCCGATGAGGATGCCGATGAACGCCATGCCG
ATGGACATGGCGAGCCACGGCATGAGGGAGGCCTGCGCCGGCTT
>TCONS_00085685 geneID=XLOC_017115 scaffold_28:1164428-1165085(+) Intergenic
TCTCATCCTGCATTAAGAACCAACTCTTTATTCTCTGATGGACCAGGGCCTGTGGTCATCCAACCAGTC
ATCGTTGAGTTTATTAGGTGGCCGAAAATTGACTCTTTGAAGAAAGCGCCTAAAGTAGAAGTGCCTATA

GAAGTAGTGCCTTTCAAGGAGGAGGTTACGCTCAATGATTTCGGAAACGTAGGAGGAGACTGTTTTTA
CGATTACTATGCATAAGATTAAATACTACCATTTTAAAATATAATGTAACCCCATAGTAATAATATTTTATA
AGTACAGTTGTAAGGAATGAAAAAGTTTTTAAGTTCCAACTGACCCAAATTATTCAAAAC
>TCONS_00085687 geneID=XLOC_017116 scaffold_28:1180872-1181431(+) Intergenic
GTGTAATTGTGCATGGAACATATAACAGAAACGTAAAAATAATGATAAATTTAGTGACTCTACATTTTAA
GATTCACATACTATCATGTGCAATCACATCCATCCTTTGTACAATAGACGGAGTAAGTTACATATCCGCC
AGCCCTGGGACGCCAAAAGGCTACATAACATCTGTGGACGGTTCACAACGCAGGAAAGACGACCCGT
GCAACTCGCTGTACTGGTATCCTAAGCATGTGCCGGACTATTGCTACAACCGGACT
>TCONS_00085264 geneID=XLOC_017141 scaffold_28:1192623-1213650(-) Exonic overlap (+)
GTGCGCTATTGTCACGCACACATCGACAACTCTACCTGCGCGAGAGTCACATTCCGTCGTCCACCTCA
GTGATTGGCCGAGCCTCCGAAGGCTTCGAATTCGAAATTTAAATTTAGAAAGTACCTATTACGTTAGTG
CCGTTCTGTGTATTATATTGAAGTGCAATAATAATGGTGAAAAAGTTATAGCAGGCTCAGGCATGCATG
GTCTCCCACTATGCCTTCTGGCGCTGGCCGCGTCAGGCTCCGCCCAACTGACCCTCACCGGGGTCAAG
TGTGGTCAGCTCAAGTGTGCCGCAGATGACTACTGCTCCCCGGACACTGATAGATGTGCGCCCTGCAG
C
>TCONS_00085481 geneID=XLOC_017120 scaffold_28:1346157-1348337(+) Intergenic
CCCTTGAGTTGTAGTATCTTGAATAGTAGTGTAAGTAGGTACTGTTGTCCAAGAACCGTCGGTAGTTTG
GATAGTTGCGTCAGTATCAGGATATTCTGTTGTGTTTGAATTTGCGGTTGTCAAGTCAACTTCAGCAGT
CGTTGTTTGTGTGTGTGAAGTAGTCTCAACATCTGGTTGAACTGTTGATGGAAGATTAACGATCGTAGT
AGGGTAAGCTTCTGTAGTAGTCAATGTTTCTAATGTAGTCAACTCTTCGTCAGTAGTTGTACCATTTACT
AAAGCTTCCGTCGTCGGTACTTGGGTAGTAACTTCAATATCTGGTTTAATAGTTGTTGTTAAATCCGCAA
TTGTAGTGGAGGTATCATTTGTTTCTACAGTTGTGAATTCGTTTTCTGTTGTAGTTTCAAGGTTTCCAGT
AGTAGGAACACTCACGGTTGTAGTGGATACGTCTGTTGTACTCTCTGTAACATTAACAGGTAGACTTTC
ATCAGTTGTAGGGTAGTTTTCTTCAGTTGAGTTATTTGTTTCTGTGGTCGTCCATGCGTTTGTGGTTGAT
GAAGAGAAGGAACTCTCAGTAGTTGTTATTTCATCCGTTGTTGTTGTTGCTTCAGTAGGAAATTCGATT
GTTGTCAGATTTTCCGTCGTAGGTTCTACAGAGGAAACTGTCGTCAATATAGTCGTAGGATCCGTTTCA
ATTGATGCAGTGGTCAGTTCAACAGTTGTAGGTTGTTCAGTCACACTACTGTCAGTTGTAGAAATAACT
TCTGCAGTAGTAGGAGAACTTGTATTTACATCAACTAGACCCTCAGTTGTCTTTATATTTTCGGTCGTTC
CCGTAAAATGTTCAGCCGTGCTAACCACTTCAGTTGAATTTTCAACCGTTTCAGTTGTAGAAATTGATT
GTGTGGTTCCTTCTGGAACACTTGTGTAACTTGTGGTTTCGACTTCTGCTTCTGTTGAGTTTACAGACG
GTGTTACTTCATTAGTGCTCGGTTCTTCTGTCGTCGATACAACTATTTCAGTGGTCACTGTACTGAATTC
GGTGGTTTTAATATCTTCTGTAGTGGTTTCAGTAGCCTCAGTTTCGGTTACGATATCTGTCACCGCTACT
GTAGTGCTTTGAACAGTGTCAGTAGATTCTGGTTCCTTTGTAGTGTTGGCTACGTCTTCCGTGATGGAA
GATGTAGTAGTAGTAGGTTCAGTCGTAACTTCTATAGGAGCTTCAGTTGTGTAAACCTCCGTGGTGACA
CTTGGTGTGGTAGTTTCTTCTATAGGTTTGGTTCCTTCAGTTACTGAACTGGTTTCAACAGTTGGTTCGT
TTGTAGTAGTGTAAACCTCGGTGGTAACACTTGGTTTGGTAGTTTCTTCTATAGGTTTGGTTTCTTCAGT
AACTGGACTGGTTTCAATAGTTGGTTCGCTTGTAGTAGTGTAAATCTCGGTGGTAATGCTTGGTTTTGT
CGTTTCTTCACTGGGTTGGGTTTCTTCAGTTACTGGACTGGTTCCAAGGGTTGGTTTGTTTGTAGTAGT
GTCCTCAGTGGTGACACTTGGTGTTGTCGTTTCTTCACTGGGTTGGGTTTCTTCAGTTACTGGACTGGT
TTCAAGGGTTGGTTTGTTCGTAGCCGGGTTCACAACACACTCGGTCGCCCACGGCCAGTTGCAAACCT
GCAGCTGAAAATCGAAGATCAAGCCGGCAGGACAGTCCTTCTCAACCTTGTTACCAAAATTACACTGA
TAGAACTTGCTACAGTCATGGTCGTGCGTCAACAACGCATGCTCGTGAATGTTCACAGGACAACCATT
GGGTAGCACGCCATCTGGCGGCGTGTACGGAACTTCTTCGTGCGTAGTCCACTCTACGGGGGGAGGTG
TTGGTTGGGTAGTGGGAGCGGGTGTCGTGGTGGTTGTGGTGGTAGTAGTGGTGGTAGTCGG

>TCONS_00086001 geneID=XLOC_017268 scaffold_281:76396-79733(-) Exonic overlap (-)
ATAAAAATATGGAGTCAGTGCAAATGAAATGTCTTGACACAAATAATACCCTACTTCTACAACTGAGTG
ATTATTTTGAAGTTTTTGGCTTTGTTTTTGTATGTGATGTGTTACAACCATCTTGTTCATATATCGTTAATC
TAAGGCAATCTAATATGCACAAAATGATTGAAGTCTTGTTACTTACCAAAAGGGTAATTCATTACACAA
CTTTATCATAGTTTTCACTGCTTAACTGTATGTTTATATTGTATTATAGTAGGTACAATTGATTGTCTGTAG
GTATACAAAAATATATACATATAAGCCTTACCTACATAGTACATAGCATTCCATACAATAAACTTATGAAT
GAAATTTTGATAGTAGTTAGCCA
>TCONS_00085921 geneID=XLOC_017260 scaffold_281:139432-142175(+) Exonic overlap (+)
ACACGATTAGTGTGACTATGGTGTTCACAACGTTTATTTTTATCCAACGTTGAATTCCTGATTTATAAAA
GTGGCAAACACAGAGTACTTTTTTAATAAAAGTGGCAAGTGACATTCGATGATTGTTGTCATTGTTTTA
GGTTATGTTTATGTTTTGGAAACTCGCAAATATTCATAGAAAAACACATAAAAAATAATTATTTGGTTCT
ATGAATACGTACAAATTTGAAGACATGAACATGATTGCGCATTAAATAAATATAGTCAGTGTTTAGAAC
CTACACCTTCAAAATAACAGTTCTTTGTATGAGTCACCAAGATGTTGCAAACCCTAGGTTCGCTAGTCC
TGGTGGGCGCGCTGTGGGGCGGCACGAACCCGGGGCTGCGGCGCGGCGCGCGGGGGCTGCGGGCCG
TGCGCGGGGG
>TCONS_00085924 geneID=XLOC_017260 scaffold_281:142819-144665(+) Unclassified
GGGCGAGGAGCGGCTTACTGGCAAATCAATAGTGGGCATAGTGCTGATCGTGCTAGGAACCACGCTGT
GCTACACGGACAAAATGGACGCCGAGAGTGAGGTGTGAGTCCGCAGAGTATTTACCTAGTCATGTAAT
TCGTGAATACATTTTTATCATTCCCAAATTTAATATAGTTGGTACCTACCCTACAGTCCATCTATAAAGCC
AAGAAAATAAAGAGTGCTATTTAGCAAGGACTTTATGGCATTGTCGCTATTTACTTAGCGTACATGAAA
AGTAAAATAGTATTTAAAAAATCCACCGATTAAAAAAAACACCGCAAAAACTCAACTTTATAGATGGAT
TGATTGTACCTACGTTTAGCCGAGTTATGTAAATTTTAAAGCTCACATTTCACACATGTTAATTATGAAG
GAGAGTGGATGTGTGAAATTATGTGTGTAAACCTCATTTAGCGTCGAAATTAATTCAAAGAAATCAGTA
TTTTTGTTAAATAATTTAATATTATTATTATAGGTGTAAGTACTTTGTTATTTTATACATTGCTACGCTGACT
TAAACAGTAAATACCTAGTTAAGTACATTTTCCATCTAGGTATTAAAATGATAACAGGCACCACTCGTTA
TCAACAGCAAATGTAAAAGAAACACTTCACTGTAAATTAATATGCAACGTAAATCATGTTTTATATTATA
AAAAAGTGACTAGGTAGTTAGTAGATAAACTAAATAATGGTGTTTTCTACATTCAGCAAACTTATTTTAA
TCAAATTTAATGTAAACTATTCAAACACAGTCAGTCATAATACCTTCAAAGGTAGGCCAGGGTAGTTTT
ATGTTAGGCACTGTATTTTGCACAGATCCAAACTCAGGGCGGCAAGCGTCTGGTGTTTATTAATTAGTT
TATAACTAGGGCGAACAAATAGGGGTAAGTATTAGGTGTATGATTGCAATATTTTACTGACACTGTTAAC
TGTATAAATACCTAATGTATTAACTTTAGTATTGATTAATTACTTTGCGAGTTTTACTCGCGCTTTATGTTA
CCTACTAAATATGTTACATAATTTTACTTTAAAATACTAGTTATAATACAGGTATAAATAGTAGATGGCTTA
ATTTTAAATAAACCTATTTTAATACAAAAATTGCACTCTATATTTGAAGTTCTTATTTAAATGTTTTTATAT
GCTAAAATTAATTAAACCTTTTTTTAATAAATATATTTACACTAACTAAATAGGTAATTAATATATTTGGCC
TTCTAATGTCAAGGAGGGTCAACCAGGGTGCAAAGTATCGCTGACAATAACCACAGAAGAGGCGGTA
AAAAAT
>TCONS_00086017 geneID=XLOC_017271 scaffold_281:218077-223679(-) Exonic overlap (+)
CGGGACTGATAAGAATTAGAACCCGCTACACCGCAGTCTTGGTGTGACCGTCGCGGAGAGCGGACGC
CTTTTACGCATGAGATTGTATAGTCTTACGAAACTTTCTCAAATAAAACAGTGTTTTTGTAGACTGCAA
AGTAATGATGGGTCCATAATAGCCAGGCAGTGTTGTGTGCTACTGAGTGACGTATAAAGAGTTGTTTGA
AGTGACAAGTGAAGTGAGAGTGACGGACGATGTTGGATCGCAGTGGAGTGAAGGAGGAGCGACTGT
CGCCGGTGGATTATAGGATGCGCGGGGTCCCGTGCGCAGAACGCGTGTACAGCCTGCGCAGCCGCCC
GCCC
>TCONS_00086092 geneID=XLOC_017305 scaffold_282:231844-233042(+) Unclassified

AAGGACGTGGAGTGTCCCTCTCAGCATACTTCTCTGGTATAAGACGATGACGCTGCCGCCTGACGTTT
GCAGGAAACTGCTAAGATTGTACGAGCCGACATCTCAAGCACCGCTCTAGAATATAAACCACCCGATA
GTCAAGAGGATAATAATTTACGTGATAATAAGTATTTCTTTGCACCAAAGATGTAAGTAAATGTACATTC
CAACAATATATTTTTTTACGTTGGCATTCACCACTTTATAGTAAATGTGGTGCTTCTTTTGAAAAGCTATA
ATTGTGTAGTTTCTTATTTATGTCAAACAGTTTGTATGTTACTCACACGATAAACGAAGCTATGTGATTG
GTGGAATAGAAAGTGATAGCAATAGCACGAATAAAGTAACTGTATAACAATGGCGGAATAAGTGTCTA
GAAATAGACAATCATACTAATACGAATGAAGATGTGTCTGCCATTATTAATTATAGTTTCTTTAATTGCGC
GCCTTATAAGCACCTACTTGTTTTTTGCGAAGTATGTAATGGCGGGGTTACGCATTTTATTCTAGGATGC
ACTAAAATGGGTCCTAGAGTTCAGAGTGAGAGGTAATAAACATCCCAAATGACTGCATTGTATAGAATT
CTACTTCTAGTCTAAGGAAGTTTCTAGGATTATCGATGCCATTCTTTTGAAGGGCTGATCGATAATGAAT
GACTAAGGGTGAGGCCACACGAGCGTAATTTTGTGAGTTGTGTGTCGCGTATTCCGGCAGAAATATGT
GCGCGGGGACGAGGGGGGCGGGGAAGTTACTCGCTCCCCTCGTCCCCGCGCACACTTTTCTGCCGCA
CACAACTCACAAAATTACGCTCGTGTGGCCTCACCCTAAGGCTGCATTTCCAGTAGGAAAACAGTTCG
CTACAAACTGAGTTGACGGCCTATGGGTTAACACAGTTCAGCTAGTTTTCTGCTCACTCTACCTACACA
ATTTTGCTAACAGTTAGCGTACTGGGGACGCAGCCTTATAGACCATACCGAC
>TCONS_00086197 geneID=XLOC_017319 scaffold_282:317898-333202(-) Unclassified
AGTAGATTGGCGATAGAACGCACGCGCGTGAATGTTCGCTTCTATCGTCGCGCGGAATTATTTACGATC
GTAACCGCTTTTTAGAACGCCTTTATAAATTTTTGGTTGCAGTGGCAAGGAACAGGGTGTGAATTATCG
CCATCCCAACTGAACTAGAACTTGATAATGATAACACTCACGAACTACCCGCTCGCGTAATCACAC
>TCONS_00086295 geneID=XLOC_017321 scaffold_282:400514-401851(-) Intergenic
AAGATCAGAAAATAAAAGTTAGCGGCCCAAGATTAACATTTTGTATGGGGGCACCAAGATGCCATAGA
CCTGCCAGCATCTCACCTGGTTTATTATATAAAATGACTTTTGGTGAATATTCAAGGGGAAGATCAGAA
AATAAAAGTTAGCAGCCCAAGATTAACATTTTGTATGGGGGCACCAGGATGCCATAGACCTGCCAGCA
TCTCACCCGGTTTATTATGTGTGTTGACTCATTATAAAACACTGACAGAAGTTTCATCAACATGGAAAC
GGTATAGCAGGTGTCCAGGAGCCACTTTCTGACAGATTTTGGGTAAAAATACACAATATTTCACGAATA
ATCTAGTTTGCAGGTGGAACCTGAAGGAAATCAGCTTAAAATGATAGTTCATATGAAAGATATTATTAAT
ACCTAGAAACGGTTTTCAGCAATTTCAGATAAAATGACTTTTGGTGAATATTCAAGGGGAAGATCAGA
AAATAAAAGTTAGCAGCCCAAGATTAACATTTTGTATGGGGGCACCTGCCAGCATCTCACCTGGTTTAT
TATGTGTGTTGACTCATTATAAAACACTCACAGAAGTTTTATCAACATTGAAACGGTATAGCAGGTGTC
CAAGAGCCACTTTCTGACATCAGTTATCATCATACAGTTTTTTTTGTACTTACGACAAAAAAAACTGTA
CTTATAGAAAAGGACGATACTCTTATCATTATCACAAAAGGTCATCTTTTCAGCCTTACTCTTTCCGCCA
TACAATACGCATGACGCAGGTGTGAGCCTTTGATGGCATGCCGACTAGTGTTATTTTATAGTTGGGAAC
CCATTTACCTACCTGCCTAAAACAAAGAGTTTATCACACCACAACAATAGAATGTCCTAACTTATTACCT
GTTGCGAACCTAAAGAATTGATTATGGTTATTTTTTTGTGCGTTAGTATACATAATATTTTTGTACCTTTG
ATCATAAGCCGACTTGCAAAAGTTTTTATTGACAAGACGTGCACCTGGCAAAATAATGCTATTGTCTCT
CATAAAACATAATAGAAACATAATAAAAATCAACCAACAATTAAAAAATACAATAAAACTGCACATTGT
ACCAAAAGATGTCG
>TCONS_00086182 geneID=XLOC_017321 scaffold_282:401444-402387(-) Intergenic
TAAGGTGTGTGAAGTTAGCAGCCTAAAGTTAGCAGCCTTTGAATAGCCGACCAAATTAAGATGGCCAC
TTCAGTCATTGCATACTTTATCAATTAGAACACGTAAAAAGCACCAAATACATGGGAATAATAATGTTAG
ATATTCATATAAACATTTCATATGTTCTAAAATAAACAAATTACTAAAAAATACGACGTTTACTTACTGAC
GCCCTTGTTGGTTAATGGATATTTTGTATGGGGGCACCAGGATGCC

>TCONS_00086436 geneID=XLOC_017355 scaffold_283:186839-189324(+) Intergenic

AACAATGATACCAAAACCGTATATCTACGTCAAGGCAGTCAAGAATTATTGAAAGTTCAAACACACCT
AACGGAGCTTATCTAAAATATAATAATTCATACATCAAAGAAGTTTCCTAATTATACAATAATGATAATAC
CGCAGGTCAAGCTGCGTCAAAGACTAAAACATTAATAAAACTTCAGATCTTAAAATAATAGAAGACAT
TAATGGGTGATAATTGGACGGTCATCGAGATATAACGCAACTACACAGCAAATAACATGAAAATACTAC
CGAGGCGGTGAATGGAGCTATGTTGACGCAGCACAGGACACAAAAACCCGAAAAAAAACTGGAATT
ACTTAGGTATGCTTAAAAATTGCTTTTCTAGATATTTTTTTGTTAAAGTAGCGTAGACCGCATACTCTATA
ATTTTGTGTCCAGTAGTTAGCAATGTATAATGATGGAATTAAAGACAGTTATGAATGGCAAATGGCACA
GTTTTCTTGTTATATTGGCCGAAATAACAGCGTCGAACGTAGCTCCAATACCGCTCCACAGTGCAAAGA
ACACTTCAAAAAAAGTGACAAAAAGATCAACTAACAAGTTCGGAAAAACCACGATTAAATTTAAAAA
AACACCGATCTGATTATGTTCACAACGATTGCTTAGATTGTACAGTCAGTGTTCACATATATCTCTACGC
TAATATGGCAAAAAATATACCGACTTCCGTCATGATATGCAACGAGTGGGACAGAGACAGTTATATGTC
TCCTAGCTCCCGCCGTAAGCGATTAAACAGAGGAAATTAAAAAGTAAAAACTTAACTAAACTTACAAA
CTAGGTTTTGGACATTGATCCATTAGTTGAAGTTATTTTCCGAAGTATTTTGATATAATTCTTCACATGTA
TAGCCACTTAGTACCTAGTTGTTAGTAAAAATCCCTTAACGCATTCAAAATATATATATAATTATATAAGT
TCACTATATATAGTGGCTGAACGTAGACAAACACAAACTGTGTTATGTAAATATCGTACATCTATATGTTT
TTCAAATTACTCTGTGCCTAATATACATTTAAGCCGTTTTTTTAAAAGCTCGTAAGGGTAAGGTGTTTGG
ACATTTTATAAATTTTATTCTACAGGAGAAAATTGTACAAAGCTATTTATTTGTACTTTAACAAAATAATT
AAATGCTGTTTTGAACATAACCTTGCGTACTACTAATCCTTTAAAGTGTCAGAAAAAAATTGCGTCCTT
AGTCCTTAAAAGAAATACAGTAAGAAAAGCGACCTTAGAGTGTATATAATATACATTGTAAGTATATAAT
ATCTTGCTTGCTTACTTACTAATCTTATTTACCGATTCCATACGAAATTACAACGACTACTATTTAGTACA
TACAGTTTTTTTTTCAATTATTTCTTAATTTTCAACGAATAGAAAATGACATCTTTTCTATAGGGGTATCT
CAAGATATATTTGTCCTTAGACAGCAAGCAAGGAAACGACTCTAAATAATGTTTGTTACGTATAAATTGA
TATGATCTTAAACCTTACAATTAGGAAATAACTACAAATTATAAACAATGCTGAATATTTTTGTGCCACG
CGTGGATAATTTGTGCAAAAAGCGTATTTTTTTCTTTAGAATGCAACTCGGAAGTGAAATGGGAATAGG
AAGGCAGTTGGGTTCAAGATATTTCTCATCTTATTAAGTACATCCGCATTTGCGTATCTAGATTCATTGGA
TGTTACTATTTATTTCACACATCAGCTATATTTTTACAATTACAATGATTTTTTAACCAATGTTTTAGTGCC
TATAATCCTCTATAATATAATTCCATCTGTTATACAATTTCAACATACCAAATCCAATACGACACATATCAA
CCTCAAGTTAAAACAATTCTACAATAAAATTTGTGACACGCCAAACGAAACCCAACTGCCTAGTTATAC
CCGATATAGCGACAGAACAACTCAATTCCTTAGTTAAAATGCAACGGTATATAAACCCCGTAAAATGCT
GAATGC
>TCONS_00086310 geneID=XLOC_017355 scaffold_283:186898-189537(+) Exonic overlap (-)
AACACACCTAACGGAGCTTATGTAAGTTGATTTCATGTACAATCAGAACTCTTTCACAAAAAAATATGA
ATTATGAAAGAAACACCAAAAAAAGTGGTTCCACCAGACAGATATGTTGCCAGTAATATTGGTAACTAT
GATAGTCTCCTGTTCTTCAATATGAAGATAGTCAAGCAATATGGCTTATGATGATGATGATAAATCTACC
CCAGCCAAGTAGGTACCACTGCAGTATGATTCAAGTCTGTCCTTCAGGTCTAGCCTACTTAATTATAGTT
CGGTGGTAGGTCTTCCCTAAGCCACAACAAATGAAACGATGATAAGTAATTTTAAACCAACTTTATTTT
TAGCTAAAATATAATAATTCATACATCAAAGAAGTTTCCTAATTATACAATAATGATAATACCGCAGGTCA
AGCTGCGTCAAAGACTAAAACATTAATAAAACTTCAGATCTTAAAATAATAGAAGACATTAATGGGTGA
TAATTGGACGGTCATCGAGATATAACGCAACTACACAGCAAATAACATGAAAATACTACCGAGGCGGT
GAATGGAGCTATGTTGACGCAGCACAGGACACAAAAACCCGAAAAAAAACTGGAATTACTTAGGTAT
GCTTAAAAATTGCTTTTCTAGATATTTTTTTGTTAAAGTAGCGTAGACCGCATACTCTATAATTTTGTGTC
CAGTAGTTAGCAATGTATAATGATGGAATTAAAGACAGTTATGAATGGCAAATGGCACAGTTTTCTTGT
TATATTGGCCGAAATAACAGCGTCGAACGTAGCTCCAATACCGCTCCACAGTGCAAAGAACACTTCAA
AAAAAGTGACAAAAAGATCAACTAACAAGTTCGGAAAAACCACGATTAAATTTAAAAAAACACCGAT

CTGATTATGTTCACAACGATTGCTTAGATTGTACAGTCAGTGTTCACATATATCTCTACGCTAATATGGCA
AAAAATATACCGACTTCCGTCATGATATGCAACGAGTGGGACAGAGACAGTTATATGTCTCCTAGCTCC
CGCCGTAAGCGATTAAACAGAGGAAATTAAAAAGTAAAAACTTAACTAAACTTACAAACTAGGTTTTG
GACATTGATCCATTAGTTGAAGTTATTTTCCGAAGTATTTTGATATAATTCTTCACATGTATAGCCACTTA
GTACCTAGTTGTTAGTAAAAATCCCTTAACGCATTCAAAATATATATATAATTATATAAGTTCACTATATAT
AGTGGCTGAACGTAGACAAACACAAACTGTGTTATGTAAATATCGTACATCTATATGTTTTTCAAATTAC
TCTGTGCCTAATATACATTTAAGCCGTTTTTTTAAAAGCTCGTAAGGGTAAGGTGTTTGGACATTTTATA
AATTTTATTCTACAGGAGAAAATTGTACAAAGCTATTTATTTGTACTTTAACAAAATAATTAAATGCTGT
TTTGAACATAACCTTGCGTACTACTAATCCTTTAAAGTGTCAGAAAAAAATTGCGTCCTTAGTCCTTAA
AAGAAATACAGTAAGAAAAGCGACCTTAGAGTGTATATAATATACATTGTAAGTATATAATATCTTGCTT
GCTTACTTACTAATCTTATTTACCGATTCCATACGAAATTACAACGACTACTATTTAGTACATACAGTTTT
TTTTTCAATTATTTCTTAATTTTCAACGAATAGAAAATGACATCTTTTCTATAGGGGTATCTCAAGATATA
TTTGTCCTTAGACAGCAAGCAAGGAAACGACTCTAAATAATGTTTGTTACGTATAAATTGATATGATCTT
AAACCTTACAATTAGGAAATAACTACAAATTATAAACAATGCTGAATATTTTTGTGCCACGCGTGGATAA
TTTGTGCAAAAAGCGTATTTTTTTCTTTAGAATGCAACTCGGAAGTGAAATGGGAATAGGAAGGCAGT
TGGGTTCAAGATATTTCTCATCTTATTAAGTACATCCGCATTTGCGTATCTAGATTCATTGGATGTTACTAT
TTATTTCACACATCAGCTATATTTTTACAATTACAATGATTTTTTAACCAATGTTTTAGTGCCTATAATCCT
CTATAATATAATTCCATCTGTTATACAATTTCAACATACCAAATCCAATACGACACATATCAACCTCAAGT
TAAAACAATTCTACAATAAAATTTGTGACACGCCAAACGAAACCCAACTGCCTAGTTATACCCGATATA
GCGACAGAACAACTCAATTCCTTAGTTAAAATGCAACGGTATATAAACCCCGTAAAATGCTGAATGCGT
CATAGAAAGTCAAATAACAGATGTAATCACGTCAACATTAATTCGACAAGGTTCCTGCGCAATAGTATT
TTACGGGGCTTATGCTACGCCTGCGTTAGAGCGAGACGCGTGGCGCGAGCGGGATGGCGGTATGTTTG
TCGCTTACATCGTGCTGTTGTCCCACTCTAACTGGCTGATGTTCCGGTATTTGTGTTGCTTGCTCGACAC
CACT
>TCONS_00086394 geneID=XLOC_017355 scaffold_283:187282-193822(+) Exonic overlap (-)
CCTAATTATACAATAATGATAATACCGCAGTAAGTAATACACAGAATAAGTGAGTTGTAAAAAAGTAAA
ATAATTAAAACATAAGTTTAAGTAGAAATAGCTACCACATGTAATACTTTTAAAAGAGTGGGTTAAAAG
TTTAAATGCCTGCATTTTAGGGTCAAGCTGCGTCAAAGACTAAAACATTAATAAAACTTCAGATCTTAA
AATAATAGAAGACATTAATGGGTGATAATTGGACGGTCATCGAGATATAACGCAACTACACAGCAAATA
ACATGAAAATACTACCGAGGCGGTGAATGGAGCTATGTTGACGCAGCACAGGACACAAAAACCCGAA
AAAAAACTGGAATTACTTAGGTATGCTTAAAAATTGCTTTTCTAGATATTTTTTTGTTAAAGTAGCGTAG
ACCGCATACTCTATAATTTTGTGTCCAGTAGTTAGCAATGTATAATGATGGAATTAAAGACAGTTATGAA
TGGCAAATGGCACAGTTTTCTTGTTATATTGGCCGAAATAACAGCGTCGAACGTAGCTCCAATACCGCT
CCACAGTGCAAAGAACACTTCAAAAAAAGTGACAAAAAGATCAACTAACAAGTTCGGAAAAACCAC
GATTAAATTTAAAAAAACACCGATCTGATTATGTTCACAACGATTGCTTAGATTGTACAGTCAGTGTTC
ACATATATCTCTACGCTAATATGGCAAAAAATATACCGACTTCCGTCATGATATGCAACGAGTGGGACAG
AGACAGTTATATGTCTCCTAGCTCCCGCCGTAAGCGATTAAACAGAGGAAATTAAAAAGTAAAAACTT
AACTAAACTTACAAACTAGGTTTTGGACATTGATCCATTAGTTGAAGTTATTTTCCGAAGTATTTTGATA
TAATTCTTCACATGTATAGCCACTTAGTACCTAGTTGTTAGTAAAAATCCCTTAACGCATTCAAAATATAT
ATATAATTATATAAGTTCACTATATATAGTGGCTGAACGTAGACAAACACAAACTGTGTTATGTAAATATC
GTACATCTATATGTTTTTCAAATTACTCTGTGCCTAATATACATTTAAGCCGTTTTTTTAAAAGCTCGTAA
GGGTAAGGTGTTTGGACATTTTATAAATTTTATTCTACAGGAGAAAATTGTACAAAGCTATTTATTTGTA
CTTTAACAAAATAATTAAATGCTGTTTTGAACATAACCTTGCGTACTACTAATCCTTTAAATTTAAACGG
>TCONS_00086560 geneID=XLOC_017392 scaffold_284:50065-51249(+) Unclassified

TGTTAATGTTATGCAAGTTAGGTATTTGTGTGGTCAAATGTGTAGTAGATATTATTGAAAATTATTTTGTG
TGTTGTGAATGGAGAGTACCCTAAATGAAGAGTTATATAGTAAGTAGTTCCCTCAGTTATCAATGATTCT
AAATACGAATATTGTTTATGTCGGCAGGACGACAGCAAGTCCGCGGAGTGCACGCCGCAGCGCTGGG
CGCTGCCGCACCACGTCACGTCCCTGCCAGGCACCCCCCTCTCCCAGTCAAGCGTGCGTTCCTCGGGC
GAGTCGTACAGCTCGACCTCCTCCACGCGTCAGCTGCTGACCCAGCCGGTGGCGCACGCGCCGTGCT
AGCGACTAGCGCCCGTCGCGGAGGCAGTGCGCAGCGACCAATCACGGGACGATGTTGAGTGCGGAG
GTTAAGTTGGCGACCGCCTCGACCAATCACGGGACGATGTTGAGTGTTGATGTTGAAAGACAGTGGC
GGTTTGGTGTACATTTGGTTTTGTCGAAAAATCTGTAATGTTTATGTAATTTATAGATAGTCAAGTTAGG
TTACGTAGGTATATCGTCCCCTGTATGGAGAAAAAAGATATAAATCCATATAACTATAACACTATCATATT
GGCAAACTAGCGATAGATTTAGCTGCTAAGTAAAATAGATCATCGAAGATCTTGTGTACTATTATACTCT
TGATAGAGTGTTGCTATGCATTTGTATGGTGCACGTATTGAATTTGTGATTAGTAGGTATTCGTCCAGTC
GTGCAGATGATTCATAAACAATTTTTAGATCTGATGGGAAATTTATGCGAATTTGTTGTTCTTTCCAAGA
AAATTATTGTAAAAATTATAGTTATTAAAATAAAGTTATATAATTTAGTAAGCTAGGTATCTAGGTATCCAT
AAGACAG
>TCONS_00086750 geneID=XLOC_017393 scaffold_284:53868-62042(+) Exonic overlap (+)
GTTTAAAAGTACATAACTGGTATATTTTCAGGATCTTTTAAATCTTAGTTTTGATTCCTAAAGTGCTCATT
GTTGCATGTAACAGAATGATCTCTAGCGAAATCGAAACTTAGGAATGAACGTCTCATTCCGAAAATAAT
GAACATTGAACAGTACAAGGTACTTAGTACTTAGGTTGATCTTAACCTTTCTTACACGGAGTAGCATAA
ATTATTACTATAAATAAATAGCTTTTGATTCTAATAAAAGAAGTTACATATTTTTGACATAATATTACAAG
AGGCATCCTCTTTTTGACATAGGTACTAGTTCTTCACTCTCGAAATTTTAGATGGCTTGAGAGCTTTGAT
AAAATAAGTACATTATTTCCACACTAGTAAAAATGCGCATATTCGTATGAACATATTTTTTCATATTATTTT
GTTATAAATATTCATTTATGAGGTTTACAAATTGGTTATTTTTATTTCATAACAAATACAGTTTTATAAAAC
AGACTTACGTATTTTTTTCAACTTATTTTTAAGAGAATGTGTTTGTTTCTAGGATGAAAATGTGCAAGCG
AGGGTTTTCCCCACAGACATACATAGCATAGATGCCGCAAGTGCGTGTAGTCGGCGTGCCACTTCGCG
GCCAGATAAGTGTATTATGCTAAATCTTGACATGGGCGGGCACGGAAAGGGTGAGAATTTGAGTAAAT
GTACCTACTACTTAGATGTTAAGGTTACAACACAAATATTGGTGTTGTATTGTTGCAACGTAATTTTGGT
GTTATCTATATCTGTGTGGTTTTCCCTCCTATCCTGTTCAACTCGAAATGTTCCTCCTACTTTCTATTATGT
TTATAAAAGATTTGTAAGAAGAAATGAGAATGTTCTAATGCAGATAATAGTTATCTGTTAGTACCATAAG
TGTGACAGCTAAATATTTTCTTAAATAAGAAGTACAGTATTCATTAGAGAAGCTAGCTGCCCTCATACCC
TTCATATCAAATATTTACAGTATTTCTGCAATACAATATGATTAATGTGATTTCTAGTTTAAGTGAGAG
>TCONS_00086499 geneID=XLOC_017398 scaffold_284:101467-103979(+) Exonic overlap (+)
TCATACGACTTTTTCACGATTTTTAGTAAAATATTACATAAATTATAGCTTTCTAATCAGAAATTTAGCTT
AGTCAACTATTTGAAATACCGAGCTCTGTAACTAATCCCAAAACTCAAGTGAAAAGTGGTAATTTTACG
AGTAATAAGTGTAAGTTGTGTAGGCAGTGGATTAGTGTCTCCGTATTTTATAAATACAGCAAATTCCAA
GTGATTCGCGTAAGCTAAATTGTTTATCAGACACATAGACAGTTAACTGCAAAGTTTGTGACCTTTCAA
CCGGTGAATTAGCTGCTCCAGTGATGTGTTTACAACTATAACGACTCCCACCGTGCAGTGCATCAGCCG
AGATGGATCCATCGCGGCGGCAGCGCACTCAGATCAAGGCATCTGCCGAAGAACAAGCTCTAGATGC
CATAGCCAAGGAGGTATGTGTCTTTAAACTATTGAATATCCTTATTAATTTTTATTTATGG
>TCONS_00086929 geneID=XLOC_017445 scaffold_285:44-2652(+) Exonic overlap (-)
ATACCTATAGGTACTTACTGTATCACCCTACAGGTGTCCACTTCGTCCTATTAATTTGCCGGCTATCACTA
ACCGAGATAAATCTTACACGTGCTGTTAATTCTTGTGATTAGTTGATCCGCTGTCAGATTACTGATTACA
CAGCTCCGCTAGAGATAGCAGAAAAATACTATTTCGATCTATCCGAACTACCTACCGGCTTATCGTATAC
TATTTTACTAATGAGATATGTATTTAATTTACAGAAAACATAAAAAGAAACACAAGAGTGACGAGAGTC
TTTTGGAAATGCACGAAAAGAAGCTGAAGAAAAAGAAGAAGAAGGAGGAGCGTGAAGACGGCAAG

CAGGAGCGGCGGCCGTTCAGCCGCGACGTGGACCTCGCCGTCAACCGCTTCGACGAGGCGCAGAAG
AAGTCCATCATCAAGAAGGCGCAGGCTCTGGACACGCGCTTCTCACGGGGGGACGCCAAGTATTTATA
GTAGTGTTGTTTGTGAGCAATAGATGGCGCTGATACTGGGTTTGTTTCATGATGTTGTGGGAAGTTGTT
GTTCTGAGGATGAGGTATATAAAATTGGATTTTATAGTGAGAGTTTACACGATTGCAACTGCCATGGTTG
AAAAGTGCCTTCGTCGAAACGTGCTAGCCGAGAAGGATAATTCGAACGATTTGAAGCCTTGTGCTGCT
GAAGTTCCAAACAGGAAGCTACCTCATAAAACTACTTTATTGAGCATCAAATGTATCATTGTTTTTTCT
GAACAATAGGGTTCTTTAATTCAGCATTGTGAAACAGACACTTGGTATTTAGTATTTGGTTAAAACAGT
GAACGATAAAATAAATGAACGTGCTAAAGTCAGATAACTTTTAACTATTATGATGAGTCTGAGTGCATT
TTTAACAAATGTATTTATTTCTTTAAGTATCCATTCTGATTGTATCGCGCAAGAGTTACTTATCGTCACTG
CTTTATCTTTTAACATCGATTTTAAATATGGCGCCGCTCTTTTTACCTCTTATATGAAATTGTCCATAAAAA
TAAGTTCAATTTAGTTTTAGAATTCGGTAATAACACTTAGCGAGTGCATTTTAAGTAGTTATTTAGTATGC
ACACTATCCAAATAAAATTATGTCGTCTTCATAAAAAATAACATATGAATTTTCATTGTATTATTTAATATT
TGCATTTACTCAGGTTTTACATTTCAAAAGCAAAATATGCGTATTTATAAACACTACTACCAAGAAACAC
GTATAATACTTCACTGGTGGGAATTAGCACGGTACTATGTTTACATAAACACTGTGTTATGGTATCGTGT
GGTATACACAGCTGATTACATTGGTCAATGTATGGACTAATTGTTACCTACACAAGTACCAAATAATAAT
AATAACATTAACCTAGCTCTAATACATAAGCTCTAATGTATAAATCAATTTAAAAGTCACCTCTTACGTG
AACACATATTATTTATCAATCATTATTTTACTACCTAATATCAAAATCAATAATACTCGTAAGTAAAACTT
GCTTCCTAACTACCATAGCTAATTCAAAAGCTAGTACATCTATAACGGGCTAATTAAACCGCTGCCGCT
GGTGGGGTTACTCCTACGGTGGTGGCGTTAAGGGCGACGGCTGAGGCGCAGACCTTGACCTTGCCGG
CCTCACAGCAGAGCGGCGCCTCGGCAGTCATGCCGGCGTACAACCACGGCCGGCTGCCCACCTTGAA
GAACGCTGACAGC
>TCONS_00086755 geneID=XLOC_017445 scaffold_285:127-2653(+) Unclassified
CTTACACGTGCTGTTAATTCTTGTGATTAGTTGATCCGCTGTCAGATTACTGATTACACAGCTCCGCTAG
AGATAGCAGAAAAATACTATTTCGATCTATCCGAACTACCTACCGGCTTATCGTATACTATTTTACTAATG
AGATATGTATTTAATTTACAGAAAACATAAAAAGAAACACAAGAGTGACGAGAGTCTTTTGGAAATGC
ACGAAAAGAAGCTGAAGAAAAAGAAGAAGAAGGAGGAGCGTGAAGACGGCAAGCAGGAGCGGCG
GCCGTTCAGCCGCGACGTGGACCTCGCCGTCAACCGCTTCGACGAGGCGCAGAAGAAGTCCATCATC
AAGAAGGCGCAGGCTCTGGACACGCGCTTCTCACGGGGGGACGCCAAGTATTTATAGTAGTGTTGTTT
GTGAGCAATAGATGGCGCTGATACTGGGTTTGTTTCATGATGTTGTGGGAAGTTGTTGTTCTGAGGATG
AGGTATATAAAATTGGATTTTATAGTGAGAGTTTACACGATTGCAACTGCCATGGTTGAAAAGTGCCTT
CGTCGAAACGTGCTAGCCGAGAAGGATAATTCGAACGATTTGAAGCCTTGTGCTGCTGAAGTTCCAAA
CAGGAAGCTACCTCATAAAACTACTTTATTGAGCATCAAATGTATCATTGTTTTTTCTGAACAATAGGG
TTCTTTAATTCAGCATTGTGAAACAGACACTTGGTATTTAGTATTTGGTTAAAACAGTGAACGATAAAAT
AAATGAACGTGCTAAAGTCAGATAACTTTTAACTATTATGATGAGTCTGAGTGCATTTTTAACAAATGTA
TTTATTTCTTTAAGTATCCATTCTGATTGTATCGCGCAAGAGTTACTTATCGTCACTGCTTTATCTTTTAAC
ATCGATTTTAAATATGGCGCCGCTCTTTTTACCTCTTATATGAAATTGTCCATAAAAATAAGTTCAATTTA
GTTTTAGAATTCGGTAATAACACTTAGCGAGTGCATTTTAAGTAGTTATTTAGTATGCACACTATCCAAA
TAAAATTATGTCGTCTTCATAAAAAATAACATATGAATTTTCATTGTATTATTTAATATTTGCATTTACTCA
GGTTTTACATTTCAAAAGCAAATTAATCAAGTGAATCATTGAAGACACTTCGAAGACTAACGATATCTG
TAACTTTCCTCTCGAATTTCTTTAAAAATATCCAGTTTCTATTGATATGATAAGGTGATCTGCATAATCTAT
TTCATGTAGATAAATATATGCCTAAATATAAATAAAATAAGATTTCTCCTCAAATCGGATTGTAAGAATAT
ACATCTCTGATAAGTAATGTACCGTAGTTTTATTCATAAATTAAAATAAGTAATCGTGATAAATTATAATA
GTAATACTCGTAATGAACTAAACGCAAAAATATAAAACTTCCGACTATCCTATGAGACAATATGCGTATT
TATAAACACTACTACCAAGAAACACGTATAATACTTCACTGGTGGGAATTAGCACGGTACTATGTTTACA

TAAACACTGTGTTATGGTATCGTGTGGTATACACAGCTGATTACATTGGTCAATGTATGGACTAATTGTT
ACCTACACAAGTACCAAATAATAATAATAACATTAACCTAGCTCTAATACATAAGCTCTAATGTATAAATC
AATTTAAAAGTCACCTCTTACGTGAACACATATTATTTATCAATCATTATTTTACTACCTAATATCAAAAT
CAATAATACTCGTAAGTAAAACTTGCTTCCTAACTACCATAGCTAATTCAAAAGCTAGTACATCTATAAC
GGGCTAATTAAACCGCTGCCGCTGGTGGGGTTACTCCTACGGTGGTGGCGTTAAGGGCGACGGCTGAG
GCGCAGACCTTGACCTTGCCGGCCTCACAGCAGAGCGGCGCCTCGGCAGTCATGCCGGCGTACAACC
ACGGCCGGCTGCCCACCTTGAAGAACGCTGACAGCT
>TCONS_00086877 geneID=XLOC_017448 scaffold_285:31738-32876(+) Intronic
TTTTTTCAGACATACCCTATTCATTGTCTATATATAGTATGTATATATAGTTATATTATGTTTATTTATATAAC
ATTTCGTAAAGTTTTCAAGAACTACCTTCAATATTTACAATATAAATAAATACACATGCAATGTCATTACA
TGATACTGGCAGAAATGGTTGCCACACCCGGCAGATTGCCATAAATAAAATTTCAAAGAAGAACACAT
AGATAATATTAGGTATGATAGGCCTAGGCACCTAGTTTTTTAGGATTGCTGAATTAGTAAAATGAATAAG
TGAATAATCTATACTCGTCGAAATATAATAAGCCCGTTTGGACAGCTACAAAATGTATGGTTGCTGGTGT
CAAATCTAAATCATAAAAAAATCTCAACAATAAGTAACTTTTCGTGCTTTAAAAGTTCTTTTTCCTTTCAG
CCGTTAAAAAATAAGCCAGATTATGTTCTTTTTATATATTTCATCAAGTAAATGAAGAGAAAATAGCAAA
TTTGTGTATCTGTCCAAACGGGCCTATTGTATTTCGACGAGTATAAGGTGTTTGGCGGAGAAGCAGACT
AGCGAAGTGTTGGTCGATAAACAAGATTAATTATGAAGAAAAATCGATTAACACAGAAGAATTGCGCA
AGATGAAGATTAGATTTGTCCAATTGAAGGAGTACAACAGAAGCCATATTCTCAATGAGACAATAAAAT
TATTAATGGGAAAATTTCGATATGAACAAAGAAACCTGGATAATAATTATGGTGGAAAGCTTACATGAT
GGCTTGGGGTTTCTCTGATGGATCGTATCAGAACAGAAATGAGGTTATCCGTCAGAGGACTAAGGTTA
CCAACATAGCTGTCAAAATATGCAAGCTGAAGTGGCAGTGGGCTGATCATATGTGTGGCTGAGGATAG
AGTGTTGTGGTGCTCCTTGGGAGAGGCCTTCAGTAAGAGAATGATGTGGTGCGATCATGTATATTTTAA
TTTAATAAAATAAGTTCAGTTAAAATATATTTTTTATTTATAAACAATTTAATCACAGAC
>TCONS_00086835 geneID=XLOC_017464 scaffold_285:440707-441332(-) Splice junction overlap
GTGTCATCAGGCAGGGCGGCGCTCCCGCTGGCGCTGTACGGCGCGCAGCTGCTGCTCAACTGGGCCT
GGACGCCCATCTTCTTCGGCAAGAGGGACTTCAAGCTGGCCATGATCGACATCAGCCTGCTCCTGGCG
ACGTCGACTGCCACGACGGTCGCGTTCTGGCGCGTGTCGCCGGCGGCGGGGGCGCTGCTGCTGCCGT
ACCTGGCCTGGCTCGCCGGGGCCG
>TCONS_00086937 geneID=XLOC_017467 scaffold_285:535773-567374(-) Splice junction overlap
CTCAAAATATCGGATTTAGTGCTAATTTCTCTCATTAACGTGTTTCTAAACCATAAAATCGTGTGTTCATA
GTGAATTTTGATGATTTTTTACAGTGTTTACAAGTGTATTGAAGAATGCTTTTGATAAAAAATCTTGTCA
GTGTAAGCAAACACTCTGTGAATGCTACAGAAATATCATAATGATAGAGTGTAAGGAATGTCACCAAA
CACCGATTTGATGGCGCGGTGATGGTGGTTGGGCCGGTGATGCGGTGGCGACATGTGGCGCGGGTGG
CGCTGGCTGGTGCTAGCTGTGAGCGCGGTGGCGGCCGTGTCGCCGGTTGTTAAGATTGGCGCAATCTT
CACCGAGGAAGCTCGTGGAGGCAGTGTCGAGTTAGCCTTCAAGTATGCGGTGTACAGGATAAACAAG
GAGCG
>TCONS_00087002 geneID=XLOC_017508 scaffold_286:145748-148499(-) Unclassified
CAAGAATCAGTCTTCAGTCTACGAGTACTTTGTATAAACTTCAGTCCTTCAGTCTTGTCCGCACCACTG
CCGTGGCAACATGTGCCGTCAGTCGCTGTTCCAAATATATATCGCACGAAACTTTCGCATACCGGGGTC
GCGTGATTTGCGTGCTTAAAAAGTAACGACTCGAAATAAAATATTGTAGTGGTGGAATCAAATAAATGG
GCAAAATGTTATAGTGATGGTCAACTTTATGAAACTTTCAGCAAGGTCCCCATCAGATGCTGGCACGCC
ATGCATGGTGCGGCCAGTGACCCCGTGACGTCATGTGCCAGGGAATGAAGGTGCAAT
>TCONS_00087099 geneID=XLOC_017504 scaffold_286:344519-348440(+) Unclassified
TACCGGTACCAGTGCAAAACTAGTAAGTAATATTACTGATATTTGTACATAGTTTTGTAGTTAGGACCCT

GAATTAAAAATAAAAGTCAATATAAGGATAAAAATAAACCATTCTTACTTATGATTTAACAGACGTTTTA
AATCAGGGATGCACAGACCGCGGCCCGCGGGCCGCATGCGGGTGGCCCGCCGACAGCAAAAATAATA
CTCCCGTACATTCGAAAAATATTTAAGTAGGTAATTTTGAAAAAATATGTTCGAAAAGTACTCTGCATAC
TCGTATACATACTGCAATTAACAAACAATTCCTTGATAACATCGAAAGCGAATATAGAGATTTATTGTAC
CTACTATACAGAAATAAGGTGGCTAAGTCATGGATTGACAGTGGAACGATTTCTTAATAAGTAGAAAAA
AAATATTTTTTCTGGAAAAGCTGATCAATGCAGTTAGAAATTATCGACATTAAAAACGACCAAGATTTA
CGAAAGAAAATTACTGAACTTTTACCGCTGCATTGATAAAAATACGTACCTACCTTCAAAGAGTTGCGT
ATAAACTCTGCTCTAAAAATGTGTCAGTTTATTTGGTTGTACCTTTATATGTGAACAAACCTTTTCAATA
CTAAATCGACAAAAAAAATCGAAACCGCCTAGCAAACGAATGGTTGGAAGCAGTTTTACATGTAAATTT
AAGCCAAATAAAAAAATTTAAGCACTATGCAGTGCCACGCTTCAAAATAAATCAATTCTTTTTCAATTTT
AACATTATTATCAATTCTATGGCGGCCCGCCATCAGTTTATAATTTGTCCGAGTGGCCCCTAGTCTTCATA
TGTCTGTGCATCCCTGTTTTAAATTGTTTTCGAGTAGCCAAAGTTTAGCTCTTAACATTTTTTGTGTTGT
TATATCAGTTAGTAATTAGAAATAATTACGCTATAGGTAAATAAAATATATGAGTATCAGTATAATTTCAC
AAAAATAATCATGCCATTATGTAAATAAATACTTTAAACATTAATATAAAATTATACTTCAAAGGTACGTT
ATCTTAAATTAAATAAATTGTTGACAAAATAAAGTCAAATCTATTATACAGGCTGCTGCAAAATTGGTAT
AGTAAGAATGACACACTAAATCACCCCCTTCGCCCTTTTTGCAACACCTGAATATATTTTCCTACTAGG
AGATTCACTATTAATAAGATCAGAACTTTGTACCAGTTAAATACCTACTTTCAAGCAGAAAAATACGAA
TCTTTATGTTCCTTGCATAAAATATATCGTTTTTCTCGCCATCCTATAAAACTTTTGTTATAAAAGTTTCG
GAACTTTGCTAGAAAAGGGCATTATTTAGAATTAGTTAGGTGAGCATAGCCTTTATGTAAGTATTATCCT
TTTTTAATCACTAGTTATTTGAATTTACGCCTCATACATAATTTTGAAATAAATTCTTTCAAGTAAATTAGT
ATTTTCAGTTTTATACAACCATTGCAACAAATTTCAACATAAAAATGTACCTAATCTAAGTAGATATATTA
ACTTTTAGAAATAACATTTCATTGCTTATTTTCAAAATTATATTTTAAAAGAACATCACTTAGGTACTTTG
TTTCTATATGGGAAGACGCACTACTTATTTCTACATTATCTACTAAACTACGTACGTTAATATGCAGAGA
AACCTGACCCCCCTCTCATAGTAACAATGCTTCTGAGAGGGGTCAGATTTCTCTGCCTTGTACATGGAT
TTGCATGCTGCAACATAAAGCGGATTCAAACATGCTTTTACACATACCACATTCAAGATAAGTACGGGA
CATTGACATTGATTTAAATAAATAATTATTAATCAAACATTGCGTTCATTAATATTGGTAAGGCCTGTTGG
TATTTTATGCCATCATATTATACACTCGCGAGCAATGCTAACGTTTAACTGCCAGTTTCTATATCTCTATCT
ATCCATAACTGGTAGTTACTTTCATACGATTTTGACATAATCAAAAGTAACAATCTGTCAAAATCGTATG
AAAGTAACTACCAATTATGGATAGATAGAGTTATAGAAACTGGCAGTTAGTGGCAACAAATTACTCCTA
AACGGAAAAGGCTAGCCGTCGCCGTATATTGAAATTTGAAGTGCATATTAATCAAGAACATTTATCAGC
GCATCAGAACAAAAAACAAATTCAGTATCTCGTGAGTGTATAAAAATAACTTGATTTTTTTTGTCGCTT
ACAGCGGGTGCAGTGTAAATGCGCGGGCGTGAATGCGAAATTAACATAACGTGCTATCCCTGACAGGG
TAGGCAAATTAACTTAGATGAGAATGCACCCGGCACATACTTTTGAATTTATTTATGTTACTGGCCAAGT
TGAAAAATAAATAAGGTGTGGGAGAGAATTATTTGCGAGTGACTTTTGACTCATATGGCATCCCGAATA
ATCTAGGTAAATCACTGACTAAGCTACTGAAGAGTAATCAAGCAGTGACAGTGTAAATAATTTTTTCGT
AAAAGCATTTAATCAGCTAGTAGAATTACTTAAAGTATGTACATTATAAATTACTAATTTAGTAACAGTTT
ATACTCATTTGTTGTAGATTCTCTCCCACACCAATGTAACAACTCACCAGACAAAATTATCAGCATTTTA
TCATGAGTGATATGTTACATTAGATGAATACCCTCTTTATAGCTAATCACAATTAGTAACAGTCCATTAGT
TGTTTAGTCAAAATACGTAAAGAATTTGAGTATTCCCTCTGTTTCGTCTCAGTCTAATAGTCGCATTTAT
GGTTTCATTGAAAGCGATAAGCTAATAAAGCAGTACATACTAACATTGCTATAAACGCCACATGACATC
CGCATTCTAACTGTCATTGAACAAGTATACATAAGAACAACAATTCAAGAATTTATTTATGATACGAATG
GCTGTGATGATGATTAATAATCATAATTTAATTTCCATTCGTGGAAGTAAAAGTACCTTCCATATAAAATA
TATCGCACGCCAAGCCTCAATAGGCACACAACCTGAGATTGGTTTATAGCATTAAATTTAGTCGATGGA
TTCCATGAAATAAACAGTATTTGCTACAGTCAAAGACAAATTGAGTATAGTCTAAGGTTTACCCTACTG

TACCTACCTGGCGGTAGTCATAGTCAAAGTAGTCAAAGTCAAGTCAAGAAAACATACGAGTCTAGTCT
ATGGTTAGTTCAGGACAGCACCTAGCTGCATGACTGCCGCAGTCTTTACACCTGTCAAAGTCAAATTA
AGAAACAAAAACAGTCCAGTCTATGATTACTTCAGGGCGGCACCTGGCTGCATAACAGTCGCAATCTT
TGCAAGTCAAATTAAGAAACAAAAACAGTCTAATCTATGTTTAGTTAGCGGAACTTGGCTGCAAGACA
GACGCAATCTTTACATCTGTCAAAGTCAAATTAAGAAACAAAAACAGTCTAGTCTGATTACTTCAGGG
CGGCACCTGGCTGCATAACAGTCGCATTCTTTGCAAGTCAAATTAAGAAACAATAACAGTCTAGTCTAT
GGTTAGTTAGCGGTTCTTGGCTGCAAAACAGTCGCAGTCTTTACATCTGTCAAAGTCAAATAAAGAAA
CAAAAACAGTCTAGATAGTCTATGGTTAGTTGCACCTTGCGGCTTGCTCGGGCGGCGCGAGCGCGGTG
GCATCGAGTCTCGCCGGAAACGCCCTGTAGTAGGCCGCTCTATGGCCGAGGCGCTCCGAGGGGCCAAT
GACCTCCAGCTCGGCGGGTACTTGAACACTATTGGAGAGGGTCGACACGTTGTAGCCCCGACTCGAC
CAGCCTTCGCCGAGACGCCGTC
>TCONS_00087090 geneID=XLOC_017504 scaffold_286:347281-347822(+) Intronic
CCTCTTTATAGCTAATCACAATTAGTAACAGTCCATTAGTTGTTTAGTCAAAATACGTAAAGAATTTGAG
TATTCCCTCTGTTTCGTCTCAGTCTAATAGTCGCATTTATGGTTTCATTGAAAGCGATAAGCTAATAAAG
CAGTACATACTAACATTGCTATAAACGCCACATGACATCCGCATTCTAACTGTCATTGAACAAGTATACA
TAAGAACAACAATTCAAGAATTTATTTATGATACGAATGGCTGTGATGATGATTAATAATCATAATTTAAT
TTCCATTCGTGGAAGTAAAATCAAGAAAACATACGAGTCTAGTCTATGGTTAGTTCAGGAC
>TCONS_00087467 geneID=XLOC_017546 scaffold_287:186960-187677(+) Intronic overlap (-)
CCTCTTATTATGCTTCGCTGGCGGCGGAGAGGGTGACCGCTTGCGCTTCCCTTTGGCCTTGCCCTTCCC
GGCCGGCTCCGCGCCCGGCATCAGGTCGTCCACTGATAAACGCAGCTTGTGTACCTTCTTCTTGCCGG
CGGCGTCCACCTCGTAGTCCTCCTCGTTCATCCACTCGTTGTACTGCAGCAGGTCCAGCGCCCACGTG
GCTGATACTAGGTGCGGCCCGGGACGCTCCCACTGCGCCG
>TCONS_00087372 geneID=XLOC_017560 scaffold_287:208683-211609(-) Splice junction overlap
CTAATTTACATTATTAATGGAGTGTATTTGTGTTCCTTGATAAAGACTCATCTTTTACATAAAGATAGGAT
ATTTTAACAGTTCGCAAAAAATGGGTAATGTGGCGTTTAGCAGAACTGTGTGAAATTGCAAATATATTT
TTTTGTGACACCAAAATGTATAAAGTTTTCAGTGTTTTATAGCAGTGTTTAATGCGGAGACTATATTTAA
AATATTTTGGATCCTGTGCAAATTACTGAACGACCAGAAATCTCCTTGTAAAATTTAACAGTCAACAAA
CTTTTGTTTATTTCGTTGGAGTGCTATTTGTTAATTTCTGCATTGAATACTTGATTATGAGCCACCGATAC
GCTTCCTGAATTAGTGGATTAAGGACTGACGTCGATAATATAGGTTATGGCAAACTTGAATTTCCAACA
AGCGCCTAGAAGCCTCACAAGTGGCGGGGTGGGGGGCGAGTGGGCTCCGGCCTAGTGGGGGGCGT
GTCCGGCCATGTGACGCCCACGTTCGGGGGCGCCCTGTCCCCGGGGCGCAGTTCGGCATCCATGCCCT
CCGCGCCACCCTCCATGGCCTCTAGAACAACACTATTTGGACAGCGAGCATTCGCTGACCGGAGAGTA
CCCATTCCTACACCCTTATCTCAAGCTAATTCTAATGCTATGTCAAGTATGAGCGG
>TCONS_00087226 geneID=XLOC_017564 scaffold_287:484475-486901(-) Splice junction overlap
TTGTCGCATCTTCAACAGGAACCAACGAAGGGCCGTCCACAACCAACTGATCAGCCTCACAAACCTC
ACCTTTAAACCAGTTACCATAGAGATCACTTTCCCTTACCATTACATAGAATATAAGAACAACTAAATTG
GATGAAACACCCCAATTGGATGCATATGAACCCTGGAGTGTGAACGGCATGGTCATGTTTTTTTGCATT
TTGTTTTTAAAGCTTTAGAAGAGATGCCGTCACAAGACAACTGCAACGATTGGGACGACCGTTCACCG
GACGTTGAGCTGCCGATTTACCAACAACGCGACAACTTCTTCACATAAATTGATATCGAATGTACTTTC
CATAGCGTCACTTGACTTATTTACAAGCTCAATATTAAAACATCATCTGACGTCAGAGATTGTGGAAATA
GACACTTTTTCCTACATGATGTACCTACTTATCCTTATTGTAGAGGTAACTATATGTATCAAAGATTGTGC
AAATAGACTCGTTTTTCCATTACGAATGTTATTGGATACTCTTATATATGGTTACTTACTTAGATATAATCA
AACATTTGACATATTGATTTCGATTGTTGTTTAAAACAAGTGTGTAGATTAACCAAAACTAACACGTTT
GTAATACCTACAGCACGTTTAGATGTGTTGTTTTGTTATACATATTACCGATATTATGTGTATGATAGTGTA

TGTCAGTGTTAATAATATTGGCTTCAATATCTATGACACGGCATTGGATAGAACAAACAGTTTATTATTA
ACGTTCCCCAGTTCCCACCTCTTGGAGATTTTCAGAGACGTAAAAGTCTCGGAAAATAATATTGCTGA
GTGTCATTTGTTTTGCAACAAACATATCAATGATATTATTACTTATATTAATGAATATTTTGCAATAACAAT
AACAAATACTTAAATAAAATGATTTGCCGATGCAGATTTAAATTTTTAAAAAGGATGCCATAATAATTTT
CCGTGATAGCCTGAAATGTGTGATGCTTACAACATATCCTGACAAATATCGTTACTGCACATTTGCAGTT
AAATGCTAGGATATTTTAGATTTTTTACTACATACATTATATGATATCTAGCTATAGTATGCTATTTCAGTTA
CTATAATTGAACTTGAGTTAGTATCTATCTATTAGCTGTACTTGAAATACATTCCTGTTTATAGTGGTTTTT
ATCTTATTTATTGCGGCCAGTTTTTGTATTCATTTTCTACTTAGATATGTAGGTACTTAATAATGTATTTAAT
GGAAGTCTTCTGAACAGGCCTTGAACTACTTAACTTAGTGGGTAATATATTAATAATAGAAATGTAGATG
ATAAAATAAACGATGCCATTTTAATCAAATATTTACTTAGATTTAATGATAAAAGCACAGTTTAATATTAA
GTTTGGACGAATTATAATGCGTTTTAGTATTTTTCGTTTTGTGTACTTATTATTATTTGTATCTATTGTATTG
TACTATATTTAGTTTTAAGTTGAACCTGAGAGATCAAAATATGTTGTAATTTACTAAGTTATTATAAATAT
CTTTAGTTTGTTAGATGTTAGGTTGAATAGAAACTTACAAATATATTATGTATACTTACCATATAAATTGT
GTACTTAGATAATAACCTACACCTTAGTTTGTCAATTGTACTCAAATATTGGATCTTATGAAAAAACGTT
CGATAAATAATCAAAGTAAAAAGTGACTAATTATAATGTAATGTTGCTTCTCTTGTTATCCTAGATTAGCT
GGAATGAGACTGAAATATAGACCTTATTATATACATAACTAAAAAGTTTTTTACTAATCAAAAAACTATG
ACGGCTCGAAAATCAGGCAAAACATCTCAGATCCAGCGATAGATGCATTGATGTAGCAAAAGTTATCAT
ATTTGTCTAGATAAATGGCTGTATTTATAAGCATAGAGTGCAATGTGGTGGTAAAATCCAAAATATGTTT
GGACATCGTCCCTATAGGCGTTTTTCAAAATATGTATCCACTTATATCTACTCATACCAGTATAATGCAAG
TGTATTAGAATTTGTGCCTAACGTAAAGGTTCATAATAATATAATGTACTCTATTCTATTCTATTCTCTGTG
GGGGTGTAAGTACCTGCACCTGGCTCTCTCGAGTGGAACCTTTGTGCATAG
>TCONS_00087335 geneID=XLOC_017564 scaffold_287:486733-487531(-) Splice junction overlap
AGAGCCCTGATGATGAGGAGAAGAAGCAACGCGTGCACCTGCTGCAAATGAATCAGATCTTGGCTAA
GCAGGTTACCGAGATGAGCAAAATCATTGCAGGAACCAACGAAGGGCCGTCCACAACCAACTGATCA
GCCTCACAAACCTCACCTTTAAACCAGTTACCATAGAGATCACTTTCCCTTACCATTACATAGAATATAA
GAACAACTAAATTGGATGAAACACCCCAATTGGATGCATATGAACC
>TCONS_00087470 geneID=XLOC_017554 scaffold_287:511330-511837(+) Intronic overlap (-)
TGGTATCTTGTAAGAAAGGATTGGCGGTGCGTGCTTGTCGGTCGTATGTTTGTTCCTGGGGGTTTTCCG
GTCTTTGTTGCGATTGGCTTCGGCTCCTATTATCAAAGTAAGGTGACTGAGGATTACTTCTGTACATAGC
TGACGTGCCGGGTTGGTTGTTCTCTAGTCGTTCTGTGACTGTGTTGGTTAGGGGCAATGATAGGTTGTG
AGTGTCGTATGGTCGTTTGGTTTGATACTGCTGTGGAGGATGTTGTGGCGGCGGTTGCGGCGGTGGTT
GGTTTCGAGAGTACATGCTCTCAACGTCTTGTCTTGTCTGATATGTTGAGTTTTCTTGCCGTGTGTGAGT
AGTGCCCTGGCTGTAGTGTGATGCTGGCTTTTTGAGTAGGTCGTAGTTAGGGTCTTACAT
>TCONS_00087732 geneID=XLOC_017590 scaffold_288:14862-15844(+) Intergenic
TGTAATCGGAAATTTTGATTTGCAGGTTCATCCAGCAGTTTTGACTACAATACAAAAAGTATCCAGTTC
CATTGAGGTCGAAGCATTAAAAGGTGTCACTCACCCCAATTTGATAAACGCGGGACAACACTGACGTC
TTTGCGATACAAGATAAACCAATATGGTAAGCCTCAAATATCAATCTTTCCGTATGGTTTAGGTGTTTTG
TCAGCTTGTTTATAGTGTTAGATGATGATAAAACTCCCCCTGATGCCACCATGACGGTTTTTACAAGCTC
AACAGGACTGAATAATTAGCAACTACAAAATAAATAAATACAGGAATTATACTAGTATAAAATAACTAA
GTACAA
>TCONS_00087698 geneID=XLOC_017590 scaffold_288:15185-15789(+) Intergenic
CTATTTATAGCTCTTACTCATACTGTATCTAAAATGATGATCTATCACAGACCTGTTCTGAATTTCTTTGA
GGACATCTTTAAATTATTACCTGATTATTTTTTAATTCAAATTTCAAACACATTTTAAAGATTTATCAATC
ACTAAAACTAAGTTTATTTTCGTTTACAGTATCCAGTTCCATTGAGGTCGAAGCATTAAAAGGTGTCAC

TCACCCCAATTTGATAAACGCGGGACAACACTGACGTCTTTGCGATACAAGATAAACCAATATGGTAA
GCCTCAAATATCAATCTTTCCGTATGGTTTAGGTGTTTTGTCAGCTTGTTTATAGTGTTAGATGATGATAA
AACTCCCCCTGATGCCACCATGACGGTTTTTACAAGCTCAACAGGACTGAATAATTAGC
>TCONS_00087699 geneID=XLOC_017590 scaffold_288:15185-16305(+) Intergenic
CTATTTATAGCTCTTACTCATACTGTATCTAAAATGATGATCTATCACAGACCTGTTCTGAATTTCTTTGA
GGACATCTTTAAATTATTACCTGATTATTTTTAATTCAAATTTCAAACACATTTTAAAGATTTATCAATC
ACTAAAACTAAGTTTATTTTCGTTTACAGTATCCAGTTCCATTGAGGTCGAAGCATTAAAAGGTGTCAC
TCACCCCAATTTGATAAACGCGGGACAACACTGACGTCTTTGCGATACAAGATAAACCAATATGGTCG
CATCTGCCAAACGGAATCATTTGAAGACACGATTTACTGTGAAGCTAAAAGCGGTCCAAGTTAATGGT
AAGTTTCATGGTCCTCATAGTTTTTAAGTTAGCATTTAATTAGAAGATCCATTTTCATTTAAGAATTCCAA
CGTAAAAATACATATCTAGAAATTAATTTATACGATGATGATAAAATTCCCCCTGATGCCTTCCATGACG
GTTTTTACAAGCTCAACAGGACTGATTTAATTCTTTTATTATTTTAAATGAAATATTTATGGTTATGATGG
AATGGTTTTAAAAATAACATTATTTTCTTACAGACTTATCTGGGTGCAACATCGGTCCCAACTTCACAAT
TTTGCACGTGGTAAGGCATTTTACTAATTTATTACATTAATTAACTTAACATTCTTATGATGATCAACACA
GACCTGTTCTGAATATTATTGAGGAC
>TCONS_00087571 geneID=XLOC_017590 scaffold_288:15226-16433(+) Intergenic
CTATCACAGACCTGTTCTGAATTTCTTTGAGGACATCTTTAAATTATTACCTGATTATTTTTAATTCAAA
TTTCAAACACATTTTAAAGATTTATCAATCACTAAAACTAAGTTTATTTTCGTTTACAGTATCCAGTTCCA
TTGAGGTCGAAGCATTAAAAGGTGTCACTCACCCCAATTTGATAAACGCGGGACAACACTGACGTCTT
TGCGATACAAGATAAACCAATATGGTCGCATCTGCCAAACGGAATCATTTGAAGACACGATTTACTGTG
AAGCTAAAAGCGGTCCAAGTTAATGACTTATCTGGGTGCAACATCGGTCCCAACTTCACAATTTTGCA
CGTGATTCCGCTGTCATCATGTGAGGGAATTTAGTTGTAAA
>TCONS_00087700 geneID=XLOC_017590 scaffold_288:15282-16437(+) Intergenic
ATTTTTTAATTCAAATTTCAAACACATTTTAAAGATTTATCAATCACTAAAACTAAGTTTATTTTCGTTTA
CAGTATCCAGTTCCATTGAGGTCGAAGCATTAAAAGGTAAGAATCTATCTATATCCAAATGTTTAAAATG
AAATATATTGTTTACCTAGTTTGTATGATGATAAACTTCCCCCTGATGCCACCATGACGGTTTTTACAAG
CTCAACAGGACTGACAAATTATTACAGTCAACTTTATCCTAAACCAATGTAACATCAATTACATGTTATT
AATGTTATAATAACTCTTCCAGGTGTCACTCACCCCAATTTGATAAACGCGGGACAACACTGACGTCTT
TGCGATACAAGATAAACCAATATGGTCGCATCTGCCAAACGGAATCATTTGAAGACACGATTTACTGTG
AAGCTAAAAGCGGTCCAAGTTAATGGTAAGTTTCATGGTCCTCATAGTTTTTAAGTTAGCATTTAATTAG
AAGATCCATTTTCATTTAAGAATTCCAACGTAAAAATACATATCTAGAAATTAATTTATACGATGATGATA
AAATTCCCCCTGATGCCTTCCATGACGGTTTTTACAAGCTCAACAGGACTGATTTAATTCTTTTATTATT
TTAAATGAAATATTTATGGTTATGATGGAATGGTTTTAAAAATAACATTATTTTCTTACAGACTTATCTGG
GTGCAACATCGGTCCCAACTTCACAATTTTGCACGTGATTCCGCTGTCATCATGTGAGGGAATTTAGTT
GTAAATTGC
>TCONS_00087619 geneID=XLOC_017590 scaffold_288:15348-16420(+) Intergenic
GTTTACAGTATCCAGTTCCATTGAGGTCGAAGCATTAAAAGGTGTCACTCACCCCAATTTGATAAACGC
GGGACAACACTGACGTCTTTGCGATACAAGATAAACCAATATGGTAAGCCTCAAATATCAATCTTTCCG
TATGGTTTAGGTGTTTTGTCAGCTTGTTTATAGTGTTAGATGATGATAAAACTCCCCCTGATGCCACCAT
GACGGTTTTTACAAGCTCAACAGGACTGAATAATTAGCAACTACAAAATAAATAAATACAGGAATTATA
CTAGTATAAAATAACTAAGTACAAACTTTTACAGGTCGCATCTGCCAAACGGAATCATTTGAAGACACG
ATTTACTGTGAAGCTAAAAGCGGTCCAAGTTAATGACTTATCTGGGTGCAACATCGGTCCCAACTTCAC
AATTTTGCACGTGATTCCGCTGTCATCATGTGAGGGA
>TCONS_00087596 geneID=XLOC_017590 scaffold_288:15525-16433(+) Intergenic

TTTATCCTAAACCAATGTAACATCAATTACATGTTATTAATGTTATAATAACTCTTCCAGGTGTCACTCAC
CCCAATTTGATAAACGCGGGACAACACTGACGTCTTTGCGATACAAGATAAACCAATATGGTCGCATCT
GCCAAACGGAATCATTTGAAGACACGATTTACTGTGAAGCTAAAAGCGGTCCAAGTTAATGACTTATC
TGGGTGCAACATCGGTCCCAACTTCACAATTTTGCACGTGATTCCGCTGTCATCATGTGAGGGAATTTA
GTTGTAAA
>TCONS_00087572 geneID=XLOC_017592 scaffold_288:27478-28712(+) Exonic overlap (-)
CCGCTCCCAGCCGCCGCACGCAGCCCCCGCTGTAGGTTGACACTGGGCTGTCCGCGTATTTTGTTAGG
CCGACGACCTCGAGCCAGGCGTCCGTCTCTGCGCGGACGTCGCGCGCGCGCAGCCCCGCCAGCGTCA
GCAGCAGCCGCAGCGTCTGGCGCCCCGTCAGGAACTCGTCGAGACCGAGGAGTTGGGGGCAGTAGG
ATACCGACCATAGGTACTGGAAGTGAGGAATAAAAGGCATAATTAGTCAGGGTTTTCTTCTTATAGTGC
CTCTCCATCATTAGAGGTTGGCAGTCAGCTCTTTGCAGCGCTCTCTATTCCTGGCTGTTTTAAAGATTTC
TTCCACGGTTTTTACACCGG
>TCONS_00087933 geneID=XLOC_017636 scaffold_289:116988-117914(+) Intronic
CTCTGTGATAAGAAATTTGGCATGTTTTCTATTTGATTTTGTAATAAACTAAAAAATCTAAAAATAACGA
ACATGGATAAAAAAAGCTTGAGTTACGAAATCTCTGATGAAACAAGTGTAGCATCGAGAAAACCAATG
GGATTTTGCTGTGTCTGTTTAGCAACTCCGTGCCAACTCAGAAAATACTATAGCCACCAATTTTGTTCT
GACTGCCTTTATAGATACATAAAGGTCCCTGATAGAAACAGAAGTCAAATTTAGCAAGCAAAATGTAA
AAAATAATATAACGGAGTTAATTTTTTTTACTATGAAGTTAAGTATTGCTTCTCAGAAATAAAGTATCAAT
GATCTAAGTACCT
>TCONS_00087979 geneID=XLOC_017638 scaffold_289:175313-183598(+) Intergenic
CGAAACATTCCTAATTCAAAATAACAAAATAGAACAATGCGTTCTTAATTTAAAAATAGCGGTTCACGT
GCAAATATCAAAATAGGACCAATACCAAAAAGGATGCATATTATTTTTCAAATAGTGCCGTTGTTGTAG
AAATATTTTTATACTTAACGAAATATATTGTATTAATTGTTAGAAATATACAACCCCGTAGTGTAGACGTG
TTAGAAATTGGTAGTTTATATTGAATGTTTCATGTTGTGAGATTGACGGTTTTTTTACACCCAGAAATATT
AACTAAACCAAACCAGTGTACCTACTCTAGTGAACCCTATATCAGCACAACGTAGTGGTCCAAATACG
AATTTTATTTAAAACCCATTTATAAAGGAAAAGTGATTTGAACAAAGACTGAGGACTTGAGTGGACTG
AGTGAAACGAAATTGGCTAATATCCGATAGCCGAGTAATAGTTGACCTTTATTGGCGGAGAGCTCTTGG
GGATTCCTGTTTGCCTGGTACCTACCCCATACTAGTGTGCTTGACCACTCATGTGTGGCGAGTGCTGTG
CCCTTTCATTCTTCTTATATCCTGGTAAATTAAATCACGGATAGGCGAAAAAACCAATTACTTTTGATCA
AATTAAGAAGAAAAAAACATTCGTCTTATATGACAGTGACTAAGTTCGAACTAAATTAAGTTCTACTAA
ATTTTTAAAAAGAGTTCTTAATTAAAATTCAGTGATGCGTAAAGTTTCTGCTAAGAAGAAGTGAACTTA
AAATTAAAGAAGTAAAGGTTATTATGGTGTGACAGATAAGACGTTAAGTACGGTCTGCAAACATAACTT
GAGCTTCAAATCTCTTTGTAAGAAGGTCCCAAATGGAACAGAATAACGGTCCCAGTTGAAAACTCTAC
TTAATTGAATTGAAAAATGAGAG
>TCONS_00087756 geneID=XLOC_017639 scaffold_289:212871-213883(+) Intergenic
CAGGAGATTATTAAGTTTACGCATTATTGCTTAATATCGTAGGATTATCTTTTCAATTAATAAACCCTATA
AAAACCTATGATACGTTCAAGTAATATTGACTTGGACATGATGTTAAAAAAAATATGTTGGTTTGTGCCT
CTGTTATTTTTTCTAAATGAAACAAGTTCTCAACTAACTCATGGTAATGACTTGGATAAAATGTACCCTT
GGACGCGGTTGGACCATATCAGTCGAGCCAGAAGACAGAGTTACGATATCACTTGCTCTGATGAATAC
TGCACGAAAATGTGTATTACAGAAGAGAGAAAACGAATTGGAACTTGTATTTTATTTGTGTGCCATTGT
CAGTGAAATATGTTTTAGACCATAGAATTCGTTAAGCTTCACTAGATTGGATTTCCTAGAAAGCAAAAT
CGGTCAGTGAAAATGTAGGTAATGCAGGTAGATCAGCAATTTTAGCCAAACAACTATTATTTCGAAAAT
GTTGATGAAATAGTTTTGTTTGGTAACTTTGCAACAAGCAATAGTATTGAAAATCAGAGAAATTCGACA
TTATAGCCGATAAACTTCGTCTCTGCCTTTAGTCTCTTCGCTGGGTATCAATATCTAGACAGGGTTTGTC

ACCGTGGTGACAGGATTAGTCACTAAGTTTAGTTCACACAGTCACTGACTGCCAAGGGTGCGCCCTAT
TCTTTG
>TCONS_00087824 geneID=XLOC_017653 scaffold_289:298655-308401(-) Unclassified
GCTAAAGTGTCCTGCAATGAGCCACAAGGAAATCCCAATGGGCATTTGCACCATACAGGATAAACAAA
ACGTGTTGCTTCACTTGTGTAAATAGTAAACTTTAGTCGTTCTCTGACTGTTAACCGCGGTATACTGGAT
AAAGAGTGGTTATTTTTAGCGTATCTTCAAAATCTAAAAACCCTTTAACTCAGAGAAGAGCAAACGTT
GTGGTATCCTGTTCCTACAAGTGTACATTCCGTAATTAATAAAATATATCTTAACAGAGTAAACATAAAT
CTAAAAGTGAAATAATATAATACCATAAATATTATATGAACCGCGTCTCATTATCGAGAATATTAATAATA
ATAATTGATTACACGGTGCGACCCAGAGTAGGCGCGACGTGAATTTACAGAGCTATATTGTCGTTCTGT
TCATTTCATAGTAACCAAAGAGAGAAAATCGATTTCTGTACGAAAGATGCCGGCTGTAAAATGAGAAA
TCTAATATTTTTATTCTGCCTTGTGGTATTTATACTCGTGTCAAATTTAAACATTTCAAATGCCAAATATAT
TCGCTCCAAAGGACAATGCGGAGACTGTGACGAGTACTGCCATCTACGAGGCAAGAGCGCCGGGACT
TGCATGAGGAAAATATGCTACTGCCGAAGAGATAAGTGGACCAGCTGAGCTTTCAAAATAAAGATGGT
TGAGAATGGA
>TCONS_00087878 geneID=XLOC_017644 scaffold_289:445968-447751(+) Exonic overlap (+)
GGAACTTTTTCATTGGCTGCACTGGCATAAAGATGGGGCGTGGATATGCAGCTCTGGAGTATCATGGAG
CTCCTTAGGACATGTCTATGTACAGCAATGGATGATTGTTGGCTGATGATGGATCACTATTCACGACCAA
TTTTGCGAGCCAAATGTTCCAATCCCAGGACAGTTTTTTGTTCGCTTTGATGTCGGATGCCAGATGTAT
GCGTGTGTAGAGGGATATTTCATATCTGCCAACCAAGTGAATAAATTTAGCATAGGGAGCTTTCTCATA
GTAAAGCATTTTAGAATATCCAGCCTTAATACCCTATTTGTTTGACCCATACCTTCTGGGACAGCCTGTG
TATATCCCCTAAAGAGTTTGCTAAAAGCTTTCCAAACTATCATCACTTATTTATTCACGAGAAAAACATA
ACAAGTAGGCGCAACTATCGGTCAGTCCTGCCGACTCTGCCGATAACTCGTGTAAAAAACTAAACTTG
GACGTAGTGACCAAACTTCACGGAATCTCAAGCCGGCAATAAAACTAAAACACTAGTTTTTTAAGGTT
CGAAGTTTTTCTATAAATAACCCAACGCTGGAAAAAGTGTTAAAAGTTGGTTAAACTTTGTAACCCCA
GCGGAGTATCATATTGTCGCGAGACTTTTAAACTGGATACGGAAAAAATATAAATAATAGTAGCCTTTTA
AATGTTTATGAACAAATAAATCATTGAATGACTAAGTTAAATGTATTTTCCTGCTACAGATATCCTCTATT
ACACAATAATCATTGAATAGTAGATCAATTTTTCCTAAATTCTTTCAAATCACTTGGTGATTTGGGCTAG
CTCAGTTCAGAATCACAATCAGAGTCTTGGATTCGGGATACGATTGCCATAGATTTCCATAAATTAATTT
GAGATTATCTATCTTAGAACCTAACGAAAAGAGGATGTTTCTCATTAAGATTACCGCCTGGTGATAGATA
CCTATATAACTGGTGGTATTCAGATAGAACGCTGTGTACCGGCCGATCTGAGCTTCATACATTTGACGA
ACCAGCGTGAAAATAATCCATAGAAAATGCTACCGTCTTCGCTGGTTACCTTTATAATTATGGTTACCTT
TACCTTTATACCTAAAAAGGGGTATACAGAGAGAAAATACCTGAAGCCTTGTGAATCTCATTGCTTTAA
ATTAAAATCTTACCTTCTTGATCTTTAAGGCTCCAACATCACACTCGTTTTCTAAAACTAAAATTAAAAT
CCTTACTAAAACAGATCGTTGTCTTTTTCAGTTCAACATTCCAGAAATGCTAGTCGTATTCGCCGTGTC
GGTGCTGGCCACGGTCGCCATTGAGATGCCTTTCAACGAGGTCTACAGAATATACTTCGGAAAATCAC
AAACGAAATTAAAGGACAAATGAAACAAACAAAATCAAGTATGACTGTGAAAACTGTATTATATTTAG
TTGTAAGATTTTATGAAGCTTAAAGAGCC
>TCONS_00087859 geneID=XLOC_017646 scaffold_289:473521-475518(+) Unclassified
TTATAAGTTCGATTAAGATACTAGAACACAGATCCGAAATGGGGCAAAGAATCAAACAAAAAATATATT
TGACTAAAAATACCTGTGCAATGCGGTTAAAACAAACCAAAATAGTAGGTAGGTATGTATTTTGTCTCG
CCATATTATACATGTGTATTGCCAGCACAGAACACATTGCCAGATAATCCAAAGGTTTTCTAAACGAGC
CTGATTTGCCGGCTGTAAAAATTGTATGGCATAGTACAAAATATCTGAACTCATAAAGCAGTTAACAGA
ACTATAATTTGCCATAGTATAGTATGGTATTCGAAGCCATTTTTAAGTAAGTATTTAAAAGAGCTACTAGC
TTTGTAATATAATATCGAACACAGATCTTAAAACTAGTATTTTAAACGTAACTAGGTATAGCCTTATCACA

TCCAAACCATCACATTTTTATTTCTATTCAACTTACAAAAACTCTGACTGTGTTATGGCAAAAAGCAATA
GGTACCTATAAAACGATTTTTTTATGGTGAAATTTAACGTAATTAATTTATTGTTCATCATGCAAGTAGTA
AAAAAAACTACGGTTCCTTAAAAATAATTAACTAAAATTGAAACTATTATAGGTACTTATACAGGATGG
AATGTTTCGTATTCCTGAATGATAAAGTGACCGCCATAATGGAAGTTGTTCAGTACATCATCAGGTAGAT
TGACCTGACCCGGAAGGTCAGTAATG
>TCONS_00088609 geneID=XLOC_017699 scaffold_29:252078-258414(+) Splice junction overlap
CGCCCCCAGCGAACGATGAAGCACCGACCCACGACACAAACAACTACGCCGAGTCTGTAGGCGGTGG
CAACCCTAACGGAAGGCCAATGAACTTACAGGGCGTCCAATCAACGATGCCCATGAACAACAGCCGT
TAGTGCGCGCGACTGTGAGCCTCAACGCTCTGCAAATAGCGTCTAGTTCCGCTCGTGGGGAGATGCCG
CCATTTTGAAAAAGTTTGAACTGTCAAATTTGAAGTAACGGATTTCGAATGTCATCAAAGCGCGTTTTC
TCACTTTAGCGGAATTAGTTTAAGTTATGTTTAATTTAAATTATGTACGCATTTAATTACAAAATAACTTT
GTTTGTACGCAAACAAAAGATCGCAAAAGTAATGACAAATATATTTGAACCATATCACGTGGGAAAGC
AATGAAAATGTCTTGGCCGGTCGACGTTTAGCCATTATTTTAGTAAATAATAAAAATATATATAGCACAA
GCCTATTTTTACGACTGGTAGCGGTAGAACTCAAGCACTGATGAATGTGGTATTTATAAAAATACTTGTA
TCTATATAATTTGGATTATCTTTAAGTGGCCGCTTTTGTGTGACCGTGGTTAGACTTTTATCCCAATAATG
ATAAGGCCTAAAATCGTATAAATACTACAGTGCATTCGTTTTAAATGCCATGTTCTGTTTGTTTCACAAT
AAAATGTCAATTCCCTATGTAAATAGTCGTGCCATTTGTGTATTTTAAAACGAGTAATTAAGGAATCACA
AGAATGTTATTTATTAATATAAATAATACGGACTAAAACTGAATCGTTGAGGCTCACTGTGTAAAAGTTA
TTAAAAACTTGAAAATGATTAGAGAAATAAAAAAAAACTATGAAATTTGTATCCAACGGATGACACAC
AAAACGTGAACCGTGTAGCGAATTCATAGATGCAATAGGACATTATTTTAGCATGAATCAATGCAATCG
TTTGAAAATTATAGCATATTTCAGCTGGTTTATGTAAGTGTAATAAGTGCATAACATAGATTATACAACAA
CAGCGTTCAGTTTTATAAAAACAGCTAAAATAAGCTATTTAAAACCATCAAATGAAAGGTCTTAAAATA
TATATAAATAATATAATGATTATACAGTTTTATGCCTTTCTCATAAGCAATATCTCTCGAGTATGACAAGTG
GAAATAGGTGGAAAGCTTCTGTTGAAAAGAATTGAAACAAACACTTAGTATAATGTCATTTAAAACTTT
ATCTAAAACTAATCTTCATGACATTTCAAACCTCGTGGAATAATATCGATTACTATTATTTTATAATACATA
AATTAGGACATGTAGTTTACACCTGATAGCTTGTAAAACTACGGTATTTATATAGAAAAAAAACTTTTGT
AATATTTTGTATCCATTCCATAAACCTTTATGATAATAGGTGTCTAACGTCTTCTAGTATGAATATACCTAT
ACTATACTGAATATCCGTCTCTGTAGTTACGATTGTAAGATAGACTGTGTGAGGTTCCGTCGAGCGAAG
ATGAAAATCCGCCGCCATTTTGTTTTGAATACAAGATGGCCGCTCCTGTCAAATTCTTATTAATTTATTTA
AATTTGTTAGTTTTTGTGTCTAGTTCTGTTATTCTGAACTGAATGGTGTTTTGATAGTTATTAACATACCA
ACTGTTTGTAGTTAAAGTTATACATATTTTTTGTATAAGTAATATTTTACTGAATTAATTTAAGTTACTTAT
TTTTTGCAGTAATCGTAGCCAGCCGGAATTTCACGCATTCCATATGAATACTTAACCTTAAGC
>TCONS_00088231 geneID=XLOC_017720 scaffold_29:680969-684823(-) Intronic
CGAAATCATTAAAAATTTGTTTGACAGTGCAGCAAAAATTAATTTAGATTTTATCTGCGAATTTGCGTGC
GTCACAACTTGAAGGTTGCAAGTGCCTGCGCGTGCGCTTGTTGCAAAATCGATTCTAAGTGGTGGCGA
TGAGGTTGAGATTGTTGCAAATTTGATGAGCACATTATTCTGTACCGAATATGTGAGTCGAGTGAAACC
CACGCTTGGGCTAGGGGCATCTTTAGAGCAGTCTGATTCAGGCGGCAATCCGGGGCGGGTGTCCAAA
AATTTTCAAAGATGGCCGCTGCGAATTCCGAATTTGAACTTTTGAATTTCGAACGGAAGCGAGGGCGG
AAACGGCGGAAACTCTGGGCGGCGGGGGGTGTCGGGTGAAATGCAGTTAAAAGAAAGTGTTATCTAA
CAAAAAAATAAAAATCGCGGTTGCAAATTGGGATAAGCTGAGGGCGTCAGCTGAATTCCCTGATGCAT
CAACACTTATTGACGAAAACATAAATTGCGTTGTCCTCAAGCATTTGGAATAATGTATAAATTTTCCATA
AAGGCAATCCAAAACCATTGCGATTAGGCTGAAGATAATAAAATCGTGCAATGGGACTACAGTGTATTC
AAATATTGTCAACAAGTTTGGAAGGTGACGCATGGC
>TCONS_00088338 geneID=XLOC_017703 scaffold_29:906088-927030(+) Exonic overlap (-)

GATGAGACTAGACTTCTCTTCTAGAAGGTTCTCGGAGCTTCGCTTCTCGTCCTCGCCGTCGCCCTCATC
CTTGAACACCTTGACCTCGTCCGTGGAGCCCAGGTCATCGCCCCCCGAGCTAGCAGCTGAACTCGAGT
GCGCATGCGGCATTTTCAAACACCTACACCCATTAACCCATCCCTAAACACCGAAAAACCCGCACTATC
TGTCATCCATGACATTTCAGTCGCGCGCCATCTTGTTTCCCTTCCTACACTGGGGCTTCTGTCATGTATG
CATTAGTTCGTCACGTTACACTAAAACTAGGGCTTACGGAGTTTTGTTTCCTAAATTACAGAGATCACA
GATAACTTTTGTCAGGTTGTTTTTATGGTTTTATTCTTGCGGGCCGCTTCACGTGTTTCCTGGTTATACCT
AAACTCAACTGGCATGATCACCGCGGTCCAGGCCGCGATCCCACTCCAAAATGCACGTTCCAAAACCC
ACTTTAGTTAGCACATCACGCACGGTACCTTCAGGTTATCACACAAATAGTTGCACACGCGTTTGATGC
GATTCACTTGACATGTTTTGTTTATCGCCGGCGGAATTTTTGGTTTGCGAGAGCGTCGACGTGCCGCGC
TCGGTCG
>TCONS_00088534 geneID=XLOC_017704 scaffold_29:1035954-1037896(+) Unclassified
CATCACAGATCCTTCTACAAAACCCTTCCTTCATTGTAAGAAGACCCGTGCACCCAGTGGGGACGTGA
TGGGTCGCGATGAGAATGTCCGGTGGTGGTAACCCCTCTGTCTCTCCGCAGGAGGCGAACACGATGGC
GGAGCGCGACCTCGGCTCCACCTTCCTCCTCCCGCACGTGCACCTCTATAAACCAGACCTTACCAGCG
ACGTGTCAGAATTTGATTCGCTATAAGGATCAATCTAAATGTAAAAGTAATCAGTCACAAATTCAAATG
TAAAAGCATAATAGTGCCTATTGGCTTTCATGTGTAGGGTTATTATAAATGTGTACCTAATTTAATGTTCA
AAATACATTGTACCTTTGAATCACGAGGCTTTACTGTCTCTGTCTGAAGCCGGTATTATAATTTCATAATT
CAATTTGTGATTGGACGCTTCACTCCCAAACTTGCACCTAATTGGTTAGTTACAGTACCAAACCGCACA
TGATAAGCGTATGACCTCACTTGATCATCTTAATAGGTGCTTAAGTAGGTACTTACTTATTTCTTAAAATA
TTCTACACACAGCTTCGATTAAGTAACACTTATATGAAATATTCACATCCCCGAAAGCTGTGAATGAGAT
ATAATCTAGACTTAATCACCAAAGTGAAAACTTATTTATTTTTAATTTAAGTTGCTTATTTTATCTGTTATT
TATCTATGTACTTACCAATATTATGGAAATATTGCTTACAGTGATGAATCACTCCTACCAAGATTTTTG
>TCONS_00088452 geneID=XLOC_017707 scaffold_29:1214210-1219953(+) Intronic
CTGGCCGCGCTGCATGCGCACTCTGCGCGACCGCCGCACGCGCAGGTGGGAAGCACGGCCGACTACG
ACGAGGCTCCACTGAACCTAGTCGCGAGCCACGTCACGGGCCGACTACGACGAGGCTCCACTGAACC
TGGTCGCGAGCCACGTCACGGAGGAGACGCACTGACTGTCCTCCGCGCCCCGCACCCCGCCCCCCGG
GCCGCCCGAGGAGGATGCACGCAACACGCACCGGCCTCCTTTGACAGTTCCATAGACAAAAAGCCCG
CCAACAAGTACGTGAGATAAGCGACGTTGTCTTTTGTTTTGTTGTTCGAGATTCAAACTTTCATTTTCT
GCCAGTTTATTTTTAATTCCCCCGTTGGAAGGATATACAAACTCCGTATTTAATTTCTATTTCAATCAGTT
TGATTGATTTATGAATCGTCGTAATTAAAATAACAGTTTTATATTTTATAGTGATAAGTGAGGCCAGTGTT
TATTACCCAGTCCGTTTCGAAATCAAGTAAAGAATGAGATTACAGTTTTATCGAATAAAAATAAAAATCT
ACGCACATAAATATAAATATTTCATCGTATTACTGCAGCCTGAAGTTGAATGAAAGTTGCCTGCTTGCTT
TTACACATTCAAGTAATAGTAAATAACCATTCTTTATTGGAGATCGAAACTGACTGAGGGAATCATCAAT
GTTTATTTTTGGGTATATTAATGTGACGGGCATCCGTTAGCAGGAGATACAGTCGTTGTACGTAGTGCCT
ATTGAAACTAAAAGCAAATAATAAAATAAATAATCGATATACTTATCTGTGGTAGTAAAGTTAAATGGAA
CTTGGGATATTATACTATTTAGTAATCTCTTGTAATTGGCTAAGTTATTTAAAGTATGGATAGTTTATTCAT
GCTGAAAACGAGCGATGAATAAAAAGTTTGTAAGATAAAGTTCTGAAGCTACACAATCATACTATGATA
GAGTGATTCAAGAGTGTAGAAATAGGACGACACTTTAGGGATAGAGATTCGTTCTATTCCTTACTCCTT
CAATAACTATATTATTGAATATAGGTCGAGTCGGATATGATATTGTGAATGATGACCGAGAATCGTGATTC
GATGAATGGATGCCAATTCTAATTAAGTGAGGTAAAATCTCAAAGTAGAGTACGCGTAAGGGTAGAGT
AACGAGAATAGAAACCTATTAAGCCTTGCCTTATAATGCTCTCTTGATAAAATGATGAAATTATATTTAC
GGTTACCTTTTTCAGGAAGCAAGTATTCATTTAGGATTAGGACTTTTTAAATTGTATATTATTTACTTAGT
ATTAACGTCGTCGGTACGGTAGTTGTCTAATTTATTTCCCTTGTCGGTCAGTATTTAAAGTGTAAGTATTT
TCGTTCAAGTATTCGTATTTAGTTACTTAAAACTCTACCTGAAGTTATCAACTACCTGTTGCAATAATTC

GTCGATGTTAAGCTTCCATTTGTTTTGCCCCAAAACACATTACCAGTTATATTTGCCTTCCAATACACATA
TTTTTACACCTTTACTGTGTATTGATCTATGCCTTTATAATATAAACGTATAGCAATAAGAACATGGCATTA
TTTGAATAGATATTAGTTAACATATTTATATAAGAAGCATATTTTCCAATGCATTAAAAATAGTTTTATATA
ATCGCATTTTTGAGATGAAACATTCAATTTAGTTGTTGAATAACATAACCAAATCACTGTGGCATTATGT
ATTTTGGTTTTTTATAATTTATATAGTACACCTCAGAATGGTCAGTTTTTATGTATTATTAAAGCAAAAAG
CATTTACTTGTATCGTTGTCCTGTACTTGGCTGTTTGTTGTTGCTTCCATCCGAATTTTTGTTTAGTTTCG
CCGTCCTTACTTAACGTTAGTTTTAGTTTATTAGTGTCGAACCCGACGGGCCAGTAAAGCAAGTCTCTG
AGACATTGTGATAGTATTTATGTAGACAATGCTCACCTTCCATACACTTGGCTATTATTATGTATTTAGTTA
TATGCCCTACAACAAAATAACAAATCGTGTTGATATTTCGTAAGCTTACCATATTTAGAAGCTTTAAAAT
TTGAGATTATTTATTTATTGGGATCATTAATGTATATATTTTTAAGACAACCCTGTGTTTTTGTTTTTCTTT
GTAATAAAGGAGCAGTACCCGTGACAAAATGATTGTCTCATGTGGTAACGTGTGATTCTCAGAATATGC
CATCCATGTCTTTGCTCTCTATTGATAATTTATTATGTATTGTGATTTATACACGAATTCCCTTTCTCCTCT
GAAATTGTAATCTCATTAGCTGTACCTATATGTATTATTTAATTATTACTTCCGGTTTTTGAGGGCTGAGT
CCGCCAGCTAAGTTATCTCTAGCGACATTATTAAAATGGTCGACTATTGTGAAACCGGGGGTTTGTATCA
TCGATTGATACGTATTGTAATTATTTTGCCAACCTTTTTGGTTGATATCAGTTTATTATGTGTGTCTACGTA
ATGTCCATTGCACTATTCACTAATATTGTCAGTGGGAATAGTGATACAGTATTATTTTTAAATTATAACAG
TGTAAATTTGTGTTAGGGATACTACTTTAATTATATGTTTGTAGTTTTTATCTAGTTGACTATCGAATTTAA
TTTCTTAATTTCCTAAACCATGGTATTTAATGCAGAGGTTAATTGAATTTTCGTTTCTTACCAACTACTTA
TAGATATCAGTATGATTTATTAAAACAATTTTGATTATAAATATTAAAAATTGTGACCTTGTACTTCTGTC
GTGGCAATATGTATGCTATTTGATCTTCCACATTGTTTATTTATTTACTTTAGGTTAAATCTAATTAACTTT
TGTATTAGAGTTGATGAACAATTATTGATATTAAGTTACTAACAAACCGGTTGTGTTGTGTGTGCTTACG
CCCTAACAAGCATGTGTTTGTGATTATATTGTAATGGTATGTATCGGGCATCTCGATTCAGCGCCAGTCG
ACACCGTGGCCGAACCTCGATGGGGACGTGGCGGCCGGCTGGTCCTGAATCCCGTAGCCCCGCGCAG
CTAATTTGTGTTAAATGTTTATAATTTATGCTGAAATATTGGCATTAACGTTATGAAATTTCATTCCGCTTT
CACCGCACTACTGAATGAATACTAAGACTGATGATTGTTCAGCTATTTTGAAGTCTTTTCCTACAATACA
AAGCTCTCAATATTTATCTGTATAGTAATCGAGATATAACAAAAGTATTTAATTAAAATGTGATATTTGTT
TGAGAATGTAAATGTGTGTTTTTCAGAAAGCAATAATGTTGTGATAAGTGGCGACGGGCCTTGTACTGT
ACGCCTGAAGAGATTTATTCTGTAATAATGCAAAATATTGTGGTCAATTTTCAAGTAAGTAGATTCACTA
TTAGTACGGCCAAACTTTTAAAATCGTATTAATAGTCTGCAAAATTCAAGATGAGCCAGGACAATATTA
GTAATTAATTAGACTTACCTAAATAAAATAAGTATTTACTTACAGCAAAATAAATATGTTTCATACTTGAA
AATAGCTAATTACATACGTGAAGACGACCTATACTATAATTAACTAAATCCAATATCTGCATGATGTTGA
AATATTTCTTGTAATGATACTAATAATTGCAACCTTTGCAAACGCGAATATTGTTAACGTGTACAGGTAG
TATTGTCCTGTCGATCATCTTGATCTAGTTCATAGTAACGAAAGCAATAATAATTGTAACTTATAGATTCT
AAAATAGCTTACTGAAATAAGCAAGGTCGCTTCGGATTAGATTGCAGATTTATTATCTGCTCTCCTAGGA
ATAACCGATACTAATTCATAAAAAATTAAGAGTTTTGAAATAATAAAAATGATTTACACATAGCTATAGC
ATAGCATACCTATAATATGTAATATTTTGTTTATTAACTTTGTCTATAAAATGACGGTTACCTACAAATTAC
TGCACTTATTGGTATTTTAATCTAAAATATGCTTGAAAGTCAGCAGTCTAATTTAAATTGATCCTAAATTA
CAAGTAACATAAAACAAAAAAAAATCTTTCAACATTATTGCATTAGTGTGGTTGTGAAATATCAAGAGTT
CTTGGTGATATTCCGATAATGTTAGGCCACACGCCTCGAATTTAAAAACTTTACTATTCCTAAAGTACAT
AGATTCCTTATAAGGATAAAATTGCTCTATCTTTGTAATTTCAGCGCTATATCTTGGTGTTTTATTGTAAA
AAAAGCTCAGATTGTATGTACCTAGGTTTATTTTCAGTATACAGACTTACCATTCGGACCACTGTTACCT
TATTGTTATTTCAATAGTTAGAAGTTTCAGCATAAATTATCTGTTAATTGAGAATGTGAACATGTGGCTG
GAATACTCATTGAAAAAAAATACATTAATTATTGTTGTATAGATGATAATAATGTGACTTACGCAGGGATG
GATACTTAAATTATTAAAATTTTAACTGATATATTGGCTATGTAGTGAGTAGAATATTTTTTTACAAATAAA

TTATAGCAGCTAAACAGTTTGTTATTATTTAGTTTCCCTTATAATAACT
>TCONS_00088626 geneID=XLOC_017707 scaffold_29:1215453-1219911(+) Intronic
CGACGAGGCTCCACTGAACCTGGTCGCGAGCCACGTCACGGAGGAGACGCACTGACTGTCCTCCGCG
CCCCGCACCCCGCCCCCCGGGCCGCCCGAGGAGGATGCACGCAACACGCACCGGCCTCCTTTGACAG
TTCCATAGACAAAAAGCCCGCCAACAAAAAGCAATAATGTTGTGATAAGTGGCGACGGGCCTTGTACT
GTACGCCTGAAGAGATTTATTCTGTAATAATGCAAAATATTGTGGTCAATTTTCAAGTAAGTAGATTCAC
TATTAGTACGGCCAAACTTTTAAAATCGTATTAATAGTCTGCAAAATTCAAGATGAGCCAGGACAATATT
AGTAATTAATTAGACTTACCTAAATAAAATAAGTATTTACTTACAGCAAAATAAATATGTTTCATACTTGA
AAATAGCTAATTACATACGTGAAGACGACCTATACTATAATTAACTAAATCCAATATCTGCATGATGTTG
AAATATTTCTTGTAATGATACTAATAATTGCAACCTTTGCAAACGCGAATATTGTTAACGTGTACAGGTA
GTATTGTCCTGTCGATCATCTTGATCTAGTTCATAGTAACGAAAGCAATAATAATTGTAACTTATAGATTC
TAAAATAGCTTACTGAAATAAGCAAGGTCGCTTCGGATTAGATTGCAGATTTATTATCTGCTCTCCTAGG
AATAACCGATACTAATTCATAAAAAATTAAGAGTTTTGAAATAATAAAAATGATTTACACATAGCTATAG
CATAGCATACCTATAATATGTAATATTTTGTTTATTAACTTTGTCTATAAAATGACGGTTACCTACAAATTA
CTGCACTTATTGGTATTTTAATCTAAAATATGCTTGAAAGTCAGCAGTCTAATTTAAATTGATCCTAAATT
ACAAGTAACATAAAACAAAAAAAATCTTTCAACATTATTGCATTAGTGTGGTTGTGAAATATCAAGAGT
TCTTGGTGATATTCCGATAATGTTAGGCCACACGCCTCGAATTTAAAAACTTTACTATTCCTAAAGTACA
TAGATTCCTTATAAGGATAAAATTGCTCTATCTTTGTAATTTCAGCGCTATATCTTGGTGTTTTATTGTAAA
AAAAGCTCAGATTGTATGTACCTAGGTTTATTTTCAGTATACAGACTTACCATTCGGACCACTGTTACCT
TATTGTTATTTCAATAGTTAGAAGTTTCAGCATAAATTATCTGTTAATTGAGAATGTGAACATGTGGCTG
GAATACTCATTGAAAAAAAATACATTAATTATTGTTGTATAGATGATAATAATGTGACTTACGCAGGGATG
GATACTTAAATTATTAAAATTTTAACTGATATATTGGCTATGTAGTGAGTAGAATATTTTTTTACAAATAAA
TTATAGC
>TCONS_00088667 geneID=XLOC_017735 scaffold_29:1393242-1393657(-) Splice junction overlap
CAACTGGAATATCTAAAGGAGTTTGGATTGAAAAATGACATATTGCTTTTAAATATGTACAAACAGGGG
AAATGTACTATAGCTATAGATGAGCGAGATGTTGAAATAAAAACTCTTAAAAGTTGGTATCGCCACTAT
GTGAATATTGTTAAATGTAGCAAGAAAACGAATGACGTTTTTGGAACACCGATGACACTATCGATCGGC
TTGACGCTAATGTGCAATATTATTTTGTTATACGTCATTGTATGTGAAAATATTAAAACGGT
>TCONS_00089013 geneID=XLOC_017833 scaffold_290:310085-319579(-) Exonic overlap (-)
AGGGGTTCTGGTGGGGTGAGATAGGCATCCTGAGCATTGCGCTGCCACCTATCTAGGAGAAGGACAAC
TCCAAAATTAAACCATGGGTTGTAGTGGGGTGAGATGGGGTTGTGATGGGGTGAGATAGGCATCCTGA
GCATTGCGCTGCCACCTATCTAGGAGAAGGAAAACTCCAAAATTAAACCAGGGGTTTTGGTGGGGTG
AGAAGGGGTTATGGTGGGGTGAGATGGGGTTGTGGTGGGGTGAGATAGGCATCCTGAGCATTGCGCT
GCCACCTATCTAGGAGAAGGACCACTCCAAAATCAAACCAGGAAAGTCCACGCGTGACGAAATTTGC
CCATGGACTAACCAGTCCTAGCCGGTTCAAAGAGAGAAACTGAAGACAGCCCGTGTTGTAAAAACAA
AGCGATGAAGTCGGCTACCTGAATGGTAGGTCAGACTAGAAGATGGCTAGTATCGGGATACGAATCAC
TCGGAGAGACCACTGGTCTTTTAATAACGGAAAGGCAAATATTTTTTTGTCTTGTAGTGATGGTTGCCA
CAGCATTATAAAAACAATAGAAATAAACAGTATATTATACTGGTGTTGCAAATGTGATAGATTAGTCGTA
GAATAAGATGTTTAGAAGACCCCCTTAGTCATAGTCACCCCCTTTCCGCTTATTGTACCCTTTTTGCAAC
ACCTAGTATACTATATTCTCAAATCATACTATCGCATATTATAACTACGCTCGACAATAGATAACAAATATA
TTGTAAAAACTTTTTTTTTCTATTGGTTCTTTTTTGTACAAAGGACTGTGGCATTTTAACGAAACGTTTC
GAGAACAAAGTGAGTCGTACGTCTTATTACTAGTAATAGTTTAACAGCCCAGAGAGATTTTTGGCCGCT
TCGTCTCTCCTAGTCATATGGGCGCCTAGTGAAGCGGTGGTAGATCCATAGTATGTGCCAACATACTGG
GTGGTATCCGGTGATATTTTGACCCGTTTTCCTCCATAGGGTGAGATAGGCACCCTAAGCATTGTGCTG

CCACCTATCTAGGAGAAGGACAACTCCAAAATTAAACCAGGAGTTTTGGTGGGGTGATAAGGGGTTAT
GGTGGGGTGAGATAGGCATCCTGAGCATAGCGCTGCCACCTATCTAGGAGAAGGACAACTCAAAATTA
AACCAAGGCTTGTGGTGGGGTGAGATGGGGTTGTGGTGGGGTGAGATAGGCATCCTGAGCATTGCGC
TGCCACCTATCTTAGGAGAAGGAAAACTCCAAAATCAAACCAGGGGTTGTGGTGGGGTGAGATAGGC
ATCCTGAGCATTGTGCTGCCACCTATCTAGGAGAAGGACAACTCCAAAATTAAACCAGGTCCGGCCAG
TCCCTGATTCACGTAGTACCAAATTATCAACAACATCTCAGGACTAGTCTTGTCAAGTGCTTACCATCG
AGCGAATATTGCTGTCAATCTGCACCTAATTGTGACAGTTTTTGGAAAATAAAAGTCGTTGTCGGTAAA
GAAAATATGAGAACGGATCAAGCCGTCTGCCTGTAAGGTAGGAGCGGCTAGCTGCTGGACAGTATCGA
GATACGAGTCACTCGGGAGACCACTCTGGTCTTAGACGAATTGGTAATCGAATGTGAAATAGACAACA
ACAAAAGTACATACAATCAAAACAATCAATAGATCGTGAAAAATAAAGAAAAAAAAATCCTAGTGAA
AGTCAAACAAACAACTATACCTATTCCGAAAAGCTTACCCCGGATATACATAATGATAGATGACAAGAT
GTTCTGATGAGGCGAAAGCGTCGGCTTGAGAGCGGGAAAAGGATATGCGGACTCGTAACATATTCTAA
ACAGTAGTAATTAGGTGGCCAGAGAGTTTGTTAGACGTTTCTTCTCTCTTAGTAGAATGGCCTAGCGAA
ACGGAGGTAGATAAGTAAG
>TCONS_00089014 geneID=XLOC_017833 scaffold_290:310085-319600(-) Exonic overlap (-)
GGAAAACTCCAAAATCAAACCAGGGGTTATGGTGGGGTGAGATAGGCATCCTGAGCATTGCGCTGCC
ACCTATCTAGGAGAAGGAAAACTCCAAAATTAAACCAGGGGTTTTGGTGGGGTGAGAAGGGGTTCTG
GTGGGGTGAGATAGGCATCCTGAGCATTGCGCTGCCACCTATCTAGGAGAAGGACAACTCCAAAATTA
AACCATGGGTTGTAGTGGGGTGAGATGGGGTTGTGATGGGGTGAGATAGGCATCCTGAGCATTGCGCT
GCCACCTATCTAGGAGAAGGAAAACTCCAAAATTAAACCAGGGGTTTTGGTGGGGTGAGAAGGGGTT
ATGGTGGGGTGAGATGGGGTTGTGGTGGGGTGAGATAGGCATCCTGAGCATTGCGCTGCCACCTATCT
AGGAGAAGGACCACTCCAAAATCAAACCAGGAAAGTCCACGCGTGACGAAATTTGCCCATGGACTAA
CCAGTCCTAGCCGGTTCAAAGAGAGAAACTGAAGACAGCCCGTGTTGTAAAAACAAAGCGATGAAGT
CGGCTACCTGAATGGTAGGTCAGACTAGAAGATGGCTAGTATCGGGATACGAATCACTCGGAGAGACC
ACTGGTCTTTTAATAACGGAAAGGCAAATATTTTTTTGTCTTGTAGTGATGGTTGCCACAGCATTATAAA
AACAATAGAAATAAACAGTATATTATACTGGTGTTGCAAATGTGATAGATTAGTCGTAGAATAAGATGTT
TAGAAGACCCCCTTAGTCATAGTCACCCCCTTTCCGCTTATTGTACCCTTTTTGCAACACCTAGTATACT
ATATTCTCAAATCATACTATCGCATATTATAACTACGCTCGACAATAGATAACAAATATATTGTAAAAACT
TTTTTTTTCTATTGGTTCTTTTTTGTACAAAGGACTGTGGCATTTTAACGAAACGTTTCGAGAACAAAG
TGAGTCGTACGTCTTATTACTAGTAATAGTTTAACAGCCCAGAGAGAGATTTTTGGCCGCTTCGTCTCTCCT
AGTCATATGGGCGCCTAGTGAAGCGGTGGTAGATCCATAGTATGTGCCAACATACTGGGTGGTATCCGG
TGATATTTTGACCCGTTTTCCTCCATAGGGTGAGATAGGCACCCTAAGCATTGTGCTGCCACCTATCTAG
GAGAAGGACAACTCCAAAATTAAACCAGGAGTTTTGGTGGGGTGATAAGGGGTTATGGTGGGGTGAG
ATAGGCATCCTGAGCATAGCGCTGCCACCTATCTAGGAGAAGGACAACTCAAAATTAAACCAAGGCTT
GTGGTGGGGTGAGATGGGGTTGTGGTGGGGTGAGATAGGCATCCTGAGCATTGCGCTGCCACCTATCT
TAGGAGAAGGAAAACTCCAAAATCAAACCAGGGGTTGTGGTGGGGTGAGATAGGCATCCTGAGCATT
GTGCTGCCACCTATCTAGGAGAAGGACAACTCCAAAATTAAACCAGGTCCGGCCAGTCCCTGATTCAC
GTAGTACCAAATTATCAACAACATCTCAGGACTAGTCTTGTCAAGTGCTTACCATCGAGCGAATATTGC
TGTCAATCTGCACCTAATTGTGACAGTTTTTGGAAAATAAAAGTCGTTGTCGGTAAAGAAAATATGAG
AACGGATCAAGCCGTCTGCCTGTAAGGTAGGAGCGGCTAGCTGCTGGACAGTATCGAGATACGAGTCA
CTCGGGAGACCACTCTGGTCTTAGACGAATTGGTAATCGAATGTGAAATAGACAACAACAAAAGTACA
TACAATCAAAACAATCAATAGATCGTGAAAAATAAAGAAAAAAAAATCCTAGTGAAAGTCAAACAAA
CAACTATACCTATTCCGAAAAGCTTACCCCGGATATACATAATGATAGATGACAAGATGTTCTGATGAGG
CGAAAGCGTCGGCTTGAGAGCGGGAAAAGGATATGCGGACTCGTAACATATTCTAAACAGTAGTAATT

AGGTGGCCAGAGAGTTTGTTAGACGTTTCTTCTCTCTTAGTAGAATGGCCTAGCGAAACGGAGGTAGA
TAAGTAAG
>TCONS_00089015 geneID=XLOC_017833 scaffold_290:310085-319600(-) Exonic overlap (-)
GGAAAACTCCAAAATCAAACCAGGGGTTATGGTGGGGTGAGATAGGCATCCTGAGCATTGCGCTGCC
ACCTATCTTAGGAGAAGGAAAACTCCAAAATCAAACCAGGGGTTGTGGTGGGGTGAGATAGGCATCC
TGAGCATTGTGCTGCCACCTATCTAGGAGAAGGACAACTCCAAAATTAAACCAGGTCCGGCCAGTCCC
TGATTCACGTAGTACCAAATTATCAACAACATCTCAGGACTAGTCTTGTCAAGTGCTTACCATCGAGCG
AATATTGCTGTCAATCTGCACCTAATTGTGACAGTTTTTGGAAAATAAAAGTCGTTGTCGGTAAAGAAA
ATATGAGAACGGATCAAGCCGTCTGCCTGTAAGGTAGGAGCGGCTAGCTGCTGGACAGTATCGAGATA
CGAGTCACTCGGGAGACCACTCTGGTCTTAGACGAATTGGTAATCGAATGTGAAATAGACAACAACAA
AAGTACATACAATCAAAACAATCAATAGATCGTGAAAAATAAAGAAAAAAAAATCCTAGTGAAAGTCA
AACAAACAACTATACCTATTCCGAAAAGCTTACCCCGGATATACATAATGATAGATGACAAGATGTTCT
GATGAGGCGAAAGCGTCGGCTTGAGAGCGGGAAAAGGATATGCGGACTCGTAACATATTCTAAACAGT
AGTAATTAGGTGGCCAGAGAGTTTGTTAGACGTTTCTTCTCTCTTAGTAGAATGGCCTAGCGAAACGG
AGGTAGATAAGTAAG
>TCONS_00088984 geneID=XLOC_017834 scaffold_290:416104-424242(-) Intronic
CTTCCTCTTTTTGGAGGAATTTGAGAAGAAAGACTGAAAGATGTCGAGCACCTCATGCGGCTGTAAGA
GGTGCGAAATCGCGTAAGCTGTAAGATGCTGACAGTCCACCACCACCATGGACTATGGATAGAACGAC
ATCACGCCTGTCAATTTTTTCTTCTGCAACACGGCCTCATCTACAACACCGACGGATCCATCCGCCGTC
CTTTATTTGTTTCCCGCTTGATTTCATCGTACTACATCCTGCATCAAACCTGTACTGAACTGCATTTCATA
TATTTTTAGGTAAAATTTACGATTTTAAAGTTAACCTTTGCGTTTTGAAGGATTTTGAAATGACTGTACG
TTTTATACCTGCCCGTATTTACAATTTGTAAGAATTGGTTTTTTCCTACCGGACTTTCGATATATTGAATG
ATATTACATAAATATTAATTTATACACAAATTTCAAACTCCTGTAAGTATACCTATTCGACTTGGTGCTCT
ACCTACACAATTCTTGTTGTGATTTATGTAAAAATACTTTAAATATTTTTTTATACATCTTATTATCTGTATT
TTTTGCTAAAGTGCTACAAACGTACCTACCTATAATTTAAAAGAAAACCAATGTTACGTATAACGATGTA
TACCAGTTTTGCTTTATGTCAGATTGATGGCAACGTTTGCCTATCTTATTTCCGTTTAAGATTTTTAGAAG
TGGTAAAAACTTTATTCATTTCAATACTGAAACATTTTTTGCAAAACCACAATTCTATTTTATTCTATTTA
AATTTATTAAGTGGCTTAAATTATTTTCTTACAGAGTACATTGATTTTTAATTTTATTTATTAAAACATGAT
ATGTATTTCCATCAAATTACTTTAGATACATATAGATACATATTGATAGATATAATAATATTAATAAAAATAT
GTATTAGGTATCTGCTTATTGTCATAGAATTTTTCGAGGTATACAGACGATGTAATAGTTTCGCTAATAGT
TAGGTCTTGTTTCCGGGTAGGATAGTAGTTATTTATTAGACGGTTATATTCCAGTTTAACGTGATAAGGG
ATGTGATAAAAAACGCAGGAATAAAGGTTAGTGTGGCTGAGGGCAGGAGCTAGATGAGGGTAGTATTAA
AAATTATATTCTCTAATGTTCAGTCAATCTGGTTGTTTAGAATAATTACTCATTAGACTTACAGTTATAATT
ACAGAAATCATCATACAGTAATATATCCATTTGTTCAAACGTAGGTATTTTATTTATATAACATTATATCTT
AATATATTGCACTTAGGTATAAGCATTTTAACC
>TCONS_00088985 geneID=XLOC_017834 scaffold_290:416104-426211(-) Intronic
GTTTTTCTTTCTCCTGGATGATTTTGTTGAGAAGATGTCGAGCACCTCATGCGGCTGTAAGAGGTGCGA
AATCGCGTAAGCTGTAAGATGCTGACAGTCCACCACCACCATGGACTATGGATAGAACGACATCACGC
CTGTCAATTTTTTCTTCTGCAACACGGCCTCATCTACAACACCGACGGATCCATCCGCCGTCCTTTATTT
GTTTCCCGCTTGATTTCATCGTACTACATCCTGCATCAAACCTGTACTGAACTGCATTTCATATATTTTTA
GGTAAAATTTACGATTTTAAAGTTAACCTTTGCGTTTTGAAGGATTTTGAAATGACTGTACGTTTTATAC
CTGCCCGTATTTACAATTTGTAAGAATTGGTTTTTTCCTACCGGACTTTCGATATATTGAATGATATTACA
TAAATATTAATTTATACACAAATTTCAAACTCCTGTAAGTATACCTATTCGACTTGGTGCTCTACCTACAC
AATTCTTGTTGTGATTTATGTAAAAATACTTTAAATATTTTTTTATACATCTTATTATCTGTATTTTTTGCTA

AAGTGCTACAAACGTACCTACCTATAATTTAAAAGAAAACCAATGTTACGTATAACGATGTATACCAGT
TTTGCTTTATGTCAGATTGATGGCAACGTTTGCCTATCTTATTTCCGTTTAAGATTTTTAGAAGTGGTAA
AAACTTTATTCATTTCAATACTGAAACATTTTTTGCAAAACCACAATTCTATTTTATTCTATTTAAATTTAT
TAAGTGGCTTAAATTATTTTCTTACAGAGTACATTGATTTTTAATTTTATTTATTAAAACATGATATGTATT
TCCATCAAATTACTTTAGATACATATAGATACATATTGATAGATATAATAATATTAATAAAAATATGTATTAG
GTATCTGCTTATTGTCATAGAATTTTTCGAGGTATACAGACGATGTAATAGTTTCGCTAATAGTTAGGTCT
TGTTTCCGGGTAGGATAGTAGTTATTTATTAGACGGTTATATTCCAGTTTAACGTGATAAGGGATGTGAT
AAAAACGCAGGAATAAAGGTTAGTGTGGCTGAGGGCAGGAGCTAGATGAGGGTAGTATTAAAAATTAT
ATTCTCTAATGTTCAGTCAATCTGGTTGTTTAGAATAATTACTCATTAGACTTACAGTTATAATTACAGAA
ATCATCATACAGTAATATATCCATTTGTTCAAACGTAGGTATTTTATTTATATAACATTATATCTTAATATAT
TGCACTTAGGTATAAGCATTTTAACC
>TCONS_00089026 geneID=XLOC_017835 scaffold_290:460601-467347(-) Intergenic
GACAACCCCGTAAATACCTTTTCTTTTCAACACTAATCAAAAATTCGTTTCTTGAAGAATTTACTAATTA
AATTAACGAAAATACACCGTTTTTACAAATTTATCTTCTTTAAATACTGATTTAAGTACCGGATTAGTGAT
CAATAATCTACGGTGTCGCCAGGCCTACAATGTTGGAATATTAATAGTTAATTATATGCCATGGAACGAT
TCCCACTGAAGTCGTCAACTCCGAAAATGAGCACTACATATCAATCAGTCTACAAAAAGGGCGCGACA
AACGGGCGCGGCCACGGCTCCACGACCCCCGACGACTCGGTGGACATCAGCCCGGGCGTGAGGGAC
GACGGCTCCACGCCCACCAGCCACGGCACGTTCCGGCTGTACGAGCATCCGCACCC
>TCONS_00089022 geneID=XLOC_017824 scaffold_290:504849-517371(+) Exonic overlap (+)
CCGGCGTCCGCCCGCTCTGTACGTCCGCGACACCGCGCGAACCTTGCCCGCTCGCCGGCTCCGATCGA
ATCACGAACACGATCGATTGCCAACTTAACCGTTTATTTACATGTTTATAAACAGTCTATATCTGTGACA
GTGTTGGGAAACTTCTCGGAAGATTCTGTATGCACCGGTACAACTACGACAACAATCAAATGGAAACA
AACGTTTCACACACTGAATAATACACTACCAAACACGGTATTGCCACACCAACCTTGATAGCGAAAAC
CGGCGGAGACAACTGATATTGATATCTATCTGAAGATAAGAACCTTGGAACAGAGACATTTATAATCAA
AACGACAGTGTGACATTACAAAACGATATTTAGGACTAGTGCAAAACGAAGCCATTCCATAAAGTGCC
GTGAAAGAGTAAGGTGAAGGTGAAACACAAGGCCGCTTACATAACCCTTGAATAGAAAAAAAACAAC
ACAATCTTTCCGATCTCCGATAAAAAAAAACCGTTCAATGTGTTTGCGAGCGAAGTTTCTCTTTCGAGTT
ACGCTTCTAGATGTGAATATTTAGAGTCCTTGCCACGGCAGCAATGCTGTCTTACATGGTGCCCGTGGA
AGAGAAGCCGCCCCCAGTTCCTGTCCAGGGTCGGTTGGCTCTGGAGCTGGTGGCAGCTCCGGCGAGC
CCCGAGCCGACGG
>TCONS_00089155 geneID=XLOC_017868 scaffold_291:186612-189412(-) Splice junction overlap
TAAAGATCCGCCCAAATTAATTCATAGTTGAATATATGAAATTTGAAAAGGTCGAAATGTTCTTTATTTT
TCGTTATTCCTAAGTAAAAGTATACCTATAAACAATATTTAAAATTTTACAGGTTCTCGACCATTCGTATG
AAGAAGTGGACGTTGACTTATCAAAATCAGTGAAATTGGATGTTTCATTATCAGAGGACTATGAGATTC
CAGCCAAGAGAGCAAAAACAAACTACATTTATCAGCCAGTGTCAACTCAGTCACACACAAGTCGACG
AAAAACACCACTTGTCAATCGAGTAGCACTGATGTAAGAAGGAAATCTGCTGAAGTGCAACGCAATC
TTGTGACCCCAGTGATAGCGTCGGCGTCGCTCTCGTTGAAGATTCATCAATTGTGTAAGTGATCAGTGA
TTTTATAAACTTAAAAAATTCAAAAGTCTGTACCAGATAAGTGTATTTACTTTTTTTACAAACTGACACT
TGTTACATATCTATCACTGTAAATAGTGTTTATTTTAGTTTCGGTTGGTAGACATTCCTTGATTAGGGAAT
GTTGAATTGACTTCTAAAACGAAGTTTCTTGTGATTTGAACATGTTTCTAATTAATTTTTAATAAAAATC
AAGCTTATGAACTAGGCACGTAAAGTTATAAGTTTTAAGTATCAATTTCCACCTAGTTTGATTTATTAAG
TAGATAAATTAGATTTAAGATCATCTTGTCAGTACAAAAATATGATGTTTCTGAATCGCATTTTTTTGTAA
TTAAAAGTATGTAGGTATATATAAACTGTAATGAGTCGAAAATATCATGACTTCTCAATTGGTTTTATAAA
TTGGGAACATTGATATTTTCTTAAGCATGGGACTTTGTGTTTTCGTTGGTTGACATAAAAATCCGATTCA

GTTACATCAGTCAGAGCGGGTATATTAGGTAAATGTCCCACCGCTGATTAGACACTTGTTTATTCTTAGT
AATGTTTAGCTATGGAGAAAAAATGGGCCGCTCTACTTTAATCTCTAATATCTAACAATTCGTTATATTCT
GAAGTTATCATAAGTTTAGGGTGGGTTGCACCATTTAACTTTAACTATTACAAACGTCAAATCTCTTGA
AGATTGATCTCTCTCGTCAAGGGATTTGACGTTTGTAATAGTTAAAGTTAAATGGTGCAACCCACACTT
AGTGTATTAATTACGATTTTTACCGTTTAAACTGATAGAAACGTACCTTACTCCATATATAGGGAAGTTAT
TGATATTTAATGATAAACTAGGCACATTCAATTTTATACCCGGTATAATTTTTAGGAGGAAGTGATTGCG
GCGACTGAGCGATTATTTAGGTATTTCGTTACTTTAATCACAAACCTACTGTAATATCATTCATCAGAAA
GATTAATTGTCTAATATTGTTTGTGTTTATGACTATCTCGAATAAATTAGTTATTATTGATTCTTTTCGATT
GCAATATCCTTTCATATTGAAAGTATGTTTCAATTGACAGAAAAAAGTGAATAACATTTGAGAATATAAT
TGTATGTTAGTGAAGATTGCTAATTAGAGGCATTGTAAGTTCTTAATTGATTCTGTCGACAAATAGATAG
ATTAGGTTGTGATAGTAGATCTCTTCAACATTTCTTTGTTACTAAAACTGGAAATACATTGAAAATTGTA
TAAGAATGGAATGTAATAAAAAATATTGGCCACATGTAGGTAGTTTAGAATGTGTATTTAAAAGTGTATC
CTGAAAGAGAAACAAGCAAATATGTGAATAATGATTTAAGCACAGAATGCCAATATACATTTTAGATGA
TTAATCGCAATAATAATTGAAAGCTTCTGTAATTACATAGAAATACTGTTTTAGGAATGGTTATTTGTTAT
TAATCGTTTAAATGAAACTGTGATATCAGATAAAATTGCTGCCTGTACATATATTTGCTTGATTACTCTAT
TTAATACATTGGAACAAAATATAAGTGTGAGCCTCACTTATTGACGTGTTTTGTGCTTGTGAACTTTATG
GTATCGCGAATTTGTAAATGTATTCTAAATATAATAAGGGTTTTACTTAATGACAGTGTTATTAATTCTTA
GGATTTGGACCATAGTTTTGAGCACTGTTATAATTTAGTGTCAAGTAAAATACACCTTAATTCACCAAG
AACCAATAAGTCTATAAATACACTTATAGAAGTTTGAACTCGGTCGGTAGGAAAAAATATTTTTAAGTTT
AGTACTATACATATTCTTAAATTTAGGTCGTCTTCTCTTTAACTGACTAGAGTAGCGAATTAATTTAGGTG
CTTGCGACGTATACTAATGCGAGATATACATTTGTGCAATCATATAGTACGATTACCTACGTCAGTCGTTA
CTATTTGCCCTTTTAATCATAACACCAATGAAATCTGCCTCATTTTGTATAAATTGAAATTTCATTCAAGA
ATAAAATCTCAATATAAGTGAATGGTTATTGATATTCACTTTTAAATCTGGTAGCTATGTAATCATCTGCT
TCCTATTTGTGATTCTAAATATGGAGCATAGTTTAAAATAAATAAATC
>TCONS_00089365 geneID=XLOC_017907 scaffold_292:19563-35137(-) Unclassified
CTCAGAACACAATCCACAGGAACGGTTGACGCGCGCACGCACAAGCATTTCAAACAGCCGTCAAAAG
AATCCGAGCAAAAAGGCGCGAGCAAAAAGTTTTAAAAACAAAATTCTAAACTGCCAGTATTAATAAA
ACCAAAAACTTTTTAGTGAAAAAGTAGGTTGTAAAAGAAAAAGTGTGGACGAAGTTCAAAACTAATT
CGAAACTTAAATTGAAATTGTGGTGCAGCGATAGTTGCAAAACACTAAGGACTAGGCGTCCAGATTCG
AAAGGTGATAATGATAGTTTATATCCAGTAGTGGCGATAATTGGACCCTTGGAATCTATTGGACTGGAG
GCGGCTGCATGACTCCTGGTACTCACGATGCTGCGGTTCGAGGAGATAGCCTTCAAGGCGGAGTTGAA
CCGGGACGAGGACCCGGAGAGCGACCGGGACGCGTGGCTGCTGGGCGGCGAGG
>TCONS_00089489 geneID=XLOC_017908 scaffold_292:75914-77536(-) Intronic
ATTACGTCCCTTACTATCCCAGGATCACACAAATAGTGCTATCCTTGTTTATGTGACCGCCGCCGCGCGC
CGCCGCCGGTGAAGTTGGGATTAGTTTTTTGCATGTTTTGTTTGTTCATTTGTTTACAGTTTAGTGCAAA
CCATGGCCACGATCGGTGTTATTGTTGTTTGTTAAAATATTGTGAGTGCTCTAAAAGCAAATGAATATTC
AAGATCTTATATTCTAAAAGATATTAGTGAATGCAGATAAGAGTGTTTAGTGAGTGCTATGTTTGAATAT
TGCAAGTGAAAACACAGCCCATCAATGTAATGCGCTAAAAAACTGCAATGGCTCCGAACACATGATTA
CCAGATAACAAATCCTGGCGCAGCTTACGTGTGTGCTTCTATAACGTCGCAAGCTACAGTGGCTGTTCA
TAATTTTTGCAAGTTCTACACCTACAGGATTCAGTTATCTGGGCATTATAGATACCCTATTATCACCTAAG
AAGTTATTTAGGTATATTTATTAAAACCATTAAATAGAGGTGAGTGATTGAGTTAA
>TCONS_00089333 geneID=XLOC_017898 scaffold_292:89018-92235(+) Intergenic
CTGTGCCGAGACCCACGGCGGAGAATAATGCTGAGGTGCTGGAAGACTTTGCGGCCATGAACCTCTTC
CCAAAGGAACTGCTCCTGTGATCTAATCCAGAAGTAAAAGTAAAATGTAAGAACAAGTCAATCTCCTA

GTGATACCGACAGTCGCGTTAGTTTCGTTCACTCATTCAGTCATTATTTAGTTCACCTTGTTAGAACGC
AAAGAATGAAAGGAACTCTGTTACAAT
>TCONS_00089456 geneID=XLOC_017898 scaffold_292:89641-92795(+) Intergenic
CCGAGACCTACGGCGGAGAATAATGCTGAGGTGCTGGAAGACTTTGCGGCCATGAACCTCTTCCCAA
AGGAACTGCTCCTGTGATCTAATCCAGAAGTAAAAGTAAAATGTAAGAACAAGTCAATCTCCTAGTGA
TACCGACAGTCGCGTTAGTTTCGTTCACTCATTCAGTCATTATTTAGTTCACCTTGTTAGAACGCAAA
GAATGAAAGGAACTCTGTTACAATATTTTTACTACTTGAAGGCTTTTATAGAATATTTTCTTCAAGCAAA
TTTCAAATTATGTGCAACTAATTGTATTGGTATTTATTTTTACCATTACGTTGACGATTCATTTTCTTAATC
GAAAGTTACGATTTTATATTTTTATTGCAAAGTATATTTACTGTGATAGCTCTAAGCTAGTATGAGTATGT
CTCACTAAGAAAAATACTATCTTATGAAAAATTCCGCCGATTTTGATAGAATTAACCTATTTGGAGACTA
ATACGACAATAAAAAGATGTAGGATACATTAAAATTAACTTTTTAAAGAAAACTTAACTTTATATGTAAG
GAAATCAAGACTGCAGAGACCAAAAAGCAATTCCTTCCTTGCTTTTTTACTAAAATCTTCCAATATGTT
GACTAAAAGTAATATCCAGACACATTCCTATAACAATACTTACCCTAAGACTTATATTCCTGTCTTGCATT
TACAACTTAAATATATTACCATGTTAATAGTCAAATGTGACTAGTCAACCCAACAACTGATAGGTTAGGT
TGCAAACCGTATCGACGAGGT
>TCONS_00089540 geneID=XLOC_017898 scaffold_292:89792-92825(+) Intergenic
ACCGTGCCGAGACCCACGGCGGAGAATAATGCTGAGGTGCTGGAAGACTTTGCGGCCATGAACCTCT
TCCCAAAGGAACTGCTCCTGTGATCTAATCCAGAAGTAAAAGTAAAATGTAAGAACAAGTCAATCTCC
TAGTGATACCGACAGTCGCGTTAGTTTCGTTCACTCATTCAGTCATTATTTAGTTCACCTTGTTAGAAC
GCAAAGAATGAAAGGAACTCTGTTACAATATTTTTACTACTTGAAGGCTTTTATAGAATATTTTCTTCAA
GCAAATTTCAAATTATGTGCAACTAATTGTATTGGTATTTATTTTTACCATTACGTTGACGATTCATTTTCT
TAATCGAAAGTTACGATTTTATATTTTTATTGCAAAGTATATTTACTGTGATAGCTCTAAGCTAGTATGAG
TATGTCTCACTAAGAAAAATACTATCTTATGAAAAATTCCGCCGATTTTGATAGAATTAACCTATTTGGA
GACTAATACGACAATAAAAAGATGTAGGATACATTAAAATTAACTTTTTAAAGAAAACTTAACTTTATAT
GTAAGGAAATCAAGACTGCAGAGACCAAAAAGCAATTCCTTCCTTGCTTTTTTACTAAAATCTTCCAAT
ATGTTGACTAAAAGTAATATCCAGACACATTCCTATAACAATACTTACCCTAAGACTTATATTCCTGTCTT
GCATTTACAACTTAAATATATTACCATGTTAATAGTCAAATGTGACTAGTCAACCCAACAACTGATAGGT
TAGGTTGCAAACCGTATCGACGAGGTTATAAAAGTACTAAGTACATACTGATGAAG
>TCONS_00089368 geneID=XLOC_017911 scaffold_292:118293-120652(-) Exonic overlap (-)
TAGCGCTACTTATGAAGCTAACCGTATACATATAATGTGTTATTTGTATTTTTGTATAGTCTTATGAAGACA
TTGTAAAAAGAAAATTAGGATTGGTCTTCCAAAATTATCCTGTGATGCACAAACAATGATAGCTACTAT
GTAGGTGTAAGTGCAGTTTTTTTGTTATTGATGATATCCATAATTTTTATGTATATTTTGTATAGTGTTGAT
TATTTCATGATAAGTATTTTCAGTAATTTTGGTCATCTAAACGCATTTACTAGATACTGTGCCATTCATTG
TCTTATTTTTCATAATTTTTTAACTGCCATAGTTTGGTGCTCAATATTATTTTTATATGTGTATACAATACAA
TAAAAATCTAAAATAAGCAATATAAAAAGTTTTATTTTATAAACGTTTAGTCTAAAATCAACTTTGTGTT
CAAGAAACGAACTAATAATTAAACACCTAATATAAAAGTCTTTAAAATCAACTTATTCAGTTTTAGAAA
CTAATCATAAAGATATTAAAATAGATAGAACAATTTCCGGGATTTTGAATCCAACAATAACAGAAAGTA
GACGTAAAATTAGAAAGAGAAATTTCGGAACCTAATGGTTTCAGCAGCAGTTCCGAATAACAAGCAAA
TGATAACTTACAAATAATGCCATGTAAGATTGATAAAATTCTAAAATCGACTTAAAAGTGTGCACTTCC
AAAAATAACATGTAAGTACCTAATAATTAATAATAAGTAAAATGTGCTCTCGGATTATACGTAATCTTGTA
CCGACGTGAGGAAACACTAATCTAAATCACAACAGTGGAATGTTAACATCTCTATAACAAATCACATAG
CCTTTTAGTCCTAAAATATGTATTTGGTTATAAATACAATGGACGGTTTACAAGTTTCATTCAGTTACAAT
CTTTTCTTAATCGCATTAGCATATAACCCGAAAATAAATAAGTAAACAAAAGGCAAGAAAACCTTAAAT
TATCTTTGGATTTTCTTACTAAGTACGAATATTTAATTGTGATCGTTCCTAATGTTATTTTAACTTTAGTTA

TTAAAAATAAATGTTACATAAATATGAGGTACACAAAATATAGTAACCGATCTGTTATAATCTACCCTGAT
TATCTATATAAAGCCTACTGACAATACCATTGTCTAATCACAGCTACAAATTATTGTACTTAGTACCACAG
GTATAGGAATGTTTTCAGATCTGTAAATCTTGTATTATATCATACTTATTAGTCTAAAAGACAGTTTTCTC
TTCAGCTGTCTTGAAAGTACAGTATGTGAGTACTGAACTGGCCATTTAAAATATTAGTGTAGCGAGCAC
GAAATAGTGAAGTAAAGTTAACGTATCGCCACCGAGTAAAAATTAAGTTATTTAGTAGTTCTAACACAC
GAAGTGCGCTGATCATTATTTGCATACATTATTTCTCAGTGACGCTACTAACATGTTTTCTGCTTGTTACA
CAGTACCTACCTCAGGTAACCATGAGTTGCTAAAATTACCAAACGTTTTCTTCTCCCTTTTGCATACGTT
TTCTGTCAAAAGTTTCCTGAAAAATGCCGCTAGAGGCGCCACATTGTATGTGAGAAATCCGCGAAAGA
CACGACAGAAGATACCGTTAGGCAATTTACTATAGCAATCCATGGTTCAGGCACAGTTCACTCACCCCT
TCACACTCTTGGCCTCATTCTCTGAAGACTCCTCGTTGATAACCGACAGGTTTTCCTCCGAGATCTTCT
CGAGACAGTTGCAGGCGAGGCAAGTCATACTCGCGTTGGCCACGTCTTCCTCTATCTCCTTCTCCAAC
TGCTCAAAGTCATCATTCTCAACTTGACACTTCAAGCATTCGTCAGACGACGAGTCCTGATAACACATA
ACATTCTCAATATTCAATTCCACTTCGTCACTCCTATCATCTTTGGTGTATAAAGACGGGCTGCTGTTGC
TTAGAACCCTGGATTCTCTGCTTATCTTTTGGACGATGACTTCGGACTTTGGTCGTGTTATAGGTACATC
TTTGTGTACATTTCGTCTCTGCACGGGCGTGTTGGCTCGCTTGTCCTTGGGGTAGACTCTCTGTATGCG
TTCCTCTACTAGCGAAATGCTTCGCTTGTACACGACGGTGTCTTGAGGGCCGTCGATGGCGTCTCGCG
ACTCCGTGTCGGAGTTGTCGCATTTGAAATAGATGCTGTCGCAAC
>TCONS_00089472 geneID=XLOC_017902 scaffold_292:186737-195363(+) Intergenic
GTTATGAAAATTGTTATGGATATAACAATATGCAGTTTCTATGATTACTGAAAAGAAATTTACGTATTATT
AACCACTATCTTAAGAAATTAATATCACGTAAAGTCCAAATAAGGTTATAAAAGGTAAAAACGCGATCA
TCACGCTATAACTATTTGGCGAAAATTTTATCATTGTGATTAATTTCATTATCGAAAAGTGCAAAAAACA
GCTTTATAAGGTAGATTTGTATTAAAACTGTTGTCTTGCAAACAGCAACAGTGAATGCAAAGTACTTTA
CTTTACTTTATTTGAAATATCGCGTGCGTATTAGGCCTAAATGCAAAATGGAGAATTCGCAATTCCTCCC
ATTATTGTGAATTAGCAGTAGCAACTGTAGCAAGTCAATAATTAATAATCATAAAGTTTATTTCTCTTTGA
TTTAATTGATCAAGTAATAACTGAATTTTTAGCATGAACTGACGATGTAATATCCTACAGAAAAAATCTT
AATACCGCGATGTAATATCCACCATGGAATGCCAACCCTACTTTAGAGTATTGTACTCTATATCATTTAAG
CGTGATCTATAATCTATAACACTAATAGAGTACGCTAAAATAGCCTTTTTTTTACACATTTTTACTAGTCA
TATAATATTACGCAAAAACACATAATCAAAACCAAAAAAGTCGACTATTTACTTTGAACGATCTTGAAC
GCAGCCACTTATGTCAAACGTCAAATGTCACATGTGACAAGTTATTTTGAGAAGTTTTTGAGGTTTGTT
TTGTTTGTGACTTTGGTTTTGTTTGTTTGGAATACTTTTATCCATTCGTTATAACGTGATTGATGAACATT
GATAACGCCGATCAAGCATCCTGTACTGATAATAATAACGCAAGTACCCCATCTAAAAATCGAAAAGTT
TTGACGAAGAAAAAAAGGACACAGAAGTACACACAACTTTGGGAAAATGAGTTTTCATGGCTAAGAA
AAGACTCAGAAAGTGATACAAATGCAAAATCCATAATCTGTGGCGAAGTTTTCTCTCTATTCCATCAGC
TGGAGTTGTACACATTCTGAGGAACAATGAACATACAGTGTAGCCGACCAAAAATTCACCTAGCAACG
TTGCAGCGTAGCAGACCAGCGTCAGACCAGCATTAAGTACATCAATAAGAACAGCCGGAATATGGTTT
ACTAATTACTTATAGTCTCTTTGAGTTTTGTTTTAATTATGATTGCTAAGTGCATTTAATATCACCGCATAG
ATATAATAACTTTATTAATTTTGCTAATTCATTCAGTTTGTTAACATAAATTTAGAACTTGTTAGTTAGCGT
TCGAATGGCCGACAGGGAATGGGGACATTCCCAATAACAGGACGGCCGACTGTTAGCGTTTAAATTTA
CCAATGCTATTCTTTACTAATATCAAGCTACTTATTTCATCATAGGTCCGGCCGGATTACTAGATGTAGCT
AGGAGTTGTTCTTTAGTTCATTTAGTTTCTATATTGTCTGTGATTCGCATTGACTGGCCGGTCACTGGAT
TCATGCGGATTCATTCAATTCAAGATGGCAGAAAAAACAAGAATAACTTTTAAAAGACTTCTGTTTGTT
ATTAGATTCCTTTTTGAATAGTTTGTGTTCCTTATAAAAGTATTTTACTTCAAACTTCCTGAGTGTCTTAT
TTTCAGCATCTTCTATAGTACCAAGCTAACATACGGAACCCTATAAACAACACATATCACAAAAAAAAC
AATGCGAGTAGAGTCTATTTAGGAGGGGCAATGATTTAAAGATTAATTGAATTCATACGTCTTAGCATAA

ATCAAGGTTAATATTAATAAAAATACTCCAAGGTTGCACATATTGATGATATAAATTAGCCATCTTCATAG
TAACGTAATAAGAGGATGTAGAGATTCTAGAGTCAGGTTTTCCCAACGTTATAAGACCTACTTATGGGA
ATATTATAGCTATCTGCTAACTAAGTAGGTTGGTATGTACGGTAGCCTGCGCCTAAAAGTATACAGGCGG
AGTTTTTAAAATAGCGATCTCAAGCTCACATGCTGCTCAAGCACATCCACATAAACACCACTGCTACAC
ATATCACACACAACACTTTCCCAGATTTATAACAGATGTAGCTGGTCCCTTCATCATCAGGCATCGATAT
TTTAAAAACCCCGCCTGTATACTTTTAGGCGCAGGCCACCGTACCTACAGTATGTAGTTAATCAAATTAC
ACTGACGGGCAATGAAAAGGTTCCACTGAGAAAATCACCAAATTACTTCTAATCGGAAAAGGCTAGCT
TAACGCCGTCTTCTGCAACATTAAAGTACATTTAACAGAGCATCATCAGGATATTACCAACTAAAATCG
GTAATATTTTGTTCCAATTTGAAATTGAATCTTTGAAAAAATTGGGATCGATCGTTGTCGGCATTTTGTG
AGTGGAACTTTTTCATTGCCCGGCAGTGTATAGGGTGAAATCGCCAAAATTTGTACGTAACAAAAACG
TAACAAAAGGGAAACGTTACAAAAATGTTACAAAAAGGAACTATACGGACTCGATCACACTGCTATGA
ATAAATAAAACAATTTTTATTTAATCAGCACTACGGCGAAGTGAAAAGGATGGTTATGATTTTGACCGG
TACCTATTCAATAATTAAAACACGAAC
>TCONS_00089491 geneID=XLOC_017913 scaffold_292:213039-214207(-) Exonic overlap (-)
GACTTGCAGTAAGTATACTCGTTTTTAATCTATTCAAAGCTAAATATTAAAAAATTAAAATATAGAATAG
TTATTTGTTACTTTTATTTTATCTTATAGATTACGAAATTCAAATTATGATAACCCTAACCTTATTTAAATTT
AAATATAATTTCTTTCTACATACTAACCCTTATTAAAATAAGGAAAAATCACAAAACATTCTTATCATTAA
ATCTATTCTTATCAAAAATAAATATTTAGAGTTTTAGCTAACATTTTCAAAAATACATATCGTATATTACAT
CAAACAAAACAGTTATGTAGCTAGAACTTACAGCGTTGAACTCTTTTGTACGAAAAACTATTTGTGCTA
CGAACCGCTACGCCCCCTGTCATTGCCCAAACGTATTTTTTAAACATCATCATCTTTATTAGCCTGTAAA
TTTCCACTGCCGGACATCGGCCTCCGCCTATGCATCGCAAAACCCTGTCATCAGCCTTCATTTTTTTTAT
AAATTGTTGTATTAAACAGCACATACGAGTAATATGTATATCCTTCTTTTTACACAAGGAGTTATAGATAT
TGTAATCCACAAAGGGTTATATAAAATATTAAAAAAGGTAACCTCCAACTCCAATTCATAGAATAGACT
GTCAGCTTTCGAAGGCTTGCAATTTGTTCTATTATCCTTGTTACACCTACCGAGTAGAGTGTCCGATATA
GTTCTCTCGGCTGAGCACTAGGTCAATTGCTAGTTGTTCATTGGTCGAGGATCGGCCGAGCATACTGTC
TCGGCCACTGTACTCGGTAGGTGTAACAAGGGTACATGAGTATAACACATCAGTCTAGCGCGAGTGCG
CGGAATGACCCGGGCACATCCTTCAGTTTTTCAGCGCGCGCATCGCTGTGCGCGGATGAACGCAGCTT
TTCAGTCACCTTCCAGCGCTGGTCGTTCTCC
>TCONS_00089494 geneID=XLOC_017916 scaffold_292:293144-297928(-) Intergenic
GTTAACTGTTAACAAATATTTTCGTATTTTTGAACAGGTTGTGACCTTAACTGAATACAAGTCAAATAGC
GTATGCAGATACCATACAATCTTACAGAAAAAATAAACACTGCCAACTGTTTCTATGAATATTAATATTTA
TTTTTATACTTACAATTCAATTCAGTTTCGAAAGCATAATGTATTACAGTATAATACATAAAGTTAATAAC
TATAGCTCAAAGTCAGCGTGCTTCGCAAGCCGGGACCGACCGCTCTGCAAGCGAATATTACAAATTATA
ATTTAAAATTAACTACACAGTACTTTCACAAGACCAGAGCCAGCGCCACACGCTCGCTGCAGGCGCGG
CCACGCTGCAGGCCAACCAACGTAGAACGGGGTAAATGGGAATTTCAGGATAAATAGGTACAATATTC
TGACCAATCAGAACGTCTAATAAGACCTATTAGGTTTCACGATATACCAATGCTTATAAAATACATGGAA
ACATAGATTCTTGTTCCTTTTGCCCTGGAATTCCCATTCATCACCTTTTATACTTAGAACTTATCAAATGT
ACACTTAAAAGTATTCCGCCCCTGGTCCCTGGCAGCTTGGTCGCGCAGCTCACGGAATGACGACTCCG
CGTCGCGGTAAACGCTTCTACATTTTTGGTGCAGCCTTCCTTTACAATAAATAAACTTTCCCACAATACT
TTCATTTTGGTAAAACAACAGTGCTCGATAGAAATGGCTTTAAATGGTGTGAAAAAGTTATGAACGAC
CTTAATAGCTGTAAGAACCGTAAAAATGTGTTGTCGGCCCGAGTCCGTCGACTCTGTACTATACATTGA
TAACTTTTATGTTACACAACAATTAAAACATTATTGCAATCTTAAATAACCACAAATTTGTCTTCAATGA
TTTAATTACACTTTCAAATATTCGTAATCTAATTCGATTATCCTATGTTGCAAATAGAAAGTAACAAATAT
AAAGTAGTAACGTGTGGCCGAGAGTACTGCCGGCACTCGCCGGGCAGTGGCGGCGACACGGCATGCA

ACCTTCAGGTGTCGGATCTATTGTACATACAATAAGTTATCCATCAACGCGGAGAGTTATCAAATTTGTT
CACAATATATAATAATTTTATACAAATTATGGCTAATGAATTAATTTTAAAACTCCCCCTCAACATATGCA
CATCATTAGATATTTGACATTATTCGCATTATCTATACTTTTTCCTCTAAAAATATGAAAACGACACAACA
AGATGTTTTTAATGATACAAAACACTTCAAAATGTACTTAGCCGAAACGAAGAAAGTTCTAACGCTGG
GGATACAATTTTGTGATGACTAAATATTCGTGACTAATCCATAGTACCTTATTCATGGGGTGAGACCATC
ACACACGCGTCGCGCGACGGTCGCACCCACGCCTTTACTTACGTCTACGCAAAATCACTTGCCTTTCC
TGCCCAAGCAAAAATATACAAATGTGTTTCTCCTAAGCTGTACAATTTTATACACCGACAATGGAGCGA
TCGCCTGCTGTCGTGAAATGTAACGAAATGAATAGTAATGAGTGTAAAAATGTAGTTTAGACCCAACAT
TTAAGTTCAGAGGACACATTTATCTTGAGAGTTTATGACAAGCCGGTGGTCTTGTCAATTGTTGTCTGC
ATTTATTATATTATTTGTTGTATAAACTGAATAGTGCGGGAAGATAATCCGTATCCTTTTTGAGTTTCAAA
TGCACGATTGAATAGCAAAAGCTATCGAGATAAAAAAGCAACATTGTGGAATAGACTTCCCTTTCACT
CTATTACCCGTCAATCATACCTTTTCCTCGCTACGTAGCTCTAGCTTCAATAGTAAATTTAGCTGCTAAGA
TGACCTAATCAAAATGATTTTGCGAATATTTGAGTATCTAGCGGTATGAACTTAAATTGCTTCTGGAATA
GTGGAAAGTAGCTGCATTCGTTACCCTTAGTGACTGCGAGCACACATAAAACAGTTATTATAATGTATAT
TTCAACTATGATATCGCCTCGTTCGCTTGAACGTAGAATTGCAACAATATAAATTGACATCACCCAGAG
ACGCGCATTTTGTAAATGACGTGAAATGTCCAATCAGTCTTGAAGATACCCGAACAGCGCCGGCGAGC
GCCGCCGTCGGTTCAAAACAATATCATTAACTTTACGCTTTCTATAATATTTATATTTGTTGTATTGGCCA
TTACATAAATTATTGCACGTTATATTCTGTCGTTCATTTAACCAATCATAAATAAGACATTTGCCATGTCA
CAAATATTACTCAATGCATGACTTAAAACTGTTTATTACTTCATTTTTAAATACAATTATACAATACGGAC
ATAAAATTAAGATAGGAAAGTGTGGCTTCACTAGGAACGGAGCCAATTATATGTAATCATATTATAAATA
TTATACATGTATTCATACGTGAATATCCCATAAAATGAACTTAGGTTTTGAAAATGACTAAATAAAATTAT
ATTCTAGAATATGCACATTTTCCGATATGTTGTACTCTATACATGCGGTTTCTAATTCTCTCATTGTACATT
GACACGACTCGGGCGCTTGGCCCCGTCACAACCTGATACAAATAACTGTACGCAAGTATGTAAAATAC
AACATATCTCGAACAAAGATAAGTTCAACAAACACACATAAACCTAGTACAAAAGTAAGTCACGGTAT
TTCCTACTTTATGGTAATTCATTACTTATGTGATTAACAAAACCGAAGGATACAACACTGCATTCACAGA
AAATAAACAAACTCACAGGACGTAAAAAAATAATATTTGACTGCTTCACTAATGGGGAGAAAGCGTAC
ACTACTCGATAAGTATTATTATAACTAGTGCTGTTTATAATAAATGATAATAATGTTATGATTCTAATGTAC
AAATCGATTGACTTGCTCGAGCGCAATGTGTTTGGAATGGACTCGATCAAAGTAAATTAAATCTCAATT
CAAACTCCGCACACCCCTAGCCCGCCCACTCAACTGCACTCAATCCTTAATCAGTGTAAATAAATATAT
ATTAGGTATAATTTGTTCTATAACACATGGTATGAATCCAAGGCGACGTTTGAAAAATCGGTTCAGTAGT
CTTTCTGCAGTATAAGAAGCAGTGAAGCGGGCGGTCGGAAGGTAGAAAGTCACTTACAAATTGAGCT
CACATAAATTTGGTGAAAAAACTAACCATGTTTCGCTACGGACGGGAACACAAAATGAATATTACTTC
GCCATCCCCACCCCGCCGCCAAGTATACACAAACTAAAGGTCCCACGGCTTATAACTTTCAGCATCGCT
GGACTACCTAGAATAACTATATCGAACAATGCTAAAACGTTATAAGTGACCACTCAGAACGCCATATTC
GCCCCAAATATTACATAGGTACGACGAATATACATTACTTTAAAATCACATGAACCGAAAACAGCAAAA
TTTTAAAAATATTTAACAATTTTATTCATTTGTTATACTGCATTAATTACAAATGTATATACATATTATGTAC
TTGGAGCCGTGTTTGCCAACATTTTCTTTGCGTGAAAATGCTGCGAACATGAATCGGTATTTAAAATGG
AGACTGCCACATCGATCTATTAGTTCACCGTAGGTTCAACAGAAAATATAGTATTGAGCTAACATTAAG
GTTTAATTAGAGGCTCGTTTTCTTTAGTTATTCGGATCGATATGAAGTTCGTTCGATAGATTGAAGCTTT
GAATATAACAATGTAACTTGTAATTAGAAAACTAGACGAAACATATGTTTGTGAATGTTTTTTAAAATGT
TCCTTGTTCTTTGCTATTACGTTAGCATCGTCTCAATACTAAAATTCTTAGTTTTTTTCTGTCAAACACTA
CTAAAATGATAGGCCATGAAATGGAACAGACCAAGATCATAAGTAATCATTCAAGTCCGTTCTACGATT
TATATTTCAACATCTTTACCAATGGCAGTGTGGTTTCAGTTAACACATATTCTTCGTGTTCATAATTTAGA
GCAACCATCGTACGGATTGATTTAAAACCAAAATTATATTTTGTTAATTTATGTACGAAAATATCCAAAC

ACAAATTGTAAACAGAACAGTTTAAAAACATGCGCAAACGAAGGTTTCTCGCGCAGATACGTTTAGGA
TTGTGGCCGAAAACGGAACAATTTCACTATCCAACCTCACCAGCCAATCGATACAAAACCACTCTGAA
AGCCACAGCACTGATGTATAGAGCTAAATTGATCCCCATCCCATATAGAACCTATAGATCGATTTCTTTA
CAAATGAAAGTCAGACAACTACCCACTGCCTCAACTCACCTAGAAATCCATCAACAGATTCTACAAAA
CCCCCACAATCCTAAACGCATCCACTAAGGCTTAGAAAGACAGTAAAAGCACAGTACAGTAATACGCA
GCGTAGAAGTTAAGCGCCAAAGTTATGTTATCACGCCGTCTCTAGTATCGTTTCTACGCTTATTACACGG
TAGCAGTCCAAGTGAGATATACAGGGTGGTGGGGGGGCGACTCGGTCACACCACACGTCACTTGCCG
GCGCGGGCGGCGCACTTCTTCTTTTTGCGTTTGAAGAAACAACAGCAT
>TCONS_00089739 geneID=XLOC_017941 scaffold_293:222560-224417(+) Exonic overlap (-)
GTCTGGTCGTGGCTTTCCACCTGCTTCTTGGGTATCTGAGGGTATAAAAGTAATATTTCGATTTAGGCCG
CTAAATACCTATTTACCTATAACTGAAGAGGTACTCATGTGAGCTGAAAATCCTGAAAATTGTTTTCAGT
AATTATCATTGTCAATCATATAAACTAGCAGACGTATTGGTCTCATCTGCATTTTCAGCTGAAAATTTAA
AAAAACTTTTTGAAATATGTATTTTTTCCCCAGAACTCAGCATTCTTCCACTTACTGACACCTGTAATCC
TCTTTCAATGTTGATGAAACTTCTTTCAGCTGTTTGTACATAACCAACCTTCGATTGAGACTAGGTCTG
GTCGTGGCTTTCCACCTGCTTCTAGGGTATCTGAGGGTATGAGGGTATTATTTCGACTTAGGCCGCTAAA
TACCTATTTACCTATAACTGAAGAGGTACTCATGTGAGCTGAAAATCCTGAAAATAGTTTTCAGTAATCA
TCATTGCCGATCATATAAACCAGCAGACGTATTGGTCTTATCAGCATTTTCGGCTGAAATTTTTAAAAAA
CTTTGTGAAATATGTATTTTTTCCCCAGATTTCAGCATTCTTCTTCTTACTGACACCTGTAATCCTCTTTC
AATGTTGATGAAACTTCTTTCAGGTTGTGTTACTTAACCAGCCTTCGATTTAAACTAGGT
>TCONS_00089648 geneID=XLOC_017942 scaffold_293:228062-231239(+) Intergenic
ATATATACTTACCTACCTAGTTACTTATACAATATGTAAGATATATTTGAATTTTAAACCCTGTTTAAATCG
AAGGAGGGTACACAGGGGGTAATTGTGAACTTTGGAAAATTCGCGAAAAATAACTCCATCAAAACTG
TGAAAAAGTAACTTTTTTCATGAACTTCTAGAAAGTCTTGATTTGATTACATAGTCTACCTACCTTCCGG
CATAGGAAATGAAAAAAGACATTAATTTTTATGAAGTAGGTATTTCTTTATTGTTGTAAGTATACATACGT
ACAATCTGGGACATATAAATCTTACCCCTCAGAGTGACCATACTTACAGGGAGTCAACACAAAAAATA
AGTACTTACCTATAAGATAATTATTATTAGGTACAAAATCTAAAACTCTAAATAGCATTTGCAAGGTACTT
AACTAGCTAATTATGAATACTAATATTTTTTTCAAACAAAAAAGAGTTTTGTATGAGGTCAACTTGATAA
ACGATTTTTTTTATTTTATTTCTTGTAAATATGTAGTCACTAAAATGGTCAGAAACATTGCCAAGTCACA
AACATGTATTGTTCTTACTTTGGTTAAACATATTATAATATGTATTAATTGTTACTAAAACATAATGATAGA
ATAATAACAAAATAAATTATACCTATGTATTTAATTATGTTAAAATTATAATTTTACCTAACTACATAGTAA
TATAAAAAGTATTAATTTCACAGTTTGGTCCCTTGGTTTAATGAATAAACTTGTACCTAGATATATATGCG
ATTTACACGTTTTCATTGTTGGTCAAAAATACTTGAATATAATGCATGAGTCTAATTAAATCGCTTAAAA
CGTTTTGATACTCCGGTGAAGCCGGTCTGTCGCCAAGTACGGTTAGAGCCTTACATATTTTCTTAAAGC
CTGCCATGTGTATATTATTGACGTAAGAAAACCGAGGTTTCTTCAAAAACAATAATTGTTCTTGACTCCA
TGACGAGTTGTCTTCACTGGTCTCACAAGCACACAGAGCATTGAGCAACCGGAACGTGGTTTGATGG
TGTATTTTTATATTATCATGATATAAATTGTTTCTGAAAAACAAAAAGAAGGTACTTATCT
>TCONS_00089708 geneID=XLOC_017956 scaffold_293:307379-324688(-) Unclassified
GGAGACCGCGGGCGGGGCGGCACGTGCGGCGCGCTCGTTGCGCCTCGCGACTGTCATTGTTATTGCAA
TGTGCACCATTGTTCGAATCCATCCCAGCTCAATCAATACCATATTTTAGTGAAACCACTAGGCCTATTA
CAAGCGTTTGCCTACAGTATTTATTGTGCTAAATTAACGTTAGTGTTTTATACAACGCATAGGTACACAA
AGTACCGGTGACTTATTGACAAGTGAAACTCTAATCGTTTTATTCATCCGTTGGTGGATCGTGTTTATAC
CTACTGATGTTTATATACTAAGTGAATGTGATTTTTTATACGGGAGGATGCAAATCAATAACTGAAGATA
CCCTGGAGCAAGTTGCCAACATCTCTTGTGTGGGTTATACCACACTCTGCTGCATATTGATCAGGACCT
CTCCGTTGGGACTTCACAGAATTGGCATTATTGCTTGGGAACTACGTTCGGGCTAAATCCCTAACCGTC

GGTCGGCCGACCAAGAAGAATATATACCTGCTAATCGCCCCCGTGAAGAAGGTCATCCCAGCACAAAT
CCCACGTACACACGGCTGGAGATACCATTCCGAGGGAAGTGCGGTCAGGTGTTAGATTTGACGGAATC
GAACTTATTTGGCCCG
>TCONS_00089709 geneID=XLOC_017956 scaffold_293:313805-324689(-) Unclassified
TGGAGACCGCGGGCGGGGCGGCACGTGCGGCGCGCTCGTTGCGCCTCGCGACTGTCATTGTTATTGCA
ATGTGCACCATTGTTCGAATCCATCCCAGCTCAATCAATACCATATTTTAGTGAAACCACTAGGCCTATT
ACAAGCGTTTGCCTACAGTATTTATTGTGCTAAATTAACGTTAGTGTTTTATACAACGCATAGGTACACA
AAGTACCGGTGACTTATTGACAAGTGAAACTCTAATCGTTTTATTCATCCGTTGGTGGATCGTGTTTATA
CCTACTGATGTTTATATACTAAGTGAATGTGATTTTTTATACGGGAGGATGCAAATCAATAACTGAAGAT
ACCCTGGAGCAAGTTGCCAACATCTCTTGTGTGGGTTATACCACACTCTGCTGCATATTGATCAGGACC
TCTCCGTTGGGACTTCACAGAATTGGCATTATTGCTTGGGAACTACGTTCGGGCTAAATCCCTAACCGT
CGGTCGGCCGACCAAGAAGAATATATACCTGCTAATCACCCCCGTGAAGAAGGTCATCCCAGCACAAA
TCCCACGTACACACGGCTGGAGATACCATTCCGAGGGAAGTGCGGTCAGGTGTTAGATTTGACGGAAT
CGAACTTATTTGGCCCGGTATTTTTTTAAATTAATAAACTTATTTTTTTTAACTGGACTCTGCCTCGCCTA
CTTTTTTTTTACCTACACTAAAACCCTTAGGTCTAGGCAGGTAGCACGTCACACTGGCGCCCAACGTGG
TTAAAAATAAGTTTGGTTCCGTTCTACTCCTGTAGTAGGTAGTTGTGCATGTTGGTGTGTTGTTTTTTTT
TTATGTTAAAACGCTTATTCTATGGTTTTGATTTTTTAAACAGCTAATGTGTACATGGTTGCAAATTTAAG
AATTACTTATATTTATTTGTTTTAACTTGTGACATTCTATCCATTGTAATTTTATTTTAT
>TCONS_00089948 geneID=XLOC_017993 scaffold_294:52894-60927(+) Splice junction overlap
CATCCTTTGAAGACGTATCGGACATACGCAGCCATGAAGACCTTTGTGACCGTCCTCTGCTCGCTGGC
GCTGGCCTCGGCGGGGCAGGTGCGTGAGAAGCGCGGGTTCCTGAGCGGCCTGCACAGCTACGCACCC
GCTGCCAGCTACTCCGCCCCGGCTGTGAGCTATGCGAGCTACTCCGCCGCGCCTAGCTACAGCTACTC
CGCCGCGCCTAGCTACAGCTACTCCGCTGCGCC
>TCONS_00090051 geneID=XLOC_017999 scaffold_294:131378-132180(+) Splice junction overlap
AACACTCTGGCGAAAAGTGGGTGCAGCAATGCACCTCTGCCTACCCCGCAAGGGAGTACAGTAGTAC
AAGGCGTGAGTGCGTGTTTTTTTTTATTATTCATACTTCATGTCTTGTAAATAAGATGATCATACTGTAAC
TTGTAACAAAGTCATTAAATCCATACTACAGTTGCGCGGAGTCGGCGCGGCACTACGTGAACCAGACG
CCGCCGCCGCGCACGCCGCCCACGGCGCTGCTCGCCAACCACCACACAGATATCTTTGACATGCAGCC
ATTCACAGCGGCCCC
>TCONS_00089968 geneID=XLOC_018013 scaffold_294:232306-233262(-) Unclassified
AAATAAATCACTGCATATTTTAATAAAGTTTTGCTTTCGCGCCGAAGACACGGTTGTGTTTTAGGGTGT
TTCGATTGCGATGCTACTAAGCACTCGTAAACAAGAACCCCTGAGAACCCCCATGATGGAATCCGCTT
CTTTGAGGCTCTGCTATACTAGCGATCAATAGCAAATACCGAGAGAGTCTTCATACAGCCTTATCCCCT
GAGCCTCTGCCTCAGCCACAAGATCAAGAAGAATACTATATCTGCAAGACATGAATATTCAATACCTAC
AGATTCTTAAAAGAAGGATTTTCGCTTTCATATAGAAAATGTTTATGATTCTCACAGAACAATACAGAA
AGGTGGTGCCAAACATCAACACATCAACAAAACATCAACAACAACAACACAAATTCTTCACATCACA
CACCTCAGCCTCATGACCTCACACTTCACCAAAACATGTAACCAGGAAACCGGGTCCTTTTTCACCCA
CACAGCCCATCCAATGTGCACGTAATAATTTACATACCAGCAGCGCAGTCGCTGCATACGCAGTTTATA
CTTTACAGGCATACAGGATGTCTCGGACACAAATCCTGGATGATGTCGGGGAACCGCCGGCAAAACTT
GAAGCCCCGAAGCTGGCACATCACTTCCCCCCCTACCCCCCTCACTCCGAGGAGCCCCCGACCGTGG
CGGAGGCAGTGGACCGCGGCAACGAGGTGCGGCGCACAGAGAAGAACAAACGACATAAACCCATTC
GGAAACCGCCCAGGATGGCTAAACCCGGGCAAGTATTTTTTTATTCATTTAGATTTTTTGGTAAAAAGT
GTTAAA
>TCONS_00089955 geneID=XLOC_018003 scaffold_294:289741-290615(+) Intronic

CAGAAATAATTAAGCTTTACGTTTCAAGCGTTTTAGAATAAAAATATTTATATTTACGTCGTTTTATAAAT
TGAAAAATGGGTGATCTAATTGAAATTCCAACATATTCTCAAAGAAATTGCTTGTTTTATAAAGTTACA
AGCAATACACTGGTAATTGAAATCAAAGGATCAAACAATGCAGCTATAGGACTAGCCACCCGGCCAAG
CAGCAACTGTGAGATAGAGATTTATATTGGAAATGAGTTTTGTTGGATTAAAGTGGAAAAACAACCCA
GAAAAAAGTTAAACAGAAGTTTACTAGCTCGAC
>TCONS_00089803 geneID=XLOC_018003 scaffold_294:289999-290628(+) Intronic
AAAAATGTCTGTTTCCAGAAATTCCAACATATTCTCAAAGAAATTGCTTGTTTTATAAAGTTACAAGCA
ATACACTGGTAATTGAAATCAAAGGATCAAACAATGCAGCTATAGGACTAGCCACCCGGCCAAGCAGC
AACTGTGAGATAGAGATTTATATTGGAAATGAGTTTTGTTGGATTAAAGTGGAAAAACAACCCAGAAA
AAAGTTAAACAGAAGTTTACTAGCTCGACACTCATTCAGAAA
>TCONS_00089854 geneID=XLOC_018020 scaffold_294:393961-395302(-) Unclassified
GCTGTGTGTGGGTGCGCGTGCGCGTTGCGCATGTGTTTGCACAGAGCGGCGCGCGCGGGGAGGCAGC
GGGGGGCGGGCAGCGCGGCGCAGGGGGGCTCGCCCTGGCAGCCGGAGCGCCGGCACTGGTTCTGCA
GCCCGTTCCTGCGCAGGCAGAAGCAGTCCGTGTACAGGATCCCGCCGCGCAGCTCCCGGCTCCTGCA
GGCTTGCAGGTTCATGGCGACGGTTGATGTCAGAGAAATCTAGCTAAAGTCCCGTTTACACAGGATAT
AATAATAAAGTTAGTTTTTTTGTATTTTCACAAAGAATTTATAGTAAAAACATAATGATATCTGAAAACT
GATGTTAAATCATTGTGAGTAACTTTTTTAATTTGTATTTTTTATATTTTCACGAATAATTTTGATAATAATA
TACGATGATGACTGTTACTGAATCATGTTAGGGACTGAATTTTTCACATTTCTAGTAGGCTACATAAAAT
AATATCTAGTTGGCTAAGCGCAGTTCTTTAGTAATGGCGATAAGGGATATACCTCCTTACTATTGCTACAT
TTATAACATAACACAGCCTAAGCTAGGAGCTTGCCCCGCACATTTTCACTATTCACGAAACATCGCGTA
AAATGTCGCGTTTTATAACACCCTGTCTAAATAAAGATACTTTGTACAAAGTACTTGAAAGCCATCTACT
TGGCATCGGGCATGAACTAAAATAAAAAAGAAGGTGGTAAAATATTTGGCAGTGTGTGAAGTAGGTAC
ATTAGCCAATATATTGATACTGGCATATAATATTCTTTCTGAAAAGAATATTTCAAATACATTTTCAAAATT
ATTCCTCATTTGAAGCCGGCAGAAGTGTTCTGTAGAAGAGAACGCATTGAAGAAATCAGTTCATAAAA
ATTCTTCAAGTGAAAACAACTAAAAACGTCAAAAAAACATTAATTTAAATCATCAAAAATAATTTAATA
TACTCCTATACCGTATATTACTTGAAATACCTACTTTTCCAGACCTTCCGGTTTCCAGATCCAGTTTACGA
AATAGCATTAGCTATTTCTAATTTCTACATTTTATTTTAAGAGTAATCTTCACCAAACCCTAAAATGTCTT
GCGGAGGATGTGGCCCATGCTGCGGCCCCTGCGGCGGGTGCGGCCCGTGCTGCGGCCCCTGCGCCCC
GCTCTGCTGCCCCCCCC
>TCONS_00090232 geneID=XLOC_018050 scaffold_295:21713-23746(-) Intergenic
GTTTGGAATAGCTTTGTATTCTAAATTTAAATTAATTTTGTTTTAGAATCATGACATTCTTTTTACAAAGT
CAGTCCGTTGCTTAGTAAACGTCGTAGTCTGTGGTGCTGAGGCAACCCTAAGGTGGCTTCTTTTTCAAT
GCGTATAACCACGTAACTTATTATGCATCGTAGTCCAGTGAAATAAGGCGCATATTCCCTCAAAAATAAA
TTGATAGGTAAGTAAATAAAAACGTGTGCGGCTGGAACGCGATCTAAATAAGATCTTATTGCTTATGCTA
TTGGAGTCATATTTCTTTGAAAGCCATTGCGAAGATGGACTTCTATACTGGTACTAGTTATTATTCTGTG
GCATGGCGCGACAGCAATAGATCTTATGAAAACCTACTTTTGAATTCACGCTCTCACTTGAATCGCTCT
TAAGTCTTTTTTATTTTTCTCATTATATTTCTACCACTTTTTCAGAATATTTGAAAAAGTATATCGCAAAA
AAAGAAAAGCAAACGAATTACAAAATTAAATGAAAACGAGTGAAGTAGAAAATAATTCAAGAAAACG
TAACTATGATGAGAACTCAATAAAATTACCAACGTAATAAAAAAT
>TCONS_00090146 geneID=XLOC_018059 scaffold_295:405527-407727(-) Unclassified
GACGTGGCGCGCTCGCTGCCCATCAACATGCCGTTCCCGCTTGAGCGAGCCGACCATCGCGACAGGG
ATGATGACGAGGAGCCGTCACAGGACATAGCGGCGAGCATCAAGGCGCTGGCCAAGTCGGTCCACGG
CGACGTGTTCGAACTGCCGCGCCCGCGCTTCTCCACGCAGATCTAGCCTCACCCCTCTCACTATACATA
TACACACATACAAACTACTTATAAGCGACTATATACATACTCTATCATACGCTATCTCTCTCACACGTATG

CGAGTGCTTGAGTTTTGTTGCGCGAGCCGCTTTCTTGGTTAAGAGTTTTACGGGTAGTGTGGCGGATTT
AAACTGAGGCGTCAGGCCGTAAGTTATTGTTCAACTGTCTCGTCTGCATTCATTATATTGTGTAGACTC
GTCGTTATGTGTAAACGAGAAATTGCTATGTGCGTGTATGGCCTAATGGCTCAGTTTAATACTCTCCTGC
GGTTAACGGTGGCAATGCCATGTTGAAAAGCTATGAGTATCAGATGAGTGAAACTCTTAATCGGAGAA
GCCGGCTTCAGTCACGATATGGTGCTTAGTCCGGGTCACAGTTGCAGCCAACATTTTCATGTTTCAGCC
AAAGTTGATTCTATACAGAACGGTTCTAACTGGACTCTAGCGTACATACTGTGATCAATACAATGAAGA
TGACTGTTTACATAAATGAGGCTATACAGAGTTGTTATTCCATAGTTTACTGTCCCACCCTCGCAGCCAT
GTTTACCGCTGTTTCATAAACATTATGTTTCATTAATATTAAGCGCCGCACGCAAGTTTACATATTGCGA
GGCGCAAAGTAAGTAAGGGTTATGAACGTGTTGCATCATCAGATATATTTGTGTAGTATGTAAATAAGAT
TACGTGAATGACATGTCAAATCTATAAATCATTATGCATTGTTAACATCTAAGTCATATCTCCGTTAATTA
ATAGAGACTATAGAGTTTTCTATTATTTTATTATCTGGTGTTATCTTAATGATAAGAGAAAAAGTGTACAA
AAATATTAATACTTGCATTTCTTACTAGTCTTTTAGGGTCACGAAATATAATGTTTCATTTATTAATTTTAA
TTTTTCGGAAGTTTTTTCCCTTTGAACGCATGGTCAGTTAATTAGGGTATTGCACGCCCGTCGCCATCG
CCGAGCCGTGGTCATGAACTGTACATAGAATCATTGTTACTCAAGACGAGGATGTGAAAACGTTTTGG
AAGAACTTTATTTTTATACCCAATATTTTGGCAATCAGGAATGAGTAATGCTGGCATTGGAAAGGTTTCA
GAGATGGAGGTTTCGTTTTGTTTACCTACTGTATTGAATGTTAAAAATAAGTATAAGTAAACAAGTAATA
ATTATAACTTTAGTTGTTATTGTCTGCTCGAGAGAACAGTGCAGCTGTCGTAACGGTTAATTGTCTTCCA
GCCCCTCTTACCGACATAAATATTATACTTATCTGTTTAGTTCTGTGATGTAAATTTTCAGTCATTTTCCTA
AGCCATGGTTAAGAGTTTAGTTTCATTGGAGCGTTGAGATTTAAGAAGGAAAGACCTAGAATGTAATG
AATATTAGAAATCATCAATAAATATTGACTGTGAAA
>TCONS_00090176 geneID=XLOC_018059 scaffold_295:405527-408184(-) Unclassified
CAGGCAGCGACGACGAGGGCATCCACATCGCCCGCGCGCGCGCGCCCAGCGCCGCCGACGTGGCGC
GCTCGCTGCCCATCAACATGCCGTTCCCGCTTGAGCGAGCCGACCATCGCGACAGGGATGATGACGAG
GAGCCGTCACAGGACATAGCGGCGAGCATCAAGGCGCTGGCCAAGTCGGTCCACGGCGACGTGTTCG
AACTGCCGCGCCCGCGCTTCTCCACGCAGATCTAGCCTCACCCCTCTCACTATACATATACACACATAC
AAACTACTTATAAGCGACTATATACATACTCTATCATACGCTATCTCTCTCACACGTATGCGAGTGCTTGA
GTTTTGTTGCGCGAGCCGCTTTCTTGGTTAAGAGTTTTACGGGTAGTGTGGCGGATTTAAACTGAGGC
GTCAGGCCGTAAGTTATTGTTCAACTGTCTCGTCTGCATTCATTATATTGTGTAGACTCGTCGTTATGTGT
AAACGAGAAATTGCTATGTGCGTGTATGGCCTAATGGCTCAGTTTAATACTCTCCTGCGGTTAACGGTG
GCAATGCCATGTTGAAAAGCTATGAGTATCAGATGAGTGAAACTCTTAATCGGAGAAGCCGGCTTCAG
TCACGATATGGTGCTTAGTCCGGGTCACAGTTGCAGCCAACATTTTCATGTTTCAGCCAAAGTTGATTC
TATACAGAACGGTTCTAACTGGACTCTAGCGTACATACTGTGATCAATACAATGAAGATGACTGTTTAC
ATAAATGAGGCTATACAGAGTTGTTATTCCATAGTTTACTGTCCCACCCTCGCAGCCATGTTTACCGCTG
TTTCATAAACATTATGTTTCATTAATATTAAGCGCCGCACGCAAGTTTACATATTGCGAGGCGCAAAGTA
AGTAAGGGTTATGAACGTGTTGCATCATCAGATATATTTGTGTAGTATGTAAATAAGATTACGTGAATGA
CATGTCAAATCTATAAATCATTATGCATTGTTAACATCTAAGTCATATCTCCGTTAATTAATAGAGACTATA
GAGTTTTCTATTATTTTATTATCTGGTGTTATCTTAATGATAAGAGAAAAAGTGTACAAAAATATTAATAC
TTGCATTTCTTACTAGTCTTTTAGGGTCACGAAATATAATGTTTCATTTATTAATTTTAATTTTTCGGAAGT
TTTTTCCCTTTGAACGCATGGTCAGTTAATTAGGGTATTGCACGCCCGTCGCCATCGCCGAGCCGTGGT
CATGAACTGTACATAGAATCATTGTTACTCAAGACGAGGATGTGAAAACGTTTTGGAAGAACTTTATTT
TTATACCCAATATTTTGGCAATCAGGAATGAGTAATGCTGGCATTGGAAAGGTTTCAGAGATGGAGGTT
TCGTTTTGTTTACCTACTGTATTGAATGTTAAAAATAAGTATAAGTAAACAAGTAATAATTATAACTTTAG
TTGTTATTGTCTGCTCGAGAGAACAGTGCAGCTGTCGTAACGGTTAATTGTCTTCCAGCCCCTCTTACC
GACATAAATATTATACTTATCTGTTTAGTTCTGTGATGTAAATTTTCAGTCATTTTCCTAAGCCATGGTTA

AGAGTTTAGTTTCATTGGAGCGTTGAGATTTAAGAAGGAAAGACCTAGAATGTAATGAATATTAGAAAT
CATCAATAAATATTGACTGTGAAA
>TCONS_00090112 geneID=XLOC_018059 scaffold_295:405528-408127(-) Intergenic
ACGTGGCGCGCTCGCTGCCCATCAACATGCCGTTCCCGCTGGAGCGCGCCGAACACCGCGATAGGGAT
GATGACGAGGAGCCGTCACAGGACATAGCGGCGAGCATCAAGGCGCTGGCCAAGTCGGTCCACGGC
GACGTGTTCGAACTGCCGCGCCCGCGCTTCTCCACGCAGATCTAGCCTCACCCCTCTCACTATACATAT
ACACACATACAAACTACTTATAAGCGACTATATACATACTCTATCATACGCTATCTCTCTCACACGTATGC
GAGTGCTTGAGTTTTGTTGCGCGAGCCGCTTTCTTGGTTAAGAGTTTTACGGGTAGTGTGGCGGATTTA
AACTGAGGCGTCAGGCCGTAAGTTATTGTTCAACTGTCTCGTCTGCATTCATTATATTGTGTAGACTCGT
CGTTATGTGTAAACGAGAAATTGCTATGTGCGTGTATGGCCTAATGGCTCAGTTTAATACTCTCCTGCGG
TTAACGGTGGCAATGCCATGTTGAAAAGCTATGAGTATCAGATGAGTGAAACTCTTAATCGGAGAAGC
CGGCTTCAGTCACGATATGGTGCTTAGTCCGGGTCACAGTTGCAGCCAACATTTTCATGTTTCAGCCAA
AGTTGATTCTATACAGAACGGTTCTAACTGGACTCTAGCGTACATACTGTGATCAATACAATGAAGATG
ACTGTTTACATAAATGAGGCTATACAGAGTTGTTATTCCATAGTTTACTGTCCCACCCTCGCAGCCATGT
TTACCGCTGTTTCATAAACATTATGTTTCATTAATATTAAGCGCCGCACGCAAGTTTACATATTGCGAGG
CGCAAAGTAAGTAAGGGTTATGAACGTGTTGCATCATCAGATATATTTGTGTAGTATGTAAATAAGATTA
CGTGAATGACATGTCAAATCTATAAATCATTATGCATTGTTAACATCTAAGTCATATCTCCGTTAATTAAT
AGAGACTATAGAGTTTTCTATTATTTTATTATCTGGTGTTATCTTAATGATAAGAGAAAAAGTGTACAAA
AATATTAATACTTGCATTTCTTACTAGTCTTTTAGGGTCACGAAATATAATGTTTCATTTATTAATTTTAAT
TTTTCGGAAGTTTTTTCCCTTTGAACGCATGGTCAGTTAATTAGGGTATTGCACGCCCGTCGCCATCGC
CGAGCCGTGGTCATGAACTGTACATAGAATCATTGTTACTCAAGACGAGGATGTGAAAACGTTTTGGA
AGAACTTTATTTTTATACCCAATATTTTGGCAATCAGGAATGAGTAATGCTGGCATTGGAAAGGTTTCAG
AGATGGAGGTTTCGTTTTGTTTACCTACTGTATTGAATGTTAAAAATAAGTATAAGTAAACAAGTAATAA
TTATAACTTTAGTTGTTATTGTCTGCTCGAGAGAACAGTGCAGCTGTCGTAACGGTTAATTGTCTTCCA
GCCCCTCTTACCGACATAAATATTATACTTATCTGTTTAGTTCTGTGATGTAAATTTTCAGTCATTTTCCTA
AGCCATGGTTAAGAGTTTAGTTTCATTGGAGCGTTGAGATTTAAGAAGGAAAGACCTAGAATGTAATG
AATATTAGAAATCATCAATAAATATTGACTGTGAA
>TCONS_00090415 geneID=XLOC_018096 scaffold_296:267584-292984(-) Exonic overlap (+)
ACCGGTTGTTTCGATACAAACGAACAGTCAGTGAAGCCGCGAACGCTATACGAACTCCTTGTGTGACC
GCGCGAGCTGGCCGTTAGTAAACTCACCAGTTTAAAAATAAAAACGCACGTGGACTGTCAAAATCATT
TATTTTTTTAATTTAAAAATAGAACTTAGAAGCTGCGTTTGTAATCCTATTTCGGTGTGCTCGGTGAAAT
ATTTCGGTGGGAAAAGTTTTAGTAACTATATTAGAAATAGGAAAAAATATCAGTGATAAGGAAGTGATA
ACGTAAAAGAGGTCGATAGTGAAATGTTTGGATGATAGTGAATATTTTGTGCAGTGATGCGGCAAGGA
GTTTTGGATATACATCTACCATCAATCTTGTTTTGACATAAAACAGCCTGCAGCGGCCGGTGTCGGT
>TCONS_00090445 geneID=XLOC_018096 scaffold_296:267809-292984(-) Splice junction overlap
ACCGGTTGTTTCGATACAAACGAACAGTCAGTGAAGCCGCGAACGCTATACGAACTCCTTGTGTGACC
GCGCGAGCTGGCCGTTAGTAAACTCACCAGTTTAAAAATAAAAACGCACGTGGACTGTCAAAATCATT
TATTTTTTTAATTTAAAAATAGAACTTAGAAGCTGCGTTTGTAATCCTATTTCGGTGTGCTCGGTGAAAT
ATTTCGGTGGGAAAAGTTTTAGTAACTATATTAGAAATAGGAAAAAATATCAGTGATAAGGAAGTGATA
ACGTAAAAGAGGTCGATAGTGAAATGTTTGGATGATAGTGAATATTTTGTGCAGTGATGCGGCAAGGA
GTTTTGGATATACATCTACCATCAATCTTGTTTTGACATAAAACAGCCTGCAGCGGCCGGTGTCGGT
>TCONS_00090475 geneID=XLOC_018091 scaffold_296:410631-411269(+) Intergenic
GCGCCATTTTGTTTTTTCAATTTTTCGTCGCGTCTCGGCTCGGTGCGGTGTGCTTGTGCGTGAATTTTTC
CTTGTGATAAATACAGAAATAGAGTGATTTCGTGTTGTTTTAGTTTTGTTAAACAAATTGCAGAGCATCA

TGTCCGACCGAGAGAGAAGCCGATCTAGGACTCGCAACGGGTCGAGGGAGGGCCGTGAGGGCCGCG
AGGTGGCTCCGAAGCCCCCGACCATGTCCCGCGGACACTCTAGGAGCCGGTCGCGGACGCCGCCCCC
GCCTAAATCTAGTAGGAGGTATAAAACCCCGTGAGTATTCAAGCTTTACTAACTAAAGACCTATCAGCT
ATTAAGAATTGAAATCATATAGAAGGTCATAACTTGATATTAGCTTGCATGGGAGAGTAACTCCTAACCT
CGCAGCCAAGGTCACTTTTTGTGTTTTCCGCGTTGCCTCGGTATTTAATATAGAGGTGTGATTGTGAAAT
AGGCA
>TCONS_00090509 geneID=XLOC_018122 scaffold_297:126209-167294(+) Exonic overlap (+)
GCCTTTTGATCGTTACAATGAACTCAGTTATTTTTTAGTTTTATTCGATGACAGTAATGCGCAACTTTTT
GAAGACAACCTGTTGCGATTTCCTCTTCTAGCCCATGTGGGTTCGTAAATAAGAAATAATACAGTACAT
AAATTTTGAAACAAATTAAACGTTTAAAGCGAAATGATGATTGTTTATGCAGATGACCTCATCGTCGTC
AGATATTTTGGCTATAATTACAAGTGCTTGTTACGTATCCGAGATGCCGCGACGAGAATAGACGCTGCG
TCGGACCGAGCGGCGACAGCGGCGCGTCACCTGACCGTGGCACCCGGAGCATCATCACACTCGTCTT
CCTCTGCAATTAAAATAATTACCACGTTAAAAAAAACGAACAAAAAAACACACGAAAAGAACAAACA
TTTTCTGACACCAATCATAATTTTAACCCAACCGTGCTATTATATCGATTGACCTAAATCTTTTCATCGCC
TAATGAGTGGAACATACACGGAAATAAAACTAAACAGAGGCTCGCATGCAGCGTGTCTGCAGGTGCAT
GCTGGAGCAGCAGGTTGGTCGCGTGCCGCCCGCGACCAGTCGTGAGCCGAGCGTCGCGCCGCCCGCC
CGCGCCACCGCCGCGCCGCCCGCCCAGCGACCGATGCTCCCCGACCGACCAAACGAAACTTTTACTA
ACCACTTCACCTCAACTCCGCGACCACAAATACGAACGAACCCCTACCGCGACCCTTAAGTGCACAAT
TTTTATTTACTAATTTTAATTTCGACAGCACCAACGTAACGCACTTTCAAAATGCACGTCAAATCAATGT
TGTACCAAAACCTATTTTTACACAAAAAACGACTGAAAGTTTCTATTCGTAACCTTTGAAAGTTTACAC
TGACTTTCTAACTGTTTATAACTACGATTTTCGATGTGGTTTTGAACGTTTTTTTTATCCTTCCTATTCCTA
TTGTTAAAAAGTTTGATACCCTACAGTTAAGTGTGACCAAAAGTTCAGTGAGATTTTAAATTTTACCCC
AGACAATGTGTGCCCTACCCAAGTGATCGAAAGTTTAAACTTGTCTAGCTGTGTTTACTAGTGTTAGTG
TAAGTTCACACGAGATAAGAAATTGAAGAAAACATCATTGTTATCAATAAATTGGAAAATCAGCATTTC
ATGGACTGACATCACAAGAAGAAGTACCCACCCTACCTCCTATAAATGGCTCAAAATATCCTTCCTGCA
ACATTCCTCAATCGATCGTCCTCGTAAAAAGTGACTTTACTACTGACATTCCAAAAACATCTTGATTTTT
AAACAGTCACTATATTCTTCAATGATTTCTAAACTGGAGTAGTATCTACGGGACTGTGAGCCGGAGCAG
CGGGAGACATCGAGGACGAAGCGTGATCCTCCTCGGAGGGCGCGAGAGGCCGGCGCGATGCGGTCTC
GCCAGGGATGTATGTAATATACCCAGCGGAAGAAGTAGTGTCCCTGACGTACTGTTCACAAACGCTGC
CCCGCGTGAGGCAGCGGCCCACACTAGACAACCTCCTCCCAC
>TCONS_00090844 geneID=XLOC_018159 scaffold_298:194748-258578(-) Splice junction overlap
CGGGCAGGCGGCCAGTGCGCACGCGCGAACGTCCACCATCTTGAATTAACTGTCACTCTTCCCGCGCT
ACGAACTGTGTGTGCACAGCTTTGAAAAGTGTTTATTTTTTTAATTTTATTGTTTTTTTTATAAGTACATA
ATAAGTTAATTTTATGGTGTTGCGTTTTTGAGTTTTTATTAAGTTAAGTGTTTGACTGTGCAGAACTTTG
TGTTGTGACTTTTTATTCGTGTTCATTTGGGATTTACTTAAACATTGTGTGAGCAAAGAGTAGTTTTAAC
CATTACAATGGACCTCGACGGGGGCGACCTGATAACTTGAGGCACAGAGGATGGACCTCAGATCTTCC
GAGCTCAACAAGAAAATACGTCCTGATCAGGGGAAGGAGAAAAGCAATCGAGAAGATTTGAAAACA
GACATCCAACACAGAAAAGAGACCTTTTATCGGGTG
>TCONS_00090820 geneID=XLOC_018159 scaffold_298:209172-258580(-) Exonic overlap (+)
CGCGGGCAGGCGGCCAGTGCGCACGCGCGAACGTCCACCATCTTGAATTAACTGTCACTCTTCCCGCG
CTACGAACTGTGTGTGCACAGCTTTGAAAAGTGTTTATTTTTTTAATTTTATTGTTTTTTTTATAAGTACA
TAATAAGTTAATTTTATGGTGTTGCGTTTTTGAGTTTTTATTAAGTTAAGTGTTTGACTGTGCAGAACTT
TGTGTTGTGACTTTTTATTCGTGTTCATTTGGGATTTACTTAAACATTGTGTGAGCAAAGAGTAGTTTTA
ACCATTACAATGGACCTCGACGGGGGCGACCTGATAACTTGAGGCACAGAGGATGGACCTCAGATCTT

CCGAGCTCAACAAGAAAATACGTCCTGATCAGGTTTGTTTTTGTTCATTACTTGAAAGTGTTAACCAAT
AATCGAAAACGTGAG
>TCONS_00090767 geneID=XLOC_018162 scaffold_298:321706-326874(-) Exonic overlap (+)
GAAAATATTGCTGCGATCCGAAATCGAACATGTTTACGTGGTTCCTTCTTTATTTTACGCTTAATTAAAA
CCTATATCATTAACTTTAAAATACTCTGAAGCACCTTCTCTAGTCATAGTTTAAATTTTAACGTAATTAAT
CCCTAAACCACGTAACAACTCTACCATTCTCTACACTCTTGCTCAGCATGGATCCTTTCATCATAGCAGC
TATTATCAGTGCCATTATCATTGTTGTGTTGTCAGTAACCTTCCTCAGACCGTCCCAAGTGAAACAACTA
CCTGGAGCA
>TCONS_00090999 geneID=XLOC_018200 scaffold_299:294251-294833(-) Intergenic
GATTCTCTGGCTTTCCCTTCTGCTGATAACCGAGGTTTTCCTTCTGCTGATAATCTAGGTTTTCCTTCTTC
TGATAATATAGGTTTTCCTTCAGCTGATAGTCTGGGTTTCTCTTCTGTCGACTCCATGGCTTTCCCTTCTG
TTGATAATCTTGGTTTCTCTTCTACCGATTCTATGGCTTTTTCTTCTGCCGATTGCTGAGTATCAG
>TCONS_00091821 geneID=XLOC_018222 scaffold_3:178281-179159(+) Splice junction overlap
ATGTTCTGTGTCTAACAATATGTTTTTCAGTTGACCCTTTCATATTAGTTTAAGTACACACAAATAGTTTT
ACAGAGCATGATCTTATTATCCCTTTCATTGCTGTACAAATAGAAATGCCTATTATCATCTGTAACAGTTT
CCTTATAATGTTTTTCATACTGTAGCTATCATTACACTAACCTAGCACCCTCTCATTATCAGTAATCATCAT
CATCATCGTCATCATCATCATCACCTGTAGTTAATGGACTATTGCCCGTGTACCACTAGACCACTAGCCA
CTAGTCACCCCGTACCACGCACGTCCTGACCTCTCCCGTTCATTTCAGTACCAGCGTCGGGACAAGCC
CATTAACACCCGGCGGCCACATTTAATTAGCGGAGGACCCACAGGTGGGCGCGGGCGCGCGGCGCGG
CCGGAGCGGCTGGAGGAGGAG
>TCONS_00091613 geneID=XLOC_018231 scaffold_3:523473-566480(+) Splice junction overlap
ATAAACACTACTGGATCTGAATCATCATATCGCAGTCGTTTTCAAGTTCCTATAAAGTGGCCTAATTATA
CACGTCCTAAAAACGCAACATCCGCGCGTCAAGCTGTGAAAAACATCAGACAAACAAAGATGGCGGC
GGAGATTGCGCGCCAAATCTCACGTCAAAACCAGCGCCATCTGTCCGCGAACGGCGACAACCACTTC
CGCAGTTTGAATCACGCGGGCCGTTCGAAATAGTCATTTGTGATTTAACTGTGACTGTGTCGTGATGTG
AATGATTAGATGATTACGTTTGTTTTTGTGTATTAGTGTTGTGTTTTGTGAAGTGTTAAGATGTCGGAGG
ATCGTTCGCGGCGGTTGTCGCAGATCTTCGACCCGCTGTCGAGGTTCAGTGACCTCAGCGGCGCGGGG
CAGTCCGTCAGCACCCCCAACAGCCCCCGGCTGCTGCCGCGGCGGCAGCGGGAGGCGCCGCCACCG
CCGCCGAGGCCTCACCACCCGCGCGAGGAGGCAGAGGCCCCCAACTGGCAGGAGAGGTGT
>TCONS_00091632 geneID=XLOC_018260 scaffold_3:772333-803710(-) Exonic overlap (+)
CGCCGGCCTGGCGACTCGTGCTCCGCGCACTCCGTCACTTTCTCCACACTCTCGCGAGCCCTGTGCGT
AGTCTCTCGCTTCTTCCGATTTAGTTTCCCGCACTTTTGTGGCGCTCCTCACCTGTGCCCCCACGAGTG
TCCACTGTCGGCTCGACGCGCCAAGTTTCAACTTTGAAAGTTTTGAGTTCCCGCGTTCGCAAGTTCGC
GCGCGATCCCCTGCGGGTGTCGGAGGCCGCGGATGCGGTAGTGCGAGGGTCGTGAGGGCCTCCAGGA
GGCCGCCGCCACGCTGTTCCCACCGCAGCTCGGGTGCGTGTCCTACGTGCCGCCGTGCGCCTGCTGCC
CGCCGCTGTTCCTGCAGCCCGCACTGCCCCCGCCGCTGGCGCTCCCGCTGCCTCTCCCGCTGCCCCTT
GCCCTCCCGATCCACTCGCTCGACCCCCAAGCTAAGCCCCAGCGGCTGCTGCTCTCAAACATGGTGAG
TCCGACTTCACTACTAGACTGCAACAACGGTCGCTGCATTGCGTCCGAAACGGTGAGTTGAACACTAC
TTTTTACTATTATAATTACATATTAATTATTATAATTAGATATTAATTAATTACCGTTATCCATTCCTAGCTTT
ATACCTTTAATAAGCCTCGCGCTCGCAGAAAAATGCAAAAAGAGCAATAAAAAGGCCGGC
>TCONS_00091572 geneID=XLOC_018261 scaffold_3:882621-924232(-) Exonic overlap (+)
CTCTTATTCGCGGTTTTCTGCCAAGCGTGCGCTACACGTGTCGGCGAAACAACACCACTCCAGCTATTT
ACGATGTACTGTGCACCTGTGACTCCGCAAATATTGTAACGCGATGTGAAAGACCATTGTTGGTTTATC
ATCAAGTGAACTGTATTGGTGTATTGTGGGATTGTTTATCGAAACTACTGTCGGAATCATGAATTCGTTG

GAGATGCTGTGTGCATAGTGTGGTGTTTGTTATATACATTTGTGAAGTTTGGACAGTGTTGTAAGTTTG
GCTCGCTTTGTTGGAGTAGTTGAAGCGGCGCAGACTGATCGTGCACTAACTGTACCGATATTTCCGAAT
ACGAAAATTCGGCGATAAGCAGAGCGGCCAGCACAGTTGGATGAACGCCAACCCTCCTGCTGCTAAG
GTAAACACCGATTATGCAACTTCCGTGAGAAATCAACCGCGC
>TCONS_00091795 geneID=XLOC_018235 scaffold_3:1134081-1137043(+) Unclassified
GTTTATTTATGAAAAAAACATATTTGAGCTTAGCAAATAATTATTCGGTAATTATTTATTTATACAATATTT
TAATTATAATGAAATACCTTTTACATTTCGATACGAGTACGATTAAAAATGACACATGACAAAGGAACTA
TTTGTATGCAGACTTGAGATGATGTATGAACCCTACAGTTAAATATGATTTAGCAATATGGCTATCATAAC
TATTATAAATGTAAACTTATGCTCTATTTGACAATAACATTGCGAATAACATACAACAAACCTTGATTCGT
ATTTAATTTATATTTATTATTTGGTGAATTATCTTCTATTCGGCCAGGCGTTATAGACCTGTTCCTATTCCT
AGGTACTTACTAAGTATGTTTTCTTAGTTATTTTATGTTATTGCCGCCTAACCAAGCGTCCGTAGTTCGAC
GTACTTACACGCAATCGAAGCGTTTTAACTAAGACCGTTTCATTCATAATGTTAATCTAATAAAACAATA
ATTTAATTACACATTAAACATCAGTAGCAAAGCGTATTTAGGTATACGTTTCGTTTATTCAATCGGTTACT
TACATAAGCGAATTAACCTCGACCATTCCCATGAACATATTTTTTATTTTATTTTATTCGTTTAGGTAAGA
AAGTATAATATTAAATACGATAATGGTATCTATTTACATTGCACAAAAAGATAATCTTTTTTAACGATGGT
TAACAACTAGAAGTAATATTAGTTATTTAGCACAAACATAGGAAGCTTTTCATGAATTTAATAGTCCTTA
TAACCGTAGTAACCTAAATTAATGGTTTAATTTATTAGCATAGGTACATACTAATGTAGTTTATCATATATT
TTGGCTATATAATTTATTGATTTGATGATACCTACATGTGTTTTGTCCAAAAGGTTTAGATTGTAGCGATT
GAATGCTTTGCAAAAAAAATAATATCAAATCTTTATTTTTTATGACCAGTAATGGTATTTTAAAATACCTT
TGAGCTATTATGAAATGTTGTATGACTCGCTTATTTTAATTAGCATTACTTAATTATACCTCCTTACACAGT
GTGGCTTTTTTATTAGCAAATGAATATAGAAGATATTCAAACTAGTTATGTTAGTTAATGTTATGTCAAAT
AAATGGATCATATTGGACCTATATTGAACGTAAATATTTGCTTTACCATACCTACTTGATAAGGGAACAA
AAAATTTAATTCCTACATAAAGTGACTACCTACAACACTATGGATTTATTTCTAAAGTTCTCACTATTATT
CATTGTGATATAATGGAACATCGGTAAAGTTAAATCTGGTTATTTTGCTAAATTACATCTGTAAGTAGAC
TGTTTCATAATTATTATAATGGTGGAACTGTATTTGTCTTCATTTAGTTTCAATCACAATGCGGGGCCGCT
CAGTCTATGGTTCAATAATTGGCGGTGTTTATGGATTTGTTCAATGCTACGTATGCAAATAAAAATGTAC
CGTAGTGACGCTTACAGAAGAATTAAATAGGATTTTAAACTGATGCCTAAGATACGATAAGACAGTTTT
ATGCATTTTAGCCATTTTAAAATATATAAAAAGTAGCGTGTTCTATTCGAGGAAATAAAATAATATTGTAC
GGCGAGGAATGGAAATGTCATCAGCCTCAAAGCAAGATACAATCTAATTGGACAAGTTTTATGAATGT
CGTAATTAGATATGTCTGGTATTTTTATTCACATTAAAATAAACGATACGAGAATTCAATAGGTACAAATG
ATTAGAAATCGCTTTTTGTAATGTGAATTGATATAAAGACTTCTAGTGAATAAAATATCTAGATTTAATTA
GTGGGTCGGCGGCTAAACCACCGACATCGACATTCGACTCCACGGCTGAGGGACATGTGTTGTAGGCT
GCATAGGCCATACCACTATGGTGTAGGTTTCTGATAATTATTACCGGCATCTGATTATTTTGAGTGGTATC
AAACCCCCCGTTCTTTCAACTATTTTAGGCACCTTATTGAATTTTATAAAAATTAGAGTAGGTACTGTTT
TACAAGTACAAATTTCACTGTATTCCAATAACTACATTAGCTACGCGGTCTGGATTCTATGTCATATGCCT
AAAACAGATGTGTATAAGGACAACATTCAAACTGTACGTGTATCAAATATGTTCACGCTTCAATACTCT
CTAAATTAGGCACAACTGTCGTGTATAAAAAACTAACCCTACAAAGTATTCATAAGGCAATTCTCAATC
ACTAAAATATTAAAATATGTTTCAGGGATGCAGTGCGTGGCAAATTATAATCACTTTGCTGATTTATTGA
TTAACATTCATAGTCATACATCATAAAAAGAGGTTCTACGCATAAAATAAGTTCACAGTTTCGAGAACC
AACCCCCAAAATTGCGCATCGCATCCCTGAAGACAACAGTTACACGGAAACAACACTGTATATTTTTTA
CTAAGACGTGCTAAGTTTACTAATAATTACTTGCTAGTTCCAATTGTCATCTAAAAACGCCGTATGGCGG
CCACAAGTAACACCGAAGGCTTTTTTAACAGCATGCGGATTTAATAACGCACGCGGGTTTGCCTCGCA
CGCGTTCATATAAGTATTATTAGTTAAGTATATATATGCACGCGAGACGCGGGAAAATGGCGTGCAATCC
CGCGTGCGTGATGAAATCCGCATGCTGTTAAAAGCACCCTCAAGGTGAGTGCAGGGAGCGCGGGCCG

AGGCGGTGCAATTGGGTAATTATGTATCAATTAATTGGCAATCCTCGCACGATCTCCGCTGGCTCTCCTC
TCAAAATCGCGCGAGCCTGCAGACTTCCAATTAAAATATTATAATTTACCTCGCCCGCTCGGCTCAAGC
TACCCCACGCTGATCAAACTGCAC
>TCONS_00091225 geneID=XLOC_018265 scaffold_3:1134081-1139401(-) Unclassified
CGACGTGACGCTTGCGTGCCGATGACGCTGTAGGAGATAGGCGAGGACTTGGCCCTTTGTACTTTGGA
AAATATAGCATGAGCCCCATGACTATTTATTTTAATTTAAATTGAGTTTTTTTCAAAGGAAAATGAAAT
TTACCAGGCATAAGGATCTGTCAATCAAATGTCATAGGACTCACGCTATATTTTACAAAGGGTAGTAAA
AGTTCCGAAAGCATGATCCCGGCTACGTTCACGGATTTTAAGACATGGCGTTAGAAAATGATACCCTCT
AGGGCTCTTTATGGACATTGTCACTCGCCCGATACCTCCCGGTCACGTTGTAGGCTGGATATGGCAATA
TGTATGCCCTAGGCTCTATGTCCACCAGTGCGTCCCACTCATGCTAGGCCCCGGCCCTATAGGCCTAGG
ACAGTTTACACAAACGTGGCGCCTCTATAGGAAACTATAATCAAACGCAATCAAAAAGTTCCATCTGT
CATTGAGGTTTCATATGTCACTGAAACGTTTTTCATTTATACCTAAATGAAAAAACGCATTTGCTGAGA
GTTATTGCAACGTTTCAATCGGCAATAGAATCTAGAGTTATTGTAATTTCACTGGAGGATTGTTTCGAAG
CGTGATCGGGACTGTACTGCCCAAAGACTGATAGTATTATAAACTGGGCACCTACCACCGCCGGCGCC
GGGATTTAGTAACACGTTATGTATAGACTTATTCACTACTATTGTTAATAATAATATGTACCTATGTATGTA
TAATCAAGTTTTCTCGTATCCCCGAATAAGCGTAGCACTGTTTAAAATTCAGAATTTCTTTAAAGGACTC
CGTTGTTGTAGAAGTTTTGAATGAAATTGTTAAGATTTCATTGATAATTCTGATCAAACGCGAAGCATG
ATGTATTTATTGGCTATGAGAACTATTGAGATACAAATATTGATTCGATGAAATATTTGTTGTAAACATTC
GGGGATGCGTGAAATTCGAGTATCATTTAATTTAATAAAAAGATAAGGAGTGTAGGCCGCAGCGTCGTA
TTGGCTGTGATATTAAGTATGGGCGCTCAGTATGAGCTAGCTAATAGGTCAGGTTTTATAAATACTATTTC
GTATGGTGTGTAGGAATTTACCTATTAACTTTAATAAATAGCTTGGACTTTACGTGCACAGCGGCAGCGT
TCGTTCTGTTGGAAAGCCAAAAAATCATAAGCACTGTTTCAAATATTAAAAAAAAGTTTGTGTACTCA
GTCGTTATTGTAACATTTTTTATTGATACCAGTTTTAATAGTATACTATACACGCTTTATTATAAAAAAGAT
TCTGAGTGCTGAAATAACGCTAAAAGCGAGGCAACGTAAAAGATAAATTTAGCGGTAATTCTAGGTAA
ATTGTTTGGGATAAAGTCCGTTTGAAGGTATCAAAATTAGCAATTTGGAATCATATGAAATATGGTGTTG
TAATAATTGTAATTGAATTTGGATAACGTTTAGATATGGGTTCGGAATTCATGTATGGTTTAGCGCAATCG
TTAACTAGCTTTAGGCTCGTGTATACTCTTTAGTTAAATATATGCATAAGTGTAACTGTTAGGGGTGATTC
TGTTGTATCGGAGAGAGTAACGAGATGTGGACGACTGCTGCTGGCCTAACTCCTTAGACTAGTAATGT
ACCTACTATAAGAACATTGCATGCTATTGTAAAAGAATATTGCCGACAAGACCACCGTGAAAATTCACA
GTTTATGAAATGATTTTAATATGATGCGGCGGCTTTAAGAATGTAAGGAAAGCGGGAGAATGTATAGCG
ATTGGAATATCGTGCTTTATGTATAACGGCTCAAATATTGTAGATAGTGGGCAATGCATTGTCACTTAAC
AGATGCTTGCAAGTACTGAGGGTTAATGAGTTGGTAATCATCCGGATGTAATTTTTATGAGTGTTTATTT
AATTTGAAAAGGTTAGCTCCTCAGCAAATGTTGTTAGATACACTTGTATGTGAACGAGTTGAGCAGTGT
TGTACATTCTAGTTTTTTTATCAGATCCAATAGCCTCTGGTGGGATATATCATAGTTTAAGCAGTATTATAT
CGTCGACCATCAGAAGATTTCGAGGATCAATTTAGTTTGTTTCATGAATTATCAGGCTACCTCAACACG
GCTTTGTTTTCACCATAGTTATTCCACCAATCTCCATGTTTGTTTTTTAATTCTTTTTGTAGTCAGTTTGA
ATATTATGAGAATGCACGAATGTGTTTACGTTCCCACACGAGTCATGAAGTCGAAATCAAATTCTGCTA
AACAAATAAGTTAATTACTCGACAGTAAATTCAAACTACTAATTTTGTAAGTTGTGTTAAGGTGCAGTT
TGATCAGCGTGGGGTAGCTTGAGCCGAGCGGGCGAGGTAAATTATAATATTTTAATTGGAAGTCTGCAG
GCTCGCGCGATTTTGAGAGGAGAGCCAGCGGAGATCGTGCGAGGATTGCCAATTAATTGATACATAATT
ACCCCATTGCACCGCCTCGGCCCGCGCTCCCTGCACTCACCTTGAGGGTGCTTTTAACAGCATGCGGA
TTTCATCACGCACGCGGGATTGCACGCCATTTTCCCGCGTCTCGCGTGCATATATATACTTAACTAATAAT
ACTTATATGAACGCGTGCGAGGCAAACCCGCGTGCGTTATTAAATCCGCATGCTGTTAAAAAAGCCTTC
GGTGTTACTTGTGGCCGCCATACGGCGTTTTTAGATGACAATTGGAACTAGCAAGTAATTATTAGTAAA

CTTAGCACGTCTTAGTAAAAAATATACAGTGTTGTTTCCGTGTAACTGTTGTCTTCAGGGATGCGATGC
GCAATTTTGGGGGTTGGTTCTCGAAACTGTGAACTTATTTTATGCGTAGAACCTCTTTTTATGATGTATG
ACTATGAATGTTAATCAATAAATCAGCAAAGTGATTATAATTTGCCACGCACTGCATCCCTGAAACATAT
TTTAATATTTTAGTGATTGAGAATTGCCTTATGAATACTTTGTAGGGTTAGTTTTTTATACACGACAGTTG
TGCCTAATTTAGAGAGTATTGAAGCGTGAACATATTTGATACACGTACAGTTTGAATGTTGTCCTTATAC
ACATCTGTTTTAGGCATATGACATAGAATCCAGACCGCGTAGCTAATGTAGTTATTGGAATACAGTGAAA
TTTGTACTTGTAAAACAGTACCTACTCTAATTTTTATAAAATTCAATAAGGTGCCTAAAATAGTTGAAAG
AACGGGGGGTTTGATACCACTCAAAATAATCAGATGCCGGTAATAATTATCAGAAACCTACACCATAGT
GGTATGGCCTATGCAGCCTACAACACATGTCCCTCAGCCGTGGAGTCGAATGTCGATGTCGGTGGTTTA
GCCGCCGACCCACTAATTAAATCTAGATATTTTATTCACTAGAAGTCTTTATATCAATTCACATTACAAAA
AGCGATTTCTAATCATTTGTACCTATTGAATTCTCGTATCGTTTATTTTAATGTGAATAAAAATACCAGAC
ATATCTAATTACGACATTCATAAAACTTGTCCAATTAGATTGTATCTTGCTTTGAGGCTGATGACATTTCC
ATTCCTCGCCGTACAATATTATTTTATTTCCTCGAATAGAACACGCTACTTTTTATATATTTTAAAATGGCT
AAAATGCATAAAACTGTCTTATCGTATCTTAGGCATCAGTTTAAAATCCTATTTAATTCTTCTGTAAGCGT
CACTACGGTACATTTTTATTTGCATACGTAGCATTGAACAAATCCATAAACACCGCCAATTATTGAACCA
TAGACTGAGCGGCCCCGCATTGTGATTGAAACTAAATGAAGACAAATACAGTTCCACCATTATAATAAT
TATGAAACAGTCTACTTACAGATGTAATTTAGCAAAATAACCAGATTTAACTTTACCGATGTTCCATTATA
TCACAATGAATAATAGTGAGAACTTTAGAAATAAATCCATAGTGTTGTAGGTAGTCACTTTATGTAGGAA
TTAAATTTTTTGTTCCCTTATCAAGTAGGTATGGTAAAGCAAATATTTACGTTCAATATAGGTCCAATATG
ATCCATTTATTTGACATAACATTAACTAACATAACTAGTTTGAATATCTTCTATATTCATTTGCTAATAAAA
AAGCCACACTGTGTAAGGAGGTATAATTAAGTAATGCTAATTAAAATAAGCGAGTCATACAACATTTCA
TAATAGCTCAAAGGTATTTTAAAATACCATTACTGGTCATAAAAAATAAAGATTTGATATTATTTTTTTTG
CAAAGCATTCAATCGCTACAATCTAAACCTTTTGGACAAAACACATGTAGGTATCATCAAATCAATAAA
TTATATAGCCAAAATATATGATAAACTACATTAGTATGTACCTATGCTAATAAATTAAACCATTAATTTAGG
TTACTACGGTTATAAGGACTATTAAATTCATGAAAAGCTTCCTATGTTTGTGCTAAATAACTAATATTACT
TCTAGTTGTTAACCATCGTTAAAAAAGATTATCTTTTTGTGCAATGTAAATAGATACCATTATCGTATTTA
ATATTATACTTTCTTACCTAAACGAATAAAATAAAATAAAAAATATGTTCATGGGAATGGTCGAGGTTAA
TTCGCTTATGTAAGTAACCGATTGAATAAACGAAACGTATACCTAAATACGCTTTGCTACTGATGTTTAA
TGTGTAATTAAATTATTGTTTTATTAGATTAACATTATGAATGAAACGGTCTTAGTTAAAACGCTTCGATT
GCGTGTAAGTACGTCGAACTACGGACGCTTGGTTAGGCGGCAATAACATAAAATAACTAAGAAAACAT
ACTTAGTAAGTACCTAGGAATAGGAACAGGTCTATAACGCCTGGCCGAATAGAAGATAATTCACCAAAT
AATAAATATAAATTAAATACGAATCAAGGTTTGTTGTATGTTATTCGCAATGTTATTGTCAAATAGAGCAT
AAGTTTACATTTATAATAGTTATGATAGCCATATTGCTAAATCATATTTAACTGTAGGGTTCATACATCATC
TCAAGTCTGCATACAAATAGTTCCTTTGTCATGTGTCATTTTTAATCGTACTCGTATCGAAATGTAAAAG
GTATTTCATTATAATTAAAATATTGTATAAATAAATAATTACCGAATAATTATTTGCTAAGCTCAAATATGT
TTTTTTCATAAATAAAC
>TCONS_00091384 geneID=XLOC_018242 scaffold_3:1242503-1291953(+) Exonic overlap (+)
GCCGTCAGTGATACCAATCGAAGCATCTAAAATATTAATGAAACTTCGAGTTTTTCAAGCAGTTTTTTT
GCAATTTTGTGCCAATATTTATGCTTGCTTTAGTGATTTGTTGATTTGCAATTTGAAGTGTATTCTTCGTC
CTTGTGCCACGGAAGCTGATGACCTAATTCTCCACAAAATACACCATGGACGACTTGGACGCCCTGCT
AGCAGACCTGCAGAACTCCATCAACCCTGGCCACAGAGTGGGCTCTCCCATCGGCCGGACATCGTCTC
CGGGCTTCAGAACCGCGTCCCCTGGCGGCAGAACTGCTTCCCCTGGAGGGAG
>TCONS_00091879 geneID=XLOC_018244 scaffold_3:1381641-1382324(+) Exonic overlap (+)
TAGTATATTTCCTTATAATATAATAAATGACTTCATTCATCATCAACATTTATATCTAAGAGCCGTGTATTC

ATTTGTCATAAACGCCCAATTAGAGCACCGGCATATTCTGCACGTTCTTCCTTGCTCACTCCATCACTAA
GAGACGGAGGGCTTCCATGAGGAACTCCACGCTGCGGGAGTCCACCTTCGCCAGCCCCGACGACTCG
CTGCTGGCCGCGCCCACCGCGCCCAGCGCCACCCTGCCAAAGTATTAGACCGGAGATCAGCGATTCCT
GAACGCTTACGCTACGGAAGCTGCTGTTGATGGTCGCGTCACAAGATTAGGTTAGGTACTACTGAAAG
CCTAGCAAGATGCGCGTGACTCTCGAAGCCGCGCGATGTAAGTGTGCGAAACGC
>TCONS_00091253 geneID=XLOC_018272 scaffold_3:1434044-1439251(-) Splice junction overlap
CCTGGACGTGATGCTGGCCTCCAGCGACTCCTCGCCTTCCTCATGCGGCTCCTCGCCCGCCGAGCCGC
CGCCGCCGTACTCCCGCCTGCATCGCCTGCACCGCTCGCGGAGGCAGAAGACTGCCGCCGCGCTGCG
CAGTCGCGGCCTCGGCGTGCTGCGCTTCCCGCGCCGGCGACGACCCTCCATCATCGACCGCGACGGCT
TCTACGTCCGCTACGGCGACCGCGAGCGCCAGGCCGTCGCCAGCTTCGACTTCCTCGATGACCTCTGC
AAAGACTGCAACTAGCAATAACCTAGACTAACTATATTTTTGTATCGGCCGACTGAGTGGAACGTGAA
AGTTCCGACGTGTGCTTAAATTGTATCTCGCTTGTAGCTGATAAATTGATAAGCCAATAGATTGTATGAT
TTGTTAACGTAGCTGTTATTTGTATTGCATTTTGGGCGAAGGAGCTGCATGTGTGCCCTAAGTGACTGT
GGTGAGCTGCAGCTTGGGAAGTTTCGGTTAGCGAAAACGGCGTGTTTTCCTTTTTACTTAGAAAGACA
ATTGTCGTTGTAGACTCTCACTCACATGTGTATATTTCGAACTTGGTCACCTCTAGATCTTCCTAGATTAT
TTCCTATATGGTAGGTAGTCTACCCTTCTCTACTGGGCTGACAGCAATTAAGAGAATAGTTAAGCTGTAT
GTGTTTTCGAATATATTTATGTTCTTGTTAATCTGGCAGTGGAGTGGCCGCAATCTCAGCTTTGCTTCAC
GCAACCAATCGAATCGCCACAAACAAACGGCAAAACCCATATAATATGCTTCTGTTTGAGAAACCCGC
GTTAGCTTAAATTGCGCCAATTGCATCGAAAACTTAGGTAAGAAAACGAAAATAACTAATGACTAATAA
AGAAAGCTGCGGCCTCAAAGTAATGTGATATTTTTGTATATACTGTAAGTCCTACTTATTTTCATATACAT
TTAAATGGAATCCTGTTTTATTTCGAGACGAACTATCGTTGGTTGTTAAGGGTAGGTGGTCTAGGATAG
AAGGACTAGTGGTATGTCTACCTACGACTACTTAATCGTATGTTATGTAAATATTTTGAAATAATTATGAT
CGTACGTTATAGGTAATAGTGAGATGTCGGTGTATTGTTTAACTATAGTCATTAACTGAAGAACTCATTT
AACCTAACTTGGTAAGTTCATTAACATGAAGGGTACTAAAGTATATAAGATGTGTATATAATGGTGTCGT
TTAGTTATTTATTGAGTGATTTCTTTGCAATTTGTGGTCAGTTGTGTAATTAATTAGGTAACATATCATTG
CATTTTCCTACTTAACTCTCAAATAGTTTGTCTTTTGCTCTTATGGTTATAAACCTTATATAATTATAGTTA
TTATCGTCATTAGATTACAGTTGCGCCTGTAATATTGAGTTCTTCTTAATAATGATTCTTGAGAATAACTT
AGCTTAACAATGAAACTCTGAAATTCGCTTTTAGATCTTAAATTAGAGACCAAAGCAATTTTTAACAAA
CTAAAACTTAATTTCATATAAATATTGCCACCACTTGTTAAGCTGCAACACTTTTGATTTGGATAAGTATA
TGTAAGACTTTTGTTTTTATCTTGTGAAGTGGTAAAGGTTTAAGACAATAAAGGAAGATTATACCTATAA
TATACCTATACATAAACGAAACGACAATTCGATAATTAATATTAACGATGACCTAAAAGTACAGATATGG
ACAAAGAAATTAGGATTCGAGATCCCATACCTTGAAAATTTAACATTTGAGACTTATCTCATGTATTTTA
CTGCCAGTTTCAATAACTATCTATAATTACATACTTGTCATCTGTACCTAGATAGAGTTATGGACACTGGT
GGTTTATATTCTGAATCCTAGTATCTTTGCTCTCATGTACTTATGTATTTAACTCGAACATTGTGTAAAAA
ACGGTTCTACCTTTTTTGGCAGAAGATTTTTTTGCCTAATCTCGTATTGCATAGTAACGTTTGGTCAAAG
TCTCGTTACGCCGAAAATCGTATGGCATAAATCTCGTTTAGTAAAAAGTTATTTCGCATAACATTGTTTA
GCCTAATAATGGTATGGCCAAATCTTGAATAGCCTAATAATGCTATGGCATAGGTTTATACAAAGTAATAA
TATTCGTTTGGCTTTAACTTTGACGGAGCGTCTCCCTACATAGCGCAAACTGGTGCCTTGTATTGTTTCC
GCTGTTTGGAAAACGCTCCGCTCCGCTTCGCTGCGCTCCGCTTTGGTTTTGATGAACATGTGCACCTAA
CACGCTCCTCCTCGCTTTGCTCGTCGTCGCACCTATTTTTAGGTTTCGATCTCATGGGGTTTGTAATAAT
TATATTGGTCGTTAACTTTCGATTTTTTGATCATACAATATCGTGATTTTCGGGATGTAGGAGAAAAATAC
CACAATTTGTACATTTACTACATACTTAATATATTATTTATTAAGATAACATTAGAAGAAACAAGATTATA
CATAGGTATAGACGAACATAAATTTGAGCAAACGAAACTTAGTTGTTTAAAGATTGTGCCTAAAGAGTT
TTAGACGAAACGAGCCTTATGCCACATAAGTCTTGGCAAAGTGATGGTTCGGCCAAAAAAGTTTAGAC

CATACGAGCTATGCGAAATGAGTTTGGTCAATAAAGATTATGCCAGATGAGCGGAACCCGTAAAAAA
CGTCCTTAGATATAAACTTCGCCTAGGTATTTTGGAGTCATTTAATTACGATGTCATATATAAGTAGGTAC
CTACCGATTAATCAGACTTTAGACTTTCCATCGTTTTACAATATGAATCATAAATTATTGTACTGTCAATT
AATATACAATGTTAATTTTAAAATCATCTCATCCTCATTAAATTTAAATTAAATAATGAATGAAATAGAAA
ATAACATTAGTTTTAGGTACTTACGTAAGTAGGTTCCATCTTGATTTTTATTTATGTATCGAATAAATAAA
AACTGAATTAAGAAAAAAAAACTGATTAATCATAAAACCTAAGACCTATTGCAGTACCTACCGTGATTA
GCGAGTAGAAAATCATGAATCAATTTGATTTTTGTATAATAAAAAACTTAAACAATGATTGTACGAGTAT
CCCTTTGAATTGGTGCATTTGTTTGACTTCCTAACCTAAGACATATTTATGTAGATATTTTGATTAGGTAT
GTCCTTACTTTATAGCTTGATTAGACATTTCTTTTGACTAAAGTGTATTGTAAATTGTAAGACATCCTTCG
GTAAATTCGCCACTCATAAAACAACTTTAGTTTGTAAACCAGACTTTCTATATGTTGTTAAAACAGTTTT
TGTTATAAGACAGGTATTAAGTATGTTAAATAGAAAAGAGCAATACTAATCAAGCTATAATTTGAGGAGT
ATAGGTACTTGAACAGTACAAAAATGTAGATTAACTGTATTATTTAATTATATTCTACTGTAACTGGAAAT
TGTCACGGGTTCATTACACGATTCCGTTCCCAAAT
>TCONS_00091258 geneID=XLOC_018273 scaffold_3:1660250-1689759(-) Unclassified
CAAAATAAAATAACAGTCCGTTCATTACGTTGCATACGCAAGAAAAATCTCGCGATCCGTCCTCGGCCC
GCGATTACCGTGTGCGTCGGCCACCTATCTTTCATTGCACGTACAGCGGGGCGCGCGGGGGGGGTCCG
ACTGAGCGCGGAGCCATCTTGCCGGCAACCGTCGAATCGCGCGCGTCTCTGCCCAACAAATCGTTCAA
TTCCCACTAATTAACCATTAAAACACACAAAAACAATCGAAAACTGTGCGGAAACATCGATTAGAGTG
CTTGTGCCGTGATAACGAGACACTGGAGTGGATTATTGTGGTGTAACGGTGTTGTTCTGTGCGTGTACA
TTCCTAGTCGGTTTGAGATTACCTATTCTGGATTGCCCGCCTGATGATGTGCTAGTGTTGTAAAGTGAA
AGTGAAAATATAGTGTTCGCAAGTGTAAAGTGAATTCCAAAGTGATATCTCAGTGATAAAGTGTGATCA
TGCCGCCCTGGAATCACTGTTCTGAATGGTAGCTAATAATTTAGAAGCGGGATGGAGAAGGAGAACGG
CGACGGCGACGCCGGCGGGGGGGCG
>TCONS_00091415 geneID=XLOC_018273 scaffold_3:1665504-1689597(-) Unclassified
CAACCGTCGAATCGCGCGCGTCTCTGCCCAACAAATCGTTCAATTCCCACTAATTAACCATTAAAACAC
ACAAAAACAATCGAAAACTGTGCGGAAACATCGATTAGAGTGCTTGTGCCGTGATAACGAGACACTG
GAGTGGATTATTGTGGTGTAACGGTGTTGTTCTGTGCGTGTACATTCCTAGTCGGTTTGAGATTACCTAT
TCTGGATTGCCCGCCTGATGATGTGCTAGTGTTGTAAAGTGAAAGTGAAAATATAGTGTTCGCAAGTGT
AAAGTGAATTCCAAAGTGATATCTCAGTGATAAAGTGTGATCATGCCGCCCTGGAATCACTGTTCTGAA
TGGTAGCTAATAATTTAGGTGAGTTCTACTTCATATAAAATATTTACTTAGATTAAGAATATTGGCACAAG
CTGGTGTGTCACATACAAAAACAAAGC
>TCONS_00091260 geneID=XLOC_018274 scaffold_3:1789194-1791871(-) Intronic
TCGTGACACAAGCGAAGTGAAAAACTGTGAAGTGTCATCCGCTGTTAAAATTGAACACACAAAATAA
ATAATGCGACTGTAAAAATTTCAAGTGCCGCCAAAAAAATATGAGCATTCAACATGTTGTGTATGTCCG
TTGGTGCTATGATAATTGCCGGAGCTCTAGGATGTATCGCAAGTTGGCCCTGCCTACTATCATGCTGCCT
GACACTGCCGTTCCCTATCCCGTTACTCGTGGTGCAGATGCCGGGCCGCAAGCCAAGATGCGACAAGA
AGGCACACGACTACAGCGACTCTGACCTCTCTGTCGAAGAATCTGCAGGCTAAACCAGCCACTACCTT
CGGCTTCGGCGGCACCACTACAGTCCAGTTCTCCACTGTGAAGCACAGAACCTGTGCCGAGAATGAG
ATTGAGTTCTACGGCACCGATGGCTCCAAAAGACGTCCCGGCTTCATACGCAAATTGTGAGTGACCGA
TTTGGTTACGAAATTCCTTGTGCTGCCTGTGTTTTTAACTATTTTGTTTCTACAATCTTTATTTATACTGAT
TAAAAAGAAGAGCAAAGCAACTGGCTGTTTTACTACGCTACAACGGGACAAGAC
>TCONS_00091259 geneID=XLOC_018274 scaffold_3:1789237-1790553(-) Intronic
AGACAAGTAGACTTGACAACTGCTAGGCGACAAACAGAAAAATAATTTGACGATGTGACAGCACTCA
AATTTTAAACAAGACGTGTCTTGCAAAAAAATGATGAATATTTGAAACAAAATGATACAAATTTTTGCA

CCTTTAACGGTCGTAGGCCTTGGTGTTTTGGCCTGTTGCTGGCCCTGCCTACTATCATGCTGCCTGACA
CTGCCGTTCCCTATCCCGTTACTCGTGGTGCAGATGCCGGGCCGCAAGCCAAGATGCGACAAGAAGGC
ACACGACTACAGCGACTCTGACCTCTCTGTCGAAGAATCTGCAGGCTAAACCAGCCACTACCTTCGGC
TTCGGCGGCACCACTACAGTCCAGTTCTCCACTGTGAAGCACAGAACCTGTGCCGAGAATGAGATTG
AGTTCTACGGCACCGATGGCTCCAAAAGACGTCCCGGCTTCATACGCAAATTGTGAGTGACCGATTTG
GTTACGAAATTCCTTGTGCTGCCTGTGTTTTTAACTATTTTGTTTCTACAATCTTTATTTATACTGATTAA
AAAGAAGA
>TCONS_00091591 geneID=XLOC_018274 scaffold_3:1789675-1791877(-) Intronic
AGTCATTCGTGACACAAGCGAAGTGAAAAACTGTGAAGTGTCATCCGCTGTTAAAATTGAACACACA
AAATAAATAATGCGACTGTAAAAATTTCAAGTGCCGCCAAAAAAATATGAGCATTCAACATGTTGTGTA
TGTCCGTTGGTGCTATGATAATTGCCGGAGCTCTAGGATGTATCGCAAGTTGGCCCTGCCTACTATCATG
CTGCCTGACACTGCCGTTCCCTATCCCGTTACTCGTGGTGCAGATGCCGGGCCGCAAGCCAAGATGCG
ACAAGAAGGCACACGACTACAGCGACTCTGACCTCTCTGTCGAAGAATCTGCAGGCTAAACCAGCCA
CTACCTTC
>TCONS_00091192 geneID=XLOC_018250 scaffold_3:1799923-1803890(+) Splice junction overlap
GTTCGTTTCACGTTTGGAAATTGTCTAGTTTCTAACCGTTATTTGACCCAAGAAGTCGCGTTTTTTTTAT
TATTTTTTATTATGTGAAGTTCTCTCTCTATTGAAGCAGTTAAAACAGATTGAACAATGGCGGTGTTACA
TTACGTGCGAATGTTCGCCTACACCGCCACCATCGTCGTGCATGTCACTAACATCGCCTACATGGTAAG
TTGAGTACCTTGCCTACCCACTAAGGGACATGCCTCGAATGGTGCGAATACACTCCCGAGCTATGACA
AAGTTTCAGTGACATATAGAATGGTATAGGGAGGTGAAACTTTGTCATTGCTCGCGACGTTATCAATTTA
TCTGTCGTTTGAAATTAGTATAAAGAATAAATATTTATAATTCCCATTTCAAAAGATCTTGCTTAGAAGA
AGAATGAGGGAAACAAAGTATAAAGACGCGTTCACAAAATCTGAAATTACTGAGTACTGAGCTGACC
CAACGACATTAGGTCAAGCATTGTATGTAGATAAATTAATTTCATCTTGCCCAAGTTCATACCAAGCCAT
GCATTGCGAATTGGGAAGCATACTTGAAGCTAATGTCGGCATTAAATCAATGTCTGTCTCTTTCTGTCC
GTATACTATCAAAGCAAGGC
>TCONS_00091356 geneID=XLOC_018275 scaffold_3:1806801-1807780(-) Exonic overlap (-)
ATATTGATAAACGTAATAATATCCTTAACCACATGTACTTGCAAGACTTGTAAAGTAAGTTGTGAGGTTT
TCATAGCTCGATGACAAACAATGTCGGTAAACATACTTATATTAAAAAAATACAGACCTCCTTTTTTAGA
AGTCGGTTGACAAGGCCATATTTTGGTCAAATTCAAGACAACTAATACACAACCTCAGCACAATATAAC
TTATAGGAAAGGAACAATACATAATATTTTAATTCATTTACAAAAATCATGAAATCTTTTTAGTTTTTCTC
TTGCTTGTCTCCTTATTCCACAGAATTCTGACGACAATCCACTGCATGTGATAGAAGCCTAGACAAGCG
AATACTATGATTAGTATTAGTACCGGGAAGTATATGGTTTTGTACAGAATTTTGAATATTGGGTAGATCCA
TATTCCTCGTTCGTAGTATGTGTACAGGAG
>TCONS_00092093 geneID=XLOC_018452 scaffold_30:484759-486442(-) Splice junction overlap
GTTTATTTGAAAACCAACAAAGACACATACAAATTGTTGCAACCTTGGTCCTCAAACTAGGATTCCCT
GTGCTATGAGGACCAGGACCCGTCGTTGATTATTTATCTAATTTAAAAGTGCCTATTAGAAGCATATATA
AATTACAAAAGTTAGATGCTTACTTGCTAATTAGGGATATACTTTAGATCTGATGAGATTACATCTGGTA
GGTACATACTTAATGAATAAATTTAAAAAATAACAGTTATTGAACATTTAACTTTGTAGGTACATTTTATG
AAAGTAGAATATAAAAAGTTAAAAAATAAAAACCAAATCATGTATGATAGTGAGTGTTGTGTGTGAGC
TGTGTGTATGTGTGTCAGGTGTGTTTGTGTGTGTGTGTGTGTGTGTGTGTGTGTGTGTGTGTGTGTGTG
TGTGTGTGTGTATGTGATTGTGTGTGTTTTCACATTAGAGCCCACTTAGCCCTGCGACTAAGTCTTGGG
ATCGAGGGACCATAATTAAGGTATCACTAAGTGGGCATATTATATACTTACATATTGTTGAAAACAGCAA
ATTATTTATTTATTTACTTTAATGACTTTTTCAGTGTCGTCGTAATTAAGTGTAAGTAACCAGGAGTTAAT
AATTTTTTTACACACAAAGCGTGTTTTTTTAAATATGCAATGAGATTGATCATTAAAGTAAATGTATTTTA

TTTTGTAGATTCTCAACGGATAGCTCGAAACGAAGTTACGAATACTCAAGAGATTGCTACAGAAAGTA
AAGAATACGAATGCACCGATAAACTCAACAACTGTGGTTTGGCCTTGCAAGCGCGCTTATGCCACTAC
AAGTATTACACACAAAACTGTTGCTTCTCCTGCAGAGGAAGATAAACTCAATTTTGGTAAACATAAAG
TTTATTTAACAAAAACAGCAAACGAGTTCTCGGATAAATTACACTGCCAGTATTACCTACTGTTTTAATT
TATTTTTGTATTGACTCTCTTTATATTATTTATGATAAGCTATAATGATCAGTTTAAACGCTTTCTTAAAGT
TATAATATATGTACCTACCTATATCAAACAATGTATTTTTATTCTGCTTTTAATTCTTAAAAAAATATACCTA
CTTATTCAGTTTAACATTGTTATTTGGTGTACTAAATTTGGCTTACTTTTAGCGATTTATAGTATAAATAAG
TCAATCCATGACTTTAATTATATTTAAACCTAGGTTAATACTATGTACATAGATAGTAATAGATTGAATAA
ACATTGTAAACATAAAGTTTTTTAATTAAAATATTCAGTAGCTCTACATTTATTTAGAAGAGTGCCGGA
GTATCAAAATATTTCTTAATTTGTTTTCACTGCAAAAACATAAATAAGAAATCAGTGCCCTGAGTGCGA
GTTTTTCCACCTATCGAG
>TCONS_00093091 geneID=XLOC_018458 scaffold_30:681944-727775(-) Splice junction overlap
GTAGTATCCCCGTGCTTTACTCACACCTCAGTGACGCGGGCACAGGTGTATTCCACCTACTCGTCATTT
ATCGTACACACGCTGAGCGAGACAGTGCGCCCCGCTTGGTACCTCCACGGAACTCTCGATCGTGCGAA
AATCGAGACATAGAATCGATACTCTGTCGATTATAATTGTAACAGCACCGCACTAGTTTCCCGCGCGTT
CTTTTTTCAAAATTAGAGAATGAACTTTGTTTTTTTCTCGCGTGTTGTGTTTCATGTTTAATGCCAGTTT
AAAACTATTAATATTTTTCTCTTAATTAATTTAATGTGAAGTGGTGTAAAGATTACAGACTTGCTGCCTAT
TGTCAAAACAATGTATTGTTTAAGAATTTGAGAAGTTCACACGCTGATTCCGGCTGCAATTACTTGATA
GACATCGAAATGCAACCCCAAAGATGAAGCTATAGCGTGTGAGAGCGATGTGCGCGGCGGGTGCGAG
CGGGTGGGGAGGGGCCGCGGGCTGACATGGCCTCGCTCCTTCTGCTGCTGCTCTCGGCGGTGGCGGC
GCAGCGGCTCGGTGACGTCACCAGGCGGGACGCGGGAGACACCTTCACTTTGACCAGCGGGAGTTG
CGAGGCAGCAGAATGCGCGGAGCACGGAG
>TCONS_00092283 geneID=XLOC_018392 scaffold_30:787913-790952(+) Splice junction overlap
GTCGGCGGCGCTGGCGGGCTACACGCCGAGCAAGACGCGCGCCGAGGAGTTCCGCCTCGGGCTGTC
GCGCCAGGACCCCGCGCCGCCGGCCAAGACACCTGCCGTGGAGACAGCTGAAGAAGAAGAAGACGA
GGATCTGGGCGACGTGCGGCCGTGCGGGGGCGCCGCNNNNNNNNNNNNNNNNNNNNNNNNNNNN
NNNNNGCGGAGCTGGAGCCAGGCGCGCGCCGCCCGCACCGACCTGCTGTTCTAGTGCGAGCGAGAC
AGGCGCAGTGCAGGTTGTTGAAACCGACACGGATGGAGACGCGGTGCCCAGCGCCGCATGGACTTGT
GTAAGCTAAGCTTTATTAGACATAGCCACTATTACATATTAATATTATATTGTTATTTTCACATTCCAAAAT
ATACATTCGATTAAAGAAAGAGAAATATATTCCAAAAGACTTGACCTGTGTCAATTTTTGTACTGCGAA
ACTGTAACATAAAACTGTATTTTATAAATTATACATTTTGAGAAAATTTTGTACTATTTCATAACTTTACC
AAAACATTTACATTGTATCGTCATTATAGAGCTCACATTTCGTTTGTAATTAAATTAGTAATTTACTTATTG
TCATATTGAGTATCCTAAATTAGTAATAACTATGTGTAAATGATTTAAACTCAGATAAAAGGGTCTTTGA
GTAGCGTCGAGGCTCGCCGAGTCTAAGCTAAATGTAGTAGGTACTTGCTAGTTATGTATTAATGTACTTG
AAAAGGTGTTTCCACATGTGTTCTTAATCTATGACTGACCATAGACAAATAGACAAAACGAAAAGTATT
ATTATGTAAAGGTGGCGTTGGTTTACGACGACCATTCCTGATATGATATCATTCCTATATTTTGTACAATT
TGTATTTTGTAATAATATTACTATACATGTTTGTACTGGCGATCACGCGGCCCGAAGTGCCTCGGTGTTC
CTTCCATTGTGATCGTGAACTGTGAAGTATATAACTCTTCTATAAATATATAATGTTTATTACTGCTAAGA
AACAATGTATACTCTATCTATGGTCTACTGCCTTATTGCATATGTACTGAGCGCTTTCTGTGGTAGGGAG
GCGTTGTTTGCGTCACTCATGGTCGTTATAAGGGACCAAAGCTTCTTATAATATTATGGTGTCATACATG
AGTGCTGGCGCGGCGCGGCGC
>TCONS_00091957 geneID=XLOC_018392 scaffold_30:788824-791342(+) Splice junction overlap
CGGAGCTGGAGCCAGGCGCGCGCCGCCCGCACCGACCTGCTGTTCTAGTGCGAGCGAGACAGGCGC
AGTGCAGGTTGTTGAAACCGACACGGATGGAGACGCGGTGCCCAGCGCCGCATGGACTTGTGTAAGC

TAAGCTTTATTAGACATAGCCACTATTACATATTAATATTATATTGTTATTTTCACATTCCAAAATATACATT
CGATTAAAGAAAGAGAAATATATTCCAAAAGACTTGACCTGTGTCAATTTTTGTACTGCGAAACTGTAA
CATAAAACTGTATTTTATAAATTATACATTTTGAGAAAATTTTGTACTATTTCATAACTTTACCAAAACAT
TTACATTGTATCGTCATTATAGAGCTCACATTTCGTTTGTAATTAAATTAGTAATTTACTTATTGTCATATT
GAGTATCCTAAATTAGTAATAACTATGTGTAAATGATTTAAACTCAGATAAAAGGGTCTTTGAGTAGCGT
CGAGGCTCGCCGAGTCTAAGCTAAATGTAGTAGGTACTTGCTAGTTATGTATTAATGTACTTGAAAAGG
TGTTTCCACATGTGTTCTTAATCTATGACTGACCATAGACAAATAGACAAAACGAAAAGTATTATTATGT
AAAGGTGGCGTTGGTTTACGACGACCATTCCTGATATGATATCATTCCTATATTTTGTACAATTTGTATTT
TGTAATAATATTACTATACATGTTTGTACTGGCGATCACGCGGCCCGAAGTGCCTCGGTGTTCCTTCCAT
TGTGATCGTGAACTGTGAAGTATATAACTCTTCTATAAATATATAATGTTTATTACTGCTAAGAAACAATG
TATACTCTATCTATGGTCTACTGCCTTATTGCATATGTACTGAGCGCTTTCTGTGGTAGGGAGGCGTTGTT
TGCGTCACTCATGGTCGTTATAAGGGACCAAAGCTTCTTATAATATTATGGTGTCATACATGAGTGCTGG
CGCGGCGCGGCTGGCAAAGCATTTATTATCCATTAAATATTCGGTAACTGTAGGGTAACTAGACTAGCC
TGCACTAGGGCTACGTAGCTGTAAGGGCACGCTGTAAGAGTGTCATATTCATTTTGCGTATGTTTAAGG
TTGCTTACATATTTTACCGTGTTTTGTTTTGTATTTGTACACTCAGCGGGGCAGGGAGGGGGATCGCAA
CTTAGACCGAGTAAAATATGGAAGCGAGGCCGCCCGGCCGCCA
>TCONS_00092284 geneID=XLOC_018392 scaffold_30:789421-792096(+) Unclassified
GGGCTTTCACAGCATTATTATTATTGGGGCTAAGAGGAGAGACGCTCCGCTCCGCTTCGCTGCGCTCCG
CTTTGGTTTCGACGAACATGTGCACCTAACACGCTCCTCCTCGCTTTGCTCGTCGTCGCACCTATCTTT
AGGTTTTGGTGCTAGGGGGTTTGACGGTGTTGGGTTAACACAGATTATGGGTTAATACAGGTTAAACA
CAGATTATGTTATATGTTAGTATGTTTTATGAACTTTATACTAAATGATAGTATATATTTTGAACGATATACG
TAATGTATGTTATACGAATTGATATTAGTCCTCGTAAGTATTATATATTTTGATATTATATCAAATGTACGTT
ATACCAATTGAATTTTATACCTTATGAAAATATAGATTTTGTTGTTATACGTAACGTTCATTATATGTTGTG
AACGTTATTAATGTGAAATTATACATTTCGTTTTTATACGTCGCAATACGCACCCGTTCTGCCCTCGCCC
GCTCCTCTTAAGAAACCTTTTTAAATTGCTTTCTGCAGTGTACTTCTACAAGTAATTTCAGCTTCACAAT
AATGTAGCATTTTTCATTTTATTAATTCATGTTAATTTTTATCGACAGGTTGTTGAAACCGACACGGATG
GAGACGCGGTGCCCAGCGCCGCATGGACTTGTGTAAGCTAAGCTTTATTAGACATAGCCACTATTACAT
ATTAATATTATATTGTTATTTTCACATTCCAAAATATACATTCGATTAAAGAAAGAGAAATATATTCCAAA
AGACTTGACCTGTGTCAATTTTTGTACTGCGAAACTGTAACATAAAACTGTATTTTATAAATTATACATT
TTGAGAAAATTTTGTACTATTTCATAACTTTACCAAAACATTTACATTGTATCGTCATTATAGAGCTCACA
TTTCGTTTGTAATTAAATTAGTAATTTACTTATTGTCATATTGAGTATCCTAAATTAGTAATAACTATGTGT
AAATGATTTAAACTCAGATAAAAGGGTCTTTGAGTAGCGTCGAGGCTCGCCGAGTCTAAGCTAAATGT
AGTAGGTACTTGCTAGTTATGTATTAATGTACTTGAAAAGGTGTTTCCACATGTGTTCTTAATCTATGAC
TGACCATAGACAAATAGACAAAACGAAAAGTATTATTATGTAAAGGTGGCGTTGGTTTACGACGACCA
TTCCTGATATGATATCATTCCTATATTTTGTACAATTTGTATTTTGTAATAATATTACTATACATGTTTGTAC
TGGCGATCACGCGGCCCGAAGTGCCTCGGTGTTCCTTCCATTGTGATCGTGAACTGTGAAGTATATAAC
TCTTCTATAAATATATAATGTTTATTACTGCTAAGAAACAATGTATACTCTATCTATGGTCTACTGCCTTAT
TGCATATGTACTGAGCGCTTTCTGTGGTAGGGAGGCGTTGTTTGCGTCACTCATGGTCGTTATAAGGGA
CCAAAGCTTCTTATAATATTATGGTGTCATACATGAGTGCTGGCGCGGCGCGGCGCCGCGGGGGGGGC
GCGGGGGGGCGGCGGCGGCCGCTGGGAGGGGGGGGGCTGCGCGACGGCACGCGATGGCGACCACC
GCCCGGGCGCGGCGAGGCTGGCAAAGCATTTATTATCCATTAAATATTCGGTAACTGTAGGGTAACTAG
ACTAGCCTGCACTAGGGCTACGTAGCTGTAAGGGCACGCTGTAAGAGTGTCATATTCATTTTGCGTATG
TTTAAGGTTGCTTACATATTTTACCGTGTTTTGTTTTGTATTTGTACACTCAGCGGGGCAGGGAGGGGGA
TCGCAACTTAGACCGAGTAAAATATGGAAGCGAGGCCGCCCGGCCGCCAGGGGGAGGCCGCCCGGCC

GCCACTTGCCAATGCCTAGGTATATTGTACTTTACGCGTATGACTATAAGATATATTGTAATGGGTTACTG
TACTTATCCGTTTAATGGTGAGCGAATTTTAAAATCGCATAAATATCTTGTTTACAATAAGTCATGACAA
GTAAAATACTCAAGTGTTTTGTTATATAATAATTTTGCTCAACTATTTCGTGAAACGCCAGGAAGGCTAT
TGTTTGATTCGCTCACCGCCGCCGTCGCCGCCGCGAGGGCGCGGGGAGTGGGGCGCGGGGCGCGGGG
AGGCGGCGGCGACAGGTGGGGTGTGTTGGGAGGCGACGGCTACAGTTTAGTTTTGTTTAGTTAACATT
GTGATGTCCACTTAAGTTTTTGGGTACAAAATGGAGGGCAGAGCCGCAGGCTTTTATATAGTCATTTTT
TATTGTAATTTGTAATGTTTAGTTTATATTTAGTTGAATTACGTTATTGATATGGAACCCTTTTAATCAGGA
TATAGCTTGTAATTTGGAAAGAAATATAAGTTTTAGTTGGTAGTATAATGTAACCAGAAACTATAGCTAT
TGGATCGACGGTTCAGCTAGCTACATATTCACAGCGCACGAGCGCGCGACGATGAGTATTTATGATATG
GATAGTAATTATTTGTACCTATTTATGTTATTATATGTATGTATGAAGGTCCATTTATTATGACATGTAAATG
TTCTGTACCTACTGATGCTGAAATTGCAATAAAACAC
>TCONS_00093093 geneID=XLOC_018461 scaffold_30:792344-793550(-) Exonic overlap (-)
CTCCAGGCGCGCCGCGCCGCGGGCCGCGCGACTACACAGGTTCAACATGACGATGAGCAGCTCCTAC
CTTCTGGGACGGGAGGGTTTCGCCTACGATTACGTAAATATCTCGGAAGCGGGCGAGGTGCGTAAACA
ACTCGGAGACTAATACTGATACGTGTCACGGCAGTTGGCAACAGACTAGACTAGGTGACCTTCACGAA
TTTACATTTTGCACTCGCCGAATAGACGAGAATCGCACACAAAAACAAATTGAAGATCCAATCAAATG
ATGGCAGACTTTGCGTGTTCTGTCAGCTGACGCGATGACAGCATTATACCATAGACAATGTAAAAAAGT
GGACCAGTGCCGGTCGATAATGTATTCCCTTTTATTCAAAAGCCTAACTTTTAACCTAGGGGGATGAATA
TAAAATATTTACAAAAATTCTCAGAATAAATGATACCTACCTAAATAGAGAAAAGGCATATTTTTACTTA
ACTTTCGTTTCGCAGGTTATCGATTTTCATATAATTTTGCTTTTGCGGTTTGTAATACGCAAGCGCGGGT
AATACGAATTTGGAAATCTATACAGGGAATGTGACATGTGGACATGCGTTAATGGCCAGCAAGAACCA
CGGGTCACACCGGTTGTAGTATTAACTCCTACCTTTATTGTAGGGTTATGAAACGAAGTTATGAAAACG
AAAATGGATTTTTCGGTGGTTAATCGATGTGTTACCTACCTTTTTATGTTTCAGAG
>TCONS_00091964 geneID=XLOC_018394 scaffold_30:823397-824493(+) Intronic
TAACTAATTATTGGACGATCTTTTGTTCCATATTATTTTAGTCATGTTTTGGTGCTCAATTTTACACATAGT
TGATGTTAAACCTACCTATAGTACCTTTATTTAATCACTTTACTGTACTTATAATAATAAAGCATTATATTG
AAGCAGTTGCCTACTTATTACTCCAACATCCTTTATTATTTGTTATTCCTAATAAATATACAGACATGTGC
TTTTAAAATTTTGTTTTGAAACTGATGTACCTACTTACCTGTCTTAGTTATCTTACACTGTGATACCTACA
CATTTATTTGTTTTTAAAATGCATGCGATACACAAAACTAGGACTAGGGACATTTCTAACACAGTGCTG
AGGAAAGAAATTTTGTAGAATAGAAACGCATGAAGCCATCATCAATCATCTTCATTTAATTGAACTGCA
TTGCAACTATGTAAGTATTAGCTATGTAAGTAATTATAGAAGTCGTGGTGAATGCGGAAGAAGCATGTC
ACAGCTAAAATTAGCGGTAAATTCAGTTTTTACTCGATTATAAGTGAATAATTGAGCCATGTCATGATAC
ATTTCCGTGTGTATAAATAAGTGATTTCAACAATAATTAACGTCAACTATCTTTTTAAGAAAGCATTGTG
TAGTACTTAGTACGGTCAGGTTTCTCTGCTTCTTACTGTACCTTCCCTGAGGAAAAATACTCAAGGTAC
ATGGACTACAGGAGGGTAGAGTTTTATTACACACTTTTCTACAGCATCAGTTTCTGGACTGTCTATTCA
ACTCTATTCTAATATATGATATTATGTATGTAGGTACTGAAGTATTAATGGCCATAGATGTCGGTACTATAA
ACTGATAAGCTTCAAGTACCTGAGTTATTAGTAAATAAAGTATCTAGATATGATTAAATATCGCTTGGCA
ACAAAGCAGGTGTTTACCACAACAGTTCAATCTTCCTATTTGTTTTTGGTAAATACGTGTCTACCTATTG
TTTTTACATGGGCTGGAGTGACATTAAAGTAAGTACTTATAGTAGTTTAAATCCTTCTCC
>TCONS_00093044 geneID=XLOC_018397 scaffold_30:929352-931643(+) Exonic overlap (+)
CCCCCAATTGTCTCTGACCTCCTCCGTCGCCTGCATGTGTAGTAATGTCCTGTTGTAGTCGGTGGTGGT
GACGAGCGGGCCGGCCGCGCCGCAGAAGGTGGTGGTGGTAGGCGTGCCGCCGCAGCAGCAGCTCAG
CCTGCAGCATCTGCAGCACTCGCAGGCGCCAGTGTCCGTGGTCTCCAAGCCGGTGTTCGCGCGCGCG
CCCGCCGCCGGCCCCCAGCAGCCGCCGGAGCTGGACGACTTATCGCACCTCGCCTGACGGCCGACCA

GGTTCCTCCTCATCAACCAGAAGCGGCCGCAGTGATGAGGGTAGCCAGTGAACGGTGTAGGGGTGAC
GGGACTTTGACCAATGAGAAGAAAGTTTTTAGGAGTTGCGGATGGACTGTGTTAAAATCGCGCGATGT
GATTGGTGCGCGCTATGCCGCTCTGTCGCTATTCGTCTATTGATGAACGAATTGTATGAGAGGATTCCTC
ATATCATTCATTCTTGAATGCAAAATAGCTGACGTTTTGGTTAAGTTACGTGGTTGGTTATATCGCTTGG
CATCGTATTCGTAGTGATATATTTGAATACGGTTAACGTGGCGTAATATAGCAGTAAAAGGATAATTTAG
ATTCATGTAGCACTGAAGAGTGATAATCTTAAAAAATATTGAGATATTTACTTTTCATGGAGCTTTCTGA
AGTGGTTGATACTGGGTGGCAAATTTAAACGGGTTGGTTGGGAGGACTTTTGAATTATACGAATACTAA
ATAAATAGGTATCTATGTATAGCATCTAATGTAATTACCTAAGTGTACGTAGAATACCTTTATATCTGAAAT
ACCCCTTCCCTAAGTTATCTCAGCCACCATAATCACAATCCTACATCGATCGTGATGCAATTCTCATGCA
AATAAATGGCATACATTTGTACCAAGTAACAGGTTTCCAGGCATAATTCGAGCAACTTTGAGTCGCACT
AATAGACCTACTAACGATCTAGGTTACCTACCGGGCGCACTTAAGATAAATTAACCAATCACAGATCAA
GATTTGTGCATTTGTTATTTGAGTGTGAGTGGTGCATTAGTGCAACTTTAGTTTTACCTAAAACTTGGTT
GAAATGACGGTGATTGACGACATAAATATCATATGTATTTCATACATTTATTATAAGTATATTATTATAAGA
TTTGCATATAAAGTGTAGTTATTTTTAACAATAATTAAGTTCAGATAGAGCTTAAGAATAATAATTACTTT
TTACTCTTTCTTTAAGTATTCGATCGTTGTTTTCTTCAGTGGCGAATGTGCTCACGTATACAATCAATTGC
TATATTCTATACGCAGGGATTTTATAAGTGACGATCCCGTTACCAGTCCAATAGACTCGATGTATCGTATA
AGTAATAACACCCTGTAAAAGTTAGCCTATATACCCCGTCGACCAAAAAATACCGAGTACGTGAATAAC
GAACATCGCCATTTCTAAACGGGCGCTGTTACACTTTGTTCATCTTATTTCTTTCGTGAAATTTCCAAAA
CCCTCATACCTTATATGGTAGTCATAGTCGTGTCAACTAATGAAACCTATTTTCCATTTTCTTATTGGTGT
TCTCTACTGTGTCGATGTCGTCTAAATGTAAATCATATCTAAATGTTACTTGGTGTCGTGAAATAACTTAA
TGTAATTTTCAATGAAATAGTGTTATGCAAACTTGTTTTGTATTATAATTTAATTAAGAATTAAACAG
>TCONS_00092927 geneID=XLOC_018400 scaffold_30:987354-988237(+) Unclassified
CACACTCGTTAGCGCTGCTGTCGTTACTAGATTTATCTGAAAATGCATTTGAAATGGATAAAATTAATTA
TATTTAAAAAAAGACTGTGCTTTGAAGCTTTAGTATAGTGAAAAGTATTATAAAATGTGTAATAAGCTTA
TTGTGTTGTGTTTTTGTGATTGATGTAACAAACCTGTGTACGGCAAGATGGACGAATTGTCTAAGGGCA
TGACACAAACAGGAAGCGCCGCTATAACCGACAAAATTCAAGAAAACGATGCAAAGCATTGAGGGGA
TTTGTTACATAAAAAGAATGCCAGAGATACTAATAACAAATGCTACAGGTTGACTGGAGTACTATATATT
ACCTTTGGTTAGCCTCTCAGATACCACAGGGTGGTTAAATGAAAGAAGTTTGGAATGAGAGCTGTGAC
CAGAGAG
>TCONS_00093316 geneID=XLOC_018400 scaffold_30:987354-991335(+) Intergenic
CACACTCGTTAGCGCTGCTGTCGTTACTAGATTTATCTGAAAATGCATTTGAAATGGATAAAATTAATTA
TATTTAAAAAAAGACTGTGCTTTGAAGCTTTAGTATAGTGAAAAGTATTATAAAATGTGTAATAAGCTTA
TTGTGTTGTGTTTTTGTGATTGATGTAACAAACCTGTGTACGGCAAGATGGACGAATTGTCTAAGGGCA
TGACACAAACAGGAAGCGCCGCTATAACCGACAAAATTCAAGAAAACGATGATGCGGAGGCGTGCGT
GTCGCGTCCTGCGCCCCGCCTAGCCCC
>TCONS_00093103 geneID=XLOC_018471 scaffold_30:1131100-1133484(-) Intergenic
ATTTATTTCATAAGTATGTAATGATTACGTTGTTCTACGGTAACTATCTATACTGTCTATTTACCTTATAGT
ACTATAATTCTTTATGTTTTGATTACTAAGCGACTATTTTACTATTGTTCTATCATAATCGGGAAGAAAAC
AAAACAAAGGTTTAGCAGCTCAAGGTATCAAAAATGCCGTGATGATTTATTTAACCTGACTATCACACT
GGAGTACCTACACAGTCATGTACTAGAGAAAAAAATACAGAAATCTCATAGAAGAAGACAAGAGCTAA
ACTCCGCCAGTAAGATTAAATGTATTTTTGGATTTTTGCCTCTGATCTCATTGATCTGGGTTCTAAAAGG
TTATTAAACAGTATCTTATTGTACTTATGTACAGTGTGGTCCTACACGGTTATGAGGACGCTGCCGACCT
CATCTGCAAAATGATTTGTGTTTCTTTTCTTCCCCATTCTTTATTGTTAAGTAAATTGTTTATATTATTAAT
ATTAAAATGGGCCACGTTAAAAGTTTATTTAGTTGTTATGTAACTAATAGGTATACATGTAGATCCACA

CATAACACTAGGATTTTATTGACACTTATTTACACATGGCAAGGTAAGCTAAATTTATAAAGATAATGGA
TTGTGGACGTTATTAACAAAATAGTAGTTTGTAACACTTGAGTTAATTAATGTAAATCACAGAACATGTC
ATATCTCACATTGTGAAATAAAATACCTACCTGTATGTACCATGAGTACTTTGAAACTAAGAATAAGAAG
CT
>TCONS_00093321 geneID=XLOC_018403 scaffold_30:1134345-1136026(+) Exonic overlap (-)
GATTGAAATGTAATCCATACTAGTACCGTTAAAAAACTAGGCATGATATATGTTACCATATAAACAAAAC
TTTGGCTGTTGATAAAACACAATCATAAACTCTTACAAATCTAAACAAGGACGATAAACAATAAGAAC
CATAACATAAAATATTACTATCTATACCTAATGCTTAGCATAAAACTAAAGACAACGCAAACAACGTCA
GTCACTTTCCTGATGGTCTCACGCCACTAGGAGGCATATTCATTAAACTAATCATAAAACTCTTCTGTTG
GCCTTTCGTGTATAAACGATTTGTGAGGCCACGCAGGGCATGCCACTGCACTGCAAGATACTACAACC
GAAACAGAAATTCTTTTTTAAAGATTTTCGAAACTAAAATCATAAACATCCTTGAGCTTTACATAAATC
GTTTTACAGACGCATTTTAATGAAGAGCTCAGGATGGAGTTTTTACCCCTGTCCTAATTAGGTCCTTTAA
ACCAGATACATTTTTAACAGTGAGGTCAAATTGAACCCACCATAATTCATGTTTTTATATATAAATTCGT
GCAACATTCACACGTTTACTGATTTGTCATTAAACATTATGCTTTAGACAATGTCTTCAACCTAACTACT
TAGTCAAAATATTTCTTTACACCATAAATTCCATTTCATCTTTTAAAATTCAACCATATTATAACTTACAA
ATTTCACTATCGCACATTTATATTTGCCAATGAGCCGTATTTGTCTCCTTTTTATTTGGCACCAGGTTCGT
TATTCTGCGGCCACTGTGCTCTGTGCCTCAGACTCACCTTGCTTGAATGCGGAGTGTGAGTTTTTACAC
CGTTCGTTCCGTGGAAAATAAACCCCAAACGCCACCTATTGGTCCACGCGCTGTGCTACCTAACTATTC
ATTGGTCGAACGGTTCAAACCCACTCCCACCGGCGTCTGTGATTGGTCGATTACTTGTTGAGGTGCGC
TTGCAGCGTGTCCTGCCCCTCCACGGAGGTCCAGTAGGCGGCGACCGTATCCTGCTCGGCGGCCAGGC
GGCTCTGGAACGAGGACTCGGGAGCGGAGGTCGCCTTCAGAAGCTGCTTCTTGATCAACAGCGTCTG
GAAATTAGGAGGGCGGCGTATGATTGGCTGTAAAATCGACGAACCGACTACTTACTACTACGGCTTAGT
GAGTACCTATTCGATTATAATACGTTGAATGTTTATC
>TCONS_00092856 geneID=XLOC_018474 scaffold_30:1216286-1217413(-) Intronic
GCGCTAACCTCGTCGTTCGCCAAGGATAACGACCAGATGATCCTACAGGGCGTGGCGGGGATCTCTGC
GCACGCGGCCGCGCGGCCCGCCTCGGCGGCTCCGAGCGACCTGCGCAGCCCCGCGCTCATGCCGCTC
GACCACATGATCACGCTGCTGGTGGAGCTTATTAGTTCAACAACAGGGCGCGTGCAAACCGCCATCAC
CC
>TCONS_00093115 geneID=XLOC_018477 scaffold_30:1301729-1304631(-) Splice junction overlap
CTTCAAAAATACACATACCTAAGTATTTAAATAAAAAAGAATCACTATTCTTACAAAATTTACTACGTGA
CCGACACTCTAGTTGGAGAAAGTTATAGGTATAGCTTGGTAGTCTAGATCTCTAGGCCCCCTGCAAATT
GGGGCTACTTTTTGTTAACTTCACGTGCTGTAGCGGGGAATCCCCGTGGTTTTCGCAATGGACTCTACA
TATACTCATGTTTGCCATCAACCCACCATTCAGTATCTTTGCTTTTCGTGTTTACATACGATGTATTGATT
AGTCGAGCTCTTGAATAGTAATTAGTTCAATGTACAAAAATATCGTACATTTAGTGTGTGTGTTAGTGAA
TGGAAGCAGATCTGTGATGTGAAAACATACTCAATTACTTATTAACTATAATATTAACGTAGTGCTTAATT
AATTACATTACTACTTTATAATTACTTGGGCCGTGCCAAAAACAATAAAAAAGACTGGAATCTGTGGAT
TTAATACAGAATTGTTTAATAGAAGATATTACAAAATAATGTAAACCTCCATCAACGAAGTCCAAGACC
ACTTCGCCTACACCCCGCGGC
>TCONS_00092396 geneID=XLOC_018479 scaffold_30:1314734-1317208(-) Unclassified
GCATGCTGGACAGCGAAAATTGAATTTGTTTATAAATATAGATTTGTAATCTATCTCAGTATTATCATTCA
TAACATTAAATTTAATAAATAAATACATGCTTAATTATAGCAGTTACACTTAAGTTAAAGTTTTAACGGCA
TAACAAGTGATAACAAGTGACTAAGGTTAGTTTCGAGAGCATAACGTTTCAATGATGGTGCCGTCTTCC
AGTATGGTGGTTGTGGCTCTGAGTCTACTTGGATTCGTGAGCGCGCTGCCGCGTGTTTTGAAACTATAC
AAGGCCAGAACGCAGAGGGTGGGACTGCTCACGTATAGTTTCAAATTAAAATGGTGCCATGATTTACT

TGAAAGTATACAAGGCCATTACGCACTAATCGCGTATATTTTCAAGTAAGTTGGGCCAAAATTATGGCTT
GAAAATATACTGCAGCAGTTACCACTGCGTACTAATCGTGTATATTTTCAAGTGCCAAGCGGCAATGCG
AGCACTTGAAACTATACGTGATTAGTTACCAGGTAGTAGGTTTTTATATTTAATACCCATAGATAGATAG
GGTAGGCTCCCCAAAACTTGATGAAAAATATGTCTCTAATAGGTCTATAGCGTTATACACTCACGAGCA
ATCAATAAGTCCAAGTTGCAAAATGTCAACACCAAATTTATTTTAAGAGTTTTTTTTTTAAGATTTTATG
ATCACTCCACAGACTTTATGTCCGTGCCTTTTTGAGAGATCAATATATTGTGAGAGTTTGGTTGTTTTTT
TTTATCTTCATCTTAATATTTTAAGAGCTTACATTATATACAACCGATAACTACTAACCAAAGGTTGGCTG
GTAGAAACTGCATTTTATCAATAAGTCCGCCTTTGTGCGCAATTTATTGACTATGTTTTAAAATGCATCT
GTGTAAAATTTGATTTTTGTGGGCAATAAAGTGAATATAAATAAATATATAAATGACCCCAATTTTTTAA
AACATTTAATTTAGAATTTGATCAAAATTAACGTTTTTAGTTGGTTGAGCTGTTAAATGTACATAATATTG
CCGACGGCGTCATTAAGCTAGTCTTTTCCGTTCAGGAGCTATCGTCACTGGAACTTTTTCATTTCTCGT
GAGTGTAGTAGGTAGGTAGGATCTTAATTCAAATAAATAATAAGATATTAGCAGGTACTTACTATTAGTAT
TACTTGCAAGTATAATTGCTATAAATATCAATTTATCAATCAAATGAGCTTGTTCATTTTTATTTATTAACA
TTCTATATAGGTATCACAGGCAGAACAAGTAAAGTAGTCATGTACCTACATTTACCGAAATAGTTTAGTA
AATCAGAATCAGCATGTAGGTATAAATGATATGCGACAAGTACGCCAATCAAGCAATGTGCGTAATAGC
AATGTATATTTTCAAGAACGATTCAGTAAATCCCAACTTGAAAGTATACGCGATTAGTACGCACAAATC
GTGTATATTTTCAAGTAAGATATTATTTTTTTTCACTTGAAAGTATACAGTGCTAGTACACTATGGGTGCG
TTCTGGCCTTGTATAGTTTCAAAACACGCCGCGC
>TCONS_00092615 geneID=XLOC_018479 scaffold_30:1315322-1317209(-) Intergenic
CGCATGCTGGACAGCGAAAATTGAATTTGTTTATAAATATAGATTTGTAATCTATCTCAGTATTATCATTC
ATAACATTAAATTTAATAAATAAATACATGCTTAATTATAGCAGTTACACTTAAGTTAAAGTTTTAACGGC
ATAACAAGTGATAACAAGTGACTAAGGTTAGTTTCGAGAGCATAACGTTTCAATGATGGTGCCGTCTTC
CAGTATGGTGGTTGTGGCTCTGAGTCTACTTGGATTCGTGAGCGCGCTGCCGCGTGTTTTGAAACTATA
CAAGGCCAGAACGCAGAGGGTGGGACTGCTCACGTATAGTTTCAAATTAAAATGGTGCCATGATTTAC
TTGAAAGTATACAAGGCCATTACGCACTAATCGCGTATATTTTCAAGTAAGTTGGGCCAAAATTATGGCT
TGAAAATATACTGCAGCAGTTACCACTGCGTACTAATCGTGTATATTTTCAAGTGCCAAGCGGCAATGC
GAGCACTTGAAACTATACGTGATTAGTTACCAGGTAGTAGCGTTATACACTCACGAGCAATCAATAAGT
CCAAGTTGCAAAATGTCAACACCAAATTTATTTTAAGAGTTTTTTTTTTAAGATTTTATGATCACTCCAC
AGACTTTATGTCCGTGCCTTTTTGAGAGATCAATATATTGTGAGAGTTTGGTTGTTTTTTTTTATCTTCAT
CTTAATATTTTAAGAGCTTACATTATATACAACCGATAACTACTAACCAAAGGTTGGCTGGTAGAAACTG
CATTTTATCAATAAGTCCGCCTTTGTGCGCAATTTATTGACTATGTTTTAAAATGCATCTGTGTAAAATTT
GATTTTTGTGGGCAATAAAGTGAATATAAATAAATATATAAATGACCCCAATTTTTTAAAACATTTA
>TCONS_00092688 geneID=XLOC_018491 scaffold_30:1599995-1602538(-) Intergenic
CTTTGTAGTATGTTTCAAAGTAAATTTTTACCTTGTTGTAAAGTTACAATACGGTCTAAGAATTTAAGGG
TAAGTCTTGGTTGTGAAATTATTACAAAAAAACTAAAAACTGTAAAAAAAAGAAACCTATTACTAACC
AACCTATTTTTTAATTAACATTTTGAAGTCGATAAAAATGTGATATCACTCTATTATGATGAAGTATAGGT
ATGCTAAACTAAGTAGGTAAGTAACTATACTTAATTAAAATGATTCTAAATTTCATGTCATACCAGCGTT
TTTATTATCTTCAAACTACATGCCTAAAAGATATTGAAAAAAAAACTATTATTATTTACTTTTTAAATAGT
TTTTATTTTCTTCGACAAACAGGTGATTTTAGTGACTGTGTATAATAAAATCACACGAGATCTAGGCACC
ATCATAAATAAAATTATTCTAAGTACTATTTCACCTCTTCTACTTATACATGATTATAGTAGAAATTTTCTA
GTAAGTAGTAGAAAGTACCTATGTGTTTGCCTATTACTTAATTAGGTATATATTAATTATGTAAATATTGAC
AATGATGCCGATTCTGAAGGAACAAAATTCAATTTTTGCGATG
>TCONS_00092043 geneID=XLOC_018426 scaffold_30:1729089-1753764(+) Exonic overlap (+)
GTTCTCGGCAGTGATCAGCTGGGCGGATCCACGCGCGCAGCTCGCTTCCCGCCGCCGCCGCCAGTCCA

CCACGCGGCGCCACTGAGCACCCACTCACCACTCCAACTCCTGAAAGAACCCCGTCAATTACTGCCTT
CCACTCCTACTATCCACACCTTTATTAAAAAAGACTACAGTTTAAAAAAATATGGCGAAAATGTGCAAA
GATTTTAAGTGTTAAGTGTGTTGTGTAGAGGCAGGGGCGACAAACCTTTTCGCCGGGCGTTACGTAAG
CGCGTTCCGGGCAAAATCAATATCCTCGGCGTATAAATAGCACCTATAGTTGAGAACAGCTGGCGTCCA
ATATGCAGCCTGCTATGCCGCCTATGAGGATGCGCCGGCCGCGCCGCCACCGCTTCCTGACTCTATCCG
TGTTCTTCATCACGTACTCGGTGTTCCAC
>TCONS_00092057 geneID=XLOC_018430 scaffold_30:1926828-1927543(+) Intergenic
TGTAAACTTAAACAGATAGGAAGATGAGCTGATAGAATCAGTATTATTAAAAACTATAGCAGTAGGAAC
TTCAAAGTTCCTATGTTAATTCGAATTTTACTAGGACATTCAATTAGTGTTTGGTTGAGTTGTTTGTGGG
TATTTTATAGGCTTCAATTTATATTTTACTTAACAAACACTTAAAATAAGCATATCCTTTGCATATAGATAT
ACATGGAAGTACATGTACAAATGTACATTCACTTATGATGGAATTGTCTATTAATTCTAGCAGCAGTAAA
TACTGATCATTAAATTGATTGTTTATGAAATGTGAAACAGCAGTTACCTATATACTGATCATTTAATTGAT
TGTTCCTTTATGGAACATAGCAGTGAGAAACTGATCATTAAATTGATAACAGCAGTTACCTATATACTGA
TCATTTAATTGATTGTTCCTTTATGAAACATAGCAGTGAGAAACTGATCATTAAATTGATAACAATAGTA
AGTTATTATTTACAAAACAAAACGGCAGTTACATACTGATCATTTAATTGATTGTTTATATAAGGAAGAT
AGCAGTGAGGAACTGATCATTAAATTGATAACAACAATAAGTTAT
>TCONS_00093409 geneID=XLOC_018655 scaffold_300:36573-40436(+) Unclassified
GAGCGGCTGGACTGGTGGCCCAGGCCGCTCGTCAGGATGCTGGGAGCGGCGCGCTCGTGGCTGTACA
ACCCGAAGTCGATCCCCGAGGCCGTGGTGTGGTCGCTGGTGCCGGCCAGACACGGATGCTGAACACA
TAGGTATCCTAGGACTCAAGTGGTGTATTATTCATCGCAGAGGTAGGTAGTAATTTGTTAAACATCGATT
TTCGGAACTTAATTTTACATAGGGTATTTTTAAGCAATTTATTTTAGTTTATAGTTTAGTTTTTATTTCTAA
GGTAGTTTTAACTGGTGAGTATTATCTAATAAAAGAGGTGTTAAATTATTAATTTGTTTATTTCAGTCTTA
ATTCCTAAGCAACAACGTCTCCTACAATATCAACAATGGAATGATTTTCTATGTCTAAAACATGTTATGG
AATTGAAATTCAGTATCACGCTCTTCTCTAAATTATTGAGCTACAAGCGAGATTTGTAGCGCCAGCGCA
TCGCAATCGAGAATAAACAGTAGATATTTTCACCTAGACATCATCTGTGGGGCAATTTTACTATCGAATG
CTGTTTGCCTCTATTATCTTACGATGGCGCTATGTAGTCTAAAAGATCTTTTAAATTACGACTTTAAGAG
ACCAGGCTTGACTTGACTATGATCTATGATACTGAATTTCAGATAATATTCTAATCTCAAAGTGTGAATG
GAATTTTAGACACGACACCAACAAGTAATTTTTCTATTGTTTACTTTCAGTGTTGGTTTTCATTGGTGTT
TACAAGTGTTAATAAATTTATTACAACTTTGAGATTGAATTTAATTCTACTTGGTAGGTAACTACCGTCTA
ATGTTAAAGATTTCAATTTATTTACAAATAAATAAACAATTTATTATATTCATTAAAATATAAAATCTGCAT
AATATTTGACTAGAAATAAAAGATTCACAGATAGAGATTAACAATGGTGTTTTTGATAGGCCTGAGTTA
GTTTGAATGGCGATTTCATCAAGGGTGACATTACACTGCATACACTTAAGAAAGCTAAAGTAGAATAAT
TTGATTGTAAAATTGGTTCATTTCTCAATATAGTCTCAGTGTAACTACACCCTAATACTTAAGAAGAGTT
CTAGATTATTCCTACTTATCTAATAGTATCTACGATACACGAGAGGTATTTGTTTATTTACATATAAGATAA
TATTAATACAATAAAGTACTTGAGGGAGACACAACTTCCGGTCCATTTCTTTTCAAAAGTAAATAATTTC
CTTTTTCATCACTAGTTGTGAATAATTTACATATGAATTTTCCTGCAATGTAGCCGGCACTAACCAAAAA
CTATAACGGCTATGGCGATCTTATCTTACACAACCAGTTGACAATTTCACGGATAGATTTTATTGTCATAC
ATTTTCAATTCCATATTCAACCACAAAGGTAGAAATAGGCAGGAGTTAGCATTATTTTAGCCTCAACAAT
TTAATCGATTAATAAAGAGACTAGAACGAGATCCATTAATTAGTAATTCGAGCATTTTTATAAATACAAA
CTTGTCCTAGTTACGATGCTAGACTATACCTACTAGTGATGTAACGAATATTCGCATTCGCATTCGCATTC
GCGAATATTCGCATGTTTTTGGATATTCGCATTCGCATTCGCATTCGCAAAATTTTGTGCGAATGTTTTG
CGAATGTCAGTATTAATTAGAAAAAAAACTATTTGAAAATAATGGTACGTTAAAAAATTCAAAATCCAT
TCGTTTATAACCTTTTTATTACAAGTAACCTTGTTAATCACATTATCACCCTTACTTTATCATTTGGTATCC
TAACTAATATTATAAATGCGAAAGTAACTTTGTATGTCTGCCTGTCTGTCTCTTTCTTTCACGCCAAAAC

TACAGAACGGATTTGAATGAAATTTGGTATACATACGGTCTAGACCCTGGGAAAGAACATAGGCTACTT
TTTATCCCGGAATTCCCACGGGAAAACTTTTTATGGCGAAGCGAAGCGCGCGGGAACAGCTAGTTATT
TATAATGATCAGAATGTCAGATGAGTTCGTATCCACATGGCAAATCTCGGAAAATTGAGACAGCCTTCC
GATACGCACTGAAATCTATTAGAGCACCCGTAGGTAATCGTGTCAGTGTCACTGTCAGCCTTATTATTAT
ATTGTGTGAGAACTGTTACACACTCAGAAACCGTACCGCACACAGTCGCAATATGTCGTAACAGTTAC
GGGGCTAAATATAGTGTCATCATCATCATCATCACGACCCATTACGTCCTCATTGCTGGGGCACGGGTCT
TCTTCCAATTATGGAAGGGGTTTACGCCTAGTCCACTACGCTGGTTTAGTGAGGAAGTTGTCGGGTAAG
GGCCGGCGCCCCCCTACCCCCCGCCAGGCCGGCGGCGCCCACTGCCGGCACGGCATGGCATGGCACG
GAAATTAGTGCAGTGGCATCCCGCGGCGTGCTGCGACATTCCGCGGCATTCAGTGGCATATTCCGCAG
CATTCAGTGGCATGCCTTGGCAAAGCCATCAGGCATCCATCAGTTTTGCATAAGTTTCAACCTTATTTC
AACTAGCTAGTAATCATAATTCCTCGATCCCAAAGTGACGCATAAGCCTCGCGGGATGCAATGGCGCGC
CGCGACATCCTACGGTGTGCCGCGTCGTGCTGCAGCATCCTGCGACACGTCGCGGCATCGTGGCATCG
AAATAAAATAACTTGAATACTAAACTGATTGAAATAAGAATCAAAATAGCTTCATTAGTAATAAAAAAC
TTTGCCCTGCTATGCCGGCAGTGGGCGCCGCGGGCTCTAAGTGGGCAACCCGACTTTTCAAGGTGCCC
GAAGGCCTCTGACTAGGCTTAACGAATGCTGCCGAAGCAGCAACTGGGACCCACGGCTTAACGTGTC
GTCGGAAACAAGGAAGCGTCCAGAAAAAAACACTTGAAATCAATCACCCATCCAATCGCTGTCCGTG
CCATGTGTTGCTTAACCTCAGTAATCAGTTACGATCACTAAAGCTAGCTCGACTAAAGAGGCTTCAAAA
GGTGTAACTACTTTTTAAAAAATTGTGACTAATGTTCTGTCTATAGTAGTCTGATTCGGAATGCACCTCG
AACATAACGTAGGTACCTCGTTCAGTATGAGACTGCCACTAAACCATACAAATGGCGCCACATTTGAAC
ACAAACTGAATATTCGCATTCGCATTCGCATTCGCGAATATCGATTTCAGATATTCGCATTCGCATTCGC
ATTCGCGAATGTTCAAAAACACACATTCGTTACATCACTAATACCTACTACACTTATAAAAAAATAATTT
TTTTTTTTATAAGTTAATGCAAGCCAACGACGACGAAGGTAAATAATAGAAAGTATATTTTATAATATAC
TTTTTACCTGAAGTTTGGTTCTTAACATCCAGCACATCCATGAACTTTCGAACTACGATTTTAAATTTTC
GACGATGTTTCTGTAACAAAAAAATATACGTAATTTATATAGGTTTACTCATTCAACAAC
>TCONS_00093656 geneID=XLOC_018655 scaffold_300:49865-52124(+) Unclassified
TAGGGAGCGGCTGGACTGGTGGCCCAGGCCGCTCGTCAGGATGCTGGGAGCGGCGCGCTCATGGCTG
TACAACCCGAAGTCGATCCCCGAGGCCGTGGTGTGGTCGCTGGTGCCGGCCAGACATGGCTGCTGAA
CACATAGGTATCCCAGGATTCATTGTAGCGGTGTATTATTCATCGCGGATACGGTAGGTAGACGAATTTG
TTAAATAACGTTTTTCTGAAGTAAATTTTACATAGGATATTTTTAAGCAATTTATATTTAAGTTTTTATTTT
TAAGGTAGTTTTAACTGGTGAGTAATTATTTAATAAAAGAGGTGTTAAATTATTAATTTGTTTATTTCAGT
CTTAATTCCTAAGCAACAACGTCTCCTACAATATCAACAATGGAATGATTTTCTATGTCTAAAACATGTT
ATGGAATTGAAATTCAGTATCACGGTATTCACCAAATTATTGAGCTACACGCAAGACTTGTAGCGCCAG
AACGTAGCGCAATCGAAAAAAAACATAAACAGTAGGTATTTTTACCTAGCTGTATCATCTGTGGGGCAA
TTATTTTATTATCGAATGCTCTATTACCTTACTATGGCGCTATGAAGTAAAATCTGTAAGAAGTCTTTTAA
ATGACGATTTTGAGAGATCAGACTTGACTTGAATATAATCTATGATACTGAATTTCAGATAATATTCTAAT
CTCAAAGTTAGAATGGAATTTTAGACACGATACCAACAACAAATTTTTCTATTGTTTACTTTAGTGTTGG
TTTTCATTGGGGTTAACAACAGTTACGTGTTGATAAAATTACAACTTTGAGATTGAATTTAACTCTACTT
GGTAGGTAACTACCGTCTAATGTTAAAGATTTCAATGTATTTACAAATAAATAAACAATTTATTATATTCA
TTAAAATATAAAATCTGCATAATATTTGACTAGAAATAAAAGATTCACAGATAGATATTAACAATGGTGT
TTTTTGATAGGCCTGAGTTAGTTTGAATGGCGATTTGATCAAGGGTGACATTACACTGCATACACTTAA
GAAAGCTAAAGTAGAATAATTTGATTGTAAAATTGGTTCATTTCTCAATATAGTCTCAGTGTAACTACAC
CCTAATACTTAAGAAGAGTTCTAGATTATTCCTACTTATCTAATAGTATCTACGATACACGAGAGGTATTT
GTTTATTTACATATAAGATAATATTAAATACAATAAAGTACTTGAGGGAGACACAACTTCCGGTCCATTT
CTTTTCAAAATTAAATTATTTCCTTCTTCCAATAAAATTATTAGTTGTGAATAACTATCTTATGAATTTTCC

TGCAATGTAGCCGGCACTAACCAAAAACTATAACGGCTATGGCGATCTTATCTTACACAACCAGTTGAC
AATTTCACGGATTGATATTATTGTCATACATTTTCAATTCCATATTCAACCACAAAGGTAGAAATAGGCA
GGAGTTA
>TCONS_00093657 geneID=XLOC_018655 scaffold_300:50322-52124(+) Intergenic
TAGGGAGCGGCTGGACTGGTGGCCCAGGCCGCTCGTCAGGATGCTGGGAGCGGCGCGCTCATGGCTG
TACAACCCGAAGTCGATCCCCGAGGCCGTGGTGTGGTCGCTGGTGCCGGCCAGACATGGCTGCTGAA
CACATAGGTATCCCAGGATTCATTGTAGCGGTGTATTATTCATCGCGGATACGGTAGGTAGACGAATTTG
TTAAATAACGTTTTTCTGAAGTAAATTTTACATAGGATATTTTTAAGCAATTTATATTTAAGTTTTTATTTT
TAAGGTAGTTTTAACTGGTGAGTAATTATTTAATAAAAGAGGTGTTAAATTATTAATTTGTTTATTTCAGT
CTTAATTCCTAAGCAACAACGTCTCCTACAATATCAACAATGGAATGATTTTCTATGTCTAAAACATGTT
ATGGAATTGAAATTCAGTATCACGGTATTCACCAAATTATTGAGCTACACGCAAGACTTGTAGCGCCAG
AACGTAGCGCAATCGAAAAAAAAACATAAACAGTAGGTATTTTTACCTAGCTGTATCATCTGTGGGGCAA
TTATTTTATTATCGAATGCTCTATTACCTTACTATGGCGCTATGAAGTAAAATCTGTAAGAAGTCTTTTAA
ATGACGATTTTGAGAGATCAGACTTGACTTGAATATAATCTATGATACTGAATTTCAGATAATATTCTAAT
CTCAAAGTTAGAATGGAATTTTAGACACGATACCAACAACAAATTTTTCTATTGTTTACTTTAGTGTTGG
TTTTCATTGGGGTTAACAACAGTTACGTGTTGATAAAATTACAACTTTGAGATTGAATTTAACTCTACTT
GGTAGGTAACTACCGTCTAATGTTAAAGATTTCAATGTATTTACAAATAAATAAACAATTTATTATATTCA
TTAAAATATAAAATCTGCATAATATTTGACTAGAAATAAAAGATTCACAGATAGATATTAACAATGGTGT
TTTTTGATAGGCCTGAGTTAGTTTGAATGGCGATTTGATCAAGGGTGACATTACACTGCATACACTTAA
GAAAGCTAAAGTAGAATAATTTGATTGTAAAATTGGTTCATTTCTCAATATAGTCTCAGTGTAACTACAC
CCTAATACTTAAGAAGAGTTCTAGATTATTCCTACTTATCTAATAGTATCTACGATACACGAGAGGTATTT
GTTTATTTACATATAAGATAATATTAAATACAATAAAGTACTTGAGGGAGACACAACTTCCGGTCCATTT
CTTTTCAAAATTAAATTATTTCCTTCTTCCAATAAAATTATTAGTTGTGAATAACTATCTTATGAATTTTCC
TGCAATGTAGCCGGCACTAACCAAAAACTATAACGGCTATGGCGATCTTATCTTACACAACCAGTTGAC
AATTTCACGGATTGATATTATTGTCATACATTTTCAATTCCATATTCAACCACAAAGGTAGAAATAGGCA
GGAGTTA
>TCONS_00093635 geneID=XLOC_018657 scaffold_300:176966-180401(+) Splice junction overlap
GCCACTTCAAACAGCGCCAACGGCTGGGCTCGGAACACGGCGTTCAACGGACTCACCGACGACATTG
ACGACGCAGAGAGGCGCGAGCGAAAATCACCGCATGCCTAGTCTTGTGCACGTTGAACCTTCCGTCT
GTTGTCATTAGAGTCATTTTAGATTGTCGCTTTCAGATATTTGGGTCCGTCATTTTGAGATAAATGTCAA
AATCGACGACATTGTTTGGTCCATTGTGCTGACATGTAGCGGTTTCAATAGTAAACTCTTTGAATTGGT
TTATGAAAAGGGCTGTCTTTTCAATGGTCAGATGAAAGTTATATGGGGAATAATTCTGATGCCTTCGCGT
ATGTATTAAACCGTGACAGCAACCATGTGATGTCTCAGATAACATTTATCTTACTATCGCATAATCAGATA
TCTGAAAACGACAGTACAATTAATTATGATTGTTTTTCTAGTGTTACATATAGCTATTCGTTATAATTTGC
ATCTGCTGTCGCTTCACGGGTTTGTTATCGACGTAGATAAACGGCACTATATCACGATTCGTCATAGATA
CGCCGCTCTGCCCCTGTAGCACAGCGCGCTATTAAGACGTGACAAGAGTTCTCCGTCTCGCTCGCTCT
TGAGGCTGCGCGATGTGAGTGAACGCGATGCAAAACTCTTGTCACGTCTTAAGTGCGCCCCGTACTAG
CCCTTCTGATTGCTGGAGCACGCATATAACGCGTTTATCGACGTCGATAACAGCCCCGTGCCGCGGCCG
CTGTGTTGTTTATATGGGATGCATGAATACAGGATGTTGTTCAAGATTTGTTATTATTATGCCGTAAAAG
AATGGAAAGTATTACAATGTACTTATTTAAAAGTAAATATTCTTAACATATTTTTAACAATTTAGATTAAT
TTAACAACATTTTGTAAAAGGACCCGTTTTTTAATATGGAAAGATGATTAAATCACTAGCACGAATAAG
CAGCCTTATGGTCTAAAACACCAATTTCAGCGTGGCAATATGTTATATCTTCTTTTTTTTTTAACTGGCCC
GAATTGAATTTAATTACATATTTTTATTGTATTATTCTTTCGCAAATGTTCAAGCAAGGCATAAATTTTAA
AAAAAAAATACTTCTATAAATAGTTTAGAAAGTGAATAGTTGTATATAAATTCATAATTTTAGAATATCAC

GAATAAGACATTCCATTGTGTTTATTTGTTTTTTTTTTTATAACACTTTATAGTTTTTATTTATATTGGTTT
GATTTACTGCTTTGTTTGTTCTTCTGTATGGTTTTTTACATAAATGAGCTTAGTAAATTCACAATATAGGT
TGGAATATTATCAGTGGACGTACTCAATCCCATGTGTATTATTATGTGTGGCACATTTGTCACGCAAATC
AATCTGTTACGAGTCGAGCACAGATAATATTTCACCCTATATCATATAGGATCATTATTGAGGAGAATGA
CAATCAAAATTTAGTTACAATAGTAAGGGCCCGACTGGGCCTATTGACTAAATGACTATTTAGCATTGTA
TGTATAAGGCATTTGATAAAAAAAACTTACAATACTGGGAGAAACTACAAATAGATGTCCGAAAATTAT
TTTTTATTCATATCAGTGCTAAAAATAAATGAATATTTTTTGACAATGTTACTACGCAATCTGATATTGAG
TTACACAAAACCACAACTGAAAGTGACAGCTATCAACGAATGAGTAAAAAAAAATCGAATGATTAATA
TTTTTGACACATCATGATAAAATTGATTGGATTTTATTAGTAACTATGTTTTAGTTCTTTATTTTTATAACC
TATACTTTTAACAGTAGTTTTGTATATGGTTTATTTATTTTTTAGTTATACACTGCCAGTTTTTTAGCATTT
CAGCATAAAAAAAATATCTTTACTCGTAAAACCAGTAAAGAAATATCACTAATGTAAGGTTTTTACGTAT
GAAAATTGAATAATAAGCTGTATTTTAAAAATATTATAATTATGTTCTAATGAATCTCAAATTTTTATATTT
GATTACCAATTTTCGTTATTTTTTATGGTATGAAATTCTTTTTTATTTTAATTTTTTGACCCAGTG
>TCONS_00093528 geneID=XLOC_018657 scaffold_300:184527-186199(+) Intergenic
GATTTAGGTAGGACTAGCCAATAAATAACGTTGAAAAACAGTGTGATAGTATTATATAGGTCTATATATC
ATGCAATGTTCAAAAATCTTTTCGATTTTACGAAAAATACTACGATCAGTGAGATATAGGACATAATAAG
TACCGATTAAGTATCGCTGTTATGTATGAAACCACTATCTTTAACGTCTTCGTAATAAGTGCTACTTTTCA
ACGAATGCCGATTTTATTTTCTTAACTGCGGTTTTAAAATGCAGTTTTAAATATTGAGTGACCGCACTAC
ATAATAGAACTGTAAACCAGACATCTAATTTATAATCTCCTAAGGGTGAGGCCCCATTGGTGCGTTATGT
CGTCGCTGCCATACATTTATTTGACCGTTCCTTTAGGACGTCGCGACGCAACGCAACAATGGGGCCTTG
AGGTAATATTTTCTACCTACACAATGATTTGTATTGCATTGAAACCGTTTATTTATTTAACTATGGGCCTG
ATTCACAATGTCTGGTTAGTGGCTACCTGTGAGATAAAATAAATGCTGTCACTGTCTAAAAAATATTAC
AGAGAGTGACAGCATATATTTTATCTCACATGTAGCCACTAACCAGACGTGAAACAGGGCCTTAATGTT
ATTATTTCATAGTTATAATATATACACCTTGTACTAATGCACTCCCTTGCGGGGTAGGCAAAGGTGCATTG
CTGCACCCACTTTTCGCCAATTTCATAGTTATCTTGATTTTAACTGTGTGAATTAGAATGATATATCAAAA
TAATACCCTCAATACTTTACACAAGTTACACCATTGACCAGAAAGCAGCCAGGTTGCCAAGTTAATTCA
CTCATGTTGTACGAGTAGGTAAGTACCTTTTGTTTGTATTAAGTACATTCTTAATTTTCGCTCGATACCGA
AACATACACGATCAGTATTTTTATCTTTACTCATACCTAATTGTGTTACATCACAGTAAATATGAATTTGT
AAACAATAGGTAGGCATTACCGAAAATACATACCCCCTTATTCATAGGAAAGTTATAGGACTTTTTAGCT
ATTGTACTGTTTTGTCCCCCTCTGTCAAAGAACAAATTGTTCATTCAAGGTGGATAGCATTCGGTTCCT
CGATTTACACCTATCTATATATAATTTTATAGATATAGAATATATTTTTTCAATATTTATATTAAGCTGTACA
ATATTTTGACCGCCTGTTTCACAATGTCTTGATAAGGGTTATCTGTGGCATAAAACTTAAGCTGATACTG
TCTAACATCATAAGTATTTTAAACAGTTTAAAATACTTGTTTTGCTTTTTATCCCAGAAATAACTCTTATA
TGCACATCGTGAAAGAGCATTGTAATCTTAATATTCTTAGGGCCTGTTCCACTAACCAGACATTGTGAA
ACGGCCCTTAATGTATTGTATACCTACATAATTTGATGACACAAGGACTTATGACACTTGCAATACTAAT
CTATTTAATAATTACTGTGAAGAATATTTATAAGTAAATAAAATTGTTCTAGTAATAAGTAGTATCAACTG
TAAACAAGGAGAGCGGGA
>TCONS_00093691 geneID=XLOC_018658 scaffold_300:187857-191217(+) Splice junction overlap
AGAATTTCTATGATTAAACCTACTCGTCTGTTAGCCATGAAGGGGTTAGGTGTCTTGTTTGTACTGGCTG
CCCTGTTCGCGTTGGTTGTGGCTAAAGAGGCCGGCGGCGATACGGCTCGCAACAAGGACTACACGGC
CAAGTACGCCGAGATCGCGGCGCCGGTGCCGGCTGGCGTGAATGTGGAAAAACTGGTCAAAATATTG
AAGGAGGCGAGTCGCAGGAATTATG
>TCONS_00093539 geneID=XLOC_018669 scaffold_300:206250-212815(-) Splice junction overlap
CGAAGGTCCCGGGTTCGATTCCCGGCTGGGGCAGATATTTGTTTAAAGACAGATATTTGTACTCGGGTC

TTGGGTGTTGATATTTATATTTAATATGTATCTATCTATGTATTTGTGTAGATATATCAGCTGTCCGATACCC
ATAACACAGGCTCTGCCTAGCTTGGGGTCGGATGGCCGTGTGTGAGATGCCCCCACATATTTATTTATTT
ATTATGTGTACATTTCCAGCCTGACCACGGCGCTGACGACGGGCGGCGCGGCGGCGCGGCGCGACGT
GCCCAACCCGCTCGTGTTCCCGAGGAATGTGCCCTCTAGGTCCACCTTCCACTCAGGTCAGACGCGGT
CGCGCGGCTCGTCGGCCGGCGGCGCGTACGGCGACGGCGGCTCCCCGCACGCCGCGCGCCCCTCCTT
CTTCAGCAAGATCTCCTCCAGGTTCAGTAAACGGTTGAATCAGAATCCATGAGACAAGATGGCGGACG
GCGGCGGCGAGAAGAAGAATTAAACAACGTGCTTGACGCTGATTTTTCTTTGACTTTGATAGTTTTAA
ACTTACGTAATGATCGTTATTATTGTTACTATAGTAACGGCTCATAATAACGGTCATCATCACCTTAGTGT
TTTTTATTAAATTGAATTTATGGACTCGAAACTTGCATCGATGCAAAAATCATTATTTTATCCATATCTCA
GTAAGTTATTATTAGCATTCATACCTACGTGAGTACTTACTTTGTTTTTCGTTTCATAAAAAATCAGCTGCA
AGTACGTTAAAAGAGCTTAGAACGTTATAAAAATTACATTGTAAAAAGAACTTGTACATCTTTAACTTAT
ATGAATAATCTTATTTCATATAAAAAATATAATTATATATAAATGTTGCATTGTATTATATTTTAGTCGTAAG
TGGTCCATTCGTAGGTAGTGTTCTAACTGTATTAATATTATTCATTGTGTAAAATATGTGGGAATGACTAA
ATATGGCGGCGGTTAAATCTGCGTGTTGTTTATTTTTTTAATGTGGCACATAATAATTATTATAAAGAATG
CATACTTTTAAATTTATATATAATGCAAATTATTTGCTATCATCAAATTCATCTATGTGTTTAGCCAAAATA
CAAAACTCAGCTATTATGTAAAATAACTGCCTTAAATATTTTAAATTAAAAAAAAAATAATTAATCAAAAC
AACACGTGGTTTTGGGTGATCTAGTCGCTCCGCATCGCATTTCAATGTTTGTAGTTTAATAAGAAAATG
CAGGTAAAAACTATTGAATTTCAACTCTTGTGTCTACATTCCAGGGTCCCAATATTAGGGTTCTTCTGTT
AATTAATCTATTATGGTACTCTTCAATCGTGGAGGTTAACAAAAAATCTATTATGTGATGTAGAAAATTTT
CTTTTTTGATCTGGGCAATTTCATTTAATTTCTGAAGTTTAAAGAAAATATATGTGATTCGAACTTTGCC
CTAAATCGGGCATAAACTCGATGACTGAAAATATTTTGTAAAATTAAAATCTAATGGTAGTTTTACTAAG
ATCTATCTATAATATACCCCTTTATATTTTAATATTTCTTAGAGAAATCTACAAGTAAATTAAAAACATCAT
TTCGACTGCGAATGTGTGAGTGAGAACTATGTATGTGTGGTTTCGTCTTAGCGTGTAGATGTTTAACTTA
TTTGCTAGGCAGGTTGTGAAAAAACGTCTGTGACTCGCACGCGCTGCGTTCTAGTTTTCAAATTTTGA
CAAAAAGCCTAGGCCGATCGGTGAATCTTAGCGCTAAGTTACAACGACATGTAATTTATGTTAACAGTA
GTTAGATTAATGTTTTTAGTGTGTGACTGGTCGATCCCTATGCTTTGGTACAAATACTCACATAGTGTTA
CCAATTTAAGTGAAAGCAAGTATGGCAGCTCATAAGACTAGAACACATAAGCCCCCTTCTGACGCGGC
CGGGTAAAATATCATTGAGCATTTTAGTTATATTTTTATGCGTTTATCAAATGTGCTTTGGAACCTACTTG
TATAGAAATACTAACTATCTGTCTGAAAATATAATTATGATGATTTTGAAAAATGGGTATTTATATTTGAG
TCTAAATATTGAATAAATAAAGGGTTAAGTAAGTAAATATGGTAAAAAATCTTTCTGTACATACAAACAA
ACAAATGTCTATTTTCATTTCGTATAAAGTAGACCCCCGTATTTTCCTATAGCCCATATTGTATCAGTAGA
GAAATAAAGATGCATATATAAAATAAAATATATGTATATGGAAGGAGTTGTGTGTTTGTCTGTCGATTTTT
ACTCATATGCCTTAATGATTTTTTTTTCTTATTCTAATTAATCATAATGTTCTAACACTACAACCACAATAC
TTTGCTTATGTGCAGATTTAATAAACGAAATAACTATTCTAAGATTTAATAAGCAGACACAGGATGTTAG
AAAAATTAAACAAAATTATTTATACACTATTGACATAGTTCCTGTTTTTGTTTCTACAATGTATATTGTAG
AAACAAAAAATACTATCACTATCTGTTAGTTTTGACAAGAGGTGCAGGAAGTTAAAAAGAGGAGTTCCA
AAAGTCGTAGAACAAAACTTGTTTTGATTGACCCTCTGAGTCACCCCTTTACGGCTCAGTAGGTACCTA
TACCACTTACACGTACCTACACAGTACCACCCCGCACTAGGCCAGCGTGGTGGACTAGGCCTAAACCC
TTCCTTCATTGGAAGGAGACCCGTGCCCCAGCAGTGGGGACGTAATGGGTCGCGATGACCTATACCAC
TTTGGCTGC
>TCONS_00093452 geneID=XLOC_018669 scaffold_300:245357-303767(-) Exonic overlap (-)
CTGGAATCCGATCCGCCATCTTGAGAGTGAATTCAAGAATGTGGGGACTTTTTATTGAGATTAGGGTGT
AAATTTAGTTATATTCATATTAAATGGATGTGTATTCATCCACGGCGCGTCTCTATAAAACACCCGCGAC
ACCAACATCAGTCAGCCGCGTCGCGGTAAACGGCTAATCGCGAATCCTTATTAGTGTTTATGTGACAGC

AGTCAGCACAGGGTGCGCTTGCTACCCATTCGAGTGAGATTTCTGGGTGAGCCCTCGGCTATAGGCAG
GGTTGTGACCGCGGCGGGAGTAGAGGATGCGAGGGATCACCGTAGGCTAGCTGGGTGTTAAGTGTTG
ACGTGTTAAAGATGTCGACGACGATGCGGCCTACGCTGCAAACAGTGCCGGAATCTCTGCCGGCGGA
CCATGTCACGTCGCCAGCGCCCACCCCGGTCACCACCACCAGCACCACGGTCGCGCCGCGCCACCCC
CGGCCCATCTTCAAGATAACCCCATCGTCGGCCATCTGCTGTCCCGTTCGCACCCAGGATTTCGGGATT
ATGCTGTCTTAGATTATCTGTTTGCTTTGACACTTGGTCATCGTGGCTCTTTTGGAAACGTCAATAGGCG
TTATCATTGATATTCGGGTCCTCCTTCAAAGCTGCTCTTTTTAATTTCTTTAGTCTCTACGGTTTGC
>TCONS_00093700 geneID=XLOC_018670 scaffold_300:271740-286967(-) Intergenic
TGTACATGTGGTGTAATCGCAGTCATATACAGGACCGAAAGCCACATTATCATCTTGATTAAACACTAA
CATAGACAGTTCAATATTGACTCCCGATAATACCATATCAACCACATATCAACGCACAGCTAAACAGTTC
ACTAAAGCAACAACATTTCTGATCAGTGGTTCCGTGCAGTGGAAAAATGAAGTGTGAGTGATTGTACA
AACAGCGAGCGGTATGTACGAAGTGTCGTGTGTGGCCGGCCACAAAGTCGATGCAGCTCACGCCGTC
AAATCCCTGTCCAACGTCGCCCCACCAGCCCTGGGCCATACGGTGTCGGT
>TCONS_00093701 geneID=XLOC_018670 scaffold_300:271740-287165(-) Intergenic
ATTCGCCCCGTACGCCGCACGGGCCGTCACGGTTAAACGAAAATTCCTATTTCGATTTCTGCGTTCGAT
TTTTAAATGTGAAACACATTGGATGTGAGGTGCTTTTGTTTCCTTTTTGTGGAATTTCTGGATGTGCGTG
CCAGTGCTTTTTGGATTGATAGTGTACTCAAGGAGGATTTACACTCGGACAATATTCAGTGTACATGTG
TGGTTCCGTGCAGTGGAAAAATGAAGTGTGAGTGATTGTACAAACAGCGAGCGGTATGTACGAAGTG
TCGTGTGTGGCCGGCCACAAAGTCGATGCAGCTCACGCCGTCAAATCCCTGTCCAACGTCGCCCCACC
AGCCCTGGGCCATACGGTGTCGGT
>TCONS_00093650 geneID=XLOC_018671 scaffold_300:308698-310323(-) Intergenic
AGGATCGGCTGGAGAATGAGCTGGCTTTGTTTTTTCAGGCCATCGGGTTGGTGTTGCCAGAGCTGTCA
ATGTTCGCATCGAGCGCCAGTTGAATTTGCAGCTGGCAACATTTGACAGCTAGTTTTTTTTCTGGAGAA
GGTTTTGGCAAAGGTACAACTGGGGATCTCCAGTCTACATATTTTTTCTTCGGTGTAATGTAGTTAGGG
ACGTTATATAGTGCCAGAAGAAAAGACACTGTTACATCGACAGTGGTCAAAAGTTGTATTGTAATCCG
AAAGTGGATATCTGAGCTCGTCATTCTCATCAATTTCAGTTATTTTAAACTTTAAAGGATTGGAAATAAA
AGTATGGCCATGCTGAAAAGATGCTTAGATATCATTAACCTAGCCAACAAAGTTTATAATTATTAAACTC
TATGGCACACAAAAACGTATAGTGCCACAATCTTATCTGTTCCGGTGGTCAAAATGTGCAACTAACGA
GACGTCATTGTCAAACGTCATTTCATACTCTGTGCGTTATTTGAGTTGTCAATTTCTGCGAAGAGGCTG
ACGCTACATTATTTAATTTGTTTGTGATTTTCTGCGTGAAAATAATGCCAAAAGCGCTAAAATGTGATGC
AAGAAAAGTAATACTAGATATATTGGCTTTTATGCAGCAAGAATAACGTATTCAGGCGCCTTTAATTCCG
TTTGGAAAAATTGGAAGAACGAGTTGCAACGGCTACAGGTTAGTGTTGCCAAATATGACGAATAATTT
TACCCATATACTCACATGTAATTTTATTAATATTTTCCCGAGAGACCCGTACCCCAGCAGTGGGGACGGG
ATGGGTCATGATGAAAAGTATATAATCTTTATTTTTCTTTGATTACAGGTGTGACATGAC
>TCONS_00093603 geneID=XLOC_018671 scaffold_300:308784-310944(-) Intergenic
CGCGACCGGAGCCCCAGCAACGTAGATGTGGAGTCGTCATCAGACGAATCATCAGAGGACTGGGAGA
ACAGCGTGACCAGTTCCGAGCTCGATGTGCAGTACCAGAACGATGAGGATCGGCTGGAGAATGAGCT
GGCTTTGTTTTTTCAGGCCATCGGGTTGGTGTTGCCAGAGCTGTCAATGTTCGCATCGAGCGCCAGTTG
AATTTGCAGCTGGCAACATTTGACAGCTAGTTTTTTTTCTGGAGAAGGTTTTGGCAAAGGTACAACTG
GGGATCTCCAGTCTACATATTTTTTCTTCGGTGTAATGTAGTTAGGGACGTTATATAGTGCCAGAAGAAA
AGACACTGTTACATCGACAGTGGTCAAAAGTTGTATTGTAATCCGAAAGTGGATATCTGAGCTCGTCAT
TCTCATCAATTTCAGTTATTTTAAACTTTAAAGGATTGGAAATAAAAGTATGGCCATGCTGAAAAGATG
CTTAGATATCATTAACCTAGCCAACAAAGTTTATAATTATTAAACTCTATGGCACACAAAAACGTATAGT
GCCACAATCTTATCTGTTCCGGTGGTCAAAATGTGCAACTAACGAGACGTCATTGTCAAACGTCATTTC

ATACTCTGTGCGTTATTTGAGTTGTCAATTTCTGCGAAGAGGCTGACGCTACATTATTTAATTTGTTTGT
GATTTTCTGCGTGAAAATAATGCCAAAAGCGCTAAAATGTGATGCAAGAAAAGTAATACTAGATATATT
GGCTTTTATGCAGCAAGAATAACGTATTCAGGCGCCTTTAATTCCGTTTGGAAAAATTGGAAGAACGA
GTTGCAACGGCTACAGGTTAGTGTTGCCAAATATGACGAATAATTTTACCCATATACTCACATGTAATTT
TATTAATATTTTCCCGAGAG
>TCONS_00093645 geneID=XLOC_018663 scaffold_300:360167-361268(+) Splice junction overlap
CGGCTGAGTTTGAGATATCTGCACCGCGCTAAAACGTCTCCGGAGCCGGAGGGGCTCCGGGTCTCGG
GTTCGAAGCACGACAAGGGTGACTTTGCGCAGGCGCTCAACTATTTGCGTCAGAAAGGCCAAACTCA
GTGATAGCGGTTTATAAGAGATCGCACAGTAAGATGGACACTTCGGTATAATATAGAAGAATGTGATGC
CCTTTTTCAGTTATGATTCTAGGTTTTCAGTCAGGAAATAGGTAGGTATATATATCAGTTTATATACAAGG
ATGTATGCAACGAATTTAAATAGGGAATACTGTAATACGTCTAGAGGTAATAAGTATATTTATTTTAATTT
TGTTTTTATGTGAAATTATTGTAGGTACCTACTTGCATTTAAACTAGACTAGGCTACGTTAGTTAAAACC
TAGTTATAAAAACTGTTATAGTAAAATGTGTACTAGAGGTA
>TCONS_00093828 geneID=XLOC_018710 scaffold_301:11370-13191(-) Intergenic
ATTTTAAATATTCCGAAAATACAAATTCTCGTATGTTTTTAATGAACAAGAAATAGATATATATTAGGAAT
AGTAGGTTTATTAACAGCATAATCCGTATACAAACAATATTTTTTTAGCTATATTATATGAAACGCTCCGC
TCCGCTTCGCTGCGCTCCGCTTTGTTTTTCGATATCTATGTGCACCTAACACGCTCCTCCTCGCTTTGCT
CGTCGTCGCACCTATCTTTAGGTTTAGGTCCTAAGGTTTTTAAGGTTAAACACCGTAAAAATCCGAGCA
ATTGCATTGCTTTGTTAACTTAACAACATATAGTTTACTATTTAACAATATTTATGCTCTATATTTGAAACG
CTCCGCTTTGTTTTTCAATATCTATATGCACCTAACACGCTTCTCCTCGCTCTGCTCGTCGTCGCACCTAT
CTTTAGGTTTAGGTCCTAAGGTTTTTAAGTTTAAACACAATAAAAATCCTAACAATAGTTTTGCTCTATA
TTTGAAACGCTCCGCTCCGCTTCGCTGCGCTCCGCTTTGTTTTTCAATATCTATGTGCACCTAACACGCT
CCTCCTCGCTTTGCTCGTCGTCGCACCTATCTTTTGGTTTTGGTTCTAAAGTTTTAAAGACGTTTATTTT
TTTTGTCCAAATTACGAAATATCATATTTTCGATCGGGATTTCACTTTTTCGTGATAATAATAAATCGGTA
TTTTATTTTTCGTATATTAAAGTAATCGTATTTTTTAACGTTCGTATATTTAACAATCGCAATAACAAATAT
TCGTGATTATAATATTCGAAATTATAATTTTCGTGATTTTTATAATTCGGTATTTTAAAAAATCGGTATTGC
AATTTTTCGTAAATTACATATTCGGTGTTATCAAATTCGGAAAAAAGTTTTTCGGAATATTTGGGTGTTC
CCAGGGGGCGGGCCTATTGCCACAAATATCTGCCCCAGCCGGGAATCGAGCCCGGGACCATCGGCTCA
GTAGTCAGGGTCACTAACCACTACGCCATTCGGTCGTCGTAGTTAACTCGACTTACATAAATAATAATGT
ATTTATTTTCAAAGCCAAAAACATAAGAAAAGGAACAATCTTACAAAGGTTCATAAAATAAGTAGGCT
GCTGAATATGCTTAAATCGTGTCCCCTGATTCCCAAACTTGGTAATAACCTGAAGGTACCTACGATGAG
GATCAGCGGTCACTCACCGTGATAACATACCTAGGTACATATATGCATAGGTACATACATAATTATTATAT
TTATGCTCACGACGGTAATCCCCGAAGGGGTAGTTAGAGTTAGAGGTGACTCGCATGCGAGTCACCCA
CTTTTCGCTGAACATTTGTACTCACGTGATAGGTCGCGTAGCCGTTTTTGAATGACTACAATGCACTAG
TGTCACGTAAGTGGGCATCCCGACTGTCAGATGTTTTCAAGCTGCCCAAAGGCCTCTGAGGACCCACG
GGTTAACGTGCCGTCCGAAGCACGGAAGCGTCCAGAAAAGAACCACCTGAAATCGGTCCGCCGCAC
GGTCGACCGTGCACCGTGCCAGTTGTTGCTTAACCTCAGTGATCAGTTACGTACACTGAAGCCAGCTC
GAGCTCTTGTACATAGCTTTTCTGTAAGTAAGTAGTGTGCTCCAAATTATACAACAG
>TCONS_00093715 geneID=XLOC_018706 scaffold_301:171249-175644(+) Unclassified
CGGAAGCGCTGAAATAGCCGCGCGCATCGGCCCGCGCGCCGAAACTAAATACCCAAAACCGTCTGAC
AGCGCGCGCCTGCCAAATATGGCACGAATCTCAAGTTTCCCGCGCGCCCGCTGGCACGCCACCAGCC
GCACTGCAAAAACGCTCTCTATTCCCGTCTAATGCTCGTGAAAAACACGTAAAAACGTTTTTATTTCGG
TAGCGAGCGGCCTCTAAATTTAGGCCGTATGGAAACCGAAAGTAATACGAGTGATTTTGGTCCTGAGC
GAGTTGTCTGTATTTAGATTATAGGGGCTGGCGGCGGCCGACGGGCAGTAGGCTCGCGCCTGTCGCGA

CCTCTGTCTGATAAGGAGCTGCCGCGACCCGCCGCGCGTGACAGTGACTAGTGAGTGCCGTGCTTAGT
GACCACCACAGGACTTGGATGTCACAGATGTTGCCCGAAGGCGGAGAAGTGATGCGTCGCGTGGCGT
GCGGCGACGTCTGCCCCGCGCCGCCCGCCCC
>TCONS_00093756 geneID=XLOC_018708 scaffold_301:344635-346599(+) Unclassified
GGACGCTGGGGTCGCGGGGGTCGCCGGGGGCGCGGCGTGGACCGACCCCGGCTGTGCTGGCCGCC
GATGATGTCCCCATACGCGGCTGCCTACAGACAGGCGTATAGCTTCGAGCCAGGTCTATGGTGGCCCG
GCCCCGACTCCCGCGCCTCTCTGCCGCGGCGCGCAGGCGGGCAGGTGCGCTTCACGGCGGCGCAGGC
GGCGGCGCTGGAGCGGCGCTTCCTGGCCACCAGGTACCTCAGCGCGGATCAGAGGCG
>TCONS_00093823 geneID=XLOC_018719 scaffold_301:370429-376073(-) Unclassified
GTGCCTAGTCTAGTACACCACACGAATATCATTACACACTCGCTTTTACGCTTCCACCGAACAAAGCCA
CAACCATACCGATCAAAGTGTTCACACTACACCGCGTTTTTTTATTACAACTGTAATATATCCTAACACC
GCCACCTAGCGGCAGAGTTGGTAACTAGGTGGTAGTCGGGCACATTATTTCCGCCACGCCTTAGCAAG
CTCGCATGACACGACACGTATTGCCCAGACCCATGCAATGGAACTCTCCGAAGAACGGCAACAAAGG
GGGCGCCACTTGGAGCCCGCAGCCGATGCAAGCCACCACCGGGGTGGGGTACCGGCCTATGGGCCCC
GGCATGCAAATGCAGCCCACGCCGCATACCCTGCACACATACCACCCCCCGCACTATGTCATGCAACA
GGGTGGGGTAATGGTGATGGGCATGCAGCAAGGACCGGGGATGGCGATGGCAGCGCCGATGCGGCCC
GCGATCCCGCCACAACCCACCCAAAACCAATTCAGCTAAGCTCTTCGTAACACTGTAGAACTCTGGAC
AAAATCACACCTAGTGGTCGTATGCACTACCCCGCAAATTCACTCTTACATATTTTACATTCGTGCCTGG
TTTAGGGGTTTCTAAATTTATGTTTACATGTATCGCGTTATTTGTTTTTAAATAATTGGTCTCATGTTTAAG
TTAACGTTTTATTATGGTAGTGCATACTTTTAGCTGACTTGTATACGAGGTAGGTTCGCAATGGAACCGT
GAGGTGTATTTTAATGTTTATTGGCGGTTTGTAATGTTTGCAGTTACCAATGAGTTTGTATCCGCAATGA
AGTATATTAGTCAATGTGGCCAGGATTTTATTAGGAAATTTCATACTTCTATTTCTAAATTTACTTATGAA
AATATTTGTTGCGATGTAGGAAACTTCATGCAGGTACCTTAAATTTAATAGGTGTTTTTAATAATTTAGTT
TAATCATGTTCCATATTCGTTATGTTTTGTAACGATTTATTTGTACAATTTATGCAACGCTATCAAATGAA
ACAATAATGTTTTTATTTTTAATAATTCGTCTTATGAAATGGTGCGTATAAATCGTTGAATAAAATTTAATA
TTGTTTCAAAATTATTCAGACATCACATTCCATAACTGTACAAAGCAATCAATTTTAATTATCGCTTTAGT
ATTACATGTGTTCGTCCTACCGTATGGCATAGTCAGCATTTAGCAGGATGGGAATCAATAACTAAGTTAC
AGTAAACTTATAGTGGTTTTGTAATAAGTTACAAGAATGGCATCTATTTAATAGTAGTTTTCATATAAAA
AATAAAAATACAACTATGAAAAATCTATGTGTGTTCGAAATATCAAATAATACTGGCAATTTAAATATGT
ATGTTGCCTATGAAAATACATCGGTATTGCTAGTATTTAAAACTGATAGTGTAAAAAAATATTCCACATATT
TCTATTATGTATAGCGAAATTACTCTTGAAAAGTAAAACATATATCTATGTATCTACATATTTATCTTTAGA
CGTAACACATACCTTACTGTATGTAAAAATGAGTGTATGTATGTCATAGAGAAATTTAAAATACCTAAAT
GTATAAGCTATTCTTTAGATTGTTAAAATTAGCTGTTACTGATGTATATTTTGTTATTAAAACGGCTGGTT
TTCACGCGCCCTCTATGGAACAATTAGAAATTTCAAAATGAAAGTCCTCTATTTTAGACAAAATAATGG
GTACCTATGGTTTTATTAAGTACTAACTTGAACATTTATGTGATTACGAATAATTGCTTTCCGTGTGGGAC
AAAGAGGCCTAGATGACAAATCCTTGATAGAAATTCATAATGCTAGAAAGTGTAATGGCTAGATCTTGC
AAGAATTGGATAGTGATATCGTATATTTTAAGCATCCTTACAGTTTTAATTGATTCCATCTCTCGTTGACC
GAAGATCCTACCAGCATATTCACAGCTTGTAAAAATTGTTGTGTATGAAATATTATTTCTTTGTATATTTG
TCTAGATTACGTGGTTTAATTAGATCTATGGTACTTATAACTTATTATGCCTTTTATGGTAGAATGTGAAG
TTTTGAATGGTATCGCATATTCACTGCTGTCATTAAATTGTTACTTAGAAGTGAGCATATATCTGTATATT
CCTGTAGTAATCATTGTACCTACAAAGTAGTTGTTGCGCAGAATCAAGTACTAGTTCTTATTACCTGTTG
AAAACTTGCAAAAATACTGATAAACAGCGTGTACATAATATTGTAAGAACATATAAGTTGATGTGACCA
AGCATTACAATAAAATGCTTTTTAATTAGTCAAGTTGATTGAAATTACAAATTAATGGTATGTAATATCAT
TTGCAGTTATTTTTTATGAGATCTGTAAATCTCGGCTGAGATTTTGACGAAAATCAATAGTATTTTTTCGT

GCAATACGTTTTTAAAATTTGCGTGACTATGCGCATATTACATTCTGTTCTTTAATAGTCAAGTATTATTT
TGGTTACTTTAATTATAGCAAGATTAGCAAGCTAAATATATTTGTACATTGAAGTCTTGTGCTTAGACTTG
CCAGGTTTATGTTATGCTCAGTAGATGTTGGTAGAAGGCTGGCGTCGTGGCTGGCAACACCACTGTCA
GTGTTGCTATTTGCAGTGTTTTTGTCGACTGAGTTATTTGAAAGCAATTAGTGGGTTTTGCAATTAGTGT
TTTACATTAAGGTTTAACTCCGTATCAATTTTAAGACCTGTTTTAAATTCCCTGTAGGATTTTTATTATTTT
TTACGTTGCAATCCTATTTTGATTGCCCCTAGTTTTAGAGTAATGCAATTATTAAATCTTCTGTATTTTTTT
TACTCCTAGTGGCCTTGTATTTTTTGAAGTTGTAAATTTTAATTCTATTATAAATAACTCAATCATATGGCT
TCTTTTGTTTCTTGTAAATATTCCAACTATGTTATTTTCGAGCCTTCGCCTTTAGATATTCATTTTAGTTTA
TTTATAAAAAATAAATCAATTTAAATATATTTTTTCGTTTTTGATTTGTTTCCATTTTTTGCGTTCACCTGT
GGGTCCACTCTGAGCCTTTGGCTACATAAGTTCAAATAGACTTGTCTAATATGTATAATTTCAATAATTTA
CTCATAATTAATTTTAGTTTTGAAATCTATCTGTAGTTCCAATGACAATACTTTTAAAGAACAAAATATGA
AATAATATCTATTCGTATGTAAATGTTACTCTATTGTGTGTATCGAGATTTTCTTTTGTTTTGAGCAGTTTT
TAATACTTATCTAAGTACCTTGTACAAGACAGAAAATAATGGTGTTTATATCTACATCAGTTTCATCACA
CTGCACTTCAGAAATCTATTCGCAAAATAAATATAAAGTGTAGAACAAGCTAAATCATCTATAAATAGGT
ATATATAGCATTATATTATATTCAATACGATCTCAGTATTATATATGACATATGACAGACCTCCCTAATGATA
TATTTTATAAAGCGTGTTACTAACATTCGCCGATTTTGAGTAGGGGCTTCAATATGTTGAAATAATTATTG
TAATTAATAAGTATATCATAGGAGTGTTTAGCGGAGTCAAAATATATTGAAGAATAATATATATTTTTGTT
GTCGTGGCCAGATATGAGATATGTAGCTACCCTCAATAGTTGGTAGCGTTTGTGAGAGATGTAAGTGTT
CTGCTTTCCAACCTGTGTATCTTGTTAAAACGCTAGCTGTTACGATTGAACGATATCTATAAACACAATA
AAGAGGATAAACTTGCTATTGGAGTTTCATTACCATCCACTACACGCA
>TCONS_00093836 geneID=XLOC_018719 scaffold_301:372980-376080(-) Intergenic
AAATAATGTGCCTAGTCTAGTACACCACACGAATATCATTACACACTCGCTTTTACGCTTCCACCGAAC
AAAGCCACAACCATACCGATCAAAGTGTTCACACTACACCGCGTTTTTTTATTACAACTGTAATATATCC
TAACACCGCCACCTAGCGGCAGAGTTGGTAACTAGGTGGTAGTCGGGCACATTATTTCCGCCACGCCT
TAGCAAGCTCGCATGACACGACACGTATTGCCCAGACCCATGCAATGGAACTCTCCGAAGAACGGCA
ACAAAGGGGGCGCCACTTGGAGCCCGCAGCCGATGCAAGCCACCACCGGGGTGGGGTACCGGCCTAT
GCAACAGGGTGGGGTAATGGTGATGGGCATGCAGCAAGGACCGGGGATGGCGATGGCAGCGCCGATG
CGGCCCGCGATCCCGCCACAACCCACCCAAAACCAATTCAGCTAAGCTCTTCGTAACACTGTAGAACT
CTGGACAAAATCACACCTAGTGGTCGTATGCACTACCCCGCAAATTCACTCTTACATATTTTACATTCGT
GCCTGGTTTAGGGGTTTCTAAATTTATGTTTACATGTATCGCGTTATTTGTTTTTAAATAATTGGTCTCAT
GTTTAAGTTAACGTTTTATTATGGTAGTGCATACTTTTAGCTGACTTGTATACGAGGTAGGTTCGCAATG
GAACCGTGAGGTGTATTTTAATGTTTATTGGCGGTTTGTAATGTTTGCAGTTACCAATGAGTTTGTATCC
GCAATGAAGTATATTAGTCAATGTGGCCAGGATTTTATTAGGAAATTTCATACTTCTATTTCTAAATTTAC
TTATGAAAATATTTGTTGCGATGTAGGAAACTTCATGCAGGTACCTTAAATTTAATAGGTGTTTTTAATAA
TTTAGTTTAATCATGTTCCATATTCGTTATGTTTTGTAACGATTTATTTGTACAATTTATGCAACGCTATCA
AATGAAACAATAATGTTTTTATTTTTAATAATTCGTCTTATGAAATGGTGCGTATAAATCGTTGAATAAAA
TTTAATATTGTTTCAAAATTATTCAGACATCACATTCCATAACTGTACAAAGCAATCAATTTTAATTATCG
CTTTAGTATTACATGTGTTCGTCCTACCGTATGGCATAGTCAGCATTTAGCAGGATGGGAATCAATAACT
AAGTTACAGTAAACTTATAGTGGTTTTGTAATAAGTTACAAGAATGGCAT
>TCONS_00094082 geneID=XLOC_018746 scaffold_302:1548-2007(+) Intergenic
TCGCTCAGCTAAATATGATATCATCAGCAATGTATAATATTGTCTATGCTCTGCAACGCTAGACGCCAGC
AATGGATCAACATTAGTTTTTATAGAAGCTATAAATTTAACTCTCCTAGCAATATACATTTGATAGGAAAC
ATATTCGATTAAGACTAGTATGTTCCTCTCAGGTGCTATATCAGTGCCCTAACTGCAGTCTTATTTATACA
GCACTTTGAGCTTTATTCACACTAAAATTACTTACTTAGTAAGCTTTTCAACTATTCTAATACGTTTTTTC

ATCTATTTCTTCCCACCCATTTTTGCCACCACCCATCTACCTAAAATACTAACAGACAAGCCCAAAAGT
GTTGTGTGGCCCCCCACGAACCCCCTTGAAGGCGAAGTTTCGAAGATGTC
>TCONS_00094012 geneID=XLOC_018754 scaffold_302:6681-62520(-) Exonic overlap (+)
ATTAGAAGTGGGAATGTTTTTGTATGAATTAGAATTGTTCATGTAGGTTTCAGTTTTGTCCATTATCACA
GTATTAGAAGTGGGAATGTTTTTGTATGAATTAGAATTGTTCATGTAGGTGTCAGTTTTGTCTGATATTA
CAGTATTAGAATTATCCATTGTCAGTTTTGTCTGATATTACGGTATTAGAACTGTCCATGTTACCATTGGA
TATATTATCTTGTCTTTCAAACAGTTTCCTTTTGTTAAGCGTGTTGGAATAACCGTTATTAATTTTATTATC
CGGCTTGAAAGCTTCTTGTTTAGGTGTAACATTTTCACTATGATCATTTACTTTTCCCATGAAATTATTTT
TCTTTAAAGTGTTTAAGTAGAAGCTGTTGTCTGATTTATTTTCTTTCTTGTAATCTTCATATCCATTTCCG
TTTCGCTCGGAGGACTTATTGTAGCTGTTGTCAAGAGAGTAGTTCAACTCTTTTGGTGTTTCTTCTGCA
CGTTTCAGTATGTCATTGATGACGGAATGTATGGGCGTGCTGNN
>TCONS_00094153 geneID=XLOC_018754 scaffold_302:6717-62428(-) Exonic overlap (+)
CATTGGATATATTATCTTGCCTTTCAAACAGTTTTCTTTTGTTTAGCGTGTTGGAATAACCGTTGTTAATT
TTATGGTCCGGCTTGAAAGCTTCTTGTTTAGGTGTATTATTTTCACTATGATCATTTACTTTTCCCATGAA
ATTATTTTTCTTTAAAGTGTTTAAGTAGAAGCTGTTGTCTGATTTATTTTCTTTCTTGTAATCTTCATATCC
ATTTCCGTTTCGCTCGGAGGACTTATTGTAGCTGTTGTCAAGAGAGTAGTTCAACTCTTTTGGTGTTTC
TTCTGCACGTTTCAG
>TCONS_00094149 geneID=XLOC_018747 scaffold_302:18764-75610(+) Exonic overlap (+)
CTGTCTTCCAAATCTCTGGTTTTAATCTGGAAGCCTTCACATCCGGCCCGTAGTTGGAGACAAACTCAG
GATTCTTTATAAATGGAAATTCTGAAGTTTCTCTGCCGTTGCTGAGATTATAGCTGAGGTGAGAGAGGT
TTGTGCCGAGGAAATTAATGTTTGTGGAAGGGTTGCTGAGCACGTAGTCCTCGGACAGGCCGAGCTCG
AACATGGCGCTGCGGTTGTTCTTCTGCAGCTTCTTCACCAGCAGGATGAAGCGCTTGCGCTCGCCGAG
GGTCGTGCTGCCGTTGTGAGCTTCTTCACCAGCAGGATGAACCGCTTGCGCTCGCCCAGCGTCGTGCT
GCCGTTGTGGCTGAACGGGTACTCGTAGAAGTCACGGTCCACCAGCGACAGCAGGCACTGCCCGTCC
ATGTCATGCCGTTTACAAATATCTATAATTTCCTTACATATTTTCTCTTTGTGTAACACATCTAATACGTCT
TTGTTATTCCAGTTAACAATATCGGTTGCCATTTTTCACGATTAAAACTACACACTGCCACCAGTTTTAT
TTTAACACATAGCTTTAGTGTCTAGTTATAACTTATAACATTCAATTCTAGAGAAATAATTAATAAATTCT
TCTAAACACGAGAGGAATTTTCTATTTTGTGAAATTTACTGAGCTCTTCACTGAC
>TCONS_00094150 geneID=XLOC_018747 scaffold_302:18987-75610(+) Exonic overlap (+)
GTTCTTCTGCAGCTTCTTCACCAGCAGGATGAACCGCTTGCGCTCGCCCAGCGTCGTGCTGCCGTTGT
GGCTGAACGGGTACTCGTAGAAGTCACGGTCCACCAGCGACAGCAGGCACTGCCCGTCCATGTCATG
CCGTTTACAAATATCTATAATTTCCTTACATATTTTCTCTTTGTGTAACACATCTAATACGTCTTTGTTATT
CCAGTTAACAATATCGGTTGCCATTTTTCACAATTAAAACTACACACTGCCACCAGTTTTATTTTAACAC
ATAGCTTTAGTTTCTAGTTATAACTTATAACATTCAATTCTAGAGAAATAATTAATAAATTCTTCTAAACA
CGAGAGGAATTTTCTATTTTGTGAAATTTACTGAGCTCTTCACTGAC
>TCONS_00094083 geneID=XLOC_018746 scaffold_302:36193-37038(+) Splice junction overlap
TGGATATCTATTGTTTTCGCAGATGATGAAACTGGTGAAAACCACACTATGGTGCGTCCCAACCCGTCG
TCTTCGGCGACTACTAATGAGTACATGGACATGATATACAGGGCGGCCGCCATTCCGGGCCAGCCCCA
GCCGGGGACGTCGGCCTCCTCGTCTGCACCGCCGCCGACTACTACGAGCAACACTGAGCCCTCCACA
AGTCAAGCATCAAGGCGCCCACAAGCCA
>TCONS_00093902 geneID=XLOC_018748 scaffold_302:81522-83475(+) Unclassified
AAAAAACGGGTGTCATTTAAACGATCGCCAATTAAATCCGTTTTGAATTTGCTAAATAATAGGTTTGAA
GGCAAAATAGTAGATTTGCCAGCAAATTTTTGCCCCCAAACAATTTTAAATCAATTCAACAATTAATCA
CTTAAAATTGCCGGGATTTATGATCAAGTGGCAAGTTAACATTTATGTATATATTTTAATAGGTATGTCCA

CATAACTCGATGTTATGTTTAATATTTAAATGAAACAAAAAATTAAGTTTAAATCATCAATATTCTTAATT
AAAAACAACATAAACAACCTTCTTCTAATTATAACTTAAACCTTAATCTTAGAACTCGTCTACCCCAAC
GGTTATCGGTTCTTCGGCAGATATGACCAGCCCACTGCCTCTTCAGCTTGCATATTTTGACAGCTAAGTA
TGTCGGTAACCTTGGTCCTCTGACGGATAATCTCATTTCTGATACGATCCATCAGAGAAACTCCAAGCA
TAGCTCTCTCCATAGCACCGTCTCCGCTAATTTGTAAACTCATGTATTAAATTGCTAACGAATTATAATGT
TTTAATAACTGGACCAATCATAATGTAACTACTGCAATGCTGATAAAATATGAATTAAAAAGAAATCGCA
ACATGCGACCTAGAGTCAATATATTTGCTGGCAAATCTACTATTCTGCGGGCGCAGTTATTATTTGAGGA
GCAAAAATAATATTCTGCATACAAAGTTTTTATTTATGTAATGAACTCAAATTTTTTGGCAATCCATCTAT
TATTTTAGATTTTTGTATTGATATTATTTGCTGATTCATACCAGAGGACACTTTTTATTTATTTGAGGACAA
AAATATATTGTTGCGGTTGATCTATTAATTTGCTGATACAAGTTGATGACAAAAATAAACTTTGCTGGCA
AGAAATATAGTTCCCAAAAAAAAACGGTCCTTAAAAAATGACACTGCAAGAATCGAATATGACATTTCG
TTCCCCTCCTCGTACTTCACCCCCTCCAATATTCGGGCGCCATTGAGAATTTGCATGACAGTGACAGCC
AGCACGCCAGGCCGCCATTGCTTATTGTTGTTGAGAAGTGCTTGCGTCGATTAATTTCCGCCTCTTTAA
ATCCACAATTTGTGCAATATCGTGTAAATTTTCGGAAATGTTTCGAAAGTGTTGTGTGCCGTTTGCAATA
ATAAACGACAGGACGTTAAATTGCATAAATTACCCGCTGCAGAGGACAGCAAAAACCGGTGGTTAAA
GTGCATAGGTTACGAAAATATTGGTGAAATAATCACCACGAACATGTTTGTGTGCCATAAACATTTTGA
ACAGAGGTTTGTTAACCCGATGACTAATCGTTTGAAGGGTGATGCCTATCCATCCTTGTTTACTGACTT
GGAGATTTCCTCAGGACATCCGTCACCGGTCCTTACAGAAACAGATGCCAAGAAGACAACAGATCATA
GTTATTCCAAGAGCGCCACATTGACCATTCATATGCACAACCACCAAATAAAATGGCATGCCTTGAAGT
TAAGGGTATGTAGTAGTATTTTAGTGAAATAAAAAACTATTTAAATAATCATTTTGTTTTAATTATATCTCC
CGTAATGTATTTATTTTTGAATGGGCCGTATTTTCAATCGCCGGACAGCACATTTATTCCTAGGAACAAA
TTAATGTCTGGCGATTGGAAATATTGGCCATAAGGAAAATGCCCGATTTCAAACCGTAGGGTGCTCGGT
TATATATTAAGGCTGAAAATTTTGATTCCTCAGCCAAAAAAAAAAAAAACCGAGCACCTTAAAAA
>TCONS_00094135 geneID=XLOC_018750 scaffold_302:183821-184863(+) Exonic overlap (+)
CATACATTCAAGCTTATCTTTCCAGGTCACAGCACTGGCTACACACAATTCAAGTATTCAAGCAGCTGA
TAGCAAAATTAACTATGGCTTATTCAGAGGAGAACTGGAACTGAACATAGTTCTTACGCCTACATACCA
TACGTTGTACCTGACATGTGTGTTCGACGAGGGCGCATGCGCAGTCAGCTGCAAGACCAACCCCGAG
GCTCGCAAGGAAGAGGTGAGTAGTATTTGGAATTATTAATTATTCAACGTCTTCGTCTTCTTCTCAAGG
GGCGTATCCACAGGCGAAGAAATCGCGATGAAATGACGTTTAGCCGCACTCAACTAACCTCAGCAATA
ATATGGCCGTTGGGAGACCAATTATTTAGAATTATATCACTGTCACCCCCTATGGAGATGACTTCCAATA
CCCTTGTTACACTACTAGGTGTAACTAGGGTATTAGTGTGTCGGTGACTAACACTCGAGCGGG
>TCONS_00093942 geneID=XLOC_018761 scaffold_302:188023-195686(-) Exonic overlap (-)
GGAAAACTTTTTGGGCCGAAGCTCGCGGGAAAAGCTACATAGGCATTAACCGTTTAAATCCCGTGAAA
TCAGACACAACGAAAATCTATTGCGTCTCGCGCTATATCGCTCACACGGGTTAATATAAAAAGATGGTT
CCGTCAGAATGGTTCTCCACTTTGATGGTGGGGGGCGGCGGGTCCCGCTCCAGCAGGTACTTCCGGGT
CTCCAGTAGCGCCACGTTGAGGGCTGAACACAGGATGGAACCAAATAG
>TCONS_00094103 geneID=XLOC_018762 scaffold_302:204856-206612(-) Unclassified
GTTTAATATAATTTAAGTAAATAATAGACACACAGACATGTTAAACTTATAACATCTCCCTTTTTACATCC
AAGTCACAAGTAAAAATTTTGCTCAGACTTAGAATAAGGATTGTAAATATTTGAACGCAGCCCAATACG
TTACTCTGTGAACGTACACAGGTTAGAATTTTTGTTGTCAAGTCAATGTCAAGTGTCAAACCGAGAAA
ATTTCAGCGCAAGGGCACATGCCGTATAAATTTTATGTTATTTGCAATGTAAATAATCTGATACCGAAAA
GCTGAAACCGTATACCTAATGTAATTACCAGGAAAATGTATATCAGTACTCAATAAGTTGTACATACATA
GCCTACAATGTAAAAATTCTCCTGCTGAGGGGGGAGAATGTAACGAGCCGTTACCAAACCATCCACCG
CACCTACCCACATACCGAAGCCACAAGAGCGCCCGCGAGTCGAGGTGGGGCACCACACAGGCGACTC

CGGAGAATACAAGGGGCGCGGGGCAACCCGCTTATAAATAGCCGCCGGACCGCCGCCCGGCCACCAT
TCGTCACCTCCTGCTCCGGCGAGTGATCAGTCCGTTCCCGAGATCCTTCCCTGCACCTCTATCTATTTAA
GTATCTATTCACGCACACTATTCTCACTACACCGTCCTCCCTACGCTGTGGGTCGCCTACTAGCGGATCG
AGGTTCCCCGGAATACACGGACGCCTGGGTCGCCTAGTTTAAAGCGGCCGTGTACCGAACTATACAAC
CGCTGAACACACACCGGCCCTCAGCCACACGTTCTTACCAACACAAATACACTCCACAAATCCTGGAA
CAAGTCCACACGTACCACCGGTATTCGCCCGACCTCCTGTACCCTGTATTCCTTCCCTTTATTAAGATAG
GAACATCCGCGTAGGCCCCTTCAGGGCCCGACCAATATAAATACCCATAACCGTGATAAGTATTTGTGC
ATTTGTGTAGTGTGTTAATATTAAGTACATAAGCTGTAAGTACCCTAGATATATAAGTTAACCTGGTTGTA
AATAAAGCGGGTCCAGTGCCGCCTGAATTGTGTTACACTAATTAATCGGCATTATTAATTCTCACCCCGT
ACACCTACCGAGGTTATTTCCCTCCAGGGGCCTCAGGATCAAGATCAGGATAGTTAGGACCGCTTCAC
GGTCATTGTAGATCCCCCAGGCGACCAGGCACTCCTTCAGAGGCCGGCGTCAAGCTGAGCTAGTCTAA
AACCGCTTCCCGGTACATATTAATTGAGAGTTATTAGGTAGGTACCAGCCCTAAGCACTAGGGACGTTA
GTCATAAGGGTAAGTATCACATTACAATATAGCACCCAACCCCTACTCTCGGGGGAGGCCAGGCAGGT
CGATTCGCCCAGGGGTCGCTCC
>TCONS_00093925 geneID=XLOC_018753 scaffold_302:284957-342335(+) Exonic overlap (+)
GGGAAACTGATGTTCGATTTTCAAACTGGGGTCGGAAAGCCAGCTACAGGTGCGCCAGTTTCCATTTT
CATTTTTCGCCGAGCGCCGAAGCGCAGAATGGCAGTTGGCATCGAGACGTCGATACGCACGCACGTC
AGTCGCCGCGCCATTTTCGACATGTAACCGAAAAAAATATCCCGCAAAAAACCAAACTTCGAACAAA
GTGTTTCGGCGGAAAGTGAAGTTCACTTTTTTTTTGTTGTGATGTGGAGTGTATCAGAATTTAATGTGA
CTTACTTGTAAGTTATAAAAAATAAAATGTATCTTTAAGATTTAAGATATCGTAAGTCTTATCTTATAGCT
ATTATTTTAGATGCGTCATAAGGACACAGTGTTTCAAACTGATAGTGATAAGATTGAATGTAAAGTTACA
TCGTGTATTAAAAACATATATTTTGAGTTTGAAAAACTGTTACAAGTTGTGGTGAAAGTGTTGTCTCAG
AACTTTCAGGAGAAAAGTTTGAAGAACTGTTCAGGCGTATCGCTTCTGCTGCTCGCTCGAATCCACAA
AATATCATGTTCCGAATACAAAAAGCTAATTAGTTCAAGTTGGTGAGTACAAAATCTATTTAATCTTTTT
CATTAACTGAAGCCAGATATTTCAATACCCCATTTCATCGGTAGGCATTTTAATAAAAGTGAAACGCAA
ATTTTCAATTGAATCGGTTTTCTGTGGATCTCTTTCTTATTTGTTCC
>TCONS_00094123 geneID=XLOC_018753 scaffold_302:285199-390867(+) Exonic overlap (+)
GATGTGGAGTGTATCAGAATTTAATGTGACTTACTTGTAAGTTATAAAAAATAAAATGTATCTTTAAGAT
TTAAGATATCGTAAGTCTTATCTTATAGCTATTATTTTAGATGCGTCATAAGGACACAGTGTTTCAAACTG
ATAGTGATAAGATTGAATGTAAAGTTACATCGTGTATTAAAAACATATATTTTGAGTTTGAAAAACTGTT
ACAAGTTGTGGTGAAAGTGTTGTCTCAGAACTTTCAGGAGAAAAGTTTGAAGAACTGTTCAGGCGTA
TCGCTTCTGCTGCTCGCTCGAATCCACAAAATATCATGTTCCGAATACAAAAAGCTAATTAGTTCAAGT
TGATGATAATGATGAAACATTCGATTTATAATCAAAC
>TCONS_00094172 geneID=XLOC_018790 scaffold_303:216852-219524(+) Unclassified
ACCAGGCGGCCGCGCAGGGCGCCGCGGCCTACCAGCAGCAGTACGCCGCCGCCGGCTTCGACCCCTA
CGCCACTGCTGCAGCAGCGGCGGCGGCGGCGAGCGGCGCGGGCTACATGTCGCCCTACTACACGCTG
CCGTCCGGCGGGGTGCAGGCCCCCCTCCTGCCCCCGCTTTCCTACCCCCCACAGCTGCAAGAGGCCC
GCATGCAGTAACTCCTTCACAATCTGCTACCCTAGTACTTCTCGAGGACTCGCGGACTCGTTGAACCAT
CGATCGATTGGCATAAGTTCGAATTAGATTGACAAGTTTCACATTATTTTAAAGGTGACGGTAATTTAAT
TTATATTTAAACTTGTCAATTGAATCCCAGCTTATGGTATTACTTAAGGCATATTCAGGTACATAATTGCA
TAAATTTTAGCCGCGAGTCTCAATACATTAAGCAAATATAAATAAGGTAACTGTGAAAACGGGAAATTA
AGTCCATATTGTTGATGTGCAACGGCGCATATCATAATGTGGTTGTGTCCACATATTATGGGAATTTAAC
AGAACTATCGAGAGTTTAGATATTATTATCGTTACCGCAAGATAGCCAAATAATTGTATAGTGAAAAAGC
TAAATAGATGGAATATACAGTTAAAGATGCAACCAAAAATATTAGCAATTTGACCAAAGCTGAAAGCTT

TTGCTTTAGAATAGTTACACAAAAAGTGTAAAGTTTAGCGTCAAATAAACTTAATTTAAGGAAACTTTA
TCCTTATCTATAATCATTATACTTGAAGATAGTTATTGTACAATGCCTTCTTTCTTGCCTTCAATACCTTCA
CTCTTTTAAATTCAGCAATATTTTATTATATTTAAATAGTTTAATGAAAGCCTTAGAAACAGCTGAAGAG
ACAATAAAATTGAACACTTGAAAGGTCGATGACTGGTATCCACGATAGAGTTGGTAATCGTATTTTTAA
GGCAGCTCCTTATCATTACACTGTACTTCCTGTATAATGTACCTCCTTCGTTAGGCATCTTAAAGTACTAT
TTTAAGTATAACTTGGCGGTCGCCTATTCTTTCCTAGGTCTGTAATATGATCGTATTGCTCAATCTGTCTA
CTTAAGGGATTCGGTTTAAGCCAGACGGGGAGCAATAAGGGCTATATATTATTAGTTTAAGTTTAAGTAC
GTACTATTCTAGTAAATCAAAGTATATTTTGTATTATACATGCCCTTATATAATTTAGATTTGTTCTCCAGA
AGCGGTATAGTACTTACTAGGTAGAATAATTCAATCACTAGTCCATGTAATTTTAATATAATGAGTTTAGG
AGGTAATAAGAGATGTGCGCTGGCTGTGGTGTCCAGTATCAAGACTATTTTATGTATTTTTTTATTGAAA
ACGATCGTATAATTTAATTACTTGATGTTTCACAAAAAAAGTGACTTTAGAACTATACGATATATATAATA
TTTACTAAAAAATTTGTAAAAAAACAAGGAAAGCAGCCAGCGCCGTCAATACATGTTAGATAAGTACT
AGAAGACGGAGTTTAGCTCACTGAATCTTGGTTTAGATCGTAAGTTAATTATAATAATTATTGTATAATG
CTGTTATTGGTAATTTATTTTGTTGATATTTGTAATTCTTCATGTTGATAACTATTTATTATGTCGAGGAAT
AATAATAAAATTTGTTAAATGTTTGAAGATCGGGCCGCGGTCCCGAGACCCGGCGCAAGAGGGGGTTC
GATCTAATATATTGAATTATACACAATAATATAAGTAGATTAATGAACATTAATTATAAGTACGCACCAAAT
AATGAATCATATATATGGTTTAGCCATCGTTACGGCCATCTTGCCTTATATAGACGTACATACATATATACA
TAACATATTGTATTGTTTATATACAAATAGTAACGGATAGACATATCTTTAAAATAATAAAATATAATTCGG
TTTCATCAAGTTTACCGAGAGTCCATAATAGTGCATTTCATTTGCAGTAATTTAAGCACAATCATATTTAT
TTATATGTACTTAATACAATAATAC
>TCONS_00094339 geneID=XLOC_018805 scaffold_303:258170-259236(-) Splice junction overlap
TAATAACAACAACAACTACAGGAATCCTAACAACAATGGTGGCACAACAGTAGTGGTTGTAGAAGAA
CAAAACTCCAGATACCCGACCGGACGGACCGGCTACGGCTACTACCGCAGCACGCGTAGCACAGTAT
GGCTGCGACAAGATATAAATGACAGCTAGTTGCAACAAATTGTGTGATACCAGTTGTCCTCACAAACG
CTAACATGGCCAACACGAAATAATCACTACAAGATGTGAGTTACAATTTTGACAGCGAGATTGGAAGA
TTTGTCTTATTTCCTAACATGGGTGTACCAATTTATCCACACATGTCTCAGTTTGACATTTGTTTAGGTAG
CATTATATCACTCAAGTTCGAAGGTGGTGATAACATGTGACACAAATAATTATTTGTTTAGTTTTTGTTA
ATTTCATCCTTGCTCTACAAGAAGAGATGCAGGAAAGGAAAATTACTATAATACTTATAGGGAGGATGA
TCCGGGACTCTAAAAACCCGTTGAAAATGCTTTAAAAACAACATTTGTCAAAACACACATCCTCTTTT
TTATTTGACTAAAATTATACATTAAGTATATTACTTATATGTAGCGGTGTTAGCTCAGACGGGTAAGCGCC
CGCCCCTCACG
>TCONS_00094340 geneID=XLOC_018805 scaffold_303:260076-263334(-) Splice junction overlap
CATTGATTTTAGTTCCCAACATTGTATAACACCTATATACTTTGTACAATTATTATGTACTTAGGTATAGGT
ACTAGGTAGTTAAAAGGAAGGTAAGTGTACGCATACGTAATAAGTACGTTTCCATGGGATGATAGTGTC
ACACCATGAAACATAATAATTATAATTGCTGAAATTAACGTGCACCTAACTACTCAGCATCCTCCATATAT
AAGGGCGCCTTGACGAAGTGTCCGGAACAATTCTATCTTACTTTGTACTGAAATATTTAGAAAATAAAG
TAAAAAATGGCAGCAATTCAGAACAGTGAAAGTTTTATTATTCTTATTATTTTCGCAGTGATTGCTCAAC
TGACTTGTTTTCCCTTGGAAAAGCCGTCTGATGATGATCACACCTTAGTTCTCCGCTTCAAAAGGCAGT
TGGGGAATAGCCATATTGCAGATCATGCATTAGTCTCAGCTCATGAGTTGAAAGGAGCC
>TCONS_00094227 geneID=XLOC_018791 scaffold_303:285093-286987(+) Unclassified
CAATAACACTGTACTTGCACGCGTTACTTAAATATGAAATGAAAAAATACTGATTCAGTATATTTTTGAC
TGTTACGGTTGTAATACATATATAGAAGGGATAGAAGTGTTTAAGAAAGTAAATAATTTGCCATATCATG
GCATTCATACAGCTGTTTGTAGTCGTGATGTTCGTGGGCGCACAAGCTCAGTTCCACAACATGTTTGCT
GGGTAAGTGGTTTTTACTAAAATTTATAATCTAATTTATACTTTAATCTAATCTGACAGTACCTAGGTACA

CTACCTTTTATATGTACTTTGATTTAAAAATTATCAAATAAATATATTTGAATTTGAAAAAAACGCTAGGT
ACTTCAGGAGAATACAAAAAAAATATATAGATAAATACTACTGAACTAAATTGTATATATGACCCTGCAA
GCTTGGTCCCACATTCTACGAGTAAAAAATCATGAAG
>TCONS_00094409 geneID=XLOC_018844 scaffold_304:279945-282526(+) Splice junction overlap
TACCCGCTAGTGAGGACCCCCAGTCGCGAGCGCGAAGAGCGGAACAACACGGCGACGAAGAAGAAG
AAGAAGCGGAGCGCGTCTCCTGAAGGCAAGAAGTCCAAAAAGAAAAAGTCGAAGAAGAATAAAAA
GGAAAAACAAGACGGCTCCAAGAAGAAGAAGAAGCGTTCGAAATCCGTAGACAGCGACTCTGAAAG
CAGTTCCGAAGCATCGCAGGACTCCGACAGCGGCAGCTCCAACGACGAACGGCAGAAGAAGAAGAA
AAAGGATAGCCATCTTTATCTGAAGAAATAACGTTTACTGTAAGTATATCACAAAACAATCACACTCAG
TAAGAACACTTATGTTAGGAGAGATACTAAAAAGTTGTACTGGGGTATATATTTTGCGACTAACTCTGTA
ATTTTAGTTTAGTTTTGGGTGGCAGTGTGTCTATTGACATGCATGGGCTTGTATATCCTCTTATGACAATG
GTGATATATCCTTGCTTGCGCGCGACGTGTGACTAAGCTGGTATATACTTGAAACTAAAGTACGTCTTTT
GATTAGAAAAGTCGTACTATAGACACGCACACGGCTGTCGAACGATTCTGTGTCACACGTCCTGTGCA
GCAGCGTCCACAATTGATTGATGTATGGTTGTGAAACTCTTGACTAATCCATAACATTCTCCCTCTTCTC
ACTTTACCCAAAACTTGTTAAGGATGAACATCTTTTGATGGTATTCTGTACCAACACCGTTAGTCGAAG
AAGGTACGTGCCATCGGTGTTTACTCATTTGTTTTATTGTGTACTTACATTATGTATTCTATGCATTTACTT
ACATAACTTATACATGTGTTACTAAACATAGAACCTTGAAGGAGGCAAGCACTGGTGCCAAGAAC
>TCONS_00094511 geneID=XLOC_018844 scaffold_304:287247-288794(+) Exonic overlap (+)
TGCGCGGCGCCCAGCGCTCGGTCGTCGCGCTCGCCGTCCATCCCGCGGCGCGCCGGCTCGCCCTCGTT
CCTGGACCGGCGCCGCATCACCAGCGCATCGGCAATAGATTTAAAGTATTCGCCAACGCAAAAAAAAT
ACAAGTATGTTAGAAAGAAGCCGAAAAAAGAAGATTACTATTACTAGAGTTTTGTGAAATGAAGTAGT
ATTTTTACAAAATAATTAAGTTAAAAGTGATGGACGCCCGTCTTAGATATTGTATAAAGGACTTGTTTAG
TTCCAATAGTGTTTCCGTTTCCACCAATGTAGAATGTTTTACGAAATAAAAGCTATTTGT
>TCONS_00094797 geneID=XLOC_018892 scaffold_305:120725-121147(-) Exonic overlap (-)
CTCGTTTATATTTACAGTTTTATTATCACAGCACACATCTTTTTTAACATCATGGCTTAGTACAGTTTGGA
GTATCCCAAGTGAGCGCCGTAGGCGGCAGGGGCGGCGTACGCTCCGTAGGCGAGGGGCGCGGCGTGG
GACGCGTAGGCGAGAGGAGCAGCGTGAGACGCGTAGGCAAGGGGCGCGGCGTGAGCTCCGTAAGTC
AGGGGTGAGACGGCAGCGTACGACAGAGGAGCGGCGTGAGCGCTGTAGACGGCAGGCGCGGCGTGG
GCCACGACGGGAGTGTGGGCGACCAGGGCGGGGTCCTTGCGCACGACGGCGTTGAAGCCGGAGTGC
GCGTCAGCGGCGTAGTCGACGGTGCGGCGCGAG
>TCONS_00094871 geneID=XLOC_018892 scaffold_305:120824-121148(-) Exonic overlap (-)
TCTCGTTTATATTTACAGTTTTATTATCACAGCACACATCTTTTTTAACATCATGGCTTAGTACAGTTTGG
AGTATCCCAAGTGAGCGCCGTAGGCGGCAGGGGCGGCGTACGCTCCGTAGGCGAGGGGAGCAGCGTG
AGACGCGTAGGCAAGGGGCGCGGCGTGAGCTCCGTAAGTCAGGGGTGAGACGGCAGCGTACGACAG
AGGAGCGGCGTGAGCGCTGTAGACGGCAGGCGCGGCGTGG
>TCONS_00094936 geneID=XLOC_018897 scaffold_305:207582-209192(-) Intergenic
GTCGTGTTCATATATCTATGTGATTTATACCTAAAAAGGTACAAGTTGCAGGTAGGTAGTAGGTATCTTT
GATATAATCAACTATTTTATTTAAACTAAGCATTTAGTAAGGTAAGTTAGTGCATGTCCGACAGTTAATT
GGAAATATAAATAAATGTAACACATATTAGATTAAGCTAAACGTAACGAGAAAATGTTCTACTTTACTCG
ACACGAGTCTAAGGAAATATGCCAAAAATAATGAAAATTTCGCTAGGAAAGAATAAACTTATTTGTAG
GTACCCCAGTACTTTTGTCATGAAAGTACAAGCCCTGAAAATGTCACTGAAGATGTAAATTGTAATTTT
GAAGGACCATTTCATGACTGGTTGTATGTAGATATTATGCACCATAGTTTCAGATGCATAATTTTGAATC
AAACAAGCATCAAGAGGCTTAATTTCAGATTAGGTTCATCTTCAGTGACACACATCAGCAGTTACTGTA
CATTTCCAGTACCTGAACTAATTTACTTATTAGTACTTCACATTTATACACATAATAAATCCAACCCGCTC

AACCGTTCCTAAACTATAAAATTCAAGTTTCAAAAAATGAAGTCTAAAATATGAAACTAATAAAATGTC
TGATGTAAAATCACAAATAGTGCTATATACTTAGTACGTACTGAATTATACTTTTCATCGTAATATATATTA
AAAAAGTCGCTTAATAATCACAGACAATCACTTCCCTTTTACAGACACAGACCAACGAACGTTTTACT
AAGCTTCAAAATTCTGCTACGTACAAAAACCTACTTAGTTTTACAACCATCTGGTTTACAAAAATTTATA
CTTATCCGACCTATGTCCCGTCTAATAATTCTTATGTTACCTTCCGTTATTTACATGCTGACATCCTCATCA
TACTTTACCCTTGTTCTCCATTACTTACCAAAGAATAATATATAAAAACTAAAGCTTAAACATATACATAG
TTTTACATAGAGAAATACATCCCCTTGCATGTTCGCTCGCGGGTGGACCACTCACTCGGTATACTTTGTA
CTCTCCCACAGTGCCTAAATCTAAAATTATATACTTTAGTGATGTACCAAATTTTGGCAACATTTTACATA
AATTTCGATACAATAATTTTAATTTGTACTGGCAGTAACTCTTAAGACTTTCAGAATTTGTACTTTAGTTC
TTGAGGTAAGAGAGTGCTCGTTGTCTTTTACTGTATTTTTTTTTTATAAATTTTATAATTTTTTAGTGTAA
GTACACGCACACAAAGATGACACCGAGACCAGATAACGACACCGAGATACGCGATCAGAACTTAACA
AGAGATGAGTAACGTTAAACACGTAGCTGCCCTCCGTCCCGAGCTCAACTCCAG
>TCONS_00094781 geneID=XLOC_018883 scaffold_305:344447-388732(+) Exonic overlap (-)
CTTGGCCTAAAACGAATATGTATAATGTATTACAGCTAAATATACAACATAATACAGTGTTACAGAAATC
ATAAACTTTTAACTACTTTCTAGTATAAATTCCCTCAGCAAAGCAACATAGCTCATCTCATAATAACGAA
AGGTTCAAAGCTAATAATAAGGGTAGTAAGATACGTACTGAATTTAAATACTTTAAGGAATGTCTAATC
GGTCTAAATGTTTAAACCTATGTACATTTGTTATAAAATCATAGCAGGTAATAGTTTTATAATATTATCTTT
TGATCTATGTGTGATATCACTTCAACGATTATGGTTGATATCTCAGAAGTATAAGGTAGGTAATAAATTTA
ATGGAATTGTTTATTTTACATGGATTACAAAATACCTCATAAATAATAAACATAGTAACTAAATTCTCATT
AACAAACACCTAATTACTTAACTGGTTTTTATTAACTAGAGTTCTCGAATACTTGCACCAGCTTTTTAAA
ATATAAGGCGTCATCCTAGTTGTCCTATTTTTTATAATTAAAACCTTCTGAATAAATTAAATCACTAAAAT
ATAGCTACAGTTTTATGTAATGTAGTACAAATTGGTGTTATGCTTTTCAATACAGAGTGAGTTTGTGACC
CAATTTTATCGCACCGTTTCTCACTAAATGTTACCTAACAGGTGTTACAAAAAGGTGTAGAGTGAATTT
ACAAAAGTCGCAGAGAAAAAGTTATTTAGAATGACCCCCGAGTCACTCCTTTTCGGTTTCCTATACCA
CTTTTGCAACACCTTGTATTATCATCAGCTATTACAGTAAATACATATCTAATCTGCATCTCAATCGCACT
CTTACGCAATAACATAACCCTTCTTTTTGCTTCCGTTTTACATTCACATGATAGAATAATATATTTATGTAT
TA
>TCONS_00094564 geneID=XLOC_018884 scaffold_305:349776-393982(+) Exonic overlap (-)
CATATAAATCTCAGTCACCAATTCATATAATTTATACATTAGGCAAGCAATGATTCCTAGAACTTACCCTG
ATTATTACTGTATGTTACAGAAGAGTCCACTCCCTGTCCTGATTGCATGTAAGTCGACTGATTCATATTG
GCCCCATAGTTGGTGTTGACTGTTTGTGAATTTGGGACCACTTCATTTGGCTGCATAGTCTGGTACCCG
TAAGGGTTGTTGTAGTATATTTGGTTGTTCGGTTGCTGTGCCGGCATTTGAGGCGGCTCTCCCGAAGGC
GACTGTACTGTTTCATGTGTCCCAGCACTGGTATAAACTCCTTGAGCGGCCCAATTAGGATCTTGACCT
GTCTGAGGGTCTATATTTCCTTGCACGGAGTTGTTATATGAAACAAA
>TCONS_00094568 geneID=XLOC_018887 scaffold_305:358712-402712(+) Exonic overlap (-)
GGAGACGGGAGGAGAGATCGTGTACAATGGTGATCAGTTCACGGCTTACCAGCTGCTTGGAAAGCAA
CCTGTTCTGAAGGACTTGGAGAAGCAGAGTGCGGACATCATCAGGTCTCGCTCCAGGTCTCTCTCAAT
ATCCAAGGAATCATCATCAGGCAAAGGAAAGAGGGGTCGGCCACGTAAGAACCGCGAGGAAGACCG
CGACTACTCGCCGTCGCCGCCGCCGTCGCCCGACGCAAGGCACAAGAAGAAACACAAGAAGGACAA
AC
>TCONS_00094865 geneID=XLOC_018889 scaffold_305:485972-488705(+) Splice junction overlap
CCGCGGCTCCTGCAAGACCTCGCGAGAGCGGGTAGAAGAGCCACCGGCCCCGCAGTCGGTCGCGATAC
GGCACCTACCAGAATAACTTTAAGGCTGGCCGGAAGTCCAGTTCACTCATGAACCTGTGCGGTAAGAG
AGAGAACACCCTGTATATATAAAGTGGAGCTTTTGGTCTAGTGGCGGGTTCGGCTTGGTTTATCTTGGT

GCGTTGGGTTGGTTTATTTTTGGTTGCGGTCTCGTTTTGGGTGTTTTTGGACGGTTTTATGGATTAGGAA
TGTTTAAAATAAGGTTAAAATCATTATAGATGGGACGGAAAAAAAAGTTTCATCTAATCCAGATAAATAT
TAAAATAGCAAACACTACCTAGTCAATAACACTGTCCAAGAACATACCTCTTCTATGCATATATTTATTTT
GTCTCGTGCACTTTCTGTTCGGCGTTCGTCTAACACGAAATACGTCTTTATTTATTGGTGTCTAAGGGTC
AATTTCGGGTATTTAATAAGGTTCAATGTTAGAGCATGTTTTAAATATATTGGTGTTGGTATGACATAGTA
TGACATTTAATTTTATACCTATAGC
>TCONS_00094985 geneID=XLOC_018953 scaffold_306:328294-329767(-) Intergenic
CTTTGAAATCCTTCAAATGGTCCCGTAACGTTTTAAGATGACTATTTAACTGTATGTACATATAATTAGCG
TTAATACCTAATATATTAATTATTTGAGTTAAAATGTTGTTTTTTTTGCAAACCACGCGTTTGCCAAACCG
TATGATAAGAAATTCATAGATGTGTTGTTTTAATTCAGTACGGTAGGACTTAGGGTAGAGTTTGTTTGTT
TTATTTAATTAGAAGTTATCGTTAAATATATTTATTATAAAGTAAATTTTAGCCTTTTGCTATTAATATACCA
GGAGCCAGGACACAACTAGCTAGGAATAAATAAGCATATAATTTAGTATAGCTCAGAATAACGACAGG
ACGCCTTTACATTAGTCGCCAGAAAATAAGACTGTGTTTAGGAGTCAGAATTGTTTCTGAAGGTTTTTA
ATGTATTTTTTTGTTTAGTTTAGTCGAGGGTTATTACTGTAATGTTTAGTCCTACCGTCTCTTACTATGCC
GTGGAGCGTTTTTCGTTTCTTCTAGCATCTAAAACTAAGAATGACGCAATACAAATAATATTTGTAATTA
ACACATAAGATTGTATGTATATGAGTTTATTAATATTACACTTAACCTGCATATATTTACCAAGTAACTAC
GATTCTCTAAATACCTACCTACAATATTGCATAGCAGTCAAATAATAATATGTAATTTTACTATATGGATCT
ATAATAAGTTATGGGCTCTAATAAGTAGCTGTATATTTTGATGCTACACTTCATCTAATATTTCACAATGTA
TTTATTAACTTCATCACAATCATCCATCAAATCATCACACAGAACATTTTTAAATGCTTAAGGTATGTATT
GCTAATTCCTATATATTGTATCACTAATTTACAATAATCATAATTCATAACCCGCTATCATTATGAAAAACA
CACCAATAAAGCAGAATATACCTACTTAATTACAAGTAGATGTATAAATACTTTATTTAGCAGAAGTTTA
GTCATCTAGAATGTTGTAATCCTTTATTAGTAAATAGAACTTGGAGGGTGACTAGGGGTTTAGGAATAGT
ATACTTCCCGTGTGACAGTATGGGGTTAGTTAGCCCTTCTTATCTTGACAAAACAATACAACGAGATAG
AATAGTGGTTAGTTACGCTCCGAAACCAGGTTTTACAGTGGCCCATTTTAGCAGTCATGAAAAGTACTG
TAATGAATTCCTAAGTATCTAGTTTCCTAGACTAGGTACCTACTTGAAAATTTATTAATTATAAGTATCGC
TATTCACCTACGGCCACGTAACTGTTGCGTATGTATGTAAGTTTTTAATTTGAATTAAATAATTTCACG
>TCONS_00095042 geneID=XLOC_018953 scaffold_306:333583-334492(-) Intergenic
GGCAAATATTTTATAAACTCCACCTTACATAATATATTGATGTTGATAAACATATTATATACATTTATTTACC
GGCTATCGGTTTATCGTGTAGTTACATTTATTCATATTGTAAATGCAGTAATATGTCACAGCCGACAGGAT
ACGGGCCGCCGGGCGCGTCCGGGATGCCCGAGGACGGCGAGGTCAAGAACTTCGACTTCACCGACC
AGTCCATACGCAGGGCCTTCATACGTAAGG
>TCONS_00095153 geneID=XLOC_019008 scaffold_307:3603-4955(-) Intergenic
TACGTTATTCCGGTAAAAACATGAGCCACACCATAAGTAAGATGAGTTGGGACAAGAGCGCCTACGAG
ATATAGAATCAGCATGGAACATGTGCCTGGTACGAGCAATACCAGCGTATGCTGTCCCGTCTCAGGAAC
CCCAGCATCACGGACTCGGCCATCGCCTGTATGTGAATCCCAGTGGAAGCATAATAATTATAAGTGTGC
GTTTTGACTGCTCGGTGAAAAATACATTTAGGAGAAAAGATATTTCATACATTTTGTTATACTATCCTAAT
GTTTACAAATTGAAACAGATAAGTCTGAAATGTTACTACTTAGGGAAGATAAATTTGTTCTTTTTTATTT
ATTTAATTCTATTCTAGTTTATATTAATACGCACAATTTTCAATTTCAGGTTTTAAACCATAATAAACATCA
GACAAGGATGGACTGGCCGTTTTACTAAAGTAAATAGGCAAGTCATTAGTTATATGAATCAACATGTTA
ATTAACTAGAAATAAGATAAATTCTTCTAACCTCAGTAACAATAACATCATTAACACATTGGCTTACCCA
TAATTGTTAATAATTAATTTAATTATTTAACAAAATTACTAAAATTTTATACAAATGGAAAAAGAAAAATT
ACTATAAGTAATATAGATAATATCGCCATGATATCTTACGTCCATCCATGTCTGAATTAAAAGACTTTTCA
TCACTAAAATATCACAACATTGTTGCACCAATCAAAGTTAAATAGTCAGTCGCAAAACGCACTCTGTTTC
GACGATGCTGGGCCCTGAGTGTGAGGATCGGTTAGACAATTTTCTTCGCCACCTTCCGACACCTCATCC

CAGCAGCACGCAAGTGAACCCGTACGGTTTGTAGGGATATATTATGTGCTGTGGCCGTAGAACGGGTG
CTGCAGAACGGATCAGCGCTATGTGCTGCTACGATTTCTCGGTGGCGTGGTGATGCATCCGTATAGGTA
GCAGTAGGTTTCCGGCCACGAC
>TCONS_00095160 geneID=XLOC_019010 scaffold_307:48908-52132(-) Splice junction overlap
CTGGTATCAACAGGGCCAGTAATCTTGGGTTTCCCAAGATGCGCAGACGCAATGAGTGTGCCTCGTTT
TAAAACAACAATTAAAAACAAAACAAAACTAAAATCCTTAACTTTTTAACTCTAGTGAAGTTTCGTTTA
GAAATATTTGGACATAGACGTTAGTGATATAGTGAATATTGTGATATTATTATTTTAAGTGGTTTTAGTTAT
GAGTGCATATAAGGATAAAATGTTACTGTTTATTATTGTATTGTTTACGGCTACCTCACTAACACAAGCG
TCAGTTGTGAAGAATCGAGTGGATTCGTGCGGTTTCGACGAGTTTGCGTGCACAGATGGCGCG
>TCONS_00095469 geneID=XLOC_019037 scaffold_308:124332-189791(+) Exonic overlap (+)
GCGAGTAAAGGAGTGGTGATTAGGCTGTGTTTCGCTAAAAATGACGTTTTTTATATGGTCCCAGGCCTC
CTCGGACCCTGTGCTCGGAACTAGTGCTAGATGAAGTCGGCCCACAGGTGAGATATCGTTCGCAGCTC
CTCAAACGAGCTAAAAAGTGTTAAAAATCGGTAGTGAATGTTGGTTATGACGTTTACGCGTGTTTGTGT
TGGTGTGCGTGAGTTCCGTCGGTGTTGTCGCGCCGTGTGCTGAGAATACTTGTAACGGGATTGCTGTAT
GGTGTTTTGTTGAAACGCTCCAATAACGCGATAAAAGATAAACACTACTGGATCTGAATCATCATATCG
CAGTCGTTTTCAAGTTCCTATAAAGTGGCC
>TCONS_00095741 geneID=XLOC_019136 scaffold_309:184913-185666(+) Splice junction overlap
GGGAAGGAGTGGAGGGCGACGCACCCGACGACCAGCCTGAGCCTGCCCTCGCGCTCGCCCAGCCCG
CCACACCACACCTACGTTTGGGCCCCGATAGGTGGTGAGAGGGAGAGGGAGAGGGAGTGGCGGGAG
TGGCGCGAGCGGTCGTGGGAGCGAGAGGGCGCGAGGCGCGGGCGGGGGAGGCAGCTGCCGCCGAC
GCCCACCAAGCCGTCCACGCTGCAGGTCAAGCAGGGCGCCTACCCGCAGACCAAACTCACACATAGC
CCGTCGCACG
>TCONS_00095626 geneID=XLOC_019138 scaffold_309:237608-241627(+) Splice junction overlap
CAGATGCACAGCCCGCACTAGTGTTCACCGCACAGAGCATATCAAAGCTGCGCCGGCGCACGACCGC
GCTCACACAGCCAATAGCAAACCGCCTCTGAAACAAACCGCACGCCGACAACCAAAAATATTTAAATA
TAATAGATATTCTAAATTCAAAAATGAAGAAAGTACTAGTGCTGTGCGTAGCCCTTTTTGGAACCGTTC
TCGCTCAGGAGTTCGATTGCCCAGACAAATCTGGATTCTACCCGGATCCGTACCAATGTGACCTTTACT
ACAAATGCTCTAAAGGTCAG
>TCONS_00095897 geneID=XLOC_019142 scaffold_309:365515-370478(+) Exonic overlap (-)
CGAAGCCTGTGCTTGTGCCTTGAGCGAACCTATTCCAGACGGTGAGCTAAGCGTCACCGCCTCCACCC
GTCTAGTATAAACGATTGCTCTATAAATTTTGCCACTTAGTTAGGTATATTTACTGTCGCTCCCGAGTCTG
ACCCACAGACGAAAAATCCGCCGGAAAACGTGTCATTCCAATTGCGACCGTCGCGGGCTACACACGT
GTGCAGATGCATGTAAAAAAATATACAAACAAAATTTTCAAGTGTTAATAGAATCGGTGATATAAATATT
ATCGTGCGAAAAACTGCGCGTGAGTTCGTAGCAGGTTTCCCTCGTAGCAGTGTGCGGCCGCGGCGGA
CGACGCGTGCGCGTTTTAGTGCATTCGAATTTCGACTTTTTAGTGAAGTAATTAACGAACTTTCTGCGA
TAAAAAGCCATGGCCACCAAGAATGTAAACAATGTAGAGTCGGGGGCTCCGGCGGCACAGGCGGAGG
CCGAGGCCCCAGCCCTGGGCCCGAAGGCGCCCAAAAAGGG
>TCONS_00095755 geneID=XLOC_019146 scaffold_309:528370-530281(+) Splice junction overlap
GTTCATTAATCGAAAATGTTAACATTTAACGATAGCGTATCACCTATATGGTCCTGCCGTAAATTATACCA
TTATTTCGTGAGATTGTTAATATCGAAATGTCGTTTATTGTAGGTCATTAGCAAGACAAAGAAATCCAGT
TAAAAGGTTCCATCATGGAGGCGGCGCAGAACCGGCCAACGTTCCAGACGTACTTGACAGAGCAGAG
TCCACCAATCAGGAGGCAGAGTTCGTTCTCCAACTCGCACGGAAGTCAGCCGCAATTGGACCGCCGA
GTCTCCGAGCCGTATAGCCCTGACGTTAGGAGGAAGCGGGACCAACCGGCGAAACGTCACTCTATGAT
CGTCGAATCAACTCTGGCAAAGGACGCACAGGAAATCACCCGAAGGAGCCAGGAGCTCACGCCCGC

CAACATGGTCAAAAACCTATCAATGGGTTGACAGTATTCTTTTTTTGAATTTTGGTTGGCGTAAGTTTTT
AAGCGTGACTCGAAATTTTAAGAATGTTCAGGTTTGGCTTTTTGCGTCCGATCTTAAGGTGATTGAAAG
TTTAAGCTAACTAATGTTTTCTATGAATCTCCGTTCAGCAGCTGTGTAGAGTTGAAGTGACGTTTGTATT
CTACAGTCTGAAGTGCCGTTACACTAGTCAAGCGATAAAAAATCTGTTTTTATTTATCTACCGCTTTGTT
GTGCAATATAACTTGTGATTTGATTACCTTGCGAACTGCGAATGTTCTGGATCATTACTAAGTTCTGGAC
TTGTATATTGTCATATAGATACTTAACTGGTGTCAGAATGTAAGGGTTGTTAAGATAGGCTTTGTCTTAG
GCGGACTTATTGCCATAGAATGAGTGCCTTGAAATCGACTCTATGTTTAGAAAACGACTCAGATACTTG
GTGATTGGTAGAGGTTTGATAGGGCTACTGCAGCGACCAATCAGCGTCAAGTAAATTGACCAATCGCT
GAGCAGTCAGTCTCCGATTCGTTCTCTGTCTCAAAGTCGATGTAAGAGGATATTCGGAAAGTATGTGCG
GTAAATATTCGAAGATGGGACCTAATGTAATAAAAATATAATATAGTAAAGTATGTAAATGATTTATCGAA
CAGTACAGTGCATTAGTGCCAGTAACGATAACTATGTCTTGAAAGGCGATACAAATATAATTTAATGTTT
CTCGCCCATTGAATGATGAGTTTAATTCATGTTCATTCAATAGTATTTTTCCTAGATGTTACTTGACTTTA
GAGCACTGACTGTGGCGATACATATTGTATTAATGTCGTGTGTGATTTGTGAGCAACGAATCGCTAGAG
GAGGCAAAGGTATTTATACTATAAAATTGATATTAAATAACATACATTACTATAATTTTTAGTTTAAATGT
GTGTTTTAAAATATATGATTGTTTTGTAGAGTAGTAACTGTACTAATGGATTTGTCGTAAACTCTACTTAA
TGGAAGTTGTAATCGTAGATTATGTATTTGTTCTTTTCACGATAATAATCGTTGGTTAAATATTAATTATGA
GTAGTGAAATTATAGTAAAAGTA
>TCONS_00095964 geneID=XLOC_019165 scaffold_309:647159-649011(-) Intergenic
GTCCCTTATGTGACCGCCGCCGCGCGCCGCCACCAGTGAAGTTGGGGTTAGTATTTTGCATGTTTTGTT
TGATAATTTGTTTACAGTTTAGTGCAAACTATGGTCACGATCGGTGTTATTGTTGGTTGTTAAAATATTG
TGAGTGCTATATATGCAAATGAATATTCAAGATGTTATATTCTAAAAGATATTAGTGGATGCAGATAAGA
GTGTTTATAGTGAGTGCTATGTTTGAATATTTGCAAGTAAAAACACAGCCTATCGATGTAATGCGCCGA
AAAACTATCATGACTGCATTGATGTAATGCGCTAAAAAACTGCAATGGCTCCGAATACATGATTACCAG
ATAACAAATCCTAGCTCAGCTTACATGTGTTCTTCAATAACGTCGCAAGCTACACTGGCTGTTCATAATT
TTTGCAAGTTCTACACCTACAGGATTCAGTTATCTGGGCATTATAGATACCCTATTATCACCTACGAAGA
AAAATGTTTCTTTCTTTCTTTCTTTAAATCAGAATATAAATATTCGCGAAAAAAATATATATTTTTTTATAG
AAACTTAGTTGGTCACGTGACTTTTTACTATGGAAAATAAAAATAATTAAATCCTAATCTACCTAAAGAT
CAATGAAGGAATGAATAACTTAAGTATAAATAAAAGTACTTTCGGCCTGTGATTGTTATTAAATAAGTTA
TATTTCATTATGACAATAACCGTGATTATACTTACCCAAACCTAATAATGAACAATCGGTGATTACTTTAA
CTTAAGCTTATTATGATATAGGGCTAGAAAATAATTATTTTTAAATAGTGAAATAACTACATATTTAAGTT
CATA
>TCONS_00095963 geneID=XLOC_019165 scaffold_309:647469-649388(-) Unclassified
CACCGTAGCATTGAAGTACTCATGTCGAATTTATCTATAAATCATTTATTTCGTCATACTTTGCATCAATA
GTATCTAAACTAATCTTTGGTATGAGATTTTATTGCTTTTGCCTTTTACCTTACTGTGTACAGAGAAAAC
GTATGCCTATACACGCACACATTTATCTCTGTTATCTTGACAAACGATAGCGACGATGACGACTGCGGC
GATGTAGTACCTAGGTCAGTTATTGTCATCCCTTACTATCCCGTGATCAGACAAATATTGCTATCCTTGTC
TCCTTTCTAACCGGCATGCATCGCACGCATAGAACGCGTGAGCGACTGCGTCACCGGTAACATCTTGA
CAAGCTATAGCAAACCGGTATTTATTACGTCCCTTATGTGACCGCCGCCGCGCGCCGCCACCAGTGAAG
TTGGGGTTAGTATTTTGCATGTTTTGTTTGATAATTTGTTTACAGTTTAGTGCAAACTATGGTCACGATC
GGTGTTATTGTTGGTTGTTAAAATATTGTGAGTGCTATATATGCAAATGAATATTCAAGATGTTATATTCT
AAAAGATATTAGTGGATGCAGATAAGAGTGTTTATAGTGAGTGCTATGTTTGAATATTTGCAAGTAAGTA
TCCTTTTTAATATTCAATATCAAAGCAAGCCATCCCATTTTGTTATGCTCAGCTTATTTATTCACATGATTT
TTTATTGAAGTATCGTAACCAAATTATTTTTTAATATCTATGCTTAATATGTATGGGGATTGTTATTCAATT
AGCAATTTTACACTATGAGCTAGTAAATTGTGCATACATGCAATCAAGGCTGTATGTACATATCTTAGTA

GATACGAAAATATGTGGGTATCATCGATTACCTACATATGGTAGTCCTGGTTAAATGTGGATATTTACCCC
TCAATAGCAAAAAAAGGCGTTATTGTTTGTATTTCTGAATGAAACTAGGCAAATAAATACCTATATGTAT
AAAAATAAATTATGTGTAACCTACCTATAAGCCTACCCATTATTATTTCAGGTAAAAACACAGCCTATCG
ATGTAATGCGCCGAAAAACTATCATGACTGCATTGATGTAATGCGCTAAAAAACTGCAATGGCTCCGAA
TACATGATTACCAGATAACAAATCCTAGCTCAGCTTACATGTGTTCTTCAATAACGTCGCAAGCTACACT
GGCTGTTCATAATTTTTGCAAGTTCTACACCTACAGGATTCAGTTATCTGGGCATTATAGATACCCTATTA
TCACCTACGAAGAAAAATGTTTCTTTCTTTCTTTCTTTAAATCAGAATATAAATATTCGCG
>TCONS_00096115 geneID=XLOC_019151 scaffold_309:677838-678224(+) Intronic
CGTTCGAACAAGGCCGCAGCAAGTCGCGTCTCCCGCGCCGTCGCCAGCCCTACGTGTCTTGTACACA
AACACAGCTCTTCAAGTTTACAAATTGTTTTTTTTCTTTCTTTTCAACTCGGTGCAAAGGATTGAGCAT
GAATTACGGTCTGCGAAGATGCTGGTTTCATCTTCGGTGAATGATCGTGGCGGCTGCATGGCCTTGCTG
CGGGTCAGCGCGCGAGCCGGCC
>TCONS_00096535 geneID=XLOC_019243 scaffold_31:368796-370114(-) Unclassified
ATTGCAACAAAAGCACCGTAGTCGTATACTCTCCTTCTTTTATAATATTTTCAGTCTAAAAACCTCTTGT
TAAATGCAATACTCAAAATAAGAGTACTCAATTTAAGTCGGTAATCAGCTAATTAGTGGACTTGCAGGG
ATTCATGCTTGCGGTGAATCCAAAGACCTAAGTGAAAAGTGATGGACCATTCGTTAGCAGATCTCATTA
TTGTGGTCAGTCAAATAGAAACTAATATAAAATGGGGAGATACAGTATTGGCAGTTTAACAGAGTGATA
TTGGCTCATCCAGTAGAGGACAAGACCTACAACAGGACAAGAGTATTAAGTAGATGACGAGTACTAAG
CAGAAGAACTCATGGACTCTATTTTGATTTGATAGTGATGAGTGATAATAATATACATG
>TCONS_00096727 geneID=XLOC_019202 scaffold_31:633832-634495(+) Exonic overlap (+)
CCAGGACTGGACCGAGGAAGTAGAGAAGAGCATGAAGCTGGAGTCGGGCGGCTCCTCGTCCAGCTC
ACCGACGCCGCTCGCGTCCGCCTCCNNNNNNNNNNNNNNNNNNNNNNNNNNNNNNNNNNNNNNNN
NNNNNNNNNNNTGTAGCGGTGCGCCCCGAGACCAAGGAGGCCAAGAGAAGCGCCCGGTCTAAGCGG
AGGGACAAGAGAAGTTCAAGCCGCGGTCAGAAGAAGAGCGACCGGCGGCGGGACAACGCGCAGTC
GGACATCN
>TCONS_00096818 geneID=XLOC_019203 scaffold_31:674845-677420(+) Intergenic
CTGTCTCATTTCACTTTCAGCTAACTATTATTCAATTATTTTGTTGTATTTCCACTCTTACTTTTGTCACAA
TGGTGTTTTATTGATAGTGCCGCCAGCACCATGCAGCATGCAGCATGCACCAGGCACCAGCAGCATCC
GGCTCGAGACCCAACATTTTTGGCTCGAAGAAAACTATTTTCTTGTTGGTTTTTTCTCTAAGATAACTG
AAGGAGGAGGTCATCAGCTGGTGGTTTTTGTTGGGGTTCTATCGAAGACGACCTTTCGCGATTATGAC
TCCGAGGGAGACTGACGGCGCGCTCCTCGCATGAGCTGCGGCGTGGGACTCAACCCGATAGCCAATT
AATATTGCTATGTAACTTGTTTGCAAGCCATCGTCGAAACCTCACACAAACACTCATAAATACTCATAAT
AGATGAAAGCAAGCC
>TCONS_00097022 geneID=XLOC_019203 scaffold_31:674929-678339(+) Intergenic
GATAGTGCCGCCAGCACCATGCAGCATGCAGCATGCACCAGGCACCAGCAGCATCCGGCTCGAGACC
CAACATTTTTGGCTCGAAGAAAACTATTTTCTTGTTGGTTTTTTCTCTAAGATAACTGAAGGAGGAGGT
CATCAGCTGGTGGTTTTTGTTGGGGTTCTATCGAAGACGACCTTTCGCGATTATGACTCCGAGGGAGAC
TGACGGCGCGCTCCTCGCATGAGCTGCGGCGTGGGACTCAACCCGATAGCCAATTAATATTGCTATGTA
ACTTGTTTGCAAGCCATCGTCGAAACCTCACACAAACACTCATAAATACTCATAATAGATGAAAGCAA
GCCAAAGATAAAAAGAACCGTAACAGCAATGTCTGTCAGGGGTTCAAGGGAACTTCCTTGTTGGGCG
AGTTGTGCGAGAACACAGCTCATCACTCGGCACAGCCGGCTGACTGCCGCGATGACGACTGATGATG
GATGACCGACGACCTTCGCAAGGACGCCGCACAAACACGTACAACTACTCGCCGGCGCGAGCACTCG
ACGAGGTAATGCGACGGTTAACTACCATAATTGAGTTATTTTAGCTATTTACTGCGTTACTAGCTTCATT
ACGTACCACTTGTATGCGGCGGGAGTGCGCGAGGCCCTTGCTGCTCGAGTGCGAGCTAGTAGAGGTA

AGCGACGGCCGGCGCCACCTCGATGCATACCACCACGCCCATTGAAACTCTACAAATTAATTAATATGT
AACAAATACGTATCTAGTATTAAAGGTATATAAATAGATAAGTAAATATAAATCATATAGTTACTTAGAAG
TTTTTAAAAACTGAACAAAACAAGCATCATGTCTATGAATAAAAATCTCGAAAATGCGCCGCGCAATTA
TTACATTATCCGACATCAATGTTGTTAAGAAAACGTGAGTAAATACAAATGCAAATAGGCTCTCTTAAC
TTCATTCAATTACAGTCAATATTGATGGATATTGGAAAATCTAATAATAATGGATAGTTGCAAATGATCCG
>TCONS_00096245 geneID=XLOC_019255 scaffold_31:797904-799042(-) Unclassified
TTTTTATTAAAAGAGCCGTTTACTTTTTTAAATTAGCATAAGATACAAACAAAATAACAGTATTTAAACT
GCAGTCGGTGGTCTAAGAGTCACTCTCATCCTCTCTCTACTTCTTGCAACATGAAGTTCGCTACAGCAG
TCGTGATCGCTATCTACGCCATGGTGGCATATGCCGCGGCCGCCGTGCCCAGGGAGGGCGGCGGCGGC
GGCGGCGGCGGTGGCGGCAGCTCCGACAGCTAAGTGTCAAGCTGACATTTCAAGTACTGAGACCTGA
AGACTATCGTGCTGCCATCTGCCATCTTCTCGTCAACAACGTTAAATAAATTATTAATGATGTACTAGTA
CACAAATAAATAATTT
>TCONS_00096180 geneID=XLOC_019218 scaffold_31:1196557-1197556(+) Intergenic
ACCAGTTAGCGCATAGAGCGCCGCCGCCGCCGCCAAAGTATTAGCACGGGTTAAAATATAAGATTCCTA
TCACCTTGTGATAAGGCATAAAAGGAATTCAGTTCACACGTTGAAACGTGAAGAATAATGTAGAAGGA
GCCGTTAAAAGCAAAAGTATGTAGATTCAGAAGAGCCGACGACAGGATATAAGCCCTTCGAGATACGC
GTATGAAGATTACCTTAAATATACGTGAGTATCTAATTATTTAAATGACTCACGGTTGCTGCTATTTACAT
GAGATATACAGATGAAAGTTTAAGCATTTGTTGTACCAAAG
>TCONS_00096183 geneID=XLOC_019220 scaffold_31:1339446-1345030(+) Unclassified
AAAACACACACCTCCCACGCAACACCACACCACCAAATCTTCACACAAAAAACGCAACCCCCACGCA
ACACGCAAAACAAAATGGCGGCGGCACAGCTACGTGAGCAACCACGACACTCTGATGGACGAGACA
TTCTTCGAGCACCTCCGGGCACTGAACCCGGAGCAGTTTGATGAGCTGGACTTCTACGGGACTAACAG
CATGGAGGGGTAATCAAAATATACGCATATTATGGGAAACACCATTATTTTTATTATTATTATGTGAATTA
ATGTAAGTATAAATGTCTTCTTTTCAATTTGACAAATTCTATTCTGTACCTAGAAGTTTTTTTTATGAATT
TTGTAACTGTGCCATACATATTTCTTTCTTCATAAAAACATATTATATTTTTTGTTGTATAAATAATAATCG
AAGAAATATATAAAATATACATTTTGCTTTCTATTGATGTTCGTATGGATTCATTACAGTTTTTTTTTGCAC
AATTATTGTATATTTTTAAGATTGAATTTAATTTTTTTTGGGGAAAATCATAGCAGTTTCTCTGTCGTATTT
ATGATAGTATGGTGCCATTTATTCGTGAATATTTAAACGAATTAAATTTAAAAGCTATGACATTATGACAT
GATGTATTTTCAAAAATCATGTTTTTATTTGTGGCGACCAAAAATATGCTTTTCACAGAATTAACTAAAT
ATAATAGTGTTATGTGCATTTAAAATTTAATATTATTAGAACTATGACATTTAAAGAGTTATTGTGAATGA
AAACACAAGAAAAATACATTTATATTCAAATAAACAACTTTGATATACAAATGTATTTTTGTCACAATAT
ATTACTGTATATGTCATGTGTTGTGAAATACAGGGTGTTTAAATAAAATGGGTCTGTACTTATTTATTTTG
AGAAAATCGATCATGTGGTTGAATTTTCCAGGAGCATCGCTACCATAACACCCTGTATACAATATTTAGT
ACGGATGTGTTCGGAAAAGGGTGGCTTTATGAAATGTATTAAAATTTAAATCATAGTTATATTTACATCG
TCGTTTTAGGATCAAAAATGCGTATAAGTATGAAATTATTTTAGAAAACTAGGCTTCTATTTATCTACTCT
TCGGAAAAAGCGATAAACATGTTTTTCACTGTCGAAAAAATATTTAGCTTTGCTTTTTGCTACTTACTG
AATATTTAGCTGCCTTATTGACAGAATGAAATATCTTAATTTGATAAAAGGTTGCGTATAGAAACCAGAA
TTCACTATGCTAAAACAAATGACAGAAGATGATTGTAAGCATGACATATAGATACTGCATTTTAAACGA
ACAACCTTGAATGCCAATTTCCCCATAGTCGGTTAGAGCATAACCAGGTATTATAGACTTTTGACACATC
TGTCAAGAATTTTATATGGAGATGACATAAATATATGAACCAATCCTTGGTCGATTAGATAACCGACTAT
GGAGAAATTGGGGCTTAACCATGTTTATAGAATTTGACATGGTATTTGACAGATACATAAGGTATTTGAA
GAAATGTTGTTTTACGTGTCTACTTAATAAAACCGATAGCTTCCAAATCTTCGAGTTTTTATGTTCGATG
GCAGATTTATACAGGGTGTTACAGAAAGAGTATACTAAGCTGAAACCTACATGTGCAGCATGGTATATC
TAAGCCCAAAAATTAAATCAGAATTTCAAAATTCCACTCCATAGTAAAAAGTTTCGTGACCGACATAAG

TAGTTTCTATGGAAAATGTATTTTTTTTCTCGAATTTTGAAATTCTGATTTCAGTTTTGGGCTAAGATATA
CCATGCTGCACATGTAGGTTTCAGCTTAGTATACCCTTTTTGCAACACCCTGTAGATAGATCCTCTATTTA
TAAAGCTATGTTTTAGCTGTGTGTAACCTTTGACTGCTTTTGGTGTCATTTTATTTAATTATGTATGTGAT
AAACGCTGCCTTATAAAGTCCCTGTACATTGTGTTGAAATTTACACCTAAATGTTAACATAAATAAAGGT
TGTTTCTGAAAAATATGGTATTGATACAAGGTGAAATGCCATCCATGAAAATTAGTAATCTGATGAAGG
CTTAGATTAACAATATACAGGGTGTTACAAAAAGGGTATACTAAGCCGAAAGGGGGTGAATTTCCAAA
ACTCGTAGAACAAAAGTTGTTCCGAATGACCCCCTGAGTCACCCCCTTTCGGCTTAGTATACCCTTTTT
GCAACACCCTGTATATAATATACGAACAAGATGGAGTGTCAGTGCAAAAGAAACGTTTCGGCAAATAA
CACCCCACTTCTGCCACCGAGATCTTATTTTGAATTAATTCGGTTAGAAGATTCGTGACCTTATTCGTTA
TTATTATTAAACTCTTTATTGAACAAAATTAATTGTTACAAAGGCGAACTTAATGCTAGCAGCATTTTCT
GGCAGTTAACCTTTGGTGATGTTGAAAAAGTGATTGGTAGTGCTTACGATAGAAGATGGTCTAAATTGA
GTACCTAACCAAAATATTAATATAACTATACCTATACAATATAAGTAAATAATTATATACATGATACATACA
TACATACACATATACATACCTAAACTTAAAATTACTATCATTATCATCACATAGATAGGGATCAGTTAAGC
TGAATGTTCTGCTCTTGGAGGTAGTGGAGACGAACTTTAGATTTAAAAAGATCTATAGACTTAGTTTGC
CTTATATCCAGTGGTAACTTGTTCCACAAGCGAACCGCTGTGACAGTGATGTTCGTTGGTGCTATGGTT
GAACTTAAAACGGTTTGAAGGTAAACAATGCGGTCAGTTTTTTTTTGTTTATGGATGTGACGCTCCATC
TTGTTGCTATATGGTTAATCTAAGGAGTAAATACAGCGACGATTAGGAGAAAACGTAGACATTTACTTAT
ATAAATATGGACAAAAGTAGCTTTACTTGAGCTTATTAATTTTAACCTAACCTAAATTTTTAATTGGTGAA
TATTTATAATTGTTAGTATAATTATGCACCATGAAAGTTTGAACTACACGGGCAATGAAAAAGTTCTATT
CTCAAAATGCCGACACCAAACGACAACGATTTTATAAAACATTTAATAAAAAAATTGAACAATATTGTA
TTGTTTTTAGTTGATAAAATCCTGATGCTCTGTTAAATGTACTTCACTATTGCAGAAGGCATCATTAAGC
TAGCCTTTTCCGTTTAGAAGTAACTTGCTATTTTTCTCAGTGGAACCTTTTCATTGCCCGTGAGTGTATA
ATGTTATGTTATCAAACTGCCTGTGGCACTAACGCTAAGGATTACCTGGATGCCTTTTGAAATTAAATGG
AACGAAATATATTGATTTCATTGTAAAATTTTATTAATTGAAGAAGTTTAAAGTAAACTACCCTCCAATC
TGAAAGACCTATTTCTCTAACTCTAGTTTCTATTAAAAAAAACACGTTTTCAGTCTTGTCAATTTAAAAA
TAAATCACGATAAGTGTTTTAATTGTAGGATAAGACCCATGAAAGGTTAGTGTAAAGGCTAAGTGCCAA
TTTCTCCATAGTCGGTTAGAGCATAACCAGGGAGTAGTGGTAAAATTTTGACACATCTGTCATGAATTTT
ATATGGAGATGACGTTTAATTTATAAATCAATCCCTGACGTTAGATAACCGACAATGGAGAAATTGGCA
CTAAAGGCCGGTTTTATGATCCGTAGTCAATTTTAACCATTAATCAAGTTTTATTATTGTGATTTTTGACC
ATTTTAAACTATCTATGGATCTAAAAACCGGCCCTAAGCCAAGTTAACATGAGTTGAAGTTATATGTAAG
ATTGTTAAGGAAGGAATAGTCAAACATATTTGGAGTTTACCTAGTATTATTGCATTAGTATACCTATTGTG
AAAGTAGGATAAAAACAGAATTTATGCTGGCAATGTCCTAGCCAATCAGAGCTTGACTTGACAATTTGA
TTGACCAATGAGAAGAGAGCTTAATGGCTGAACGCCAGTGGCGATATCGAGATTTTTAGGCCATCCAAT
AATTCATATTTTACTTAAATCTTATTCAATGTTGACTGTGTAAAATGTGTAATTTAGGATTTAAAGAGAAA
TAGCACATATATTATGACACCACCCTAAGTTTGAAATAAATTGTGTGTGCTTATAGATTTTATGCAATATA
TTATGTACTTATAAATATAAAGTAGTTAGCACCATCTGCAACTAACAGTCTATAGGAAAAAATTATATGA
ATTGGTCACCAAAATATGAAATTACATTATGAATTTTAAAAGTTAACATGTAAGGTTGAGAATAGTTTTA
GTCCTTATTACCAAGTGTCTAAACTTAAGTTCTTATAGTGTTTTAATTTTATACAGGATGCATTTTAACCA
GTTGTCGACCCGACAGACGTATATGCCCCAAAGCAACTGAAAAAGTTCCGATGCTAACAAAAAAAAA
ATTGTCATAAAGAACTATAATTAGTTACTTGTTTGTACTTTTTAACACCATTTTGTCCACCAACCCGCAC
TAGGCCAGCGTGGTGGACTAGGCCTAAACCCTTCCTTTAATGGAGACCCGTGCCCCAGCAGTGGGGAC
GTGATGGTTCGGATGATGACTTTTAAACAAACCTTCGGGTTGACGACTAGTTTAGAT
>TCONS_00097060 geneID=XLOC_019269 scaffold_31:1345304-1346280(-) Intronic
TCGCTGCAGCGGCAACGACACGTGGCTCGGCCACAGCTGCAGCCAGGACACCCGGGGACTGTTCCTT

TACAGGGCCGAGTCTTCGCATAGAAGAAGGATATCAGCTGATAAACAGTACAACTCAAACGTAAGAAC
CTTTAATCCCTTCGATCCGTTCTCGTGGTTTGGAAGATGAAGGCGCATCATCAGAAAGCTAAAGCTATC
GTCCACTGCTGGATATAGGCCTCTTCTAAGGAACACAACTCTGTCTTCCTTAGCCAGCCACTACCGGCT
GACTTCAGTGATCATACCTAGAATATTTTTATAGGTCAAAAGTTTTTGTTACTATTAGCCAAGACGAAAT
ATGGCTATCGTATTTGGAACTGTTTGCCATTGAATGAATGTCCAAAACCATAACTTTATTTTAAGGGGTT
AAGTGCCAATTTCTCCATTGTCGGTTAGATCGTAACCAGGGAAAGTTTGATCAATTATGCGTATGCGTC
ATCTCTATATTAAATTATTGACAGATGTGTCAAAAGTCACGCAATAATCCCTGGTTATGCTGTAACCCAC
TATGGCGAAATTGGCACTAAGTATTGTTTTTGCCATAAGCCACAAACTTCTGATTTATTCAAATACATTT
ATGTGTATACATGTATGGCATGTTTAAGTAAGAGAGACCCATAGTGAGTTATATTAGTATGTGTCAAAGA
ACAAATTAATACTTTTAGTTGAAATACCTAGTTGTTAAAAAACTGTACAATAAC
>TCONS_00096274 geneID=XLOC_019272 scaffold_31:1551038-1551630(-) Intergenic
GAAATATATACGAGAAGGTATATACTGCCGGAGCCGGATTTTATGCAAAAACTGTCGGAAGGGGTTGGT
TAGGCGGTACATGCTCGGCTCGTGGAGCTGGAGGAGCGTCGGCTCCACCAGACGGATCGTCGCATCG
ACGTTGAGGAGCGTCGGCTGGCTGTGGAGACGCAACAACTCGCCGTGGAGGAGCGACGACTCGCTTT
TGAGCAGGAACTAGGAACGCCAACTCGCCCCCTAAAACATGATATAATATACTTACTGTGATTTTTATTA
TTTAATTGTAATATTGCTATAATATACTGTGATTTTTATGTAGATCCATGGTAAATTATTATAATGACTGTTT
AGATAATAAATGCA
>TCONS_00096472 geneID=XLOC_019234 scaffold_31:1971067-1972458(+) Unclassified
TACTTTATTAAATACCATTAGTAAATAAATATCATAAGTATATATACATAATGTAATAATATTGAATAAAAG
TAGCATACATTTTTTATAAATAAAATTAGAGAGAAATTGTGGGTATGAATAGAAAAGAACGAATAGCCT
TCGGCGTTTCAGCGCGTGAGCGAGACCGGTCGCCATCGCTGACTTGCTTCAGCGCTCAGACCAGCCC
GTGCCGTCCGGCTGAACACAACTGTAAGACAGTTTCATCATAATATATACACAACTGTGAGTGAAAAA
CGGACACAATGCACACTACATAAGACGCATTTTCCAATGGCGGACTATAAGAAGAAAAAATTTAATAAA
ATAACATACAAGGTAAGAAATTTATGCTGAGAACTATCTTAAATACATGTAATAATAATATAAACGAATA
AATTAAGTATAAATTAAATTATATTGAAGCAATCTTCATTAAAAACTACCTGTGTTGTTTTAATTTTATTA
AATTCATTCATTTTGTTTAGGATTTTCACTTCGTGTGTACAACAACATGATATGAAAAGTATTGAAGGTG
AAGATTAGACTAGTGGACCATCAACACAAGGACTGATGGCCTGATTTCTCGGGTTTGAGGGGGAAACT
AGCACCAACACGTGTCGTGACGAAAATGCCAGTTACGCAAGCTTGGGTCTAGGCGGTTCCAACAGGA
AGTAGCATTGAAGGAAAATGATGCTTTGAATATGGCGGTCTTGTGCTGAGGAGTGAGCAGCAGGTACG
AGGAGGCTCGCGTTCGGTGCATGTTGAAGTCGCGGGACCACCTTAGTTTATTATAAAGATATTCAGGTA
TGTGTCTATGTACTATGCCAAATAATAAACATGCAAAATGTAGGTACCTTCGACTTGCTATTTTCAACAT
ATTACCTTTATTTATGTAAGGTGTCACATGCGTGCGGGGGGGGATGTCATAGCAAAATCTAGTGCAGGC
ATTCTGTACTCGCTGCAGTAGGCGCTGTGTTTTGGCATAAAGGCAAGGTCCATAGACTGTATCTGCATA
ATTTAATTTAGATAGTACTAAGCTCTCACATAGTTTTATACGTACCTCAGTTTTAATATAAGGTCTAATAC
GGTATAATACTTTTAGCCGGTAGAAGCAACTAGAG
>TCONS_00096767 geneID=XLOC_019281 scaffold_31:1983508-1987614(-) Exonic overlap (+)
GCCGCCGCTGGGCGGGCTGGCAGTGGCGTCGGCGCTGGTGACCCTCGGCCAGCACGCGCACAAGCA
CGCCAAGCCGATACGACACTGAGATACAAAAGTGGCGGCAGTGTAAATAGGAACTCTTATAATATGAT
GTACGTACACAATGTATATTGTATTGTATATAGACTGTGGTTCGCGTATTGCTCAACACTGGTCGCCTGT
CGGCGTACATTAATACTCACGTATGTTCATTTGTAACGTTATTTAATAAATGGAACTCGTTCTAAGGGTG
AAAATTGATGTCCGACGACATGAGGTGGCAGCACACGACATGTAGTTTTTAGGTACTTGTTTCTACAG
GGTGTTGCAAAAAGGGTATAATACGCCGAAACCTACGTGTGCAGCAGTGTGTGTAATATCTAAGCCCG
GAAATAAAATCTAGATGCAAACTTTCACTATAGAGAACAAATTATGGTTTTCGCGAATTTCCTATACACG
TAGAACAAAAGTTGTTCACAATGAGCCCCTCAGTCCCACCCCCTTTCAGCTTAGTATACCCTTTTTGCA

ACATCCTGTATGTCCTTATTTCTATAAACGTATCGTCGAGCATTATATCGCTAGCCGCCTAATGAGTCGTT
TATAAATATTGCATAGAAACCAGTATAGACGTTGCATCACGTCTGCTGCAACCTTTTGTCGCCACCGAAT
TGCACCCTAATGACTAATATTTTAATATTAGTGGCACTGTCAAAACTTATAAAGTTCCACGGGTTTTGT
CAGCTCTAAAATTAAGAAGACAGTCTTCAAGATGAGTTCAGTAGCTAAGTAGTTGATCGCTGGCGATG
TTTTTGACTCGGCTTTGTGACGCCATGATTTTCGAATAAGAAACAGTATTAATAAACGGTCAATATCAC
AAAATCAATAGGAGGTTCGAAGATTAAATTTCAAGATAGTTCCTATTTCTGCCGCTAGTTGCGTTGTTCT
AACTCTGCAGTTTGTGAATTTTCTTGTCAGTTTCTGAACCTCCTATCTGTAGCTATACAAGCATAAAAA
ACACACCTAAATACTTGTAATATTTGTACAATAAAAAGTTAAATAAATAAATAAATATAGTTTCCATGCTA
TTATGTAGAAGAAACGTCAAGTTTTATTAAACTACACAGATCTGAAAACGCAGTCTTTCAAAAATAACA
TTTAAACTTAAGTTAAACTTAATAAAAATGAAACCTTCCGTTTACTATGTTGCCAAAATAGACACACTG
TATAATTGTATATTGTACTTTAATTGATCAAAATTATACTGATTACGCCTGTCTATTGATGGGTAAGCATTT
ATAAAGGAACAGGCCCTAACAGATTGTAGGATTTTGTTCACATAACTGTCAACCCGCCTATGAATTCGC
TACGGGGTAGGCAAAGGTGCGTTGCTGCACCGAGTGTTTCACCTATAGCGGTATTGCTATTTTTAATATT
AAGAGATCACCCATTCATTACGTGACTATCACATCAGTTGCTTCTCTTGGTAATTGCCACATACTTGTAA
TGGAAGCCTGTTTCGTTATAAATGCATTTTAGTCGCACTATGGTATAAGTAGGTGTAAATTTTGTAAAAG
GATTTAGAATTATAATATTCGCTCACTCTCGTATTCATAGAACTGGCTTTGTAGTTTTTTTTCATGATTCG
TTAAGTACCTATACGAATGTTGACTTTTTGACTAATACATAGATTCGAAATTTGGGTGCTTTGGATCGGC
CTTATGGCACGACAAATCTTGCATTCAAGCAATTCTTGCAGGTTAATGTTGCCAGTTAAGTAACTTATAT
TAATTTATAAAAGCTTAATAACATTTTATACAATTTAAGAAGTTACTTAATTGCAACAATATGTATGAAAG
TCAACTGTTCTATGAATACGGGGGTAAGATTTTGTAAGTATATAATTGTGTTGCCTGCCAGTAAAGTTTA
GCTTCTGTGTATAATATAAATGGATAAGCGAGTAGTTGTAAAGTAAAAAAAATTGTACCAGCGTGACGT
TCTGCAATAGGTTTGTTTTTTTATAGCATGTTGACCACTAGTCAATTGAAACGAACACTTATTTATGTAA
CAGATACGTGTATTCAATAATGAATATATTTACAGTCGATTCCATAGTAAAATTATGTGTACACTTTTGTA
CATTGTCAAAATAAAAATTCCGGAAATCTAATCTTATTTGTTAGATTGGTGGTTTGGAAGTTTTATTTTG
ACAAAGATCTACACATACTGAAATCATACGGTCGACATATCCTGTATGCCACATTCTCTTTCAACTAATA
ACACCTTTAAATCGTTTAGTACTAATTTCTCCTTAGTCGGTTAGAGCATAACCAGGGAGTAGTGGTTGA
CTTTTGACACGTCTGTCATGAATTTTATATGGAGATGATGTTTAATTTGGTTCTACCTTTTTTGGCATAAG
ATTTTTTTGCCTAATCTCGTATTGCATAGTAACATTTGGTCAAAGTCTCGTTACGCCGAAAATCGTATGG
CATAAATCTCGTTTAGTACAAAGTTATTTCGCATAACATTGTTTAGCCTAATAATGGTATGGCCAAATCTT
GAATAGCCTAATAATGCTATGGCATAGGTTTATACAAAGTAATAATATTCGTTTGGCTTTAACTTTGACGG
AGCGTTACTAAGTAATAATATTCGTTTGGCTTTAATTTTGACGGAGCGTCTCCCTACATAGCGCAAACTG
GTGCCTTGTATTGTTTCCGCTGTTTAGAAAACGCTCCGCTCCGCTTCGCTGCGCTCCGCTTTGGTTTTG
ATGAACATGTGCACCTAACACGCTCCTCCTCGCTTTGCTCGTCGTCGCACCTATTTTTAGGTTTCGATCT
CATGGGGTTTGTAATAATTAGGTATATTGGTCGTTAACTTTCGATTTTTTGATCATACAATATCGTGATTTT
CGGGATGTAGGAGAAAAATACCACAATTTGTACATTTACTACATACTTAATATATTATTTATTAAGATAAC
ATTAGGAGAAACAAGATTATACATAGGTATAGACGAACATAAATTTGAGCAAACGAAACTTAGGTGTTT
AAAGATTGTGCCTAAAGAGTTTTAGACGAAACGAGCCTTATGCCACATAAGTCTTGACAAAGTGATGG
TTCGGCCAAAAAAGTTTAGACCATACGAGTTATGCGAAATGAGTTTTGGTCAATAAAGATTATGCGAGA
TGAGCGGAACCCGTTTAATTTATAGATCAATCCCTGTCGTTTAGATAACCGGCTATGGAGAAATTGGCA
GTTATTGTACCACATATCATATCAAATTCATTGATCCATGAAAAACCTGGCTACATCGACATCTTTCACAT
AAATCTTATAAACAAAACTATTTCTGTAAACAAAAAAGTGTACTTTAGAAATTACTAGTGGATCTAGTAT
ATTCGTTCGTTTAAATGGAAAGTTATAATTTAATTACAATGATACGACTGAAAAGATTTTTATAGTTCTAT
ATATATAACGCATTTATTTGCAAGTATATAGCTTGAGTATGAATTTTGCTAGAGTTTTCATATTTTTTGGAA
ACAATAATATAGTTTTACAGTTATGTATGTTACATTGTATTCGTGCGTCATGTAAAGCCTTAACTATTTTA

AGTGTTAATTGTATTGTAAGTATTTTTTAA
>TCONS_00097297 geneID=XLOC_019444 scaffold_310:5995-7385(+) Splice junction overlap
CCCGAGGCCTCTCTGCACGCATTATAACTCGGTCGGTTCCCTGCGAAGGTCCACGTCCTACGTCCTGA
AGGAGCCGCGAGACACTGCCAAGAAACACGTTCAAATTATTGCGTAGCCCAGCGAGAAACACTTCGT
TGAATGTGTTACGTTGTTATAGTGCGCCTGTGGAATGCATACTCAACCTGGAGATCAATTTACTTGCATA
AGTAGGTACAATAAGGGGCAGCGAAACCTGACTGCTCTTAGAATGACAGTGCTAGCTTAGAGGGGTC
AGTTTTATTTGCACCTGTCTGTACGATTACATACTTATGTTTGCCAATATTAGTGCCTCTGATTTGGACGA
AAATTCAGTCATGAAAGATTGGTCATGACTGAATTTTTGGGATTTTTTTCCAGGATGAATATGAATTCCG
AGAATGAACTTTATATTAGCAGCGCCATCTAGTGCCGTGCGGTTGAACTACGAAAGTGTAGTTTTTTTG
ATGTGTGTACTTTTATAGACGTTTAGGATAATAAAATGTGAAAAAAGTTACGATACTATTTTATAATAATA
TGTAAAAATGATTGCATGTATTTTTCCAGCTTGAATTAAATTATTGGTGAAGAAAGTGCCAAGCTATGAT
TGTGCCAATGATTGTGCAAAAAAGTGCCAATTTTATAAAAAAAAGGATGGAAATTTATCAGTGAACCC
ACCAGGACAGTGAATGTATGAATTTACAATAAAAAAAGAGTTTGTTTGCGTGGTATGATTTTTAAACAT
ATTTTTTCACTATTCCAGTAAATTGGTCCAGTTTGACATGCAGTGACAGTCCACTGATAAAAATTGCAC
GATGTATAAATTGTTAATATGTACCTACCTATACTGAAAACTCGAACCTGGTCAGGTGGTTAGATTACTC
TTACTTATTATATAGTTGGTACTATTATTTTCATATTTAATTTGGTTAAGTATATACAGGAATTTTGCAAAAT
TGGTATGGTAAGCTAGAAGGAGTGACTAGTGACTAAAGGGGTCATCTCAATAATTTCCAAG
>TCONS_00097305 geneID=XLOC_019456 scaffold_310:396666-430417(+) Splice junction overlap
TGAAAAGTGATGTACAGAGTGAAATTACAGTATCCTGTATAAGTTTCAGTGCATCTGAAAGTGTAAATT
TTGTGTTTTTCTGTGAAAGTGTGTTTATTTATGTGTAGGGCGTTGGTGCCGACTTTTGTGTCTATTTTTA
AACACAATGAGGTGTTTTAAGCCGCTTATTTATGCATTGTGAACGGGTGCAGTGTGGAGCCACCAGCA
TGCAGGTGCAGCGGGAGAGCCTGGCACTGAGCAGCGAGGCCAGCTACTGTGAGCGGGAGCCATGGG
ACGAGTGGGGGAGTCGCTCCTCGCTCACGCAGTCGCAACCGGACGAGCTGTCCTCGCCCCGTCCCCA
AGACTCCGTGTCGGCTCTGAAGGCGGAGCGCGCGGCGCTGGCCCGCAGCCTCGCGGCGGAGCGGCA
GC
>TCONS_00097741 geneID=XLOC_019497 scaffold_311:153963-156158(+) Exonic overlap (-)
GCCACCCCAGTAGTAGTTGTATGAGGGCGGGCTCTGGGCGGAGACTGTAAAGCGAACTGTTGCATGTG
TCCGGCAGAAGAAGATCATGATCCTAGTGTGCCTGCTGATTCTGGGGCTGGTGATCGTGGGCTACCTCA
TCAGTTACTTCAAGTAGCGGCGGCGCGCGCAGAAACTACAAACAAAAGAAGAAACTGTTGGAGTTAC
GAAGGCTGCACCCTCGCTGCACCAGCAAGGCGAATCATCGACTACACTTTACTTTCGTTATAACCCCA
ACGTTTATGTAACGTTGCTATATTTCGTTGCTGTCAGAGTGCGTTGCCGCTCTTACCGTAGAGCTACTTT
TCAGGCGTGATACGTTCGTGCTATTTATAATTTAATTTAAATTTATTGCATCTTGCAATTTGCTGTTCAAA
GAATAACTAGTACTAAATGTTTGATAGTCTATTTTTTTAGCAATCCCAAGATTAACATTTTGCTACTGCGC
TAATTTTGATGTTAAGTTTAAGACGATGCGCTACATTGCTATAATGGCAAAACATGAGCAAATACATCGG
AGGCAAAGATGGTCAGTGCCATCAAAAATACTTAACCGCTTTGAAATGCAACACTTTCTCAATGTTAA
CTATAGAATTCAGGGGAATTCAAACAAGCGGTTAAGCATTAGTGATGGAACTGCAGATTGTATTATCAG
TATTCGCCTCGTGGTGACTTTGTTTTGTGGTATTTAAGTATAAGGAAAAATAACGTATATCATTTTAATCA
CGGGAG
>TCONS_00097764 geneID=XLOC_019507 scaffold_311:361425-363908(+) Intronic
CTGCAGGCTGATAACCCGATGCATTTAAAAGCCCTGGAGCAAGCGAGAGCCGTGATAGCTGAGGCAA
AAGCATGTCAAAAACTAGAAGAACAAAAATAGCATTAGCCAACAATATTATGGAAGCAGTGATGTTAA
TTTATAAAGAAGTATGCCTTGACTAGCACCAGCCTTATATAATGAAGTTCTAAATAATCATAATGGACAA
CATTTCAAAAAACTAAAGCTGCTACAAATCGAAAACCATTTCGAGATTTAGCTCTAAAGACCACAAAA
AAAATGTTTTTGTATTTTTTGGATGTATCATTTTCGAGAAAATCATAAAATAGTAAAAAAGTGCTGATGA

CGTCATGCATCAGGTGCGATGCATTTTGATGATCAAAGCCTATAGATTTAAAAACAAAGTAACTGTCAC
TTTCATTTTTGATTGACGTTGACGTTTTATCGTGACGTTGTTGTTTATTTGGGTCATTTTCATTAATTCTG
TTCTGACATTTTCTGACTATTTTTTTCATTTATTATTAAGAGATGACCTCTTTATAATATGTCCCAGAGCAT
GCCACCGCCTACACAGCTGAAAAAATGCTTCTGCTTATTTTTTTACATGATAAGTACCTACTTTCCATCA
TTAGAAATATCAAGGTCATTTGAAAATGACCCATTTGTTGGTTGGCATGTAAACAAAGTTTAAATGTCA
CTTGTCAGTGTCATTTATGTTTTTTTTCGAGACTCAGGCAATAGACAAAAATAAAAATGGAGCCTAATA
TGTGACGTCACTTCCGGTTTTAAAATAAAGTTAAAAATAACATGCAAAAATTTATGTATATACAAAATAA
AAAAATACGGGTCCACTAATTTTCTATAAACTTTCCGAATAACTAATAAAAATATAGGGTAAAGAAAAT
GAAATGTTGTCCATTCTGCATTCATGTAAACATTTTAAGAATCAATTAATGATTGCGGTAACAGCATACG
AGGAATAAACTTGTGGTTGTAATTAAAGTGCTTTCTTTCTTCCAAGATGTTTAAAATCTCGTCATAATTA
TTATGAATCAGGTTAAATGGTGTTGTATATGTATGTATATACCTACTTAATGCAATTTCAATGTGAGAG
>TCONS_00097670 geneID=XLOC_019507 scaffold_311:361437-363848(+) Intronic
AACCCGATGCATTTGTAAGTGTGTTAACGCTATTAGTACGGCCATATAGTAGCTCCTGCGATGCGTCGTA
TAGAGGTGCTAGTCGAGCTGCATTCAGACAACCAGCTGCATTTTTGCTACTTTGGGGATAAGCTCTAGA
ATCACAAAACGCTATTAGCGGCCTATAACCGGCCCCCTGCGCGCCCCCGCGCTGCGCCGTATAGCCGG
GCTAGCTGAGCTGGCGGTTAGCAAGCTGCAGGCTGATAACCCGATGCATTTAAAAGCCCTGGAGCAAG
CGAGAGCCGTGATAGCTGAGGCAAAAGCATGTCAAAAACTAGAAGAACAAAAATAGCATTAGCCAAC
AATATTATGGAAGCAGTGATGTTAATTTATAAAGAAGTATGCCTTGACTAGCACCAGCCTTATATAATGA
AGTTCTAAATAATCATAATGGACAACATTTCAAAAAACTAAAGCTGCTACAAATCGAAAACCATTTCGA
GATTTAGCTCTAAAGACCACAAAAAAAAATGTTTTTGTATTTTTTGGATGTATCATTTTCGAGAAAATCAT
AAAATAGTAAAAAAGTGCTGATGACGTCATGCATCAGGTGCGATGCATTTTGATGATCAAAGCCTATAG
ATTTAAAAACAAAGTAACTGTCACTTTCATTTTTGATTGACGTTGACGTTTTATCGTGACGTTGTTGTTT
ATTTGGGTCATTTTCATTAATTCTGTTCTGACATTTTCTGACTATTTTTTTCATTTATTATTAAGAGATGAC
CTCTTTATAATATGTCCCAGAGCATGCCACCGCCTACACAGCTGAAAAAATGCTTCTGCTTATTTTTTTA
CATGATAAGTACCTACTTTCCATCATTAGAAATATCAAGGTCATTTGAAAATGACCCATTTGTTGGTTGG
CATGTAAACAAAGTTTAAATGTCACTTGTCAGTGTCATTTATGTTTTTTTTCGAGACTCAGGCAATAGA
CAAAAATAAAAATGGAGCCTAATATGTGACGTCACTTCCGGTTTTAAAATAAAGTTAAAAATAACATGC
AAAAATTTATGTATATACAAAATAAAAAAATACGGGTCCACTAATTTTCTATAAACTTTCCGAATAACTA
ATAAAAATATAGGGTAAAGAAAATGAAATGTTGTCCATTCTGCATTCATGTAAACATTTTAAGAATCAAT
TAATGATTGCGGTAACAGCATACGAGGAATAAACTTGTGGTTGTAATTAAAGTGCTTTCTTTCTTCCAA
GATGTTTAAAATCTCGTCATAATTATTATGAATC
>TCONS_00097701 geneID=XLOC_019507 scaffold_311:361892-363908(+) Intronic
GCTGAGCTGGCGGTTAGCAAGCTGCAGGCTGATAACCCGATGCATTTAAAAGCCCTGGAGCAAGCGA
GAGCCGTGATAGCTGAGGCAAAAGCATGTCAAAAACTAGAAGAACAAAAATAGCATTAGCCAACAAT
ATTATGGAAGCAGTGATGTTAATTTATAAAGAAGTATGCCTTGACTAGCACCAGCCTTATATAATGAAGT
TCTAAATAATCATAATGGACAACATTTCAAAAAACTAAAGCTGCTACAAATCGAAAACCATTTCGAGAT
TTAGCTCTAAAGACCACAAAAAAAAATGTTTTTGTATTTTTTGGATGTATCATTTTCGAGAAAATCATAAA
ATAGTAAAAAAGTGCTGATGACGTCATGCATCAGGTGCGATGCATTTTGATGATCAAAGCCTATAGATT
TAAAAACAAAGTAACTGTCACTTTCATTTTTGATTGACGTTGACGTTTTATCGTGACGTTGTTGTTTATT
TGGGTCATTTTCATTAATTCTGTTCTGACATTTTCTGACTATTTTTTTCATTTATTATTAAGAGATGACCTC
TTTATAATATGTCCCAGAGCATGCCACCGCCTACACAGCTGAAAAAATGCTTCTGCTTATTTTTTTACAT
GATAAGTACCTACTTTCCATCATTAGAAATATCAAGGTCATTTGAAAATGACCCATTTGTTGGTTGGCAT
GTAAACAAAGTTTAAATGTCACTTGTCAGTGTCATTTATGTTTTTTTTCGAGACTCAGGCAATAGACAA
AAATAAAAATGGAGCCTAATATGTGACGTCACTTCCGGTTTTAAAATAAAGTTAAAAATAACATGCAAA

AATTTATGTATATACAAAATAAAAAAATACGGGTCCACTAATTTTCTATAAACTTTCCGAATAACTAATAA
AAATATAGGGTAAAGAAAATGAAATGTTGTCCATTCTGCATTCATGTAAACATTTTAAGAATCAATTAAT
GATTGCGGTAACAGCATACGAGGAATAAACTTGTGGTTGTAATTAAAGTGCTTTCTTTCTTCCAAGATG
TTTAAAATCTCGTCATAATTATTATGAATCAGGTTAAATGGTGTTGTATATGTATGTATATACCTACTTAAT
GCAATTTCAATGTGAGAG
>TCONS_00097702 geneID=XLOC_019507 scaffold_311:362136-363908(+) Intronic
GCTGAGCTGGCGGTTAGCAAGCTGCAGGCTGATAACCCGATGCATTTAAAAGCCCTGGAGCAAGCGA
GAGCCGTGATAGCTGAGGCAAAAGCATGTCAAAAACTAGAAGAACAAAAATAGCATTAGCCAACAAT
ATTATGGAAGCAGTGATGTTAATTTATAAAGAAGTATGCCTTGACTAGCACCAGCCTTATATAATGAAGT
TCTAAATAATCATAATGGACAACATTTCAAAAAACTAAAGCTGCTACAAATCGAAAACCATTTCGAGAT
TTAGCTCTAAAGACCACAAAAAAAATGTTTTTGTATTTTTTGGATGTATCATTTTCGAGAAAATCATAAA
ATAGTAAAAAAGTGCTGATGACGTCATGCATCAGGTGCGATGCATTTTGATGATCAAAGCCTATAGATT
TAAAAACAAAGTAACTGTCACTTTCATTTTTGATTGACGTTGACGTTTTATCGTGACGTTGTTGTTTATT
TGGGTCATTTTCATTAATTCTGTTCTGACATTTTCTGACTATTTTTTTCATTTATTATTAAGAGATGACCTC
TTTATAATATGTCCCAGAGCATGCCACCGCCTACACAGCTGAAAAAATGCTTCTGCTTATTTTTTTACAT
GATAAGTACCTACTTTCCATCATTAGAAATATCAAGGTCATTTGAAAATGACCCATTTGTTGGTTGGCAT
GTAAACAAAGTTTAAATGTCACTTGTCAGTGTCATTTATGTTTTTTTTCGAGACTCAGGCAATAGACAA
AAATAAAAATGGAGCCTAATATGTGACGTCACTTCCGGTTTTAAAATAAAGTTAAAAATAACATGCAAA
AATTTATGTATATACAAAATAAAAAAATACGGGTCCACTAATTTTCTATAAACTTTCCGAATAACTAATAA
AAATATAGGGTAAAGAAAATGAAATGTTGTCCATTCTGCATTCATGTAAACATTTTAAGAATCAATTAAT
GATTGCGGTAACAGCATACGAGGAATAAACTTGTGGTTGTAATTAAAGTGCTTTCTTTCTTCCAAGATG
TTTAAAATCTCGTCATAATTATTATGAATCAGGTTAAATGGTGTTGTATATGTATGTATATACCTACTTAAT
GCAATTTCAATGTGAGAG
>TCONS_00097703 geneID=XLOC_019507 scaffold_311:362380-363908(+) Intronic
GCTGAGCTGGCGGTTAGCAAGCTGCAGGCTGATAACCCGATGCATTTAAAAGCCCTGGAGCAAGCGA
GAGCCGTGATAGCTGAGGCAAAAGCATGTCAAAAACTAGAAGAACAAAAATAGCATTAGCCAACAAT
ATTATGGAAGCAGTGATGTTAATTTATAAAGAAGTATGCCTTGACTAGCACCAGCCTTATATAATGAAGT
TCTAAATAATCATAATGGACAACATTTCAAAAAACTAAAGCTGCTACAAATCGAAAACCATTTCGAGAT
TTAGCTCTAAAGACCACAAAAAAAATGTTTTTGTATTTTTTGGATGTATCATTTTCGAGAAAATCATAAA
ATAGTAAAAAAGTGCTGATGACGTCATGCATCAGGTGCGATGCATTTTGATGATCAAAGCCTATAGATT
TAAAAACAAAGTAACTGTCACTTTCATTTTTGATTGACGTTGACGTTTTATCGTGACGTTGTTGTTTATT
TGGGTCATTTTCATTAATTCTGTTCTGACATTTTCTGACTATTTTTTTCATTTATTATTAAGAGATGACCTC
TTTATAATATGTCCCAGAGCATGCCACCGCCTACACAGCTGAAAAAATGCTTCTGCTTATTTTTTTACAT
GATAAGTACCTACTTTCCATCATTAGAAATATCAAGGTCATTTGAAAATGACCCATTTGTTGGTTGGCAT
GTAAACAAAGTTTAAATGTCACTTGTCAGTGTCATTTATGTTTTTTTTCGAGACTCAGGCAATAGACAA
AAATAAAAATGGAGCCTAATATGTGACGTCACTTCCGGTTTTAAAATAAAGTTAAAAATAACATGCAAA
AATTTATGTATATACAAAATAAAAAAATACGGGTCCACTAATTTTCTATAAACTTTCCGAATAACTAATAA
AAATATAGGGTAAAGAAAATGAAATGTTGTCCATTCTGCATTCATGTAAACATTTTAAGAATCAATTAAT
GATTGCGGTAACAGCATACGAGGAATAAACTTGTGGTTGTAATTAAAGTGCTTTCTTTCTTCCAAGATG
TTTAAAATCTCGTCATAATTATTATGAATCAGGTTAAATGGTGTTGTATATGTATGTATATACCTACTTAAT
GCAATTTCAATGTGAGAG
>TCONS_00097482 geneID=XLOC_019535 scaffold_311:634771-636701(-) Splice junction overlap
GGTGACGATCACGAACGTGGGCGTGGACGGCACGGTGCGCGTGACGCCGAGCATCGTGTCCGGCATC
GCCATCCGCGAGCTGCTCATCTACTTCTACATCGACGGACTCAACTCGGAGATCGATGAGCTGAACATC

CTGGGCGTGGAGATCTCCTCGCAGGTCAACGACTTCGTCAACGACCCGCTGCTGGCCGCCATCATCGC
TCACAACAAAGACATCGGTGAGATGATTGCCATCATCGTTGAGAAACTCATTGAAAGATTTGCGTAAG
CTCTCTTTGTTCATGACTACTGCGCGAAAACATACTGAAATTGCAACAGTGCTTGATCTTCTATCTGTAT
AATTTATTTTAAGTTAAGTGTCCACATCAATTTAACAAATAAAATATTAATTTCGAAACTGAT
>TCONS_00097804 geneID=XLOC_019535 scaffold_311:634808-635701(-) Splice junction overlap
TTATGGTCCCCATGTTCTCTAATGATAATATGACGTCTATTAATCTAATTAAGTAATTAAGAAAAAGTTCC
AGTGACATAATGGAACGCCAATGGCATATGGCAGGTGGACCTTTTTATCGCTCGTGAGTGTACTAACAT
TGGGCGGTACACCCGAGGCCGCGTTGTCCTACGAGTATGTACCTACTAACAAGAATGTTCTGTTTCCAG
TCGGAGATCGATGAGCTGAACATCCTGGGCGTGGAGATCTCCTCGCAGGTCAACGACTTCGTCAACGA
CCCGCTGCTGGCCGCCATCATCGCTCACAACAAAGACATCGGTGAGATGATTGCCATCATCGTTGAGA
AACTCATTGAAAGATTTGCGTAAGCTCTCTTTGTTCATGACTACTGCGCGAAAACATACTGAAATTGCA
ACAGTGCTTGATCTTCTATCTGTATAATTTATTTTAAGTTAAGTGTCCACA
>TCONS_00097491 geneID=XLOC_019538 scaffold_311:722675-724061(-) Unclassified
CCACCGTCGGACTGTATATGATGAAGTAGGCATGCGCGAAGTAGGTAGGCTTGCGCTGCCACTCCGCC
ACCCAGACCCTCGACCATGTTGTCAGGTTATTGCTTCAATCTGCCGCCGGCCATGAAGGTTACTGTGCG
AGCGTGAGGGCACTATTACTGTGCGCGTGCGACAGAGGCATAAAAGATAGAGATAGAGATATTTCAGA
GATATAAAAAGGGGAATGCCCGATTTTCGCTGTGCTTAGCGACCTTATATGTATGATGATGTTATGTAGT
GGAGAGTAGCATTTCGAAACGTTCTACCAGCCCACGCAAGAAAAACTCCCCTGAAACTAACGTTAAAT
ACATTGTAGTTTTAGACCAGTGAGAGAAGCACCGCCCCCTCAGCCGCCGCCGCCGCCGCCGCCGCGG
CCCGAGTCGCCCGAAGACGAAGTTGGTGATTTGTTATTGTATTGGCTAAGGCGTGATAGAGAAGCACA
AGAAGCTGAGTTGCGGCTGGTGGCGGCTTACAGGCATCATGAAGCACGGGCCGCAGCAGCCGCAGCC
GGGCGGCGAGGTGAGCCGCTCG
>TCONS_00098225 geneID=XLOC_019623 scaffold_313:27117-31320(+) Intronic
GGTACTGTGATACCAAAGAACTGCGTGGAGCCGCTGAAGGAGGCGCTCTCCTCCGACGCAGTTTCCG
TCAACGAGCAGAAGTACGACGAGACCTAGGCGCGACCCTACCGCCCTAAGAATAAAGCCTAGTTTAG
CGCGGGAGCCGACCCGTTCACAGTTCCTCTATTACTCTATAGTCGCTTCTACACTGAACTAGAAGTTCT
ATCGAATTGTTTATGTTCCGAGAGGTGAGTCAGAGCAATAGACCCCACATAGTCTTTTATCATTTACGG
AAATATAAATTAAGAGCTGATTTTTTACAGTAGGATAGAAATGGGACATAAATATGTTGTGACTATCTAA
ACTTTTGTTTTAGACAAATATAACACACTTATTTCCTAGATATTTTATCCGACTGTAAAAAGAACCCGTA
TTTGTAAGTAAATAATTGGATTAAATATAAATAAATATGGCGGATGCCTCTATCGATGTTTTAGCTCACAC
GTTTTATTAATCAGAAAGAGATATAAATAGTTGTTCAAAAAGCCAAATAGCCCATTCAATATAAAAATTC
TGAAGCCTTTTTACTCTATATTTCCAAATAGATTTCAGTAAAGATATATTTAAACTTTATATATATAACTAA
ACTAAATAACCGTAGTGTATGTCTGTGAGAAAGTATTTTTTATATGTTACTGATGTGTGCGTAGGATTAAT
CTTCACTAGTGATAATTAAACATTAATATGTATTTTTTCTCATGGACATTACTATTCACCATCGATTATTTA
TGATTTGTTACCGACAGTCGCATTGCTGATGGCTCGTATTCAAATAATCCAATGTGCAGGTTAATTCCGA
TGATATCGTTTTATAATAATAATGAATGAAACATTAGTAGTGCCTAATCCAAACAGGGAAAACATATAAA
TGTGTACCTTTGTAAATGATTTTGAACTTAACAAGGGAGCTTTTTGCCCATTGCCGCCATCTAGTTATGA
TAATAAATTGATCCACAGATTCGATTATTTATTAATACCAGCCGTGAGTCAATCCAAGTGAAGCTGTAGG
TTTACAACTTACAAAATAAGTAGACACAAGTTAACTTGAATAGCTTATCAAATTCGATACTGTATAAAG
GTCACGGCACATATAAGCGTAACTGGTTGGCAGAAGAAAAACAGGCGACCCCTTTACGTTACGGTTAT
ATGTGCAGTGACCATAACTCTCGAGTGTCATAGTCAATTGATATAATTTAATGGAGAGCCATTTAGTATA
CAATGATTTTATGATCATTATATTAATCATTTATGTTTTCTTGTTGACTTACCAACACAGGCGTATCTATCC
CAGACTTATCAAGATTGAAGAAAATAGTATGTAAAGAAATATTTTGTGATTACAAATACACTATGTACTT
GCCTGTTCACTTTTGCACGTTTACTAAACTAGAAATATAATTGTATGTATCAAGCAAGATCTAATTTTGT

ACATGACTCGTAGGCACTACGATAAAAGATCTTCTATTGGTAAAAAAATGTGATGTATAATTGCATCCCT
GCATGGGTGGAGCTGCCGATGATTTGCCGCCGTGAGCCGATCCAGCGGCCGCTACACGGGTTCGTTAT
CTACGTCGATAAACGCGTTTAATGCGCGTTCCACCAATCACTGCGCCGTATTTATATCCAACTAGCTGTT
CCCGCGCGCTTCGCTTCGCCTTAAAAAGTTTTCCCGTGGGAATTCCGGGATAAAAAGTAGCCTATGTTC
TTTCCCAGGGTCTAGACCGTATGTATACCAATTTTCATTCGAATTCGTTCAGTAGTTTTGGCGTGAAAGA
GTAACAGACAGACAGACAGACACAGTTACTTTCGCATTTATAATATTAGTTAGGATAGCTATATAAGTAC
ATAATATATTGATGTTTCTTTACGTCCGTCGACAACGAACCCGTGCATCAGCAGCTGTAAGATAAGTTTA
TGGCAGATGCGATTTATAGGGTGTTAATGATTCACTGTTATTAATTCGTGCGTCGATTGGGTTTGACTCT
ATGCACCTATTTAAAATGTATGTCTTGCAAAATATGCCAAGATATAGACTAAAGTAACTTTATGATAGAG
TTGGACTTTAGTCAGAGAGCAAGTTTATTTTGATGGACGTGTCCTAAAAATAAAAATAACGGAATAACT
CCAACAAAAATATTGTACGTCCCTAAAATAAAATTGTTCTCAACCTGACTTTATCAATGTTCAAACGAA
TTGTACCTTTCTCTATTTAATTTGTGTATATGCTGTTTTAGTTAATATTATTTTGTTATGAATCGAATTGCTT
GTTTTTTTCTGTAGATTTGATATTTTAAATTAAGTAGCTGGATTTTATAATCAATTTGCCAAAATTAAAAC
CTTAGTGCAATAAAAACACTTTCCGTGACAAAAGAAATGTATATTTAATGTCTCAAAAATATTTGCATAT
TGAAATCGTAGTTTGCAGAACGTATTGGATTTTTTGTTTTTAGATTAGCTTCGAATTCGACTGTGCTGTG
ACCTGTTGTAACAGCTGTTTATTTTTGTATTTTATGCATTAATTTAAAAGTTTTTTACTATAATAAATATCT
TCACAAAGGTCCATCTAGATATTGACTGAATGATATTACAGGAATGGAGGCGAACTTATATTACCGCGG
GGTAATATTACTGCTAGTATGATTAATATTAATCACCTTTCAATGCTGTTTAGTGTATAAAGAGAAAATAA
ATGTATATAACATAGAAT
>TCONS_00097924 geneID=XLOC_019624 scaffold_313:31885-34241(+) Intronic
CCCTTAAATTTAAAGAAAGCATAGTGCAATGTTCTAACTTCATAACTAATTATATTGAAATAAATAAGCG
ATTTACCACTTCATTTAAAGGAAAATTTAATAACATAGGCACATTTTACATCAGTTTAATAAAACGAAAT
ATCTTACAATTTTATTCTTAGATATTACTACTCACTTATGAATACTAACTGTAAAAAGTTTTTTTAGGCTA
AAAATATGTTGAACACGTTTCACAAAATGCAAGTTTACAGGAAGGGTGGATAATACAAATGTGTAACA
ATACTTTATTAATTTAATATTGGGGTGATAGGAAGGCGACACACTACATGCGCCGTTAGCTTATTTTTTTT
ACTAAAGCACATTTAGAAATAGATACAAATTTGTGGCTTCAAATAATTATGTCAAGAATGTAAATTCATC
GAAATACCTACCAAACAACTTTTGTTCTGCAAAATTGTATGTAATTAAGGCACATTTTAATCTCCTATAA
AAATAAATTATAATGTCATAAGTGTAAAGTATTAACTATGAATTAAGAGCGCACTTATCGATTACTTATGA
AAGAAGTTAGTTATGGCAACACTTAAAAATATAAGGACGAAATAAAAGAGCGTACTTCGCTCACTCCT
CTGTCGCTTAACTTCTAAAAATACAATAAATAAACTGTTCACAGTAGAATTCAAGCTTGCAGCAGCGTG
AAAACCTACGTGAAAAACGAAAGGCTTAGTTTGAGCAAAATCTATATTACTCACTATTAATCTCCTATC
AAAAACTAATAAAATCTAGAATTTAACAAAATATATTATGTATTTATTAATCATTTATCAATTACCTATTAA
GAGTAAAAGTACCAGTGTACCTATAGTACTGTTCTTAAGATTTGTTTAATTACGTTATTTTAACCCTGTT
GCTATAAAAATATGCAGATATCTGCGTAAAATTTAATACTGATACTAAAAATATAGATTAATAAAAAATAT
GTGCGTATGTATTATTTTGGCATCAAAAAATTTATAGTAGAAGCAATCGATCTAGAGAGTCTATTTAGAT
AGCTTTAAAAGACGTATCTTTTGTGTATCACTGTTAATAAAATTGACAATTTGTCCCACTACGTCGACTT
AGGCGTAGTGAGCGAAAAAATAATATATTACTGGCAATAGGCGGGTGTAAAATTCAAAATTTGTGCCCT
CACAACCCGAAAGTGCCTATTGGCAAAAGCACACATACATATTGTATCTCTTAAGTATAACATTTCCTTT
CGCATATCTTACAGTCTTGGATGGAGTAGGCAAGAGAGACCACTTTAGTGACCCAGAACGTAGAACAG
ACATAGTAAAGACGTATTTTAAAATTACCATTATTATCCATACAAAATTTTTAGATCTAGAGAGTTTTGCG
TTAGTTAATGAATGTCACACGAACCTTCTTTCGCACTAGTTCTACAGCTGTGTTCAATGATTAAACTTCA
CATACATATTTACTGTCACTGTTCGGCAATATCAGGAATACTAGGACGTTTACTCTAAGCTTGGACGAGA
CTTGTCTTTGGCAATCTTTGAAATGAACCGCCTATGCAAAATTGCGGGTCGTTCAATTAGACCTACTAT
GTAGTTCCGGAAATAAAACGATACAAAAGTCGCCACGGACATGTCTCATCCACGTTCTGACACTACAT

ACAGTAGGGTCAATATAGTAATTATATTGTTCATGGCTCACATTTAAAATATCTCAAGAATCTCACTCTAA
TTTCAAATACATCGTTGCTGCTCTCAAACCTTAATTATTATACTAATAGAACCATTTACAAAACTGTAAA
CACGACACTCGTTCACCATCCTGGGTTACTCTAACCTGACAAAAACGAACGAGCTATCATGCAAAGAG
ATCTTAATTAGCGCTCTGAAACTTCGTTTTCGCAACCTACAGTAACCCTGCTCTCAAATGTCACCTATTA
CACTAAAATACATAACGACAAACATTACACTCGTTCACCGCGCGCCCACAAAGTGTTCCTTCCACAGT
ATATTGGCTAATACACCGCCGGCCATGAGCGCGAAGTACAGCAGCACGGCCGCCCACCACAGCCGCTT
CTCCCACTTGCGCTTCTCCAGGTTGCGGAGGACGCCGATGCCGACTAG
>TCONS_00097966 geneID=XLOC_019635 scaffold_313:75926-77432(-) Exonic overlap (+)
GTGCTGCTGCGGAACGACCGCGAGTGGAGGCAGGCCGAGTAGACACCAGATCCCACACATCAAGAA
GCAGTAATCAACATAGCTCAATAATTTATAAGGATATTCCGACCAATATCTTGGTATTTAAAGATTTCATC
ATATTTTAACAATGTGTTATAACGTTTTGTCAAATCGCGAATAATGATTCCGTATCTCGATCTCTTTTCTAT
ACGTAACTATACAGTATTAAAATGGTTCCGTCGATTTTATGAATAATAATTGTAAAAAATACTTTGAAATA
CTGCAAGGAATACGTATTTTTGAAATTACTGATAATTACTAAGGTTTAAACAAGCTAGGATTGAAGCAA
TAATTTTATAATATTTTTAAATATGATATTACAATTTGTAAAGTATTGTTGTAGAGATGGAATTGAAATGAA
TAACTATTTTGTATGTCTTTTAATGATATTGTAGATTTAATATTATATTTTAATTTCTACTACTTATAGAGTAT
TTTTAATATAATTATATTTTCATAAAAAATATTTGTATTTTATAAGAATATACAATCTTGCTAAGACTTTTCA
GTTGGAGAGAATCTCGCTCCGCATCTTTTTGTATTGTATTCTTACTTTTATTTTTACTATAATACGTAATGT
TAACCAATTTAGATTTTATATTAATGTTATTTTAGTATATTTATTTTGTAATTTTAACTTTACACCGAATTAG
CTATACTCCATTCCAATTTCGCATTTAATTATTCTTCCCTGTGCACACAGGACACCTATTTTATAATAATTT
TTTTAAAACTTAATCCAATATAAGTACCTACATTTATTTTAGAGATCGTATGTGAACAAGGAACATAAGT
ACTTGTACGTGAATCTCTGAACTATAAAGGTGAATCAGCAATAAAAAAATATTTGTTTACGTACAATATA
TAATT
>TCONS_00098139 geneID=XLOC_019631 scaffold_313:318447-320998(+) Intergenic
AAATAATAAGATACCTATTAGGATTTTTACTTGCAAGTATGATTGCTATAAATATCAATTTATCAATCAAAT
AAGAGTTTGTTCATCTTTATTTATTAACATTCTATAGCTATGAGGGGCAGAAGAAGTAAATATTGTAGTC
ATGTACATTTACCGAAATAGTTAAATAAATCAGAATCAGCATGTAGGTATAAATTATATGCGACAAGTAC
GCCAATTAAGTAATGTGCGTAATAGCAATGTATATTTTCAAGAACGATTCAGTAAATCCCAACTTGAAA
GTATACGCGACTAGTACGCACAAATCGTGTATATTTTCAAGTAAAATTTTTTTTTTTTTTTGAAACTATAC
AGTGCTAGTACACTTCACTTGAAACTATACAGTGCTAGTACACTATGGGTGCGTTCTGGCCTTGTATAG
TTTCAAAACACGCTGTTTTGACCCCTGCTATCCCCTGGACTCCGTTTTCGACTACTTCTCTACTGACTT
GTGCATACTGTGATTAATTGACTCCGATTTACGCCCAACGCAATAAAATGACAAAGTGCGCATATCTTAA
TTGTGAAAATACGCATCGAGACATTTCAATGCATCCGCCGCAATACATGAAATGCTTTTATTTATGAATT
AAATTATTACCCACTAATGTTCAGCAGTTAACGTGCGTTGGTTGTTGATGATAGTGATGATAACTAGGTA
CTTAATTTACCAAAATACATTAAGAGGGAAGTAGGTACACCACTTAAAACCGAAAACTTTTTCTTAGAA
AAAAGTTCATACAAAAAGTAAGTATTTAGTTAGTGTAAAACGTTTTCTCCCAAAACCAGAATTTCATTC
AGAAAAGTAGGTACTTACCTATTGTCAGTCGCGAGCTGCAGTATATCTCTGTCGCACCCGTGCCCGGGG
CATGAGAGTACAAGCGATAGCAAATGTTTTCTTAAATACCTAACTATTTCGCTAGAATCGCAAAGGTAT
GTATACTCTCCTCTTAAGTAGGTACTAGTATAGTACATACGTATAAGTACATCGATAAACCGTTTATTTCA
GTTTCAGGTAAGAGCTAAAATAATGAGAAGGGTAAATTACTTTTCACTGTTACTGAGGTTAAACTTTGG
CTAGTTATACTATTCTATGAAATAATAATCATTTTAGTCAACATATTTCTAAAAGTTAAGTGATCATCACTT
TATATGCAATCAATTAAGAAATGCAAAAACCTTTTCTGTCGATTGTACTTTGAGATACGGAAAAAACAC
AAACAAAAATGAACCTTATCAAAATTGTGAAATTCGGGGCGAATGTGACAGTTTTGTGGCGTGCTTTG
GGTGAGGCCTATGGTCAGCAATGGACGTGCGTTAGCTGTTGATGTTGATGATGATGATAACTAAATACT
TAATTTACCAAAATACATTAAGTAGGTACCTACTCGTTCTATGATACAAGATTGACTCGATGGTGCGAGG

GCGGCGACCGCGCACATCAGCGAGCAAAGTATACTTACGCTAGTTAACTTGACACCTGAAAAATGGTC
CTATCCCTTTCACTCCATTCTATTATCACGTGAGAGTGAAAGAGACGGGTATAGGTAGCGGGTCTCAAG
TTGACTCTCGAAGGTACGAGCAAAATAAATATCTTCTTACAAAGTTGACAATGATATAGTACCTAAGTA
GGTACAGCATTATTTTGTTTCCATAAGTTTATGAGTGCAATATTTACCAAACTGCATGCACGTTAGATGA
AGAGAATAATATTAATTTCAAAGATTTATATTTTATGCTTAAAATGCCATCTATTGGCCGTTTCCAATATTA
CGACGACCATCGAATAGATAGATGTCATTGCATTCATCCATAGATAGTGGTGCCACCTAGAAACCGCTT
AACGAAACGTATAGCGCCATCTGGAAACTGCTTAAATTTTCAAGCTTTTTTATGGCTACGATTCCGTACT
ATGACATTGTCATTCTTTAGTACTTAGTTATTCTATGTCTGAACGCACTACACACGCAGTGTTCACTCTT
ACCACAAGCGGCGTAAGGACGCTGTTTGAACAACGCCCGGGTAGATCTGCCGTCTCTTTCTACTCGGG
AATATTCTAAAAAGAAACAGTCTGGTGCGGAGATTCATGAGATGTTATACTCGTTATGTAAGTTAGATT
GTAAGTTAATTTGATCAGTATTTTGAATGAAAAAAGTTTAGTTATAGTTTTATAGACACTGTAGTCTTGT
TTTTAGAAGATTGCACAAGCACTCGAGGTCTTAGTTACACTGTACTTAGGTACTATTTTCATCAGGACT
TGCTTCTAAACTGCAATGTTTTTTCACATCGCGTTATTGTCTCGTTGACTAAGTTATTTCTATTAGAACA
GAATGTTAATTAAGTTCAGATACTATTTATGCATTTATTTATAAACGTTGTTATTTTTAAATCGTTAACCCA
TTTGTTTTTGTTGAAATCTATGCAATAAATTTTATATGATA
>TCONS_00098395 geneID=XLOC_019679 scaffold_314:28602-31283(-) Splice junction overlap
GTCCGACGACTCGTTCGGCTACGACGACTACTCTGACGACGGCGGCTCATCCTCCGGCGCAGAATCCT
GCTCCCGTCTCCGCGACGCTCTCGAAGCCCGTCGGCGGGAGGTGCCAGCCGAGCCCCGAGCCCCCAC
CGACAGCAGCGAGGTGGAGTCAGACGACGACAGTTCATCGAGCAGCAGCGAGGAGGATGTATCATCA
GGCACGGGCGTGTCCACCGACAGCGAGTTCGCTTGCGAAGAACCAGCTGATAGTACCCCCGCCCCCG
CGATCACC
>TCONS_00098363 geneID=XLOC_019679 scaffold_314:31361-32133(-) Splice junction overlap
CCATGACGCCCGAACGCATCAGTCTGGAGGCTTCGGACCACTGTGCGAGGGAAATAGACGAAGACGA
GTGGACTGTGAGCAACTTTCTGGCGTCCGAGACTTCTGGCGCTGGCGGGCTTAGTGACGAAGACGAG
TCAAGCTCCGACGAGTACACGGACGTGGAAGAGAGCTCGGCGGAAGCGGAAGCGGAGGCGGAGGC
GGGGCCGGGCGCGCCGCGGCTGTCGCCCGTGTACCTGTCCGACGACTCGTTCGGCTACGACGACTAC
TCTGACGACGGTGA
>TCONS_00098451 geneID=XLOC_019669 scaffold_314:110586-113206(+) Intergenic
TTCAATACCGGTATTGAGTTCCGGTATTACAACTCAATACCGGGATCCCGGTATTAATACCGGTATTAGA
CTTCCTTGTAATAAATGGCATATACTGTGTTTAAAGGTCGTATACGACAAAATAAAACAAGAAAATGCA
ATCATATTCGTATTTTTCTGTAGATTTTTACGAGAATTAAGTTTTAGAGTTTAAATTTTAGAATAAATTATT
TTTTTACATCCGGTGTCGGTTTACGCATGGTCAACAGTAGATTTCTAGCAGCCGAAAACTGCATTAAAC
AGTTGGGAACAAGTGCTCTTTCACAGTATATAGGAACACAAGGCTAACTAAATCTTAATTGCTTTATTTA
TAGCCTAAAAAAGTCCGCTTCCGGTATTACTTCAATACCGGTATTAACTTTCAATACCGGTATTGAAAAA
AGCGGGAATTTTGTCCGGGATCCCGGTATTACGGATACCGGTATTGCGGTATTGCATACCCTACTTGGGA
TACTAGGGAAAGTTATTTCCGCTATGGAAGCCGCCTAAGTCACCACCGCCCGCCCCGCCGCGCCGCCC
GCATACGAAACCGCCCCGCCGCTCCATTGACCGCTGTTGCTCCACGGACTGCTGTAGCCACCGCTGAC
TGCTGTAGCCACCGCTGACTGCTGTAGCCACCGCTGACTGCTGTAGCCACCGCTGACAGCTGTAGCAA
CCGCTGACTGCTGTAGCTACTCTTCCGCCTACTTCGGACTCTTCCGCCTACGATCACACCCTACATTGA
ACACTGAAAGCTTAGTTTCCAAGAAAATTTATTCCAAGTATGAACTGGGAAGCTAAACACAGTGTCTAT
AAAGCCC
>TCONS_00098407 geneID=XLOC_019669 scaffold_314:112177-113235(+) Intergenic
AAGCGAAGCGCGCGGGAACATAGATATATATACCTACTCACGAAGTACGTAGATGCATAGATTTGTTAA
ACTGTATTGTTATTGAAAACATAATAGAATATTTAATTGTTTTTCGTATCCTTAATATTTCGAAGCGTATCT

TATTGTATTTATGAACAAATCGCAATTTTAAAACGCCTACTTATTTTTGCAGTTATTTCCGCTATGGAAGC
CGCCTAAGTCACCACCGCCCGCCCCGCCGCGCCGCCCGCATACGAAACCGCCCCGCCGCTCCATTGAC
CGCTGTTGCTCCACGGACTGCTGTAGCCACCGCTGACTGCTGTAGCCACCGCTGACTGCTGTAGCCAC
CGCTGACTGCTGTAGCCACCGCTGACAGCTGTAGCAACCGCTGACTGCTGTAGCTACTCTTCCGCCTA
CTTCGGACTCTTCCGCCTACGATCACACCCTACATTGAACACTGAAAGCTTAGTTTCCAAGAAAATTTA
TTCCAAGTATGAACTGGGAAGCTAAACACAGTGTCTATAAAGCCCGTCACAGACTTTGTGTTAAGTAA
GTTAGT

>TCONS_00098382 geneID=XLOC_019669 scaffold_314:112413-114487(+) Intergenic

CGCCGCGCCGCCCGCATACGAAACCGCCCCGCCGCTCCATTGACCGCTGTTGCTCCACGGACTGCTGT
AGCCACCGCTGACTGCTGTAGCCACCGCTGACTGCTGTAGCCACCGCTGACTGCTGTAGCCACCGCTG
ACAGCTGTAGCAACCGCTGACTGCTGTAGCTACTCTTCCGCCTACTTCGGACTCTTCCGCCTACGATCA
CACCCTACATTGAACACTGAAAGCTTAGTTTCCAAGAAAATTTATTCCAAGTATGAACTGGGAAGCTA
AACACAGTGTCTATAAAGCCCGTCACAGACTTTGTGTTAAGTACTTAGGCAAAAGTTTGCGGGGCATT
AGACCGGAAACATCTAAAGGAAATCTATTTTTGTTAAATCTAGTCACCATAATACTTGTCAAGACAAAC
CCTTCAGGTTTAATGGCACGTTAATTTAAAAACCTAACTTAGACCTGGTTTCTCATGATTCTGTTAGTTA
CAGTCTCAATAACGGATTTGCTGACGACGCGTTTAAGGATTATTACCCTTGTTAATTGGATGAGTCCCA
CGACGAAAATCTAATAGACCATTCATAAATCAAGCATTATGTGAATCATTCACTCACACTTCATTCAATA
TTCTGTCATATTCATTGAAATTGGTGTCTATGTCTCTATAATGTCAAACAACCTCAAACAACCAAACTTT
TTGGTTTTGAGGTTGCCATGGTTACTATTTCTATCGAGGTTGTTTTTTGACATCCGTCATTGAAAATAATA
TCATTACGCCATCATTACGCCATGCCCTTTGAAAAACAATACAACGAACTAGATGGAGTGTCAGTGCAA
AAGAAAGGACTCAACAAATAACACCTCAATTTCTAAGTAAGGTCTTGTTTTAAAATATGTTGTTAGAAG
ATTTTTCACTTAATTCAATAGTTCTCATTAAGTCATTAGGTTTAACTTCTTATATCTACTTCTGTGGTCTAG
TGGTAGAAGCTCAGCTTCATCACCCAGGGATCCGATTCGATTTCCAGGTAGGGCCATCAATTTGTGTTT
CTATATTGCGGTTCCAAGTTAGGTTAGGACATTAAATTTAATCTTTTATCTCGGTTTGACATTATATAAAA
CCCAGACAGTATTTCGCTTAATTTTTTATGTCACACAACATCTAGTTCGTATATTGTTTATCAAAGGCTCT
GTTCATTTTTCCATCTTGAATCTTGACAGAATGAGTGACAAGTAAACAACCCGTTGCCTGGTTACCGGT
CTTAACCAAGTCTTAGCGGAACCCCACAGCAGCAATCAAGTTTATCACCCGATCAAGGGAGTTAACCC
CGTGATTTAGCATGGTGGGACACTCACTAACTTTAACGGTCCA

>TCONS_00098284 geneID=XLOC_019676 scaffold_314:220182-227346(+) Exonic overlap (+)

GTCGTGGGTCGTGTGTGAAACCGCGCCGTCATTTGTTCTTAACAATTGTGACGCTTCCGCGAGTGATAC
GCAGTGCTGGTTGTATGAGTGTGTGCGGTAGGCAGGCGGTGCAGGGTAGGCGGGGTCCACGTGAGCG
CCGAAACAGTTGCCACCGCAGGCCGTCGACCCGCGCCCGCGCACGTCACCGGCCTTCTAGACTATTCA
CCCATTATGCCACCTACATTTCGCTGATGCGATAAGTGACGACATCAATTAACGACCTCAGTGTTCCAG
TGACTATTAAAATGAAATACAAATTGAAATTCTAATCACAGTCGAGCGGTTTGATCGAGAACAATGCAC
GGCATATCGCAGTTCGTGCACAAGCACGAGTCCCGGGAATCGACGCCGCCGCTCGGCAAGGAGGGCA
GCGGGGAGA

>TCONS_00098285 geneID=XLOC_019676 scaffold_314:220399-227346(+) Exonic overlap (+)

CTGTGGCCACCACATGGTCGCGAGTGAGCGCCGAAACAGTTGCCACCGCAGGCCGTCGACCCGCGCC
CGCGCACGTCACCGGCCTTCTAGACTATTCACCCATTATGCCACCTACATTTCGCTGATGCGATAAGTG
ACGACATCAATTAACGACCTCAGTGTTCCAGTGACTATTAAAATGAAATACAAATTGAAATTCTAATCA
CAGTCGAGCGGTTTGATCGAGAACAATGCACGGCATATCGCAGTTCGTGCACAAGCACGAGTCCCGG
GAATCGACGCCGCCGCTCGGCAAGGAGGGCAGCGGGGAGA

>TCONS_00098374 geneID=XLOC_019676 scaffold_314:262297-265436(+) Unclassified

AATTTCTTTAAAATTTACTGTACCTATATGAAAAAATATACTGTAGGTTTACATAACTATATTATACTAAAA

TTATAACCATTACATGTATACATAACCATGGTAAAGAATTTGATAAGGGAACTTTATAATTAAATATTTATT
GTAGCATGTAAAATTAAACAAACACTTCCTTTACTAATGTTCTTCCTTGCGGGATAGGCTGTGGCGGTA
TCGTGTATTTATAAATTATGCGGGTAGACCTAGATACTCATTAGCGTTGCTACAACTTGCAACGTATTTA
GTGTGTAGTGAATAGCCCGCTTTATAACGTGTTTAGACATTGTGAAACAGGTGCTTAGTATTTATGGTCA
ATTCAATCTCCTCCCCCACTTTAAATTTTAAATATGATAAATTGAACCATATCTTCATTTTCTACAATTGTT
TCAAGTATAAAATATTATGCTCTTAGCAGGTATGTTCATGTCTATTGTGTAATCACATATTTATAATTCGTA
ATCTTATATTCCCATTCTCATAGAGTATTTGGCGAACTAAGGGTGAACCCTAACAATATAAGAACTGTTT
ACATATTAACTAATATCTTAAGAACGAGTAAAGTAGTAATGTTGGCCAATTAAAGCCTAGATATATGTGT
TAGTTTCAATGTATGTAGTATGATCAAAGTATGTTGGCCAGAGATCGGATAGTCCGGTGTTATAGTCTAA
ATACTTAAAGCTACTTTTATCTACATCACCAAAGCTAATTTTAGTTAGCGAAACACTTAATTTATTTTATT
CGGGATGCCTATTTTACTATGTCATAGCCACACTTCAACTCCGAAGTGTTAAATTAGGACTACTGGCTAA
AAGTCAATACAACAATACGTGCCTACTCTGAAAGGGAATTAATGGCGTGAATGTAAAAAAAATATGTA
AGAATGCATCCGAATATCCGATCTTTGTTGATTGCGCTAGTTTCTAAACGCCATACCCATAAATATTGCT
CAGACTTTTCGCAAGTATTATAATTGTGTTAATTTATTTATAAATAGTTATTGATCACTAAAATAGTTATCT
ACATAAGCGTTTTAACCATGAGCGGAATATGTTCGTGTACTAAATATATTTATGTCTATTGTAGTTTTAGT
AGTATCTTTGGGACATTGTGTAAACTCATCATCATCATAATTGTAAATACACTTAAATATGTAACGGCTGT
AGTGAGTGAAATAATCCCCTAATTATTGTGTAAATGAAACTCGATTTGGTTAGACTAAAGTCGGGTTGT
ACTATTCATATTATTTTGCACAAATATGTATGTTATCGTAAAACGAGTGCATTTAAACACATTGTACATTG
ACGTTTGAGAATCTTAGAAAGACGTAGCCCTGTAGGTTATTTCTAGATTGTGAATATTTACCACAACTTA
CGTCGATACTCCAGACACGAATTACCTTATATCCTTGCTATTTATATACAATATACGAACCTACTTATGTG
TTTCTTAAATTGTACGTCTGATGTGCACAGACTATTGAAAATGCCTTTATGGGCAAACAAAAAAATACCT
ATGTAATACCTAACACGCACAGGATGATTAAAAGCATGATACATTTCTAGAAATAAAACATGTTTCGAG
TATTGTTTTTGTTTTATTTTTAGGCTACAAACACAATCCGTCTGTTCGCTCCTTCAGGTATAAACAAACA
TCCATGAGAAATATATTTGCTGTTGCTTGACTTTCCTTTACAGATAGGTACATTTATTAGGCGTTTTTTTT
TCTGTTGCGTTGACAGATATCGGACATAATGACGACAATAGTATTATCCTCGCGTTTTACCATTTGCAGA
ATTGTTCAAGTGTTATTAGGTTATAGGGCTCGTAAATGCTATTGCACCGCAAGCAATACCAGTGTAATTG
ACGGTGTTACGACGCTGGCGTAAACTTTATTATTACTTCGACTAAGCTTCAGCCTCAATAGTCAATAGCA
TCGGGGATTATGCTTGAACGTTTCTGATCTGTTTCATATTACTTGTTTGAAACCACCACATATATCGTGTC
TTTTATCTGTCATCGTAACAAAAATAATAACGACCTGTACATTAACTAAGATACATTATCACAATGACTT
GATATCATAGCAGAGACGAATATTTAGCGAGTAAGTATATAGAATAGCTACAGTCTTGGTCCAGAGAAT
CGTTAACTAAAATACTTAATCAACTTAAATACTAAACAAGTTGTTTAGTAATCTGTATTAATAATCAAAG
CAAAGCAGTTTGCTGGTAACAGTACAGGAATCTGTTAAATTATGGTAGCTAGGTTCATACTTAATTCGA
GAACAACAAGGAATAATGTCAGGATATAAAATATGCAAAATAACAATGTCTCTGCTAAGACGACTAAGT
AGATAAACAGCGATTTACACATTTATATCGTTAAATTGATAATATGCAAGTAGTCGGCGGAACAATACAT
TTATTAATAGACTAAATAAAAACTGGGTCATTAACTAAAATAGTACCTACTAAACAAAATATAAAAAATG
GAAGTTTTTTAATTCTACTGATAAAATGGTGCCTTTCTTGCGTTAATGTATAATATAGGTTGAATTGTGAT
TTTTTTAAATAGGTATAAGTACATAGTCACAATTTCATTTCTTTCTATTGAAGGGATACAATTCGTATTCAT
ATCTAACATAATCCTTCGATAAATTTTACTCTCAGTGGGTTTAATCTTTTGGGATTGATAGGTCACCCAA
CAAAATTACAAAATAATCAAAATTTCCTACTTAGTAAGAATATGAAACGTTTCAGTAAGAAGTCCTAAT
AAATAGTAGAAAATGTAAGCATACTTGGGCGTAATCCTTCCATACAAATACATTTATACAATAACAATTG
GAACGATTATTCGGTTACATAATATGCATTATACGAGTACCTACTACAATTATATCAACGTTACACTTATT
AATACGTAGTAACAATTTGAAAATATTAAAACTTTGGTTTCTATCTAAAAAAGTAAAAATGGT
>TCONS_00098493 geneID=XLOC_019709 scaffold_315:283795-287233(+) Unclassified
ATTTATTTGAGGATGAGTTTAAGTTTTTAATATTAATCACAGCTATTACTGTACATAAACATTTATTTTAAT

ATTTTTGCTGAGTTACGTTCGGATATTTCAATTTCGGATTTGTATTATTTTTGAGAACGATTTTGAACGAA
TCAAAATGGATGCTGGGTGTGAATGAACTACTGAAAAACGAATTTAAGTTGTGTCGTGGGCTCTAATA
AATCGGTAATTAATCCGGCATAGAAGTTTTGTTTTATAACTATTTAGTCAGTGACAAGCCCACGAGTGC
CCTACAGTAAACAAGTGCCCGTGTTCACTCGTCGATTTGATGGTTGCCGTGTCGGAATGTGTGTAGTG
GAAGGTTTCGACGGAGCTTTTTACACTCGCAGGTGGCTCTCGTAAGATGAGGACTCGGGTGGTTCTAT
TGAATGTTGATCCGAGTGCGGGAGTGGTGCGAGAGCAGCCGCCGCGCCCGCAGGGCCCGACATGGCG
AGTGTACGCGAACTAAAGTGTCATTTACGTACTGTTTGAGTGGTCAGAAATGAAATGCATATACAGGA
AGGAACAAACACAACTCTGACAACATGTACCAAAATGACAGAGGAAGTAGACAGAAGACATGGCGT
GAATGGTAACAGCGGCAGCAAGGGCGGCGAGGAGCCCGCGCCCTCCGCGGCGGCGCCTGGCGGCAG
GCTCAAGTTCTTCAAAGATGGCAAGTTTATCCTGGA
>TCONS_00098808 geneID=XLOC_019745 scaffold_316:6-996(+) Intergenic
GCGTCTAGTACGGGCTTGCAATATATGTCAACGTTGATAGAAATCGTAAGCACGCCAAACTATTAGTTT
CCAAGTTAATTGGTAGTATGAGTTACATTTTTTTATTATAGTTCATGCGATATCGTTTATGCTACAACTCG
ATTGTGCGAACTTCACTCTAGATACTTTACCTCTTTCTAGCGCTTGTAAAAAGAAAGGGCAAACACACA
TGTCATTTTGGCGTCTTGATTTTCGTATATTATATGTCAATGACTCTAACCAAAATATATATGTCAATCAAT
GTCAATGACTCTAACAAAAACAAACTGAAATTTGGTTTACAAGCTTTTATTTATAAAAATTATAGAATTA
AGAATGATCCTCACAAGTGCATCGCAATTCGAAGTACCTAATTGTAGATTTTATGGAAAGGAATGGGGA
CCTAAACAAGCCTGCCTATGGACCCCACAGGTTAACTGCCATAAATAAATGTCCGTACATGGTGTAGAA
TCTCCTACGACCTATTCTGCACATGTGGGGAGCCCAAGACGCTCTGTCGTTGGACGAAGCCCAAGAAG
CCCTGTCGTCGGAAGAAGCCCAATACTTGTTGCCCCACTGAGACCAAGGCAGAGTCCTAGGACAACC
CGTCGACGCGTGATTCGTCCGAGGCAGTCAACGCCGCGACGCTATCGTCCTCTCGTGGGGATTTAGGC
CATTTATGTAATGGATAACTATATAAGTAATAAATGATTATTTTTAAACATTTATTTTTCATTTCTAAGGGT
GCTTACGTAAAGAATCGGGTTCCCTGTATCTACCCGTACCGGTAACTTGATTAGGTATCGCTCACTGAG
AAATTTAAAACCGTGAAATGCTCTAGCGCACTCGCTGAAAGATAAAC
>TCONS_00098688 geneID=XLOC_019748 scaffold_316:93768-96610(+) Exonic overlap (+)
AAACATCTTTGTAGGTTATGTTATGCAGGGTAAAATATTATCAGTGAACTTGCCGTAGGCAGTGCAACC
TGACATACCTGGTACTGAACTTTTATCAAACTCTGAAACCCCAATCAAACTGATGTTCATTCATACTAA
CGTCAAATGGGCAAAGTATCTACCTACCTAACTACTCTTCCAAATTGTACGGAACAGCGGGATCTTCGT
ACCTACTTTCGATATTTAGTTGCTAATGGAAATCAGGTGTTATGGCGCGCGTAAAAGTGGAACAGGGGT
TGCTGGAAGGCGCTTGGTGCGAGAATGCGGAAGGGAAATACTTCGCGTTCAGAGGAATACCGTACGC
AAAGCCGCCTGTTGGAG
>TCONS_00099086 geneID=XLOC_019782 scaffold_317:62010-65918(-) Unclassified
GCCGACCCGGCCGCGCCCCCAGGTGACTCACACACACACACACACACAAATTACCTTAAGTTACCGA
CAGGGGCGCTGAATCCAGATGTCGTACAAATTGCTATCGACTTGAGTCGACGATCTTCGTGATTTATTTA
TTCTTTTATTTAATGCACAATATACAAAAGTAATTTTGTACAATCAGGTGGACTTAATGCTAAACGTATTT
TATACCAGTCAACCTACATGAGGTACAAGGAGGAAAGAAAAATCTGTATTTGAAGTATTGTTTACTTTG
ATTGCAGTGAAGGCGGCGGAGGGCGCGGGCTCGGGCGGCGACTCGTCGGGCGACGACGACAGCGAC
GACGACGATGACCGCGTCGCGCAGGAGAAGCACGATGAGCCAAAGTTCTACAGTGACGGTGATAAGA
CTGAAGAGTCCAACAATCATGCTGCGAAGGAGGCCGGCGGCAAGTGAGACCACGAGCGAGTAACAA
CATCTTCATAACGACCTGTTGCTTGAAAGCAGAATGACGGAACAAAGGTATTGAATTTCGACAAGTAT
TCATCCGTACATACTAATATTGTAAAAGCAAAAGATTTTTTGTTTGACTATGTCTCATGCAGAAGGGTTA
GTACGGGGCGCATTTACGGACGGAGAATTCTTGTCACGTCTTAATGGCGCCCTGTGCTACACGGCCAG
ATCAGAATTCACAGTATATTTTTTTATTTATATCCTGTACTTCCTAGGAAAATCTGCAAATATATCAGGGT
AAAACTTGTCAATTGATCTTTTTTTGTCATCTGCTTTTATACAACAGATCCTATGTTAGTTCTGGCAGATT

GTCTACGGTTTCCCATATCAATACATGATAATTTTTCATTATTTGTAAATGGTAAAATTCCATTCCATTTTT
GACGAAAGTTCTATTCTGATAATCTTCACGACGTTTTTTTATAACTGATCATGTTTTTTCAAACCAAAGT
ACTGCCTTTATACAAATGGTCGATTTTCAAAATAAATCTTATCTTTGAGGCCTTGGGAAATAACTGGATG
ATTGAAAATACTATTTTTACCAAACCAGTAAAATATGTATGTAGATAACGAAGACACTACCACGATTGCC
TTACGTTTTATAAAGTAACTTGTAATTAGGAACAATATTTCAATTTAAAAGCTTTTAAATTTATATTTCTTA
CAAAATTAGGTTCATAGATTTAAATTGGTATTAATTTGGTTAAGTTACTTAAATAATGCCTAATATTTAAA
GCCTTTTTGAAACGTCTGAAGCGGTTTTAGTGTTTTAATACAGCTCAATCGTAAATTACAAAATATTAAA
GTGATTTTTCTTAACTTAATTAATCGCATAGGGTTGTAAATATCATGAGAAGTGTCAAATATAGATTGAG
TGCCTGTTTCACAATGTCTGGCTACAGTGCGGACGCATCATATAAAGTATTCTTGACATTTAACTTCTGT
CAAGTGGCTCATCAAAGTCATCAAAGATTGCCCGCGGCCGCGTCTGTACAATAACGAACATGGCCTTT
CGTAGTACTATGATTATTATTTGAATGACGAAGTTGTGGTCATCATATAACCACAAATAAACTATTCAGG
ACTTATGCAAGTATACATTGTGAAACAGAACCTTAGTGCAGTTATTTTTAGATCGAACTGAGTGTGAAA
CTTTTTATGAATTCTATTTATTGAAGGCCTACTAACAAATTGATACCTAAAAACGGTACTAACTTTTAAAT
ATTAACCTTAGTTTTATAATCGTAAATTTTATGTCAACATAATATTTAATATTTACATGAGTTTATTATAAAC
TGTCATAGTTTATATTTTGCTATTTTAATTGTTAAACTCTGGTGATTAGTTTACGGGTGAACGTGCAGAGT
TGGTTTGTAAACTATCACCGGTTGTATGTATATTAGTTCCGTAGGTTATAGGAAGGTGCATTGTGTAGCA
TGTAAAAGTTAAAAATATATTTTATATTACGGGCTAGCTTATTTCCAAATATGTTAAAAGTATTAATTAAC
TGGAATGCATCTTATTTGCTTTAACGGCGCTTTATTTCAACAACAACAGTCTGTACTTCTCCGATATTCTA
TAAATCTTATTTTTTCCGACTGATTTTGTCACTACTGCATGATTGGCATCAAAATGTCTGGAAAAATAGG
CATAAGTACATTTACAGCCCTATCAGTAAAGTTGACATAAAGTTCAGAGCTTTATATCATAACATAATTTA
TAGTGACAACATGGCAAATATGTCAATTGTTAACCTAATAACTTATTCCATATTCTCATATATTGCTTTTCT
ATTCAATTCGGCTACTGCTGTGCCAATCGTGACAATCGGTCTATTCTATATCTGATGGATTTCCTTATTGT
GAAACTCTTATTTTCTTTGTAATGATTAAACAGCAGTTATTAGCTAAAATAACAATAAGCCTAAATGTTA
ACAAGTTACAATGAATAGCGTTTCTGTTATATTTCCAAAATATTGCTAGGCAAATTAATTAGGTTTAAGAT
ATTGTTTAATTTCAGTTGACAAATATAC
>TCONS_00099106 geneID=XLOC_019782 scaffold_317:62010-65918(-) Splice junction overlap
GCCGACCCGGCCGCGCCCCCAGGTGACTCACACACACACACACACACAAATTACCTTAAGTTACCGA
CAGGGGCGCTGAATCCAGATGTCGTACAAATTGCTATCGACTTGAGTCGACGATCTTCGTGATTTATTTA
TTCTTTTATTTAATGCACAATATACAAAAGTAATTTTGTACAATCAGGTGGACTTAATGCTAAACGTATTT
TATACCAGTCAACCTACATGAGGTACAAGGAGGAAAGAAAAATCTGTATTTGAAGTATTGTTTACTTTG
ATTGCAGTGAAGGCGGCGGAGGGCGCGGGCTCGGGCGGCGACTCGTCGGGCGACGACGACAGCGAC
GACGACGATGACCGCGTCGCGCAGGAGAAGCACGATGAGGGCTTTACAAGCTATAATGGTGTGAGAA
ATGGATTTGGAAATAAAGTCACAACCGTATAGTACAAATTACAAATGTCCAAAGTTCTACAGTGACGGT
GATAAGACTGAAGAGTCCAACAATCATGCTGCGAAGGAGGCCGGCGGCAAGTGAGACCACGAGCGA
GTAACAACATCTTCATAACGACCTGTTGCTTGAAAGCAGAATGACGGAACAAAGGTATTGAATTTCGA
CAAGTATTCATCCGTACATACTAATATTGTAAAAGCAAAAGATTTTTTGTTTGACTATGTCTCATGCAGA
AGGGTTAGTACGGGGCGCATTTACGGACGGAGAATTCTTGTCACGTCTTAATGGCGCCCTGTGCTACA
CGGCCAGATCAGAATTCACAGTATATTTTTTTATTTATATCCTGTACTTCCTAGGAAAATCTGCAAATATA
TCAGGGTAAAACTTGTCAATTGATCTTTTTTTGTCATCTGCTTTTATACAACAGATCCTATGTTAGTTCTG
GCAGATTGTCTACGGTTTCCCATATCAATACATGATAATTTTTCATTATTTGTAAATGGTAAAATTCCATT
CCATTTTTGACGAAAGTTCTATTCTGATAATCTTCACGACGTTTTTTTATAACTGATCATGTTTTTTCAAA
CCAAAGTACTGCCTTTATACAAATGGTCGATTTTCAAAATAAATCTTATCTTTGAGGCCTTGGGAAATAA
CTGGATGATTGAAAATACTATTTTTACCAAACCAGTAAAATATGTATGTAGATAACGAAGACACTACCA
CGATTGCCTTACGTTTTATAAAGTAACTTGTAATTAGGAACAATATTTCAATTTAAAAGCTTTTAAATTTA

TATTTCTTACAAAATTAGGTTCATAGATTTAAATTGGTATTAATTTGGTTAAGTTACTTAAATAATGCCTA
ATATTTAAAGCCTTTTTGAAACGTCTGAAGCGGTTTTAGTGTTTTAATACAGCTCAATCGTAAATTACAA
AATATTAAAGTGATTTTTCTTAACTTAATTAATCGCATAGGGTTGTAAATATCATGAGAAGTGTCAAATAT
AGATTGAGTGCCTGTTTCACAATGTCTGGCTACAGTGCGGACGCATCATATAAAGTATTCTTGACATTTA
ACTTCTGTCAAGTGGCTCATCAAAGTCATCAAAGATTGCCCGCGGCCGCGTCTGTACAATAACGAACA
TGGCCTTTCGTAGTACTATGATTATTATTTGAATGACGAAGTTGTGGTCATCATATAACCACAAATAAAC
TATTCAGGACTTATGCAAGTATACATTGTGAAACAGAACCTTAGTGCAGTTATTTTTAGATCGAACTGA
GTGTGAAACTTTTTATGAATTCTATTTATTGAAGGCCTACTAACAAATTGATACCTAAAAACGGTACTAA
CTTTTAAATATTAACCTTAGTTTTATAATCGTAAATTTTATGTCAACATAATATTTAATATTTACATGAGTTT
ATTATAAACTGTCATAGTTTATATTTTGCTATTTTAATTGTTAAACTCTGGTGATTAGTTTACGGGTGAAC
GTGCAGAGTTGGTTTGTAAACTATCACCGGTTGTATGTATATTAGTTCCGTAGGTTATAGGAAGGTGCAT
TGTGTAGCATGTAAAAGTTAAAAATATATTTTATATTACGGGCTAGCTTATTTCCAAATATGTTAAAAGTA
TTAATTAACTGGAATGCATCTTATTTGCTTTAACGGCGCTTTATTTCAACAACAACAGTCTGTACTTCTC
CGATATTCTATAAATCTTATTTTTTCCGACTGATTTTGTCACTACTGCATGATTGGCATCAAAATGTCTGG
AAAAATAGGCATAAGTACATTTACAGCCCTATCAGTAAAGTTGACATAAAGTTCAGAGCTTTATATCATA
ACATAATTTATAGTGACAACATGGCAAATATGTCAATTGTTAACCTAATAACTTATTCCATATTCTCATATA
TTGCTTTTCTATTCAATTCGGCTACTGCTGTGCCAATCGTGACAATCGGTCTATTCTATATCTGATGGATT
TCCTTATTGTGAAACTCTTATTTTCTTTGTAATGATTAAACAGCAGTTATTAGCTAAAATAACAATAAGC
CTAAATGTTAACAAGTTACAATGAATAGCGTTTCTGTTATATTTCCAAAATATTGCTAGGCAAATTAATTA
GGTTTAAGATATTGTTTAATTTCAGTTGACAAATATAC
>TCONS_00098995 geneID=XLOC_019782 scaffold_317:62012-65911(-) Splice junction overlap
CGGCCGCGCCCCCAGTGAAGGCGGCGGAGGGCGCGGGCTCGGGCGGCGACTCGTCGGGCGACGACG
ACAGCGACGACGACGATGACCGCGTCGCGCAGGAGAAGCACGATGAGGGCTTTACAAGCTATAATGG
TGTGAGAAATGGATTTGGAAATAAAGTCACAACCGTATAGTACAAATTACAAATGTCCAAAGTTCTACA
GTGACGGTGATAAGACTGAAGAGTCCAACAATCATGCTGCGAAGGAGGCCGGCGGCAAGTGAGACC
ACGAGCGAGTAACAACATCTTCATAACGACCTGTTGCTTGAAAGCAGAATGACGGAACAAAGGTATT
GAATTTCGACAAGTATTCATCCGTACATACTAATATTGTAAAAGCAAAAGATTTTTTGTTTGACTATGTC
TCATGCAGAAGGGTTAGTACGGGGCGCATTTACGGACGGAGAATTCTTGTCACGTCTTAATGGCGCCC
TGTGCTACACGGCCAGATCAGAATTCACAGTATATTTTTTTATTTATATCCTGTACTTCCTAGGAAAATCT
GCAAATATATCAGGGTAAAACTTGTCAATTGATCTTTTTTTGTCATCTGCTTTTATACAACAGATCCTATG
TTAGTTCTGGCAGATTGTCTACGGTTTCCCATATCAATACATGATAATTTTTCATTATTTGTAAATGGTAA
AATTCCATTCCATTTTTGACGAAAGTTCTATTCTGATAATCTTCACGACGTTTTTTTATAACTGATCATGT
TTTTTCAAACCAAAGTACTGCCTTTATACAAATGGTCGATTTTCAAAATAAATCTTATCTTTGAGGCCTT
GGGAAATAACTGGATGATTGAAAATACTATTTTTACCAAACCAGTAAAATATGTATGTAGATAACGAAG
ACACTACCACGATTGCCTTACGTTTTATAAAGTAACTTGTAATTAGGAACAATATTTCAATTTAAAAGCT
TTTAAATTTATATTTCTTACAAAATTAGGTTCATAGATTTAAATTGGTATTAATTTGGTTAAGTTACTTAAA
TAATGCCTAATATTTAAAGCCTTTTTGAAACGTCTGAAGCGGTTTTAGTGTTTTAATACAGCTCAATCGT
AAATTACAAAATATTAAAGTGATTTTTCTTAACTTAATTAATCGCATAGGGTTGTAAATATCATGAGAAG
TGTCAAATATAGATTGAGTGCCTGTTTCACAATGTCTGGCTACAGTGCGGACGCATCATATAAAGTATTC
TTGACATTTAACTTCTGTCAAGTGGCTCATCAAAGTCATCAAAGATTGCCCGCGGCCGCGTCTGTACAA
TAACGAACATGGCCTTTCGTAGTACTATGATTATTATTTGAATGACGAAGTTGTGGTCATCATATAACCA
CAAATAAACTATTCAGGACTTATGCAAGTATACATTGTGAAACAGAACCTTAGTGCAGTTATTTTTAGAT
CGAACTGAGTGTGAAACTTTTTATGAATTCTATTTATTGAAGGCCTACTAACAAATTGATACCTAAAAA
CGGTACTAACTTTTAAATATTAACCTTAGTTTTATAATCGTAAATTTTATGTCAACATAATATTTAATATTT

ACATGAGTTTATTATAAACTGTCATAGTTTATATTTTGCTATTTTAATTGTTAAACTCTGGTGATTAGTTTA
CGGGTGAACGTGCAGAGTTGGTTTGTAAACTATCACCGGTTGTATGTATATTAGTTCCGTAGGTTATAGG
AAGGTGCATTGTGTAGCATGTAAAAGTTAAAAATATATTTTATATTACGGGCTAGCTTATTTCCAAATATG
TTAAAAGTATTAATTAACTGGAATGCATCTTATTTGCTTTAACGGCGCTTTATTTCAACAACAACAGTCT
GTACTTCTCCGATATTCTATAAATCTTATTTTTTCCGACTGATTTTGTCACTACTGCATGATTGGCATCAA
AATGTCTGGAAAAATAGGCATAAGTACATTTACAGCCCTATCAGTAAAGTTGACATAAAGTTCAGAGCT
TTATATCATAACATAATTTATAGTGACAACATGGCAAATATGTCAATTGTTAACCTAATAACTTATTCCATA
TTCTCATATATTGCTTTTCTATTCAATTCGGCTACTGCTGTGCCAATCGTGACAATCGGTCTATTCTATATC
TGATGGATTTCCTTATTGTGAAACTCTTATTTTCTTTGTAATGATTAAACAGCAGTTATTAGCTAAAATAA
CAATAAGCCTAAATGTTAACAAGTTACAATGAATAGCGTTTCTGTTATATTTCCAAAATATTGCTAGGCA
AATTAATTAGGTTTAAGATATTGTTTAATTTCAGTTGACAAATAT
>TCONS_00098982 geneID=XLOC_019782 scaffold_317:63088-64830(-) Splice junction overlap
GGGCTTTACAAGCTATAATGGTGTGAGAAATGGATTTGGAAATAAAGTCACAACCGTATAGTACAAATT
ACAAATGTCCAAAGTTCTACAGTGACGGTGATAAGACTGAAGAGTCCAACAATCATGCTGCGAAGGA
GGCCGGCGGCAAGTGAGACCACGAGCGAGTAACAACATCTTCATAACGACCTGTTGCTTGAAAGCAG
AATGACGGAACAAAGGTATTGAATTTCGACAAGTATTCATCCGTACATACTAATATTGTAAAAGCAAAA
GATTTTTTGTTTGACTATGTCTCATGCAGAAGGGTTAGTACGGGGCGCATTTACGGACGGAGAATTCTT
GTCACGTCTTAATGGCGCCCTGTGCTACACGGCCAGATCAGAATTCACAGTATATTTTTTATTTATATCC
TGTACTTCCTAGGAAAATCTGCAAATATATCAGGGTAAAACTTGTCAATTGATCTTTTTTTGTCATCTGC
TTTTATACAACAGATCCTATGTTAGTTCTGGCAGATTGTCTACGGTTTCCCATATCAATACATGATAATTT
TTCATTATTTGTAAATGGTAAAATTCCATTCCATTTTTGACGAAAGTTCTATTCTGATAATCTTCACGACG
TTTTTTTATAACTGATCATGTTTTTTCAAACCAAAGTACTGCCTTTATACAAATGGTCGATTTTCAAAATA
AATCTTATCTTTGAGGCCTTGGGAAATAACTGGATGATTGAAAATACTATTTTTACCAAACCAGTAAAAT
ATGTATGTAGATAACGAAGACACTACCACGATTGCCTTACGTTTTATAAAGTAACTTGTAATTAGGAACA
ATATTTCAATTTAAAAGCTTTTAAATTTATATTTCTTACAAAATTAGGTTCATAGATTTAAATTGGTATTAA
TTTGGTTAAGTTACTTAAATAATGCCTAATATTTAAAGCCTTTTTGAAACGTCTGAAGCGGTTTTAGTGT
TTTAATACAGCTCAATCGTAAATTACAAAATATTAAAGTGATTTTTCTTAACTTAATTAATCGCATAGGGT
TGTAAATATCATGAGAAGTGTCAAATATAGATTGAGTGCCTGTTTCACAATGTCTGGCTACAGTGCGGA
CGCATCATATAAAGTATTCTTGACATTTAACTTCTGTCAAGTGGCT
>TCONS_00098896 geneID=XLOC_019771 scaffold_317:79928-80631(+) Intergenic
AAAATTGTCATTCACAGTCATTCACTGTTTTGTGGGTTCGCTGTGCATTAGTGTTTTCTGTTTCCGATTT
CTACGCTTTTGCATTCTCATTTTGGACTTTTGTTTTGCTTTACGATTTGGCTGGACATATTTTTCTTGGAA
GGACGACCTTTGCACGGCATCACGGACTTTGATATAGCATACGATTAGTATTATTTCTCAAGGAGTAATC
AGGATCAGGAGGTTATAATGAGCGAGCCGCAGCCAGGTTCGTCGGGGACTGCTCCGCCGCCGCCGGT
CAAAAAACCAAAATTGGCTGTTAAAAAACAGTTATCGCAAAGGGAGAAAAAAATTTGGTGAATATTT
ATAATCACTTGGAGAAAACCTCTCCTACATACCCATCAAAAATGGAATTATATAAAACCACCTCAAAAA
TAATGGACGTCTCAGAATATATTGTTCGTGACGTAATGAAACATTACACACAAAATCAAGAAGTACCCC
AGCCTAAGGTATTTATAATATTAACTTAGCCAGCCTTTTTTTTACTCCTTTGAGCTTTACTTTATGTTATGA
TTTGTGCCAT
>TCONS_00098990 geneID=XLOC_019773 scaffold_317:94025-94704(+) Exonic overlap (+)
TATTACTTTTTCTAAAAAAAGATTTGTAGCAAGTAACCACAGAGTAAAATTACGAATCCTTGACTTGAA
CCACAGACAAATAAAAAAAATGAAATTTTTCCTCATTTTGGTTTGCGTTAATAATTTTGTAAACAAATAA
TTAACTTTAAGCTTTCTTCAGAAAAGATGGAAAAATAAACAGGCAAGACGGTACAGATGTATTCTAGC
CTACAGTTTAAACTATTTTTACTGATATTCGTGTGCATTTGTTGGTTACACGTCGTCTATCTGAGTTATTC

ATTTGCTAAAATATCCTACAAATATGCCGCTGACGGCTGATGTTGCTGCCCAGCAAGTACAGGTGAGTA
AATGTTTATAAATTACTTACAATTACCGGCCTCCGCCGGAATACCCACATCCTTTCTCTCTTGTATTCAAT
ACTGTTTAACCCATCATACCCGGCTTATCTTAAAGAGCGCTTTCAGTTCCGGTACGAATCTCACAACCG
TACACTGCGCTCTGCCCACAACTTACAACTAAATATTCCTCCCCATTCCACAAAATTCTACAGTAACTC
GTTTACTATACAGGCGATCACTTTGTGGAATGATCTCCCATTAG
>TCONS_00099038 geneID=XLOC_019777 scaffold_317:179110-180942(+) Intergenic
CCTTTCCATTGTGTCCTAAAAAAGAATCACACTTTCTTATACAAAGTGTTATAGAAAGTTTTCTTAAAAT
ACTATTGCAAGAAGATGGTAATTTTAAATATTTATTTTAGTAGACACAATGAAAAGATACATTTTAAGTG
GTATTTAATATATCCTGCCCTAATGGTTTTGTACATTTTTATATGCCGTAGGCGTCTTATTTCGAGACAACT
TATATAGCAGTTAGATCAATTTTACAAAAATATACATTTTGAGTTTAGTTACTTCGAGATAAATAAGTATC
TGAGTACATAGGTTCATTTTAAGCTGCATTTTACAAAAATAAAGTCCTTTTTAAGAGATCATGTGGCTGT
TTAAATGGAATGTTTATTTTGTCATCATGATTAAGTACTTATAACATTCTTAAATAAATAAATATAAAAATA
GTGATATGAAATCAAGGATTGCTTTACGAGTGGTCCATTTTTACATCTACAGTCTACAAATAAATGCATT
ACTAGGAGCGTCTTAAAAGCAGCTAATTATACAATACTGATAAGCTGTCTTGAACTTATAAAATAAAGC
TAGAAAATGGTTATATTACCTAATATGGAGCCCTTTATATAAAGTGATTTTCATAAACATGTCGTGTCTAG
CATTTAGTATCAGCAATGTGGGTTTTTTACTGTTTTAGTTAAATCTGCATAAATCTGTGTTGAAAATGGC
GTACAATTAGTGGCACAGATATCGCAACGAAGAATTATTTTCATTTGAAGCTATTGAACATAGGTCACT
ACTTAGATACGGGACGAAACTTTATCAATTTTTTTAAGCTGACTACAGGATGTTGCAAAAAGGGTATAC
TAAGTTTCTATGGCTTTTTTTTCACGAATTTTGAAATCCTGCTTTCAGTTTCGGGCTTAGATATAACCTG
CTGCACATGCTGGTTTCGGCTTAGTATACCCTTTTTGCAACACCCTGTATAATGAAAGCTCGACATCCA
GAAAATAGTAAGTAAGATTTCTAACCTTTCTGGTTTTGGAGCACTTAGATGGGTGGTTTCTAAATCAGC
CTTATTATGACTTTTTAAGTAATCAAGTACTATATAGGGCAGCATACTAAGAACGTAACCTTTCTTACATG
CGTAGACGTTAAATTCCGTTTGAATGTACCAGGACACCCTAGACCGGGTTTTTTTGGCTGCATATGACC
GGTTTTTTTTCAACTATAAAATACGATTTTGCCTTACAAAATACTAGACTTCTCAAAACTTTTCGGAAGA
TTAATATTTAATCGAGGTTAGATTGGAGTAAGTGTTATAGTCAGAGTGTGTCCACCAAAAGTCTACTAG
GTTGCAAAATAAACCAGCAGCCAGCATTATGATATACGATAGTCCGAGTGTGCTTACTAAAGATAAGCG
CCCACCTATCAGTATTGTAAGTATCGGACAAAACGGATTTTCACATATTTGGAGAAACGGCGTCAGCAA
TACCAGGCAGTGAGTGCGATGCGTCCGTTGAGTACGATGCTGAAAGACCTCAAAGTACCTACAAAGC
AAGTGAAAGTACCAGGAGCGCACAAAACAGCAGAAGGCAGCCTATTATCCAGTCTAGTTGATAATCCG
AATCTTCTCAAAGTACCAGGGTCGCACAAAACACTAGGAGGCAGCATATAATCTAGTCTAGT
>TCONS_00099050 geneID=XLOC_019787 scaffold_317:179213-180820(-) Intergenic
TGGTGTTTTGTGCGATCCTGGTACTTTGAGGTCTTTCAGCATCGTACTCAACGGACGCATCGCACTCAC
TGCCTGGTATTGCTGACGCCGTTTCTCCAAATATGTGAAAATCCGTTTTGTCCGATACTTACAATACTGA
TAGGTGGGCGCTTATCTTTAGTAAGCACACTCGGACTATCGTATATCATAATGCTGGCTGCTGGTTTATT
TTGCAACCTAGTAGACTTTTGGTGGACACACTCTGACTATAACACTTACTCCAATCTAACCTCGATTAA
ATATTAATCTTCCGAAAAGTTTTGAGAAGTCTAGTATTTTGTAAGGCAAAATCGTATTTTATAGTTGAAA
AAAAACCGGTCATATGCAGCCAAAAAAACCCGGTCTAGGGTGTCCTGGTACATTCAAACGGAATTTAA
CGTCTACGCATGTAAGAAAGGTTACGTTCTTAGTATGCTGCCCTATATAGTACTTGATTACTTAAAAAGT
CATAATAAGGCTGATTTAGAAACCACCCATCTAAGTGCTCCAAAACCAGAAAGGTTAGAAATCTTACTT
ACTATTTTCTGGATGTCGAGCTTTCATTATACAGGGTGTTGCAAAAAGGGTATACTAAGCCGAAACCAG
CATGTGCAGCAGGTTATATCTAAGCCCGAAACTGAAAGCAGGATTTCAAAATTCGTGAAAAAAAAGCC
ATAGAAACTTAGTATACCCTTTTTGCAACATCCTGTAGTCAGCTTAAAAAAATTGATAAAGTTTCGTCCC
GTATCTAAGTAGTGACCTATGTTCAATAGCTTCAAATGAAAATAATTCTTCGTTGCGATATCTGTGCCAC
TAATTGTACGCCATTTTCAACACAGATTTATGCAGATTTAACTAAAACAGTAAAAAACCCACATTGCTG

ATACTAAATGCTAGACACGACATGTTTATGAAAATCACTTTATATAAAGGGCTCCATATTAGGTAATATAA
CCATTTTCTAGCTTTATTTTATAAGTTCAAGACAGCTTATCAGTATTGTATAATTAGCTGCTTTTAAGACG
CTCCTAGTAATGCATTTATTTGTAGACTGTAGATGTAAAAATGGACCACTCGTAAAGCAATCCTTGATTT
CATATCACTATTTTTATATTTATTTATTTAAGAATGTTATAAGTACTTAATCATGATGACAAAATAAACATT
CCATTTAAACAGCCACATGATCTCTTAAAAAGGACTTTATTTTTGTAAAATGCAGCTTAAAATGAACCTA
TGTACTCAGATACTTATTTATCTCGAAGTAACTAAACTCAAAATGTATATTTTTGTAAAATTGATCTAACT
GCTATATAAGTTGTCTCGAAATAAGACGCCTACGGCATATAAAAATGTACAAAACCATTAGGGCAGGAT
ATATTAAATACCACTTAAAATGTATCTTTTCATTGTGTCTACTAAAAT
>TCONS_00099297 geneID=XLOC_019817 scaffold_318:199075-206758(+) Exonic overlap (-)
GTCCAATAAAGTAAACTGAGTGCGAAACTTGGTCACAATCGCATTAATTGTTTGCTCACTTGGTCGATT
ATGTAGACCATAAATCGGGCGTAAAGCGCGAAACACATTTCGAACCGAACACTGATTTTGGTAATAAA
ATTCAATGATTTGCAAGCGTTGCTCGTTAGTAAGTCTATTCATGATGAAATGTCAAAGCATACTGAGCAT
CTTTCTCTTTGACACCATGTCTGAAATCCCGCGTGATCTGTCAAATACTAATGCATGAAAATCCTAACCT
CAAAAAAATCACCCTTTACTATGAGGATATATAATTAATAAATATTCTGAGCAATTTGTCCGTGACATCTT
GCACTTCATCAATATGTTAGTGAATTACATATTGAGCGTTAGACACTTCAAACTGTTTTTCGCCTCACGA
ATCGAACACTTCGACTTAAATAAATCGCTGTGCCTTTTTCCGATGAGACGTTGAAAATCTAGTTATTGT
GGACTTAACAATAACCACGTGAAGCCACGAGTTGCTTCAACTCGGATTCAGACATCCTGACTTGTTTTT
AGCACTATAACACTAGAGTTGTTAAAAATAGTTTCAACTAAATTTACTGTCCACTAATTTTAAACTGTAT
GGTTGAGATATAATTTTAAACAACAAAAAAAATACTTTTAAGCGTGACTGCTCAAACCAAAGCGTACA
AAATGGCGGTTGCACACGGGAAACTTTGCTTGTTATTATCAATGACCTCAGGTACATAGTGAAGTTTCG
TGCCGTCTCGGGGAGGTTTTTAATGAAACTTGGAGATTTGATACTCGATAAAGATTATATTATTAACTTC
ACAATTTAAAATGTTAAATATAGTTTTAACAAAAACAAACTGAGTTGACTATGAGGACCGGAGGTGGA
GACAGAAGTGCCTGACTCTAGCTCCTGCGCCTGTTATACAGGCATGCATTGCGTTTTACTTGTACAATAT
CACACATTAGCACTACTCAGGAATG
>TCONS_00099175 geneID=XLOC_019826 scaffold_318:205603-221463(-) Unclassified
CACCTTATTACCTATTTTTCCTGTACCTCCTGCGGGTTGACTGGCAGAAAATGCTTTTAGCGTTAAGTCC
ACCCTTTGTACTTTTATTTGTAATATTTGTACAATAAAGAGTTAAATAATAATAATAAATAATCCGCTGGC
AAATTAAAATGTCGTAAAAGTCAGCAAATATTTTTGTTTATGAGCTTTCTAGAGCTAAAACGTGCCTAC
AGAGGTCAAAACACAGTTTAGTAACGCACCCATGCATTTCGTGATGATAAGGAAGACCATTCATATATA
AGAGGCGCAGGAGCTAGAGTCAGGCACTTCTGTCTCCACCTCCGGTCCTCATAGTCAACTCAGTTTGT
TTTTGTTAAAACTATATTTAACATTTTAAATTGTGAAGTTAATAATATAATCTTTATCGAGTATCAAATCTC
CAAGTTTCATTAAAAACCTCCCCGAGACGGCACGAAACTTCACTATGTACCTGAGGTCATTGATAATAA
CAAGCAAAGTTTCCCGTGTGCAACCGCCATTTTGTACGCTTTGGTTTGAGCAGTCACGCTTAAAAGTA
TTTTTTTTGTTGTTTAAAATTATATCTCAACCATACAGTTTAAAATTAGTGGACAGTAAATTTAGTTGAA
ACTATTTTTAACAACTCTAGTGTTATAGTGCTAAAAACAAGTCAGGATGTCTGAATCCGAGTTGAAGCA
ACTCGTGGCTTCACGTGGTTATTGTTAAGTCCACAATAACTAGATTTTCAACGTCTCATCGGAAAAAGG
CACAGCGATTTATTTAAGTCGAAGTGTTCGATTCGTGAGGCGAAAAACAGTTTGAAGTGTCTAACGCT
CAATATGTAATTCACTAACATATTGATGAAGTGCAAGATGTCACGGACAAATTGCTCAGAATATTGTAA
GGTTTTCTAATAATTACCCTAAATTATTGGGTAAATGGATTTTCTGTGAACTAGCCACACTGGTGGCCGC
TTGGTGGGGCGCGGCATTCGGAGCGCCAGTCTCAGACTAGAGCTCGAAGCGTTTGGACGTGTAAAGC
TAGCCACTTGTTATTAATAAATAAATTAGTGATTAACTGAGTTGAGTTTGAGTCTTGTTAAGAAAAATAA
AATAGTTCTTGCCAGTTTAACCTGAGTTTTGTTACAACCTTCGTCGAGACCAACAGATATAAAAGGAA
GTTCACATCTCAGAAGCCAGGGATAAACCAATTAAAATGTAAGTACACGCCTACCATTTAATTATGACC
TTACAAGCTGACTGTTAAGTTTTGTAATTTGTTTTTTTTGATAAAAAAATATTTATGTCACCCTAACCTAA

CAAAAAAATAACGGAAATGTCAGACTACCCACTTTTAAAGCTCGAAAAGGCATGAAAC
>TCONS_00099309 geneID=XLOC_019818 scaffold_318:206969-209018(+) Intergenic
GTTTCATACCATTTTGGAAACTGCATTAAATGAGCAATCTTTTCAAATAAGGTGCCAGGTTTGCGTGGT
ATATTTTTTTTTACAGTATGTAGAGTCAAAGTTGTTTATAAAAAATACGATTACCTTTTATGACTTTGAAA
ACCGTGTTTTTTTTAAACATACAGCATTACTCGATATTTTTTTGACTGCGATCAATAGGAGGGCTATACA
GGATGATGCAACACCAAACAAATTCTAACAATATGGATTTTTGTACCGGCGGCACGCAAAAAAACTGAT
CCAAGGATTTCGATTTTCAGCAAAGTTATAAAAGTGTCAATATCTCGAAAATTGTCGAGTTTTTACTAA
GGTCATATTGTGATATTCTGGCCTCTCATGAGCTGACCTATCGGCCCTAGAGCCAGTCCACACGGCGCG
TTGCGTTGCGTTGCGTCGTCGCAACGCAAATATTTTGACGCATAGCCGGCCACACGGGCGCTGCACCA
TCGCAGCGTTACTATGACGCAGTCTTAACGTTCCGTCGGCGCAGCGGCGAGGCACAGTTGCGGCAAG
CATTTTGAAAAAAAATCGCAGTCAGTCTATAGCAAATCATGGATCGGAAAAGACGTCTAGCATTGCTC
CTATTACTACGTCACCGCCGAAATCGACGCAAGATCACAAGACGGTACTGGATCAGTCCTTTCATTTCA
TTAAGAAATCGTGATGGACAGTTTTTTTTTTAGAATATCGGGAGTTGCTATTAGACGAAAAGAAGTTTT
ATAATTTTTTTAGAATGAGTGTATCCAGCTTCGAATGTTTATTGAAGTCCTTAAAACCACACATACCTAC
GAAAAAACTATACTAATAAGAGGAATCCAGTGGAACCAGTAGAAATGCTGGGAATTACTTTAAGATAC
CTACGAAGTGGAAATTCAATAACTGATTTACATTTCAAATTCAAACGGGG
>TCONS_00099390 geneID=XLOC_019887 scaffold_319:146114-156332(-) Splice junction overlap
GTTTGACAATCAGCTGTGAGTGGGGAGGAAAAGGCAAACTCTTGTGTGTTATGTAGTTGATGTCCTCA
AGCGTATCGTTGCAGTTGTAGCTATGGCGTGTTTAAGTCCGAGTAGGATCTGTGCTCGTGTTCAAGATA
AGTTATTGTGTCATTTCGGACATAAAGTTCAAGTTCGGAGGCAAACTGTATTTGTGAAACTTAAACCTC
CTGGACCCTCCCCATTGCGGATATTTGGAAAGACGTGCCAGCGCGTGACAACCTGCGGGCCAGCATGC
GGCTCGGCCGGCGGCGCCCCCTCCCC
>TCONS_00099582 geneID=XLOC_019946 scaffold_32:66909-87041(-) Splice junction overlap
GAAAAAATGTCGTCTCGTTTGGAAAAACATCGATTTCTCTAATTTTGGGACATTTATTTATTTTATTCAG
TTCTCACTTATTTTTACACTTTTATTTCGGTTTATTAATATCAAGTGACATAAAATTAGTGATCAGTTGTA
AACGGTATTCGTCCCAATCGTTTTTAGGGCAGTGATTTATAGTCGTTTACTTTCGGATCCGTTTTTCTTTT
ACAAACAAAAACATTTTACCGTAGCCAGCGATTAATATTTGCTTTTGATCGTATTGTGTTCTAGTGTGTC
GTGCAAGTTTACGTTGTGAAGAAATCTCCACTATTCCAAATAACTTTGGACGCCATGTTGATGTTTTGA
TTGTAATCGTGGGGTGTCGTGATATGGGTTATTGTTTCCGTGGCTGATCTTGCAGTGTGAGCATGTTGG
AGGATGTCCCTGTGCACGGCTGCAGGGGTGAAGGTGGAGGCAGAAGCGCCAAAGTGGCCACCCACT
CGAGAAATCTCAGCCTTGACTTCAGTTCCTAACATCGCTGACGACAATAGGGCGTCGTCT
>TCONS_00099721 geneID=XLOC_019954 scaffold_32:603667-604528(-) Splice junction overlap
ATTTAATAAAAAATATAAATGTGTTCACAAAAGAAATAACTGCATAATTTTATCATGAAATTTATAGCCC
AATAAAATAGTCCTCAAAGATAATCCAAGCTATTTCAATCCGACAAAAGTCAAATGACGGTAAAGATAA
TAAGGATTCCGACAAAAAAGACAAAGAAGAAGAAGCAAAAAGAGTCTTAGAAGAATGTTTTGGACC
CAAAAAACCAATTCTTGATGTTACTAAAGCACATCATGAATACTTCTTGAAATATTCCAAGCCACCTGA
AGTGATACAGCAAGAATGTAATGTTGTAATTAAAGAGGTATTTGGAGAAGAACAACATAATACGGCTG
TTACACATTCATCAATTAACAAAGACACCGACGAAAAATTGATAACTAAAATTGAAACTGTAGAAGAT
ACAGACACAGTAGATGTAACGAGAAACAACTTGGCTGATCAAAACCAACCACTAAAAATAATTGATG
GTAAGAAAGTTGAAAATTTAAATGAAAACAGTCCAGTTAATAAATTATTAAACCATAACTTAGATAACA
TTAAATTGGAAAACAATAAGCGAGTAGTACCCAAACAATATTT
>TCONS_00100120 geneID=XLOC_019958 scaffold_32:766187-770407(-) Unclassified
CAGCATTTATTGAATATTGTCATATTCCACATTAAGAACCCACAATAGGGCAGGGACCAAACGAATAAA
TGTAAAAATTAAACTAACAATGCTGGTTGGTTACCCGCAACCCGGCCAGCAAATACATTCCATTCTGAA

TCTTATAACAATGTGCTAGAGTTCAAAATCAAAATACCGTAACCGGGTTTAGGTTAACTTTGATTACCAT
TGAGTCACTACTGAAAATCCCGATAGGGTAAGTCAGAGGTGACTAATAGTAACTACACCGTACCCACA
CATGAACCTACGCCTCTCCTACACGGGTAGGCATAGACTATGGATATCCACTTACAGTAGTTCTGAAGT
AGACAGCGAATGTGCTTTTGTACCTTGTCAAACTCACAAACCAACTGAAGGACTAGCTGTTGGTCAGT
TGGACAAAGCACAAAAGAACTATCAAGTTATTTGGACCGAACTGTACTTCGTTATCGCCATCAGCATAC
ATTAACATCGATCGGCCGCCAATCCTCCACTACTGGACATAGGGCTCTCCCAATGAGCCAACACTCTGT
CCTTGGCCTTCCTCATCCAGCCACTACCGGCCGCCCGTCTGAGGTTTTATGTCTCACGCTTATATTAACT
ACAGGGTGTTGCAAAAAGGGTATACTAAGCCGAAACCTACATAAAATATTATTTTTAATCGAAACTATG
TTCGTCACGTGAGAATGACTTTTTTTTTCGCGAACTTTGAAATTCTGATTTCCGTTTCGGCTTAGTATAC
CCTTTTTGCAACACCCTGTATATACGAACAAGATAGTGCGTCAGTGTGTCGCATACAAAACAAGGCAG
ACAAAATATCATATCCGCACTTTTGTTTACTGTCAAACCGTTTTAAGTTCAACCTAATGACAACCATTGC
ACCGATGAATAAAGTTACAAATCTTCTAATCGAACTAATATTATAAATGCGAAAGTAACTGTGTCTGTCT
GTCTGTTACTCTTTCACGCCAAAACTACTGAACGTATTTGAATGAAATTTGGTATACATATGTTCTAGAC
CCTGGGAAAGAACATATGCTACTTTTTATCCCGGAATTCCCACGGGAAAACTTTTTAAGGCGAAGCGA
AGCTCGCGGGGACAGCTAGTTCAAAATAAGAGCTCAGTGGAACAAGTGGGTTATTTGTCGAGACGTT
ACTTTTGCACTGATACTCCATGTAGTACGTATTACAATATCTTTGCGTGACACATAATTTTAACAAACTTT
ATTATATTTATAGCAACATTACTAAGGCACGTTAAAGTGAAACTTGATTACACAACAATATTTTACAGTC
ACCCTGTTTCCGTTATGCCATTCAAGGATAAATCAATGATTAAAAATATACTGAACGTCCTTTTAACTAG
TTTTGCTAGAAAGAGAACTTTAAAACAGTATCTTTTGAAATCCCCAAATAGTATACGAAAACTAAGGAC
AGTATGAGCCAAAAAAGATGAGAATTTTGATAATTATTCATATTTTAAATCATTAGGAATGCATCTAAAA
TTAACAAAGTCCACTTTTCAAACAAATAATAAAAAATTACAATGCATAGATGAACAAATTATTTCCATA
GAGACCTGTGAAAGATTTTTCCATACAAAATGTTTGGCTTTTAAATGAATTTTGGATATGATATTCTCAA
TTTTTGTGTGAAACTGTGTTCACTTCATAGCAAAACAATGTTGTCTATGGAATTTGACATAAAATGGAC
AAAATGCATATTATATAATAATGTATGTGTATGTATGTACATTCTTTAAATAATACAATTAATAATAATAGA
ATTAACTGAATATATCTTGATATACAGGATGTTTAAACCAAGAATCGCGGTAAAATATATAACAAAAGGA
CACACACCCTCCAAATCGCTCAAAATAGACTACATATATAATATATAAAATACAATATATAATATAATGTC
AGTTCTTTCCTTTTGACGCTCCTGTGCGGTTTCAAAATTAACATTAAAATATGAAAAACGTAATAATAAA
CATGCTTGCGACTTTATAAATAAATGATTGTTTTAAAAAGATATTTTGTGCTGTTCGTAAGTTGTCAAAA
ACGTGTACCTAATGTTACCCCAAGTGATTTTAGTCTTATTTTATCAAAATCGGCTGAGCAGTTCAAAAGT
TACGCTACTTTGACTGCACTGCCACAAAAAACTTTAAACAAGTGTTTAAAACGAAATTTTACAGTTTTT
GAAGGCGTTTGACAGCGCTAAACACCTGTTTAATACTGTCTTGAGTTTTTGATGTAAGGCTTGTTGAAT
TTTTGGGGTTTTTACCGTTAGTTATGTTATCACCGGTGTCTTGTCAGGTTATCATCACCTACTATACAGGG
TGTTGCAAAATTGGTATACTAAGCCGAAACCTACATGTGCAGCATGTTATATCTAAGCCCGAAACTGAA
ATCAGAATTTGGAAATTGGCGAAAAAAAACAATTTTTTTCCATAGTAAAAGTCACGTGACCAACAAAG
TTTCTATGGAAAATGAAAATTTTTTTTCGCGAATTTTCAAATTCTGATTTCAGTTTCGGGCTTAGATATA
ACATGCTGCACATGTAGGTTTCGGCTTAGTATACCCTTTTTGCAACACCCTGTATAAAGGCATTTTGACG
AAACAGCACAAAACATACAATTTATTTATATGGGTCGACAACATATACAATACAAGAATTAACCTATACT
TTCATCCACATTTCGTGTTGAAATCACCAACCATTGCTTTTAAGTGACCAATTTCAGCAAAATATATATC
TTTATATAATAATTTTATATATTTTTAGATGTATTACTCAACTAATCCAAAAATATAAAGATTATTTACTCTA
CTTTATTTAAAAAACGAAATATCGCGAGGTTTTGGTAATAGTCGTTTTTATAAACAAAGATCTTATTTAT
GCTAATTTCATTTTTATAAAATAAAACGACGTTATTAGGCTCACTTAATTAGCTCACCTGGATCTTATATT
ATTTAAAGAAAGCAAGCAAAATTCACCGCATACGACGCGTGTGATTGGTCAAACCCAACAGTCTTGTG
CTGCCATCTAGCGGTACAAAATTGCTTTCAAATATATCACGATATTTCGTTTTTTGTGCGAGTATTTTGGG
AACTTCTGTTCAATAATGTCGATGGGCCACTGTACACAAACATAGGATGTTACATACCATTGCATAAAGT

ATATACTCATTCAGTAATTCAAGCAACCACTATTTTCATCACGTCTTTACATCAGATGACTGACTTACTTA
TTATATTACTAGCTAGGGACAGGGCCAACCGCTCAAGACATTGTTCTGACGGGCGCATCCTTCCTTTTG
AAATCATTGACTTTAGTAGGCAATTTATGTAAGTCCAGAACGGCATAAAAGTGAACTAAAACATAATCC
CTCACGTGATGATAAACAACTATAATTACTTCGAATATATACCTCATCGCAATGGCACAAAGCGCTTGAC
AAATGTTACCACAGTGGCGCCATCTAGTTCCCTACAAGACGAGGCGCTGTCCCTCCACGACGAGTCGC
TGCGGCCGAGTGGCGCCATCTAGTAGTCGTCGATGTCGTACAGGTTGTCGATCTCCTCGTCGTCCAGTT
GCATGGACTGGAAGTCCACTTTGTAGCGCAGAAGACCTGCGGAGAAGGAATTTAGGTTTATATCTGGT
TTTTAGCTGAATGGTTATTGCAAAAAAAAACGGTGATATGAGTCTTTTGACTTTGAAGTAAGTATTTTTA
GAGATATTTTTCTATACCCTGTTTTACGGGGAAAGACATCTGACAGAACCCTTATTACATTCGAATAAGG
GTAGTTTTTGTTTGTATCAGATGACTTTCCCTGTGAAATGGAATATAGCTTTCAATTTAATTAAACAACT
TTTATTAAAATACAAAATAATAAAAAATCTTTCTTTTAAAAAAGTATTATAAAATTCACACATCAAGCAA
TGAAATATTTAGAAATTTAGATGAATAAAGCTCACGCAAAATATTGGTAGCGATTCTGAAGGGAACAAC
ATACACCAGAGGAATAGCACCAAAAATTGTAAAAAAAAACA
>TCONS_00100239 geneID=XLOC_019958 scaffold_32:766187-770407(-) Exonic overlap (-)
CAGCATTTATTGAATATTGTCATATTCCACATTAAGAACCCACAATAGGGCAGGGACCAAACGAATAAA
TGTAAAAATTAAACTAACAATGCTGGTTGGTTACCCGCAACCCGGCCAGCAAATACATTCCATTCTGAA
TCTTATAACAATGTGCTAGAGTTCAAAATCAAAATACCGTAACCGGGTTTAGGTTAACTTTGATTACCAT
TGAGTCACTACTGAAAATCCCGATAGGGTAAGTCAGAGGTGACTAATAGTAACTACACCGTACCCACA
CATGAACCTACGCCTCTCCTACACGGGTAGGCATAGACTATGGATATCCACTTACAGTAGTTCTGAAGT
AGACAGCGAATGTGCTTTTGTACCTTGTCAAACTCACAAACCAACTGAAGGGCTAGCACGGGGCGCT
ATTAAGACGTGACAAAAGTCATCCGTCGCGCTCGCTCTTGAGGCTGCGCGATATGAGTGAGCGCGATG
CAAAACTTTTGTCACGACTTAAATGCACCCCGTGCTAGCCCTTCTGTTCATTCTTTCAAAGACTAGCTG
TTGGTCAGTTGGACAAAGCACAAAAGAACTATCAAGTTATTTGGACCGAACTGTACTTCGTTATCGCC
ATCAGCATACATTAACATCGATCGGCCGCCAATCCTCCACTACTGGACATAGGGCTCTCCCAATGAGCC
AACACTCTGTCCTTGGCCTTCCTCATCCAGCCACTACCGGCCGCCCGTCTGAGGTTTTATGTCTCACGC
TTATATTAACTACAGGATAGTGCGTCAGTGTGTCGCATACAAAACAAGGCAGACAAAATATCATATCCG
CACTTTTGTTTACTGTCAAACCGTTTTAAGTTCAACCTAATGACAACCATTGCACCGATGAATAAAGTT
ACAAATCTTCTAATCGAACTAATATTATAAATGCGAAAGTAACTGTGTCTGTCTGTCTGTTACTCTTTCA
CGCCAAAACTACTGAACGTATTTGAATGAAATTTGGTATACATATGTTCTAGACCCTGGGAAAGAACAT
ATGCTACTTTTTATCCCGGAATTCCCACGGGAAAACTTTTTAAGGCGAAGCGAAGCTCGCGGGGACAG
CTAGTTCAAAATAAGAGCTCAGTGGAACAAGTGGGTTATTTGTCGAGACGTTACTTTTGCACTGATACT
CCATGTAGTACGTATTACAATATCTTTGCGTGACACATAATTTTAACAAACTTTATTATATTTATAGCAAC
ATTACTAAGGCACGTTAAAGTGAAACTTGATTACACAACAATATTTTACAGTCACCCTGTTTCCGTTATG
CCATTCAAGGATAAATCAATGATTAAAAATATACTGAACGTCCTTTTAACTAGTTTTGCTAGAAAGAGA
ACTTTAAAACAGTATCTTTTGAAATCCCCAAATAGTATACGAAAACTAAGGACAGTATGAGCCAAAAA
AGATGAGAATTTTGATAATTATTCATATTTTAAATCATTAGGAATGCATCTAAAATTAACAAAGTCCACTT
TTCAAACAAATAATAAAAAATTACAATGCATAGATGAACAAATTATTTCCATAGAGACCTGTGAAAGAT
TTTTCCATACAAAATGTTTGGCTTTTAAATGAATTTTGGATATGATATTCTCAATTTTTGTGTGAAACTGT
GTTCACTTCATAGCAAAACAATGTTGTCTATGGAATTTGACATAAAATGGACAAAATGCATATTATATAA
TAATGTATGTGTATGTATGTACATTCTTTAAATAATACAATTAATAATAATAGAATTAACTGAATATATCTT
GATATACAGGATGTTTAAACCAAGAATCGCGGTAAAATATATAACAAAAGGACACACACCCTCCAAATC
GCTCAAAATAGACTACATATATAATATATAAAATACAATATATAATATAATGTCAGTTCTTTCCTTTTGACG
CTCCTGTGCGGTTTCAAAATTAACATTAAAATATGAAAAACGTAATAATAAACATGCTTGCGACTTTATA
AATAAATGATTGTTTTAAAAAGATATTTTGTGCTGTTCGTAAGTTGTCAAAAACGTGTACCTAATGTTAC

CCCAAGTGATTTTAGTCTTATTTTATCAAAATCGGCTGAGCAGTTCAAAAGTTACGCTACTTTGACTGC
ACTGCCACAAAAAACTTTAAACAAGTGTTTAAAACGAAATTTTACAGTTTTTGAAGGCGTTTGACAGC
GCTAAACACCTGTTTAATACTGTCTTGAGTTTTTGATGTAAGGCTTGTTGAATTTTTGGGGTTTTTACCG
TTAGTTATGTTATCACCGGTGTCTTGTCAGGTTATCATCACCTACTATACAGGGTGTTGCAAAATTGGTAT
ACTAAGCCGAAACCTACATGTGCAGCATGTTATATCTAAGCCCGAAACTGAAATCAGAATTTGGAAATT
GGCGAAAAAAAACAATTTTTTTCCATAGTAAAAGTCACGTGACCAACAAAGTTTCTATGGAAAATGAA
AATTTTTTTTCGCGAATTTTCAAATTCTGATTTCAGTTTCGGGCTTAGATATAACATGCTGCACATGTAG
GTTTCGGCTTAGTATACCCTTTTTGCAACACCCTGTATAAAGGCATTTTGACGAAACAGCACAAAACAT
ACAATTTATTTATATGGGTCGACAACATATACAATACAAGAATTAACCTATACTTTCATCCACATTTCGTG
TTGAAATCACCAACCATTGCTTTTAAGTGACCAATTTCAGCAAAATATATATCTTTATATATAATTTTATA
TATTTTTAGATGTATTACTCAACTAATCCAAAAATATAAAGATTATTTACTCTACTTTATTTAAAAAACGA
AATATCGCGAGGTTTTGGTAATAGTCGTTTTTATAAACAAAGATCTTATTTATGCTAATTTCATTTTTATAA
AATAAAACGACGTTATTAGGCTCACTTAATTAGCTCACCTGGATCTTATATTATTTAAAGAAAGCAAGCA
AAATTCACCGCATACGACGCGTGTGATTGGTCAAACCCAACAGTCTTGTGCTGCCATCTAGCGGTACA
AAATTGCTTTCAAATATATCACGATATTTCGTTTTTTGTGCGAGTATTTTGGGAACTTCTGTTCAATAATG
TCGATGGGCCACTGTACACAAACATAGGATGTTACATACCATTGCATAAAGTATATACTCATTCAGTAAT
TCAAGCAACCACTATTTTCATCACGTCTTTACATCAGATGACTGACTTACTTATTATATTACTAGCTAGGG
ACAGGGCCAACCGCTCAAGACATTGTTCTGACGGGCGCATCCTTCCTTTTGAAATCATTGACTTTAGTA
GGCAATTTATGTAAGTCCAGAACGGCATAAAAGTGAACTAAAACATAATCCCTCACGTGATGATAAAC
AACTATAATTACTTCGAATATATACCTCATCGCAATGGCACAAAGCGCTTGACAAATGTTACCACAGTG
GCGCCATCTAGTTCCCTACAAGACGAGGCGCTGTCCCTCCACGACGAGTCGCTGCGGCCGAGTGGCG
CCATCTAGTAGTCGTCGATGTCGTACAGGTTGTCGATCTCCTCGTCGTCCAGTTGCATGGACTGGAAGT
CCACTTTGTAGCGCAGAAGACCTGCGGAGAAGGAATTTAGGTTTATATCTGGTTTTTAGCTGAATGGTT
ATTGCAAAAAAAAACGGTGATATGAGTCTTTTGACTTTGAAGTAAGTATTTTTAGAGATATTTTTCTATA
CCCTGTTTTACGGGGAAAGACATCTGACAGAACCCTTATTACATTCGAATAAGGGTAGTTTTTGTTTGT
ATCAGATGACTTTCCCTGTGAAATGGAATATAGCTTTCAATTTAATTAAACAACTTTTATTAAAATACAA
AATAATAAAAAATCTTTCTTTTAAAAAAGTATTATAAAATTCACACATCAAGCAATGAAATATTTAGAAA
TTTAGATGAATAAAGCTCACGCAAAATATTGGTAGCGATTCTGAAGGGAACAACATACACCAGAGGAA
TAGCACCAAAAATTGTAAAAAAAACA
>TCONS_00100044 geneID=XLOC_019958 scaffold_32:766776-770439(-) Intergenic
TAATAAATAATACACAAGTAAAATTTTATATACAGCATTTATTGAATATTGTCATATTCCACATTAAGAAC
CCACAATAGGGCAGGGACCAAACGAATAAATGTAAAAATTAAACTAACAATGCTGGTTGGTTACCCGC
AACCCGGCCAGCAAATACATTCCATTCTGAATCTTATAACAATGTGCTAGAGTTCAAAATCAAAATACC
GTAACCGGGTTTAGGTTAACTTTGATTACCATTGAGTCACTACTGAAAATCCCGATAGGGTAAGTCAGA
GGTGACTAATAGTAACTACACCGTACCCACACATGAACCTACGCCTCTCCTACACGGGTAGGCATAGAC
TATGGATATCCACTTACAGTAGTTCTGAAGTAGACAGCGAATGTGCTTTTGTACCTTGTCAAACTCACA
AACCAACTGAAGGACTAGCTGTTGGTCAGTTGGACAAAGCACAAAAGAACTATCAAGTTATTTGGAC
CGAACTGTACTTCGTTATCGCCATCAGCATACATTAACATCGATCGGCCGCCAATCCTCCACTACTGGA
CATAGGGCTCTCCCAATGAGCCAACACTCTGTCCTTGGCCTTCCTCATCCAGCCACTACCGGCCGCCCG
TCTGAGGTTTTATGTCTCACGCTTATATTAACTACAGGATAGTGCGTCAGTGTGTCGCATACAAAACAA
GGCAGACAAAATATCATATCCGCACTTTTGTTTACTGTCAAACCGTTTTAAGTTCAACCTAATGACAAC
CATTGCACCGATGAATAAAGTTACAAATCTTCTAATCGAACTAATATTATAAATGCGAAAGTAACTGTGT
CTGTCTGTCTGTTACTCTTTCACGCCAAAACTACTGAACGTATTTGAATGAAATTTGGTATACATATGTT
CTAGACCCTGGGAAAGAACATATGCTACTTTTTATCCCGGAATTCCCACGGGAAAACTTTTTAAGGCGA

AGCGAAGCTCGCGGGGACAGCTAGTTCAAAATAAGAGCTCAGTGGAACAAGTGGGTTATTTGTCGAG
ACGTTACTTTTGCACTGATACTCCATGTAGTACGTATTACAATATCTTTGCGTGACACATAATTTTAACAA
ACTTTATTATATTTATAGCAACATTACTAAGGCACGTTAAAGTGAAACTTGATTACACAACAATATTTTAC
AGTCACCCTGTTTCCGTTATGCCATTCAAGGATAAATCAATGATTAAAAATATACTGAACGTCCTTTTAA
CTAGTTTTGCTAGAAAGAGAACTTTAAAACAGTATCTTTTGAAATCCCCAAATAGTATACGAAAACTAA
GGACAGTATGAGCCAAAAAAGATGAGAATTTTGATAATTATTCATATTTTAAATCATTAGGAATGCATCT
AAAATTAACAAAGTCCACTTTTCAAACAAATAATAAAAAATTACAATGCATAGATGAACAAATTATTTC
CATAGAGACCTGTGAAAGATTTTTCCATACAAAATGTTTGGCTTTTAAATGAATTTTGGATATGATATTC
TCAATTTTTGTGTGAAACTGTGTTCACTTCATAGCAAAACAATGTTGTCTATGGAATTTGACATAAAAT
GGACAAAATGCATATTATATAATAATGTATGTGTATGTATGTACATTCTTTAAATAATACAATTAATAATAA
TAGAATTAACTGAATATATCTTGATATACAGGATGTTTAAACCAAGAATCGCGGTAAAATATATAACAAA
AGGACACACACCCTCCAAATCGCTCAAAATAGACTACATATATAATATATAAAATACAATATATAATATAA
TGTCAGTTCTTTCCTTTTGACGCTCCTGTGCGGTTTCAAAATTAACATTAAAATATGAAAAACGTAATAA
TAAACATGCTTGCGACTTTATAAATAAATGATTGTTTTAAAAAGATATTTTGTGCTGTTCGTAAGTTGTC
AAAAACGTGTACCTAATGTTACCCCAAGTGATTTTAGTCTTATTTTATCAAAATCGGCTGAGCAGTTCA
AAAGTTACGCTACTTTGACTGCACTGCCACAAAAAACTTTAAACAAGTGTTTAAAACGAAATTTTACA
GTTTTTGAAGGCGTTTGACAGCGCTAAACACCTGTTTAATACTGTCTTGAGTTTTTGATGTAAGGCTTG
TTGAATTTTTGGGGTTTTTACCGTTAGTTATGTTATCACCGGTGTCTTGTCAGGTTATCATCACCTACTAT
ACAGGGTGTTGCAAAATTGGTATACTAAGCCGAAACCTACATGTGCAGCATGTTATATCTAAGCCCGAA
ACTGAAATCAGAATTTGGAAATTGGCGAAAAAAAACAATTTTTTTCCATAGTAAAAGTCACGTGACCA
ACAAAGTTTCTATGGAAAATGAAAATTTTTTTTCGCGAATTTTCAAATTCTGATTTCAGTTTCGGGCTTA
GATATAACATGCTGCACATGTAGGTTTCGGCTTAGTATACCCTTTTTGCAACACCCTGTATAAAGGCATT
TTGACGAAACAGCACAAAACATACAATTTATTTATATGGGTCGACAACATATACAATACAAGAATTAAC
CTATACTTTCATCCACATTTCGTGTTGAAATCACCAACCATTGCTTTTAAGTGACCAATTTCAGCAAAAT
ATATATCTTTATATAATAATTTTATATATTTTTAGATGTATTACTCAACTAATCCAAAAATATAAAGATTATT
TACTCTACTTTATTTAAAAAACGAAATATCGCGAGGTTTTGGTAATAGTCGTTTTTATAAACAAAGATCT
TATTTATGCTAATTTCATTTTTATAAAATAAAACGACGTTATTAGGCTCACTTAATTAGCTCACCTGGATC
TTATATTATTTAAAGAAAGCAAGCAAAATTCACCGCATACGACGCGTGTGATTGGTCAAACCCAACAGT
CTTGTGCTGCCATCTAGCGGTACAAAATTGCTTTCAAATATATCACGATATTTCGTTTTTTGTGCGAGTAT
TTTGGGAACTTCTGTTCAATAATGTCGATGGGCCACTGTACACAAACATAGGATGTTACATACCATTGC
ATAAAGTATATACTCATTCAGTAATTCAAGCAACCACTATTTTCATCACGTCTTTACATCAGATGACTGA
CTTACTTATTATATTACTAGCTAGGGACAGGGCCAACCGCTCAAGACATTGTTCTGACGGGCGCATCCT
TCCTTTTGAAATCATTGACTTTAGTAGGCAATTTATGTAAGTCCAGAACGGCATAAAAGTGAACTAAAA
CATAATCCCTCACGTGATGATAAACAACTATAATTACTTCGAATATATACCTCATCGCAATGGCACAAAG
CGCTTGACAAATG
>TCONS_00100262 geneID=XLOC_019924 scaffold_32:768775-770408(+) Intergenic
AATTATCAAAATTCTCATCTTTTTTGGCTCATACTGTCCTTAGTTTTCGTATACTATTTGGGGATTTCAAA
AGATACTGTTTTAAAGTTCTCTTTCTAGCAAAACTAGTTAAAAGGACGTTCAGTATATTTTTAATCATTG
ATTTATCCTTGAATGGCATAACGGAAACAGGGTGACTGTAAAATATTGTTGTGTAATCAAGTTTCACTTT
AACGTGCCTTAGTAATGTTGCTATAAATATAATAAAGTTTGTTAAAATTATGTGTCACGCAAAGATATTGT
AATACGTACTACATGGAGTATCAGTGCAAAAGTAACGTCTCGACAAATAACCCACTTGTTCCACTGAG
CTCTTATTTTGAACTAGCTGTCCCCGCGAGCTTCGCTTCGCCTTAAAAAGTTTTCCCGTGGGAATTCCG
GGATAAAAAGTAGCATATGTTCTTTCCCAGGGTCTAGAACATATGTATACCAAATTTCATTCAAATACGT
TCAGTAGTTTTGGCGTGAAAGAGTAACAGACAGACAGACACAGTTACTTTCGCATTTATAATATTAGTT

CGATTAGAAGATTTGTAACTTTATTCATCGGTGCAATGGTTGTCATTAGGTTGAACTTAAAACGGTTTG
ACAGTAAACAAAAGTGCGGATATGATATTTTGTCTGCCTTGTTTTGTATGCGACACACTGACGCACTAC
CCTGTAGTTAATATAAGCGTGAGACATAAAACCTCAGACGGGCGGCCGGTAGTGGCTGGATGAGGAAG
GCCAAGGACAGAGTGTTGGCTCATTGGGAGAGCCCTATGTCCAGTAGTGGAGGATTGGCGGCCGATC
GATGTTAATGTATGCTGATGGCGATAACGAAGTACAGTTCGGTCCAAATAACTTGATAGTTCTTTTGTGC
TTTGTCCAACTGACCAACAGCTAGTCTTTGAAAGAATGAACAGAAGGGCTAGCACGGGGTGCATTTA
AGTCGTGACAAAAGTTTTGCATCGCGCTCACTCATATCGCGCAGCCTCAAGAGCGAGCGCGACGGATG
ACTTTTGTCACGTCTTAATAGCGCCCCGTGCTAGCCCTTCAGTTGGTTTGTGAGTTTGACAAGGTACAA
AAGCACATTCGCTGTCTACTTCAGAACTACTGTAAGTGGATATCCATAGTCTATGCCTACCCGTGTAGG
AGAGGCGTAGGTTCATGTGTGGGTACGGTGTAGTTACTATTAGTCACCTCTGACTTACCCTATCGGGAT
TTTCAGTAGTGACTCAATGGTAATCAAAGTTAACCTAAACCCGGTTACGGTATTTTGATTTTGAACTCTA
GCACATTGTTATAAGATTCAGAATGGAATGTATTTGCTGGCCGGGTTGCGGGTAACCAACCAGCATTGT
TAGTTTAATTTTTACATTTATTCGTTTGGTCCCTGCCCTATTGTGGGTTCTTAATGTGGAATATGACAATAT
TCAATAAATGCTGT
>TCONS_00099543 geneID=XLOC_019927 scaffold_32:848061-849125(+) Intronic
CCTTTATTAAAGTCCCTATGTATCTTCACATCGACCACATCTCTGTCCTGGTTCGGGTACAACTCCTGCT
TGGTCTGCGTGTCCCACTCGCCGGCTCGAGCGCGGATGCTCTTGGTCTGCCAGTCTCGGTTCACGTAG
TGGGATGCCGTCAGCGAGTGTTGTGACCCCCATGAGCCTAACTAGTTACCTGGACTTTTTGCGACATCC
TGAAGAAAACCTCTATTCCGCAAATATCACTATAATGATTCCTCTTTGTCTTAGGGTGCCGTTGTAACAT
CAGAGCTGAATCAGGGTTTGTTACCGTGTTAAAAAGATTAGCCAGGACACGATTTGTCAATAGATTACC
GGACAGACTCAAGGTCAAGGCTTCTTGTGGCACCAATCTCCATGCAACACAGCAACAACCCACAAAC
CTTTATTAAAGTCCCTATGTATCTTCACATCCACCACATCTCTATCCTGGTTCGGGTACAACTCCTGCTTG
GTCTGCGTGTCCCACTCGCCGGCTCGCGCGCGGATGCTCTTGGTCTGCCAGTCGCGGTTCACGTAGTG
GGCGGCTGTGAGGACCACACTCGGGTGGATGAGGGAGCCTCCGCCGACGTACACGTTGAGCTTGGAG
CCTTCGGGCTCCGCTTCGTTTATTGGCTCGAT
>TCONS_00100012 geneID=XLOC_019928 scaffold_32:893146-893743(+) Intronic
TGACAGATTCTGATTATCGACAGCAGGACAATCGACTGTTGATGAACCGTTCAGAATCTGTCAATTTAG
TATCTGACATTAAAATACAGACTTTAGGTACATTCGACAGGTGTTTAAAAGTGACATATCTCGTATGAAT
TTGACAGGCTTTACAGCGCTATACAGCTAAAGTGGTATTGTGTGCAGAAAGAGGGTCACATAACTTGT
CATTTTTAACAATTTGACATACCAACTACAGTGAGCGATTAACTAAACAGATTTTACGGATA
>TCONS_00099746 geneID=XLOC_019970 scaffold_32:1356560-1365157(-) Unclassified
GTGCGGGTCGCTACTCTACTTTGATGTGATTGATAAAATGATAGTCCGGTAAAAGTTTAACACTATTTAT
TTATACCGAAAATACTATGCTATGATAATTCTAATGATTTGATGTGAATGCACCCGATTTGTATGATTGCT
AAAAGTACACTGATTTATGCCGCGCGTGTAAGTACCGCTACGTCACAGTGACGCCGACAGAGGGTGTT
CGGTGACACACAGTGGCCCGAA
>TCONS_00099629 geneID=XLOC_019971 scaffold_32:1366421-1367398(-) Intergenic
TTCTGAACCGGTGGAATAGTCTTTTTTTTCCTCCTCTGTATTTTTTTGAACTAAATCTAAAGAAAAATAA
TAATAGCGAATATGCATGTATTTTTATTATATTCTTATTCGACAGTTTTACATCGCTTTATTATAGTAAAAA
AAATTCAACCCCAAAAAAAATAATTTAAGGTATTTTAAAGAAAGTAAAAAATTTATAACCGACACATCC
ACTATAGACGCCCAAAACTTTGTGTGAAGAAGTTTGACGTCATCGTCGACGTTTCACGGCTTAGAATA
ACGAGTCTCGAAAGTCGCGCCGGCGCTCAGTTCTATGGGCATAGATTATTATGAGATTGATATATCGTCT
ACCTCTTCTTTTTCTTCGTTTTGTTTTTTGTTTGTGTTAGCTAACTCGTGTACGTGCACGGTCAGGTGCA
AAGAAACCTGACCCCCCTCTCATAGTAACAATGCTTCTGAGGGGGGTCAGTAGGTTTCTCTGCTCCTTA
CTGTACGTGACTGAGGTCACTTAAAAGAACCAGCTGAAAATCAGCGCTGTGGCCTTACTGTCCACGTG

TCTTCTTTCATTCCTTGATGGGGCGGAAAACTACAGGAGCCCAGGGCGCGCAATCTTTAAATACGCCC
CTGGTGGACACTGATGGTGGGACTGGTCCACCGATTTAAAGTCACTCAGCGTGCTATGGAGAGAGCTA
TGCTTGGGGTTTCTCTAATGGATGGTATCAGAAAAGAGGTTATACGTCAGAGGACTAAGGTTACCAACA
TAGCTGTCAAAATATGCA
>TCONS_00100052 geneID=XLOC_019974 scaffold_32:1414127-1417053(-) Unclassified
AATGGTTTGTTTTTGTTCCAGGATTTACTAGTTGATGCCAAAAAGTCCCAAAAAGATGTAAAGAAATTC
ATCAACGATTTGTTAGATAAGGCAAAGACTCTGTAAGGAGCGATGGGCGCAGGAACCAAATACCACG
AGGCGAGATTGTATCCGCCATTTTGAGTGCCACGTCAGGACAAGATGGCCGCCGCTGGTTGGCACGTG
AAGGATTGTGCGAGCCGTCTTCACGCTCAGTTTATTAACGAGATTCGATTTGTGTAGATGGACTTATAC
ATATTTAGATTAATTTGATAATCATTGATAAATAGGTAATCTTATGTGTGTACGTTTTTGTGTACCCTAGGC
TTCGCATTGCTGTAAACCCGATATGTCTCGAGATGCGGGATTTGTGCCGGTATTTCTAAGTAGAATTTCA
TGAAATTTGTTTTTTTAAGTTACGATATTAGATAAAGAACTGCCTATATTGGAGTAGTTCCATGCCTAATG
CGTGATGTCGATTATTGTATTATACATAGAGCACAGACTGTGTTTGTGATAATCCACATAGGGTTATTTCA
AAAGTGGCTGTCCATATTTACTAAATCCAATAGCGTAAAGTCTACATGGACTGGATGGCCGCTTGAAGA
AGCCTACTAATAGACTGGCCACAAAGATAATGAGCGCTTGTGTAACGCGTTGCGTACGTCAATGTTGGT
ATGTGATGTGACTTTGCTAAAGGTTTTGTGAACTAAGCTGTACTTTTTGTATGGGATTATTAGTCAATGT
GTAATTTTAAACGTAAATAGCTAGTGCCTCGACAGGGGCCGTGTTTTAGCAGTAAAAGTTCAACTAGTT
GTAAGCATCTTTTTGATTTAGGTATACATTTTACCATTTTATGAACCGTTTCGGAGGCGTTTCCACAAATA
TAAATTGTCAGTTATTCCCAGTCAACTGGGAACAAATATAATGCAATCCACATATCATTGTCCATTGAAT
TCGTACTGAGGAGCACGTCTGTTCATAAGAGTAATGTACTTCTTAGTATAAAAATGTGATGATCGCCAA
GTAAATGGTAAGTAAGGTATTGAAATCTCATAAAATCACGAATAATGACAACGCGTTAACAAAATTTAT
AAGCCATATTGCATTAATTGGTAGTCGAATCGTAATTAATTACATACGTAACCGATAATTCAAATAGTTAT
ACAATAATACCACAGCTATTTACGCTGTCCTAGAACAAGAAACCATTGATTGGTATTTGTGAAATGAAA
TTACGACGATGCCATCGTAGTCTCAAGTTACAAATTAGTAGGTACTATAGGAATGTGAAACAATATAAGT
ACAAATGTAAGGTTAAACTGATAGCCAAAATATAGATGGTCATATCTAACGTATCACGGTATGCTTTTTC
TAGATACTGCGGGTCAGGTTCATTATTGTCAGTGATAAAAACAATCAACTGGGGGTTTTAACCATGGAT
GGAAACATATTGGTCAATAACATTTCGGCCTAATTCGATGTTACAGATCTTTGTAAAAATACTAATAGTA
CCTACGATTTGTGATTTGTATGACTAACTTGGCCGCGTCTTAATCAAATAGTGAATTAATGAAATAAAAG
TAAAATTGTATTGCGACGCTCTGGCGGGCGGCGACTGAGGTCATTCTAGTTTAAGAAAAAAATATTTCA
GACGACTCGTCTCTATCTCTTTACATCTAATTTTCAAAGGAGTGTTTCCAAAAAGTATAAAACAATAATG
TTCATCGCGCTAATTAAATGTTTAGGCCACAAACAACTATATTTTCAAAAGGAATCGATTACGACTGTTT
TGTAAGCTCGAGTGACCCTCCGGAACTACTCGCCGCCCGTCGACATGCACTTTTATAATATACCATTTTA
ATAGAAAAATATCATTTCTTGTTTTATTTATCCCAATGTAAATGATGCCCTATACCAGGGAACTTAATAAT
AACTTAACCCCTTTATAGAACGATATCCCCTTATGGATTCATAATATGTGACCTATCCAGAGTTAAATTAA
AGAAAAACGTAAAAGTAAATTAAATGATTTTATATTTGTATCTTTTTAAAAGTATCAAATGATTCAGTCT
TAAATATAAAAGTATTAACAAGTACAAAAATATGTTTTAAACGCTATTGTGCAGATTAACAGTGTCATTA
TCACATGGCGGATGCCCAGTTTTACTTACACATACATTTAGGTTAAAAAAATATCTAAATCTAAAACAA
ACATCCTTGCGCCTTGAATTATCTTACAGACGGCGACTTCACTAAAATATACTTAAAACATGCTTCGCCA
AATCAGATTTGTTTTGTGAATAATGAATCTGACTTGGAACATTCCACTACAAACACATCTCATTATGGCT
TCACAAGCCATGGCAGCTTCACAAACGTGGAAGCTCTAATCTACTATTTAAAACAATCACACTATTCAA
AATCAGACCATTTAACTTGAACTTCAATTTAACAGCTCTTACGATTACAAACTTTAACCTAAGGCTACTT
TCCCAGTAGGCTAACTGTTCCCTCGTTATTAGGGGCGTTAAACGCTGCAAAAGTGCCTTGAGTCTGCAT
AAATTATCAAACTGTGAACACTCGGTATACTAAACGATACGTCACCAACAGTTTGCCTACTGGGAACG
CAGGCTAACATCTCATACACAAAAAACCCCACGTCACGT

>TCONS_00100132 geneID=XLOC_019980 scaffold_32:1541368-1544180(-) Unclassified
GCCATAAATTAAAGACTTGTTGAAATTTACACATAGGTAGCCCCAAAGTATCTGTCTAGAACTAGATAG
ACTTTCTTATTGTTTTCCCCGAATAATATTGATTCATTTAATTTACAGAAAATCGCGTTTGCGTTAATTTTT
TTTACTTTATTTTAATTATTTTTAAGGCACCGCGCAATATGTTGTCCGCGACCCGGAGCCGATGATAGTT
GGTGGTGGGTGAAATACGCTATCGGAGGCGTGGTCATCGCCATAATAGGAGGCCTGGTCTACCGCAAG
CTGTCTTCACCCTCGCCCCCCGAGCCCCTCATAGAGCCCGCCGCGCCCGCCGCGCCGGCCGCCACGTA
GACCACCAGGCTGGGACTCGATACACGGAAGTAAATGAAGATGGTGTTTTAGCGACGAGCATTTTTAC
AGAAGAATTCCTAAAGGATAATGCGTTTTACCCCATTAAAAAAGAGGCAAGGGACAACCTTAATGGAA
AGATGTTTTAGGGCTCATTAAAAAATAACTAAATCGTAGATGACACAGGTACACGGTAGATTAGTTCAT
TCAAACTAACCTTTTATTTTAAAATCGGATGGGATAAAAATGCAGGTCGCTAAAATGCCGCTGTCGTTT
TGTTTCGTGAAATGAGGGGCAGACGTAGCAATAAGAGAATGAGCTCTGAACAAACACTGCGTATGCA
AGGGTTTTGCAGTGCCACCGTATCGCCGTCGAGAGGTCTAGCATTTTGTTAATAGATATACATATTTAAC
TCACAGAAATTACAATTACGGTTTAACTTATGTCTATAAAAAATTTCAGTTCGTCATTAAGTTCTCCTTT
AATATAGTGGACGTTTAAAATCCCTACTTACATTAATATTTATTCTCGATGGCGATACGAACCCGATACTA
ATCTGATGCTCATGGCGCGTCGACAATCACAAATCTTAGTTCTAAATAAACAGGGTGGATTTTTAAACA
TTGTAAACTTTGTGAAAATTGCCCATTCATAACATTTACAATGCTTGCCCTTTGAGAATCCACCCTGTTT
AATCCACAGGCTCACAACTATCAGCACAAAAGGTTCCTGAGGTTGAATATTGAGTGAAGGGTCTCATT
CATTCATAATTTCACTCATAATTACCTACAGATGCAAATGAATGAATGAATCGCTCCCACACAGGTCATG
CCTTTTAATTTAAAGAGCTAAGGGCTCATTATTTCCAATATTGTGATCTTGAAATACTTAATTTCGCATTG
TTAAATGTGCTTTTAATAGTTTATATTTTATTCCTTGTTATTTGATACCTTGAACTTTTGTACCTTGTATCTA
ATATTTGAGGTGTTATTTTTATGCCAATGTACAAAAGTAGATGTGGATGTAGAATTATGTCTGTACGACA
ATAAAATATACTTTGTTATACATATTTCATCTTTATTTTAACTAACATGAACATTTAAAAATATCTAAAATA
TAAAATAGGAATAATTTAGTAACAGCCTGTCTAAAACTTACCTAAGTCTTAAAATACATAAAAAATATAA
ATAGATACAAAAATACACTTTATGCTTACAGCAGAAAGCAATAAGCATAAGCTTTAGCTTCTTTAATGC
AGTCGTTCCAACCTATTGTGATTAGACTGACTTGATTTGGTACCCATAGAAAAGGTGCAAATAGCTAGC
GGTCGAAATTAGCAACATGTTACTGGCTGCACAGAAACCAGTGTTTTTTTTTCTCTGCGCGCTATGCTA
ACAATTTGCACCTCAAACACTAATCACAATACGCAGTTCGAACTCCTTTGCAATGACCTCCTTGCTAGC
TTCCGCCTCACACCACGGAGTTCCGGCGCGAAGGCGGTGGCACACTATCGGGCGAGCAAAGCGGTCG
CGAACAAAATGGCTGTCAGCACGCAGCTCGCACG
>TCONS_00099971 geneID=XLOC_019980 scaffold_32:1541368-1544304(-) Unclassified
GTGCCCGCCAAGGCACCGCGCAATATGTTGTCCGCGACCCGGAGCCGATGATAGTTGGTGGTGGGTGA
AATACGCTATCGGAGGCGTGGTCATCGCCATAATAGGAGGCCTGGTCTACCGCAAGCTGTCTTCACCCT
CGCCCCCCGAGCCCCTCATAGAGCCCGCCGCGCCCGCCGCGCCGGCCGCCACGTAGACCACCAGGCT
GGGACTCGATACACGGAAGTAAATGAAGATGGTGTTTTAGCGACGAGCATTTTTACAGAAGAATTCCT
AAAGGATAATGCGTTTTACCCCATTAAAAAAGAGGCAAGGGACAACCTTAATGGAAAGATGTTTTAGG
GCTCATTAAAAAATAACTAAATCGTAGATGACACAGGTACACGGTAGATTAGTTCATTCAAACTAACCT
TTTATTTTAAAATCGGATGGGATAAAAATGCAGGTCGCTAAAATGCCGCTGTCGTTTTGTTTCGTGAAA
TGAGGGGCAGACGTAGCAATAAGAGAATGAGCTCTGAACAAACACTGCGTATGCAAGGGTTTTGCAG
TGCCACCGTATCGCCGTCGAGAGGTCTAGCATTTTGTTAATAGATATACATATTTAACTCACAGAAATTA
CAATTACGGTTTAACTTATGTCTATAAAAAATTTCAGTTCGTCATTAAGTTCTCCTTTAATATAGTGGACG
TTTAAAATCCCTACTTACATTAATATTTATTCTCGATGGCGATACGAACCCGATACTAATCTGATGCTCAT
GGCGCGTCGACAATCACAAATCTTAGTTCTAAATAAACAGGGTGGATTTTTAAACATTGTAAACTTTGT
GAAAATTGCCCATTCATAACATTTACAATGCTTGCCCTTTGAGAATCCACCCTGTTTAATCCACAGGCTC
ACAACTATCAGCACAAAAGGTTCCTGAGGTTGAATATTGAGTGAAGGGTCTCATTCATTCATAATTTCA

CTCATAATTACCTACAGATGCAAATGAATGAATGAATCGCTCCCACACAGGTCATGCCTTTTAATTTAAA
GAGCTAAGGGCTCATTATTTCCAATATTGTGATCTTGAAATACTTAATTTCGCATTGTTAAATGTGCTTTT
AATAGTTTATATTTTATTCCTTGTTATTTGATACCTTGAACTTTTGTACCTTGTATCTAATATTTGAGGTGT
TATTTTTATGCCAATGTACAAAAGTAGATGTGGATGTAGAATTATGTCTGTACGACAATAAAATATACTTT
GTTATACATATTTCATCTTTATTTTAACTAACATGAACATTTAAAAATATCTAAAATATAAAATAGGAATAA
TTTAGTAACAGCCTGTCTAAAACTTACCTAAGTCTTAAAATACATAAAAAATATAAATAGATACAAAAAT
ACACTTTATGCTTACAGCAGAAAGCAATAAGCATAAGCTTTAGCTTCTTTAATGCAGTCGTTCCAACCT
ATTGTGATTAGACTGACTTGATTTGGTACCCATAGAAAAGGTGCAAATAGCTAGCGGTCGAAATTAGCA
ACATGTTACTGGCTGCACAGAAACCAGTGTTTTTTTTCTCTGCGCGCTATGCTAACAATTTGCACCTC
AAACACTAATCACAATACGCAGTTCGAACTCCTTTGCAATGACCTCCTTGCTAGCTTCCGCCTCACACC
ACGGAGTTCCGGCGCGAAGGCGGTGGCACACTATCGGGCGAGCAAAGCGGTCGCGAACAAAATGGC
TGTCAGCACGCAGCTCGCACG
>TCONS_00100385 geneID=XLOC_020068 scaffold_320:98551-104311(-) Unclassified
GTACTAATGAAAATTTCATCATCTGATGAACTGAAAAAGTTGTCGAGCGATTATAAAGCGACTTGAAAT
CGCTCGTTTAGGCTAGAGTTATATCATGGCTAATGTTATTATTACTTGAAAATTTACGGCTATATTCGTAAT
AAATAATGTAAATTAGAGCAAATAAAGTAAATTTAATAGCAGGATTTAAAATCGAATTCATGGTACTTAT
GTACTTCGAGTAATACCTATTGTTATGAATATGATTTACAATGACAAGCTTATTTCTTGATGATTTTTTTTT
TATCCATAATTTTATACGCATTCAATTTTTGTACCGGTATTTATTGGCCAGTTACCTTTATCAAAATTAGGA
AAGTGAAAAGTTTATATTTCTATCCTAACAATGATTTTTAGATTCAATATAGTATTATTTATTGTACCTAAC
TTGTTTTTTATTAGAAATGATTTACAGGAATCTTTACTGAAGCTGCATTGTAAAAACCCTGTAATATTTTA
TTTTTATTTGAACTTGTTGAACTTAAGTATAACTTTTTTATTCAAGCAAGAATTTGAAATCATCTGCCATC
CAGTACAACATTTTTTTTAAATTTCAGGTTAATTTAACTTATAAAATAGAGTGGCAATACCAATCAAATA
TCTTTCATAGCCCCCTAAAAGTTAACCAACTTAATACTTGCCCCGTACCAAACACAATCAATTTACACC
TCTTATTCGAGTGACAACCAGTCATCTTTACACAAAAGACTGCACTCAGCCGTCTCCAGAAACCTGCA
CTATGTACAATTAATTTTGAGCTTCAGGCAGGCATCGCAACGACAGCGACAACACAACCACATCGACA
TTTTGTCGCTGCCACAACGCAACTGCAAAAAGCCGCTGCCACACTATTCACATGTAACCACAGCTTGA
CCACATTATGTCGCTGCGACGTGGTGTGGTTATGGTACGTGTGAATTGACGCTAAGTATATGATGCTGTA
TGTTTAGTATATGATTCTGCTGGGAGCGGTGGCGGTGTTGGAGCTCATGGCCTGCTCCAGTGTGTCTAG
C
>TCONS_00100378 geneID=XLOC_020059 scaffold_320:99359-106521(+) Intergenic
CATATTCATAACAATAGGTATTACTCGAAGTACATAAGTACCATGAATTCGATTTTAAATCCTGCTATTAA
ATTTACTTTATTTGCTCTAATTTACATTATTTATTACGAATATAGCCGTAAATTTTCAAGTAATAATAACATT
AGCCATGATATAACTCTAGCCTAAACGAGCGATTTCAAGTCGCTTTATAATCGCTCGACAACTTTTTCAG
TTCATCAGATGATGAAATTTTCCTTTGAAACCCCATATTTTTGCCCTTTTAAAAAGGAGGGGGAATTCAT
TTAAACTGTTAGCTGAACTACCTATGCCCCGTATACTGTATGTTCGTCATCAAATTGAGTACAATATTAGT
AAGTAATCTAATTTAATATAATAATTATTTATTTACTTACATTATTTTAGATGAAACTATGGTGTTAGATTC
ACGTTTCATGGGAATAATGTGACCACTGAGATCTCTGACTAACATTTCAAGAAGTATTTTTATGGAAATT
ATCTGTTGTATCTATTCCAAATTGTGGAAAAAGTACATTATAATTATGTTTTGTTAATATTCATATTTAAAA
AAAACGTTTGTTAATGTATATTCCAACAAAAAAATACGTGCAAGTAGTATTTAATTACACCTAAAATAAA
ATGTGGCTTTCTTTCTTGCTGCATAGACCTTCAATTTCGAGGCATGATTATCTTGGACTATCAAACTATA
GTGTGATATGATAACTTACGTTTCACCCAAATTCAAATACTTATAAACTGTTGATTGGTATAAAGGTAAA
CTCGGTAAAAAGATCAAGAATCATGCTCAAAGGTTATTATGTAATGATCCTTCCTAAACATAAACCGAT
GCTTTAGTCTACCGTGTACCTGTGCTTTCTCATCGAAACGCTATTCGCTTTTAAATTTTGCTCTGTCTCAT
TTATTTCCTAAATGAAGAAAAGAGACAATTGTTTCAAATTGACGTCACAGGCGCAGTAGCTATTCAGA

AAAGCGTCTCAGGTACGACGATTGATAGGAATACATCATACTTAATACACCTACATCGCATGTAGTTCCT
CTGATATTCCTGAAACAACTGCATGATCACTAGTAGAAAATGATATTTGTACTAAGTCCACAC
>TCONS_00100442 geneID=XLOC_020088 scaffold_321:174018-176124(+) Splice junction overlap
TGCTGGCGTGGCGGCACGTGCACGAGCGCGCCTTCTGGACCGAGCTGCGGCTGGCATTGCCGCTGCC
ACCGAGACACATTGAAAAGGAAGTGAAAGTAGAGGTGGACGAATCGAAGAACTCTCGGTCGGGCAG
CCTCACGGCGCTCGTGTGAGTCGCTCGCGACTCGACGCAACACAAGAACTGAACTCTGCCGTGTTATT
GTAAACAACACGCGGAATAGTGTATAACACTTCGGGGTCAGTGGCCGGCTAGTTCCCGCGGCCGCCAC
GGCAGCACTGTGCTAGTGTCCCCCGCGCCCGCCACGGCAGCACTGTGCTAGTGCGACCCACATGTAGA
TATCAAGACTGTGAATAAATTAGTTCTAAGATTTTAATTATTATTCTTATTCTCATTACGTATGTATCACAC
TAAGTTGTAATTTTTTTTATTTTCTATTTATAGCTTGTAAGGCAGGCTTGTTTTGTTCATGGTTTTCTAAGA
TCTCAATTTTGATTTTCTTTCTTCTTTATTTGGAATCTGGAATTCTATTTCTACTCTACCGCTTTACTGTGC
CAAAAATATGGTTTCATGACAATTTATTGTAATAATGTTTCGTGTTCTGAATATTGTTATTGTATTCACTG
ATGATTTCTTAGCATGCTAATGAAGAGGAAAGTGCATGGTTTTATTTTGTTATATTTTCTGTTTCTGAATT
ATTTCGGTGTATTTTGCTAAGAAGTATGTAAGTACAAAGTACTCCACTTTTTTATTGTGTAATGAATGATT
TAATTTAAAAAACACCTGTCAATGTTGCATGTTTCTGCAACCGCTTTGTGGCGTCAATTTCATTTCCTAA
AGATGTAAATGTATAGGTACTATTATTAGTACCTAATTATGTAATTAACATTGTATAAACACTCTTGAATGT
ATACGATATCTTTAAATTAGTTTACTGTACAGTTGGCTCGACATGCAAGTAGAAAGATTTGTCTAGTTGC
GAGATTTTTTCACCTTTTCTATGACCTGGTGGTGAACTTCAAAATATCTACCAGGAAATGCAATGTACA
GTCAGCTGCATAAGTAGCTACACACTGTTGGACCTTGTCAATCTGAAACCGCCTATCCATTCATTGAAA
CAATGACGGTTCGTCAAATTCTAAACCCGCCTATCCATTCATTGAAACAATGACGGTTCGTCAGTTTGA
CAATGTACAGAAGCGTATAGCTACTTATGCGGCTGACCGTACCTTCTTGGTTGAGGATTGTGATAAATT
GATAATATCAAGAGTGGGTATTCTGGAAACTGTTCTCAATAGCCTATACTTACTTCTAAATGTAACCAAA
ATGTAAATATTATGTGGGAATAGTTGATTAGTGACAAAGAAATTGTAGTTTTTGTATAATAATTAAAATAA
TGATTCTTATACTAATTATTAATAATTATGATTACTTTGATACATTCCGGCTTTCTTCGTTAAAGTTTTTCTT
TCAATACCTACATATTTATAAAATTTCTAATAACGC
>TCONS_00100443 geneID=XLOC_020088 scaffold_321:174366-175692(+) Splice junction overlap
GAGCGCGCCTTCTGGACCGAGCTGCGGCTGGCATTGCCGCTGCCACCGAGACACATTGAAAAGGAAG
TGAAAGTAGAGGTGGACGAATCGAAGAACTCTCGGTCGGGCAGCCTCACGGCGCTCGTGTGAGTCGC
TCGCGACTCGACGCAACACAAGAACTGAACTCTGCCGTGTTATTGTAAACAACACGCGGAATAGTGTA
TAACACTTCGGGGTCAGTGGCCGGCTAGTTCCCGCGGCCGCCACGGCAGCACTGTGCTAGTGTCCCCC
GCGCCCGCCACGGCAGCACTGTGCTAGTGCGACCCACATGTAGATATCAAGACTGTGAATAAATTAGT
TCTAAGATTTTAATTATTATTCTTATTCTCATTACGTATGTATCACACTAAGTTGTAATTTTTTTTATTTTCT
ATTTATAGCTTGTAAGGCAGGCTTGTTTTGTTCATGGTTTTCTAAGATCTCAATTTTGATTTTCTTTCTTC
TTTATTTGGAATCTGGAATTCTATTTCTACTCTACCGCTTTACTGTGCCAAAAATATGGTTTCATGACAAT
TTATTGTAATAATGTTTCGTGTTCTGAATATTGTTATTGTATTCACTGATGATTTCTTAGCATGCTAATGAA
GAGGAAAGTGCATGGTTTTATTTTGTTATATTTTCTGTTTCTGAATTATTTCGGTGTATTTTGCTAAGAAG
TATGTAAGTACAAAGTACTCCACTTTTTTATTGTGTAATGAATGATTTAATTTAAAAAACACCTGTCAAT
GTTGCATGTTTCTGCAACCGCTTTGTGGCGTCAATTTCATTTCCTAAAGATGTAAATGTATAGGTACTAT
TATTAGTACCTAATTATGTAATTAACATTGTATAAACACTCTTGAATGTATACGATATCTTTAAATTAGTTT
ACTGTACAGTTGGCTCGACATGCAAGTAGAAAGATTTGTCTAGTTGCGAGATTTTTTCACCTTTTCTAT
GACCTGGTGGTGAACTTCAAAATATCTACCAGGAAATGCAATGTACAGTCAGCTGCATAAGTAGCTAC
ACAC
>TCONS_00100508 geneID=XLOC_020098 scaffold_321:223585-224156(-) Intronic
CCAACCTGCACTGGTCGCCCAAGATTTCCCTCCAAGATGGCCTTCAAAGGACTATAGAATATTTCCGAG

AAGAATTATCCAGACATACATTTTCTCATGATCAAACGTATATGAATGTAATAAAAAGTCCACCAAAAA
CATGATCTAATAAACAGTTAACCTGCACAAATGTGATACCTAGTAAGTTATTAAAAGTATGTAAATTTTA
TTTTATGTTTAAAATACAATGATG
>TCONS_00100509 geneID=XLOC_020098 scaffold_321:223585-224297(-) Intronic
CCAACCTGCACTGGTCGCCCAAGATTTCCCTCCAAGATGGCCTTCAAAGGACTATAGAATATTTCCGAG
AAGAATTATCCAGACATACATTTTCTCATGATCAAACGTATATGAATGTAATAAAAAGTCCACCAAAAA
CATGATCTAATAAACAGTTAACCTGCACAAATGTGATACCTAGTAAGTTATTAAAAGTATGTAAATTTTA
TTTTATGTTTAAAATACAATGATG
>TCONS_00100544 geneID=XLOC_020090 scaffold_321:266185-268676(+) Intronic
CGCCCCGCCGCCGCCCGCGCCCGCCCTCGCGTAATCAAATAACGTTTTTCATCTATAATACCCAGGTTTT
AACTAGGCAGATATCACTTGATACAGAATTTTTTAGAGCACCGTATACCTATATGAAAAATTTTCGATTA
CTTGCAGTAAAATATAGCCATTGGGTGAGTTAAGTAATGAATAACTTTAAATCGGTGGACCAGTCTCAC
CTTAGCTTTGCCTGAAAGTCTAAAGGTCTTCAATCATTGCATTCTGCCGGTGATGACGTGTAGTGCAAA
AGGTGGGTAGCATAAATTTCTATATTTTATAATCGTGGACTATTCTGTAAGAATTTGAATTCGAGATTTTG
GATAGTTTTTCAACTGAAGCCGTTTATTATCTCCGTATGTATCAACAATCGATGTCTACTGGATTTGGTG
CCTTATAAATGTAAATATTATTTAAGTATTTATACGTTAGCCCAGCCTAATCAGAGCAAGCTTTTATTCTG
TCTAAGCTCGAATAAAACAGGAACGATATTAAATAATAGTATTACTACGAATAAGTCTGTATTATAGTTTT
GCGAACTGATTTAGAAATATTATACATGCAGTATTACAGCAAATACGATTGATATTTATCTATAGCGACTC
ATTGAACCAGATTAAACTTTGTTGTGCAAGACAACCCTTAACTTTTTCAGCATGGGTAGTGCGAGTTTT
TACTCCGTTCGAGAAGAGGCAAGTGGGTTCACGAAAACTTACACTTGGCCCTCAGATCTAGATCTTAT
TCTTGTTCAATGACTCTACGAGGTTGTGTATAACAAATGCCGGTGCTAATTTAATTATTTTATATCTTCCA
TACATGCATCTGTTACCATAATATTCGCTCACCATTAGAACAGTACATAATATTATGTGTATTAAAAATGA
TTTCTTTCGTGTCATAATATTCGTGTCATTGTTTGCTGATTCGCAGGTGTACCTACTTTGTGAATCTTTTC
AACGGAAAGCTTCATATATTATTGTAAGTGATTAGGATAAGTTAGAATTTATTGATTGTATAAGAATACTC
TTGTGACATAAGTTTATAAATGGTTCAGCGACATCTATGAGAAATAATTTGAAAATAATATAAGTTAAGT
GATTGCTAATATTCATTAGATGATGCTCAAAAAAAACTTTTGTATAATAGCCCACTATTGCGGAAATACA
AGCAATAGTGTGTCTAATTATCATGTACCGCTGAGTGCAATTTCATACAACTTGCCAACGACAAGTCTG
ATCTATTTGTTTGTATATGAGAAGAGAGGGGTAGTTATAAGATAGAGTGTATGTGTGTATTAGGTAAACG
TACAAAATAATATAAGAATTTATTTTAAAATATCCATATATTTACAATAGTTAAGTTGTACCACAAAATATT
ATGTATACGTACTACGGAATGTGGGGACATTGTGTATAGCGACGCGCATCATTTACAAAACTACAGTTTT
TGGGCAGATATTTAGATTTTTATCACTTTCTTCAAATCATGAATATTGAACTTTGCTTAAAATTTATTTGA
AATTATGTTTTGTATTATTTATGCGCGTGCGATAGTACAATGTACTTAATACATTTTATATATTTTTATAACT
GACATAGAGATTGTAGAGATTAGAAACAATATACAATGTTCAAATGTTTATAATATTGAAAATACATCAC
AAAGTATGAAAACGGTATTTTATTTCAAAATGTTACGTCCTACACATTTCTACGAACGAGTGAGAATATA
GTTAGTAATAATTAACTAGCATAAGTTGCATCCAACTCTATAGTAAATGCTTTATTTGAACACAATTAGC
AAAGACACGTATATTGTAATAATGCACTAAGATCCGTTTAGGCAAAAGGCAGTGTTTGTCTTTCCAAGA
AATCGACGACATGAAAACCAACCTTAACAACTCAGTGTATAAGGTATAAAGTAAAAAAAACGGTGCGA
TCCAGATCTTAACCACATGTCGTGTTATGCCATACAATTTCACTAACAAATACTTTATGAAATAACGATAT
CTCTACACATCTAACTACACCACAGAATAATAACTAGTACCAGGTACAGAAGTCCATCTTCGCAATGGA
TTGCAAATAAATATGACTCCATCCCATAAGCCAGTGTTTTTCAACCTGTGGGTCGCG
>TCONS_00100699 geneID=XLOC_020120 scaffold_322:41739-42082(+) Intronic
CCCCTGTCGAGCGCACGCCCGTCCGCCATCTTGAAAAACCTTAAAGGAAACAAAAGTCAACAAAAAC
GTCCATGACAACGCAATCCTGCCTCCTTTTTAAACTTTGTAGTGGCAGCTTGTGTTAACGTTCTTTTGTT
TTGTGTGTTTAATACAGTTCGGATTGTTTGGAAAAAGTGGTTAAAATTGTCGGTTTTCAAGTGAAATCG

AGTGCAGACCCAATTGAC
>TCONS_00100741 geneID=XLOC_020128 scaffold_322:161186-163828(-) Exonic overlap (-)
CAGCGCCTAGCCAGGCCAGCGCCAGGTATACAGGCCACCGTACCTACAGGTACACGATATTTCATGTC
AACAAACAATTTCTGAATATATCAAGAACGATTTCTTCAGCGAATCTCTCGTGGGACAACCCCAATCAC
GGTGAAAAATACAAAATATACATACAAAGTACAAATAATTCGGACGAGAAGATACGTATTTACAACTAA
GACTGATTAATGAAAAAAAACATTGTTTATTTGATTCTATACATATTTTTAGCCTGTTTTTTATCGCTTGT
AACTTCAGCTCGCTGCACGGGTCCTTATCGATTCACCCGGCGCTGTGATTTTTGGAACGCGCATTAGAA
GAGTTTATCAGTGTCGATAACTGATCCGTGAAGCGGAGACTGAAGACAAATTATTCTCTTGGCAAGCTA
TCAATCTCATAACAAACAAAACGCACCATTTCATACTATTTCAGATATCATTAAAATCTAAAGTATCGAC
ATTACCAGTTTATTTATATCATAACGAATTCAATTGATGATAATTTTGATGAAGATGTGAAATATAATAGTT
ACACAAGAAACAGTAAAATGTCGATGAGATGTTGATTAATAACTGTTAAGTAGCGAGTTTAACAACAT
CATTGGAATACCTACTAACTAATTTGATTCATTTTAAAATTGAGTGAACATTAATTTAAATACTCATTTAA
CGAGTGAAACTTTTTATTTGTAATTTTATGGAATAATGTAGCAGGAATNNNNNNNNNNNNNNNNNNN
NNNNNGAATGATTAAATGGAATATACACCATACATGGTCATTTTACATAGTTTCAGAATATGATCTCGCT
ACAACATGACAACAGAATGACGTCGCAGTGATCAAACTTAACGTAACATCAACAACACCAACGTTAA
ATTCGAGTAAAATTGTAAAAACTTCGAAAAAATCGCTACAAACATTTCCTATAACGGAACATTTTATAA
AAATGAACACAGCAAATCACTACCCTTGAATACATTAGTAGAATACGTCCCTAATATTATTTTAGTAGAC
ATCACGTTTACCGTTACCTAATGATTGAGGAACAAGACGCAATGCCAATGTAGATGGCACCACCGTCG
ACTGTCATTGCAAGCTTGCTTGTGATTGGCTTATTATCAAGCCGTAATTAAACATATTTATCGCCAAAGA
CCTCGAAGTTCATTACAATATTGTTGAGCAGTCTCACATCGGTGCAATCATAGATAAGTGCTTAATACAA
ATAATTTACTTCACAATTTCATCTGAAACCAGATCAAAATTTGATCTCAAAGCTTGGCAAGGCAATTTTT
ATTAATACACATACTCTTTGTATTTAAGAAAATTTTCGGCACAGTCAACACTTGGAATGTAACCTTATTC
CCAAAGCAGACAGTACAAAATCCATAAAATTCTAGTCACATGACAACACCGATCTTATCCATAAGGCTT
TAATTACAGTCCATACAATTTTAAAATCAGTACTTTTGACATAAGTTTGTTAAAAAGTCATTATTTAGGT
CACTTCTGATATCTCATTTTCTTTACATTTTTAGTACACAAAGGTTACATTTGTCATGAAGACCTTTTCCA
ATAGTAAATGGCACTGATGCATACATATAAAAACCTTCCTTCATTGGAAGGAGACCCGTGCCCCAGCAG
TGGGGACGTGCTGGGTCGTGAGTCGTGATTACTGATGCATACAGTTTAGTACACTAATCTGTGCCAAAT
TGATTTTTTCAGTTTGCAAATTATAATCGTAAAATCGGTCTTTTGTTTTAATGAGCATTACAAATTGGTG
GTTTGTTGTATTTCCTCTTATTCATATATATTCTTTAACTCATGTTAGTCAAATTGGCACAATAAACTTAGA
TGATTCTTGATAAGATTATATGTATATGGGAAATAAAATAATAAGAATGGCATAAACCACCTCCAGTACC
AGTTTAACTTGTTAAGATCACTGCAAGATAGTCTAATAATAAGAAATTTCATTAGACCACAACTTTTAAC
CTAATTATTTTTTTAAATCTTACATAAGAGATTCAGTATGATTCTTCTTTTTTCTGCCGCCCCTAGCAATA
AGGCTAGAGGACAAATCACAAAGACATTAAACAACGAAAATTTGTCAGTTTTGTCACATTAAGTTTTA
GCTAATAACGATAAAGAACTTCAGGTCCCAATTATTAGAAACATGTCTTTTACGTGTCTCGTTAAACAC
TTACAAGTATATGTATAAAAATGATACAACCCAGTTATGCAAAAGTATGCAATGTCTTTTCGGGACACA
GATATTAGATTTAAAAGTGTCAATTTAAATATTACTCAAAGGAAATTCACTCAGCCCCAATGGTGTTAGT
CCAAGCATCATATTCACATGATATATTTTGAGATTATAACATCTCCAATTGCAATTGTTATGCACTTAGAT
TTTATGATAAAAAAAAAAAAAAAAAACACGC
>TCONS_00100729 geneID=XLOC_020128 scaffold_322:161230-163914(-) Exonic overlap (-)
TGCAGTTACAGTAATAATATCTACTGATATATAGTTGCTTTCAATAGCTTTCTGCATTCTCAGAAATCCAT
CTGTTGTACGGTGGCCAGCGCCTAGCCAGGCCAGCGCCAGGTATACAGGCGGAGTTTTCAAAATAGCG
ATCCCTGATGATGAAGGGACCAGCTACATCTGTTATAAATCCGGGGACGTGTTGTGTATGGTATGTGTA
GCAGTGGTGTTTATGTGGATGTGCTTGAGCAGCATGTGAGCTTGAGATCGCTATTTTAAAAACTCCGCC
TGTATACGTTTAGGCGCAGGCCACCGTACCTACAGGTACACGATATTTCATGTCAACAAACAATTTCTG

AATATATCAAGAACGATTTCTTCAGCGAATCTCTCGTGGGACAACCCCAATCACGGTGAAAAATACAA
AATATACATACAAAGTACAAATAATTCGGACGAGAAGATACGTATTTACAACTAAGACTGATTAATGAA
AAAAAACATTGTTTATTTGATTCTATACATATTTTTAGCCTGTTTTTTATCGCTTGTAACTTCAGCTCGCT
GCACGGGTCCTTATCGATTCACCCGGCGCTGTGATTTTTGGAACGCGCATTAGAAGAGTTTATCAGTGT
CGATAACTGATCCGTGAAGCGGAGACTGAAGACAAATTATTCTCTTGGCAAGCTATCAATCTCATAACA
AACAAAACGCACCATTTCATACTATTTCAGATATCATTAAAATCTAAAGTATCGACATTACCAGTTTATTT
ATATCATAACGAATTCAATTGATGATAATTTTGATGAAGATGTGAAATATAATAGTTACACAAGAAACAG
TAAAATGTCGATGAGATGTTGATTAATAACTGTTAAGTAGCGAGTTTAACAACATCATTGGAATACCTAC
TAACTAATTTGATTCATTTTAAAATTGAGTGAACATTAATTTAAATACTCATTTAACGAGTGAAACTTTTT
ATTTGTAATTTTATGGAATAATGTAGCAGGAATNNNNNNNNNNNNNNNNNNNNNNNNNGAATGATTAA
ATGGAATATACACCATACATGGTCATTTTACATAGTTTCAGAATATGATCTCGCTACAACATGACAACAG
AATGACGTCGCAGTGATCAAACTTAACGTAACATCAACAACACCAACGTTAAATTCGAGTAAAATTGT
AAAAACTTCGAAAAAATCGCTACAAACATTTCCTATAACGGAACATTTTATAAAAATGAACACAGCAA
ATCACTACCCTTGAATACATTAGTAGAATACGTCCCTAATATTATTTTAGTAGACATCACGTTTACCGTTA
CCTAATGATTGAGGAACAAGACGCAATGCCAATGTAGATGGCACCACCGTCGACTGTCATTGCAAGCT
TGCTTGTGATTGGCTTATTATCAAGCCGTAATTAAACATATTTATCGCCAAAGACCTCGAAGTTCATTAC
AATATTGTTGAGCAGTCTCACATCGGTGCAATCATAGATAAGTGCTTAATACAAATAATTTACTTCACAA
TTTCATCTGAAACCAGATCAAAATTTGATCTCAAAGCTTGGCAAGGCAATTTTTATTAATACACATACTC
TTTGTATTTAAGAAAATTTTCGGCACAGTCAACACTTGGAATGTAACCTTATTCCCAAAGCAGACAGTA
CAAAATCCATAAAATTCTAGTCACATGACAACACCGATCTTATCCATAAGGCTTTAATTACAGTCCATAC
AATTTTAAAATCAGTACTTTTGACATAAGTTTGTTAAAAAGTCATTATTTAGGTCACTTCTGATATCTCAT
TTTCTTTACATTTTTAGTACACAAAGGTTACATTTGTCATGAAGACCTTTTCCAATAGTAAATGGCACTG
ATGCATACAGTTTAGTACACTAATCTGTGCCAAATTGATTTTTTCAGTTTGCAAATTATAATCGTAAAATC
GGTCTTTTGTTTTAATGAGCATTACAAATTGGTGGTTTGTTGTATTTCCTCTTATTCATATATATTCTTTAA
CTCATGTTAGTCAAATTGGCACAATAAACTTAGATGATTCTTGATAAGATTATATGTATATGGGAAATAA
AATAATAAGAATGGCATAAACCACCTCCAGTACCAGTTTAACTTGTTAAGATCACTGCAAGATAGTCTA
ATAATAAGAAATTTCATTAGACCACAACTTTTAACCTAATTATTTTTTTAAATCTTACATAAGAGATTCAG
TATGATTCTTCTTTTTTCTGCCGCCCCTAGCAATAAGGCTAGAGGACAAATCACAAAGACATTAAACAA
CGAAAATTTGTCAGTTTTGTCACATTAAGTTTTAGCTAATAACGATAAAGAACTTCAGGTCCCAATTATT
AGAAACATGTCTTTTACGTGTCTCGTTAAACACTTACAAGTATATGTATAAAAATGATACAACCCAGTTA
TGCAAAAGTATGCAATGTCTTTTCGGGACACAGATATTAGATTTAAAAGTGTCAATTTAAATATTACTCA
AAGGAAATTCACTCAGCCCCAATGGTGTTAGTCCAAGCATCATATTCACATGATATATTTTGAGATTATA
ACATCTCCAATTGCAATTG
>TCONS_00100797 geneID=XLOC_020160 scaffold_323:71082-71877(+) Splice junction overlap
AAAAAAAAAAACACGCACTCACGCCTTGTACTAATGTACTCCCTTGCGGGGTAGGCAGAGGTGCATTG
CTGCACCCACTTTTCGCCAGAGTGTTATGTTAGTCCCAATGTAATAGGGGGCGGGCCTATTGCCATTTTA
CGGGCACATCCAAGACCCGAGAACAAATATCTGTGTTTAAACAAATATCTGCCCCAGCCGGGAATCGA
ACCCGGGACCATCGGCTCAGTAGTCAGGGTCACTAACCACTACGCCATTCGGTCGTCATATTATGGCCG
TATTATTTCAGGTGCTGAAAAAGCTGAAACTAAACTTGTGCTACGCCACCATGGAACAGCTAGCCAAC
GCCACACCCAACATTATTGAGCGAGTCCTTATTATGGTCCGTGAGAAGATCCTCCGTGATGAAGAGGC
GAGCCGAGCCAAGGAGGAGCAGAACCTGTCGAG
>TCONS_00100815 geneID=XLOC_020171 scaffold_323:215993-219718(-) Intergenic
GTAAACAAAACAAACTTAGAAACACGGAATGGCAACGAAATTATGTTTGAAATGTATTTATTATAAATT
ATGTTTTTCCCGATTTCCTGTAATAGTTTTGTGAGAAAACTGACCAAAGCACTCTTAATACAATGAATG

CGTTTGCCCTGGCTTGCTCTACGGTTGTTACTGGGTATAATTAAGTACGGAAGACTCCACACATCGCAA
AAAAATAACGCTAAATTATGATCAAAATATCGAATACAAACATGTGTGATTGCAAGTAAACAAATAGGT
GACCATAAAGACATCTTATGAT
>TCONS_00100867 geneID=XLOC_020166 scaffold_323:358665-359048(+) Exonic overlap (-)
CTTTGATTGATTCTCTGGCTTTCCCTTCTGCTGATAACCGAGGTTTCCCTTCTGCTGATAATCTAGGTTTT
CCTTCTTCTGTTAATATAGGTTTTCCTTCTGCTGATAATCTAGGTTTTCCTTCTTCTGATAATATAGGTTTT
CCTTCAGCTGATAGTCTGGGTTTCTCTTCTGTCGACTCCGTGGCTTTCCCTTCTGTTGATAATCTTGGTT
TCTCTTCTACCGATTCTATGGCTTTTTCTTCTGCCGATTGCTGAGTATCAG
>TCONS_00100940 geneID=XLOC_020199 scaffold_324:18913-19899(+) Unclassified
CAAACAGAAAGACAAAGATCTCCACGAGTCCCTGGTGGGATTCATCTCGTTACAAGTGAAAGCTGCA
AAAAAGAATTTGCAGGCGTGGACCCAAATCAAAGAATGCCTTCAAAATATGCCTTGAGCGCCCTTGCC
CCACATATACGGGTTTTTATACCAAATTGTACAGATTTGTCTATTTATTATTACTTTTACCTTTTTAATACTT
AGTCTGTCCTACATTTTTTACCCAAGAAAAATGATATTGATATTACCTACCTGATTAATGATTATAGATCT
AGTTATTGAATAATGCTTATCAATACTTTAAAAACTTAATTTAAATCAATAAATACTTTTATGTATCTTTGT
TATATTTTAGCTATTTTAAGTCATTGTAAAATAAGTTCCCTAATCGAAAAATGCCTTGAATTTAAAGTTAG
CCATTATATAAATAATAGAACAACATTTTATGTAGCTTTGATACATAATTAGGAATATGTAAGAAATATCA
AGTTTCCGTTGGATGAAAATGCTTTGTATTGTAGCTAAAGAAATAGTTAGCGTTCGCTAACACTCGAGT
GTAATAAACTTATAAAAAACAAACATATCTTGAAATATATCACGAGACTACGTTTGAAGTTGAAAATTT
GGAAAATCCTCAATGTGTTTGTATACTTCGGTCTGAGATTTTTATTATTATCTATATTATAACAGATTGTTC
AAAATCTATATACATACTTATAGGTAATTTGTAAAGTATTTACCTACCGCATGCTGACAAGATAAGATATG
AAACACTTTTCAATGCAGACTGACAAAACATCCTGATATTTTGTCTCAAACTAAACAGCTAACTTGCCT
TATTGAAATACATAAACAATAATGTTTAGAAAGCCTTTTAT
>TCONS_00101068 geneID=XLOC_020222 scaffold_324:183577-194972(-) Exonic overlap (-)
AACGGTAGCGAAGTAAGCTTGGACTTATTCGTATAGCTATGGGGCTGATTTCATAAACAATACACATACT
ACATTAAATTAACACTGTCGTAGTTTATAGTAGTAATTTAAAAAACCCAACTAAACTATATTGAAAGTTA
GTTTATCGGGTATCGCTTGTTTAAGTATTTTGGTTGTATTCCAATTATACGAAATAAATAATTATAATATGT
ATATAGTAGTAAAT
>TCONS_00101122 geneID=XLOC_020210 scaffold_324:252714-253236(+) Splice junction overlap
ATTGTCTTATTTTTTCAGGACGGTGTACAAGCAGCTATCTGTCTCTGGTCCAGGTAAGTGGGACACAAA
ACCAGAAACATAAAAAAATATTGTATAAGTAATTTAAAATAATAATTTTGTCAATTGGAATTCAATGTTA
TTATTTTTCAGTATGTAAGTATACATAGTTATAAGTGCTGGTTTTGATTTCAGGATATTTGGGAGACAATG
AAGCCAGGAGAGCCCTTCTTTCGCGATTTTTGGCTGCGTTCTTTGAGCGCGCGCTGGACGGCATGAGA
TGGGTGGGCTGTGAGCGGTCGCTGCTGGAGCGTCTGCTAGACGTGGAGGTGACGTCACGGGAGGCCG
GCGCTACTAGCGTCCTACTGTACGACCCTCAAGACAAC
>TCONS_00101284 geneID=XLOC_020265 scaffold_325:387768-437985(-) Unclassified
GTCGTCCTCACAACTTATCTACTAATCGTAAACGGCTATAAGTACACTAATAATAATAACTCATCTATGAT
TTAATTCAAGGCTCCAAACATTTTTCATAGTATACGAAATGAGATTTCACGTGTATTTCGCTACGCATGA
AAAAGTATCACCCACATTGTGAATTGTGAGCTACAGCGGCCAGTCTGATAGAACCGCCCGGCCCGCTC
GGTCGCGCCGCGACTCTGCTGGATAATAAAAAAAATATACCTGAATTAAAATAATTCAAATATCGAACA
CTTTTACTTCTTCATCGACCTACGAGGTCACTTTTGTGAAACAAGTGATTTTTCGTGGATGTGTAGAGC
CTCCGCGAGGCGTGCGCCGGCCGGCACCACGTGATAGCAAGGAGAGCTGCAGAAAAGTTAACTGGG
AGCAAAATTTATTCGGGTCTAAACCATCAAAATGTGTGAAAGATAAAACACAGTGATAACTTAGAAAT
GATTCTCAAAATCAGAAGCTTTTATATGAGTGAACCGTCAGTGAAACCCCAGTGTTAATCAGTGTTAAT
TTCTTTGATTAGTGAAACGGATATTAGATAACCTTATTCAAAATGGAAGCTTTGGAAAGGCGGGG

>TCONS_00101767 geneID=XLOC_020337 scaffold_327:144427-144922(-) Exonic overlap (+)
ATTTAAATATGAAAATTGTTTTTTTATTTAGCTTTTATTCTTATTTTGAAATTATAGAAGGGCATAATAATA
AACCAATCAATTATGCAGACTTGGAGTCAGCTCTCATGCCACAGGAGTTGGACCCACAATTCACCCCG
GATAAGGTAACTAATTTATTTTTTTTCCTCTAAAATGATAAATTTGAATCATTTACACTTGTAATGCAAAC
ACCGCCTCTTGACTAACCTATAGTACCACCTAACACTATAGTAGCTTCAAAGACTTACAATGAATAGCA
CCTTGTGCAATACGCTCATGACTTAATTAATAGAAGTTTAAAGACTACGCGTTTAGTGCGAGCAG
>TCONS_00101760 geneID=XLOC_020333 scaffold_327:279646-280879(+) Splice junction overlap
CAATTGGTCATGCATGCCACATAACTACTTACGTGAGTTTCACGACTCTTCAAACCTCCAAGTCCAAAA
CAATGATCAATGCGAAAACACGCAAGCGTTCACACTACAACGAATTTTAAAGGTCGTTATTATTCTGAG
ATACATTCGGGTTTCTGTCAAAACCAAACTCTGTGAAGTGAGTCTCGCTAATCTGTGGTCGAAAAAAA
AAAGAGCAGTCATAATTTTGTCTTTGAAGGAGTTTATACTCCCTGCGAGTATTTTCTGTTTATAATATTTT
ATGTGAAGTTAAATGGATATTTGGTGAATAAAAACTTAACTCTAGAATACAGTTACACCGGAAGAAAC
GTCCACTGTACTTGAAATTGTGATGAACGACCAGAAAATGAAGTTTTAGTGAATTGTTTTGGTACTCAT
TCAACTATATTGATTTATCTCGTATGCCAGTGACCTCTTTTTCAGATTTGTAACGTGTTACTCGTAAATAA
TTAATTTCCTCATTTAGGAGGAATTACACTGATGATGTAGATAAGTGCGTGTTAATTGTGGGACACAGA
GAGATGTGCGGCTAATGAGTTTTTGCTAATTAAGTGGACAGCCGTGGAGTGCAGTGTGTTCGTTATGAT
CTGCTTATTATTTACAAAATGATGACGCCGCTAGAGGAAAAGAAAGATGAAAAGATCCGCATGGGCAT
GGTGCAGGT
>TCONS_00101946 geneID=XLOC_020350 scaffold_328:81750-84780(+) Unclassified
GTCTAAATTAAGTTAAATATTGCACGTTTAATGATTATGATTAGTTGACTAAGGTGTCACCAATGCAAAA
CAATCAAAAAAGTATTTAAAAAATCCTTAGTTTTTGAAATATTTTGCTTTTAAACATACATTTTGGCAAA
ATTCCTAAAAAAACTTAAAAATTAAATAACTCAGAAATGGTTGACTTTCGGATAATGCATTATGGGGTT
CAATCGACTGGAAATGAGTTCGACTACAACCCATTTAAAGGAATACATCGACTTACCAATCACCCTGTA
TACGGGCGCGGGCGGGAAGAGACAGTCATAGATTTAATAATGCCGACATTAGCTTTAAAGTTCCTTTCT
GCAACTCAGTGATTGAGTCATGGTCAGCGAAGCGCTCCGAAGCATATACAAAATTAAACCGCTACGCT
GAATATGACCCTATTGCGTTGCAGTCACCGTACCGATTGCGTGACAGTGACAACTCTCGTTCGTTCATA
GGGTGCTTACAGGGAACCCAGTCTCTCTATGTAAGCACATATAGAGACCCCCTTGGATTCGGTAGACAC
ACGATATTCTGATAATATGATCTGTACGACGAACCCCAAACGATCAATACGATGTATTATCGATCTAATTA
GGATATAATATACTTATTGTATGTGTGTGTTGCCCGAGCACAGGATTCGAAACCTCCGTTTACGTTGCGT
ATCGTCAAATGTCACTGTCAAAATGTAAGGCAAATTTGTTAGTTCCAAAAGTGGCATTTGTTTTTTCCA
TGTTAGGTGAAATTTCACCAAACGCTAAACGTATTTAGTAGCCTGTCATACGTCTGTCAGTTGGTACAA
AATGTGTTTAAAATTTGATTTAAATACGTTTAGCGTTTGGTAAAAGCGACCCTTCGTGTAGATTCGGAA
ATAGTATCGAATCCTATGCTCGCGCCTCTGGTCTTTATTTGCGAAAACTACACAGGAGGAGGAATACTT
AAATACTTAAGTAACCAATCCGTCGTAATATCAAATTTTCGTAAATCAACGAATGTTACTTAACGATTGT
GCCTTACATTATTAATGTTACTTAAATATATTAAGGAAGTAAATGAAATTATTATTAATACTTTATGAATTT
TAATCAAAATGATGTTTATATTCTAAGTATCTTTTAAGGCTCGAATACATGTATATTTCGATGTTATTCTTT
ATTTTTAACAATTGTGTGTTTTTCATTAATTTTAATTAATTGTTAACATCAAATGATTGTACTCCAAATGG
CATTTCAGATACTTATTATTTTTATGTATATTGGAATTGAAGTATTCATGGTATGTTTTGGTACTTTTTCAG
GTAGAAAGGTATACTTATCTCAATGTTTAAAAACGTTATACTAATACTAATAACAACCCTTAAATGAAAT
TTTAATTTTACGATGTTTGATTTGACTCTACAACATTTATTATTTTGGCTGCTATTAGGATATTAATCTTTTT
ATACCTATTTAATTAAGTAGAAGTAATTTGGGTAATGTTAATTACTTTCTATGTATATTTGAGATAAAATAT
TACAACGTCTTCAAACAAGTTGGTTTTAAAACCACTAAATAGTCTAAGTGCCAATTTCTCCATAGTCGG
TTATCTAACCGACCAGGTGTGATTTCTTAATTACACGTCATCTCCATATAAAATTCTTGACAGATGTGTC
AAAAGTCAAGCAATAATCCCTGGTTATACTCTAACCCACTATGGAGAAATTGGCACTAAATAGCGCGTT

AAGAACAACTAAATAGTTTAAATACCTTTAAACCAAATTGTTAACTTGATTTCGATGTTACTCAGCAGTA
TGCCAATATTGAGATTAAACATATTATGATAATTAGCAGTATTTACTATACTGTACCAACATCGCACATTA
GCGATCAACAATCTTTAAGCAGCATAGCTTATTGCATTGATTGCTAATGTTTCGGTGGTGGTAAGGCAG
CTGGCCCTTATTTTACTCAAGTGAAATAAAATAGGCCAGCTGATTTTTAATTCACGGTTGGCGGTCAGC
CTAACCCTAAAAGTATTTTTTAATTTTTATCCCAAAGAAAATGTGAATTCAAATGTGCCAAATTAGATTT
ACGTTTTTTTTTATTTTTTCGTTAACCTTATGTTTAAGTTTTGCTACATTATGATATTTACTTAAGTATTAG
TTAAGTATAAGAGAGTTTTAAGACACATGATTTCTTTTTACTTCGAGTCGCTGAATCAGAGGTGCTTATT
TTTAGTGATATTAGGTACTGCATTGAGTGTCTTCAGAAAATATGTCACATGCATTAGCTTCCTAGTATTTT
TAATCATTGTAAAACACTACATTACATACCTATTCCTATATATGTTATAATCCACGCCAATAATGAAAAAA
TCATCAAATCAGTTTTCAGAACAATGAGTGCTTATTTTTATACAAGTTGGAAATGTATTTTGAAATTACT
GACATACCCTATGCATATTTTTGTGACAAAGTACTTATTAAGTTAGCCCAAAGATTTGCTTACCAATTTAT
TAATTGTTTACTGGTTTTGGGTGTATCCGCGGGTCTCTCTAGTTCACAAAAAACTAATTTTCAGATCGTT
GCTGCTCTAACCCCGACTCTATCTCGGTGCATTACTGTACACGTTGCGACTGCAACTCTCGTTCATTAG
TTTTTTATCAAGCTAGAAAGATCCTCCTGAACTTTATATAAGTAGTAAAACGAAACCAGATTTGTGCCTT
GTAATTAAGTGCTAAGGAGGATTCATTAAATTTTTCAGAAATTTGTATTAAGTAAGTACTTATTAAAATTA
TGTTAGTGACCGATGAAACTAAAGAAAATTATTATTTACTTAATCGGTAACGTGAGAAAAATATTTTATA
TTAAATTTATTTACCTGCCTT
>TCONS_00102001 geneID=XLOC_020363 scaffold_328:83445-84779(-) Intergenic
AGGCAGGTAAATAAATTTAATATAAAATATTTTTCTCACGTTACCGATTAAGTAAATAATAATTTTCTTTA
GTTTCATCGGTCACTAACATAATTTTAATAAGTACTTACTTAATACAAATTTCTGAAAAATTTAATGAATC
CTCCTTAGCACTTAATTACAAGGCACAAATCTGGTTTCGTTTTACTACTTATATAAAGTTCAGGAGGATC
TTTCTAGCTTGATAAAAAACTAATGAACGAGAGTTGCAGTCGCAACGTGTACAGTAATGCACCGAGAT
AGAGTCGGGGTTAGAGCAGCAACGATCTGAAAATTAGTTTTTTGTGAACTAGAGAGACCCGCGGATAC
ACCCAAAACCAGTAAACAATTAATAAATTGGTAAGCAAATCTTTGGGCTAACTTAATAAGTACTTTGTC
ACAAAAATATGCATAGGGTATGTCAGTAATTTCAAAATACATTTCCAACTTGTATAAAAATAAGCACTCA
TTGTTCTGAAAACTGATTTGATGATTTTTTCATTATTGGCGTGGATTATAACATATATAGGAATAGGTATG
TAATGTAGTGTTTTACAATGATTAAAAATACTAGGAAGCTAATGCATGTGACATATTTTCTGAAGACACT
CAATGCAGTACCTAATATCACTAAAAATAAGCACCTCTGATTCAGCGACTCGAAGTAAAAAGAAATCAT
GTGTCTTAAAACTCTCTTATACTTAACTAATACTTAAGTAAATATCATAATGTAGCAAAACTTAAACATA
AGGTTAACGAAAAAATAAAAAAAAAACGTAAATCTAATTTGGCACATTTGAATTCACATTTTCTTTGGG
ATAAAAATTAAAAAATACTTTTAGGGTTAGGCTGACCGCCAACCGTGAATTAAAAATCAGCTGGCCTAT
TTTATTTCACTTGAGTAAAATAAGGGCCAGCTGCCTTACCACCACCGAAACATTAGCAATCAATGCAAT
AAGCTATGCTGCTTAAAGATTGTTGATCGCTAATGTGCGATGTTGGTACAGTATAGTAAATACTGCTAAT
TATCATAATATGTTTAATCTCAATATTGGCATACTGCTGAGTAACATCGAAATCAAGTTAACAATTTGGTT
TAAAGGTATTTAAACTATTTAGTTGTTCTTAACGCACTATTTAGTGGTTTTAAAACCAAC
>TCONS_00101810 geneID=XLOC_020365 scaffold_328:109779-111490(-) Intergenic
CGGGTCGCGGCGAGGCGAGGTGAATATAATATTTTTGGTCTCTAAAATATTTGATACTGTGTTTGAGTTC
CTTGTTGTTGTGTATCCGAATTTCATGACCATTCTTGTTATTGTGCAGACTAGGAGTAGTGGTGAGAGT
GTGATCCATTGATTAGGTACTTATTTGCGATTGTTATAAACTTACCTTACTAACTAAGTACTTACTTGTTAT
ACATCTACTTAGTTACTAATGAGAGTTGAATATTATGCCGCATTGTCATTCGGTCATTTTATTATTATCAAA
TTAAGTAAGTAAGTACCTACCTTATTTATCTACAAATACCTAAATGGCTGTTGCAGATTGTGGCTAACAG
TGCAGTGTTTCATATAGAAAAATATATGTATATTTTCGGTAAATTCGTCATTGATACTTTATTTACCTAGTT
ATGTACAGGCTGAGGCAAGGAAACCTGACCCTCTCATGTATACAGTTCGGTCCGTAGCATTTTTATTCT
AAGAAATGTTTCTTTGTCTCAGACTGTATTTAATAGACTGTAGACTACTGTAGGTGTAATGATTTATGAG

AACAGCTGGGTGGCTTTAT
>TCONS_00101916 geneID=XLOC_020358 scaffold_328:273358-278401(+) Splice junction overlap
GTTGGACGGAGCATGATGAGGCGGCTGAGGCGAATATTGAGGAGGCATTCGGCATGACCAAAGAAGA
GTTTGCAGCGCTGCCGCCGTGGAAACAAACCAACATGAAGAAAGAAGTGGGCCTCTTCTGACACTTT
TGCCTTACAAGTAACGATATATCTCTATCCCTGTCCCGCCCGCCGCTGTCTCATAAGGGCGGGTGCGAT
GGCAGTAAGCTGCCCGTATTTTACTACTAAACGTTTTTGTACACTCAACAATATTTCGTACTTTTTTACC
GACGACGTTTAAGGATTTGATACCATAGTGTTAAAGCACGCTTTAAATTCATTAGTGATTATTGTAATAG
TCTTTTTGTACTAACGGATCATAAACTATGTATTCATAATCGTGATATTTACATTGTAACTTTCAGCGTAG
AGCGAGCTAAGTGAAGGGTTTTCGAATGGACTATAATTATGTATTGAGGATTTGTTCAGCGGAATTCCT
AGGTGTCCGCTATTTTTCTCATCCACGGCTCTCAAATGTTTGTATATAGTTGTTTTGTTTTTAATTTATTGT
TTATTTCTTTCTCTCTATTGCGGAATTCGAGTCTGAGCCCAGTTTTAGAATTTAGGACTTCAGTTTGAGT
TTAAGAAGAGTAAATGAAAACGTTGGTACAATTGCTCTATTTGAGCTCTAAAGAAATCTTAATTTTGAA
ATTGGGTATAAATTTCCGGTAAGCTCATGGTAGCTGATGAAGAATAAACTAGTTTTTAGACATTTAAGTT
TTGTTTTAATCTTGCAAATATTTATTTCGGGCATGTGGATGCGAAAAAACATAAGAGTATAGGTAGAAGT
ACTTTTTCTAGTGTCATTTTTAGAGAAATTGCCAACATCGTGGTTTTATATTATTCAAGACAAGATATCC
AATCAGTGAAAATGTAACAGTACACAGGCAACATTTGTTGAGCAACTACTTACAACAAATTAGTTGCA
TTACCCATTCAATTCTCTATACCGACCACTTGATATCAAATAAAAGTTTGACTTACAAATAAATACGACA
TGTTTAATATGACCCACAGTGTGTTAGTTAAAACAAAGTCTACCATAGTTGCCACTTGCCAGTGTACTG
AGAGGTTATTCACGACGGCAAATTGTATTGATCAAGGTTGACTAATGGTTAAAGTTGCTAGTGTTACAA
TTATACACGTATTTACGCTTTAGGGTAGTTTTATACAGTTCGGACATCAGCTGAGAGCCAAACAGGATAT
TACTTTGTGATATCGGCAAATCTTCCCGCCATTGTTATACTTCCAAATGTTGGTCCAACTGTTGGCTCTC
TGCTGGAATCCGAACACCAATATCTGAGAAAAATAACACATCACATATCTAATCTACTCATTAGCCATTT
TTATACTACGCATTACTGTATTTTTATATTGTTTATGTGGTAAAGTTGACCGTCGCGCCATATCGACCTGA
CATCACTGAGACTGAAGTTCGTTTATTATGGTTTCGTTTGGTTAAATTTAAAAGATTGCCTTTAAGATTA
CGCCGGCGCTCTTCGCCACAAAGTCTTTGTTTAAAGAGCACTTGAAACCAATTTATATGGCTTCCGTTA
CGTATAAATTAAAACAATAATTACGCTCTCAAAATGGCGGTAGGTACTTGCGTGACGATCAACAGTGCC
ACGTTATCGCGATCACCTTAGCTTGGTAGTGAAAATACATTTTTATTGGACTTACTTATGTGACTTAACC
TGACACTTCGATGGACTTTTTGTTGCCAAACTTGACTCAATGCGACTCTCAATAAAATGTGGTTTGTAC
GTTTTATATCCCGACCTTTCTATGTTGTCTACAATTTTAGATGTTTATTTTTTATAATCCTTATTTTAATGTC
ATTTAGGCTCATTAAATTGTAATGTTAGTATGAATTTTTGAATTGCCAGTATTGAAAATGTGGGGTGAAC
TGCCAATGAGTAGAAAAGTACTTTTTGTTAATGAAAAGTCATTTAGTTATTTCTGAATATTAATCACACG
CCATTAACTGTGTAACTTCCACTTCGATGTGTCCCGAGCTGCATTTAGGGGCGTCCCGCGCCGGGCGA
CATAAATTATCTCGAATCCATTGTTTAATGTTTGCTTTGGTTGTGTTACTTATAACTATAATTGAGATTTAA
TTTTGTATAATAATGGTG
>TCONS_00101804 geneID=XLOC_020359 scaffold_328:366959-367938(+) Unclassified
GGCTCATAGGACATAGATATTTGCTTCTTTCATTTAAAAACAACAATAACTTGTCTATGGTATAAAAATA
ATGCCTGAACATCCGGCTACAGTACCAACATTATAAAATTACAAATCGTCTATGGTTACGCTGTCTATGG
TGTAAATACTGCGTTTCTATTGGCTGGTTACCGATATTGAAAATGGCGCGGCTTTTGTCACATCCTGTTA
TGTGTTTTTGGGTGAATGCATGGCCGCTCTGTTGATTCCGATCCTAATTTTGTCTCCTTCTCAATCTCAG
TCGCTGTATTTATGTGATGTTAATGCAATATAATTCGTGATGGTGTTCATAATAAGGCCATGGATTTGATG
AAAACGAAATTGTGTGCACGTGGGAGGTAAGTTATCATAGTTATCATTGATTAGATGGTGTCCAGTTTC
GGGTTTTCACGATATAATCACTTGTGTGTTGATTTGTCAATACAATTACGGCACCCGGCAACTAGCCACT
AACCCGAGCGTTATTACGCCATCTTATCCACCAAAAGTTCCGCATATTTCGCTTTTTCCGATAGAACTAA
TGAATATCTTACGATGGAGATGATTGTTAGTTGAAAATTATTATAATAGGAGTTGAATTTGGCCAACAGC

AGTAGGTATGGCGTTTTTCATGTTGCTTGCACCCCGTTTCTTCATTATCATGACTTGACAAACCACATGT
GCATAGTCGAATCCGACGCTAATTTGCACTTAAAACGACGAAATCGTTGGTTCACGATGTCAAAGGAT
GGTAATTTGTAGAAAATACATGTACTTGTGCTTTGTAATATGGATTGAAAGTAGGCCTTCAAATTATAAC
CTTAAAATTTGACAGTCTCCATGAAGTCCAAT
>TCONS_00101968 geneID=XLOC_020360 scaffold_328:369131-370273(+) Intergenic
GTTTTTTTTGTTGTACTCTTTTGATGAACATATTCATCTCTGTTAAGTATTATAAAAGTACAGTAGAAACA
AGAAATTGGTTGGAATTATAGCTAGATTTTGAACTTACAGTATTTACATAACGATAACTCAGATCTATAA
CGGTGGGATGTGTCTATCAAAAACTCTCAATATGAGTTATTGAATTTCTAGACAAATTCAATTGTAGCG
GGATGAGTAACTTGATAAATTTAAAACTCAGGTAAAAGTGAATGTTGTTCTTTTAATAAATAGTGCACTA
TCATGCAATGTCACCTGCAGACAGAGAGCAGGTGAAAGTAGCTTGTAAGGAATTATCATTACCAACTG
TTTTCAATAAGTAAGATTTACCATATTGCTATCTCTACATATAATGTACAAATGCAATAATTATGTTTGCAA
CCTTGTATGTTAGTGATTGCTGGAC
>TCONS_00102293 geneID=XLOC_020415 scaffold_329:143400-149011(-) Intergenic
AACTTCGCGTCCATAAACAAGTTTACTCGGTCGTTAAGTTAGTTATGTTTTGAATAGTTTGTGTACAAGT
AAAATGGAATTTAAGTAAAGAAAACCTCAACGCTTTAAAGTGCACTATTAAGGCGAAATGAAGGCAGT
GATTACAAAATTCCGTGTTTTGTGCGAACTTTGTTTGGATAGGATCCAGTGAAAGTGCGGTTATGTTGA
GTGCTGACAGTTAAACGAATTATTTCGTGCGCTTTAAAGCTCTCCTTGGAATATGGTTTTAAGAGGCCC
GCATTCGCGCTCGCGCCGCCCGGTACAGTGAACGTGCAGGTGAACCCACGGCCACGGCTCG
>TCONS_00102302 geneID=XLOC_020405 scaffold_329:244495-245346(+) Exonic overlap (+)
ATTCATAGGAGACAGTCATACTCATGGCATTGCTATTGTCAACAAAACTACGTCAGACAAAGTGATGCC
TGTTTATTGACTCTGCTATTGATAAGGGTTTCATTTGCGTTAGGTAAGTTAGTTTCAGTGTAAGGTAAGG
TAGGCACCATAATATCACGTCTCTTTTTTCACAGTTATAAACTCTAACCACTATAGAAGTGCGTTCAGAA
GCATGTGCCACGGAACCTTGAACGAACAATATCCTGAAACCTCGCAACACGGCCGCAGCTCAACATCT
GATAACATATCAGTGTTATTTATGCACAACAGACGAACGGAATTATGTAGCCTCAAATAACTATCATACT
AACATATCCACCTAGTCATTAGGTACTTACTTTCACAATGTTGATTCTTTTGTGAGTGAAAATGCAGTTT
TGCATCTCTACTTCTGTGCTGTGCCTTCTTCTTGTGCTGGTGGCCGGGCAGACGCACCATGAAGGT
>TCONS_00102429 geneID=XLOC_020493 scaffold_33:195606-196446(-) Exonic overlap (+)
TTCTACCAGGTGAAAACAAATATAAAAATATTAAAAATTATAAACCATTCTGTAAAACTAAAAAACATT
TCTCCTTATTAATTAATTTTTAGGTCATCAGTTTCTTTTCTTACTATACCTGAACAATGGAATCCTGCAAA
GTCGACTGCGCCCACTGCCAGCGCTCGGTGGAGGGGTGCGCGAGCATCAAGTGCAGCCTGTGCTGCT
CCCAGTTCCACCTGGAGTGCCTCGGCAGTGGCCTGCCCCCCTCCAACTCCTGGCGCTGCAGGAGGTG
CAGCCAGTGTGAACAGGCCCTAACGCCGCAAGCGCTCGAGTCTACTGTAGTTCGTGCTTAAGGAATTT
TCTAGTGTCTTTTGACTGTATCGTTTGTAATAAATATTAAGTGGATGTTTTAATTTTGGTTTTTTATTTTGT
TTTGTTTAGGAATTGATAGAGTAAGGCCACGTTCAGATGACAAGCGCTCAACGCGCGTTTTGGTAACG
CGCGTTTACAACTACATACATTTTAT
>TCONS_00103114 geneID=XLOC_020505 scaffold_33:666365-667908(-) Intronic
CATGATGATGATTTGTGAAACACTGATCAGTTCATAACTAGCTATACACTTGGGTTCCTTGTCGAAATAA
CAAACCGAAAATGTATATTTCAATAAAATGTCAGTTGTGAGTTTGACAACGACAAGGCATAGAGCTAC
ATAATTATAATATTATGCTTTCGATTAATAATAATATGTGGGGACATCTCACACACGGCCATCCGACCCCA
AGCTAGGTAGAACCTGTGTTATGGGTGTCGGACAGCTGATATATCAACACAAATACATAGATAGATAGA
TACTAAATATAAATATCACCACCCAAGACCCGAGAACAAATATCTGTGTTTAAACAAATATCTGCCCCA
GCCGGGAATCGAACCCGGGACCTTCGGCTCAGTAGTCAGGGTCACTAACCACTACGCCATTCGGTCGT
CAAACCTCATGACTCACCTCTGACTACCCCTTCAGGGGATGAAAGTCGTGACTGTCGTGAGCAATTTG
TAAGGTAGGGTAGGGTTCATCACCAGTATCTTTGAAGGTATGAAAGTGGATAAGTTCTTATAGTTTCAG

CTATACATTCTCCAAGGAGTAGGTACATACATCTTTAGTTATGAATTAATACCTGGGTGAAATCGCCAAA
ATTTGTACTTTCAATTTTTGTTCATTTATCTTGGTTGCACTAAAACCTACTAAACGAGAAAAATATAACA
ACTTCACATAGAGAATCACGTAGGTCTACAAGAAAATCATTAGTAGTCACAGCCACAGGCACAGAAGT
CAAAGGAATTAAAAAATTCAAGCTTAGATCGCCGGATACAAAAATAATGTACAATCTCGGGCTCAACG
CTAAATTATTTCAGACTAATGTAAAACTTTTTACTACATGACCTTCACACATCGTAAAACCACAATATGA
TCTACAATAGGTAGTCTTCTGATTTGTGCCATACACCGCGATTACATACAATATTTAAAAAGTAACGACT
GCTTGACAACATATTGTGTCATACTATACTATAACTATAAAGGCTTGATGATACTAGGATAGTTTGTTAGC
TCCTAATAGGCGAGTTTGAAAGTGAGCGAGAGAGCAATTTTAAGTTTTTTTCAATCGGATAGTTTAATA
ATCAGATTTTCAATAAAAGCTCAATAACACAGAGCCATTCTACAAGCTACTCGTGATCTAGTAAACTAG
CCTGCTTGACAACATCAAATGACATACTATAACTTTATTAATAAACAAATAAATCTTCCATGTCCATTGG
ACGTGTAGCGACCTTGACACGGGATTCTCCAAACATTATCCTTTTGTCTCAATTGACTGGTAATTGCCG
GAGACAACTCGTTCTAGCCCATATCAGACATTTTACACTAAGCACACGGCTCCAATTGCCTTATCTGATA
TTTCGTGGGGAT
>TCONS_00103123 geneID=XLOC_020511 scaffold_33:984255-984601(-) Intergenic
TCACCTGTTAATGGGTTTCTACAGCATGTGCTGATTTTACACAAAGAGTTTGTAAAAACACCTCTCTCC
TGAACGAACGTTAAAGTAATTGTTTTTTATTTCACGCGCGATGAAAAACTTTAGAACAAGCAATGGCA
ACTGCTTCAACAGTTTACAAAAACGCAACGGCCGAGGGTTATTTCCCCGAGACGCAATGCATGGTGGT
CATAATCAGGTGTATTTTGCTGGGTACAGTTCGTTTAACGGGAGATGACTCGGTGGAGGGGCGCGGTA
GTGGTGGCGCCGCGCGGG
>TCONS_00102775 geneID=XLOC_020465 scaffold_33:1044367-1047142(+) Unclassified
CAACATTGTATCTAAGTCATATTTTGTTGAAGATACAGTTTACTGACTGAAATTAACTGATGAAACTTCA
TTTCAGAGGGTAGCAGTTCCCTGCTGGCCATCGTGTGGACCGCCACCCTAGTCCTCTGCTTCTACGGA
GCGTTCATTTTGTACCGTGGAGTTATTGCTTGTCTCCGAAGCCGAAAGCTAAAATCTATTACCAAGGTC
TAAACTGTATAGTACCCACCACTCATTTTTGAGAGCAAAGTCTAGTCATAATCACCAAAGAATTGGAAT
TTTGTTGAATTGTAACAGTCATTACTAATATTTTATTCATAGTATTATTGGTAATCTGTTAATTTAAGACAA
TGTTAAAGTTAAATCACAAACAGATTTGAAGAATACCCGTTTTGCTCATATTAACCTTTTCTAGTCAAAT
AACGTGTACCAAAGCTTAAATAATATCACTGCAGCTGTAAGCGACCATATCATAGATTTCCAGATTCTAC
TTTTACCTAAATACAATAGTTCTATTATGATATTCACCCTGTTTGTAAAAGATTAGTGACGTATTTTATGG
AATCAAACAAACAAAAAATAGTCATAAAACAGTCTGTCCGCTGGAGTGCTTCCAATTTTTACGGCATA
ATGCTACTTTAGACGGTTTTATAAATATTCCAACTAGTGACACCTATTGACTATTGGCATCTGTAGGTAC
GGTTAGGGAAGGTGTCTATAAATTGAGCAGTTACTTATTGAATTAGGGCTTGAGGATTGACAATATTTTA
TTGGATATACGCGGGATTCGTTGCATAAGGTTAAAATGTGCTGAGTTTTTCAACTAGTGAAAGATGGAT
TATCGCCAAGTACCTACAACAGATTTAAAACGGGCGAAACTAATTTTGGTAACTAATTAGGTACAAATA
GCAGTTTTAGGTGTCCCTCCCGTCGGAACTAATTCAGAAATGTCCTATTAATGAGCTATTGATAACCCA
GACGTCCATTTTATAATATTAGACTAATCGATGGGATTGATATTTTTATGGACGGCTAATATTAGCTCAGC
ATTGATATTAAGTTTATTTAAACGTAAATTGTTATAACTATGATGATATAACTTGATGTTTTGTTAACTCAG
AACTTTTATGATAATTTAGCGCACAATATTACAACTCCCAAAACTATGAAGCATGAATTGGTGTACCCTA
GTATACTTCCTATGAATAATGGCGTAACTAATTATACTTAAATTGAAAAGTCAAAGAATGTTGAGGAGCT
ATAACTATTGACTAATGAATGACCAATTGGACGTGACACATCAAGGCACATATCAGTGTATTTAATTAAA
AATTATGACGGTGACGATGATGATTGATGATCTTTTTGCAACTGTAGAATAATCGTAACACATCTGGCAA
CTTTGATTCAGAATCTAAGAACGGGTAAGTGTATACAGCTATACCCACAGTATGATAATGTAAAAAACT
CATTATCAGTTGCATGAGCATCAAAATCAGACTTGTCAATGCTTAACAATATATAAACTACTGTAGAGCT
TTGACGCACAAATACTGAGAGTACTAACTACCAACATTATTTACGTATAGCTTTAAGACCATTTATGATA
AAATATAAAGCATTCCGATAATTATTATATCGCGTATAACAATTTTTTGTAGGTTTTACATTTAATCCCATG

TATCGTGATGAGTATTTGTATAACGTGAATGTGTTCTATTTCTTGAACCCACTGCATTTCTAATCTCTTAC
CTTTTTTTCAAAAGTGTGGACTGTCTTCATTCTTGAACTTGAACTAACCTTCTTATACATTTCCTATCTTC
ATGGAATCAGCGAATCTAGACTTAATAAGAAAAGTAAGAGACTTTTCGACTGTATACTTATATTTTCGAT
ATGCTAAGCCTAATTTACCTGTTTTAAATATATTTTAAAATGTTTCATACTTTTTTTAAATCAGAACTTTTG
TATATTTTTAAACTGAAGTATTTTTCTATGCAGACGCTTGCATTTTGGGTGCAATAAGAACAACCTTCTC
GTTCCTTTGTGACAATATATTATATTTATCAGTATCTCAATACCTAAATCATGTAATAAGGAGTACTATAAT
ATCTCTATGATCTACGAGTATACTCTAGTTATTTCCTAGATTCGCTATATTTGTCACTCAACTGTTCTATCC
TTTGATTTCAATGCATACTTGCTGCATACCTACTTATATTCCTTCATATATTTGTAACCGTATGTTATTATGC
CATTACAGTATATTGATGTGAAACATAATACCTAGTTAAGGAATCGTGTTAGACAATACAATATTTGATAT
TTAATAAATTTGATTGTGTTTTATACATATGACTTTTAATTATTTAATCCATATAACAATCATCTCCTATAAC
CAGTTATGTCAATTTATATTGAATTTCCTTTCAAGTAGCCCGACATTTTTCCTTGAATCCTACTTTCTTTT
ACTTGTGAAGTGGCTTAGGTTCATAAATATAGTCCCCGAGCCCCTGCTTCACTATCGCGTTGATCCTCG
CGGCGCGCAGCTCTCTTCTACACTTCGCCTCGTCCTTCTCGGCCACAGACAGGTTCTCCAGGTTGCTC
AGTAGAACCTAAACATGACAAGTTTCACGGATTACAAGCAAATGTGAC
>TCONS_00103321 geneID=XLOC_020519 scaffold_33:1198891-1200781(-) Exonic overlap (+)
GCGCGGGGAGCGCGGGGGGGACGGGGGTGGACTCCGGCGACGAGGACTCCGGCATCGAGAGCAGC
GCCAAGACCACGCTCGAGCGCAAGCAACACTCGCCCAACGTGTCGTAAGGATAGGGTTGTTTATTGTA
TAAAACTTATAGATCTGTTTACGTATCGTGTACGAGTTTAGAATGAGCCATTAGATTAGACGTGAAGTAG
TATTACCGCGAGGTTAACCGTATTATTGTTGCTACATCGTGTTTGTTATGAGCATTATACATATCCGAGCA
CCGCCGGCCGCGCATCTACAGGTTATTTATTCGTTCAATAATTACAAAACTAAGGTACAAAATAGCCGT
GACAGTAAGGTGATTTATAGCGAAGTAGTTGTAGGGCTTTCACGTAATTAATTATACATAATTATGCTAA
ATATACACAGAATATGTATATCGTAGAGCTTCGATGAAGTTTACAATCTCATGGATGCTGGTGCCTAATTT
TTATCAACTCTAGGCCTATCCGACCATATGACACTTGAAGCCTATGTTAATGAAATGCTAGTTTTTGTCAT
ACGAACCATAGAAAGTAATACATTGCCTAAATTATAGTAAAGAATATTATAGTCAATAGATACGTGCAAT
AGCAAAATGAACCACTTACTGCAGTAAAAATCTATTCATAAGTTTATACGTATACAATCAGTCCTCGAAT
CTCCGCTTTAGAAAATCATATATTTATCTCTGTTTATCTTTACTCGATGAATCGAGATGGCGAGTCTGAAT
GATTTACTAAGACGGTGAATGATAGAATGTTTTCTTTAAAATCTGAATTATAAAACAGGAATTACTCAAA
TGAATTGTCCAATATTTACTGTATTTATTGTTTGTAAGTTGTTATTGATTGTGTATGAGCCCTTGGCAGTA
TTTAGTGAATGTGAATGC
>TCONS_00103208 geneID=XLOC_020519 scaffold_33:1229581-1231162(-) Exonic overlap (+)
GGTATCCCTAATTTAGAAAAATGGCGTTCTGTACAGCTGTGGGTTGCGCAATTTTTATGTTAATGTGAAT
TGAATTTATAGTAATATTAGCGTTATAAGCGTAGTAAATCGTAAGGACTAAAGTAATGCCCTATATAAATA
ATTTACAGTGTGGTTTTTGATTGGTTAACCTGTGTTTTTTGTGAAGTGCAAAAACTGTCATGGACCGTG
TATTTCTTGTGTTATGGTGATGTATCAAACCGAATTATGATGCCGAAAACATGATACGGCTGTTTAAATA
ATGTTATGATGGGTTTGTTATGGGTGGTGTTGTTCGTGGCCTTGACCGGCTCACAGGTGGGACATGGAT
TACCGATCGGATCCTCGCCGCCAAGCTACCCGCACGAATCCCCTCAAGATG
>TCONS_00102400 geneID=XLOC_020484 scaffold_33:1521922-1537504(+) Exonic overlap (+)
CCTCAATCTCTGGACTCTTTTGTGGCACCAGCACTTCTACAACCTCCTTCTTCTCCGGAGACTTTGGTT
TTACCTCAACCACCAGCTCTGGCGGTAACTCAGGTCCAATAGGCACCGTATACTCCTGGTTGGGAGGA
AGTGGAGGTCCATACTCCACTGGTTTGGTCCTCTGCTTCTTGAATGGGTTCTTGGCAGGCCTGGGTGTG
TCTGGCTGGGGTTTTGGCTTGCTGGTGGGTTTGGGAGTGGGTGCTGCAGCTGGGGCTGGTGGCTTCTT
CACAGGGCTGGTGGTTTCGTCGTTGGAATCAGAG
>TCONS_00102805 geneID=XLOC_020532 scaffold_33:1638343-1641237(-) Unclassified
TATTTCCAGACGGCCGCGGTGGTGACTGAGAAAGCTAAGCCGAAGGCGACGCCCAAGCGGACGCCG

GCGAAACAGGCCACGCCCAAACCCTCCCCCGCCGCCACGCCCAAGGGAAAAAGGAGGGCGCCTGTT
AAAGAAGTGAAACCCATCGTGCCCACCCCCTCCACATCGACGGGCAAGGCCACACGCACGCCGAGG
AAACCGCCCGCGAAGAAATAATACTAGAGTGAACCGGACATTGTTGACTAGTTCCAGTGACTGTGCGC
GGCTGTAATACACGCTTCTACTATTTATTAGTCACGTGCCACGGGGCAGATCTTACTGGTTCGGGTAAAT
AAGCTCGTTATGTCGGGCCGCAGCGCTAAAGTCGCTCCAATGAGTTTTAAGGTCTAGGCACATATAAGC
GTAACTTAAAGGGATCGTCTTTTATTCTTCTATTAACCAGGCGTTCGCGAGCACGCGTTCTACTGATTGT
TAGAAGAAAAAGAGGCGATACCTTTATGCCTCGGCACAGTCTTATAGAGCTCATTGGAGCGAGTCTTG
CGCTGCGGTCTGACATAATGTGCACGAACCTCTAGGGTCGGTTTGGTGTAGGGTTTTGGACAAATGCA
AAGAGTGTAGTAAGGAGCGGTAATGCTGCGTAGGTAATGATAATGAAGTGTGTTCGTGGAACTACGGG
CCAGCTAGTGAGTAGGTATGTAATGCATCTGTTCAACGGTCTACGTTAAACAAACTAGTGTGGAGGGAT
TTGAAACATGTGACTGGGGGTATAACGTAAATTAATGTGGGTTGATGCGTGAACGACACCTTTATATTA
CTTTTAATTTTTAACCCTGTTCCATTCAGCATAATCATGTTTTTTAAATACTGTATAGGTTTACGGGAGTC
TTGAACTTTGTTTTTTTTATAAATAAGTATAATATATTTTATGTTTACTTTGGTAACACATTACTTACAATAT
GCAGAATTGCAGATACAATGTTTAGCGTAATATGGTTTTGTTGTATTTAGTTAATGCAATTTTCAAAATAA
TAGTTTTAGGTCTCAGGTCTCCTGGACAACTTTATAACAGCGAAACAAGTATGATTTAGTCATTTATATA
TTATTTTCACAAATATTTTATTTTATAAGTGTTTTCGTGTTATGAACGTACACTGTAAAACAGTTTGAAAT
CTAAACATTAATTTATATGGAAATTTTCGAAATTTTTGAAGCAATAATAAATCTTTTCTTCTTGATGAAAA
TTATAAGAAATCTTTAGTATAAAACATCACACATTATATTTTTTAAGATCTTTTATATGAAATCATATTTGA
GGAAAGAATATAAAATATATTATAGTTGTAGACACAACTAAAGATTAAACAATCATAAATCTTAAATTTT
TTACTGTAAATACATATTAATATGATGATTTCAAGTTTTTCTTGTTTATAGTGTTACAGTGAAATTAAGATT
TGTGAAGAATCAATTAGGCAATAGATTTGTTTAGATTTTTATAAGATTTAGGCTAAAATTGGGATTAATTT
GGAGATATTTGGAATGCAAATCTTTTATATCAGTACAAAGAATTTAAAATTTATCAAATAAAATTCTGAG
ACATTGGGCCGTTTGAGTTACATAACATAAGAGTATATTTTGATACAATTGCAAATTTGTGCTGCGCTGT
TGTGATTTTCAATATATTTGTATCTGACTAAACTCAATGTTCAATATCAGTGTAAGATGTTATGTACTAATT
ACATAATAATATTATAGATCAACATTCACCAGAACTCTTAATCTGTATTCTGTTCTATTGTATCGATGCTG
AATCGGTACTAAAGTTTACTTATAAACCTCAGACGGAATAACTTTCTGCAACTTTTTGTTGAACAGTTT
TTGTGACTTATTTTTAGAGAATGGCATGTTTAGTACACCAATTTCTTTTTTGTTAAATTGTTTTCAATGA
ATATTCGTATGTTGTCGTTTTTATAAAACTGGTTCGCCAGTGAGGAGTGTGTGTGCATTCATGATAGGCA
AATTAAGTACCTAAAGTTTAGGAGGTATTTTTAAAACTAGAGTAATTCTAAAGAGAATAAATAAGCTTA
GGATATAAATTATTGTGTGTGTATTTTCTGTTTATGGTGTACCAATTGAGAGAGAATGAATGTGAAGTAG
GTTCTCTGGTACAATAGTGGTTAACTATTTTAGCGTTTTATACAAAATGATATCAGACGAAGAGTACTAT
TGTAACACAGAAAGTCCACAAATTACAAGATTTATATGCAATTCAGAATTAAAATATAATAATTTAAGAA
ATAAATGTGTGTTTTATTAAAGCTTTGATTATTATGCAAATACAGGCAGTTCCAAGTAGGCAAGATCATC
TATGGGCTTGTACACAAAACTGCGTTGCGACGACGCATCGCAAGAACCTGCGACAGTAATCAAGCATT
TTCCCTGAAAAACTGTCAAAACAGACGCAGATTTTTGCGATGCGACGTCGCAACGCAGTTTTGTG
>TCONS_00103424 geneID=XLOC_020639 scaffold_330:5199-5907(+) Unclassified
GTAGTTAATAGTAGTTGTGGTTTTCGCCATAGTCCTGTAGGAAGGTACTACTAATGTCAAAGTTTGTACA
GTCTAGGTATTTTGTAAGTACTTACCTACCTAACTACTTTCTCATAGCAATACCATGTCCTATTAATACAA
TACGTTTCAGACATGAACTCTACACAGCGGACTGAAAGTTTCACAGCTTCAATGGGGGATCACGCGGC
TTTGGAGAGAGCCGTTGTACCTGACGTAGAAAATCCATCTCCGACAGACACTGCGCGCGGCACTGAC
GAGTCTGGGTCGTTTATTCAAGTAGTTGTTCACAAACATCATCAGAAG
>TCONS_00103450 geneID=XLOC_020639 scaffold_330:5648-6620(+) Intergenic
CGCGCGCTTCGCTTCGCCTTAAAAAGTTTTCCCGTGGGAATTCCGGGATAAAAAGTAGCCTATGTTCTT
TCCCAGGGTCTAGACCGTATGTATACCAAATTTCATTCAAATCCGTTCAGTAGTTTTCGCGTGAAAGAG

TAACAGACAGACAGACAGACAGACAGACAGACAGACAGACACAGTTACTTTCGCATTTATAATATTAGTAAG
GATAAGTACTTACTGCTCACCCTACAGGTAGTTGTTCACAAACATCATCAGAAGAAGGAGTCGCAGCC
CGCGGCCGCGCAGACCTCGCCGCGCCTGCCCGCAGCCTCCAGCCCCATCGCTAAGACGGAGCATCCT
TCCAAGGATCCGGTGCTAGCGGGATGCGGCGATGGGCCGGCGCCAGGGCCGCCCCCCGCCCCCGGTG
TGTACATGTTGCCGCCCCCCCTGCTCCTCACCTACCCTGGCATGGCCCAGCAGCTGGACGGGCGTGAT
GGCTACTCGTGAGTACTCCTGTTATACCTATCCTAAAACGAGAAGGTAGTCCAAAAACTTTGGATTTTA
CGAATATTTAGGCAATGCAAAATAAAACAAGTTGCAAGTAGGTAGGTAACTTATTATATTCTCTAAATAG
TAAAAATTAGTTTAAAATTTTACGTAGGTACTTTATGTATGTAGTTAACGATATTTTGTAATAAGTATTTGT
ACAGTTGGTATCCCGGGCGGCGCACGATGGTTGAGGTGTCCACGAGTGCGCAGAGCTCGTCCTCGTTC
AAGAAGCATGTCCGTGCTGCGCGCCCCCTTGCACCCACCCGCAAGGCGAAGGTGCCGCCATCTACCC
GTCC
>TCONS_00103521 geneID=XLOC_020639 scaffold_330:6248-6696(+) Intergenic
CCCAGCAGCTGGACGGGCGTGATGGCTACTCTTGGTATCCCGGGCGGCGCACGATGGTTGAGGTGTCC
ACGAGTGCGCAGAGCTCGTCCTCGTTCAAGAAGCATGTCCGTGCTGCGCGCCCCCTTGCACCCACCC
GCAAGGCGAAGGTGCCGCCATCTACCCGTCCTGCCGCGGGGGGCGCTGGCGATGGCGGCGACATGGA
GGATGATGGCCCTGTGGGGATTTTTTTTTTCATATCGAAC
>TCONS_00103428 geneID=XLOC_020644 scaffold_330:161523-167426(+) Exonic overlap (+)
CGACCACTACGCGCTATTCGCCCTGATCGCCTGCCTAGAGTTCTTGTTCTGAGATCACGCCGCCGGCGG
CGAACGTCGGAGCTCGCGGCGTCGGTCCCGGCGCGTTAAGGCGCTGTGCGGGAACGGTGCCTCGTGC
CGTCACCTCACACACTCTGACACTGAAGTCCCGCTCTCTATCCAGCCTCGTCAAGTGACCGCCTGACA
GACCCCCAACGAGGTGTACTAGAAACTACTAGAACATTCGACGGTAAATTGCATCCATGTTTAGTACG
AGTTTCTCCCGATAGATGGCGTTGAAAGCGAAATTTCTGTTTAGGTTCAGTATTACTATAGTAGATTCGT
TCTAATACTTCACATTGGTAGATATAAATTCGACGGTAATGCACGCATGTCTTAACCAATGGGGAAGAA
AACCAAGTGAAACGTTGGCAGCACTATCGGGTAAGCCTTTCAGGTTCTGACCAATTATATTATTGGTGA
GAAAAATGGCCACTGAACCAACCAGTTAGAACTGGGGAATCGGGCCTGCACGACAGTGGCGACATCT
GAAATAAGCTTTGAGTTTTCCTCCCCATTTGATGTTTAGTACGAGTTGCTCGCGATAGATGGCGTTGAA
AACGAAATTTCTGTTTAGGTTCAGTATTACTATAGTAGATTCGTTCTAATACTTCACACTGGCAGCTAAA
CTAGATGTAACTTATAAATTAACGTGGTTTTGACAGCTGTCAAGTGTGACCTATTGGATTGATCCTACTA
CTTATACTACATTTTATCTTAACTATGTACTGTCGCTGTAGTAAAGTTTCTCAGATATAGTGTGAGTACTT
TATTCATGTAAGTCATTAAAGATTAATCTCAGAAGTTTACAATAGATGGCGCTGAACCATCGATCGTTCA
ATCGCAACCTCAATACCTATCAGTTTCCAACGAATTTTATCGCATACAGTTAAATTCGTGTTGATTCGTG
TTAAACTAGGTTTTAGATAGGTAGATTGTTCTAAACATGTATTATTGGTGTATTAGCTAAATAATTAACGA
TTATTAAAAAAAAAATTGAGTTAAAATTGGGATAAAATATTTCGGAGAGCATAGCATTTGAAATTGAAAC
ACGCACATCCGATATTTTAAATAATTGTACGGTGTAAATTGTTAATGAATTTAGTTACTAAATTAGTTAAA
ACAATACCATCTCTCAGCTCTCTAATAATCCGAATTAATGCGATCAAAATGTTTGTTATATCTCGGAAGG
ACTAAGTATGGTGCCTTTTGTCATTGATTTATATTAACAAGGCACCAGCTTCCTTAGTTAACACACTATC
AGCATTTCATTACAATGATTAGATTGGGATAAATGTGCCATGGTTGAAAAACCGAGAAATTGCCAGCAC
ACACTTTTATAACATAACGGCTACTAGTGCCTAACACGCACATAACTAGTAAATAAGTTGTTAAACATTC
CCGTATTGATGTTATTTTAATATTTAGCACGTTTATTATCTAAGGTAGTTGCTCCCTTATTCATAAATACCT
CTTGCTTAATAGTGCGTACAGTTTGGTATCTTTTTATTGGGAATGGAACAAAGATTTCTGCACTGAGCTA
ATGAGGTTATATATAATCTATTTATGAGGGTAGGGGGTAGTTTGTATAGTACGTATGTTTTAAATAAGTCT
CAAATCATTGAGGGTTTTTTTTAATTTGACTGATCTATCACCAAGCATTTCGATTTCACTACCTCAATTG
ATTCCATGTAACTTTGAAACATTTCATCGGCATTTTAGTATTTTTGGGAGGACATACAAGTATTATCACCT
TCAAATCTTATAACGTCGTTAATTAATATTGAAACAAGAGGTACGTGTGTATCTACTTTTGACACTAACT

TCTTAAGCCAAAATATGTAGCCAGGTTTTACATAAAGACTGAATTATCTTTTTATAGTAATTTTAGCAAA
GAAACTTACGAAACAACCTGAAACGAATATTTGCGATGGTAATTTGGAATCTATTTATCATAGTACAAAT
AATTTTTGCTAGAGCTTATGTGTAATCGTTGTCTTATTATTGGTTGCTTTAGAGGACAACTTTGATTGGTC
TGTGAGAACTTACGGTGGAAATGTTTATCATTGTTTGAAAACTGCACCTTTAAGAGTGTATTCCTTGTT
CTTTGCAATACGTAACCAAAATGAGTAACTAGGTACAAAAATGTGAAAGTAAATAAATATAAATTGCTT
ATGTTTTTTTAATATACAGATTATTGCAATAGTGGATTAGAAAGCCGAAAGGGTTCAGAGTGTGTTCATT
TGAACAACTTTAAGTTAAACTTTAACTTTCAGCCCCTAAGGGTCCGTTTAGCATAGTCCGGCTCTCCTC
TCCAACAGGTATGTGTGAATATACCTTATGTCACGCCTTTTGACTTACTATACTACTTTTGCCCCTGTATA
CTCTAGAATAACTTATTAGTATGGTGTACAATTAAAAAGTTATTTAAAACTTTTCTGCCTCTTTCAACCTT
ATACCAAAAGGTAAGGGAGCTAGATTTGAAAAAAAAACCATAGGTGCTAGTTTCATTAACGAGTCTCT
TATGAAATCTCTTGTAAAATATTACACTTCTCTTTATAATTTTTATATTGGCTATATTGACGAAAACTATAA
CTAAACCTATTTCTTCCTGTTTGTTACATTTGTATTTGTAGTTATAAAATTAGTAATTATATGAAAATATTT
ACATACATAGGTATTATATTCGTTTATTTATGTAAATGGTGAATTGATGTAACGTTTAATATCGTCAATTGT
TATGCACATCGGTTTCGCACATTGTTTTTCATATACTTATTGTTAGGTTTGTTAAGACTGTTACTTTACATT
ATTTAACCAATCGAATGCAAGTCATGAGTATTTGTACTAGAAATAATTATTAAATTATTGTTGAGGGCGG
AATGCTGCTCAGTATTTATTAGTATGCATATTGTCATTAATGTTGATAGAGAAATACCTTAAAAATAAGAT
GATAGGGATACGAAGTTACCAACAACTTGATGATTCCCAGTGCTCTATAATAAATTTATTGAAAACTACT
ATATCACTATGAATACTATAATTCATAGTTTTGAACGTTGTGGATACGATTTCGGGTACAGGGGCTGCATT
AAATGACCATAAGTAGTCGCCCTTCGCTCCCGTAGATAGAATAGGGTATGCTTATGATATATAAAGTATAT
AGAGGTGTTTTAACGAATTTGGCTTGCATTTCTTGGACGAAGTGTCCAAGTCAGCACTGTGTTCATTTC
TTATTACTAGGGTGTAAATGAAAATTTATTAAATTAGCTGACGAGAATTCCGTGTTCCGTTCTTTGTATG
AGAGAAAATTATATATATTTTAATGTTAAAATATATAACGTAGTTTTAATATTGTATCTTCATCTGTATACA
AATTGCAAACGTCCTAATTTTACTAAAAAGGTAACTAGTGTTTTTAAAGTTTAACATTTATTACTCTGAA
TACTAAATACCTTTTTGTACAATATTGTAAGATAGATAACCTAACCCTAGCTAGAATAATAATTTGTTTTAT
AAGACTGTTTTATGTTATTCGTTAATTTATAGATATCGTTTTAGTTAGTTTAATATAATTTTAATTCACTATA
TTCTGAGATTTGCTCACAGAATAAATAAATATCCAGGGCATACTTTCTAGATTTATTGAACCATATACTGT
ATTTCTGTAATGTAACGATTTAATATTATTTACGGCCCATGCATAACCTTTATATTAATGTAATAAAAGTAT
GCCCATATCCTTAATCTCAGAATATTTAATCATT
>TCONS_00103530 geneID=XLOC_020646 scaffold_330:211308-212796(+) Intronic
CCCAATTTTCTCAAACATTTACCCCGACTTGCACAAACATATTAAATCTAGATTTAGTGTTAAATCTCGA
TTTAGTGTAAAATTTTATGGGCGGCTTGCAGAAAAGAAACCCTAAGCATAGCTTTACTAAACGTTTCTT
AAGACTAAACTGATTTTTATTTAAAACGATTTAAAACTGTCTTGCGCCATGGTTTTTCTAAAGCGATTTA
ACAAAACCTAGTTTATGATATCATTCAATTTCTCATTCGGTCTATATACGATTCGTTATACACTATTTGATT
TAAAATTTGATAGTTTCCAATGAAGTGGGCTAATATTTTGGGTGAAATGAAATATACTTGTCGTCACATG
TCAGTCCAGTCACCGACTGTTCCTGTCATTTGATAAACAAACAACCGTGTGCTAGAAATATAAATACCT
AATTGATAAATTATAAAAATGGAAGGTAAAAACCACCAACGGGCACCCAATTATACATGTGACGAAAG
TATGCTTTTATGTAACCTCGTAGACAAATATAATTTCATAATTGAAAATAAAAAAAACTGATGGTTAGAC
CGTAGCCGAAAAACAGAAGGCTTGGAAGATGCTGAGTAAAGAATAATATAACGTCAACACCACTTATG
TACCCAGAACTACTGAGAATTTACTGGTAGGTACAAAAACCTGAAAACCAAGACGAAAAACAGCACT
CAAGTGAAAAAATGCAGATCTCAAATATACCTAGAACCTAGATAGCAAAGTTTTACACGCGTCTGTAGT
TCAGACAGTGGAGCTCCATGGACAAGACTGGGAAACGGTGTCGGCTGATGTTAATCTCCCCCCAGAG
GAATCCCAAACAACATTCAATATTTTAAGTGAAGAAGCGAAACAATCATCAAGCAAAATAAAACAACA
AGCCATATTTTAAGATTTAGGTATATAATATACAGTGGTACTTAATTAATAATAATAAACATGTTTTTAAAT
CAACTGGTGTTTGGTACTGGTGTCGCTATAACCTTGCAAAATAGTTATTGATCAGCGAAGTTCTTGCAT

TGTTCCCAGACTGTAAGGCACGAGGAGGTTGGTCTATATAGATTGTTGTGGTATTGTTTGTAAGGCGAA
AGCTTTTAATAAATTCATCATCTCGATAAGCACGAAACAAATCTGTTATTTTAGTAGTTAACCCTCCTATA
AAAGTAAGGGGTATTCAAATAATCAATCATTTCTTTATCGTCGTCTGAGTAATACATTTTTGATCTTCTTC
TTCTTCTATCGTGTAAATGCACTAAGCTAACTTCCTCCAGTGTTTTGCTACTCTTATTGCGAGGTCATTG
GCCTTCATCAGGTCTCCTTGACTG
>TCONS_00103591 geneID=XLOC_020688 scaffold_331:16448-18289(+) Splice junction overlap
CGCACAGTCGTAGCCGATCTAGATCTGGCTCACGAAGGTCCAGATCAGGGTCCAGGCGTTCCAGATCT
GGCTCTAGACGGTCCCGCTCCGGCTCTCGCTCCCGATCCGGCTCCCGACGCTCCCGGTCCGGATCTAG
ACGATCCAGATCTGGCTCTAGGAGGTCTAGGTCTGGATCTGGGTCAAGGAGATCTAGGTCTGGGTCTA
GACGGTCTAGGTCTGGATCTAGGAGGTCTAGGTCCGGCTCACGCAAGTCTGGGTCTCGGTCGCGATCA
CGATCAAAGTCTGTTGGTGCTGATGGAGGTCGCCGCAAGCGCTCTCGTTCCGGTTCAAAATCTAGATC
TAGGTCCGGATCTAGAAAGTCTAGGTCTAGGTCTCGATCTCGCTCCGGATCTCGCTCCAGATCTCGCTC
GGGCTCCGGCGAACCTCAGACCAAGAAGCGTCGCACAGTCCGTCTGACGTCAGAGTCGGGTGATGAG
GCTCCCC
>TCONS_00103593 geneID=XLOC_020688 scaffold_331:16448-83633(+) Splice junction overlap
CGCACAGTCGTAGCCGATCTAGATCTGGCTCACGAAGGTCCAGATCAGGGTCCAGGCGTTCCAGATCT
GGCTCTAGACGGTCCCGCTCCGGCTCTCGCTCCCGATCCGGCTCCCGACGCTCCCGGTCCGGATCTAG
ACGATCCAGATCTGGCTCTAGGAGGTCTAGGTCTGGATCTGGGTCAAGGAGATCTAGGTCTGGGTCTA
GACGGTCTAGGTCTGGATCTAGGAGGTCTAGGTCCGGCTCACGCAAGTCTGGGTCTCGGTCGCGATCA
CGATCAAAGTCTGTTGGTGCTGATGGAGGTCGCCGCAAGCGCTCTCGTTCCGGTTCAAAATCTAGATC
TAGGTCCGGATCTAGAAAGTCTAGGTCTAGGTCTCGATCTCGCTCCGGATCTCGCTCCAGATCTCGCTC
GGGCTCCGGCGAACCTCAGACCAAGAAGCGTCGCACAGTCCGTCTGACGTCAGAGTCGGGTGATGAG
GCTCCCCCCGCGGGGGACCCCCCGGCGGGGGAGGGGGCGGCCCCC
>TCONS_00103726 geneID=XLOC_020688 scaffold_331:18662-19200(+) Exonic overlap (+)
CAGTCCGTCTGACGTCAGAGTCGGGGGATGAGGCTCCCCCCGCGGGGGGCCCCCCGCGGGGGGGG
GGGGGGGCCCCCCCCGCCCCCCGCCGATGATAGTTCTGATGATGAAGTGGCTCCGGACTCTAAGAGTA
ACGAGATGGCGGGCGGCATGACCGACTTCGAGATCATGTTGGCTCGCAAGCGAGACGAGCGCAAGGG
ACGGAGACGCAGAA
>TCONS_00103544 geneID=XLOC_020689 scaffold_331:132971-160556(+) Unclassified
CGATCCTTCGCCGCGAAAGCGCGCGCCAACGATTCCAAATTTAAAATTTCCGAACGTGATTCGAACGT
CGCGTGTGTTTTTAAAGTGCGTTTTCTGAGGATTAACTCCCGAATACAGTGCAGTGAATACTCTTTGGA
TTACTGTTGTGACGTCGAATGCGTATAATTGACCCGAAAGAAGAAAACGACTAATCATGTTATAGTTTA
AAAATACTAATTTTAAAAAGTGCAAAAAAGTAATACTGCAATCCTAAAATAGTCTTTACAGTGTTGTGT
TTAAGTGTGATTTTGTAATGGTGTGACATACGAGTGGCGAGCGCTGTGATTGGTCAGACGCTTCAAGAT
GGCGTCTGTAATCTTGACAGTTCTCTGTCTTTTGGCGGGAATATCCGGCGCGGTCTACGCTGAAACAG
GT
>TCONS_00103877 geneID=XLOC_020740 scaffold_332:82794-84443(-) Intergenic
TACGATTCGTTATACACTATTTGATTTAAAATTTGATAGTTTCCAATGAAGTGGGCTAATATTTTGGGTGA
AATGAAATATACTTGTCGTCACATGTCAGTCCAGTCACCGAGTGTTACTGTCACCTGTCACTTGATAAA
CAAACAACCGTGTGCAGTTACCGAAATATAAATACCTAATTGATAAATTATAAAAATGGAAGGTAAAAA
ACACCAACGGGCACCCAATTTTACATGTGACGAAAGTATGCTTTTATGTAACCTCGTAGACATATATAAT
TTCATAATTGAAAAAAAAAAAAACTGATGGTTAGACCGTAGCCGATTAACAGAAGGCTTGGAAGATGCT
GAGTAAAGAATAATATAACGCCAACACCACTTATGTACCCAGAACTACTGAGAATTTACTGGTAGGTAC
AAAAAAAAACAGCACTCAAGTGTAAAAATGCAGATATCTCAAATATACCTAGATAGCAAAGTTTTAAA

CGCGTCTGTAGTTCAGACAGTGGAGCTCCATGGACAAGACTGATGTTAATCTCCCCCCAGAGGAATGC
CAAACAACATTCATCAATATTTTAAGTAAAGAAGCGAAACAATCATCAAGCAAAATAAAACAACAAGC
CATATTTTAAGATTTAGGTATATAATATACAGTGGTAATTAATAATAATAAACATGTTTTTAAATCAACTGG
TGATGCTATAACCTTGCAAAATAGTTATTGATCAGCGAAGTTCTTGCATTGTTCCCAGACTGTAAGGCA
CGAGGAGGTTGGTTGTTGTGGTATTATTTGTAAGACGAAAGCTTTTAATAAATTCATCATCTCGATAAGC
ACGAAACAAATCTGTTATTTTAGTAGTTAACCCTCCAATAAAAGTAAGGGGTATTCAAATATTCAATCAT
TTCTTTATCGTCGTCTGAGTAACACATTTATGATCTTCTTCTTCTTCTATCGTGTAAATGCACTAAGCTAA
CTTCCACCAGTGTTTTGCTACTCTTATTGCGAGGTCATTGGCCTTCATCAGGTCTCCTTGACTGCACTTT
GGTGCATAAGGTGTGACGTAGTCTGAGTAATACATTTTTGATCTTCTTCTATCGTGTAAATGCACTAAGC
TAACTTCCTCCAGTGTTTTGCTACTCTTATTGCGAGGTCATTGGCCTTCATCAGGTCTCCTTGACTGCAC
TTTGGGGCATAAGGTGTGACATAGTCTGGAGGCTTCAGCAACTGCATAGAGTGCTGGCATTGGCTGGG
AGCATATCGCATCGCCCCAAACATTTTTGATATTACCGAGTAAGTTAACTAGAAATTCTAATTACTAACA
ACAAAACAACCAAAAAGAATACTTCAAAGATTGCTTTTGACATTTGACAAAGAAGTTAAATCTCGATT
TACGCGTAAAACCGAGATATTGCCGGTTTAATTTTGACGTTTCTCTAAATCGAGATTTGACACTAAATCG
AGATCGTGCAAGAGGATTTTGCTGATTTACGCTTAAACTGAG
>TCONS_00103878 geneID=XLOC_020740 scaffold_332:82794-84443(-) Unclassified
TACGATTCGTTATACACTATTTGATTTAAAATTTGATAGTTTCCAATGAAGTGGGCTAATATTTTGGGTGA
AATGAAATATACTTGTCGTCACATGTCAGTCCAGTCACCGAGTGTTACTGTCACCTGTCACTTGATAAA
CAAACAACCGTGTGCAGTTACCGAAATATAAATACCTAATTGATAAATTATAAAAATGGAAGGTAAAAA
ACACCAACGGGCACCCAATTTTACATGTGACGAAAGTATGCTTTTATGTAACCTCGTAGACATATATAAT
TTCATAATTGAAAAAAAAAAAAACTGATGGTTAGACCGTAGCCGATTAACAGAAGGCTTGGAAGATGCT
GAGTAAAGAATAATATAACGCCAACACCACTTATGTACCCAGAACTACTGAGAATTTACTGATATACCT
AGATAGCAAAGTTTTAAACGCGTCTGTAGTTCAGACAGTGGAGCTCCATGGACAAGACTGATGTTAAT
CTCCCCCCAGAGGAATGCCAAACAACATTCATCAATATTTTAAGTAAAGAAGCGAAACAATCATCAAG
CAAAATAAAACAACAAGCCATATTTTAAGATTTAGGTATATAATATACAGTGGTAATTAATAATAATAAAC
ATGTTTTTAAATCAACTGGTGATGCTATAACCTTGCAAAATAGTTATTGATCAGCGAAGTTCTTGCATTG
TTCCCAGACTGTAAGGCACGAGGAGGTTGGTTGTTGTGGTATTATTTGTAAGACGAAAGCTTTTAATAA
ATTCATCATCTCGATAAGCACGAAACAAATCTGTTATTTTAGTAGTTAACCCTCCAATAAAAGTAAGGG
GTATTCAAATATTCAATCATTTCTTTATCGTCGTCTGAGTAACACATTTATGATCTTCTTCTTCTTCTATCG
TGTAAATGCACTAAGCTAACTTCCACCAGTGTTTTGCTACTCTTATTGCGAGGTCATTGGCCTTCATCAG
GTCTCCTTGACTGCACTTTGGTGCATAAGGTGTGACGTAGTCTGAGTAATACATTTTTGATCTTCTTCTA
TCGTGTAAATGCACTAAGCTAACTTCCTCCAGTGTTTTGCTACTCTTATTGCGAGGTCATTGGCCTTCAT
CAGGTCTCCTTGACTGCACTTTGGGGCATAAGGTGTGACATAGTCTGGAGGCTTCAGCAACTGCATAG
AGTGCTGGCATTGGCTGGGAGCATATCGCATCGCCCCAAACATTTTTGATATTACCGAGTAAGTTAACT
AGAAATTCTAATTACTAACAACAAAACAACCAAAAAGAATACTTCAAAGATTGCTTTTGACATTTGAC
AAAGAAGTTAAATCTCGATTTACGCGTAAAACCGAGATATTGCCGGTTTAATTTTGACGTTTCTCTAAAT
CGAGATTTGACACTAAATCGAGATCGTGCAAGAGGATTTTGCTGATTTACGCTTAAACTGAG
>TCONS_00103764 geneID=XLOC_020732 scaffold_332:143222-170001(+) Exonic overlap (+)
TTCAGGACGTATCCCGGGCGAATTAACCAAGCTGAAAGTATCTGGAATGAGCCAACCACAAATTCAGC
ATTACAGACCACCAGACCGAATGTCAGGGTCGCGGGAGTAACATGGCGCGCGCGGGCATGCTGCAGA
GAGGAGGGAAAAATGGAGCAGAGTGAGTAATCGATTGTTTTTTTTCTAGACAGAATACTAACACATCT
ATTGGCAATTAAACCGGGCTCGGGGCTGGTTTGCGCACAGCTTCTGACCTCCATTATCAAAGCCGGCG
TCGGATCGGGCGGTCAGATGTTGATTGCTTCTAATAGGCTGCACAATGCACATCCGTGAACTACTACCC
CAGTTTCAGGTTTCCTGACGCTGGTGTTTTGTTTTTATTGGTTACTCAATCAAGCTATTGTTCGTATCTA

CTACATTTTCAGGGCGTATTGAAAACTCGTACTCAATCAACGTCTCTATTATGTTCTGAAAGTGCAATAA
A
>TCONS_00103897 geneID=XLOC_020732 scaffold_332:197957-199017(+) Splice junction overlap
GTTTTATTCGCTTATGCAGTATTTTCTGAAACGAAAATATGCACATTCGATTGTCCGACGATAAACGGAC
CAGCAAACTCAGTTCACGACGATGCAATAGCGTGCGGCTATGACCCTCAGTAAATACAGTACAAGTTG
TTCCCCAATCAATGCTCTCTGGAGTCGTACAATCACTGCCATGGAACCAATTTCACCGTAACTGCCGTG
AAATTTTGCGTCATAGAGCACTTTCTGTCCCGTCGCATGTACGGTGACGTCCCGTGTCCGGTGTTCTGT
CCGGGACATTACCGGCCGGTCTGCGCCTCGTCGCGGACCCGGTCCAACAAGTACCAGACCTTCAACA
ACAGATGTGTAATGGACATATTGAATTGCCATGGTGTTGAATATAATGGTATGTACCAGTGTACCTACGT
ATTA
>TCONS_00103898 geneID=XLOC_020732 scaffold_332:197957-199795(+) Splice junction overlap
GTTTTATTCGCTTATGCAGTATTTTCTGAAACGAAAATATGCACATTCGATTGTCCGACGATAAACGGAC
CAGCAAACTCAGTTCACGACGATGCAATAGCGTGCGGCTATGACCCTCAGTAAATACAGTACAAGTTG
TTCCCCAATCAATGCTCTCTGGAGTCGTACAATCACTGCCATGGAACCACTTATGTTGAAGTTTCCATG
AGTTTTTGTCCAAACGCCTTGAAAAACATATTCAAGGAACAAGTCGCCGTCGAGGGCTAAGGCAACT
GGGTCGGCTTCGATAACAATAACAATAATTATACGTGAAATACC
>TCONS_00103830 geneID=XLOC_020743 scaffold_332:271864-274126(-) Intronic
ACATCAGCCCCGCCAAACAGGCGGAAGGCAAAGACGGCGCAGAGTCCACGCCACTGCTCGTGAACG
GCGGAGTGAAGAGGCGGGTCAGCGGGTTCCGAGTTATATAAAAGCGACTCGGAGATAGCAAGGTGGA
CTAAGCGCCTGGTTCACGACGACGCCTGGGTTAAGGATATCTGTTGTGTAAGTTTTACAGTCTTAGGGA
ATCGGGTTCCCTGTACCTAACTGTACCGGTAACCTGATTATGAGAAAAGCGCTCACTCACGGAGCATTT
CCCGGATTTTTAAATGTCATGAGCGCTTTCGCATTATCAGGTTACCGGTACAGGTAACGGACGCATCGC
ATTCAGTATTCTTTGTACATAAATTCACACACGTGCGTCCACTTTATCGGCACTGCCGGCGCCGTTTTTC
CATACATTTATGACAATCCGTTTGGTGCGGTAGGGTAAGCACCCTACTGCCAAACCAAAAGAATACTGA
ATGCGATGCGTCCGTTGCGTACGATGCTGAAAGGTTGACGCTTATTTTTAGTTAGACTTTTATTCAACA
GATTAATCTTGATCCAGATAAACATATATTGTGAAACAGAGGGTACTTACCTAAGTCACTTTGCTATACT
TAACTATACATATAATACCGATTAGTTTTCCTATGAGATATAAATACAGATTAGGATATTATCAATGGACAT
CTTGAAGACATCAATGAAACCACATAGTCACTGGCAAATTTTATTATGGAATAATCATGAGAGTCTATTA
GTATTTCGGATGCATTGCTATTTTGAGATGGTTTCGTTAAAGTCATTAATAAAATTCTACTCCGTTTTGTA
TAAAGCAGTACCTACTTAATTATATGTAAGTACATACATACATAATATATAAACCAGTGAAC
>TCONS_00104169 geneID=XLOC_020804 scaffold_334:63065-88480(+) Exonic overlap (+)
CGACCGCCGGCCGCGACACACGTGCGTGACTCCCCAGACTGCGCCGCGACATCATAACTGACTCGAA
CTCTTTCGTTGTTTCAAGTTTTAAACTCGGTTTTCGTGCTCCGTACTTTCTTTTCGACCGGACTGAGGA
CAGTTTTGCACTCATCAGTTTTTGTTGATTGAATTGTTTTGTGTAGTGATCTGTAAGTGAGTGCTCGGTC
GAGTTGGAGGTGGATTGCGCGGCCACGCTGACCGGCGACAGCAGGGCGTGCGTCGGCGCCCGCGAC
CATGTGAGCCGCGCGGCC
>TCONS_00104176 geneID=XLOC_020809 scaffold_334:123164-137839(-) Exonic overlap (+)
AAAAAGTACATCTATGCCGAGTAGACGTGTTTTTGATTTTGGAACTGAATCTTTGGTTTTGCAACGAAC
TTAATGTTTTTGTGTGCTTGTCTATGTGTGCGTGTAACTAGTGACAGATTTAAACTTAGAACAAGATATC
TCTATGATCATCACTTCAGTGCATGACGCTCCTCTGGAGAGTTATTCCATAATCCCCTCGCTGGGCAGG
CCGGTTGGACATCGCAGTATCGGCAAAAAAGCAAACCCTAAGTTACAGACTAGTCCTAGATTTAGTTAT
TAGTCCTGTTGTTACGAGTACCTACAATTTTTTTGGACTTATTGCCTAACTATGGTGTATGTTGTAGTTCA
TAACTAGATTACTCATGATTTATGTTCATCTAAAGGTTATCTGGTAGAGATTGCTTTAAGCAATAAGAAC
ACCTTTATATGCTGTTTTATTTATAAGTTGTTATTTTTTAAATCACTCACTCAATCACTTTCATGGTGCAA

AATAAATAGTATTCTATTATAAATGTAAAACACCTGTTACAGTTATCTGTGTAACGTTAAACGTAACGAG
TGTTATTATAAGTTCCCACATATTAACCCAAGGTTAACCCACGGGTATTTAGGAAACCACATACAAAATA
CCAATGCTTTGCAACAAGAAAAGAATGGGTACATTCAATAATTTAAAATTACAAGTTGTATTGTTACCG
TATTTGAATAAATTATTTACACAATGAATATTATTCAAATGTGCCCTCCCAAGTTTATGCAATTATAAAGA
AGTAATCTTAAGAAAACATTTTTAATATAAAATGGCGCCTTTGTGTAATAGTGACAGACTGATATTATAA
TCGACAAATAATTGTAAAATCTTAAATCGAATAAAATAATAATATATTTTAAAAGGATTACCTTGTTTCTC
TGTTGATATTTTTTTATTAATGTTTAGGGTATAGAGATATGATAGTGATAAGGAGTACCTATGGAATAAAA
GTAAAATGCTATTAATAAATGCTACCCACTTGAGTGAGTTTGATTTTTCCTAATACATACACTCCTTTACC
TTTTTACTGTTAATAATGATGTCGATTCTGAAGGCAGAAATTCTAATTTTAACGCTGTCAAAACCTTAAT
TTTTTTGTTTAAAATTCGACTCTATGATTGTTTCCGTAAATGTCATTTTATGCTAGCAAATTTTTTAGTTT
GACAGCGTTAAAATTAGAATTTCTGCCTTCAGAATCGACATCAAT
>TCONS_00104177 geneID=XLOC_020809 scaffold_334:123164-173008(-) Exonic overlap (+)
GAAACGGCTGTTGATTTTTTAAGTGTTTTTGTGATTGGAAAGTGTAACTGAGACTTGGCACGTGAAAC
TTAGTAAATATTGCGTTTGTAAAGAAAGAGTTTTTGTACTAAAGTTTTTTTAAACAAAATATTTCGCTT
TGGTGCCAGATCTAGATGCTGAACTGGATAGATGAAATTATTAAAGATGAACTAATGGAGAATCAAAAC
CGATTAGGACTTGATGAAAATTAAAATGATAAATTTATATCTCTATGATCATCACTTCAGTGCATGACGC
TCCTCTGGAGAGTTATTCCATAATCCCCTCGCTGGGCAGGCCGGTTGGACATCGCAGTATCGGCAAAA
AAGCAAACCCTAAGTTACAGACTAGTCCTAGATTTAGTTATTAGTCCTGTTGTTACGAGTACCTACAATT
TTTTTGGACTTATTGCCTAACTATGGTGTATGTTGTAGTTCATAACTAGATTACTCATGATTTATGTTCATC
TAAAGGTTATCTGGTAGAGATTGCTTTAAGCAATAAGAACACCTTTATATGCTGTTTTATTTATAAGTTGT
TATTTTTTAAATCACTCACTCAATCACTTTCATGGTGCAAAATAAATAGTATTCTATTATAAATGTAAAAC
ACCTGTTACAGTTATCTGTGTAACGTTAAACGTAACGAGTGTTATTATAAGTTCCCACATATTAACCCAA
GGTTAACCCACGGGTATTTAGGAAACCACATACAAAATACCAATGCTTTGCAACAAGAAAAGAATGGG
TACATTCAATAATTTAAAATTACAAGTTGTATTGTTACCGTATTTGAATAAATTATTTACACAATGAATATT
ATTCAAATGTGCCCTCCCAAGTTTATGCAATTATAAAGAAGTAATCTTAAGAAAACATTTTTAATATAAA
ATGGCGCCTTTGTGTAATAGTGACAGACTGATATTATAATCGACAAATAATTGTAAAATCTTAAATCGAA
TAAAATAATAATATATTTTAAAAGGATTACCTTGTTTCTCTGTTGATATTTTTTTATTAATGTTTAGGGTAT
AGAGATATGATAGTGATAAGGAGTACCTATGGAATAAAAGTAAAATGCTATTAATAAATGCTACCCACTT
GAGTGAGTTTGATTTTTCCTAATACATACACTCCTTTACCTTTTTACTGTTAATAATGATGTCGATTCTGA
AGGCAGAAATTCTAATTTTAACGCTGTCAAAACCTTAATTTTTTTGTTTAAAATTCGACTCTATGATTGT
TTCCGTAAATGTCATTTTATGCTAGCAAATTTTTTAGTTTGACAGCGTTAAAATTAGAATTTCTGCCTTC
AGAATCGACATCAAT
>TCONS_00104178 geneID=XLOC_020809 scaffold_334:123164-178061(-) Exonic overlap (+)
CAAATTATAGGAAAGCGAGTAGGATTAAGTACCTACATATTTTTTTCATGTGTCGCTGGATTGTTAAGAT
GAACTAATGGAGAATCAAAACCGATTAGGACTTGATGAAAATTAAAATGATAAATTTATATCTCTATGAT
CATCACTTCAGTGCATGACGCTCCTCTGGAGAGTTATTCCATAATCCCCTCGCTGGGCAGGCCGGTTGG
ACATCGCAGTATCGGCAAAAAAGCAAACCCTAAGTTACAGACTAGTCCTAGATTTAGTTATTAGTCCTG
TTGTTACGAGTACCTACAATTTTTTTGGACTTATTGCCTAACTATGGTGTATGTTGTAGTTCATAACTAGA
TTACTCATGATTTATGTTCATCTAAAGGTTATCTGGTAGAGATTGCTTTAAGCAATAAGAACACCTTTATA
TGCTGTTTTATTTATAAGTTGTTATTTTTTAAATCACTCACTCAATCACTTTCATGGTGCAAAATAAATAG
TATTCTATTATAAATGTAAAACACCTGTTACAGTTATCTGTGTAACGTTAAACGTAACGAGTGTTATTATA
AGTTCCCACATATTAACCCAAGGTTAACCCACGGGTATTTAGGAAACCACATACAAAATACCAATGCTT
TGCAACAAGAAAAGAATGGGTACATTCAATAATTTAAAATTACAAGTTGTATTGTTACCGTATTTGAATA
AATTATTTACACAATGAATATTATTCAAATGTGCCCTCCCAAGTTTATGCAATTATAAAGAAGTAATCTTA

AGAAAACATTTTTAATATAAAATGGCGCCTTTGTGTAATAGTGACAGACTGATATTATAATCGACAAATA
ATTGTAAAATCTTAAATCGAATAAAATAATAATATATTTTAAAAGGATTACCTTGTTTCTCTGTTGATATTT
TTTTATTAATGTTTAGGGTATAGAGATATGATAGTGATAAGGAGTACCTATGGAATAAAAGTAAAATGCT
ATTAATAAATGCTACCCACTTGAGTGAGTTTGATTTTTCCTAATACATACACTCCTTTACCTTTTTACTGT
TAATAATGATGTCGATTCTGAAGGCAGAAATTCTAATTTTAACGCTGTCAAAACCTTAATTTTTTTGTTT
AAAATTCGACTCTATGATTGTTTCCGTAAATGTCATTTTATGCTAGCAAATTTTTTAGTTTGACAGCGTTA
AAATTAGAATTTCTGCCTTCAGAATCGACATCAAT
>TCONS_00104173 geneID=XLOC_020806 scaffold_334:199031-225008(+) Intergenic
TTCGAAACGAACATTTTACAAGATTTGGCGCGCACTTTTCAGTCTTGCAACTTCTTATAAGATAACATTT
TTTGAAAGTTTTTTTTAAATGGAATTTAAAAAAAAAGTGTTGGTTTTTGGGGAACTCAACTGAGCGTA
GAGAAAGCGATTATGTCCAATAACTATAACACTTGTATCAAATACGAAGAAATACTTTTTGAACCTTAC
GCTGAAATAGAAGATACAATTAAAGAATAAGGATTAAAATATTTTATTGTGAAAGTTTAAATTAATGAAC
TTACAAGTGAGTAGCTACTTAGTGCTTCAGTGATGGAATCATGCGATGCATGGAAACAAGACTTTGGC
GCCCAAATAGTGCCAACGAGGCAATCGCCGCCAAACGTTGAGAAATCAATAAAGAACTCGAGTTTAC
CATCCAGTGTGCACTTCACATGGAGAAATAAACCGGATTTTGATGTGATGCTGATTAGAGCTGTCAAAT
TATAATTTGACTGCAAACAGCCAGTGACAGTTGAAAAATGTGTGGAAATCTCAGAATACTGATAAAGT
ACGAACATCAGAAGCAAAAATACGGATTACAGATGGCAAAGAAAAGTGAATGCACAGAAGCGACAC
CAAAGAAGAACGAAGTTGCAGAACGTCATCAAAGAAGAACGAAGATTGCAGAACCGTCATCGATAG
GTATTAACC
>TCONS_00104240 geneID=XLOC_020846 scaffold_335:59016-61805(-) Intronic
ACCCGTGTCGCCGCTGCCGAGGCTGCTGCAGGACGTGTCGACGAGTGAGCGGCAGGCCGTGCTCGCG
AAACCGGGGATACTGAATGAGGACCGGGATTTGATAACGAATGCAGTGAAGCGCGCGGCGGCGGCCA
TGTCGGAATTACACGTGGAACACCACGAGGAGCTGGTGGTTCCGTTCCGGGTTCCATAGCGACGGAC
CCCGTCCGACCACCTGTAGGCGAGGGTTCCGTAGTCCCGACCAGACTGAGCTGAGTACACGATAAGTT
TGAACCTTTATGCGGCGGAGAGTGGAAACTATGGCAGCACTTGGTGTAAAGTTTCCGTGTCTGTGATT
GGTTGCTGCAGGAGACATTTTGTAAGACTTTGAATTGGCCTAACACATATTTTTCATATTGATGATTTAA
AAAATGGCAACCGAAGCAACTAATCTCAGATGCGATAAGAAAGAATTAAACTGCCATTAGCTTAGCGT
TCTGTTCCCCATAAAGGTAATTTTTGCTTATTTCGACGCGCAAACTTATCACAAGCGCAGCTTTTAGCGT
GAACGGACAGTTTTTGGACACAGATTCGAGTAACTACCTCAATTAAAAGACTAATCTCTGAAACTAGA
GATGACATCCGTGGAAGACATCAGACAAGCGTTGGACAATGACCCCTAAGGGTGAAAATTGGTAGCT
AGCGACACAAGGTTGCAGTGGGCGACGCGTAACAAAGTTACTTCTGGTTTCTGGCGACATATCATTCG
ATAACAGGTTTATATAAATACCTAGAAATAGGTAGAGACTTTGAGACACGAAGTCTGATGCAACCTTTT
GTCGCCAGCCACCAATTTTCACCCTTAGTTTCCGACTTGACCTGCAAATTTTCCGGTGACTAGCTGAGA
CAGAAGGCCAGTAATGGCTGAAAGAGGAAATTAGAGGACAAACACTCAATAAAAATCTAAATTAATAT
ATTCTCCTATCTGTGTTCAATTCAAACCCTTAATAGGTTTAGTGTTTAAGATATTGTGATCGTTTGTTTTA
AAGTTGGCACTTTTATGAGGTCTTAACGGCTGCAAAAAGTGTTGCTTTGAAATGGATTTGTAAGAGCTT
TCATAAAAATATTAAATTAGAATTATGTTTGTGTTATATAAACTTTAAATTGGTTCATTCAACATGACTGA
TTCAGCTTTGGACCAAAAATTGTTATGATTTGATTGGTTTCGCTTCTACAGTGTAAATATGTTTCGCCAT
AGGTTTTTATAAATAATAATATTTTATTACTATGTCATTGATGAATAGAGTTAACTAATTAGTTGATTGTAA
TTAATTAATTTCCATTCAAATTAGGTACAGTATAAAATCATTTTAAAACATAGTAACTGCTATAAGTTTAT
CTCGTTCTTTCTCATTGCATGGCTATTCGAAAGAGCGAGTAAAAATATAATAGATATCTAACTCAGTCAT
TATACCAGTATTGTTTATCCCAGTAGCCATTTTATGTTATACTTACTTACATTATACTACTTACTTATATTAT
ATTATTTTTACATTACTTATGTATAGTTTTGTGTTACATAATTTGAAGGCAAAATGAAATTTCGACTTTTG
CACAATATTGGACTCTCACAAACCAATCGACAATAACTACAATAAAATTGACAATAGACACCATATAGG

GTAGGTAGTAGTGCACTATATCTATAGATAGCTAAAGAGTACCCATCCATACCCAGCGCTTAATAAAATG
TACTGATGAATTATTGCTCATTCGAGGAACACTGGATAGGTATTACCATTGGGGATAACTTACATATATCT
CTTTTGTTGACCGTACATCTGCCTATGATAATTTGTTTGACCGGAGTTACCAACAGGTGACATTTTGTAC
CTAATTAGGTATTTCACTTTAGTGTACAGCTGTTATACATAGAGTTATTCCTAAAATGAAAAGTGCTCTG
TAATTCGGATATAAATGATACAATTTCATCCTGCATGAGTTTAACTCAATGAAATATGGGAAATCTTGCC
AGAAACATGAGAACCTTTAAAATGGTTATTGACAAAAAACTTAAAATATTATTATGTTGCCTTCAAATTT
GCTAAAATTTGTGTTATCTAGTTGTGTAAAAACTATTACCTTAAAATGATTAATTTTGTTTCAACATGAGT
TTCAATTTTAAGTTTATCCGGAGTAAATTTAAAATTTGACGCTTTT
>TCONS_00104316 geneID=XLOC_020841 scaffold_335:70327-124656(+) Unclassified
CAACAATTTATTCAACAGTGTAAAACAGGTGTTAGCTGAACTTCCCCTTAAATTTTGCATGCGTTTTGA
GCACGCGGGGCGAGGGGGGCTTTGTAAACGGTTCTGAGTTTGCATCCCTTTCTCTCCTGTTGTTTTTGT
CGTGTCGTCCCGCCCGCACGCCCGTCTCGCCCGCCGCCGCCATTCGCGCGGCGCCCTCGCCACTGCAC
GGACGTCCGCCACGCTCCCGACGCCGGCAGCATCCGTGTAAAGTTTCCCAACTTTTGCACCACCGGCC
ATTACCGACACAAAACTTTACCCCTCCCAGGAATCAACTTCAGTGTGGTGTTTAGTGAATAAATAGCTA
CTTCAGTGAGTTTTGGTGTTTTGCGTGTACTTTTTTTGCGATCACGATTTACCGTTTCAATTGAACGTTA
CTGTTTCGTTCGATAAAAAGCGCGTAAGTAGAGTTGGTCGCTCTTCGCTAGAGAGGGCCGTATATGGTT
GGATTTGTTCTCGGGTCCGGTGAAAGTACACCTTTGTTGAGTTCGTAAGGACACGCCGCAGCCGCAAC
TAGCTCGATCCGACTGGCTGTTGCACTCCGTGCGGATAAGGTCATCGAGGTCATTGCCATGGCAACGG
AAATGAAAACATACTGAAACACCCAG
>TCONS_00104353 geneID=XLOC_020841 scaffold_335:146821-154822(+) Intergenic
TATCAGAGTCGTGTGCTAAACTTTTTTTGCAAACTCTTTACTCTAACCTCCATCAAGTTTGAGATGAATC
ACGCGGCCAGTCGGCAGCGCCGCGCGGCCGCACCAACCCGCCTGTTCCATATTCAAAAACGGAATTC
GAAAAACATAATTCCTGTTCTGTGTTTATTTATCAGTCTAGATATTGTGATAAATAGCTAGTAAAAGTGA
AATATTCCTAAATGGTGCCAAGTTGAGTGAGTGATATGAGCCGAACATGGAGGAAGAGATTCTGTTGG
ATAAAATTTTGGGCGGAGCTAGTGGCGGCGGTCCGTCGACGCGGCCGCGGAGACAGCGACGATGATG
AAGACCTAGGGCTGCCGAGGAGTCCCCCCGCTTCGCCGCCCACTGACAAGGCTGA
>TCONS_00104223 geneID=XLOC_020841 scaffold_335:163603-164044(+) Unclassified
AAAATTAAGAACATCAAATTATAACCAATAAAAAAGTAATGTTTGTAAACAAAATTCTAGCATAATATAG
TTAATAATTAAATTAAACGAACAAAACTCGTGAAGACTCAACTTTTTCGCAAGCCATTTCAATTGTATC
TCTACTACTAAAATCCGATCATAAATATTCTCAATCTTTGAACATGTGAACATGGCTATAATACAAAGAG
GCGAGGTGATCGGCAATAGCTGGATGTCTGGAAACAAGGTTAGGTTCCTGATAGGTTGTGCTATTTGTG
TAATTTTTGTTTCGATTGTGTTAGTCGCTATTTTCGCGTTTGTAGAGGTATAATGAATAAAGACATGGATA
ATTGATTTTTAT
>TCONS_00104320 geneID=XLOC_020843 scaffold_335:228632-231479(+) Exonic overlap (-)
TGACCGGCCGCCGCGCCTCCTGAGCCGAGGATAGCGCTACTCCTGTTGATGAACATACAGAGAAGGTC
CACCACCGGCACCTGCAGTCGCCGTCGGCGTCGCCAGAACGAGAGTAGCGCCACCATCTTCACTCCA
CACTTCAACCAGCCGTTTAGTGCAGTTACTTTTGTATGCAACGTTTGAATCAACAGCCGTAAAGTGTAC
CATCCAACGTTTGAAGCAACCGTTTAGTGCAGTTAATTTTATATCCAACGTTTGAATCAGCCATTTAATC
CAAGATGTTCTATGCTGTTACCAACTTCTATATGCAACGTAATAAGCATTTAAGAAACTACATACATCGT
TACATTCAAATCTGCGCTTGTTTACTTTATAAGTACGTTTGATCAGGCGGTTTACAGCTGAGTTGCTTTA
TTACCTACGTTAGATGAAACTGAAATTAAGGTAGGTTAGGTAAAACAGGTAAATTTATGATGATGCCTA
CCTGTACCTATCTACCTCTAATTTAAAAAATATATATATATTAACTAATGTTGGCAAGAGTTTGAAACAGT
GTTTAATTAAGTATAAAATTTTCATTCACCATCCACCATCGTATCAAACCGGTATAACCATTCTACAAAA
CGGCTAAACAGTTAATTTGCGATGATTTGCAAATTATTGCCAGTATTCCACTATTTATCTTATAATTTGAA

CATAAGACACAAACTGTCTTGACTGCATCAACATATCTATTAGTTTTTACACAACACAGTCATAGTCAT
AGCTCACTGTATAGATGCGGCTTTTAGGTGTGTCACTGTAATTTCAACCTTATTGGACAAAAAGTAGGT
TAATATTGTTAACTCGCTCTGTCTATTTGCTGATATGGCATGGGAAAGAGACGGGTAGGATCTAGGAAC
ACTATCTTGAGTTTTGACAATAAGATTGGTCTTGTTTAGTCAGTAGGTGTTCTTCTAAATGTTTTCACTA
TTAGAAGTTTCATTTGCATTATCACCATTGAATGATATGGGTGTAGATACTGTAGATGTCACCTAAACAT
ACATTTTCCTCTTATTTATAATACCTTCAGGCATGATGTGTTATTATTTACTTACATCCAGTATGTTCGTAT
GGTACAGCTAGAAATATAACCAATTACCTACTGAATGTTATTACATTAAGTACATCGCTACTGTATATTTC
AAATATTCCGTAAACAAATTCTAAATCAATAATACCAATGGGCAAATTGGTATATTTGTACTATCAATATG
ATATACCCAAGATAATAATAATTTGAGTAAGTACATATTTATATTAGTGAATTAAATTGTTATAATTTTTAA
GTTAGTACCTACATTGTTATTTTTTAACAGAGCCATCTAGTGTGTTATTTGATGTATGCATGTGATGGTTT
TTGAAGTAAGTTTCACTGATCTTATTTTATTAATTTTAACAAGAAAATATATTTACTTTATTGTATTTGTTG
TGTGTTACCTTAACCCTAGTCGGTAGAATAAATTTTGCTTGTGTAAATGGCCGCCATACAATATTGTATT
CGTTAAATTACGTTATAATACTGCGGTTTTAGCTAATAAAGATAAATGGTAAATTAGAATCGTTGTAATTT
AGATAAATTGACGTTTAGCATTATTCAAATGGTTATTGAATGTCGGTAATTTTAAGGTGCATCTTAGTGA
AAATTGTAAATAATTGTATTGTATTTATTACTAAAATTGAATGGTTTATTATGGTTAACATTGGCTGCAAA
ATCTGTTAACTACATAGTTTTTCATACTAAGGATTTCTTAAAGGTTAAACAGATTTTGTAGAAATAGTTA
CAGCCAGGCATTGGAGTATTTCAAAGAAAATTAACTAAAAATTAATTTTGGAAGACGAGCACGGTATG
AACTAGTGTAAGAATAGAACTAATTCACAAATTGGCATAATAACGAGGTCTCTTTCTAACGGTAACGCT
AAATAAGAGACGCCATTATGGTAAATATATATTTAAAAAAAACTCGTTAATAGGGCACTATAATAATGCT
AAAGCTTATAAGAAAAATGGGTGTTGTATTTGTAAATTATATTGCAAAAGAAATTCTATTTTGTATGATAT
TTCGATTAGGCTTTGAGAATATTTAAAATCGATTGGTAAGTTCTTTTTTCTGCGATTTGTTTTGATATGAT
CCTTGCACTTTCCCAGTAAATCTAATAACTTAATAATCACAAACCTCACCACATAATAAGGGTCACATAT
TGAGCTTTCATAGTACTATAATATTATATTTTTTCGCCATTTAATAGCTAAGATATATAATAATACCGAAAG
GCAGAAGATTAAAATGTTCAATAGATGTCGCTACGGGTCCTAATGTTATATATTATAAAATTTATTTTCTG
>TCONS_00104555 geneID=XLOC_020884 scaffold_336:65980-92190(-) Splice junction overlap
TACAAATAAACACCTGCTAACATAAAATACAGCGTTCAGTCCTATACTTTAACATTATGAGCACATGTTA
AAGTGAACTATGTACCTTAATTATGTAATTATTGGATTCGCCCCAAAAAGAACTAAACACTCAGCAAAA
AATTCAGATCAAGTTCAAAAATGCACACTTGTACCAGATTAGCACAGGGAATTCTGCCGATTTACAAAT
AATACGTTTAAAATTGGGGTCAATCACAGAGTTTGACTCCATTTCGCTAACAAATTGTTAAAACAAAGG
CCGTTTTGTGGCTGACGCGGCGAAACTTTGACATTGGCGCGAGACGAGGAAGTCAGTGATCAGAAAA
TGCACTCATTTAATTATTATTCTTAATCTCGAGCTGCCAACTTGACGGTTGCACCTTGCACTTGCGCGCT
TTCCGCTAGTTTCCAGTTTTTTTTTTTCTGAAAAACTTCTCGCGCGACCATAAATAACTGAATATTTCGA
GTGTTTTGTTTAAAAATGTGTTGATTAAAATATGAGTGAACGTTAAAAACGGGTTTGTTTTGTGAATTT
GTTTGTGTGTGAATTATAGAAAAATCATCATGAAAATGTCAGCCAGCCGCAAGGAGGCCAGGCACTCG
CCGCTCGACTCGGCCGCCATTATGAAAGAGAAAAG
>TCONS_00104556 geneID=XLOC_020884 scaffold_336:65980-101975(-) Splice junction overlap
CTCATTTTGACATCACCTCAAACTGTCGCTGGAGCTGTGAGATTATCCCCCCTCCCGTTCCCTCCTTTTC
CCTCTTCCCTCTGGATGAAGAGGGAGCGAGAGCGCACGTCAGTCAGCCGGTGACAGGCGCGTCGAGA
TAACGCCACGGAAAAGGATTTAGCGGCTATGTCTTTGCATCAAACTGTGTTTTTTAATTAAGTGAAAA
AAGAAAGCGCGTTTTTTTTTTAAATTCTCGGACTTTGTGGCCTTTGTAGAAATAGTTTTTTTCAATAAGG
TACTTTTTATGAATATTTATGTGGCTTTGAAGAGGCTCAAACAATTTTCAGGACTGTTTAAAACAAAATT
TAGTTTTTTTATATAATTTTGGCGGCAAGCATAATTAAAACCAAATCAACATGTCATTACCGATTATTTTA
TTTTTTCTCGTGTGCCTAGTGGTTCTATTGATTTGAAACTACAAAATGTGTGTTCTTAATGGGACAGTTG
ATAGTGTTGTGCTTCTGAAAGTGTTGTTCGGTGGATTCTTATGAGAAGTCCAGTCAGGATGCGTTGGAA

TGCGCGCTGCAGTAAGCGACGGGGACTGGATTCTACTCTTGGACTGCTCTAAGCCAGCCGCAAGGAG
GCCAGGCACTCGCCGCTCGACTCGGCCGCCATTATGAAAGAGAAAAG
>TCONS_00104462 geneID=XLOC_020879 scaffold_336:212278-221243(+) Exonic overlap (+)
TAAAAATATAAACCTTAACCTCAAAATAGATTATGCATGTAACGAAACACGATTCGTCATACTTCCTTCG
ATTTTGACAGCTCACCAACCTTGGGGCGAAAACGGCTTCTGAAATCGAAAATAAATGTAGATCTTGGG
GCCCCAACACCTGAAAACTATACTCGCAGCTCGTGAAACTAAAGTGAGATATCAGTTTATTTCATTACG
TGCACTGTTAGGCTATTTGCTTTTGAGTACGCTTGCAAAATGTGCTATGTCTTGGTCACGATTATACTGT
CATAAGACTCCGTCTTATGTAACATATCTGTCTGTGAAATAACAATTTTGTGCTGTGATAACGCTTGTTT
ATATCATCCCGAGGAACCCGTT
>TCONS_00104467 geneID=XLOC_020879 scaffold_336:212327-221312(+) Exonic overlap (+)
CGATTCGTCATACTTCCTTCGATTTTGACAGCTCACCAACCTTGGGGCGAAAACGGCTTCTGAAATCGA
AAATAAATGTAGATCTTGGGGCCCCAACACCTGAAAACTATACTCGCAGCTCGTGAAACTAAAAACCC
GTTTCTGCACCGGGAAGCAACACCCTTGCTCATTTTCCTTTACCTTTTTTTATTATTTGTTTTATTATTTA
>TCONS_00104441 geneID=XLOC_020879 scaffold_336:212348-213028(+) Intergenic
ATTTTGACAGCTCACCAACCTTGGGGCGAAAACGGCTTCTGAAATCGAAAATAAATGTAGATCTTGGG
GCCCCAACACCTGAAAACTATACTCGCAGCTCGTGAAACTAAAGTGAGATATCAGTTTATTTCATTACG
TGCACTGTTAGGCTATTTGCTTTTGAGTACGCTTGCAAAATGTGCTATGTCTTGGTCACGATTATACTGT
CATAAGACTCCGTCTTATGTAACATATCTGTCTGTGAAATAACAATTTTGTGCTGTGATAACGCTTGTTT
ATATCATCCCGAGGCCAGCAATAAAATAAAATATAATTGCAAAAGAACAGCATTTATT
>TCONS_00104730 geneID=XLOC_020926 scaffold_337:58273-59850(-) Intergenic
TTTTTAAAACTACTAACTTAATAATACGTGGGGATATCTCACACACGACCATCCGACCCTAAATTAGGCA
GAGCCTGCAATATGGGTATCGGACCGTTAGTTAGTAGTGTGCAAATTGGTTCAAGTAATTTACACACCA
TACACTCTTAGTCTTGCTTTTGTAGTAAATTGGCGAAGAAAGGCGGTTCTACTCGGGCACAAACGTGG
TACAAACGCTTTAGTCGATTTATTATTGAAGTTAGCTATGCACGCATTAACACATGAGACGTCTATACAG
GCTATAGATGTTTTTACAAAGTACCTTTTACAGACTCCACACGACACACTACACTTTACACAAAAAAAA
AATACAATTACTTTGAAACAATTTGCTTTAGAAAAATATCTACATAACAAAAGTTGATCATGACGGAAA
ACAAATACACTTTACACTAAAAACACATTCTGATCGAAGTGTAAATAAACTAGATGTATTTTCTTTTGAA
TTTAGGTAGGTAAGTTTTAAGTTTGAACTGGGGGTTGAATATTAAAATAAAATTAATAATTAGATACATT
ATATTTTATTGATTTAATCAGTGGGGAGGAAGAAGGCCTTGATGTTTATTAGAGTGGCGTAAATCAGAG
AAAGAGGATGAGAGTTGTGGCGAATTGAATATCTAAGAATCTAAGTATTGGAAAAAATAATACATAGTT
ACAAACATAAAATATAGTTGAATGAAAGTGGCTTTAGTAAAATATCATGGCTGTTCTCCTGCACTGAAG
GAAAGATTCCGATTTCACCTTAATCACACAACACTATACAAAAGAGAATATTTATGAAGATTTTGGTGTT
AGGTTAGGACAGCATAGTCAAATTATACTGTTGTATGTACAGTATCTTCATAATATTCTATACTCAACATG
TACTTACTTTTGTCATTTTGCTGGATTGGAGGGGGATTGAAAAGTTGTGAATCAGTTGTAAACATGAAT
GCAATAACGGTTCACAATAATAATTCTCTTTCTAGTTTTGGTAAAGTGATACTTGATATTTCTACACTCTA
CATCAAGCTCGGAGATTGGTGGACATTGTTCCTGGGGATTGTTAGAATGAGACTTGAACTTTATTAATT
TCTATTGATGATGCATCGTCGAAAACATAGGGCGTGGGAGAGTTCGTGCTCTAAGAGGGCGTGCTAAA
GATGCCATAGAATTCTTAAAAATAAATTTAAGGTTCTGTGGTTTCTCATTTAGTATTGGATCCTTCTGATC
GACAGGGTATCCCCTTGTTCCCGGTTTATTGGAGCATGAATAATCTACTAAGTATTCGATCAATAGTTAT
GTAAATAATAAATAATAATAGGGATGGTCGTTGATTTTTGATAATTTCTGGGGATCTTGTTGGCTAATCAT
CTGAGTCATTTTACACTCACACACAGACATATTGAGGTGATGATCGGAATTTCCTTTTACAAATT
>TCONS_00104754 geneID=XLOC_020929 scaffold_337:215804-216678(-) Splice junction overlap
GTTTGCGATAATTCCCAAAATAGCGATGCCGAAATACAGTGGCTATAATACAGCGGTGCCACATAGAAG
GTCATCTATGAATACTAAATGGCGCTGTTTTGTAGATTGCCCAATAGACAGCGAAAAGTCAGCTGTTGG

CTTGTGGAAAAAACTATCCTCCTTTGATTCTTACTATATCTTTTACTCCTTATTTTATACAAGTATACAAA
CAAACAGTATTTTTTAAAATTTTAACTAATATCCCCTATCTCCCCCACAGCGGGCCTAACCCCTGACAAG
AGCGACCGTCGCTCCTCCGACAAACTGTCGCCTTCAGCCGCCGCCGCGTTACTGCAGCAACAGTCGC
TGCGACTGCACTCCACCCCGCGCGACAAGCACCACTACACCGACGACCACAAGCTCACAGACTTCCT
CAGGG
>TCONS_00104784 geneID=XLOC_020930 scaffold_337:237078-242697(-) Splice junction overlap
TTGTTGTGCTCCAGAAGCATTGCAAAACATAGAACAGTGATGTGGATTACACAGTGTTACCGTTTTCAT
AAGTGCATTTGAAAAGTTCGCTTGGTGATTAAAAGTTTGCATGTTCAGTGGCCATGTTTATTCAGTTGT
GGTGAATTCGCAACGTGCCCCTCGGCCGTAGCCCCGGGTATCCGAGCCGGCTCGCGTCTTCTAAATAA
CACATTCATTGAGCAGCGTCTCGACCCGGCTGGCGATAAAATATTTATCTCAACGATAATACTCTGACG
TTCTCCCTTTTTCGCTATCTAAGTACGGCTGCCCGGGAATACTGAACTAAATTATAATCACAGAACCCTA
GTATAGCTTCGCAAGAGCAATAAAACCGACAGATATGTCGGTTCCACATTCCTCTAAAATATTTTCGTTT
CGCCTTATCTTTGGCGTATTGTAAATATTTAGCAAAGAATATATCTGAATTTCTGTGTTATACCCGACAGA
TGTAGTACACGTTCAGTGATTTGTTGTTGTATTTCGGTCTGGTCGGCTCGTCTAGGTGCGGTGCCAATTT
GCGGAGACGTTGTTTGTTTACAAGCCGCGCCCGCGCACCGGACATCGGGCATCGGCGCCCGCGCACC
GGACATCGGGGATCGTTAACTTTTTTTTAGTTTTACTGCTGGTGCCCGGATAGTAGTTATCTTTCTGCGT
GCTGGATTAGATCTGTGCTAGCAGTGTACGGTGTAGTCAAGACTCAAGATACGTCGTTGCGAGGTGCG
CGTGGTGGTGCGGGCTGGGGGACAGCCATGGCGCCGCCGANNNNNNNNNNNNNNNNNNNNNNNNC
GGGGGCGCGCGCTCGAGGCAAGCGGCACCTGGACATGCGCCTCTGCGNGCTGCTGCCTCGTGGGTGG
AGGCAAGTCCCCGGAGGCGGACCGCGGCTCCCGCGCGTCCAGCGTGTCGGACGAGGAGGGCGGGTT
CAACGAACCC
>TCONS_00104606 geneID=XLOC_020931 scaffold_337:256411-261047(-) Unclassified
ATTTGTACGTCGTTCGGGCCGCAACTCGCTTTTTTATTATTTTCTAAATTAATTGTATTGTACATGTAGAA
ATGGACAGAACAGTGTTGGTTCAGTAATCAAATTAACATCGTAGCGCCGCCTTAAACTTAATTCAAGAT
AATGTTGTTACCGTAATAGTAGGAGACACCCCGAGTTGTATTCTCAATCAGCGTTTTTATTGACGTTTCA
TTCATTACTTTTCGTCCCGAGGATGGTGGGTATAGCAAATAAATAGACTGATCTTTACACTTGTAAAAGT
AAATTGCTGTGTAAATACGAACGAAGTAAACGTGCTAAGACGCTTGGTGTTTAAAAGTGGTGTGTGTT
GATAGTGATAGTGAGCATACCTACTTTGGATTGATGTGGCACACTGGATCAGCAGGCACCGGCAGAGG
AAGTGGATATGTGATCAGCTCTTAAGATGGGTGAGCGGGGAGCGGGGGCAGGGGGTCGCCGGGCGTC
GGGTGTCGAGCGCGGCCGGGCCTACCGCCCGTACCCACTAGTCCGGTGAGACAAGACGCGAAACTCC
GACAGAAACCAACGCTTTCTCACATGAGTGCATATTACTG
>TCONS_00104609 geneID=XLOC_020932 scaffold_337:268371-270363(-) Unclassified
CCCCCCGAGCGACCCCGGCGGCTTCGCTGCCCCGGGAGCTCCGGCCGGAATGTCGAGCAATGATGCT
AAGGCTCGCGAGGCGGCCGCCAGTGCGTACTACGATCCCAACCGACCCCAGACAGCCTACGTGCCCC
CGCCCTATGACCAGCCCCCGACCTATCAGGAGTCTACTTCAAAGAAACAAAACTAGGCCCTTGTCCTC
ATTTCCAAATGCAACAAAAAGCTGAGGAAATGAAACCGAGCAAGTAATATACGTTCTACATTTATTTTC
AGTACGTATTATTATTAGTCAAATAGCACATCTACACCGTGTGATGTTTTAATTTACGTATTATAAGATAC
ATTCCTTATCTAAACTCGGTCTTATATGCATAATTATATTCTTGGAAGGAAATACATAGCTATCCGAGACG
AATAAAATAACAGAGTGTACTGAAGAAAGTTGACACTCATTTCTAAATTTAATAATGTTAGCGTACCAA
GTAGCAAAGAAAGGCCATGCCACATGTAATCGAAATAAATTAATTCTTATGCGAAATATACTTCTTAAAT
TAAGTAATGTAATGCGAAATTTGGAATTACGAAGATATCAATGGACTGAATCCTTAGTCGCAAGACTAC
TTGTTTGTTGATAAAACACAAAAAATATAAATTATAATTATATTATAAAGATTATTAATGAATTTATATTTA
AATAAGAAGCTTTTTAGTATTAATTATTGCAAGCTTTAGATAAATTGTAAAGCGCATTTCTTCAATATAAC
AAGCCCTACTTCCAGTAAAATTACATAATTTGTTTGTTTATTCTAACTTAGTTAATTAAAACATATTGGAG

TCAGCATAACAGTCGATATTTTTAAAATGATCTCTTCTAGATTTTTCGCACATTCATGACGGTAACATATT
TCTCAGTATTAAAATTCAATAAGTTATTAAAAAATGTACCTAATAGATTTGTTATACTGAAAAAATGCATT
CTAACTTCTGTTGTTGCTGTGTGTAGTTTTATTATTGCTTATAAAATTTGATATTTTAAGATGTATATTGTT
ATATAAAATAAA
>TCONS_00104880 geneID=XLOC_020962 scaffold_338:6144-9478(-) Unclassified
TCCGGGAAAGCTGAAAATTTCATTTTGACACAATTCTACTAGTGTCAGACTAAGCTAACTTTGCAAGC
AACAATAATTATTTAAAGTACTTTTTGAAACTGACGTGGAACGTCTTACTCTTATTCATTCTTCCTACCC
TTTTATGGAAATGTGTGGAAGTAGTGTAGGTACGCGTCAATATAGACGTCTTAAAAGGTTCTAAAGAAG
TGGAAGGCAAAGTACTTATCGAAAATGGCTGATGATAATAATTTCAGCTCAATGACCTAATTGGTCAGA
TTGTAATGTACATATTTTAGGCTTTAATAGTAAGCGTGCTGAGTGACAAAGCTGCACCAACAATAGATG
ACAACAAACAACGAAGTAACGTTATGTCTCGAGCCGACACAAAATGAACCGTAAAATTTGAATATTTA
AATAACTAACTTATCCTTACCATAGAAACTATACTAGTTTGAATTTTTACGACTAGACACAAAATCGACC
AATAAATCGGTCAAAATGTGCTGAATGACTGTTCTCGTCTGCTCTGGTTTCCCGACAAATGTTGAGTAT
TCGACCGCATCTCAACAACTTGTCCGAGTTCGTCCTCTCATGTCGTCGAACTGGCAAAGGACACCGAG
ACGTTAACCTACTTGACGGGTGTGTCGAATGAGTTTGAATCAGGTGCCTCGATTGTTGCAGCTGTATTG
TGGCCGGCGGGCGTGGGCTGGCCATATATAAGACCCTCACGAGCCATCACCGCCGTATTCTGTCCATCC
CCGGGAACTGATCAGGACCTCACTATGAAGATCTTGCTACTCTGTATGTCAGTGGCGGCGGCGCTCTC
CTGCGTCCAGGCTGGAGCACTGCTGTCTCGGGGGCTGCTCCCGTACCGCTCCCTGCAGCCCTACCAGA
GCGTGGCCTGGCCGGGGGCCCACACCGCACAGTATGTCAGCACGGTAAGGCATAATATGGGAAATTAC
TTAAGTCTCTTTCTAGCTGCTGTAGACGTCTACTCTATTATTTTTGGGGGATGCAAGTATATACCTGGCTC
TTCAAACTGGTCTAATCTGCCTTAATAAAGATCCCGGTATAGATTTCCCACCACGCTGGTCTACTGCTAA
TTGGTGGGTTCACACATGTAGTTGTGCCGAATCTTTATGTTCTGGCTGACGATGTTTTCCTTCACCGTAA
GAGCGATGTGCTTTGATTCCAAAGAGAAGGCAAGATTTCATTGGAAATATAATGTGAAAAAACATTTA
CATTCATCTGTAATGAAGCAAGGCACAATCGAAGCTGCGATAATAGAAAATGTATTTTGCTGCCTTTATT
ATTAGATAGTTATGAAATAAGAAAGTACTTAAAATCACGATAGGTAATTCTATCTACATCTTTACATATAT
TCTAAACTAGTTTCGTGGAAGTTTAAAGTAGGAAGGAATGTACCTACTAATACGTCTCCAAAATAGGTC
AACTTGAAATATTATATTCGATTTGATATTTAATACTTACTTATCCTTTAACTGCCGACGTGGTCTAAACG
CGCCACTAACAACGAGGTTACGTCTGAGAGACGTAGATATTTAAAAAATCATTTAAAAATAAAGTATTC
GTTTTATTTAATTTATAACAACCCATAAAAATACTATAGTTAAATGGCTTATAATATATGGATAATCAATAA
AACAATTAGTTAATTTAACAATAAATCAGCCTGTATTGATGAGACCCCAAGTTTTATTTGAACATTTTTA
GGAACTTTTATTAGACATATATTTTTATTTATACACATATCAGTAGATTTTTTTAAATAATTTTACTCTTTTA
TAACTAATAAAGTAAAATAAAAAGGAAACAGCACAAAAACTTAACGAAAAAAAAAAACTGACACACAA
TCAAGTTAGCATATTTATGGCTTGCAGCTATTGCATATTTGTAGGCAACATGGGTTTCATGCAGAGACAA
CATGAATTTTTTGTGCGTCTTTTTAGTCGACTTGGACAAATTGCACAGGTCTTCCGAATACCGAGCTATT
CTTGACCATATGCTCGGGCAGGAGGCGGTGAGGGCATATCTGTAGGGCCAATAACTCGTTCAATGGAC
CACCACGAGAACGTGGGTGGTCTGATATTTCATCGCCTCGCACGTCCTGTCTCCTTTCTCTCAGTTTCC
TATGATTATCAATTGCTTGATTTTTATAATCACTAGGTAGTGTCCTCTTTTTTGACCCTGAACTACCACTT
TCAGACATTCCGTAGGAAAAAAAC
>TCONS_00104941 geneID=XLOC_020967 scaffold_338:188454-192384(-) Unclassified
TGTCATTTTAATTAGGGAGAATTTATTACTCCCTCGCTTCATTTTGTAAGTAATCGGGAGTATTTAAATTA
ATTTTCGCCTTACTTAATAGGATTATAATCTGATTTATTGAGTCTTCGTCAATAGTACAAATATTTTTACTA
TAAATTAAAAAAAACGTTCCTCAGCCGTCGTCATGTAGCCACTAAATGTTAGTTGTTTACTTTAGAGGA
CATGACGGCATTAAATAGTAATAGAAACGTTCCTTATCGTGATAATAATATCTGATCACGGGGAATTTCA
AAATTAAACCTAAGATGAATGATGGGCTTTCGAAATAGAAACGATGTAAAAATGCCAGATACACTCTTG

CTTTGCACGGTACGTCCTTCCAAAAGTTTAAGGTCTGCCAATACTTTTTCTTACGAGATTTGCTTGAATT
TCACGTTTTGTATGTTAGCTGGAACTGCTTCCGAGATCCATGCTACTGGTAAACACCCGGACAATGCCG
TCAAAAACATAAATCTCCATTTCACATAAAAATGAACTCCAAAAGTGGCAACAACAACGCGCCCTTTA
AAGCAAAGCTCAGTTTTTGTGTGTCCGTCATCATTTAGGGGGGCCGCTAACTTTCAGCGCATTTGTGAA
CGGATCGCACCGGAGCATTACTCTATATTGACGAGAAATCAACGACCCAGAGCTGTAATAAAATCGTCA
TCTTTAGAACGACGAGATAGAGTCGTGGCTCGCGTAGAAACGCTCCGAAACTTGGTTATTCGTCGTATA
GAGTGAGCCCCCTGACCGTCACACTGTCACCGCGATCCGGTGTTTTTTATTTACCTTTTTTTTCTTTACC
CCGCCTTTGTTTGTCCCGGTGCAGGCTTGGCAGCGATTTTAGCAATTTCCCGCGCGAAATGGACACGTT
ATGATAGTCTCTCCGTGAACGCAAAAATACGGACCGACTGCCGGTAAAAACTAGTTGGATACGACGCG
TCGGTTCGGAAAGCCACGTTTTTAGGGTTCCGTAGCCAAAATGGCAAAAACGGAACCCTTATAGTTTC
GTCATGTCCGTCTGTCCGTCTGTCCGTCTGTCCGTCTGTCCGTCTGTCACAGCCGATTTACTCGGAAAC
TATAAGTACTACAGAGATGAAATTTGATGGGAATATGTGTTGTATGAACCGCTACAAAAATATGACACT
AAATAGTAAAAAAAAGAATTGGGGGTGGGGCCCCCATACATGTAACTGAGGGATGAAATTTTTTTTT
TCGATGTACATACCCG
>TCONS_00104873 geneID=XLOC_020971 scaffold_338:326414-327015(-) Unclassified
TGCGTCGCGCACGGCCCGGGGACGCCCCGCGCGTGGTCCGCTTCGTTCGCGAGCACGCGGGCAGCTG
GCCCGGAGCGCCGCCCGCGCGCGCCGCCGCCGTCGCGCTGTCGGACTTGGTGGCCCGGGCGCTGGCG
CAAGGCCACTCGATGCTAGCGGAGCAGCAGGAGCGGCAGGCGGGCTGGGCTCGAGTACGCGGGGTG
GCCATCGGCGCCGCCGCCTGCCCGTGGGACGCCGCGCTGCT
>TCONS_00105111 geneID=XLOC_021017 scaffold_339:246709-255203(-) Unclassified
CTGGTTAAATACCAAAAATTATATCGATCCTTCCTCTGTTTAGGATAGGATTAAGTGTTTCTTATGAAAA
ATACCAACTGTTTACCTTCCACAGTTCCACACTTGAGTACTTTTAAAAGGGGTATATTAAAGTTGCAGC
AATCAACTTAAGCCACAGACAGACAAGTCGATTATTTACCTTAAAAGTAAACTTTGTGTAAAGGCACAT
AGAGCAGATTGTACTTTGTACAATAATTACAGTGGGTTCAAAGAATCGCCTACGATTTCCACGTCAACT
GAACTCAGTCATCATTCTGAGGTCACAGTAAGCGCACGCGCAATAAACAGTTTATTTTGTTTAACTGAC
GGTTATGTGTGTTTAACGGACAGTTAAGTTTGTTTAACTGTCATTTGCGTGTAATTAGTAAGTGTTTAGA
TGTGTTTGTTGGATTTCGTGCATGTCTACTGTCATGGGGCAGCTGAAGCGCCTGCTGCCGACCCTGTCT
CGCAAGGAGAAGGGGCCCCGCACCAGGACATCATGGTATGCGGAGTGTGGAGACGAGACGCCTCCGT
CGCAGCCCTCGCCGCGGCCGAGCACCACCTCTTGGTACGCAGAAGCGGGCCTCTACCAAGCCGGCAG
TGTGACGTCATCGAGCGGCGCCTCCTCCGCCTCCCCCCCCCCCCCN
>TCONS_00105275 geneID=XLOC_021017 scaffold_339:246710-252841(-) Unclassified
AACGATTGTATTTAACGGTTCGTTTCGAATTGAACCGAGTTTGGAATTTAAATAGCAATCTATAGTGTAG
TGGTGATATTTTAATTTTTATTTGAGTGCAGCAGTGATTTGGATTTTAGTTTTGCTCCGTTTTTTTAAAAG
ACATATCCTATTTACCTACGTGTAACTAAAGTGTATTTAATTCAAGTACGCAGAAGCGGGCCTCTACCAA
GCCGGCAGTGTGACGTCATCGAGCGGCGCCTCCTCCGCCTCCCCCCCCCCCCC
>TCONS_00105723 geneID=XLOC_021041 scaffold_34:110220-111586(+) Intergenic
GGAAGCGGTGGCCGATCGGTACTTTCGGCTTACGGTGTAGTTTTTAATGTCTAACTTTTCTGGTGGAAA
TGGTGAGATTTTGGTGGATGTTTATATTTTTTGCGAAGACGGCGTCGGCTAGTGACAATGCACTCCCGG
ATCCTGAAGCTGGAAAAGAGTCCCGTTTCCTGCCTCTATTCGAGAAGCGCTACGACCGCCCGCTCGGG
CCGGGGGGTCTACCACCACTGACCTC
>TCONS_00105649 geneID=XLOC_021062 scaffold_34:469836-470319(-) Unclassified
CGGCAGGCCACGCCATGCCCCAACTTTCATTTCATATAGTGCTCTCAAATTAATCGATATATACGTGTGG
CTTTTTCAATTAATGAAAACGTTCAATTGTCAATGTGTATGTAGAGTTGCTGGATTGTTATCTATCCATCT
TGGTTGGAATAATGGATTTCCATCAAGTGAATGGTTGGCTTTTGACTCCTCGGATACGATAAGGATCCA

ATCCTGCATGCCTTTCCCGTCCCTGAAGATCACCCACATCCCTGAAATAACCCTTAAAGCTTAATGTATT
TATTATACCTAGTAGAAACGCAATACCTACAAAGCAATTATAAATACACTATTGATGTAAAATTGACTTTT
AATACTGAC
>TCONS_00105366 geneID=XLOC_021062 scaffold_34:469859-474417(-) Unclassified
GTTCGGAACGCACCTGCATATGGCGGACGTAGAGTTTGTGTTTATGTCAATAAGCATCTGATTTTTAAC
AATTGCGCGGGTGTGGAATTAATGAAAAACAATTCGTGATGTGCTAGTGGATCTTGTGAACTGTGAAA
ATTATTTGTGATAATGTCCGCCGTGGCCGGGCTTGAAAACTGTTATAAATGTGCCCTGTACTTTTAAGGA
CTAATATCTAACATACTGCACTGAGGATGGTGTACTATATCAGAGCCATCCAATCCTGCATGCCTTTCCC
GTCCCTGAAGATCACCCACATCCCTGAAATAACCCTTAAAGCTTAATGTATTTATTATACCTAGTAGAAA
CGCAATACCTACAAAGCAATTATAAATACACTATTGATG
>TCONS_00105367 geneID=XLOC_021062 scaffold_34:473981-474396(-) Intergenic
GGCGGACGTAGAGTTTGTGTTTATGTCAATAAGCATCTGATTTTTAACAATTGCGCGGGTGTGGAATTA
ATGAAAAACAATTCGTGATGTGCTAGTGGATCTTGTGAACTGTGAAAATTATTTGTGATAATGTCCGCC
GTGGCCGGGCTTGAAAACTGTTATAAATGTGCCCTGTACTTTTAAGGTATTCGTCCTGTTATAACAATTT
GGAACAGAGGTTATCAATTAATTTTCACCAGATGACTGAAGAATTTATGTTTCTTGCGAAATTATCA
>TCONS_00105381 geneID=XLOC_021073 scaffold_34:785668-786557(-) Splice junction overlap
GGAAATCTCTGATAACTTGGTTGATAAGATAGTTATGGTTTTTCCGAATCCAAGTTGATAAAGTTCATTC
ATAATTAGTATAAATGACACTACTGTGCCAAAGTTTATAACATAACAATGAGGTGTGTCATCATATTGAC
GGTGATCAGCGTTGCATTAGCGGCGTCGGTGCCTGCGCCGGCGCAGCAGCGTATAGTCGGCGGCCGAC
CCACCACAGTCGACAAGTACC
>TCONS_00105564 geneID=XLOC_021054 scaffold_34:1017997-1043495(+) Unclassified
ATGCAAATGGCCAAGCAATGTGACTAAAATTTTAGTTAATATTTTGTGTTGTGTGTTTTATTTTTATATGT
AGTATTTTTTGAGTCGTATTTGATTTTGTACAAAATTTCCGTGTTTTAGTATCATTTATTATCGGTGTCTTA
ATTTTTTTAATGGTTTCGTGAGTGGATCCGTCCAAGTCCAACCTTTCCATGTCGGACCAAATGTATTATG
GGGAGATCCCCGTGAACGTCAATAACACCCTGGCACATTTAGATTTCACACAATTCATGGGGGACGTC
GAGATCCAAGACTTCACGATGATGCCGGGAGGAGCCGAGAGGTGAG
>TCONS_00105871 geneID=XLOC_021123 scaffold_340:96756-101912(+) Exonic overlap (-)
CTAAGAAATATGATATACATTGTGATTAGAAGTAGTCGTGGAATCAATTTTATGTAGCGTACTCGACGTG
GGGAGTGATAAAGTATTGTGAGTTTTAAATGATCGTATTTTGTTTTAACTTGGCGTCGAAATGTAACGA
AACGAGTACTTTTCTTATGTAGGTATTTTTCGTGGTCTACAAAAACATATTGGAAATTCGTACACGTCGT
TTTGCTATTGTTTTAAGTCATGAAATCTCGCCGTTGTTATTGCAATTATCTAATGAACAACTTATTATGTC
TATGCCAACCATTACCTACCTATATCCTTTTCTATAATTGGAACCTTGGTATATGTTCAGCTTAAAAAACT
ACAATAAAAAGGTGTTTTGGTCCGTTGTTTGCCGTATCGGCTCAACCGATTTAAGGAACCTGTGAGAA
ATAATTATAATATTACACTGTATAACCTGCATTCAACCCAATTTGTTTCGAATAAGTACCTTATATCATAAC
TTTTGGGTTAAATACATAAAGCGTTGATCCCCTTCAGATATTATTTACGATATAGGTATCTTTACATAGCTA
TTATACGACCTAGAACCTTCAATCGTCGTTTGAAATTATATATTTTATCACATGCAAATAAAAGTAGGAC
GCGAACGACTTGGGTATATCAAAGATCGTATAATCTCTTCTGATAAGGTTGCCCTACAAAAGGATTTATT
GTAATTTGCAAACGTTTACGTACCCTGTCTATTTGAGGCGGTTGATCTGTTATTTAGCAATTACCATTTC
AACATTATTGGTCTTATTTTAGCCATGACAAACACATTGTGTAGGAACCGTAAAGATATTTTAAAAATAT
TTGCAGTCAATCAATAATTTCTTAGTTTTCAAAAAAAGTTAACATTATTTATACATTATTTTGTTTTGTATC
TTAAACGCACAAGTACGTTTTTAACTAACTGTACATTAATGTTATGCAATCTGAAGGATTTTTTAAAACT
ATTCCATAATTCATACAGTGGGACACCAAGTATGAAAAGACTGATTTGAATATCGACTTGATCTTATATT
GGTTCTAATTCTAACTACACAATGGCAGGAGATCTTGTCTTATTCTTGCTATGATTCTGAATATTTTAAAA
CTACTAAAATATTAGTCTTTTCATACTTGACCGAGCAGTCAAATTAATTTTAAGAATGTCAATTAAATAA

GTTTTTGAAAAGTTTGTGTTATAAAATGTAGTTGTTACGTAAACTAATAGGTAGGGCTAACAAATTTATA
TCTTGGAATATTGGAAGACGAAGGAGATCTATTATTGCTTGTCGACAGAACGAATGCTAACACACTTGT
TTGATTAATTAACTTTTTGCTCCAACAAACTTTTAAAATTTTACAGTATTTTATATTTCAGTGTTAGTTAAT
AGAAAACATTGTAATATGCACTATTAAATTGTATTTGTGGGAATGATCAAATTAAGACAGGATGTTAGG
ACATTCTGCATCATCTAAAAGTTACAACCAACACAGGTTTATTAGGTCAATGCGACGAACTTACACTTA
AAAAATAACGTCATAGCAGTAATTAAGCTAAAAAATACTGCAAGCGAGTCAATAATTTGTAATTGTTCA
GTTAGTATATTGAAATGTGGTGTTAAGGTACTAGTTGGACTTCTTGGAATCCTTGCTGGGAGAGCGGGA
TCTTGAGCGCGACATGGAGCGCGCCTTGGAGCGAGACCTGGAGCGCGAGCGGGAGCGAGAGCGCGA
GCCCGAGCGCGCCGAGCGGGAGCGCGAGCGTCTGCGCGACCTGGAGCGGCTGTAAAATAACATAGAG
CTGTTAGTTATGTGCATTGCTTGGAGGCACATCCCCACCCCCGCTACATTACTGTACTATCTTCTGAAGG
AGCAGCTCCGTCCCACTAAATTGTTGGTATCAAAGGATTCAACACCTGTAAACTATTTAAATAATTTTTG
GTCCTGGTGCAGATTAAAGTTGATATAATTGTGGTCTGTAGCGGCATGTCGATCTTTCCGAGGTCCCGT
GTAGATGAGTGATGACTTAGTCTATACTGCGAAATTACTTATACTTAATTAACCTATTACACTACTGTACA
TAATAAAAATACAAGTCAACATATCGTTAGCATATGACTGCAACACAAATATGGGTAAACTCACTGAGT
GCACGCAGTGATTACTTTAGGAGTGCGTAGCGGACGCGCAGAATACGTGACACTCAATGTAGTTGTAG
TAGTCGACTATTGCCGTGCGCACGGAGTAACGAAGTACGCAGTTTCACAGAGCGCAGTTACCGAAGC
GGCCGACTCAATTGGAGTGCAGACCGCGAGGTGTAAGCTACGTAGCGCAGAGTATAAGAAGTGCAGA
CACTGACGAGTTTGTTCTCGGCGCACAGGCCAATGATAACCTATAACATAGTTATCATTGGTTTACGTTC
AGTATAGATGTTTGTATAGGGACCTTTGGAAATTAATTGTTCAGTTGTTACTTACATTTAATGAAATTTAT
TCAAACTACCATCTCATTGAAAGCTTGGATAGACTTGGGTTAGTGAGGGGTAACTTTAATAATAAAAAG
ATTTATACATCAATTGTTAAGGGAAATCATAGTAGGTAACTTAACACCCTACCTACCCACCCATATTTAA
CTTGAATGTCTAGAACCTTCATCTCAAAAAGGTTTTTACTTTTGTTTGCTTGTTGTTTTCG
>TCONS_00105781 geneID=XLOC_021134 scaffold_340:100643-101866(-) Splice junction overlap
TTCTAGACATTCAAGTTAAATATGGGTGGGTAGGTAGGGTGTTAAGTTACCTACTATGATTTCCCTTAAC
AATTGATGTATAAATCTTTTTATTATTAAAGTTACCCCTCACTAACCCAAGTCTATCCAAGCTTTCAATGA
GATGGTAGTTTGAATAAATTTCATTAAATGTAAGTAACAACTGAACAATTAATTTCCAAAGGTCCCTATA
CAAACATCTATACTGAACGTAAACCAATGATAACTATGTTATAGGTTATCATTGGCCTGTGCGCCGAGAA
CAAACTCGTCAGTGTCTGCACTTCTTATACTCTGCGCTACGTAGCTTACACCTCGCGGTCTGCACTCCA
ATTGAGTCGGCCGCTTCGGTAACTGCGCTCTGTGAAACTGCGTACTTCGTTACTCCGTGCGCACGGCA
ATAGTCGACTACTACAACTACATTGAGTGTCACGTATTCTGCGCGTCCGCTACGCACTCCTAAAGTAAT
CACTGCGTGCACTCACCGCTCCAGGTCGCGCAGACGCTCGCGCTCCCGCTCGGCGCGCTCGGGCTCG
CGCTCTCGCTCCCGCTCGCGCTCCAGGTCTCGCTCCAAGGCGCGCTCCATGTCGCGCTCAAGATCCCG
CTCTCCCAGCAAGGATTCCAAGAAGTCCAACTAGTACCTTAACACCACATTTCAATATACTAACTGAAC
AATTACAAATTATTGACTCGCTTGCAGTATTTTTTAGCTTAATTACTGCTATGACGTTATTTTTTAAGTGT
AAGTTCGTCGCATTGACCTAATAAACCTGTGTTGGTTGTAACTTTTAGATGATGCAGAATGTCCTAACA
TCCTGTCTTAATTTGATCATTCCCACAAATA
>TCONS_00105759 geneID=XLOC_021125 scaffold_340:187922-190393(+) Exonic overlap (-)
CACACACACAATTTTAAGTAAATTAATCGTAAAATAGAAATAATAAATAATACACATGCTTGCAGTATTA
AATACGATAAAATAAGGTACTGTGTACATTGAAAAGCCTCAACCAACTGTAAATATAGCTAAAAATACC
ATTATACAGGAAGAAGATACTTGACATAAAATATAAGTAGAAATAAAGTTTGACAAGACTTAAAGGTTC
CACCGGCTAATAAGATTTCAAATATCTATTAATCATTTCGAAAATAAATAACAGTATTAAAGAAGTAAGC
CGGTTGAACCTAGAATCTATGTAAAGTTATGCCATTTTAAAAGATAAACAGTACTGAATAGAAAAAAAT
GAGAAGAAAATAATTGAATTAAATAATGATAATTCTTTCGGGGCTGCTCTAGTTGTAAGAAAAGAACGC
AAGTAAAAACCGATACAACCGTACCACATTCACGCTCTTTACACAATCACGCATGCTCTCTAACATTTA

TAATAACAACTAAGTATGTTTTTTTATAATTTACGACACATTACCTATTACCGAATAGATAATATTGTATGA
AATTAATATTACGAAGCGATCATTTGGATCGGCGCTTGACTATCATCCCGAAAGAGTATAATTATAGCTC
TTAATATCCGTATTACAAATCATGTGTTTCATTTATTACACAGTAGACTCGACATACCGGTGGAACTATGT
ATAGCCAAGCGTCAATACTCCCGAGATATCCTTTGAACCTTATCCAAGCAGTTAAATATTTTGACATAGA
GTCATCTCGTAAACTAGGCTTCAATATAAATATCGCTATCGCTTATTATGTTTAGTTAATACTACCGCACTT
ATCATTTATTGATTACTCTAATCATATACATTGAAACTGCTAATTGCATGAAACAGGGAATGGAACTAAG
TGTTGATATACAGGGTGAAACGCAAATATCTGACCTTTCTACGTAGGTATTTCAATTTCGGTCAGATATC
TGCATTGCTGCCTGTACTTAACTTGTAGCTATTTTTATTTGAAATAGACTTATGTTAAGATGAAAGTAGC
TTGCAACTGATCAGAGTTAAACAATCAGGACTTCAAGTAGGCAATTTACGTTGAAATCCTCATTATTAG
TTTAAACTAGCTCTGCAGTAATATCTTACGATACAAATTTGCTTCTTTCAATTCACAAGAGATGGTGCTT
TTTATTTTGAATTGTGTACATATTCCATCATCTATTGGTTCTAGTATTTGTGTTTCAGATACAATTTTAATAT
ACTTTTAAATTGTATTCATTTAACACTTACAATATTTTATTGTACGTTGTTACTGTCTCTTAAGTTTCAGTC
AGTAAAATTAAAAAGTATTTATTTTCAACCTTATTTAACAAATCTATCATACATGTTGAATGATTACACCT
AGTTACATCTAGTCATTGCACTAAAGTTGAATAAATTACATGTGTCTTATAAACAAATTATCATTATTGAA
GATCACATAATTCTAATGAACGTAAAACTAATGTATGATTGATGATTGTTTGTTCGAAGTGATGATGACG
TTGATGATGATAAACAGTTTGTGCTGCAAACAGGAATCATTAGGAACTAAATTCAACAGACACCGGTG
AAGGGACTAGTGGACGAGGGATGTTTCCGAAAATCAACTCCAAAAGCAGTCTTTCTTCAATTCCAACC
ATTTGAATCAATTTGATGTTTTCAATTAGATTTTTGAACACGAAATTATGATATGTATGTACATAAAATAT
TTATATTTAAAGACCAAGATATTATATCCTCGGTACATAGATACGCGATAAACTGAAAAAGATTGCTTAT
GAAATAAATATGGCATTTATATTTAAAGGTACTTCACATGAATAAAATGATTAAAACTGTCCGACAAACG
CCTACCGCGGACGAGACCGTAACTAACGAAGAAAAATATTTACAAGTTACAATAATAATTATCTAATTA
CATTTTCACTATGGAAGAAGTGTCCCCGCCGTGGCGCACAAAATACTACTTCACTATCGATAACAATAT
TTACATTAGCGTAATAAAACGACACTTAGTACGACGTCGTCGAGATCTGTTATAAGAAAAACGATTATAT
AAAAACGAACCGTCGCTTGCTACCTGCCACTACTTGACTCTGAACTCTGGCTTGACGCTTATGATGGG
TTTCTTGTCTTCCTTGTCTTGGTCGGGGAAGTCCCCGGCGGCGGGTAGCTAAGTGGCGGAGCTGAAGA
GCACGGAGTTCTTGTAGTACGGCGCCCGCTCCGACAGAGGGCTCTTCACGTCGCGGTCCAGCGCCTG
CTGCCGCGACTTGCGCTCCATCTCCAACTCTTGGGCCAGGGATTCTGAG
>TCONS_00106339 geneID=XLOC_021197 scaffold_342:171164-171594(+) Splice junction overlap
GTGGAACCTAGACCGATATTCGGGATAAGAAGACCAGTAGAAAGACCTGGAGGATACCGTGGAAGTT
ACCCGCCTCCAGAGCGTCCTATTGGTGCACCAGCTTACGGGGGCTACGAGCCTGATGGACCAATTGGG
CCAGGGGCGCCTATTCCAGTAGGTGGACCAATAGGAGGCCCGCTCGGTGGACCAGTGGGAGGCCCTC
CAATAGGTCAGAGGCCATGGCAAGGGTCCCGCTGTGAAGATGACAGCTTCAGACAAGTGGGGAGACA
GCGCATGCAGAGGAGATTCGTGCGAAGATTTACCACTGCCCAGTCTTTGGCACAATGCCAGAGGGAGT
GCATCGAGGCA
>TCONS_00106311 geneID=XLOC_021200 scaffold_342:352669-369424(+) Exonic overlap (+)
TTATTTTTTTGAAATACTAAAAACATAGTTTATTATAAATTTTATCGAGTCTGAAATTACAAAATGGGCAC
ATTGCTCATCTAATAATATTGAAGTTCTACACATTTTTACAAATAAATAAGTAATATTATAATATGGTTTCA
TCCCGTGGCGGCATCCATTTCGTGACGAGTCCAGCGTTTCAGCGGACAATCCTGATGTTGGTTCGGAA
GCAGAACAGGTCGGCCCCGCCCCTCTCCCTCACCCTGAACTCGATGTTAGCAGAAACACCCTGTACAA
ATGAAAGTTTTACTCCATACGGATTCCACATCTATAAGTTCTAGCAGCGTCAAACGGTTAGTTTCTCTAA
ATTACCTAATATTTTATACTATCCTAACCCACGAGCAACATACAAGTCTTT
>TCONS_00106312 geneID=XLOC_021200 scaffold_342:353791-369424(+) Exonic overlap (+)
TGTTTTTTTTGAAATACTAAAAACATAGTTTATTATAAATTTTATCGAGTCTGAAATTACAAAATGGGCA
CATTGCTCATCTAATAATATTGAAGTTCTACACATTTTTACAAATAAATAAGTAATATTATAATATGGTTTC

ATCCCGTGGCGGCATCCATTTCGTGACGAGTCCAGCGTTTCAGCGGACAATCCTGATGTTGGTTCGGA
AGCAGAACAGGTCGGCCCCGCCCCTCTCCCTCACCCTGAACTCGATGTTAGCAGAAACACCCTGTACA
AATGAAAGTTTTACTCCATACGGATTCCACATCTATAAGTTCTAGCAGCGTCAAACGGTTAGTTTCTCTA
AATTACCTAATATTTTATACTATCCTAACCCACGAGCAACATACAAGTCTTT
>TCONS_00106259 geneID=XLOC_021211 scaffold_342:388891-389409(-) Exonic overlap (+)
CTGTCAACTGTCAAATCAAATTAGCGTGTGTGTGTGCCATTGTGAAAATGCCGGATTTATTTTAGAAAT
GAATAACATTTGAATTGTTATGAAGCAAACGTTACTGTAATTTATACCTATAACAATACTATCATAATGTC
TAATCCATCACTTTCAAGCTCGAGCATAGCTGCTCAGAACCAGTTGGTGACATGGCTGAACGAGGCGG
GGCTGGCGGAGGGCGGGCGGCGCGCGGAGCTGCTGCGCAAGGCGGCGGAGGTGCTGCTGCACCAGC
TGCCGGCGCTGCTGCCCGCGCACCTGGACCTCGTGCTGAGCCACGCCGCCGACAAGCACACCGACGT
CAAGAAGGTGGTCATTGGGTTCATTGAGCAAATCAGGTCAGTGCCAGCGACATGCTCTGCATAGTAAC
>TCONS_00106424 geneID=XLOC_021231 scaffold_343:175139-175723(+) Intronic
CAACAACACAACAACCAAAATCAGACCCAGAAAAACAGCAACAATTCGCCCTCCTAACCAGTCCCAG
CCAAGGTCTTCAGCAACCTTTGAAAACTGTCGCCGTGAACTCTTGCATGGCCCCTCGTATGGACTTTG
GCTGCGCGCCGTCCTACTGCCAGCAGCCCAACCCCATGTCTGGGATGGTGGAGATGTGCTGCGGACCC
TGCATCTTCTGCTGCAACTGCTTGATGAGTCTCTGCTCCTCTTGCATGGCCTCGTGCATGTCTTGCTTCA
CCTACCAGCGGTGCTATTGATGGGATTGACAAAATCTAAAAAAAAAAATACTTATGAAATATCAGGATT
TAAAAAAAATGCAAAATAAAGTGAAGTTTTAATAAACGAAGCTTCCATAATGAAGAACGAATAGCTAC
TTATATTTATTTGTGTGTTATTCAGTGAAAATGTCT
>TCONS_00106425 geneID=XLOC_021231 scaffold_343:175168-176153(+) Intronic
GAAAAACAGCAACAATTCGCCCTCCTAACCAGTCCCAGCCAAGGTCTTCAGCAACCTTTGAAAACTG
TCGCCGTGAACTCTTGCATGGCCCCTCGTATGGACTTTGGCTGCGCGCCGTCCTACTGCCAGCAGCCC
AACCCCATGTCTGGGATGGTGGAGATGTGCTGCGGACCCTGCATCTTCTGCTGCAACTGCTTGATGAG
TCTCTGCTCCTCTTGCATGGCCTCGTGCATGTCTTGCTTCACCTACCAGCGGTGCTATTGATGGGGACG
ACCAAAAACAAGCAACTGAGCAGACCAAAACCATTACCCAAACAGCAGACAAAGAATACGCAGCAT
ACACATGCACGAATCAACCGATCACGGTCCCTACTGGCCCCAACTACACCCAAGTCCTGCACTTCAGC
TGCGGGCCTCTGGACCCCTCCTGCCCGGCGAGCCCCTACATGACTATCTTTGA
>TCONS_00106514 geneID=XLOC_021233 scaffold_343:190742-193055(+) Intronic
GTAAAAAGCCCTAAAAACATTGGAATAATAATGCTAGGTATTTATATAAACACTAAAATATGTTCTAAAA
TAAATAAATTCAAAAAAATACGACGTTTACTTACTGACGCTCTTGTTGGTTAATGGATATTTTGTATGGG
GGCACCAAGATGCTATAGACCTGCCCGCATCTCACCTGGTTTATTATGTGTCTTGTGTCATTAAAAAAC
ACTGACAGAAGTTTCATCAACATTGAAACGGTACAGCAGGTGTCCAGGAGCCACTTTCTGACAGATTT
TGGGAGAAAAATTTACAATATTTCACGAATATTCAAGTTTGCAGGTGGAACCTGCAGAGATTCAGCTG
CAAATGATAGTTCATATGAAAGGTATTATTAATACCTAGAAATGGTTTTCAGCAATTTCAGATTAAATGA
CTTTTGGTGAATATTCAAGGTGAAAACCAAAAAATAAAACATAGCAGCCCAACATTAACATTTTGTATG
GCGGCACCAAGATGCTATAGACCTGCCCGCATCTTACCTGGTTTATTATGTGTGTTGTGTCATTAGAAAA
CACTGACAGAAGTTTCATCAACATTGAAACGGGATAGCAGGTGTCCAGGAGCCACTTTCTGACGGATT
TTGGGTGAAAAATTTACAATATTTCACGAATATTCAAGTTTGCAGGTGGAGCCTAAACAGGTACAGCTG
CAAATGATAGTTCATATGAAAGGTATTATTAATACCTACAAACGGTTTTCAGCAATTTCAGATTAAATGA
CTTTTGGTGAATATTCAAGGTGAAAACCAAAAAATAAAACTTAGCAGCCCAACATTAACATTTTGTATG
GCGGCACCAAGATGCTATAGACCTGCCCGCATCTCACCTGGTTTATTATGTGTTTTGTGTCATTAGAAA
ACACTGACAGAAGTTTCATCAACATTGAAACAGTATAGCAGGTGTCCAGGAGCCAATTTCTGA
>TCONS_00106528 geneID=XLOC_021233 scaffold_343:190765-191919(+) Intronic
AATAATAATGCTAGGTATTTATATAAACACTAAAATATGTTCTAAAATAAATAAATTCAAAAAAATACGA

CGTTTACTTACTGACGCTCTTGTTGGTTAATGGATATTTTGTATGGGGGCACCAAGATGCTATAGACCTG
CCCGCATCTCACCTGGTTTATTATGTGTCTTGTGTCATTAAAAAACACTGACAGAAGTTTCATCAACATT
GAAACGGTACAGCAGGTGTCCAGGAGCCACTTTCTGACAGATTTTGGGAGAAAAATTTACAATATTTC
ACGAATATTCAAGTTTGCAGGTGGAACCTGCAGAGATTCAGCTGCAAATGATAGTTCATATGAAAGGTA
TTATTAATACCTAGAAATGGTTTTCAGCAATTTCAGATTAAATGACTTTTGGTGAATATTCAAGGTGAAA
ACCAAAAAATAAAACATAGCAGCCCAACATTAACATTTTGTATGGCGGCACCAAGATGCTATAGACCTG
CCCGCATCTTACCTGGTTTATTATGTGTGTTGTGTCATTAGAAAACACTGACAGAAGTTTCATCAACATT
GAAACGGGATAGCAGGTGTCCAGGAGCCACTTTCTGACGGATTTTGGGTGAAAAATTTACAATATTTC
ACGAATATTCAAGTTTGCAGGTGGAGCCTAAACAGGTACAGCTGCAAATGATAGTTCATATGAAAGGTA
TTATTAATACCTAGAAACGATTTTCAGCAATTTCAGATTAAAT

>TCONS_00106546 geneID=XLOC_021249 scaffold_343:190768-192948(-) Intronic

CAGGTCTATAGCATCTTGGTGCCGCCATACAAAATGTTAATGTTGGGCTGCTAAGTTTTATTTTTTGGTT
TTCACCTTGAATATTCACCAAAAGTCATTTAATCTGAAATTGCTGAAAACCGTTTGTAGGTATTAATAAT
ACCTTTCATATGAACTATCATTTGCAGCTGTACCTGTTCAGGCTCCACCTGCAAACTTGAATATTCGTGA
AATATTGTAAATTTTTCACCCAAAATCCGTCAGAAAGTGGCTCCTGGACACCTGCTATCCCGTTTCAAT
GTTGATGAAACTTCTGTCAGTGTTTTCTAATGACACAACACACATAATAAACCAGGTAAGATGCGGGC
AGGTCTATAGCATCTTGGTGCCGCCATACAAAATGTTAATGTTGGGCTGCTATGTTTTATTTTTTGGTTTT
CACCTTGAATATTCACCAAAAGTCATTTAATCTGAAATTGCTGAAAACCATTTCTAGGTATTAATAATAC
CTTTCATATGAACTATCATTTGCAGCTGAATCTCTGCAGGTTCCACCTGCAAACTTGAATATTCGTGAAA
TATTGTAAATTTTTCTCCCAAAATCTGTCAGAAAGTGGCTCCTGGACACCTGCTGTACCGTTTCAATGT
TGATGAAACTTCTGTCAGTGTTTTTTAATGACACAAGACACATAATAAACCAGGTGAGATGCGGGCAG
GTCTATAGCATCTTGGTGCCCCCATACAAAATATCCATTAACCAACAAGAGCGTCAGTAAGTAAACGTC
GTATTTTTTTGAATTTATTTATTTTAGAACATATTTAGTGTTTATATAAATACCTAGCATTATT

>TCONS_00106529 geneID=XLOC_021236 scaffold_343:219137-220984(+) Splice junction overlap

CCTGTGTACATTTTATTCACATTCCGGGAAATCAGAGTTAATAATAAATCTATTGTGTCTGGTTAACCAA
GGGGTCACCGGGTGAGTGGTTAAGTTACATTGATGATGTTGTTGTATTCCAGCTGACGACGTTCGAGC
AGGAGCAGCTCCACCCACGGCTGCAGGCTGTATTGCTGAAGAGCCTCAGCCAACTCACATTGCCGATA
GAGGAGGAGTCACCGGACGAGGACGAGTCTTCCATCTCGTTCAGTGAAGGCAGCAAGAGGAAGAAG
AAGAAGATGAAGAGAAAAAGAGGGAAGAGCTCAGGCGACTCTGACTCAGACTCGTCAACGACATCA
AAGAAGAAGAAAAAAACTAAGAAGAAAAAGAGTAAAAAGACGAAGTCAACTTCCGATGACGAATCA
GACGCCTCAAAAAAGAAAAAAAAGAAAAAGGGAAAGAAAACAAAAGGAAGTAAATCCGAATCAAC
AGACAGTGATTACGAATCAGATTCGTCTACGAAAAAGAAGAAAAAAACAAAGAAGGAAAAGAAAAA
GTGAAAGTTAAAGAATCGGTGGAACACTCGTCCACAAGTTCCTCAAAGAAGAGCCGGTGGTAGTTTA
AACAGCCAACAGACGTCCACTACATAGGCTACTCACAAACAAACACTCACAGTAAAAAGCCTTTGGT
CCGATTTACATCAAATACTTTGCTAAATCATGTTCTGTCTGAATTTTTAAGAATATTACGTTTGTTAAACC
AGTATGAAGTAAATCAAAGTTAGTAATAATTAAATTATGTTTTTAATTATTATATAATAATATATCCTTTTA
GCTACGAACTAGCTTTTTCTAAATCGTGTAGGCACCCGTAGGAATTCTACGGGAGCAAATTTATGAATT
TATGAATCTTAAAATAAGCATGTTAGTAGGGCTCGTTAGCATCAAAGATTAGTTCTGATTTTTATCTGAC
ATTATGACAAACCAAACATTTTGTCTATGACTAACCTAAAATAATAATATAAATCTAAATCAAATTTATAA
ATCGCAAAAAAAAACAAAAACACTTAATTTCAAGTACAGGATTTACAATGATTACCGACAACCAACTA
ACAAAACAACAGAAATACGACCAATGCGAGCTGATGCCCGTGATCCAGCACATCGACATCTGCGGAC
GAGAGCGGAGAATGAGCTGTGAGAAGTATCACCACTTGCAGTCGGACCTCCCCGTGAGTGTGACGGC
GGTGGCGCTGGCCCCGACCAGGAGTGGGGGACACCAGGAGGCCAGAACGCTGCAGAAGCTGCAGAC
ATTTAGCGTCGTGAAGAGACACACGAGTGTTTGTCCCGAACGCGACTGCAGACGGACGAAGACCAAG

GC
>TCONS_00106501 geneID=XLOC_021236 scaffold_343:220335-220996(+) Intergenic
GAATTTTTAAGAATATTACGTTTGTTAAACCAGTATGAAGTAAATCAAAGATTTACAATGATTACCGACA
ACCAACTAACAAAACAACAGAAATACGACCAATGCGAGCTGATGCCCGTGATCCAGCACATCGACATC
TGCGGACGAGAGCGGAGAATGAGCTGTGAGAAGTATCACCACTTGCAGTCGGACCTCCCCGTGAGTG
TGACGGCGGTGGCGCTGGCCCCGACCAGGAGTGGGGGACACCAGGAGGCCAGAACGCTGCAGAAGC
TGCAGACATTTAGCGTCGTGAAGAGACACACGAGTGTTTGTCCCGAACGCGACTGCAGACGGACGAA
GACCAAGGCATTCAACAAAGC
>TCONS_00106510 geneID=XLOC_021252 scaffold_343:237844-238733(-) Splice junction overlap
GGCCCCGCAGGTCCCACAGTCCACTTCCAGCCACAACCATGAGGGTCTTTCTGCTCGTCTTCGTCGTT
ATGATGCTATTCGCTGCTGCGTTCGGTGACGGTGAAACCGAGGCCTCAGACGCAGCTGGTATGACCGA
AGCAAGAACTGACCCCACCCTCGCCGATGTTGACCCCGACACTACCAAGCCTAGTAGCGGTTCATACT
TCGATAAGGTAAATGACCGAGTTAAAGCTGAGCAATACACTCAGACGGGCTAACGGCCTCAGCCACC
ACCGCCCCGGTTGATACATCGACGTGGAATCTGACCCTCAAACCACTACGGGGTGGAGCCCCGTGCAT
AAATAAAAGGTTTTTGTCATTAAGCAAAACTGTCTTCTTTTTTAATTATAATTAAATTTGTGTTACAATAA
TAATATAACTACTACACTCTCTATCACTCGA
>TCONS_00106511 geneID=XLOC_021252 scaffold_343:237844-243394(-) Splice junction overlap
CGGGGGTCCCACAGTCCACTTCCAGCCACAACCATGAGGGTCTTTCTGCTCGTCTTCGTCGTTATGATG
CTATTCGCTGCTGCGTTCGGTGACGGTGAAACCGAGGCCTCAGACGCAGCTGGTATGACCGAAGCAA
GAACTGACCCCACCCTCGCCGATGTTGACCCCGACACTACCAAGCCTAGTAGCGGTTCATACTTCGAT
AAGGTAAATGACCGAGTTAAAGCTGAGCAATACACTCAGACGGGCTAACGGCCTCAGCCACCACCGC
CCCGGTTGATACATCGACGTGGAATCTGACCCTCAAACCACTACGGGGTGGAGCCCCGTGCATAAATA
AAAGGTTTTTGTCATTAAGCAAAACTGTCTTCTTTTTTAATTATAATTAAATTTGTGTTACAATAATAATA
TAACTACTACACTCTCTATCACTCGA
>TCONS_00106512 geneID=XLOC_021252 scaffold_343:237844-243394(-) Exonic overlap (+)
CGGGGGTCCCACAGTCCACTTCCAGCCACAACCATGAGGGTCTTTCTGCTCGTCTTCGTCGTTATGATG
CTATTCGCTGCTGCGTTCGGTGACGGTGAAACCGAGGCCTCAGACGCAGCTGGTATGACCGAAGCAA
GAACTGACCCCACCCTCGCCGATGTTGACCCCGACACTACCAAGCCTAGTAGCGGTTCATACTTCGAT
AAGGTAAATGACCGAGTTAAAGCTGAGCAATACACTCAGACGGGCTAACGGCCTCAGCCACCACCGC
CCCGGTTGATACATCGACGTGGAATCTGACCCTCAAACCACTACGGGGTGGAGCCCCGTGCATAAATA
AAAGGTTTTTGTCATTAAGCAAAACTGTCTTCTTTTTTAATTATAATTAAATTTGTGTTACAATAATAATA
TAACTACTACACTCTCTATCACTCGA
>TCONS_00106486 geneID=XLOC_021252 scaffold_343:242317-243519(-) Unclassified
CGCACGCGGGCTCAGCCTCGGGTGTCGCTTCACACACTCATCGGTGGCCAGAATACAACTCCGAGAC
ATACAGTATCACTTCCGGGATGTGTTTGTACGAGTACCTACATATACCGGCGCGGCCCCGGGGGTCCCA
CAGTCCACTTCCAGCCACAACCATGAGGGTCTTTCTGCTCGTCTTCGTCGTTATGATGCTATTCGCTGC
TGCGTTCGGTGACGGTGAAACCGAGGCCTCAGACGCAGCTGGCGTGACCGAAGCCAGAACTGACCC
CACCCTCGCCGAATCTGACCCCGACACTACCAAGCCTAGTAGCGGTATATACTCCGATAAGATAAATGA
GCGAGTTGAAGCTGAGAAATACAGTCAGACGGGCTAACATCTGCCACGATCTCCGCCGCCCCGGTTGA
TGCATCGACGTGGAATCTGACCCTCAAACCACTTCCTCCACCCTTTGCATAAATAAAAGAGGTTTTTGT
CATTAGTAAGAATAAGCAGCATAATTGTCTTCTTTTTTAAATATTAAATTTGTGTTGCAATATAACTACTA
CCTACACTCTCTATCACTCGAACAATTTAATAAGTTCCATTTGCCATCGACATTCCATAATGTCACTGGA
ACTTTCGAATTGCTCGTAAAGTGTATAACTAATAGTAACACTAATTGTTTGTAACTTGTTAGCTAATACC
TTATTGATGTGTAGAACAATGACAAGACAGTGAG

>TCONS_00106537 geneID=XLOC_021239 scaffold_343:244318-252519(+) Intergenic
CACAACCATGAGGGTCTTTCTGCTCGTCTTCGTTGTTGTGATGCTATTCGCTGCTGCGTCCGGTGACGC
TGCCCCCTCCCCCCTCCTCGCCCCCCCGAGGGCGTCACCACCTGCCGCCCCACCGCCGGCGACCAAC
AACTCAGGAAGATATTGAATGATCGACTTGCAGAAATTAACAAATCCAATGATCAAAATCTAGAATAAT
CTAACGGTGGCTAAAATGGGTCCTGCCA
>TCONS_00106455 geneID=XLOC_021253 scaffold_343:253326-254519(-) Unclassified
CCAACAGTTGGGTCTCCCCAACGACTCTTCAGTAGTTTCCCACATACCTCTGTGCCCAGAATACCACCC
CTGGGTGTGACGCTATCAGTTCCGGAGTGCGCGTGTGTATGCGTTTGTGTGTGTGTGTGTGTGTGTGCG
TGCGTGTGTGTGTGTGCGTGCGTGTGCTATATAGCGGGTCGGCGGGCCTAGCTCCTCACTGCACCATGA
GGTCCACACTCCTTCTCTTCGTCGTGATCGTGATGATGATGTGGGCGCTGGCCCTAGGCGAGGGCGAG
GCGTACGTGCAGCCCGACAACACCGAAACCGGAACGTACCTGCAGCAGATCCTCGACCGACTGGAGG
CCAACAAGGACAACATGACGGGCTAGGCCATGAACACCGCCACCTACTTCGACGACTTCTTCTTCTCC
TTGCTAGGCCTCTGAACAACGCTACCGACCGAGCGACTCTGTTCCTCAAT
>TCONS_00106730 geneID=XLOC_021316 scaffold_345:14752-16359(-) Unclassified
GCTCGGCTGCCCGAGCGGCAGCCGTTCATCGCTCAGGTGGTGAACGAGGAAGAGGGCCAAACAAAAT
AAACATCGACTTCTTGGCCACAATTTAATTATATTTCTGTATTATTTTTTTGAAATGACATTCTCATAGAC
ATTCAATTATTTTATTTGATATTATTATTTAGCCATCATATATTTTTTGCCCAACAATAATTTTTCATGGATT
GTTTTTCAAAATTATCCTTTGTTAAAGTCAATTTTGTATGAATTTTATTGTTTTTTTAATTGTTGACTCAA
GATCAGTTGTTATTTACCCTAATTATACCTATTGTTTTGAGATATAGATACCTACAGTCAAACTATAAAGT
CCTGACATCAAAGAGTGCGATTTTGCTAAGACTTTTTATGATTATAAGGTATAAATTTACTTACTTTTACA
TACATTTTATAAATAAAATACCGATTGATTATGGATTTTTATTGGATTTGTCCTTTAATAAATATAACAGAT
CGATTAAATACGATCTTAAACAATTAGTTGGGATCCACTTCCGTTTTCACGACTTTATAGTTGGACTGTA
TAACTGTTAACTGTACATGCTATTGCTAGAAATGTTTCGATCAAGCGCAGATCCTAGTTTGGTATTACTT
TTAGTAGGTCGTATAGGTGTGTACCTAGTACCTACATAGAACAACAAGTACAGTAGTATGTTATTCTATG
TAGTGTACAACGTATCTATACTATGTCTAGTGTAACAATTTATTTTCATATAAAAGTAACTACTCGTACTA
AAAGAAGCGC
>TCONS_00106716 geneID=XLOC_021306 scaffold_345:18929-20082(+) Intergenic
CGCGCTGCACACTCACTACGTGCTAAATTACAGATTGAATAAACATACGATGTGAAAAATGTGAAATGT
TGATTAATAAATGATAATAAGAAGTTATGTGTATGAGAATTTGTGCTGTGTGCAGATAACGAACTACCAC
TTATGTAGTAATTACACAAACATGGAGGGATGTTATTTGAAAAGTCTGTCTGCACGTCGCGAAGAACA
CTATTGTCGCGCAGACTTGGGTCTAATGTGTTCCAACAATGAGTGGAGTTGAAGGAGAACGAATGCTG
CAGACTTGTGTTGTGGAGTCAGGAGCAAGTACGAGGACGCCCTAGTGCGGTAAGAGTTAAAGTCACG
TGACCACCGTAATTTGTTATACTTATAAATATTCAGGCAGCCTTTTTTACCCGACTGCCGAAGGAGTAGA
GGAATATTTTTCGATTGTATGTATGTATGTATATTTGTCTGTTTCTTTGTTCCCTCCTGTAACCTAAACGGC
TTGATAGATTTTGATGTATGAGGTATCGTTAGAAGCGTATTAATAGCGCAATGGTTATAGGCTATGTGAC
GTTACATTAAAATCTACATAGCGCCCTCTAGAGGACATAACGTGATGATCGAAAAAATGATATTTTTCCA
ACTATGCCGTGTGGGGTATCAAATGAAAGGGCCTGATTTGCACATTACAAA
>TCONS_00106859 geneID=XLOC_021347 scaffold_346:33277-42646(-) Exonic overlap (+)
CGCGACTCGGGTGCTGACTGAACGCACTCTGCCAGCGAGTATATAAGCGCCGGCTTTATCACTGGCCC
ATCAATATATTGCAGGCAGGCACAGCGTACACACCATTCCAAATTCAAATAATATTTTTTTCTTTTTTCA
AATCTCGTGTGTGTTGGTGGAAAAGAAAGCCGAGAGCTTTTGTTTTGTTGTCTTCAGGAGCTAAATCA
GAATGAAGCTGTTATTATTATGCATGGCGCTGGCGGCGGGGGCGGCCGCCCAGCCG
>TCONS_00106934 geneID=XLOC_021348 scaffold_346:109004-109661(-) Intergenic
CACAGGCCCCGCCTCACTCGTCTTCACACTACTATTTCATTCATCATTCTATTAAAAAAAACTCTATTAA

ATAACAATCCGATTCCTTCCAACCGCATCCAGTGACCCCATGCCGGTTCAACGTTAAAAAGCCAAAAG
TGTTATGAACTGGCGATAGTTGCGTGGTTAAAGTGACAGTGTTTGTGTTTGTTAACAACTATATCAGCT
GAGTGGCAAGCAGTCAAGCAAAGAGCACGAGATGACTATCACAAGTATGAACGTCACTGAAGCGGGC
GCTGTTTCAGCAAAAAAGTGCTGGCCGAGGT
>TCONS_00106863 geneID=XLOC_021349 scaffold_346:112249-114767(-) Exonic overlap (+)
TTTTTTTCTGTGTTTTTTTAATATAAACTTTTTACTGCCCCACCCTGTATATCAGTAGGTACATTTGCAGA
ACATTTTGTAACAATGTGTATTTCTATCAAGTACCTACTTAAGCAAGTCAATGTATGAAAAATGAACAA
GTAATGAAGTAGCCAATCAAATGTATTTAATAATGAATATTACTTATTTACAAGAGGAAGAATGAAATGA
ACTAATTATTTAAGTACTTGATGTATTGGTAGACTGTTATTTGAATCACAAATGTTTATTTATATTTGAAA
GCGAAGTGTTATTGGTTAATAAAATTATTCTAGTCATGTTAAAAATATAAAAATACCGAGGATTACAGAG
TGTTTATTTTGTTTTATAGAAAAGATCTATACATATTAATTAGTATAGGTACGTATTATGTGTACAGTTTGT
AATAACGTGTGTGAGTTCATATATTGTACAGACCGTCCAATCAAGGTGCAATCTCAAAGAACATCTCGG
GATTTTTGTCAGAGAAGTACTTATGTTCCTTGGATTCGGGTGATGCCTTGCTTACCACTCAAGACCGTG
GATTAAAACCCCATGTATGTGAATCCTTATTCTAAGCTTATGGAGTACAAGAACAAAAAAAAAGACAAAT
CCCACCCACAGACCCATTGTAGATTTTGTTTTTACAGAAAGTCATCACCAAGTAAATTTTTATTATTATC
CATATCCATATTAATAAATAAACATATCGTAATTAGGTACTGTGCGTATTTTGAAAATCATTTTATGAGACT
AATAACATGAAGATTATCTTGAAGATTATGACATGTTTTATTATATTTTAACTGAGATATGTGCCTATTTAT
TAAGTGTCTCTGCATTGTATCGCCGGTGATCGTGGTCATCATGATTTTCGGCTTGTACTTCTACGACAAG
ATGATAATCAAGAAGCCTTGCTACTTCCCTTGATTGAGATTAGCTCATTAGTGATATTCGCTGCTGCGGG
TCTAGAACTATTTACAATGGTGGACTATGTAGGCGGACTTGGCTGCTTCATCAACTTAATAGCATTACTG
TTTCTACTATACTCACCACTACATATAATATTACCATAAGCCCTTCTACGACTTTACTGCGACCTACTGGG
ACACTACCGAGTCTACCGAGTTACCGACATGGCATAGATTATTACTCTTTGCCACCACATTTCTGGCTTT
TGCAGATGTATGCCAACATTGCCAACCAAACATTATATTGAAGACTACGTGCCAAAGTATGACTCTATA
GGCGAATAGGACTTTATAGTTTTACTGTACTTAGAAACACTGAAGACAGACTTGAAGAAGATGAGGCG
GCGGAA
>TCONS_00106864 geneID=XLOC_021349 scaffold_346:112510-114767(-) Exonic overlap (+)
TTTTTTTCTGTGTTTTTTTAATATAAACTTTTTACTGCCCCACCCTGTATATCAGTAGGTACATTTGCAGA
ACATTTTGTAACAATGTGTATTTCTATCAAGTACCTACTTAAGCAAGTCAATGTATGAAAAATGAACAA
GTAATGAAGTAGCCAATCAAATGTATTTAATAATGAATATTACTTATTTACAAGAGGAAGAATGAAATGA
ACTAATTATTTAAGTACTTGATGTATTGGTAGACTGTTATTTGAATCACAAATGTTTATTTATATTTGAAA
GCGAAGTGTTATTGGTTAATAAAATTATTCTAGTCATGTTAAAAATATAAAAATACCGAGGATTACAGAG
TGTTTATTTTGTTTTATAGAAAAGATCTATACATATTAATTAGTATAGGTACGTATTATGTGTACAGTTTGT
AATAACGTGTGTGAGTTCATATATTGTACAGACCGTCCAATCAAGGTGCAATCTCAAAGAACATCTCGG
GATTTTTGTCAGAGAAGTACTTATGTTCCTTGGATTCGGGTGATGCCTTGCTTACCACTCAAGACCGTG
GATTAAAACCCCATGTATGTGAATCCTTATTCTAAGCTTATGGAGTACAAGAACAAAAAAAAAGACAAAT
CCCACCCACAGACCCATTGTAGATTTTGTTTTTACAGAAAGTCATCACCAAGTAAATTTTTATTATTATC
CATATCCATATTAATAAATAAACATATCGTAATTAGGTACTGTGCGTATTTTGAAAATCATTTTATGAGACT
AATAACATGAAGATTATCTTGAAGATTATGACATGTTTTATTATATTTTAACTGAGATATGTGCCTATTTAT
TAAGTGTCTCTGCATTGTATCGCCGGTGATCGTGGTCATCATGATTTTCGGCTTGTACTTCTACGACAAG
ATGATAATCAAGAAGCCTTGCTACTTCCCTTGATTGAGATTAGCTCATTAGTGATATTCGCTGCTGCGGG
TCTAGAACTATTTACAATGGTGGACTATGTAGGCGGACTTGGCTGCTTCATCAACTTAATAGCATTACTG
TTTCTACTATACTCACCACTACATATAATATTACCATAAGCCCTTCTACGACTTTACTGCGACCTACTGGG
ACACTACCGAGTCTACCGAGTTACCGACATGGCATAGATTATTACTCTTTGCCACCACATTTCTGGCTTT
TGCAGATGTATGCCAACATTGCCAACCAAACATTATATTGAAGACTACGTGCCAAAGTATGACTCTATA

GGCGAATAGGTACTACAGACCAACT
>TCONS_00107155 geneID=XLOC_021372 scaffold_347:42037-42635(+) Intronic overlap (-)
GTCTTCAGCCCCTCAAAGTAGTCCCGGCTCCACTTCTCCCGGGCGATGGATCGTATGACGGCGGGCGC
GTGCAGCTCTGTGTCGAAGTCGAAGTGCCTGGTCGTGGCCGTCCACTCGCCCTGGAAGTCTACTTCTA
CCGTCTTTGAGGGGTCGTTTTGATACCACATAGGTCCCTTGTACTGCAGGGTCCACTTCTTCATGGGCT
CGACGGGAGTCATCTTGAGACCCTCAGCCCCAAATTCTCCCACTTCTGCTCCGAACAACACCGTGTCC
GGAATCTTCTTACTGCATAGCAGGCCTTTACCTGGT
>TCONS_00107094 geneID=XLOC_021385 scaffold_347:111435-134615(-) Intergenic
GCTCAGTCTGACAACACCACGCGGGCCGTCCGAACGAATCGACTCCGAAAACAGGACGCGATAAAA
AAGAAACTAATAAAAAATATAAACAAACTTCTCACTGGCCGGAAATAAAAGAAAAAGAAAACCCAAT
GCCAGGGTCGTAGGACGCGTTCCAATTTAGAATTCTAAATTCGTGTGCGCGGGAATCGCGGTGTTTCG
AGCGGTTTTGTTTTTTTTATTTTATTTAATAATAATTATGTGGTATAGTGGATGAATTTGTGTGTCGGAGT
GAATGATAATAATCGTTATTAAAAATAAATAAAAATGTTATCAAATAAATAACTTGTACCAAACAACAAA
CAAAACCTTCCAAAACATCCACCCAATCACCCAACACTGCCAACCATAAAACCTCAAACCAAG
>TCONS_00107228 geneID=XLOC_021389 scaffold_347:329697-341667(-) Unclassified
CGAAAGGAAACTAGGCAGGTACAAATTGCGCGTGTGTTAGTGTATGCGTACACAAAATTTCACGTTTG
ACCACTCTGGACACCTACACAAGCATACAAGGCGGGGGTGCGCGGGGCGGAGGGAGTTACGTCCGTG
AAGTTGTGTCAGCATATGCGCAAACGAGGTGCGCATTCGTCGCGTGCGTTGTCGGGATTCTTTGTGCCA
TTCTTCGCATACCAATAAGAGTATATTCTTAAAAATTACTGTTAGATTAACAACTAATCAAAATTCGCCC
CTTCACTTCATCTTCTGGGACCGTAATAGGTAAGTCAATATTGATTTCTGTGTATTTTTCATACAATCAAA
GTGATATTTCGACGATTTTTATTCGGGAATGTGAAAAATGCGATCGCGATCCTGCAATATTTTACATAGT
TGTCGTGTTTGCTTCGTTTCGACTTCCGGTTGTTGGGGTGTCGGCATTTCGCGATGTAAACATTATCTAG
TTTTAGGGTTCGATTGCATTTAGGCGAGCTGTCAGGAATGAAGTGTGCTCAAGGTGCTTTGTGTTGTTA
ATTTCGCTTGAGTGTGCTTACTGGGATGGCGCAATTTTCGAAAGACGCGGTATGGTATCGTTCGCCTGC
GATACACTTCAGTCGCGTTGTTAGCTGCATGCGATTTGACGTATTCTGCGCGTGGTGATGTCACTTGTC
GGCTTATTTTTATGAAATGTCAAACGATCTTCATGTAATGTGGGAGTGAATGATATAAACATATTGACAG
TGTAATTAATTGATCGGTGAAATTATAATCGTGTGATATGGACTGTTATTATTCGAAATTTTATCCAATAC
GTAAGTGACAAATGGAAAAGTTACTTGAACGGTGATCGCAGCACATACGATGCATTCCGCCGGATATT
ACGCGAACCTCGGTAAGACAAACAACAAAAGGTTGGCTGGTAGAAAATGAAATACTAAAATTAAGCC
CACCTTTTGTACATTTACCTATATTCT
>TCONS_00107197 geneID=XLOC_021391 scaffold_347:389393-392678(-) Unclassified
GAGCTTTCTTAAATATGTATTTTGTAAACATACAAATTATTGTACTATCACAATCGACAAGTCAAATTGTA
TTTATAAATATGGGTTTATATTACGAGATTATTCAAAGTACTCGTAATTATACCATTGTGCACTGTGTTTTA
CTCCCTGTTTTAATATATTTTTTACATCAATTTTAAATCATGTAAAACATCTCATCTCAATCTTTGCAAGA
CAAGAGGTAGTTTTCATTGACACCCAATATTACAGCATAATTGCATATACAATGATATCGATTCTGAAGG
CAAAAATTTTAAATTTAGCGATGTCAAAACCTTAATTTCTTTGTTTAAAATTCGACTCTATGATTGTTTC
CGTAAATGTCATTTTATGCTAGCAAATTTTTTAGTTTGACAGCGTTAAAATTAGAATTTCTGCCTTCAGA
ATCAACATCAATAAACTGTTTATAAGATAAAAACTGTGTTTTACTAATATCCCCGAATACTAGGAAACTT
ATAGACAGTTAATTTTGTATGTGGTGCGAGTTCGATAATTTAGCCGTTTTGCATCGTTTTTCTCATATACA
GGGTATAGGAAGATGTTATAATCCACTGGGAACAATGGGAATAAATGGAAACATTTGGGAATAAATACA
TTCACGTTTGAAGAGATTCGAATAAAAACGAACAACAATACGATAGTTTCCATGGACGCACCTTTGCA
GTAAGAAAAGCGGTGGTGTTATCTTATTTTTCTGCTATCGCTATCACTTTAACCGTTTGTATGTCAGAAG
CGCGGATTCACGCGAGACGCAATTTCTATACTATAACTGCATTCAAGCTGTTAAATCAAGGGCCCCATC
GCGAAGTCAAAACGAAGTAAGTACCTACCTAATGAGTGTCAAATTCAATACAATTTGAATGCACAAAA

TACAATTTAGCACATCTAGATATGTGAACCCACCACCCGCAGTGGACCATCGTGGTGGGAAATGGTCC
AAGCTTAGGAAGGAAATTTAGACCTTGGGGATATGCACAAAGGTTCCACTCGAGAGAGCCAGGTGCA
CGTTCTTACACCCCCACAGAGAATAGAATAGAAGTTTGTACGGGAGCCCAAGGAGCTGTACAATTAAA
TCCGCAATCAATTAAATAAAGCGATAGTGACAGCAGAAAAATAAGATTATAATACCAGCCTGTTTCCAT
GTATAGCAAGCATCGCTATAAATAGTTATATCGACCGAAATAGATCAATACTGAAGAGACCTGCAACTTT
TTACTCTATGAGGGGTTCGAATGTGTACAAACACAGACCATAATTATAAGTAATCTGAAAGACAACGCA
TAAGCAATTTCCATATGAAAATCACTGAATGATGGTGCCAGGTATGAAATCTTGTTTACATTGGCTCGTT
CCAGGAAAAGTGAAAATCTTCGTTCTAATGAGTATCAGGGTGTCACTCTATATGAAACACGAAGTTGC
GGAGCTCTGATGCCCGAGCCACGACTCTACAGGGTGTTGCAAAATTGGTATACTAAGCCGAAACCTAC
ATGTGCAGCATGGTATATCTAAGCCCGAAACTGAAATCAGAATTTGGAAATTCG
>TCONS_00107400 geneID=XLOC_021424 scaffold_348:198461-198935(+) Intergenic
ATTTATTTGGCTATTAGGTAGGAATCGGAACTGTGGAATCGTTTAAATGTTTTACTTAAATTAAAGATAAT
ATATTTTACCCTTGATCAAAAAGGGGATTGTACTAAGTACCTATATAAATTTAATCATGAATGTGATACAC
CTTCTGATTTGTACCGCATTACTAACTATTTTTAGTGCAGCTGCAATACCGACTGGAAATAAAAACAAA
CAAACCAAACTTCTGTTTATCAGGTACCCCGAATACAAACCTGGTTACAAATATTACTATCCAACGACG
TACAATATCTACTTCAATACAGA
>TCONS_00107518 geneID=XLOC_021447 scaffold_349:145175-148510(+) Intergenic
GTGGAGTCAGGCCGCGAGGAGCTGAAGAAGGAGCGGGAGATCATGCAGCTGGTGGCGGGGCTGCAG
CTCGGGCCCGACGAGCTCCAGCGGCGCGAGGGGCCGCCACACATGATGCGCGCCAAATAACACCACG
CCACGGGTACTCTTAGCCTTCCCTTGAAGGAGACTGGAGCCGGGGGGCTACTCTTGCTACGCTGAACA
TGAAGTTTCGGGTCATGCAATCCGTACCTACGCTTAATACAACGAGATTTGCGCAAGCCAGAGTGAAC
CTACAGGTGCTCTCAAGAGTTACGAAATATTGCAGGAGATTGTCATACTGCCGAATACAGTAAATCACA
CTGTAAATTACAAACTACATTTTATGAGAATCGAAAATAACCTACGGTCTTTTACTAATAACAGAGAAC
TTCAAAAGACATTGAATTATTCGAAAGAAAGACAAAAAATAGAATTTCGTGTTTTACCCCCTTTACAAA
TCTTGTATTCACATTGCACACCTTTGAAAGTTATGAACAGTGTAATGATAAGTAAACAGGATGGTACGG
TCAGATGCAAAAAAACCTGACCCCCCTATCATACTAACAATGCTTCTGAGGGGGGTCAGGTTTATCTGC
CCCTTACTGTGTACCTATTGTGTGACTTTATTGTTCGGTATTATGACTTTTATTAAGATTAGTTATATCTAC
GTTTGTACAGAACAATATCATAGGTTATAAGTAATTATGAATTAATTACGCCATGTGTGTCGCATGATGTA
GGAATTGATGTAATCTCCATTTATATTGTTGTATATTCACATAATAACAAATTACCTACTTAGGAATTGTAT
ATTTTTTACATGTAAGTACCTACCTATTTACTTGTATCAAAAACTTAAGGTTTTAAAAAAGAAATTGTTA
GTATCAAACCTCTATCAATATTTCAAAACCATAGGCTATGTTGTTTTATGTTTTACTTAGGTAGTTACATT
TTATGCTATAGATCAAAGTAAAACTTATTAAGTATCAACTTAAGTTCATTTTCCACTGCATACAAATAATA
ATGAACAAAGCGCAATTTAATAGTTTAAATGAAAAATTATCATTACTCGAATGTTATCGTGAGATTGTGC
GTCGTAATGGTTCAGAAATACGTCATTCAGTACCTAAGGAAGTAAATAGTTTCGATACACATTAATCAA
ATTATCCGTGTACCGAGTGTGAGTTTTGTACACCAAATCACAGAAATCCAATAAAAAAATACTTCAAAA
CTACCTATAAAAGTAATCTGGGGTACAATTTTATTCAGATGTTGTGGCGTCTGAATGTTTGTATTAGACA
GTAACTGCCACCATTATTATTTTTTTCATTCATCAAGAAACATTTTTCGAACTATAAAAAAAAAGTTAAA
TTAAGTCCTATATGATGTAAATATATTATTTTTATCATAGGATAGGTACTATTTAAAATAATGGGCAAGAAT
GTAGTCAGTAGTTAAATTGTATTTTAAATGTGCCTTAATAAGTAACAAATAGGAAAAGTTAATAGGCTTA
ACTTTTTTGTCAACAAGTTGTACTATACCCATTTGAAAAGATTTTTAAAATGTATACTATTAATTGGTATT
TACTACAAGATTTTGATGAAAAAATAAAGAGAAAATTTATTAGTAACTACCTAAACAGCCACAACTTTA
TACGTTGTCAAAAAAATATCGTATTAAGTAGATAGTACAACTTGTTTTCAAAAGAAAAAGTCTCTAGGT
ATTAGGTAAATAAATACAATTGTACAAAAATACTAGAATGGTTAGTCAAAGAAGGAAATATTACATATAG
GTATTATACATACGAGTGGAGTTTTTCTATAAAATATGGAATAGTTTGTATAAGGTTTATATGGAGATAAG

TTCGTTGATAAAAATGCATTTGTTATACTGTACCACACAACGAAACTTAGATAGGTATATATTTTTTATAA
TGTGAAGTATAAATAAAAACTTATAGTAAAAGTTTCTATGACAATAAATCTAACTATTTTTGGATTTTTAT
GGTCACCGAGTGCCATCTTTCCTTTTATAAAAAAATATTGATGAACAGTTTTTCAACTATGATGGAAACT
ATAAATTGTGTACGTATTACTTATAATTTTATTATTAATTTAATTAGGTATGGCTAGACAACGATGTATCCA
ATTCTTATTGTCATATTTTTTTAAATAAATATAGGCCG
>TCONS_00107486 geneID=XLOC_021447 scaffold_349:145179-148813(+) Unclassified
AGTCAGGCCGCGAGGAGCTGAAGAAGGAGCGGGAGATCATGCAGCTGGTGGCGGGGCTGCAGCTCG
GGCCCGACGAGCTCCAGCGGCGCGAGGGGCCGCCACACATGATGCGCGCCAAGTACGCTCCCAGATA
ACACCACGCCACGGGTACTCTTAGCCTTCCCTTGAAGGAGACTGGAGCCGGGGGGCTACTCTTGCTAC
GCTGAACATGAAGTTTCGGGTCATGCAATCCGTACCTACGCTTAATACAACGAGATTTGCGCAAGCCA
GAGTGAACCTACAGGTGCTCTCAAGAGTTACGAAATATTGCAGGAGATTGTCATACTGCCGAATACAG
TAAATCACACTGTAAATTACAAACTACATTTTATGAGAATCGAAAATAACCTACGGTCTTTTACTAATAA
CAGAGAACTTCAAAAGACATTGAATTATTCGAAAGAAAGACAAAAAATAGAATTTCGTGTTTTACCCC
CTTTACAAATCTTGTATTCACATTGCACACCTTTGAAAGTTATGAACAGTGTAATGATAAGTAAACAGG
ATGGTACGGTCAGATGCAAAAAAACCTGACCCCCCTATCATACTAACAATGCTTCTGAGGGGGGTCAG
GTTTATCTGCCCCTTACTGTGTACCTATTGTGTGACTTTATTGTTCGGTATTATGACTTTTATTAAGATTAG
TTATATCTACGTTTGTACAGAACAATATCATAGGTTATAAGTAATTATGAATTAATTACGCCATGTGTGTC
GCATGATGTAGGAATTGATGTAATCTCCATTTATATTGTTGTATATTCACATAATAACAAATTACCTACTTA
GGAATTGTATATTTTTTACATGTAAGTACCTACCTATTTACTTGTATCAAAAACTTAAGGTTTTAAAAAA
GAAATTGTTAGTATCAAACCTCTATCAATATTTCAAAACCATAGGCTATGTTGTTTTATGTTTTACTTAGG
TAGTTACATTTTATGCTATAGATCAAAGTAAAACTTATTAAGTATCAACTTAAGTTCATTTTCCACTGCAT
ACAAATAATAATGAACAAAGCGCAATTTAATAGTTTAAATGAAAAATTATCATTACTCGAATGTTATCGT
GAGATTGTGCGTCGTAATGGTTCAGAAATACGTCATTCAGTACCTAAGGAAGTAAATAGTTTCGATACA
CATTAATCAAATTATCCGTGTACCGAGTGTGAGTTTTGTACACCAAATCACAGAAATCCAATAAAAAAA
TACTTCAAAACTACCTATAAAAGTAATCTGGGGTACAATTTTATTCAGATGTTGTGGCGTCTGAATGTTT
GTATTAGACAGTAACTGCCACCATTATTATTTTTTTCATTCATCAAGAAACATTTTTCGAACTATAAAAA
AAAAGTTAAATTAAGTCCTATATGATGTAAATATATTATTTTTATCATAGGATAGGTACTATTTAAAATAAT
GGGCAAGAATGTAGTCAGTAGTTAAATTGTATTTTAAATGTGCCTTAATAAGTAACAAATAGGAAAAGT
TAATAGGCTTAACTTTTTTGTCAACAAGTTGTACTATACCCATTTGAAAAGATTTTTAAAATGTATACTAT
TAATTGGTATTTACTACAAGATTTTGATGAAAAAATAAAGAGAAAATTTATTAGTAACTACCTAAACAGC
CACAACTTTATACGTTGTCAAAAAAATATCGTATTAAGTAGATAGTACAACTTGTTTTCAAAAGAAAAA
GTCTCTAGGTATTAGGTAAATAAATACAATTGTACAAAAATACTAGAATGGTTAGTCAAAGAAGGAAAT
ATTACATATAGGTATTATACATACGAGTGGAGTTTTTCTATAAAATATGGAATAGTTTGTATAAGGTTTATA
TGGAGATAAGTTCGTTGATAAAAATGCATTTGTTATACTGTACCACACAACGAAACTTAGATAGGTATAT
ATTTTTTATAATGTGAAGTATAAATAAAAACTTATAGTAAAAGTTTCTATGACAATAAATCTAACTATTTT
TGGATTTTTATGGTCACCGAGTGCCATCTTTCCTTTTATAAAAAAATATTGATGAACAGTTTTTCAACTA
TGATGGAAACTATAAATTGTGTACGTATTACTTATAATTTTATTATTAATTTAATTAGGTATGGCTAGACAA
CGATGTATCCAATTCTTATTGTCATATTTTTTTAAATAAATATAGGCCGTAATATAGCGTTAACTTGTTTTA
TTTACGATTCTTTTAGCACGGTCACAGTAAATTTTCATAGATCTGAAACTATAGAGTTGGACTAGCCTCT
TTATTCGTAGCTTTTTGGCGGTAGGAACCGGTGATAGTTGGAATCTAATTAGTGACTTATTAACAACAA
ACAATATAAATCTTACTGAAAAAAATATTAAATAAATGTATTATTATTATTATGTTGTGTCTTTACCATCAC
GGGACCATGGGGTCCCGGGCATTTGGAAGGCGTGCATGGCGCCGAAGCCAACACGTAGAGGCCCGTT
TG
>TCONS_00107487 geneID=XLOC_021447 scaffold_349:145179-148813(+) Unclassified

AGTCAGGCCGCGAGGAGCTGAAGAAGGAGCGGGAGATCATGCAGCTGGTGGCGGGGCTGCAGCTCG
GGCCCGACGAGCTCCAGCGGCGCGAGGGGCCGCCACACATGATGCGCGCCAAGTACGCTCCCAGATA
ACACCACGCCACGGGTACTCTTAGCCTTCCCTTGAAGGAGACTGGAGCCGGGGGGCTACTCTTGCTAC
GCTGAACATGAAGTTTCGGGTCATGCAATCCGTACCTACGCTTAATACAACGAGATTTGCGCAAGCCA
GAGTGAACCTACAGGTGCTCTCAAGAGTTACGAAATATTGCAGGAGATTGTCATACTGCCGAATACAG
TAAATCACACTGTAAATTACAAACTACATTTTATGAGAATCGAAAATAACCTACGGTCTTTTACTAATAA
CAGAGAACTTCAAAAGACATTGAATTATTCGAAAGAAAGACAAAAAATAGAATTTCGTGTTTTACCCC
CTTTACAAATCTTGTATTCACATTGCACACCTTTGAAAGTTATGAACAGTGTAATGATAAGTAAACAGG
ATGGTACGGTCAGATGCAAAAAAACCTGACCCCCCTATCATACTAACAATGCTTCTGAGGGGGGTCAG
GTTTATCTGCCCCTTACTGTGTACCTATTGTGTGACTTTATTGTTCGGTATTATGACTTTTATTAAGATTAG
TTATATCTACGTTTGTACAGAACAATATCATAGGTTATAAGTAATTATGAATTAATTACGCCATGTGTGTC
GCATGATGTAGGAATTGATGTAATCTCCATTTATATTGTTGTATATTCACATAATAACAAATTACCTACTTA
GGAATTGTATATTTTTTACATGTAAGTACCTACCTATTTACTTGTATCAAAAACTTAAGGTTTTAAAAAA
GAAATTGTTAGTATCAAACCTCTATCAATATTTCAAAACCATAGGCTATGTTGTTTTATGTTTTACTTAGG
TAGTTACATTTTATGCTATAGATCAAAGTAAAACTTATTAAGTATCAACTTAAGTTCATTTTCCACTGCAT
ACAAATAATAATGAACAAAGCGCAATTTAATAGTTTAAATGAAAAATTATCATTACTCGAATGTTATCGT
GAGATTGTGCGTCGTAATGGTTCAGAAATACGTCATTCAGTACCTAAGGAAGTAAATAGTTTCGATACA
CATTAATCAAATTATCCGTGTACCGAGTGTGAGTTTTGTACACCAAATCACAGAAATCCAATAAAAAAA
TACTTCAAAACTACCTATAAAAGTAATCTGGGGTACAATTTTATTCAGATGTTGTGGCGTCTGAATGTTT
GTATTAGACAGTAACTGCCACCATTATTATTTTTTTCATTCATCAAGAAACATTTTTCGAACTATAAAAA
AAAAGTTAAATTAAGTCCTATATGATGTAAATATATTATTTTTATCATAGGATAGGTACTATTTAAAATAAT
GGGCAAGAATGTAGTCAGTAGTTAAATTGTATTTTAAATGTGCCTTAATAAGTAACAAATAGGAAAAGT
TAATAGGCTTAACTTTTTTGTCAACAAGTTGTACTATACCCATTTGAAAAGATTTTTAAAATGTATACTAT
TAATTGGTATTTACTACAAGATTTTGATGAAAAAATAAAGAGAAAATTTATTAGTAACTACCTAAACAGC
CACAACTTTATACGTTGTCAAAAAAATATCGTATTAAGTAGATAGTACAACTTGTTTTCAAAAGAAAAA
GTCTCTAGGTATTAGGTAAATAAATACAATTGTACAAAAATACTAGAATGGTTAGTCAAAGAAGGAAAT
ATTACATATAGGTATTATACATACGAGTGGAGTTTTTCTATAAAATATGGAATAGTTTGTATAAGGTTTATA
TGGAGATAAGTTCGTTGATAAAAATGCATTTGTTATACTGTACCACACAACGAAACTTAGATAGGTATAT
ATTTTTTATAATGTGAAGTATAAATAAAAACTTATAGTAAAAGTTTCTATGACAATAAATCTAACTATTTT
TGGATTTTTATGGTCACCGAGTGCCATCTTTCCTTTTATAAAAAAATATTGATGAACAGTTTTTCAACTA
TGATGGAAACTATAAATTGTGTACGTATTACTTATAATTTTATTATTAATTTAATTAGGTATGGCTAGACAA
CGATGTATCCAATTCTTATTGTCATATTTTTTTAAATAAATATAGGCCGTAATATAGCGTTAACTTGTTTTA
TTTACGATTCTTTTAGCACGGTCACAGTAAATTTTCATAGATCTGAAACTATAGAGTTGGACTAGCCTCT
TTATTCGTAGCTTTTTGGCGGTAGGAACCGGTGATAGTTGGAATCTAATTAGTGACTTATTAACAACAA
ACAATATAAATCTTACTGAAAAAAATATTAAATAAATGTATTATTATTATTATGTTGTGTCTTTACCATCAC
GGGACCATGGGGTCCCGGGCATTTGGAAGGCGTGCATGGCGCCGAAGCCAACACGTAGAGGCCCGTT
TG
>TCONS_00107513 geneID=XLOC_021447 scaffold_349:145179-148813(+) Unclassified
AGTCAGGCCGCGAGGAGCTGAAGAAGGAGCGGGAGATCATGCAGCTGGTGGCGGGGCTGCAGCTCG
GGCCCGACGAGCTCCAGCGGCGCGAGGGGCCGCCACACATGATGCGCGCCAAATAACACCACGCCAC
GGGTACTCTTAGCCTTCCCTTGAAGGAGACTGGAGCCGGGGGGCTACTCTTGCTACGCTGAACATGAA
GTTTCGGGTCATGCAATCCGTACCTACGCTTAATACAACGAGATTTGCGCAAGCCAGAGTGAACCTAC
AGGTGCTCTCAAGAGTTACGAAATATTGCAGGAGATTGTCATACTGCCGAATACAGTAAATCACACTGT
AAATTACAAACTACATTTTATGAGAATCGAAAATAACCTACGGTCTTTTACTAATAACAGAGAACTTCA

AAAGACATTGAATTATTCGAAAGAAAGACAAAAAATAGAATTTCGTGTTTTACCCCCTTTACAAATCTT
GTATTCACATTGCACACCTTTGAAAGTTATGAACAGTGTAATGATAAGTAAACAGGATGGTACGGTCAG
ATGCAAAAAAACCTGACCCCCCTATCATACTAACAATGCTTCTGAGGGGGGTCAGGTTTATCTGCCCCT
TACTGTGTACCTATTGTGTGACTTTATTGTTCGGTATTATGACTTTTATTAAGATTAGTTATATCTACGTTT
GTACAGAACAATATCATAGGTTATAAGTAATTATGAATTAATTACGCCATGTGTGTCGCATGATGTAGGA
ATTGATGTAATCTCCATTTATATTGTTGTATATTCACATAATAACAAATTACCTACTTAGGAATTGTATATT
TTTTACATGTAAGTACCTACCTATTTACTTGTATCAAAAACTTAAGGTTTTAAAAAAGAAATTGTTAGTA
TCAAACCTCTATCAATATTTCAAAACCATAGGCTATGTTGTTTTATGTTTTACTTAGGTAGTTACATTTTAT
GCTATAGATCAAAGTAAAACTTATTAAGTATCAACTTAAGTTCATTTTCCACTGCATACAAATAATAATG
AACAAAGCGCAATTTAATAGTTTAAATGAAAAATTATCATTACTCGAATGTTATCGTGAGATTGTGCGTC
GTAATGGTTCAGAAATACGTCATTCAGTACCTAAGGAAGTAAATAGTTTCGATACACATTAATCAAATTA
TCCGTGTACCGAGTGTGAGTTTTGTACACCAAATCACAGAAATCCAATAAAAAAATACTTCAAAACTA
CCTATAAAAGTAATCTGGGGTACAATTTTATTCAGATGTTGTGGCGTCTGAATGTTTGTATTAGACAGTA
ACTGCCACCATTATTATTTTTTTCATTCATCAAGAAACATTTTTCGAACTATAAAAAAAAAGTTAAATTA
AGTCCTATATGATGTAAATATATTATTTTTATCATAGGATAGGTACTATTTAAAATAATGGGCAAGAATGTA
GTCAGTAGTTAAATTGTATTTTAAATGTGCCTTAATAAGTAACAAATAGGAAAAGTTAATAGGCTTAACT
TTTTTGTCAACAAGTTGTACTATACCCATTTGAAAAGATTTTTAAAATGTATACTATTAATTGGTATTTAC
TACAAGATTTTGATGAAAAAATAAAGAGAAAATTTATTAGTAACTACCTAAACAGCCACAACTTTATAC
GTTGTCAAAAAAATATCGTATTAAGTAGATAGTACAACTTGTTTTCAAAAGAAAAAGTCTCTAGGTATT
AGGTAAATAAATACAATTGTACAAAAATACTAGAATGGTTAGTCAAAGAAGGAAATATTACATATAGGT
ATTATACATACGAGTGGAGTTTTTCTATAAAATATGGAATAGTTTGTATAAGGTTTATATGGAGATAAGTT
CGTTGATAAAAATGCATTTGTTATACTGTACCACACAACGAAACTTAGATAGGTATATATTTTTTATAATG
TGAAGTATAAATAAAAACTTATAGTAAAAGTTTCTATGACAATAAATCTAACTATTTTTGGATTTTTATGG
TCACCGAGTGCCATCTTTCCTTTTATAAAAAAATATTGATGAACAGTTTTTCAACTATGATGGAAACTAT
AAATTGTGTACGTATTACTTATAATTTTATTATTAATTTAATTAGGTATGGCTAGACAACGATGTATCCAAT
TCTTATTGTCATATTTTTTTAAATAAATATAGGCCGTAATATAGCGTTAACTTGTTTTATTTACGATTCTTTT
AGCACGGTCACAGTAAATTTTCATAGATCTGAAACTATAGAGTTGGACTAGCCTCTTTATTCGTAGCTTT
TTGGCGGTAGGAACCGGTGATAGTTGGAATCTAATTAGTGACTTATTAACAACAAACAATATAAATCTT
ACTGAAAAAAATATTAAATAAATGTATTATTATTATTATGTTGTGTCTTTACCATCACGGGACCATGGGGT
CCCGGGCATTTGGAAGGCGTGCATGGCGCCGAAGCCAACACGTAGAGGCCCGTTTG
>TCONS_00107488 geneID=XLOC_021447 scaffold_349:145400-147503(+) Unclassified
GGGGCCGCCACACATGATGCGCGCCAAGTACGCTCCCAGATAACACCACGCCACGGGTACTCTTAGCC
TTCCCTTGAAGGAGACTGGAGCCGGGGGGCTACTCTTGCTACGCTGAACATGAAGTTTCGGGTCATGC
AATCCGTACCTACGCTTAATACAACGAGATTTGCGCAAGCCAGAGTGAACCTACAGGTGCTCTCAAGA
GTTACGAAATATTGCAGGAGATTGTCATACTGCCGAATACAGTAAATCACACTGTAAATTACAAACTAC
ATTTTATGAGAATCGAAAATAACCTACGGTCTTTTACTAATAACAGAGAACTTCAAAAGACATTGAATT
ATTCGAAAGAAAGACAAAAAATAGAATTTCGTGTTTTACCCCCTTTACAAATCTTGTATTCACATTGCA
CACCTTTGAAAGTTATGAACAGTGTAATGATAAGTAAACAGGATGGTACGGTCAGATGCAAAAAAACC
TGACCCCCCTATCATACTAACAATGCTTCTGAGGGGGGTCAGGTTTATCTGCCCCTTACTGTGTACCTAT
TGTGTGACTTTATTGTTCGGTATTATGACTTTTATTAAGATTAGTTATATCTACGTTTGTACAGAACAATAT
CATAGGTTATAAGTAATTATGAATTAATTACGCCATGTGTGTCGCATGATGTAGGAATTGATGTAATCTCC
ATTTATATTGTTGTATATTCACATAATAACAAATTACCTACTTAGGAATTGTATATTTTTTACATGTAAGTA
CCTACCTATTTACTTGTATCAAAAACTTAAGGTTTTAAAAAAGAAATTGTTAGTATCAAACCTCTATCAA
TATTTCAAAACCATAGGCTATGTTGTTTTATGTTTTACTTAGGTAGTTACATTTTATGCTATAGATCAAAG

TAAAACTTATTAAGTATCAACTTAAGTTCATTTTCCACTGCATACAAATAATAATGAACAAAGCGCAATT
TAATAGTTTAAATGAAAAATTATCATTACTCGAATGTTATCGTGAGATTGTGCGTCGTAATGGTTCAGAA
ATACGTCATTCAGTACCTAAGGAAGTAAATAGTTTCGATACACATTAATCAAATTATCCGTGTACCGA
>TCONS_00107519 geneID=XLOC_021447 scaffold_349:145408-148510(+) Intergenic
CACACATGATGCGCGCCAAATAACACCACGCCACGGGTACTCTTAGCCTTCCCTTGAAGGAGACTGGA
GCCGGGGGGCTACTCTTGCTACGCTGAACATGAAGTTTCGGGTCATGCAATCCGTACCTACGCTTAATA
CAACGAGATTTGCGCAAGCCAGAGTGAACCTACAGGTGCTCTCAAGAGTTACGAAATATTGCAGGAG
ATTGTCATACTGCCGAATACAGTAAATCACACTGTAAATTACAAACTACATTTTATGAGAATCGAAAATA
ACCTACGGTCTTTTACTAATAACAGAGAACTTCAAAAGACATTGAATTATTCGAAAGAAAGACAAAAA
ATAGAATTTCGTGTTTTACCCCCTTTACAAATCTTGTATTCACATTGCACACCTTTGAAAGTTATGAACA
GTGTAATGATAAGTAAACAGGATGGTACGGTCAGATGCAAAAAAACCTGACCCCCCTATCATACTAAC
AATGCTTCTGAGGGGGGTCAGGTTTATCTGCCCCTTACTGTGTACCTATTGTGTGACTTTATTGTTCGGT
ATTATGACTTTTATTAAGATTAGTTATATCTACGTTTGTACAGAACAATATCATAGGTTATAAGTAATTATG
AATTAATTACGCCATGTGTGTCGCATGATGTAGGAATTGATGTAATCTCCATTTATATTGTTGTATATTCAC
ATAATAACAAATTACCTACTTAGGAATTGTATATTTTTTACATGTAAGTACCTACCTATTTACTTGTATCAA
AAACTTAAGGTTTTAAAAAAGAAATTGTTAGTATCAAACCTCTATCAATATTTCAAAACCATAGGCTATG
TTGTTTTATGTTTTACTTAGGTAGTTACATTTTATGCTATAGATCAAAGTAAAACTTATTAAGTATCAACTT
AAGTTCATTTTCCACTGCATACAAATAATAATGAACAAAGCGCAATTTAATAGTTTAAATGAAAAATTAT
CATTACTCGAATGTTATCGTGAGATTGTGCGTCGTAATGGTTCAGAAATACGTCATTCAGTACCTAAGG
AAGTAAATAGTTTCGATACACATTAATCAAATTATCCGTGTACCGAGTGTGAGTTTTGTACACCAAATCA
CAGAAATCCAATAAAAAAATACTTCAAAACTACCTATAAAAGTAATCTGGGGTACAATTTTATTCAGAT
GTTGTGGCGTCTGAATGTTTGTATTAGACAGTAACTGCCACCATTATTATTTTTTTCATTCATCAAGAAA
CATTTTTCGAACTATAAAAAAAAAGTTAAATTAAGTCCTATATGATGTAAATATATTATTTTTATCATAGG
ATAGGTACTATTTAAAATAATGGGCAAGAATGTAGTCAGTAGTTAAATTGTATTTTAAATGTGCCTTAAT
AAGTAACAAATAGGAAAAGTTAATAGGCTTAACTTTTTTGTCAACAAGTTGTACTATACCCATTTGAAA
AGATTTTTAAAATGTATACTATTAATTGGTATTTACTACAAGATTTTGATGAAAAAATAAAGAGAAAATT
TATTAGTAACTACCTAAACAGCCACAACTTTATACGTTGTCAAAAAAATATCGTATTAAGTAGATAGTAC
AACTTGTTTTCAAAAGAAAAAGTCTCTAGGTATTAGGTAAATAAATACAATTGTACAAAAATACTAGAA
TGGTTAGTCAAAGAAGGAAATATTACATATAGGTATTATACATACGAGTGGAGTTTTTCTATAAAATATG
GAATAGTTTGTATAAGGTTTATATGGAGATAAGTTCGTTGATAAAAATGCATTTGTTATACTGTACCACA
CAACGAAACTTAGATAGGTATATATTTTTTATAATGTGAAGTATAAATAAAAACTTATAGTAAAAGTTTCT
ATGACAATAAATCTAACTATTTTTGGATTTTTATGGTCACCGAGTGCCATCTTTCCTTTTATAAAAAAATA
TTGATGAACAGTTTTTCAACTATGATGGAAACTATAAATTGTGTACGTATTACTTATAATTTTATTATTAAT
TTAATTAGGTATGGCTAGACAACGATGTATCCAATTCTTATTGTCATATTTTTTTAAATAAATATAGGCCG
>TCONS_00107514 geneID=XLOC_021452 scaffold_349:312183-342354(-) Unclassified
CCGCGGCCACACGTGTGTCGCGCTCGCCAATAAACCGACGAGATTCGTGATCAAATTATACATTCGATT
CATCGGGAAAAGGGACGCATTTGCATATAAATGTAATCGTGTAAACTGGTACCAGATTAACCGGTTGTC
AAATTAACAGTGTGGTTTGTGAAAGTAATTTATTATTGAAATTTGCCTTTGAGTGAGTGCGGCGTTTCTT
GTTGATTGTGAACGGGAATTGGGATTTTGATTGTTCATCAAGGAAGCCAATCTACTCATGGATGCATGC
TGGAAATGTCTGAATACATAACAATGATAATCCGTTAAAAAGTGAGAAACATAATATTTCAAACAATGA
AAGAGTCAAAGTTTACAATTTGTTCTTTTTATAGTGTCAAGTGAAATCATCGCATAAAAATGCAGAAAT
TCAAAAGTGAAAACAGTAAATAATAATGTGCAATATGACGTCACTGGGGTTGCTCCACTTTTTCATCGT
TTGCATCACTCTGCATGTATTCGGT
>TCONS_00107528 geneID=XLOC_021481 scaffold_35:8723-9783(+) Unclassified

CCTCTCTCTACATCTTGCAACATGAAGTTCGCTACAGCAGTCGTGATCGCTATCTACGCCATGGTGGCAT
ATGCCTCGGCCGCCGTGCCCAGGGAGGGCGGCGGCGGCGGCGGCGGCGGCGGTGGCGGCAGCTCCG
ACAGCTAAGTGTCAAGCTGACATTTCAAGTACTGAGACCTAAAGACTATCGTGCTGCCATCTGCCATCT
TCTCGTCAACAAAGTTAAATAAATTATTAATGATGTACTAGTACACAAATAAATAATTTTATTAATTTGAC
TAAA
>TCONS_00107635 geneID=XLOC_021517 scaffold_35:166640-174068(-) Intergenic
CCGCAGCACGCGACGCGCGCACGACGCGTGCTCGACATATTTGCGCCGCGAAATTCGGTTTCCGAAC
GCAAACTTGGCTGCAAAGGGACCGACCCTTTGCCGGCCCAAACTTTTTTCGCAAACGAAAAATTTTCC
CGCTAAAAAGTTTTAATCGAAAAAGTTTAGTGAAAAATACGAGTAACAAATTAAATGACCACTCAAAA
TATACTGTAACGAATTTTATTTACTTTCTTTTAGATTCG
>TCONS_00108131 geneID=XLOC_021517 scaffold_35:200174-214083(-) Exonic overlap (+)
CATCTCATCTCACCTCACTAGTCTAGTGACAACTGTCAGTCAGCCACGCAGCCTGCGCAGGCGCCGCT
TCAGCGCAAACCAACCAATAACAACCTTAATACTAAACAGCAAAGTTGACTTGTGATTGGTGCAACCT
AAGGTTATGCTTTGAACAAACTGAATTTCAAGTGGTTCTAAGTCAAAAGTGATATAAAGTGTGGAAAG
TGTATTTTAATTGTTTCAAAGTTAATGAGTGTAGAAGTTTTGATGTTATGTTGGTTGAATAAAAGTAGGT
AAGAGCGAAGAAAAACAGTAAATAAATCATAAAAATGTGACAATATGAAGATTATGAAGTGATATCAA
AATAAAATTGTGATGTGAACCCTTTGGAACTTCCTCGAATCTTGTGACATTTAAATGTGAGAAAGTTAC
AACCGGCACCGCTGACCTATTCTATTATTGGGAGTCTCCAAGATGCCAGACACCTTGTCTCTCACGACC
GGAGTCAGCCTGGGCCTGCTGGCTGGCATCGTTTCTGGAGTGTGCTACATCTTCGCTAATGGACTTAAT
AAGCCGAG
>TCONS_00107638 geneID=XLOC_021518 scaffold_35:274834-276494(-) Unclassified
GTACTTACTTTCTAAAGTAAACTTATGTTAGCTAAATAATAAACAATTTATAATACTGGGAACGTATTGCA
TAAATAATATTGTAAAACAATTCACAAGCCTTTACTAAATAACAAAAGATGTATATTCTCAAAATTCATT
TGGGGCGTAAAAGCGACATTGTATAGCTTGTAAATATAAAATTTGGAAACTGTTTTATTAAAATTTGCTT
TAAATATCAATTATTAAATTACGCATGCATAAAAATACTTGAAGTAAATTGGATATAAAGTATTTTTTTCT
CATCGATACATAAAACATTTCACTTTATCTTAATTAAATTATAATCACAGTGATCTATGTATAATCTCAATT
TATTTATTCAAATAATACGCAATGCCAGATTTTAAAATACTTATATTATATTGATTTATGTAAAATACGTTG
GTTCAAATGCATAACGGTAGCTTTTAGCAGTGGGCAGCGGATAATAATGAACGGTTAGCTTTTGCCTCT
AATCCACTTGTTGCTGAATATATAGCATCGTACATTGCGTTCATAATGTGATAGACATCTGCAAGTCAGC
AAGCCATTTCTGAAAAATCTGGAAAAGCTGAATCTAG
>TCONS_00108113 geneID=XLOC_021492 scaffold_35:293113-297061(+) Intergenic
CGGCGGACGACGCACGCACCTCGCGCGCGACCGAAATACATCACGATCGGCGATCGGACCCGTTTCC
GTTCGGGGAGTGGTTGAGTCGGTGCGGGGTGCCGGCAGTGCTGCGGGGTCCCAGCCGGGGTGCCGA
AGGTGCCGAGCGGGGCGGGCGGTGGAGCGAGCGGCGCGCGCGCGCTCGCACTCTGCGGCTAGCCAT
GGAGATGGAGG
>TCONS_00108012 geneID=XLOC_021493 scaffold_35:365589-366030(+) Splice junction overlap
GCCAACAGCTTGAAGCACAATTTTCTCGCATTATGTTGCATGTTTTAACCTGTGTTTTAACAACCCCTG
TGTGATTTGTTTGTTGTCTAGGTAAAGTGTTCCAAACTGTAACCACATTCAAGACAGGCGACAATGGC
TCGATCATTGCGTCCAAAGTAAGCAGCACGAGGTCTCACGGCGCTATCATTACAGGTCCGGCTGAAAC
GCATACTATTCACAAAATGAC
>TCONS_00108035 geneID=XLOC_021521 scaffold_35:443857-447180(-) Exonic overlap (+)
CGCGGCAGGTGAGGCGCCGAACGTCTCGCCATCCACGGTTCCAATTTTAAATTTAAAACTCGAACTCG
AATCACGACCGGACGCTTTCTCTCTCTCTTCCACTTTTACTTTCGCACACGTTTCTGGATGTTGCATCAC
TGTCGTGCACGGATTTCGTAATTCATCATTAATTTTAAGTTTTTTTTTTAATAATGTTAATGGTGTAGTGC

AAAGTGATTGCCTGGATATAGGCTGTTCAAATAAATGTGTTTAATAATAAAGTGCGTTAAGGTGATGAA
ATGAGTTTGTGACCTCAAGTGTGTGGCTGACAACCGCTGTTTAATTATAAGCATGTCTCCACCTGAAGA
CCTGCAGGGCGCGGGGGTCGTGAACCGCGGGTTCGAGGGCGCCGACGACGGCTTCCAGACCATCGA
CCTGCGCACGCACGGGAAGG
>TCONS_00108135 geneID=XLOC_021521 scaffold_35:445734-447178(-) Intergenic
CGGCAGGTGAGGCGCCGAACGTCTCGCCATCCACGGTTCCAATTTTAAATTTAAAACTCGAACTCGAA
TCACGACCGGACGCTTTCTCTCTCTCTTCCACTTTTACTTTCGCACACGTTTCTGGATGTTGCATCACTG
TCGTGCACGGATTTCGTAATTCATCATTAATTTTAAGTTTTTTTTTAATAATGTTAATGGTGTAGTGCAA
AGTGATTGCCTGGATATAGGCTGTTCAAATAAATGTGTTTAATAATAAAGTGCGTTAAGGTGATGAAAT
GAGTTTGTGACCTCAAGTGTGTGGCTGACAACCGCTGTTTAATTATGTTGCTTGGATGAATTTGCGCAT
TAGGCAGAGGCACCGGCAATTTTCCACCATGACACGGCGCCCGCCCCATCAGCGCACTGTTATGGAAA
ATTGACTTCTCTGAAAGCTACATGATTCCCGGCATCAACAAGACTG
>TCONS_00107583 geneID=XLOC_021497 scaffold_35:474667-475949(+) Unclassified
CCTTACCTACTCGTAGATAGACTATTCCTCTTGGACCTTAGTCAACGTCATTAGTACCTTAAAACCTTAT
GTAATTAGTATTTAGTACTATCAAAACAATTATTCTTTGATCACGGGAATTGCATAGACTTTATCTATGTT
TTCTGCTTCCATATAATTATCTATCACTATAAATAAATATTTAACTAAAAGTAAACATATTAAAATTCAAA
ACACCATTGTCGTCCTGTTTTCAACGATGATATTAACACTTTATTATTTTATATGTTACCCAATTTGTAAAT
TGACACAAACCAAAAGTCAAAAGTCCCCTCGCCTCGGAGCTGTGGCTTGAAGATTTGCCTCGCGTCT
GGCGCCGCCACCGACCACTCCGCAATGTACACATGATTCCCAGGCGGAGTCGGAGCCCGAGCTGCCG
CCGGCCGAGCCCGCGCCGCG
>TCONS_00107934 geneID=XLOC_021501 scaffold_35:527180-528546(+) Splice junction overlap
GCTCAGTGTCTTTAAACAGTCGTTTATGAAAATAAATACTTTTCTTCTAGTGAAATAGTAAGCTCAAATA
GTGTCCAAAAATGAAGTTTAACAAAAGACCTCTCGGTCGAATAAAAAGTGAAATATTGGATAAGTTTT
ATTCATCTTTTTCTGGTGGTATGTCTTCTATGTTGAGCACTGATTCCCGCGCGGAGGCGGACGGCG
>TCONS_00107745 geneID=XLOC_021503 scaffold_35:569524-588958(+) Exonic overlap (+)
AAGGGGTTAGTATCTACAATTTATTGTCATTATCTCGGAGTGGATAATCCTAATAACTACGTAATTTTCTT
TCAGTTGGTTCATTCATTAATATCACAATTTCTTATTATTAATTACCTACTTAAGCTACTTGGGTATTGTTC
TGAAAGAGTATCAAAAGACTATTATTACCACAAAGTAATTTGTGATTATTACAGTAGTGAAGTGGAAAA
CTAAAATCTTCAGTTACGAACATTACATTTCAGATGAAAGATGATAGTGACAAAAACAGTATCGGCATA
TTCTAGAATTTGTTAACAAATTTCTAGTTTGTACTTAATTACTTGTGCTTCCTCCATTAATATTATGGTCA
CCATATTTATAATGTATCAATTTGACCACCGTTATAAGCAATGATGGAATTTGATTACGTTTTTATAATATT
CAAAAATGCGTGAGTAACCTGAATGACGCTATTCGAAATCTACATATAAATAAATAAGTACCTACGTAAT
TCATAATTTTAGTTATTTAGTAGTGAAACTTATTTCTTAACATAATGAACTTCTTTAAAGATAAACTCCAA
TTTAAGAATCTACAGACTTTTTCTGATGAAAAGTTATGTTTTTCGCATGTTACAATGGTTCTGGTGCCCT
AAAATGAAGCTAAATATTATATTATTTTTTCACAGCCCTGTATGCAAGTACATTATGAAATTCCTTGTTTA
TTTCACACTATGTTTAGTTAAAAAAAAATAGCTACAGACGGATGAAATCCTCTTACAAAAAGGAACTTAT
ATTTATCTCTACATCTCAAACATAACGACATAATTACTAAGTACTTACGAATTAAGTTATTACTGGACTTT
TATAGCTAAGTGCCAATTTCTCCATTGTCGGTTATCTAACCGACAGGGATTGATTTGTAAATTAAACGTC
ATCTCCATATAAAATTCATGACAGATGTGTCAAAAGTTAACCACTACTTCCTGGTTATGCTCTAACTGAG
AATGGAGAAATTGGCTCTAAGTATTTTAATCTAACTACGGCCCATGCCTACATAGTGTTAGATGTAAACT
AATGAAAATGCCTCAATTATTTCGTGCCTTATTCTAAAATAAATACAAATCTATTTACAAATCACTGTAA
GATAGTTCAGGTTTTATTATGACTGTCCAAAATCGGAAAAGTCACAATATTACTCGTATATTTTAATTATC
AATTAATTTTAAACACAGCGTATCATAACATATTTTCGTTGTTTTATAATTAGGTACCTATTCATCACGATA
ACGCATATAAAACGATTTTATCGCCATCGATACCGAACCCATGCCGCGTATCCAGATTCGTGGTGTAACG

TGTGCAGTTGTAATTTGGCATGACGTCCTAATCCTAACCAGTCACGCATCTGACATGGGACCTTTACCA
CGTTATGTTCCAACGAATCCACAGCGACAGAGGCTAGATTATTTCCAAAAGCATATTTTTTCTGGGAAA
CCACGCCATTTTGTTACCACAGCCAATTTTCTAGAATATTAGAAAACTATGCGGCTTTAATGCCACTGTA
CATCAGATGCGTAAGAAGCAGCGACTAGCTTTTTGACGCTTTTATAATAAGCACAGATAACAAAAGAA
AATAATCTAGCTATCATTACGCTGACGTTTTGATACGTGAATTGAAAGGCACTGCTAGGCGTCTTCTTCT
AATCTCCTGTGCCGCTACGCATCTACCGTCACAGCCACCTCCGCCTTCTTGCTCATGATGCGGTGGTAC
ACGACGGCGTCGTGCACGGTGGGGAACACGTCCAATTGAAGCCGGTCTGCTTCTAGCGAGTTGTAGC
GCGCTATCATCTCTAGCATCGGACCT
>TCONS_00107950 geneID=XLOC_021530 scaffold_35:624426-624936(-) Exonic overlap (-)
CTCACCCAAATACCGCACTTTGGTGTCACGAAAGAGGTCTGTCTCTCCATGAACCATCTTTGTAAATTG
CAATAGCTAAATCGCTACTGCTCTCGCTATGTATCACGACCAAATCAACGAACACACTAAACTAAATAA
ACAGTAAAATCTTATCTCAATCTTGTTTATTGCTCCGCCCAAAGAGGTTATAATTTTAGAACTGAGTTCA
TTATTTTATGAATGACAAATATAATTTTGCACGTTTGTCTAGACTAAAACAAATTACGATTAACGTATGTT
TTTGTTGTTTTCATGATTAATTTTAGCCCGAGATTTGAGTATACGAATTTATTTACGAAATAACATGAACA
TGACAGTGAGCCGAGT
>TCONS_00108140 geneID=XLOC_021532 scaffold_35:701999-704218(-) Intergenic
ATTATATTTACTTTAGATTATAAATACTGTGTCTAAAGTCTTCTTCTAATATAAATGGGACTTATGCGGTCA
CCATCCTCCAAAAAGTGATTTTCTTGATTTCCATAGGATTTATGAAATGAGCTTATAAGTAAATTGAAAT
ATTTAGATATGGTGGTTTATTAGTGTTACGCTGCGTAAGCACAATATTAGGGTTGCCATACTCACCTCAG
CTTTATTAAATAAATAAGAAGGTAATAAAATCAGTGTGGTTTTATGGCCAAAGCAAAAGTAATCCATTG
TTGCAAGGGTTTCTGTTTGATTCTCTCGATACATTCACGTTATTACATAGTTATTGAAAATCAATGCTCAT
TTGAATATGGCAGCTCTAAACATATTTAGATCTATGGTGTTGTTTGTTATGTAAAATGTATTGCACAAAA
CTAGGCAGGTATGTTCAATTTATCACATTGGAAATGATTACATAAGATTCACCGTACCTATGGCTCAATT
CTGATAAAATAATGTGAGTGTGCAATTCATACATCGGACATGCCTCTACACTACATGTCTAAGAGGTTTA
TTGAAATTAAATTAAGGTTGCAGTTTGCACCCACTTATATTAAGGTTGTTCCGACTTCTTCCATACAGTA
CACTTCTATCCCTACATGTGCTATGTTTATTGTTATTACTGCAAAATTGCAGGTATTTTGAATTTAGCTAA
GTCAAACGCGAAAAAATATAGCAATAGGTAATATTGAGCATGCCTTCCTAGATTGCCGATTTATGGCCTA
ACCAATGTTTAAAATATATACGATGTGTAGTGGTTTTGAATCAGTAAAAAAGTAGATAGTACTTTATTTT
TTATTAAAAATGTTATTTGAAAAAATATTGATGTGATTTTGAGAATTCCAGTGCTATAGGGAGTTATGAA
CTCTATACTTTACTAAGAAGAAGGTATAAAATTATTTTGTTTATTTATTTTGACTGGCCTGTGTATCGATAT
TGCTTATTAAACTAATTTAGGACTTTGTGATGCTTCCTTAATGTACCTATATTTAGTGTAATTTTTGAATGC
TTATATTTTTTTATTGTCTAAACTTCTAAAGGGTATTGCAAATTACTCGAATTCTGTGTAATTAAGTGATG
ATGTTTTGCTAAAAAAACCTTTGACTTTTAGGTATATAAATTGCAATACCCTATATGATACAAACATCATA
CCAATTATGTAGGTTTCTATGATGGGGTGGGGCAGAAGATCTGAAATTGACGTCATAAAATCGACGTAT
TATCGTTACAAAATAAGATCAATAGTAAATATTACACATCTAAGGCATGGCATGTTCTTAAAGCCTATAC
AAGCCACGACAAATAATGAAACAATAGCCCTTCTGCACCGCCCAATTACAAAACAAGTTGTAAAACAG
CGGATAACTATCTCAACGTTTAAAAAAATATGTTGTTAGCCTGATTAGATCAGTACGTGATTTTTATTTG
AATAAGTAATTACTTAAGTATATAAAAAAAGTTCTCTCTGACTCTGACTATCATTCAACAAAAAGTACTG
ATAAGACAATGGCCTAACTTACTTTTAAGAAAAAAAGGTGAAAAATATCAGTGAAATTCTCAGTATTGT
TGATGTTG
>TCONS_00108206 geneID=XLOC_021602 scaffold_350:29409-29793(+) Exonic overlap (-)
GAGATCACTTATCGTGGTGGTAGTGTTGATTCTGAGATATTGTTGAAACAACTGCACGTATGGATCGCT
ACTATAGTCTACGCTAGCACAAGATGCACTAGTATTTAGGAAAATTATCAAAATATGGCACGAAACCAA
ACTCCATATTTTGGTCATTTTAATTTAGGTGTACTGTAAATCTTGTTACCATATACTTTTACGATCTTAAC

>TCONS_00108359 geneID=XLOC_021631 scaffold_351:253533-260419(+) Unclassified
CATTTCTTCCCTCATTTGTTTGCAATATGTTAAATTATTCATGTATTTTTTTCAATGTTACTAAAATACTTA
CTGTAATATTTTTTTGCTACCCAAATTTTGCAAGTTTTTCAGGTTTTGCTACGAATTGAATTTATGTACAA
TGACATCATGGAATCATTAAAATTTCTGAGAATACCTACCTGGCTAGCGCGCTGACGCTATCTACTGGC
AGAAGATCCATTCTTCATGAAAATCTTACAATGAAACAAAAAGTGAAATCAGACGGGCCAGAGTCGG
GCAGACACGATGCACCGAGTGGAAAACGAGGGAAGGTGTCTTGTTTTTAAGATGGTGTGCCGCCGGA
ACAGGGTAGCTTGAAGCAATAATCAGAAACTTTTTTACAGGACTTTTCAATATATTTGCAAACAGAC
>TCONS_00108305 geneID=XLOC_021632 scaffold_351:254643-255995(+) Unclassified
CGATAGAGACCTACAATCTACACTCACGGGCAATGAAAAGGTTCCACTGAGAATTACTCCTAAACTGA
AAAAGCTAGCTAAATGATACCTTCTGCAACATTGGAGAACATTTAACAGAGCATCGGGATATTACCAAC
TTAAAACCGTAATATGTTGTTAATTTTTTTTATTAAATGTTTGAAAAAATTAGGTTCGTTTGGTGTCTGCT
TTTTGTTTGTGGCACTTTTTCATTGCCCGTGAGTGTACTAGTGGTGTGCTATTTATTTACTAGACTACTAC
CATGTCTACCATACGTAGTTTACGACGCTGGAGACTAAACGAAGTGACGATGTCACAAATATAGCACTG
AACATAAACCTTCCCATTCGTTGCGGATGTCAGTTTATTTATATTTTACTGCCTGAAGGCTCAAATTGAG
GAAACCATACTGTAGTAGGTATTTGTATTCGGTGATGTTATTGTGTCCTACATATTATAATTACTTACGCTA
TTCTTTATAAAAAACATCCAAAGACTAAAATCAATTTAGGTTTAGAGACCTCCTTCTTACAGAATATTAT
ACTTACGCTATTCTTTATAAAAAACATCCAAAGACCAAAATCAATTTAGGTTTAGAGACCTCCTTCTTAC
AGAATATTACTGAAAAACGTATAATTACACACAT
>TCONS_00108462 geneID=XLOC_021664 scaffold_352:20348-22339(-) Unclassified
AAATACAAAATATATTTATCAATTATGTATTTAAATCAAAAATTATTTATTGTAAATTCGTAAATATTTATAC
AAAATTGGCATAATAAATAATCTAAACCACCGCGCCTGCTCACAAGGCGCTTAAGCGTAGAAGACGAA
GACCCTGAACCTCATGAAGCAGTTGACGGCGGCGGCGTTGAGCGCGATGATGGCGAGGAAGCGCGG
GCTGCGGTGCTATGGCGCTGCGGCTTAGCAACGCGGGCCAGCGGTTACAGACGGAGCAGCAGCACGG
CGGTCGACCGACCGGCATAACCAGAATACCAACGGATGATCTGTCGGTCGTTCCGGTAAGCGCGCTGC
ACTCGCTCTTCGCCAGGGTCGTCCAGCTTCACTTACAGGGCGCTGGCGTAGTACAACACCACATGGAT
GCCCATGTACCACATGATGAGGTATGGAATCCCGAAGGCAATCCCGCGCGCCGGCTGCGGCAGCGGCG
TGACGG
>TCONS_00108411 geneID=XLOC_021665 scaffold_352:64447-112960(-) Exonic overlap (-)
GTTGTGTAAGACTATCAAAAAAAGTAAAAAAAAGTGATAATGGTAAAGCATGGTCTGTAACACCAGAC
GAGTGACTATCGAGTAATTTCGGCCGGTTTCGGCGCCAGCGACCGCCACGTCTACGCAATGCTACGAG
ATACGTGCAGTCCGCACCGGACAGGCCGCACAGTATGGACTTCGCTCTCTGGATCTACGGGAAGTTCG
TATTAAAAGACCGCGCAACGGAGTAGTGGGCCTGCTGCAGAGCGCGGCGCGGCCCGCCCTGTGACTA
GCGCGCCTGCTCGCCGGCGGCACGGGCGGCGGATTGTGAGTACAACTGCCTTCCTTCTAAAATAATCT
AGAAGATATAGTTACAGCGTGCCTTCCTTCTAAAATATAGTCATAATGTAATTATAATATGCAAAGGGCA
AATGAAAAGGGCATGCATCCGACCCCAAACTAGGCAGAGCCGTCAGAGACAGCATATTAACACCCAA
GACCCGAGTACAAATGTCTGTCTTTTAACAAATATTTGCCTCGGCCGGGAATCGAACCAGGGACCTTC
GGCATAGCAGATAGGGTTACTAACAAATACACCATTTGTTCGTCATAGTTTACACCGTGTCATCCTTCTA
GAAGATACAGAGATGGTACACTGTGTTATGATCTGGCCAAGCCTTGGCTGAGCTACGGAAGACTATCT
CCTGTGTTTTTTACCTATTGGCAAATCATATACATAAACAATGTGTTATTGTGCATGTAAGGGCGTCAAT
GCCATCGTTTTTAATACTCAC
>TCONS_00108437 geneID=XLOC_021675 scaffold_352:288828-307293(-) Intergenic
AGGCGCGATCACCATAGCAACCGCAAACGGAAAATCGGAGACAAGACAAAGCGCTGAACTGTTGAC
GGATCTCCATGGCAACGGCTACTTGTTACTTTGAGGCAAACAACAACATCCGCGGACAGCCACCTTGA
CCGCCGCTGGCCGAGCGATGACACCGCGCTCCGTGATCGATTGTTCCGGCGTTCGACGTTCGAAAGTG

ACAGTAGTTGTCGTGGCGTCCCGCAGGCTCCGCGGGGTGTGATGTGCCGCGGGCTGCTGCCGTGTGTG
TGGTGTGTGCAGTGTGTAGCGTGTGCAGGGTGTGCCGCGTGCAGGCATGTCGGTGTCGGTGCGCGTG
GTGGAGGCGCCGCGGCCGCGCGGCCCTACACCTACTCCAGCTCCCCGCGCGCGCTCTATAACAACGC
CAAGCTGAAGAGGGACCCGCCGTGCGGCTACGCGAACGTGATGCACGAGCGGCGCGTGGCGCGCGG
CAGCACCGTCGCC
>TCONS_00108578 geneID=XLOC_021662 scaffold_352:332227-335237(+) Intronic
TTCGAATCAGTAGGGTCTGTCCAACCGTCAGTGTTCATCCACCGCCGCGCCGCCGCCCTGCTACCGCC
CCGGCTACCACTACCGCCACTGCCTCCGCTCCACGCCGCCGCCGCCGCCGCCGCCGCGACGCTCGCGA
TGCCGCGCGCCACATTCCCACAGCGTCGTCTTGTGAATACAAAATGCCTCCTGATACGGCACTGACTA
CTACTGAGGGCCAACTGCAATGCTCAGATTATTCTACTG
>TCONS_00108609 geneID=XLOC_021676 scaffold_352:524476-525241(-) Intergenic
CTCTCATCCAATTCTTTCAAATAATTGCCTAATCTTTCCATTTTAAATTACGAATCTTCAATCTTTCCACTT
AAATGTCCAGTACGCTTGGATCTTTTTTAAAACTATGGTGCACTTGCTTTACTTGCAGTGTGGGAGGTC
ACGATAGCCTGGTTACAATATCATAGCACCCTCGAGTAATAAGGCAAGGTGGCAGCGAACTAACCGCC
CCTGAAATGGTTATTTTTCTAAAGTTCCCGTTATAAGCCGGAGACACTCTTACTCTGGCGCATGCAAAT
CCATGATAGCAAAAAGCGCACTCGTGTGTAAGTGCAGCACATTCGTCACGCCACCGCGACACATGGCT
AGTATGGCTCCAGCCACTACTCGACCTTCACCCACTTTTTCTTGAATTCAACCGCATAATTTGCACATAT
AAAAATTTACATTAACATCATTGCTCAGT
>TCONS_00108682 geneID=XLOC_021712 scaffold_353:56647-56995(-) Splice junction overlap
CTACGCTACGTAGCTAGATACCAATATTAGATATTTTTTGATAAGTTGATAAGGTGCTTTGATATTCGAAT
CTAATAAAACGATATCAATTTTAAGTTACATCAATATAAAACGCTAAAAGTATAATCGCATTCTCTTACAT
CTTTAGGCAGCGCGTGCCCGGGTTGGGTACGTGGCTTAACAATGGGTGACTCCGTGAAAGTAGCAGA
CGCTGGAAACGACA
>TCONS_00108760 geneID=XLOC_021707 scaffold_353:94106-102749(+) Exonic overlap (-)
TTGGTCCGCGCGTGGCGTAAGAGCGAGAAGACCAATACTCTATCCTTCTTTGGCACAGAAGTCGAGTT
CTAATAGGGACGTTTAATTTGTACGCTAACTATGTATGATTGTGCGTTGATCTAGGAATAATTTGTTCTAT
TAAAATGTGTGTGTGAGAGGTTTAAACAGTTCAAACACATTTATTCAACTTGAAGCTTTTTATTAACAT
TTTCACTTATAACTATGCAGGAAAAATAATCTGTTCAACATATTATTATAGATACAAAATAAATAAATAAA
TGTACACTATATACAAAATAGGAGTAAATATCAACTTAGTACAGTTTTCTTTTTAATCGATTTGGTATTTA
ACAGGGACACATCGATGTCCCAGTGTTTTGCTAGGGAGGTTAGGGAATTGAGTAGTTTATCTAGTCTAT
GGTTGTTGTCATAGAGGTCGCTAGAGTGTAGAAGTGACAGGTGGAAGTGCACTTGGTGTAATACTTCT
AGTCTCTTGGAGATCGCGTCTCTTAGTTTGTAGGTCTGTGCTCGTCGCCAGTATTTAAGGTTGCGGCCG
ATGACTGAGGTGATGTGGTCTCTTACGTTGCGAAGACACGCCTTCTTGC
>TCONS_00108671 geneID=XLOC_021707 scaffold_353:94282-102747(+) Exonic overlap (-)
CATTTATTCAACTTGAAGCTTTTTATTAACATTTTCACTTATAACTATGCAGGAAAAATAATCTGTTCAAC
ATATTATTATAGATACAAAATAAATAAATAAATGTACACTATATACAAAATAGGAGTAAATATCAACTTAG
TACAGTTTTCTTTTTAATCGATTTGGTATTTAACAGGGACACATCGATGTCCCAGTGTTTTGCTAGGGAG
GTTAGGGAATTGAGTAGTTTATCTAGTCTATGGTTGTTGTCATAGAGGTCGCTAGAGTGTAGAAGTGAC
AGGTGGAAGTGCACTTGGTGTAATACTTCTAGTCTCTTGGAGATCGCGTCTCTTAGTTTGTAGGTCTGT
GCTCGTCGCCAGTATTTAAGGTTTCGGCCGATGACTGAGGTGATGTGGTCTCTTACGTTGCGAAGACA
CGCCTTCTT
>TCONS_00108629 geneID=XLOC_021707 scaffold_353:94312-102748(+) Exonic overlap (-)
ATTTTCACTTATAACTATGCAGGAAAAATAATCTGTTCAACATATTATTATAGATACAAAATAAATAAATA
AATGTACACTATATACAAAATAGGAGTAAATATCAACTTAGTACAGTTTTCTTTTTAATCGATTTGGTATT

TAACAGGGACACATCGATGTCCCAGTGTTTTGCTAGGGAGGTTAGGGAATTGAGTAGTTTATCTAGTCT
ATGGTTGTTGTCATAGAGGTCGCTAGTGTGTAGAAGTGACAGATGGAAGTGCACTTGGTGTAATACTTC
TAGTCTCTTGGAGATCGCGTCTCTTAGTTTGTAGGTCTGTGCTCGTCGCCAGTACTTGAGGTTTCGGCC
GATGACTGAGGTGATGTGGTCTCTTACGTTGCGAAGACACGCCTTCTTG
>TCONS_00108764 geneID=XLOC_021713 scaffold_353:94331-103966(-) Exonic overlap (+)
CAGCGGACCAAGGCGTCAGCAGGCAAGAAGGCGTGTCTCCGCAACGTAAGAGACCACATCACCTCA
GTCATCGGCCGCAACCTTAAATACTGGCGACGAGCACAGACCTACAAACTAAGAGACGCGATCTCCA
AGAGACTAGAAGTATTACACCAAGTGCACTTCCACCTGTCACTTCTACACTCTAGCGACCTCTATGACA
ACAACCATAGACTAGATAAACTACTCAATTCCCTAACCTCCCTAGCAAAACACTGGGACATCGATGTGT
CCCTGTTAAATACCAAATCGATTAAAAAGAAAACTGTACTAAGTTGATATTTACTCCTATTTTGTATATAG
TGTACATTTATTTATTTATTTTGTATCTATAATAATATGTTGAACAGATTATTTTTCCTG
>TCONS_00108635 geneID=XLOC_021709 scaffold_353:138871-142871(+) Unclassified
CGCGACTGTATGCGGCATCTCTTGGAATTCCAAGTATTTTAAACACCCGACTGCACGCTAGCCGCAGTC
TGGTCCTTCGGACCGGATCTATAGATACAAATGGCGACACAATGGGGGTACTACGTGGGCGGGCGCGA
GCTGGACCGCCGCGCGCGCCGCCACCGCGAGAACGGCCGCCCCGACCGCGACCGCAGGAAGGACGA
GAAGGACAAGGAGCGGGACAAGGACAGGGATAAGAAGCGCAGTCGCTCGCGCTCGCGATCTCGCTC
GAAGCGCTCGCGTTCGCGCAACCGCTCGCGCTCGTCGCGCCGCGAGCGACACCGCTCCGGCTCCCGG
GAGCGCCGTCGCGACTAGACACCCGCAGGAAGTGCCGTGAGGATCATCATCGATGTGGCTTGGAGAC
TTCTGGTGACCTTTTGTTTGCGACTCAAAGTTTACTTTAACCAACTTATCGCCGCTGTTATTGCAAAATG
TTCTATGTCATCGAAAATAGATTTGAGGTTTTGATCTTTATAAGTAAGTACTCTATAGAATTGATCCAAA
GTTTTGTCCTGAAATCTATATTCGATGACATAGAACATTTGCAAAACAGTGACGTTATAGGATTTAGGGT
CTCATCAGCACATATAAGAGTAACGTAAAGGGATCGCCTTTTTTCTCCTGTCAACCAGGCGTTCGCGAG
CTCGGGTTCTACTGGTCGACAGAAGAAAATGGGTCCCGGATCCCGTTACGTTACGCTTATATGGTCGGT
CACCTTTAGCGTCATCTATATATTATGGTAATAAATTGTAAATGGTGGTTCAGAACAACTGGGACCATGG
GACATGAATGTTGTGGTTAAATGACTGAGTAGCAGTTGTTTGTTGAATGTTTGGCGCATTAATGCAGTG
CAACCTTCCATAGTACACATTTGTTATGATCCTCGTCGCAGTGTATGTAAACTGGGCTCAGTTTATACCT
GACAAAGGCCCTTGTAGCGATTAGTTGGATACCATTCTGGAAACTATCGCACGCCTCCATATATTGATG
GCTCATCATGTCATTTTTATTTCAGGGTTTCCACGTAAGAGTATGTTAGGTCATAGCCAGTTGTATGGAA
TAAACGATAGATCTGAAACATACTCGTATCGTGAAATGCCTGCTAAAAATAGGATCCCTATACCCTAGAT
ACTCCAGATATAGTTAGGACTAGACGCATGTTGTCTAGACCTGGCAAATTGTAAGAAAACACCAATTTG
TTAATAGAGGGGAATGTATTTATATATTATCTTAATTATTGTACTGTGCATTGAGACTAGAATATATTTTAT
GTACTAGCGTCGTCATTCATTATCCGTAAGGTACTTTTATTGGCATGTGTCGTTTCCTTTCCTTTTTGGTT
CACTATTGCAATCCATGCAAGACCGAGAAAGCGTCAACTTCCGATCGGATATCAAAGTCATTAAAATGC
AAAAATTTACCCCTCATCCATCGCTTATTTATATCATCCTACATACACTTGATACACTGTGTTTTTAATGTA
ATGCTTGTTTCCCTTATTAGCTATTTTATTTTTTCCTGTACTCTATTAAGTGTACAACTCTTCTAAGCTGCA
TTCAACCATTACATTAACCTCTTTGAAGAGCAGTTATAGAGCTCTTATTAATATCTGACATATTGTTCATA
ATCCTTTCAAGTCTACATTAGTAAGGCTTAGAGGTACTTCCCATTTATAAAAAAATACATTTCAAGCATG
TTGTGATAAAAGCGCTAGTTTTACTGACATGTAGCCTAAGATATTGTAATTTTTATCCACCCTAAAATGG
ACCGCCGCCGTTTAAATGTACACAATTATGTTTTCTTTATTTATGATTTCCACACATCACAACTTTAACA
GTTTATGTAATAATTGAATGTTTACGTTTCATATTGTTTTGATTTAGTCTGATGTTCAACAAAATAGCAAA
AAAGACGTATACCATGTTTGGTTTTGATAATCCATTCATAACGTTGTTGAGTCCAGTTTGTATAAAAGTT
TAGAAATCTCCAACTGAAGCGATGGGCCAAACTCTGATATGGCATGAAAAGTTAAAATATTTAGGAGA
CTCATACAAATAGTATGTAGCGATGAGTTTGTGGTGCGTTTTCTGAGTAGTGGGTACAACATAGTCATA
AGTATCTGCAGTTTTGTATAAAGTAAGGAAAATAATTATATATTATTGTTTTAAGATTGAATTTGTATGAG

ACTAGACGAGCCTATGAAAGGTTGTCAAAAGTAGCCGATAATTTTATTTCTAAGATTTTTAATTTACGTA
CAGTTATGGGAGAAATCGTATTGTTTTGTTTTGAGTAAAATGAAATTTCATGGTATGGAAATTTCTAGCA
CAAACTAATTTTGTTTTGCGTTATCAGAATAGGTAGGTCTGTGTGCCAGTCGTTTTAGGAGCGCGAGTT
TTCTTTTAATTGTAAAATGTAAACTATGAAGTTGAATTGTTCTCTACTTTTGTTGATAGTTTTAATAAATA
CATTCATGATATTAAGAAAATTACTGGTTTTATTATGTCCGAATGAAAAACAAACATGGATAAATTCATA
ATCTTTTATATTTGCTAATAAGTAGTAAGTCTTGTATTTAACATCTAACAATGTAGGTTCCTATAGCTGTTT
CTCAAACGGAAAGATTTAATAAAGTTCGTCTACTGAAGCTATCAAGGGAAAGATTTATCTTTTCCATCC
TGTATATTATGTACAAAGGTATTTGTGTAAATGCTCTTTTAACGAAAATTATTCTTAATTTGATCTGAATTT
AATCTAAAATTTCTTCAATACAACAAAGCCTTTTGCTGTGTGCTCATATTAAAATTTGTCGTAGAGTAAC
AGATTGAAAGTGTAACATTTTCGTGTTATAATGATGGTCATTTAAGCACCAACAATTAGGCGGCCCCTA
GGTGACCATTGACAATGACGCTTACAAAGCGTGGTAAGGCAATATATTATGTTATTGTGGGCCCGCCCT
AAAAATATATCTAGGTTCACACAAAATAAAATATATAAGTAGTACGACTTCTTGTTTTGTCTAACAATAA
AAGTAAATCTAAGTAGTTAAAAAAATATAAAAAACCAACGTTTGTACAGTATTATGCTATACGACTTTG
GAACTTCACACCTAATTTTCAATTAAAAAATTATAACTAAAATCGTTATACATAATTTAAACATTTCAATA
TATGTTCACAATTACATACAATGTTATCACAATTCGATATTATTCTACTATTCTTTGCCAAATTAATTTCAA
CACATAAGCCCCTTAATGGTACCTACATATTGCTTACTACCTGCGCCCGAAGCTCAGGGGCCGTCTCCA
CAAACGAGTAAATCTCGTATGCAGATAACTTGGCGCTAATATCGTGTGGAGACACTTACAAACTTTACT
GAAAATACCATTGATTTATGCATATACGTAACACGTAAGTTTAGGTATACCTAAACTCACGATTCACGAA
AGATGACCAAAATGGCGCCACAATACTGTCAAAAGCAGCTAACTACGTTACATTTTATTAATGTAATTT
AACTTAAACAAGCCGATTAACTTAAGGTTTAAGTAAATAATAGGTAAAATGACACACGAAAGTTTACG
TGAATCGAACTCTTACGTAGGTATTACTTGACTGTATTTCATATTTATCAAGGCTTTGCAATGGCATTGCT
ACAAG
>TCONS_00109160 geneID=XLOC_021767 scaffold_355:142038-144173(+) Splice junction overlap
ACCAACGCGCTCATGTACAACGGCAGCTCGCACCGCGTGCACCACATGGCGCGCGAGATGCTGCGCG
AGGCGGAGACCCAAATCAGCTTGCTGGTGGCTGCCCAGACGGCGTCCCCGCCGCCCGCGCGCCGCCG
CCGCCGCGACCAGCCCCGGGCTGACCAGCCGCCCGCTAAACGACAGCACTCGCTTTAGAAACATTTA
AACGGCTAAGCCGATTTTGATGAAATATGGCTAAGGACGCTCGGAACGGAACATTACCTAAGTGTATG
AAAAAAAAAATGGTTTGGGAAGGTAGCTCCCACCATAGACATACACAAAGATAAACAGACTCGTTAA
ACTTATAACACCCCTCTTTTTTGTCGGGGTTAAAAAGGTCACTAACCAGACGTCATATTCCTACGAAGA
TTTGATCGAGATTTCATTTAAAAAGCTTCCATCCCCTAAAAACCGTGAAATAAATAGGTATCCTGTTTAA
ATTTATCGTAGAAACCTACTAAATATACATTTTATTCCGGATTACCGCGACTAGCCAATAAATAGGTGTCC
GGATTAACGAGCAGAAGAAAAACAAAAAATAATAATGATATCATGTCACTTTAAATTAAAATCTATGTG
CAAATGCAAATAGTACATGCGCTCATTAATTGTAAGAATAATTTTAAAAGTAAATAAAATTAAGTACAAA
CTACTCTAAAATGATTGTTTCATTTTGTTGAATTTAATTTATTTAGTCCACCCATAGTATAATCAAACGTC
CACTTGTACGTAACTTTCCTCAGCCTTCCCTATCTATTGACCACTGGTTACCTATTTAAGACAGTCTACG
TTCTATTATATTCTAGTCTAGGTTATAATGGTGTCTTTCTGAGGAAACAAATTATATACTTTTAATGAACCT
ATCTCATTTTCCAGTGGTTTTTTTATATATTTTTAACTTTTAT
>TCONS_00109118 geneID=XLOC_021774 scaffold_355:348408-349393(+) Splice junction overlap
ATCTGATATCAACATTTAATTGATGACTGTCATCAATTTCCTTTGACAGTAAAAAGTAGACGAAATACGT
TTAATGTTTATTAAAACCTTTCTGCCACTGGGTGATAGAAAACCATTGTTAAGTAGGTAAAGAAAAATA
GTAAGAATGAAGCAAAGAAATACGAGCGACTTCCATTATACCTAAATCCAAACGCGACGACGCCAGA
AATGGAAGAGCTGAACAGTAAATTTAAAACAAAGATGGAGGATTTACTGTACGTGGAGGACGACGAA
GAGGATCTAAAGTAAATATTTGTAGTATTTCTAATTACTAATCACGATTTAATAGCAGTGTAAATATTTTT
TGACATATTTTTTAGTCAACATTTTGTGATC

>TCONS_00109209 geneID=XLOC_021783 scaffold_355:372931-374580(-) Exonic overlap (-)
GGTTCATCTCCCTATACATTTTAGGTCATTACATAATTTTTAAAACACATTGCTTATCTGAGATAAAAGGA
CACTTGGACACTTATTTACGTTTAACATCAGTACATAACTAAGTAAATAGGAAATATATCTACGAGTGAC
GCAATTAATTTATAAATACCTACTACATTCGTATACGATTATGTTCCATTGGGGGTCCGCGAATACATAAA
AGTAGAGACAAATAATTAATTTAGGCACTTACATAAAATAATTATGGTCACAAACCGAAGCACTGGTTT
AAAAAAGTTGAATGTATACTTACCTATACATTGTAGATAGAAATGGCAGCAATATAAACAAACTTCTATA
ATTTCAGATATAATTTTATTATCCTCTAATCTTTTTAGAAAGCTATTTAGTTTTCGGGTGGACATCTTCCTC
AGATTCTACTTGTGTCTCAGTTTTCATACAACTTTCTAATGCACCCAATTTAATCGGATAAAAAAACAG
ACTTCGAGTAGGTAGTTTTTGCCAGCAACTTTTCAAGTGTATTCGGAATCAAATTACCAACATACAGAC
GACAGGGCAGTCTATCGGACATTCATATTAGTCATGAATTTGACGCTCCGAAACTACAGACCCTCTTCG
AGAAATAATTCGTCGGGTTTATATCATTCCTCATACACTTGAGAGTCCGATTTCAAAGACAGTATGAGAT
AAAGAATAAATGCTATGAGAAGAGTTTTCCGTAGGTCTGTTTTCACACGCGTGTAAAAGTAATAGAAC
TCGATGCCTGTACAATGAATTGATATTGGTAAGATTATGTCGTGGTAGTGGATATAAAATAAATTCTAAG
AGCTTCCTTTCTAAGGTTAGACAAATTACACGTGTATTTTTTAAGGGTCAGAAATATTATCTTTCGAATT
ATGAAACATGGTTGGCAGTTTAAAAGTACTTAAGTTAAATATATTATGCTAAATATAAATATGTATAATTA
TTCTGTTATAATAATTCTTGGTCTATAGGCATTGTAGGACAGATTAAAATAAATCCAGAGGCAGACATGT
TAGTTAGATATTCATTAAATATTGCCAAAATGGTAGTAGATTCCTACATTTTACGACGTTACAAACTAAC
CAAGTTAATTATTTACTTTTTGCACAAAAAGAAAATCTTATATGGAATCATCTGCATAATTTTACAAAGA
AATTAGCATCCGGTCAATACGAATCACCAAAACAGTTTAGCTCAGCCCTGAACAAAATCCTTCTACAGT
TTGTCTAAAGCCACCCGGTCTTGGGCTGCCTCTTGATAGTCCTGAGTTCCTCGATGGGCTTGACAGCAT
TTTACAAAGTCATAAGCCAATCGAATCTCCAAAATAAGCAACAGTCTCCCTAAAGCCATCCGGTCTTGG
GTTGCCTCTTGATAGTCCTGACTTCCTCGATGGGCTTGCCGGCTCTCAGCGGCGTCGGCGACGGCGGG
ATCAGCTGTTCTGTACTAGAGGTGGACGACAGGCGCGCCGGCCCG
>TCONS_00109617 geneID=XLOC_021861 scaffold_357:14521-15244(-) Intronic
TGGCGCCTTAATTAATTGGGATAGTTTTTAGTCTATGGTTTTGGTGACAATGACAAAGGAAGCGCACGC
TACAGTCAAGAAAATCCGTAAAAAACGCTTGGACAAAGACACCTGAGTCATCTTCAGAATGCTGCTAA
TGCTACTTATGCTTCTCTCGGCCCAGCCAGCCTTCCCTAGTGGGGGCTTACACCTAGGGGGGGTGGTG
AAACACGTGGACCAGGAGTGCCTGACAGGATCTATGACGGACGTACAGTCCTGGGTCGGTCCTGACG
GTCACCTGTTGATGGACGTCGGTATGTCGATTTTTATTTTAAGATTTGGTTCGCTGTACTCCCTACTAGA
TGGCGTTGCTAGACTTGGTTTACTTCGCGGCGTGTTTAGGATGCCCTCCGGCTAGATGGAGAGAAG
>TCONS_00109508 geneID=XLOC_021852 scaffold_357:219002-221449(+) Intergenic
ATCGCGATAGGGATGATGACGAGGAGCCGTCCCAGGACATAGCAGCCAGCATCAAGGCGCTGGCCAA
GTCGGTCCACGGCGACGTGTTCGAACTGCCGCGCCCGCGCTTCTCCACGCAGATCTAGCCTCACCCCT
CCTCCCTCTCACTATATACACACATACAAACTACTTATAAGCGACTATATACATACTCTATCATACGCTAT
CTCTCTCACACGTATGCGAGTGCTTGAGTTTTGTTGCGAGAGCCGCTTTCTTGGTTAAGAGTTTTACGG
GTAGTGTGGCGGATTTAAACTGAGGCAACAGGTCGGTAGTAATGCCATATTGAAAAGCTATCAAGGTAT
CAGATGAGTGAAACTCTCAATCGGAGAAGCCGGCTTCAGTCACGATGTGGTGCTTAGTCCGGGTCACA
GTTTCAGCCAAGATTTTCATGTTTCAGCCAAAGTTGATTCTATACAGAACGGTTCTAACTGGACTCTAG
CGTACATACTGTGATCAATACAATGAAGATGACTGTTTACATAAATGAGGCTATACAGAGTTGTTATTCC
ATAGTTTACTGTCCCACCCCCGCAGCCATGTTTACCGCTGTTTCATAAACATTATGTTTCATTAATATTAA
GCGCCGCACGCAAGTTTACATATTGCGAGGCGCAAAATTAAGTAAGTAAGGGTTATGAACGTGTTGCA
TCATCAGATATATTTGTGTAGTATGTAAATAAGATTACGTGAATGACATGTCAAATCTATAAATCATTATG
CATTGTTAACATCTAAGTCATATCTCCGTTAATTAATAGAGACTATAGAGTTTTCTATTATTTTATTATCTG
GTGTTATCTTAATGATAAGAGAAAAAGTGTACAAAAATATTAATACTTGCATTTCTTACTAGTCTTTTAG

GGTCACGAAATATAATGTTTCATTTATTAATTTTAATTTTTCGGAAGTTTTTTCCCTTTGAACGCATGGTC
AGTTAATTAGGATATTGCACGCCCGTCGTCCGTCGTCGAGCCAAGGTCATGAACTGTACATAGAATCAT
TGTTACTCAAGACGAGGATGTGAAAACGTTTTGGAAGAACTTTATTTTTATACCCAATATTTTGGCAAT
CAGGAATGAGTAATGCTGGCATTGGAAAGGTTTCAGAGATGGAGGTTTCGTTTTGTTTACCTACTGTAT
TGAATGTTAAAAATAAGTATAAGTAAACAAGTAATAATTATAACTTTAGTTGTTATTGTCTGCTCGAGAG
AACAGTGCAGCTGTCGTAAAGGTTAATTGTCTTCCAGCCCCTCTTACCGACATAAATATTATACTTATCT
GTTTAGTTCTGTGATGTAAATTTTCAGTCATTTTCCTAAGCCATGGTTAAGAGTTTAGTTTCATTGGAGC
GTTGAGATTTAAGAAGGAAAGACCTAGAATGTAATGAATATTAGAAATCATCAATAAATATTGACTGTG
AAA
>TCONS_00109509 geneID=XLOC_021852 scaffold_357:219149-221449(+) Intergenic
CACCGCGATAGGGATGATGACGAGGAGCCGTCCCAGGACATAGCAGCCAGCATCAAGGCGCTGGCCA
AGTCGGTCCACGGCGACGTGTTCGAACTGCCGCGCCCGCGCTTCTCCACGCAGATCTAGCCTCACCCC
TCCTCCCTCTCACTATATACACACATACAAACTACTTATAAGCGACTATATACATACTCTATCATACGCTAT
CTCTCTCACACGTATGCGAGTGCTTGAGTTTTGTTGCGAGAGCCGCTTTCTTGGTTAAGAGTTTTACGG
GTAGTGTGGCGGATTTAAACTGAGGCAACAGGTCGGTAGTAATGCCATATTGAAAAGCTATCAAGGTAT
CAGATGAGTGAAACTCTCAATCGGAGAAGCCGGCTTCAGTCACGATGTGGTGCTTAGTCCGGGTCACA
GTTTCAGCCAAGATTTTCATGTTTCAGCCAAAGTTGATTCTATACAGAACGGTTCTAACTGGACTCTAG
CGTACATACTGTGATCAATACAATGAAGATGACTGTTTACATAAATGAGGCTATACAGAGTTGTTATTCC
ATAGTTTACTGTCCCACCCCCGCAGCCATGTTTACCGCTGTTTCATAAACATTATGTTTCATTAATATTAA
GCGCCGCACGCAAGTTTACATATTGCGAGGCGCAAAATTAAGTAAGTAAGGGTTATGAACGTGTTGCA
TCATCAGATATATTTGTGTAGTATGTAAATAAGATTACGTGAATGACATGTCAAATCTATAAATCATTATG
CATTGTTAACATCTAAGTCATATCTCCGTTAATTAATAGAGACTATAGAGTTTTCTATTATTTTATTATCTG
GTGTTATCTTAATGATAAGAGAAAAAGTGTACAAAAATATTAATACTTGCATTTCTTACTAGTCTTTTAG
GGTCACGAAATATAATGTTTCATTTATTAATTTTAATTTTTCGGAAGTTTTTTCCCTTTGAACGCATGGTC
AGTTAATTAGGATATTGCACGCCCGTCGTCCGTCGTCGAGCCAAGGTCATGAACTGTACATAGAATCAT
TGTTACTCAAGACGAGGATGTGAAAACGTTTTGGAAGAACTTTATTTTTATACCCAATATTTTGGCAAT
CAGGAATGAGTAATGCTGGCATTGGAAAGGTTTCAGAGATGGAGGTTTCGTTTTGTTTACCTACTGTAT
TGAATGTTAAAAATAAGTATAAGTAAACAAGTAATAATTATAACTTTAGTTGTTATTGTCTGCTCGAGAG
AACAGTGCAGCTGTCGTAAAGGTTAATTGTCTTCCAGCCCCTCTTACCGACATAAATATTATACTTATCT
GTTTAGTTCTGTGATGTAAATTTTCAGTCATTTTCCTAAGCCATGGTTAAGAGTTTAGTTTCATTGGAGC
GTTGAGATTTAAGAAGGAAAGACCTAGAATGTAATGAATATTAGAAATCATCAATAAATATTGACTGTG
AAA
>TCONS_00109510 geneID=XLOC_021852 scaffold_357:219206-221449(+) Unclassified
CAACACAAGTGAGTCACTCGCGAGTCACAGCGCCGCCGACGTGGCGCGCTCGCTGCCCATCAACATG
CCGTTCCCGATGGAGCGCGCCGACCATCGCGATAGGGATGATGACGAGGAGCCGTCCCAGGACATAGC
AGCCAGCATCAAGGCGCTGGCCAAGTCGGTCCACGGCGACGTGTTCGAACTGCCGCGCCCGCGCTTC
TCCACGCAGATCTAGCCTCACCCCTCCTCCCTCTCACTATATACACACATACAAACTACTTATAAGCGAC
TATATACATACTCTATCATACGCTATCTCTCTCACACGTATGCGAGTGCTTGAGTTTTGTTGCGAGAGCC
GCTTTCTTGGTTAAGAGTTTTACGGGTAGTGTGGCGGATTTAAACTGAGGCAACAGGTCGGTAGTAAT
GCCATATTGAAAAGCTATCAAGGTATCAGATGAGTGAAACTCTCAATCGGAGAAGCCGGCTTCAGTCA
CGATGTGGTGCTTAGTCCGGGTCACAGTTTCAGCCAAGATTTTCATGTTTCAGCCAAAGTTGATTCTAT
ACAGAACGGTTCTAACTGGACTCTAGCGTACATACTGTGATCAATACAATGAAGATGACTGTTTACATA
AATGAGGCTATACAGAGTTGTTATTCCATAGTTTACTGTCCCACCCCCGCAGCCATGTTTACCGCTGTTT
CATAAACATTATGTTTCATTAATATTAAGCGCCGCACGCAAGTTTACATATTGCGAGGCGCAAAATTAAG

TAAGTAAGGGTTATGAACGTGTTGCATCATCAGATATATTTGTGTAGTATGTAAATAAGATTACGTGAAT
GACATGTCAAATCTATAAATCATTATGCATTGTTAACATCTAAGTCATATCTCCGTTAATTAATAGAGACT
ATAGAGTTTTCTATTATTTTATTATCTGGTGTTATCTTAATGATAAGAGAAAAAGTGTACAAAAATATTAA
TACTTGCATTTCTTACTAGTCTTTTAGGGTCACGAAATATAATGTTTCATTTATTAATTTTAATTTTTCGGA
AGTTTTTTCCCTTTGAACGCATGGTCAGTTAATTAGGATATTGCACGCCCGTCGTCCGTCGTCGAGCCA
AGGTCATGAACTGTACATAGAATCATTGTTACTCAAGACGAGGATGTGAAAACGTTTTGGAAGAACTT
TATTTTTATACCCAATATTTTGGCAATCAGGAATGAGTAATGCTGGCATTGGAAAGGTTTCAGAGATGG
AGGTTTCGTTTTGTTTACCTACTGTATTGAATGTTAAAAATAAGTATAAGTAAACAAGTAATAATTATAA
CTTTAGTTGTTATTGTCTGCTCGAGAGAACAGTGCAGCTGTCGTAAAGGTTAATTGTCTTCCAGCCCCT
CTTACCGACATAAATATTATACTTATCTGTTTAGTTCTGTGATGTAAATTTTCAGTCATTTTCCTAAGCCAT
GGTTAAGAGTTTAGTTTCATTGGAGCGTTGAGATTTAAGAAGGAAAGACCTAGAATGTAATGAATATTA
GAAATCATCAATAAATATTGACTGTGAAA
>TCONS_00109607 geneID=XLOC_021874 scaffold_357:505772-507746(-) Intergenic
AAGCCGTTTTATACGCAGTACCGTTAGGCGATCGAGTGCGGGGACGTCTGTTGCCATTCTTATTTTTTC
CTAAGTTGATTTAATTTATTTACATACGTGGTAATGAGATGGGGGTGACAAGTTTGCTACGTGAAGAAG
AACCTGAATAAGTAAAGATTTTTATAAATACCTACATACAGGATAATAAGTAAGGCAAATTAAAGCAGT
ACCTATGCAGTAGTTCAATAAGTAAATTTACTTGACAAGGATTCTACTGTCCAACACTTCATGCAAAAA
TGTGACCAACTGGACAATATCATTCATTGTATTCTGCGATACAATATATTTGACCATATTTACAGGGAACA
TTACTGCTGCAGACCAGTTAGTTCGCCTGTAAGATTTCACACTGATGTCTTTTGAAGTATGTCGAAGCA
TATAAATTGCCTACTTACTTAATTATTTAGTTTATAAAATAAGTTTTTAATTTTGGTTTATAATTTTATACAG
CAACAGACTTTCTCGCACATCACACAACAGTCGCGTAGTGCATAATAATGCAACAAATAAGTACATCA
GATGCATACTCTGCACAGTATTTCTACTTGCGAGTATAACTCGAGGTTATGCCAGCAACAGACGATGGT
TTCATATCATTCATGTTAGGTGTTAGGATTAAGTGAGGATGTCTTTATTTACTAAGATTATATTTGAATCTC
AGGTTTGCACATTCCGTGTTGTTCTTGTGAGGGTCTATAATAAAGAATTTATGGGGGAGATTTATAAATT
TAAAATGTCTTGTGATTTTTCGTACAATCGTTGTATTAAAGTATAAACCAGAACCAAGATTGAACATACA
TATTTTATTGTTATGAAAGTAATAACTTGGAGAATTATGTGGCCCACACGAAATGTATTCATATTTTTTTAT
ATTGGCAAACCTAAACAATGTTGACTGAAGTGTATTTTTGAAACGCAGTATTATTTTGCATTTACCTACT
ATACATTATTCATTGTTAAGATTAGATTAAAAACGTGTGAACATTTTGCTTTGAATCGTTGTTTCCATTCC
ACCTAGATCACAGTTTCACCATTCAAGTGGAAAAAGGTCCATTGTTATATCCAGAACGATCCAAAAAAT
GGTAAATGTTTATTTTACTATTTCCACTAGTAACTATCACTCATTCACTACTACTCGTTGATCAGACATCC
CAACTAGAGCCCCACTTGTTGTAACTAGCTGCAGATGTACTAGTAGCGCCGCGCCGAGGCGCACCCAC
TCTCGAACCGAGCTCATGGTAACAGCTTCATCATCTGTCCCTAACTCCCATTAACACTGGGCCCCATCG
ACTCACTGCCCGATCTATTATCGAATCGAAACACTGTCAAGAGTAAAATGTGATTTTGATGTGCCATCC
GCACTATCGGAGGATCGGAATGAAAATCAGCGGTAATTTTATTCACATTCCTTAATGGATTTGCTTATTG
TAGTAGCTCTCTTATATTACTGTCTCGATTTTCTAGTAGTGTCATATAAAATGTGACTTTTTGTCGGTTTAT
GGGTGGGTACACTAATATTATATTTACGTACAGTAGTTTGTCGCTTTACAATGTGCATGAACCGAACAGT
TACTAGTCGTTGGCTGAGTTGGATCTACGAGACAGTCTCACACCGGATACATGCATGACATTACGGATA
CGATATACTATCGCCGAGTATTTTGGACGTGTGATTTTTCATGTGAATTATATTTATCCTACCTACCTGTGT
TCCTTCGCTTATACTGCACCTATAATGTCGCCACACTGGAGTGCCGAGTGCTCCCGGGGCGGGTGACA
GGCGATTGTGGATCAGTCACAACGGCTGGGCCGCTTGTTGGACCTCGGAGTCCCCTGTCGCGACGCG
CGACGCGCCGGCCCGC
>TCONS_00109729 geneID=XLOC_021907 scaffold_358:128818-130435(+) Intronic
GCTCCTTTAGTTCTGACCAAGATTCAGACCCGCGCGTAGTGTGGAATAATAAACTGTTAAAAATCTTTT
CCAACGAAATCATATAACATTTTAATGCCGTTTTGTGGTGTAAAGTGGTGTGGGAAGTCTTCTAAGACT

TCAAATTTCCAAAAAGATGGAATTACTTTCCACCGGTTTCCGCAATGCCCTGAAAGAAAAAGCAAATG
GATAGATGCAGCACATCGTGGCGACCTTTGGTGGCCAAATTAATCCATTTTAAAAATCAATAGTAATGCT
ATTTCTTCGCGTGTACTGATTATTGCTGCCCACTTCGTCGGCCTATGATTATGTATCGTCATAATTTTAGC
CTATTTTTTCAATTATGTAAAATTTTATCTACTTATAATAGGTAAGTCAAGAATAGCTTCTAATACGTACCT
TTGTACCCTCATAATGTTATGGTTTTTTAAGTGTGTTTTTGATTAAATTGTAAGTATTAATTGTAAATATTA
AATTGTAATTGTAAATTGTAAACTACACTACACTACCTTATACTACCCTACCCTACACTACACGACCCTA
CCCTACCCTACACTACACTACACTACCCTATCCTACCCTACCCTACCTTTCTTACCTTTTAAACCTTCCCT
GGAGTTCCACGAACATTTCAAGACCAAAATTAGCCAAATCGGTTCAGCCGTTCTCGTTTTTGCGAGAC
TAACGAACAGCAATTAATTTTTATTATACAGGGTGTTGCAAAAAGGGTATACTAAGCCGAAACCTTCAT
GTGCAGCATTGTATATCTAAGCCCGAAACTGAAATCAGAATTACAAAATTCGCGAAAAAAATATTAAAT
TTTCCATGGAAACTATGTTGGTCACGTGACTTTTTACCATGCAGAATGACTTTTTTTTTCGCGAATTTTA
AAATTCTGTTTTCAGTTTCGAGCTAAGATATACCATGCTGCACATGTAGGTTTCGGCTTAGTATACCCTT
TTTGCAACACCCTGTATAGACTAGCTGACCCGGCGAACTTCATACCGCCTCTTACCCTACACTACCCTA
CCCTACCCTACACTACCCTACACTACCCTATACTACACTACCCTACCCTACCTTTCTTACCTTTTAAACCT
TCCCTGGACTTCCACGAATATTTCAAGACCAAAATTAGCCAAATCGGTTCAGCCGTTCTCGAGTTTTAG
CGAGACTAACGAAGAGCA
>TCONS_00109748 geneID=XLOC_021908 scaffold_358:157408-221928(+) Splice junction overlap
GTCTTCGTCGACCACCGCTCGTCGCGGGCGCATCTTCACCGCTTCCAAATTCAAACTTGCGTGTTTCA
GGAGGCTAGTGAGTGGTTATTGACTATATTGTGAACTGTGAGCGGGAGCCAGCTGCGACGGAGATCTA
ACCTCCATTGGCTTTAGGAAACGCAGTTTGGCTGATAATCAGAAACTGATGAAGCGGAAGTGGAGTGG
TACCGGAAACAATATGGCGACGACACGGCAGATCGCCATCACATTACTGTTATTATGCATAGCGAGTGC
GTGCGACGGCTCACGGCGGACTGCTCAGCGAAGAAAGGTAAGCGTCCACTTTACAGCATCGTACACA
ACAATCGTAAAGAAATTCACACAGG
>TCONS_00109750 geneID=XLOC_021908 scaffold_358:157906-221928(+) Splice junction overlap
CGCAGACTGTCGCGGAATCGCTGACGATTAGTTTGATACATATTTTTATTGGTTATTGTTAGGCGGTTGA
GTTGCGTGGCGTGTTTAGATAATACGCGTGCGGTGAACGCTCGGCGAGTGCAGTTTGAAAGTACGCGG
CTGAAAGTGGGTTTGCTTCAGTGTTTCAGGAGGCTAGTGAGTGGTTATTGACTATATTGTGAACTGTGA
GCGGGAGCCAGCTGCGACGGAGATCTAACCTCCATTGGCTTTAGGAAACGCAGTTTGGCTGATAATCA
GAAACTGATGAAGCGGAAGTGGAGTGGTACCGGAAACAATATGGCGACGACACGGCAGATCGCCATC
ACATTACTGTTATTATGCATAGCGAGTGCGTGCGACGGCTCACGGCGGACTGCTCAGCGAAGAAAGGT
AAGCGTCCACTTTACAGCATCGTACACAACAATCGTAAAGAAATTCACACAGG
>TCONS_00109642 geneID=XLOC_021911 scaffold_358:283673-284265(+) Unclassified
CAGAGACATCGTGTTCTTCACAGCCACCTGACGGGCTAGCACAGGGCGCAATTAAAACCCCTTCTTAC
GAGTACTCTCGAAGCAAGGAGTCCATTGAGCCATGAGGACACTCAAAGAACAAAGTACATTGCGAGA
TAGATGATGATCTCCTTTCTAGGCCACTCAGGCAATCGTTCATATCGGACAAAAACAGCCATCGCCATC
ATAAATAGTGATCTAATAGCTAACTAATAATTTCAGTTTTTCTAACATCTCGATATTTTTGTATGAAATCCC
GTACTTACATACATCACATTGATGATGCCCGATAAGTCCTAATAAAACAACCTATGGTAAATTACATCAT
TTGAATTTTTTGTGTGAGCTCTGCCGCTATTTTTATGCGAAGGTGATTATCGTTTTGGTCAAGTTTGGTG
GTTCCTGCTGTAAATAGGTACCTACTTACCCACATCTAGGCTTGTAGCACAAGGCGCATTTAAGACGTG
ACATAAGTTTTGCA
>TCONS_00109833 geneID=XLOC_021943 scaffold_359:5390-14213(+) Intergenic
GCCGCACGCACGCATTCGCGCGCGAGAAAGTTCGATTCGAGATTCGAAATTTAAATTTGGCGGCAACG
TCAAAAATTAAAAAAATATCCCTTGAAGGACAATGAACTGTTTTTAGCGTTCCATTTTTGAATAAGGGA
TTGGATTATATAACTGGCTGGTTGAGGTGTAAGTTGTCGTTGGTTGCACTTGTGTCAAGTGAAGTGACA

AGGTGTGCAGTGTGTGAGTGACTTTCGCCGTTGTACAGGATGACTAGCAAACACAGCTTGAAGCTCTG
GATATGCGTGGCGTGCCTGGCCACCTCGCTGGCGGCGCCGGCGCTGCAGCAGTACCAGTACCAGCCCC
TCGAGATGGACATCATCGACCTGGCCGCCATCAGCCGCATGTCCCCGCAGCAGCTCGAGAGCCTGGCC
GAGGGTCTGGCGGCAC
>TCONS_00109842 geneID=XLOC_021955 scaffold_359:229137-236512(-) Unclassified
CTCAATTTAGAAAGAACTCTGATCAAAATACTTACATTCAATTACATACACATTAATTAATTCCCAATAA
AATTATCATAATTTACTACACCTACTCATTTACTACCGCATCATCGCCTCATCGAATAGGCAAAGGTGCTT
TGCTGTATCCACATATCATGTTCTGCTTTCCAATCAATTTTATAAATATTTTGAACGACTCACTGACACCA
CATCTCCATACACACATAAAACCAAAAACTAAGCATTTACATACACCAATGATACATCGAACCATGCGC
TAACCAGCTATTGCAAATCAGAGCTCTATCTAACACGGGACCATCCATCCGCTAATTACGTCACACAAA
TGGTGAACTAGGGGGTCGAAGGACCATTTTATGGCAAGGCAATTGTTTTACTACTAAAGATGCAATATT
ATGCCAAACATTGCCACACTTTACACGGGATCTCACAAATGTGCCTAATTAAATAGGGCTGTCCAAAAT
CACAATGAGAAAAAAGTGTGCATTGCCAAACAACAAACGTCAAACTAGGGTTGTCCAAAATCCCAAC
GACAAACAAGTGTGACATAATTAGCGGGTAGCCCTCACAAAACCCATGCCACTAGCGCCGCCGCGGC
CGTCGGACGCCGTGGAGCGGGACGCCATGCAGCTGTGGTTCGAGGCTCGCTGGGCGCGCCTCATGTC
GCAGCGGCAGGACGACCGCCGGCTCGCCCGGCTGGAACGCAGGCTTAGGCGACCGCTGTCAGCGGA
GCAGCAGGCGGCGGCGGTGGCGGAGCTGGTGCAGGTCTCGGCGCAGAGGGAGGCGCACGAGGCACT
CATGTCCGCTGACAGGAGGCGATGTATGACGAGCGGCAGTGCCTGCGAGTAGGCCGCGCCAACAGTC
CGGTCTCAACCCTGTATGGAAATGCTTCAGAAACTACCAAACACCACGCCAGAGCTTTCGTAAGTGCC
CTTCGGAAATCCGGAACCAATTTTACTTTCGCCAAAACACAGTCATCGAGGCAACAGGAATTGATTGA
TATAAATAATTGTTAACTGGTGATTTAAACTTAATATTGCTGCTAATAATATATCATCTAGTCCTTTGGATT
GTAGAGTTGTAAAGGTTGAGATGATATTAGGGTAGGCAGGTTTGTTGTTAAACATTTTCAAGTAGGCAG
TTGTTATGATGTTACATTCCTCAATATTAAAAAGGAATAATGTTTAAATTTAATAAAACATTCATTGAACT
TATCCTCTGTTAGCTGCTCCTCTGTACAGAAATCTAAACGGATTTATTTAGGGAAATTGTTTATGATTTTT
CGCTGTCGTTTTCAAGTTACAAACGAATTGTCAGGTGGATTTGCTTCAATCCACTCGCGATGCGATAGA
CTGTGTGGTCCCGTGTAGTTAAAACGGCAAGGTTACATTGTCACAAATTATTTTTAAGTAATTGCTCCC
AGTGCGATCTGGAATGTTTTTAATTTACAAAATAGCGAGTTTTCCCGCAATATTCTCGGTGTAGGTGCG
ACTGTCTCATCTATAATAAGCTAATGAGCGACATAAACACACTATTCTGGTTTTAGTGCTGAGTGAGCAT
GATTTAGTGTCTCGTTGGGAACATTCCAGACTGCGCTCGGACTACAACGGTGTGCTAGTAATGCCAAAT
GTATTAGTATATTGTATTTAGACGGGCTCCTCCTGCCCAATGACTTATTTCGTAGGCTAAAAATATACTTA
ACAAATCTAAGTTTAAATGTTTAAATATACCTATTATGTCTTTTCGCTACTTCATGTACTCAAGTGCGGGT
CAAACTTGCACATAAGCTTCTTATGTAAGGTTTACTGACATTTATATTACTATAAAACATATAGTGAACAT
TGACTTCTACTGGGTATCATTTAGGGTAAGACAAGTATTAGCCTAAAATCATTATTTTTGTATGCGATGA
GGAAAGAGGGTTGTAAAGATTAAATGAATTTAAAAGTGATTGCGGTCTAGCTTTCTGCTATTTAGTTTT
TATATACTGTGCGAGTAAATAGTCTTCTAAAGATTTCTTACCGAGAGTTGAGGTGAACACTAGATTTATC
ACGCATTCTGAGGGTAGATTGTTACGCGTTGACTCTAGTCGTACATATATTTATGCTACACTTGTAATATT
TCTTCCCGTACAAACATGCTAAATCTAGTAGTTATTAACACTAGAGTAGGCGCGCTCATTTAACTCCTGG
GATGGTCGCGCGGGCTACATATGTAGCCCATTTGTAATGCCTCATAAGCACAGATATTTGATTGAAAAGT
AAAACACTTTGCTATATTTCAACTATCATATTTATTTTTAAACTTTAAAGTAAAAAACGTATGAATATTTT
CTAATTTTAACAAAGTTATTAACAAACAAAAAGATCCTTCTAAATATCGATATTATTTGGGACCATCGCA
CAAATTAGTTTTTTTTTTAGTTTAGGACACAAAACTTATAAACTGATCATAGTTTCCTACAATATAGGATA
CAATATAGTAGCAGATGGTGGAAATTTACTCCATTTCATACCATTCCGGGACTTGTAGCTGATCAAAACT
GATGATGATGGTGGTACCTTCACATCACATCATCTTCGTCCAAATCTTCTACTTATTGTTCACTCTCCGAT
AGATGAGAATTATCGGAAAGAATTAACGGTTCCATAGGACCTTCTTCTTCGCTTTCCGTCAAGTTATCA

CTATTTTCCTCGTCTTCCATAAGCCATTTCCGAATTTGACCTTCGTTCACCGCCATTATACCTAAAATGAT
TCAAATGTTTTTTTTTGTGTTTTTAGTCCAAAATAGAACTAACAAAGTAAAAAAAACAATGAAGTAATA
TGATTATACATTTCATATTAACGTAAATGTGCACAAAAAATATTTTATCTAAGAAATAAAACAGAAAATC
AGAAAAAAAACACTTACCTAGAACGGTCGCGCGGGCTACATACGTAGCCCAGCAACTTTAGCCGATGT
GCTCCTCAGGTTCCCGTGTGCCGGGGCAGTGGCACGCTGCTGGTGGACGCGGGGGTTCATAAGGAA
GGAATACATGGGGGGAGGGAGCGCTAGTGCCGTGCGTTCCAGATATTTACATCAAAATGATACCCTGG
TCTACACCTGTAGCCCACGCGCCCACTCTAGTGTTAATAGTCATCTTTGTTTGTACAGTAAGTAGGCTAT
CGATAAAATATCCGGACACTCTTCTGGTTGCAACTTACAACGATGATTCGATCTGTCGGAGATACCTTA
CAGTATATTTCAAGCAGTGTTCGACTGTAAAGACCTGTGAGATGCCGTCCCAAATGAAATATGTACGAC
TCATATTTAAATCCCAGTATTCATATATTTGATGATTATCTGATATTATCTGACATAGGCAGGCCCAGTATT
CATAAGTAAGTAGACTGTTCCATCAGTCTCACAGCCCAGTGAGACATGAGTGGGGTTCTTGTGAAGAA
AGTTTGTTAAGCAAACCCCTTGAAGACACCCTCTGGGAAAGCCGAATACAAAAAATATTGTGTTTTTC
TCTGTCTACTAAAACAATAAATTTTGTATCAAATATATTTTTATATCAAAATTAAACGGATCCTATTAGGT
TTAGCAAATATGTATGTAGCAATAATAAGCTGCCGAGGCCAGACCGCGCTTACCACAGTAGTGACTAAA
GAATCTTCCAAGTTTAGTGGTTAAAGATAAATAATATATATTTTGTGTTGCTATGTTTACACGCCCCCCCA
AAATTACACTAAAATGTATATATAACATATAGCTGTAGTATAGCGTAGCGATAAATTGTTGACACATCTCT
TGACCTGCAAAAGTTAATTCTATTTCAATAGACGAAGAGTTATAGCCACAGTTTATACGCTTATGAATAG
ATGTTGTACAAATCGTTTATTAGGTACATTATGTACAGTAGGTAGTTGACAGAAGTGCACTTTAATATCT
GGAATAGTGTTCCTGAAATTATTTTTATAAGAAACTTTTTGTGAGTAAATAATGTTTGCCTCAACTCTCG
CTAATATATATAAGTTAGACAACTTTTATATTATATATTCTAATCAGGTGAGGAACCAGACATCAAAATAT
GTGATGAGCAAGTATAATAAAACTAAAGACCAAGTTGGATTTTGAACTAGCTTATGAGTCCCGACTCAT
TCCCACTCAAAGCCTATTGAACTCCAACTTGCACTTGGCCGATTTTCAAGTGATTAGACTTGCTCATCA
CCTATTTTGAGGTCTGGATCCACTCCTAAAGCCGATTCAATGGCACATGATAGACAAAATGAGTATGTA
TGAATTATGACTTATTTACTTGCTTCTATATTCTATACCATTATAATATTAATATATGTGTTATTGGTATATAT
TTTCTCTTCGGAGAGACTAGGAAAGTGGTTCTATGGCGATTTCGAAAACCCCAAGTTTATTTCAATACT
TTTTACATAGAATCATATTTTTGCATGCACAAGACTATCATTTAGATAAAATATACTTACTTCTTAGTTAA
AACTTAAGCTATGAAATATTATAGAAACCGATCGGTTGTTCAGTATTTTTGTTTTGTAATACTCAATATTT
TTTATACTTACACGTAATCATGTTGATTTGTAAGCTCTTTACTATTTTTTTGTTTAAAGGTGTGTTTTTCTA
AATTACTCTAAGCATTAACTTAAGTAAGAATGTATTAAGACGAGTTCTATTATTTTTGTAAAAGGATAAC
AATTTATAAAATCTGTTATATCGCCTACTTGGCTTCCGGCTAACTAACTTGCATTAAAGTAAATATACTAG
TACCTGAGTTAAAAGCAGATATCTAGTGAACTAGCCGTGCCATGATGCCGCCACGGCACTCATAGTACA
ATACAACAGTCAGTCTGCTCTCAAATATAATAACATTAACTATAGCGTAAACGAAGTCGAGGTATAGCG
GCGAGGGAGAATGCTATGTTTTAAATACCTACCTGAAATATTTACTTATATAACACTAACCAAGTAATAT
GGCACAAGACTTCGTACGAGTTCTTACATGAACAGTTTGAATGATGCTATCCAAAAGATTTTTACTTTC
AATAAAATCCGCAATGGTAGCCCATCTTATATAAATGCAGCTGTTACAATTTGTCTAGTAGTATTTTGTTA
TCTTTATCATAATGAAACATATTATATCGACTTTATAGTTATAAAAATAAAAGTTAACATTGTAAGTAGAC
TGTAGTGTGTATCTTAAATTACACCTCTAGTTTGCGTCGCCACCGTCGCGGCAAACTTCGCGTCAACAA
ATTATTGTTTAAAGGTTTTTTATTATTTTGTTTTGATTCTAACAAGTTTATGTTATTGAAAGTGTAAACAG
CAGTTATATTTACGCTGTATATTATTTCGGGATTAAGTGGTTTTTTTTTTAAGTTTATAGTTAACATTTGAG
ATTATATTTAGATGCAATGTGTTACACAAAGCTATGTACCTCTTTTTGTATGCGTAATTCAACCTAGAAG
AACT
>TCONS_00109849 geneID=XLOC_021955 scaffold_359:229141-235384(-) Unclassified
GTTAAATGAAGTGTGACTACGTATAGTTGTTCACAAGCCTTAATTTGACCTTTCATCTGATGCCATTTGG
AATGAAGTGAAAGAAGATATGGTGTAATGGATGATAAATATTTTGGGCTGGCTTAAAAAACATCAGTAA

AATATGACTGTCTGTACCATATCTGTCCAATGGTTTATCCAAAAACTTATAATAATAGAGCCACTTCTGA
CTTTCACCTCTAATAATATAGTAAGTACGTATACAAATTAGTTTAATAGATCATCGACACAGGCTCTCAA
ATCACCGTGTCTGCACTACAGTACCAAGTGGGTTATTCACAATACCATAGTTAATGTAATAGCAATTAAA
CTTGCACATCTATCACCCATTCGTAACTTCATCACAGATTCACTTCATTTCATAATATGGATGCAACCTGC
AGCTAGCCTTCCCTGCTTTATGCACCACTCCTAGTAATCTTTTTTTGCCATATCGTCATAATTATCTGATA
ATAATATATAATAGCTTCTTCTTCAAGACCGTTGTATGATTAAGGTAACAACTGGACTGTGTGGTGAAGA
TGAATAACGATATATACTTGTGTTAGATTGCAATGTAGGTACATACATGTCAATACAACCTGTAGGTCTA
CCAAAGATGGGGCCATTTAGGGCCATCCATCAACTGTTTATCCAAAGGACATAACTTGTATGGAATTGA
CTGCATATAAACGTCATGACGCAAGGAGCCAAGATGCATCACAACACTCTATCCTGAACTATCCTCTCT
CTGCCTCATGATATAATGATGCTATTGATAGACATGATTTTTTAATGATAATTGCGTGTCGGTGACAAACA
CTATAGCTGAACTAGGGATTGTTACCTTGGTCAAATGAGTACCCAATCTGTTACATTTCGCCATATATATG
ACTGGCTGCCTTCTGATGATGATGACGATGATGACTAGGCTTTGTTATCTTGGTAAAAAGAGTACCTAG
TTCGATTACGTTCCGTCATGTGTATGACTGGCTGCCCAGCCCACTGATGATGATGATTATGATGATATAA
ATGGTGATGATGATGATGATGCCATTTCAGCGGAGCAGCAGGCGGCGGCGGTGGCGGAGCTGGTGCA
GGTCTCGGCGCAGAGGGAGGCGCACGAGGCACTCATGTCCGCTGACAGGAGGCGATGTATGACGAGC
GGCAGTGCCTGCGAGTAGGCCGCGCCAACAGTCCGGTCTCAACCCTGTATGGAAATGCTTCAGAAACT
ACCAAACACCACGCCAGAGCTTTCGTAAGTGCCCTTCGGAAATCCGGAACCAATTTTACTTTCGCCAA
AACACAGTCATCGAGGCAACAGGAATTGATTGATATAAATAATTGTTAACTGGTGATTTAAACTTAATAT
TGCTGCTAATAATATATCATCTAGTCCTTTGGATTGTAGAGTTGTAAAGGTTGAGATGATATTAGGGTAG
GCAGGTTTGTTGTTAAACATTTTCAAGTAGGCAGTTGTTATGATGTTACATTCCTCAATATTAAAAAGGA
ATAATGTTTAAATTTAATAAAACATTCATTGAACTTATCCTCTGTTAGCTGCTCCTCTGTACAGAAATCTA
AACGGATTTATTTAGGGAAATTGTTTATGATTTTTCGCTGTCGTTTTCAAGTTACAAACGAATTGTCAGG
TGGATTTGCTTCAATCCACTCGCGATGCGATAGACTGTGTGGTCCCGTGTAGTTAAAACGGCAAGGTTA
CATTGTCACAAATTATTTTTAAGTAATTGCTCCCAGTGCGATCTGGAATGTTTTTAATTTACAAAATAGC
GAGTTTTCCCGCAATATTCTCGGTGTAGGTGCGACTGTCTCATCTATAATAAGCTAATGAGCGACATAAA
CACACTATTCTGGTTTTAGTGCTGAGTGAGCATGATTTAGTGTCTCGTTGGGAACATTCCAGACTGCGC
TCGGACTACAACGGTGTGCTAGTAATGCCAAATGTATTAGTATATTGTATTTAGACGGGCTCCTCCTGCC
CAATGACTTATTTCGTAGGCTAAAAATATACTTAACAAATCTAAGTTTAAATGTTTAAATATACCTATTAT
GTCTTTTCGCTACTTCATGTACTCAAGTGCGGGTCAAACTTGCACATAAGCTTCTTATGTAAGGTTTACT
GACATTTATATTACTATAAAACATATAGTGAACATTGACTTCTACTGGGTATCATTTAGGGTAAGACAAG
TATTAGCCTAAAATCATTATTTTTGTATGCGATGAGGAAAGAGGGTTGTAAAGATTAAATGAATTTAAAA
GTGATTGCGGTCTAGCTTTCTGCTATTTAGTTTTTATATACTGTGCGAGTAAATAGTCTTCTAAAGATTTC
TTACCGAGAGTTGAGGTGAACACTAGATTTATCACGCATTCTGAGGGTAGATTGTTACGCGTTGACTCT
AGTCGTACATATATTTATGCTACACTTGTAATATTTCTTCCCGTACAAACATGCTAAATCTAGTAGTTATTA
ACACTAGAGTAGGCGCGCTCATTTAACTCCTGGGATGGTCGCGCGGGCTACATATGTAGCCCATTTGTA
ATGCCTCATAAGCACAGATATTTGATTGAAAAGTAAAACACTTTGCTATATTTCAACTATCATATTTATTT
TTAAACTTTAAAGTAAAAAACGTATGAATATTTTCTAATTTTAACAAAGTTATTAACAAACAAAAAGAT
CCTTCTAAATATCGATATTATTTGGGACCATCGCACAAATTAGTTTTTTTTTTAGTTTAGGACACAAAAC
TTATAAACTGATCATAGTTTCCTACAATATAGGATACAATATAGTAGCAGATGGTGGAAATTTACTCCATT
TCATACCATTCCGGGACTTGTAGCTGATCAAAACTGATGATGATGGTGGTACCTTCACATCACATCATCT
TCGTCCAAATCTTCTACTTATTGTTCACTCTCCGATAGATGAGAATTATCGGAAAGAATTAACGGTTCCA
TAGGACCTTCTTCTTCGCTTTCCGTCAAGTTATCACTATTTTCCTCGTCTTCCATAAGCCATTTCCGAATT
TGACCTTCGTTCACCGCCATTATACCTAAAATGATTCAAATGTTTTTTTTTGTGTTTTTAGTCCAAAATA
GAACTAACAAAGTAAAAAAAACAATGAAGTAATATGATTATACATTTCATATTAACGTAAATGTGCACA

AAAAATATTTTATCTAAGAAATAAAACAGAAAATCAGAAAAAAAACACTTACCTAGAACGGTCGCGCG
GGCTACATACGTAGCCCAGCAACTTTAGCCGATGTGCTCCTCAGGTTCCCGTGTGCCGGGGCAGTGGG
CACGCTGCTGGTGGACGCGGGGGTTCATAAGGAAGGAATACATGGGGGGAGGGAGCGCTAGTGCCGT
GCGTTCCAGATATTTACATCAAAATGATACCCTGGTCTACACCTGTAGCCCACGCGCCCACTCTAGTGTT
AATAGTCATCTTTGTTTGTACAGTAAGTAGGCTATCGATAAAATATCCGGACACTCTTCTGGTTGCAACT
TACAACGATGATTCGATCTGTCGGAGATACCTTACAGTATATTTCAAGCAGTGTTCGACTGTAAAGACC
TGTGAGATGCCGTCCCAAATGAAATATGTACGACTCATATTTAAATCCCAGTATTCATATATTTGATGATT
ATCTGATATTATCTGACATAGGCAGGCCCAGTATTCATAAGTAAGTAGACTGTTCCATCAGTCTCACAGC
CCAGTGAGACATGAGTGGGGTTCTTGTGAAGAAAGTTTGTTAAGCAAACCCCTTGAAGACACCCTCT
GGGAAAGCCGAATACAAAAAATATTGTGTTTTTCTCTGTCTACTAAAACAATAAATTTTGTATCAAATAT
ATTTTTATATCAAAATTAAACGGATCCTATTAGGTTTAGCAAATATGTATGTAGCAATAATAAGCTGCCGA
GGCCAGACCGCGCTTACCCACAGTAGTGACTAAAGAATCTTCCAAGTTTAGTGGTTAAAGATAAATAATA
TATATTTTGTGTTGCTATGTTTACACGCCCCCCCAAAATTACACTAAAATGTATATATAACATATAGCTGT
AGTATAGCGTAGCGATAAATTGTTGACACATCTCTTGACCTGCAAAAGTTAATTCTATTTCAATAGACGA
AGAGTTATAGCCACAGTTTATACGCTTATGAATAGATGTTGTACAAATCGTTTATTAGGTACATTATGTAC
AGTAGGTAGTTGACAGAAGTGCACTTTAATATCTGGAATAGTGTTCCTGAAATTATTTTTATAAGAAACT
TTTTGTGAGTAAATAATGTTTGCCTCAACTCTCGCTAATATATATAAGTTAGACAACTTTTATATTATATAT
TCTAATCAGGTGAGGAACCAGACATCAAAATATGTGATGAGCAAGTATAATAAAACTAAAGACCAAGT
TGGATTTTGAACTAGCTTATGAGTCCCGACTCATTCCCACTCAAAGCCTATTGAACTCCAACTTGCACT
TGGCCGATTTTCAAGTGATTAGACTTGCTCATCACCTATTTTGAGGTCTGGATCCACTCCTAAAGCCGA
TTCAATGGCACATGATAGACAAAATGAGTATGTATGAATTATGACTTATTTACTTGCTTCTATATTCTATA
CCATTATAATATTAATATATGTGTTATTGGTATATATTTTCTCTTCGGAGAGACTAGGAAAGTGGTTCTATG
GCGATTTCGAAAACCCCAAGTTTATTTCAATACTTTTTACATAGAATCATATTTTTGCATGCACAAGACT
ATCATTTAGATAAAATATACTTACTTCTTAGTTAAAACTTAAGCTATGAAATATTATAGAAACCGATCGGT
TGTTCAGTATTTTTGTTTTGTAATACTCAATATTTTTTATACTTACACGTAATCATGTTGATTTGTAAGCTC
TTTACTATTTTTTTGTTTAAAGGTGTGTTTTTCTAAATTACTCTAAGCATTAACTTAAGTAAGAATGTATT
AAGACGAGTTCTATTATTTTTGTAAAAGGATAACAATTTATAAAATCTGTTATATCGCCTACTTGGCTTCC
GGCTAACTAACTTGCATTAAAGTAAATATACTAGTACCTGAGTTAAAAGCAGATATCTAGTGAACTAGC
CGTGCCATGATGCCGCCACGGCACTCATAGTACAATACAACAGTCAGTCTGCTCTCAAATATAATAACA
TTAACTATAGCGTAAACGAAGTCGAGGTATAGCGGCGAGGGAGAATGCTATGTTTTAAATACCTACCTG
AAATATTTACTTATATAACACTAACCAAGTAATATGGCACAAGACTTCGTACGAGTTCTTACATGAACAG
TTTGAATGATGCTATCCAAAAGATTTTTACTTTCAATAAAATCCGCAATGGTAGCCCATCTTATATAAATG
CAGCTGTTACAATTTGTCTAGTAGTATTTTGTTATCTTTATCATAATGAAACATATTATATCGACTTTATAG
TTATAAAAATAAAAGTTAACATTGTAAGTAGACTGTAGTGTGTATCTTAAATTACACCTCTAGTTTGCGT
CGCCACCGTCGCGGCAAACTTCGCGTCAACAAATTATTGTTTAAAGGTTTTTTATTATTTTGTTTTGATT
CTAACAAGTTTATGTTATTGAAAGTGTAAACAGCAGTTATATTTACGCTGTATATTATTTCGGGATTAAGT
GGTTTTTTTTTTAAGTTTATAGTTAACATTTGAGATTATATTTAGATGCAATGTGTTACACAAAGCTATGT
ACCTCTTTTTGTATGCGTAATTCAACCTAGAAG
>TCONS_00109850 geneID=XLOC_021955 scaffold_359:229141-237608(-) Unclassified
GCGCACAAGTCCTGCGGAGGGCTGGAGCAGGCGCCGCCCGTGCCCCCGCCCCCCGCCAGCTACTACC
CCCCGGAGCCGTTGAGGGAACCGCCGCCGCGGCCGTCGGACGCCGTGGAGCGGGACGCCATGCAGC
TGTGGTTCGAGGCTCGCTGGGCGCGCCTCATGTCGCAGCGGCAGGACGACCGCCGGCTCGCCCGGCT
GGAACGCAGGCTTAGGCGACCGCTGTCAGCGGAGCAGCAGGCGGCGGCGGTGGCGGAGCTGGTGCA
GGTCTCGGCGCAGAGGGAGGCGCACGAGGCACTCATGTCCGCTGACAGGAGGCGATGTATGACGAGC

GGCAGTGCCTGCGAGTAGGCCGCGCCAACAGTCCGGTCTCAACCCTGTATGGAAATGCTTCAGAAACT
ACCAAACACCACGCCAGAGCTTTCGTAAGTGCCCTTCGGAAATCCGGAACCAATTTTACTTTCGCCAA
AACACAGTCATCGAGGCAACAGGAATTGATTGATATAAATAATTGTTAACTGGTGATTTAAACTTAATAT
TGCTGCTAATAATATATCATCTAGTCCTTTGGATTGTAGAGTTGTAAAGGTTGAGATGATATTAGGGTAG
GCAGGTTTGTTGTTAAACATTTTCAAGTAGGCAGTTGTTATGATGTTACATTCCTCAATATTAAAAAGGA
ATAATGTTTAAATTTAATAAAACATTCATTGAACTTATCCTCTGTTAGCTGCTCCTCTGTACAGAAATCTA
AACGGATTTATTTAGGGAAATTGTTTATGATTTTTCGCTGTCGTTTTCAAGTTACAAACGAATTGTCAGG
TGGATTTGCTTCAATCCACTCGCGATGCGATAGACTGTGTGGTCCCGTGTAGTTAAAACGGCAAGGTTA
CATTGTCACAAATTATTTTTAAGTAATTGCTCCCAGTGCGATCTGGAATGTTTTTAATTTACAAAATAGC
GAGTTTTCCCGCAATATTCTCGGTGTAGGTGCGACTGTCTCATCTATAATAAGCTAATGAGCGACATAAA
CACACTATTCTGGTTTTAGTGCTGAGTGAGCATGATTTAGTGTCTCGTTGGGAACATTCCAGACTGCGC
TCGGACTACAACGGTGTGCTAGTAATGCCAAATGTATTAGTATATTGTATTTAGACGGGCTCCTCCTGCC
CAATGACTTATTTCGTAGGCTAAAAATATACTTAACAAATCTAAGTTTAAATGTTTAAATATACCTATTAT
GTCTTTTCGCTACTTCATGTACTCAAGTGCGGGTCAAACTTGCACATAAGCTTCTTATGTAAGGTTTACT
GACATTTATATTACTATAAAACATATAGTGAACATTGACTTCTACTGGGTATCATTTAGGGTAAGACAAG
TATTAGCCTAAAATCATTATTTTTGTATGCGATGAGGAAAGAGGGTTGTAAAGATTAAATGAATTTAAAA
GTGATTGCGGTCTAGCTTTCTGCTATTTAGTTTTTATATACTGTGCGAGTAAATAGTCTTCTAAAGATTTC
TTACCGAGAGTTGAGGTGAACACTAGATTTATCACGCATTCTGAGGGTAGATTGTTACGCGTTGACTCT
AGTCGTACATATATTTATGCTACACTTGTAATATTTCTTCCCGTACAAACATGCTAAATCTAGTAGTTATTA
ACACTAGAGTAGGCGCGCTCATTTAACTCCTGGGATGGTCGCGCGGGCTACATATGTAGCCCATTTGTA
ATGCCTCATAAGCACAGATATTTGATTGAAAAGTAAAACACTTTGCTATATTTCAACTATCATATTTATTT
TTAAACTTTAAAGTAAAAAACGTATGAATATTTTCTAATTTTAACAAAGTTATTAACAAACAAAAAGAT
CCTTCTAAATATCGATATTATTTGGGACCATCGCACAAATTAGTTTTTTTTTTAGTTTAGGACACAAAAC
TTATAAACTGATCATAGTTTCCTACAATATAGGATACAATATAGTAGCAGATGGTGGAAATTTACTCCATT
TCATACCATTCCGGGACTTGTAGCTGATCAAAACTGATGATGATGGTGGTACCTTCACATCACATCATCT
TCGTCCAAATCTTCTACTTATTGTTCACTCTCCGATAGATGAGAATTATCGGAAAGAATTAACGGTTCCA
TAGGACCTTCTTCTTCGCTTTCCGTCAAGTTATCACTATTTTCCTCGTCTTCCATAAGCCATTTCCGAATT
TGACCTTCGTTCACCGCCATTATACCTAAAATGATTCAAATGTTTTTTTTTGTGTTTTTAGTCCAAAATA
GAACTAACAAAGTAAAAAAAACAATGAAGTAATATGATTATACATTTCATATTAACGTAAATGTGCACA
AAAAATATTTTATCTAAGAAATAAAACAGAAAATCAGAAAAAAAACACTTACCTAGAACGGTCGCGCG
GGCTACATACGTAGCCCAGCAACTTTAGCCGATGTGCTCCTCAGGTTCCCGTGTGCCGGGGCAGTGGG
CACGCTGCTGGTGGACGCGGGGGTTCATAAGGAAGGAATACATGGGGGGAGGGAGCGCTAGTGCCGT
GCGTTCCAGATATTTACATCAAAATGATACCCTGGTCTACACCTGTAGCCCACGCGCCCACTCTAGTGTT
AATAGTCATCTTTGTTTGTACAGTAAGTAGGCTATCGATAAAATATCCGGACACTCTTCTGGTTGCAACT
TACAACGATGATTCGATCTGTCGGAGATACCTTACAGTATATTTCAAGCAGTGTTCGACTGTAAAGACC
TGTGAGATGCCGTCCCAAATGAAATATGTACGACTCATATTTAAATCCCAGTATTCATATATTTGATGATT
ATCTGATATTATCTGACATAGGCAGGCCCAGTATTCATAAGTAAGTAGACTGTTCCATCAGTCTCACAGC
CCAGTGAGACATGAGTGGGGTTCTTGTGAAGAAAGTTTGTTAAGCAAACCCCTTGAAGACACCCTCT
GGGAAAGCCGAATACAAAAAATATTGTGTTTTTCTCTGTCTACTAAAACAATAAATTTTGTATCAAATAT
ATTTTTATATCAAAATTAAACGGATCCTATTAGGTTTAGCAAATATGTATGTAGCAATAATAAGCTGCCGA
GGCCAGACCGCGCTTACCACAGTAGTGACTAAAGAATCTTCCAAGTTTAGTGGTTAAAGATAAATAATA
TATATTTTGTGTTGCTATGTTTACACGCCCCCCCAAAATTACACTAAAATGTATATATAACATATAGCTGT
AGTATAGCGTAGCGATAAATTGTTGACACATCTCTTGACCTGCAAAAGTTAATTCTATTTCAATAGACGA
AGAGTTATAGCCACAGTTTATACGCTTATGAATAGATGTTGTACAAATCGTTTATTAGGTACATTATGTAC

AGTAGGTAGTTGACAGAAGTGCACTTTAATATCTGGAATAGTGTTCCTGAAATTATTTTTATAAGAAACT
TTTTGTGAGTAAATAATGTTTGCCTCAACTCTCGCTAATATATATAAGTTAGACAACTTTTATATTATATAT
TCTAATCAGGTGAGGAACCAGACATCAAAATATGTGATGAGCAAGTATAATAAAACTAAAGACCAAGT
TGGATTTTGAACTAGCTTATGAGTCCCGACTCATTCCCACTCAAAGCCTATTGAACTCCAACTTGCACT
TGGCCGATTTTCAAGTGATTAGACTTGCTCATCACCTATTTTGAGGTCTGGATCCACTCCTAAAGCCGA
TTCAATGGCACATGATAGACAAAATGAGTATGTATGAATTATGACTTATTTACTTGCTTCTATATTCTATA
CCATTATAATATTAATATATGTGTTATTGGTATATATTTTCTCTTCGGAGAGACTAGGAAAGTGGTTCTATG
GCGATTTCGAAAACCCCAAGTTTATTTCAATACTTTTTACATAGAATCATATTTTTGCATGCACAAGACT
ATCATTTAGATAAAATATACTTACTTCTTAGTTAAAACTTAAGCTATGAAATATTATAGAAACCGATCGGT
TGTTCAGTATTTTTGTTTTGTAATACTCAATATTTTTTATACTTACACGTAATCATGTTGATTTGTAAGCTC
TTTACTATTTTTTTGTTTAAAGGTGTGTTTTTCTAAATTACTCTAAGCATTAACTTAAGTAAGAATGTATT
AAGACGAGTTCTATTATTTTTGTAAAAGGATAACAATTTATAAAATCTGTTATATCGCCTACTTGGCTTCC
GGCTAACTAACTTGCATTAAAGTAAATATACTAGTACCTGAGTTAAAAGCAGATATCTAGTGAACTAGC
CGTGCCATGATGCCGCCACGGCACTCATAGTACAATACAACAGTCAGTCTGCTCTCAAATATAATAACA
TTAACTATAGCGTAAACGAAGTCGAGGTATAGCGGCGAGGGAGAATGCTATGTTTTAAATACCTACCTG
AAATATTTACTTATATAACACTAACCAAGTAATATGGCACAAGACTTCGTACGAGTTCTTACATGAACAG
TTTGAATGATGCTATCCAAAAGATTTTTACTTTCAATAAAATCCGCAATGGTAGCCCATCTTATATAAATG
CAGCTGTTACAATTTGTCTAGTAGTATTTTGTTATCTTTATCATAATGAAACATATTATATCGACTTTATAG
TTATAAAAATAAAAGTTAACATTGTAAGTAGACTGTAGTGTGTATCTTAAATTACACCTCTAGTTTGCGT
CGCCACCGTCGCGGCAAACTTCGCGTCAACAAATTATTGTTTAAAGGTTTTTTATTATTTTGTTTTGATT
CTAACAAGTTTATGTTATTGAAAGTGTAAACAGCAGTTATATTTACGCTGTATATTATTTCGGGATTAAGT
GGTTTTTTTTTTAAGTTTATAGTTAACATTTGAGATTATATTTAGATGCAATGTGTTACACAAAGCTATGT
ACCTCTTTTTGTATGCGTAATTCAACCTAGAAG
>TCONS_00109817 geneID=XLOC_021955 scaffold_359:229179-237615(-) Unclassified
GCCGACGGCGCACAAGTCCTGCGGAGGGCTGGAGCAGGCGCCGCCCGTGCCCCCGCCCCCCGCCAG
CTACTACCCCCCGGAGCCGTTGAGGGAACCGGAGCAGCAGGCGGCGGCGGTGGCGGAGCTGGTGCA
GGTCTCGGCGCAGAGGGAGGCGCACGAGGCACTCATGTCCGCTGACAGGAGGCGATGTATGACGAGC
GGCAGTGCCTGCGAGTAGGCCGCGCCAACAGTCCGGTCTCAACCCTGTATGGAAATGCTTCAGAAACT
ACCAAACACCACGCCAGAGCTTTCGTAAGTGCCCTTCGGAAATCCGGAACCAATTTTACTTTCGCCAA
AACACAGTCATCGAGGCAACAGGAATTGATTGATATAAATAATTGTTAACTGGTGATTTAAACTTAATAT
TGCTGCTAATAATATATCATCTAGTCCTTTGGATTGTAGAGTTGTAAAGGTTGAGATGATATTAGGGTAG
GCAGGTTTGTTGTTAAACATTTTCAAGTAGGCAGTTGTTATGATGTTACATTCCTCAATATTAAAAAGGA
ATAATGTTTAAATTTAATAAAACATTCATTGAACTTATCCTCTGTTAGCTGCTCCTCTGTACAGAAATCTA
AACGGATTTATTTAGGGAAATTGTTTATGATTTTTCGCTGTCGTTTTCAAGTTACAAACGAATTGTCAGG
TGGATTTGCTTCAATCCACTCGCGATGCGATAGACTGTGTGGTCCCGTGTAGTTAAAACGGCAAGGTTA
CATTGTCACAAATTATTTTTAAGTAATTGCTCCCAGTGCGATCTGGAATGTTTTTAATTTACAAAATAGC
GAGTTTTCCCGCAATATTCTCGGTGTAGGTGCGACTGTCTCATCTATAATAAGCTAATGAGCGACATAAA
CACACTATTCTGGTTTTAGTGCTGAGTGAGCATGATTTAGTGTCTCGTTGGGAACATTCCAGACTGCGC
TCGGACTACAACGGTGTGCTAGTAATGCCAAATGTATTAGTATATTGTATTTAGACGGGCTCCTCCTGCC
CAATGACTTATTTCGTAGGCTAAAAATATACTTAACAAATCTAAGTTTAAATGTTTAAATATACCTATTAT
GTCTTTTCGCTACTTCATGTACTCAAGTGCGGGTCAAACTTGCACATAAGCTTCTTATGTAAGGTTTACT
GACATTTATATTACTATAAAACATATAGTGAACATTGACTTCTACTGGGTATCATTTAGGGTAAGACAAG
TATTAGCCTAAAATCATTATTTTTGTATGCGATGAGGAAAGAGGGTTGTAAAGATTAAATGAATTTAAAA
GTGATTGCGGTCTAGCTTTCTGCTATTTAGTTTTTATATACTGTGCGAGTAAATAGTCTTCTAAAGATTTC

TTACCGAGAGTTGAGGTGAACACTAGATTTATCACGCATTCTGAGGGTAGATTGTTACGCGTTGACTCT
AGTCGTACATATATTTATGCTACACTTGTAATATTTCTTCCCGTACAAACATGCTAAATCTAGTAGTTATTA
ACACTAGAGTAGGCGCGCTCATTTAACTCCTGGGATGGTCGCGCGGGCTACATATGTAGCCCATTTGTA
ATGCCTCATAAGCACAGATATTTGATTGAAAAGTAAAACACTTTGCTATATTTCAACTATCATATTTATTT
TTAAACTTTAAAGTAAAAAACGTATGAATATTTTCTAATTTTAACAAAGTTATTAACAAACAAAAAGAT
CCTTCTAAATATCGATATTATTTGGGACCATCGCACAAATTAGTTTTTTTTTTAGTTTAGGACACAAAAC
TTATAAACTGATCATAGTTTCCTACAATATAGGATACAATATAGTAGCAGATGGTGGAAATTTACTCCATT
TCATACCATTCCGGGACTTGTAGCTGATCAAAACTGATGATGATGGTGGTACCTTCACATCACATCATCT
TCGTCCAAATCTTCTACTTATTGTTCACTCTCCGATAGATGAGAATTATCGGAAAGAATTAACGGTTCCA
TAGGACCTTCTTCTTCGCTTTCCGTCAAGTTATCACTATTTTCCTCGTCTTCCATAAGCCATTTCCGAATT
TGACCTTCGTTCACCGCCATTATACCTAAAATGATTCAAATGTTTTTTTTTGTGTTTTTAGTCCAAAATA
GAACTAACAAAGTAAAAAAAACAATGAAGTAATATGATTATACATTTCATATTAACGTAAATGTGCACA
AAAAATATTTTATCTAAGAAATAAAACAGAAAATCAGAAAAAAAACACTTACCTAGAACGGTCGCGCG
GGCTACATACGTAGCCCAGCAACTTTAGCCGATGTGCTCCTCAGGTTCCCGTGTGCCGGGGCAGTGGG
CACGCTGCTGGTGGACGCGGGGGTTCATAAGGAAGGAATACATGGGGGGAGGGAGCGCTAGTGCCGT
GCGTTCCAGATATTTACATCAAAATGATACCCTGGTCTACACCTGTAGCCCACGCGCCCACTCTAGTGTT
AATAGTCATCTTTGTTTGTACAGTAAGTAGGCTATCGATAAAATATCCGGACACTCTTCTGGTTGCAACT
TACAACGATGATTCGATCTGTCGGAGATACCTTACAGTATATTTCAAGCAGTGTTCGACTGTAAAGACC
TGTGAGATGCCGTCCCAAATGAAATATGTACGACTCATATTTAAATCCCAGTATTCATATATTTGATGATT
ATCTGATATTATCTGACATAGGCAGGCCCAGTATTCATAAGTAAGTAGACTGTTCCATCAGTCTCACAGC
CCAGTGAGACATGAGTGGGGTTCTTGTGAAGAAAGTTTGTTAAGCAAACCCCTTGAAGACACCCTCT
GGGAAAGCCGAATACAAAAAATATTGTGTTTTTCTCTGTCTACTAAAACAATAAATTTTGTATCAAATAT
ATTTTTATATCAAAATTAAACGGATCCTATTAGGTTTAGCAAATATGTATGTAGCAATAATAAGCTGCCGA
GGCCAGACCGCGCTTACCACAGTAGTGACTAAAGAATCTTCCAAGTTTAGTGGTTAAAGATAAATAATA
TATATTTTGTGTTGCTATGTTTACACGCCCCCCCAAAATTACACTAAAATGTATATATAACATATAGCTGT
AGTATAGCGTAGCGATAAATTGTTGACACATCTCTTGACCTGCAAAAGTTAATTCTATTTCAATAGACGA
AGAGTTATAGCCACAGTTTATACGCTTATGAATAGATGTTGTACAAATCGTTTATTAGGTACATTATGTAC
AGTAGGTAGTTGACAGAAGTGCACTTTAATATCTGGAATAGTGTTCCTGAAATTATTTTTATAAGAAACT
TTTTGTGAGTAAATAATGTTTGCCTCAACTCTCGCTAATATATATAAGTTAGACAACTTTTATATTATATAT
TCTAATCAGGTGAGGAACCAGACATCAAAATATGTGATGAGCAAGTATAATAAAACTAAAGACCAAGT
TGGATTTTGAACTAGCTTATGAGTCCCGACTCATTCCCACTCAAAGCCTATTGAACTCCAACTTGCACT
TGGCCGATTTTCAAGTGATTAGACTTGCTCATCACCTATTTTGAGGTCTGGATCCACTCCTAAAGCCGA
TTCAATGGCACATGATAGACAAAATGAGTATGTATGAATTATGACTTATTTACTTGCTTCTATATTCTATA
CCATTATAATATTAATATATGTGTTATTGGTATATATTTTCTCTTCGGAGAGACTAGGAAAGTGGTTCTATG
GCGATTTCGAAAACCCCAAGTTTATTTCAATACTTTTTACATAGAATCATATTTTTGCATGCACAAGACT
ATCATTTAGATAAAATATACTTACTTCTTAGTTAAAACTTAAGCTATGAAATATTATAGAAACCGATCGGT
TGTTCAGTATTTTTGTTTTGTAATACTCAATATTTTTTATACTTACACGTAATCATGTTGATTTGTAAGCTC
TTTACTATTTTTTTGTTTAAAGGTGTGTTTTTCTAAATTACTCTAAGCATTAACTTAAGTAAGAATGTATT
AAGACGAGTTCTATTATTTTTGTAAAAGGATAACAATTTATAAAATCTGTTATATCGCCTACTTGGCTTCC
GGCTAACTAACTTGCATTAAAGTAAATATACTAGTACCTGAGTTAAAAGCAGATATCTAGTGAACTAGC
CGTGCCATGATGCCGCCACGGCACTCATAGTACAATACAACAGTCAGTCTGCTCTCAAATATAATAACA
TTAACTATAGCGTAAACGAAGTCGAGGTATAGCGGCGAGGGAGAATGCTATGTTTTAAATACCTACCTG
AAATATTTACTTATATAACACTAACCAAGTAATATGGCACAAGACTTCGTACGAGTTCTTACATGAACAG
TTTGAATGATGCTATCCAAAAGATTTTTACTTTCAATAAAATCCGCAATGGTAGCCCATCTTATATAAATG

CAGCTGTTACAATTTGTCTAGTAGTATTTTGTTATCTTTATCATAATGAAACATATTATATCGACTTTATAG
TTATAAAAATAAAAGTTAACATTGTAAGTAGACTGTAGTGTGTATCTTAAATTACACCTCTAGTTTGCGT
CGCCACCGTCGCGGCAAACTTCGCGTCAACAAATTATTGTTTAAAGGTTTTTTATTATTTTGTTTTGATT
CTAACAAGTTTATGTTATTGAAAGTGTAAACAGCAGTTATATTTACGCTGTATATTATTTCGGGATTAAGT
GGTTTTTTTTTTAAGTTTATAGTTAACATTTGAGATTATATTTAGATGCAATGTGTTACACAAAGC
>TCONS_00109856 geneID=XLOC_021955 scaffold_359:232389-240856(-) Intergenic
TTTTCAGTGGGTTTCCGAGCCAGACCTGTCACAAAACCAGGAGCCCCGCCCCGCCTCGTCCAGACCG
CCGACGGCGCACAAGTCCTGCGGAGGGCTGGAGCAGGCGCCGCCCGTGCCCCCGCCCCCCGCCAGCT
ACTACCCCCCGGAGCCGTTGAGGGAACCGGAGCAGCAGGCGGCGGCGGTGGCGGAGCTGGTGCAGG
TCTCGGCGCAGAGGGAGGCGCACGAGGCACTCATGTCCGCTGACAGGAGGCGATGTATGACGAGCGG
CAGTGCCTGCGAGTAGGCCGCGCCAACAGTCCGGTCTCAACCCTGTATGGAAATGCTTCAGAAACTAC
CAAACACCACGCCAGAGCTTTCGTAAGTGCCCTTCGGAAATCCGGAACCAATTTTACTTTCGCCAAAA
CACAGTCATCGAGGCAACAGGAATTGATTGATATAAATAATTGTTAACTGGTGATTTAAACTTAATATTG
CTGCTAATAATATATCATCTAGTCCTTTGGATTGTAGAGTTGTAAAGGTTGAGATGATATTAGGGTAGGC
AGGTTTGTTGTTAAACATTTTCAAGTAGGCAGTTGTTATGATGTTACATTCCTCAATATTAAAAAGGAAT
AATGTTTAAATTTAATAAAACATTCATTGAACTTATCCTCTGTTAGCTGCTCCTCTGTACAGAAATCTAA
ACGGATTTATTTAGGGAAATTGTTTATGATTTTTCGCTGTCGTTTTCAAGTTACAAACGAATTGTCAGGT
GGATTTGCTTCAATCCACTCGCGATGCGATAGACTGTGTGGTCCCGTGTAGTTAAAACGGCAAGGTTAC
ATTGTCACAAATTATTTTTAAGTAATTGCTCCCAGTGCGATCTGGAATGTTTTTAATTTACAAAATAGCG
AGTTTTCCCGCAATATTCTCGGTGTAGGTGCGACTGTCTCATCTATAATAAGCTAATGAGCGACATAAAC
ACACTATTCTGGTTTTAGTGCTGAGTGAGCATGATTTAGTGTCTCGTTGGGAACATTCCAGACTGCGCT
CGGACTACAACGGTGTGCTAGTAATGCCAAATGTATTAGTATATTGTATTTAGACGGGCTCCTCCTGCCC
AATGACTTATTTCGTAGGCTAAAAATATACTTAACAAATCTAAGTTTAAATGTTTAAATATACCTATTATG
TCTTTTCGCTACTTCATGTACTCAAGTGCGGGTCAAACTTGCACATAAGCTTCTTATGTAAGGTTTACTG
ACATTTATATTACTATAAAACATATAGTGAACATTGACTTCTACTGGGTATCATTTAGGGTAAGACAAGTA
TTAGCCTAAAATCATTATTTTTGTATGCGATGAGGAAAGAGGGTTGTAAAGATTAAATGAATTTAAAAG
TGATTGCGGTCTAGCTTTCTGCTATTTAGTTTTTATATACTGTGCGAGTAAATAGTCTTCTAAAGATTTCT
TACCGAGAGTTGAGGTGAACACTAGATTTATCACGCATTCTGAGGGTAGATTGTTACGCGTTGACTCTA
GTCGTACATATATTTATGCTACACTTGTAATATTTCTTCCCGTACAAACATGCTAAATCTAGTAGTTATTAA
CACTAGAGTAGGCGCGCTCATTTAACTCCTGGGATGGTCGCGCGGGCTACATATGTAGCCCATTTGTAA
TGCCT
>TCONS_00109889 geneID=XLOC_021955 scaffold_359:236888-240963(-) Intergenic
CCGAGGGAGCAAGGAAATTGGTGTAAAAACAGCTCTGCTGCTCTGTAAATAAGATTGAATTTTTCAAA
ATTGTGTGGGGAAACGATCGTTTGATTAACACTAGTTCATTTTCAGTGGGTTTCCGAGCCAGACCTGTC
ACAAAACCAGGAGCCCCGCCCCGCCTCGTCCAGACCGCCGACGGCGCACAAGTCCTGCGGAGGGCT
GGAGCAGGCGCCGCCCGTGCCCCCGCCCCCCGCCAGCTACTACCCCCCGGAGCCGTTGAGGGAACGT
AAGTGTCTAGAGAGTGAATGATTTGAGAAGTGAATGAATGAATGAATGAGTTGTTGAAGGTTGATTGA
ATGATTGAGAGGAATGAATAATTGAGGATGAATGAATGATTTGAGGATCAATTAATGATTGTACGAATG
TTTATACTGTTTATGGTTATCTGGACATCAAACTGTTGGTAGTAGGATAGGTACTCTTACATTCATGATGT
AAGGGTTTACGATAAGACGATAGAAACTGAAAAACAAATATTTTATTCGCTATTGTAATAAATAATCATT
GAAAGATTTCATGATCAAATCAAGTATCATAAATGTTGTAACTGGATTTCATCAAAGTATAAGTATGATA
ATTAGCATGTTTGTTGTCGTAAATAAATATCACAATTAGCTGCTATCCTAGCCTCTGCCAGTTATGACCG
CAAAGGCTTCTGATCTTACGTTGAAACAACCAATATAGTTGAAACTGTTACTCCTGACCCGCAATTATG
TAATTCATATTGATTGTTTGAACATTCCCCCGTTCATGAAAAAGACAATGAGTAAAAATATACTGAATAA

TTCTGGTTTCAGAAAGTAAAACAAGATTTTTTACCCATTGTCATTTGCTTACTGTGGCTTGCGATTCCC
CCGT
>TCONS_00109843 geneID=XLOC_021956 scaffold_359:251068-254367(-) Splice junction overlap
CACGAGACTGAGAATGTGTTCGATCAGATGAAGCAGAACATTCAGAAGGAGAAGCACGCCCGGACCG
CACCGGACCAGTCTCTCTACATGTATGCAACTGCCGTGGAGTCGGACAAATCTCACTCGAGCTACAGG
AAGAAGCGCCGCGGACAGAGGGCGCCACAAGAGGAGGGTCGTATAGTGGCGGGCGGCGGGCGCAGC
GCCGGCGGCGGCGGCGCGAGGGTGGCGAGACTCGCGGCCGAACTGGC
>TCONS_00109808 geneID=XLOC_021949 scaffold_359:312085-313157(+) Unclassified
GGAAGTTTTAGATCACAGTGATGTTAAAATCATGGAGGAGCAGCTAGCGAACGCGATAGAAAGTATAG
TTAATTGTCCATCTTGTGCGTTGGGCACAAATGCGATAGTTTGGAGCACGGATGTTTGAATCAGCCATT
CGTTTTATTTAATGTGTTTTAACCACCACCGACCTCACCCTGGTGCCTTGTTCTAGTGTAGCCAGTTTAT
AATAACGACTGTAAATATACGATTAATGTGTAAATGATACTCTTAATACCTACGTAGTAAGATCAAATATC
GAACAACTTGTAACGTTCTTTATAATCTAAGCGTACCGTTTTGTTTTGAATGTATTATCTATCAATTTTCC
GTGCCAATAATGTTTTACTTAATAGATTTGTTGTAAATAATTGACTTTTTCGATAAATTATAACGATTTTG
ATTAATTTTTAGAATTATTTCTAATCGACATTCACAAATTAGCGATGGTATGTTTATTTTCATTCTTGTCTA
TTTCGATGGCGTAATTTAACGATAAA
>TCONS_00109965 geneID=XLOC_021992 scaffold_36:446857-475579(+) Exonic overlap (+)
GTCAACCCACCAAGCGAGTTTGGCATAACTCAGTTATGCGCAGACGCCCGTGCGGGTAAGACGTATGT
CAGCCCACTCCGTTTTAAATTCAAAAATTCGAACGCGCGTTCCAAAAGTGCTCTGTGAACTTGACGTG
TTAAAAAAATCAGTTAATCGGACAGTGACACATGCCAACTACACGAGGATAAATGCGATTTGAGCTGG
ACCAGACGGAATCTTTAAAGGTGTCGACCGGCTCGATGACCTGACGATGTTCAGGACGGGGTCAGCC
GAGGGC
>TCONS_00110249 geneID=XLOC_022027 scaffold_36:884536-888956(-) Splice junction overlap
TACAGGGTAAAGAAACCTCATGAGTTCAATCGTCCGAAGCTGCCGGCATCAAAAAGGAGTCTACAGCT
ATCAAATACTCTTTCGAAAAAGCCAGCAATAGGATCTTCTTCAATGACAACCAGAAGGAGACGAGGCA
TCCTAAAAAACAAGCCCACAATAGTCAAAGGCAAGAAGATGACCCGACTGGAGTGACGTCACAACCC
TAGCCGTGCGGACACCTAGCCGTTTAAGATGTTACTATAGGTGTAGCGCACTCAAGGGCTGCCTTAGGC
AGGAGAGCGGACCGGGACGGGACTTATGACGCAATAATATTGGAGCTGTGAATAGGTTTGTGTGCAGT
TATAGGAAGAGGTCGCGCCGTCCCCACCGCCCCACTGCTTAAGGTATCTTACGAGACACAGATTGATTT
ACACAATGCATTTATAAGCTACTTAGAACAATATGGGTTAGTGTGCGGACAGTTTTAACTTGTATGTGCA
TTGTGTATCTGTTTATTACTTGCGCCGTATAGCTTGAGAAGTCGATAATCTGTATTTAAATTGGCTGTCAG
GAAAATATTGTATTCTGCCAAAAATGTCGAAAATCTTTGTAAAACAAGTAATAGAAAAAAGATATAACG
ACTCTTTCAAGCTATGCGGTGCTAGCGCCAGCTGGCATGAGCGATTATAACATATTTATAAATAACGGCT
ATGTAAAAACGAATGAAATTGAATTCTCGAGAAAGCTATTTAAAAAGCAAATAATCGTTCGTGCCAAC
TGGATTTAGATTCAAACTAATTGATACAAGATAATATTTCGTCGTATTAAGTCTTGTTCAAAACCGTGAT
CCTATTATATCTTGTATCAATATATTTGGATGATTTTTAGTTATCAATCAGTGGTAACATTGCAATGATTTG
TGAGGTATAGTGTATAATATATTATGTATCGTATGTATCTATTTATTTTCCTTGGTGTACCTATTCATTCTTA
CCCGGTGTGTATTTCCTGTAAGTACTTGTACGCTAGTAACCTTACCTTAAAGATTTAAAATTTTTACTCA
GTGATTTATTCTATACTAGAGTGTTGTTGTGTAAACTTAGAATTTTATCAGAGGTCATTTGAAATTGCATT
TATTCCGGAGCACAAATGTTCAAATTATCAGTGTGATTGATGATGTGTTTATTTAGACTTACGGAGTGTG
CCGTTGGTATGGGGCGTCATGGGAAAGGGCTATTTTTATACGCATACAAATTTTATTAACAGTTGATGAA
TTTGTATAGCTCTGCGGTAGAGGATGTAGGTTACTATTTGTAAATCACTAGTTCTGCATGTGTTAGAAAG
TGCTAGGTATGATCATTGCGTTCCCTGCTAAAGTTGGGAATAGCAATAATTAGTTCATTCCTATAATACGT
TTATCTACAATTCAGCAAACTTACCGAAGTTCAATTTATCTGAGCTGTGTTTGCATTTTGGAGTCCTTAA

TGAGCGACATATCCATAGACGTGACCTCCGCCGCGCCCGCCCGCCTTGCATTCTTACCACTGACAATAA
CCTGACGAACGACTGACACCAATTATAATGTATTGCGTATTTAGTCCAGTAAAATATAGTTTGTTGAAAT
TTCCCTTTTAGTTGATGTTTTGTCTCTGCGATGTCATTATCAAGGGAGTGTAGGAAACAGGCTGCCAAC
TGTTTTAAGATCCCGTTCTTTTAGATTCAAAATGAAATGACAATACACTGGTTTGATTGCACACTGGAG
TGGTCTGTGCTGCTACGGCCTTTTCTATGACATCGGTTTTAAAGAGAATTTTGTAATTTAACGTCCATTT
TAATATTTAATAAGTAGTTTAAAATAATATGTCTAGCAATATTGGTCAAGCAAGTGTTTACTTTGCACGAA
TGGGATGGAGAAGGGAGATAATTAATTTTGTTTGATGGAAATATTGTGGTTTGATTTTATAAGAGACTGT
TTCGCCCCAAATGCTTATTTCTTATCACAATTTTTGTCACACTGTTATTTTCATACGTGTTGAATGGAGA
GTATGGTTACTCTAAAATGTGTTCTAAACAGTGATTTTTTATCTAAAATGGACGATTGTATAAACAACCG
GTGTGGAAGTCCATTTGAAATGTAATTTATTGTATTGTTGCACTGCACTTTGTTTTTCATATTACTTGACA
GAGTTTAATTAATCTGTAGATTATGCATTGTGAGTTGACAATGTTCTAAATAAACAATTGAAGAGTAATT
TAATGTCTGTTTTTGTTATACCTACATTTTCATGCAGCGTTAGGACTTTTTGAGTGACGTAACACAGTCG
ACCGTCATGATCATGTTTGAACGTCGCCGATTTACGCACCTCTTACAAAACTTTGTAAGTTCTATACGG
CAGAAAGTAATACTTAAAATAGCAGGGCCGTACATACTAGAGAGGAAAACTAATAATGCAAAACGATT
TGACAAAACCTTCACTATATATTTATTAATAAAGACAATCTAAAAGATCACGTAATAGTCATTGTCTTAA
AATTAGTTAGCCTAATAAATTTATCAATATATATATAATAGATTATTTATTTAATTACTTATTGTTTCAAGGA
TATGCGAAATTTGTGAAGTCGTCGGAAAAGAACGGCTTCCTAATCGTTGTTCACACGAGCTGTCACAC
TGACACTGAATATAACAATGAAACTAATTTATATTACCGTTTACACTTAACACCTCCAAATTATGTCTAAT
TCATGTCTACGAAGAATGGTGCTAATCTAAAAAGGAGTCTACAAATTTGTCAAAGACTGGTATTTTACA
GAACATTTAAAATCACAACACAGAGTGTTATATTCATTATCGTGCCATACGCACGAGTATTACATCTATG
TCATGTTACAAATACGTATTTAATTAAGTACATCGAACGTGCAGCGCCGCCTAGCGGCGACCCACGGGA
CTAGGCTACAGTTGGACTCTGGAAACAGCAATTCTATTATATAATAGTCCACCGTTCCATTATCTGCAGG
TACTTATCCATTTAGGAATCTCATAGAAATATAGTGGATGCAGTAATGCCACCTTGCAATGCAGTACATT
ACAAGACGTGACGCAAGATTCTTTCTTGAGGTATAATTATGGAAGTTCCTACATGGATCTGAAAAGTGC
GTTTTACATACATATTCTAGAATCCAATACAAAATTAGAAGAATATATATTATTATAATCCATCCATTTACT
TATAGACTTAGACATAAGTATTAATAATACTTTACCATAATATTCAATTTAAGCCGCTGTGACACAATTTG
TGTTATGTACATAAAGTGCTCAATAGTATATAACAAAGTAAGACAGAATACAACTATTAACTTGATAACA
TTTAAGTAGATAAGTAAAGAAATACAGAATAATAATATTGTAATTAAATGCTACGAGTGGCTACGGGCTA
CGAAATTTCGTAGCGCTACAAGCTACGTCGTAGCGAGTCGTAGCGAAGCCGCTACGAACCGGCGGCGT
AGGCTCTAAGATTACAGGAAATTTCGATGAGTGATTAATTTAGGCATTCATGGCTTTAAAAATATTCTTA
GTATAAAGTATAACTAGGTTTGTTACGTCATCAGATGAAATAATGAAATATTTTCGTCTAATTATTTTTTTA
TTTAAATAGAATTGACTTTCAGTTATTATCATTTCATGATCATTAAAATGGTTATTGCGAGGAGTATCTGG
ATATTTGAAACTCTAAATTTTAAGAATGTAAG
>TCONS_00110366 geneID=XLOC_022027 scaffold_36:884536-888956(-) Unclassified
TACAGGGTAAAGAAACCTCATGAGTTCAATCGTCCGAAGCTGCCGGCATCAAAAAGGAGTCTACAGCT
ATCAAATACTCTTTCGAAAAAGACAACCAGAAGGAGACGAGGCATCCTAAAAAACAAGCCCACAATA
GTCAAAGGCAAGAAGATGACCCGACTGGAGTGACGTCACAACCCTAGCCGTGCGGACACCTAGCCGT
TTAAGATGTTACTATAGGTGTAGCGCACTCAAGGGCTGCCTTAGGCAGGAGAGCGGACCGGGACGGG
ACTTATGACGCAATAATATTGGAGCTGTGAATAGGTTTGTGTGCAGTTATAGGAAGAGGTCGCGCCGTC
CCCACCGCCCCACTGCTTAAGGTATCTTACGAGACACAGATTGATTTACACAATGCATTTATAAGCTACT
TAGAACAATATGGGTTAGTGTGCGGACAGTTTTAACTTGTATGTGCATTGTGTATCTGTTTATTACTTGC
GCCGTATAGCTTGAGAAGTCGATAATCTGTATTTAAATTGGCTGTCAGGAAAATATTGTATTCTGCCAAA
AATGTCGAAAATCTTTGTAAAACAAGTAATAGAAAAAAGATATAACGACTCTTTCAAGCTATGCGGTGC
TAGCGCCAGCTGGCATGAGCGATTATAACATATTTATAAATAACGGCTATGTAAAAACGAATGAAATTG

AATTCTCGAGAAAGCTATTTAAAAAGCAAATAATCGTTCGTGCCAACTGGATTTAGATTCAAACTAATT
GATACAAGATAATATTTCGTCGTATTAAGTCTTGTTCAAAACCGTGATCCTATTATATCTTGTATCAATATA
TTTGGATGATTTTTAGTTATCAATCAGTGGTAACATTGCAATGATTTGTGAGGTATAGTGTATAATATATTA
TGTATCGTATGTATCTATTTATTTTCCTTGGTGTACCTATTCATTCTTACCCGGTGTGTATTTCCTGTAAGT
ACTTGTACGCTAGTAACCTTACCTTAAAGATTTAAAATTTTTACTCAGTGATTTATTCTATACTAGAGTGT
TGTTGTGTAAACTTAGAATTTTATCAGAGGTCATTTGAAATTGCATTTATTCCGGAGCACAAATGTTCAA
ATTATCAGTGTGATTGATGATGTGTTTATTTAGACTTACGGAGTGTGCCGTTGGTATGGGGCGTCATGGG
AAAGGGCTATTTTTATACGCATACAAATTTTATTAACAGTTGATGAATTTGTATAGCTCTGCGGTAGAGG
ATGTAGGTTACTATTTGTAAATCACTAGTTCTGCATGTGTTAGAAAGTGCTAGGTATGATCATTGCGTTC
CCTGCTAAAGTTGGGAATAGCAATAATTAGTTCATTCCTATAATACGTTTATCTACAATTCAGCAAACTT
ACCGAAGTTCAATTTATCTGAGCTGTGTTTGCATTTTGGAGTCCTTAATGAGCGACATATCCATAGACGT
GACCTCCGCCGCGCCCGCCCGCCTTGCATTCTTACCACTGACAATAACCTGACGAACGACTGACACCA
ATTATAATGTATTGCGTATTTAGTCCAGTAAAATATAGTTTGTTGAAATTTCCCTTTTAGTTGATGTTTTGT
CTCTGCGATGTCATTATCAAGGGAGTGTAGGAAACAGGCTGCCAACTGTTTTAAGATCCCGTTCTTTTA
GATTCAAAATGAAATGACAATACACTGGTTTGATTGCACACTGGAGTGGTCTGTGCTGCTACGGCCTTT
TCTATGACATCGGTTTTAAAGAGAATTTTGTAATTTAACGTCCATTTTAATATTTAATAAGTAGTTTAAAA
TAATATGTCTAGCAATATTGGTCAAGCAAGTGTTTACTTTGCACGAATGGGATGGAGAAGGGAGATAAT
TAATTTTGTTTGATGGAAATATTGTGGTTTGATTTTATAAGAGACTGTTTCGCCCCAAATGCTTATTTCTT
ATCACAATTTTTGTCACACTGTTATTTTCATACGTGTTGAATGGAGAGTATGGTTACTCTAAAATGTGTT
CTAAACAGTGATTTTTTATCTAAAATGGACGATTGTATAAACAACCGGTGTGGAAGTCCATTTGAAATG
TAATTTATTGTATTGTTGCACTGCACTTTGTTTTTCATATTACTTGACAGAGTTTAATTAATCTGTAGATTA
TGCATTGTGAGTTGACAATGTTCTAAATAAACAATTGAAGAGTAATTTAATGTCTGTTTTTGTTATACCT
ACATTTTCATGCAGCGTTAGGACTTTTTGAGTGACGTAACACAGTCGACCGTCATGATCATGTTTGAAC
GTCGCCGATTTACGCACCTCTTACAAAACTTTGTAAGTTCTATACGGCAGAAAGTAATACTTAAAATAG
CAGGGCCGTACATACTAGAGAGGAAAACTAATAATGCAAAACGATTTGACAAAACCTTCACTATATATT
TATTAATAAAGACAATCTAAAAGATCACGTAATAGTCATTGTCTTAAAATTAGTTAGCCTAATAAATTTAT
CAATATATATATAATAGATTATTTATTTAATTACTTATTGTTTCAAGGATATGCGAAATTTGTGAAGTCGTC
GGAAAAGAACGGCTTCCTAATCGTTGTTCACACGAGCTGTCACACTGACACTGAATATAACAATGAAA
CTAATTTATATTACCGTTTACACTTAACACCTCCAAATTATGTCTAATTCATGTCTACGAAGAATGGTGCT
AATCTAAAAAGGAGTCTACAAATTTGTCAAAGACTGGTATTTTACAGAACATTTAAAATCACAACACA
GAGTGTTATATTCATTATCGTGCCATACGCACGAGTATTACATCTATGTCATGTTACAAATACGTATTTAAT
TAAGTACATCGAACGTGCAGCGCCGCCTAGCGGCGACCCACGGGACTAGGCTACAGTTGGACTCTGG
AAACAGCAATTCTATTATATAATAGTCCACCGTTCCATTATCTGCAGGTACTTATCCATTTAGGAATCTCA
TAGAAATATAGTGGATGCAGTAATGCCACCTTGCAATGCAGTACATTACAAGACGTGACGCAAGATTCT
TTCTTGAGGTATAATTATGGAAGTTCCTACATGGATCTGAAAAGTGCGTTTTACATACATATTCTAGAAT
CCAATACAAAATTAGAAGAATATATATTATTATAATCCATCCATTTACTTATAGACTTAGACATAAGTATTA
ATAATACTTTACCATAATATTCAATTTAAGCCGCTGTGACACAATTTGTGTTATGTACATAAAGTGCTCAA
TAGTATATAACAAAGTAAGACAGAATACAACTATTAACTTGATAACATTTAAGTAGATAAGTAAAGAAAT
ACAGAATAATAATATTGTAATTAAATGCTACGAGTGGCTACGGGCTACGAAATTTCGTAGCGCTACAAG
CTACGTCGTAGCGAGTCGTAGCGAAGCCGCTACGAACCGGCGGCGTAGGCTCTAAGATTACAGGAAAT
TTCGATGAGTGATTAATTTAGGCATTCATGGCTTTAAAAATATTCTTAGTATAAAGTATAACTAGGTTTGT
TACGTCATCAGATGAAATAATGAAATATTTTCGTCTAATTATTTTTTTATTTAAATAGAATTGACTTTCAG
TTATTATCATTTCATGATCATTAAAATGGTTATTGCGAGGAGTATCTGGATATTTGAAACTCTAAATTTTA
AGAATGTAAG

>TCONS_00110129 geneID=XLOC_022008 scaffold_36:1336302-1339275(+) Splice junction overlap
GCACCCCAACGACATACGCGAGGGCGTCGCCAGCGCGTACCAAGTTATGAAGGAGGGCTTCGCCGAA
ACAGCAGCAACAATAACCATAGCGGCGCGCAGCGAGAGCGGCGTCGGCGTCCTGCGCCACCTGCCCG
GCGCGGCCGTGTCGCCGCTGGCGCTGGCCGCCGCCGGCGCCGCCGACGTGTTGGGCGGCGTGCGCGC
CTCGCTCGCGCCCACCGCCCAGCGGGATCATAAGGACAAGTACCGATACTCGCAAGATAAATCGTCGG
AGTGAGTGAATTTCACAAATCTTTGACTCAACGAGTGAACGCAATATCTCTCATATAATACAAATAAATT
AACTAGATTAATAACCTATTACCTATTGGTAACAAAAATGCTCTATATTCAACTTCAACTACAGGTTCAT
AATTGAGTTAATTATTTTGTACTGTTTTATCGGGTGAAGGTGTTCAAACAAATGCTCAGTCTAAGCTAAA
TAGAGGTCTAAAATAAGTCTTATTCTTATGTAAATAATGGTTATATCAACTCGCTGTTGTAGGCAAAGAC
TAAATAATGGTTGTAGACACAGGATGTCACAAAAATCATCTAGTACTTATATACAGGATGCGATTGAATT
TATAGTTTAGAACATTTTTTATTTTAGTTAGTTATTTTACTATTGACGGGACAACGCAAAAAAAAAACTT
CAAGAAGTGGTAGTCTGTGATTTGAAAATTGTAAAGAATAAAGAAATGGATAAGATAACTATAATGATA
ATATAATAGCTAAAGCACAGCTTCTATTGTAACTTCTTACAATAATTAATTTTGATTTAATAGTAGATATAC
GTTTCAAGAATATAAACTCAATCTAGATCAATCGCTTTGTTAACCCTTACTTGGACATAATGATTATAGAT
CCCACATTGTTTTGAACTTCATCCCATTGAGTTAAGAACGAAAACCAATGAAAAATATCGAATAGACAT
GCCCAATTAAGGGTTAAGCGGCGTGGAAGTGGGAGGGCGTCCCACGCTACATTGTCCCACTCTAAGA
ACTCACTAAAAGGCTAATCTACACTGAATGAGAAGCGCCGCGAAAAGCAGGGCCCTCTGCCCAACCG
TTTTTACACCTTCAAACATTGCACATACAGTTCATAATGTGTAGTTATTAAAACTGCTCTTCCTTTCGCTT
CCCTTTCAGTAAAGATTGGCAAAAGCAAAAGGTCGTTCATCGTTTTTATCGTTCGATAGTTAGATGAAA
GTTGTGGTCTACCTTAGTTAGTAATTTTTCATAGGTACCAAGTAGCATTTAAATTTAAAAATTCGTAGTC
CTACGTAACGCAGTTTTTTTCTTTCTTTCAGTAAGATTTAGCTGTGAAACTATCGTACTTGCTATTTTAAT
AGTATACACATGTTTGCAATATTATTTAGTATGAATATTATGTAGCAAGAATCAATCGCTATGCTTTTGTTT
CAAAAAAAATATGGTGCTTTATATTTGTAAGAGTTTTAGCATTTTATAAAAAGCATTTACCGTCGTAGGT
GTCGATGATAATTTAACTCGAAATCATAATTGTATAATTTAATTATCAACAAACTTGTTTATTCCTACGAC
GGATTCGCTTTTGTAGATTTTATATAAAGGTGTAAATATTTATTTAAAAATAGAATAATGGAATGATCCAG
GGGTGCCATGACAAGAAATGTAAAAAGAAAATTATATTTTGGATATTGTGATTTCTGCTTTTTAAAATGT
ATGCCGTTTAATAACTTTTAATCACCTATTTTGAAAACTGTAATCGTAATTCAAATCTTCCTAGAA
>TCONS_00110193 geneID=XLOC_022008 scaffold_36:1336903-1339326(+) Splice junction overlap
CCCAGCGGGATCATAAGGACAAGTACCGATACTCGCAAGATAAATCGTCGGAGTGAGTGAATTTCACA
AATCTTTGACTCAACGAGTGAACGCAATATCTCTCATATAATACAAATAAATTAACTAGATTAATAACCT
ATTACCTATTGGTAACAAAAATGCTCTATATTCAACTTCAACTACAGGTTCATAATTGAGTTAATTATTTT
GTACTGTTTTATCGGGTGAAGGTGTTCAAACAAATGCTCAGTCTAAGCTAAATAGAGGTCTAAAATAAG
TCTTATTCTTATGTAAATAATGGTTATATCAACTCGCTGTTGTAGGCAAAGACTAAATAATGGTTGTAGA
CACAGGATGTCACAAAAATCATCTAGTACTTATATACAGGATGCGATTGAATTTATAGTTTAGAACATTT
TTTATTTTAGTTAGTTATTTTACTATTGACGGGACAACGCAAAAAAAAAACTTCAAGAAGTGGTAGTCT
GTGATTTGAAAATTGTAAAGAATAAAGAAATGGATAAGATAACTATAATGATAATATAATAGCTAAAGCA
CAGCTTCTATTGTAACTTCTTACAATAATTAATTTTGATTTAATAGTAGATATACGTTTCAAGAATATAAA
CTCAATCTAGATCAATCGCTTTGTTAACCCTTACTTGGACATAATGATTATAGATCCCACATTGTTTTGAA
CTTCATCCCATTGAGTTAAGAACGAAAACCAATGAAAAATATCGAATAGACATGCCCAATTAAGGGTTA
AGCGGCGTGGAAGTGGGAGGGCGTCCCACGCTACATTGTCCCACTCTAAGAACTCACTAAAAGGCTA
ATCTACACTGAATGAGAAGCGCCGCGAAAAGCAGGGCCCTCTGCCCAACCGTTTTTACACCTTCAAAC
ATTGCACATACAGTTCATAATGTGTAGTTATTAAAACTGCTCTTCCTTTCGCTTCCCTTTCAGTAAAGAT
TGGCAAAAGCAAAAGGTCGTTCATCGTTTTTATCGTTCGATAGTTAGATGAAAGTTGTGGTCTACCTTA
GTTAGTAATTTTTCATAGGTACCAAGTAGCATTTAAATTTAAAAATTCGTAGTCCTACGTAACGCAGTTT

TTTTCTTTCTTTCAGTAAGATTTAGCTGTGAAACTATCGTACTTGCTATTTTAATAGTATACACATGTTTG
CAATATTATTTAGTATGAATATTATGTAGCAAGAATCAATCGCTATGCTTTTGTTTCAAAAAAAAATATGGT
GCTTTATATTTGTAAGAGTTTTAGCATTTTATAAAAAGCATTTACCGTCGTAGGTGTCGATGATAATTTAA
CTCGAAATCATAATTGTATAATTTAATTATCAACAAACTTGTTTATTCCTACGACGGATTCGCTTTTGTAG
ATTTTATATAAAGGTGTAAATATTTATTTAAAAATAGAATAATGGAATGATCCAGGGGTGCCATGACAAG
AAATGTAAAAAGAAAATTATATTTTGGATATTGTGATTTCTGCTTTTTAAAATGTATGCCGTTTAATAACT
TTTAATCACCTATTTTGAAAACTGTAATCGTAATTCAAATCTTCCTAGAATGATAATGTACCTAATGTTTC
TAGTAAAGAATATACACACATGTATCATCC
>TCONS_00110656 geneID=XLOC_022096 scaffold_360:126050-127128(+) Intronic
AGAAGGTCTATTGTTACCCTCCATGACATAACATTTTATTACTTAGATCTCTTAAATAGTATGATTTTTATG
TTGATAATTACTATTAATTACATAGGTAATTATTTTTATTGAGTCTCAAAAGTTTATACATTTATGTAATAG
CATAACCTAATATACCAGTGATAGTGTAAGCAACGTATTTGCCATAACAAGACCACAGGATTAGTAATG
CATTTATAATTTTGCTGAGTGTAACCCGCACTAGGCCAGCGCGGTGGACTAGGCCTAAAAAACCCTTCC
TTCATTGGAAGGAGACCCGTGCCCCAGCAGTGGGGACGTGATGGGGCGTGATGATGATACAGAGTGT
GCGGAAAGTGGCACTTAGATACCTTAATACAGTAATCATGTGGTCATTTTTTTCTGTACTTATCTTTCGAT
ATTTTAATGGTAAGTATGATTATTACTTCAAAAAAGGAGGAGGTTCTCAATTCGTAGGGATCTTTTTTTA
TGTATGTTCACTGATTACTCCGCCGTTTATGGACCGATTTTGAAAATTCTTGGACTCCCAGGTGGTCCCA
TTTTTTTTAGTTAGTAACGTGTAGAGTCGGACAGCGCTAACCTTAACGTATCAACACCAGAAAAAAGG
GTTCTACTATTCGTGCGAACTAACTGTGCACCTTGCCAACAGAGGTCACGCCGGGTACTCCAAAGCTA
GTTAATATGGCCTCTGGTTTTTGTCAGTCGTAGTACCATAGTTTTTACCCGTGTTAAAGATAACATAGTC
TGACTAGTGCTAAAGATAGGAAAAGAATACTATTTTTTTTCTATATTTTTCGTTTTCAATCTTAGATGTGG
ATTGTTTAGTTGTTTATGTATGTTATAAAGTTATACTTGATACTCTTTAATTTAGTTTCATAGAATACTTATA
TACATTTTGTGATCAAATAAAATCATTTTG
>TCONS_00110865 geneID=XLOC_022126 scaffold_361:162179-162518(-) Unclassified
GGCGACGGGCGCAGAGTGGCTGGAGATGGACTGGTAGGAGACGGGCGCCACGGACTTGGTCACTAC
TGGGGCGGCGGCGTAGTGAGCTACAGGAGCAGCAGCGTAGTGAGCTACAGGAGCAGCGGCGTAGTG
GGCTACTGGAGAGCTGTGGGAGGTGTGAGAGGAGTAGGAAGAGACTGAAGACACGGCGGGGGTGAT
GGTCTTGGTCACGACGGGGGCGGTGGCGTAGGTGGCGACGGGGGCGGCGTAGTGCGCCACGGGGGC
G
>TCONS_00110908 geneID=XLOC_022163 scaffold_362:39137-40673(-) Intergenic
GCACATTTATTTTTAAAAGACATACACCGTCCTTTGCACAAAAACATTTGACAACATATTTACAAAAAC
TTAAAAAACTAAGTGAAGAGAATTAGTGTTTAATGCTACGGAAATATAAAATTATTAATTATAAATGATA
TTATAAAATAATATAATGTAATGAAATGAAAACTACCGGTGACTAGGTTATCATTATTAGAAGGTTGAGT
TGGGTCGCGAAATTATTAGCAATATCTTGCAACATAAAAATAGAAAAAGGCGAATATGAAAACTAAGAT
CAATAAAACCGTATAGCAGCTAAATACCAAAACTTGGTGAATAAATTGATCTTATAGGACAAAAAAGTG
CCACTTCAGTGTTATATTCTTAAAATTATGCTTTTAAATAAGCTCCAATCACATCATAAAATGCTTGCAAT
TAAACACTATGCCTATTAATTACTACTTTTCGCAATCAATATAATAAAAATGCACTATAATGCAGTAAGTA
ACTAAACTTAAAATCCTTCATAATTTCTTTGTACAAATAATTGGACGAAATTCAGCATTGTATCTGGGAC
GCAAGTAATTTAGTTCAAACGAAGCAAGCCTTCAGTATGAATCGAAATAATATTTCACTAACTTACACA
GAAACTTGTAAAAAATACCGTTTCTATTATAAAATTGGGGTCCATTCATATCACAACGCTTCAATTCAGA
CAATGTAATCCGCCACTACATACTACGTTGATAGCTACGTTTAGTGCTTGTGCACTTAATAAGAAGGAAT
GTAATTATTCCATAAAAGTTTACAGTATGATTATTTAAATAATTTATTAAAACTGCCTTACTACCCGTATG
GACTAAGGCTACTAGTTATAATTTGACAATATTTTCTTCTATTATCCCCAATTCTATTTGTCTCTACAGATA
CTTTGTACCTGGTATGGAATGACACAAGTTTTCTAGGTCTACATTCTAAATACAATGTTCTCAAATCGGG

CATTTCGGTCAAACAAAATAAATTGATGTCCAGAAGATTTGTACTAACATATTAAATATAACTACGTTAG
TTACAATTTACAAAACAGTAGGTACATTCTGTATTGACTGCAGATAATTAACTGAAAATTAGTTACGATT
ATTTATACATTTGTACTTTCATAATGTCTGTATTTTGTTGACCCAAACACCACTGTATCTTACGAAGATCG
CTATAAATAACGAATCATAATAATATACCTAGCTGATTCTGAAGCAACAGAACTGAAATGAAGAAACGC
CAAAACGGGCTCTAACTTTAGGTAAACAGAAGTTTCCAGTTCGTTA
>TCONS_00111154 geneID=XLOC_022163 scaffold_362:43752-44089(-) Splice junction overlap
GTCATCTGCCATGGACGGGATTTACGAATGGGTTTTCTGAATTGCAATAATTATAATTTCCTAGATTTATA
GCCCATAAAATAAAGTTAAACATCTAAAGTGACATAAATAAAACTGCAGTGCTTTGTTTAGAAACCCA
GTGTTTTCAGAAATGCATGCCTGCCGACAGTTGCTCTACTCGCCGGCGCGGTGGAAAAGTGCTGTACC
AGTCAATTTCAAAACAACAGTCTTGAACACCAAAAATGAACAGGTACA

>TCONS_00111044 geneID=XLOC_022153 scaffold_362:44914-45710(+) Unclassified
CTTTATTATTCTAAGAATTTGTTTTTTATGGAACTTATTTCCGTTTCCTGTGTATTCATTTCCTTTTGCCAT
TTTCAAAACTGATTTCGATTGTTCATTCGTGATTCGGTATTAAAAATCTTTAAAAGTATTGTTCCTCAGA
GATTTGTGGCTTAAAATTAAGACATGAAATATTTATCTGATAATTATTTTAGTTTAAAACAATGCGTTAAG
CTCAATAATATATTTTACGAAAAATTATATCGATGAAAAATGTTTACATCCGGTGTGAAGTTAGTACCAG
AAACATAAGAAAGTGTCCATTTTCATTTATTTTCTGTCATCATTTTTACCAAGTCAACGTGCTCGTGTTT
GATCAATTTATCATTTTATTTTACGTTAATTTCCTATTGATTGTGAAAGTGGTTAAAGACTATCCTTCTCC
GTGTCCCGGAGGAGTTACACGCGGTTCTTTTTACTACTACAGTGGAAGGTCGCAGCGGCCGGCTAGCG
TGGAGGTCCTGCGGAGCCCCCAGTGAGAGTTCGGTTCAGCTTTTCATCGCGTTGGTAATCTCCCGT
>TCONS_00111021 geneID=XLOC_022153 scaffold_362:75365-77594(+) Splice junction overlap
GTCCAAGAAGTACGCTCGCAAGGAGGTCGCGGCCGAGGTGCGCGCCGCCGCCCAGCCATTCTTGGAC
TGGCTTCAGCAGGCGGAAGAAGATGACAGTGATGAGGAGGATGAAGAAGATATTGAGATCGAGTACG
ACGACCGCGCCAAGGCGACGCCGATCAAGGCGGTGGCGGCGCCGGCCGCGAAAAAAGACGCAGACG
AAGAGGAGAATGACGTTGACATCGACGCCATCTAGACGTCGACGTCGCCTTGTTGACGTCGACGTCG
ACTTGCGGACGAGACGTCAGTCGTCTACGGATAGGGCGCGGGTTGAATGCAG
>TCONS_00111159 geneID=XLOC_022154 scaffold_362:106902-107554(+) Splice junction overlap
ATGGCTATAGCTGTCGTGTCTGTTGAGTTGGTGGCAGTGGTGTTTGCGCTGTGCCTGGCGAATAGGGC
GAGGAACTACCAGCGGCGGTCCCGCTACTGAACCAGTTATGCACTGTACTGTGCCTATCAGTTAAATAC
ACGTACAAAATAGTCTTAAGATTATGTTGAACTAAGTAAATTCTAATAAAAATATGTTTTTAATTAATTAT
CTCAGTACTAAGAGCGCGTTCAGGGTTTTCAGCCAG
>TCONS_00111201 geneID=XLOC_022214 scaffold_363:75109-76226(-) Intronic
TACATATCTTATTTATGATATTGTACCTAAGGATAACCTAGGATAAGTGATAACAGTGCGAAATCAAAAT
ACGACCTACCTGTTTAACTCCCTTGGGGATAACATAGATAGGTGTTATAAGTTTGGTGCGTGTGAACTT
TCATAGATCTGTGTCTGTGATACATTTGAACTATCTGTCGCATATAGATACTATCTAATATGGTCAGACTG
TATGTATAAATAATTTATGAAGGATGTCAGTTGAATAAGTATAGTAGTAGTTTTTGAATTAAGAATACATG
GATTCTAGGATGTGATGTCTATTCACTACTGTTAAACCGTATTGAACATTTCGATCCGGCTCGAAGGACC
ATGAAAAGGAAGTGTAGTGATAATTTCACAGTCGCCTGGATCGTTGGTAGACGCAATGTTTATTAAAAT
TAGTAGTAGGTAGTAAGGAAAGCACTAAATACGGGTTTATAGTGAAATCTCTTGATACATATTGTGATGT
AATAACTGAAAGATTTTCAGTAGGTACGGTATTTCAGGTACATTGACCTACCGACGGCTAGTAAGTACC
TATTACCTTCTGATAAAATTCCATTCTATAATCTTAATGTGCCTTCTTTTTAATTGATTACAAAATAATGCT
GGCTATCTATAATTAACTTTATTGATTAATAAATGGCGGATAATTATCAGTCGCAAATTCGCAATCCAACC
GAATTTGGAAGTGTTTTATAG
>TCONS_00111203 geneID=XLOC_022215 scaffold_363:87644-91539(-) Exonic overlap (-)

GACGGAATCAAGAACGTTGCTGCTATAGCTATACGAACGAAAGAGACAGAACTAAAAATCAAAGGTT
GTTTTGTTATGGTGGGAAGGCAATAAATAAAATTTTATGCAAAATGTAATAAAAAATGTGAAGCAAACT
TTGTTATAGATATAAATCTTTTCACACAACATTCCGGATACACTTGCATTTCAATCTGTGGGGACCAGTT
GACTGTTGCTGTTGTTATTCGCTAAAAATAATAAGTCGGTAGATAGAAACGCTATGGCTTCCTGTCAAG
TAAAAGTAGAACAAGGCATTCTGCAGGGCAAAACAACAAGCACGCACAATGGCCGACAATACTACAG
CTTCGAAGGCATTCCGTATGCAAAGC
>TCONS_00111430 geneID=XLOC_022254 scaffold_364:259738-260860(-) Unclassified
ACAGACTTATCAAGACTAGGGGCCACTCGGACAAATTATGAACTGATGGCGGGCCGCCAGAAGTACTT
ATAGGTAATTAAAGACAATCAAGAATTGAAAATAAGTACTTAATGTTAAAATTTAAAAATAATTGATTTA
TTTTGAAGCTTGGCAGGGCTTAATAATCTTTTATTTATTTATTTGGCTTCAATTTACACGTAAAACTGCTT
CCAAACATTCCTTTGCAAGGCGGTTTCGATTTTTTTGTCGATTTAGTATTGAAAAGTTTTGTTCACATAT
AGGTATAGGTACAACCAAAAAGCTGGCATATTTTTAGAGCAGAGTTTATAGGCAACTCTTTGTACGTAT
TTTTATAAATGCAGTGGTAAAAGTTCAGTAATTTTCTTTCGTTGAATTAGCTGCGTAAATCTTGGTCGTT
TTGAATGTCGATAATTTCTAACTGCATTGATCAGGATTTTTAAGTTCCTGGACATCCCTTTGCTTTTCTG
CCAGAAAAAAATCGAATTTCTTCTTATTAAATTAAGAAATCGTTCCAGTGTCAATCCACGACTTAGCCAC
CTTATTTCTGTATCCTTAATACAATAAATCTCTATATTCGCTTTCAATGTCATCAAGGAATTGTTTGAATTG
CCTGTGATTAACTACTTTTCGAACATTGTTTTTAGAAATAATATTTTTCGAATGTACGAGGGTAACTATTA
TTTTCGCCGTCGGCGGGCCACTAGTATTCGACC
>TCONS_00111587 geneID=XLOC_022288 scaffold_365:297883-298630(+) Intergenic
TGGGAAGTTGAGTTCTCAATGGTCGATTTGAATGAAGTGACGGTTAGTGTAGTACCTACCGCACAACC
AAAGCACAAAACGTACTTAGTTTATAATTAGACGATTTGCACAAAACAAATATATCTTCAACAGTCAGA
AATTAGATGTTATTTAGTTTAAAAAGTGCTTATAACACACCTTTAAATATATATTTATGGAAAGGTCAGCA
TTTAGAAAATGTATATTTACTAGTTTTAACTGGAGGAGGGATTTTTATTTTATACAATGACATGGGATTTA
TAATTATTTTTGCGTAATATTTTTTATAATTAATGCATTTAATTAAGAGCATAGAATTTGTGATTGAAAAAT
TAAT
>TCONS_00111727 geneID=XLOC_022302 scaffold_366:133698-135644(+) Unclassified
AGGAGCCTAGCCAACATGCGGTCGGGATTCGTTTTAGTTCTTGTGACTATCGCCATAATTCTTCAATGG
TCTTCTGTCGAATGTCACCCGCCGCCTCAAACCGTCTCTACGACCAGTCGACCACCCTGGACTACTAG
GATTAAGAATGGAGTGGGTCGGTTTCTCGAAGGGGCGGCCGTCGTGAGTGCAGTGCAGAACGCGTAT
CATAGTGGCAAGTCTGGCAGACCGAAGTCCTATCCAGATGTGCCGTTGAAGAAACATTAGCATAGTATT
TATGATATTTCTTAAATAGTATTTACTTGAAGATATAACTTGTAGAGAAATGTCTGACTGGTGAAGTGTA
CCTAGGAAGAAAGAAGTTGTTAAAGGATATACCTACCTTATAATAAAATGTAATGTTATAGTATGCAGTT
AAGTCAACATTACTTATAAATAACTAATCGTCATCAATCAATCACCATTCTTCAGACTTTTATTTCTTACT
TACAACAGTTGTTACAACTAAACATTTCATAACCGATTCATAACCGATTACATAACTTCCTGCTTATTTTC
ACAAACATATTTTCTTTTCTAACACGAAATAAACAAATACCGAACGTTCTTTCAAGCGAAAACATAAAT
ATTGTTATAAAAATAAAAAAGACGTCTAAGTGCTGGTTGGACGGACCTGTATTTTTCATTTTTCATACAG
CTTTCGGGCAGCTTTTGTATTTTGTTCTACAATACGGCCTGTTCACTATCTCTTGTCACGTAACTGTTTTA
CTCTTTTTCACGGTACTTGTTGGTATATCAGTCGGCGGATTAAGCAAAATTTGCAGGATTATTTCTGAGC
GAATAGGACATTAGGTGACAAGTTAAACTGAACAAATTGTATGGTGAACCTGGAATGCAGCCAACA
>TCONS_00111888 geneID=XLOC_022305 scaffold_366:177117-180235(+) Intronic
GACCGTGCTACGAGCTCTAGCCGTCGCATCAGCTGTTCAAGGAAGTAAGCGGGGAGGCGCGATCCAA
CATATCATATCAACAGCCAGTTAGTGTTCGCCCAACCGCCGATACAACCGTATTGTATAGCCGCGCTAAT
ATTTGCCTACCATACACACACGATATTATAAGTACCTTATCGTGATCAGTGATTAAGTGATAGCCGTGAC
AGTGAAAAGTGTGTTCTGTAACACAGCCGTCTCCAGAGTGATATTGTGTGCTCGTATAGTGTTATTAGT

GTTGTGATTAAACCGAGAC
>TCONS_00111889 geneID=XLOC_022305 scaffold_366:177117-180235(+) Intronic
GACCGTGCTACGAGCTCTAGCCGTCGCATCAGCTGTTCAAGGAAGTAAGCGGGGAGGCGCGATCCAA
CATATCATATCAACAGCCAGTTAGTGTTCGCCCAACCGCCGATACAACCGTATTGTATAGCCGCGCTAAT
ATTTGCCTACCATACACACACGATATTATAAGTACCTTATCGTGATCAGTGATTAAGTGATAGCCGTGAC
AGTGAAAAGTGTGTTCTGTAACACAGGTGACAAGGCCGAGGCCGTCTCCAGAGTGATATTGTGTGCTC
GTATAGTGTTATTAGTGTTGTGATTAAACCGAGAC
>TCONS_00111842 geneID=XLOC_022308 scaffold_366:263362-265320(+) Intronic
CACGCGCGCCTCATAATGCTTGCATAATAATAATGACGGATGCCGATCGCGCGGCGGCTCCTTTGATCT
CCGGCGCCTTTGATGTTGCCGCCGGAGCCGGTCCCGCTGCCCCTGCCTCACAAACGAGCCGCCTGCTT
ACCTTTATTCGGTCCGACGCCGCGTAGCGAGTCGTTCGGCCCTTCGCCCTCATTCTAAATAGAGGCTTT
ATTTTGTAAACAGCGGGAGCGCTACTGTGCGTTGTGCGGGTGTTGTGGGGCTGTGCGTTGAGTCAGTG
GCGTTTGCGTCGTGAGCAGTATGGGGTGTGCTGGCTGCCCCTCGGCCCGCAGTCTCCGTGATGAGTGA
ACCCTGGCGCGGCAGCCTGCGCCGCCATCGCACCTGGAACTTCATCACCTGCACCATTTGTGTTCTGT
TGGGCGCCGCGAGTGATGGCGCGGCCCCGAGTGAGGGCGCGGGGGGT
>TCONS_00111987 geneID=XLOC_022350 scaffold_367:70492-73148(-) Intronic
GTGCGCGATCCATTATAAGTCTATTTCAAATTCCCTCTAAAATAGCTTCGTGTTCACTTTACACTAAGCG
AATTAGGATTTTAAGCTTTATTACAAACCCACTTTGAGCTACGCGAAAGTAAACTCTATTTTAGATTGTG
TTAAATATAAAGCGTCTTGTTCATCCGGCCAACAAAAGTTTTTCCAAATATAACAAGACAACCTATCAA
AAGCTCTTATAGAAATTTAATCTCTACTTTTGTTATTTTTGTAACTTTTCATTGTTATTCGTGCGGTATTTA
TTGCTGTATTGGGATGAAGTTTTACAAGTGAGTTGTGACCATGTGAACTGTGTTTCAGTGTTGCACCAA
CCACAGAATTAAATGCACCAGCTGTACAATCAGTAGCCATTCATCTCAGCGCTAACTCACTCATGAAAC
CTTCAATATTTGCTAACTATCTAGGCGCTTATTTATGTCGCACTTAATTCAAAGTAACCTAAGTGTAAAA
ATAATCTAAAGTTTTAAATTGTCATTTATATATGTAATATTACCATCAGATACGAACATGCGCATCACCATC
GCATACACGAATACACCTAAACCTATACAACAAAAACACGATAACTTCCCCACTAATTAAATTACATTA
CCATTAGTCTAAAGGGAAATACACAAGGATTAATATTGTTTTCCATCACGCACAGGTGACCGGGCCGCT
ATAGCGACAGAGTCCCGCCCTACTGCGGGTGGTCGAGGAGAGCATCATATAGCATGACGAAAATGCAC
GTGCTCTCTATTTCTTAATTACTGATCTGTATTTATATTGTAAATAGTAGTCTGTAAAGTTAAATCGAATAC
GTAAGTACCATATTTAAGAAATTAATTAATGGGTACCAGCTGTAAATAATAGGTCTTATAATGTAAGCAC
ACTTGTTTGTAAATTTAATTAAAATAACTTAAAATTGACGTTTATTTGGTAAAGTAATGCCCGTTTTCAC
CAAGACATCCTAAATTTTAGGAAACTTTTAACTCATTTTAAGGATATCCTAAGCATAGAATGGTTTTCAC
CACTGACGAACTGTCACTTAAATCCGTTAGGGACTTCGTAAATTTAGGAGGTGGAATTTCGGCCTCTTA
AAGTTAAGTAACAGTTTTTTGTTTGCAAATATACTTCTAGCTACTCAAAATTAGATATATTGATGCTATTG
AATTAATCACAGATCGTGTATTCGACCACGTAATTACGTGAGAGATGTCGAGAGACAGCATTTTACCAA
AAAAATATGGAGTAACAACTTGGATACTTGAACTTTTTTCGAAGGTTCATATTAACTAAAGAAAGTAAC
CTTTTCCTGCTTACTAGGTACTTAAATATTGAGTTCCAGGATTAATAATACCATTGTGAATTGGGTGAAA
ATATTTATTTGATCTAAATTAAAACATATTATTTTATTACACTCATCAACACTCTTCCACCAATGAATCAGT
TGCTAATGATATTGCGCTCTGCCACTTCTTCAAGTTCTGTCTCCTAGCGGAACAGAGCAAAGCGAAGG
TGGATTTCTTCAAAATAGTCAAGTTTCAAGAAAATAATTGTCCGCGTTTTCTATTTTCCGCCATTGCTTC
CTGCACTTTTTAAAATTATACTTCTTCTCTTTTGGAAGGATAAAATACAATTTTCGGTTTCTTATTTACAA
TCAAAACGTAATAGAATAAGACGATGTCAACAAATATTATACTTTTAAAAGCATAGACAATTGACAAAC
AGCCAATGTTGACATCAAAATGAATGAATAAAATTGAATCGGTAAAAAACATCACTAGTATTTAGGTGA
CGGATTGCTAGGGAGCTCTTAGCTTGGTGAAAATGAATTTACGTAGGGAGTGGATTTAAGAAGTCTCTT
AGCTTAAGAAACTTTTAGTACGGTGACTGTTTAGGATGTCTTGGTGAAAACGGGCATAAGTCCCACAA

TGACACAAATACTGTGTACTGTGTAAATTTTCATTCAGTCCTAGATTTTGCGTTTATTGAGCCATAACGC
AGAATCTAGGTAATGTTTATAAAGTATTTAAGATTAGATTATCGATCTACTTTCAATTTACCATAAATTAAT
GTAATACAA
>TCONS_00111913 geneID=XLOC_022351 scaffold_367:93967-101960(-) Exonic overlap (+)
ATTTGAGCATAAAACCAAAAAGACAGAGAGTGTATGGCAATATCAAGTAAAGGCGCGGACAGCTAAA
TCCAACTCCGTCCATTTCGCCCGACATTTCGAAGCACAAAATTAAATGTCGTGTCAAATCCTTTGGAGA
CCTACAGAGAGTGTAGGTGTCAATGAGTGAGCCACATCAAAAGCAGTTGTCACGTCGCCTCGCGTTAG
CAATTTGGGGTGTCGAAGAGGCTCAAGTTGTCGCGCCATGTCGGCTCTCCGAGTTGCCGACAGCGATG
CCGGCAGTGTCTCCACCCCGGCCGCGGCGCCCGCACGCCTCGCCTTCCCGTCCCGGCCCAGTCCCGC
GCGTACCTACGTATTTGTGTCCCGGCGAGATTGTGTCCTTTCTGATTTGATAACAATGATGAAGTGCCC
GCTGGACCCTCAATAACCTTGGTCGTGATGGAGGCAG
>TCONS_00111956 geneID=XLOC_022345 scaffold_367:257049-259291(+) Splice junction overlap
GGACTGCGCCATCGCCGCCTCCATCGCCAGCAAGCGAGCACAAAGACACAGCGAGTCAAACAGCTCT
TCAATGAAGTCGGTTGAAGTTGTGGAGACACCTAAGAAGAGTAAAGTGAAGGACAAAATGAAGAAA
CAGTTCAAAAAAGCTAAGCACACACTCATTCCGAAGAAAAAGGACTCAACCCCAGAGAAGAAAGAC
GGCGAAAAGAGCCCCGAAAAAGATAAGAAGTCTAAAAGCAAAGAGCTAGTACACGCCTAAATTGTAA
GAGAAAACTGACATGAATCTATTTGTCGATCTGTTTCATAATATCTACCAAAGTTAAGGTAAACTACCTA
AAATAATAACGCACTTTCAGAGACCGAATGCAAATTGTATTGAACGGTTAGCCAATTTTGAATGGCCTT
AGTAGGTAAATGATATACAAAATTAGGTCCGTCTTGGTTCTCCCCTTGCTTATACTAGGTAAGTACCTAT
GATGCACACAGTATTTTTAATTGTTCAGTTAACTGACACATCCACAAAGAAACACGGGATGATTGGATA
AAACAGGTTTTCTCAGAAAATCAGATACTAAAAGCACTTTTAACAAGCAGTAGGTACACACTCAAGAT
CAAACGTATCATTATTATTCATCAGAAAAAAAAATCAACAATCATCAAATAAATTACATTATTAATTTCAT
AGTTGATTTATTTACTACAACTAGACTTGTTGATCTGATGTACCTAAGTACGTAACAACACTATTAGATAT
TTTTTTACTAAGGAAAGACATTTATAAATTACCGATTTGGATATTATTTTAGGATTAGTTAAAATATGTATT
AAAGCGGCTCGAAATTCTTATAAAAATGTACTTATTAAGACTGATACATTCCACTAATTGTGTCATAAAA
TTGTTGAACAATAATGTTGACGTCAATTTATACTTATTGTAGTAGTAAAGTCCACATGTGCCAGGATTCA
TTTTGCTGTAATGCGAAACTTCTAAAGCCATATTCGCTTAGTCCAGTTTGGGCGATGTCAGAATTCTAA
GTCTTGTTTTAGCTATAGCTACTGTATGTTCATTTAACCACGCACAAACTAATCTACACGGTCGTGCAAG
TTTTTCTGCACTTTTACACTTATATTTTTTTAAATATTCACAGTATTATTATAATCATGCTGAAGCTTCGAA
ACACCAGCAGATGATTATGTATATTTATTATTATTCGAATTTATTACCGTATTTTTGCGGGTTACATATATTT
TAATACTCTTTGTCTTCCGAATTTAAGCATATCTTATAGGCTTATTTTTTATTGTTATTCACTAAAGCAAAA
TACTTACTTATTGACTTATAAACACAGGGTTAGCATAGCTTAGCTCGCTGAATGTAAAGATAGATATAAA
AGAAATATTCATACCTACTAGTTAACATTTACTTGTTATTTTCATAAGAAAAAATATTATTTTAATATATAT
AAACCACTTAAGCGCACGT
>TCONS_00111963 geneID=XLOC_022345 scaffold_367:257147-259286(+) Splice junction overlap
CTGCGCCATCGCCGCCTCCATCGCCAGCAAGCGAGCACAAAGACACAGCGAGTCAAACAGCTCTTCA
ATGAAGTCGGTTGAAGTTGTGGAGACACCTAAGAAGAGTAAAGTGAAGGACAAAATGAAGAAACAG
TTCAAAAAAGCTAAGCACACACTCATTCCGAAGAAAAAGGACTCAACCCCAGAGAAGAAAGACGGC
GAAAAGAGCCCCGAAAAAGATAAGAAGTCTAAAAGCAAAGAGCTAGTACACGCCTAAATTGTAAGAG
AAAACTGACATGAATCTATTTGTCGATCTGTTTCATAATATCTACCAAAGTTAAGGTAAACTACCTAAAA
TAATAACGCACTTTCAGAGACCGAATGCAAATTGTATTGAACGGTTAGCCAATTTTGAATGGCCTTAGT
AGGTAAATGATATACAAAATTAGGTCCGTCTTGGTTCTCCCCTTGCTTATACTAGGTAAGTACCTATGAT
GCACACAGTATTTTTAATTGTTCAGTTAACTGACACATCCACAAAGAAACACGGGATGATTGGATAAA
ACAGGTTTTCTCAGAAAATCAGATACTAAAAGCACTTTTAACAAGCAGTAGGTACACACTCAAGATCA

AACGTATCATTATTATTCATCAGAAAAAAAAATCAACAATCATCAAATAAATTACATTATTAATTTCATAG
TTGATTTATTTACTACAACTAGACTTGTTGATCTGATGTACCTAAGTACGTAACAACACTATTAGATATTT
TTTTACTAAGGAAAGACATTTATAAATTACCGATTTGGATATTATTTTAGGATTAGTTAAAATATGTATTA
AAGCGGCTCGAAATTCTTATAAAAATGTACTTATTAAGACTGATACATTCCACTAATTGTGTCATAAAAT
TGTTGAACAATAATGTTGACGTCAATTTATACTTATTGTAGTAGTAAAGTCCACATGTGCCAGGATTCAT
TTTGCTGTAATGCGAAACTTCTAAAGCCATATTCGCTTAGTCCAGTTTGGGCGATGTCAGAATTCTAAG
TCTTGTTTTAGCTATAGCTACTGTATGTTCATTTAACCACGCACAAACTAATCTACACGGTCGTGCAAGT
TTTTCTGCACTTTTACACTTATATTTTTTTAAATATTCACAGTATTATTATAATCATGCTGAAGCTTCGAAA
CACCAGCAGATGATTATGTATATTTATTATTATTCGAATTTATTACCGTATTTTTGCGGGTTACATATATTTT
AATACTCTTTGTCTTCCGAATTTAAGCATATCTTATAGGCTTATTTTTTATTGTTATTCACTAAAGCAAAAT
ACTTACTTATTGACTTATAAACACAGGGTTAGCATAGCTTAGCTCGCTGAATGTAAAGATAGATATAAAA
GAAATATTCATACCTACTAGTTAACATTTACTTGTTATTTTCATAAGAAAAAATATTATTTTAATATATATA
AACCACTTAAGCG
>TCONS_00111949 geneID=XLOC_022345 scaffold_367:257251-259354(+) Splice junction overlap
CGCCGCCTCCATCGCCAGCAAGCGAGCACAAAGACACAGCGAGTCAAACAGCTCTTCAATGAAGTCG
GTTGAAGTTGTGGAGACACCTAAGAAGAGTAAAGTGAAGGACAAAATGAAGAAACAGTTCAAAAAA
GCTAAGCACACACTCATTCCGAAGAAAAAGGACTCAACCCCAGAGAAGAAAGACGGCGAAAAGAGC
CCCGAAAAAGATAAGAAGTCTAAAAGCAAAGAGCTAGTACACGCCTAAATTGTAAGAGAAAACTGAC
ATGAATCTATTTGTCGATCTGTTTCATAATATCTACCAAAGTTAAGGTAAACTACCTAAAATAATAACGC
ACTTTCAGAGACCGAATGCAAATTGTATTGAACGGTTAGCCAATTTTGAATGGCCTTAGTAGGTAAATG
ATATACAAAATTAGGTCCGTCTTGGTTCTCCCCTTGCTTATACTAGGTAAGTACCTATGATGCACACAGT
ATTTTTAATTGTTCAGTTAACTGACACATCCACAAAGAAACACGGGATGATTGGATAAAACAGGTTTTC
TCAGAAAATCAGATACTAAAAGCACTTTTAACAAGCAGTAGGTACACACTCAAGATCAAACGTATCAT
TATTATTCATCAGAAAAAAAAATCAACAATCATCAAATAAATTACATTATTAATTTCATAGTTGATTTATT
TACTACAACTAGACTTGTTGATCTGATGTACCTAAGTACGTAACAACACTATTAGATATTTTTTTACTAA
GGAAAGACATTTATAAATTACCGATTTGGATATTATTTTAGGATTAGTTAAAATATGTATTAAAGCGGCTC
GAAATTCTTATAAAAATGTACTTATTAAGACTGATACATTCCACTAATTGTGTCATAAAATTGTTGAACA
ATAATGTTGACGTCAATTTATACTTATTGTAGTAGTAAAGTCCACATGTGCCAGGATTCATTTTGCTGTAA
TGCGAAACTTCTAAAGCCATATTCGCTTAGTCCAGTTTGGGCGATGTCAGAATTCTAAGTCTTGTTTTA
GCTATAGCTACTGTATGTTCATTTAACCACGCACAAACTAATCTACACGGTCGTGCAAGTTTTTCTGCAC
TTTTACACTTATATTTTTTTAAATATTCACAGTATTATTATAATCATGCTGAAGCTTCGAAACACCAGCAG
ATGATTATGTATATTTATTATTATTCGAATTTATTACCGTATTTTTGCGGGTTACATATATTTTAATACTCTTT
GTCTTCCGAATTTAAGCATATCTTATAGGCTTATTTTTTATTGTTATTCACTAAAGCAAAATACTTACTTAT
TGACTTATAAACACAGGGTTAGCATAGCTTAGCTCGCTGAATGTAAAGATAGATATAAAAGAAATATTCA
TACCTACTAGTTAACATTTACTTGTTATTTTCATAAGAAAAAATATTATTTTAATATATATAAACCACTTAA
GCGCACGTACATGTAGTGTAGTAAAATAGTTATGTATTTTATGTTTACAGATTTAAATTAATAAACTTTGG
>TCONS_00111992 geneID=XLOC_022348 scaffold_367:296671-302012(+) Intergenic
CATCACTATGTCGGCCGGTCGTGTCGATTGTGTTGTTGTGATAAAATTTATTCTGTCAAATAAAAGCGAA
TGTTGTGGTTCTTTGCTATCGTGCGCTCGGTTTGTTTGTTAAATTTCGGCTAAATCGACGATACTGTTTT
TGTTATTGTGCCACGCGCCCCAAAATGAAAACACTGAATAAATTATTTCATTAATTCTTTAGTGTCGTCC
CGATATCCACAGCTGTGGACGTGAGAAGGTGCAATCGTGCAAAATTTTGTGTTGAAATATCTCTTTTGG
ATTTGCAAAATAGATCGCTTCGCAAGGTCGTGATAACAGTCCGAAATGTGAACTGATATTTAGATACAC
TCTTACACCAAGAGTTGTTTTCTAAGTTGAGTGATTAGTGAGTGAGTGCAAGATGGGGTCCTATGGGG
TGACCCTGGCAGACTTGCCGAATATCTTCATCATGATAGCGGCCCTCGTGTCCCTGTTCATTGTGCTGG

TCATATTCCTCAGGAACATGGAGGTGATCGGCATAGTGGGTGAGGGTGCCCGGGAGCCGTGGCCGCGG
G
>TCONS_00112119 geneID=XLOC_022370 scaffold_368:1417-2141(-) Intergenic
CTGCATTACATACCTACTTAAATTATAAGACAGATACAGACTATTTGGATGTAAACAAACTGTACGATGA
ATGTCTGACGTATTTAGAAACAGTTAAAGCGGCGTATGTACAGACATTCAACACAAACATCAATAGAA
AACTATTTGAAACGGACGAAGATGATGATTATGATGAAATAGATTCGCCTGGATATGAAACTGACAAGA
GAAAGAATGATTTTACTAATGAGAGAAGCGAATCATTTGTTTATGAAACACCTAAAGCAGATTTCAAA
GTTGAAAATGAAATAATAAAACCACACGAAAGTAAAACACAAAGTACAGATGAAGTTTCAAAAACCG
ATAACGAATTATGTCATTTAACTAAAAAACAGAGTCTTGATATAAAACCACAAAAAGAAGCAATAAAAT
CAGTTAGCAACGAAGAAATACGTTCGGAG
>TCONS_00112191 geneID=XLOC_022403 scaffold_369:158507-159238(-) Intergenic
ACAAAATTAAGCTTTAACCATTACACGGATTGTATTTCCAAAGGAATATATTCATTCGTCAAAAATATAA
CACCTTATTACGTCTATCGTAAGTTTATATATATATTATAGCATATTATTATGAGACGTCCGTAACTTTATTG
TATAACGTGGACTGTATTGTAAAAACTCAATTGAACGTATGTTGTGACTGAAAAGCAATGTTCGTCGTA
ATGAAAATGTAACGTTTCTGTAAATACATTGTGTCTATGTTTGTGTAATAATCGGAGATATAATTTTACGT
ATGTGCTATATGCTTATATAATATCTCGGTGTTTTGAAAGTTTTCGGTTTTGTTTCCATAACTAAAACAAA
CTTTTTTTTAACGTAGAATACAGAGCCCCTAGGATACTATCGTAAATATTGTAATACAGATGACAGTACG
TCTAGTACAGGTTTGATATTTTGTTGATAACTATAAAAGTTTACGTTTTCCCGCGTACAAAGTGGAGGTT
AGATAGTTAGTCGAAAACTATAAAATATTACGGTTTCCCATACAAAGTGGCGCCTCTCGCCGCATTTTGT
ATGAACATAAGAAGGCATTGAATGTATAACTACCGTAAATAAAGAAACCATTATGAAGATGGCGACTGC
TACATAAGTGGGA
>TCONS_00112129 geneID=XLOC_022397 scaffold_369:159877-160819(+) Exonic overlap (-)
CAAACTAACTTAGACACATTACTAGATGTTATGTATATGTCTATGAGTGCAATAAATTATTATCAAGTGCC
TATATTATGTACGAGTACTAAATATTTAAGAAGTAATTAATGAAAATGTTAAACTTTTATAAGATAAAGAT
ATTTGTTTTTAAATAAATCATCATAAATGTCTCTTGCATATCCATATTATCAATTTATAAAGCTGCCTACAT
AAATACTCATAATATTTTTTCGCTACTTGGCATGTTTTATTTAATGGCATACATATCTGATAAAGGTACTGC
TATTTTGACCCAGCATATACCTATAATTATGTAGAAGGATTACAATATTTACGTTCTTTCTCTCTCATATAA
TCCATAATATACAATATCCTACAAGCACAATTTTTTTATCCCGTAACTACACCATCTAAACTGAGTTTCAA
ATAAGACCACTTTTTTAACTATTCATACATTTTAACAAACGCGAAATTCTCAATTTTATCAAATCGAACT
CAAAAGTCAATCCAACCTCTCAAAAAGAACCTAAAATTCGTTCCAACCTCTCAAAAAGAACTCAAAA
CTTATTCCAACCTCTCAAGAACTCAAAACTTAATCCAACTTCTGTTTCTTATTATCACTTTTGCCGTAAA
TTTCGTCATCCAGAGCACTGAAACTCAAAGAGCGTTTATTAATCCTAGTGCCGGGTACATTTAGCTCGC
TAGTTTTAGCCAAATTCCTAATCTCTTTAGCGAAGTCCCTGATTTTCTCCATAGTGGTTTGAGCGCGGTT
TTTGACCGTGCCTTGTGAAGTGGAGGGGGTAGGGCTAGGCTGTTTTTGATGAGTTTCCTCGCTCTTTTG
CTTCTTTGGCGGGGGCTCGCTGTCTTCTGAGAAACTTGG
>TCONS_00112185 geneID=XLOC_022397 scaffold_369:160505-160816(+) Exonic overlap (-)
GCCGTAAATTTCGTCATCCAGAGCACTGAAACTCAAAGAGCGTTTATTAATCCTAGTGCCGGGTACATT
TAGCTCGCTAGTTTTAGCCAAATTCCTAATCTCTTTAGCGAAGTCCCTGATTTTCTCCATAGTGGTTTGA
GCGCGGTTTTTGACCGTGCCTTGTGAAGTGGAGGGTGTAGGGCTAGGCTGTTTTTGATGAGTTTCCTC
GCTCTTTTGCTTCTTTGGCGGGGGCTCGCTGTCTTCTGAGAAACT
>TCONS_00112644 geneID=XLOC_022452 scaffold_37:322284-323318(-) Splice junction overlap
CCCTTGCAGTCTGCCCGCGATCTCCGCTGCAAACATACTAAGCGCCACTAATCCGCGGAAATTAATACC
TTTTTTATCAACCACTTGTGACTATAATCGTGCGTCTCACCGGCTCCTTGCCACGCTGTAAAAAACAGG
CATATCATTGAGTACGAGGAGGAGGGCCGGCGGTCGCGGCGCCACTCGTCCCCCTGCAACTCGCGAG

AGGGCAGCACCAACGCGCTACACACGCAACACTACGGCGACG
>TCONS_00112735 geneID=XLOC_022435 scaffold_37:693313-697580(+) Intergenic
CTCAACTCCGACGCGAAGCCTGTGCCGCTTTACGCTAGCCCGCTACTGCTTACGTTTACGCGATCTTTC
TGCTGTGTACCTAGCTTGCTTAATTACTAGCTTACGCTTTATACCTAAGTGCTAAATAGTATAAACTCTGC
TGTGTTATTGGCTTGTGCATTCTCCTTCTAAATTTCTATAGACTTTGTGTGTGCTTGTTCAATATACAAAC
CTGGACTGAACTATGCCGTCCAGAGAACAGATTTGCAGAATTAGAAGGGAAATGGTTGAAGCCGACA
CTTGGTTCAGACAGGG
>TCONS_00112370 geneID=XLOC_022435 scaffold_37:697239-697580(+) Unclassified
ATTTTTGTAGATTTTTGTATATGTAGTGGGAATTCATGTTGTGAAATATTTAAATTTAACTGGATTATAGTT
ACCTAAATTCAAAATTATAAAACCACAAGGAAAATGTCTTTTTTAACTGAATATTTTGACGAGTACCGA
CCGCATCCCTTAATAAAATCTAAACAATGCCGTCCAGAGAACAGATTTGCAGAATTAGAAGGGAAATG
GTTGAAGCCGACACTTGGTTCAGACAGGG
>TCONS_00112382 geneID=XLOC_022440 scaffold_37:819251-821106(+) Unclassified
AGTAGAGGGATCTTAGCGGCTGTTGGCTTGGTGTGTCGTATGGCGCCGCCCATGGAGGGTCAGCAGTA
AAGAAAATAAATTGGCTATGCTGCAACGTCGGACTACCCAGAAGTTGATGCCAATTATAACGTAATTTG
TGAGGAAAGATACGTTTCTCCACAATATTTGAAACACTGGATGTGACTTGTGCATAAAACAAAGCAAT
AAAATATGGGCGGGGAAATACCTGACGCCACCTTTTGGAATAAAAGTTTTTGTACCATACCCTCAATAA
TGCAGTACAAATGTGCAAGTATTGTGAGCAAAGCAATTTTTACCGTAAAATTTATACATTGATAATGCCA
TTATTGTTCTTCTAAGGTTTCTAAAATGGAACTTATACATCAGAAAATGGAACCGCGGTATTTATTTTAC
TGTTTATATTTAAACTAGTATTATACACTGTATTATTGTTCGTACAATTTTTAGCTCAACTATTGTTCTTTC
TTTTTGTATATAGGTACAAGTATTAAGACCAGTGAACAAAATTACCGTCATATCAGAAACATTATTATTTT
AGGCATTTTGTACTGTTCATTGTGTTTTTACTGTCTCACGAATGAAGTTTTAACTTGTGATTTTAAACCA
CTACTGAAGCAATGCATTCATTATTTGCCGACAGCAATAGTATTTACTATTTAACTAAACGGGAAGTTAA
TATGGTATTCATTTAAGTTTAATATTATTTTACGAGGAGGTATGAACAACTTGGTCCAATTTTGTTTAGAA
TTGTAAGGAAACATAGTCTAATATATAAGCATGTTGTATATTTTTTATTTATTTCACCTCAATTTCATAAAT
TGTAATGTCTAAAATGGTTGTCTTTGCATTTTGAATAGTCACAATTTATTTTTAGTACCTATGTCATTCCA
ACACACCTTGCATTAAATTTTGAAAAGATTGAATAAGCAAAATAATGTTTTTGTATGTTACAAACTTTTT
TTTACATCGAATACTTATTGTGAAATGGCGAGCTACCCTGTATGTGTTAGTTATAAATGCACTTGGTTTT
GGGTGATATGTAGTCAGGATTTTTAATAAAACGTTATAAACTA
>TCONS_00112508 geneID=XLOC_022442 scaffold_37:863634-867794(+) Exonic overlap (+)
GTCACATCGGACGTGGTCTATCGAGAAAATATTGTCAAAATCATTTATAGAGTTCCCTTCAAGTATTTTT
TTCAACACAGACCTTTATTTTCAGGCCTTACCTAGGCCTTAATGTATAATAATATCACAAAAATTAAGGA
CACTGTCAAAATAAGCTCGGCCTGGTGAGAGATGGCAGAGTCATCCGCGTAAAAGTAACGTGTTTACA
TGAGCGACAGTGATGCGTTTATAAAACCACAGTTCGCGGCAAAAAACTGTTTCAGTCGGAGCGACCT
GCGCGCGCGCAAGGTCAAGAGCACGCCACGAACGAATCTTTTGAAAATCGAACACACGCCCCGGCAA
CCCGGCCCGAAAGATGGTGTGTTAAAAAGAAAACACCTCGTGTTTTTTGTTTACCTATCTATGAGAAA
AAAAACATTGACTGGAAATAAAACTGGTGAATTTGAGGTGCTTGAAAAAAGAAACAATAATATGCTCA
AAGTGGTGGAATCTTGAAAACTTTTTACAATTAGAATGAGTTCGCAGTTTCCCAAGCTGCTGGCTGAA
AATTAAAAAAAGAAAATCCCGTGTATTCGCACGTGAATTCCAAATTTAAACGCGTAAGCTCGCCCGCA
CGTCCTCCGCGCTTACAACTGATTATTGTGTTGGAATGTTTAAAGTTCAAAATAAGAACCTGAAATCTA
CAAAGTGCCAGTGAATAAGCTGCCAAATTGGGGGGCTATAACGACTCACAGTCAGGCGCGGGCGCGG
GGCGCTGCGGCGCACGCAGCATGCTCTCCAGCATCAAGGGCTTCTGGAAGGTCTGCCGGTATGGGGA
CCTGTACATGGTGAGTGACTAGCTTCTTATCATAGATATAGTGAACATATCACGTAAGAACAATGAAGG
GAACTGTTGATACTAGAACCTGGAGGGTTACTCTATACTGACCTAAAACGAACGAACGAGAGCTGTCG

TGCAATCGGTAGTCGTGGCACAACTTCGTTTTTGTCATGTCGAGTAACAAATAAGTGTATTGTGATTTA
TTTATTTTATTTTTATAAACAGTGGTAATACTGAATGCTGAATTTGCTTGTAGCACCATGTAAGACCTAAA
TATATCTCTCGATCTGAGAAGGGTTTAAATCCTGGAAATTTATAGAAATTCAGTAATAACAGCTGTTATT
CTAATTATTAGCAATATTTACTGCTGAGTAAAACTTTGTTGCTGTTGTTGCTAGATAGAAATTCTGAGTA
AGATGAAGTTAATGTGAAGAGCTTGGATTGGGGTCACATGAATAGGTAGAAACCAGCCGTTGGAGTCT
AGAATTTTCTTCACAGCTCCACACACGTGGTAATACAGTTACGTAACTAATTTATTCGCGAATCCTTTAC
GAACCGGTCGGGGCTGCTGTCCTTTTCTCATAATTTATTGCACTTGAAAGATATCAGTGACTCAGACAT
TGGCTGGCAACTTGTAGAAAGCAAGCACTTTAACATTTTCT
>TCONS_00112786 geneID=XLOC_022524 scaffold_370:79281-80522(+) Intergenic
GTTCTACAACATCGATCGCACGAAGAAATCTAAAGTTTACCCGATTAAACTTACGAAACCCTAAAATAA
TGAGTGAATCTTACAGGACCAAACATAACAATGCAAACCCAACTACACTCTTTGTTGAACTAAAACCC
ATGATAAACCACTGATCAAACCATCCATTGTGACAAAACATTCATAATGCCTCACTATTTATAGACCAAA
GTTACTATTCTTCAATAAGATATGACATACTTTTTGAATGCTGATGATGATGTTCTATATTTGACAACTAA
ACTCTGAGACTATACATATACTATGAAGTAAACATCCTGAGTCAGGTGAAGAAGCGGATTGCAATGGCA
CTCAAAATAAGAACAACTAAACTACCCACATAATTCTTAACTTACAAAGAATATAATAATATTAATACTA
AATCATTTTGGATAAAAGATTACAATCAAACTCCTAGCCTACTTTACCTGGTCTAACACTTTATCAACAA
CAGTAACTTGCTCTACATTCATTGTTTTCTAAATTTATTATTATGTCTGACCGCTACCCTTTCTTCACTAA
CATCTTTTTAAAATGAGATTATTGTTATATTTTCTTTTCTTTATTAACTGTAACTCATACTT
>TCONS_00112788 geneID=XLOC_022525 scaffold_370:81968-82517(+) Unclassified
AACTTTTGAAACTTTCTTCAAGTTTCTTCTAGCCATAACATTAAAAGAAATGCCTTTATGTGAAAACCG
CCATATTAAGTAAATATTTACTTACTAATCGCAAAGGCAAACAACAAAACTTAATGTTTTTTCGAGACA
AACATAAAAGAACAGAAAACTAGCTGCCTGACACGTAACATGAAGAGATGAAGCAAATCTAATATCCT
GGTCTTTCGGTACCAAATTGTAACGTTTCGCCCTACCTCCTGCCTACCTAACGGATTTACTAAGAAGATA
TTTGAAAACACCTAACAAAACTAACTTGAGTGAAATAAGAGTGAAACATATACTACTTTAGCTTGGCC
ATGAAACATCCAGGGAACCCCTACATTGTGTAGTGTAACCCAATAAACGAAAGCTAATGAATTTAATAA
AACTATGAACGTGAATTGAATACAACCTTCCTAACTACCAGC
>TCONS_00112798 geneID=XLOC_022526 scaffold_370:222427-223950(+) Exonic overlap (+)
ATTTTTTACAAACGCACTTTTAACGTGTTTTGAGGTGACAATTTCCATAAAAATATAGAAACAAGATGG
CTGCAAAGGTTTTCCTGATAATTTGCGTCGTGGCAGTCCTGATTGCAGTGTCCTGCGGACAGAATGAG
ACTACAGATTCTACTACAAAATGCTCTGGCTTCGGTGCGCCGTGCAACACCACTGAGGAATGCTGCCG
AAGATTCAAATGTCAACCTCAGTTATTGTTTCCAACATCAACTACACGTAATGTGTGTATGATAGCATTT
TGAATCTGAATTCTTCATTCGCCATTGTATGCTTTTACTGTCTTTTTCAATTGTTGTGTATTAATATTATGT
TTCAGTGTACAATAAAGTGTTTAAATAAATTCTTATTAAAGGAAATGAAATATAGTTACC
>TCONS_00112795 geneID=XLOC_022526 scaffold_370:222928-223951(+) Exonic overlap (+)
TGAGACTACAGATTCTACTACAAAATGCTCTGGCTTCGGTGCGCCGTGCAACACCACTGAGGAATGCT
GCCGAAGATTCAAATGTCAACCTCAGTTATTGTTTCCAACATCAACTACACGTAATGTGTGTATGATAG
CATTTTGAATCTGAATTCTTCATTCGCCATTGTATGCTTTTACTGTCTTTTTCAATTGTTGTGTATTAATAT
TATGTTTCAGTGTACAATAAAGTGTTTAAATAAATTCTTATTAAAGGAAATGAAATATAGTTACCT
>TCONS_00113013 geneID=XLOC_022580 scaffold_372:153413-186290(-) Exonic overlap (+)
ATTGAAACGGTCTCACAATTTGTCAATATTCATAATTGCAGTGAATATACAAAGAACCATATTATAAATAT
ACGATTTAAATCTGTTTATCCAGGGCTTGTTTGAAAACATTTTACTGTGCTTTGTTCTCACTATCCTTGA
GTTAAAAGGATTTTGTCGAAATCAAGAGTAAGATATTACAAAGTTTCCTTCCTGAATATTCTTCGAAAC
ATGGACGAGCGAACACGTAGTGCCAACTTAACCAACGCAATGTCGATCAAATTTTTCTAATTTTAATAT
TTTTACAAAACACTTTCAGAGTGTAAAAGCAAACAACCAGTAAAATATCTGTGATTTTTATACAAATAA

GAATTTAAAGTGATCTCTATACATTTTTAACTAAACCAGCCCAAAGTTGAAATTATATCTGCAGTATATG
TGAAGAATCGAGATTAATCATCCCTCAATCGTGGTATACGCAAAAAAAGGACGTAACTCATAAATTATC
ATATTTTATTTTTTTATAAAAAATTAATAAAAGAAAATGCCAGCAATACTATCAAAGATCAGGGAGCAGG
CGTCGCGGGTGCAGCCGACGGTGGCACTGGGGGGGGGGGTTGGGGTGGCGCTGGCTGCGTGCGCCC
TCTACGGGACACGGGAGAAGAAACAACCTCCGCCCTGTCAGAACAACAATAACTATAAGGT
>TCONS_00112920 geneID=XLOC_022580 scaffold_372:153414-163314(-) Unclassified
AGCGCCATAGTGCTCGTCGCTCATTGGTCGGGGACGGACGCACGCCATTGGCCCTCGATCTCCAATT
CGATTTTTAAATCCGAAAATCTTTTTGGCCAGTGCTTTGTGCGTTTGAGTGCATTTTTTGTGACAGTGTG
GTTCTTTGTGGATTATTTTTGTCACGAAACATGGACGAGCGAACACGTAGTGCCAACTTAACCAACGC
AATGTCGATCAAATTTTTCTAATTTTAATATTTTTACAAAACACTTTCAGAGTGTAAAAGCAAACAACC
AGTAAAATATCTGTGATTTTTATACAAATAAGAATTTAAAGTGATCTCTATACATTTTTAACTAAACCAGC
CCAAAGTTGAAATTATATCTGCAGTATATGTGAAGAATCGAGATTAATCATCCCTCAATCGTGGTATACG
CAAAAAAAGGACGTAACTCATAAATTATCATATTTTATTTTTTTATAAAAAATTAATAAAAGAAAATGCC
AGCAATACTATCAAAGATCAGGGAGCAGGCGTCGCGGGTGCAGCCGACGGTGGCACTGGGGGGGGG
GGTTGGGGTGGCGCTGGCTGCGTGCGCCCTCTACGGGACACGGGAGAAGAAACAACCTCCGCCCTGT
CAGAACAACAATAACTATAAGG
>TCONS_00112921 geneID=XLOC_022580 scaffold_372:153414-186395(-) Unclassified
GTTGTAGTCGTACTGTCACCTGTCAGGTTATGTTGCATGTTTTGGTTTTGTTATTTTCCTCAAATTGTGA
CTTTTATTTGACAGTTCTCCGTCGGCCGTGGCGTGATTGAAACGGTCTCACAATTTGTCAATATTCATAA
TTGCAGTGAATATACAAAGAACCATATTATAAATATACGATTTAAATCTGTTTATCCAGGGCTTGTTTGA
AAACATTTTACTGTGCTTTGTTCTCACTATCCTTGAGTTAAAAGGATTTTGTCGAAATCAAGAGTAAGA
TATTACAAAGTTTCCTTCCTGAATATTCTTCGATTTCACTTGATCAGAAGATTTTTAAAAACATGGACGA
GCGAACACGTAGTGCCAACTTAACCAACGCAATGTCGATCAAATTTTTCTAATTTTAATATTTTTACAAA
ACACTTTCAGAGTGTAAAAGCAAACAACCAGTAAAATATCTGTGATTTTTATACAAATAAGAATTTAAA
GTGATCTCTATACATTTTTAACTAAACCAGCCCAAAGTTGAAATTATATCTGCAGTATATGTGAAGAATC
GAGATTAATCATCCCTCAATCGTGGTATACGCAAAAAAAGGACGTAACTCATAAATTATCATATTTTATT
TTTTTATAAAAAATTAATAAAAGAAAATGCCAGCAATACTATCAAAGATCAGGGAGCAGGCGTCGCGG
GTGCAGCCGACGGTGGCACTGGGGGGGGGGGTTGGGGTGGCGCTGGCTGCGTGCGCCCTCTACGGG
ACACGGGAGAAGAAACAACCTCCGCCCTGTCAGAACAACAATAACTATAAGG
>TCONS_00113083 geneID=XLOC_022574 scaffold_372:226880-234158(+) Exonic overlap (+)
ACACAAGTCACCACTACCAGTCTCCAAGCCTCTAAGCGTCAGTTGGCAGTTGACCGCTCTACAACGCG
GAACCAAGTGCCTAAGCTGAAATACTCTTTCACGCATTCCGGGAGTAATTTCTCTGAAATCCTGAATGA
CATTTATTGCGATAACAGTCAAGTTATAAATGTGAAAAGTGGTGATGCAAATTATGTGCGGAATCAGCG
GTCGATAAGTGATTTTTTATCAAAACACGGCAGTACCGAGTGATGAACAGCTGCTAGCGGACCGAAGA
CCTCATCATGAGTGAGAAGCGAGTGGACCTCGGCGCCGCCATCGACCGGGCCGGCTCCCCGTTCTTGA
GACAGGTC
>TCONS_00113066 geneID=XLOC_022581 scaffold_372:240510-244902(-) Splice junction overlap
GGAATCCTAAACGTACCATGGATTTAGCAGAATCTGTGAAAGCTGAAGCGACAGTGGCGCCATTTAAT
ATTCATTCAGTGTGTAGGATAATCGAATGTACCTGTTTATAATCAAATAACTAAAGGTGCTAACGATAGA
ATGAAATCTATGGACTGGTGAAATGAATGTGATATTTTGCATCTAGCAAATGACATTGAAGAATAAAGT
GTATCAAAATGGATGACCTAACAGTTCCCGAGAAAAAAAGTATGGTGCCCTTTCTGAGGCAGCTGTAC
ATCACGACGGCAGTCAGCGTGAACATCATGGGCCACGGCTGCGTCATCGGCATCTCCGCCATCCTGAT
CCCGAGCCTGCGCCGCCCCGAGTCGAGCATCCACGCCACCCAC
>TCONS_00113068 geneID=XLOC_022581 scaffold_372:240510-244902(-) Splice junction overlap

GGAATCCTAAACGTACCATGGATTTAGCAGAATCTGTGAAAGCTGAAGCGACAGTGGCGCCATTTAAT
ATTCATTCAGTGTGTAGGTCGTGTTGACAAAATTATGTAGTTTTCATTTACAAACGTTACAAAGTGGAA
GGTCATATTATATTTGTTGTATAAAATATACGTGTAACTGAATTAATAATATTTATAAGCTGTTACGGAAAT
GTTTTCACGTTCTTTAACAGTTACGGAGTGTTAAAGGTTTAACTTATTGGTGCCATGTTCATGATGTGTT
TGGTGATTAAAATTCCTACTTTGGGCGCTCCTGCTACAGTGCAGTATTTATTTTATAAGTATTAATTAATT
AGGATAATCGAATGTACCTGTTTATAATCAAATAACTAAAGGTGCTAACGATAGAATGAAATCTATGGAC
TGGTGAAATGAATGTGATATTTTGCATCTAGCAAATGACATTGAAGAATAAAGTGTATCAAAATGGATG
ACCTAACAGTTCCCGAGAAAAAAAGTATGGTGCCCTTTCTGAGGCAGCTGTACATCACGACGGCAGTC
AGCGTGAACATCATGGGCCACGGCTGCGTCATCGGCATCTCCGCCATCCTGATCCCGAGCCTGCGCCG
CCCCGAGTCGAGCATCCACGCCACCCAC
>TCONS_00113067 geneID=XLOC_022581 scaffold_372:240524-245037(-) Splice junction overlap
ACACACTATAATAAGAAATAGCTGTAAAATTTATTCAAATATACCCTAAAAAATAGACGACTTTTTAGTA
CCCATCTGTGCTGTCATATATTCTATGACGCCAGCCTGTTTGATTGGTTTGTTAAAATTCATGGAGGAAT
CCTAAACGTACCATGGATTTAGCAGAATCTGTGAAAGCTGAAGCGACAGTGGCGCCATTTAATATTCAT
TCAGTGTGTAGGTCGTGTTGACAAAATTATGTAGTTTTCATTTACAAACGTTACAAAGTGGAAGGATAA
TCGAATGTACCTGTTTATAATCAAATAACTAAAGGTGCTAACGATAGAATGAAATCTATGGACTGGTGA
AATGAATGTGATATTTTGCATCTAGCAAATGACATTGAAGAATAAAGTGTATCAAAATGGATGACCTAA
CAGTTCCCGAGAAAAAAAGTATGGTGCCCTTTCTGAGGCAGCTGTACATCACGACGGCAGTCAGCGT
GAACATCATGGGCCACGGCTGCGTCATCGGCATCTCCGCCATCCTGATCCCGAGCCTGCGCCGCCCCG
AGTCGAGCA
>TCONS_00113069 geneID=XLOC_022581 scaffold_372:240524-245037(-) Splice junction overlap
ACACACTATAATAAGAAATAGCTGTAAAATTTATTCAAATATACCCTAAAAAATAGACGACTTTTTAGTA
CCCATCTGTGCTGTCATATATTCTATGACGCCAGCCTGTTTGATTGGTTTGTTAAAATTCATGGAGGAAT
CCTAAACGTACCATGGATTTAGCAGAATCTGTGAAAGCTGAAGCGACAGTGGCGCCATTTAATATTCAT
TCAGTGTGTAGGTCGTGTTGACAAAATTATGTAGTTTTCATTTACAAACGTTACAAAGTGGAAGGTCAT
ATTATATTTGTTGTATAAAATATACGTGTAACTGAATTAATAATATTTATAAGCTGTTACGGAAATGTTTTC
ACGTTCTTTAACAGTTACGGAGTGTTAAAGGTTTAACTTATTGGTGCCATGTTCATGATGTGTTTGGTGA
TTAAAATGTGAAGTTTCGGACGACTCAGAAAGATAATCGAATGTACCTGTTTATAATCAAATAACTAAA
GGTGCTAACGATAGAATGAAATCTATGGACTGGTGAAATGAATGTGATATTTTGCATCTAGCAAATGAC
ATTGAAGAATAAAGTGTATCAAAATGGATGACCTAACAGTTCCCGAGAAAAAAAGTATGGTGCCCTTT
CTGAGGCAGCTGTACATCACGACGGCAGTCAGCGTGAACATCATGGGCCACGGCTGCGTCATCGGCAT
CTCCGCCATCCTGATCCCGAGCCTGCGCCGCCCCGAGTCGAGCA
>TCONS_00113138 geneID=XLOC_022581 scaffold_372:240524-245037(-) Splice junction overlap
ACACACTATAATAAGAAATAGCTGTAAAATTTATTCAAATATACCCTAAAAAATAGACGACTTTTTAGTA
CCCATCTGTGCTGTCATATATTCTATGACGCCAGCCTGTTTGATTGGTTTGTTAAAATTCATGGAGGAAT
CCTAAACGTACCATGGATTTAGCAGAATCTGTGAAAGCTGAAGCGACAGTGGCGCCATTTAATATTCAT
TCAGTGTGTAGGTCGTGTTGACAAAATTATGTAGTTTTCATTTACAAACGTTACAAAGTGGAAGTCCTA
CTTTGGGCGCTCCTGCTACAGTGCAGTATTTATTTTATAAGTATTAATTAATTAGGATAATCGAATGTACC
TGTTTATAATCAAATAACTAAAGGTGCTAACGATAGAATGAAATCTATGGACTGGTGAAATGAATGTGAT
ATTTTGCATCTAGCAAATGACATTGAAGAATAAAGTGTATCAAAATGGATGACCTAACAGTTCCCGAGA
AAAAAAGTATGGTGCCCTTTCTGAGGCAGCTGTACATCACGACGGCAGTCAGCGTGAACATCATGGGC
CACGGCTGCGTCATCGGCATCTCCGCCATCCTGATCCCGAGCCTGCGCCGCCCCGAGTCGAGCA
>TCONS_00113125 geneID=XLOC_022581 scaffold_372:240547-244572(-) Splice junction overlap
GATTGTGCCAAAAGTGATAATGATAAAAGTTTGGTAGTAAAATGTGTATTTAATTTCTCAGTCCTACTTT

GGGCGCTCCTGCTACAGTGCAGTATTTATTTATAAGTATTAATTAATTAGGATAATCGAATGTACCTGTT
TATAATCAAATAACTAAAGGTGCTAACGATAGAATGAAATCTATGGACTGGTGAAATGAATGTGATATTT
TGCATCTAGCAAATGACATTGAAGAATAAAGTGTATCAAAATGGATGACCTAACAGTTCCCGAGAAAA
AAAGTATGGTGCCCTTTCTGAGGCAGCTGTACATCACGACGGCAGTCAGCGTGAACATCATGGGCCAC
GGCTGCGTCATCGGCATCTCCGCCATCCTGATCCCGAG
>TCONS_00113039 geneID=XLOC_022586 scaffold_372:350837-355396(-) Unclassified
CCCGATGTTGTGTTCGTCGCCGACGCGGCAGCTGTGTCTGAGCGAGATGGTGGCCGCGGCCGCCGAC
GACAAGTTTCTACACGACCCCGAGCTGCTGGGCAGAAATAATGGCTCATCCTCCCTACTAGACGCCCC
TCACCTACTCCTCGGCGAGTCCTGCCTGCCGTCGCTACTGCTCGGGGGGCCGCACCACCACGCCCCGC
ACCCGCACCACCACCCGCAGACGCACTGCTTCGACATGGACCTCGGCGGGTAGGGATTGTTCCGGCG
TTTGCCCGGACGCGCGGTTGAAACTGCCGCGGAGGTTGGAAGTGCTTTTTTGTTTTTCTGGTTTACTGT
TTGGAGTTTGGAGCAGATTACTGGATGGAGAATATTATTTCATACTTCAGGCAGCGTTCCACAAGTTAC
ACAATTTCCAGCGTTACAGTTGCAATACAACAGCAATGATTAGGGTAACTAATGTTTGTGAAAATCAAA
GAAGTACTTTTAATTTTGCTATCACCTTGAGTCTTTGGGAACGACTAGGTTTCTTGAGGTTGACATTCTC
AGAATTTTGTTAATGTTTTCGATATTACAGTTAGCAAGATTTATACATATAATTATATACTTAACATATATAC
TTATAAACTCAATCATGTATAAGTAATGTTTTTAACCTAAGGTACTGTAATCCGTTAAACCATGTTTACTT
CCAAAGACTCCGCGGCCGCGGCATGGGCGAGTGTGTTTGTTATATTTTTATATCTGACGAAACAATAAA
TAGTTCATTTGAGAAACGAGACATATTTTCATATACTTTCTTACATATTTGGTGCTCAGTTCTATGAGATT
TATAGGTGCAACATTTTTAAACAGAGCAGAATTTTTTTAAACAATAAGACCTGCTTAGTGTATTCTCAA
GTCTTCTGTTAATGAATCAAATAGCTATTTGGTTATGTGAAAGTAGAGCGACATACGTATTTTTAGTACC
AGTTTGAATGAAAGTAAATTAAATACAAAACCAAAGAGTTCATTAACATACTTGGATGAAATGGGATG
AAAGAAAAACAATGATTGATGTCACTCCACTAAAATAACATTATTTAAACTTATAGCTAGTATTTTTTTC
CTATAGCTTCTTAGGCTCTGTATTTTTAGAATTTTTTGTAGTTATCGTGATACTTTATAGTTACCATGCTGT
TGTATTCATTTGTTTTAGTATCATGTTTGATTTTAGTTACAGTACATTTTAGTTCAAAAACTTAGGGAAAA
GTAAAAATATACCTATTCATTTACTAAATATTAAATAATAGAAATATTTGTATGGACTCAGTATTTTTCGTT
CTAAAATGTGCTTCAGTTGGCTTCAATTCGTGAATAATTTTGTATTATCCATTCGTAAGTTCAGTATACCA
CGCTGATATCTGAACTGCCACGTAACTATATTTAGGTTCTTCACTGGTTTCTGATAGCTGCACATCCCTA
AATTTTATAAACGTCATTCAAATACCTTAACCCGTGGGTCAGATATCTGCGTGGCAGAATGTACAGTATG
TAGGGTAACGTAACGTACCTTGGGACGCTTGTACCTCGGGACATTGATTGTATTTTTAAAACCGGCTGA
ATAAACACATTAAAATTCTACCCTAGGCATAGTATTTTACTCGCTTGACAAAACTAGTGAGTCTCATGAT
CATACCAGCATCGAGTGTGTGGTGAAGACAATATGAAGTTTTTTGGAGTCAAAAGTAGCTTTTGTATAG
TTTTTTGAGTTGCGTTATCTCATTGAGGCATGCTCAAAATAGAGACATGGTTGTCACGTACAAATTTTC
AGTTATTTAATTTTATGACATGGCAATTATCTGAAAGATTTCTAAATTAAAAAAAAAGATATTTAAATTTT
GGTTAAATATTGCTTTTTTGTACCTTGGGACACGATTTAATTTGTACCTTGGGACACTGTTTCCTATGGC
TTTATACTATGGAAAATGCAAACTTCTAAATAACGCCTTATATCTCTGTTCTTATTACTTCTTATTAGTGG
CAGAGTGAAACTGGACAGAAGAGAGATGCAGTCAATCAATAAGAACAAAGATATAAGGCGTTATTTA
GAAGTTTGCATTTTCCATAGTACAAAGCCATAGGAAACAGTGTCCCAAGGTACAAACGTGTAACGTAC
CTTGGGTCAAAACGAGCATTTTTACTTTTATCTTCATTTAATAAAATCTGGCCACGCCAAAGCTCCAGC
ACAAATACTAGTGATAATAGATTATATATCCTACCGAATAAAGAAAATTTCACATTAATATCTTAGTTGTA
CGCTGCGATAGCTTCATTCAAAGTTGGGGTAGTCGAAAATGTCCCTAGGTACGTTACGTTACCCTACGT
ACGTACAGTAAATGATTGATCTTATCGGAGATTTACTTAACTTTGCTGTCATTCCCTCCCTGTCAGAGGC
TTACGATGATGATGGCGAATGACAGAGAAGGAGTATAGAAACATTCAACTTTACTTACATTATAAATAA
AATTTACCCATATTATTTATATCTAAGAGAATTACAAAAGTCTTAAAAAGTATTACACACATATTTTAATA
ACAGAATTTCAATATTTAAGATTTAAGTAATGAATTATGCTTTTAAAAACGTATATATTATAATAAAATATA

ATAATATTTTTGATATGCATGCCGACATAGTTAATTTATTTTTCTAAATTTAGAAATCTGTAATTGTACATA
ATATTTCATATTTATATGTATAACTATTTTTGTGAAACAAAGCAGAGTGCTATAGTAGGTATTTCTAATGG
ACCATTATATTAACAGAAATGTGATAAAATAAATATATTTTTAGAAGATATTGTACCTATACATAATTATTT
GAGAAAACCTTTTCTTTGTACATACAATTTATTTAGGCATATTATTGCTAAAAAGCTATCATATTATGCAT
ACAGCATGATCCAACTCGTACCTAATACGACTTGGAAATAAAATATGAGCTATTCGATTTACCTACCTGA
CTTTGTCATTTTCTTTATGACTTTGAAGTCAAATTACTAAAATTATATATACCTATTTTATATTGTGACGAT
TCTTTCTGTAAATATATCCTTATCCGCTTTTTAGCTTAGAGCTTGGAAGACACTGGTTAATAAATCACAA
AATTATTTATATAAGACGATCAATGTACTCTAGGAGGGTAGGCAGAGGTGCGTTGCACCCACTTATGTC
CAGTGTTATGTTTATCTATTGTTGCAAGTTAAAATTGCATTACATCCGGGATTTAATAAAACCGATAAAAT
AATATACCACGGTATATTTATTTCTTGAGTGCAATTGTTTTTATTGTAAAAACATAATTTATGGGTTCATAA
TTTTTTTTAATATTTCAATAACAAAAACCAAGTCAACAAATTAAGCATTTTCTTACTTTACAGGAGCAAA
AAGTCACTCATTTGACCCGAACAAAAAAATATTAATGTAGTAAATTTTAGGTTAGGTCGTTTCATATTCA
TAGCAGTGAATTGATATAGAAAATAAAGTTTTTGTTGAGACATAATTTGTTATTGTGTGTTATTTTAGTAA
AATATTTGCACACTCTGTATCTTTCTTATTAGAATTTTTGGATTTAACTTTTTTGTCTACAGCACATACTTA
AGCGTATTTCTGCTCATTAAATTTGTTATCTACTATTGTGTTCCTAAAAAGAGCACTACGATTAAAATATA
GTACATTCTCTACGTATCTATAATACGACATAAAGGTTCCATTGTCTTATGATCGAACTGTTGCGCCAGG
GATGGTAACTTATCTCTTTTATTGGCTGTACTTAAATGACATTCACCACTTCACAAATCTCATACAGAGC
CTTTAGGTTGTTTTGTAGATAGGTGATTTGTATTTTAATAAGTGTTAAATGATTATTGTTATTAAAATTGTG
ATATTCATGAAGTGCCTTAACAAAGCAATAAATAATAGTAATATTTATGTAGCAAATTCATTTTTACCTAC
TTTTAAATAGGTAAGCGAAAAAAAAAGTTGGATACATTAACAAACATACACATTGTTATGTATAAATGTTA
TAATAATGATGTTGAAAATAGAAT
>TCONS_00112910 geneID=XLOC_022578 scaffold_372:436517-567282(+) Unclassified
TAGTACAGCGCTCAGACAGAGTATCGGGCGCGCGGCTTTGCAGCCGACGACCTTATCTTTATATTCTTC
GTTTATTTTCTTTTCTATTCGTTTAATTTGTCGCACGGACCAAACTACGTTCCGGGAACGAAATGGATTG
TCTTCTAGTGATTTACAAGTGTTCCAGTGTTTACCGGCGAGTTTAGTTTTGACAGAATCACGTGTGGAA
CCGAATTGTTGCCGGTTCCGGAGTTTTCAAAGTGTTTATATACGCTGGCGACCTTCAATTACCACAGGT
AGAGCTGACGGATCGTCAAAAACGTGCTCAGTGTCAAGTTTAAAGAATTTTGTATACTGTGAATTGTTT
TGAATTGTCGTGTGAACAATTCTCCCAGTGCCTATCGTGTGGTCGATGCCTTACGTCCGCGGCAGGAG
ACGGCTCGGTTCGCTCCAATAGGATTCCGCTTGGAGACCAGTGATCATCGCGTGTGCTAGTGAATTAAT
AACAAAGGTGCTCACAACTTAAACATAACCTTTTTGAACCGGCGCGTGTTTTTAGGACATGCGCAGTA
AAGGTCACGGAATTATTAATAAGTTACATATAATTTAACAAATAAACAGTGTGCCGGTGTGGATATAGTT
GAAGTGTTGTGGTTAGTTGCTAATAGGAAAAGCACGAGCCCCCTCAAACCTCTTTGGATTTCTGCTTC
AATGCTTGTTGACAGTTTTTGGATTTGAATCCAGGATAACGTTCACCTGTGAGATTCAACAAATTTACC
TGCGCGCGCACGAAGTTTCTTTACAAAGGTTGGCGCAACTATGATGTCCTGTCCTCTACCTCTGCCGCT
GCTTCCAGCCCTGCACCCTCAGCCAGCTCTAGCGTCAGACTTCCCTATACACATTGGAATAAAGGGAC
CGTCCTCTTCCTGAGCGACTTGAAAGGGACAAGTGATAAAAACTTTACCGAAAACTAGTGACTTATCG
TCACTTGTTAATTAATATAATTTGGACGTGTGTACCGCCGGCCCTCGGTCGCATTTCGTGA
>TCONS_00113085 geneID=XLOC_022578 scaffold_372:436540-557388(+) Intergenic
TCGGGCGCGCGGCTTTGCAGCCGACGACCTTATCTTTATATTCTTCGTTTATTTTCTTTTCTATTCGTTTA
ATTTGTCGCACGGACCAAACTACGTTCCGGGAACGAAATGGATTGTCTTCTAGTGATTTACAAGTGTTC
CAGTGTTTACCGGCGAGTTTAGTTTTGACAGAATCACGTGTGGAACCGAATTGTTGCCGGTTCCGGAG
TTTTCAAAGTGTTTATATACGCTGGCGACCTTCAATTACCACAGGTAGAGCTGACGGATCGTCAAAAAC
GTGCTCAGTGTCAAGTTTAAAGAATTTTGTATACTGTGAATTGTTTTGAATTGTCGTGTGAACAATTCTC
CCAGTGCCTATCGTGTGGTCGATGCCTTACGTCCGCGGCAGGAGACGGCTCGGTTCGCTCCAATAGGA

TTCCGCTTGGAGACCAGTGATCATCGCGTGTGCTAGTGAATTAATAACAAAGGTGCTCACAACTTAAA
CATAACCTTTTTGAACCGGCGCGTGTTTTTAGGACATGCGCAGTAAAGGTCACGGAATTATTAATAAGT
TACATATAATTTAACAAATAAACAGTGTGCCGGTGTGGATATAGTTGAAGTGTTGTGGTTAGTTGCTAAT
AGGAAAAGCACGAGCCCCCTCAAACCTCTTTGGATTTCTGCTTCAATGCTTGTTGACAGTTTTTGGATT
TGAATCCAGGATAACGTTCACCTGTGAGATTCAACAAATTTACCTGCGCGCGCACGAAGTTTCTTTACA
AAGGTAATTCAGAAACGAAAGCGTAATTTCAATTCACCAAGAAACTCAAAATGCTGGTTGGCGCAACT
ATGATGTCCTGTCCTCTACCTCTGCCGCTGCTTCCAGCCCTGCACCCTCAGCCAGCTCTAGCGTCAGAC
TTCCCTATACACATTGGAATAAAGGT
>TCONS_00113060 geneID=XLOC_022578 scaffold_372:436546-477705(+) Intergenic
GCGCGGCTTTGCAGCCGACGACCTTATCTTTATATTCTTCGTTTATTTTCTTTTCTATTCGTTTAATTTGTC
GCACGGACCAAACTACGTTCCGGGAACGAAATGGATTGTCTTCTAGTGATTTACAAGTGTTCCAGTGT
TTACCGGCGAGTTTAGTTTTGACAGAATCACGTGTGGAACCGAATTGTTGCCGGTTCCGGAGTTTTCA
AAGTGTTTATATACGCTGGCGACCTTCAATTACCACAGGTAGAGCTGACGGATCGTCAAAAACGTGCT
CAGTGTCAAGTTTAAAGAATTTTGTATACTGTGAATTGTTTTGAATTGTCGTGTGAACAATTCTCCCAGT
GCCTATCGTGTGGTCGATGCCTTACGTCCGCGGCAGGAGACGGCTCGGTTCGCTCCAATAGGATTCCG
CTTGGAGACCAGTGATCATCGCGTGTGCTAGTGAATTAATAACAAAGGTGCTCACAACTTAAACATAA
CCTTTTTGAACCGGCGCGTGTTTTTAGGACATGCGCAGTAAAGGTCACGGAATTATTAATAAGTTACAT
ATAATTTAACAAATAAACAGTGTGCCGGTGTGGATATAGTTGAAGTGTTGTGGTTAGTTGCTAATAGGA
AAAGCACGAGCCCCCTCAAACCTCTTTGGATTTCTGCTTCAATGCTTGTTGACAGTTTTTGGATTTGAA
TCCAGGATAACGTTCACCTGTGAGATTCAACAAATTTACCTGCGCGCGCACGAAGTTTCTTTACAAAG
GTAATACTCTCCATTATTCCGGCTTTCCACAAGA
>TCONS_00113033 geneID=XLOC_022578 scaffold_372:436549-567215(+) Intergenic
CGGCTTTGCAGCCGACGACCTTATCTTTATATTCTTCGTTTATTTTCTTTTCTATTCGTTTAATTTGTCGCA
CGGACCAAACTACGTTCCGGGAACGAAATGGATTGTCTTCTAGTGATTTACAAGTGTTCCAGTGTTTAC
CGGCGAGTTTAGTTTTGACAGAATCACGTGTGGAACCGAATTGTTGCCGGTTCCGGAGTTTTCAAAGT
GTTTATATACGCTGGCGACCTTCAATTACCACAGGTAGAGCTGACGGATCGTCAAAAACGTGCTCAGT
GTCAAGTTTAAAGAATTTTGTATACTGTGAATTGTTTTGAATTGTCGTGTGAACAATTCTCCCAGTGCCT
ATCGTGTGGTCGATGCCTTACGTCCGCGGCAGGAGACGGCTCGGTTCGCTCCAATAGGATTCCGCTTG
GAGACCAGTGATCATCGCGTGTGCTAGTGAATTAATAACAAAGGTGCTCACAACTTAAACATAACCTTT
TTGAACCGGCGCGTGTTTTTAGGACATGCGCAGTAAAGGTCACGGAATTATTAATAAGTTACATATAATT
TAACAAATAAACAGTGTGCCGGTGTGGATATAGTTGAAGTGTTGTGGTTAGTTGCTAATAGGAAAAGC
ACGAGCCCCCTCAAACCTCTTTGGATTTCTGCTTCAATGCTTGTTGACAGTTTTTGGATTTGAATCCAG
GATAACGTTCACCTGTGAGATTCAACAAATTTACCTGCGCGCGCACGAAGTTTCTTTACAAAGGTAATT
CAGAAACGAAAGCGTAATTTCAATTCACCAAGAAACTCAAAATGCTGGTTGGCGCAACTATGATGTCC
TGTCCTCTACCTCTGCCGCTGCTTCCAGCCCTGCACCCTCAGCCAGCTCTAGCGTCAGACTTCCCTATA
CACATTGGAATAAAGGGACCGTCCTCTTCCTGAGCGACTTGAAAGGGACAAGTGATAAAAACTTTACC
GAAAACTAGTGAC
>TCONS_00112978 geneID=XLOC_022578 scaffold_372:436663-557382(+) Intergenic
GTGATTTACAAGTGTTCCAGTGTTTACCGGCGAGTTTAGTTTTGACAGAATCACGTGTGGAACCGAATT
GTTGCCGGTTCCGGAGTTTTCAAAGTGTTTATATACGCTGGCGACCTTCAATTACCACAGGTTGGCGCA
ACTATGATGTCCTGTCCTCTACCTCTGCCGCTGCTTCCAGCCCTGCACCCTCAGCCAGCTCTAGCGTCA
GACTTCCCTATACACATTGGAAT
>TCONS_00113437 geneID=XLOC_022648 scaffold_374:139206-140737(-) Unclassified
CCTACGTGCCGCCCGGGCTTCGGAAGCGCTAGATTCCAGCAGTGAAGCTTTAATATCCTTCGACTTGCT

AGGTCGTAGTTGAGTAGCAAACGGCAGCCACGTTATTATGTATTATTCTTATAGAGAAGTTGATGAAAC
AACACCTTTTCCTAGATATATATTGTAGTTGTGATGAAATCGAGAGCTTAATGTACTGTGCCTTCAAGGG
TTCCTGCGATTTTACAGTGCAATAAATTATAAATAGTTACACATATGTAACTAAATAATAATATTTATTAAA
ATGAAAACCTACAAAAAACAAAAGTTAGGAGTATTTACATGCATAATAAAAATAGATAATTATTTTAGT
GTAGTTCAAACAAAGCATGAAATTTGTAAGTGCTATACAGATTTAATATATTTTATGGGTATAGAACGAA
ACGTAGCAATAACATGATGTACAATAAAATTATGTTTCCTGTTTTGTCTAGAGGATAAATATATAATTGAT
GCATTGATATTCTTATATTGAACATCGATTTAAATACACTTTAGGTACAAATAAAAGCACAGTGTGATGA
AGTGCTCTTTTCAACACCCACAATGCTTGTTTATTCTACATTATGTAGTAGAACTTAGAGCAATTAGATG
AAGTTTTTTTACTGTGATTAATGAACTTGTTAAGGATTATTTATTTATATTCCTTCCGGTTTTCACATGGT
CAATTTAAATGTATTATTGTTTGCCACATTCCTATTGTGAGACTGTTTATAACAAAGTATTTGAGTAACAT
AATACAGTATTTTATCAGCATTCCATGTTTACAAGTGTGAAGTGTTATATGAATAGGCATTAGGTATACAT
TGTACATGTACCTACAATATAAAAGATTTTTGGATGTGTATACTTACCTACCTAGACAGAGTTGAAGTTA
ATCCAGTGCAGTGCCTTATAAGTACCTTATATTGTCAAGTACAGAGTTAATATCGCTTTACCA
>TCONS_00113374 geneID=XLOC_022653 scaffold_374:275026-292135(-) Exonic overlap (+)
CGACTCCGCCCCGGTACAGACGTCTAGGGCGGCCCGCCCGCCCAAACATCGCGATCAACTCTCGCAA
CAATTAGTGCACGCGAGCTTACTTCCACTACCGACGGAAATTGGATTGGGTTCGAATGTTGTGCAAGT
GACTCGGTGATCGGAGGTGCTGTGCTTAGTTCGGCTTGTGTGGTTTGAACGGAACTAGGTACACAGTG
GTTAAACTTGTTGCGAAAGATTAAAAGTGATTAAAAGTGCTACGTGGGATATTTAAACATGTGAACTCT
AATTTTCCCTTGAAGAGCTGCAGCTTCAGCCATGTGAGCGTTCAAGACTTCATTCCACATTATGTTGGA
GACTGATTATGGCGAATCATACAACGAGTTCGACAGATGGGGCAACTGGTCGCTGGGCAGCACCGAG
G
>TCONS_00113585 geneID=XLOC_022669 scaffold_375:84334-85828(+) Intronic
AGAGTAGGTACGCTACGATATGAAACCGTTCACAAGTACGAAGATGTGACACGCTGTCGTCGCAAAGT
AACTTCCCGACACGGCGGCAGTCGCGCGACGGCCCATAGCATAGCAGTATAATCTAGCACGTATTCATA
GCAAACATCCTTCCACAGCACAGCTCGTGAAATGGCACGATTACAAGTTACAAACCGTATCGCCCGAT
GAACCTTTTTGGAGGGTTAAGTATTTATTACATGCAACATTCTATTTTAACGTATAATTTTTAAACATGGC
AACTCCAAGAGTCAGCGGCCATTTCTAAACGTCGTCCATCCGTTTTTCGGGATTGCGGAATTTTATACA
AATTACGGACTTTTAGAACAATTTAGTGATTATTGGACGCTGAGGAGTTTGTGCAGTGAAACCTGATGT
GATTTATTCCGTTCTTGTGTGAATAAAGACTGTAATAAGTGCTTAAATCTGCAAAATCGTAGCCAATGTC
AGCAGGCGTGTCAGATCAGCGGATGAAAGGGCAAGGGAACCAAGTGACAAATGCACCAAAAGAGCA
TCAGTTAGAGAGTGGAGGGGGTG
>TCONS_00113593 geneID=XLOC_022672 scaffold_375:264873-287098(-) Unclassified
CAGGGACGCGCCTCGACGCAATGCGCCTGCACAACCCGACGACATAACCTCACAGCGAAAACAGCCG
GCCTTCCAAATTCAAAACAAGATCCTGATTACAGATAGGTGTCTACGACGGGCTGAGCAGCGGAGTGG
TGAAACAGCGACTGCGGAGCAGATCGGCACTACGGCAGCGGAGTGGCAGCGCAACGGCAGCGGAGT
GGCAGAGCGACGGCAGCAGAGTAGCAGATCAACGGCAGCGGAGTGGCAGAGCAGCGGCAGCGGAGT
AGCAGATCAACGGCAGCGGAGTGGCAGAGCGACGGCAGCGGAGTAGCTGTTCAGCGGCAGCGGAGT
AACAGCGACGCAGGCGACATGATGTGACGCCACCTCCACGCAAAACACTGCAGAAACATACTCACTT
GCTCTTTTTC
>TCONS_00113803 geneID=XLOC_022698 scaffold_376:62942-63450(+) Splice junction overlap
TCGGCAATCACGGAATTAAAATATTTAAATTTCCAGTGATGTGTTGACATACAAAAAAACCGTACAAAA
TATGAAATTATAAAACAAACATTGTTTTAAAGAAACAGAGAAATGGCAAGTAGTAATTGCGCTGGTCTA
TCTAGACTAGACAAGTCTAGACTACCACAAAGTCCGTGTGTGGAGCGTCTCTCGACGCGGCCTATCCC
GCCCCCTGCCAATATGAATTGTGACAAGAGGT

>TCONS_00113719 geneID=XLOC_022710 scaffold_376:241281-254831(-) Splice junction overlap
CAGTCTGATGGAGGCGCTACAGAGCGGGTCGGCGTTCAGCAGAGAGCGGCCGCGTAAGAAGGCCAA
CCCGAGGGTAGCGGGAGCTGAAAGAAGAGCTCAACTCAGTCGGTCTCGATCCCGCTCCGGTCTGACG
GGGCCTTTATCCACCATGGACCTAATGACCAACGAGCTGCTCAGCAATGCGTGATAGCTTCACGCACC
AAGTGATATTAGTGATGTAATTATTGTCATTTAGAATAAACCGTAAGCTGACTACTTAAAACGTATTTAC
ACAGCCATGAGCGCGTCAAACGAGAATGAACCCTGTTGTTGTAATCTATACGGCCTAAAACGCAATGA
GAAAATGGCAGGTGCCAATAATTGAAAAGACAATTCGTAAAAGTCAAAATATGCTTGCGTAAAAAATA
TAGACTTTAGTGGCCTTTCCACAAAAACTTAACGCCTCAAAATCTGTCAAATTTCATTTTAAACACTTT
TTAATTTAAA
>TCONS_00113720 geneID=XLOC_022710 scaffold_376:251222-254831(-) Splice junction overlap
CAGTCTGATGGAGGCGCTACAGAGCGGGTCGGCGTTCAGCAGAGAGCGGCCGCGTAAGAAGGCCAA
CCCGAGGGTAGCGGGAGCTGAAAGAAGAGCTCAACTCAGTCGGTCTCGATCCCGCTCCGGTCTGACG
GGGCCTTTATCCACCATGGACCTAATGACCAACGAGCTGCTCAGCAATGCGTGATAGCTTCACGCACC
AAGTGATATTAGTGATGTAATTATTGTCATTTAGAATAAACCGTAAGCTGACTACTTAAAACGTATTTAC
ACAGCCATGAGCGCGTCAAACGAGAATGAACCCTGTTGTTGTAATCTATACGGCCTAAAACGCAATGA
GAAAATGGCAGGTGCCAATAATTGAAAAGACAATTCGTAAAAGTCAAAATATGCTTGCGTAAAAAATA
TAGACTTTAGTGGCCTTTCCACAAAAACTTAACGCCTCAAAATCTGTCAAATTTCATTTTAAACACTTG
TTTAACAATATTTTTTTTGGTAGTATAGTATCAGAGCTTAGGAAAATTACAATGTATAATTTGAGAAAGT
GTTTGTTTTTTTTTCGGACTGTAGAGAACTTGCTTCCGCGATGTAGGTTCGGCTAAGCGTGTGCTGCCA
TCTATTGAACCGTGTCGCAATCCTGAATCTGGGTCTATATTTATTTACACAAGCATTAAAATGTTTGTATA
TAAAGTG
>TCONS_00113964 geneID=XLOC_022745 scaffold_377:132011-135743(-) Splice junction overlap
ATAAATTCTTATACATGGTGTTTTATTCGATTTATGTAAAGACATTCTCAGTGTCAATTTAATAACACTTT
TGACGTCGGCTTTTCAGGTTAGGAGTAGTATTCGTCATCAGTACTATCTAATGCCAAGCTTATTTTTAGA
AAGAAAATCGAATTAACGTAGCAAATACAGTGCTCCTACAATTATTTGCCTAATACCTACAATTCGGTG
ACCGCTGGCAGTCAAAATGGGTCTTGGACTAGGATGGCTTATATCCAGATTTCGACGCAAAAAGACCA
CGAACGAGATTCTCGAGAAACTAGAGACCAACATAAAGCGAATAGAGCAGGACGGAGCGAACAAAG
AGCAACGAC
>TCONS_00113851 geneID=XLOC_022746 scaffold_377:156997-166455(-) Exonic overlap (+)
TAACGATAAAATCGATTGTATAAATAGATTATACTATCTATTGTTTGATTAAATTGAACATTATCATTATCA
AATGGGAGTCTGTGATTGGTTAAATTTTGTCCGACCTCCATTACAAGCATGTGATTCAAAATTTTCAAG
ATGGCTGAACTGAAAATGACTGCAAGTTTAGAAACGTTACAGGCGCGGCTGACGGTGAATACTACAGT
TGCACCTGATCCAGAGGGGCCAGAGTGCAAAATTAGACTCCTCTCATCACATCCATTTGAAAATGGCT
CCTACCCCAATCAACGAGCAACTGCTGGACATAGGACTCTTCCAAACAGCGCCACAACACTCTGTCCT
CAACCTTCCTAGTCAAACCGCCACCGGCTGCCGGCTCCGAGGGCATCCCACGCTACATGGCAGCCCGC
GCGAGAGCGTCCCACGCTTCATGCAGCCTATAATCTGTTAATGAAGAGAGCTATCCGTCGACAGTATTG
ACCATAAAAACTGTCATGGAGTTGCTCTCTTTATGAACGGTTGCTGGCCCAACGTTGGATTATGAAAAC
CGATGGATTCTTACTGAAGAAGCAGAAGATTACTACGGCAGATGCTGAGCTGGTGCCTTTTTGATGCTC
TGGACAGAAACCTGTTGTGTAATTATTGTGTTTATTTTATTGTTGTTTTGTACTATCATTTTCTGTTTTTTT
GTTGTTGTTGTCAGTGTGTCAAATAACTGAATCTTTCTTTCTATTTCTTTACCTAAGGTCTAACAACTGG
AAGAATTAGAAGTAAAGAGCTGAAGAAACTCGCTGAAGGCTTTTGTAGTACACTTAAACAGGAAGAG
GATGATCAGGGATGTTTCACTTACGACCAATGAAAAAGTTCCACTTAGAAAATATCGGCTCCAAATTCA
TTATAGGTATTCCTCAATTTTATAAAATAATTAATTTAGAATTTGATCAAAATATTTACTTTTTTAGTTGAT
AACATTCTGATGCGCTCGCTGTTAAATGTACCTACACTTCAATACTGCCGACTGCGTGAACTTTATCATT

GCTCGCGAGACTGTGCCAATTTCAGCATAGTCGGTTAGAGCAAAACCAGGTAGTAGTGGTTGACTTTT
GACACTTCTGTCAATAATTTTATATGGAGATGACGTATAATTGATTAACCAATCCCTGTCGGTTAGATAA
CCGACTATGGAGACATTGATACTAAAACCCGTGGGTGGCCCCTCAGAGCCGACAGTCACGGACGGCA
GGTATGGACGACAATGGTAGATGAAATATTTGATATACCTATGTATGACTGCCTCTGTGGTCTAGTGGTA
GAAGCTTCGCTTCATGACCCAGAGGTCCCGGGTTCGATTCCCGGGTGGGACCATCAATTTGTGTTTCTA
AATTGTGGTTCTAAGTTTGGTTAGGACATAGAAGGCTGATCACCTGATGTCCGAAACAGTGAAACGAT
CCATGCTGTCGGATGGGCATGTAAAGCAGTCGGTCCTGCGCCTAGCTCTCTTCAGTCGTGTCGGTCAAT
CCGTCCCATTGGGTTATGAGAGTGATGGAACAGAGAGTGCTCCTGTGTACTGCGCACACACTTGGGCA
CTATAATCTACTCCTGCGTAGAT
>TCONS_00113899 geneID=XLOC_022738 scaffold_377:169909-184742(+) Unclassified
ACATAACAACACAAAATTTCTTGACTTTTCTTTCATTCGCGAAACTCTGCGGGTGCCTCGAACGAACC
ACCAGGCCAGCATGAGAACCACACAAATTGCCCAGTACGCGTTAAGGCTCACATATTCGCAGCCAGCG
AATCGTGAGAAGATGATGTCGAGATCCGCAGCAGTCCTCGCGGCCGTCGGCCTCGGCTTGTCCACCTT
CAGCGTCCGCCAGATGGTCATCAGCCAGACCAGAAGGATCTAATATCCTCACTAGTAAACGCTACAAT
CAATTGTCACCAACACCCGACAGTATGAACATCACCTGTCAAAGTTAGCTGTCAGTTAAAGCTCTGTT
CCCATTGGTAGATGGGTTTCAAGGTTGTTCCACTGTATGGCGGAAAGAGCTGAGCTGTTTAATAGTTGA
AGCGAGTAAAGTGTTATCTGTTGTCCGCTGTTTTTTTCGGCAGACGCTTTATGTACGCTTGACGCTATC
GCTCGCTCGTTTCAGAGGATGCAACGCTAGTATATCTTCGCTACCAGTGGAGGCATGACTTCTTCAAGT
AGTTAAATGTTAGAGAGCTGTTGTATATGTTTGTCTGCTATCGCCTCTCAAATTCGATGTTTATTTGTGAC
TTACGTGACGCATCGAATGTAATATGTTAAATTTTGTGTGCCGACCGCTCTAATGTTGAATTTTTCAGGC
GATTTATTTAAATTCTTATCTTTAATGCGTGTTTCTGTCTGACTTTGAATGTGAAATGTACATGTGTTTTG
AATAATTATGGGAAATCTTTGATTTGTATAATAATGATAGTTGAAATCCGAATGTTTTGGTCTATTTTTTTA
ATTATATAGTGAATTAAGTTAAGTAGTCACCATTTTCATGAAACTAAGGACTTAACTATTATAATTTAGAA
TGGAAGATCGCGCGTGGTCGATTCGTGGATGTACTTATTGTGATGGCAACCAAATACAAGTAGATCCAT
ACCGTA
>TCONS_00113833 geneID=XLOC_022738 scaffold_377:169964-184745(+) Intergenic
CCTGTACGTAATTAAAAAATATATTTAATTTCTCCTTTCTCCGGTCGCTATACAAACCAAACAGTTTGTTT
TTATTAGCAATGTTCTTTTTATAAATTCTAGTGTACTTTTTTCTTAATGCCAGTGAAATTTCAAAAGTTAT
TCGTAGGTTATGTAAAGGGTGTGAAATTGTTCAAAAATGTGCATTCTGGATCGATTTTTGAGTGGTTTT
CGATTGATTTACCAGATTTTGTAGCGTGACGTTTTATTTGCAGCGAACGAACCACCAGGCCAGCATGAG
AACCACACAAATTGCCCAGTACGCGTTAAGGCTCACATATTCGCAGCCAGCGAATCGTGAGAAGATGA
TGTCGAGATCCGCAGCAGTCCTCGCGGCCGTCGGCCTCGGCTTGTCCACCTTCAGCGTCCGCCAGATG
GTCATCAGCCAGACCAGAAGGATCTAATATCCTCACTAGTAAACGCTACAATCAATTGTCACCAACACC
CGACAGTATGAACATCACCTGTCAAAGTTAGCTGTCAGTTAAAGCTCTGTTCCCATTGGTAGATGGGTT
TCAAGGTTGTTCCACTGTATGGCGGAAAGAGCTGAGCTGTTTAATAGTTGAAGCGAGTAAAGTGTTAT
CTGTTGTCCGCTGTTTTTTTCGGCAGACGCTTTATGTACGCTTGACGCTATCGCTCGCTCGTTTCAGAG
GATGCAACGCTAGTATATCTTCGCTACCAGTGGAGGCATGACTTCTTCAAGTAGTTAAATGTTAGAGAG
CTGTTGTATATGTTTGTCTGCTATCGCCTCTCAAATTCGATGTTTATTTGTGACTTACGTGACGCATCGAA
TGTAATATGTTAAATTTTGTGTGCCGACCGCTCTAATGTTGAATTTTTCAGGCGATTTATTTAAATTCTTA
TCTTTAATGCGTGTTTCTGTCTGACTTTGAATGTGAAATGTACATGTGTTTTGAATAATTATGGGAAATC
TTTGATTTGTATAATAATGATAGTTGAAATCCGAATGTTTTGGTCTATTTTTTTAATTATATAGTGAATTAA
GTTAAGTAGTCACCATTTTCATGAAACTAAGGACTTAACTATTATAATTTAGAATGGAAGATCGCGCGTG
GTCGATTCGTGGATGTACTTATTGTGATGGCAACCAAATACAAGTAGATCCATACCGTAATT
>TCONS_00114174 geneID=XLOC_022778 scaffold_378:258442-275562(+) Intergenic

ATTTCAAGGAATCTGTGAAATTCTAAAATTCACACAGGGTGAAGCATATGTTTAGCCGCGGTTCCGATT
ACAACGTGTGGCGTAGTGGCGAAACACCGAAGTTCCAGGTCCCGCCCAGTGGCACGCGATGGAAGCT
ACGGTTTAAGGAAGATTTACTGTCCCTAGAACTCCCCCTACAATCTTTTGAGTAGACACACCTCCTACT
ATGTTGAGTGGTGAAGATCCTGGGCGTAGCGGCAGCCCGCGGATTGATTGTGGTGATAAGGGTGCAGG
TGAACAATGTTGAAGCACTTGAAATTTGTTTTTAT
>TCONS_00114183 geneID=XLOC_022800 scaffold_379:45308-49149(+) Splice junction overlap
GTACTTTCCATGCTACCAGATAGAAACTGGCAGTAAGGAGTAAGTATTTTTCTCTATGGATGAAAAGTA
GACGAAAACTAAAGTCTTCAAAATTAAACTGCAAATTACGACAGCAAAAATAATATCAACAATATGCAT
TATTCACTAACATTTATTATTTTGACATGGATCTGCAGCTATAACTATTACAAACCAGTATTTGGATTACC
CATTGATGATGGAACATCTTCTGGCCAGGCTAACTTGCTCCCTAGTAGTGAGACTGATTCCACCGACGA
TATCAAACCCGAAAGACGTGTAGACAAAATTGTACGGGAAGCCGAAAAAGCGGTAAGTACCTATCGT
AAGTCAAAAATGGGTGACCGTTGTTATACGTTCATAATGAACTCGTGGGTCACCTCCTTGCGGCGTACG
T
>TCONS_00114314 geneID=XLOC_022818 scaffold_379:287123-287528(-) Exonic overlap (+)
CGCCGCCCCCGTCGCCAAGGTCGCCTACGCGCCCGCCCAGGTCCAGTACCACGCTGCCCCGGTCGCTA
AGGTCGCCTACGCGCCCGCCCAAGTGCAGTACCACGCCGCGCCCGTCGCCAAGCTCGCCTACGCCGC
CGCCCCGGCCCAGGTCTACACCCAGAACCTCGGACACGTCTCCTACTCCTCCCCGTCCGTCTCCTACC
ACCACTAGATGCCATGTTGTTAATATTTAATATCTATTTATTATGTTAAATAAAAACATACAGTCATTTGAA
TTATAAATATTACTGCCTTTACTTTTCCC
>TCONS_00114245 geneID=XLOC_022818 scaffold_379:287146-287552(-) Exonic overlap (+)
CGCCGCTGCTCCCGTCCACTACGCCGCCGCCCCCGTCGCCAAGGTCGCCTACGCGCCCGCCCAGGTCC
AGTACCACGCTGCCCCGGTCGCTAAGGTCGCCTACGCGCCCGCCCAGGTCCAGTACCACGCTGCCCCG
GTCGCTAAGGTCGCCTACGCGCCCGCCCAAGTGCAGTACCACGCCGCGCCCGTCGCCAAGCTCGCCTA
CGCCGCCGCCCCGGCCCAGGTCTACACCCAGAACCTCGGACACGTCTCCTACTCCTCCCCGTCCGTCT
CCTACCACCACTAGATGCCATGTTGTTAATATTTAATATCTATTTATTATGTTAAATAAAAACATACAGTCA
TTTGAATTATAA
>TCONS_00114357 geneID=XLOC_022818 scaffold_379:287210-287528(-) Exonic overlap (+)
CGCCGCCCCCGTCGCCAAGGTCGCCTACGCGCCCGCCCAGGTCCAGTACCACGCTGCCCCGGTCGCTA
AGGTCGCCTACGCGCCCGCCCAAGTGCAGTACCACGCCGCGCCCGTCGCCAAGCTCGCCTACGCCGC
CGCCCCGGCCCAGGTCTACACCCAGAACCTCGGACACGTCTCCTACTCCTCCCCGTCCGTCTCCTACC
ACCACTAGATGCCA
>TCONS_00114380 geneID=XLOC_022834 scaffold_38:20158-21432(+) Intergenic
AATTTAATTCTTTCTAAGACTTTTCACTAACTTCAACTGATGATGAATCTCCGTAAATCATTCCAATTATA
AATTCTAACAATAATGCTGTAGAATAACTGAGTTTACTGTGTTTACAATGTGCGCAACTCATCCGGTGTT
AGTTGGAGTGTGTCTGCTGTTGGTGGTGGCTGCCGGCGTCGCACTCGTTGTGACTCTAGTACTTGTCTT
GTGAATTAAGGTCCAAAAT
>TCONS_00115193 geneID=XLOC_022837 scaffold_38:34421-34930(+) Exonic overlap (+)
ACTTTGTTATCAGCTCCGTATCTTTACGCTCGGATCCAAACATTCCCTACTCAAGCGAGCTGAAAGTTA
CATGTTACTTATTAAAAATACAGCACCAAAACTGAAAAAACTGTTGGTCGAAACCAGTTGTCAAGTGC
CGTGTAAAGTAAACAACATACCTCTAGTTTAAAGGATAAAAGTGAAAATGGGAAATTCTAAAAGTAGA
AAGATGAGAGTGACCGAGATTAATTCGGAGAATACGAAACCAGAGGTGTCGAGAGAAAGGATAAACT
CTTTGAAAACTGACATACCAGATGCGGCAAATGACAGTGGTAACCTGAAAGCTGGTGGGGGAATCATC
GATGATGACAATGCCAGCAGTGCCTCCACCGTAGAAGTGTTGGATCAAAAGTGATATCATGATGACAT
GACC

>TCONS_00114733 geneID=XLOC_022873 scaffold_38:79981-83219(-) Splice junction overlap
CTTTGTTTACCCGAACGCAGGCATTCTGCTGCACTAAACACGACTCTATCACGTTGGATTAAAGCTATC
GATAGTATGGCCAATAAATATTATATCCAAAAATATCCAAAAAATATCGTTTTCCCAGGAATCTCGGTATC
TTATCGTGTTGTCGGAGCAGGTAGGCAGTGACGAATCTTCATAGGTGCAGACAGTATATCAGTATAGCT
TCGACTATTGCTTAGTACTTAGGTTGAATAATATTTCATGAATACTTTAGTCTAACCGTGTATACCGGTCG
TAACGTACGGCAAAGTGGCGTGACTTTAAATACAATAAAAATACAAAAACAAAATGTGTCTGGGCTGT
GTGTGCAATCTGATGTGCAAGCTGATCAGCTTAGCGATATCCATCCTTTTCATCATAGCAGTAGTGTTGC
TTATTCTTTGGGGTGTCGGAGTTATATTCCAAAAAGAAGGAGAATCGGGCAGCCCAAGATTTTATGAAA
ATAATAAGTTTCTTACTTCTTAGTTAAGTATTAGTATTAGATTAGTAAGTACCTATAGGTAGTTAATAATAA
TTTATTTTTATATATGTAAGTTTGTACCTATGAAATTTATCATAAGTAATTTATTTACTTACTTATTGTCAGT
ATTTTAAAGATTAATCAAGCA
>TCONS_00114442 geneID=XLOC_022873 scaffold_38:80586-83012(-) Splice junction overlap
CTTCGACTATTGCTTAGTACTTAGGTTGAATAATATTTCATGAATACTTTAGTCTAACCGTGTATACCGGT
CGTAACGTACGGCAAAGTGGCGTGACTTTAAATACAATAAAAATACAAAAACAAAATGTGTCTGGGCT
GTGTGTGCAATCTGATGTGCAAGCTGATCAGCTTAGCGATATCCATCCTTTTCATCATAGCAGTAGTGTT
GCTTATTCTTTGGGGTGTCGGAGTTATATTCCGTAAGTACTTACTTAAAATATATTTACTTACTTCTTCAA
TGGTTTCAGCCGAGTTTCACAATAGACGTGGTAATGGTAATAGGACTGACCGTGAGTCTCCATGCACG
GGTCAAAAGACACTACGTATACAAACTATACAAATACATAGACATAGTTACCTAATCCTATTCGTAAACA
CGTGAGTTATAGAAGAAAGGATGTTCTTAGACAGAATTAGGTAGGCTCTATTTACTTGCGAGCGATCAT
GAAAACATGGCTTTAGATTTAAGTACCTACTTTACTATAAGTACCTACTGATTCTGATCTAAAGGTGGCC
CTTAAATCGGGGGTCTAGTTACAACATCATAATAATATCCTTACCTCGTCTGTGGTTGGAACTAACAAAA
CACTTAGTGGAATGAGC
>TCONS_00114443 geneID=XLOC_022874 scaffold_38:84160-86575(-) Exonic overlap (+)
AATTTCTGTTAATTTCTAAATGGACGACGGCTGCTCATGTGGTGGTGGAACTTTTCACTCTTGTAACCA
GACGGGTACGCTTTCGATAAATATGTGTCAACTCATATGGAACATACTAACGTGTATTGCGTTCATGCTG
ATTATATTTGCAATTATACTGCTCATTCTGTGGGCGGCTGGAGTAGTTAAGGTTGAAAAGGTGAACAAA
ACCGATTCTAACTATTAGCATGTACTTAATGTTTATGTAAAGCGTGTAGTTATTTTATTCTGTTAATTAAAT
AAAG
>TCONS_00114444 geneID=XLOC_022875 scaffold_38:88020-90698(-) Intronic
GTTTTACTTATTTAGATTTTGCAAAATGATTACTGTAAGTCTGTAGGTAATTATTATGTTTACCAATTCTC
ACAAAAAAAAAAAAATTGACAATATATTATTATTATTTTACATAATATACTGAAATCAATTTTATGCATTTAT
GAATACTATTTTATAAAAAGTTAGAATGAGTTGTGCATGTCTAGCCGACATCCTCTCGTGTATATGTGTC
ATGGCAATTTTAATGGGCATCATAATCGTCCTCCTGTTAGCTGAAGGATGTCTCAAAATTAAGGCTCCCT
CTCCAATAAACGGTTCTATCAACAAATAATGATTTGGTGACTATATTTTACTGGTACTTTTACTGCACGAT
GTATTAATGACGCAGGGATAAAATATACCAAATTTTAATCCAAGGAGTAAATCCGTTTCGAATGGATTAC
CAACTGGTAACCAGTCTATAAGTAACTATTTGGAAGTTATGCGTGGGTGAAGGTCCCATCTACAATAAT
TTAAGCATTCTTTAGCATTAGTATTATTAATTTATTAATAACTTGGTATTGAGTTCTTCCAATACTGCTCAA
AATGAGTTGCCAGTTTCTATGGAGCATTATGAGTTGCCTTATATGTGTAATAATTATATTGGTTATCATTG
GATTGATACTATGGGCGATAAGAAAAGCATAGTCTGGG
>TCONS_00114445 geneID=XLOC_022875 scaffold_38:89058-90705(-) Intronic
GTTCATAGTTTTACTTATTTAGATTTTGCAAAATGATTACTGTAAGTCTGTAGGTAATTATTATGTTTACC
AATTCTCACAAAAAAAAAAAAATTGACAATATATTATTATTATTTTACATAATATACTGAAATCAATTTTAT
GCATTTATGAATACTATTTTATAAAAAGTTAGAATGAGTTGTGCATGTCTAGCCGACATCCTCTCGTGTAT
ATGTGTCATGGCAATTTTAATGGGCATCATAATCGTCCTCCTGTTAGCTGAAGGATGTCTCAAAATTAAG

GCTCCCTCTCCAATAAACGGTTCTATCAACAAATAATGATTTGGTGACTATATTTTACTGGTACTTTTACT
GCACGATGTATTAATGACGCAGGGATAAAATATACCAAATTTTAATCCAAGGAGTAAATCCGTTTCGAA
TGGATTACCAACTGGTAACCAGTCTATAAGTAACTATTTGGAAGTTATGCGTGGGTGAAGGTAAACCTC
GTTAAGTAACCTGAGTAATTATACAACCTACTACTTAATTTTCATATATCGGAACCCACCATGGTTAGAT
GAGTAAAGAAAAGGACAGCTGTCTCGCTAGAGTTCTGTGGCGCTTTGGGAGCGGCATATGTGCCTATT
TTATACCTGAGTGTGAGTGTTCTATCGTTACGTATACCGTGCCATGAGAAGCAGAAGAAAATAGGCCAC
ATTTAATGTAGCAGAAAAGCAAATCTCTAATAATTATAATAATCGAATGGA
>TCONS_00114394 geneID=XLOC_022845 scaffold_38:465966-466901(+) Intronic
TTTGTCAGTTACAGTTGGTGTCGCAGGCAGTGAGGTCTCTGGCAGGTGGATCTGTCACAGTGGGATCT
ATGGCAGTGGGATCTACGGCGACGGCGGCTGCTGGCTGCTGCGTCAGGGCAAGCTCATCGAGTTCTCC
TATGCTAGATCGTACATTTACCGTGGTACCAAGAGGACTGTATATGTACGGTCGGCGGGCCATTACCTC
GTACCGCTGGACCCCCCATCCGGACACCGAGCGTCACACTGGGACTATCAGTGAGGTGTTCCAAAAA
GTGTTGTGTTTATTGTACGGTCGGAACTCTGCACGAGAAGCCACGATGACCTGAGGGTGTAACACACA
GAACAACCGTGTGAAAACCTAACGTGAGAGTGCAATAAAATTGCATTTTTTGGAAGCTGGTTACATAC
TGGAGTGAATATCATCAAGGAAGAAACCAATTGACTTTTAGTCAACTTCTGGGACTAAGAACTTGTAA
TTGCCCGTCTAAAAAGGATTTAGAGTAAGGTAGTTATGTAATTTAGGATTCACTCTATATTTAAAGTTCT
AATAAAGTTTTAGCATCAAACTAATATTTAAGCCTAAGTAGATAATAAAACAAAGGTAAACAAAAAAA
AATGCAAAAAATAAAATCAAAGAGAAAATAATAAAAATAAAAAACAAAAAAAATTAGGTATAATCTTA
ATAGCTTAGATGTACATGCTTTATTATATAATCTTTAACCTGAC
>TCONS_00114471 geneID=XLOC_022882 scaffold_38:495559-497116(-) Intronic
CGCTTGGTGATAGCTTGTGCTTATGCGGTTGTGGACTGACCTGCCAGTTTACAGTCGGCGGTTATCGAG
GGCACAAGCCTCAACCGCGCTAGTGCCGTGCTACTGCGCCGCAAGTGACAATCTAACTGGAATTTAAA
AAAAAAATAGTGATTTAATTATTGCATTTAGTTAAATCAAGGAAAGATATGAGGCAGTACGTGGGAGTG
GAATGTTCTTTCATTACAAGTGAATAATTGTGCCTAGCGCAGTGAAGATGTTCAGTTTCGACAAGGACT
TATGGCACGTCGGACGCACCATGCAGGAGCCGCGGCAAGAGACCCCTCCGACCCCTCATACCAACC
>TCONS_00114867 geneID=XLOC_022882 scaffold_38:495559-497116(-) Intronic
CGCTTGGTGATAGCTTGTGCTTATGCGGTTGTGGACTGACCTGCCAGTTTACAGTCGGCGGTTATCGAG
GGCACAAGCCTCAACCGCGCTAGTGCCGTGCTACTGCGCCGCAAGTGACAATCTAACTGGAATTTAAA
AAAAAAATAGTGATTTAATTATTGCATTTGTGAGCTTTTGAGTTAAATCAAGGAAAGATATGAGGCAGT
ACGTGGGAGTGGAATGTTCTTTCATTACAAGTGAATAATTGTGCCTAGCGCAGTGAAGATGTTCAGTTT
CGACAAGGACTTATGGCACGTCGGACGCACCATGCAGGAGCCGCGGCAAGAGACCCCTCCGACCCCT
CATACCAACC
>TCONS_00114675 geneID=XLOC_022849 scaffold_38:536971-543692(+) Unclassified
CTTTATTATTACATCGTATTACAATTATCAATTATATCCTAAAACCAAAACCAATGTACAAAACGACACA
GTAAGATATAATAAAATAATGCTATGCGGCTGCTCGTAACAAAAATATGTAGGTCTCAGGCCATCCATAA
CTTCTTAATAAAAAAATGGCTTATTTTATAACCCAAGACCTTAGGACGTAATTTAAAGGACTAACTCGTT
ACATTTAAAACCTTCCCCAAACATTAGTCCGACCTGGGAGTTGGAAAATAGCAGACTTATGGCACTAA
ACTGATAACGCAAGTTAAAACAATCCTCTTTATAAACATTGATAATAATAATATGAGCAGAAGGGAAAG
GAGTGGCCAATGCGTGCGAGCGAGACAACTATACGTCCCCACCGCCGTCGTCAGAGTGAGCGTACCAT
AAACAACAAAATACCTTTTATAAATCATTTCAAAATAAGGGCCACACTTTACCAATATAGGGGAAACTT
GGGATAAATGTAGCCCGATCTTGAACAAACACACGAGCACTCTGGCGACGCTTCAACCAGCCACGCA
GGCGACAACACACACATATCTATATTAATACAAAACAACAATAAATAACTATTTATATAAATTCAACAAA
AAATACAATATCCTACATTGAAAATTGAGCATATGGCGTGAAATTAGCTAAGAAACGGGCTTGAATGCT
GGGCTGCTATTGGTCGAACCGGCCAAGCTTTTAACCAATAAGCGGCGTGAGTCGCACACGGCGGCAA

AGTTAGAAAACATTAAGTAGATCTATGAATACACTTAAAACAAGATTAGAAAACATCCAAAAATACAC
AAGACCATGATTTCATCAGGAAAATAAATGTTCTAAGGTTTTCTAACAGACTAACTAAAACTACCAAGT
AAGAGTGATAAAATAATATGTTTTAATAAAATTAAACTACATAAATATTTAAATTTTACACGGATATCCCA
TGTGTTTAGTCAATTATAATTGCCATGAAATAGAATAATACAGTGAAATAAAAGTAGTGTAAAAGTAAG
ATAAAGCCAAAAGGTACAGAATATTGTCATGTAGCAGTCGATGACTAGCTTAAAATTACTCTTATTGCG
AAGTAAGTTATCGAGCTCACTTATATGGACAATTTAATTTTAAAGTTTTAAATTCGTCTGTTTAAATATTT
AGACACATCAATGAGAGAAGCATTAACGCTCGAGTTTAAGAAATAAAATGCCAGATGTTTTTAAACAA
GTATTACATTTTAGGAAAAATCTTGGTACAAAGATTTGGCACACAGAGAAACATGCAGTTTTTACACAC
AACGAAATTTATTTAAAATGAAGATACTTTAGGCACTTGTGGCTTTTATTTACACATTGTTACAATTATAA
TAGTAAAACATCGATATTTGAAGATTCTATTTAAAAACGCTTTAAAACAAACCGATTTTCTCACAACCA
CTTAGTAATGCCCGAATATAAAAATACAATAGAAGAGTAAAATAGATTCAACAACAATAATATCCTCTCA
ATCATAAAAAAAGTGAGGGAGTGAAGATACTATACTTGAGAAGGCCTTTAAGAGGACTATTTTATGAAT
AAGGTTTACGGTTTTGAATTAGTGTGGCAAGACATTGCACATGCATCAGTAGGCACTAGATAGACCGG
GCTTTGTGAGATGTAAAACAAAAACAAACACCAGAATTTAAAACAAAATTTTAAATCAAATTTAACAA
AATAAACTCTTCAATCAATAACATATTTGATCCCTGATTACCTAATGATTTCTAAAACTGGGACAGATAC
TATGATCGTAGGTAGTGGGTACGGTGAACGTTATAAATAGTATTAAATGTGTATTTGAAACTGGTAAAAA
CTAACCGGTATATATTACAGTAATCATGATCACAAGAGACAAATTTCATAACACAGGTTGATGAACACT
TGGCACGATTTAGGACAATCCAACATTATATAATTATTATGTATTATATCAAACAAGATATGTAACAAATT
TAAATACAGTATATACAATTTATTTACGGGGGCTATTCACCTGATATGGGGTTAATTTAGACATATTTTAC
ACACACACACACACCAAAAATAATTTATGATTCTGATTGAAGTTTACTTTTGATTCACATATTTTACTTT
ACGCGGTGTAGGAAAAATATAGTACATAATTTATAAATTTTTCAGTGCTACATAACAAGTTGCTTTTTATA
TAATATCTATATCAAATGACAAAGTGGTTTATAATGTGGGAGCACAAGTATAATTTACTAGGTCTTTATTT
ATAAATACATATTATATACATTGTTTTACAGTCCACTAAACACATAACACACTAGATAAACGATACAACA
CTGCCCCGATTATAATTAGAAAACCCCATTTAGTTATTGATATTAAAACTGTTTTATCTCTTTCACACAAC
CTTTGAAAACACTTGTAAAGCCAGAAACAAAACTGTTGCGTTATTAGTAACATGTATTGTGTCATTTTA
GTGTGAAGTAATTAATTTAGCATATGATTATGAACTGCACAGACCATTAGTCATTACAGAACAGAAGTG
ACCACAGATATTTTATTTACCAGACTTTTTAGACATAATAGTATAGATTTTAGTTAAAACCCTAGTTAACT
CTACCGTAAACTCATTTAGATAATAATATATTTATAACATACATACATATATACGATTTGTAGTTATATTTTG
TCGGTTGCAAGTAAAAAAAACAATATCATTAGTACTATCAACTAGACAATTTTGATTTGAACCCTTACT
GTAACTTTCCTAAGATTTAGTTTCACCCAGCATTGCACTGTGGCATTTTATATCGACTCGAGTCATAAGT
TTTTACAAATATTGTCAAACTACCATTGACCATAAATATACAAAGTAATCTAACCTAATATTCTCACCTTC
AATGTTAATCGTTCGGGTGGAAGGACAGATTTCACTATACAATTTGAGTAACAGTAACATAACATTAAA
AAGTTTTGTTATTTACACCGCGTGACACACCTGACATTGCTTTACACGCGTAGTGCTCCGGGGTGCCCC
AAGGTAGTATGTTAGGACCTATGTCTTTTCTAATTAATATTATGGTTGCGGTGCTTGAAACGAGCAGGAA
AGTAGGGGAGGGCAAACTTTCGGTTAGATGGTTTTATAGGTATATTTAAATTGACCTACTACAACATGAT
AGCACTATCTATAATTTAGAATCATTGTGACCGGGTCAGTCTAATAAAAACTGCTAGCTTAAATCGTGCC
ACTTTTCGCTTCACCTCGATCAAAACATTTCAACATTCACCCACAGACCAACCATCCAACCAACATGG
CTACACTATACGCCCACGATTACAATGGATGAGGAAACGCTTACACCAAACCACCTAACTCAGCAGCA
AGTTACTTAAATGAATGACCTCAGCCACTTACAAACCAACAGCGTACTATAATCACATCAGCCGCGAAT
TCTAGAAGTTTTACACCCCCACGCCCCCTCGCCTTCCCCGCTAGCGCCGCGCGTGGGCCCATGCGTTG
GCCCGCGCGAGTGTGCGTGCGTCGTGCATCACAACTGCTTGTGCTTCCCGTTGGCGGTGGGGGGGCC
AAGCGCGCGCTCCGCCGCCGCCGCCGC
>TCONS_00114748 geneID=XLOC_022887 scaffold_38:605266-613895(-) Exonic overlap (+)
GTACGTAACGCCATTCTCAAGTGAGAAACTGGGCATAAAAGAACATAATTATTGGTTAATCCGTTTCAT

TGCTACGAACGAGTCCATCGGTTGATAAACACACACGTTAAATTTGTATAATAGCATCATTATCAGCAG
AGACTGGGGTTAAAAAGCTAGCTTCTACTCACTGAGCACGTTAAATCTAACGTGTTGAATGTCACAGT
GGCTGGCTATAGCAATAGCAAGCGACGACATTGACATTGAGTTTATGTATCTTTGAACTGATTTAACTTT
ATGATGGCGAGGTTAAGTTAATCTTGCTTGATTGTGCTGTGTTGATATATCTTCTTCGAACAAGACCGGT
AGCATTTTCTCGTTATACCTACATACATATATTATGTAGTAGGTATTTATTTAGAACATGTTATTATGATAA
GAGTTTATGTGCCGTTCACGAACTTCAAAAAGGAGAACTTGAGTTTGCAATGACGCCACCTTTAACCT
CACCTGGTCATCCATAGCATCTTGCCATTAATATATTAGAATTAGGGAATATGCAAAAATTCCAGTACCTA
TC
>TCONS_00114726 geneID=XLOC_022853 scaffold_38:609822-614644(+) Unclassified
CTCCGGGACAGGTACTACTAACAGTGAATGAACTGAGTGATCTCAATCAGCTGCTTCTCATGGGCCAC
ATTCCTCACATGTTTGCCTTGATCAGATATTTCTTTTAAAATAAGCTTTATACCTGTTTTTATAATTGACTA
CTGAGGGTGCAATCCACGGAGCGGAGCCACCATCTGAGCTCCATGTTCAAACCCTCACTTCATGTTTA
AGTAAGCGATACGCTGAAAATGTATGATATGTACAAATTGTAATATAATAATTGAATGAATTTTAAAGCA
ATTGATGTTGCTTGAGTCCCACGCTTAGAGTCATGGAGACGAGAGTGAGCTGATCACCGCATCACACT
GAAGTGAGCCGATCATCCTGCATCTTGCATGAATTGGGCTTTCTGCACAACTATCATGTGATCCTCGAT
GAGACCATCACTACTTAGTCACCCTTAATGGTTTCATGGCATTTATTCATCAAAGATAATCATAATAATAT
AGTTTTAAATTATTGTATTTATATTTCTTGTTAAGGCTTGTTCCACAGTGCCCAAAATCTAGTTACATCAA
AAAAGCTACTTCTAGCAAATATATAATTGTAAGTGTTAAATCAAATAAGAACCCTTGTTATTGTGAAACA
AGCCCTAGGTCAACGAACTCTAGGTTAACTGGACAGTATTCCAACCAAAAAGGAAATTGAGAAAATTA
GACAATTTACTAAATAATGCAGGTCTGTGATGGATAGTTTCCCAAAATGAGATTTTTGATATCTGTGCCA
GTGTGCACTGACGAGTGATGTGCGCTAGCACAGAAAGAATTTTAGCACTCTACTCATCCAAAAAAAAGG
TCTAAAACTTTGAGAAGTATCCATCTACAGTCTACACACTTATAGAAGGTATTTCTAGAAGCCATAACT
GTGTCAATTTCCATAATTGTGTTTAATTAACAATTAATAGTTTATTATTAAAGGTTTTTTGGATGAGTAGC
AATGTTTTGAATCTTAAGTTCAAGCTACAAAGATCTCAGGTGCGGTAAATAATTTAGATTGTGTTGTTAC
ATTTAGGTTATTTAATAAATAAATTAATATCTGCCCAAACTGTCTAAATATAAAATAGATTTAAAAGTTAG
AAATACGAAAGTCAGGCGACATGAAGCTGTGAACTATATTTTTAACCAAAATAAAAGCCGTCTGCAAA
TACTATGTGGCAGAAACATACCAGTTCTTTTAATTTATTCCAAAAATATTTATATCTTCATAGTTACCTCAT
CTCATTAATTATTATAACATGAAACTGGTACCAATGTCATGACTGCCGCCATTCCGGCCGCCTGGCTATC
TACAAGTCGTCGGCATTCTACAAGTCGGACTTCACATTTCAAGATCAATCGGTTACATCAGCATAAACC
ATGCATAAACACAACATTGAGAAAACTAAGTTAGAACATTTTAGGTTCCTTTTATTTAAAGTGATAAAG
TGGTAATATGATAAAGTAGGTGTGTTATCATCACCTGCAACTGGCTCACTCGAATGGAATCTTTGTATAC
TTTTCCCTGAGGCCTAAACTCCTTTCCTAAGCTTGGACCATTTGCCACCACGCTGGTCCACAAAGAGTT
GGTGGGTTAACCCATCTAGACATAGGTAGGTTTCACTATGTTTTCCTTCACTGTAATACGTGGACTTGGT
ATATTAAATAAAATAATAATACATACAATTGAAGGTAAAATCCTTAATTTTAAAGAATTCATTATTTGAAC
CCACGTCCTCTCAAATGAGACGACGAGTCCTTATCCGCTAGGTCATCCACCACCATGGCTCATATCAGG
TATATTATATGTAGTGAAATTATGCTGTTGTTTATATTCAGCTGTCGTTCAGTTCAGCCACCCTACAACCA
ACATGTATTAATTAACTATGACACATTTAAATGTATCGCACTTACCTACGCGTAGTGTGGTGTCCTATTTA
GATAATTAATGTATAGCCCACGCCTCTGTGTGTTAAACCATAGTTATAGGTAGGTACTTGTTGAACATTG
TTAACAGGGAACCTGCGAAAGTAAGATTTGCCGATGTATCAATAACTAGCTGTTCCCGCGATCTTCGCT
TCGCCTTAAAAAGTTTTCCCGTGGGAATTCCGGGATAAAAAGTAGCCTATGTTCTTTCTCAGGGTCTAG
ACCATATGTATACCAAATTTCATTCAAATCCGTTCAGTAGTTTTGGTGTGAAAGAGTAACAGACAGACA
GACAAACACAGTTACTTTCGAATTTATAATATTAGTTAGGATAAATTAATTAGGATACCATCTCTAGATAT
AACTAGGTACCTACTGCATGGAAGCTATAGGGAATGGTACAGTTCGGTGGTGATGGTATGAAGTTGGC
AGTGAATGCACATTTTAACTTTGTCAAACTAACCTACCGCCTATCCATTCTTTCATAGACTGACAGTTGG

TTAGTTTGACAAAGCACAAAAGTGTTCATCAGGAACTATCAAGTTGTTTGGGACCGACTGTAACTTGT
ACCGTTCTTAATTTGTGCGTTACGCTTGATGGTAACTTCCTGAGCCTAGCGAATTGCGATACCCTCTAGT
CTAGTGTATTGCTCACCGAAGGCAGGAGGCTTTTAGTTCCATGCAATACAAATATTTTACCTAAATACCT
ACCTAGGTTATTCAGTATTATTTAAACATTAAATACCTAGTATTTCCTGGCGTCTTTTTATCGCCAAGTCG
AGTCACATTTATCAAGTCTAGAAGGAAACACAGTATTTTTCTTCATACCTTTGTTTGGACTAGTCAAAA
GTTGTCAAAGTTATCCCTCGGCTCGTTGCCACAGTTAGGCTCGGTGACTAAAGTACGGGATGGAGTAC
TGTGCTTGGCCGGATTGTCTACGAGTAGGACTTAGCGCAACGGGGTCTAAGGCCTAGTTTCTCATTAGT
ACCTATATCAGTTCATGAACTTAATTCAGACAATCACAGCGCAACATAGCATAGTTACTTAACGTAGTTA
AGTCAAGTTTAACTTGATGTTCTCATAATCTTTAGGAAGGGAACTCAATAATAATAGTAATAAATGTAAG
TAACTTATTTTATATTTCGAGCCAGTTCTGTGTCTGAAAGTCATGGTTTCATGAAAACCTTGCTCACGCG
ATAAATTTGTGACGCTTATATCTTCACACTATTTACATAAAATAAAACGTACATACATATGCCTACATTGT
GTAAACATTGGAACACAAATAGCACTGAAAGAAGTTTAATCAACCGTACAGAGGCATACTAATACTGG
TGTGTAGGATTAATTGCTGAAAATAATATTTTTATAACTGTATTGATTGAGCCATGCGGTCCATGAGTGA
CCATTGACTATGATATTTTACTAGCGTTGTTTACTAAGTGCTCTGAATGGCAATTATAATAGAAGCGAAG
CAGCCCACTCGGTAGGGTTGACGTCACTATTCCAATATGCCAGAGCAATATGAATGAAGGCATCTCTTA
TGGCATTTTACTTGCCAAGTATATTTTGTTTTAATATGTTACTGGAATTTTTGCATATTCCCTAATTCTAAT
ATATTAATGGCAAGATGCTATGGATGACCAGGTGAGGTTAAAGGTGGCGTCATTGCAAACTCAAGTTCT
CCTTTTTGAAGTTCGTGAACGGCACATAAACTCTTATCATAATAACATGTTCTAAATAAATACCTACTAC
ATAATATATGTATGTAGGTATAACGAGAAAATGCTACCGGTCTTGTTCGAAGAAGATATATCAACACAGC
ACAATCAAGCAAGATTAACTTAACCTCGCCATCATAAAGTTAAAAACAGCAGCATGTACTAATCATACG
ATGGGGAAAGGCATAACTTAAAAATAATGAGTGGTGATTAAAAATGGCTTTTGTTAAAAAAAAATTATT
GCACTTCACAGTTTAAAAACAATACAAAAAAAGACAATGATATCAAATAAGTTTTTCATTATTTTCTTTC
GAGATTTTAAATTTGCATAGCTAATCATGAATTAACTAGGTATATTTTCGTATGAAATATCTTTCATAAGTT
CTGAC
>TCONS_00114699 geneID=XLOC_022890 scaffold_38:681676-682349(-) Splice junction overlap
TTAACTGTCAAAATCTGTAAATTATTAGATTGGCTGGGTTGATGATGTGTTTCGATCAACGAAATCCTTA
ATTAAATAGTTTAACTAGCATGTAAATAACTTCCTAGCAATCCCTGCACCATACCAATAATGAATTTCAA
CGCGTCAAGGAACGTGGGTTCGTCGGGAGGGCTGCGGAAGGCGAAGCGCGTGAGTGACGTGGCGGC
GATGGGTGCGCCGTCGCCCACGCCGGGGTTCAGCCCGGGCCCGGCGCCCGCCTACAGCGCGGGGCCC
GCCGGCGCGCCGCCGCCGTACTCAGAGGGCATCCCGCTGGACCACAGCCAGGGCTTCGGACCCGCCG
CGCCCGGAGGAGATTTGGGTTTGTGCCCGGATTTCCGCA
>TCONS_00114502 geneID=XLOC_022892 scaffold_38:724515-726200(-) Splice junction overlap
CCGGTCAAGCTGGCGCGGATTATGAACAAGTTGCTAGACCGCGAGATCCACGCACTAACGAAAGGCC
CGATGGTGTCCATGTTGTATAGCAACAACCCGCGCGCGCTGTACCGCGCCGAGCCCGCGCCGCCCGTC
GTCTCACCACCCGCGATGTCGTCCATGTCCGGCGGCAGCGGGCAGCTGTCGCGCTCGCTGTCGGGCG
ACATGGAGCGCCTCTCCGTGAGCGAGG
>TCONS_00114423 geneID=XLOC_022858 scaffold_38:1008191-1008959(+) Intergenic
GTTGTTGCGTGCTACTGACCAACAGATGGCGTTACTGGTAGCTTAAATGGCTATTAGCTTCACATTTCAT
TAGCGGCGGTATGCCAAATAAAATGTATGAGGGTACTTCTGGTCTACCAGTGCAAAGGTACGACTAGTC
TTTTTGTGAGAAATTGTAACATTATCGACATCTTTGGTGTAAGATTGATGAGATTTAAGTTTAATCAACT
CTGCGTATTGTGGTTGAATTGTAGTTTTTGTGCATCTTCCGGAATAATAGGGCCTAAGGAAACGCCCTTA
TTATACACGGAGATATTATTTACGGATCTATTAAGGAAGACTTGGCCGTATATAGTGTTGGACATTTTCAC
ACGGTCACTTATCAGCACAACAGCCTAGTCGGTGGAAGCTCGGACTGTGGGCCTTCGATCGGGAGTTC
GAGTCCCGCGGAAGTCAATTCTTTTTGTTTTTTTATTTTTGCCTTACAAGCATTATTTATTTGCTCATCA

TTTTAAATGGTGTATTGAACGGTAATAAATAGGTTTTAGGAATTAAGTTAGATTAAGGTTAGTTTTAATTA
GAGATAGGTTTACACCGGGTAAATGTCGGTTTCGTTAGGTTAAGGCTTTAGCAATATATACCATTGGAGT
GACTGCTATATTCACTTGTATCATTTAAGAACCCC
>TCONS_00114428 geneID=XLOC_022861 scaffold_38:1134929-1135540(+) Exonic overlap (+)
CTAGGCGAGCCACGGACCGCGCCTCGCGAGCACGCACACACTGCAACCACGGCCTTTCTCATCGTTAT
CTTTATCATTATCGACACCATTGACTAGCAAACTAGTCCCGCGGGTTTTAGAATCGCCGCACAGTGAAC
TAGTGCCGGCAACAGTGAACCAGTGACAGCTCCCGTTTTAGAACTGTTTGCGGTACACATACCGATCG
GAAGATGAACACCGTTCGGGCGCTTCTTTTTGCGTTCCTTTTGTGTGTGGTGTCGGTGAGCGGGCGGC
ACCACGGGTCAGACGCGGGGGAGGTGGGCGCGCGGGAGGAGCCCGCGGAGGCGGGGAGGAGGGCG
CGGGAGGGGCATCTGCGCCGGCGCCGGAAGCGCTGGCAGGCCGGCTTCGG
>TCONS_00114545 geneID=XLOC_022900 scaffold_38:1264349-1264771(-) Intergenic
AAAAGTTTTAAAGTGTTACTAAATACTATTTGTTTGACAGAAATTAATAGTTTCGGAATGTAGCTTTTAA
AATTGAATAGTGGACAAATCGGTATGATGCTCGTAGAATAAACACAAAAACACCTGATAGATAATCAAT
GCCTTCCAAATAGAAATAGTGCCTTTAACTTTAGAGTATTGTGTTCACAATAAGCTCCATAACGGGCAG
TTTTAAGTGTGCAGTGTCCCTTTAATCGTCAAAGATTGACTCCGTGTGGGGCTGGGCTTCGCCCCCGCT
GCGCCTGCAGCTGGGAACCTCGCGCGGAC
>TCONS_00115375 geneID=XLOC_023009 scaffold_381:25911-27711(-) Exonic overlap (+)
GAGGCGGTGACGCGACTCGGGTGTGTGCTTTGCATTTCGAGTTATTTCGATCGAATTTTCGTGAAAATA
TTACAAAATTAGTGATCATTGTGCGATAATCTACGACATATTTATATTTTAGTTGAGTTGACGCAACCATC
ATGGAGCAGATCCCGCCTCCCAAGGAGGTGAAGATCCTCGAGACAGCTGAGGATATTCAGGAGCGCC
GTGAACAGGTA
>TCONS_00115496 geneID=XLOC_023004 scaffold_381:44827-52699(+) Intronic
AAAAAACAAGAATAACTTTTAAGGATTTCTGTTTGTTATTAGATTCCTTTTTGAATAGTTTGTGTTCCTT
ATAAAAGTATTTTACTTCAAACTTCCTCAGTGTCTTATTTTCAGCATCTTCTATAGTACCAAGCTAACATT
TCAGAATTGAGATGAGTAAGTCTACGCCCACCGCATCCTGTACTGATAATAATAACGCAAGCACCCCAT
CTTAAAATTGAAAAGTTTTGACGAAGAAAAAGGACACAGAAGTACACACAACTTTGGGAAAATGAGT
TTTCATGGCTAAGAAAAGACTCAGAAAGTGATACAAATGCAAAATCCATAATCTGTGGCGAAGTTTTC
TCGCTACTCCATCAGCTGGAGTTGGACACATTCTGAGGAACAATGAACATACAGTGTAGCCGACCAAA
AATTCACCTAGCAACGTTGCCGCGTAGCAGACCAGCGTCAGACCAGCATTAAGTACATCAATACGAAC
AGCCGGAATATGGTTTACTAATTACTTATAGTCTCTTTGAGTTTTGTTTTTAATTATGCATATCACCGCATA
GATATAATAACTTTATTAATTTTGCTAATTCATTCAGTTTGTTAACATACATTTAGAACTTGTTAGTTAGCG
TTCGAATGGCCGACAGGGAATGGGGACATTCCCAATAACAGGACGGCCGACTGTTAGCGTTTAAATTT
ACCAATGCTATTCTTTACTAATATCAAGCTACTTATTTCATCATAGGTCTGGCCGGATTACTCTAGGAGTT
GTTCTTTAGTTCATTTAGTTTCTATATTGTCTGTGATTCGCATTGACTGGCCGGTCACTCCATCCATTCAT
TCATTCATTCAAGATGGCAGAAAAAACAAGAATAACTTTTAAGGATTTCTGTTTGTTATTAGATTCCTTT
TTGAATAGTTTGTGTTCCTTATAAAAGTATTTTACTTCAAACTTCCTCAGTGTCTTATTTTCAGCATCTTC
TATAGTACCAAGCTAA
>TCONS_00115498 geneID=XLOC_023004 scaffold_381:51179-52699(+) Intronic
AACACACTGTTGTTATTATAATAAATGATGATTTAAATATAAAAATACAAGAATATTATATATAAAATAGA
AGAATTGGAATATATTTTGATACTAAGATACCTGCATTATAAATATATTATGAGAATGGATGACCGAACAG
TATTGTTCCGATAAATGAAGTTGAATGATATGCTGAAGTGCAAGCGGTGCTCACACAAGGATGTGATTG
GTATGCCGAAGCACAAGCGGTACTCACTGAATGATAATACACACACTTTAATATTGTTATAATAAAAGG
TGAATGGAATGCCGAAGTGCAAGCGGTGCTCACACTGGGTTGTGAATGGTATGCCGAAGCACAAGCG
GTACCCACTATACTATTTATCTGATAAAGAATTCTGAGGAACAATGAACATACAGTGTAGCCGACCAAA

AATTCACCTAGCAACGTTGCCGCGTAGCAGACCAGCGTCAGACCAGCATTAAGTACATCAATACGAAC
AGCCGGAATATGGTTTACTAATTACTTATAGTCTCTTTGAGTTTTGTTTTTAATTATGCATATCACCGCATA
GATATAATAACTTTATTAATTTTGCTAATTCATTCAGTTTGTTAACATACATTTAGAACTTGTTAGTTAGCG
TTCGAATGGCCGACAGGGAATGGGGACATTCCCAATAACAGGACGGCCGACTGTTAGCGTTTAAATTT
ACCAATGCTATTCTTTACTAATATCAAGCTACTTATTTCATCATAGGTCTGGCCGGATTACTCTAGGAGTT
GTTCTTTAGTTCATTTAGTTTCTATATTGTCTGTGATTCGCATTGACTGGCCGGTCACTCCATCCATTCAT
TCATTCATTCAAGATGGCAGAAAAAACAAGAATAACTTTTAAGGATTTCTGTTTGTTATTAGATTCCTTT
TTGAATAGTTTGTGTTCCTTATAAAAGTATTTTACTTCAAACTTCCTCAGTGTCTTATTTTCAGCATCTTC
TATAGTACCAAGCTAA
>TCONS_00115360 geneID=XLOC_023005 scaffold_381:87421-144746(+) Exonic overlap (-)
GATAACTATTAATAAGCAGCATCGTCTGCGGCGGCGTGGACCGGCGGTGGGCGCGGGAAAGTCGCGA
GCGCGCCGCGGGCCCCGCACGTGCGCCATGCCCCTCCACTCGCTCTGTGCACTCGGACATCACAAAA
CTACAAACAACAACTATTTACGCGACTTTTATGCAAACCTAAATCCTAAGAAATGAAACGGTGAATTGA
AATGGAATTTCCCAAAAGTTTTCGAGACGCGGAAAGATCGCGCGAAATGTAGAACGATGGGTGGTTTT
AATGTTTACCTCTTCTGTGTTGTGAATAAAACATAATTTAAATGTAGTGAATAGTTATTAGAGAGTGACG
AAAATGTAGGTGTTGAGGAGGAATGTGAGTGGAGGAACAGCCGTGGGGTAGCAGAGTGCGGAATTCT
CCAAAGTGCCAACGATCTGTCCGTTCTTCTGGATAGACTGTACAGTAAAGGTTGTAACATACTCATAAG
TTGTGATTAAGAGCTAAAAGATCTATTTATACGACGAAACCTAATATTTAAATTCTTATATTTCTTGATGT
TAATATAAAGTACCTGTCCCGGTGTTCCGCTGGGACTTTGTTTTCGCTCGCTGATGCTGAGAGGTTGTG
AATATTTGCAATAAGACATTGACTAGTACAAGTAACTGTTGGTTGTGTCCGGTTGTAAGTGTAGTGTAT
GTGCCGGCCCGGCACCAGGACTCCTTGTGTCTACTTGTATATATGTGTGTTACTCGGACCGATCTCGAC
GAGTGACTCGGATACCATTTTATCAGCCGGTAGTCTAACTACCAACGTCGAGGCACCGATGACGTAATG
GCTGCAGTTAATTCGGT
>TCONS_00115385 geneID=XLOC_023005 scaffold_381:87493-197863(+) Exonic overlap (+)
CCGCGGGCCCCGCACGTGCGCCATGCCCCTCCACTCGCTCTGTGCACTCGGACATCACAAAACTACAA
ACAACAACTATTTACGCGACTTTTATGCAAACCTAAATCCTAAGAAATGAAACGGTGAATTGAAATGG
AATTTCCCAAAAGTTTTCGAGACGCGGAAAGATCGCGCGAAATGTAGAACGATGGGTGGTTTTAATGT
TTACCTCTTCTGTGTTGTGAATAAAACATAATTTAAATGTAGTGAATAGTTATTAGAGAGTGACGAAAAT
GTAGGTGTTGAGGAGGAATGTGAGTGGAGGAACAGCCGTGGGGTAGCAGAGTGCGGAATTCTCCAAA
GTGCCAACGATCTGTCCGTTCTTCTGGATAGACTGTACAGTAAAGGTTGTAACATACTCATAAGTTGTG
ATTAAGAGCTAAAAGATCTATTTATACGACGAAACCTAATATTTAAATTCTTATATTTCTTGATGTTAATAT
AAAGTACCTGTCCCGGTGTTCCGCTGGGACTTTGTTTTCGCTCGCTGATGCTGAGAGGTTGTGAATATT
TGCAATAAGACATTGACTAGTACAAGTAACTGTTGGTTGTGTCCGGTTGTAAGTGTAGTGTATGTGCCG
GCCCGGCACCAGGACTCCTTGTGTCTACTTGTATATATGTGTGTTACTCGGACCGATCTCGACGAGTGA
CTCGGATACCATTTTATCAGCCGGTAGTCTAACTACCAACGTCGAGGCACCGATGACGTAATGGCTGCA
GTTAATTCGGTGGCATCATCGCGACTGCCGCTGCCGTCGTTCTCGCCGCGCGCGGTGCTGATGTACAGC
GCGGCGGTGGGCGGCGAGGGCGCGGTGGCGCTGCAGCTGCGCGAGGCGCG
>TCONS_00115465 geneID=XLOC_023005 scaffold_381:87550-198147(+) Exonic overlap (+)
CAAAACTACAAACAACAACTATTTACGCGACTTTTATGCAAACCTAAATCCTAAGAAATGAAACGGTG
AATTGAAATGGAATTTCCCAAAAGTTTTCGAGACGCGGAAAGATCGCGCGAAATGTAGAACGATGGGT
GGTTTTAATGTTTACCTCTTCTGTGTTGTGAATAAAACATAATTTAAATGTAGTGAATAGTTATTAGAGA
GTGACGAAAATGTAGGTGTTGAGGAGGAATGTGAGTGGAGGAACAGCCGTGGGGTAGCAGAGTGCG
GAATTCTCCAAAGTGCCAACGATCTGTCCGTTCTTCTGGATAGACTGTACAGTAAAGGTTGTAACATAC
TCATAAGTTGTGATTAAGAGCTAAAAGATCTATTTATACGACGAAACCTAATATTTAAATTCTTATATTTC

TTGATGTTAATATAAAGTACCTGTCCCGGTGTTCCGCTGGGACTTTGTTTTCGCTCGCTGATGCTGAGA
GGTTGTGAATATTTGCAATAAGACATTGACTAGTACAAGTAACTGTTGGTTGTGTCCGGTTGTAAGTGT
AGTGTATGTGCCGGCCCGGCACCAGGACTCCTTGTGTCTACTTGTATATATGTGTGTTACTCGGACCGAT
CTCGACGAGTGACTCGGATACCATTTTATCAGCCGGTAGTCTAACTACCAACGTCGAGGCACCGATGA
CGTAATGGCTGCAGTTAATTCGGTGGCATCATCGCGACTGCCGCTGCCGTCGTTCTCGCCGCGCGCGGT
GCTGATGTACAGCGCGGCGGTGGGCGGCGAGGGCGCGGTGGCGCTGCAGCTGCGCGAGGCGCGCCC
GGGGGAGCGAGGCGCCGCGCCAAGGTTCAACGAGG
>TCONS_00115422 geneID=XLOC_023007 scaffold_381:272148-273387(+) Splice junction overlap
GTTGGATACTCGAGCTCATGACGTGGACTCCAGTGACTGCGAGCCACACCAAGAGACGCAGCTGACG
CGGCACTGCGACGAAATGGAAACGCAGACCGACACGTGATCAAATGGAATATTCCAACGAATCGGGA
GTCCAAACCATTTGACTTGTCAATAAAATATCAGTATTAAAACCGTGATATCCATGCGAACCAAAACGA
ACAAACATTTGCAGAGGCAAATAACAAAACAGAGTGCATGATCCGAGTTAATTTCTGCGAAACTTAGG
TACCGCGAGTAGTTGACATGGCCAGCTATTTGTGTTCACATGAGGGGAATAAATTTAAACTTGCAGGTT
ATGGAATGTTATCGTGTGTACACCTTGTTTCATAGGAACGACTGCTATACAAGCTAAAAGTAATCTTATC
CAGTAGCATAGAGGGTTTTGTTTCGAAAGTTCTTACCTGGAATAGGCTATAGTGAACGTCTTGAGACAG
CCGATGAAATATTGTTTCGATACCATCACAGACGTAGTCGGCAGACATTCATGGGCGACCAATAACCAT
TGAGTTTTCAGGTGACTTATGATCGGGGGCTATTCTGTAAGCATTCTCTGAGGAAGTGTTACCTCAACA
TCTTCGGTAGTAAGCTTTGTAAATTGCGTAGTAAAATTCGAATAAATTTAATGTTTAATTCTAGATAATTA
TAACCATTTAGGATTGGCAAATTTCAGTACGAAATAATTTTTGATGCTGCGATTTATAAGTTATAAGTTTA
TTATCAACAATATTTTTATATCTATAACTATCATCACGAAACAGAAC
>TCONS_00115640 geneID=XLOC_023037 scaffold_382:133062-139714(-) Splice junction overlap
TTCAGTTTCAGGTCGTGCCACAAAATCGGAGCTGAAAATGGTGAAGGATAAAATATGTATCTTAAAAC
AGTTCTGGGTGGCGATGGGCATCGTGTTCAACATCGTGGCGGTGGGCGTGACGCTCAGCTTCCCCTCG
AGTCTGCTGCCAGCGCTGAGGCAGCCGGACTCCCCCATACAGGTCGACCTGAATACTGCCTCTTTGCT
TGCTTCAAGTCCGGGCATCGCATCCCCAATCGGCTTCATC
>TCONS_00115675 geneID=XLOC_023038 scaffold_382:168204-168701(-) Intergenic
AGGGCGGCGGCGCGCGCTCGAGCGGTCAGCCCATCCCGCTCCCCACAGATATAGACGTGTTTTCAAGC
GGCGCGACTCACAGGATTTGATGCGAGGGCGATGACCTCTTCTACTGAACTGACGACTACCCCCACCC
ACACACTCACACTCACCCACACAGTCGCAGCTCTATAGAACCCCGTCGCACTCTCGCGAGTTATGTAT
GTATATGTGTATATATCTATGTATGTAGTAGGTGTATGTTAGCCGCTATCCGTTTCGGG
>TCONS_00115558 geneID=XLOC_023039 scaffold_382:231509-232446(-) Intergenic
TTGGTGAGAGCTGTGGAGGCGACGACACGCAAGGCTCCACGTCTGAGTTGGACAGGGGATCTAGACA
GTTCGCAGCATACTTCTGCCAGATCTAACTTTCCAGCTCACGCGTGGAGCCAACCCACGGACGCGCCA
CGCCGCCATCGCCAGACGACAACCACCGCCAATCACCACACGCCATCATCGCTCACGTGACGCTACTT
CATCTAGACGCATGAAGGGTTAAGTGGAGGTGTGATCTCTTCACTTTTTTACATTAAAGTGAGATTCAA
CTCCTCCAACTTAATCCGGATTCGCACATTCGTCGAAAATTGGAACATTGGAGCCATCGTCAGCGTTTT
TATTAACAACTTGTATGACTTATTATAATGTTGTATGCCTATATTTTTTATGAAATAAAAA
>TCONS_00115600 geneID=XLOC_023039 scaffold_382:231529-232523(-) Intergenic
TTAGAATAGACTGTGGCCTTCTGGTGATTGTGGATCCTGTTCAGGAGTAGCTGGTAAAATTAGAGCTGT
GGAGGCGACGACACGCAAGGCTCCACGTCTGAGTTGGACAGGGGATCTAGACAGTTCGCAGCATACT
TCTGCCAGATCTAACTTTCCAGCTCACGCGTGGAGCCAACCCACGGACGCGCCACGCCGCCATCGCCA
GACGACAACCACCGCCAATCACCACACGCCATCATCGCTCACGTGACGCTACTTCATCTAGACGCATG
AAGGGTTAAGTGGAGGTGTGATCTCTTCACTTTTTTACATTAAAGTGAGATTCAACTCCTCCAACTTAA
TCCGGATTCGCACATTCGTCGAAAATTGGAACATTGGAGCCATCGTCAGCGTTTTTATTAACAACTTGT

ATGACTTATTATAATGTTGTATGCCTA
>TCONS_00115874 geneID=XLOC_023075 scaffold_383:269129-281972(+) Exonic overlap (-)
TTATTCGCCGTCACGTTCTAAACTAATTTGGTCTACGTGAGCCACAGAGAGGTGAATAAAATATCTGCA
TGTTATTCTTCTCACAGTATTATTTAGTTAGAGACTGAAGGTAAGCGTCCACCTTTCAGCATCGTACGCA
ACGGACGGTATTTGGTCCGATAAGTACGATACCGACGATAGGTGGACGCTTACTTCAAGGGATCGTACC
GATTAGGCTCGTTAAATAGAACGTAGCCGACAATATGTTTTAGGAGAAGTTAGTTAGATCTTTGGCTG
CATGTCTAGGCCAAGACAAATATTTTTTATGTAACAATGAGTTCTTTGAATTTAAGTACAATGTACGTAG
GTGGTCTGAAAAGTTTCCGACCTCAACGTGAAGATGGCAGCACACATCAATAAAAGTCAGGGAATGT
AACTGTGCATCTTTTGACAGCAACACTTCAAAGTTTCAGCCATTTTTGGCGCGTGGTTTTTAATTTACA
GTCGTTTGAGTGAGTCAATGTGAGCATTTCT
>TCONS_00116251 geneID=XLOC_023130 scaffold_385:168510-182975(+) Intergenic
CGCGCGCCGAGCAGTACGCCGCGCTGCGCCTGCGCAACACGACCCGCAGCGGATCCGCGCTTCCGAC
GGTTAATCGAATGTTCCTCTTTCAAATTTAAAAGAATTATCTTACGAAAGTGCTGGTATGAGTGAAGTG
AAGGACAAACAGTTTGTTTTGGATCCGTGACCGTGTTAACTGGATTGTGCTTTTGTTAGATTATGGCGG
CAGAAGACAGCTAAACAGAAAAAGGAAATTAAACGCAACGGAAACATCATGGCTGAAAAGGAAGTG
TGACTGAAGATTTACGTTAATAAAGATGGCGCCACTGGTCTTTCTCATATTGGCGCTTGGCCTGGCGCG
AGTTTCCACGGCCGGCGCAGAGATGGCGCTCACAAACATTACGAGATTTAATTTCGGCGGAGGAGATC
TTTTCTCCATTCCTG
>TCONS_00116255 geneID=XLOC_023139 scaffold_385:257186-259327(-) Unclassified
GAAAGGTTCACAAACCACTACACTATATGGTCATCAACAGTGCAGAATACCCATCTCGTAACCTCTCGT
ATTTGTCCACAGCATGGAGACATCAGGCGGCGCCGTGTGGACGTGTCCGCACTGCACCTTCCACAACC
CCCCGCAGCGGGACAACTGCGACATGTGCGCGCTGCCCAGAGGCAACGCTTAATTATGGAACCGAGT
AGTGAGCAGAATAAAACGGACTATAACTCAATACTTTTTATACTAAAATTTAATATCACACTACTATTTTT
GTCAGCAGAGAATGATTTTATGATTGACAACAGATAGCTGGAGATCTGTGACGGTGGCTGTTGGAAAC
TATTTTGACCTCAGAGGGAAAGTAAAGTTATTGCCAGTATGATCTCTAAACTTGTCGATATTTGTGGAA
AATGGTTATCTGTCACAGTACCTACCAATTTTATACAAAACATACCTAAAAAGAGATTTCTAAAGTTCCG
TCGAGTTCCCGCTAAGGAATAGAGGCGCACTTTAGTTATTAGTACTTATTCAATTCTTACCCACTGAAAA
TCGCAGTAGGTACATATTTGGCCGAACCCTAACGGTATTTTTCATTTTATAATGCCTTATATGATTTGTATC
ATAAATAATCCATATCTGTGAATTAAAATTTACTTATTACATTTAATTTCCTGAAAAAAATAGTTTGTACT
TGTCACAATCCAAATGCATTTTGTGCGTGCTGAAGCATTTTGCTTTCTTTATTGTTTGTTCGTACCTTCAT
ATAGCGCAATGTGCCTTACGCATGTTTAGATAAGGCGATAATAATGTCATATACTTACATAACTACCAATT
GTTTTTTAATCATAGCTCCAGAAAAGTTTACAACGTTCCATGTTTTATTTCAACAGTTTTATGAGCGACT
GACATAAATGGCTGTTGGTTTCTAAACTCGATAACTACTATATTACTGCCACATAATTTAAGATATCTGTT
TAAATCTCTAAGTTTGGAATGCCGGACCTTTTAATAATACATCCATTCCTTCAAATACCTACCTAAATAA
ACAAATGGTTCCAATTCGCTGAGAGTGACACGCTTTTATATGTAATTTTAGCAATGAAGAAATTCTATG
AATATAATTATTAGTAACATAAAAAAATATTGTATGTCCCGCTCTCACTGTTAATTTGCGGGAAAGGATC
TTAAAGTATATTACATTTAGAACTGTCTAAAATGGAAAGTAGTCGCTTTTATGATTGGCCGGTGAGAATT
ACGTTTGGACAGTTTTTGATTGAATATTTGTGTCCTCAGAGTTAGCCCTATTGTATGTATAGTTTATGTAT
GATCTAATTTGTATTTATTGGTTAATTTACGGTTTTAATTTACGGCTACGGGCGGCTACCGCAAAACACT
CCCATGTTACTTTTACGTGTTGATTGTATATTTCTGACGGGTTTGTTTTTAGTTCCGATTTTGGCCGCGTG
AATGACGCCATCTTGTGGCGCTGCAGTCGCCCTCGGCTCAATGTCTGCTGGTACAGCTACTGTTAGATT
TTATATGAGAGCTTGGAAATGGCGAGAAATACAGTGTGCATTATACACGAGGAATA
>TCONS_00116270 geneID=XLOC_023132 scaffold_385:276056-278234(+) Intergenic
ATTAATCCTTATGCGTCCCGATTCTGGTTTCTGCTCCTGCATTGGATCTAGAAAAACAGCATCACAAATC

ACAAGTGGTCTGTTGAACTAGAAAATAAATTTGGTAAATATGAATCAATGGAGGAACAAGCATGAAGT
TCTCCGGGACCTTGCCGAGGTCTCCGACTACGGTCCACGATGCACACTGAACTTGAACCCAGTTACTG
ACAAACCACACATATATGCCACTGAGGATACAGATTTAAATGAAATAGCAG
>TCONS_00116246 geneID=XLOC_023132 scaffold_385:277052-278315(+) Intergenic
GCATTTTGAGCTAATCTAGATATTTTATTAAATTTTTTATTTGCAGCTCCTGCATTGGATCTAGAAAAACA
GCATCACAAATCACAAGTGGTCTGTTGAACTAGAAAATAAATTTGGTAAATATGAATCAATGGAGGAA
CAAGCATGAAGTTCTCCGGGACCTTGCCGAGGTCTCCGACTACGGTCCACGATGCACACTGAACTTGA
ACCCAGTTACTGACAAACCACACATATATGCCACTGAGGATACAGATTTAAATGAAATAGCAGACTACT
ATGGTTTCCCGAAAAATACTTCATCAGAAAGATATGTGAAGCCTAATCTTCGTTGTATCACACAGATGAT
AGATA
>TCONS_00116280 geneID=XLOC_023140 scaffold_385:314944-315725(-) Intronic
GCCATAATTTTTAATTTCACCATTGTTTCACCTAAAACTTATTCACTCAGACTTACAGTTGAAAATGCAG
TAAATTTAAAAACATGGAAGGTGGAGTTTACTTAGTACGTAATGGCGCTTTGATATAGCACAGGCGACG
AGCTTTGAAAGTCACTTAATTAAAGCCTTCCATGTTTGTGTTGGCTCGTGTGACCAGTGTGTGTACAGG
GGAGTGGGGATGGTTTACTGTTGGGGAAAGTGGACTAAGCTGCAATAAAATTCCAGTATCACGTGGTC
TGAAAGTCTGCCATGAATGGCCGAGTGTCCCCTGTTGTACCTTTTTGAGAAAAAACTACTTGACGGTA
TTCTCATCTTGGGAAAATCTAATTATGTTTTATTTATTTCGGACTTCCCATACTGAACAATATTATCCATCT
CAAAAACTCCGTACAGTTTCTAAATGTATGTAGTCGGTATTTATATCAGGATGTGACGCCAGAAATCGAT
AACGGAGCATAATTTACCTCGTCTCTGAAACTGGGGTAAAAGGGAAACAAAATGTACCTACTCACAAG
TAAGTAGGTAAGTGTAAAAGTACTTAAACTCTTTATTGCCTTAATCCCCGGAGACTAATAGGGGTGTAA
AATACGAGTACACTCTGGTGAAGTGGAAAGACGACGAAGCGACCTTAAAATATCGCAATTTTTCCCGA
TC
>TCONS_00116366 geneID=XLOC_023154 scaffold_386:95269-100339(+) Exonic overlap (+)
GCATTCTACATTTGCCGATCCGTAACATAACCTCAAAAATTTGGTTTTCAGGCAAATGTATCAATTTGTT
TGCTGTAATCCAAGTTTTCCACGAAATTCTCGTGAAAAAAGTGTATTAATGTGTACATTTATATGAAATT
CAAATGGGGAAGTGACAATTTGTTGTGACTTAAGTTTTGGGGGACTTTTCTGTGATGTTGGGTGTGTA
AAATGGATTTAAGTGGTAATAGTGGATTTGTTGGATTATGTAGAGTGAACTAACAGGTTATTTCGTATTG
TTTGTAATGACAATGCACTTAGCGGGCCTGTGGTTGTGGTATTCAAACTATGTGGTATAACGGAATACTT
AGTTGACTTCAAAATGAGTCACATGGCAACAGAGGACAGCATGAGCGATGACGTGTTCGAGGAGGAG
CAACTGCGCGCGCC
>TCONS_00116503 geneID=XLOC_023186 scaffold_387:240536-244657(+) Splice junction overlap
GTGGAGGTCGCTGCGCCTAGCAGTGCTCCTCCCGGTGAGCGTGTGCTTCATGAGCACCATCCTCACCA
TCCTCACGCTGTTCCACATCTACACGTAGGCGTAGCATCGTGAACGGCATTGGCTATAGCCGCGTGCGG
TACGGTTTAAACATGTGGAGGCCAGAAGTTGGAGCTGTAGTAAAGTCTGATTTTTAATCTGAGATACTG
AACGGTTCATCAACAGTCGATGGTCTGGCGATTAAGTGACGTATCGTTCTATTAGCGGGACAATCGACT
GTTGATGGACCGTTAAGAATTTGAGACTAAAAAAATAGCACACTTTTGTTAATTGTCAAACTAACCAAT
CATCATCAATCTTTGCAAGAATGAACAGATTGTGTGTTAGTTCGACAAGGTACAAAAGTGTTAGCTTCA
ACTTCAGGACTACCTACTTGCTTGGACCGAAATGTATCAATCTACTACGCAATAGGTGAGTCTTAAGAT
CGGTTTCTGAGTAGCGTATTTTTGGGCACGTATACGGTTGTATTATGCTACTTTTCATGTTGCATCCCAAT
GAATGAACTTGAAATTCATAACTACAATCATACAAAAATTATCAAATGTATGGACTGTTAAGGAAATTA
AAATTGCTAATTTAACTCTACCGAGCTGTAACTTGGATAAGAGTGTAATTTGGAAAGTCAATATTGTAA
CCGACTCTCAAGTAAAATTGAGGATTTAATGCTTTTGTGACTTGTGATTTTGTATGCTAAAGACATTTTA
ATATCATCTATGTCATTATTTTAATTTCCAAATAAAATAAATACTCACAAACATTCATTTGAGACTTGTAAT
AAAGTTAATTCTCACATTTTATTAAGATAGTAGGTGTACCTATTTATTGTTTGTCAAATAAGTAGTAAGTA

CGTATAAGCCCCTGTTCCACAATGTCTGGTTAGTCGCTACCTGTCGGATAAAATACATGCTGTCACTGTC
TAAAAAATAATAACAGAGAATGACAGCATGTATTTTATCCGTCAGGTAGCCACTAACCAGACATTGTGA
AACAGACCCTTAGTAAACTATTTTTTTATGAGATGAAACGAACATCACGTTTGCAGTACTCTGTAGGTA
ATATTTTTATATAAAAACTTTGTATTTTATTCACACAGAGATAATTACATATTTTTTAAAATTATGAATGAT
ATATATGTTGCTTTTTTATTTACTTAATCTATTTATACAGGGTGTCCCAAAAAGGGTAATCCGCAGTGGAG
TCAAACAAATTGAAACTTTTTGTTTGCAATATTATTATTTTTTTTGCACCACCCTGTTTATGCACGGGTT
GTAAATGTATTTAACAGGTCACAATCTTCAACTGCTGGACTTAGGCCTCTGTCTAGGAAAGCCATAACT
CTTAGTCTTCAGCATTCGAATCTGTATTTTCTCTATACTTAGCTTTTTTAATATTTGAAAATCTAATCCGTA
TTAAATTTTATACAGGGTGTTAACTAGAATGCGTTTGAGCGGGAAATGGGGTTTAGTATTAGTCTAACC
AAGCACGTTTCTAAGAAAAAAAAACATCAGAATCTCGAATATTTATGTTTAAAGTCTAAAGTAAAGTGG
AAAGTTTTACCTTAATCAAAAACATGGTCACCCTACACAAACACATAACCAATGAGACATTCGATTGCA
AAAGAACACACACTGTAAATTTGTTATTAACTTCACTTTTCATAATTTATTTGATGTAACTATGATTGTAA
TTAATTACACAGTAGGTAATAACAATTATTAAATAAAATTTAAATTATTCATGGTGATTAATGACATACTA
ATCAACAATTAAACAGCTGATTGTCATAAAGTTTGATAGATCGTACAGAATATAATTTATGACTTTCTAG
GAGCTCAGAACAATAATTAAACCTTTTTACTCAGAATAAGGTAAGCGTTTAGTATTCACCCTTGATACTT
TCACCACATTTTATTTTTCTTTTATTACCTAAAGATGCGTACAGACCGGCCCAACGAACGCCCACCGAT
GGACATTTATCATACATAATACACGGGCAGATTGAGCAACGAAGGTCAACAAAACCCGTTCGTTGGCG
TTCGTTTGGCCGGTCTGTATGCAGCTAAAGATAAAAGACCACAATGCATAACAGGATGATAACAGGGT
CTACTTCAATGACGAATTGGTTTACTTACACTTGCTCATCTCGATTTGTGTTGTGTACTTGCGATTGAGA
CGTCTTTGATGACGAATAGTACAGAAATCCTCATAATAAGTATGGCCTCACTTTAAATACCAGTAGAATA
ATAACAATCCCGCGTATCATAACCATCGAGTATTTATGATAGTCATTACCTTCTCTGTGGATCTAGTCTGT
CAGTCAGTCATTGTTGTTTACTTGTTTACAATATTTTTTTTACAATTATTACCTAAAAGGGAAAAGGTAC
CTAAAATTCTATCTTTTGACTACCAAGCCTTGTTCCTCATTTCGTTTTATGTTGTGCTGTGCTGTGAATAC
GTGTGACCTATGTTATGAAGCATAAAATAAAAATGTTCTGTATTCATAAAAGATAATTGTGTATCAATAA
AGTGATACTTATGGAGTAAAATGAAATGTCTACAATGGATTGGGTACTTTGAGTACCCAAAATCTCGGT
AGGTACCTTGACGATGTGGATCCACGAGTGTGGTGCAGATGCACGCATACTTTTTTATTGACCTTGGCT
TTGAATTGTTTGGCCTTGATCTTGGCTTTCAAGTTCATGGTCATTCTGTCAAACGTACCTAAACCTGAAT
AGGGTGACTATGAAATTTTAAAATGTACTAAATAAAACTGTTTTATGTTGGTCAGCTTAAAGCAAATAA
AATATGTTTTTTAATGTCTTATCGTGTTCAGTATCATCAGCTGTATCTACCATTTCCCGCTCCAACGCAAT
TAAGTTAACACCCTGTATAATGATTTATTTACTCATAAATTATTCACATTCCGTTATTTGTTATTTATAATTA
TTAATTAGTTATGATAATATGACACTATCTTTAAGTATAATTAGTATTAACTGCTATATATTATTTTAATGAT
TCACATGTACCTAAGTATTATTTTAGATTTGTAATACAAAGTCACTCATCAAAATAATTTTAATAAAAAG
ACAGAATATTATACTGCCAAATCTAAGAATAGAATAGTGATTTTGAAAGTTATAGATGCTCGTTGAATTT
GCACTTTATATGGTCGTGTTAGAGTTCGAATTTAAGCGTTGGTTTAGTACGTTTTACGACGATGCACTTG
GTATAAAGAAGATAACTACCTAGATGATTGATTGTAAATGCAAAATTAAGCTTATTGCATTCTACATAAG
TATTATTTCAAAATTGCGATGAAGAAGGAAAACCATATGAAAATGGCGGACAGACTCTTGCTATGTCAC
TCGTTTTGTAGGTCTGTCAAAGTATTGTTTTCAAAAGTACATGTACGTAAAGTCTGTTTTTATTTATTTC
AGAAACTAAATTGATAGATTCTGAACGGCT
>TCONS_00116595 geneID=XLOC_023231 scaffold_388:79247-81700(-) Exonic overlap (-)
TTGATTTCCCGTCACTTTGCGCGCAACATTTGAACTCTCTTCAAGTCAACCCATTTTCCATTACAAAAA
ATTTGTGATAAAAATGAAACTAATAACCCGTGTCTATATTTGAGTGTTCTTAAATAATCTTATATTAATAA
AACTTGAACATATCGAGCTAACAGCTTATTAAATTATTGTTGATCAATTTAAAAGCACTCAACCACAATG
TCATCCGACGACCCCGCGCCGCCGCCCGCCGCGCC
>TCONS_00116802 geneID=XLOC_023256 scaffold_389:246800-250665(+) Intronic

CATCTACATCAACTAAAAATAATTGCCGGGTTCGGTAAAAATAGAACTTATACATAAACATTATCACATT
TAAACAGTTGGTTGTCAAAAAACGCTAATTTATTTTTACGAGAACATTTTGTTTGTTTAAAGTTAAGTCA
TCACGTCAATAATACTCATACGTAGGCAAAGGTGTAATAATTACAGTAAGTATACATATTATTAACATAG
GTATGTATAAAAATATAATAAAGTAAACAAGTAGTATTCTTCTTCAGGTTACTGCACGATACATATCTCAT
TCAAGATTCATTTGCATCTTTTCACTTGTATCTATCCTAAAAAACTGTTAATTTGAACCCGGGGTGTCGA
AACTCAGTCAGAGTACTAACACCTGGTCTATTTAATGATATTATGAATATCTTTAGTTTTATTTTTAAAAA
GTGGTTAACGCTTACGACTGTCATCCCCCAATGGGTAGCCAGAGGTGATTCCCAAAAGAGCCATCCGC
TTTTCTCTGTACGGTCAGCTGCATTTGCACTCACGTGATGAATCGCGAGCCGTATCGCCAATATAGGCA
AATATGAGAGCTAAATAATATTATAGGCACTTAATCACAACGACTATTTGGTCTGTCTATCTGAATGTCTT
TATTGAGGGAGATAGCGAGTTCCTATGACGGATGCCTTGTTTGGTGCCTCTTGTTCTCACATGACGCAG
ATAAAGAGCTACTGTTTGTGATCGAATCAAAACATGACCATTACACGTTGTGATTACGTTCTGATATGCT
TTATTGATGATGCTTATGACGATAAGAGTAGCCAAGGTTCACTTTATAGTATGGAAATAATGCGTTAGTC
GTACATTGATAATGATAGCCAAATGTATGAAGCAATTGTCTCAAAAAACAAAGGAGATCAGACTAGAC
GTTGTTGCATGGACTCTACTCGTATCTCGAAAGCCAGCAACACGCAACTATCCAAGTACGCAGTGAAT
CTACAGCCATTTTGCAACACCCTGTAGAGAAGCATTAGCAAAGTCCTAAGTATAGTTATTTTGAGCCTC
CTGGCATGTATACTTGATGGCTGATGCCTGAAAGGTAAGCTGGTCCAAATTTTTTTGTTCGATGGTACAT
ACATCATCATCACGACCAGCAGTGGGGACGTAATGGGTCGTGATGATGGTACATACATCATGAGTTATT
TGTAAGGTTTGCACAACACCTACAAGACAACACAGTGCAAAAAGTATCAAAAGGTCAGCAACTCAAA
ATTAAGAACTGAAATTCATACTGGGTTAATTATGAGTATTATTTATTACCCAATGTGGGCTTGTAAGGTA
AACCTGCTTACAAAACAATATACATAATTTTCACCAAGAACAAAAATAAAATAAATAAATTAGTATATAC
AAAGAGGCGGCATTATTGCTTATAGCAATCACTACCGGACAACCCTCATTGGAGTCACAAAACTGTTA
ACGTGTTAAATAACAACATGGACCGCCGGTCACTATCAAGGTTGCCGATGATAAATATTGATATAGAATA
ATAAAATAACTTAGAGCAAAAAGTTAAATAATTCGTTGGTAATTATCTGTATCCCATTTACTGTGGCGAA
TTAAATAAAAACACACACAAAATGTACAGCGTCATTATGTTGCTATTACACTGAGATAATTGGAAATATC
AGTGCTTTTTTGTGACTGAAAAAAACGTTACTGAATAAGTCTGCCTTCAACCCGCCAACATGCGTCTA
CTATAGGTACTGGACATAGGCCTTAGTACAGATGGGGGGTATCACAGCACAATCAGACTGAAGGGCTA
GTACGGGGCGCATCAAGAGTTTTGCATCGCGCTCACTCACATCACGCAGCCTCAAGAGCGAGCGCGA
CGGAGAACTTTTGTCACGTCTTAATAGCGGCCTGTGCTATACTGGCTGAGCAATGTAAAAGATCTACTT
AGGTACAAAATGTCGACAAAGGGCCCAATATTAAATATTAAATAACAATTTGAACATAGTATTTACGTTT
CAGTTGATAGTATTTTGATGCGCTGTATAGGTATACGTACTTCTATACAGCAGACAGCGTCATTAAGCAG
CAATTTGGTGCTAATGTCACTGGAACTGTTTCATTGCTCGTGAGTGTAGAATACAATAAGGAAGTGCCA
CTAATTTCATTGAATGGTCACATAGAATGATGGGAAATAAAATGGTACAATTTATGTAACCGCTCATTGA
TGGATGTGAATACTTGTACATAAACGAAAACCGCAATATAAGTATATATTTTATAGACCAAATGTTAATTT
TGGAATTTGTACTAACAGTGGATGTTGGTTTCTTTGTTAAAGCAATGCAGTCGTGTGTAATTTTTCAGG
CACATCCAGGACTACAGAACAAATACTCGTAGGTACTTATTTAAAGAAGAACCTGGGACCTTGATGTC
TTAAGTTTACGCACCGCATAATGAACCGTTGTTATCAGTCGTTCCTCTTTAACAAGGTTACTAATTATATA
TTTTTTAAGTTTTTATTATACAAATCACAATTTCTTGAGTGGTAAGCAGAAAATGTTAGTGCCTATAGATA
TTAAATTTATTTAGATATTCCCTTATTTTTATTATTTATCCTACACAACCTATGACTTGGACTAAGGTGAAC
CCGTTAATCCATAGCATGTAATTATTTTGCATAAAAACCGGCGGACAGAATTCATTGCGAATAACAATAC
TGAGCCCCGCAAATAGATAAACAGATACACGGAAAGATTATGATGTTGATACAAGATCTATTGAAACAA
TAAGTGTAATATTCCACAGATGTGTTGATGCGTTCAAGGCTAAAGGTACTAGGTATGACTCTGTATGTAT
GTATGGTGTTTATTTCATTTTCATCAGGTCACATAGTATTACTACTAGCTGTTTTATTGTCATTGATGAAAT
TCCGGTTTTTACCCGAAAAACGGGGTTTGGGGTGACCTTATGTTGACTTGTGTTCTGATGTGTGTGTCT
GTGTATGTGTTGTGTGTGGTATCGTACCGTAGACACTCTTTTTAAGGGACTACCATAATCAAATGCTTTT

AGAAATATTATAAATACTGCAGTTAAGCAGGCATACACGCAAGTTTTCACTTAATCAAAATCTACTGTC
AATACCACGGAAGTCCGTTTATTTCAGGCCAGCTCATGATTATACCTACATTAGTTTACTATCTACACTTA
AAATCAATTCCGAACTTAATAGCTGAAGCCTTGAAAACGACATAACCAATAACCGCATCAAAGCTCCC
GAGTCAAGTCTATTCATATTTTAATAAACTTCAGGTACGAGGTGGGGCTATATTTAGCTGCCCCCCCCTT
CGTGCCCAGTTGGCGCCCCCTTCGTCCCCAGATGCCGCCCCCGCCCGGTGACATTGGTAAGGTTGCAC
GCAACAGGCCAGGCGTTAAGCGACAAAACGCTGCTACGCTTATAAAACTCGTTTAACGCTTTGCATGT
AGGTATGTAGGTAGGTTCTATGTTTTACATTCGTTTTAATTCATGGGAAACATACAGGTATATAAAGTAA
GAGATTGGATGGCCCATTGGTTAGCATCGGCGACTTTTAGCCCGTTGATCCCTGTTGGTTTCTTGGTTC
GAATATGGTTTAAATGTGTATTTATGATTGTTTTCTAGGATTCTATGTAAATATGGCAATAGAATTCATTTT
ATTACTTTGGTATTAATATGATTTCAGTTACAGTAGGAGAGTTTTAACCCTAGGGCTAGTA
>TCONS_00117391 geneID=XLOC_023302 scaffold_39:46569-48165(-) Intergenic
TACGTTTTCAACTTGCTTATTCAAAAGTAGCTAACAAGATGAAGGTTTTTCTGATTATGGCGCTCCTGAT
GGGAGCATGCTACAGCGACGACGATGAGTCTGACTATGAGTCGCTGATGCCATGGAATTCTGGTACAG
GGAAATGCTCACACTTCGGTATGCCGTGCAACACAACTCAGCCATGCTGCCCATTACTAACCTGTCGA
CGTCAATTTTTGCCAATGTTTCGTAAAGATATAAATGCGAAAAAATGCCTGCCGTAAATGGGATACCTA
CTTAGGAATGGGAAGGAAAACTGGAAAAATAATCTTCAATGCTTTTTAAATACTTATAATTTATAAATGT
AGGTATGCCTCTATCGCTTTATACAGGTTGTTGCAGAAAGGGTATACTAAGCCGAAACCCACATGTGCA
GCATGGTATATCTAAGCCCGAAAATGAAATCAGGATTTCAAAATTCGCGAATAAAAATATTAATTTTTCA
TAGAAACTATGTCGGTCACGTGACTTTTTACTATGGAGAGGTTTAGTATACCCTTTTTGCAACACCCTGT
ATAAATAAAACTATTTAGGTATAGTATATTTTACCCCTTCCATTCCTACTTATACACTGTACATCAAATGTT
AACAACTAAAGAGTGCGAAGACCTAACTGCTTAAAAATCTTAAAATACAATGAC
>TCONS_00117392 geneID=XLOC_023302 scaffold_39:46569-48165(-) Intergenic
TACGTTTTCAACTTGCTTATTCAAAAGTAGGTATGATTATAGTTTAAAATATTATAAAATAATTTTTAGTGT
AATACCGGAACTTAGCCGATTTTTTACTGCAGCTAACAAGATGAAGGTTTTTCTGATTATGGCGCTCCT
GATGGGAGCATGCTACAGCGACGACGATGAGTCTGACTATGAGTCGCTGATGCCATGGAATTCTGGTA
CAGGGAAATGCTCACACTTCGGTATGCCGTGCAACACAACTCAGCCATGCTGCCCATTACTAACCTGT
CGACGTCAATTTTTGCCAATGTTTCGTAAAGATATAAATGCGAAAAAATGCCTGCCGTAAATGGGATAC
CTACTTAGGAATGGGAAGGAAAACTGGAAAAATAATCTTCAATGCTTTTTAAATACTTATAATTTATAAA
TGTAGGTATGCCTCTATCGCTTTATACAGGTTGTTGCAGAAAGGGTATACTAAGCCGAAACCCACATGT
GCAGCATGGTATATCTAAGCCCGAAAATGAAATCAGGATTTCAAAATTCGCGAATAAAAATATTAATTTT
TCATAGAAACTATGTCGGTCACGTGACTTTTTACTATGGAGAGGTTTAGTATACCCTTTTTGCAACACCC
TGTATAAATAAAACTATTTAGGTATAGTATATTTTACCCCTTCCATTCCTACTTATACACTGTACATCAAAT
GTTAACAACTAAAGAGTGCGAAGACCTAACTGCTTAAAAATCTTAAAATACAATGAC
>TCONS_00117393 geneID=XLOC_023302 scaffold_39:46569-48166(-) Intergenic
ATACGTTTTCAACTTGCTTATTCAAAACTAACAAGATGAAGGTTTTTCTGATTATGGCGCTCCTGATGGG
AGCATGCTACAGCGACGACGATGAGTCTGACTATGAGTCGCTGATGCCATGGAATTCTGGTACAGGGA
AATGCTCACACTTCGGTATGCCGTGCAACACAACTCAGCCATGCTGCCCATTACTAACCTGTCGACGTC
AATTTTTGCCAATGTTTCGTAAAGATATAAATGCGAAAAAATGCCTGCCGTAAATGGGATACCTACTTA
GGAATGGGAAGGAAAACTGGAAAAATAATCTTCAATGCTTTTTAAATACTTATAATTTATAAATGTAGGT
ATGCCTCTATCGCTTTATACAGGTTGTTGCAGAAAGGGTATACTAAGCCGAAACCCACATGTGCAGCAT
GGTATATCTAAGCCCGAAAATGAAATCAGGATTTCAAAATTCGCGAATAAAAATATTAATTTTTCATAGA
AACTATGTCGGTCACGTGACTTTTTACTATGGAGAGGTTTAGTATACCCTTTTTGCAACACCCTGTATAA
ATAAAACTATTTAGGTATAGTATATTTTACCCCTTCCATTCCTACTTATACACTGTACATCAAATGTTAACA
ACTAAAGAGTGCGAAGACCTAACTGCTTAAAAATCTTAAAATACAATGAC

>TCONS_00117050 geneID=XLOC_023312 scaffold_39:423307-424833(-) Unclassified
CCTACGTGCCGCCCGAGCTTCGGAAGCGCTAGATTCCATCAGTGAAGCTTTAATATCCTTCGACTTGTT
AGGTCGTAGTTGAGTAGTAAACGGCAGCCACATTATTATGTATTATTCTTATAGAGAAGTTGATGAAAC
AACACCTTTTCCAAGATTTATATTGTAGTTGTGATGAAATCGAGAGCTTATGTACTGTGCCTTCAAGGGT
TCCTGCGTTTTACAGTGCAATAAATTATAAATAGTTACACATCTGTAACTAAATAATAATATTTATTAAAA
TGAAAACCTACAAAAAACAAAAGTTAGGAGTATTTACATGCATAATAAAAATAGATAATTATTTTAGTGT
AGTTCAAAGCATGAAATTTGTAAGTGCTATACAGATTTAATATATTTTATGGGTATAGAACGAAACGTAG
CAATAACATGATGTACAATAAAATTATGTTTCCTGTTTTGTCTAGAGGAAAAATATATTATTGATGCATTA
ATATTCTTATATTGAACATCGATTTAAATACATTTTAGGTACAAATAAAAGCACAGTGTGATGAAGTGCT
CTTTTCAACACCCACAATGCTTGTTTATTCTACATTATGTAGAAGAACTTCGAGCAATTAGATGAAGTTT
TTTTTACTGTGATTAATGAAGTTGTTAAGGATTATTTATTTGTGTATTCCTTCCGATTTTCACATGGTCAA
TTTAAATGTGTTATTGTTTGCCACATTCCTAATGTGAGACTGTTTATAACAAAGTATTTGAGTAACATAAT
ACAGTATTTTATCAGCATTCCATGTTTACAAGTGTGAAGTGTTATATGAATAGGCATTAGGTATGTACATT
GTACATGTACAATATAAAAGATTTTTGGATGTGTATACTTACCTACCTAGACAGAGTTGAAGTTAATCCA
GTGCAGTGCCTTATAAGTACCTTATATTGTCAAGTACAGAGTTAATATCGCTTTACCA
>TCONS_00117205 geneID=XLOC_023313 scaffold_39:448193-448647(-) Intronic
TTGCACAACACTGGTATCACTGATCCGTTAGGAAACGGATCCGGCTCGAAGGACCAAACTGTTGAGG
ACAGCAAGTGGACTGAGCGGTGAATAAAAAATACCGTTTCGATTCTACTCTCCTCAACAAGAGGAATT
TATTTACACAATCACACGGAACACAAAGATTAACACAAATACATAAAAACACAGGCGTAAAAGGTTCA
CTGAAGGACGGGTGTCCATATCACTGTTTGTAAACACTTTCACTTAGTCACTGTTCTTTATACTGGTGTC
ACG
>TCONS_00116909 geneID=XLOC_023318 scaffold_39:665658-669572(-) Intergenic
CTTTTGGTAGATAAATCACGTTCGGAGGGCAATTTTGCAGTGTGTCAGTCTATAACTAGCAGTAAAGTT
TGTCGGGTGGGTTCCGCCGCATCTATTTACAACGTGTGCAACATCGGAATAATAATCAGCTTTTATCCTT
TAAACCGTGCCGACAAATGATGGCATCTCAACTTTACTCACAAGCCTATTACTAACTACTCTTACTCATA
TTATACATTTTTATTATTTTTATCAAGTTCTTACCATTGGAAAGTTGGAAAACATTAGGAAGAAAAAGTA
TTGCAAGCCATACAACTCATAAATTTTGATTCTGATAATTTTGGTTTTATACTGTCAAAAATATTCTGTCT
ACATCTTACATAGGTATTTACTCACAGTGACATGACGAGAGGAGGTTATTTATTATGACCATTTAATAAT
GTTAATAATAGAAATGTCCGGTTTAAATAACGAGTTGAATTATTAAGTTGAAGGGGCGACGAAGATTCC
AGCACTTGACTAATTTGCTTAGGAGTTAATGGGGCGTTCCTAGTCGGGATTACGGAATGTCGGAGGCC
GACAATTTATGTTATTTACGAAGTCGCACTTAGTTTCACACAGACTCTGAGGCATTTCTCTATACCGACT
TGTTTCAT
>TCONS_00117282 geneID=XLOC_023319 scaffold_39:837546-851651(-) Exonic overlap (+)
TGCCTCCGTCCACGGCGACCCCACGCCGCTGCCGAGACCAAAACAACCCTTGCACGTGACACCAAAC
GGAAAACACTCGAAACGAGAACCCACCTGAAAAACTCATTCCTCTAACAGTGTAGTGTGTGTACGCA
ACAGTTCGCCAGGACCGACCGTCTAAAGGGTATCCTCGGCGTAGTTTCAGGAGGACTGGTTGTAGTGC
AGTGCTTATTACCGGGCCGTAAGGACTATGTGCAGTGACAGGTGACGTGAACTTGTGGGTGACTGTAG
CGAGATGTTGCTGTGGAGTGTAAGGAACTCCGGACGGCGGAAACAAATTGGATGGTAA
>TCONS_00117413 geneID=XLOC_023321 scaffold_39:909497-910785(-) Exonic overlap (+)
CCTGTACTGGAAGCTGAAGCTGGTGCTGGTGCGCAAGGACGAGCACAAGTCCGCGTCGCTGCTGGCG
CACCTCGGCCGCATCGACGCGCTGCTCGCCCGCCGCGCCACCAGGTACCGAAAGTGACCGGTAGATG
GCGCACCTCGACCACCAGGTACCGCCCAGAGCCGCTAGATGGCGCACTTCGACCACCAGGTACCGCC
CACCGCCGCTAGATGGCGCCATGGACGCCACCAGGTACCGCCCACCGCCGCTAGATGGCGCTGATTGG
AGTTTTTAGTTTTTATTTGGGTGGCGATATATGGCGCTGATTGGAGTTTTAATGAAAAGAAAGAAAGAA

AACATTTAATTACCATACTGACAAAAACAAAAATGATACAAAACAAAATACAGAAAATAATACAATACA
AAACAAACAAAGTAAAACGAAACAAACAATGGTTGCTGTCCAAAGCGTCGTTTTGTTTTGGCTTGAG
TGGCGATAGATGGCGCTGTGAGAGGTTTTGGGTATTCAATGTTAGTTTTATAGAAAGGCTATCTAGAATT
ACCAGCCTTTCTCGCCCACCTCGCCGCCAGGTACCGAAGCCAGCCGAGAGATGGCGTTTATTGGAGTT
ATTTTTTTGGG
>TCONS_00117286 geneID=XLOC_023324 scaffold_39:1016565-1017910(-) Unclassified
AAATTTAAAAAAAATACCACTCAGCCACCTGTCAGACCCAGCATTGTGATATTGTGTGTCACTTTCAAT
CTTAATAATACTTAATAATGTTTTTGTTACCAACTTAATTTACGAGTTTTCAATGAAATTATTTGCAGTAG
TATCATAAATTGTTTGACCAATAACAGGAAAAAGGCACACATCACAATGTGCGACCCAGGACGCTGCA
ACTTATGATACGCAAGATCCAGCCGAGCTCCCTGAATGGCCAGCGCATTTCCCGAAGGTCGTAAAACT
ACCTAAAGAGCGGCTACGGAAGAATACCAGTCATACCAACAACTCAACACTAAACTCAAGCGTTATAC
TCTGTTGTCGTGAAAGTCCAGTGTTTCGTGATCGCCACCGGAAGCCTACTAGATGGGACCGAATTTGG
GCTGTATTAAACACGACATTCATTGGCAGAATCCATGATTATTTGAAGAAAATTGTTCCTCATCTTGCTC
AAGATTCTTTGGAGTACTTATTATAAGAGTTTATAATCGGAATTAGCTGAAAAATGTATGTGTATTGGGA
ACAAAATTGACTGTTTATGTTTATTTTGCTGATTACTCACAACACAACAATGATGCTCGATCGTGATATG
TGGTGTATTAGTTGTTCTGGTTATAGTAGCGTGTTCTACTGAGAGGGGGACGAATTGCTTTCCAAAGGC
ATGGAACGACATTTTGAAGATATACCATAGTTTTGGCGTAAAAATGTAATGTTTATGCTTACAAAAATCG
GTAGTTTATTTTTAATTTGCTGATTAGTCACAATCATGATCGCATTGTTAATAATAGTTCTGGTGGTACTG
GCGGGGTACTTGTACTGCAACCAGCAACAGTCGATCAGGGATCAGCTGGATCCGTCAAGGAACCTTC
AGAACAACTTCCCGTGCAACTTCTTCAGCAGGGTCAGTCAATGGTTCTCAGACACGTGGAACAGTATC
TTCAAATGATTAACTTGGGATAAGGAATATTCATAGGCGCAAAGCTCTCAATCATGTACTAAATTTATGA
GGGTTCTTATACTTATACATTTACAGGGTTCTTTTATACTTATAAAAAGTATTATTTTTATCATAAAAAAAT
AACCCTAGGTATATTATATTTATACAAACTTTATATTTTCTTGGCAAGACGGTGT
>TCONS_00117476 geneID=XLOC_023400 scaffold_390:93531-95413(-) Exonic overlap (+)
CAAGGAGCAGGACAAGGAGCAGGGCAAGGAGCAGGACAAGGAGCAGGGCAAGGAGCAGGACAAG
GAGCAGGGCAAGGAGCAGGACAAGGAGCAGGACAAGGAGCAGGACAAGGAGCAGGACAAGGTGC
AAGGCAAGGCGAAGGCGGCCCAGGCGGGGGTCAAAACTGACAATGCCGGATTTGGAGCCAATCAAG
AGCCAATACAGTCGCTG
>TCONS_00117482 geneID=XLOC_023401 scaffold_390:213041-228405(-) Exonic overlap (+)
CGATGATGTCGCATGAAATGGGGGTGCGGTGGTGGCGGCGCGCGGAGCTGCCGGCCGGCTGCAGTAC
CGACGAGCTCTCGGCGGCGACAGGTGCCGCGCGGTCCCTCGCCTGGCTCAAGTTTCCCGCGACGCGA
AACACCTCCTTTTGTTTGCAACACTTGTACTATCACAACTCAGCCATGTGAGTTTCTACGATCGGAACT
ACACTCAAAGTTCTGAATAAAATGTGAACTTGTTAAAGTGCTGTGAAGTGTTTCGAGATTTGGGAATA
CCGGCTCCAAGTCTTGTACAATTTAAGTGCAATATTTCTGTATAAAACTTCGCTGACGATGAGCTGCAT
GTGTGTGGAGTGAAACTGCTGGAGTTTGTCTCCGCTACAGTTAACTTTGTGTGTGTGTGTGGGCGCCA
TGTTGGACTGCCTGTGCCCGTCGCCGCGCACGCTGGCGTGCCGCGTCGTGCTGCTGGACGACCGGGA
GCTCATGCACGAGATACAGG
>TCONS_00117495 geneID=XLOC_023407 scaffold_390:384822-387380(-) Unclassified
CGCTTCTCAAGACCCAGGGAGAATGTGGATCGGTCGCGGCCTATAATAGCGTACAACGATCTCGACTT
CCCGGACAGTGGCGATATATTCTAAATTGTGCCGTAAACACGAATTTCGTCCTTATGACAACAGCTGCC
TTAAGCATTCCCTACAGTACATTATGTATATTATTATGACAATGAGATATGTAAAATGAACTATATATTTTT
AATATTATGGTTAGGTAATATCAAAACAGATCCGCTTAAAATAGTGTTCATCATTAATGTCATTCCAAGT
GCCTGAGTCTTAAATTAACTTTTGAGTATTTATGTAATTACCTCGTCATTTAACTGTGGAAAGTCACATC
AACTATATTTGCTAATATAAATATGACACCTAGATTATACAGGTAATATCGATATTATGTTCAATATTTTAA

GAGTTTTTATCTTGTTACCTATTTATGTAAGATGTCGTAATGATAAATGGATAGTCAGTACATTTGTAAAC
TGTACTTCCTGCTATAGTAAACGTATTTTGATTGTTTCTCGCAGTGATGTCATGTTTCCTTTAGTAGATAT
TTTACAGAAATATGACAAAGAGTCGATGTTGTACACGCACGCACACATCTATTTATCTTGTAAACCGTT
CTTGACATACTTATGTATTAACAGAATTTAAATAAACTGTCGGACCTTATGCTGTTGCACTTTTTCTGCA
CCTCTTCTCTTTCCTTTCGACTTTTGTGACCGGGAAGCAGAGATTCGCTTTGAAGAGTATTCTATGTGAT
TTGTGTAGATTAGATGTGATAGCTGCATCTGCGGCATAGCCTAATATACGCCTAATGTCGTTGTTGTACA
AACACTTTCCAACTAAAAACGTGGCATGAGAATATGAGGCATTATACATTCACAGTCAGCAAGCCAGG
AGTTACTAAACTGCTAGATGTGTTTGCCTGAGAGTGTTGCCCCGCATGAGCCGTCTATTAGTGTTATGC
CGTGGTCGGCGGCTACTAACGGAGGTGTGGTTGCTTGTTGAAGGGGTGGCGGCGGCGGCGGCGAGTT
CCGTCCCGTCATTCACTACCGGGACCTGGACGCGCCGCGCGAGCCCGACGAGTTCGGTTAGCACCTCC
GCCGTGGGACCGCCCGGGACCCACACCAATGTAGACTAGTGTAACTTACATAATATAAAAGAATATTGT
ACTAAAACGGTTATGGTAGCTCAACATGTTAGGACATTTTATGACCTTGTGATTGAAACTAAATGTATTA
AAAGGAACAATTAACTTATGAAATTATTTCATTGATAATTTTAGTGACCTTGACGTTAAGTAGGTTTAGA
GATGATCAATGTATAA
>TCONS_00117464 geneID=XLOC_023398 scaffold_390:409818-418212(+) Unclassified
TCGCCGCCCACTCCTCGATCGCCCGCGCGACTCACTCAGTCTTCCACCACGCAGCGCTGCGACTGCTC
GCCGCACTCATGCTACACCGACTAGTTTCTCCCGCGACTATTAATATATTATGTGAATGCAACCGTTTAA
TTCACCCAACACGAATATAAGTGAAATGTTAGTGCTCGTCGGCTGATCTGCGAGACGTAGCGACGCTT
TTCACTCGCAAACTTCATGGTACCTGCACGATTTGTTTATAATAAGGAATCCTCTTCCTCTTCCACTACA
CAGTTATGATTATCAGAATACCATGGCTTCTGCCATTGCTGCTGAGTACATGCACATTCACCTTCACCTC
AGGTGGCCCCTTATTCCTGGAGAGATCGAACAAGCCGCTGATACAATGGAGCCAGCCCA
>TCONS_00117610 geneID=XLOC_023398 scaffold_390:409821-417964(+) Intergenic
CCGCCCACTCCTCGATCGCCCGCGCGACTCACTCAGTCTTCCACCACGCAGCGCTGCGACTGCTCGCC
GCACTCATGCTACACCGACTAGTTTCTCCCGCGACTATTAATATATTATGTGAATGCAACCGTTTAATTC
ACCCAACACGAATATAAGTGAAATGTTAGTGCTCGTCGGCTGATCTGCGAGACGTAGCGACGCTTTTC
ACTCGCAAACTTCATGGTACCTGCACGATTTGTTTATAATAAGGAATCCTCTTCCTCTTCCACTACACAG
TTATGATTATCAGAATACCATGGCTTCTGCCATTGCTGCTGAGTACATGCACATTCACCTTCACCTCAGG
T
>TCONS_00117765 geneID=XLOC_023459 scaffold_391:2401-7147(-) Splice junction overlap
CAACTTAGTCATCAACATACCTATTATGTATATTTAGATGATCTCTTAGTTAATAATCTTGTGCCTTTTCAC
TAACCGAATAGAGTGAACTTATATTTTAGCGACAAACTAAAGATATTGACAATTAAGATATGTACTTGGT
AAAGTTCAGTGAACTCATTACCTTGGAACTAGGTACATATATCAAATATTTTACCGCCAATTTATGTTTTT
TTCCACTATGCTTCATTATACCTTACTTAAATAGTATGGCTTTCTAAACTATACCTTAATACTATGTGCCAA
GTCTTTATGGATTAGCGGAGTGCTTAATGGTAGATAGTTAAATAGCGGTTGTATGTTGCAGGGGCCGGT
GGGCAAGCGCGGGCGCAAGGGCGACAAGGGCGACAAGGGCGAGCAGGGCGTGCCCGGGCTGGACG
CGCCCTGCCCGCTCGGCCCCGACGGCCTGCCGCTGCCCGGCTGCGGCTGGCGACCCAGCAAGGAGGC
GCTGCTACAGTGGGTGCGGCGGAGGCTCGACATCGACAACGACCTGCGAGTGTGACCGCCGCCGCCG
CCGCTTCTTCACCGTGGTGCAACATGCACTTGAAGCTTCAATAGCATTCAAAACTAATCAATGAAGCG
CCTAGTGAAATGTATATAGCAATGGAAACGTCTGTCACTATTGCTAATAATTTACTTGCAGGCGTTTATTT
AGTATGGATTCTGTACGTAACATTACGTAATCTATCTTTTCCGCCATAAAGGCATGCACTATCCACGTAC
GTATGAATACTTTAGCTGACATATAAATGGCAACAACATGGTAATGTAAAAAAACCTAAACTTACCTAC
CCGGCAGGATTTTATTTATACTCTGTACTTTACTTGACAAGGACCTTTGGTACTGGATGATTTTATCTTTA
ATTAAATTTTTACCATCGACATGACACATTTTTGCCGTAAATAAGACATATCAAGTTATCTGTCGTCAGT
AAATAATTACCTACTATAAATAAGTATAATTAGTTTTCTTAAAAGGAAAACCAAGGTGACGTCATAGATC

TTTGGGAATTACCGACTACTTCAACGCGTCGGCTAAATTAAACCTGTTTGTTTGGGTTATTCTCAATAAG
AGTAAGCATTACACTATAATCGTGTTAACAGGCATATTGTACAAAAACACGCGAGTTGAAGGTCGAGC
TCCGCCGCCGACCACACATACTGCGGCTTGCGATATTTATAGTGACACGAAATCAAGTATAGATATTTG
GGTCAAAGCTATACCAATATGCACTTTGCGGATGTGGCTAACCGGATGTAACCATAAAAAATATGATTAT
GTAAATTTTACATCAATTTATTACCTTATATCTAAAAATTTATAGAAAAACATAAAACGACATTGATATAA
AAGGTAAAGTTTATATAAAAAATATAATTATAGTTAGGTATAGCGAGCGCAGATAAAGAAACAATATGA
AAATAGTGAACGTACTCATTCTTTCTATTCGTAAGTCTTTTAACTCTCCTTCCTAGTGCCACACTTGCAA
CAGCCGCTATCTTGGATTTGATTCTTTATCTGCGCACATTACACTCGCGGTTTATGTGTACCTCTGAAAA
GTTTCTGGTTATTTATTCATTTAAATTTGAATCTATAATTTGTAGCAATAAGTAAACTGTTTTGGTGTGAC
AATAGACCGGTGTTTATCAAAACGTAACATTTACCTACTAACAGTTGTAGTAAAACTTGTTAACAATAAT
GTACCTATTACATGACCTTAGCAGAAATAGTTTACGTTTTGATGAACACCGTGCACATCGATAAATGATT
TGTTTCAACCATATGTAACAGTTTGCTGTGAGTACACATTAGGCTAGGGAAGCTCACATTCACTGTCTT
ACTTTAAATAGTTAGTAAAAAACAATGCAGCACCGATTTGTATGGCACTTGTTGTTCTATAATTATTTTG
TAAATACATTTCTTAATATTATTTCATAACGTATTCTAATTATAAGAGGAACAATGCGCAGGGCGCGAGT
AGCCAGCGAGCCACTATAGAGAAATAAACAGACAGGCCCACATTATTTAGTTGTTTAAATTATGCAATG
TGTATGCTGCCAAATGAGTTATACAGTCAACTTCATAAATATCAATACTTTTGTACTCAAAATTTCGATTC
AGTAGGACTTTGGTTTTGGGATATTTATGGAGTGGACTGTACGTGGGGCTGCGCTGACTGTACGCCCCT
GACACTTGCTGTGTGGAAGAATTACTGCTGTTACATTTTGTTACCTATTTATTAAACTTTTGTAAATATTA
TCTGATAATTTAAGTTACCTCTATAAGTCTTCAGTAGCATTATAAATTCGATTTAGCACATTAAAATGGGT
CAATGAATAGTGCAAAAATATAAAATTAAGAAAATAATTGATTTTGGAACATAAAATACAAAATGTTTAT
AAAAAAATTCACATTTTCATTGGTCTGTTTTATCCCACAGTCTGTGTTAGTAGCAGTAGAACGCGCATT
ACGCACAGAGTGTGGGCCACAGACTGTAGAATGTATGTTTATAATAAGGATGTATTATTTTCTTACCGCT
CAGTGTATGTTTTAGTCTAAATAACATAAGATTGTGTAAGATCTATTGTGGCACGGCATAAAAACTAAAT
TAAGTATATCAGATGTTATACTTAAATGCCTTCTTTTTACAAATGCATCAGTCTCGATATAATTAGGTTGA
GTTTACTATGTAACAATCTTAGTGCCGATTTCTCCATTGTCGGTTATCTAACCGATAGGGATTGATTTATA
AATTAAACGTCATCTCCATATAAAATTCATGACAGATGTATCAAAAGTTAACCACTACTCCCTGACACC
GTTAAACAAAAAACAATGTATAATAATATAGTGGCACGGTAGTCCCCCACAGAGATGTAAATATTGTCA
ACAGAACATGTACAGAGCGCGACGGAGACAGATTATATTGAATATACTCATTTTTTACAAACCATCTGC
TTTTACTATACCTCTATATGTATATTTACCAATGTTTATACCTATCCTAGCTATCCCAAACTAATTACTTAAC
TAAAGAGAGTATAAATTTCATTTGTAACATTCTCATGGTCATGGTTGGTTTAAAAGGAATTTACATAGAT
ATTTTGCTATGAAAAGAGATTATTTCTAATACAATCAAAATAGGAGTATCAATGAACGGCCATGTTCAAA
ATAAACTCACCAGAATATAAAGTAAGTAAATAGATCTACACTCTCTGGAATGTGTAAGTACCTAGGAAT
ATCCGCCTATAATCTATGTATTTACGAACATATAAGGCACTCAGTCAATGTTAAGATCAGGACCAGATCT
AACGAAATGATGGCATTTCAGCAGTAGCAATATCATTAACATTAACAAACAAAATATCCAATGACTACT
TTTCGGTTGCACTCATTTCTTTTCTAATCTTTACAAAACCAATTATGTTGGTGAGTACACATTATTTATTA
CTATGAGTCATATAGTGAGTTGCAGTGTCATCCATTGCTCGACACAGTTT
>TCONS_00117843 geneID=XLOC_023459 scaffold_391:6602-8091(-) Splice junction overlap
ATAGAATACTGGAATGTGTATAACATACTGTGAGTGTGTGCGTGTAGTGACGCGGTGTATTGTTGCAGG
GCCCGCCCGGACTCGACGGCATGAAGGGGCCGGTGGGCAAGCGCGGGCGCAAGGGCGACAAGGGCG
ACAAGGGCGAGCAGGGCGTGCCCGGGCTGGACGCGCCCTGCCCGCTCGGCCCCGACGGCCTGCCGC
TGCCCGGCTGCGGCTGGCGACCCAGCAAGGTAACATTCACTTTCAATCGTTTGTAATTACTTACATTTT
TGGTTATTGATTGCCCCGTTAATAG
>TCONS_00117754 geneID=XLOC_023453 scaffold_391:172408-174727(+) Intergenic
GTTTTAATTAAAATGTTATTTATTTTCACAAATGTAGCACTTAAAAGCTAGGCATTAATTTTATGATAAAA

TTAATAATCTTTACAATATACAAATTCCTTAAAAAATCCATAAAATATTGGTTTATTTAAAAACATATGTA
CATTACAACTATTGATACATAACATAGTCCTACGTGATATGGGCATGGGCCGTAGCTAAGGCACGTGGGT
AGTATATTGCAGTGTGTGGGAACAGCCTACACACACAGAAACAACAATGACTCAATTCCTTACCATATA
TCCATGTACGAAATAATAGATTCATCGATATTATATTATTAACATAAAAATATACCCACTAACTATAAAATT
GTTATACAAAATGAACTATCAGCAACAAGCTACTTGAGAATATCTTTACAGATTATGAGAAGCTAGCTA
AGTTAGGAACTTACAACTGTAGCTGATAAATAACGTATATAAATGTAATGAAATATGTAGTAATAAATAA
TTTACATAAACATGATTTCGCACAGTTTATTATAATTATAGACAAACACGTTTTGGAAGTATTTTAGCTTC
ATTTGGCAGTTTATTATATTATAGACAAACACGTTTTGGAAGTATTTTAGCTTCATTTGCAATATTGCATT
CCATCAAAAATACTTTCAACAACGAAATGTATAAAACGAATTAATTATGCGTAGTAAGTATTAGATTAAC
ACCAAAAAATAAATGGAAGATAAAAGTAATATGAACTCTATTAAATTCGAAACAAATTGTAAATTTCGT
CAATTCTGAATTTCAATAATATCTTTGGCAAACAAACCATTTCAAACAAATAAGACAGATACACAAAGG
AATGAGAATGATGATGAGTGAACAAATCAAAACACATAGCTGCCCTAAAAATAAATTTCAAAATGCAT
CTCAAAAAATAAGCGTAGGCAGAACTACGATGGACTGCTAATTTTCCAAAACTTAAGTACGTACGTAAT
GTTGAAATTCCTATATCGATACATAAATCAGAGATTGTTTGAACTTAAACACTACAAAAATAAAAGCATT
TACACAAAACAAGTGAAGGCTCACTCCCGATCTACCGATCGGGGCGGGCTCGACTTGAGTCGATATAA
AACTACGATAAACAAAACTCAACATAGCTTTCGTTGGAGGAATGTAAACAGTGATCATGGAGCAATAA
TAAACAGCGCGTATAACTTTTAACGAGAACAAGAAGTATAAATAACAACAAGCGTCGGAGGCCCTTCC
GCCTAGCACCACGGGTGTCGCCTACCTTTGTACTTCACCATCACAAGGCGTGCCACCTTATGTACAGCC
ACGAGTTATATACCAGCGACACTATAATATGCGTAGTAACTACGATCCCAGATATCGGCGTCCAATAACA
ACAATGTTGCAGTCTGCACGAGTTCTAGTAAAAAACATTCACTTGCAGTTCTTTAATTTTGTAAGATATA
AAATTTTGGCACCTAATCGTGATTTTTTACTAGTTATTATCTACGTTTTATATAATTACAAGTATGCGGTTA
AGACACTGGTTTTATAACACTTTGATCCTAGGGACCCGTTAGTGTGTAACTACAAGAACGTGGAAATA
AGCTCAAAAAATTACACTATACTACGAAAAATCATGTGATTTGGGGAAGCTCATTAATCGTTAGGAAAG
TCTTTTCAAGTTTAAGTAACACGCGAGAGTCGTGCGGGATTGAGCAAGGGTGGACCGCGGGGTCGGT
GTGGATATGGCACGGTATTCGATTTATTGCGATGAGCAAAACTTGGTCGATCATGAAGCAGCCACCGCG
CTGTACACTACACTAGTGCCGCTTATCAACTAAACTGGTGCTGCAATAGGAATTCATTTTAAAGTGACA
AAATATTCGTTTCTCTCTTAGAAGAATCTTATTTTACTCAAACTGGTGTGATTTAGTAGGAAAATGCAAC
TAGAAATTTTAATAAGTGTCATTAAAAACAGTGCATCAGTGAACATGTGATGAGGGGCGCCGCCGTGG
CCACCATTATTAACTGTGAATCTACATTTAACAAATCGGAACATCTAAACTTGGAAAGAAGTTCTATTC
ACTACGTTTGTACGTTTTTGAGTTTTGTACATTAGGCAGGTAACTTTATTAGGCATCAACACAGTCAAC
GTGGCGTTCCATTAAATAAATCTATGTTATTTTGGAAAACACAATATTTATATTACAAAAATAAATTCTCT
GACGTTTCGTACATTACATGATGTCGAATCCGC
>TCONS_00117806 geneID=XLOC_023453 scaffold_391:173034-174838(+) Unclassified
ATTGCATTCCATCAAAAATACTTTCAACAACGAAATGTATAAAACGAATTAATTATGCGTAGTAAGTATT
AGATTAACACCAAAAAATAAATGGAAGATAAAAGTAATATGAACTCTATTAAATTCGAAACAAATTGTA
AATTTCGTCAATTCTGAATTTCAATAATATCTTTGGCAAACAAACCATTTCAAACAAATAAGACAGATA
CACAAAGGAATGAGAATGATGATGAGTGAACAAATCAAAACACATAGCTGCCCTAAAAATAAATTTCA
AAATGCATCTCAAAAAATAAGCGTAGGCAGAACTACGATGGACTGCTAATTTTCCAAAACTTAAGTAC
GTACGTAATGTTGAAATTCCTATATCGATACATAAATCAGAGATTGTTTGAACTTAAACACTACAAAAAT
AAAAGCATTTACACAAAACAAGTGAAGGCTCACTCCCGATCTACCGATCGGGGCGGGCTCGACTTGA
GTCGATATAAAACTACGATAAACAAAACTCAACATAGCTTTCGTTGGAGGAATGTAAACAGTGATCATG
GAGCAATAATAAACAGCGCGTATAACTTTTAACGAGAACAAGAAGTATAAATAACAACAAGCGTCGGA
GGCCCTTCCGCCTAGCACCACGGGTGTCGCCTACCTTTGTACTTCACCATCACAAGGCGTGCCACCTTA
TGTACAGCCACGAGTTATATACCAGCGACACTATAATATGCGTAGTAACTACGATCCCAGATATCGGCGT

CCAATAACAACAATGTTGCAGTCTGCACGAGTTCTAGTAAAAAACATTCACTTGCAGTTCTTTAATTTT
GTAAGATATAAAATTTTGGCACCTAATCGTGATTTTTTACTAGTTATTATCTACGTTTTATATAATTACAAG
TATGCGGTTAAGACACTGGTTTTATAACACTTTGATCCTAGGGACCCGTTAGTGTGTAACTACAAGAAC
GTGGAAATAAGCTCAAAAAATTACACTATACTACGAAAAATCATGTGATTTGGGGAAGCTCATTAATCG
TTAGGAAAGTCTTTTCAAGTTTAAGTAACACGCGAGAGTCGTGCGGGATTGAGCAAGGGTGGACCGC
GGGGTCGGTGTGGATATGGCACGGTATTCGATTTATTGCGATGAGCAAAACTTGGTCGATCATGAAGCA
GCCACCGCGCTGTACACTACACTAGTGCCGCTTATCAACTAAACTGGTGCTGCAATAGGAATTCATTTT
AAAGTGACAAAATATTCGTTTCTCTCTTAGAAGAATCTTATTTTACTCAAACTGGTGTGATTTAGTAGGA
AAATGCAACTAGAAATTTTAATAAGTGTCATTAAAAACAGTGCATCAGTGAACATGTGATGAGGGGCG
CCGCCGTGGCCACCATTATTAACTGTGAATGTGAAGCTACATTTAACAAATCGGAACATCTAAACTTGG
AAAGAAGTTCTATTCACTACGTTTGTACGTTTTTGAGTTTTGTACATTAGGCAGGTAACTTTATTAGGCA
TCAACACAGTCAACGTGGCGTTCCATTAAATAAATCTATGTTATTTTGGAAAACACAATATTTATATTAC
AAAAATAAATTCTCTGACGTTTCGTACATTACATGATGTCGAATCCGCGAGCATGGGTCTCGCAGTTAG
AACACCATCGACAGTACAAATATGTACTTACGTTACAATTCGTTATCAAAATGCACTTCGATATCAAGCT
GGCAGACAAACAATTCAAAT
>TCONS_00117800 geneID=XLOC_023455 scaffold_391:265305-266882(+) Intergenic
ACAGGCCCTTAATGTCATTACAAAATTAACTTGCATTACGTATTTATTGTTAGGGAATTTGAACTATTGAT
TTTAGTAATTTCTCAATTAATCATCGCTAAAGACCAGTTCAAACGATTTGCCTCTTCATAATAATTGACA
CAAGACATTAATTTCATATTCACAGAGCAAAAATAATTGAAAATGACCAACACTTCCATGATTCTTTAC
AGAGTGTTACCAAAGTGGTGGAGAAAGCCGGAAAGGGGGTGACTCAAAGGTCATTCTACCGTGCTAG
GGCTAAAGTTTTGCATCGCGCTCACTCACATCGCGTGGCCTCAAGAGCGAGCGCGACAGAGAACTTTT
TTCACGTATCGACGTAAGCGTCTTGCGTCCCTTGCAACGCTATCTATCCTTGAAAGATTGCTATTTGTTT
TGCTTCTCCATAGTCTTTGTTGGTCACGTGATTTTCACCATGAAGAAGCAAAAAAAAAAAGCGTAGAA
TCAAAAGTTGTTCAGAATGACCTCTAACCCACCATAAACATAGGTCACTGCTTAAATACCTAGGCTTCT
CCCGAGTTAAGGCTAGAACTATAAAAATATCTAGGGTACTTATAGTTTCACACTAAATACATTAACTGTT
GTTTTTCGCAAGTACAATCAGACATATGTGATCAAATCACTATAATACAGGATGTTACAAAAAGGGTATT
CTAAGCCGAAACCTACATGTGCAGCATGGTAGGTAAGGTATATCTAAACCCGAAACTGAAATCACAATT
TGAAAATTCTCGAAAAAATCACATTTTCCATAGAAACTTTGTTGGTCACGTGACTATTTTACTATTGAG
AATGTTTTATTTCGCCAATTTTGCAATTCTGATTTCAGATTCGGGTTTACATATAACATGCTGCATATGCT
GGTTTCGGCTTAGTGTACCCTTTTTGCAACACCCTGTATACCTTCAGCATACATAAAACAATGTGTGTAT
GCTGAACAAGACAGTCGAATGCGAAAAACTCCAGCCATTTTCATTTACAATGAAAATTTACAATAGCT
AAGAAGTAAAATCGGTATTAGAACAACTTTTAACACGAGAAATACGTAAACGCGTAAGGTTTTGAGGG
TGTCAGCCCATTATCTAATATCTACCCTTTTAAAGCCATCCACATCCCCTCCCTGTATTCAGCCTAGGTCT
TAGGGTGCTTACATAGAGGAGAGTCTGGGTTCCCTATCCCGTACCGGTAACCTGCTGTTTATCTTCTAGA
TCATTTCTCGGATTTAAATCTCTCAGTGACGCATACTTAATCAAGTTACCGGTACGGGTAGATACAGGG
AGCCCGATTCTTTATGTAAGCACCCATAGAAACGACACTTAGGGTGACTTGCACAAGACATTTAAACGT
ATTTAAGGCTTGCTTTGTCAGTATAGTGACTCTGTC
>TCONS_00117761 geneID=XLOC_023455 scaffold_391:265702-267321(+) Intergenic
AAAGATTGCTATTTGTTTTGCTTCTCCATAGTCTTTGTTGGTCACGTGATTTTCACCATGAAGAAGCAA
AAAAAAAAAGCGTAGAATCAAAAGTTGTTCAGAATGACCTCTAACCCACCATAAACATAGGTCACTGC
TTAAATACCTAGGCTTCTCCCGAGTTAAGGCTAGAACTATAAAAATATCTAGGGTACTTATAGTTTCACA
CTAAATACATTAACTGTTGTTTTTCGCAAGTACAATCAGACATATGTGATCAAATCACTATAATACAGGA
TGTTACAAAAAGGGTATTCTAAGCCGAAACCTACATGTGCAGCATGGTAGGTAAGGTATATCTAAACCC
GAAACTGAAATCACAATTTGAAAATTCTCGAAAAAATCACATTTTCCATAGAAACTTTGTTGGTCACGT

GACTATTTTACTATTGAGAATGTTTTATTTCGCCAATTTTGCAATTCTGATTTCAGATTCGGGTTTACATAT
AACATGCTGCATATGCTGGTTTCGGCTTAGTGTACCCTTTTTGCAACACCCTGTATACCTTCAGCATACA
TAAAGCTGCGTACACACCGGGCCAACGAAGGCCAACGAACGGGTTACGTTGGCCTTCGTTGCTGCAA
TCTGCCCTGTATTATGTATGATAAATATCCATCGTTGGGATAACCGTTGGCGTTCGTTGGGCGTTCGTTG
GCCCGGTGTGTACGCAGCTTAAGACAATGTGTGTATGCTGAACAAGACAGTCGAATGCGAAAAACTC
CAGCCATTTTCATTTACAATGAAAATTTACAATAGCTAAGAAGTAAAATCGGTATTAGAACAACTTTTAA
CACGAGAAATACGTAAACGCGTAAGGTTTTGAGGGTGTCAGCCCATTATCTAATATCTACCCTTTTAAA
GCCATCCACATCCCCTCCCTGTATTCAGCCTAGGTCTTAGGGTGCTTACATAGAGGAGAGTCTGGGTTC
CCTATCCCGTACCGGTAACCTGCTGTTTATCTTCTAGATCATTTCTCGGATTTAAATCTCTCAGTGACGCA
TACTTAATCAAGTTACCGGTACGGGTAGATACAGGGAGCCCGATTCTTTATGTAAGCACCCATAGAAAC
GACACTTAGGGTGACTTGCACAAGACATTTAAACGTATTTAAGGCTTGCTTTGTCAGTATAGTGACTCT
GTCACCAGCCAGCAATATGTTTAAATGTCTTGTGCAAGTCACACTTAAAGAGTCCGACAGTGCAAACT
TTGCAACTGTGGTACATTAGTTAGCTTACAGTGGTACAATATTA
>TCONS_00117770 geneID=XLOC_023461 scaffold_391:270239-271429(-) Splice junction overlap
GCTTTCAGAAGTGTCAGCGGACGCCACAGACGGTGCACAAGCGGTGTCGAAGGCGAAAGAACAGAC
GAAGGGGAAAGACAAACGAACAGACAAGAAGAAACCTGAAGTCAGGCCCGCCAAGAGTGATAGCA
AGGTACCTGCCAAGAAGGAGGCGAAGAAAGAAGTAAAGTCAAGTAAAAGTGAAACGAAAGTGGAA
CCCGCAGAGATTGAGGTTATGGAAGAATCGGCCTTCCCTGCGCTAGAACCTGTCAAGGTA
>TCONS_00117818 geneID=XLOC_023462 scaffold_391:327620-330953(-) Splice junction overlap
TGGTATCAAAATTTCTGTAATTTTGTTTGTTTATTTCTGCGATTATTCTAGAAAATACATTAATTACGGGT
TTAACTATCGGTTTAATGAGTAATACGGACATCTTATCATTGTAAATTGTCTATATAACTATTTACAGTCAT
GATTACTACCAAGCTGGAACTTTTGGTAGATTTTGGTGATAAACCTGAACAAGCCCTAGTGAACAAAA
CTGTGCAGGCACTGAAAGCCAAAGAAGGTGTAAAAGAGGT
>TCONS_00117831 geneID=XLOC_023463 scaffold_391:331621-333575(-) Intronic
GTTCTAAGGGCGGGACAATCGACTGTTGATGAACCGTTCAGAATCTGACAATTTAGTATCTGAGATTAA
AAACAGAGTTTACTTTGCAACATAAACAATAAAAAAATGATTTATTATTCTCTGTAATTTCTAAAACTTT
CATGGTACGTCTTCCTCTTCTTCTCGTATAGCTACGAGATATACAGGATGCCACCAAAAGGCAACACCT
GTAGAAAAAAAACTATCGTTACAAAGTTAGCGTTGTTAGACTCTATAACCCTTATGTCAAGTTGCACAT
ATGAACTTTAAACTAAGGTATGTACTATCACTTTCTGTCTGTTCACTATCAAAATAAGGCAGCAATACAT
TTAATTTCGACCTTACTTTAAAGTTTCATGCAAAGACAATAAAATTAAGATAAGTCGTTTGTATTTGTAG
CTGTCTAATTTGGCAATAGTGCCAAAAGTACACCTCAAATCTATGTGATACATACTTTTCTATGCATTTTT
AACACCTAAACTAAGAGTTAATTGTAAAATTTCCGCTTTGATTTGTTATAATCGCAATAACTATGTTAGT
GTTAACGACTGCTTATGTGTAAAGATATAATTGAAGTTGTATATTTGTGATTGATCGATGATTTATTTGTC
GGAAACAGCGGGTAATTTAGAACCATTTAAGGTCTATCTGTACTAACAATGTACGGTCAGCTGCATAAG
TAGCTATACACTTTTGTACCTTGTCAAACTGACTACATAACAAACCGTCAATGTTTGAATAGGCAGTTT
GTTAGTTTGACAAGGTACAGAAGTGTACAGCTGCTTATGCAGCTACTTATACATATTCATCAAGACCCAT
CACGTCCCCACTGCTGGGGCACGGGTCTCCTTCCAATGAAGGTCCACCACGCTGGCCCAGTGCGGGT
TGGTGGATATACATATTCGTATGATTCTTATGTGTGTTTGAATGGGGCTATAAATGATTAATCATCATCATC
ATAATAAAACTGATGTTTAACTTAGATAGGTTTATTAAAAAGGGGGGTAAATCAATTTAAAAATGTACTT
TACGAAATAATAAGGGTTGAAAGAAATGGCAAAAACTATTACAATAGATATAAGAATCGATTGATATTTT
TTATAGCTATTATAAGAGTATTATAAACGTAAGCATAGGTTATTTAGTTTTAAGAAATAAATATTTTTTCG
>TCONS_00117764 geneID=XLOC_023458 scaffold_391:346169-378885(+) Exonic overlap (+)
AACCAATAAGAGCTACGCATTATAAATACCACTTACATCTCATTGGCCGGTCATAGTCACGTGATCAATA
ATCTGTGTAAGAAACTACGCTATTTCCGGTTGTCATACGTCGTAAATTGACGACATTGTAGAGAAATCA

AAACAGAAAACAAAGTGTGCTGGCGACTGGAAAAACTCCTCGCCTATCTCATCGACGCGAACTAAAT
CGACAAAAGTGACTGTTTTGTGTAGTTAAAGACATACACAGTGAAGTGATCGCATCTTGAGTCCACGA
TGACCACTCGGAAACTGAATACGCTGGAAGATTTGAGCGATATGTTTGATGAGCAGAAACCGAAGGAT
GAGCCTAGCGCACTGATATCCAGGAAGACCCTTAACGCTATCCAAGAATCGTGGGTGGAGCTGAACTC
GGCGGGCGGGCTGGCGGCGGAG
>TCONS_00117785 geneID=XLOC_023458 scaffold_391:346169-378885(+) Exonic overlap (+)
AACCAATAAGAGCTACGCATTATAAATACCACTTACATCTCATTGGCCGGTCATAGTCACGTGATCAATA
ATCTGTGTAAGAAACTACGCTATTTCCGGTTGTCATACGTCGTAAATTGACGACATTGTAGAGAAATCA
AAACAGAAAACAAAGTGTGCTGGCGACTGGAAAAACTCCTCGCCTATCTCATCGACGCGAACTAAAT
CGACAAAAGTGACTGTTTTGTGTAGTTAAAGACATACACAGTGAAGTGATCGCATCTTGAGTCCACGA
TGACCACTCGGAAACTGAATACGCTGGAAGATTTGAGCGAATCGTGGGTGGAGCTGAACTCGGCGGG
CGGGCTGGCGGCGGAG
>TCONS_00118096 geneID=XLOC_023510 scaffold_393:202122-203134(-) Intronic
GTCGTTGTCGATGGTCGTCGTCTCGCTGTCGCTGTTGTTTGTTGGTGTTTTGTGCTTTTTAAATTATTGG
TTTTCATCGAAGTTTTACAAGCTTTCTACATGTAGTGTGTTTAATAATTAGTATTTTATTATTTTTTTTTCC
TGAAAGTTCCCTTGTGTAGTGTAGGATCCGAAGAGCAATCAAAATGGCATCAACTACAATGATAGAGA
CAGTCACCATCACCCGACCACTGAAGGTATGTAAATATGTAGATAATACCAGATAATTAATTCCTAAC
>TCONS_00117976 geneID=XLOC_023512 scaffold_393:266039-268069(-) Unclassified
AGTATTTCTCAGCTATCGAGTAATTTGAACTACATATATTATAGAAACCGTACTTGCTAACTTTCTGTCGC
TCTTGGAATTATTATGAACAAGTATATATTTTTTTATAATTTTAAAGAATTGGCTTATTATGGTTTGACCGA
AATTAATTTGAAAATATAATTTGGGTCAATAAAAAAAAACACGAGCAAATGTTAGCCCTACATAATTTAA
ACTGAAAGCAATTTACAATAACGAATCTAACAATACTAACATAGATAGAGCAGCTTGCCAGGACAGTG
ACACCCTAAGAGTAAACCACATAAAGTATGTACATAATATCTATAAAAATCGTTTTATTTGTCTTTTCTAT
TTGAATGTAACTACATATTTTAAGACAAATTACATAGTACGTTTTGAGCAAGGCTCAAAACATTATACTC
ACTTATTAGTTAGAGTACTTACTAAAAATTTTAAAAATATATTTATAGACATCCATTAATTCTTTAAATTGA
TCTGCTATTCATCGCCAAAGTTAACTATTTCTAAACAACTATAGGACAACACCAGATAATACAACTATTG
CACTGTATCATGCGTCAGAAATTGGGGGATGTGCAAGCTCAGAAATCATGTTTACATTAACTACACTTT
GGTATTCATACTGGCGACTCGTTCATAGCCGTACACAAATACCCATACCTTGTTCAACTTTAGTTTATGG
CAATCTGTCGGTTTTGCCAACAATTTGTGCCCATACCTTGTTGCAAACCTACATTAGTGAACACATCTT
CAGTCCTTTTCCGTTTCACCATTATTTGACCAAACAAAATGCATCTACACTGTGCCCTCCCTGTCACTAA
TGAGTAAAAGGGTACCCACAGCATATAAAAAATATTATTTGTGGATGAACGTGATTGAATTTAACATGG
AAAGGAACAAGCGCATATCGATAGGGGACAATAGTATACAAAGACCAAACGATTCCACCTGCCATCAA
AGCAAGAATTATGATTTTGTAGCTAAAATCTATTTCGTAGCCACATTGGGAGTGATTCTGTAGAAATACT
ATACGAGCATAAGATTAAAACACTCTTAAAAATCTGTTTTACGTGTTGAAAGACGAACGAATAAAAGTA
TGCGAATGAACGCACATAGACAAGTAGGATATAGGAGACAGATTATAACAAAGATCACAGACCAAGGT
TCTTGGAGCATATTGTGTAGCTACTACCAATTTCGTAGCAACATTTACCACAATTCTGTGAAAATACTGT
ATTACCAGAAGTTTAAAACACTCTTAAAACGTCTGTGGCAAGCGCAATCAAATAGAAGTGCTTTTAAA
GACAGCTACAGCTGAAACCTAAAATTGCCCTCACTTATTAGCTACATTATGTTGCCCTCTGTTTCCACTA
GCGGTGGTGCCTCTTACACTCGTTACTTTCGCTGCCTGTGTTCACTAGCGGTGCAGCCTGCGCCGCAG
TCGGGCGACGAGGCGGCCGGTGCGCCCCCGCGGTCAGATGTGCACCGGCACGTGTAGTAGCAACTCG
TCGCGTCGCGGGAACGAAAGCGCGCACATTTCACAGCGGAATCCGCCGCCGGGCCACTCTTCACTCT
ACGGGAGGGAAATTATTATATTTTTTTAGGCTGCCTGTCGAACAGAGCGGAGCGGCGTGTGAGGTAAA
AACCGACCAATGACACTGCATAAATACTCCGAGCGTGCAATTTTATTGGTCTATTTTTACCTCGCAAGC
CACTCCGCTAGCCCGCCCCGCTCTGTGAACAGGCAGGGGTGCATGTAAGGAAGTGGATGATAGTTTAA

CATATTCTGCTAGCATGCCCA
>TCONS_00118243 geneID=XLOC_023530 scaffold_394:156656-162426(+) Intergenic
CGCGAACGCAGCCGACGCGTCCCGGTTCCAAATTCGAAAAACACTGGCCATAAAGAAAGAATATTCT
GCATGCTGGCCGGAAAAAATAAAAAAGAAACAATAAGTACTTTTAATACCAAAATCTCCCGGGTTCGA
TTTGTAAAATTCGAACTGTGGGCGCGAATGTTTCTTTTTTCTGTGTAGTTCTTTTGTTTTTATTGAAAAA
TACAAGGGTCCGTTGTTTTCTCGATGGTCTATCAATAAATGTGCAAGCGTGCTGGAACTAGGTCACTGT
ACTTAGGTAACCTGTTGCAGTGAACTGCGTAGGTGGAAGCGTACTATAAAGCGTTCACGGCGTGAAAA
ATTGTGTGCTAAAAAACTGAGAAGCGGCGAAAACTATTGTTGTGTGTGAATTAACATGTAAAAAATCG
TGTGATGTCGGAAGGGCCGTCCCGGTTGCCGTCTTTGACGAACCCAAAATGCAAAACGCATGAGATGT
GGGCAGCCATGACTAGGTCCATCGAGATTTTGGTTGCCTTCTTCGAGTACTATGCACAGAGGTTTGCAG
CGCCGCTGCTATCTCTATTCGTGGAAAAAGTGCTCACTTGCTTGGGCTTGGCTAATTAATGGACTTCAT
CATCTCAAATTCAC
>TCONS_00118170 geneID=XLOC_023530 scaffold_394:160927-162477(+) Unclassified
CGATATTGACTTATGGTAAATTTATAATTATGTTTGTAGAATTGTGTTTTTACGATTTTGTATATAAGGTTC
CACTTTCTCAGAGGTTGCATCTGTTACTATATTGTCACTGAAACTCGTTGTGTAAAATGAAAATCTCTTC
GGCAATTATTGTCCTCGTGGCCCTGGTTTGCAGCGCCGCTGCTATCTCTATTCGTGGAAAAAGTGCTCA
CTTGCTTGGGCTTGGCTAATTAATGGACTTCATCATCTCAAATTCACAACCCATCACATTTCTACTGCAC
CCACGGCACGAGTCTTCTTCCAATGAAG
>TCONS_00118191 geneID=XLOC_023546 scaffold_394:162242-162884(-) Intergenic
TGCTGTGTAGCAAAAGTGATTTTGTGCGGTATTCCTACCACATTACATTTCAACATTATTTTCAAGAAAA
AAGATGACTACGGAAGACTTTTAAGAAACACGCTTCATTGGAAGAAGACTCGTGCCGTGGGTGCAGT
AGAAATGTGATGGGTTGTGAATTTGAGATGATGAAGTCCATTAATTAGCCAAGCCCAAGCAAGTGAGC
ACTTTTTCCACGAATAGAGATAGCAGCGGCGCTGCAAACCTGAATAAAAACATTATAAGATAATAATAT
GTAGTGACATTTTACACACGGCCATCAGACCACAAAACTCGGCATAGCCTGTAATGTAAGTATC
>TCONS_00118254 geneID=XLOC_023533 scaffold_394:192624-197630(+) Splice junction overlap
ATGAGTTCATAGCACTAGTGAAGTAATATTGACTGGTCACTCGATCTACGTACATTTTGCAGTACAATTA
TACATTTTACAGTACAATAATTTTACTTGCATATTCTGACTGTCTAAACTGTTTAATTTATTATTTAATACT
TATAACTTTATCTTTTCACAGTTCAAGTAGCCGATGGCTCAGGAGTTCAAGTGGCCGATGCCAGTGCTT
CAGGACCTGGAGTCCAAGTTTCGAACGGCGGCGGGGGAGTTCAAGTAGCGGATGCCTCAGGAGTTCA
AGTGGCCGACGCCAACGCGGCAGGACCAGGAGTTCAAGTAGCTGACCAAGGATTTTATGTCGGTAAG
AAATAAAGTTACCTGAAGAGACATAATTACTTCGTACAATTCTAACATTAGGACAAGCCGCTGTTAAAC
AAGTATCGGATGATTATTTTATTATTTAAAATACGAAGACCTTTGGAAAAGGCTTTCGAGAAACCCTTTT
ATTTGGAATTCAGAAAACCTTGACATTGGATCGCTAACATAGCAACTAGCAAGCTGTAAAATAGTATAA
AATATTTGAGGGCTGAATGAATTTGTGTTGACTTTTGACTGACTTTTTGTAACTTTCAGGTGGCCAACC
TCGCGGCGCACGGTGAATTATATAAAAAAATTATAGAAATTTATTAAATAAATTACCTTACATTATTCCTA
CTAAATATATATGTTTTCTAAAATAATTTATTTAAGTGTACAATTATTCACTCCCACATCTTAAAAGTAAA
ATAGGTAGTCAGATATTTTTTGTACATATACTCACGAATTTCTATAAAATACAATGTATCAGATATAAAAT
AATCATTGACCTGCACACTCCAGTTTTTTTTTCTAATCTGCGTTACAAAAAATACAAAAACATTATCAAT
ACTCAATGCAAAATAAGCTATAAGGATTTACTCACAGCAACCTTAGTTCAGGCATCCATAGTACTTACAT
AGCAAAGAAGAATAGGACTATAGTCAACTATTACTTACCTATAAGTGAAGGTAGCGCGACATTGGTGCC
CAAATAAAGCACATATTGACCTCTGTATTTCGGCATTGAGGTCAAGACTGCTCTGCATCCGTCCTCAAT
AGTTATATTTGAATTGTATCTGATATATTATTATTATTATATGTATGTATAAAATATTAGGCTCTTTACACTC
ACACAAAAAATCTTAAAAAGTTTACGTTAGTAAAGTTGGATGTCGTAGAAAAGTTCAGTATGCTGTAG
ATTTCAGGTTATTCTTTTATTCAAGTCATATAATAGGTACGGTCAGGTGCAGAAAAACCTGACCCCCCTC

TCATAGTAACAATGTTTCTGAGAGGGGTCAGGTTTCTCTGCCCCTTACTGTACCTATCATTAGTATCATG
CAAGTCATTAGAAAGGAGCTGATCATTTTCAACCAAAATAACTAAGGACCAGCAATAAAATTTTCATTA
AGTAGATTTAAATTTGTAGACCTGCATACAATAGGGAAGAATATGTTATTTGGTTTGCTTATATCGATCA
GAGATTTTATTGGTGGATGATACCTACTGATTTATTTATTTATTTATTTATTTTACTATATTAGGAAAACTTA
ACTAATTTATTTTGTAGATAACAATGTCAGGGTAGCTTATGTTTCACAAGTTTCCACCTCTAGGTAGATT
AGTTAGGTAGTATAGAGAGAGTTAACTGAAGCTATAATTAGACTTAGGGCCGATTTTATTTTCTATGACA
TGTTTGAATTTATCTACCAAACTCACTACACAGTAACTTCAACATTATTAAAATTCGCTAAACGCTCTCT
TTTTTGTGAATCCATTGTGATTCAAAAATAACAAATTTAAATACCAAAATCAAAACAACCAAACAGTCA
GTGAGGCTTGTGGCTTGATTGCTCTTTGCTTGGCAGTGATAGTTGACACTGACAATTTAAATAGTAGTC
TGTCCTGCTTTAGCACTAGTGTCTGGATATACTTGTTTCCATGGAAGTAATAATACAACAGGAATGCGC
ACTCACCAAAATGATGCAAACAAAAATAGACCTAACGAATTCACCTTAAGATTTATATTTGAAGTAAAA
GAGGCCGCGAGCCGATAGAGAGGGTTACCACAGAGCCCTTCCTCTCTTTCTAACAATTGCCAGAATAG
TTGTGTAAACTTTAGTAGATTATGTACGAAGAGAGCTATGCAAAAAAAAATATTAAATTAACTCATAGA
CTACTGTATATCATATCATTTTCAAAAACAAATTTATAATAAATCATTATAAACATGTAATTTTAATGAAAT
ATTTGGTATTTGTATGAATAATAATTTCATAAAAAATATTGGTAGTTTTTCTAAATACTTTA
>TCONS_00118218 geneID=XLOC_023533 scaffold_394:192785-196113(+) Splice junction overlap
AGTTCAAGTAGCCGATGGCTCAGGAGTTCAAGTGGCCGATGCCAGTGCTTCAGGACCTGGAGTCCAA
GTTTCGAACGGCGGCGGGGGAGTTCAAGTAGCGGATGCCTCAGGAGTTCAAGTGGCCGACGCCAACG
CGGCAGGACCAGGAGTTCAAGTAGCTGACCAAGGTAAATATTGTTTTTGTTTATAATTAATACTACCTA
AGTAATCAAAGCAACTTATCACCTAGCTGTAGCTATAACATAACATTCAAGATAAAACGTGGTTGGTTT
TTCTTACCAAGTTTACAACAACTTTCCATTTGTTTCAGGATTTTATGTCGGTAAGAAATAAAGTTACCTG
AAGAGACATAATTACTTCGTACAATTCTAACATTAGGACAAGCCGCTGTTAAACAAGTATCGGATGATT
ATTTTATTATTTAAAATACGAAGACCTTTGGAAAAGGCTTTCGAGAAACCCTTTTATTTGGAATTCAGA
AAACCTTGACATTGGATCGCTAACATAGCAACTAGCAAGCTGTAAAATAGTATAAAATATTTGAGGGCT
GAATGAATTTGTGTTGACTTTTGACTGACTTTTTGTAACTTTCAGGTGGCCAACCTCGCGGCGCACGGT
GAATTATATAAAAAAATTATAGAAATTTATTAAATAAATTACCTTACATTATTCCTACTAAATATATATGTTT
TCTAAAATAATTTATTTAAGTGTACAATTATTCACTCCCACATCTTAAAAGTAAAATAGGTAGTCAGATAT
TTTTTGTACATATACTCACGAATTTCTATAAAATACAATGTATCAGATATAAAATAATCATTGACC
>TCONS_00118255 geneID=XLOC_023533 scaffold_394:194242-197630(+) Splice junction overlap
AGTTTATAACACTAGTCAAGTAATGATTTATGGCTCTATCTAAGTGCACAATTCTACATGCATACTAGCT
GCCCAGAGAAGTACTTATAAATGTTCCTTGCGAGTAAGTAACTCGGCCATTTTTATATTTAGATGTTATT
TTAGTTATTTTGGATAACTCTTATTTTTAGTGTGTTAATTTCATAATAATCATATAATGTTAATCATAATAAA
AATATTATAATGTCACTTTATCTATTCACAGTTCAAGTAGCGGATGCCTCAGGAGTTCAAGTGGCCGAC
GCCAACGCGGCAGGACCAGGAGTTCAAGTAGCTGACCAAGGATTTTATGTCGGTAAGAAATAAAGTT
ACCTGAAGAGACATAATTACTTCGTACAATTCTAACATTAGGACAAGCCGCTGTTAAACAAGTATCGGA
TGATTATTTTATTATTTAAAATACGAAGACCTTTGGAAAAGGCTTTCGAGAAACCCTTTTATTTGGAATT
CAGAAAACCTTGACATTGGATCGCTAACATAGCAACTAGCAAGCTGTAAAATAGTATAAAATATTTGAG
GGCTGAATGAATTTGTGTTGACTTTTGACTGACTTTTTGTAACTTTCAGGTGGCCAACCTCGCGGCGCA
CGGTGAATTATATAAAAAAATTATAGAAATTTATTAAATAAATTACCTTACATTATTCCTACTAAATATATA
TGTTTTCTAAAATAATTTATTTAAGTGTACAATTATTCACTCCCACATCTTAAAAGTAAAATAGGTAGTCA
GATATTTTTTGTACATATACTCACGAATTTCTATAAAATACAATGTATCAGATATAAAATAATCATTGACCT
GCACACTCCAGTTTTTTTTTCTAATCTGCGTTACAAAAAATACAAAAACATTATCAATACTCAATGCAA
AATAAGCTATAAGGATTTACTCACAGCAACCTTAGTTCAGGCATCCATAGTACTTACATAGCAAAGAAG
AATAGGACTATAGTCAACTATTACTTACCTATAAGTGAAGGTAGCGCGACATTGGTGCCCAAATAAAGC

ACATATTGACCTCTGTATTTCGGCATTGAGGTCAAGACTGCTCTGCATCCGTCCTCAATAGTTATATTTG
AATTGTATCTGATATATTATTATTATTATATGTATGTATAAAATATTAGGCTCTTTACACTCACACAAAAAA
TCTTAAAAAGTTTACGTTAGTAAAGTTGGATGTCGTAGAAAAGTTCAGTATGCTGTAGATTTCAGGTTA
TTCTTTTATTCAAGTCATATAATAGGTACGGTCAGGTGCAGAAAAACCTGACCCCCCTCTCATAGTAAC
AATGTTTCTGAGAGGGGTCAGGTTTCTCTGCCCCTTACTGTACCTATCATTAGTATCATGCAAGTCATTA
GAAAGGAGCTGATCATTTTCAACCAAAATAACTAAGGACCAGCAATAAAATTTTCATTAAGTAGATTTA
AATTTGTAGACCTGCATACAATAGGGAAGAATATGTTATTTGGTTTGCTTATATCGATCAGAGATTTTATT
GGTGGATGATACCTACTGATTTATTTATTTATTTATTTATTTTACTATATTAGGAAAACTTAACTAATTTATT
TTGTAGATAACAATGTCAGGGTAGCTTATGTTTCACAAGTTTCCACCTCTAGGTAGATTAGTTAGGTAGT
ATAGAGAGAGTTAACTGAAGCTATAATTAGACTTAGGGCCGATTTTATTTTCTATGACATGTTTGAATTT
ATCTACCAAACTCACTACACAGTAACTTCAACATTATTAAAATTCGCTAAACGCTCTCTTTTTTGTGAAT
CCATTGTGATTCAAAAATAACAAATTTAAATACCAAAATCAAAACAACCAAACAGTCAGTGAGGCTTG
TGGCTTGATTGCTCTTTGCTTGGCAGTGATAGTTGACACTGACAATTTAAATAGTAGTCTGTCCTGCTTT
AGCACTAGTGTCTGGATATACTTGTTTCCATGGAAGTAATAATACAACAGGAATGCGCACTCACCAAAA
TGATGCAAACAAAAATAGACCTAACGAATTCACCTTAAGATTTATATTTGAAGTAAAAGAGGCCGCGA
GCCGATAGAGAGGGTTACCACAGAGCCCTTCCTCTCTTTCTAACAATTGCCAGAATAGTTGTGTAAACT
TTAGTAGATTATGTACGAAGAGAGCTATGCAAAAAAAAATATTAAATTAACTCATAGACTACTGTATATC
ATATCATTTTCAAAAACAAATTTATAATAAATCATTATAAACATGTAATTTTAATGAAATATTTGGTATTTG
TATGAATAATAATTTCATAAAAAATATTGGTAGTTTTTCTAAATACTTTA
>TCONS_00118208 geneID=XLOC_023539 scaffold_394:255029-255715(+) Unclassified
ATTCAAAAAAGACAGAATGAACATTATAGTTTTGGGAGGTGTGTTGGTGGCGTTGTGCGTTGTGGCGT
CGGCGGTTCCCGCGGGCCCGGCGGAGGGTGCTGGGGTGCCTGTGGAGTCCCTGCAGGCCGCTGAGAC
TCGCGGCTACGGACACGGGCACGGTGGACATGGCCACCACGGTGGCTACGGCGGCGGATACGGTGGC
AGCTACGGAAGTCACCATGGCGGACACCACGGCGGCTACGGTGGAGGCTATGGCGGTCACCATGGATA
TCGCCGCGGCTGATACGCTAAATTAACTGAGTAGCTAATGTGAAGCTGTGTGATTAACGATATGGATTG
TAGTTTTAATTTTAAATAGAATTGACGTCCATTCGTTGAAACCTAAGATAATTTGCCTGTGATTAATGTA
AAAAATTAAAAAAATATTAATGTAAGTAACTAAGTACAGCCAAATAATAAAACCTGTGC
>TCONS_00118237 geneID=XLOC_023541 scaffold_394:267910-269852(+) Splice junction overlap
GTCGATATTTATATTTAATATGTATCTATCTATGTATTTGTGTAGATATATCAGCTGTCTGATACCCATAACA
CAGGCTCTGCCTAGCTTGGGGTCGGATGGTCGTGTCACACGACATCTCACACACGACTCGTGTGTGAG
ATGTCCCCACATATTTATTTATTTATAATCCATGTTCTGCTCTCCCCCAGGTGATTCTAACAACGCTAGGC
CCTCGCGACGACTGCGCCGCGATGCTGGCGGCGCTGGGCGCGGCGGGCTGCTACGCGCTGCACCTCG
CGCTGCTCCGGAGAGAGCTGAGGAAGGCCGAGGGGATCAACTCACCGCTGCTGGCTGGTCTAGTAGG
CTGCTTCTGCCTGGCGGGCGGCTGGGCGCTTGGGGGGGCGCTGGCGGCGGCGGGG
>TCONS_00118446 geneID=XLOC_023594 scaffold_396:90507-105788(-) Exonic overlap (+)
ATAATATTTTTAGTTTCTTTGGAGTATATTACTATAGAATTCGTTATGATTTGGATATTTGAATTATAAGTAC
ATGTCCATCAGCCTAAAAGCCATCTCGGTAAACCTAATTTTTATTTAGATATATTACGACTTTAACACGCT
TCTAAATCCTTTTCGCCACAACAGTACCTGTTGAACATCATCTTTTTTATTTCTCCGGCCCGAATCGAAA
ACGTTCGAATACTAAAAATCGAATAAATCAAAAATAACTGATCTCACACAAAAGTCCATTCACAATTAA
AACCAGTTTTTTTATTATCATTTTCTAAAAACCCGCAGCATCCGCGCTTTTGGATTCATCTTTTGTGAGC
TTTTATGGTGAAAAGACAAAGTACGATTTTGATAACCGGTTTCTTTTTGTATTTTTAAAACGCGTATTTA
GGTCAGCAACGGCCAGTAGCTGGGATTCTGAACAGTCCTCCTTGGTCTGTGAGTCGGGATACTCGGGA
TAAACTGTGATGTTCGATTAAAAAAGTTATGTTAATTAGTTTTCTTTTTGGAGCCGCGAGGAAAATGAG
CGGTAGTTTCCGGCGGATGTGACGTCACGCCGCCGCAGCGCCCCCTCACGCCCAGTGGTCGCAACTA

AATATTCAAAAGTTAAGAGAACGAAGCTGGCTCCCAGAAAGAAAATGGCGAGAACTCTAAGAAGCAA
GAGGACAACTAAACACCTGATGGATCCTTAATAGTCCGTGTAGTACCTATGTAAATTAATATTGTAAAAA
TAATAATATATAAGCAGTTAACACCATGAAATTACGTAAATCTATAAATAAGTACTATGACATTATGAATT
TAACAAAGTGCCTTTTAATTAATAAATTATTCCTAGTACTTATCTAGGTTCAAAATTAG
>TCONS_00118488 geneID=XLOC_023594 scaffold_396:90555-98302(-) Exonic overlap (+)
CAGTCACTAAATTTCAAGTTACTAAATTATTTTAAAAACTAATATGTTATCCATGAGGACTAGCTACATT
GAATATACTATACACGTGTATTTATTTCCACTTTATATGTATGTATGTATGTATGTATGTGTGTTTACAGGA
GCGGTAGTTTCCGGCGGATGTGACGTCACGCCGCCGCAGCGCCCCCTCACGCCCAGTGGTCGCAACT
AAATATTCAAAAGTTAAGAGAACGAAGCTGGCTCCCAGAAAGAAAATGGCGAGAACTCTAAGAAGCA
AGAGGACAACTAAACACCTGATGGATCCTTAATAGTCCGTGTAGTACCTATGTAAATTAATATTGTAAAA
ATAATAATATATAAGCAGTTAACACCATGAAATTACGTAAATCTATAAATAAGTACTATGACATTATGAAT
TTAACAAAGTGC
>TCONS_00118429 geneID=XLOC_023595 scaffold_396:115807-135236(-) Unclassified
CGCGAGATCATCATGGGGCAACATGGGAAAGTTCCGTTTTTGAAACCGTTCAGCAACGCTTAAGTTTT
GGTGGAATTCTGACAGCTGCAGCAAAAACAATAAATCATATCTCATCATCATTGAACAAATTACAATCG
TCAAGGCTGTTCTACTGGAGATGGCGGAACAAGGAGACTCTAAACAGGGCTACAGTTGCATGCTGTA
GTATTTAAACATGAAACAAACCTCTATCCCGCACTTTCAAGCTAAAATTACTACGATTAGATAAATTAAA
AATAAAAACAAACTAACTTTGAGAAGAATGCAGCTGAATGAAAATTCCAAACAGGATTCTTAAATCCA
AATCGGTCTTGCTCCCGATTTTTTATATCTTATCTTTCACACCTGCCACTAATACGAAAAAATATAAGTG
AGCCAGAAAAAAGCAGATAAGCTACAGTCCAAGACAAAATAAAACTACCAGAACACAAACCTAACG
CCATAAAACGAGCAGTAAACTAAATACCAGCCCGTAATCTTCGTCGCATAAAGTTGCTTTTACATCGCT
TTTCATAATAAAAAATGTTTTAAAGCAAATAATTTTCCATCTCATTAGAATGCAGCCAGTTATCTGGACG
CCACTTTATGCTGCACTTCTGCAGTCGCGCATAATTCCACAAGTGCAGACTTATTGTTCAAAAGAAAAT
TGTTACGGGCAAGCGCTCTTCGGAATTTTAATTCTGCGAGTTTGTCAATGCTTGGCTTCTTTGTAAGGT
TGCTTTTACAGCAAAAAAAAACGTTTTTCGTATGAAAAATAAGCTATATTTTCGTGGTAGATAGATATAG
GAGTTTTCTGTTTTCTCAGCTTAAATGTATTTGAATTATTCTTATATTCTAAATTTGAAAATCCCTAAGTA
CCTTCTGTTTGAACCCCAGCTCAACACATGATTCTCAAATTAAATATGCAGATAAAATCAAGCGCATATA
TAATTGGCATAAAAGAAGAAACGTACAGGCTGTCACAAAAAAAGAAAAAACAAAAAAAAAACTCGT
CGTCCGTCGCATTAGCACGCGGCGTCTTCTTGCAGGAAGATAATTTCATGCAAATCTTTTTCCGCTACT
TTAGC
>TCONS_00118467 geneID=XLOC_023597 scaffold_396:265457-266213(-) Intergenic
CAAATATCTGCCCCAGCCGGGAATCGAACCCGGGACCATCGGCTCAGTAGTCAGGGTCACTAACCACT
ACGCCATTCGGTCGTCTATCAAGCGTGCCGGTTGCGTGATAATTTTCATTCGTTCGTTCGTCGATTTAAA
GAGTAACCCCCAGGTCCCTATGCCTTGGTAATAGGTTAGGTGGCACGAGCCGTCTTACTTCGTTGGGTT
TAAGAAAGCACAGGATGCACATTAATGAGGGTAGGTAGGTATGTATGGAAGCTTTCTTAGCCGACTGC
CGAAGGAGGAGGGTAATGTTTTTACAAACTCAGTTTCTGTAAGCACATTCATTATTTTACCTCGATGTC
AATAGAAGCAATTTTGCGTTGGACAATTTTGTGGAAACCATGTTAGGAATAGGATAGTAATTTAGGCAC
AATACAACATTATAAACGGCACAGAAAAAACCAAGATATTTTTAATGTAGTTGTTCTGTTACTGCAGAC
CTGCAGTAGACCCAAATCTTTGACCTCTACACTCACGAGCAATGAAAAATTTCCAGTGACATATGGTAA
GTAAGTAAAGTACGTCAATGGTAGGTGGAACTTTTTCATTGCTTGCGAGTGCATTAATTAGG
>TCONS_00118441 geneID=XLOC_023590 scaffold_396:270578-272710(+) Intergenic
GTCCAATTTAATAAAAAATCAATATAATTTTAAAACGCCGAGCGGAGACACACCAAACAGCTTGTATAG
CAAAGAACCCACGTACCATATCGTCATTGAGACTTGGGAACCAACGAAGGGGATCGAAGGGGTCCCT
AACGTCTTTTGTCCCTAACAATAAGACGCCGAGTTCCTTACCGACCGCCGCGCGGGCCGCATGCTCGC

CTCCCCTGGCTTAGCGCGCCAAACCTGACAGTTCCAAATTTAAAAACCTACAACAGTTCCAAATTTAA
AAATCCCACGACCGTTCCAAATTTTAAAAACGCTTCGTCTGTGCTCTAGTCTTCTTCTTTTTTAGTGTAG
TTTGTTTCTTAAAGTGGCCAGTGATATTCTAGTGTGCAATTATTATAGTGATTATTAAGTCTGATTAAATG
TGCCTTACTTTTGTGTATGTATACTCTTGGATTGCAATTCAATCGTATTGATTGTACCTACTGCATGGTAT
GCAGCCTGCCGGTTGCATCCATTCATAAAAAGCAGAGGACAGTGACGTCATTATATTGTCGATTGGAG
GGAAGCCCAACCTTCACCCAATAATTGAAGGCAAACGCGTCACCATAGTATAAGGTAGGGCTGAAGAT
AAATCATAAATTATGTTTATAATTATTATTTAATACCTACTTTATTGGATAAATATAAAATATAAGTGTCAA
AAGATGGATTTACTGGTAAAAGCATTTTCTGCCAGTCTTCGTTCAAGAGGTACAGAAGAACCAGTAGA
AAGGTGTAGAAAAGGTATATGTATAAAATAAATAATTCGTTTGGACCGTTTAGTAGGCACAGCGAGATA
GAGTCGTGTTAGACACAAATTTGTTTTTGGTAAGTAGTGGCCCCCAGAGTGAATGACTTTCTAAGGTTA
AAATTTGGTTCTCGTTTCATTAAATTAAGTGGGTCGTTAGAGTAATATTCTAATACTCTTATTACATCTGC
GTAATAGTTAGATTCACGTCAACTAAACAATTTTATCAAGTGCATTTTTTTAATTTTTACTGTTTACCAAA
GCTGTCATTAGGATTATTCAGATATTCCTTTTTTATTACACACATAAAATAACTTAGATGTTACCTTACAC
GCTTACAGGGTTTACAGGCAAGTACAGACCATCGAACTTTTATACATGCAACGTGGACCAAATGATTTA
TCTTCTCGTCGCATATACTGTGACTCGCCGTACACAAAAAGCTGTTATTTTTAAATGAAATAAAAAAAG
TAATGAGTTATCGATTCGAACAGGGTTAATGCACAGCGCTGAATGCCGCCGTAGTAATTCGTATTACCG
GCCAGTGTCAAGGCCGCCCTCCATGTGTTTACATTACCGTGTTTTTAAAAATATTCTTTTTTCAGCGAAC
CATTCAGTTTGACCCTGTATTTAAATTTACTGTTCGCATTTTAAACTTCAGTCTTTTTGGTACTATTGCTC
CAATAACTGTACCTATCTGCATGTTTTGAGCCGTCCATACCTCAGAATACGGTATCCTAAATTAATTGAG
TTAGAAACTCCTTACGTCCATAGGCAGGGGTGACCAACCGCAGGTTCACAAGAAGCCAGAGCGTTTA
ATTGTCGCGCAGATTTACGAGCGGGGGCCGTGTTCGCCAAATTGAAAATTAACTATAAATTAGAGCAAA
TACCGGCCTTCGGACAGAGCTAATGTATAG
>TCONS_00118528 geneID=XLOC_023618 scaffold_397:203184-206244(+) Splice junction overlap
CCTGGAGCGCAGCGTGGAGTCTCTCTCCGCGAAGCAGGACTCCTACTGCGTGTCCCCGCTGCGGTAC
GAGGGCGGGGGCGCGGGGGGCGCGGGGGGTGTGGGGGGCGAAGACGAGCCCGACATCATACCCGGG
AACTATGACAAGCTGCCGCCGCCGCCGGAGTACCTGCGGCTGCGGCCCGCGCCGTACCACCCCGCAG
ACATGTACAGTGGGACGCAGTTCACAGACAACTGTCTGTCGCCGGGCTCGCTCAGCCGCGGGGTGTC
GGCGCGCGCGGCGGAGCTGGCGCCGCGGCCCGAGGTCGTCACCACCGCCCGGAAGGTCAAGGAGAG
CTGCATCTGAAGGTCAAGGTCACCCCGTGGCCACTCACGCAGGACTAGCATTTGACTGTGAAGAAGG
GAATTTAGG
>TCONS_00118599 geneID=XLOC_023641 scaffold_398:115458-119058(-) Unclassified
TAAAATTATATTGAAATAAAGGTATTTTTCTGTGATGGATGCAGTTTCAAGTAAAAATCTGTAATCAATG
ACCTATAGAAATCAAATAAAACTATTATAACCAATAAAATTCATTGTAACACGTTCATTAGCTATCTGAC
AATAAATAACAATCGATACATTATTGAGTACCAAACAATAATACCCAAATATTCATATAAATGTGATTCCC
GCGCGGGCGCTCTCTGCTGTCAAAAAGCGCCGGCAGACGGCAGTTACATGTGTGCGCGGCTGTTCGG
CATAGGAAAGGTTAATGTTCTGTTATGACGTTAACGCGGGGACAGCAGTAACCTATGGCGTGTCCGCTT
CATGTTTGTTGTTGTGTCAAATAACAAGTTACGTTACATTTGGCTTGTGATACAGTGTTACCCCGTTGAG
GAAGCTTCATATCAGGTATAGGTACCTAATGTTAGTTAGTGTTGCACATAGTTTCTTGGCAATAAAAAGT
GCTCAAGTGTAAAACAGTTGTGCCCGCTTTGCGATACTTCGGAGATCACGCACAGATTACAAGGAGAA
ATAAATATTATCTTTTACGAAGAGACATTTTTATAAAAAAATAATACATTACTTATATCTTGTAGGATTATA
ATCTAATAAATGCTTGAATATAAAATACATTCCAAAAGAAGAAAAATATGTGAAATGTTCAGTTTAAAC
AAAAATGTTATTTGGTTCAGTATTAATCGACAAATTTATGACTTTTAAGAGTGGTTAAGGCGCTATGAAA
TGTATTAAAATGTATATTTGAATTATGTATTTTACTTAAACTGTTACAATTATATTACGGGCTATGTAATTA
AAATTAAAGATTGATTTGTAAATGTATCACTGAACGCTTAGGGTAGCGTCGGTCGAGGAGCGGTGAGA

AACGATCCTGTCTACGATATATTATGTAGTACCGGACGGTACGGTAGGAGGCAATCTGCATATCACGTTA
TTGTTTTGTAAATGTGCTAGCGTTGTATGTACACGATGTATTAGTGGCCGTGCGCGCGGGCGCTCGTTG
TTGCGCACGCACACAAGTTGCCGTAGACTCGTAGTCACGTTGTTATAAACAGCCACACTCGCGCGGTA
GATGTTCCGGGTAGAGTAGCGCCCCCCCGCGGCCGCAGCCCGACTCACCTCGGTAGGACGCCACACA
TCAGATAGATTCCAATACAGGTTATCAACTAGAAATTTTAACAAACAATTTTGAATGTTAACTTGCACAA
TTGATATAAAATTGAATGTAAAATTATTGTAATTAATATAATAGCAATAGTAATAACCGAAGCTGTCTTCT
CTTGTACTGTTGCTGGTAGCAATATGTTTGGTCAATTATACTTATTAGAAAGTTTCATTATCGTGATTATAT
GTATTTGTTATTGATTATAACATGCCATTAGGAAAATGTCGCTGTTTATCTACGACCATTGTTATCATGTAT
TGGACATCATTATTCATTGCGCGGATACATTTAGACATTATAAAACTATTTTAATTTCAATAAAATGTCGA
TCGAACTCGTAGTCGATGTAAGTAGTTGGGCCGAGGACACCCGAGTTCCTATTATACACATCATTTCTA
GTTGTTCCATCGCTATTTCAATATTATGGTTAAAACTTAACGAAAATAATGATGTAAAGTTTTGCTCTCTT
GTCTTGTTAATATAGCGTTAGTACCATCGAAAAAGGTATAGACAGTGATTGTTGGATTTGATATAGTTTAT
GAATATTGTTAATCTTATGCTGTATAAATAGAGTAATTAAATGCTATATGTGTACTAAAATGTTTGATTTCA
GTAGTGTCGTACTTATATTGCCTATAGGCTATAACACAGCCCCACTAACAGTTTCCCTAACGATAAACCA
TTGGAAATGATAGGTAAGCTTAGTTTAAAATATTGTAAGCAGTATTTGACTCTAGTGTAATTTCTGTTATA
TTTTCTTCTTGGAATGTATCTTCTGTCCTCTCTGCTTGTAATCCAAACTGAAATTAAATAAATCCTGCTTG
AGCACCCGTCCGGTGCCGAATGTCCCTCCGAAGCTTCCCGAGCGACCACAGAACGGGCGGCCATATT
GAATCCACCGCCGCCATCTTGAATTAAAATAATTATCTGTAAATACGAAGCAAATTCAAGTGTACATTGC
TTTAAATTATGTTTATGCTTAGCATATTAGTTCGTCATAGACTTTGAGTCAGTTGAAAACTAAATACAATA
ATAGCTAGACGATACGGTTTCAGTATTCTGGATTATGTTGCATTATCATTATCACAATCACACGATTGGCA
GGATGCCTTCATTGTAGTGCATGTCGATAAGAAATTTATTTAAAATTTACCTAATTGATAAATTATTGTAT
AACATTAATTTAAGTAGACACACTTGTAACCAATAAATTTAACTACATTGAACATTATAATGAATGCTAC
CACACAAAGGGTAACACAATACATTATAATGTATATTGATAAATTTAACAAATGAACGTGTTATTCCTAG
TATATCAAGCGATATCTATTGAATGTATTTTGCATGGTTCCTAAAGAACCTTTAAACATTAAACAATGGTT
TTACTCATTTCCAGTCATTCAGTAGTCAAATGGACTGATATTACATAAAAGTATTTTAGATTACCTTGTGA
TAGATTGGAGGCACCTATTCAAAGAGCCGTGGACATGAGATTGTTAAGAGAAATGTAAACATTATAGTT
GTTAGTAAGCGATGGTGTAATGTAATGTGGCATTTAGTAGTAAATCTGGCTGGTGAACAACTGCGTAAT
CTTTATGTATATTGTCAAGGACGATTCATTTCATGGCCAGATACTTCAGATAAATAATTATAGTCTTAATT
AACAGACCGGTAGGTAAATATTTGTCGGATAATATTATTTAGATCAATTTAATAAAATACTATTTATGAAA
TAATTCAGTTACGATAATCTAGCTCTACGATACAAAGTTCTACATCATTATGTTAACTTATAATGATTTTAA
TGATTTTTTTGTAATTAATTATGGACCTTGTTGTTTGAAATAAAATGATTATTATTATAAGATGGAAACTA
AGATGATTCTTACACCCATAAAACTATTGTGACTACAAAATAGATCAAGTTTTATGTCACATCATATACTT
TCCTATTCAAATTCAAATTATTTTTAATTAAATTGGTATCATACGGTTTTTTAACTGTATTTGTATTTATTTC
TTACATTCCCTTAGCGATAGTTGTTCACTTGCCAG
>TCONS_00119611 geneID=XLOC_023682 scaffold_4:123784-140966(+) Splice junction overlap
GGTGAATGGCGATGCTGCGAGCTTTACCAGTTAGTTATTATTCCGAAGCCTGCGCCTGCGCACGCGACC
TACACTTGGAGTTGTCGGCTTCTCTTTGAATTGTAATTTAGGGAAGTTTTGTTTTGGTGGTTTTTGAACT
GTGCTGTTTGTAAAATTCGAGTTATTTATACAGGCGAATTAAAAAACATTGAGGCAACATGAAGCGGG
CTACGCTTTTCATAGCTTTTGCTATGATTGTGTCCCAAGTTTACTCGGCGCCGCAGTTCATCACGTTCAA
GGACGGCAAGCTCGGAGTCAACTTCGGCGGCTACCACGCCGGCGTCGGCATCGGGGGACTCCTCGG
>TCONS_00119522 geneID=XLOC_023682 scaffold_4:123836-129126(+) Exonic overlap (+)
CGCCTGCGCACGCGACCTACACTTGGAGTTGTCGGCTTCTCTTTGAATTGTAATTTAGGGAAGTTTTGT
TTTGGTGGTTTTTGAACTGTGCTGTTTGTAAAATTCGAGTTATTTATACAGGCGAATTAAAAAACATTG
AGGCAACATGAAGCGGGCTACGCTTTTCATAGCTTTTGCTATGATTGTGTCCCAAGTTTACTCGGTA

>TCONS_00118896 geneID=XLOC_023688 scaffold_4:542907-544586(+) Splice junction overlap
CTTTTATGTTAGGTAATATAAAACTTCCCTATTTTAAAAATTATGACTTTTTAACAAAAAGACTCCGGAT
GATTTAAATGCATTTTATTTAGCTCTTAAAGCCCCTTTACACCGGCACTTAACCCTGACCGTGTGACCGT
CCCGCCCGGTATTACTCTTTTATTACATACTTTACATAAAATTTCAGGCTTCGTGCATAACAACGGCACG
TGGCTTTGCCATTAATATGAAACTCCACTGTACATAATAAATATAATTATTATTAATGCTTCATCAAAATA
AGTTACTTACAACAAATATAACTTTTCTGCATTTAAAAATACCTATATAATTATTTATTTGCGACCGTAAT
GGTGTAATGGAGTTAGCGAGACTTGAGCAAGTAAAAATATACCGACAGTTAACCGCAAAAAAACAGT
CGCTGTTCGGATGTGTACACTGGTCTTAATGATTTTCCCATTCAAACTGTAAATGGTTTTTTAAATACCT
CCCTTAATTGGAATATGTTTGTTTAAAAGACGAAAATTGAATAAAACGCGCGTTTTAAAAATGTTTTGAT
AAAAGTATGAATAAATGGAAGAGGTTTTAATTCAATAATAAAATTATGTCCATCTCCACAGAGTTTGTTC
TTTGCCGTTTAATGTGGGAAATGGGAAACAAAAATAGAGCGAAGGTTTTCAAAGTTCTCATGACCAGT
ATAAATATGAATGTTTTCTTATTTAAAGCAATAATACAAACGTTTCACCGATAGACAAAACCTTTATGGG
GCAATAATTATCATGGCTTTTTGGTTTGTCCGCTCATCTCTTCAGTGTAGTAAACCCTCTCCCGTGTGTC
CGCAGACGGACCCGGCGGACTGGCGCAAGGTGTTCTACCTGGCGTCGCTGGTGTACCTGCTCAGCAA
CGGCTTCTTCCTCATCTTCGGCACCAGCGAGCGCCAGCGCTGGAACGACCCACCCACTCACGAGCAC
AACGAAACTTCCACTCAGCATGCATCAGGGAAGGTGGTTTAAAATATTTAAGTTTCTTACCAAGTATTT
ATTGTTAGTCCTATAGTGTAATTATTTATTGAAAATAAAATGTTAATATTAAAACACTTTTTTTTTCATTTG
CATATTTTTTTGATTCAAAGCCTGTCTGGTAGACCATTTTGCTCTAATTAATAAGGCTGCCTTTTTGGCA
CCATCTTATTTTAATGATGCAAATTATAAATATTTTATTGCAAAGGCAATTCATAGAAGGTTAAGTTTAAA
C
>TCONS_00118911 geneID=XLOC_023699 scaffold_4:642039-651939(+) Unclassified
GCGCAGGCTGCGCCGGTTCGCCTCAGCAGTGACGTAAGTGAGCGGAGTTTTCGGTGCGTTCGGGGCC
CCCACCAGCCGTTTTGTTTACGCGACGGACGCCGAGCACGCCACTCCGTAGCCGGCCCTCGCGCCCG
ACGCGCCGCCTGTCCTCGCCGATCTCTGTTCACGACGAGCACTACACGTGATACCACCAGCCCGCCGC
CGACGGATCCCGGGCTCTCCCTCGCCCGCCCCTGCACCCCGGATCTACCTCCACCACCATCACCACTC
CCGCTACGTTCGACAAGTGAACGTCTAAGGCACCGACTGCATCTCACCCAAATTCTTTGGCTCAGGGC
AGCGCGGAGACGGTCGCGGGCACGCCGGGCGCGGACGCGGGCACCATGAAGGGCGCGGAGGGCAC
GGCGGAGGAGCAGGAGCGGCTGGCCACCGGCAACAACAACNN
>TCONS_00119392 geneID=XLOC_023699 scaffold_4:668494-671376(+) Splice junction overlap
CTGTGCGGCGTGTTCGGCTCCTGCGTCTCTTACAACAACCAGGCGTTGCGATACAACTTCTTAGGTCTA
TCAGCCGGCCTCATGTGCCTCGCCTTCATAATGGACATGCTAGTATGGAAGGCGGCGTCTCGCATCGAC
ATGAACCCCGAGGACGAGGACCTGCCCGCGCCCGACGCCGCGCCCCTCGCGCCCCTCCCCGCCCCCG
CGCCCGCCTCCGACACGCAGCTGTGACACACGGCGTACGAGAGCCATTTCTAAACGTTCCAATTTTGA
AACGACATGAACATTTTTAGGCGGAATGTGACTAGCGTTTGGGGGTTTCTGAACGTTCCACTTTTGAA
ACCGTGAACATTTTTAGCCGGAACTGTGACTATTTGAGGGCCATTTCTAAACGTTCCACGCAAGACGC
CATGTAGGATGTAGGTGGATTGATTGATATTTAGAGTGTAGTGTGTGACGAGGATCGTCGAATGTGAGC
GGCGCTAGACGGCGCTAGTGTCTATGTGGACGTAGCAAAGGATATGTGACTTTTGGTTGGTAGTTTCGC
CGCGTTCGATCGATCCTTGTTATTTGAGTGTATGTTTGAGTGTAGTGTAATGATTGATTTTTGATTGGTTT
AGAAATATTACTGCATGGGGAGTGTGTGTGGGAGCTGGAACAAGGCCAGTGTGTATTGTGTGAGATAA
GCGCGATCTTATGGCATTGTACCTATTTAGAGAGCAAAACATTGAAAGCTTTATACTATTTATACTTTTAC
AATCGAATTAACTAACGATACTATACAACGTGACAATATTTTAAAGGCAGATCATCTTTAGTTTTCTACT
CTGACTAAATATATCTGAATAAATAATATTTTTCGCATTTCGTCACATAAACAAGACTCAATTTTTACTTT
CTTTTGAAATATCTCGAAACCACTAAATGAAGTGTTCATAATGATTTTTTTATCTACGGTTTTTATTTCTT
TACACATTTACTTTTTTACCTATGTAGGTACTCTCGACATTTTTAATTACACAAATGTCTAACTCGAAAGT

TAATGATATTAGACATTATGATCACGAACCAAAAAGCATAATAATAATAATCATAATATATTTTACATTCTA
ATACAATTAATCTACGTTTTGTTCATGTAAGTACAACAGGTAGTGCGTAAAAGCATTTGTGGTGACGAA
ACTATGTAGTGTTCATTCCATACAAATCATAAATACTTACTGAGAATGACAAATTCTTTATATTGCTACAA
TTTTTTTATCATAAAATTGGGGCAGCTTTACTGCTTGATTTATATCAGCTATATTTAATACCTACAAATAGT
TAGGAGGTATACGTTGTTTTGTTAAGGTATGTATGAAGTTCAAATCTTACAAGGTTCCTAGGCCTGAGT
AATGTTACCTAACTGGGCTACAGGGTTAACCTTTACATCACTCTGCCTCACTGAAAGACTAAATAAAAT
TAATACATCACAACAAATCACAATATATATTGGGACTGAAATAAATATATCTGATATCGGATAAAGCGAAT
AAATGGCTAGTGATTAGTGAATTATTTTAGTTTAATATTAAACGTATTTAGGTTTTATTACAAGTAATGTT
ACAATTTGTAAATACGCTAATGAATTATACCCTATTACATATTATATTCTATCAAATTTATGTTCAATCACA
ATTTATACATAATTGTATTACAACTTCTATGTAAGTAATTCTTGAACCTATTGTTCTTGTTATTTTTTACTGT
AATATAATATACTATAAACATATTAGACACAAATTAAGGGAGGTGCATATTATATCTTCACAAAGGCTGG
GTCACGTGTAAAATTAAGATATAATGTTTAGGAAAATTAAGTACAATTTATTGTAGGTTATCCTCGGTTA
AATGAATCTTGATGAATTTGTGTATCGTAGAACCAGTGTTATTACTTAAATTCGCCTAAAATTAATGAAA
TGCATATTAGGACTAATTAACGCAATATCTGATTCCTACTTATAAAACATGTGCATTATACTGTGCAATTT
ATTGTTCGCGTTGAAGCGTTTTTGTATTAATTACAAAGTTTTCT
>TCONS_00118970 geneID=XLOC_023733 scaffold_4:676554-677265(-) Intronic
NNGTTACAAAGGCGGATACGGACGCTAAACCTGAAAATACTCAAAGATGACCCTGAAAACCCCAATG
TGATATTATTTCTTACTAGGAACCTTTGTAATTGGCAACATTATGAAGAAAATCATGTATCCTATGCGTAT
TAATAAAAGTTAAGCTTTTAAACAAATAAAACTTTACTTTTCTCCTCATTTAAGTCATCGCGACCCATTA
CCTCCCCACTGCTGGGGCACGGGTCTCCTTCCAATGAAGGAAGGGTCTAGGCCTAGTTCACCACGCTG
GCCAAGTGCGGGTTGGTGGACCCCAACACAAGCAAGCTTGTGCTGAGAGAGTTGTCGGGTAAGTGGG
CAACCCGACTGTCAGATGTTTTCAAGCCGCCCGAAG
>TCONS_00119541 geneID=XLOC_023735 scaffold_4:701265-701872(-) Splice junction overlap
TGCGAGGCTGACCCCCTTATAAACACGGGCAGCCAGCAGTTCATCGAGGAGGTGGTGGTGGAGTCGG
TCCTGCAAGTCCGCAGCCCCGCCGCCGGTCGCCAGGCCCGCACCAACACGCAGACTTCGCTGAAAGA
TGGCGCTAGGGTCGCCGCACTTAAAAGCAATTGATAGTATATTGGGTTAGCAGGAGATTAGTTGTATTTT
AGATTTAAGGAATAAAATGTATTGAATTGTATATTCATAACAAATCCACCGAGTTTTATACTACAAAGTC
AGATAATAAAGAG
>TCONS_00118977 geneID=XLOC_023739 scaffold_4:722124-722783(-) Unclassified
ATTTAACAAATATTGACGACCATCGAACATGTGAGGTCGTTTTAGTAGGTACTATCAGCCTCATTGGAG
CATCTAGTTCTGATTTAAAGCCTTATTTGGTTGAACAAAGGACAGATATTTCATGTTTACGAGTAATTCC
TGAACTATTGTTAGCATTGACTGTACTACGATTCACACTTATCTGCAAATTAACAAATGGCCATACGGAT
GTATATATTGAAAGGTTGTGAGGATATCCATCAGTGCTGAAGTATTCGGGTCCACAAAATTACATAAAAT
GGGTCGGATAAGTGTCCTCGTGTTGGCCGTAGTGGCCGCAGTTTGTTTAAGTTTAGTAAGCTCAGCCC
GTATAGCAAGGTCTCCTCAATTCGGTGGTGGCTCGTTCTCCCAGGCCAACTCCCAGTCTGGCACCTTC
GGCGGTGGTCCCTTCCAGCCGCAGTTCGGCCCCCGCCCCCCGTTCAATAACCCAAC
>TCONS_00119646 geneID=XLOC_023745 scaffold_4:895842-900516(-) Exonic overlap (+)
GTTTATCGAAAATGAGATTCGGAATTTAAAATTTGAGTTGTGTTCTGAATTTTAAATTATTGTGATACATA
AACTGGTATAATGCGGAGATAACGAACGGACCGCTTAAAATATAGTTTGATCTAATAGTGAACATGTTA
AAAGTGTGATAATTAAAATAAGTGTAACAAAAAGTTATCCGATAGTGCAGAGTGTGTAAACAGTAAAC
GGAAACAGTGAATATAGTGTCACAAAGTGTAATCTCATAATCACTAAGTGCAATCCTAGTGCAGCAGGT
AAAGTGAGATCTCAGTGCCGCAAATTGTAATCCCAGTCACACAAAGTGTGAGCCTATACTGCCGCAAA
GTGCAATATTAGTGCCTCAAAGTGCGATCCTATTACCTCAAAGTAAGATCTCTGTGCCGAAAAGTGAGA
ATATATTGCCATAAAGTGAGATCCTTGTGCCGCAAAGTGATATCTCGGTGTCGCAAAGGGAGATCCTAG

TGCCGCAAAGTGCCGCATTGCCCCGCCGAGCGTGCCCGGAGTGCGCCGCTCGGCTAACATGCGTTGAT
ATTATGGAGACAATATCCGAAGATGCAGAGGCT
>TCONS_00119490 geneID=XLOC_023746 scaffold_4:972664-981801(-) Splice junction overlap
GTTTTTTTCGCGGCAAAGAAACCATCTGAAACAGCCAAACAATTTCAGCCAAAATCGTCCTTTTTAAA
TTAAAGTTTACCCGTTTTTGTGTGTGGACAAATTAAATTGCCTTGTTTGTTGTGTTTTTAATTTATTTTTT
GTGTTTTTGCGTCGTGTTGGAAAATTACTGGTGTTGTTTTCGACTTGGTGGGATAAAAGTGGCTGGCA
AGACGAAAGAACATAAGTGCTGTGAAAAAGAAACATACGTCAATAAATAAAATTTGAATTTGGATCGT
TATAAAATGTGCGACGCGACCGTAGTGTCTATGGTTCCGGTCGGGAGGTGCTCGGAAGCATTCGGCTG
TTTCCGTGAGAGCTGTCAGTGAGAAAACAAAATGGCGGAGAAAGTCAGCTGATCGGAAAAGCGTCG
CGCGCGCAGGGAACACAGCGAGCGGCGACAAAAACGAATCATGCAGGTGTTCGGTGAATACGCCCA
AGTGGAAGTGGTGG
>TCONS_00119632 geneID=XLOC_023708 scaffold_4:1273962-1275162(+) Intronic
AGCTGATCCCGATACCTGCGCGCGCGCAGACGCGACCCAGTTCCACAGTTTACGTTACTGATATAGGG
AATATACTCTTGAGGAGTAGGGGCGGGGTTGCCGTGTGCTGGTGGCGGCTATATCTGGGCGGCTGACG
TCAAGCCGCGGCGCGCAGTCGCTCGACGCCGGTGCTGGAGTGCGGAGCGCTCAAATTCAAATTAAAT
AATAAATAAAGAAATGATTTAGGCGAAACATTCAGAAAATAATTCCGTTAGTGACGTAGTAAGTGACGT
TCAGGATTGCACGCCGTTGCGTTTTTA
>TCONS_00119555 geneID=XLOC_023757 scaffold_4:1310957-1313708(-) Exonic overlap (+)
TGTGGGCCTGGAGCCAGCTTCTAGACCCAGGCTGGGGCTAGCCTGGCGCACGGAACTGATGCAACTG
TAAGGGAGACACTGGGACCTGAGAGGTTTTCTACCAGGATGCTCCGGAAGTAAGGGACCCACGTGTA
GTAGAAGCCTTTGTGACCGGTTAGACCCTGGCATTTAATGGATCTATCATGGAGATTTACAACCCTCCG
CAGGGAACAAAGATATCTGCAGACGTAAGGGTTTGGTCTAGAGTAGGAGAAGATTTGTTCTAGTTAAT
TTGCTGTACAGAGTATTCTAGACCCTTAAGACAAGGGTCACTATGGATTCTTATGTTTAGAAGTGTCTG
GTCAAATCGTTTGCTTACCTGGACAAGGATGAACGATACCTATTTGTATCTCTAAATGTCTTCTATGATG
TCTTCTGAGATGAAGAAGGCATGGCTTTCTTTCATCCAGTGTTGTTAGGAACTTGGGATGGTGTTTTAT
GGCCATAGCAAAGTTCGACTGTCGGTTACGACGTCTGTACGGGTTTTCTTTTAGTTATTAATAATGTATG
AATGCGTTGACAGTTCTTGAATTGAACAAAATTATTAATTGTTACATAAATAATGCAAATAGAAAATACT
CAGTGAAAGATGGCGATAGAAACTGTTAGACAAATAACAGTCATACATACTATGTGTAGTATTGTGTAG
GTACTGACATAAATCCAAATTAAAGAGCTGCATGTGAATGAAGTGGTTATTTCGTTTCTATTACATGTGT
TGTAATGTTTACCTAGATATAAGTACATAGTAGATAGCTGTGTTATTTTATGTATAATTTGTAAATAAGTCA
TTCTCGACTGGGCACTGTACTTGGCTGTGTGTGTAGTCATAGCATTTATTACTTTAAATATTTTGTCTCG
AAAATAATATTATAATGTTTTCCTGAAAGCTAATACCTTCATTCACTTTTTATAAGAATTAAATAAGGTTT
TTCATCAATTTCGCTATCGAAATGCTCAAAACCGCTTGCCGGCGTGTCACTTTTTCTTATCTCTATTAATA
CTTCAACAGTCTGAAATTTTCCTAACATACTACCTATGTGTAACATTCGTAAAGCTCTCATTTTAGCATT
TCTATACCGTCACATATCCACACAACACAGCATTTTAAATAAAGATTTTGAATCTATTTTGTGAATTAGG
GATTTTTGTGCCATGTGAATGATCTATACAACATTTATTATGTATTTCAATACGAATACGATTGATAGGTA
ACTTTAGATGACTATCTATAAAATAAAATGCGAAGAATTTAACAAGTCGAAACTGCACAATGCAACTCC
TGTGTACAGTATTCCAATAGTTGTAGATAGGGATTTAGATGTAGTGAATTTTAAAACATAAAATTGATGA
GACTCCCATCATTTTCTAGACTCCAGTGAACTAAACACTTAAATGTTAAATTAATATTTCTTACTGTGCG
TAAACTCTTGTAGGTAACTGCCCTACGAGGCTTGGCCTATAATCAGATCCGAAGTGTTTTAGGCTTCAG
GCAGGGATCAGGGTCCTAATACCCGGCTTAGCTAACCATCAACCAATGCATTTCTTATATCCTTTATTTTT
ATATTCCTCTGTGTGATGAGAGTCGGTGTAAATAAGTAGTTGGAATGAGCATAAAATGTTTGTGTCGGA
GGATTCCCTAGATAGTCTTTGAAATTCTAATGGAAGGCGGTGAAGTTTATTGTACTTGTATTATTTTTATG
TAAGTATAACGAGTGCACAGGGGTATAATCGTGACAGTTCTGACATTGCGAAAAAGAGACGCTTAGCT

ACATTCTCGCTACATTAAGCGTAAGAAAGAAACGTTAAGCTGTAAAAGTTTTTTGTCCTTTTTGCTGTG
GCGCCTGTTTACGTCAGATCTCTGTGCCAAACAATGAACTCTATGCCAAACAAAGACTGACAGTTAAA
AACAATATTGGAAAAAAGATGGAAAAACTGATATTATTGCATAAATGGTTTCCGAAAGAAC
>TCONS_00119362 geneID=XLOC_023758 scaffold_4:1331649-1332308(-) Unclassified
CGTTCAGTCTGCATATAAAATTCATCACTTTATTCTTATTTTTCCCCTATGTTCAAACTATTCCCAATATAT
ACAACTTCAACTTTAAAATATTGATAAGCGACTAGATAAGTATTTACTATGATCAGTTTGATTTACATCCC
TTATGTCGAGTTACATTATGTACACATTAAATCATCGCTAAATGCCATCTGAAATCTGATTCACCAACTTA
CTGTAAACTCAATACACATTAACCTCTAGAATGTCGTGTGCCAGATCTGTCCCAACCCCCCTTGTATGC
[truncated: 471,688 more chars]
